# Characterization of Quasispecies of Pandemic 2009 Influenza A Virus (A/H1N1/2009) by *De Novo* Sequencing Using a Next-Generation DNA Sequencer

**DOI:** 10.1371/journal.pone.0010256

**Published:** 2010-04-23

**Authors:** Makoto Kuroda, Harutaka Katano, Noriko Nakajima, Minoru Tobiume, Akira Ainai, Tsuyoshi Sekizuka, Hideki Hasegawa, Masato Tashiro, Yuko Sasaki, Yoshichika Arakawa, Satoru Hata, Masahide Watanabe, Tetsutaro Sata

**Affiliations:** 1 Laboratory of Bacterial Genomics, Pathogen Genomics Center, National Institute of Infectious Diseases, Tokyo, Japan; 2 Department of Pathology, National Institute of Infectious Diseases, Tokyo, Japan; 3 Influenza Virus Research Center, National Institute of Infectious Diseases, Tokyo, Japan; 4 Department of Bacterial Pathogenesis and Infection Control, National Institute of Infectious Diseases, Tokyo, Japan; 5 Department of Clinical Laboratory, Nagano Red Cross Hospital, Nagano, Japan; 6 Department of Pathology, Nagano Red Cross Hospital, Nagano, Japan; National Institutes of Health, United States of America

## Abstract

Pandemic 2009 influenza A virus (A/H1N1/2009) has emerged globally. In this study, we performed a comprehensive detection of potential pathogens by *de novo* sequencing using a next-generation DNA sequencer on total RNAs extracted from an autopsy lung of a patient who died of viral pneumonia with A/H1N1/2009. Among a total of 9.4×10^6^ 40-mer short reads, more than 98% appeared to be human, while 0.85% were identified as A/H1N1/2009 (A/Nagano/RC1-L/2009(H1N1)). Suspected bacterial reads such as *Streptococcus pneumoniae* and other oral bacteria flora were very low at 0.005%, and a significant bacterial infection was not histologically observed. *De novo* assembly and read mapping analysis of A/Nagano/RC1-L/2009(H1N1) showed more than ×200 coverage on average, and revealed nucleotide heterogeneity on hemagglutinin as quasispecies, specifically at two amino acids (Gly_172_Glu and Gly_239_Asn of HA) located on the Sa and Ca2 antigenic sites, respectively. Gly239 and Asn239 on antigenic site Ca2 appeared to be minor amino acids compared with the highly distributed Asp239 in H1N1 HAs. This study demonstrated that *de novo* sequencing can comprehensively detect pathogens, and such in-depth investigation facilitates the identification of influenza A viral heterogeneity. To better characterize the A/H1N1/2009 virus, unbiased comprehensive techniques will be indispensable for the primary investigations of emerging infectious diseases.

## Introduction

In April 2009, an H1N1 triple-reassortant swine influenza virus (A/H1N1/2009) was detected in humans with febrile respiratory illness in North America [1], and the virus has rapidly spread worldwide by human-to-human transmission. According to the disease outbreak news from the World Health Organization, at least 14,711 people died from A/H1N1/2009 between April 2009 and January 2010 (http://www.who.int/csr/don/en/). Fatal cases from A/H1N1/2009 viral infection were summarized in a report by Gill *et al.* [2].

The genome of influenza A virus (family Orthomyxoviridae) consists of 8 single-stranded negative sense RNA molecules spanning approximately 13.5 kb. The segments range in length from 890 to 2341 nucleotides (nt) and encode a total of 11 proteins [3]. Genetic diversity in influenza virus results from a high mutation rate associated with replication using a low-fidelity RNA polymerase and the reshuffling (reassortment) of segments among coinfecting strains. Multiple-reassortant influenza viruses from avian, human, and swine origins emerged as major pandemic influenza viruses (i.e., 1918 H1N1, 1957 H2N2, and 1968 H3N2) causing significant mortality in humans in the 20^th^ century [4]. Such an “antigenic shift” by multiple reassortant drives the emergence of pandemic influenza viruses, with their severity and clinical outcome always unpredictable [5].

Influenza A virus can evade antibodies specific to its attachment protein, hemagglutinin (HA), by the accumulation of amino acid substitutions in HA epitopes [6]. This “antigenic drift” in HA epitopes [7] affects recognition by antibodies that neutralize viral infectivity by blocking the interaction of HA with sialic acid residues on host-cell membranes. The H1 subtype HA has four antigenic sites recognized by monoclonal antibodies with high neutralizing activity, designated Sa, Sb, Ca, and Cb [8]. In addition, 8 continuous B cell/antibody epitopes for human H1N1 HA proteins have been experimentally defined by the Immune Epitope Database and Analysis Resource (IEDB: http://www.immuneepitope.org/) [9]. Immune epitope analysis of HA epitopes in A/H1N1/2009 is also summarized in the Influenza Research Database (http://www.fludb.org/brc/homeExtraPage.do?decorator=influenza&extraPage=separate) [10].

To better predict a future pandemic of influenza A virus, the characterization of possible antigenic drift will be indispensable. Igarashi *et al.* and Shen *et al.* reported that a structural comparison of HAs could predict probable future antigenic changes during the evolution of A/H1N1/2009 in the human population [11], [12].

In addition to this prediction, extensive investigations on viral quasispecies will be required to reveal the actual appearance of those antigenic changes. Nakamura *et al.* demonstrated the direct detection of potential pathogens, including influenza virus, using *de novo* pyrosequencing [13], but the detection appeared to have insufficient redundant sequencing reads to reveal the genetic variation of the viruses. Ramakrishnan *et al.* demonstrated the discrimination of quasispecies in mixed HA subtype infections of influenza A virus using the same pyrosequencing approach [14]. However, it was shown that the influenza viral RNA sample should be enriched through sequence-specific oligonucleotide capturing prior to pyrosequencing, indicating that such enrichment might represent a biased result.

Here, we performed *de novo* sequencing using total RNAs extracted from an autopsy lung of a patient infected with A/H1N1/2009, and detected potential pathogens such as bacteria in addition to A/H1N1/2009. Extensive DNA sequencing using the Illumina Genome Analyzer II (GA II) revealed that quasispecies for the HA sequence were generated in single patient. Such heterogeneity demonstrated the antigenic drift of HA, implying the existence of a mechanism to escape pre-existing human immunity to the virus.

## Results

### Summary of sequencing reads and detection of potential pathogens

**Figure 1:**
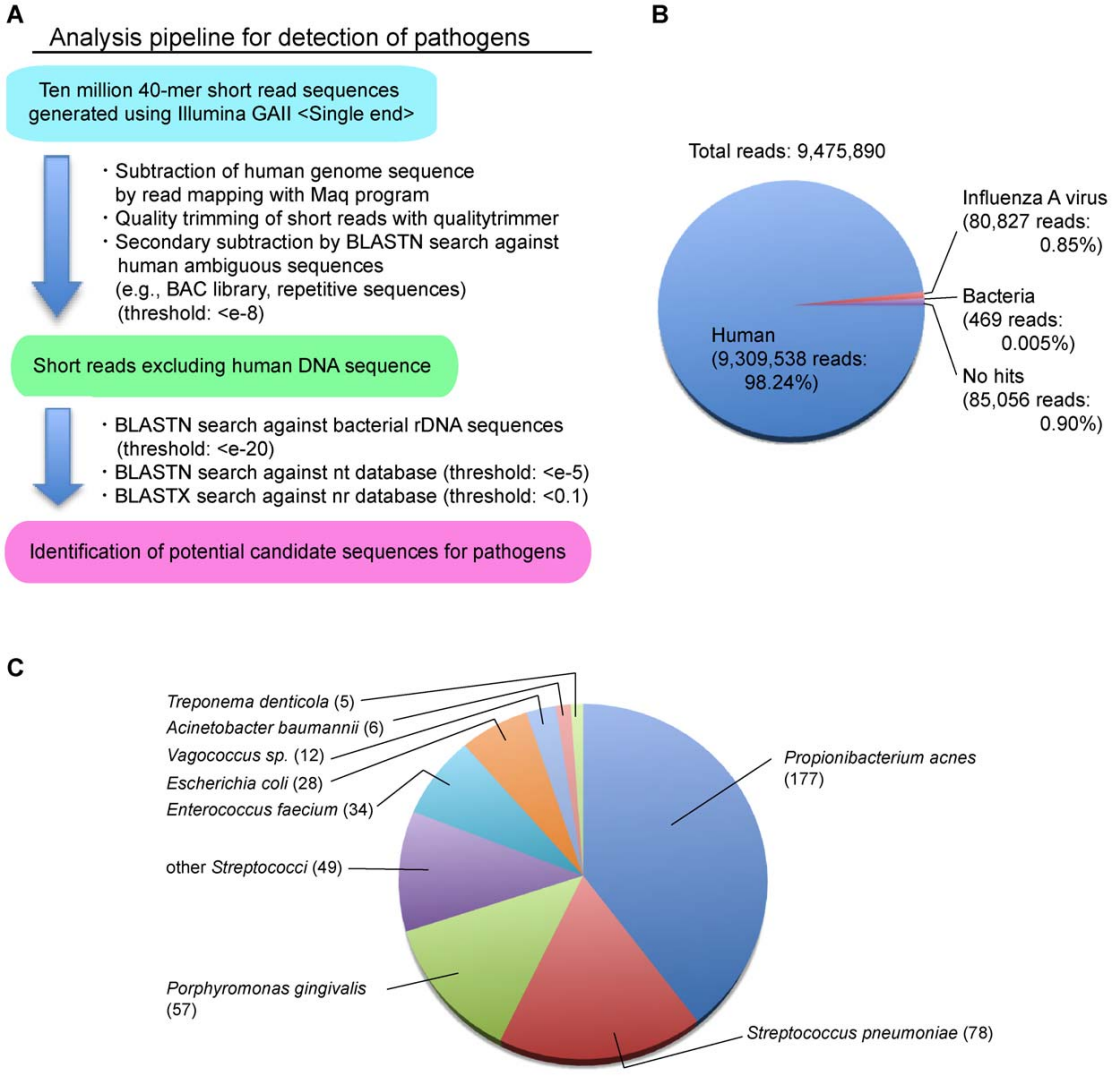
Detection of potential pathogens by comprehensive *de novo* sequencing. (**A**) Schematic representation of the analysis pipeline for the detection of pathogens from comprehensive sequencing of human clinical specimens. After excluding human-derived DNA sequences using Maq software and a BLAST homology search against human genomic DNA and human ambiguous sequences extracted from the nt database, the remaining short reads were subjected to a BLAST search to detect potential pathogens. (**B**) Pie chart of the homology search results for the 40-mer short reads. Read numbers and their percentage to the total reads are shown in parenthesis. (**C**) Pie chart of identified bacterial hits. Number of hit reads is shown in parenthesis. Bacteria with less than 5 hit reads were excluded.

To determine the influenza A virus sequence and other potential pathogens, we performed *de novo* sequencing of double-stranded cDNA from total RNA extracted from the autopsy lung of a patient infected with the A/H1N1/2009 virus (A/Nagano/RC1/2009(H1N1)) in August 2009 in Japan. The patient was found to be positive for A/H1N1/2009 by real-time reverse transcriptase-polymerase chain reaction (RT-PCR); histopathological findings were also reported [15]. GA II produced 9.4×10^6^ 40-mer reads from the cDNA library (Fig. 1B). To exclude the human-derived read sequences, we performed an analysis pipeline as follows (Fig. 1A). Initially, all 9,475,890 reads were aligned to a reference sequence of human genomic DNA, followed by quality trimming to remove low-quality reads and excluding reads with similarities to ambiguous human sequences, resulting in 9,309,538 reads (98.24%) with a possible human source (Fig. 1B). The remaining 166,352 reads (1.75%) were further analyzed using a BLAST search against non-redundant databases, revealing 80,827 (0.85%), 469 (0.005%), and 85,056 (0.90%) reads as influenza A virus, bacteria, and no hits, respectively (Fig. 1B).

Regarding the bacterial hits, species classification was determined based on the results of a BLASTN search against the nt database (Fig. 1C). The most abundant bacterium was *Propionibacterium acnes*, but our other sequencing trials for clinical specimens suggest that this species is always detected (data not shown). Therefore, the presence of *P. acnes* could be the result of contamination at some point from the autopsy to the preparation of the cDNA library. In addition to *P. acnes*, *Escherichia coli* and *Acinetobacter baumannii* were frequently detected as possible contaminants. Suspected bacterial pathogens were identified as *Streptococcus pneumoniae* and *Porphyromonas gingivalis*. Specific PCR using 16S-rDNA and the *lytA* gene was performed for further verification of the presence of *S. pneumoniae* (data not shown). Although *S. pneumoniae* was not sufficiently abundant to conclude a coinfection with A/H1N1/2009, the severity of the A/H1N1/2009 infection could be correlated with *S. pneumoniae*, as reported by Palacios *et al* [16]. The other detected bacteria, such as *Streptococcus* sp., generally constitute the normal human oral flora.

### *de novo* assembly of the A/H1N1/2009 virus

**Figure 2:**
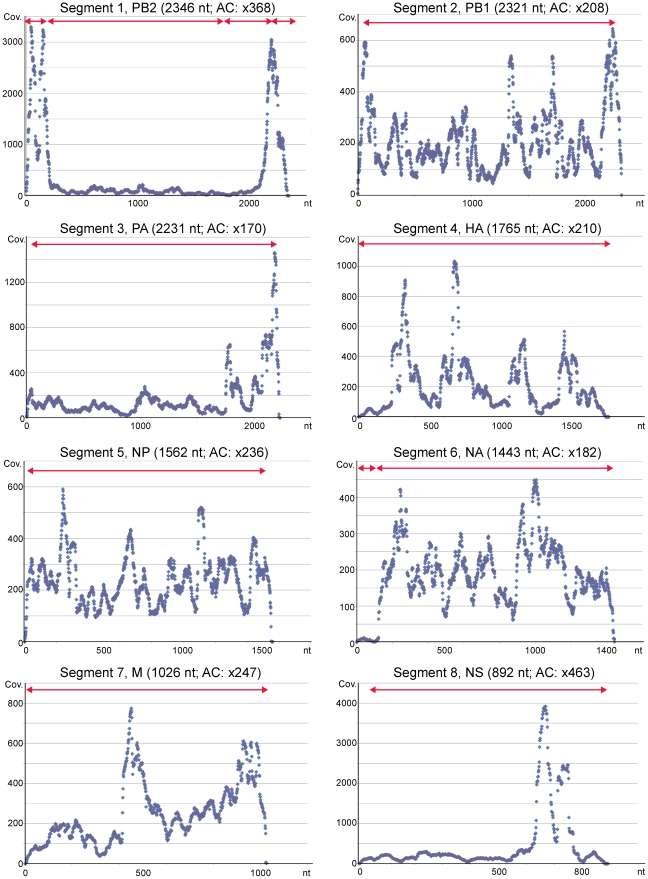
Dot plot of short read coverage (Cov.) at every nucleotide for the 8 segments of A/Nagano/RC1-L/2009(H1N1). To obtain the consensus sequences for the respective 8 segments, 40-mer short reads were aligned to the complete segment sequences of A/Tronto/T0106/2009(H1N1) (gb|CY045951.1 – .8). Short read sequencing was performed using total RNA including human RNA, and also vRNA, cRNA, and mRNA from influenza A virus; thus, coverage bias was detected throughout the segments, but the average coverage (AC) is likely to be similar at approximately ×200 or more. The horizontal red arrows show the location of the contigs obtained by *de novo* assembly, as shown in Table 1.

Whole 40-mer short reads, including human-derived reads, were assembled using Euler-SR or the Velvet *de novo* assembler. The resultant contigs generated using Euler-SR had longer extended sequences than those generated using Velvet (data not shown); thus, all further analyses were performed using the contigs generated using Euler-SR (Texts S1 and S2). All contigs showed high similarity to the sequences of A/H1N1/2009 (Table 1). Among the 8 segments, almost the whole lengths of segments 2 (2321 nt), 3 (2231 nt), 4 (1765 nt), 5 (1562 nt), 7 (1026 nt), and 8 (892 nt) were correctly assembled as single contigs of 2204, 2198, 1761, 1514, 1019, and 834 nt, respectively (Table 1 and Fig. 2). Segments 1 and 6 were divided into several contigs, but were correctly aligned to the known sequences (Table 1 and Fig. 2).

**Table 1:** BLASTN search results of *de novo* assembly contigs against database of Influenza virus sequences.

Euler-SR_contigs	Contig length (bp)	Virus segment	Top hit of accession number using BLASTN search against databse of Influenza virus sequences	Length of subject (bp)	Identities	Contig location for A/Toronto/T0106/2009(H1N1)
>826 183 2835	183	1	gb|GQ328865|INFLUENZA A virus (A/Finland/553/2009(H1N1)) segment 1 polymerase PB2 (PB2)	2345	167/168 (99%)	5–168
>324 1558 136	1558	1	gb|GQ365425|INFLUENZA A virus (A/Fukushima/1/2009(H1N1)) segment 1 polymerase PB2 (PB2)	2280	1556/1558 (99%)	201–1758
>1194 239 112	239	1	gb|GQ894926|INFLUENZA A virus (A/Delaware/03/2009(H1N1)) segment 1 polymerase PB2 (PB2)	2280	214/214 (100%)	1894–2107
>887 174 3294	174	1	gb|GQ894833|INFLUENZA A virus (A/Rhode Island/08/2009(H1N1)) segment 1 polymerase PB2 (PB2)	2280	156/156 (100%)	2145–2300
>890 2204 4651	2204	2	gb|GQ894924|INFLUENZA A virus (A/New Mexico/04/2009(H1N1)) segment 2 polymerase PB1 (PB1)	2274	2200/2204 (99%)	41–2244
>696 2198 3968	2198	3	gb|GQ866924|INFLUENZA A virus (A/Thailand/CU-H106/2009(H1N1)) segment 3 polymerase PA (PA)	2238	2152/2155 (99%)	54–2208
>868 1761 3831	1761	4	gb|CY045503|INFLUENZA A virus (A/Bayern/66/2009(H1N1)) segment 4 sequence	1754	1750/1754 (99%)	1–1741
>897 1514 1710	1514	5	gb|GQ502907|INFLUENZA A virus (A/Toronto/R8557/2009(H1N1)) segment 5 nucleocapsid protein	1558	1511/1514 (99%)	36–1549
>224 101 9	101	6	gb|GQ502908|INFLUENZA A virus (A/Toronto/R8557/2009(H1N1)) segment 6 neuraminidase (NA)	1458	101/101 (100%)	3–103
>1206 1302 2468	1302	6	gb|GQ906584|INFLUENZA A virus (A/Stockholm/49/2009(H1N1)) segment 6 neuraminidase (NA)	1447	1299/1300 (99%)	124–1423
>750 1019 1128	1019	7	gb|CY045957|INFLUENZA A virus (A/Toronto/T0106/2009(H1N1)) segment 7 sequence	1026	1017/1017 (100%)	9–1025
>809 834 4399	834	8	gb|GQ485660|INFLUENZA A virus (A/Ekaterinburg/01/2009(H1N1)) segment 8 nuclear export	877	828/830 (99%)	52–881

Schematic representation of contigs is shown in Fig. 2.

### Read mapping analysis of the A/H1N1/2009 virus

To obtain whole sequences and identify single nucleotide polymorphisms (SNPs) for the 8 segments, the 40-mer short reads were aligned to the sequence of A/Tronto/T0106/2009(H1N1), which was found to be the most similar to the A/H1N1/2009 virus using a BLASTN search. Figure 2 shows dot plot images of the coverage at every nucleotide of the segments. Read coverage was observed at ∼×200 on average for all segments, indicating a sufficient redundancy to identify the viral sequences and SNPs. The obtained viral sequences, designated as A/Nagano/RC1-L/2009(H1N1), were consistent with those from A/Nagano/RC1/2009(H1N1) passaged in the Madin-Darby canine kidney (MDCK) cell line, except for 3 possible heterogeneous nucleotides in HA.

The coverage plot curves were not flat throughout the segments. Intriguingly, both ends of segment 1 (encoding PB2), the 3′–end of segment 3 (encoding PA), and approximately 700 nt of segment 8 (encoding NS) showed significant abundant coverage greater than ×1000.

### Genetic population analysis of the A/H1N1/2009 virus

**Figure 3:**
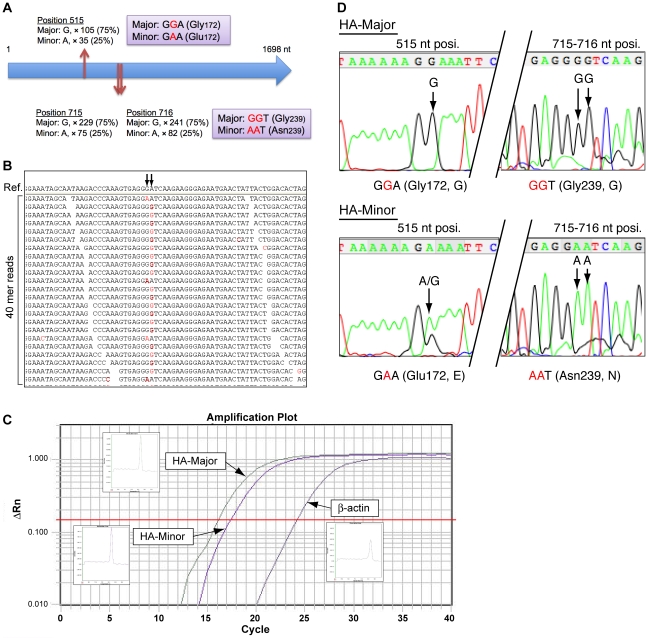
Genetic variations of the HA nucleotide sequence. (**A**) Schematic representation of 3 nucleotide variations (positions 515, 715, and 716 nt) in the HA coding nucleotide sequence. Three variations were classified as Major (75% appearance) or Minor (25% appearance) by read coverage (×), and the coding amino acids are also shown. (**B**) Arrows indicate positions 715 and 716 nt of the HA sequence, and the alignment image of the 40-mer reads. Nucleotides shown in red are the mismatches to the reference sequence of A/Tronto/T0106/2009(H1N1). Every read suggested that either the GGT or AAT sequence was present, but not the GAT or AGT sequence. (**C**) An amplification plot for HA-specific qRT-PCR. (**D**) Validation of genetic variation by Sanger capillary sequencing. HA-Major or HA-Minor PCR products were obtained by qRT-PCR using HA-Major- or HA-Minor-specific PCR primers. HA-Major PCR product shows G nucleotides at positions 515, 715, and 716 nt, while HA-Minor shows A nucleotides.

To identify heterogeneous populations, alignment results were screened using MapView software (Fig. 3B). Three potential heterogeneous genetic populations were found in segment 4 (encoding HA) at the 515, 715, and 716 nt positions (Fig. 3A), but not in other segments. The read alignments shown in Fig. 3B indicate that either the GGT or AAT sequence appeared at the 715–717 nt position, but not the GAT or AGT sequence. In addition, the read coverage implied that the major (GG; HA-Major) or minor (AA; HA-Minor) nucleotides were detected at the frequencies of 75% and 25%, respectively. To validate these variations, HA-Major- or HA-Minor-specific quantitative RT-PCR (qRT-PCR) was performed for the preparation of specific PCR products between the 434 and 738 nt in the HA coding sequence (Fig. 3C). qRT-PCR demonstrated that the expression of HA was ∼40,000-fold greater than that of human β-actin, and the expression ratio of HA-Major/HA-Minor was 4.05, suggesting that it corresponds to the read mapping shown in Fig. 3A. Furthermore, HA-Major and HA-Minor sequences were verified by Sanger DNA sequencing of the specific PCR products (Fig. 3D). Taken together, these results suggest the following amino acid substitutions of HA: one nucleotide alteration causes Gly_172_Glu and the other two alterations cause Gly_239_Asn (Fig. 3D).

### Epitope analysis of heterogeneous HA

**Figure 4:**
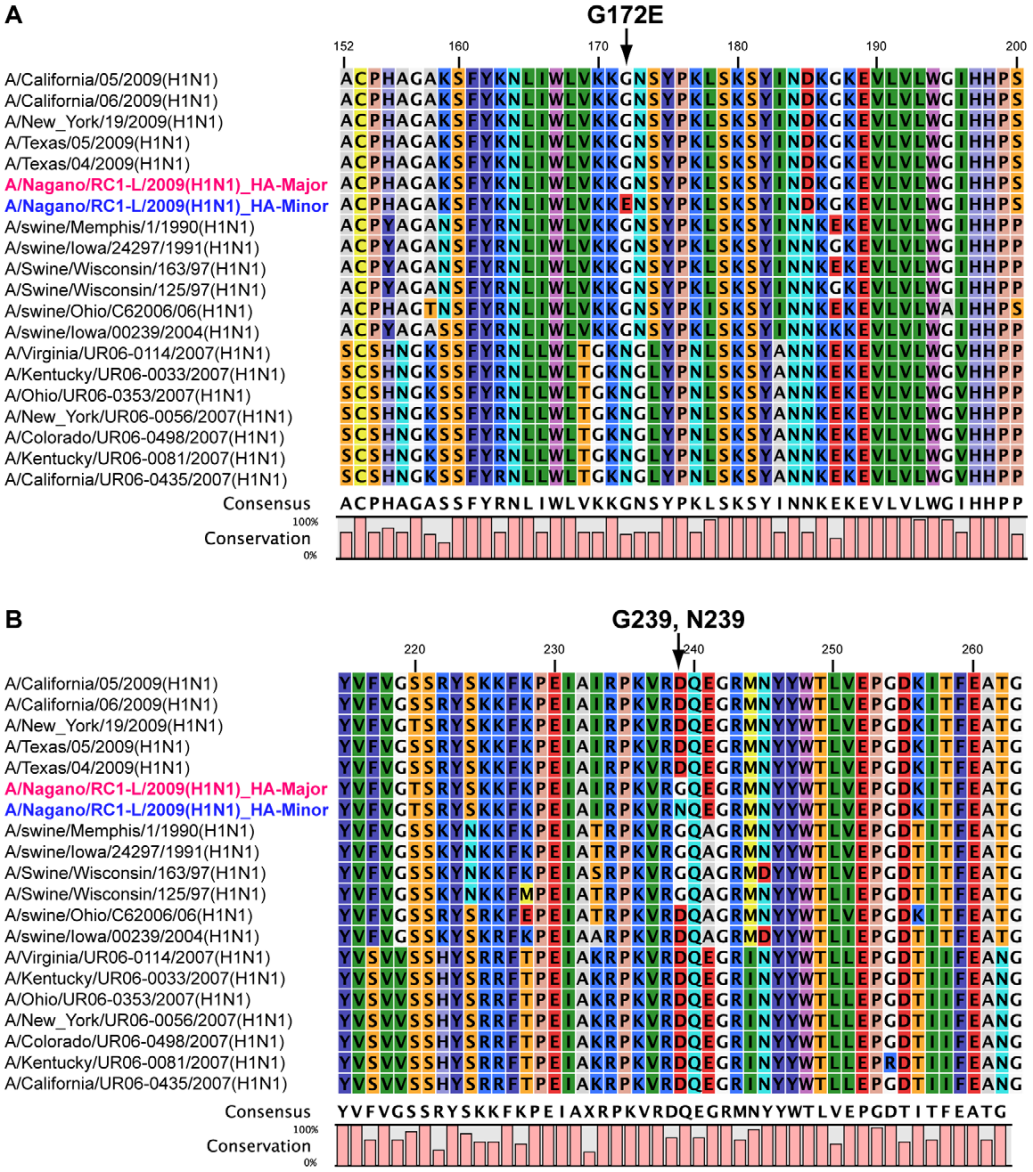
Alignment of HA amino acid sequences in influenza A virus around the identified mutations in A/Nagano/RC1-L/2009(H1N1). (**A**) Genetic variation at position 515 nt causes the amino acid substitution Gly_172_Glu; HA-Major: Gly172, HA-Minor: Glu172. (**B**) Genetic variation at position 715 and 716 nt causes the amino acid substitution Gly_239_Asn; HA-Major: Gly239, HA-Minor: Asn239.

To elucidate whether the Gly_172_Glu and Gly_239_Asn amino acid substitutions in the HA sequence could be associated with antigenic drift, they were compared to known potential epitopes [8], [9]. Representative HA amino acid sequences of the H1N1 influenza A virus were aligned with the heterogeneous HA-Major and HA-Minor sequences. The Gly_172_Glu substitution (corresponding to Gly158 in the mature HA lacking a signal peptide) was located on the Sa antigenic site (Fig. 4A).

**Figure 5:**
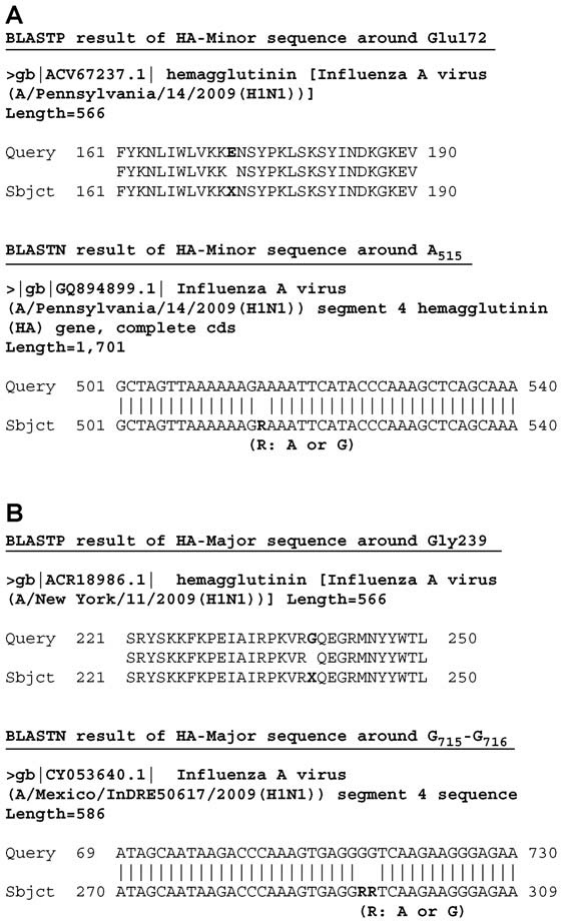
BLAST homology search of the HA sequences against the nr or nt databases. (**A**) Glu172 of HA-Minor. (**B**) Gly239 of HA-Major. R: A or G.

The HA Gly_172_Glu substitution is likely to be rare thus far because a BLASTP search against the non-redundant nr database revealed only two identical hits, A/Bayern/62/2009(H1N1) in Germany and A/Catalonia/S1207/2009(H1N1) in Spain (data not shown). One intriguing hit was to A/Pennsylvania/14/2009(H1N1) isolated in the US, whose HA sequence has an Xaa amino acid at position 172 due to the presence of the heterogeneous nucleotide R (A or G) (Fig. 5A). This deposited sequence completely coincides with our observation, suggesting that two variants of HAs are likely to coexist in the human lung, further implying that such heterogeneous populations might frequently be generated during infection.

Furthermore, HA Gly_239_Asn was located on the Ca2 antigenic site that contributes to binding with the host's sialic acid receptor [17]. Asp239 (corresponding to Asp225 in the mature HA lacking a signal peptide) was frequently distributed in H1N1 HAs (Fig. 4B), but Gly239 and Asn239 were found to be minor amino acids among HAs; a BLASTN search found 18 and 5 hit entries on the nt database, respectively. As was observed for Gly172, Xaa239 was found in the nt database (Fig. 5B), suggesting that HA heterogeneity of both minor amino acids may affect its binding affinity to the sialic acid receptor.

## Discussion

In this study, we demonstrated the detection of potential pathogens using a next-generation DNA sequencer. We speculated that, in addition to influenza A virus, additional potential pathogens such as *S. pneumoniae* could contribute to the severity and fatality of infection with the A/H1N1/2009 virus [2], [16]. In this case, the amount of bacteria detected was small (Fig. 1B and 1C), and they were considered to be the result of contamination during the course of the experiment, from autopsy to short read sequencing. The clinical outcome of the patient and histopathological examination suggest that this was a case of influenza viral pneumonia rather than bacterial infection [15], although *S. pneumoniae* coinfection has been reported to play a crucial role in the severity of influenza virus infection in some cases [16], [18].

In the present autopsy lung sample, very significant viral copies (∼40,000-fold greater than β-actin) were determined using qRT-PCR, but this was not always observed in autopsy samples from other patients (less than β-actin) (data not shown). Such abundant read sequencing enabled us to obtain almost full-coverage contig sequences for the viral segments using *de novo* assembly, suggesting the importance of this result in terms of being able to evaluate uncharacterized emerging infectious agents using an unbiased sequencing technique at the outbreak of a pandemic. Indeed, this study demonstrated that whole contigs can be identified as A/H1N1/2009, but not seasonal H1N1 or other subtypes (Table 1).

The read coverage profile generated by mapping was very indicative for segment 1 encoding PB2 (Fig. 2). Both ends were highly redundant with up to ×3000 coverage. The coverage is reflected by the amounts of vRNA, cRNA, and mRNA of influenza A virus, implying that the coverage bias may detect a more stable region as it is dependent on the expression level or manner of replication.

Contrary to the viral sequences obtained for A/Nagano/RC1/2009(H1N1) isolated from passaging in MDCK cells, *de novo* sequencing revealed the presence of A/Nagano/RC1-L/2009(H1N1) HA quasispecies in the autopsy sample (Fig. 3). Despite the fact that immunity to A/H1N1/2009 viruses is supposed to be limited among the general human population [19], we detected the amino acid substitution Gly_172_Glu in the HA Sa antigenic site in A/Nagano/RC1-L/2009(H1N1), and this appears to be a very rare event among A/H1N1/2009 viruses to date.

We also observed another substitution of Gly_239_Asn in the HA Ca2 antigenic site of A/Nagano/RC1-L/2009(H1N1). This antigenic site plays a crucial role in conferring specificity to galactose of the human α2-6 sialylated glycan receptor [20]. Interestingly, Asp239 (corresponding to Asp225 in the mature HA that lacks a signal peptide) is highly prevalent in known H1N1 HAs, indicating that both Gly239 and Asn239 appear to be very minor amino acids among all HA sequences.

Thus far, amino acid substitutions in the HAs of A/H1N1/2009 have been identified compared with seasonal H1N1 HAs. Homology-based structural investigations [17], [21] suggest that A/H1N1/2009 HA has the necessary residues to provide optimal contacts for high affinity binding to α2-6 sialylated glycans present in the human upper airway [22], while it apparently shows minimal contact with α2-3 sialylated glycans present in the human lower respiratory tract [23]. Indeed, the recombinant A/H1N1/2009 HA has been characterized to exhibit lower binding to the alveolar tissue of the lower respiratory tract [17]. However, we previously detected abundant viral nucleoprotein of A/Nagano/RC1-L/2009(H1N1) in pneumocytes expressing α2-3 sialylated glycans in autopsy lung tissue sections [15], suggesting that the above substitutions could affect the binding affinity to both types of sialylated glycans.

Very suggestive reports predicted the possible future antigenic drift of A/H1N1/2009 viruses from viral sequence and structural comparative analyses [11], [12]. Prior to the initiation of the current study (September 2009), Igarashi *et al.* predicted possible substitutions and these included the two amino acid substitutions presented here (Gly_172_Glu and Asp_239_Gly) [11]. Furthermore, Shen *et al.* suggested that host-driven antigenic drift based on evolutionary trends appeared to favor Asp239 (corresponding to Asp225 in the mature HA) in swine HAs and the 1918 pandemic, while Asp204 (corresponding to Asp190 in the mature HA) was favored in seasonal H1N1 HAs [12]. These predictions are very attractive and our experiments demonstrated one of them *a posteriori*. Furthermore, recent study has shown that receptor-binding avidity can influence antigenic drift [24]. HA antigenic sites Sa is the membrane proximal region, therefore, the identified variations on both Sa and Ca2 might contribute to the alteration of antigenicity and receptor-binding avidity by synergistic effect. The newly identified Asn239 substitution could be a probable candidate for further investigation of the manner of viral binding to sialic acid on the host receptors.

In conclusion, this study demonstrated that *de novo* sequencing can comprehensively detect pathogens, and such in-depth investigation facilitates the identification of influenza A viral heterogeneity during infection. The possibility of mixed infections with variants remains to be elucidated in this case, but worldwide sequencing efforts suggest that quasispecies of the A/H1N1/2009 virus evidently appear and are observed. To better characterize the currently emerging A/H1N1/2009 virus and prevent worse pandemics in the near future, unbiased *de novo* sequencing techniques will be indispensable for the primary investigations of emerging infectious diseases.

## Materials and Methods

### Ethics Statement

The study protocol was approved by the institutional medical ethical committee, National Institute of Infectious Diseases, Japan (Approval No.236), and the study was conducted according to the Declaration of Helsinki Principles. In the autopsy case, a written consent for autopsy was obtained from relatives.

### Total RNA and cDNA preparation from autopsy human lung

Information for the patient was previously reported [15]. Briefly, in August 2009, a 33-year-old male patient with chronic heart failure due to dilated cardiomyopathy, mild diabetes mellitus, atopic dermatitis, asthma, and obesity (BMI: 38) died from respiratory failure and multiple organ dysfunction syndrome. A diagnosis of pandemic influenza A virus (A/H1N1/2009) infection was determined using RT-PCR testing in a clinical laboratory. Total RNA was prepared from a 5-mm cube of the autopsy lung tissue using ISOGEN (NipponGene, Japan), followed by Ambion TurboDNase treatment (Ambion, Austin, TX USA). Double-stranded cDNA was prepared from 5 µg of total RNA using the random priming method with SuperScript Choice System for cDNA synthesis (Invitrogen, Carlsbad, CA, USA). cDNA was purified using a QIAquick PCR Purification kit (QIAGEN, Hilden, Germany).

### Short-read DNA sequencing using the Illumina Genome Analyzer II

An approximately 300-bp length cDNA library was prepared using a genomic DNA sample prep kit (Illumina, San Diego, CA, USA), and DNA clusters were generated on a slide using a Cluster Generation kit (ver. 2) on an Illumina cluster station (Illumina), according to the manufacturer's instructions. To obtain ∼1.0×10^7^ clusters for one lane, the general procedure as described in the standard recipe (Illumina) was performed as follows: template hybridization, isothermal amplification, linearization, blocking, denaturation, and hybridization of the sequencing primer (Illumina). All sequencing runs for 40 mers were performed with GA II using the Illumina Sequencing kit (ver. 3). Fluorescent images were analyzed using the Illumina base-calling pipeline 1.4.0 to obtain FASTQ formatted sequence data.

### Homology search analysis

The obtained DNA sequences were investigated using a BLAST search as shown in Fig. 1A. The results of the BLASTN search were analyzed and visualized using MEGAN v3.7.4 [25] with the following parameters: minimum support, 5; minimum score, 35.0.

### *de novo* assembly of short reads

Prior to *de novo* assembly, all obtained 40-mer reads were trimmed based on the *phred* quality value obtained using the Euler-SR *qualitytrimmer* command [26]. Such trimmed read sequences were assembled using Velvet v0.7.55 [27] or Euler-SR v1.0 [26] with the default parameters (Velvet: hash length, 25; Euler-SR: vertex size, 25).

### Read mapping

To obtain consensus sequences for the respective 8 segments of influenza A virus, 40-mer short reads were aligned to A/Tronto/T0106/2009(H1N1) sequences (gb|CY045951.1 – .8) as reference sequences with Maq software (ver. 0.7.1) [28] using the *easyrun* Perl-command. The consensus sequences were extracted as a “cns.fq” file for each segment, and deposited in the DNA Data Bank of Japan (DDBJ; accession numbers: AB538386 to AB538393 for the 8 segments of A/Nagano/RC1-L/2009(H1N1), and AB538394 for segment 4 encoding the HA-Minor sequence). Read coverage at every nucleotide was obtained using Maq software (ver. 0.7.1) with the *pileup* command. Read alignment for the validation of SNPs was performed using the MapView graphical alignment viewer [29].

### qRT-PCR analysis

qRT-PCR was performed using 100 ng of total RNA, HA variant-specific primers (forward common primer: pdmFlu09-HA-F, 5′–CGAACAAAGGTGTAACGGCAGCAT–3′; HA-Major-specific reverse primer: pdmFlu-HA-R_Major, 5′–ATAGTTCATTCTCCCTTCTTGACC–3′; HA-Minor-specific reverse primer: 5′–ATAGTTCATTCTCCCTTCTTGATT–3′), and the SuperScript III Platinum SYBR Green One-Step qRT-PCR kit with ROX (Invitrogen), and analyzed using the ABI PRISM 7900HT Real-time PCR System (Applied Biosystems, Foster City, CA, USA). The following qRT-PCR program was used: RT reaction, 50°C for 3 min; initial denaturation, 95°C for 5 min; 2 steps of amplification (×40 cycles), 95°C for 15 sec and 60°C for 30 sec. The human β-actin gene was used as the internal control. PCR products were resolved by 5% polyacrylamide gel electrophoresis, followed by Sanger sequencing using the BigDye Terminator v3.1 Cycle Sequencing kit (Applied Biosystems).

### Virus isolation

The A/H1N1/2009 virus was isolated from MDCK cells passaged once with trypsin.

## Supporting Information

Text S1 Fastq file of the 40-mer short reads with similarity to influenza A virus extracted from whole obtained short reads.
(11.19 MB PDF)

Text S2 De novo assembly of the influenza A virus using Euler-SR v1.0 [26] with the default parameters (vertex size, 25).
(0.04 MB RTF)

## References

[1] Dawood FS, Jain S, Finelli L, Shaw MW, Lindstrom S (2009). Emergence of a novel swine-origin influenza A (H1N1) virus in humans.. N Engl J Med.

[2] Gill JR, Sheng ZM, Ely SF, Guinee DG, Beasley MB (2010). Pulmonary pathologic findings of fatal 2009 pandemic influenza A/H1N1 viral infections.. Arch Pathol Lab Med.

[3] Ghedin E, Sengamalay NA, Shumway M, Zaborsky J, Feldblyum T (2005). Large-scale sequencing of human influenza reveals the dynamic nature of viral genome evolution.. Nature.

[4] Kilbourne ED (2006). Influenza pandemics of the 20th century.. Emerg Infect Dis.

[5] Nelson MI, Viboud C, Simonsen L, Bennett RT, Griesemer SB (2008). Multiple reassortment events in the evolutionary history of H1N1 influenza A virus since 1918.. PLoS Pathog.

[6] Knossow M, Skehel JJ (2006). Variation and infectivity neutralization in influenza.. Immunology.

[7] Air GM, Laver WG, Webster RG (1987). Antigenic variation in influenza viruses.. Contrib Microbiol Immunol.

[8] Caton AJ, Brownlee GG, Yewdell JW, Gerhard W (1982). The antigenic structure of the influenza virus A/PR/8/34 hemagglutinin (H1 subtype).. Cell.

[9] Bui HH, Peters B, Assarsson E, Mbawuike I, Sette A (2007). Ab and T cell epitopes of influenza A virus, knowledge and opportunities.. Proc Natl Acad Sci U S A.

[10] Squires B, Macken C, Garcia-Sastre A, Godbole S, Noronha J (2008). BioHealthBase: informatics support in the elucidation of influenza virus host pathogen interactions and virulence.. Nucleic Acids Res.

[11] Igarashi M, Ito K, Yoshida R, Tomabechi D, Kida H (2010). Predicting the antigenic structure of the pandemic (H1N1) 2009 influenza virus hemagglutinin.. PLoS One.

[12] Shen J, Ma J, Wang Q (2009). Evolutionary trends of A(H1N1) influenza virus hemagglutinin since 1918.. PLoS One.

[13] Nakamura S, Yang CS, Sakon N, Ueda M, Tougan T (2009). Direct metagenomic detection of viral pathogens in nasal and fecal specimens using an unbiased high-throughput sequencing approach.. PLoS One.

[14] Ramakrishnan MA, Tu ZJ, Singh S, Chockalingam AK, Gramer MR (2009). The feasibility of using high resolution genome sequencing of influenza a viruses to detect mixed infections and quasispecies.. PLoS One.

[15] Nakajima N, Hata S, Sato Y, Tobiume M, Katano H (2010). The first autopsy case of pandemic influenza (A/H1N1pdm) virus infection in Japan: Detection of high copy number of the virus in type II alveolar epithelial cells by pathological and virological examination.. Jpn J Infect Dis.

[16] Palacios G, Hornig M, Cisterna D, Savji N, Bussetti AV (2009). *Streptococcus pneumoniae* coinfection is correlated with the severity of H1N1 pandemic influenza.. PLoS One.

[17] Maines TR, Jayaraman A, Belser JA, Wadford DA, Pappas C (2009). Transmission and pathogenesis of swine-origin 2009 A(H1N1) influenza viruses in ferrets and mice.. Science.

[18] Louie J, Jean C, Chen T-H, Park S, Ueki R (2009). Bacterial coinfections in lung tissue specimens from fatal cases of 2009 pandemic influenza A (H1N1) - United States, May-August 2009.. MMWR Morb Mortal Wkly Rep.

[19] Greenbaum JA, Kotturi MF, Kim Y, Oseroff C, Vaughan K (2009). Pre-existing immunity against swine-origin H1N1 influenza viruses in the general human population.. Proc Natl Acad Sci U S A.

[20] Gamblin SJ, Haire LF, Russell RJ, Stevens DJ, Xiao B (2004). The structure and receptor binding properties of the 1918 influenza hemagglutinin.. Science.

[21] Soundararajan V, Tharakaraman K, Raman R, Raguram S, Shriver Z (2009). Extrapolating from sequence–the 2009 H1N1 ‘swine’ influenza virus.. Nat Biotechnol.

[22] Chandrasekaran A, Srinivasan A, Raman R, Viswanathan K, Raguram S (2008). Glycan topology determines human adaptation of avian H5N1 virus hemagglutinin.. Nat Biotechnol.

[23] Shinya K, Ebina M, Yamada S, Ono M, Kasai N (2006). Avian flu: influenza virus receptors in the human airway.. Nature.

[24] Hensley SE, Das SR, Bailey AL, Schmidt LM, Hickman HD (2009). Hemagglutinin receptor binding avidity drives influenza A virus antigenic drift.. Science.

[25] Mitra S, Klar B, Huson DH (2009). Visual and statistical comparison of metagenomes.. Bioinformatics.

[26] Chaisson MJ, Pevzner PA (2008). Short read fragment assembly of bacterial genomes.. Genome Res.

[27] Zerbino DR, Birney E (2008). Velvet: algorithms for de novo short read assembly using de Bruijn graphs.. Genome Res.

[28] Li H, Ruan J, Durbin R (2008). Mapping short DNA sequencing reads and calling variants using mapping quality scores.. Genome Res.

[29] Bao H, Guo H, Wang J, Zhou R, Lu X (2009). MapView: visualization of short reads alignment on a desktop computer.. Bioinformatics.

