# Supplementary material for: Characterization of Quasispecies of Pandemic 2009 Influenza A Virus (A/H1N1/2009) by De Novo Sequencing Using a Next-Generation DNA Sequencer
Source: PLoS One. 2010 Apr 23;5(4):e10256. doi: 10.1371/journal.pone.0010256 (PMC2859049; doi:10.1371/journal.pone.0010256)
Supplement: Text S2 — De novo assembly of the influenza A virus using Euler-SR v1.0 [26] with the default parameters (vertex size, 25). (0.04 MB RTF) [file pone.0010256.s002.rtf]

>826 183 2835
CGCAAGACGGACCAGAGCGAAAGCAGGTCAAATATATTCAATATGGAGAGAATAAAAGAACTGAGAGATCTAATGTCGCAGTCCCGCACTCGCGAGATACTCACTAAGACCACTGTGGACCATATGGCCATAATCAAAAAGTACACATCAGGAAGACAAGAGAAGAACCCCGCACTCAGAATG
>324 1558 136
AGCAGACAAGAGAATAATGGACATGATTCCAGAGAGGAATGAACAAGGACAAACCCTCTGGAGCAAAACAAACGATGCTGGATCAGACCGAGTGATGGTATCACCTCTGGCCGTAACATGGTGGAATAGGAATGGCCCAACAACAAGTACAGTTCATTACCCTAAGGTATATAAAACTTATTTCGAAAAGGTTGAAAGGTTGAAACATGGTACCTTCGGCCCTGTCCACTTCAGAAATCAAGTTAAAATAAGGAGGAGAGTTGATACAAACCCTGGCCATGCAGATCTCAATGCCAAGGAGGCACAGGATGTGATTATGGAAGTTGTTTTCCCAAATGAAGTGGGGGCAAGAATACTGACATCAGAGTCACAGCTGGCAATAACAAAAGAGAAGAAAGAAGAGCTCCAGGATTGTAAAATTGCTCCCTTGATGGTGGCGTACATGCTAGAAAGAGAATTGGTCCGTAAAACAAGGTTTCTCCCAGTAGCCGGCGGAACAGGCAGTGTTTATATTGAAGTGTTGCACTTAACCCAAGGGACGTGCTGGGAGCAGATGTACACTCCAGGAGGAGAAGTGAGAAATGATGATGTTGACCAAAGTTTGATTATCGCTGCTAGAAACATAGTAAGAAGAGCAGCAGTGTCAGCAGACCCATTAGCATCTCTCTTGGAAATGTGCCACAGCACACAGATTGGAGGAGTAAGGATGGTGGACATCCTTAGACAGAATCCAACTGAGGAACAAGCCGTAGACATATGCAAGGCAGCAATAGGGTTGAGGATTAGCTCATCTTTCAGTTTTGGTGGGTTCACTTTCAAAAGGACAAGCGGATCATCAGTCAAGAAAGAAGAAGAAGTGCTAACGGGCAACCTCCAAACACTGAAAATAAGAGTACATGAAGGGTATGAAGAATTCACAATGGTTGGGAGAAGAGCAACAGCTATTCTCAGAAAGGCAACCAGGAGATTGATCCAGTTGATAGTAAGTGGGAGAGACGAGCAGTCAATTGCTGAGGCAATAATTGTGGCCATGGTATTCTCACAAGAGGATTGCATGATCAAGGCAGTTAGGGGCGATCTGAACTTTGTCAATAGGGCAAACCAGCGACTGAACCCTATGCACCAACTCTTGAGGCATTTCCAAAAAGATGCAAAAGTGCTTTTCCAGAACTGGGGAATTGAATCCATCGACAATGTGATGGGAATGATCGGAATACTGCCCGACATGACCCCAAGCACGGAGATGTCGCTGAGAGGGATAAGAGTCAGCAAAATGGGAGTAGATGAATACTCCAGCACGGAGAGAGTGGTAGTGAGTATTGACCGATTTTTAAGGGTTAGAGATCAAAGAGGGAACGTACTATTGTCTCCCGAAGAAGTCAGTGAAACGCAAGGAACTGAGAAGTTGACAATAACTTATTCGTCATCAATGATGTGGGAGATCAATGGCCCTGAGTCAGTGCTAGTCAACACTTATCAATGGATAATCAGGAACTGGGAAATTGTGAAAATTCAATGGTCACAAGATCCCACAATGTTATACAACAAAATGGAATTT
>887 174 3294
GTGGAGATAAATCATATTATGGCCATCCGAATTCTTTTGGTCGCTGTCTGGCTGTCAGTAAGTATGCTAGAGTCCCGTTTTCGTTTCATTACCAACACTACGTCCCCTTGCCCAATTAGCACATTAGCTTTCTCTCCTTTTGCAAGATTGCTCAGTTCATTGATGCTTAATGCT
>1194 239 112
AAGGACAAACCCTCTGGAGCAAAACGCTGCTCCACCAGAACAGAGTAGGATGCAATTTTCCTCATTGACTGTGAATGTGAGAGGATCAGGGTTGAGGATACTGGTAAGAGGCAATTCTCCAGTATTCAATTACAACAAGGCAACCAAACGACTTACAGTTCTTGGAAAGGATGCAGGTGCATTGACTGAAGATCCAGATGAAGGCACATCTGGGGTGGAGTCTGCTGTCCTGAGAGGAT
>890 2204 4651
CTAAAAATTCCAGCGCAAAATGCCATAAGCACCACATTCCCTTATACTGGAGATCCTCCATACAGCCATGGAACAGGAACAGGATACACCATGGACACAGTAAACAGAACACACCAATACTCAGAAAAGGGAAAGTGGACGACAAACACAGAGACTGGTGCACCCCAGCTCAACCCGATTGATGGACCACTACCTGAGGATAATGAACCAAGTGGGTATGCACAAACAGACTGTGTTCTAGAGGCTATGGCTTTCCTTGAAGAATCCCACCCAGGAATATTTGAGAATTCATGCCTTGAAACAATGGAAGTCGTTCAACAAACAAGGGTAGATAAACTAACTCAAGGTCGCCAGACTTATGATTGGACATTAAACAGAAATCAACCGGCAGCAACTGCATTGGCCAACACCATAGAAGTCTTTAGATCGAATGGCCTAACAGCTAATGAGTCAGGAAGGCTAATAGATTTCTTAAAGGATGTAATGGAATCAATGAACAAAGAGGAAATAGAGATAACAACCCACTTTCAAAGAAAAAGGAGAGTAAGAGACAACATGACCAAGAAGATGGTCACGCAAAGAACAATAGGGAAGAAAAAACAAAGACTGAATAAGAGAGGCTATCTAATAAGAGCACTGACATTAAATACGATGACCAAAGATGCAGAGAGAGGCAAGTTAAAAAGAAGGGCTATCGCAACACCTGGGATGCAGATTAGAGGTTTCGTATACTTTGTTGAAACTTTAGCTAGGAGCATTTGCGAAAAGCTTGAACAGTCTGGGCTCCCAGTAGGGGGCAATGAAAAGAAGGCCAAACTGGCAAATGTTGTGAGAAAGATGATGACTAATTCACAAGACACAGAGATTTCTTTCACAATCACTGGGGACAACACTAAGTGGAATGAAAATCAAAATCCTCGAATGTTCCTGGCGATGATTACATATATTACCAGAAATCAACCCGAGTGGTTCAGAAACATCCTGAGCATGGCACCCATAATGTTCTCAAACAAAATGGCAAGACTAGGGAAAGGGTACATGTTCGAGAGTAAAAGAATGAAGATTCGAACACAAATACCAGCAGAAATGCTAGCAAGCATTGACCTGAAGTACTTCAATGAATCAACAAAGAAGAAAATTGAGAAAATAAGGCCTCTTCTAATAGATGGCACAGCATCACTGAGTCCTGGGATGATGATGGGCATGTTTAACATGCTAAGTACGGTCTTGGGAGTCTCGATACTGAATCTTGGACAAAAGAAATACACCAAGACAATATACTGGTGGGATGGGCTCCAATCATCCGACGATTTTGCTCTCATAGTGAATGCACCAAACCATGAGGGAATACAAGCAGGAGTGGACAGATTCTACAGGACCTGCAAGTTAGTGGGAATCAACATGAGCAAAAAGAAGTCCTATATAAATAAGACAGGGACATTTGAATTCACAAGCTTTTTTTATCGCTATGGATTTGTGGCTAATTTTAGCATGGAGCTACCCAGCTTTGGAGTGTCTGGAGTAAATGAATCAGCTGACATGAGTATTGGAGTAACAGTGATAAAGAACAACATGATAAACAATGACCTTGGACCTGCAACGGCCCAGATGGCTCTTCAATTGTTCATCAAAGACTACAGATACACATATAGGTGCCATAGGGGAGACACACAAATTCAAACGAGAAGATCATTTGAGTTAAAGAAGCTGTGGGATCAAACCCAATCAAAGGTAGGGCTATTAGTATCAGATGGAGGACCAAACTTATACAATATACGGAATCTTCACATTCCTGAAGTCTGCTTAAAATGGGAGCTAATGGATGATGATTATCGGGGAAGACTTTGTAATCCCCTGAATCCCTTTGTCAGTCATAAAGAGATTGATTCTGTAAACAATGCTGTGGTAATGCCAGCCCATGGTCCAGCCAAAAGCATGGAATATGATGCCGTTGCAACTACACATTCCTGGATTCCCAAGAGGAATCGTTCTATTCTCAACACAAGCCAAAGGGGAATTCTTGAGGATGAACAGATGTACCAGAAGTGCTGCAATCTATTCGAGAAATTTTTCCCTAGCAGTTCATATAGGAGACCGGTTGGAATTTCTAGCATGGTGGAGGCCATGGTGTCTAGGGCCCGGATTGATGCCAGGGTCGACTTCGAGTCTGGACGGATCAAGAAAGAAGAGTTCTCTGAGATCAT
>696 2198 3968
CAATGATCGTCGAGCTTGCGGAAAAGGCAATGAAAGAATATGGGGAAGATCCGAAAATCGAAACTAACAAGTTTGCTGCAATATGCACACATTTGGAAGTTTGTTTCATGTATTCGGATTTCCATTTCATCGACGAACGGGGTGAATCAATAATTGTAGAATCTGGTGACCCGAATGCACTATTGAAGCACCGATTTGAGATAATTGAAGGAAGAGACCGAATCATGGCCTGGACAGTGGTGAACAGTATATGTAACACAACAGGGGTAGAGAAGCCTAAATTTCTTCCTGATTTGTATGATTACAAAGAGAACCGGTTCATTGAAATTGGAGTAACACGGAGGGAAGTCCACATATATTACCTAGAGAAAGCCAACAAAATAAAATCTGAGAAGACACACATTCACATCTTTTCATTCACTGGAGAGGAGATGGCCACCAAAGCGGACTACACCCTTGACGAAGAGAGCAGGGCAAGAATCAAAACTAGGCTTTTCACTATAAGACAAGAAATGGCCAGTAGGAGTCTATGGGATTCCTTTCGTCAGTCCGAAAGAGGCGAAGAGACAATTGAAGAAAAATTTGAGATTACAGGAACTATGCGCAAGCTTGCCGACCAAAGTCTCCCACCGAACTTCTCCAGCCTTGAAAACTTTAGAGCCTATGTAGATGGATTCGAGCCGAACGGCTGCATTGAGGGCAAGCTTTCCCAAATGTCAAAAGAAGTGAACGCCAAAATTGAACCATTCTTGAGGACGACACCACGCCCCCTCAGATTGCCTGATGGGCCTCTTTGCCATCAGCGGTCAAAGTTCCTGCTGATGGATGCTCTGAAATTAAGTATTGAAGACCCGAGTCACGAGGGGGAGGGAATACCACTATATGATGCAATCAAATGCATGAAGACATTCTTTGGCTGGAAAGAGCCTAACATAGTCAAACCACATGAGAAAGGCATAAATCCCAATTACCTCATGGCTTGGAAGCAGGTGCTAGCAGAGCTACAGGACATTGAAAATGAAGAGAAGATCCCAAGGACAAAGAACATGAAGAGAACAAGCCAATTGAAGTGGGCACTCGGTGAAAATATGGCACCAGAAAAAGTAGACTTTGATGACTGCAAAGATGTTGGAGACCTTAAACAGTATGACAGTGATGAGCCAGAGCCCAGATCTCTAGCAAGCTGGGTCCAAAATGAATTCAATAAGGCATGTGAATTGACTGATTCAAGCTGGATAGAACTTGATGAAATAGGAGAAGATGTTGCCCCGATTGAACATATCGCAAGCATGAGGAGGAACTATTTTACAGCAGAAGTGTCCCACTGCAGGGCTACTGAATACATAATGAAGGGAGTGTACATAAATACGGCCTTGCTCAATGCATCCTGTGCAGCCATGGATGACTTTCAGCTGATCCCAATGGTAAGCAAATGTAGGACCAAAGAAGGAAGACGGAAAACAAACCTGTATGGGTTCATTATAAAAGGAAGGTCTCATTTGAGAAATGATACTGATGTGGTGAACTTTGTAAGTATGGAGTTCTCACTCACTGACCCGAGACTGGAGCCACACAAATGGGAAAAATACTGTGTTCTAGAAATAGGAGACATGCTCTTGAGGACTGCGATAGGCCAAGTGTCGAGGCCCATGTTCCTATATGTGAGAACCAATGGAACCTCCAAGATCAAGATGAAATGGGGCATGGAAATGAGGCGCTGCCTTCTTCAGTCTCTTCAGCAGATTGAGAGCATGATTGAGGCCGAGTCTTCTGTCAAAGAGAAAGACATGACCAAGGAATTCTTTGAAAACAAATCGGAAACATGGCCAATCGGAGAGTCACCCAGGGGAGTGGAGGAAGGCTCTATTGGGAAAGTGTGCAGGACCTTACTGGCAAAATCTGTATTCAACAGTCTATATGCGTCTCCACAACTTGAGGGGTTTTCGGCTGAATCGAGAAAATTGCTTCTCATTGTTCAGGCACTTAGGGACAACCTGGAACCTGGAACCTTCGATCTTGGGGGGCTATATGAAGCAATCGAGGAGTGCCTGATTAATGATCCCTGGGTTTTGCTTAATGCATCTTGGTTCAACTCCTTCCTCACACATGCATTGAAGTAGTTGTGGCAATGCTACTATTTGCTATCCATACTGTCTCAAAAAAAATAAAAAATAAAAAAAAATTAGCCAGGCATGGTG
>868 1761 3831
AAAGCAGGGGAAAACAAAAGCAACAAAAATGAAGGCAATACTAGTAGTTCTGCTATATACATTTGCAACCGCAAATGCAGACACATTATGTATAGGTTATCATGCGAACAATTCAACAGACACTGTAGACACAGTACTAGAAAAGAATGTAACGGTAACACACTCTGTTAACCTTCTAGAAGACAAGCATAACGGGAAACTATGCAAACTAAGAGGGGTAGCCCCATTGCATTTGGGTAAATGTAACATTGCTGGCTGGATCCTGGGAAATCCAGAGTGTGAATCACTCTCCACAGCAAGCTCATGGTCCTACATTGTGGAAACAACTAGTTCAGACAATGGAACGTGTTACCCAGGAGATTTCATCGATTATGAGGAGCTAAGAGAGCAATTGAGCTCAGTGTCATCATTTGAAAGGTTTGAGATATTCCCCAAGACAAGTTCATGGCCCAATCATGACTCGAACAAAGGTGTAACGGCAGCATGTCCTCATGCTGGAGCAAAAAGCTTCTACAAAAATTTAATATGGCTAGTTAAAAAAGGAAATTCATACCCAAAGCTCAGCAAATCCTACATTAATGATAAAGGGAAAGAAGTCCTCGTGCTATGGGGCATTCACCATCCATCTACTAGTGCTGACCAACAAAGTCTCTATCAGAATGCAGATGCATATGTTTTTGTGGGGACATCAAGATACAGCAAGAAGTTCAAGCCGGAAATAGCAATAAGACCCAAAGTGAGGGGTCAAGAAGGGAGAATGAACTATTACTGGACACTAGTAGAGCCGGGAGACAAAATAACATTCGAAGCAACTGGAAATCTAGTGGTACCGAGATATGCATTCGCAATGGAAAGAAATGCTGGATCTGGTATTATCATTTCAGATACACCAGTCCACGATTGCAATACAACTTGTCAGACACCCAAGGGTGCTATAAACACCAGCCTCCCATTTCAGAATATACATCCGATCACAATTGGAAAATGTCCAAAATATGTAAAAAGCACAAAATTGAGACTGGCCACAGGATTGAGGAATGTCCCTTCTATTCAATCTAGAGGCCTATTTGGGGCCATTGCCGGTTTCATTGAAGGGGGGTGGACAGGGATGGTAGATGGATGGTACGGTTATCACCATCAAAATGAGCAGGGGTCAGGATATGCAGCCGACCTGAAGAGCACACAGAATGCCATTGACGAGATTACTAACAAAGTAAATTCTGTTATTGAAAAGATGAATACACAGTTCACAGCAGTAGGTAAAGAGTTCAACCACCTGGAAAAAAGAATAGAGAATTTAAATAAAAAAGTTGATGATGGTTTCCTGGACATTTGGACTTACAATGCCGAACTGTTGGTTCTATTGGAAAATGAAAGAACTTTGGACTACCACGATTCAAATGTGAAGAACTTATATGAAAAGGTAAGAAGCCAGTTAAAAAACAATGCCAAGGAAATTGGAAACGGCTGCTTTGAATTTTACCACAAATGCGATAACACGTGCATGGAAAGTGTCAAAAATGGGACTTATGACTACCCAAAATACTCAGAGGAAGCAAAATTAAACAGAGAAGAAATAGATGGGGTAAAGCTGGAATCAACAAGGATTTACCAGATTTTGGCGATCTATTCAACTGTCGCCAGTTCATTGGTACTGGTAGTCTCCCTGGGGGCAATCAGTTTCTGGATGTGCTCTAATGGGTCTCTACAGTGTAGAATATGTATTTAACATTAGGATTTCAGAAGCATGAGAAAAAACAC
>897 1514 1710
GAAGCCATGGCGTCTCAAGGCACCAAACGATCATATGAACAAATGGAGACTGATGGGGAGCGCCAGGATGCCACAGAAATCAGAGCATCTGTCGGAAGAATGATTGGTGGAATCGGGAGATTCTACATCCAAATGTGCACTGAACTCAAACTCAGTGATTATGATGGACGACTAATCCAGAATAGCATAACAATAGAGAGGATGGTGCTTTCTGCTTTTGATGAGAGAAGAAATAAATACCTAGAAGAGCATCCCAGTGCTGGGAAGGACCCTAAGAAAACAGGAGGACCCATATATAGAAGAATAGACGGAAAGTGGATGAGAGAACTCATCCTTTATGACAAAGAAGAAATAAGGAGAGTTTGGCGCCAAGCAAACAATGGCGAAGATGCAACAGCAGGTCTTACTCATATCATGATTTGGCATTCCAACCTGAATGATGCCACATATCAGAGAACAAGAGCGCTTGTTCGCACCGGAATGGATCCCAGAATGTGTTCTCTAATGCAAGGTTCAACACTTCCCAGAAGGTCTGGTGCCGCAGGTGCTGCGGTGAAAGGAGTTGGAACAATAGCAATGGAGTTAATCAGAATGATCAAACGTGGAATCAATGACCGAAATTTCTGGAGGGGTGAAAATGGACGAAGGACAAGGGTTGCTTATGAAAGAATGTGCAATATCCTCAAAGGAAAATTTCAAACAGCTGCCCAGAGGGCAATGATGGATCAAGTAAGAGAAAGTCGAAACCCAGGAAACGCTGAGATTGAAGACCTCATTTTCCTGGCACGGTCAGCACTCATTCTGAGGGGATCAGTTGCACATAAATCCTGCCTGCCTGCTTGTGTGTATGGGCTTGCAGTAGCAAGTGGGCATGACTTTGAAAGGGAAGGGTACTCACTGGTCGGGATAGACCCATTCAAATTACTCCAAAACAGCCAAGTGGTCAGCCTGATGAGACCAAATGAAAACCCAGCTCACAAAAGTCAATTGGTGTGGATGGCATGCCACTCTGCTGCATTTGAAGATTTAAGAGTATCAAGTTTCATAAGAGGAAAGAAAGTGATTCCAAGAGGAAAGCTTTCCACAAGAGGGGTCCAGATTGCTTCAAATGAGAATGTGGAAACCATGGACTCCAATACCCTGGAACTAAGAAGCAGATACTGGGCCATAAGGACCAGGAGTGGAGGAAATACCAATCAACAAAAGGCATCCGCAGGCCAGATCAGTGTGCAGCCTACATTCTCAGTGCAGCGAAATCTCCCTTTTGAAAGAGCAACCGTTATGGCAGCATTCAGCGGGAACAATGAAGGACGGACATCCGACATGCGAACAGAAGTTATAAGAATGATGGAAAGTGCAAAGCCAGAAGATTTGTCCTTCCAGGGGCGGGGAGTCTTCGAGCTCTCGGACGAAAAGGCAACGAACCCGATCGTGCCTTCCTTTGACATGAGTAATGAAGGGTCTTATTTCTTCGGAGACAATGCAGAGGAGTATGACAGTTGAGGAAAAATACC
>224 101 9
TTTCCAATTTGTAATATTAAGTTAGCCATTCCAATTGTCATACAGACCGAACCAATGGTTATTATCTTTTGGTTTGGATTCATTTTAAACTCCTGCTTTTG
>1206 1302 2468
TCAATGGTAAATGGCAACTCAGCACCGTCTGGCCAAGACCAACCCACAGTGTCACTGTTTACACCACAAAAGGATATGCTGCTCCCGCTAGTCCAGATTGTGTTCTCTTTGGGTCGCCCTCTGATTAGTTCAACCCAGAAGCAAGGTCTTATACAATCCAGCCCTGTTAGTTCTGGATGCTGAACAAAACTCCCGCTATATCCTGACCACTCATTTATTCCTACGATATCTTGCTTTATTGAGAAGTTATTGTCTGTCCCAGTCCATCCGTTCGGATCCCAAATCATCTCAAAACCGTTTCTTGAACTAATGCTTTTAGTTCTCCCTATCCAAACACCATTGCCGTATTTGAATGAAAATCCTTTTACTCCATTTGCTCCATTAGACGATACTGGACCACAACTGCCTGTCTTATCATTAGGGCGTGGATTGTCTCCGAAAATCCCACTGCATATGTATCCTATCTGATATTCCAGATTCTGGTTGAAAGACACCCACGGTCGATTCGAGCCATGCCAGTTATCCCTGCACACACATGTGATTTCACTAGAATCAGGATAACAGGAGCATTCCTCATAGTGATAATTAGGGGCATTCATTTCGACTGATTTGACTATCTTTCCCTTTTCTATTCTGAAGATCTTGTATGAGGCCTGTCCATCACTTGGTCCATCGGTCATTACAGTAAAGCAAGAACCATTTACACATGCACATTCAGACTCTTGTGTTCTCAATATATTGTTTCTCCAACTCTTGATAGTGTCTGTTATTATGCCGTTGTACTTTAACACAGCCACTGCCCCATTGTCTGGGCCAGAAATTCCAATTGTTAGCCAATTGATGCCATCATGACAAGCACTTGCTGACCAAGCGACTGACTCAAATCTTGAGTTGTATGGAGAGGGAACTTCACCAATAGGACAGCTCATTAGGGTTCGATATGGGCTCCTGTCTTTAATGGTTCCATTGGAATGTTTGTCATTTAGCAAGGCCCCTTGAGTCAAGAAGAAGGTTCTGCATTCCAAGGGGGAGCATGATATGAATGGTTCCCTTATGACAAACACATCCCCCTTGGAACCGATTCTTATACTGTTGTCTTTACTGTATATAGCCCATCCACTAACAGGGCAGAGAGAGGAATTGCCCGCTAATTTCACGGAAACCACTGACTGTCCAGCAGCAAAGTTGGTGTTGCTGATGTTAACATATGTCTGGTTTACCCAAGTGTTGTTTTCATAAGTAATGACGCTTTGATTGCATGTTTCAATCTGATTTTGATTCCCAAGTTGAATTGAGTGGCGT
>750 1019 1128
TCGCAAAAGCAGGTAGATATTTAAAGATGAGTCTTCTAACCGAGGTCGAAACGTACGTTCTTTCTATCATCCCGTCAGGCCCCCTCAAAGCCGAGATCGCGCAGAGACTGGAAAGTGTCTTTGCAGGAAAGAACACAGATCTTGAGGCTCTCATGGAATGGCTAAAGACAAGACCAATCTTGTCACCTCTGACTAAGGGAATTTTAGGATTTGTGTTCACGCTCACCGTGCCCAGTGAGCGAGGACTGCAGCGTAGACGCTTTGTCCAAAATGCCCTAAATGGGAATGGGGACCCGAACAACATGGATAGAGCAGTTAAACTATACAAGAAGCTCAAAAGAGAAATAACGTTCCATGGGGCCAAGGAGGTGTCACTAAGCTATTCAACTGGTGCACTTGCCAGTTGCATGGGCCTCATATACAACAGGATGGGAACAGTGACCACAGAAGCTGCTTTTGGTCTAGTGTGTGCCACTTGTGAACAGATTGCTGATTCACAGCATCGGTCTCACAGACAAATGGCTACTACCACCAATCCACTAATCAGGCATGAAAACAGAATGGTGCTGGCTAGCACTACGGCAAAGGCTATGGAACAGATGGCTGGATCGAGTGAACAGGCAGCAGAGGCCATGGAGGTTGCTAATCAGACTAGGCAGATGGTACATGCAATGAGAACTATTGGGACTCATCCTAGCTCCAGTGCTGGTCTGAAAGATGACCTTCTTGAAAATTTGCAGGCCTACCAGAAGCGAATGGGAGTGCAGATGCAGCGATTCAAGTGATCCTCTCGTCATTGCAGCAAATATCATTGGGATCTTGCACCTGATATTGTGGATTACTGATCGTCTTTTTTTCAAATGTATTTATCGTCGCTTTAAATACGGTTTGAAAAGAGGGCCTTCTACGGAAGGAGTGCCTGAGTCCATGAGGGAAGAATATCAACAGGAACAGCAGAGTGCTGTGGATGTTGACGATGGTCATTTTGTCAACATAGAGCTAGAGTAAAAAACTACCTT
>809 834 4399
AAGGGTGTTTTTTATCATTAAATAAGCTGAAACGAGAAAGCTCTTATCTCTTGTTCTACTTCAAGCAGTAGTTGTAAGGCTTGCATAAATGTTATTTGTTCGAAACTATTCTCTGTCGCTTTCAATCTGTGCCGCATTTCTTCAATTAACCACCTTATTTCCTCAAATTTCTGTCCCAATTGCTCTCGCCACTTTTCATTTCTGCTCTGGAGGTAGTGAAGGTCTCCCATTCTCATCACAGTTTCTCCAAGCGAATCTCTGTATATTTTCAGAGACTCGAACCGTGTTACCATTCCATTCAAGTCCTCCGATGAGGACCCCAACTGCATTTTTGACATCCTCATAAGTATGTCCTGGAAGAGAAGGTAATGGTGAAATTTCTCCAACTATTGCTCCCTCCTCAGTGAAAGCCCTTAGTAGCATCAAGGTCTCTAATCGGTTAAAGATTACACTGAAGTTCGCTTTCAGTACTATGTTCTTTTCCATGACCGCCTGGTCCAATCGCACGCAAAGAGGGCCTATTATCTTTTGCCTAGGCATGAGCATGAACCAGTCTCGTGACATTTCCTCGAGGGTCATGTCAGAAAGGTAGCGCGAAGTAGGTACAGATGCAATTGTCATTCTAAGTGTCTCGCTGGATTCCTCTTTCAAGATCCATTCCACGATTTGTTTCCCACCAAGAGTGGCTGTTTCGATATCGAGGCCAAGGGTGTTGCCTCTTCCTTTTAAGGACTTTTGATCTCGGCGGAGCCGATCAAGGAATGGGGCATCACCCAATCCATTGTCTGCAAATCGCTTGCGGATATGCCAAAGGAAACAGTCTACCTGAAGCTT
