# Supplementary material for: Characterization of Quasispecies of Pandemic 2009 Influenza A Virus (A/H1N1/2009) by De Novo Sequencing Using a Next-Generation DNA Sequencer
Source: PLoS One. 2010 Apr 23;5(4):e10256. doi: 10.1371/journal.pone.0010256 (PMC2859049; doi:10.1371/journal.pone.0010256)
Supplement: Text S1 — Fastq file of the 40-mer short reads with similarity to influenza A virus extracted from whole obtained short reads. (11.19 MB PDF) [file pone.0010256.s001.pdf]

```
@HWI-EAS443:3:24:33:2036#0/1
AGATGGCCATCCGAATTCTTTTGGTCGCTGTCTGGCTGTC
+HWI-EAS443:3:24:33:2036#0/1
^Z_¥ZZ[a```_^XZNQTQVZXTY]_`_WZY]ZWV][]J[
@HWI-EAS443:3:74:1367:921#0/1
AGATGGCCATCCGAATTCTTTTGGTCGCTGTCTGGCTGTC
+HWI-EAS443:3:74:1367:921#0/1
aabb_bbabbabbbbbaabbbbb]`__^ab`¥_b`ab[_G
@HWI-EAS443:3:32:1050:283#0/1
GGGTAAACCAGACATATGTTAACATCAGCAACACCAACTT
+HWI-EAS443:3:32:1050:283#0/1
aba[bbb`abaa`baaabaaa`_aa[aaaaa]a^`_Y_a`
@HWI-EAS443:3:32:1685:830#0/1
AGTTGGTGTTGCTGATGTTAACATATGTCTGGTTTACCCC
+HWI-EAS443:3:32:1685:830#0/1
Y```__^``]W^a`¥Vb]`^`_]aabb_]^`XNZ`[Y¥]L
@HWI-EAS443:3:18:1139:1572#0/1
AGTTGGTGTTGCTGATGTTAACATATGTCTGGTTTACCCA
+HWI-EAS443:3:18:1139:1572#0/1
Va^abaaaa`]`ba_aaaaaaa`abaa```aUUab`U[aZ
@HWI-EAS443:3:91:1157:1910#0/1
AGTTGGTGTTGCTGATGTTAACATATGTCTGGTTTACCCA
+HWI-EAS443:3:91:1157:1910#0/1
abWYX``b_b_``]ab__aa]^^baa_FP_`XPSaW¥]^a
@HWI-EAS443:3:31:325:729#0/1
GTTGGTGTTGCTGATGTTAACATATGTCTGGTTTACCCAA
+HWI-EAS443:3:31:325:729#0/1
a`ab`Ya_aa`aa^`a_^UVYT]Y]^^__]]U_a^Z``_]
@HWI-EAS443:3:64:1370:1771#0/1
TTTGGGTAAACCAGACATATGTTAACATCAGCAACACCAA
+HWI-EAS443:3:64:1370:1771#0/1
^b`a`[F]abab¥b]^¥a_¥b_a^__aaY^`a_`__a]aa
@HWI-EAS443:3:11:1050:1121#0/1
CACTTGGGTAAACCAGACATATGTTAACATCAGCAACACC
+HWI-EAS443:3:11:1050:1121#0/1
aaaaa`_a¥aaa_aaaaaaaaaaaaa`_aa_aa^a`_a_]
@HWI-EAS443:3:12:1561:1041#0/1
TGGTGTTGCTGATGTTAACATATGTCTGGTTTACCCAAGT
+HWI-EAS443:3:12:1561:1041#0/1
a```babbabb^b_aba^]`aaaa__aaa[aa]W]Y`__Q
@HWI-EAS443:3:14:1602:823#0/1
CACTTGGGTAAACCAGACATATGTTAACATCAGCAACACC
+HWI-EAS443:3:14:1602:823#0/1
`aa`^bb_abaa]]aa_bababaa^[`_ab¥`b_a`Wa^U
@HWI-EAS443:3:14:498:532#0/1
ACTTGGGTAAACCAGACATATGTTAACATCAGCAACACCA
+HWI-EAS443:3:14:498:532#0/1
a`__aa`^a_aaa__X^a`^_a]^`V]^¥X_^]a_Z^XZ^
@HWI-EAS443:3:36:824:786#0/1
ACTTGGGTAAACCAGACATATGTTAACATCAGCAACACCA
```

```
+HWI-EAS443:3:36:824:786#0/1
ab`_`ba¥^_`a[`a_a``aab^]`_^^a`[_^aI^a`V`
@HWI-EAS443:3:53:594:2036#0/1
CTTGGGTAAACCAGACATATGTTAACATCAGCAACACCAA
+HWI-EAS443:3:53:594:2036#0/1
abaa]bV]`b`_aa^a`a`aaaaabaa_`aa^a`]aZ`aa
@HWI-EAS443:3:63:331:41#0/1
CTTGGGTAAACCAGACATATGTTAACATCAGCAACACCAA
+HWI-EAS443:3:63:331:41#0/1
aaaaaaU^`a_aaa_a^a[aaa`_a`_aW_a^`_^a_W`J
@HWI-EAS443:3:68:430:1714#0/1
CACTTGGGTAAACCAGACATATGTTAACATCAGCAACACC
+HWI-EAS443:3:68:430:1714#0/1
Y]ba][[`W_bb^S`_Ubbaa[aaabbbb`a`¥bbbbb_b
@HWI-EAS443:3:79:1038:1961#0/1
CACTTGGGTAAACCAGACATATGTTAACATCAGCAACACC
+HWI-EAS443:3:79:1038:1961#0/1
aaabaaa_Sbbb`abaaaaaaaa__baaaaaa¥_ba`a`_
@HWI-EAS443:3:83:138:1228#0/1
CTTGGGTAAACCAGACATATGTTAACATCAGCAACACCAA
+HWI-EAS443:3:83:138:1228#0/1
aaZV[]G`Y_aa`Y_aa``aaa_¥`a`_aa_ZT]a``__^
@HWI-EAS443:3:8:1691:246#0/1
ACTTGGGTAAACCAGACATATGTTAACATCAGCAACACCA
+HWI-EAS443:3:8:1691:246#0/1
aZbaa`aaa_a][^`]_aa]aaaaa]X`a]¥_Ua_U_QLY
@HWI-EAS443:3:93:442:34#0/1
CACTTGGGTAAACCAGACATATGTTAACATCAGCAACACC
+HWI-EAS443:3:93:442:34#0/1
aaa_a``a^`__`[]aa_aaaaa¥¥a_¥]`_`a]`__`]`
@HWI-EAS443:3:50:948:300#0/1
CTGCTTTTGCTGGAAAGAACACAGATCTTGAGGCTCTCAT
+HWI-EAS443:3:50:948:300#0/1
abbabbbbb`Y`b`babbbaaabaababbbaaba``ZQTS
@HWI-EAS443:3:82:1782:211#0/1
CTTCATTACTCATGTCAAAGGAAGGCACGATTGGGGTGGT
+HWI-EAS443:3:82:1782:211#0/1
ab]ZbbabX^^^a¥¥U^`VL`ba_XR`BBBBBBBBBBBBB
@HWI-EAS443:3:55:1706:1447#0/1
AGTGACCACGGAAGCTGCTTTTGGTCTAGTGTGTGCCACT
+HWI-EAS443:3:55:1706:1447#0/1
ab_bb_^]RO¥b_`¥]TY]¥__X`Q_]_Z[[]a][SY[_`
@HWI-EAS443:3:89:1675:996#0/1
ACGAGAGGATCACTTGAATCGCTGTATCTGCACTCCCATT
+HWI-EAS443:3:89:1675:996#0/1
aab_aabaaa_`_babab`Y__a__^_]_a[^^__^__]]
@HWI-EAS443:3:80:988:258#0/1
CATCCTAGCTCCAGTGCTGGTCTGAAGGACGACCTTCTTG
+HWI-EAS443:3:80:988:258#0/1
``ba]ab`]XX`¥OS_X¥]XXS^W_NJ`]FGJOZ][M[_T
```

@HWI-EAS443:3:20:987:1160#0/1
GTCGGATTGACCTCCATTCAAATGGTTTGCCTGCTTTTCC
+HWI-EAS443:3:20:987:1160#0/1
a`aaaaa`aa]¥Z_Z`__]Sa]]]^X__ZZW^ZW_^]]ZW
@HWI-EAS443:3:72:1043:387#0/1
CGGATTGACATCCATTCAAATGGTTTGCCTGCTTTTCCTA
+HWI-EAS443:3:72:1043:387#0/1
aaabaa¥a_bb^S`]Zbb`a`aaIZKa]__ZW]`a`^¥¥_
@HWI-EAS443:3:6:318:1784#0/1
GCCCATCCGAATTCTTTTGGTCGCTGTCTGGCTGTCAGTA
+HWI-EAS443:3:6:318:1784#0/1
I_X¥aaZaaabaZbb^[`bb]a_aa`_]bbb_`b¥WX`UB
@HWI-EAS443:3:44:773:453#0/1
TAGCATACTTACTGACAGCCAGACAGCGACCCAAAGAATT
+HWI-EAS443:3:44:773:453#0/1
OR_¥U_Q^_¥aaa^¥Y^_I_¥aSWV]a_QRNG[O¥Q¥[JJ
@HWI-EAS443:3:92:1551:480#0/1
ATTCTGTTTTCATGCCTGATTAGTGGATTGGTGATAGTAG
+HWI-EAS443:3:92:1551:480#0/1
aba_bb]Y^^a_`U`]_XY¥a_aaab^`^aaPLXP[a_[W
@HWI-EAS443:3:84:730:1940#0/1
GAACAGGCAGCAGAGGCCATGGAGGTTGCTAATCAAGCTA
+HWI-EAS443:3:84:730:1940#0/1
_`^^b^W_¥P]Z[Za¥QZVOKXXOXBBBBBBBBBBBBBBB
@HWI-EAS443:3:59:322:631#0/1
AATTGGAAAATGTCCAAAACATGTAAAAAGCACAAAATTG
+HWI-EAS443:3:59:322:631#0/1
^baabb`b`aa```aa`aa]UT_Z^UX]VZWSZZNMMRXL
@HWI-EAS443:3:58:1127:519#0/1
AAAGAGTTCAACCACCTGGAAAAAAGAATAGAGAATTTGA
+HWI-EAS443:3:58:1127:519#0/1
aaaaab_aaaa`aa__aaa`___``b`aa`aaa``aa¥G_
@HWI-EAS443:3:59:1328:894#0/1
AAAGTACACATCAGGAAGACAAGAGAAGAATCCCGAACTC
+HWI-EAS443:3:59:1328:894#0/1
^DQVV][R]N¥ZXX]PT]Q¥Z^`Z_SYZTMXSUUXDKY]Y
@HWI-EAS443:3:4:437:1272#0/1
GACCATATGGCCATAATCAAAAAGTACACATCAAGAATTC
+HWI-EAS443:3:4:437:1272#0/1
_b¥]]aaa_Z`W^aaaaa_`[`[`Ra_aVTRQTa¥ZS[TR
@HWI-EAS443:3:62:354:225#0/1
ACCATATGGCCATAATCAAAAAGTACACATCAAGAAGCTT
+HWI-EAS443:3:62:354:225#0/1
a¥b¥baabaa[I]`^_^a[][`aN^__`__a_]_]][_]Z
@HWI-EAS443:3:31:1446:1068#0/1
CTTCTTGATGTGTACTTTTTGATTATGGCCATATGGTCCA
+HWI-EAS443:3:31:1446:1068#0/1
_¥aZ_X`Y]a]_]Z_bYbbaaabaaa^S_^[JR`HXYNBB
@HWI-EAS443:3:100:50:1040#0/1
ACCGGTTGGAATTTCTAGCATGGCGGAGGCCATGGTGTCT

```
+HWI-EAS443:3:100:50:1040#0/1
[^^Z_W¥b```YW^`aZ_^Z_aZG[DV]VZ¥]UP¥BBBBB
@HWI-EAS443:3:37:1699:1609#0/1
AATTTCTAGCATGGCGGAGGCCATGGTGTCTAGGGCCCGG
+HWI-EAS443:3:37:1699:1609#0/1
aaa]aabXa¥`_aZGa^]SU`a]]^MIJVU^W`X¥X^]BB
@HWI-EAS443:3:82:672:1710#0/1
GTTGGAATTTCTAGCATGGCGGAGGCCATGGTGTCTAGGG
+HWI-EAS443:3:82:672:1710#0/1
^^]`a_Xa]ba_¥]`aab^aQbbaa`ZX`RTP^HIaU]aZ
@HWI-EAS443:3:43:681:1973#0/1
AGAGGACTTCTTTTTGCTCATGTTGATTCCGACTAACTTG
+HWI-EAS443:3:43:681:1973#0/1
^[`KZa^baaYSVSTOQaaa]`X]Jb`¥IYIbR`a`V]S_
@HWI-EAS443:3:73:1169:1660#0/1
CTGATGGCCATCCGAATTCTTTTGGTCGCTGTCTGGCTGT
+HWI-EAS443:3:73:1169:1660#0/1
aa`ba``babaaa]``aaaaaaa¥^Taa`Ya_`Y_X`Z^U
@HWI-EAS443:3:38:1492:591#0/1
CTGATTAATGATCCCTGGGTTTTGCTTAATGCATCTTGGG
+HWI-EAS443:3:38:1492:591#0/1
`ZbVababbaUa_^a^b`babbaV]a_Yb[I`KZaa^bZF
@HWI-EAS443:3:62:106:1042#0/1
CGCCTGGGCTTTGCTTAATGCATCTTGGTTCAACTCCTTC
+HWI-EAS443:3:62:106:1042#0/1
ab^a_X^aJa]]]]^Z`aaaaaa`a__XU[``_a]V][R`
@HWI-EAS443:3:87:744:1078#0/1
GCCTGATTAATGATCCCTGGGCTTTGCTTAATGCATCTTG
+HWI-EAS443:3:87:744:1078#0/1
a^ab`_VZ`V`T]a_ba¥TROFOO_]Y_X[]a]WS`_aW^
@HWI-EAS443:3:96:1313:697#0/1
GACCATCGTCAACATCCACAGCACTCTGTTGTTCCTGTTG
+HWI-EAS443:3:96:1313:697#0/1
`aPDX^__QU_WT[YVH]W_]OPN[U`NFR¥^[QG`aZBB
@HWI-EAS443:3:63:1071:373#0/1
GACAAAATGACCATCGTCAACATCCACAGCACTCTGTTGT
+HWI-EAS443:3:63:1071:373#0/1
a``X]a]ab`WXY[]YSY[PLXZXRW^___BBBBBBBBBB
@HWI-EAS443:3:51:1710:228#0/1
CTAATGGATGGCCATCCGAATTCTTTTGTTCGCTGTCTGG
+HWI-EAS443:3:51:1710:228#0/1
^XXQ_H]QaXYYP``SS`NP¥b[TWb`WFPHLFTBBBBBB
@HWI-EAS443:3:98:578:1309#0/1
TCCGAATTCTTTTGGTCGCTGTCTGGCTGTCAGCAAGAAG
+HWI-EAS443:3:98:578:1309#0/1
`^R_abZ`]_ba_ba__ba^W_L`X_a_ZDIOVFFX[FYQ
@HWI-EAS443:3:29:483:1907#0/1
CATCCGAATTCTTTTGGTCGCTGTCTGGCTGTCAGAAAGA
+HWI-EAS443:3:29:483:1907#0/1
^a``¥UaZ]bbab`[S^VUY`ZUGWY_BBBBBBBBBBBBB
```

```
@HWI-EAS443:3:42:539:1214#0/1
TGTCAGTAAGAATGCTAGAGTCCCGTTTTCGGTTCATTAC
+HWI-EAS443:3:42:539:1214#0/1
ZS`Q_``W¥RDTQQZPZZ`b`aSRRIW¥U]]H[PDVUMYN
@HWI-EAS443:3:12:115:1801#0/1
TTTTGGTCGCTGTCTGGCTGTCAGTAAGAATGCTAGAGTC
+HWI-EAS443:3:12:115:1801#0/1
[`aa`[Pbab]`^baTR_aXXTWXDMXHDXWDZQ_Y_XU_
@HWI-EAS443:3:19:662:350#0/1
ATCCGAATTCTTTTGGTCGCTGTCTGGCTGTCAGTAAGAA
+HWI-EAS443:3:19:662:350#0/1
ab_]¥[aaaa`aaa^]L^a```[¥]a`[YaLGW`YBBBBB
@HWI-EAS443:3:36:911:1681#0/1
CTGGCTGTCAGTAAGAATGCTAGAGTCCCGTTTTCGTTTC
+HWI-EAS443:3:36:911:1681#0/1
^aa`a`_]a¥WXa__LVb]V]YUYa^¥UV`X]a```¥aaa
@HWI-EAS443:3:51:1102:762#0/1
TCCGAATTCTTTTGGTCGCTGTCTGGCTGTCAGTAAGAAT
+HWI-EAS443:3:51:1102:762#0/1
aX_Z_Yb]S_aa^QQV]a[_aX[aBBBBBBBBBBBBBBBB
@HWI-EAS443:3:83:794:866#0/1
GACAAAATGACCATCGTCAACATCCACAGCACTCTGCTGA
+HWI-EAS443:3:83:794:866#0/1
X^aaYMU^X_ab`^¥^R``_¥_aaYQ`_]V`SY`OQ]JVD
@HWI-EAS443:3:71:194:1040#0/1
ACGCGAGATACTCACTAAGACCACTGTGGACCATATGGCC
+HWI-EAS443:3:71:194:1040#0/1
aaQWRF¥WT___`XY]WYYY[[]¥WYWTNTZZVYOQXYZZ
@HWI-EAS443:3:18:1382:1667#0/1
AGTGTCTTGTGAATTAGTCATCATCTTTCTCACAACATTT
+HWI-EAS443:3:18:1382:1667#0/1
aa^b^ab_U[LP_aaX`_Z_^^`__aaa^_[_Yaa]_¥Y^
@HWI-EAS443:3:11:1484:1720#0/1
AGTCCTCGTGCTATGGGGCATTCACCACCCATCTACTCTC
+HWI-EAS443:3:11:1484:1720#0/1
aa]^^aaaaa¥`a_¥X[`a_^^Y]]S`SXU^^U]^UZM^¥
@HWI-EAS443:3:76:503:1088#0/1
CATGAACCAGTCTCGTGACATTTCCTCGATGGTCATGTCA
+HWI-EAS443:3:76:503:1088#0/1
aaVRVZa_HP¥RQ_aQ`R`a_`[_`[^SXH[ZTVUY_W¥V
@HWI-EAS443:3:38:714:578#0/1
CTCGCCACTTTTCATTTCTGCTCTGGAGGTAGTGAAGGTT
+HWI-EAS443:3:38:714:578#0/1
aaa`_abb]abaaaa_aaa`aaa`aa`a`Y]_O^¥^`]JF
@HWI-EAS443:3:78:1212:1214#0/1
CTCGCCACTTTTCATTTCTGCTCTGGAGGTAGTGAAGGTT
+HWI-EAS443:3:78:1212:1214#0/1
aaaa`aaaaaab`abaa`aa``^`a_^__S`]V[]^[YIM
@HWI-EAS443:3:18:1511:1244#0/1
TTAGCCACAAATCCATAGCGATAAAAAAAGCTTGTGAGTT
```

+HWI-EAS443:3:18:1511:1244#0/1
aaabaaaaaa`aa`_a``_a_`______^`¥a^`Y_ZK[^
@HWI-EAS443:3:78:687:1271#0/1
ATTTGAACTCACAAGCTTTTTTTATCGCTATGGATTTGTG
+HWI-EAS443:3:78:687:1271#0/1
aaaa`]aaabaa`aa__a_`_````_^`__a__^^___Y]
@HWI-EAS443:3:53:669:495#0/1
AGCTGGGCCATATCTCTTGTCTTCTTTGCCCAAAATGAGA
+HWI-EAS443:3:53:669:495#0/1
aba_ababaab`aa_aba_`aaaaaaba`_a`_a_a`aa`
@HWI-EAS443:3:13:1224:1406#0/1
CAAGAACCCCGCACTCAGAATGAGGTGGATGATGGNAATG
+HWI-EAS443:3:13:1224:1406#0/1
H]WaW__^^_b^]]a``baab`[b`¥b]aa`b__UDY`]]
@HWI-EAS443:3:73:897:706#0/1
GAAGAACCCCGCACTCAGAATGAGGTGGATGATGGCAATG
+HWI-EAS443:3:73:897:706#0/1
aaababbbaaaaaabaaa¥_aaXSZXa`^aa`aa_``_`a
@HWI-EAS443:3:17:858:734#0/1
ACTGTGCCCATGGAGTATCCTGTTCCTGTTCCATGGCTGT
+HWI-EAS443:3:17:858:734#0/1
_`baa_VZX_]b]Mb_aabV]b`YHQ^^JaYYGX___BBB
@HWI-EAS443:3:96:419:527#0/1
GTCGCTTTAAATACGGTTTGAAAAGAGGGCCTTGTTCGGA
+HWI-EAS443:3:96:419:527#0/1
aUaXNWVX]]`XN^aWR[_`]UGV]R^LQ¥NX^GMHLXBB
@HWI-EAS443:3:68:155:1066#0/1
CGCGGGAAGTCTTCGAGCTCTCGGACGAAAAGGCAACGAA
+HWI-EAS443:3:68:155:1066#0/1
a¥]T¥KIPDIXON`XKR[XZ¥^VZZZUKPPJPRTRXBBBB
@HWI-EAS443:3:11:1653:958#0/1
TGCCTGAGTCCATGAGGGAAGAGTATCAACAGGAACAGCA
+HWI-EAS443:3:11:1653:958#0/1
_¥`]`^`aa_RS[bNP`]O`aTP`]XXP^OZY¥[Z^¥ZKY
@HWI-EAS443:3:68:846:731#0/1
CTCTGCAAAAGCAGGTAGATATTTAAAGATGAGTCTTCTA
+HWI-EAS443:3:68:846:731#0/1
abbaa`bb_abaabb[baa_^`baa_¥aaaa`^_aba`aa
@HWI-EAS443:3:43:1159:1907#0/1
GGGTTTTTTTTATCATTAAATAAGCTGAAACGAGAAAGCT
+HWI-EAS443:3:43:1159:1907#0/1
__^Wa`baaaaa`¥]ba`aa`aa^W^^_```_``_^_ZOY
@HWI-EAS443:3:73:464:494#0/1
CTGCAGCAAATATCATTGGGATCTTGCACCTGATATTGTG
+HWI-EAS443:3:73:464:494#0/1
abbbbaba``baaaabaaYa`aaaaaaa`aba`aaaaa]a
@HWI-EAS443:3:5:550:967#0/1
CTCTCGTCATTGCAGCAAATATCATTGGGATCTTGCACAT
+HWI-EAS443:3:5:550:967#0/1
a^``a¥HY^baY_b]a_TabbaS`Va_U`_aZ^`aaW^BB

@HWI-EAS443:3:100:899:992#0/1
ATTGGGACTCATCCTAGCTCCAGTGCTGGCCTGAAAGATG
+HWI-EAS443:3:100:899:992#0/1
]¥[SaW¥P`U_aZ]aSWUa]aabT¥ZaZUH_]ba`a¥aaB
@HWI-EAS443:3:74:483:1529#0/1
GGTGTATCCTGTTCCTGTTCCATGGCTGTATGGGGGGTGT
+HWI-EAS443:3:74:483:1529#0/1
a`R`aYP¥QXXOQZUZ]^O^^ZBBBBBBBBBBBBBBBBBB
@HWI-EAS443:3:3:61:966#0/1
ATTGAACTAATCAGAGGGCGACCCAAAGAGAACACAATCT
+HWI-EAS443:3:3:61:966#0/1
`^]]aa[U[[U_R]ZWEY`^[YU[¥[P`XXV^[`]ZFY¥S
@HWI-EAS443:3:18:1199:1748#0/1
CAACACTTGGGTAAACCAGACATATGTTAACATCAGCAAC
+HWI-EAS443:3:18:1199:1748#0/1
aab^b`ab_`]O_ab``a[aabaaa`[aaaXaaaa_Y`aZ
@HWI-EAS443:3:1:677:1923#0/1
CAACACTTGGGTAAACCAGACATATGTTAACATCAGCAAC
+HWI-EAS443:3:1:677:1923#0/1
`bbWbaaabb`Y¥bbRJ¥bb_baabbbbbbbb_abbMabb
@HWI-EAS443:3:21:1313:1573#0/1
CAACACTTGGGTAAACCAGACATATGTTAACATCAGCAAC
+HWI-EAS443:3:21:1313:1573#0/1
a]VRXUY[YTOLVZ`XSMZR^a^]XMXR]¥PU[PZGSQZK
@HWI-EAS443:3:25:325:1061#0/1
CAACACTTGGGTAAACCAGACATATGTTAACATCAGCAAC
+HWI-EAS443:3:25:325:1061#0/1
a¥_]]]X]Z^_N[`aaaa^aa`_Q^`Xa`]`a`_aaa^¥S
@HWI-EAS443:3:29:663:258#0/1
ACACTTGGGTAAACCAGACATATGTTAACATCAGCAACAC
+HWI-EAS443:3:29:663:258#0/1
aa`a`aa`a[_aa_``Z]``_a``X_`_X___`^_a`]_]
@HWI-EAS443:3:42:698:1406#0/1
TGTTGCTGATGTTAACATATGTCTGGTTTACCCAAGTGTT
+HWI-EAS443:3:42:698:1406#0/1
^RV]]`a[X`a[[]_]W¥^¥`[`XYUUZ^Y[_`¥Q¥L]S^
@HWI-EAS443:3:66:1266:45#0/1
CAACACTTGGGTAAACCAGACATATGTTAACATCAGCAAC
+HWI-EAS443:3:66:1266:45#0/1
abbababbbba_bab`aaba`ababbaaa`_aa]aa`aa^
@HWI-EAS443:3:66:1389:388#0/1
GTTGCTGATGTTAACATATGTCTGGTTTACCCAAGTGTTG
+HWI-EAS443:3:66:1389:388#0/1
a]_`aba[a``_V]]_a_a`[[__`Y_`^[¥^¥^ZVaY^Y
@HWI-EAS443:3:6:899:1943#0/1
CAACACTTGGGTAAACCAGACATATGTTAACATCAGCAAC
+HWI-EAS443:3:6:899:1943#0/1
`ab_b_aab`a_`¥aa^aaa]^_aaa_aaa_a¥_b_`bXB
@HWI-EAS443:3:75:426:56#0/1
CAACACTTGGGTAAACCAGACATATGTTAACATCAGCAAC

+HWI-EAS443:3:75:426:56#0/1
`bab`_bbbbbabaaaa`aababa`a]aaaaabaaaaaa_
@HWI-EAS443:3:87:695:732#0/1
CAACACTTGGGTAAACCAGACATATGTTAACATCAGCAAC
+HWI-EAS443:3:87:695:732#0/1
abbbbabbbab_bbbbbbabbbbbbbabb_abbabbabba
@HWI-EAS443:3:89:1370:370#0/1
AGGGGAGTCTTCGAGCTCTCGGACGAAAAGGCAACGAACC
+HWI-EAS443:3:89:1370:370#0/1
```^abbbaab`bab_bababbaaba_aW]a]aa¥abaW¥
@HWI-EAS443:3:83:77:89#0/1
CTGGCTGTCAGTAAGCATGCTAGAGTCCCGTTCTCGTTTC
+HWI-EAS443:3:83:77:89#0/1
aaZY]]U_YOW¥ZT]FPY]QVJX[BBBBBBBBBBBBBBBB
@HWI-EAS443:3:50:1256:1454#0/1
TGGGGTTTGCTTAATGCATCTTGGTTCAACTCCTTCCACA
+HWI-EAS443:3:50:1256:1454#0/1
Sa¥_I_U`b_[_``Z_YH]Ya[`¥K_ST]Y[SHYGUQFSZ
@HWI-EAS443:3:17:1675:608#0/1
CCCTGGGGTTTGCTTAATGCATCTTGGTTCAACTCCTTCC
+HWI-EAS443:3:17:1675:608#0/1
Xa`^b`_Lbbbabbbab`a[SbabbbbaZFYaa_W[a[I]
@HWI-EAS443:3:20:1454:729#0/1
CTGGGGTTTGCTTAATGCATCTTGGTTCAACTCCTTCCTC
+HWI-EAS443:3:20:1454:729#0/1
`baRQGUUaXa]]]WWa``SQ``bWTQ_a`H`MV`[UFTS
@HWI-EAS443:3:49:888:1321#0/1
CCCTGGGGTTTGCTTAATGCATCTTGGTTCAACTCCTTCC
+HWI-EAS443:3:49:888:1321#0/1
`ababbbHbbb]`a`]b`bbba^bba[_ab`]U__`baaY
@HWI-EAS443:3:13:1764:488#0/1
AGCCCATCCACTAACAGGGCAGAGGGAGGAATTGCCAGCT
+HWI-EAS443:3:13:1764:488#0/1
a[^`_^MX^a]^aa¥`ZS[GMS]WPa¥WW¥WWU[RRH_BB
@HWI-EAS443:3:77:930:83#0/1
ATTGTCAACATCCACAGCACTCTGCTGTTCCTGTTGATAT
+HWI-EAS443:3:77:930:83#0/1
V`bb]``a]^b`Vb^ba[aG[]b]^baaaaR^a]Y`Z]_]
@HWI-EAS443:3:62:356:565#0/1
CTCGGGACCACTAGATTTCCAGTTGCTTCGAATGTTATTT
+HWI-EAS443:3:62:356:565#0/1
¥¥U]RF¥W]WQY_a_XQW¥¥X^VW]]TX_]TYZSZ[SWVV
@HWI-EAS443:3:6:833:61#0/1
AAGTGATCCCAAGAGGAAAGCTTTCCACAAGAGGGGTCCA
+HWI-EAS443:3:6:833:61#0/1
`aa`b_a]Y[aa_aaa`_Y`¥_`YTVZY[¥_W^¥W¥UUQB
@HWI-EAS443:3:39:822:2019#0/1
AAGAAGCAGATACTGGGCAATAAGGACCAGGAGTGGAGGA
+HWI-EAS443:3:39:822:2019#0/1
aaSYaa^b^]^ba[`QTNK_Za^^QbWS`[YLLU_]^ZZV
```

@HWI-EAS443:3:49:617:338#0/1
AGCGAGAGCAATTGGGACAGAAATTTGAGGAAATAAGGTG
+HWI-EAS443:3:49:617:338#0/1
`bab^baba_]_b_aa`aba^aaaaaaabb__`aa_a`Ob
@HWI-EAS443:3:72:1186:1866#0/1
ACTACCTCCAGAGCAGAAATGAAAAGTAGCGAGAGCAATT
+HWI-EAS443:3:72:1186:1866#0/1
`R__RS[_a`_][Z^YPY[`¥]^_^^YJY]^^^X]XW^_Y
@HWI-EAS443:3:44:703:680#0/1
GGGAGACCTTCACCACCTCCAGAGCAGAAATGAAAAGTGG
+HWI-EAS443:3:44:703:680#0/1
__Z¥aba¥I_`_`P^]_`_[aaZb[L]_¥Za_^a^[`^^Q
@HWI-EAS443:3:10:1444:337#0/1
GTTATCTTTTGCCTAGGCATGAGCATGAACCAGTGTCGTG
+HWI-EAS443:3:10:1444:337#0/1
P[_¥`T[Z`a]PSVP__Va^_GLRZZaBBBBBBBBBBBBB
@HWI-EAS443:3:44:191:1533#0/1
GCCTTATTGAATTCATTTTGGACCCAGCTTGCCAGAGATC
+HWI-EAS443:3:44:191:1533#0/1
```ba[[aab^`[ba`b]_¥U_baa_Raa`^a^`U`^a[F
@HWI-EAS443:3:40:233:602#0/1
AACGACTTCCATTGGTTCAAGGCATGAATTCTCAAATATT
+HWI-EAS443:3:40:233:602#0/1
Ya`_Y_ZZ___U]_GUX¥_a`S_`¥_FZ[]UWYV¥PL¥N]
@HWI-EAS443:3:81:1584:1772#0/1
AATTGTTTTCCCAAATGAAGTGGGGGCAAGAATACTGACA
+HWI-EAS443:3:81:1584:1772#0/1
`abbba`aabXb¥aJbaa_^O^Sa^a]`a_aaaaaa^VS^
@HWI-EAS443:3:71:927:1101#0/1
TGATGTCAGCATTCTTGCCCCCACTTCATTTGGGAAAACA
+HWI-EAS443:3:71:927:1101#0/1
X`V__K`[`RXQ_`aYZa¥XP]Taaa`Y`aWP_[`aa__`
@HWI-EAS443:3:69:1499:647#0/1
GAAAGTGATTCCAAGAGGAAAGCCTTCCACAAGAGGGGTC
+HWI-EAS443:3:69:1499:647#0/1
W^__X^b_U¥_H]aa_a_ba`V_P_`Z]YY__aaa_a_YY
@HWI-EAS443:3:3:369:63#0/1
ATCCCACTGCATATGTATCCTATCTGATATTCTAGATTCT
+HWI-EAS443:3:3:369:63#0/1
a_^]`aT^b¥_^_aa[_aRORX_Y]aW_]_H]HZ`YZ^BB
@HWI-EAS443:3:1:1250:376#0/1
CTAAGATCAAGATGAAATGGGGCATGGAAATGAGGAGCTG
+HWI-EAS443:3:1:1250:376#0/1
UNRRZP^JOXVQ^aaSS`[MNaa_aWRJH^^a_a`L[_BB
@HWI-EAS443:3:47:1641:469#0/1
GAATAAGTGGGAGACCTTCACTACCTCCAGAGCAGAAATG
+HWI-EAS443:3:47:1641:469#0/1
aaab`_bYabb]b_]^`__`V__]^`]U]^Y^_^_^Y^`_
@HWI-EAS443:3:10:704:678#0/1
GAGTGGGAGACCTTCACTAGCTCCAGAGCAGAAATGAAAA
```

```
+HWI-EAS443:3:10:704:678#0/1
]UHQ[Za¥¥R¥NVUXX[^[LS[X[Y_]_TY^T¥[G^W¥VW
@HWI-EAS443:3:34:19:1333#0/1
AAAATGACCATCGTCAACATCCACAGCACTCCGCTGTTCC
+HWI-EAS443:3:34:19:1333#0/1
]X^b^_aabaaa_T`aa_`[_¥V_`^^`a^`a¥¥WJOZQX
@HWI-EAS443:3:74:825:862#0/1
ATTCGAACACAAATACCCGCAGAAATGCTAGCAAGCATTG
+HWI-EAS443:3:74:825:862#0/1
abba`aa`aabaaaba]^]^^`Z__^_]^[_Y_`^¥_^[R
@HWI-EAS443:3:42:520:709#0/1
CGCTGCCGGTTGATTTCTGTTTAATGTCCAATCATAAGTC
+HWI-EAS443:3:42:520:709#0/1
abb]baabbOaS¥W`aaaa¥``]]]a__^`_^`_^`a_G`
@HWI-EAS443:3:48:182:1525#0/1
CGCTGCCGGTTGATTTCTGTTTAATGTCCAATCATAAGTC
+HWI-EAS443:3:48:182:1525#0/1
^[R_¥[`Xaa`QRHYS_T¥VaZTV^^SR^a_^[Q`V_U[O
@HWI-EAS443:3:56:1759:152#0/1
CGCTGCCGGTTGATTTCTGTTTAATGTCCAATCATAAGTC
+HWI-EAS443:3:56:1759:152#0/1
ababa`abbaa¥]^abXa`[ba`a_¥W¥¥`aaa_a]_Y[Y
@HWI-EAS443:3:82:1272:64#0/1
AAAACTAGACCAAAAGCAGCTTCTGTGGTCACTGGTACCA
+HWI-EAS443:3:82:1272:64#0/1
^QHQ_PQaZUU¥_R¥ZQV__RZ¥UVUK^FMY^_BBBBBBB
@HWI-EAS443:3:10:1149:67#0/1
CGATGCTGTGAATCAGCAATCTGTTCACAAGTGGCACAAA
+HWI-EAS443:3:10:1149:67#0/1
¥a^a_ab_b`Y_a[bb`aJ`Z[aabbaGb`UR]_G`^aGa
@HWI-EAS443:3:37:1566:233#0/1
GAATCAGCAATCTGTTCACAAGTGGCACAAACTAGACCAA
+HWI-EAS443:3:37:1566:233#0/1
aa¥^^`¥SYa`Xa`HT[QQ]^^VZT¥]W]HZXX¥WVXYXZ
@HWI-EAS443:3:37:258:158#0/1
CTGTTCACAAGTGGCACACACTAGACCACACGCAGCTTCT
+HWI-EAS443:3:37:258:158#0/1
a¥`Yaaa]¥XQZP]a`_Y[a^Y^`ZZEYLVFV`^VZUSOP
@HWI-EAS443:3:37:1071:180#0/1
CAAAAAGTACACGTCAGGAAGACAAGAGAAGAACCCCGCA
+HWI-EAS443:3:37:1071:180#0/1
abbabbb`bbbbbaabbbaabaabbba`RP[aa`a_aaaW
@HWI-EAS443:3:51:1612:58#0/1
CAGGAAGACAAGAGAAGAACCCCTCACTCAGAATGAAGTG
+HWI-EAS443:3:51:1612:58#0/1
aab[a_b[ZXbbaa`[b[[`UaaIbbaaa_]abbbaaVRa
@HWI-EAS443:3:66:56:878#0/1
CAGGAAGACAAGAGAAGAACCCCTCACTCAGAATGAAGTG
+HWI-EAS443:3:66:56:878#0/1
aY_TFMU__WU[[T]_¥V[Z__]GTUWUYMZLTY^P[VUV
```

```
@HWI-EAS443:3:40:870:2014#0/1
CGTCGCTTTAAATACGGTTTGAAACGAGGGCCTTCTACGG
+HWI-EAS443:3:40:870:2014#0/1
`bbabab_^aba^a^ba__ba`bWO__b_UD¥FRa^ZIYX
@HWI-EAS443:3:37:172:1603#0/1
GGGGTGGGGAGTCTTCGAGCTCTCGGACGACAAGGCAACG
+HWI-EAS443:3:37:172:1603#0/1
`a][S[[XX`¥^`a^`[`Y^¥`¥`¥V]^XPDYMNS^^][P
@HWI-EAS443:3:62:1028:551#0/1
CGGCCATCCGAATTCTTTTGGTCNCTGTCTGGCTGTCAGT
+HWI-EAS443:3:62:1028:551#0/1
abababbbbbbbabbbbbbb`VDZ`b`aaa_aaaY]]^P
@HWI-EAS443:3:32:629:659#0/1
CACGGCCATCCGAATTCTTTTGGTCGCTGTCTGGCTGTCA
+HWI-EAS443:3:32:629:659#0/1
abba_[baaab^aaaa_`aaa]a¥¥__aS_¥_¥]`WaHMP
@HWI-EAS443:3:42:939:1589#0/1
TCGGCCATCCGAATTCTTTTGGTCGCTGTCTGGCTGTCAG
+HWI-EAS443:3:42:939:1589#0/1
`b^aaba`aa¥Z_aa¥aaaa¥a_^`a_a]Y`WZBBBBBBB
@HWI-EAS443:3:5:1094:807#0/1
CGGCCATCCGAATTCTTTTGGTCGCTGTCTGGCTGTCAGG
+HWI-EAS443:3:5:1094:807#0/1
abb[]__a^baabaYbbba[bSaba¥Va`a[^^baXZ^aB
@HWI-EAS443:3:15:413:499#0/1
CGGCCATCCGAATTCTTTTGGTCGCTGTCTGGCTGTCAGT
+HWI-EAS443:3:15:413:499#0/1
abababaa`baaabaaaba``__aXaa_`aa¥_^RULRTU
@HWI-EAS443:3:21:988:731#0/1
CGGCCATCCGAATTCTTTTGGTCGCTGTCTGGCTGTCAGT
+HWI-EAS443:3:21:988:731#0/1
a_Y][]WSWXN[[¥_a_W_`PUY_W`_SR`Z_XVYBBBBB
@HWI-EAS443:3:24:250:1544#0/1
CGGCCATCCGAATTCTTTTGGTCGCTGTCTGGCTGTCAGT
+HWI-EAS443:3:24:250:1544#0/1
aaYbb[¥abXOQZU_`¥`[a`P_a`][^a^]ZYYBBBBBB
@HWI-EAS443:3:33:973:1266#0/1
CGGCCATCCGAATTCTTTTGGTCGCTGTCTGGCTGTCAGT
+HWI-EAS443:3:33:973:1266#0/1
_]¥a`__][U_S¥aaaaY```Ta``aa¥baaXR¥ZDRQUH
@HWI-EAS443:3:34:27:1344#0/1
CGGCCATCCGAATTCTTTTGGTCGCTGTCTGGCTGTCAGT
+HWI-EAS443:3:34:27:1344#0/1
aa[b^a]abb_Zaa`aba^`a_aZ``a_a]`_a_¥Q[UYS
@HWI-EAS443:3:37:640:841#0/1
CGGCCATCCGAATTCTTTTGGTCGCTGTCTGGCTGTCAGT
+HWI-EAS443:3:37:640:841#0/1
VOV_baS__a_aba`Uaa__¥Ta¥_aa[b[J`[[BBBBBB
@HWI-EAS443:3:45:1329:1928#0/1
TTGACGGCCATCCGAATTCTTTTGGTCGCTGTCTGGCTGT
```

```
+HWI-EAS443:3:45:1329:1928#0/1
aba¥`]YT]a`VV`¥`^^LX¥`^R_SDU^BBBBBBBBBBB
@HWI-EAS443:3:46:103:1008#0/1
CGGCCATCCGAATTCTTTTGGTCGCTGTCTGGCTGTCAGT
+HWI-EAS443:3:46:103:1008#0/1
aaaaa`abbbaZaaaaaaaaa`aa^a`^_a]¥Z^]Y¥[UP
@HWI-EAS443:3:56:1785:709#0/1
CGGCCATCCGAATTCTTTTGGTCGCTGTCTGGCTGTCAGT
+HWI-EAS443:3:56:1785:709#0/1
_]¥_^bbaab`[_aaaaa_W_X`a`^`]]][U][]BBBBB
@HWI-EAS443:3:60:930:210#0/1
CGGCCATCCGAATTCTTTTGGTCGCTGTCTGGCTGTCAGT
+HWI-EAS443:3:60:930:210#0/1
a^Z_a`__baa`aaaaabb`aG_aa_bV[¥aa``BBBBBB
@HWI-EAS443:3:64:907:352#0/1
CGGCCATCCGAATTCTTTTGGTCGCTGTCTGGCTGTCAGT
+HWI-EAS443:3:64:907:352#0/1
`aabaaaaab_aabaaaaa]aYaa]aa][`a`a`^UQOTR
@HWI-EAS443:3:71:38:826#0/1
CGGCCATCCGAATTCTTTTGGTCGCTGTCTGGCTGTCAGT
+HWI-EAS443:3:71:38:826#0/1
_babbbaa_V_b_aabbbbb`¥aTaab_bZ]aa__TXLSQ
@HWI-EAS443:3:88:1502:1857#0/1
CGGCCATCCGAATTCTTTTGGTCGCTGTCTGGCTGTCAGT
+HWI-EAS443:3:88:1502:1857#0/1
_a_UZ_WZ`[^^a_aa`a][a¥QaG¥TXVaaRS_[RLPNN
@HWI-EAS443:3:8:153:306#0/1
CGGCCATCCGAATTCTTTTGGTCGCTGTCTGGCTGTCAGT
+HWI-EAS443:3:8:153:306#0/1
aaba¥]_``]QI`a__aaaaaV`____U^]^Y^ZBBBBBB
@HWI-EAS443:3:56:1738:1572#0/1
AGCCTTTTCCGCAAGCTCGACGATCATTGGATTGAAGCAT
+HWI-EAS443:3:56:1738:1572#0/1
a`QWYbaba^ababa_]`b]Va¥ZX__XF[DX_BBBBBBB
@HWI-EAS443:3:8:1484:778#0/1
AGCCTTTTCCGCAAGCTCGACGATCATTGGATTGAAGCAT
+HWI-EAS443:3:8:1484:778#0/1
aa]^baab``a`a^Z]aGaYNb^`WS_^]_¥__]]^VFV]
@HWI-EAS443:3:65:1621:1657#0/1
AAAGCAGGTACCGATCCAAAATGGAAGGCTTTGTGCGACA
+HWI-EAS443:3:65:1621:1657#0/1
aa¥QYaaa_baa`aa_aa^a^aYR`^^____]XZ]Sa_[`
@HWI-EAS443:3:26:296:769#0/1
TAAGCAAAACCCAGGGATCATTAATCAGACACTCCTCGAT
+HWI-EAS443:3:26:296:769#0/1
abaaab`a__aabaaa`_aaaaa_aaa]J¥a`aaa_aa`_
@HWI-EAS443:3:2:1306:197#0/1
GGGGATGTGGACGACAAACACAGAGACTGGAGCACCCCAG
+HWI-EAS443:3:2:1306:197#0/1
^__I`b¥`]^UV_aIX``S¥[_``_OFOaaG_BBBBBBBB
```

@HWI-EAS443:3:75:592:975#0/1
CAAAGCGTCTACGCTGCAGTCCTCGCTCACTGGGCACGGG
+HWI-EAS443:3:75:592:975#0/1
a[`_`a__a_`a^Z^`^_ZU^^Y¥Z`ZXY^KKR`aQUZ^N
@HWI-EAS443:3:4:276:635#0/1
CGTAGCGAAAGCAGGTACTGATCCAAAATGGAAGGCTTTG
+HWI-EAS443:3:4:276:635#0/1
ab`ababa`abaaaaUaa`a^__aaa```a[a_a_Taaa_
@HWI-EAS443:3:45:923:332#0/1
GAAAGCAGGCAAANCATTTGAATGGACGTCAATCCGACTC
+HWI-EAS443:3:45:923:332#0/1
abbaababbbaab^D[`aa`baaaaa__b]^aa`]U_¥Z_X
@HWI-EAS443:3:3:349:1958#0/1
AGCAAAAGCAGGGAAGATAATCACTCAATGAGTGACATCA
+HWI-EAS443:3:3:349:1958#0/1
_`baWaaU[JU[`WaI^]WaaYHT^`T^J_aFPH¥I]`ZG
@HWI-EAS443:3:28:849:1541#0/1
CAATTGCTCTCTCCACTTTTCATTTCTGCTCTGGAGGTAG
+HWI-EAS443:3:28:849:1541#0/1
aaba^`baZ_aab``aaaa``aaaaaa`]]^Z_[_aX]¥X
@HWI-EAS443:3:32:1696:2017#0/1
GTCCCAATTGCTCTCTCCACTTTTCATTTCTGCTCTGGAG
+HWI-EAS443:3:32:1696:2017#0/1
aHZN¥X_a]VPNYaODTY¥^ZX_SUXaWLPVR^UWU_^]B
@HWI-EAS443:3:44:1382:837#0/1
CCTTCACTACCTCCAGAGCAGAAATGAAAAGTGGAGAGAG
+HWI-EAS443:3:44:1382:837#0/1
`b^¥^XU`]XWa_YPV_aa^]¥`a`bXY^]`Z`]G`V¥^]
@HWI-EAS443:3:74:778:211#0/1
CAGAAATGAAAAGTGGAGAGAGCAATTGGGACAGAAATTT
+HWI-EAS443:3:74:778:211#0/1
_[aaabb_bbbba[__I__¥T^T_]^^bbb^HW`^Y¥`_a
@HWI-EAS443:3:42:913:1857#0/1
GAAAAAGGAGAGCAAGAGACAACATGACCAAGAAGATGGT
+HWI-EAS443:3:42:913:1857#0/1
`YYaa^`_`aa^M_bYa[`Zaa_aX_`]^__a_^U^¥`^V
@HWI-EAS443:3:67:750:650#0/1
CTGGCTGTCAGTAAGTATGCTAGAGTCCCGGTTTTGTTTC
+HWI-EAS443:3:67:750:650#0/1
ab`^^aa````IYUUFRUX¥TOW[¥F¥^`HFXaaQIH[`¥
@HWI-EAS443:3:34:1667:88#0/1
GTCGCTGTCTGGCTGTCAGTAAGTATGCTAGGGTCCCGGG
+HWI-EAS443:3:34:1667:88#0/1
aXQ`a]a]_¥LNVX`PQUVSSUBBBBBBBBBBBBBBBBBB
@HWI-EAS443:3:1:624:96#0/1
GTCGCTGTCTGGCTGTCAGTAAGTATGCTAGAGGCCCGGT
+HWI-EAS443:3:1:624:96#0/1
a[Ua]^_WYUY_W¥¥HTL¥MRVVUWSUERWTTSBBBBBBB
@HWI-EAS443:3:38:1454:1987#0/1
GGTCGCTGTCTGGCTGTCAGTAAGTATGCTAGNGTCCCGG

```
+HWI-EAS443:3:38:1454:1987#0/1
aa]aaa_a`a`__^aa]^`]Y`a^W`[^_VYKDPOVVVPD
@HWI-EAS443:3:86:1187:1157#0/1
CGCTGTCTGGCTGTCAGTAAGTATGCTAGAGTCCCGGTTT
+HWI-EAS443:3:86:1187:1157#0/1
abaab__`b_a`b^aabV^bb`baa`b]]ba``bbaGaaa
@HWI-EAS443:3:92:1284:1367#0/1
CGCTGTCTGGCTGTCAGTAAGTATGCTAGAGTCCCGGTTT
+HWI-EAS443:3:92:1284:1367#0/1
aaaab[^R`_a[a_WJRNZUZaUa_PQNZLQQ¥Ta_GZ^`
@HWI-EAS443:3:15:110:482#0/1
GTCGCTGTCTGGCTGTCAGTAAGTATGCTAGAGTCCCGGT
+HWI-EAS443:3:15:110:482#0/1
`[bbaa¥R]aaaa_ZTPYUN_`]U¥V]¥ZX¥_]LS¥ZTKV
@HWI-EAS443:3:57:1770:1335#0/1
TCGCTGTCTGGCTGTCAGTAAGTATGCTAGAGTCCCGGTT
+HWI-EAS443:3:57:1770:1335#0/1
aaaa]Z]`^`W`ZXFWV_HOOX[[NNN^BBBBBBBBBBBB
@HWI-EAS443:3:77:465:909#0/1
GTCGCTGTCTGGCTGTCAGTAAGTATGCTAGAGTCCCGGT
+HWI-EAS443:3:77:465:909#0/1
a`_`a^`[_W[U^]^XTRZKXWDNRYQYTL[WYN[L[ZFV
@HWI-EAS443:3:9:1419:1610#0/1
GGTCGCTGTCTGGCTGTCAGTAAGTATGCTAGAGTCCCGG
+HWI-EAS443:3:9:1419:1610#0/1
]aH_bY`a¥abbaab`U`Ta^X`¥¥[]YZ[a`¥`[]NR¥D
@HWI-EAS443:3:9:209:50#0/1
ATGAAAAGAAGGCCAAACTGGCAAATGTCGTGAGAAAGAT
+HWI-EAS443:3:9:209:50#0/1
abbba`_baaaabaaaaabaa_`a`aaYJ[Za_a_]]a__
@HWI-EAS443:3:81:1464:381#0/1
TGTTGGTAATGAAACGAAAACGGGACTATAGAATACTTAC
+HWI-EAS443:3:81:1464:381#0/1
YZ]U[`_a^MZ`b`Rb`^aYMaa]WZVG^X]IZLZG_X_R
@HWI-EAS443:3:76:1649:1700#0/1
GGTCGCTGTCTGGCTGTCAGTAAGTATGCTATAGTCCCGT
+HWI-EAS443:3:76:1649:1700#0/1
a[WX_[WaW_^]YT[IMU^`BBBBBBBBBBBBBBBBBBBB
@HWI-EAS443:3:68:1311:54#0/1
CGGGGACCGGTTGGAATTTCTAGCATGGTGGAGGCCATGG
+HWI-EAS443:3:68:1311:54#0/1
]¥^``ZV^`a¥```^`a`_a^```[``[OaX[a`MFV¥`[
@HWI-EAS443:3:17:1646:725#0/1
CCTGGGATGATGATGGGAATGTTTAACATGCTAAGTACGG
+HWI-EAS443:3:17:1646:725#0/1
X¥``abYN^bb¥`U`_W¥a_`V]]X_`bbb_bab`H`]_J
@HWI-EAS443:3:73:1524:1778#0/1
CTGGGATGATGATGGGCATGTTTAAGATGCTAAGTACGGT
+HWI-EAS443:3:73:1524:1778#0/1
X^a_^T[^_`¥[]WWab¥X_SWaabVbb_R_aZYL[_VP[
```

```
@HWI-EAS443:3:84:905:118#0/1
TACTTAGCATGTTAAACATGCCCATCATCATCCCAGGACT
+HWI-EAS443:3:84:905:118#0/1
aaaa_aa_aaa``a_`¥_a__]`__¥`a¥^^_]Z^_^]O^
@HWI-EAS443:3:70:1674:111#0/1
GTCCTGGGATGATGATGGGCATGTTTAACATGCTAAGTAC
+HWI-EAS443:3:70:1674:111#0/1
`aa`bbbb`baabbabbba^`bbabaa_^aaaXa__a^aU
@HWI-EAS443:3:91:650:548#0/1
GTCCTGGGATGATGATGGGCATGTTTAACATGCTAAGTAC
+HWI-EAS443:3:91:650:548#0/1
aUZX]`_]GX_UY_W¥_VXWVY^YYVX^UX]]WOUS]GVV
@HWI-EAS443:3:14:1039:1173#0/1
ACCTGGGATGATGATGGGCATGTTTAACATGCTAAGTACG
+HWI-EAS443:3:14:1039:1173#0/1
aaaaaaa]`baab``a``__aa]``a_]`__]_^]`X`Y]
@HWI-EAS443:3:20:847:841#0/1
TCCTGGGATGATGATGGGCATGTTTAACATGCTAAGTACG
+HWI-EAS443:3:20:847:841#0/1
``JW^b]V[]Ybbaaa[XZaabaaaa`_¥V^a__VVVZXa
@HWI-EAS443:3:52:601:197#0/1
CGTACTTAGCATGTTAAACATGCCCATCATCATCCCAGGA
+HWI-EAS443:3:52:601:197#0/1
abZbbbbbbabbbbbbb]abab_a`abaabaaaa_`aaa]
@HWI-EAS443:3:23:1101:1549#0/1
CTGGGATGATGATGGGCATGTTTAACATGCTAAGTACGGT
+HWI-EAS443:3:23:1101:1549#0/1
abbb`baa_bbabaa`babaaab`aabababb`a`aa_aX
@HWI-EAS443:3:46:1318:86#0/1
ACCGTACTTAGCATGTTAAACATGCCCATCATCATCCCAG
+HWI-EAS443:3:46:1318:86#0/1
aaabU[[```_^[]a_____]__[[¥^Z_ZZ[TU^VVRV]
@HWI-EAS443:3:47:21:1457#0/1
CCTGGGATGATGATGGGCATGTTTAACATGCTAAGTACGG
+HWI-EAS443:3:47:21:1457#0/1
aa`baaV¥`[a___WOLY¥UaZ_]VVRQ]¥S[`ZRNZSWN
@HWI-EAS443:3:58:1508:1658#0/1
CCTGGGATGATGATGGGCATGTTTAACATGCTAAGTACGG
+HWI-EAS443:3:58:1508:1658#0/1
Saa[[X_a^^a`_bb[]X`^`]_S^QZ^_ZW___¥]]QX_
@HWI-EAS443:3:59:511:193#0/1
CCTGGGATGATGATGGGCATGTTTAACATGCTAAGTACGG
+HWI-EAS443:3:59:511:193#0/1
`]Zaa[QZa`_b`¥¥`_¥_Y_R¥V[[^S]a[¥^¥]WS^[Q
@HWI-EAS443:3:62:1262:1109#0/1
CTGGGATGATGATGGGCATGTTTAACATGCTAAGTACGGT
+HWI-EAS443:3:62:1262:1109#0/1
ab`bbabbabbaabbaabbbabaa_]abbabaaa_a`aa[
@HWI-EAS443:3:74:451:1354#0/1
CTGGGATGATGATGGGCATGTTTAACATGCTAAGTACGGT
```

```
+HWI-EAS443:3:74:451:1354#0/1
abb`^Zb`abbaa``_aaaa[aaaa_aa`a_`[]^a`][W
@HWI-EAS443:3:81:269:1119#0/1
CTGGGATGATGATGGGCATGTTTAACATGCTAAGTACGGT
+HWI-EAS443:3:81:269:1119#0/1
abbaa]abaab^baXaaXaaaa`aa`aaa_`_WX]a`a_X
@HWI-EAS443:3:95:1411:860#0/1
CTGGGATGATGATGGGCATGTTTAACATGCTAAGTACGGT
+HWI-EAS443:3:95:1411:860#0/1
Vab`_^bX¥[_bb_b_S_baabbb¥XaabY¥`b_b^a`aP
@HWI-EAS443:3:95:820:241#0/1
CCTGGGATGATGATGGGCATGTTTAACATGCTAAGTACGG
+HWI-EAS443:3:95:820:241#0/1
abbabb`abbbbbbaababbb`bbba_bbb]baab`a]a_
@HWI-EAS443:3:87:296:693#0/1
GTCTTATTCATATAGGACTTCTTTTTGCTCATGTTGATTC
+HWI-EAS443:3:87:296:693#0/1
a_aaaaaaaaa_]Z```aaaaa`aaa`a``a`_[^`_`^`
@HWI-EAS443:3:4:239:1748#0/1
ATCCGAATTCCTTTGGTCGCTGTCTGGCTGTCAGTAAGTA
+HWI-EAS443:3:4:239:1748#0/1
a[a^`]aa`XU__^`_W`a¥¥ZT]_]YZ¥^N¥NSG¥BBBB
@HWI-EAS443:3:52:205:1941#0/1
AATTGATGGCCATCCGAATTCCTTTGGTCGCTGTCTGGCT
+HWI-EAS443:3:52:205:1941#0/1
^`bbba``_``aaaa[]aaaXG]`aaaS_a]`a¥_ZXRLV
@HWI-EAS443:3:65:1114:1301#0/1
ATTGATGGCCATCCGAATTCCTTTGGTCGCTGTCTGGCTG
+HWI-EAS443:3:65:1114:1301#0/1
^^]a]a¥ZZZaa[__S_^`]`P¥NZa_``TU_L[]RSRX^
@HWI-EAS443:3:63:755:186#0/1
GTAGTTTTTTACTCTAGCTCTACGATGACAAAATGACCAT
+HWI-EAS443:3:63:755:186#0/1
a^`a_`aaa_]]`a_``^_^_``ZDV_____^^[^¥[U¥W
@HWI-EAS443:3:35:1152:1398#0/1
GAGGAGTCTTCGAGCTCTCGGACGAAAAGGCAACGAACCC
+HWI-EAS443:3:35:1152:1398#0/1
``¥`b^aaab^ab`aaaaaaaa`aa`a``_aa`a^a``Qa
@HWI-EAS443:3:34:716:1967#0/1
CCCTGGGTTTTGCTTAATGCATCTTGGTTCAACTCCTTCA
+HWI-EAS443:3:34:716:1967#0/1
aba`^¥`P_a```abbb_S`a¥aa`a_]^bba__^_a]]F
@HWI-EAS443:3:37:1593:61#0/1
CGGAGAACAAACCTGTATGGGTTCATTATAAAAGGNAGGT
+HWI-EAS443:3:37:1593:61#0/1
]U¥UISK]TZ¥a`_`R_aaZ___^aa[UaGTXR^KDDIHQ
@HWI-EAS443:3:65:247:301#0/1
CTTTCTCTTTGACAGAAGACTCGGCCTCAATCGTGCTCTC
+HWI-EAS443:3:65:247:301#0/1
¥a]aabb¥b__]`abaababaab]O_Z]]]K]WOZRVIXQ
```

@HWI-EAS443:3:46:1574:882#0/1
TGTGGTAATGCCAGCCCATGGTCCAGCCAAAAGCGTGGAA
+HWI-EAS443:3:46:1574:882#0/1
__T__M_``_`^]^ZWZ¥a]]^__Z_Z]^U¥ZZN_Gaa__
@HWI-EAS443:3:85:152:1737#0/1
CACGCTTTTGGCTGGACCATGGGCTGGCATTACCACAGCA
+HWI-EAS443:3:85:152:1737#0/1
_XX_baaUU¥Z^aZX]^_O[TVW^GWZ¥U¥]]VXX``[ZU
@HWI-EAS443:3:26:1540:779#0/1
GTGGTTTTTAAACAATTCGACACTAATTGATGGCAATCCG
+HWI-EAS443:3:26:1540:779#0/1
a¥H_ab`T`YQXH`T_`a[RNWYaY[XQVW_a¥FEZ_XKQ
@HWI-EAS443:3:49:599:2024#0/1
GTGGTTTTTAAACAATTCGACACTAATTGATGGCAATCCG
+HWI-EAS443:3:49:599:2024#0/1
aWF^_`aaaa_`Va`a__aYO¥VY¥¥¥]Z[[]ZRF¥[XRS
@HWI-EAS443:3:18:1444:197#0/1
GTCGTTTTTAAACAATTCGACACTAATTGATGGCAATCCG
+HWI-EAS443:3:18:1444:197#0/1
_X_^[W^_X_UOL[^ZWXZ[Z¥S]`WYSUY[[WKDURUYW
@HWI-EAS443:3:57:373:923#0/1
CTAGTGGTCACTGTTCCCATCCTGTTGTATATGAGGCCCA
+HWI-EAS443:3:57:373:923#0/1
abb__aa`aaa___a]^`^^`__a`aa^aaa_]_YUV¥_a
@HWI-EAS443:3:96:671:1981#0/1
AACAAGGACAAACCCTCTGGAGCAAAATAAACGATGCTGG
+HWI-EAS443:3:96:671:1981#0/1
aaTaTYYOaT]a__a_Y_YZZa]Wa[¥¥]Y^W`_OWZPBB
@HWI-EAS443:3:73:1133:1256#0/1
GTTCTGCACACTTTCCCAATAGAGCCTTCCTCCACTCCCC
+HWI-EAS443:3:73:1133:1256#0/1
a``aaaaaaaaa^aa_aa[[``XP¥Y^`]aZ``V]Z_^`]
@HWI-EAS443:3:8:1482:1558#0/1
AAATTTCTGGAGGGGCGAAAATGGACGGAGGACAAGGCGA
+HWI-EAS443:3:8:1482:1558#0/1
`ba`^[[Q_aUa]]`GYSUX``a¥¥[W^]`XREVQ¥OGXB
@HWI-EAS443:3:80:528:59#0/1
CAATGAATTGAGCAATCTTGCAAAAGGAGAGAAAGCTAAT
+HWI-EAS443:3:80:528:59#0/1
a``_¥]aZ_]¥[^a[__aXa_]^S^YVXaa`_¥]]Qa]^Z
@HWI-EAS443:3:35:1491:893#0/1
CATGGGCCTCATATACAACAGGATGGGAACAGAGACAACA
+HWI-EAS443:3:35:1491:893#0/1
a`Y]aYSX_Z¥U]^]`_`^`¥UXZ_U¥Z¥N¥]FZXDD^VZ
@HWI-EAS443:3:30:104:1615#0/1
TCGCTGGATCAGACCGAGTGATGGTATCACCTCTGGCCGT
+HWI-EAS443:3:30:104:1615#0/1
`aa_VWOTU___W_```U]SWVZRFWV¥GMTQ`_`XT`BB
@HWI-EAS443:3:17:1406:548#0/1
CGGGTTCATTATAAAAGGAAGGTCTCATTTGAGAAATGAT

```
+HWI-EAS443:3:17:1406:548#0/1
abba^aaaaaab^aa_a_a``a^_a_aaaaa`a^_``a`_
@HWI-EAS443:3:98:1412:1696#0/1
CGAGGGGGAGGGAATACCGCTATATGATNTAATCAAATGC
+HWI-EAS443:3:98:1412:1696#0/1
Kb[bbbbbab]a¥bbaa[Wa_baa[aaPDHbabWaab[bP
@HWI-EAS443:3:39:214:919#0/1
GGAGTTGAACCACGATGCATTAAGCAAAACCCAGGGATCA
+HWI-EAS443:3:39:214:919#0/1
aSOZZ]`¥Za^YR¥YZ`¥a^¥]V_WDR¥__TJFM^_^U]M
@HWI-EAS443:3:57:545:572#0/1
CGTCTTTCTCTTTGACAGAAGACTCGGCCTCAATCATGCT
+HWI-EAS443:3:57:545:572#0/1
aa`ba^`b`aaaaba_abaXOXaaaaa_]HVGZZ[^WZ¥X
@HWI-EAS443:3:63:200:362#0/1
CTTTTTTGGACAGCATGGATAGCAAATAGAAGCATTGCCA
+HWI-EAS443:3:63:200:362#0/1
abaaaaaU[`aXH[aa¥W¥]IU]QY¥NL_LS[¥RYPV^WX
@HWI-EAS443:3:10:1306:318#0/1
GTACTTTTTTGGACAGTATGGATAGCAAATAGCAACTTTG
+HWI-EAS443:3:10:1306:318#0/1
aU[__[`_TZSRBBBBBBBBBBBBBBBBBBBBBBBBBBBB
@HWI-EAS443:3:88:272:1230#0/1
GTACTTTTTTGGACAGTATGGATAGCAAATAGCAGCATTG
+HWI-EAS443:3:88:272:1230#0/1
_UWWUZYU`]P]Z[SXYQ[`ZSPIO[Y[BBBBBBBBBBBB
@HWI-EAS443:3:63:1609:1370#0/1
ATCCTGTTCCTGTTCCATGGCTGTCTGGAGGGTCTCCTGT
+HWI-EAS443:3:63:1609:1370#0/1
a_]^WZ¥`^^ab^XSXPX[`U]BBBBBBBBBBBBBBBBBB
@HWI-EAS443:3:2:456:1963#0/1
GGTGTAGCCTGTTCCTGTTCCATGGCTGTATGGAGGATGT
+HWI-EAS443:3:2:456:1963#0/1
`bVbX_FRa``VaXa``^WSPaaWV¥abBBBBBBBBBBBB
@HWI-EAS443:3:6:271:1210#0/1
CTGTGAATCAGCAATCTGTTCACAAGTAGCACACACTCAA
+HWI-EAS443:3:6:271:1210#0/1
aaaababaaaaaaa`_aa_``a`aa_]aa_`^```___`_
@HWI-EAS443:3:32:312:1863#0/1
AGGTGCCACAGGGGAGACACACAAATTCAAACGAGAAGAT
+HWI-EAS443:3:32:312:1863#0/1
_]WXXTU]V]ULH[J[aWa_]R^^SUU^]M]P]TNRKS]Y
@HWI-EAS443:3:67:302:1346#0/1
TGGAGAAGTTCGGTGGGAGACTTTGGTCGGCAAGCATGCG
+HWI-EAS443:3:67:302:1346#0/1
`bb[_a`¥]aa[aUR[_R_``a]UV]U^_^a``[TF¥SY_
@HWI-EAS443:3:87:1555:543#0/1
CTTTTTTGGACAGTATGGATAGCAAATTGTAGCATTGCCA
+HWI-EAS443:3:87:1555:543#0/1
]¥¥U¥^a_ba]````a[aYa_aV^`_a^^_bba^aa`L¥a
```

```
@HWI-EAS443:3:25:1165:705#0/1
CAAATAGTAGCATTGCCATAACTACTTCAATGCATGTGTG
+HWI-EAS443:3:25:1165:705#0/1
^bbbbb_abbb_aababaJ`bbbbbbb_ababbbbbbbab
@HWI-EAS443:3:90:1277:1983#0/1
GACACTAATTGATGGCCATACGAATTCTTTTGGTCGCTGT
+HWI-EAS443:3:90:1277:1983#0/1
`_^_P]a`Z`b¥^a`¥]^_QaaaTXZ¥`]``__RG]Y¥ZB
@HWI-EAS443:3:77:1295:1617#0/1
CAGTAAGTATGCTAGAGTCCCGTTTTCGTTTCAACACTAC
+HWI-EAS443:3:77:1295:1617#0/1
`b_abababbbabbbb__babbbbbbaaabbbbbbababbb
@HWI-EAS443:3:76:1682:1254#0/1
CTGATGTGTACTTTTTAATTATGGCCATATGGTCCACAGT
+HWI-EAS443:3:76:1682:1254#0/1
aa``a`^bbabbbbbbbbbbbbbaaa`aaZGXX``_H_][
@HWI-EAS443:3:45:830:934#0/1
CCTCGTTTGAATTTGTGTGTCTCCCCTATGGCACCTATAT
+HWI-EAS443:3:45:830:934#0/1
aabaX¥ba`Waaab`a¥¥_aaaXa_`[Y`¥]TX^`_^_]`
@HWI-EAS443:3:11:81:1325#0/1
GAAGGCACGATCGGGTTCGTTGCCTTTTTGTCCGAGAGCT
+HWI-EAS443:3:11:81:1325#0/1
aXaaaa]baa¥[KJSM^¥XXXMTX[]a[BBBBBBBBBBBB
@HWI-EAS443:3:80:1277:1858#0/1
CACAAATTCAAACGAGAAGATCATTTGAGATAAAGAAGCT
+HWI-EAS443:3:80:1277:1858#0/1
abb]M]aaba_a_`Yb`U^a]]`YYY¥R`F¥[a`^a]_XS
@HWI-EAS443:3:16:704:1117#0/1
CAGGATGTGATTATGGAAGTTGTTTTCCCCAATGAAGGGG
+HWI-EAS443:3:16:704:1117#0/1
`^abbabJbb[`aaab_b[[[^TQY^U¥`GFQM]JLYBBB
@HWI-EAS443:3:69:1078:263#0/1
GAGAATTCATGCCTTGAAACAATGGAAGTCGTTCAAAAAA
+HWI-EAS443:3:69:1078:263#0/1
a`¥¥^aa¥``YJ[a_a_Sa`bX_`a`^_X¥RLWBBBBBBB
@HWI-EAS443:3:96:1642:659#0/1
TGAGAATTCATGCCTTGAAACAATGGAAGTCGTTCAAAAA
+HWI-EAS443:3:96:1642:659#0/1
aa_ZaKaaZb`VYa¥b`WRVPaJa]I_]WLFFT_HUY[U_
@HWI-EAS443:3:37:1682:967#0/1
CATGCCTTGAAACAATGGAAGTCGTTCAAAAAACAAGGGT
+HWI-EAS443:3:37:1682:967#0/1
aaP[[abbbb^aaa_b`^^aaWaa^aaT_Q`_[PY_aaaV
@HWI-EAS443:3:3:1672:795#0/1
AATTTCTAGCATGGGGGAGGCCATGGTGTCTAGGGCCCGG
+HWI-EAS443:3:3:1672:795#0/1
aa_X_X_aaW¥]aaSb```^^UZ^]^M^¥KPX^WWSMG[B
@HWI-EAS443:3:62:1366:341#0/1
ATTTCTAGCATGGGGGAGGCCATGGTGTCTAGGGCCCGGA
```

+HWI-EAS443:3:62:1366:341#0/1
a^VZI_¥UaaaZQDPaX`a]POSQ]UZWZOO[^^FK¥``S
@HWI-EAS443:3:36:881:902#0/1
TTTCTGGACAGTATGGATAGCAAATAGTAGCATTGCCACA
+HWI-EAS443:3:36:881:902#0/1
aaZO_aba`aa^¥_aa``_aY_aa`^]S`_aT]X[_aSX]
@HWI-EAS443:3:92:26:1709#0/1
AAAGTACACATCAGGAAGACAAGAGAAGAACCCAGCACTC
+HWI-EAS443:3:92:26:1709#0/1
^¥¥V_`abaVa_a`]`]T[^a^`a`¥__]RZX¥F¥__¥W_
@HWI-EAS443:3:65:233:1109#0/1
GTGGATTGTCCCCGAAAATCCCACTGCATATGTATCCTAT
+HWI-EAS443:3:65:233:1109#0/1
RUYaSSa_N]L`b_Za¥U¥W^^MWW]HPY¥ZJ]`TV[]`^
@HWI-EAS443:3:90:1397:2041#0/1
ATTTCTAGCATGGCGGAGGCCATGGTGTCTAGGGGCCGGA
+HWI-EAS443:3:90:1397:2041#0/1
_¥aXSO[W_Q^VbJ_U_WHL¥ZY¥aFGKQJR[VWHX]a`^
@HWI-EAS443:3:12:1081:2036#0/1
ATTTCTAGCATGGTGGAGGCCATGGTGTCTAGGGGCCGGA
+HWI-EAS443:3:12:1081:2036#0/1
Z]ba]^`^Tba`[DUVTZN]VQ^^[TFJZWS_`LEMN_BB
@HWI-EAS443:3:87:1190:79#0/1
GGGGGACGTANTGTTGGGAATGAAACGAAAACGGGACTCT
+HWI-EAS443:3:87:1190:79#0/1
abaaa^_bTJDRa]`a_LP]`VO^`Z_]]_XU`_^XXYNU
@HWI-EAS443:3:31:1595:280#0/1
GGGGGACGTAGTGTTGGTAATGAAACGAAAACGGGACTCT
+HWI-EAS443:3:31:1595:280#0/1
_bbba_abY¥¥R_`aaa^¥__Q^`_NWN_]_LRZZ[HWTT
@HWI-EAS443:3:33:947:1147#0/1
GGGGGACGTAGTGTTGGTAATGAAACGAAAACGGGACTCT
+HWI-EAS443:3:33:947:1147#0/1
a]a_]_`^NYW_a¥a`¥N_`aX^__V]U]Z]SSMRWQV[_
@HWI-EAS443:3:40:343:1291#0/1
GGGGGACGTAGTGTTGGTAATGAAACGAAAACGGGACTCT
+HWI-EAS443:3:40:343:1291#0/1
aQ^]XRF^MYLNNWBBBBBBBBBBBBBBBBBBBBBBBBBB
@HWI-EAS443:3:62:159:540#0/1
GGGGGACGTAGTGTTGGTAATGAAACGAAAACGGGACTCT
+HWI-EAS443:3:62:159:540#0/1
```X^M^_V_]Z^R]]^UW]]`U[`Y^ZPZUR[Z]RRX¥W
@HWI-EAS443:3:64:1776:755#0/1
GGGGGACGTAGTGTTGGTAATGAAACGAAAACGGGACTCT
+HWI-EAS443:3:64:1776:755#0/1
ababaa`baa_`aab`^R]^_`aa]N^[aa^G[Y`¥Z[^^
@HWI-EAS443:3:69:928:1256#0/1
GGGGGACGTAGTGTTGGTAATGAAACGAAAACGGGACTCT
+HWI-EAS443:3:69:928:1256#0/1
`Y___]__PH`Q_T__[UT¥UN[Y_YTGX__TZ¥WHTN¥W
```

````
@HWI-EAS443:3:75:692:1059#0/1
GGGGGACGTAGTGTTGGTAATGAAACGAAAACGGGACTCT
+HWI-EAS443:3:75:692:1059#0/1
`aaba`aa_aa[a_aaa¥__`X`_a^^]a¥_Z[__[Z[_¥
@HWI-EAS443:3:77:415:768#0/1
GGGGGACGTAGTGTTGGTAATGAAACGAAAACGGGACTCT
+HWI-EAS443:3:77:415:768#0/1
aaaaa_a_Zaa_a]`a[W^¥¥]_`_^_X^_][¥W¥]_]_Y
@HWI-EAS443:3:85:296:1786#0/1
GGGGGACGTAGTGTTGGTAATGAAACGAAAACGGGACTCT
+HWI-EAS443:3:85:296:1786#0/1
`^]Waabb_`a]b_aaaT`a^LXbb[]_aaaLNU^`W^`]
@HWI-EAS443:3:93:913:1765#0/1
GGGGGACGTAGTGTTGGTAATGAAACGAAAACGGGACTCT
+HWI-EAS443:3:93:913:1765#0/1
a]¥P]Y[^]a]N`^X]RNN_R`H`RLOJV_^XZ^_LSTVB
@HWI-EAS443:3:95:1137:1241#0/1
GGGGGACGTAGTGTTGGTAATGAAACGAAAACGGGACTCT
+HWI-EAS443:3:95:1137:1241#0/1
abaab_]aaa`_baab]YaYaaaa]Q]aaaaVZ^`[HZUY
@HWI-EAS443:3:95:1439:131#0/1
GGGGGACGTAGTGTTGGTAATGAAACGAAAACGGGACTCT
+HWI-EAS443:3:95:1439:131#0/1
aabba]RaYYa_aP```VY_a]Y[^TY]__¥QYa`GPVIX
@HWI-EAS443:3:9:165:19#0/1
GGGGGACGTAGTGTTGGTAATGAAACGAAAACGGGACTCT
+HWI-EAS443:3:9:165:19#0/1
abaab^_a^]aT`V]aaPY[^_TZ^PYT¥][]V^]XXXVY
@HWI-EAS443:3:59:326:1810#0/1
GTCTGCTGTAATTGGGTATCTCATTGCCATCATCCATTTC
+HWI-EAS443:3:59:326:1810#0/1
aWUY_a`RY]Z_SQVQSSQ[^][`ZV_a¥X[NQT]BBBBB
@HWI-EAS443:3:5:963:221#0/1
GTGGAACGAAAATCAAAATCCTCGAATGTTCCTGGCGATG
+HWI-EAS443:3:5:963:221#0/1
a]baa`^b^_`___aa`a__V_^]^^_`Z_ZSZ`_O^^BB
@HWI-EAS443:3:27:566:1562#0/1
CCCGTTTTCGTTTCATTACCAACACTACGTCCCCCAGCCC
+HWI-EAS443:3:27:566:1562#0/1
a^_HPQXV¥RKM[ZV¥X^__]UX_PQNRHDT^^BBBBBBB
@HWI-EAS443:3:32:1573:340#0/1
GGGGCGGGGGACGTAGTGTTGGTAATGAAACGAAAACGGG
+HWI-EAS443:3:32:1573:340#0/1
_``abbbbb[Ra``]]R``_T]RV`[aX^]J¥[V`OW`a`
@HWI-EAS443:3:4:845:731#0/1
GGGTGGGGGACGTAGTGTTGGTAATGAAACGAAAACGGGA
+HWI-EAS443:3:4:845:731#0/1
`aaGYa[]`LV_W[aWa¥X¥`RU^Y_RV^SYGV^^SX^YB
@HWI-EAS443:3:83:1736:1401#0/1
GGGGGACGTAGTGTTGGTAATGAAACGAAAACGGGAGTCT
````

```
+HWI-EAS443:3:83:1736:1401#0/1
U__W^b_^U_¥_a]bbbWOZRF¥ZNP[^U`_M_bbUFWPV
@HWI-EAS443:3:35:1121:734#0/1
GTCNCGTTTTCGTTTCATTACCAACACTACGTCCCCCTGC
+HWI-EAS443:3:35:1121:734#0/1
aa¥D^baaaaabaaa]aaaaaaaa``a`aaa]`a]a¥VVa
@HWI-EAS443:3:17:1666:580#0/1
TGGGGGGACGTAGTGTTGGTAATGAAACGAAAACGGGACT
+HWI-EAS443:3:17:1666:580#0/1
[abaX_b`IaXabQaT_`^H¥_Z]WaZG_`JUQG_S^[FU
@HWI-EAS443:3:26:915:1209#0/1
CGGGGGACGTAGTGTTGGTAATGAAACGAAAACGGGACTC
+HWI-EAS443:3:26:915:1209#0/1
aa_bbb]]baab¥`_a`]I[¥¥a```_`_aaa]a_a^PVF
@HWI-EAS443:3:4:123:667#0/1
CGGGGGACGTAGTGTTGGTAATGAAACGAAAACGGGACTC
+HWI-EAS443:3:4:123:667#0/1
a]bbb`]Ra_``_bIbabT_[Saa¥¥U`YX``YSVRZU__
@HWI-EAS443:3:52:543:2017#0/1
GTCCCGTTTTCGTTTCATTACCAACACTACGTCCCCCTGC
+HWI-EAS443:3:52:543:2017#0/1
]TQTNZW_`]SUZ¥aaa`]b`R`a`YIKRGWXV][BBBBB
@HWI-EAS443:3:59:866:615#0/1
TGGGGGACGTAGTGTTGGTAATGAAACGAAAACGGGACTC
+HWI-EAS443:3:59:866:615#0/1
`aa_Z_XaaVV¥V`¥aa¥KW^[]W_a^`¥[[BBBBBBBBB
@HWI-EAS443:3:66:259:1029#0/1
GTCCCGTTTTCGTTTCATTACCAACACTACGTCCCCCTGC
+HWI-EAS443:3:66:259:1029#0/1
aaba`_aaabab`aabbbaaa``baaaabbaabaa`a_aa
@HWI-EAS443:3:80:1043:1907#0/1
CGGGGGACGTAGTGTTGGTAATGAAACGAAAACGGGACTC
+HWI-EAS443:3:80:1043:1907#0/1
a`b]_aa[aSa_Z`S`abNF`baaa[_[aaaaZYa^_ZZ[
@HWI-EAS443:3:55:1729:857#0/1
TTATGATTGGACATTAAACAGAAATCAACAGGCAGCATGC
+HWI-EAS443:3:55:1729:857#0/1
¥]F[Ta[_ZWTUS`Z]_[N¥^S_ZZ_H[XP_YY^]VFMVM
@HWI-EAS443:3:17:1041:1547#0/1
GATTGGACATTAAACAGAAATCAACAGGCAGCAACTGCAT
+HWI-EAS443:3:17:1041:1547#0/1
QWRT[¥_ZZaaZT`[¥`TZ^_bb`^JFM¥]ZMU_OGMHSN
@HWI-EAS443:3:100:700:568#0/1
GTCTGAGTTCTTCAATGGTGGAACAGATTTTCATGATCTC
+HWI-EAS443:3:100:700:568#0/1
a`YY_]ZWR][a_[WUa^Ya`[¥_`_UVDU`¥_^_QRX^B
@HWI-EAS443:3:71:1057:444#0/1
GCCGTCTGAGTTCTTCAATGGTGGAACAGATTTTCATGAT
+HWI-EAS443:3:71:1057:444#0/1
Ka_¥Xaab]LFY_aa[W[__UM[¥_TNTWYWIN[WY]ZJZ
```

@HWI-EAS443:3:40:939:130#0/1
AAGCACCACATTCCCTTATACTGGAGATCCTCCGTACAGC
+HWI-EAS443:3:40:939:130#0/1
aYaa`]]a_a¥`[_¥]a`^b[_b`¥¥PV_[[^LDKV[Z^S
@HWI-EAS443:3:59:819:72#0/1
AAGATTGGTCTTGTCTTTAGCCATTCCATGAGAGCATCAA
+HWI-EAS443:3:59:819:72#0/1
``b`abba`abab`abbaaaa_a`aa_aaa`P^_XDK]YR
@HWI-EAS443:3:8:1304:823#0/1
ATGAAAATCAGAATCCTCGAATGTTACTGGCGATGATTAC
+HWI-EAS443:3:8:1304:823#0/1
Z[]TRW¥YTZO¥U_]aYM[UW_`SUIMYF¥^aYQJM^ZTB
@HWI-EAS443:3:47:1421:971#0/1
TGTAATCATCGCCAGGAACATTCGAGGATTCTGATTTTCA
+HWI-EAS443:3:47:1421:971#0/1
Sa]SL_`GS¥_`¥``XO^XP_Q_`UaaOX¥bR[OVOY]YV
@HWI-EAS443:3:93:1591:682#0/1
CATTCGAGGATTCTGATTTTCATTCCACTTAGTGTTGTCC
+HWI-EAS443:3:93:1591:682#0/1
aa`aY]`ZY[_`HYR¥a^aaJ`bbXGSGa^_bSaWa^G_B
@HWI-EAS443:3:43:1187:1430#0/1
GCCCAGACTGCTCAAGCTTTTCGCAGATGCTCCTAGCTAA
+HWI-EAS443:3:43:1187:1430#0/1
a`UOH¥_Z[WHOOXXYNXOX`W`][GTOLFVTLVW[WVTU
@HWI-EAS443:3:13:1628:170#0/1
CGCAGTTGCTGCCGGTTGATTTCTGTTTAATGTCCAATCA
+HWI-EAS443:3:13:1628:170#0/1
[ba_babb`bbaU_b_b[¥[a`^bb_bbaaba``_a_aGa
@HWI-EAS443:3:10:1015:727#0/1
GTTCCGTTTTCGTTTCATTACCAACACTACGTCCCCTTGC
+HWI-EAS443:3:10:1015:727#0/1
aZ]NN_S^OSX_WWXNQYWZWZZMHOZWXZOZZZSXXXWX
@HWI-EAS443:3:40:44:705#0/1
GTTCCGTTTTCGTTTCATTACCAACACTACGTCCCCTTGC
+HWI-EAS443:3:40:44:705#0/1
a__Yaa]^^_]`U_Z[OZ¥YZ]^YWZWROTVV^W¥WVYV[
@HWI-EAS443:3:80:105:382#0/1
CGTCTGAGTTCTTCAATGGTGGAACAGATCTTCTTGATCC
+HWI-EAS443:3:80:105:382#0/1
ab^a``ab`aaaaaa`bba^bZR_Z]]_^^a`__¥`aX^_
@HWI-EAS443:3:36:123:913#0/1
GTTCTTCAATGGTGGAACAGATCTTCATGATCCCAGAGAA
+HWI-EAS443:3:36:123:913#0/1
a``a`_aa`___X`a`aaa`_^^_^^`^]`^__]]^MS_S
@HWI-EAS443:3:91:927:437#0/1
GACAAGAGATATGGCCCAGCATTAAGCATTAATGAACTGA
+HWI-EAS443:3:91:927:437#0/1
aWUXJa`aa_aa]NS¥^_a^[Y]aW_S]^___]___^S^^
@HWI-EAS443:3:30:1462:1959#0/1
AGATTGCTTAGTTCATTGATGCTTAATGCTGGGCCATATC

+HWI-EAS443:3:30:1462:1959#0/1
aa^HZbaQVZMN¥`Z_J_X^][^PVY]NRW_WTGT¥S_Ga
@HWI-EAS443:3:90:859:43#0/1
TGCAAGATTGCTTAGTTCATTGATGCTTAATGCTGGGCCA
+HWI-EAS443:3:90:859:43#0/1
aaXTb`T^a`a_H]^``^b`ZaaP`I^aZaabY[a_ba^a
@HWI-EAS443:3:43:530:276#0/1
GAGCAATCTTGCAAAAGGGGAGAAAGCTAATGTGCTAATT
+HWI-EAS443:3:43:530:276#0/1
a]b[Y_bb]bb`T]aabbbZabaa_baab[a^¥a[aa_a]
@HWI-EAS443:3:77:311:1134#0/1
CTTCATACCCTTCATGCACTCTTATTTTCAGTGTTTGGAG
+HWI-EAS443:3:77:311:1134#0/1
a_]b_U_^[`TPX_`YHWbaaS¥]^a^¥¥QY_aO[Za_Y_
@HWI-EAS443:3:80:1158:1548#0/1
AGGCAAACCATTTGAATGGATGTCAATCCGGCTGTACTTT
+HWI-EAS443:3:80:1158:1548#0/1
a[``a`aY¥^`]VWX_]aUX]aHSMRSZVWIVVFT¥X_U^
@HWI-EAS443:3:14:554:368#0/1
GCGAAAGCAGGCAAACCATTTGAATGGATGTCAATCCGGC
+HWI-EAS443:3:14:554:368#0/1
a]XFU__Y_aa_]UPRWZW^XYOOZYY¥WZPSSUNXUGFQ
@HWI-EAS443:3:80:769:1689#0/1
GCCGGATTGACATCCATTCAAATGGTTTGCCTGCTTTCGC
+HWI-EAS443:3:80:769:1689#0/1
YG]_¥aZ¥¥]_RR^W__ZXZU¥[¥_X_XVXZZ__]^]a_Q
@HWI-EAS443:3:75:252:1113#0/1
CGGTCCTGTTCCTGTTCCATGGCTGGAAGGAGGATCTCCA
+HWI-EAS443:3:75:252:1113#0/1
Z¥_^ab_¥WTba^WOQ`¥R_]]`Z]FGFNOOX]PHNR]]K
@HWI-EAS443:3:12:1721:221#0/1
GGTGTATCCTGTTCCTGTTCCATGGCTGTAAGGAGGATGT
+HWI-EAS443:3:12:1721:221#0/1
a_TVYa`a^WOPZQ^MBBBBBBBBBBBBBBBBBBBBBBBB
@HWI-EAS443:3:72:297:599#0/1
CATTCTGTTTTCATGCCTGATCAGTGGATTGGTGGGAGTA
+HWI-EAS443:3:72:297:599#0/1
aaba^`babaaaaaaa`aa```[`W``_[V`¥BBBBBBBB
@HWI-EAS443:3:55:558:88#0/1
CGTGGGGGCAAGAATACTGACATCGGAGTCACAGCTGGCA
+HWI-EAS443:3:55:558:88#0/1
a_S¥aaa`F`_a[QZaR^a^R^[YG_¥_QVY¥¥_PR¥BBB
@HWI-EAS443:3:96:685:857#0/1
ATATATTCAATATGGAGAGAATAAAAGAACTGAAAGATCT
+HWI-EAS443:3:96:685:857#0/1
aa`b_aa`aaa`aaa_a_a_`aa`]]_¥_S__`I`__]_`
@HWI-EAS443:3:24:1344:1524#0/1
CGGTTGGAATTTCTAGCATGATGGAGGCCATGGTGTCTAG
+HWI-EAS443:3:24:1344:1524#0/1
]aaX`bbb]bbb`bbbababbaabbb]aWab``T]^^^aa

@HWI-EAS443:3:62:1442:560#0/1
CCGGTTGGAATTTCTAGCATGATGGAGGCCATGGTGTCTA
+HWI-EAS443:3:62:1442:560#0/1
abaa_ab`b]bbbabbbaabbaaab`ba`abbb```]_Xa
@HWI-EAS443:3:74:1418:1008#0/1
ACCGGGCCCTAGACACCATGGCCTCCATCATGCTAGAAAT
+HWI-EAS443:3:74:1418:1008#0/1
a_^aa`a`^aaaaaa^^aaa`]Y`]¥a`Ua___`¥a`_^]
@HWI-EAS443:3:4:1213:435#0/1
TAGGGGGGAATGAAAAGAAGGCCAAACTGGCAAATGTTGT
+HWI-EAS443:3:4:1213:435#0/1
UQU[ZY_ROY¥^X^b^¥R_¥XWX¥]UV_ULIPRPLWRBBB
@HWI-EAS443:3:49:1465:1077#0/1
GGTGGAGGAAGGCTCTATTGGGAAAGTGTGCAGGACCTTA
+HWI-EAS443:3:49:1465:1077#0/1
a__aaaaa`¥baaa_a_aab`a_]aaV]_aV]aWY^^`a_
@HWI-EAS443:3:39:729:571#0/1
CATTAAGCATCAATGAGCTGAGCAACCTTGCAAAAGGAGA
+HWI-EAS443:3:39:729:571#0/1
aaW[Q[V`_^^^a[TLHZ]¥]^V^SGM`ULP[]PTQ_W]Y
@HWI-EAS443:3:12:133:578#0/1
GGGGGAGGAGTTCTCTGAGATCATGGAGATCTGTTCCACC
+HWI-EAS443:3:12:133:578#0/1
T^]_a^X^ab]]^¥a]_]¥]^ZZZY_Z[^^_X[TZZ^_^R
@HWI-EAS443:3:86:59:344#0/1
GTTTCTGTTTAATGTCCAATCATAAGTCTGGCGACCTTGA
+HWI-EAS443:3:86:59:344#0/1
aaaaa_b`^`aaa``a`X`]_^`_``Z]]_[Z_]_[]]]Z
@HWI-EAS443:3:90:1302:766#0/1
CACATATAGGTGCCATAGNGGAGACACACAAATTCAAACG
+HWI-EAS443:3:90:1302:766#0/1
a`bbbbbabb]b]abbb[D^b¥`_^b_babb`b``abb`a
@HWI-EAS443:3:56:114:658#0/1
ATTAGAACACATCCAGAAACCGATTACCCCCAGGGAGACT
+HWI-EAS443:3:56:114:658#0/1
abbb¥S^_`]b¥]¥Xa]K_a[`^]PRY`Z[V¥`P[^[X_[
@HWI-EAS443:3:84:534:498#0/1
AAACAATTCGACACTAATTGATGGCCAACCGAATTCTTTT
+HWI-EAS443:3:84:534:498#0/1
J_`ZVWa]`bab`__U_`__Z^[¥¥`__X¥_Y^`_V]``V
@HWI-EAS443:3:64:902:517#0/1
CATCAATGAACTGAGCAATCTTGCAAAAGGAGAGATGGGA
+HWI-EAS443:3:64:902:517#0/1
ab^``bbb]bbaa`aababababaaaa`baab`baab_`_
@HWI-EAS443:3:96:593:1015#0/1
AAGCATCAATGAACTGAGCAATCTTGCAAAAGGAGAGATA
+HWI-EAS443:3:96:593:1015#0/1
WL_¥X¥_b¥¥]^a`^ba_TaSM]_]_aaY]Wa`¥¥X`Z^]
@HWI-EAS443:3:79:885:907#0/1
AGCTAATGTGCTAATCGGGCAAGGGGACGTAGTGTTGGTA

+HWI-EAS443:3:79:885:907#0/1
aaaZ_baa_aaaYaaaaa`aaaaaa`^_a^`a[`X_a`X_
@HWI-EAS443:3:96:662:1102#0/1
GTGCTAATCGGGCAAGGGGACGTAGTGTTGGTAATGAAAC
+HWI-EAS443:3:96:662:1102#0/1
_U`Z]``[FXW`_N[aZ__WV_NWa]a`[VXU][XQBBBB
@HWI-EAS443:3:100:860:236#0/1
CTCAGGCACTCCTTCCGTAGAAGGCCCTCTTTTCAAAGCG
+HWI-EAS443:3:100:860:236#0/1
aaaaaaaa`a_]aa``a_aa`]aaSVRa]_a_aY_`[GLa
@HWI-EAS443:3:94:1624:855#0/1
ATCAAACTTTGGTCAACATCATCATTTCTCACTTCTCCCC
+HWI-EAS443:3:94:1624:855#0/1
aaZ¥RaabZSSSU¥`ababbb`YJ¥`_`baaS¥S_a_ZFT
@HWI-EAS443:3:35:1201:1784#0/1
TCTCCTTTTCATTCTGAGTGCGGGGTTCTTCTCTTGTCTT
+HWI-EAS443:3:35:1201:1784#0/1
_ZIV^[`Y¥Wa[[]a¥WOMY`]UTNG¥XZR`¥X[LZL`¥Y
@HWI-EAS443:3:68:675:506#0/1
CTTCCTGATGTGCACTTTTTGATTATGGCCATATGGTCCA
+HWI-EAS443:3:68:675:506#0/1
a¥TZZ`Y[`Y`[GWUa]Yb¥]¥`abTELZYRGTYGWXW_Z
@HWI-EAS443:3:92:177:1893#0/1
GGGCCTCATATACAACAGGATGGGAACAGTGACCACCGAA
+HWI-EAS443:3:92:177:1893#0/1
T`^b¥Za[`][T__¥aYF[^P¥P¥^aaVQJUaX]SYFF¥X
@HWI-EAS443:3:62:1681:1009#0/1
GGCGATCTTAACTTTGTCAATAGGGCAAACCAGCGACTGA
+HWI-EAS443:3:62:1681:1009#0/1
aaaaa_UXGQa_¥aaZ]^`aXZZ]`V^aa[XZ]X`SUY]V
@HWI-EAS443:3:31:177:1403#0/1
ATAAAACTTATTTCGAAAAGGTTGAAAGGTTGAAACATGG
+HWI-EAS443:3:31:177:1403#0/1
aaaa¥Ya`][^_`a[ZR¥___S_^a`Y`]]_`]Y`_`¥^^
@HWI-EAS443:3:47:1050:201#0/1
ATAAAACTTATTTCGAAAAGGTTGAAAGGTTGAAACATGG
+HWI-EAS443:3:47:1050:201#0/1
abbba^`babbbb_b`aaabbabb`a`ab^`aa¥a_aaa_
@HWI-EAS443:3:64:161:150#0/1
ATAAAACTTATTTCGAAAAGGTTGAAAGGTTGAAACATGG
+HWI-EAS443:3:64:161:150#0/1
abb`a``a]J``a`a[¥``Z]Za``^^``_Z^U`V^`^`_
@HWI-EAS443:3:9:728:1296#0/1
ATAAAACTTATTTCGAAAAGGTTGAAAGGTTGAAACATGG
+HWI-EAS443:3:9:728:1296#0/1
aYbaaa`^^aaa^SaS^a_`WOU_^_¥^PMTWQX^Z`X]B
@HWI-EAS443:3:70:525:1954#0/1
TATAAAACTTATTTCGAAAAGGTTGAAAGGTTGAAACATG
+HWI-EAS443:3:70:525:1954#0/1
ababbbbaaaabaaaaabaaa_Waabaaaa^__baa`b_`

@HWI-EAS443:3:78:509:1622#0/1
TATAAAACTTATTTCGAAAAGGTTGAAAGGTTGAAACATG
+HWI-EAS443:3:78:509:1622#0/1
ab¥^`_aba_Z^ba`]aa_[]_Yaaaa`Y`^a`baaaa__
@HWI-EAS443:3:7:753:613#0/1
TATAAAACTTATTTCGAAAAGGTTGAAAGGTTGAAACATG
+HWI-EAS443:3:7:753:613#0/1
`bb`a^``^`aa_`Ta]a_``a`Z][`¥`b__a`]]`_`a
@HWI-EAS443:3:74:634:950#0/1
TTTCAACCTTTCAACCTTTTCGAAATAAGTTTTATATACC
+HWI-EAS443:3:74:634:950#0/1
^_W_]`ab[WZ¥^_^V^Z^W^UO¥TZ^Z`T`__aa__ZTT
@HWI-EAS443:3:76:1084:2026#0/1
GAATTTTAGGATTTGTGTTCACGCTCACCGTGCCCAGCGA
+HWI-EAS443:3:76:1084:2026#0/1
TZ][``XTXX]]POTQYFMV_aVNVX¥QU¥V_VTX¥BBBB
@HWI-EAS443:3:44:1206:1961#0/1
ATTAGCTCATGTTTCAGTTTTGGCGGGTTCACTTTCAAAA
+HWI-EAS443:3:44:1206:1961#0/1
a^¥N¥TXV_WIY_`P[RWa`Z[HHR]]XX]OGOSVFU[^Y
@HWI-EAS443:3:99:462:1927#0/1
CTAGAGTCCAGTAATAGTTCATTCTCCCTTCTTGACCTCT
+HWI-EAS443:3:99:462:1927#0/1
aba^V_`TaaaaV]bb[]baba_[SZVSXVXbbbaaSQ`K
@HWI-EAS443:3:17:495:1054#0/1
GTGGTAATGCCAGCCCATGGACCAGCNAAAAGCATGGAAT
+HWI-EAS443:3:17:495:1054#0/1
a]Y_LaUV¥aaU¥`[[TZ_[NS_`SWDESY¥V¥_Q¥¥YY¥
@HWI-EAS443:3:63:1203:1771#0/1
AATCAATGACCGAAATTTCTGGAGGGGAGAAAATGGAACG
+HWI-EAS443:3:63:1203:1771#0/1
ZYV]¥¥¥T¥]aaa`Yba_QW_¥X^baYDVP[_a^[UYX`a
@HWI-EAS443:3:25:1534:227#0/1
CTGGGCATGAGCATGAACCAGTCTCGTGACATTTCCTCGA
+HWI-EAS443:3:25:1534:227#0/1
`abbb_bbbbbabbbbabbbbb_bab_a_Wbbbbaaa`ba
@HWI-EAS443:3:100:1260:763#0/1
TGGCTCTTCAATTGTTTATCAAAGACTACAGATAAACATA
+HWI-EAS443:3:100:1260:763#0/1
¥X¥Xb``]¥]`]^X¥^IU]V^]`^SYMQFWaP^_H^T^_B
@HWI-EAS443:3:77:1684:1679#0/1
ATCTCATTGCCATCATCCACTTCATTCTGAGAGCGGGGTT
+HWI-EAS443:3:77:1684:1679#0/1
`aXYQ[b]_VW[YP[[_UXHZ[UL[QK¥WKUD[HW_BBBB
@HWI-EAS443:3:75:1751:1567#0/1
CTCATTGCCATCATCCACTTCATTCTGAGAGCGGGGTTCT
+HWI-EAS443:3:75:1751:1567#0/1
][YQNQ^[]_aVXUHYaZS^PXa`aaaZ_G_a]`SaLQOY
@HWI-EAS443:3:79:679:376#0/1
GCGGGGTTCTTCTCTTGTCTTCCTGATGTGTACTTCTTGA

```
+HWI-EAS443:3:79:679:376#0/1
ababbbabbbbabbbbbbaabbaaababbabaaabaabbab
@HWI-EAS443:3:29:601:1786#0/1
AGCAAATAGCAGCATTGCCACAACTACTTCAATGCATGTG
+HWI-EAS443:3:29:601:1786#0/1
¥aa¥[¥]_WFY`_U¥Z[Y]_U]`a``a_X_^[^aaa`XFO
@HWI-EAS443:3:57:598:1980#0/1
GCAAATAGCAGCATTGCCACAACTACTTCAATGCATGTGT
+HWI-EAS443:3:57:598:1980#0/1
aaUNTHa]MYU]VZH[_aRQ]TOJW][RPT`¥aRW_^U`O
@HWI-EAS443:3:62:1034:1918#0/1
GCAGCATTGCCACAACTACTTCAATGCATGTGTGAGGAAG
+HWI-EAS443:3:62:1034:1918#0/1
[D]aa_aa`_`a````_b_a`_a_]V[`_`V`Xa^^^`]_
@HWI-EAS443:3:81:1008:1942#0/1
CAAATAGCAGCATTGCCACAACTACTTCAATGCATGTGTG
+HWI-EAS443:3:81:1008:1942#0/1
abb`]a[H¥ba`aa`Y`^a_baab[_Y]a¥S_`]aa]^Xa
@HWI-EAS443:3:43:1039:1840#0/1
ATGGATAGCAAATAGCAGCATTGCCACAACTACTTCAATG
+HWI-EAS443:3:43:1039:1840#0/1
[aa_aa`a_b^SZ]ZJ¥TKPWHU^aY]aa`_aYa_[`]Q_
@HWI-EAS443:3:64:312:1189#0/1
ATGGATAGCAAATAGCAGCATTGCCACAACTACTTCAATG
+HWI-EAS443:3:64:312:1189#0/1
[a`aa_[aTab`Va[D[[]_Ya[_[¥[Y¥]_a`^Y`_]YY
@HWI-EAS443:3:40:1533:150#0/1
CAAATATATTCAATATGGAGAGAATAAAAGAACTGAGGGA
+HWI-EAS443:3:40:1533:150#0/1
abbababbbbbbbabbbbabaaabbbabbbbb`abbbaab
@HWI-EAS443:3:13:222:1166#0/1
AATATATTCAATATGGAGAGAATAAAAGAACTGAGGGATC
+HWI-EAS443:3:13:222:1166#0/1
_`^Ya]UI_`Z^a]YR[Y[^VT_]Z]T^_`a_aZaX`[UO
@HWI-EAS443:3:16:795:730#0/1
CGCCGAACTGTTGGTTCNATTGGAAAATGAAAGAACTTTG
+HWI-EAS443:3:16:795:730#0/1
aaaaa_aaaa]aaaZ_ZDZ`aa_]__a_a__a`__]`^^_
@HWI-EAS443:3:4:66:238#0/1
CGCCGAACTGTTGGTTCTATTGGAAAATGAAAGAACTTTG
+HWI-EAS443:3:4:66:238#0/1
abbaba]abbabab_a_babb]a```aaa`a`¥`a¥``a_
@HWI-EAS443:3:23:355:1190#0/1
GCCATTACTTATGAAAACAACACTTGGGTAAACCAGACAT
+HWI-EAS443:3:23:355:1190#0/1
]S^IYSW_R¥TY`]]S_a]YZ^¥YYX[TR]X`RW[SHY[¥
@HWI-EAS443:3:68:163:1238#0/1
AAGGAGTTGAACCAAGATGCATTAAGCAAAACCCAGGGGA
+HWI-EAS443:3:68:163:1238#0/1
_`^Y_]Qa`aaa^`a[_^__`Y^Z[_^____T]¥_^Y¥¥S
```

```
@HWI-EAS443:3:1:1566:1074#0/1
CTGGGTTTTGCTTAATGCATCTTGGTTCAACTACTTCCTC
+HWI-EAS443:3:1:1566:1074#0/1
VYba_FW`_`[]ba^PbW`bWb^]X¥WIa`_¥GGa]S^aB
@HWI-EAS443:3:33:1498:1219#0/1
CTGGGTTTTGCTTAATGCATCTTGGTTCAACTACTTCCTC
+HWI-EAS443:3:33:1498:1219#0/1
^`R`b^¥_ab¥`a__Zab^a`^][_Z`__```NW]^[UaV
@HWI-EAS443:3:7:192:263#0/1
ATGGGCCTCATATACAACAGGATGGGAACAGTGACCGCAG
+HWI-EAS443:3:7:192:263#0/1
aaaVV^`ba^]b`VW^___``R`^^___WXKRUTUQBBBB
@HWI-EAS443:3:11:142:1246#0/1
CGCCTTCTACGGAAGGAGTGCCTGAGTCCATGAGGAAGGA
+HWI-EAS443:3:11:142:1246#0/1
^`]¥TTXR`TQV[U_Z]`¥`a_aa]`[`__]_KBBBBBBB
@HWI-EAS443:3:51:68:375#0/1
GGGCCTTCTACGGAAGGAGTGCCTGAGTCCATGTGGGAGA
+HWI-EAS443:3:51:68:375#0/1
abbbabaabaab]aaab_aYa¥Yaa[a_aY^^aY^aaX`X
@HWI-EAS443:3:70:1362:918#0/1
CTTTAAATACGGTTTGAAAAGGGGGCCTTCTACGGAAGGA
+HWI-EAS443:3:70:1362:918#0/1
abbbababbbaa`bbabbbb`^aabaaaabbaaba`aaa`
@HWI-EAS443:3:31:342:1938#0/1
CAAATATATTCAACATGGAGAGANTAAAAGAACTGAGAGA
+HWI-EAS443:3:31:342:1938#0/1
aaa^^aR[¥U_R¥JNY^TPZMUMDV_[YYI]WPXYZR^QZ
@HWI-EAS443:3:25:1061:529#0/1
CAAATATATTCAACATGGAGAGAATAAAAGAACTGAGAGA
+HWI-EAS443:3:25:1061:529#0/1
¥a`YV`aX^ZZa_PS¥`baZ_Wab]aT``b¥OFRQ¥a[^`
@HWI-EAS443:3:3:595:1241#0/1
GTGATGAGAATGGGAGACCTTCACTATCTCCAGAGCAGAA
+HWI-EAS443:3:3:595:1241#0/1
`^[V[aa]¥^Z[]`_ZFSZWTLZUYVEKUTW¥]]]LHUZW
@HWI-EAS443:3:76:1022:1368#0/1
ATGAGAATGGGAGACCTTCACTATCTCCAGAGCAGAAATG
+HWI-EAS443:3:76:1022:1368#0/1
aaaa`_aaa_aaa``a`a_a`_a[¥^__aa__^^__^^_`
@HWI-EAS443:3:16:953:1826#0/1
GACGTAGTGTTGGTAATGAAACGAAAACGGGACTCAGCAT
+HWI-EAS443:3:16:953:1826#0/1
aaa__bb_b[a`aS¥a`¥aab`[__abW_X¥aRXIU[O``
@HWI-EAS443:3:29:591:1946#0/1
CTTGGCGAAAATATGGCACCAGAAAAAGTAGACTTTGATG
+HWI-EAS443:3:29:591:1946#0/1
aaX`]D¥V¥R_Y^`WQ_`L[]Y^Ra¥¥Z^a_^__]_[X]O
@HWI-EAS443:3:31:789:1845#0/1
CTCGGCGAAAATATGGCACCAGAAAAAGTAGACTTTGATG
```

+HWI-EAS443:3:31:789:1845#0/1
a[ZX^I]_`a`K][___]X[]aY[WK]UUa_[__MR^_VY
@HWI-EAS443:3:1:321:517#0/1
ACTCGGCGAAAATATGGCACCAGAAAAAGTAGACTTTGAT
+HWI-EAS443:3:1:321:517#0/1
¥_bab`Ja_`a¥`¥ab¥_aS[Z`^aaa¥YV[_a_a_SVBB
@HWI-EAS443:3:35:127:1910#0/1
ACTCGGCGAAAATATGGCACCAGAAAAAGTAGACTTTGAT
+HWI-EAS443:3:35:127:1910#0/1
_ba`^ZHXb`bba_Vaaba__b^b¥baa_Ga_`ba[__aW
@HWI-EAS443:3:61:329:877#0/1
ATTCGACACTAATTGATGGCCATCCGGATTCTTTTGGTCG
+HWI-EAS443:3:61:329:877#0/1
a]aaab^`¥]]_¥Z_]Z`X]^UMX][XFV[QU^_^YPHRQ
@HWI-EAS443:3:26:829:328#0/1
CGCTAAGAGTCTCGTTTTCGTTTCATTACCAACACTACGT
+HWI-EAS443:3:26:829:328#0/1
ababb`b`a¥abab]bbbab`b`abaaba_aaYa`aa`_]
@HWI-EAS443:3:20:49:473#0/1
GAGTTGGTGCATAGGGTTCAATCGCTGGTTTGCCCTATTG
+HWI-EAS443:3:20:49:473#0/1
a``^_^ZV`^]``aa_R^aYLX_¥]¥¥]U]XY_¥¥¥¥¥__
@HWI-EAS443:3:55:1250:182#0/1
CTCGGCGGAGCCGATCAAGGAATGGAGCATCACCCAATCC
+HWI-EAS443:3:55:1250:182#0/1
ab`aaabb[aXY`[RZZRW[LV]S[BBBBBBBBBBBBBBB
@HWI-EAS443:3:62:1046:1105#0/1
GGCCAATCGAACAAGTTTGCTGCAATATGCACACATTTGG
+HWI-EAS443:3:62:1046:1105#0/1
abb`bababbabbbb_ab`a_abbbbaaabbab`¥aa_ba
@HWI-EAS443:3:12:220:725#0/1
CGCCATCATCCACTTCATTCTGAGTGTGGGGTTCTTCTCT
+HWI-EAS443:3:12:220:725#0/1
aaabaabbabaabaa`aab`b[T¥]`^b^aa^aaaaa``a
@HWI-EAS443:3:84:873:430#0/1
CACGCCATCATCCACTTCATTCTGAGTGCGGGGTTCTTCT
+HWI-EAS443:3:84:873:430#0/1
__`ZUaa`b`ZR[¥]__X¥b^aaa`_XZ¥b__aS`aaaaa
@HWI-EAS443:3:44:1356:898#0/1
GAGAAGAACCCCGCACTCAGAATGAAGTGGATGATGGCGG
+HWI-EAS443:3:44:1356:898#0/1
a]ba^Z^a^`_ab_a``aaa]¥ab[TaYaa][a__a`^`W
@HWI-EAS443:3:27:867:214#0/1
CGCCATCATCCACTTCATTCTGAGTGCGGGGTTCTTCTCT
+HWI-EAS443:3:27:867:214#0/1
___aababaaaa`aa``b`aaaXa^`^`a`a[^^^__[Z`
@HWI-EAS443:3:32:605:322#0/1
CGCCATCATCCACTTCATTCTGAGTGCGGGGTTCTTCTCT
+HWI-EAS443:3:32:605:322#0/1
`U`YQ]a_a`b`aabbaaa`^TUa`a`a_aaV`Tb_baa`

```
@HWI-EAS443:3:60:1100:1788#0/1
CGCCATCATCCACTTCATTCTGAGTGCGGGGTTCTTCTCT
+HWI-EAS443:3:60:1100:1788#0/1
aa`bbbabab`aa_aabaaaaaa_]a_aaa____]]Za_a
@HWI-EAS443:3:87:1018:170#0/1
CGCCATCATCCACTTCATTCTGAGTGCGGGGTTCTTCTCT
+HWI-EAS443:3:87:1018:170#0/1
U¥[_[__W_XVU[a`]^`_aabbaZ`^¥bbaY¥UU]R_]]
@HWI-EAS443:3:95:392:1041#0/1
CGCCATCATCCACTTCATTCTGAGTGCGGGGTTCTTCTCT
+HWI-EAS443:3:95:392:1041#0/1
`bbbbaab`baaaaaaaaa^ab`a_a]aaa^__aa`Sa¥a
@HWI-EAS443:3:98:1546:374#0/1
CGCCATCATCCACTTCATTCTGAGTGCGGGGTTCTTCTCT
+HWI-EAS443:3:98:1546:374#0/1
`bYbbbab`^Y`_bb^abbaaaaa_`aa¥ab[a`b_RaWa
@HWI-EAS443:3:83:1384:97#0/1
AAGCAAACTTGCAAAAGGCGAGAAAGCTAATGTGCTAATT
+HWI-EAS443:3:83:1384:97#0/1
[U[OW]OaZ]bPV_aWa_SYWa_aTb_]IG_aa`_aaY_a
@HWI-EAS443:3:28:1052:701#0/1
GCTTTGCGCGACAATGCTTCAATCCAATGATCGTCGAGCT
+HWI-EAS443:3:28:1052:701#0/1
_U]`YZGUT]a^_^_aYW`M¥P¥RRQPI[[[WUNRSS^[S
@HWI-EAS443:3:29:901:488#0/1
TTGCCGTCTGAGTTTTTCAATGGTGGAACAGATATTCATG
+HWI-EAS443:3:29:901:488#0/1
`¥a`aa_XW`b`W¥DI`aT^]W_SVG^[MXaX[FP^[BBB
@HWI-EAS443:3:83:1102:230#0/1
CCGGCTTCCAAGATCCAAGGTTCCAGGTTCCAGGTTGTCC
+HWI-EAS443:3:83:1102:230#0/1
aab_`a_¥Z`W`a[Z_a`a^VaVDY`a_]VV]_¥T]`VOO
@HWI-EAS443:3:42:1706:1161#0/1
ATGGATAGCAAATAGTAGCACTGCCACAACTACTTCAATG
+HWI-EAS443:3:42:1706:1161#0/1
_``¥bbbb`aZ^_^Z_^aZ```aaa``ba`aba`bb^`a`
@HWI-EAS443:3:69:1430:1085#0/1
GTTCCATGGCTGTATGGTGGATCTCCAGTATAAGGGAATG
+HWI-EAS443:3:69:1430:1085#0/1
a_aaa`aaaaaaQYWJ]R``RY_^^]VSYZ[S^_^`G]^]
@HWI-EAS443:3:33:895:1510#0/1
AAACGAAAACGAGACTCTAGCATACTTACTGAGAGCCAGA
+HWI-EAS443:3:33:895:1510#0/1
aaba^babb__a]a_aa_a_]``a^_`a^a`_G[RZ^`X_
@HWI-EAS443:3:74:545:1992#0/1
GCTGTCAGTAAGTATGCTAGAGTCTCGTTTTCGTTTCATT
+HWI-EAS443:3:74:545:1992#0/1
aa_aT`TT]_a`WZYaaWaKa__]W]EDM_¥]HF[_WYWN
@HWI-EAS443:3:77:632:1030#0/1
AATATGGAGNGAATAAAAGAACTGAGAGACCTAATGTCGC
```

```
+HWI-EAS443:3:77:632:1030#0/1
_a_aa_¥`^D¥b`b]_Vaa]``abbaY`¥W]aaaaa_`]^
@HWI-EAS443:3:34:1748:1153#0/1
GAGATACGGCCCAGCATTAAGCATCAATGAACTGAGCAAT
+HWI-EAS443:3:34:1748:1153#0/1
¥R_WW_RUab`ZSZ`a¥Y`Z]¥]aa_¥_]]`UZNS_Z_RJ
@HWI-EAS443:3:16:103:1518#0/1
GGAGCCACTCAAATGGGAAAAATACTGTGTTCTAGAAATA
+HWI-EAS443:3:16:103:1518#0/1
a_SQa`¥^[^aa_RJXYZ]YY_]_]W[U]X]^__UXW^Y[
@HWI-EAS443:3:62:470:511#0/1
CTGGAGCCACACAAATGGGAAAAATACTGTGTTCTAGAAA
+HWI-EAS443:3:62:470:511#0/1
abbbbb`aaababaabbbbabaaaaa_baa`aaaaa`a_Y
@HWI-EAS443:3:16:1439:1241#0/1
ATTTCTAGAACACAGTATTTTTCCCATTTGTGTGGCTCCA
+HWI-EAS443:3:16:1439:1241#0/1
`¥aaa_^a_ba`Yba_ba`abb]]``^a[aaa`ZY[aQaX
@HWI-EAS443:3:23:1105:2035#0/1
GGAGCCACACAAATGGGAAAAATACTGTGTTCTAGAAATA
+HWI-EAS443:3:23:1105:2035#0/1
_¥`b¥`Z]TP[bbba`[^^_^`[_[^]Q¥Za]X`aaY¥^a
@HWI-EAS443:3:47:1383:1839#0/1
ATTTCTAGAACACAGTATTTTTCCCATTTGTGTGGCTCCA
+HWI-EAS443:3:47:1383:1839#0/1
`baabb`aaaaa`baWa`baaa]__`aV_a^`Y`U]ZZU]
@HWI-EAS443:3:52:239:1508#0/1
ATTTCTAGAACACAGTATTTTTCCCATTTGTGTGGCTCCA
+HWI-EAS443:3:52:239:1508#0/1
`aaaabaaaaaaaba_abaaaa`a`aaaa__a``__a_``
@HWI-EAS443:3:70:1009:573#0/1
GAGCCACACAAATGGGAAAAATACTGTGTTCTAGAAATAG
+HWI-EAS443:3:70:1009:573#0/1
abaaaaaa^_^¥_```]aa__aa_aa^a^`a`aa`__^`X
@HWI-EAS443:3:87:1727:680#0/1
GAGCCACACAAATGGGAAAAATACTGTGTTCTAGAAATAG
+HWI-EAS443:3:87:1727:680#0/1
]`ab`bbb_aabbb¥abb_`^¥_^baabaa`bb```abab
@HWI-EAS443:3:76:1138:987#0/1
AAACGATCTGAGCAATCTTGCAAAAGGAGAGAAAGCTAAT
+HWI-EAS443:3:76:1138:987#0/1
abaaa`aaaaa`aa_a`a_a_a_`[__^_``_``a]_^_`
@HWI-EAS443:3:8:1744:1087#0/1
CACGATCGGGTTCGTTGCCTTTTCGTCCGAGATCTCGAAG
+HWI-EAS443:3:8:1744:1087#0/1
]bb]]Ua]baVb`bXbaXGbabaGa¥_HRXBBBBBBBBBB
@HWI-EAS443:3:42:844:80#0/1
TGGGGGCAATGAAAAGAAGGCCAAACTGGCAAATATTGTG
+HWI-EAS443:3:42:844:80#0/1
ab^^aaa]_aaUM[__[^`]^__]_^_aa]`^Y_Y_]aZa
```

@HWI-EAS443:3:94:659:1598#0/1
TGGGGGCAATGAAAAGAAGGCCAAACTGGCAAATGGTGTG
+HWI-EAS443:3:94:659:1598#0/1
X¥ab¥_]I_^¥U¥a]QZ¥aYYUbWTXX_HR]ba¥XGUbYX
@HWI-EAS443:3:46:330:2022#0/1
TGGGGGCAATGAAAAGAAGGCCAAACTGGCAAATGTTGTG
+HWI-EAS443:3:46:330:2022#0/1
T`bbba``^bbT^^TN]RJ[a[[a[¥VbM`U[_`^R]b[a
@HWI-EAS443:3:51:417:1240#0/1
ATCAGACCGAGTGATGGTATCACCTCTGGCCGTGAGAGGA
+HWI-EAS443:3:51:417:1240#0/1
_babbbaaaba_baa`b]aa`a`_aa`aa__bZa_`__`^
@HWI-EAS443:3:9:700:473#0/1
ATCAGACCGAGTGATGGTATCACCTCTGGCCGTGAGAGGA
+HWI-EAS443:3:9:700:473#0/1
`a`abaaabbaabaaaa¥aaaa^__]a_b__aY`¥a`aaR
@HWI-EAS443:3:37:433:1447#0/1
CACGGCCAGAGGTGATACCATCACTCGGTGTGATCCAGCA
+HWI-EAS443:3:37:433:1447#0/1
ab`_Z`ba[a^_UT]Y`Yaa^^^^[^YUPMQRNS^Y¥S[U
@HWI-EAS443:3:34:1572:1851#0/1
CTGGATCAGACCGAGTGATGGTATCACCTCTGGCCGTGAG
+HWI-EAS443:3:34:1572:1851#0/1
a``bbabbb``[[`bWaa_baUa¥]aU_`a[¥]¥¥_SVU¥
@HWI-EAS443:3:17:1612:998#0/1
GCTGGATCAGACCGAGTGATGGTATCACCTCTGGCCGTGA
+HWI-EAS443:3:17:1612:998#0/1
^R^a`aa`a^W]_a]¥_aTV[¥G_aY¥^¥ZZT]VRR_KPB
@HWI-EAS443:3:14:1340:1839#0/1
AAGACAGGAGATATGGCCCAGCATTAAGCATCAATGAACT
+HWI-EAS443:3:14:1340:1839#0/1
`ab```bbabaaaa[a`a`_^SW__aa^Z^`__^``_`Y_
@HWI-EAS443:3:42:1530:286#0/1
GAAGACAGGAGATATGGCCCAGCATTAAGCATCAATGAAC
+HWI-EAS443:3:42:1530:286#0/1
a^Z_aaXE¥V`]a`aa_`[[]_[_]__]^W¥]]YTZ]S^Z
@HWI-EAS443:3:57:811:317#0/1
AAATTGCATCCTACTCTGTTCCGGTGGAGCAGCAGCAAAG
+HWI-EAS443:3:57:811:317#0/1
`XY_ab``Xa__aaaaa]a_^^`_[`]_`Z^¥¥_`Z_V]`
@HWI-EAS443:3:55:258:1832#0/1
CATATTCTTTCATTGCATTTTCCGCAAGCTCGACGATCAT
+HWI-EAS443:3:55:258:1832#0/1
a[b_ababb_ababbbGbbb`aa_aaaSa_a]`_a^_Z¥^
@HWI-EAS443:3:25:1140:1696#0/1
CCCTGGGTTTTGCTTAATGCATCTTGGTTCAACTCGCTCC
+HWI-EAS443:3:25:1140:1696#0/1
``ba`^`aab_W¥aa_QYX¥]aV¥X]^U__XbaabU^F^]
@HWI-EAS443:3:69:1340:584#0/1
GCAGCGTTTTGCTCCAGAGGGTTTGTCCTTGTTCATTCCT

```
+HWI-EAS443:3:69:1340:584#0/1
aW¥a[`R^``_]`]¥`aV```Vaaa___a_a^a[U]^[[]
@HWI-EAS443:3:83:866:31#0/1
CAATTATTGCCTCAGCAATTGACTGCTCGTCTCGCCCACT
+HWI-EAS443:3:83:866:31#0/1
a`Q]Z^Z`aZ_WZ^a¥^VPZ]WS[ZWRRO^^YBBBBBBBB
@HWI-EAS443:3:98:232:2019#0/1
ATCAATGAACTGAGCAATCTTGCAAAAGGAGAGAAGGCAG
+HWI-EAS443:3:98:232:2019#0/1
^Xa[`bU_W¥Y^H¥][bY[U`_aab`TNT¥_XSWYFMX__
@HWI-EAS443:3:52:1453:1432#0/1
ACCAACACTACGTCCCCTTGCCCTATTAGCACATTAGCTT
+HWI-EAS443:3:52:1453:1432#0/1
aa____a^]`_^^¥]¥]OU_YXX]_]¥]VGZG^]O]YH]V
@HWI-EAS443:3:65:383:1411#0/1
GACGCTGTCTGGCTGTCAGTAAGTATGCTAGAGTCCCGTT
+HWI-EAS443:3:65:383:1411#0/1
¥F[ab[_`b``ab¥Z^_RPY]ZZJX[][V[NRVLa^XVFO
@HWI-EAS443:3:81:1636:1404#0/1
GTACTTTTTTGGACAGTATGGATAGTAAATAGTAGCATTG
+HWI-EAS443:3:81:1636:1404#0/1
a[S^[Z^__V[`TSF]__Yb_U¥TUDN¥_^VMKQ[QOZZB
@HWI-EAS443:3:85:1645:1138#0/1
CATCAGGAAGACAAGAGAAGAACCCAGCACTCAGAATGAA
+HWI-EAS443:3:85:1645:1138#0/1
`_Z^a]ba[SMaaabbb[ZW¥bP`bIbSaa]]aabUbb_T
@HWI-EAS443:3:99:898:1727#0/1
GAAGAACCCAGCACTCAGAATGAAGTGGATGATGGCAATG
+HWI-EAS443:3:99:898:1727#0/1
Y¥X^bSS¥ZDY`¥[[^G^_aba_]ZH_¥X[a[I[``RQU]
@HWI-EAS443:3:36:1767:774#0/1
GGGGCGGGGAATCTTCGAGCTCTCGGAAGAAAAGGCAACG
+HWI-EAS443:3:36:1767:774#0/1
`^_`¥X`¥¥PPFRXBBBBBBBBBBBBBBBBBBBBBBBBBB
@HWI-EAS443:3:48:912:259#0/1
CAAATATATTCAATATGGAGAGAATAAAGGAACTGAGAGA
+HWI-EAS443:3:48:912:259#0/1
_LY_¥KQS]W^a¥`QYa[Q^¥^a_`POQGZJLF¥Q[MP[W
@HWI-EAS443:3:8:375:2030#0/1
ATTTCTAGCATGGCGGAGGCCATGGTGTCTAGGGCTCGGA
+HWI-EAS443:3:8:375:2030#0/1
]ZWV]a^X¥a^_TGSW`Xa_BBBBBBBBBBBBBBBBBBBB
@HWI-EAS443:3:22:1580:33#0/1
GAAAACAAGACTTGGGTAAACCAGACATATGTTAACATCA
+HWI-EAS443:3:22:1580:33#0/1
aYY¥`Z]WLMR]P][¥SY^_[Z[ZYY[PV^`Z]]XOV^WX
@HWI-EAS443:3:46:262:180#0/1
GAAAACAACACTTGGGTAAACCAGACATACGTTAACATCA
+HWI-EAS443:3:46:262:180#0/1
aa`aaaaa_a_```__U`a_]]]a_^_^_^a]_`]]^_]_
```

```
@HWI-EAS443:3:22:176:1869#0/1
TATGAAAACAACACTTGGGTAAACCAGACATATGTTAACA
+HWI-EAS443:3:22:176:1869#0/1
_¥``a`_V`SSa`a]_a`a¥_¥_b`_^_bb`b^^]`b_`]
@HWI-EAS443:3:2:753:202#0/1
TATGAAAACAACACTTGGGTAAACCAGACATATGTTAACA
+HWI-EAS443:3:2:753:202#0/1
aaabaaaa_a`[`^a_`a^]``a^Y``_]_^_^_]__^[^
@HWI-EAS443:3:33:26:528#0/1
TATGAAAACAACACTTGGGTAAACCAGACATATGTTAACA
+HWI-EAS443:3:33:26:528#0/1
Z¥]`_Z`^^[Y_`aaaaV_^`[^a¥J_V[P[aaa¥aaZa^
@HWI-EAS443:3:44:14:1116#0/1
TATGAAAACAACACTTGGGTAAACCAGACATATGTTAACA
+HWI-EAS443:3:44:14:1116#0/1
aabba_S¥_b`aS¥¥]RY[Wa]ZR_Z]`]_[a¥¥_^`^^`
@HWI-EAS443:3:4:203:436#0/1
TATGAAAACAACACTTGGGTAAACCAGACATATGTTAACA
+HWI-EAS443:3:4:203:436#0/1
¥b¥[[a_``Ybab^X_TQa[^^¥`UV``aa^^aaXSYa_a
@HWI-EAS443:3:66:1051:752#0/1
TATGAAAACAACACTTGGGTAAACCAGACATATGTTAACA
+HWI-EAS443:3:66:1051:752#0/1
aaabaa``aaaaaaaaaa`W`aa`aaa_a``U`a_`a`aa
@HWI-EAS443:3:68:1364:1647#0/1
TATGAAAACAACACTTGGGTAAACCAGACATATGTTAACA
+HWI-EAS443:3:68:1364:1647#0/1
_a_abbaaa_baaZ_ab`aW`_baa`aa`aaaa`a`aa_a
@HWI-EAS443:3:68:639:1802#0/1
AANCAACACTTGGGTAAACCAGACATATGTTAACATCAGC
+HWI-EAS443:3:68:639:1802#0/1
a]DT`a^W[RLPZSTV`_aR¥Z[X^Z_WP¥_Y[LXXP`S[
@HWI-EAS443:3:90:56:1862#0/1
TATGAAAACAACACTTGGGTAAACCAGACATATGTTAACA
+HWI-EAS443:3:90:56:1862#0/1
U_RZ_YRUa_WZ^``¥]_]Paaa^¥Z^aaaTa[QYX`_aa
@HWI-EAS443:3:92:964:109#0/1
TATGAAAACAACACTTGGGTAAACCAGACATATGTTAACA
+HWI-EAS443:3:92:964:109#0/1
a¥_`__XXX]`[RITaa^WLZ¥VTJ`WSOaWTT_[XTM[^
@HWI-EAS443:3:93:1541:303#0/1
TATGAAAACAACACTTGGGTAAACCAGACATATGTTAACA
+HWI-EAS443:3:93:1541:303#0/1
[Y__^``]b``]_`aaa`aIaaa_U¥a__Y````___]V`
@HWI-EAS443:3:99:1778:1190#0/1
TATGAAAACAACACTTGGGTAAACCAGACATATGTTAACA
+HWI-EAS443:3:99:1778:1190#0/1
U¥¥^bb¥`ab_Z]PW_J^¥Vbbb``baa`]a]`baaZaV_
@HWI-EAS443:3:13:133:661#0/1
ATGAAAACAACACTTGGGTAAACCAGACATATGTTAACAT
```

```
+HWI-EAS443:3:13:133:661#0/1
a^a^aaa]W[^`[_[^[^Z_^Z__`_]^^¥`^`X[[^]]]
@HWI-EAS443:3:18:733:1313#0/1
ATGAAAACAACACTTGGGTAAACCAGACATATGTTAACAT
+HWI-EAS443:3:18:733:1313#0/1
aa_a^aaa¥^``]^[]a[U`¥a````^___a`a^]¥^^_^
@HWI-EAS443:3:19:1591:1828#0/1
ATGAAAACAACACTTGGGTAAACCAGACATATGTTAACAT
+HWI-EAS443:3:19:1591:1828#0/1
aabaaab_a``aVaaa_[]aaa^Va``^``aa_^``^^`_
@HWI-EAS443:3:23:498:1201#0/1
ATGAAAACAACACTTGGGTAAACCAGACATATGTTAACAT
+HWI-EAS443:3:23:498:1201#0/1
a``aaabaa`a^^_``ZOM[aa``_`^`a_`__`_`a[__
@HWI-EAS443:3:28:85:326#0/1
ATGAAAACAACACTTGGGTAAACCAGACATATGTTAACAT
+HWI-EAS443:3:28:85:326#0/1
aaaaaabaaaa`aa]Y^`Xaa`aa```aaaaaa_^V¥`a_
@HWI-EAS443:3:32:655:213#0/1
ATGAAAACAACACTTGGGTAAACCAGACATATGTTAACAT
+HWI-EAS443:3:32:655:213#0/1
aaa[b`_abaaa^aa_aZWaaaaaa`__]^a_a]``X¥]^
@HWI-EAS443:3:62:669:205#0/1
ATGAAAACAACACTTGGGTAAACCAGACATATGTTAACAT
+HWI-EAS443:3:62:669:205#0/1
a_`T`[`_aXX`O_¥a`ZGa__ST_RP`^[^Z_X]^XY_U
@HWI-EAS443:3:69:1188:870#0/1
ATGAAAACAACACTTGGGTAAACCAGACATATGTTAACAT
+HWI-EAS443:3:69:1188:870#0/1
ab`baabaaaaaa`aaa^Waaa`_a``aaa__a__a^_a_
@HWI-EAS443:3:75:1033:752#0/1
ATGAAAACAACACTTGGGTAAACCAGACATATGTTAACAT
+HWI-EAS443:3:75:1033:752#0/1
aY_¥abb`a¥NS¥a]a``WZ_[RIa_]W`[X`aa_]aS`a
@HWI-EAS443:3:80:996:1089#0/1
ATGAAAACAACACTTGGGTAAACCAGACATATGTTAACAT
+HWI-EAS443:3:80:996:1089#0/1
aa¥]babaaaa`a`^aPaP_```]]_`aa`^a_```]^]^
@HWI-EAS443:3:81:770:1483#0/1
ATGAAAACAACACTTGGGTAAACCAGACATATGTTAACAT
+HWI-EAS443:3:81:770:1483#0/1
T]¥_`_US`^^``¥_NGOW]a^aa`_aaaa`¥HT^_[]a^
@HWI-EAS443:3:82:478:289#0/1
ATGAAAACAACACTTGGGTAAACCAGACATATGTTAACAT
+HWI-EAS443:3:82:478:289#0/1
_``abab^X`bab^ab_a_aaa^ab`aaa_aaa_`[_Q_`
@HWI-EAS443:3:84:504:905#0/1
ATGAAAACAACACTTGGGTAAACCAGACATATGTTAACAT
+HWI-EAS443:3:84:504:905#0/1
aab_]a¥b__]aaaaaa_N^]^``S]aab_a_aZ[a¥aa_
```

@HWI-EAS443:3:8:1751:41#0/1
ATGAAAACAACACTTGGGTAAACCAGACATATGTTAACAT
+HWI-EAS443:3:8:1751:41#0/1
¥a_VbbaabYP^SXW___OZ``LP]^^N_`[]a¥SX_SWB
@HWI-EAS443:3:90:311:1343#0/1
ATGAAAACAACACTTGGGTAAACCAGACATATGTTAACAT
+HWI-EAS443:3:90:311:1343#0/1
a_XVYR__UU`¥Qa]UTUO]_^]]`_aaaa]WQ^^a`[][
@HWI-EAS443:3:91:187:49#0/1
ATGAAAACAACACTTGGGTAAACCAGACATATGTTAACAT
+HWI-EAS443:3:91:187:49#0/1
aaW¥V]abba^TY_T^^^X]S]]Y^]ONQ[TU^XRTXVYQ
@HWI-EAS443:3:93:105:1573#0/1
ATGAAAACAACACTTGGGTAAACCAGACATATGTTAACAT
+HWI-EAS443:3:93:105:1573#0/1
a`aaaaaaaa__]^^W_VaV_`a``^`__^^`]]`_X`U
@HWI-EAS443:3:14:352:1719#0/1
TGAAAACAACACTTGGGTAAACCAGACATATGTTAACATC
+HWI-EAS443:3:14:352:1719#0/1
abbbbabbbbaaaaa_^_ababa`^baaaaaa^_baab__
@HWI-EAS443:3:39:998:124#0/1
TGAAAACAACACTTGGGTAAACCAGACATATGTTAACATC
+HWI-EAS443:3:39:998:124#0/1
ab`]aaaaaa`___``aZ____`^`^Z_^Y]Y]]^_]]YX
@HWI-EAS443:3:66:401:901#0/1
TGAAAACAACACTTGGGTAAACCAGACATATGTTAACATC
+HWI-EAS443:3:66:401:901#0/1
a[``ab__¥aaaa_[R^_aa]a_`_aa]^a``_^TY_`¥[
@HWI-EAS443:3:100:1555:411#0/1
ACAACACTTGGGTAAACCAGACATATGTTAACATCAGCAA
+HWI-EAS443:3:100:1555:411#0/1
¥K`aaa`aaYYVXa``^]baa[aa`Z`^_aaO`[Y__ZBB
@HWI-EAS443:3:16:1622:1874#0/1
AAACAACACTTGGGTAAACCAGACATATGTTAACATCAGC
+HWI-EAS443:3:16:1622:1874#0/1
`b_^baaa[b``]_S_aY¥G_ab^a]a`X]`aYZ¥^W___
@HWI-EAS443:3:17:1194:1205#0/1
AAACAACACTTGGGTAAACCAGACATATGTTAACATCAGC
+HWI-EAS443:3:17:1194:1205#0/1
[^`aaaa_`a`X`aG__`]P]__^]^__`]X`^P^¥¥^_S
@HWI-EAS443:3:23:1453:904#0/1
GAAAACAACACTTGGGTAAACCAGACATATGTTAACATCA
+HWI-EAS443:3:23:1453:904#0/1
aababaab`a`aaaaa^`aaW_a^`_`aaba`a`_Yaa[a
@HWI-EAS443:3:24:1583:1129#0/1
GAAAACAACACTTGGGTAAACCAGACATATGTTAACATCA
+HWI-EAS443:3:24:1583:1129#0/1
`babbbabab`bba_a_bbb`]aabbbbbbbabaabbbaa
@HWI-EAS443:3:24:81:292#0/1
GCTGATGTTAACATATGTCTGGTTTACCCAAGTGTTGTTT

+HWI-EAS443:3:24:81:292#0/1
aa`a``a_a]aaaa]``]__aaWa_Z__^Z^`U`[^_W[^
@HWI-EAS443:3:26:661:936#0/1
AAAACAACACTTGGGTAAACCAGACATATGTTAACATCAG
+HWI-EAS443:3:26:661:936#0/1
aa`baba``aaaaa`]b``a``a`aaaaaa_`a_```_a`
@HWI-EAS443:3:28:1048:1405#0/1
AACAACACTTGGGTAAACCAGACATATGTTAACATCAGCA
+HWI-EAS443:3:28:1048:1405#0/1
_`b^a^aaaaa^UUbaaaaaaaa``_]^S`aa`a``aa¥a
@HWI-EAS443:3:28:1209:1102#0/1
GAAAACAACACTTGGGTAAACCAGACATATGTTAACATCA
+HWI-EAS443:3:28:1209:1102#0/1
`babbb_babbabba``bbba_baaaaabbaabbaaaaaa
@HWI-EAS443:3:30:323:967#0/1
GCTGATGTTAACATATGTCTGGTTTACCCAAGTGTTGTTT
+HWI-EAS443:3:30:323:967#0/1
aa`_TY^]`Z_]Va^_¥^`¥¥XR]W_aa_S[TMZU^_V^^
@HWI-EAS443:3:36:192:1868#0/1
AACAACACTTGGGTAAACCAGACATATGTTAACATCAGCA
+HWI-EAS443:3:36:192:1868#0/1
aaaTGV]`_^^WUO`a[aa_^^_a_`]¥XZI¥W_Wa[XS[
@HWI-EAS443:3:36:412:291#0/1
TGCTGATGTTAACATATGTCTGGTTTACCCAAGTGTTGTT
+HWI-EAS443:3:36:412:291#0/1
YU¥`a[Z[`b__[R^`V[]]^a`Y^``X`]H^]QUOOW[¥
@HWI-EAS443:3:37:614:1488#0/1
AAACAACACTTGGGTAAACCAGACATATGTTAACATCAGC
+HWI-EAS443:3:37:614:1488#0/1
abbaaaaa`____]U_aaaaa_`a__`]YVT^Z`a_]_]¥
@HWI-EAS443:3:38:757:1217#0/1
TGCTGATGTTAACATATGTCTGGTTTACCCAAGTGTTGTT
+HWI-EAS443:3:38:757:1217#0/1
baa^JaaVaQZ`¥¥babQab`aQb`aaa`^`ZV_Y_`^
@HWI-EAS443:3:40:1099:212#0/1
GCTGATGTTAACATATGTCTGGTTTACCCAAGTGTTGTTT
+HWI-EAS443:3:40:1099:212#0/1
a```^^_]aZ^_^]]_`[^^__X]^[¥Y^VQ¥X¥V¥¥YY^
@HWI-EAS443:3:45:1046:1060#0/1
GAAAACAACACTTGGGTAAACCAGACATATGTTAACATCA
+HWI-EAS443:3:45:1046:1060#0/1
aab`[^a^ab`a^[``]a]`^`aaa_a`aaa^^``__`aa
@HWI-EAS443:3:45:509:1905#0/1
AAAACAACACTTGGGTAAACCAGACATATGTTAACATCAG
+HWI-EAS443:3:45:509:1905#0/1
aYVa_aaa]ZYYZR¥^_]aa]Z`¥O`aaa]^X``^a^MZY
@HWI-EAS443:3:48:741:740#0/1
AAACAACACTTGGGTAAACCAGACATATGTTAACATCAGC
+HWI-EAS443:3:48:741:740#0/1
aaaaaaaaaab]aa[aa`_a^_^``_¥_b^¥a_`]aQab[

```
@HWI-EAS443:3:49:986:932#0/1
GCTGATGTTAACATATGTCTGGTTTACCCAAGTGTTGTTT
+HWI-EAS443:3:49:986:932#0/1
aa``aaa`a¥`¥__T[`VY[aaXW^_]WUX]`]`V^_V^[
@HWI-EAS443:3:50:1196:1006#0/1
AACAACACTTGGGTAAACCAGACATATGTTAACATCAGCA
+HWI-EAS443:3:50:1196:1006#0/1
a`bbbb``bbbbba`bba¥bbaaabaab_ba]a`baaaaa
@HWI-EAS443:3:51:1280:1126#0/1
ACAACACTTGGGTAAACCAGACATATGTTAACATCAGCAA
+HWI-EAS443:3:51:1280:1126#0/1
aXWaSa[QY]P]XaTaaaZIY]__T¥aT_`_^aaZ`RGa_
@HWI-EAS443:3:53:347:1961#0/1
GCTGATGTTAACATATGTCTGGTTTACCCAAGTGTTGTTT
+HWI-EAS443:3:53:347:1961#0/1
X_¥]¥¥_H_^__Xbb^^^^^¥_`b`^__a_XVG^Q`¥aab
@HWI-EAS443:3:5:345:712#0/1
GCTGATGTTAACATATGTCTGGTTTACCCAAGTGTTGTTT
+HWI-EAS443:3:5:345:712#0/1
abbb`baaaba``baabaaaba_aa`]a]^`aNa_aa`aa
@HWI-EAS443:3:61:649:336#0/1
TGCTGATGTTAACATATGTCTGGTTTACCCAAGTGTTGTT
+HWI-EAS443:3:61:649:336#0/1
aa`aa__aa`¥^_``_`aY^V¥¥P[_`_[WVYYQ^Z]^[]
@HWI-EAS443:3:63:1367:358#0/1
GCTGATGTTAACATATGTCTGGTTTACCCAAGTGTTGTTT
+HWI-EAS443:3:63:1367:358#0/1
T__b`_]¥_WT_```b_Y¥``]V__^[]_aY``baab_ab
@HWI-EAS443:3:66:1181:248#0/1
AAACAACACTTGGGTAAACCAGACATATGTTAACATCAGC
+HWI-EAS443:3:66:1181:248#0/1
ab`bbba`aaa]aaSab`a`aaa_`__aa_aa_]_`[_a^
@HWI-EAS443:3:66:275:580#0/1
GAAAACAACACTTGGGTAAACCAGACATATGTTAACATCA
+HWI-EAS443:3:66:275:580#0/1
]]H^`^___aa```]YWT^aa`a`¥`aWW]a¥_¥SQZ^_`
@HWI-EAS443:3:67:211:1603#0/1
ACAACACTTGGGTAAACCAGACATATGTTAACATCAGCAA
+HWI-EAS443:3:67:211:1603#0/1
abbV`b_^^^_^Zbaa_a__`aL^`__]`a_[_^___Yaa
@HWI-EAS443:3:73:397:1796#0/1
CTGATGTTAACATATGTCTGGTTTACCCAAGTGTTGTTTT
+HWI-EAS443:3:73:397:1796#0/1
aaX]_¥PV¥^`]``aa¥a``__^`aa`a^_]Za¥^¥V___
@HWI-EAS443:3:7:111:1403#0/1
AAACAACACTTGGGTAAACCAGACATATGTTAACATCAGC
+HWI-EAS443:3:7:111:1403#0/1
a]aaa^a`Xa^aa]Z____a`_^___a_¥]`a^¥^__`Y_
@HWI-EAS443:3:81:725:761#0/1
TGCTGATGTTAACATATGTCTGGTTTACCCAAGTGTTGTT
```

+HWI-EAS443:3:81:725:761#0/1
a`ab^V__aaaa`aa^^b_aa`a^aaa_aa__bXa]aa__
@HWI-EAS443:3:82:1001:1325#0/1
GAAAACAACACTTGGGTAAACCAGACATATGTTAACATCA
+HWI-EAS443:3:82:1001:1325#0/1
]abbaabbbaabb^PYZbabYba`_`ababa_a_aaaaaa
@HWI-EAS443:3:83:303:1177#0/1
AAACAACACTTGGGTAAACCAGACATATGTTAACATCAGC
+HWI-EAS443:3:83:303:1177#0/1
a`__V__a``]][Y¥¥Za]`_a¥`_^__`__Y^^¥^_`Z^
@HWI-EAS443:3:88:53:1455#0/1
ACAACACTTGGGTAAACCAGACATATGTTAACATCAGCAA
+HWI-EAS443:3:88:53:1455#0/1
ababaaaa``a`V`]aaaa__a¥a`_`]_``a_^]]Z___
@HWI-EAS443:3:93:1374:130#0/1
GAAAACAACACTTGGGTAAACCAGACATATGTTAACATCA
+HWI-EAS443:3:93:1374:130#0/1
aX_aXU¥^_[`]_OV`^S^VZXRZ[SZ¥^S_S_^ZXZ]WZ
@HWI-EAS443:3:93:765:786#0/1
GAAAACAACACTTGGGTAAACCAGACATATGTTAACATCA
+HWI-EAS443:3:93:765:786#0/1
`bbbbabbabaabbaa^aaaa`aaaaaaaaaaaa`ba¥a
@HWI-EAS443:3:94:1528:784#0/1
GAAAACAACACTTGGGTAAACCAGACATATGTTAACATCA
+HWI-EAS443:3:94:1528:784#0/1
]_^Maaa`]a[a¥U_aMZ]a[`a_a`a`a`b¥a_`P^¥¥a
@HWI-EAS443:3:90:293:979#0/1
CACAGCCCCATTGTCTGGGCCAGAAATTCCAATTGTTAGC
+HWI-EAS443:3:90:293:979#0/1
_Z`ba`bb_bbbb_aa^b_Z`a`a`aaa`a`a_aa]a^`_
@HWI-EAS443:3:72:1045:1116#0/1
GCTCTGGATGCTGAACAAAACTCCCGCTATATCCTGACCA
+HWI-EAS443:3:72:1045:1116#0/1
^aab`aaaaa``_aa_bb`aa`_a_aZa`_ba_^__`a`Z
@HWI-EAS443:3:24:443:166#0/1
CTTCCCTCATGGACTCAGGCACTCCTTCCTTAGAAGGACC
+HWI-EAS443:3:24:443:166#0/1
]OZ__a`aU]`_]Z`_ROYYR`¥VYBBBBBBBBBBBBBBB
@HWI-EAS443:3:69:822:713#0/1
CTTTCTCCTTGACAGAAGACTCGGCCTCAACCATGCTCTC
+HWI-EAS443:3:69:822:713#0/1
abb]aUa]]]TM]_]X`XUS¥¥UOSZJT_OIDOPW¥MFVX
@HWI-EAS443:3:40:1184:803#0/1
CAATGGTGGAACAGATCTTCATGATCTCAGAGAACTTTTC
+HWI-EAS443:3:40:1184:803#0/1
¥```bbabbbb[a`a`a`ba^_]][Y_``^^`ZYaa`a`¥
@HWI-EAS443:3:27:738:1109#0/1
AGGGCGCCTTGCTAAATGACAAACATTCCAATGGAACCAT
+HWI-EAS443:3:27:738:1109#0/1
^a`_X`ba^Y^aaa]a`R][XU¥W[^]aa]aaa_`^Ya

```
@HWI-EAS443:3:55:1126:1320#0/1
GGGGGCCTTGCTAAATGACAAACATTCCAATGGAACCATT
+HWI-EAS443:3:55:1126:1320#0/1
a`_aa__``__`a`_a^a_``_^^`a_¥]_``]__^Z_]a
@HWI-EAS443:3:73:344:925#0/1
GCATCCTACTCTGTTCTGGTGGAGCAGCAGCAAGGGGAAA
+HWI-EAS443:3:73:344:925#0/1
`Z[Z`a¥___]W[YZ¥[^_W]V¥¥]_¥Q_^WURUT¥WWW[
@HWI-EAS443:3:94:425:213#0/1
CCCTCTTCCCTTTGCTGCTGCTCCACCAGAACAGAGTAGG
+HWI-EAS443:3:94:425:213#0/1
a`a`^bbaa_aaaaaaa_aa^_V[_W_``^^Z__¥^Q_^_
@HWI-EAS443:3:3:533:667#0/1
GCCGGTTGATTTCTGTTTAGTGTCCAGTCATAAGTCTGGT
+HWI-EAS443:3:3:533:667#0/1
W``U_][`P^abZ]VRX^VGVa[SLOH[_[_X`XO^]_BB
@HWI-EAS443:3:58:1143:740#0/1
TGCCGGTTGATTTCTGTTTAATGTCCAGTCATAAGTCTGG
+HWI-EAS443:3:58:1143:740#0/1
a_aabb`b_V^aaaaaaaaaaaaT¥`_a^aaaa]]U^`a¥
@HWI-EAS443:3:75:808:660#0/1
TGCATTCGCAATGGAAAGAAATACTGGATCTGGTATTATC
+HWI-EAS443:3:75:808:660#0/1
¥baTa¥ZaaaUS``abb_Ybb`aY_a[ba_aYSO]__aaa
@HWI-EAS443:3:86:771:286#0/1
GAAATACTGGATCTGGTATTATCATTTCAGATACACCAGT
+HWI-EAS443:3:86:771:286#0/1
aaba`]``[Y]]a^^aPZ_W_b[ba`^¥_aa`a_aaaa`]
@HWI-EAS443:3:73:859:680#0/1
AGAGAATAAAAACTGAGAGATCTAATGTCGCAGTCCCGCA
+HWI-EAS443:3:73:859:680#0/1
aa`baabbabbbaaababbaaaababb]aa_aa_a_a_`_
@HWI-EAS443:3:37:622:228#0/1
GCAAACTAAGAGGGGTAGCCCCATTGCATTTGGGCAATTG
+HWI-EAS443:3:37:622:228#0/1
a]Q[X]^Y[XDTVRUMT^Y_Y_WVBBBBBBBBBBBBBBBB
@HWI-EAS443:3:67:1522:79#0/1
AATGTAGGACCATGAGCTTGCTGTGGAGAGTAATTCACAC
+HWI-EAS443:3:67:1522:79#0/1
a]aaZaa``YW___]a[aX_]__TZZ[^^`U]]^]UUX^Y
@HWI-EAS443:3:37:958:730#0/1
CTCCTGGGTAACACGTTCCATTGTCTGAATTAGTTGTTTC
+HWI-EAS443:3:37:958:730#0/1
abbbaYab^R__[baaa```aba_^^^``G[aa_a``]a]
@HWI-EAS443:3:76:1738:516#0/1
GAGTGTGAATCACTCTCCACAGCAAGCTCATGGTCTTACA
+HWI-EAS443:3:76:1738:516#0/1
`_aVG[b`aaR`_a]_Y`_¥a_Ya]_X`O^RR_[WGY¥G^
@HWI-EAS443:3:38:259:281#0/1
ATGTTTTTGTGGGGACATCAAGATACAGCAAGACGTTCAA
```

```
+HWI-EAS443:3:38:259:281#0/1
X¥`[YU¥TZ^bVbZ¥QQ_aa]aUDOY¥N_b]YNGYFGSbX
@HWI-EAS443:3:91:1709:1060#0/1
GGCTGGTTAAAAAAGGAAATTCATACCCAAAGCTCAGCAA
+HWI-EAS443:3:91:1709:1060#0/1
a`[WDWUXX_[VQ[]aQ___[TXW_TQO]_[^RT^Z]TS_
@HWI-EAS443:3:81:1208:308#0/1
CAGGGAGACTACGAGTACCAATGAACTGGCGACAGGTGAA
+HWI-EAS443:3:81:1208:308#0/1
V`¥XQ_¥¥UbaZH]b`_`UX[_a^^VaaaabV[a]FQa_R
@HWI-EAS443:3:65:1767:1938#0/1
GATTGCCCCCAGGGAGACTGCCAGTACCAATGAACTGGCG
+HWI-EAS443:3:65:1767:1938#0/1
a^aaaba^R_[DZ[Y[`a``¥ZaVP_XQ]^a[_`WV^Y]B
@HWI-EAS443:3:91:439:1047#0/1
TGCCCCCAGGGAGACTGCCAGTACCAATGAACTGGCGACA
+HWI-EAS443:3:91:439:1047#0/1
abbabbb_aba`aba_a]^¥a¥aaZ]_aa___^_`Ya_¥¥
@HWI-EAS443:3:10:1229:1712#0/1
AAGAAATAAGACCCTTCATTACTTATGTCAAAGGAAGGCA
+HWI-EAS443:3:10:1229:1712#0/1
a^a`aba`b_b_^_^_]aa``^_Wa^bW[_a]_^_S`ZYa
@HWI-EAS443:3:73:1658:412#0/1
CAAGTAGTAGCATTGCCACAACTACTTCAATGCATGTGTG
+HWI-EAS443:3:73:1658:412#0/1
abZH¥ba[aaa^aXa`O`^[Sa¥a¥]aK¥a]a^`ba]aUa
@HWI-EAS443:3:89:1078:968#0/1
CTCGGCCTCAATCATGCTCTCAATCTGCTGAATAGACTGA
+HWI-EAS443:3:89:1078:968#0/1
Q^__[S_ba^aa¥`bbaaZ_abb_bbb_bb]ababbbbb`
@HWI-EAS443:3:40:641:1019#0/1
CTGTGATGAGAATGGGAGACCTTCACTACCTCCAAAGCAG
+HWI-EAS443:3:40:641:1019#0/1
abb]``a`^^¥Sabaaabaaababaa]aa`_aa`UH¥_¥`
@HWI-EAS443:3:33:900:1972#0/1
CTTTGTTAGTAATCTCGTCAATAGCATTCTGTGTGCTCTT
+HWI-EAS443:3:33:900:1972#0/1
a^`Waaa_Y¥_Y^¥Z]_QS``PEXY_Ya^YYWRVU_OYMQ
@HWI-EAS443:3:87:1070:1323#0/1
TTTTGAGATGATTTGGGATCCGAACGGATGGAACAGGGAC
+HWI-EAS443:3:87:1070:1323#0/1
ababa`aaaaaabba`a`a``a__`_`a`_VG^]]a`Y]¥
@HWI-EAS443:3:93:1735:640#0/1
AAACGGGTTTGAGATGATTTGGGATCCGAACGGATGGAAT
+HWI-EAS443:3:93:1735:640#0/1
WUb`¥PH¥b``¥ZGSSN`¥]a`TT`_ZTU_YW`[^DMUBB
@HWI-EAS443:3:4:475:1019#0/1
ATTTGGGATCCGAACGGATGGAATGGGACAGACAATAACT
+HWI-EAS443:3:4:475:1019#0/1
a__b]P_VYYX`]Z_aa¥bb]MHbbba¥_YUa^``^``RY
```

@HWI-EAS443:3:37:672:304#0/1
CTCCTGTTTTCTTAGGGTCCTTCCCAGCACTGGGGTGCTC
+HWI-EAS443:3:37:672:304#0/1
`abb`a`bbbbbbabbababbbbabbabGaa`ab_baa_
@HWI-EAS443:3:22:115:1872#0/1
GCGGATCATCAGCCAAGAAAGAAGAAGAAGTGCTAACGGG
+HWI-EAS443:3:22:115:1872#0/1
ab]TUT]^Z_¥VHTXZ¥SMZ^W¥][`a`ZOFV]^X]^¥SV
@HWI-EAS443:3:8:155:375#0/1
GCTGATGATCCGCTTGTCCTTTTGAAAGTGAACCCACCAA
+HWI-EAS443:3:8:155:375#0/1
_ZOZQXUT¥aaYV¥VQ_NZ^a¥_aP¥`aY]VZZFYX`W]N
@HWI-EAS443:3:82:258:360#0/1
AACCAGAAAGCTCTTATCTCTTGTTCTACTTCAAGCAGTA
+HWI-EAS443:3:82:258:360#0/1
T[WMRXONZb¥WQ_WS^]^aZXZWWZXZX[`a]RT`a_OS
@HWI-EAS443:3:72:751:262#0/1
GAACAATTGAAGAGCCATCTGGGCCGTTGCAGGCCCAAGG
+HWI-EAS443:3:72:751:262#0/1
abaaa`]``_bb¥¥¥¥a^]__aaa¥a^_aZTa`N_V^[^^
@HWI-EAS443:3:76:1282:743#0/1
GAAGAGCCATCTGGGCCGTTGCAGGCCCAAGGTCATTGTT
+HWI-EAS443:3:76:1282:743#0/1
`[baN]]^`]]U^]_UG_TKLVGVTUXOUUYNWUUUUUU]
@HWI-EAS443:3:18:156:648#0/1
ATGCAATTGTCATTCTAAGTGCCTCGCTGGGTTCCTCTTT
+HWI-EAS443:3:18:156:648#0/1
_]Y_VX]YaZ^]W]H^``_ZFGSaVNYO[FFN_NXOMY^Q
@HWI-EAS443:3:7:764:1612#0/1
CAGATCGCCCCTAATTGCCTTGATCATGCAATCCTCTTGT
+HWI-EAS443:3:7:764:1612#0/1
abaaa`a_`aa`b`_aa`Qaa__^Ya_W_aa_[______V
@HWI-EAS443:3:42:1488:131#0/1
AACAACTCGACACTAATTGATGGCCATCCGAATTCTTTTG
+HWI-EAS443:3:42:1488:131#0/1
aaaaaaa_a`_a^a```^^^^__]Y__Y_`YYY]Y]_^^S
@HWI-EAS443:3:62:439:2001#0/1
GCCAGAAAGGTAGCGCGAAGTAGGTACAGATGCAATTGTC
+HWI-EAS443:3:62:439:2001#0/1
UK_]U[_a`^`_SUZaY]aYLZWOZ¥NYS]^W^]VTPLDW
@HWI-EAS443:3:68:681:1190#0/1
CCAGTCTCGTGACATTTCCTCGAGGGTCATGCCAGAAAGG
+HWI-EAS443:3:68:681:1190#0/1
a¥OZ]_¥XRX¥`XX`aa_`]]W¥][NNaaaYG^YOUUWMQ
@HWI-EAS443:3:21:1522:75#0/1
GAAGCCAGTTAAAAAACAATGCCAAGGAAATTGGAAATGG
+HWI-EAS443:3:21:1522:75#0/1
aL]W[]]`QZ`_`_^WNY_¥¥PXIYYYMSPUBBBBBBBBB
@HWI-EAS443:3:10:547:372#0/1
ATCCCCTTGCCCAATTAGCACATTAGCTTTCTCTCCTTTT

+HWI-EAS443:3:10:547:372#0/1
a`aaaa``bX`a^_aa`X`aa`aaaa`_aa``^a^`a_`a
@HWI-EAS443:3:33:337:1451#0/1
ATCCCCTTGCCCAATTAGCACATTAGCTTTCTCTCCTTTT
+HWI-EAS443:3:33:337:1451#0/1
a`abbbba``^a`^^b`_bb]X_^`U_X_aa^Ja_S¥Y¥^
@HWI-EAS443:3:39:1073:1920#0/1
GAGAAAGCTAATGTGCTAATTGGGCAAGGGGATGTAGTGT
+HWI-EAS443:3:39:1073:1920#0/1
a`^`__S¥`[PaaQVZaa_a_][^__`Y]ZSMNKRY^PPP
@HWI-EAS443:3:81:1344:993#0/1
TGGCTGGATCCTGGGAAATCCAGAGTGTGAATCACTTTTC
+HWI-EAS443:3:81:1344:993#0/1
a``a^b]]aaaRa_Z_]]_J`ZUUa`]HTaa_a^``GSFV
@HWI-EAS443:3:66:339:1834#0/1
ACAAAAGCAACAAAAATGAAGGCAACACTAGTAGTTCTGC
+HWI-EAS443:3:66:339:1834#0/1
abZ`ba_b_a``^¥^`aaa]__ba^F]_[a_[aZGQ_XS`
@HWI-EAS443:3:51:883:1191#0/1
ATCATTTGAAAGGTTTGAGATATTCCCCAAGATCCTGCTT
+HWI-EAS443:3:51:883:1191#0/1
aa``bbab^bab`Uaaabbbaaaaa^aa````a][aa_a_
@HWI-EAS443:3:1:1455:1181#0/1
CTAGAACACAGTATTTTTCCCATTTGTGTGGCTCCAGTTT
+HWI-EAS443:3:1:1455:1181#0/1
aab_bbabbbbabbbabba]]abbaaab_a_Ya¥`_`VaB
@HWI-EAS443:3:46:1006:926#0/1
GTGGGCTCTATGTTGACAAAATGACCATCGTCAACATCCA
+HWI-EAS443:3:46:1006:926#0/1
```b___^X__b]X`__aa^ZY]`[Z_S`]WR_[Q¥X¥Y_
@HWI-EAS443:3:66:764:470#0/1
GTTCTTGGAAAGATGCAGGTGCATTGACTGAAGATCCAGA
+HWI-EAS443:3:66:764:470#0/1
a[^X]aa`UV[SJQPT]_[RNOJOM[[¥TVKT¥ZNSTZUP
@HWI-EAS443:3:12:857:1602#0/1
ATCAGCGAAAGCAGGCAAACCATTTGAATGGATGTCAATA
+HWI-EAS443:3:12:857:1602#0/1
aaYWW`QUZ[PZYJ¥[`[WDTWY¥RTOSX__G^_UHXXBB
@HWI-EAS443:3:33:129:803#0/1
CAGCGAAAGCAGGCAAACCATTTGAATGGATGTCAATCCG
+HWI-EAS443:3:33:129:803#0/1
aaab`aaaaaa``abaXaaaaaaaSaaa]_`¥U__`¥S¥Y
@HWI-EAS443:3:91:1696:1935#0/1
CAGCGAAAGCAGGCAAACCATTTGAATGGATGTCAATCCG
+HWI-EAS443:3:91:1696:1935#0/1
XbbXbbabbbb_aabababb`bbbbabbbbbbabbbbOab
@HWI-EAS443:3:36:1438:1647#0/1
ATGACTTTCAGCTGATCCCAATGGTAAGCAAATGTAGACC
+HWI-EAS443:3:36:1438:1647#0/1
a_``abbab_`]`aaaaa`_a`¥`V``^^``¥^ZW`]_S`
```

```
@HWI-EAS443:3:86:637:439#0/1
CTTTGGTTCTACATTTGCTTACCATTGGGATCAGCTGAAA
+HWI-EAS443:3:86:637:439#0/1
abbbbb^V_babbabbbbbabbabbabbbabbbbaaaaa_
@HWI-EAS443:3:78:566:153#0/1
AGGGGTTCAGGGGATTACAAAGTCTTCCCCGATAATCATC
+HWI-EAS443:3:78:566:153#0/1
[aZ_H[`¥a¥aaa_^¥Z_a^`_JO__aV¥¥XU]_VZHZPR
@HWI-EAS443:3:11:1673:651#0/1
GGGGTTCTTCTCTTGTCTTGCTGATGTGTACTTTTTGATT
+HWI-EAS443:3:11:1673:651#0/1
aa_WOJM¥]X_QY`[SKVWRTWVO[Y[^TKR]`ZLEU_^B
@HWI-EAS443:3:42:1753:76#0/1
CGGGGTTCTTCTCTTGTCTTGCTGATGTGTACTTTTTGAT
+HWI-EAS443:3:42:1753:76#0/1
abbabbbbbbbbabbbbbb_G]bb`bbabaa`bbbbbbab
@HWI-EAS443:3:69:1246:1359#0/1
GTCGGAACTATGCGCAAGCTTGCCGACCAAAGTCTCCCAC
+HWI-EAS443:3:69:1246:1359#0/1
`Z`]ZF^ab`ZbbaJ`bbbaaaaa`aaab`WaWLVaYaaa
@HWI-EAS443:3:18:1539:163#0/1
GTGTTTTTTCTCAAGCTTCTGAAATCCTAATGTTAAATAC
+HWI-EAS443:3:18:1539:163#0/1
a¥a]]``a_^`__R]]`_]^¥^^[_Y]`_Y]_[^_XZ[^Y
@HWI-EAS443:3:47:669:1731#0/1
TGTGTTTTTTCTCATGCTTCTGAAATCCTAATGTTAAATA
+HWI-EAS443:3:47:669:1731#0/1
H__`[]¥abba_¥babZbbbbbaabbWI`bbabbbbbbbb
@HWI-EAS443:3:10:75:1807#0/1
GTTTTTTCTCATGCTTCTGAAATCCTAATGTTAAATACAT
+HWI-EAS443:3:10:75:1807#0/1
aSHUU[_^VX_YPSZ¥[^[^W[HVP[NFLPH]KKO[[Z]Z
@HWI-EAS443:3:18:314:978#0/1
GGTGTTTTTTCTCATGCTTCTGAAATCCTAATGTTAAATA
+HWI-EAS443:3:18:314:978#0/1
^aRaX]bbbbbabbbbbaabbbb`bbbaaaaba_aaaba`
@HWI-EAS443:3:32:401:1944#0/1
GTGTTTTTTCTCATGCTTCTGAAATCCTAATGTTAAATAC
+HWI-EAS443:3:32:401:1944#0/1
¥`a]_aaabbabaabb`abaaabbaababaa^_`bbaabb
@HWI-EAS443:3:35:885:458#0/1
GTGTTTTTTCTCATGCTTCTGAAATCCTAATGTTAAATAC
+HWI-EAS443:3:35:885:458#0/1
aab^`]]^aaa``]`¥]`T`a`X[`Z`[XW___¥[`^^`a
@HWI-EAS443:3:38:1231:1304#0/1
GGTGTTTTTTCTCATGCTTCTGAAATCCTAATGTTAAATA
+HWI-EAS443:3:38:1231:1304#0/1
T_O_US_^¥`^Z¥`a^_abaZ`^_¥^][^KY_aaa[]`aY
@HWI-EAS443:3:38:316:390#0/1
GTGTTTTTTCTCATGCTTCTGAAATCCTAATGTTAAATAC
```

```
+HWI-EAS443:3:38:316:390#0/1
a¥b__````a`aa``a__``_[Q]^a``a_^`]_a¥[X¥P
@HWI-EAS443:3:42:291:278#0/1
GTGTTTTTTCTCATGCTTCTGAAATCCTAATGTTAAATAC
+HWI-EAS443:3:42:291:278#0/1
aab`aaabaaaaaaaaaaaabaa_aa`ab`aa_`a`a`__
@HWI-EAS443:3:43:968:69#0/1
GGTGTTTTTTCTCATGCTTCTGAAATCCTAATGTTAAATA
+HWI-EAS443:3:43:968:69#0/1
ab]b^abaab`a_aaa_aa`aa__`a_¥aa¥aa`a_]^a`
@HWI-EAS443:3:58:1690:82#0/1
TGTTTTTTCTCATGCTTCTGAAATCCTAATGTTAAATACA
+HWI-EAS443:3:58:1690:82#0/1
ab_aabb_N_a`ba_aa`aa___`[_aa`ab_a_^`a]¥^
@HWI-EAS443:3:59:960:1557#0/1
TGTTTTTTCTCATGCTTCTGAAATCCTAATGTTAAATACA
+HWI-EAS443:3:59:960:1557#0/1
abaabbaaaaaabbaba`aa_a`baaaaaba]aa_]H`_¥
@HWI-EAS443:3:64:1077:1636#0/1
GGTGTTTTTTCTCATGCTTCTGAAATCCTAATGTTAAATA
+HWI-EAS443:3:64:1077:1636#0/1
^`Xa^ababbaaaaaaaa`]abaaa````aaaa`aa`a`b
@HWI-EAS443:3:66:1267:1398#0/1
TGTTTTTTCTCATGCTTCTGAAATCCTAATGTTAAATACA
+HWI-EAS443:3:66:1267:1398#0/1
abY[abbaab¥a_¥¥aaab^`aaa]`aa`aaaa_`aaa``
@HWI-EAS443:3:67:1576:1783#0/1
TGTTTTTTCTCATGCTTCTGAAATCCTAATGTTAAATACA
+HWI-EAS443:3:67:1576:1783#0/1
aaaaabbaaaabb`aaaaaaaaaa]aaaaabaaa`aaaaa
@HWI-EAS443:3:67:730:495#0/1
GTGTTTTTTCTCATGCTTCTGAAATCCTAATGTTAAATAC
+HWI-EAS443:3:67:730:495#0/1
a¥`¥`aaaaaa`aa`aaaaaa`aaaa_aaaaa``a_a`a_
@HWI-EAS443:3:89:1071:874#0/1
GGTGTTTTTTCTCATGCTTCTGAAATCCTAATGTTAAATA
+HWI-EAS443:3:89:1071:874#0/1
a^Ya[_aaaaW]a]`aaa__¥a¥ZW^_^`]X`a__^^_a`
@HWI-EAS443:3:94:1103:741#0/1
TGTTTTTTCTCATGCTTCTGAAATCCTAATGTTAAATACA
+HWI-EAS443:3:94:1103:741#0/1
aa__a^_aaa_^a]]aa_ba¥^Ya^_ba_aa`a_a_a_^`
@HWI-EAS443:3:95:1772:1077#0/1
GTGTTTTTTCTCATGCTTCTGAAATCCTAATGTTAAATAC
+HWI-EAS443:3:95:1772:1077#0/1
]F_¥¥aa`aaa`b_aaa_^aa_aWa^_aaa^¥`aaa_`_W
@HWI-EAS443:3:99:100:1510#0/1
ACTCACTAAGACCACTGTGGACCATATTGCCATCATCCAC
+HWI-EAS443:3:99:100:1510#0/1
UWY^¥TabV[[[YV^b¥HH_bb]Y^ZT^JaaTUU`W[U_N
```

```
@HWI-EAS443:3:5:1575:1905#0/1
ATTGAAGCACCGATTTGAGATAATTGAAGGAAGAGACAGA
+HWI-EAS443:3:5:1575:1905#0/1
`ZV[JLPZTWT^a_aWbSbaaU^__^Y_a_P^`¥BBBBBB
@HWI-EAS443:3:91:1696:887#0/1
GAGCGAAAGCAGGCAAACCATTTGAATGGATGTCAATACG
+HWI-EAS443:3:91:1696:887#0/1
a¥IWZ^[^[G^_XT_^``QYZKZ_ZJXVPTRTDMMWYFQS
@HWI-EAS443:3:95:1398:1010#0/1
GCGAGCGAAAGCAGGCAAACCATTTGAATGGATGTCAATC
+HWI-EAS443:3:95:1398:1010#0/1
Z_aT`bbb_¥aa^GWYVb_¥`abbb`]ZW``_]`YZ¥]WF
@HWI-EAS443:3:93:159:1511#0/1
CGGAGCGAAAGCAGGCAAACCATTTGAATGGATGTCAATC
+HWI-EAS443:3:93:159:1511#0/1
ab_ZX]aY]a_a¥aaaaZ[Za_``aa`___`__`]]``]¥
@HWI-EAS443:3:23:976:519#0/1
GAGCGAAAGCAGGCAAACCATTTGAATGGATGTCAATCCG
+HWI-EAS443:3:23:976:519#0/1
]]_Q^`ba]^aaa`]a`_aaaaaa_a]aXYa`]____W[a
@HWI-EAS443:3:45:1503:168#0/1
GTTTGTTTTCCGTCTTCCTTCTTTGGCCCCACATTTGCTT
+HWI-EAS443:3:45:1503:168#0/1
aY]X`_``aSQZNR[_VWV[UVUV]^RWVVRV]]XYYYWW
@HWI-EAS443:3:6:451:1216#0/1
TCTGATGTGTACTTTTTGATTATGGCCATATTGTCCACAC
+HWI-EAS443:3:6:451:1216#0/1
aaX¥U]aZ¥aaZ`Yba^^[`_aaRT^]_TV¥GP[[_BBBB
@HWI-EAS443:3:24:415:16#0/1
ATTACACTGGAGTTCGCTTTCAGTACTATGTTCTTTTCCA
+HWI-EAS443:3:24:415:16#0/1
^`^Y¥``b_GU_aaa^aYW`aaa]aaa]ab[^]Za`^[]¥
@HWI-EAS443:3:12:1789:393#0/1
ACCGGCTTGAAATTCTTGCTGTATCTTGATGTCCCCACAA
+HWI-EAS443:3:12:1789:393#0/1
aS^`^^a`ON[Y^_O_]OXVMVMBBBBBBBBBBBBBBBBB
@HWI-EAS443:3:80:924:423#0/1
ACCGGCTTGAAATTCTTGCTGTATCTTGATGTCCCCACAA
+HWI-EAS443:3:80:924:423#0/1
a`aaaaaa[W¥GX^V]_NTW]SZ_S^_RXVKZSY_[¥]]_
@HWI-EAS443:3:8:1416:996#0/1
GCTATTTCCGGCTTGAAATTCTTGCTGTATCTTGATGTCA
+HWI-EAS443:3:8:1416:996#0/1
`aYa^^_`[[a`Y`^UXDU¥NN^W^]]Z_QGY^¥¥V^LOG
@HWI-EAS443:3:37:1487:1043#0/1
GACTCGGAGTCGACCCTGGCATCAATCCGTGCCCTAGACA
+HWI-EAS443:3:37:1487:1043#0/1
aaaa`b`ab`a_baQZYXMDLNYa`¥VMYDYOG]UYXaY^
@HWI-EAS443:3:1:1182:1880#0/1
ATGACCGAAATTTCTGGAGAGGTGAAAATGGAGGAAGGAC
```

```
+HWI-EAS443:3:1:1182:1880#0/1
a_[UNZa`aa¥[U]aab`_`¥^Nb^Y__`a_^Ga_G`BBB
@HWI-EAS443:3:12:83:1234#0/1
CTTGTCCTTCGTCCATTTTCACCTCTCCAGAAATTTCGGT
+HWI-EAS443:3:12:83:1234#0/1
``_^¥aa]Z__¥^R_P]]`aaWVV¥T¥ZTX^XNXTYUUBB
@HWI-EAS443:3:22:995:775#0/1
CCTTGTCCTTCGTCCATTTTCACCTCTCCAGAAATTTCGG
+HWI-EAS443:3:22:995:775#0/1
abbbb``abbaaa``abbbbaa```_a``_`a_^aaa]`Y
@HWI-EAS443:3:31:683:248#0/1
GTCCATTTTCACCTCTCCAGAAATTTCGGTCATTGATTCC
+HWI-EAS443:3:31:683:248#0/1
aaa``abbaaaaaaaaaa``aa`a`¥^a^]_`_`__]_X
@HWI-EAS443:3:68:1023:1426#0/1
GATCAAACGTGGAATCAATGACCGAAATTTCTGGAGAGGT
+HWI-EAS443:3:68:1023:1426#0/1
_`¥a_R]`__[LW_a]Y[a^aba`_^aaaa`a_]]¥W¥`T
@HWI-EAS443:3:75:231:496#0/1
CAATGACCGAAATTTCTGGAGAGGTGAAAATGGACGAAGG
+HWI-EAS443:3:75:231:496#0/1
^WaaaaZY__Q¥]_]bab]Z`a][L^URXZY___`[]][L
@HWI-EAS443:3:81:1128:1275#0/1
GTCCCCCATCTGATACTAATAGGCCTGCCTTTGATTNGGT
+HWI-EAS443:3:81:1128:1275#0/1
a¥TTFVU`¥]_a¥`X[_`^RaWQOVNFXR]MSXV_VDMRV
@HWI-EAS443:3:37:984:228#0/1
GGGGGCTATACGAAGCAATCGAGGAGTGCATGATTAATGA
+HWI-EAS443:3:37:984:228#0/1
a][HY[]``]`a`a]ZY[[L]]UUXXFX¥PKTRSQNUQTQ
@HWI-EAS443:3:52:261:1975#0/1
GTTCCCTTGATGGTGGCGTACATGCTAGAAAGAGAATTGG
+HWI-EAS443:3:52:261:1975#0/1
a``aY__X]]]``Za]^a]YZ[]__^V¥R]][T¥¥¥W^^V
@HWI-EAS443:3:19:1481:1427#0/1
GCATGTCCCTTGGGTTAAGTGCAACACTTCAATATAAACA
+HWI-EAS443:3:19:1481:1427#0/1
_O]GXI]PXRb_aaMU[T`]a_`ZN^^aZT^[]_``ZYR[
@HWI-EAS443:3:31:803:455#0/1
GTCTCATATACAACAGGATGGGAACAGTGACCACAGAAGC
+HWI-EAS443:3:31:803:455#0/1
`^baaa`[`a_`]ZM]a__¥YR¥a^]]¥aa]¥UW`X[]YS
@HWI-EAS443:3:31:1366:695#0/1
AGGAATTGCCCGCTAATTTCACGGAAACCACTGAATGTCC
+HWI-EAS443:3:31:1366:695#0/1
Y`a¥bb`_ZZ¥ZYHI_V_Y`X_¥W_]a[BBBBBBBBBBBB
@HWI-EAS443:3:30:1254:816#0/1
CCGCATTTCTTCAATTAACCACCTTATCTCCTCAAATTTC
+HWI-EAS443:3:30:1254:816#0/1
XbbZ``¥`bba^_a`bbbaba^¥^`b`aa[ab`ab]bWaa
```

```
@HWI-EAS443:3:31:518:1819#0/1
GCCGCATTTCTTCAATTAACCACCTTATCTCCTCAAATTT
+HWI-EAS443:3:31:518:1819#0/1
a_`Y¥_Y¥T¥YXbba`¥R¥_PZa_X_J_H_Y`_a]ZO[¥Y
@HWI-EAS443:3:40:698:355#0/1
GCCGCATTTCTTCAATTAACCACCTTATCTCCTCAAATTT
+HWI-EAS443:3:40:698:355#0/1
a_TXLQVTLR[XXLNTVQLLW^ZVVVRSTTQ]VYQFOYRT
@HWI-EAS443:3:69:667:176#0/1
GCCGCATTTCTTCAATTAACCACCTTATCTCCTCAAATTT
+HWI-EAS443:3:69:667:176#0/1
aa`aaa`_Ya_aa_a_¥[¥TY^_`^Z^]]]__]^[YY]VV
@HWI-EAS443:3:12:74:1186#0/1
CAGGGCCGAAGGTACCATGCTTCAACCTTTCAACCTTTTC
+HWI-EAS443:3:12:74:1186#0/1
a]YT^aaa[RUTP`_OR]¥DU[Y_[]aW``_^YU¥XZ]¥^
@HWI-EAS443:3:35:1406:736#0/1
GGTACCATGCTTCAACCTTTCAACCTTTTCGAAATAAGTT
+HWI-EAS443:3:35:1406:736#0/1
ababbabbbbbbbbbbbbbbabbbabbbbbbbbbbbaa_b
@HWI-EAS443:3:70:143:175#0/1
TTCAGTCAATGCACCTGCATCCTTTCCAAGAACTGTCATT
+HWI-EAS443:3:70:143:175#0/1
`_XQY[`]JSJZY__VV^¥^^TTVJZFW^UFS^IUVVR]]
@HWI-EAS443:3:27:1570:654#0/1
GCAACATTGCTGGCTGGATCCTGGGAAATCCAGAGTGTGA
+HWI-EAS443:3:27:1570:654#0/1
_H_aaaaaaaaa`W^X`¥W`aXZZaS``_[LSVV_W^VZ[
@HWI-EAS443:3:38:841:280#0/1
GCCCAATCATGACTCGAACAAAGGGGTAACGGCAGGATGT
+HWI-EAS443:3:38:841:280#0/1
aaaaa_ZZ¥]¥^_]aa^U]`_¥]ZDVUBBBBBBBBBBBBB
@HWI-EAS443:3:69:988:446#0/1
GGCCCAATCATGACTCGAACAAAGGGGTAACGGCAGCATG
+HWI-EAS443:3:69:988:446#0/1
abaaa_`babbbb_ab_a`[`baaaH][aba``Z_RBBBB
@HWI-EAS443:3:80:879:124#0/1
CACGCCAGCTGTGACTCTGATGTCAGTATTCTTGCCCCCA
+HWI-EAS443:3:80:879:124#0/1
ababaab`bab`ba^``aa__a¥Uaa[`aa[[aYS]U¥V¥
@HWI-EAS443:3:18:1256:993#0/1
ATCAGGCACTCCTCGATTGCTTCATATAGCCCCCAAAGAT
+HWI-EAS443:3:18:1256:993#0/1
X_[`UW_b_^ZSU_VYO`]aa`VYQ[a__`I`^OF]XV¥I
@HWI-EAS443:3:19:1637:89#0/1
CATTAATCAGGCACTCCTCGATTGCTTCATATAGCCCCCA
+HWI-EAS443:3:19:1637:89#0/1
`a_bKb`b[ab_]U_`^a_]b`WJJ]Z_bV``a^_OIZ^G
@HWI-EAS443:3:22:1042:122#0/1
TGGGGGCTATATGAAGCAATCGAGGAGTGCCTGATTAATG
```

```
+HWI-EAS443:3:22:1042:122#0/1
`bbaab_`aa]``^¥b`aQ`Z`VZYWY__US[a]V`¥]`Y
@HWI-EAS443:3:2:518:443#0/1
TGGGGGCTATATGAAGCAATCGAGGAGTGCCTGATTAATG
+HWI-EAS443:3:2:518:443#0/1
]a`^a[¥Z`b`a]b__J]]UY_]`aKaYbU`U_X[[aaab
@HWI-EAS443:3:37:1495:1644#0/1
TGGGGGCTATATGAAGCAATCGAGGAGTGCCTGATTAATG
+HWI-EAS443:3:37:1495:1644#0/1
ab`_]Z_aabba[b_`Xaa_aaaY[a_U¥H]]`_a_aa`T
@HWI-EAS443:3:50:21:1166#0/1
TGGGGGCTATATGAAGCAATCGAGGAGTGCCTGATTAATG
+HWI-EAS443:3:50:21:1166#0/1
Zbaa^^]`b]S^`]a]aaaaaa`^^^`X_[[S^QZN_`_]
@HWI-EAS443:3:71:303:882#0/1
TGGGGGCTATATGAAGCAATCGAGGAGTGCCTGATTAATG
+HWI-EAS443:3:71:303:882#0/1
aa^^^abb_aZa```_baaaaaaaaY]_`_`aa_a``Y]a
@HWI-EAS443:3:92:1241:1682#0/1
AAATGAGCAGGGGTCAGGATATGCAGCCGACATGAAGAGC
+HWI-EAS443:3:92:1241:1682#0/1
T[_¥X_GL¥HaVXQa^`P`aa`a^aZUMbaGGXX[_¥^^_
@HWI-EAS443:3:74:1460:1351#0/1
GGCAACACCCTTGGCCTTGATATCGAAACAGCGACTCTTG
+HWI-EAS443:3:74:1460:1351#0/1
^]_bbbbaab`^_b_W¥¥`abaaTa``]WbbQDYX`__aa
@HWI-EAS443:3:48:956:1260#0/1
CAGATCTTATGATCTCAGAGAACTCTTCTTTCTTGATCCG
+HWI-EAS443:3:48:956:1260#0/1
Zb[Y_baab^[^bbbaa^Xbbbbbabaa_baabba`b`a^
@HWI-EAS443:3:34:258:1523#0/1
GACGGATCAAGAAAGAAGAGTTCTCTGAGATCATAAGATC
+HWI-EAS443:3:34:258:1523#0/1
abbb`baabbbbaa`abab`_bbaba`a`aa`aaaa¥aa`
@HWI-EAS443:3:94:1737:49#0/1
ACTTGGGTAAACCAGACATATGTTAACATCGGCAACAGCA
+HWI-EAS443:3:94:1737:49#0/1
aX]aYU```a`^_`a`_Y_aa`^_aXP`_¥]Z[^YFUDHZ
@HWI-EAS443:3:32:36:614#0/1
TAGTATTCTCACAAGAGGATTGCATGATCAAGGCAGTTAG
+HWI-EAS443:3:32:36:614#0/1
aab_aaababaaaaa_a]]`_aaaa`^^^`Z`^]^_OXVX
@HWI-EAS443:3:92:645:794#0/1
CAAGTGGCACACACTAGACCAAAAGTAGCTTCTGTGGTCA
+HWI-EAS443:3:92:645:794#0/1
_¥_`]^__a`][VXaaZRF¥a``Z_^abb`_Z`Z¥^]Y¥_
@HWI-EAS443:3:18:41:1718#0/1
AACCGGTTCATTGAAATTGGAGTAACACGGAGGGGAACAT
+HWI-EAS443:3:18:41:1718#0/1
`aaa¥UP[]]UU]XGR^```^`UY____`Z[]UR[TWT^Z
```

```
@HWI-EAS443:3:22:284:1737#0/1
TGGCCGGGAGACAAAATAACATTCGAAGCAACTGGAAATC
+HWI-EAS443:3:22:284:1737#0/1
a^_bbaa`aaaaab`babbab`aaaaaYaaaa¥_a`a__¥
@HWI-EAS443:3:82:1231:2032#0/1
CGGCCGGGAGACAAAATAACATTCGAAGCAACTGGAAATC
+HWI-EAS443:3:82:1231:2032#0/1
ab__V]b`_`[]bbb`abbabb__`aa__ab_b`_aaaaa
@HWI-EAS443:3:23:1498:444#0/1
GGCCGGGAGACAAAATAACATTCGAAGCAACTGGAAATCT
+HWI-EAS443:3:23:1498:444#0/1
__^_b]babbababbabbbaaaaaa`b]aa_`a`aaa`Na
@HWI-EAS443:3:62:1690:1820#0/1
GGCCGGGAGACAAAATAACATTCGAAGCAACTGGAAATCT
+HWI-EAS443:3:62:1690:1820#0/1
abaaabb`^b`aabbbba_bba^aa`b]aa`bbaa_`baa
@HWI-EAS443:3:77:1468:330#0/1
CGAAAATCGAAATTAACAAGTTTGCTGCAATATGCACGTT
+HWI-EAS443:3:77:1468:330#0/1
`b``ba_bbPbbKbb`X_V`^b_YaWbb`bbbba_¥``Xa
@HWI-EAS443:3:30:1747:1935#0/1
ACACGTTCCATTGTCTGAACTAGTTGTTTCTACAATGTAG
+HWI-EAS443:3:30:1747:1935#0/1
`]_YWSX`¥Z]WORZV¥OTJYW[UX_BBBBBBBBBBBBBB
@HWI-EAS443:3:25:103:391#0/1
CTGTAATGAGAATGGGAGACCTTCACTACCTCCAGAGCAG
+HWI-EAS443:3:25:103:391#0/1
abb`_V`bbb_R_b`¥_ba`aaabbbba¥_^`a`a`a```
@HWI-EAS443:3:46:881:1494#0/1
GTCTCTGAAAATATACAGAGATTCGCTTGGAGAAACTGTA
+HWI-EAS443:3:46:881:1494#0/1
a]a[_aa^XNL¥]a]``Z_^^_[X¥XZ[]]¥`^^¥U]`¥Z
@HWI-EAS443:3:73:807:1869#0/1
CTGTAATGAGAATGGGAGACCTTCACTACCTCCAGAGCAG
+HWI-EAS443:3:73:807:1869#0/1
aa_YF]aaaa^a``___a`aa___a]N_a_``a__^^`__
@HWI-EAS443:3:35:1218:238#0/1
GCCTGCGGATGCCTTTTGTTGATTGGTATTCCCTCCACTC
+HWI-EAS443:3:35:1218:238#0/1
]`a¥¥]a¥Za_ZZb]V^^Z_```]UZYZZ_a^¥ZPW]WKa
@HWI-EAS443:3:96:933:248#0/1
GGAATGCTCCTGTTATCCTGATTCTAGTGAAATTACATGT
+HWI-EAS443:3:96:933:248#0/1
aaaaaaWa`a^aX¥aaa`[]R¥`O`Z^Y]U¥V`_^Z][]O
@HWI-EAS443:3:55:1512:1275#0/1
ACTAATTGATGGCCATCCGAACTCTTTTGGTCGCTGTCTG
+HWI-EAS443:3:55:1512:1275#0/1
`_aa][R¥a`baaZ`¥[¥_]_WSQW``a__[UVUa_]W¥[
@HWI-EAS443:3:68:1174:1527#0/1
ATTCGACACTAATTGATGGCCATCCGAACTCTTTTGGTCG
```

```
+HWI-EAS443:3:68:1174:1527#0/1
aba_R¥¥aba`aa`X``_XDZaa`W``aJ`X``aaaR[¥[
@HWI-EAS443:3:54:350:951#0/1
GAAGGTCGGACATCCGACATGCGAACAGAAGTTGCAGTGA
+HWI-EAS443:3:54:350:951#0/1
U`_WTL]PUPa`]¥_^]SM`XZaVVQGWXWSJQ`[J_N_M
@HWI-EAS443:3:38:1232:1048#0/1
CACCTTCATTGTTCCCGCTGAATGCTGCCATAACGGTTGC
+HWI-EAS443:3:38:1232:1048#0/1
_¥_S[[abb^_GOQ]_YabaaUabS_Z`_ZHZ[```[a[D
@HWI-EAS443:3:91:418:698#0/1
GAGGAAATACCAATCAACAAAAGGCATCTGCAGGCCAGAT
+HWI-EAS443:3:91:418:698#0/1
^Z¥`_Y¥Z]__]___`[R[V`_¥VY^¥UUUTUVRSUY¥WW
@HWI-EAS443:3:71:205:956#0/1
CGTTGGGCCATTCCTATTCCACCATGTTACGGCCAGAGGT
+HWI-EAS443:3:71:205:956#0/1
aa``aa`aa^aa`aaa`aaaaa`^^a¥^aa^^a^¥¥^^VM
@HWI-EAS443:3:40:523:966#0/1
TTCCTGGCGATGATTACATATATTACCAGAAATCACCACG
+HWI-EAS443:3:40:523:966#0/1
XS¥¥_¥`XZWY^__[OWYaWX^¥^XEYYDRX__`TIUH]T
@HWI-EAS443:3:89:262:1330#0/1
CATATATTACCAGAAATCAACCCGAGTGGTTCAGACTGAA
+HWI-EAS443:3:89:262:1330#0/1
aaaaab]a``aa¥^aaa_¥]aa]___V^[Y[_a_```___
@HWI-EAS443:3:25:1602:1433#0/1
GAACCACCCGGGTTGATTTCTGGTAATATATGTAATCATC
+HWI-EAS443:3:25:1602:1433#0/1
a_ZH[_aDP[S¥EM[IVT^I`_BBBBBBBBBBBBBBBBBB
@HWI-EAS443:3:50:766:1438#0/1
TATTACCAGAAATCAACCCGAGTGGTTCAGAAAAATCCTG
+HWI-EAS443:3:50:766:1438#0/1
`bab_¥bba`abbb_ba_^b^]`aa^^a]_IU¥F]GQ^J^
@HWI-EAS443:3:74:520:1538#0/1
TATATTACCATAAATCAACCCGAGTGGTTCAGAAACATCC
+HWI-EAS443:3:74:520:1538#0/1
YXQT_aY`Z¥HP[_Sa¥Z^abaWTS^a`ab`_aZ_bb`aa
@HWI-EAS443:3:90:1251:1372#0/1
CTGAACCACTCGGGTTGATTTCTGGTAATATATGTGATCA
+HWI-EAS443:3:90:1251:1372#0/1
`b``b``aba^`b_F_bb¥bb`bba]``aaaabaXSab`]
@HWI-EAS443:3:3:766:705#0/1
GTCGGGTTGATTTCTGGTAATATATGTAATCATCGCCAGG
+HWI-EAS443:3:3:766:705#0/1
a_]]_aXaa^aaa]aaa]`aa`aa```a_`¥a_^a[X_aY
@HWI-EAS443:3:13:1186:684#0/1
ATGTTCCTGGCGATGATTACATATATTACCAGAAATCAAC
+HWI-EAS443:3:13:1186:684#0/1
a^aWX¥`^aV]a___^`¥ZX`__^_``^ZW_O]_^_]`]W
```

```
@HWI-EAS443:3:13:840:1347#0/1
GAACCACTCGGGTTGATTTCTGGTAATATATGTAATCATC
+HWI-EAS443:3:13:840:1347#0/1
a`bb`baabaaa_baababaabb_aaaaababaaaa^aaY
@HWI-EAS443:3:1:954:391#0/1
TCCTCGAATGTTCCTGGCGATGATTACATATATTACCAGA
+HWI-EAS443:3:1:954:391#0/1
abb¥]_`bbaaa`[`a_Ra^S`]^^`_[`]baa_¥OYaBB
@HWI-EAS443:3:22:1216:1924#0/1
CTGGTAATATATGTAATCATCGCCAGGAACATTCGAGGAT
+HWI-EAS443:3:22:1216:1924#0/1
abba^bbbbbbbbabaabbaaaa_a_`aa`a___]aa__a
@HWI-EAS443:3:23:792:845#0/1
CAAAATCCTCGAATGTTCCTGGCGATGATTACATATATTA
+HWI-EAS443:3:23:792:845#0/1
_ZLZ[abbaS`ab]_Q^^UQ_aaZaU[¥]YWKFNX]`]__
@HWI-EAS443:3:24:1251:1428#0/1
ATATTACCAGAAATCAACCCGAGTGGTTCAGAAACATCCT
+HWI-EAS443:3:24:1251:1428#0/1
aabaa`T[baab___a_[`VaW_U`_Y_]`Y[^_U_^YR`
@HWI-EAS443:3:25:1336:1643#0/1
AGGATGTTTCTGAACCACTCGGGTTGATTTCTGGTAATAT
+HWI-EAS443:3:25:1336:1643#0/1
^^_a^[Z_bZ``_aa_VP`¥``[Qaa``a^Taba¥^_a_a
@HWI-EAS443:3:2:176:1652#0/1
GTTTCTGAACCACTCGGGTTGATTTCTGGTAATATATGTA
+HWI-EAS443:3:2:176:1652#0/1
aQWYX^_^ZI¥¥_Y]a`aGSa][Z^V_a¥OJ¥XY]__¥ZX
@HWI-EAS443:3:30:107:403#0/1
GATTACATATATTACCAGAAATCAACCCGAGTGGTTCAGA
+HWI-EAS443:3:30:107:403#0/1
a`aa_`]aaaaaaaa`aaa^Q_`aa_`a__`S`_]_^`¥Q
@HWI-EAS443:3:33:1176:1390#0/1
CAGGATGTTTCTGAACCACTCGGGTTGATTTCTGGTAATA
+HWI-EAS443:3:33:1176:1390#0/1
a`]__]^]aaa_V_aXaa```^STUXVV_`^]YYOY¥]_¥
@HWI-EAS443:3:33:297:965#0/1
CTCGAATGTTCCTGGCGATGATTACATATATTACCAGAAA
+HWI-EAS443:3:33:297:965#0/1
aab`W`T^N]aa^___``Z__]¥W¥QW^YWZZWVU[UW¥W
@HWI-EAS443:3:33:836:403#0/1
GGTAATATATGTAATCATCGCCAGGAACATTCGAGGATTT
+HWI-EAS443:3:33:836:403#0/1
_Z[`^`¥_`[XW¥Z__a``^]a]VUSU¥X]QWW¥¥TYXRQ
@HWI-EAS443:3:36:897:1391#0/1
ATGTTTCTGAACCACTCGGGTTGATTTCTGGTAATATATG
+HWI-EAS443:3:36:897:1391#0/1
`^_W^bb_Z___bbbba¥¥¥Z_aaa^`[```Y`[^a`aa`
@HWI-EAS443:3:38:1217:1408#0/1
CAAAATCCTCGAATGTTCCTGGCGATGATTACATATATTA
```

```
+HWI-EAS443:3:38:1217:1408#0/1
ababbabaaba`aabaaab``^aa`_a`a`]Y^`__`_a¥
@HWI-EAS443:3:3:609:1830#0/1
AACCACTCGGGTTGATTTCTGGTAATATATGTAATCATCG
+HWI-EAS443:3:3:609:1830#0/1
`a]^_Y`¥aaa[^__Y_a``]_Z`Yaa_a]`^`Z_Ua^^_
@HWI-EAS443:3:40:740:127#0/1
ATGTTCCTGGCGATGATTACATATATTACCAGAAATCAAC
+HWI-EAS443:3:40:740:127#0/1
a_aYZM^_XZY^`_][[__¥ZZVYZUHX^[[SYJPTXYPN
@HWI-EAS443:3:43:276:188#0/1
ATATTACCAGAAATCAACCCGAGTGGTTCAGAAACATCCT
+HWI-EAS443:3:43:276:188#0/1
abbbaaababbbaaaaba_`baa_aa`^X`a^¥a``_]_^
@HWI-EAS443:3:44:298:430#0/1
CAAAATCCTCGAATGTTCCTGGCGATGATTACATATATTA
+HWI-EAS443:3:44:298:430#0/1
aababXaaa_Z¥a_b_aa]_a[¥ZW¥a`X]HLZNXUUZ_W
@HWI-EAS443:3:45:1434:944#0/1
CTGAACCACTCGGGTTGATTTCTGGTAATATATGTAATCA
+HWI-EAS443:3:45:1434:944#0/1
`¥ZW¥^[^`ZZ__[EY_V¥_][^`ZOWWZX_`]_X]X¥W_
@HWI-EAS443:3:45:1604:493#0/1
CACTCGGGTTGATTTCTGGTAATATATGTAATCATCGCCA
+HWI-EAS443:3:45:1604:493#0/1
`Z¥OR``bSa`[¥_X]aba^``a]aa`aa`_a`]`YaTM¥
@HWI-EAS443:3:47:118:1278#0/1
CTCGGGTTGATTTCTGGTAATATATGTAATCATCGCCAGG
+HWI-EAS443:3:47:118:1278#0/1
abaabb_bbabaaab^b_aabbaaab^bbaaaaaa`aa^a
@HWI-EAS443:3:47:998:820#0/1
CTGGCGATGATTACATATATTACCAGAAATCAACCCGAGT
+HWI-EAS443:3:47:998:820#0/1
a_¥]X``^b`ab`^¥babaaaaa`b]W`]_^`a]UXa¥XL
@HWI-EAS443:3:53:389:23#0/1
GGATGTTTCTGAACCACTCGGGTTGATTTCTGGTAATATA
+HWI-EAS443:3:53:389:23#0/1
a_U¥^W__V`UY]QUX[¥Z]a`X^]TZ_]S]Y_WSU[]]X
@HWI-EAS443:3:56:1760:950#0/1
GAACCACTCGGGTTGATTTCTGGTAATATATGTAATCATC
+HWI-EAS443:3:56:1760:950#0/1
aaa`_aaa`aaa]a¥U^aa^a^ZV__`_a`a_`__[O[]V
@HWI-EAS443:3:58:1208:631#0/1
GAACCACTCGGGTTGATTTCTGGTAATATATGTAATCATC
+HWI-EAS443:3:58:1208:631#0/1
aaba_a``aaaa^aa_aaa_aaa__aa`aaaaa``a_``[
@HWI-EAS443:3:61:1335:695#0/1
CTCGGGTTGATTTCTGGTAATATATGTAATCATCGCCAGG
+HWI-EAS443:3:61:1335:695#0/1
abbb`bKbbbabbbbbbaabbbbbbbbaabbabbbaXa`a
```

```
@HWI-EAS443:3:63:898:357#0/1
CATATATTACCAGAAATCAACCCGAGTGGTTCAGAAACAT
+HWI-EAS443:3:63:898:357#0/1
abbaabaaa__baaaaaaa```aa_aZa_^_^a_]``YaT
@HWI-EAS443:3:68:580:294#0/1
GAACCACTCGGGTTGATTTCTGGTAATATATGTAATCATC
+HWI-EAS443:3:68:580:294#0/1
_^a``a]]aaa_U_a^aa`_aa`U`__a_a`a^`_]W__`
@HWI-EAS443:3:6:198:420#0/1
GGATGTTTCTGAACCACTCGGGTTGATTTCTGGTAATATA
+HWI-EAS443:3:6:198:420#0/1
aa``aab]aa^^bIaL[Waaa_N_[VS`_¥a`aN¥_`___
@HWI-EAS443:3:71:106:1500#0/1
CAGGATGTTTCTGAACCACTCGGGTTGATTTCTGGTAATA
+HWI-EAS443:3:71:106:1500#0/1
abbaa]a`^``¥]]aab_a_`[_`M_]^^``_a]_Y`_^Y
@HWI-EAS443:3:71:36:634#0/1
CAAAATCCTCGAATGTTCCTGGCGATGATTACATATATTA
+HWI-EAS443:3:71:36:634#0/1
aaa]UYab[ba`Q^_Y_aa__`_]_][SY¥]^M¥^^_][Q
@HWI-EAS443:3:72:1784:123#0/1
GTTCCTGGCGATGATTACATATATTACCAGAAATCAACCC
+HWI-EAS443:3:72:1784:123#0/1
aaaW_aba^b`^aO^_S]^]^_Wa`UGX^`VSW_X¥ZWSX
@HWI-EAS443:3:72:524:225#0/1
TCTGGTAATATATGTAATCATCGCCAGGAACATTCGAGGA
+HWI-EAS443:3:72:524:225#0/1
a`aaa^`_aaa_aa_aa``a_]`Y[_`_^_]_`^Z`^`^Z
@HWI-EAS443:3:79:277:1353#0/1
TGTTTCTGAACCACTCGGGTTGATTTCTGGTAATATATGT
+HWI-EAS443:3:79:277:1353#0/1
UN[Za_^V^^`]]_Y¥¥[U¥VQ]UZX`^ZXOR^Z^a_¥YL
@HWI-EAS443:3:7:1375:86#0/1
TCAAAATCCTCGAATGTTCCTGGCGATGATTACATATATT
+HWI-EAS443:3:7:1375:86#0/1
XY^`Y_baa_Yb_a^``aXY`aUPa¥X]R_`]UXFNZ[_X
@HWI-EAS443:3:7:1691:1357#0/1
CATATATTACCAGAAATCAACCCGAGTGGTTCAGAAACAT
+HWI-EAS443:3:7:1691:1357#0/1
a`a^aaaba_`baaaaaaaa`XUa_a]a_T_Y`_^^_X_`
@HWI-EAS443:3:7:922:1444#0/1
CGAATGTTCCTGGCGATGATTACATATATTACCAGAAATC
+HWI-EAS443:3:7:922:1444#0/1
`bbbabab``aaaab]aaXZ``W_a`_aaa[MSa`_^`__
@HWI-EAS443:3:82:1780:396#0/1
CAGGATGTTTCTGAACCACTCGGGTTGATTTCTGGTAATA
+HWI-EAS443:3:82:1780:396#0/1
Za_`bbbbbbbbbbba¥a^```bb[`a`bbbbbbS]^abb
@HWI-EAS443:3:84:400:1372#0/1
CTGGCGATGATTACATATATTACCAGAAATCAACCCGAGT
```

```
+HWI-EAS443:3:84:400:1372#0/1
aaa`aaa_a`a_``a_]_aa_a``a_`a`_]]a^`_`^]Y
@HWI-EAS443:3:84:468:512#0/1
CTGGTAATATATGTAATCATCGCCAGGAACATTCGAGGAT
+HWI-EAS443:3:84:468:512#0/1
abb`^abbbbabbabbabbabab`bba`aabaa`a`a`_a
@HWI-EAS443:3:90:43:2018#0/1
ATATATTACCAGAAATCAACCCGAGTGGTTCAGAAACATC
+HWI-EAS443:3:90:43:2018#0/1
a[[]W^_]aa`]a``a_b^a_a`¥^O¥SU[¥[^XTZ[YT_
@HWI-EAS443:3:92:1144:448#0/1
CAGGATGTTTCTGAACCACTCGGGTTGATTTCTGGTAATA
+HWI-EAS443:3:92:1144:448#0/1
abbaabbabaabbaaa_a`aabba¥U^`aab_aba_a`aa
@HWI-EAS443:3:93:1402:1954#0/1
GAACCACTCGGGTTGATTTCTGGTAATATATGTAATCATC
+HWI-EAS443:3:93:1402:1954#0/1
aZa]ab_b¥bbaIaaa_abXb¥bWb_aaa¥ab_aaYY_Ja
@HWI-EAS443:3:94:462:1959#0/1
CAAAATCCTCGAATGTTCCTGGCGATGATTACATATATTA
+HWI-EAS443:3:94:462:1959#0/1
a¥ZZ¥UFWUa]]RU[VQZ`Y`VZ[V[WSTQMMSUa`^WWV
@HWI-EAS443:3:97:467:441#0/1
CAGGATGTTTCTGAACCACTCGGGTTGATTTCTGGTAATA
+HWI-EAS443:3:97:467:441#0/1
aabb`babbb`baabaaaaa_abbaababaa`aab^aaaa
@HWI-EAS443:3:24:875:343#0/1
TCGTCCTTCATTGTTCCCGCTGAATGCTGCCATAACGGTT
+HWI-EAS443:3:24:875:343#0/1
aabaaaaaaaaab`aaa`]^_^]^_`^_`^`a``aW`_Q_
@HWI-EAS443:3:84:893:464#0/1
TCGTCCTTCATTGTTCCCGCTGAATGCTGCCATAACGGTT
+HWI-EAS443:3:84:893:464#0/1
``b`a^aa`aa_aaa`_T[Xab``a`VVYZT`a¥^X¥]YX
@HWI-EAS443:3:50:1236:1523#0/1
AGTAGTGCTAGCCAGCACCATTCTGTTTTCATGCCTGATT
+HWI-EAS443:3:50:1236:1523#0/1
Q¥YWaU_H__bW^`b`NaUSb_Sa`^^Y`OHJYM]``[aG
@HWI-EAS443:3:83:1099:1376#0/1
TCTTGATGTCCCTACAAAATCATATGCATCTGCATTCTGA
+HWI-EAS443:3:83:1099:1376#0/1
a`bba¥``_^`¥a_^^aa^a`a```^_a_`_]^]_`^``]
@HWI-EAS443:3:18:325:159#0/1
CCGGCTTGAACTTCTTGCTGTATCTTGATGTCCCTACAAA
+HWI-EAS443:3:18:325:159#0/1
ZS¥]YLXYROLMXV¥]][HKXSXX¥^VOSZSSUYFJOLV[
@HWI-EAS443:3:4:1016:1382#0/1
CAAAAATGCAGTTGGGGTCCTCATCGGAGGACTTAAATGG
+HWI-EAS443:3:4:1016:1382#0/1
^Z¥aQX_b]Ia``Yabaa_``a_ZOaa`aZ]^aaZ`a]a]
```

```
@HWI-EAS443:3:97:347:1255#0/1
ATCGGAGGACTTAAATGGAATGGTAACACGGTTCGAGTCT
+HWI-EAS443:3:97:347:1255#0/1
a_aa`__a][`a`_a_a`______a_¥`W_]W^¥_^_X]^
@HWI-EAS443:3:32:713:170#0/1
AGAGAACTCATCCTTTATGACAAAGAAGAAACAAGGAAAT
+HWI-EAS443:3:32:713:170#0/1
aaa__aa]a`^_Y^__a^a__`WY_`a¥___¥a`aXTWQR
@HWI-EAS443:3:67:1649:1200#0/1
GAATAATGAGAAGCAATTTTCTCGATTCAGCCGAAAACCC
+HWI-EAS443:3:67:1649:1200#0/1
^a`ZXbb^OaS_W`bRUaTaUaZ]Xa[`Ta__`[T_[``^
@HWI-EAS443:3:58:735:1199#0/1
GGCGGGGGAGGTATATCTTTCGCTCCGAGTCGGCATTTTG
+HWI-EAS443:3:58:735:1199#0/1
``^a`a`YXV_K¥_Z¥`]``X^VXX¥Y]]PV[XQX]^^`S
@HWI-EAS443:3:82:818:1912#0/1
GGGGGAGGTATATCTTTCGCTCCGAGTCGGCATTTTGGGC
+HWI-EAS443:3:82:818:1912#0/1
``_`__[_Xa__a¥^`]]V¥¥a]``]¥`O¥`¥`¥_VKPP¥
@HWI-EAS443:3:97:1691:1759#0/1
CGGGTGTCTGACAAGTTGTATTGCAATCGTGGACTGGTGT
+HWI-EAS443:3:97:1691:1759#0/1
Kb```_bbbbW``]bbab`bbbbbbab_b^b`aaaba`ba
@HWI-EAS443:3:20:1770:723#0/1
ATCTTTCGCTCCGAGTCGGCATTTTGGGCCGCCGGGGTAT
+HWI-EAS443:3:20:1770:723#0/1
a^Ra`¥¥]a]`¥`U¥Z¥`ZZZ^[]^GV¥QMQRRPUZBBBB
@HWI-EAS443:3:70:280:1602#0/1
TATCTTTCGCTCCGAGTCGGCATTTTGGGCCGCCGGGTTA
+HWI-EAS443:3:70:280:1602#0/1
^W__R_a___]^_X^R¥b``]T]``a[GJ`_¥`ZXVQTM`
@HWI-EAS443:3:80:488:938#0/1
CCCTGGGTCTTGCTTAATGCATCTTGGTTCAACTCCTTCC
+HWI-EAS443:3:80:488:938#0/1
abaaaaa]aW`WZ`S^`Z[]_Uaaa]`]`_a]``a__`^^
@HWI-EAS443:3:15:244:1307#0/1
AGTGCTGGGAAGGACCCTAAGAAAACAGGAGGGAGATGCA
+HWI-EAS443:3:15:244:1307#0/1
abWa^]ba``aaa`a`aabaa`a_`aaa_abaa`aa^_`a
@HWI-EAS443:3:36:1119:1908#0/1
GGGCCCTCCTGTTTTCTTAGGGTCCTTCCCAGCACTGGGA
+HWI-EAS443:3:36:1119:1908#0/1
`aabbbbabbbabbbbbbbba``baaaaaabaaaaaaa_a
@HWI-EAS443:3:83:38:1927#0/1
GCCCTCCTGTTTTCTTAGGGTCCTTCCCAGCACTGGGATG
+HWI-EAS443:3:83:38:1927#0/1
YK`_X`_R]TYXa]ZR_Wa]Ya``Z]_^`[`Ya[XWS]V]
@HWI-EAS443:3:86:1245:974#0/1
ACGCTGTCCTGAGAGGATTTCTCATTTTGGGCAAAGAAGA
```

```
+HWI-EAS443:3:86:1245:974#0/1
aab`Z]WOW_¥Jaa[`W^]a]`S^a`]Z_aaRX]_`aa`]
@HWI-EAS443:3:61:1161:424#0/1
AAAGAGTTCAACCACCTGGAAAAAAGAATAGAGAACTTAA
+HWI-EAS443:3:61:1161:424#0/1
aaa^`b^a`aa][`^aaba`WKV¥_a_`_`a[`^_X_`]Q
@HWI-EAS443:3:60:1048:679#0/1
GGGCCATCCTAGCTCCAGTGCTGGTCTGAAAGATGACCTT
+HWI-EAS443:3:60:1048:679#0/1
aZX`aaW^aYSTT_HWX¥Xa¥[a[SJUaTX[]TLNQP^NS
@HWI-EAS443:3:67:246:1199#0/1
CGGGCCCCTCCAATCTGTGTGCTGTGGCACATTTCCAAGA
+HWI-EAS443:3:67:246:1199#0/1
a`XV_baaaaa`[aaaaU[[a^]X^`¥¥]V]__^^`a_^V
@HWI-EAS443:3:63:1124:1998#0/1
GATTTGTCCTTCCAGGGGCGGGGAGTTTTCGAGGTCTTGG
+HWI-EAS443:3:63:1124:1998#0/1
abaaaYaa^^^`]`Z¥``b]a¥BBBBBBBBBBBBBBBBBB
@HWI-EAS443:3:80:44:1371#0/1
GGGGTGGGGAGTTTTCGAGCTCTCGGACGAAAAGGCAACG
+HWI-EAS443:3:80:44:1371#0/1
__`¥S_^[`^_Waa```a^_`_¥`¥`_`a¥aI[`T¥¥a`[
@HWI-EAS443:3:52:452:1600#0/1
GGGATCGGGGTTGAGGATACTGGTAAGAGGCAATTCTCCA
+HWI-EAS443:3:52:452:1600#0/1
aab_aaaaaa_abab`^``XaX`^Z^aZ_aWNX]ZJV^[]
@HWI-EAS443:3:62:1643:506#0/1
AGCCCCGCACTCGCGAGATACTCACTAAGACCACTGTGGA
+HWI-EAS443:3:62:1643:506#0/1
][FT[``bY__`Ra_^Y_Z[_]X_U_N]___Y`SPZ¥X`U
@HWI-EAS443:3:37:168:464#0/1
ATGCCACAGAAATCAGAGCATCTGTCGGCAGAATGATTGG
+HWI-EAS443:3:37:168:464#0/1
_`a_a_`b¥a¥RWaaa]aa`a`a_Y^][FQRYLY^Y¥VUW
@HWI-EAS443:3:94:271:606#0/1
TAATCACTCAATGAGTGACATCGAAGCCATGGCGACTCAA
+HWI-EAS443:3:94:271:606#0/1
`aa_[```_¥U]a]_OX^]¥SRMPWJ^^¥]`^XYFVNQTY
@HWI-EAS443:3:42:966:602#0/1
AGTAGAAATGAAAAGTGGCGAGAGCAATTGGGACAGAAAT
+HWI-EAS443:3:42:966:602#0/1
_aP^`¥abbbbababababaab¥`[`¥a]`a_aaa_aaaaa`
@HWI-EAS443:3:94:632:1623#0/1
CAGAAGTGGAGGAAATACCAATCAACAAAAGGCATCCGCA
+HWI-EAS443:3:94:632:1623#0/1
a`[^]¥Xa^__]¥`aaaY```_aa__``^]¥___^]___`
@HWI-EAS443:3:15:1368:816#0/1
ATCCCTTTTGAAAGAGCAACNGTTATGGCAGCATTTAGCG
+HWI-EAS443:3:15:1368:816#0/1
¥¥`]¥a`b`baaa`¥b^aaWD^aa`aaaZ`]IMLWFQKKH
```

```
@HWI-EAS443:3:81:107:1213#0/1
GGGGGCGATCTGAACTTTGTCAATAGGGCAAATCAGCGAC
+HWI-EAS443:3:81:107:1213#0/1
aaa`ab[_ZVTNX[ZX^]¥T`]X[XX`_^ZTRUUTT][UM
@HWI-EAS443:3:2:1190:688#0/1
CATTCCTGAAGTCTGCTTAAAATGGGAACTAATAAATGTT
+HWI-EAS443:3:2:1190:688#0/1
aa_¥¥[]TUQ]XRTXQ¥[[Ya]a``___Y^N`_ZU`Y_YT
@HWI-EAS443:3:33:1591:1202#0/1
CGCCGTCTGAGTTCTTCAATGGTGGAACAGATATTCATGA
+HWI-EAS443:3:33:1591:1202#0/1
]SaaXHV`¥_a`]¥`a_OU]`]Ua^Y¥aa_WUPWOSSSZ^
@HWI-EAS443:3:53:254:1285#0/1
CGCCGTCTGAGTTCTTCAATGGTGGAACAAATCTTCATGA
+HWI-EAS443:3:53:254:1285#0/1
ababbabbbabaaabaabaaa``a`aa`^W^aaabaaaaa
@HWI-EAS443:3:16:950:1813#0/1
CGCCGTCTGAGTTCTTCAATGGTGGAACAGATCTTCATGA
+HWI-EAS443:3:16:950:1813#0/1
aaUJXX^^a_X]Za^[`]UR]RN[VW]Y]¥XSH¥W[¥]¥W
@HWI-EAS443:3:22:1336:882#0/1
CGCCGTCTGAGTTCTTCAATGGTGGAACAGATCTTCATGA
+HWI-EAS443:3:22:1336:882#0/1
aba```_ab^a[aba^abaa_YS[a_a`_^^^_a`_a_`_
@HWI-EAS443:3:30:43:863#0/1
CGCCGTCTGAGTTCTTCAATGGTGGAACAGATCTTCATGA
+HWI-EAS443:3:30:43:863#0/1
a^ZaZYaa_L`^`a_`a]TWXYZ[ST[¥¥TN¥_[[S¥Y]_
@HWI-EAS443:3:40:1444:262#0/1
CGCCGTCTGAGTTCTTCAATGGTGGAACAGATCTTCATGA
+HWI-EAS443:3:40:1444:262#0/1
abbbbbbbbbbabbbbbbbbbb`ba_Z_ab_a_aaaaaa`
@HWI-EAS443:3:42:1656:1068#0/1
TCGCCGTCTGAGTTCTTCAATGGTGGAACAGATCTTCATG
+HWI-EAS443:3:42:1656:1068#0/1
aa`_]``^aaa`^a`aa`a`aaaWaaaa``a^`[_aaa_]
@HWI-EAS443:3:47:745:709#0/1
CGCCGTCTGAGTTCTTCAATGGTGGAACAGATCTTCATGA
+HWI-EAS443:3:47:745:709#0/1
aba_`^baaYb`a`abaaaaaa^Y`aa`a__^_aa`a_]`
@HWI-EAS443:3:48:320:553#0/1
CGCCGTCTGAGTTCTTCAATGGTGGAACAGATCTTCATGA
+HWI-EAS443:3:48:320:553#0/1
Z`babbabaaabbbbbbbbbb`Tbbbbabbaba`^¥bbbb
@HWI-EAS443:3:50:367:549#0/1
CGCCGTCTGAGTTCTTCAATGGTGGAACAGATCTTCATGA
+HWI-EAS443:3:50:367:549#0/1
`b¥_a^baa^a`bbbaabaa¥^]ab`bW_`ab`a``baa`
@HWI-EAS443:3:62:958:1723#0/1
CGCCGTCTGAGTTCTTCAATGGTGGAACAGATCTTCATGA
```

```
+HWI-EAS443:3:62:958:1723#0/1
`aaaa^bbaaa__abaabaaa[^a`¥aaaa`_a`aZa`^a
@HWI-EAS443:3:78:521:141#0/1
CGCCGTCTGAGTTCTTCAATGGTGGAACAGATCTTCATGA
+HWI-EAS443:3:78:521:141#0/1
abaab`aa``b`a]a`a```a`]_a____`^^[____Z^[
@HWI-EAS443:3:84:644:624#0/1
CGCCGTCTGAGTTCTTCAATGGTGGAACAGATCTTCATGA
+HWI-EAS443:3:84:644:624#0/1
aabbbababaa_aaaa`aaa`_]`_`aaba_aaaa^a``_
@HWI-EAS443:3:95:990:160#0/1
CGCCGTCTGAGTTCTTCAATGGTGGAACAGATCTTCATGA
+HWI-EAS443:3:95:990:160#0/1
abb]aabab`b_b_babbbabb_bbabababaaa^bb_aaba
@HWI-EAS443:3:96:38:1457#0/1
CGCCGTCTGAGTTCTTCAATGGTGGAACAGATCTTCATGA
+HWI-EAS443:3:96:38:1457#0/1
`_bbbbb`bb¥HZ_`Z`_Z_bbX^a¥R`_]PYaaaaa_^H
@HWI-EAS443:3:31:1094:2036#0/1
GCCGGTTGATTTCTGCTTAATGTCCAATCATAAGTCTGGC
+HWI-EAS443:3:31:1094:2036#0/1
]`_P_Vb``^Xa^a`S`Y^abb``]`_^P_ab`PZ_aZ_`
@HWI-EAS443:3:97:732:272#0/1
TGCCGGTTGATTTCTGCTTAATGTCCAATCATAAGTCTGG
+HWI-EAS443:3:97:732:272#0/1
a`a[RW[Z__¥a_ZH_ZS[PP¥ZR`^PX[]`¥TVSVR]_B
@HWI-EAS443:3:25:219:1113#0/1
AGAATTTGGCGCCAAGCAAACAATGGCGAAGATGCAACAG
+HWI-EAS443:3:25:219:1113#0/1
aaabaaaaabaa_`a_`aaa]_a¥¥^____¥¥XR__]W_[
@HWI-EAS443:3:3:1416:1401#0/1
TGCTGTTGCATCTTCGCCATTGTTTGCTTGGCGCCAAATT
+HWI-EAS443:3:3:1416:1401#0/1
`bbbaa`[``b__Y¥VORGUaNUa``]X[HYZY_OLV]BB
@HWI-EAS443:3:21:259:172#0/1
CGATGCTGGATCAGACCGAGTGATGGTATCACCTCTGGCG
+HWI-EAS443:3:21:259:172#0/1
[a¥_`ab`_IT]_¥aaa_]_Y^BBBBBBBBBBBBBBBBBB
@HWI-EAS443:3:98:312:1703#0/1
GGGCAGCAATAGGGTTGAGGATTAGCTCATCTTTCAGTTT
+HWI-EAS443:3:98:312:1703#0/1
]_Z]_¥`a__^__WQ_bbbZaUSW[]ba`baba]b_b^bb
@HWI-EAS443:3:29:1185:1142#0/1
GGCATGAGCATGGACCAGTCTCGTGACATTTCCTCGAGGG
+HWI-EAS443:3:29:1185:1142#0/1
_]ba`_a`baa`aaaaa__aa_]JYP^^`^`_a_[X^`^X
@HWI-EAS443:3:28:424:390#0/1
GAAAGTGATTCCCAGAGGAAAGCTTTCCACAAGAGGGGTC
+HWI-EAS443:3:28:424:390#0/1
`^a¥b[a`aabaGb_aaWaaa^abaa__aa``_aZ_Y`^_
```

```
@HWI-EAS443:3:99:1497:754#0/1
CTCCAATATGTGTGCTGTGGCACATTTCCAAGAGAGATGC
+HWI-EAS443:3:99:1497:754#0/1
a`T_a`bJ`bT[¥]abbb_[b]a^a^a_FWab¥bYbG_aZ
@HWI-EAS443:3:1:299:1318#0/1
GGGCTGCACACTGATCTGGCCTGCGGATGCCTTTTGTTGA
+HWI-EAS443:3:1:299:1318#0/1
`bb`^bUbZa^_a_b^aba_X`U`a^]a_V_aaa_a¥`U¥
@HWI-EAS443:3:89:927:729#0/1
ACCCTGTAGAATCTGTCCACTCCTGCTTGTATTCCCTCAT
+HWI-EAS443:3:89:927:729#0/1
RGV¥b`_¥bbaaaUa``b_NXR_a`^aaS^bb_a¥`^aW]
@HWI-EAS443:3:22:726:1416#0/1
CTAACTTGCAGGCCCTGTAGAATCTGTCCACTCCTGCTTG
+HWI-EAS443:3:22:726:1416#0/1
[^ba__S_Z^[[Y]aa¥Q^U``^_PJONP``[¥¥UULT_X
@HWI-EAS443:3:61:1258:1305#0/1
CGTCGCTTTCAATCTGTGCCGCATTTCTTCAATTANCCAC
+HWI-EAS443:3:61:1258:1305#0/1
_bbab_abaZab`abb`bbbbbaabbabbabaaa^DFY_S
@HWI-EAS443:3:31:41:191#0/1
GGCGTCGCTTTCAATCTGTGCCGCATTTCTTCAATTAACC
+HWI-EAS443:3:31:41:191#0/1
V_ba[¥_aaba¥`_``K¥U[¥``WO[]¥RY^WW_W[O^Y]
@HWI-EAS443:3:51:86:137#0/1
CTCGTCGCTTTCAATCTGTGCCGCATTTCTTCAATTAACC
+HWI-EAS443:3:51:86:137#0/1
abbbbbabbbb_baaabb`baaba^bababb``^ba``Za
@HWI-EAS443:3:1:1614:256#0/1
CGTCGCTTTCAATCTGTGCCGCATTTCTTCAATTAACCAC
+HWI-EAS443:3:1:1614:256#0/1
`baaaa`baaaaaab`aaa¥a__aaa^`a]¥Saa``W]_B
@HWI-EAS443:3:27:52:1635#0/1
CGTCGCTTTCAATCTGTGCCGCATTTCTTCAATTAACCAC
+HWI-EAS443:3:27:52:1635#0/1
`Zaaa`aa__¥_a^`^^`abaa__a`a``a^S_`X^a__a
@HWI-EAS443:3:30:356:737#0/1
CGTCGCTTTCAATCTGTGCCGCATTTCTTCAATTAACCAC
+HWI-EAS443:3:30:356:737#0/1
aZUab_`U]ZU¥__`_L]_a¥a`_a_T_`^^Z__^_ZT^_
@HWI-EAS443:3:32:237:1574#0/1
CGTCGCTTTCAATCTGTGCCGCATTTCTTCAATTAACCAC
+HWI-EAS443:3:32:237:1574#0/1
a¥_a_a^_`a^]`__^W__^^`_^_Z_^^^`_^^__a`_a
@HWI-EAS443:3:35:1468:334#0/1
CGTCGCTTTCAATCTGTGCCGCATTTCTTCAATTAACCAC
+HWI-EAS443:3:35:1468:334#0/1
abaabababaaaaaaa_baabaaaaaaaaba_aaaa_b`a
@HWI-EAS443:3:48:963:737#0/1
CGTCGCTTTCAATCTGTGCCGCATTTCTTCAATTAACCAC
```

+HWI-EAS443:3:48:963:737#0/1
ababbbbbbbabb`bbabaab_bbbaaabababbaa_^a_
@HWI-EAS443:3:54:175:21#0/1
CGTCGCTTTCAATCTGTGCCGCATTTCTTCAATTAACCAC
+HWI-EAS443:3:54:175:21#0/1
ab`abbabbaaaaaab_a``b]¥_aa`aaa`¥aa]U]___
@HWI-EAS443:3:55:349:1930#0/1
CGTCGCTTTCAATCTGTGCCGCATTTCTTCAATTAACCAC
+HWI-EAS443:3:55:349:1930#0/1
aaTaaa`aaa`]^__`_^[_]_____^_^_]^_^^^Y]]]
@HWI-EAS443:3:62:1131:1166#0/1
CGTCGCTTTCAATCTGTGCCGCATTTCTTCAATTAACCAC
+HWI-EAS443:3:62:1131:1166#0/1
Z_`a`_ba_bbbabba`^`_Z¥abW``aa__abbaa_aaa
@HWI-EAS443:3:74:1519:462#0/1
CGTCGCTTTCAATCTGTGCCGCATTTCTTCAATTAACCAC
+HWI-EAS443:3:74:1519:462#0/1
abaababa_Wa]baaa_baab__aaaaa_a]_aa^b^^^W
@HWI-EAS443:3:74:424:1185#0/1
CGTCGCTTTCAATCTGTGCCGCATTTCTTCAATTAACCAC
+HWI-EAS443:3:74:424:1185#0/1
a¥SVT¥UZ]^TR]YNY]P__TW¥QHRUQQ^YVYSBBBBBB
@HWI-EAS443:3:77:308:262#0/1
CGTCGCTTTCAATCTGTGCCGCATTTCTTCAATTAACCAC
+HWI-EAS443:3:77:308:262#0/1
^b`bbaa_aaaaba`aYa`_aaaa_a_a`a``_^aa__Z_
@HWI-EAS443:3:79:1033:421#0/1
CGTCGCTTTCAATCTGTGCCGCATTTCTTCAATTAACCAC
+HWI-EAS443:3:79:1033:421#0/1
`b]¥_^aaa`^_`a]b_a^Xa^]`````]¥a`aaa_YN__
@HWI-EAS443:3:7:957:132#0/1
CGTCGCTTTCAATCTGTGCCGCATTTCTTCAATTAACCAC
+HWI-EAS443:3:7:957:132#0/1
ab]aa^_a_aa`a__a_aZ]a]Z`_^_a^^^^a_][^Z_X
@HWI-EAS443:3:81:1001:1379#0/1
CGTCGCTTTCAATCTGTGCCGCATTTCTTCAATTAACCAC
+HWI-EAS443:3:81:1001:1379#0/1
ab[¥^W`a¥Y¥LXa]XWZaab_Y_]Z^`_S]^`]^`^N][
@HWI-EAS443:3:81:275:1762#0/1
CGTCGCTTTCAATCTGTGCCGCATTTCTTCAATTAACCAC
+HWI-EAS443:3:81:275:1762#0/1
a___Wa_X[[_^_aNHZ_``_a_Ya]]__a^Z^^_`¥^`]
@HWI-EAS443:3:87:333:1346#0/1
CGTCGCTTTCAATCTGTGCCGCATTTCTTCAATTAACCAC
+HWI-EAS443:3:87:333:1346#0/1
ab_babaaba`Waa``]`aa_b_aaa`aa`__a```^_`]
@HWI-EAS443:3:89:1374:1714#0/1
CGTCGCTTTCAATCTGTGCCGCATTTCTTCAATTAACCAC
+HWI-EAS443:3:89:1374:1714#0/1
ab`bababb`a^ba``]b¥Naaa^b`^bb_a^aaaa__a]

@HWI-EAS443:3:89:683:1162#0/1
CGTCGCTTTCAATCTGTGCCGCATTTCTTCAATTAACCAC
+HWI-EAS443:3:89:683:1162#0/1
]baaa`¥ba`b`_a^b[aa_abbbaabbaabbbbabaWab
@HWI-EAS443:3:8:1166:1402#0/1
CGTCGCTTTCAATCTGTGCCGCATTTCTTCAATTAACCAC
+HWI-EAS443:3:8:1166:1402#0/1
ab``bTaaa^baa`a`Zbaa``aaaa¥aa]a`aa_a]Sa^
@HWI-EAS443:3:17:663:1236#0/1
TGGGCTATTAGTGTCAGATGGAGGACCCAACTTATACAAT
+HWI-EAS443:3:17:663:1236#0/1
`bbaaaa`Wb_JHZ`a¥Sa``UX_]VYHL]HQ`_¥`^¥¥`
@HWI-EAS443:3:26:40:1084#0/1
CCTGTGCCGCATTTCTTCAATTAACCACCTTATTTCCTCA
+HWI-EAS443:3:26:40:1084#0/1
`aW`ab]_^_R^`][_Yb^`[WTWYa^a``ZJYVQ`X]¥^
@HWI-EAS443:3:79:429:1850#0/1
GTCGCTTTCAACCTGTGCCGCATTTCTTCAATTAACCACC
+HWI-EAS443:3:79:429:1850#0/1
[O]Z[PORaTLWYXZMO¥¥Q_]Z^]S_Y^[YWPKT[W[W`
@HWI-EAS443:3:5:1606:1699#0/1
ATCTGGAGTAAATGAATCAGCTGACATGAGTATTGGAGTA
+HWI-EAS443:3:5:1606:1699#0/1
abaab]`Taaa^b`aa_a_bYbb`[a`a`b`ba`[`_a]Y
@HWI-EAS443:3:23:61:322#0/1
CACAAATCCTAGAATTCCCTTAGTCAGAGGTGACAAGATT
+HWI-EAS443:3:23:61:322#0/1
a^^[_`[[aY[T_R^^Y`a^^W^OX]]^]SU]PVSW]]WS
@HWI-EAS443:3:67:864:399#0/1
GGATGCAGGTGCATTGACTGAAGATCCAGATGAAGAATCA
+HWI-EAS443:3:67:864:399#0/1
ab_aaaaaaT]^baaaaaaaaaa__a__a`_a__a`____
@HWI-EAS443:3:36:523:1301#0/1
GCCGCATTTCTTCAATTGACCACCTTATTTCCTCAAATTT
+HWI-EAS443:3:36:523:1301#0/1
aba_a¥^]]a```^R_¥M¥`_aaaa_]aa`a`aa^ZQaa^
@HWI-EAS443:3:85:1222:574#0/1
CCCAGGAATATTTGAGAATTCATGCCTTGAAACAATACGG
+HWI-EAS443:3:85:1222:574#0/1
ababb`Yaa_ababbaa^aa`aaaa^aaaa_`^aa_aY^_
@HWI-EAS443:3:51:137:1638#0/1
AAAGAAATCTCTGTGTCTTGTGAATTGGTCATCATCTTTC
+HWI-EAS443:3:51:137:1638#0/1
abbbaa]bbbaab_a``b_XXa¥^_a^YSVWXZ^`^a`XP
@HWI-EAS443:3:64:1158:683#0/1
AAGAAATCTCTGTGTCTTGTGAATTGGTCATCATCTTTCT
+HWI-EAS443:3:64:1158:683#0/1
a`aZ^bbbaaa^R_`bbaa^aTaaa]`UZZ`[]a`aaa¥a
@HWI-EAS443:3:5:311:1086#0/1
AAAGGCGTAAATCCCAATTACCTCATGGCTTGGAAGCAAG

```
+HWI-EAS443:3:5:311:1086#0/1
[U`¥YPG]_Rba]¥SKaXO^aaJ]I__`X¥PW[BBBBBBB
@HWI-EAS443:3:52:956:377#0/1
CGCCTTTCTCATGTGGTTTGACTATGTTAGGCTCTTTCCA
+HWI-EAS443:3:52:956:377#0/1
¥_^R__^^aa`a[Sa^``b`XSb`^aS^Z]ZV_Z¥baX¥O
@HWI-EAS443:3:70:79:974#0/1
TCCTTTTGCAAGATTGCCCAGTTCATTGATGCTTAATGCT
+HWI-EAS443:3:70:79:974#0/1
aabaa`aa`]``a___aa`Z`]`a``^`_^^_`__[^]]]
@HWI-EAS443:3:95:1226:235#0/1
CAGACTGTTCGAGCTTTTCGCAAATGCTCCTAGCTAAAGT
+HWI-EAS443:3:95:1226:235#0/1
Z``a¥^b^``abbababb_^aX`_b`N`X^`bbZbUaa_L
@HWI-EAS443:3:61:808:1538#0/1
CTGGCTGGATCCTGGGAAATCCAGAGTGTGAATCACTCTA
+HWI-EAS443:3:61:808:1538#0/1
abb¥bbV^]bbb__^]b`bbbb[bbbXb`baa__`Y_`¥H
@HWI-EAS443:3:53:226:666#0/1
AGCCAATTGAAGTGGGCACTCGGTGAAAATATAGCACCAG
+HWI-EAS443:3:53:226:666#0/1
a``_V¥aa¥`VUGZ[]VW[T¥aSV¥RXV^[¥[X¥V¥Z[X]
@HWI-EAS443:3:76:534:1199#0/1
GTTAAGTGCAACACTTCAATATAAAGACTACCTGTTCCGC
+HWI-EAS443:3:76:534:1199#0/1
a]UEMPWTJRWJQ]QZXY]YW^[]ODUZIDNWTSTXPLPB
@HWI-EAS443:3:33:156:901#0/1
GTCCAGGCCATGATCCGGTCTCTTCCTTCAATTATCTCAA
+HWI-EAS443:3:33:156:901#0/1
aab``abb`W]]b^Q__XYb_ba`aa`a``[``R`aa]`a
@HWI-EAS443:3:92:947:1730#0/1
CGTCCATCATAATCATTGAGTTTGAGTTCAGTGCACATTT
+HWI-EAS443:3:92:947:1730#0/1
ab`abaaababaa`abaaabaaaaaa`ab`a_a_a_aaaa
@HWI-EAS443:3:13:1126:501#0/1
GCTCCCGCTATATCCTGACCACTCATTTATTCCTACGATA
+HWI-EAS443:3:13:1126:501#0/1
a_NXaaaX]__¥¥]]]_^]¥XZ_a`___]_^OZ]]^a_Z^
@HWI-EAS443:3:23:402:1370#0/1
AGAGAGGATGGTGCTTTCTGCTTTTGATGAAAGAAGAAAT
+HWI-EAS443:3:23:402:1370#0/1
a_QQ]a^a^]XRX`V]¥V¥`R¥`_]FZZ`V]Z[Y__`_^R
@HWI-EAS443:3:36:70:1027#0/1
GGGCCATGTCCATTATTCTCTTGTCTGCTGTAATTGGGTA
+HWI-EAS443:3:36:70:1027#0/1
¥`_baX¥`Z¥_W`_¥^¥aa_`]¥]ab_V^_^Wab¥¥Z_VZ
@HWI-EAS443:3:95:1582:1221#0/1
GGGCCATGTCCATTATTCTCTTGTCTGCTGTAATTGGGTA
+HWI-EAS443:3:95:1582:1221#0/1
abb_¥¥``^babbbb_YYTXbbXL]Yb]_b]_]a_Q_aP¥
```

```
@HWI-EAS443:3:21:1284:308#0/1
GCCATGTCCATTATTCTCTTGTCTGCTGTAATTGGGTATC
+HWI-EAS443:3:21:1284:308#0/1
aa`[_a__aaaa_aa]``a__^^a_]]aZZ^``_`_W^YV
@HWI-EAS443:3:58:365:1473#0/1
GATACCCAATTACAGCAGACAAGAGAATAATGGACATGGC
+HWI-EAS443:3:58:365:1473#0/1
a`[aaaab]a`a__a__a`_^_a__[Z____]^a[^Z]¥^
@HWI-EAS443:3:27:466:303#0/1
AGGGGACGTAGTGTTGATAATGAAACGAAAACGGGACTCT
+HWI-EAS443:3:27:466:303#0/1
aba``]bb[aaZW^b___¥a`T^aaY[`aa_ZY`^^^__^
@HWI-EAS443:3:63:1429:1618#0/1
CAATCAACAAAAGGCATCCTCAGGCCAGGTCAGTGTGCAG
+HWI-EAS443:3:63:1429:1618#0/1
a[Y¥]Q```abSaVTXSSGLIa`¥SS¥BBBBBBBBBBBBB
@HWI-EAS443:3:25:401:1728#0/1
GAGGAAATACCAATCAACAAAAGGCATCCGCAGGCCAGGT
+HWI-EAS443:3:25:401:1728#0/1
aa[aa_^^aaab¥]aaZ_b__][_a``WJX¥aaZ]aaVSI
@HWI-EAS443:3:6:828:1885#0/1
AGGGCCTCATATACAACAGGATGGGAACAGTGACTACAGA
+HWI-EAS443:3:6:828:1885#0/1
aa_baa^Z_a¥XJR`Ra¥V]YMU]PM]Pa_LVaTDK^ZX_
@HWI-EAS443:3:33:1775:823#0/1
GCCTGTCCATCACTTGGTCCATCGGTCATTATAGTAAAGC
+HWI-EAS443:3:33:1775:823#0/1
S`^¥baW¥`a`XP¥bbbaaab_¥aaYH``aTFJ`MZa^PG
@HWI-EAS443:3:91:157:596#0/1
CACCAATTGACTTTTGTGAGCTGGGCTTTCATTTGGTCTC
+HWI-EAS443:3:91:157:596#0/1
`[b`Va[^aabba`]`[a]aaaa]`G___¥`]U[¥PX]XZ
@HWI-EAS443:3:100:868:538#0/1
GCCTGATGAGACCAAATGAAAACCCAGCTCACAAAAGTCA
+HWI-EAS443:3:100:868:538#0/1
a_aaa__aaa_`_a_aaa_`a_]_]]a[_W_Z__]Z_Z^B
@HWI-EAS443:3:14:748:1478#0/1
CAGCCTGATGAGACCAAATGAAAACCCAGCTCACAAAAGT
+HWI-EAS443:3:14:748:1478#0/1
a`aa_a^aa`b`aaab^abaa__ba`_aa^_`]`aaa_NR
@HWI-EAS443:3:16:831:598#0/1
GGTCAGCCTGATGAGACCAAATGAAAACCCAGCTCACAAA
+HWI-EAS443:3:16:831:598#0/1
aa_abaabab`abaa`a`a`aaa``aaaZ]_`^_]a_a_]
@HWI-EAS443:3:17:658:998#0/1
ACTTTTGTGAGCTGGGTTTTCATTTGGTCTCATCAGGCTG
+HWI-EAS443:3:17:658:998#0/1
`bbaaa`ab`aaaaab_a__`aaaa`a¥aaab`_aa`_a`
@HWI-EAS443:3:17:798:1212#0/1
GCCTGATGAGACCAAATGAAAACCCAGCTCACAAAAGTCA
```

```
+HWI-EAS443:3:17:798:1212#0/1
aaaaaa`a___aaa]aaa``_a`_^_a_`^__^_]_a]_Z
@HWI-EAS443:3:18:1190:654#0/1
CACCAATTGACTTTTGTGAGCTGGGTTTTCATTTGGTCTC
+HWI-EAS443:3:18:1190:654#0/1
WOV^T`bbb[^SXVX`_````ab__Y^`]][aW[`aX_`T
@HWI-EAS443:3:1:164:1217#0/1
GATGAGACCAAATGAAAACCCAGCTCACAAAAGTCAATTG
+HWI-EAS443:3:1:164:1217#0/1
a_aaaaa_a`aaaa``_a]Y_`_a`¥^^a`__`Z^^]`BB
@HWI-EAS443:3:21:1720:304#0/1
CTTTTGTGAGCTGGGTTTTCATTTGGTCTCATCAGGCTGA
+HWI-EAS443:3:21:1720:304#0/1
abbbbbabbba`baa_aab[aaaaaa_aa`ab_`a`]aa`
@HWI-EAS443:3:23:1430:310#0/1
CTTTTGTGAGCTGGGTTTTCATTTGGTCTCATCAGGCTGA
+HWI-EAS443:3:23:1430:310#0/1
`bbbbbabbaabbaa^bbaZ`babb]`aaaaaaaaUb`a
@HWI-EAS443:3:25:316:785#0/1
CTGATGAGACCAAATGAAAACCCAGCTCACAAAAGTCAAT
+HWI-EAS443:3:25:316:785#0/1
abbb_bbbbbabbabbbba`bbbabbaabbbb_bbYaaba
@HWI-EAS443:3:26:915:1227#0/1
GCCTGATGAGACCAAATGAAAACCCAGCTCACAAAAGTCA
+HWI-EAS443:3:26:915:1227#0/1
``b```_]¥Q]abbbbaabZ_¥a]]ab]aa_baaa_b]aW
@HWI-EAS443:3:27:66:1172#0/1
TGATGAGACCAAATGAAAACCCAGCTCACAAAAGTCAATT
+HWI-EAS443:3:27:66:1172#0/1
``]¥]a^S`b_I_bbb__¥a``__a_baaa^MUSF____a
@HWI-EAS443:3:28:152:1324#0/1
ACTTTTGTGAGCTGGGTTTTCATTTGGTCTCATCAGGCTG
+HWI-EAS443:3:28:152:1324#0/1
`baaaaa__^`ba``_^`aab_aa`a]Ya`aa_aa]^aW_
@HWI-EAS443:3:2:940:1287#0/1
GACTTTTGTGAGCTGGGTTTTCATTTGGTCTCATCAGGCT
+HWI-EAS443:3:2:940:1287#0/1
aZP]Z]__I¥O`a`_X]NFV[SV]Z¥^`NT]VZUYUOBBB
@HWI-EAS443:3:30:59:524#0/1
GATGAGACCAAATGAAAACCCAGCTCACAAAAGTCAATTG
+HWI-EAS443:3:30:59:524#0/1
aaaaaaaaaba`aaa_aa`aa¥aa___`_X[¥]Z`¥__^^
@HWI-EAS443:3:33:622:252#0/1
GCCTGATGAGACCAAATGAAAACCCAGCTCACAAAAGTCA
+HWI-EAS443:3:33:622:252#0/1
_¥^RTXPXIRX^[X]`S¥X[Xaa^aVZaT_[_UNSY[S^R
@HWI-EAS443:3:35:1139:328#0/1
CACCAATTGACTTTTGTGAGCTGGGTTTTCATTTGGTCTC
+HWI-EAS443:3:35:1139:328#0/1
abbbaaabbaabbbbb]babababbba¥baab`baaaa_a`a
```

```
@HWI-EAS443:3:35:1720:1424#0/1
CTGATGAGACCAAATGAAAACCCAGCTCACAAAAGTCAAT
+HWI-EAS443:3:35:1720:1424#0/1
¥b[Kbbb``^^a¥bbab_bbaaabbU_WY^_Saba^aYZ[
@HWI-EAS443:3:35:483:1570#0/1
TTTGTGAGCTGGGTTTTCATTTGGTCTCATCAGGCTGACC
+HWI-EAS443:3:35:483:1570#0/1
`ba``aa`^^^W¥T]]aaa]_¥^XYW]^LU_YY_a]¥¥Za
@HWI-EAS443:3:37:949:1463#0/1
GACTTTTGTGAGCTGGGTTTTCATTTGGTCTCATCAGGCT
+HWI-EAS443:3:37:949:1463#0/1
a`aaaabba_^a`aba`aaaaabaaaa]]Uabbabaa_a`
@HWI-EAS443:3:39:1754:1266#0/1
CACCAATTGACTTTTGTGAGCTGGGTTTTCATTTGGTCTC
+HWI-EAS443:3:39:1754:1266#0/1
abbababbbaabbbba_bab_abab_`bb`a^``a_Z`a¥
@HWI-EAS443:3:45:1024:486#0/1
CACCAATTGACTTTTGTGAGCTGGGTTTTCATTTGGTCTC
+HWI-EAS443:3:45:1024:486#0/1
^bb]aab`bbabbbbbababXbbbb_`bbaabaabaaaaa
@HWI-EAS443:3:47:1501:942#0/1
GCCTGATGAGACCAAATGAAAACCCAGCTCACAAAAGTCA
+HWI-EAS443:3:47:1501:942#0/1
a]]_aaa`X`I_a¥Y_aa^^_`_¥_``^`[_YX[[W_WZV
@HWI-EAS443:3:52:1779:693#0/1
GCCTGATGAGACCAAATGAAAACCCAGCTCACAAAAGTCA
+HWI-EAS443:3:52:1779:693#0/1
aaabYaaWQTUa`¥U[XTUPTY]UPYVNWVZY]^¥VQ]¥]
@HWI-EAS443:3:54:1064:1556#0/1
ATTGACTTTTGTGAGCTGGGTTTTCATTTGGTCTCATCAG
+HWI-EAS443:3:54:1064:1556#0/1
aaaa`^baaaa_`[a`aa_aVaaa__a``aa]¥__`_^_`
@HWI-EAS443:3:56:1314:647#0/1
ATTGACTTTTGTGAGCTGGGTTTTCATTTGGTCTCATCAG
+HWI-EAS443:3:56:1314:647#0/1
aa`R`abbbb`_aaa__bba]aaa^aaaaa]_`a_aa^a`
@HWI-EAS443:3:57:305:1606#0/1
GGTCAGCCTGATGAGACCAAATGAAAACCCAGCTCACAAA
+HWI-EAS443:3:57:305:1606#0/1
ZQWT^[`b^_OT^^YX¥M]SVZ]ZaZVQY]aZOV]BBBBB
@HWI-EAS443:3:60:86:1372#0/1
TTTGTGAGCTGGGTTTTCATTTGGTCTCATCAGGCTGACC
+HWI-EAS443:3:60:86:1372#0/1
a`b`_a^baaaa_Zaaaaaaaa`_]a`b``__^]^```_^
@HWI-EAS443:3:62:1430:1574#0/1
GTCAGCCTGATGAGACCAAATGAAAACCCAGCTCACAAAA
+HWI-EAS443:3:62:1430:1574#0/1
_T_`_^¥a_aaa_a```a`[_aa_a`__¥^`W^M^^_`_`
@HWI-EAS443:3:63:1012:1939#0/1
GATGAGACCAAATGAAAACCCAGCTCACAAAAGTCAATTG
```

~~~
+HWI-EAS443:3:63:1012:1939#0/1
a]a`a_aaZba`Z`aababba[aaaba]`]_a]Ta`aa`]
@HWI-EAS443:3:63:882:1738#0/1
GATGAGACCAAATGAAAACCCAGCTCACAAAAGTCAATTG
+HWI-EAS443:3:63:882:1738#0/1
``Z¥_aaabbabbaaaaba``aZ`aba^aa__^^`_`a``
@HWI-EAS443:3:64:1081:1878#0/1
ATTGACTTTTGTGAGCTGGGTTTTCATTTGGTCTCATCAG
+HWI-EAS443:3:64:1081:1878#0/1
`aaa`___X_a¥__a``aa^O_aa`__]`a_W]_^_Z[a[
@HWI-EAS443:3:64:1324:1024#0/1
CTTTTGTGAGCTGGGTTTTCATTTGGTCTCATCAGGCTGA
+HWI-EAS443:3:64:1324:1024#0/1
abbbb_bab`bbaba_bbb_bbabaa`aaaaabab``abb
@HWI-EAS443:3:64:552:196#0/1
GGTCAGCCTGATGAGACCAAATGAAAACCCAGCTCACAAA
+HWI-EAS443:3:64:552:196#0/1
aa]aab_aabaaaaa`^^a__`a`_a`^[V^`__``__^]
@HWI-EAS443:3:65:1431:1761#0/1
GACTTTTGTGAGCTGGGTTTTCATTTGGTCTCATCAGGCT
+HWI-EAS443:3:65:1431:1761#0/1
aaaabaaaUaaX]aabbQ^a_V^_][a_Jaaaa_^[^S[[
@HWI-EAS443:3:67:1212:1425#0/1
CACCAATTGACTTTTGTGAGCTGGGTTTTCATTTGGTCTC
+HWI-EAS443:3:67:1212:1425#0/1
``ababba^_¥¥^aaWZb^Y`ab`b_``b¥^ba_a]aab_
@HWI-EAS443:3:6:587:1215#0/1
GACTTTTGTGAGCTGGGTTTTCATTTGGTCTCATCAGGCT
+HWI-EAS443:3:6:587:1215#0/1
aY[X[PZHM^`XV]aY_U_ZYYYSTR[_¥WV^]^S]__U_
@HWI-EAS443:3:70:1354:938#0/1
CACCAATTGACTTTTGTGAGCTGGGTTTTCATTTGGTCTC
+HWI-EAS443:3:70:1354:938#0/1
^a[aa¥__a```bb``W`R`]``Wa`a]b]abbb^V]TZa
@HWI-EAS443:3:71:1540:1874#0/1
GTCAGCCTGATGAGACCAAATGAAAACCCAGCTCACAAAA
+HWI-EAS443:3:71:1540:1874#0/1
aa`__^_]ZW[`X[[`[```````¥`^¥XZ¥[Y¥[Q_ZVT
@HWI-EAS443:3:71:798:1617#0/1
CTTTTGTGAGCTGGGTTTTCATTTGGTCTCATCAGGCTGA
+HWI-EAS443:3:71:798:1617#0/1
aaa`^TWOZ`^a]a_P_Z[^X][_`^N_]b_P^a¥`aa_a
@HWI-EAS443:3:73:224:315#0/1
GACTTTTGTGAGCTGGGTTTTCATTTGGTCTCATCAGGCT
+HWI-EAS443:3:73:224:315#0/1
a^_a`aaa`a``^aa^aU_aa`_a_^``W_`_a^Z^`^X_
@HWI-EAS443:3:74:1170:1788#0/1
GACTTTTGTGAGCTGGGTTTTCATTTGGTCTCATCAGGCT
+HWI-EAS443:3:74:1170:1788#0/1
abbbbabbV_abaabbb¥`bbbbbabba`ab`baab__a`
~~~

```
@HWI-EAS443:3:74:483:1256#0/1
GCCTGATGAGACCAAATGAAAACCCAGCTCACAAAAGTCA
+HWI-EAS443:3:74:483:1256#0/1
ab¥]^][aZ]XU]]X_^Q^^``aaS^[SYaaaWR]¥XRZU
@HWI-EAS443:3:75:1026:988#0/1
TTTGTGAGCTGGGTTTTCATTTGGTCTCATCAGGCTGACC
+HWI-EAS443:3:75:1026:988#0/1
aa`a_b`¥`aa``]aaa`aaaaa_Z^a_a`_a`^`_a_`a
@HWI-EAS443:3:75:94:284#0/1
GACTTTTGTGAGCTGGGTTTTCATTTGGTCTCATCAGGCT
+HWI-EAS443:3:75:94:284#0/1
a_a^Yaa``_Ka^Z_]aYaa````a_^^Ta_^_Ya__[^]
@HWI-EAS443:3:76:102:1818#0/1
CTTTTGTGAGCTGGGTTTTCATTTGGTCTCATCAGGCTGA
+HWI-EAS443:3:76:102:1818#0/1
aa¥aa``]`aaa^Z¥Maa¥_O[aaQDP__aa__aVXV_^_
@HWI-EAS443:3:7:118:343#0/1
GACTTTTGTGAGCTGGGTTTTCATTTGGTCTCATCAGGCT
+HWI-EAS443:3:7:118:343#0/1
a[Za[baa__`aaaaaaSV¥^`Y_``a^^`^_`]`_]R[^
@HWI-EAS443:3:7:154:418#0/1
GCCTGATGAGACCAAATGAAAACCCAGCTCACAAAAGTCA
+HWI-EAS443:3:7:154:418#0/1
aa]]YSW]]aYaa_TZYWRWPL[ZYZ^LRU[^SPVW^RZB
@HWI-EAS443:3:80:495:1561#0/1
CTGATGAGACCAAATGAAAACCCAGCTCACAAAAGTCAAT
+HWI-EAS443:3:80:495:1561#0/1
a_baaba]aaaaa`aaaaaaaabaaa__a_a[aaaU_^_`
@HWI-EAS443:3:81:1312:1681#0/1
GATGAGACCAAATGAAAACCCAGCTCACAAAAGTCAATTG
+HWI-EAS443:3:81:1312:1681#0/1
aaabaaaaaaabaaaaab`_`aaa_[`]a`__aX_`]`_`
@HWI-EAS443:3:83:1147:322#0/1
GCCTGATGAGACCAAATGAAAACCCAGCTCACAAAAGTCA
+HWI-EAS443:3:83:1147:322#0/1
`a^SQGZZU¥Y_^]U^a`Z___Y^_^``_¥]XUYQV`RY]
@HWI-EAS443:3:83:1541:1114#0/1
ATTGACTTTTGTGAGCTGGGTTTTCATTTGGTCTCATCAG
+HWI-EAS443:3:83:1541:1114#0/1
a`W]VaV]P``_WMVSQZ^PTZ[U^S¥UW_ZOM[R^]WOT
@HWI-EAS443:3:84:650:1726#0/1
GACTTTTGTGAGCTGGGTTTTCATTTGGTCTCATCAGGCT
+HWI-EAS443:3:84:650:1726#0/1
aabaaaaa_a`a`_^a^Taa`a`_`¥QQR__`aXa_ZV¥_
@HWI-EAS443:3:85:1623:1386#0/1
GGTCAGCCTGATGAGACCAAATGAAAACCCAGCTCACAAA
+HWI-EAS443:3:85:1623:1386#0/1
`[]aaaY[`a`aa]a^¥S__^a^`_^YO[`^a^]]^_`XY
@HWI-EAS443:3:88:803:592#0/1
GCCTGATGAGACCAAATGAAAACCCAGCTCACAAAAGTCA
```

+HWI-EAS443:3:88:803:592#0/1
_aabbabbbaa^`a^`bb_R_baa`[a_^``ZW_[¥_U`_
@HWI-EAS443:3:91:1154:415#0/1
GATGAGACCAAATGAAAACCCAGCTCACAAAAGTCAATTG
+HWI-EAS443:3:91:1154:415#0/1
¥]V`^aaYZ_WL¥¥X_`]^ab`^S`a`^SS]UXQ^_[__¥
@HWI-EAS443:3:91:468:1714#0/1
GCCTGATGAGACCAAATGAAAACCCAGCTCACAAAAGTCA
+HWI-EAS443:3:91:468:1714#0/1
aaa][`Z[^_`_]``_a]_a^a]]`^____Y[_^Q]^V¥_
@HWI-EAS443:3:92:157:1025#0/1
TTTGTGAGCTGGGTTTTCATTTGGTCTCATCAGGCTGACC
+HWI-EAS443:3:92:157:1025#0/1
aaaa_`aa__a`a]`aaa_a__a_]a`_`a_a`__^__^]
@HWI-EAS443:3:98:179:342#0/1
ACCAATTGACTTTTGTGAGCTGGGTTTTCATTTGGTCTCA
+HWI-EAS443:3:98:179:342#0/1
aR`__aba`V[a_a_N^_`_aaa`V[`ZW¥^^^^[T]_]]
@HWI-EAS443:3:98:582:80#0/1
GACTTTTGTGAGCTGGGTTTTCATTTGGTCTCATCAGGCT
+HWI-EAS443:3:98:582:80#0/1
a_abbbbbababbbbb[aba__aaaaa_`_]aa]aa_^a
@HWI-EAS443:3:86:453:1332#0/1
GGTGAAAATGGACGAAACACAAGGGTTGCTTATGAAAGAA
+HWI-EAS443:3:86:453:1332#0/1
`b¥aaabaaba^aaa]GSYT^¥]]_X]___]^___WYY]]
@HWI-EAS443:3:2:417:879#0/1
GAACAAGGACAGACCCTCTGGAGCAAAACAAACGATGCTG
+HWI-EAS443:3:2:417:879#0/1
a___`¥aaTW`^[UUY¥[^_UV_¥XX[VLY_US^S[¥^BB
@HWI-EAS443:3:24:1767:420#0/1
CCCATGGCTGTATGGAGGATCTCCAGTATAAGGGAATGTG
+HWI-EAS443:3:24:1767:420#0/1
`baababababba`a_aZabQ[Q¥U_^a^__Z_aabQ^[ZU`
@HWI-EAS443:3:4:293:1443#0/1
GTGGGTTGGTCTTGGCCAGACGGTGCTGAGTTGCCGTTTA
+HWI-EAS443:3:4:293:1443#0/1
`Za]¥HVNHEV_¥YXRU_`_S`^KVNV_^_W¥¥W[PU^BB
@HWI-EAS443:3:97:1442:191#0/1
AAAAATTCGACACTAATTGATGGCCATCAGAATTCTTTTG
+HWI-EAS443:3:97:1442:191#0/1
]XF^aXLYa]]ZV_b`a_[_``KPXRJKDOYY`XFY___B
@HWI-EAS443:3:49:1493:51#0/1
CAACACTACGTCCCCTTGCCCAATTAGTACATTAGCTTTC
+HWI-EAS443:3:49:1493:51#0/1
abbabbb`_b_]__ba_Z_OW[a]^SXGNaaa^]_PXaa]
@HWI-EAS443:3:93:60:402#0/1
AGCTAATGTACTAATTGGGCAAGGGGACGTAGTGTTGGTA
+HWI-EAS443:3:93:60:402#0/1
`bb_ZZZ`SaZa][ba]`a^X¥`¥aaXaa__ZOZZ][`[]

```
@HWI-EAS443:3:61:555:259#0/1
GGAGGCGCTGCCTTTTTCAGTCTCTTCAGCAGATTGAGAG
+HWI-EAS443:3:61:555:259#0/1
aaX`_`a^^QTW¥WHR^`V^UZ¥_W¥QQRTN[TYYWWWQU
@HWI-EAS443:3:34:571:1608#0/1
CATTAATCAGGCACTCCTCGATTGCTTCATATAGCCCCAC
+HWI-EAS443:3:34:571:1608#0/1
]_Z___^]XSL_¥_XY`UQZOVF[YZ]WZS¥[[MDPBBBB
@HWI-EAS443:3:69:419:485#0/1
TGGGGCTATATGAAGCAATCGAGGAGTGCCTGATTAATGA
+HWI-EAS443:3:69:419:485#0/1
UXT]aZZQ^``^`Z¥_Zaa`_I_]O_Pa^[`¥R_`a`]_Z
@HWI-EAS443:3:77:178:1373#0/1
TGGGGCTATATGAAGCAATCGAGGAGTGCCTGATTAATGA
+HWI-EAS443:3:77:178:1373#0/1
aaabababaaaaba`a^`_`a]]aW[X_a_`[^^]`_Y__
@HWI-EAS443:3:18:1389:876#0/1
TGACCAAAATTTGATTATCGCTACTAGAAACATAGTAAGA
+HWI-EAS443:3:18:1389:876#0/1
abaa___YV]baa]aaa`_a[^MW`^_]^`_`a^]]]^`_
@HWI-EAS443:3:58:874:908#0/1
CTCCGATTGGCCATGTTTTCGATTTGTTTTCAAAGAATTC
+HWI-EAS443:3:58:874:908#0/1
a¥a[a^aaaY]`^``P]]FWZX^a^aaaaaR`]V¥WR]a¥
@HWI-EAS443:3:23:964:385#0/1
AATCACTCAATGAGTGACATCGAAGCCATGGCACCTTAAA
+HWI-EAS443:3:23:964:385#0/1
aaaaa_aaa^aa_`Zaa^a`^Z`^^U]^___UUWW__]W]
@HWI-EAS443:3:54:778:787#0/1
CTCATTTTGGGCAAAGAAGACAAGAGATATGGCCCTGTCA
+HWI-EAS443:3:54:778:787#0/1
aabbbaababab`aaaaaba_bbbabaaaaaa``¥a`__G
@HWI-EAS443:3:81:1188:800#0/1
CATCAGTTAAGAAAGAAGAAGAAGTGCTAACGGGCAACCT
+HWI-EAS443:3:81:1188:800#0/1
aZa^W¥RJ__^V``¥UY^_]]]^X¥¥R¥_`VZ¥ZG]XVHV
@HWI-EAS443:3:3:645:134#0/1
GGGGGGGTGTATGAAGCAATCGAGGAGTGCCTGATTAATG
+HWI-EAS443:3:3:645:134#0/1
_[]_a]GXaSP[]SG¥¥SUSS]S^PU^WWUUW¥WTPUBBB
@HWI-EAS443:3:45:30:514#0/1
CAGGGATCATTAATCAGGCACTCCTCGATTGCTTCATACA
+HWI-EAS443:3:45:30:514#0/1
aa]VYZ_^W_¥^SZSU^ZTZZVURZUT[UYRH[ZWZ^BBB
@HWI-EAS443:3:2:1022:254#0/1
GGGGGGCTGTATGAAGCAATCGAGGAGTGCCTGGTTAATG
+HWI-EAS443:3:2:1022:254#0/1
ab`a]¥a_a_]_¥[[JFX_S[_ZX_UQF¥¥^¥[XSRPVXB
@HWI-EAS443:3:1:745:1027#0/1
GGGGGCTATATGAAGCAATCGAGGAGTGCCTGGTTAATGA
```

```
+HWI-EAS443:3:1:745:1027#0/1
R_Y``Jaabaa¥_`a`]aaa`_ba``UZbZZ_I¥bba]TB
@HWI-EAS443:3:65:1402:1236#0/1
GGGGGCTATATGAAGCAATCGAGGAGTGCCTGGTTAATGA
+HWI-EAS443:3:65:1402:1236#0/1
^M[_U`WaY_Z¥UYZW]W¥PYFU]RPVaRYNSDM¥Za^_[
@HWI-EAS443:3:27:442:1866#0/1
GGGCTATATGAAGCAATCGAGGAGTGCCTGGTTAATGATC
+HWI-EAS443:3:27:442:1866#0/1
TVV`bbb_]`T`Zab]abY_a[a_abaaa_[a``^ZHRT_
@HWI-EAS443:3:6:757:820#0/1
GGGGTTCTTCTCCTGTCTTCCTGATGTGTACTTTTTGATT
+HWI-EAS443:3:6:757:820#0/1
aTaa^^aa_`__Z_aZ]a__Y``__^]]X^V^aa___Z¥Z
@HWI-EAS443:3:37:457:1027#0/1
TCTTGGAATCACTTTCTTTCCTCTTATGAAGCTTCATACT
+HWI-EAS443:3:37:457:1027#0/1
M`bUU``[`¥^b`ab^`]]aabba_aaaGJFL^MFU¥]VY
@HWI-EAS443:3:23:1010:1291#0/1
CTCTTTACCTACTGCTGTGAACTGTGAGTTCATCTTTTCA
+HWI-EAS443:3:23:1010:1291#0/1
a]aYO_aYb¥JS¥^^¥^U¥XY`Y_Y]EDS^XO[Z_R¥YV`
@HWI-EAS443:3:84:259:1269#0/1
CAGCACTAGAAAAGAATGTAACGGTAACACACTCTGTTAA
+HWI-EAS443:3:84:259:1269#0/1
aa^a¥^W¥_^`__[T^]XU_]Z_YXZ_Z_VZWX^]]UYUY
@HWI-EAS443:3:55:125:28#0/1
CAGTACTAGAAAAGAATATAACGGTAACACACTCTGTTAA
+HWI-EAS443:3:55:125:28#0/1
abb`bab`baaa`b`^bL`baaba_a`aa^__```a[a_¥
@HWI-EAS443:3:14:739:618#0/1
ACAGAGTGTGTTACCGTTACATTCTTTTCTAGTACTGTGT
+HWI-EAS443:3:14:739:618#0/1
abaaab_b_b_abaaa_aa`^^a_`a_`^``_^````_a^
@HWI-EAS443:3:26:809:156#0/1
ACAGTACTAGAAAAGAATGTAACGGTAACACACTCTGTTA
+HWI-EAS443:3:26:809:156#0/1
abab_^YYabaaaab¥abb^baaaaWa`V`[`_a]_^VW[
@HWI-EAS443:3:40:1055:975#0/1
CAGTACTAGAAAAGAATGTAACGGTAACACACTCTGTTAA
+HWI-EAS443:3:40:1055:975#0/1
^b_¥bbb___VV``R]ba`ab_baSaV^a`a^_]_`W`a`
@HWI-EAS443:3:7:1710:356#0/1
ATTCTGGGATCCATTCCGGTGCGAACAAGAGCTCGTGTTG
+HWI-EAS443:3:7:1710:356#0/1
_]aWWWK``aHX^T]]aaQNSY¥X¥[_M¥GOFWNFX`RBB
@HWI-EAS443:3:43:685:75#0/1
GCGCTTGTTCGCACCGGAATGGATCCCAGAATGTGTTCCC
+HWI-EAS443:3:43:685:75#0/1
aab`^^b`a]]_]U_aaZ`_^U^Z[``_`^^¥]Q`¥^RFR
```

@HWI-EAS443:3:86:343:1593#0/1
ACTCCAGATTCTGGTTGAAAGACACCCACGGCCGATTCGA
+HWI-EAS443:3:86:343:1593#0/1
XHW_b`T`WVb_`VU``b`_Xb`bb^]`aa[D[]MYaTW¥
@HWI-EAS443:3:86:388:1813#0/1
AAGACACCCACGGCCGATTCGAGCCATGCCAGTTATCCCT
+HWI-EAS443:3:86:388:1813#0/1
^`^a_Z]b_`_¥OH^]SWQ_¥Z_]^JJWaPQORKT¥^^`M
@HWI-EAS443:3:30:1328:1503#0/1
CGATCTCGGCTTTGAGGGGGCCTGACGGGGTGATAGAAAG
+HWI-EAS443:3:30:1328:1503#0/1
aa_JW]]__a^`[[R`Z_a^[`XMXM_``IYUSDVTOUTB
@HWI-EAS443:3:59:115:491#0/1
CGCGATCTCGGCTTTGAGGGGGCCTGACGGGGTGATAGAA
+HWI-EAS443:3:59:115:491#0/1
`aaa_aaaaa_`aa`a¥aa_`_^`¥XXZ`_¥K__OFX^[Z
@HWI-EAS443:3:48:333:1722#0/1
GGATGATGATGGGCATGTCTAACATGCTAANTACGGTCTT
+HWI-EAS443:3:48:333:1722#0/1
ab``^aWRZa[``^Z`aYXa[[XZ^¥^ZYXDH¥SUZV¥]W
@HWI-EAS443:3:42:1446:1808#0/1
TGATGATGGGCATGTTTAACATGCTAAGTACGGTCTTGGG
+HWI-EAS443:3:42:1446:1808#0/1
aaWa]`aaaa^aaa_aaa_^_`aOa^a_]aZa_R¥a_``^
@HWI-EAS443:3:9:581:1634#0/1
TGATGATGGGCATGTTTAACATGCTAAGTACGGTCTTGGG
+HWI-EAS443:3:9:581:1634#0/1
]_¥aaaaa[a^ba``aa`aaa^a_aaaa_`_a_]a_a[Y_
@HWI-EAS443:3:9:960:156#0/1
ATGATGATGGGCATGTTTAACATGCTAAGTACGGTCTTGG
+HWI-EAS443:3:9:960:156#0/1
abbababababaaa_aa]````^___]`_^_Z_Z`^ZZ^^`]
@HWI-EAS443:3:26:235:947#0/1
CAAGACCGTACTTAGCATGTTAAACATGCCCATCATCATC
+HWI-EAS443:3:26:235:947#0/1
a¥]`aaaa``a`_a^aaaa``aaa_a^^a_a__``^a__a
@HWI-EAS443:3:50:24:1962#0/1
GGATGATGATGGGCATGTTTAACATGCTAAGTACGGTCTG
+HWI-EAS443:3:50:24:1962#0/1
]_X]aZ¥Wa_XYT^aa¥WU]`_]Z_^a^]X¥]`_]UTY_[
@HWI-EAS443:3:64:1134:488#0/1
CGGGATGATGATGGGCATGTTTAACATGCTAAGTACGGTC
+HWI-EAS443:3:64:1134:488#0/1
`aaaHZU`_b`[a[¥^[T¥_aaa_]a``Ya^aaP^VZUPH
@HWI-EAS443:3:93:1292:2027#0/1
GACCGTACTTAGCATGTTAAACATGCCCATCATCATCCCA
+HWI-EAS443:3:93:1292:2027#0/1
aaab`_]`a_aa_aaaaaa`a_a¥`_YJ¥a_`^^_¥VYQ]
@HWI-EAS443:3:17:1617:1730#0/1
GGGATGATGATGGGCATGTTTAACATGCTAAGTACGGTCT

```
+HWI-EAS443:3:17:1617:1730#0/1
aba`b`^a]`aa`a`aaa`aa^a[a_a^aa_^]`Y`aXR_
@HWI-EAS443:3:18:1624:509#0/1
GGGATGATGATGGGCATGTTTAACATGCTAAGTACGGTCT
+HWI-EAS443:3:18:1624:509#0/1
`¥`][`]Y^][O][SV[^SUBBBBBBBBBBBBBBBBBBBB
@HWI-EAS443:3:24:843:432#0/1
GGGATGATGATGGGCATGTTTAACATGCTAAGTACGGTCT
+HWI-EAS443:3:24:843:432#0/1
a`¥]Z_Z_aa`a`[UTM^^`U`aU_^^X¥]]aS^R`_U[_
@HWI-EAS443:3:27:646:71#0/1
GGGATGATGATGGGCATGTTTAACATGCTAAGTACGGTCT
+HWI-EAS443:3:27:646:71#0/1
`bbabbabbbabaaa`aa_aba`]a_a``a`_]a_a_WZ`
@HWI-EAS443:3:55:145:108#0/1
GGGATGATGATGGGCATGTTTAACATGCTAAGTACGGTCT
+HWI-EAS443:3:55:145:108#0/1
aaa^_a__a`_`^`^_^aY^^`]]^]_V^]X^X¥Y__YY^
@HWI-EAS443:3:61:647:323#0/1
GGATGATGATGGGCATGTTTAACATGCTAAGTACGGTCTT
+HWI-EAS443:3:61:647:323#0/1
a_Waa`¥`][¥HL_Y_ZU_^¥VYXXQNM^MUWVTQMJWYY
@HWI-EAS443:3:70:52:655#0/1
GGGATGATGATGGGCATGTTTAACATGCTAAGTACGGTCT
+HWI-EAS443:3:70:52:655#0/1
W`_RX`abb^^^^^_ab`Zba^I]`_X_[Y`_¥aaaYY`Y
@HWI-EAS443:3:71:1632:1921#0/1
GGGATGATGATGGGCATGTTTAACATGCTAAGTACGGTCT
+HWI-EAS443:3:71:1632:1921#0/1
aaZW`a_aa`]a_]]`a`W`___^[_``a_Z_U`^[UQU[
@HWI-EAS443:3:77:638:1898#0/1
GGGATGATGATGGGCATGTTTAACATGCTAAGTACGGTCT
+HWI-EAS443:3:77:638:1898#0/1
N[`[bbb`^¥`a`aabb`Ybb`ab`a_b_ab``baba^_Z
@HWI-EAS443:3:94:1256:430#0/1
GGGATGATGATGGGCATGTTTAACATGCTAAGTACGGTCT
+HWI-EAS443:3:94:1256:430#0/1
aba``aaab`aa__`_ba_```a_a_`W`_`a]a_aWHV`
@HWI-EAS443:3:94:933:515#0/1
GGGATGATGATGGGCATGTTTAACATGCTAAGTACGGTCT
+HWI-EAS443:3:94:933:515#0/1
[X]_aa`bbaaa_a¥aaaaaa_`]`_a^aa_`Ra]a_Y]a
@HWI-EAS443:3:96:1306:1815#0/1
GGATGATGATGGGCATGTTTAACATGCTAAGTACGGTCTT
+HWI-EAS443:3:96:1306:1815#0/1
a``_a``[`a_]`T^aa``^_]]¥_^U`__[W_U_]ST^_
@HWI-EAS443:3:96:1745:796#0/1
GGATGATGATGGGCATGTTTAACATGCTAAGTACGGTCTT
+HWI-EAS443:3:96:1745:796#0/1
`]X`_aX¥aXUU^_UQ]QX__Y[¥]W¥V¥¥WS_¥S¥BBBB
```

```
@HWI-EAS443:3:97:336:973#0/1
GGGATGATGATGGGCATGTTTAACATGCTAAGTACGGTCT
+HWI-EAS443:3:97:336:973#0/1
aa`__`_`a`X`^^¥__aZ`__¥¥¥_]¥^_¥_W^W_¥WU^
@HWI-EAS443:3:57:1319:1351#0/1
GCGGGGAGTCTTCGAGCTCTCGGACGAAAAGGCTACGAAC
+HWI-EAS443:3:57:1319:1351#0/1
a`_W]¥XYZaa`^aY`^`Y^Ya[¥]aRY^¥X]XX¥S¥VBB
@HWI-EAS443:3:80:1377:1883#0/1
GACCATCGTCAAAATCCACAGCACTNTGCTGTTCCTGTTG
+HWI-EAS443:3:80:1377:1883#0/1
O[UaZ[YUFH]ND[PTOWO[¥PUUMDU^DDUYU^OXYKPT
@HWI-EAS443:3:18:1203:2004#0/1
GACAAAATGACCATCGTCAACATCCACAGCACTCTGCCGT
+HWI-EAS443:3:18:1203:2004#0/1
^`^`_`bbba[a¥]X¥S¥Yb]`a`P`b``[`]ab]XRDXZ
@HWI-EAS443:3:24:995:354#0/1
CACAAATTCAAACGAGGAGATCATTTGAGTTAAAGAAGCT
+HWI-EAS443:3:24:995:354#0/1
ababb`bbbbbbbabbaabaaaabbbababb`aabaa`a
@HWI-EAS443:3:95:1318:975#0/1
CGAAAGCAGGTCAAATATATTCAATATGGGGGGAATAAAA
+HWI-EAS443:3:95:1318:975#0/1
[Z`RQ]UP[^¥YI¥[ZVX_]Y[Y`a[TTZJYD[Z¥SVS^P
@HWI-EAS443:3:8:449:373#0/1
GCAGGTCAAATATATTCAATATGGGGAGAATAAAAGAACT
+HWI-EAS443:3:8:449:373#0/1
a_W`aY`Zaa`_Y¥`^_^X^Z```]_OaPN¥`]]^_[^S`
@HWI-EAS443:3:34:1305:2020#0/1
AGCAGGTCAAATATATTCAATATGGGGAGAATAAAAGAAC
+HWI-EAS443:3:34:1305:2020#0/1
aa_`aa[abbbaaabaaa`aaaa^RG¥_aa_^__^_[^U[
@HWI-EAS443:3:80:1783:910#0/1
GAAAGCAGGTCAAATATATTCAATATGGGGAGAATAAAAG
+HWI-EAS443:3:80:1783:910#0/1
a`GKMX^^`S¥a^_W[^``^__^_¥¥U]_]W_]V_Y^__Z
@HWI-EAS443:3:58:1620:658#0/1
AAAGCAGGTCAAATATATTCAATATGGGGAGAATAAAAGA
+HWI-EAS443:3:58:1620:658#0/1
_bb_H_^Z[bb^_X`b`bbaa_aP[^NGSZ_]¥`aa`][a
@HWI-EAS443:3:16:1774:1694#0/1
AACAATTCGACACTAATTGATGGGCATCCGAATTGTTTTG
+HWI-EAS443:3:16:1774:1694#0/1
a`^bb^¥WZ]bb`a`¥`[^aYWRD¥Za]Z`ZS_WDT`^[K
@HWI-EAS443:3:77:75:117#0/1
GTCTGGCTGTCAGTAAGTCTGCTAGAGTCCCGTTTTCGTT
+HWI-EAS443:3:77:75:117#0/1
_NW_Y`[U_QTLYPQWYDDK¥QWY^W¥QYYY¥GTYYWSUT
@HWI-EAS443:3:48:399:358#0/1
AACGAAAATGGGACTCTAGCATACTTACTGACAGCCAGAC
```

```
+HWI-EAS443:3:48:399:358#0/1
`aU]^Tb`abb`]^^`RD]T_aY_a^a¥_b`RHb_UT`ZF
@HWI-EAS443:3:84:659:1379#0/1
TGGGACTCTAGCATACTTACTGACAGCCAGACAGCGACCA
+HWI-EAS443:3:84:659:1379#0/1
XX¥W¥¥a^^_S]]a¥_QU¥`^a]_a¥aZX[¥YXP]¥WUZ¥
@HWI-EAS443:3:27:1074:1259#0/1
CGGCTGTATGGAGGATCTCCAGTATAAGGGAATGTGGTGC
+HWI-EAS443:3:27:1074:1259#0/1
^_^aaZYYXY_U]_U[Z_^XGWW[X]^a_aSYUXX^[JJL
@HWI-EAS443:3:66:924:213#0/1
CGGCTGTATGGAGGATCTCCAGTATAAGGGAATGTGGTGC
+HWI-EAS443:3:66:924:213#0/1
aabbbbZ^a]_Z]`X[UV_ZH^X]_[]]``YX`W¥_`NRL
@HWI-EAS443:3:83:582:1561#0/1
CGATTCGACACTAATTGATGGCCATCCGAATTCTTTTGGT
+HWI-EAS443:3:83:582:1561#0/1
ab`]]bYX]W`¥]L[_a_[_Saa[_a[a_`___``UZ^]G
@HWI-EAS443:3:61:1491:1295#0/1
CAGCACCGTCTGGCCAAGACCAACCCCCAGTGTCACTGTT
+HWI-EAS443:3:61:1491:1295#0/1
]]`bbaab_^I[]UP`_[Xaa]Vaa_L]V`PWGZLGL_GW
@HWI-EAS443:3:46:249:861#0/1
CACGATCGGGGTCGTTGCCTTTTCGTCCGAGAGCCCGGGC
+HWI-EAS443:3:46:249:861#0/1
``a_¥UXX_XJDTNLTNVYBBBBBBBBBBBBBBBBBBBBB
@HWI-EAS443:3:44:285:345#0/1
AGGCGGGGAGTCTTCGGGCTCTCGGACGAAAAGGCAACGA
+HWI-EAS443:3:44:285:345#0/1
`aa_a_]O__¥UW¥`XNTPX¥XRR_JGW¥`U`^XP[¥TZ]
@HWI-EAS443:3:19:630:1052#0/1
CGGGGAGTCTTCGGGCTCTCGGACGAAAAGGCAACGAACC
+HWI-EAS443:3:19:630:1052#0/1
aR¥Iaa`aa^[[VFTWa`¥U```_^RZ_¥U[¥`X`^X¥^U
@HWI-EAS443:3:76:523:1997#0/1
GAGCAATTGGGACAGAAATTTGAGGAAATAAGGTGGCTAA
+HWI-EAS443:3:76:523:1997#0/1
`Y_bb¥[VaZ]Rbb```a`^]V¥aaO__a^``PH¥VJJS^
@HWI-EAS443:3:56:1079:1326#0/1
AGCCAACAAAATAAAATCTGAGAAGACACAGATTCATCTT
+HWI-EAS443:3:56:1079:1326#0/1
aaa`aaaaabaabaaaaaaaaa_aaa`a_aaa`a``a^``
@HWI-EAS443:3:3:593:1464#0/1
CTGTTCGCATGTCGGATGTCCGTCCTTCATTGTTTCCGCC
+HWI-EAS443:3:3:593:1464#0/1
abab_HTabaaUXZa_bb¥S_b^ZHa`R^]_bOUIRG_BB
@HWI-EAS443:3:78:65:1228#0/1
CAGGTCCAAGGTCATTGTTTATCATGTTATTCTTTATCAC
+HWI-EAS443:3:78:65:1228#0/1
aa_a^bba_aa¥aaa_P[a`_^aaa_^_[`_aaa__`^^¥
```

```
@HWI-EAS443:3:32:23:907#0/1
AAAAAGTACACATCAGGGAGACAAGAGAAGAACCCCGCAC
+HWI-EAS443:3:32:23:907#0/1
`]X_]_X^_abaaaaa_K]a_^`a`^``a`¥a`^][]a[a
@HWI-EAS443:3:81:375:1642#0/1
TGCGGGGTTCTTCTCTTGTCTCCCTGATGTGTACTTTTTG
+HWI-EAS443:3:81:375:1642#0/1
ab`ba__Sab]]`abb`__aab`b___W^]Y^_b_^a`]X
@HWI-EAS443:3:75:1660:170#0/1
CAAGATTGGTCTTGTCTTTAGCCATTCCATGAGAGCGTCA
+HWI-EAS443:3:75:1660:170#0/1
J_^`Za^``^aa¥a¥Q_bb¥]]_¥Z_I_aaa¥b`_MG[Q_
@HWI-EAS443:3:18:1177:655#0/1
CTTGAGGATGAACAGATGTACCAGAAGTGCTGCCATCCAT
+HWI-EAS443:3:18:1177:655#0/1
abab aaa_`a`b[`a_ba]`_a_`aaa^a__`^_`__^``
@HWI-EAS443:3:83:1486:746#0/1
GCGAAAGCAGGTCAAATATATTCAATATGGAGAGAGTAAA
+HWI-EAS443:3:83:1486:746#0/1
a¥a[ZZ`XR¥¥P^_`¥RTRNY]OVU^SY]NT[N`WI^WS[
@HWI-EAS443:3:99:1386:1215#0/1
GCGAAAGCAGGTCAAATATATTCAATATGGAGAGAGTAAA
+HWI-EAS443:3:99:1386:1215#0/1
`XU[`ab]^]a]^_`U`ba_]aU^_`]P^`_b`aIG¥Z__
@HWI-EAS443:3:4:1268:1733#0/1
AATTGATGGCCATCCGAATTCTTTTGGGCGCTGTGTGGCT
+HWI-EAS443:3:4:1268:1733#0/1
^`_]b_abaTT[a]Y_aa``[aaab_XFXYO_¥DDZ`ZBB
@HWI-EAS443:3:57:342:2039#0/1
AATTCGACACTAATTGATGGCCATCCGAATTCTTTTGGGC
+HWI-EAS443:3:57:342:2039#0/1
Z¥XXW¥_S¥`_PT]`^]NJFUX^XWTTNY[VR]T^_BBBB
@HWI-EAS443:3:9:439:1335#0/1
ATTGATGGCCATCCGAATTCTTTTGGGCGCTGTCTGGCTG
+HWI-EAS443:3:9:439:1335#0/1
aaaaa^X`aaa_V[__^a¥R]`a]aMDH¥JVaU__¥VZ]B
@HWI-EAS443:3:75:1019:833#0/1
TACGGTTTGAAAAGAGGGCCTTCTACGGCAGGAGTGCCTG
+HWI-EAS443:3:75:1019:833#0/1
a¥a]TRQYW_SWYb¥]b`TQLR_TQ]Z`HW¥PV[GZYV^`
@HWI-EAS443:3:9:1051:1197#0/1
ATCTAATGTCGCAGTCCCGCACTCGCGAGATACTGACTAA
+HWI-EAS443:3:9:1051:1197#0/1
]]bb`^ab`]aa]^`_Uaa]X_a_`X]`a_^^_[EY[_``
@HWI-EAS443:3:25:1193:252#0/1
GGCTGGAGAAGTTCGGTGGGGGACTTTGGTCGGCAAGCTT
+HWI-EAS443:3:25:1193:252#0/1
abaaa_L_WLXRaaa^Ua]FFWSRS___aBBBBBBBBBBB
@HWI-EAS443:3:3:1368:1735#0/1
ATCTAATGTCGCAGTTCCGCACTCGCGAGATACTTACTAA
```

```
+HWI-EAS443:3:3:1368:1735#0/1
aa`aI[[^]U`V]GQPV_UY]BBBBBBBBBBBBBBBBBB
@HWI-EAS443:3:80:1177:208#0/1
GCGCGATCTCGGCTTTGAGGGGGCCTGAGGGGATGATAGA
+HWI-EAS443:3:80:1177:208#0/1
aabWa]a^`^^`SZ¥]¥M^`_¥WNNVTQOO^[PP[BBBBB
@HWI-EAS443:3:77:1344:1899#0/1
GGGTCGCTGTCTGGCTGTCAGTAAGGATGCTAGAGTCCCG
+HWI-EAS443:3:77:1344:1899#0/1
T`_WYabbaXbb_H_a]_aaaab^`O`MMFH]X`X]Q`bS
@HWI-EAS443:3:54:880:125#0/1
CGGTCGCTGTCTGGCTGTCAGTAAGTATGCTAGAGTCCCG
+HWI-EAS443:3:54:880:125#0/1
abb`bb`abaaaab_ba`^_b¥YaaSU^_[a¥aZ_OQ]]^
@HWI-EAS443:3:72:937:891#0/1
CGGTCGCTGTCTGGCTGTCAGTAAGTATGCTAGAGTCCCG
+HWI-EAS443:3:72:937:891#0/1
aba`abbbbaabaaaaa[]_^_aba[a][]^a]Y¥PT[^^
@HWI-EAS443:3:74:701:1288#0/1
CGGTCGCTGTCTGGCTGTCAGTAAGTATGCTAGAGTCCCG
+HWI-EAS443:3:74:701:1288#0/1
abb_baaba`ba^aaa^P`[`Y]^`Wa¥`^^_`¥¥M^a]Z
@HWI-EAS443:3:33:624:1404#0/1
CACAAAGCCTTCCATTTTGGATCAGTACCTGCTTTCGGAA
+HWI-EAS443:3:33:624:1404#0/1
]WaaY_aba^Za]J]a]____[_[NRV]W__^ZU¥QNUZ^
@HWI-EAS443:3:54:1548:1398#0/1
GAAGAAATAAGGCCCTTCATTAGTCATGTCAAAGGAAGGC
+HWI-EAS443:3:54:1548:1398#0/1
a[`_MZ`_]`UNOFT¥XWaX^UDY^___QTVLXV_ZS^XN
@HWI-EAS443:3:13:1553:842#0/1
AACAGATGTACCAGAAATGCTGCAATCTATTCGAGAAATT
+HWI-EAS443:3:13:1553:842#0/1
aZSS¥YOL[OX_]a_[DUaU^`V_a_``U]aPaa^Y¥_aW
@HWI-EAS443:3:71:386:667#0/1
CTGGGTTTTGCTTAATGCATCTTGGTTCAACTCCTTCCTA
+HWI-EAS443:3:71:386:667#0/1
ababbXabb`b`^bbbbbababbbabb_a_a`abab`NOF
@HWI-EAS443:3:63:844:565#0/1
GAACAGTGACCACAGAAGCAGCTTTTGGTCTAGTGTGTGC
+HWI-EAS443:3:63:844:565#0/1
H]__YY]¥`]YU[`a¥^¥a¥^^W`aZ^UH_¥BBBBBBBBB
@HWI-EAS443:3:51:298:930#0/1
GGGGTTCTTCTCTTGTCTTCCTGATGTGTACTTTCTGATT
+HWI-EAS443:3:51:298:930#0/1
WY`]XZ^aZ`a]aY`a`aaa^[]^¥]YaZ¥^a`Z`X^]Q]
@HWI-EAS443:3:73:473:1467#0/1
TTGGTGTGTTCTGTTTACTGTGTCCATGGTGTATCCTGTC
+HWI-EAS443:3:73:473:1467#0/1
Z]Y^ZQG[b^QRNT[WZaaUNT[TZ`V^GOZRSS]W^OQF
```

```
@HWI-EAS443:3:53:1618:497#0/1
TCGAACCGTGTTACCATTCCATTCAAGTCCTCCGATGAGT
+HWI-EAS443:3:53:1618:497#0/1
`_¥aSaV_a_W__K_V^]`N]_^bb`Z]¥V_GN]XOWV_J
@HWI-EAS443:3:39:989:97#0/1
CTTCGACTTTCGGACGAAAAGGCAACGAACCCGATCGTGC
+HWI-EAS443:3:39:989:97#0/1
abbbbbbbbbabbaabaaab`b_abaaa`a[Ua_`¥a¥aW
@HWI-EAS443:3:3:1005:1257#0/1
TCCTGGGTTTTGCTTAATGCATCTTGGTTCAACTCCTTCC
+HWI-EAS443:3:3:1005:1257#0/1
_]`_abb]baaaa`]aaaa`a_aa_a`¥`__aZWVOW`X]
@HWI-EAS443:3:40:12:1301#0/1
TCCTGGGTTTTGCTTAATGCATCTTGGTTCAACTCCTTCC
+HWI-EAS443:3:40:12:1301#0/1
aaa`]ab]aaaaaa`aa__aaaaa``__Ta`[`_aa__a_
@HWI-EAS443:3:76:284:548#0/1
TCCTGGGTTTTGCTTAATGCATCTTGGTTCAACTCCTTCC
+HWI-EAS443:3:76:284:548#0/1
abaabba^aaaaaaaaaaa_`aaaa_`]`a`_`a`]a`__
@HWI-EAS443:3:99:556:872#0/1
TCCTGGGTTTTGCTTAATGCATCTTGGTTCAACTCCTTCC
+HWI-EAS443:3:99:556:872#0/1
a]Uabbb^baba]ab_aab`abaaaa^_]aaaXa__a][_
@HWI-EAS443:3:76:1357:198#0/1
ATAGGATACATATGCAGTGGGGTTTTCGGAGAGAATCCAA
+HWI-EAS443:3:76:1357:198#0/1
]``aaVaWH¥`QXSNZXJ`_^IS__YBBBBBBBBBBBBBB
@HWI-EAS443:3:54:364:1135#0/1
CAAGAAATGGCCAGTAGGAGTCTATGGGATTCCTTTCGCC
+HWI-EAS443:3:54:364:1135#0/1
aaaa_aaaa```aaZ_`a[`]__^__`_]^_Y¥_¥^Q^WV
@HWI-EAS443:3:35:331:577#0/1
GATCGAAGGTTCCAGGTTCCAGGCTGTCCCTAAGTGCCTG
+HWI-EAS443:3:35:331:577#0/1
aZ[_]X^]ZN^a]P¥VM^a]`^YFQKK^`YBBBBBBBBBB
@HWI-EAS443:3:65:754:657#0/1
TTTTCGGTTCTTCCCCATATTCTTTCATTGCCTTTTCCGC
+HWI-EAS443:3:65:754:657#0/1
aabb`b]N[Waa]a`aaaOab_babaabaaa`babaa^`¥
@HWI-EAS443:3:32:144:51#0/1
CAGTGGGTTGGTCTTGGCCAGACGGTGCTGCGTTGCCCTT
+HWI-EAS443:3:32:144:51#0/1
TJXVXHWWW[]WZZ_W_ba`W_W`YTYFTYFYITXBBBBB
@HWI-EAS443:3:14:141:1507#0/1
TCTGAGTGCGGGGTTCTTCTCTTGTCTTCCTGATGTGTAC
+HWI-EAS443:3:14:141:1507#0/1
a`bbWWUaba^a_X^^a_`_aa`a_]aa^a``_`[¥`¥_]
@HWI-EAS443:3:22:511:1547#0/1
GTACACATCAGGAAGACAAGAGAAGAACCCCGCACTCAGA
```

```
+HWI-EAS443:3:22:511:1547#0/1
`_`_ab`_b^U[aa`a_aaaaa_b^aaaNa`ba]]_`Z`a
@HWI-EAS443:3:37:540:1154#0/1
GTACACATCAGGAAGACAAGAGAAGAACCCCGCACTCAGA
+HWI-EAS443:3:37:540:1154#0/1
`O^XX¥ZSOSZT`^VNV^`Ha_SVOJQ]QGYO_SNUXNFL
@HWI-EAS443:3:86:709:99#0/1
GTACACATCAGGAAGACAAGAGAAGAACCCCGCACTCAGA
+HWI-EAS443:3:86:709:99#0/1
a¥a_aa[¥aab_T¥aWQ_P_a_^NYa_WMXX_]LRUSUZM
@HWI-EAS443:3:17:312:1634#0/1
AAGCATCAATGAACTGAGCAATCTTGCAAAAGGAGAAAAA
+HWI-EAS443:3:17:312:1634#0/1
`a`aa`a`aaaaaa_^`a_`XY`_^_a^`^]___`^_^^^
@HWI-EAS443:3:32:1107:804#0/1
TTCGGATTTCCATTTCATCGACGAACGGGGTGGATCAATA
+HWI-EAS443:3:32:1107:804#0/1
aaaa``aaaa`Y]aa_a]_a`[aXXQ____Raa^WVUSYX
@HWI-EAS443:3:40:119:936#0/1
CGGACAGTATGGATAGCAAATAGTAGCATTGCCACAACTA
+HWI-EAS443:3:40:119:936#0/1
abb_`¥a`bbbbaaab`¥a`^ba]a`_[]aYa]Z_V¥Y`b
@HWI-EAS443:3:45:1570:1193#0/1
GGCTGGTGTTTATAGCACCCTTTGGTGTCTGACAAGTTGT
+HWI-EAS443:3:45:1570:1193#0/1
aaa`bb_``bbaa][[a__¥a¥H_]BBBBBBBBBBBBBBB
@HWI-EAS443:3:43:1316:2016#0/1
GATTGGGCCATGAACTTGTTTTGGGGAANATCTCAAACCT
+HWI-EAS443:3:43:1316:2016#0/1
^XT`X_XZ`aa`]]¥W^a]FSZb¥aa]JD[a_]P]_aWZa
@HWI-EAS443:3:39:162:1614#0/1
CATATCTCGGTACCACTAGATTCCCAGTTGCTTCGAATGT
+HWI-EAS443:3:39:162:1614#0/1
aaaa¥]_b`aVaa_O`Y]HX]aa[aa_VaU`]^Z`]OY¥M
@HWI-EAS443:3:76:1467:1076#0/1
GGGGGATGGAAATGAGGCGCTGCCTTCTTCAGTCTCTTCA
+HWI-EAS443:3:76:1467:1076#0/1
a^POFYRFON_QN_W[¥S_U^_UWRUNQ¥GVZWQSPPPUV
@HWI-EAS443:3:48:1583:1266#0/1
GGCGAATCAATAATTGTAGAATCTGGTGACCCGACTGGCA
+HWI-EAS443:3:48:1583:1266#0/1
a_QWZ]a_UOZZ`aaa¥¥]WWUT]ZWQ`_`ZPZRRX^_]Z
@HWI-EAS443:3:62:585:557#0/1
GATTCTTGCCCTGCTCTCTTCGTCAAGGGGGTAGTCCGCC
+HWI-EAS443:3:62:585:557#0/1
P_VZ^ZP_X¥aa[a_`R^a`a`XQ][ZSZEYGGVGWa`BB
@HWI-EAS443:3:19:481:1366#0/1
GGGCGAATCAATAATTGTAGAATCTGGTGACCCGAATGCA
+HWI-EAS443:3:19:481:1366#0/1
^a`P`^IZbba`aba]TZbbabaaaaaWa_G_aa]aVa__
```

```
@HWI-EAS443:3:21:1429:384#0/1
TAGCCCCCCAAGATCGAAGGCTCCAGGCTCCAGGTTGTCC
+HWI-EAS443:3:21:1429:384#0/1
`bZa]X]aa``bXa]`_WaYTKTWQ]^MVHMLRY¥BBBBB
@HWI-EAS443:3:13:1067:775#0/1
CAAACCATGAGGGAATACAAGCAGGAGAGGACAGGTTCTA
+HWI-EAS443:3:13:1067:775#0/1
]bb[aZ[b_Z^``a_aa_¥JS``[a[aG¥N_HNWGYLV[_
@HWI-EAS443:3:84:767:902#0/1
GTCCACTCCTGCTTGTATTCCCTCATGGTTCGGTGCATTC
+HWI-EAS443:3:84:767:902#0/1
a¥__aa`aa`aaaa]Yaaa``a`a^^]]¥__a`Ya^____
@HWI-EAS443:3:95:172:1718#0/1
CAGGAATGTGTAGTCGCAACGGCATCATATTCCATGCTTT
+HWI-EAS443:3:95:172:1718#0/1
^aabXbaa]b_aaLH[aa¥a^UW``aaa_``a]a¥__]]^
@HWI-EAS443:3:44:1092:379#0/1
GACCATATGGCCATAATCAAAAAGTACACATCAGGGAGAC
+HWI-EAS443:3:44:1092:379#0/1
aZY]RW__a`V`[EZQ^T_a_`^XYS[aNOFL^`TDR_NG
@HWI-EAS443:3:51:732:152#0/1
GGCCATCCGACTTCTTTTGGTCGCTGTCTGGCTGTAAGAA
+HWI-EAS443:3:51:732:152#0/1
^[a[`Z^^X]HR_YURRQ`^O]_SV[_]^U¥BBBBBBBBB
@HWI-EAS443:3:92:299:1119#0/1
ATTCGACACTAATTGATGGCCATCCGACTTCTTTTGGTCG
+HWI-EAS443:3:92:299:1119#0/1
a`ab^]aaaa_N¥S`^TV[`aa]XYU¥H¥`W``a___ZZa
@HWI-EAS443:3:39:1754:1449#0/1
TTTTTGGACAGTATGGATAGCAAATAGTAGCGTTGCCACA
+HWI-EAS443:3:39:1754:1449#0/1
]b]^`[^a_W`[TQ¥_PX¥]]`_]¥^MFMa¥GKSYZ_¥TQ
@HWI-EAS443:3:86:151:1559#0/1
GGCACTTGGTGAAAATATGGCACCAGAAAAAGTAGACTTT
+HWI-EAS443:3:86:151:1559#0/1
``bb__P]¥]]PVHWa]a`]]a`__[_a`]ULGaSZT^^X
@HWI-EAS443:3:59:1357:359#0/1
GAAACAATGGAAGTCGTTCAACAAACAAGGGTAGAAAGAT
+HWI-EAS443:3:59:1357:359#0/1
a^XY^`aaaa¥`_QLYV¥MU]I__¥U[YY_]VV¥¥YNRSZ
@HWI-EAS443:3:38:686:1047#0/1
CCTTGAAACAATGGAAGTCGTTCAACAAAAAAGGGTAGAT
+HWI-EAS443:3:38:686:1047#0/1
aa_`_a]a`bR__[_a^X¥OT]ZLN[[`YGFXUWZGVQGT
@HWI-EAS443:3:3:998:126#0/1
GCCTTGAAACAATGGAAGGCGTTCAACAAACAAGGGTAGA
+HWI-EAS443:3:3:998:126#0/1
¥_a_ba^a_[`a`baa`¥DI[X]UY_O¥U^G¥]R`_QZ]T
@HWI-EAS443:3:100:736:1369#0/1
TTGAAACAATGGAAGTCGTTCAACAAACAAGGGTAGATAA
```

```
+HWI-EAS443:3:100:736:1369#0/1
a`^``a]`ba_]_aZOWXZ¥X¥¥U__XQZa`_`Y^_VZ^B
@HWI-EAS443:3:61:1670:38#0/1
TTGAAACAATGGAAGTCGTTCAACAAACAAGGGTAGATAA
+HWI-EAS443:3:61:1670:38#0/1
aabaaaaa`abaa`a^_a`^¥XXPUZ`J^]^^¥PQYT]XZ
@HWI-EAS443:3:34:869:1455#0/1
CTTGAAACAATGGAAGTCGTTCAACAAACAAGGGTAGATA
+HWI-EAS443:3:34:869:1455#0/1
a`X`ab`abaa`X[a¥V[VXWSPYa_aa^^^QJSY]UWBB
@HWI-EAS443:3:58:684:870#0/1
CTTGAAACAATGGAAGTCGTTCAACAAACAAGGGTAGATA
+HWI-EAS443:3:58:684:870#0/1
aaaa```ab_b_a_a^WV¥Y_S^_W[_`__`]_¥SZ^¥V_
@HWI-EAS443:3:64:323:630#0/1
CTTGAAACAATGGAAGTCGTTCAACAAACAAGGGTAGATA
+HWI-EAS443:3:64:323:630#0/1
abaaWX`aa[aaaa]^Y]ZX_[]]S_`UR_¥VZ[OOSXP^
@HWI-EAS443:3:71:1162:1755#0/1
CTTGAAACAATGGAAGTCGTTCAACAAACAAGGGTAGATA
+HWI-EAS443:3:71:1162:1755#0/1
YS^`Y^bbbbab`^a¥]a`^aS[_H]_`¥``^WWT_X_UY
@HWI-EAS443:3:85:1218:1054#0/1
CCTTGAAACAATGGAAGTCGTTCAACAAACAAGGGTAGAT
+HWI-EAS443:3:85:1218:1054#0/1
aabbbaababaab^aab¥[]``[[a_a`_Q[``aaS__Z^
@HWI-EAS443:3:97:1404:709#0/1
GTTGTGAATTGGTCATCATCTTTCTCACAACATTTGCCAG
+HWI-EAS443:3:97:1404:709#0/1
X__¥`a`_a_S`SS[_]aXab¥^V`ababbXXX]_a¥HH_
@HWI-EAS443:3:9:452:1502#0/1
ATCTACCCTTGTTTGTTGAACGACTTCCATTGTTTCAAGG
+HWI-EAS443:3:9:452:1502#0/1
a_`a_]Y_a``V_^^[_`Y[`^P`]_]__ZP_T[SZSOW[
@HWI-EAS443:3:17:847:832#0/1
GCCTTGAAACAATGGAAGTCGTTCAACAAACAAGGGTAGA
+HWI-EAS443:3:17:847:832#0/1
aba`aba^aa_]_aa```¥]`^][¥`a^aZLZS[`_X^][
@HWI-EAS443:3:20:114:2040#0/1
TACCCTTGTTTGTTGAACGACTTCCATTGTTTCAAGGCAT
+HWI-EAS443:3:20:114:2040#0/1
abbbbaaa`a`]_aaabba¥aaaaaba`aXa`aaaa_aa_
@HWI-EAS443:3:28:1162:1323#0/1
TGCCTTGAAACAATGGAAGTCGTTCAACAAACAAGGGTAG
+HWI-EAS443:3:28:1162:1323#0/1
¥_ba^a]¥Y¥a]WO]Z[T]a_XOSRS^MW]^M^ZT[OJ^L
@HWI-EAS443:3:46:1084:406#0/1
TGCCTTGAAACAATGGAAGTCGTTCAACAAACAAGGGTAG
+HWI-EAS443:3:46:1084:406#0/1
abbabb`_`bbbabbbaba[`a¥`Y^_ZZ``^_aa`aO^_
```

````
@HWI-EAS443:3:4:740:851#0/1
ATGCCTTGAAACAATGGAAGTCGTTCAACAAACAAGGGTA
+HWI-EAS443:3:4:740:851#0/1
aaaaabab`aa^a_aaaaabUR¥^_^]^^_``WY^Y`_P^
@HWI-EAS443:3:59:657:551#0/1
TGCCTTGAAACAATGGAAGTCGTTCAACAAACAAGGGTAG
+HWI-EAS443:3:59:657:551#0/1
aaaaabbaababaaa``aa[_a^__^[P]a`G_`_YXO^N
@HWI-EAS443:3:92:1068:768#0/1
TGCCTTGAAACAATGGAAGTCGTTCAACAAACAAGGGTAG
+HWI-EAS443:3:92:1068:768#0/1
ab`^`b`_^babab¥b`]]`QZaaa¥aba[^a]a^Y_a
@HWI-EAS443:3:89:88:704#0/1
CAGGCAAAAGCAGGGCAGATAATCACTCAGTGAGTGACAT
+HWI-EAS443:3:89:88:704#0/1
a`a]aT^Z¥_a[ab]Ga]X`a__a^__UX^]`_`Z_`[`¥
@HWI-EAS443:3:38:1113:175#0/1
AAGAGAAGAACCCCGCACTCAGAATGAAGTGGGGAGGGGC
+HWI-EAS443:3:38:1113:175#0/1
a]bbbabaa_QZ^^bb_a`_`a[_a`Z^_MPP]^¥_^W_Q
@HWI-EAS443:3:4:268:610#0/1
AAGAGAAGAACCCCGCACTCAGAATGAAGTGGGGCGATGG
+HWI-EAS443:3:4:268:610#0/1
a]`VO¥¥a`[[`¥TT^ZX¥NQZPJ[aNSVGY[¥YU]P^VP
@HWI-EAS443:3:33:1018:1729#0/1
CAAGAGAAGAACCCCGCACTCAGAATGAAGTGGGTGAGGG
+HWI-EAS443:3:33:1018:1729#0/1
_XR_N]ZT]ZSVRXHZ]RVHT[_YU]]X^_R]NFRUXF[[
@HWI-EAS443:3:73:223:643#0/1
CCCAGCACTAAGCATCAATGAACTGAGCAATCTTGCAAAA
+HWI-EAS443:3:73:223:643#0/1
abaY`a_I_a[[baabb¥`ZZa_aaTaY]Uaa`a_`__¥O
@HWI-EAS443:3:43:526:1226#0/1
TTGACATCCATTCAAATGGTTTGCCTGCTTTCGCTAAAAA
+HWI-EAS443:3:43:526:1226#0/1
aabbaabaa]aaaZV_a_``aaaa```Za``_^¥^```a_
@HWI-EAS443:3:21:1376:963#0/1
GATTGACATCCATTCAAATGGTTTGCCTGCTTTCGCTATT
+HWI-EAS443:3:21:1376:963#0/1
a_``_`__]^_[a^X__^``_V__]YX^^W^_aXaX]Z^^
@HWI-EAS443:3:91:1124:1365#0/1
ATTGACATCCATTCAAATGGTTTGCCTGCTTTCGCTAAAA
+HWI-EAS443:3:91:1124:1365#0/1
aababaa``_aaa^aa¥aa__`aa_Xaa`aa_`aZ[aa``
@HWI-EAS443:3:36:339:920#0/1
GATTGACATCCATTCAAATGGTTTGCCTGCTTTCGCTAAA
+HWI-EAS443:3:36:339:920#0/1
aa__`````]X]`[a`a__a_Z_``^^]^__`^^`]]^¥Q
@HWI-EAS443:3:86:517:1967#0/1
GATTGACATCCATTCAAATGGTTTGCCTGCTTTCGCTAAA
````

+HWI-EAS443:3:86:517:1967#0/1
]_aaab`aY^ab``^bab`a`Sa_`_Y]_`a^`__`abaa
@HWI-EAS443:3:8:1513:572#0/1
GATTGACATCCATTCAAATGGTTTGCCTGCTTTCGCTAAA
+HWI-EAS443:3:8:1513:572#0/1
aaa`aa_aa[_`aa]___a`_Xa__XX^`X`^_U[X`___
@HWI-EAS443:3:13:1711:817#0/1
CGGATTGACATCCATTCAAATGGTTTGCCTGCTTTCGCTA
+HWI-EAS443:3:13:1711:817#0/1
_aa_a_a¥aZ_^`Za¥Z¥^]``_Z^_^ZG_[LZ_`]`]]_
@HWI-EAS443:3:29:1125:453#0/1
CGGATTGACATCCATTCAAATGGTTTGCCTGCTTTCGCTA
+HWI-EAS443:3:29:1125:453#0/1
]ab]b``^b`b¥Z^baaaaaab[]a¥S]aaab¥¥_Ybaa`
@HWI-EAS443:3:2:507:58#0/1
CGGATTGACATCCATTCAAATGGTTTGCCTGCTTTCGCTA
+HWI-EAS443:3:2:507:58#0/1
abaaaaa__aaS^Z`_S^[a_]JN__a[U_`O_]^U^W^^
@HWI-EAS443:3:90:964:1486#0/1
ATCCGGGCTCTCCTTCCGTAGAAGGCCCTCTTTTCAAACC
+HWI-EAS443:3:90:964:1486#0/1
a_bb_b``aWZUQ_]a[V[aaaa_aU]GY_a¥aa_¥R^M[
@HWI-EAS443:3:19:1166:851#0/1
GGGCACTCCTTCCGTAGAAGGCCCTCTTTTCAAACCGTAT
+HWI-EAS443:3:19:1166:851#0/1
a``¥`]a`Vaa]aa`aa]]`]]X^`__`a`[a_aR]_R[[
@HWI-EAS443:3:35:78:1647#0/1
CGGGCACTCCTTCCGTAGAAGGCCCTCTTTTCAAACCGTA
+HWI-EAS443:3:35:78:1647#0/1
a[S[abb`bbaaa`XZ^U¥UZY]¥`^a_^W^a[M]`]YRO
@HWI-EAS443:3:58:34:1277#0/1
GTCGTCGCTTTAAATACGGTTTGAAAAGAGGGCCTTCTAC
+HWI-EAS443:3:58:34:1277#0/1
a`a``a^`]_a`Y][VK_[N^YUW^`[ZTHWSOJMWBBBB
@HWI-EAS443:3:48:706:455#0/1
GCATCAATGAACTGAGCAATCTTGCGAAAGGAGAGAAAGC
+HWI-EAS443:3:48:706:455#0/1
a`aa_babaaa`aa_baaaa_aaa_b``_b`]aaa`__a^
@HWI-EAS443:3:5:27:512#0/1
GAACTGAGCAATCTTGCGAAAGGAGAGAAAGCTAATGTGC
+HWI-EAS443:3:5:27:512#0/1
]`ba``_aaa_baaabaS_[X_W^^``[_XaUa`]`a_a_
@HWI-EAS443:3:27:294:1562#0/1
TGTGTTGGTAATGAAACGAAAACGGGACTCTAGCATACTT
+HWI-EAS443:3:27:294:1562#0/1
aa`[_aaa[aaa]abbaaaZaa]_`__a_a]_`_a^a_¥]
@HWI-EAS443:3:50:1088:514#0/1
TGTGTTGGTAATGAAACGAAAACGGGACTCTAGCATACTT
+HWI-EAS443:3:50:1088:514#0/1
ab_b`bba[[``a_`_V]¥_`^Yaaa^^`__¥]V[_[T¥`

```
@HWI-EAS443:3:86:867:1325#0/1
CCAGCATTAAGCATCAATGAACTGAGCAATCTCGCAGAAG
+HWI-EAS443:3:86:867:1325#0/1
aab[_aa`[ab]aZba]aW^baa`_ab_X`aa¥_aaHZW`
@HWI-EAS443:3:68:1410:1380#0/1
CTCATGTCAAAGGAAGGCACGAGCGGGTTTGTTGCCTTTT
+HWI-EAS443:3:68:1410:1380#0/1
Zbba_aXaa^_PQTX^^a`Xb[DW`^`IQF¥OJHFUBBBB
@HWI-EAS443:3:32:418:1522#0/1
GAGTCTTCGAGCTCTCGGACGAAAAGGCAACAAACCCTGA
+HWI-EAS443:3:32:418:1522#0/1
`_]abbab]__`a`]`¥WabaaabaPT¥aaaaa`a[b__X
@HWI-EAS443:3:34:162:1614#0/1
TGGCCCATATCTCTTGTCTTCTTTGCCCAAAATGAGAAAT
+HWI-EAS443:3:34:162:1614#0/1
R`[H]_S_```aaXW^^Yaaa]`abb^a``aaa[aZ]W`[
@HWI-EAS443:3:67:369:1180#0/1
ACCGGTTGGAATTTCTAGCATGGTGGAGGCCATGGTTTCT
+HWI-EAS443:3:67:369:1180#0/1
Y^TR]P^`^ZZa[]^^WT`TXR_N_YQWXaBBBBBBBBBB
@HWI-EAS443:3:60:1176:2027#0/1
TTTATCGTCGCTTTAAATACGGTTTGAAAAGAAGGCCTTC
+HWI-EAS443:3:60:1176:2027#0/1
U^a[L`bTSUXX`Y]V^XZR]_LU[OM`a]U]HTaUGV¥R
@HWI-EAS443:3:29:198:660#0/1
GGGGCTCTAGCATACTTACTGACAGCCAGACAGCGACCAA
+HWI-EAS443:3:29:198:660#0/1
]a`bbabYYbbabaaabbbb`abbbbbbabbb`bbaaabb
@HWI-EAS443:3:91:534:1514#0/1
GAGGGACTCTAGCATACTTACTGACAGCCAGACAGCGAAC
+HWI-EAS443:3:91:534:1514#0/1
H^_¥¥]¥[__`bbb_`b_``abbabb^^abUbb]abVQH[
@HWI-EAS443:3:100:1160:1378#0/1
AGGGACTCTAGCATACTTACTGACAGCCAGACAGCGACCA
+HWI-EAS443:3:100:1160:1378#0/1
``_aaaaXa__^aaaaaaWI¥`a¥`_Raa`HHV^U_XIBB
@HWI-EAS443:3:25:836:366#0/1
AGGGACTCTAGCATACTTACTGACAGCCAGACAGCGACCA
+HWI-EAS443:3:25:836:366#0/1
_UN`_aW[aX[¥Za_W___Y_V[^]Z`X]]_^__^[ZPS[
@HWI-EAS443:3:30:1757:923#0/1
AGGGACTCTAGCATACTTACTGACAGCCAGACAGCGACCA
+HWI-EAS443:3:30:1757:923#0/1
a¥T`ZW_ba]baab`_abb_aba_aa¥Xa]aaaaGZ^THV
@HWI-EAS443:3:45:1236:1507#0/1
AGGGACTCTAGCATACTTACTGACAGCCAGACAGCGACCA
+HWI-EAS443:3:45:1236:1507#0/1
ababa`baaaaaaaaabbb`aba_ab_a_`a_a___[_`[
@HWI-EAS443:3:68:78:685#0/1
AGGGACTCTAGCATACTTACTGACAGCCAGACAGCGACCA
```

```
+HWI-EAS443:3:68:78:685#0/1
S`_^`aaa`aaba`_aaa^aa`aa``aaaaa¥]`a__`ZJ
@HWI-EAS443:3:88:537:809#0/1
AGGGACTCTAGCATACTTACTGACAGCCAGACAGCGACCA
+HWI-EAS443:3:88:537:809#0/1
aa]a_a_]^a^a^`[^`aa]`^¥^aa_`^a`_``___^UX
@HWI-EAS443:3:98:1628:588#0/1
AGGGACTCTAGCATACTTACTGACAGCCAGACAGCGACCA
+HWI-EAS443:3:98:1628:588#0/1
ab`aa^_aabbbbb]R^ab_aaaabb]aa_[XYR__aJDY
@HWI-EAS443:3:9:1036:1608#0/1
AGGGACTCTAGCATACTTACTGACAGCCAGACAGCGACCA
+HWI-EAS443:3:9:1036:1608#0/1
a]aaaabY^TS]abaa[F[UZ¥a_``]Ta^_____aWRVZ
@HWI-EAS443:3:34:1595:1926#0/1
CCCGTTTTCGTTTCATTACCAACACTATGTCCCCTTGCCC
+HWI-EAS443:3:34:1595:1926#0/1
_¥]^K¥bba¥Z[a`aa^b[abb]TZ]ba`¥YV[PPMTHFF
@HWI-EAS443:3:37:1203:751#0/1
GCCCCGTTTTCGTTTCATTACCAACACTATGTCCCCTTGC
+HWI-EAS443:3:37:1203:751#0/1
]JRa^[Z^¥`WYaYZZa[ab_^`]`^]¥XFYUa`Z]UPRU
@HWI-EAS443:3:83:1577:1167#0/1
ACCCGTTTTCGTTTCATTACCAACACTATGTCCCCTTGCC
+HWI-EAS443:3:83:1577:1167#0/1
a^¥Zb`aa`Xa]``_aaa_[Z`^Va^_XS[]R^[^]¥^RX
@HWI-EAS443:3:43:1205:958#0/1
GTCCCGTTTTCGTTTCATTACCAACACTATGTCCCCTTGC
+HWI-EAS443:3:43:1205:958#0/1
]FUZa^a[¥TT[`Y`[N_¥^LSZUPZR_TFI¥GN_WMHGG
@HWI-EAS443:3:52:1288:988#0/1
CGCCGTCTGGGTTCTTCAATGGTGGAACAGATCTTCATGA
+HWI-EAS443:3:52:1288:988#0/1
a``abaab_I^abbbba[aaabYb_]IW]a^RHa`[]``a
@HWI-EAS443:3:31:812:1341#0/1
GCCGTCTGGGTTCTTCAATGGTGGAACAGATCTTCATGAT
+HWI-EAS443:3:31:812:1341#0/1
abb`PbaaaaZaaaaaaa]a]^a_]``aaa`^_`_`[^``
@HWI-EAS443:3:73:63:1098#0/1
CTGGGTTCTTCAATGGTGGAACAGATCTTCATGATCTCAG
+HWI-EAS443:3:73:63:1098#0/1
`]`W¥[Va_^`RQ_baX^QU[ab_TX`]¥``¥[R¥]^ZXS
@HWI-EAS443:3:81:1110:1426#0/1
TGCCGTCTGAGTTCTTCAATGGTGGAACAGATCTTGATGA
+HWI-EAS443:3:81:1110:1426#0/1
[¥_T_^^`a`aW`aMZbaX^`bIabaaPX^`[a`^F¥J^R
@HWI-EAS443:3:43:1525:954#0/1
ACGCCGGTTGATTTCTGTTTAATGTCCAATCATAAGTCTG
+HWI-EAS443:3:43:1525:954#0/1
a_ba^bb`ba_bba_abaab_^aa]_]a`a^_`_`_]]_`
```

```
@HWI-EAS443:3:24:535:1872#0/1
CGCCGGTTGATTTCTGTTTAATGTCCAATCATAAGTCTGG
+HWI-EAS443:3:24:535:1872#0/1
aaab`]Xaa`aaa`aa`a`aa_a^``a`¥_a_`^[U_^[^
@HWI-EAS443:3:36:1027:1520#0/1
CGCCGGTTGATTTCTGTTTAATGTCCAATCATAAGTCTGG
+HWI-EAS443:3:36:1027:1520#0/1
aaaa`a^abaaa`_`^`aaaa__^`_^Taaa^a_a]a__]
@HWI-EAS443:3:49:1019:800#0/1
CGCCGGTTGATTTCTGTTTAATGTCCAATCATAAGTCTGG
+HWI-EAS443:3:49:1019:800#0/1
`a^^`babbabb````abbaab_TV^aa]^`aa[a_`aa^
@HWI-EAS443:3:93:1164:960#0/1
GCGGGGGAGTCTTCGAGCTCTCGGACGAAAGGGCAACGAA
+HWI-EAS443:3:93:1164:960#0/1
_a`_¥Z^]a[```]`_^¥`¥a^`a`X`[¥¥FN`¥¥^P^]X
@HWI-EAS443:3:38:1757:1885#0/1
GGATAGCAAATAGTAGCATTGCCACAACTAATTCAATGCA
+HWI-EAS443:3:38:1757:1885#0/1
`ab`b`bab_ba_Sbaabaab_ab_bbT][D[[KVY`^EO
@HWI-EAS443:3:6:1751:454#0/1
ATGGATAGCAAATAGTAGCATTGCCACAACTAATTCAATG
+HWI-EAS443:3:6:1751:454#0/1
abab_[^_¥a[¥aaaXa¥WXa`NQL¥]__H]XEUW^a__]
@HWI-EAS443:3:97:328:971#0/1
ATAGCAAATAGTAGCATTGCCACAACTAATTCAATGCATG
+HWI-EAS443:3:97:328:971#0/1
^Z¥bVb_H_a[W`a]Y^b`]^``aaIa]F]T¥[¥`^UP]`
@HWI-EAS443:3:79:881:229#0/1
ATGGATAGCAAATAGTAGCATTGCCACAACTATTTCAATG
+HWI-EAS443:3:79:881:229#0/1
abbaaa`]aa`aaa`^aa_____^a`_a`^_`a_aW_]__
@HWI-EAS443:3:86:1586:686#0/1
GGATAGCAAATAGTAGCATTGCCACAACTATTTCAATGCA
+HWI-EAS443:3:86:1586:686#0/1
_a`aa^]_aaaY]TZa`a^aa]Y¥V¥_^^aHa_¥_`¥_R^
@HWI-EAS443:3:96:1276:1892#0/1
TGGATAGCAAATAGTAGCATTGCCACAACTATTTCAATGC
+HWI-EAS443:3:96:1276:1892#0/1
aaa__ab_aaaaa¥G_Va]_`_`¥¥Wa^Z_]Gba¥aW_`_
@HWI-EAS443:3:13:691:242#0/1
CAAATTGATGGCCATCCGAATTCTTTTGGTCGCTGTCTGG
+HWI-EAS443:3:13:691:242#0/1
^M`ababbabb_abb`abaaaa`babbb`__aaab```T`
@HWI-EAS443:3:34:875:2034#0/1
CACTAATTGATGGCCATCGGAATTCTTTTGGTCGCTGTCT
+HWI-EAS443:3:34:875:2034#0/1
a^a`aa^XYU_^Z`_]N[ZZZ^[]^]^^`NFQVWTBBBBB
@HWI-EAS443:3:11:1207:1566#0/1
GAGCAATCTTGCAAAAGGAGAGAAAGCTAATGTGCTGATT
```

```
+HWI-EAS443:3:11:1207:1566#0/1
]b`bab_Z[`b`_Y_abaabaaaa``aaa^Ya[aH_`^aZ
@HWI-EAS443:3:9:457:1177#0/1
CCTTGTCCTTCGTCCATTTTCACCTCTCCAGAAATTCCGG
+HWI-EAS443:3:9:457:1177#0/1
S_aab^a`a]abb_X__]b¥Q_``Yaa]bY_^]`]ZGYaa
@HWI-EAS443:3:90:177:1828#0/1
GGCCGGGCCATATCTCTTGTCTTCTTTGCCCAAAATGAGA
+HWI-EAS443:3:90:177:1828#0/1
^`ab_X_aabbabbbbabab`bbbbb`^babbbbbaabab
@HWI-EAS443:3:61:1639:1235#0/1
TTGGCCATCCGAATTCTTTTGGTCGCTGTCTGGCTGGCAG
+HWI-EAS443:3:61:1639:1235#0/1
TRWWHT___TQ__b[P^[¥ZWa[[[[OUZ[^WTT^XFOW_
@HWI-EAS443:3:53:149:577#0/1
CGGCTTGTTTTCTTCATGTTCTTTGTCCTTGGGGTCTTCT
+HWI-EAS443:3:53:149:577#0/1
ZZV`_aV]XHY_QUSXVX_a`]ZZFQXNWJX[YFNFXSMU
@HWI-EAS443:3:27:1195:1116#0/1
CGCGTCACTAATTGATGGCCATCCGAATTCTTTTGGTCGC
+HWI-EAS443:3:27:1195:1116#0/1
ab`_WVW_[aW`[T_XVNS_]Z_`aRK[H¥^]ZZa¥HV__
@HWI-EAS443:3:94:1662:465#0/1
CCACTTTTCATTTCTGCTCTGGAGGGAGTGAAGGTGTGCG
+HWI-EAS443:3:94:1662:465#0/1
a^TaZZUU]aa`]HUQM]O¥^GWYWS`UNYXBBBBBBBBB
@HWI-EAS443:3:12:243:938#0/1
TCTCGCCACTTTTCATTTCTGCTCTGGAGGGAGGGAAGGG
+HWI-EAS443:3:12:243:938#0/1
^VTWTa_YWXPK_a_VU]^^R^BBBBBBBBBBBBBBBBBB
@HWI-EAS443:3:17:1734:195#0/1
GCTCTCGCCACTTTTCATTTCTGCTCTGGAGGGAGGGAAG
+HWI-EAS443:3:17:1734:195#0/1
aa_^Y[¥ZYUN¥R^[VV[¥¥WNPPVUUMRUW¥BBBBBBBB
@HWI-EAS443:3:3:1240:92#0/1
AATTGCTCTCGCCAGTTTTCATTTCTGCTCTGGAGGGAGT
+HWI-EAS443:3:3:1240:92#0/1
a^bVYZaT`^^YZQGQ`ZaU¥_]]Y__WYW¥BBBBBBBBB
@HWI-EAS443:3:6:481:556#0/1
CTTCACTCCCTCCAGAGCAGAAATGAAAAGTGGCGAGAGC
+HWI-EAS443:3:6:481:556#0/1
abb`b`aS¥``_a_a__¥_aZ^]]`¥Z__TMX_W`^YY^B
@HWI-EAS443:3:45:434:35#0/1
TATGGGCCTCATATACAACAGGATGGGAACAGTGACCACA
+HWI-EAS443:3:45:434:35#0/1
abbabaaaba_baaaaaa^`aa^^aaa___¥a]aa__``_
@HWI-EAS443:3:79:323:1274#0/1
TATGGGCCTCATATACAACAGGATGGGAACAGTGACCACA
+HWI-EAS443:3:79:323:1274#0/1
Z_S]_`a¥¥a[¥`Tab[V¥¥W¥¥^¥UWS]a¥]LUbbaa_Z
```

```
@HWI-EAS443:3:33:1461:1821#0/1
GCTGGTTGATTTCTGTTTAATGTCCAATCATAAGTCTGGC
+HWI-EAS443:3:33:1461:1821#0/1
aa_`_I[X¥`a`aababaaaaaV¥aa[`[``_SZRRO^]Y
@HWI-EAS443:3:54:751:588#0/1
TGGCGGGGAGTCCTCGAGCTCTCGGACGAAAAAGCAACGA
+HWI-EAS443:3:54:751:588#0/1
aba`^a^_]]MV¥PZXJX]¥^T[QYQNYT¥a[ETNZXBBB
@HWI-EAS443:3:82:483:1548#0/1
GGGGCGGGGAGTCCTCGAGCTCTCGGACGAAAAAGCAACG
+HWI-EAS443:3:82:483:1548#0/1
¥^a_aaa[VDY``a`¥Y`_RX_`aUa`VTW`a_FP``a_T
@HWI-EAS443:3:72:626:1799#0/1
CGGCGGGGAGTCTTCGAGCTCTCGGACGAAAAAGCAACGA
+HWI-EAS443:3:72:626:1799#0/1
a^¥aWYPKLEK]R¥XQOIDT¥RUMUQFQOU]ZDNUPXVSB
@HWI-EAS443:3:17:735:45#0/1
TCCAGGGGCGGGGAGTCTTCGAGCTCTCGGACGAAAAAGC
+HWI-EAS443:3:17:735:45#0/1
]aaRX_`_Y_Z]X`XIRRZNUJXPZLQYUXUU¥MY_YPBB
@HWI-EAS443:3:64:1755:153#0/1
GGAGACCTTCACTACCTCCAGAGAAGAAATGAAAAGTGGC
+HWI-EAS443:3:64:1755:153#0/1
ab`ba__aa_a_`aW¥^YX¥__aGW_U]_^`]_X`_X^]Y
@HWI-EAS443:3:28:568:744#0/1
GCTGGACCATATCTCTTGTCTTCTTTGCCCAAAATGAGAA
+HWI-EAS443:3:28:568:744#0/1
]aab``ab`_aaaaa`aa^baaa`aaaaaaa]a``aaaaa
@HWI-EAS443:3:34:1223:269#0/1
AGCGACCAAAAGAATTCGGATGGCCATCAATTATGGAGAG
+HWI-EAS443:3:34:1223:269#0/1
`abYUZa]]aaa_aa`¥_aa`a`HUW[```Y]¥^PPBBBB
@HWI-EAS443:3:72:648:676#0/1
CGAGGCCATCCGAATTCTTTTGGTCGCTGTCTGGCCGTCA
+HWI-EAS443:3:72:648:676#0/1
aaabaabbabbaaaaaaaaaaa`_aa_`_``_a_`ZaS_Z
@HWI-EAS443:3:59:1602:1553#0/1
AGGCCATCCGAATTCTTTTGGTCGCTGTCTGGCTGTTAGT
+HWI-EAS443:3:59:1602:1553#0/1
^b]S]bb`a`^[b^R_aa`]][`ZZabaaBBBBBBBBBBB
@HWI-EAS443:3:85:1147:890#0/1
AGGCCATCCGAATTCTTTTGGTCGCNGTCTGGCTGTCAGT
+HWI-EAS443:3:85:1147:890#0/1
a_``V]aaabRX``a`]`a``]ZaSDZ`a_`a_`aV¥XaN
@HWI-EAS443:3:19:485:582#0/1
AGGCCATCCGAATTCTTTTGGTCGCTGTCTGGCTGTCAGG
+HWI-EAS443:3:19:485:582#0/1
W[¥¥`ba_¥T_¥W^T_ZQVPP[`aU]aXRZ`BBBBBBBBB
@HWI-EAS443:3:100:1039:608#0/1
AGGCCATCCGAATTCTTTTGGTCGCTGTCTGGCTGTCAGT
```

+HWI-EAS443:3:100:1039:608#0/1
ab__`¥a`]b_^`aS_aaba`a]]N^]`F`_Q[R[LOBBB
@HWI-EAS443:3:22:1147:325#0/1
AGGCCATCCGAATTCTTTTGGTCGCTGTCTGGCTGTCAGT
+HWI-EAS443:3:22:1147:325#0/1
a_```aaaaaaaaa_abaa`¥^_X`aa^W``_^`aZU¥¥V
@HWI-EAS443:3:28:146:893#0/1
AGGCCATCCGAATTCTTTTGGTCGCTGTCTGGCTGTCAGT
+HWI-EAS443:3:28:146:893#0/1
a``bab`aabb`aaaa_a_aa¥aa`aa^``[___`[[[YG
@HWI-EAS443:3:43:845:489#0/1
AGGCCATCCGAATTCTTTTGGTCGCTGTCTGGCTGTCAGT
+HWI-EAS443:3:43:845:489#0/1
aaaa`baaabaaaa_aaaaaa^`a_a¥`_aa[Za`TZU¥F
@HWI-EAS443:3:46:1232:1485#0/1
AGGCCATCCGAATTCTTTTGGTCGCTGTCTGGCTGTCAGT
+HWI-EAS443:3:46:1232:1485#0/1
^U¥aabbb`bbaaaabaab¥^^bbaaaa`]]]`aaV[__V
@HWI-EAS443:3:46:284:245#0/1
AGGCCATCCGAATTCTTTTGGTCGCTGTCTGGCTGTCAGT
+HWI-EAS443:3:46:284:245#0/1
aa_babaaabaaaa_aaaaaa_`a^`a¥`aaa_`aRUYXT
@HWI-EAS443:3:48:837:201#0/1
AGGCCATCCGAATTCTTTTGGTCGCTGTCTGGCTGTCAGT
+HWI-EAS443:3:48:837:201#0/1
]`_`abaa¥b`^aaaaaaaaa^`a_`a^``a_Q`a¥_[[R
@HWI-EAS443:3:6:1085:1899#0/1
AGGCCATCCGAATTCTTTTGGTCGCTGTCTGGCTGTCAGT
+HWI-EAS443:3:6:1085:1899#0/1
a]__aa^aab`^a`_a_a]a_^aaZ[`^]]_]^^_UUXBB
@HWI-EAS443:3:73:19:853#0/1
AGGCCATCCGAATTCTTTTGGTCGCTGTCTGGCTGTCAGT
+HWI-EAS443:3:73:19:853#0/1
_]O_`a¥a_ZX¥_¥_^]_a^TY_]_XZ]Z[a]BBBBBBBB
@HWI-EAS443:3:8:922:1807#0/1
AGGCCATCCGAATTCTTTTGGTCGCTGTCTGGCTGTCAGT
+HWI-EAS443:3:8:922:1807#0/1
`^^b`bba`aXa]`_aa]aa¥^`a__bSX_`_^]YLBBBB
@HWI-EAS443:3:91:296:653#0/1
AGGCCATCCGAATTCTTTTGGTCGCTGTCTGGCTGTCAGT
+HWI-EAS443:3:91:296:653#0/1
aaaabbaaabaZa``ba`baa^aaaa`__a_b``a_T^VT
@HWI-EAS443:3:9:236:1531#0/1
AGGCCATCCGAATTCTTTTGGTCGCTGTCTGGCTGTCAGT
+HWI-EAS443:3:9:236:1531#0/1
^^]bb]]_b_WT¥_b^b]`a`^_NZ``QZaa`aUaR_QBB
@HWI-EAS443:3:51:793:711#0/1
CTGCCGGTTGATTTCTGTTTAACGTCCAATCATAAGTCTG
+HWI-EAS443:3:51:793:711#0/1
ab_b]bb`bbZbaaa`baaba[G_[aaU``a_]a_a^X`a

```
@HWI-EAS443:3:54:1158:933#0/1
GTTCCCTTGGCTGTATGGAGGATCTCCAGTATAAGGGAAT
+HWI-EAS443:3:54:1158:933#0/1
aba`aaabaaaab_^XG_S¥a[VXZY]RaUQUN]^a]G¥X
@HWI-EAS443:3:17:1035:809#0/1
TGCAGGAGCATTGACTGAAGATCCAGATGAAGGCACATCT
+HWI-EAS443:3:17:1035:809#0/1
]ZJ__[N_`QPZ_`PXbbaYX_Z^aa_a_PXbZX_H_`Z_
@HWI-EAS443:3:50:1487:399#0/1
ATCTCCTGGTTGCCTTTCTGAGAATAGCAGTTGCTCTTCT
+HWI-EAS443:3:50:1487:399#0/1
aa`a``bb``bba`abababababaabaaaaa]`^W_]`_[[
@HWI-EAS443:3:99:1184:1912#0/1
GGAAGAATGATTGGTGGAATAGGGAGATTCTAAATCCAAA
+HWI-EAS443:3:99:1184:1912#0/1
aaW`aTa[ba_ab`Z_aa`YG^]a^ba_a`a]U[]U_W_a
@HWI-EAS443:3:94:534:1037#0/1
TGTCTTCGAGCTCTCGGACGAAAAGGCAACGAACCCGATC
+HWI-EAS443:3:94:534:1037#0/1
aa`b]_aa`¥aaaaaba^a````¥V¥aaaa`a_`¥XUX`X
@HWI-EAS443:3:16:1060:301#0/1
ATGAGAATGAGAGACCTTCACTACCTCCAGAGCAGAAATG
+HWI-EAS443:3:16:1060:301#0/1
aaaUa``a`Y_^a^¥^__`a[`_]___]__^a]^`Y^]]`
@HWI-EAS443:3:21:1578:262#0/1
CTAGCTTTCAGGTAGACTGTTTCCTTTGGCATATCCGCAA
+HWI-EAS443:3:21:1578:262#0/1
aaaa__`aaaaa^aa_¥a_]__¥Y__^X^Y¥^¥V]V]XX[
@HWI-EAS443:3:33:1029:453#0/1
GAATGAGAGACCTTCACTACCTCCAGAGCAGAAATGAAAA
+HWI-EAS443:3:33:1029:453#0/1
^[b]bbaaa``bba_babb_Qaaa^`abZabXIXaa][a^
@HWI-EAS443:3:73:1773:1709#0/1
CCTTCACTACCTCCAGAGCAGAAATGAAAAGTGGCGGGAG
+HWI-EAS443:3:73:1773:1709#0/1
^QN]aaTRR[QOUQU^ORPHWWI]YXBBBBBBBBBBBBBB
@HWI-EAS443:3:89:746:1894#0/1
GAGAATGAGAGACCTTCACTACCTCCAGAGCAGAAATGAA
+HWI-EAS443:3:89:746:1894#0/1
a¥O¥aa^N_Z¥]_]_^¥[`[`a_ab`__¥¥^`aY__a_[_
@HWI-EAS443:3:76:1242:922#0/1
TGGTGGGCTATATGAAGCAATCGAGGAGTGCCTGATTAAT
+HWI-EAS443:3:76:1242:922#0/1
UaSP]]`¥¥bbaa`a`OU]W``Z[a_`aU`GL]a^ZL¥WS
@HWI-EAS443:3:77:1275:53#0/1
CATTAATCAGGCACTCCTCGATTGCTTCATATAGCCCACC
+HWI-EAS443:3:77:1275:53#0/1
`bbbbbbaabbaaabbbbb`babbbbaabbaaab_`[G_a
@HWI-EAS443:3:8:1352:1172#0/1
ATTAATCAGGCACTCCTCGATTGCTTCATATAGCCCACCA
```

```
+HWI-EAS443:3:8:1352:1172#0/1
a[POZWIUX`_XIXMLYSZ[YVTW¥UDN[XX[[ZTBBBBB
@HWI-EAS443:3:49:1055:1796#0/1
ATTGGGAAAGTGTGCAGGACCTTACTGGCAAAATCTGTGT
+HWI-EAS443:3:49:1055:1796#0/1
`]^a^a`bb^[b_`aa``]``aa`a`_```_`_`^]aMRN
@HWI-EAS443:3:8:1318:804#0/1
CCGATTGGCCATGTTTCCGACTTGTTTTCAAAGAATTCCT
+HWI-EAS443:3:8:1318:804#0/1
`a^^a_aX`RUa]_a^]Z`LE¥`^``ba]``[`X]aaT¥a
@HWI-EAS443:3:78:780:1168#0/1
TATGGTGTTGGCCAATGCAGTTGCTGCCGGTTGATTTTTG
+HWI-EAS443:3:78:780:1168#0/1
aa_b`UaXVYa`Z^T`b^WQWP[^]`WNX[OO[OGUTONS
@HWI-EAS443:3:47:1699:19#0/1
GCGGGTTTGTCCTTGTTCATTCCTCTCTGGAATCACGTCC
+HWI-EAS443:3:47:1699:19#0/1
TMZb`P_T_^TMTV``WTNTHL]URULLYQFTTSPDRYXB
@HWI-EAS443:3:37:1070:1231#0/1
CATTAAGCATCAATGAACTGAGCAGTCTTGCACAAGGAGA
+HWI-EAS443:3:37:1070:1231#0/1
a¥¥O[]Y__^OaZWQIa^]]¥a]_X^^ZHNbWHRV^TS^[
@HWI-EAS443:3:27:1487:801#0/1
GCATCAATGAACTGAGCAGTCTTGCAAAAGGAGAGAAAGC
+HWI-EAS443:3:27:1487:801#0/1
a^_bU`a]b_a¥`_P`R`G`R_Z`S_¥¥W`^Ra]`W^Z^X
@HWI-EAS443:3:18:40:603#0/1
ATCCGTATTCTTTTGGTCGCTGTCTGGCTGTCAGTAAGTA
+HWI-EAS443:3:18:40:603#0/1
VbaTQHX^U]aaUaa[Zaa_aa]aa[[`__RHVXSGVPG_
@HWI-EAS443:3:15:460:1300#0/1
TCAGGATGTTTCTGAACCACTCGGGTTGATTTCTGGTAAT
+HWI-EAS443:3:15:460:1300#0/1
ab`_YX_VO_ab]Yaa_aa`_Z]¥XDJY^Z]Y]___XWMW
@HWI-EAS443:3:1:135:112#0/1
GCTCAGGATGTTTCTGAACCACTCGGGTTGATTTCTGGTA
+HWI-EAS443:3:1:135:112#0/1
[ba^`aaaXZPYZZ[^`aHIa_W_[]`¥ZZS^[ZW`[aBB
@HWI-EAS443:3:26:1452:471#0/1
CTCAGGATGTTTCTGAACCACTCGGGTTGATTTCTGGTAA
+HWI-EAS443:3:26:1452:471#0/1
aabaab[ab_aa`ababa___`_aa_Xaa]_``^```]]^
@HWI-EAS443:3:40:1104:1222#0/1
GCTCAGGATGTTTCTGAACCACTCGGGTTGATTTCTGGTA
+HWI-EAS443:3:40:1104:1222#0/1
`__]Z`a[`aX_a]_XDVW_QVWVUQYVW_][]]a¥ZOOX
@HWI-EAS443:3:45:492:987#0/1
CTCAGGATGTTTCTGAACCACTCGGGTTGATTTCTGGTAA
+HWI-EAS443:3:45:492:987#0/1
abbbbb`bbabbbb^abaaa[a`baa_aaaaaaab¥_]aa
```

@HWI-EAS443:3:60:593:305#0/1
GCTCAGGATGTTTCTGAACCACTCGGGTTGATTTCTGGTA
+HWI-EAS443:3:60:593:305#0/1
a__`[^^VZ_V`_]__¥^WV^W^V_^¥XZ_Y]YYX[Y[YZ
@HWI-EAS443:3:61:768:248#0/1
GCTCAGGATGTTTCTGAACCACTCGGGTTGATTTCTGGTA
+HWI-EAS443:3:61:768:248#0/1
a]^ZVY^XY_Z^^RUTZ]WRXXOXWX^OYOUVZZZXZXUZ
@HWI-EAS443:3:65:1632:1468#0/1
GCTCAGGATGTTTCTGAACCACTCGGGTTGATTTCTGGTA
+HWI-EAS443:3:65:1632:1468#0/1
a`_`aaSXXZERXKHTSYZRT_YYPXPDP[XUQPSNYKDM
@HWI-EAS443:3:86:244:1837#0/1
GCTCAGGATGTTTCTGAACCACTCGGGTTGATTTCTGGTA
+HWI-EAS443:3:86:244:1837#0/1
ab`aT¥a^a_^`aaa_aa`_aa^_]Y_Z__^^]]W^]WZ^
@HWI-EAS443:3:88:466:1238#0/1
GCTCAGGATGTTTCTGAACCACTCGGGTTGATTTCTGGTA
+HWI-EAS443:3:88:466:1238#0/1
aaaabbaaabaabab`a^`¥`a`aaaaWa__a_`_a[a]`
@HWI-EAS443:3:88:896:213#0/1
GCTCAGGATGTTTCTGAACCACTCGGGTTGATTTCTGGTA
+HWI-EAS443:3:88:896:213#0/1
ab_a]¥^]aYZ_a^WOF]WU`PRU_`^V¥¥UZ[aU¥Y_NS
@HWI-EAS443:3:94:675:166#0/1
GGCAACTCAGCACCGTCTGGCCAAGACCAACCCACAAGAT
+HWI-EAS443:3:94:675:166#0/1
VHT]VOP_`Y¥_MX`Y¥R[RZSMU^YYV¥XRRRYUXBBBB
@HWI-EAS443:3:63:958:588#0/1
TATGGCCTCTGCTGCCTGTTCACTCGATCCAGCCATCTGT
+HWI-EAS443:3:63:958:588#0/1
HZZbbba_abb^T[abZZWabX¥b_WW`JbLWTWTZ`Uaa
@HWI-EAS443:3:40:1328:668#0/1
CACCCTCTCGTCATTGCAGCAAATATCATTGGGATCTTGC
+HWI-EAS443:3:40:1328:668#0/1
abbbbbbbbbababbbaba`aaaa`aa`^aZ^`[_aa`_X
@HWI-EAS443:3:37:1486:806#0/1
TACCATTCTGTTTTCATGCCTGATTAGTGGATTGGTGGTA
+HWI-EAS443:3:37:1486:806#0/1
aa`a`a`aab`aab[aa`a^ba_aa_aWaZaa`_____¥]
@HWI-EAS443:3:97:356:378#0/1
AGCAGGCAGATATTTAAAGATGAGGCTTCTAACCGAGGTC
+HWI-EAS443:3:97:356:378#0/1
UUS_S_KbXQW^^TUZTTYYU_aRT]]SL]LY]HaT¥[BB
@HWI-EAS443:3:10:463:629#0/1
TGCAAAAGCAGGCAGATATTTAAAGATGAGTCTTCTAACC
+HWI-EAS443:3:10:463:629#0/1
`bX¥`aaa¥b_`P^a¥P¥b]a]b`VZ`ab¥_Vab¥_^aaa
@HWI-EAS443:3:100:745:876#0/1
GAAAGATGACCTTCTTGAAAATTTGCAGGCCTCCCAAAGT

```
+HWI-EAS443:3:100:745:876#0/1
_Y`[a[```]W``aaaaabaaaaaa[aa`_Ya_UPY¥Za_
@HWI-EAS443:3:39:848:1381#0/1
CTTCCCTCATGGACTCAGGCACCCCTTCCGTAGAAGGCCC
+HWI-EAS443:3:39:848:1381#0/1
a_Z_``_[[aUU^]_`^RY_a[GXa]aa__a¥UVIMR^[Q
@HWI-EAS443:3:5:533:1869#0/1
GGTGCCTGAGTCCATGAGGGAAGAATATCAACAGGAACAG
+HWI-EAS443:3:5:533:1869#0/1
ab_bb]abbaU`[bababbaa_ba`a___b^]aaZ_]N`^
@HWI-EAS443:3:93:543:371#0/1
CGGTCTTGTCTTTAGCCATTCCATGAGAGCCTCAAGATCT
+HWI-EAS443:3:93:543:371#0/1
aab`a_``U[abaaabaaa_a`Xaa``_`¥_^Z^[a_`^`
@HWI-EAS443:3:36:1669:970#0/1
CAGCACTTTGCTGTTCCTGTTGATATTCTTCCCTCATGGA
+HWI-EAS443:3:36:1669:970#0/1
`bbb`]abbaaaaR`_abbQ`aXaTaaNa`aT__a`a[F_
@HWI-EAS443:3:31:1662:1099#0/1
GCTGGATCGAGTGAACAGGCAGCAGAGGCCATTGAGGTTG
+HWI-EAS443:3:31:1662:1099#0/1
`^ZZ_^b`b`^¥ba]QVU^Y¥¥KXGWY`BBBBBBBBBBBB
@HWI-EAS443:3:14:359:1845#0/1
GCCTGAATCCATGAGGGAAGAATATCAACAGGAACAGCAG
+HWI-EAS443:3:14:359:1845#0/1
SZb]a_Q__VVZbZOHX]bU__]SP_¥SXX`aS`T`RbIB
@HWI-EAS443:3:37:1741:1964#0/1
TGGGAAACAAATTGTGGAATGGATCTTGAAAGAGGAATCG
+HWI-EAS443:3:37:1741:1964#0/1
abb`bV`aaba`S`[bb^a_ZZW]XYY_ZG_¥abb`^_`O
@HWI-EAS443:3:32:170:1445#0/1
CCCACTGCAGGGCTACTGAATACATAATGAAGGCGGTGTA
+HWI-EAS443:3:32:170:1445#0/1
ab`^_^¥¥SLKR`QTT_XQPVZ¥PV_TGN[J]REIYPXQB
@HWI-EAS443:3:15:1645:776#0/1
ATTGGCCATGTTTTCGATTTGTTTTCAAAGAATTCCTTGG
+HWI-EAS443:3:15:1645:776#0/1
abbbabab`V^b_HUaY^bbVXabbUZaa_`a^_]__aa_
@HWI-EAS443:3:58:166:121#0/1
CCTGGTTAATGATCCCTGGGTTTTGCTTAATGCATCTTGG
+HWI-EAS443:3:58:166:121#0/1
aaWb`Ua```]`T^a_aaaZRX__^^`_[YW_VR]¥]O]W
@HWI-EAS443:3:46:1060:643#0/1
GAGTGCTGCTGTCTTGAGAGGATTTCTCATTTTGGGCAAA
+HWI-EAS443:3:46:1060:643#0/1
a`a`aaaaab``aaa`aaaa]aba^a`_aaaa_a_]_`a
@HWI-EAS443:3:65:1061:851#0/1
CTAAGGTATATAAAACTTATTTCGAAAAGGTTGAAAGGTT
+HWI-EAS443:3:65:1061:851#0/1
a`a¥ab¥bbab`¥baba_abbaaa]a``_a[a``XT`a]a
```

```
@HWI-EAS443:3:29:1788:836#0/1
GCATAAATGAACTGAGCAATCTTGCAAAAGGAGAGAAAGC
+HWI-EAS443:3:29:1788:836#0/1
a]PZLUNQ]H_Y¥_Y¥_^S`W][]]Z[V¥P[^SS_^]BBB
@HWI-EAS443:3:44:1716:1827#0/1
CAAGGACAAACCCTCTGGAGTAAAACAAACGATGCTGGAT
+HWI-EAS443:3:44:1716:1827#0/1
``^]^[`Z_][`b`aaVba]G]_YXQX[aYW`VQDQ]GUV
@HWI-EAS443:3:50:14:1407#0/1
ATCTCTTGTCTTCTTTGCCCAAAATGAGAAATCCTCTCAA
+HWI-EAS443:3:50:14:1407#0/1
`abbbba]_`aaaaaaaa`aaabaaaaaaaV__^aaa_]a
@HWI-EAS443:3:9:560:567#0/1
AAGAGATATGGCCCAGCATTAAGCATAAATGAACTGAGCA
+HWI-EAS443:3:9:560:567#0/1
a¥¥[R]a`[O[SGS]`T]]Z`^X[aTFQWTVMQRZX`^`_
@HWI-EAS443:3:67:1020:919#0/1
ATTCTTTTGGTCGCTGTCTGGCTGTCCGTAAGTGTGCTAG
+HWI-EAS443:3:67:1020:919#0/1
V¥¥X¥``Y^[OXUVab_ba_TWJOOYF[I[JOQGW¥^`YJ
@HWI-EAS443:3:16:877:256#0/1
CATATTTTCACCGAGTGCCCACTTCAACTGGCTTGGTCTC
+HWI-EAS443:3:16:877:256#0/1
a¥[OQ¥_[¥Z¥a``a]T`aWUaa`]¥¥L^aaBBBBBBBBB
@HWI-EAS443:3:24:387:1217#0/1
ATGGAAATGAGACGCTGCCTTCTTCAGTCTCTTCAGCGGA
+HWI-EAS443:3:24:387:1217#0/1
¥ZZb__bb^aZH__`]W]¥WTPaab]aHGQXUU_ZHHH]P
@HWI-EAS443:3:60:582:579#0/1
AGCGTCTCATTTCCATGCCCCATTTCATCTTGATCTTGGA
+HWI-EAS443:3:60:582:579#0/1
[^bb]aaZO^aaaaa`aaaaaaaa`^`_`a_^^a``aaNY
@HWI-EAS443:3:13:939:899#0/1
TCTGTTTTCATGCCTGATTAGTGGATTGGTGGGAGTTGTC
+HWI-EAS443:3:13:939:899#0/1
aa_aW^a^`]aa][^_Ua^JX_a_U_BBBBBBBBBBBBBB
@HWI-EAS443:3:100:702:920#0/1
TTCTGTTTTCATGCCTGATTAGTGGATTGGTGGTAGTTGC
+HWI-EAS443:3:100:702:920#0/1
aXZZX]^aa]a`_Y`a]YXVa¥Tb`_aBBBBBBBBBBBBB
@HWI-EAS443:3:24:1377:1750#0/1
GTGTTTTTTCTCATGGTTCTGAAATCCTAATGTTAAATAC
+HWI-EAS443:3:24:1377:1750#0/1
a^a]`baaaaaaba_SVa_aa`^`_]`aaaaa_``a__a]
@HWI-EAS443:3:60:146:633#0/1
GTGTTTTTTCTCATGTTTCTGAAATCCTAATGTTAAATAC
+HWI-EAS443:3:60:146:633#0/1
a_^Z``a_a^`a_^]S_]_`^^_^^^______Y_YOY^`X
@HWI-EAS443:3:97:648:422#0/1
AGATGATCACTCAATGAGTGACATCGAAGCCATGGCGTCT
```

```
+HWI-EAS443:3:97:648:422#0/1
_`^`H]a``a]]¥aY]¥[[`]^aa____PTJX¥Z]L_FOZ
@HWI-EAS443:3:33:1119:1703#0/1
GCCCAGATGGCTCTTCAATTGTTCATCAAAGATTACAGAT
+HWI-EAS443:3:33:1119:1703#0/1
aabbaa[_aa]a_`bba``aaU_aa_N]a``VDP_]^]a^
@HWI-EAS443:3:10:498:641#0/1
AACCATTTGAATGGATGTCAATCCGACTCTACTCTTCCTA
+HWI-EAS443:3:10:498:641#0/1
a`_aaaaab`aaaa__`^`a_^_]^UZ^^]^[^[^_[^^_
@HWI-EAS443:3:19:891:1854#0/1
GTTCGGATCTTCATTCTTTTACTCTCGAACATGTACCCTT
+HWI-EAS443:3:19:891:1854#0/1
]TQ[]ITTQ]]^Z_YYU_¥V^]][VOYW^]WOSUWLY^^T
@HWI-EAS443:3:82:760:716#0/1
GCGAAAGCAGGCAAACTATTTGAATGGATGTCAATCCGAC
+HWI-EAS443:3:82:760:716#0/1
^a`a¥TXa[^b¥Z_aaba`]]__`a_`]Z_W¥Q¥___YWZ
@HWI-EAS443:3:30:824:621#0/1
CACAAATTCAAACGAGAAGATCATTTGAGCTAAAGAAGCT
+HWI-EAS443:3:30:824:621#0/1
a_UXYWW_][_Y^`VYZYa`[_]^Y¥]SXFYU_N¥^OXFO
@HWI-EAS443:3:99:64:858#0/1
ACGCAACGGCATCATATTCCATGCTTTTGGCTGGACCATG
+HWI-EAS443:3:99:64:858#0/1
[[bbbbb_bbbb`bbbbbbb_`b]babaaaaaaa_aYXYP
@HWI-EAS443:3:81:441:383#0/1
CAGAAATCAACCAGCAGCAACTGCATTGGCCAACACCATA
+HWI-EAS443:3:81:441:383#0/1
aa^]]Z_```X`aa^^]]_[YX`XXVSY_¥`^[UWV[XW]
@HWI-EAS443:3:93:1419:1121#0/1
AACCATTTGAATGGATGTCAATCCGACTTTACTTTTCCTA
+HWI-EAS443:3:93:1419:1121#0/1
aZQ`aT[`b__b^]^^V^`TaYVQTSTXFORSaa_YBBBB
@HWI-EAS443:3:13:945:613#0/1
CTCGATACTGAATCTTGGACAAAAGAAAGACACAAAGACA
+HWI-EAS443:3:13:945:613#0/1
a`ZaV]¥NY^_Xa]Y]]PPW][WSS[]UDJFV[GWNXPPZ
@HWI-EAS443:3:31:950:817#0/1
CACGCCAGACTTATGATTGGACATTAAACAGAAATCAACC
+HWI-EAS443:3:31:950:817#0/1
a`V^WQ_JWSVaYS_aaXQ¥SYaaa`]`X_H__W`U__¥[
@HWI-EAS443:3:3:616:1035#0/1
CACGCCAGACTTATGATTGGACATTAAACAGAAATCAACC
+HWI-EAS443:3:3:616:1035#0/1
abbbb`aaaabb`baabbbbaabbbbbb_baabaaaba^[
@HWI-EAS443:3:46:134:744#0/1
CACGCCAGACTTATGATTGGACATTAAACAGAAATCAACC
+HWI-EAS443:3:46:134:744#0/1
`aabaaabaabaabaaaaaa``aaba_aa``aa`a^aa`]
```

```
@HWI-EAS443:3:64:1490:415#0/1
CACGCCAGACTTATGATTGGACATTAAACAGAAATCAACC
+HWI-EAS443:3:64:1490:415#0/1
abbbbbabbbabbbbaabaaa`aabaaaWbaabaa``aX`
@HWI-EAS443:3:92:1322:1489#0/1
CACGCCAGACTTATGATTGGACATTAAACAGAAATCAACC
+HWI-EAS443:3:92:1322:1489#0/1
abbb`_bbaYba_`b``bba`LG_a]bb^b`bba_Taa^]
@HWI-EAS443:3:66:1479:917#0/1
ACGCCAGACTTATGATTGGACATTAAACAGAAATCAACCG
+HWI-EAS443:3:66:1479:917#0/1
aaaa`aaaaa`]`]`aaaa^[^aa__`_`¥]T_``_`W_X
@HWI-EAS443:3:43:596:1861#0/1
GAAGAATCCCACCCAGGAATATTTGAGAATTCGTGCCTTG
+HWI-EAS443:3:43:596:1861#0/1
a]bbb`¥]_aaa[Z`aa`a`a`^_]_aaaaWO^Ya[``_a
@HWI-EAS443:3:54:1764:587#0/1
CCAGCTCACAAAAGTCAATTGGTGTGGATGGNATGCCAAT
+HWI-EAS443:3:54:1764:587#0/1
`abbababab baaa_a__aa^_Y]¥^a`abVDZOHEVWFY
@HWI-EAS443:3:63:587:1238#0/1
ACAGAGTGGTATGCCATCCACACCAATTGACTTTTGTGAG
+HWI-EAS443:3:63:587:1238#0/1
Ubabbaaab`bbb`ba`aa_abbaaaaa`^abaaaXaaaa
@HWI-EAS443:3:40:1624:1332#0/1
GAGGGGCATGCCATCCACACCAATTGACTTTTGTGAGCTG
+HWI-EAS443:3:40:1624:1332#0/1
a[¥FYWX^Z[R]RLNURV]_¥^[UWNGRW¥SWYSRUURUR
@HWI-EAS443:3:45:1665:1510#0/1
ACCGAGCTCACAAAAGTCAATTGGTGTGGATGGCATGCCA
+HWI-EAS443:3:45:1665:1510#0/1
]^[FZS[¥]]T_SS]]]__`a_a¥NT]]VBBBBBBBBBBB
@HWI-EAS443:3:19:1038:1667#0/1
CCCCAGCTCACAAAAGTCAATTGGTGTGGANGGCATGCCA
+HWI-EAS443:3:19:1038:1667#0/1
R_a_K_`^TaYaJbZ_GR]R[¥aa¥b¥bbYD[]X]I¥U`B
@HWI-EAS443:3:11:1418:2045#0/1
CAAATGAAAACCCAGCTCACAAAAGTAAATTGGTGTGGAT
+HWI-EAS443:3:11:1418:2045#0/1
[``aab``]_baYaaa`SaaaUaYa[IVIb`]`[`_a_BB
@HWI-EAS443:3:13:887:392#0/1
CAAATGAAAACCCAGCTCACAAAAGTCAATTGGGGTGGAT
+HWI-EAS443:3:13:887:392#0/1
a`b^bbab__URaaba`a_T^[aZaMUZL]¥`YF[¥a_^Q
@HWI-EAS443:3:15:569:1631#0/1
CAGCTCACAAAAGTCAATTGGTGTGGATGGGATGCCACTC
+HWI-EAS443:3:15:569:1631#0/1
`b[ababaaaaa[HZaa``Ya][`a`^^_`HYaY¥aa`^`
@HWI-EAS443:3:15:912:441#0/1
GGCATGCCATCCACAGCAATTGACTTTTGTGAGCTGGGTT
```

```
+HWI-EAS443:3:15:912:441#0/1
aab`a`_XQaa^YaILT^[`]_aa^a_aaGPW_QZZN]OX
@HWI-EAS443:3:19:1687:1585#0/1
CATGCCATCCACACCAATTGACTTTTGTGAGCGGGGTTTT
+HWI-EAS443:3:19:1687:1585#0/1
a_`b_]¥^aVa_a]abbb`¥^abbab]Ma[baQ_ab`aab
@HWI-EAS443:3:58:226:562#0/1
ACCAAATGAAAACCCGGCTCACAAAAGTCAATTGGTGTGG
+HWI-EAS443:3:58:226:562#0/1
¥`_^XW`a^_`R¥_[R]]]_`]`XR¥`Za^Y^]]_TZ[_Y
@HWI-EAS443:3:66:164:550#0/1
GCAGAGCGGCATGCCATCCACACCAATTGACTTTTGTGAG
+HWI-EAS443:3:66:164:550#0/1
aaXOX^G[U_`_[[[OZZ^^Z[¥PRWFNZMRWZ^^RQQRW
@HWI-EAS443:3:77:80:661#0/1
ATGAAAACCCAGCTCACACAAGTCAATTGGTGTGGATGGC
+HWI-EAS443:3:77:80:661#0/1
Za`Q_UW_`a_a_`]Z`¥H¥U]Z]```^]¥[`Z`XTZ^VB
@HWI-EAS443:3:88:1002:190#0/1
CACGCCATCCACACCAATTGACTTTTGTGAGCTGGGTTTT
+HWI-EAS443:3:88:1002:190#0/1
a_``_VU^V[[X_¥__¥aa^^]]^aa`]Y]]XaaaaUZU_
@HWI-EAS443:3:59:331:1543#0/1
GTGGCATGCCATCCNCACCAATTGACTTTTGTGAGCTGGG
+HWI-EAS443:3:59:331:1543#0/1
`a^_aa^a`a``a¥DR^`aa]aa```aaaa`U[`¥WT_`^
@HWI-EAS443:3:72:767:1309#0/1
GAGNGGCATGCCATCCACACCAATTGACTTTTGTGAGCTG
+HWI-EAS443:3:72:767:1309#0/1
a`XD^ab]babaaaaaabaaa`[a`aa`aa`a¥^a_`_aa
@HWI-EAS443:3:26:1374:631#0/1
TGAAAACCCAGCTCACAAAAGTCAATTGGTGTGGATGGAA
+HWI-EAS443:3:26:1374:631#0/1
a``YSQZF[]_T¥QSU]LQXWLUPUZS^WBBBBBBBBBBB
@HWI-EAS443:3:39:551:570#0/1
GCGCCATCCACACCAATTGACTTTTGTGAGCTGGGTTTTC
+HWI-EAS443:3:39:551:570#0/1
T_aa`UK`Zbbbab`a]_I`bQb_aXa__¥b`babNSVV_
@HWI-EAS443:3:46:196:1570#0/1
CCAGCTCACAAAAGTCAATTGGTGTGGATGGCATGCCAAT
+HWI-EAS443:3:46:196:1570#0/1
a`YbWH``a`¥ZP_NXa]a]a]Z_aaYS``aXYBBBBBBB
@HWI-EAS443:3:59:1530:657#0/1
GCGCCATCCACACCAATTGACTTTTGTGAGCTGGGTTTTC
+HWI-EAS443:3:59:1530:657#0/1
aXD¥¥a_TQU^]YXVT`WXVU_a`aa^`¥`[^`YULSUYP
@HWI-EAS443:3:61:478:1106#0/1
AGCCCAGCTCACAAAAGTCAATTGGTGTGGATGGCATGCC
+HWI-EAS443:3:61:478:1106#0/1
]]bba]_babbbb```a__PY__aa^`Za``[VUUXYXNR
```

```
@HWI-EAS443:3:6:853:184#0/1
TGAAAACCCAGCTCACAAAAGTCAATTGGTGTGGATGGGA
+HWI-EAS443:3:6:853:184#0/1
`b]]`ba_`aaaaaaaba_`aY_`Va]_`____^__BBBB
@HWI-EAS443:3:99:1646:1913#0/1
ACACCAATTGACTTTTGTGAGCTGGGTTTTCATTTGGTTT
+HWI-EAS443:3:99:1646:1913#0/1
aaaaIabbaaWIa`Y^`[b`XZaaS]L``a^S_¥^¥`LFZ
@HWI-EAS443:3:13:394:533#0/1
GACCCAGCTCACAAAAGTCAATTGGTGTGGATGGCATGCC
+HWI-EAS443:3:13:394:533#0/1
abbabbbaababaaaab]baaaba`_a_`aa_^aZ__XW]
@HWI-EAS443:3:34:1244:1149#0/1
CGCCATCCACACCAATTGACTTTTGTGAGCTGGGTTTTCA
+HWI-EAS443:3:34:1244:1149#0/1
abaababaab`b`]]aaaaaaaaaa_a`aaaa``]^__T[
@HWI-EAS443:3:35:88:2044#0/1
GCCCAGCTCACAAAAGTCAATTGGTGTGGATGGCATGCCA
+HWI-EAS443:3:35:88:2044#0/1
[bbbbbbabbab`]bb_babbabTX[`a_a`aaaa_^bTI
@HWI-EAS443:3:40:150:1869#0/1
CCCCAGCTCACAAAAGTCAATTGGTGTGGATGGCATGCCA
+HWI-EAS443:3:40:150:1869#0/1
abbbb`baabbbb]IT]baaaaaIXPN_^a_aP__QX[_`
@HWI-EAS443:3:43:479:542#0/1
GCCCAGCTCACAAAAGTCAATTGGTGTGGATGGCATGCCA
+HWI-EAS443:3:43:479:542#0/1
abbbbbbbbb`a_`bb_bbabbba]baaaaab`Y^ZT[``
@HWI-EAS443:3:51:883:83#0/1
GCCCAGCTCACAAAAGTCAATTGGTGTGGATGGCATGCCA
+HWI-EAS443:3:51:883:83#0/1
a`aabbabaaabba`a`aabbbbbababaaabbXaa^SY_
@HWI-EAS443:3:52:1725:1956#0/1
GAAAACCCAGCTCACAAAAGTCAATTGGTGTGGATGGCAG
+HWI-EAS443:3:52:1725:1956#0/1
```aa[WX¥`VXT^R^__ZT[Y^]_^[ZM`Y[PU[^BBBB
@HWI-EAS443:3:57:811:354#0/1
TCCCAGCTCACAAAAGTCAATTGGTGTGGATGGCATGCCA
+HWI-EAS443:3:57:811:354#0/1
aaa`ab`aa`]¥Ya_aWaW^a`a_¥_^^`^_``V^[Z[¥X
@HWI-EAS443:3:64:1310:1143#0/1
GCCCAGCTCACAAAAGTCAATTGGTGTGGATGGCATGCCA
+HWI-EAS443:3:64:1310:1143#0/1
aaaaabaaaaababaa¥a_`babaNa¥aa`]a`U_Y__^¥
@HWI-EAS443:3:70:1013:34#0/1
GCCCAGCTCACAAAAGTCAATTGGTGTGGATGGCATGCCA
+HWI-EAS443:3:70:1013:34#0/1
aabb]^¥`_]^aa]R[QZRZbb]¥YT¥_`R^`YQBBBBBB
@HWI-EAS443:3:70:1772:984#0/1
GCCCAGCTCACAAAAGTCAATTGGTGTGGATGGCATGCCA
```
```

```
+HWI-EAS443:3:70:1772:984#0/1
abbbbbbbabbbbbbbabbbbbbb]Y__``aa_[_`Z[_a
@HWI-EAS443:3:92:684:1036#0/1
TCCCAGCTCACAAAAGTCAATTGGTGTGGATGGCATGCCA
+HWI-EAS443:3:92:684:1036#0/1
`¥_aba``]^aaaZ^^¥_a]a`¥`G`X[aaT^VZBBBBBB
@HWI-EAS443:3:100:644:1041#0/1
GCCATCCACACCAATTGACTTTTGTGAGCTGGGTTTTCAT
+HWI-EAS443:3:100:644:1041#0/1
aa[a``aa__YWaa``a]__a`a_T_Z]¥aaa`Z_a`]^`
@HWI-EAS443:3:10:1074:1036#0/1
AGCAGAGTGGCATGCCATCCACACCAATTGACTTTTGTGA
+HWI-EAS443:3:10:1074:1036#0/1
aaabbab_aabba_a_aa`^`_a^^a_a``_Y`aa`_]`^
@HWI-EAS443:3:10:816:574#0/1
TGAAAACCCAGCTCACAAAAGTCAATTGGTGTGGATGGCA
+HWI-EAS443:3:10:816:574#0/1
abaaaaa`a_aa_a_]`_a_`X_[^]`__Y^Y_YYX_¥RV
@HWI-EAS443:3:13:1198:2013#0/1
GAGTGGCATGCCATCCACACCAATTGACTTTTGTGAGCTG
+HWI-EAS443:3:13:1198:2013#0/1
]^aVa__`aaa_b_a_a_a`^[]_[a`__aa_]T__`^R]
@HWI-EAS443:3:13:133:768#0/1
AAATGAAAACCCAGCTCACAAAAGTCAATTGGTGTGGATG
+HWI-EAS443:3:13:133:768#0/1
a`baaaa]`aa_aa``aa_aYNQa]``^a__`X`]`_^^`
@HWI-EAS443:3:13:762:37#0/1
CAAATGAAAACCCAGCTCACAAAAGTCAATTGGTGTGGAT
+HWI-EAS443:3:13:762:37#0/1
ababbbbaa^`_aabaa```a```aX]__a_a`Xa]`^Y]
@HWI-EAS443:3:15:1453:1608#0/1
GAGTGGCATGCCATCCACACCAATTGACTTTTGTGAGCTG
+HWI-EAS443:3:15:1453:1608#0/1
¥¥`aa`a`aa¥_^b_ba^`Z`_`a`b`aab¥b^¥a`Y¥aa
@HWI-EAS443:3:15:1491:1226#0/1
ATGAAAACCCAGCTCACAAAAGTCAATTGGTGTGGATGGC
+HWI-EAS443:3:15:1491:1226#0/1
abb`bbba_[_a[a_^_[aXWaR[^^a_^]]`_[a]XTXF
@HWI-EAS443:3:15:270:1353#0/1
GTGGCATGCCATCCACACCAATTGACTTTTGTGAGCTGGG
+HWI-EAS443:3:15:270:1353#0/1
aUbb]b[[a`[aaZPNba`_`ZZ[Z]``b_aH__¥_¥a[_
@HWI-EAS443:3:15:697:126#0/1
CAGCTCACAAAAGTCAATTGGTGTGGATGGCATGCCACTC
+HWI-EAS443:3:15:697:126#0/1
H_bb[bbab``¥a``__babbaabb[bbb[ZUaaaa_IUR
@HWI-EAS443:3:16:1461:1213#0/1
CACACCAATTGACTTTTGTGAGCTGGGTTTTCATTTGGTC
+HWI-EAS443:3:16:1461:1213#0/1
a`X`^¥_ab`ba_bbaa]^_W_¥`]V_^aT`XP``^aW¥_
```

```
@HWI-EAS443:3:16:44:1487#0/1
GAGTGGCATGCCATCCACACCAATTGACTTTTGTGAGCTG
+HWI-EAS443:3:16:44:1487#0/1
`a¥_aabaaaaa]aaa``aaaaaa`_`_`a`_a_`V^`_`
@HWI-EAS443:3:18:752:1209#0/1
GGCATGCCATCCACACCAATTGACTTTTGTGAGCTGGGTT
+HWI-EAS443:3:18:752:1209#0/1
``¥]T`aaaabaaaa``[a_a_``aY`a[_S_¥[Sa_[MU
@HWI-EAS443:3:19:1728:344#0/1
ACCAAATGAAAACCCAGCTCACAAAAGTCAATTGGTGTGG
+HWI-EAS443:3:19:1728:344#0/1
aaaba[ababba___aa_a_a^a_``a_]_^aaaXW`V__
@HWI-EAS443:3:19:1789:601#0/1
CAAATGAAAACCCAGCTCACAAAAGTCAATTGGTGTGGAT
+HWI-EAS443:3:19:1789:601#0/1
a`bbaababaaababbbababb`abUabaaaaa`aa```_
@HWI-EAS443:3:20:1384:756#0/1
AGCAGAGTGGCATGCCATCCACACCAATTGACTTTTGTGA
+HWI-EAS443:3:20:1384:756#0/1
aaaaa`aXb`aa`a`______R`]Z__`_`^X`^a^VW^Y
@HWI-EAS443:3:21:249:49#0/1
AGCTCACAAAAGTCAATTGGTGTGGATGGCATGCCACTCT
+HWI-EAS443:3:21:249:49#0/1
aa_`a_aaY__aXa_¥````X_^__^X_`NRSSY]]XYYX
@HWI-EAS443:3:22:1584:896#0/1
GACCAAATGAAAACCCAGCTCACAAAAGTCAATTGGTGTG
+HWI-EAS443:3:22:1584:896#0/1
abZK¥]`abZ`a``OXUa_`U^Y_^Y]`UV]Y__][Y_X_
@HWI-EAS443:3:23:1678:602#0/1
ATGCCATCCACACCAATTGACTTTTGTGAGCTGGGTTTTC
+HWI-EAS443:3:23:1678:602#0/1
¥b^^]`b[U^_[_`_aaab_K^aaZU[baa^_`baP^[^G
@HWI-EAS443:3:25:1463:346#0/1
CAAATGAAAACCCAGCTCACAAAAGTCAATTGGTGTGGAT
+HWI-EAS443:3:25:1463:346#0/1
H__b_`Yab]```````U¥bbb]``W`_bb¥`¥X``bbaa
@HWI-EAS443:3:25:761:516#0/1
AGCTCACAAAAGTCAATTGGTGTGGATGGCATGCCACTCT
+HWI-EAS443:3:25:761:516#0/1
aabbbabbbabb`baaaaaa_aaaa_`ab^a_`aVYX`]`
@HWI-EAS443:3:26:967:1919#0/1
ATGAAAACCCAGCTCACAAAAGTCAATTGGTGTGGATGGC
+HWI-EAS443:3:26:967:1919#0/1
`aba`a`_¥Pa_Y`_`__a`_[Wa`_`_]XUYTT]^YZXU
@HWI-EAS443:3:27:501:375#0/1
CAGCTCACAAAAGTCAATTGGTGTGGATGGCATGCCACTC
+HWI-EAS443:3:27:501:375#0/1
abb_bbbbb`bbb[baabaab_aaba_`b_O[[]`W[YZ]
@HWI-EAS443:3:27:523:954#0/1
TGAAAACCCAGCTCACAAAAGTCAATTGGTGTGGATGGCA
```

```
+HWI-EAS443:3:27:523:954#0/1
a`]^__aabaaaaaaaa``_W`_```__¥aW¥Y¥^`^UZ
@HWI-EAS443:3:28:1176:368#0/1
CCCAGCTCACAAAAGTCAATTGGTGTGGATGGCATGCCAC
+HWI-EAS443:3:28:1176:368#0/1
`a]_b]_a`ab`aaaYZa_abb¥YaUa`[[b]G]RXQM^N
@HWI-EAS443:3:28:178:392#0/1
ATGCCATCCACACCAATTGACTTTTGTGAGCTGGGTTTTC
+HWI-EAS443:3:28:178:392#0/1
aaaaaa_aaa`U`]_a___a`a`_`_^¥^a___`_W¥__`
@HWI-EAS443:3:28:478:140#0/1
GAGTGGCATGCCATCCACACCAATTGACTTTTGTGAGCTG
+HWI-EAS443:3:28:478:140#0/1
]Za]]VbaYaba¥_a[a_XWS[`PTaQ]OR_Z^HIMS¥QQ
@HWI-EAS443:3:28:532:886#0/1
CCCAGCTCACAAAAGTCAATTGGTGTGGATGGCATGCCAC
+HWI-EAS443:3:28:532:886#0/1
abb`aaaa`aaa_W_Z_`aaa`]X^WWa__a^QY¥[]`XP
@HWI-EAS443:3:29:1444:98#0/1
CAAATGAAAACCCAGCTCACAAAAGTCAATTGGTGTGGAT
+HWI-EAS443:3:29:1444:98#0/1
ab`b]aaa]bUaa^`]a_^YZa__^]¥[Va`_¥Y_]aBBB
@HWI-EAS443:3:2:1125:1862#0/1
GTGGCATGCCATCCACACCAATTGACTTTTGTGAGCTGGG
+HWI-EAS443:3:2:1125:1862#0/1
_QYWZ^YZVYX_¥SYL`WPS___¥GG]_[Y[V]Y]UU]]^
@HWI-EAS443:3:2:1445:1136#0/1
AAATGAAAACCCAGCTCACAAAAGTCAATTGGTGTGGATG
+HWI-EAS443:3:2:1445:1136#0/1
a`XV_TZ]¥a]YZZZYGPPUU[_````]]SZSQ^P]`^[Z
@HWI-EAS443:3:30:743:1234#0/1
GCCATCCACACCAATTGACTTTTGTGAGCTGGGTTTTCAT
+HWI-EAS443:3:30:743:1234#0/1
abba`aaba`bbb[a_`_aaaaaa_a^a_aaa_V_a__Z_
@HWI-EAS443:3:31:1291:731#0/1
CAGCTCACAAAAGTCAATTGGTGTGGATGGCATGCCACTC
+HWI-EAS443:3:31:1291:731#0/1
W^`¥[_`_Waa`¥O]QQZZ^S^`__U]S``[PTPO[V[PR
@HWI-EAS443:3:31:787:1098#0/1
GCCATCCACACCAATTGACTTTTGTGAGCTGGGTTTTCAT
+HWI-EAS443:3:31:787:1098#0/1
a``^]Y]]`a`Z]_V]____[_]LZWNN]¥VULSYXRN^R
@HWI-EAS443:3:33:144:1198#0/1
ACCAAATGAAAACCCAGCTCACAAAAGTCAATTGGTGTGG
+HWI-EAS443:3:33:144:1198#0/1
]abb_Ma¥O_^aabaaaaa`aa^Z`W_[aX`__[VP_Q¥S
@HWI-EAS443:3:33:972:1639#0/1
ATGAAAACCCAGCTCACAAAAGTCAATTGGTGTGGATGGC
+HWI-EAS443:3:33:972:1639#0/1
aa`aaa```aaa___aaa`__aXa^_`_`[YZOO^^V]UN
```

```
@HWI-EAS443:3:34:460:1483#0/1
ACACCAATTGACTTTTGTGAGCTGGGTTTTCATTTGGTCT
+HWI-EAS443:3:34:460:1483#0/1
aaaabaa_`Taaa`^`_^`a_Xaa^`Z^_`aa^ZWZ¥ZXU
@HWI-EAS443:3:35:1643:1698#0/1
GCCATCCACACCAATTGACTTTTGTGAGCTGGGTTTTCAT
+HWI-EAS443:3:35:1643:1698#0/1
aUM[[YR^`^¥__VV^ZXV]]_]]RU^G___a`X_XRHPZ
@HWI-EAS443:3:35:673:1200#0/1
AACCCAGCTCACAAAAGTCAATTGGTGTGGATGGCATGCC
+HWI-EAS443:3:35:673:1200#0/1
]aba_``^T_a¥Z]QOVN]_¥Q]aURPQUTPX_XTTSLLY
@HWI-EAS443:3:37:1139:1791#0/1
GCCATCCACACCAATTGACTTTTGTGAGCTGGGTTTTCAT
+HWI-EAS443:3:37:1139:1791#0/1
a`^aaaaa_`__``Z_U[][_`__T_^`_Ua_aUN[¥U]Y
@HWI-EAS443:3:37:140:1343#0/1
ACCCAGCTCACAAAAGTCAATTGGTGTGGATGGCATGCCA
+HWI-EAS443:3:37:140:1343#0/1
aabb]]b¥aaa_KZ^_Ya``____R]V^TSS`[]NZU^^X
@HWI-EAS443:3:39:1355:172#0/1
CCCAGCTCACAAAAGTCAATTGGTGTGGATGGCATGCCAC
+HWI-EAS443:3:39:1355:172#0/1
abbbbbaba_baaab_a`^aa`¥_a_¥U¥__HJVYRPZYB
@HWI-EAS443:3:42:1015:584#0/1
ACCAAATGAAAACCCAGCTCACAAAAGTCAATTGGTGTGG
+HWI-EAS443:3:42:1015:584#0/1
aa_b`aaba`aaaa_abaaaa_`_`^a]_`_a``_]`^__
@HWI-EAS443:3:43:724:45#0/1
CAAATGAAAACCCAGCTCACAAAAGTCAATTGGTGTGGAT
+HWI-EAS443:3:43:724:45#0/1
`aa`¥_ababbb`bbb``baba`_aRR`a`aa]`b]a`a¥
@HWI-EAS443:3:44:1713:725#0/1
CCAGCTCACAAAAGTCAATTGGTGTGGATGGCATGCCACT
+HWI-EAS443:3:44:1713:725#0/1
abbbbbbabbaY¥a_`a^ab`^^a`a___a^U[^^[X_RU
@HWI-EAS443:3:44:604:145#0/1
AAAACCCAGCTCACAAAAGTCAATTGGTGTGGATGGCATG
+HWI-EAS443:3:44:604:145#0/1
a^U``a^Y]a`__^a`^__YY`_`_a^W^Y`UMX]_WQOS
@HWI-EAS443:3:45:485:782#0/1
ATGCCATCCACACCAATTGACTTTTGTGAGCTGGGTTTTC
+HWI-EAS443:3:45:485:782#0/1
a`U¥YY[a_]]XVWVPNUZX]]_]`^URUYV]Ra`GG^^V
@HWI-EAS443:3:4:148:521#0/1
GCAGAGTGGCATGCCATCCACACCAATTGACTTTTGTGAG
+HWI-EAS443:3:4:148:521#0/1
a_Q_WZW¥a_UZ^¥[P[^Q[VQYX[]_[^JNX[a[TY[UZ
@HWI-EAS443:3:51:457:1312#0/1
AGCAGAGTGGCATGCCATCCACACCAATTGACTTTTGTGA
```

+HWI-EAS443:3:51:457:1312#0/1
aabba_a_aaaaaa_`aaa`a¥a_^b]_aa`V_`¥``Y_]
@HWI-EAS443:3:51:721:1829#0/1
TGGCATGCCATCCACACCAATTGACTTTTGTGAGCTGGGT
+HWI-EAS443:3:51:721:1829#0/1
VaaaaabaaYba[aaaaa`aaab_Va¥^`aVVU`Q]_a__
@HWI-EAS443:3:51:859:1455#0/1
AGCTCACAAAAGTCAATTGGTGTGGATGGCATGCCACTCT
+HWI-EAS443:3:51:859:1455#0/1
`bbaaa^`babbY`aabbab[VYa`V^`XV]W^S[__]R¥
@HWI-EAS443:3:52:752:1459#0/1
GTGGCATGCCATCCACACCAATTGACTTTTGTGAGCTGGG
+HWI-EAS443:3:52:752:1459#0/1
a¥`a`^]``[¥]```_aYY_`_^^¥Y`aaaaX[Y^^__a`
@HWI-EAS443:3:53:641:793#0/1
AGCAGAGTGGCATGCCATCCACACCAATTGACTTTTGTGA
+HWI-EAS443:3:53:641:793#0/1
a_aaba`Xaaaaaa`aa___aZ_``a__``_V``aa_^`Y
@HWI-EAS443:3:53:787:744#0/1
ACCCAGCTCACAAAAGTCAATTGGTGTGGATGGCATGCCA
+HWI-EAS443:3:53:787:744#0/1
a``bab`aaaaaaaab[a^¥aab`Y^¥aa_aaa]WYW]ZZ
@HWI-EAS443:3:54:1565:1825#0/1
ATGAAAACCCAGCTCACAAAAGTCAATTGGTGTGGATGGC
+HWI-EAS443:3:54:1565:1825#0/1
`bbabab_YRb`]^Z]`bbaab^XaaaYaaX_^aTa_[VW
@HWI-EAS443:3:54:386:1615#0/1
CATCCACACCAATTGACTTTTGTGAGCTGGGTTTTCATTT
+HWI-EAS443:3:54:386:1615#0/1
ababbbbbbbbabbbbabab`b``^]`abbbaaba_aaaa
@HWI-EAS443:3:54:498:1809#0/1
ATGAAAACCCAGCTCACAAAAGTCAATTGGTGTGGATGGC
+HWI-EAS443:3:54:498:1809#0/1
]W_[a`a_U]`_a^`]S]SYTJT_YZ`_]^NQ¥_^]Ra_Z
@HWI-EAS443:3:54:615:1253#0/1
GAGTGGCATGCCATCCACACCAATTGACTTTTGTGAGCTG
+HWI-EAS443:3:54:615:1253#0/1
WY_[¥`_baZab¥TZbb```a^`a]ba[aaaa^`T¥`a]a
@HWI-EAS443:3:55:1158:913#0/1
ATCCACACCAATTGACTTTTGTGAGCTGGGTTTTCATTTG
+HWI-EAS443:3:55:1158:913#0/1
aa_aaaa``baa`a__aabaa_a_a¥abaaVaaa__a__a
@HWI-EAS443:3:55:150:1960#0/1
ATGAAAACCCAGCTCACAAAAGTCAATTGGTGTGGATGGC
+HWI-EAS443:3:55:150:1960#0/1
Ua_X][`_a`_P]a_a_a]_^_Z`ZM^^VGTUU¥X^Z¥XS
@HWI-EAS443:3:55:923:740#0/1
CCCAGCTCACAAAAGTCAATTGGTGTGGATGGCATGCCAC
+HWI-EAS443:3:55:923:740#0/1
___UaYZSWG¥Q__aVNa_Y^``NRY^`VUa¥NJOLYVQW

```
@HWI-EAS443:3:57:147:1150#0/1
GAGTGGCATGCCATCCACACCAATTGACTTTTGTGAGCTG
+HWI-EAS443:3:57:147:1150#0/1
_ZZ]ababW_¥a¥`a_YDN]bMa``aZ_``a__T¥[YYa_
@HWI-EAS443:3:57:1562:423#0/1
GCCATCCACACCAATTGACTTTTGTGAGCTGGGTTTTCAT
+HWI-EAS443:3:57:1562:423#0/1
a`SYUZT[KKX¥UP[[]U[UXa_[]`S¥SUT`ZW^_¥V^]
@HWI-EAS443:3:59:1564:1272#0/1
GAAAACCCAGCTCACAAAAGTCAATTGGTGTGGATGGCAT
+HWI-EAS443:3:59:1564:1272#0/1
`bbaab`abU¥`aabbbbbaVYb`abb[XS_X][^VNLTK
@HWI-EAS443:3:5:103:1607#0/1
GCCATCCACACCAATTGACTTTTGTGAGCTGGGTTTTCAT
+HWI-EAS443:3:5:103:1607#0/1
a`XQT[a^[¥aYWYXMW¥]`]ZU[S[[TPX¥ZTQU^BBBB
@HWI-EAS443:3:5:604:444#0/1
GCCATCCACACCAATTGACTTTTGTGAGCTGGGTTTTCAT
+HWI-EAS443:3:5:604:444#0/1
_¥_[b_aaU`¥M[]YX[VS^`U^¥V^W¥Y__Y¥XXZ^UBB
@HWI-EAS443:3:62:1471:75#0/1
ATCCACACCAATTGACTTTTGTGAGCTGGGTTTTCATTTG
+HWI-EAS443:3:62:1471:75#0/1
aa_`a^a__`_a_`__`a``a]a]_¥_`a`[_`_Y¥`]]^
@HWI-EAS443:3:63:635:396#0/1
GGCATGCCATCCACACCAATTGACTTTTGTGAGCTGGGTT
+HWI-EAS443:3:63:635:396#0/1
`XS`Xa__]X]V]_Ta`]_]¥`VW__][`]`]`^Za_`UR
@HWI-EAS443:3:63:991:1787#0/1
ATCCACACCAATTGACTTTTGTGAGCTGGGTTTTCATTTG
+HWI-EAS443:3:63:991:1787#0/1
a_aa`aaa]ba`aaa`_aaa`]```aa__a]_`a¥_a^_a
@HWI-EAS443:3:64:343:179#0/1
GGCATGCCATCCACACCAATTGACTTTTGTGAGCTGGGTT
+HWI-EAS443:3:64:343:179#0/1
¥__bb`_`b__Y`a]b]ba`¥a[_[¥aXTRa¥_]_baaY^
@HWI-EAS443:3:64:759:369#0/1
GCCATCCACACCAATTGACTTTTGTGAGCTGGGTTTTCAT
+HWI-EAS443:3:64:759:369#0/1
`ba]abaaaaaa_`a`a___`a__^____`a`aY_``_Ya
@HWI-EAS443:3:66:469:141#0/1
GAGTGGCATGCCATCCACACCAATTGACTTTTGTGAGCTG
+HWI-EAS443:3:66:469:141#0/1
_U_Uaaaa`a]]Y^^`X]]^[¥N¥_^WZ`_^^¥W^RNRW`
@HWI-EAS443:3:67:237:932#0/1
GACCAAATGAAAACCCAGCTCACAAAAGTCAATTGGTGTG
+HWI-EAS443:3:67:237:932#0/1
`_bbbabbb__b[bb`SZ¥abaaaW_baXaa[aa`¥YZ]`
@HWI-EAS443:3:68:1118:524#0/1
ACCAAATGAAAACCCAGCTCACAAAAGTCAATTGGTGTGG
```

```
+HWI-EAS443:3:68:1118:524#0/1
]`a`¥K^bW[`]¥[^Za^a_aXYY]``_``_Y¥][SOTV]
@HWI-EAS443:3:69:1456:571#0/1
GACCAAATGAAAACCCAGCTCACAAAAGTCAATTGGTGTG
+HWI-EAS443:3:69:1456:571#0/1
`bbbba`baaabbaa`abaaaaaaa`ab_aaabaaa_aaa
@HWI-EAS443:3:6:1340:199#0/1
GCAGAGTGGCATGCCATCCACACCAATTGACTTTTGTGAG
+HWI-EAS443:3:6:1340:199#0/1
TZb`YTGV_R`aba`aa]R_Q`]```]aa^X[aaa¥`BBB
@HWI-EAS443:3:6:437:704#0/1
GACCAAATGAAAACCCAGCTCACAAAAGTCAATTGGTGTG
+HWI-EAS443:3:6:437:704#0/1
abaab`aabaaba`aYa`[a___a_a_aV___aa_WOWY`
@HWI-EAS443:3:70:1303:642#0/1
AGCTCACAAAAGTCAATTGGTGTGGATGGCATGCCACTCT
+HWI-EAS443:3:70:1303:642#0/1
a^aaaaaa``Y`]a`aaaaaYaUa_^^_^Y[^ZW^^_]Y^
@HWI-EAS443:3:70:1624:868#0/1
ACCCAGCTCACAAAAGTCAATTGGTGTGGATGGCATGCCA
+HWI-EAS443:3:70:1624:868#0/1
aaa`abaaaaaaaa^aZaa_aaa`^a_a__a`]R[WZMV[
@HWI-EAS443:3:71:1712:552#0/1
CAAATGAAAACCCAGCTCACAAAAGTCAATTGGTGTGGAT
+HWI-EAS443:3:71:1712:552#0/1
abbbbb`a`_a_aabbbaaabbaab]aaaba^aU`aba_[
@HWI-EAS443:3:71:63:780#0/1
AGCTCACAAAAGTCAATTGGTGTGGATGGCATGCCACTCT
+HWI-EAS443:3:71:63:780#0/1
Uba]^a_X_a^VR[aa^aa`_a[]NPT^¥R[WT^]PQLVT
@HWI-EAS443:3:72:1090:1797#0/1
GTGGCATGCCATCCACACCAATTGACTTTTGTGAGCTGGG
+HWI-EAS443:3:72:1090:1797#0/1
`XX]bbHaaa¥VY^_]_a¥a[_[_[^`VJ^a^_UBBBBBB
@HWI-EAS443:3:72:1405:2039#0/1
ATGAAAACCCAGCTCACAAAAGTCAATTGGTGTGGATGGC
+HWI-EAS443:3:72:1405:2039#0/1
aba]aaaaaaa_``^a``a^[`W__]`^[UG]][T[Y]]G
@HWI-EAS443:3:72:1750:1737#0/1
ACCCAGCTCACAAAAGTCAATTGGTGTGGATGGCATGCCA
+HWI-EAS443:3:72:1750:1737#0/1
a^`^[V]T_UURX^X`W`[U]¥]]LV[YGY_BBBBBBBBB
@HWI-EAS443:3:72:975:744#0/1
AGCTCACAAAAGTCAATTGGTGTGGATGGCATGCCACTCT
+HWI-EAS443:3:72:975:744#0/1
]bbaaaa``^`aaaaabab__a_a`aaaZX[¥]^a_Y_`[
@HWI-EAS443:3:73:445:1766#0/1
GAAAACCCAGCTCACAAAAGTCAATTGGTGTGGATGGCAT
+HWI-EAS443:3:73:445:1766#0/1
_``[W`b_ZW^¥a`¥HRU¥PZa_]```RWYUZXX]UBBBB
```

@HWI-EAS443:3:74:1105:287#0/1
GAAAACCCAGCTCACAAAAGTCAATTGGTGTGGATGGCAT
+HWI-EAS443:3:74:1105:287#0/1
a`a_a]_`_a__^_¥^¥]_aX^_^^_`_U`Y^¥UX_]VVV
@HWI-EAS443:3:74:620:660#0/1
GAGTGGCATGCCATCCACACCAATTGACTTTTGTGAGCTG
+HWI-EAS443:3:74:620:660#0/1
a_]_a]abaaa_a_aaaaaaaa_aa`]`aaaa]Ta]_^^`
@HWI-EAS443:3:74:902:200#0/1
GAGTGGCATGCCATCCACACCAATTGACTTTTGTGAGCTG
+HWI-EAS443:3:74:902:200#0/1
a`X_abaZ]`UQ¥aZW¥V^]V^]VVVVJU[___¥UYYPV¥
@HWI-EAS443:3:74:930:1000#0/1
CAGAGTGGCATGCCATCCACACCAATTGACTTTTGTGAGC
+HWI-EAS443:3:74:930:1000#0/1
aaaa`_a`ba`a_aa^a_aaa__`_]a__]`_a^_U_X_Q
@HWI-EAS443:3:74:946:325#0/1
GCATGCCATCCACACCAATTGACTTTTGTGAGCTGGGTTT
+HWI-EAS443:3:74:946:325#0/1
aa`^^Y_¥]^^`V^[JZ¥_a]VRV^¥]WS[PXM^^V[Z_¥
@HWI-EAS443:3:75:1009:857#0/1
GAAAACCCAGCTCACAAAAGTCAATTGGTGTGGATGGCAT
+HWI-EAS443:3:75:1009:857#0/1
aabbbb``aa]`Z``baYa`PaaaabZU`_Y]aaaBBBBB
@HWI-EAS443:3:75:384:710#0/1
CAGCTCACAAAAGTCAATTGGTGTGGATGGCATGCCACTC
+HWI-EAS443:3:75:384:710#0/1
`bbbabaabaa`a_``aab`aXa_`__`_``[]^____]¥
@HWI-EAS443:3:76:1235:352#0/1
GTGGCATGCCATCCACACCAATTGACTTTTGTGAGCTGGG
+HWI-EAS443:3:76:1235:352#0/1
a^aa^`^aYY`_[[^¥aX^aa__Z^O__¥`_Z_Z`Z]_]_
@HWI-EAS443:3:76:1327:1580#0/1
AGCAGAGTGGCATGCCATCCACACCAATTGACTTTTGTGA
+HWI-EAS443:3:76:1327:1580#0/1
a[^`aa_[baaaa_a^a`Ya`__Y]_^_`V¥Xaa`^¥Y^^
@HWI-EAS443:3:77:188:1003#0/1
GTGGCATGCCATCCACACCAATTGACTTTTGTGAGCTGGG
+HWI-EAS443:3:77:188:1003#0/1
a¥QORX¥`a`][_X¥¥RXZOU[SLKSXRUQUSLKRW[WZQ
@HWI-EAS443:3:77:232:966#0/1
GCCATCCACACCAATTGACTTTTGTGAGCTGGGTTTTCAT
+HWI-EAS443:3:77:232:966#0/1
a`XQU`a¥]U^¥ML[Z_]]UYLY`RUTVY[`^RUUXXPPS
@HWI-EAS443:3:78:1293:503#0/1
GCATGCCATCCACACCAATTGACTTTTGTGAGCTGGGTTT
+HWI-EAS443:3:78:1293:503#0/1
a]^`a^W___`]RX`Y[¥_aa¥V_``_aX_]_Q``_aU][
@HWI-EAS443:3:79:127:662#0/1
GACCAAATGAAAACCCAGCTCACAAAAGTCAATTGGTGTG

```
+HWI-EAS443:3:79:127:662#0/1
aa[aaaaaaa`]_a^aaa_`]`_`X_TTN`^W__`]TWV^
@HWI-EAS443:3:7:1276:1233#0/1
AATGAAAACCCAGCTCACAAAAGTCAATTGGTGTGGATGG
+HWI-EAS443:3:7:1276:1233#0/1
aa_aba]b`]``b``]aaaaa¥a]aaSaaaX[ba^Z``a`
@HWI-EAS443:3:7:787:997#0/1
TGAAAACCCAGCTCACAAAAGTCAATTGGTGTGGATGGCA
+HWI-EAS443:3:7:787:997#0/1
ab_aa[`_]_aaa_a^aa`_`X_`¥_]a_Y`^a¥Y]`BBB
@HWI-EAS443:3:80:885:785#0/1
AACCCAGCTCACAAAAGTCAATTGGTGTGGATGGCATGCC
+HWI-EAS443:3:80:885:785#0/1
`X_aaaaa^^a``[`Xa[a_`aaa_T`¥[RZaa`V[`SML
@HWI-EAS443:3:81:532:1052#0/1
ATGAAAACCCAGCTCACAAAAGTCAATTGGTGTGGATGGC
+HWI-EAS443:3:81:532:1052#0/1
aaa¥aaa``]¥Z_]_]aa^`][]a^^^^[RV^U^Z]]^]R
@HWI-EAS443:3:82:1152:1448#0/1
CAGCTCACAAAAGTCAATTGGTGTGGATGGCATGCCACTC
+HWI-EAS443:3:82:1152:1448#0/1
`[```^aa`a`a]Waaabaa`WaX`a__a^Q[][V`XT][
@HWI-EAS443:3:83:1388:932#0/1
GAGTGGCATGCCATCCACACCAATTGACTTTTGTGAGCTG
+HWI-EAS443:3:83:1388:932#0/1
``aabbabbab]bbabbba`ababbb_aaabbb`a_b[ab
@HWI-EAS443:3:83:271:580#0/1
CAAATGAAAACCCAGCTCACAAAAGTCAATTGGTGTGGAT
+HWI-EAS443:3:83:271:580#0/1
aaM^bba`baaaaaaaaaaaaaa_a[a_aaaaU¥``a___
@HWI-EAS443:3:83:72:362#0/1
GCCATCCACACCAATTGACTTTTGTGAGCTGGGTTTTCAT
+HWI-EAS443:3:83:72:362#0/1
V]ab¥Waa¥a_¥_U`bbaaUZaa[P[WQH`[VYG[IZW^V
@HWI-EAS443:3:83:904:439#0/1
CAAATGAAAACCCAGCTCACAAAAGTCAATTGGTGTGGAT
+HWI-EAS443:3:83:904:439#0/1
abbbbbbbbbbaaaabbaabab`]b_aaababa`baab`a
@HWI-EAS443:3:83:986:1888#0/1
AGAGTGGCATGCCATCCACACCAATTGACTTTTGTGAGCT
+HWI-EAS443:3:83:986:1888#0/1
_`^[N`VWaVT[LYFV_¥ZYP^^V¥Z¥QSZ^][RKNUKHL
@HWI-EAS443:3:84:627:1427#0/1
CCAAATGAAAACCCAGCTCACAAAAGTCAATTGGTGTGGA
+HWI-EAS443:3:84:627:1427#0/1
aababbaaa_aaaaaaaaa`aa`^TZUaa_`a¥^W`^_W_
@HWI-EAS443:3:85:1361:1067#0/1
GCCATCCACACCAATTGACTTTTGTGAGCTGGGTTTTCAT
+HWI-EAS443:3:85:1361:1067#0/1
a¥[^a`¥ZP^`[_Z]^][_ZV`]`W_U¥]]``]RWVZV^Y
```

```
@HWI-EAS443:3:85:687:1587#0/1
AGCAGAGTGGCATGCCATCCACACCAATTGACTTTTGTGA
+HWI-EAS443:3:85:687:1587#0/1
a`aa__a]`a`b^S_`a_¥aaaa`]]___Z^^_`_^_VWX
@HWI-EAS443:3:86:213:1680#0/1
AGCAGAGTGGCATGCCATCCACACCAATTGACTTTTGTGA
+HWI-EAS443:3:86:213:1680#0/1
Yaa^aZYQa[ba]OZZ¥Y``a]``]]^^YVNY¥^`_NSZU
@HWI-EAS443:3:87:813:748#0/1
CAAATGAAAACCCAGCTCACAAAAGTCAATTGGTGTGGAT
+HWI-EAS443:3:87:813:748#0/1
abaa`^]Yaa_]aaa`a_a`]]¥]`_``]aa__]aX`UV_
@HWI-EAS443:3:89:184:1302#0/1
GAGTGGCATGCCATCCACACCAATTGACTTTTGTGAGCTG
+HWI-EAS443:3:89:184:1302#0/1
aa¥U]aababaaa`aab]X_a^aa_[¥]`aa``]YR^__`
@HWI-EAS443:3:8:1141:1423#0/1
GACCAAATGAAAACCCAGCTCACAAAAGTCAATTGGTGTG
+HWI-EAS443:3:8:1141:1423#0/1
aaa[aa`aaa`a_]_Qa_aaaa]aX^`a^S_]____R_X`
@HWI-EAS443:3:90:890:423#0/1
GAGTGGCATGCCATCCACACCAATTGACTTTTGTGAGCTG
+HWI-EAS443:3:90:890:423#0/1
abaaa```bb_`bbaab`aaabbaaaaaaaaabYa`a_aa
@HWI-EAS443:3:91:771:338#0/1
CATCCACACCAATTGACTTTTGTGAGCTGGGTTTTCATTT
+HWI-EAS443:3:91:771:338#0/1
^`a_Z^^^^_`aa^¥]`¥JT_a¥aVa`_a]`S_Y[R¥¥YY
@HWI-EAS443:3:92:650:1778#0/1
AGCAGAGTGGCATGCCATCCACACCAATTGACTTTTGTGA
+HWI-EAS443:3:92:650:1778#0/1
a`a^_aaRZY¥_¥Z`_]W_a]X`aaU]`^`^_`aaYUPX^
@HWI-EAS443:3:93:1140:1546#0/1
AAACCCAGCTCACAAAAGTCAATTGGTGTGGATGGCATGC
+HWI-EAS443:3:93:1140:1546#0/1
``a_`_aaaa____a]L`¥[``a`__U_YYJY^a^PMWWB
@HWI-EAS443:3:93:1507:1717#0/1
TGAAAACCCAGCTCACAAAAGTCAATTGGTGTGGATGGCA
+HWI-EAS443:3:93:1507:1717#0/1
Z]a_a`_T^[baa`a_a`Xa¥LZY_ZO[aX^BBBBBBBBB
@HWI-EAS443:3:95:591:736#0/1
GAGTGGCATGCCATCCACACCAATTGACTTTTGTGAGCTG
+HWI-EAS443:3:95:591:736#0/1
`ab_aa`baba_aa_aa_a_¥aa```__aa_a_]a_a^`a
@HWI-EAS443:3:97:140:415#0/1
GAGTGGCATGCCATCCACACCAATTGACTTTTGTGAGCTG
+HWI-EAS443:3:97:140:415#0/1
a`__`aa^a``_aa¥]Z_`¥^_]_[_Z^^^]X]Y_W_X]_
@HWI-EAS443:3:97:939:468#0/1
CAGCTCACAAAAGTCAATTGGTGTGGATGGCATGCCACTC
```

+HWI-EAS443:3:97:939:468#0/1
abbaaababaaab[aabbb`aab`a]a`bb]`XaZ``V[[
@HWI-EAS443:3:98:1283:720#0/1
GAGTGGCATGCCATCCACACCAATTGACTTTTGTGAGCTG
+HWI-EAS443:3:98:1283:720#0/1
aaZJ]__bbba]bb``b^`Q]JYX]]`[Saa_ZUb¥_S``
@HWI-EAS443:3:9:312:514#0/1
GCCATCCACACCAATTGACTTTTGTGAGCTGGGTTTTCAT
+HWI-EAS443:3:9:312:514#0/1
aXS][aa_`]a`_YNW`¥V]^_^`Z_X]^^_][NXZ]W[Z
@HWI-EAS443:3:29:1757:1458#0/1
CTTAGCAAAAGCAGGGTAGATAATCACTCAATGAGTGACA
+HWI-EAS443:3:29:1757:1458#0/1
ab`VZZbaaabaaaWQU`a_`aa]P`__X[[`a`aW``_`
@HWI-EAS443:3:76:948:1068#0/1
AGGGAGTCTCGATACTGAATCTTGGGCAAAAGAAATACAC
+HWI-EAS443:3:76:948:1068#0/1
aaabaaabbabaaa`aaaaaaaa`_`_aaa`a_aa``^`^
@HWI-EAS443:3:6:471:67#0/1
GGGAGTCTCGATACTGAATCTTGGGCAAAAGAAATACACC
+HWI-EAS443:3:6:471:67#0/1
a^`VPT^ZW_SWOGSYXOXZWU^XSYWZ^ZZV]XUBBBBB
@HWI-EAS443:3:15:201:1709#0/1
CATCAATGAACTAAGCAATCTTGCAAAAGGAGAGAAAGCT
+HWI-EAS443:3:15:201:1709#0/1
^_]_a`^^^^Z^aaa`¥K_b[^Vaaaaaa_a`a_a``_a_
@HWI-EAS443:3:78:77:445#0/1
CATCAATGAACTAAGCAATCTTGCAAAAGGAGAGAAAGCT
+HWI-EAS443:3:78:77:445#0/1
aa^a`a]^¥^`Y¥Ya_aa[`_[`^aZUU`_Wa]_¥WP^_^
@HWI-EAS443:3:96:416:703#0/1
AGCTAATGTGCTAATTGGGCAAGGAGACGTAGTGTTGGTA
+HWI-EAS443:3:96:416:703#0/1
a`aaaaaa_a_a`aaaa_a_`_a_^`_^a[]a]`U___SW
@HWI-EAS443:3:91:512:1446#0/1
GTTGCAAAAGCAGGGTAGATAATCACTCAATGAGTGACAT
+HWI-EAS443:3:91:512:1446#0/1
_```^[U^_`_]`TY]]_]_`PW]_U^_]^^UV^UZX^^X
@HWI-EAS443:3:52:713:194#0/1
GTGCAAAAGCAGGGTAGATAATCACTCAATGAGTGACATC
+HWI-EAS443:3:52:713:194#0/1
a^a^```¥a_][^aT`¥a`___[_Z^Z_^_¥Y_S]^Z[]Z
@HWI-EAS443:3:66:624:1962#0/1
TGCAAAAGCAGGGTAGATAATCACTCAATGAGTGACATCG
+HWI-EAS443:3:66:624:1962#0/1
abbbaaYSa¥ZYRGba¥[`]¥^aaZZ_^MZaaNGaTMY_U
@HWI-EAS443:3:67:1749:1673#0/1
TGCAAAAGCAGGGTAGATAATCACTCAATGAGTGACATCG
+HWI-EAS443:3:67:1749:1673#0/1
ab`aaaa````_`N_Ya```___^]Xaa_¥X^V^]^a]_R

```
@HWI-EAS443:3:69:1704:218#0/1
TGCAAAAGCAGGGTAGATAATCACTCAATGAGTGACATCG
+HWI-EAS443:3:69:1704:218#0/1
^`bW[aa`b`aa]Rbbaaa]aT`Pa_`_aa_a^a`]__VI
@HWI-EAS443:3:20:172:1618#0/1
GAAAAGTGGCGAGAGCAATTGGGCCAGAAATTTGAGGAAA
+HWI-EAS443:3:20:172:1618#0/1
^^b`bb`X^¥ab_`¥_S¥a_Z]NFL`S_b`PWbY_aaR_Y
@HWI-EAS443:3:56:1304:1705#0/1
TGCGGGGTTCTTCTCTTGTCTTCCTGATGTGTACTTGTTG
+HWI-EAS443:3:56:1304:1705#0/1
]`QV__`¥`T^KH¥T^ab_aZPP[TV^¥XOZVXW¥GGNRY
@HWI-EAS443:3:92:356:2001#0/1
AAATGTACACATCAGGAAGACAAGAGAAGAACCCCGCACT
+HWI-EAS443:3:92:356:2001#0/1
_aaaa]``a¥a_`aWO_a^_^a`[W]_a_`a]¥SXU^`Z^
@HWI-EAS443:3:16:736:211#0/1
TATGGCCATAATCAAAAAGTACACATCAGGAAGAAAAGAG
+HWI-EAS443:3:16:736:211#0/1
a^aaa^a_YX`[[^b`aYLO¥_b¥^Y¥[[^a_aSFU__TB
@HWI-EAS443:3:19:798:1909#0/1
TGCGGGGCTCTTCTCTTGTCTTCCTGATGTGTACTTTTTG
+HWI-EAS443:3:19:798:1909#0/1
aab^babV`ab`Ubb^`b¥`a`b[b``aaXaXaa_``___
@HWI-EAS443:3:21:1272:145#0/1
AAAAAGTACACATCAGGAAGACAAGAGAAGAACCGCGCAC
+HWI-EAS443:3:21:1272:145#0/1
WV¥¥I_ZV_¥U_¥^_a`W¥aW_ZS¥TUVV_ZRMDDZ^PLT
@HWI-EAS443:3:21:396:1400#0/1
CAAAAAGCACACATCAGGAAGACAAGAGAAGAACCCCGCA
+HWI-EAS443:3:21:396:1400#0/1
aaaZ_[TFT_a_a_`__aaa__a¥^Za_UNV`_^]_]]X[
@HWI-EAS443:3:24:1229:1393#0/1
TGCGGGGGTCTTCTCTTGTCTTCCTGATGTGTACTTTTTG
+HWI-EAS443:3:24:1229:1393#0/1
V^Yb¥`¥FZ^aV]I]`a`ZIa_T`YQ[ab^a__`aa__a`
@HWI-EAS443:3:28:1300:1512#0/1
AAAAGCACACATCAGGAAGACAAGAGAAGAACCCCGCACT
+HWI-EAS443:3:28:1300:1512#0/1
``ZZ¥G]¥_aba``¥]S[bY^¥b¥Q_XaaaaSQFY`]RWT
@HWI-EAS443:3:36:1690:1672#0/1
AAAAAGTACACATCAGTAAGACAAGAGAAGAACCCCGCAC
+HWI-EAS443:3:36:1690:1672#0/1
Ub]b_]XbaV`baa`VJXaaaaa`aaaO_ab_WOa^ba^a
@HWI-EAS443:3:3:201:1181#0/1
TGCGGGGCTCTTCTCTTGTCTTCCTGATGTGTACTTTTTG
+HWI-EAS443:3:3:201:1181#0/1
aba]_^_Tbbba_a``__]aabTZa¥Y]`W_bJ^baaaa[
@HWI-EAS443:3:40:842:549#0/1
TGCGGGGTTCTTCTCTTGTCTTCCTGCTGTGTACTTTTTG
```

```
+HWI-EAS443:3:40:842:549#0/1
aaZa^R]aZUYYZa[UWWa]SOU_[]HTaHDHYb`V[HVR
@HWI-EAS443:3:42:913:614#0/1
TGCGGGGCTCTTCTCTTGTCTTCCTGATGTGTACTTTTTG
+HWI-EAS443:3:42:913:614#0/1
ab¥`aY```_baaa__`a`VXW¥]_a_a__ZTXU[^¥a`a
@HWI-EAS443:3:43:1686:1765#0/1
CAAAAAGTACACATCAGGAAGACATGAGAAGAACCCCGCA
+HWI-EAS443:3:43:1686:1765#0/1
aaZ^VZX^b_aKb[a¥¥`_b_bbTH_ab_aaaaQaS]bW[
@HWI-EAS443:3:43:1745:1912#0/1
TGCGGGGGTCTTCTCTTGTCTTCCTGATGTGTACTTTTTG
+HWI-EAS443:3:43:1745:1912#0/1
WRUPWWXH[Z_XQGM]]aVQ]PV[Y]V¥PVJO¥SZZ[^XL
@HWI-EAS443:3:47:261:1306#0/1
GTGCGGGGTTCTTCTCTTGTCTTCCTCATGTGTACTTTTT
+HWI-EAS443:3:47:261:1306#0/1
aXT¥ba_RJWY_[N]N¥_aPUVZ_[VFK^_XYVVX_]]^[
@HWI-EAS443:3:4:1731:135#0/1
AAAGTACACATCAGGAAGAGAAGAGAAGAACCCCGCACTC
+HWI-EAS443:3:4:1731:135#0/1
abbba^Yaaaa_Sba^a]XL`WaWa_`]``V^XXa^BBBB
@HWI-EAS443:3:4:707:124#0/1
CAAAAAGTCCACATCAGGAAGACAAGAGAAGAACCCCGCA
+HWI-EAS443:3:4:707:124#0/1
aabbbSbH[L__S`[_Q``Za]^a]aX`___]TLLWSaXN
@HWI-EAS443:3:50:621:1480#0/1
AAAAAGCACACATCAGGAAGACAAGAGAAGAACCCCGCAC
+HWI-EAS443:3:50:621:1480#0/1
a`ab_bRb_babbabab_ba^aab[aaababbb`aabYb_
@HWI-EAS443:3:63:460:1229#0/1
CAAAAAGTACACATTAGGAAGACAAGAGAAGAACCCCGCA
+HWI-EAS443:3:63:460:1229#0/1
T_XV_¥Z`NTba``_TV^`_ZX¥`¥R]_[^_U_`a_^ab_
@HWI-EAS443:3:68:508:1056#0/1
CAAAACGTACACATCAGGAAGACAAGAGAAGAACCCCGCA
+HWI-EAS443:3:68:508:1056#0/1
aa[T[JYP_aa`WZ^aaa``^]a__a]`_Z¥^`_a_Y_]`
@HWI-EAS443:3:69:360:1514#0/1
GTGCGGGGCTCTTCTCTTGTCTTCCTGATGTGTACTTTTT
+HWI-EAS443:3:69:360:1514#0/1
aZ^_``SWIY¥ZZ]]aa]]Yaa[a`PNMZ```V[a`a^ZX
@HWI-EAS443:3:6:286:1239#0/1
TGCGGGGTTCTTCTCTTGTTTTCCTGATGTGTACTTTTTG
+HWI-EAS443:3:6:286:1239#0/1
abaabba`a_aaW`_bab^`a`^_aaaab_`_a_`aa`_`
@HWI-EAS443:3:71:939:1187#0/1
TGCGGGGGTCTTCTCTTGTCTTCCTGATGTGTACTTTTTG
+HWI-EAS443:3:71:939:1187#0/1
^baUaTWG_bUaVZ_R_aaZb__a]aZ^`XZWP_abba^K
```

@HWI-EAS443:3:76:269:1983#0/1
TGCGGGGTTCTTCTCTTGTCTTCCTGATGTGAACTTTTTG
+HWI-EAS443:3:76:269:1983#0/1
a``aKZ¥Naaaaa`]]_Y`XY`_[a`_a^P]HW_Z^_^`X
@HWI-EAS443:3:78:1348:1939#0/1
TGCGGGGTTCCTCTCTTGTCTTCCTGATGTGTACTTTTTG
+HWI-EAS443:3:78:1348:1939#0/1
abaaa`^ZaZS]G`V```[^^^XR^]]__VTLSUV]¥TPB
@HWI-EAS443:3:81:438:992#0/1
TGCGGGGTTCTTCTCTTGTCTTCCTGATGTGCACTTTTTG
+HWI-EAS443:3:81:438:992#0/1
]]_RW¥XT_b`abaaaaU[aaZ]]]]]``X[FNZX¥PNZ]
@HWI-EAS443:3:89:1058:570#0/1
CAAAAAGCACACATCAGGAAGACAAGAGAAGAACCCCGCA
+HWI-EAS443:3:89:1058:570#0/1
abUOZTZG_V_X_baaYZW]_XXHOZaa`P`¥S_UFSaNT
@HWI-EAS443:3:8:367:1742#0/1
TGCGGGGTTCCTCTCTTGTCTTCCTGATGTGTACTTTTTG
+HWI-EAS443:3:8:367:1742#0/1
ababaa`_aaaa^a]aab^_aa_a`^aaa_a¥_^a_``a_
@HWI-EAS443:3:94:872:437#0/1
CTCTTGTCTTCCTGATGTGTACTTTTTGATTATGGGCATA
+HWI-EAS443:3:94:872:437#0/1
a_Vb]XUbb¥a]_aU^aHVGba¥_T`¥PSa^RabQHW[JT
@HWI-EAS443:3:95:1226:26#0/1
CAAAAAGTACACATCAGGAAGAGAAGAGAAGAACCCCGCA
+HWI-EAS443:3:95:1226:26#0/1
ababbbbabbbbbbabbbaab_Qabbababbaa`_S¥b_a
@HWI-EAS443:3:95:1606:557#0/1
TATGGCCATAATCAAAAAGTACAAATCAGGAAGACAAGAG
+HWI-EAS443:3:95:1606:557#0/1
ab`a`a_bUa_]O[abaa_]aSaG`^O_aa]_Wa^`^UW^
@HWI-EAS443:3:97:1632:580#0/1
TATGGCCATAATCAAAAAGAACACATCAGGAAGACAAGAG
+HWI-EAS443:3:97:1632:580#0/1
aa`b`S_^]^SYG^_aa`_DHU]¥__VUDVZ_^XH^SZ][
@HWI-EAS443:3:98:1496:1120#0/1
CAAAGAGTACACATCAGGAAGACAAGAGAAGAACCCCGCA
+HWI-EAS443:3:98:1496:1120#0/1
Z_[`KVb^][`babbb_`Y^b__aaaa`ba]ZU__Ha`[b
@HWI-EAS443:3:99:291:1594#0/1
TGCGGGGTTCTTCTCTTGTCTTCCTGAGGTGTACTTTTTG
+HWI-EAS443:3:99:291:1594#0/1
[[_[QTZPY_Z`_`XU]QGT¥ZTaa_QFZ]]HDLRR_TBB
@HWI-EAS443:3:15:346:204#0/1
TGCGGGGTTCTTCTCTTGTCTTCCTGATGTGTACTTTGAT
+HWI-EAS443:3:15:346:204#0/1
abbbbba^bbababaaa```aaaaaa_aa_a^^X``aaaa
@HWI-EAS443:3:20:1132:23#0/1
TGCGGGGTTCTTCTNTTGTCTTCCTGATGTGTACTTTTTG

```
+HWI-EAS443:3:20:1132:23#0/1
^babbaa`]`baa^D¥ab__a_`^aa``]¥`[_]a`^`Z`
@HWI-EAS443:3:24:1539:58#0/1
CAAAAAGTACACATCAGGAANACAAGAGAAGAACCCCGCA
+HWI-EAS443:3:24:1539:58#0/1
ababbabbbbbbbb`bbbb[DWabab`b`abaaaO[Zb`_
@HWI-EAS443:3:31:1722:1446#0/1
TGCGGGGTTCTTCTCTTGTCTTCCTGATGNGTACTTTTTG
+HWI-EAS443:3:31:1722:1446#0/1
aba_`aa`b`bbaa`b`¥```a__a``¥VD¥^_]a`aaa]
@HWI-EAS443:3:35:1767:1129#0/1
CTCTTGTCTTCNTGATGTGTACTTTTTGATTATGGCCATA
+HWI-EAS443:3:35:1767:1129#0/1
ab`ba^``a`[D¥`_``]_W^abaaa_Z`baa`R_[]`]`
@HWI-EAS443:3:35:251:1136#0/1
CTCTTNTCTTCCTGATGTGTACTTTTTGATTATGGCCATA
+HWI-EAS443:3:35:251:1136#0/1
a`baUDW]a_aaa`_]_YYS^``__aa___^_YOU`^XSS
@HWI-EAS443:3:37:1586:361#0/1
AAAAGTACACATCAGGAAGACAAGAGAAGNACCCCGCACT
+HWI-EAS443:3:37:1586:361#0/1
]TW__O¥Y_U¥_]`¥W[UUQQ^aWTOWMTDR[NPWOTXJL
@HWI-EAS443:3:39:1291:1668#0/1
CAAAAAGTACACATCNGGAAGACAAGAGAAGAACCCCGCA
+HWI-EAS443:3:39:1291:1668#0/1
Y``]`]¥Sa`¥baa]D][a``b_aaaYbaaa]____`a_N
@HWI-EAS443:3:56:1350:1497#0/1
CAAAAAGTACNCATCAGGAAGACAAGAGAAGAACCCCGCA
+HWI-EAS443:3:56:1350:1497#0/1
abbb`ababWD[ab_bbbbaba`aababababbbba_[^b_a
@HWI-EAS443:3:59:615:174#0/1
CAAAAAGTACACANCAGGAAGACAAGAGAAGAACCCCGCA
+HWI-EAS443:3:59:615:174#0/1
^¥abb`aO^_bb[D[aaa_ab`ababaa[R¥¥`[]NRaa[
@HWI-EAS443:3:62:1574:1282#0/1
TGCGGGGTTCTTCTCTTGTNTTCCTGATGTGTACTTTTTG
+HWI-EAS443:3:62:1574:1282#0/1
aaaaa`a_¥abbaaaaa`TD^`aaaa]`a_a_^^aa`aa[
@HWI-EAS443:3:78:594:1177#0/1
TGCGGGGTTCNTCTCTTGTCTTCCTGATGTGTACTTTTTG
+HWI-EAS443:3:78:594:1177#0/1
aaaaa`a[aYD¥`a`aaa^a`aa^`_`_a]a^``aaaaa_
@HWI-EAS443:3:95:1390:677#0/1
AAAAAGTACACATCAGGAAGACNAGAGAAGAACCCCGCAC
+HWI-EAS443:3:95:1390:677#0/1
abb`ab`bababb¥abbaaaa¥D[babaaaaa_``Xa^a_
@HWI-EAS443:3:19:836:1280#0/1
CGTGCGGGGTTCTTCTCTTGTCTTCCTGATGTGTACTTTT
+HWI-EAS443:3:19:836:1280#0/1
`b_bbbbba_aabaaabaab_abaaaaaaaa_a^`_aa`_
```

```
@HWI-EAS443:3:32:1459:1435#0/1
CAAAAGTACACATCAGGAAGACAAGAGAAGAACCCCGCAC
+HWI-EAS443:3:32:1459:1435#0/1
aab`_aabbbbabbbabbbaaababaaaaabba_a_bbab
@HWI-EAS443:3:49:1417:427#0/1
ATGCGGGGTTCTTCTCTTGTCTTCCTGATGTGTACTTTTT
+HWI-EAS443:3:49:1417:427#0/1
aababbbb`babb`aabba_`aaa`b`]a__`_^^aaaaa
@HWI-EAS443:3:63:843:1178#0/1
CTGCGGGGTTCTTCTCTTGTCTTCCTGATGTGTACTTTTT
+HWI-EAS443:3:63:843:1178#0/1
ababbab^`babbaaabbbaabbabbaaaa_a`a`baaaa
@HWI-EAS443:3:96:224:458#0/1
AAAAGTACACATCAGGAAGACAAGAGAAGAACCCCGCACA
+HWI-EAS443:3:96:224:458#0/1
XSYP^Wbaabaa_Vaa_]ZYWUY_aa_Sba[QRYMa[_]_
@HWI-EAS443:3:100:1460:75#0/1
TATGGCCATAATCAAAAAGTACACATCAGGAAGACAAGAG
+HWI-EAS443:3:100:1460:75#0/1
a_aaaYU__[]UQV_``¥YG¥MXN[YTY^_¥[^WSZZBBB
@HWI-EAS443:3:100:204:114#0/1
TGCGGGGTTCTTCTCTTGTCTTCCTGATGTGTACTTTTTG
+HWI-EAS443:3:100:204:114#0/1
aaabaa^^a`a__a`a_a_`a`a_`a]a¥Y`Z^Z``_^`]
@HWI-EAS443:3:100:772:1874#0/1
CAAAAAGTACACATCAGGAAGACAAGAGAAGAACCCCGCA
+HWI-EAS443:3:100:772:1874#0/1
`babaa[Xb_[G^bYaba^YZWNab_bY[^`ZN¥SHD`BB
@HWI-EAS443:3:10:1283:695#0/1
CTCTTGTCTTCCTGATGTGTACTTTTTGATTATGGCCATA
+HWI-EAS443:3:10:1283:695#0/1
abbbbabbbbbbbbbabbbab_bbbbbabab`baa`]a_a
@HWI-EAS443:3:10:1370:1846#0/1
TGCGGGGTTCTTCTCTTGTCTTCCTGATGTGTACTTTTTG
+HWI-EAS443:3:10:1370:1846#0/1
ab¥U[^a_aabaGaQaaa__``]^a____^[[^¥____`Y
@HWI-EAS443:3:10:608:1909#0/1
TGCGGGGTTCTTCTCTTGTCTTCCTGATGTGTACTTTTTG
+HWI-EAS443:3:10:608:1909#0/1
_bbaab`_b¥^U^_[TabTab[aKaab]a_aGa[ab`[aa
@HWI-EAS443:3:10:80:104#0/1
CAAAAAGTACACATCAGGAAGACAAGAGAAGAACCCCGCA
+HWI-EAS443:3:10:80:104#0/1
abbbbab```bbbbbabbbabaab`a`aaaaaa_[[Ya_`
@HWI-EAS443:3:10:842:1165#0/1
CAAAAAGTACACATCAGGAAGACAAGAGAAGAACCCCGCA
+HWI-EAS443:3:10:842:1165#0/1
abbabbb_b`bbaaaabaaaaa`b`a_aa`a`a_W^¥a_`
@HWI-EAS443:3:11:1225:1401#0/1
TATGGCCATAATCAAAAAGTACACATCAGGAAGACAAGAG
```

```
+HWI-EAS443:3:11:1225:1401#0/1
aba`_^a_`a`_W]``a¥Y[aY¥Z¥_`_a`__`TP`_YZB
@HWI-EAS443:3:11:1545:1946#0/1
AAAAAGTACACATCAGGAAGACAAGAGAAGAACCCCGCAC
+HWI-EAS443:3:11:1545:1946#0/1
`bab`_RbYaaaa^]b^a``^^a__^____`_^UOGXHXW
@HWI-EAS443:3:11:1648:650#0/1
AAAAAGTACACATCAGGAAGACAAGAGAAGAACCCCGCAC
+HWI-EAS443:3:11:1648:650#0/1
]bb]^_G_`a^ab[`aa`b`_¥^``aba``_`TXGNa]`_
@HWI-EAS443:3:12:1312:1807#0/1
CAAAAAGTACACATCAGGAAGACAAGAGAAGAACCCCGCA
+HWI-EAS443:3:12:1312:1807#0/1
aaaba`aaba`bbZQ`bab`a_a``aabaa_^b[_^V_X`
@HWI-EAS443:3:12:1490:521#0/1
AAAAAGTACACATCAGGAAGACAAGAGAAGAACCCCGCAC
+HWI-EAS443:3:12:1490:521#0/1
a^`]]^W^^^`__V]a`_`_UVW¥^W[Z]_¥]UYVTYQWZ
@HWI-EAS443:3:12:166:1635#0/1
TGCGGGGTTCTTCTCTTGTCTTCCTGATGTGTACTTTTTG
+HWI-EAS443:3:12:166:1635#0/1
aaab]^a_aabb`aaaab_aaaaaaaa`a^_]``a_aaa^
@HWI-EAS443:3:12:424:1853#0/1
CTCTTGTCTTCCTGATGTGTACTTTTTGATTATGGCCATA
+HWI-EAS443:3:12:424:1853#0/1
a_RXZ`Y^P_aa````_OUU`V```__Sa]_¥^^V¥__QV
@HWI-EAS443:3:12:842:1144#0/1
CAAAAAGTACACATCAGGAAGACAAGAGAAGAACCCCGCA
+HWI-EAS443:3:12:842:1144#0/1
abbbbYbabbb`abababb`baab_baaabaaba[__`aa
@HWI-EAS443:3:13:1006:1638#0/1
TGCGGGGTTCTTCTCTTGTCTTCCTGATGTGTACTTTTTG
+HWI-EAS443:3:13:1006:1638#0/1
aabaTZZJ`X^^_a_a_XQ_abW¥a_aa`SaR]aa``]¥]
@HWI-EAS443:3:13:1184:1161#0/1
CTCTTGTCTTCCTGATGTGTACTTTTTGATTATGGCCATA
+HWI-EAS443:3:13:1184:1161#0/1
aba_ab]`ba_a``_]¥aa`aaaaaab[aaaaa[U¥^aW]
@HWI-EAS443:3:13:1427:1675#0/1
TGCGGGGTTCTTCTCTTGTCTTCCTGATGTGTACTTTTTG
+HWI-EAS443:3:13:1427:1675#0/1
`baaaUaRa`a`WaH^Ya[]_]R^¥a_aaH^]_¥aaWW_¥
@HWI-EAS443:3:13:417:274#0/1
GTGCGGGGTTCTTCTCTTGTCTTCCTGATGTGTACTTTTT
+HWI-EAS443:3:13:417:274#0/1
a`_`aaaY]a_``_]`a_a][_¥`]__V^_Y`VYS]^_]Y
@HWI-EAS443:3:13:534:1719#0/1
AAAAGTACACATCAGGAAGACAAGAGAAGAACCCCGCACT
+HWI-EAS443:3:13:534:1719#0/1
a`X_UZaa`aa_aa`__a_^_`__]_^`_`_WZ^]^_`VX
```

```
@HWI-EAS443:3:13:721:985#0/1
CAAAAAGTACACATCAGGAAGACAAGAGAAGAACCCCGCA
+HWI-EAS443:3:13:721:985#0/1
a_]aaaaYbabaaa`abaaaaaa[U[aaaaa_a``]^a^a
@HWI-EAS443:3:13:76:1231#0/1
AAAAAGTACACATCAGGAAGACAAGAGAAGAACCCCGCAC
+HWI-EAS443:3:13:76:1231#0/1
X_V`^[[`b`baaaa^`a`aaa_`aaa_`a`^`_^____a
@HWI-EAS443:3:13:792:1559#0/1
GTGCGGGGTTCTTCTCTTGTCTTCCTGATGTGTACTTTTT
+HWI-EAS443:3:13:792:1559#0/1
_X`_X¥ZOSVQU_[]T¥^][TY¥X`^Q`XaYSWVUYX]__
@HWI-EAS443:3:14:1045:1011#0/1
CAAAAAGTACACATCAGGAAGACAAGAGAAGAACCCCGCA
+HWI-EAS443:3:14:1045:1011#0/1
aabb^aa_baaaaaa`^`aaa`aa`aYa```a_`[S`a^a
@HWI-EAS443:3:14:1302:150#0/1
TGCGGGGTTCTTCTCTTGTCTTCCTGATGTGTACTTTTTG
+HWI-EAS443:3:14:1302:150#0/1
a^`a_¥^Z``a```[__a]___[Y``[_aZ[]XO]_^^_]
@HWI-EAS443:3:14:1576:1642#0/1
TGCGGGGTTCTTCTCTTGTCTTCCTGATGTGTACTTTTTG
+HWI-EAS443:3:14:1576:1642#0/1
aa`aa_]ZaaaaV_X`aa^]_`¥Z`_^]`]_]]Za_```^
@HWI-EAS443:3:14:1681:874#0/1
TGCGGGGTTCTTCTCTTGTCTTCCTGATGTGTACTTTTTG
+HWI-EAS443:3:14:1681:874#0/1
aababbb_`a_aaa^bab]aaa___]`_a_^^[^aaaa`a
@HWI-EAS443:3:14:1683:1061#0/1
CAAAAAGTACACATCAGGAAGACAAGAGAAGAACCCCGCA
+HWI-EAS443:3:14:1683:1061#0/1
a`aa__ZLaaa`a`U^aV`_]`L[Xa^_^_`]¥V]YV_]]
@HWI-EAS443:3:14:176:1679#0/1
GTGCGGGGTTCTTCTCTTGTCTTCCTGATGTGTACTTTTT
+HWI-EAS443:3:14:176:1679#0/1
a`S¥R__a^a`aaa_Ua`J[ba_a_`Y`a`][O`aaaaaZ
@HWI-EAS443:3:14:413:1060#0/1
CAAAAAGTACACATCAGGAAGACAAGAGAAGAACCCCGCA
+HWI-EAS443:3:14:413:1060#0/1
aaYU^ab_baaaaaa``baaa_ab_a`a_aaa``_]`a_a
@HWI-EAS443:3:14:569:1921#0/1
AAAGTACACATCAGGAAGACAAGAGAAGAACCCCGCACTC
+HWI-EAS443:3:14:569:1921#0/1
¥aba_abbbbabb_aa`aa`ba`aaaaaab]^`_`_`___
@HWI-EAS443:3:15:1339:974#0/1
CAAAAAGTACACATCAGGAAGACAAGAGAAGAACCCCGCA
+HWI-EAS443:3:15:1339:974#0/1
abbaaa[K_ab_a_a_`ba]b`__a``aX¥[a```[^aU`
@HWI-EAS443:3:15:1644:56#0/1
AAAAGTACACATCAGGAAGACAAGAGAAGAACCCCGCACT
```

+HWI-EAS443:3:15:1644:56#0/1
aUY`bUbZZW`LV]GW_ab___Y]__W^[a`][VGaWVVU
@HWI-EAS443:3:15:559:2017#0/1
TGCGGGGTTCTTCTCTTGTCTTCCTGATGTGTACTTTTTG
+HWI-EAS443:3:15:559:2017#0/1
WbabaaWaaa]¥_a¥W¥b_ab_aa_aYaa]TTa__`aaTT
@HWI-EAS443:3:15:780:544#0/1
CAAAAAGTACACATCAGGAAGACAAGAGAAGAACCCCGCA
+HWI-EAS443:3:15:780:544#0/1
ababbbbaba`_b`ab_bbbab`b_bbb[``a^_S`^b]b
@HWI-EAS443:3:16:1014:866#0/1
AAAAAGTACACATCAGGAAGACAAGAGAAGAACCCCGCAC
+HWI-EAS443:3:16:1014:866#0/1
Ua[S_``b]`]bbbaaa_^b_ab]¥`a^]``b¥a¥_aaa_
@HWI-EAS443:3:16:168:1088#0/1
TGCGGGGTTCTTCTCTTGTCTTCCTGATGTGTACTTTTTG
+HWI-EAS443:3:16:168:1088#0/1
JXa`a^_WR`_a`bb¥bbbbbbbabbb`ba``b_bbb`^`
@HWI-EAS443:3:16:315:938#0/1
TGCGGGGTTCTTCTCTTGTCTTCCTGATGTGTACTTTTTG
+HWI-EAS443:3:16:315:938#0/1
`]a__ba`_baa_``b_a`a`_bS_`a^a¥bZ`^aa`_WY
@HWI-EAS443:3:16:960:280#0/1
CAAAAAGTACACATCAGGAAGACAAGAGAAGAACCCCGCA
+HWI-EAS443:3:16:960:280#0/1
aa`aaaa^baaaaa`abaaaaa`a```a_`a`a_]]__Z`
@HWI-EAS443:3:17:1055:1225#0/1
TGCGGGGTTCTTCTCTTGTCTTCCTGATGTGTACTTTTTG
+HWI-EAS443:3:17:1055:1225#0/1
abab_a`P``bbab``ab^aaaa``a_`b^a[_Yaaaaa^
@HWI-EAS443:3:17:1230:1335#0/1
CAAAAAGTACACATCAGGAAGACAAGAGAAGAACCCCGCA
+HWI-EAS443:3:17:1230:1335#0/1
a¥abbbO`bbbbbba_bb`bab^bab[_G_bO`baabaaa
@HWI-EAS443:3:17:309:80#0/1
AAAGTACACATCAGGAAGACAAGAGAAGAACCCCGCACTC
+HWI-EAS443:3:17:309:80#0/1
aaabXbaa`a`[_bb_aa_]a__Ya^`__]]]X]a_R]_[
@HWI-EAS443:3:17:385:39#0/1
CAAAAAGTACACATCAGGAAGACAAGAGAAGAACCCCGCA
+HWI-EAS443:3:17:385:39#0/1
]ab_a_b¥bbba`aaY`ba]ba`a_a_aa_aaa__[`aa_
@HWI-EAS443:3:17:778:807#0/1
CAAAAAGTACACATCAGGAAGACAAGAGAAGAACCCCGCA
+HWI-EAS443:3:17:778:807#0/1
aabba`aS¥abba``abaaab``aaa`ba`a`a[__`__`
@HWI-EAS443:3:17:822:229#0/1
AAAAGTACACATCAGGAAGACAAGAGAAGAACCCCGCACT
+HWI-EAS443:3:17:822:229#0/1
a_]ab_aa`a_aaaaaaaa````a``^`a``^^]]a¥^XO

```
@HWI-EAS443:3:17:832:279#0/1
CAAAAAGTACACATCAGGAAGACAAGAGAAGAACCCCGCA
+HWI-EAS443:3:17:832:279#0/1
[]Ybb_bbb]baba```b_bba`aabaab^aaaQGNPa_`
@HWI-EAS443:3:17:978:407#0/1
CAAAAAGTACACATCAGGAAGACAAGAGAAGAACCCCGCA
+HWI-EAS443:3:17:978:407#0/1
`]S^`a`Va^`^`^]aaaaaa_aa`aaa^_a`]YOU`aX`
@HWI-EAS443:3:18:234:1672#0/1
CAAAAAGTACACATCAGGAAGACAAGAGAAGAACCCCGCA
+HWI-EAS443:3:18:234:1672#0/1
aabbabW]babaaa``aaaa^aaa`_¥_`Y]aa`a`a`aa
@HWI-EAS443:3:18:305:96#0/1
GTGCGGGGTTCTTCTCTTGTCTTCCTGATGTGTACTTTTT
+HWI-EAS443:3:18:305:96#0/1
a^a_a¥a_U____``_a[_]]__ZX¥¥Y]_XYYX]^^^]Z
@HWI-EAS443:3:18:488:1582#0/1
CAAAAAGTACACATCAGGAAGACAAGAGAAGAACCCCGCA
+HWI-EAS443:3:18:488:1582#0/1
_]¥QXW_SbaUa`aa^W_VTa`^`Z__X^Wa_^^GPU¥`]
@HWI-EAS443:3:18:493:2019#0/1
AAAAAGTACACATCAGGAAGACAAGAGAAGAACCCCGCAC
+HWI-EAS443:3:18:493:2019#0/1
^aa``_Ya_a`a``aYY_a_^___¥`___^_`_¥X^_X_S
@HWI-EAS443:3:18:499:678#0/1
TGCGGGGTTCTTCTCTTGTCTTCCTGATGTGTACTTTTTG
+HWI-EAS443:3:18:499:678#0/1
aaZa]_a]`_aa`a_aaa_a``__`a_``^`^_^`a_`__
@HWI-EAS443:3:18:603:337#0/1
TGCGGGGTTCTTCTCTTGTCTTCCTGATGTGTACTTTTTG
+HWI-EAS443:3:18:603:337#0/1
abbbbbb`abbbabbbabaaaaaaa_a`b`a_aaba``aa
@HWI-EAS443:3:18:971:1702#0/1
TGCGGGGTTCTTCTCTTGTCTTCCTGATGTGTACTTTTTG
+HWI-EAS443:3:18:971:1702#0/1
ababbaa_ab`aaabaaaaa`aa_aa`aa``Z`_aa``aa
@HWI-EAS443:3:19:1069:655#0/1
AAAAGTACACATCAGGAAGACAAGAGAAGAACCCCGCACT
+HWI-EAS443:3:19:1069:655#0/1
aa`^`]bba_abaabaa]aaaaaa``_aaa_]^R`a``^`
@HWI-EAS443:3:19:170:747#0/1
TGCGGGGTTCTTCTCTTGTCTTCCTGATGTGTACTTTTTG
+HWI-EAS443:3:19:170:747#0/1
abaaa`a^b`aaa`abaa_`aaa_a_`_aZa¥¥¥`_a_a_
@HWI-EAS443:3:19:631:905#0/1
AGTACACATCAGGAAGACAAGAGAAGAACCCCGCACTCAG
+HWI-EAS443:3:19:631:905#0/1
Zaaab¥aZ`ab_baabba¥[ab`aa`aaaYa`aaaaa[ab
@HWI-EAS443:3:19:64:1145#0/1
AAAAAGTACACATCAGGAAGACAAGAGAAGAACCCCGCAC
```

```
+HWI-EAS443:3:19:64:1145#0/1
^aa¥aaSbaaaa^`abaaaaaabaa_a_a`]aa_a_a`aa
@HWI-EAS443:3:1:1124:1495#0/1
CAAAAAGTACACATCAGGAAGACAAGAGAAGAACCCCGCA
+HWI-EAS443:3:1:1124:1495#0/1
_bbabbaababbbbaba`aababbbbbbbbbbaa`H_aa`
@HWI-EAS443:3:1:1451:318#0/1
TATGGCCATAATCAAAAAGTACACATCAGGAAGACAAGAG
+HWI-EAS443:3:1:1451:318#0/1
`bZW]`¥_¥^]`^W]a_``^R[a_a`VY_a]__UJY]BBB
@HWI-EAS443:3:1:149:47#0/1
CAAAAAGTACACATCAGGAAGACAAGAGAAGAACCCCGCA
+HWI-EAS443:3:1:149:47#0/1
aaaaaaaYaaaaa`aaaa_ab`_a`a^b_aa`_`^]VaBB
@HWI-EAS443:3:1:939:400#0/1
CAAAAAGTACACATCAGGAAGACAAGAGAAGAACCCCGCA
+HWI-EAS443:3:1:939:400#0/1
``babbb`aY¥_abX_a_`aa¥_aab^aaa][N^UUV_BB
@HWI-EAS443:3:20:1485:319#0/1
CAAAAAGTACACATCAGGAAGACAAGAGAAGAACCCCGCA
+HWI-EAS443:3:20:1485:319#0/1
^P_^`_b]bba^¥^^abbabbabbabbbabba`]][_b_`
@HWI-EAS443:3:20:1618:1428#0/1
TATGGCCATAATCAAAAAGTACACATCAGGAAGACAAGAG
+HWI-EAS443:3:20:1618:1428#0/1
a_^]_aa_VYa¥S¥_`Y^XRaWa]]]OW`a¥__GS^[^^a
@HWI-EAS443:3:20:242:401#0/1
TGCGGGGTTCTTCTCTTGTCTTCCTGATGTGTACTTTTTG
+HWI-EAS443:3:20:242:401#0/1
ZZaaa`_TZba[`^P[abab```ab_R]a¥`V]]^`]]¥R
@HWI-EAS443:3:20:422:433#0/1
GTGCGGGGTTCTTCTCTTGTCTTCCTGATGTGTACTTTTT
+HWI-EAS443:3:20:422:433#0/1
`R_`a_]¥JYa]a]a[¥[`TVU^^V[[_¥YN_UU^_¥^`^
@HWI-EAS443:3:21:1636:99#0/1
CAAAAAGTACACATCAGGAAGACAAGAGAAGAACCCCGCA
+HWI-EAS443:3:21:1636:99#0/1
`aaaa_b_a^a_`a^_aa__a_]__a]]]^]]^OLRW_VX
@HWI-EAS443:3:21:1779:117#0/1
CAAAAAGTACACATCAGGAAGACAAGAGAAGAACCCCGCA
+HWI-EAS443:3:21:1779:117#0/1
aa[baaU¥b`babbaaba_a_]`b`aab^a``a][[O`U^
@HWI-EAS443:3:21:1785:1485#0/1
TGCGGGGTTCTTCTCTTGTCTTCCTGATGTGTACTTTTTG
+HWI-EAS443:3:21:1785:1485#0/1
aa^a_`a]a¥aa^`_a_`X^a_`X___]U]_Z]Z_`^_^X
@HWI-EAS443:3:21:506:1267#0/1
AAAAGTACACATCAGGAAGACAAGAGAAGAACCCCGCACT
+HWI-EAS443:3:21:506:1267#0/1
aaaaaYbba`aaa`^`a`aa`aX¥__`]``a]]_]`]_^^
```

```
@HWI-EAS443:3:22:607:1414#0/1
CAAAAAGTACACATCAGGAAGACAAGAGAAGAACCCCGCA
+HWI-EAS443:3:22:607:1414#0/1
a¥[`aaaZbaaaa`aa`]aaaa```a`a`aa`a`]^a`_`
@HWI-EAS443:3:22:769:1828#0/1
AAAAAGTACACATCAGGAAGACAAGAGAAGAACCCCGCAC
+HWI-EAS443:3:22:769:1828#0/1
a]a`aaWaaa`a]]`a_`a^`_a`_^____``]]U^_^_^
@HWI-EAS443:3:22:870:229#0/1
GTGCGGGGTTCTTCTCTTGTCTTCCTGATGTGTACTTTTT
+HWI-EAS443:3:22:870:229#0/1
aa[[^UVRNY]^[K_[a_aZN`]RNR_Y`[V_[R[]¥^[W
@HWI-EAS443:3:23:1323:1200#0/1
AAAAAGTACACATCAGGAAGACAAGAGAAGAACCCCGCAC
+HWI-EAS443:3:23:1323:1200#0/1
abaaa_Vba``ba`abaaba_ab]aa_abaaa`¥¥_b_a`
@HWI-EAS443:3:23:175:1391#0/1
TGCGGGGTTCTTCTCTTGTCTTCCTGATGTGTACTTTTTG
+HWI-EAS443:3:23:175:1391#0/1
W_`a^^b^H_bY`bbbbb`^^a¥bZW]`URWQ[`^^_`aa
@HWI-EAS443:3:23:183:751#0/1
TATGGCCATAATCAAAAAGTACACATCAGGAAGACAAGAG
+HWI-EAS443:3:23:183:751#0/1
aa[a_aaa¥_a_`_``a`XT_M^Y^ZZ¥_a___VKV`a]^
@HWI-EAS443:3:23:259:172#0/1
TGCGGGGTTCTTCTCTTGTCTTCCTGATGTGTACTTTTTG
+HWI-EAS443:3:23:259:172#0/1
abbbbba_aaabbb_aab_`aa`aaaaaa^a^a__a`_a`
@HWI-EAS443:3:23:311:1926#0/1
GTGCGGGGTTCTTCTCTTGTCTTCCTGATGTGTACTTTTT
+HWI-EAS443:3:23:311:1926#0/1
aPPZOW¥HT`X_`]aULN]O]VXNZ^VFGTGOUVUSM^G^
@HWI-EAS443:3:23:479:711#0/1
CAAAAAGTACACATCAGGAAGACAAGAGAAGAACCCCGCA
+HWI-EAS443:3:23:479:711#0/1
_bbb_bb^aabbab^baa`bbabb`babbbbbaa`b_a¥a
@HWI-EAS443:3:23:878:1469#0/1
CAAAAAGTACACATCAGGAAGACAAGAGAAGAACCCCGCA
+HWI-EAS443:3:23:878:1469#0/1
``[T_bZabUU¥bXGRZa`aa[aZ`aa_¥¥^]_``XU`^I
@HWI-EAS443:3:25:1107:1770#0/1
TGCGGGGTTCTTCTCTTGTCTTCCTGATGTGTACTTTTTG
+HWI-EAS443:3:25:1107:1770#0/1
a`R`Tab^VY¥¥QUaZaW`PVQV`_WaUHGSNPGOZIQNB
@HWI-EAS443:3:25:1205:1599#0/1
AAAAGTACACATCAGGAAGACAAGAGAAGAACCCCGCACT
+HWI-EAS443:3:25:1205:1599#0/1
abbbaZbbbabaabaaaaaa`aaaaa```aba__^a``__
@HWI-EAS443:3:25:1383:227#0/1
TGCGGGGTTCTTCTCTTGTCTTCCTGATGTGTACTTTTTG
```

+HWI-EAS443:3:25:1383:227#0/1
a`abbab`aabbaa_aab`_a`^Xaa]a`_a_Y]```aa_
@HWI-EAS443:3:25:1658:810#0/1
CAAAAAGTACACATCAGGAAGACAAGAGAAGAACCCCGCA
+HWI-EAS443:3:25:1658:810#0/1
abbbbb_G_bbaab`bbabaa]a_Z_a`a]aaa`RYSa]_
@HWI-EAS443:3:25:180:861#0/1
TGCGGGGTTCTTCTCTTGTCTTCCTGATGTGTACTTTTTG
+HWI-EAS443:3:25:180:861#0/1
ZI_b]_bUa[`baU^a]aYa`]b``]X_[Wa¥S^`_``aU
@HWI-EAS443:3:25:377:1393#0/1
TGCGGGGTTCTTCTCTTGTCTTCCTGATGTGTACTTTTTG
+HWI-EAS443:3:25:377:1393#0/1
]aa````aabbbbabaab`a`babaaaaa^a_Vaa_a``a
@HWI-EAS443:3:25:905:1993#0/1
TGCGGGGTTCTTCTCTTGTCTTCCTGATGTGTACTTTTTG
+HWI-EAS443:3:25:905:1993#0/1
abbb_``^abbbabaabb_aa`aabaa_a_a`aaaaaaaV
@HWI-EAS443:3:25:913:1072#0/1
CAAAAAGTACACATCAGGAAGACAAGAGAAGAACCCCGCA
+HWI-EAS443:3:25:913:1072#0/1
^^_b_]b[bbbabbaa^aa]bb`bbaaaa``abaJ][a`a
@HWI-EAS443:3:26:1008:1868#0/1
CAAAAAGTACACATCAGGAAGACAAGAGAAGAACCCCGCA
+HWI-EAS443:3:26:1008:1868#0/1
a`Y`bbaUbabbbaaaa¥_aaa``aaa``aaba`^__`_a
@HWI-EAS443:3:26:1052:1511#0/1
TGCGGGGTTCTTCTCTTGTCTTCCTGATGTGTACTTTTTG
+HWI-EAS443:3:26:1052:1511#0/1
VaaX[YH^RU[WPP¥]YZ[T_a`S]`RZ]M_UT``T`¥HV
@HWI-EAS443:3:26:1090:834#0/1
CAAAAAGTACACATCAGGAAGACAAGAGAAGAACCCCGCA
+HWI-EAS443:3:26:1090:834#0/1
abbbbbbIb`bababaZb`bb_aa¥aaaaabab`]__`^a
@HWI-EAS443:3:26:1299:499#0/1
CAAAAAGTACACATCAGGAAGACAAGAGAAGAACCCCGCA
+HWI-EAS443:3:26:1299:499#0/1
abbbaababbbbbbbbabbbababbbbaabbbab[[aba^
@HWI-EAS443:3:26:499:1512#0/1
CAAAAAGTACACATCAGGAAGACAAGAGAAGAACCCCGCA
+HWI-EAS443:3:26:499:1512#0/1
aaaabaaQbaaaa_aaa`ab``a]``_a]_¥_`__`_a_`
@HWI-EAS443:3:27:1758:153#0/1
TGCGGGGTTCTTCTCTTGTCTTCCTGATGTGTACTTTTTG
+HWI-EAS443:3:27:1758:153#0/1
ababb]aPaa[a¥`[[`_U^_`PTY^[UXU_WUZ^_]]^[
@HWI-EAS443:3:27:590:265#0/1
AAAAAGTACACATCAGGAAGACAAGAGAAGAACCCCGCAC
+HWI-EAS443:3:27:590:265#0/1
abba`X_bbbaa[aaaa`]a_[^X_^Y[Za_`U¥ZW_`^U

```
@HWI-EAS443:3:27:602:1920#0/1
AAAAGTACACATCAGGAAGACAAGAGAAGAACCCCGCACT
+HWI-EAS443:3:27:602:1920#0/1
a[`_RTa`aaa`^_]Saa[`_a_^`^`a]`_ZV^_]_ZKU
@HWI-EAS443:3:27:991:639#0/1
TGCGGGGTTCTTCTCTTGTCTTCCTGATGTGTACTTTTTG
+HWI-EAS443:3:27:991:639#0/1
S_^_W¥aW`¥¥ZR_YaX_]^`_ZZU¥W_a`bYS]aabb¥X
@HWI-EAS443:3:28:1291:789#0/1
AAAGTACACATCAGGAAGACAAGAGAAGAACCCCGCACTC
+HWI-EAS443:3:28:1291:789#0/1
aaab^baaaab_¥aaa`baaaaa_aaaa`aZ]WXa^_]`^
@HWI-EAS443:3:28:1296:851#0/1
CAAAAAGTACACATCAGGAAGACAAGAGAAGAACCCCGCA
+HWI-EAS443:3:28:1296:851#0/1
a`bbaab_baaaaa`a`bba_a`a_aaa`aaaa`___aa`
@HWI-EAS443:3:28:1396:1937#0/1
TGCGGGGTTCTTCTCTTGTCTTCCTGATGTGTACTTTTTG
+HWI-EAS443:3:28:1396:1937#0/1
abababbZ`a`babYa_`_^_`]`aa`]aZ^LLZa___a_
@HWI-EAS443:3:28:1558:602#0/1
CAAAAAGTACACATCAGGAAGACAAGAGAAGAACCCCGCA
+HWI-EAS443:3:28:1558:602#0/1
U_b]bbaUab¥Y_a]``aa]baa`W`abbabb[PIHRb_]
@HWI-EAS443:3:28:168:827#0/1
TGCGGGGTTCTTCTCTTGTCTTCCTGATGTGTACTTTTTG
+HWI-EAS443:3:28:168:827#0/1
abaZY_[^`_^babaaab^ba]bbba]a]`a][[ababa^
@HWI-EAS443:3:28:650:1265#0/1
TGCGGGGTTCTTCTCTTGTCTTCCTGATGTGTACTTTTTG
+HWI-EAS443:3:28:650:1265#0/1
V¥b[S_¥T_`_X`W[^V¥`^aa`aaYSZ]N_N_H`]aXPG
@HWI-EAS443:3:29:1097:137#0/1
AAAAGTACACATCAGGAAGACAAGAGAAGAACCCCGCACT
+HWI-EAS443:3:29:1097:137#0/1
_¥`aaP^abbaa`Tb``a``a_aa_a_aa_a_V_[aX^``
@HWI-EAS443:3:29:1353:1328#0/1
AAAAAGTACACATCAGGAAGACAAGAGAAGAACCCCGCAC
+HWI-EAS443:3:29:1353:1328#0/1
`bbb¥RYb]ba]]aa_Q`T[`¥____a````a___aa^`O
@HWI-EAS443:3:29:1625:966#0/1
AAAAAGTACACATCAGGAAGACAAGAGAAGAACCCCGCAC
+HWI-EAS443:3:29:1625:966#0/1
a_a]^b¥_aaab_Ya`_aaa_^aaa``_aa``__]]a^_]
@HWI-EAS443:3:29:1759:1361#0/1
AAAAAGTACACATCAGGAAGACAAGAGAAGAACCCCGCAC
+HWI-EAS443:3:29:1759:1361#0/1
abbba`Yaabbaa__`__aaa_a`^^a`^a`a`O^^a^`]
@HWI-EAS443:3:2:176:249#0/1
CAAAAAGTACACATCAGGAAGACAAGAGAAGAACCCCGCA
```

+HWI-EAS443:3:2:176:249#0/1
abbabb`Rbaaaaaaaaaaaaa`a_a`aaaaaa]T^^`__
@HWI-EAS443:3:2:437:1459#0/1
CAAAAAGTACACATCAGGAAGACAAGAGAAGAACCCCGCA
+HWI-EAS443:3:2:437:1459#0/1
`bbbbabZa``abbabaaa_b`aaab_`baaaa_^ZVbY`
@HWI-EAS443:3:2:906:1206#0/1
CAAAAAGTACACATCAGGAAGACAAGAGAAGAACCCCGCA
+HWI-EAS443:3:2:906:1206#0/1
a^]¥¥^`V^]^HOVZ_^^`aa]]a``_b_[aaa]UG[aX]
@HWI-EAS443:3:30:1165:691#0/1
TATGGCCATAATCAAAAAGTACACATCAGGAAGACAAGAG
+HWI-EAS443:3:30:1165:691#0/1
`a_b]]_aWV[[_a^a`_`YGNX¥SVQ]ZTOY_SVYNXV_
@HWI-EAS443:3:30:1207:991#0/1
TGCGGGGTTCTTCTCTTGTCTTCCTGATGTGTACTTTTTG
+HWI-EAS443:3:30:1207:991#0/1
abab]¥_[Y]Zabaaa_a¥^^aa_a_¥¥b[a¥`^aa`_`O
@HWI-EAS443:3:30:15:1911#0/1
GTGCGGGGTTCTTCTCTTGTCTTCCTGATGTGTACTTTTT
+HWI-EAS443:3:30:15:1911#0/1
a¥Y`aaaa¥abbab`aaaa`ba`ab]ZU[XQT¥[`Y[`_^
@HWI-EAS443:3:30:40:639#0/1
TGCGGGGTTCTTCTCTTGTCTTCCTGATGTGTACTTTTTG
+HWI-EAS443:3:30:40:639#0/1
abbbbba_babaababbb`abaab`aaab_aZ]`aa`aa`
@HWI-EAS443:3:30:451:1849#0/1
TGCGGGGTTCTTCTCTTGTCTTCCTGATGTGTACTTTTTG
+HWI-EAS443:3:30:451:1849#0/1
ab`Y`_T_ab`bbb_QTZabbaabXW[ab_bYaaaaa``S
@HWI-EAS443:3:30:765:42#0/1
TGCGGGGTTCTTCTCTTGTCTTCCTGATGTGTACTTTTTG
+HWI-EAS443:3:30:765:42#0/1
abaabb`H^aabaa`bbb_aa^``a``a``a[___aa^aa
@HWI-EAS443:3:31:1591:931#0/1
TATGGCCATAATCAAAAAGTACACATCAGGAAGACAAGAG
+HWI-EAS443:3:31:1591:931#0/1
aab^abaa]]a_¥¥`baaZP[M^¥^a¥T]a__a_N_Z]][
@HWI-EAS443:3:31:1744:1048#0/1
CAAAAAGTACACATCAGGAAGACAAGAGAAGAACCCCGCA
+HWI-EAS443:3:31:1744:1048#0/1
]bbabbb]b`ba`aaaababbaababaaaabaaa]`ab__
@HWI-EAS443:3:32:189:1431#0/1
TGCGGGGTTCTTCTCTTGTCTTCCTGATGTGTACTTTTTG
+HWI-EAS443:3:32:189:1431#0/1
a_b`bab_¥b^]bba]Z__a_aaa]^a``Ya`¥b_`Za^X
@HWI-EAS443:3:32:434:1903#0/1
TGCGGGGTTCTTCTCTTGTCTTCCTGATGTGTACTTTTTG
+HWI-EAS443:3:32:434:1903#0/1
a^babYU_a^a`aaaa_a]baaa`__a`Y]_Wa`]a^___

```
@HWI-EAS443:3:32:518:527#0/1
TGCGGGGTTCTTCTCTTGTCTTCCTGATGTGTACTTTTTG
+HWI-EAS443:3:32:518:527#0/1
`a```baaa_abaaa`Z¥aaaaaa`]¥^a_a¥U¥a`_``W
@HWI-EAS443:3:32:605:1367#0/1
AAAGTACACATCAGGAAGACAAGAGAAGAACCCCGCACTC
+HWI-EAS443:3:32:605:1367#0/1
`^VT[`ab_^^`Z`]]_Z]`a`ZUX[`^Z_a_`_[]T¥[a
@HWI-EAS443:3:32:763:1586#0/1
CAAAAAGTACACATCAGGAAGACAAGAGAAGAACCCCGCA
+HWI-EAS443:3:32:763:1586#0/1
ababb_a`aaa^`a__aaaaaaab``aaaa`aa_`_bXaa
@HWI-EAS443:3:32:977:1155#0/1
TGCGGGGTTCTTCTCTTGTCTTCCTGATGTGTACTTTTTG
+HWI-EAS443:3:32:977:1155#0/1
`abaa^a_aaabaa`a`aaaaaa_a``aY^a^_^aa_a__
@HWI-EAS443:3:33:1391:478#0/1
TGCGGGGTTCTTCTCTTGTCTTCCTGATGTGTACTTTTTG
+HWI-EAS443:3:33:1391:478#0/1
aaaaaaa]a^aa`a_aa_`^a`^Ua_]`_]_YO¥__`^__
@HWI-EAS443:3:33:1671:346#0/1
CAAAAAGTACACATCAGGAAGACAAGAGAAGAACCCCGCA
+HWI-EAS443:3:33:1671:346#0/1
aa[R_^`N_Z`^[`¥Ub_a``^P]]aOa___NNSFV][_^
@HWI-EAS443:3:33:463:98#0/1
CAAAAAGTACACATCAGGAAGACAAGAGAAGAACCCCGCA
+HWI-EAS443:3:33:463:98#0/1
abbbbbb_bbaabaaabbabaaaaaa`a`aaa^`]__`]_
@HWI-EAS443:3:33:980:963#0/1
AAAAAGTACACATCAGGAAGACAAGAGAAGAACCCCGCAC
+HWI-EAS443:3:33:980:963#0/1
`bb__babbbbbbababbbba`baa`aaabbaOa^aa_a^
@HWI-EAS443:3:34:502:154#0/1
TGCGGGGTTCTTCTCTTGTCTTCCTGATGTGTACTTTTTG
+HWI-EAS443:3:34:502:154#0/1
``baba__Ubbaaabbaaaaaaba`a__a_`]___a__a]
@HWI-EAS443:3:34:960:1407#0/1
AAAAAGTACACATCAGGAAGACAAGAGAAGAACCCCGCAC
+HWI-EAS443:3:34:960:1407#0/1
aaaaaa]ab`aba_`baab_aaaa`_`aa`aa`^Z_Z]_O
@HWI-EAS443:3:35:109:1043#0/1
CTCTTGTCTTCCTGATGTGTACTTTTTGATTATGGCCATA
+HWI-EAS443:3:35:109:1043#0/1
abbba_Zbaabba[`^a_a[[aaaaa`a]_]Z]^]a_QON
@HWI-EAS443:3:35:1467:307#0/1
TGCGGGGTTCTTCTCTTGTCTTCCTGATGTGTACTTTTTG
+HWI-EAS443:3:35:1467:307#0/1
abba`aa¥a`ba[b_abb`¥aaaaaba`aaa`a`aaaa^b
@HWI-EAS443:3:35:288:1082#0/1
CAAAAAGTACACATCAGGAAGACAAGAGAAGAACCCCGCA
```

```
+HWI-EAS443:3:35:288:1082#0/1
abbbUbbRZ`ba_ababaabb]ab[a_a`aa`a]b_aaaa
@HWI-EAS443:3:35:506:964#0/1
TGCGGGGTTCTTCTCTTGTCTTCCTGATGTGTACTTTTTG
+HWI-EAS443:3:35:506:964#0/1
aaa`_aa_aabaaaaa_a_^aaa```a_UN_V___`_Va^
@HWI-EAS443:3:35:964:39#0/1
CTCTTGTCTTCCTGATGTGTACTTTTTGATTATGGCCATA
+HWI-EAS443:3:35:964:39#0/1
abbbbbabbbaabb`bbabaaabbbbb_`baaaa_`_]S^
@HWI-EAS443:3:36:1104:1351#0/1
AAAGTACACATCAGGAAGACAAGAGAAGAACCCCGCACTC
+HWI-EAS443:3:36:1104:1351#0/1
^abZQ¥_]a``Y```]Z]][a]aaY]VR^^`¥_X_]a`_a
@HWI-EAS443:3:36:316:1095#0/1
AAAAAGTACACATCAGGAAGACAAGAGAAGAACCCCGCAC
+HWI-EAS443:3:36:316:1095#0/1
aaaZaa]aaaaa`aa__a`aaaa_`_¥[a[]`__`aZa_`
@HWI-EAS443:3:36:383:2000#0/1
AAAAGTACACATCAGGAAGACAAGAGAAGAACCCCGCACT
+HWI-EAS443:3:36:383:2000#0/1
aaa__Vaa`aaZ___^_a]W__^]_^_]]_[_¥^_^a^ZZ
@HWI-EAS443:3:37:1205:1318#0/1
AAAAGTACACATCAGGAAGACAAGAGAAGAACCCCGCACT
+HWI-EAS443:3:37:1205:1318#0/1
^URSPXV_]^_aX¥U^Q^_¥T¥¥`aa`SMHRT]Z]X`ZTU
@HWI-EAS443:3:37:1732:547#0/1
TGCGGGGTTCTTCTCTTGTCTTCCTGATGTGTACTTTTTG
+HWI-EAS443:3:37:1732:547#0/1
_```aa``aaaaX`Ubaba¥_bb`ba_abOb[bbb`aa`S
@HWI-EAS443:3:37:291:658#0/1
CTCTTGTCTTCCTGATGTGTACTTTTTGATTATGGCCATA
+HWI-EAS443:3:37:291:658#0/1
aaaa`a_aaaaaa`_`a]_]`aa`a`aY`]Za¥W^`^_¥_
@HWI-EAS443:3:37:780:106#0/1
CAAAAAGTACACATCAGGAAGACAAGAGAAGAACCCCGCA
+HWI-EAS443:3:37:780:106#0/1
abbbbbb_bbbaaaababbabaaaaba`IS`aaa_[`a`_
@HWI-EAS443:3:37:794:361#0/1
TATGGCCATAATCAAAAAGTACACATCAGGAAGACAAGAG
+HWI-EAS443:3:37:794:361#0/1
`ba___aa`^a]]¥[W[^ZL`¥`a]]Y_]QMWZTG_Y_ZP
@HWI-EAS443:3:38:1280:840#0/1
GTGCGGGGTTCTTCTCTTGTCTTCCTGATGTGTACTTTTT
+HWI-EAS443:3:38:1280:840#0/1
T[U¥^abaaa]ba_ab]b`Ybb`baZO^XRUaTRW^¥_a`
@HWI-EAS443:3:38:356:252#0/1
AAAAGTACACATCAGGAAGACAAGAGAAGAACCCCGCACT
+HWI-EAS443:3:38:356:252#0/1
ab_R][aba`a`_``a`ab^_a^`_aZ_Y^_WX^_`UZVX
```

@HWI-EAS443:3:38:903:716#0/1
AAAAAGTACACATCAGGAAGACAAGAGAAGAACCCCGCAC
+HWI-EAS443:3:38:903:716#0/1
aZ]aaaQbbabaaabb_a]a_a```_aaaa_a[Z[[a]`S
@HWI-EAS443:3:39:1053:669#0/1
GTGCGGGGTTCTTCTCTTGTCTTCCTGATGTGTACTTTTT
+HWI-EAS443:3:39:1053:669#0/1
a^``ZTa^P[Y`^Sa_¥UaJSa[WX[`Q_[NSYHYaXZWB
@HWI-EAS443:3:39:111:1046#0/1
TGCGGGGTTCTTCTCTTGTCTTCCTGATGTGTACTTTTTG
+HWI-EAS443:3:39:111:1046#0/1
aba__a^Y]¥`[__^Za`Paa]]Z]`aaZU^^`X[`]QZ¥
@HWI-EAS443:3:39:1618:1694#0/1
TGCGGGGTTCTTCTCTTGTCTTCCTGATGTGTACTTTTTG
+HWI-EAS443:3:39:1618:1694#0/1
aa_a_^bXUaaaYa_a`ZU]_]Y_aa]^¥]_Y]]`]_^XX
@HWI-EAS443:3:39:432:729#0/1
AAAAAGTACACATCAGGAAGACAAGAGAAGAACCCCGCAC
+HWI-EAS443:3:39:432:729#0/1
aabbUb]aabab_ab_`aaa]ba_a_``a^a`^_a`a_a]
@HWI-EAS443:3:39:956:1250#0/1
CTCTTGTCTTCCTGATGTGTACTTTTTGATTATGGCCATA
+HWI-EAS443:3:39:956:1250#0/1
a`b`abaa^abb`]MZb[`Xaaaa`__]¥``__[`a`¥ZY
@HWI-EAS443:3:3:1007:996#0/1
CAAAAAGTACACATCAGGAAGACAAGAGAAGAACCCCGCA
+HWI-EAS443:3:3:1007:996#0/1
``bba[`Xbabbaa]O_aaaaaaa``Zaaaaa`ZQ_Y`]¥
@HWI-EAS443:3:3:1315:895#0/1
CAAAAAGTACACATCAGGAAGACAAGAGAAGAACCCCGCA
+HWI-EAS443:3:3:1315:895#0/1
¥¥TW¥¥aZaYJYa_]`ba`Zbaa¥`][babb`SYN^Mb_a
@HWI-EAS443:3:3:1332:1273#0/1
TATGGCCATAATCAAAAAGTACACATCAGGAAGACAAGAG
+HWI-EAS443:3:3:1332:1273#0/1
`abaa]`a`a`]W¥`a``_S_W_[^]X]_`¥___V^_]]^
@HWI-EAS443:3:3:1450:1704#0/1
TGCGGGGTTCTTCTCTTGTCTTCCTGATGTGTACTTTTTG
+HWI-EAS443:3:3:1450:1704#0/1
aaaaa_aUaaaa`a_aa_`^`__]aa__a]_^^X_a^`__
@HWI-EAS443:3:3:1479:1390#0/1
TGCGGGGTTCTTCTCTTGTCTTCCTGATGTGTACTTTTTG
+HWI-EAS443:3:3:1479:1390#0/1
`a]a]Za¥XO__]_a]ab_Y`aM[]`_[aRaO`WYaW[_a
@HWI-EAS443:3:3:295:1919#0/1
AAAAAGTACACATCAGGAAGACAAGAGAAGAACCCCGCAC
+HWI-EAS443:3:3:295:1919#0/1
`ZZa``TSa_aa`[aaaa^a__baaa``aa`V_]SRa^_]
@HWI-EAS443:3:3:398:47#0/1
CAAAAAGTACACATCAGGAAGACAAGAGAAGAACCCCGCA

```
+HWI-EAS443:3:3:398:47#0/1
abb`aaa¥^a```[__aaa_a_^`Sa`a_S`_SMN^¥a_]
@HWI-EAS443:3:3:618:578#0/1
GTGCGGGGTTCTTCTCTTGTCTTCCTGATGTGTACTTTTT
+HWI-EAS443:3:3:618:578#0/1
a_aabaa`SaabaTa_baa`__`^Sb`[a[^aS__a_a_a
@HWI-EAS443:3:3:696:1569#0/1
AAAAAGTACACATCAGGAAGACAAGAGAAGAACCCCGCAC
+HWI-EAS443:3:3:696:1569#0/1
_aa`aa^[Uaab_F`baaabTF]S[__[_aaaQ_[^_U_O
@HWI-EAS443:3:3:977:743#0/1
TGCGGGGTTCTTCTCTTGTCTTCCTGATGTGTACTTTTTG
+HWI-EAS443:3:3:977:743#0/1
ab`ba`a]a_ba^a`aab`]aa_^aa_``Z`^^]```aa_
@HWI-EAS443:3:40:169:509#0/1
GTGCGGGGTTCTTCTCTTGTCTTCCTGATGTGTACTTTTT
+HWI-EAS443:3:40:169:509#0/1
aYY`_Z]^OW]^^YXY^[^QYVYXXYWSRURSLHVXYYXV
@HWI-EAS443:3:40:29:921#0/1
CAAAAAGTACACATCAGGAAGACAAGAGAAGAACCCCGCA
+HWI-EAS443:3:40:29:921#0/1
a``_`]a^baa`^`aaaa]Ya_``_`X`Y`^_```__`__
@HWI-EAS443:3:40:350:676#0/1
TATGGCCATAATCAAAAAGTACACATCAGGAAGACAAGAG
+HWI-EAS443:3:40:350:676#0/1
abaaabbaaaaaa_aaa]aN__aa`]WY^`_`]^__`ZX[
@HWI-EAS443:3:40:533:1143#0/1
AAAAGTACACATCAGGAAGACAAGAGAAGAACCCCGCACT
+HWI-EAS443:3:40:533:1143#0/1
_W]X^X_`¥```aa]]aa`Y`_QY_ZH]N__V]^^]]`a_
@HWI-EAS443:3:40:550:1122#0/1
TGCGGGGTTCTTCTCTTGTCTTCCTGATGTGTACTTTTTG
+HWI-EAS443:3:40:550:1122#0/1
``aabab_`aabba`aab_`aaaaa]H[_¥_Z¥a`__a¥¥
@HWI-EAS443:3:40:781:1736#0/1
TGCGGGGTTCTTCTCTTGTCTTCCTGATGTGTACTTTTTG
+HWI-EAS443:3:40:781:1736#0/1
a¥aa¥_`Xaaa_`a]aaa_aaaaa`LH^_]Y]aaa_`a^X
@HWI-EAS443:3:40:977:979#0/1
AAAAGTACACATCAGGAAGACAAGAGAAGAACCCCGCACT
+HWI-EAS443:3:40:977:979#0/1
aabb¥T_abbaa_aa`abba`aaa__V``aa``_`aaa`_
@HWI-EAS443:3:42:1255:371#0/1
TGCGGGGTTCTTCTCTTGTCTTCCTGATGTGTACTTTTTG
+HWI-EAS443:3:42:1255:371#0/1
ab`_a`aXaab`_babb^^^ab]S^`]a_U`]_Obaa_b`
@HWI-EAS443:3:42:1754:1576#0/1
TGCGGGGTTCTTCTCTTGTCTTCCTGATGTGTACTTTTTG
+HWI-EAS443:3:42:1754:1576#0/1
[b^^_a`¥]`aUZ_Y`a`[^]`¥_a^P]a[a[[U_]`]_[
```

```
@HWI-EAS443:3:42:28:634#0/1
TGCGGGGTTCTTCTCTTGTCTTCCTGATGTGTACTTTTTG
+HWI-EAS443:3:42:28:634#0/1
_``abaaabbbbaabbbbaabbbaa^Qab_a__abaaab_
@HWI-EAS443:3:42:326:676#0/1
TGCGGGGTTCTTCTCTTGTCTTCCTGATGTGTACTTTTTG
+HWI-EAS443:3:42:326:676#0/1
abaa``aG_aaaaaTa^a_`a`a^`a^^`X`OY_b¥a`a_
@HWI-EAS443:3:42:428:490#0/1
AAAAAGTACACATCAGGAAGACAAGAGAAGAACCCCGCAC
+HWI-EAS443:3:42:428:490#0/1
ab`a`b_bbbbbbbbbbaabaaaaaaaaaa`aa__aaaa`
@HWI-EAS443:3:42:621:1360#0/1
TGCGGGGTTCTTCTCTTGTCTTCCTGATGTGTACTTTTTG
+HWI-EAS443:3:42:621:1360#0/1
aaaaaa`Xa`aa_aaaaa_a`_a_]¥``a_a]``aaa``Z
@HWI-EAS443:3:43:1067:292#0/1
TGCGGGGTTCTTCTCTTGTCTTCCTGATGTGTACTTTTTG
+HWI-EAS443:3:43:1067:292#0/1
^abb]]bYaaa`aaab_bW[a`a`aa`]Z]`]__aa`a__
@HWI-EAS443:3:43:1362:207#0/1
TGCGGGGTTCTTCTCTTGTCTTCCTGATGTGTACTTTTTG
+HWI-EAS443:3:43:1362:207#0/1
abbbaabababb_a`ba``aaaa`baa``_a___a`a`a_
@HWI-EAS443:3:43:140:1379#0/1
TGCGGGGTTCTTCTCTTGTCTTCCTGATGTGTACTTTTTG
+HWI-EAS443:3:43:140:1379#0/1
[¥a^Q]a[_`a_`a_aa`___aa_YP¥`a¥[_^U`Z`a[M
@HWI-EAS443:3:43:33:1439#0/1
TGCGGGGTTCTTCTCTTGTCTTCCTGATGTGTACTTTTTG
+HWI-EAS443:3:43:33:1439#0/1
aaabaa`_b`aa_baaaa_`aaa`a``_]_aX_^a`aaa_
@HWI-EAS443:3:44:1576:1199#0/1
TGCGGGGTTCTTCTCTTGTCTTCCTGATGTGTACTTTTTG
+HWI-EAS443:3:44:1576:1199#0/1
abbaaaa]a`aa`baaaaa`aa_^aa^ab_aZ^¥a``a``
@HWI-EAS443:3:44:527:467#0/1
CAAAAAGTACACATCAGGAAGACAAGAGAAGAACCCCGCA
+HWI-EAS443:3:44:527:467#0/1
aaa`^]_Zaaaaaa`aba``ba^a]aYaY_a_`^^]_aW^
@HWI-EAS443:3:45:1109:1567#0/1
AAAAGTACACATCAGGAAGACAAGAGAAGAACCCCGCACT
+HWI-EAS443:3:45:1109:1567#0/1
WTOOGRa`a`X¥`^YZ]aa`^¥baSZ`^a_a]XV``]]]Y
@HWI-EAS443:3:45:122:154#0/1
TGCGGGGTTCTTCTCTTGTCTTCCTGATGTGTACTTTTTG
+HWI-EAS443:3:45:122:154#0/1
_S_¥¥_b]a]_]Sa]bab_a`a`aaaU^ZU_^]a^_`a]]
@HWI-EAS443:3:45:1248:935#0/1
CAAAAAGTACACATCAGGAAGACAAGAGAAGAACCCCGCA
```

```
+HWI-EAS443:3:45:1248:935#0/1
abbbbab^ababbaabbbbaba`aabbbaabaaa][aa_`
@HWI-EAS443:3:45:1595:732#0/1
TGCGGGGTTCTTCTCTTGTCTTCCTGATGTGTACTTTTTG
+HWI-EAS443:3:45:1595:732#0/1
WGUY_Z^]aX`WZ]aT_WW__[R]ZW¥K]ZZ[HY^V]aa]
@HWI-EAS443:3:45:1759:771#0/1
TGCGGGGTTCTTCTCTTGTCTTCCTGATGTGTACTTTTTG
+HWI-EAS443:3:45:1759:771#0/1
ababbbb]babaaaabba_abaa`aa```^a___baaaaa
@HWI-EAS443:3:45:256:12#0/1
TATGGCCATAATCAAAAAGTACACATCAGGAAGACAAGAG
+HWI-EAS443:3:45:256:12#0/1
ab`]`a_Z^SYXQ]^¥XZZKUFXOVXXK_`XVaQMVVX]_
@HWI-EAS443:3:45:258:1526#0/1
CAAAAAGTACACATCAGGAAGACAAGAGAAGAACCCCGCA
+HWI-EAS443:3:45:258:1526#0/1
abaaaa]`aaa`ba`aaa_^___a^aa`aaaa^a__``_`
@HWI-EAS443:3:45:285:925#0/1
TGCGGGGTTCTTCTCTTGTCTTCCTGATGTGTACTTTTTG
+HWI-EAS443:3:45:285:925#0/1
ababaa`Va^ba]b]ab`_aaaa_baa`a_a_a_aaaa`a
@HWI-EAS443:3:46:1431:102#0/1
AAAAAGTACACATCAGGAAGACAAGAGAAGAACCCCGCAC
+HWI-EAS443:3:46:1431:102#0/1
¥Z^UVXU`YSaaa_^`a^[b__¥Y_W_[^a]ZTOUVaN_]
@HWI-EAS443:3:46:631:1565#0/1
CAAAAAGTACACATCAGGAAGACAAGAGAAGAACCCCGCA
+HWI-EAS443:3:46:631:1565#0/1
a_b^`bbaaabbaaaabbaaab`bbbaa`abbba^_ab_b
@HWI-EAS443:3:46:782:972#0/1
TGCGGGGTTCTTCTCTTGTCTTCCTGATGTGTACTTTTTG
+HWI-EAS443:3:46:782:972#0/1
aaaba_bTbaab`a^_aa``aaaaaa_aa_`S_]a`a_a`
@HWI-EAS443:3:46:833:325#0/1
AAAAGTACACATCAGGAAGACAAGAGAAGAACCCCGCACT
+HWI-EAS443:3:46:833:325#0/1
```ba_]`ba_a_[`aa_a_^aa`¥aa_`Z^W_^¥a^_Y]
@HWI-EAS443:3:46:902:677#0/1
CAAAAAGTACACATCAGGAAGACAAGAGAAGAACCCCGCA
+HWI-EAS443:3:46:902:677#0/1
aba`¥W^¥bb]a``a^`a_ab^``]aab¥ab]_a_`]a]^
@HWI-EAS443:3:46:91:320#0/1
TGCGGGGTTCTTCTCTTGTCTTCCTGATGTGTACTTTTTG
+HWI-EAS443:3:46:91:320#0/1
ab_Z_bb`bbaaababbbaabaaaab`bb]b_a`bbaa`a
@HWI-EAS443:3:47:1004:853#0/1
TGCGGGGTTCTTCTCTTGTCTTCCTGATGTGTACTTTTTG
+HWI-EAS443:3:47:1004:853#0/1
SW``¥¥¥M¥a¥X_U`¥ROM¥bbbabXRQ`Q^]a^`Mbab]
```
```

```
@HWI-EAS443:3:47:1475:206#0/1
TGCGGGGTTCTTCTCTTGTCTTCCTGATGTGTACTTTTTG
+HWI-EAS443:3:47:1475:206#0/1
ab^a``a_babaaaaa`ba_a][_a`__a]`YVSa`a__S
@HWI-EAS443:3:47:1553:468#0/1
CAAAAAGTACACATCAGGAAGACAAGAGAAGAACCCCGCA
+HWI-EAS443:3:47:1553:468#0/1
aabab`aP`bbabbaabb`a_aaa`baaaaaabXRR[b^_
@HWI-EAS443:3:47:506:90#0/1
CAAAAAGTACACATCAGGAAGACAAGAGAAGAACCCCGCA
+HWI-EAS443:3:47:506:90#0/1
Tabb_¥a`bbb_baaabb^bbbabbbbbbbbbb_V_`b``
@HWI-EAS443:3:48:1456:1031#0/1
CAAAAAGTACACATCAGGAAGACAAGAGAAGAACCCCGCA
+HWI-EAS443:3:48:1456:1031#0/1
]Y]`QWWHRR_TT^SXabaaa`T_`][`[`a__`XPW¥ZX
@HWI-EAS443:3:48:1625:866#0/1
TGCGGGGTTCTTCTCTTGTCTTCCTGATGTGTACTTTTTG
+HWI-EAS443:3:48:1625:866#0/1
_bbb]ab_abbaaaaaaa__aaa_ab_ab_a¥`_aaaaaX
@HWI-EAS443:3:48:1628:49#0/1
CAAAAAGTACACATCAGGAAGACAAGAGAAGAACCCCGCA
+HWI-EAS443:3:48:1628:49#0/1
abbbbababbbbabbbbaabbbabbbbbb_baa`^_aa_a
@HWI-EAS443:3:48:1758:1981#0/1
TATGGCCATAATCAAAAAGTACACATCAGGAAGACAAGAG
+HWI-EAS443:3:48:1758:1981#0/1
]K_U`]_a^Za_W[W]a^ZH]SX[WNV`aa¥^Y^GX`ZUG
@HWI-EAS443:3:48:74:1302#0/1
AAAAAGTACACATCAGGAAGACAAGAGAAGAACCCCGCAC
+HWI-EAS443:3:48:74:1302#0/1
aaa`]ZW_`a`````__]``]Z_[``a_``a^]U]¥a[_W
@HWI-EAS443:3:48:854:234#0/1
AAAGTACACATCAGGAAGACAAGAGAAGAACCCCGCACTC
+HWI-EAS443:3:48:854:234#0/1
Ya]_QUX^^Ya[`b_`Ra[aaab`b^^^aR[][ab[YR^X
@HWI-EAS443:3:48:882:491#0/1
TGCGGGGTTCTTCTCTTGTCTTCCTGATGTGTACTTTTTG
+HWI-EAS443:3:48:882:491#0/1
abaaaab_aaaa`aaaaa__aa`_aaaaa_a[__a`aaaa
@HWI-EAS443:3:49:1298:1683#0/1
TGCGGGGTTCTTCTCTTGTCTTCCTGATGTGTACTTTTTG
+HWI-EAS443:3:49:1298:1683#0/1
¥¥U_aaRG_]¥^bbb`Y^_baaaa]WGHZ[¥TX¥¥ZW^]Z
@HWI-EAS443:3:49:546:618#0/1
CAAAAAGTACACATCAGGAAGACAAGAGAAGAACCCCGCA
+HWI-EAS443:3:49:546:618#0/1
abbaabbJ`b_a`babb`bbab¥`W^a]abbaab`_aaab
@HWI-EAS443:3:49:553:234#0/1
TGCGGGGTTCTTCTCTTGTCTTCCTGATGTGTACTTTTTG
```

```
+HWI-EAS443:3:49:553:234#0/1
a^bbbb`M`¥aa_aa`^YZ`b_]]aaa_b¥T¥`__a_`aa
@HWI-EAS443:3:49:909:1754#0/1
AAAGTACACATCAGGAAGACAAGAGAAGAACCCCGCACTC
+HWI-EAS443:3:49:909:1754#0/1
`¥b`Yaabbba`^T`bbba`baa`aba`bb_Y¥_a`a_aW
@HWI-EAS443:3:4:1189:185#0/1
AAAAAGTACACATCAGGAAGACAAGAGAAGAACCCCGCAC
+HWI-EAS443:3:4:1189:185#0/1
aa_``a^aa]_`]_^aa^`a_Z^^X^^_^ZXTMMZUWZBB
@HWI-EAS443:3:4:1421:939#0/1
TGCGGGGTTCTTCTCTTGTCTTCCTGATGTGTACTTTTTG
+HWI-EAS443:3:4:1421:939#0/1
aa]`]_`^``baaaZaaba^b`a`aa_aa_[]LXa`a_`S
@HWI-EAS443:3:4:1496:55#0/1
CAAAAAGTACACATCAGGAAGACAAGAGAAGAACCCCGCA
+HWI-EAS443:3:4:1496:55#0/1
Ybbbaba`baaaaaa`ab_ab_`a^a`a[^aa_`UXWaZ_
@HWI-EAS443:3:4:1523:1508#0/1
AAAAAGTACACATCAGGAAGACAAGAGAAGAACCCCGCAC
+HWI-EAS443:3:4:1523:1508#0/1
aaa¥``V`[`a_a^`aaaa`a^¥Z`Z_U`__XV¥FX`_`^
@HWI-EAS443:3:4:1778:1848#0/1
TGCGGGGTTCTTCTCTTGTCTTCCTGATGTGTACTTTTTG
+HWI-EAS443:3:4:1778:1848#0/1
abba]baYUR]a_¥aa`aZV`YT[````aY`W]_a____[
@HWI-EAS443:3:4:765:548#0/1
CAAAAAGTACACATCAGGAAGACAAGAGAAGAACCCCGCA
+HWI-EAS443:3:4:765:548#0/1
ab^`baa[aaa_aa^aab_aa`_a]a_a__a``_]^^a]^
@HWI-EAS443:3:4:842:340#0/1
CAAAAAGTACACATCAGGAAGACAAGAGAAGAACCCCGCA
+HWI-EAS443:3:4:842:340#0/1
aabbbab^bababaaabbaaa_aa`baaaaaa_aR`Ya_`
@HWI-EAS443:3:50:849:2000#0/1
AAAAGTACACATCAGGAAGACAAGAGAAGAACCCCGCACT
+HWI-EAS443:3:50:849:2000#0/1
abba_Pab`[[`aa¥¥aaa_abaaa`aa_aa]ODWbRaQ]
@HWI-EAS443:3:51:1692:1269#0/1
CAAAAAGTACACATCAGGAAGACAAGAGAAGAACCCCGCA
+HWI-EAS443:3:51:1692:1269#0/1
Z`bababUaab[baa_ab`ab[`[`aT_a`P`[`abZK^`
@HWI-EAS443:3:51:210:436#0/1
CTCTTGTCTTCCTGATGTGTACTTTTTGATTATGGCCATA
+HWI-EAS443:3:51:210:436#0/1
a__]``a_a^`_]¥^_aY`__X_aaa`_a_^_^a_`YPPV
@HWI-EAS443:3:52:1113:1322#0/1
CAAAAAGTACACATCAGGAAGACAAGAGAAGAACCCCGCA
+HWI-EAS443:3:52:1113:1322#0/1
Z¥[RW__VbW¥^b¥`bba```_]`X_a`ab`aba`^Sa`a
```

```
@HWI-EAS443:3:52:1320:1967#0/1
AAAAAGTACACATCAGGAAGACAAGAGAAGAACCCCGCAC
+HWI-EAS443:3:52:1320:1967#0/1
X``a`b^b¥a[aZ^``aaaa]S`_a____`a_^_¥]aX¥O
@HWI-EAS443:3:52:743:281#0/1
CAAAAAGTACACATCAGGAAGACAAGAGAAGAACCCCGCA
+HWI-EAS443:3:52:743:281#0/1
abbbbbbababababababbbbba_abaabaabab`a__b_a
@HWI-EAS443:3:53:30:1403#0/1
CAAAAAGTACACATCAGGAAGACAAGAGAAGAACCCCGCA
+HWI-EAS443:3:53:30:1403#0/1
a[bbUbb]`]]b_[ba]aa`b`bbab`b^baaaaaa_baa
@HWI-EAS443:3:53:58:273#0/1
TGCGGGGTTCTTCTCTTGTCTTCCTGATGTGTACTTTTTG
+HWI-EAS443:3:53:58:273#0/1
abbaabaaa_abaa`aba_aaa_aaa`aa_aXX]`_``_[
@HWI-EAS443:3:53:75:2018#0/1
TGCGGGGTTCTTCTCTTGTCTTCCTGATGTGTACTTTTTG
+HWI-EAS443:3:53:75:2018#0/1
Vb`]X__Rb`bYYa]]ba_X`¥]_bbY_Y_^OY]^¥b]_T
@HWI-EAS443:3:53:821:1698#0/1
AAAAGTACACATCAGGAAGACAAGAGAAGAACCCCGCACT
+HWI-EAS443:3:53:821:1698#0/1
^bbbb_bb^]]b_]b]][__aab_b`b¥abaYX[SbW``_
@HWI-EAS443:3:54:1177:1281#0/1
CAAAAAGTACACATCAGGAAGACAAGAGAAGAACCCCGCA
+HWI-EAS443:3:54:1177:1281#0/1
abababaabbbbbbbbabbbb`abbbbabbbbbbababab
@HWI-EAS443:3:54:255:659#0/1
CAAAAAGTACACATCAGGAAGACAAGAGAAGAACCCCGCA
+HWI-EAS443:3:54:255:659#0/1
¥[`¥UO]LWa_W^¥VTZa_]¥_`YZ[T¥MW_^ZS]YZa`[
@HWI-EAS443:3:54:27:1377#0/1
CAAAAAGTACACATCAGGAAGACAAGAGAAGAACCCCGCA
+HWI-EAS443:3:54:27:1377#0/1
a`b`b_a^U^baaaa`aa`aaaaa]aa_]]_``^S]`b``
@HWI-EAS443:3:54:761:282#0/1
CAAAAAGTACACATCAGGAAGACAAGAGAAGAACCCCGCA
+HWI-EAS443:3:54:761:282#0/1
abbb_¥_`b``a``ba^aaabaaaabaaaaaba`^`¥aaa
@HWI-EAS443:3:54:823:148#0/1
CTCTTGTCTTCCTGATGTGTACTTTTTGATTATGGCCATA
+HWI-EAS443:3:54:823:148#0/1
abbbbbbbbbaababbbabbbabbbbbabbbababa_b`a
@HWI-EAS443:3:55:140:1821#0/1
AAAAGTACACATCAGGAAGACAAGAGAAGAACCCCGCACT
+HWI-EAS443:3:55:140:1821#0/1
aaaa^^a_aa`__`a__`_`_`a`__`_¥]a__]__W^]^
@HWI-EAS443:3:55:1433:1329#0/1
CAAAAAGTACACATCAGGAAGACAAGAGAAGAACCCCGCA
```

+HWI-EAS443:3:55:1433:1329#0/1
a`[W`Zb]bbaab_Xabbbaa`Wb[bbbbabbba¥X`aT`
@HWI-EAS443:3:55:1649:1212#0/1
GTGCGGGGTTCTTCTCTTGTCTTCCTGATGTGTACTTTTT
+HWI-EAS443:3:55:1649:1212#0/1
¥NWQ[_b]RRaa¥QZUa¥¥]L_¥KU`_TTYX_[MDP^X¥`
@HWI-EAS443:3:55:750:521#0/1
CAAAAAGTACACATCAGGAAGACAAGAGAAGAACCCCGCA
+HWI-EAS443:3:55:750:521#0/1
`b``__a^aabaaaabbb__a`ab_b`b``baa[¥Zaa_a
@HWI-EAS443:3:56:1281:475#0/1
AAAAAGTACACATCAGGAAGACAAGAGAAGAACCCCGCAC
+HWI-EAS443:3:56:1281:475#0/1
¥ZZ]X¥ZX¥_V_XSW]`^`]^¥a_aa]aaa[a]XYO^U_^
@HWI-EAS443:3:56:525:1581#0/1
AAAAAGTACACATCAGGAAGACAAGAGAAGAACCCCGCAC
+HWI-EAS443:3:56:525:1581#0/1
[`TWababb_`baa¥Z¥a`Ta^[a`]abaW¥[¥a_Yaaaa
@HWI-EAS443:3:56:751:826#0/1
TGCGGGGTTCTTCTCTTGTCTTCCTGATGTGTACTTTTTG
+HWI-EAS443:3:56:751:826#0/1
a]bbabaH^aab_a`aabaaaba`aa_¥aZa_]`aa`aaa
@HWI-EAS443:3:57:778:219#0/1
AAAAAGTACACATCAGGAAGACAAGAGAAGAACCCCGCAC
+HWI-EAS443:3:57:778:219#0/1
abbbababaabbaabbbbbbaaaab`a`abaa`]a_b_`_
@HWI-EAS443:3:58:1087:1725#0/1
TGCGGGGTTCTTCTCTTGTCTTCCTGATGTGTACTTTTTG
+HWI-EAS443:3:58:1087:1725#0/1
a_Z`]a``]J¥a]aaaaa^¥aa]U`a_aY^]ZZF¥aaaa`
@HWI-EAS443:3:58:148:490#0/1
TATGGCCATAATCAAAAAGTACACATCAGGAAGACAAGAG
+HWI-EAS443:3:58:148:490#0/1
Vaabbbb_[¥[^Z[^Z]TXV]W_¥XYXW^]W]`[ZY_¥UX
@HWI-EAS443:3:58:1492:1560#0/1
AAAAAGTACACATCAGGAAGACAAGAGAAGAACCCCGCAC
+HWI-EAS443:3:58:1492:1560#0/1
]aZab`Paaaa__V]^Yaa`]Na_a_a`aaaaW_ZS__a[
@HWI-EAS443:3:58:1502:1309#0/1
AAAAAGTACACATCAGGAAGACAAGAGAAGAACCCCGCAC
+HWI-EAS443:3:58:1502:1309#0/1
aaaa]]^`_b^ab``aaaab__a^_`aa`___]X_¥`__^
@HWI-EAS443:3:58:1644:957#0/1
TATGGCCATAATCAAAAAGTACACATCAGGAAGACAAGAG
+HWI-EAS443:3:58:1644:957#0/1
aaaaa]`__N`]TVY_`¥RKOUXZ^ZMX^_Y]_XX]ZZW_
@HWI-EAS443:3:58:212:753#0/1
AAAAGTACACATCAGGAAGACAAGAGAAGAACCCCGCACT
+HWI-EAS443:3:58:212:753#0/1
a_XW¥Vaaaa``aa_^^aa_``S`Za_``_``W¥____^]

```
@HWI-EAS443:3:58:738:915#0/1
TGCGGGGTTCTTCTCTTGTCTTCCTGATGTGTACTTTTTG
+HWI-EAS443:3:58:738:915#0/1
abbbbba]babbabbbbbaabba`bb`b`_[_a`babab^
@HWI-EAS443:3:58:828:687#0/1
TGCGGGGTTCTTCTCTTGTCTTCCTGATGTGTACTTTTTG
+HWI-EAS443:3:58:828:687#0/1
aZ``^_aaZU¥a¥`Taab[V]`_UaaV]R]a^¥U_aaa]U
@HWI-EAS443:3:58:8:989#0/1
GTGCGGGGTTCTTCTCTTGTCTTCCTGATGTGTACTTTTT
+HWI-EAS443:3:58:8:989#0/1
aU[a^`T¥X_a`a_aa``^]aa___a][]ZRZ___a`_`V
@HWI-EAS443:3:59:1009:904#0/1
TGCGGGGTTCTTCTCTTGTCTTCCTGATGTGTACTTTTTG
+HWI-EAS443:3:59:1009:904#0/1
[baa`a_T]Q`_SZ[V^_RZ]]`]`_US`W^QOLVWZX]X
@HWI-EAS443:3:59:363:1139#0/1
AAAAAGTACACATCAGGAAGACAAGAGAAGAACCCCGCAC
+HWI-EAS443:3:59:363:1139#0/1
a`a`]^O[_aa]]_`_a`aa_``Z]`a]`aY^]]XY]_XX
@HWI-EAS443:3:59:577:962#0/1
TGCGGGGTTCTTCTCTTGTCTTCCTGATGTGTACTTTTTG
+HWI-EAS443:3:59:577:962#0/1
]b^_a_^a`][_abbbba]]_bbbbaa_a`b]aaa^`^a^
@HWI-EAS443:3:59:617:216#0/1
TGCGGGGTTCTTCTCTTGTCTTCCTGATGTGTACTTTTTG
+HWI-EAS443:3:59:617:216#0/1
aabbbb_Qaaaaaaaaab_aaaa_aaaaa_^V_[a^^_a_
@HWI-EAS443:3:5:1454:709#0/1
TGCGGGGTTCTTCTCTTGTCTTCCTGATGTGTACTTTTTG
+HWI-EAS443:3:5:1454:709#0/1
¥b_b`Z`ab]ab¥babb`¥`ba¥[ab_aaaaL¥]b]OZaa
@HWI-EAS443:3:5:440:1841#0/1
AAAAAGTACACATCAGGAAGACAAGAGAAGAACCCCGCAC
+HWI-EAS443:3:5:440:1841#0/1
aa`aaa¥ba`aa¥¥aaaaaa__ba``¥aaYa^^^^_`¥_B
@HWI-EAS443:3:60:1038:1547#0/1
TGCGGGGTTCTTCTCTTGTCTTCCTGATGTGTACTTTTTG
+HWI-EAS443:3:60:1038:1547#0/1
aaUW_`XFY]a^[[`]a^]Y^aa_a]]`a[`]WYa¥`a_V
@HWI-EAS443:3:60:1185:1000#0/1
TGCGGGGTTCTTCTCTTGTCTTCCTGATGTGTACTTTTTG
+HWI-EAS443:3:60:1185:1000#0/1
aaaaaaa]aaaa`a_aab_aaa`^aa`_`]a]]Xa___`_
@HWI-EAS443:3:60:271:1112#0/1
AAAAAGTACACATCAGGAAGACAAGAGAAGAACCCCGCAC
+HWI-EAS443:3:60:271:1112#0/1
_a`a]_P^ab```]`baa^baaba]abb]aa]aY^^]`a`
@HWI-EAS443:3:60:785:1139#0/1
TGCGGGGTTCTTCTCTTGTCTTCCTGATGTGTACTTTTTG
```

+HWI-EAS443:3:60:785:1139#0/1
`bb`baaTZ_bb¥`]bbaUa`X`_ba]aa]a_`_baaaaV
@HWI-EAS443:3:61:1285:710#0/1
TGCGGGGTTCTTCTCTTGTCTTCCTGATGTGTACTTTTTG
+HWI-EAS443:3:61:1285:710#0/1
a^_bbb``baba`aaabbaabbba``^aa^`^^^aa^^^[
@HWI-EAS443:3:61:1618:1560#0/1
TGCGGGGTTCTTCTCTTGTCTTCCTGATGTGTACTTTTTG
+HWI-EAS443:3:61:1618:1560#0/1
`aabaaa¥babb`aabbbaabbbabba_aa^`]]aa_a_`
@HWI-EAS443:3:61:1677:772#0/1
AAAAGTACACATCAGGAAGACAAGAGAAGAACCCCGCACT
+HWI-EAS443:3:61:1677:772#0/1
abaa_¥b_aba_]^_Z^bbabaabab_abaa`RYS_X^Y^
@HWI-EAS443:3:61:170:416#0/1
AAAAGTACACATCAGGAAGACAAGAGAAGAACCCCGCACT
+HWI-EAS443:3:61:170:416#0/1
aabab^aaaaaaa`aaaaaa_WW`P````Z`_]_¥_^`^^
@HWI-EAS443:3:61:206:235#0/1
TGCGGGGTTCTTCTCTTGTCTTCCTGATGTGTACTTTTTG
+HWI-EAS443:3:61:206:235#0/1
ababab`_abbbaaaaaa`a`]`a_`[`a¥`U_¥a^__^Y
@HWI-EAS443:3:61:666:902#0/1
TGCGGGGTTCTTCTCTTGTCTTCCTGATGTGTACTTTTTG
+HWI-EAS443:3:61:666:902#0/1
`baab]a^aaaaaaaaaa`aa_ba`_]a`]`Y]^a_^_YQ
@HWI-EAS443:3:61:917:649#0/1
TGCGGGGTTCTTCTCTTGTCTTCCTGATGTGTACTTTTTG
+HWI-EAS443:3:61:917:649#0/1
abaabab_aaaaaaaaba`aa_baaa_aa_`Z__a__`]N
@HWI-EAS443:3:62:1733:1782#0/1
GTGCGGGGTTCTTCTCTTGTCTTCCTGATGTGTACTTTTT
+HWI-EAS443:3:62:1733:1782#0/1
a_V_¥ZWTWZVa`^YU``]]_`XX[a`Y[O[ZGLRZ]_`X
@HWI-EAS443:3:62:370:857#0/1
GTGCGGGGTTCTTCTCTTGTCTTCCTGATGTGTACTTTTT
+HWI-EAS443:3:62:370:857#0/1
aQQU¥^[RQT¥U_¥OaUOOXXYYT^T`[U`GXFQ¥Z[PLR
@HWI-EAS443:3:62:944:217#0/1
CTCTTGTCTTCCTGATGTGTACTTTTTGATTATGGCCATA
+HWI-EAS443:3:62:944:217#0/1
abbbbbbbabbbbbbbb`b^a`bbbbbbabbaaa^^_aU[
@HWI-EAS443:3:63:120:685#0/1
GTGCGGGGTTCTTCTCTTGTCTTCCTGATGTGTACTTTTT
+HWI-EAS443:3:63:120:685#0/1
_Z¥_]X_TMT]^``aa`^a`aaZa¥WQR¥XRRP¥`]YBBB
@HWI-EAS443:3:63:192:1438#0/1
AAAGTACACATCAGGAAGACAAGAGAAGAACCCCGCACTC
+HWI-EAS443:3:63:192:1438#0/1
aa_K[aaaaa]a]]Yaa_``a`_]_____]Z]]]_]_YX_

```
@HWI-EAS443:3:63:628:1975#0/1
AAAAAGTACACATCAGGAAGACAAGAGAAGAACCCCGCAC
+HWI-EAS443:3:63:628:1975#0/1
aaa]]¥Xaab`a[_aa]aa¥^`]^`_^_^``__`_V^^_`
@HWI-EAS443:3:63:745:1149#0/1
CAAAAAGTACACATCAGGAAGACAAGAGAAGAACCCCGCA
+HWI-EAS443:3:63:745:1149#0/1
aaa^__aXa]aaa`_aaaba_a_a]a^`aaYaaaa]`^aa
@HWI-EAS443:3:64:1078:411#0/1
GTGCGGGGTTCTTCTCTTGTCTTCCTGATGTGTACTTTTT
+HWI-EAS443:3:64:1078:411#0/1
a]a`¥_a_^a]aa`a^a`a^a`_¥__[¥_`S`ZW^a^_^_
@HWI-EAS443:3:64:113:1967#0/1
CAAAAAGTACACATCAGGAAGACAAGAGAAGAACCCCGCA
+HWI-EAS443:3:64:113:1967#0/1
aS`YZZSYbaTSZ^____`_NN]__OYX`_W_]]¥[¥WUY
@HWI-EAS443:3:64:1203:477#0/1
TGCGGGGTTCTTCTCTTGTCTTCCTGATGTGTACTTTTTG
+HWI-EAS443:3:64:1203:477#0/1
aba__ab]_W_a`_Y]ba]`aaa`a``aa_a_[J^aa___
@HWI-EAS443:3:64:1628:1062#0/1
TGCGGGGTTCTTCTCTTGTCTTCCTGATGTGTACTTTTTG
+HWI-EAS443:3:64:1628:1062#0/1
aa`_`bb^a`bbab`bba`^baa_aa_aa_a^a^baaaaY
@HWI-EAS443:3:64:204:1391#0/1
CAAAAAGTACACATCAGGAAGACAAGAGAAGAACCCCGCA
+HWI-EAS443:3:64:204:1391#0/1
aa_¥¥¥aX^a``a`_`a``__`_````____`__]X]^^^
@HWI-EAS443:3:64:605:1032#0/1
GTGCGGGGTTCTTCTCTTGTCTTCCTGATGTGTACTTTTT
+HWI-EAS443:3:64:605:1032#0/1
aZa`¥`aa^`aa`a`aa`_`a`aa_a`__]XZ[_]a___a
@HWI-EAS443:3:65:1131:24#0/1
TGCGGGGTTCTTCTCTTGTCTTCCTGATGTGTACTTTTTG
+HWI-EAS443:3:65:1131:24#0/1
aaababb[aaaa_a_aaaa`aa`^`aaaa^a^^]aa_a``
@HWI-EAS443:3:65:1783:37#0/1
TATGGCCATAATCAAAAAGTACACATCAGGAAGACAAGAG
+HWI-EAS443:3:65:1783:37#0/1
aaaaaa[aVYa]X[aa`]UF¥X_UWUXZ_`U]WZNXTMVZ
@HWI-EAS443:3:65:259:273#0/1
TGCGGGGTTCTTCTCTTGTCTTCCTGATGTGTACTTTTTG
+HWI-EAS443:3:65:259:273#0/1
aa_¥¥`^Taaaaaaaab^`]`aa_aa```^a]a``_a_^V
@HWI-EAS443:3:66:1292:306#0/1
TGCGGGGTTCTTCTCTTGTCTTCCTGATGTGTACTTTTTG
+HWI-EAS443:3:66:1292:306#0/1
U`]ba__ababb_b¥abaaabaa]ab_bb_b``_b`aa_Z
@HWI-EAS443:3:66:1546:1177#0/1
CTCTTGTCTTCCTGATGTGTACTTTTTGATTATGGCCATA
```

```
+HWI-EAS443:3:66:1546:1177#0/1
aaa`¥ZNT^aaW`Y][bZ][V```aa`]^]Z[BBBBBBBB
@HWI-EAS443:3:66:642:407#0/1
GTGCGGGGTTCTTCTCTTGTCTTCCTGATGTGTACTTTTT
+HWI-EAS443:3:66:642:407#0/1
a`aaaaaa]`aaaa``aaa__a__`aa_``_a]^_`a_a`
@HWI-EAS443:3:66:875:1940#0/1
TGCGGGGTTCTTCTCTTGTCTTCCTGATGTGTACTTTTTG
+HWI-EAS443:3:66:875:1940#0/1
Z]bbY`aP`aabb`Y^_aa`a`^]_]¥_a_UT]`a`aa[M
@HWI-EAS443:3:67:684:1581#0/1
CAAAAAGTACACATCAGGAAGACAAGAGAAGAACCCCGCA
+HWI-EAS443:3:67:684:1581#0/1
aaaaZaaG^``ba^]]L^a`__`a___XYYW_]^_X__`_
@HWI-EAS443:3:67:935:526#0/1
TATGGCCATAATCAAAAAGTACACATCAGGAAGACAAGAG
+HWI-EAS443:3:67:935:526#0/1
aaaaaaaaa`a`_aaa^a_O_S^_`_^_a__`a_N^a`_a
@HWI-EAS443:3:68:1520:1923#0/1
TGCGGGGTTCTTCTCTTGTCTTCCTGATGTGTACTTTTTG
+HWI-EAS443:3:68:1520:1923#0/1
]`aab`¥U]`aaa__aaa__a^a`a^[aaX`^_aa`]]a^
@HWI-EAS443:3:68:1709:231#0/1
TGCGGGGTTCTTCTCTTGTCTTCCTGATGTGTACTTTTTG
+HWI-EAS443:3:68:1709:231#0/1
]_]]P¥a]_aba_`]a^SYU]a[L¥_Y__V¥W¥N¥^]]]B
@HWI-EAS443:3:68:412:368#0/1
CAAAAAGTACACATCAGGAAGACAAGAGAAGAACCCCGCA
+HWI-EAS443:3:68:412:368#0/1
abb¥^a`T`bbT^_a`a]a¥`Vaba_]_Y`aaa_a^_aaa
@HWI-EAS443:3:68:634:1337#0/1
CAAAAAGTACACATCAGGAAGACAAGAGAAGAACCCCGCA
+HWI-EAS443:3:68:634:1337#0/1
abbaaa_[]aaaaaaa`^aaaaa`[aa_a`^aa__a`_aa
@HWI-EAS443:3:68:698:75#0/1
CAAAAAGTACACATCAGGAAGACAAGAGAAGAACCCCGCA
+HWI-EAS443:3:68:698:75#0/1
Yabaaab_baaa_bb]aa_ab`bb`b`bUaaaYa`^]_`^
@HWI-EAS443:3:68:930:366#0/1
GTGCGGGGTTCTTCTCTTGTCTTCCTGATGTGTACTTTTT
+HWI-EAS443:3:68:930:366#0/1
aX^`aa^¥P``aaa^aa^¥XT¥aZ[a]Z`a__]¥P``_`a
@HWI-EAS443:3:69:1677:220#0/1
TATGGCCATAATCAAAAAGTACACATCAGGAAGACAAGAG
+HWI-EAS443:3:69:1677:220#0/1
Xaaaaaa`_R_YO[QYYZTHHQJGVTSXF¥MVZUOPYBBB
@HWI-EAS443:3:69:396:458#0/1
TGCGGGGTTCTTCTCTTGTCTTCCTGATGTGTACTTTTTG
+HWI-EAS443:3:69:396:458#0/1
abbbbbb`babbaaaaaa`aaabaa`U_a`a_aaaaaaa`
```

@HWI-EAS443:3:69:500:155#0/1
CAAAAAGTACACATCAGGAAGACAAGAGAAGAACCCCGCA
+HWI-EAS443:3:69:500:155#0/1
abbabbbaba_bbaaabaabbabb`bbbab`bba`a_aaa
@HWI-EAS443:3:6:131:685#0/1
GTGCGGGGTTCTTCTCTTGTCTTCCTGATGTGTACTTTTT
+HWI-EAS443:3:6:131:685#0/1
a¥^baa`a^a_a``a`aaa_`a`¥¥a__^]V`]^¥`_a`a
@HWI-EAS443:3:6:1581:815#0/1
CAAAAAGTACACATCAGGAAGACAAGAGAAGAACCCCGCA
+HWI-EAS443:3:6:1581:815#0/1
ab]b_`WV`a_a`bT^aY`babYbaaa`a```aa[[_a`a
@HWI-EAS443:3:6:231:245#0/1
CAAAAAGTACACATCAGGAAGACAAGAGAAGAACCCCGCA
+HWI-EAS443:3:6:231:245#0/1
`baaaa`^b`ba`aabba`aaa`a`aaaa_aa_`a]_a__
@HWI-EAS443:3:70:1102:1634#0/1
AAAAGTACACATCAGGAAGACAAGAGAAGAACCCCGCACT
+HWI-EAS443:3:70:1102:1634#0/1
aba`Z[aababa_aaaaaaa`b`baaaaaaa___`_aa]¥
@HWI-EAS443:3:70:1271:1003#0/1
CAAAAAGTACACATCAGGAAGACAAGAGAAGAACCCCGCA
+HWI-EAS443:3:70:1271:1003#0/1
abaaWWUL_`^^``^`a]_]b`a`X]`a¥Y_``^¥Y]aS`
@HWI-EAS443:3:70:1306:214#0/1
CAAAAAGTACACATCAGGAAGACAAGAGAAGAACCCCGCA
+HWI-EAS443:3:70:1306:214#0/1
abaaZ`baba`b`_aabb`ababbbbabbbbbaYOTaba_
@HWI-EAS443:3:70:1497:204#0/1
TGCGGGGTTCTTCTCTTGTCTTCCTGATGTGTACTTTTTG
+HWI-EAS443:3:70:1497:204#0/1
ab[aabb^a`ba`a_aaba_aa_`a`^aa`a]^V``a``]
@HWI-EAS443:3:70:250:1769#0/1
CTCTTGTCTTCCTGATGTGTACTTTTTGATTATGGCCATA
+HWI-EAS443:3:70:250:1769#0/1
aaaaa`[a_`baa`_`]X_^aa````a_`_]]_[X__`VY
@HWI-EAS443:3:70:59:1956#0/1
AAAAAGTACACATCAGGAAGACAAGAGAAGAACCCCGCAC
+HWI-EAS443:3:70:59:1956#0/1
a^][aYRa_aaa``a]U]UY__W^__^[ZUS`]^_]XVZ^
@HWI-EAS443:3:71:1033:56#0/1
TGCGGGGTTCTTCTCTTGTCTTCCTGATGTGTACTTTTTG
+HWI-EAS443:3:71:1033:56#0/1
ababbbb_aaba_b`baba`bb`_abaabaa`a_aaaaaa
@HWI-EAS443:3:71:1250:1447#0/1
TATGGCCATAATCAAAAAGTACACATCAGGAAGACAAGAG
+HWI-EAS443:3:71:1250:1447#0/1
aaaaXabaYVab`Xa_bbWJHZ`U``__ba_^VS___YOS
@HWI-EAS443:3:71:1381:1374#0/1
TGCGGGGTTCTTCTCTTGTCTTCCTGATGTGTACTTTTTG

```
+HWI-EAS443:3:71:1381:1374#0/1
]^`a`a]Xaaa`^_`a`VXZaaa]_a`^]Z`X^_]`_^``
@HWI-EAS443:3:71:1717:1804#0/1
AAAAGTACACATCAGGAAGACAAGAGAAGAACCCCGCACT
+HWI-EAS443:3:71:1717:1804#0/1
aaaabaaaabba`abbaaba`aaa`baabbb``a`a`a_`
@HWI-EAS443:3:71:298:690#0/1
TGCGGGGTTCTTCTCTTGTCTTCCTGATGTGTACTTTTTG
+HWI-EAS443:3:71:298:690#0/1
aabbbbb`bbbbbbbbbbabaaaaab]ab_a^abaaaaa¥
@HWI-EAS443:3:71:354:2023#0/1
CAAAAAGTACACATCAGGAAGACAAGAGAAGAACCCCGCA
+HWI-EAS443:3:71:354:2023#0/1
a]aZ`YXZabaaa_aa``a`^]a``]_¥_`^a_`_``]_^
@HWI-EAS443:3:71:399:574#0/1
AAAAAGTACACATCAGGAAGACAAGAGAAGAACCCCGCAC
+HWI-EAS443:3:71:399:574#0/1
aaaaa`[``aaaa`aaa`]`_aa`aaa`aaaaa``_a]_^
@HWI-EAS443:3:71:954:321#0/1
AAAGTACACATCAGGAAGACAAGAGAAGAACCCCGCACTC
+HWI-EAS443:3:71:954:321#0/1
[ab_Sbab`J`aa]``aXa^aaa_W_XbaaZaa_bX`]__
@HWI-EAS443:3:72:1035:61#0/1
AAAAAGTACACATCAGGAAGACAAGAGAAGAACCCCGCAC
+HWI-EAS443:3:72:1035:61#0/1
aaaaa_`baba^baaa¥a^b__a`b¥a_aaaaa^¥^a]^U
@HWI-EAS443:3:72:1606:145#0/1
TGCGGGGTTCTTCTCTTGTCTTCCTGATGTGTACTTTTTG
+HWI-EAS443:3:72:1606:145#0/1
ab_a]_YVYaaa^a]_aa`Za_WX_Z[`_U`Z_Y`a``_N
@HWI-EAS443:3:72:19:679#0/1
AAAAAGTACACATCAGGAAGACAAGAGAAGAACCCCGCAC
+HWI-EAS443:3:72:19:679#0/1
`^`]Z_]a^T^`aa`]aa]a__a_``^W[^`_^^X__``_
@HWI-EAS443:3:72:529:772#0/1
CAAAAAGTACACATCAGGAAGACAAGAGAAGAACCCCGCA
+HWI-EAS443:3:72:529:772#0/1
a`^`YT`]¥¥a]¥U`Za`R¥¥S¥Za`]a[``]aZa``aY[
@HWI-EAS443:3:72:612:579#0/1
CAAAAAGTACACATCAGGAAGACAAGAGAAGAACCCCGCA
+HWI-EAS443:3:72:612:579#0/1
abbbbabRbabbaaaaaa_aa_aaaaaa`_aa```__a_a
@HWI-EAS443:3:73:128:1055#0/1
TGCGGGGTTCTTCTCTTGTCTTCCTGATGTGTACTTTTTG
+HWI-EAS443:3:73:128:1055#0/1
aabbb``^`aaabaaaaa`aa`aaaaaaaZa]a`a_a_a`
@HWI-EAS443:3:73:1550:451#0/1
CTCTTGTCTTCCTGATGTGTACTTTTTGATTATGGCCATA
+HWI-EAS443:3:73:1550:451#0/1
aaY¥¥¥¥X`_P]_J_^^][T_aa_b`aaaaa]_^PMEQ]^
```

```
@HWI-EAS443:3:73:1607:1223#0/1
GTGCGGGGTTCTTCTCTTGTCTTCCTGATGTGTACTTTTT
+HWI-EAS443:3:73:1607:1223#0/1
aV`^W^X`Zaaaa]`__a___¥a^^_WU][W[Y][^Z^V`
@HWI-EAS443:3:73:261:1287#0/1
AAAAAGTACACATCAGGAAGACAAGAGAAGAACCCCGCAC
+HWI-EAS443:3:73:261:1287#0/1
VWVQ_aNWZ__S¥a_^[F]`a__VWU^¥QUY`_^^[Xa¥^
@HWI-EAS443:3:73:394:965#0/1
AAAGTACACATCAGGAAGACAAGAGAAGAACCCCGCACTC
+HWI-EAS443:3:73:394:965#0/1
_[XZ[ab_aaaa`aaabaaaa[[`a`X``Z]^__a`a__a
@HWI-EAS443:3:73:892:1927#0/1
TGCGGGGTTCTTCTCTTGTCTTCCTGATGTGTACTTTTTG
+HWI-EAS443:3:73:892:1927#0/1
aab`a``_abbaaabaaa_aaaaaa_aaa_`]`aa_a`aZ
@HWI-EAS443:3:74:571:924#0/1
CAAAAAGTACACATCAGGAAGACAAGAGAAGAACCCCGCA
+HWI-EAS443:3:74:571:924#0/1
abbaaba_bbbbababbaaab`aaa`aaa`aaaaa`aaaa
@HWI-EAS443:3:74:788:169#0/1
TGCGGGGTTCTTCTCTTGTCTTCCTGATGTGTACTTTTTG
+HWI-EAS443:3:74:788:169#0/1
aa¥baabXb`aa_a^aaa_^aaZ_a`_`a]`]`_``__]_
@HWI-EAS443:3:75:1210:1987#0/1
AAAAAGTACACATCAGGAAGACAAGAGAAGAACCCCGCAC
+HWI-EAS443:3:75:1210:1987#0/1
`bbab`_bbbabaaabaabbaaaabaaaabaa__a_a`aa
@HWI-EAS443:3:75:805:238#0/1
AAAAAGTACACATCAGGAAGACAAGAGAAGAACCCCGCAC
+HWI-EAS443:3:75:805:238#0/1
aa`abaZb_aaaa`abaaa``Ya`__a___`a_^^V_R_Z
@HWI-EAS443:3:76:1188:1610#0/1
CAAAAAGTACACATCAGGAAGACAAGAGAAGAACCCCGCA
+HWI-EAS443:3:76:1188:1610#0/1
[`a`abb`Z^bb`babbbbb]`bba`aa_baaabbXZ_^b
@HWI-EAS443:3:76:271:898#0/1
TGCGGGGTTCTTCTCTTGTCTTCCTGATGTGTACTTTTTG
+HWI-EAS443:3:76:271:898#0/1
]a`YOR`_aba_`ZX`_aXaaa`^`a_]ZS[V_``_^^`_
@HWI-EAS443:3:76:275:1401#0/1
TGCGGGGTTCTTCTCTTGTCTTCCTGATGTGTACTTTTTG
+HWI-EAS443:3:76:275:1401#0/1
aab``a]¥`abaa`aa`a^aaaaa^_]][WaW_a_a___^
@HWI-EAS443:3:76:435:1298#0/1
CAAAAAGTACACATCAGGAAGACAAGAGAAGAACCCCGCA
+HWI-EAS443:3:76:435:1298#0/1
abb`]`_^aaaaa``a``]R^`__U_^^_`_`a`[^_]`Z
@HWI-EAS443:3:76:526:166#0/1
CAAAAAGTACACATCAGGAAGACAAGAGAAGAACCCCGCA
```

```
+HWI-EAS443:3:76:526:166#0/1
a`__aaa`b`aba_aaba`_b[]a_a_^_^`^__YWW_[^
@HWI-EAS443:3:76:592:1183#0/1
AAAAGTACACATCAGGAAGACAAGAGAAGAACCCCGCACT
+HWI-EAS443:3:76:592:1183#0/1
^a_aa]baaaaa`_aaaa_``a``____a``___a``]__
@HWI-EAS443:3:76:594:679#0/1
AAAAAGTACACATCAGGAAGACAAGAGAAGAACCCCGCAC
+HWI-EAS443:3:76:594:679#0/1
`[_ba_QZ`aa`aaaaa]a`aab`a`[`a_aa`a_ab]`a
@HWI-EAS443:3:76:685:966#0/1
TGCGGGGTTCTTCTCTTGTCTTCCTGATGTGTACTTTTTG
+HWI-EAS443:3:76:685:966#0/1
abbb]`^]aaaaaaa`aa_aa^^a_]Y[YWaY__```^^`
@HWI-EAS443:3:76:907:1453#0/1
TGCGGGGTTCTTCTCTTGTCTTCCTGATGTGTACTTTTTG
+HWI-EAS443:3:76:907:1453#0/1
ZNX]W`a]YI]a_`]W]]¥`_a[_]`^_ZV^Y]^a^_^_^
@HWI-EAS443:3:76:965:1077#0/1
AAAAAGTACACATCAGGAAGACAAGAGAAGAACCCCGCAC
+HWI-EAS443:3:76:965:1077#0/1
ZXX¥¥Za^_``a`¥X¥`]aW¥abb`^`W]``a`[`^aX`a
@HWI-EAS443:3:77:1052:794#0/1
CAAAAAGTACACATCAGGAAGACAAGAGAAGAACCCCGCA
+HWI-EAS443:3:77:1052:794#0/1
Zab`bbbbabbabbbbbbbbbbbabbbbbbbbbbaa^bbbb
@HWI-EAS443:3:77:1096:1735#0/1
TGCGGGGTTCTTCTCTTGTCTTCCTGATGTGTACTTTTTG
+HWI-EAS443:3:77:1096:1735#0/1
ab___^YZbaab`aaabZYaaa^aaa_aaW^W¥Q_[Xaa`
@HWI-EAS443:3:77:1749:451#0/1
AAAAGTACACATCAGGAAGACAAGAGAAGAACCCCGCACT
+HWI-EAS443:3:77:1749:451#0/1
^abYX¥_b`]Y]T_bab`a]_a`ba`b]bb]Q[`¥_]T^`
@HWI-EAS443:3:77:294:898#0/1
CAAAAAGTACACATCAGGAAGACAAGAGAAGAACCCCGCA
+HWI-EAS443:3:77:294:898#0/1
abb^a^_`aa_`aaaab`Vab]aaaa`aXVa_a`]_aa_a
@HWI-EAS443:3:77:314:701#0/1
TGCGGGGTTCTTCTCTTGTCTTCCTGATGTGTACTTTTTG
+HWI-EAS443:3:77:314:701#0/1
aaaaabaXaaaaaaaaab_a`aaaaaaaa_aR`]a````a
@HWI-EAS443:3:77:336:1989#0/1
GTGCGGGGTTCTTCTCTTGTCTTCCTGATGTGTACTTTTT
+HWI-EAS443:3:77:336:1989#0/1
aGNXLU[QVIZU`YPYTVYMYPOTSNNRP^WXPU¥TOZ^U
@HWI-EAS443:3:77:513:53#0/1
AAAAAGTACACATCAGGAAGACAAGAGAAGAACCCCGCAC
+HWI-EAS443:3:77:513:53#0/1
a[FYXNL]a__]`NP`XX_W`[SX^U^RP`[^_XTZ_]SL
```

```
@HWI-EAS443:3:78:106:1037#0/1
TGCGGGGTTCTTCTCTTGTCTTCCTGATGTGTACTTTTTG
+HWI-EAS443:3:78:106:1037#0/1
ababaaa_a`aaaabaaa`a`a`a`^¥_a^`WW_a`_``^
@HWI-EAS443:3:78:1073:907#0/1
AAAAAGTACACATCAGGAAGACAAGAGAAGAACCCCGCAC
+HWI-EAS443:3:78:1073:907#0/1
^Y^VXa¥bababa^bba^aa_`b¥``a``_b`^]_[`a_^
@HWI-EAS443:3:78:1081:1570#0/1
TGCGGGGTTCTTCTCTTGTCTTCCTGATGTGTACTTTTTG
+HWI-EAS443:3:78:1081:1570#0/1
Za`TX`R[baa^Y`aaaa[_]____a_aT`a^aaaR`a`S
@HWI-EAS443:3:78:1172:1161#0/1
GTGCGGGGTTCTTCTCTTGTCTTCCTGATGTGTACTTTTT
+HWI-EAS443:3:78:1172:1161#0/1
a^a_a_a`]^^a_¥a_a_`^Z`_ZW__^^_Z`^_V___^_
@HWI-EAS443:3:78:462:127#0/1
TATGGCCATAATCAAAAAGTACACATCAGGAAGACAAGAG
+HWI-EAS443:3:78:462:127#0/1
^aa````][]`]Y`a]aXUH[T]_`WZY``^^YXPY¥NY[
@HWI-EAS443:3:78:483:1434#0/1
TGCGGGGTTCTTCTCTTGTCTTCCTGATGTGTACTTTTTG
+HWI-EAS443:3:78:483:1434#0/1
abbbbbb`bbbababbba`bbbaaaaaaa_b`aabaaaa_
@HWI-EAS443:3:78:943:995#0/1
CAAAAAGTACACATCAGGAAGACAAGAGAAGAACCCCGCA
+HWI-EAS443:3:78:943:995#0/1
`W¥_P¥^U`aaaa`^_¥_VU^^`````aa`aaa`XUP`Z_
@HWI-EAS443:3:79:1194:1050#0/1
TGCGGGGTTCTTCTCTTGTCTTCCTGATGTGTACTTTTTG
+HWI-EAS443:3:79:1194:1050#0/1
abW]bab`b[bV_b`a_[PaaZ]]b_]P_X]X[X`aa^a`
@HWI-EAS443:3:79:1570:602#0/1
CTCTTGTCTTCCTGATGTGTACTTTTTGATTATGGCCATA
+HWI-EAS443:3:79:1570:602#0/1
ababba^abbaa`bbb]ab`bbbbbbbbabbbb`b]aaaa
@HWI-EAS443:3:79:420:1326#0/1
AAAAAGTACACATCAGGAAGACAAGAGAAGAACCCCGCAC
+HWI-EAS443:3:79:420:1326#0/1
aba¥KZZYbaaaaa`aaaa^S`aaaa_^Z]aaa`_aa_`^
@HWI-EAS443:3:79:867:1137#0/1
TGCGGGGTTCTTCTCTTGTCTTCCTGATGTGTACTTTTTG
+HWI-EAS443:3:79:867:1137#0/1
`baa¥_`Y`aaaababbb_abaaaa_aaa]aaZ¥aaa_aa
@HWI-EAS443:3:79:965:250#0/1
TGCGGGGTTCTTCTCTTGTCTTCCTGATGTGTACTTTTTG
+HWI-EAS443:3:79:965:250#0/1
ababbbb]baba_a_bab_Yaaaaaa`aa`a__]aaaaaa
@HWI-EAS443:3:79:968:518#0/1
CAAAAAGTACACATCAGGAAGACAAGAGAAGAACCCCGCA
```

```
+HWI-EAS443:3:79:968:518#0/1
a`bb`ab[bbbbbb¥bbbbbbaab_aaabbbbaaa_[aaa
@HWI-EAS443:3:7:1372:29#0/1
AAAAAGTACACATCAGGAAGACAAGAGAAGAACCCCGCAC
+HWI-EAS443:3:7:1372:29#0/1
aaaaaaWa```_a_aaaaaaa^a_]^``__^a]RZVaX¥W
@HWI-EAS443:3:80:1464:1578#0/1
AAAAAGTACACATCAGGAAGACAAGAGAAGAACCCCGCAC
+HWI-EAS443:3:80:1464:1578#0/1
``_b¥Z_b^b`[_V]^`_^Y¥¥bb`a`]_]`^^ZXRWaa^
@HWI-EAS443:3:80:613:800#0/1
AAAAGTACACATCAGGAAGACAAGAGAAGAACCCCGCACT
+HWI-EAS443:3:80:613:800#0/1
`_aa`_b_Z_`aaaaa¥aaa`_a``aaaaaa_`a`a_`__
@HWI-EAS443:3:80:637:1464#0/1
TATGGCCATAATCAAAAAGTACACATCAGGAAGACAAGAG
+HWI-EAS443:3:80:637:1464#0/1
abaaabbbaa_]_]_``a¥Q_a`__]Y_a__a^[a_a^``
@HWI-EAS443:3:80:793:884#0/1
AAAGTACACATCAGGAAGACAAGAGAAGAACCCCGCACTC
+HWI-EAS443:3:80:793:884#0/1
`_]NLW¥^]X]]```a_¥ZZ``baaZGXZ`ZTVa_[¥_ZR
@HWI-EAS443:3:80:908:961#0/1
GTGCGGGGTTCTTCTCTTGTCTTCCTGATGTGTACTTTTT
+HWI-EAS443:3:80:908:961#0/1
a_^Ya]`a__¥aa^``aaa]_a__^``]``Y____`_a^`
@HWI-EAS443:3:81:1107:1521#0/1
AAAAAGTACACATCAGGAAGACAAGAGAAGAACCCCGCAC
+HWI-EAS443:3:81:1107:1521#0/1
aaaa_a_baa^aV`¥`Y``a`_a`aaa_[`aa^^XW]^a[
@HWI-EAS443:3:81:1159:1329#0/1
AAAAGTACACATCAGGAAGACAAGAGAAGAACCCCGCACT
+HWI-EAS443:3:81:1159:1329#0/1
Z[^]WQ``[a_baabaX^b^W_ab`_a`X`U¥]^a`]U_`
@HWI-EAS443:3:81:187:1719#0/1
GTGCGGGGTTCTTCTCTTGTCTTCCTGATGTGTACTTTTT
+HWI-EAS443:3:81:187:1719#0/1
a_abaa`a_aabaaaaa`__aaaaa^¥^_a^aQ_aa_`_a
@HWI-EAS443:3:81:313:226#0/1
CAAAAAGTACACATCAGGAAGACAAGAGAAGAACCCCGCA
+HWI-EAS443:3:81:313:226#0/1
aaaa_T_Waaaaaaa_aa``aa`aZ]`a___]_]]^^_^^
@HWI-EAS443:3:81:805:780#0/1
GTGCGGGGTTCTTCTCTTGTCTTCCTGATGTGTACTTTTT
+HWI-EAS443:3:81:805:780#0/1
aRSX___ZFOa`_`_^¥W`R^`]_]__P¥XV_W]_[U]^_
@HWI-EAS443:3:82:1106:1101#0/1
TGCGGGGTTCTTCTCTTGTCTTCCTGATGTGTACTTTTTG
+HWI-EAS443:3:82:1106:1101#0/1
_^b`]bb_baabbab_babbaba`b[aabbbaa`bbbbaa
```

@HWI-EAS443:3:82:1637:1831#0/1
AAAAGTACACATCAGGAAGACAAGAGAAGAACCCCGCACT
+HWI-EAS443:3:82:1637:1831#0/1
U]]OVUZZYN^YN[JYY]W^M]Z[R^WT]]]W]FY]U[S^
@HWI-EAS443:3:82:1674:104#0/1
CTCTTGTCTTCCTGATGTGTACTTTTTGATTATGGCCATA
+HWI-EAS443:3:82:1674:104#0/1
ab_aabbaab`bba_ba_bb`bbbbbaS¥bbbabb_O__S
@HWI-EAS443:3:82:1707:1653#0/1
AAAAGTACACATCAGGAAGACAAGAGAAGAACCCCGCACT
+HWI-EAS443:3:82:1707:1653#0/1
aaabaa[a``aa[abba`¥a_bbaabbaaaa[a¥[aRaa`
@HWI-EAS443:3:82:53:233#0/1
GTGCGGGGTTCTTCTCTTGTCTTCCTGATGTGTACTTTTT
+HWI-EAS443:3:82:53:233#0/1
a_aab_`a]`aa`aa`a__]`_[`a__^__^aWZ^_____
@HWI-EAS443:3:82:843:52#0/1
TGCGGGGTTCTTCTCTTGTCTTCCTGATGTGTACTTTTTG
+HWI-EAS443:3:82:843:52#0/1
ababaab_aaaaa``aa_aaaa__ab`_a_`_[Raa`aaa
@HWI-EAS443:3:83:1276:307#0/1
TGCGGGGTTCTTCTCTTGTCTTCCTGATGTGTACTTTTTG
+HWI-EAS443:3:83:1276:307#0/1
```bbbaW`abb_aababa]ba_[abaa`_`_aZaa``a^
@HWI-EAS443:3:83:1554:705#0/1
AGTACACATCAGGAAGACAAGAGAAGAACCCCGCACTCAG
+HWI-EAS443:3:83:1554:705#0/1
abaabbaa_`b[]_aY]aa`a]`_aa_a_Y]_aYbOa_^_
@HWI-EAS443:3:83:169:480#0/1
GTGCGGGGTTCTTCTCTTGTCTTCCTGATGTGTACTTTTT
+HWI-EAS443:3:83:169:480#0/1
a_aaaaaa¥aaaaaa`aaa_`aa_`a`]aa_a]__`a__a
@HWI-EAS443:3:83:1754:750#0/1
GTGCGGGGTTCTTCTCTTGTCTTCCTGATGTGTACTTTTT
+HWI-EAS443:3:83:1754:750#0/1
`[_N¥b`X_aaaa_`[aa^WPa`^aaS__YG`^^SP]`__
@HWI-EAS443:3:83:370:1191#0/1
TGCGGGGTTCTTCTCTTGTCTTCCTGATGTGTACTTTTTG
+HWI-EAS443:3:83:370:1191#0/1
aa`a``aZaa`aaaaaa`^`a`a_a¥¥__^^]^^¥__a`¥
@HWI-EAS443:3:83:39:563#0/1
CAAAAAGTACACATCAGGAAGACAAGAGAAGAACCCCGCA
+HWI-EAS443:3:83:39:563#0/1
X``a¥`_M_^Z^b_`X`b_[_aaaZ^]_X__H^O``a`YR
@HWI-EAS443:3:83:631:480#0/1
CAAAAAGTACACATCAGGAAGACAAGAGAAGAACCCCGCA
+HWI-EAS443:3:83:631:480#0/1
^a^_X]IRa_b`baabbbabba¥a^`aaa`b`YTa^^ba^
@HWI-EAS443:3:83:813:682#0/1
CAAAAAGTACACATCAGGAAGACAAGAGAAGAACCCCGCA
```

```
+HWI-EAS443:3:83:813:682#0/1
aba`bb__bbaaaaaaba`aa`aaab`a`aaa`a__`a_^
@HWI-EAS443:3:84:752:409#0/1
TGCGGGGTTCTTCTCTTGTCTTCCTGATGTGTACTTTTTG
+HWI-EAS443:3:84:752:409#0/1
[[a`abbaa¥ab`aabbbaaaa`[baa_b__VDWaaaaa]
@HWI-EAS443:3:85:1650:1142#0/1
CAAAAAGTACACATCAGGAAGACAAGAGAAGAACCCCGCA
+HWI-EAS443:3:85:1650:1142#0/1
`¥ababbbaab]a_`a¥HQaba_ba``aab¥ab_`W]aaa
@HWI-EAS443:3:85:87:1446#0/1
GTGCGGGGTTCTTCTCTTGTCTTCCTGATGTGTACTTTTT
+HWI-EAS443:3:85:87:1446#0/1
a^a^^W^[Ra_M`a`[a_WZ[PR^VOWGNHGJ[^Ua`_UO
@HWI-EAS443:3:86:504:166#0/1
AAAAGTACACATCAGGAAGACAAGAGAAGAACCCCGCACT
+HWI-EAS443:3:86:504:166#0/1
^baba[[_abWbbbbba[b_bbWbSbaaaaWH`SIa[aa¥
@HWI-EAS443:3:86:557:751#0/1
TGCGGGGTTCTTCTCTTGTCTTCCTGATGTGTACTTTTTG
+HWI-EAS443:3:86:557:751#0/1
aY¥Z`__W``a`_aa``a]_Z¥```¥U[_Y`P^^_^`[_S
@HWI-EAS443:3:86:670:1210#0/1
TGCGGGGTTCTTCTCTTGTCTTCCTGATGTGTACTTTTTG
+HWI-EAS443:3:86:670:1210#0/1
a``baabOaba``a`aa__ba`a__a`Ya]aY_Ya^`aZZ
@HWI-EAS443:3:86:671:279#0/1
CTCTTGTCTTCCTGATGTGTACTTTTTGATTATGGCCATA
+HWI-EAS443:3:86:671:279#0/1
ab`bbbabbba`b``ab``]^_bbbb``aaaaa`^``]``
@HWI-EAS443:3:87:1391:1987#0/1
AAAAAGTACACATCAGGAAGACAAGAGAAGAACCCCGCAC
+HWI-EAS443:3:87:1391:1987#0/1
abaUb]F]ZWa`Ta[_^YVa`]_a]]baa^W_WHQ¥aaY`
@HWI-EAS443:3:87:1735:342#0/1
TGCGGGGTTCTTCTCTTGTCTTCCTGATGTGTACTTTTTG
+HWI-EAS443:3:87:1735:342#0/1
a_K_``aab_baUaaabba_aa`^a]^a``aX]Xaaaaaa
@HWI-EAS443:3:87:585:1584#0/1
CAAAAAGTACACATCAGGAAGACAAGAGAAGAACCCCGCA
+HWI-EAS443:3:87:585:1584#0/1
abaaabaRba_aa`^]XaSa`aYa`__Y`^ba]G[O__^¥
@HWI-EAS443:3:87:621:911#0/1
CAAAAAGTACACATCAGGAAGACAAGAGAAGAACCCCGCA
+HWI-EAS443:3:87:621:911#0/1
`^¥_Z¥`P_`aZaZ`_`_]Z_Z_`^`]^aaa]a]V]`^¥Z
@HWI-EAS443:3:87:928:844#0/1
TGCGGGGTTCTTCTCTTGTCTTCCTGATGTGTACTTTTTG
+HWI-EAS443:3:87:928:844#0/1
ab`aaaaVaaaa`aaaaa^aaaaaaa]aaZa^__aaaaaa
```

@HWI-EAS443:3:88:104:1111#0/1
CAAAAAGTACACATCAGGAAGACAAGAGAAGAACCCCGCA
+HWI-EAS443:3:88:104:1111#0/1
a]bab`]_bbbb¥`b`]a]ab`aa_a_`a]^aa[_]```_
@HWI-EAS443:3:88:1508:1554#0/1
CAAAAAGTACACATCAGGAAGACAAGAGAAGAACCCCGCA
+HWI-EAS443:3:88:1508:1554#0/1
aaaabbbabbbabbbbbbbbbbaabbbbbbbbaa_^a]Q[
@HWI-EAS443:3:88:841:1120#0/1
CAAAAAGTACACATCAGGAAGACAAGAGAAGAACCCCGCA
+HWI-EAS443:3:88:841:1120#0/1
`aaa_ab]a`a`a^^Z^aWabaaa`aa^_`aaa_^R```a
@HWI-EAS443:3:89:1069:1314#0/1
CAAAAAGTACACATCAGGAAGACAAGAGAAGAACCCCGCA
+HWI-EAS443:3:89:1069:1314#0/1
``¥`a__O_aaZ^ababba]aa``aa`aaabab][LZ_`a
@HWI-EAS443:3:89:135:750#0/1
CTCTTGTCTTCCTGATGTGTACTTTTTGATTATGGCCATA
+HWI-EAS443:3:89:135:750#0/1
ababbbbbbababbabb_b`aabbaabaaaabaaaaaa__
@HWI-EAS443:3:89:1600:710#0/1
TGCGGGGTTCTTCTCTTGTCTTCCTGATGTGTACTTTTTG
+HWI-EAS443:3:89:1600:710#0/1
a`_ba`]`ba`a_`aab```aba_aY_aa_a^a_`_``a[
@HWI-EAS443:3:89:1652:445#0/1
CAAAAAGTACACATCAGGAAGACAAGAGAAGAACCCCGCA
+HWI-EAS443:3:89:1652:445#0/1
aabbbbbabbb_aabbaabbabbaabbbaabbaa[a¥bab
@HWI-EAS443:3:89:222:976#0/1
CAAAAAGTACACATCAGGAAGACAAGAGAAGAACCCCGCA
+HWI-EAS443:3:89:222:976#0/1
`bab`_``bbbababbbbbab`b`]`^bb^]baaaaabaa
@HWI-EAS443:3:89:528:1535#0/1
TGCGGGGTTCTTCTCTTGTCTTCCTGATGTGTACTTTTTG
+HWI-EAS443:3:89:528:1535#0/1
]a_b¥_VR_`¥WZ_aa¥S```]a`bb^_b`a``aaba`aa
@HWI-EAS443:3:89:996:1429#0/1
AAAAGTACACATCAGGAAGACAAGAGAAGAACCCCGCACT
+HWI-EAS443:3:89:996:1429#0/1
`a]aaabbb`b_b`b_^Z¥abb`^bbbbb```b[abaaa_
@HWI-EAS443:3:8:1353:658#0/1
CAAAAAGTACACATCAGGAAGACAAGAGAAGAACCCCGCA
+HWI-EAS443:3:8:1353:658#0/1
ab_bba``baabb`abbbbbbb`ab`abbabaa`[PXb^a
@HWI-EAS443:3:8:352:51#0/1
GTGCGGGGTTCTTCTCTTGTCTTCCTGATGTGTACTTTTT
+HWI-EAS443:3:8:352:51#0/1
aZaab¥X]Q]X_`¥`_a`a[^`]YY[a^_`XaYTY]]_]]
@HWI-EAS443:3:8:440:1338#0/1
AAAAGTACACATCAGGAAGACAAGAGAAGAACCCCGCACT

```
+HWI-EAS443:3:8:440:1338#0/1
aaaababa]ab¥¥ab¥[]a`HY_T¥aaaaK]`¥XS__^a^
@HWI-EAS443:3:91:1405:99#0/1
CAAAAAGTACACATCAGGAAGACAAGAGAAGAACCCCGCA
+HWI-EAS443:3:91:1405:99#0/1
abbab]b`b[b_b`H_aba`baXa_bYbaabaaU^XQ¥NZ
@HWI-EAS443:3:91:1489:790#0/1
TGCGGGGTTCTTCTCTTGTCTTCCTGATGTGTACTTTTTG
+HWI-EAS443:3:91:1489:790#0/1
QV^``S_a`W_ZL_^b_R¥^^_OJW_[¥^^a¥TXabaa`Z
@HWI-EAS443:3:91:273:14#0/1
TGCGGGGTTCTTCTCTTGTCTTCCTGATGTGTACTTTTTG
+HWI-EAS443:3:91:273:14#0/1
ababbba_aaaa`aabaa_`aa^_aaV``]_YYY_`]___
@HWI-EAS443:3:91:480:1410#0/1
AAAGTACACATCAGGAAGACAAGAGAAGAACCCCGCACTC
+HWI-EAS443:3:91:480:1410#0/1
abaa]baaaaaa`a`aaa_aaaaaaaa_`a`¥]]a_a^^_
@HWI-EAS443:3:91:536:1453#0/1
AAAAAGTACACATCAGGAAGACAAGAGAAGAACCCCGCAC
+HWI-EAS443:3:91:536:1453#0/1
^XTY[UV[¥XR`OWbaZ^¥¥__OO]W`¥aS``T[aab`[_
@HWI-EAS443:3:91:608:1368#0/1
TGCGGGGTTCTTCTCTTGTCTTCCTGATGTGTACTTTTTG
+HWI-EAS443:3:91:608:1368#0/1
`a^_¥_¥X`U_a`aa_`b__aa___ZZ]b[a^S[a]`Y_V
@HWI-EAS443:3:91:743:1711#0/1
CTCTTGTCTTCCTGATGTGTACTTTTTGATTATGGCCATA
+HWI-EAS443:3:91:743:1711#0/1
]_b_bbbabbaabbbbbab_babbbb_abbbbb_abbbaa
@HWI-EAS443:3:91:810:1376#0/1
CAAAAAGTACACATCAGGAAGACAAGAGAAGAACCCCGCA
+HWI-EAS443:3:91:810:1376#0/1
Vbababbababbbaababbbaabb_baabbbbbb`]babb
@HWI-EAS443:3:92:1556:755#0/1
TGCGGGGTTCTTCTCTTGTCTTCCTGATGTGTACTTTTTG
+HWI-EAS443:3:92:1556:755#0/1
`ba[TaX`_^abUa`bba`Va`]UD¥`aa`a^_[ab`^aa
@HWI-EAS443:3:92:334:1162#0/1
TGCGGGGTTCTTCTCTTGTCTTCCTGATGTGTACTTTTTG
+HWI-EAS443:3:92:334:1162#0/1
aaaabaa[aaa``a`abb^aaaa_a``_a¥a^`^_aaaa^
@HWI-EAS443:3:92:619:1047#0/1
TGCGGGGTTCTTCTCTTGTCTTCCTGATGTGTACTTTTTG
+HWI-EAS443:3:92:619:1047#0/1
a]aaaY_[a`_^W]aaa]Xa]]__`^_Ta_a^^]]¥][_X
@HWI-EAS443:3:92:688:1200#0/1
CAAAAAGTACACATCAGGAAGACAAGAGAAGAACCCCGCA
+HWI-EAS443:3:92:688:1200#0/1
`¥^`bba[`¥```ba`abaa`a¥a`abba_`a___a^``a
```

@HWI-EAS443:3:93:1174:1729#0/1
CAAAAAGTACACATCAGGAAGACAAGAGAAGAACCCCGCA
+HWI-EAS443:3:93:1174:1729#0/1
a¥abaaa^``b`_Uabbbaabaab_a_aaaaaa^WU¥a]]
@HWI-EAS443:3:93:157:107#0/1
TATGGCCATAATCAAAAAGTACACATCAGGAAGACAAGAG
+HWI-EAS443:3:93:157:107#0/1
aaababb`V]aX__Vba¥RM_[IY`[O[aa[a¥^S`_W[`
@HWI-EAS443:3:93:1762:207#0/1
GTGCGGGGTTCTTCTCTTGTCTTCCTGATGTGTACTTTTT
+HWI-EAS443:3:93:1762:207#0/1
a[^HN]aYQP[UT]¥[P``VWWOOGY_YWYOQTWN]X^ZZ
@HWI-EAS443:3:93:845:171#0/1
TGCGGGGTTCTTCTCTTGTCTTCCTGATGTGTACTTTTTG
+HWI-EAS443:3:93:845:171#0/1
YQOZWaa[XPY_P]`]`bT`aaaaa^^_aZa]]]__a[_`
@HWI-EAS443:3:93:984:1207#0/1
AAAAAGTACACATCAGGAAGACAAGAGAAGAACCCCGCAC
+HWI-EAS443:3:93:984:1207#0/1
STV_T]TY[`b_]VG`b`¥a^S]a___]IYUPXUU_^UW_
@HWI-EAS443:3:94:983:844#0/1
TGCGGGGTTCTTCTCTTGTCTTCCTGATGTGTACTTTTTG
+HWI-EAS443:3:94:983:844#0/1
abaaa`aXa¥ba`baabaa_aaa^a`Ya`^a_¥`aaaaa`
@HWI-EAS443:3:95:402:1582#0/1
TGCGGGGTTCTTCTCTTGTCTTCCTGATGTGTACTTTTTG
+HWI-EAS443:3:95:402:1582#0/1
]bbbbbb¥ab`bbaaaab`aabaaa]aaa¥a]`_aaaa`a
@HWI-EAS443:3:96:1133:344#0/1
GTGCGGGGTTCTTCTCTTGTCTTCCTGATGTGTACTTTTT
+HWI-EAS443:3:96:1133:344#0/1
a_ba`abaaa_aaaa_aba_^ba^_aa_aa^a`_Za```a
@HWI-EAS443:3:96:1141:1906#0/1
GTGCGGGGTTCTTCTCTTGTCTTCCTGATGTGTACTTTTT
+HWI-EAS443:3:96:1141:1906#0/1
a[^Q`__a[]^P[_YPV]WPW_PR[¥^WX]X]U]O^ZXXB
@HWI-EAS443:3:96:373:296#0/1
GTGCGGGGTTCTTCTCTTGTCTTCCTGATGTGTACTTTTT
+HWI-EAS443:3:96:373:296#0/1
a^a`aa`[GT^a_^a¥`^`^]__X]_`Y^`]`Y^Y__^^_
@HWI-EAS443:3:96:660:456#0/1
CAAAAAGTACACATCAGGAAGACAAGAGAAGAACCCCGCA
+HWI-EAS443:3:96:660:456#0/1
abaabab`a^aa`babbbaabaaaab_aaaaaaa]__a]a
@HWI-EAS443:3:97:1008:41#0/1
TGCGGGGTTCTTCTCTTGTCTTCCTGATGTGTACTTTTTG
+HWI-EAS443:3:97:1008:41#0/1
ababab__b`a_^^V]ba[_aaTTa__`X`a]YY`aa^a_
@HWI-EAS443:3:97:1611:687#0/1
CAAAAAGTACACATCAGGAAGACAAGAGAAGAACCCCGCA

```
+HWI-EAS443:3:97:1611:687#0/1
_babab`Yb_bbba_bb`abaW__ab`]abbb`_I_a[ab
@HWI-EAS443:3:98:111:1836#0/1
CAAAAAGTACACATCAGGAAGACAAGAGAAGAACCCCGCA
+HWI-EAS443:3:98:111:1836#0/1
`bbY_b`^baa_aa`aaaaaaaaa^aa_aa`aa`_[___`
@HWI-EAS443:3:99:1351:436#0/1
TGCGGGGTTCTTCTCTTGTCTTCCTGATGTGTACTTTTTG
+HWI-EAS443:3:99:1351:436#0/1
a`XG][b[babbS^Yabba¥baI^b```baa_``aaa]_]
@HWI-EAS443:3:99:261:1835#0/1
CAAAAAGTACACATCAGGAAGACAAGAGAAGAACCCCGCA
+HWI-EAS443:3:99:261:1835#0/1
ab`]aZ^P^_a__a`¥XMMZa][a]¥```aY`]PV^R¥S_
@HWI-EAS443:3:99:395:333#0/1
AAAAAGTACACATCAGGAAGACAAGAGAAGAACCCCGCAC
+HWI-EAS443:3:99:395:333#0/1
abb`^b`a¥^_a`_aba_^a]_a^a^_``a_a^]X_a_^_
@HWI-EAS443:3:99:968:1652#0/1
TGCGGGGTTCTTCTCTTGTCTTCCTGATGTGTACTTTTTG
+HWI-EAS443:3:99:968:1652#0/1
ab¥babaJ^ab`^a`abaSaaaWaba^a__b`_aUa_`ab
@HWI-EAS443:3:9:1323:2011#0/1
TGCGGGGTTCTTCTCTTGTCTTCCTGATGTGTACTTTTTG
+HWI-EAS443:3:9:1323:2011#0/1
`bY``[ZSXS`_aa]_aa][a`^Z`U]]a_a]^^___`WO
@HWI-EAS443:3:9:152:881#0/1
GTGCGGGGTTCTTCTCTTGTCTTCCTGATGTGTACTTTTT
+HWI-EAS443:3:9:152:881#0/1
aYY[Z[[_N_a[`W^_]Z`UV^XZQ_]LX_VYQGU_Z[]T
@HWI-EAS443:3:9:1531:83#0/1
AAAGTACACATCAGGAAGACAAGAGAAGAACCCCGCACTC
+HWI-EAS443:3:9:1531:83#0/1
^_abab`b[`b¥aa`baa`_baa`a]¥a^a]R]Ra¥`R^]
@HWI-EAS443:3:9:395:1958#0/1
TGCGGGGTTCTTCTCTTGTCTTCCTGATGTGTACTTTTTG
+HWI-EAS443:3:9:395:1958#0/1
a`a_a`aJbaaaaa[a`¥`_a_aa`aaaYSa^_``_]a]U
@HWI-EAS443:3:9:407:1144#0/1
TGCGGGGTTCTTCTCTTGTCTTCCTGATGTGTACTTTTTG
+HWI-EAS443:3:9:407:1144#0/1
a_¥^aaa[_aaa_aaa`b___aa`aa^^aWaZ_]a_`a`_
@HWI-EAS443:3:9:481:223#0/1
TGCGGGGTTCTTCTCTTGTCTTCCTGATGTGTACTTTTTG
+HWI-EAS443:3:9:481:223#0/1
^¥a`_`b_baba_aaa```aaa`_`a___Ga^_X_a``aa
@HWI-EAS443:3:9:630:1083#0/1
AAAAAGTACACATCAGGAAGACAAGAGAAGAACCCCGCAC
+HWI-EAS443:3:9:630:1083#0/1
a^¥Z_a]]`a_][Ya]_`_a_`_`a`a`_a_`XZX_aYa_
```

@HWI-EAS443:3:9:682:1325#0/1
TGCGGGGTTCTTCTCTTGTCTTCCTGATGTGTACTTTTTG
+HWI-EAS443:3:9:682:1325#0/1
a`bbabb_]_baaa_aba`¥aaT_aa`_b_``]]aa`aa_
@HWI-EAS443:3:9:748:1034#0/1
TGCGGGGTTCTTCTCTTGTCTTCCTGATGTGTACTTTTTG
+HWI-EAS443:3:9:748:1034#0/1
a_`_`a¥R^aa¥___a_aYa_aa^a`ZP__bYY[a_^[]_
@HWI-EAS443:3:9:749:1608#0/1
CAAAAAGTACACATCAGGAAGACAAGAGAAGAACCCCGCA
+HWI-EAS443:3:9:749:1608#0/1
abbbb_bPbababbbab_bbb]b__abbababb__`Saaa
@HWI-EAS443:3:29:1758:767#0/1
CTCATTTTGGGCAAAGAAGACAAGAGATATGGCCCCAAGT
+HWI-EAS443:3:29:1758:767#0/1
abbbbbbabbbbaaababba_bbaaaaaaaaa`_[_a`[Q
@HWI-EAS443:3:51:1358:1250#0/1
AGAATGGCCCCGCATTAAGCATCAATGAACTGAGCAATCT
+HWI-EAS443:3:51:1358:1250#0/1
ab`]]ab^_^HS_`a`aZPSX`[S[__^US^XZ_¥^VMUS
@HWI-EAS443:3:35:255:1930#0/1
AGATTGCTCAGCTCATTGATGCTTAATGCTGGGCNATCTC
+HWI-EAS443:3:35:255:1930#0/1
a``aaaa_a`aa_^a_^``^^``^a^_^`VZU¥XDXY^]^
@HWI-EAS443:3:19:1728:1931#0/1
GAAGACAAGAGATATGGCCCAGCATTAAGCATCAATGAGC
+HWI-EAS443:3:19:1728:1931#0/1
ab`¥a^_^aaaa^a__aXQV_]W^_^]_¥W__U_^XTRUW
@HWI-EAS443:3:91:243:1145#0/1
CAGACAGCGACCAAAAGAATTCGGATGGCCATTGTGTATA
+HWI-EAS443:3:91:243:1145#0/1
aabba[bbbabab]a[abbbbba`^abaZ_^ba^^ba_a_
@HWI-EAS443:3:72:1780:1975#0/1
CACAAATACCAGCATAAATGCTAGCAACCATTGACCTGAA
+HWI-EAS443:3:72:1780:1975#0/1
Z`^V¥Z_b^_YSWYHXVY]TaWX^[`]G¥¥`aT^_`UJZZ
@HWI-EAS443:3:67:627:914#0/1
ACAAAATGCAGCGATTCAAGTGATCCTCTCGTCATTGAAG
+HWI-EAS443:3:67:627:914#0/1
a_XZYT[¥`]`__`^___^`O]`¥`_^]]]¥Z^_¥]S]_X
@HWI-EAS443:3:19:520:1612#0/1
GGATACACCATGGACACAGTAAACAGAACACATTCGAGAA
+HWI-EAS443:3:19:520:1612#0/1
aaabaaabba`baabaabaaa_`a``aab`^aT]aaa_aa
@HWI-EAS443:3:3:41:1167#0/1
CGCGAGATACTCACTAAGACAACTGTGGACCATATGGCAA
+HWI-EAS443:3:3:41:1167#0/1
U`S``[^aa^`abbbbaa`TF]__`Z^YJ[FXWb`a]BBB
@HWI-EAS443:3:29:1182:18#0/1
CTCACTAAGACCACTGTGGACCATATGGCAATAATCAAAA

```
+HWI-EAS443:3:29:1182:18#0/1
Q_^`¥Y[[a__aa`XaWZW`YPZT[`XOFG¥X_S]TXBBB
@HWI-EAS443:3:54:393:291#0/1
CCACTGTGGACCATATGGCAATAATCAAAAAGTACACATC
+HWI-EAS443:3:54:393:291#0/1
aa]¥ab^a_`[¥PZ_^`_¥G[]]¥OUWZ][W[HX]]YQXY
@HWI-EAS443:3:47:1270:1190#0/1
CGAGGGATAAGAGTCAGCAAAATGGGAGTAGATGAATACT
+HWI-EAS443:3:47:1270:1190#0/1
R_]`___TY_ba`abI]¥^¥UWa`^^a[RY^]_¥¥U__]]
@HWI-EAS443:3:69:1058:148#0/1
GGGTTTAGTCGCTGGTTTGCCCTATTGACAAAGTTCAGAT
+HWI-EAS443:3:69:1058:148#0/1
ab__a`[a__a_`a`Q_[ZTX^_^_aVUW^¥Z[U_TZ[^Y
@HWI-EAS443:3:23:1699:222#0/1
AACTAATTCAATGCATGTGTGAGGAAGGAGTTGAACCAAG
+HWI-EAS443:3:23:1699:222#0/1
`a[aaIa[M_]¥b_L_aMaXY_aa]^^^X`_UZZ^X^[BB
@HWI-EAS443:3:75:359:1490#0/1
AGATACTGGGCCATAAGGACCAGGAGCGGAGGAAATACCA
+HWI-EAS443:3:75:359:1490#0/1
S[[^`_^ZU`_a`]KQa`^`aa`ZVWF¥¥]`YX]^V`aaa
@HWI-EAS443:3:86:1017:1636#0/1
CAAAAGTCAATTGGTGTGGATGGCATGCCACTGTGCTGCA
+HWI-EAS443:3:86:1017:1636#0/1
abU__Y¥`aabVIYXa`aaa_aaJZ_QQG][PKU_XRQPG
@HWI-EAS443:3:86:1674:436#0/1
CACAAAAGTCAATTGGTGTGGATGGGATGCCACTCTGCTG
+HWI-EAS443:3:86:1674:436#0/1
U^T__PYU_ba`a`_[^bbaa¥bb_HO_aa]UabY¥TL¥_
@HWI-EAS443:3:95:924:280#0/1
ATGCAGCAGAGTGGCATGGCATCCACACCAATTGACTTTT
+HWI-EAS443:3:95:924:280#0/1
aab]aa]aa[aSb]IY`YQO]_Ia`RUR[_S_¥a`Q[^__
@HWI-EAS443:3:48:679:700#0/1
CACAAAAGTCAATTGGTGTGGATGGCATGCCACTCTGGTG
+HWI-EAS443:3:48:679:700#0/1
[a`a`aa^_`aba`bbaaa`^Za¥YHRRW[NRWRRYWGY^
@HWI-EAS443:3:25:1111:1637#0/1
ATGCAGCAGAGTGGCATGNCATCCACACCAATTGACTTTT
+HWI-EAS443:3:25:1111:1637#0/1
aba_`babaa`_bbaaa¥D^a`_aa]a_`__]¥[Ya]`__
@HWI-EAS443:3:99:1730:1598#0/1
CAAAAGTCAATTGGTGTGGATGGCATGCCACTCTGCTGTA
+HWI-EAS443:3:99:1730:1598#0/1
`[___Waa``¥¥^abba]b^``[^HYQH``bbX`^XYYMZ
@HWI-EAS443:3:19:344:243#0/1
ACAAAAGTCAATTGGTGTGGATGGCATGCCACTCTGCTGG
+HWI-EAS443:3:19:344:243#0/1
`VSa^W`¥`YX`b_`Wa]a¥S`a]Za^__^`P[___VXTF
```

```
@HWI-EAS443:3:4:1104:1660#0/1
ACAAAAGTCAATTGGTGTGGATGGCATGCCACTCTGCTGT
+HWI-EAS443:3:4:1104:1660#0/1
a_[`_^a]]`bV`a_¥`WO[P]aaU[W`ZZYGQa^FFQRB
@HWI-EAS443:3:75:775:2047#0/1
NCAAAAGTCAATTGGTGTGGATGGCATGCCACTCTGCTGC
+HWI-EAS443:3:75:775:2047#0/1
DNVVRSOMSSXYSRRSYXTSVSQQVQULUWWWTVVQMQTL
@HWI-EAS443:3:12:655:88#0/1
CACAAAAGTCAATTGGTGTGGATGGCATGCCACTCTGCTG
+HWI-EAS443:3:12:655:88#0/1
ababaa^bV`_`aaba^a_a```aaXZU^U_^[]X^QUZ^
@HWI-EAS443:3:19:1294:961#0/1
CAAAAGTCAATTGGTGTGGATGGCATGCCACTCTGCTGCA
+HWI-EAS443:3:19:1294:961#0/1
a¥]bba__a`ba`baaab_`aaa^YXY`a_Wa``aZ^`W]
@HWI-EAS443:3:19:1410:809#0/1
ATGCAGCAGAGTGGCATGCCATCCACACCAATTGACTTTT
+HWI-EAS443:3:19:1410:809#0/1
a_aa`^a_a_^W`aZ_a^YZ¥_¥¥¥QZ¥VN¥_WVSQ¥W^_
@HWI-EAS443:3:23:1326:698#0/1
CAAAAGTCAATTGGTGTGGATGGCATGCCACTCTGCTGCA
+HWI-EAS443:3:23:1326:698#0/1
ab^]¥ZXba]bbbb_]Z[ZXR`XLY[bRUa]WS]WNWZ[^
@HWI-EAS443:3:23:766:504#0/1
CAAAAGTCAATTGGTGTGGATGGCATGCCACTCTGCTGCA
+HWI-EAS443:3:23:766:504#0/1
¥_ZZS_]]_abXaaROYZ]V¥[LHVT`NMV[^YOZW]XZY
@HWI-EAS443:3:23:773:1895#0/1
AAATGCAGCAGAGTGGCATGCCATCCACACCAATTGACTT
+HWI-EAS443:3:23:773:1895#0/1
abbababababaa`[HP]abU[`_[U]Qa___^^_UZR]RXZ
@HWI-EAS443:3:24:42:202#0/1
ACAAAAGTCAATTGGTGTGGATGGCATGCCACTCTGCTGC
+HWI-EAS443:3:24:42:202#0/1
abaZZV_^a]S````Z`^``[[a][PJV]a¥SZ[S[XWUZ
@HWI-EAS443:3:26:737:1787#0/1
CAGCAGAGTGGCATGCCATCCACACCAATTGACTTTTGTG
+HWI-EAS443:3:26:737:1787#0/1
Z_a`bbba_bZbbb_bbbabba[baab_Z_a_`ab_b^a_
@HWI-EAS443:3:27:993:1107#0/1
CAGCAGAGTGGCATGCCATCCACACCAATTGACTTTTGTG
+HWI-EAS443:3:27:993:1107#0/1
ab`aa[aaYaaaa^_`_a]__[Y^_^a_^VUY_]`^_[]]
@HWI-EAS443:3:2:714:1497#0/1
CAAAAGTCAATTGGTGTGGATGGCATGCCACTCTGCTGCA
+HWI-EAS443:3:2:714:1497#0/1
aba]]]Wa^b]ab¥OaUaY_Ra_ZYUG[]aU¥P¥¥_a¥GW
@HWI-EAS443:3:33:1678:1082#0/1
ATGCAGCAGAGTGGCATGCCATCCACACCAATTGACTTTT
```

```
+HWI-EAS443:3:33:1678:1082#0/1
aabaabaaba_^_a_aaa_Y__]]_]Y^Y^^XW^YVYX_Y
@HWI-EAS443:3:33:967:966#0/1
CAAAAGTCAATTGGTGTGGATGGCATGCCACTCTGCTGCA
+HWI-EAS443:3:33:967:966#0/1
aa[[aaSbWb`ba_Wa_``aab`Z]¥OU_a[[FZ[`Z]WV
@HWI-EAS443:3:35:902:1279#0/1
GCAGCAGAGTGGCATGCCATCCACACCAATTGACTTTTGT
+HWI-EAS443:3:35:902:1279#0/1
abbabb``b_]aaa`aaaUZ`a¥`a_b`a^ZVN__`a__Y
@HWI-EAS443:3:36:145:1681#0/1
ACAAAAGTCAATTGGTGTGGATGGCATGCCACTCTGCTGC
+HWI-EAS443:3:36:145:1681#0/1
`abaYR_^baba_]]¥`^]^^^[¥a]XO]_Y]^^VT^¥Z[
@HWI-EAS443:3:36:495:1751#0/1
CAAAAGTCAATTGGTGTGGATGGCATGCCACTCTGCTGCA
+HWI-EAS443:3:36:495:1751#0/1
aa_^Z_¥baaa``_V_]`]_^_a[UQQTa[_XXZQZYUXX
@HWI-EAS443:3:39:563:1109#0/1
AAAAGTCAATTGGTGTGGATGGCATGCCACTCTGCTGCAT
+HWI-EAS443:3:39:563:1109#0/1
]^`Y^Ia`a_aa_]___]_¥`Z_WX`Z]YJQV]Y¥HVT]Z
@HWI-EAS443:3:3:328:1815#0/1
ATGCAGCAGAGTGGCATGCCATCCACACCAATTGACTTTT
+HWI-EAS443:3:3:328:1815#0/1
a_a_aX_``]]¥_RRaY`^Z¥SG_YMWN_XWQUVTBBBBB
@HWI-EAS443:3:42:938:168#0/1
CAAAAGTCAATTGGTGTGGATGGCATGCCACTCTGCTGCA
+HWI-EAS443:3:42:938:168#0/1
a_¥_^a_baaaaab_]Vaa`aab`_UTQ¥[ZRX^^^UVVF
@HWI-EAS443:3:49:1553:818#0/1
ACAAAAGTCAATTGGTGTGGATGGCATGCCACTCTGCTGC
+HWI-EAS443:3:49:1553:818#0/1
a^^X_`_XZZ_[`a_F[TaaZ`]a^¥ZTVTWR]Y[XWX[K
@HWI-EAS443:3:4:32:1015#0/1
ATGCAGCAGAGTGGCATGCCATCCACACCAATTGACTTTT
+HWI-EAS443:3:4:32:1015#0/1
`_Z_aba`a^_Q_a`aZSRZ`T[YXZXWRRRYQSQ___BB
@HWI-EAS443:3:50:888:1421#0/1
ACAAAAGTCAATTGGTGTGGATGGCATGCCACTCTGCTGC
+HWI-EAS443:3:50:888:1421#0/1
aabbabbabaabbab_b`aa`aa`^^]Z^a`¥^^`[Y¥[Z
@HWI-EAS443:3:54:318:1289#0/1
AAAAGTCAATTGGTGTGGATGGCATGCCACTCTGCTGCAT
+HWI-EAS443:3:54:318:1289#0/1
a]a_a]a]Pa`aaV^RN_¥^_]QMSOOZXXTRZ]XZYSKT
@HWI-EAS443:3:55:1436:962#0/1
ACAAAAGTCAATTGGTGTGGATGGCATGCCACTCTGCTGC
+HWI-EAS443:3:55:1436:962#0/1
aK^_`__Q_`Y`a_Z`[__[a¥^b]X^`¥Y]FLR^[YQRW
```

@HWI-EAS443:3:57:1751:756#0/1
AATGCAGCAGAGTGGCATGCCATCCACACCAATTGACTTT
+HWI-EAS443:3:57:1751:756#0/1
__`b_bb^^^¥]]a^a`XZOPPWV][U[Ua`XJWYQLXZ^
@HWI-EAS443:3:57:918:1165#0/1
CACAAAAGTCAATTGGTGTGGATGGCATGCCACTCTGCTG
+HWI-EAS443:3:57:918:1165#0/1
ababa_^babbabbba^b_aaaab`]¥``^]`_`]Y]V¥Y
@HWI-EAS443:3:60:814:1952#0/1
CAAAAGTCAATTGGTGTGGATGGCATGCCACTCTGCTGCA
+HWI-EAS443:3:60:814:1952#0/1
abaaaa^aaaaa_aV`^a`aaa^Q^YF[T`YUXXXWY[Y^
@HWI-EAS443:3:62:1435:729#0/1
AAATGCAGCAGAGTGGCATGCCATCCACACCAATTGACTT
+HWI-EAS443:3:62:1435:729#0/1
aabababbaaaa[Zaaababa__`_a`^__^_a`_[V[`a
@HWI-EAS443:3:65:710:657#0/1
ATGCAGCAGAGTGGCATGCCATCCACACCAATTGACTTTT
+HWI-EAS443:3:65:710:657#0/1
_X^aa¥a[^¥]U^P^a`_aa`[WPNVV_a]`___^_^__]
@HWI-EAS443:3:67:1279:414#0/1
CAAAAGTCAATTGGTGTGGATGGCATGCCACTCTGCTGCA
+HWI-EAS443:3:67:1279:414#0/1
`aba^b`aaa_abaYY`aa_ab^J[Za[^`XTXX[X¥aWX
@HWI-EAS443:3:68:1252:693#0/1
ACAAAAGTCAATTGGTGTGGATGGCATGCCACTCTGCTGC
+HWI-EAS443:3:68:1252:693#0/1
abbabbb¥_abbabaab_baaaa^XaZS`]_`]¥]WLVZP
@HWI-EAS443:3:69:949:720#0/1
CACAAAAGTCAATTGGTGTGGATGGCATGCCACTCTGCTG
+HWI-EAS443:3:69:949:720#0/1
abab^Z___baaaab_¥a]aa_aaa[`ZWZ`ZUY_[_Z^_
@HWI-EAS443:3:6:485:1962#0/1
CACAAAAGTCAATTGGTGTGGATGGCATGCCACTCTGCTG
+HWI-EAS443:3:6:485:1962#0/1
ababbbaaabbbbbb_^aab_baba`aaaaab_`]`a]a^
@HWI-EAS443:3:71:1430:882#0/1
CAAAAGTCAATTGGTGTGGATGGCATGCCACTCTGCTGCA
+HWI-EAS443:3:71:1430:882#0/1
H_bZZbS[a`b``¥Uab``^^a_O¥aY__PX`a`_Z_WDP
@HWI-EAS443:3:72:920:1076#0/1
CAAAAGTCAATTGGTGTGGATGGCATGCCACTCTGCTGCA
+HWI-EAS443:3:72:920:1076#0/1
a]_a`_[`_[`a``X__`T`]_^X]^VQ¥]][^]Z]ZZWW
@HWI-EAS443:3:76:994:1745#0/1
CACAAAAGTCAATTGGTGTGGATGGCATGCCACTCTGCTG
+HWI-EAS443:3:76:994:1745#0/1
`_bbbbbbabbaaba_aba^aaaaY[_YZaaa[aa_]Y]`
@HWI-EAS443:3:78:771:161#0/1
ACAAAAGTCAATTGGTGTGGATGGCATGCCACTCTGCTGC

```
+HWI-EAS443:3:78:771:161#0/1
aa`a^[_G_`_b_a_^aVUZ[`_^K[Z[QW`¥T[XVRTXN
@HWI-EAS443:3:82:912:115#0/1
ATGCAGCAGAGTGGCATGCCATCCACACCAATTGACTTTT
+HWI-EAS443:3:82:912:115#0/1
abbabbaabbaUba`b_aY]¥aa``]`Y¥I`^`aWV````
@HWI-EAS443:3:87:398:997#0/1
ACAAAAGTCAATTGGTGTGGATGGCATGCCACTCTGCTGC
+HWI-EAS443:3:87:398:997#0/1
`bba[[_]aaaaa_a]`]aa`^a_a_]W_a__[_^_]^YS
@HWI-EAS443:3:89:1250:419#0/1
CACAAAAGTCAATTGGTGTGGATGGCATGCCACTCTGCTG
+HWI-EAS443:3:89:1250:419#0/1
aaabbbaaaba`bbbb_a_bbbaaba_aaaaa`a`aa¥`a
@HWI-EAS443:3:92:1215:996#0/1
ACAAAAGTCAATTGGTGTGGATGGCATGCCACTCTGCTGC
+HWI-EAS443:3:92:1215:996#0/1
`abaaab_aaabbaa_baaa`ab`¥a`[[`a^]Z`ZS[UO
@HWI-EAS443:3:95:1471:88#0/1
AAAAGTCAATTGGTGTGGATGGCATGCCACTCTGCTGCAT
+HWI-EAS443:3:95:1471:88#0/1
a_a`aWa`_bbbb¥aaaZZX`a]_a_[`_Y^W`_]]SNX`
@HWI-EAS443:3:99:1030:629#0/1
AATGCAGCAGAGTGGCATGCCATCCACACCAATTGACTTT
+HWI-EAS443:3:99:1030:629#0/1
a]_b_Z¥_^Y¥]`]``a]aa^a_NY[P¥WX_WOZ`YS¥ZZ
@HWI-EAS443:3:64:459:227#0/1
AAGCCGGTTGATTTCTGTTTAATGTCCAATCATAAGTCTG
+HWI-EAS443:3:64:459:227#0/1
]]X`aa`ZbbY_^b_b_aaaaaYaXV^a^aabaaaa^_`a
@HWI-EAS443:3:15:959:1544#0/1
AGCCGGTTGATTTCTGTTTAATGTCCAATCATAAGTCTGG
+HWI-EAS443:3:15:959:1544#0/1
¥bbY[aJ^ba____]`X^aa]`_PZTaa`_aaa__¥__a]
@HWI-EAS443:3:2:1205:1580#0/1
AGCCGGTTGATTTCTGTTTAATGTCCAATCATAAGTCTGG
+HWI-EAS443:3:2:1205:1580#0/1
`^¥^bbabb`abb¥baabbaaba_a^aaaaaaa`a[Zaa_
@HWI-EAS443:3:46:305:1925#0/1
AGCCGGTTGATTTCTGTTTAATGTCCAATCATAAGTCTGG
+HWI-EAS443:3:46:305:1925#0/1
a_a`ababa^U]`¥^Xbaa`a__H_`]MONUYaBBBBBBB
@HWI-EAS443:3:67:1425:230#0/1
AGCCGGTTGATTTCTGTTTAATGTCCAATCATAAGTCTGG
+HWI-EAS443:3:67:1425:230#0/1
abba^^[[^Yaab``baabaab``_aaa]R_`a_a¥^a_¥
@HWI-EAS443:3:66:1453:1093#0/1
ACCCAGCTCACAAAAGTCAATTNGTGTGGATGGCATGCCA
+HWI-EAS443:3:66:1453:1093#0/1
aabbaaaaa`a`¥`[b_a_``]D^_aabaaaa`H[^`U¥L
```

```
@HWI-EAS443:3:13:1406:280#0/1
CTATGGTATTCTCACAAGAGGATTGCATGATCAAGGCAGT
+HWI-EAS443:3:13:1406:280#0/1
abbbbbaabbabaaaaab_bb_aaaaaaaaa¥`a_aX_aX
@HWI-EAS443:3:30:707:1196#0/1
CCCTGGGTTTTGCTTAACGCATCTTGGTTCAACTCCTTCC
+HWI-EAS443:3:30:707:1196#0/1
ababba[¥a`_aba`b_`ab_a_aa]`_^aaba_¥DX_]`
@HWI-EAS443:3:57:982:267#0/1
CGTTGAGTTCTTCAATGGTGGAACAGATCTTCATGATCTC
+HWI-EAS443:3:57:982:267#0/1
abZVYGYY`bb`Za_ba¥NX``_QO`¥T]bWT]X^aa[¥S
@HWI-EAS443:3:98:1408:1761#0/1
GGGCCATATCTCTTGTCTTCCTTGCCCAAAATGAGAAATC
+HWI-EAS443:3:98:1408:1761#0/1
aba^``_¥[V__aabZUaZW]`aa]_ab`ba^a]bbabb`
@HWI-EAS443:3:63:1681:2016#0/1
GATAAGAGATATGGCCCAGCATTAAGCATCAATGAACTGA
+HWI-EAS443:3:63:1681:2016#0/1
`b__PYWa^¥Y_`Y]`^_aQ_`a_]`UU^[[]¥_^^¥[_S
@HWI-EAS443:3:45:590:1403#0/1
GCCCCATTTCATCTTGATCTTGGAGGTTCCATTTGTTCTC
+HWI-EAS443:3:45:590:1403#0/1
a_`aYV¥aaYV]X_[XY]I`_a`^aBBBBBBBBBBBBBBB
@HWI-EAS443:3:40:389:1490#0/1
CACCGGATTCTACAATTATTGATTCACCCCGTTCGTCGAT
+HWI-EAS443:3:40:389:1490#0/1
abbb_aabaaab]_aaaaaa[U_``aaaa^YW__]Xa^]^
@HWI-EAS443:3:73:1730:1319#0/1
CAATGCTACTATTTGCTATCCATACTGTCCAAAAAAAAAA
+HWI-EAS443:3:73:1730:1319#0/1
a`bab``baaababaab`aaaa_bbbbVbabbaa`Ybabb
@HWI-EAS443:3:70:714:1679#0/1
GGTGTATCCTGTTCCTGTTCCATGGCTGTACTGAGGATGT
+HWI-EAS443:3:70:714:1679#0/1
_PXbTT_UR`YLaab[_R]aa_TQ¥Q_BBBBBBBBBBBBB
@HWI-EAS443:3:100:802:1161#0/1
ATTCGATATGGAGAGAATAAAAGAATTGAGAGATCTAATG
+HWI-EAS443:3:100:802:1161#0/1
^a^QG_b¥`bbX`ISZ¥Y¥Zb`_]¥F[¥^aaba^W^a_BB
@HWI-EAS443:3:33:948:1754#0/1
CAAATATATTCAATATGGAGAGAATAAAAGAATTGAGAGA
+HWI-EAS443:3:33:948:1754#0/1
`[I_]_YS`Xaa`¥`]YXQ^^][]W]ZTZ_`WFYZ[_]Y_
@HWI-EAS443:3:38:533:1889#0/1
GAGAAGAACCCCGCACTTAGAATGAAGTGGATGATGGCAA
+HWI-EAS443:3:38:533:1889#0/1
`bab_^_`aa`_bSba`¥a```a¥_`¥HURIPXTKN^^ZH
@HWI-EAS443:3:88:375:1362#0/1
AGCAATCTTGCAAAAGGAGAGAAAGCCAATGTGCCAATTG
```

```
+HWI-EAS443:3:88:375:1362#0/1
^^`]b]baab`aa``aa[aU`aa_a_H`_a`_LZ_^aaW`
@HWI-EAS443:3:15:1079:1240#0/1
AATGAACTGAGCAATCTTGCAAAAGGAGAGAAAGCCAATG
+HWI-EAS443:3:15:1079:1240#0/1
[aabZ_a``^^`bbYbba`^`aa`ba_babbb_`aXW[ab
@HWI-EAS443:3:46:209:1951#0/1
ATCTTGCAAAAGGAGAGAAAGCCAATGTGCTAATTGGGCA
+HWI-EAS443:3:46:209:1951#0/1
aaaaaaaaa^_a_`__a`a¥MU__]a_][]^^____^S][
@HWI-EAS443:3:53:955:1638#0/1
GGCTTTCTCTCCTTTTGCAAGATTGCTCAGTTCATTGATG
+HWI-EAS443:3:53:955:1638#0/1
a^^[`baa_[aa]VYaY[aSa¥[```_]W___KZTa`_]_
@HWI-EAS443:3:88:1640:288#0/1
ATCTTGCAAAAGGAGAGAAAGCCAATGTGCTAATTGGGCA
+HWI-EAS443:3:88:1640:288#0/1
`b`bbbbbabab]abbbabba`_[`bba_X``abb]]]G^
@HWI-EAS443:3:100:929:1600#0/1
TGTGAGACCGATGCTGAGAATCAGCAATCTGTTCACAAGT
+HWI-EAS443:3:100:929:1600#0/1
`b^^¥_^]bU`[a_b`^b^aaaa`aaaaS`a___]G``]B
@HWI-EAS443:3:47:1689:1648#0/1
CATCAATGAACTGAGCAATCTTGCAAAAGGAGAAACAAAG
+HWI-EAS443:3:47:1689:1648#0/1
aaa¥`baS_aaa]aa_b¥a__`a_`Z^__a`____V^^YZ
@HWI-EAS443:3:95:1444:591#0/1
GGTCTTCTTACCTTTTCATATAAGTTCTTCACATTTGAGT
+HWI-EAS443:3:95:1444:591#0/1
`_GLPPKZO_`Qa[U]NPaQ``aYPNR_UDRMX_^V]BBB
@HWI-EAS443:3:14:222:1771#0/1
GTCAAAAATGCAGTTGGGGCCCTCATCGGAGGACTTGAAT
+HWI-EAS443:3:14:222:1771#0/1
aQaba[``ab`aaY_aa__GZa¥aX_``_W_^^^a__¥¥_
@HWI-EAS443:3:44:594:1672#0/1
GGATGTCAAAAATGCAGTTGGGGCCCTCATCGGAGGACTT
+HWI-EAS443:3:44:594:1672#0/1
V`^bb^Q¥abaM`X`W_]___SWPXbX`_RXPT]XHVaa^
@HWI-EAS443:3:32:969:1148#0/1
GGGGCCTATTATCTTTTGCCTAGGCATGAGCATGAACCAG
+HWI-EAS443:3:32:969:1148#0/1
_Za``bbaaa_`aaabaa```aabaaabaa`[aaaaa```
@HWI-EAS443:3:14:755:61#0/1
GCTCTCGCCACTTTTCATTTCTGCTCTGGAGGCAGTGGAG
+HWI-EAS443:3:14:755:61#0/1
ab_ZZ^QXa¥a]a]¥VSM]_[XW]a]YTYBBBBBBBBBBB
@HWI-EAS443:3:75:225:223#0/1
CGCCACTTTTCATTTCTGCTCTGGAGGGAGTGGAGGTCTC
+HWI-EAS443:3:75:225:223#0/1
_`a[ab¥YNV]]]XYX[THRV[a¥BBBBBBBBBBBBBBBB
```

```
@HWI-EAS443:3:16:262:915#0/1
CATCGGGGGACTTGAATGGAATGGTAACACGGTTCGAGTC
+HWI-EAS443:3:16:262:915#0/1
ZUaa^]bbb``a]ba[ab]V_`_XV`Za_aa_]Y[[Z]¥a
@HWI-EAS443:3:69:986:798#0/1
GCTCTCGCCACTTTTCATTTCTGCTCTGGAGGTAGTGGAG
+HWI-EAS443:3:69:986:798#0/1
a`Va^aY^aaR^b[aWYabaaW^RW]a]Z^UZGZBBBBBB
@HWI-EAS443:3:48:208:469#0/1
AGAATGGGAGACCTTCACTACCTCCAAAGCAGAAATGAAA
+HWI-EAS443:3:48:208:469#0/1
aaaaababaa_^a`¥^a`ab]_a`Taaa`a_b^a`aa_`_
@HWI-EAS443:3:48:1581:383#0/1
AGAAGGCCAACAAAATAAAATCTGAGAAGACACACATTCA
+HWI-EAS443:3:48:1581:383#0/1
P__^F[Z^_¥`]SY¥¥]`aab[ba`^WO_SQXV`ZN¥]XU
@HWI-EAS443:3:66:846:266#0/1
CTTTGGTCCTACATTTGCTTACCATCGGGATCAGCTGAAA
+HWI-EAS443:3:66:846:266#0/1
ababbaaabbbbbbbbbabbb_abbaaa`a¥_aa_]`aaa
@HWI-EAS443:3:19:180:1653#0/1
ATGCCTGATTAATGATCCCTGGGTTTTGCTTAATGCATCT
+HWI-EAS443:3:19:180:1653#0/1
H]abbabbbbbbb¥bbbbaabbabbba_ababbbb`aaab
@HWI-EAS443:3:91:975:464#0/1
GGAATTCTTTGAAAACAGATCGGAAACATGGCCAATCGGA
+HWI-EAS443:3:91:975:464#0/1
a__]a_]ZW^`TXQRaUDM^_`ZTWY][X__^^U]YU^]X
@HWI-EAS443:3:94:1418:477#0/1
GGAGAGGAGATGGCCACCAGAGCGGACTACACCCTTGACG
+HWI-EAS443:3:94:1418:477#0/1
a`]]]a¥ZZQ^[`ZZ¥WXUM]]`_NYX[[Q`ZWNRX[GJ]
@HWI-EAS443:3:55:1514:1899#0/1
GGGGGCTATATGAAGCAATAGAGGAGAGCCTGATTAATGA
+HWI-EAS443:3:55:1514:1899#0/1
[_`a^Y[b_U``bb`V`_HS_SZKabIbUX_aH`a]abba
@HWI-EAS443:3:24:95:1264#0/1
GCCTGGACAATGAGAAGCAATTTTCTCGATTCAGCCGAAA
+HWI-EAS443:3:24:95:1264#0/1
aaYU]FZZNZ[WOX`aR`^SYQYQaPZPUBBBBBBBBBBB
@HWI-EAS443:3:51:776:156#0/1
GGGGGCTATATGAAGCAATAGAGGAGTGCCTGATTAATGA
+HWI-EAS443:3:51:776:156#0/1
`aba`bbaba`_]abaaaaaaabaaa[a`¥`aaaaa`aa_
@HWI-EAS443:3:76:851:1868#0/1
AGAGGAGTGCCTGATTAATGATCCCTGGGTTTTGCTTAAT
+HWI-EAS443:3:76:851:1868#0/1
a`aaa`a_`aaaaaaaabaaaaa`a`aaaZa_`_``aaa_
@HWI-EAS443:3:1:644:88#0/1
TGGAAATGAGGCGCTGCCTTCTTCAGTCTCTTCAGCAGGT
```

+HWI-EAS443:3:1:644:88#0/1
abb^`a`aaaZ_aV^a¥¥a_Va^Z]a[Q_N`¥NVY¥BBBB
@HWI-EAS443:3:55:1202:40#0/1
ATGGAAATGAGGCGCTGCCTTCTTCAGTCTCTTCAGCAGG
+HWI-EAS443:3:55:1202:40#0/1
[_ba]a¥]^`U_^b¥a`H¥]_VZa]^`a``H^`KQXLW¥F
@HWI-EAS443:3:36:347:1303#0/1
GGCTCATGTTGATTCCCACTAACTTGCAGGTCCTGTAGAA
+HWI-EAS443:3:36:347:1303#0/1
abbabbbb_bbbbaab`^`abb`aa`baa^¥aaaaZS_`K
@HWI-EAS443:3:90:96:1600#0/1
TGCCGGTTGGTTTCTGTTTAATGTCCAATCATAAGCCTGG
+HWI-EAS443:3:90:96:1600#0/1
a``S]aa_YFZaababaa¥__a`^^aab¥`bZ_JZG_``Y
@HWI-EAS443:3:86:1028:783#0/1
TGCCGGTTGGTTTCTGTTTAATGTCCAATCATAAGTCTGG
+HWI-EAS443:3:86:1028:783#0/1
Ub]aa¥bWXH]][^`¥PY¥SV_aU]Y[HY^W_XOZP^`_[
@HWI-EAS443:3:46:201:1080#0/1
AGCCGTCTGAGTTCTTCAATGGTGGAACAGATCTTATGAT
+HWI-EAS443:3:46:201:1080#0/1
_Z^`]ZS`¥ab`aaaaab`]`XFP[^`a`]X^]aa]aaaR
@HWI-EAS443:3:48:1482:1717#0/1
TGCCGTCTGAGTTCTTCAATGGTGGAACAGATCTTATGAT
+HWI-EAS443:3:48:1482:1717#0/1
`_```[`aaaaZa`a_`aaa`aZ___`]``Z^^_`____Z
@HWI-EAS443:3:72:1143:2013#0/1
TGCCGTCTGAGTTCTTCAATGGTGGAACAGATCTTATGAT
+HWI-EAS443:3:72:1143:2013#0/1
[Y]V¥babZ^a``aKV_bb_aaU_a`aIZaKR_a^_SX_Y
@HWI-EAS443:3:91:1130:1958#0/1
TGCCGTCTGAGTTCTTCAATGGTGGAACAGATCTTATGAT
+HWI-EAS443:3:91:1130:1958#0/1
^a_]aaabbab]aa`b_aaaaa_baab_aa``_`_b`__^
@HWI-EAS443:3:37:864:1354#0/1
GAGAATTCATGCCTTGAAACAATGGAAGCAGTTCAACAAA
+HWI-EAS443:3:37:864:1354#0/1
`[W[Z¥aa___^a^`aaa`Xba_aY``XFSTTN_UYT[`^
@HWI-EAS443:3:36:21:66#0/1
TTGCCGTCTGAGTTCTTCAATGGTGGAACAGATCTTATGA
+HWI-EAS443:3:36:21:66#0/1
aaabba[`abY_¥`__``_a_a`T][NZ[Ya_¥]^`[VYY
@HWI-EAS443:3:100:1254:1467#0/1
CAATGGTGGAACAGATCTTAATGATCTCAGAGAACTCTTC
+HWI-EAS443:3:100:1254:1467#0/1
abbbb¥^a`ababb¥]`a[D^abaaaaJYaaWa`PSY^^B
@HWI-EAS443:3:1:537:1331#0/1
GAGTTCTTCAATGGTGGAACAGATCTTAATGATCTCAGAG
+HWI-EAS443:3:1:537:1331#0/1
aNb^[DP`a[_Taa[T¥ba]a[__X`SI`a_^]H_^BBBB

```
@HWI-EAS443:3:27:105:1921#0/1
CATACCCACTTGGTTCATTATCCTCAGGCAGTGGTCCATC
+HWI-EAS443:3:27:105:1921#0/1
a`TZ`bb^`aT^aZ`aZ¥W_`aaW^MXYFI[WLSF]XJKV
@HWI-EAS443:3:62:800:493#0/1
GCATACCCACTTGGTTCATTATCCTCAGGCAGTGGTCCAT
+HWI-EAS443:3:62:800:493#0/1
aabbba``_ababa_baaaaa_a`a_aaaa[XS^¥ZW^ZS
@HWI-EAS443:3:80:1791:325#0/1
CCGGCAGCAACTGCATTGGCCAACACCATAGAAGTATTTA
+HWI-EAS443:3:80:1791:325#0/1
Vabb¥aV^``]XSXaab__]b]a^`Yaaa^`^a]SL_ba]
@HWI-EAS443:3:10:1122:321#0/1
TGGGCTGGCATCACCACAGCATTGTTTACAGAATCAATCT
+HWI-EAS443:3:10:1122:321#0/1
``_^WYaa`aZFY_X`_aaZ__`a_`_¥W`__^¥S_]YW]
@HWI-EAS443:3:43:1382:1215#0/1
CTCTCTGCATCTTTGGTCATTGTATTTAATGTCAGTGCTC
+HWI-EAS443:3:43:1382:1215#0/1
aabbabbbbb`baabb`aaaX_^abbbaaba`a``ZY^`P
@HWI-EAS443:3:65:304:1263#0/1
GGGTACTTTTTGATTATGGCCATATGGTCCACAGTGGTTT
+HWI-EAS443:3:65:304:1263#0/1
_F]Yaa^_V¥ZQX`[O¥XQ¥_XVSOOJPZYBBBBBBBBBB
@HWI-EAS443:3:37:776:2035#0/1
AATGTCGCAGTCCCGCACTCGCGAGATACTCACTAAAACC
+HWI-EAS443:3:37:776:2035#0/1
ab¥TZaa``a^`Wa^_X_]YX`¥`aZU_aVa¥aZa]VWXY
@HWI-EAS443:3:49:379:767#0/1
CTAATGTCGCAGTCCCGCACTCGCGAGATACTCACCAAGA
+HWI-EAS443:3:49:379:767#0/1
`abbbbbabbbbbbbbba]abbbbbbbabaabb¥DZXa_¥
@HWI-EAS443:3:34:798:239#0/1
AGCAACCGTTATGGCAGCCTTCAGCGGGAACAAAGAAGGA
+HWI-EAS443:3:34:798:239#0/1
aba^^aa]¥¥a_a]V`aBBBBBBBBBBBBBBBBBBBBBBB
@HWI-EAS443:3:60:65:1094#0/1
CGCACTCGCGAGATACTCACTAAGACCACTGTGGACCACA
+HWI-EAS443:3:60:65:1094#0/1
a_¥P_`aaaaa^^`^^`]_^^¥^^^__[_ZZP[TV_[V¥Z
@HWI-EAS443:3:57:220:578#0/1
CACGGGCATGAACCAGTCTCGTGACATTTCCTCGAGGGTC
+HWI-EAS443:3:57:220:578#0/1
NGP[U[OUYa_`WSQ`_a`aaRaXa¥L[]b_HIWRb`aGZ
@HWI-EAS443:3:22:1600:1449#0/1
GGGGAGCGCCAGGATGCCACAGAAATCAGAGCATCTGTAG
+HWI-EAS443:3:22:1600:1449#0/1
^[a`[a_a¥SVW__¥[¥¥RP]PY_]UUM[UUUVUVVVRGV
@HWI-EAS443:3:97:974:285#0/1
TGGGGAGCGCCAGGATGCCACAGAAATCAGAGCATCTGTA
```

+HWI-EAS443:3:97:974:285#0/1
a`Xaa_b_aTU[_WSU^V^_^`_¥```LVYYUT][N¥VBB
@HWI-EAS443:3:7:1753:415#0/1
AATTTCTAGCATGGTGGAGGCCATGGGGTCTAGGGCCAGG
+HWI-EAS443:3:7:1753:415#0/1
¥_¥]TRR¥_QQab]MZ¥`Z[ZQ^Y]_FZPKNY¥RBBBBBB
@HWI-EAS443:3:59:502:687#0/1
ATTGACAAAATGACCATCGTCAACATCCACAGCACTCTGC
+HWI-EAS443:3:59:502:687#0/1
^^aba`abbbbbbaaabaaab]b_baab`b`^^aaWH_[Q
@HWI-EAS443:3:95:537:653#0/1
ATATTGACAAAATGACCATCGTCAACATCCACAGCACTCT
+HWI-EAS443:3:95:537:653#0/1
`b_]^`aa]]_b]`]][`a]^]_`V[___a`a`aZ]X^V^
@HWI-EAS443:3:73:1335:433#0/1
AGGGCCTTCTACGGAAGGAGTGCCTGAGTCAATGAGGGGA
+HWI-EAS443:3:73:1335:433#0/1
aWaUFPTPU^¥M]_X^XTQ_W_HQ`¥^¥RFKYONPS^^^U
@HWI-EAS443:3:53:1626:1640#0/1
AAGAGGGCCTTCTACGGAAGGAGTGCCTGAGTCAATGAGG
+HWI-EAS443:3:53:1626:1640#0/1
_^aaaabaabb^baaabYaaa`a^[``_a`a[P¥]ba[a^
@HWI-EAS443:3:25:137:1926#0/1
TTGACACTAATTGATGGCCATCCGAATTCTTTTGGTCGCT
+HWI-EAS443:3:25:137:1926#0/1
]GUYb`WUW`a¥T^XZUZYRL[XZ`TXW[YYZ[[[NVXUB
@HWI-EAS443:3:55:313:858#0/1
AGGGGGGTTATATGAAGCAATCGAGGAGTGCCTGATTAAT
+HWI-EAS443:3:55:313:858#0/1
abb_bbbaabbbaaabaa_¥_``aba_a_b]Z_`]aa``¥
@HWI-EAS443:3:43:1552:1800#0/1
GGGGGGTTATATGAAGCAATCGAGGAGTGCCTGATTAATG
+HWI-EAS443:3:43:1552:1800#0/1
aa`XQOL[]a_P¥P^_PR^_VaSLSVUQYX¥X_VW^VVYY
@HWI-EAS443:3:59:1277:1082#0/1
CAAGATCGAAGGTTCCAGGCTCCAGGTTGTCCCTAAGTGC
+HWI-EAS443:3:59:1277:1082#0/1
_aa````aZ``b^^]__X[aaaa_Z]Paa[[J[`¥NGQXT
@HWI-EAS443:3:72:1270:243#0/1
CGGGGTTCTTCTCTTGACTTCCTGTTGTGTACTTTTTGAT
+HWI-EAS443:3:72:1270:243#0/1
aabb]Ybbb_¥V¥V[YDY_aa¥`[NZ]QLMLMP`LYFONT
@HWI-EAS443:3:66:992:1530#0/1
TGGGTCACCAGATTCTACAATTATTGATTCACCCCGTTCG
+HWI-EAS443:3:66:992:1530#0/1
]Z[_[`aa_a^¥`aaa[`a`aaaaaaaa`W`aaY`_KU[]
@HWI-EAS443:3:3:1364:612#0/1
CGTGCCTTCATCTGGATCTTCAGTCAATGCACCTGCAGAG
+HWI-EAS443:3:3:1364:612#0/1
a`]_`a[`_`¥W_^_^]bb_T¥Z_aba¥WSXLGROG[__^

```
@HWI-EAS443:3:83:491:240#0/1
TGCCCATAATCAAAAAGCACACATCAGGAAGACAAGAGAA
+HWI-EAS443:3:83:491:240#0/1
a]G]_SRNYT_YR][_ZVIZaX]W^Wa_YQZHU¥WRNYWV
@HWI-EAS443:3:100:807:1578#0/1
GCCATAATCAAAAAGTACACATCAGGAAGAAAAGAGAAGA
+HWI-EAS443:3:100:807:1578#0/1
a^RH^]a¥OZa¥SU¥`aWaV[[S]XPGa^NG_YYJBBBBB
@HWI-EAS443:3:23:1531:1714#0/1
GGCCATAATCAAAAAGGACACATCAGGAAGACAAGAGAAG
+HWI-EAS443:3:23:1531:1714#0/1
[aabVababVb]ZabaI]`b`bb[Zabb`b`ab[`aaaa^
@HWI-EAS443:3:32:1527:952#0/1
CCATAATCAAACAGTACACATCAGGAAGACAAGAGAAGAA
+HWI-EAS443:3:32:1527:952#0/1
a`^`¥PMZWONJT[KWJO[PX^SWVSTX[PUTHOZG¥XZB
@HWI-EAS443:3:53:1187:539#0/1
GTTCTTCTCTTGTTTTCCTGATGTGTACTTTTTGATTATG
+HWI-EAS443:3:53:1187:539#0/1
]a`aa`abbaab_bb`aab_abb_b`a_bbaaaZ_ba`aQ
@HWI-EAS443:3:73:238:1929#0/1
GCCATAATCAAAAAGAACACATCAGGAAGACAAGAGAAGA
+HWI-EAS443:3:73:238:1929#0/1
abaaaVUTZV^OQLOFW`__a_]a__a]U_YYXGaZ``Y]
@HWI-EAS443:3:78:1740:576#0/1
AAAAGTACACATCAGGAAGACAAGAGAAGAACTCTGCACT
+HWI-EAS443:3:78:1740:576#0/1
]aaa`ZTT_U_^YTZa]¥`WR_a]]UZ]a_`XKUOSR[_`
@HWI-EAS443:3:79:171:941#0/1
GCCACAATCAAAAAGTACACATCAGGAAGACAAGAGAAGA
+HWI-EAS443:3:79:171:941#0/1
aa_U``T^a¥XJUU^F^^][Y^`[_]NS[]Y¥FYUXW^ZZ
@HWI-EAS443:3:82:210:166#0/1
TTCTTCTCTTGTCTTCCTGATGGGTACTTTTTGATTATGG
+HWI-EAS443:3:82:210:166#0/1
aaaaa_a`aaa__aa```a^_`U`ZZ^_^`^`_¥`^]`^]
@HWI-EAS443:3:94:324:983#0/1
GCCATAATCAAAAAGGACACATCAGGAAGACAAGAGAAGA
+HWI-EAS443:3:94:324:983#0/1
aYZYa`aaaa``XJ]GS¥OQ^[`¥a^^^a^^_^_^[[__^
@HWI-EAS443:3:98:115:1572#0/1
CTCTTGTCTTCCTGATGTGTACTTTTTGATTATGGCCCTA
+HWI-EAS443:3:98:115:1572#0/1
]b_HO`ZT^^SW^^UNU_WKZaYaba``Taa^aWISBBBB
@HWI-EAS443:3:42:1090:548#0/1
GCCATAATCAAAAAGTACNCATCAGGAAGACAAGAGAAGA
+HWI-EAS443:3:42:1090:548#0/1
aaaabaaaaaaaaaa]a[DWaaaaaaaaa``a_a_aaaa`
@HWI-EAS443:3:54:1275:1670#0/1
AAAAAGTACACATCAGGAAGACAAGAGAAGAACCCNGCAC
```

```
+HWI-EAS443:3:54:1275:1670#0/1
aab`¥b`baaaa_`a`b_aaaa_ab¥ba`a``_^TDV__^
@HWI-EAS443:3:87:981:1554#0/1
GCCATAATCAAAAAGTACANATCAGGAAGACAAGAGAAGA
+HWI-EAS443:3:87:981:1554#0/1
`ab`bbbb`ababbbabb^D]babbbbbaabbbbababbb
@HWI-EAS443:3:23:1678:1034#0/1
GNTCTCTTGTCTTCCTGATGTGTACTTTTTGATTATGGCC
+HWI-EAS443:3:23:1678:1034#0/1
¥D]]]S]W_YT_`WN_Z_`ZL]Z¥M¥a`_^`_`[]X_SBB
@HWI-EAS443:3:76:759:547#0/1
GCTCTTCTCTTGTCTTCCTGATGTGTACTTTTTGATTATG
+HWI-EAS443:3:76:759:547#0/1
¥DO¥N^U[TX^¥NQVZUYU]¥¥[N^XRZ_[]_Y[VZWZU_
@HWI-EAS443:3:91:539:1267#0/1
ATGGCCATAATCAAAAAGTACACATCAGGAAGACAAGAGG
+HWI-EAS443:3:91:539:1267#0/1
aaa`bbababb¥`aaZaaZb`a`aa[``^`]]`_a]aa]H
@HWI-EAS443:3:10:1631:1201#0/1
CATAATCAAAAAGTACACATCAGGAAGACAAGAGAAGAAC
+HWI-EAS443:3:10:1631:1201#0/1
]bbbbbabba^`baaba`aa`bbbbabb_babbbbbb`b_
@HWI-EAS443:3:11:198:1303#0/1
CATAATCAAAAAGTACACATCAGGAAGACAAGAGAAGAAC
+HWI-EAS443:3:11:198:1303#0/1
a_`a__a_`Y__[Va_`a`^]__^]`_^__^_X]]^^_^]
@HWI-EAS443:3:15:288:1436#0/1
TCTTCTCTTGTCTTCCTGATGTGTACTTTTTGATTATGGC
+HWI-EAS443:3:15:288:1436#0/1
]_¥S]¥aa¥O[YUWQS]YLO`H[Z]^[RZZWMMVURR__`
@HWI-EAS443:3:15:422:993#0/1
GGCCATAATCAAAAAGTACACATCAGGAAGACAAGAGAAG
+HWI-EAS443:3:15:422:993#0/1
_Va_¥W_`]_[^`aT¥QW`bba``^`abYaaaaaa`a`]a
@HWI-EAS443:3:16:968:836#0/1
TGGCCATAATCAAAAAGTACACATCAGGAAGACAAGAGAA
+HWI-EAS443:3:16:968:836#0/1
abbbabaYF^bab_`^`SX`baa_a¥baaabb¥a_`aa_R
@HWI-EAS443:3:17:1170:1319#0/1
GTTCTTCTCTTGTCTTCCTGATGTGTACTTTTTGATTATG
+HWI-EAS443:3:17:1170:1319#0/1
aaaaaaaaaab]^aaaa`a``aa`aXa_aaaaaaaaa_`_
@HWI-EAS443:3:17:285:51#0/1
ATGGCCATAATCAAAAAGTACACATCAGGAAGACAAGAGA
+HWI-EAS443:3:17:285:51#0/1
aWW¥`a_a[TTJVbaa¥_V]]Z]ZVOV]]`Q][^aXWW`]
@HWI-EAS443:3:18:1121:1627#0/1
GCCATAATCAAAAAGTACACATCAGGAAGACAAGAGAAGA
+HWI-EAS443:3:18:1121:1627#0/1
a___YYYa^U[ZL¥[Y]T^VW]Y¥ULU][RY¥^]V_VY_Y
```

```
@HWI-EAS443:3:18:1440:422#0/1
CCATAATCAAAAAGTACACATCAGGAAGACAAGAGAAGAA
+HWI-EAS443:3:18:1440:422#0/1
`aaaa^aaaaa__a¥aZ_`_¥^_b^]``S¥a_a]_^__``
@HWI-EAS443:3:18:52:1998#0/1
GCCATAATCAAAAAGTACACATCAGGAAGACAAGAGAAGA
+HWI-EAS443:3:18:52:1998#0/1
_abaaa[````_]``Xbaaaa`a_aW`a_`_a`aa`aa`a
@HWI-EAS443:3:19:795:84#0/1
TTCTTCTCTTGTCTTCCTGATGTGTACTTTTTGATTATGG
+HWI-EAS443:3:19:795:84#0/1
`bb_b]a`_a]PHZ_]`ab`YbYa`]V^aaaaa`__ZUP_
@HWI-EAS443:3:21:1087:1412#0/1
CATAATCAAAAAGTACACATCAGGAAGACAAGAGAAGAAC
+HWI-EAS443:3:21:1087:1412#0/1
aba``aabaaaaa[a`aaa[`aaa``a_`a_aa`_a_a`W
@HWI-EAS443:3:21:204:421#0/1
CATAATCAAAAAGTACACATCAGGAAGACAAGAGAAGAAC
+HWI-EAS443:3:21:204:421#0/1
abbaabbaabbab_abbbaaaaaaa`a_aa^^`aa``aa]
@HWI-EAS443:3:21:645:1362#0/1
GGCCATAATCAAAAAGTACACATCAGGAAGACAAGAGAAG
+HWI-EAS443:3:21:645:1362#0/1
ab`]RI_Z_a¥^^¥Z]Q^OO`]a¥YM^ZW]`M``[UNNaU
@HWI-EAS443:3:22:1544:1400#0/1
CTTCTCTTGTCTTCCTGATGTGTACTTTTTGATTATGGCC
+HWI-EAS443:3:22:1544:1400#0/1
]a`a^aaa¥aa^X^b^_UaU`TYZXb¥`b^Y^^¥^b]^MH
@HWI-EAS443:3:22:225:1560#0/1
GCCATAATCAAAAAGTACACATCAGGAAGACAAGAGAAGA
+HWI-EAS443:3:22:225:1560#0/1
`bbb[`^X`]aaa]¥Ha`a`aa¥RWa]Z¥_aa`_aa_a_]
@HWI-EAS443:3:23:1201:1413#0/1
CTTCTCTTGTCTTCCTGATGTGTACTTTTTGATTATGGCC
+HWI-EAS443:3:23:1201:1413#0/1
¥baa¥bbbbbabbb_^^a^M^___aaaaa``ababa`TT_
@HWI-EAS443:3:23:1589:352#0/1
TTCTTCTCTTGTCTTCCTGATGTGTACTTTTTGATTATGG
+HWI-EAS443:3:23:1589:352#0/1
]W_¥_ZZP_ab_``O¥PZ]ZS¥V^Y`^^]]Y[`^_¥W]UT
@HWI-EAS443:3:23:721:373#0/1
GTTCTTCTCTTGTCTTCCTGATGTGTACTTTTTGATTATG
+HWI-EAS443:3:23:721:373#0/1
`UVSU]U^`X_YLYTTW¥]RRWVO¥PNGNX^_]VYUS[_B
@HWI-EAS443:3:23:75:1667#0/1
GTTCTTCTCTTGTCTTCCTGATGTGTACTTTTTGATTATG
+HWI-EAS443:3:23:75:1667#0/1
aaaaaabbba`_`ab¥^aaa^`Z]]]`V`^aa¥Y_`_TFU
@HWI-EAS443:3:25:23:1701#0/1
CATAATCAAAAAGTACACATCAGGAAGACAAGAGAAGAAC
```

```
+HWI-EAS443:3:25:23:1701#0/1
_bbZV¥bab`¥`^_bbbbbab^QQ]bbaaba_a¥¥baa``
@HWI-EAS443:3:26:1089:525#0/1
CATAATCAAAAAGTACACATCAGGAAGACAAGAGAAGAAC
+HWI-EAS443:3:26:1089:525#0/1
`bbabbbbbbbababababbaaabbbbabaaa_bababbaba
@HWI-EAS443:3:26:508:1987#0/1
GCCATAATCAAAAAGTACACATCAGGAAGACAAGAGAAGA
+HWI-EAS443:3:26:508:1987#0/1
a_ab````aaaaa_XX^^`a¥TZ`][`_a_aaa__^aa_`
@HWI-EAS443:3:27:110:711#0/1
GCCATAATCAAAAAGTACACATCAGGAAGACAAGAGAAGA
+HWI-EAS443:3:27:110:711#0/1
^¥W__RWRa^YP[Z_O¥`_]Zaa`]`_[_`^`aa^`¥R¥¥
@HWI-EAS443:3:27:399:173#0/1
ATGGCCATAATCAAAAAGTACACATCAGGAAGACAAGAGA
+HWI-EAS443:3:27:399:173#0/1
_TV]bbaUU]Y`Ya`aYaJ``a¥^`_^___YYXY_X]VOY
@HWI-EAS443:3:28:1784:2005#0/1
TGGCCATAATCAAAAAGTACACATCAGGAAGACAAGAGAA
+HWI-EAS443:3:28:1784:2005#0/1
aaa`__¥_b`_`aa^_UZaaa_aaZaaaa^aaa`_aa]_`
@HWI-EAS443:3:28:237:741#0/1
GCCATAATCAAAAAGTACACATCAGGAAGACAAGAGAAGA
+HWI-EAS443:3:28:237:741#0/1
`b`^^`^^ba[]``RTPaa][]aa`Y^`a]a_a[_`a__a
@HWI-EAS443:3:28:351:2045#0/1
CCATAATCAAAAAGTACACATCAGGAAGACAAGAGAAGAA
+HWI-EAS443:3:28:351:2045#0/1
aaZ^aa¥aaaa`YZZaa^aa_aa_¥aa_`a]]Z`¥`^^``
@HWI-EAS443:3:28:401:685#0/1
CATAATCAAAAAGTACACATCAGGAAGACAAGAGAAGAAC
+HWI-EAS443:3:28:401:685#0/1
aa^¥a[aY^_¥^YYbaa]_aYa]_Zaaaaab`^``aaa_
@HWI-EAS443:3:28:826:1776#0/1
TGGCCATAATCAAAAAGTACACATCAGGAAGACAAGAGAA
+HWI-EAS443:3:28:826:1776#0/1
HX^^¥^R````ab```Yabbbbbbb[[abbbababba``b
@HWI-EAS443:3:29:168:490#0/1
ATGGCCATAATCAAAAAGTACACATCAGGAAGACAAGAGA
+HWI-EAS443:3:29:168:490#0/1
a_aaaaa_a_[aaa````X___``___a_]_`]^_]`]_^
@HWI-EAS443:3:29:284:1308#0/1
CATAATCAAAAAGTACACATCAGGAAGACAAGAGAAGAAC
+HWI-EAS443:3:29:284:1308#0/1
aaa]aab]aaaa`]baaaaaaa`_aa_`]aa``a_`_aa_
@HWI-EAS443:3:29:642:881#0/1
GGCCATAATCAAAAAGTACACATCAGGAAGACAAGAGAAG
+HWI-EAS443:3:29:642:881#0/1
`babaaaab`b`a`aa]baaa``aaa_a`a_aaaa^a^`^
```

@HWI-EAS443:3:2:1315:402#0/1
ATGGCCATAATCAAAAAGTACACATCAGGAAGACAAGAGA
+HWI-EAS443:3:2:1315:402#0/1
aa[MX`a]]_aU`a``aa^a][U]_[]a`]a_Z]X``X`Z
@HWI-EAS443:3:30:1304:712#0/1
GCCATAATCAAAAAGTACACATCAGGAAGACAAGAGAAGA
+HWI-EAS443:3:30:1304:712#0/1
a]P]Z_R_]W^STZ^U]_aa``RUYSQLZZYWX`W_]V^T
@HWI-EAS443:3:30:491:554#0/1
GTTCTTCTCTTGTCTTCCTGATGTGTACTTTTTGATTATG
+HWI-EAS443:3:30:491:554#0/1
a`aababaaaba_aaabaaaa`a_a^``a`a`a¥``___Z
@HWI-EAS443:3:30:631:198#0/1
GCCATAATCAAAAAGTACACATCAGGAAGACAAGAGAAGA
+HWI-EAS443:3:30:631:198#0/1
^]aUOHQZV``bJ_bXaaaaaXaa]X`U[``W`a_a_¥_`
@HWI-EAS443:3:31:1360:413#0/1
GCCATAATCAAAAAGTACACATCAGGAAGACAAGAGAAGA
+HWI-EAS443:3:31:1360:413#0/1
YZP_`aa¥ba`a_W_Raa]Yaa__a^Saa`_aabaa``^^
@HWI-EAS443:3:31:398:203#0/1
GCCATAATCAAAAAGTACACATCAGGAAGACAAGAGAAGA
+HWI-EAS443:3:31:398:203#0/1
aabb_``b`_ababb^aaaba`aab`aaa^aaY``_``_a
@HWI-EAS443:3:31:522:983#0/1
GTTCTTCTCTTGTCTTCCTGATGTGTACTTTTTGATTATG
+HWI-EAS443:3:31:522:983#0/1
aaaabaaaaaaaaaaabba_a_]Z_¥aaaaaaa_`a`a_Y
@HWI-EAS443:3:32:1462:311#0/1
GGCCATAATCAAAAAGTACACATCAGGAAGACAAGAGAAG
+HWI-EAS443:3:32:1462:311#0/1
`bSZZZ]W[[a]VUUZF]]_^^aT[]^_aaXU^V]QZO[a
@HWI-EAS443:3:34:1202:796#0/1
CTTCTCTTGTCTTCCTGATGTGTACTTTTTGATTATGGCC
+HWI-EAS443:3:34:1202:796#0/1
abbbbbbbbaabbbbbbabaaaaaaabbbba`aaa¥JWU_
@HWI-EAS443:3:34:405:799#0/1
GTTCTTCTCTTGTCTTCCTGATGTGTACTTTTTGATTATG
+HWI-EAS443:3:34:405:799#0/1
`abbbbbbbaaaabbaaaa^aaa_a_aaaaaaa`aa_a__
@HWI-EAS443:3:35:1466:1833#0/1
GTTCTTCTCTTGTCTTCCTGATGTGTACTTTTTGATTATG
+HWI-EAS443:3:35:1466:1833#0/1
a`_S]_^X`[¥`[[`X^_]QTXXU[V]U_aaaaYa`_aZ_
@HWI-EAS443:3:35:1755:185#0/1
GCCATAATCAAAAAGTACACATCAGGAAGACAAGAGAAGA
+HWI-EAS443:3:35:1755:185#0/1
]a__b`]b``_aa]b_aZ^VU`[a^P_[`]_aab^a^aWN
@HWI-EAS443:3:35:224:982#0/1
TGGCCATAATCAAAAAGTACACATCAGGAAGACAAGAGAA

```
+HWI-EAS443:3:35:224:982#0/1
aaababa]baaaaa]aa]bbaaa`aaa]Y`a_aaYa`a`a
@HWI-EAS443:3:35:586:263#0/1
ATGGCCATAATCAAAAAGTACACATCAGGAAGACAAGAGA
+HWI-EAS443:3:35:586:263#0/1
a_`aa^_¥W__`_^X]`^X_U^]Y¥^]]T]`^XZ]^^X^Z
@HWI-EAS443:3:36:1399:968#0/1
GTTCTTCTCTTGTCTTCCTGATGTGTACTTTTTGATTATG
+HWI-EAS443:3:36:1399:968#0/1
aX¥b`_aaaaaaM``aa__JZ[MUaV¥T```]`^U`][PK
@HWI-EAS443:3:36:662:1247#0/1
CTTCTCTTGTCTTCCTGATGTGTACTTTTTGATTATGGCC
+HWI-EAS443:3:36:662:1247#0/1
abababaaa`baaaa[]aa^¥^¥[^aa`aaa_`^^_¥Z_a
@HWI-EAS443:3:37:764:1503#0/1
GCCATAATCAAAAAGTACACATCAGGAAGACAAGAGAAGA
+HWI-EAS443:3:37:764:1503#0/1
aba``a^_a^___Y^Ra`aaaaa_^a``_^b`Q_`]Z]__
@HWI-EAS443:3:38:794:654#0/1
TCTCTTGTCTTCCTGATGTGTACTTTTTGATTATGGCCAT
+HWI-EAS443:3:38:794:654#0/1
aabaaaa_aaa[]_]`_a^`]__a`a``_¥__Z_^]VX¥O
@HWI-EAS443:3:38:858:58#0/1
CATAATCAAAAAGTACACATCAGGAAGACAAGAGAAGAAC
+HWI-EAS443:3:38:858:58#0/1
`abaabababaaa_b^baa`aaaaaa`ab`a_Ua^a`^`a_X
@HWI-EAS443:3:39:1740:457#0/1
GCCATAATCAAAAAGTACACATCAGGAAGACAAGAGAAGA
+HWI-EAS443:3:39:1740:457#0/1
aaa_`Z]a_a^XXV`P^]]_X[_Xa`¥¥_USXW^]_YZ_X
@HWI-EAS443:3:3:193:444#0/1
TTCTCTTGTCTTCCTGATGTGTACTTTTTGATTATGGCCA
+HWI-EAS443:3:3:193:444#0/1
a``baaba^aaa`_aa^`b^`___a^a^aa`__a_]^_]¥
@HWI-EAS443:3:3:700:1493#0/1
GCCATAATCAAAAAGTACACATCAGGAAGACAAGAGAAGA
+HWI-EAS443:3:3:700:1493#0/1
a_][WXZ]]]TX`^_I`¥`_]_]X^[Y_^_ZRU_]_[BBB
@HWI-EAS443:3:3:723:707#0/1
CATAATCAAAAAGTACACATCAGGAAGACAAGAGAAGAAC
+HWI-EAS443:3:3:723:707#0/1
abbabbbbbb`bb`baabbbaabaaabaababababaabaa_
@HWI-EAS443:3:3:831:623#0/1
CCATAATCAAAAAGTACACATCAGGAAGACAAGAGAAGAA
+HWI-EAS443:3:3:831:623#0/1
aa_^][]Za[]aa¥F`aa`ba]^]baab¥`a_a`b__`aa
@HWI-EAS443:3:40:1630:1321#0/1
GCCATAATCAAAAAGTACACATCAGGAAGACAAGAGAAGA
+HWI-EAS443:3:40:1630:1321#0/1
aVSYN]`[TZ_^VRRTYLYW]VQZ`PYRWVLXT[ZZVV_]
```

@HWI-EAS443:3:40:396:1807#0/1
TGGCCATAATCAAAAAGTACACATCAGGAAGACAAGAGAA
+HWI-EAS443:3:40:396:1807#0/1
[bbabY`b`^¥a]¥a_WYabbabba^[`S_`]^b``[¥K^
@HWI-EAS443:3:42:1050:287#0/1
CATAATCAAAAAGTACACATCAGGAAGACAAGAGAAGAAC
+HWI-EAS443:3:42:1050:287#0/1
abaaababbab_b¥babaaaaabaaabaaaaaaa_`a`a`
@HWI-EAS443:3:42:1572:690#0/1
ATGGCCATAATCAAAAAGTACACATCAGGAAGACAAGAGA
+HWI-EAS443:3:42:1572:690#0/1
Y^bb^_Y^IW¥QT¥ab_¥R]aZa¥LUS¥a^YXR^J_aW_[
@HWI-EAS443:3:42:653:1261#0/1
ATGGCCATAATCAAAAAGTACACATCAGGAAGACAAGAGA
+HWI-EAS443:3:42:653:1261#0/1
a__]a_aa_a__aa`a`_W_¥`_`_``_a_a`^___¥U^^
@HWI-EAS443:3:42:796:670#0/1
GCCATAATCAAAAAGTACACATCAGGAAGACAAGAGAAGA
+HWI-EAS443:3:42:796:670#0/1
aaa¥]S]]¥]aY¥¥aS[¥_^^^]_a`_Ya^_^_`Ya[Y]_
@HWI-EAS443:3:43:1342:979#0/1
GGCCATAATCAAAAAGTACACATCAGGAAGACAAGAGAAG
+HWI-EAS443:3:43:1342:979#0/1
]M_a]]LOYZ]XUUS^^Y^`YY]^^`[[]_QO[ZX[_[S[
@HWI-EAS443:3:44:1236:444#0/1
GTTCTTCTCTTGTCTTCCTGATGTGTACTTTTTGATTATG
+HWI-EAS443:3:44:1236:444#0/1
a¥a`a`_a^aaa_`a_^__a]_aZ_WO¥`a`_`[U^Z^[M
@HWI-EAS443:3:44:757:1757#0/1
GGCCATAATCAAAAAGTACACATCAGGAAGACAAGAGAAG
+HWI-EAS443:3:44:757:1757#0/1
aababbbaaabbabbaYaaa`a``a^aaaaaaaa_aa`a`
@HWI-EAS443:3:44:970:1434#0/1
CATAATCAAAAAGTACACATCAGGAAGACAAGAGAAGAAC
+HWI-EAS443:3:44:970:1434#0/1
aabaababbabba^aabaaa^abaaaa`aa`baaaaaaa^
@HWI-EAS443:3:45:1377:1419#0/1
CATAATCAAAAAGTACACATCAGGAAGACAAGAGAAGAAC
+HWI-EAS443:3:45:1377:1419#0/1
`abaaa``aa]`a^a_a^``Z_a^^```]`_a_a`^`_]W
@HWI-EAS443:3:48:1330:658#0/1
TGGCCATAATCAAAAAGTACACATCAGGAAGACAAGAGAA
+HWI-EAS443:3:48:1330:658#0/1
RY`_X]]WWZQ]aa`XZKZQ]]a_]^baYZ`QL[Wa]a_X
@HWI-EAS443:3:48:133:676#0/1
TTCTCTTGTCTTCCTGATGTGTACTTTTTGATTATGGCCA
+HWI-EAS443:3:48:133:676#0/1
aaab`aab^`baaaa```a¥¥[a`aaaaaa_``a^¥]_^^
@HWI-EAS443:3:48:1598:455#0/1
CATAATCAAAAAGTACACATCAGGAAGACAAGAGAAGAAC

```
+HWI-EAS443:3:48:1598:455#0/1
`bbVQ_T_bbb^`ababab_`aab`bbaabababa]ba`]
@HWI-EAS443:3:49:1718:813#0/1
GTTCTTCTCTTGTCTTCCTGATGTGTACTTTTTGATTATG
+HWI-EAS443:3:49:1718:813#0/1
a]babbbbbbbba_aaa````aaZ___¥aaaaa]^`a`_]
@HWI-EAS443:3:49:679:118#0/1
CTTCTCTTGTCTTCCTGATGTGTACTTTTTGATTATGGCC
+HWI-EAS443:3:49:679:118#0/1
abbabbbbbbbbbaa```bbabababbbbb^bbbbbaZ``
@HWI-EAS443:3:4:1240:1800#0/1
TGGCCATAATCAAAAAGTACACATCAGGAAGACAAGAGAA
+HWI-EAS443:3:4:1240:1800#0/1
abb`a^a`b`]bababa_b]bab_NabaaaaZJ]aaaa`a
@HWI-EAS443:3:4:154:408#0/1
CTTCTCTTGTCTTCCTGATGTGTACTTTTTGATTATGGCC
+HWI-EAS443:3:4:154:408#0/1
abb_b^bbb^aaaaaaaaaa^aY``ba``a_^¥Yaa`Y`_
@HWI-EAS443:3:50:249:721#0/1
GTTCTTCTCTTGTCTTCCTGATGTGTACTTTTTGATTATG
+HWI-EAS443:3:50:249:721#0/1
__aaaaaa_aaa^[abaa`a_`¥RaZ__baaa`X[a`_a_
@HWI-EAS443:3:50:391:440#0/1
GTTCTTCTCTTGTCTTCCTGATGTGTACTTTTTGATTATG
+HWI-EAS443:3:50:391:440#0/1
T¥_ab]¥¥`_b`b^_`^`][Y]_H_a]bbbbb```bbab`
@HWI-EAS443:3:50:700:1046#0/1
CATAATCAAAAAGTACACATCAGGAAGACAAGAGAAGAAC
+HWI-EAS443:3:50:700:1046#0/1
¥bbbabb]b_V_b]ab`[ab[a_aaaba`bab_baa`[`a
@HWI-EAS443:3:50:853:1637#0/1
TTCTTCTCTTGTCTTCCTGATGTGTACTTTTTGATTATGG
+HWI-EAS443:3:50:853:1637#0/1
[aa^a``Z^``[a`aa``X]_¥[_YaSaaaa^WZ_]^[NZ
@HWI-EAS443:3:51:1327:2038#0/1
GCCATAATCAAAAAGTACACATCAGGAAGACAAGAGAAGA
+HWI-EAS443:3:51:1327:2038#0/1
aaaaaa`b`aaaZ`_]aa_[aa_`b[aaaa_a][[_Y_`_
@HWI-EAS443:3:51:690:312#0/1
ATGGCCATAATCAAAAAGTACACATCAGGAAGACAAGAGA
+HWI-EAS443:3:51:690:312#0/1
aab]aa__`]^W_aaaaaL_^`U]]¥`baV_a¥Za]aZa_
@HWI-EAS443:3:52:1512:835#0/1
ATGGCCATAATCAAAAAGTACACATCAGGAAGACAAGAGA
+HWI-EAS443:3:52:1512:835#0/1
`a_Y_a^a^`a``X`__]_a¥a^`a_Ya]Ya¥¥__aa`a_
@HWI-EAS443:3:52:899:364#0/1
GTTCTTCTCTTGTCTTCCTGATGTGTACTTTTTGATTATG
+HWI-EAS443:3:52:899:364#0/1
aababaababbaaaaa_aaa`aa_a___aaa`a__a``_^
```

```
@HWI-EAS443:3:54:1060:1029#0/1
GGCCATAATCAAAAAGTACACATCAGGAAGACAAGAGAAG
+HWI-EAS443:3:54:1060:1029#0/1
aaaaabbab^baabba_bbbaababbaa`b`_b``aaaaa
@HWI-EAS443:3:55:884:659#0/1
GCCATAATCAAAAAGTACACATCAGGAAGACAAGAGAAGA
+HWI-EAS443:3:55:884:659#0/1
a]TFQ``_TRU^aU^Za`P¥P¥PP`aYJ[XWZ__X__X[[
@HWI-EAS443:3:55:93:1310#0/1
TTCTTCTCTTGTCTTCCTGATGTGTACTTTTTGATTATGG
+HWI-EAS443:3:55:93:1310#0/1
aaaaaabaaaa`aaa`aa__`aX`^__aaaaaZ_````_Y
@HWI-EAS443:3:56:1126:457#0/1
TCTTCTCTTGTCTTCCTGATGTGTACTTTTTGATTATGGC
+HWI-EAS443:3:56:1126:457#0/1
a`aW_`^¥^[V_a¥_`^_^Z_X[YZ^_aa^aZ^_^_WW^Y
@HWI-EAS443:3:56:1765:1173#0/1
GCCATAATCAAAAAGTACACATCAGGAAGACAAGAGAAGA
+HWI-EAS443:3:56:1765:1173#0/1
abaaaaabab`^aa__aaa^a`]``]]`¥]aaaa_``U¥_
@HWI-EAS443:3:56:381:1849#0/1
GCCATAATCAAAAAGTACACATCAGGAAGACAAGAGAAGA
+HWI-EAS443:3:56:381:1849#0/1
aa`bbbababbaaaaXbaaaaa`aa`abaaaaa`a``a^_
@HWI-EAS443:3:57:119:503#0/1
GCCATAATCAAAAAGTACACATCAGGAAGACAAGAGAAGA
+HWI-EAS443:3:57:119:503#0/1
`aaaa_`aba`aa]a¥`a_aa`aaaaaaa¥_a```a¥Z_a
@HWI-EAS443:3:57:1232:993#0/1
GTTCTTCTCTTGTCTTCCTGATGTGTACTTTTTGATTATG
+HWI-EAS443:3:57:1232:993#0/1
^[a]a`aa¥¥^a`]`a¥TZ_S¥_S¥W¥Za`aaaX`_`[_¥
@HWI-EAS443:3:58:566:1924#0/1
GGCCATAATCAAAAAGTACACATCAGGAAGACAAGAGAAG
+HWI-EAS443:3:58:566:1924#0/1
abbab`_bbababb^bZbbbabb_bb`bbbbabbabaab^
@HWI-EAS443:3:59:1741:567#0/1
GGCCATAATCAAAAAGTACACATCAGGAAGACAAGAGAAG
+HWI-EAS443:3:59:1741:567#0/1
ababbabaaa]]_aa``a`a]a`^aaaaba`G```NH_¥Z
@HWI-EAS443:3:59:674:381#0/1
GTTCTTCTCTTGTCTTCCTGATGTGTACTTTTTGATTATG
+HWI-EAS443:3:59:674:381#0/1
aab`bbaaabbaaabbaabbaaa_bZT^baaaaaa_^aa[
@HWI-EAS443:3:61:1232:83#0/1
CATAATCAAAAAGTACACATCAGGAAGACAAGAGAAGAAC
+HWI-EAS443:3:61:1232:83#0/1
abbbababbbbbbabbababbbaabbbb_bab`aaa`a`^
@HWI-EAS443:3:61:1713:253#0/1
GCCATAATCAAAAAGTACACATCAGGAAGACAAGAGAAGA
```

+HWI-EAS443:3:61:1713:253#0/1
a_]aaaaaX¥aaa`aWa_a]Y__abaa__[R`^^]_][][
@HWI-EAS443:3:63:111:1264#0/1
GTTCTTCTCTTGTCTTCCTGATGTGTACTTTTTGATTATG
+HWI-EAS443:3:63:111:1264#0/1
]OTZM^ba^]__T``aa_¥OXW^GYYJRS^aa_H]XaBBB
@HWI-EAS443:3:63:389:1200#0/1
CTTCTCTTGTCTTCCTGATGTGTACTTTTTGATTATGGCC
+HWI-EAS443:3:63:389:1200#0/1
aaababaaa_aaaaaaa^`a^a^¥`aaa``T`a`a_S[_a
@HWI-EAS443:3:64:439:1006#0/1
TGGCCATAATCAAAAAGTACACATCAGGAAGACAAGAGAA
+HWI-EAS443:3:64:439:1006#0/1
[aWaa_`__[bbbZZRP[bab_¥¥XH]Z`]``aXXTN`ZN
@HWI-EAS443:3:65:593:1900#0/1
CATAATCAAAAAGTACACATCAGGAAGACAAGAGAAGAAC
+HWI-EAS443:3:65:593:1900#0/1
aaa``_aa_aZQ[Ua]_]`Y`aa__ZQ]a`[U`Z[ZXZ^a
@HWI-EAS443:3:67:1378:901#0/1
GCCATAATCAAAAAGTACACATCAGGAAGACAAGAGAAGA
+HWI-EAS443:3:67:1378:901#0/1
^``ab_abaabba`XYbabaabbaabaaaa`bababaaaa
@HWI-EAS443:3:67:163:1829#0/1
GGCCATAATCAAAAAGTACACATCAGGAAGACAAGAGAAG
+HWI-EAS443:3:67:163:1829#0/1
¥ab_X__`b`bbb`aZa`bbb_b`a`a_JTba_a^¥]`ba
@HWI-EAS443:3:67:1699:474#0/1
CCATAATCAAAAAGTACACATCAGGAAGACAAGAGAAGAA
+HWI-EAS443:3:67:1699:474#0/1
ab``aaa`a]^baa_baaaaaaaaaaabaaaaa^b_aaa`
@HWI-EAS443:3:68:1695:793#0/1
GCCATAATCAAAAAGTACACATCAGGAAGACAAGAGAAGA
+HWI-EAS443:3:68:1695:793#0/1
^a```_`^_``^`^_S^Wa`_`]]a``aaa]^¥X]a`¥^^
@HWI-EAS443:3:68:399:820#0/1
GGCCATAATCAAAAAGTACACATCAGGAAGACAAGAGAAG
+HWI-EAS443:3:68:399:820#0/1
aabbba`aba]baaaa]baaabaaaaaabaa`a_aa`_a`
@HWI-EAS443:3:68:456:1162#0/1
TGGCCATAATCAAAAAGTACACATCAGGAAGACAAGAGAA
+HWI-EAS443:3:68:456:1162#0/1
aa`bbabbbbbabaaa^bbaa`a_aa`abaaaaaa```a
@HWI-EAS443:3:68:527:1484#0/1
ATGGCCATAATCAAAAAGTACACATCAGGAAGACAAGAGA
+HWI-EAS443:3:68:527:1484#0/1
a``abaaaa`a]``a`a_Za_aaa_^^^_aa_¥__Y^_]^
@HWI-EAS443:3:69:738:1479#0/1
GTTCTTCTCTTGTCTTCCTGATGTGTACTTTTTGATTATG
+HWI-EAS443:3:69:738:1479#0/1
_abbbbabbaaa_aaa`aa_`aa`_^aaaaaaa]a````_

```
@HWI-EAS443:3:69:985:1266#0/1
ATGGCCATAATCAAAAAGTACACATCAGGAAGACAAGAGA
+HWI-EAS443:3:69:985:1266#0/1
a_[X___]]^Z]_^__ZWR^VZV^Z]ZW]Z^¥XX]OZZXZ
@HWI-EAS443:3:6:304:1848#0/1
GGCCATAATCAAAAAGTACACATCAGGAAGACAAGAGAAG
+HWI-EAS443:3:6:304:1848#0/1
JTa`baS_a`bbb`baJabaVaa]ba^baa`baabaa`aa
@HWI-EAS443:3:6:956:177#0/1
GCCATAATCAAAAAGTACACATCAGGAAGACAAGAGAAGA
+HWI-EAS443:3:6:956:177#0/1
ab]___]`__]]_]aX^U^Y]^YX^X]V_YY^^^]^BBBB
@HWI-EAS443:3:70:1690:1230#0/1
CCATAATCAAAAAGTACACATCAGGAAGACAAGAGAAGAA
+HWI-EAS443:3:70:1690:1230#0/1
abbbbbbabbbbbbababbbbababbbbbbbbbbbbbbab
@HWI-EAS443:3:70:185:191#0/1
TGGCCATAATCAAAAAGTACACATCAGGAAGACAAGAGAA
+HWI-EAS443:3:70:185:191#0/1
a`_`_^_¥_Zb__``[_F_a`U[^^^X_]]]^aa`_]a`¥
@HWI-EAS443:3:71:199:628#0/1
CATAATCAAAAAGTACACATCAGGAAGACAAGAGAAGAAC
+HWI-EAS443:3:71:199:628#0/1
ab_aabaabaabb¥baaa_aba`aa^``aWaa^`__`aaZ
@HWI-EAS443:3:72:1361:928#0/1
TGGCCATAATCAAAAAGTACACATCAGGAAGACAAGAGAA
+HWI-EAS443:3:72:1361:928#0/1
`_SZ`aaaa]b]ab^SVI_HaYaZNSb`]a`_`]a`a[a]
@HWI-EAS443:3:72:1427:318#0/1
TTCTTCTCTTGTCTTCCTGATGTGTACTTTTTGATTATGG
+HWI-EAS443:3:72:1427:318#0/1
]a`]Z][OW`aZVX^¥aaZ¥```a^MLZ`aaa^[a^_`BB
@HWI-EAS443:3:72:515:804#0/1
TGGCCATAATCAAAAAGTACACATCAGGAAGACAAGAGAA
+HWI-EAS443:3:72:515:804#0/1
a][^^a_^`_Z_]][^]V^aa`aa]]``^^_aa``a^`_a
@HWI-EAS443:3:72:727:1526#0/1
CTTCTCTTGTCTTCCTGATGTGTACTTTTTGATTATGGCC
+HWI-EAS443:3:72:727:1526#0/1
a`^^a`]aa^]```[``[¥VT^UZ¥aa`][[][]¥YKDW_
@HWI-EAS443:3:73:537:763#0/1
ATGGCCATAATCAAAAAGTACACATCAGGAAGACAAGAGA
+HWI-EAS443:3:73:537:763#0/1
¥^_ab_S`^Wba_baZZQZ¥X[aaa_W¥``baa_bY]Y[Z
@HWI-EAS443:3:74:318:887#0/1
GTTCTTCTCTTGTCTTCCTGATGTGTACTTTTTGATTATG
+HWI-EAS443:3:74:318:887#0/1
`¥``a`[_aaa^LX_a]aa]S]]P]YQT][`_Z^a__U]R
@HWI-EAS443:3:74:905:1044#0/1
GGCCATAATCAAAAAGTACACATCAGGAAGACAAGAGAAG
```

+HWI-EAS443:3:74:905:1044#0/1
abaabbaabaab`baa_bbaaba`aaaaab`abaaaaaa_
@HWI-EAS443:3:75:822:990#0/1
TGGCCATAATCAAAAAGTACACATCAGGAAGACAAGAGAA
+HWI-EAS443:3:75:822:990#0/1
`bb`bbbabb`^`b¥X`aabba_[ba``bb`Y````aa`a
@HWI-EAS443:3:75:829:406#0/1
GTTCTTCTCTTGTCTTCCTGATGTGTACTTTTTGATTATG
+HWI-EAS443:3:75:829:406#0/1
aaabbbabbba_`aaaabaa`aaaaXa_b`aabaaaaaaa
@HWI-EAS443:3:77:344:1962#0/1
GCCATAATCAAAAAGTACACATCAGGAAGACAAGAGAAGA
+HWI-EAS443:3:77:344:1962#0/1
]bbbbaXRZ`V^aV_Rb_U_a_b^ZZ_SZ`Yb]a]aaa]Z
@HWI-EAS443:3:79:909:1759#0/1
GCCATAATCAAAAAGTACACATCAGGAAGACAAGAGAAGA
+HWI-EAS443:3:79:909:1759#0/1
_^]_`]^]]¥ZU]_^V_aa`[[a`aa`[]_a¥S^__`[`_
@HWI-EAS443:3:7:1493:357#0/1
TGGCCATAATCAAAAAGTACACATCAGGAAGACAAGAGAA
+HWI-EAS443:3:7:1493:357#0/1
]b_YW¥bRW¥aaab_O]QZ¥aY]_V_aaa`aZLZ_a_``]
@HWI-EAS443:3:7:539:926#0/1
TGGCCATAATCAAAAAGTACACATCAGGAAGACAAGAGAA
+HWI-EAS443:3:7:539:926#0/1
aba`aaba[^aa`_aba^a`baa^_aaa_a`a]a`aa``a
@HWI-EAS443:3:7:587:590#0/1
GGCCATAATCAAAAAGTACACATCAGGAAGACAAGAGAAG
+HWI-EAS443:3:7:587:590#0/1
Z_U```X`_R_aa`_U]`WaVa]aabb`bba]aXa^baab
@HWI-EAS443:3:82:1242:625#0/1
CCATAATCAAAAAGTACACATCAGGAAGACAAGAGAAGAA
+HWI-EAS443:3:82:1242:625#0/1
``aa]_babX]_]WS^Z]Za`a^ba`aaa`b`aaa`___a
@HWI-EAS443:3:82:296:1964#0/1
GGCCATAATCAAAAAGTACACATCAGGAAGACAAGAGAAG
+HWI-EAS443:3:82:296:1964#0/1
[^bbbb`babbbbbbbabb]bbabbbbbb__bbbbbbbbb
@HWI-EAS443:3:82:981:1289#0/1
GGCCATAATCAAAAAGTACACATCAGGAAGACAAGAGAAG
+HWI-EAS443:3:82:981:1289#0/1
`a_baaabaaa``aba_baa`aaaaaaaaaaabaa`a`aa
@HWI-EAS443:3:83:1678:1134#0/1
GGCCATAATCAAAAAGTACACATCAGGAAGACAAGAGAAG
+HWI-EAS443:3:83:1678:1134#0/1
`aR]`a^Ya```aaWaYb]aY_`^aWa_[a`_`¥a]a__a
@HWI-EAS443:3:84:37:592#0/1
CTTCTCTTGTCTTCCTGATGTGTACTTTTTGATTATGGCC
+HWI-EAS443:3:84:37:592#0/1
abaaabaaa_aaaaaa^Q^aY`XT]`a`a^`]`]S[a^`_

@HWI-EAS443:3:84:771:500#0/1
GCCATAATCAAAAAGTACACATCAGGAAGACAAGAGAAGA
+HWI-EAS443:3:84:771:500#0/1
a]baa[aa¥ZaaT_a`baa¥aXU_]aY_a_Za_`]`__Y]
@HWI-EAS443:3:85:327:695#0/1
GGCCATAATCAAAAAGTACACATCAGGAAGACAAGAGAAG
+HWI-EAS443:3:85:327:695#0/1
abaabaaba`[`[a_a^aaaaaaaaba]aaa¥a¥aaa_a`
@HWI-EAS443:3:85:446:1390#0/1
GCCATAATCAAAAAGTACACATCAGGAAGACAAGAGAAGA
+HWI-EAS443:3:85:446:1390#0/1
]`b`bbbb`aU^`aa[b]`aaabaa`aaaaaa]]Zaaaaa
@HWI-EAS443:3:85:592:611#0/1
TGGCCATAATCAAAAAGTACACATCAGGAAGACAAGAGAA
+HWI-EAS443:3:85:592:611#0/1
[`[^¥`_ORV_^b[¥WWW¥a_`bb`Yb^a¥a^W`a`X_Za
@HWI-EAS443:3:85:925:1737#0/1
CATAATCAAAAAGTACACATCAGGAAGACAAGAGAAGAAC
+HWI-EAS443:3:85:925:1737#0/1
a`^aX^_aL_U`_Naa`¥`aaaa_^_]_]baa]]Yaa``^
@HWI-EAS443:3:85:93:1148#0/1
GGCCATAATCAAAAAGTACACATCAGGAAGACAAGAGAAG
+HWI-EAS443:3:85:93:1148#0/1
[^aaWZababbaW¥¥_]_ZS`]]`]`bbaaZaaaa`aaa`
@HWI-EAS443:3:86:864:509#0/1
GCCATAATCAAAAAGTACACATCAGGAAGACAAGAGAAGA
+HWI-EAS443:3:86:864:509#0/1
a`__a`[aaa^aa_a^a`a_aa`a`a^aa`^__a]`YUZ¥
@HWI-EAS443:3:87:1509:372#0/1
CCATAATCAAAAAGTACACATCAGGAAGACAAGAGAAGAA
+HWI-EAS443:3:87:1509:372#0/1
aaaabbbababbaa`baaaba^¥aaa_aaababbabb^aa
@HWI-EAS443:3:88:1034:1241#0/1
GGCCATAATCAAAAAGTACACATCAGGAAGACAAGAGAAG
+HWI-EAS443:3:88:1034:1241#0/1
abab_Y_abbbb`ab]_aaabbaaaa`bbb`ab``aaaaa
@HWI-EAS443:3:88:218:771#0/1
GTTCTTCTCTTGTCTTCCTGATGTGTACTTTTTGATTATG
+HWI-EAS443:3:88:218:771#0/1
aaaaaaaaaaaaaaaaa`aa`a`_a^`a``aaaa_a_``^
@HWI-EAS443:3:88:411:973#0/1
CATAATCAAAAAGTACACATCAGGAAGACAAGAGAAGAAC
+HWI-EAS443:3:88:411:973#0/1
aba^X^abaa`ab`ababbb``b`bbbabbbbbaaababa
@HWI-EAS443:3:8:1287:325#0/1
TTCTTCTCTTGTCTTCCTGATGTGTACTTTTTGATTATGG
+HWI-EAS443:3:8:1287:325#0/1
]¥]^aZ^[^`aZH`¥RV^a_abT[WPW`Y``ZZZT`XTYB
@HWI-EAS443:3:8:399:1367#0/1
GGCCATAATCAAAAAGTACACATCAGGAAGACAAGAGAAG

```
+HWI-EAS443:3:8:399:1367#0/1
aaaaab^W`aa`¥^[^Va``aa`^aa¥Z]aa[_`___Za_
@HWI-EAS443:3:90:1043:1536#0/1
TTCTTCTCTTGTCTTCCTGATGTGTACTTTTTGATTATGG
+HWI-EAS443:3:90:1043:1536#0/1
_bbababaabb_`baaaa__ab`]]``]J]_a_^____UY
@HWI-EAS443:3:90:1136:1043#0/1
CTTCTCTTGTCTTCCTGATGTGTACTTTTTGATTATGGCC
+HWI-EAS443:3:90:1136:1043#0/1
abbbaabbb`baabaababbab^``babbbbabaaa¥aa_
@HWI-EAS443:3:90:1138:550#0/1
GCCATAATCAAAAAGTACACATCAGGAAGACAAGAGAAGA
+HWI-EAS443:3:90:1138:550#0/1
ab`abbabbaa`aaa]baaaaaaa`a_`aa_aa``aaa_a
@HWI-EAS443:3:92:1491:760#0/1
ATGGCCATAATCAAAAAGTACACATCAGGAAGACAAGAGA
+HWI-EAS443:3:92:1491:760#0/1
aa_``a_`a_Ya__a`aaMZ]_O_`^¥aa__^^_[Z_]U]
@HWI-EAS443:3:93:113:390#0/1
TTCTCTTGTCTTCCTGATGTGTACTTTTTGATTATGGCCA
+HWI-EAS443:3:93:113:390#0/1
aaba^a`[[`abaaa_X_aUa_aaaaaaa_`a__`]^_^^
@HWI-EAS443:3:98:469:1622#0/1
ATGGCCATAATCAAAAAGTACACATCAGGAAGACAAGAGA
+HWI-EAS443:3:98:469:1622#0/1
`aaba_b_]`__`aaaa^Taa``a____`^_``¥a_]___
@HWI-EAS443:3:98:787:1376#0/1
CATAATCAAAAAGTACACATCAGGAAGACAAGAGAAGAAC
+HWI-EAS443:3:98:787:1376#0/1
a_abababab`bbabbabbb_bbbbabbabbbbbbbbbb`
@HWI-EAS443:3:99:215:172#0/1
GCCATAATCAAAAAGTACACATCAGGAAGACAAGAGAAGA
+HWI-EAS443:3:99:215:172#0/1
abaa^¥Z^`baa`a]D]VVZ`^Y]ab`V``^`Xa]¥]`aa
@HWI-EAS443:3:9:1456:970#0/1
GTTCTTCTCTTGTCTTCCTGATGTGTACTTTTTGATTATG
+HWI-EAS443:3:9:1456:970#0/1
aabaabaaaaba`]aa[^aaa`a_`__]a`a`a``a`_a_
@HWI-EAS443:3:9:1472:1479#0/1
ATGGCCATAATCAAAAAGTACACATCAGGAAGACAAGAGA
+HWI-EAS443:3:9:1472:1479#0/1
aaaa__aa`_^Q]`aa_a[a_`^_`__a_a]a^V^_a_a^
@HWI-EAS443:3:9:407:170#0/1
ATGGCCATAATCAAAAAGTACACATCAGGAAGACAAGAGA
+HWI-EAS443:3:9:407:170#0/1
a`aaaaaaa`aaaaaa_aZa__Q__^]``^__¥T¥__]_^
@HWI-EAS443:3:84:602:1639#0/1
GGCCATGTACCATTCTGTTTTCATGCCTGATTAGTGGATT
+HWI-EAS443:3:84:602:1639#0/1
aab`[_¥Oaabba`^ab_aaaaaaaaaaaaaaaZaa_a`
```

```
@HWI-EAS443:3:14:56:978#0/1
AAGCAGGTCAAATATATTCAATATGGAGAGAAAAAGATGT
+HWI-EAS443:3:14:56:978#0/1
_a_bbaa[abbab`aaaa`a`aaab`a`]aaaa]¥aa`a[
@HWI-EAS443:3:10:1733:328#0/1
TAGGGCTACTGAATACATAATGAAGGGAGTGTACATAAAT
+HWI-EAS443:3:10:1733:328#0/1
aaba`bbbaabb_baQ`bbabbaa`]baa_baa_]aaab`
@HWI-EAS443:3:88:1390:1178#0/1
GGCCGTCTGAGTTCTTCAATGGTGGAACAGATNTTCATGA
+HWI-EAS443:3:88:1390:1178#0/1
ab_[b_U]^R`V```bba[bb`aaaW]]bb_¥D^bVbaab
@HWI-EAS443:3:89:270:1739#0/1
AGGGCCGTCTGAGTTCTTCAATGGTGGAACAGATCTTCAT
+HWI-EAS443:3:89:270:1739#0/1
^QOS¥^[S_XTY¥Y¥[_V^]]R]VTRSZ^UV[Z[__[[`S
@HWI-EAS443:3:47:1455:1552#0/1
TGGCCGTCTGAGTTCTTCAATGGTGGAACAGATCTTCATG
+HWI-EAS443:3:47:1455:1552#0/1
ababab_a`]_a_a``aa`a`a[Ua_`_N_]]`]`a¥``]
@HWI-EAS443:3:57:110:1099#0/1
GGCCGTCTGAGTTCTTCAATGGTGGAACAGATCTTCATGA
+HWI-EAS443:3:57:110:1099#0/1
aabba]abba_Ya_aaaVaa`bYaa`a^a_a_aaa``a__
@HWI-EAS443:3:60:1563:1950#0/1
GGCCGTCTGAGTTCTTCAATGGTGGAACAGATCTTCATGA
+HWI-EAS443:3:60:1563:1950#0/1
ab]``GYaaZ]P[¥Ya_Wa_¥TLSZP[U^^X¥RPW^ZYMU
@HWI-EAS443:3:30:1721:1269#0/1
CTCTGGAGCAAAACAAACGATGCTGGATCAGATCGAGTGA
+HWI-EAS443:3:30:1721:1269#0/1
ab`bbabbbbb`_bbbbXH^bbT`bba^L`N^HDY]aWaU
@HWI-EAS443:3:77:475:23#0/1
CAAGGTACTTTTTTGGACAGTATGGATAACAAATAGTAGC
+HWI-EAS443:3:77:475:23#0/1
aabbaW`bbbbbbbbbaabb_baba`a_P]`a^``aWaaU
@HWI-EAS443:3:38:1066:1410#0/1
AGAAATTCCAACCGGTCTCCTATATGAGCTGCTAGGGACA
+HWI-EAS443:3:38:1066:1410#0/1
aa``_a`aa_aW¥__]___¥_^_]^^^[^VWWU¥UUWWWU
@HWI-EAS443:3:9:304:395#0/1
GCCTGGGTTTTGCTTAATGCATCATGGTTCAACTCCTTCC
+HWI-EAS443:3:9:304:395#0/1
a]__aa_TX_¥`^^¥a`¥a_[^[L[^ZU][[RX¥_V^VU¥
@HWI-EAS443:3:82:1114:306#0/1
AGGTTCCAGGTTGTCCCTAAGTGCCTGGACAATGAGAAGC
+HWI-EAS443:3:82:1114:306#0/1
aZTJa`_a]aQXaZa_UaX¥FU¥`]_[I^^_`]a^a]__^
@HWI-EAS443:3:89:1181:726#0/1
TTCAAATACGGTAATGGTGTTTGGATAGGGAGAACTAAAA
```

```
+HWI-EAS443:3:89:1181:726#0/1
_aaaa`baabaP^a`^b]`_abaa^`_aaa`aaaa`_a^`
@HWI-EAS443:3:34:211:1230#0/1
ATTAAGCATCAATGAACTGAGCAACCTTGCAAAAGGAGAG
+HWI-EAS443:3:34:211:1230#0/1
```a__`__a`Ya`_``_^`_]¥^¥_^_^__]]]]X]^][
@HWI-EAS443:3:56:1089:30#0/1
GATATGGCCCAGCATTAAGCATCAATGAACTGAGCAACCT
+HWI-EAS443:3:56:1089:30#0/1
aaaa`aaa`a`b_aa`_`a[^aV__a_]_¥_`^`]^ZJO`
@HWI-EAS443:3:44:1087:1148#0/1
CGCCGCTTTTCATTTCTGCTCTGGAGGTAGTGAAGGCGTT
+HWI-EAS443:3:44:1087:1148#0/1
`a`]a[`b]___a[ZH^b][`Z¥¥Q`BBBBBBBBBBBBBB
@HWI-EAS443:3:48:1321:262#0/1
TGCAACCTCCATGGCCTCTGCTGCCTGTTCACTCGATCCA
+HWI-EAS443:3:48:1321:262#0/1
aa^a^¥^a]¥_a```_a]``¥^a¥¥``W^U_S¥U`W¥UUW
@HWI-EAS443:3:65:696:504#0/1
TGGGGTCTCTTCTCTTGTCTTCCTGATGTGTACTTTTTGA
+HWI-EAS443:3:65:696:504#0/1
abZM^`abaaaa`a`aa_aa`aaa`````a`]_aaaaa_^
@HWI-EAS443:3:3:669:919#0/1
GCGGGGATCTTCTCTTGTCTTCCTGATGTGTACTTTTGAT
+HWI-EAS443:3:3:669:919#0/1
a``Z]¥S```ZG^¥`]]¥`a^^^a`]__R]WXZ_]]_W_^
@HWI-EAS443:3:9:1403:1221#0/1
TGAGGGGTTGTTCTCTTGTCTTCCTGATGTGTACTTTTTG
+HWI-EAS443:3:9:1403:1221#0/1
abIY_b_RaI_X¥I]^]aWUbaPa^b`aaT_Y_Z_a_aa_
@HWI-EAS443:3:100:1516:1298#0/1
AAAAAGTACACATCAGGAAGACAAGAGAAGAACCAAGCAC
+HWI-EAS443:3:100:1516:1298#0/1
`¥]YH_^ZYXRXa[`b`aaaaQ_]b^__aUaSUBBBBBBB
@HWI-EAS443:3:12:374:1799#0/1
GGGGGTTTCTTCTCTTGTCTTCCTGATGTGTACTTTTTGA
+HWI-EAS443:3:12:374:1799#0/1
aaa`bY_a`aaaaba`_`a_`__a^__a_PL[V``]]`__
@HWI-EAS443:3:14:874:1591#0/1
GGGGGTTTCTTCTCTTGTCTTCCTGATGTGTACTTTTTGA
+HWI-EAS443:3:14:874:1591#0/1
_`aaWQ_^U[ZJS_a__YUa_`Z`[¥`WT_S_T¥¥UYWO]
@HWI-EAS443:3:36:106:1029#0/1
GGGGGTTTCTTCTCTTGTCTTCCTGATGTGTACTTTTTGA
+HWI-EAS443:3:36:106:1029#0/1
^`_`b]abbabaaaaababbaba¥ZY`a]`Z``a]a``VJ
@HWI-EAS443:3:51:602:962#0/1
AAAAAGTACACATCAGGAAGACAAGAGAAGAACCCGCACT
+HWI-EAS443:3:51:602:962#0/1
VW¥`bb^Y``[YUUP_Z^a^`b`¥RU¥RURZG]`BBBBBB
```
```

```
@HWI-EAS443:3:73:1140:835#0/1
GGCTCTTCTCTTGTCTTCCTGATGTGTACTTTTTGATTNT
+HWI-EAS443:3:73:1140:835#0/1
a_FW[aa[a_`aZW_`__R``]_a^_]_Za``_[Y¥`WDR
@HWI-EAS443:3:19:78:1476#0/1
CTGGGTTCTTCTNTTGTCTTCCTGATGTGTACTTTTTGAT
+HWI-EAS443:3:19:78:1476#0/1
aa¥[ZQaa^¥a[D[a`[]_¥aa]]_XaZaT____^_XN¥`
@HWI-EAS443:3:67:1667:1124#0/1
GGGCGGGGTTCTTCTCTTGTCTTCCTGATNTGTACTTTTT
+HWI-EAS443:3:67:1667:1124#0/1
a`ZQ`]baYaZ__N[Ua¥`UZ^WQQZJWVDU¥X^U_ZUV¥
@HWI-EAS443:3:12:861:302#0/1
CGCGGGGGTCTTCTCTTGTCTTCCTGATGTGTACTTTTTG
+HWI-EAS443:3:12:861:302#0/1
^bbaXZVGT¥_U__ZU¥_R_URaaa`PXaa]HG[`]GVZ^
@HWI-EAS443:3:35:113:1480#0/1
TGGGGCTCTTCTCTTGTCTTCCTGATGTGTACTTTTTGAT
+HWI-EAS443:3:35:113:1480#0/1
¥¥ZRXR`b]H¥U¥`^`Y`aba`[[ZMXT[LV^W¥WHVQ^Y
@HWI-EAS443:3:79:1280:276#0/1
AGGGGTTGTTCTCTTGTCTTCCTGATGTGTACTTTTTGAT
+HWI-EAS443:3:79:1280:276#0/1
`baabaaSZ^YV¥aZ__^^ba_V]N¥b¥b``]b[aaa]__
@HWI-EAS443:3:85:196:418#0/1
CGCGGGGCTCTTCTCTTGTCTTCCTGATGTGTACTTTTTG
+HWI-EAS443:3:85:196:418#0/1
aba_¥_aH^aZZR]b`aaU`a¥^a[]J_bY`Z_Z_`_a`H
@HWI-EAS443:3:10:593:2013#0/1
CGGGGTTCTTCTCTTGTCTTCCTGATGTGTACTTTTCGAT
+HWI-EAS443:3:10:593:2013#0/1
a_`bb`bbbbbbbabb`abbbabbbaa_b_aa`_aa¥`aa
@HWI-EAS443:3:16:586:1752#0/1
GCGTGGTTCTTCTCTTGTCTTCCTGATGTGTACTTTTTGA
+HWI-EAS443:3:16:586:1752#0/1
a`a`a_[_]aa¥`aaaaWa^_¥`_¥]WTTX__X`a[_`_`
@HWI-EAS443:3:25:508:1424#0/1
CGCGCGGGGTTCTTCTCTTGTCTTCCTGATGTGTACTTTN
+HWI-EAS443:3:25:508:1424#0/1
S_```a`IXJYa_¥`Va^_][]^^a]IMOZUFQQH`¥]WD
@HWI-EAS443:3:28:907:299#0/1
CGGGCTTCTCTTGTCTTCCTGATGTGTACTTTTTGATTAT
+HWI-EAS443:3:28:907:299#0/1
Z_a`a_baba``bZZabbab`ab]]aa`S`bb`_bbbaa¥
@HWI-EAS443:3:59:1254:464#0/1
GGCGGGGGTTCTTCTCTTGTCTTCCTGATGTGTACTTTTT
+HWI-EAS443:3:59:1254:464#0/1
abaa^_¥_¥_``a]````^]Ya``````_^WMZ^USV`^_
@HWI-EAS443:3:71:1584:1430#0/1
GGGCTCTTCTCTTGTCTTCCTGATGTGTACTTTTTGATTA
```

+HWI-EAS443:3:71:1584:1430#0/1
a¥M]a`b¥^[```Q`b]]__b`RUV^][^^aabXKY]Z
@HWI-EAS443:3:75:1733:798#0/1
CGGTGTTCTTCTCTTGTCTTCCTGATGTGTACTTTTTGAT
+HWI-EAS443:3:75:1733:798#0/1
I^a`¥bb`b_bbbaba`bbbabb`aba_``babbbba_ab
@HWI-EAS443:3:7:441:450#0/1
CGGGGTTCTTCTCTTGTCTTCCTGATGTGTACTTTTGATT
+HWI-EAS443:3:7:441:450#0/1
_babaRb`baab`abb¥[bb``]`abVV`aa_abbaa_bb
@HWI-EAS443:3:82:614:1796#0/1
GCGAGGTTCTTCTCTTGTCTTCCTGATGTGTACTTTTTGA
+HWI-EAS443:3:82:614:1796#0/1
ab`U_ZM¥ba]_]ba^]Za^Zab_U_`]P^LU`__aY_]]
@HWI-EAS443:3:88:783:1935#0/1
GGGTTCTTCTCTTGTCTTCCTGATGTGTACTTTTTGGTTA
+HWI-EAS443:3:88:783:1935#0/1
aa`Wbbbbbbbbbbabbbbb``abaa_`aaabbbba]a`b
@HWI-EAS443:3:89:1787:1287#0/1
GAAGAATCAAAAAGTACACATCAGGAAGACAAGAGAAGAA
+HWI-EAS443:3:89:1787:1287#0/1
[`bbbbbabbbabbT^ababbbba]a`baaaaba_aa]`a
@HWI-EAS443:3:94:865:354#0/1
GGGTGGGGTTCTTCTCTTGTCTTCCTGATGTGTACTTTTT
+HWI-EAS443:3:94:865:354#0/1
aaZG]UU_^a]]aW`_`^`^Za_YRYS]^aSWZ]X___^a
@HWI-EAS443:3:98:641:1645#0/1
CGGGGTTCTTCTCTTGTCTTCCTGATGTGTACTTTTAGAT
+HWI-EAS443:3:98:641:1645#0/1
abbKYU_^VIQUa^^_S_[U¥TQUXZ]Pa^`_``¥WK[_S
@HWI-EAS443:3:36:1398:1892#0/1
ACGGGGTTCTTCTCTTGTCTNCCTGATGTGTACTTTTTGA
+HWI-EAS443:3:36:1398:1892#0/1
aabaaZ]abaaaa^a`Saa¥DYa`Z_a^_^]_^aaaa^N_
@HWI-EAS443:3:56:370:1816#0/1
GGGGGTTCTNCTCTTGTCTTCCTGATGTGTACTTTTTGAT
+HWI-EAS443:3:56:370:1816#0/1
`a¥abT_^MDM¥]_UQXUaS`Y¥TT[[S[[WU¥W``^W[[
@HWI-EAS443:3:84:842:869#0/1
TCGGGGTTCTTCTCTTGTCNTCCTGATGTGTACTTTTTGA
+HWI-EAS443:3:84:842:869#0/1
abbbba]bbaaaa_ab__ZD]aaaa^_Y_a__aaaaaa_a
@HWI-EAS443:3:10:398:654#0/1
CGGGGTTCTTCTCTTGTCTTCCTGATGTGTACTATTTGAT
+HWI-EAS443:3:10:398:654#0/1
aabbaabbbbbbbbbbaabbabab`aa`b^aabaaabbab
@HWI-EAS443:3:11:937:1204#0/1
GGGGATCTTCTCTTGTCTTCCTGATGTGTACTTTTTGATT
+HWI-EAS443:3:11:937:1204#0/1
ab_U`aababa_aa`^`aaa__NY`a_`^_^`_a_aa^`a

```
@HWI-EAS443:3:12:1054:297#0/1
GGGTTCTTCTCTTGTCTTCCTGATGTGTTCTTTTTGATTA
+HWI-EAS443:3:12:1054:297#0/1
aaaSa`abaaa_a___aa`_aa^`a^a__]aaaa`Y_`^_
@HWI-EAS443:3:12:1421:2033#0/1
CGGGGTTCTTCTTTTGTCTTCCTGATGTGTACTTTTTGAT
+HWI-EAS443:3:12:1421:2033#0/1
abbbb^bbaa`[HWbaa_bba_abaaa_aX`_bbaaa`a_
@HWI-EAS443:3:14:655:950#0/1
CGGGGCTCTTCTCTTGTCTTCCTGATGTGTACTTTTTGAT
+HWI-EAS443:3:14:655:950#0/1
a^X`HDXW^]¥_^SVSU_¥`__[_Y][¥^BBBBBBBBBBB
@HWI-EAS443:3:15:1212:451#0/1
GGGGTTCTTCTCTTGTCTTCTTGATGTGTACTTTTTGATT
+HWI-EAS443:3:15:1212:451#0/1
abaaVa`aaXa¥b]`^_a`TF¥`X^¥Za]a__a_a_`aa`
@HWI-EAS443:3:18:1693:1491#0/1
AGCGGGGGTCTTCTCTTGTCTTCCTGATGTGTACTTTTTG
+HWI-EAS443:3:18:1693:1491#0/1
¥HVV__aR`^aY[ba`a_Z_^a_]a¥]_aO`^^]a___aU
@HWI-EAS443:3:1:676:867#0/1
GCGGGGCTCTTCTCTTGTCTTCCTGATGTGTACTTTTTGA
+HWI-EAS443:3:1:676:867#0/1
aYT]^XFY_`aY```]R]V]Y[Y`[^¥VG^WIDV]^^BBB
@HWI-EAS443:3:20:709:1401#0/1
GCGGGGGTCTTCTCTTGTCTTCCTGATGTGTACTTTTTGA
+HWI-EAS443:3:20:709:1401#0/1
Z_[Z_YLaaa[b``bb¥^¥STaUU`WJWQV_[R`U`¥`I`
@HWI-EAS443:3:21:562:1379#0/1
GGGTTCCTCTCTTGTCTTCCTGATGTGTACTTTTTGATTA
+HWI-EAS443:3:21:562:1379#0/1
a_`RY[a_a]a^W`H__`^a_a`]aHaW]]__`^__W¥W_
@HWI-EAS443:3:21:947:343#0/1
GGTTCTTCTCTTGTCTTCCTGATGTGTACTTTTTCATTAT
+HWI-EAS443:3:21:947:343#0/1
a_Ya^aa_a```a_]__`]_a^[`S_YYY_¥^][FV_]_^
@HWI-EAS443:3:22:1003:820#0/1
TCAAAAAGCACACATCAGGAAGACAAGAGAAGAACCCCGC
+HWI-EAS443:3:22:1003:820#0/1
VXX¥`X_XF]`aaZ¥YX^`aab[][Z_¥`_Y][^OR[[Z[
@HWI-EAS443:3:22:1315:568#0/1
GCGGGGTTCTTCTCTTGTCCTCCTGATGTGTACTTTTTGA
+HWI-EAS443:3:22:1315:568#0/1
a``aaa_a`a`[`_aaa_[¥Z_T__U¥[W[V]Z^YTY¥WZ
@HWI-EAS443:3:22:677:1051#0/1
GCGGGGTTCTTCTCTTGTCATCCTGATGTGTACTTTTTGA
+HWI-EAS443:3:22:677:1051#0/1
aaaa¥^[aa_a]aWaa`S^a__U`^__a]`X]^_^__^¥X
@HWI-EAS443:3:24:1251:367#0/1
CGGGGTTCTTCTCTTGTCTTCGTGATGTGTACTTTTTGAT
```

```
+HWI-EAS443:3:24:1251:367#0/1
¥QQ_YZW[]YW[`_Y^_Z_¥PN]YY_X_WWZT_aZ]ZZ[S
@HWI-EAS443:3:26:1372:1970#0/1
GGGGCTCTTCTCTTGTCTTCCTGATGTGTACTTTTTGATT
+HWI-EAS443:3:26:1372:1970#0/1
]RXaT¥[aa_[YPY^_aW¥I¥__]Q`X`ZZ[PZ]]_VZXB
@HWI-EAS443:3:29:238:1669#0/1
CGGGCTTCTTCTCTTGTCTTCCTGATGTGTACTTTTTGAT
+HWI-EAS443:3:29:238:1669#0/1
U_`TIPR^Z^_¥aZ¥¥``a¥ab_TVbXHWRGY_`[`¥H^B
@HWI-EAS443:3:32:1680:1857#0/1
CGGGGTCCTTCTCTTGTCTTCCTGATGTGTACTTTTTGAT
+HWI-EAS443:3:32:1680:1857#0/1
aa^a__HX``Y^]ZQbX]b`]ZV_Xb]^aMOab]_`a^aa
@HWI-EAS443:3:32:233:393#0/1
AGCGGGGTTCTTCTCTTGTCTTCCTCATGTGTACTTTTTG
+HWI-EAS443:3:32:233:393#0/1
^`abb_]`ab``a`aaab¥aaaaaYHHUUMNFT_^][^_]
@HWI-EAS443:3:33:1393:1924#0/1
ATCAAAAAGTACACATCAGGAAGACAAGAGAACAACCCCG
+HWI-EAS443:3:33:1393:1924#0/1
abaab`aaYD]aaaa^^aa]Y¥OR`a__a``TDT_WLHLV
@HWI-EAS443:3:33:501:1120#0/1
CGGGGTTCTTCTCTTGTCTTCCTAATGTGTACTTTTTGAT
+HWI-EAS443:3:33:501:1120#0/1
a```a[`_`aaa]a``^aa]aa^O^¥¥V_¥_^^_`^^P^^
@HWI-EAS443:3:34:686:211#0/1
GCGGGGTTGTTCTCTTGTCTTCCTGATGTGTACTTTTTGA
+HWI-EAS443:3:34:686:211#0/1
aaa]XbPZGMTa_YRR^RP¥WQXLUNW]]SM_YQTXXV[B
@HWI-EAS443:3:35:1539:259#0/1
GCGGGGGTCTTCTCTTGTCTTCCTGATGTGTACTTTTTGA
+HWI-EAS443:3:35:1539:259#0/1
a^a``[DY`XUYTP_Z_]V^UZ_[UWY`^aYXYZ]]Y[YZ
@HWI-EAS443:3:36:834:1302#0/1
CGGGGATCTTCTCTTGTCTTCCTGATGTGTACTTTTTGAT
+HWI-EAS443:3:36:834:1302#0/1
aaS]¥F]`]_``[`Ta`]P`[[Yaa]¥LaZ^U`¥Y`¥UV_
@HWI-EAS443:3:40:1229:1087#0/1
GGGGTTCTTCACTTGTCTTCCTGATGTGTACTTTTTGATT
+HWI-EAS443:3:40:1229:1087#0/1
a]__^^a_^`]X__aY]Y^`]WXPVYV¥V]TP¥^_VO^^Y
@HWI-EAS443:3:42:586:480#0/1
CGGGGCTCTTCTCTTGTCTTCCTGATGTGTACTTTTTGAT
+HWI-EAS443:3:42:586:480#0/1
ab_Y]JTa`aUQQ¥JaQTZWYaa¥[X^FTUHU^__a`[V[
@HWI-EAS443:3:4:1376:79#0/1
GCGGGGTTCTTCTCTTGTCTTCCTGATGGGTACTTTTTGA
+HWI-EAS443:3:4:1376:79#0/1
a_bab_[_abaabU`aYZ```¥_ab^a_H_]^Vaaaaa_`
```

@HWI-EAS443:3:51:732:1225#0/1
GCGGGGATCTTCTCTTGTCTTCCTGATGTGTACTTTTTGA
+HWI-EAS443:3:51:732:1225#0/1
]XX`_ZDY_ba[a_a]aa^a`¥S_¥O[]S¥RZZ`aba`Y¥
@HWI-EAS443:3:53:1348:1793#0/1
AGCGGGGTTCTTCTCTTGTCTTCCTGATGGGTACTTTTTG
+HWI-EAS443:3:53:1348:1793#0/1
`aUab[a_a[_[HRaba_[]aa[`[`a``G¥UaYaaa_a_
@HWI-EAS443:3:56:210:1523#0/1
GGTTCTTCTCTTGTCTTCCTGATGTGGACTTTTTGATTAT
+HWI-EAS443:3:56:210:1523#0/1
[VSaabbaaaba]^¥V`aaaaa`a`¥G`_aa`aba_aa`a
@HWI-EAS443:3:58:720:1244#0/1
GCGGGGGTCTTCTCTTGTCTTCCTGATGTGTACTTTTTGA
+HWI-EAS443:3:58:720:1244#0/1
]`U_a]IY_aS`¥¥a¥WQI`X]_ab_bb[[[K`ZZUNZTS
@HWI-EAS443:3:58:821:879#0/1
GGGTTCTTCTCTTGTCTTCCTGATGGGTACTTTTTGATTA
+HWI-EAS443:3:58:821:879#0/1
]OOFYVHWVYTPR]X__a]NHQSFYHTTPG`VV[`VN[^[
@HWI-EAS443:3:58:902:627#0/1
GCGGGGCTCTTCTCTTGTCTTCCTGATGTGTACTTTTTGA
+HWI-EAS443:3:58:902:627#0/1
aaaaa]Uaa]aaa_a``PXXZUXMOU]]VaSZTWaa_X[Z
@HWI-EAS443:3:59:1628:1735#0/1
CGGGGTTCTTCTCTTGTCTTCCTGATGAGTACTTTTTGAT
+HWI-EAS443:3:59:1628:1735#0/1
a_QW`US^ZO]Y¥]U]¥ZZYUYL]NFXRTUGRPX¥TR]MQ
@HWI-EAS443:3:5:601:979#0/1
GGGGTTCTTCTCTTGTCTTCCTGATGCGTACTTTTTGATT
+HWI-EAS443:3:5:601:979#0/1
abaa]a`aa_`_aa_`_`a_a^a^_`G`Z_W___```_^_
@HWI-EAS443:3:5:874:1435#0/1
GGGGTTCTTCTCTTGTCTTCCTGATGAGTACTTTTTGATT
+HWI-EAS443:3:5:874:1435#0/1
abbWR^^Y__baaaXMSS[a^ab[EVG[ZaG^_^X¥``YB
@HWI-EAS443:3:61:1394:161#0/1
CGGGGTTCTTCTCTAGTCTTCCTGATGTGTACTTTTTGAT
+HWI-EAS443:3:61:1394:161#0/1
abbbbababbbbbbababbbbbaaQab`b`a`aaaaa``a
@HWI-EAS443:3:62:313:1710#0/1
CGGGGTTCTTCTCTTGTCTTCCTGATGTGTGCTTTTTGAT
+HWI-EAS443:3:62:313:1710#0/1
abPQ¥`bbbbb```b]a`b_ba_TT^Waa``Xabbab]b_
@HWI-EAS443:3:63:1673:1903#0/1
ATCAAAAAGTACAGATCAGGAAGACAAGAGAAGAACCCCG
+HWI-EAS443:3:63:1673:1903#0/1
aaaab_aa]V`N`F¥aabba__baabaaaa¥ba__RaW`a
@HWI-EAS443:3:63:296:887#0/1
GGGGCTCTTCTCTTGTCTTCCTGATGTGTACTTTTTGATT

+HWI-EAS443:3:63:296:887#0/1
abaaJaa`VaaabW[U`abaaa_aa^L^a]Yaa`aa_¥_¥
@HWI-EAS443:3:64:85:1098#0/1
ATCAAAAAGTACACACCAGGAAGACAAGAGAAGAACCCCG
+HWI-EAS443:3:64:85:1098#0/1
abaSVVRUXT]_a^YF^[XY_TZY[][^[[^`]ZVZLRRY
@HWI-EAS443:3:67:131:1114#0/1
GCGGGGTTCTTCTCTTGTCTTCCTGCTGTGTACTTTTTGA
+HWI-EAS443:3:67:131:1114#0/1
`^^_¥¥M¥a`X`[`Y]_^]HZaa`]FW]Y]YT]_Z`_UW[
@HWI-EAS443:3:67:1472:885#0/1
AGCGGGGTTCTTCTCTTATCTTCCTGATGTGTACTTTTTG
+HWI-EAS443:3:67:1472:885#0/1
aa[ab`aG__`aWaSa_^aZ``^`a`Z___aZ¥W`aX`__
@HWI-EAS443:3:68:878:819#0/1
GGGGCTCTTCTCTTGTCTTCCTGATGTGTACTTTTTGATT
+HWI-EAS443:3:68:878:819#0/1
^Z_¥G_a]V`¥][YPOZ¥[¥TZRTPXNaM[[_[__`VZ_¥
@HWI-EAS443:3:70:614:584#0/1
CGGGGTTCTTCTCTTGTCTTCCTGATGTGTACATTTTGAT
+HWI-EAS443:3:70:614:584#0/1
abbbaYbababaaaababaaaaaaaaaZa_aZL]a``¥`a
@HWI-EAS443:3:71:1699:889#0/1
CGGGGTTCTTCTCTTGTATTCCTGATGTGTACTTTTTGAT
+HWI-EAS443:3:71:1699:889#0/1
TQ_a¥PY_^]N]Z__[YHRPZ[O]]X]F[GWY¥^W]]Q[M
@HWI-EAS443:3:72:854:707#0/1
CGGGGTTCTTCTCTTGTCTTCCTGATATGTACTTTTTGAT
+HWI-EAS443:3:72:854:707#0/1
`bbbb^¥¥`b¥bbbb`aaab_bba]`aaaVaa`a`a`Yaa
@HWI-EAS443:3:73:689:1627#0/1
CGGGGTTCTTCTCTTGTCTTCCTGATGAGTACTTTTTGAT
+HWI-EAS443:3:73:689:1627#0/1
Z¥baaWV]bWbWa¥LX__bZZb``bb_I¥Paaa_J¥aaa_
@HWI-EAS443:3:78:1472:44#0/1
AGCGGGGTTCTTCTCTTGTCTTACTGATGTGTACTTTTTG
+HWI-EAS443:3:78:1472:44#0/1
a_SW`abaa_b_Z¥V``b^Z[VFW__[ZNSaP_]____^a
@HWI-EAS443:3:78:90:1421#0/1
GGGGTTCTTCACTTGTCTTCCTGATGTGTACTTTTTGATT
+HWI-EAS443:3:78:90:1421#0/1
aa¥QS[^X]¥T_^¥_O¥`aab_XPT¥^^WVW[^[[^_[UR
@HWI-EAS443:3:7:1565:340#0/1
GCGGGGTTCTTCTCTTGTGTTCCTGATGTGTACTTTTTGA
+HWI-EAS443:3:7:1565:340#0/1
aZOX^UI¥_W__`KKXZRHLZJMMIM^ZTXVOPUTQP¥BB
@HWI-EAS443:3:83:853:1257#0/1
GGGTTCTTCTCTTGTCTTCCTGATGAGTACTTTTTGATTA
+HWI-EAS443:3:83:853:1257#0/1
Ya_NUM`IU`a]SaZ[SYWR`a^¥UIZWXL[bY^aBBBBB

```
@HWI-EAS443:3:87:1419:1168#0/1
TTCCTGATGTGTACTTTTTGATTATTGCCATATGGGCCAC
+HWI-EAS443:3:87:1419:1168#0/1
^TQ[U`Y^`RMDU]_X_^]XWZ`aZDT[WUFS¥Q^NQRNO
@HWI-EAS443:3:88:1615:2038#0/1
CGGGGTTCTTCTCTTGTCTTCCTGATGGGTACTTTTTGAT
+HWI-EAS443:3:88:1615:2038#0/1
`bba[NN[b`Z]]a`]¥`^^YYX[¥Z]G`ZMN[aaa]_[]
@HWI-EAS443:3:8:1463:1640#0/1
GGGTTCTTCTCTTGTCTTCGTGATGTGTACTTTTTGATTA
+HWI-EAS443:3:8:1463:1640#0/1
X_¥H[a_S]YUYX_[V]^YWWVU[UOaURRZ]XZ]__YBB
@HWI-EAS443:3:90:353:1637#0/1
CGGGGTTCCTCTCTTGTCTTCCTGATGTGTACTTTTTGAT
+HWI-EAS443:3:90:353:1637#0/1
a[bab`bbbbb_bbbaabbbbbaaaa`Za_abaaaaaaaa
@HWI-EAS443:3:91:240:406#0/1
CGGGGATCTTCTCTTGTCTTCCTGATGTGTACTTTTTGAT
+HWI-EAS443:3:91:240:406#0/1
aa_^¥G`bb^aa¥XW]V`^^_Z]_W]^TUQTX¥]]`aa¥`
@HWI-EAS443:3:92:1468:1354#0/1
GGGGTTCTTCTCTTGTCTTCCTGATGTGCACTTTTTGATT
+HWI-EAS443:3:92:1468:1354#0/1
abaa_bbbaSbaba`ba`bWY^aa]`ab_a[bbabbaaba
@HWI-EAS443:3:94:1207:1816#0/1
CGGGGGTCTTCTCTTGTCTTCCTGATGTGTACTTTTTGAT
+HWI-EAS443:3:94:1207:1816#0/1
`bba[D¥abaababbba¥b`babb]ab^b_a^abaaaaaa
@HWI-EAS443:3:94:509:1278#0/1
CGGGGGTCTTCTCTTGTCTTCCTGATGTGTACTTTTTGAT
+HWI-EAS443:3:94:509:1278#0/1
aa^OZL_YY`ZZVT[PMUTW`QZ[OT_FZVGUPPa^RRO^
@HWI-EAS443:3:96:1181:1720#0/1
CGGGGGTCTTCTCTTGTCTTCCTGATGTGTACTTTTTGAT
+HWI-EAS443:3:96:1181:1720#0/1
ab]`aGaaaa_a_b__VZa`_[a_¥_`W`YaRa__^__]a
@HWI-EAS443:3:97:1085:257#0/1
GCGGGGTTCTTCTCTTGTCTTCCTGCTGTGTACTTTTTGA
+HWI-EAS443:3:97:1085:257#0/1
a_]a`aSK[aa__^a`a_VQaOW[HDT_Xa]V]`_^_OJQ
@HWI-EAS443:3:98:339:229#0/1
CGGGGTTCTTCTCTTGTCCTCCTGATGTGTACTTTTTGAT
+HWI-EAS443:3:98:339:229#0/1
ababb_babbbbbbaba^Ia]abaabb_b_a_abbaa_ab
@HWI-EAS443:3:17:1315:1879#0/1
TGTGGGGTTCTTCTCTTGTCTTCCTGATGTGTACTTTTTG
+HWI-EAS443:3:17:1315:1879#0/1
ab____a_aaaaaa`aaa`_ba`^a_a`a^_^_Xa__aa¥
@HWI-EAS443:3:19:1264:127#0/1
TGTGGGGTTCTTCTCTTGTCTTCCTGATGTGTACTTTTTG
```

```
+HWI-EAS443:3:19:1264:127#0/1
aa`aaaa]`_aa_a_`aa^_```]a`_`_^_Z]W``_U]^
@HWI-EAS443:3:24:1213:870#0/1
CGGGCGGGGTTCTTCTCTTGTCTTCCTGATGTGTACTTTT
+HWI-EAS443:3:24:1213:870#0/1
abbabb`]baa^ba_a`baa_aabaabb``b`]VZaaaaa
@HWI-EAS443:3:28:786:1354#0/1
GGCTTCTTCTCTTGTCTTCCTGATGTGTACTTTTTGATTA
+HWI-EAS443:3:28:786:1354#0/1
`RFY_a_^WZ¥`ZZU__]]]`^M¥_]_Z^_]QW__^][Y]
@HWI-EAS443:3:32:641:76#0/1
CGGGGTTCTTCTCTTGTCTTCCTGATGTGTACTTTTTATG
+HWI-EAS443:3:32:641:76#0/1
ababbabbbbabaa``b]b`X[aba`a`aaa¥aaab_```
@HWI-EAS443:3:36:673:955#0/1
CGTGGGGTTCTTCTCTTGTCTTCCTGATGTGTACTTTTTG
+HWI-EAS443:3:36:673:955#0/1
`b_bbb`abbabb`aaaa]a`aaaaaTZaVaY__`a__aR
@HWI-EAS443:3:37:760:595#0/1
CTCGGTTCTTCTCTTGTCTTCCTGATGTGTACTTTTTGAT
+HWI-EAS443:3:37:760:595#0/1
a]H^__bbbaaaaa`aaaabaaa`aa^[aY__`aaaa``a
@HWI-EAS443:3:40:980:573#0/1
TGCGCGGGGTTCTTCTCTTGTCTTCCTGATGTGTACTTTT
+HWI-EAS443:3:40:980:573#0/1
Maab]bU]`baP_a]_aabbUa_b``__^bVYb`aI_bbb
@HWI-EAS443:3:5:879:842#0/1
CGCGCGGGGTTCTTCTCTTGTCTTCCTGATGTGTACTTTT
+HWI-EAS443:3:5:879:842#0/1
abbb`bbba_aaaa`b`babaaaa`_ba`ab_b`a¥b`ab
@HWI-EAS443:3:61:950:1101#0/1
CGGGGTTCTTCTCTTGTCTTCCTGATGTGTACTTTTTATG
+HWI-EAS443:3:61:950:1101#0/1
aabb_Y_bba__ab`bb_bbbabb[abaaaaaaaabaaaa
@HWI-EAS443:3:71:123:445#0/1
CTCGCGGGGTTCTTCTCTTGTCTTCCTGATGTGTACTTTT
+HWI-EAS443:3:71:123:445#0/1
aaaaaV]UWQ[_^`YN__`_]_Z__`]VTR]W¥YNTSLO^
@HWI-EAS443:3:79:1304:405#0/1
GCGGGGTTCTTCTCTTGTCTTCCTGATGTGTACTTTTGAT
+HWI-EAS443:3:79:1304:405#0/1
a¥^`a_WWZ_[`aZ`^]G¥`[UU`NEPXG_OJZa]UXSVX
@HWI-EAS443:3:99:456:1508#0/1
CACCGGGGTTCTTCTCTTGTCTTCCTGATGTGTACTTTTT
+HWI-EAS443:3:99:456:1508#0/1
`¥`ab_`_Ya_Y``^aa_b_a`^[]``[`a^`P¥_a_aUa
@HWI-EAS443:3:17:1083:1892#0/1
CGGGGNTCTTCTCTTGTCTTCCTGATGTGTACTTTTTGAT
+HWI-EAS443:3:17:1083:1892#0/1
a`^^¥D[bb¥_``aZb_a`aaaaaa_`_aZ_]``_aa`aa
```

```
@HWI-EAS443:3:17:1321:702#0/1
CGGGGTTCTTCTCTTGTCTTCCTGATGTGTACTNTTTGAT
+HWI-EAS443:3:17:1321:702#0/1
abba``abbbababb``_aa``a]_a__a^aa^D^aaV``
@HWI-EAS443:3:17:1622:175#0/1
CGGGGTTCTTCTCTTGTCTTCCTGATGTGTACTTTNTGAT
+HWI-EAS443:3:17:1622:175#0/1
ab`a[_a_U_Z¥U]aa_^`aVW¥MS^`YaX]W^`XD^NZ^
@HWI-EAS443:3:25:41:1847#0/1
GCGGGGTTCTTCTCTTNTCTTCCTGATGTGTACTTTTTGA
+HWI-EAS443:3:25:41:1847#0/1
a`ba`_Uaaaaaaaa[DR_a_aa_Z[ZNZXV^a_Y¥a[R[
@HWI-EAS443:3:28:799:56#0/1
CGGGGTTCTTCTCTTGTCTTCCTGATGTGTACTTTTNGAT
+HWI-EAS443:3:28:799:56#0/1
]bbbbS[Vaa_aa`aa`_a`]`]b__¥RZRX]aaa^DMYa
@HWI-EAS443:3:3:858:1029#0/1
CGGGGTTCTTCTCTTGTCTTCCTGATGNGTACTTTTTGAT
+HWI-EAS443:3:3:858:1029#0/1
aaabb_bbbbbbabbbbbbbbabb`a]D]_a`bbbaaaaa
@HWI-EAS443:3:40:1252:526#0/1
CGGGGTTCTTCTCTTGTCTTCCTGATNTGTACTTTTTGAT
+HWI-EAS443:3:40:1252:526#0/1
ababa`abbbabZbb`aab`aa`^][DU`_^`a`^aaZ]`
@HWI-EAS443:3:48:1208:1027#0/1
CGGGGNTCTTCTCTTGTCTTCCTGATGTGTACTTTTTGAT
+HWI-EAS443:3:48:1208:1027#0/1
abbb¥D^bbbab¥bba_`ba_aba_aa^a`__aa_aa]`_
@HWI-EAS443:3:52:1125:860#0/1
GCGGGGTTCTTCTCTTGTNTTCCTGATGTGTACTTTTTGA
+HWI-EAS443:3:52:1125:860#0/1
aaabba_bbaaab_aa_^D¥a__a`_aa^a^_^aabaa[_
@HWI-EAS443:3:53:872:1368#0/1
AGCGGGNTTCTTCTCTTGTCTTCCTGATGTGTACTTTTTG
+HWI-EAS443:3:53:872:1368#0/1
abaab]DZbaaa`baaaaa^baaaaaaab_b^`_abaaaX
@HWI-EAS443:3:55:575:1220#0/1
GGGGTTCTTCNCTTGTCTTCCTGATGTGTACTTTTTGATT
+HWI-EAS443:3:55:575:1220#0/1
aaaa[aaaaYD[aaa_`aa`^aa`a`^_^^^aa_a_T[__
@HWI-EAS443:3:63:260:1246#0/1
CGGGGTTNTTCTCTTGTCTTCCTGATGTGTACTTTTTGAT
+HWI-EAS443:3:63:260:1246#0/1
``_ab`¥DW^_`^]]Z¥b`aZ[Z]S]QF]__X]ba`aRZ_
@HWI-EAS443:3:65:499:1487#0/1
CGGGGTTCTTCTCTTGTCTTCCTGATGTGTNCTTTTTGAT
+HWI-EAS443:3:65:499:1487#0/1
`]HZ`^b`aV¥_]_TXX_]_aW]YUR_YRFDY_`aab`^a
@HWI-EAS443:3:77:569:1561#0/1
GGGGTTNTTCTCTTGTCTTCCTGATGTGTACTTTTTGATT
```

+HWI-EAS443:3:77:569:1561#0/1
a`a`]¥D[abaaaba_baa`b`a[a]Wa_a`baa``_aaa
@HWI-EAS443:3:82:1588:696#0/1
CGGGGTTCTTCTCTTGTCTTCCTGATGTGTACTTTNTGAT
+HWI-EAS443:3:82:1588:696#0/1
¥[[_baabbbbbab``Zabbabb¥__QXb^b^bb]D[^a`
@HWI-EAS443:3:89:62:179#0/1
CGGGGTTCTTCTCTTGTCTTCCTGATGNGTACTTTTTGAT
+HWI-EAS443:3:89:62:179#0/1
a`aba`aaaaaaaaa`aabaaaba[aWDY___a`aaa__a
@HWI-EAS443:3:91:1519:1227#0/1
GCGGGGTTCTTCTCTTGTCTTCCTGATGNGTACTTTTTGA
+HWI-EAS443:3:91:1519:1227#0/1
aZa[W]GY^^^R[_^]YRHT`ZP[^]TDDJTRWZU]SSBB
@HWI-EAS443:3:93:1353:658#0/1
GCGGGGNTCTTCTCTTGTCTTCCTGATGTGTACTTTTTGA
+HWI-EAS443:3:93:1353:658#0/1
``baaYD¥abbababba``abaaaaaaa`a_a_aaa_a`a
@HWI-EAS443:3:16:1704:560#0/1
GCGCGGGGTTCTTCTCTTGTCTTCCTGATGTGTACTTTTT
+HWI-EAS443:3:16:1704:560#0/1
a_PY^`a_X¥`PNVPVQTVGXNSPP¥QHY[URMHDHTBBB
@HWI-EAS443:3:16:710:585#0/1
CAGGGGTTCTTCTCTTGTCTTCCTGATGTGTACTTTTTGA
+HWI-EAS443:3:16:710:585#0/1
a`abba¥__bbbaaaaa_aaa]``^`aa]a_^^`a```_`
@HWI-EAS443:3:22:925:1231#0/1
ACGGGTTCTTCTCTTGTCTTCCTGATGTGTACTTTTTGAT
+HWI-EAS443:3:22:925:1231#0/1
a_a`aVaaa]a¥`^aaaaaa_``]_^^UaURUa``_aZ^`
@HWI-EAS443:3:23:911:1722#0/1
GTGGGGTTCTTCTCTTGTCTTCCTGATGTGTACTTTTTGA
+HWI-EAS443:3:23:911:1722#0/1
WBBBBBBBBBBBBBBBBBBBBBBBBBBBBBBBBBBBBBBB
@HWI-EAS443:3:24:354:190#0/1
GAGGTTCTTCTCTTGTCTTCCTGATGTGTACTTTTTGATT
+HWI-EAS443:3:24:354:190#0/1
aaaa_aaaaabaa``Zaaaa_______aZ__``aa___`^
@HWI-EAS443:3:24:967:1590#0/1
CAAAAAGTACACATCAGGAAGACAAGAGAAGAACCCCGNA
+HWI-EAS443:3:24:967:1590#0/1
aaba]aa^b^abaa`abaabbaabba`bbaab_]NXa[D¥
@HWI-EAS443:3:30:1135:150#0/1
GTGGGGTTCTTCTCTTGTCTTCCTGATGTGTACTTTTTGA
+HWI-EAS443:3:30:1135:150#0/1
a_aa`_Za_baaa_aaaa_^`ZX`__``^_^_Sa```]Z_
@HWI-EAS443:3:31:125:701#0/1
GTGGGGTTCTTCTCTTGTCTTCCTGATGTGTACTTTTTGA
+HWI-EAS443:3:31:125:701#0/1
a^aaaaY_aa```a_¥]X]`_aa_XNY_P_Y__ZX_Z]LZ

```
@HWI-EAS443:3:32:378:1045#0/1
GCGCGGGGTTCTTCTCTTGTCTTCCTGATGTGTACTTTTT
+HWI-EAS443:3:32:378:1045#0/1
`babbaaa[aaaaaaaaaa]aaaa]````a]a___a^`__
@HWI-EAS443:3:34:1185:696#0/1
GGGCGGGGTTCTTCTCTTGTCTTCCTGATGTGTACTTTTT
+HWI-EAS443:3:34:1185:696#0/1
^bbabbbbab`bbbbabbb`abbaabaaaa`aaaZa`aa`
@HWI-EAS443:3:38:1565:1619#0/1
TTCTTCTCTTGTCTTCCTGATGTGTACTTTTTGATTATCG
+HWI-EAS443:3:38:1565:1619#0/1
¥[^_bb^bbaaPDW]Z¥`^P[aXaT^a``Wa[Y[U]`WFH
@HWI-EAS443:3:3:491:1407#0/1
GTGGGGTTCTTCTCTTGTCTTCCTGATGTGTACTTTTTGA
+HWI-EAS443:3:3:491:1407#0/1
a``^aa_aaaaaaabaaa`aaaaa_`a`^aYVY^_``a¥^
@HWI-EAS443:3:43:1594:547#0/1
GCGGGGTTCTTCTCTTGTCTTCCTGATGTGTACTTTTTAA
+HWI-EAS443:3:43:1594:547#0/1
aabbbb]aaba`aabaaa`bba`a`]_a_a_`_aabaaa_
@HWI-EAS443:3:46:892:348#0/1
GACGGGGTTCTTCTCTTGTCTTCCTGATGTGTACTTTTTG
+HWI-EAS443:3:46:892:348#0/1
aa`aaa]Q]a``__^a_]¥``a__[[X¥]S_WY¥_``_¥R
@HWI-EAS443:3:47:388:188#0/1
CCGCGGGGTTCTTCTCTTGTCTTCCTGATGTGTACTTTTT
+HWI-EAS443:3:47:388:188#0/1
abb`bbbaV_aab`aaaba_^aa__aa]a____`]aaaa¥
@HWI-EAS443:3:56:410:1529#0/1
TGGCGGGGTTCTTCTCTTGTCTTCCTGATGTGTACTTTTT
+HWI-EAS443:3:56:410:1529#0/1
Xbaa_a]]DYZKaX_[aaRYYPY]__WSX]X¥JSZa`a`a
@HWI-EAS443:3:56:842:985#0/1
GCGCGGGGTTCTTCTCTTGTCTTCCTGATGTGTACTTTTT
+HWI-EAS443:3:56:842:985#0/1
a^Q[_WQ^V_``SFZ__`_QZ[^YX_UQY_N[N[][YVZZ
@HWI-EAS443:3:59:145:1317#0/1
CAAAAAGTACACATCAGGAAGACAAGAGAAGAACCCCGTC
+HWI-EAS443:3:59:145:1317#0/1
a_V_b`[^b¥baabb`aVZK¥aab^^^b```SaYMG]UZ]
@HWI-EAS443:3:59:1476:58#0/1
CGGGGTTCTTCTCTTGTCTTCCTGATGTGTACTTTTTGGT
+HWI-EAS443:3:59:1476:58#0/1
`bbbbabbbbbbbbbbabbbbbbbbbabb`Ubbbaabaa
@HWI-EAS443:3:61:1042:1641#0/1
GCGCGGGGTTCTTCTCTTGTCTTCCTGATGTGTACTTTTT
+HWI-EAS443:3:61:1042:1641#0/1
`_`JNV_[W¥L[[U¥T`Y_T^__]U]WLPYW[TWY¥GU¥U
@HWI-EAS443:3:63:1144:360#0/1
GTGGGGTTCTTCTCTTGTCTTCCTGATGTGTACTTTTTGA
```

+HWI-EAS443:3:63:1144:360#0/1
aSWRO^QYZQZV^U[^^NXXZNTZ`¥V_RZQNLW__^Z_X
@HWI-EAS443:3:65:1055:1313#0/1
TCGCGGGGTTCTTCTCTTGTCTTCCTGATGTGTACTTTTT
+HWI-EAS443:3:65:1055:1313#0/1
a_WU`aV¥^`¥Ia`a`aaa_`a]MZYPW^UR__a``__YW
@HWI-EAS443:3:65:1108:533#0/1
GCGGGTTCTTCTCTTGTCTTCCTGATGTGTACTTTTTGAT
+HWI-EAS443:3:65:1108:533#0/1
aa_R_F]a]]aaa_]aVY^^W_a`ZU_N]VYGZ`___HV_
@HWI-EAS443:3:66:1254:889#0/1
GCCGGGGTTCTTCTCTTGTCTTCCTGATGTGTACTTTTTG
+HWI-EAS443:3:66:1254:889#0/1
^¥_Z^_`¥_X¥baa]b`[Y^`a[a^XZ_bZ`ZZ[`¥]U_¥
@HWI-EAS443:3:66:513:1155#0/1
TACGGGGTTCTTCTCTTGTCTTCCTGATGTGTACTTTTTG
+HWI-EAS443:3:66:513:1155#0/1
^aaZ]^XDY]```Z_[Q_¥^]aa]aa[`aZ_Z`_`a]_¥_
@HWI-EAS443:3:67:1626:830#0/1
CTGGGGTTCTTCTCTTGTCTTCCTGATGTGTACTTTTTGA
+HWI-EAS443:3:67:1626:830#0/1
ab_``^abbababababbabbbabbbbbb]`]aabbbbaa`
@HWI-EAS443:3:69:711:756#0/1
GTGGGGTTCTTCTCTTGTCTTCCTGATGTGTACTTTTTGA
+HWI-EAS443:3:69:711:756#0/1
`X^a`^¥aaaaaa`aaa_`aaaaa`ZT^¥^X¥_`_a__[_
@HWI-EAS443:3:75:607:798#0/1
TGGCGGGGTTCTTCTCTTGTCTTCCTGATGTGTACTTTTT
+HWI-EAS443:3:75:607:798#0/1
a_^`aa_[¥_X`WORR__^WVZ^`XUMRPYNVX[VU^^_`
@HWI-EAS443:3:81:1489:262#0/1
CTGGGTTCTTCTCTTGTCTTCCTGATGTGTACTTTTTGAT
+HWI-EAS443:3:81:1489:262#0/1
ab`][_`abb`bXbbab_`b__`]]¥U_b_a_b_`aba^b
@HWI-EAS443:3:85:352:611#0/1
GTGGGGTTCTTCTCTTGTCTTCCTGATGTGTACTTTTTGA
+HWI-EAS443:3:85:352:611#0/1
a]_`XV^_[_Z`X]]V`ZX]UYW^]]ZTR]UWYTRVWWUW
@HWI-EAS443:3:8:1009:1576#0/1
GACGGGGTTCTTCTCTTGTCTTCCTGATGTGTACTTTTTG
+HWI-EAS443:3:8:1009:1576#0/1
aXa_aV_RaaaaU]¥`^`PT^ZWP[TW__X`SYW¥^a]W]
@HWI-EAS443:3:93:102:63#0/1
CGGGGGTTCTTCTCTTGTCTTCCTGATGTGTACTTTTTGA
+HWI-EAS443:3:93:102:63#0/1
abbbaaabaaa]]Zaaba_aaaaaa`ab_a^WSZ`aaaa_
@HWI-EAS443:3:95:80:1962#0/1
GTGGGGTTCTTCTCTTGTCTTCCTGATGTGTACTTTTTGA
+HWI-EAS443:3:95:80:1962#0/1
aYaaaaT_`aaaaaaaa^ba``aa___`]]NW]]_```Z_

```
@HWI-EAS443:3:99:732:1520#0/1
GTGGGGTTCTTCTCTTGTCTTCCTGATGTGTACTTTTTGA
+HWI-EAS443:3:99:732:1520#0/1
``baa`^a`_a_a]`b``_aaa`a]_`W]__^_`aaa`_^
@HWI-EAS443:3:9:314:220#0/1
CAGGGGTTCTTCTCTTGTCTTCCTGATGTGTACTTTTTGA
+HWI-EAS443:3:9:314:220#0/1
a_bbab^babababbb`aa`abbb]aaZ_``a]`aaaa_`a_
@HWI-EAS443:3:100:1402:1692#0/1
AGGGGTTCTTCTCTTGTCTTCCTGATGTGTACTTTTTGAT
+HWI-EAS443:3:100:1402:1692#0/1
aaJab¥bTa__a``_a_L``_Saa_^aQ[[__`aSU[P_a
@HWI-EAS443:3:100:1611:908#0/1
TGGGGTTCTTCTCTTGTCTTCCTGATGTGTACTTTTTGAT
+HWI-EAS443:3:100:1611:908#0/1
abaaJZb¥`aa_aa__US[aOGa_`]V]NMYJWW_`WNBB
@HWI-EAS443:3:10:1520:176#0/1
TGGGGTTCTTCTCTTGTCTTCCTGATGTGTACTTTTTGAT
+HWI-EAS443:3:10:1520:176#0/1
a_`_^V_X__]_Z_^]YZ`^]Z]]ZX^Z_ZZZ^^Z^^ZZZ
@HWI-EAS443:3:10:420:475#0/1
TCGGGGTTCTTCTCTTGTCTTCCTGATGTGTACTTTTTGA
+HWI-EAS443:3:10:420:475#0/1
a^aa]]L]a`aa`a`a`LL]a^^]^`_`Za`[_aa_a___
@HWI-EAS443:3:11:751:581#0/1
CGCGGGGTTCTTCTCTTGTCTTCCTGATGTGTACTTTTTG
+HWI-EAS443:3:11:751:581#0/1
ababbba_babaaaabaa``aaaaaaaaa_a^`_aaa_aa
@HWI-EAS443:3:12:1243:996#0/1
TGGGGTTCTTCTCTTGTCTTCCTGATGTGTACTTTTTGAT
+HWI-EAS443:3:12:1243:996#0/1
abbaa`a`aa`aW`aaX_aa_¥`a[_a]a]^^__a^`___
@HWI-EAS443:3:13:1211:1463#0/1
CGCGGGGTTCTTCTCTTGTCTTCCTGATGTGTACTTTTTG
+HWI-EAS443:3:13:1211:1463#0/1
abaaa]a`aabaaa``bba`aa¥[baaaa_b``_a`aaaa
@HWI-EAS443:3:13:1302:825#0/1
GGCGGGGTTCTTCTCTTGTCTTCCTGATGTGTACTTTTTG
+HWI-EAS443:3:13:1302:825#0/1
U^_bba_Na]a^Z^^¥`[_Z¥aZ¥^]`aaab^aaaaaaaL
@HWI-EAS443:3:14:1319:343#0/1
GGGGGTTCTTCTCTTGTCTTCCTGATGTGTACTTTTTGAT
+HWI-EAS443:3:14:1319:343#0/1
aabba]a_ab`a`aaa_aaa`[aa_aa`a`__aaaaa`_a
@HWI-EAS443:3:14:346:1220#0/1
AGGGGTTCTTCTCTTGTCTTCCTGATGTGTACTTTTTGAT
+HWI-EAS443:3:14:346:1220#0/1
`abaa_babbaaaaba`aaa`aaaaaa___aaaaaaaW_a
@HWI-EAS443:3:15:877:1779#0/1
CGCGGGGTTCTTCTCTTGTCTTCCTGATGTGTACTTTTTG
```

```
+HWI-EAS443:3:15:877:1779#0/1
abbabbbabbbbaaabbaaaaaabbaaaa`a_aaabaaa`
@HWI-EAS443:3:16:1053:1233#0/1
TGGGGTTCTTCTCTTGTCTTCCTGATGTGTACTTTTTGAT
+HWI-EAS443:3:16:1053:1233#0/1
a`_`[Yababaaabaa``aaaaaaa``_a_]_aa_aa__a
@HWI-EAS443:3:17:162:1254#0/1
CGCGGGGTTCTTCTCTTGTCTTCCTGATGTGTACTTTTTG
+HWI-EAS443:3:17:162:1254#0/1
aa_^a``Gbaa_aa`a_`[`aa_^aa¥`^T^W]_a```_^
@HWI-EAS443:3:17:1726:259#0/1
TGGGGTTCTTCTCTTGTCTTCCTGATGTGTACTTTTTGAT
+HWI-EAS443:3:17:1726:259#0/1
X^TbaSbbbaUa`abaa^aa__[^[_a_^R`[a__`]Z_a
@HWI-EAS443:3:18:1375:1249#0/1
GGGGGTTCTTCTCTTGTCTTCCTGATGTGTACTTTTTGAT
+HWI-EAS443:3:18:1375:1249#0/1
aaaa_Ua^aa_``aa___aa]__`]`^Z`]^Z_____[W^
@HWI-EAS443:3:18:1657:1042#0/1
ACGGGGTTCTTCTCTTGTCTTCCTGATGTGTACTTTTTGA
+HWI-EAS443:3:18:1657:1042#0/1
^Y¥_W[Ja_`_`baa^P]aaa]UUXD¥YYSQY]aaa^^LW
@HWI-EAS443:3:18:322:1716#0/1
TGGGGTTCTTCTCTTGTCTTCCTGATGTGTACTTTTTGAT
+HWI-EAS443:3:18:322:1716#0/1
a_a`YQaba]aaaa_`Ibaa_aQPP_^¥¥¥UY`__¥][ZT
@HWI-EAS443:3:18:576:1493#0/1
CCGGGGTTCTTCTCTTGTCTTCCTGATGTGTACTTTTTGA
+HWI-EAS443:3:18:576:1493#0/1
a^[W^_LX`_`_a_¥YV]a`^[][RZ]^UXW_T^`[^XRZ
@HWI-EAS443:3:18:67:402#0/1
GGCGGGGTTCTTCTCTTGTCTTCCTGATGTGTACTTTTTG
+HWI-EAS443:3:18:67:402#0/1
a_aaa_aH__`a__^_a^_]_`_^[[]^_V^V]Y^`_]_S
@HWI-EAS443:3:1:1616:314#0/1
CGCGGGGTTCTTCTCTTGTCTTCCTGATGTGTACTTTTTG
+HWI-EAS443:3:1:1616:314#0/1
VbbV^]Y]b_Yaa`Yaaa`Ha_S_aaVa`^_]¥Xaa_BBB
@HWI-EAS443:3:1:913:1304#0/1
ACGGGGTTCTTCTCTTGTCTTCCTGATGTGTACTTTTTGA
+HWI-EAS443:3:1:913:1304#0/1
`_a`b¥G¥WaU_`U¥a^VW_VPMYa_YMS_XWW_]XBBBB
@HWI-EAS443:3:21:1086:1460#0/1
TGGGGTTCTTCTCTTGTCTTCCTGATGTGTACTTTTTGAT
+HWI-EAS443:3:21:1086:1460#0/1
aaaa`R_PV_`a_`YX[aaa__``[¥GU_Z^_a___ZYU^
@HWI-EAS443:3:21:265:1155#0/1
CGCGGGGTTCTTCTCTTGTCTTCCTGATGTGTACTTTTTG
+HWI-EAS443:3:21:265:1155#0/1
aa```a`]aWaaa_^aa`_aaa`X`[Y^a¥_W_aa^a¥`_
```

```
@HWI-EAS443:3:22:976:452#0/1
TGGGGTTCTTCTCTTGTCTTCCTGATGTGTACTTTTTGAT
+HWI-EAS443:3:22:976:452#0/1
aabaaTa__a^a`aa``aaaXM`a__a]aZU^_a^_`a_^
@HWI-EAS443:3:24:1281:164#0/1
GGCGGGGTTCTTCTCTTGTCTTCCTGATGTGTACTTTTTG
+HWI-EAS443:3:24:1281:164#0/1
H_bababbb_aba`Xa``¥b`bab[babb]bbbbabbb`b
@HWI-EAS443:3:25:1180:1598#0/1
AGGGGTTCTTCTCTTGTCTTCCTGATGTGTACTTTTTGAT
+HWI-EAS443:3:25:1180:1598#0/1
_^_]__babbbbaaba^``a]`aaaa``a_`Waa_a_[]`
@HWI-EAS443:3:26:1096:826#0/1
ACGGGGTTCTTCTCTTGTCTTCCTGATGTGTACTTTTTGA
+HWI-EAS443:3:26:1096:826#0/1
a`babb_a`bbababaa`aaaa`aa_aa__``]aaaaa]`
@HWI-EAS443:3:27:423:281#0/1
ACGGGGTTCTTCTCTTGTCTTCCTGATGTGTACTTTTTGA
+HWI-EAS443:3:27:423:281#0/1
aabbbbaabbaaa^aa`aaabaaaa`aa_`__`aa_a`_a
@HWI-EAS443:3:27:701:611#0/1
GGCGGGGTTCTTCTCTTGTCTTCCTGATGTGTACTTTTTG
+HWI-EAS443:3:27:701:611#0/1
aabbbab]`aab_aaabaaaab_a`¥_`_T`^a``aabbU
@HWI-EAS443:3:28:1577:1473#0/1
TGGGGTTCTTCTCTTGTCTTCCTGATGTGTACTTTTTGAT
+HWI-EAS443:3:28:1577:1473#0/1
aa[¥PQP_^]Z]XZZW_QW¥QNa_HS_OUN_L`Y^Xaaa]
@HWI-EAS443:3:28:938:270#0/1
TGGGGTTCTTCTCTTGTCTTCCTGATGTGTACTTTTTGAT
+HWI-EAS443:3:28:938:270#0/1
abaaaYbaaaaa`aaa_`aa_]a^[_`^¥___a`a``U]`
@HWI-EAS443:3:2:196:562#0/1
CGCGGGGTTCTTCTCTTGTCTTCCTGATGTGTACTTTTTG
+HWI-EAS443:3:2:196:562#0/1
`b_aa^]__a`aI`U^_¥¥¥`Y`¥X_Y^a]^Z^U^UW^¥`
@HWI-EAS443:3:2:623:1346#0/1
CGCGGGGTTCTTCTCTTGTCTTCCTGATGTGTACTTTTTG
+HWI-EAS443:3:2:623:1346#0/1
a[P_¥YLQZ¥[a[_`ZY^¥`_a¥R``[[bYUGQG`Z]¥a]
@HWI-EAS443:3:30:1173:298#0/1
TCGGGGTTCTTCTCTTGTCTTCCTGATGTGTACTTTTTGA
+HWI-EAS443:3:30:1173:298#0/1
`aba^^_`aaba`VZabY`_a¥_aa____aW`^a`____]
@HWI-EAS443:3:30:505:1236#0/1
TGGGGTTCTTCTCTTGTCTTCCTGATGTGTACTTTTTGAT
+HWI-EAS443:3:30:505:1236#0/1
S_TV_V¥^¥¥¥^^bab]_b_a`^^[W_`Ua^b`^abbb_G
@HWI-EAS443:3:32:956:233#0/1
TGGGTTCTTCTCTTGTCTTCCTGATGTGTACTTTTTGATT
```

+HWI-EAS443:3:32:956:233#0/1
^`b]_]¥aa]aa`aa_aa`_`ZSP_aZ_]`^`_```a__^
@HWI-EAS443:3:34:1371:92#0/1
CGCGGGGTTCTTCTCTTGTCTTCCTGATGTGTACTTTTTG
+HWI-EAS443:3:34:1371:92#0/1
``_ababbbbbbab``bbbaba`abb`ab]ba`_aaYa_a
@HWI-EAS443:3:34:495:1580#0/1
CGCGGGGTTCTTCTCTTGTCTTCCTGATGTGTACTTTTTG
+HWI-EAS443:3:34:495:1580#0/1
`]aa]][Q]aZW`_`]][N^YYTXSPRQWLVQMYZ[^XZV
@HWI-EAS443:3:36:187:1436#0/1
CGCGGGGTTCTTCTCTTGTCTTCCTGATGTGTACTTTTTG
+HWI-EAS443:3:36:187:1436#0/1
aabaaab]abaaaaaaa¥_baabaaaS[^^```aaa`_a_
@HWI-EAS443:3:37:162:1976#0/1
TGGGGTTCTTCTCTTGTCTTCCTGATGTGTACTTTTTGAT
+HWI-EAS443:3:37:162:1976#0/1
[]F[XUba]`baaaZ¥¥a`abba_^X_aXKLY¥]]`]J]a
@HWI-EAS443:3:37:217:191#0/1
TGGGGTTCTTCTCTTGTCTTCCTGATGTGTACTTTTTGAT
+HWI-EAS443:3:37:217:191#0/1
ab^Ya¥baaabaaaaa[aaa`a`]R[a_[GV^__``^VUZ
@HWI-EAS443:3:37:236:825#0/1
TGGGGTTCTTCTCTTGTCTTCCTGATGTGTACTTTTTGAT
+HWI-EAS443:3:37:236:825#0/1
a^aXaJabaa`b``a`Waa``aa_``a_XWP^_`__a_W_
@HWI-EAS443:3:38:1357:1328#0/1
TCGGGGTTCTTCTCTTGTCTTCCTGATGTGTACTTTTTGA
+HWI-EAS443:3:38:1357:1328#0/1
`bb]V_R[]aba¥`^^¥_aaa```a^`WY[Y[]_```_a]
@HWI-EAS443:3:39:834:1575#0/1
GGCGGGGTTCTTCTCTTGTCTTCCTGATGTGTACTTTTTG
+HWI-EAS443:3:39:834:1575#0/1
a^aaa_aYaa]aa_aaba`^_b^_`YRYY^¥VZ^_a`a_J
@HWI-EAS443:3:3:345:321#0/1
GGCGGGGTTCTTCTCTTGTCTTCCTGATGTGTACTTTTTG
+HWI-EAS443:3:3:345:321#0/1
a^Y^baa]^Z``[]]T_aYY]_]`]_V^_X_YYGX^^Y_Q
@HWI-EAS443:3:3:773:469#0/1
CGGGTTCTTCTCTTGTCTTCCTGATGTGTACTTTTTGATT
+HWI-EAS443:3:3:773:469#0/1
UaabaaU_a_b¥^a`¥Tabaaaa`[a]b_^_b_aa^b[`a
@HWI-EAS443:3:3:884:610#0/1
GGGGGTTCTTCTCTTGTCTTCCTGATGTGTACTTTTTGAT
+HWI-EAS443:3:3:884:610#0/1
ababb_bYaaaba¥Z¥Sa_`^aa][]`a^T^¥_Ya]aa^¥
@HWI-EAS443:3:40:1487:1665#0/1
CGCGGGGTTCTTCTCTTGTCTTCCTGATGTGTACTTTTTG
+HWI-EAS443:3:40:1487:1665#0/1
aabbbaaabbbaababba`aabaaa`U`a`]_aaa_a_aa

```
@HWI-EAS443:3:43:1132:711#0/1
GGCGGGGTTCTTCTCTTGTCTTCCTGATGTGTACTTTTTG
+HWI-EAS443:3:43:1132:711#0/1
aaaaaabU_^`a][_aa``[_a____[U`_TV]]]]`_^T
@HWI-EAS443:3:43:11:1285#0/1
CGCGGGGTTCTTCTCTTGTCTTCCTGATGTGTACTTTTTG
+HWI-EAS443:3:43:11:1285#0/1
abab`ab_bbbbaaaabaaabaaaaaa`__`^¥^`aaaa`
@HWI-EAS443:3:43:72:1217#0/1
ACGGGGTTCTTCTCTTGTCTTCCTGATGTGTACTTTTTGA
+HWI-EAS443:3:43:72:1217#0/1
`ab```^bbbbbbbbbbaabaaa]U_abab_aaaabaa``
@HWI-EAS443:3:43:848:841#0/1
ACGGGGTTCTTCTCTTGTCTTCCTGATGTGTACTTTTTGA
+HWI-EAS443:3:43:848:841#0/1
aba`aa^aaaaaaaabb__aa_aaa``a_a__^aaaaa`^
@HWI-EAS443:3:45:1380:930#0/1
TCGGGGTTCTTCTCTTGTCTTCCTGATGTGTACTTTTTGA
+HWI-EAS443:3:45:1380:930#0/1
a``aab¥aaaaa][_ab[`_YY¥`^R¥Y¥]V[]``_^a_]
@HWI-EAS443:3:45:736:524#0/1
CAAAAAGTACACATCAGGAAGACAAGAGAAGAACCCCGCG
+HWI-EAS443:3:45:736:524#0/1
aabaaaaR`b`]a``_bbaa`_a^]`abaabaa]a[`a]O
@HWI-EAS443:3:46:611:1639#0/1
CGCGGGGTTCTTCTCTTGTCTTCCTGATGTGTACTTTTTG
+HWI-EAS443:3:46:611:1639#0/1
Q`[`_^`L[bbb]b]][¥[``bb]RN[a``_PZ]bbb``a
@HWI-EAS443:3:46:835:299#0/1
GGGGGTTCTTCTCTTGTCTTCCTGATGTGTACTTTTTGAT
+HWI-EAS443:3:46:835:299#0/1
a`ba`]aa_aX`_`a`^`a_`_a_[__^_V^^a_a``_¥]
@HWI-EAS443:3:48:1037:1731#0/1
CCGGGGTTCTTCTCTTGTCTTCCTGATGTGTACTTTTTGA
+HWI-EAS443:3:48:1037:1731#0/1
G[S_XYXbbb_PO_b]bb_a_ZW¥aYa¥Y_XVX]Y`b^[Z
@HWI-EAS443:3:48:1071:1714#0/1
GGCGGGGTTCTTCTCTTGTCTTCCTGATGTGTACTTTTTG
+HWI-EAS443:3:48:1071:1714#0/1
``_a___Z^[__]]^`_¥_X¥_`]]¥VY`ZZNXX[`^a[Q
@HWI-EAS443:3:48:316:1932#0/1
AGGGGTTCTTCTCTTGTCTTCCTGATGTGTACTTTTTGAT
+HWI-EAS443:3:48:316:1932#0/1
_`_b_aaabaa``b^_a`baa_a_`aVWT`]¥`a^_`SZ_
@HWI-EAS443:3:49:298:1890#0/1
AGGGGTTCTTCTCTTGTCTTCCTGATGTGTACTTTTTGAT
+HWI-EAS443:3:49:298:1890#0/1
_a`a^Ta]Y]`a]aa^Sa^_[aaZ`]a_aW^Oa__`¥PZa
@HWI-EAS443:3:4:1515:769#0/1
TGGGGTTCTTCTCTTGTCTTCCTGATGTGTACTTTTTGAT
```

```
+HWI-EAS443:3:4:1515:769#0/1
a^^``Za__`]_T`_`]_`^PR^UW^_X`XZZ^_]_]^]B
@HWI-EAS443:3:50:1661:1234#0/1
CAGCGGGGTTCTTCTCTTGTCTTCCTGATGTGTACTTTTT
+HWI-EAS443:3:50:1661:1234#0/1
a_`abaZ_]`U_```abab`Yaa_X`Z[a`__[^_``aaa
@HWI-EAS443:3:50:1689:359#0/1
ACGGGGTTCTTCTCTTGTCTTCCTGATGTGTACTTTTTGA
+HWI-EAS443:3:50:1689:359#0/1
aabbbaQa_a^Va`bbbaab_T¥aa`]^TbY`Zbabab¥Z
@HWI-EAS443:3:50:866:1462#0/1
GGCGGGGTTCTTCTCTTGTCTTCCTGATGTGTACTTTTTG
+HWI-EAS443:3:50:866:1462#0/1
a`^_a]^PWV¥UXZT_¥WRVYVQV¥XU]WFZQRRZRZ^ZB
@HWI-EAS443:3:53:730:1031#0/1
CGCGGGGTTCTTCTCTTGTCTTCCTGATGTGTACTTTTTG
+HWI-EAS443:3:53:730:1031#0/1
`bababb`baababababab``bba`b`aa]_a]``baaaa`
@HWI-EAS443:3:54:201:831#0/1
AGGGGTTCTTCTCTTGTCTTCCTGATGTGTACTTTTTGAT
+HWI-EAS443:3:54:201:831#0/1
abbbb`baabbaababaaaba`aa`a]^b`__aaa``]Xa
@HWI-EAS443:3:54:292:1684#0/1
TGGGGTTCTTCTCTTGTCTTCCTGATGTGTACTTTTTGAT
+HWI-EAS443:3:54:292:1684#0/1
Xa__bZ`bb`]aab`¥`babb¥a]^aWHWXa`bbbaaQ[b
@HWI-EAS443:3:54:332:1446#0/1
TGGGGTTCTTCTCTTGTCTTCCTGATGTGTACTTTTTGAT
+HWI-EAS443:3:54:332:1446#0/1
]]YV]P]_bb[aa`aaYaaa`a]YTZbY^XPTa¥^___S`
@HWI-EAS443:3:54:649:1966#0/1
ACGGGGTTCTTCTCTTGTCTTCCTGATGTGTACTTTTTGA
+HWI-EAS443:3:54:649:1966#0/1
aa`^__Ua_abUaZa`__``aWT]H]_¥`W__Zaaa``W_
@HWI-EAS443:3:54:664:425#0/1
CGCGGGGTTCTTCTCTTGTCTTCCTGATGTGTACTTTTTG
+HWI-EAS443:3:54:664:425#0/1
_^aaaaXN_W`Y[_Z¥```]ab]^¥^`b`Z_XSM^ba^[R
@HWI-EAS443:3:55:551:541#0/1
TGGGGTTCTTCTCTTGTCTTCCTGATGTGTACTTTTTGAT
+HWI-EAS443:3:55:551:541#0/1
Y_V¥bUUa``_IW`aY[_Y``WXYZ[_Q_¥_bbabaa¥X¥
@HWI-EAS443:3:56:1587:1547#0/1
TGGGGTTCTTCTCTTGTCTTCCTGATGTGTACTTTTTGAT
+HWI-EAS443:3:56:1587:1547#0/1
aa`a`]a`ba_bW_aaaaaa¥O¥XYaX¥a`^WaaaaaT_a
@HWI-EAS443:3:56:892:443#0/1
TGGGGTTCTTCTCTTGTCTTCCTGATGTGTACTTTTTGAT
+HWI-EAS443:3:56:892:443#0/1
aab`aZa`aa`aaaa__aa`_¥QYV[`_aZ^¥_a_a_[^_
```

```
@HWI-EAS443:3:57:1128:953#0/1
AGGGGTTCTTCTCTTGTCTTCCTGATGTGTACTTTTTGAT
+HWI-EAS443:3:57:1128:953#0/1
¥`a`bVbbbbabbbba_Zbaabbabbba`abbbbbba`ab
@HWI-EAS443:3:59:1410:69#0/1
TGGGGTTCTTCTCTTGTCTTCCTGATGTGTACTTTTTGAT
+HWI-EAS443:3:59:1410:69#0/1
ab`^`_a¥a`_a^aaa_Za_ZWaa]`a]a_VHZ^`ZXZTZ
@HWI-EAS443:3:59:827:637#0/1
TGGGGTTCTTCTCTTGTCTTCCTGATGTGTACTTTTTGAT
+HWI-EAS443:3:59:827:637#0/1
abbba^aaaaaaaaaa`aaa``aaZ`a^a^_Waaa^Y¥¥_
@HWI-EAS443:3:59:948:897#0/1
TGGGGTTCTTCTCTTGTCTTCCTGATGTGTACTTTTTGAT
+HWI-EAS443:3:59:948:897#0/1
aaa_a_a^a_`aaaaa_Za`aaaa_``_a^WVaaaYWVZ]
@HWI-EAS443:3:60:227:1062#0/1
TGGGGTTCTTCTCTTGTCTTCCTGATGTGTACTTTTTGAT
+HWI-EAS443:3:60:227:1062#0/1
aba[aSaaaaaa`a]^U`aaaa]`[a`[[]``____`[¥_
@HWI-EAS443:3:60:461:647#0/1
GGCGGGGTTCTTCTCTTGTCTTCCTGATGTGTACTTTTTG
+HWI-EAS443:3:60:461:647#0/1
a]`aaa^P_[]a^¥^_]MS`Y`_]UXNUVT_RQTY]__ZP
@HWI-EAS443:3:61:339:107#0/1
TGGGGTTCTTCTCTTGTCTTCCTGATGTGTACTTTTTGAT
+HWI-EAS443:3:61:339:107#0/1
aaaa`Z`_a```__`a^__``___W]aZaZWZ^_^^^ZZ]
@HWI-EAS443:3:62:601:895#0/1
GGCGGGGTTCTTCTCTTGTCTTCCTGATGTGTACTTTTTG
+HWI-EAS443:3:62:601:895#0/1
_`Ya`S`G_ZPU_`_RYSSXHXRXTBBBBBBBBBBBBBBB
@HWI-EAS443:3:63:1307:252#0/1
GGGGGTTCTTCTCTTGTCTTCCTGATGTGTACTTTTTGAT
+HWI-EAS443:3:63:1307:252#0/1
a^YZ`NJ¥]]]XNY]aU```¥VZ`Z`¥Z^VUS[__`^[`^
@HWI-EAS443:3:63:1475:432#0/1
GGCGGGGTTCTTCTCTTGTCTTCCTGATGTGTACTTTTTG
+HWI-EAS443:3:63:1475:432#0/1
]ab¥bb^Jbb[b_aaaaaa_aaaa`b_aaXYM^Y`aaaa]
@HWI-EAS443:3:63:169:41#0/1
AGGGGTTCTTCTCTTGTCTTCCTGATGTGTACTTTTTGAT
+HWI-EAS443:3:63:169:41#0/1
abaaa¥aaba`a`aba_baa```a_aa`a]_]`aaaaPY_
@HWI-EAS443:3:63:730:748#0/1
GGCGGGGTTCTTCTCTTGTCTTCCTGATGTGTACTTTTTG
+HWI-EAS443:3:63:730:748#0/1
aYbaaa_R`aab^aa`a¥_`aaaaaa`¥Y__[[____a_R
@HWI-EAS443:3:64:23:498#0/1
TGGGGTTCTTCTCTTGTCTTCCTGATGTGTACTTTTTGAT
```

```
+HWI-EAS443:3:64:23:498#0/1
^^b`¥Zbbb__bba_¥¥baaaaa_Uaa_b`XaaaaaaaXa
@HWI-EAS443:3:64:44:2037#0/1
GGCGGGGTTCTTCTCTTGTCTTCCTGATGTGTACTTTTTG
+HWI-EAS443:3:64:44:2037#0/1
aab^Y^]V``X^aa`^`Y_`W`aa_XXOLV_ZWV¥Z`^¥H
@HWI-EAS443:3:65:1394:1567#0/1
TGGGGTTCTTCTCTTGTCTTCCTGATGTGTACTTTTTGAT
+HWI-EAS443:3:65:1394:1567#0/1
Y[b^LNab``]`^^^¥_aa^¥[^aaa`[a_[_aaaa`_W`
@HWI-EAS443:3:65:673:1681#0/1
CGCGGGGTTCTTCTCTTGTCTTCCTGATGTGTACTTTTTG
+HWI-EAS443:3:65:673:1681#0/1
aaaYbaaU[`a`¥VYaab^b[`]LYUa`]WV[^Z_^a^¥V
@HWI-EAS443:3:65:944:1384#0/1
CGGGTTCTTCTCTTGTCTTCCTGATGTGTACTTTTTGATT
+HWI-EAS443:3:65:944:1384#0/1
a`YXYbbababababba_baa`aa`[`a`aa]`baaa`O_aa
@HWI-EAS443:3:66:1387:1220#0/1
CGCGGGGTTCTTCTCTTGTCTTCCTGATGTGTACTTTTTG
+HWI-EAS443:3:66:1387:1220#0/1
¥¥``^`b^`bbb^babbbaaa]a¥`a_ab`b`¥V``baa]
@HWI-EAS443:3:66:553:595#0/1
GGCGGGGTTCTTCTCTTGTCTTCCTGATGTGTACTTTTTG
+HWI-EAS443:3:66:553:595#0/1
aabbaaaPa`Yaaa`a`Q¥^``Y^¥V¥T`PZ]^Z_`_a^P
@HWI-EAS443:3:66:627:1334#0/1
CGGGTTCTTCTCTTGTCTTCCTGATGTGTACTTTTTGATT
+HWI-EAS443:3:66:627:1334#0/1
]V__GUa_^aR^ZXNO_XPYT¥RNOOLRPDWTPWIOZXXB
@HWI-EAS443:3:67:1622:733#0/1
CGCGGGGTTCTTCTCTTGTCTTCCTGATGTGTACTTTTTG
+HWI-EAS443:3:67:1622:733#0/1
abaabbaababaab_bbba^bbaaababb^b``_bbbaba
@HWI-EAS443:3:67:443:1315#0/1
AGGGGTTCTTCTCTTGTCTTCCTGATGTGTACTTTTTGAT
+HWI-EAS443:3:67:443:1315#0/1
W_bbb`bbbbbbabbabbabb`^_aa`aZaaaaaabTa`
@HWI-EAS443:3:67:885:844#0/1
GGGGGTTCTTCTCTTGTCTTCCTGATGTGTACTTTTTGAT
+HWI-EAS443:3:67:885:844#0/1
aa_]RT`ab`a`baaa_aa`aaa¥^``^a_aaaa_a`aaa
@HWI-EAS443:3:68:105:1428#0/1
CGCGGGGTTCTTCTCTTGTCTTCCTGATGTGTACTTTTTG
+HWI-EAS443:3:68:105:1428#0/1
abbbaaa[abb`baaab[`aaa`aa`_aa]`^aaaaaa`X
@HWI-EAS443:3:69:1025:253#0/1
GGGGGTTCTTCTCTTGTCTTCCTGATGTGTACTTTTTGAT
+HWI-EAS443:3:69:1025:253#0/1
_Nab^XabbaaabaTSaaZTQ_XTWSR`YTQGVSYZWSWZ
```

```
@HWI-EAS443:3:69:1394:400#0/1
AGGGGTTCTTCTCTTGTCTTCCTGATGTGTACTTTTTGAT
+HWI-EAS443:3:69:1394:400#0/1
abab_N_bbbb`aaaaaaaba`aa[aa`a]__aaa`b`_a
@HWI-EAS443:3:69:1395:1725#0/1
AGGGGTTCTTCTCTTGTCTTCCTGATGTGTACTTTTTGAT
+HWI-EAS443:3:69:1395:1725#0/1
Zabb``a^a`ba_]¥_Y[a`Ya_a^¥_Ya[a^aaa`_[[]
@HWI-EAS443:3:69:1773:1972#0/1
AGGGGTTCTTCTCTTGTCTTCCTGATGTGTACTTTTTGAT
+HWI-EAS443:3:69:1773:1972#0/1
^``b_abbbbbabb]WYaaaaaaaaa[^a_a^aaaaa^aa
@HWI-EAS443:3:72:887:1102#0/1
CGCGGGGTTCTTCTCTTGTCTTCCTGATGTGTACTTTTTG
+HWI-EAS443:3:72:887:1102#0/1
aba]Za]S`_¥X_`aa¥]¥``aX^[X^`aU`Ua_^¥U_XZ
@HWI-EAS443:3:73:793:2035#0/1
CGGGTTCTTCTCTTGTCTTCCTGATGTGTACTTTTTGATT
+HWI-EAS443:3:73:793:2035#0/1
a`_[KZaaaa¥__^a¥^___]_YJ¥^O_Y[]^__^^__^Z
@HWI-EAS443:3:74:907:624#0/1
TCGGGGTTCTTCTCTTGTCTTCCTGATGTGTACTTTTTGA
+HWI-EAS443:3:74:907:624#0/1
Y¥¥YUTF¥^aZV_[`W`Z[^YTYaVHY¥¥VGSP]a[Y^QZ
@HWI-EAS443:3:76:401:708#0/1
TGGGGTTCTTCTCTTGTCTTCCTGATGTGTACTTTTTGAT
+HWI-EAS443:3:76:401:708#0/1
a_XHXR_]VL^R^]]XR]]W]QX^ZOMGPVVQNTTZ]U]]
@HWI-EAS443:3:76:49:830#0/1
AGGGGTTCTTCTCTTGTCTTCCTGATGTGTACTTTTTGAT
+HWI-EAS443:3:76:49:830#0/1
abaab`bbbbbbbabb`bbabbaaaaa_a_a`aaaaaWa`
@HWI-EAS443:3:76:641:1904#0/1
TGGGGTTCTTCTCTTGTCTTCCTGATGTGTACTTTTTGAT
+HWI-EAS443:3:76:641:1904#0/1
_]a[RY]``aa``__]]a__a_`^_UV[Z¥W^__^__]`_
@HWI-EAS443:3:76:901:93#0/1
CGCGGGGTTCTTCTCTTGTCTTCCTGATGTGTACTTTTTG
+HWI-EAS443:3:76:901:93#0/1
`b`aabbaaaabaaaWbba_ba`_`a_aa``^_]abaab`
@HWI-EAS443:3:77:705:791#0/1
GCGGGGTTCTTCTCTTGTCTTCCTGATGTGTACTTTTTGT
+HWI-EAS443:3:77:705:791#0/1
`^`WI]GQ_I_YaSG]`G^a_RY[[R]_`aUU]^_[]BBB
@HWI-EAS443:3:79:220:1032#0/1
ACGGGGTTCTTCTCTTGTCTTCCTGATGTGTACTTTTTGA
+HWI-EAS443:3:79:220:1032#0/1
``_]_bV]aaa``aa_a^aa`aa__W_aZ`Y``a```_^a
@HWI-EAS443:3:79:945:1078#0/1
AGGGGTTCTTCTCTTGTCTTCCTGATGTGTACTTTTTGAT
```

+HWI-EAS443:3:79:945:1078#0/1
V__¥`Yabb¥_^__``_bb¥X^abbaOY]`aX¥_`a_¥¥a
@HWI-EAS443:3:83:177:581#0/1
CGCGGGGTTCTTCTCTTGTCTTCCTGATGTGTACTTTTTG
+HWI-EAS443:3:83:177:581#0/1
a[`a¥¥aO^^`]_aY^a`]_____`VNN^Z[U_YV[]U_^
@HWI-EAS443:3:83:242:607#0/1
TGGGGTTCTTCTCTTGTCTTCCTGATGTGTACTTTTTGAT
+HWI-EAS443:3:83:242:607#0/1
babY`b`a`a_[_a]`YVaa_]V]a^a_Q]``^^___`
@HWI-EAS443:3:84:227:1165#0/1
ACGGGGTTCTTCTCTTGTCTTCCTGATGTGTACTTTTTGA
+HWI-EAS443:3:84:227:1165#0/1
aaaa_aYZaab_aaa`a]a_aa_KP]``Z^Wa^]___]]_
@HWI-EAS443:3:85:359:1109#0/1
ACGGGGTTCTTCTCTTGTCTTCCTGATGTGTACTTTTTGA
+HWI-EAS443:3:85:359:1109#0/1
[a`ZT]Y`_¥^aaba¥V[aa`ZW_]Z_YPWWQ^``a`[T[
@HWI-EAS443:3:86:71:587#0/1
TGGGGTTCTTCTCTTGTCTTCCTGATGTGTACTTTTTGAT
+HWI-EAS443:3:86:71:587#0/1
`bbb]Vaa`aaa]a_Z[aa]_a]WY_a^aZ[Z_```a_Z_
@HWI-EAS443:3:87:119:1238#0/1
TGGGGTTCTTCTCTTGTCTTCCTGATGTGTACTTTTTGAT
+HWI-EAS443:3:87:119:1238#0/1
aaaaX^aaaaaaaaa`aaa`aa`_¥_a^a]]^aa_`a``a
@HWI-EAS443:3:87:1579:580#0/1
ACGGGGTTCTTCTCTTGTCTTCCTGATGTGTACTTTTTGA
+HWI-EAS443:3:87:1579:580#0/1
abaaaa``RaaYbabbbY¥b``aabaab`ba__aabbaa`
@HWI-EAS443:3:87:722:871#0/1
CGGGTTCTTCTCTTGTCTTCCTGATGTGTACTTTTTGATT
+HWI-EAS443:3:87:722:871#0/1
ababZabbaaaabaa`aaaa`a``aa`a`a`aaaaaaaa^
@HWI-EAS443:3:88:1101:1621#0/1
CGCGGGGTTCTTCTCTTGTCTTCCTGATGTGTACTTTTTG
+HWI-EAS443:3:88:1101:1621#0/1
`b`a`_`J^aba]__bba_^`ababaaababab Tbbbaba
@HWI-EAS443:3:88:1236:399#0/1
TAGCGGGGTTCTTCTCTTGTCTTCCTGATGTGTACTTTTT
+HWI-EAS443:3:88:1236:399#0/1
a`¥YaQ]a^a_aT`]]a]aaWaa]Zaaa_aaaTFMa_aaa
@HWI-EAS443:3:88:169:1186#0/1
TGGGGTTCTTCTCTTGTCTTCCTGATGTGTACTTTTTGAT
+HWI-EAS443:3:88:169:1186#0/1
aba]^_abaaaaaaa]Yaaaaaa`¥a`]a]``aaaaa_[a
@HWI-EAS443:3:88:61:907#0/1
AGGGGTTCTTCTCTTGTCTTCCTGATGTGTACTTTTTGAT
+HWI-EAS443:3:88:61:907#0/1
aab``]`ab```abab[a¥aaa`aa_¥_¥]__`a`a`_^_

```
@HWI-EAS443:3:91:226:302#0/1
TGGGGTTCTTCTCTTGTCTTCCTGATGTGTACTTTTTGAT
+HWI-EAS443:3:91:226:302#0/1
a¥aaUZ[_a[]a_a``¥YP¥__`_^UZOa]_Z]Z_^__V^
@HWI-EAS443:3:91:505:461#0/1
CCGGGGTTCTTCTCTTGTCTTCCTGATGTGTACTTTTTGA
+HWI-EAS443:3:91:505:461#0/1
WaaabaX`aa^`aT]Q__b_Z^¥a`]Va`b[]abb````a
@HWI-EAS443:3:92:584:1928#0/1
AGGGGTTCTTCTCTTGTCTTCCTGATGTGTACTTTTTGAT
+HWI-EAS443:3:92:584:1928#0/1
__^ab]]`aaaabaa``a^abab^Z^aW`_]aaT_`aSaJ
@HWI-EAS443:3:93:821:1558#0/1
CGCGGGGTTCTTCTCTTGTCTTCCTGATGTGTACTTTTTG
+HWI-EAS443:3:93:821:1558#0/1
aaaa¥]a¥`aaaZ]`ba___aaaaaa_]a]`Y_Vaaa`aa
@HWI-EAS443:3:94:1413:100#0/1
CGCGGGGTTCTTCTCTTGTCTTCCTGATGTGTACTTTTTG
+HWI-EAS443:3:94:1413:100#0/1
W^`bbb[[^YZ]¥[bbbb[S^bYR]^Y[[[[Y[I]bbbb¥
@HWI-EAS443:3:94:391:345#0/1
CGCGGGGTTCTTCTCTTGTCTTCCTGATGTGTACTTTTTG
+HWI-EAS443:3:94:391:345#0/1
abbbbba`b`bbaaaaaba_a`a`aa[aa^a__]aa`a`a
@HWI-EAS443:3:94:912:847#0/1
ACGGGGTTCTTCTCTTGTCTTCCTGATGTGTACTTTTTGA
+HWI-EAS443:3:94:912:847#0/1
aa^[baT`bV__bba_H[^^¥¥ba]P]aYbWWZa`b¥`a`
@HWI-EAS443:3:95:44:1611#0/1
AGGGGTTCTTCTCTTGTCTTCCTGATGTGTACTTTTTGAT
+HWI-EAS443:3:95:44:1611#0/1
]S[X¥^bab_`O_`b_Za]a`aTbZX`T]P]a__¥_¥XYa
@HWI-EAS443:3:96:26:1290#0/1
CGGGTTCTTCTCTTGTCTTCCTGATGTGTACTTTTTGATT
+HWI-EAS443:3:96:26:1290#0/1
aa`_W^MVXR]YWSXYURWZ[_`_YLMTPSSTRLTVVRBB
@HWI-EAS443:3:96:789:793#0/1
TCGGGGTTCTTCTCTTGTCTTCCTGATGTGTACTTTTTGA
+HWI-EAS443:3:96:789:793#0/1
`bbba`L^b_a[`b`aaa`a[Z[aaYa`¥[Xaab`a¥XR_
@HWI-EAS443:3:97:535:344#0/1
AGGGGTTCTTCTCTTGTCTTCCTGATGTGTACTTTTTGAT
+HWI-EAS443:3:97:535:344#0/1
ababa¥a]bbaaaaaa`_ba[_aaa_a^a___a_``a]_`
@HWI-EAS443:3:98:1734:1979#0/1
GGTTCTTCTCTTGTCTTCCTGATGTGTACTTTTTGATTAC
+HWI-EAS443:3:98:1734:1979#0/1
aa^WW`a¥`SXU^XLN_ZPT]RGPWP]QLQZ][_BBBBBB
@HWI-EAS443:3:9:976:2041#0/1
TGGGGTTCTTCTCTTGTCTTCCTGATGTGTACTTTTTGAT
```

```
+HWI-EAS443:3:9:976:2041#0/1
`[ab_W`¥^aT_[a`a_^``[]a`^_^]`]ZLW`a]`Y_`
@HWI-EAS443:3:100:1176:356#0/1
CGGGGTTCTTCTCTTGTCTTCCTGATGTGTACTTTTTGAT
+HWI-EAS443:3:100:1176:356#0/1
abbZ``babbSb_baaa`ba_Sabaababaa^babbaRBB
@HWI-EAS443:3:100:1731:91#0/1
CGGGGTTCTTCTCTTGTCTTCCTGATGTGTACTTTTTGAT
+HWI-EAS443:3:100:1731:91#0/1
aaba`^a_a[NYabTbb_b_RK^^[]b_a^a^baba`UBB
@HWI-EAS443:3:100:1756:686#0/1
CGGGGTTCTTCTCTTGTCTTCCTGATGTGTACTTTTTGAT
+HWI-EAS443:3:100:1756:686#0/1
aba[baaabbbbabaa`_baa[a_`a`a^^`Zba`bYBBB
@HWI-EAS443:3:100:183:1571#0/1
GCGGGGTTCTTCTCTTGTCTTCCTGATGTGTACTTTTTGA
+HWI-EAS443:3:100:183:1571#0/1
_aabba[[b`a]ZVaab`¥_`a`aa[aa``_Y__¥^^a^_
@HWI-EAS443:3:100:259:1222#0/1
CGGGGTTCTTCTCTTGTCTTCCTGATGTGTACTTTTTGAT
+HWI-EAS443:3:100:259:1222#0/1
abbbaRabbba`bbbbabbaaab^`aa_``aaaa`a¥Z]a
@HWI-EAS443:3:100:50:2001#0/1
GGGGTTCTTCTCTTGTCTTCCTGATGTGTACTTTTTGATT
+HWI-EAS443:3:100:50:2001#0/1
`¥]`MZSa`Y]aaa__aa_aa`]XX_Z^¥Y_`]_Y`VTBB
@HWI-EAS443:3:100:690:984#0/1
GCGGGGTTCTTCTCTTGTCTTCCTGATGTGTACTTTTTGA
+HWI-EAS443:3:100:690:984#0/1
a]`a`^N__a[Z_`a]VQYa_^```R^aV`Z]^`¥`Y`_B
@HWI-EAS443:3:10:128:1983#0/1
CGGGGTTCTTCTCTTGTCTTCCTGATGTGTACTTTTTGAT
+HWI-EAS443:3:10:128:1983#0/1
abbabPab]aaa```S^b`]`]`_aLXWQYJa`_a]aU`a
@HWI-EAS443:3:10:1688:304#0/1
AGCGGGGTTCTTCTCTTGTCTTCCTGATGTGTACTTTTTG
+HWI-EAS443:3:10:1688:304#0/1
aW`_`ab`ba`]a_[abaa`aaZYa_¥_`^a_¥X^_[``a
@HWI-EAS443:3:10:1707:1431#0/1
CGGGGTTCTTCTCTTGTCTTCCTGATGTGTACTTTTTGAT
+HWI-EAS443:3:10:1707:1431#0/1
a``baI^bbbbaH`T_]`ab¥`ab[ab`a_aW^`a`a¥aa
@HWI-EAS443:3:10:307:1140#0/1
ATCAAAAAGTACACATCAGGAAGACAAGAGAAGAACCCCG
+HWI-EAS443:3:10:307:1140#0/1
`bbabb`ab_babbbaabbaaababbab_aaaaa`a^_``
@HWI-EAS443:3:10:312:1011#0/1
GGGTTCTTCTCTTGTCTTCCTGATGTGTACTTTTTGATTA
+HWI-EAS443:3:10:312:1011#0/1
a`]NY`_Y]_`TTZXQ[V]___WXWUYQ[WZW_]]¥UXZU
```

```
@HWI-EAS443:3:10:379:1031#0/1
GGGGTTCTTCTCTTGTCTTCCTGATGTGTACTTTTTGATT
+HWI-EAS443:3:10:379:1031#0/1
a`XT]QO_W]QOWYYUY]¥ZFO[OOQLVYOXG[[_TRS¥Q
@HWI-EAS443:3:10:476:470#0/1
CGGGGTTCTTCTCTTGTCTTCCTGATGTGTACTTTTTGAT
+HWI-EAS443:3:10:476:470#0/1
`[`b`_bbbabb`bbbaaaaaabbaa`]b`a`baaaa_`a
@HWI-EAS443:3:10:522:861#0/1
AGCGGGGTTCTTCTCTTGTCTTCCTGATGTGTACTTTTTG
+HWI-EAS443:3:10:522:861#0/1
Yaaabbbabaaa[_b`bb``ab_[aaT[]ab^a`a]__ab
@HWI-EAS443:3:10:889:1708#0/1
CGGGGTTCTTCTCTTGTCTTCCTGATGTGTACTTTTTGAT
+HWI-EAS443:3:10:889:1708#0/1
abb`¥aa``baXHZZ^^]¥__¥aY]Z]^a]a`^W_V`]``
@HWI-EAS443:3:10:894:1607#0/1
CGGGGTTCTTCTCTTGTCTTCCTGATGTGTACTTTTTGAT
+HWI-EAS443:3:10:894:1607#0/1
ab]_^Uaabbaaa`a`_aaaa]a^__b^]]`^a_a_`]_a
@HWI-EAS443:3:11:1077:860#0/1
AGCGGGGTTCTTCTCTTGTCTTCCTGATGTGTACTTTTTG
+HWI-EAS443:3:11:1077:860#0/1
[W¥U_W^SX_[[]^¥]aa[^a]_Y_^]]^RaUaZ^[`_`]
@HWI-EAS443:3:11:1330:587#0/1
CGGGGTTCTTCTCTTGTCTTCCTGATGTGTACTTTTTGAT
+HWI-EAS443:3:11:1330:587#0/1
]]`bb]_bYSHY]UZaRbaaYVab]`aQQ]YHa]a`X[__
@HWI-EAS443:3:11:1470:820#0/1
GGGTTCTTCTCTTGTCTTCCTGATGTGTACTTTTTGATTA
+HWI-EAS443:3:11:1470:820#0/1
a]ZFVWWX[^aY`]LQ]ZVJZXY¥]LVXXR¥Y¥_]WVBBB
@HWI-EAS443:3:11:1525:685#0/1
CGGGGTTCTTCTCTTGTCTTCCTGATGTGTACTTTTTGAT
+HWI-EAS443:3:11:1525:685#0/1
[bba_Sa`aaaaa__a]X_a``]]T^bV¥R_N_aa___]]
@HWI-EAS443:3:11:1538:805#0/1
GGGTTCTTCTCTTGTCTTCCTGATGTGTACTTTTTGATTA
+HWI-EAS443:3:11:1538:805#0/1
aab¥a`bb_a`aba_^aa`^a`_`a^a_^Zaaa`a___a`
@HWI-EAS443:3:11:1685:651#0/1
CGGGGTTCTTCTCTTGTCTTCCTGATGTGTACTTTTTGAT
+HWI-EAS443:3:11:1685:651#0/1
abba^YaXab]a_bab`]baa``^`bb_``_T`bb`b]]b
@HWI-EAS443:3:11:21:538#0/1
GGGGTTCTTCTCTTGTCTTCCTGATGTGTACTTTTTGATT
+HWI-EAS443:3:11:21:538#0/1
a]I[MZ`]]a_¥a__P]`[[a]¥U¥`UYSY[[]_Z___¥B
@HWI-EAS443:3:11:271:1205#0/1
GGGGTTCTTCTCTTGTCTTCCTGATGTGTACTTTTTGATT
```

```
+HWI-EAS443:3:11:271:1205#0/1
a`aaQaa`abab_aa[^a`aaaa_`[^a]`^`^```___^
@HWI-EAS443:3:11:274:627#0/1
AGCGGGGTTCTTCTCTTGTCTTCCTGATGTGTACTTTTTG
+HWI-EAS443:3:11:274:627#0/1
aba^bbb^bbab_aaaba`aaaaaaa^aa^a^_`a_aa^P
@HWI-EAS443:3:12:1158:455#0/1
GGGTTCTTCTCTTGTCTTCCTGATGTGTACTTTTTGATTA
+HWI-EAS443:3:12:1158:455#0/1
a`WEVaab_a`]a___`a____¥_`T^]^Y__``a[¥`¥_
@HWI-EAS443:3:12:1322:465#0/1
AGCGGGGTTCTTCTCTTGTCTTCCTGATGTGTACTTTTTG
+HWI-EAS443:3:12:1322:465#0/1
abbaa`b]]X]aaaaaaaa_aa`a``__a__[`]_`a_`a
@HWI-EAS443:3:12:1537:1016#0/1
GGGGTTCTTCTCTTGTCTTCCTGATGTGTACTTTTTGATT
+HWI-EAS443:3:12:1537:1016#0/1
abbaP^^a]^]^aX]XXaPLOa`^_aSa]]Ra^WN__¥_B
@HWI-EAS443:3:12:1600:1469#0/1
AGCGGGGTTCTTCTCTTGTCTTCCTGATGTGTACTTTTTG
+HWI-EAS443:3:12:1600:1469#0/1
WV_¥^a`X]O¥]R`^^ZXXW[___¥[X`[[_^HZ``^_]B
@HWI-EAS443:3:12:175:1359#0/1
GGGGTTCTTCTCTTGTCTTCCTGATGTGTACTTTTTGATT
+HWI-EAS443:3:12:175:1359#0/1
a]UaYX__W_a_^XRS__^]a[XY]I^¥XWIY^^]VSBBB
@HWI-EAS443:3:12:1787:607#0/1
GGGTTCTTCTCTTGTCTTCCTGATGTGTACTTTTTGATTA
+HWI-EAS443:3:12:1787:607#0/1
a_¥R¥^]`]¥_a[[[SX¥_X^`X^]U_OXUUU¥W]X]]WB
@HWI-EAS443:3:12:259:1910#0/1
CGGGGTTCTTCTCTTGTCTTCCTGATGTGTACTTTTTGAT
+HWI-EAS443:3:12:259:1910#0/1
aa_]a_`bbbaaaa`a_ababaa^_aa`[_aaaaaba^aa
@HWI-EAS443:3:12:327:219#0/1
CGGGGTTCTTCTCTTGTCTTCCTGATGTGTACTTTTTGAT
+HWI-EAS443:3:12:327:219#0/1
abbbb_a[_O`]__W^J^_TPYYZS__NOJU]_`_^_[NB
@HWI-EAS443:3:12:716:551#0/1
CGGGGTTCTTCTCTTGTCTTCCTGATGTGTACTTTTTGAT
+HWI-EAS443:3:12:716:551#0/1
^TZa_aa`bbb_babb_a`aa`aba`bIaa_aa_bab__a
@HWI-EAS443:3:12:72:251#0/1
AGCGGGGTTCTTCTCTTGTCTTCCTGATGTGTACTTTTTG
+HWI-EAS443:3:12:72:251#0/1
`abbaaa_aaaaaaaaaa`aaa``a`__a^a]___`_``_
@HWI-EAS443:3:13:1125:360#0/1
GCGGGGTTCTTCTCTTGTCTTCCTGATGTGTACTTTTTGA
+HWI-EAS443:3:13:1125:360#0/1
a``TW¥GTHS¥¥XONVUURNXTQVROGUQNWNPRWBBBBB
```

```
@HWI-EAS443:3:13:115:231#0/1
GGGGTTCTTCTCTTGTCTTCCTGATGTGTACTTTTTGATT
+HWI-EAS443:3:13:115:231#0/1
abba¥ababaaaaaa_aaaa`aa`aa]a_``aaaaaa`a`
@HWI-EAS443:3:13:1307:509#0/1
CGGGGTTCTTCTCTTGTCTTCCTGATGTGTACTTTTTGAT
+HWI-EAS443:3:13:1307:509#0/1
abbbbababaababbbaabba_baaaa`b^a_aaabbaaa
@HWI-EAS443:3:13:20:1689#0/1
CGGGGTTCTTCTCTTGTCTTCCTGATGTGTACTTTTTGAT
+HWI-EAS443:3:13:20:1689#0/1
a``]_]aaaaaaaaaa_aaa_``_`]XS^Z`]`a`_aW_`
@HWI-EAS443:3:13:222:320#0/1
CGGGGTTCTTCTCTTGTCTTCCTGATGTGTACTTTTTGAT
+HWI-EAS443:3:13:222:320#0/1
aaba`^b_aaaaaaab`bbaaaaa`aa_a^__aaa_aa`a
@HWI-EAS443:3:13:23:1515#0/1
GGGGTTCTTCTCTTGTCTTCCTGATGTGTACTTTTTGATT
+HWI-EAS443:3:13:23:1515#0/1
a``_Q_aaaaaaaaa^aaa`^_[Z_`¥]Z]`]¥__a¥^_[
@HWI-EAS443:3:13:259:1593#0/1
GGGGTTCTTCTCTTGTCTTCCTGATGTGTACTTTTTGATT
+HWI-EAS443:3:13:259:1593#0/1
`¥S_U¥Z_¥a`_aaaabZb`_]]a[NVJPb^aWaa]]``B
@HWI-EAS443:3:13:374:1328#0/1
CGGGGTTCTTCTCTTGTCTTCCTGATGTGTACTTTTTGAT
+HWI-EAS443:3:13:374:1328#0/1
aaU]aO^]_aa`_]X[P[^``___^[[X`ZZ^^]¥¥^^X^
@HWI-EAS443:3:13:510:126#0/1
GCGGGGTTCTTCTCTTGTCTTCCTGATGTGTACTTTTTGA
+HWI-EAS443:3:13:510:126#0/1
aab```Q`aaaaa[a``_a`_a____`a]`Y[]_^^_`[_
@HWI-EAS443:3:13:520:252#0/1
GGGGTTCTTCTCTTGTCTTCCTGATGTGTACTTTTTGATT
+HWI-EAS443:3:13:520:252#0/1
aaaa_aaaa_aaaab_aa`a`a`_`a]`^a_aaa```_a_
@HWI-EAS443:3:13:641:1407#0/1
CGGGGTTCTTCTCTTGTCTTCCTGATGTGTACTTTTTGAT
+HWI-EAS443:3:13:641:1407#0/1
Za¥^aZ]`R[X^[b`_¥b_b]W``P¥RQbITX[a_aa_Tb
@HWI-EAS443:3:13:814:459#0/1
GCGGGGTTCTTCTCTTGTCTTCCTGATGTGTACTTTTTGA
+HWI-EAS443:3:13:814:459#0/1
a_abWVYX_I]a`S^_NGP¥SW^^TO`ZSJGO_`Wa^`]B
@HWI-EAS443:3:14:118:1272#0/1
CGGGGTTCTTCTCTTGTCTTCCTGATGTGTACTTTTTGAT
+HWI-EAS443:3:14:118:1272#0/1
abaabXaa`abaUaa_`aaa``a]]_a__R```aa``]]_
@HWI-EAS443:3:14:1238:166#0/1
CGGGGTTCTTCTCTTGTCTTCCTGATGTGTACTTTTTGAT
```

```
+HWI-EAS443:3:14:1238:166#0/1
abbbbababa_baabaaaaba]a``ab_a`^^aaaabaaa
@HWI-EAS443:3:14:1320:1353#0/1
CGGGGTTCTTCTCTTGTCTTCCTGATGTGTACTTTTTGAT
+HWI-EAS443:3:14:1320:1353#0/1
aabba[aabaababbbZaaa^a`ba_bVa``[baaab``a
@HWI-EAS443:3:14:229:474#0/1
GGGGTTCTTCTCTTGTCTTCCTGATGTGTACTTTTTGATT
+HWI-EAS443:3:14:229:474#0/1
`aaa]__aa```_`aWZ]`_`_`__a_aWPW_`_Z[[__`
@HWI-EAS443:3:14:29:1857#0/1
GCGGGGTTCTTCTCTTGTCTTCCTGATGTGTACTTTTTGA
+HWI-EAS443:3:14:29:1857#0/1
aaa_]_N__a`aa`__a_````a`¥V¥VU_^^bX^_]]XY
@HWI-EAS443:3:14:516:180#0/1
CGGGGTTCTTCTCTTGTCTTCCTGATGTGTACTTTTTGAT
+HWI-EAS443:3:14:516:180#0/1
aaaaa]a`a_`a`_aa_``_`Z^`^^aW`X^Z^_]^`_Z_
@HWI-EAS443:3:14:590:357#0/1
GGGGTTCTTCTCTTGTCTTCCTGATGTGTACTTTTTGATT
+HWI-EAS443:3:14:590:357#0/1
aaaaUa`aaaaaaaa_```_^`_aaaZ`__[`a`a`_a_`
@HWI-EAS443:3:14:627:201#0/1
GGGGTTCTTCTCTTGTCTTCCTGATGTGTACTTTTTGATT
+HWI-EAS443:3:14:627:201#0/1
_QYHJ__^Z^]^_]RT^W^[__ZNX_X_R^Z_^^^YYYYY
@HWI-EAS443:3:14:898:412#0/1
CGGGGTTCTTCTCTTGTCTTCCTGATGTGTACTTTTTGAT
+HWI-EAS443:3:14:898:412#0/1
]YSa`Pa_ZW]`W_¥]^`b_a^]SSRa[a[]N_^Ya`]Z[
@HWI-EAS443:3:15:203:979#0/1
AGCGGGGTTCTTCTCTTGTCTTCCTGATGTGTACTTTTTG
+HWI-EAS443:3:15:203:979#0/1
TZ_[_aa^U`_``a_bab_Y`[a`]ba`_`[`¥¥Z¥`aaa
@HWI-EAS443:3:15:317:345#0/1
CGGGGTTCTTCTCTTGTCTTCCTGATGTGTACTTTTTGAT
+HWI-EAS443:3:15:317:345#0/1
abbbUSaba_[a`abXJ^a`aa^`_a`]`Q]S`[`aa_`a
@HWI-EAS443:3:15:328:587#0/1
GGGGTTCTTCTCTTGTCTTCCTGATGTGTACTTTTTGATT
+HWI-EAS443:3:15:328:587#0/1
aab__bbbaabaaaa__baaaa`aa[¥a]a^aaaaaaaa_
@HWI-EAS443:3:16:1558:280#0/1
GGTTCTTCTCTTGTCTTCCTGATGTGTACTTTTTGATTAT
+HWI-EAS443:3:16:1558:280#0/1
aa_`N__R^]aaWPR_`[``]¥`a^[]QO[`¥]TR¥^`]¥
@HWI-EAS443:3:16:1567:261#0/1
AGCGGGGTTCTTCTCTTGTCTTCCTGATGTGTACTTTTTG
+HWI-EAS443:3:16:1567:261#0/1
ababbab_a`ba_baaaa`_aaaaaa_aa^`___`aa``_
```

```
@HWI-EAS443:3:16:205:413#0/1
GGTTCTTCTCTTGTCTTCCTGATGTGTACTTTTTGATTAT
+HWI-EAS443:3:16:205:413#0/1
a¥PZ`ab¥X¥ZaZ]aa¥[XS][]SR¥W]U][]a_YR]a`X
@HWI-EAS443:3:16:214:413#0/1
GCGGGGTTCTTCTCTTGTCTTCCTGATGTGTACTTTTTGA
+HWI-EAS443:3:16:214:413#0/1
Vbb__ba^bb`[_babb__``baaa^_a_aW[^aab`b_a
@HWI-EAS443:3:16:509:143#0/1
AGCGGGGTTCTTCTCTTGTCTTCCTGATGTGTACTTTTTG
+HWI-EAS443:3:16:509:143#0/1
¥`]_X_a[_a]YQ^_a^¥``__[^`[[]`UaYX[^]_^]_
@HWI-EAS443:3:16:547:800#0/1
GCGGGGTTCTTCTCTTGTCTTCCTGATGTGTACTTTTTGA
+HWI-EAS443:3:16:547:800#0/1
Zaaaa^^_`aa[][a_`]`aa[a_]`a`IJZ^`_W¥`a`a
@HWI-EAS443:3:16:650:1389#0/1
GGGGTTCTTCTCTTGTCTTCCTGATGTGTACTTTTTGATT
+HWI-EAS443:3:16:650:1389#0/1
aaaa_ababaa^aaa_aaaa_a``aa_a__`aaaaa_`a_
@HWI-EAS443:3:16:653:268#0/1
TCAAAAAGTACACATCAGGAAGACAAGAGAAGAACCCCGC
+HWI-EAS443:3:16:653:268#0/1
S¥¥`^¥]VT^]b^_aaabb^X`a`a^a_a`a_`]¥VZN``
@HWI-EAS443:3:16:660:134#0/1
CGGGGTTCTTCTCTTGTCTTCCTGATGTGTACTTTTTGAT
+HWI-EAS443:3:16:660:134#0/1
abaaa^`aaa¥`¥_a`_a``_^__^^a]aZ]Z`__^a_^a
@HWI-EAS443:3:16:70:49#0/1
GCGGGGTTCTTCTCTTGTCTTCCTGATGTGTACTTTTTGA
+HWI-EAS443:3:16:70:49#0/1
aaa¥^YPY^^Z[ZY[[YN¥Y]]]V]YV]WVQUQWSUBBBB
@HWI-EAS443:3:16:748:698#0/1
AGCGGGGTTCTTCTCTTGTCTTCCTGATGTGTACTTTTTG
+HWI-EAS443:3:16:748:698#0/1
a`aaaaa^aaaa_a^aa`aa`_a_`___aZa_^___a`_Z
@HWI-EAS443:3:17:1125:249#0/1
CGGGGTTCTTCTCTTGTCTTCCTGATGTGTACTTTTTGAT
+HWI-EAS443:3:17:1125:249#0/1
ab]ZXPbaa]RZY¥_]_aa^PY]_Uab_VKGGR^_[^¥PW
@HWI-EAS443:3:17:114:1172#0/1
CGGGGTTCTTCTCTTGTCTTCCTGATGTGTACTTTTTGAT
+HWI-EAS443:3:17:114:1172#0/1
abbb¥_bbaabbbabaaab`aab`aaa`^^aaaaa^aaaa
@HWI-EAS443:3:17:1296:1593#0/1
CGGGGTTCTTCTCTTGTCTTCCTGATGTGTACTTTTTGAT
+HWI-EAS443:3:17:1296:1593#0/1
```b^Qabbaababa_`bba^`aaa`aX^^`aaaa`aYaa
@HWI-EAS443:3:17:166:1034#0/1
GGGGTTCTTCTCTTGTCTTCCTGATGTGTACTTTTTGATT
```
```

```
+HWI-EAS443:3:17:166:1034#0/1
aa^¥Vabbaaaaaaa_a`aa`_`Y^`[a]`aa_a__]_^_
@HWI-EAS443:3:17:1680:1350#0/1
GGGGTTCTTCTCTTGTCTTCCTGATGTGTACTTTTTGATT
+HWI-EAS443:3:17:1680:1350#0/1
a`b__aabbabbbba_abaaaa```^_a_`aaaaaaaaa`
@HWI-EAS443:3:17:220:1427#0/1
CGGGGTTCTTCTCTTGTCTTCCTGATGTGTACTTTTTGAT
+HWI-EAS443:3:17:220:1427#0/1
aa`XEUaaaaaaaaaa_aa``_a_Z`a]]Y_^`````[a_
@HWI-EAS443:3:17:262:1659#0/1
GGGGTTCTTCTCTTGTCTTCCTGATGTGTACTTTTTGATT
+HWI-EAS443:3:17:262:1659#0/1
a_X¥F_aa^bba_aaH`_a¥_`NVYST_^`X`_]`]a`SY
@HWI-EAS443:3:17:392:306#0/1
CGGGGTTCTTCTCTTGTCTTCCTGATGTGTACTTTTTGAT
+HWI-EAS443:3:17:392:306#0/1
abbab^babbbbaabaaabaabbbaab`a_a`baaaaaaa
@HWI-EAS443:3:17:429:423#0/1
GCGGGGTTCTTCTCTTGTCTTCCTGATGTGTACTTTTTGA
+HWI-EAS443:3:17:429:423#0/1
aaaaaa]aa___[a[`¥¥a_aa^V[[_PFXYTW^____Z]
@HWI-EAS443:3:17:433:1932#0/1
GGTTCTTCTCTTGTCTTCCTGATGTGTACTTTTTGATTAT
+HWI-EAS443:3:17:433:1932#0/1
aa¥aaaaaaa``a_aa_aaa_a__^^^a``_]Y_]a^_a_
@HWI-EAS443:3:17:536:659#0/1
GGGGTTCTTCTCTTGTCTTCCTGATGTGTACTTTTTGATT
+HWI-EAS443:3:17:536:659#0/1
abaaXabaaaa`aaa^_a___aaa`a_`]_]a`_`_[]^_
@HWI-EAS443:3:17:77:462#0/1
GCGGGGTTCTTCTCTTGTCTTCCTGATGTGTACTTTTTGA
+HWI-EAS443:3:17:77:462#0/1
aaba¥WV^`a^aaa`^_V_a`]```YPYG¥WUY[`^¥^[_
@HWI-EAS443:3:18:1050:74#0/1
AGCGGGGTTCTTCTCTTGTCTTCCTGATGTGTACTTTTTG
+HWI-EAS443:3:18:1050:74#0/1
abababaa_aaabaa_aba`^aa_]aa__a]a]^Va__aa_
@HWI-EAS443:3:18:1310:1332#0/1
GGGTTCTTCTCTTGTCTTCCTGATGTGTACTTTTTGATTA
+HWI-EAS443:3:18:1310:1332#0/1
¥`b`ab`_````_a]]]aa`aa[^WH¥IZ^a`]```PY_]
@HWI-EAS443:3:18:1463:1122#0/1
GGGGTTCTTCTCTTGTCTTCCTGATGTGTACTTTTTGATT
+HWI-EAS443:3:18:1463:1122#0/1
_`]UP^`aZW_^b^`[U^__```^_]YaYZXa`a`a````
@HWI-EAS443:3:18:163:2027#0/1
GGGTTCTTCTCTTGTCTTCCTGATGTGTACTTTTTGATTA
+HWI-EAS443:3:18:163:2027#0/1
`__TL^^aaWW¥Ya¥``¥b`^QTVV_a]__^`aa_Z^a_a
```

@HWI-EAS443:3:18:653:1037#0/1
ATCAAAAAGTACACATCAGGAAGACAAGAGAAGAACCCCG
+HWI-EAS443:3:18:653:1037#0/1
WZWWU_WTVYb^b_a^`aba^^a_a]P^`_XZ_^]]`R``
@HWI-EAS443:3:19:1129:280#0/1
CGGGGTTCTTCTCTTGTCTTCCTGATGTGTACTTTTTGAT
+HWI-EAS443:3:19:1129:280#0/1
Zaaabaa```bbbabaaabb_````bba`_``babbbaab
@HWI-EAS443:3:19:1161:1071#0/1
GCGGGGTTCTTCTCTTGTCTTCCTGATGTGTACTTTTTGA
+HWI-EAS443:3:19:1161:1071#0/1
aa`^`ZT][a`_aa`_aU`aa_^_`]__]`]_`_``a]]_
@HWI-EAS443:3:19:1239:1956#0/1
CGGGGTTCTTCTCTTGTCTTCCTGATGTGTACTTTTTGAT
+HWI-EAS443:3:19:1239:1956#0/1
aa_bb___aaabababaaaaaaaaaaaa^a^`_aaaaa_aa
@HWI-EAS443:3:19:1279:1514#0/1
CGGGGTTCTTCTCTTGTCTTCCTGATGTGTACTTTTTGAT
+HWI-EAS443:3:19:1279:1514#0/1
a`bbb_aa`bbbaaaa^[aaaaaaa[`^b[¥Vaaaaa_``
@HWI-EAS443:3:19:1624:1495#0/1
GGGGTTCTTCTCTTGTCTTCCTGATGTGTACTTTTTGATT
+HWI-EAS443:3:19:1624:1495#0/1
ab`aRbbbbabZ`Ybaaaaa^b`a`baa`a^bba`aWXa_
@HWI-EAS443:3:19:327:377#0/1
GCGGGGTTCTTCTCTTGTCTTCCTGATGTGTACTTTTTGA
+HWI-EAS443:3:19:327:377#0/1
a_`a_]X]aa^^^^__a¥_^¥^]]_ZZ^W]WOW]]]_^XZ
@HWI-EAS443:3:19:399:1297#0/1
CGGGGTTCTTCTCTTGTCTTCCTGATGTGTACTTTTTGAT
+HWI-EAS443:3:19:399:1297#0/1
aaaaYRYaaa``a`a`_aaa_`a`TTWXXX_^___`_]_B
@HWI-EAS443:3:19:447:2027#0/1
CGGGGTTCTTCTCTTGTCTTCCTGATGTGTACTTTTTGAT
+HWI-EAS443:3:19:447:2027#0/1
`X]__M¥_a_aaa__¥Y]^^_a`_a]`R_X[W]_`^^[Q_
@HWI-EAS443:3:19:46:1812#0/1
CGGGGTTCTTCTCTTGTCTTCCTGATGTGTACTTTTTGAT
+HWI-EAS443:3:19:46:1812#0/1
a[FY_KYaa¥W_aa`¥X]_`]a_]ZP[Ra]_]__`__HWY
@HWI-EAS443:3:19:663:15#0/1
GGGGTTCTTCTCTTGTCTTCCTGATGTGTACTTTTTGATT
+HWI-EAS443:3:19:663:15#0/1
aaaaZaaaa_a¥a`a^_``_a__^^_^`^^X_^_]^_^^U
@HWI-EAS443:3:19:695:132#0/1
GCGGGGTTCTTCTCTTGTCTTCCTGATGTGTACTTTTTGA
+HWI-EAS443:3:19:695:132#0/1
a_¥_a^R]X¥_]]]_^_S]^`WZ]]VV_Z¥WWOXZ]ZVRX
@HWI-EAS443:3:19:760:1853#0/1
CGGGGTTCTTCTCTTGTCTTCCTGATGTGTACTTTTTGAT

```
+HWI-EAS443:3:19:760:1853#0/1
aaaaa[_a^`a`U¥]XUa`[[_a___^^`SXT[_^^]UY^
@HWI-EAS443:3:19:793:1367#0/1
GCGGGGTTCTTCTCTTGTCTTCCTGATGTGTACTTTTTGA
+HWI-EAS443:3:19:793:1367#0/1
abaaaaWaaaaaaa`aaYaa`_aa`__^Z____^a__^S^
@HWI-EAS443:3:19:795:225#0/1
CGGGGTTCTTCTCTTGTCTTCCTGATGTGTACTTTTTGAT
+HWI-EAS443:3:19:795:225#0/1
abbab_bbbbabaaab^aaaa_aaaab^a_a^aaa_a``a
@HWI-EAS443:3:19:815:1367#0/1
CGGGGTTCTTCTCTTGTCTTCCTGATGTGTACTTTTTGAT
+HWI-EAS443:3:19:815:1367#0/1
aa`aa[b`baba`baa_aa_aaaa[aa^a[^]a`a^`a`a
@HWI-EAS443:3:1:1186:238#0/1
GGTTCTTCTCTTGTCTTCCTGATGTGTACTTTTTGATTAT
+HWI-EAS443:3:1:1186:238#0/1
aa¥_aa`^a]a`a]¥`__^_a_`a^a^]]_a_a`¥]BBBB
@HWI-EAS443:3:1:1297:431#0/1
GCGGGGTTCTTCTCTTGTCTTCCTGATGTGTACTTTTTGA
+HWI-EAS443:3:1:1297:431#0/1
aaa`aaWa```_a^aaa_^aa_]a`_``_a]^Z`_a__`B
@HWI-EAS443:3:1:1400:427#0/1
GCGGGGTTCTTCTCTTGTCTTCCTGATGTGTACTTTTTGA
+HWI-EAS443:3:1:1400:427#0/1
`aaa`[Zab`b__]aaa_^aRXYaa_]aZaZYZ_`a`a_B
@HWI-EAS443:3:1:1411:1204#0/1
GGGGTTCTTCTCTTGTCTTCCTGATGTGTACTTTTTGATT
+HWI-EAS443:3:1:1411:1204#0/1
[¥X]baaabbbabbbaabb]Waa¥]a_b``Yabaaaa`aB
@HWI-EAS443:3:1:1543:1302#0/1
CGGGGTTCTTCTCTTGTCTTCCTGATGTGTACTTTTTGAT
+HWI-EAS443:3:1:1543:1302#0/1
`a[`^T^aba]aIb_b]^b`Y`abYaaZ``]Oa`SXaa`B
@HWI-EAS443:3:1:161:1934#0/1
CGGGGTTCTTCTCTTGTCTTCCTGATGTGTACTTTTTGAT
+HWI-EAS443:3:1:161:1934#0/1
a_b_bM`[ab_a_b_¥``bb¥VaaObbKa`aa]^a[bV`B
@HWI-EAS443:3:20:1287:1189#0/1
GCGGGGTTCTTCTCTTGTCTTCCTGATGTGTACTTTTTGA
+HWI-EAS443:3:20:1287:1189#0/1
`R]]^¥LX_[[SYRG[XVWYZURQVWV^SQTKINVVRWNV
@HWI-EAS443:3:20:1440:452#0/1
GCGGGGTTCTTCTCTTGTCTTCCTGATGTGTACTTTTTGA
+HWI-EAS443:3:20:1440:452#0/1
a^STZZ^`O_a_aR_a`[UX`[X`aZXTT]XLV¥_^¥]UB
@HWI-EAS443:3:20:1459:60#0/1
GCGGGGTTCTTCTCTTGTCTTCCTGATGTGTACTTTTTGA
+HWI-EAS443:3:20:1459:60#0/1
_Uabaa¥`Za__a_aaa`V`^UU_`¥]_]_]YY^_`__RU
```

```
@HWI-EAS443:3:20:145:988#0/1
CGGGGTTCTTCTCTTGTCTTCCTGATGTGTACTTTTTGAT
+HWI-EAS443:3:20:145:988#0/1
ab_Z_J]baaaaba`][baaa`[``]]UZSY¥_`W]^RY_
@HWI-EAS443:3:20:249:1547#0/1
GCGGGGTTCTTCTCTTGTCTTCCTGATGTGTACTTTTTGA
+HWI-EAS443:3:20:249:1547#0/1
a`aa`^QZbaaa_X[aaZ[[a_``a`_a]a_`a[`]_Z_]
@HWI-EAS443:3:20:271:360#0/1
CGGGGTTCTTCTCTTGTCTTCCTGATGTGTACTTTTTGAT
+HWI-EAS443:3:20:271:360#0/1
Z_X_``b`_a`__a¥¥a¥aa_^a_`¥ZZb¥``]`aabZ¥a
@HWI-EAS443:3:20:309:273#0/1
CGGGGTTCTTCTCTTGTCTTCCTGATGTGTACTTTTTGAT
+HWI-EAS443:3:20:309:273#0/1
abbbb`bab`baaaaa]baa`aa_a`a`a`_`aaaaa^¥]
@HWI-EAS443:3:20:580:543#0/1
GCGGGGTTCTTCTCTTGTCTTCCTGATGTGTACTTTTTGA
+HWI-EAS443:3:20:580:543#0/1
aaOY_aZaaa_a_a]X]X`a^^[[T[_a^aZ^PU`]]^`]
@HWI-EAS443:3:20:630:280#0/1
CGGGGTTCTTCTCTTGTCTTCCTGATGTGTACTTTTTGAT
+HWI-EAS443:3:20:630:280#0/1
aa`¥¥R[SY¥a¥_T_YY`]^]_^¥RZMNXVZ^ZX]ZZ^Z]
@HWI-EAS443:3:20:802:516#0/1
GGGGTTCTTCTCTTGTCTTCCTGATGTGTACTTTTTGATT
+HWI-EAS443:3:20:802:516#0/1
a`ZUFN¥]]Y_L[¥bWY[U]`_`¥_^X[X_¥_`^^Z^Z^[
@HWI-EAS443:3:20:865:1049#0/1
CGGGGTTCTTCTCTTGTCTTCCTGATGTGTACTTTTTGAT
+HWI-EAS443:3:20:865:1049#0/1
aa^aa^aZWWa`a_`aSaa_aYa_]]a^`U___a__^^][
@HWI-EAS443:3:21:1606:122#0/1
CGGGGTTCTTCTCTTGTCTTCCTGATGTGTACTTTTTGAT
+HWI-EAS443:3:21:1606:122#0/1
abaaa_a^b`_b^aaa_¥aaZ_`^^_`]_UXZa^_`_¥Z^
@HWI-EAS443:3:21:1621:117#0/1
CGGGGTTCTTCTCTTGTCTTCCTGATGTGTACTTTTTGAT
+HWI-EAS443:3:21:1621:117#0/1
abbab`babb`babbbaabbaabaaaa_`_a^bbbbaV_a
@HWI-EAS443:3:21:1650:1367#0/1
CGGGGTTCTTCTCTTGTCTTCCTGATGTGTACTTTTTGAT
+HWI-EAS443:3:21:1650:1367#0/1
a]a]a_a^bbaa]`a___a[[^a`__`]a_¥]a```W]`_
@HWI-EAS443:3:21:1736:1225#0/1
CGGGGTTCTTCTCTTGTCTTCCTGATGTGTACTTTTTGAT
+HWI-EAS443:3:21:1736:1225#0/1
a_]^YU_¥aa`a`aaa__a__]aa^^]Za__Xaaaa¥PYa
@HWI-EAS443:3:21:244:1112#0/1
CGGGGTTCTTCTCTTGTCTTCCTGATGTGTACTTTTTGAT
```

```
+HWI-EAS443:3:21:244:1112#0/1
a`¥^UPR`ZYY¥___]PYV_aa_]_`¥V_W_]___^XR^^
@HWI-EAS443:3:21:568:1638#0/1
CGGGGTTCTTCTCTTGTCTTCCTGATGTGTACTTTTTGAT
+HWI-EAS443:3:21:568:1638#0/1
aaa`a[aaaaa`b``^[`aaaaaS^_`X[Y_^a_a_a_``
@HWI-EAS443:3:21:649:38#0/1
GCGGGGTTCTTCTCTTGTCTTCCTGATGTGTACTTTTTGA
+HWI-EAS443:3:21:649:38#0/1
`[_^]N^_ZZQVGW¥`ZLLWGQ_X_JSY[VXNT]¥Z¥`¥X
@HWI-EAS443:3:21:813:107#0/1
GGGGTTCTTCTCTTGTCTTCCTGATGTGTACTTTTTGATT
+HWI-EAS443:3:21:813:107#0/1
aaaa_aaa`_a_```]¥`a^X^_]Z_Z`WXW_^_]__^]^
@HWI-EAS443:3:21:865:1057#0/1
AGCGGGGTTCTTCTCTTGTCTTCCTGATGTGTACTTTTTG
+HWI-EAS443:3:21:865:1057#0/1
¥_b```b_`bbabaab`b_b`^^a`aXaaOb__``a``a[
@HWI-EAS443:3:21:877:1963#0/1
CGGGGTTCTTCTCTTGTCTTCCTGATGTGTACTTTTTGAT
+HWI-EAS443:3:21:877:1963#0/1
__``b[bbabaab`````aabab`aa_^bZa_aaa_a`ba
@HWI-EAS443:3:22:1101:878#0/1
CGGGGTTCTTCTCTTGTCTTCCTGATGTGTACTTTTTGAT
+HWI-EAS443:3:22:1101:878#0/1
]R]VSX_]GZ]ZUZZ]N[V]U[ZZLZZMP[X^`¥^]¥UY_
@HWI-EAS443:3:22:1161:295#0/1
AGCGGGGTTCTTCTCTTGTCTTCCTGATGTGTACTTTTTG
+HWI-EAS443:3:22:1161:295#0/1
a_b^_aaab]bbababbaaV_ba_ba`aa`bW_`babaaZ
@HWI-EAS443:3:22:1181:807#0/1
GCGGGGTTCTTCTCTTGTCTTCCTGATGTGTACTTTTTGA
+HWI-EAS443:3:22:1181:807#0/1
aa_aba]___`__¥``_^^a__Z`^X]_Z_ZXX^_`X`^]
@HWI-EAS443:3:22:1223:1796#0/1
GCGGGGTTCTTCTCTTGTCTTCCTGATGTGTACTTTTTGA
+HWI-EAS443:3:22:1223:1796#0/1
```aa`_aaabaaaaa_a_aa_^aaaa]]a^aZa`a``Y_
@HWI-EAS443:3:22:1247:1123#0/1
AGCGGGGTTCTTCTCTTGTCTTCCTGATGTGTACTTTTTG
+HWI-EAS443:3:22:1247:1123#0/1
abbbaba`aaba`a`bbbaaab_aaa`aa`a_`]aaaaaU
@HWI-EAS443:3:22:1247:96#0/1
GCGGGGTTCTTCTCTTGTCTTCCTGATGTGTACTTTTTGA
+HWI-EAS443:3:22:1247:96#0/1
RTNOb_T[U]¥`a]_aa_¥_]__aRNOZV^XYN_a`aaY`
@HWI-EAS443:3:22:159:1237#0/1
CGGGGTTCTTCTCTTGTCTTCCTGATGTGTACTTTTTGAT
+HWI-EAS443:3:22:159:1237#0/1
a`a_`Y`aaa``[_``_a````_^_^`Z`Y]_a_``]¥`_
```
```

@HWI-EAS443:3:22:264:529#0/1
GGGGTTCTTCTCTTGTCTTCCTGATGTGTACTTTTTGATT
+HWI-EAS443:3:22:264:529#0/1
a]ZVK_aa_¥_YWVO[____``XP_aTZUNY_^]^WRWBB
@HWI-EAS443:3:22:27:1022#0/1
GCGGGGTTCTTCTCTTGTCTTCCTGATGTGTACTTTTTGA
+HWI-EAS443:3:22:27:1022#0/1
aa`_`a_baababaaaaaaaaaaaa`aa_a^]^aaaa^U[
@HWI-EAS443:3:22:552:931#0/1
CGGGGTTCTTCTCTTGTCTTCCTGATGTGTACTTTTTGAT
+HWI-EAS443:3:22:552:931#0/1
aaaa`]aaaabaaaaaabbaaa`Y^`a^a^`_a_a_a`a`
@HWI-EAS443:3:22:632:1132#0/1
ATCAAAAAGTACACATCAGGAAGACAAGAGAAGAACCCCG
+HWI-EAS443:3:22:632:1132#0/1
]babaa¥_aYbbbaaabb`aaaaaa`Z]aaaa_`aaa__a
@HWI-EAS443:3:22:64:693#0/1
CGGGGTTCTTCTCTTGTCTTCCTGATGTGTACTTTTTGAT
+HWI-EAS443:3:22:64:693#0/1
`b_aa_baa]bbabbb`bbba]]``aa`b_`¥abbaab`b
@HWI-EAS443:3:22:764:60#0/1
GGGGTTCTTCTCTTGTCTTCCTGATGTGTACTTTTTGATT
+HWI-EAS443:3:22:764:60#0/1
aaa`^`aaa`a_aaa`_a`_X```_`Za]^^a^`^`^___
@HWI-EAS443:3:22:778:424#0/1
GGTTCTTCTCTTGTCTTCCTGATGTGTACTTTTTGATTAT
+HWI-EAS443:3:22:778:424#0/1
¥¥XWV[¥^TGZ_a¥Y¥^_``¥V¥`TaXTG]aaaaJJ]^^a
@HWI-EAS443:3:22:790:743#0/1
GGTTCTTCTCTTGTCTTCCTGATGTGTACTTTTTGATTAT
+HWI-EAS443:3:22:790:743#0/1
^_X`aa`aa__`a`]aaaaa`]]^_`^]Uaaa``aZ¥aa¥
@HWI-EAS443:3:22:812:456#0/1
CGGGGTTCTTCTCTTGTCTTCCTGATGTGTACTTTTTGAT
+HWI-EAS443:3:22:812:456#0/1
ab`_aa[_abababbbabbbabaaaabaZa`abababaaaa
@HWI-EAS443:3:22:988:867#0/1
GGTTCTTCTCTTGTCTTCCTGATGTGTACTTTTTGATTAT
+HWI-EAS443:3:22:988:867#0/1
aaR[^^aa`_``a]Uaa]_]WT¥_YaX`X``^_`XV^`^^
@HWI-EAS443:3:22:998:765#0/1
GGGGTTCTTCTCTTGTCTTCCTGATGTGTACTTTTTGATT
+HWI-EAS443:3:22:998:765#0/1
a_¥_R^X_][]SW`STV`_TRZ_U_]Y^]SQY`X_`]¥¥Y
@HWI-EAS443:3:23:1059:245#0/1
GGGTTCTTCTCTTGTCTTCCTGATGTGTACTTTTTGATTA
+HWI-EAS443:3:23:1059:245#0/1
aaa[aaaaaa`aaa_^aa__`__^_]_]^^_aa^``__]_
@HWI-EAS443:3:23:1230:1510#0/1
CGGGGTTCTTCTCTTGTCTTCCTGATGTGTACTTTTTGAT

```
+HWI-EAS443:3:23:1230:1510#0/1
abbbbababb`babb_aabb_ab¥`aa^_`a_baaa_``a
@HWI-EAS443:3:23:1396:168#0/1
CGGGGTTCTTCTCTTGTCTTCCTGATGTGTACTTTTTGAT
+HWI-EAS443:3:23:1396:168#0/1
aaZRY¥¥TY__aY_]XLZ``Y^[ZR[[[aUQF[_]_[NR]
@HWI-EAS443:3:23:1428:1210#0/1
CGGGGTTCTTCTCTTGTCTTCCTGATGTGTACTTTTTGAT
+HWI-EAS443:3:23:1428:1210#0/1
abba`abbbab_bb`[ababa_a`_aa^b^_Za`aaaa_a
@HWI-EAS443:3:23:1749:245#0/1
GGGTTCTTCTCTTGTCTTCCTGATGTGTACTTTTTGATTA
+HWI-EAS443:3:23:1749:245#0/1
ab]__aaa_aba_ba¥aa`^a_WaZXa__^_aaZ]O[a^B
@HWI-EAS443:3:23:1767:210#0/1
GCGGGGTTCTTCTCTTGTCTTCCTGATGTGTACTTTTTGA
+HWI-EAS443:3:23:1767:210#0/1
ab`bababbbbabaaaa`abbP^_X_a_`a_`_`a^bb`_
@HWI-EAS443:3:23:442:1358#0/1
GGGGTTCTTCTCTTGTCTTCCTGATGTGTACTTTTTGATT
+HWI-EAS443:3:23:442:1358#0/1
aaa^Gbaaaa``aaXV[aa``W_[a^^`]¥Z_^`^`_^_^
@HWI-EAS443:3:23:496:86#0/1
GGTTCTTCTCTTGTCTTCCTGATGTGTACTTTTTGATTAT
+HWI-EAS443:3:23:496:86#0/1
abZaa__aa_`aa`[`^^_`^]`^^^XX^_`^^_]^^^]Z
@HWI-EAS443:3:23:742:868#0/1
GGGTTCTTCTCTTGTCTTCCTGATGTGTACTTTTTGATTA
+HWI-EAS443:3:23:742:868#0/1
_b`_aabbbbaaaaaaaaaaabaaa_a]¥]aaaa`_Za_`
@HWI-EAS443:3:23:747:1493#0/1
GGGGTTCTTCTCTTGTCTTCCTGATGTGTACTTTTTGATT
+HWI-EAS443:3:23:747:1493#0/1
_T¥EPT_Z]Y[``__]a`]UWTUXYLFPL]W_¥_]Y`_ZY
@HWI-EAS443:3:23:759:241#0/1
GCGGGGTTCTTCTCTTGTCTTCCTGATGTGTACTTTTTGA
+HWI-EAS443:3:23:759:241#0/1
]aaY[b]aZY`¥`Zaaa``bVN¥ZaY^aabMYaaa_^aaa
@HWI-EAS443:3:23:914:1697#0/1
GGGGTTCTTCTCTTGTCTTCCTGATGTGTACTTTTTGATT
+HWI-EAS443:3:23:914:1697#0/1
aba[P`L`]O]Z`_aX`YQ^SU_^[VF[R^_W[U_]_]_X
@HWI-EAS443:3:24:1014:1356#0/1
AGCGGGGTTCTTCTCTTGTCTTCCTGATGTGTACTTTTTG
+HWI-EAS443:3:24:1014:1356#0/1
aaa__a`Waaaaaa^_```_^__]`]_`_^]Z_^`_`a_Q
@HWI-EAS443:3:24:1037:778#0/1
ATCAAAAAGTACACATCAGGAAGACAAGAGAAGAACCCCG
+HWI-EAS443:3:24:1037:778#0/1
aa_`a_b]bKbbaa]`babaaba`a`ba`aaaaaba`UWb
```

```
@HWI-EAS443:3:24:108:525#0/1
GCGGGGTTCTTCTCTTGTCTTCCTGATGTGTACTTTTTGA
+HWI-EAS443:3:24:108:525#0/1
abaaaaabbbabbabab`abbaaba_aaaa^aaabaabaa
@HWI-EAS443:3:24:1144:1859#0/1
ATCAAAAAGTACACATCAGGAAGACAAGAGAAGAACCCCG
+HWI-EAS443:3:24:1144:1859#0/1
^¥¥`b`bb_G_abb`bb^XY^]Z]]ba^a`^^^`bbXN_a
@HWI-EAS443:3:24:434:1690#0/1
CGGGGTTCTTCTCTTGTCTTCCTGATGTGTACTTTTTGAT
+HWI-EAS443:3:24:434:1690#0/1
ab`_a_bbb```bbaaaaaabaaVa`a^¥^a`a``_a]aa
@HWI-EAS443:3:24:830:599#0/1
GGTTCTTCTCTTGTCTTCCTGATGTGTACTTTTTGATTAT
+HWI-EAS443:3:24:830:599#0/1
¥``b¥bbb_[b`Y]Ybaa`XS^_a`a¥`aaaaba^a___a
@HWI-EAS443:3:25:1003:99#0/1
CGGGGTTCTTCTCTTGTCTTCCTGATGTGTACTTTTTGAT
+HWI-EAS443:3:25:1003:99#0/1
_bbbbba`_babaabbaabb[__babbaaa_T`abbb[bb
@HWI-EAS443:3:25:1181:1209#0/1
GCGGGGTTCTTCTCTTGTCTTCCTGATGTGTACTTTTTGA
+HWI-EAS443:3:25:1181:1209#0/1
abaaa]U]^aaaaaaa]_T^`[_`a^[UV`[SS]a``aV¥
@HWI-EAS443:3:25:1313:1489#0/1
CGGGGTTCTTCTCTTGTCTTCCTGATGTGTACTTTTTGAT
+HWI-EAS443:3:25:1313:1489#0/1
[`¥abUbbaabbabbbbbababbbbaa[aa^bbbbbbabb
@HWI-EAS443:3:25:1581:1471#0/1
ATAATCAAAAAGTACACATCAGGAAGACAAGAGAAGAACC
+HWI-EAS443:3:25:1581:1471#0/1
a^_^`b``a[a`aa_ba_aaa_O¥aX_RY]aa`^¥[a_^U
@HWI-EAS443:3:25:1585:231#0/1
AGCGGGGTTCTTCTCTTGTCTTCCTGATGTGTACTTTTTG
+HWI-EAS443:3:25:1585:231#0/1
aabbbab`babb`b_bb``_aa__ba_a`^`a_Wabaaa[
@HWI-EAS443:3:25:1686:1388#0/1
CGGGGTTCTTCTCTTGTCTTCCTGATGTGTACTTTTTGAT
+HWI-EAS443:3:25:1686:1388#0/1
a]a`babbabbbabbab`bbaabb¥bbaa]`Ybbbbbbab
@HWI-EAS443:3:25:382:964#0/1
GCGGGGTTCTTCTCTTGTCTTCCTGATGTGTACTTTTTGA
+HWI-EAS443:3:25:382:964#0/1
aba_[_PPX[___^__`U__¥Y_^[WZZZ^Y¥¥¥¥^¥[VT
@HWI-EAS443:3:25:460:806#0/1
GGGTTCTTCTCTTGTCTTCCTGATGTGTACTTTTTGATTA
+HWI-EAS443:3:25:460:806#0/1
aa__ba]__WTPaUWT]__]_S`Q`T_G`][^_]_XUV¥^
@HWI-EAS443:3:25:549:427#0/1
CGGGGTTCTTCTCTTGTCTTCCTGATGTGTACTTTTTGAT
```

+HWI-EAS443:3:25:549:427#0/1
`aa`aXab_bbbbb_b`abbaabba`_`b`Z_b`bab[^`
@HWI-EAS443:3:25:568:162#0/1
CGGGGTTCTTCTCTTGTCTTCCTGATGTGTACTTTTTGAT
+HWI-EAS443:3:25:568:162#0/1
aaabb]abbb`]`babab bb`aaaaa`]ZZa_a`_ba`a`
@HWI-EAS443:3:25:688:1039#0/1
GGGGTTCTTCTCTTGTCTTCCTGATGTGTACTTTTTGATT
+HWI-EAS443:3:25:688:1039#0/1
a[a`Yaa`a^a`_a`^]a`____^^_Y_V_Y^_^__^]^^
@HWI-EAS443:3:25:839:248#0/1
GGTTCTTCTCTTGTCTTCCTGATGTGTACTTTTTGATTAT
+HWI-EAS443:3:25:839:248#0/1
a[SZ^X_]_X__^Q^]¥Z]^WIX]N]UWOZZ^Z]SWZZZX
@HWI-EAS443:3:25:928:816#0/1
GGGGTTCTTCTCTTGTCTTCCTGATGTGTACTTTTTGATT
+HWI-EAS443:3:25:928:816#0/1
a`^XXa_aSD¥_```_`]¥RY`VU[_U^O]]_^_W_YU`_
@HWI-EAS443:3:26:1063:1498#0/1
GCGGGGTTCTTCTCTTGTCTTCCTGATGTGTACTTTTTGA
+HWI-EAS443:3:26:1063:1498#0/1
^``[¥Y_V¥a_X_O¥[¥Y¥_`I_a¥W¥_T_TU¥^_ZY¥^[
@HWI-EAS443:3:26:1121:959#0/1
GCGGGGTTCTTCTCTTGTCTTCCTGATGTGTACTTTTTGA
+HWI-EAS443:3:26:1121:959#0/1
a_S]XTKXY__a]]_``YY^¥`YW]]_`X_W[_[_]`]PZ
@HWI-EAS443:3:26:1144:1681#0/1
CGGGGTTCTTCTCTTGTCTTCCTGATGTGTACTTTTTGAT
+HWI-EAS443:3:26:1144:1681#0/1
VbaZTJbaSY`ab¥aaXab``XVVbb_Jaa^bbabb_¥a`
@HWI-EAS443:3:26:1183:876#0/1
CGGGGTTCTTCTCTTGTCTTCCTGATGTGTACTTTTTGAT
+HWI-EAS443:3:26:1183:876#0/1
abbbbab`babbabbaa`bba`baabaabaa`bbabb]ab
@HWI-EAS443:3:26:1373:755#0/1
CGGGGTTCTTCTCTTGTCTTCCTGATGTGTACTTTTTGAT
+HWI-EAS443:3:26:1373:755#0/1
a_]Y¥]__^aZ^aYa^WU_a[a`a`a```R_`^a`ab_]a
@HWI-EAS443:3:26:1691:615#0/1
GGGTTCTTCTCTTGTCTTCCTGATGTGTACTTTTTGATTA
+HWI-EAS443:3:26:1691:615#0/1
abaW_Wa^[]]aaa]X``YL_a__aYa]_U_a_^_¥^_^Y
@HWI-EAS443:3:26:279:580#0/1
GGGGTTCTTCTCTTGTCTTCCTGATGTGTACTTTTTGATT
+HWI-EAS443:3:26:279:580#0/1
``aaYaa_[aabXbb[YVXbaVWZVNY]PVYa[aa_`___
@HWI-EAS443:3:26:691:738#0/1
CGGGGTTCTTCTCTTGTCTTCCTGATGTGTACTTTTTGAT
+HWI-EAS443:3:26:691:738#0/1
a`Z¥[[_Zb^Xbbaaa_[ba]]a``a`Ya_^`ba`baWa_

@HWI-EAS443:3:26:814:836#0/1
CGGGGTTCTTCTCTTGTCTTCCTGATGTGTACTTTTTGAT
+HWI-EAS443:3:26:814:836#0/1
`a_T]a[aabbbaaa]aX]]ab]Xa`^Waab`a`ba_X]b
@HWI-EAS443:3:26:899:1046#0/1
GGGTTCTTCTCTTGTCTTCCTGATGTGTACTTTTTGATTA
+HWI-EAS443:3:26:899:1046#0/1
aa`^baaaabaaaa``baaaaa^aa_a^_^aaaaa_a``a
@HWI-EAS443:3:26:926:1329#0/1
GGGGTTCTTCTCTTGTCTTCCTGATGTGTACTTTTTGATT
+HWI-EAS443:3:26:926:1329#0/1
aaa_Z``aa`aa``___```a`^a]_X_]`^``__a^__^
@HWI-EAS443:3:27:1167:1855#0/1
CGGGGTTCTTCTCTTGTCTTCCTGATGTGTACTTTTTGAT
+HWI-EAS443:3:27:1167:1855#0/1
a_GN^X_aHY^UbYa`IZS^a_XNGQ`^^SUUXS¥ULL^]
@HWI-EAS443:3:27:1187:1073#0/1
GGGGTTCTTCTCTTGTCTTCCTGATGTGTACTTTTTGATT
+HWI-EAS443:3:27:1187:1073#0/1
aXYaS¥[a]¥[^_WYW[``]``_UZ_TZY_^```^^]]`W
@HWI-EAS443:3:27:1296:715#0/1
GGTTCTTCTCTTGTCTTCCTGATGTGTACTTTTTGATTAT
+HWI-EAS443:3:27:1296:715#0/1
`UYa_aa]a`aa^V`aaaS]^^_^^Y[`^_``aaa^_^_^
@HWI-EAS443:3:27:167:1178#0/1
GCGGGGTTCTTCTCTTGTCTTCCTGATGTGTACTTTTTGA
+HWI-EAS443:3:27:167:1178#0/1
`baabYVY`b`b`aaa__`aab`a_a`_^`]aa`aa_a[_
@HWI-EAS443:3:27:274:1646#0/1
CGGGGTTCTTCTCTTGTCTTCCTGATGTGTACTTTTTGAT
+HWI-EAS443:3:27:274:1646#0/1
a_P_Q¥````^``a`]P_[`aaV]`[RY_Z^T]a]]_V_`
@HWI-EAS443:3:27:397:1130#0/1
CGGGGTTCTTCTCTTGTCTTCCTGATGTGTACTTTTTGAT
+HWI-EAS443:3:27:397:1130#0/1
aabaaYabaaa`^aaa`aaaa_a__^¥^a_``a```a_``
@HWI-EAS443:3:27:900:1291#0/1
ATAATCAAAAAGTACACATCAGGAAGACAAGAGAAGAACC
+HWI-EAS443:3:27:900:1291#0/1
^X[Z¥a]ab``U`ab``_]baaaaba_baV``aaaa`a_W
@HWI-EAS443:3:28:1489:208#0/1
GCGGGGTTCTTCTCTTGTCTTCCTGATGTGTACTTTTTGA
+HWI-EAS443:3:28:1489:208#0/1
a^a]aaXa[_a]a^``a_^`__^`_^]aX_Y^^_`^`_^]
@HWI-EAS443:3:28:1647:552#0/1
CGGGGTTCTTCTCTTGTCTTCCTGATGTGTACTTTTTGAT
+HWI-EAS443:3:28:1647:552#0/1
abbb¥O^UUTX_V^]QHI`]WZa`T`aZbS_¥aX_a_NNY
@HWI-EAS443:3:28:382:1177#0/1
GCGGGGTTCTTCTCTTGTCTTCCTGATGTGTACTTTTTGA

```
+HWI-EAS443:3:28:382:1177#0/1
ab]`aaaaaa_aabaa`X`^]aaa_`]]TZT^Za_`¥a]`
@HWI-EAS443:3:28:784:1834#0/1
CGGGGTTCTTCTCTTGTCTTCCTGATGTGTACTTTTTGAT
+HWI-EAS443:3:28:784:1834#0/1
[_R¥WR`aZ_bbaa`Jaba__[U`Y`a``_]`Va][`S¥_
@HWI-EAS443:3:29:1049:781#0/1
TCAAAAAGTACACATCAGGAAGACAAGAGAAGAACCCCGC
+HWI-EAS443:3:29:1049:781#0/1
abaaaa``^baaaaaaaaa`aa``a`a_a_aaa`aZZ_a]
@HWI-EAS443:3:29:113:1507#0/1
CGGGGTTCTTCTCTTGTCTTCCTGATGTGTACTTTTTGAT
+HWI-EAS443:3:29:113:1507#0/1
a`¥`_¥`baaaaaa``_a`aaa_``^[VaX¥^`_`_`]`^
@HWI-EAS443:3:29:1154:354#0/1
GCGGGGTTCTTCTCTTGTCTTCCTGATGTGTACTTTTTGA
+HWI-EAS443:3:29:1154:354#0/1
`TW¥W^¥aa]b^aaa¥ZQ`¥^[Y`a_Y¥TVR`[Y¥Y`_Y`
@HWI-EAS443:3:29:1240:488#0/1
GCGGGGTTCTTCTCTTGTCTTCCTGATGTGTACTTTTTGA
+HWI-EAS443:3:29:1240:488#0/1
a`abb`H`abbZa¥ba]Vaabaaabaaa[^N^^aaaaa]a
@HWI-EAS443:3:29:1299:2025#0/1
GCGGGGTTCTTCTCTTGTCTTCCTGATGTGTACTTTTTGA
+HWI-EAS443:3:29:1299:2025#0/1
a¥X_OHXbaa]_a_¥WMP]X^`__[__]WYT__]_]`_Y^
@HWI-EAS443:3:29:1674:626#0/1
CGGGGTTCTTCTCTTGTCTTCCTGATGTGTACTTTTTGAT
+HWI-EAS443:3:29:1674:626#0/1
_b¥bb¥X_bbO¥`¥bX¥_baVab_abbb`bbbbbbbb`b_
@HWI-EAS443:3:29:239:479#0/1
CGGGGTTCTTCTCTTGTCTTCCTGATGTGTACTTTTTGAT
+HWI-EAS443:3:29:239:479#0/1
aba``^bbbbbbbbaa`abaaaabaaa^^¥a_aaaab]`a
@HWI-EAS443:3:29:262:1209#0/1
CGGGGTTCTTCTCTTGTCTTCCTGATGTGTACTTTTTGAT
+HWI-EAS443:3:29:262:1209#0/1
aaaa]^aaaabaa``a^a_`a^^^P[_Y_UV]`^`__^a_
@HWI-EAS443:3:29:444:136#0/1
CGGGGTTCTTCTCTTGTCTTCCTGATGTGTACTTTTTGAT
+HWI-EAS443:3:29:444:136#0/1
a`¥`aO]^abaaaaaa]`_``^Y__¥_V_PTY[___^[^B
@HWI-EAS443:3:29:48:659#0/1
GCGGGGTTCTTCTCTTGTCTTCCTGATGTGTACTTTTTGA
+HWI-EAS443:3:29:48:659#0/1
abbaaa]aaaaaaaaaa]_a^`a`a_^`V[][]`_a_`Y^
@HWI-EAS443:3:29:634:21#0/1
AGCGGGGTTCTTCTCTTGTCTTCCTGATGTGTACTTTTTG
+HWI-EAS443:3:29:634:21#0/1
a]]aaY`O]]a`^¥``]]X^W`_]]aZX^¥`U_W^^a]_B
```

@HWI-EAS443:3:29:768:858#0/1
GGGTTCTTCTCTTGTCTTCCTGATGTGTACTTTTTGATTA
+HWI-EAS443:3:29:768:858#0/1
abaUaaaaaaa`aa^aaaaaaa_`aX_^_^aa`aa`^``_
@HWI-EAS443:3:29:896:86#0/1
CGGGGTTCTTCTCTTGTCTTCCTGATGTGTACTTTTTGAT
+HWI-EAS443:3:29:896:86#0/1
abbb^Va`bbbbababaabaa]bbaababaaaabbbb^aa
@HWI-EAS443:3:2:1001:432#0/1
CGGGGTTCTTCTCTTGTCTTCCTGATGTGTACTTTTTGAT
+HWI-EAS443:3:2:1001:432#0/1
abbba^aaba_a`aaa`aba_``a``a`a_`Zaa`aa_a`
@HWI-EAS443:3:2:1052:2004#0/1
AGCGGGGTTCTTCTCTTGTCTTCCTGATGTGTACTTTTTG
+HWI-EAS443:3:2:1052:2004#0/1
aaXabZ[[aX^Y^]SN^a^_]YN_]`__a^a__V_^^¥YB
@HWI-EAS443:3:2:1125:326#0/1
CGGGGTTCTTCTCTTGTCTTCCTGATGTGTACTTTTTGAT
+HWI-EAS443:3:2:1125:326#0/1
abb^`[b]abaa¥aaa]`a_aY``¥_a]a]__`a`^^]]_
@HWI-EAS443:3:2:1167:1460#0/1
GGGTTCTTCTCTTGTCTTCCTGATGTGTACTTTTTGATTA
+HWI-EAS443:3:2:1167:1460#0/1
aXOPX^_VPYI]YP]Q¥`_^W[U]_W]S^U`^__U]BBBB
@HWI-EAS443:3:2:1769:360#0/1
CGGGGTTCTTCTCTTGTCTTCCTGATGTGTACTTTTTGAT
+HWI-EAS443:3:2:1769:360#0/1
¥`XZb]bbbaba`bb]`abbaab¥``Y`b_a^baaa_Q`a
@HWI-EAS443:3:2:178:693#0/1
CGGGGTTCTTCTCTTGTCTTCCTGATGTGTACTTTTTGAT
+HWI-EAS443:3:2:178:693#0/1
abbaaRaaaaa_a_^a_aa__aa_``_^`U^Za]``a`a_
@HWI-EAS443:3:2:315:923#0/1
GGTTCTTCTCTTGTCTTCCTGATGTGTACTTTTTGATTAT
+HWI-EAS443:3:2:315:923#0/1
a``a_aaXa]__aX_`a_Y_`^_aW`X_Z___X__^^_]Z
@HWI-EAS443:3:2:722:1220#0/1
GCGGGGTTCTTCTCTTGTCTTCCTGATGTGTACTTTTTGA
+HWI-EAS443:3:2:722:1220#0/1
a_`^[[Y[Ya`O`Za___Z___]a__`^]_Z]X]`]]a]]
@HWI-EAS443:3:2:750:967#0/1
GGGGTTCTTCTCTTGTCTTCCTGATGTGTACTTTTTGATT
+HWI-EAS443:3:2:750:967#0/1
a`_]Ya`_a`a_aaa¥^aaXX_`__aU_I¥W_^aaa__Y`
@HWI-EAS443:3:30:1120:1375#0/1
GCGGGGTTCTTCTCTTGTCTTCCTGATGTGTACTTTTTGA
+HWI-EAS443:3:30:1120:1375#0/1
ababa_Xaaaa`aaaba`aaaa_aa_`^V`]`__a_``[_
@HWI-EAS443:3:30:1320:1355#0/1
GGGGTTCTTCTCTTGTCTTCCTGATGTGTACTTTTTGATT

```
+HWI-EAS443:3:30:1320:1355#0/1
aba_Za[a`aa`aaQ¥aa``^_`__`X^U`S``Z``]^^U
@HWI-EAS443:3:30:1359:1585#0/1
GCGGGGTTCTTCTCTTGTCTTCCTGATGTGTACTTTTTGA
+HWI-EAS443:3:30:1359:1585#0/1
_bbbbb`bbb```aabb_abba_a`_aa^Z`aaaaaaa`a
@HWI-EAS443:3:30:1550:1024#0/1
CGGGGTTCTTCTCTTGTCTTCCTGATGTGTACTTTTTGAT
+HWI-EAS443:3:30:1550:1024#0/1
]bbaT`a`^``abb`bbbbabY^ba]]aaaa`bb`a_RYa
@HWI-EAS443:3:30:1552:159#0/1
ATCAAAAAGTACACATCAGGAAGACAAGAGAAGAACCCCG
+HWI-EAS443:3:30:1552:159#0/1
abaaaaa¥¥Xb`ba``_aaa`aa_^a_^_^]`__XXRUP¥
@HWI-EAS443:3:30:282:1396#0/1
CGGGGTTCTTCTCTTGTCTTCCTGATGTGTACTTTTTGAT
+HWI-EAS443:3:30:282:1396#0/1
Z`^^XWaa`YYZb^a^aaa`YaZYY]`U¥Taa]Z¥a_]a[
@HWI-EAS443:3:30:339:1457#0/1
GGTTCTTCTCTTGTCTTCCTGATGTGTACTTTTTGATTAT
+HWI-EAS443:3:30:339:1457#0/1
W]aZa^¥``¥¥V^[`Ja`b^YSaYH_abbVb¥¥QV¥aaaB
@HWI-EAS443:3:30:434:1858#0/1
ATCAAAAAGTACACATCAGGAAGACAAGAGAAGAACCCCG
+HWI-EAS443:3:30:434:1858#0/1
a`b_abbV^_baaba`ba``aa_`ba__a_`a^aa`¥``^
@HWI-EAS443:3:30:480:1060#0/1
CGGGGTTCTTCTCTTGTCTTCCTGATGTGTACTTTTTGAT
+HWI-EAS443:3:30:480:1060#0/1
aaa^ZWaaaabaaa`a_aa``a`^a_a]_X]_a__aa¥__
@HWI-EAS443:3:30:627:922#0/1
CGGGGTTCTTCTCTTGTCTTCCTGATGTGTACTTTTTGAT
+HWI-EAS443:3:30:627:922#0/1
abbb`Saaabbbaa]bTbb_ba]Ya_Y[_U``ab`[]TY`
@HWI-EAS443:3:30:876:1237#0/1
GCGGGGTTCTTCTCTTGTCTTCCTGATGTGTACTTTTTGA
+HWI-EAS443:3:30:876:1237#0/1
a_¥]``[]aa`aaa¥Z^¥a``aaTNVY[SPZ[]^¥¥`^¥^
@HWI-EAS443:3:31:1204:292#0/1
GCGGGGTTCTTCTCTTGTCTTCCTGATGTGTACTTTTTGA
+HWI-EAS443:3:31:1204:292#0/1
a_baaa^aaa`_a_`aa¥^_`S[a`_[]]_]_^`__a`Y]
@HWI-EAS443:3:31:1527:1017#0/1
GCGGGGTTCTTCTCTTGTCTTCCTGATGTGTACTTTTTGA
+HWI-EAS443:3:31:1527:1017#0/1
aab`b_G``aaa`[aa``_a[`a_^]_`Ya]_^aW`^a_U
@HWI-EAS443:3:31:1610:1068#0/1
GCGGGGTTCTTCTCTTGTCTTCCTGATGTGTACTTTTTGA
+HWI-EAS443:3:31:1610:1068#0/1
abaa^YR`a`]baaaab^``aaa`a^^[`a]][_^]_aa^
```

```
@HWI-EAS443:3:31:1629:330#0/1
GGGGTTCTTCTCTTGTCTTCCTGATGTGTACTTTTTGATT
+HWI-EAS443:3:31:1629:330#0/1
`_b¥Lb[aaP^abb`a`b`UaaaUaaUa[aWa_aaaa_a[
@HWI-EAS443:3:31:178:490#0/1
GCGGGGTTCTTCTCTTGTCTTCCTGATGTGTACTTTTTGA
+HWI-EAS443:3:31:178:490#0/1
abaaba^aabaaaaaaa`aa`a`aXaa^V]^^_a`a_a]_
@HWI-EAS443:3:31:516:489#0/1
CGGGGTTCTTCTCTTGTCTTCCTGATGTGTACTTTTTGAT
+HWI-EAS443:3:31:516:489#0/1
¥^Y``La^^UZYU_`_Y_ab^a]V¥T_YQ[GV]aZN¥I`_
@HWI-EAS443:3:31:669:563#0/1
GGGGTTCTTCTCTTGTCTTCCTGATGTGTACTTTTTGATT
+HWI-EAS443:3:31:669:563#0/1
aaab_baabbbaaaaaabaaaaaa`_]a^__aaa`a`_a_
@HWI-EAS443:3:31:701:1882#0/1
CGGGGTTCTTCTCTTGTCTTCCTGATGTGTACTTTTTGAT
+HWI-EAS443:3:31:701:1882#0/1
aa`^¥¥^a^_a_a_``R``^_`¥¥RU]Z^X_Z^^]]XX_Z
@HWI-EAS443:3:31:726:556#0/1
AGCGGGGTTCTTCTCTTGTCTTCCTGATGTGTACTTTTTG
+HWI-EAS443:3:31:726:556#0/1
a_aaaaaOaaaaaa`a`a^a`_``__`_`^aY]^_a^_Z]
@HWI-EAS443:3:31:761:1893#0/1
GGGTTCTTCTCTTGTCTTCCTGATGTGTACTTTTTGATTA
+HWI-EAS443:3:31:761:1893#0/1
````aba`bb_bbb^abbaa`^abaaTL]a`aaab`aaa[
@HWI-EAS443:3:31:78:530#0/1
CGGGGTTCTTCTCTTGTCTTCCTGATGTGTACTTTTTGAT
+HWI-EAS443:3:31:78:530#0/1
ab^^^S]][aa_a]¥Uaaaaaa_PW`UN]X^Xa_UXZFP[
@HWI-EAS443:3:31:819:1171#0/1
CGGGGTTCTTCTCTTGTCTTCCTGATGTGTACTTTTTGAT
+HWI-EAS443:3:31:819:1171#0/1
`V[``^aaaa^aaaa_`aaaa``_`a`W]U^¥a_a_```^
@HWI-EAS443:3:31:83:1764#0/1
CGGGGTTCTTCTCTTGTCTTCCTGATGTGTACTTTTTGAT
+HWI-EAS443:3:31:83:1764#0/1
aa`¥]¥aa``aaa`a`^ba_aa`____]]¥[aa^``_Xa^
@HWI-EAS443:3:32:1081:675#0/1
CGGGGTTCTTCTCTTGTCTTCCTGATGTGTACTTTTTGAT
+HWI-EAS443:3:32:1081:675#0/1
abb^`YbbbabbbbbabbaabaaXaaTP]]aaaabaa``
@HWI-EAS443:3:32:1120:344#0/1
GCGGGGTTCTTCTCTTGTCTTCCTGATGTGTACTTTTTGA
+HWI-EAS443:3:32:1120:344#0/1
`aX¥ababbbab_baa^P`b`[R_MU¥YR]SX_`aaba``
@HWI-EAS443:3:32:1208:377#0/1
CGGGGTTCTTCTCTTGTCTTCCTGATGTGTACTTTTTGAT
```
```

```
+HWI-EAS443:3:32:1208:377#0/1
abbb^¥babb`b`bba`^b`O`bba`b_a^a]babaa_aa
@HWI-EAS443:3:32:1483:1465#0/1
GGGGTTCTTCTCTTGTCTTCCTGATGTGTACTTTTTGATT
+HWI-EAS443:3:32:1483:1465#0/1
a]¥_Z_]^¥QZa]_`XT_`U[Z^Y¥ZVXTXT^]X^^_[_]
@HWI-EAS443:3:32:1605:121#0/1
GCGGGGTTCTTCTCTTGTCTTCCTGATGTGTACTTTTTGA
+HWI-EAS443:3:32:1605:121#0/1
a`a`aa[a```¥_]__`^]``TX_^Z__]`WZZ^_^`_[Y
@HWI-EAS443:3:32:359:686#0/1
GGGTTCTTCTCTTGTCTTCCTGATGTGTACTTTTTGATTA
+HWI-EAS443:3:32:359:686#0/1
S^`V`Z`ab`baY`aaa]b_]baaaVa`ba^abbaaXaa`
@HWI-EAS443:3:32:442:1980#0/1
GCGGGGTTCTTCTCTTGTCTTCCTGATGTGTACTTTTTGA
+HWI-EAS443:3:32:442:1980#0/1
ab_a¥_G_b`aa`a_a`]a`aab__a_`__^aa````_I_
@HWI-EAS443:3:32:596:674#0/1
CGGGGTTCTTCTCTTGTCTTCCTGATGTGTACTTTTTGAT
+HWI-EAS443:3:32:596:674#0/1
^_^_S^_a``Z`baababba^X^WU^``^W]X[a]a`Z¥^
@HWI-EAS443:3:32:669:1309#0/1
GGGGTTCTTCTCTTGTCTTCCTGATGTGTACTTTTTGATT
+HWI-EAS443:3:32:669:1309#0/1
a`V`SRaa^`^b[`]J[Z_XZWWa_aVVSXX^_]]ZS[^T
@HWI-EAS443:3:32:707:1313#0/1
GGGTTCTTCTCTTGTCTTCCTGATGTGTACTTTTTGATTA
+HWI-EAS443:3:32:707:1313#0/1
aaa^bbbababbbb^a`abaaa_a[^^^``aaaaa_¥]_a
@HWI-EAS443:3:32:841:1070#0/1
CGGGGTTCTTCTCTTGTCTTCCTGATGTGTACTTTTTGAT
+HWI-EAS443:3:32:841:1070#0/1
^^aZZQ]^^]`b¥¥]`[`V_¥V`PWVXN_Taa^UY]UQ[Z
@HWI-EAS443:3:32:842:343#0/1
GGTTCTTCTCTTGTCTTCCTGATGTGTACTTTTTGATTAT
+HWI-EAS443:3:32:842:343#0/1
a`T`aaaaa`aaa_`aa_a`_`_][`]`__`a_^^¥`a__
@HWI-EAS443:3:32:860:1974#0/1
ATCAAAAAGTACACATCAGGAAGACAAGAGAAGAACCCCG
+HWI-EAS443:3:32:860:1974#0/1
¥W¥aX^`^[Ua`Y`^Pa^OV_^X]^_]W_`a_WWaW^X^R
@HWI-EAS443:3:32:91:797#0/1
CGGGGTTCTTCTCTTGTCTTCCTGATGTGTACTTTTTGAT
+HWI-EAS443:3:32:91:797#0/1
aba`_P]babbaaaaa_aa]aaa``_a^`PS_`aaa¥_`_
@HWI-EAS443:3:32:930:1275#0/1
GGGGTTCTTCTCTTGTCTTCCTGATGTGTACTTTTTGATT
+HWI-EAS443:3:32:930:1275#0/1
aaaa_aaab`baba`__bba_`a```^a_``aaa`baaa_
```

```
@HWI-EAS443:3:33:1063:648#0/1
GCGGGGTTCTTCTCTTGTCTTCCTGATGTGTACTTTTTGA
+HWI-EAS443:3:33:1063:648#0/1
aabaaa^aabb`a_aaa^_`a`]``]]a]a^MHXYa_a[^
@HWI-EAS443:3:33:1468:738#0/1
GGGGTTCTTCTCTTGTCTTCCTGATGTGTACTTTTTGATT
+HWI-EAS443:3:33:1468:738#0/1
abaa[aaaa_a`abb¥aaaa_aaZ_bXa[_^_Y__`^^`a
@HWI-EAS443:3:33:1512:1758#0/1
CGGGGTTCTTCTCTTGTCTTCCTGATGTGTACTTTTTGAT
+HWI-EAS443:3:33:1512:1758#0/1
ababbabbbbababbbbaaba`bbbaa`aaaM^aaab_`a
@HWI-EAS443:3:33:360:603#0/1
GCGGGGTTCTTCTCTTGTCTTCCTGATGTGTACTTTTTGA
+HWI-EAS443:3:33:360:603#0/1
aaaaa]Q`aaaa`aaa`Za__a``__]_[^SYS^`[_][_
@HWI-EAS443:3:33:434:584#0/1
GGGTTCTTCTCTTGTCTTCCTGATGTGTACTTTTTGATTA
+HWI-EAS443:3:33:434:584#0/1
^Y^]a`babbbbbbaaaaaaa¥a[[ZaXX`aaa_``_a_a
@HWI-EAS443:3:33:89:1326#0/1
GGGGTTCTTCTCTTGTCTTCCTGATGTGTACTTTTTGATT
+HWI-EAS443:3:33:89:1326#0/1
a`^¥`_b¥_b_ba¥V]U`^_``YJY_]_[Y__Y]]_Q]_]
@HWI-EAS443:3:34:110:958#0/1
GCGGGGTTCTTCTCTTGTCTTCCTGATGTGTACTTTTTGA
+HWI-EAS443:3:34:110:958#0/1
aa`X`WPU_^]b_aaYTV_Z[^¥TZVY]HQQ^^`^¥^TBB
@HWI-EAS443:3:34:1213:967#0/1
CGGGGTTCTTCTCTTGTCTTCCTGATGTGTACTTTTTGAT
+HWI-EAS443:3:34:1213:967#0/1
aaaab_a^b_`b_bbaaba_a^aaa`^_a``^`_`_^BBB
@HWI-EAS443:3:34:1215:1226#0/1
GGGGTTCTTCTCTTGTCTTCCTGATGTGTACTTTTTGATT
+HWI-EAS443:3:34:1215:1226#0/1
^a``^abbaabbbb_`abaa_ba^aa`a^`^aaa`^R]`_
@HWI-EAS443:3:34:1218:1669#0/1
GGTTCTTCTCTTGTCTTCCTGATGTGTACTTTTTGATTAT
+HWI-EAS443:3:34:1218:1669#0/1
`a_abbbababbaaaabbaa^a``^a]a`aaaa_^a^^]T
@HWI-EAS443:3:34:1238:183#0/1
GGGGTTCTTCTCTTGTCTTCCTGATGTGTACTTTTTGATT
+HWI-EAS443:3:34:1238:183#0/1
Yaa_M]ba_`bb[```S`_¥Zab_]^a^]WTZ^a`_P__B
@HWI-EAS443:3:34:1417:1028#0/1
GCGGGGTTCTTCTCTTGTCTTCCTGATGTGTACTTTTTGA
+HWI-EAS443:3:34:1417:1028#0/1
aabbba`aaaaab`baa`aaa`aaa_`a`]__W_aa__[^
@HWI-EAS443:3:34:1791:1928#0/1
CGGGGTTCTTCTCTTGTCTTCCTGATGTGTACTTTTTGAT
```

```
+HWI-EAS443:3:34:1791:1928#0/1
abbbbababb`bbabb`bbba_bab`b¥b¥_M`a`a]]]`
@HWI-EAS443:3:34:196:1509#0/1
CGGGGTTCTTCTCTTGTCTTCCTGATGTGTACTTTTTGAT
+HWI-EAS443:3:34:196:1509#0/1
`[Yaa_bbaababbaa`baa`baaaa_]^_aaaaaaa_aa
@HWI-EAS443:3:34:519:288#0/1
CGGGGTTCTTCTCTTGTCTTCCTGATGTGTACTTTTTGAT
+HWI-EAS443:3:34:519:288#0/1
abab`Yabbaaaaaaa_aaaaaa_`_a_`Xa^_^a`_]_]
@HWI-EAS443:3:34:692:829#0/1
GCGGGGTTCTTCTCTTGTCTTCCTGATGTGTACTTTTTGA
+HWI-EAS443:3:34:692:829#0/1
aa]]_a_aa]ab`a`a````_`a_XP¥[S_X[Y__^^a]Z
@HWI-EAS443:3:34:768:1775#0/1
CGGGGTTCTTCTCTTGTCTTCCTGATGTGTACTTTTTGAT
+HWI-EAS443:3:34:768:1775#0/1
^VUXMYPaZ¥YV`_[_WZTZ¥¥^^^Z[OWOWWTV^ZBBBB
@HWI-EAS443:3:35:1176:2031#0/1
CGGGGTTCTTCTCTTGTCTTCCTGATGTGTACTTTTTGAT
+HWI-EAS443:3:35:1176:2031#0/1
ab]b``baaaaaa`_T_a_aa`^`[]`]a]]_`__`]^]_
@HWI-EAS443:3:35:1184:1297#0/1
GGGTTCTTCTCTTGTCTTCCTGATGTGTACTTTTTGATTA
+HWI-EAS443:3:35:1184:1297#0/1
a[aY[a]Y^a_a__QS__`a__¥[ZVaOa__`__]V^_VY
@HWI-EAS443:3:35:1328:1162#0/1
GGGGTTCTTCTCTTGTCTTCCTGATGTGTACTTTTTGATT
+HWI-EAS443:3:35:1328:1162#0/1
a`[a[T]aa_aaa`a_[a[]¥__¥U_S¥SR¥`__]_`V¥^
@HWI-EAS443:3:35:1371:1549#0/1
GCGGGGTTCTTCTCTTGTCTTCCTGATGTGTACTTTTTGA
+HWI-EAS443:3:35:1371:1549#0/1
abaabaZabbabbabab`aaaaaa`_aa`a`aabaa`a]a
@HWI-EAS443:3:35:347:1251#0/1
CGGGGTTCTTCTCTTGTCTTCCTGATGTGTACTTTTTGAT
+HWI-EAS443:3:35:347:1251#0/1
aa]a]^aaaaaaaaa`Z``aaaa^Y^_Y¥TS_`__`a^__
@HWI-EAS443:3:35:357:1888#0/1
CGGGGTTCTTCTCTTGTCTTCCTGATGTGTACTTTTTGAT
+HWI-EAS443:3:35:357:1888#0/1
aa^U]Z`aa_baaaaa^aa`aaa^a_`Y_]aa`_`_aS``
@HWI-EAS443:3:35:511:708#0/1
GCGGGGTTCTTCTCTTGTCTTCCTGATGTGTACTTTTTGA
+HWI-EAS443:3:35:511:708#0/1
aaXL^__`¥Taaaaaaa]_`a^^WY^WXW]R¥a^Z^_VHR
@HWI-EAS443:3:35:519:725#0/1
CGGGGTTCTTCTCTTGTCTTCCTGATGTGTACTTTTTGAT
+HWI-EAS443:3:35:519:725#0/1
aaaaaY`aaaa_aa`a`_a_a_aa_a_]`VZ_`_a`a¥]_
```

@HWI-EAS443:3:35:622:762#0/1
CGGGGTTCTTCTCTTGTCTTCCTGATGTGTACTTTTTGAT
+HWI-EAS443:3:35:622:762#0/1
a___aXaa`a`a``a`_aa`_```_`_X]YX_`_^_a_^]
@HWI-EAS443:3:35:656:1741#0/1
CGGGGTTCTTCTCTTGTCTTCCTGATGTGTACTTTTTGAT
+HWI-EAS443:3:35:656:1741#0/1
a¥W]^[aaaab`aaaaZaa`aa^^¥__^_V^`_]`_^SUY
@HWI-EAS443:3:35:725:287#0/1
GCGGGGTTCTTCTCTTGTCTTCCTGATGTGTACTTTTTGA
+HWI-EAS443:3:35:725:287#0/1
^babba_abbbbbaaa``bbaaabaaab_`R`aaaaaa_a
@HWI-EAS443:3:35:998:75#0/1
CGGGGTTCTTCTCTTGTCTTCCTGATGTGTACTTTTTGAT
+HWI-EAS443:3:35:998:75#0/1
ab`_WZaaa`bbYa_aaaa_RX`b_aaZaZQ^abaa`YWa
@HWI-EAS443:3:36:105:111#0/1
CGGGGTTCTTCTCTTGTCTTCCTGATGTGTACTTTTTGAT
+HWI-EAS443:3:36:105:111#0/1
aba¥aNaaaa`aa_aa__``a^`____[aYMYaa^]]_X_
@HWI-EAS443:3:36:1271:388#0/1
CGGGGTTCTTCTCTTGTCTTCCTGATGTGTACTTTTTGAT
+HWI-EAS443:3:36:1271:388#0/1
abaab`babbbabbbb_bbb`abb`ab`bZUUaaba`¥_a
@HWI-EAS443:3:36:1459:903#0/1
GCGGGGTTCTTCTCTTGTCTTCCTGATGTGTACTTTTTGA
+HWI-EAS443:3:36:1459:903#0/1
aa^__`aaaab_a`aaaaaaa^_aa`a`^a_[U`a_`aXW
@HWI-EAS443:3:36:1528:1687#0/1
GGGGTTCTTCTCTTGTCTTCCTGATGTGTACTTTTTGATT
+HWI-EAS443:3:36:1528:1687#0/1
ab``V`bbbabaa```aaaaaaa_`_^`]aaaa`aa`a`_
@HWI-EAS443:3:36:411:473#0/1
GCGGGGTTCTTCTCTTGTCTTCCTGATGTGTACTTTTTGA
+HWI-EAS443:3:36:411:473#0/1
aa¥RZ_N_Z^Z¥¥a_aaX]^^aYSOT]aTTZ_YXU¥`YLS
@HWI-EAS443:3:36:431:1390#0/1
CGGGGTTCTTCTCTTGTCTTCCTGATGTGTACTTTTTGAT
+HWI-EAS443:3:36:431:1390#0/1
`aa``Ybb`abababaaa_baabbaXaaa]^^a_aa^`^U``
@HWI-EAS443:3:36:534:1103#0/1
GCGGGGTTCTTCTCTTGTCTTCCTGATGTGTACTTTTTGA
+HWI-EAS443:3:36:534:1103#0/1
a_TXZ^LSS[W^^^W[P[_WY_ZW]]UJSXSVZ[BBBBBB
@HWI-EAS443:3:36:850:615#0/1
CGGGGTTCTTCTCTTGTCTTCCTGATGTGTACTTTTTGAT
+HWI-EAS443:3:36:850:615#0/1
ababb]`bbaa_aa``aaaaa_a`¥`a^a¥[Ya`aa`a`^
@HWI-EAS443:3:36:883:1936#0/1
GGGTTCTTCTCTTGTCTTCCTGATGTGTACTTTTTGATTA

```
+HWI-EAS443:3:36:883:1936#0/1
Z¥_T_`[^a^V¥_aT¥`][M]ZY`RQ]¥a`___]^X[ZVT
@HWI-EAS443:3:36:980:342#0/1
GCGGGGTTCTTCTCTTGTCTTCCTGATGTGTACTTTTTGA
+HWI-EAS443:3:36:980:342#0/1
a`U^`aR]`aa_a`aaa_aaa__aa^`_[]WZY`_`^`XZ
@HWI-EAS443:3:37:1036:1828#0/1
GCGGGGTTCTTCTCTTGTCTTCCTGATGTGTACTTTTTGA
+HWI-EAS443:3:37:1036:1828#0/1
`ba]`a^aaaa`aa`aa¥`_a`_a^``]NU¥`aa_``aS_
@HWI-EAS443:3:37:1138:519#0/1
CGGGGTTCTTCTCTTGTCTTCCTGATGTGTACTTTTTGAT
+HWI-EAS443:3:37:1138:519#0/1
abbab^baabb`babbaabbabab^b_^¥HOaaabbbaab
@HWI-EAS443:3:37:1165:716#0/1
GCGGGGTTCTTCTCTTGTCTTCCTGATGTGTACTTTTTGA
+HWI-EAS443:3:37:1165:716#0/1
abba`a^a]aaaaaaaa``aaaa`aaa¥UZY_aa`aa`__
@HWI-EAS443:3:37:1246:890#0/1
CGGGGTTCTTCTCTTGTCTTCCTGATGTGTACTTTTTGAT
+HWI-EAS443:3:37:1246:890#0/1
aabab`b`baaaaaaaabab_aaa_aa``Xa`aa_aa`a`
@HWI-EAS443:3:37:1665:506#0/1
CGGGGTTCTTCTCTTGTCTTCCTGATGTGTACTTTTTGAT
+HWI-EAS443:3:37:1665:506#0/1
aaaaaH^a``a`[aab__aa]a`a_`a¥aV]Z]aaaaXa_
@HWI-EAS443:3:37:618:290#0/1
GGTTCTTCTCTTGTCTTCCTGATGTGTACTTTTTGATTAT
+HWI-EAS443:3:37:618:290#0/1
aaT¥¥aa`]][¥¥SW[^_`WWWW^V_T]_UM_R__]]]_]
@HWI-EAS443:3:37:625:985#0/1
CGGGGTTCTTCTCTTGTCTTCCTGATGTGTACTTTTTGAT
+HWI-EAS443:3:37:625:985#0/1
aba_aVabaaaa`aaa_aaaaaaa_^¥Z`S_`_`aa_^[_
@HWI-EAS443:3:37:678:185#0/1
GCGGGGTTCTTCTCTTGTCTTCCTGATGTGTACTTTTTGA
+HWI-EAS443:3:37:678:185#0/1
aaaaVVRaWW_]XP[_a¥U^XV¥[OWRWPOMSP]__]TO[
@HWI-EAS443:3:37:853:700#0/1
CGGGGTTCTTCTCTTGTCTTCCTGATGTGTACTTTTTGAT
+HWI-EAS443:3:37:853:700#0/1
aba_a]`aaaaa`a`a_`_aa`a¥__a[aVT]_`_a``__
@HWI-EAS443:3:38:1018:854#0/1
GGGGTTCTTCTCTTGTCTTCCTGATGTGTACTTTTTGATT
+HWI-EAS443:3:38:1018:854#0/1
H¥abUba_aabbababa``bababbaV]`a`bbbba_aaa
@HWI-EAS443:3:38:1251:377#0/1
CGGGGTTCTTCTCTTGTCTTCCTGATGTGTACTTTTTGAT
+HWI-EAS443:3:38:1251:377#0/1
]```aL^aba_aabbb_baa`bababb_b]Y[aaaaa__a
```

```
@HWI-EAS443:3:38:1690:1766#0/1
CGGGGTTCTTCTCTTGTCTTCCTGATGTGTACTTTTTGAT
+HWI-EAS443:3:38:1690:1766#0/1
Saba^IX¥¥¥Ub`][a[^a^`[ab`[bRZLNVV[]_aX`¥
@HWI-EAS443:3:38:1698:1906#0/1
CGGGGTTCTTCTCTTGTCTTCCTGATGTGTACTTTTTGAT
+HWI-EAS443:3:38:1698:1906#0/1
aa]aaSa``aY^^_baW`_]__a^¥``V¥NTSZZ^[^[]V
@HWI-EAS443:3:38:1781:1547#0/1
GCGGGGTTCTTCTCTTGTCTTCCTGATGTGTACTTTTTGA
+HWI-EAS443:3:38:1781:1547#0/1
a^a_Z_Y^]¥_Z^]`__^^^^R^¥^¥^]^WVXU]ZX^ZZZ
@HWI-EAS443:3:38:264:1843#0/1
AGCGGGGTTCTTCTCTTGTCTTCCTGATGTGTACTTTTTG
+HWI-EAS443:3:38:264:1843#0/1
a_aaa`aZabaa_aa_a_^_a_a`_W__^XUZ]]_^___I
@HWI-EAS443:3:38:324:1370#0/1
GCGGGGTTCTTCTCTTGTCTTCCTGATGTGTACTTTTTGA
+HWI-EAS443:3:38:324:1370#0/1
]¥__a_N^bbb]¥R^]VF[¥[Zba[NR^]`MZZZ[[Xa^^
@HWI-EAS443:3:38:343:985#0/1
CGGGGTTCTTCTCTTGTCTTCCTGATGTGTACTTTTTGAT
+HWI-EAS443:3:38:343:985#0/1
`bb`^^abaab`aa`baaaabaa[aaa^`[]^`_`aa_Y_
@HWI-EAS443:3:38:437:1220#0/1
GCGGGGTTCTTCTCTTGTCTTCCTGATGTGTACTTTTTGA
+HWI-EAS443:3:38:437:1220#0/1
abb`]]Zaba`aaaaaa_aaaaaa`U^`XaY^]a`a`a¥Z
@HWI-EAS443:3:38:712:1652#0/1
CGGGGTTCTTCTCTTGTCTTCCTGATGTGTACTTTTTGAT
+HWI-EAS443:3:38:712:1652#0/1
aaa[`Q_a``_a__][^a``aa`[P`¥]SZZZ__`_¥V^V
@HWI-EAS443:3:38:715:673#0/1
CGGGGTTCTTCTCTTGTCTTCCTGATGTGTACTTTTTGAT
+HWI-EAS443:3:38:715:673#0/1
ab`a^L_aaaa``_aa_aaaa````[_]_WYW_`_aZGZ^
@HWI-EAS443:3:38:726:364#0/1
GCGGGGTTCTTCTCTTGTCTTCCTGATGTGTACTTTTTGA
+HWI-EAS443:3:38:726:364#0/1
a`[`a]NRVLUXQJY[VUWKDR`]]]YBBBBBBBBBBBBB
@HWI-EAS443:3:38:853:337#0/1
GCGGGGTTCTTCTCTTGTCTTCCTGATGTGTACTTTTTGA
+HWI-EAS443:3:38:853:337#0/1
aabaa`UX__XZ_V^^`WW]ZT¥^^ZYUVXOEN^Z^Y]ZV
@HWI-EAS443:3:39:1413:406#0/1
GGGGTTCTTCTCTTGTCTTCCTGATGTGTACTTTTTGATT
+HWI-EAS443:3:39:1413:406#0/1
aa_a]a__`_a]`__^_a^^Z__^^_X^X]XZ_^a^]]^Z
@HWI-EAS443:3:39:1695:1450#0/1
GGGTTCTTCTCTTGTCTTCCTGATGTGTACTTTTTGATTA
```

```
+HWI-EAS443:3:39:1695:1450#0/1
¥_a]bbbbbbabbb^_bbbbbba_ZV]a`ba_`baaaaaa
@HWI-EAS443:3:39:314:716#0/1
CGGGGTTCTTCTCTTGTCTTCCTGATGTGTACTTTTTGAT
+HWI-EAS443:3:39:314:716#0/1
_b_bb`[ZZ^`]b^Zab[aaaa]¥I[^I^`]aab`_aKKa
@HWI-EAS443:3:39:405:20#0/1
GCGGGGTTCTTCTCTTGTCTTCCTGATGTGTACTTTTTGA
+HWI-EAS443:3:39:405:20#0/1
a]`aZ_VX__[S[VXKVFP^¥VPWQFS]WSSVV]VXXXQS
@HWI-EAS443:3:39:480:423#0/1
GGGGTTCTTCTCTTGTCTTCCTGATGTGTACTTTTTGATT
+HWI-EAS443:3:39:480:423#0/1
aa]^_a^`aaa_[`¥[_`_a__¥R[VZXTIS[]_^¥RU¥¥
@HWI-EAS443:3:39:672:1541#0/1
CGGGGTTCTTCTCTTGTCTTCCTGATGTGTACTTTTTGAT
+HWI-EAS443:3:39:672:1541#0/1
aaaaa_bbb_]`baba_baaab``_aa_a]_Taa`aaSa_
@HWI-EAS443:3:39:939:346#0/1
TCAAAAAGTACACATCAGGAAGACAAGAGAAGAACCCCGC
+HWI-EAS443:3:39:939:346#0/1
a`^`aaaaZ__``aaa_aa`^a_^a`__`__]_`a]^]`W
@HWI-EAS443:3:3:108:1680#0/1
CGGGGTTCTTCTCTTGTCTTCCTGATGTGTACTTTTTGAT
+HWI-EAS443:3:3:108:1680#0/1
a`_WXHb_b[¥`^¥RQ¥a`XKR__Qba]Z_¥`aa^___``
@HWI-EAS443:3:3:1419:731#0/1
GGTTCTTCTCTTGTCTTCCTGATGTGTACTTTTTGATTAT
+HWI-EAS443:3:3:1419:731#0/1
aa^¥_aaaa`aa_]^``ZWa^Y`YYa]¥M`___`_^_^_]
@HWI-EAS443:3:3:460:1560#0/1
GGGTTCTTCTCTTGTCTTCCTGATGTGTACTTTTTGATTA
+HWI-EAS443:3:3:460:1560#0/1
¥[aPaabbbaabbba`bbbbaaba`_a`b[bababaaaaa
@HWI-EAS443:3:3:829:859#0/1
GGGGTTCTTCTCTTGTCTTCCTGATGTGTACTTTTTGATT
+HWI-EAS443:3:3:829:859#0/1
aaa`Ga[aa`b`^aa__`^USVa__`W_XZY__^___]¥B
@HWI-EAS443:3:40:1237:1395#0/1
GGGGTTCTTCTCTTGTCTTCCTGATGTGTACTTTTTGATT
+HWI-EAS443:3:40:1237:1395#0/1
_XY¥XTTZ_QRHXXTVN¥^[UW[]YRHTRNSPQZYSVPUS
@HWI-EAS443:3:40:1326:1617#0/1
CGGGGTTCTTCTCTTGTCTTCCTGATGTGTACTTTTTGAT
+HWI-EAS443:3:40:1326:1617#0/1
abbbbbbbbbbbabbbabbbbab_`bbabaaabbbbb]bb
@HWI-EAS443:3:40:1452:538#0/1
ATCAAAAAGTACACATCAGGAAGACAAGAGAAGAACCCCG
+HWI-EAS443:3:40:1452:538#0/1
a`bba`aababaaa`aaabb]abaa^Q_Ya`abbab`U`a
```

@HWI-EAS443:3:40:1732:168#0/1
GCGGGGTTCTTCTCTTGTCTTCCTGATGTGTACTTTTTGA
+HWI-EAS443:3:40:1732:168#0/1
a]baaa^[_`[SX^^_`U[[_]]ZOJO[[YLVV¥_]_]]X
@HWI-EAS443:3:40:1758:1494#0/1
ATCAAAAAGTACACATCAGGAAGACAAGAGAAGAACCCCG
+HWI-EAS443:3:40:1758:1494#0/1
aa`aaaa`a]aaa_a``aba`aaa_]¥^`_^a_baa^V__
@HWI-EAS443:3:40:586:91#0/1
CGGGGTTCTTCTCTTGTCTTCCTGATGTGTACTTTTTGAT
+HWI-EAS443:3:40:586:91#0/1
abbab]b`a^a_aa_a_[¥`a_``[^^^_UPU^X¥^__^[
@HWI-EAS443:3:40:66:1664#0/1
GGTTCTTCTCTTGTCTTCCTGATGTGTACTTTTTGATTAT
+HWI-EAS443:3:40:66:1664#0/1
a`Z_aa__^a]^UX_`^`a¥W¥XWSVMQZa^_^YRVYYO¥
@HWI-EAS443:3:40:761:186#0/1
GCGGGGTTCTTCTCTTGTCTTCCTGATGTGTACTTTTTGA
+HWI-EAS443:3:40:761:186#0/1
aSU^P]X^_XVFSUZ][WVWV^_RVYVRTTWQUQRXYYOX
@HWI-EAS443:3:42:1020:1666#0/1
GGTTCTTCTCTTGTCTTCCTGATGTGTACTTTTTGATTAT
+HWI-EAS443:3:42:1020:1666#0/1
aaababbbbabba_`ba`]a[¥^_]b`aabaaab_baaaa
@HWI-EAS443:3:42:1072:572#0/1
GCGGGGTTCTTCTCTTGTCTTCCTGATGTGTACTTTTTGA
+HWI-EAS443:3:42:1072:572#0/1
aabbbb]aaab`aaaba_`aaa`a]`aa_a]V¥a`aa_^`
@HWI-EAS443:3:42:12:690#0/1
CGGGGTTCTTCTCTTGTCTTCCTGATGTGTACTTTTTGAT
+HWI-EAS443:3:42:12:690#0/1
aabbbabbbbbbbbbbaabbabbaaaa]`__^`aaa`^_a
@HWI-EAS443:3:42:1374:43#0/1
CGGGGTTCTTCTCTTGTCTTCCTGATGTGTACTTTTTGAT
+HWI-EAS443:3:42:1374:43#0/1
abbab^a`ab`b`aaa]`aa`aa_¥``[a``_a`aaaa[a
@HWI-EAS443:3:42:1423:1825#0/1
GGGTTCTTCTCTTGTCTTCCTGATGTGTACTTTTTGATTA
+HWI-EAS443:3:42:1423:1825#0/1
__b^bbbbababababa^aaa`aaaab`a^a]bbaaaY`ba_
@HWI-EAS443:3:42:1439:415#0/1
CGGGGTTCTTCTCTTGTCTTCCTGATGTGTACTTTTTGAT
+HWI-EAS443:3:42:1439:415#0/1
abbbb`bbbbabaaba`aba^`bb_ab`b^`]aaaaaba_
@HWI-EAS443:3:42:1452:1034#0/1
CGGGGTTCTTCTCTTGTCTTCCTGATGTGTACTTTTTGAT
+HWI-EAS443:3:42:1452:1034#0/1
ababbababbbbbbbbaabaaaabaaaaaa_abababaaa
@HWI-EAS443:3:42:237:2034#0/1
AGCGGGGTTCTTCTCTTGTCTTCCTGATGTGTACTTTTTG

+HWI-EAS443:3:42:237:2034#0/1
[Q¥_SPY``a^^]``^`aZ_`Y_O^[a[PTZR^]ZU^`_X
@HWI-EAS443:3:42:273:784#0/1
GCGGGGTTCTTCTCTTGTCTTCCTGATGTGTACTTTTTGA
+HWI-EAS443:3:42:273:784#0/1
abbbab_bab`aaa`aa_a_a^aaa`_a^aY]aaaa_]R_
@HWI-EAS443:3:42:310:1430#0/1
GCGGGGTTCTTCTCTTGTCTTCCTGATGTGTACTTTTTGA
+HWI-EAS443:3:42:310:1430#0/1
`bbaba¥abbaba_a^a[`aaab`a[[a_b^`a`a`aa_`
@HWI-EAS443:3:42:611:437#0/1
CGGGGTTCTTCTCTTGTCTTCCTGATGTGTACTTTTTGAT
+HWI-EAS443:3:42:611:437#0/1
abbab`b`baab`bbb_abaa`aaa`b^`^__aaaa`a_a
@HWI-EAS443:3:42:654:690#0/1
CGGGGTTCTTCTCTTGTCTTCCTGATGTGTACTTTTTGAT
+HWI-EAS443:3:42:654:690#0/1
abbb]P`_a]bba`[SJ^^^aa`a`ab^b^[`a```aa``
@HWI-EAS443:3:42:750:857#0/1
GGGGTTCTTCTCTTGTCTTCCTGATGTGTACTTTTTGATT
+HWI-EAS443:3:42:750:857#0/1
abab_b`bbaa`bba^aaa`aaa_aaaa_a^aa`aa_aaa
@HWI-EAS443:3:42:950:366#0/1
GCGGGGTTCTTCTCTTGTCTTCCTGATGTGTACTTTTTGA
+HWI-EAS443:3:42:950:366#0/1
aababa_baba`aaaaa_aaa^aaa_aa_a`_^aaa`a[`
@HWI-EAS443:3:43:1241:289#0/1
AATCAAAAAGTACACATCAGGAAGACAAGAGAAGAACCCC
+HWI-EAS443:3:43:1241:289#0/1
]^_UWU¥TRSY¥GWa^a^]aaYX]`a_Y_W¥QNZ[XVNRQ
@HWI-EAS443:3:43:1273:1284#0/1
GGTTCTTCTCTTGTCTTCCTGATGTGTACTTTTTGATTAT
+HWI-EAS443:3:43:1273:1284#0/1
aaWa_`aa`aaaa``aaa[`a]_aYaXQ^aa`aaWY_a^`
@HWI-EAS443:3:43:1553:1072#0/1
GCGGGGTTCTTCTCTTGTCTTCCTGATGTGTACTTTTTGA
+HWI-EAS443:3:43:1553:1072#0/1
a``_ab]``aaaa]a``^_`a]]_`X`]T`WP[`a^a`¥]
@HWI-EAS443:3:43:157:1428#0/1
AGCGGGGTTCTTCTCTTGTCTTCCTGATGTGTACTTTTTG
+HWI-EAS443:3:43:157:1428#0/1
aabaabb]abaaa__ba_^abaaaaa``_]`]`_a`baa^
@HWI-EAS443:3:43:1676:32#0/1
GCGGGGTTCTTCTCTTGTCTTCCTGATGTGTACTTTTTGA
+HWI-EAS443:3:43:1676:32#0/1
S`a^R¥J[bba_`]Y_b¥a`WX¥]¥T`aR_`YV]``a[`]
@HWI-EAS443:3:43:283:254#0/1
CGGGGTTCTTCTCTTGTCTTCCTGATGTGTACTTTTTGAT
+HWI-EAS443:3:43:283:254#0/1
_[X_^Z_OZa_Z``a¥P`¥¥T¥`XRU]X`QH¥[a][]W_]

@HWI-EAS443:3:43:367:1182#0/1
GGGGTTCTTCTCTTGTCTTCCTGATGTGTACTTTTTGATT
+HWI-EAS443:3:43:367:1182#0/1
ababS``[a^]b_a]a`Raaaa_SUaTVQ]]`][Z^``^Y
@HWI-EAS443:3:43:57:1361#0/1
CGGGGTTCTTCTCTTGTCTTCCTGATGTGTACTTTTTGAT
+HWI-EAS443:3:43:57:1361#0/1
aa_a]aaaabbbab`aaaa`a_b`ab[`_`a_abaaa_`a
@HWI-EAS443:3:43:661:846#0/1
CGGGGTTCTTCTCTTGTCTTCCTGATGTGTACTTTTTGAT
+HWI-EAS443:3:43:661:846#0/1
abbb_Yaababaabbb`aab^aaa_ab[_J]Yaa`[^a`a
@HWI-EAS443:3:43:857:691#0/1
CGGGGTTCTTCTCTTGTCTTCCTGATGTGTACTTTTTGAT
+HWI-EAS443:3:43:857:691#0/1
`bbba_bbbaababbbaa`bba_ba`ba`aUa`bbaabaaa
@HWI-EAS443:3:44:106:608#0/1
CGGGGTTCTTCTCTTGTCTTCCTGATGTGTACTTTTTGAT
+HWI-EAS443:3:44:106:608#0/1
aba¥S¥^aa_MQVYZRR_]Z`_VV^L^G]JSNZX^SSTK_
@HWI-EAS443:3:44:1131:616#0/1
GCGGGGTTCTTCTCTTGTCTTCCTGATGTGTACTTTTTGA
+HWI-EAS443:3:44:1131:616#0/1
aaaaa_]a]aaaa_aa^`aaa`_aa_`a_a^`]aaa_aa_
@HWI-EAS443:3:44:1322:1517#0/1
GCGGGGTTCTTCTCTTGTCTTCCTGATGTGTACTTTTTGA
+HWI-EAS443:3:44:1322:1517#0/1
a`_Z_a^a_a]aa^`aV^]_]]Za`X¥^VVZ^^_aa_a¥¥
@HWI-EAS443:3:44:150:800#0/1
GGGGTTCTTCTCTTGTCTTCCTGATGTGTACTTTTTGATT
+HWI-EAS443:3:44:150:800#0/1
aaa^Ya_aa^``aaaaZaa^Sa]V^XS[V_X`a`_UQT`]
@HWI-EAS443:3:44:1591:1203#0/1
CGGGGTTCTTCTCTTGTCTTCCTGATGTGTACTTTTTGAT
+HWI-EAS443:3:44:1591:1203#0/1
^UaYHOb[bTLY¥^^]Taa]]TVaT^H^V`a`]aW__¥_^
@HWI-EAS443:3:44:337:333#0/1
GCGGGGTTCTTCTCTTGTCTTCCTGATGTGTACTTTTTGA
+HWI-EAS443:3:44:337:333#0/1
aabbbbabaaa_abbabaaab¥[^[`_a_a¥__aaaaaa`
@HWI-EAS443:3:44:421:812#0/1
GCGGGGTTCTTCTCTTGTCTTCCTGATGTGTACTTTTTGA
+HWI-EAS443:3:44:421:812#0/1
aabaa]Y_aa``a_a__``aaZ`¥[^__^_Y[Y_`a``__
@HWI-EAS443:3:44:564:914#0/1
GCGGGGTTCTTCTCTTGTCTTCCTGATGTGTACTTTTTGA
+HWI-EAS443:3:44:564:914#0/1
abbabbababbabbabbaabbaabaa`a¥a``]baaaa`a
@HWI-EAS443:3:44:59:865#0/1
GGGTTCTTCTCTTGTCTTCCTGATGTGTACTTTTTGATTA

+HWI-EAS443:3:44:59:865#0/1
aaa_aaaaa`a`aa_``____W]_`¥aRXYa`a__a___¥
@HWI-EAS443:3:44:812:19#0/1
CGGGGTTCTTCTCTTGTCTTCCTGATGTGTACTTTTTGAT
+HWI-EAS443:3:44:812:19#0/1
``bbaZabbbababbb`abaaaab`ab`b__aaaa`baaa
@HWI-EAS443:3:44:873:1270#0/1
GCGGGGTTCTTCTCTTGTCTTCCTGATGTGTACTTTTTGA
+HWI-EAS443:3:44:873:1270#0/1
aU¥babXW^^_aa_aaa]aaa^Q¥`¥]^ZXYRL[__aa_^
@HWI-EAS443:3:44:953:973#0/1
GGGGTTCTTCTCTTGTCTTCCTGATGTGTACTTTTTGATT
+HWI-EAS443:3:44:953:973#0/1
NUKKY`X]SU^GNDP_Z^]RLTQYNURQR[[[RV]UZB
@HWI-EAS443:3:45:1167:39#0/1
ATCAAAAAGTACACATCAGGAAGACAAGAGAAGAACCCCG
+HWI-EAS443:3:45:1167:39#0/1
abbbbaa`b_aaaaabaabbaaba`a`a`a``a``^Z_¥a
@HWI-EAS443:3:45:128:898#0/1
AGCGGGGTTCTTCTCTTGTCTTCCTGATGTGTACTTTTTG
+HWI-EAS443:3:45:128:898#0/1
abb`U```````]a`aa^W]_`_X]a_]^[aYWY``_Z¥Q
@HWI-EAS443:3:45:1345:1667#0/1
GCGGGGTTCTTCTCTTGTCTTCCTGATGTGTACTTTTTGA
+HWI-EAS443:3:45:1345:1667#0/1
a_b_`WNa]a^[_WabZP_aa_a``[aaR]LW[ab__a[_
@HWI-EAS443:3:45:1347:1990#0/1
CGGGGTTCTTCTCTTGTCTTCCTGATGTGTACTTTTTGAT
+HWI-EAS443:3:45:1347:1990#0/1
ab^baVaV_a_aaaab__aaY^`_W_¥Y^W¥V_¥`^`]__
@HWI-EAS443:3:45:1535:124#0/1
AGCGGGGTTCTTCTCTTGTCTTCCTGATGTGTACTTTTTG
+HWI-EAS443:3:45:1535:124#0/1
aabbaab`babaaaaabaaaaaaaaa^aa_a][_aaaaa]
@HWI-EAS443:3:45:1679:352#0/1
GCGGGGTTCTTCTCTTGTCTTCCTGATGTGTACTTTTTGA
+HWI-EAS443:3:45:1679:352#0/1
a]aaaaY`aaa^a^``___`a^]^X]_aZ_Z]Z`__a_[Z
@HWI-EAS443:3:45:173:1059#0/1
GGTTCTTCTCTTGTCTTCCTGATGTGTACTTTTTGATTAT
+HWI-EAS443:3:45:173:1059#0/1
``abbbbbbbbbbaabbaaaaaaa`aaaabbaaba`baaa
@HWI-EAS443:3:45:215:107#0/1
CGGGGTTCTTCTCTTGTCTTCCTGATGTGTACTTTTTGAT
+HWI-EAS443:3:45:215:107#0/1
abaa`Hbbab^baab^a`aaa_ab`aa`aa__abbb_S]a
@HWI-EAS443:3:45:271:205#0/1
GCGGGGTTCTTCTCTTGTCTTCCTGATGTGTACTTTTTGA
+HWI-EAS443:3:45:271:205#0/1
aWa¥__U]YG]^[N__XRV]_^RZUWLRHPNSS^ZY_VWM

@HWI-EAS443:3:45:516:1132#0/1
CGGGGTTCTTCTCTTGTCTTCCTGATGTGTACTTTTTGAT
+HWI-EAS443:3:45:516:1132#0/1
`baaaabbbbaa^bbb`_ba`_ba]a`¥`_a^baaaa^`a
@HWI-EAS443:3:45:619:1004#0/1
CGGGGTTCTTCTCTTGTCTTCCTGATGTGTACTTTTTGAT
+HWI-EAS443:3:45:619:1004#0/1
abbbb_aba``a_a[abababWaVMaaW__aWaa`a^^Ub
@HWI-EAS443:3:45:731:389#0/1
CGGGGTTCTTCTCTTGTCTTCCTGATGTGTACTTTTTGAT
+HWI-EAS443:3:45:731:389#0/1
ababaabbb_]a_bbb_abb]Za`]`aab_`_baaabaaa
@HWI-EAS443:3:45:826:1586#0/1
GCGGGGTTCTTCTCTTGTCTTCCTGATGTGTACTTTTTGA
+HWI-EAS443:3:45:826:1586#0/1
¥_^^_XQ¥a`[Kbaab_bbaS_¥bbaaabaa_X¥b]aaa_
@HWI-EAS443:3:45:858:1320#0/1
GCGGGGTTCTTCTCTTGTCTTCCTGATGTGTACTTTTTGA
+HWI-EAS443:3:45:858:1320#0/1
a_a`_`]aaa__a]`__]^_`]_`_V__X_XXUaa_^^R[
@HWI-EAS443:3:45:883:453#0/1
GGGTTCTTCTCTTGTCTTCCTGATGTGTACTTTTTGATTA
+HWI-EAS443:3:45:883:453#0/1
_ba_b]baabaabaaaaa`_aa_`a`a_``aaaa_]]aa_
@HWI-EAS443:3:45:941:41#0/1
CGGGGTTCTTCTCTTGTCTTCCTGATGTGTACTTTTTGAT
+HWI-EAS443:3:45:941:41#0/1
aabbbbbbbbbbabbbbabbabab`bbabb^abbbbabab
@HWI-EAS443:3:46:1204:1838#0/1
ATCAAAAAGTACACATCAGGAAGACAAGAGAAGAACCCCG
+HWI-EAS443:3:46:1204:1838#0/1
abaaaaaaa_aa`T`a_^¥[aa``^a]__^Z__aa¥TN¥`
@HWI-EAS443:3:46:1204:710#0/1
CGGGGTTCTTCTCTTGTCTTCCTGATGTGTACTTTTTGAT
+HWI-EAS443:3:46:1204:710#0/1
abbab_bbbbababbb`aabaabbaab_b__`aaabbaaa
@HWI-EAS443:3:46:1225:971#0/1
CGGGGTTCTTCTCTTGTCTTCCTGATGTGTACTTTTTGAT
+HWI-EAS443:3:46:1225:971#0/1
`baXUG^ba`^a^`Z^_a`¥UU^WZWX[`]T^^Z]^^]SW
@HWI-EAS443:3:46:1280:477#0/1
ATCAAAAAGTACACATCAGGAAGACAAGAGAAGAACCCCG
+HWI-EAS443:3:46:1280:477#0/1
aaaaa`aaaWaaaaaaaaab`]``^aaa`a``aaa__^^a
@HWI-EAS443:3:46:1613:1090#0/1
CGGGGTTCTTCTCTTGTCTTCCTGATGTGTACTTTTTGAT
+HWI-EAS443:3:46:1613:1090#0/1
abbab_bbbbaaababa`bba`aa`aa`a__`ababaa`a
@HWI-EAS443:3:46:1658:640#0/1
CGGGGTTCTTCTCTTGTCTTCCTGATGTGTACTTTTTGAT

```
+HWI-EAS443:3:46:1658:640#0/1
a`aababababbabbbbb_abbb`bb¥`ba^^aabbbbb^aa
@HWI-EAS443:3:46:226:102#0/1
GGGGTTCTTCTCTTGTCTTCCTGATGTGTACTTTTTGATT
+HWI-EAS443:3:46:226:102#0/1
aabaVa`aa_a_`aa_`a___`_Z__^_]^^`_a^`^]_]
@HWI-EAS443:3:46:308:119#0/1
CGGGGTTCTTCTCTTGTCTTCCTGATGTGTACTTTTTGAT
+HWI-EAS443:3:46:308:119#0/1
N^bbbbaabbababbbabbbabbbWababaT¥bbbbbbb
@HWI-EAS443:3:46:354:1295#0/1
GCGGGGTTCTTCTCTTGTCTTCCTGATGTGTACTTTTTGA
+HWI-EAS443:3:46:354:1295#0/1
_]``]ZJ_VD[]YY__a__a__`ZW[L^ZaW^^^a¥^][¥
@HWI-EAS443:3:46:42:715#0/1
CGGGGTTCTTCTCTTGTCTTCCTGATGTGTACTTTTTGAT
+HWI-EAS443:3:46:42:715#0/1
^_X]¥P^]]^]_]_^`^W_W[]_¥VSZ[WVXZ_]]a¥V[]
@HWI-EAS443:3:46:487:323#0/1
GCGGGGTTCTTCTCTTGTCTTCCTGATGTGTACTTTTTGA
+HWI-EAS443:3:46:487:323#0/1
a`baaa^`_a__a_aaa]`a`_]a`Y^a]a]_Y_`a`a_]
@HWI-EAS443:3:46:572:1514#0/1
GCGGGGTTCTTCTCTTGTCTTCCTGATGTGTACTTTTTGA
+HWI-EAS443:3:46:572:1514#0/1
aaa]`YUa`aaaaYa_aa_a_]a`_S_aV_X^Ya``a`W_
@HWI-EAS443:3:46:623:1066#0/1
CGGGGTTCTTCTCTTGTCTTCCTGATGTGTACTTTTTGAT
+HWI-EAS443:3:46:623:1066#0/1
aa]bZaabbaaaabaaaaa`[`aa```Y`¥a_`aaaa__^
@HWI-EAS443:3:46:657:132#0/1
CGGGGTTCTTCTCTTGTCTTCCTGATGTGTACTTTTTGAT
+HWI-EAS443:3:46:657:132#0/1
Xa]`¥_b¥bbV`bbb`T]b]`Jbb[ababa[``abbaY]`
@HWI-EAS443:3:46:681:1237#0/1
CGGGGTTCTTCTCTTGTCTTCCTGATGTGTACTTTTTGAT
+HWI-EAS443:3:46:681:1237#0/1
abaaa`babbababaa[abaaabaaaa¥_^__aaaab_`a
@HWI-EAS443:3:46:783:1319#0/1
GGGTTCTTCTCTTGTCTTCCTGATGTGTACTTTTTGATTA
+HWI-EAS443:3:46:783:1319#0/1
aa_[bab]abYbaa`[[`bb]]Ta`V]X_`a`U^_a_^XJ
@HWI-EAS443:3:46:836:1830#0/1
GCGGGGTTCTTCTCTTGTCTTCCTGATGTGTACTTTTTGA
+HWI-EAS443:3:46:836:1830#0/1
a`aa_`^aaaa_`^aaaa`ba__a```¥]_]^Ya``a^X]
@HWI-EAS443:3:46:892:619#0/1
GCGGGGTTCTTCTCTTGTCTTCCTGATGTGTACTTTTTGA
+HWI-EAS443:3:46:892:619#0/1
a^¥T¥_FV_aaa[]_`a__¥^VX^`_]aX_NTU[^``__[
```

@HWI-EAS443:3:47:112:1997#0/1
GGGGTTCTTCTCTTGTCTTCCTGATGTGTACTTTTTGATT
+HWI-EAS443:3:47:112:1997#0/1
aa]VP]``a¥a^``_Y_a_¥^_`]]]OaX]]^`]_YTZ_]
@HWI-EAS443:3:47:1206:1741#0/1
CGGGGTTCTTCTCTTGTCTTCCTGATGTGTACTTTTTGAT
+HWI-EAS443:3:47:1206:1741#0/1
aa¥_^`]```a^abaaaaba]H]aaa`_aXOO`a^T]`[`
@HWI-EAS443:3:47:1261:229#0/1
CGGGGTTCTTCTCTTGTCTTCCTGATGTGTACTTTTTGAT
+HWI-EAS443:3:47:1261:229#0/1
Ya`YVV`¥VZQ_¥V_TQaabL``VNY`[`TP]babaa``a
@HWI-EAS443:3:47:1681:325#0/1
GCGGGGTTCTTCTCTTGTCTTCCTGATGTGTACTTTTTGA
+HWI-EAS443:3:47:1681:325#0/1
aab]aaV__a``^]__`X^]_^^__]__W[RVY¥__^[[[
@HWI-EAS443:3:47:1773:1952#0/1
GGGGTTCTTCTCTTGTCTTCCTGATGTGTACTTTTTGATT
+HWI-EAS443:3:47:1773:1952#0/1
abaW_a[ba_a¥a]a]]_`YHa_]RaR_W_X^`__^^^_¥
@HWI-EAS443:3:47:1776:1408#0/1
CGGGGTTCTTCTCTTGTCTTCCTGATGTGTACTTTTTGAT
+HWI-EAS443:3:47:1776:1408#0/1
[abb``bbbb`babbabbbbaabaabaaba^abbbbb¥bb
@HWI-EAS443:3:47:312:1808#0/1
GGGTTCTTCTCTTGTCTTCCTGATGTGTACTTTTTGATTA
+HWI-EAS443:3:47:312:1808#0/1
aaaYaaaaab¥aa_`_aa_^__a_a¥_^^_a````_a`_]
@HWI-EAS443:3:47:497:296#0/1
CGGGGTTCTTCTCTTGTCTTCCTGATGTGTACTTTTTGAT
+HWI-EAS443:3:47:497:296#0/1
`bbabZ``baW^Z`bb¥¥b]_X_a[^¥Vaaa_b^babba]
@HWI-EAS443:3:47:600:534#0/1
CGGGGTTCTTCTCTTGTCTTCCTGATGTGTACTTTTTGAT
+HWI-EAS443:3:47:600:534#0/1
aba`a¥a`bab`aaaa^_a`¥^a]`^`X[¥YZaaaaa^_`
@HWI-EAS443:3:47:661:681#0/1
CGGGGTTCTTCTCTTGTCTTCCTGATGTGTACTTTTTGAT
+HWI-EAS443:3:47:661:681#0/1
[bbbbabUbbabbabaaWbb`_baVa_V`^`__aabaa`T
@HWI-EAS443:3:47:685:444#0/1
GGGGTTCTTCTCTTGTCTTCCTGATGTGTACTTTTTGATT
+HWI-EAS443:3:47:685:444#0/1
abbaab`abababababa`bb__ba]a`^a]X^aaaaaW¥`a
@HWI-EAS443:3:47:731:116#0/1
AGCGGGGTTCTTCTCTTGTCTTCCTGATGTGTACTTTTTG
+HWI-EAS443:3:47:731:116#0/1
ab]bbbbV`abb`baabaa_aa_``^YXaXaW^W]a`a`¥
@HWI-EAS443:3:47:989:1509#0/1
GCGGGGTTCTTCTCTTGTCTTCCTGATGTGTACTTTTTGA

```
+HWI-EAS443:3:47:989:1509#0/1
a_¥```R`aabaaVaaa`_aaZaaa``a_a_`_aaaaaY¥
@HWI-EAS443:3:48:145:1629#0/1
CGGGGTTCTTCTCTTGTCTTCCTGATGTGTACTTTTTGAT
+HWI-EAS443:3:48:145:1629#0/1
ab`bbabbbbbbabbabbbbbabbab¥`baaabbbbb]ab
@HWI-EAS443:3:48:158:1345#0/1
GCGGGGTTCTTCTCTTGTCTTCCTGATGTGTACTTTTTGA
+HWI-EAS443:3:48:158:1345#0/1
abbaaa`aabb`a`bbaaaba`aaaa`^_a`a`aaaaa`_
@HWI-EAS443:3:48:277:288#0/1
GCGGGGTTCTTCTCTTGTCTTCCTGATGTGTACTTTTTGA
+HWI-EAS443:3:48:277:288#0/1
aaa_]¥O`aa`]a`a`Z^____^]ZW¥^S]TVQ[^^_]W]
@HWI-EAS443:3:48:388:543#0/1
CGGGGTTCTTCTCTTGTCTTCCTGATGTGTACTTTTTGAT
+HWI-EAS443:3:48:388:543#0/1
abbba]a_baba`abaabbba_a^`aaa__a_bbaaa^ab
@HWI-EAS443:3:48:461:196#0/1
GCGGGGTTCTTCTCTTGTCTTCCTGATGTGTACTTTTTGA
+HWI-EAS443:3:48:461:196#0/1
a_a_^[HZXST[MZ_]aRUZWVV[_XV]V_QVV_`^^[TY
@HWI-EAS443:3:48:489:778#0/1
GGGGTTCTTCTCTTGTCTTCCTGATGTGTACTTTTTGATT
+HWI-EAS443:3:48:489:778#0/1
aaaa]b`aaab`aaaa_aa`aaa^``X`^]^``aaaa^``
@HWI-EAS443:3:48:496:910#0/1
CGGGGTTCTTCTCTTGTCTTCCTGATGTGTACTTTTTGAT
+HWI-EAS443:3:48:496:910#0/1
_baab¥abbaababaaababaaaaaa__a]a_abaaaaaa
@HWI-EAS443:3:48:512:1708#0/1
GGTTCTTCTCTTGTCTTCCTGATGTGTACTTTTTGATTAT
+HWI-EAS443:3:48:512:1708#0/1
`bY]_ZXXabXbaK`aaabb`_VaJbaa__aa`aa^^``b
@HWI-EAS443:3:48:981:1956#0/1
GCGGGGTTCTTCTCTTGTCTTCCTGATGTGTACTTTTTGA
+HWI-EAS443:3:48:981:1956#0/1
a_WX]`P``a`____``U^^VJ[]^[]_Y_U^S__]]]]]
@HWI-EAS443:3:49:1032:797#0/1
GCGGGGTTCTTCTCTTGTCTTCCTGATGTGTACTTTTTGA
+HWI-EAS443:3:49:1032:797#0/1
abb`bbabbbbababbbaabb_aaaaab_a`a`babba¥a
@HWI-EAS443:3:49:1191:1270#0/1
AGCGGGGTTCTTCTCTTGTCTTCCTGATGTGTACTTTTTG
+HWI-EAS443:3:49:1191:1270#0/1
aa_a`bbYbaba`a`bbb`aaa`_bb__aY`_a`aabaaa
@HWI-EAS443:3:49:1262:1641#0/1
CGGGGTTCTTCTCTTGTCTTCCTGATGTGTACTTTTTGAT
+HWI-EAS443:3:49:1262:1641#0/1
`bbab]baa]__ab`ba^b_a`bWFTb`b]W`bbab_`ba
```

```
@HWI-EAS443:3:49:1378:349#0/1
GCGGGGTTCTTCTCTTGTCTTCCTGATGTGTACTTTTTGA
+HWI-EAS443:3:49:1378:349#0/1
aQX^`aV^ZVZY[¥QYPDQJYYSUNUVNML[YO^ZNZRTR
@HWI-EAS443:3:49:1387:1323#0/1
CGGGGTTCTTCTCTTGTCTTCCTGATGTGTACTTTTTGAT
+HWI-EAS443:3:49:1387:1323#0/1
abab^aaabbaa_abaa`ba_a`^Taa`a```aaaabV_a
@HWI-EAS443:3:49:139:80#0/1
GGGTTCTTCTCTTGTCTTCCTGATGTGTACTTTTTGATTA
+HWI-EAS443:3:49:139:80#0/1
`¥_QU_W¥aX¥[[X]Y[`Z`a]`[_Y¥WZWaa_b_SVbb^
@HWI-EAS443:3:49:1702:1810#0/1
GGGTTCTTCTCTTGTCTTCCTGATGTGTACTTTTTGATTA
+HWI-EAS443:3:49:1702:1810#0/1
_N^IRMW_X^[U]SO_^_O]]OMPYGWOVGTS¥^ZSUZWW
@HWI-EAS443:3:49:216:735#0/1
CGGGGTTCTTCTCTTGTCTTCCTGATGTGTACTTTTTGAT
+HWI-EAS443:3:49:216:735#0/1
abbbbbaabbbbbbbabbbababbbabbbababbbbbbab
@HWI-EAS443:3:49:330:612#0/1
CGGGGTTCTTCTCTTGTCTTCCTGATGTGTACTTTTTGAT
+HWI-EAS443:3:49:330:612#0/1
abbbb]baab`_`abaPY_aa_b^R]][a[[V^aaa]`_b
@HWI-EAS443:3:49:36:611#0/1
CGGGGTTCTTCTCTTGTCTTCCTGATGTGTACTTTTTGAT
+HWI-EAS443:3:49:36:611#0/1
a^[YU[]babb_bbbbbb_[`aaba[aX`[a_aabb]Y_b
@HWI-EAS443:3:49:756:1133#0/1
GCGGGGTTCTTCTCTTGTCTTCCTGATGTGTACTTTTTGA
+HWI-EAS443:3:49:756:1133#0/1
aa]``^_X¥aaaa_`bb``[ZV_`VZ¥U¥^^^`]a`aa]_
@HWI-EAS443:3:49:888:75#0/1
CGGGGTTCTTCTCTTGTCTTCCTGATGTGTACTTTTTGAT
+HWI-EAS443:3:49:888:75#0/1
ababbab¥bbba_bbZ`abbKVbbabbaaTaZbbbbbaab
@HWI-EAS443:3:4:1084:1277#0/1
CGGGGTTCTTCTCTTGTCTTCCTGATGTGTACTTTTTGAT
+HWI-EAS443:3:4:1084:1277#0/1
abbbbababbaa`bab``ba`abb_aaab_a_aaa`aaab
@HWI-EAS443:3:4:1519:527#0/1
GGGGTTCTTCTCTTGTCTTCCTGATGTGTACTTTTTGATT
+HWI-EAS443:3:4:1519:527#0/1
abbb``aa_^`a]a]¥]aa]^_aZ_`^aX]^_`a___]_]
@HWI-EAS443:3:4:1532:871#0/1
CGGGGTTCTTCTCTTGTCTTCCTGATGTGTACTTTTTGAT
+HWI-EAS443:3:4:1532:871#0/1
ab]bb]aa`bUaX_`a_]`_R_¥WYaTN__ZQXa__]¥Y^
@HWI-EAS443:3:4:1594:93#0/1
CGGGGTTCTTCTCTTGTCTTCCTGATGTGTACTTTTTGAT
```

```
+HWI-EAS443:3:4:1594:93#0/1
]bbbaab`bbb`_bbba`ba`bbb^bbabb`^bbbbaabb
@HWI-EAS443:3:4:275:928#0/1
GGGGTTCTTCTCTTGTCTTCCTGATGTGTACTTTTTGATT
+HWI-EAS443:3:4:275:928#0/1
[ba^K`a`[Ua_`aaa[a[aV_aaa_WaYaH`aU`_`¥``
@HWI-EAS443:3:4:432:324#0/1
CGGGGTTCTTCTCTTGTCTTCCTGATGTGTACTTTTTGAT
+HWI-EAS443:3:4:432:324#0/1
abbba^a`aa`a_aabX_aa_^`YZ`a_a]_^a`aaaa^a
@HWI-EAS443:3:4:559:842#0/1
CGGGGTTCTTCTCTTGTCTTCCTGATGTGTACTTTTTGAT
+HWI-EAS443:3:4:559:842#0/1
abbaZbbabb_aaabbabbb`aabaab_b`¥a_aaab`aa
@HWI-EAS443:3:4:721:982#0/1
GGGGTTCTTCTCTTGTCTTCCTGATGTGTACTTTTTGATT
+HWI-EAS443:3:4:721:982#0/1
abbaK[Xa`T^Ra]]aW`_V¥a^T^]Xa^_X_``_]a¥_B
@HWI-EAS443:3:4:829:1417#0/1
CGGGGTTCTTCTCTTGTCTTCCTGATGTGTACTTTTTGAT
+HWI-EAS443:3:4:829:1417#0/1
]`a_^FY^Zb]baZV_``aa`_aba^b`a_a`^ab`a`aa
@HWI-EAS443:3:50:1041:2030#0/1
GCGGGGTTCTTCTCTTGTCTTCCTGATGTGTACTTTTTGA
+HWI-EAS443:3:50:1041:2030#0/1
_Taa^WOWa`¥`XQT``W^_^^^[[^^]W`VW]^[V^[R[
@HWI-EAS443:3:50:1065:427#0/1
GCGGGGTTCTTCTCTTGTCTTCCTGATGTGTACTTTTTGA
+HWI-EAS443:3:50:1065:427#0/1
a^Q_aZFR^Y]VSO[]]]V]TX^XTRX¥L^UQLMVY[^BB
@HWI-EAS443:3:50:1098:603#0/1
GCGGGGTTCTTCTCTTGTCTTCCTGATGTGTACTTTTTGA
+HWI-EAS443:3:50:1098:603#0/1
_X`Z¥aV¥[__WY_X^a^VO_Q¥TPPZGW]VJOWQSZTBB
@HWI-EAS443:3:50:1105:598#0/1
GCGGGGTTCTTCTCTTGTCTTCCTGATGTGTACTTTTTGA
+HWI-EAS443:3:50:1105:598#0/1
abbabb_`Y`babbbbbaaab¥^`[`baaa`_a]abbb_a
@HWI-EAS443:3:50:1106:303#0/1
CGGGGTTCTTCTCTTGTCTTCCTGATGTGTACTTTTTGAT
+HWI-EAS443:3:50:1106:303#0/1
`a```^aaaa]aaaa`a^abaaa^``b`ba]^_bbbaU_a
@HWI-EAS443:3:50:1117:1623#0/1
CGGGGTTCTTCTCTTGTCTTCCTGATGTGTACTTTTTGAT
+HWI-EAS443:3:50:1117:1623#0/1
ababbabbbbbbabbbbabba_baababababbabbbbbaab
@HWI-EAS443:3:50:1153:1262#0/1
GCGGGGTTCTTCTCTTGTCTTCCTGATGTGTACTTTTTGA
+HWI-EAS443:3:50:1153:1262#0/1
aaa`^]Paaa``aaaba]_a`a__WVaa¥]R][`_aa`_]
```

@HWI-EAS443:3:50:1307:517#0/1
GCGGGGTTCTTCTCTTGTCTTCCTGATGTGTACTTTTTGA
+HWI-EAS443:3:50:1307:517#0/1
a_baa`_¥Taa``__aa^¥_RU`_`¥`aNaW_^``_a]^Z
@HWI-EAS443:3:50:1328:1578#0/1
GGGTTCTTCTCTTGTCTTCCTGATGTGTACTTTTTGATTA
+HWI-EAS443:3:50:1328:1578#0/1
abb_babbbbaabb`bba`aaaaba`b_a^a`aaaaaa`a
@HWI-EAS443:3:50:1336:2025#0/1
GGGGTTCTTCTCTTGTCTTCCTGATGTGTACTTTTTGATT
+HWI-EAS443:3:50:1336:2025#0/1
ab``[[[a`I__ORaST¥PLS``V``W`MZHYYa___[[^
@HWI-EAS443:3:50:1481:291#0/1
GCGGGGTTCTTCTCTTGTCTTCCTGATGTGTACTTTTTGA
+HWI-EAS443:3:50:1481:291#0/1
a^`aab_]aa_^aaaa^[_a`[^__[`aZ^[[^a_`a`_^
@HWI-EAS443:3:50:1605:494#0/1
CGGGGTTCTTCTCTTGTCTTCCTGATGTGTACTTTTTGAT
+HWI-EAS443:3:50:1605:494#0/1
`bbba_bbbb_babbbaabba`b`aab_aaa`bbbbbaaa
@HWI-EAS443:3:50:1791:1618#0/1
GCGGGGTTCTTCTCTTGTCTTCCTGATGTGTACTTTTTGA
+HWI-EAS443:3:50:1791:1618#0/1
`^`a_]]]U`aaa_b_b`_aa__``Z¥a_]TV¥_aaa_NU
@HWI-EAS443:3:50:666:1211#0/1
CGGGGTTCTTCTCTTGTCTTCCTGATGTGTACTTTTTGAT
+HWI-EAS443:3:50:666:1211#0/1
^`bbaab[bbbb_baaaaabaaba_bb_b_¥Ubabbba_`
@HWI-EAS443:3:50:694:926#0/1
GCGGGGTTCTTCTCTTGTCTTCCTGATGTGTACTTTTTGA
+HWI-EAS443:3:50:694:926#0/1
aabbbb_babbababbaa^ba_`b^_aa`_^`_aaaaa`a
@HWI-EAS443:3:50:697:1852#0/1
CGGGGTTCTTCTCTTGTCTTCCTGATGTGTACTTTTTGAT
+HWI-EAS443:3:50:697:1852#0/1
`ababbbababbbbbabbbbbabbba_aaababbbbbabb
@HWI-EAS443:3:50:801:1875#0/1
CGGGGTTCTTCTCTTGTCTTCCTGATGTGTACTTTTTGAT
+HWI-EAS443:3:50:801:1875#0/1
abbbb_bbbb`bababaabba`bbaabab^a`bbbbbaaa
@HWI-EAS443:3:51:1112:540#0/1
GCGGGGTTCTTCTCTTGTCTTCCTGATGTGTACTTTTTGA
+HWI-EAS443:3:51:1112:540#0/1
[^_`_babbbbabaabba`bbaaaa`b`_``a`abbaa^`
@HWI-EAS443:3:51:1332:289#0/1
GGGTTCTTCTCTTGTCTTCCTGATGTGTACTTTTTGATTA
+HWI-EAS443:3:51:1332:289#0/1
abb`b`bb`babbba^bb__b]aab_aa]]babbbaaaaa
@HWI-EAS443:3:51:1407:916#0/1
CGGGGTTCTTCTCTTGTCTTCCTGATGTGTACTTTTTGAT

+HWI-EAS443:3:51:1407:916#0/1
¥abb_ZbXb¥`bX_`b¥¥aba¥¥a¥_VVbabaa`bbbbb^
@HWI-EAS443:3:51:1435:819#0/1
CGGGGTTCTTCTCTTGTCTTCCTGATGTGTACTTTTTGAT
+HWI-EAS443:3:51:1435:819#0/1
UX``ZSa_aa¥babbbaSZ`baa``aba]a`^b_bba^ab
@HWI-EAS443:3:51:1582:1187#0/1
GGGGTTCTTCTCTTGTCTTCCTGATGTGTACTTTTTGATT
+HWI-EAS443:3:51:1582:1187#0/1
Sabb`babbababbZbbbbbaaabb]`aa_abbbbbXbba
@HWI-EAS443:3:51:297:694#0/1
CGGGGTTCTTCTCTTGTCTTCCTGATGTGTACTTTTTGAT
+HWI-EAS443:3:51:297:694#0/1
abbbbababbbbabb`abbbaabaabbab`a^bbabbaaa
@HWI-EAS443:3:51:7:1552#0/1
CGGGGTTCTTCTCTTGTCTTCCTGATGTGTACTTTTTGAT
+HWI-EAS443:3:51:7:1552#0/1
aa`ab_bbbaaaab`[T`aaaX`]_Y`_a_a_aaa``Z^a
@HWI-EAS443:3:51:7:1958#0/1
GCGGGGTTCTTCTCTTGTCTTCCTGATGTGTACTTTTTGA
+HWI-EAS443:3:51:7:1958#0/1
aa^Za_V¥_`_Y]^R^a^^Za^Z^]]__[^[_ZY`][¥Y[
@HWI-EAS443:3:52:1031:978#0/1
GCGGGGTTCTTCTCTTGTCTTCCTGATGTGTACTTTTTGA
+HWI-EAS443:3:52:1031:978#0/1
a_a`¥a`aaaa^a_a`a]¥``^Z`][_]V`^___¥]_a[^
@HWI-EAS443:3:52:1378:1290#0/1
GGGGTTCTTCTCTTGTCTTCCTGATGTGTACTTTTTGATT
+HWI-EAS443:3:52:1378:1290#0/1
a`a`Y__ab^a[b`¥[_a`_]]_^__ZRU]X_a__]XX__
@HWI-EAS443:3:52:1476:178#0/1
CGGGGTTCTTCTCTTGTCTTCCTGATGTGTACTTTTTGAT
+HWI-EAS443:3:52:1476:178#0/1
H_¥_Z¥WS`^¥aa]``Xab`[ba^a^aaa]``bbbb`O_b
@HWI-EAS443:3:52:1536:313#0/1
GGGGTTCTTCTCTTGTCTTCCTGATGTGTACTTTTTGATT
+HWI-EAS443:3:52:1536:313#0/1
aaa__a^baaa_b`a^_aaa_a`a`a]a]_]ab`aa`]_`
@HWI-EAS443:3:52:199:319#0/1
CGGGGTTCTTCTCTTGTCTTCCTGATGTGTACTTTTTGAT
+HWI-EAS443:3:52:199:319#0/1
a_``ba¥[`b`¥_aababab`aa^Qa]Q___abbaaaZ¥a
@HWI-EAS443:3:52:284:730#0/1
CGGGGTTCTTCTCTTGTCTTCCTGATGTGTACTTTTTGAT
+HWI-EAS443:3:52:284:730#0/1
abaa_^`aba^]Y^XaZ[`a¥]aYOSWI`]VS]aa`¥LQa
@HWI-EAS443:3:52:419:838#0/1
CGGGGTTCTTCTCTTGTCTTCCTGATGTGTACTTTTTGAT
+HWI-EAS443:3:52:419:838#0/1
aaab]^a`aaaaaa`a_aaa_a`a_a]Za]`_aaaa`X^a

@HWI-EAS443:3:52:520:669#0/1
AGCGGGGTTCTTCTCTTGTCTTCCTGATGTGTACTTTTTG
+HWI-EAS443:3:52:520:669#0/1
abbbaa_[aaaaaaaaaa_aaa__abVaa_a__^a`a^aa
@HWI-EAS443:3:52:651:836#0/1
CGGGGTTCTTCTCTTGTCTTCCTGATGTGTACTTTTTGAT
+HWI-EAS443:3:52:651:836#0/1
abaab_bbbbbbabbbaabbaabbabaaa`a`bbbbbaab
@HWI-EAS443:3:52:863:588#0/1
GCGGGGTTCTTCTCTTGTCTTCCTGATGTGTACTTTTTGA
+HWI-EAS443:3:52:863:588#0/1
aaaaa_Saaaa`aaaaa`_aa__`_]`a]_W¥[a`^a`]Y
@HWI-EAS443:3:53:1036:599#0/1
AGCGGGGTTCTTCTCTTGTCTTCCTGATGTGTACTTTTTG
+HWI-EAS443:3:53:1036:599#0/1
ababba_`baaa_babab]]baaaaa^ab[a]`_baaaa_
@HWI-EAS443:3:53:1327:200#0/1
GCGGGGTTCTTCTCTTGTCTTCCTGATGTGTACTTTTTGA
+HWI-EAS443:3:53:1327:200#0/1
a`X`bb_babbaa_bbb`aaa`aaa``V`b_^^babaa¥Z
@HWI-EAS443:3:53:1545:1783#0/1
GGGGTTCTTCTCTTGTCTTCCTGATGTGTACTTTTTGATT
+HWI-EAS443:3:53:1545:1783#0/1
`ab_O_S¥¥baaaba`bab[a^_Y[b[b¥aaabbbbaaba
@HWI-EAS443:3:53:169:1967#0/1
GGGGTTCTTCTCTTGTCTTCCTGATGTGTACTTTTTGATT
+HWI-EAS443:3:53:169:1967#0/1
aa^_[a_`b`aaa`¥V_a_T__]aa[Z]^aWa_`aa_aa^
@HWI-EAS443:3:53:1784:1149#0/1
GCGGGGTTCTTCTCTTGTCTTCCTGATGTGTACTTTTTGA
+HWI-EAS443:3:53:1784:1149#0/1
^bbaT`^bbbba^bbb`bbbb`ba¥`b[`_`a`bbbb`R_
@HWI-EAS443:3:53:226:1422#0/1
CGGGGTTCTTCTCTTGTCTTCCTGATGTGTACTTTTTGAT
+HWI-EAS443:3:53:226:1422#0/1
_baab]babbababababa`bbbbbbbbbab`aabbbbb`ab
@HWI-EAS443:3:53:337:78#0/1
GGGGTTCTTCTCTTGTCTTCCTGATGTGTACTTTTTGATT
+HWI-EAS443:3:53:337:78#0/1
aa`^LV^[`[a]_[`^^__]_^`UZZYaOZ^__aZaZY_V
@HWI-EAS443:3:53:696:105#0/1
GCGGGGTTCTTCTCTTGTCTTCCTGATGTGTACTTTTTGA
+HWI-EAS443:3:53:696:105#0/1
¥]aba`a``aba`X_bbaaabZY_`X`aYa`W[a`]`aXY
@HWI-EAS443:3:53:964:306#0/1
GCGGGGTTCTTCTCTTGTCTTCCTGATGTGTACTTTTTGA
+HWI-EAS443:3:53:964:306#0/1
``abbbababa_``abba`aa_aaa_aa`b`^_aabaaa^
@HWI-EAS443:3:54:1369:1710#0/1
GCGGGGTTCTTCTCTTGTCTTCCTGATGTGTACTTTTTGA

```
+HWI-EAS443:3:54:1369:1710#0/1
`]W¥aa`baab[a_aaa_¥aa_]`a`_a_a^]]aaaaa^U
@HWI-EAS443:3:54:1374:604#0/1
GGGTTCTTCTCTTGTCTTCCTGATGTGTACTTTTTGATTA
+HWI-EAS443:3:54:1374:604#0/1
]QLKXX]a_```X^QQ^`PM`^X^aNUETQW]_a]_]^_U
@HWI-EAS443:3:54:1555:1067#0/1
CGGGGTTCTTCTCTTGTCTTCCTGATGTGTACTTTTTGAT
+HWI-EAS443:3:54:1555:1067#0/1
a`baaZaabb`_^bbb¥bbabaababba`aa_b_`b^aab
@HWI-EAS443:3:54:229:1103#0/1
CGGGGTTCTTCTCTTGTCTTCCTGATGTGTACTTTTTGAT
+HWI-EAS443:3:54:229:1103#0/1
`aaaa]baa`aaV[¥a__a`YV`[`]QSY[_¥`^_a^NZ_
@HWI-EAS443:3:54:331:1632#0/1
GGTTCTTCTCTTGTCTTCCTGATGTGTACTTTTTGATTAT
+HWI-EAS443:3:54:331:1632#0/1
ab_baababaaa`]_aa`aaa^a__a^_^`aa_a`aa__`
@HWI-EAS443:3:54:373:953#0/1
GCGGGGTTCTTCTCTTGTCTTCCTGATGTGTACTTTTTGA
+HWI-EAS443:3:54:373:953#0/1
a_`aa_U`_aa_a^a_`^_``_^``U[^]_X]]_^^`]VV
@HWI-EAS443:3:54:615:1282#0/1
AGCGGGGTTCTTCTCTTGTCTTCCTGATGTGTACTTTTTG
+HWI-EAS443:3:54:615:1282#0/1
aa`baaaUaaba_aaabbaaaa__aY_¥^Y^V`Ya`aa_U
@HWI-EAS443:3:54:669:1851#0/1
ATCAAAAAGTACACATCAGGAAGACAAGAGAAGAACCCCG
+HWI-EAS443:3:54:669:1851#0/1
ababbbbba]baa_baaaaaaaaaaaaaaaaa`a``^`]a
@HWI-EAS443:3:55:1115:869#0/1
ATCAAAAAGTACACATCAGGAAGACAAGAGAAGAACCCCG
+HWI-EAS443:3:55:1115:869#0/1
a`aa`Y`baZ[aaa``_`aaa`Z`a`aa¥b`a`ba`a[[b
@HWI-EAS443:3:55:1354:134#0/1
GCGGGGTTCTTCTCTTGTCTTCCTGATGTGTACTTTTTGA
+HWI-EAS443:3:55:1354:134#0/1
aabaab_b_bb_a^abaa^ab^]aa^aaa`_^]aaaaaa_
@HWI-EAS443:3:55:1407:470#0/1
GCGGGGTTCTTCTCTTGTCTTCCTGATGTGTACTTTTTGA
+HWI-EAS443:3:55:1407:470#0/1
`_`aab_a_aa_a^aaa__aa__a¥]aaaa`]^aaaa`]_
@HWI-EAS443:3:55:1502:1224#0/1
CGGGGTTCTTCTCTTGTCTTCCTGATGTGTACTTTTTGAT
+HWI-EAS443:3:55:1502:1224#0/1
abb`¥¥babbbb`bb`]^`bTS¥babb]`Y`_bbbbb`bb
@HWI-EAS443:3:55:1719:192#0/1
GGGGTTCTTCTCTTGTCTTCCTGATGTGTACTTTTTGATT
+HWI-EAS443:3:55:1719:192#0/1
a`aa`babbab_bbaa_bb¥]`U¥a]aa`^[aaaaba_ba
```

```
@HWI-EAS443:3:55:621:530#0/1
GCGGGGTTCTTCTCTTGTCTTCCTGATGTGTACTTTTTGA
+HWI-EAS443:3:55:621:530#0/1
aaaa_a_a_aa_a]`]a^_`a_ZZ_Z[¥ZaY]]```YaW[
@HWI-EAS443:3:55:640:440#0/1
GCGGGGTTCTTCTCTTGTCTTCCTGATGTGTACTTTTTGA
+HWI-EAS443:3:55:640:440#0/1
aaa`aa_b¥bb_a_a`a__aa`]_a¥`_^aZZ^^a^a___
@HWI-EAS443:3:55:787:1150#0/1
GCGGGGTTCTTCTCTTGTCTTCCTGATGTGTACTTTTTGA
+HWI-EAS443:3:55:787:1150#0/1
aaaa`aUY¥_]`[]VU_GY^^SWT[Y^_Y_Y]X^`_]Y_^
@HWI-EAS443:3:55:92:1093#0/1
GGGTTCTTCTCTTGTCTTCCTGATGTGTACTTTTTGATTA
+HWI-EAS443:3:55:92:1093#0/1
aa]N]^^¥[``¥__UXXLPXVYYZ[GZTLQS[WXU_X^¥X
@HWI-EAS443:3:55:952:1239#0/1
AGCGGGGTTCTTCTCTTGTCTTCCTGATGTGTACTTTTTG
+HWI-EAS443:3:55:952:1239#0/1
Ma_]]SUX]_S_Y_ababW]a^R_`_`YQZS][_aaaa_a
@HWI-EAS443:3:56:1037:444#0/1
CGGGGTTCTTCTCTTGTCTTCCTGATGTGTACTTTTTGAT
+HWI-EAS443:3:56:1037:444#0/1
aaR``^b`aZ^aYZ]aV_aUN^a`^_aVZSXXa```_U¥_
@HWI-EAS443:3:56:1057:689#0/1
CGGGGTTCTTCTCTTGTCTTCCTGATGTGTACTTTTTGAT
+HWI-EAS443:3:56:1057:689#0/1
aabba_aa`Zabaa¥XW]aaY_`RPaaTP__^aaab_I]]
@HWI-EAS443:3:56:1327:1231#0/1
CGGGGTTCTTCTCTTGTCTTCCTGATGTGTACTTTTTGAT
+HWI-EAS443:3:56:1327:1231#0/1
``aaaS_¥_aaaS`aaaaaaaXaa¥aa`a`a^aaaaba^a
@HWI-EAS443:3:56:1387:1491#0/1
CGGGGTTCTTCTCTTGTCTTCCTGATGTGTACTTTTTGAT
+HWI-EAS443:3:56:1387:1491#0/1
]bbb_P`W¥¥J¥]`bb__]ba``[Y_¥J¥YXOWa]aaK]_
@HWI-EAS443:3:56:1452:857#0/1
CGGGGTTCTTCTCTTGTCTTCCTGATGTGTACTTTTTGAT
+HWI-EAS443:3:56:1452:857#0/1
abbab]bIbb_b`bab__bb__aaa``_b_a^bb`abb`b
@HWI-EAS443:3:56:1700:565#0/1
CGGGGTTCTTCTCTTGTCTTCCTGATGTGTACTTTTTGAT
+HWI-EAS443:3:56:1700:565#0/1
```b`_babbababa^¥abaaa`Z`b¥``[a]baaa_Q^a
@HWI-EAS443:3:56:506:342#0/1
CGGGGTTCTTCTCTTGTCTTCCTGATGTGTACTTTTTGAT
+HWI-EAS443:3:56:506:342#0/1
[bb[TNabaaW`a_^^I_`a]_aaaa¥Z`SYW¥a¥]ZZY]
@HWI-EAS443:3:56:834:450#0/1
GGGGTTCTTCTCTTGTCTTCCTGATGTGTACTTTTTGATT
```
```

+HWI-EAS443:3:56:834:450#0/1
aaa^RXX`Kaa_aa^VRRaXQ[¥P]_N`ZYYZU¥BBBBBB
@HWI-EAS443:3:56:930:826#0/1
TCAAAAAGTACACATCAGGAAGACAAGAGAAGAACCCCGC
+HWI-EAS443:3:56:930:826#0/1
aaaaaaab]babaaa_abbaaba`aaa`aaaaaa_¥^¥a`
@HWI-EAS443:3:57:1074:1121#0/1
CGGGGTTCTTCTCTTGTCTTCCTGATGTGTACTTTTTGAT
+HWI-EAS443:3:57:1074:1121#0/1
abbbaZ`a_b``aaaaaa_a`aaa_a_[aZ¥_`^aaa_`a
@HWI-EAS443:3:57:1113:1656#0/1
CGGGGTTCTTCTCTTGTCTTCCTGATGTGTACTTTTTGAT
+HWI-EAS443:3:57:1113:1656#0/1
aa^Va_`aa^aa]`aaX_abaaba]ab`¥¥aYa^bbbYaa
@HWI-EAS443:3:57:1242:228#0/1
GCGGGGTTCTTCTCTTGTCTTCCTGATGTGTACTTTTTGA
+HWI-EAS443:3:57:1242:228#0/1
aabbbb_b_bb_baabbaaab_`ba_aa_a_a`aaaaaaa
@HWI-EAS443:3:57:1346:767#0/1
GCGGGGTTCTTCTCTTGTCTTCCTGATGTGTACTTTTTGA
+HWI-EAS443:3:57:1346:767#0/1
IX___^VW_a^¥a_]_a]a^^aaaaY_a]b]Y^^`a``¥^
@HWI-EAS443:3:57:1605:1113#0/1
GCGGGGTTCTTCTCTTGTCTTCCTGATGTGTACTTTTTGA
+HWI-EAS443:3:57:1605:1113#0/1
[^^`^abbaab[_ab]]bNbbaX^]abb^Ta_¥bbbbaaa
@HWI-EAS443:3:57:1707:1319#0/1
AGCGGGGTTCTTCTCTTGTCTTCCTGATGTGTACTTTTTG
+HWI-EAS443:3:57:1707:1319#0/1
`_```ba]aab``a_a``a^a_ZZ`a_a`ZX¥__a```¥V
@HWI-EAS443:3:57:410:1319#0/1
GGTTCTTCTCTTGTCTTCCTGATGTGTACTTTTTGATTAT
+HWI-EAS443:3:57:410:1319#0/1
]`_bbaabb`bbabbbbbbb`aaaa`Y_ab_bbbaaabaa
@HWI-EAS443:3:57:628:581#0/1
CGGGGTTCTTCTCTTGTCTTCCTGATGTGTACTTTTTGAT
+HWI-EAS443:3:57:628:581#0/1
abb_``baaaaaaaaa]aa`aaa```a_a]__aaa`a[Va
@HWI-EAS443:3:57:888:1014#0/1
GCGGGGTTCTTCTCTTGTCTTCCTGATGTGTACTTTTTGA
+HWI-EAS443:3:57:888:1014#0/1
`_baaa`bbbbababaaaabaaabaaaa`¥_aaaaaab`a
@HWI-EAS443:3:58:1004:296#0/1
GCGGGGTTCTTCTCTTGTCTTCCTGATGTGTACTTTTTGA
+HWI-EAS443:3:58:1004:296#0/1
a`baaa_a_aa`a_aabaaaaX[¥a_``[a^__`aa_`__
@HWI-EAS443:3:58:1094:319#0/1
GGGGTTCTTCTCTTGTCTTCCTGATGTGTACTTTTTGATT
+HWI-EAS443:3:58:1094:319#0/1
aaa_T``^]^a¥```^__`_^_^¥_`X`Y]]a`__`_^_^

```
@HWI-EAS443:3:58:1145:936#0/1
GGGGTTCTTCTCTTGTCTTCCTGATGTGTACTTTTTGATT
+HWI-EAS443:3:58:1145:936#0/1
a][¥V`aa`^^^a``W^a]^^^WZ__YV[ZZa`^a¥U¥__
@HWI-EAS443:3:58:1302:1537#0/1
ATCAAAAAGTACACATCAGGAAGACAAGAGAAGAACCCCG
+HWI-EAS443:3:58:1302:1537#0/1
aaaaabb[bYba]X_R^aa`¥__aYa¥W^ba`ba`^Y[Wa
@HWI-EAS443:3:58:1383:686#0/1
GCGGGGTTCTTCTCTTGTCTTCCTGATGTGTACTTTTTGA
+HWI-EAS443:3:58:1383:686#0/1
a¥]`]aOaPZ`[¥[¥[aN_aW[Z`^U^_^_T^^_^¥__`V
@HWI-EAS443:3:58:1486:606#0/1
CGGGGTTCTTCTCTTGTCTTCCTGATGTGTACTTTTTGAT
+HWI-EAS443:3:58:1486:606#0/1
abbb__baabababbbaabaa`a`_abaa_a_bbaab`aa
@HWI-EAS443:3:58:719:964#0/1
GGGGTTCTTCTCTTGTCTTCCTGATGTGTACTTTTTGATT
+HWI-EAS443:3:58:719:964#0/1
a_V^GP_N[UW¥a`XU`X`]QNVU¥^T[XJO_`W__UN]`
@HWI-EAS443:3:58:745:201#0/1
CGGGGTTCTTCTCTTGTCTTCCTGATGTGTACTTTTTGAT
+HWI-EAS443:3:58:745:201#0/1
a`a``Y¥`a_U_`]^a_a```_aaV`b`aZ¥`a`aaa]__
@HWI-EAS443:3:58:986:1119#0/1
GCGGGGTTCTTCTCTTGTCTTCCTGATGTGTACTTTTTGA
+HWI-EAS443:3:58:986:1119#0/1
aaa¥`bUbbbbabbbbbaabbaabbaaaaaaaabbbbb`a
@HWI-EAS443:3:59:1186:1175#0/1
CGGGGTTCTTCTCTTGTCTTCCTGATGTGTACTTTTTGAT
+HWI-EAS443:3:59:1186:1175#0/1
Zbb¥OOb¥^`bbbabb`aaaaa`aUaa`aa]Tbaa]^a`T
@HWI-EAS443:3:59:1567:1439#0/1
GGGGTTCTTCTCTTGTCTTCCTGATGTGTACTTTTTGATT
+HWI-EAS443:3:59:1567:1439#0/1
aa`^¥babbaaabababa_baaa``b`a]aaaa_]_a¥
@HWI-EAS443:3:59:1569:903#0/1
CGGGGTTCTTCTCTTGTCTTCCTGATGTGTACTTTTTGAT
+HWI-EAS443:3:59:1569:903#0/1
abbbbabbbbababbbbbbbaabbab`a`ab]bbbZZaaa
@HWI-EAS443:3:59:1580:1799#0/1
GGTTCTTCTCTTGTCTTCCTGATGTGTACTTTTTGATTAT
+HWI-EAS443:3:59:1580:1799#0/1
ab^aaba_a¥aaa_]baO^aa_aa_a_`_aa``a_]X`VV
@HWI-EAS443:3:59:560:698#0/1
CGGGGTTCTTCTCTTGTCTTCCTGATGTGTACTTTTTGAT
+HWI-EAS443:3:59:560:698#0/1
abbaa`abbaabaaaa^aaaaab`^ab_a_]Yaba^YZ]a
@HWI-EAS443:3:59:566:1199#0/1
CGGGGTTCTTCTCTTGTCTTCCTGATGTGTACTTTTTGAT
```

+HWI-EAS443:3:59:566:1199#0/1
abbba`bbbbababbaaababaabaabaa_a]aaa^_aaa
@HWI-EAS443:3:59:576:109#0/1
GTTCTTCTCTTGTCTTCCTGATGTGTACTTTTTGATTATT
+HWI-EAS443:3:59:576:109#0/1
a_aW`]aaN¥`R`_¥_aRZY^R^Qa]X`a_`^_XKSYBBB
@HWI-EAS443:3:59:819:380#0/1
GCGGGGTTCTTCTCTTGTCTTCCTGATGTGTACTTTTTGA
+HWI-EAS443:3:59:819:380#0/1
aaOR^¥P¥^_]]Z[Y]]X`U_W`a^TV`HaUZ^_^NYTLJ
@HWI-EAS443:3:5:1423:476#0/1
CGGGGTTCTTCTCTTGTCTTCCTGATGTGTACTTTTTGAT
+HWI-EAS443:3:5:1423:476#0/1
^_ba`^b^ZT`bba¥¥_`abb]ab`_b^bab^`_`_`aBB
@HWI-EAS443:3:5:1480:471#0/1
GCGGGGTTCTTCTCTTGTCTTCCTGATGTGTACTTTTTGA
+HWI-EAS443:3:5:1480:471#0/1
aab`_^^a[aa^aaa`]¥Zba]_``]aa]a__Za`aa`_B
@HWI-EAS443:3:5:1574:1615#0/1
CGGGGTTCTTCTCTTGTCTTCCTGATGTGTACTTTTTGAT
+HWI-EAS443:3:5:1574:1615#0/1
a_bbbbabbbbb`bbbbbbbbbbbbbababbaababbabB
@HWI-EAS443:3:5:1661:1486#0/1
GGGGTTCTTCTCTTGTCTTCCTGATGTGTACTTTTTGATT
+HWI-EAS443:3:5:1661:1486#0/1
ab`aXaYYZP`VYaa_a``^^`a[_a_`M¥PY````[W¥B
@HWI-EAS443:3:5:220:1181#0/1
GGGGTTCTTCTCTTGTCTTCCTGATGTGTACTTTTTGATT
+HWI-EAS443:3:5:220:1181#0/1
^`baYbabb`bbaab`ababaa`_ab_b__``aaaaaaa¥
@HWI-EAS443:3:5:302:1683#0/1
CGGGGTTCTTCTCTTGTCTTCCTGATGTGTACTTTTTGAT
+HWI-EAS443:3:5:302:1683#0/1
[baa[Kb``¥b_Vab`Uaba_bb[b[baab`_Wbabaaab
@HWI-EAS443:3:5:476:1208#0/1
CGGGGTTCTTCTCTTGTCTTCCTGATGTGTACTTTTTGAT
+HWI-EAS443:3:5:476:1208#0/1
_abbaababb^b`bbb`abaabb`abaaaaa_bbbba`BB
@HWI-EAS443:3:5:575:935#0/1
GCGGGGTTCTTCTCTTGTCTTCCTGATGTGTACTTTTTGA
+HWI-EAS443:3:5:575:935#0/1
[`b`¥]OQ¥Y_UQIP^^Z][SN][_HG_W`OJOPT¥WBBB
@HWI-EAS443:3:5:719:1238#0/1
CGGGGTTCTTCTCTTGTCTTCCTGATGTGTACTTTTTGAT
+HWI-EAS443:3:5:719:1238#0/1
abbbbabbbba`bbab`bbba[bbabbab`a`abbbbbaa
@HWI-EAS443:3:5:813:1205#0/1
CGGGGTTCTTCTCTTGTCTTCCTGATGTGTACTTTTTGAT
+HWI-EAS443:3:5:813:1205#0/1
abbbb_babaababab`abaa`baaab_b^`^aaaa_a`a

```
@HWI-EAS443:3:5:823:657#0/1
CGGGGTTCTTCTCTTGTCTTCCTGATGTGTACTTTTTGAT
+HWI-EAS443:3:5:823:657#0/1
`bb_b`bbbbab`bbbbbbba_bb`bbabaaabbbbbbab
@HWI-EAS443:3:60:118:1245#0/1
CGGGGTTCTTCTCTTGTCTTCCTGATGTGTACTTTTTGAT
+HWI-EAS443:3:60:118:1245#0/1
aaa¥^¥aaaaaaaaaa^aaa_¥aa_`a]_Y_^``a`_Z^_
@HWI-EAS443:3:60:1622:704#0/1
GCGGGGTTCTTCTCTTGTCTTCCTGATGTGTACTTTTTGA
+HWI-EAS443:3:60:1622:704#0/1
a``a_`]a`ab`a^aa]`_`_]_____aZa^_Z`a`a_Z_
@HWI-EAS443:3:60:1689:224#0/1
CGGGGTTCTTCTCTTGTCTTCCTGATGTGTACTTTTTGAT
+HWI-EAS443:3:60:1689:224#0/1
`ab`aZb`bb`b`aaba`bb__baaaaaa^_^aabaa_aa
@HWI-EAS443:3:60:1708:806#0/1
GGGTTCTTCTCTTGTCTTCCTGATGTGTACTTTTTGATTA
+HWI-EAS443:3:60:1708:806#0/1
^VU¥a`Y^¥_]]]YR]Y[YTZWRXXOMLY]ZYYYBBBBBB
@HWI-EAS443:3:60:460:408#0/1
GCGGGGTTCTTCTCTTGTCTTCCTGATGTGTACTTTTTGA
+HWI-EAS443:3:60:460:408#0/1
]abbb`[````^aaabaaaaa`]a`]``]a___aaaaaX]
@HWI-EAS443:3:60:512:438#0/1
CGGGGTTCTTCTCTTGTCTTCCTGATGTGTACTTTTTGAT
+HWI-EAS443:3:60:512:438#0/1
aaaaa_aaaaaaabab`abaa`a¥`a`^a^`_aaaaa``a
@HWI-EAS443:3:60:78:1779#0/1
CGGGGTTCTTCTCTTGTCTTCCTGATGTGTACTTTTTGAT
+HWI-EAS443:3:60:78:1779#0/1
ab^[_Za`aaaaaaaa_aaaaaa_a_`]_^^^a__`_¥`_
@HWI-EAS443:3:61:1150:630#0/1
GGGTTCTTCTCTTGTCTTCCTGATGTGTACTTTTTGATTA
+HWI-EAS443:3:61:1150:630#0/1
`b^H`]a`¥Z[a`_N^]a`¥`¥QNYR_LF[ZTUYXRPYGQ
@HWI-EAS443:3:61:1211:1381#0/1
CGGGGTTCTTCTCTTGTCTTCCTGATGTGTACTTTTTGAT
+HWI-EAS443:3:61:1211:1381#0/1
]baS¥JbWbbb]_abaabbaabaT^a_`aU_`^U^¥aTYY
@HWI-EAS443:3:61:1224:1179#0/1
CGGGGTTCTTCTCTTGTCTTCCTGATGTGTACTTTTTGAT
+HWI-EAS443:3:61:1224:1179#0/1
aa^bb]aabbbabbaabbbbbbbbbaa`aaaaabbaa[_a
@HWI-EAS443:3:61:1263:339#0/1
GGTTCTTCTCTTGTCTTCCTGATGTGTACTTTTTGATTAT
+HWI-EAS443:3:61:1263:339#0/1
a[W^U]_[^R^^YWX[ZU[ZZPS^R`X¥SWU¥X¥_YXWPU
@HWI-EAS443:3:61:1437:1041#0/1
CGGGGTTCTTCTCTTGTCTTCCTGATGTGTACTTTTTGAT
```

+HWI-EAS443:3:61:1437:1041#0/1
abbbabbabbbbbbbbbbbabbbb^abbbbbbbbabb`_a
@HWI-EAS443:3:61:1685:175#0/1
CGGGGTTCTTCTCTTGTCTTCCTGATGTGTACTTTTTGAT
+HWI-EAS443:3:61:1685:175#0/1
ababbababbbbbbbabbbabbbbbbbbbaa`aabbb_[a
@HWI-EAS443:3:61:182:201#0/1
CGGGGTTCTTCTCTTGTCTTCCTGATGTGTACTTTTTGAT
+HWI-EAS443:3:61:182:201#0/1
a`bbb[aabbb^bbab_aaaaa^^T^a_a```aaa`a¥^`
@HWI-EAS443:3:61:196:367#0/1
GGTTCTTCTCTTGTCTTCCTGATGTGTACTTTTTGATTAT
+HWI-EAS443:3:61:196:367#0/1
ab¥aaa_`aaaaa[`a`¥]a¥FX_Y`UX[_`]_`^^_XTZ
@HWI-EAS443:3:61:239:516#0/1
CGGGGTTCTTCTCTTGTCTTCCTGATGTGTACTTTTTGAT
+HWI-EAS443:3:61:239:516#0/1
aaaaa]`bab`aab_a`aaaa`_ZI¥a_aY]^___``_¥`
@HWI-EAS443:3:61:349:212#0/1
AGCGGGGTTCTTCTCTTGTCTTCCTGATGTGTACTTTTTG
+HWI-EAS443:3:61:349:212#0/1
abbbbba¥aabaaaaaaa`a`_aa¥ZZ`a_a[¥¥``__`_
@HWI-EAS443:3:61:767:903#0/1
GCGGGGTTCTTCTCTTGTCTTCCTGATGTGTACTTTTTGA
+HWI-EAS443:3:61:767:903#0/1
``SZ`UG^¥Z]^Y^`__Y_Z`_a]YXZ[VWGNX^^YXVFQ
@HWI-EAS443:3:61:919:1705#0/1
CGGGGTTCTTCTCTTGTCTTCCTGATGTGTACTTTTTGAT
+HWI-EAS443:3:61:919:1705#0/1
ababbababababbab`_abbbbbabaaaab_aaaaaaaa_a
@HWI-EAS443:3:62:1001:514#0/1
GCGGGGTTCTTCTCTTGTCTTCCTGATGTGTACTTTTTGA
+HWI-EAS443:3:62:1001:514#0/1
`TWT¥WYOL^WNT`TP__WT[ZWSQQYaNFLRN`¥BBBBB
@HWI-EAS443:3:62:1261:276#0/1
GCGGGGTTCTTCTCTTGTCTTCCTGATGTGTACTTTTTGA
+HWI-EAS443:3:62:1261:276#0/1
a¥^a_aWYU`^__¥a_`[¥a^Z]_[O]]ZaXZXa``^`Z]
@HWI-EAS443:3:62:1333:1052#0/1
AGCGGGGTTCTTCTCTTGTCTTCCTGATGTGTACTTTTTG
+HWI-EAS443:3:62:1333:1052#0/1
`]a]^¥ZUZ¥bb__UZS¥]a`aZ]NV[]VPY``P^¥XR`P
@HWI-EAS443:3:62:1420:1618#0/1
CGGGGTTCTTCTCTTGTCTTCCTGATGTGTACTTTTTGAT
+HWI-EAS443:3:62:1420:1618#0/1
¥abbbbbbbbab_`baaabbabb]`ba_bab_bbaabbbb
@HWI-EAS443:3:62:158:240#0/1
CGGGGTTCTTCTCTTGTCTTCCTGATGTGTACTTTTTGAT
+HWI-EAS443:3:62:158:240#0/1
ZY`¥`[____[`a`_aU¥aZ`a`W]_a_aYU¥a_aa`[`[

```
@HWI-EAS443:3:62:279:1449#0/1
GGGGTTCTTCTCTTGTCTTCCTGATGTGTACTTTTTGATT
+HWI-EAS443:3:62:279:1449#0/1
aa]`]aaaaaaaa^DWaaa````_`aYUY^a_`a``U¥`^
@HWI-EAS443:3:62:333:973#0/1
CGGGGTTCTTCTCTTGTCTTCCTGATGTGTACTTTTTGAT
+HWI-EAS443:3:62:333:973#0/1
a_bab_bbbbbaaabaabaaaaa`_aa``_¥_aaaaa[Ta
@HWI-EAS443:3:62:625:576#0/1
GGTTCTTCTCTTGTCTTCCTGATGTGTACTTTTTGATTAT
+HWI-EAS443:3:62:625:576#0/1
aaO^bbbaaab`^_abbaaaa_a`_a``_a_]a[a_``a_
@HWI-EAS443:3:62:674:983#0/1
CGGGGTTCTTCTCTTGTCTTCCTGATGTGTACTTTTTGAT
+HWI-EAS443:3:62:674:983#0/1
aV]_NF``[XRXL[ZHUa_aa_VOOMMO¥G[RVS^]_XP]
@HWI-EAS443:3:62:737:723#0/1
GGGGTTCTTCTCTTGTCTTCCTGATGTGTACTTTTTGATT
+HWI-EAS443:3:62:737:723#0/1
`[^YW]Y__YXN¥_aL¥VV_^[QNU`V^BBBBBBBBBBBB
@HWI-EAS443:3:62:803:912#0/1
GGTTCTTCTCTTGTCTTCCTGATGTGTACTTTTTGATTAT
+HWI-EAS443:3:62:803:912#0/1
`_LYbba_abb^```^a]b``][_¥a`aaaaa]_Q]a_VY
@HWI-EAS443:3:63:1144:247#0/1
AGCGGGGTTCTTCTCTTGTCTTCCTGATGTGTACTTTTTG
+HWI-EAS443:3:63:1144:247#0/1
ababbaa`baabaaaaaaa_bba_aa```]a_`Zaaaaa^
@HWI-EAS443:3:63:1666:163#0/1
CGGGGTTCTTCTCTTGTCTTCCTGATGTGTACTTTTTGAT
+HWI-EAS443:3:63:1666:163#0/1
abbbb_b^^bab[babbab^aabba]_T^]aababbb]ab
@HWI-EAS443:3:63:320:890#0/1
CGGGGTTCTTCTCTTGTCTTCCTGATGTGTACTTTTTGAT
+HWI-EAS443:3:63:320:890#0/1
abaaa¥aaaa`aa`aa^aaaa`_```]]aY]]aa`a``_^
@HWI-EAS443:3:63:333:799#0/1
AGCGGGGTTCTTCTCTTGTCTTCCTGATGTGTACTTTTTG
+HWI-EAS443:3:63:333:799#0/1
¥¥aba`]aa`^ZU_[``aab`abaaXY¥a[`STRa^L[VO
@HWI-EAS443:3:63:563:219#0/1
GCGGGGTTCTTCTCTTGTCTTCCTGATGTGTACTTTTTGA
+HWI-EAS443:3:63:563:219#0/1
`abbbb`baaaabaaaa`aaaaaa`¥`a^a_``aa`a`__
@HWI-EAS443:3:63:667:532#0/1
CGGGGTTCTTCTCTTGTCTTCCTGATGTGTACTTTTTGAT
+HWI-EAS443:3:63:667:532#0/1
abbb`[bab```_a]aa`]_¥WW_V¥YO][W]^]]`a¥TZ
@HWI-EAS443:3:63:757:900#0/1
AGCGGGGTTCTTCTCTTGTCTTCCTGATGTGTACTTTTTG
```

```
+HWI-EAS443:3:63:757:900#0/1
aaa^a`a_`aaa`aa`a_``aaaaaaa`_]_[_^aa`a__
@HWI-EAS443:3:63:797:1449#0/1
CGGGGTTCTTCTCTTGTCTTCCTGATGTGTACTTTTTGAT
+HWI-EAS443:3:63:797:1449#0/1
`abbb`bbbbbbbbba]abbabb`^`b`¥`aabaaaaab`
@HWI-EAS443:3:63:807:1109#0/1
GCGGGGTTCTTCTCTTGTCTTCCTGATGTGTACTTTTTGA
+HWI-EAS443:3:63:807:1109#0/1
`aaa`a^`aaaa__aa`^_a_``a_^`a]`Z^_`a__aV`
@HWI-EAS443:3:64:1098:795#0/1
CGGGGTTCTTCTCTTGTCTTCCTGATGTGTACTTTTTGAT
+HWI-EAS443:3:64:1098:795#0/1
abaa`R_abbababaaaabaa_a``aa_b]]`aaaa`[`a
@HWI-EAS443:3:64:1216:1292#0/1
CGGGGTTCTTCTCTTGTCTTCCTGATGTGTACTTTTTGAT
+HWI-EAS443:3:64:1216:1292#0/1
aaaaaTbaaaaaa[a[`aa`__aWV__]]]_aa_aaa]^a
@HWI-EAS443:3:64:1312:1580#0/1
GGGGTTCTTCTCTTGTCTTCCTGATGTGTACTTTTTGATT
+HWI-EAS443:3:64:1312:1580#0/1
`aaa[babb`aabab_ab`_`aa_^V^``_^aa`aa[aa_
@HWI-EAS443:3:64:1764:737#0/1
CGGGGTTCTTCTCTTGTCTTCCTGATGTGTACTTTTTGAT
+HWI-EAS443:3:64:1764:737#0/1
_^a`_¥^a_aa``b^_]ab_¥^bba_^¥a`_Wa`baa^a`
@HWI-EAS443:3:64:23:266#0/1
CGGGGTTCTTCTCTTGTCTTCCTGATGTGTACTTTTTGAT
+HWI-EAS443:3:64:23:266#0/1
abbb_U`baaabaaab`aaaa_a_P_a[a]T¥^a``a^^a
@HWI-EAS443:3:64:255:951#0/1
GCGGGGTTCTTCTCTTGTCTTCCTGATGTGTACTTTTTGA
+HWI-EAS443:3:64:255:951#0/1
ababb¥Z^baab]a_aZY__aY`_aZ¥a]_RP]`_a_^T¥
@HWI-EAS443:3:64:535:324#0/1
CGGGGTTCTTCTCTTGTCTTCCTGATGTGTACTTTTTGAT
+HWI-EAS443:3:64:535:324#0/1
ZabXS`bbaba_bbb`bbbbbbbab]a]_¥bbabab^Y_b
@HWI-EAS443:3:64:542:722#0/1
CGGGGTTCTTCTCTTGTCTTCCTGATGTGTACTTTTTGAT
+HWI-EAS443:3:64:542:722#0/1
abbab_babbbbabbbabaab`a^aaa`b_a`ab`abaaa
@HWI-EAS443:3:64:576:1789#0/1
CGGGGTTCTTCTCTTGTCTTCCTGATGTGTACTTTTTGAT
+HWI-EAS443:3:64:576:1789#0/1
a^TWXMWa]]W]a]__^___]aUFWYVW_N____aXXPZZ
@HWI-EAS443:3:64:698:299#0/1
CGGGGTTCTTCTCTTGTCTTCCTGATGTGTACTTTTTGAT
+HWI-EAS443:3:64:698:299#0/1
aa`ba[a_aaaaaaa^_aaaaaaa¥^a]^_^_aaaaa``a
```

```
@HWI-EAS443:3:64:744:1191#0/1
CGGGGTTCTTCTCTTGTCTTCCTGATGTGTACTTTTTGAT
+HWI-EAS443:3:64:744:1191#0/1
a`^]]R]V`aX_WVZ^`]Z`_RYJT][G[P^]aaW_a`__
@HWI-EAS443:3:64:76:99#0/1
CGGGGTTCTTCTCTTGTCTTCCTGATGTGTACTTTTTGAT
+HWI-EAS443:3:64:76:99#0/1
aba_aGa]]`^_X_]^]X^`^_]_V^_]]YRW]]^]^]W^
@HWI-EAS443:3:65:1361:199#0/1
CGGGGTTCTTCTCTTGTCTTCCTGATGTGTACTTTTTGAT
+HWI-EAS443:3:65:1361:199#0/1
_bbbba]aabbababa[b`b][¥aaabQa`aZaaaabY¥_
@HWI-EAS443:3:65:350:1876#0/1
GCGGGGTTCTTCTCTTGTCTTCCTGATGTGTACTTTTTGA
+HWI-EAS443:3:65:350:1876#0/1
V_`Z[aaababbbaaOY]W^abbaab`[a_Uabaaabb_b
@HWI-EAS443:3:65:38:63#0/1
GCGGGGTTCTTCTCTTGTCTTCCTGATGTGTACTTTTTGA
+HWI-EAS443:3:65:38:63#0/1
abaaaaYaaaa_a`aa`^_a`^`___YaX`]^Y`___a]]
@HWI-EAS443:3:65:55:1972#0/1
AGCGGGGTTCTTCTCTTGTCTTCCTGATGTGTACTTTTTG
+HWI-EAS443:3:65:55:1972#0/1
Y[ba]Z_Va^]_a`^ab^`_W¥^U^`aYZ[^QW^_aaa_]
@HWI-EAS443:3:65:571:1556#0/1
CGGGGTTCTTCTCTTGTCTTCCTGATGTGTACTTTTTGAT
+HWI-EAS443:3:65:571:1556#0/1
ab``^[abaabbba`__bbabbbaaabaa[T_baba````
@HWI-EAS443:3:65:592:865#0/1
GGGTTCTTCTCTTGTCTTCCTGATGTGTACTTTTTGATTA
+HWI-EAS443:3:65:592:865#0/1
aaa¥aaaaabaaaa]aaaaaa][_a_aZ^`aaaaa¥]a_a
@HWI-EAS443:3:65:833:1758#0/1
GCGGGGTTCTTCTCTTGTCTTCCTGATGTGTACTTTTTGA
+HWI-EAS443:3:65:833:1758#0/1
aa¥`aaZbbababaabb`a]aabaaa`[`a_a_aaaa`[a
@HWI-EAS443:3:65:871:66#0/1
GGGTTCTTCTCTTGTCTTCCTGATGTGTACTTTTTGATTA
+HWI-EAS443:3:65:871:66#0/1
abaYaa]a¥a`_aa[___`_a_^]a]a]WX_````¥]__¥
@HWI-EAS443:3:65:89:887#0/1
GCGGGGTTCTTCTCTTGTCTTCCTGATGTGTACTTTTTGA
+HWI-EAS443:3:65:89:887#0/1
abaa[aP`a``¥W_a]`Y`aaa]_^PY`Q_YQ^_^a[_[_
@HWI-EAS443:3:66:1130:790#0/1
GCGGGGTTCTTCTCTTGTCTTCCTGATGTGTACTTTTTGA
+HWI-EAS443:3:66:1130:790#0/1
I_¥[__Q_`^`_bb``¥]a```^ab`S`¥X^¥__Z¥¥_¥_
@HWI-EAS443:3:66:1230:1716#0/1
ATCAAAAAGTACACATCAGGAAGACAAGAGAAGAACCCCG
```

+HWI-EAS443:3:66:1230:1716#0/1
ab^bbbaaa^baa`aaaaaaaaaa`aaa`aaaaaa_U]Ya
@HWI-EAS443:3:66:1233:665#0/1
GGGGTTCTTCTCTTGTCTTCCTGATGTGTACTTTTTGATT
+HWI-EAS443:3:66:1233:665#0/1
abb`]aabbababbaaabaaaaa_ab_a___aaabaa`a`
@HWI-EAS443:3:66:1240:1580#0/1
GCGGGGTTCTTCTCTTGTCTTCCTGATGTGTACTTTTTGA
+HWI-EAS443:3:66:1240:1580#0/1
aba_^¥Yaaa¥_`a`_aWRYHU`aaWP`X_PX_a[[`^ZU
@HWI-EAS443:3:66:1258:1112#0/1
CGGGGTTCTTCTCTTGTCTTCCTGATGTGTACTTTTTGAT
+HWI-EAS443:3:66:1258:1112#0/1
aaaa¥]aabb`aaaaaaaba¥_a_``]_`_Z_aa_a`]^`
@HWI-EAS443:3:66:1596:448#0/1
GCGGGGTTCTTCTCTTGTCTTCCTGATGTGTACTTTTTGA
+HWI-EAS443:3:66:1596:448#0/1
a^aab`]`__`_`^__a^_``__^_[__Y`]YXY]^a_¥^
@HWI-EAS443:3:66:188:55#0/1
GCGGGGTTCTTCTCTTGTCTTCCTGATGTGTACTTTTTGA
+HWI-EAS443:3:66:188:55#0/1
aabaaa]aaaaaaaaa`¥_a`^aa_^^_]`^¥]^`_`_X^
@HWI-EAS443:3:66:198:476#0/1
CGGGGTTCTTCTCTTGTCTTCCTGATGTGTACTTTTTGAT
+HWI-EAS443:3:66:198:476#0/1
aaaa[G]aa`a^P`_a_````[`[^X¥VaYSV`___Z^Z]
@HWI-EAS443:3:66:303:863#0/1
CGGGGTTCTTCTCTTGTCTTCCTGATGTGTACTTTTTGAT
+HWI-EAS443:3:66:303:863#0/1
aa`^^Q]aaa[aaa`_NY_[Z]a_J¥¥TS[___a`[`Y__
@HWI-EAS443:3:66:597:609#0/1
GCGGGGTTCTTCTCTTGTCTTCCTGATGTGTACTTTTTGA
+HWI-EAS443:3:66:597:609#0/1
[bb`b`Xbb`a_^b`aa]aba_WaaWaa_a¥a`a_aaab^
@HWI-EAS443:3:66:651:419#0/1
GGGTTCTTCTCTTGTCTTCCTGATGTGTACTTTTTGATTA
+HWI-EAS443:3:66:651:419#0/1
a^^X_```]^a`a¥[^a`__^a^__U]S¥Z_Y__`_]^]^
@HWI-EAS443:3:66:73:653#0/1
CGGGGTTCTTCTCTTGTCTTCCTGATGTGTACTTTTTGAT
+HWI-EAS443:3:66:73:653#0/1
aaba_^abaaaaa`aa`aaa_``a`_`]aYX¥`a_`^[[`
@HWI-EAS443:3:67:1016:228#0/1
GGTTCTTCTCTTGTCTTCCTGATGTGTACTTTTTGATTAT
+HWI-EAS443:3:67:1016:228#0/1
``SaY^babaaab_[a_U]a¥aa`W]P^`a`aa[JX_`_W
@HWI-EAS443:3:67:1203:577#0/1
CGGGGTTCTTCTCTTGTCTTCCTGATGTGTACTTTTTGAT
+HWI-EAS443:3:67:1203:577#0/1
abaaa¥bbbbabab_`_aba_``aaaaaaaa¥bbaaaaaa

```
@HWI-EAS443:3:67:1274:1862#0/1
GCGGGGTTCTTCTCTTGTCTTCCTGATGTGTACTTTTTGA
+HWI-EAS443:3:67:1274:1862#0/1
a^WQ_XRV^`a^[WaaaY^_`X¥_VYX_Z[U]V_^_W`^_
@HWI-EAS443:3:67:1733:1737#0/1
CGGGGTTCTTCTCTTGTCTTCCTGATGTGTACTTTTTGAT
+HWI-EAS443:3:67:1733:1737#0/1
``¥UXUO^¥¥V^aaaa]X`a`^][NXUG[L[[XXY¥]_TY
@HWI-EAS443:3:67:232:1072#0/1
GCGGGGTTCTTCTCTTGTCTTCCTGATGTGTACTTTTTGA
+HWI-EAS443:3:67:232:1072#0/1
aaaa_ZHY`Z[_^``_^R`^^_`YRRPYU]SQWR[X_UVZ
@HWI-EAS443:3:67:23:826#0/1
ATCAAAAAGTACACATCAGGAAGACAAGAGAAGAACCCCG
+HWI-EAS443:3:67:23:826#0/1
¥ab`¥aZ__Zbbbaababbbbabaababababababaaaa`a
@HWI-EAS443:3:67:268:776#0/1
GCGGGGTTCTTCTCTTGTCTTCCTGATGTGTACTTTTTGA
+HWI-EAS443:3:67:268:776#0/1
abaaba^baabbbabbb`aaaaabaaab_a_`aaaa`aa`
@HWI-EAS443:3:67:445:64#0/1
CGGGGTTCTTCTCTTGTCTTCCTGATGTGTACTTTTTGAT
+HWI-EAS443:3:67:445:64#0/1
abbbb_bbbbababbbaaaaa`aaaaa_a_`_aaaaaaaa
@HWI-EAS443:3:67:455:738#0/1
CGGGGTTCTTCTCTTGTCTTCCTGATGTGTACTTTTTGAT
+HWI-EAS443:3:67:455:738#0/1
]W]YH[_Za]Y`__aa`aa`_YaSY[¥^]N[]`[a_a__^
@HWI-EAS443:3:67:494:1102#0/1
CGGGGTTCTTCTCTTGTCTTCCTGATGTGTACTTTTTGAT
+HWI-EAS443:3:67:494:1102#0/1
a`_Z¥HbbaaaaaaZa[aa`aaaZU]]¥aR¥`___``¥^`
@HWI-EAS443:3:67:968:616#0/1
CGGGGTTCTTCTCTTGTCTTCCTGATGTGTACTTTTTGAT
+HWI-EAS443:3:67:968:616#0/1
abbabaaabaaaaaabaaba`a```_a¥`¥``aaaaa[`a
@HWI-EAS443:3:68:1074:768#0/1
GGGTTCTTCTCTTGTCTTCCTGATGTGTACTTTTTGATTA
+HWI-EAS443:3:68:1074:768#0/1
aa]Qaa¥[`]TY[TN¥`UTL[[SW¥HWHSW__Y_YY]_Z[
@HWI-EAS443:3:68:1159:1277#0/1
CGGGGTTCTTCTCTTGTCTTCCTGATGTGTACTTTTTGAT
+HWI-EAS443:3:68:1159:1277#0/1
a``aa`babbbbbbba]bbababY`a`[aaa]babbaaa`
@HWI-EAS443:3:68:1412:1068#0/1
GCGGGGTTCTTCTCTTGTCTTCCTGATGTGTACTTTTTGA
+HWI-EAS443:3:68:1412:1068#0/1
aabb]]Ya`ba``^b`a`_a``_][X`Y_a_^¥^`_`^]`
@HWI-EAS443:3:68:1557:310#0/1
CGGGGTTCTTCTCTTGTCTTCCTGATGTGTACTTTTTGAT
```

```
+HWI-EAS443:3:68:1557:310#0/1
S¥bbbU¥^``bbbbabb[bbaJbabbbabaaN`__bbaaa
@HWI-EAS443:3:68:1621:1834#0/1
CGGGGTTCTTCTCTTGTCTTCCTGATGTGTACTTTTTGAT
+HWI-EAS443:3:68:1621:1834#0/1
`b`Za[aaba[bbabb`Sabaabbaab]aT``aaaaaaaa
@HWI-EAS443:3:68:183:903#0/1
GCGGGGTTCTTCTCTTGTCTTCCTGATGTGTACTTTTTGA
+HWI-EAS443:3:68:183:903#0/1
abbbbb`bbaaaaaaaa¥baaaaa]X^`^a[V_a`aaaaa
@HWI-EAS443:3:68:447:1691#0/1
GCGGGGTTCTTCTCTTGTCTTCCTGATGTGTACTTTTTGA
+HWI-EAS443:3:68:447:1691#0/1
aa^QVXH[b__aaa_ZRUb_^a_RUX¥UY_VZ[_WZ^_QN
@HWI-EAS443:3:68:452:1558#0/1
GCGGGGTTCTTCTCTTGTCTTCCTGATGTGTACTTTTTGA
+HWI-EAS443:3:68:452:1558#0/1
abba]_¥abbbabbbba_bbabba``aa¥^]`aaaaba[a
@HWI-EAS443:3:68:812:1405#0/1
GCGGGGTTCTTCTCTTGTCTTCCTGATGTGTACTTTTTGA
+HWI-EAS443:3:68:812:1405#0/1
`aa]aaZab`a```aaa^_`a_a_[a``X_^a__^`^_R[
@HWI-EAS443:3:68:996:1415#0/1
GCGGGGTTCTTCTCTTGTCTTCCTGATGTGTACTTTTTGA
+HWI-EAS443:3:68:996:1415#0/1
`bab`a_abaaa`aaaa`aaaaaa`a``_aYa``aa_`¥`
@HWI-EAS443:3:69:1092:1888#0/1
CGGGGTTCTTCTCTTGTCTTCCTGATGTGTACTTTTTGAT
+HWI-EAS443:3:69:1092:1888#0/1
aaaaa`bb`aabbba`abbbaababba_^aa_bbabbaba
@HWI-EAS443:3:69:116:107#0/1
GCGGGGTTCTTCTCTTGTCTTCCTGATGTGTACTTTTTGA
+HWI-EAS443:3:69:116:107#0/1
aabaaaY``aa^a_`aa___a^__`]`]X_X]Y__a`][]
@HWI-EAS443:3:69:1727:1079#0/1
AGCGGGGTTCTTCTCTTGTCTTCCTGATGTGTACTTTTTG
+HWI-EAS443:3:69:1727:1079#0/1
`^abbab`babbaaabbaaaaaa_`aa```a_a_aaaaa`
@HWI-EAS443:3:69:233:524#0/1
CGGGGTTCTTCTCTTGTCTTCCTGATGTGTACTTTTTGAT
+HWI-EAS443:3:69:233:524#0/1
a`[¥_Q¥aaaaa`a`a__aaba``[]UU_]`_```aa```
@HWI-EAS443:3:69:305:153#0/1
CGGGGTTCTTCTCTTGTCTTCCTGATGTGTACTTTTTGAT
+HWI-EAS443:3:69:305:153#0/1
abbbbababbbbabbbaabaaaaaa`a_a¥`Zaaaaa^]`
@HWI-EAS443:3:69:439:323#0/1
GGGGTTCTTCTCTTGTCTTCCTGATGTGTACTTTTTGATT
+HWI-EAS443:3:69:439:323#0/1
aaaa[aaaa`aaaa`¥aa`a]ZZZV_Y_YYV]]^X[ZY]]
```

```
@HWI-EAS443:3:69:538:1982#0/1
GCGGGGTTCTTCTCTTGTCTTCCTGATGTGTACTTTTTGA
+HWI-EAS443:3:69:538:1982#0/1
ab¥]`]Rab]baabba]`b^¥bba^S[WTa[ba`a`a_X_
@HWI-EAS443:3:69:606:225#0/1
GCGGGGTTCTTCTCTTGTCTTCCTGATGTGTACTTTTTGA
+HWI-EAS443:3:69:606:225#0/1
]a`a`Z]abb_¥a_bab_Z`aY___^]_¥`Yaababaaaa
@HWI-EAS443:3:69:613:1260#0/1
CGGGGTTCTTCTCTTGTCTTCCTGATGTGTACTTTTTGAT
+HWI-EAS443:3:69:613:1260#0/1
abbba]_`]_a^U^`b^aaa_a^V`aa_a^¥Za_a¥_a`^
@HWI-EAS443:3:69:654:271#0/1
CGGGGTTCTTCTCTTGTCTTCCTGATGTGTACTTTTTGAT
+HWI-EAS443:3:69:654:271#0/1
aba_a`b`aaa`_`]RY]a_a`a[Vab]a]a_``a_aJ_`
@HWI-EAS443:3:69:919:347#0/1
GCGGGGTTCTTCTCTTGTCTTCCTGATGTGTACTTTTTGA
+HWI-EAS443:3:69:919:347#0/1
aaa`a`^_aaaa_`aaa^_`_^_`a`_`_`^__a`a`_P_
@HWI-EAS443:3:69:929:696#0/1
GCGGGGTTCTTCTCTTGTCTTCCTGATGTGTACTTTTTGA
+HWI-EAS443:3:69:929:696#0/1
aab`bbRbabbaaaabab`abaabbab`aa]ababbbaa^
@HWI-EAS443:3:69:978:1124#0/1
AGCGGGGTTCTTCTCTTGTCTTCCTGATGTGTACTTTTTG
+HWI-EAS443:3:69:978:1124#0/1
abbbaaa]babaaabba`a`baa`ba`aa]]^a]a`aa_`
@HWI-EAS443:3:6:109:1736#0/1
CGGGGTTCTTCTCTTGTCTTCCTGATGTGTACTTTTTGAT
+HWI-EAS443:3:6:109:1736#0/1
aaaabRabaaaa_aaa_[a`a`aTH_ZWa^[Ya]¥ZaZa`
@HWI-EAS443:3:6:1319:238#0/1
CGGGGTTCTTCTCTTGTCTTCCTGATGTGTACTTTTTGAT
+HWI-EAS443:3:6:1319:238#0/1
_abbb]``ba`bPbbba`a`a^b__abaa_`_bbaabb`a
@HWI-EAS443:3:6:1404:939#0/1
GGGTTCTTCTCTTGTCTTCCTGATGTGTACTTTTTGATTA
+HWI-EAS443:3:6:1404:939#0/1
aa`OaRa`Va^aaa_]a`¥^__Z_`X^]]Zaa```]_a^^
@HWI-EAS443:3:6:372:488#0/1
CGGGGTTCTTCTCTTGTCTTCCTGATGTGTACTTTTTGAT
+HWI-EAS443:3:6:372:488#0/1
abb[`_bb`[X`_a¥]__aaa]aZ¥a[SYR¥U[_aa_``^
@HWI-EAS443:3:6:702:821#0/1
CGGGGTTCTTCTCTTGTCTTCCTGATGTGTACTTTTTGAT
+HWI-EAS443:3:6:702:821#0/1
Vb_baTaaabbab[ab[a`b_`ababb^b^a`bb`aabaa
@HWI-EAS443:3:70:1004:103#0/1
CGGGGTTCTTCTCTTGTCTTCCTGATGTGTACTTTTTGAT
```

```
+HWI-EAS443:3:70:1004:103#0/1
abb`b`bbbbbbabbbaabbbbbbbb_bbb_bbbbbba`b
@HWI-EAS443:3:70:1281:388#0/1
GCGGGGTTCTTCTCTTGTCTTCCTGATGTGTACTTTTTGA
+HWI-EAS443:3:70:1281:388#0/1
a`abababaabaa`bbb_abaaabb`bbYaaaRaaabbaa
@HWI-EAS443:3:70:1372:286#0/1
GCGGGGTTCTTCTCTTGTCTTCCTGATGTGTACTTTTTGA
+HWI-EAS443:3:70:1372:286#0/1
aaaaaa]a_aa_a^aaa``b`_aa_^`a^a^_]``_``X^
@HWI-EAS443:3:70:1611:86#0/1
CGGGGTTCTTCTCTTGTCTTCCTGATGTGTACTTTTTGAT
+HWI-EAS443:3:70:1611:86#0/1
abbaKGb]aaa_Ta_a_a`a[a`Y``XUa]^^a```a]Z_
@HWI-EAS443:3:70:345:75#0/1
GGGGTTCTTCTCTTGTCTTCCTGATGTGTACTTTTTGATT
+HWI-EAS443:3:70:345:75#0/1
aa_aV_a___`````]^`__^`_]]_^_YR[^_^^`_]]^
@HWI-EAS443:3:70:384:875#0/1
CGGGGTTCTTCTCTTGTCTTCCTGATGTGTACTTTTTGAT
+HWI-EAS443:3:70:384:875#0/1
a`bbb]aaaaaaaaba`abababaaa_`_a]aaaaaaa`aa
@HWI-EAS443:3:70:445:928#0/1
GGGGTTCTTCTCTTGTCTTCCTGATGTGTACTTTTTGATT
+HWI-EAS443:3:70:445:928#0/1
`_aa^aaabaaaa_a_aa`a_aa¥`a^`]]]aa`a`^]__
@HWI-EAS443:3:70:487:674#0/1
CGGGGTTCTTCTCTTGTCTTCCTGATGTGTACTTTTTGAT
+HWI-EAS443:3:70:487:674#0/1
abZZ^U]ba`baaa^`aaaaaba``aa^`]X]aaaa_P^a
@HWI-EAS443:3:70:57:105#0/1
CGGGGTTCTTCTCTTGTCTTCCTGATGTGTACTTTTTGAT
+HWI-EAS443:3:70:57:105#0/1
ababaY`^¥]Y_``a¥L¥a`]]`[QQYUYWUUa`_a_Y_T
@HWI-EAS443:3:70:614:1417#0/1
CGGGGTTCTTCTCTTGTCTTCCTGATGTGTACTTTTTGAT
+HWI-EAS443:3:70:614:1417#0/1
aa¥a`Waab_baa`ba`aabaaa```a`RUTaa_aaa_aa
@HWI-EAS443:3:70:653:1251#0/1
GCGGGGTTCTTCTCTTGTCTTCCTGATGTGTACTTTTTGA
+HWI-EAS443:3:70:653:1251#0/1
abbaaa]aa^`aaa``Xaa``baa¥Y_]Na^`¥a_a_`^`
@HWI-EAS443:3:70:737:198#0/1
CGGGGTTCTTCTCTTGTCTTCCTGATGTGTACTTTTTGAT
+HWI-EAS443:3:70:737:198#0/1
a_baa_baab`bbab`]bba`aaaa_b`a___aaaaaa_a
@HWI-EAS443:3:70:82:453#0/1
AGCGGGGTTCTTCTCTTGTCTTCCTGATGTGTACTTTTTG
+HWI-EAS443:3:70:82:453#0/1
aTa]ab__aa`aWNW`a[Z_aR^_a__¥][YX_^a_X¥[Y
```

```
@HWI-EAS443:3:70:919:1323#0/1
CGGGGTTCTTCTCTTGTCTTCCTGATGTGTACTTTTTGAT
+HWI-EAS443:3:70:919:1323#0/1
`_UZbaabbbbbbbba`bbaaab__aa`a``aaaabaXaa
@HWI-EAS443:3:71:1015:1009#0/1
CGGGGTTCTTCTCTTGTCTTCCTGATGTGTACTTTTTGAT
+HWI-EAS443:3:71:1015:1009#0/1
a^^_TF^ba`aa_ab_S]a`aaaa`_]]][W_]`^``UZ¥
@HWI-EAS443:3:71:1032:934#0/1
GCGGGGTTCTTCTCTTGTCTTCCTGATGTGTACTTTTTGA
+HWI-EAS443:3:71:1032:934#0/1
abbbaZXa`b_L[aba]_`b^[H¥Y^a`QaGPaaa_aaP[
@HWI-EAS443:3:71:14:249#0/1
GGGGTTCTTCTCTTGTCTTCCTGATGTGTACTTTTTGATT
+HWI-EAS443:3:71:14:249#0/1
a¥¥VLY^TR][`Y[_Y¥Z¥W¥^ZW¥_W¥NUU¥Y[V[YXXU
@HWI-EAS443:3:71:1513:1970#0/1
CGGGGTTCTTCTCTTGTCTTCCTGATGTGTACTTTTTGAT
+HWI-EAS443:3:71:1513:1970#0/1
ababbZbbaba^aabaaabaaa`aba]`b`a^baaa``aa
@HWI-EAS443:3:71:1577:1625#0/1
CGGGGTTCTTCTCTTGTCTTCCTGATGTGTACTTTTTGAT
+HWI-EAS443:3:71:1577:1625#0/1
X_¥¥WY_¥_`a___R_aa`a_VZ¥aY[S_VL]^^a`]`Z]
@HWI-EAS443:3:71:1585:1224#0/1
GCGGGGTTCTTCTCTTGTCTTCCTGATGTGTACTTTTTGA
+HWI-EAS443:3:71:1585:1224#0/1
_OaXaaVZ¥]_G]^V[a]`X[WZ``X[^M`QU[Z^_Z]YV
@HWI-EAS443:3:71:177:90#0/1
AGCGGGGTTCTTCTCTTGTCTTCCTGATGTGTACTTTTTG
+HWI-EAS443:3:71:177:90#0/1
aaaaaa_Xaa`a``_`aa^a`a`___X^aZY^^^^_a¥`^
@HWI-EAS443:3:71:302:466#0/1
GCGGGGTTCTTCTCTTGTCTTCCTGATGTGTACTTTTTGA
+HWI-EAS443:3:71:302:466#0/1
_^¥`YMTa_YTYYZORWZTWGOXYV^]LEYRV`XVPY_X^
@HWI-EAS443:3:71:340:218#0/1
GGGTTCTTCTCTTGTCTTCCTGATGTGTACTTTTTGATTA
+HWI-EAS443:3:71:340:218#0/1
a_[U_a`[W]NRZ]R¥^QPY[RNR_X_XMOXX¥T^_]YTV
@HWI-EAS443:3:71:347:1660#0/1
GCGGGGTTCTTCTCTTGTCTTCCTGATGTGTACTTTTTGA
+HWI-EAS443:3:71:347:1660#0/1
ab_aZFIU``[`aa`YUU_Yaba¥_]¥]HW[^aY]YX__[
@HWI-EAS443:3:71:593:699#0/1
CGGGGTTCTTCTCTTGTCTTCCTGATGTGTACTTTTTGAT
+HWI-EAS443:3:71:593:699#0/1
ab_aaWaa]]`aaaab_]aaa]baTaZY]Z[_`aaaaa`_
@HWI-EAS443:3:71:868:1038#0/1
GGGGTTCTTCTCTTGTCTTCCTGATGTGTACTTTTTGATT
```

+HWI-EAS443:3:71:868:1038#0/1
¥aZ]S_a`babbabbaba`a^b^_]babaaabbbbbaaab
@HWI-EAS443:3:71:893:579#0/1
GCGGGGTTCTTCTCTTGTCTTCCTGATGTGTACTTTTTGA
+HWI-EAS443:3:71:893:579#0/1
aa`ba`Yaaaaaa_^ba__aa^`____aVaY]a`a`_a^_
@HWI-EAS443:3:71:984:836#0/1
GCGGGGTTCTTCTCTTGTCTTCCTGATGTGTACTTTTTGA
+HWI-EAS443:3:71:984:836#0/1
``b]Qa`aaaaa`_]U^[Zaa]Q[X[¥aQ`Ra_]aY_`YZ
@HWI-EAS443:3:72:1775:1076#0/1
CGGGGTTCTTCTCTTGTCTTCCTGATGTGTACTTTTTGAT
+HWI-EAS443:3:72:1775:1076#0/1
abaaa]bbbaababaa_aaa^_`[`_a^a_`_aabaa¥`a
@HWI-EAS443:3:72:215:833#0/1
GGGGTTCTTCTCTTGTCTTCCTGATGTGTACTTTTTGATT
+HWI-EAS443:3:72:215:833#0/1
ab_a]abbaaaaaaa`aaa_aa][]a__^__aa`a`[[`^
@HWI-EAS443:3:72:291:430#0/1
GGGGTTCTTCTCTTGTCTTCCTGATGTGTACTTTTTGATT
+HWI-EAS443:3:72:291:430#0/1
ab``]aaaaaaaa`a^a`a_a_VX^Y^aX__`_``a____
@HWI-EAS443:3:72:380:667#0/1
AGCGGGGTTCTTCTCTTGTCTTCCTGATGTGTACTTTTTG
+HWI-EAS443:3:72:380:667#0/1
aaaa_aaUaaaaaa^aaa]aa``aaa``a]a]U^a`__`[
@HWI-EAS443:3:72:442:1754#0/1
GGGGTTCTTCTCTTGTCTTCCTGATGTGTACTTTTTGATT
+HWI-EAS443:3:72:442:1754#0/1
aaaa]bbabbababaabaabba`_aa`a`abaaa`a_```
@HWI-EAS443:3:72:457:290#0/1
GGGTTCTTCTCTTGTCTTCCTGATGTGTACTTTTTGATTA
+HWI-EAS443:3:72:457:290#0/1
R__J_^YXXa_QVT_W¥__^_WUUWW_GW`Y``ab]`b_^
@HWI-EAS443:3:72:481:559#0/1
GCGGGGTTCTTCTCTTGTCTTCCTGATGTGTACTTTTTGA
+HWI-EAS443:3:72:481:559#0/1
`¥`a_`^aaaaaa`aa`P`aaaa`aa_a^]]`_a`a``U]
@HWI-EAS443:3:72:646:1069#0/1
GGGTTCTTCTCTTGTCTTCCTGATGTGTACTTTTTGATTA
+HWI-EAS443:3:72:646:1069#0/1
`]_Z``baa`aaaa^aaaaaaaa]]^_¥aaaaaaa_````
@HWI-EAS443:3:72:659:692#0/1
CGGGGTTCTTCTCTTGTCTTCCTGATGTGTACTTTTTGAT
+HWI-EAS443:3:72:659:692#0/1
ab`b^Wb_aaaaaaaa`aa[^_`]^aaM]Z]¥aaaa``a_
@HWI-EAS443:3:72:701:134#0/1
GCGGGGTTCTTCTCTTGTCTTCCTGATGTGTACTTTTTGA
+HWI-EAS443:3:72:701:134#0/1
aab]aZU]`aa[a`a_a]¥__^__aYH]¥_Y^^__`_^_]

```
@HWI-EAS443:3:72:860:1700#0/1
CGGGGTTCTTCTCTTGTCTTCCTGATGTGTACTTTTTGAT
+HWI-EAS443:3:72:860:1700#0/1
aaa¥aZZbabb``b¥b_aaab`_W_`__[[aaaa_`a]a_
@HWI-EAS443:3:72:886:1322#0/1
GGGGTTCTTCTCTTGTCTTCCTGATGTGTACTTTTTGATT
+HWI-EAS443:3:72:886:1322#0/1
`ba_Wbbb_abb``___a_`aaa``a_a^__`a`aa_aa_
@HWI-EAS443:3:72:908:342#0/1
AGCGGGGTTCTTCTCTTGTCTTCCTGATGTGTACTTTTTG
+HWI-EAS443:3:72:908:342#0/1
^S`a¥Z¥Q¥ba^a`[[a`[[aaaa``W`a^]`YL`^_``¥
@HWI-EAS443:3:73:1061:146#0/1
GGTTCTTCTCTTGTCTTCCTGATGTGTACTTTTTGATTAT
+HWI-EAS443:3:73:1061:146#0/1
a`W`_aa_`]_`_]^``^Z__^`_]_]XX_]_^_^]^^Z]
@HWI-EAS443:3:73:1193:1249#0/1
CGGGGTTCTTCTCTTGTCTTCCTGATGTGTACTTTTTGAT
+HWI-EAS443:3:73:1193:1249#0/1
aa`_][aba_`a^aaa]aa`_^a[`_`_a]_Z`a``[_``
@HWI-EAS443:3:73:123:361#0/1
CGGGGTTCTTCTCTTGTCTTCCTGATGTGTACTTTTTGAT
+HWI-EAS443:3:73:123:361#0/1
a_WYXQ[`¥_^_[_^]T¥[]^[Z¥VWYUZXUW^[^[[LW]
@HWI-EAS443:3:73:1311:361#0/1
CGGGGTTCTTCTCTTGTCTTCCTGATGTGTACTTTTTGAT
+HWI-EAS443:3:73:1311:361#0/1
Y_aab]a¥_ba`_Y`b[Wa`Ya`¥^ba_]aaRZ`babb]_
@HWI-EAS443:3:73:1322:1635#0/1
CGGGGTTCTTCTCTTGTCTTCCTGATGTGTACTTTTTGAT
+HWI-EAS443:3:73:1322:1635#0/1
^a_bbabbaaa¥L_aaU``YbWbbaaa^b`aba`aa`aab
@HWI-EAS443:3:73:1419:169#0/1
GGGTTCTTCTCTTGTCTTCCTGATGTGTACTTTTTGATTA
+HWI-EAS443:3:73:1419:169#0/1
`YFMa`[[Sa_a]XZ]aa`U_`¥_]X[O¥Q]`a`a_]`^]
@HWI-EAS443:3:73:1620:1300#0/1
CGGGGTTCTTCTCTTGTCTTCCTGATGTGTACTTTTTGAT
+HWI-EAS443:3:73:1620:1300#0/1
a`^`b`aabbab`baaY`b`Y[_b``]JXX^a_aa]¥V^`
@HWI-EAS443:3:73:312:234#0/1
AGCGGGGTTCTTCTCTTGTCTTCCTGATGTGTACTTTTTG
+HWI-EAS443:3:73:312:234#0/1
^`a_aaa^a``aa`a__a`W__```[]_`[`NJV]`a¥`_
@HWI-EAS443:3:73:430:1331#0/1
GCGGGGTTCTTCTCTTGTCTTCCTGATGTGTACTTTTTGA
+HWI-EAS443:3:73:430:1331#0/1
aaa[aaWY_[_aQ[aSa_aX`]YYO^XYO`GG`_SS¥[GR
@HWI-EAS443:3:73:432:303#0/1
GGGTTCTTCTCTTGTCTTCCTGATGTGTACTTTTTGATTA
```

+HWI-EAS443:3:73:432:303#0/1
a_aW`aa__a_a_a__a`__a¥_`_W_Z^_`_^``__^^_
@HWI-EAS443:3:73:515:248#0/1
AGCGGGGTTCTTCTCTTGTCTTCCTGATGTGTACTTTTTG
+HWI-EAS443:3:73:515:248#0/1
[_Y_``_T¥_U_aZ]ab__S_`]Y[ZW]`URYZ__YY]_Y
@HWI-EAS443:3:73:668:765#0/1
GCGGGGTTCTTCTCTTGTCTTCCTGATGTGTACTTTTTGA
+HWI-EAS443:3:73:668:765#0/1
Y`babbZbbbabaabb_``ba`aaa`a``a__``aaaa]`
@HWI-EAS443:3:73:758:471#0/1
CGGGGTTCTTCTCTTGTCTTCCTGATGTGTACTTTTTGAT
+HWI-EAS443:3:73:758:471#0/1
Waa`_O_YOX¥b`_Ja[XVVV]b^[^bXaH¥baa][aa`_
@HWI-EAS443:3:73:828:1862#0/1
CGGGGTTCTTCTCTTGTCTTCCTGATGTGTACTTTTTGAT
+HWI-EAS443:3:73:828:1862#0/1
a_TYIJ_a__`^^TQNO^KT[ZSX]UUQ_UVUWU^ZXZZZ
@HWI-EAS443:3:73:849:1488#0/1
CGGGGTTCTTCTCTTGTCTTCCTGATGTGTACTTTTTGAT
+HWI-EAS443:3:73:849:1488#0/1
`a_a]`abbbababbaaabaabaaaaa_baa_abbab^aa
@HWI-EAS443:3:73:84:595#0/1
CGGGGTTCTTCTCTTGTCTTCCTGATGTGTACTTTTTGAT
+HWI-EAS443:3:73:84:595#0/1
aaaaaXaaaa``a__a]a`^____ZVWV^X]^^Y^]]]]_
@HWI-EAS443:3:73:911:1244#0/1
GGGTTCTTCTCTTGTCTTCCTGATGTGTACTTTTTGATTA
+HWI-EAS443:3:73:911:1244#0/1
aabObbaaba_ba^aaaaaaaaaa]_`X`_aaa`a````a
@HWI-EAS443:3:74:1116:674#0/1
GCGGGGTTCTTCTCTTGTCTTCCTGATGTGTACTTTTTGA
+HWI-EAS443:3:74:1116:674#0/1
a`aa`]_a_aa_a`aa]`_a`]^a_^_a]a_^Za``__]]
@HWI-EAS443:3:74:1288:1069#0/1
CGGGGTTCTTCTCTTGTCTTCCTGATGTGTACTTTTTGAT
+HWI-EAS443:3:74:1288:1069#0/1
abba¥^ba_bbbbbbbVaabaab`babV_a¥Y`baa`bbb
@HWI-EAS443:3:74:157:47#0/1
CGGGGTTCTTCTCTTGTCTTCCTGATGTGTACTTTTTGAT
+HWI-EAS443:3:74:157:47#0/1
ababbHPP_[a]YY^]`V^^aYbaR]XX`MLFY^aWaBBB
@HWI-EAS443:3:74:1587:1421#0/1
GCGGGGTTCTTCTCTTGTCTTCCTGATGTGTACTTTTTGA
+HWI-EAS443:3:74:1587:1421#0/1
U¥_Z`a_a`a__^¥b``]Vab``aa^^`V`a]`bbba¥``
@HWI-EAS443:3:74:1594:1044#0/1
GGGGTTCTTCTCTTGTCTTCCTGATGTGTACTTTTTGATT
+HWI-EAS443:3:74:1594:1044#0/1
ababaabb`babababb`baa_baaa_a`bbbab`aba

```
@HWI-EAS443:3:74:1624:1426#0/1
GGGGTTCTTCTCTTGTCTTCCTGATGTGTACTTTTTGATT
+HWI-EAS443:3:74:1624:1426#0/1
a_QY[`_`^]a_`a_X__a`_aaM]`^_YYVZ_a]_V¥`^
@HWI-EAS443:3:74:174:408#0/1
CGGGGTTCTTCTCTTGTCTTCCTGATGTGTACTTTTTGAT
+HWI-EAS443:3:74:174:408#0/1
_b`abX`aa_``aa]a_`X`__]_`aa^`[U¥_aa^`^_`
@HWI-EAS443:3:74:353:1901#0/1
CGGGGTTCTTCTCTTGTCTTCCTGATGTGTACTTTTTGAT
+HWI-EAS443:3:74:353:1901#0/1
aba^Q[¥^Z_ZZ][UTNb`_]a__WXFNJSPY_RZSY_`Z
@HWI-EAS443:3:74:476:828#0/1
CGGGGTTCTTCTCTTGTCTTCCTGATGTGTACTTTTTGAT
+HWI-EAS443:3:74:476:828#0/1
^a`^`VH^baa`_^aa`aa[aba^WSVWaH¥_bba``Y[`
@HWI-EAS443:3:74:524:724#0/1
GGGGTTCTTCTCTTGTCTTCCTGATGTGTACTTTTTGATT
+HWI-EAS443:3:74:524:724#0/1
`_VFDP_[a^RT¥__YK^T^aY`RZU¥[^J__]ZY`TK[]
@HWI-EAS443:3:74:603:1971#0/1
GGGGTTCTTCTCTTGTCTTCCTGATGTGTACTTTTTGATT
+HWI-EAS443:3:74:603:1971#0/1
^FZSL`ba_aab`aa_ab_bb[`X^[SaY```a_`¥T[`]
@HWI-EAS443:3:74:724:571#0/1
AGCGGGGTTCTTCTCTTGTCTTCCTGATGTGTACTTTTTG
+HWI-EAS443:3:74:724:571#0/1
`baU_bbZ¥``Z_]_``b_`Z_]a`aaaaX^PV^aaa]]Y
@HWI-EAS443:3:74:747:1426#0/1
CGGGGTTCTTCTCTTGTCTTCCTGATGTGTACTTTTTGAT
+HWI-EAS443:3:74:747:1426#0/1
aaaaa¥ab`abaaaa_S_aaaa`a_a`][]_`a_``a[a`
@HWI-EAS443:3:74:843:272#0/1
GGTTCTTCTCTTGTCTTCCTGATGTGTACTTTTTGATTAT
+HWI-EAS443:3:74:843:272#0/1
a`]a^aa^a_aaa]^a_a_`a¥¥XT`^^]aa__`^__^[^
@HWI-EAS443:3:74:876:680#0/1
CGGGGTTCTTCTCTTGTCTTCCTGATGTGTACTTTTTGAT
+HWI-EAS443:3:74:876:680#0/1
abba`abbaabaaaa_aabaaaaa``aaa¥a^`aaaa]aa
@HWI-EAS443:3:74:88:54#0/1
AGCGGGGTTCTTCTCTTGTCTTCCTGATGTGTACTTTTTG
+HWI-EAS443:3:74:88:54#0/1
_Z_a_`]]aaa`¥[^aabW_`a]^`Y]^`H^QXZ][^_`S
@HWI-EAS443:3:75:1120:1667#0/1
AGCGGGGTTCTTCTCTTGTCTTCCTGATGTGTACTTTTTG
+HWI-EAS443:3:75:1120:1667#0/1
Sabbb_WG¥`^]a`b]`]N¥baba^¥[`^R]H^^_a]^`Y
@HWI-EAS443:3:75:1336:1614#0/1
CGGGGTTCTTCTCTTGTCTTCCTGATGTGTACTTTTTGAT
```

```
+HWI-EAS443:3:75:1336:1614#0/1
ab`a^abbbbabbbabbabbbbbbabbaa]]ababbbbbb
@HWI-EAS443:3:75:1552:976#0/1
GCGGGGTTCTTCTCTTGTCTTCCTGATGTGTACTTTTTGA
+HWI-EAS443:3:75:1552:976#0/1
aa`a_b^babbab¥abba`bbaaa`aabaaaa[aaaaa^a
@HWI-EAS443:3:75:53:619#0/1
CGGGGTTCTTCTCTTGTCTTCCTGATGTGTACTTTTTGAT
+HWI-EAS443:3:75:53:619#0/1
aabba_bbbbb`a`ab`baabaaaa`b_`^_aaa`aa[aa
@HWI-EAS443:3:75:674:1386#0/1
GCGGGGTTCTTCTCTTGTCTTCCTGATGTGTACTTTTTGA
+HWI-EAS443:3:75:674:1386#0/1
aa_`[^[abaaaaaa_`_a`X`aa___`Y`W`_`[a``^Y
@HWI-EAS443:3:75:687:202#0/1
GGGTTCTTCTCTTGTCTTCCTGATGTGTACTTTTTGATTA
+HWI-EAS443:3:75:687:202#0/1
¥^a]^_`^XU[aa^Uaba^U¥¥aa`VaN]a_abaaa___^
@HWI-EAS443:3:75:717:397#0/1
GCGGGGTTCTTCTCTTGTCTTCCTGATGTGTACTTTTTGA
+HWI-EAS443:3:75:717:397#0/1
abaab_N_ba_]_`a``aZaa¥`a`aab__^¥Wba`ab^`
@HWI-EAS443:3:76:1038:1658#0/1
GCGGGGTTCTTCTCTTGTCTTCCTGATGTGTACTTTTTGA
+HWI-EAS443:3:76:1038:1658#0/1
a^^SS^U^]^^Z[U[^`[¥_^__YSW]]Z^VZX^^ZV^WZ
@HWI-EAS443:3:76:105:255#0/1
CGGGGTTCTTCTCTTGTCTTCCTGATGTGTACTTTTTGAT
+HWI-EAS443:3:76:105:255#0/1
Zab^^Xabbba_b`ab`__aaba`Y_a`^VaZ^`_`^`aa
@HWI-EAS443:3:76:1396:696#0/1
CGGGGTTCTTCTCTTGTCTTCCTGATGTGTACTTTTTGAT
+HWI-EAS443:3:76:1396:696#0/1
`[aa^^`a`bb_[``babb`_a`baa``aa_X_a`ba_ab
@HWI-EAS443:3:76:1414:156#0/1
GGGGTTCTTCTCTTGTCTTCCTGATGTGTACTTTTTGATT
+HWI-EAS443:3:76:1414:156#0/1
a¥]^Xa]aaY[X¥Z_¥T^¥VW¥VRVZV_QUZZ^__]^S]Z
@HWI-EAS443:3:76:1433:451#0/1
CGGGGTTCTTCTCTTGTCTTCCTGATGTGTACTTTTTGAT
+HWI-EAS443:3:76:1433:451#0/1
ababbababbbbbbbbabbb_bbbaababaa^bbbbbaba
@HWI-EAS443:3:76:148:65#0/1
CGGGGTTCTTCTCTTGTCTTCCTGATGTGTACTTTTTGAT
+HWI-EAS443:3:76:148:65#0/1
ab``a_babaaaabaa^aaaaaaa__a_^_¥Va`aa__`a
@HWI-EAS443:3:76:1552:1959#0/1
ATCAAAAAGTACACATCAGGAAGACAAGAGAAGAACCCCG
+HWI-EAS443:3:76:1552:1959#0/1
_][_aaa``Oa¥`V[¥]`^`_[_]_aU]__^_a_]YRVO_
```

```
@HWI-EAS443:3:76:1783:1282#0/1
CGGGGTTCTTCTCTTGTCTTCCTGATGTGTACTTTTTGAT
+HWI-EAS443:3:76:1783:1282#0/1
S¥`bbUbabbbbab_ba`babaa__baba``Zbbbbb_aa
@HWI-EAS443:3:76:224:900#0/1
GCGGGGTTCTTCTCTTGTCTTCCTGATGTGTACTTTTTGA
+HWI-EAS443:3:76:224:900#0/1
aaba_`EMNV[YOTX¥¥`XXQYU¥RLW^P_WY_Z_[__]^
@HWI-EAS443:3:76:249:1124#0/1
CGGGGTTCTTCTCTTGTCTTCCTGATGTGTACTTTTTGAT
+HWI-EAS443:3:76:249:1124#0/1
aaabbabbbbabbbbaababaaaa`ab_a^``aaaaaZaa
@HWI-EAS443:3:76:762:1391#0/1
GCGGGGTTCTTCTCTTGTCTTCCTGATGTGTACTTTTTGA
+HWI-EAS443:3:76:762:1391#0/1
abZa`aOZ`a__`____¥]__^^WU_^^V_^TQX¥^][XY
@HWI-EAS443:3:76:854:325#0/1
CGGGGTTCTTCTCTTGTCTTCCTGATGTGTACTTTTTGAT
+HWI-EAS443:3:76:854:325#0/1
^`aba^`b`b_aaa`a¥_ab`^^¥_^a]`[_]_`^_`^^a
@HWI-EAS443:3:76:889:598#0/1
GCGGGGTTCTTCTCTTGTCTTCCTGATGTGTACTTTTTGA
+HWI-EAS443:3:76:889:598#0/1
a^__VVO_W_SKN[_¥RO¥¥TTKTSUTLQOFSNPWX]¥FB
@HWI-EAS443:3:76:944:740#0/1
GGGGTTCTTCTCTTGTCTTCCTGATGTGTACTTTTTGATT
+HWI-EAS443:3:76:944:740#0/1
`abbUbaaa``aa`b_aaabaab^¥a_``aaaaaab]¥a`
@HWI-EAS443:3:77:1416:281#0/1
CGGGGTTCTTCTCTTGTCTTCCTGATGTGTACTTTTTGAT
+HWI-EAS443:3:77:1416:281#0/1
a`¥`b]b[babbKab`bXb`aabba_aabbaXbbbbb_bb
@HWI-EAS443:3:77:1742:886#0/1
GGTTCTTCTCTTGTCTTCCTGATGTGTACTTTTTGATTAT
+HWI-EAS443:3:77:1742:886#0/1
a¥F^`_babaaaa_`bb``a]aabYW¥a¥`aaaa]_aa`]
@HWI-EAS443:3:77:1757:1016#0/1
CGGGGTTCTTCTCTTGTCTTCCTGATGTGTACTTTTTGAT
+HWI-EAS443:3:77:1757:1016#0/1
IV^bb_babbbbabbaabbbaba``^X`bb^bbbbb`I[b
@HWI-EAS443:3:77:415:673#0/1
CGGGGTTCTTCTCTTGTCTTCCTGATGTGTACTTTTTGAT
+HWI-EAS443:3:77:415:673#0/1
abbaa¥`a``^bbba^^aabbbaaaaZ[a[a_aaaaa_Z^
@HWI-EAS443:3:77:506:789#0/1
GGTTCTTCTCTTGTCTTCCTGATGTGTACTTTTTGATTAT
+HWI-EAS443:3:77:506:789#0/1
aa_a]baaaaaa`aaaaaaab`ab`]_``babaa`_aa``
@HWI-EAS443:3:77:839:1526#0/1
GGTTCTTCTCTTGTCTTCCTGATGTGTACTTTTTGATTAT
```

+HWI-EAS443:3:77:839:1526#0/1
a_^baaa`aaa_```a][aaa`_a_aX`Za_`_`^a__`_
@HWI-EAS443:3:78:1091:567#0/1
CGGGGTTCTTCTCTTGTCTTCCTGATGTGTACTTTTTGAT
+HWI-EAS443:3:78:1091:567#0/1
]¥[a`]`a`babaW_b_]ZaY¥`aT`bSa]_¥¥aaaaX¥a
@HWI-EAS443:3:78:1182:49#0/1
CGGGGTTCTTCTCTTGTCTTCCTGATGTGTACTTTTTGAT
+HWI-EAS443:3:78:1182:49#0/1
¥abbbab__b_`]bbababb_Xa``bbY`¥aabbaab`]a
@HWI-EAS443:3:78:1205:898#0/1
GGGTTCTTCTCTTGTCTTCCTGATGTGTACTTTTTGATTA
+HWI-EAS443:3:78:1205:898#0/1
a`a^a`aa_a_a___]a`_a`_W_a^_^_]a_aa`^^`__
@HWI-EAS443:3:78:1539:1109#0/1
GCGGGGTTCTTCTCTTGTCTTCCTGATGTGTACTTTTTGA
+HWI-EAS443:3:78:1539:1109#0/1
a^a]ZPM¥SYa_]YZ``[Ra_]QVQW^¥[[PXZ`][`¥[[
@HWI-EAS443:3:78:1648:1572#0/1
GCGGGGTTCTTCTCTTGTCTTCCTGATGTGTACTTTTTGA
+HWI-EAS443:3:78:1648:1572#0/1
__aaa]aYL[[_a_`aaIUa_]]_`LY`_^¥aR`a_`YR¥
@HWI-EAS443:3:78:1666:1845#0/1
CGGGGTTCTTCTCTTGTCTTCCTGATGTGTACTTTTTGAT
+HWI-EAS443:3:78:1666:1845#0/1
^baab_bVaa_b^abaa]ba[_a`]¥b`a]a_bbaaa`]b
@HWI-EAS443:3:78:1723:1261#0/1
CGGGGTTCTTCTCTTGTCTTCCTGATGTGTACTTTTTGAT
+HWI-EAS443:3:78:1723:1261#0/1
abab`_baaaaa_aab`aabaW`]]``aZ¥Y^aaaaa``a
@HWI-EAS443:3:78:252:471#0/1
CGGGGTTCTTCTCTTGTCTTCCTGATGTGTACTTTTTGAT
+HWI-EAS443:3:78:252:471#0/1
aa]_[¥aaa¥aK^a_a¥aaYa_]Z¥aaWaG[¥aX_____^
@HWI-EAS443:3:78:522:1168#0/1
GCGGGGTTCTTCTCTTGTCTTCCTGATGTGTACTTTTTGA
+HWI-EAS443:3:78:522:1168#0/1
`bb`baRabbab`baba`ababa`Z`aa_a___aaa`aa_
@HWI-EAS443:3:78:662:1652#0/1
ATCAAAAAGTACACATCAGGAAGACAAGAGAAGAACCCCG
+HWI-EAS443:3:78:662:1652#0/1
aabaaaa_a^aaaaaaaa```a`aaa____a`W``_`]_^
@HWI-EAS443:3:78:69:481#0/1
GGGGTTCTTCTCTTGTCTTCCTGATGTGTACTTTTTGATT
+HWI-EAS443:3:78:69:481#0/1
`_R¥ME[`Ra¥MRVYJ[ZRVUXPNSULTFQVVY[]VWZZP
@HWI-EAS443:3:78:725:802#0/1
GCGGGGTTCTTCTCTTGTCTTCCTGATGTGTACTTTTTGA
+HWI-EAS443:3:78:725:802#0/1
a`bbababbbbbabababaaa_abbba`baa_`abaabbaa

```
@HWI-EAS443:3:78:853:1842#0/1
GGGGTTCTTCTCTTGTCTTCCTGATGTGTACTTTTTGATT
+HWI-EAS443:3:78:853:1842#0/1
Vb]RWbbaababaaa_a_ab`aW`aa[a¥[aa`a`_V`_^
@HWI-EAS443:3:78:893:458#0/1
GGGTTCTTCTCTTGTCTTCCTGATGTGTACTTTTTGATTA
+HWI-EAS443:3:78:893:458#0/1
]OSWaaa][``^`¥]Xaa`aa_V_a]`]`_a`_^`^_^]^
@HWI-EAS443:3:79:1042:1486#0/1
CGGGGTTCTTCTCTTGTCTTCCTGATGTGTACTTTTTGAT
+HWI-EAS443:3:79:1042:1486#0/1
a`^aaZ^b`ab_ab`b`bbaaa`a_`b_aaa]aaaa_Y_a
@HWI-EAS443:3:79:128:522#0/1
CGGGGTTCTTCTCTTGTCTTCCTGATGTGTACTTTTTGAT
+HWI-EAS443:3:79:128:522#0/1
[_OY[T]X_ZY]`PP[LT¥RZYXW^G^Q¥L[]Z_a`YYVX
@HWI-EAS443:3:79:1344:309#0/1
GCGGGGTTCTTCTCTTGTCTTCCTGATGTGTACTTTTTGA
+HWI-EAS443:3:79:1344:309#0/1
a`abbb`babb_aabbbbabba`bbaaa]b`^[bbaaa[_
@HWI-EAS443:3:79:1448:149#0/1
CGGGGTTCTTCTCTTGTCTTCCTGATGTGTACTTTTTGAT
+HWI-EAS443:3:79:1448:149#0/1
abbaSK`Ya`bb`bbbbbaZaaa`aaa^aaa_b^baa`aa
@HWI-EAS443:3:79:1474:1662#0/1
ATCAAAAAGTACACATCAGGAAGACAAGAGAAGAACCCCG
+HWI-EAS443:3:79:1474:1662#0/1
abb_ba_`^¥ba]aaaaabaa_`a`a^aabaaaaaYS_Ya
@HWI-EAS443:3:79:1643:749#0/1
CGGGGTTCTTCTCTTGTCTTCCTGATGTGTACTTTTTGAT
+HWI-EAS443:3:79:1643:749#0/1
aaabb_bbbbababbbabbaaabbbb`bbababbbbb^bb
@HWI-EAS443:3:79:1655:1651#0/1
GGTTCTTCTCTTGTCTTCCTGATGTGTACTTTTTGATTAT
+HWI-EAS443:3:79:1655:1651#0/1
a`W_Ybbb_TbabT_K[Y_aSW^bXaPYaa^bJb¥a[[Z¥
@HWI-EAS443:3:79:265:1517#0/1
GCGGGGTTCTTCTCTTGTCTTCCTGATGTGTACTTTTTGA
+HWI-EAS443:3:79:265:1517#0/1
ab^^X¥X^babaabaa`aaY^]aa_T¥a_^W]aa_a`a[W
@HWI-EAS443:3:79:265:2019#0/1
GCGGGGTTCTTCTCTTGTCTTCCTGATGTGTACTTTTTGA
+HWI-EAS443:3:79:265:2019#0/1
abZ^^[]aa^`aaaa`_]aT`aaZW_U¥Z_W[^[¥`¥SV^
@HWI-EAS443:3:79:507:1853#0/1
GCGGGGTTCTTCTCTTGTCTTCCTGATGTGTACTTTTTGA
+HWI-EAS443:3:79:507:1853#0/1
ab`aa]]``aaaaba_a^aaaaa`_``_]_^]a_`a_`_a
@HWI-EAS443:3:79:750:717#0/1
GCGGGGTTCTTCTCTTGTCTTCCTGATGTGTACTTTTTGA
```

```
+HWI-EAS443:3:79:750:717#0/1
abbab`^babbbbbbbb`bbabbbbba``baaaa`aab``
@HWI-EAS443:3:79:909:1640#0/1
CGGGGTTCTTCTCTTGTCTTCCTGATGTGTACTTTTTGAT
+HWI-EAS443:3:79:909:1640#0/1
a_¥_babbb`a`Vab_`bbba`aaabb]^`aabbbabSbb
@HWI-EAS443:3:79:951:1430#0/1
CGGGGTTCTTCTCTTGTCTTCCTGATGTGTACTTTTTGAT
+HWI-EAS443:3:79:951:1430#0/1
a`aab]bbbbaba`a`Zabab`abZba`a_`aaaaaa[a¥
@HWI-EAS443:3:79:982:1736#0/1
CGGGGTTCTTCTCTTGTCTTCCTGATGTGTACTTTTTGAT
+HWI-EAS443:3:79:982:1736#0/1
ab`aaaaabbababaa`bbaaab`aa`_b^a_babaa[ab
@HWI-EAS443:3:7:1148:251#0/1
GCGGGGTTCTTCTCTTGTCTTCCTGATGTGTACTTTTTGA
+HWI-EAS443:3:7:1148:251#0/1
a`babaQ`¥aa^a^aaa¥T`aUY`aX_`]`]^X__^`^X[
@HWI-EAS443:3:7:1162:1599#0/1
GGGTTCTTCTCTTGTCTTCCTGATGTGTACTTTTTGATTA
+HWI-EAS443:3:7:1162:1599#0/1
a_¥ZaSa]_a[a^a___a_Z^Z¥ZZXa[[^__[^^_`VXB
@HWI-EAS443:3:7:138:49#0/1
GCGGGGTTCTTCTCTTGTCTTCCTGATGTGTACTTTTTGA
+HWI-EAS443:3:7:138:49#0/1
aaSQ¥_GWNWZVWUZ]`NXPVRRZ[YS¥W]UJGS][STPB
@HWI-EAS443:3:7:1403:532#0/1
CGGGGTTCTTCTCTTGTCTTCCTGATGTGTACTTTTTGAT
+HWI-EAS443:3:7:1403:532#0/1
abbab`aba`aba`aaaaba^aa^^aa]a]]]aa`]aa`_
@HWI-EAS443:3:7:1431:1144#0/1
GCGGGGTTCTTCTCTTGTCTTCCTGATGTGTACTTTTTGA
+HWI-EAS443:3:7:1431:1144#0/1
`ab`aaY_]`bab_baba]a`YM^a_aaaa`_[aaaaa[_
@HWI-EAS443:3:7:1438:1601#0/1
CGGGGTTCTTCTCTTGTCTTCCTGATGTGTACTTTTTGAT
+HWI-EAS443:3:7:1438:1601#0/1
_bbb¥O`Zaba__aaY^b_abVbbaabab]a_bbbbb_aa
@HWI-EAS443:3:7:292:1050#0/1
GGGTTCTTCTCTTGTCTTCCTGATGTGTACTTTTTGATTA
+HWI-EAS443:3:7:292:1050#0/1
a_aZaaaa`ba`aa_`aaa_a^`__]_W^Sa_``_¥`___
@HWI-EAS443:3:7:53:455#0/1
ATCAAAAAGTACACATCAGGAAGACAAGAGAAGAACCCCG
+HWI-EAS443:3:7:53:455#0/1
aaba`]X^a^aaaaa`a_baaab^`__a_a_aaa`_`__`
@HWI-EAS443:3:7:881:1098#0/1
GCGGGGTTCTTCTCTTGTCTTCCTGATGTGTACTTTTTGA
+HWI-EAS443:3:7:881:1098#0/1
a```[YFT]`a_UUTO¥N]¥W_]^¥FXaW]VRHY_^]__Y
```

```
@HWI-EAS443:3:7:964:672#0/1
GCGGGGTTCTTCTCTTGTCTTCCTGATGTGTACTTTTTGA
+HWI-EAS443:3:7:964:672#0/1
a`]_aaI`^aa_a¥aa`ZXa_OX`aZ_`^`^]W^X`[_^]
@HWI-EAS443:3:7:980:1227#0/1
CGGGGTTCTTCTCTTGTCTTCCTGATGTGTACTTTTTGAT
+HWI-EAS443:3:7:980:1227#0/1
abb_`SWbababa`bb_ab`_¥a_aab_b`a_bbbabY`a
@HWI-EAS443:3:80:1328:1370#0/1
AGCGGGGTTCTTCTCTTGTCTTCCTGATGTGTACTTTTTG
+HWI-EAS443:3:80:1328:1370#0/1
abbbab`]a`bb_a`baaa`aa``aa`a`^a_`^aaaaa_
@HWI-EAS443:3:80:135:2012#0/1
GCGGGGTTCTTCTCTTGTCTTCCTGATGTGTACTTTTTGA
+HWI-EAS443:3:80:135:2012#0/1
ab]Z`a`_aa]bbaabaTV_aba`_`_`G`Z]^`__aaYa
@HWI-EAS443:3:80:1736:1613#0/1
CGGGGTTCTTCTCTTGTCTTCCTGATGTGTACTTTTTGAT
+HWI-EAS443:3:80:1736:1613#0/1
aaba`ab`bbba_bbbaabba`b`aaaaa_aaabbbb_aa
@HWI-EAS443:3:80:304:206#0/1
CGGGGTTCTTCTCTTGTCTTCCTGATGTGTACTTTTTGAT
+HWI-EAS443:3:80:304:206#0/1
Va`¥WT¥`¥¥a^abbaX[a_Z]_VX]_I]VR[]``]^]]]
@HWI-EAS443:3:80:460:898#0/1
CGGGGTTCTTCTCTTGTCTTCCTGATGTGTACTTTTTGAT
+HWI-EAS443:3:80:460:898#0/1
a`bba__`_aaaaaaaaaaaaaaaaaa^a_``aaaa`]`a
@HWI-EAS443:3:80:821:901#0/1
GCGGGGTTCTTCTCTTGTCTTCCTGATGTGTACTTTTTGA
+HWI-EAS443:3:80:821:901#0/1
abaaaa_aabaaa`aaa`aaaaaa`a```a^`_aaaaa`a
@HWI-EAS443:3:80:966:1134#0/1
GGGGTTCTTCTCTTGTCTTCCTGATGTGTACTTTTTGATT
+HWI-EAS443:3:80:966:1134#0/1
_```]__^Y¥a¥`¥ZU¥b]V_`^X``]^]Z¥]a¥^^ZZ`V
@HWI-EAS443:3:81:1083:174#0/1
CGGGGTTCTTCTCTTGTCTTCCTGATGTGTACTTTTTGAT
+HWI-EAS443:3:81:1083:174#0/1
ab`ba`baba]b_abbaa`aaab¥aab_a``[aaaab^aa
@HWI-EAS443:3:81:1144:109#0/1
GGGTTCTTCTCTTGTCTTCCTGATGTGTACTTTTTGATTA
+HWI-EAS443:3:81:1144:109#0/1
aba[a`ab_a`baa`_aba`aaaa__a]^Xaaa`a^]``_
@HWI-EAS443:3:81:1179:695#0/1
GGGTTCTTCTCTTGTCTTCCTGATGTGTACTTTTTGATTA
+HWI-EAS443:3:81:1179:695#0/1
aa¥J¥]VYSR]ZZ¥QTVW^X^aZ^^GWNPL[aSY[NUX]N
@HWI-EAS443:3:81:1276:254#0/1
GGGGTTCTTCTCTTGTCTTCCTGATGTGTACTTTTTGATT
```

+HWI-EAS443:3:81:1276:254#0/1
`bba[baab]b`aaba^bb__bba``_baa_aaaaaa`aa
@HWI-EAS443:3:81:1336:644#0/1
CGGGGTTCTTCTCTTGTCTTCCTGATGTGTACTTTTTGAT
+HWI-EAS443:3:81:1336:644#0/1
abbbaabbbbababaaabbbaabbaaa`aaa`bbbab`ab
@HWI-EAS443:3:81:1355:910#0/1
GGGGTTCTTCTCTTGTCTTCCTGATGTGTACTTTTTGATT
+HWI-EAS443:3:81:1355:910#0/1
abWP^^T_b[RR^[]_[W[VVb`aWaG[V^PNVP_^_]aB
@HWI-EAS443:3:81:147:115#0/1
GGGTTCTTCTCTTGTCTTCCTGATGTGTACTTTTTGATTA
+HWI-EAS443:3:81:147:115#0/1
aaa¥aaaa`a`aaa^aa`_```_`a^a^_^__aaa_^a[¥
@HWI-EAS443:3:81:1492:198#0/1
GCGGGGTTCTTCTCTTGTCTTCCTGATGTGTACTTTTTGA
+HWI-EAS443:3:81:1492:198#0/1
a_^aa^Wb^]a__U_a^_R]]TT]^Z]Y¥X¥YY_a_^¥RW
@HWI-EAS443:3:81:1576:880#0/1
CGGGGTTCTTCTCTTGTCTTCCTGATGTGTACTTTTTGAT
+HWI-EAS443:3:81:1576:880#0/1
ab_a^¥¥[_¥_b]_¥_Zaaa^ZZb^a`T¥[ZI_]`^YSY`
@HWI-EAS443:3:81:158:619#0/1
GCGGGGTTCTTCTCTTGTCTTCCTGATGTGTACTTTTTGA
+HWI-EAS443:3:81:158:619#0/1
aa_Z^a_aa``_^`]a]_a```a`_WY`P]PQY`¥^_^YS
@HWI-EAS443:3:81:1637:1585#0/1
CGGGGTTCTTCTCTTGTCTTCCTGATGTGTACTTTTTGAT
+HWI-EAS443:3:81:1637:1585#0/1
]bb`V_baba^a_abaVXb_bbbbbab`b^]abaaaaV`a
@HWI-EAS443:3:81:212:1328#0/1
GCGGGGTTCTTCTCTTGTCTTCCTGATGTGTACTTTTTGA
+HWI-EAS443:3:81:212:1328#0/1
aba_aa_^a_aba]a`_N`¥`bb`XaV[V__a^¥^V__^_
@HWI-EAS443:3:81:438:1137#0/1
CGGGGTTCTTCTCTTGTCTTCCTGATGTGTACTTTTTGAT
+HWI-EAS443:3:81:438:1137#0/1
a¥```[aaaa_`_a``]``_````^_XV[^]___^`^¥_^
@HWI-EAS443:3:81:457:453#0/1
CGGGGTTCTTCTCTTGTCTTCCTGATGTGTACTTTTTGAT
+HWI-EAS443:3:81:457:453#0/1
a```aYaaaaaaaaaa^_aaaaaa``a]a]_^````a__`
@HWI-EAS443:3:81:926:796#0/1
CGGGGTTCTTCTCTTGTCTTCCTGATGTGTACTTTTTGAT
+HWI-EAS443:3:81:926:796#0/1
aa`a¥¥a_a]Zbaa`^`a_]]ZZZ[]a]a__[aa[``[^a
@HWI-EAS443:3:82:1012:1083#0/1
GGGGTTCTTCTCTTGTCTTCCTGATGTGTACTTTTTGATT
+HWI-EAS443:3:82:1012:1083#0/1
aWXVPTV_¥_aa^_NN¥_a[]__XT]]_U^[`_aa`[Z_^

```
@HWI-EAS443:3:82:1077:1259#0/1
CGGGGTTCTTCTCTTGTCTTCCTGATGTGTACTTTTTGAT
+HWI-EAS443:3:82:1077:1259#0/1
abaa`^abbaaaaabbaaaa_`aa`aa^a__aaaaaa_`a
@HWI-EAS443:3:82:277:1121#0/1
GCGGGGTTCTTCTCTTGTCTTCCTGATGTGTACTTTTTGA
+HWI-EAS443:3:82:277:1121#0/1
]a`]`[D^``baabbba`aa`bbaba__Wb^aabaaabaa
@HWI-EAS443:3:82:391:1873#0/1
CGGGGTTCTTCTCTTGTCTTCCTGATGTGTACTTTTTGAT
+HWI-EAS443:3:82:391:1873#0/1
_b`_a]`aaaaabaaaabaaabaaaaa_[_aaaaaa_Zaa
@HWI-EAS443:3:82:615:560#0/1
CGGGGTTCTTCTCTTGTCTTCCTGATGTGTACTTTTTGAT
+HWI-EAS443:3:82:615:560#0/1
`b`VaJ]_Z]^aaaab_X``_baa¥a`]^a`aa_``^Y__
@HWI-EAS443:3:82:636:748#0/1
CGGGGTTCTTCTCTTGTCTTCCTGATGTGTACTTTTTGAT
+HWI-EAS443:3:82:636:748#0/1
^`abaababbaabbbb]abbbbba`[]baaabab`baba
@HWI-EAS443:3:82:691:254#0/1
GCGGGGTTCTTCTCTTGTCTTCCTGATGTGTACTTTTTGA
+HWI-EAS443:3:82:691:254#0/1
aabaaa_a_bb_aaabb_aaaaaa]``a_a``^aa`aa]_
@HWI-EAS443:3:82:70:1512#0/1
CGGGGTTCTTCTCTTGTCTTCCTGATGTGTACTTTTTGAT
+HWI-EAS443:3:82:70:1512#0/1
`¥VWFU`aXabY_bY`_b`]`a`V`W_T_[]aa_a]`[^_
@HWI-EAS443:3:83:1004:1310#0/1
GCGGGGTTCTTCTCTTGTCTTCCTGATGTGTACTTTTTGA
+HWI-EAS443:3:83:1004:1310#0/1
_abbaaabbbbbbbbbbaabbbUbabab`]ab`bbbbb`a
@HWI-EAS443:3:83:1050:87#0/1
GGGGTTCTTCTCTTGTCTTCCTGATGTGTACTTTTTGATT
+HWI-EAS443:3:83:1050:87#0/1
aa]WS`^__]_Z_^XQY_^YV_]RW^W]WSP^[]`YVYZZ
@HWI-EAS443:3:83:1054:64#0/1
GGGGTTCTTCTCTTGTCTTCCTGATGTGTACTTTTTGATT
+HWI-EAS443:3:83:1054:64#0/1
ababJ^aa[S^Laa[]aa`RU``W___]PVP__a`_[Y__
@HWI-EAS443:3:83:396:461#0/1
GGGTTCTTCTCTTGTCTTCCTGATGTGTACTTTTTGATTA
+HWI-EAS443:3:83:396:461#0/1
aba`bbbaabbbbbaab_aaabaaa_a^`_aa``a__``a
@HWI-EAS443:3:83:396:517#0/1
CGGGGTTCTTCTCTTGTCTTCCTGATGTGTACTTTTTGAT
+HWI-EAS443:3:83:396:517#0/1
ab_aa_aaaaaa`aab_aaaa`aa_`aW_^`¥aaaa_``a
@HWI-EAS443:3:83:518:678#0/1
ATCAAAAAGTACACATCAGGAAGACAAGAGAAGAACCCCG
```

```
+HWI-EAS443:3:83:518:678#0/1
`baa^aaa^O^abaa_a_aaaba``]Zaaa¥aa`b_a__a
@HWI-EAS443:3:83:627:1004#0/1
GCGGGGTTCTTCTCTTGTCTTCCTGATGTGTACTTTTTGA
+HWI-EAS443:3:83:627:1004#0/1
aababaYOX^aaa¥a_a_a`a]_a]_]^]][_aaa_aaZ[
@HWI-EAS443:3:84:1036:1232#0/1
CGGGGTTCTTCTCTTGTCTTCCTGATGTGTACTTTTTGAT
+HWI-EAS443:3:84:1036:1232#0/1
`aa`bX__abbbbbbbaabaababbaaaaa`aaabb`]^a
@HWI-EAS443:3:84:1211:320#0/1
CGGGGTTCTTCTCTTGTCTTCCTGATGTGTACTTTTTGAT
+HWI-EAS443:3:84:1211:320#0/1
abbbb_baba^b_aaa`]aa_aaaaaa]a_`^aaaaaa_a
@HWI-EAS443:3:84:1465:696#0/1
CGGGGTTCTTCTCTTGTCTTCCTGATGTGTACTTTTTGAT
+HWI-EAS443:3:84:1465:696#0/1
abba`ababbababbba`ab_aa`aaa^b_a`bbbab``b
@HWI-EAS443:3:84:1474:1456#0/1
GCGGGGTTCTTCTCTTGTCTTCCTGATGTGTACTTTTTGA
+HWI-EAS443:3:84:1474:1456#0/1
aa```YL[ZZ]WSOQ¥ZXTR_¥`¥]¥P¥NRRXS[`Y^[XN
@HWI-EAS443:3:84:212:734#0/1
GCGGGGTTCTTCTCTTGTCTTCCTGATGTGTACTTTTTGA
+HWI-EAS443:3:84:212:734#0/1
aa`aaaYaaaaa``_aaW```aa``]_`]^]_`a____``
@HWI-EAS443:3:84:409:829#0/1
CGGGGTTCTTCTCTTGTCTTCCTGATGTGTACTTTTTGAT
+HWI-EAS443:3:84:409:829#0/1
ab_bZJb^S_W_Yaaa]UH[aW[QUY_M[S[]U__]aaYZ
@HWI-EAS443:3:84:412:1081#0/1
CGGGGTTCTTCTCTTGTCTTCCTGATGTGTACTTTTTGAT
+HWI-EAS443:3:84:412:1081#0/1
a]_]_U`aa¥TYaa`XP^U[Y¥OXRTVRTLKTWYSXWVS^
@HWI-EAS443:3:84:425:1841#0/1
GGGTTCTTCTCTTGTCTTCCTGATGTGTACTTTTTGATTA
+HWI-EAS443:3:84:425:1841#0/1
abaY`abbbba`aaabb`babaa^a^`^aaaba`a`aaba
@HWI-EAS443:3:84:827:349#0/1
GGGTTCTTCTCTTGTCTTCCTGATGTGTACTTTTTGATTA
+HWI-EAS443:3:84:827:349#0/1
aa^R^^^`^aaa_a[_aY`^aY]ab]`^_Uaa`_a`[___
@HWI-EAS443:3:85:1106:1028#0/1
CGGGGTTCTTCTCTTGTCTTCCTGATGTGTACTTTTTGAT
+HWI-EAS443:3:85:1106:1028#0/1
_a`b]W```_`bbaabY[a_b^baa]aXb_X_babbbaba
@HWI-EAS443:3:85:272:1101#0/1
AGCGGGGTTCTTCTCTTGTCTTCCTGATGTGTACTTTTTG
+HWI-EAS443:3:85:272:1101#0/1
a]`WPY][RV``^`````Y_]YVXZ]T]_¥_[[___[[WK
```

```
@HWI-EAS443:3:85:573:1048#0/1
CGGGGTTCTTCTCTTGTCTTCCTGATGTGTACTTTTTGAT
+HWI-EAS443:3:85:573:1048#0/1
abbbb_bbbbabbbbbabbbaabaaaa_[_aaaaaaa_`a
@HWI-EAS443:3:85:642:247#0/1
CGGGGTTCTTCTCTTGTCTTCCTGATGTGTACTTTTTGAT
+HWI-EAS443:3:85:642:247#0/1
aaaba]aaaa`aa`aaZ`a`^aaa`_a¥a^^_`_`_`___
@HWI-EAS443:3:85:709:605#0/1
CGGGGTTCTTCTCTTGTCTTCCTGATGTGTACTTTTTGAT
+HWI-EAS443:3:85:709:605#0/1
abba]F]aa[aaaa`a_`a`_a`^^^aYa]XWa_ba`aaa
@HWI-EAS443:3:85:838:521#0/1
GCGGGGTTCTTCTCTTGTCTTCCTGATGTGTACTTTTTGA
+HWI-EAS443:3:85:838:521#0/1
_T¥^WXNVW^`X[Q_UTHaaa`]W]NST_R[aU`[_]]SX
@HWI-EAS443:3:86:1164:511#0/1
GGGTTCTTCTCTTGTCTTCCTGATGTGTACTTTTTGATTA
+HWI-EAS443:3:86:1164:511#0/1
aba_b`bbababbbaabbaaaaaab`a_aaaaaaa_aaa
@HWI-EAS443:3:86:1733:489#0/1
CGGGGTTCTTCTCTTGTCTTCCTGATGTGTACTTTTTGAT
+HWI-EAS443:3:86:1733:489#0/1
abaaXaaU]`X`]b¥`YbbO^Ua^S^aa]b_Ubbbbb^b]
@HWI-EAS443:3:86:252:618#0/1
AGCGGGGTTCTTCTCTTGTCTTCCTGATGTGTACTTTTTG
+HWI-EAS443:3:86:252:618#0/1
^HWbb`_Sa`aaaa``ba``aa_a_^_¥a_`W]a`Y_a__
@HWI-EAS443:3:86:375:940#0/1
CGGGGTTCTTCTCTTGTCTTCCTGATGTGTACTTTTTGAT
+HWI-EAS443:3:86:375:940#0/1
abb`b`aabbbbaab`aaaa`abaaaa_a_aaaaaaa`a`
@HWI-EAS443:3:86:565:206#0/1
CGGGGTTCTTCTCTTGTCTTCCTGATGTGTACTTTTTGAT
+HWI-EAS443:3:86:565:206#0/1
abbbb_aaabababbbabbbaabaaaa_a`aVbaaabaaa
@HWI-EAS443:3:86:708:1287#0/1
CGGGGTTCTTCTCTTGTCTTCCTGATGTGTACTTTTTGAT
+HWI-EAS443:3:86:708:1287#0/1
abb[babbbbaaabba`bb`abbbaaaab]`_b`aba¥ab
@HWI-EAS443:3:86:75:1328#0/1
AGCGGGGTTCTTCTCTTGTCTTCCTGATGTGTACTTTTTG
+HWI-EAS443:3:86:75:1328#0/1
a`a___ZG¥^^_¥a____Y]a]^`__]^_]]^XX_]YX_S
@HWI-EAS443:3:86:772:60#0/1
GGGGTTCTTCTCTTGTCTTCCTGATGTGTACTTTTTGATT
+HWI-EAS443:3:86:772:60#0/1
abaa]aaa¥`a_a^^Z^aaW]`a^`^_¥]UY^]`]__^_]
@HWI-EAS443:3:87:1005:1192#0/1
GCGGGGTTCTTCTCTTGTCTTCCTGATGTGTACTTTTTGA
```

+HWI-EAS443:3:87:1005:1192#0/1
a[aXLZPP]]UG_W_[¥SRNTR__OR__S`QQ^¥_V_^O[
@HWI-EAS443:3:87:1081:614#0/1
GCGGGGTTCTTCTCTTGTCTTCCTGATGTGTACTTTTTGA
+HWI-EAS443:3:87:1081:614#0/1
aabba]Yaaaa^b_baa``aa_`aa_aa]`__`aaaaaV^
@HWI-EAS443:3:87:1130:1340#0/1
GGGTTCTTCTCTTGTCTTCCTGATGTGTACTTTTTGATTA
+HWI-EAS443:3:87:1130:1340#0/1
`aOO^¥_WYXV^_ZT¥`YQO¥¥^Y¥ONMGQX^SRW_^Y[]
@HWI-EAS443:3:87:1205:592#0/1
CGGGGTTCTTCTCTTGTCTTCCTGATGTGTACTTTTTGAT
+HWI-EAS443:3:87:1205:592#0/1
UabbbH``b_ab]aab`_aa_bbaaaabaaaGbabbbaab
@HWI-EAS443:3:87:127:1454#0/1
GGTTCTTCTCTTGTCTTCCTGATGTGTACTTTTTGATTAT
+HWI-EAS443:3:87:127:1454#0/1
a_^bbab[bb_baaabbb_ab```_[]]bbUb_b`a_`^a
@HWI-EAS443:3:87:1532:773#0/1
CGGGGTTCTTCTCTTGTCTTCCTGATGTGTACTTTTTGAT
+HWI-EAS443:3:87:1532:773#0/1
ab`aaabbbbba[`bbbabb`abbbb`bbbbabbbbbbbb
@HWI-EAS443:3:87:209:502#0/1
CGGGGTTCTTCTCTTGTCTTCCTGATGTGTACTTTTTGAT
+HWI-EAS443:3:87:209:502#0/1
abaab_bbbbab`bbb`ababbb_P_b_bY__bbaaaaaa
@HWI-EAS443:3:87:701:1505#0/1
GCGGGGTTCTTCTCTTGTCTTCCTGATGTGTACTTTTTGA
+HWI-EAS443:3:87:701:1505#0/1
``aa`_X[aa``__a``O_`_^a__¥^_WaZ_``^^_^R^
@HWI-EAS443:3:87:717:539#0/1
GGGGTTCTTCTCTTGTCTTCCTGATGTGTACTTTTTGATT
+HWI-EAS443:3:87:717:539#0/1
abab]aaaaaa`aab``aaa`a`aaa`a^a^aa`aaaa`_
@HWI-EAS443:3:87:904:1966#0/1
GGGGTTCTTCTCTTGTCTTCCTGATGTGTACTTTTTGATT
+HWI-EAS443:3:87:904:1966#0/1
aZ^]WR¥[__ZX_Z^UZZW¥YZ^WR^W]UZ^WZXXWUZZB
@HWI-EAS443:3:88:1177:1204#0/1
GCGGGGTTCTTCTCTTGTCTTCCTGATGTGTACTTTTTGA
+HWI-EAS443:3:88:1177:1204#0/1
`aabbaba`ab`bbbbbbabbZ[^abbb`_aaababbbab
@HWI-EAS443:3:88:1224:1134#0/1
CGGGGTTCTTCTCTTGTCTTCCTGATGTGTACTTTTTGAT
+HWI-EAS443:3:88:1224:1134#0/1
a``]aR`ab``baaab_`b_`XaaZa^¥aY[[babaaU_`
@HWI-EAS443:3:88:126:294#0/1
GCGGGGTTCTTCTCTTGTCTTCCTGATGTGTACTTTTTGA
+HWI-EAS443:3:88:126:294#0/1
aZS_WV__`^NPI[O][RX_]aVR`]ZZU_NW_]ZUY^¥^

```
@HWI-EAS443:3:88:1350:1932#0/1
CGGGGTTCTTCTCTTGTCTTCCTGATGTGTACTTTTTGAT
+HWI-EAS443:3:88:1350:1932#0/1
ab¥Z_FO^aK_`ba_QOT`Ta^a`UIb_b_a¥]`[babaa
@HWI-EAS443:3:88:1517:1222#0/1
CGGGGTTCTTCTCTTGTCTTCCTGATGTGTACTTTTTGAT
+HWI-EAS443:3:88:1517:1222#0/1
abbbb_bbab¥ba`bbaabaaabbabb_¥aa¥aabbb¥aa
@HWI-EAS443:3:88:200:868#0/1
GGGTTCTTCTCTTGTCTTCCTGATGTGTACTTTTTGATTA
+HWI-EAS443:3:88:200:868#0/1
WZYHabaa``ZV]`IaaY^a_aa__ZX[S[aa]__aQQP^
@HWI-EAS443:3:88:410:365#0/1
GCGGGGTTCTTCTCTTGTCTTCCTGATGTGTACTTTTTGA
+HWI-EAS443:3:88:410:365#0/1
abbabaPbbbbabababaabaaaaaaab`a_a`aaaaa[a
@HWI-EAS443:3:88:699:1436#0/1
CGGGGTTCTTCTCTTGTCTTCCTGATGTGTACTTTTTGAT
+HWI-EAS443:3:88:699:1436#0/1
_^ab`]_`bb_babaa`aaaaaa^`]a^a^``aaaaa[a]
@HWI-EAS443:3:88:793:81#0/1
CGGGGTTCTTCTCTTGTCTTCCTGATGTGTACTTTTTGAT
+HWI-EAS443:3:88:793:81#0/1
abbba_b`bbababbba_baa`baaab`b``^abaaa``]
@HWI-EAS443:3:89:1062:261#0/1
CGGGGTTCTTCTCTTGTCTTCCTGATGTGTACTTTTTGAT
+HWI-EAS443:3:89:1062:261#0/1
a`XW_Haaaa`^]^a`¥``^_^]`Y_a[_]^Zaaaa]¥]a
@HWI-EAS443:3:89:1604:379#0/1
GGGTTCTTCTCTTGTCTTCCTGATGTGTACTTTTTGATTA
+HWI-EAS443:3:89:1604:379#0/1
aba_a`baab^aaa`^aa_^aa__`W`___aabaa``a^_
@HWI-EAS443:3:89:214:1056#0/1
CGGGGTTCTTCTCTTGTCTTCCTGATGTGTACTTTTTGAT
+HWI-EAS443:3:89:214:1056#0/1
abbabW`a`XYaaaUZSa`_]]Y`aZS_[Y^a]^`^RP^_
@HWI-EAS443:3:89:468:1536#0/1
GGGGTTCTTCTCTTGTCTTCCTGATGTGTACTTTTTGATT
+HWI-EAS443:3:89:468:1536#0/1
]baaU``aWb`_]]bVb[RKP]ZYZ`S_Y_ba]aa_a]a_
@HWI-EAS443:3:89:550:1443#0/1
CGGGGTTCTTCTCTTGTCTTCCTGATGTGTACTTTTTGAT
+HWI-EAS443:3:89:550:1443#0/1
a`b_bPbaa`aa]baa``b`baa``]aY`NT[aaa____a
@HWI-EAS443:3:89:563:264#0/1
CGGGGTTCTTCTCTTGTCTTCCTGATGTGTACTTTTTGAT
+HWI-EAS443:3:89:563:264#0/1
`aaaa_b`aaaaa`aa^aaaaaab`_aZaXX`a_`_a`_`
@HWI-EAS443:3:89:650:262#0/1
AGCGGGGTTCTTCTCTTGTCTTCCTGATGTGTACTTTTTG
```

```
+HWI-EAS443:3:89:650:262#0/1
ababab]Zba`aaa`aaa`_aa__`_]a`^_^_^a````Z
@HWI-EAS443:3:89:674:1290#0/1
CGGGGTTCTTCTCTTGTCTTCCTGATGTGTACTTTTTGAT
+HWI-EAS443:3:89:674:1290#0/1
aba_a`bbbbabbbbaaaababbbaaa`[`a`babbbaba
@HWI-EAS443:3:89:836:858#0/1
CGGGGTTCTTCTCTTGTCTTCCTGATGTGTACTTTTTGAT
+HWI-EAS443:3:89:836:858#0/1
a_ab`Z]bYaabbba`Z_bbb_bbb``[b`babaabbaaa
@HWI-EAS443:3:89:876:1580#0/1
CGGGGTTCTTCTCTTGTCTTCCTGATGTGTACTTTTTGAT
+HWI-EAS443:3:89:876:1580#0/1
^abba][`ab_abbbb_abbbbbbabaaaabaababa`ab
@HWI-EAS443:3:8:1144:2041#0/1
AGCGGGGTTCTTCTCTTGTCTTCCTGATGTGTACTTTTTG
+HWI-EAS443:3:8:1144:2041#0/1
a^G`aa[Y]]aa^aWaaa`R``S_^¥`_[HX]UX_YU___
@HWI-EAS443:3:8:1216:1541#0/1
CGGGGTTCTTCTCTTGTCTTCCTGATGTGTACTTTTTGAT
+HWI-EAS443:3:8:1216:1541#0/1
`aab[H__b__b^b_bVbba^[`^aaaZb¥[_`a¥Z¥ZU]
@HWI-EAS443:3:8:1660:782#0/1
CGGGGTTCTTCTCTTGTCTTCCTGATGTGTACTTTTTGAT
+HWI-EAS443:3:8:1660:782#0/1
a^`ab`_bbbbbabbbaa_baVbbbbba``_`bbbbbM`b
@HWI-EAS443:3:8:1682:950#0/1
CGGGGTTCTTCTCTTGTCTTCCTGATGTGTACTTTTTGAT
+HWI-EAS443:3:8:1682:950#0/1
Za_W_H_b``^T_`abbZbbTZ¥a`a]Y_aaZabaabVab
@HWI-EAS443:3:8:234:1793#0/1
CGGGGTTCTTCTCTTGTCTTCCTGATGTGTACTTTTTGAT
+HWI-EAS443:3:8:234:1793#0/1
aaba_]aaaa`[`¥V^W_^_T¥¥QHTDM]H_``¥]_FV`S
@HWI-EAS443:3:8:281:844#0/1
GCGGGGTTCTTCTCTTGTCTTCCTGATGTGTACTTTTTGA
+HWI-EAS443:3:8:281:844#0/1
abbaba`babbaaaaab`_aaa`aaa_a_a__`aa_aaa`
@HWI-EAS443:3:8:353:1513#0/1
CGGGGTTCTTCTCTTGTCTTCCTGATGTGTACTTTTTGAT
+HWI-EAS443:3:8:353:1513#0/1
aVaaa_a``VTaZTVa_a`a`_a``Ya^YU_X`]a__NY`
@HWI-EAS443:3:8:41:427#0/1
ATAATCAAAAAGTACACATCAGGAAGACAAGAGAAGAACC
+HWI-EAS443:3:8:41:427#0/1
``[_`_`aYa`a¥_aa^^`_a`a_`a`^a`_^a_`aa__[
@HWI-EAS443:3:8:498:47#0/1
GCGGGGTTCTTCTCTTGTCTTCCTGATGTGTACTTTTTGA
+HWI-EAS443:3:8:498:47#0/1
a`TP_TGGY_YGUPUU]JMO^TQVSSRPS¥ZWTRSBBBBB
```

```
@HWI-EAS443:3:8:567:658#0/1
GGTTCTTCTCTTGTCTTCCTGATGTGTACTTTTTGATTAT
+HWI-EAS443:3:8:567:658#0/1
a`X`_`__^_`]_]^_]^^^^]^_Z_V_Z^_^[^_]YVZ]
@HWI-EAS443:3:8:830:424#0/1
CGGGGTTCTTCTCTTGTCTTCCTGATGTGTACTTTTTGAT
+HWI-EAS443:3:8:830:424#0/1
a_Za_]^^a_Sba_V`a]aaVY_a``b[a_^YaaaaaXSa
@HWI-EAS443:3:8:944:1265#0/1
GCGGGGTTCTTCTCTTGTCTTCCTGATGTGTACTTTTTGA
+HWI-EAS443:3:8:944:1265#0/1
aaa`_a¥aaaa_a`_`a`^`a__a`^^a^a]OYa`¥_a__
@HWI-EAS443:3:8:980:490#0/1
GCGGGGTTCTTCTCTTGTCTTCCTGATGTGTACTTTTTGA
+HWI-EAS443:3:8:980:490#0/1
``bb_bab¥bb^bba`baaab¥bba`a`aa_aXaabb``a
@HWI-EAS443:3:90:1051:824#0/1
GGTTCTTCTCTTGTCTTCCTGATGTGTACTTTTTGATTAT
+HWI-EAS443:3:90:1051:824#0/1
aabbabbababbbbbbbbbab`abaab`a`ababaaaaaba
@HWI-EAS443:3:90:1134:2022#0/1
GCGGGGTTCTTCTCTTGTCTTCCTGATGTGTACTTTTTGA
+HWI-EAS443:3:90:1134:2022#0/1
aa`ab`]Y_bbbaaabb^aaa``aa_a`_`^`aa_b`aaa
@HWI-EAS443:3:90:1160:1871#0/1
GGGTTCTTCTCTTGTCTTCCTGATGTGTACTTTTTGATTA
+HWI-EAS443:3:90:1160:1871#0/1
^a^a[bVab]`Y¥^Hbb¥GYb`bW`_a^Tab_a_`Y_[aa
@HWI-EAS443:3:90:1204:653#0/1
GGGGTTCTTCTCTTGTCTTCCTGATGTGTACTTTTTGATT
+HWI-EAS443:3:90:1204:653#0/1
abaa_aaaa]a`aaa]_a`_`aa`_a]a]__aa_`_a__`
@HWI-EAS443:3:90:1312:315#0/1
ATCAAAAAGTACACATCAGGAAGACAAGAGAAGAACCCCG
+HWI-EAS443:3:90:1312:315#0/1
^¥Kabb]¥WY_U`^Raa`X^^b_QT^a_S`a^V_]^LDD¥
@HWI-EAS443:3:90:1635:951#0/1
CGGGGTTCTTCTCTTGTCTTCCTGATGTGTACTTTTTGAT
+HWI-EAS443:3:90:1635:951#0/1
a`bbb`bbbbbbabbbaabbbabbaab_b`a[babbbaaa
@HWI-EAS443:3:90:432:448#0/1
CGGGGTTCTTCTCTTGTCTTCCTGATGTGTACTTTTTGAT
+HWI-EAS443:3:90:432:448#0/1
aba`aR[abaab`a`a¥a]¥b``a`¥`Va¥YOY``a_a`^
@HWI-EAS443:3:90:451:1949#0/1
GCGGGGTTCTTCTCTTGTCTTCCTGATGTGTACTTTTTGA
+HWI-EAS443:3:90:451:1949#0/1
^b`^V¥Q[aaba`b`b`a`aaabYaa_a_[]aa`_aa`R]
@HWI-EAS443:3:90:494:271#0/1
CGGGGTTCTTCTCTTGTCTTCCTGATGTGTACTTTTTGAT
```

```
+HWI-EAS443:3:90:494:271#0/1
ababbababbabaabbaaaaabaaa`][a]a`b`aaaaaa
@HWI-EAS443:3:90:496:873#0/1
GCGGGGTTCTTCTCTTGTCTTCCTGATGTGTACTTTTTGA
+HWI-EAS443:3:90:496:873#0/1
aabaaa^a`aaaaaaaa_`a`aaa_^`_Xa^`^^a]aa__
@HWI-EAS443:3:90:564:184#0/1
GGGTTCTTCTCTTGTCTTCCTGATGTGTACTTTTTGATTA
+HWI-EAS443:3:90:564:184#0/1
aba_abaaababab_`aa_aabaaa^a_a`aaaaaa``aa
@HWI-EAS443:3:90:691:559#0/1
GCGGGGTTCTTCTCTTGTCTTCCTGATGTGTACTTTTTGA
+HWI-EAS443:3:90:691:559#0/1
aa``__abab`^`b^a`aabaaaa]`[`U_[aa_a[abaa
@HWI-EAS443:3:90:741:900#0/1
GCGGGGTTCTTCTCTTGTCTTCCTGATGTGTACTTTTTGA
+HWI-EAS443:3:90:741:900#0/1
aaab`bO`aaaa`_a^HR_aaaa``_`VYa]aW`__aa__
@HWI-EAS443:3:90:753:1875#0/1
CGGGGTTCTTCTCTTGTCTTCCTGATGTGTACTTTTTGAT
+HWI-EAS443:3:90:753:1875#0/1
aa`^`Zaaaaaa_`]a_`a`aa_```_¥`]__`aaa`¥a`
@HWI-EAS443:3:90:972:635#0/1
GGGGTTCTTCTCTTGTCTTCCTGATGTGTACTTTTTGATT
+HWI-EAS443:3:90:972:635#0/1
aabaV```a``Xa^]S_aaa`a`¥^`X_P¥¥`a__[][[[
@HWI-EAS443:3:90:981:1492#0/1
CGGGGTTCTTCTCTTGTCTTCCTGATGTGTACTTTTTGAT
+HWI-EAS443:3:90:981:1492#0/1
abaaa]a[^aIYW[_]]^aW_Y[aXa`Jb^^Oa`Raa_aa
@HWI-EAS443:3:91:1032:930#0/1
ATAATCAAAAAGTACACATCAGGAAGACAAGAGAAGAACC
+HWI-EAS443:3:91:1032:930#0/1
M¥aab_a``a]¥]bRZ^aJa¥b¥V_aLXb]_b_^bbbb_`
@HWI-EAS443:3:91:1128:1685#0/1
AGCGGGGTTCTTCTCTTGTCTTCCTGATGTGTACTTTTTG
+HWI-EAS443:3:91:1128:1685#0/1
abb¥Z]bZ¥T¥aX[XS`SI^aVVTTQYZaVaSVHV`_QZP
@HWI-EAS443:3:91:1259:1044#0/1
TCAAAAAGTACACATCAGGAAGACAAGAGAAGAACCCCGC
+HWI-EAS443:3:91:1259:1044#0/1
a`aa^a_aOba``a_^aa`a_aaaa_a_a__``__O]Z`Y
@HWI-EAS443:3:91:1278:2013#0/1
CGGGGTTCTTCTCTTGTCTTCCTGATGTGTACTTTTTGAT
+HWI-EAS443:3:91:1278:2013#0/1
abbbabaabbbbbbaa`bbabbbbbaaaba`abbaab_a^
@HWI-EAS443:3:91:1293:739#0/1
GGTTCTTCTCTTGTCTTCCTGATGTGTACTTTTTGATTAT
+HWI-EAS443:3:91:1293:739#0/1
a_GZZ¥_`__`_WXX`_[ZZOX^aZ][YO___`^¥R__]]
```

```
@HWI-EAS443:3:91:1518:1790#0/1
CGGGGTTCTTCTCTTGTCTTCCTGATGTGTACTTTTTGAT
+HWI-EAS443:3:91:1518:1790#0/1
abbab`babb_b¥babaaaa`baaaa`^aaa_aaa`baa_
@HWI-EAS443:3:91:178:502#0/1
CGGGGTTCTTCTCTTGTCTTCCTGATGTGTACTTTTTGAT
+HWI-EAS443:3:91:178:502#0/1
I[abb`aaab`¥abbb`^ababb```babaaabab`baZ_
@HWI-EAS443:3:91:474:1333#0/1
CGGGGTTCTTCTCTTGTCTTCCTGATGTGTACTTTTTGAT
+HWI-EAS443:3:91:474:1333#0/1
ab¥R¥]bbbaba]aaa_ba_a```a_a^a]__`aa_a_`^
@HWI-EAS443:3:91:587:412#0/1
GCGGGGTTCTTCTCTTGTCTTCCTGATGTGTACTTTTTGA
+HWI-EAS443:3:91:587:412#0/1
a`]]a^DZQ_ZX[Ua`YZY_`_]UXUZTZNSU]Y¥S_UU[
@HWI-EAS443:3:91:827:887#0/1
ATCAAAAAGTACACATCAGGAAGACAAGAGAAGAACCCCG
+HWI-EAS443:3:91:827:887#0/1
abba^aaab]`_a`aa[aa]^bb`aa`baaaabaaX]NWa
@HWI-EAS443:3:92:1024:904#0/1
GCGGGGTTCTTCTCTTGTCTTCCTGATGTGTACTTTTTGA
+HWI-EAS443:3:92:1024:904#0/1
aaaaaa0aaaaaa_aaba`a``_aa_`a_a^_]aaa`aa`
@HWI-EAS443:3:92:1087:177#0/1
ATCAAAAAGTACACATCAGGAAGACAAGAGAAGAACCCCG
+HWI-EAS443:3:92:1087:177#0/1
a[`^``NRWZYTMV]a]Y_Y¥X_RZ`^aXa]WZ]_TQPF^
@HWI-EAS443:3:92:1638:999#0/1
CGGGGTTCTTCTCTTGTCTTCCTGATGTGTACTTTTTGAT
+HWI-EAS443:3:92:1638:999#0/1
abbbb_aabbbb_bbbaabba_baabbabaa_bbaba`ab
@HWI-EAS443:3:92:1770:412#0/1
ATAATCAAAAAGTACACATCAGGAAGACAAGAGAAGAACC
+HWI-EAS443:3:92:1770:412#0/1
abaaa`aaaaab^a_a`aa_aa`a`a_`aaa_a`a__`[Q
@HWI-EAS443:3:92:326:663#0/1
GGGGTTCTTCTCTTGTCTTCCTGATGTGTACTTTTTGATT
+HWI-EAS443:3:92:326:663#0/1
aa]aZ[Y¥_`a^¥_]Z__]_`__]W_U_ZR^_]]_UWZ¥]
@HWI-EAS443:3:92:377:1142#0/1
CGGGGTTCTTCTCTTGTCTTCCTGATGTGTACTTTTTGAT
+HWI-EAS443:3:92:377:1142#0/1
abaaa_aaaaaaabaaaaba_baa]aa_a___baaaaaaa
@HWI-EAS443:3:92:404:996#0/1
GGGTTCTTCTCTTGTCTTCCTGATGTGTACTTTTTGATTA
+HWI-EAS443:3:92:404:996#0/1
K`^^`_`bbba^_`abb_Xa¥^_abab]Y`V¥ba^Y^^X_
@HWI-EAS443:3:92:516:52#0/1
CGGGGTTCTTCTCTTGTCTTCCTGATGTGTACTTTTTGAT
```

```
+HWI-EAS443:3:92:516:52#0/1
abbbb`b`abaaaaab^]ab_aaa_a__a^^_a_`aaa__
@HWI-EAS443:3:92:681:990#0/1
GGTTCTTCTCTTGTCTTCCTGATGTGTACTTTTTGATTAT
+HWI-EAS443:3:92:681:990#0/1
aaWa`a`^a`aaa^^`___`a_a¥X`]_a_`a_a_____`
@HWI-EAS443:3:92:725:176#0/1
GCGGGGTTCTTCTCTTGTCTTCCTGATGTGTACTTTTTGA
+HWI-EAS443:3:92:725:176#0/1
a`aa`aW]]``]`X__`]Y`_Y¥^Z¥^^X_X]Z]_^_^]Z
@HWI-EAS443:3:92:919:391#0/1
GCGGGGTTCTTCTCTTGTCTTCCTGATGTGTACTTTTTGA
+HWI-EAS443:3:92:919:391#0/1
aaaaaa]aaabbb`bab`aaaaaaa`a`_a]_]aaaaaa`
@HWI-EAS443:3:93:1055:1270#0/1
CGGGGTTCTTCTCTTGTCTTCCTGATGTGTACTTTTTGAT
+HWI-EAS443:3:93:1055:1270#0/1
ab`abXaaa_aa_aaa_^aa_`^a__a_a]^^_aaa`[^`
@HWI-EAS443:3:93:1147:1428#0/1
GCGGGGTTCTTCTCTTGTCTTCCTGATGTGTACTTTTTGA
+HWI-EAS443:3:93:1147:1428#0/1
]L¥_JQJ_¥ZR^REX`aPZZSP]]PQT[U]FPK¥_UHJ¥B
@HWI-EAS443:3:93:1245:162#0/1
AGCGGGGTTCTTCTCTTGTCTTCCTGATGTGTACTTTTTG
+HWI-EAS443:3:93:1245:162#0/1
_a_b^aa¥b_bb_a_bab`_ba``aa_`aZa^_Z`__a_`
@HWI-EAS443:3:93:1331:951#0/1
CGGGGTTCTTCTCTTGTCTTCCTGATGTGTACTTTTTGAT
+HWI-EAS443:3:93:1331:951#0/1
abaa``baba`aTabaa_bba^abaaa^aZaaaaaaaaaa
@HWI-EAS443:3:93:1630:870#0/1
GCGGGGTTCTTCTCTTGTCTTCCTGATGTGTACTTTTTGA
+HWI-EAS443:3:93:1630:870#0/1
`]ba]WLY__aa]^baa]V`aa``Y¥_[_]P^¥]`^][G[
@HWI-EAS443:3:93:1679:521#0/1
GGGGTTCTTCTCTTGTCTTCCTGATGTGTACTTTTTGATT
+HWI-EAS443:3:93:1679:521#0/1
a^¥`Xa_`a]a]`_a^_``]_aa_`_^_Z]]a`___]]^^
@HWI-EAS443:3:93:42:1433#0/1
GGGGTTCTTCTCTTGTCTTCCTGATGTGTACTTTTTGATT
+HWI-EAS443:3:93:42:1433#0/1
`KZ^[aaaba^R_`]_^]`_]`NYa`R^^_Ra_`_a_`_[
@HWI-EAS443:3:93:432:329#0/1
CGGGGTTCTTCTCTTGTCTTCCTGATGTGTACTTTTTGAT
+HWI-EAS443:3:93:432:329#0/1
aa¥ab_``ba`baaaba`a^aa_a`aa_bZa^aaaaa[X_
@HWI-EAS443:3:93:716:733#0/1
CGGGGTTCTTCTCTTGTCTTCCTGATGTGTACTTTTTGAT
+HWI-EAS443:3:93:716:733#0/1
abaab^baaaabaabba`aaaa`]`aa`a¥]aaaaaa__a
```

```
@HWI-EAS443:3:93:963:651#0/1
CGGGGTTCTTCTCTTGTCTTCCTGATGTGTACTTTTTGAT
+HWI-EAS443:3:93:963:651#0/1
abbbbX^_a`baaa`ba`aaa`ab_`bSa]a`aaaaa_a`
@HWI-EAS443:3:94:1278:1649#0/1
CGGGGTTCTTCTCTTGTCTTCCTGATGTGTACTTTTTGAT
+HWI-EAS443:3:94:1278:1649#0/1
abaaa^aaaaa¥_ab`__baSaba^`a_b``^ba`abaaa
@HWI-EAS443:3:94:146:60#0/1
GGTTCTTCTCTTGTCTTCCTGATGTGTACTTTTTGATTAT
+HWI-EAS443:3:94:146:60#0/1
^ZVVG_`[_Z]aa^TYZL¥`a^^aX^¥ZZ_^`_^]Y[]VZ
@HWI-EAS443:3:94:1580:1022#0/1
CGGGGTTCTTCTCTTGTCTTCCTGATGTGTACTTTTTGAT
+HWI-EAS443:3:94:1580:1022#0/1
abbba`b]bbabaabba`bba^ab_aa_b]a_babaaa`a
@HWI-EAS443:3:94:165:1339#0/1
CGGGGTTCTTCTCTTGTCTTCCTGATGTGTACTTTTTGAT
+HWI-EAS443:3:94:165:1339#0/1
aaZ_aYaa`Xaaaa_a_`_[`V]]^¥[V¥¥^¥____ZMX^
@HWI-EAS443:3:94:213:128#0/1
AGCGGGGTTCTTCTCTTGTCTTCCTGATGTGTACTTTTTG
+HWI-EAS443:3:94:213:128#0/1
`aaaa``Zaa`_`aa`_a^``a_Y]_]^]¥^Y^]`]___^
@HWI-EAS443:3:94:233:1780#0/1
GGGTTCTTCTCTTGTCTTCCTGATGTGTACTTTTTGATTA
+HWI-EAS443:3:94:233:1780#0/1
aaaXR¥`UZYX¥aVX`_X_¥aa_ZRMT__H]`GWaSVFK]
@HWI-EAS443:3:94:417:1866#0/1
CGGGGTTCTTCTCTTGTCTTCCTGATGTGTACTTTTTGAT
+HWI-EAS443:3:94:417:1866#0/1
abbbb_bbbbababaaabaaaaaaaaaXa_a`baa`a^aa
@HWI-EAS443:3:94:556:1324#0/1
AGCGGGGTTCTTCTCTTGTCTTCCTGATGTGTACTTTTTG
+HWI-EAS443:3:94:556:1324#0/1
a``a_``Z___a_a^`_aW__^a^__^__RXMRR^__^ZS
@HWI-EAS443:3:94:790:1874#0/1
GGGGTTCTTCTCTTGTCTTCCTGATGTGTACTTTTTGATT
+HWI-EAS443:3:94:790:1874#0/1
a``_O__aa`^X¥a`]aa_`__^^^`W^[¥bY`^^VW¥]]
@HWI-EAS443:3:94:909:366#0/1
TAATCAAAAAGTACACATCAGGAAGACAAGAGAAGAACCC
+HWI-EAS443:3:94:909:366#0/1
aa`a_a`aa_a]``a_aa__aaa`a^]_`a^a_aa``]^X
@HWI-EAS443:3:94:951:67#0/1
CGGGGTTCTTCTCTTGTCTTCCTGATGTGTACTTTTTGAT
+HWI-EAS443:3:94:951:67#0/1
abbbbabbbb¥babbbbabba^bbabbabaa_bbbbbbab
@HWI-EAS443:3:95:1086:1604#0/1
CGGGGTTCTTCTCTTGTCTTCCTGATGTGTACTTTTTGAT
```

```
+HWI-EAS443:3:95:1086:1604#0/1
abbabab^bbbbbbaaaabbaabbbbbbbbbabbbbbabb
@HWI-EAS443:3:95:1144:332#0/1
CGGGGTTCTTCTCTTGTCTTCCTGATGTGTACTTTTTGAT
+HWI-EAS443:3:95:1144:332#0/1
`bbababbbbbbbabbbbab`bbbbabbb`babbbbbabb
@HWI-EAS443:3:95:1549:1782#0/1
GGGGTTCTTCTCTTGTCTTCCTGATGTGTACTTTTTGATT
+HWI-EAS443:3:95:1549:1782#0/1
M¥ba`ba_a¥abaaaaX¥aab_RIba]aXb_aU`T^`b^a
@HWI-EAS443:3:95:1586:1183#0/1
CGGGGTTCTTCTCTTGTCTTCCTGATGTGTACTTTTTGAT
+HWI-EAS443:3:95:1586:1183#0/1
aba`aWaS_`¥bbbbbbabbaabb`a__bbbVabbbbaba
@HWI-EAS443:3:95:31:139#0/1
GCGGGGTTCTTCTCTTGTCTTCCTGATGTGTACTTTTTGA
+HWI-EAS443:3:95:31:139#0/1
Y^bba^`U_b_^¥Za^_`abb]aa¥Qaaaa`__aaaaa__
@HWI-EAS443:3:95:773:1610#0/1
GGGTTCTTCTCTTGTCTTCCTGATGTGTACTTTTTGATTA
+HWI-EAS443:3:95:773:1610#0/1
¥a`K¥¥bVbabbaaabbaK_aVba[GV_bZ_bb_b`aabb
@HWI-EAS443:3:95:939:35#0/1
GCGGGGTTCTTCTCTTGTCTTCCTGATGTGTACTTTTTGA
+HWI-EAS443:3:95:939:35#0/1
_`a`ab^b`bbababbba[ba_Yabaab`b_`Sbb`aaa`
@HWI-EAS443:3:96:1739:499#0/1
ATCAAAAAGTACACATCAGGAAGACAAGAGAAGAACCCCG
+HWI-EAS443:3:96:1739:499#0/1
aa`a_aaaa`a_bZaa`a`^`aaa_Ya__a]_a^^^ZZWB
@HWI-EAS443:3:96:248:1656#0/1
GCGGGGTTCTTCTCTTGTCTTCCTGATGTGTACTTTTTGA
+HWI-EAS443:3:96:248:1656#0/1
`a¥^aa^abb`aaaabbaaaa`aaa`aa]][`aa`__a__
@HWI-EAS443:3:96:339:719#0/1
CGGGGTTCTTCTCTTGTCTTCCTGATGTGTACTTTTTGAT
+HWI-EAS443:3:96:339:719#0/1
abbbb^babbaa]aabaaaa`aa`aaa`b]a_bab_b`aa
@HWI-EAS443:3:96:403:927#0/1
CGGGGTTCTTCTCTTGTCTTCCTGATGTGTACTTTTTGAT
+HWI-EAS443:3:96:403:927#0/1
aba``]abababababab`baa`aa`¥ab^a^_^aa`aa```
@HWI-EAS443:3:96:409:1490#0/1
GCGGGGTTCTTCTCTTGTCTTCCTGATGTGTACTTTTTGA
+HWI-EAS443:3:96:409:1490#0/1
ab_b`[FZ``aaaa`ba_a`]_a_]]^]QZY`]_`aa_[]
@HWI-EAS443:3:96:49:1216#0/1
GCGGGGTTCTTCTCTTGTCTTCCTGATGTGTACTTTTTGA
+HWI-EAS443:3:96:49:1216#0/1
VVTP¥X¥^a`YZ`¥X_aX]Q^]`]_XUZXYXT^aa_]¥]B
```

```
@HWI-EAS443:3:96:556:1380#0/1
CGGGGTTCTTCTCTTGTCTTCCTGATGTGTACTTTTTGAT
+HWI-EAS443:3:96:556:1380#0/1
ab]Q_]a`bV_¥^__`NVZ_`_``a_aXaW_^`^_`aUaB
@HWI-EAS443:3:96:563:804#0/1
GGGTTCTTCTCTTGTCTTCCTGATGTGTACTTTTTGATTA
+HWI-EAS443:3:96:563:804#0/1
a`aYaaabaaab_Z`_aa¥Z`]``a_b_TP_a[```^``B
@HWI-EAS443:3:96:679:1878#0/1
CGGGGTTCTTCTCTTGTCTTCCTGATGTGTACTTTTTGAT
+HWI-EAS443:3:96:679:1878#0/1
aa`b¥Saabababbbb]bb`bbb_bb``baa_ababb`bb
@HWI-EAS443:3:96:96:478#0/1
CGGGGTTCTTCTCTTGTCTTCCTGATGTGTACTTTTTGAT
+HWI-EAS443:3:96:96:478#0/1
abbabYaaaaab`abbaba_aaaa^ab]a^_`aba``^_`
@HWI-EAS443:3:97:1027:612#0/1
ATCAAAAAGTACACATCAGGAAGACAAGAGAAGAACCCCG
+HWI-EAS443:3:97:1027:612#0/1
_babb`bababbbabba_bbabbbabbb^bbabaa`a^aa
@HWI-EAS443:3:97:146:608#0/1
GGGGTTCTTCTCTTGTCTTCCTGATGTGTACTTTTTGATT
+HWI-EAS443:3:97:146:608#0/1
`Z`a¥WW][^[[__¥Ta___]X_XU_X_VY]__^_¥YZ]]
@HWI-EAS443:3:97:1513:1236#0/1
GGGTTCTTCTCTTGTCTTCCTGATGTGTACTTTTTGATTA
+HWI-EAS443:3:97:1513:1236#0/1
P_X^^a`_b]M_b^b]abaabbZV^Xb_¥^aR``a`aa_a
@HWI-EAS443:3:97:1602:1561#0/1
CGGGGTTCTTCTCTTGTCTTCCTGATGTGTACTTTTTGAT
+HWI-EAS443:3:97:1602:1561#0/1
`bab]Z]bbb¥aabbbbbbb¥ababbb`]Z¥_bbbbbS]]
@HWI-EAS443:3:97:222:1321#0/1
CGGGGTTCTTCTCTTGTCTTCCTGATGTGTACTTTTTGAT
+HWI-EAS443:3:97:222:1321#0/1
abbaba`abbbbbaaa_aaba]bba``aa`aabababT¥`
@HWI-EAS443:3:97:306:1975#0/1
GCGGGGTTCTTCTCTTGTCTTCCTGATGTGTACTTTTTGA
+HWI-EAS443:3:97:306:1975#0/1
abb¥b_G¥a_a[`b¥__Z^_[`aY]U_a¥XFWP¥¥¥__SZ
@HWI-EAS443:3:97:431:1753#0/1
GCGGGGTTCTTCTCTTGTCTTCCTGATGTGTACTTTTTGA
+HWI-EAS443:3:97:431:1753#0/1
ab____`a`]]Sa`_Z^Saba]Q]a_Z[UbSYa````_`a
@HWI-EAS443:3:97:580:1353#0/1
CGGGGTTCTTCTCTTGTCTTCCTGATGTGTACTTTTTGAT
+HWI-EAS443:3:97:580:1353#0/1
`bbba_babbababbb`abaaabbaabaa_a`baaabYaa
@HWI-EAS443:3:97:707:1969#0/1
CGGGGTTCTTCTCTTGTCTTCCTGATGTGTACTTTTTGAT
```

```
+HWI-EAS443:3:97:707:1969#0/1
aa``_]a`aa`a`aaa]a`aa``¥__`^aXa^a_a¥a^aa
@HWI-EAS443:3:97:737:1275#0/1
GCGGGGTTCTTCTCTTGTCTTCCTGATGTGTACTTTTTGA
+HWI-EAS443:3:97:737:1275#0/1
abbbbb_babbbbabaa`abb_`baaaaabaaab_a`b_a
@HWI-EAS443:3:97:978:1391#0/1
GCGGGGTTCTTCTCTTGTCTTCCTGATGTGTACTTTTTGA
+HWI-EAS443:3:97:978:1391#0/1
aabbabYb^bb_babb_a^a``^b`]ab^b`^Waaaaaa`
@HWI-EAS443:3:98:1104:580#0/1
GGTTCTTCTCTTGTCTTCCTGATGTGTACTTTTTGATTAT
+HWI-EAS443:3:98:1104:580#0/1
aa^`^a`_a¥aaa]`a``__`]``]_]^Xaa]_`]_^`]^
@HWI-EAS443:3:98:1306:424#0/1
CGGGGTTCTTCTCTTGTCTTCCTGATGTGTACTTTTTGAT
+HWI-EAS443:3:98:1306:424#0/1
ab_a_Vbaa`J_`bbb`b[bZIbbbbaa__`_bbbbaaa_
@HWI-EAS443:3:98:1591:42#0/1
CGGGGTTCTTCTCTTGTCTTCCTGATGTGTACTTTTTGAT
+HWI-EAS443:3:98:1591:42#0/1
aaa]J^bbb_¥bXbbZbaa`aW`baba]a^]I¥baa`abb
@HWI-EAS443:3:98:1685:1863#0/1
GGGTTCTTCTCTTGTCTTCCTGATGTGTACTTTTTGATTA
+HWI-EAS443:3:98:1685:1863#0/1
abW_^O^a^b_`a^aXa^_Waaa_a^b^_Vaaaba`_baa
@HWI-EAS443:3:98:266:312#0/1
CGGGGTTCTTCTCTTGTCTTCCTGATGTGTACTTTTTGAT
+HWI-EAS443:3:98:266:312#0/1
aaaaa_aa`a_a]aaa`]``_^``]_]]_¥_Z_``_a__`
@HWI-EAS443:3:98:457:63#0/1
GCGGGGTTCTTCTCTTGTCTTCCTGATGTGTACTTTTTGA
+HWI-EAS443:3:98:457:63#0/1
^G^W]aVZ[`TV[OLO¥LVPUWKHKSR[LXNJOSVQBBBB
@HWI-EAS443:3:98:460:982#0/1
CGGGGTTCTTCTCTTGTCTTCCTGATGTGTACTTTTTGAT
+HWI-EAS443:3:98:460:982#0/1
]ba^a]_`V`bb]aabbaXba`bb]ab]a__aab`baaa`
@HWI-EAS443:3:98:584:1569#0/1
CGGGGTTCTTCTCTTGTCTTCCTGATGTGTACTTTTTGAT
+HWI-EAS443:3:98:584:1569#0/1
RTR_aR`__¥Q^^X^`a^S__T¥_W_^WVRW_^`[`a[a¥
@HWI-EAS443:3:98:689:1148#0/1
GCGGGGTTCTTCTCTTGTCTTCCTGATGTGTACTTTTTGA
+HWI-EAS443:3:98:689:1148#0/1
aabaa^QZ[a`¥__a`¥X[`^^_`]]__^`W]V`_]`^TZ
@HWI-EAS443:3:98:689:585#0/1
GCGGGGTTCTTCTCTTGTCTTCCTGATGTGTACTTTTTGA
+HWI-EAS443:3:98:689:585#0/1
abbbbb_aaabaaaabaaaaaa`aa_aaS_^XG[b`aaX^
```

@HWI-EAS443:3:98:745:1074#0/1
CGGGGTTCTTCTCTTGTCTTCCTGATGTGTACTTTTTGAT
+HWI-EAS443:3:98:745:1074#0/1
aaaa`S]aa`_a```¥X^``[Qaa__aR`U¥Z__^X^]^^
@HWI-EAS443:3:99:1007:396#0/1
GCGGGGTTCTTCTCTTGTCTTCCTGATGTGTACTTTTTGA
+HWI-EAS443:3:99:1007:396#0/1
^`Y¥_a¥aaaa``Xa`ba_`b¥_aaa]YWaZ`^aaaaa^a
@HWI-EAS443:3:99:1097:1146#0/1
CGGGGTTCTTCTCTTGTCTTCCTGATGTGTACTTTTTGAT
+HWI-EAS443:3:99:1097:1146#0/1
`bbbbU`]bbaaaaaa``ababaaaaaaa]aababa^`ab
@HWI-EAS443:3:99:120:208#0/1
GCGGGGTTCTTCTCTTGTCTTCCTGATGTGTACTTTTTGA
+HWI-EAS443:3:99:120:208#0/1
aWaa_XF¥_UY__R[O`PL¥]]V]_XQ_V^ZVZ]VYXU^Z
@HWI-EAS443:3:99:1694:793#0/1
GGGTTCTTCTCTTGTCTTCCTGATGTGTACTTTTTGATTA
+HWI-EAS443:3:99:1694:793#0/1
a`__`Rabab`bb`a`bb`_bb`a^^Yaa`bbabba`_aa
@HWI-EAS443:3:99:352:1542#0/1
CGGGGTTCTTCTCTTGTCTTCCTGATGTGTACTTTTTGAT
+HWI-EAS443:3:99:352:1542#0/1
ababb_aabbbababaabbbb`b`aa`_¥_aaaaaa_aa
@HWI-EAS443:3:99:676:1285#0/1
GGGGTTCTTCTCTTGTCTTCCTGATGTGTACTTTTTGATT
+HWI-EAS443:3:99:676:1285#0/1
a]¥^WY[[[ZZYZXRUSZPVX[_^YSQ^SW]WMY^Q]BBB
@HWI-EAS443:3:99:791:1410#0/1
GGGGTTCTTCTCTTGTCTTCCTGATGTGTACTTTTTGATT
+HWI-EAS443:3:99:791:1410#0/1
WX``]`^`_aba`abbZb`]a]a]a`__X__``^babba_
@HWI-EAS443:3:99:981:1652#0/1
GGGGTTCTTCTCTTGTCTTCCTGATGTGTACTTTTTGATT
+HWI-EAS443:3:99:981:1652#0/1
ab``¥SVYbba]abb_`_a]]baa`babYbb`Waab`a__
@HWI-EAS443:3:9:114:1154#0/1
GGTTCTTCTCTTGTCTTCCTGATGTGTACTTTTTGATTAT
+HWI-EAS443:3:9:114:1154#0/1
^aTa]``a_aaba¥]aabaab`^b_`[_`aaaaa_`a_``
@HWI-EAS443:3:9:1184:1194#0/1
GCGGGGTTCTTCTCTTGTCTTCCTGATGTGTACTTTTTGA
+HWI-EAS443:3:9:1184:1194#0/1
abbaaa`babbababbbbbbbabb_aaabbaaabbbbaab
@HWI-EAS443:3:9:1288:1596#0/1
CGGGGTTCTTCTCTTGTCTTCCTGATGTGTACTTTTTGAT
+HWI-EAS443:3:9:1288:1596#0/1
^a`Y^T__V[_]Ua^[X``V¥FZ[R`aX_`^T`ab`aaba
@HWI-EAS443:3:9:1317:963#0/1
GGGGTTCTTCTCTTGTCTTCCTGATGTGTACTTTTTGATT

```
+HWI-EAS443:3:9:1317:963#0/1
ZbbaVaaaa_a_a_b_[`a_aa_[_^^aUUQaa_a``_`]
@HWI-EAS443:3:9:1370:824#0/1
ATCAAAAAGTACACATCAGGAAGACAAGAGAAGAACCCCG
+HWI-EAS443:3:9:1370:824#0/1
ab]a`]]_a_aabaa``abaaaa^aaa`[aa``aa`^]aa
@HWI-EAS443:3:9:1469:66#0/1
CGGGGTTCTTCTCTTGTCTTCCTGATGTGTACTTTTTGAT
+HWI-EAS443:3:9:1469:66#0/1
abbaa`bbbbabbbbbabbbbabbabbbbbbabbbbbabb
@HWI-EAS443:3:9:1505:912#0/1
CGGGGTTCTTCTCTTGTCTTCCTGATGTGTACTTTTTGAT
+HWI-EAS443:3:9:1505:912#0/1
aa_a]_^¥ba^bababbabba^aaaaaYZ_`^aaaaa_aa
@HWI-EAS443:3:9:165:639#0/1
GCGGGGTTCTTCTCTTGTCTTCCTGATGTGTACTTTTTGA
+HWI-EAS443:3:9:165:639#0/1
aaa¥PUGV[`^`__YW]U_`a]_UQZQLJLTVQV_YY^BB
@HWI-EAS443:3:9:228:1217#0/1
GCGGGGTTCTTCTCTTGTCTTCCTGATGTGTACTTTTTGA
+HWI-EAS443:3:9:228:1217#0/1
`bbaaZF_aaba]^aaaXaaaaaa```a_a[_`aaaaa`_
@HWI-EAS443:3:9:405:1932#0/1
CGGGGTTCTTCTCTTGTCTTCCTGATGTGTACTTTTTGAT
+HWI-EAS443:3:9:405:1932#0/1
aaaaa_`bb`ababbb_a_ababaaaa^a_aba``aaaba
@HWI-EAS443:3:9:490:1383#0/1
CGGGGTTCTTCTCTTGTCTTCCTGATGTGTACTTTTTGAT
+HWI-EAS443:3:9:490:1383#0/1
aaba``aabbabaaa``aaaa`]^`_aX__aaaaaaa^__
@HWI-EAS443:3:9:717:363#0/1
CGGGGTTCTTCTCTTGTCTTCCTGATGTGTACTTTTTGAT
+HWI-EAS443:3:9:717:363#0/1
abaaaWa`aa_a[aaa¥]a`^aaa__`WaW_`````Y¥_^
@HWI-EAS443:3:9:800:226#0/1
AGCGGGGTTCTTCTCTTGTCTTCCTGATGTGTACTTTTTG
+HWI-EAS443:3:9:800:226#0/1
aaab`b_]aaaa`a_aaa`_a`]``]]aa]a^^^aa_`a_
@HWI-EAS443:3:9:807:1251#0/1
AGCGGGGTTCTTCTCTTGTCTTCCTGATGTGTACTTTTTG
+HWI-EAS443:3:9:807:1251#0/1
a]abbaYG_W^a__^`_a`aaaY¥aaaRaX`]_¥a__aa¥
@HWI-EAS443:3:78:155:429#0/1
CTGGCATGGCTCGAATCGACCGGGGGTGTCTTTCAACCAA
+HWI-EAS443:3:78:155:429#0/1
aaa_ba_a___`^VX^][Y_[YFSZ¥FWJNJ^[QSZ[BBB
@HWI-EAS443:3:20:684:1714#0/1
CTGGCATGGCTCGAATCGACCGTGGGTGTCTTTCAACCAA
+HWI-EAS443:3:20:684:1714#0/1
`aa^]_XY_^WQOLZRBBBBBBBBBBBBBBBBBBBBBBBB
```

```
@HWI-EAS443:3:58:354:397#0/1
TCCATTTGTTCATATGATCGTTTGGTGCCCTGAGACGCCA
+HWI-EAS443:3:58:354:397#0/1
`a]Z^`bbaaabb^ab`baa`aaba^aa`a`a`a]__a_a
@HWI-EAS443:3:38:1035:1602#0/1
CTACTGAATACATAATGAAGGGAGTGCACATAAATACGGC
+HWI-EAS443:3:38:1035:1602#0/1
a_XWV¥]_^^]NNQZ^a¥]]_QX¥[]Z^]ZQT^RZ]_ZMF
@HWI-EAS443:3:40:415:1827#0/1
GTCGCTGTCTGGCTGTCAGTAAATATGCTAAAGTCCCGCT
+HWI-EAS443:3:40:415:1827#0/1
[K[aa__[a¥¥¥ZNWRVPWSWWEI¥NR`HTDHJY^a[SUR
@HWI-EAS443:3:92:936:1926#0/1
CCAAGATCGAAGGTTCCAGGTTCCAGGTTATCCCTAAGTG
+HWI-EAS443:3:92:936:1926#0/1
aaa``Z[]W_aa`]^aa_`¥]SSQ¥_]N`a^NQKV]]NTQ
@HWI-EAS443:3:4:370:647#0/1
ACTGATCCAAAATGGAAGGCTTTGTGCGACAATGCTTTAA
+HWI-EAS443:3:4:370:647#0/1
¥aaIY^ZM]]_Y[_Z¥ZWOR`]ZQXa`a`¥^JUWP[TDWU
@HWI-EAS443:3:54:1381:1641#0/1
GAAGAGCATCCCAGTGCTGGGAAGGACCCGAAGAAGATCT
+HWI-EAS443:3:54:1381:1641#0/1
R]]Z]Z]bbabb]babMaab`a]b^a`ba_GW]^ZH^_PI
@HWI-EAS443:3:69:602:1977#0/1
CTGTCTTCTTAGGGTCCTTCCCAGCACTGGGATGCTCTTC
+HWI-EAS443:3:69:602:1977#0/1
a``UJ^bbbbbaa¥_baa`baaa``^`_aa]aa_a^a`_a
@HWI-EAS443:3:25:1566:1237#0/1
TGCTGGGTTCTTCTCTTGTCTTCCTGATGTGTACTTTTTG
+HWI-EAS443:3:25:1566:1237#0/1
a__aaaa``[__]__aa``^`_[Za`Z^a]]Y]]^`_^_^
@HWI-EAS443:3:33:447:967#0/1
CAGAATGCCATAAGCACCACATTCCCTTATACTGGAGATC
+HWI-EAS443:3:33:447:967#0/1
ab]aa`aaaaa]a`abababaaaa`a_a`^a_a^¥¥¥_¥^Y^
@HWI-EAS443:3:39:1136:335#0/1
TGATTCCAGAGAGGAATGAACAAGGACAAACTCTCTGGAG
+HWI-EAS443:3:39:1136:335#0/1
^aaaaS[]a^_a]ZU^^S^T¥YZ]]P[aXSPDRa`Ta`RU
@HWI-EAS443:3:60:389:956#0/1
TGTGGATCATATGGCCATAATCAAAAAGTACACATCAGGA
+HWI-EAS443:3:60:389:956#0/1
aaZaa¥_¥L¥¥[¥`aaV¥]`XXZ_a^_PP¥XXZZUZ[X¥Z
@HWI-EAS443:3:19:1182:41#0/1
AGAGATATGGTCCAGCATTAAGCATCAATGAACTGAGCAA
+HWI-EAS443:3:19:1182:41#0/1
aaaaaaabab]^a_b_^aaa`a`_a`a_aa^^^a___O_^
@HWI-EAS443:3:22:809:867#0/1
GAAGACAAGAGATATGGTCCAGCATTAAGCATCAATGAAC
```

```
+HWI-EAS443:3:22:809:867#0/1
^]Z]^a_aa¥a_¥]_[a]__Z_[[SU][]Z^_^^V_]__[
@HWI-EAS443:3:79:566:1757#0/1
AGATTGCTCAGTTCATTGATGCTTAATGCTGGACCATATC
+HWI-EAS443:3:79:566:1757#0/1
abaaa`baaX`Xaaaa^R`[^^a`````a```]]_^a__`
@HWI-EAS443:3:2:353:202#0/1
AATCGCTCCTAACTGCCTTGATCATGCAATCCTCTTGTGA
+HWI-EAS443:3:2:353:202#0/1
aaa^a__[``__U]_Y_]_[]^V`]_V^`¥Y]YY_^]Y_Y
@HWI-EAS443:3:79:648:1698#0/1
ATGGAAATAAGGCGCTGCCTTCTTCAGTCTCTTCAGCAGA
+HWI-EAS443:3:79:648:1698#0/1
a``a]aa_``__a`a``aa``aa`a_`[`aa__`aZ_^Z_
@HWI-EAS443:3:82:428:1462#0/1
GTTAAACCAAGATGCATTAAGCAAAACCCAGGGATCATTA
+HWI-EAS443:3:82:428:1462#0/1
a^aab`ab`b`a`aaaaaaaaaUaaa````_]_]``a¥a
@HWI-EAS443:3:64:708:521#0/1
CCTGCTGTCTGAGTTCTTCAATGGTGGAACAGATCTTCAT
+HWI-EAS443:3:64:708:521#0/1
abab^a_a`^`V__aaaa_`]]a`Xa]```_`^^a``a__
@HWI-EAS443:3:71:489:1374#0/1
CGGGATTCTTCTCTTGTCTTCCTGATGTGTACTTTTTGAT
+HWI-EAS443:3:71:489:1374#0/1
_¥`[Z``bbb^]ab^`]Z`Ta^ZZ]¥¥V`^_`aa_`_`a]
@HWI-EAS443:3:18:581:1764#0/1
CAAGGACAAACCCTCTGGAGCAAAACAAACGATGCTGGGG
+HWI-EAS443:3:18:581:1764#0/1
^[^_`V`]¥T^Y]^]]TQ_`L^R_]b]Wa¥a_R`Z__a_a
@HWI-EAS443:3:99:1000:1707#0/1
TATGGCCCAGCATTAAGCATTAATGAATTGAGAAATCTTG
+HWI-EAS443:3:99:1000:1707#0/1
_`a_Y_bbZU`bbb^^`abaJ^a__N_G[KaUG^VYJaaa
@HWI-EAS443:3:40:1048:444#0/1
CGTGTCCATGGTGTATCCTGTTCCTGTTCCATGGCTGTAT
+HWI-EAS443:3:40:1048:444#0/1
[_¥_``b¥`_b_`]^[a_aba`````U_^ZW¥`a`a`GFJ
@HWI-EAS443:3:37:162:1501#0/1
AGCAGGGTAGATAATCACTCAATGAGTGATATCGAAGTCA
+HWI-EAS443:3:37:162:1501#0/1
`¥_^WXPD^``[ba]`U[_`¥U¥]]Z_]NFOa__]YQP¥Q
@HWI-EAS443:3:22:738:1211#0/1
GGGAGCAAAAGCAGGGTAGATAATCACTCAATGAGTGACA
+HWI-EAS443:3:22:738:1211#0/1
abb__]bbabZabbaa¥bab]]aabaaaa`_`aa_G_``a
@HWI-EAS443:3:69:964:2021#0/1
GGGTCTTATTTCTTCGGAGACAATGCAGAGGAGTTTGAGA
+HWI-EAS443:3:69:964:2021#0/1
[`bSbb`^_^[ba`ba^]Xaaba`_aaaTb[X]T¥aWQ¥[
```

```
@HWI-EAS443:3:75:416:706#0/1
TTTCAGACCAGCACTGGAGCTAGGATGAGTCCCAATCGTT
+HWI-EAS443:3:75:416:706#0/1
`^_a__aaab¥a][LaZZ`NN]`X_LXX¥V[_UTZXGVTZ
@HWI-EAS443:3:15:1479:356#0/1
TCCATGGTGTATCCTGTTCCTGTTCAATGGCTGTATGGGG
+HWI-EAS443:3:15:1479:356#0/1
]SZ^^a¥XSW^SNS¥_Z^¥]]bXXQHRZabLKYFJHXYDW
@HWI-EAS443:3:10:626:1280#0/1
GTTCCTGTTCCATGGCTGTATGGGGGATCTCCAGTATAAG
+HWI-EAS443:3:10:626:1280#0/1
a_ZZ[_^UX_[PWa¥TXZGTUGVQ^_GWGSWQBBBBBBBB
@HWI-EAS443:3:95:934:641#0/1
TCCATGGTGTATCCTGTTCCTGTTCCATGGCTGTATGGGG
+HWI-EAS443:3:95:934:641#0/1
a_QQX^`UZYXZ^L][```Va_`Y^PLS`]BBBBBBBBBB
@HWI-EAS443:3:64:1721:1020#0/1
TCACAAGACCACTGTGGACCATATGGCCATAATCAATAAG
+HWI-EAS443:3:64:1721:1020#0/1
abaaa`^a^aaY__Q_a__YV¥]_]`]]Y]Y_UH`BBBBB
@HWI-EAS443:3:66:405:1733#0/1
AAGACCACTGTGGACCATATGGCCATAATCAAAAAATACA
+HWI-EAS443:3:66:405:1733#0/1
aaPP¥abX¥_K¥a`a^]Ya_a]aSZR]UDMNH^`]GFW_a
@HWI-EAS443:3:5:1532:1493#0/1
TGCCATCTTCAGTCAATGCACCAGCATCCTTTCCAAGAAG
+HWI-EAS443:3:5:1532:1493#0/1
ab_Zb_^bbSba_M^`a_Qb^U^TRWZHX]abYUaabaaG
@HWI-EAS443:3:1:1606:918#0/1
AAAAGCTTGTGAATTCAAATGTCCCTGTCTTGTTTATATA
+HWI-EAS443:3:1:1606:918#0/1
ab`]`V_Q^a`^Vb^¥a]IZa_THYY^__`¥I_aa`_BBB
@HWI-EAS443:3:9:597:183#0/1
TGTACACTCCAGGAGGAGGAGTGAGAAATGATGATGTTGA
+HWI-EAS443:3:9:597:183#0/1
Z]]aaaa``a_aa^aaX^aW_UXU]V]]^`]Z^X]^X]¥]
@HWI-EAS443:3:14:1045:297#0/1
CTTTGTGCGACAATGCTTCAATCCAATGATCGTCGAGCTA
+HWI-EAS443:3:14:1045:297#0/1
abbbb`babbaaaabaabaaaa_aaaaaaa^`]_a_`__`
@HWI-EAS443:3:58:791:933#0/1
AAAGTTTTCAAGGCTGGAGAAGTTCGGTGGGAGGCTTTTG
+HWI-EAS443:3:58:791:933#0/1
`aaa[aaaa_`a^a_aa¥a¥_¥U¥S_]TY__NSKPW``HZ
@HWI-EAS443:3:60:492:653#0/1
AAGCCTCCCACCGAACTTCTCCAGCCTTGAAAACTTTAGA
+HWI-EAS443:3:60:492:653#0/1
a]]G¥[Y_QW]a_^^^_^W]__UXSLEJZU[XSX]T[`ZN
@HWI-EAS443:3:70:317:522#0/1
CCCTAATTGATGGCCATCCGAATTCTTTTGGTCGCTGTCT
```

```
+HWI-EAS443:3:70:317:522#0/1
QI_T_RPZ]¥a`¥¥^VZa^aYY_``]a^X^^X[T^ZOZQ_
@HWI-EAS443:3:36:548:601#0/1
AAGAGAATAATGGACATGATCCCAGAGAGGAATGAACAAG
+HWI-EAS443:3:36:548:601#0/1
aaaaa^^a``aaaaaa_aa]_aaV[^]X^_^_^[[_Q__]
@HWI-EAS443:3:52:720:1813#0/1
CTTTTGCAAGATTGTTCAGTTCATTGATGCTTAATGCTGG
+HWI-EAS443:3:52:720:1813#0/1
aYQ_][MX^a_YSUF¥L]ZQVaTSN_WWY[VRYZZSDTYO
@HWI-EAS443:3:58:1408:46#0/1
CGGTGGTGCTTATGGCATTTTGCGCTGGAACTTTTAGGAA
+HWI-EAS443:3:58:1408:46#0/1
abbabbaaaabbb`_Y_bbbbZZ_^aab``V`bbbabb¥a
@HWI-EAS443:3:45:1788:1789#0/1
CATGAGCAAAAAGAAGTCCTATATAAATAAGACAGGAAAC
+HWI-EAS443:3:45:1788:1789#0/1
^]aS¥a_PPY_¥PXDL[UV]]aS]X`NUYXa[OFI^GZXW
@HWI-EAS443:3:38:1087:1793#0/1
ATCGTCTTCCTTCTTTGGTCCTACATTTGCTTACCATTGG
+HWI-EAS443:3:38:1087:1793#0/1
aab_^YRZa_TYMQY][^GST^X`][_[ZMEMUTMZ^TGP
@HWI-EAS443:3:98:1221:1090#0/1
TCGTCTTCCTTCTTTGGTCCTACATTTGCTTACCATTGGG
+HWI-EAS443:3:98:1221:1090#0/1
aa_[_ba]_aa^aaaa`__]a___`a_`[`a`Y]a_^__`
@HWI-EAS443:3:59:1108:1151#0/1
ACAATGGATTGGGGGATGCCCCATCCCTTGATCGGCTCCG
+HWI-EAS443:3:59:1108:1151#0/1
U`^TQb`__baUQGVa[b`bbba__Ya]aWXN^G]UQYTY
@HWI-EAS443:3:89:943:1175#0/1
GACCTTCACTATCTCCAGAGCAGAAATGAAAAGTGTCGAG
+HWI-EAS443:3:89:943:1175#0/1
abaTaaTTa_^GQT`aa]PRSZ`W``^_¥N^Y]H[HN^_^
@HWI-EAS443:3:36:750:1797#0/1
AGCAATAGTTGGAGAAATTTTACCATTACCTTNTCTTCCA
+HWI-EAS443:3:36:750:1797#0/1
a^baa_b`TRS^_Wa`[`__I`^``]]X`_]MDT_V]_S`
@HWI-EAS443:3:57:347:692#0/1
CTGCCTTGCATATGTCTACGGCTTGTTCCTCCGTTGGATT
+HWI-EAS443:3:57:347:692#0/1
a_bbb`_ba^_`a`^a_aaa^aa`H¥`VTJZBBBBBBBBB
@HWI-EAS443:3:80:1693:1843#0/1
AGAATTGCCTCTTACCAGTATCCTCAATCCTGATCCTCTC
+HWI-EAS443:3:80:1693:1843#0/1
aa^``aa_a`X^¥[R¥aaO_]XY`Q]`YYW__^_]N¥XW^
@HWI-EAS443:3:97:723:767#0/1
GGACCTTCGATCTTGGGGGGCTATATGAAGCAATCGAGGA
+HWI-EAS443:3:97:723:767#0/1
a`_`^aVa`¥`Uaa``^a`[___a^aa___U_¥UY`^`¥¥
```

@HWI-EAS443:3:64:121:1997#0/1
AGCCAATTGAAGTGGGCACTTGGTGAAAATATGGCATCAG
+HWI-EAS443:3:64:121:1997#0/1
a`b`V]_`X¥NTT_T_``U¥_PWP¥X]R]X]RTQT¥]^¥Y
@HWI-EAS443:3:66:72:780#0/1
CCATATTTTCACCAAGTGCCCACTTCAATTGGCTTGTTCT
+HWI-EAS443:3:66:72:780#0/1
abbbbabbbabbb_aV[bbaabbbaa[`a`]^]a`OZ^^`
@HWI-EAS443:3:20:904:1387#0/1
AGCCAATTGAAGTGGGCACTTGGTGAAAATATGGCACCAG
+HWI-EAS443:3:20:904:1387#0/1
``aaba^aa]XaWaaa_b_aa`b^a_[aa__aaaaa]_aa
@HWI-EAS443:3:49:1000:1518#0/1
GTGCCATATTTTCACCAAGTGCCCACTTCAATTGGCTTGT
+HWI-EAS443:3:49:1000:1518#0/1
a[¥¥¥a`W`aa`W_`aa`aZa_XP^]`_Wa]__a]Y]_OH
@HWI-EAS443:3:79:74:833#0/1
GCCAATTGAAGTGGGCACTTGGTGAAAATATGGCACCAGA
+HWI-EAS443:3:79:74:833#0/1
aba]baaa¥aaXaaaaba``aaZ[S[[`^___^^_^`__^
@HWI-EAS443:3:49:1553:233#0/1
ATTGATGCCATCATGGCAAGCACTTGCTGACCAAGCGACT
+HWI-EAS443:3:49:1553:233#0/1
abba`ab```a``a][_YY^¥WSZ_ZU_Q[WS^^UO_]O¥
@HWI-EAS443:3:92:798:1839#0/1
AGGATTTTCATTTAAATACGGCAATGGTGTTTGGATAGGG
+HWI-EAS443:3:92:798:1839#0/1
aZUOMMVYTSPXT_[U¥`¥]ZVWUWXUGVVQZWSZ^QJQY
@HWI-EAS443:3:7:1639:1360#0/1
NAAGAAGAGTTTTCTGAGATCATGAAGATCTGTTCCACCA
+HWI-EAS443:3:7:1639:1360#0/1
DIPPPWROVNXRTPTZZTPSTXTQTNQRXWOZSZSZZOMQ
@HWI-EAS443:3:44:1034:909#0/1
ATTTATCGTCGCTTTAAATACGGTTTGAAAAGAGGGCCTG
+HWI-EAS443:3:44:1034:909#0/1
abbbaY[VRUa_aaaVT_`IQ¥aSTPSLOY¥WJY¥`KFLF
@HWI-EAS443:3:87:1449:573#0/1
ATTTATCGTCGCTTTAAATACGGTTTGAAAAGAGGGCCTG
+HWI-EAS443:3:87:1449:573#0/1
[bababa`U]S_bbV¥]aaa]b¥_`a[a`aY]RR]_`MJ^
@HWI-EAS443:3:80:1423:104#0/1
GTCAGGATATAGCGGGAGTTTTGTTTAGCATCCAGAAATA
+HWI-EAS443:3:80:1423:104#0/1
a[^`aa^_aa[a]_XaXa¥_a_SXWFGTDIYPUU_NTL]O
@HWI-EAS443:3:96:96:1691#0/1
CGCGCGAAAACAGGCAAACCATTTGAATGGATGTCAATCC
+HWI-EAS443:3:96:96:1691#0/1
YT¥¥_UR]^W]]SO_RVW_`]]Y[]ZU[W[¥]_OOHHD¥B
@HWI-EAS443:3:36:702:574#0/1
GGGACGCGAAAGCAGGCAAACCATTTGAATGGATGTCAAT

+HWI-EAS443:3:36:702:574#0/1
aa]R^a`¥LVY¥TZWZ``^a¥¥¥X`[XW^[`[Z¥^WYVYR
@HWI-EAS443:3:21:665:963#0/1
AGCCGCGAAAGCAGGCAAACCATTTGAATGGATGTCAATC
+HWI-EAS443:3:21:665:963#0/1
aaaa]¥a[W]_Z`[^a¥R^^^[_^^_[[Z_]XX`UYUVRL
@HWI-EAS443:3:29:1335:65#0/1
CACGCGAAAGCAGGCAAACCATTTGAATGGATGTCAATCC
+HWI-EAS443:3:29:1335:65#0/1
[baba_aaaaaaaa`a`a]^a_aaa``aa__aa_^``_]R
@HWI-EAS443:3:32:492:144#0/1
CCCGCGAAAGCAGGCAAACCATTTGAATGGATGTCAATCC
+HWI-EAS443:3:32:492:144#0/1
abaaabaa]aa`]`aa`^]__^__^_`^_[_X¥WVVTS_Z
@HWI-EAS443:3:13:956:1377#0/1
CGCGAAAGCAGGCAAACCATTTGAATGGATGTCAATCCGA
+HWI-EAS443:3:13:956:1377#0/1
_``baba¥baaa^a`_a`a_aa^_TWa`a_`SPW^_U^¥W
@HWI-EAS443:3:23:1639:1192#0/1
CGCGAAAGCAGGCAAACCATTTGAATGGATGTCAATCCGA
+HWI-EAS443:3:23:1639:1192#0/1
aa[aYbaaabaa_bbaaaab[ab]aaaa_]b_]`a^[U`[
@HWI-EAS443:3:23:18:706#0/1
CGCGAAAGCAGGCAAACCATTTGAATGGATGTCAATCCGA
+HWI-EAS443:3:23:18:706#0/1
abbb`WJ_baaaa_a``b]aa`b_aaaa__a[``a]_^__
@HWI-EAS443:3:25:605:887#0/1
CGCGAAAGCAGGCAAACCATTTGAATGGATGTCAATCCGA
+HWI-EAS443:3:25:605:887#0/1
`bb_Wa`aabaaabb`bbbbbabb_`babab¥UXZ_Y]`_
@HWI-EAS443:3:2:902:715#0/1
CGCGAAAGCAGGCAAACCATTTGAATGGATGTCAATCCGA
+HWI-EAS443:3:2:902:715#0/1
aabbbbbbbbbbbbabaabbbbbab`bbbaba^aaa^_a`
@HWI-EAS443:3:32:1583:1992#0/1
CGCGAAAGCAGGCAAACCATTTGAATGGATGTCAATCCGA
+HWI-EAS443:3:32:1583:1992#0/1
`aa¥]W_`ZH[^]Y_a``aS_a¥U^IR]]GaGS[aW^LSB
@HWI-EAS443:3:36:109:1239#0/1
CGCGAAAGCAGGCAAACCATTTGAATGGATGTCAATCCGA
+HWI-EAS443:3:36:109:1239#0/1
abbbabaabaaabab^bbaaaaaaa``ab`a_aaaaaa]P
@HWI-EAS443:3:38:522:344#0/1
CGCGAAAGCAGGCAAACCATTTGAATGGATGTCAATCCGA
+HWI-EAS443:3:38:522:344#0/1
abaaY``abaa_``_¥_`_``_a`a^a_`]^ZU__UW[^W
@HWI-EAS443:3:69:161:1130#0/1
CGCGAAAGCAGGCAAACCATTTGAATGGATGTCAATCCGA
+HWI-EAS443:3:69:161:1130#0/1
aaaa]TP_a]a``a^]_¥`_aa__`Y_]]_XSUMUSPYRX

```
@HWI-EAS443:3:82:1377:757#0/1
CGCGAAAGCAGGCAAACCATTTGAATGGATGTCAATCCGA
+HWI-EAS443:3:82:1377:757#0/1
`bbbb`aaaabaabbbabbbbbbbbbb`bbba`abaXa`a
@HWI-EAS443:3:98:136:1226#0/1
CGCGAAAGCAGGCAAACCATTTGAATGGATGTCAATCCGA
+HWI-EAS443:3:98:136:1226#0/1
aa``]]]`a`_a[WLT¥Y^^_YV[Y¥_^UYZOTX¥VTULW
@HWI-EAS443:3:5:330:1897#0/1
AGGCGGGGAGTCTTCGAGCTCTCGGAGGAAAAGGCAAGGA
+HWI-EAS443:3:5:330:1897#0/1
YGQM``aZa[^¥Z`a_R¥T¥a^^_[VQZ¥¥^¥QVRBBBBB
@HWI-EAS443:3:63:149:123#0/1
GGGGGCGGGGAGTCTTCGAGCTCTCGGACGAAAAGGCAAC
+HWI-EAS443:3:63:149:123#0/1
a_R_`¥^[]XP]R`RU``PSSXTTZ[ZZRYPZNPTTRTST
@HWI-EAS443:3:55:626:2020#0/1
GCCATAATCAAGAAGTATACATCAGGAAGACAAGAGAAGA
+HWI-EAS443:3:55:626:2020#0/1
aaT[]WYTab_K][^UYT``_^¥RRZNU[TSGY`VZ`¥`¥
@HWI-EAS443:3:2:1389:1185#0/1
CTCTTGTCTTCCTGATGTGTACTTNTTGATTATGGCCATA
+HWI-EAS443:3:2:1389:1185#0/1
Q_bbbbba]bb`bZV]bVbbUbb[D^bbabab`_`[aaab
@HWI-EAS443:3:34:1310:223#0/1
ATGGCCATAATCAAGAAGTACACATCAGGAAGACAAGAGA
+HWI-EAS443:3:34:1310:223#0/1
a]]_O]`^]^`¥Z¥FR_[[`T_X¥_XZ`YU]YXXW^¥¥BB
@HWI-EAS443:3:38:360:986#0/1
GGGTTCTTCTCTTGTCTTCCTGATGTGTACTTCTTGATTA
+HWI-EAS443:3:38:360:986#0/1
_aYG]bYX_U_`¥`Z^`aYUY¥^T]ZT[YO[_P¥_X[XWS
@HWI-EAS443:3:100:1437:425#0/1
CGGGGAGTCTTCGATCTCTCGGACGAAAAGGCAACGAACA
+HWI-EAS443:3:100:1437:425#0/1
ab^RbJa`aaZ`¥`IRIZM^Zbbbb_I_S_UJQUG_YBBB
@HWI-EAS443:3:96:1146:111#0/1
GGGAGTCTTCGAGCTCTCGGACGAAAAGGGAACGAACACG
+HWI-EAS443:3:96:1146:111#0/1
aP^^`RLRXM`S^DDMUUBBBBBBBBBBBBBBBBBBBBBB
@HWI-EAS443:3:10:405:24#0/1
AGCATGTTAAACATGCCCATCATCATCCCAGGACTCAGTG
+HWI-EAS443:3:10:405:24#0/1
]_^Y^`]^b`^]a__¥X^`ZYZ]Z[ZXWWW^^^]TDN_GY
@HWI-EAS443:3:26:1648:566#0/1
CTGAGTCCTGGGATGATGATGGGCATGTTTAACATGCTAA
+HWI-EAS443:3:26:1648:566#0/1
`a`Yb^bUa[aaaaaabb^aa`_aabaaaaa^Zb_a_aa`
@HWI-EAS443:3:32:130:1559#0/1
TGAGTCCTGGGATGATGATGGGCATGTTTAACATGCTAAG
```

~~~
+HWI-EAS443:3:32:130:1559#0/1
aabb_bbb`aaaaa`aaaa_``aaaaaaaaaaa__a`ba[
@HWI-EAS443:3:48:247:1118#0/1
ACTGAGTCCTGGGATGATGATGGGCATGTTTAACATGCTA
+HWI-EAS443:3:48:247:1118#0/1
a¥__]b`^]b]a^[a^a`b``a`[`aaa`aaa^Vaaa_aa
@HWI-EAS443:3:50:1196:902#0/1
CACTGAGTCCTGGGATGATGATGGGCATGTTTAACATGCT
+HWI-EAS443:3:50:1196:902#0/1
`bbbaabaaabbbbbbbbbbabaaaaabbbbbbaabaaaa
@HWI-EAS443:3:69:1060:1064#0/1
CACTGAGTCCTGGGATGATGATGGGCATGTTTAACATGCT
+HWI-EAS443:3:69:1060:1064#0/1
aVaZVa`_b``a[^`b^[`a]^¥^aaaaaR^a]a[a[`b`
@HWI-EAS443:3:87:470:881#0/1
AGCATGTTAAACATGCCCATCATCATCCCAGGACTCAGTG
+HWI-EAS443:3:87:470:881#0/1
a_aaaaWaaba^`__`_`__aa`_a```_]_¥^]`___X^
@HWI-EAS443:3:89:1510:1221#0/1
CACTGAGTCCTGGGATGATGATGGGCATGTTTAACATGCT
+HWI-EAS443:3:89:1510:1221#0/1
aa^_bU`¥Yab¥a`ab`aba_baaaaabbaabba¥_a_[Z
@HWI-EAS443:3:98:704:930#0/1
AGCATGTTAAACATGCCCATCATCATCCCAGGACTCAGTG
+HWI-EAS443:3:98:704:930#0/1
]V]aabaabaaab```aaabbaa`aa_a^``a_T_^a_^a
@HWI-EAS443:3:20:928:1288#0/1
CGTCGCTTTAAATACGGGTTGAAAAGAGGGCCTTCTACGG
+HWI-EAS443:3:20:928:1288#0/1
abaa_b¥_]^`b¥a_ZTGYa^S_a]QS]¥`XOMS]^aKTH
@HWI-EAS443:3:54:604:530#0/1
CAATTTCAATGAACCGGTTCTCTTTGTAGTCATACAAATC
+HWI-EAS443:3:54:604:530#0/1
ab_bb`b¥]^a__^^bb`a_aaaabaa^a]aa`a¥aa^a`
@HWI-EAS443:3:76:1392:1363#0/1
GCCTGAGTCCATGAGGGAAGAATATAAACAGGAAAAGCAG
+HWI-EAS443:3:76:1392:1363#0/1
a^V`P][JSQSWTTQY^^]]Y]XVUGRYUTVYUZHVUQSV
@HWI-EAS443:3:33:1095:262#0/1
AGGAGGGCCTTCTACGGAAGGAGTGCCTGAGTCCATGAGG
+HWI-EAS443:3:33:1095:262#0/1
a`]Z¥_aba^aWa_¥_]W`_[QXTYGJ]]V_MPY[^¥P^X
@HWI-EAS443:3:29:930:1461#0/1
ATCCCAGTGCTGGGAAGGACCCTAAGAAAACAGGAGGCAG
+HWI-EAS443:3:29:930:1461#0/1
]^]`_WTU``Z[YUa]RX^`aa]a¥P`[X^aaXO^Z¥a`a
@HWI-EAS443:3:96:77:518#0/1
AGGGGCCTCCTGTTTTCTTAGGGTCCTTCCCAGCACTGGG
+HWI-EAS443:3:96:77:518#0/1
`bb]OZaaa`W``]a``¥]¥G]]N]^`QTJS[^^YRX[R¥
~~~

@HWI-EAS443:3:82:1075:343#0/1
GCCTCCTGTTTTCTTAGGGTCCTTCCCAGCACTGGGATGC
+HWI-EAS443:3:82:1075:343#0/1
aaaaa_ababbbabaa_aa_^abaa```a[a_b`[aa`b¥
@HWI-EAS443:3:83:1074:66#0/1
GCCTCCTGTTTTCTTAGGGTCCTTCCCAGCACTGGGATGC
+HWI-EAS443:3:83:1074:66#0/1
a``a_aab`abb¥bbaaa]_`_aa_^]___^Y`aa^^``V
@HWI-EAS443:3:53:132:1714#0/1
ATCCCCAGTGATTGTGAAAGAAATCTCTGTGTCTTGTGAA
+HWI-EAS443:3:53:132:1714#0/1
^^b]_¥ZX[¥`][WVU^a]JMU__[S¥O]_WLX[T]OMVW
@HWI-EAS443:3:61:953:1112#0/1
CTCGAATCGACCGTGGGTGTCTTTCACCCAGAATCTGGAA
+HWI-EAS443:3:61:953:1112#0/1
M`abbbaa_b_aVa¥aWZ_]bb¥b`^HWb`ZW`Z[_K_Z^
@HWI-EAS443:3:4:1643:2023#0/1
TGGCTGATTTGAATTTTACCACAAATGCGATAACACGTGC
+HWI-EAS443:3:4:1643:2023#0/1
aabbb_HQa]b_`L_a[VTa_V^b^_VDSYa^ZYTMTBBB
@HWI-EAS443:3:91:235:752#0/1
TGGCTGCTTTGAATTTTACCACAAATGCGATAACACGTGC
+HWI-EAS443:3:91:235:752#0/1
aabaabYaaa^_aa```¥Z[_a_^a]]_`Z^__[^^`Z^Y
@HWI-EAS443:3:29:1640:1817#0/1
ATTCTGTGTGCTCTTCAGGTCTGCTGCATATCCTGACACG
+HWI-EAS443:3:29:1640:1817#0/1
_Z_Z__SUZb`b_`a_a¥_U`UXHU[Qa^aZRP¥UBBBBB
@HWI-EAS443:3:38:285:1679#0/1
ATCAGTTTCTGGATGTGCTCTAATGGGTCTCTACAGTGCC
+HWI-EAS443:3:38:285:1679#0/1
aaa``^`aaZ^Z]]`WSa[U_`XVU^XX]NNWYUXYVV]V
@HWI-EAS443:3:38:194:1902#0/1
GAACTGAGCAATCTTGCAAAAGGAGAGAAAGCTAATGTGT
+HWI-EAS443:3:38:194:1902#0/1
UXaba_Y_]aa`a`ab`¥¥aa]]Z_bb_HHYbaa_¥¥GOF
@HWI-EAS443:3:83:857:519#0/1
GTGGTTTTTAAACAATTCGACACTAATTGATGGCCATCCG
+HWI-EAS443:3:83:857:519#0/1
]PFR]¥TYXXR]QWO¥a]VUUYZXZVTV]X^a¥[`QX[`[
@HWI-EAS443:3:87:1296:209#0/1
AGCATCATTACTTATGAAAACAACACTTGGGTAAACCAGA
+HWI-EAS443:3:87:1296:209#0/1
`b`bbb`bbbab`¥^`aaaaaaa``Z`bbaaT`]a]WZaa
@HWI-EAS443:3:14:1222:1622#0/1
CTCCAGCGCAAAATGCCATAAGCACCACATTCCCTTATAC
+HWI-EAS443:3:14:1222:1622#0/1
aba`ZZaaV]`]aX_aaaba`a_`S]_S`_`NTS[]_abV
@HWI-EAS443:3:68:1625:455#0/1
CTCCAGCGCAAAATGCCATAAGCACCACATTCCCTTATAC

+HWI-EAS443:3:68:1625:455#0/1
aY[`S_`^_aWaaa^J`]`¥¥baaaV`UH^]UDE_ZUY[Q
@HWI-EAS443:3:81:1040:1687#0/1
TTTGCATTTTCCATCATTCTTATAACTTCTGTTCGCATGT
+HWI-EAS443:3:81:1040:1687#0/1
aTXa]¥I^aYYQVSTSb_SWJ^bXaX_^ON__a_`Y`__]
@HWI-EAS443:3:61:1020:1078#0/1
AACAGATCTTCATGATCTCAGCGAACTCTTCTTTCTTGAT
+HWI-EAS443:3:61:1020:1078#0/1
¥`_¥^^abba¥aa`aba]aSRK_¥a^``aa[b^`a___`]
@HWI-EAS443:3:85:293:530#0/1
GCGAAATCACATGTGTGTGCAGGGATAACTGGCATGGCTC
+HWI-EAS443:3:85:293:530#0/1
YE[^_OX``XZXW¥¥PUK¥YSXRKQXONPZ¥KWBBBBBBB
@HWI-EAS443:3:98:1756:835#0/1
ACGGCCATATGGTCCACAGTGGTCTTAGTGAGTATCTAGA
+HWI-EAS443:3:98:1756:835#0/1
^]RZ[baWa``XRYZZR^_N¥HMDT`__SR]`WaVLBBBB
@HWI-EAS443:3:38:1660:1558#0/1
ACGGCCATATGGTCCACAGTGGTCTTAGTGAGTATCTCGC
+HWI-EAS443:3:38:1660:1558#0/1
aaX]`¥Z]X¥]TDM`¥ZQZMW[FV¥¥RBBBBBBBBBBBBB
@HWI-EAS443:3:86:929:1207#0/1
CTCCTGGGTAACACGTTCCCTTGTCTGAACTAGTTGTTTC
+HWI-EAS443:3:86:929:1207#0/1
a`aaa`aa_baaaab`aaWPT^`NZ_]U_X_[_Y^__Y_R
@HWI-EAS443:3:79:72:1605#0/1
ATTCGCAAGCGATTTGCAGACAATGGATTGGGTGATGCCC
+HWI-EAS443:3:79:72:1605#0/1
a``a_a]`_a``^__¥`[ZZ_¥[ZXUWVUWQSQWWSSWW^
@HWI-EAS443:3:54:100:504#0/1
TCGGGTCTTCCCCATATTCTTTCATTGCCTTTTCCGCAAG
+HWI-EAS443:3:54:100:504#0/1
ab¥_[X[[UO`a¥HUQT^_b]b`¥b]YX]abaOQL_F[¥¥
@HWI-EAS443:3:54:198:1368#0/1
CGGGTCTTCCCCATATTCTTTCATTGCCTTTTCCGCAAGC
+HWI-EAS443:3:54:198:1368#0/1
a¥LLU^W`[a__aaa`aaaa[ZVZ`UXN`aa]a_aa]MQT
@HWI-EAS443:3:51:612:185#0/1
GACTCAGGCACTCCTTCCGTAGAAGGCCCTCCTTTCAAAC
+HWI-EAS443:3:51:612:185#0/1
`XYaa`IPU`RT`aRPV[aaTbTaaZZ_YJJP_`[XZ_a_
@HWI-EAS443:3:33:910:1855#0/1
ACTTCAAGCAGTAGTTGTAAGGCTTGAATAAATGTTATTT
+HWI-EAS443:3:33:910:1855#0/1
a`VaaaXL_¥`L`a[`_Za_a[__[[G]Q_[W[^S^Z[V^
@HWI-EAS443:3:36:121:1479#0/1
GTCCCCAGTGATCGTGAAAGAAATCTCTGTGTCTTGTGAA
+HWI-EAS443:3:36:121:1479#0/1
aabbba^aaaa^F¥_a`¥a_Z^baaab``_a^aa]XZ`a`

```
@HWI-EAS443:3:99:152:1392#0/1
GAGTTCTTCAATGGTGGAACAGATCTTCATAATCTCAGAG
+HWI-EAS443:3:99:152:1392#0/1
a_`IY[`a`]^a``]^a___ab^¥¥^`a`aYa_¥`^`]_a
@HWI-EAS443:3:77:1741:501#0/1
ATTCATTTTGGACCCAGCTTGCTAGAGATCTGGGCTCCGG
+HWI-EAS443:3:77:1741:501#0/1
^`]XWU^[a^`¥OMSab_aWLUXWR^ZQFVWT_[RT¥GZB
@HWI-EAS443:3:46:636:1254#0/1
GCTTCCGGCAGATGCTCTGATTTCTGTGGCATCCTGGCGC
+HWI-EAS443:3:46:636:1254#0/1
_abaaa`ba`b``_¥___a__`a^a_X`__[_¥^[XX]^]
@HWI-EAS443:3:56:1472:521#0/1
CCGGCAGATGCTCTGATTTCTGTGGCATCCTGGCGCTCCC
+HWI-EAS443:3:56:1472:521#0/1
aaba`bb_Uaaab``a]]babb^aa_a[[[S`aR`[¥¥¥`
@HWI-EAS443:3:7:1049:1563#0/1
CTTCGTCCATTTTCACCCCTCCAGAAATTTCGGTCATCGA
+HWI-EAS443:3:7:1049:1563#0/1
ab¥]___b[a^_ZZ_^O_`ZYa¥_ZJS]`_W_a^¥SSDZB
@HWI-EAS443:3:31:920:962#0/1
GAGACAATCCACGCCCTAATGATAAGACAGGCTGAGGCAG
+HWI-EAS443:3:31:920:962#0/1
R]_QZ__R]HVVQ[V¥X]¥¥_W]XYZX]¥X^YW]JPZ[QS
@HWI-EAS443:3:14:141:1041#0/1
AGCCTGTCTTATCATTAGGGCGTGGATTGTGTCCGAAAAT
+HWI-EAS443:3:14:141:1041#0/1
¥¥`ZLYM_baaV`Y^^b¥S^````_a]Y^WDY]]__``]_
@HWI-EAS443:3:89:365:1138#0/1
AGCCTGTCTTATCATTAGAGCGTGGATTGTCTCCGAAAAT
+HWI-EAS443:3:89:365:1138#0/1
_`aSXQ^^`YI^X¥_X__OZTQM]^Z_MNXaa¥`_XNFZ_
@HWI-EAS443:3:23:883:1286#0/1
AGCCTGTCTTATCATTAGGGCGTGGATTGTCTCCGAAAAT
+HWI-EAS443:3:23:883:1286#0/1
aaba```ba_`a¥aa`_]Z^^_YZ¥]^]^TYYVQXX_^_^
@HWI-EAS443:3:3:872:899#0/1
AGCCTGTCTTATCATTAGGGCGTGGATTGTCTCCGAAAAT
+HWI-EAS443:3:3:872:899#0/1
aba_``b^aa`a^`a`aaa_aaXa]^_`]X_]R[_^__`B
@HWI-EAS443:3:97:1178:1836#0/1
CGTCTTTTTTTCAAATGTATTTATGGTCGCTTTAAATATG
+HWI-EAS443:3:97:1178:1836#0/1
[^a]`¥a_b_bba`aba_¥¥bba]G[``^^Wb¥ba_b_Mb
@HWI-EAS443:3:11:1305:1557#0/1
AAAGAACTGAGAGATCTAATGTCGCAGTCTCGCACTCGCG
+HWI-EAS443:3:11:1305:1557#0/1
_]_¥a]]a]_V]U^`[aaW^`R]¥X]XWUDTLV¥NR[BBB
@HWI-EAS443:3:2:47:1756#0/1
GAGAATAAAAGAACTGAGAGATCTAATGTCGCAGTCTCGC
```

```
+HWI-EAS443:3:2:47:1756#0/1
T_bbbZ`abb]aababaaab`b`aabbaaaaRba]WDX`¥
@HWI-EAS443:3:49:993:809#0/1
GAACTGAGAGATCTAATGTCGCAGTCTCGCACTCGCGAGA
+HWI-EAS443:3:49:993:809#0/1
a[a`b_aabaaaaaa_ab_aa_a_¥^a_a_V``a`^`¥`¥
@HWI-EAS443:3:66:763:906#0/1
GCCGGTATTTCTTCAATTAACCACCTTATTTCCTCAAATT
+HWI-EAS443:3:66:763:906#0/1
abb__SXP¥ZS^¥¥[^`[Q¥_WYSMY]]_`_]Z]YXT_`Z
@HWI-EAS443:3:34:1133:1689#0/1
GTCGTATTTCTTCAATTAACCACCTTATTTCCTCAAATTT
+HWI-EAS443:3:34:1133:1689#0/1
¥J``Sab^Yaba^]`Vaaab`aa^`XaaaaaV`__`^`_^
@HWI-EAS443:3:4:828:1107#0/1
CGCGCTACCTTTCTGACATAACCCTCGAGGAAATGTCACG
+HWI-EAS443:3:4:828:1107#0/1
aba]a_`_abba`aaa_baaa``^__aab^S_a`_¥Ra¥a
@HWI-EAS443:3:50:1161:604#0/1
AGGGGACGTAGTGTTGGTAATGAAACGGAAAGGGGACTAT
+HWI-EAS443:3:50:1161:604#0/1
^``bbU¥_UXZa]_^a`V_X_[V¥`J_GZ]UG[b_GOZDQ
@HWI-EAS443:3:14:1646:20#0/1
ATTGGGCAAGGGGACGTAGTGTTGGTAATGAAAAGGAAAC
+HWI-EAS443:3:14:1646:20#0/1
a[``_Z¥¥M]`aa^^`R]a^BBBBBBBBBBBBBBBBBBBB
@HWI-EAS443:3:100:1083:570#0/1
CGTCCCGTTTCCGTTTCATTACCAACACTACGTCCCCTTG
+HWI-EAS443:3:100:1083:570#0/1
`b``]Z_a¥Z[]T[a_¥UaU¥_a¥W[]U^`TWZ[QBBBBB
@HWI-EAS443:3:17:1572:1312#0/1
ACCGTTTCATTACCAACACTACGTCCCCTTGCCCAATTAG
+HWI-EAS443:3:17:1572:1312#0/1
^_¥a`aa`¥a¥_S¥[W[a`a]`a]YQZVT[Z¥RV^[[UTT
@HWI-EAS443:3:37:79:420#0/1
CCCGTTTCCGTTTCATTACCAACACTACGTCCCCTTGCCC
+HWI-EAS443:3:37:79:420#0/1
abbaW^``ab]`a`Z[M]PXa_`W]N_`VL_a_`YV^[[V
@HWI-EAS443:3:50:1766:1013#0/1
GTCCCGTTTCCGTTTCATTACCAACACTACGTCCCCTTGC
+HWI-EAS443:3:50:1766:1013#0/1
a``^Q_aaa[^a^]__aa^`¥¥]XW[^^`X]S¥TZVZTXS
@HWI-EAS443:3:55:510:1386#0/1
ATATCAACAGGAACAGCAGAGTGCTGTGGATGTTGNCGGT
+HWI-EAS443:3:55:510:1386#0/1
Q¥_XW_aab`_[W__TY_WR_V¥W``_`BBBBBBBBBBBB
@HWI-EAS443:3:83:358:603#0/1
CGTGGGTTGGTCTTGGCCAGACGGTGCTGAGTTGCCATTT
+HWI-EAS443:3:83:358:603#0/1
aaZ_]]NV[XS^_ZUY^_]UYZ[YFZ^[V[]LSRRU[SSZ
```

```
@HWI-EAS443:3:90:1018:533#0/1
CGTGGGTTGGTCTTGGCCAGACGGTGCTGAGTTGCCATTT
+HWI-EAS443:3:90:1018:533#0/1
aaaa^`ZabaabZbaa`aabaaa^[a_a][a[`aXYUXaa
@HWI-EAS443:3:44:162:552#0/1
GGCCAACCGAATTCTTTTGGTCGCTGTCTGGCTGGCAGTA
+HWI-EAS443:3:44:162:552#0/1
_V¥_]¥XU]W¥]_aU¥__WY]_`X`XM¥^RZTVTFTUVJF
@HWI-EAS443:3:55:1159:1619#0/1
CTAATTGATGGCCAACCGAATTCTTTTGGTCGCTGTCTGG
+HWI-EAS443:3:55:1159:1619#0/1
`bXXZ¥P_S`P`G`PZTOYV_[[PYTTN`G`a]PaHTV__
@HWI-EAS443:3:61:976:1947#0/1
CAATGCTTCAATCCAATGATCGTCGAGCTTGCGGAAAAAG
+HWI-EAS443:3:61:976:1947#0/1
abb]`aaabb`a``aaaabaaa_aaaa^^]Y^_^^Z]YY^
@HWI-EAS443:3:63:1487:519#0/1
TTGTGTATTCATCTTTTCAATAACAGAATTTACTTTGTTA
+HWI-EAS443:3:63:1487:519#0/1
aa`_a^_a`a_`^aa`_^¥_`^`_aa`^_^_^^a`_a¥_`
@HWI-EAS443:3:49:1637:1516#0/1
GAATCCCACCCAGGAATATTTGAGAATTCATGTCTTGAAA
+HWI-EAS443:3:49:1637:1516#0/1
a¥bbb`a¥a`baab_ababb`aaaaabaYaaa__aaa¥aa
@HWI-EAS443:3:37:1024:1756#0/1
GCTGGCTGGATCCTGGGAAATCCAGAGTGGGAATCACCCT
+HWI-EAS443:3:37:1024:1756#0/1
^_¥_a_¥¥XQWWRYXXZQ]X]_¥_[BBBBBBBBBBBBBBB
@HWI-EAS443:3:19:414:246#0/1
AACAGTGACCACAGAAGCTGCTTTTGGTCTGGTGTGTGCC
+HWI-EAS443:3:19:414:246#0/1
aaaaaU^aaa^aaabaaaV[¥_¥__^¥VUTZ^¥_SSN¥UZ
@HWI-EAS443:3:48:217:258#0/1
GCCCAATCATGACTCGAACAAAGGTGTAACGGCAGCGTGT
+HWI-EAS443:3:48:217:258#0/1
aa`a[aaTG``_`YYZT^[_^__QTHZ_`[_BBBBBBBBB
@HWI-EAS443:3:61:1724:651#0/1
CGCTGCCGTTACACCTTTGTTCGAGTCATGATTGGGCCAT
+HWI-EAS443:3:61:1724:651#0/1
ab^bb`bbbbb_a_^bbbababb`b`baab`a^b``U¥S_
@HWI-EAS443:3:53:15:852#0/1
GCCGGTCAAATATATTCAATATGGAGAGAATAAAAGAACT
+HWI-EAS443:3:53:15:852#0/1
a`L_[Vba¥¥__a_aaa``a^`^SO¥Z_R]a`a`a^[`[^
@HWI-EAS443:3:34:1157:1336#0/1
ATAAATCCCAATTACCTCATGGCTTGGAAGCAGGTTCTAA
+HWI-EAS443:3:34:1157:1336#0/1
abaaaa_`bb]a[[ST``TYKS`aWBBBBBBBBBBBBBBB
@HWI-EAS443:3:90:218:227#0/1
AGGTCTGGTGCCGCAGGTGCTGCGGTGGAAGGAGGTGGGA
```

```
+HWI-EAS443:3:90:218:227#0/1
V^¥GXaS^RZa_X¥X^`T`ZZ][S_BBBBBBBBBBBBBBB
@HWI-EAS443:3:60:1122:1090#0/1
ACTTCCCAGAAGGTCTGGTGCCGCAGGTGCTGCGGCGGAA
+HWI-EAS443:3:60:1122:1090#0/1
aa_aa]]¥ZY```Ua_[XXZR]`¥XS]P^`Z`QYWBBBBB
@HWI-EAS443:3:68:1690:1199#0/1
CTTCCCAGAAGGTCTGGTGCCGCAGGTGCTGCGGGGGAAG
+HWI-EAS443:3:68:1690:1199#0/1
]W[^[a[¥OG]]ZWSY¥GOVYLYNRVVMPPRF¥BBBBBBB
@HWI-EAS443:3:88:1783:574#0/1
ACTTCCCAGAAGGTCTGGTGCCGCAGGTGCTGCGGGGGAA
+HWI-EAS443:3:88:1783:574#0/1
Z¥a^Z[a^a_RT[QX¥]_M_[¥_V_¥LJSNYXJ[]MBBBB
@HWI-EAS443:3:99:1567:1320#0/1
AAGGTCTGGTGCCGCAGGTGCTGAGGTGGAAGGAGTTGGA
+HWI-EAS443:3:99:1567:1320#0/1
a]]aXab_aaaTL_U^^ZNHP_SGa_YaP¥_M^BBBBBBB
@HWI-EAS443:3:25:1712:1792#0/1
CTTCCCAGAAGGTCTGGTGCCGCAGGTGCTGCGGTGGAAG
+HWI-EAS443:3:25:1712:1792#0/1
abbaab¥`_bbaaabbb]b_]aababJb_aa¥a`SaHZ]T
@HWI-EAS443:3:45:366:12#0/1
ACTTCCCAGAAGGTCTGGTGCCGCAGGTGCTGCGGTGGAA
+HWI-EAS443:3:45:366:12#0/1
aT[_]_`_^U^`_XZ¥a]N]XLT]TM[DXZQY¥^XBBBBB
@HWI-EAS443:3:86:715:51#0/1
GCCGCAGGTGCTGCGGTGGAAGGAGTTGGAACAATAGCAA
+HWI-EAS443:3:86:715:51#0/1
aa]``_a]V`WPa]ZaUYGVU`[WVH[[VLVS]]ZVUVWT
@HWI-EAS443:3:78:1419:1406#0/1
ATTTCTTCGGAGACAATGCAGAGGAGTATGACAGTTGAGA
+HWI-EAS443:3:78:1419:1406#0/1
ababab``_U]a_abaabWb^`a_X[[aaTa]^YF`a``_
@HWI-EAS443:3:56:1381:747#0/1
GGGCCATGAACTTGTCTTAGGGAATATCTCAAACCTTTCA
+HWI-EAS443:3:56:1381:747#0/1
a¥OV^_¥`_`U`U`O`_a`XT_ZZ¥¥_OWU¥ZGZ_a__R¥
@HWI-EAS443:3:27:261:1972#0/1
GACCAAATGAAAACCCAGCTCACAAAAGCCAATTGGTGTG
+HWI-EAS443:3:27:261:1972#0/1
O__[aS¥QZ^R¥_a]a`V_U_`_[Z_L[H^S[abb_PY`Z
@HWI-EAS443:3:50:675:1423#0/1
AGAGCACTGACATTAAATACGATGACCAAAGACGCAGAAG
+HWI-EAS443:3:50:675:1423#0/1
`YQ]Yaabba`^bba]]aL^¥aaaaVT`aaZVa_S_^^O^
@HWI-EAS443:3:69:569:1313#0/1
TCATTGTTCCCGCTGAATGCTGCCATAACGGTTGCCCTTT
+HWI-EAS443:3:69:569:1313#0/1
a``_`_`ab`^`baaabWZ_X`aVOU_O^]]XV¥TDW¥]T
```

@HWI-EAS443:3:14:629:1843#0/1
TCACCATCCACCTACTAGTGCTGACCAACAAAGTCTCTAT
+HWI-EAS443:3:14:629:1843#0/1
abbaaa_`aaaa_b`aaYKPaa_aW`aa_a`a^XS`__`_
@HWI-EAS443:3:83:300:1272#0/1
GGGCCTTCTACGGAAGGAGTGCCTGAGTCCCTGAGGGAAG
+HWI-EAS443:3:83:300:1272#0/1
_]`aa`aa`[a_V^`[]X`[^aa_]V]U^[HY^W`^ZNLX
@HWI-EAS443:3:99:1299:569#0/1
CAGCGATTCAAGTGATCCTCTTGTCNTTGCAGCAAATATC
+HWI-EAS443:3:99:1299:569#0/1
U_`¥_¥aS`¥¥OX_a`]_a]]M[OYD^_]¥__LUSYU]IX
@HWI-EAS443:3:14:491:1767#0/1
AAGGGAAAGTGGACGACAAACACAGAGACTGGTGCCCATA
+HWI-EAS443:3:14:491:1767#0/1
^T`_`X]]aUaaaSba[X_a]aba`[_^_^^_U``W`^Z^
@HWI-EAS443:3:34:1093:607#0/1
GGGTTTTTTATCATTAAATAAGCTGAAACGAGAAAGCTCT
+HWI-EAS443:3:34:1093:607#0/1
¥JYQU^^_U_b]Z`_TT_ZQVW¥Ya`SPR]¥¥T^MWPZYB
@HWI-EAS443:3:70:1558:791#0/1
GGGGTTTTTTATCATTAAATAAGCTGAAACGAGAAAGCTC
+HWI-EAS443:3:70:1558:791#0/1
abIbYVaaab`]W_aa_a```aV_aY[aWaa__]¥S`^_]
@HWI-EAS443:3:52:1586:2023#0/1
ATTGTTTCTCCAACTCTTGATAGTGTCTGGTTTTATGGCG
+HWI-EAS443:3:52:1586:2023#0/1
```b]`ZTSXEVaZ]^^aZ`Y]a^BBBBBBBBBBBBBBBB
@HWI-EAS443:3:59:336:799#0/1
TGTTTCTCCAACTCTTGATAGTGTCTGGTATTATTTCGTT
+HWI-EAS443:3:59:336:799#0/1
aa_aa`aaa`aaaaaaaaaaa_a_aaBBBBBBBBBBBBBB
@HWI-EAS443:3:93:508:1385#0/1
AATATATTGTTTCTCCAACTCTTGATAGTGTCTGGTTTTA
+HWI-EAS443:3:93:508:1385#0/1
`Z^¥]Zbab_a`a`aa_V[V`_```ZX`[a]aBBBBBBBB
@HWI-EAS443:3:66:795:1835#0/1
GATAGTGTCTGGTATTATGCCGTTGTACTTTAACAAAGCC
+HWI-EAS443:3:66:795:1835#0/1
abaa^[a^b_¥NMVM[TNHW^WLUSXVHV[ZPYD¥FSGOV
@HWI-EAS443:3:95:429:696#0/1
CAATATATTGTTTCTCCAACTCTTGATAGTGTCTGGTTTT
+HWI-EAS443:3:95:429:696#0/1
]Tabb_V¥T^H¥[b^[VWWY`Y`Y[¥VWIRZU_^BBBBBB
@HWI-EAS443:3:100:496:172#0/1
TCTCAATATATTGTTTCTCCAACTCTTGATAGTGTCTGGT
+HWI-EAS443:3:100:496:172#0/1
aaa`b`ba_a^_bPYa`Ya`_¥Va¥`aa_^a`[aBBBBBB
@HWI-EAS443:3:10:998:1149#0/1
CTCAATATATTGTTTCTCCAACTCTTGATAGTGTCTGGTA
```

```
+HWI-EAS443:3:10:998:1149#0/1
abbbbbbbbbbbbabbabaaab_aaaaaaaaa]`^_BBBBB
@HWI-EAS443:3:14:742:1151#0/1
AATATATTGTTTCTCCAACTCTTGATAGTGTCTGGTATTA
+HWI-EAS443:3:14:742:1151#0/1
_aa_``aaaR_a___a_a`Y`_`¥_^`a¥^^BBBBBBBBB
@HWI-EAS443:3:57:1025:401#0/1
GTTTCTCCAACTCTTGATAGTGTCTGGTATTATGCCGTTG
+HWI-EAS443:3:57:1025:401#0/1
a`aaabaaaa`aaaaaa`aa^a`_`BBBBBBBBBBBBBBB
@HWI-EAS443:3:86:619:1052#0/1
ATATTGTTTCTCCAACTCTTGATAGTGTCTGGTATTATGC
+HWI-EAS443:3:86:619:1052#0/1
ababbaa`aaaa_aaaaaaaaa_``]__aaBBBBBBBBBB
@HWI-EAS443:3:36:1098:916#0/1
GGGCCCCTTGAGTCAAGAAGAAGGTTCTGCATTCCAAGGG
+HWI-EAS443:3:36:1098:916#0/1
¥a_b`bb]b_S_^bab`abbabaaRaa_aa[`_`aZ_a``
@HWI-EAS443:3:62:139:1094#0/1
GGATTTCTCATTTTGGGCAAAGAAGACACGAGATGGCCCA
+HWI-EAS443:3:62:139:1094#0/1
_]]^]aba`¥a`a]Z_W`]P_]¥^[ZaXH[V¥_Z^Y_SVF
@HWI-EAS443:3:21:427:1763#0/1
GAGAGGATTTCTCATTTTGGGCAAAGAAGACACGAGATAT
+HWI-EAS443:3:21:427:1763#0/1
UHUS¥T`bYb_VXURa]¥RTb`bWabbaaZM_HH¥`a`UP
@HWI-EAS443:3:71:791:1012#0/1
GCATCAATGAACTGAGCAATCTTGCAAAAGGAAAAAAAGG
+HWI-EAS443:3:71:791:1012#0/1
a__¥W`Z]`baVZ_Z_^^OS_¥_aa`Y^W[_XSXa`_ZX¥
@HWI-EAS443:3:68:1486:1320#0/1
ATTAAGCATCAATGAACTGAGCAATCTTGCAAAAGGAAAG
+HWI-EAS443:3:68:1486:1320#0/1
_``aa]]`a_`_aa_^¥`a]_``R`P`_`]Z^___aXX]`
@HWI-EAS443:3:81:952:241#0/1
TGCGAGAGTCCCGTTTTCGTTTCATTACCAACACTACGNC
+HWI-EAS443:3:81:952:241#0/1
ababZb¥a_^[`bWaba`b^aa_aaaa]_a_¥a^_a_UDK
@HWI-EAS443:3:37:318:565#0/1
GTTGGTAATGAAACGAAAACGGGACTCTCGCATACTTACT
+HWI-EAS443:3:37:318:565#0/1
a^aaa^_a`a_]``_^``]X_^QU^]]YHW]^X_[_WUW]
@HWI-EAS443:3:92:530:1678#0/1
ATCGACGAACAGGGTGAATCAATAATTGTAGAATCTGGTG
+HWI-EAS443:3:92:530:1678#0/1
aUZWZaa`¥FY_``Ob`__`^V__W`_aN_¥]_]_Y]XX^
@HWI-EAS443:3:40:733:1882#0/1
GTGCTGACCAACAAAGTCTCTATCAGCATGCAGATGCATA
+HWI-EAS443:3:40:733:1882#0/1
a]¥G]_`^`a_S`_`^U[OaW`W[]OFYWOO`SPMR_WUY
```

@HWI-EAS443:3:11:441:1119#0/1
AGCGAAAGCAGGCCAATTATATTCAATATGGAGAGAATAA
+HWI-EAS443:3:11:441:1119#0/1
a``_Z_aZZ¥`]L_aUF_a[aP`aaOa]VLXJVUaaTPaa
@HWI-EAS443:3:62:1264:420#0/1
GCCTCATATACAACCGGATGGGAACAGTGACCACAGAAGC
+HWI-EAS443:3:62:1264:420#0/1
`aabaabaaa`a`¥H`a]_aaa`aV]WZ`aa[_Z^`^]`_
@HWI-EAS443:3:30:1652:287#0/1
CTCTCATGGAATGGCTAAAGACAAGACCAATCTTGTCATC
+HWI-EAS443:3:30:1652:287#0/1
]QbaaX^^`a_bbbZbbaabbb`b_b`[Y`aabb_XPZK[
@HWI-EAS443:3:60:1171:1068#0/1
AACAAATCGTGGAATGGATATTGAAAGAGGAATCCAGCGA
+HWI-EAS443:3:60:1171:1068#0/1
aXPYa_RPbPYU`[U`QPNGL_YR]X¥]X[XUYXBBBBBB
@HWI-EAS443:3:35:732:446#0/1
CGGCTCTACTAGTGTCCAGTAATAGTTCCTTCTCCCCTCT
+HWI-EAS443:3:35:732:446#0/1
a_b_aa¥¥O[]]NZLY`S`GZX[Z¥Y^ZHT`^Y[`^FRJP
@HWI-EAS443:3:28:1120:53#0/1
GGGGAGAATGAACTATTACTGGACACTAGTAGAGCCGGGA
+HWI-EAS443:3:28:1120:53#0/1
]a¥[Yb_aab`aa`a_a`ab```^^Za^__`a_a¥Zb¥_X
@HWI-EAS443:3:20:433:702#0/1
CGTCAATCGCATTCTGTGTGCTCTTCAGGTTGGCTGCATA
+HWI-EAS443:3:20:433:702#0/1
aaa`_X]^__]]W`_a^_RZZ^Z`_V[VZ¥¥_^_NZNX¥a
@HWI-EAS443:3:37:644:1801#0/1
ATCGCATTCTGTGTGCTCTTCAGGTCGGCTGCATATCCTT
+HWI-EAS443:3:37:644:1801#0/1
U_HXbbbabXS]_Qbb_¥X¥b`RNOb]Rb¥`T]PaS]aTH
@HWI-EAS443:3:30:667:1978#0/1
ATCGCATTCTGTGTGCTCTTCAGGTCGGCTGCATATCCTG
+HWI-EAS443:3:30:667:1978#0/1
]]R_bbR`a`WH_Xab^ba_SaZ_GaZY`^abaaaSa_NV
@HWI-EAS443:3:70:947:1189#0/1
AGCCTTGCTAAATGACAAACATTCCAATGGAACCATTAAA
+HWI-EAS443:3:70:947:1189#0/1
Q___ZbT`YTQ¥__bb`_^¥_`_abaTY]¥_Z^a_`a^UP
@HWI-EAS443:3:73:263:1939#0/1
ACCTGGAGCTCTTCTTTCTTCTCTTTTGTTATTGCCATCT
+HWI-EAS443:3:73:263:1939#0/1
Y¥aOQ[^`aX¥ZHa]¥_a]XS_a`^_U_S]Q¥WMMTTFPV
@HWI-EAS443:3:48:277:1195#0/1
CAAGAAACGGTTTTGAGATGATTTGGGATCCAAACGGATG
+HWI-EAS443:3:48:277:1195#0/1
abaa_a`a[_Ra_`b[^]`a`^a_X]`^TZ^_^aYa_]_[
@HWI-EAS443:3:92:1684:1027#0/1
GGACCAGCATTAAGCATCAATGAACTGAGAAATCTTGCAA

```
+HWI-EAS443:3:92:1684:1027#0/1
``K^¥`bVa_bbabba_baXbb¥aab[aXJb_aabTbY[`
@HWI-EAS443:3:20:1416:896#0/1
AAGAGATATGGCCCAGCATTAAGCATCAATGAACTGAGAA
+HWI-EAS443:3:20:1416:896#0/1
¥UPZbaYY[VVa`__[aaaZZT[WUaYYXX]_¥I^UWXFW
@HWI-EAS443:3:19:1150:1375#0/1
TTAAGCATCAATGAACTGAGCACTCTTGCAAGAGGAGAGA
+HWI-EAS443:3:19:1150:1375#0/1
a`_]_Q[UZ[RV```]TYXZ_XFP^U`a^LOFJ`a]a^a^
@HWI-EAS443:3:11:263:192#0/1
GCATCAATGAACTGAGCAATCTTGCAAGAGGAGAGAAAGG
+HWI-EAS443:3:11:263:192#0/1
`a[R¥SH``__^YTNa¥]GY]]LZX[ODH]`U¥W¥]UBBB
@HWI-EAS443:3:67:772:319#0/1
GCATCAATGAACTGAGCAATCTTGCAAGAGGAGAGAAAGC
+HWI-EAS443:3:67:772:319#0/1
a`VU`WXaW`b`aZ]Ya``a_aaZWVRFSa`]aaaa_I¥Q
@HWI-EAS443:3:72:852:678#0/1
CAGCATTAAGCATCAATGAACTGAGCAATCTTGCAAGGGG
+HWI-EAS443:3:72:852:678#0/1
`bbbbbbbbbbbababbbbaba]]bab`aabab`[_bbaa
@HWI-EAS443:3:34:1025:314#0/1
GCATTAAGCATCAATGAACTGAGCAATCTTGCAAGAGGAG
+HWI-EAS443:3:34:1025:314#0/1
abbbabbbabababbbabaaaab`a`aaaaa[a_Q_a_^`
@HWI-EAS443:3:59:444:173#0/1
CAGCATTAAGCATCAATGAACTGAGCAATCTTGCAAGAGG
+HWI-EAS443:3:59:444:173#0/1
^T¥^WaXaa`^¥]a__ba_]a¥W_`_a_a_`^^^^LGL_]
@HWI-EAS443:3:21:1765:632#0/1
TGTCTTTAGCCATTCCATGAAAGCCTCAAGATCTGTGTTC
+HWI-EAS443:3:21:1765:632#0/1
[^^_aaaa]]Y_aVQM___OFYWXX^[ZVXGWN^_VXXXV
@HWI-EAS443:3:99:907:1969#0/1
ATAGGTTTTTACTCTAGCTCTATGTTGACAAAATGACCAT
+HWI-EAS443:3:99:907:1969#0/1
a¥b`IYbbababbS`b`bbaa`a^`ba``bbbbaab[Vaa
@HWI-EAS443:3:45:1420:1601#0/1
GGTTTTTACTCTAGCTCTATGTTGACAAAATGACCATCGT
+HWI-EAS443:3:45:1420:1601#0/1
ab`bbbbbab`bba`b_babaabaa_aaaaaaa^V`a^_Z
@HWI-EAS443:3:18:1405:1025#0/1
TTCTCGCCACTTTTCATTTCTGCTCTGGAGGTAGTGAAGG
+HWI-EAS443:3:18:1405:1025#0/1
¥_`XJ]WF¥Wb_a`URZYN_¥YR]Y[b_Y__Y[`BBBBBB
@HWI-EAS443:3:31:839:70#0/1
CTTCACTACCTCCAGAGCAGAAATGAAAAGTGGCGAGAAC
+HWI-EAS443:3:31:839:70#0/1
^abbb]``babb``abbbbabVbbbbbbab`baabaa`_U
```

@HWI-EAS443:3:96:1611:558#0/1
GTTCTCGCCACTTTTCATTTCTGCTCTGGAGGTAGTGAAG
+HWI-EAS443:3:96:1611:558#0/1
a[`[_`[U`]Z_`aaT`a`]^`]X`N]RVZ]]Z[PUBBBB
@HWI-EAS443:3:95:1175:977#0/1
CTGGGGTCTGAGTTCTTCAATGGTGGAACAGATCTTCATG
+HWI-EAS443:3:95:1175:977#0/1
a`bb_`a^ab`bababbbaa_a``baaaaaa`aaba_``a
@HWI-EAS443:3:51:888:227#0/1
TGGGGTCTGAGTTCTTCAATGGTGGAACAGATCTTCATGA
+HWI-EAS443:3:51:888:227#0/1
]`¥aaaabb`bbbbbbbbabbbWbbba_bb[aabbaabba
@HWI-EAS443:3:76:1353:439#0/1
GGTCTGAGTTCTTCAATGGTGGAACAGATCTTCATGATCT
+HWI-EAS443:3:76:1353:439#0/1
QQ¥^¥[ZZaaS`_¥aa`b__^a]aJa¥V[`[[^_ba^YF_
@HWI-EAS443:3:61:1032:1194#0/1
GGGCCCCCTCAAAGCCGAGATCGCGCAGAGACTGGAAAGT
+HWI-EAS443:3:61:1032:1194#0/1
`]UXV¥``_]ZURZV]]R^YX_^_``UZVZZ¥UU[URXSQ
@HWI-EAS443:3:43:1574:1694#0/1
CAACAGAATGGGAACAGTGACCACAGAAGCTGCTTTTGGT
+HWI-EAS443:3:43:1574:1694#0/1
``XZa``abbbb[_Zbb_b_S¥_abbba]a`VH_aaa]`X
@HWI-EAS443:3:75:99:1541#0/1
CCTCTCTGGAATCATATCCATTATTCTCTTGTCTGCTGTA
+HWI-EAS443:3:75:99:1541#0/1
`aabab_aa[]aaaaaaaaaa_aaaaaa``__a``a_[Z_
@HWI-EAS443:3:21:1233:717#0/1
TCCCAATTGCTCTCGCCATTTTTCATTTCTGCTCTGGAGG
+HWI-EAS443:3:21:1233:717#0/1
`a]¥TI^a`N[RRNQRNNLLL^YZTFX[GSYF¥^`¥W[BB
@HWI-EAS443:3:76:1686:435#0/1
GCCGATCAAGGAAAGGGGCATCACCCAATCCATTGTCTGC
+HWI-EAS443:3:76:1686:435#0/1
a__aS_SZ^YTQLDS¥_^M[YV_UWXVV¥WWUUSVWVUVS
@HWI-EAS443:3:98:503:19#0/1
CGAGAGCAATTGGGACAGAAATTTGAGGAGATAAGGTGGT
+HWI-EAS443:3:98:503:19#0/1
_b^``baa_aa_`^`Xbba¥aabbbYbbVD]ba_abOb^M
@HWI-EAS443:3:89:414:1071#0/1
GCCGCACTTCTTCAATTAACCACCTCATTTCCTCAAATTT
+HWI-EAS443:3:89:414:1071#0/1
aa¥Q¥_QJT__YaT_Y_`ZYM¥__[ZP_`^^Z]Z^WV__¥
@HWI-EAS443:3:25:1035:547#0/1
GCGGCACAGATTGAAAGCAACAGAGAATAGTTTCGAACAA
+HWI-EAS443:3:25:1035:547#0/1
aaaa`aY__`_]`a_`_[]_^_`¥_¥]_^^[^^Y]Z`S]_
@HWI-EAS443:3:26:940:1695#0/1
GCCGCACTTCTTCAATTAACCACCTTATTTCCTCAAATTT

```
+HWI-EAS443:3:26:940:1695#0/1
a`]Y^¥S_YY_[XLTYa_``XVZI^Y___U¥_PT]VZ¥]X
@HWI-EAS443:3:67:1595:1051#0/1
TCGAGGATTTTGATTTTCATTCCATTTAGTGTTGTCCCCA
+HWI-EAS443:3:67:1595:1051#0/1
a`aaaa`abbababbba`aa`_a`aaaaa^aa`____]__
@HWI-EAS443:3:70:1356:188#0/1
CTTGTGAATTAGTCATCATCTTTCTCACAACATTTGCTAG
+HWI-EAS443:3:70:1356:188#0/1
Y_^^`baXa`¥`_^]`I__X]aa^aX^b¥`U¥^aaaZDVa
@HWI-EAS443:3:58:1102:690#0/1
AAAAGTCAATTGGTNTGGATGGCATGCCACTCTGCTGCAT
+HWI-EAS443:3:58:1102:690#0/1
aab```aaaaaabWDXaaZ]`_YPX_]_]WZ^UWKR^VW[
@HWI-EAS443:3:50:1252:1426#0/1
GAATGATTGGTGGGATCGGGAGATTCTACATCCAAATGTG
+HWI-EAS443:3:50:1252:1426#0/1
aXRXbY]]baQb[DXb`¥¥aXaG^¥_]_¥¥]Y]LY[Z[BB
@HWI-EAS443:3:50:925:180#0/1
GATTGCTTCAAATGAGAATGTGGAAGCCATGGACTCCAAT
+HWI-EAS443:3:50:925:180#0/1
`bbbbabbbbbabbbbbabbabbab`]_aaaa^V`_¥`a`
@HWI-EAS443:3:98:1109:1545#0/1
CAATGACCGAAATTTCTGGAGGGGTGAAAATGGACGAAGA
+HWI-EAS443:3:98:1109:1545#0/1
a_abbba`bab`bbb`b`b`bbabYbaabba]abaababb
@HWI-EAS443:3:12:1546:486#0/1
GATTGGTGGTAGTAGCCATTTGTCTGTGGGACCGATGGTG
+HWI-EAS443:3:12:1546:486#0/1
a`aaab^b`Q`UZV_^V[```HUSYXUUFXHMTZZBBBBB
@HWI-EAS443:3:94:1455:1657#0/1
GATGGCTGGATCGAGTGAACAGGCAGCAGAGGCAATGGAG
+HWI-EAS443:3:94:1455:1657#0/1
^_¥^`bb`]`aaaaZ_X]]R_N_¥WOFX_^U¥BBBBBBBB
@HWI-EAS443:3:50:678:1765#0/1
ACCGAATTTCTCCAGCCTTGAAAACTTTAGAGCCTATGTA
+HWI-EAS443:3:50:678:1765#0/1
_HWX`[HRXVba^`Sa^`MMSW`X]aRX_aR¥DDIa_][a
@HWI-EAS443:3:54:859:614#0/1
GTGGCTCCAGTCTCGGGTCAGTGAGGGAGAACTCCATACT
+HWI-EAS443:3:54:859:614#0/1
`¥`a__`]OYVVX[W`BBBBBBBBBBBBBBBBBBBBBBBB
@HWI-EAS443:3:32:424:1838#0/1
ACCATATNGCCATAATCAAAAAGTACACACCAGGAAGACA
+HWI-EAS443:3:32:424:1838#0/1
aabaaa¥DUaaa[_a`aaaaaa[]a]aaaYaaXPZ^Y_a^
@HWI-EAS443:3:26:274:61#0/1
TGTCTTCCTGATGTGTACTTTTTGATTTGGCCATATGGTC
+HWI-EAS443:3:26:274:61#0/1
_Y_WQV_^PLRW]LUMOI[_TTO^BBBBBBBBBBBBBBBB
```

```
@HWI-EAS443:3:84:836:1314#0/1
GTCTTCCTGATGTGTACTTTTTGATTATGGCCAAATGGCC
+HWI-EAS443:3:84:836:1314#0/1
`^aaZ[T^L[ZX^WS¥OVY[VU]X_`]ZZS[^VG¥SSKDV
@HWI-EAS443:3:12:434:198#0/1
ATATGGCCATAATCAAAAAGTACACATCAGGAAGAAGTGG
+HWI-EAS443:3:12:434:198#0/1
aaa^`aaaaaa_a]__`___^`^^U^^^]__^__WZ_X_^
@HWI-EAS443:3:56:861:1171#0/1
ATATGGCCATAATCAAAAAGTACACATCAGAAAGACAAGC
+HWI-EAS443:3:56:861:1171#0/1
^_ba`aabaa`¥b`Z_baba]b^aa`b^a_abbba^aaba
@HWI-EAS443:3:17:245:788#0/1
GTCTTCCTGATGTGTACTTTTTGATTATGGCCATATTGTC
+HWI-EAS443:3:17:245:788#0/1
_X``abb]_aa_Za[aaa`a`aa_a[SXaaa__QYJF]Q^
@HWI-EAS443:3:2:1550:533#0/1
GACCGTCTTCCTGATGTGTACTTTTTGATTATGGCCATAT
+HWI-EAS443:3:2:1550:533#0/1
]S``ba_]^^`[_]OS]_¥¥_aa_aa`____Y`BBBBBBB
@HWI-EAS443:3:79:1325:1655#0/1
GTCTTCCTGATGTGTACTTTTTGATTATGGCCATATTGTC
+HWI-EAS443:3:79:1325:1655#0/1
^¥_^```Y¥ZU_O_HQWX_a^a^_abaa`]X_[T¥VHN[Z
@HWI-EAS443:3:100:964:1363#0/1
CGGGGGTCTTTTCTTGTCTTCCTGATGTGTACTTTTTGAT
+HWI-EAS443:3:100:964:1363#0/1
]bb_^DZ``]R]_]^_KX`a_^`baVH¥^RaT`KZ_`aBB
@HWI-EAS443:3:15:1769:483#0/1
GGACCATATGGCCATAATCAAAAAGTACACATCACGAAGA
+HWI-EAS443:3:15:1769:483#0/1
QYa]a_bbY``_aXV]`a]aWY¥X_R¥X`WUX^RT]U]`Y
@HWI-EAS443:3:20:971:183#0/1
CATATGGCCATAATCAAAAAGGACACATCAGGAAGACAAG
+HWI-EAS443:3:20:971:183#0/1
a_`¥ZMO^¥S]YaUUP__XMVOY_W]]]US^[UW`]UV[_
@HWI-EAS443:3:27:1231:1136#0/1
CGGGGTTCTTATCTTGTCTTCCTGATGTGTACTTTTTGAT
+HWI-EAS443:3:27:1231:1136#0/1
ab_]NH`b`^H]X]Yb¥`_]¥W[YYZaWYPW]`Y_a`]`[
@HWI-EAS443:3:27:1760:131#0/1
CATATGGCCATAATGAAAAAGTACACATCAGGAAGACAAG
+HWI-EAS443:3:27:1760:131#0/1
^XTXX]bYTa`_¥YLV_aTaaY_UZFVQ[R¥ZPPWXG]BB
@HWI-EAS443:3:31:1635:99#0/1
CATATGGCCATAATCAAAAAGTACACATCATGAAGACAAG
+HWI-EAS443:3:31:1635:99#0/1
`ba_abbbWbbX¥`a¥a`_`aaaa_¥a`_WH]aaa`]aaN
@HWI-EAS443:3:35:1215:1968#0/1
GACCATATGGCCATAATCAAAAAGGACACATCAGGAAGAC
```

+HWI-EAS443:3:35:1215:1968#0/1
¥WT¥ZY¥SON[a`UX_TYZ[X]VQG_a]aa¥^_`a__]P[
@HWI-EAS443:3:53:976:754#0/1
ATATGGCTATAATCAAAAAGTACACATCAGGAAGACAAGA
+HWI-EAS443:3:53:976:754#0/1
ababbbaba`_b`]`baba[WaWa_aaXabb[aa__aa¥]
@HWI-EAS443:3:60:1505:1987#0/1
CATATGGCCATAATCAAAAAGAACACATCAGGAAGACAAG
+HWI-EAS443:3:60:1505:1987#0/1
X¥T`bbbYGZ`abb]ababVaG_T_Z^HHbbaa[a`Paa^
@HWI-EAS443:3:7:513:888#0/1
CGGGGTTCTTGTCTTGTCTTCCTGATGTGTACTTTTTGAT
+HWI-EAS443:3:7:513:888#0/1
`_¥NHSaa[KVbbbbb`aVaX]a`_WaKINXGa__[SIa_
@HWI-EAS443:3:8:1739:214#0/1
CGGGGTTCTTGTCTTGTCTTCCTGATGTGTACTTTTTGAT
+HWI-EAS443:3:8:1739:214#0/1
aa_W^Oa^a`Ja[aab¥^baVN^ZQ_a_aY^]`a`_]RTa
@HWI-EAS443:3:91:1595:451#0/1
CATATGGCCATAATCAAAAAGTACAAATCAGGAAGACAAG
+HWI-EAS443:3:91:1595:451#0/1
¥b¥_bb`[UaK`_b^aaaVab[`VaF_a^a¥U¥`aa^^`_
@HWI-EAS443:3:99:480:552#0/1
ACCATCTGGCCATAATCAAAAAGTACACATCAGGAAGACA
+HWI-EAS443:3:99:480:552#0/1
a]VX¥JN_^T`_a¥¥`¥XYXX_^J[Y][UO`[^_^_]WP[
@HWI-EAS443:3:16:244:2030#0/1
GTCTTCCTGATGTGTACTTTTTGATTATNGCCATATGGTC
+HWI-EAS443:3:16:244:2030#0/1
a_`][Z]SSWZ`VaMZa`__^____[aQDO`_BBBBBBBB
@HWI-EAS443:3:32:1512:725#0/1
ATATGGCCATAATCAAAAAGTACACATCNGGAAGACAAGA
+HWI-EAS443:3:32:1512:725#0/1
aaba[`_aba^baa`aaaaaZbaa_aaTDU]]a__Ta`a_
@HWI-EAS443:3:20:720:1181#0/1
GTCTTCCTGATGTGTACTTTTTGATTATGGCCATATGGGC
+HWI-EAS443:3:20:720:1181#0/1
a_`aaZ]a`]UY_X¥U¥_____``_]_]LZ^[]W]SW¥GN
@HWI-EAS443:3:23:1367:28#0/1
ACCATATGGCCATAATCAAAAAGTACACATCAGGAAGAAA
+HWI-EAS443:3:23:1367:28#0/1
V`_R``a^`Ja`_YPYRZaa`]_Z_^UDX`U`[`][`XM¥
@HWI-EAS443:3:2:588:521#0/1
ACCATATGGCCATAATCAAAAAGTACACATCAGGAAGAAA
+HWI-EAS443:3:2:588:521#0/1
^aaY`Z^aa_N]^aaZJZa¥_Q_U`Y]R]SU_aa]``UG[
@HWI-EAS443:3:67:675:341#0/1
ACCATATGGCCATAATCAAAAAGTACACATCAGGAAGAAG
+HWI-EAS443:3:67:675:341#0/1
a_aaaa]]`W[^Y^^¥[[`__`¥U^Y__[O]^^¥QY_¥U]

```
@HWI-EAS443:3:87:1725:1005#0/1
ATGGCCATAATCAAAAAGTACACATCAGGAAGACAAGATA
+HWI-EAS443:3:87:1725:1005#0/1
¥a^`J¥^XZb]O[b[`Z¥N`M^X][QY¥]Ya¥VX_a`_HW
@HWI-EAS443:3:14:619:225#0/1
CGTCTTCCTGATGTGTACTTTTTGATTATGGCCATATGGT
+HWI-EAS443:3:14:619:225#0/1
_]]aaaaa`]¥Z_VaXY]a`a_]U_U]_XRSZ¥WWURHVB
@HWI-EAS443:3:40:1712:850#0/1
TTCTTCCTGATGTGTACTTTTTGATTATGGCCATATGGTC
+HWI-EAS443:3:40:1712:850#0/1
aaaaaa`aa``a]a_]_aa^_aa`_¥Za`UYX[YSURYPQ
@HWI-EAS443:3:87:130:1435#0/1
TGTCTTCCTGATGTGTACTTTTTGATTATGGCCATATGGG
+HWI-EAS443:3:87:130:1435#0/1
`a^`]`a`__]]YVYY]`a`aaaa``_^____aWBBBBBB
@HWI-EAS443:3:100:1202:479#0/1
ACCATATGGCCATAATCAAAAAGTACACATCAGGAAGACA
+HWI-EAS443:3:100:1202:479#0/1
a`^aba`aa^`¥aa``_`a___a]`X^S_¥W`[aX¥UBBB
@HWI-EAS443:3:100:1354:1721#0/1
ATATGGCCATAATCAAAAAGTACACATCAGGAAGACAAGA
+HWI-EAS443:3:100:1354:1721#0/1
aaa`b`aIb_aa_P_a_^`aS[K[P[KZ_aZXS^LH[WBB
@HWI-EAS443:3:11:515:1867#0/1
GACCATATGGCCATAATCAAAAAGTACACATCAGGAAGAC
+HWI-EAS443:3:11:515:1867#0/1
T^``^`bbb`aab`bb`Z`bbaVXS_U]Sa[G_baaaa^U
@HWI-EAS443:3:12:134:599#0/1
GGACCATATGGCCATAATCAAAAAGTACACATCAGGAAGA
+HWI-EAS443:3:12:134:599#0/1
Y`aba__¥P[bb]aa¥ZZ``_S¥abGN^^_PT_^QS`aa[
@HWI-EAS443:3:14:820:1386#0/1
ACCATATGGCCATAATCAAAAAGTACACATCAGGAAGACA
+HWI-EAS443:3:14:820:1386#0/1
`aa^aa_^X_aa_^____`__]_Wa]`¥^^]][^_^^XW_
@HWI-EAS443:3:15:1367:285#0/1
GTCTTCCTGATGTGTACTTTTTGATTATGGCCATATGGTC
+HWI-EAS443:3:15:1367:285#0/1
^aabbabbbabbabaa_abbbbbaabba`a`_aYa¥UaUY
@HWI-EAS443:3:15:372:1757#0/1
GTCTTCCTGATGTGTACTTTTTGATTATGGCCATATGGTC
+HWI-EAS443:3:15:372:1757#0/1
a`bbbbaaaaa`aa^aaaaaaaaa_a`a_`aaa^`OOXZ`
@HWI-EAS443:3:15:384:368#0/1
CATATGGCCATAATCAAAAAGTACACATCAGGAAGACAAG
+HWI-EAS443:3:15:384:368#0/1
a¥abaaaabb`b`aa`a`baaXaaa___S``a^aa`aaaa
@HWI-EAS443:3:16:1731:1311#0/1
ATATGGCCATAATCAAAAAGTACACATCAGGAAGACAAGA
```

+HWI-EAS443:3:16:1731:1311#0/1
`a`]R]Q]aa`^[ZUY`a^XYT`b]T^Zaa```]¥[Z¥
@HWI-EAS443:3:16:911:743#0/1
CATATGGCCATAATCAAAAAGTACACATCAGGAAGACAAG
+HWI-EAS443:3:16:911:743#0/1
aa`aa`_bbb_a[b_`ZZ^aa_a¥][`a_^S^Yaaa_ZP_
@HWI-EAS443:3:17:527:1859#0/1
CATATGGCCATAATCAAAAAGTACACATCAGGAAGACAAG
+HWI-EAS443:3:17:527:1859#0/1
¥XQS]]¥aa`ZaYT^^`__`[T¥Z^`a__]`]``__^_`_
@HWI-EAS443:3:18:1044:619#0/1
GTCTTCCTGATGTGTACTTTTTGATTATGGCCATATGGTC
+HWI-EAS443:3:18:1044:619#0/1
``abaabbbabbaaaaabbbabaaaaaaa`_`aX_¥[[VZ
@HWI-EAS443:3:18:748:485#0/1
GTCTTCCTGATGTGTACTTTTTGATTATGGCCATATGGTC
+HWI-EAS443:3:18:748:485#0/1
a_a`aaaa]`YP`YRUTa_X`_`]_^]]_Z^_]R]VWNPS
@HWI-EAS443:3:19:1492:1547#0/1
CATATGGCCATAATCAAAAAGTACACATCAGGAAGACAAG
+HWI-EAS443:3:19:1492:1547#0/1
aabbbbbbabaaaaaabbbbb¥baaaaaa``aabaaaaaa
@HWI-EAS443:3:1:907:358#0/1
GACCATATGGCCATAATCAAAAAGTACACATCAGGAAGAC
+HWI-EAS443:3:1:907:358#0/1
aaaabba`bba_ba``a]`_aa`aWa^[FV_S_`¥]a`BB
@HWI-EAS443:3:1:940:1416#0/1
CATATGGCCATAATCAAAAAGTACACATCAGGAAGACAAG
+HWI-EAS443:3:1:940:1416#0/1
`Q`]aaWHaba`Ra_]`aaaZTZ_`S_^]_aaSaa_`aaB
@HWI-EAS443:3:20:155:1241#0/1
CATATGGCCATAATCAAAAAGTACACATCAGGAAGACAAG
+HWI-EAS443:3:20:155:1241#0/1
aa¥aaaab`[X^a`aaXZa`^R_Za¥T]__`_]a`^_^`]
@HWI-EAS443:3:21:1533:632#0/1
CATATGGCCATAATCAAAAAGTACACATCAGGAAGACAAG
+HWI-EAS443:3:21:1533:632#0/1
a_YPZ_W_]TYU]¥KV`__S`UaWTFSUXW¥¥VW__^^X]
@HWI-EAS443:3:22:606:682#0/1
CATATGGCCATAATCAAAAAGTACACATCAGGAAGACAAG
+HWI-EAS443:3:22:606:682#0/1
aZ`aa^V¥^¥WZ¥WY^^ZRW]V`^`__[Z[^^_Z^UX]]_
@HWI-EAS443:3:24:224:713#0/1
CATATGGCCATAATCAAAAAGTACACATCAGGAAGACAAG
+HWI-EAS443:3:24:224:713#0/1
a`¥^_aa_abXFQaRX[^[aVJR__a__^_^]aYI`^___
@HWI-EAS443:3:25:1679:1024#0/1
CATATGGCCATAATCAAAAAGTACACATCAGGAAGACAAG
+HWI-EAS443:3:25:1679:1024#0/1
[Qa__bb[[``_a`bbbZb`_Xb]`bbbV`a`bbKaYa`T

```
@HWI-EAS443:3:26:464:854#0/1
CATATGGCCATAATCAAAAAGTACACATCAGGAAGACAAG
+HWI-EAS443:3:26:464:854#0/1
a[a]`aZ`^X__`__`a`][¥O][_Z[^_]`_]`^]^__]
@HWI-EAS443:3:27:181:509#0/1
CATATGGCCATAATCAAAAAGTACACATCAGGAAGACAAG
+HWI-EAS443:3:27:181:509#0/1
a`a]_`abaa__`_a^`aWP`Va]R^a_aaaU`aa_aaa^
@HWI-EAS443:3:27:200:1024#0/1
GGACCATATGGCCATAATCAAAAAGTACACATCAGGAAGA
+HWI-EAS443:3:27:200:1024#0/1
aaaba_aa__^aaaaaa_[aa`a_`V```^aY__a`__^X
@HWI-EAS443:3:28:1065:1118#0/1
ATATGGCCATAATCAAAAAGTACACATCAGGAAGACAAGA
+HWI-EAS443:3:28:1065:1118#0/1
a_ba_R`a`a^aa`_aaba`_b`^W__`]aa_a`a¥aX`_
@HWI-EAS443:3:28:539:102#0/1
ATATGGCCATAATCAAAAAGTACACATCAGGAAGACAAGA
+HWI-EAS443:3:28:539:102#0/1
aaaaba`a__`aaW``_a_`Z`][_`_^^]_^_^]Z^`_]
@HWI-EAS443:3:29:913:528#0/1
GTCTTCCTGATGTGTACTTTTTGATTATGGCCATATGGTC
+HWI-EAS443:3:29:913:528#0/1
aaabaaaaaaab`a_`_baaa``_a_`a`[a``Y[PFWRB
@HWI-EAS443:3:30:1379:1024#0/1
TCTTGTCTTCCTGATGTGTACTTTTTGATTATGGCCATAT
+HWI-EAS443:3:30:1379:1024#0/1
a¥a^`_¥a`a`aa_a_]a]^_a_a_a]]a¥a____U]RRM
@HWI-EAS443:3:30:312:148#0/1
GGACCATATGGCCATAATCAAAAAGTACACATCAGGAAGA
+HWI-EAS443:3:30:312:148#0/1
a`abbaab`aabaaaa_[aaaaaa`Sa`a[`[_`__^_VM
@HWI-EAS443:3:31:103:429#0/1
CATATGGCCATAATCAAAAAGTACACATCAGGAAGACAAG
+HWI-EAS443:3:31:103:429#0/1
aUZL¥aa`Z[Y_aa]^[^`¥]W^`[¥^TZ^^^__Z[]`[T
@HWI-EAS443:3:31:1136:777#0/1
ACCATATGGCCATAATCAAAAAGTACACATCAGGAAGACA
+HWI-EAS443:3:31:1136:777#0/1
abaaaZ_^aaabY¥U`[XY¥`¥VS]Waa]`G_Y[^]_PU^
@HWI-EAS443:3:32:1157:546#0/1
ATATGGCCATAATCAAAAAGTACACATCAGGAAGACAAGA
+HWI-EAS443:3:32:1157:546#0/1
abbaaa`aaaabaaaa`aaaZa`a_a`_a]Z_a``Xaaa`
@HWI-EAS443:3:32:407:1448#0/1
ATATGGCCATAATCAAAAAGTACACATCAGGAAGACAAGA
+HWI-EAS443:3:32:407:1448#0/1
_[]^_¥a`a`[a____`[YZR]S_`_]^¥PZ]_V¥ST¥SU
@HWI-EAS443:3:32:668:960#0/1
ATATGGCCATAATCAAAAAGTACACATCAGGAAGACAAGA
```

```
+HWI-EAS443:3:32:668:960#0/1
aa_`a¥^aa_R__¥```a_]T``]a¥___^a^]__¥_Z¥Z
@HWI-EAS443:3:33:191:1128#0/1
GACCATATGGCCATAATCAAAAAGTACACATCAGGAAGAC
+HWI-EAS443:3:33:191:1128#0/1
`ba¥`aabb_bb`aab__HVaS_^Q_aaaa]``aaaaaaa
@HWI-EAS443:3:33:720:552#0/1
ATATGGCCATAATCAAAAAGTACACATCAGGAAGACAAGA
+HWI-EAS443:3:33:720:552#0/1
abaaaaaaaaaaaaaba_aa^aaa_a^``aa_]a^V____
@HWI-EAS443:3:33:72:1781#0/1
GACCATATGGCCATAATCAAAAAGTACACATCAGGAAGAC
+HWI-EAS443:3:33:72:1781#0/1
[`bb`¥IXa]bbaa_QZ^X`V_^ZO]`Z_X^aVV]^]]^[
@HWI-EAS443:3:35:894:458#0/1
CATATGGCCATAATCAAAAAGTACACATCAGGAAGACAAG
+HWI-EAS443:3:35:894:458#0/1
aabb^]aa`a]RTY]a``_aaV_¥```^aYaa^YX[__^_
@HWI-EAS443:3:35:971:981#0/1
GTCTTCCTGATGTGTACTTTTTGATTATGGCCATATGGTC
+HWI-EAS443:3:35:971:981#0/1
aabaaaaaZ`aa^a_Xbaaaaaabaaa_¥aaa_SaN[SJ¥
@HWI-EAS443:3:36:1129:1226#0/1
ATATGGCCATAATCAAAAAGTACACATCAGGAAGACAAGA
+HWI-EAS443:3:36:1129:1226#0/1
aa[`aababaaaa]_aa`aaYa^`W`aR_a``_`a]^¥`[
@HWI-EAS443:3:36:1209:469#0/1
GTCTTCCTGATGTGTACTTTTTGATTATGGCCATATGGTC
+HWI-EAS443:3:36:1209:469#0/1
a`aaaa¥]aRYZWWZ_¥a```[W`__]WH[aTMK¥BBBBB
@HWI-EAS443:3:36:642:705#0/1
ACCATATGGCCATAATCAAAAAGTACACATCAGGAAGACA
+HWI-EAS443:3:36:642:705#0/1
aa_O[^Z¥LZ¥U[PT[XYQWTQYOYWHSYV_Z[Z^`¥VWY
@HWI-EAS443:3:37:764:1432#0/1
CATATGGCCATAATCAAAAAGTACACATCAGGAAGACAAG
+HWI-EAS443:3:37:764:1432#0/1
abbaT]`aa`_aa[^``aa`XUaa`a``aW_X[]^^Z¥^¥
@HWI-EAS443:3:37:847:1679#0/1
CATATGGCCATAATCAAAAAGTACACATCAGGAAGACAAG
+HWI-EAS443:3:37:847:1679#0/1
UPPPS__`a`_a`Z^^bba^ZTaYbba]bZ]a¥`a`VZa`
@HWI-EAS443:3:38:1298:767#0/1
ACCATATGGCCATAATCAAAAAGTACACATCAGGAAGACA
+HWI-EAS443:3:38:1298:767#0/1
abaa`baaa_`__]a`__a___a]`_a`a]Z]``[_a]^[
@HWI-EAS443:3:38:1587:377#0/1
ACCATATGGCCATAATCAAAAAGTACACATCAGGAAGACA
+HWI-EAS443:3:38:1587:377#0/1
a`aYaaaaa¥]XY^__^Xa^]¥_Z_W_]_ZZZX]V]^WOW
```

```
@HWI-EAS443:3:3:1179:2043#0/1
ATATGGCCATAATCAAAAAGTACACATCAGGAAGACAAGA
+HWI-EAS443:3:3:1179:2043#0/1
```abbb]baa__`b^aba_¥bXaI]_[aa^`aa`Sa^a_
@HWI-EAS443:3:40:1196:1960#0/1
CATATGGCCATAATCAAAAAGTACACATCAGGAAGACAAG
+HWI-EAS443:3:40:1196:1960#0/1
`bbbab_abbabbb]bbbbb`¥aa]DTYbaa_aab``bb_
@HWI-EAS443:3:40:159:1393#0/1
CATATGGCCATAATCAAAAAGTACACATCAGGAAGACAAG
+HWI-EAS443:3:40:159:1393#0/1
abbbbb`bb__¥a``abbb]_YaaaWGTaaa[ZU_`aaa^
@HWI-EAS443:3:42:124:195#0/1
ACCATATGGCCATAATCAAAAAGTACACATCAGGAAGACA
+HWI-EAS443:3:42:124:195#0/1
abaaa`_`_a`]][TU_]S^`X^T_aY]^W¥^__X^]T]^
@HWI-EAS443:3:42:170:1401#0/1
GTCTTCCTGATGTGTACTTTTTGATTATGGCCATATGGTC
+HWI-EAS443:3:42:170:1401#0/1
aabb^`a`a``aZ¥_]]aaa`b_`aaaaU]aa_YTYJRT¥
@HWI-EAS443:3:44:1153:1686#0/1
GTCTTCCTGATGTGTACTTTTTGATTATGGCCATATGGTC
+HWI-EAS443:3:44:1153:1686#0/1
a_aYY]^¥¥_[a^YXQS``_`[OSY^BBBBBBBBBBBBBB
@HWI-EAS443:3:44:1168:163#0/1
TGTCTTCCTGATGTGTACTTTTTGATTATGGCCATATGGT
+HWI-EAS443:3:44:1168:163#0/1
aa_`aa__aa]aa_a_^^aaaa``^a``__W_Z]X]WVYW
@HWI-EAS443:3:45:1017:554#0/1
CCATATGGCCATAATCAAAAAGTACACATCAGGAAGACAA
+HWI-EAS443:3:45:1017:554#0/1
aaaba¥b``^aa_aaWV`_aaWF]Ma_`_`_a¥``a_W_`
@HWI-EAS443:3:45:1507:1340#0/1
CATATGGCCATAATCAAAAAGTACACATCAGGAAGACAAG
+HWI-EAS443:3:45:1507:1340#0/1
aabbabaa`a`[`a_aaaaaa]a_`_a`_`a]^`¥^``_`
@HWI-EAS443:3:45:423:1837#0/1
ACCATATGGCCATAATCAAAAAGTACACATCAGGAAGACA
+HWI-EAS443:3:45:423:1837#0/1
a````a`_Y_V`_Ya]_^a`__PKU^aX_¥X_^___^]Y_
@HWI-EAS443:3:46:1341:199#0/1
CCATATGGCCATAATCAAAAAGTACACATCAGGAAGACAA
+HWI-EAS443:3:46:1341:199#0/1
aaaaaaaaaa`¥]]_^^a``aaX^ZYY_^^^`_^``ZV[^
@HWI-EAS443:3:46:1418:164#0/1
ATATGGCCATAATCAAAAAGTACACATCAGGAAGACAAGA
+HWI-EAS443:3:46:1418:164#0/1
abaaaa^a``^`]]^a`[_aX_¥`[a^__ba_`aXQ¥^`]
@HWI-EAS443:3:46:1490:1041#0/1
ACCATATGGCCATAATCAAAAAGTACACATCAGGAAGACA
```
```

+HWI-EAS443:3:46:1490:1041#0/1
aab[`[`a_aaa]Zaa^```a`a^_]_^__^_a`___]]_
@HWI-EAS443:3:48:269:1131#0/1
ACCATATGGCCATAATCAAAAAGTACACATCAGGAAGACA
+HWI-EAS443:3:48:269:1131#0/1
aaa]aa][X``aa_a`ZY^a[`aUaaU_]_¥___``_¥^^
@HWI-EAS443:3:49:1770:524#0/1
GACCATATGGCCATAATCAAAAAGTACACATCAGGAAGAC
+HWI-EAS443:3:49:1770:524#0/1
abbbab``baYaaabbbab`aa_aaaa^`_a]ab]_aa_^
@HWI-EAS443:3:49:593:933#0/1
ATATGGCCATAATCAAAAAGTACACATCAGGAAGACAAGA
+HWI-EAS443:3:49:593:933#0/1
aba]]baaabaaa]_aa`aaZa^]¥a__abaaaa__`_a^
@HWI-EAS443:3:4:1620:1054#0/1
ACCATATGGCCATAATCAAAAAGTACACATCAGGAAGACA
+HWI-EAS443:3:4:1620:1054#0/1
aaaaba`aa^^`a^aa]_a_a``V`[`]__¥_aa^^a]X_
@HWI-EAS443:3:50:237:758#0/1
TCTTCCTGATGTGTACTTTTTGATTATGGCCATATGGTCC
+HWI-EAS443:3:50:237:758#0/1
aab`aaaaaaa`a_`_bbaab__aaa`_[a^^]ZTGQWT[
@HWI-EAS443:3:51:571:61#0/1
ATATGGCCATAATCAAAAAGTACACATCAGGAAGACAAGA
+HWI-EAS443:3:51:571:61#0/1
aaZV]aY¥_¥ZR^_WZ]a``STNX[[[WPZ`N_¥NZaZ]X
@HWI-EAS443:3:52:1126:1072#0/1
ACCATATGGCCATAATCAAAAAGTACACATCAGGAAGACA
+HWI-EAS443:3:52:1126:1072#0/1
a]_Y¥`aa^__a``Z]T`__`a_]`X_^_`[_``^aa[Y_
@HWI-EAS443:3:52:1388:1072#0/1
CATATGGCCATAATCAAAAAGTACACATCAGGAAGACAAG
+HWI-EAS443:3:52:1388:1072#0/1
aab]aabaaab`ab^``aaZ`^aUaY^a][_aaaa`X`_^
@HWI-EAS443:3:53:1327:1474#0/1
ACCATATGGCCATAATCAAAAAGTACACATCAGGAAGACA
+HWI-EAS443:3:53:1327:1474#0/1
aaaabbbbaa]aa]ab`ab`a`a_`]a_a_TU_aa`a_^`
@HWI-EAS443:3:55:1018:662#0/1
GTCTTCCTGATGTGTACTTTTTGATTATGGCCATATGGTC
+HWI-EAS443:3:55:1018:662#0/1
aa_abbabb^ab[`Za]abbab``aa_a¥__`_S`[PZW[
@HWI-EAS443:3:55:386:191#0/1
CATATGGCCATAATCAAAAAGTACACATCAGGAAGACAAG
+HWI-EAS443:3:55:386:191#0/1
a^]W]a[Y¥__[¥Y[[_X]¥TQXP]U]][¥`^TW][¥]¥¥
@HWI-EAS443:3:56:1278:1244#0/1
ACCATATGGCCATAATCAAAAAGTACACATCAGGAAGACA
+HWI-EAS443:3:56:1278:1244#0/1
abbab^aa]ZaaaZ^`__`[_^ZLZ`¥`UW[_aa`_`[R`

@HWI-EAS443:3:57:1116:39#0/1
ACCATATGGCCATAATCAAAAAGTACACATCAGGAAGACA
+HWI-EAS443:3:57:1116:39#0/1
aaabbbbbb_aabaaa^aaaaab]a]`__a]_b`^^`__a
@HWI-EAS443:3:58:122:1511#0/1
CATATGGCCATAATCAAAAAGTACACATCAGGAAGACAAG
+HWI-EAS443:3:58:122:1511#0/1
a^ba`baXY]Z^¥_```bbaTT¥V_]aX]baa_aZaabba
@HWI-EAS443:3:58:1558:551#0/1
CATATGGCCATAATCAAAAAGTACACATCAGGAAGACAAG
+HWI-EAS443:3:58:1558:551#0/1
abaa]abaaba`aaO`W`X`aS¥Y_Vaa]ZabaaaaVaa]
@HWI-EAS443:3:59:1039:204#0/1
GTCTTCCTGATGTGTACTTTTTGATTATGGCCATATGGTC
+HWI-EAS443:3:59:1039:204#0/1
a``ba`aaaaab]a_`^aaa`aa`aa`a^_[U]]ZPLYVS
@HWI-EAS443:3:59:124:1055#0/1
GTCTTCCTGATGTGTACTTTTTGATTATGGCCATATGGTC
+HWI-EAS443:3:59:124:1055#0/1
aaabbaaabaab^aaaaaaba]aaaaaa__`Xa__SRYHW
@HWI-EAS443:3:59:796:173#0/1
CATATGGCCATAATCAAAAAGTACACATCAGGAAGACAAG
+HWI-EAS443:3:59:796:173#0/1
_aba_b`aab`Z]^`[^a`a]D^``abaaZ_W_aa¥UV]]
@HWI-EAS443:3:60:446:169#0/1
ACCATATGGCCATAATCAAAAAGTACACATCAGGAAGACA
+HWI-EAS443:3:60:446:169#0/1
aaaaa`aa`aa`a_aa__a`__`Y`Z_``^^^_^Z_^¥W^
@HWI-EAS443:3:61:676:169#0/1
ACCATATGGCCATAATCAAAAAGTACACATCAGGAAGACA
+HWI-EAS443:3:61:676:169#0/1
a_a`]^][a`¥VTW]¥_YZa[`YNR^_``YXY[Z^ZWVP[
@HWI-EAS443:3:61:834:943#0/1
ATATGGCCATAATCAAAAAGTACACATCAGGAAGACAAGA
+HWI-EAS443:3:61:834:943#0/1
abbabaaa`a^a^^^a^aaa[aaaaW`a^]_¥^_T¥_^_]
@HWI-EAS443:3:62:1015:40#0/1
CATATGGCCATAATCAAAAAGTACACATCAGGAAGACAAG
+HWI-EAS443:3:62:1015:40#0/1
^bb_abbbbbbba¥`_a``aaab__^`a`bbbaa]a¥^]]
@HWI-EAS443:3:62:1614:1048#0/1
CTTGTCTTCCTGATGTGTACTTTTTGATTATGGCCATATG
+HWI-EAS443:3:62:1614:1048#0/1
abbababbaabbbbbbbababbbbb¥bbbbb`baaaa`_O
@HWI-EAS443:3:62:682:406#0/1
ACCATATGGCCATAATCAAAAAGTACACATCAGGAAGACA
+HWI-EAS443:3:62:682:406#0/1
a¥^`ab`aba^OWaY`]Z]¥]a_FW]¥^WG¥W^a_`^YQ¥
@HWI-EAS443:3:64:1157:1735#0/1
ATATGGCCATAATCAAAAAGTACACATCAGGAAGACAAGA

+HWI-EAS443:3:64:1157:1735#0/1
^a`^]X_a_aabaa``]a¥_T`a¥XPUYT``_b_¥FU_`_
@HWI-EAS443:3:66:1104:1236#0/1
CATATGGCCATAATCAAAAAGTACACATCAGGAAGACAAG
+HWI-EAS443:3:66:1104:1236#0/1
`Y[`_YZ]``^^a]]]``a`_Ua__a`__``¥QY_^[Z_`
@HWI-EAS443:3:66:122:490#0/1
CATATGGCCATAATCAAAAAGTACACATCAGGAAGACAAG
+HWI-EAS443:3:66:122:490#0/1
`]`aaaaaaaa`a`[]X``_^RU[Z^SZ]^``^``[_]Y¥
@HWI-EAS443:3:66:1254:538#0/1
ATATGGCCATAATCAAAAAGTACACATCAGGAAGACAAGA
+HWI-EAS443:3:66:1254:538#0/1
[abbaaaUa`a`babbaaba_aaaab_[[`bab`a``[aa
@HWI-EAS443:3:66:362:208#0/1
TCTTCCTGATGTGTACTTTTTGATTATGGCCATATGGTCC
+HWI-EAS443:3:66:362:208#0/1
aaaaaaaa__`Za]_aaaa`]`_a_[`_^¥_VOOZWTN^Z
@HWI-EAS443:3:66:62:1630#0/1
ACCATATGGCCATAATCAAAAAGTACACATCAGGAAGACA
+HWI-EAS443:3:66:62:1630#0/1
aba]`a]__``_X¥R¥[]___a[G[[UYVYY[SY¥ZZZ_^
@HWI-EAS443:3:67:601:1842#0/1
ACCATATGGCCATAATCAAAAAGTACACATCAGGAAGACA
+HWI-EAS443:3:67:601:1842#0/1
a_a`_`a[W¥aa`_``_`a_[Z[]aa_]]^_^_X__^_^`
@HWI-EAS443:3:67:624:1429#0/1
ACCATATGGCCATAATCAAAAAGTACACATCAGGAAGACA
+HWI-EAS443:3:67:624:1429#0/1
abbaa`a``^aaaaa_`aaa]__Xa_]aa_a`_`_a[^`a
@HWI-EAS443:3:6:639:475#0/1
ATATGGCCATAATCAAAAAGTACACATCAGGAAGACAAGA
+HWI-EAS443:3:6:639:475#0/1
a^¥`abaP_abaaaaaaba_^a_^[a`aaa_aaaa_`^aa
@HWI-EAS443:3:70:468:630#0/1
GTCTTCCTGATGTGTACTTTTTGATTATGGCCATATGGTC
+HWI-EAS443:3:70:468:630#0/1
_R_a_`a]WR[aOYSGY][¥_`[W[WYUQV^ZNTXMIHRW
@HWI-EAS443:3:70:546:870#0/1
CATATGGCCATAATCAAAAAGTACACATCAGGAAGACAAG
+HWI-EAS443:3:70:546:870#0/1
a^aa`ZZ¥b]aaaa`Z`aY]^V¥aV`_^^¥`__^¥^`___
@HWI-EAS443:3:70:954:979#0/1
CATATGGCCATAATCAAAAAGTACACATCAGGAAGACAAG
+HWI-EAS443:3:70:954:979#0/1
a^^a¥_a^`]`aa``¥]]_^`W__a`a`¥`aa`aa``aa_
@HWI-EAS443:3:71:416:530#0/1
ATATGGCCATAATCAAAAAGTACACATCAGGAAGACAAGA
+HWI-EAS443:3:71:416:530#0/1
_Q¥]a`_^¥`a`_``Y`a_[TY_a]HY`_ba][`]_____

```
@HWI-EAS443:3:73:207:1405#0/1
ACCATATGGCCATAATCAAAAAGTACACATCAGGAAGACA
+HWI-EAS443:3:73:207:1405#0/1
aa]]NPLW[``ZGSWO[UTTXVONTVHPQTUZTZZWYSSW
@HWI-EAS443:3:73:66:1745#0/1
ACCATATGGCCATAATCAAAAAGTACACATCAGGAAGACA
+HWI-EAS443:3:73:66:1745#0/1
abba`a_`_aaa]W`___^Z^`^W[X^][Y__^]__^^^_
@HWI-EAS443:3:73:915:121#0/1
GTCTTCCTGATGTGTACTTTTTGATTATGGCCATATGGTC
+HWI-EAS443:3:73:915:121#0/1
aZS]aa`__Y_bZ`]`Yaa_[`¥^__^a^_VW_UXRKVQL
@HWI-EAS443:3:75:509:1250#0/1
GTCTTCCTGATGTGTACTTTTTGATTATGGCCATATGGTC
+HWI-EAS443:3:75:509:1250#0/1
a_aa`¥¥Z]^]aU]T]_aa``aZ¥`_]_W¥`[XJJMQKMX
@HWI-EAS443:3:75:58:769#0/1
TCTTCCTGATGTGTACTTTTTGATTATGGCCATATGGTCC
+HWI-EAS443:3:75:58:769#0/1
abaaaab`aaa_`^¥abaaa```a____`aa^[^¥SYY^a
@HWI-EAS443:3:76:952:1062#0/1
CATATGGCCATAATCAAAAAGTACACATCAGGAAGACAAG
+HWI-EAS443:3:76:952:1062#0/1
¥SZZ^LWa^^^XSZTYXSb`¥``baJ`V`aZY¥¥^_[KV]
@HWI-EAS443:3:79:1515:1470#0/1
GTCTTCCTGATGTGTACTTTTTGATTATGGCCATATGGTC
+HWI-EAS443:3:79:1515:1470#0/1
aa_ba[aaaaa__`_^_baaaa^_aaaa_[V]_Z_]GU]Y
@HWI-EAS443:3:79:1587:1026#0/1
TCTTCCTGATGTGTACTTTTTGATTATGGCCATATGGTCC
+HWI-EAS443:3:79:1587:1026#0/1
`b_^_ba^Waa_a`__bb]`aR^bbaa``Z__WXaYOPTL
@HWI-EAS443:3:79:589:492#0/1
TCTTCCTGATGTGTACTTTTTGATTATGGCCATATGGTCC
+HWI-EAS443:3:79:589:492#0/1
`ba_b¥¥aa`bR^R[_aa[a¥_ZaRPZJOYSVOSTQJDJ]
@HWI-EAS443:3:79:84:105#0/1
ACCATATGGCCATAATCAAAAAGTACACATCAGGAAGACA
+HWI-EAS443:3:79:84:105#0/1
a`a^aY`a`a^a__]¥]_W]^]_W_¥__]W¥]^^Z^^ZR]
@HWI-EAS443:3:7:668:341#0/1
CATATGGCCATAATCAAAAAGTACACATCAGGAAGACAAG
+HWI-EAS443:3:7:668:341#0/1
]¥W_Z_`^^OX]ZZW[[_aZWSYLWRY¥P¥_aYZ[SL[Z`
@HWI-EAS443:3:80:1073:699#0/1
GTCTTCCTGATGTGTACTTTTTGATTATGGCCATATGGTC
+HWI-EAS443:3:80:1073:699#0/1
aYXaa[a[```ZZYYa_ab_ba`]``[`_^_Z[S[ZHYPP
@HWI-EAS443:3:80:523:330#0/1
ATATGGCCATAATCAAAAAGTACACATCAGGAAGACAAGA
```

```
+HWI-EAS443:3:80:523:330#0/1
aaaaa`a``aaaaaaaa`aaXa`a_``a``aa]`_]a`a^
@HWI-EAS443:3:80:669:759#0/1
GTCTTCCTGATGTGTACTTTTTGATTATGGCCATATGGTC
+HWI-EAS443:3:80:669:759#0/1
]baabaaaaaaa`a`]abbbb_O_aaab```aa__[[_S^
@HWI-EAS443:3:81:1465:885#0/1
ATATGGCCATAATCAAAAAGTACACATCAGGAAGACAAGA
+HWI-EAS443:3:81:1465:885#0/1
Za_X[a__bbaaa^`b_aaa_bbaaab`aba¥baa_b[a`
@HWI-EAS443:3:81:1634:1895#0/1
TGTCTTCCTGATGTGTACTTTTTGATTATGGCCATATGGT
+HWI-EAS443:3:81:1634:1895#0/1
a]]`_a^aaa_^aX`¥a^aa`aaa_```___^``S]XG]X
@HWI-EAS443:3:81:241:199#0/1
CATATGGCCATAATCAAAAAGTACACATCAGGAAGACAAG
+HWI-EAS443:3:81:241:199#0/1
a_aaaaaa`]^¥_^`aa[Z_aW`¥`Z_^^`_`__`[[__]
@HWI-EAS443:3:81:618:659#0/1
ACCATATGGCCATAATCAAAAAGTACACATCAGGAAGACA
+HWI-EAS443:3:81:618:659#0/1
a`a`YbYa``aa`a_`a_^]X`a¥_]a`aX]__a_`aUO¥
@HWI-EAS443:3:82:1001:416#0/1
ACCATATGGCCATAATCAAAAAGTACACATCAGGAAGACA
+HWI-EAS443:3:82:1001:416#0/1
^]¥bbaba`_a__a_TST`a^a]N_`aT^_^ab_T¥``Va
@HWI-EAS443:3:82:1187:465#0/1
GTCTTCCTGATGTGTACTTTTTGATTATGGCCATATGGTC
+HWI-EAS443:3:82:1187:465#0/1
`babb^^bba`aa]`aabbbab_abba`aaaab^^^SPS]
@HWI-EAS443:3:82:791:1016#0/1
ACCATATGGCCATAATCAAAAAGTACACATCAGGAAGACA
+HWI-EAS443:3:82:791:1016#0/1
ab```bbb`abaa¥`aaa`a¥YV]baa_aaaaaaaaV__a
@HWI-EAS443:3:83:1267:896#0/1
ATATGGCCATAATCAAAAAGTACACATCAGGAAGACAAGA
+HWI-EAS443:3:83:1267:896#0/1
a`a]]aa^aYTaaT`[`]]bS_Ta[aYYbbb_aaY¥a_aa
@HWI-EAS443:3:83:875:1030#0/1
GTCTTCCTGATGTGTACTTTTTGATTATGGCCATATGGTC
+HWI-EAS443:3:83:875:1030#0/1
a_aabaaaaZaa_a_aaaabaa]abaa`ZZ^`_[__Q_YS
@HWI-EAS443:3:84:1363:1485#0/1
CATATGGCCATAATCAAAAAGTACACATCAGGAAGACAAG
+HWI-EAS443:3:84:1363:1485#0/1
aab`bbbaaaabb`KUaaa`a]b_`___W_b`aa[¥`b^_
@HWI-EAS443:3:84:1660:848#0/1
ATATGGCCATAATCAAAAAGTACACATCAGGAAGACAAGA
+HWI-EAS443:3:84:1660:848#0/1
aT^bbb[aXb`_bJ_ba`a`[_bb¥baaaZZ]baa_aa]Z
```

```
@HWI-EAS443:3:84:211:1852#0/1
CATATGGCCATAATCAAAAAGTACACATCAGGAAGACAAG
+HWI-EAS443:3:84:211:1852#0/1
aYXMO_^aa`V]V]_YSPX¥¥PW^_a`Za_^_WTU]^_^]
@HWI-EAS443:3:86:568:523#0/1
CTTGTCTTCCTGATGTGTACTTTTTGATTATGGCCATATG
+HWI-EAS443:3:86:568:523#0/1
¥¥T]^^XZ_WSa^^`_¥VT^¥__`a``¥Z_a]`]^]V¥TT
@HWI-EAS443:3:86:693:830#0/1
CATATGGCCATAATCAAAAAGTACACATCAGGAAGACAAG
+HWI-EAS443:3:86:693:830#0/1
]RYW]]b]Y¥^Z`^ba¥_a]aX^]_Z]¥ZX_a[aa_a^`V
@HWI-EAS443:3:87:59:1199#0/1
CATATGGCCATAATCAAAAAGTACACATCAGGAAGACAAG
+HWI-EAS443:3:87:59:1199#0/1
`S_]_`_aaa_]___TY^ZT]Sa`Z``Y`][]XV[Y_[VV
@HWI-EAS443:3:89:577:409#0/1
TGTCTTCCTGATGTGTACTTTTTGATTATGGCCATATGGT
+HWI-EAS443:3:89:577:409#0/1
abaabaaaaa`aa`a]X_aaaaa`aa`a`WT[`_VY¥YNS
@HWI-EAS443:3:89:867:1693#0/1
CATATGGCCATAATCAAAAAGTACACATCAGGAAGACAAG
+HWI-EAS443:3:89:867:1693#0/1
```aaaa`abaaabaaaaaaa¥a`aaa`¥aa[abaaXaa[
@HWI-EAS443:3:8:877:480#0/1
CATATGGCCATAATCAAAAAGTACACATCAGGAAGACAAG
+HWI-EAS443:3:8:877:480#0/1
abababaaaababaaaaaaU`Za_`^a¥`aaaSa`^¥`aa
@HWI-EAS443:3:90:1250:209#0/1
CATATGGCCATAATCAAAAAGTACACATCAGGAAGACAAG
+HWI-EAS443:3:90:1250:209#0/1
aXbaabba¥Q¥`_bYaa`¥aa_a¥`Xaa``aaa`_`_`aa
@HWI-EAS443:3:90:1646:279#0/1
CATATGGCCATAATCAAAAAGTACACATCAGGAAGACAAG
+HWI-EAS443:3:90:1646:279#0/1
`a```_b_aaa]`_I`aa`_]U`][_¥]YY^a_Xa_J__`
@HWI-EAS443:3:90:926:1889#0/1
GTCTTCCTGATGTGTACTTTTTGATTATGGCCATATGGTC
+HWI-EAS443:3:90:926:1889#0/1
`aabaabbaaaaWaXaab`aba`aaabaa__^aR____RV
@HWI-EAS443:3:91:1181:768#0/1
GACCATATGGCCATAATCAAAAAGTACACATCAGGAAGAC
+HWI-EAS443:3:91:1181:768#0/1
aa`R_]_]^a¥_^]W^ZWWZ_YWXT]H]UV]W]WXXUZZX
@HWI-EAS443:3:92:825:89#0/1
CATATGGCCATAATCAAAAAGTACACATCAGGAAGACAAG
+HWI-EAS443:3:92:825:89#0/1
a_]Y]bbaabb^Z^_]^Z]YZ[]U]N]][[a]]Z^]K[]]
@HWI-EAS443:3:93:249:1038#0/1
ACCATATGGCCATAATCAAAAAGTACACATCAGGAAGACA
```
```

```
+HWI-EAS443:3:93:249:1038#0/1
¥`a¥Z_aba^`a^^aZ__`WaZ^X``¥V^XX[]¥Q¥¥[^`
@HWI-EAS443:3:94:513:1923#0/1
ATATGGCCATAATCAAAAAGTACACATCAGGAAGACAAGA
+HWI-EAS443:3:94:513:1923#0/1
a`aa_aaa`_a_`_a_`_`aXa_a_`WX^]__a^`[`^__
@HWI-EAS443:3:95:1169:278#0/1
CCATATGGCCATAATCAAAAAGTACACATCAGGAAGACAA
+HWI-EAS443:3:95:1169:278#0/1
aabbbbbbabaabab`bbbaab_baa`ba`aabbaaa_aa
@HWI-EAS443:3:95:208:646#0/1
GCGGGGTTCTTTTCTTGTCTTCCTGATGTGTACTTTTTGA
+HWI-EAS443:3:95:208:646#0/1
a`baa¥MU`R`¥^Y_`[S`a]X[`WZ[`]a]Z]_`_]X[R
@HWI-EAS443:3:95:595:1761#0/1
CATATGGCCATAATCAAAAAGTACACATCAGGAAGACAAG
+HWI-EAS443:3:95:595:1761#0/1
aaa¥bb^b_``a[a_bbaabaGa]a_^¥_^a__b`¥¥a_a
@HWI-EAS443:3:95:695:1284#0/1
CATATGGCCATAATCAAAAAGTACACATCAGGAAGACAAG
+HWI-EAS443:3:95:695:1284#0/1
`L_b`]¥¥aX^_]¥^_]`b]a_]U]V^¥[``a_aaX_aaa
@HWI-EAS443:3:96:38:375#0/1
GTCTTCCTGATGTGTACTTTTTGATTATGGCCATATGGTC
+HWI-EAS443:3:96:38:375#0/1
a_aaaa`aa[``_b]`^aa`aaaIa`aa^V__`Y]YW^YX
@HWI-EAS443:3:99:1121:1207#0/1
TCTTCCTGATGTGTACTTTTTGATTATGGCCATATGGTCC
+HWI-EAS443:3:99:1121:1207#0/1
a^`Z`]a[ZZZRXNY¥_a`__`aaaa`^^ZXT[BBBBBBB
@HWI-EAS443:3:9:169:1257#0/1
GACCATATGGCCATAATCAAAAAGTACACATCAGGAAGAC
+HWI-EAS443:3:9:169:1257#0/1
`Z]_``]a``aaa_YW``[S]^a`TV^]^]^][[]VXYMQ
@HWI-EAS443:3:60:839:555#0/1
ATCAATGAACTGAGTAATCTTGCAAAAGGAGAGAAAGCTA
+HWI-EAS443:3:60:839:555#0/1
a`baab`¥_aa`_b_aaaaaabaa``_aa_`^a``a`a`_
@HWI-EAS443:3:94:621:1423#0/1
CAATCCGACCCTACTTTTCCTAAAAATTCCAGCGCAAAAT
+HWI-EAS443:3:94:621:1423#0/1
aaabaa¥OQGXa]a_¥aa`^abbbaa_a]^¥^`_aaaaa¥
@HWI-EAS443:3:49:390:1418#0/1
GAACATTATGGGCGCCATGCTCAGGATGTTTCTGAACCAC
+HWI-EAS443:3:49:390:1418#0/1
a`aba^a_b`¥YFX`aR`^_]¥aaY]¥PP_`]_]X_^¥_P
@HWI-EAS443:3:14:1778:634#0/1
ATTTTTGCCGTCTGAGTTCTTCAATGGTAGAACAGATCTT
+HWI-EAS443:3:14:1778:634#0/1
abba`ab[]_Z_ab]aY_`ba^]aaaaY`a_^Na__aYa]
```

@HWI-EAS443:3:31:877:1498#0/1
ATGGGCGCCATGCTCAGGATGTTTCTGAACCACTCGGGTT
+HWI-EAS443:3:31:877:1498#0/1
a_a`YFQ_aaXLZ`aSV_V]WZ_`__V¥¥V^_[S]Y¥VSR
@HWI-EAS443:3:58:1063:1417#0/1
AACATTATGGGCGCCATGCTCAGGATGTTTCTGAACCACT
+HWI-EAS443:3:58:1063:1417#0/1
O^¥``a^`¥YMDUZaab_a]a`_`L_]YZXW_¥`aaGRU[
@HWI-EAS443:3:91:1275:1552#0/1
CATTAGAGCACATCCAGAAACTGATTGCCCCCAATAGAAT
+HWI-EAS443:3:91:1275:1552#0/1
`bbbbbabbbbbbb_bbbbbbbbbbbbbabbbbbbbabb
@HWI-EAS443:3:82:1148:1799#0/1
TACCAGCCTCCCATTTCAGAATATACATCCGATCACAATT
+HWI-EAS443:3:82:1148:1799#0/1
ababba_aaa^T_aab]bbb`bbababaTbba]]aabX_R
@HWI-EAS443:3:76:1683:1721#0/1
CGATTGGCCATGTCTCCGATTTGTTTTCAAAGAATTCCTT
+HWI-EAS443:3:76:1683:1721#0/1
aab`RI^bbabYSROHb_S[ba]GYaaI[_a_[[a`S[`_
@HWI-EAS443:3:8:1003:265#0/1
CATCAAGCATCAATGAATTGAGCAATCTTGCAAAAGGAGA
+HWI-EAS443:3:8:1003:265#0/1
Xb_VZ[JWS`_aZa¥a`Jaab`Ua¥aL`_aW`ba_a`]`_
@HWI-EAS443:3:37:1599:216#0/1
GGCCCAGCATTAAGCATCAATGAATTGAGCAATCTTGCAA
+HWI-EAS443:3:37:1599:216#0/1
aaaX`U`]__Z^UVU[¥OXZ`_`XDX¥VQKDJGXZZ¥UMJ
@HWI-EAS443:3:80:1315:1011#0/1
GGGCTATCGCAACACCTGGGATGCAGATTAGAGGGTTCGT
+HWI-EAS443:3:80:1315:1011#0/1
ab`aa`_SaZa`Xa_T_][aa__aP]ZY``^Z`¥GY^R__
@HWI-EAS443:3:1:1785:1295#0/1
GGGGCATGGAAATGAGGCGTTGCCTTCTTCAGTGTCTTTA
+HWI-EAS443:3:1:1785:1295#0/1
]aY^`M`YU]aW```aIJ[H[a``]_`]`H¥aYBBBBBBB
@HWI-EAS443:3:44:1634:372#0/1
GGTTCTTCTCTTGTCTTCCTGATGTGTACTTTTGATTATG
+HWI-EAS443:3:44:1634:372#0/1
aa_`_aa`a`aaa`_aa^_aa^]]¥`^]]a_a`]]`___X
@HWI-EAS443:3:35:59:1860#0/1
GCGGGGTTCTTCTCTTGTCTTCCTGATGTGTACCTTTTGA
+HWI-EAS443:3:35:59:1860#0/1
ab__Z¥Zab`abbaaaa`baabaaXa`]V`W[`R^`a¥QS
@HWI-EAS443:3:48:922:210#0/1
GGGGTTCTTCTCTTGTCTTCCTGATGTGTACCTTTTGATT
+HWI-EAS443:3:48:922:210#0/1
ab`aV¥aaaaa¥aaa^_a_W`_`^[^Ta¥^SG]`^_¥]_Z
@HWI-EAS443:3:19:1052:1182#0/1
GGTTCTTCTCTTGTCTTCCTGATGTGTACCTTTTGATTAT

+HWI-EAS443:3:19:1052:1182#0/1
__T_`abbbbbaaabba``baa^]V_T``^^baaWZ_a``
@HWI-EAS443:3:58:508:1920#0/1
CAGGAAGACAAGAGAAGAACCCTGCACTCAGAATGAAGTG
+HWI-EAS443:3:58:508:1920#0/1
abbabbbbabbababbabaa`aY_aaaa_aaaaaa^__Ya
@HWI-EAS443:3:67:565:1157#0/1
ATTGCCATCATCCACTTCATTCTGAGTGCAGGGTTCTTCT
+HWI-EAS443:3:67:565:1157#0/1
`aaZ^a`_aa[aa`_`S`^_`^a_]_Z^_W__ZVRXU^]_
@HWI-EAS443:3:95:36:148#0/1
AGCATTAAGCATCAATGAACTGAGCAATCTTGCGAAAGGA
+HWI-EAS443:3:95:36:148#0/1
a``a``ba`bZ__ZGU¥]XWZ[NXWYSSWZX_UGYWZ^UZ
@HWI-EAS443:3:13:1618:812#0/1
TCCAAAATGCCCTAAATGGGAATGGGGACCCGAATAACAT
+HWI-EAS443:3:13:1618:812#0/1
¥Ma]Waba`a_X][`aWb]V[]V[Z`_ZG^RWUOIHLWO^
@HWI-EAS443:3:53:11:1548#0/1
GTCGCTTTAAGTACGGTTTGAAAAGAGGGCCTTCTACGGA
+HWI-EAS443:3:53:11:1548#0/1
a^_^`a`]_^aS]```Y__ZT]_¥``[¥[¥¥¥Y¥YPZ¥ZN
@HWI-EAS443:3:74:39:498#0/1
AGGCAAACCATTTGAATGGATGTCAACCCGACTCTACTTT
+HWI-EAS443:3:74:39:498#0/1
Y`a_¥aa``ba`b^UH]aaXZaWU]_a][¥_[_____Z`¥
@HWI-EAS443:3:40:1785:800#0/1
ATTGGAGTGACAGTGATAAAGAACAACATGATAAACAATG
+HWI-EAS443:3:40:1785:800#0/1
ab`a`aZab`[`aZb^b[Z^`aaX^]`a[W¥TO^XL]¥[W
@HWI-EAS443:3:25:940:1890#0/1
AGGACCGCAGAAGCTGCTTTTGGTCTAGTGTGTGCCACTT
+HWI-EAS443:3:25:940:1890#0/1
abbbbb``baba¥aY^a^^¥Z`^T`^aY[_[[[___`[V[
@HWI-EAS443:3:18:1061:218#0/1
TGGGTCCCCATTCCCATTTAGGGCATTTTGGACAAAGCGT
+HWI-EAS443:3:18:1061:218#0/1
TQ]¥FYba]`X_P]YY__ZZT¥]SYYO¥a¥^_Y^^^`UMJ
@HWI-EAS443:3:66:1274:1082#0/1
TGGCGGCTGTCTGGCTGTCAGTAAGTATGCTAGAGTCTCG
+HWI-EAS443:3:66:1274:1082#0/1
aa^YQ_Y[aYXR[[VQ[OORNLNYBBBBBBBBBBBBBBBB
@HWI-EAS443:3:24:1216:1416#0/1
CGCTGTCTGGCTGTCAGTAAGTATGCTAGAGTCTCGTTCG
+HWI-EAS443:3:24:1216:1416#0/1
aaab¥VbabbaabY^a]aaba_aaa`a^Vaa`_b_aZ_]`
@HWI-EAS443:3:38:982:595#0/1
GAAATGAAAAGTGGCGAGAGCAGTTGGGACAGAAATTTGA
+HWI-EAS443:3:38:982:595#0/1
aZ]aXa_^Y¥aFURa_[^P]QKHMXJWRUXWPU^TPVWPJ

@HWI-EAS443:3:32:1546:1013#0/1
CCGCCAGACTTATGATTGGACATTAAACAGAAATNAACCG
+HWI-EAS443:3:32:1546:1013#0/1
aaabbbbbbbbbbbabbbaa`bbbabbab`bbaYD[babb
@HWI-EAS443:3:32:760:85#0/1
GGTCCGGATTGATGCCAGGATCGACTTCGAGTCTGGACGG
+HWI-EAS443:3:32:760:85#0/1
aY_Z^_`¥_aVY__XM[^¥UYSYSP^VXYSRR^Z[[VW¥¥
@HWI-EAS443:3:72:1071:1742#0/1
GCCCGGATTGATGCCAGGATCGACTTCGAGTCTGGACGGA
+HWI-EAS443:3:72:1071:1742#0/1
^baabbaaaababbbbaabaaaaa`_aaa`_aa[a_`aaa
@HWI-EAS443:3:74:964:56#0/1
GCCCGGATTGATGCCAGGATCGACTTCGAGTCTGGACGGA
+HWI-EAS443:3:74:964:56#0/1
V_`^abIZ_bbbbbb]bb_b_bababababS]aa_VZ^aW
@HWI-EAS443:3:80:617:689#0/1
GCTGAATCGAGAAAATTGCTTCTCATTGTTCAGGCATCAA
+HWI-EAS443:3:80:617:689#0/1
`aaabaa`aab_`ab_a[a``¥a^``a_X___Y]_``]_^
@HWI-EAS443:3:91:1753:618#0/1
CCGCCACTTTTCATTTCTGCTCTGGAGGTAGTGAAGGGCT
+HWI-EAS443:3:91:1753:618#0/1
¥S_O¥¥^W``RQ[W_¥I__R_aaa`X]X__¥I[VZ]BBBB
@HWI-EAS443:3:46:1695:1137#0/1
CGGGAGACCTTCACTACCTCCAGAGCAGAAATGAAAAGTG
+HWI-EAS443:3:46:1695:1137#0/1
Ua^ZU¥¥_IUaaba]aaaZP[XaZU_¥aV¥Vb]¥_¥UaU_
@HWI-EAS443:3:85:978:1936#0/1
AAAAATTCCAGCGCAAAATGCCATAAGCACTACATACCTT
+HWI-EAS443:3:85:978:1936#0/1
_`^`^W`a[O_YaaJWI`_XSX]`a__T¥]QGHIRITUF¥
@HWI-EAS443:3:12:1670:891#0/1
AAAAATTCCAGCGCAAAATGCCATAAGCACTACATTCCCT
+HWI-EAS443:3:12:1670:891#0/1
a__a`aaVa`bX``a_a_a`_S^`^]]U_XGWZ__`OUGS
@HWI-EAS443:3:59:1567:666#0/1
AGGCCATGGTGTCTAGGGCCCGGATTGATGCGAGGATCGA
+HWI-EAS443:3:59:1567:666#0/1
a`bb]bbab[]a]ZbbabZ¥V`baab]ZbaZGa`ZDL`X^
@HWI-EAS443:3:23:847:2009#0/1
GCATCATATTCCATGCTTTTGGCTGGGCCATGGGCTGGCA
+HWI-EAS443:3:23:847:2009#0/1
ab`VZ]_aa]SOPV^¥ST¥BBBBBBBBBBBBBBBBBBBBB
@HWI-EAS443:3:37:991:245#0/1
GCCATGGTGTCTAGGGCCCGGATTGATGCCAGGATCGACT
+HWI-EAS443:3:37:991:245#0/1
abaaaa`^a^a¥`aa`a]`aa[^XKO¥_ZDFVUYUU¥VUU
@HWI-EAS443:3:18:1418:498#0/1
TGTTTATAGCACCCTTGGGTGTCTGACAAGTCGTATTGCA

```
+HWI-EAS443:3:18:1418:498#0/1
aa`aaa``a_`_^`a_a__X_]X_a]]^]^X__X]_Y¥]]
@HWI-EAS443:3:7:227:291#0/1
CCGGATGTATATTCTGAAATGGGAGGCTGGTGTTTATAGC
+HWI-EAS443:3:7:227:291#0/1
aabbabb`ababbabbaaabbaa_aaabaaQa``a``_a]
@HWI-EAS443:3:87:782:711#0/1
GACCGGATGTATATTCTGAAATGGGAGGCTGGTGTTTATA
+HWI-EAS443:3:87:782:711#0/1
aaaabbaa``aaaaa`a`[a_a`Z_[_^Z_a`¥[_^`_Z`
@HWI-EAS443:3:1:583:474#0/1
GGGGGGATATATGAGGCAATCGAGGAGTGCCTGATTAATG
+HWI-EAS443:3:1:583:474#0/1
a¥VaZ_K__]M`[SF[[G[VBBBBBBBBBBBBBBBBBBBB
@HWI-EAS443:3:22:735:110#0/1
CGCGTCTCCACAACTTGAGGGGTTTTCGGCTGAATCGAGA
+HWI-EAS443:3:22:735:110#0/1
_a```baUbbaYMbb`b`Va]aaa_[a`_[aaaVb_]Xa]
@HWI-EAS443:3:39:457:1364#0/1
ATCGAAGGTTCCAGGTTCCAGGTTGTCCCTAAATGCCTGA
+HWI-EAS443:3:39:457:1364#0/1
aaaZ_aaaMY]¥YSOMT^]VDVOYYY_`^BBBBBBBBBBB
@HWI-EAS443:3:61:1599:614#0/1
CGCGTCTCCACAACTTGAGGGGTTTTCGGCTGAATCGAGA
+HWI-EAS443:3:61:1599:614#0/1
aaaba^b]aaY[YQabbaT`baG_bb[aaJT_[ZZIV[aU
@HWI-EAS443:3:71:113:2030#0/1
CGCGTCTCCACAACTTGAGGGGTTTTCGGCTGAATCGAGA
+HWI-EAS443:3:71:113:2030#0/1
a_ba`a`b``aZa```]a`__]S_Z^^Z]]]^a_XZ]NXY
@HWI-EAS443:3:9:802:1856#0/1
CAATCACAGCAGACAAGAGAATAATGGACATGATTCCAGA
+HWI-EAS443:3:9:802:1856#0/1
ab_[FRa]a_a¥X^a_a^a]___[]_aUL]X[¥SXNS¥_^
@HWI-EAS443:3:78:1566:734#0/1
GGCCCATCCACTAACAGGGCAGAGAGAGGAATTGCCCGAT
+HWI-EAS443:3:78:1566:734#0/1
a¥^TZa_TZa`baa``X¥_¥HZ^_`_X_a¥XZ`]NKNWH`
@HWI-EAS443:3:84:168:344#0/1
GGCCCATCCACTAACAGGGCAGAGAGAGGAATTGCCCGCT
+HWI-EAS443:3:84:168:344#0/1
`baababaab^]ab_b`a_ba`_a[aaaaaa]_a`[U`__
@HWI-EAS443:3:1:170:240#0/1
CCTCCCGATTCCACCAATCATTCTTCCGACAGATGCTCTG
+HWI-EAS443:3:1:170:240#0/1
ab]a[^YaXbaa_¥a]`TUWa^¥[_[U`VU[`^][M^SaB
@HWI-EAS443:3:79:1499:199#0/1
TGGGATCCATTCCGGTGCGAACAAGCGCTCTTGTTCTTTG
+HWI-EAS443:3:79:1499:199#0/1
``]bU]UaaY[WHQ^M[`ba^[`ab_ZFP`abaU_M¥F_a
```

```
@HWI-EAS443:3:60:966:772#0/1
CTCCTGTTTTCTTAGGGGCCTTCCCAGCACTGGGATGCTC
+HWI-EAS443:3:60:966:772#0/1
```a^_X^b__ab^¥^^G__¥_b]]`aa__¥`a[_a_T¥`
@HWI-EAS443:3:30:876:1573#0/1
GTCCGAGAGCTCGAAGACTCCCCGCCCCTGGAAGGATAAA
+HWI-EAS443:3:30:876:1573#0/1
aab`[_¥L¥Y]]^_b]a`a`XRa]`bbU^_QaaQGRERZa
@HWI-EAS443:3:2:1320:1876#0/1
GCGGTGTGTTCTGTTTACTGTGTCCATGGTGTATCCTGTT
+HWI-EAS443:3:2:1320:1876#0/1
``^aUQXa^XYa^[a__Z_VQaZT¥¥N`_G_¥^ZPU`XVV
@HWI-EAS443:3:48:206:545#0/1
GCGGTGTGTTCTGTTTACTGTGTCCATGGTGTATCCTGTT
+HWI-EAS443:3:48:206:545#0/1
aaa`YaWa_`_`a_a`_`a`¥_W[Z¥_`aWaT^_ZU[_VU
@HWI-EAS443:3:86:612:1757#0/1
CGGTGTGTTCTGTTTACTGTGTCCATGGTGTATCCTGTTC
+HWI-EAS443:3:86:612:1757#0/1
^bb`b_b[aba[`bbbabbaba^aaaa][b_abba`_aab
@HWI-EAS443:3:94:735:642#0/1
CGGTGTGTTCTGTTTACTGTGTCCATGGTGTATCCTGTTC
+HWI-EAS443:3:94:735:642#0/1
abaYa_b]baab``ba`ba_a_b``a`]_a``^a_aa`a_
@HWI-EAS443:3:95:742:21#0/1
CGGTGTGTTCTGTTTACTGTGTCCATGGTGTATCCTGTTC
+HWI-EAS443:3:95:742:21#0/1
]bbUba_W_¥_bbbb`abbaa]VIX`b`]`^[_]P`bbbX
@HWI-EAS443:3:21:244:162#0/1
GCCGGCTGATTTCTGTTTAATGTCCAATCATAAGTCTGGC
+HWI-EAS443:3:21:244:162#0/1
Ja¥`^F^WS_XV`bY_VVaY[`]a¥^T[aR^aS]YSPY__
@HWI-EAS443:3:8:621:1422#0/1
ATGGGCCTGCAGTAGCAAGTGGGCATGACTTTGAAAGGGA
+HWI-EAS443:3:8:621:1422#0/1
aaaaa^HL^^aa_`aaa^^RVVa_a_^a[a__aZbZ]QRY
@HWI-EAS443:3:12:646:2015#0/1
GTACTTTTTTGGACAGTACGGATAGCAAATAGTAGCATTG
+HWI-EAS443:3:12:646:2015#0/1
^TYYa`XW]^S[^^ab_`Xbaa`YY]a^aYXNW^`VY[IY
@HWI-EAS443:3:11:1090:1647#0/1
ATGGCTCTTGCTTTACTGTAATGACCGATGGACCAAGTGA
+HWI-EAS443:3:11:1090:1647#0/1
^_b¥F]aaaa]YbaY¥[_^baa_`¥UX[``a`_X`_aXaa
@HWI-EAS443:3:69:1206:771#0/1
GTGCAGGGATAACTGGCATGGCTCGAATCGGCCGAGGGGG
+HWI-EAS443:3:69:1206:771#0/1
a_aaa]_YXaYa_]_XT^_^BBBBBBBBBBBBBBBBBBBB
@HWI-EAS443:3:50:1722:87#0/1
GTGCAGGGATAACTGGCATGGCTCGAATCGGCCGTGGGGG
```
```

+HWI-EAS443:3:50:1722:87#0/1
`V^]ZT_Z[a`TL^a]U_UBBBBBBBBBBBBBBBBBBBBB
@HWI-EAS443:3:31:108:2031#0/1
AGGGTCTTCAATCTCAGCGTTTCCTGGGTTTCGACTTTCT
+HWI-EAS443:3:31:108:2031#0/1
X_aSH]aa`^QS^`Z¥M^a¥¥T]a[NKMDTXa^H[[^¥YX
@HWI-EAS443:3:11:684:182#0/1
GCCCGAGACTGGAGCCACACAAATGGGAAAAATACTGTGT
+HWI-EAS443:3:11:684:182#0/1
abaZTSSQXZW^R¥Z¥Y¥UYVPU]¥¥¥Y^]^^WYVZZUXW
@HWI-EAS443:3:98:1107:983#0/1
AGAGGATTTCTCATTTTGGGCAAAGAAGACAAGAAGATCC
+HWI-EAS443:3:98:1107:983#0/1
]ba`[^a`YGZQ¥Y`bV¥Z¥b¥¥`bV]a¥XaaYa_a__Z_
@HWI-EAS443:3:73:278:1359#0/1
GAAAAATGGCCCAGCATTAAGCATCAATGAACTGAGCAAT
+HWI-EAS443:3:73:278:1359#0/1
abbbb¥Za`a]abaab`^``^bN_aa`a]``]_aa`a_`Z
@HWI-EAS443:3:86:789:678#0/1
AAGAAATATGGCCCAGCATTAAGCATCAATGAACTGAGCA
+HWI-EAS443:3:86:789:678#0/1
abb^``baabaaaa^``aaa``a_`a_a`aa_a_a`_a[a
@HWI-EAS443:3:97:1173:1195#0/1
CAGCATTAAGCATCNACGAACTGAGCAATCTTGGAAAAGG
+HWI-EAS443:3:97:1173:1195#0/1
Ya^aa[¥`ZbabaZD^`aZYS`bV]aYb_U`b]DY¥IRaa
@HWI-EAS443:3:28:158:671#0/1
CTTTTTTGGACAGTATGGATAGCAAACAGTAGCATTGCCA
+HWI-EAS443:3:28:158:671#0/1
aaaaaaa^a_aaa¥^`]aT¥^SWZ`YWUVS^]WVXVJYY¥
@HWI-EAS443:3:33:1091:665#0/1
ATTTTCACCGAGTGCCCACTTCAATTGGCTTTTTTTCTTC
+HWI-EAS443:3:33:1091:665#0/1
_``abbbb`a`baa^`a`abaa`aa`^^_a[DDZF¥X^`Q
@HWI-EAS443:3:61:1202:1263#0/1
CATATTTTCACCGACTGCCCACTTCAATTGGCTTGTTTTC
+HWI-EAS443:3:61:1202:1263#0/1
TM^X[a`Vb`a[X¥SZX^a__aabbb_aaKS¥a_X^^R_R
@HWI-EAS443:3:90:1322:652#0/1
CATGCTCTCAATCTGCTGAAGAGACTGAAGAAGACAGCGC
+HWI-EAS443:3:90:1322:652#0/1
[`__IU^bb^^a_W¥PZ_[`¥UW¥`[W`aaVY[HMOaTYL
@HWI-EAS443:3:99:1408:386#0/1
CAGTGGTCTTAGTGAGTATCTCGCGGGTGCGGGACTGAGA
+HWI-EAS443:3:99:1408:386#0/1
Y`abb¥b^bbKbba[_ZZ][_b[Q_KbIbbb_^V^W[BBB
@HWI-EAS443:3:34:488:20#0/1
CACCCGCGAGATACTCACTAAGACCACTGTGGACCATATG
+HWI-EAS443:3:34:488:20#0/1
`aaba`abab`abaaaa`aaaa]a`a^`]Ta[[]UT[¥ZW

```
@HWI-EAS443:3:38:196:1561#0/1
GCCTTTTCCGCAAGCTCGACGATCATTGGGTTGAAGCATT
+HWI-EAS443:3:38:196:1561#0/1
a¥[H[PTa[]]]ZU[K]]BBBBBBBBBBBBBBBBBBBBBB
@HWI-EAS443:3:44:1645:351#0/1
ATCGAAGGGTCCAGGTTCCAGGTTATCCCTAAGTGCCTTA
+HWI-EAS443:3:44:1645:351#0/1
abababaa_Ja`[S___Y__[_aKaGUUaWG_WFFWX^[FK
@HWI-EAS443:3:82:796:1701#0/1
GGAAGACAAGAGAAGAACCCCGCACTCAGGATGTGAGAAG
+HWI-EAS443:3:82:796:1701#0/1
_aab_babbbbab_abbbbaa_babbab_a`ab`baaaaa
@HWI-EAS443:3:61:1120:493#0/1
TGCTGCTGTCTGGCTGTCAGTAAGTATGCTAGAGTCCCGT
+HWI-EAS443:3:61:1120:493#0/1
abbb_Z_baaaba_ab[a`aUYY¥V¥][]][YP¥VWS]]V
@HWI-EAS443:3:90:960:670#0/1
CTGCTGTCTGGCTGTCAGTAAGTATGCTAGAGTCCCGTTT
+HWI-EAS443:3:90:960:670#0/1
abbaabbba`]```^^`b[aaaV_¥Z¥^```a`]__b]_a
@HWI-EAS443:3:72:400:893#0/1
GGGATTTATGCCTTTCTCATGTGGTTTGACTATGTTGGGC
+HWI-EAS443:3:72:400:893#0/1
aab__abb`bbbbaabbb_b_Q`]]a_aaaaaa`]a_aX¥
@HWI-EAS443:3:86:1024:1094#0/1
CCAAGATCGAAGGTTCCAGGTTCCAGGTTGTCCCTAAGCG
+HWI-EAS443:3:86:1024:1094#0/1
abbba`a^Z_^a^OZMZY_Y_W^O]VZP^]YL^V]a]OFS
@HWI-EAS443:3:98:85:110#0/1
ATCGGGTTCGTTGCCTTTTCGTCCGAGAACTCGAAGACTC
+HWI-EAS443:3:98:85:110#0/1
_a[_UX¥QSV_a`XQ]Y^^¥GVBBBBBBBBBBBBBBBBBB
@HWI-EAS443:3:30:14:1832#0/1
GCTTGCGGAAAAGGCAATGAAAGAATATGGGGAAGATCCC
+HWI-EAS443:3:30:14:1832#0/1
abaa`baV]_^GXabbaaa_S_a_Z___]Z_WW]`a^]YO
@HWI-EAS443:3:45:633:1070#0/1
AGGCTCTAAAGTTTTCAAGGCTGGAGAAGTTTGGTGGGAG
+HWI-EAS443:3:45:633:1070#0/1
aa`bbbbaa]_Tba_^a___NU]aG^H]aUXFOaZ[M_JZ
@HWI-EAS443:3:33:302:1000#0/1
CAATGCTACTATTTGCTATCCATACTGTCTCAAAAAAAAA
+HWI-EAS443:3:33:302:1000#0/1
aa]a`a`aa`a`a``a_a_aa`_``_ZXa_`¥^^____Z_
@HWI-EAS443:3:67:1741:1035#0/1
GAACTGAGCAATCTTGCAAAAGGAGAGAAAGCTAACGTGC
+HWI-EAS443:3:67:1741:1035#0/1
OTaa¥X_a¥^_a^Z_YYU¥a``a[V__]`¥Z`bY`V¥¥]V
@HWI-EAS443:3:92:141:670#0/1
TCGGATCTTCATTGCCTTTTCCGCAAGCTCGACGATCATT
```

```
+HWI-EAS443:3:92:141:670#0/1
abbb_aa_`_aabaa¥]aa`_`b_a_]^``___a[_VX^_
@HWI-EAS443:3:14:1222:272#0/1
CCGTGCGACAATGCTTCAATCCAATGATCGTCGAGCTTGC
+HWI-EAS443:3:14:1222:272#0/1
aab_aab_aa``b_aa¥]_```a_aa]a]`X_aY`]__]X
@HWI-EAS443:3:65:1094:350#0/1
ACCCTGATGTGTACTTTTTGATTATGACCATATGGTCCAC
+HWI-EAS443:3:65:1094:350#0/1
`aaaVTT`¥G¥_`Sb`a``]LXabaXDX¥TYBBBBBBBBB
@HWI-EAS443:3:52:1375:540#0/1
CCCTGATNTGTACTTTTTGATTATGGCCATATGGTCCACA
+HWI-EAS443:3:52:1375:540#0/1
aaabba[D^aaaabbbabaabaaaaa`a`J]`^¥¥`ZUP`
@HWI-EAS443:3:61:1606:1874#0/1
CCGGGCCATTCCTATTCCACCATGTTACGGCCAGAGGTGA
+HWI-EAS443:3:61:1606:1874#0/1
abaababbbbbababaabbaa^aa_aaa]`aY`PWZWJW¥
@HWI-EAS443:3:25:510:341#0/1
CCATCATCCATTTCATTCTGAGTGCGGGGTTCTTCTCTTG
+HWI-EAS443:3:25:510:341#0/1
aabbbbbbbbbbbbbbbbbbababbbbbb_bab`aabbaa
@HWI-EAS443:3:62:1000:956#0/1
CGGGCCATTCCTATTCCACCATGTTACGGCCAGAGGTGAT
+HWI-EAS443:3:62:1000:956#0/1
aZF^aa`a¥UW_aaaba^RWZYV^a]VP]_aWZXZHLWYB
@HWI-EAS443:3:68:473:1969#0/1
CGGGCCATTCCTATTCCACCATGTTACGGCCAGAGGTGAT
+HWI-EAS443:3:68:473:1969#0/1
a^FQZ_X¥_`_]¥^a``aa[a¥aa`b_YX_`YHULTXBBB
@HWI-EAS443:3:97:726:1777#0/1
TAAGCATCAATGAACTGAGCAATCTTGCAAAGGGAAGAAA
+HWI-EAS443:3:97:726:1777#0/1
]b[a]]`aba_aa^aa¥_`bbaaYa[`¥`__X_BBBBBBB
@HWI-EAS443:3:22:700:210#0/1
GTCCCCTTGCAAGATTGCTCAGTTCATTGATGCTTAATGC
+HWI-EAS443:3:22:700:210#0/1
aa_V_a`aa_a``a__aa`]]_X_``^`___`Y`__^^^]
@HWI-EAS443:3:46:1409:1165#0/1
GTCCCCTTGCAAGATTGCTCAGTTCATTGATGCTTAATGC
+HWI-EAS443:3:46:1409:1165#0/1
aZa[HQZ[X^`V_VVTQL¥``[U^Q`^[XYU`YVTYYV^O
@HWI-EAS443:3:33:1300:1856#0/1
GTTGCCTTAGGGACAAGAGACTGAAATGGTTCGAATTCCA
+HWI-EAS443:3:33:1300:1856#0/1
_TS]X¥W_Y_XNW_W^]W___`aY_YY_YQRQFOTTTOTV
@HWI-EAS443:3:49:83:1408#0/1
CAGCATTAAGCATCAATGAACTGAGCAATCTTGCAAAGGA
+HWI-EAS443:3:49:83:1408#0/1
abbbbbbbbbaab`bbb`abbbb^`^bbb_``aabaaba`
```

```
@HWI-EAS443:3:20:1029:1073#0/1
CGGGGCGATCTGAACTTTGTCAATAGGGCAAACCAGCGAC
+HWI-EAS443:3:20:1029:1073#0/1
a`T_Xa¥]^K_ZW]R¥_ZY¥[[X`^^¥_____V^[TS^[W
@HWI-EAS443:3:23:549:122#0/1
CGGGGCGATCTGAACTTTGTCAATAGGGCAAACCAGCGAC
+HWI-EAS443:3:23:549:122#0/1
abbabbbb`_`b^JYaa`a_``b¥`Z`_K``aS`_]_`^W
@HWI-EAS443:3:78:1381:156#0/1
CGGGGCGATCTGAACTTTGTCAATAGGGCAAACCAGCGAC
+HWI-EAS443:3:78:1381:156#0/1
ab^baZZ¥ZUa]_aXaaa_¥TUYXS^a¥BBBBBBBBBBBB
@HWI-EAS443:3:86:1068:534#0/1
CCAGCATTAAGCATCAATGAACTGAGCAATCTTGCAAAGG
+HWI-EAS443:3:86:1068:534#0/1
`¥]`a`^¥`a¥_a``aabbaaab``aX^]`]aaa_^UZaZ
@HWI-EAS443:3:11:1210:1585#0/1
GGGCATCGTTTGTTTTGCTCCAGAGGGTTTGTCCTTGTTC
+HWI-EAS443:3:11:1210:1585#0/1
aa`__a_b_`aaZ]S]_V`Z^^`X[^_V]]_XVV]]ZUXY
@HWI-EAS443:3:54:741:1597#0/1
AGTCCGTCATAATCACTGAGTTTGAGTTCAGTGCACATTT
+HWI-EAS443:3:54:741:1597#0/1
aa`aaa`baaaaa_a`aa_aaaa`aa_`a`_]aZaaaa`a
@HWI-EAS443:3:88:235:1611#0/1
AAATCCCAATTACCTCATGGCTTGGAAGCAGGTGTTAGCA
+HWI-EAS443:3:88:235:1611#0/1
[`bbb]_X[Yaabb[T[^ZH_]]]TT¥GRTSXTWHIOVLR
@HWI-EAS443:3:72:757:938#0/1
GATGGTTTCCTGGATATTTGGACTTACAATGCCGAACTGT
+HWI-EAS443:3:72:757:938#0/1
a][``U[[VWY¥[VWV]YYXYM[[¥[`X^[^[[[OT[][W
@HWI-EAS443:3:93:1202:423#0/1
AAGATCCAGATGAAGGCACATCTGGGATGGAGTCTGCTGT
+HWI-EAS443:3:93:1202:423#0/1
aaba¥¥`aaaaa_aaa^aZ``_`aaa_aaT^b¥P`a¥`__
@HWI-EAS443:3:43:294:1491#0/1
ATGAATGGCTCCCTTATGACAAACACATCCCCCTTGGAAC
+HWI-EAS443:3:43:294:1491#0/1
^a_a[[VQHZ]aaZ]_ZX]U[_][`OXRR]Za__][___M
@HWI-EAS443:3:53:1743:190#0/1
ATTTTGTCTCCTGGCTCTACTAGTGTCCAGTAATAGTTCA
+HWI-EAS443:3:53:1743:190#0/1
`bbbbbabb__a_Y]a[`_Pa_a]]]]V_a`[ab]_¥]QW
@HWI-EAS443:3:7:565:26#0/1
ATGGAAATGAGGCGCTGCCTTCTTCAGTCTCTTCAGCAAA
+HWI-EAS443:3:7:565:26#0/1
[F^`_ZW_b¥bbabU`[UU¥Z[QBBBBBBBBBBBBBBBBB
@HWI-EAS443:3:97:305:293#0/1
TGGAAATGAGGCGCTGCCTTCTTCAGTCTCTTCAGCAAAT
```

+HWI-EAS443:3:97:305:293#0/1
_Z_]Q``a`¥[P`]`a`__[VV¥^V`RU[¥[ZKVYSUZVY
@HWI-EAS443:3:69:1653:245#0/1
ACCAGAAATTCCAATTGTTAGCCAATTGATGCCATCATGA
+HWI-EAS443:3:69:1653:245#0/1
Maabbbabbbabbbaab`bbb_abbbbbabb`aba`bbaa
@HWI-EAS443:3:42:1709:112#0/1
CGGTCCAGTATCGTCTAATGGAGCAAATGGAGTAAAAGGA
+HWI-EAS443:3:42:1709:112#0/1
abbab_bbabbbbaabaabbb`b^a]`bb_Zb```a`aa]
@HWI-EAS443:3:72:550:284#0/1
TGTTTCTCCAACTCTTGATAGTGTCTGTTATTATTCCGTT
+HWI-EAS443:3:72:550:284#0/1
aa_a`a_aa_`¥___`]`_`_Y_]BBBBBBBBBBBBBBBB
@HWI-EAS443:3:11:1084:1844#0/1
GTGATGATTAGGGGCATTCATTTCGACTGATTTGACTATC
+HWI-EAS443:3:11:1084:1844#0/1
_V``aa_`a]aa^^[]]a_``___^__^a]][^_ZXX^^Q
@HWI-EAS443:3:79:728:1196#0/1
ACTCTTCAGGTCGGCTGCATATCCTGACCCCTGCTCATTT
+HWI-EAS443:3:79:728:1196#0/1
Vabaabbbba`bbbbbbbbbababbYbbbabbbbbbbbba
@HWI-EAS443:3:76:1569:1573#0/1
ATGGAAGTCGTTCAACAAACAAGGGCAGATAAACTAACTC
+HWI-EAS443:3:76:1569:1573#0/1
a`^`_a^HVa¥`Z`¥R_`WPZYT¥SE[a]PTW¥F[^ZFXX
@HWI-EAS443:3:22:1129:568#0/1
GAAAAAGGAGAGTAAGCGACAACATGACCAAGAAGATGGT
+HWI-EAS443:3:22:1129:568#0/1
a`____]^Za^]`__ab¥^ZYXRZ^]^XZZZXXXWZVZOO
@HWI-EAS443:3:36:452:101#0/1
GCTCCCTCATAATCGATGAAATCTCCTGGGTAACACGTTC
+HWI-EAS443:3:36:452:101#0/1
aa^[``b^¥_¥`Za_^Z`[a]_a__]Vaa^Q`V`a__Z__
@HWI-EAS443:3:3:1781:860#0/1
GGCCAGCAGGAGTCTATGGGATTCCTTTCGTCAGTCCGAA
+HWI-EAS443:3:3:1781:860#0/1
^¥a`V[O_]HYbSQ^LU[`_SU`aQ`^aZ`Z¥W`VBBBBB
@HWI-EAS443:3:92:28:903#0/1
CAGCAGGAGTCTATGGGATTCCTTTCGTCAGTCCGAAAGA
+HWI-EAS443:3:92:28:903#0/1
aa¥FTZ^[`VZ]¥¥`ZZT]UNX[Z[U^UZUXQSUUZWWWV
@HWI-EAS443:3:17:252:589#0/1
CAGTGATGCTGTGCCATCTATTAGAAGAGGCTTCTTCTCT
+HWI-EAS443:3:17:252:589#0/1
_¥WZ_`]aa`YR_baWX¥b^T¥¥^UII¥ba`Z^GWaWIHF
@HWI-EAS443:3:36:1131:198#0/1
GCTGGCAATAACAAAAGAGAAGAAAGAAGATCTCCAGGAT
+HWI-EAS443:3:36:1131:198#0/1
abbaabb_aaa_baaab`baaa``aaa`a`a]a]`aa`_`

```
@HWI-EAS443:3:82:893:2034#0/1
CAATCCAGCCCTGTTAGTTCTGGATGTTGAACAAAACTCC
+HWI-EAS443:3:82:893:2034#0/1
R[[^PGTF[^GR^WH[W[ZU^YWWPZKWY[^GZ[[WRVRY
@HWI-EAS443:3:23:234:1291#0/1
CGTGTAGGGATAACTGGCATGGCTCGAATCGACCGTGGGG
+HWI-EAS443:3:23:234:1291#0/1
``aZH_bbababa`aaaaa``]ba_TUWW¥`WS_TTO]BB
@HWI-EAS443:3:54:415:1866#0/1
CACGGGCTTCTGTCTTTAATGGTTCCATTGGAATGTTTGT
+HWI-EAS443:3:54:415:1866#0/1
`YU`ba_Y]``^L[[]aa^]a`LPSW`[VTX_a¥SNY]V¥
@HWI-EAS443:3:98:1433:1323#0/1
GGCTTCTGTCTTTAATGGTTCCATTGGAATGTTTGTCATT
+HWI-EAS443:3:98:1433:1323#0/1
abVba_aaaabababaaaab_a_`[aa`a_]`WR[bUUKV^a
@HWI-EAS443:3:37:1248:1229#0/1
GCATCGTCTAATGGAGCAAATGGAGTAAAAGGATTTTCAT
+HWI-EAS443:3:37:1248:1229#0/1
aa``aa_aa`aa`a]aa__`aaa``]``aUa^T¥^`^a`_
@HWI-EAS443:3:82:1257:610#0/1
ATATTGTTTCTCCAGCTCTTGATAGTGTCTGGTATTATTC
+HWI-EAS443:3:82:1257:610#0/1
Y`b`a_S[b¥]V__HZO¥_¥¥W_NUH`[BBBBBBBBBBBB
@HWI-EAS443:3:73:700:1565#0/1
GCTGGAGAAACAATATATTGAGAACACAAGAGTCTGAATG
+HWI-EAS443:3:73:700:1565#0/1
^L^¥Z^[¥V_a``_YU_[`_a__Y]`[ZZ`^TQ^Z_`PTV
@HWI-EAS443:3:43:1278:250#0/1
CAACTTTGCTGCTGGACAATCAGTGGTTTCCGTGAAATTA
+HWI-EAS443:3:43:1278:250#0/1
abaabbabaabaabaSa][a[[aU`_[a_a`a_`O[`a¥^
@HWI-EAS443:3:5:1326:1872#0/1
TGCAGGGATAACTGGCATGGCTCGAAAAGACCGTGGGTGT
+HWI-EAS443:3:5:1326:1872#0/1
]_bW¥KZ_aaaZZaba[a]VabaKaaUH[QBBBBBBBBBB
@HWI-EAS443:3:82:836:130#0/1
GGCCACCCGAATTCTTTTGGTCGCTGTCTGGCTGTCAGTA
+HWI-EAS443:3:82:836:130#0/1
aaaaa`aaa^Va`_Xa]ZU]^_`__`U^TZ[Y[]RNT¥TS
@HWI-EAS443:3:86:854:1284#0/1
CGCTCTTGTTCTTTGATATGTGGCATCATTCAGGTTGGAA
+HWI-EAS443:3:86:854:1284#0/1
``¥¥¥_bb^aVa`_Y`a`^^V_[_`aaaa`a_``__`_aa
@HWI-EAS443:3:24:735:1404#0/1
CTGAAATGATAATACCAGATCCAGCATTTCTTCCCATTGC
+HWI-EAS443:3:24:735:1404#0/1
abba_`b_¥babababa`a`_abaaaaa`aa_aaab^__a
@HWI-EAS443:3:78:1338:836#0/1
ATTTTGATGTCCCCACAAAAACATATGNATCTGCATTCTG
```

+HWI-EAS443:3:78:1338:836#0/1
abababYbbaaa^aaaaba_^``^`_[D¥aaaa`Za_Waa
@HWI-EAS443:3:86:1275:179#0/1
AGGGACATCAAAATACAGCAAGAAGTTCAAGCCGGAAATA
+HWI-EAS443:3:86:1275:179#0/1
¥S`¥SZaZZZa_PYb]a`U^ba]_X]`aaab¥Haaa¥^aX
@HWI-EAS443:3:19:1411:401#0/1
GTGGGGACATCAAAATACAGCAAGAAGTTCAAGCCGGAAA
+HWI-EAS443:3:19:1411:401#0/1
a¥baa^aY_aa]a^a`__aa_aaaaaaOYP^^aYQ]_]^_
@HWI-EAS443:3:45:361:2012#0/1
GGGACATCAAAATACAGCAAGAAGTTCAAGCCGGAAATAG
+HWI-EAS443:3:45:361:2012#0/1
¥ba`Ia_a_V_aababa_bbbab]T¥¥baaZYba_ab`b`
@HWI-EAS443:3:53:1166:1807#0/1
TTCTTGCTGTATTTTGATGTCCCCACAAAAACATATGCAT
+HWI-EAS443:3:53:1166:1807#0/1
aa`a`babb`abbbaaaabaZ[ZYa¥aaba^Oaaa_[Ya`
@HWI-EAS443:3:55:685:426#0/1
GGGACATCAAAATACAGCAAGAAGTTCAAGCCGGAAATAG
+HWI-EAS443:3:55:685:426#0/1
abaa_aaaa_aaaa`baaaaba`b^`_baaa^b`__`aaa
@HWI-EAS443:3:70:990:1194#0/1
ATCAGAATGCAGATGCATATGTTTTTGTGGGGACATCAAA
+HWI-EAS443:3:70:990:1194#0/1
aaabaa_Z_aaa__`a``a]`R``__aW`_]^_^__`[X_
@HWI-EAS443:3:25:395:299#0/1
ATCTAGCGAAAGCAGGTCAAATATATTCAATATGGAGAGA
+HWI-EAS443:3:25:395:299#0/1
`bb`babbba_aaaaaWaaaaaaaY`aa_`aaaaa_a_a^
@HWI-EAS443:3:8:765:1993#0/1
AGCTAGCGAAAGCAGGTCAAATATATTCAATATGGAGAGA
+HWI-EAS443:3:8:765:1993#0/1
a]YZaa_aaaaa^_¥WKT_a_^`_aURW^`^_^_RORSU^
@HWI-EAS443:3:46:1334:1122#0/1
GACTTGGAGAAACTGCGATGAGAATGGGAGACCTTCACTA
+HWI-EAS443:3:46:1334:1122#0/1
aaaaaaa_a`_[^_a`a_`a¥`ZZ[a^^X_¥[W_^Y_U^^
@HWI-EAS443:3:95:593:1569#0/1
CATACTTGGAGAAACTGTGATGAGAATGGGAGACCTTCAC
+HWI-EAS443:3:95:593:1569#0/1
S]a¥bbbbbbaaaa`a_]a[`b`b[Zaa_aaba[`_RZb_
@HWI-EAS443:3:73:276:891#0/1
CACTTGGAGAAACTGTGATGAGAATGGGAGACCTTCACTA
+HWI-EAS443:3:73:276:891#0/1
¥V^a¥¥OSZN¥XYTYV¥FFLVZOVKDRYUXLX^WVSY[YH
@HWI-EAS443:3:39:557:263#0/1
TGCCGAGATATGCATTCGCAATGGAAAGAAATGCTGGATC
+HWI-EAS443:3:39:557:263#0/1
ab`bbbb`]aW`¥ZWZ``ab_^``a_`aWP_]R`W^YROW

```
@HWI-EAS443:3:99:1010:1037#0/1
TCAGTACCAATGAACTGGCGACAGTTGAATAGATCGCCAA
+HWI-EAS443:3:99:1010:1037#0/1
ab_b[_`¥aaaa¥a_`aa`___[_Y[R]]_T_]aWYXW_^
@HWI-EAS443:3:7:219:2008#0/1
GTTGGGCCTTTCCTATTCCACCATGTTACGGCCAAAGGGG
+HWI-EAS443:3:7:219:2008#0/1
¥PVaaU_`U[`XWODX__QXa]¥P^NOUMYLNXBBBBBBB
@HWI-EAS443:3:72:36:1653#0/1
GAATTTTACCACAAATGCGATAACACGTGCATGAAAAGCT
+HWI-EAS443:3:72:36:1653#0/1
abbbbaa^abab`aaaab_`a`aaa`_^_aaa``_]`__[
@HWI-EAS443:3:66:42:291#0/1
TGCACCATCACTCGGTCTGATCCAGCATCGTTTGTTTTGC
+HWI-EAS443:3:66:42:291#0/1
`b]Z_`I¥__`^^]RO¥SZ[]^¥]_[]^]¥¥`V]SY^]W[
@HWI-EAS443:3:22:773:1993#0/1
GAAAGCAGGCAAACCATTTGAATGGATGTCAATCCGACTC
+HWI-EAS443:3:22:773:1993#0/1
a`aa`ab¥¥aaaa_`a^__`___`_`^_XZ__W__^]XXY
@HWI-EAS443:3:51:1544:423#0/1
GAAAGCAGGCAAACCATTTGAATGGATGTCAATCCGACTC
+HWI-EAS443:3:51:1544:423#0/1
aab]MYabbabaa¥ab`]_^Y^aaa`ab`Q^^`_^^RR]Q
@HWI-EAS443:3:25:1518:1906#0/1
TTTTCACCGAGTGCCCACTTCAATTGGCTTGTTTTCTTCA
+HWI-EAS443:3:25:1518:1906#0/1
`bbbbbaab`[`b`QOY]a]^`]Z]OX`UVFVVDRSZRJV
@HWI-EAS443:3:96:1594:944#0/1
TCCGAAAAAATAAGACCCTTCATTACTCATGTCAAAGGAA
+HWI-EAS443:3:96:1594:944#0/1
_HYa_^^`¥[WDH_YYOGSORRSSTUZQSUXWZWZXZZBB
@HWI-EAS443:3:71:1410:866#0/1
GTCGCTTTAAATACGGTTTAAAAAGAGGGCCCTCTACGGA
+HWI-EAS443:3:71:1410:866#0/1
a__a_aaa`a___]a`]a`^^¥_____Za[]]^_^^X]]]
@HWI-EAS443:3:42:1753:1077#0/1
TGGCAATGAGATACCCAATTACAGCAGACAAAAGAGAAGA
+HWI-EAS443:3:42:1753:1077#0/1
_¥Z``abbXW¥aaaa]aa_b`]a`aabaabbaa`_^]aa`
@HWI-EAS443:3:1:1696:1280#0/1
ATTGCTATTTCAGGCTTGAACTTCTTGCTGTATCTTGATG
+HWI-EAS443:3:1:1696:1280#0/1
a``¥aaab¥a]G_`VSaX[_X_^SO[UGPGS_^BBBBBBB
@HWI-EAS443:3:11:808:1835#0/1
TTTGGCGATCTATTCAACTGTCGCCAGTTCATTGGTAATG
+HWI-EAS443:3:11:808:1835#0/1
a^aaa`aaP^`aa____]`TNU__TPVQ[YR[^GRYBBBB
@HWI-EAS443:3:11:153:421#0/1
CTGTCTCAGTCCATCCGTTCGGATCCCAAATCATCTCAAA
```

```
+HWI-EAS443:3:11:153:421#0/1
abbaaabba_bbaaaaa`_`aaa_aa``X`_`a__`_¥Y`
@HWI-EAS443:3:46:421:1144#0/1
CTGTTTTCATGCCTGATTAGTGGATTGGTGGTAAGAGCCC
+HWI-EAS443:3:46:421:1144#0/1
abbbabbbaba_aab_bbbbaba`bb`a``aBBBBBBBBB
@HWI-EAS443:3:27:687:1844#0/1
CTGTTTTCATGCCTGATTAGTGGATTGGTGGGAATAGCCA
+HWI-EAS443:3:27:687:1844#0/1
a^ZT]^^__``¥YW```P¥_VXXBBBBBBBBBBBBBBBBB
@HWI-EAS443:3:83:1540:2037#0/1
ATTCTGTTTTCATGCCTGATTAGTGGATTGGTGGGAATAG
+HWI-EAS443:3:83:1540:2037#0/1
`_aab_aabbaa]a`aaba`_¥UHWWSaa_BBBBBBBBBB
@HWI-EAS443:3:38:1356:954#0/1
TGCTTTCGCTAAAAATTCCAGCGCAAAATGCCATAAGCAG
+HWI-EAS443:3:38:1356:954#0/1
^_bb_]^`a¥^¥a`a`]_a_abbaZZ¥a^`T^^a[Q_SQJ
@HWI-EAS443:3:37:839:1389#0/1
TTTCGCTAAAAATTCCAGCGCAAAATGCCATAAGCACCAC
+HWI-EAS443:3:37:839:1389#0/1
aba_^aababaaaaZaaaa`_a`W^`Z[aQU]HWa``a`[
@HWI-EAS443:3:10:546:322#0/1
GTGCTTATGGCATTTTGCGCTGGAATTTTTAGCGAAAGCA
+HWI-EAS443:3:10:546:322#0/1
^OVREMUSX[YWTYXQTZX¥¥¥_]RU¥IYP[SOSVTORFR
@HWI-EAS443:3:11:1001:1258#0/1
ATGGTTTGCCTGCTTTCGCTAAAAATTCCAGCGCAAAATG
+HWI-EAS443:3:11:1001:1258#0/1
_WXWLVZO]aPPMW_VZTTZ`[TZY¥RL[UGSQLTYPVUR
@HWI-EAS443:3:11:364:1116#0/1
TTCAAATGGTTTGCCTGCTTTCGCTAAAAATTCCAGCGCA
+HWI-EAS443:3:11:364:1116#0/1
aW`aaaYaX_aXU¥Y`[___Y`Za^_aa[PZR__]^¥`Y[
@HWI-EAS443:3:16:1277:555#0/1
CATTCAAATGGTTTGCCTGCTTTCGCTAAAAATTCCAGCG
+HWI-EAS443:3:16:1277:555#0/1
`Y]]b_]¥Y`^R_b`PV¥ba`[UOZPQ]M[_WYROTTKN^
@HWI-EAS443:3:19:1248:1825#0/1
CATCCATTCAAATGGTTTGCCTGCTTTCGCTAAAAATTCC
+HWI-EAS443:3:19:1248:1825#0/1
a_`_Q`a__a_Za¥^R^XU]`U¥^`a_[Y¥Ya`Za]Z¥XX
@HWI-EAS443:3:21:1698:1334#0/1
TGCTTTCGCTAAAAATTCCAGCGCAAAATGCCATAAGCAC
+HWI-EAS443:3:21:1698:1334#0/1
_b`aaaab`abaab`aa`a_a¥____^_a_W]a_a__[a¥
@HWI-EAS443:3:58:1641:1183#0/1
GACATCCATTCAAATGGTTTGCCTGCTTTCGCTAAAAATT
+HWI-EAS443:3:58:1641:1183#0/1
a[W]`]^_a`[`VY`TT]`TXTPHZSRa`Y`YX]¥SYY`a
```

```
@HWI-EAS443:3:64:673:1655#0/1
ATCCATTCAAATGGTTTGCCTGCTTTCGCTAAAAATTCCA
+HWI-EAS443:3:64:673:1655#0/1
`[_PY]`a`]]¥Y¥Z_WU][_^a``_¥_^^aa`aZ¥]]]¥
@HWI-EAS443:3:76:1126:1497#0/1
ATCCATTCAAATGGTTTGCCTGCTTTCGCTAAAAATTCCA
+HWI-EAS443:3:76:1126:1497#0/1
abbbababbbbbba`baaaaa`abaaaaaaaabaaaaY_`
@HWI-EAS443:3:77:430:602#0/1
ATCCATTCAAATGGTTTGCCTGCTTTCGCTAAAAATTCCA
+HWI-EAS443:3:77:430:602#0/1
aabb^abaa_aaab^aa¥`_``¥aY__a_aa_X^Y^[_^]
@HWI-EAS443:3:80:896:172#0/1
ATCCATTCAAATGGTTTGCCTGCTTTCGCTAAAAATTCCA
+HWI-EAS443:3:80:896:172#0/1
_a]^aab`a`^aaaXaaaa_ab```_L^^¥a`_aaa^N¥Y
@HWI-EAS443:3:82:1770:336#0/1
ATCCATTCAAATGGTTTGCCTGCTTTCGCTAAAAATTCCA
+HWI-EAS443:3:82:1770:336#0/1
]``aYR_^`_a_Y¥O_``^GVa_¥Y`][Zab^ba`]_F[Z
@HWI-EAS443:3:84:939:1161#0/1
CATCCATTCAAATGGTTTGCCTGCTTTCGCTAAAAATTCC
+HWI-EAS443:3:84:939:1161#0/1
a^aaaaaaaaaaaa`^aaa`^aa`aaaaa`aaaaa_```¥
@HWI-EAS443:3:84:987:890#0/1
CTTTCGCTAAAAATTCCAGCGCAAAATGCCATAAGCACCA
+HWI-EAS443:3:84:987:890#0/1
a`bbbbbaa]bbbab¥_bbWF]b`a_aaaaaab^a]]`U`
@HWI-EAS443:3:86:329:1348#0/1
ATCCATTCAAATGGTTTGCCTGCTTTCGCTAAAAATTCCA
+HWI-EAS443:3:86:329:1348#0/1
_P^]G¥X_`VPV^_]¥XVZ_[_`PRWWR^_`^^YXS[[NZ
@HWI-EAS443:3:88:1198:273#0/1
ATCCATTCAAATGGTTTGCCTGCTTTCGCTAAAAATTCCA
+HWI-EAS443:3:88:1198:273#0/1
abaabbbbbbab``abbb¥`ab`bbbab]bbbbbaabW[^
@HWI-EAS443:3:93:916:2044#0/1
GGTTTGCCTGCTTTCGCTAAAAATTCCAGCGCAAAATGCC
+HWI-EAS443:3:93:916:2044#0/1
aa`[Z`_WL]bU_aaa^R^aaaaa]`a_`[Ua]__PVZZ_
@HWI-EAS443:3:6:1058:1065#0/1
TTCGCTAAAAATTCCAGCGCAAAATGCCATAAGCACCACA
+HWI-EAS443:3:6:1058:1065#0/1
aYT`_`]`Y]W_a`__`_^^``^__^V[[[YW_^`YS_Z_
@HWI-EAS443:3:12:868:415#0/1
GCTTTTGCTAAAAATTCCAGCGCAAAATGCCATAAGCACC
+HWI-EAS443:3:12:868:415#0/1
Yaa¥^ZZJX¥U^XT]aa[aa_`YQR_baQ_bb]ba_]ba`
@HWI-EAS443:3:13:1584:1900#0/1
AAATGAGCAGGGGTCAGGATATGCAGCTAACCTGAAGAGC
```

```
+HWI-EAS443:3:13:1584:1900#0/1
a`aaa^_`_`_]VKM__^[_[[]_]_MIP^IMO^YX[BBB
@HWI-EAS443:3:25:851:1848#0/1
ATGGCATTCTGTGTGCTCTTCAGGTCAGCTGCATATCCTG
+HWI-EAS443:3:25:851:1848#0/1
a`[a]aaaa`aY`]`a_a___a_]W_a__¥_Z__a[_^¥^
@HWI-EAS443:3:31:1476:1545#0/1
GTCAATGGCATTCTGTGTGCTCTTCAGGTCAGCTGCATAT
+HWI-EAS443:3:31:1476:1545#0/1
a`abba`[^aaa_ba```a_aaaa`aaa_aa_¥aa]``a_
@HWI-EAS443:3:33:740:867#0/1
ATGGCATTCTGTGTGCTCTTCAGGTCAGCTGCATATCCTG
+HWI-EAS443:3:33:740:867#0/1
WT¥]]WT_]^XFSRa^aa]R^O¥aQaY_¥VSTSOMFORXO
@HWI-EAS443:3:48:515:352#0/1
GTGCTCTTCAGGTCAGCTGCATATCCTGACCCCTGCTCAT
+HWI-EAS443:3:48:515:352#0/1
a[a[^VaX`a`a¥¥N_a`aZ¥_a¥_¥¥_`^^XU^_^WNZ¥
@HWI-EAS443:3:50:84:333#0/1
GCAGCTGACCTGAAGAGCACACAGAATGCCATTGACGAGA
+HWI-EAS443:3:50:84:333#0/1
aba[abbbbb`_bbabaaaaaaa`aaaa]aaab^aaba_[
@HWI-EAS443:3:60:231:169#0/1
AGCAGGGGTCAGGATATGCAGCTGACCTGAAGAGCACACA
+HWI-EAS443:3:60:231:169#0/1
`bb_`a`aZ¥a^`S___a`X¥[_^`__Y[U]`¥_^___`^
@HWI-EAS443:3:64:1714:1670#0/1
AAAAATCCCAGCGCAAAATGCCATAAGCACCACATTCCCT
+HWI-EAS443:3:64:1714:1670#0/1
UT_W__bbUbb`a]^Z¥]`_a``a_`VWa`UZa[``_WRY
@HWI-EAS443:3:44:1001:1629#0/1
AGCAGCAAAAGCAGGTAGATATTTAAAGATGAGTCTTCTA
+HWI-EAS443:3:44:1001:1629#0/1
`]_`b^aa`_]aa^]U_a`aaaa`a^^a`__`_]_a^Ta^
@HWI-EAS443:3:54:110:96#0/1
GCAGCAAAAGCAGGTAGATATTTAAAGATGAGTCTTCTAA
+HWI-EAS443:3:54:110:96#0/1
a`^`YVQJW_ZIaZX^a]]^`__WRMVGY^U^NXSSVWY[
@HWI-EAS443:3:14:373:747#0/1
ACGTTCGAATGGGAGTGCAGATGCAGCGATTCAAGTGATC
+HWI-EAS443:3:14:373:747#0/1
a_`__a`WXZa_^G`SWQV]^N[a___`UPVZU^QPTQN¥
@HWI-EAS443:3:34:1047:1718#0/1
TAGAAGGGACATTCCTCAATCCTGTGGCCAGTTTCAATTT
+HWI-EAS443:3:34:1047:1718#0/1
Xa```abaaab_a`a¥_baaaa]MTaaa_ZBBBBBBBBBB
@HWI-EAS443:3:56:714:487#0/1
ACAGGATTGAGGAATGTCCCTTCTATTCAATCTAGCGGCC
+HWI-EAS443:3:56:714:487#0/1
XWW``]aYba__[_^b`_ZZbaa]RWTX¥YZRZ`[WO`¥^
```

@HWI-EAS443:3:62:189:1980#0/1
ACAGGATTGAGGAATGTCCCTTCTATTCAAGCTAGAGGCC
+HWI-EAS443:3:62:189:1980#0/1
a`a_VXYNN¥]RI]Y]Wa¥_PRZR]N[XTBBBBBBBBBBB
@HWI-EAS443:3:64:337:772#0/1
ATAGAAGGGACATTCCTCAATCCTGTGGCCAGGCTCAATT
+HWI-EAS443:3:64:337:772#0/1
^_]ZY^`XTX¥aa`]`]Y_`_W[^RSYZ¥SBBBBBBBBBB
@HWI-EAS443:3:81:793:1876#0/1
CTGGCCACAGGATTGAGGAATGTCCCTTATATTCAATCTA
+HWI-EAS443:3:81:793:1876#0/1
a]aa`_XY_XXFXYUUZVZ_[QPPNX_¥GJ___X]¥SMX`
@HWI-EAS443:3:97:1130:1843#0/1
CAGAGGATTGAGGAATGTCCCTTCTATTCAATCTAGAGGC
+HWI-EAS443:3:97:1130:1843#0/1
O¥Q_bbbbabb]a_bbbaaa[baabaaababbbbaaabbU
@HWI-EAS443:3:63:766:1197#0/1
GGAATGTCCCTTCTATTCAATCTAGAGGCCTATTTNGGGC
+HWI-EAS443:3:63:766:1197#0/1
``_X_^^aaa`aaaaa````[`]^]`¥Z[__^[_YD[Z^Y
@HWI-EAS443:3:64:202:1773#0/1
GAGGAATNTCCCTTCTATTCAATCTAGAGGCCTATTTGGG
+HWI-EAS443:3:64:202:1773#0/1
a`]Z]aXDUaaaaaa`a`]W^Z``]_]_^R]X_R]]¥PPZ
@HWI-EAS443:3:21:510:406#0/1
GTGGCCCCAAATAGGCCTCTAGATTGAATAGAAGGGACAT
+HWI-EAS443:3:21:510:406#0/1
aU`P^^S__[aZVPaQRVPY[¥KKU^]VTTUUGG^]PGQV
@HWI-EAS443:3:5:480:1832#0/1
CACTGGCCACAGGATTGAGGAATGTCCCTTCTATTCAATC
+HWI-EAS443:3:5:480:1832#0/1
abba^`a`b`_b_abaaabababW^``aababbba`[Y[^
@HWI-EAS443:3:69:659:1523#0/1
CGGCCACAGGATTGAGGAATGTCCCTTCTATTCAATCTAG
+HWI-EAS443:3:69:659:1523#0/1
aaabbab¥aa__`¥____a_[Zaaa^__^__¥`_^¥^W_S
@HWI-EAS443:3:11:444:1001#0/1
AAAATTGAGACTGGCCACAGGATTGAGGAATGTCCCTTCT
+HWI-EAS443:3:11:444:1001#0/1
aaaaaabaaabaa_a`a¥aa`^_`_[a`____U^[^^^]]
@HWI-EAS443:3:11:807:1775#0/1
CAGGATTGAGGAATGTCCCTTCTATTCAATCTAGAGGCCT
+HWI-EAS443:3:11:807:1775#0/1
a`aZHVU[[`^a_[YY_`aWW_Y]]¥]^RVOV`XRRXBBB
@HWI-EAS443:3:15:1624:1062#0/1
TGAATAGAAGGGACATTCCTCAATCCTGTGGCCAGTCTCA
+HWI-EAS443:3:15:1624:1062#0/1
a`_ababVa``a__aaaa`a^```_]a`Xa_YY^XLW_SZ
@HWI-EAS443:3:18:1107:36#0/1
ACAGGATTGAGGAATGTCCCTTCTATTCAATCTAGAGGCC

```
+HWI-EAS443:3:18:1107:36#0/1
`bbba_]ababbaaaaa`]aaa^^aaa]_¥`_`a___aZT
@HWI-EAS443:3:18:1301:295#0/1
GGAATGTCCCTTCTATTCAATCTAGAGGCCTATTTGGGGC
+HWI-EAS443:3:18:1301:295#0/1
abaaaa]`a^b]`b]baZUXa`a][]aaaX¥[`^_`_¥aR
@HWI-EAS443:3:1:126:585#0/1
ACAGGATTGAGGAATGTCCCTTCTATTCAATCTAGAGGCC
+HWI-EAS443:3:1:126:585#0/1
aaab]a_^UZ`aaaa`_`^Q``Ya_aa___a^aa`_S`BB
@HWI-EAS443:3:1:460:983#0/1
CACAGGATTGAGGAATGTCCCTTCTATTCAATCTAGAGGC
+HWI-EAS443:3:1:460:983#0/1
ab`[bbabbbaabbbabaa[[bbaaabb_a^a]aaa_^aB
@HWI-EAS443:3:20:703:1499#0/1
AATAGGCCTCTAGATTGAATAGAAGGGACATTCCTCAATC
+HWI-EAS443:3:20:703:1499#0/1
a`Z_YX_][¥_``W^ZS^¥¥]]XXSY]X]]XYZXZ]^ZZX
@HWI-EAS443:3:20:882:725#0/1
CACAGGATTGAGGAATGTCCCTTCTATTCAATCTAGAGGC
+HWI-EAS443:3:20:882:725#0/1
a`_[ba``ba`aaa_aa_a`^a``a_aa__¥_Y`a^¥]¥¥
@HWI-EAS443:3:21:1008:2025#0/1
TAGGCCTCTAGATTGAATAGAAGGGACATTCCTCAATCCT
+HWI-EAS443:3:21:1008:2025#0/1
¥``b_^_[Za`bbb_aZabaa]aaaU[_]]a`a]¥a]GZa
@HWI-EAS443:3:21:233:475#0/1
CAGGATTGAGGAATGTCCCTTCTATTCAATCTAGAGGCCT
+HWI-EAS443:3:21:233:475#0/1
`a`b`aa``aa¥Z_a^aaaa`_aaa``__]``Z]_``_]`
@HWI-EAS443:3:23:1375:323#0/1
ATGTCCCTTCTATTCAATCTAGAGGCCTATTTGGGGCCAT
+HWI-EAS443:3:23:1375:323#0/1
ab`aabbbbaaabaZ`Yaaaaa_`¥X]a`a`__]_`[X]^
@HWI-EAS443:3:23:647:468#0/1
AATTGAGACTGGCCACAGGATTGAGGAATGTCCCTTCTAT
+HWI-EAS443:3:23:647:468#0/1
`bababaaaaabaa`I_ab_¥aZYaaWaYI¥WaWW`_^^^
@HWI-EAS443:3:25:1024:1245#0/1
CAAAATTGAGACTGGCCACAGGATTGAGGAATGTCCCTTC
+HWI-EAS443:3:25:1024:1245#0/1
aa]Z¥¥a`]a]b__`¥aa`aa`T¥``X]`[PNYJL_^Z_W
@HWI-EAS443:3:25:1424:332#0/1
CAGGATTGAGGAATGTCCCTTCTATTCAATCTAGAGGCCT
+HWI-EAS443:3:25:1424:332#0/1
^bbbabbbbbbbbbbbbbbbbbbbbbbbbbabbaaabaab
@HWI-EAS443:3:25:178:229#0/1
ACAGGATTGAGGAATGTCCCTTCTATTCAATCTAGAGGCC
+HWI-EAS443:3:25:178:229#0/1
a^V_aX```¥aa`^``U_a_]¥]]X]XY¥[[_ZZR¥ZP[^
```

```
@HWI-EAS443:3:25:801:484#0/1
ACAGGATTGAGGAATGTCCCTTCTATTCAATCTAGAGGCC
+HWI-EAS443:3:25:801:484#0/1
aaabbabbbaba^aaaa``aaaaaaa`_aaa``a[_a__W
@HWI-EAS443:3:28:1502:1577#0/1
ACAAAATTGAGACTGGCCACAGGATTGAGGAATGTCCCTT
+HWI-EAS443:3:28:1502:1577#0/1
a``^_^a[[]`[aWJ]Y^`OOS^]Z^PYX]J`^ZBBBBBB
@HWI-EAS443:3:28:305:284#0/1
ACAGGATTGAGGAATGTCCCTTCTATTCAATCTAGAGGCC
+HWI-EAS443:3:28:305:284#0/1
abb_b``bbabba`^a`aab`_a_aaa_``_``a_`__¥W
@HWI-EAS443:3:2:1019:1478#0/1
CTTCTATTCAATCTAGAGGCCTATTTGGGGCCATTGCCGG
+HWI-EAS443:3:2:1019:1478#0/1
`baababbbaa`_ba][baa^aaaa[[ab[[Sa]aaX_aa
@HWI-EAS443:3:2:560:101#0/1
CACAGGATTGAGGAATGTCCCTTCTATTCAATCTAGAGGC
+HWI-EAS443:3:2:560:101#0/1
_a__bb_b`]^aa_^_b^aNS^_^_`a_Q^^_]__^¥]_W
@HWI-EAS443:3:2:703:407#0/1
GGCCACAGGATTGAGGAATGTCCCTTCTATTCAATCTAGA
+HWI-EAS443:3:2:703:407#0/1
N^WL[^Xa_[`b]V]b`b[aV`¥_bbZaabaaU[b`[aab
@HWI-EAS443:3:31:324:1941#0/1
CACAGGATTGAGGAATGTCCCTTCTATTCAATCTAGAGGC
+HWI-EAS443:3:31:324:1941#0/1
a__U]``[X`aaP]S¥_V^_^Y]^ZX[S_a[U_NaTWNNQ
@HWI-EAS443:3:32:1061:401#0/1
AATGTCCCTTCTATTCAATCTAGAGGCCTATTTGGGGCCA
+HWI-EAS443:3:32:1061:401#0/1
QX_ZT_Xa¥^`_¥¥_UZa```a_[_WZQ^[][WRY`a``a
@HWI-EAS443:3:32:1657:254#0/1
CTGGCCACAGGATTGAGGAATGTCCCTTCTATTCAATCTA
+HWI-EAS443:3:32:1657:254#0/1
abbb`]abbbbaabb`baaaba`¥]_aa_aaaaU`_b_ab
@HWI-EAS443:3:32:1687:1224#0/1
CAAAATTGAGACTGGCCACAGGATTGAGGAATGTCCCTTC
+HWI-EAS443:3:32:1687:1224#0/1
aY][aa^baa_`WFWY_ZM[_]MYY[QaaQ]^SQEDZ^V¥
@HWI-EAS443:3:33:1445:984#0/1
CAGGATTGAGGAATGTCCCTTCTATTCAATCTAGAGGCCT
+HWI-EAS443:3:33:1445:984#0/1
aab_P^bba`aYaaa_aaaa`_aaaaYa^`_]PUWV_N^`
@HWI-EAS443:3:34:1594:1062#0/1
CAAAATTGAGACTGGCCACAGGATTGAGGAATGTCCCTTC
+HWI-EAS443:3:34:1594:1062#0/1
abaaabbaaabbbbababa`bba_b``bbab`a¥^`O_WK
@HWI-EAS443:3:34:920:789#0/1
TTGAGGAATGTCCCTTCTATTCAATCTAGAGGCCTATTTG
```

```
+HWI-EAS443:3:34:920:789#0/1
`aa`a^Z]aa]a`aa`aa_aa^^Z^]_a[^]_]]_^^YXX
@HWI-EAS443:3:35:1212:1943#0/1
AATGGCCCCAAATAGGCCTCTAGATTGAATAGAAGGGACA
+HWI-EAS443:3:35:1212:1943#0/1
aa`a[^^^aab_F¥X¥__¥^V_ZZU^XQ_U]PY`Y¥W¥U^
@HWI-EAS443:3:35:1346:1437#0/1
CGGCAATGGCCCCAAATAGGCCTCTAGATTGAATAGAAGG
+HWI-EAS443:3:35:1346:1437#0/1
abbbb_ababbbbbabaa`aaaaa``b_aaaaaa`__a_a
@HWI-EAS443:3:35:403:383#0/1
AATTGAGACTGGCCACAGGATTGAGGAATGTCCCTTCTAT
+HWI-EAS443:3:35:403:383#0/1
_```b`b^babbbaaa___]`a]`a[``^`S^aaa¥__[W
@HWI-EAS443:3:35:725:1146#0/1
ATAGAAGGGACATTCCTCAATCCTGTGGCCAGTCTCAATT
+HWI-EAS443:3:35:725:1146#0/1
``a¥Q]]HT`__^¥`^^a^`]_aWLV[J^a_XQZV¥ZSOQ
@HWI-EAS443:3:35:796:26#0/1
ACCGGCAATGGCCCCAAATAGGCCTCTAGATTGAATAGAA
+HWI-EAS443:3:35:796:26#0/1
a`abaa_a¥_`aa_`bb^FZLYX_TVVY¥VV[[[XUUTQ]
@HWI-EAS443:3:37:515:1251#0/1
ACAGGATTGAGGAATGTCCCTTCTATTCAATCTAGAGGCC
+HWI-EAS443:3:37:515:1251#0/1
abbaaa_baaa``aaa_aa``^aaa`aXFMM]Wa]^^__]
@HWI-EAS443:3:38:1080:94#0/1
AAGGGACATTCCTCAATCCTGTGGCCAGTCTCAATTTTGT
+HWI-EAS443:3:38:1080:94#0/1
a¥^__a_]a]aaa__abaaaaTa`_``¥^YPXXX^__]]V
@HWI-EAS443:3:38:232:271#0/1
TGGCCACAGGATTGAGGAATGTCCCTTCTATTCAATCTAG
+HWI-EAS443:3:38:232:271#0/1
abbbb¥Y`[bQV_`V^ZUZ[XKUa^YHYSVQUQFL[[V`G
@HWI-EAS443:3:38:490:2004#0/1
TGTCCCTTCTATTCAATCTAGAGGCCTATTTGGGGCCATT
+HWI-EAS443:3:38:490:2004#0/1
a_Z`bbaaaaa_a``¥aa`a`a__W_aa_SKKZ¥ZQY^¥T
@HWI-EAS443:3:38:999:734#0/1
TGAGGAATGTCCCTTCTATTCAATCTAGAGGCCTATTTGG
+HWI-EAS443:3:38:999:734#0/1
aaT]^WYR^[]V_^X[`XYaBBBBBBBBBBBBBBBBBBBB
@HWI-EAS443:3:40:1122:999#0/1
ACAGGATTGAGGAATGTCCCTTCTATTCAATCTAGAGGCC
+HWI-EAS443:3:40:1122:999#0/1
aabbaaa`^]`ba`a``aaa`a``^_R_V]¥¥]a_`__aR
@HWI-EAS443:3:40:1504:377#0/1
CACAGGATTGAGGAATGTCCCTTCTATTCAATCTAGAGGC
+HWI-EAS443:3:40:1504:377#0/1
`baabb`bbba¥baaaba`a¥aa``a`aaa_a[_aaaX^Z
```

@HWI-EAS443:3:44:1779:755#0/1
ATGGCCCCAAATAGGCCTCTAGATTGAATAGAAGGGACAT
+HWI-EAS443:3:44:1779:755#0/1
a`b`a`aaaaa``_`^^`___a]__`[^_¥[^__`^TP[Y
@HWI-EAS443:3:44:780:775#0/1
GTCCCTTCTATTCAATCTAGAGGCCTATTTGGGGCCATTG
+HWI-EAS443:3:44:780:775#0/1
aabaabaaabaaaaaa`aaaaa`a`a¥a```^aa`_[^¥`
@HWI-EAS443:3:44:831:444#0/1
AGGCCTCTAGATTGAATAGAAGGGACATTCCTCAATCCTG
+HWI-EAS443:3:44:831:444#0/1
a`]_¥[bZa``_b`_K_aa¥a`ba_Y]]_`Y`X]]``_a_
@HWI-EAS443:3:45:1132:1578#0/1
CCAAATAGGCCTCTAGATTGAATAGAAGGGACATTCCTCA
+HWI-EAS443:3:45:1132:1578#0/1
S¥bbbbabbbbYZ]bbbaa`baaabbabab`W^a`Ya_U_
@HWI-EAS443:3:45:846:676#0/1
GGATTGAGGAATGTCCCTTCTATTCAATCTAGAGGCCTAT
+HWI-EAS443:3:45:846:676#0/1
_a¥][a_bb]]a_GZaS^¥T[XaaZ[PZQab_[[[ZQ][_
@HWI-EAS443:3:46:1039:20#0/1
AGATTGAATAGAAGGGACATTCCTCAATCCTGTGGCCAGT
+HWI-EAS443:3:46:1039:20#0/1
a`T]`bXVT^a[]]aa^`¥]`[S``[Y]¥_``X``ZVO[V
@HWI-EAS443:3:46:1653:117#0/1
CCCTTCTATTCAATCTAGAGGCCTATTTGGGGCCATTGCC
+HWI-EAS443:3:46:1653:117#0/1
`babaaaZbbaaabaabaabb__a_`aa`a_a_¥X^__¥¥
@HWI-EAS443:3:47:1539:1288#0/1
TAGAAGGGACATTCCTCAATCCTGTGGCCAGTCTCAATTT
+HWI-EAS443:3:47:1539:1288#0/1
aaa__a_``_``_^_`_`_a[]__]`_]X¥YY]XXXZZ]Z
@HWI-EAS443:3:48:1263:1716#0/1
CAGGATTGAGGAATGTCCCTTCTATTCAATCTAGAGGCCT
+HWI-EAS443:3:48:1263:1716#0/1
^_``Y[_¥^Z_b[aaS¥bbba`bbb¥Z]abZ`b``aaW`a
@HWI-EAS443:3:48:440:1727#0/1
CCCTTCTATTCAATCTAGAGGCCTATTTGGGGCCATTGCC
+HWI-EAS443:3:48:440:1727#0/1
aaabab_babbbaababbb_`Zaaabbb]]`a_a_`a_O`
@HWI-EAS443:3:49:268:1331#0/1
AAGGGACATTCCTCAATCCTGTGGCCAGTCTCAATTTTGT
+HWI-EAS443:3:49:268:1331#0/1
a^ZYVQbab``aaa¥`_]`a`M¥¥]S`Za_`S_U[¥aaUa
@HWI-EAS443:3:51:864:1952#0/1
CAGGATTGAGGAATGTCCCTTCTATTCAATCTAGAGGCCT
+HWI-EAS443:3:51:864:1952#0/1
abababbbbababbaaaaabbbbbbbbbbbaabab`[NWZ
@HWI-EAS443:3:53:1637:1106#0/1
CCCTTCTATTCAATCTAGAGGCCTATTTGGGGCCATTGCC

```
+HWI-EAS443:3:53:1637:1106#0/1
Z``]abbb^abbab`b_Saaaaaa_`aaVaZH]aa_aaXa
@HWI-EAS443:3:57:821:750#0/1
ACAGGATTGAGGAATGTCCCTTCTATTCAATCTAGAGGCC
+HWI-EAS443:3:57:821:750#0/1
`_aba[ab`_`^Yabbabbaa_¥`¥baa`_aa¥[XU]a_[
@HWI-EAS443:3:5:562:1885#0/1
GAATGTCCCTTCTATTCAATCTAGAGGCCTATTTGGGGCC
+HWI-EAS443:3:5:562:1885#0/1
`abaa^`]X`a``aa_Z``_Q^__¥_`ZVSZ¥__X_YBBB
@HWI-EAS443:3:60:1233:1181#0/1
CAGGATTGAGGAATGTCCCTTCTATTCAATCTAGAGGCCT
+HWI-EAS443:3:60:1233:1181#0/1
aVbb_bbb_a_aa`bbaaa`bbaa_aabQ`[a]`b`¥[a`
@HWI-EAS443:3:60:141:77#0/1
ATAGAAGGGACATTCCTCAATCCTGTGGCCAGTCTCAATT
+HWI-EAS443:3:60:141:77#0/1
`b`ba_a```TVa`]^a__``[L`^ZaXPPW`RT_WRU^_
@HWI-EAS443:3:60:289:313#0/1
GGCCACAGGATTGAGGAATGTCCCTTCTATTCAATCTAGA
+HWI-EAS443:3:60:289:313#0/1
abaa`a]aaH_aaaa_[T^a¥_¥¥¥___]¥¥ZVVW¥W^WU
@HWI-EAS443:3:62:130:63#0/1
TTGAGACTGGCCACAGGATTGAGGAATGTCCCTTCTATTC
+HWI-EAS443:3:62:130:63#0/1
aba_a^`_`a`_^_¥_`V[_^X]^WUXYXXXXXXYSKTYT
@HWI-EAS443:3:62:978:370#0/1
TGAGGAATGTCCCTTCTATTCAATCTAGAGGCCTATTTGG
+HWI-EAS443:3:62:978:370#0/1
abab``bab`aa`baaaaba`aa_aab]]`¥Xaa_^Z_a_
@HWI-EAS443:3:63:68:439#0/1
AATTGAGACTGGCCACAGGATTGAGGAATGTCCCTTCTAT
+HWI-EAS443:3:63:68:439#0/1
_a^]^WaY^_baa^^^]Q[JY`]W]_NVQSVVSZTY^ULT
@HWI-EAS443:3:64:1314:1361#0/1
AAGGGACATTCCTCAATCCTGTGGCCAGTCTCAATTTTGT
+HWI-EAS443:3:64:1314:1361#0/1
aa_```bbab_aaa``¥`Y]^U¥a_a]`Y[^^WT`a`_XV
@HWI-EAS443:3:66:1669:681#0/1
ACAGGATTGAGGAATGTCCCTTCTATTCAATCTAGAGGCC
+HWI-EAS443:3:66:1669:681#0/1
`]abaab`baba]aaa^``aa]^a_b``a`^¥aaaaa]X^
@HWI-EAS443:3:67:1128:310#0/1
CAGGATTGAGGAATGTCCCTTCTATTCAATCTAGAGGCCT
+HWI-EAS443:3:67:1128:310#0/1
aa_b_bab``baa`b`b]^^`Xa`aa_`a`_aa`a^VXR[
@HWI-EAS443:3:67:1184:1787#0/1
GGAATGTCCCTTCTATTCAATCTAGAGGCCTATTTGGGGC
+HWI-EAS443:3:67:1184:1787#0/1
_abbbb]abbbbababbabaaaabaaaaa_aaa`aa____
```

@HWI-EAS443:3:67:1221:1123#0/1
CTGGCCACAGGATTGAGGAATGTCCCTTCTATTCAATCTA
+HWI-EAS443:3:67:1221:1123#0/1
a_`b`aa`aa^_____aa^`_a__`_aa__¥¥___^¥W_`
@HWI-EAS443:3:69:663:1748#0/1
TAGGCCTCTAGATTGAATAGAAGGGACATTCCTCAATCCT
+HWI-EAS443:3:69:663:1748#0/1
ab¥`bb```baabaaabbb`aaaa_a`aaa``aaabaaa_
@HWI-EAS443:3:6:1348:247#0/1
GCCCCAAATAGGCCTCTAGATTGAATAGAAGGGACATTCC
+HWI-EAS443:3:6:1348:247#0/1
_abaaaabbb_bbabbbbbbbbbbbbbbbabbaabbaa_
@HWI-EAS443:3:70:1724:754#0/1
GAATAGAAGGGACATTCCTCAATCCTGTGGCCAGTCTCAA
+HWI-EAS443:3:70:1724:754#0/1
aaaa^a`_`_`_¥__ZSaa``aa_X¥UT¥[¥]YSOUXWWV
@HWI-EAS443:3:71:367:870#0/1
GTCCCTTCTATTCAATCTAGAGGCCTATTTGGGGCCATTG
+HWI-EAS443:3:71:367:870#0/1
aa_``a``TZS__`S`¥`¥Z[YOY_ZH[]_]¥X[^X`]¥^
@HWI-EAS443:3:73:1317:154#0/1
GAATGTCCCTTCTATTCAATCTAGAGGCCTATTTGGGGCC
+HWI-EAS443:3:73:1317:154#0/1
`TXX`]N[¥`_V[X[XPYW[V_^SVYPRUZPPUV^]WVQT
@HWI-EAS443:3:74:399:1262#0/1
CCCTTCTATTCAATCTAGAGGCCTATTTGGGGCCATTGCC
+HWI-EAS443:3:74:399:1262#0/1
aa`a`a``aaaa[`a_a][]Z`_`a¥^[TU¥__^_WY_]U
@HWI-EAS443:3:76:1027:583#0/1
ATTGAATAGAAGGGACATTCCTCAATCCTGTGGCCAGTCT
+HWI-EAS443:3:76:1027:583#0/1
abbaaabaaaaaa`_`a`aaaa_aaaaa_Y[`a_^`^VUX
@HWI-EAS443:3:76:765:646#0/1
CAGGATTGAGGAATGTCCCTTCTATTCAATCTAGAGGCCT
+HWI-EAS443:3:76:765:646#0/1
a]_``b`aa`[]b_baa``aaaa_aaa_a_a`aa_P_NS_
@HWI-EAS443:3:78:834:317#0/1
TTCTATTCAATCTAGAGGCCTATTTGGGGCCATTGCCGGT
+HWI-EAS443:3:78:834:317#0/1
aaaa_a_``a`_^a`___]a_^¥_W_^Z]]¥_^^_X¥`_^
@HWI-EAS443:3:79:1769:289#0/1
TGGCCACAGGATTGAGGAATGTCCCTTCTATTCAATCTAG
+HWI-EAS443:3:79:1769:289#0/1
`b``aaa¥bbabb]]aaaa[UaZa^^b_baaZSZaZHH^_
@HWI-EAS443:3:7:1282:1206#0/1
GCCCCAAATAGGCCTCTAGATTGAATAGAAGGGACATTCC
+HWI-EAS443:3:7:1282:1206#0/1
abaaabba]bZaaaa__aa_aaaZa^aa_____¥R__]]R
@HWI-EAS443:3:7:232:1388#0/1
AAGGGACATTCCTCAATCCTGTGGCCAGTCTCAATTTTGT

```
+HWI-EAS443:3:7:232:1388#0/1
aW[[WQ[]X][aa^`b_a__aTRX[W_JOUT¥]ZSS_ZVR
@HWI-EAS443:3:83:1021:546#0/1
GGCCACAGGATTGAGGAATGTCCCTTCTATTCAATCTAGA
+HWI-EAS443:3:83:1021:546#0/1
X_a^bbabbabbbbbaaa_bba_babbaaaba[ababbaa
@HWI-EAS443:3:83:572:368#0/1
GAGGAATGTCCCTTCTATTCAATCTAGAGGCCTATTTGGG
+HWI-EAS443:3:83:572:368#0/1
a`aaaaaa_aa_`_``aa``__`^`¥`_[ZZ_]]^X]__]
@HWI-EAS443:3:84:625:134#0/1
ATTGAATAGAAGGGACATTCCTCAATCCTGTGGCCAGTCT
+HWI-EAS443:3:84:625:134#0/1
ab``^^aaaaa`[X^^[^__Y_^[`__`_`U^[¥ZTBBBB
@HWI-EAS443:3:84:706:1011#0/1
ATTGAATAGAAGGGACATTCCTCAATCCTGTGGCCAGTCT
+HWI-EAS443:3:84:706:1011#0/1
¥_[_S]_Z_X__W_`aZYY`T^`_¥a`[WQV`QZ]`YFOR
@HWI-EAS443:3:86:738:203#0/1
CCCCAAATAGGCCTCTAGATTGAATAGAAGGGACATTCCT
+HWI-EAS443:3:86:738:203#0/1
ab_abab_]`_a^^a]aa_aaa]a`_a``a`____`^¥X^
@HWI-EAS443:3:87:139:1455#0/1
ACAGGATTGAGGAATGTCCCTTCTATTCAATCTAGAGGCC
+HWI-EAS443:3:87:139:1455#0/1
aa^aa]¥^^[Y¥¥__ZU___^¥_T[_W^Y¥Y^¥_WY¥WW^
@HWI-EAS443:3:88:1151:1038#0/1
ACAGGATTGAGGAATGTCCCTTCTATTCAATCTAGAGGCC
+HWI-EAS443:3:88:1151:1038#0/1
aa`baaaba]``aaaa]a`_aa_`_a[[`a_^_a`a__Y^
@HWI-EAS443:3:90:641:1802#0/1
CCCTTCTATTCAATCTAGAGGCCTATTTGGGGCCATTGCC
+HWI-EAS443:3:90:641:1802#0/1
`b[FW_Z_Z]aaa]_¥WR^]ZVaW]¥X^[¥YZa]Z^¥`_]
@HWI-EAS443:3:90:884:360#0/1
GAAGGGACATTCCTCAATCCTGTGGCCAGTCTCAATTTTG
+HWI-EAS443:3:90:884:360#0/1
aaaaa`aa`b``_a^`aa`aaa[_^_]_`Z]_aZ^^``__
@HWI-EAS443:3:91:1556:1295#0/1
CACAGGATTGAGGAATGTCCCTTCTATTCAATCTAGAGGC
+HWI-EAS443:3:91:1556:1295#0/1
a_baba¥b_bb`VXV_``aaa]`VVaaaY`]`¥_`^[^aR
@HWI-EAS443:3:91:687:181#0/1
CTTCTATTCAATCTAGAGGCCTATTTGGGGCCATTGCCGG
+HWI-EAS443:3:91:687:181#0/1
abbbbbbbbbabaabba`aaabaaaaa_a`a[aa___^a`
@HWI-EAS443:3:93:1338:494#0/1
ACAGGATTGAGGAATGTCCCTTCTATTCAATCTAGAGGCC
+HWI-EAS443:3:93:1338:494#0/1
[aaaZV¥W``bbUW¥UYQHH¥`ab[_`X_F¥]a_HHWZNS
```

@HWI-EAS443:3:98:1487:152#0/1
AAGGGACATTCCTCAATCCTGTGGCCAGTCTCAATTTTGT
+HWI-EAS443:3:98:1487:152#0/1
aabaV`aaa_NR_^aaa]a`a^a_YN_`XW[¥Z]_VX[GG
@HWI-EAS443:3:99:1731:1781#0/1
GGCCACAGGATTGAGGAATGTCCCTTCTATTCAATCTAGA
+HWI-EAS443:3:99:1731:1781#0/1
]^P`_][bbUa`_]baba[^a`aR`_`a]aa^aaaP]aX`
@HWI-EAS443:3:99:664:1232#0/1
GAGGAATGTCCCTTCTATTCAATCTAGAGGCCTATTTGGG
+HWI-EAS443:3:99:664:1232#0/1
aabbbbbb_aaabbbbaaba`aaaab`aYa^Yaaa`____
@HWI-EAS443:3:9:453:1641#0/1
GGAATGTCCCTTCTATTCAATCTAGAGGCCTATTTGGGGC
+HWI-EAS443:3:9:453:1641#0/1
`_ZW^aaab_TQ_a[TI`aV]Sa¥NS_ZJ[_`SZ^[¥¥_^
@HWI-EAS443:3:9:682:322#0/1
CAGGATTGAGGAATGTCCCTTCTATTCAATCTAGAGGCCT
+HWI-EAS443:3:9:682:322#0/1
ab`a_bbbabba]bbaaaaab`abb`ab]a`aab`__L[a
@HWI-EAS443:3:1:525:1574#0/1
CAACTACTTCAATGCATGTGTGAGGAAGGAGGTGAACCAA
+HWI-EAS443:3:1:525:1574#0/1
¥¥aa`]OabW[`aa`¥bb`aSbYaaaaab^_I¥_WbG^PB
@HWI-EAS443:3:43:1471:1482#0/1
CAACTACTTCAATGCATGTGTGAGGAAGGAGTTGAACTAA
+HWI-EAS443:3:43:1471:1482#0/1
abb_bbbaaaaa`_RaabJaPTZbbaaab_a^`a]a_G_a
@HWI-EAS443:3:95:821:1955#0/1
CAACTACTTCAATGCATGTGTGAGGAAGGAGTTGAACAAA
+HWI-EAS443:3:95:821:1955#0/1
¥bb`bbababb`aaaaa]_b^babaaaba`a^__aa[F]`
@HWI-EAS443:3:96:1600:1517#0/1
CAACTACTTCAATNCATGTGTGAGGAAGGAGTTGAACCAA
+HWI-EAS443:3:96:1600:1517#0/1
abbbbb`bbab`RDYbabaaaaaaabbba]`abaa¥aP_a
@HWI-EAS443:3:55:1386:1617#0/1
AACTACTTCAATGCATGTGTGAGGAAGGAGTTGAACCNAG
+HWI-EAS443:3:55:1386:1617#0/1
ab^Za`b___^VaZ¥`b_^_a^^`_W`X^`_``_^PEDZ_
@HWI-EAS443:3:94:1103:661#0/1
ACNACTACTTCAATGCATGTGTGAGGAAGGAGTTGAACCA
+HWI-EAS443:3:94:1103:661#0/1
aYD^^ab_`aaa`aa^`ab`aXb`_`__a`]a^__^a^W_
@HWI-EAS443:3:23:957:1971#0/1
CTACTTCAATGCATGGGTGAGGAAGGAGTTGAACCAAGAT
+HWI-EAS443:3:23:957:1971#0/1
aabb[]][^Z_Y¥^¥XGY[`^[`R]P_UJOR^__LLWQNSF
@HWI-EAS443:3:40:394:184#0/1
CTTCAATGCATGTGTGAGGAAGGAGTTGCACCAAGATGCA

+HWI-EAS443:3:40:394:184#0/1
WVWW_ZO_`a`a]aT^U`a^W__`TQQ^FOUZU]]PP]]Y
@HWI-EAS443:3:10:1024:1336#0/1
CAACTACTTCAATGCATGTGTGAGGAAGGAGTTGAACCAA
+HWI-EAS443:3:10:1024:1336#0/1
ababbbbbbbbbbb¥bbaababbababababab_baaa`]aa
@HWI-EAS443:3:11:108:401#0/1
CAACTACTTCAATGCATGTGTGAGGAAGGAGTTGAACCAA
+HWI-EAS443:3:11:108:401#0/1
aaaaa_^aa`aaaaa`aaW`Xa_aa]W_aX`[```a]¥ZZ
@HWI-EAS443:3:12:868:58#0/1
CAACTACTTCAATGCATGTGTGAGGAAGGAGTTGAACCAA
+HWI-EAS443:3:12:868:58#0/1
aabaaa`aaaa`abW_`b¥aUb]aa^`a]W`]^_^]XZ^Z
@HWI-EAS443:3:14:558:1785#0/1
CAACTACTTCAATGCATGTGTGAGGAAGGAGTTGAACCAA
+HWI-EAS443:3:14:558:1785#0/1
V^aabb`bbabbab[`aaabOab`_`b`^a^`aUaaa_ab
@HWI-EAS443:3:16:21:1934#0/1
CAACTACTTCAATGCATGTGTGAGGAAGGAGTTGAACCAA
+HWI-EAS443:3:16:21:1934#0/1
aa]``a_^__a`W__W^_]_R^]]X]_]ZY]V]Z^]Z_]]
@HWI-EAS443:3:18:1652:545#0/1
CAACTACTTCAATGCATGTGTGAGGAAGGAGTTGAACCAA
+HWI-EAS443:3:18:1652:545#0/1
YY¥X^[PS^WXb]__aabYaLaXbb[]`a[a_`_^VFKa¥
@HWI-EAS443:3:18:625:594#0/1
CAACTACTTCAATGCATGTGTGAGGAAGGAGTTGAACCAA
+HWI-EAS443:3:18:625:594#0/1
¥]R_Z[a]_`¥YL`¥¥¥[XQZ^aabYSZa[`^a^PU`ZXP
@HWI-EAS443:3:20:1329:498#0/1
CAACTACTTCAATGCATGTGTGAGGAAGGAGTTGAACCAA
+HWI-EAS443:3:20:1329:498#0/1
_aabb`b_abbba`ab`b^`W][bbaaaa¥`___aaa^a]
@HWI-EAS443:3:21:1112:1000#0/1
CAACTACTTCAATGCATGTGTGAGGAAGGAGTTGAACCAA
+HWI-EAS443:3:21:1112:1000#0/1
ababbbbbbabababbbb_abbababaa_ab^aabaa]ba
@HWI-EAS443:3:21:848:1564#0/1
CAACTACTTCAATGCATGTGTGAGGAAGGAGTTGAACCAA
+HWI-EAS443:3:21:848:1564#0/1
a`V`UQZ`]^^XX^¥]Y_NZTa_aV[__][WU]]Y`]XYY
@HWI-EAS443:3:23:58:481#0/1
CAACTACTTCAATGCATGTGTGAGGAAGGAGTTGAACCAA
+HWI-EAS443:3:23:58:481#0/1
abbaababaa^aaaa_aaSa]`__```aa_a]aa_a`_]Z
@HWI-EAS443:3:24:1369:220#0/1
CAACTACTTCAATGCATGTGTGAGGAAGGAGTTGAACCAA
+HWI-EAS443:3:24:1369:220#0/1
`bbb_baa^_aaaa`¥bbab]`bbb`abbaaaaabbNNaa

```
@HWI-EAS443:3:25:1034:625#0/1
CAACTACTTCAATGCATGTGTGAGGAAGGAGTTGAACCAA
+HWI-EAS443:3:25:1034:625#0/1
aaZH`bab_`Ta¥b^`Xa_a^a_a`b_```a^¥`^a^I]W
@HWI-EAS443:3:25:256:1913#0/1
CAACTACTTCAATGCATGTGTGAGGAAGGAGTTGAACCAA
+HWI-EAS443:3:25:256:1913#0/1
]a_`Ua`^R__¥QTYPVXYTX^URQOXQZOTWZUW]ZHOT
@HWI-EAS443:3:27:1143:1853#0/1
CAACTACTTCAATGCATGTGTGAGGAAGGAGTTGAACCAA
+HWI-EAS443:3:27:1143:1853#0/1
_bb`abbbabbbaa`bbb[babababbbaba^abaaa`ab
@HWI-EAS443:3:27:1187:437#0/1
CAACTACTTCAATGCATGTGTGAGGAAGGAGTTGAACCAA
+HWI-EAS443:3:27:1187:437#0/1
`b¥bbab`_bb`_`bV¥`a`a`]bbbba^ab`babYaXab
@HWI-EAS443:3:27:827:1317#0/1
CAACTACTTCAATGCATGTGTGAGGAAGGAGTTGAACCAA
+HWI-EAS443:3:27:827:1317#0/1
Z¥aZ¥a[H]¥^baYRXVSW]N^aba``]OOZTS]W`V[aa
@HWI-EAS443:3:2:1180:20#0/1
CAACTACTTCAATGCATGTGTGAGGAAGGAGTTGAACCAA
+HWI-EAS443:3:2:1180:20#0/1
a]baaa`ab`a_aaTRaaW_V_Y]Z¥]a`W_¥^a_]QN[]
@HWI-EAS443:3:33:517:1761#0/1
CAACTACTTCAATGCATGTGTGAGGAAGGAGTTGAACCAA
+HWI-EAS443:3:33:517:1761#0/1
a_``a_``aa¥^`[_aaa]`V``]^_`PY]`Z]]Z_¥PXW
@HWI-EAS443:3:33:821:1220#0/1
CAACTACTTCAATGCATGTGTGAGGAAGGAGTTGAACCAA
+HWI-EAS443:3:33:821:1220#0/1
abbaabaaaba``a_¥_b[a]a`````^¥a`]R__a]]__
@HWI-EAS443:3:34:202:1818#0/1
CAACTACTTCAATGCATGTGTGAGGAAGGAGTTGAACCAA
+HWI-EAS443:3:34:202:1818#0/1
^Xab_b¥W]_b^`H_aaXT]LQ`YUTa`S]XU`S¥YYX¥_
@HWI-EAS443:3:37:1608:832#0/1
CAACTACTTCAATGCATGTGTGAGGAAGGAGTTGAACCAA
+HWI-EAS443:3:37:1608:832#0/1
^^YP`bbbb`bbbaU`bbabV_bbbbbb]O]abbaab^_a
@HWI-EAS443:3:3:1571:970#0/1
CAACTACTTCAATGCATGTGTGAGGAAGGAGTTGAACCAA
+HWI-EAS443:3:3:1571:970#0/1
aababbabb[a```aXabab]aa`Y``a```]aa_a`Y^W
@HWI-EAS443:3:3:973:1449#0/1
CAACTACTTCAATGCATGTGTGAGGAAGGAGTTGAACCAA
+HWI-EAS443:3:3:973:1449#0/1
ababbababbabbb`bbaabababaaabaaa`aaaa_Oaa
@HWI-EAS443:3:40:1287:734#0/1
CAACTACTTCAATGCATGTGTGAGGAAGGAGTTGAACCAA
```

+HWI-EAS443:3:40:1287:734#0/1
abbbbbbbbbbbbab`abbab]baba^Sabaa_bbbbbUS`
@HWI-EAS443:3:40:1545:1725#0/1
CAACTACTTCAATGCATGTGTGAGGAAGGAGTTGAACCAA
+HWI-EAS443:3:40:1545:1725#0/1
abbabbbbbbababaaabab¥baba__`bbb^aaba`P`a
@HWI-EAS443:3:40:706:311#0/1
CAACTACTTCAATGCATGTGTGAGGAAGGAGTTGAACCAA
+HWI-EAS443:3:40:706:311#0/1
_Z¥^a_V]`aa]TTYQaa_aYaZZNQQ_]^^^^^[TZZ[W
@HWI-EAS443:3:42:116:247#0/1
CAACTACTTCAATGCATGTGTGAGGAAGGAGTTGAACCAA
+HWI-EAS443:3:42:116:247#0/1
`ba_`_aaa`a`_a^`_a^aZa_a``]a`_a]`^`_^^aY
@HWI-EAS443:3:46:1557:304#0/1
CAACTACTTCAATGCATGTGTGAGGAAGGAGTTGAACCAA
+HWI-EAS443:3:46:1557:304#0/1
a]Zab_]aaaZM]^```b_aaa_aa^`_]Xa`]`_UY[Z_
@HWI-EAS443:3:46:77:829#0/1
CAACTACTTCAATGCATGTGTGAGGAAGGAGTTGAACCAA
+HWI-EAS443:3:46:77:829#0/1
abbabbbbba`aababbbababababbbabaabaabaaa`ba
@HWI-EAS443:3:52:200:139#0/1
CAACTACTTCAATGCATGTGTGAGGAAGGAGTTGAACCAA
+HWI-EAS443:3:52:200:139#0/1
abbabbbbbaaabbaaab_b_a`ba_aaa^a_aaa`XDR`
@HWI-EAS443:3:53:170:509#0/1
CAACTACTTCAATGCATGTGTGAGGAAGGAGTTGAACCAA
+HWI-EAS443:3:53:170:509#0/1
a^Waa__a`[`^bb^^]a[_Va]a_]Za^aa_aaa_VV¥¥
@HWI-EAS443:3:55:1512:338#0/1
CAACTACTTCAATGCATGTGTGAGGAAGGAGTTGAACCAA
+HWI-EAS443:3:55:1512:338#0/1
Xb¥abbX`X]]Vabaa]a`b]aaaa`_bbaaaaaaaa^aa
@HWI-EAS443:3:55:1554:316#0/1
CAACTACTTCAATGCATGTGTGAGGAAGGAGTTGAACCAA
+HWI-EAS443:3:55:1554:316#0/1
U`abababb¥bb`b]babb¥`bababb_aaabbbbab_ab
@HWI-EAS443:3:55:1590:17#0/1
CAACTACTTCAATGCATGTGTGAGGAAGGAGTTGAACCAA
+HWI-EAS443:3:55:1590:17#0/1
¥ba`abaaa_aaaa[_ab¥a`b^aaa_aa]```a`a`G_^
@HWI-EAS443:3:55:755:20#0/1
CAACTACTTCAATGCATGTGTGAGGAAGGAGTTGAACCAA
+HWI-EAS443:3:55:755:20#0/1
`_baababa`a`aaaaab^b_babb__baaa_aa___Wa`
@HWI-EAS443:3:57:1322:2036#0/1
TAACTACTTCAATGCATGTGTGAGGAAGGAGTTGAACCAA
+HWI-EAS443:3:57:1322:2036#0/1
LYabba`b`bbabaabababababaaab`abab_b`GG_`

@HWI-EAS443:3:5:1247:1831#0/1
CAACTACTTCAATGCATGTGTGAGGAAGGAGTTGAACCAA
+HWI-EAS443:3:5:1247:1831#0/1
^abbbbbbb^aabbXabb_b`babaabab`a_`aaa_^]a
@HWI-EAS443:3:5:1588:1311#0/1
CAACTACTTCAATGCATGTGTGAGGAAGGAGTTGAACCAA
+HWI-EAS443:3:5:1588:1311#0/1
aaa_bb``¥W^aaa]`ab_b``^a___ba_bV`aa_aR_^
@HWI-EAS443:3:61:1670:879#0/1
CAACTACTTCAATGCATGTGTGAGGAAGGAGTTGAACCAA
+HWI-EAS443:3:61:1670:879#0/1
abbbbbbbbbbbaab_bbab[bbbbba[ba`aaaab____
@HWI-EAS443:3:61:484:127#0/1
CAACTACTTCAATGCATGTGTGAGGAAGGAGTTGAACCAA
+HWI-EAS443:3:61:484:127#0/1
aba``bbbb`]^a_a]aaYaV¥T`¥OU¥a^_W^_WVSPXS
@HWI-EAS443:3:63:402:719#0/1
CAACTACTTCAATGCATGTGTGAGGAAGGAGTTGAACCAA
+HWI-EAS443:3:63:402:719#0/1
a]aa`¥[Za]^a`X_Yaa_`U_Ua]_]]_Z```a_a^HYL
@HWI-EAS443:3:63:686:1184#0/1
CAACTACTTCAATGCATGTGTGAGGAAGGAGTTGAACCAA
+HWI-EAS443:3:63:686:1184#0/1
a_baabaaaab```aaaa^a]aaaa`a`_``_``aaa¥a[
@HWI-EAS443:3:64:1737:123#0/1
CAACTACTTCAATGCATGTGTGAGGAAGGAGTTGAACCAA
+HWI-EAS443:3:64:1737:123#0/1
aab`baabb`ababaabbaa`babaaaba_baba`¥X__`
@HWI-EAS443:3:64:773:683#0/1
CAACTACTTCAATGCATGTGTGAGGAAGGAGTTGAACCAA
+HWI-EAS443:3:64:773:683#0/1
abbbabbabbbba`^Q_b_aZaaaa`^a^`a_`__aa_a¥
@HWI-EAS443:3:67:610:888#0/1
CAACTACTTCAATGCATGTGTGAGGAAGGAGTTGAACCAA
+HWI-EAS443:3:67:610:888#0/1
abbaaabaaaaa_aa`aa_`^a`a__a__a`^_aa__X`]
@HWI-EAS443:3:67:963:1231#0/1
CAACTACTTCAATGCATGTGTGAGGAAGGAGTTGAACCAA
+HWI-EAS443:3:67:963:1231#0/1
abbbaaaaaaa`a``aaa`a_``a_aa_`_`]``_a_]_^
@HWI-EAS443:3:68:1767:552#0/1
CAACTACTTCAATGCATGTGTGAGGAAGGAGTTGAACCAA
+HWI-EAS443:3:68:1767:552#0/1
abbbababbabaaa_abb_a`aaaa`aaaaa^aaa_ZS^]
@HWI-EAS443:3:68:351:617#0/1
CAACTACTTCAATGCATGTGTGAGGAAGGAGTTGAACCAA
+HWI-EAS443:3:68:351:617#0/1
Y_ababbbbabZbbbbbbaaaa`a`bba_b`aabbbabbb
@HWI-EAS443:3:69:1382:794#0/1
CAACTACTTCAATGCATGTGTGAGGAAGGAGTTGAACCAA

```
+HWI-EAS443:3:69:1382:794#0/1
abbababaaabab_aabbaa`a_baa`ba_a`aaa`__a`
@HWI-EAS443:3:69:901:1434#0/1
CAACTACTTCAATGCATGTGTGAGGAAGGAGTTGAACCAA
+HWI-EAS443:3:69:901:1434#0/1
abbaabbbbabbaaa[aa]a__]a```a``_Za````[aa
@HWI-EAS443:3:6:701:1202#0/1
CAACTACTTCAATGCATGTGTGAGGAAGGAGTTGAACCAA
+HWI-EAS443:3:6:701:1202#0/1
a¥`a]bb`b¥bbbaababXb_b_bbbabb`bbaa^_^_bb
@HWI-EAS443:3:70:293:1211#0/1
CAACTACTTCAATGCATGTGTGAGGAAGGAGTTGAACCAA
+HWI-EAS443:3:70:293:1211#0/1
`b¥aabaa_ba`^__NZaYa¥b]__W¥[ZHZW``__a¥[]
@HWI-EAS443:3:70:914:439#0/1
CAACTACTTCAATGCATGTGTGAGGAAGGAGTTGAACCAA
+HWI-EAS443:3:70:914:439#0/1
a¥_`^a`_[_¥_bbVX_bY```^``aa`]Z___^ab_TZ_
@HWI-EAS443:3:76:1296:608#0/1
CAACTACTTCAATGCATGTGTGAGGAAGGAGTTGAACCAA
+HWI-EAS443:3:76:1296:608#0/1
aXb]``_abbbabb]aabY`abaaa`¥_bY``bba^¥NaY
@HWI-EAS443:3:76:542:531#0/1
CAACTACTTCAATGCATGTGTGAGGAAGGAGTTGAACCAA
+HWI-EAS443:3:76:542:531#0/1
IYbbbab`b``^^]Z__Za`^aa``_ba^aaaa^abZZ`b
@HWI-EAS443:3:79:896:1952#0/1
CAACTACTTCAATGCATGTGTGAGGAAGGAGTTGAACCAA
+HWI-EAS443:3:79:896:1952#0/1
aU`babbaaab`aTZabaS`_aa`_`]a`¥a]ab_`_G]_
@HWI-EAS443:3:81:866:2020#0/1
CAACTACTTCAATGCATGTGTGAGGAAGGAGTTGAACCAA
+HWI-EAS443:3:81:866:2020#0/1
aa]`Xaaa^]^aWW]Zaa_]USUYaX_YK_b]aYN_WF_Y
@HWI-EAS443:3:82:1358:30#0/1
CAACTACTTCAATGCATGTGTGAGGAAGGAGTTGAACCAA
+HWI-EAS443:3:82:1358:30#0/1
`bbbb_babbabaaaabb]b]_bba_abbababababaaGb`
@HWI-EAS443:3:83:39:1850#0/1
CAACTACTTCAATGCATGTGTGAGGAAGGAGTTGAACCAA
+HWI-EAS443:3:83:39:1850#0/1
aa__]L¥X^_`_[[_¥¥]VUX]^¥ZP^POX^RZX^ZZSUX
@HWI-EAS443:3:83:551:1221#0/1
CAACTACTTCAATGCATGTGTGAGGAAGGAGTTGAACCAA
+HWI-EAS443:3:83:551:1221#0/1
aaa`aabaaa]X_`aaaa^_Za_¥^```__]Y`___^W]_
@HWI-EAS443:3:83:870:1126#0/1
CAACTACTTCAATGCATGTGTGAGGAAGGAGTTGAACCAA
+HWI-EAS443:3:83:870:1126#0/1
aaa`abaaaaa`aaaaaa_a^a]]Yaaa`__Xaa``a[a_
```

@HWI-EAS443:3:84:308:1570#0/1
CAACTACTTCAATGCATGTGTGAGGAAGGAGTTGAACCAA
+HWI-EAS443:3:84:308:1570#0/1
]Zb_X¥ab¥Y``a`V¥a_F_`b_aa]`b¥]aN`a^`a[`a
@HWI-EAS443:3:85:306:211#0/1
CAACTACTTCAATGCATGTGTGAGGAAGGAGTTGAACCAA
+HWI-EAS443:3:85:306:211#0/1
aaaa]a__^aU]`a^`__X]X_XW]]]]]Y_X]^Z^W¥TW
@HWI-EAS443:3:86:175:857#0/1
CAACTACTTCAATGCATGTGTGAGGAAGGAGTTGAACCAA
+HWI-EAS443:3:86:175:857#0/1
]ab[]_TTa`a¥UZ^`ab^[S^Y^XTZ`_PaR]baX^V]a
@HWI-EAS443:3:86:274:81#0/1
CAACTACTTCAATGCATGTGTGAGGAAGGAGTTGAACCAA
+HWI-EAS443:3:86:274:81#0/1
abbbbb`baa^Qabaaaa`b]b_aaa`a`_a__a__¥S]]
@HWI-EAS443:3:87:365:197#0/1
CAACTACTTCAATGCATGTGTGAGGAAGGAGTTGAACCAA
+HWI-EAS443:3:87:365:197#0/1
abbbbabbbbaaabaabbab`aabaaaaa`a[aaaa`]a¥
@HWI-EAS443:3:88:418:970#0/1
CAACTACTTCAATGCATGTGTGAGGAAGGAGTTGAACCAA
+HWI-EAS443:3:88:418:970#0/1
a]]`VaZQW_a_X¥^XaV`a]¥X[ZXVa`V[GW`X¥VWYY
@HWI-EAS443:3:88:923:476#0/1
CAACTACTTCAATGCATGTGTGAGGAAGGAGTTGAACCAA
+HWI-EAS443:3:88:923:476#0/1
abbbbbbbbbbbbbaabbab`baaababaababbbba`bb
@HWI-EAS443:3:89:1379:1312#0/1
CAACTACTTCAATGCATGTGTGAGGAAGGAGTTGAACCAA
+HWI-EAS443:3:89:1379:1312#0/1
abbaba¥a_b¥aXb_aba^ba`]bbaaba_`^aa`^_^^`
@HWI-EAS443:3:89:375:2001#0/1
CAACTACTTCAATGCATGTGTGAGGAAGGAGTTGAACCAA
+HWI-EAS443:3:89:375:2001#0/1
_a^^babb^baaSa__a_LV¥``UW_^a`a]__a`_`Ja_
@HWI-EAS443:3:89:646:1038#0/1
CAACTACTTCAATGCATGTGTGAGGAAGGAGTTGAACCAA
+HWI-EAS443:3:89:646:1038#0/1
aV]_a^aaa`Y^^^`VZaSa_^I`XQUaP^_YX¥^^]]]_
@HWI-EAS443:3:89:917:1146#0/1
CAACTACTTCAATGCATGTGTGAGGAAGGAGTTGAACCAA
+HWI-EAS443:3:89:917:1146#0/1
aa``Zabb``aYS¥^Z`_R_¥¥__^aaa^aaX]aaa_N_^
@HWI-EAS443:3:92:961:729#0/1
CAACTACTTCAATGCATGTGTGAGGAAGGAGTTGAACCAA
+HWI-EAS443:3:92:961:729#0/1
``b`baZa^Xabbb]aab^bZbaabaaaaabaaba^RT_`
@HWI-EAS443:3:95:1276:210#0/1
CAACTACTTCAATGCATGTGTGAGGAAGGAGTTGAACCAA

+HWI-EAS443:3:95:1276:210#0/1
Vabbb^Ob`Z`aXaZ`b[Z[LbabbaaaLZba¥ab`^_ab
@HWI-EAS443:3:98:1693:153#0/1
CAACTACTTCAATGCATGTGTGAGGAAGGAGTTGAACCAA
+HWI-EAS443:3:98:1693:153#0/1
VaZ````a¥J`a`^`aab_`_^aZ¥Ya`¥¥b^aaaaRNaa
@HWI-EAS443:3:9:228:324#0/1
CAACTACTTCAATGCATGTGTGAGGAAGGAGTTGAACCAA
+HWI-EAS443:3:9:228:324#0/1
`bb`abaa`baabbbabbYb`b`babab^`a¥a_aaa_`a
@HWI-EAS443:3:29:794:978#0/1
CTANTTCAATGCATGTGTGAGGAAGGAGTTGAACCAAGAT
+HWI-EAS443:3:29:794:978#0/1
ab]D^aba``abaaa[a_a`ba_aaaab_aaa^aa_aaa_
@HWI-EAS443:3:100:527:1437#0/1
AACTACTTCAATGCATGTGTGAGGAAGGAGTTGAACCAAG
+HWI-EAS443:3:100:527:1437#0/1
aabO`^^^baaW^`]ab`¥^¥[bT¥VJ^a`Y^a_Y^Ia__
@HWI-EAS443:3:11:450:1251#0/1
AACTACTTCAATGCATGTGTGAGGAAGGAGTTGAACCAAG
+HWI-EAS443:3:11:450:1251#0/1
_ab^a¥^a_aaaa`^[`S¥Ra^`Y^`a__a^__`^_a```
@HWI-EAS443:3:11:524:1328#0/1
AACTACTTCAATGCATGTGTGAGGAAGGAGTTGAACCAAG
+HWI-EAS443:3:11:524:1328#0/1
a`^^aa]_a]a_a__aa]aUa`_S`_aYX_Xa`^a_¥]]^
@HWI-EAS443:3:15:1079:1477#0/1
AACTACTTCAATGCATGTGTGAGGAAGGAGTTGAACCAAG
+HWI-EAS443:3:15:1079:1477#0/1
^abbb`baX``VWW¥W`XZ`¥Z]^aaaa^_]``ZZa]Y]^
@HWI-EAS443:3:15:310:1424#0/1
AACTACTTCAATGCATGTGTGAGGAAGGAGTTGAACCAAG
+HWI-EAS443:3:15:310:1424#0/1
`bba^ZT¥a`[`aa`T^]a[_ZPZ¥]`]GVO]a_¥¥UU^a
@HWI-EAS443:3:17:148:773#0/1
AACTACTTCAATGCATGTGTGAGGAAGGAGTTGAACCAAG
+HWI-EAS443:3:17:148:773#0/1
`baba_aaaaaa_]aa__a__aa``aa`_`]_```_``]`
@HWI-EAS443:3:19:56:1556#0/1
AACTACTTCAATGCATGTGTGAGGAAGGAGTTGAACCAAG
+HWI-EAS443:3:19:56:1556#0/1
abb¥¥Y[`[_[L^a_VUO_Y^`aYYK^Z[YTZ]`aa]``a
@HWI-EAS443:3:29:214:1352#0/1
AACTACTTCAATGCATGTGTGAGGAAGGAGTTGAACCAAG
+HWI-EAS443:3:29:214:1352#0/1
aaaab``abaa``a```]_]_`]V_][TZX¥¥¥]`aZZ]Z
@HWI-EAS443:3:31:1494:792#0/1
AACTACTTCAATGCATGTGTGAGGAAGGAGTTGAACCAAG
+HWI-EAS443:3:31:1494:792#0/1
__bbabbbYba]b]abb¥b^babb_aba^a]bb¥V]a`aa

```
@HWI-EAS443:3:33:883:1316#0/1
AACTACTTCAATGCATGTGTGAGGAAGGAGTTGAACCAAG
+HWI-EAS443:3:33:883:1316#0/1
aaabaaaaaba_a_aa``aX`a`^`^[___W`]^]_^_]^
@HWI-EAS443:3:38:451:1995#0/1
AACTACTTCAATGCATGTGTGAGGAAGGAGTTGAACCAAG
+HWI-EAS443:3:38:451:1995#0/1
aaa`aa__aaa^]_a`_]_U]^___a_^_[W]^__Xa_`W
@HWI-EAS443:3:3:317:1734#0/1
AACTACTTCAATGCATGTGTGAGGAAGGAGTTGAACCAAG
+HWI-EAS443:3:3:317:1734#0/1
aaa`aaa`]baab_aaa^aQ__a¥^a_U`^`___`X_U^]
@HWI-EAS443:3:40:1073:137#0/1
AACTACTTCAATGCATGTGTGAGGAAGGAGTTGAACCAAG
+HWI-EAS443:3:40:1073:137#0/1
aaa`bab`^ba`a``aa]a_a]aa_]_a¥_]^aa`a`__`
@HWI-EAS443:3:42:391:2030#0/1
AACTACTTCAATGCATGTGTGAGGAAGGAGTTGAACCAAG
+HWI-EAS443:3:42:391:2030#0/1
aaa[aa__Z`a`YV_a`S_TZ¥RW^_¥^_Y¥ZY__`[`[Z
@HWI-EAS443:3:43:70:883#0/1
AACTACTTCAATGCATGTGTGAGGAAGGAGTTGAACCAAG
+HWI-EAS443:3:43:70:883#0/1
a_aabaaabbaaaa`baZa_a_baa``¥`^_^aa`````_
@HWI-EAS443:3:45:931:653#0/1
AACTACTTCAATGCATGTGTGAGGAAGGAGTTGAACCAAG
+HWI-EAS443:3:45:931:653#0/1
^Y_Z¥¥_`¥^^Xa]a^_Q_X^```ZP¥¥Y`]_`P`aaa`a
@HWI-EAS443:3:46:1792:326#0/1
NACTACTTCAATGCATGTGTGAGGAAGGAGTTGAACCAAG
+HWI-EAS443:3:46:1792:326#0/1
DOZZZZYYYYYZZZXYXYZYYXXYXXWYZZYWYZXXXXXV
@HWI-EAS443:3:51:105:134#0/1
AACTACTTCAATGCATGTGTGAGGAAGGAGTTGAACCAAG
+HWI-EAS443:3:51:105:134#0/1
[aaaa^`aaaa_b^Y^_XaRa]`a^^_^U[V_`¥a^S]¥a
@HWI-EAS443:3:51:1583:181#0/1
AACTACTTCAATGCATGTGTGAGGAAGGAGTTGAACCAAG
+HWI-EAS443:3:51:1583:181#0/1
ababbbbbaaabaaabb`]`_`^W_a`b_]_aa`a`_``]
@HWI-EAS443:3:53:604:643#0/1
AACTACTTCAATGCATGTGTGAGGAAGGAGTTGAACCAAG
+HWI-EAS443:3:53:604:643#0/1
aa`aaab_`^^^a__aa`a]a]a`__aP¥a__a`_`]^__
@HWI-EAS443:3:57:729:325#0/1
AACTACTTCAATGCATGTGTGAGGAAGGAGTTGAACCAAG
+HWI-EAS443:3:57:729:325#0/1
aa_aaaaa_a_aa__aaWaZa`a`^`aa^aZ_a_`^[^]`
@HWI-EAS443:3:59:1392:1147#0/1
AACTACTTCAATGCATGTGTGAGGAAGGAGTTGAACCAAG
```

```
+HWI-EAS443:3:59:1392:1147#0/1
__a_a`bba`__ba_`¥Xaab]bbabbbaba]aa_ZU^``
@HWI-EAS443:3:59:428:1264#0/1
AACTACTTCAATGCATGTGTGAGGAAGGAGTTGAACCAAG
+HWI-EAS443:3:59:428:1264#0/1
UU_Z]aa`^aabaaa`aS^K``ba`aba_`NNS¥SPO[]V
@HWI-EAS443:3:65:1078:856#0/1
AACTACTTCAATGCATGTGTGAGGAAGGAGTTGAACCAAG
+HWI-EAS443:3:65:1078:856#0/1
a]T]aaaaa``X_W[¥[^_Z^`XW^¥]```Y¥`¥`¥Y¥^[
@HWI-EAS443:3:70:162:906#0/1
AACTACTTCAATGCATGTGTGAGGAAGGAGTTGAACCAAG
+HWI-EAS443:3:70:162:906#0/1
a_aabaaaa`a_`a_`_]aX____^_`^^_Z__a``^`_]
@HWI-EAS443:3:72:1365:1680#0/1
AACTACTTCAATGCATGTGTGAGGAAGGAGTTGAACCAAG
+HWI-EAS443:3:72:1365:1680#0/1
aa__a]a_abaaT[_`aY_O_Y`^_[a_]`O_a_aOS_^_
@HWI-EAS443:3:72:716:369#0/1
AACTACTTCAATGCATGTGTGAGGAAGGAGTTGAACCAAG
+HWI-EAS443:3:72:716:369#0/1
abaaaaba`aaaaaaabWa]a_aa^`b__`^`_`_`_`^_
@HWI-EAS443:3:75:492:1959#0/1
AACTACTTCAATGCATGTGTGAGGAAGGAGTTGAACCAAG
+HWI-EAS443:3:75:492:1959#0/1
^bb]b`aa`^a`aaaa^Xa_`^`_^_a`¥a[``_aa_a_`
@HWI-EAS443:3:78:1609:1026#0/1
AACTACTTCAATGCATGTGTGAGGAAGGAGTTGAACCAAG
+HWI-EAS443:3:78:1609:1026#0/1
aaaaa`abaaab_aU_]VbS^aaa__ba`a^aaaa`¥_Qa
@HWI-EAS443:3:8:807:1874#0/1
AACTACTTCAATGCATGTGTGAGGAAGGAGTTGAACCAAG
+HWI-EAS443:3:8:807:1874#0/1
a`aa`a__^a_`_^_aa]a]___^`¥____X_____^_^^
@HWI-EAS443:3:97:421:1769#0/1
AACTACTTCAATGCATGTGTGAGGAAGGAGTTGAACCAAG
+HWI-EAS443:3:97:421:1769#0/1
aa_aaaa`aa__`a`aa]a]a_a_`^___aZ__``_X_^_
@HWI-EAS443:3:98:1337:1866#0/1
AACTACTTCAATGCATGTGTGAGGAAGGAGTTGAACCAAG
+HWI-EAS443:3:98:1337:1866#0/1
a`O``^`aab_aa¥_bb^b¥¥YaaSaba^a^`aaaSGaS`
@HWI-EAS443:3:21:788:352#0/1
ACTACTTCAATGCATGTGTGAGGAAGGAGTTGAACCAAGA
+HWI-EAS443:3:21:788:352#0/1
aaZ^`aab`W`ba_a`¥a_a_ba¥¥aa[^]_```_¥_Z_]
@HWI-EAS443:3:22:513:545#0/1
ACTACTTCAATGCATGTGTGAGGAAGGAGTTGAACCAAGA
+HWI-EAS443:3:22:513:545#0/1
^_`]OYR¥__U¥ZYRbUaQQ¥V__^]NZYXZ[T^Q]TOYU
```

@HWI-EAS443:3:50:448:1121#0/1
ACTACTTCAATGCATGTGTGAGGAAGGAGTTGAACCAAGA
+HWI-EAS443:3:50:448:1121#0/1
^ZVaZS^`WTSba_`aW`S]TX`U___XaY__a_RWXTY^
@HWI-EAS443:3:53:937:485#0/1
ACTACTTCAATGCATGTGTGAGGAAGGAGTTGAACCAAGA
+HWI-EAS443:3:53:937:485#0/1
__abbbaabbab__bb_bU__ba`a`a```a`_a`^__`_
@HWI-EAS443:3:100:113:1323#0/1
CAATGCATGTGTGAGGAAGGAGTTGAACCAAGATGCATTA
+HWI-EAS443:3:100:113:1323#0/1
WQ_a_U_^`R`_`[`_^aaa¥SOZ^[ZXF^Xa_WaRW``_
@HWI-EAS443:3:10:1205:302#0/1
CTTCAATGCATGTGTGAGGAAGGAGTTGAACCAAGATGCA
+HWI-EAS443:3:10:1205:302#0/1
abbbbbab_babbbababababaabWaab`aa__a__a_aS_
@HWI-EAS443:3:12:1629:1341#0/1
CTACTTCAATGCATGTGTGAGGAAGGAGTTGAACCAAGAT
+HWI-EAS443:3:12:1629:1341#0/1
abb_abaXbbbbbbbbbab`abbabbaa_aaaaaKaabaa
@HWI-EAS443:3:17:650:755#0/1
CAATGCATGTGTGAGGAAGGAGTTGAACCAAGATGCATTA
+HWI-EAS443:3:17:650:755#0/1
YVbUaY`^_]b]^^bZ^S^bZa_`a_ab^^`ab^_Xba_a
@HWI-EAS443:3:18:1088:705#0/1
AATGCATCTTGGTTCAACTCCTTCCTCACACATGCATTGA
+HWI-EAS443:3:18:1088:705#0/1
abaaa`a^baaaaaaaaaaa]aa_[a`aaaaaaa`_aaa`
@HWI-EAS443:3:19:758:233#0/1
ACTTCAATGCATGTGTGAGGAAGGAGTTGAACCAAGATGC
+HWI-EAS443:3:19:758:233#0/1
``b_¥^a`Q]aaa¥b¥_^`aa^bYY^^^]_a¥X`_¥_^_Z
@HWI-EAS443:3:1:239:1964#0/1
AATGCATGTGTGAGGAAGGAGTTGAACCAAGATGCATTAA
+HWI-EAS443:3:1:239:1964#0/1
aa`SIU_ZFY`bYabIab_J_^`aV^¥G¥_S]SU`_]BBB
@HWI-EAS443:3:21:1723:233#0/1
CAATGCATGTGTGAGGAAGGAGTTGAACCAAGATGCATTA
+HWI-EAS443:3:21:1723:233#0/1
Y^VV]X`b``aZaZSaaabaR^`aa¥]UTQYaX¥^OHa`V
@HWI-EAS443:3:21:486:719#0/1
CTACTTCAATGCATGTGTGAGGAAGGAGTTGAACCAAGAT
+HWI-EAS443:3:21:486:719#0/1
a`aa_a_][]a[T`_X_^a]`U¥__^]_]^¥^U^]_]^]^
@HWI-EAS443:3:22:835:206#0/1
CAATGCATGTGTGAGGAAGGAGTTGAACCAAGATGCATTA
+HWI-EAS443:3:22:835:206#0/1
abbbb`abbab^bab`abb``b`ababa`a_aaab_aa`a
@HWI-EAS443:3:38:262:953#0/1
ACTTCAATGCATGTGTGAGGAAGGAGTTGAACCAAGATGC

+HWI-EAS443:3:38:262:953#0/1
aaaabbaaaa`aa^aY`[`[¥`a_^`Y___]^]`__]Y_]
@HWI-EAS443:3:42:1645:1072#0/1
GCATCTTGGTTCAACTCCTTCCTCACACATGCATTGAAGT
+HWI-EAS443:3:42:1645:1072#0/1
aa`[baab¥`a^^``a`_aGX^]Z`__aaa`Y``_`U¥^U
@HWI-EAS443:3:43:285:1204#0/1
CTACTTCAATGCATGTGTGAGGAAGGAGTTGAACCAAGAT
+HWI-EAS443:3:43:285:1204#0/1
`_ba`aaTY`baV_a^`aY``[aab`]b`aaaaYQ_`aa^
@HWI-EAS443:3:43:392:353#0/1
CTACTTCAATGCATGTGTGAGGAAGGAGTTGAACCAAGAT
+HWI-EAS443:3:43:392:353#0/1
abbaaa]b`Z`aaaa[b_baa_^[^aab_aa`aa¥aa]__
@HWI-EAS443:3:44:71:2029#0/1
CTTCAATGCATGTGTGAGGAAGGAGTTGAACCAAGATGCA
+HWI-EAS443:3:44:71:2029#0/1
a_RO[R[O_a`bRUFX``[W__aYa^[aaa¥a__[RS]`]
@HWI-EAS443:3:4:1072:540#0/1
CTACTTCAATGCATGTGTGAGGAAGGAGTTGAACCAAGAT
+HWI-EAS443:3:4:1072:540#0/1
`bbabbababba_bb`b^a`_```ba¥a`aa_a`V`_aa`
@HWI-EAS443:3:4:644:1750#0/1
ATGCATGTGTGAGGAAGGAGTTGAACCAAGATGCATTAAG
+HWI-EAS443:3:4:644:1750#0/1
a`_aaab^a^_`a`^a_a_¥^^^`__U__`^]`X__^_¥^
@HWI-EAS443:3:55:1137:1133#0/1
CTTCAATGCATGTGTGAGGAAGGAGTTGAACCAAGATGCA
+HWI-EAS443:3:55:1137:1133#0/1
`^Y`Y_X¥U^bb_ZP`_bba^`]__]bb^]NX¥]]_ab]`
@HWI-EAS443:3:62:1660:1114#0/1
CTACTTCAATGCATGTGTGAGGAAGGAGTTGAACCAAGAT
+HWI-EAS443:3:62:1660:1114#0/1
_S_NU`a_Y`a]P¥b`a]UM¥_`a^`^`Y^`a`VQ[T``a
@HWI-EAS443:3:63:325:633#0/1
TACTTCAATGCATGTGTGAGGAAGGAGTTGAACCAAGATG
+HWI-EAS443:3:63:325:633#0/1
ababbb]U`ba`ababZ`aba`aabaa^aa^aaaZ`[W^`
@HWI-EAS443:3:68:1485:1540#0/1
ATGCATGTGTGAGGAAGGAGTTGAACCAAGATGCATTAAG
+HWI-EAS443:3:68:1485:1540#0/1
a[I¥U^^a`Kaa^[T]aV¥a[RUTW__aX_``UPaV[S_a
@HWI-EAS443:3:73:1074:1792#0/1
CTACTTCAATGCATGTGTGAGGAAGGAGTTGAACCAAGAT
+HWI-EAS443:3:73:1074:1792#0/1
aaaaa¥]ab_aaa_[Qa^a__¥Yaa]VS`_aaa_^`[`aV
@HWI-EAS443:3:7:834:1705#0/1
CAATGCATGTGTGAGGAAGGAGTTGAACCAAGATGCATTA
+HWI-EAS443:3:7:834:1705#0/1
aabaaa`^`Ubabaaabbba_``aaaa]_aab_`^W`a`a

@HWI-EAS443:3:90:984:967#0/1
CTTCAATGCATGTGTGAGGAAGGAGTTGAACCAAGATGCA
+HWI-EAS443:3:90:984:967#0/1
^`aa_b`abbbbabbb¥ab__aaabb^bbbbbabbbbbaa
@HWI-EAS443:3:91:1319:1237#0/1
CTACTTCAATGCATGTGTGAGGAAGGAGTTGAACCAAGAT
+HWI-EAS443:3:91:1319:1237#0/1
ab¥O_[IaTa`aX^¥``aZWaXX^`]`ba_[JYX^[^¥ba
@HWI-EAS443:3:92:1539:1694#0/1
CAATGCATGTGTGAGGAAGGAGTTGAACCAAGATGCATTA
+HWI-EAS443:3:92:1539:1694#0/1
`bbabbabb`b_b_baaaba`aaabaa``baaaaa_aaaa
@HWI-EAS443:3:9:399:1143#0/1
CTACTTCAATGCATGTGTGAGGAAGGAGTTGAACCAAGAT
+HWI-EAS443:3:9:399:1143#0/1
aab`aaa^aaaa`aa^a^aZ`^aa`a`a]`aaa_[a_a``
@HWI-EAS443:3:84:1023:1831#0/1
CTTGCCTCAGTAATTGACTGCTCGTCTCTCCCACTTACTA
+HWI-EAS443:3:84:1023:1831#0/1
aaa`aaabab_ab__aa_a^[`a`Z`aa`^X¥`___]R][
@HWI-EAS443:3:65:65:232#0/1
ATTGACTGCTCGTCTCTCCCACTTACTATCAACTGGTGAG
+HWI-EAS443:3:65:65:232#0/1
ababbbaaa]ba[a¥`¥`^[Y`W__Y¥VU¥¥_^X¥XUTTY
@HWI-EAS443:3:20:1112:770#0/1
GAAAGGCAACCAGGAGATTGATCCAGTTGATAGAAAGTGG
+HWI-EAS443:3:20:1112:770#0/1
TXZ_bbb``_¥¥bbab`¥Z¥_^RPP_Oabaa`[F_S_Paa
@HWI-EAS443:3:36:1544:1129#0/1
CTCAAGAGTTGGTGCATAGGGTTCAGTGGCTGGTTTGCCC
+HWI-EAS443:3:36:1544:1129#0/1
a¥TWX`U¥`_ab^b^`ba_X[N^a_aVD¥`a`¥NVZS[GZ
@HWI-EAS443:3:44:477:460#0/1
GCCTCAAGAGTTGGTGCATAGGGTTCAGTCGCTGGGTTGC
+HWI-EAS443:3:44:477:460#0/1
aaaba¥ZZ^bWba_G_^aa]S_bS`_VaX_a^_aZFU`_¥
@HWI-EAS443:3:72:645:1319#0/1
CAGTTGATAGTAAGTGGGAGAGACGAGCAGTCAACTGCTG
+HWI-EAS443:3:72:645:1319#0/1
aaba_X¥a``¥`^__aaa_U_aaa_[__`_V_aZIZS]ZZ
@HWI-EAS443:3:96:1283:686#0/1
GAAAGGCAACAAGGAGATTGATCCAGTTGATAGTAAGTGG
+HWI-EAS443:3:96:1283:686#0/1
XY_Z¥`VPT[I_Z`YS]`]Z`^UOO¥GY_a_^bZ[UaY]a
@HWI-EAS443:3:92:335:1792#0/1
AGTTGGTGCATAGGGTTCAGTCGCTGGTTTGNCCTATTGA
+HWI-EAS443:3:92:335:1792#0/1
a_Zaa_aUaa`ab_[Jb^W^`a`_a[`[Pa¥DLSY¥`_`^
@HWI-EAS443:3:11:41:759#0/1
CTTTTTGGAAATGCCTCAAGAGTTGGTGCATAGGGTTCCG

```
+HWI-EAS443:3:11:41:759#0/1
a`Y[Zaab_T^bba[baa[bUa``WaZa[_W^bb]]aUaa
@HWI-EAS443:3:11:1454:1497#0/1
CGGTGCATAGGGTTCAGTCGCTGGTTTGCCCTATTGACAA
+HWI-EAS443:3:11:1454:1497#0/1
abbab`abb`bbababbbbbabb`[bba_[U`aaab`[aa
@HWI-EAS443:3:31:1209:1391#0/1
CGCGACTGAACCCTATGCACCAACTCTTGAGGCATTTCCA
+HWI-EAS443:3:31:1209:1391#0/1
abaabab^^aaaaababba`aaa`aabbabaa^^aaa__a
@HWI-EAS443:3:43:845:851#0/1
GTGAACTTTGTCAATAGGGCAAACCAGCGACTGAACCCTA
+HWI-EAS443:3:43:845:851#0/1
I¥WYO^`¥W`^^ZRW[Z_bb_XXab`Z_X`K[a^WY``]S
@HWI-EAS443:3:56:1066:438#0/1
CCCCACTTACTATCAACTGGATCAATCTCCTGGTTGCCTT
+HWI-EAS443:3:56:1066:438#0/1
`aa^ZXbb^bba_^`a]_a]a^^^¥`aa`aaaa_`a_X_]
@HWI-EAS443:3:61:1342:422#0/1
GCTCTCCCACTTACTATCAACTGGATCAATCTCCTGGTTG
+HWI-EAS443:3:61:1342:422#0/1
aaaaaaaa]_X`^_`_``_a]`_a]^`_^_Z_UWXYUUSP
@HWI-EAS443:3:86:886:413#0/1
CGCCTCAGCAATTGACTGCTCGTCTCTCCCACTTACTATC
+HWI-EAS443:3:86:886:413#0/1
abbbbabbbbbaabbbbbab__]`bab^aaaabba`aaa^
@HWI-EAS443:3:89:1305:469#0/1
GGGAGATTGATCCAGTTGATAGTAAGTGGGAGAGACGAGC
+HWI-EAS443:3:89:1305:469#0/1
`b```abababababb`b_bT`ab¥baaabaaa`b`aT
@HWI-EAS443:3:100:855:493#0/1
GCAATTGACTGCTCGTCTCTCCCACTTACTATCAACTGGA
+HWI-EAS443:3:100:855:493#0/1
`[_a^`]_W_¥XV_`V^^^^`_a]Y^[UYa`_VZ^^^^VB
@HWI-EAS443:3:11:213:994#0/1
GATTGATCCAGTTGATAGTAAGTGGGAGAGACGAGCAGTC
+HWI-EAS443:3:11:213:994#0/1
a^aba``ba[aaaa]a`a]aaa¥`b`^a`_``a_``__^^
@HWI-EAS443:3:12:257:706#0/1
GAGTTGGTGCATAGGGTTCAGTCGCTGGTTTGCCCTATTG
+HWI-EAS443:3:12:257:706#0/1
a[WYaaa_`aaa_aUa]`a_aZ__^_aaY__XW___^_^_
@HWI-EAS443:3:13:1393:1531#0/1
TGCTCGTCTCTCCCACTTACTATCAACTGGATCAATCTCC
+HWI-EAS443:3:13:1393:1531#0/1
aaaaab`_b`a___`]aaa`aaa``aa`]_``U`_aUaW_
@HWI-EAS443:3:15:1201:1011#0/1
ACTTACTATCAACTGGATCAATCTCCTGGTTGCCTTTCTG
+HWI-EAS443:3:15:1201:1011#0/1
abbbbabbaabbabbaaa_`babaaaabaaaa__`a_a__
```

@HWI-EAS443:3:15:862:927#0/1
ACAATTATTGCCTCAGCAATTGACTGCTCGTCTCTCCCAC
+HWI-EAS443:3:15:862:927#0/1
aba[abZabababababaaaaaaaaa`aaZUa¥¥a]_¥a`_¥
@HWI-EAS443:3:17:1671:121#0/1
ATGCCTCAAGAGTTGGTGCATAGGGTTCAGTCGCTGGTTT
+HWI-EAS443:3:17:1671:121#0/1
aaba¥`Y``a_a_aa[W`¥]aZa__Y_^_`WW_W^¥_WVQ
@HWI-EAS443:3:17:491:224#0/1
GACTGCTCGTCTCTCCCACTTACTATCAACTGGATCAATC
+HWI-EAS443:3:17:491:224#0/1
a``a_`¥[`¥``^_____`_¥^U_]¥^[¥SWW[^YW¥W¥S
@HWI-EAS443:3:18:1303:232#0/1
CCAGGAGATTGATCCAGTTGATAGTAAGTGGGAGAGACGA
+HWI-EAS443:3:18:1303:232#0/1
aW_POMUPaQa_V_MNSXT]W¥NXZGYXURLVRaXKFLNZ
@HWI-EAS443:3:18:364:76#0/1
CAGCGACTGAACCCTATGCACCAACTCTTGAGGCATTTCC
+HWI-EAS443:3:18:364:76#0/1
aaaaa^Y^`TNZ_`__^`¥^^ZZ]PVT^]_[YY[V[V^QQ
@HWI-EAS443:3:18:581:507#0/1
CAAGAGTTGGTGCATAGGGTTCAGTCGCTGGTTTGCCCTA
+HWI-EAS443:3:18:581:507#0/1
aabbababb_ababbb]bb`ababaa_aabbYba^___aa
@HWI-EAS443:3:20:865:932#0/1
TTTGGAAATGCCTCAAGAGTTGGTGCATAGGGTTCAGTCG
+HWI-EAS443:3:20:865:932#0/1
a`]Y[W^Z`¥a]¥¥_]]aaQ`_aT``[^¥a``G[U^aUNX
@HWI-EAS443:3:21:71:1909#0/1
CTCAAGAGTTGGTGCATAGGGTTCAGTCGCTGGTTTGCCC
+HWI-EAS443:3:21:71:1909#0/1
a_a_¥___X___W]a`^^¥[YV]`^UV`¥]XZ[TVVNS]Y
@HWI-EAS443:3:21:99:110#0/1
AGGAGATTGATCCAGTTGATAGTAAGTGGGAGAGACGAGC
+HWI-EAS443:3:21:99:110#0/1
aaa^b_b_aaaaaab^aaa``a^XZaVa_`]`___R`___
@HWI-EAS443:3:22:1444:1165#0/1
GACTGAACCCTATGCACCAACTCTTGAGGCATTTCCAAAA
+HWI-EAS443:3:22:1444:1165#0/1
aaa`a_aaa`aaaa]aaa_a^aZa`]``[Za``a^^`_]]
@HWI-EAS443:3:23:117:32#0/1
ATCTTTTTGGAAATGCCTCAAGAGTTGGTGCATAGGGTTC
+HWI-EAS443:3:23:117:32#0/1
ababbbaaa``ZRaZaaaa]R_]_Yaa_]a_^`]a]`U]_
@HWI-EAS443:3:23:842:560#0/1
CAACCAGGAGATTGATCCAGTTGATAGTAAGTGGGAGAGA
+HWI-EAS443:3:23:842:560#0/1
aabbbabb`a_abb_a]``a]aa__`aY`_aUa_a^`^_]
@HWI-EAS443:3:23:861:1915#0/1
CAACCAGGAGATTGATCCAGTTGATAGTAAGTGGGAGAGA

```
+HWI-EAS443:3:23:861:1915#0/1
`aba`aaa_a`aaaa`_``a]^`^`__¥_`ZU_]_]^^_`
@HWI-EAS443:3:24:345:526#0/1
AGGAGATTGATCCAGTTGATAGTAAGTGGGAGAGACGAGC
+HWI-EAS443:3:24:345:526#0/1
abaa_abb_a`a_Y_N¥baa`aN``a^a`b_^]aa`V_`^
@HWI-EAS443:3:25:856:1431#0/1
AGGAGATTGATCCAGTTGATAGTAAGTGGGAGAGACGAGC
+HWI-EAS443:3:25:856:1431#0/1
a_Y][[^^aaZ`a¥_Z`a]a_]Q`]aT`TT`_[`_[^__¥
@HWI-EAS443:3:27:994:689#0/1
AGAGTTGGTGCATAGGGTTCAGTCGCTGGTTTGCCCTATT
+HWI-EAS443:3:27:994:689#0/1
`¥[b`b`a]bba]T_bb[baab__b_ab_V_a_S_Y`aaa
@HWI-EAS443:3:2:508:515#0/1
ACTTTGTCAATAGGGCAAACCAGCGACTGAACCCTATGCA
+HWI-EAS443:3:2:508:515#0/1
aa_aaa_[a_]aa_a_]a`Z[`_¥__W¥`^YTTZ¥_^aW]
@HWI-EAS443:3:30:806:475#0/1
CTCGTCTCTCCCACTTACTATCAACTGGATCAATCTCCTG
+HWI-EAS443:3:30:806:475#0/1
a]aaa`ba^aa]X^_aa`a_```a^`aaa`T^a`^]¥]XV
@HWI-EAS443:3:30:845:1531#0/1
CAAGAGTTGGTGCATAGGGTTCAGTCGCTGGTTTGCCCTA
+HWI-EAS443:3:30:845:1531#0/1
aZV^[¥H`aa]^ba[POX][_aaZM````_VU[_XX_]]]
@HWI-EAS443:3:31:902:666#0/1
CATAGGGTTCAGTCGCTGGTTTGCCCTATTGACAAAGTTC
+HWI-EAS443:3:31:902:666#0/1
a`aW¥_b_bbab_bbab^a`bba`ab`]`a]`aaaaX[]T
@HWI-EAS443:3:32:1159:1516#0/1
GCATAGGGTTCAGTCGCTGGTTTGCCCTATTGACAAAGTT
+HWI-EAS443:3:32:1159:1516#0/1
a`]ba^ba[abab``a`aaU¥`bY_¥aa`^a_`Za]a]_a
@HWI-EAS443:3:32:790:349#0/1
TCTGAACTTTGTCAATAGGGCAAACCAGCGACTGAACCCT
+HWI-EAS443:3:32:790:349#0/1
aab^aaaaaa`__aa_^^_aaa`a`_a`_`aU^^a_`Z__
@HWI-EAS443:3:33:1109:1009#0/1
GCATAGGGTTCAGTCGCTGGTTTGCCCTATTGACAAAGTT
+HWI-EAS443:3:33:1109:1009#0/1
a_X^`ba¥HYa_a_[``¥aZT`[V¥aW``__¥T¥¥^¥YW¥
@HWI-EAS443:3:36:1063:943#0/1
ATAGGGTTCAGTCGCTGGTTTGCCCTATTGACAAAGTTCA
+HWI-EAS443:3:36:1063:943#0/1
`ba_baXbb`baaaaab`P]a¥_a`_a_aY[Y__a_U^VU
@HWI-EAS443:3:37:808:598#0/1
GAGTTGGTGCATAGGGTTCAGTCGCTGGTTTGCCCTATTG
+HWI-EAS443:3:37:808:598#0/1
aaa[X_^[¥[_aVW[_P¥¥Z^Y``_`a[DLSR_`¥QRV¥¥
```

```
@HWI-EAS443:3:39:116:1279#0/1
ACTGAACCCTATGCACCAACTCTTGAGGCATTTCCAAAAA
+HWI-EAS443:3:39:116:1279#0/1
S_¥Sa`bbb__W]aaaba``_`a]aa¥^_[`¥aab``¥a_
@HWI-EAS443:3:39:151:247#0/1
GCATAGGGTTCAGTCGCTGGTTTGCCCTATTGACAAAGTT
+HWI-EAS443:3:39:151:247#0/1
aaa^UX]ZNW]aaUZaa`]QQXU^]Z`R]S¥NRYPMWWJM
@HWI-EAS443:3:3:885:1809#0/1
AGCGACTGAACCCTATGCACCAACTCTTGAGGCATTTCCA
+HWI-EAS443:3:3:885:1809#0/1
aa_aaUa`a_^¥Va^___a]V`a^¥^__]`^_R`]_SH[_
@HWI-EAS443:3:40:731:192#0/1
GATAGTAAGTGGGAGAGACGAGCAGTCAATTGCTGAGGCA
+HWI-EAS443:3:40:731:192#0/1
a`_`a^_`aXaa`U^V¥]^W]]]]YQT^OYY]VYYYYVXV
@HWI-EAS443:3:42:362:205#0/1
GTTGGTGCATAGGGTTCAGTCGCTGGTTTGCCCTATTGAC
+HWI-EAS443:3:42:362:205#0/1
a_aaa¥a_a`Waa_U_^¥`^_`]`a^U^]U[W^_¥^_¥^^
@HWI-EAS443:3:42:88:2002#0/1
CAAGAGTTGGTGCATAGGGTTCAGTCGCTGGTTTGCCCTA
+HWI-EAS443:3:42:88:2002#0/1
`a_^^aVWQ¥W¥W_¥¥__PXZW_¥WWZSYYHM¥PLVR^]`
@HWI-EAS443:3:42:969:1332#0/1
CTCAAGAGTTGGTGCATAGGGTTCAGTCGCTGGTTTGCCC
+HWI-EAS443:3:42:969:1332#0/1
]`bbZbZ_W]__N^```a[Q`RZ¥a_YW`a^`_N_UYG^U
@HWI-EAS443:3:43:1430:110#0/1
TCTCCCACTTACTATCAACTGGATCAATCTCCTGGTTGCC
+HWI-EAS443:3:43:1430:110#0/1
aab_`_a___X_[_^S__G__XY[XZ[¥MZVVS^VWY]WY
@HWI-EAS443:3:44:1533:1673#0/1
CTCAAGAGTTGGTGCATAGGGTTCAGTCGCTGGTTTGCCC
+HWI-EAS443:3:44:1533:1673#0/1
aaa^`[W_Z``aTTT^¥[¥_`Z¥XTRP^^^_`RIPZ]TQN
@HWI-EAS443:3:44:779:969#0/1
CTGCTCGTCTCTCCCACTTACTATCAACTGGATCAATCTC
+HWI-EAS443:3:44:779:969#0/1
abbababaababaaa_^aaaaaaaaaaaaaaaaa_`[a^a`
@HWI-EAS443:3:45:1039:778#0/1
CTTTTTGGAAATGCCTCAAGAGTTGGTGCATAGGGTTCAG
+HWI-EAS443:3:45:1039:778#0/1
abb`__`b]]aabaa__]¥b_b`aaaU_`Y[]aaaV^W`a
@HWI-EAS443:3:45:1680:270#0/1
TTGGTGCATAGGGTTCAGTCGCTGGTTTGCCCTATTGACA
+HWI-EAS443:3:45:1680:270#0/1
`aa`P_`_a¥`[aG[Raa¥TX`_`]R__^TPYRRVX[UV^
@HWI-EAS443:3:45:1758:217#0/1
AGTTGGTGCATAGGGTTCAGTCGCTGGTTTGCCCTATTGA
```

```
+HWI-EAS443:3:45:1758:217#0/1
`baaabab`aaabbaUaa_a_`a_`aaZ`ZGY¥_`__a`Y
@HWI-EAS443:3:45:728:1596#0/1
AGGGTTCAGTCGCTGGTTTGCCCTATTGACAAAGTTCAGA
+HWI-EAS443:3:45:728:1596#0/1
a`a`]aabaa[aaa]`W``]]__aaaaaa[aaa`]`^¥_`
@HWI-EAS443:3:46:912:1919#0/1
ACTTACTATCAACTGGATCAATCTCCTGGTTGCCTTTCTG
+HWI-EAS443:3:46:912:1919#0/1
aabaaabbaaaa_aaaa`aa_`_a`a_a`_``^X`_`^^^
@HWI-EAS443:3:48:1760:880#0/1
ACTTTGTCAATAGGGCAAACCAGCGACTGAACCCTATGCA
+HWI-EAS443:3:48:1760:880#0/1
aabaabaaaaaaa]aababa``a_``_a`aa_[`__aa__
@HWI-EAS443:3:48:563:1151#0/1
ATGCCTCAAGAGTTGGTGCATAGGGTTCAGTCGCTGGTTT
+HWI-EAS443:3:48:563:1151#0/1
aaaa[aaa_b_aZa`_^b`__aaaaUaU_`_^___``]_`
@HWI-EAS443:3:50:268:1643#0/1
CAACCAGGAGATTGATCCAGTTGATAGTAAGTGGGAGAGA
+HWI-EAS443:3:50:268:1643#0/1
`bbaSabbb[U¥````¥Yaabab`abbTZaaUb^TYb``b
@HWI-EAS443:3:51:261:197#0/1
GGAGATTGATCCAGTTGATAGTAAGTGGGAGAGACGAGCA
+HWI-EAS443:3:51:261:197#0/1
aba``bbbbaa`^baabaa`aW`_a^ab^[a¥a¥^a`^V_
@HWI-EAS443:3:54:1097:904#0/1
ATGCCTCAAGAGTTGGTGCATAGGGTTCAGTCGCTGGTTT
+HWI-EAS443:3:54:1097:904#0/1
a`W`^`aa_aa]]aaaTa`_a_`aaU`_`aNP^_``_¥^^
@HWI-EAS443:3:54:248:385#0/1
CAGGAGATTGATCCAGTTGATAGTAAGTGGGAGAGACGAG
+HWI-EAS443:3:54:248:385#0/1
_]`a¥W^^a`bb^_^a__baa`_U¥`^O^aWT¥ZU¥]`RT
@HWI-EAS443:3:54:696:1384#0/1
GCGACTGAACCCTATGCACCAACTCTTGAGGCATTTCCAA
+HWI-EAS443:3:54:696:1384#0/1
aaaaababaaa`abab`aa`aaaa_aaaaaa`aa_`¥^a`
@HWI-EAS443:3:55:259:1196#0/1
GAGTTGGTGCATAGGGTTCAGTCGCTGGTTTGCCCTATTG
+HWI-EAS443:3:55:259:1196#0/1
abb`ba^`_bbaaaabUaaba`ababab^aaaaaaabaaa
@HWI-EAS443:3:55:844:1736#0/1
AAATGCCTCAAGAGTTGGTGCATAGGGTTCAGTCGCTGGT
+HWI-EAS443:3:55:844:1736#0/1
a¥aUYa`_Wa`_ZZU]a_W__]_____QVQ[[]X`]^]¥X
@HWI-EAS443:3:56:1043:1463#0/1
GGCAACCAGGAGATTGATCCAGTTGATAGTAAGTGGGAGA
+HWI-EAS443:3:56:1043:1463#0/1
aa^``¥[_`¥S[Z^_a¥^UW¥_U]`¥¥]]W^V¥Q_Z^¥_W
```

```
@HWI-EAS443:3:56:1444:403#0/1
TGAACTTTGTCAATAGGGCAAACCAGCGACTGAACCCTAT
+HWI-EAS443:3:56:1444:403#0/1
aa_`^aaaaa]aa`a`[aU___Y]``V`_^aa`_]UW[`^
@HWI-EAS443:3:56:147:1273#0/1
AATGCCTCAAGAGTTGGTGCATAGGGTTCAGTCGCTGGTT
+HWI-EAS443:3:56:147:1273#0/1
aa```__`aaa_`X_`^V`X]^[[Z_Y`]][X_^VZ`QU^
@HWI-EAS443:3:56:1759:1539#0/1
CAATTATTGCCTCAGCAATTGACTGCTCGTCTCTCCCACT
+HWI-EAS443:3:56:1759:1539#0/1
Y^[¥``babJXaU[T¥R_`¥baZXVPXO`VHR_TO¥_ZQ_
@HWI-EAS443:3:56:261:865#0/1
CAACCAGGAGATTGATCCAGTTGATAGTAAGTGGGAGAGA
+HWI-EAS443:3:56:261:865#0/1
abaabbbbababbbaaaaabaab`a`a¥a`aZaa¥_aaa_
@HWI-EAS443:3:57:1397:638#0/1
AACCAGGAGATTGATCCAGTTGATAGTAAGTGGGAGAGAC
+HWI-EAS443:3:57:1397:638#0/1
Vaa````^bba]_]aaXaaabb`__a_a`b[bba^P`aa_
@HWI-EAS443:3:57:1620:249#0/1
TCTGAACTTTGTCAATAGGGCAAACCAGCGACTGAACCCT
+HWI-EAS443:3:57:1620:249#0/1
a¥bb`a`baab[Raaaaa`a_``a_[]`UT^[]`^^RT[_
@HWI-EAS443:3:57:625:43#0/1
TTTGTCAATAGGGCAAACCAGCGACTGAACCCTATGCACC
+HWI-EAS443:3:57:625:43#0/1
a][`L¥TFZ]Ya_YS¥PW[SVZ[YU[NNTSZTUTWUUVNV
@HWI-EAS443:3:58:1107:413#0/1
GCAATTGACTGCTCGTCTCTCCCACTTACTATCAACTGGA
+HWI-EAS443:3:58:1107:413#0/1
aaaaaaa``ab]`aaa`aa_a_aZYa`T^a^`Y¥]^`^a^
@HWI-EAS443:3:58:905:588#0/1
GGTGCATAGGGTTCAGTCGCTGGTTTGCCCTATTGACAAA
+HWI-EAS443:3:58:905:588#0/1
aa__``bbbb`Uaaaa^b^`b```ab[__aaaa_K_a`_a
@HWI-EAS443:3:59:1362:1294#0/1
GAAAGGCAACCAGGAGATTGATCCAGTTGATAGTAAGTGG
+HWI-EAS443:3:59:1362:1294#0/1
`bb^`abab^``baab_aaa`aba[b`ba`a^`__T]R`]
@HWI-EAS443:3:59:280:1045#0/1
AGGCAACCAGGAGATTGATCCAGTTGATAGTAAGTGGGAG
+HWI-EAS443:3:59:280:1045#0/1
VX^^`bbb`^``^[`¥^a_W_N_`]aa]aWT_]aLP]aa[
@HWI-EAS443:3:61:1001:91#0/1
TGACTGCTCGTCTCTCCCACTTACTATCAACTGGATCAAT
+HWI-EAS443:3:61:1001:91#0/1
aba`aa`a_baaa_______aa`_a]`^^_Y_]_Y^VYY^
@HWI-EAS443:3:61:1489:1305#0/1
CACTTACTATCAACTGGATCAATCTCCTGGTTGCCTTTCT
```

+HWI-EAS443:3:61:1489:1305#0/1
`bbbbbbbbbbbbabbbbbbb_abaababa]a`]^a_aX^
@HWI-EAS443:3:61:769:992#0/1
TTTGGAAATGCCTCAAGAGTTGGTGCATAGGGTTCAGTCG
+HWI-EAS443:3:61:769:992#0/1
¥b_^W`b`]Z_`V[^OZYZH]^YU_¥X]``_]QW_[XUYV
@HWI-EAS443:3:62:46:1608#0/1
GTTGGTGCATAGGGTTCAGTCGCTGGTTTGCCCTATTGAC
+HWI-EAS443:3:62:46:1608#0/1
a`a]a_abaaaaa`^aaaa¥a`a_^V^__^__`¥`_`^¥Z
@HWI-EAS443:3:62:475:773#0/1
TAAGTGGGAGAGACGAGCAGTCAATTGCTGAGGCAATAAT
+HWI-EAS443:3:62:475:773#0/1
aaV¥R]`^`aa^[a`]Y__]¥`bSaUXT]]Y_¥`a`aZV_
@HWI-EAS443:3:63:1439:182#0/1
CTCAAGAGTTGGTGCATAGGGTTCAGTCGCTGGTTTGCCC
+HWI-EAS443:3:63:1439:182#0/1
U_[[`_a_S_a[S[_O_¥b]b`_[[^UGYHXb]MY¥VNRI
@HWI-EAS443:3:63:186:1168#0/1
GTTGGTGCATAGGGTTCAGTCGCTGGTTTGCCCTATTGAC
+HWI-EAS443:3:63:186:1168#0/1
Ua_[^`]]H[^`^]]aaaaY`ba`^]Z_aI[^¥^_¥XIMX
@HWI-EAS443:3:63:368:1452#0/1
TTTTGGAAATGCCTCAAGAGTTGGTGCATAGGGTTCAGTC
+HWI-EAS443:3:63:368:1452#0/1
aabaaXVaab_`^^`¥_a¥]_`_`]aa__`WX_R_Z¥RJS
@HWI-EAS443:3:63:496:1412#0/1
AGAGTTGGTGCATAGGGTTCAGTCGCTGGTTTGCCCTATT
+HWI-EAS443:3:63:496:1412#0/1
a]]U`^a`Ra`Y^_]`]V^^]`Z^Z_`aZVXZNY`X^[Z_
@HWI-EAS443:3:66:1093:2021#0/1
ATAGGGTTCAGTCGCTGGTTTGCCCTATTGACAAAGTTCA
+HWI-EAS443:3:66:1093:2021#0/1
`aZ`Y¥G[bb[]b`_]SVF]ZS_^^aa``a`[baaUS^]¥
@HWI-EAS443:3:67:118:1333#0/1
TTGATAGTAAGTGGGAGAGACGAGCAGTCAATTGCTGAGG
+HWI-EAS443:3:67:118:1333#0/1
aN^a`_a^]Y_Y^aa`aa_[a_^Va^_Ya]aW^]a^U`W^
@HWI-EAS443:3:67:1746:101#0/1
ACTTTGTCAATAGGGCAAACCAGCGACTGAACCCTATGCA
+HWI-EAS443:3:67:1746:101#0/1
aY[`ZXaaYab^[`_X`a_P¥RaX``_¥V^¥UUN^U[QJX
@HWI-EAS443:3:68:784:767#0/1
AAGGCAACCAGGAGATTGATCCAGTTGATAGTAAGTGGGA
+HWI-EAS443:3:68:784:767#0/1
aa_]`a_aa]Y]¥a_aa`_a`_V]_ab`a`aX`Y]X___`
@HWI-EAS443:3:69:256:18#0/1
CTGAACTTTGTCAATAGGGCAAACCAGCGACTGAACCCTA
+HWI-EAS443:3:69:256:18#0/1
abb^S`a``baaa[_aaabaaa_]X``a`¥[^`V`]S]_R

@HWI-EAS443:3:69:348:195#0/1
ACCCTATGCACCAACTCTTGAGGCATTTCCAAAAAGATGC
+HWI-EAS443:3:69:348:195#0/1
a_aa¥^_a_YZXZS¥__^¥¥^`]_]WW]]WWYY]^ZXXXX
@HWI-EAS443:3:69:772:568#0/1
ATAGGGTTCAGTCGCTGGTTTGCCCTATTGACAAAGTTCA
+HWI-EAS443:3:69:772:568#0/1
`_V[ab]ZX^^a]ZN[YUW_]ZX_]RQ_]P¥]a_WWI[[]
@HWI-EAS443:3:6:1514:1432#0/1
CACAATTATTGCCTCAGCAATTGACTGCTCGTCTCTCCCA
+HWI-EAS443:3:6:1514:1432#0/1
`¥bbbbbbbbbbbbaab^ba_ab]Wbbbb`a^^babb]_a
@HWI-EAS443:3:6:250:193#0/1
GCCTCAGCAATTGACTGCTCGTCTCTCCCACTTACTATCA
+HWI-EAS443:3:6:250:193#0/1
`aaaaaaa__]]RX__a^ZUa][¥WZ]_V^U`^^¥¥^^BB
@HWI-EAS443:3:70:673:520#0/1
GAGTTGGTGCATAGGGTTCAGTCGCTGGTTTGCCCTATTG
+HWI-EAS443:3:70:673:520#0/1
a_ba`b^`aaabbaaa_aaabaaaaabaZaa``Zaaaa]U
@HWI-EAS443:3:70:926:1174#0/1
CAAGAGTTGGTGCATAGGGTTCAGTCGCTGGTTTGCCCTA
+HWI-EAS443:3:70:926:1174#0/1
aa`baaaba`_aa_baaba¥bbba_b`aaaaRaa]_aaa_
@HWI-EAS443:3:72:635:1164#0/1
TGAACCCTATGCACCAACTCTTGAGGCATTTCCAAAAAGA
+HWI-EAS443:3:72:635:1164#0/1
^bbba[Zba]bb___ZaXaUab^ab`X¥_W¥``¥`ab¥aa
@HWI-EAS443:3:73:1228:1356#0/1
CAAGAGTTGGTGCATAGGGTTCAGTCGCTGGTTTGCCCTA
+HWI-EAS443:3:73:1228:1356#0/1
a[ba`^aa]¥`abaab[`Z[]a^[^`aaa`a`aaRX`¥aa
@HWI-EAS443:3:75:58:780#0/1
CAATTATTGCCTCAGCAATTGACTGCTCGTCTCTCCCACT
+HWI-EAS443:3:75:58:780#0/1
aaaa`U`aabaaaa]a`a`a``a``_``a^_``^___^^X
@HWI-EAS443:3:75:892:110#0/1
CTTTTTGGAAATGCCTCAAGAGTTGGTGCATAGGGTTCAG
+HWI-EAS443:3:75:892:110#0/1
a``a¥`^ab``ba[_bKa`aab^aba^b_aaU`ba_aZ_]
@HWI-EAS443:3:76:489:100#0/1
AGGAGATTGATCCAGTTGATAGTAAGTGGGAGAGACGAGC
+HWI-EAS443:3:76:489:100#0/1
a_aIaSVVR__LYV`]ZSU[PSY^QZPW_^UZLQYZZUNN
@HWI-EAS443:3:79:1205:1535#0/1
CAACCAGGAGATTGATCCAGTTGATAGTAAGTGGGAGAGA
+HWI-EAS443:3:79:1205:1535#0/1
a]baM`ababV¥R_Ub[`_aaabUa]a[ba`S_abO`¥]P
@HWI-EAS443:3:7:1206:216#0/1
CTCAAGAGTTGGTGCATAGGGTTCAGTCGCTGGTTTGCCC

```
+HWI-EAS443:3:7:1206:216#0/1
aaWS^b[aRaba__`_aaa_]Na]_`Y]b__a`NT]_XVW
@HWI-EAS443:3:81:464:294#0/1
TCTTTTTGGAAATGCCTCAAGAGTTGGTGCATAGGGTTCA
+HWI-EAS443:3:81:464:294#0/1
aaaababaaaaa_aaa``_V^_a^aa_X`Z]^``__W]__
@HWI-EAS443:3:84:850:1645#0/1
TCTGAACTTTGTCAATAGGGCAAACCAGCGACTGAACCCT
+HWI-EAS443:3:84:850:1645#0/1
aa`^`b`^a_]_a``_a_[a^`aaa`_P_aaa__aa``^^
@HWI-EAS443:3:85:1119:915#0/1
AGCGACTGAACCCTATGCACCAACTCTTGAGGCATTTCCA
+HWI-EAS443:3:85:1119:915#0/1
abaaa^¥^_b`aa_aaaab`b`a¥a^aa_a``a^aaa_a^
@HWI-EAS443:3:85:529:2034#0/1
CAATTATTGCCTCAGCAATTGACTGCTCGTCTCTCCCACT
+HWI-EAS443:3:85:529:2034#0/1
^`XZ_abX^^`]aa^_`^[]]^``]X`]]N[W__aa]`¥Y
@HWI-EAS443:3:8:935:920#0/1
ATGCCTCAAGAGTTGGTGCATAGGGTTCAGTCGCTGGTTT
+HWI-EAS443:3:8:935:920#0/1
WV_[^^a`Q¥^TaaUZZ]Xa[]V]ZNU_ab¥a_Q]RWQ[^
@HWI-EAS443:3:90:226:2041#0/1
CGTCTCTCCCACTTACTATCAACTGGATCAATCTCCTGGT
+HWI-EAS443:3:90:226:2041#0/1
¥]_baab`^^[¥`]a``ba_¥__`aXa`U^b``a``_YVP
@HWI-EAS443:3:91:1787:439#0/1
CAAGAGTTGGTGCATAGGGTTCAGTCGCTGGTTTGCCCTA
+HWI-EAS443:3:91:1787:439#0/1
abbbVbababab`bbbaba]babba_a_a_]X`a[X`_aa
@HWI-EAS443:3:91:419:446#0/1
CAATTATTGCCTCAGCAATTGACTGCTCGTCTCTCCCACT
+HWI-EAS443:3:91:419:446#0/1
abbbbabbbbbbbbbabbbbbbbbbaa`baaaa`a_``aa
@HWI-EAS443:3:93:1130:1837#0/1
GGTGCATAGGGTTCAGTCGCTGGTTTGCCCTATTGACAAA
+HWI-EAS443:3:93:1130:1837#0/1
a`W`Yaa`aa¥R]S`a]``U^aaT__¥¥VN[___aYP[_`
@HWI-EAS443:3:93:1280:1185#0/1
CTGAACTTTGTCAATAGGGCAAACCAGCGACTGAACCCTA
+HWI-EAS443:3:93:1280:1185#0/1
aaa`X]]¥^``a`_^b¥_aS^W¥^SW¥S]¥Qaa_`PUM^_
@HWI-EAS443:3:93:1789:121#0/1
AGTTGGTGCATAGGGTTCAGTCGCTGGTTTGCCCTATTGA
+HWI-EAS443:3:93:1789:121#0/1
a`abaa_aaaaaaaa¥a^`b``a_`aa^`_^_^X_^^a^_
@HWI-EAS443:3:94:1288:910#0/1
GCATAGGGTTCAGTCGCTGGTTTGCCCTATTGACAAAGTT
+HWI-EAS443:3:94:1288:910#0/1
a_¥`X^^_N]_[_]V`Uaa`V`]¥WQZWS^¥¥¥N[]¥YW_
```

```
@HWI-EAS443:3:97:1109:1717#0/1
GATTGATCCAGTTGATAGTAAGTGGGAGAGACGAGCAGTC
+HWI-EAS443:3:97:1109:1717#0/1
^bbbbbb¥^``abaabaa_aaaUaaa]`aaa_aa__`][[
@HWI-EAS443:3:97:335:613#0/1
CAACCAGGAGATTGATCCAGTTGATAGTAAGTGGGAGAGA
+HWI-EAS443:3:97:335:613#0/1
a]_a`ab_H_Zaa¥^_[__aX_bV_]_Y__aV_`SW_V^V
@HWI-EAS443:3:97:651:1937#0/1
TCTGAACTTTGTCAATAGGGCAAACCAGCGACTGAACCCT
+HWI-EAS443:3:97:651:1937#0/1
aaaa`b¥`baaaa`__aaaZaaba_aaa][_W]`^`_Q¥]
@HWI-EAS443:3:97:865:1292#0/1
AGTTGGTGCATAGGGTTCAGTCGCTGGTTTGCCCTATTGA
+HWI-EAS443:3:97:865:1292#0/1
`abbbbaaaba`]bb`bbbaX`a^b_XJabb^^aaaaaa`
@HWI-EAS443:3:98:1108:117#0/1
CTTTTTGGAAATGCCTCAAGAGTTGGTGCATAGGGTTCAG
+HWI-EAS443:3:98:1108:117#0/1
a`abbbbbbbbbb_bb`aaaababba^b_bbaba^NY`b_
@HWI-EAS443:3:99:913:236#0/1
CAATTATTGCCTCAGCAATTGACTGCTCGTCTCTCCCACT
+HWI-EAS443:3:99:913:236#0/1
¥_ba__aa_[`¥S_aab__[ab`^[¥bbbaabXab_b]Qa
@HWI-EAS443:3:6:51:1575#0/1
TGTGCCGCATTTCTTCAATCAACCACCTTATTTCCTCAAA
+HWI-EAS443:3:6:51:1575#0/1
¥aaaa_Z`bbba`bb`_b_LZa^S^XX_¥_a]`¥`Y_]LB
@HWI-EAS443:3:98:651:1488#0/1
CGCATTTCTTCAATCAACCACCTTATTTCCTCAAATTTCT
+HWI-EAS443:3:98:651:1488#0/1
aaaT^abaaa_a`aa`_V¥`Y_aYaaaa][a_aX]_a_Z^
@HWI-EAS443:3:90:97:147#0/1
CCCAGTGATTGTGAAAGAAATCTCTGTGTCTTGTGAGTTA
+HWI-EAS443:3:90:97:147#0/1
a`_YWZ]WP]¥T¥YXX[[ZUPVZZUZWQRTBBBBBBBBBB
@HWI-EAS443:3:92:1680:100#0/1
CTCCTGGTTGCCTTTCTGAGAATAGCTGTTGCTCTTTTTC
+HWI-EAS443:3:92:1680:100#0/1
`]PYba_^bb]a``^]`_abaaaaa_YRP^BBBBBBBBBB
@HWI-EAS443:3:69:353:1477#0/1
AAAAGAGCAACAGCTATTCTCAGAAAGGCAACCAGGAGAT
+HWI-EAS443:3:69:353:1477#0/1
]^ZL^¥aa^```^aY^T^a^^Q[[`^UF`]``YWRPSKX¥
@HWI-EAS443:3:21:274:1342#0/1
AAAAGCTTCTACAAAAATTTAATATGGCTAAGTAAAAAAG
+HWI-EAS443:3:21:274:1342#0/1
P[`a]W`b`b_WW`a_b_¥^`¥aa]aab`_HHS`]b`abM
@HWI-EAS443:3:56:661:1228#0/1
GAAGCAATCTGGACCCCTCTTGTGGAAAGCTTTCCCCTCA
```

+HWI-EAS443:3:56:661:1228#0/1
^aaaaabbaaaY`a``Ya``_P[]X^¥_`_`^__^Z__`[
@HWI-EAS443:3:40:933:1826#0/1
GGGCTATTAGTATCAGGTGGGGGACCAAACTTATACAATA
+HWI-EAS443:3:40:933:1826#0/1
a]V_Q_NTW`X]^]NLSLTRBBBBBBBBBBBBBBBBBBBB
@HWI-EAS443:3:11:1220:1202#0/1
GGGGGACCAAACTTATACAATATACGGAATCTTCACATTC
+HWI-EAS443:3:11:1220:1202#0/1
a``_aZ_`a``[``aa^_a`_a`a^`^_^`W_`X[]^_^Z
@HWI-EAS443:3:7:141:984#0/1
GGGACGTAGTGTTGGTGGTGAAACGAAAACGGGACTCTAG
+HWI-EAS443:3:7:141:984#0/1
`aa_ab_`b_b^`baX_aRXU`aVZ¥___[^a_[]T_YZV
@HWI-EAS443:3:28:263:1224#0/1
TGGGCAAGGGGACGTAGTGTTGGTGATGAAACGAAAACGG
+HWI-EAS443:3:28:263:1224#0/1
[_]]^]X`[]_[_`V__Ya_aaaHUZZUZV_R]`[[VZ[`
@HWI-EAS443:3:46:770:912#0/1
AGCTAATGTGCTAATTGGGCAAGGGGACGTAGTGTTGGTG
+HWI-EAS443:3:46:770:912#0/1
ab`aY`bb_a_a¥aaaaba]]¥`^^a^aaY_a^aVY¥aN¥
@HWI-EAS443:3:63:963:1464#0/1
TGCTAATTGGGCAAGGGGACGTAGTGTTGGTGATGAAACG
+HWI-EAS443:3:63:963:1464#0/1
]UbM_S__WRLY_^^_¥SabaP]]N_aW¥WTHQ[X___]W
@HWI-EAS443:3:99:896:286#0/1
GTCCCGTTTTCGTTTCATCACCAACACTACGTCCCCTTGC
+HWI-EAS443:3:99:896:286#0/1
a`aa__Raa]Ra__a`_Y_`aZ``Za_[a_`]`]QL¥]ZP
@HWI-EAS443:3:34:1269:1797#0/1
CACGGCCGTCCGAATTCTTTTGGTCGCTGTCTGGCTGTCA
+HWI-EAS443:3:34:1269:1797#0/1
aZFSab`YQab``a_`Y]aa`_aY¥UR``ZT]V_]BBBBB
@HWI-EAS443:3:96:699:1225#0/1
CTCCTCTTGTGGAAAGCTTTCCTCTTGGAATCACTTTCTT
+HWI-EAS443:3:96:699:1225#0/1
a_aa^`baa_````]`_`a_^W```aa`]^`_a]`_```a
@HWI-EAS443:3:89:613:1690#0/1
CTTGGAAAAACTGTGATGAGAATGGGAGACCTTCACTACC
+HWI-EAS443:3:89:613:1690#0/1
abbbaabab^aab_aaaaabaaaa__aaa`_a^aa^aa_^
@HWI-EAS443:3:11:911:71#0/1
AGCTTCTACAAAAAATTAATATGGCTAGTTAAAAAAGAAA
+HWI-EAS443:3:11:911:71#0/1
a`^¥W_¥WUa^^^X]W`Za`BBBBBBBBBBBBBBBBBBBB
@HWI-EAS443:3:1:262:667#0/1
GAGCTTTGGGTATGAATATTCTTTTTTAACTAGCCATATT
+HWI-EAS443:3:1:262:667#0/1
a_a[Y`aa_QS¥^aYVZU[¥N]_]__`^Z[^]WPOS_¥^B

```
@HWI-EAS443:3:49:578:981#0/1
GCTTCTACAAAAATTTAATATGGCTAATTAAAAAAGAAAA
+HWI-EAS443:3:49:578:981#0/1
`a`a]bb_a`^aaabb[`a¥_a][a_JKTJJY_`BBBBBB
@HWI-EAS443:3:15:1305:797#0/1
CAGGGCTAGTTAAAAAAGAAAATTCATACCCAAAGCTCAG
+HWI-EAS443:3:15:1305:797#0/1
abbbbbb^babbbbbbbbbbabbababbbaaaaaaaaba`b
@HWI-EAS443:3:21:1122:335#0/1
GGCTTTTCTTTTTAACTAGCCATATTAAATTTTTGTAGA
+HWI-EAS443:3:21:1122:335#0/1
^RS`[]a_`[a[ZUG`a¥WR_NYZXZP`VL¥`NT[[¥SYB
@HWI-EAS443:3:30:969:249#0/1
CGGGTATGAATTTTCTTTTTTAACTAGCCATATTAAATTT
+HWI-EAS443:3:30:969:249#0/1
aa^`U`a]U__¥Z``¥`_``b_¥Zaab`_Vaa`]aaY``a
@HWI-EAS443:3:75:1244:1108#0/1
GGGGTATGAATTTTCTTTTTTAACTAGCCATATTAAATTT
+HWI-EAS443:3:75:1244:1108#0/1
abab_bbb`_bbbb`bbbbaaaaaaaaa_`aa`bb`a`bab
@HWI-EAS443:3:11:399:778#0/1
GGCTAGTTAAAAAAGAAAATTCATACCCAAAGCTCAGCAA
+HWI-EAS443:3:11:399:778#0/1
abbaab`aaaab`aaaX^T^`ab[a[_a`a^`_^`_`Y`_
@HWI-EAS443:3:16:703:623#0/1
TGGCTAGTTAAAAAAGAAAATTCATACCCAAAGCTCAGCA
+HWI-EAS443:3:16:703:623#0/1
_a^]a^]ZWa^`^[a]]`¥SZ`a_a`a_a`W``PVSZ]U^
@HWI-EAS443:3:21:1335:1131#0/1
GGCTAGTTAAAAAAGAAAATTCATACCCAAAGCTCAGCAA
+HWI-EAS443:3:21:1335:1131#0/1
_X]HSXP_YIWQ]O^[V`NUV^¥SY_YHG¥_`BBBBBBBB
@HWI-EAS443:3:26:688:169#0/1
CATTAATGTAGGATTTGCTGAGCTTTGGGTATGAATTTTC
+HWI-EAS443:3:26:688:169#0/1
abbbbbbb_ba``bbabbbbabbbbbb`a_baba`bbaaa
@HWI-EAS443:3:27:256:1586#0/1
TGAATTTTCTTTTTTAACTAGCCATATTAAATTTTTGTAG
+HWI-EAS443:3:27:256:1586#0/1
a__X_``abaaaa`aaaaaa_a_^`aaaba_a_a_[_¥^_
@HWI-EAS443:3:30:490:816#0/1
CTGAGCTTTGGGTATGAATTTTCTTTTTTAACTAGCCATA
+HWI-EAS443:3:30:490:816#0/1
aab`ababaa`^]aa`Xa`aaaaaa`aa`aaa_^_`_a^a
@HWI-EAS443:3:34:896:1485#0/1
GGCTAGTTAAAAAAGAAAATTCATACCCAAAGCTCAGCAA
+HWI-EAS443:3:34:896:1485#0/1
a`aaaaaaba]`aba_aaa_``aaa_a`aaaY¥^[_[]]^
@HWI-EAS443:3:35:236:505#0/1
GCTAGTTAAAAAAGAAAATTCATACCCAAAGCTCAGCAAA
```

```
+HWI-EAS443:3:35:236:505#0/1
aa_a_[a`_^¥RYXZa`TW_`XT]__`_WR¥`]¥X¥^Z_^
@HWI-EAS443:3:36:1159:43#0/1
ATTTGCTGAGCTTTGGGTATGAATTTTCTTTTTTAACTAG
+HWI-EAS443:3:36:1159:43#0/1
`aabbaab`b`aabbbb]aab¥Yaaaa^aaaaa__aa^a`
@HWI-EAS443:3:37:1253:1225#0/1
GGCTAGTTAAAAAAGAAAATTCATACCCAAAGCTCAGCAA
+HWI-EAS443:3:37:1253:1225#0/1
^aa`a_WX^abbb`ZYa`Y^bb¥U```VLDHY_`[X`_]U
@HWI-EAS443:3:52:678:948#0/1
GGCTAGTTAAAAAAGAAAATTCATACCCAAAGCTCAGCAA
+HWI-EAS443:3:52:678:948#0/1
aaaa`a`aa_^`a`_]`a_`aaa_a_`_``___^^_a__`
@HWI-EAS443:3:57:228:544#0/1
GTTAAAAAAGAAAATTCATACCCAAAGCTCAGCAAATCCT
+HWI-EAS443:3:57:228:544#0/1
aaaaaaY^aaaZ`¥aaaa`a``^_^¥^__¥X^_^^_]^_^
@HWI-EAS443:3:59:1046:191#0/1
GGCTAGTTAAAAAAGAAAATTCATACCCAAAGCTCAGCAA
+HWI-EAS443:3:59:1046:191#0/1
`baaabababaabbaa_aaab`bbb``aaa___`ZYV^_^
@HWI-EAS443:3:59:1397:88#0/1
AAGAAAATTCATACCCAAAGCTCAGCAAATCCTACATTAA
+HWI-EAS443:3:59:1397:88#0/1
¥b_b^babaI_ba^`U`b^b`a_a`abb_aPYZ_]LTa^¥
@HWI-EAS443:3:63:264:450#0/1
GGCTAGTTAAAAAAGAAAATTCATACCCAAAGCTCAGCAA
+HWI-EAS443:3:63:264:450#0/1
``ba]__a``baaaaaaaaa``a]a^^¥]T]ZR^[¥[R[V
@HWI-EAS443:3:64:1431:1300#0/1
ACCGGGAGACAAAATAACATTCGAAGCAACTGGAAATCTA
+HWI-EAS443:3:64:1431:1300#0/1
aa]]]¥^baaababaaaaaaa^aa`b_aa_]ab___`_aa
@HWI-EAS443:3:65:1023:1150#0/1
GAATTTTCTTTTTTAACTAGCCATATTAAATTTTTGTAGA
+HWI-EAS443:3:65:1023:1150#0/1
``Yabaabbaaaa_[^__ab__aabaaaa`aaa`a__aa¥
@HWI-EAS443:3:66:452:1883#0/1
GAATTTTCTTTTTTAACTAGCCATATTAAATTTTTGTAGA
+HWI-EAS443:3:66:452:1883#0/1
a[`aba_X_ab`a_`U``WY]^aWaa]¥VW_TZ`¥¥TaX`
@HWI-EAS443:3:66:474:1812#0/1
GCTTTGGGTATGAATTTTCTTTTTTAACTAGCCATATTAA
+HWI-EAS443:3:66:474:1812#0/1
abbabab``ab^Z_aababaaaababbaababaaabaab`
@HWI-EAS443:3:69:1107:508#0/1
AGAAAATTCATACCCAAAGCTCAGCAAATCCTACATTAAT
+HWI-EAS443:3:69:1107:508#0/1
`^`a`Saaaaaa_`abaa`_`]a__`a^_a____`__]¥a
```

```
@HWI-EAS443:3:69:1313:1296#0/1
GATTTGCTGAGCTTTGGGTATGAATTTTCTTTTTTAACTA
+HWI-EAS443:3:69:1313:1296#0/1
aabbbaaaaaaabbbbba]abaa¥bbbaaaaaaaaaa_a^
@HWI-EAS443:3:71:1068:1498#0/1
AAGAAAATTCATACCCAAAGCTCAGCAAATCCTACATTAA
+HWI-EAS443:3:71:1068:1498#0/1
`XSXaYTTWZ^aa^``b`[Y_WPQT[`Y]XYYORX_R¥GN
@HWI-EAS443:3:71:1605:1435#0/1
GGCTAGTTAAAAAAGAAAATTCATACCCAAAGCTCAGCAA
+HWI-EAS443:3:71:1605:1435#0/1
_[a`[^Z¥^PP¥_a`PK]_]UW_WTSU]a_PPVU¥XYX[]
@HWI-EAS443:3:75:359:1009#0/1
GGCTAGTTAAAAAAGAAAATTCATACCCAAAGCTCAGCAA
+HWI-EAS443:3:75:359:1009#0/1
aaa``a_a[Xaaaa_aaaaaaaa^a^a_]Sa``^_Y_`a`
@HWI-EAS443:3:77:1066:565#0/1
GCTGAGCTTTGGGTATGAATTTTCTTTTTTAACTAGCCAT
+HWI-EAS443:3:77:1066:565#0/1
`aab_aaaaaaaaY`aaR[aa``Zaab`aba`_`_a_Z`a
@HWI-EAS443:3:78:715:1682#0/1
GAAAATTCATACCCAAAGCTCAGCAAATCCTACATTAATG
+HWI-EAS443:3:78:715:1682#0/1
ababb__`baabaaab_`aa`a^abaaaaaaaaaa_aaa`
@HWI-EAS443:3:80:461:998#0/1
TTTGGGTATGAATTTTCTTTTTTAACTAGCCATATTAAAT
+HWI-EAS443:3:80:461:998#0/1
^_[¥¥RDY¥VY``[^_¥WXZPaa¥XPWXM[^S`^_QZS]R
@HWI-EAS443:3:82:1120:1050#0/1
AGAAAATTCATACCCAAAGCTCAGCAAATCCTACATTAAT
+HWI-EAS443:3:82:1120:1050#0/1
abbabaababaaa_aaaaaa_aa`_a_aa``aa`aa`_]a
@HWI-EAS443:3:82:92:429#0/1
TTGGGTATGAATTTTCTTTTTTAACTAGCCATATTAAATT
+HWI-EAS443:3:82:92:429#0/1
aaabb^Wa`¥J[baaaaaaba`V_`a_aaaa`_aa^^^``
@HWI-EAS443:3:87:918:719#0/1
AGCTTTGGGTATGAATTTTCTTTTTTAACTAGCCATATTA
+HWI-EAS443:3:87:918:719#0/1
abaababbbabbaY`bbbababbbabbaabbbaabbbbbb
@HWI-EAS443:3:88:1087:453#0/1
TTTGCTGAGCTTTGGGTATGAATTTTCTTTTTTAACTAGC
+HWI-EAS443:3:88:1087:453#0/1
`baaaba`baaaabaa^aaa`aaaaa_aabaaaaa^b_a_
@HWI-EAS443:3:88:420:888#0/1
GGCTAGTTAAAAAAGAAAATTCATACCCAAAGCTCAGCAA
+HWI-EAS443:3:88:420:888#0/1
`b`bba¥^`_a_``b¥aa`aaaa_^`aa`XR]a]`[`^]J
@HWI-EAS443:3:94:317:1770#0/1
TGCTGAGCTTTGGGTATGAATTTTCTTTTTTAACTAGCCA
```

```
+HWI-EAS443:3:94:317:1770#0/1
ab```¥baaaabb_^aa`aabaaa^aaa`aa^a_a^`S[a
@HWI-EAS443:3:97:615:2006#0/1
AAAATTCATACCCAAAGCTCAGCAAATCCTACATTAATGA
+HWI-EAS443:3:97:615:2006#0/1
a[aYaaa¥`a``a]a``^aa__`a]Y_^]^a¥___^UU]^
@HWI-EAS443:3:9:1573:144#0/1
ATGAATTTTCTTTTTTAACTAGCCATATTAAATTTTTGTA
+HWI-EAS443:3:9:1573:144#0/1
aab`abbab_babaaaaa`a__YR¥``aa``^a__`_`^_
@HWI-EAS443:3:27:106:937#0/1
ACCGCATTTCTTCAATTAACCACCTTATTTCCTCAAATTT
+HWI-EAS443:3:27:106:937#0/1
^`ba_Y_babaab```abbbb_bababbbbbbbaba¥a_]
@HWI-EAS443:3:66:1279:1316#0/1
ACCGCATTTCTTCAATTAACCACCTTATTTCCTCAAATTT
+HWI-EAS443:3:66:1279:1316#0/1
[ab]aa_aa`b]aa_a_aa_a`_``aaaaaaaaaa_``a_
@HWI-EAS443:3:81:851:506#0/1
ACCGCATTTCTTCAATTAACCACCTTATTTCCTCAAATTT
+HWI-EAS443:3:81:851:506#0/1
I`bbb`bbbabbbbbbbbbbbabbbbbbbbbbbbbbbbbb
@HWI-EAS443:3:83:735:602#0/1
ACCGCATTTCTTCAATTAACCACCTTATTTCCTCAAATTT
+HWI-EAS443:3:83:735:602#0/1
G]b``bbbbbb`^¥``Z``bbbbbbbbbbbb`bb_¥abaa
@HWI-EAS443:3:78:1591:1032#0/1
ATTCTAAACTGTAGAGACCCATTAGAGCACATGCAGAAAC
+HWI-EAS443:3:78:1591:1032#0/1
aSXZ¥bY[`V`_]_`aQUV[SLZ[TS`OVV[IDRBBBBBB
@HWI-EAS443:3:87:271:1214#0/1
GGAGATTTTTGAACAAATTACTTGTCAATGGTAAATGGCA
+HWI-EAS443:3:87:271:1214#0/1
^T[aO[``_¥a_H]bbba^VV`bb_Xa]bb^O]aa_a¥¥a
@HWI-EAS443:3:11:1276:497#0/1
AGGTTTTTGAACAAATTACTTGTCAATGGTAAATGGCAAC
+HWI-EAS443:3:11:1276:497#0/1
Z¥H[aaa`^U^`^¥Z`_`ab^``^^S_a^VW`RZa^QYa^
@HWI-EAS443:3:31:320:1943#0/1
CGGGTGGCTGGCGTTTATAGCACCCTTGGGTGTCTGACAA
+HWI-EAS443:3:31:320:1943#0/1
`_`WSPaba`aG`a`abaaTababaa^^[GKYPX`R__`¥
@HWI-EAS443:3:85:377:1896#0/1
GGCGTTTATAGCACCCTTGGGTGTCTGACAAGTTGTATTG
+HWI-EAS443:3:85:377:1896#0/1
Z_NTaK_a¥[SU¥a[VZU¥`WLYOW¥[]^¥P¥_U¥[_ZU_
@HWI-EAS443:3:35:261:419#0/1
CCCTAATTATCACTATGAGGAATGCTCCTGTCATCCTGAT
+HWI-EAS443:3:35:261:419#0/1
U__T_UOY¥U__QV_ZX]XZBBBBBBBBBBBBBBBBBBBB
```

```
@HWI-EAS443:3:63:929:1316#0/1
CGGGAATCAAAATCAGATTGAAACATGCAATCAAAGCGTC
+HWI-EAS443:3:63:929:1316#0/1
aa`a`aaa``__``abaaa_a``^a`a`aa__a_]`a`^a
@HWI-EAS443:3:2:1310:1602#0/1
GCAGGGAGAACTAAAAGCATTAGTTCAAGAAACGGTTCTG
+HWI-EAS443:3:2:1310:1602#0/1
aaaa[¥Ta_¥DXa_a[_U__`RRQ[_WZ_Z^^S_ZOL]MB
@HWI-EAS443:3:44:1514:810#0/1
CAAGAAACGGTTCTGAGATGATTTGGGATCCGAACGGATG
+HWI-EAS443:3:44:1514:810#0/1
aaabbabaab_babb`b^ababaa^aa_``_a__^a]W`a
@HWI-EAS443:3:42:958:595#0/1
AGCTTCTGTGGTCACTGTTCCCATCCCGTTGTATATGAGG
+HWI-EAS443:3:42:958:595#0/1
T¥bbabbbaWUPK]_``^¥R_J_VZWHX_Z¥DZXMUTYXX
@HWI-EAS443:3:11:872:41#0/1
TGCCCGTTAATTTCACGGAAACCACTGACTGTCCAGCAGC
+HWI-EAS443:3:11:872:41#0/1
aaaaa_F]aaaaa_aTa`^__¥__¥__^¥]_WWVUTLPWW
@HWI-EAS443:3:100:512:860#0/1
CGCTCCATTGTCTGAACTAGTTGTTTCCACAATGTAGGAC
+HWI-EAS443:3:100:512:860#0/1
abb_b[`VWa_[a]_GT[Q]W`a`aa^a^`_¥T[U^^]^B
@HWI-EAS443:3:84:406:315#0/1
AATCGATGAAATTTCCTGGGTAACACGCTCCATTGTCTGA
+HWI-EAS443:3:84:406:315#0/1
_Yabbbab^baabaaaaaaa]`[`a`ab_a]¥¥__]^a_]
@HWI-EAS443:3:44:951:1818#0/1
AATCGATGAAATCTCCTGGGTAACACGCTCCATTGTCTGA
+HWI-EAS443:3:44:951:1818#0/1
a___a_`a`__`_[^]]^^¥Za]W_]W]XWR^Z]XXXZXZ
@HWI-EAS443:3:64:567:461#0/1
CTCCTGCCTTTAATGGTTCCATTGGAATGTTTGTCATTTA
+HWI-EAS443:3:64:567:461#0/1
QV_[__H_TY¥_ZVaUR__]aZXWRYBBBBBBBBBBBBBB
@HWI-EAS443:3:97:264:422#0/1
GGAGTTTTTTTGAACAAATTACTTGTCAATGGCAAATGGC
+HWI-EAS443:3:97:264:422#0/1
ab`a_a_bbaa_Saaaa`aaa^aaaZa`_aa`P¥_^X^¥W
@HWI-EAS443:3:89:62:1457#0/1
GGAGTTTTTTTGAACAAATTACTTGTCAATGGTAAATGGC
+HWI-EAS443:3:89:62:1457#0/1
aa`a]a_aaa`¥¥aaaa]_¥[_^_`Z_a]Y_`WXS_W]W^
@HWI-EAS443:3:76:1562:1110#0/1
GGCAAAAGCAGGTAGATATTTAAAGATGAGTCTTCTAACC
+HWI-EAS443:3:76:1562:1110#0/1
a]__¥]Y^]X]OXX^]_]^^W_UX]US^XZWX]XBBBBBB
@HWI-EAS443:3:48:1170:801#0/1
GTGGGCGGGGGTTTTGTTCAGCATCCAGAACTAACAGGGC
```

```
+HWI-EAS443:3:48:1170:801#0/1
a_`]a]]`YG_`X¥¥¥NTVRNLV[^^UZ^UU[WZQNRZRS
@HWI-EAS443:3:43:692:1012#0/1
ACGGGGGTTTTGTTCAGCATCCAGAACTAACAGGGCTGGA
+HWI-EAS443:3:43:692:1012#0/1
SXX_Y`aXU^`[Mabaabbab`[_N]`Q[¥`b`aZX]``Q
@HWI-EAS443:3:32:665:264#0/1
CTGGGTCCATGAGGTAAGAATATCAACAGGAACAGCAGAG
+HWI-EAS443:3:32:665:264#0/1
a__]_Q_a]aa_aYFI__XYXW¥VYU[^VTUV¥W^S^ZYW
@HWI-EAS443:3:56:173:680#0/1
GGTCCATGAGGGAAGAATATCAACAGGAACNGCAGAGTGC
+HWI-EAS443:3:56:173:680#0/1
ab_aaa`a_a`a_aa[^`]_a_``aa___RD[`^___S_¥
@HWI-EAS443:3:22:1696:1245#0/1
GCCTGGGTCCATGAGGGAAGAATATCAACAGGAACAGCAG
+HWI-EAS443:3:22:1696:1245#0/1
aa]abba]_T¥`[NU``W``aaa^```WIT_P`QLVYFQW
@HWI-EAS443:3:22:726:1738#0/1
CCTGGGTCCATGAGGGAAGAATATCAACAGGAACAGCAGA
+HWI-EAS443:3:22:726:1738#0/1
abaabVU]^¥¥¥M]]Q_PT_]U`XV`_Z_YQX[`W[Y^Z_
@HWI-EAS443:3:30:80:1439#0/1
AAAGAGGGCCTTCTACGGAAGGAGTGCCTGGGTCCATGAG
+HWI-EAS443:3:30:80:1439#0/1
[T[^¥VU`a_ZY]]XNV¥^RHNOOQPP^BBBBBBBBBBBB
@HWI-EAS443:3:39:1393:1389#0/1
GAAAAGAGGGCCTTCTACGGAAGGAGTGCCTGGGTCCATG
+HWI-EAS443:3:39:1393:1389#0/1
`HZPKSVWZWZOQL]ZBBBBBBBBBBBBBBBBBBBBBBBB
@HWI-EAS443:3:54:162:1862#0/1
TGGGTCCATGAGGGAAGAATATCAACAGGAACAGCAGAGT
+HWI-EAS443:3:54:162:1862#0/1
aabb^a`aa]abaa_]X_baaaaabWba`aaZaa[``_aU
@HWI-EAS443:3:56:293:633#0/1
GCCTGGGTCCATGAGGGAAGAATATCAACAGGAACAGCAG
+HWI-EAS443:3:56:293:633#0/1
aaa``aa¥a`__a¥_a___]¥__^__^^¥`_¥¥¥¥¥^^Z_
@HWI-EAS443:3:60:143:1460#0/1
GCCTGGGTCCATGAGGGAAGAATATCAACAGGAACAGCAG
+HWI-EAS443:3:60:143:1460#0/1
aa^PD[aQ[a][]T¥P_UT_¥¥_W^_^X¥[^W[S[_^U^_
@HWI-EAS443:3:60:511:367#0/1
CCTGGGTCCATGAGGGAAGAATATCAACAGGAACAGCAGA
+HWI-EAS443:3:60:511:367#0/1
ababab``aQaa_aaa`abaaaaaaaaaa`a`^W`a^_a_
@HWI-EAS443:3:71:610:521#0/1
CCTGGGTCCATGAGGGAAGAATATCAACAGGAACAGCAGA
+HWI-EAS443:3:71:610:521#0/1
aaaaaa`aaaabaaaaaabaaa`_aa_aa`aab`aaa`a^
```

```
@HWI-EAS443:3:7:1299:424#0/1
GCCTGGGTCCATGAGGGAAGAATATCAACAGGAACAGCAG
+HWI-EAS443:3:7:1299:424#0/1
aa^RZb_]_SXa`P_Y_GO`]XaZa_R]¥__a^¥TXaS`a
@HWI-EAS443:3:3:612:1148#0/1
TAACATTGCTGGCTGGATCCTGGGAAATCCAGAGCGTGAA
+HWI-EAS443:3:3:612:1148#0/1
a_¥Ra¥^a_ab¥T`a_`¥]XOHU_¥`a^ZUXa_`GWWZ]`
@HWI-EAS443:3:63:227:1311#0/1
GCTGGATCCTGGGAAATCCAGAGCGTGAATCACTCTCCAC
+HWI-EAS443:3:63:227:1311#0/1
]S^aaaZ^]T^[Q[aaXa`]`¥XDWYY][ZNT¥XXL¥VTY
@HWI-EAS443:3:99:648:560#0/1
GTGCTCTCTTTGGGTCGCCCTCTGATTAGTTCAACCCAGA
+HWI-EAS443:3:99:648:560#0/1
a_a[]`_`_`]`_[T__^_W`Waa_¥_^_¥^T^¥YZN^_¥
@HWI-EAS443:3:35:539:155#0/1
TATACAACAGGATGGGAACAGTGACCACAGAAGCTGCTTC
+HWI-EAS443:3:35:539:155#0/1
Z_bb`ba]Z[SW_`R`ZYZ`]X^aaaWLZ[]P]Z¥XUR[J
@HWI-EAS443:3:64:1064:1169#0/1
GCCACAGAAGCTGCTTTTGGTCTAGCGTGTGCCACTTGTG
+HWI-EAS443:3:64:1064:1169#0/1
W^b^__[T`Ybab[`V^¥¥[T^bU¥H^¥aNYaaaaQWZXZ
@HWI-EAS443:3:51:401:190#0/1
GTCAGGATATAGTGGGAGTTTTGTTCAGCATACAGAAATA
+HWI-EAS443:3:51:401:190#0/1
¥O¥[^aab]`[`HZaaabT^[[V[]XRY¥_ZNO^_`¥DU]
@HWI-EAS443:3:28:453:1554#0/1
TGGGAGTTTTGTTCAGCATCCAGAACTAACAGGGCTGGAT
+HWI-EAS443:3:28:453:1554#0/1
]a¥¥^`]aa^a_aab]bbab`^W¥`aaaaaa`a[a`a__^
@HWI-EAS443:3:44:1636:814#0/1
GCCGGCTTGAACTTCTTGCTGTATCTTGACGTCCCCACAA
+HWI-EAS443:3:44:1636:814#0/1
aaaaa_a_VU]R]a__`Y^^`¥Y]S^_WRDR[STV^VOLT
@HWI-EAS443:3:93:1191:995#0/1
GAGAGCCTCAAGATCTGTGTTCTTTCCTGTAAAGACACTT
+HWI-EAS443:3:93:1191:995#0/1
aY]LW_`OSPOXNNZUXFX__Y]a_XTWTGTaZP`RZGV^
@HWI-EAS443:3:19:994:1215#0/1
GAGAATGGGAGACCTTCACTACCTCGAGAGCAGAAATAAA
+HWI-EAS443:3:19:994:1215#0/1
a[a_a`V`XL]YYNXRT]`WWZ[YSFWXH`YQXNRFV¥RR
@HWI-EAS443:3:12:1574:1054#0/1
GAGAATGGGAGACCTTCACTACCTCCAGAGCAGAAATAAA
+HWI-EAS443:3:12:1574:1054#0/1
aabaa`aab`ba[_aaZa`aaRZaYV^XZaV]a_ZY__^]
@HWI-EAS443:3:7:713:321#0/1
GCTCTCGCCACTTTTTATTTCTGCTCTGGAGGTAGTGAAG
```

```
+HWI-EAS443:3:7:713:321#0/1
```_``a[a_aa_aaRabaa_a`Va_]``_`_N]]U]UBB
@HWI-EAS443:3:43:772:1123#0/1
AACATTCTTTGGCTGGAAAGAGCCTAACATAGCCAAACCA
+HWI-EAS443:3:43:772:1123#0/1
H^bbbbabbbbabbbbabbbba`ababbbba`Xabbbbab
@HWI-EAS443:3:9:1455:1976#0/1
ACGCAAAGAACAATAGGGAAGAAAAAACAAAGACTAAGAG
+HWI-EAS443:3:9:1455:1976#0/1
a__L]_aa_`¥`[]_`_[]`a[_`_Y_Q__^a^V^`__a¥
@HWI-EAS443:3:10:751:1365#0/1
GCCTTGTTGTAATTGAATACTGGAGAATTGCCTCTTATTC
+HWI-EAS443:3:10:751:1365#0/1
`a_``¥`ab`aYa^_aa[G^_W][`P`Y__]W`^`^^`[[
@HWI-EAS443:3:64:729:1340#0/1
GCCTTGTTGTAATTGAATACTGGAGAATTGCCTCTTATCA
+HWI-EAS443:3:64:729:1340#0/1
aba__YDY¥F]^`_[]a``RRYa_baa__```]^a]TX__
@HWI-EAS443:3:73:674:1194#0/1
TTGTTTCCACAATGTAGGACCATGATCTTGCTGTGGAGAG
+HWI-EAS443:3:73:674:1194#0/1
¥XS]Y^a`_][WHX]bTR`_T_V`WDTXR]`a_OSDJVYY
@HWI-EAS443:3:47:1492:533#0/1
GTCCAGTATCGTCTAATGGAGCAAATGGAATAAAAGGATT
+HWI-EAS443:3:47:1492:533#0/1
aaa^`a_aa`a`Za``a`VZ`^]_^`]`X_`_^``_a¥^_
@HWI-EAS443:3:8:1220:207#0/1
TGGTCCTCCATCTGATACTAATAGCGCTACTTTTGATTGG
+HWI-EAS443:3:8:1220:207#0/1
]aSLZH[Vaa`RS¥QZ]R]__aY]XEHT¥FF¥_^OF^XBB
@HWI-EAS443:3:46:164:190#0/1
AGACCCACCAGAACAGAGTAGGATGCAATTTTCCTCATTG
+HWI-EAS443:3:46:164:190#0/1
`YH_[aYX[`^N]TD¥_YRaaXNXUVYR[UYVTQXY_UYV
@HWI-EAS443:3:72:1085:742#0/1
CCCTAAGTGCCTGAACAATGAGAAGCAATTTTCTATTGCA
+HWI-EAS443:3:72:1085:742#0/1
V_b¥`]X_W¥M¥aa_`_X^QX_[YWN]`aa^_b``aba]`
@HWI-EAS443:3:66:1559:1715#0/1
GTCCCTAAGTGCCTGAACAATGAGAAGCAATTTTCTATTG
+HWI-EAS443:3:66:1559:1715#0/1
aabbabbbb^aaaaabaaaaaabaaba`baaaaa_aaaaa
@HWI-EAS443:3:16:1358:656#0/1
ATAGAAAATTGCTTCTCATTGTTCAGGCACTTAGGGACAA
+HWI-EAS443:3:16:1358:656#0/1
ab^baaaaaaaaaa_aaa`aa_a^`_^^aZa```^`__`a
@HWI-EAS443:3:34:664:618#0/1
TAGAAAATTGCTTCTCATTGTTCAGGCACTTAGGGACAAC
+HWI-EAS443:3:34:664:618#0/1
aKaabaX]baaa`a`_`_aaUP_]a_aaV]¥a__^]ZSBB
```
```

```
@HWI-EAS443:3:89:1387:850#0/1
CACGTTCCATTGCCTGAACTAGTTGTTTCCACAATGTAGG
+HWI-EAS443:3:89:1387:850#0/1
a_`_^^X¥¥a^`W`[¥P_a_T__aa]`a^_a[aa`aaa^`
@HWI-EAS443:3:75:1107:139#0/1
GCCGGTTTCATTGAAGGGGGGTGGACAGGAATGGTAGATG
+HWI-EAS443:3:75:1107:139#0/1
a_R_aZaa_aaaa]Q`a^a^aM^_UUQ¥_¥¥_a_UVZWW`
@HWI-EAS443:3:67:1648:1643#0/1
CTCTGGGTGACTCTCCGATTGGCCATGTTTCCGATTTGTT
+HWI-EAS443:3:67:1648:1643#0/1
abbb`bb_ba`bbb`ababbaaaaa`W]aa[aZSXaa`]_
@HWI-EAS443:3:2:126:1055#0/1
CTTGCTTTATTGAGAAGTTATTGTCTGTCCCAGTCCACAC
+HWI-EAS443:3:2:126:1055#0/1
a`]`[`ab]a`R][`_bSU]ba`BBBBBBBBBBBBBBBBB
@HWI-EAS443:3:19:578:281#0/1
ATTCAAGTGAAATCACATGTGTGTGCAGGGATAACTGGCA
+HWI-EAS443:3:19:578:281#0/1
_^]ba^aYb[baa_`aYbaYb_a]^`aa`]^Za[__]]]_
@HWI-EAS443:3:82:596:1217#0/1
AGCCCGCTAATTTCACGGAAACCACTGACTGTCCAACAAC
+HWI-EAS443:3:82:596:1217#0/1
¥MUNV_RRW]PRWSYNWa^YaZBBBBBBBBBBBBBBBBBB
@HWI-EAS443:3:16:1199:87#0/1
AGCCCGCTAATTTCACGGAAACCACTGACTGTCCAGCAGC
+HWI-EAS443:3:16:1199:87#0/1
_]]]_aS`R][VZ_^R`_¥_^VP[WY^WX_¥ZTRNQGUUB
@HWI-EAS443:3:17:1037:388#0/1
AGCCCGCTAATTTCACGGAAACCACTGACTGTCCAGCAGC
+HWI-EAS443:3:17:1037:388#0/1
`a`a[aa]S__`__]_^^V`_[¥_S¥_¥W]_VRQ[YWQUW
@HWI-EAS443:3:19:1049:1597#0/1
AGCCCGCTAATTTCACGGAAACCACTGACTGTCCAGCAGC
+HWI-EAS443:3:19:1049:1597#0/1
`_[a]__```aa^`a__¥W_a¥_^W__`¥]]TZ^_¥Y¥ZB
@HWI-EAS443:3:32:602:1362#0/1
AGCCCGCTAATTTCACGGAAACCACTGACTGTCCAGCAGC
+HWI-EAS443:3:32:602:1362#0/1
¥X_aaaa`¥`aaaaa`[]``aa][[``a``^ZaYRL]TR¥
@HWI-EAS443:3:42:409:1884#0/1
AGCCCGCTAATTTCACGGAAACCACTGACTGTCCAGCAGC
+HWI-EAS443:3:42:409:1884#0/1
aH^a`a`]aa__^Ua__WTY^Y[`M`X_a¥¥TY_]Y]PDW
@HWI-EAS443:3:65:363:2041#0/1
AGCCCGCTAATTTCACGGAAACCACTGACTGTCCAGCAGC
+HWI-EAS443:3:65:363:2041#0/1
¥^_bb_[a`a`NZa]a[`aaS]a`__Y`_¥RQ`_PRSZS^
@HWI-EAS443:3:77:361:137#0/1
AGCCCGCTAATTTCACGGAAACCACTGACTGTCCAGCAGC
```

```
+HWI-EAS443:3:77:361:137#0/1
ab``ab`a__a`^^a_aa]a[S`_^^^_¥__VSTYZUUUK
@HWI-EAS443:3:7:1642:635#0/1
AGCCCGCTAATTTCACGGAAACCACTGACTGTCCAGCAGC
+HWI-EAS443:3:7:1642:635#0/1
ZOSXa_abaaaab`aQ`a_aa[a[Y_[aWaBBBBBBBBBB
@HWI-EAS443:3:87:541:1712#0/1
AGCCCGCTAATTTCACGGAAACCACTGACTGTCCAGCAGC
+HWI-EAS443:3:87:541:1712#0/1
a``b__`L[a_`_aa_ZW^a_¥]_^W[_¥¥KMLYYSZ¥BB
@HWI-EAS443:3:90:885:1665#0/1
AGCCCGCTAATTTCACGGAAACCACTGACTGTCCAGCAGC
+HWI-EAS443:3:90:885:1665#0/1
aa_abba`babb`aa_]`a_b`aaXZ`baa]¥]Naaa`_¥
@HWI-EAS443:3:19:229:1270#0/1
TGGGTCTTCAATACTTAATTTCAGAGCATCCATCAGCAGG
+HWI-EAS443:3:19:229:1270#0/1
[baaT^_`aa]`a``a[`_a^^bZ^]¥aaa]_U__`]a]Y
@HWI-EAS443:3:33:1224:1438#0/1
CTCCACAAGAAAATTGCTTCTCATTGTTCAGGCACTTAGG
+HWI-EAS443:3:33:1224:1438#0/1
aabbabaaaaaaaaaaaaaaaaaaa_``aa[R`_a_``_
@HWI-EAS443:3:63:956:360#0/1
CGTTCCCATCCTGTTGTATATGAGGCCCATGCAACTGGCA
+HWI-EAS443:3:63:956:360#0/1
a_aaaabbbbabb_abbbbbab[a`abaaaaa`aaaa`aa
@HWI-EAS443:3:86:1485:305#0/1
CATGCTCCCCCTTGGAATGCAGAAAATTCTTCTTGACTCA
+HWI-EAS443:3:86:1485:305#0/1
R``ZIWSZZYUbb`_]a¥^`_bb_FHbb¥ab^abb^_aSW
@HWI-EAS443:3:100:314:584#0/1
ATTTTCGGAGACAATCCACGCCCTAATGACAAGACAGGCC
+HWI-EAS443:3:100:314:584#0/1
[aabaaZbbaa_b`ba`b``][_aa[aaaYb`¥_^_aY`]
@HWI-EAS443:3:80:1786:637#0/1
TCAGACTAGGCAGATGGTACATGCGATGAGAACTATTGGG
+HWI-EAS443:3:80:1786:637#0/1
ab_`_^b¥¥¥ZZ^Y^Z^P^PR]XRDR^SY_`[YO¥T]RJU
@HWI-EAS443:3:2:1271:837#0/1
AAGGGTCCCCATTCCCATTTAGGGCATTTTGGACAAAGCG
+HWI-EAS443:3:2:1271:837#0/1
`a```[`Y`aaaa[]N[aa__a¥VN__a___a_¥¥¥¥_S_
@HWI-EAS443:3:47:992:1063#0/1
CAGGGTCCCCATTCCCATTTAGGGCATTTTGGACAAAGCG
+HWI-EAS443:3:47:992:1063#0/1
aba¥WW`a¥U^aa_aaa_XWa_a_X`aa^a`TV]`_aaX_
@HWI-EAS443:3:32:1487:724#0/1
AGGGTCCCCATTCCCATTTAGGGCATTTTGGACAAAGCGT
+HWI-EAS443:3:32:1487:724#0/1
^a^XXZ_aX_aVPH_^b`b]]^XST]`aa]ZUXX^Q]BBB
```

```
@HWI-EAS443:3:98:1545:613#0/1
AGGGTCCCCATTCCCATTTAGGGCATTTTGGACAAAGCGT
+HWI-EAS443:3:98:1545:613#0/1
`aZaaZaaaabb`aZabbb`][`^aaaa[baaWaaa`Haa
@HWI-EAS443:3:78:737:1325#0/1
CTGATGTTGTGGATTACTGATCGTCTCTTTTTCAAATGTA
+HWI-EAS443:3:78:737:1325#0/1
aaa_OHZV]RT¥R¥Y]ZXa`XU]OX[HZR¥¥YJHTUHHKX
@HWI-EAS443:3:16:588:381#0/1
AGCTGGAATCAACAAGGATTTACCAGATTTTGGCGATTAA
+HWI-EAS443:3:16:588:381#0/1
__aba_V^a^a]_aaba¥__a_^^aba`a`aBBBBBBBBB
@HWI-EAS443:3:52:1762:1543#0/1
GCTGGAATCAACAAGGATTTACCAGATTTTGGCGATTTAT
+HWI-EAS443:3:52:1762:1543#0/1
]`b`^`b`aa[^baab^`ab_]¥aaaaaaaT``^^^F[BB
@HWI-EAS443:3:45:515:1064#0/1
GGCGTGGACTGTCTCCGAAAATCCCACTGCATATGTATCC
+HWI-EAS443:3:45:515:1064#0/1
a_a``aa^_a]HWZ[_aZ]_SWP_]Y]^YSU^Y[VZ_]VX
@HWI-EAS443:3:43:1207:1403#0/1
GCTCCATTAGACGATATTGGACCACAACTGCCTGTCTTAT
+HWI-EAS443:3:43:1207:1403#0/1
Xa__b`Ybbaab_Yb_O_HZ^¥IZ[`]]^¥QV]HWaa]_[
@HWI-EAS443:3:37:500:1698#0/1
CGCCTGCTGTTGCATCTTCGCCATTGTTTGCTTGGCGCCA
+HWI-EAS443:3:37:500:1698#0/1
aabbbabbb`b^b`baabbabbaab^abaQba`S]a[ab]
@HWI-EAS443:3:47:1705:192#0/1
CGCCTGCTGTTGCATCTTCGCCATTGTTTGCTTGGCGCCA
+HWI-EAS443:3:47:1705:192#0/1
``aaaaabbab_]Q__]¥I`^^_V__^a^UT_¥FFMZXR¥
@HWI-EAS443:3:16:620:1294#0/1
ATGCAACAGCAGGCCTTACTCATATCATGATTTGGCATTC
+HWI-EAS443:3:16:620:1294#0/1
a`ab_OV[R^a_RM^aKaa_`a]]`Sa¥TY^_[RQ[_^]]
@HWI-EAS443:3:37:654:1375#0/1
GGCGAAGATGCAACAGCAGGCCTTACTCATATCATGATTT
+HWI-EAS443:3:37:654:1375#0/1
_K`__Z_``ZaY]`aa^[[[M^RWWa__INH_aa[a[VXT
@HWI-EAS443:3:61:1535:1537#0/1
GCGCCAAGCAAACAATGGCGAAGATGCAACAGCAGGCCTT
+HWI-EAS443:3:61:1535:1537#0/1
aY]]UWXS¥_]TT_UW_^U_]_`^SP[¥WNT¥TUWNHGTG
@HWI-EAS443:3:82:1364:866#0/1
AACTGGCATGGCTCGAATCGACCGTGGGGGTCTTTTAACC
+HWI-EAS443:3:82:1364:866#0/1
ab`baaa`ba``a`¥P[`P]_S_`R`aZH]JW^a`FP[VZ
@HWI-EAS443:3:9:462:1556#0/1
GCTCCTCATAATCGATGAAATCTCCTGGATAACACGTTCC
```

+HWI-EAS443:3:9:462:1556#0/1
^b```bbbb_b`_`b[bbbbZ`a__bbbbab``bb¥aaaa
@HWI-EAS443:3:60:119:863#0/1
AGAGCACTGACATTAAATACGATGACCAAAGATGCAGAAG
+HWI-EAS443:3:60:119:863#0/1
a^Y`abb]]aaabbaaaZaaaaaa_aaa[¥a_[_a__VHU
@HWI-EAS443:3:37:1395:1841#0/1
GTGATTCCAAGAGGAAAGCTTTCCACAAGAGAGGTCCAAA
+HWI-EAS443:3:37:1395:1841#0/1
^¥_UWT^Z^WMXWY[_¥ZY[a^URZV][WFYY]XKMSPWL
@HWI-EAS443:3:34:1441:423#0/1
TTGCTGACTCTTATCCCTCTCAGCGACATCTCCGTGTTTG
+HWI-EAS443:3:34:1441:423#0/1
_bbbabaZa_b¥```a_aaa¥b]aa_¥aRYPBBBBBBBBB
@HWI-EAS443:3:15:1605:1346#0/1
AAAGAACTGAGAGATCTAATGTCACAGTCCCGCACTCGCG
+HWI-EAS443:3:15:1605:1346#0/1
`]_Y``Zba^Yaa`aabba]WZaa¥a`KDVZ_]XYX_¥SZ
@HWI-EAS443:3:75:1471:564#0/1
GAGAGATCTAATGTCACAGTCCCGCACTCGCGAGATACTC
+HWI-EAS443:3:75:1471:564#0/1
abbbbb_abbbba``baa```_ba__aaaa_b_baa__a[
@HWI-EAS443:3:35:1766:825#0/1
GAGAGAGTGGTAGTGAGTATTGACCGGTTTTTAAGGGTTA
+HWI-EAS443:3:35:1766:825#0/1
a`a_bZaXba¥ab]UQ`XW[`YRPKDHM`aa`[VT`_[TT
@HWI-EAS443:3:42:435:1806#0/1
AAAGAACTGAGAGATTTAATGTCGCAGTCCCGCACTCGCG
+HWI-EAS443:3:42:435:1806#0/1
abNWaT_[]aW_Sb`IbJbaYORIaIba[^`SaV`¥HM^`
@HWI-EAS443:3:80:1194:1125#0/1
ATCAAAATACAGCAAGAAGTTCAAGCCGGAAATAGCAATA
+HWI-EAS443:3:80:1194:1125#0/1
abbabaabbabbaabbaaa_a`b_a¥_aa`aaaaa_`_a_
@HWI-EAS443:3:98:58:743#0/1
AAAATGGGACTTATGACTACCCAAAATACTCAGGAGGATG
+HWI-EAS443:3:98:58:743#0/1
a`aaaaa`_^aa```a```^^[_`^`¥_Z`_]__^Y`ZZ`
@HWI-EAS443:3:46:1537:2020#0/1
CCTGAGTATTTTGGGTAGTCATAAGTCCCATTTTTGACAC
+HWI-EAS443:3:46:1537:2020#0/1
HYbbbaaabbbbb``Tbbaabbbbba[abbbbbbbab]ba
@HWI-EAS443:3:100:144:946#0/1
CACTAATTTCACGGAAACCACTGACTGTCCAGCAGCAAAG
+HWI-EAS443:3:100:144:946#0/1
aaaababbb`aaa]_aaaa`_a```aa__aa_^W¥¥^]_B
@HWI-EAS443:3:39:282:658#0/1
ACTAATTTCACGGAAACCACTGACTGTCCAGCAGCAAAGT
+HWI-EAS443:3:39:282:658#0/1
a`a¥¥aaaa`_a`¥Q]____^^_^_^X_YUPW[UZRW]^X

```
@HWI-EAS443:3:18:449:1322#0/1
GGATGCTGAACAAAACTCCCGCTATATCCTGACCACTCAC
+HWI-EAS443:3:18:449:1322#0/1
aZY`]aaa`ba`b``__bba_a_a`a_^a_^aa``_]^]P
@HWI-EAS443:3:29:1615:1639#0/1
GGCAAATGGAGTAAAAGGATTTTCATTCAAATACGGCAAT
+HWI-EAS443:3:29:1615:1639#0/1
aa_a`_aaa`a¥_`aaa_]^`^`___^^^__`_]__^^]_
@HWI-EAS443:3:32:970:207#0/1
GTGGTCCAGTATCGTCTAATGGGGCAAATGGAGTAAAAGG
+HWI-EAS443:3:32:970:207#0/1
aXa`]__Z_Z^]X^ZWZU¥ZL[GUPTTV^TXM^TUX^[]U
@HWI-EAS443:3:72:1006:1087#0/1
AGGNCTCCTGTCTTTAATGGTTCCATTGGAATGTTTGTCA
+HWI-EAS443:3:72:1006:1087#0/1
aa¥D[`][X[[a`^aa`^`a]]aa_`a_]SP^]¥a`^X]U
@HWI-EAS443:3:72:115:918#0/1
AGGGCTCCTGTCTTTAATGGTTCCATTGGAATGTTTGTTA
+HWI-EAS443:3:72:115:918#0/1
`TU_aY[aa[SVYX_aa]`[QQ_IUVMPMJWW]YUTPMFS
@HWI-EAS443:3:28:1169:859#0/1
AGGGCTCCTGTCTTTAATGGTTCCATTGGAATGTTTGTCA
+HWI-EAS443:3:28:1169:859#0/1
ZWUa_^]]R_`W_baY`UQU[ORNN^^P_XaaYM]ZRZ`X
@HWI-EAS443:3:52:195:1340#0/1
AGGGCTCCTGTCTTTAATGGTTCCATTGGAATGTTTGTCA
+HWI-EAS443:3:52:195:1340#0/1
aa^_a`aaaa_]_``____`]]]]__`_]]]__]]^]YW^
@HWI-EAS443:3:71:1089:89#0/1
AGGGCTCCTGTCTTTAATGGTTCCATTGGAATGTTTGTCA
+HWI-EAS443:3:71:1089:89#0/1
aaaa`baaab__`aaaaaaa^a_[_a^aa__`a^_a`]X]
@HWI-EAS443:3:73:1460:793#0/1
AGGGCTCCTGTCTTTAATGGTTCCATTGGAATGTTTGTCA
+HWI-EAS443:3:73:1460:793#0/1
a`Kab`^b_aaaaabaa`a__b__]_a___```^`a^_^`
@HWI-EAS443:3:87:957:1619#0/1
AGGGCTCCTGTCTTTAATGGTTCCATTGGAATGTTTGTCA
+HWI-EAS443:3:87:957:1619#0/1
aa]X`ZX_]MWZHLQa`¥`_UaU]YNSFRX¥Z[PF[NK[X
@HWI-EAS443:3:95:1084:1636#0/1
AGGGCTCCTGTCTTTAATGGTTCCATTGGAATGTTTGTCA
+HWI-EAS443:3:95:1084:1636#0/1
¥^`ab]_SZS¥Vb``a^¥H]T^^V]XX]¥[]SV_``LLXS
@HWI-EAS443:3:63:19:187#0/1
AGCACAGTCAGCACTCATTCTGAGGGGATCAGTTGCACAT
+HWI-EAS443:3:63:19:187#0/1
Z^aZ__]U]OZ[^U[`X¥T[YNT^^_NFUVMXGZ^UQFMB
@HWI-EAS443:3:51:240:896#0/1
TGCACACTGATCTGGCCTGCGGATGCCCTTTGTTGATTGG
```

+HWI-EAS443:3:51:240:896#0/1
aabbbb`bbababbbaab^_aa[a``aaaaba_baV`aaa
@HWI-EAS443:3:6:1123:1506#0/1
TGATGGGGAGCGCCAGGATGCCACAGAAATGAGAGCATGT
+HWI-EAS443:3:6:1123:1506#0/1
L``Zb_¥W]b¥QQ¥T_QXWJ^`XRTaa_HYDV]abJ]LGS
@HWI-EAS443:3:61:1379:931#0/1
GATCATATGAACAAATGGAGACTGATGGGGAGCGCACGGA
+HWI-EAS443:3:61:1379:931#0/1
aZ`Y]_a`ZTRLVQQ^^VRaYT`¥N¥_¥YTWBBBBBBBBB
@HWI-EAS443:3:7:1511:373#0/1
GAACAAATGGAGACTGATGGGGAGCGCCAGGATGCGAGAG
+HWI-EAS443:3:7:1511:373#0/1
G[bUVV_XTbbaaU^``]`Ub`UV_GJ^b__THa^H¥HVb
@HWI-EAS443:3:91:1256:2013#0/1
CACCCCATCAGTCTCCNTTTGTTCATATGATCGTTTGGTG
+HWI-EAS443:3:91:1256:2013#0/1
abZaaa``abTZZ_`ZD[SP`_`aaabbbaaS`IX`aaRO
@HWI-EAS443:3:28:708:611#0/1
CTCTGATTTCTGTGGCATCCTGGCGCTCCCCATCAGGCTC
+HWI-EAS443:3:28:708:611#0/1
abba__aaa`aa_[[^aa`aaaaa_`BBBBBBBBBBBBBB
@HWI-EAS443:3:57:1680:943#0/1
TGCTCTGATTTCTGTGGCATCCTGGCGCTCCCCATCGGTC
+HWI-EAS443:3:57:1680:943#0/1
aa_b`baabbb^aa_]`]a`¥Z^V[¥^¥^^VY^¥^[Y¥Q[
@HWI-EAS443:3:63:808:1288#0/1
ATCCCCATCAGTCTCNATTTGTTCATATGATCGTTTGGTG
+HWI-EAS443:3:63:808:1288#0/1
^^baba^^a``aaa[DTaaaa`aaabaaaaa`a`a`a^V^
@HWI-EAS443:3:18:1636:106#0/1
TTCTGTGGCATCCTGGCGGTCCCCATCAGTCTCCATTTGT
+HWI-EAS443:3:18:1636:106#0/1
aa`¥PTbaSa`^¥a¥a_^GVN¥VYSGSZXNRWVUGNRWGS
@HWI-EAS443:3:20:917:1215#0/1
GCTCCTCATCAGTCTCCATTTGTTCATATGATCGTTTGGT
+HWI-EAS443:3:20:917:1215#0/1
ab^Y`[```]QRGQRSQVY¥ZRO[]]]]aXN]U]S`aSPP
@HWI-EAS443:3:21:889:1866#0/1
ATGGGGAGCGCGAGGATGCCACAGAAATCAGAGCATCTGT
+HWI-EAS443:3:21:889:1866#0/1
aZ_^]X¥]a_USJZ¥aZ`[Y`Sba^aa_S_G_¥Z__UZ]W
@HWI-EAS443:3:22:178:360#0/1
ATCCTGGCGCTCCCCATCAGTCTCCATTAGTTCATATGAT
+HWI-EAS443:3:22:178:360#0/1
aba`[`¥ab`aab_a^a]_a^_a[aZ`_GHUa__`^][[Y
@HWI-EAS443:3:33:791:1088#0/1
ATCCTGGCGCTCCCCATCAGTCTCCATTTGTTTATATGAT
+HWI-EAS443:3:33:791:1088#0/1
a[`a[aaa¥^V`__aT[_NSRYYZ¥RSZ]MMRFN¥UZMUV

```
@HWI-EAS443:3:33:923:1615#0/1
CTGATTTCTGTAGCATCCTGGCGCTCCCCATCAGTCTCCA
+HWI-EAS443:3:33:923:1615#0/1
a__`abbaa^``^bbaaaaa¥aaaX_X__RZ`¥ZNZ[[_[
@HWI-EAS443:3:50:1486:353#0/1
ATCCTGGCGCTCCCCATCAGTCTCCATTTTTTCATATGAT
+HWI-EAS443:3:50:1486:353#0/1
aabaa`_`^aY¥a^aY¥_Z]WY_^TVP`ZFX`O]`V]]V]
@HWI-EAS443:3:62:1755:522#0/1
TCCCCGTCAGTCTCCATTTGTTCATATGATCGTTTGGTGC
+HWI-EAS443:3:62:1755:522#0/1
Z]___¥ab_ZYY]`a`¥]^_X^N[`U[W^QTRWZUPNY¥¥
@HWI-EAS443:3:65:440:995#0/1
CGCTCCTCATCAGTCTCCATTTGTTCATATGATCGTTTGG
+HWI-EAS443:3:65:440:995#0/1
a`a`aaaaaaaa[PS]a`¥_`_`[___`a^_`__]U_]_¥
@HWI-EAS443:3:69:412:1356#0/1
ATCCGACATGCGAACCGAAGTTATAAGAATGACGGAAAGT
+HWI-EAS443:3:69:412:1356#0/1
a^^a`]`aa`aYV¥¥F[_aa^`[^^_^]`X¥_`__^X^VP
@HWI-EAS443:3:79:1487:1695#0/1
CTCTGATTTCTGGGGCATCCTGGCGCTCCCCATCAGTCTC
+HWI-EAS443:3:79:1487:1695#0/1
^^_bbabbbbY[F]^_`]^]aa^_aa_a^aU]]¥JGFL]Z
@HWI-EAS443:3:83:1458:1799#0/1
GATCATATGAACAAATGGAGACTGATGGGGGGCGCCAGGA
+HWI-EAS443:3:83:1458:1799#0/1
J`b^UaU_aaZbaaaTbaa]RbbaaZbJ`_Ua`bUH^b_U
@HWI-EAS443:3:84:1354:1525#0/1
CCCCATCAGTCTCCATTCGTTCATATGATCGTTTGGTGCC
+HWI-EAS443:3:84:1354:1525#0/1
`a_JYITVWSW[]`[a^¥WZQ][¥RR^N^J_GQ`[ONNT[
@HWI-EAS443:3:89:502:2021#0/1
CCCATGAGTCTCCATTTGTTCATATGATCGTTTGGTGCCT
+HWI-EAS443:3:89:502:2021#0/1
]¥`_RF[_Q_^a`_XUU¥WU_Z_Z_¥TZHVOZ¥aa_aVOL
@HWI-EAS443:3:92:939:207#0/1
TCTGATTTCTGTGGCATCCTGGCGCTCCCCGTCAGTCTCC
+HWI-EAS443:3:92:939:207#0/1
V_Z_Ja¥Z_bb^¥Z^aa``abb^^VP[U]^JHHHXP¥U[U
@HWI-EAS443:3:7:132:1348#0/1
AACGATCATATGAACAAATGGAGACTGATGGGGAGCGTCA
+HWI-EAS443:3:7:132:1348#0/1
WPZbR¥Q_XUPQY_XXT_`YT]aNTKHYY_aa^]^UBBBB
@HWI-EAS443:3:91:1141:188#0/1
GCCATCAGTCTCCATTTGTTCATATGATCGTTTGGTGCGT
+HWI-EAS443:3:91:1141:188#0/1
I_`baaab_T¥`_Z_bb`^`aa^``b`^^aa```XP_[F]
@HWI-EAS443:3:24:44:1541#0/1
GCTCCCCATCAGTCTCCATTTGTTCATNTGATCGTTTGGT
```

```
+HWI-EAS443:3:24:44:1541#0/1
ab_aba_``aY¥P_ab^T^abaWV¥b^D[aa_ab`_a¥XJ
@HWI-EAS443:3:90:1413:559#0/1
CTGATTTCTGTGGCATCCTNGCGCTCCCCATCAGTCTCCA
+HWI-EAS443:3:90:1413:559#0/1
`bbaabbabba]aUba`b[D]ba`_ZYaNSNN^]GL[ZYW
@HWI-EAS443:3:94:1000:1455#0/1
CACCANACGATCATATGAACAAATGGAGACTGATGGGGAG
+HWI-EAS443:3:94:1000:1455#0/1
aaaa[D¥aaaaaa`aba]]Xa`X``a_`_¥aa`__^`_]_
@HWI-EAS443:3:97:236:1106#0/1
CTCTGATTTCTGTGGCATCCTNGCGCTCCCCATCAGTCTC
+HWI-EAS443:3:97:236:1106#0/1
]^a¥_S_a¥W_a`a^a_^SX¥DVTY[MO]¥_XYY¥]XT[V
@HWI-EAS443:3:9:939:565#0/1
CAAATGGAGACTGATGGGGAGCGCNAGGATGCCACAGAAA
+HWI-EAS443:3:9:939:565#0/1
aa`a^X]a^aa__Uaaa^Xaa_aXDXVU]^_¥]a]aX`a_
@HWI-EAS443:3:12:1504:167#0/1
GGCATCCTGGCGCTCCCCATCAGTCTCCATTTGTTCATTT
+HWI-EAS443:3:12:1504:167#0/1
aaaaa_¥`aa__W[¥¥¥¥Y^W¥¥TGWVVV¥_¥XW¥UUW^¥
@HWI-EAS443:3:20:1350:816#0/1
TGCCCATCAGTCTCCATTTGTTCATATGATCGTTTGGTGC
+HWI-EAS443:3:20:1350:816#0/1
a```Z``a`XRTaaY____a¥PX_``_a[`Qa_a_a`U_¥
@HWI-EAS443:3:42:1028:435#0/1
CTGATGGGGAGCGCCAGGATGCCACAGAAATCAGAGCAGA
+HWI-EAS443:3:42:1028:435#0/1
a`bb`babaX`bbbabbbbabbb`aabbbZ`bbbabbbba
@HWI-EAS443:3:42:514:878#0/1
CACCCATCAGTCTCCATTTGTTCATATGATCGTTTGGTGC
+HWI-EAS443:3:42:514:878#0/1
aaaaaaaaaa`^``]R`baaa_a_aaa¥a``a``a]_WX^
@HWI-EAS443:3:58:383:891#0/1
GGCCCCATCAGTCTCCATTTGTTCATATGATCGTTTGGTG
+HWI-EAS443:3:58:383:891#0/1
abbbbbaabb]aab__]]¥ba`bUabaaa`aa`aaaaa_b
@HWI-EAS443:3:5:1682:1811#0/1
GATGGGGAGCGCCAGGATGCCACAGAAATCAGAGCATCCG
+HWI-EAS443:3:5:1682:1811#0/1
aaa```aaa]aVFYaa`Za^N^`aaZa]`¥a`YaS[BBBB
@HWI-EAS443:3:84:1240:1312#0/1
GTCTGATGGGGAGCGCCAGGATGCCACAGAAATCAGAGCA
+HWI-EAS443:3:84:1240:1312#0/1
a`a_`X``a¥^Y^]¥^TRXTX]]]X]Xa^`¥Y¥PV^U]ON
@HWI-EAS443:3:21:28:68#0/1
CTGATTTCTGTGGCATCCTGGCGCTCCCCATCAGTCTCCC
+HWI-EAS443:3:21:28:68#0/1
^^_]Ybbabaab_Z____W[_^ZOVBBBBBBBBBBBBBBB
```

@HWI-EAS443:3:25:339:299#0/1
CGGCGCTCCCCATCAGTCTCCATTTGTTCATATGATCGTT
+HWI-EAS443:3:25:339:299#0/1
aabbbbaab^aZR`SWZ¥¥]_JV``PX]RQYT¥[Y^U[V`
@HWI-EAS443:3:31:1138:512#0/1
GCTCCCCATCAGTCTCCATTTGTTCATATGATCGTTTGGG
+HWI-EAS443:3:31:1138:512#0/1
a]bX`]_`aa`_G]aZ`¥]^]]X`[_a`X_V__aVZ__¥K
@HWI-EAS443:3:38:1621:745#0/1
CGCTCTGATTTCTGTGGCATCCTGGCGCTCCCCATCAGTC
+HWI-EAS443:3:38:1621:745#0/1
abbabbbabba_baaba`baaaabaabaaUT_Sa_¥_^UU
@HWI-EAS443:3:48:848:1197#0/1
GCCCCATCAGTCTCCATTTGTTCATATGATCGTTTGGTGC
+HWI-EAS443:3:48:848:1197#0/1
____¥``[]_^]]]]a^a_a¥`a^`a`a]`]`T¥Q_`V_X
@HWI-EAS443:3:48:966:1593#0/1
CGCTCTGATTTCTGTGGCATCCTGGCGCTCCCCATCAGTC
+HWI-EAS443:3:48:966:1593#0/1
abbbabbbabbaba`aaaba_aaaa`aaa[`]Z[`^_¥PW
@HWI-EAS443:3:4:214:891#0/1
ACCCCATCAGTCTCCATTTGTTCATATGATCGTTTGGTGC
+HWI-EAS443:3:4:214:891#0/1
aaabaa`abaMU`RPH¥YaaW_Z¥a_^a_`^a[Z_[[]^B
@HWI-EAS443:3:61:1503:1096#0/1
GTCCCCATCAGTCTCCATTTGTTCATATGATCGTTTGGTG
+HWI-EAS443:3:61:1503:1096#0/1
N[b_Q^YaUUaaZbbbbYbaa`a_[¥bbbVab_`b_b][[
@HWI-EAS443:3:63:1041:1599#0/1
ACTCCCCATCAGTCTCCATTTGTTCATATGATCGTTTGGT
+HWI-EAS443:3:63:1041:1599#0/1
¥Z^`WZ¥W^`b[TLT_a]]Z]^Z¥R`]]W_aWLW_`BBBB
@HWI-EAS443:3:69:1302:1024#0/1
CGCTCTGATTTCTGTGGCATCCTGGCGCTCCCCATCAGTC
+HWI-EAS443:3:69:1302:1024#0/1
aa`aabb`abaaa^]__a````_aaa^`^____¥^ZBBBB
@HWI-EAS443:3:73:849:1174#0/1
ACCCCATCAGTCTCCATTTGTTCATATGATCGTTTGGTGC
+HWI-EAS443:3:73:849:1174#0/1
abababbb`a_a`aaV]aaa^aaaa_aa_`a`_`aaZY_a
@HWI-EAS443:3:85:600:1194#0/1
AGCTCCCCATCAGTCTCCATTTGTTCATATGATCGTTTGG
+HWI-EAS443:3:85:600:1194#0/1
`bababbbbbabaaaaaaaabbb`a¥baaa`ab_bGa_^V
@HWI-EAS443:3:100:1155:673#0/1
CTCTGATTTCTGTGGCATCCTGGCGCTCCCCATCAGTCTC
+HWI-EAS443:3:100:1155:673#0/1
T`bbbZbbbaab````bb]]bbbb`aZTVaaba`_aaS]Z
@HWI-EAS443:3:100:56:819#0/1
CTGATGGGGAGCGCCAGGATGCCACAGAAATCAGAGCATC

```
+HWI-EAS443:3:100:56:819#0/1
`b]``bab^]baaa¥_aa¥`b`a_`aaV^¥]^a_Y[TBBB
@HWI-EAS443:3:10:139:327#0/1
ACCAAACGATCATATGAACAAATGGAGACTGATGGGGAGC
+HWI-EAS443:3:10:139:327#0/1
aaaa^¥a[X¥_```__¥`^__¥`^^^`^^^_[¥`Z[^V[V
@HWI-EAS443:3:11:1043:1211#0/1
AAACGATCATATGAACAAATGGAGACTGATGGGGAGCGCC
+HWI-EAS443:3:11:1043:1211#0/1
aaa`^_[¥Pa]aW¥`_]ZVa`_Y_UM`YJ[U_^WZ[R_XR
@HWI-EAS443:3:11:1780:870#0/1
ATGGGGAGCGCCAGGATGCCACAGAAATCAGAGCATCTGT
+HWI-EAS443:3:11:1780:870#0/1
V¥`ba¥`¥`[^XH_[WY_W]aX_QY¥VW]a^^YR¥`G]RB
@HWI-EAS443:3:11:34:1089#0/1
ATGGGGAGCGCCAGGATGCCACAGAAATCAGAGCATCTGT
+HWI-EAS443:3:11:34:1089#0/1
ab`_aaabaZa_Y__`_aaa_`ba_^``¥____¥a_[_`W
@HWI-EAS443:3:11:594:1244#0/1
GCTCCCCATCAGTCTCCATTTGTTCATATGATCGTTTGGT
+HWI-EAS443:3:11:594:1244#0/1
`a_`_aa_^_a`T``^^a]^_^¥a]__a^Z¥^¥_¥Wa[[B
@HWI-EAS443:3:13:114:1167#0/1
ATGGGGAGCGCCAGGATGCCACAGAAATCAGAGCATCTGT
+HWI-EAS443:3:13:114:1167#0/1
aaa`a¥^aa`^U_[U^^¥]Z^^a`_^`¥^]Z[^_^ZV¥¥W
@HWI-EAS443:3:16:890:739#0/1
CATCAGTCTCCATTTGTTCATATGATCGTTTGGTGCCTTG
+HWI-EAS443:3:16:890:739#0/1
a]abb`Laabbababaa_abb`bb`abb^bbba_b^a`b`
@HWI-EAS443:3:17:521:859#0/1
ACCAAACGATCATATGAACAAATGGAGACTGATGGGGAGC
+HWI-EAS443:3:17:521:859#0/1
aaaaaaaa__^___a`^``_Y^`^_W`WW^`Y^_¥¥¥W¥W
@HWI-EAS443:3:17:849:1770#0/1
CTCCCCATCAGTCTCCATTTGTTCATATGATCGTTTGGTG
+HWI-EAS443:3:17:849:1770#0/1
``[aaaa]_aa^____`^__S[___^a_ZW_]]Y_¥¥MKP
@HWI-EAS443:3:18:1039:1590#0/1
ATGGGGAGCGCCAGGATGCCACAGAAATCAGAGCATCTGT
+HWI-EAS443:3:18:1039:1590#0/1
_`baaa`aa`a`a__aUa_a__aa]¥¥Sa`_`_aX[GW`H
@HWI-EAS443:3:18:1147:1965#0/1
ATGGGGAGCGCCAGGATGCCACAGAAATCAGAGCATCTGT
+HWI-EAS443:3:18:1147:1965#0/1
aa__^_aaS__aa_Z]_`¥LLNaHQX[RP[]_BBBBBBBB
@HWI-EAS443:3:18:786:216#0/1
CAAATGGAGACTGATGGGGAGCGCCAGGATGCCACAGAAA
+HWI-EAS443:3:18:786:216#0/1
abaabbb_a`abbaaaaaa_a`a¥¥^_`¥V¥¥^^S_^¥_¥
```

@HWI-EAS443:3:19:1177:1785#0/1
TCTGATTTCTGTGGCATCCTGGCGCTCCCCATCAGTCTCC
+HWI-EAS443:3:19:1177:1785#0/1
a`aaaaaaaaa]_`_a`a_`aa_^¥[]^___]]_^XU^Y]
@HWI-EAS443:3:19:674:739#0/1
CACCAAACGATCATATGAACAAATGGAGACTGATGGGGAG
+HWI-EAS443:3:19:674:739#0/1
¥RW^^ZZ_X[[_]a^__]_UQR_]Z_VU¥P`a_Y_¥_^PB
@HWI-EAS443:3:19:719:1652#0/1
GCTCCCCATCAGTCTCCATTTGTTCATATGATCGTTTGGT
+HWI-EAS443:3:19:719:1652#0/1
``aaa`]a__`aZ^a`a`_`_UU__```__]]U_X`_]RX
@HWI-EAS443:3:21:336:377#0/1
CGATCATATGAACAAATGGAGACTGATGGGGAGCGCCAGG
+HWI-EAS443:3:21:336:377#0/1
aba_a_ab[Z`aaa__a`a`a_]``ZZa^`_`a__^_]`X
@HWI-EAS443:3:21:412:1515#0/1
GCTCCCCATCAGTCTCCATTTGTTCATATGATCGTTTGGT
+HWI-EAS443:3:21:412:1515#0/1
^_K_ab`X`_]UU]____¥Z[V¥a``]_XP^_¥XD¥aMFD
@HWI-EAS443:3:21:878:1193#0/1
GATTTCTGTGGCATCCTGGCGCTCCCCATCAGTCTCCATT
+HWI-EAS443:3:21:878:1193#0/1
a_```_a`W¥[`a_^_ZWU__ZY___`UZWWXYUX[UX_X
@HWI-EAS443:3:22:637:1077#0/1
TCTGATTTCTGTGGCATCCTGGCGCTCCCCATCAGTCTCC
+HWI-EAS443:3:22:637:1077#0/1
`^X_]a[¥`]]Za`aaa]V^U[[¥^ZR^XUPGY_NGTRVB
@HWI-EAS443:3:22:772:1055#0/1
GTGGCATCCTGGCGCTCCCCATCAGTCTCCATTTGTTCAT
+HWI-EAS443:3:22:772:1055#0/1
`P]VZaa_YTDJ^¥RY[¥SXXZ_ZTNZ¥S[L[VZROOURT
@HWI-EAS443:3:22:893:1565#0/1
ATTTCTGTGGCATCCTGGCGCTCCCCATCAGTCTCCATTT
+HWI-EAS443:3:22:893:1565#0/1
`baaaaaa^[bbaa``_Z`_^]`_a^W[U[^USYVUUZ^`
@HWI-EAS443:3:23:1482:1462#0/1
ACGATCATATGAACAAATGGAGACTGATGGGGAGCGCCAG
+HWI-EAS443:3:23:1482:1462#0/1
`a_W[^¥`abbaaX^]aaaaNa_X_RP_Ya]__a]_^WGW
@HWI-EAS443:3:23:1568:1922#0/1
CTCCCCATCAGTCTCCATTTGTTCATATGATCGTTTGGTG
+HWI-EAS443:3:23:1568:1922#0/1
a`aa`bbbabbb_baabbbbaabaabbbbab`aabaaa_`
@HWI-EAS443:3:24:1015:970#0/1
ATGGGGAGCGCCAGGATGCCACAGAAATCAGAGCATCTGT
+HWI-EAS443:3:24:1015:970#0/1
^]`¥P^a^a_aa`XX^`Z[Z]Uaa_]^_R_¥^a```Z^YQ
@HWI-EAS443:3:24:254:1092#0/1
CTCCCCATCAGTCTCCATTTGTTCATATGATCGTTTGGTG

```
+HWI-EAS443:3:24:254:1092#0/1
a_bbba^a_ba`baaaaaa`a[aa[^aaaa`aa_`a_XX`
@HWI-EAS443:3:24:945:1265#0/1
TGTGGCATCCTGGCGCTCCCCATCAGTCTCCATTTGTTCA
+HWI-EAS443:3:24:945:1265#0/1
aa_a`ababba`aaaa`aaaaY[U]PT_¥Y[LU_`T]ZP[
@HWI-EAS443:3:25:1073:1361#0/1
CACCAAACGATCATATGAACAAATGGAGACTGATGGGGAG
+HWI-EAS443:3:25:1073:1361#0/1
aaaaabbbbaabbbbbbabab`abb_aa_aaa`aaaaaaa
@HWI-EAS443:3:25:31:527#0/1
TCCCCATCAGTCTCCATTTGTTCATATGATCGTTTGGTGC
+HWI-EAS443:3:25:31:527#0/1
X_b`_¥¥¥`]U[¥`_]Y`¥`¥XYOH¥W^TUW¥^aba]VV_
@HWI-EAS443:3:25:552:820#0/1
TGGCGCTCCCCATCAGTCTCCATTTGTTCATATGATCGTT
+HWI-EAS443:3:25:552:820#0/1
ab`a¥_`abbbW]_[VZa]]_¥]]aX[[P[`ZX]X^[^T¥
@HWI-EAS443:3:26:1184:489#0/1
GATGGGGAGCGCCAGGATGCCACAGAAATCAGAGCATCTG
+HWI-EAS443:3:26:1184:489#0/1
aaaaaaa_aX^`¥_``¥__¥Z^_a``_``¥``[`Z[¥T^¥
@HWI-EAS443:3:26:1459:368#0/1
ATTTCTGTGGCATCCTGGCGCTCCCCATCAGTCTCCATTT
+HWI-EAS443:3:26:1459:368#0/1
abbbabbabaabbaaabb^baa_`a^_a]]RU`_X___a^
@HWI-EAS443:3:26:462:874#0/1
AACGATCATATGAACAAATGGAGACTGATGGGGAGCGCCA
+HWI-EAS443:3:26:462:874#0/1
aaa_`aaaaaaaaaaaa[[`]]`_``[^¥a`[[Z]_Z^`¥
@HWI-EAS443:3:27:1190:1285#0/1
CTCCCCATCAGTCTCCATTTGTTCATATGATCGTTTGGTG
+HWI-EAS443:3:27:1190:1285#0/1
aabb`_Wabbb^ba`a`baa^`a_baaaaab_Yaaaa_¥`
@HWI-EAS443:3:27:692:1932#0/1
CACCAAACGATCATATGAACAAATGGAGACTGATGGGGAG
+HWI-EAS443:3:27:692:1932#0/1
abbbba`aab`abaaa`aaaaaa`aa`]¥^¥Y[a][_Za_
@HWI-EAS443:3:28:1074:1663#0/1
CTGATGGGGAGCGCCAGGATGCCACAGAAATCAGAGCATC
+HWI-EAS443:3:28:1074:1663#0/1
_W¥¥`]Y[T¥W^ZS]XZaXMXa]`¥`a`a`]Z_W_^JNUS
@HWI-EAS443:3:28:1284:1193#0/1
GCTCCCCATCAGTCTCCATTTGTTCATATGATCGTTTGGT
+HWI-EAS443:3:28:1284:1193#0/1
`a`][a`aXV^`Z¥`a`T[V^¥`[FV[a_]__^V^U__YU
@HWI-EAS443:3:28:1396:878#0/1
TCCCCATCAGTCTCCATTTGTTCATATGATCGTTTGGTGC
+HWI-EAS443:3:28:1396:878#0/1
[bb^R`W`]]J]bURa_abaR¥___[b]^^PaaaaaYL¥Y
```

@HWI-EAS443:3:28:692:823#0/1
CTCTGATTTCTGTGGCATCCTGGCGCTCCCCATCAGTCTC
+HWI-EAS443:3:28:692:823#0/1
abbb_X_]bb`[Y``aa`_aa]aaaaXaaaaWS]ZXHXYV
@HWI-EAS443:3:29:1140:382#0/1
TGATGGGGAGCGCCAGGATGCCACAGAAATCAGAGCATCT
+HWI-EAS443:3:29:1140:382#0/1
abXa]bba¥]Z^[Y^_¥OR]YP[Pa`U^VKY]^U]TSVBB
@HWI-EAS443:3:29:1232:959#0/1
CTGATGGGGAGCGCCAGGATGCCACAGAAATCAGAGCATC
+HWI-EAS443:3:29:1232:959#0/1
ab`a_T[aTO¥baa^PZHPVZUZZTS`^^MPRHRW[BBBB
@HWI-EAS443:3:2:1012:340#0/1
GGCATCCTGGCGCTCCCCATCAGTCTCCATTTGTTCATAT
+HWI-EAS443:3:2:1012:340#0/1
O__Zbb[a¥`X_aZYZaa_]YTS]]R]aaaVbZ^a[`aSZ
@HWI-EAS443:3:2:1265:933#0/1
CAGATGCTCTGATTTCTGTGGCATCCTGGCGCTCCCCATC
+HWI-EAS443:3:2:1265:933#0/1
`bb]babbbbb]bbb¥ab^_^bbb^aab_Y_¥]a^¥¥a¥^
@HWI-EAS443:3:30:158:1307#0/1
ATCAGTCTCCATTTGTTCATATGATCGTTTGGTGCCTTGA
+HWI-EAS443:3:30:158:1307#0/1
[V``Tab]a_Y`_`_^`a`_a`a`]a_¥]_^_U]]`V]]_
@HWI-EAS443:3:31:1416:1070#0/1
ATGGGGAGCGCCAGGATGCCACAGAAATCAGAGCATCTGT
+HWI-EAS443:3:31:1416:1070#0/1
]a`bb_``abbbb`ba_a`a_]¥`^_abba_`b]__abaS
@HWI-EAS443:3:31:298:919#0/1
CAAATGGAGACTGATGGGGAGCGCCAGGATGCCACAGAAA
+HWI-EAS443:3:31:298:919#0/1
`RV_¥]`[`Xaaaaaa`]_K_ba`a]UZVW_aa^^a^WW^
@HWI-EAS443:3:33:1673:1807#0/1
ATTTCTGTGGCATCCTGGCGCTCCCCATCAGTCTCCATTT
+HWI-EAS443:3:33:1673:1807#0/1
`[]a`K^_aaaaa`_VZTQ¥[a^GX`UUZ`[PPNYTVQZH
@HWI-EAS443:3:33:1764:1016#0/1
GAACAAATGGAGACTGATGGGGAGCGCCAGGATGCCACAG
+HWI-EAS443:3:33:1764:1016#0/1
a`¥aX]]aaU_^V^][Q_UUKT¥`TVT_ZRXWPWNTUQ_U
@HWI-EAS443:3:34:548:744#0/1
CGCTCCCCATCAGTCTCCATTTGTTCATATGATCGTTTGG
+HWI-EAS443:3:34:548:744#0/1
abbababbaabaa^baabaaa`a^a`abaaaa__]^`a_Z
@HWI-EAS443:3:34:603:1815#0/1
CCCATCAGTCTCCATTTGTTCATATGATCGTTTGGTGCCT
+HWI-EAS443:3:34:603:1815#0/1
`baXUa¥KGa¥aU``_aY]Z`SZYSSSQYUM_BBBBBBBB
@HWI-EAS443:3:34:64:119#0/1
CTGGCGCTCCCCATCAGTCTCCATTTGTTCATATGATCGT

```
+HWI-EAS443:3:34:64:119#0/1
`aa_aaa_aaba`MXX`¥_S__¥¥¥^SUW¥VZSS[WZY^B
@HWI-EAS443:3:35:165:841#0/1
CATCAGTCTCCATTTGTTCATATGATCGTTTGGTGCCTTG
+HWI-EAS443:3:35:165:841#0/1
`¥`aba`babbaaa``a¥`aaaaaa_a^Z^a`aWaaa^a_
@HWI-EAS443:3:35:691:580#0/1
AATGGAGACTGATGGGGAGCGCCAGGATGCCACAGAAATC
+HWI-EAS443:3:35:691:580#0/1
aaba¥Xb`baa_aaa`a]a``a^aa_Z_aaaaaa_a_```
@HWI-EAS443:3:36:1579:868#0/1
GATGGGGAGCGCCAGGATGCCACAGAAATCAGAGCATCTG
+HWI-EAS443:3:36:1579:868#0/1
aabaabaXa¥a¥[]^a_]aW¥ZUa___W¥T`WW¥¥UZUU^
@HWI-EAS443:3:37:1162:1288#0/1
AACAAATGGAGACTGATGGGGAGCGCCAGGATGCCACAGA
+HWI-EAS443:3:37:1162:1288#0/1
a```^`^baaa``_`a`]`]aXa[U^YaYL]U^]]^YaX¥
@HWI-EAS443:3:37:733:1979#0/1
CATATGAACAAATGGAGACTGATGGGGAGCGCCAGGATGC
+HWI-EAS443:3:37:733:1979#0/1
`bbbbbababaabba`aaaabaaaaa]aaG_`aa_Z`W_^
@HWI-EAS443:3:38:1719:944#0/1
ATCAGTCTCCATTTGTTCATATGATCGTTTGGTGCCTTGA
+HWI-EAS443:3:38:1719:944#0/1
abaaaaY[`VY^^aaa_VZ¥X_^^[R`[aSLSUTKNZRUP
@HWI-EAS443:3:38:494:799#0/1
CACCAAACGATCATATGAACAAATGGAGACTGATGGGGAG
+HWI-EAS443:3:38:494:799#0/1
aaa_]]^ba]V^___^^U¥U[XT^a`__]NT¥W¥`T[VGU
@HWI-EAS443:3:39:538:146#0/1
CTCCCCATCAGTCTCCATTTGTTCATATGATCGTTTGGTG
+HWI-EAS443:3:39:538:146#0/1
aa¥aab¥`bba`baba``aaa`_a``aaaa]S`^_aa^U^
@HWI-EAS443:3:39:775:667#0/1
GCTCCCCATCAGTCTCCATTTGTTCATATGATCGTTTGGT
+HWI-EAS443:3:39:775:667#0/1
Z¥J[]_a]Ya_Y_`XWZY^`[XLZWJO_[WTXDTP`_^]N
@HWI-EAS443:3:3:155:1251#0/1
CTGATGGGGAGCGCCAGGATGCCACAGAAATCAGAGCATC
+HWI-EAS443:3:3:155:1251#0/1
`bWZ]b__¥`_]^a]]a`a`a]^`Ybaaa`_]`a__S]^T
@HWI-EAS443:3:3:792:1220#0/1
ATGGGGAGCGCCAGGATGCCACAGAAATCAGAGCATCTGT
+HWI-EAS443:3:3:792:1220#0/1
`babbb]ab`X^]a`¥aaaZa_`a`aXbXa`¥aX^VH`aB
@HWI-EAS443:3:3:991:439#0/1
CTCCCCATCAGTCTCCATTTGTTCATATGATCGTTTGGTG
+HWI-EAS443:3:3:991:439#0/1
abab^abbabb^^a[^abaaa]aa`aZ`a¥__bX_`_aX_
```

```
@HWI-EAS443:3:40:1366:493#0/1
GATGGGGAGCGCCAGGATGCCACAGAAATCAGAGCATCTG
+HWI-EAS443:3:40:1366:493#0/1
_Y_a``_R¥O[ILT^ZSV¥ZS_SV_WJWDPU[^[XTPNYY
@HWI-EAS443:3:40:79:1349#0/1
AACGATCATATGAACAAATGGAGACTGATGGGGAGCGCCA
+HWI-EAS443:3:40:79:1349#0/1
_a_WT^O_aZVO_aZ^PWXPRTR^[]OGOZX_^UBBBBBB
@HWI-EAS443:3:42:1044:1146#0/1
GATGGGGAGCGCCAGGATGCCACAGAAATCAGAGCATCTG
+HWI-EAS443:3:42:1044:1146#0/1
Z[]a_¥_¥abb^b^b_aa`¥]aab`baa^aa^aa^b¥`aa
@HWI-EAS443:3:42:215:1929#0/1
ATGGGGAGCGCCAGGATGCCACAGAAATCAGAGCATCTGT
+HWI-EAS443:3:42:215:1929#0/1
a_[`a_a^^P`a[_XXS`_`aVbaa^]¥T`_YY^^X[a_^
@HWI-EAS443:3:42:225:844#0/1
ATGGGGAGCGCCAGGATGCCACAGAAATCAGAGCATCTGT
+HWI-EAS443:3:42:225:844#0/1
abbbaa]]b`b`aa``aaa_aaaaaUaa]aaaaaaa_aa_
@HWI-EAS443:3:42:615:1644#0/1
GTGGCATCCTGGCGCTCCCCATCAGTCTCCATTTGTTCAT
+HWI-EAS443:3:42:615:1644#0/1
a`aa_babbb^`aab^`aa^[[`YXN^[]^WX``JZ_```
@HWI-EAS443:3:43:355:210#0/1
CTCTGATTTCTGTGGCATCCTGGCGCTCCCCATCAGTCTC
+HWI-EAS443:3:43:355:210#0/1
a[abbaababb`abaaab`aabaa`a¥[a_`XP¥TZVXUP
@HWI-EAS443:3:43:473:312#0/1
CTCTGATTTCTGTGGCATCCTGGCGCTCCCCATCAGTCTC
+HWI-EAS443:3:43:473:312#0/1
ababbbbbbbbbababbaaaaabaaa`¥aa`[_^^¥V[]^
@HWI-EAS443:3:43:639:1181#0/1
GATCATATGAACAAATGGAGACTGATGGGGAGCGCCAGGA
+HWI-EAS443:3:43:639:1181#0/1
aaabb`bbbbbb`aaabb[ba^aa`abYaa`_``VX^a]`
@HWI-EAS443:3:45:1768:1018#0/1
AACAAATGGAGACTGATGGGGAGCGCCAGGATGCCACAGA
+HWI-EAS443:3:45:1768:1018#0/1
_a¥¥_abY`Z`Ra^ba¥]X¥aXU`]a]K[XTM[[YPH]a[
@HWI-EAS443:3:45:531:477#0/1
GCGCTCCCCATCAGTCTCCATTTGTTCATATGATCGTTTG
+HWI-EAS443:3:45:531:477#0/1
aaaaaa`aaaa^`Z^¥a_``a`a]^`V_a_^`^]¥`¥_aZ
@HWI-EAS443:3:45:657:1143#0/1
CTCCCCATCAGTCTCCATTTGTTCATATGATCGTTTGGTG
+HWI-EAS443:3:45:657:1143#0/1
aa_]¥Z¥^^][N[`[_W`a]UY_a_`_]`ZYP]Ua_`VX_
@HWI-EAS443:3:46:242:419#0/1
ATTTCTGTGGCATCCTGGCGCTCCCCATCAGTCTCCATTT
```

+HWI-EAS443:3:46:242:419#0/1
a^baab^¥YX`aa_a_aa_a`]U]a_¥Z¥_QUYY^VFLZY
@HWI-EAS443:3:47:1005:404#0/1
ATGGGGAGCGCCAGGATGCCACAGAAATCAGAGCATCTGT
+HWI-EAS443:3:47:1005:404#0/1
aa__ababbbaaaba]]aS`aZa]]a_[Va_`aV``[^_G
@HWI-EAS443:3:48:1129:1152#0/1
CTGATGGGGAGCGCCAGGATGCCACAGAAATCAGAGCATC
+HWI-EAS443:3:48:1129:1152#0/1
`bb`bbbbb_bab`bbb_`]]^`aab``a`b[ababXaa_
@HWI-EAS443:3:48:97:830#0/1
GAACAAATGGAGACTGATGGGGAGCGCCAGGATGCCACAG
+HWI-EAS443:3:48:97:830#0/1
aV`___¥`¥`T^S¥aa^^`[[^^[[_¥^W`_¥[ZSTNS¥[
@HWI-EAS443:3:4:381:715#0/1
GCGCTCCCCATCAGTCTCCATTTGTTCATATGATCGTTTG
+HWI-EAS443:3:4:381:715#0/1
Z__a]__]U_^aa^XWaSHWZU_aTWYWV^U[TYWbBBBB
@HWI-EAS443:3:50:1451:1186#0/1
CTCTGATTTCTGTGGCATCCTGGCGCTCCCCATCAGTCTC
+HWI-EAS443:3:50:1451:1186#0/1
aa`ab_bba`ab¥babab`a`b``aa¥]_`X[XZ^]T[]]
@HWI-EAS443:3:50:421:601#0/1
AGATGCTCTGATTTCTGTGGCATCCTGGCGCTCCCCATCA
+HWI-EAS443:3:50:421:601#0/1
aaba[`aaa_abb`]baUaaaaa[¥^aa``^[`_aaXVQV
@HWI-EAS443:3:51:1119:792#0/1
AACGATCATATGAACAAATGGAGACTGATGGGGAGCGCCA
+HWI-EAS443:3:51:1119:792#0/1
abbbabaabaaa`b¥`abaaa_b_`aa`a^`abYa^a`^¥
@HWI-EAS443:3:52:1713:355#0/1
GCTCCCCATCAGTCTCCATTTGTTCATATGATCGTTTGGT
+HWI-EAS443:3:52:1713:355#0/1
][¥¥¥bb`]S¥F^X¥YL^`a[ROZ]`]]]`QNZZ^[¥LSM
@HWI-EAS443:3:52:373:460#0/1
GCGCTCCCCATCAGTCTCCATTTGTTCATATGATCGTTTG
+HWI-EAS443:3:52:373:460#0/1
aaaa^`a`a`a]]_¥]`a_``_a`^_V`a`_]X`^a[`^¥
@HWI-EAS443:3:53:134:1790#0/1
GCGCTCCCCATCAGTCTCCATTTGTTCATATGATCGTTTG
+HWI-EAS443:3:53:134:1790#0/1
a`V`]_`_][[`XX[__XSOZ_[_Y`_TYU[_[¥_^U[^¥
@HWI-EAS443:3:53:31:1379#0/1
AGGCACCAAACGATCATATGAACAAATGGAGACTGATGGG
+HWI-EAS443:3:53:31:1379#0/1
aabb_a^W_baa^^`aaa``XaaaWP]aVaa__`a¥``aZ
@HWI-EAS443:3:55:1586:1756#0/1
CAAACGATCATATGAACAAATGGAGACTGATGGGGAGCGC
+HWI-EAS443:3:55:1586:1756#0/1
abababaaaababb_a_aaaaba^^a_ab_abaaa_aY`[

```
@HWI-EAS443:3:57:876:420#0/1
TCTGATTTCTGTGGCATCCTGGCGCTCCCCATCAGTCTCC
+HWI-EAS443:3:57:876:420#0/1
aaaa^aa`_aa_aa```a`_a`¥`_^Z^^`]XRX_YSYP]
@HWI-EAS443:3:58:503:809#0/1
CCCCATCAGTCTCCATTTGTTCATATGATCGTTTGGTGCC
+HWI-EAS443:3:58:503:809#0/1
_WaaS_abb]Zabaa]aa_aa`aaaba]`baabb_IV^aX
@HWI-EAS443:3:5:1589:636#0/1
CATCAGTCTCCATTTGTTCATATGATCGTTTGGTGCCTTG
+HWI-EAS443:3:5:1589:636#0/1
]`^¥¥bb`_Uab``[WUb_aaUbab`VY¥_^baTaSI]b¥
@HWI-EAS443:3:5:693:1448#0/1
ATGGGGAGCGCCAGGATGCCACAGAAATCAGAGCATCTGT
+HWI-EAS443:3:5:693:1448#0/1
ababba`bba^¥__aaaa^aaabbabaa]b_aXaaaaa_B
@HWI-EAS443:3:60:1442:33#0/1
GATGGGGAGCGCCAGGATGCCACAGAAATCAGAGCATCTG
+HWI-EAS443:3:60:1442:33#0/1
a[VaaaaWXX¥^UPLDJWZZUVNY[WWHWNWZQWXBBBBB
@HWI-EAS443:3:60:1483:378#0/1
CTGATGGGGAGCGCCAGGATGCCACAGAAATCAGAGCATC
+HWI-EAS443:3:60:1483:378#0/1
aaa]aX__b]W_aaa`_T[X]Z]^X]`]]`^[W¥Y_KV[U
@HWI-EAS443:3:61:749:499#0/1
CGATCATATGAACAAATGGAGACTGATGGGGAGCGCCAGG
+HWI-EAS443:3:61:749:499#0/1
abaaabbb_[`a^a`]bba``Q^aa_aaaba^]Z^]Y¥_[
@HWI-EAS443:3:61:889:1967#0/1
CTGGCGCTCCCCATCAGTCTCCATTTGTTCATATGATCGT
+HWI-EAS443:3:61:889:1967#0/1
^abb``^a`[]¥`][^W^]_baW_¥¥WZ`a][XXa_TRQW
@HWI-EAS443:3:62:455:535#0/1
CTGATTTCTGTGGCATCCTGGCGCTCCCCATCAGTCTCCA
+HWI-EAS443:3:62:455:535#0/1
aa_[`aaa`]_]¥`]`aa^UT]SaS¥[^[BBBBBBBBBBB
@HWI-EAS443:3:62:505:1010#0/1
CTCCCCATCAGTCTCCATTTGTTCATATGATCGTTTGGTG
+HWI-EAS443:3:62:505:1010#0/1
aabaaaaaab`Y`aaa```a`[a`a_a_a¥Z^^V_Y`UP¥
@HWI-EAS443:3:62:597:1679#0/1
CATATGAACAAATGGAGACTGATGGGGAGCGCCAGGATGC
+HWI-EAS443:3:62:597:1679#0/1
abbbbbbbbbbabbbabbaabaabaaaaa`a_baaaa_aa
@HWI-EAS443:3:63:1234:1270#0/1
GGAGACTGATGGGGAGCGCCAGGATGCCACAGAAATCAGA
+HWI-EAS443:3:63:1234:1270#0/1
aaZa`aa`Zb`aa_^aa`a¥`]a¥^a^^¥_aaa`^^^`^^
@HWI-EAS443:3:63:471:1339#0/1
CATCAGTCTCCATTTGTTCATATGATCGTTTGGTGCCTTG
```

+HWI-EAS443:3:63:471:1339#0/1
a`QXZZaa_ZGN¥aaa_aaaaaaYTY`ZS^__^U`__`^^
@HWI-EAS443:3:63:512:1733#0/1
CAAACGATCATATGAACAAATGGAGACTGATGGGGAGCGC
+HWI-EAS443:3:63:512:1733#0/1
[aaba_[¥_abba`bbabaa_V^XXZba`aa_J¥__`a`^
@HWI-EAS443:3:63:717:540#0/1
CCCATCAGTCTCCATTTGTTCATATGATCGTTTGGTGCCT
+HWI-EAS443:3:63:717:540#0/1
abba^a^_a_Q`a¥]a__[a¥a_Y`aQ^`a_`a`]V`¥_`
@HWI-EAS443:3:64:1412:636#0/1
TCTGATTTCTGTGGCATCCTGGCGCTCCCCATCAGTCTCC
+HWI-EAS443:3:64:1412:636#0/1
aab¥abaaaaWaa`a_[Z[`__TW]X_][QPRYFSVNT
@HWI-EAS443:3:64:1700:1577#0/1
CTGATGGGGAGCGCCAGGATGCCACAGAAATCAGAGCATC
+HWI-EAS443:3:64:1700:1577#0/1
abbabbababbbaaXaabbabaaa_baababK`_]`]`a_
@HWI-EAS443:3:65:1168:1160#0/1
CAAATGGAGACTGATGGGGAGCGCCAGGATGCCACAGAAA
+HWI-EAS443:3:65:1168:1160#0/1
abbZbbb_```a`aabb`^a_a_````a`][[Xa`b`aaa
@HWI-EAS443:3:65:872:470#0/1
CAAGGCACCAAACGATCATATGAACAAATGGAGACTGATG
+HWI-EAS443:3:65:872:470#0/1
a¥]_bb_``ab`ba_`aaa^abaaaaa_aa`_a`^aa`a`
@HWI-EAS443:3:66:1485:1986#0/1
TCTGATTTCTGTGGCATCCTGGCGCTCCCCATCAGTCTCC
+HWI-EAS443:3:66:1485:1986#0/1
abaa`aaa_aaZ`¥aaa_```__]¥WW_]¥]_¥^^SNUT¥
@HWI-EAS443:3:66:941:1162#0/1
CTCCCCATCAGTCTCCATTTGTTCATATGATCGTTTGGTG
+HWI-EAS443:3:66:941:1162#0/1
aa`¥GMUWb¥aUXUL¥MZZNPR[YLZXaa[¥S]ZQ[^¥MZ
@HWI-EAS443:3:67:1209:1130#0/1
GAGACTGATGGGGAGCGCCAGGATGCCACAGAAATCAGAG
+HWI-EAS443:3:67:1209:1130#0/1
^_`aaababbaaaaaaaa]WV]WYa__a`aa[]¥`^_`]`
@HWI-EAS443:3:67:534:2034#0/1
TCTGATTTCTGTGGCATCCTGGCGCTCCCCATCAGTCTCC
+HWI-EAS443:3:67:534:2034#0/1
ab`^^^_^^UXYUO]^]__WVV_X__aZ`¥[EXXYMNHQX
@HWI-EAS443:3:69:229:437#0/1
TGTGGCATCCTGGCGCTCCCCATCAGTCTCCATTTGTTCA
+HWI-EAS443:3:69:229:437#0/1
a^aaa`baaaS`^_^]N`]a]SOPWXXUVZ¥TU`XEW_]_
@HWI-EAS443:3:69:936:1023#0/1
AAACGATCATATGAACAAATGGAGACTGATGGGGAGCGCC
+HWI-EAS443:3:69:936:1023#0/1
a__`ba`__`X^]]a`]W_aa`[`^^aa^a``______ZZ

```
@HWI-EAS443:3:6:1561:147#0/1
CATCAGTCTCCATTTGTTCATATGATCGTTTGGTGCCTTG
+HWI-EAS443:3:6:1561:147#0/1
__a_bba_ba]a```a^a^`a`aZ[_Oa^_aa_Y_¥Xa__
@HWI-EAS443:3:6:189:643#0/1
CTCAAGGCACCAAACGATCATATGAACAAATGGAGACTGA
+HWI-EAS443:3:6:189:643#0/1
ababbbabaPZS_`]a_X__aaaaZ`H_W]aa¥[_LSa__
@HWI-EAS443:3:70:1025:77#0/1
AACAAATGGAGACTGATGGGGAGCGCCAGGATGCCACAGA
+HWI-EAS443:3:70:1025:77#0/1
ab^_¥X`baU``aababaa`a_a``VPV_a^X¥RVHZa`^
@HWI-EAS443:3:70:1501:1918#0/1
CTGATGGGGAGCGCCAGGATGCCACAGAAATCAGAGCATC
+HWI-EAS443:3:70:1501:1918#0/1
a_¥`^a^`Waa¥^_`^`bb]abaa_baaaa`^aaa`a``M
@HWI-EAS443:3:70:681:1111#0/1
ATGGGGAGCGCCAGGATGCCACAGAAATCAGAGCATCTGT
+HWI-EAS443:3:70:681:1111#0/1
ab`_b`_`babaaaaa^aaaaabaaaaaabaaaaaaa``Y
@HWI-EAS443:3:71:248:284#0/1
CGCTCCCCATCAGTCTCCATTTGTTCATATGATCGTTTGG
+HWI-EAS443:3:71:248:284#0/1
aabbbbbbaaab_abbbbaaaaaaaaaaaaa_`aa_aaa_
@HWI-EAS443:3:72:1463:535#0/1
CATCAGTCTCCATTTGTTCATATGATCGTTTGGTGCCTTG
+HWI-EAS443:3:72:1463:535#0/1
`bbba`]X_Va]b^bbab``ba_ba`S_`bbbbT`^¥_`a
@HWI-EAS443:3:72:358:416#0/1
CGATCATATGAACAAATGGAGACTGATGGGGAGCGCCAGG
+HWI-EAS443:3:72:358:416#0/1
U`TWOUa¥Z¥I_^[TT__[X_X¥UaTUaab_I¥`VYROOB
@HWI-EAS443:3:73:1737:646#0/1
CTCTGATTTCTGTGGCATCCTGGCGCTCCCCATCAGTCTC
+HWI-EAS443:3:73:1737:646#0/1
a¥_¥bUbaa[baZ_b]ab_`Za`]`a`]`^^^YPWZFQQJ
@HWI-EAS443:3:73:482:1271#0/1
GATGGGGAGCGCCAGGATGCCACAGAAATCAGAGCATCTG
+HWI-EAS443:3:73:482:1271#0/1
^ab_^_[^__¥Z^`]X`_aa_`ab`]]__aaa^]_ZYR]Z
@HWI-EAS443:3:73:532:1744#0/1
GATCATATGAACAAATGGAGACTGATGGGGAGCGCCAGGA
+HWI-EAS443:3:73:532:1744#0/1
aabaaaba_`aaa_aa]UV`__a^``a_]`^W_[[O^¥__
@HWI-EAS443:3:74:1203:1474#0/1
GCTCCCCATCAGTCTCCATTTGTTCATATGATCGTTTGGT
+HWI-EAS443:3:74:1203:1474#0/1
a``]Y_aaa^Z]Va`ZXZ`aaa^a_^¥^^TY[UaY_`[UN
@HWI-EAS443:3:74:97:730#0/1
CCCCATCAGTCTCCATTTGTTCATATGATCGTTTGGTGCC
```

```
+HWI-EAS443:3:74:97:730#0/1
aaa_a_b_¥[Z[Z[UZ¥W¥WZVU¥¥¥^WY_¥W^WSWUWTV
@HWI-EAS443:3:75:331:519#0/1
CAAATGGAGACTGATGGGGAGCGCCAGGATGCCACAGAAA
+HWI-EAS443:3:75:331:519#0/1
abaabbbabaaabV`baaa^aaaa_`a_`^aaaa`aa`aa
@HWI-EAS443:3:76:1365:1417#0/1
CGATCATATGAACAAATGGAGACTGATGGGGAGCGCCAGG
+HWI-EAS443:3:76:1365:1417#0/1
aba[[bbbabbbZaabbb__bbaa`^bba`aabb[PX_a]
@HWI-EAS443:3:76:286:1120#0/1
GCTCTGATTTCTGTGGCATCCTGGCGCTCCCCATCAGTCT
+HWI-EAS443:3:76:286:1120#0/1
a_`[`]SPW^U[_NHWX`]`_]Z[Q]`ZZX_ZTNYXTVWW
@HWI-EAS443:3:76:470:2036#0/1
AACAAATGGAGACTGATGGGGAGCGCCAGGATGCCACAGA
+HWI-EAS443:3:76:470:2036#0/1
^¥_^``]b_W_[^]a[]^_[ZQ¥`¥^¥X^XaWV`_[Z__W
@HWI-EAS443:3:76:607:1746#0/1
ATGCTCTGATTTCTGTGGCATCCTGGCGCTCCCCATCAGT
+HWI-EAS443:3:76:607:1746#0/1
aaaa_ba`aabab¥VZ`_aaaaa__[_¥a[W^^a_¥XTQN
@HWI-EAS443:3:76:771:1215#0/1
CTCTGATTTCTGTGGCATCCTGGCGCTCCCCATCAGTCTC
+HWI-EAS443:3:76:771:1215#0/1
ababaabbaabaaaabbaaa¥aa`aa¥aa``UJY__ZUZ_
@HWI-EAS443:3:77:1664:1716#0/1
CGCTCCCCATCAGTCTCCATTTGTTCATATGATCGTTTGG
+HWI-EAS443:3:77:1664:1716#0/1
abbabbbabbbbbaabbbbbbbbbbabbbbbbb`bbbabb
@HWI-EAS443:3:78:1238:410#0/1
CCCCATCAGTCTCCATTTGTTCATATGATCGTTTGGTGCC
+HWI-EAS443:3:78:1238:410#0/1
`ZFN`_[TL]PR¥W]Ya[U]LY_¥Q_][TL]T_`Y^W[NU
@HWI-EAS443:3:78:225:532#0/1
CTCTGATTTCTGTGGCATCCTGGCGCTCCCCATCAGTCTC
+HWI-EAS443:3:78:225:532#0/1
aa_a^a`ba`aT[_a_[_aaVZa__WNU^_^HUWTZTQW]
@HWI-EAS443:3:78:469:673#0/1
ATGGGGAGCGCCAGGATGCCACAGAAATCAGAGCATCTGT
+HWI-EAS443:3:78:469:673#0/1
aa_aa_P__aaa`]a__W_`a_a]aXVY]_``_a``]¥XK
@HWI-EAS443:3:79:1019:188#0/1
TGATGGGGAGCGCCAGGATGCCACAGAAATCAGAGCATCT
+HWI-EAS443:3:79:1019:188#0/1
ab`aaaa_`a_`¥^^`_^¥¥¥¥¥¥aa¥¥_¥Q¥]Y¥Q^WTW
@HWI-EAS443:3:79:1704:112#0/1
CGATCATATGAACAAATGGAGACTGATGGGGAGCGCCAGG
+HWI-EAS443:3:79:1704:112#0/1
aaJb`bb^bba`Ga¥aabaV^]_aa_ba`a_[aVaRZT]X
```

```
@HWI-EAS443:3:79:524:677#0/1
CCCCATCAGTCTCCATTTGTTCATATGATCGTTTGGTGCC
+HWI-EAS443:3:79:524:677#0/1
Z_bbb`baaa_bbbabbbbaabbabbb_abbabab``bab
@HWI-EAS443:3:80:1204:663#0/1
CCCCATCAGTCTCCATTTGTTCATATGATCGTTTGGTGCC
+HWI-EAS443:3:80:1204:663#0/1
a`aa]N_]ZUZY¥]P¥WKZNS[ZVZ[YJYTVU^PX^NYRU
@HWI-EAS443:3:80:222:926#0/1
CAAATGGAGACTGATGGGGAGCGCCAGGATGCCACAGAAA
+HWI-EAS443:3:80:222:926#0/1
a`¥R`^a]¥Ya^_^`^`]X¥aa¥^¥¥UU[¥TT[UU`YU^U
@HWI-EAS443:3:81:1158:1523#0/1
ATGGGGAGCGCCAGGATGCCACAGAAATCAGAGCATCTGT
+HWI-EAS443:3:81:1158:1523#0/1
abaZ_aab^`_a]^aa_`[`b]aa`_`a¥`[`a_`Y¥^aV
@HWI-EAS443:3:82:1112:1092#0/1
CCCCATCAGTCTCCATTTGTTCATATGATCGTTTGGTGCC
+HWI-EAS443:3:82:1112:1092#0/1
`a^aabbaabbba^abbbbaa_bbabbaab``bba¥[``]
@HWI-EAS443:3:82:542:293#0/1
CTGATTTCTGTGGCATCCTGGCGCTCCCCATCAGTCTCCA
+HWI-EAS443:3:82:542:293#0/1
a^aaaaaa``^_^a^^[a^[_`]_XY^Y__Y[X_YU^S¥Z
@HWI-EAS443:3:83:710:270#0/1
ATGAACAAATGGAGACTGATGGGGAGCGCCAGGATGCCAC
+HWI-EAS443:3:83:710:270#0/1
aaaaaaaa_aab^^Zaaa_`aa_____a]¥_``Y__¥_¥W
@HWI-EAS443:3:85:555:1867#0/1
CAGATGCTCTGATTTCTGTGGCATCCTGGCGCTCCCCATC
+HWI-EAS443:3:85:555:1867#0/1
`¥`_YJ^Za¥]P_bb]`a]WU^aa`_]UUaWGXYSa[¥VW
@HWI-EAS443:3:86:1157:1579#0/1
CACCAAACGATCATATGAACAAATGGAGACTGATGGGGAG
+HWI-EAS443:3:86:1157:1579#0/1
[U^[`[[WaXaV`Y[`VV¥ZRbVZ__b[bb`bab`RQR¥^
@HWI-EAS443:3:86:159:427#0/1
TGATGGGGAGCGCCAGGATGCCACAGAAATCAGAGCATCT
+HWI-EAS443:3:86:159:427#0/1
aaXY_a`_H_aa`Y[a]TX¥^]a`[PSTZT^Y[YWYP^¥¥
@HWI-EAS443:3:86:192:364#0/1
GATGGGGAGCGCCAGGATGCCACAGAAATCAGAGCATCTG
+HWI-EAS443:3:86:192:364#0/1
a`aa_]a]a]]^]U^¥¥^^¥U¥P^^Y^Z¥Y¥¥¥¥W¥WWWS
@HWI-EAS443:3:86:997:774#0/1
ATCCTGGCGCTCCCCATCAGTCTCCATTTGTTCATATGAT
+HWI-EAS443:3:86:997:774#0/1
abbaab`_]]`aaab]aXOMQ]¥LYUaa`WY_Z[a_[XZ_
@HWI-EAS443:3:87:1143:1165#0/1
TCTGATTTCTGTGGCATCCTGGCGCTCCCCATCAGTCTCC
```

```
+HWI-EAS443:3:87:1143:1165#0/1
`aabaaba_a`¥_a`aaaQ¥^]¥Z`MU``_[¥[VTU¥ZQ_
@HWI-EAS443:3:87:469:162#0/1
GCTCCCCATCAGTCTCCATTTGTTCATATGATCGTTTGGT
+HWI-EAS443:3:87:469:162#0/1
[``^`a]Y[T[^U¥`____]]`[_^^_Y]a^^[_W¥__ZW
@HWI-EAS443:3:88:1019:1125#0/1
ATGGGGAGCGCCAGGATGCCACAGAAATCAGAGCATCTGT
+HWI-EAS443:3:88:1019:1125#0/1
__^G¥Z]]T^_^`¥PUP^UHRH`^XTOKJV]Z`ZVVYVBB
@HWI-EAS443:3:88:904:366#0/1
GATGGGGAGCGCCAGGATGCCACAGAAATCAGAGCATCTG
+HWI-EAS443:3:88:904:366#0/1
a]````a¥a]a[^^`_¥^Z^^`[a[X^_`YWXU¥YR[TU[
@HWI-EAS443:3:89:1041:678#0/1
CTCTGATTTCTGTGGCATCCTGGCGCTCCCCATCAGTCTC
+HWI-EAS443:3:89:1041:678#0/1
abaab]bbbbbbaaa]`baa`bb`ba``]aa_^[Y^_¥_V
@HWI-EAS443:3:89:675:1749#0/1
GAACAAATGGAGACTGATGGGGAGCGCCAGGATGCCACAG
+HWI-EAS443:3:89:675:1749#0/1
abbbaaba]Za^^^bJaba`a`a`b¥]b`aaa^TLTaSba
@HWI-EAS443:3:91:463:671#0/1
AAACGATCATATGAACAAATGGAGACTGATGGGGAGCGCC
+HWI-EAS443:3:91:463:671#0/1
`¥]]]U_W¥aaa_aa_aa[[^aZ``Za`Y`^a`aQ_^^V¥
@HWI-EAS443:3:91:487:62#0/1
CATCAGTCTCCATTTGTTCATATGATCGTTTGGTGCCTTG
+HWI-EAS443:3:91:487:62#0/1
ab`bab`bb`Ta`b``baaabbbb`aZaabbbb_bSaaaa
@HWI-EAS443:3:91:555:1049#0/1
GATGGGGAGCGCCAGGATGCCACAGAAATCAGAGCATCTG
+HWI-EAS443:3:91:555:1049#0/1
_^aa`a`[`aa`[¥^a_Q`]`^]_X¥aZ^Xa¥^_¥^¥]]¥
@HWI-EAS443:3:92:1489:2032#0/1
GATGGGGAGCGCCAGGATGCCACAGAAATCAGAGCATCTG
+HWI-EAS443:3:92:1489:2032#0/1
a``bbaaaa]a_Saaaa]aa]aZbaa`a`_a`]_¥a]¥_`
@HWI-EAS443:3:92:1587:740#0/1
GAACAAATGGAGACTGATGGGGAGCGCCAGGATGCCACAG
+HWI-EAS443:3:92:1587:740#0/1
aba[]baabaaaa[bb^aaa_a_ba_]¥aaa^aaV_]Qa_
@HWI-EAS443:3:92:460:1043#0/1
GCTCCCCATCAGTCTCCATTTGTTCATATGATCGTTTGGT
+HWI-EAS443:3:92:460:1043#0/1
a_bababaa[aaaaa`a_abab¥baabaaaaa_aaaaa]¥
@HWI-EAS443:3:92:543:182#0/1
CTGATGGGGAGCGCCAGGATGCCACAGAAATCAGAGCATC
+HWI-EAS443:3:92:543:182#0/1
`baabaabbXbbb`a_`b``ba`^abaaaaa__aaa¥_``
```

@HWI-EAS443:3:92:892:1335#0/1
TCCCCATCAGTCTCCATTTGTTCATATGATCGTTTGGTGC
+HWI-EAS443:3:92:892:1335#0/1
abababb`^]abaaaaaaaa]aaaaa_a_a]__aaa_U`_
@HWI-EAS443:3:93:983:1170#0/1
CTGATTTCTGTGGCATCCTGGCGCTCCCCATCAGTCTCCA
+HWI-EAS443:3:93:983:1170#0/1
abbRY_a_XaTa_a`a``^¥``_`[_a_¥PN^[[UZUU¥Z
@HWI-EAS443:3:94:1508:1845#0/1
CTGATGGGGAGCGCCAGGATGCCACAGAAATCAGAGCATC
+HWI-EAS443:3:94:1508:1845#0/1
abaabbbabaaaaX_aab__a]_aGba_aaa__`_^Za`[
@HWI-EAS443:3:94:237:1191#0/1
CAAACGATCATATGAACAAATGGAGACTGATGGGGAGCGC
+HWI-EAS443:3:94:237:1191#0/1
aaa^`a]`a``aa``a``]____^`^^__^^__]]Y_]]]
@HWI-EAS443:3:94:467:620#0/1
CAGATGCTCTGATTTCTGTGGCATCCTGGCGCTCCCCATC
+HWI-EAS443:3:94:467:620#0/1
`¥`a`Z`ab`¥[b`^bbaaa`^`aaaRb^aa^¥``[`]RY
@HWI-EAS443:3:94:932:1647#0/1
CTCCCCATCAGTCTCCATTTGTTCATATGATCGTTTGGTG
+HWI-EAS443:3:94:932:1647#0/1
`¥_RG]]S`]]LRPaa^X`YU`a_ZYLX`a]P[R^_^NX[
@HWI-EAS443:3:96:1712:1289#0/1
GATGGGGAGCGCCAGGATGCCACAGAAATCAGAGCATCTG
+HWI-EAS443:3:96:1712:1289#0/1
`N```a]¥aa_^Z`_^^]^UZ]___U^RYT¥^^_UYZBBB
@HWI-EAS443:3:96:439:121#0/1
CTCTGATTTCTGTGGCATCCTGGCGCTCCCCATCAGTCTC
+HWI-EAS443:3:96:439:121#0/1
`bbbbabbbbba_ba`bba`abbaa_a^__¥_a^a__¥_`
@HWI-EAS443:3:96:750:1387#0/1
TGCTCTGATTTCTGTGGCATCCTGGCGCTCCCCATCAGTC
+HWI-EAS443:3:96:750:1387#0/1
aa_`Xa^¥baaa`a_a_aa_`aa]`_Z[`_V_a`UZ[U¥¥
@HWI-EAS443:3:97:228:451#0/1
AGGCACCAAACGATCATATGAACAAATGGAGACTGATGGG
+HWI-EAS443:3:97:228:451#0/1
Yabb`bbb_]Wbaa``bb_`ZPa`a`a`a[a¥Z]^]aaa^
@HWI-EAS443:3:97:464:1304#0/1
GAACAAATGGAGACTGATGGGGAGCGCCAGGATGCCACAG
+HWI-EAS443:3:97:464:1304#0/1
aa`¥Z_a_ab^Taab`abba]aaZaa`^_`a][a]U[¥aa
@HWI-EAS443:3:98:1213:292#0/1
TGATGGGGAGCGCCAGGATGCCACAGAAATCAGAGCATCT
+HWI-EAS443:3:98:1213:292#0/1
ab]aaaa`^aV`¥Z¥`]Z¥^UP[Wa`]^SUS^_¥¥USWM^
@HWI-EAS443:3:9:433:93#0/1
CTGATGGGGAGCGCCAGGATGCCACAGAAATCAGAGCATC

+HWI-EAS443:3:9:433:93#0/1
`bbabbbbbbbbbaa`b^aaba`a`bbbba`bbaaa```Q
@HWI-EAS443:3:4:1562:1565#0/1
CAGAAATGAAAAGTGGCGAGGGCAATTGGGGCAGAAATTT
+HWI-EAS443:3:4:1562:1565#0/1
a[aT[]]]X¥_ZbW_YLa_aGYQZTUWX]_GGJ]XOSZ^_
@HWI-EAS443:3:21:996:1008#0/1
CAAATTTCTGCCCCAATTGCTCTCGCCACTTTTCATTTCT
+HWI-EAS443:3:21:996:1008#0/1
`WZ¥[_Ua^[DUU][^¥aYY^YYXa`_Y_[[WPFYYZ]][
@HWI-EAS443:3:62:859:1431#0/1
GCAGAAATGAAAAGTGGCGAGAGCAATTGGGGCAGAAATT
+HWI-EAS443:3:62:859:1431#0/1
aaaba_bbba`]_a^a[a`a¥^``Ra`aaa_F_aaa_`_`
@HWI-EAS443:3:72:1731:132#0/1
GAAAAGTGGCGAGAGCAATTGGGGCAGAAATTTGAGGAAA
+HWI-EAS443:3:72:1731:132#0/1
a][VYa¥___a[]TUTU¥S]^PSDLQ_XYVTY¥OQWTDMY
@HWI-EAS443:3:74:361:1287#0/1
GCCCCAATTGCTCTCGCCACTTTTCATTTCTGCTCTGGAG
+HWI-EAS443:3:74:361:1287#0/1
aU]WX[R_`_aYa__ZX¥^_PFZ¥^_W^__]_PS_Q¥QW¥
@HWI-EAS443:3:85:1230:125#0/1
TGAAAAGTGGCGAGAGCAATTGGGGCAGAAATTTGAGGAA
+HWI-EAS443:3:85:1230:125#0/1
aa_a`_a^ba_a__X_WX^^`aa``X_a^^Z^a``W`_XZ
@HWI-EAS443:3:29:1779:1523#0/1
ATCGCAGCCCTCAAGAGCATGTCTCCTATTTCTAGAACAC
+HWI-EAS443:3:29:1779:1523#0/1
aa¥aa^_G[```¥T]_^W_]¥_W`Y]^Z_a_¥_^^]`R^Z
@HWI-EAS443:3:61:906:1903#0/1
GGCCTATCGCAGCCCTCAAGAGCATGTCTCCTATTTCTAG
+HWI-EAS443:3:61:906:1903#0/1
X_V]R^a_a^¥^E[UT¥___ZO_MRRTZHZGVPYVRSWUY
@HWI-EAS443:3:32:1323:1212#0/1
GGCCACCAAAGCNGACTACACCCTTGACGAAGAGAGCAGA
+HWI-EAS443:3:32:1323:1212#0/1
a`_]]`^bbbaZDZ`¥`X``aaaaa]aabXa`aYa]b]a]
@HWI-EAS443:3:33:1780:324#0/1
ATCCTGTTCCTGTTCCATGGCTGTATGGAGGGNCTCCAGT
+HWI-EAS443:3:33:1780:324#0/1
a`a^a`¥a_`aa_aTV_a]aUaBBBBBBBBBBBBBBBBBB
@HWI-EAS443:3:5:837:1146#0/1
GGGGTCTCCAGTATAAGGGAATGTGGTGCTTATGGCATTT
+HWI-EAS443:3:5:837:1146#0/1
aa`a^aa``_a__`ZW^^_]__a^a¥]a_`__^_^[^`__
@HWI-EAS443:3:52:304:1620#0/1
GATGCCAGGGTCGACTTCGAGTCTGGACGGGTCAAGAAAG
+HWI-EAS443:3:52:304:1620#0/1
a¥[]XSM]VOQWPVR[[^YXSTU^_^UW]MKNNTR[U[[Z

```
@HWI-EAS443:3:57:1005:186#0/1
CGACTTCGAGTCTGGACGGGTCAAGAAAGAAGAGTTCTCT
+HWI-EAS443:3:57:1005:186#0/1
aa_`aaaa_a]_a]W]_b``^_[Xa¥W__``_]_U`Z`Wa
@HWI-EAS443:3:87:421:1350#0/1
GATTGATGCCAGGGTCGACTTCGAGTCTGGACGGGTCAAG
+HWI-EAS443:3:87:421:1350#0/1
_SUaU_aVZ`TG^WJW^XV¥SPYU[GR¥Z`aZVYFOFSV¥
@HWI-EAS443:3:36:1337:941#0/1
AAGATGCAGAGAGAGGCAAGTTAAAAAGAAGAGCCTGAAC
+HWI-EAS443:3:36:1337:941#0/1
aaaa`bbabaaaa_`aaa`^Yaa_`_a[_aa^_Z__a]^`
@HWI-EAS443:3:33:932:619#0/1
CAAAATGNCCACCGTCAACATCCACAGCACTCTGCTGTTC
+HWI-EAS443:3:33:932:619#0/1
aa^___]D[aaa`_`_`_Zaaa`aaaZ^aa_TW_W`^V^W
@HWI-EAS443:3:64:912:533#0/1
CAAAATGACCACCGTCAACATCCACAGCACTCTGCTGTTC
+HWI-EAS443:3:64:912:533#0/1
`]]bbbb_aa¥N[^_a¥[[_aaa_V¥[Y¥`a`a`_][[a^
@HWI-EAS443:3:89:1131:30#0/1
GACAAAATGACCACCGTCAACATCCACAGCACTCTGCTGT
+HWI-EAS443:3:89:1131:30#0/1
a¥¥[_]_^`aU¥^MWa[_^¥Taa_VTT__^_SYX__XTZU
@HWI-EAS443:3:31:578:1529#0/1
AAATGATCATCGTCAACATCCACAGCACTCTGCTGTTCCT
+HWI-EAS443:3:31:578:1529#0/1
aa^VULSSXX`TT_]V¥`PX^ZUXYXZXNYUSVSXXXYXZ
@HWI-EAS443:3:86:711:1068#0/1
CAGGCACTCCTTCCGTAGAAGGCCCCCTTTTCAGACCGTA
+HWI-EAS443:3:86:711:1068#0/1
]TV_VG[¥_W[ZNPYK^QTZ^XTQ^BBBBBBBBBBBBBBB
@HWI-EAS443:3:94:1346:111#0/1
AGCTTTTGGTCTAGTGTGTGCCACTTGTGAACAGATTGCT
+HWI-EAS443:3:94:1346:111#0/1
aaabbba`_a_aaa_aaa_a_Gb]aaa^bZ^J^a][`_T_
@HWI-EAS443:3:17:1338:1523#0/1
GGCATTCCTTCCGTAGAAGGCCCTCTTTTCAAACCGTATT
+HWI-EAS443:3:17:1338:1523#0/1
aaa^]¥a¥^^`]UMU]``¥^T¥_[VU]¥^N_^ZVR^TX[_
@HWI-EAS443:3:67:642:1781#0/1
CCCCATTCCCATTTAGTGCATTTTGGACAAAGCGTCTACG
+HWI-EAS443:3:67:642:1781#0/1
a_]_aaaaa`_`_`¥PFW^Z___aRDX]``aXVSNY[_BB
@HWI-EAS443:3:92:263:2033#0/1
ATTCAGGGCATTTTGGACAAAGCGTCTACGCTGGAGTCCT
+HWI-EAS443:3:92:263:2033#0/1
T]_T]QFP^WWW]O^MO]Ta¥^aaVXZ_O^TSLG^GVYBB
@HWI-EAS443:3:30:542:1592#0/1
CCCATTCCCATTCAGGGCATTTTGGACAAAGCGTCTACGC
```

+HWI-EAS443:3:30:542:1592#0/1
abbba`bbbba]I]]Y¥aaa^```¥aaa]`S`]UW¥^¥SV
@HWI-EAS443:3:19:643:431#0/1
AGGGCGGATGGGAGTGCAGATGCAGCGATTCAAGTGATCC
+HWI-EAS443:3:19:643:431#0/1
abbbabbXbbbb^aU`aabaaa`Xa]b`_`a`aaW`a¥`^
@HWI-EAS443:3:44:30:208#0/1
GCAGGCCTACCAGAAGCGGATGGGAGTGCAGATGCAGCGA
+HWI-EAS443:3:44:30:208#0/1
aa`]a^aa_a`_`X__[^DR[_^aR^HU^PVQWXQQVKXR
@HWI-EAS443:3:8:1774:101#0/1
CAAAATGACCATTGTCAACATCCACAGTACTCTGCTGTTC
+HWI-EAS443:3:8:1774:101#0/1
``M[`baNYZ_]D[[LU_X¥^^OWGI¥OZX^OX]WXUBBB
@HWI-EAS443:3:11:1005:186#0/1
CAAAATGACCATCGTCAACATCCACAGCACTCTGCTGCTC
+HWI-EAS443:3:11:1005:186#0/1
R_`__Xa`aa[```_¥_S[¥_`[[Y¥bba[_O`b]a^EXF
@HWI-EAS443:3:97:1659:797#0/1
GGTCTCCATTTGTTCATATGATCGTTTGGTGCCTTGAGAC
+HWI-EAS443:3:97:1659:797#0/1
MWb__YZ]bab`ab_bbU`[[a_ba[aI_a][aabYJ¥_H
@HWI-EAS443:3:67:728:510#0/1
ATGCCCCATTCCCTGATCGGCTCCGCCGGGATCAAAAGTC
+HWI-EAS443:3:67:728:510#0/1
WV_`_``a`]_¥V]_VXa[]^`]V[YYRG[]¥¥Z_STZZ^
@HWI-EAS443:3:13:225:497#0/1
CACGCTCGGGTTCGTTGCCTTTTCGTCCGAGAGCTCAGAT
+HWI-EAS443:3:13:225:497#0/1
¥R`[I]_TXaO]UV`¥UaTZ¥]¥`aVW¥TX_^ZVZ^^XXP
@HWI-EAS443:3:15:238:487#0/1
CACCGAACTTCTCCAGCCTTGAAAACTTTAGAGTCTATGT
+HWI-EAS443:3:15:238:487#0/1
`bbba``aaaaabbaaaaaaa`aa``aa`aa]_F]aa`aZ
@HWI-EAS443:3:16:1531:183#0/1
CGGTGGGTTCACTTTCAAAAGGACAAGCGGATTATCAGTC
+HWI-EAS443:3:16:1531:183#0/1
__W[¥``OQUQa¥^aV`]^`¥]YS`^]Y_UVVFYTQT^UV
@HWI-EAS443:3:44:419:1573#0/1
GCCACATACCAGAGAACAAGAGCGCTTGTTCGCACCGGAA
+HWI-EAS443:3:44:419:1573#0/1
a`^[^baXGR_]a```a^W]`a`a__^]RZaa^¥¥`__^^
@HWI-EAS443:3:23:775:721#0/1
CTAATGGTACCGAGATATGCATTCGCAATGGAAAGAAATG
+HWI-EAS443:3:23:775:721#0/1
aaa__aa]baabaaaabaaaaaa`_`a```[`a_`_a__X
@HWI-EAS443:3:46:1678:1597#0/1
CAAGAAGTTCAAGACGGAAATAGCAACAAGACCCAAAGCG
+HWI-EAS443:3:46:1678:1597#0/1
a^VbMYbaaa[`_IabXUaZ_`[UU`Ga^`XZ`XSTYVD¥

@HWI-EAS443:3:26:1203:426#0/1
GGAAATTCCAATTGTTAGCCAATTGATGCCATCATGACAA
+HWI-EAS443:3:26:1203:426#0/1
JZZab`ZW_`¥`_¥WW_XVV_^Wa_`^ZK_``]Y`a[UQ¥
@HWI-EAS443:3:9:1756:1873#0/1
ATGCAGCAGAGTGGCATGCCATCCACACCAATTGGCTTTT
+HWI-EAS443:3:9:1756:1873#0/1
]`ba^a`aa^_JOa^XXVUGTTXX_X_KQ_^¥¥SSUH^I_
@HWI-EAS443:3:77:1601:426#0/1
GAGCTCTTCAATGGTGGAACAGATCTTCATGATCTCAGAG
+HWI-EAS443:3:77:1601:426#0/1
]a¥]`^aa]`b_RXU^abb__bbb]a_`_``aaaaRaV_`
@HWI-EAS443:3:38:1093:1839#0/1
GTTTTTTTGAACAAATTACTTGTCAATGGTAAATGGCAAC
+HWI-EAS443:3:38:1093:1839#0/1
a]_aa`aaa`aa`__a`a__``Z___a^¥XGUOXSYS^_U
@HWI-EAS443:3:99:1439:235#0/1
AATCCCAATTACCTCATGGCTTGGAAGCAGGGGCTAGGAG
+HWI-EAS443:3:99:1439:235#0/1
aVb_YL__baaW^U^_ba^[]a[RGX`UZTWGG_WWBBBB
@HWI-EAS443:3:86:1349:663#0/1
CTCATGGCTTGGAAGCAGGTGCTAGGAGAGCTACAGGACA
+HWI-EAS443:3:86:1349:663#0/1
abba`_b]b`¥`WaTH_`bVba_W[FUaUaQ__]ab_X[`
@HWI-EAS443:3:39:771:828#0/1
CTGGCGACAGTTGACTAGATCGCCAAAATTTGGTAAATCC
+HWI-EAS443:3:39:771:828#0/1
`ababb¥WQ_]a]XFVLRZ^^[[XOFQZNHIFQXXV]¥WV
@HWI-EAS443:3:12:483:531#0/1
AGGCACTCCTCGATTGCTTCATATAGCCCCCCAAGATCAA
+HWI-EAS443:3:12:483:531#0/1
aa`aaMQ_`_UZ^QW`_XX]^^a^_VZ`US^X`¥`^SZ¥B
@HWI-EAS443:3:27:112:1351#0/1
CAACTTGAGGGGTTTTCGGCTAAATCGAGAAAATTGCTTC
+HWI-EAS443:3:27:112:1351#0/1
a`a`a`a`a``aW`_aa`]a¥F]`^ZY_aa``a_^¥`_^_
@HWI-EAS443:3:53:1310:295#0/1
GAACAATGAGAAGCAATTTTCTCGATTTAGCCGAAAACCC
+HWI-EAS443:3:53:1310:295#0/1
aaaaaaabaa`aaaaabaaaaaaa]bbaaa_Zaa`a`]]S
@HWI-EAS443:3:54:1679:1617#0/1
GTAAAACTGGAATCAACAAGGATTTACCAGATTTTGGCGA
+HWI-EAS443:3:54:1679:1617#0/1
aaa^aaaaaaa_a_`_¥a_a`^```_]X]`Z``____^`[
@HWI-EAS443:3:35:263:565#0/1
AATAGATGGGGTAACGCTGGAATCAACACGGATTTACCAG
+HWI-EAS443:3:35:263:565#0/1
YP_Waa]aaZ^W`]DZ]`aa]^Y`¥`_^GR^YWETa_PW[
@HWI-EAS443:3:35:1375:774#0/1
AAGAAATAGATGGGGTAACGCTGGAATCAACAAGGATTTA

````
+HWI-EAS443:3:35:1375:774#0/1
aaa`a`aab`abaa_^a_¥¥^]^^ZZ^UT¥]]^^SWZ]X]
@HWI-EAS443:3:40:786:361#0/1
CAATGCCAAGGAGGCACAGGATGTGATTATGGAAGGTGGT
+HWI-EAS443:3:40:786:361#0/1
X`X[b]aba]babbbb]`^aSa¥GYZK[ZO__RW[BBBBB
@HWI-EAS443:3:73:1568:1877#0/1
CAATGCCAAGGAGGCACAGGATGTGATTATGGAAGGTGGT
+HWI-EAS443:3:73:1568:1877#0/1
ab`abababaaa]`a`aaaaa_aaaS```__^_`aYBBBBBB
@HWI-EAS443:3:91:1378:1189#0/1
GACCCTGGCCATGCAGATCTCAATGCCAAGGAGGGACAGG
+HWI-EAS443:3:91:1378:1189#0/1
```]]]`aZ^`^Z^Sa^UX``^^__UTU]¥^V^¥GWSW^¥
@HWI-EAS443:3:84:133:1859#0/1
ATCACATCCTGTGCCTCCTTGGCATTGAGATCTGCACGGC
+HWI-EAS443:3:84:133:1859#0/1
a`a_`baa_[_XZU¥Za¥ZLMVa^UHU_QYWVSN¥MDMW]
@HWI-EAS443:3:100:692:516#0/1
AGTTGATACAAACCCTGGCCATGCAGATCTCAATACCAAG
+HWI-EAS443:3:100:692:516#0/1
`YWabbaababaaYaa]aa__[__abU_Qa^_^^JS¥]_a
@HWI-EAS443:3:13:490:332#0/1
GGAGAGTTGATACCAACCCTGGCCATGCAGATCTCAATGC
+HWI-EAS443:3:13:490:332#0/1
a``b`aVaaa``_V[V___Z__]_¥¥¥[¥_ZUWW^^VWSX
@HWI-EAS443:3:14:761:707#0/1
GAGGAGAGTTGGTACAAACCCTGGCCATGCAGATCTCAAT
+HWI-EAS443:3:14:761:707#0/1
a`ba_a_a_``_Ua¥`^a^[¥^^VXVS^¥U¥_SSUWS_SU
@HWI-EAS443:3:27:1494:453#0/1
TTGGTATTGAGATCTGCATGGCCAGGGTTTGTATCAACTC
+HWI-EAS443:3:27:1494:453#0/1
aaaa]_aaaaba`]`a]^`^[_¥_a`_Z`__Y^_XUYR[W
@HWI-EAS443:3:27:575:1790#0/1
GAGGAGAGTTGATACAAACCCTGGCCATGCAGATCACAAT
+HWI-EAS443:3:27:575:1790#0/1
a`aa`baaaaaa_babaa`aaWN^Z^UZN¥^_UU¥^``aS
@HWI-EAS443:3:39:221:128#0/1
GCAGATCTCAATGCCAAGGAGGCACAGGATGGGATTATGG
+HWI-EAS443:3:39:221:128#0/1
a_S_Y^^U^_¥^_^Y^V]]¥Y]YXYX]^YV^GYGYUVYXX
@HWI-EAS443:3:76:502:1916#0/1
TGCAGATCTCAATGCCAAGGAGGCACAGGATGTGGTTATG
+HWI-EAS443:3:76:502:1916#0/1
Z_]S^[ab]^FW`^ZL[_ZV¥]]^ZN¥]ZR^BBBBBBBBB
@HWI-EAS443:3:85:1494:1066#0/1
TCCTGTACCTCCTTGGCATTGAGATCTGCATGGCCAGGGT
+HWI-EAS443:3:85:1494:1066#0/1
XaX[[X[a_aaa_bb_ZabbVZ]bb`ab[Tab[[a`bb`S
````
````

```
@HWI-EAS443:3:93:1187:1614#0/1
ATGCAGATCTCAATGCCAAGGAGGCACAGGATGGGATTAT
+HWI-EAS443:3:93:1187:1614#0/1
a_```a`____a^_^]^_`a_`a_¥_]^a]]^aGZRU]]B
@HWI-EAS443:3:8:90:1911#0/1
TGGCCATGCAGATCTCAATGNCAAGGAGGCACAGGATGTG
+HWI-EAS443:3:8:90:1911#0/1
aaaa`]^aa]]UYa`aa]¥VD¥[]__`X_]aaZ]XSU_RB
@HWI-EAS443:3:22:89:1989#0/1
CGGAGAGTTGATACAAACCCTGGCCATGCAGATCTCAATG
+HWI-EAS443:3:22:89:1989#0/1
a^O]`[WMYaaT]R^^ZORPZR_UKMY]YV[ZVBBBBBBB
@HWI-EAS443:3:38:1276:1457#0/1
ATACAAACCCTGGCCATGCAGATCTCAATGCCAAGGAGGA
+HWI-EAS443:3:38:1276:1457#0/1
`Ya]ba]_[a^M[O[]QBBBBBBBBBBBBBBBBBBBBBBB
@HWI-EAS443:3:92:1161:1213#0/1
CGGCCATGCAGATCTCAATGCCAAGGAGGCACAGGATGTG
+HWI-EAS443:3:92:1161:1213#0/1
`___`b_`]a_X¥``a[_`]Z``^`b_bba`P¥aaIa`Z¥
@HWI-EAS443:3:93:1112:1765#0/1
NGCCATGCAGATCTCAATGCCAAGGAGGCACAGGATGTGA
+HWI-EAS443:3:93:1112:1765#0/1
DOWWYWYYUYYWWWYWYWVTUWWWWYUWYYNWYWWWWBBB
@HWI-EAS443:3:11:239:698#0/1
GAGAGTTGATACAAACCCTGGCCATGCAGATCTCAATGCC
+HWI-EAS443:3:11:239:698#0/1
a```_`a`a__``a_Y`a__`_`_UX^[YRWZ¥W]KSZSR
@HWI-EAS443:3:13:1159:261#0/1
CCTTGGCATTGAGATCTGCATGGCCAGGGTTTGTATCAAC
+HWI-EAS443:3:13:1159:261#0/1
[]UU¥`U[a[ba_aT_`b]``]ZRXZbaaWa^a_a^PXU¥
@HWI-EAS443:3:13:1477:1245#0/1
TGCAGATCTCAATGCCAAGGAGGCACAGGATGTGATTATG
+HWI-EAS443:3:13:1477:1245#0/1
Waab_bbbbbbbbbbbbbbbbabaa_baabbbababbbb^
@HWI-EAS443:3:13:731:1147#0/1
GGAGAGTTGATACAAACCCTGGCCATGCAGATCTCAATGC
+HWI-EAS443:3:13:731:1147#0/1
aaZaZa_aa`a`_`a`Va_]P¥[__YZ___SUQZ¥]¥UWW
@HWI-EAS443:3:14:1086:703#0/1
GCCATGCAGATCTCAATGCCAAGGAGGCACAGGATGTGAT
+HWI-EAS443:3:14:1086:703#0/1
aaa]aa`[`aa_a_`aaa`U^_ba^a__^_^aa¥^aQ`S¥
@HWI-EAS443:3:15:56:603#0/1
AAACAACTTCCATAATCACATCCTGTGCCTCCTTGGCATT
+HWI-EAS443:3:15:56:603#0/1
U__[[¥Z]aa¥R[PUXXWT¥]_¥__U``Z_^[FSY`ZZ¥V
@HWI-EAS443:3:16:751:1077#0/1
AACAACTTCCATAATCACATCCTGTGCCTCCTTGGCATTG
```

+HWI-EAS443:3:16:751:1077#0/1
aa`_`X`a_a`_a_`]`_``]^_^^__Y^VV_U]X]^^]_
@HWI-EAS443:3:17:1182:972#0/1
TGCAGATCTCAATGCCAAGGAGGCACAGGATGTGATTATG
+HWI-EAS443:3:17:1182:972#0/1
`a`UUKaYWaWWIS¥ZZZaa`baaaPI^VTV[SaH^QXBB
@HWI-EAS443:3:17:855:552#0/1
TGCAGATCTCAATGCCAAGGAGGCACAGGATGTGATTATG
+HWI-EAS443:3:17:855:552#0/1
Z__[¥Y[___[T_`_`]¥__¥_`_[WZ__XW^W`FUUPRZ
@HWI-EAS443:3:18:348:889#0/1
CATAATCACATCCTGTGCCTCCTTGGCATTGAGATCTGCA
+HWI-EAS443:3:18:348:889#0/1
aa``]ab_aaa`a__T``a_^__^ZWZ_^^`_Z¥^_Z__[
@HWI-EAS443:3:22:1003:459#0/1
ACATCCTGTGCCTCCTTGGCATTGAGATCTGCATGGCCAG
+HWI-EAS443:3:22:1003:459#0/1
a_a`¥`_^Xa`_aa__abaY__aa_a^_[a`_VZ]`¥_¥a
@HWI-EAS443:3:22:1106:1983#0/1
CCTGTGCCTCCTTGGCATTGAGATCTGCATGGCCAGGGTT
+HWI-EAS443:3:22:1106:1983#0/1
]bab[bbb_ab`aaaaaab`aba^`[X_a_a`]_a```¥`
@HWI-EAS443:3:22:765:848#0/1
CTCCTTGGCATTGAGATCTGCATGGCCAGGGTTTGTATCA
+HWI-EAS443:3:22:765:848#0/1
aaaaaa`_a¥aaa^aaa``aaaaa_a]`a``]``aX`]]^
@HWI-EAS443:3:23:1748:936#0/1
CACATCCTGTGCCTCCTTGGCATTGAGATCTGCATGGCCA
+HWI-EAS443:3:23:1748:936#0/1
`baabbbbaab_bb_ababb_abbbaa_a`aa`_`bb_¥a
@HWI-EAS443:3:27:1048:541#0/1
ACAACTTCCATAATCACATCCTGTGCCTCCTTGGCATTGA
+HWI-EAS443:3:27:1048:541#0/1
^O^[YXSSX`_[W]U¥_RNNSX`M`QR[P_[NVTQSX[^T
@HWI-EAS443:3:28:528:1370#0/1
ACAACTTCCATAATCACATCCTGTGCCTCCTTGGCATTGA
+HWI-EAS443:3:28:528:1370#0/1
aa]I[ZP¥a`aaaaa¥aa`a`ZZYT^```HY¥[__```U]
@HWI-EAS443:3:29:332:843#0/1
ATAATCACATCCTGTGCCTCCTTGGCATTGAGATCTGCAT
+HWI-EAS443:3:29:332:843#0/1
`aaa`^`_N_``_`¥aa`]a_W_^Z`^^]¥Y_`Z_W¥¥¥U
@HWI-EAS443:3:30:1771:1292#0/1
CATGCAGATCTCAATGCCAAGGAGGCACAGGATGTGATTA
+HWI-EAS443:3:30:1771:1292#0/1
aabb_bbbbababaab_babba_aaa_`aa__a`U_R`^_
@HWI-EAS443:3:31:512:150#0/1
ATCCTGTGCCTCCTTGGCATTGAGATCTGCATGGCCAGGG
+HWI-EAS443:3:31:512:150#0/1
`abaaa^baaaaa[[`]aaaa`aaa__^^`_^¥W`^`^_¥

```
@HWI-EAS443:3:32:1459:243#0/1
CTTGGCATTGAGATCTGCATGGCCAGGGTTTGTATCAACT
+HWI-EAS443:3:32:1459:243#0/1
abbbbbbbbbabbbabb``abb`_`bbb]abbaabP``ab
@HWI-EAS443:3:32:931:683#0/1
GAGGAGAGTTGATACAAACCCTGGCCATGCAGATCTCAAT
+HWI-EAS443:3:32:931:683#0/1
_J_`YaQ^Ua_]^_a¥`[__¥U_^_Z^Q[RR[UUTW_[VB
@HWI-EAS443:3:34:978:493#0/1
AGGAGAGTTGATACAAACCCTGGCCATGCAGATCTCAATG
+HWI-EAS443:3:34:978:493#0/1
`]J¥bab^Y_b`_aaaaaX¥YY_]a]K_`ZUV^S_^^^U^
@HWI-EAS443:3:36:1310:272#0/1
AGTTGATACAAACCCTGGCCATGCAGATCTCAATGCCAAG
+HWI-EAS443:3:36:1310:272#0/1
abXb_^baa`a__aaZ_a`[YY^_U¥ZQ_Z_]VUS[`]V]
@HWI-EAS443:3:38:1260:626#0/1
ACATCCTGTGCCTCCTTGGCATTGAGATCTGCATGGCCAG
+HWI-EAS443:3:38:1260:626#0/1
_[a¥[^Z^H_Z]aVa]]Z]U^]¥a`_ZQZ`PPPRZ^^WRY
@HWI-EAS443:3:45:964:236#0/1
GGAGGAGAGTTGATACAAACCCTGGCCATGCAGATCTCAA
+HWI-EAS443:3:45:964:236#0/1
ab]a_Ubab¥aa`_a`aa[QZQ_`Z¥S`_¥[W__WW^V¥Z
@HWI-EAS443:3:47:289:981#0/1
ACATCCTGTGCCTCCTTGGCATTGAGATCTGCATGGCCAG
+HWI-EAS443:3:47:289:981#0/1
`a`_abbb``[^`UUY¥[W^[_a]^_``[aX`[a`]]W]a
@HWI-EAS443:3:4:159:26#0/1
TGGCCATGCAGATCTCAATGCCAAGGAGGCACAGGATGTG
+HWI-EAS443:3:4:159:26#0/1
abb`aZU`]R_¥VUZ_]RXa[UJQ^_W__¥ZST^^BBBBB
@HWI-EAS443:3:4:1740:204#0/1
ACCCTGGCCATGCAGATCTCAATGCCAAGGAGGCACAGGA
+HWI-EAS443:3:4:1740:204#0/1
aa^Y`bb`_^¥]¥^`^X¥`U_^¥_UW[[U[P_SUSNBBBB
@HWI-EAS443:3:50:1673:421#0/1
TACAAACCCTGGCCATGCAGATCTCAATGCCAAGGAGGCA
+HWI-EAS443:3:50:1673:421#0/1
a`^aa``]aaa]a_aab__a_^[[X_^Y¥Y[YP[ZJV^ZZ
@HWI-EAS443:3:51:988:819#0/1
TGCAGATCTCAATGCCAAGGAGGCACAGGATGTGATTATG
+HWI-EAS443:3:51:988:819#0/1
_a`WaaaW^_a¥T_RObabbXbbYJaI]bV]b_`QX[[``
@HWI-EAS443:3:55:950:366#0/1
TGCAGATCTCAATGCCAAGGAGGCACAGGATGTGATTATG
+HWI-EAS443:3:55:950:366#0/1
ab_abaa_aaa`a^``[^a`_aa`_`_ab]a_U]N¥_Q`a
@HWI-EAS443:3:56:1459:1297#0/1
ATTGAGATCTGCATGGCCAGGGTTTGTATCAACTCTCCTC
```

+HWI-EAS443:3:56:1459:1297#0/1
aa]W`a^`Z¥¥a_bb`[T^UQ]N]baY`^SX[Y__SMT[G
@HWI-EAS443:3:56:871:691#0/1
TGCAGATCTCAATGCCAAGGAGGCACAGGATGTGATTATG
+HWI-EAS443:3:56:871:691#0/1
abbabaaaabb_abaaa]b`^_a^aa_ba```Va¥`a_^¥
@HWI-EAS443:3:5:741:841#0/1
GGAGGAGAGTTGATACAAACCCTGGCCATGCAGATCTCAA
+HWI-EAS443:3:5:741:841#0/1
aaabb_bab_aaa`a¥^a_¥]¥]___W^Y_¥¥`Y]U_UBB
@HWI-EAS443:3:61:1020:1265#0/1
CCTGGCCATGCAGATCTCAATGCCAAGGAGGCACAGGATG
+HWI-EAS443:3:61:1020:1265#0/1
abbbbbbaaab_aa`aa_Z_a`baWW^a_`¥a[^_`ZZZ[
@HWI-EAS443:3:61:279:698#0/1
AGTTGATACAAACCCTGGCCATGCAGATCTCAATGCCAAG
+HWI-EAS443:3:61:279:698#0/1
ababbaabaaaa_aaaaaa`a¥^_N^_^^____¥__V[Y¥
@HWI-EAS443:3:63:1411:682#0/1
GCCTCCTTGGCATTGAGATCTGCATGGCCAGGGTTTGTAT
+HWI-EAS443:3:63:1411:682#0/1
]]]^b`a¥¥__^aabaaa`b`baaa`a¥___a^¥a_aZaa
@HWI-EAS443:3:63:177:868#0/1
TGCAGATCTCAATGCCAAGGAGGCACAGGATGTGATTATG
+HWI-EAS443:3:63:177:868#0/1
ab``baaaab_¥aaa]Y]bTH^aaaa_`U¥__Ta]X[Z^`
@HWI-EAS443:3:64:1720:1675#0/1
ACATCCTGTGCCTCCTTGGCATTGAGATCTGCATGGCCAG
+HWI-EAS443:3:64:1720:1675#0/1
abaaaaa___V]`aaa`a¥QVZ__VZZ_¥`¥_V¥___`_[
@HWI-EAS443:3:65:1427:1013#0/1
ATCACATCCTGTGCCTCCTTGGCATTGAGATCTGCATGGC
+HWI-EAS443:3:65:1427:1013#0/1
`aaa`_aaaaa[]__`aaaa^a_``aa``aaaa`_Z`^V¥
@HWI-EAS443:3:66:1535:326#0/1
GAGGAGAGTTGATACAAACCCTGGCCATGCAGATCTCAAT
+HWI-EAS443:3:66:1535:326#0/1
aabbabaaaababaaa``M`Y_a[Y`_[`[]^]¥T¥YS¥V
@HWI-EAS443:3:69:1218:256#0/1
AAACAACTTCCATAATCACATCCTGTGCCTCCTTGGCATT
+HWI-EAS443:3:69:1218:256#0/1
aa]a`_ab`a^Zaaabaa_`a]Sabaa_¥_U¥____SSaa
@HWI-EAS443:3:72:1387:1135#0/1
CCTTGGCATTGAGATCTGCATGGCCAGGGTTTGTATCAAC
+HWI-EAS443:3:72:1387:1135#0/1
abbabba^bbbabaa`bbaaa`a``abaa[aabZ_a__[[
@HWI-EAS443:3:73:1170:244#0/1
GCAGATCTCAATGCCAAGGAGGCACAGGATGTGATTATGG
+HWI-EAS443:3:73:1170:244#0/1
aaaaaa_a`aa_a]_aaa_^`a¥__^__X_aU`S¥_X^^_

```
@HWI-EAS443:3:73:823:654#0/1
TACAAACCCTGGCCATGCAGATCTCAATGCCAAGGAGGCA
+HWI-EAS443:3:73:823:654#0/1
aa^a^U`aaX_Z`_Q`a_[_U^`_Xa[X`[P¥V[ZGXaYL
@HWI-EAS443:3:74:1307:1607#0/1
ATCCTGTGCCTCCTTGGCATTGAGATCTGCATGGCCAGGG
+HWI-EAS443:3:74:1307:1607#0/1
aaaaaa``a`b_aaaaaaaaaa`a`_a`_b__a_S`_a`]
@HWI-EAS443:3:76:734:628#0/1
GGCCATGCAGATCTCAATGCCAAGGAGGCACAGGATGTGA
+HWI-EAS443:3:76:734:628#0/1
a`^_RXVa_VOP__]SYTY]TKXZSSPS^NSV[VBBBBBB
@HWI-EAS443:3:77:709:1862#0/1
CCTTGGCATTGAGATCTGCATGGCCAGGGTTTGTATCAAC
+HWI-EAS443:3:77:709:1862#0/1
K[Uabbbba]]b[baba`_baaab_baaZX^aaabbbbaa
@HWI-EAS443:3:78:842:1743#0/1
GAGTTGATACAAACCCTGGCCATGCAGATCTCAATGCCAA
+HWI-EAS443:3:78:842:1743#0/1
a^a`_b`aaa]aX^ab__aaY`_``___[`_a`[S]_S_¥
@HWI-EAS443:3:7:60:508#0/1
CATGCAGATCTCAATGCCAAGGAGGCACAGGATGTGATTA
+HWI-EAS443:3:7:60:508#0/1
aW¥WZ_VTV]]XaZ__a]_Z]`V]Za^¥HZa_XSHXMX^T
@HWI-EAS443:3:80:608:1722#0/1
AAACAACTTCCATAATCACATCCTGTGCCTCCTTGGCATT
+HWI-EAS443:3:80:608:1722#0/1
Z^Z¥WU`a¥F¥bab^[^a`a_^a[ZUXZU`UT¥FXVXVa¥
@HWI-EAS443:3:80:727:509#0/1
CACATCCTGTGCCTCCTTGGCATTGAGATCTGCATGGCCA
+HWI-EAS443:3:80:727:509#0/1
W`_b`__b``X```_bb¥YT_a_```¥]_]bab`aa]]¥V
@HWI-EAS443:3:83:747:670#0/1
GGAGGAGAGTTGATACAAACCCTGGCCATGCAGATCTCAA
+HWI-EAS443:3:83:747:670#0/1
aY`]ZP_]`^a`a^_aa`_^¥¥^X^^]^¥^YYTYWWYU[¥
@HWI-EAS443:3:84:1023:1417#0/1
TGCAGATCTCAATGCCAAGGAGGCACAGGATGTGATTATG
+HWI-EAS443:3:84:1023:1417#0/1
aba``__aaaba]a`a__aa`aa_a`_ba``aVa^__]_`
@HWI-EAS443:3:88:1288:641#0/1
CAACTTCCATAATCACATCCTGTGCCTCCTTGGCATTGAG
+HWI-EAS443:3:88:1288:641#0/1
abbabb`abb`aaababbaa`b¥a``a]_a_aa`_aaaab
@HWI-EAS443:3:91:293:1137#0/1
ACAAACCCTGGCCATGCAGATCTCAATGCCAAGGAGGCAC
+HWI-EAS443:3:91:293:1137#0/1
`aYQ^aa¥a¥`¥¥VYaVZ]]^Z[`_^RZ^_[[V[V`_`¥V
@HWI-EAS443:3:95:1715:711#0/1
CCTTGGCATTGAGATCTGCATGGCCAGGGTTTGTATCAAC
```

```
+HWI-EAS443:3:95:1715:711#0/1
__bb`V`b_bba[bb`aaUbbbab_a_babbb_bbb_a]D
@HWI-EAS443:3:96:1204:1178#0/1
CAACTTCCATAATCACATCCTGTGCCTCCTTGGCATTGAG
+HWI-EAS443:3:96:1204:1178#0/1
aa¥^Y_XWZ_]¥Z]WX¥_a``_]^ZX_G^]^^^Z¥aaa]_
@HWI-EAS443:3:97:153:790#0/1
AGTTGATACAAACCCTGGCCATGCAGATCTCAATGCCAAG
+HWI-EAS443:3:97:153:790#0/1
`babbbbbbbba^`aabb]^_aa¥aa^¥U[^YS[`¥YY[`
@HWI-EAS443:3:98:646:1497#0/1
TGGCCATGCAGATCTCAATGCCAAGGAGGCACAGGATGTG
+HWI-EAS443:3:98:646:1497#0/1
^_¥WV[T_`[^WaW_][U_`U^X_a_UZ]_XaUaZVHYSB
@HWI-EAS443:3:14:1034:884#0/1
CCCCTGGGTTTTGCTTAATGCATCTTGGTTCAACTCCTTC
+HWI-EAS443:3:14:1034:884#0/1
aaaaaa`aZaaa`_aaa^^^¥]`¥a``¥T^__Z``[W__^
@HWI-EAS443:3:19:774:612#0/1
CCCCTGGGTTTTGCTTAATGCATCTTGGTTCAACTCCTTC
+HWI-EAS443:3:19:774:612#0/1
`ba``[V]`YTbWb`Na``PJN_a_TbSHSPaa```Y`¥_
@HWI-EAS443:3:64:1181:833#0/1
CCCCTGGGTTTTGCTTAATGCATCTTGGTTCAACTCCTTC
+HWI-EAS443:3:64:1181:833#0/1
aa``UXX^QV]_^^_Z_^¥W[Z`]a_ZR¥]^a_TZY¥`]`
@HWI-EAS443:3:98:1282:990#0/1
TCCCAGTTCGTGATTATCCATTGATAAGTGTTGACTAGAA
+HWI-EAS443:3:98:1282:990#0/1
Y]_Z_Z`XPHY^Fa]Q[UO¥`_ba[VE[XaS_`SLQ¥_Ga
@HWI-EAS443:3:59:1738:1096#0/1
TGGCGCCAAGCAAACAATGGCGAAGATGCAAAAGCAGGTA
+HWI-EAS443:3:59:1738:1096#0/1
aa``aa``aY_`a`F_`RT¥O¥X`¥]][X`aG_RULT^BB
@HWI-EAS443:3:59:1290:270#0/1
TGGAGATCCAGATGAAGGCACATCTGGGGTGGAGTCTGCT
+HWI-EAS443:3:59:1290:270#0/1
[[`^[¥¥_bab^]^_^`_b]R``UKa_RX]^OL]TEM[OH
@HWI-EAS443:3:43:615:1524#0/1
GTCTGGTGCCGCAGGTGCTGCGGTAAAAGGAGTTGGAAAA
+HWI-EAS443:3:43:615:1524#0/1
`YZ_`[SQ[[X_S^VRRX¥[T]VBBBBBBBBBBBBBBBBB
@HWI-EAS443:3:3:741:1226#0/1
CGTCCATCATAATCACTGAGTTTAAGTTCAGTGCACATTT
+HWI-EAS443:3:3:741:1226#0/1
abbbbbbabbbaaababaabbb`S¥babaaa`ba¥^aaa`
@HWI-EAS443:3:55:105:611#0/1
GGTTAATTGAAGAAATGCGGCACAGATTGAAAGAGACAGA
+HWI-EAS443:3:55:105:611#0/1
ab¥XOH^UYZU^`THP]X_a`¥`a_VXX`UZTWQ¥`[TWV
```

```
@HWI-EAS443:3:14:1602:904#0/1
CATATAGCCCCCCAAGATCGAAGGTTCCAGGTTCCAGATT
+HWI-EAS443:3:14:1602:904#0/1
_`b¥_a^^_^_^^^a`^_Za``]]aa¥R_a`[`SMVaTa`
@HWI-EAS443:3:59:695:1759#0/1
TCCCGATCCAAAATGGAAGGCTTTGTGCGACAATGCTCCA
+HWI-EAS443:3:59:695:1759#0/1
S¥XQ]FUU[]`aaSGTa_YZa_`SLRZa]Y_YGYXGXNR`
@HWI-EAS443:3:2:646:270#0/1
TACTGATCCAAAATGGAAGGCTTTGTGCGAGAATGCTTCA
+HWI-EAS443:3:2:646:270#0/1
YWUT_WVW^WaX^U_VV_¥_[YP[[FPLV¥G]UUS`BBBB
@HWI-EAS443:3:56:1696:1572#0/1
TACTGATCCAAAATGCAAGGCTTTGTGCGACAATGCTTCA
+HWI-EAS443:3:56:1696:1572#0/1
aa`aa]_a``_`_^V]QUOZW^VZWVUVPYYQ][QLRWBB
@HWI-EAS443:3:95:820:606#0/1
TACTGATCCAAAATGGAAGGCTTTGTGCGACAATGGTTCA
+HWI-EAS443:3:95:820:606#0/1
¥``]R]`_^aaW``Zab^XaZ¥X`[Y`_^XI`a]_FU^`_
@HWI-EAS443:3:7:1078:1404#0/1
TACTCATCCAAAATGGAAGGCTTTGTGCGACAATGCTTCA
+HWI-EAS443:3:7:1078:1404#0/1
ab_[FWV``__ba¥_Wa¥RT]aa_a^^RbX^a`¥_SBBBB
@HWI-EAS443:3:25:399:222#0/1
CTGATCCAAAATGGAAGGCTTTGTGCGACAATGNTTCAAT
+HWI-EAS443:3:25:399:222#0/1
[`bb^abab``abbbbbabbbbaababbb_a[D]babba
@HWI-EAS443:3:13:1445:1293#0/1
ATCCAAAATGGAAGGCTTTGTGCGACAATGGTTCAATCCA
+HWI-EAS443:3:13:1445:1293#0/1
aa`¥`aYY`_]P¥¥WT_ZW]Za^]a_^Z`XHYV^_W¥BBB
@HWI-EAS443:3:14:1653:214#0/1
AAAATAGAAGGCTTTGTGCGACAATGCTTCAATCCAATGA
+HWI-EAS443:3:14:1653:214#0/1
aa`abba`^a`_^_aa]a_``[a_``^]¥X^^^][_Z`^X
@HWI-EAS443:3:25:692:1851#0/1
CGACGATCATTGGATTGAAGCATTGTAGCACAAAGCCTTC
+HWI-EAS443:3:25:692:1851#0/1
abba`a``aaaaaaaa^¥b`__¥_QRKS___a_aV^_¥^¥
@HWI-EAS443:3:100:338:938#0/1
TACTGATCCAAAATGGAAGGCTTTGTGCGACAATGCTTCA
+HWI-EAS443:3:100:338:938#0/1
[a`a````aaa^^U[^]]aa^aa`_Z[¥Z^aa`]¥SX^]B
@HWI-EAS443:3:11:1074:1470#0/1
TACTGATCCAAAATGGAAGGCTTTGTGCGACAATGCTTCA
+HWI-EAS443:3:11:1074:1470#0/1
aa_]`¥]`````_aa_a]a_^__`¥____]^_`^^X_ZX`
@HWI-EAS443:3:11:613:1421#0/1
TACTGATCCAAAATGGAAGGCTTTGTGCGACAATGCTTCA
```

```
+HWI-EAS443:3:11:613:1421#0/1
aa[^aW[a`b_^_aa_^aaba[`aaXb`__`]a^^^_^_`
@HWI-EAS443:3:13:1661:596#0/1
TACTGATCCAAAATGGAAGGCTTTGTGCGACAATGCTTCA
+HWI-EAS443:3:13:1661:596#0/1
`baa¥`a^`aab^aRFZ_[`Ya_```b^_^_S`a`¥__W_
@HWI-EAS443:3:14:1286:783#0/1
TACTGATCCAAAATGGAAGGCTTTGTGCGACAATGCTTCA
+HWI-EAS443:3:14:1286:783#0/1
``aaba^`aa]^a`aaaaaa¥``a`]¥¥aa_]`a`]`ZOY
@HWI-EAS443:3:17:959:1870#0/1
TACTGATCCAAAATGGAAGGCTTTGTGCGACAATGCTTCA
+HWI-EAS443:3:17:959:1870#0/1
ab`^aaa``babb_`Paa_a__a_WTZ`a`WaaUTXZaUa
@HWI-EAS443:3:18:1062:1067#0/1
TACTGATCCAAAATGGAAGGCTTTGTGCGACAATGCTTCA
+HWI-EAS443:3:18:1062:1067#0/1
_]a]XX^aa_OURY_aQVYa]`_Y^UY`_VDS_a¥Y]¥]]
@HWI-EAS443:3:18:1720:364#0/1
TACTGATCCAAAATGGAAGGCTTTGTGCGACAATGCTTCA
+HWI-EAS443:3:18:1720:364#0/1
abbQYW]OZaa]Y_ba`Uaa_a]_`Q`UaRN__`a[U__]
@HWI-EAS443:3:1:362:1317#0/1
TACTGATCCAAAATGGAAGGCTTTGTGCGACAATGCTTCA
+HWI-EAS443:3:1:362:1317#0/1
a^U^bb`[^¥YV[VYX`_`a^aaa¥R`S¥a^ZWabBBBBB
@HWI-EAS443:3:24:1382:755#0/1
TACTGATCCAAAATGGAAGGCTTTGTGCGACAATGCTTCA
+HWI-EAS443:3:24:1382:755#0/1
¥`[¥Y`¥SHY`aSaZZ¥^VT¥]`]XS_M_XM_aaXW[^W]
@HWI-EAS443:3:25:80:1724#0/1
TACTGATCCAAAATGGAAGGCTTTGTGCGACAATGCTTCA
+HWI-EAS443:3:25:80:1724#0/1
aba]Z__abba]^aa`aa__a___]PY__[_`_[Va^Y^a
@HWI-EAS443:3:26:1522:706#0/1
TACTGATCCAAAATGGAAGGCTTTGTGCGACAATGCTTCA
+HWI-EAS443:3:26:1522:706#0/1
`bRaaaa_``ab`a`aabY___`_aX]_a¥]``^`Y^¥]^
@HWI-EAS443:3:2:1130:60#0/1
TACTGATCCAAAATGGAAGGCTTTGTGCGACAATGCTTCA
+HWI-EAS443:3:2:1130:60#0/1
aaaabaa`_aa^_aaa``_a]aa_`¥a^aO¥^Y__]^]V^
@HWI-EAS443:3:32:1479:836#0/1
TACTGATCCAAAATGGAAGGCTTTGTGCGACAATGCTTCA
+HWI-EAS443:3:32:1479:836#0/1
aaba``babaaaZb¥[a`aa_`a^aW^Ma[Wa]_aOaa`a
@HWI-EAS443:3:32:829:999#0/1
TANTGATCCAAAATGGAAGGCTTTGTGCGACAATGCTTCA
+HWI-EAS443:3:32:829:999#0/1
]^D¥^aa^aa`[``XW`aa]_aa^V^aX]Za`aX_¥[ab`
```

@HWI-EAS443:3:3:136:722#0/1
TACTGATCCAAAATGGAAGGCTTTGTGCGACAATGCTTCA
+HWI-EAS443:3:3:136:722#0/1
ZT_aaU^U__Z^aaaa_]`^aaaaa]_Q¥]P_`^`V¥¥NX
@HWI-EAS443:3:3:1729:184#0/1
TACTGATCCAAAATGGAAGGCTTTGTGCGACAATGCTTCA
+HWI-EAS443:3:3:1729:184#0/1
a`aabaa_```aaaa^`aa^]```a__OXTM`^_]U^BBB
@HWI-EAS443:3:44:314:773#0/1
TACTGATCCAAAATGGAAGGCTTTGTGCGACAATGCTTCA
+HWI-EAS443:3:44:314:773#0/1
`aa`a_a]aba_`a`a`Sba_`aaa^a`aa]_`]a_``]a
@HWI-EAS443:3:47:1044:430#0/1
TACTGATCCAAAATGGAAGGCTTTGTGCGACAATGCTTCA
+HWI-EAS443:3:47:1044:430#0/1
^^abaX^``]baa_aaa]a`Ia``aab`aa`a`ab_Y^a^
@HWI-EAS443:3:49:1441:556#0/1
TACTGATCCAAAATGGAAGGCTTTGTGCGACAATGCTTCA
+HWI-EAS443:3:49:1441:556#0/1
^b_`¥a`a_aabbaa]a`aaXaa_]X``ba_aaaaY`a``
@HWI-EAS443:3:53:1023:1406#0/1
TACTGATCCAAAATGGAAGGCTTTGTGCGACAATGCTTCA
+HWI-EAS443:3:53:1023:1406#0/1
`aaaaaaaa`aa`aaa`aaaaaaa`¥a_aaa_`aa_Z`__
@HWI-EAS443:3:54:1608:1734#0/1
TACTGATCCAAAATGGAAGGCTTTGTGCGACAATGCTTCA
+HWI-EAS443:3:54:1608:1734#0/1
aa[abaa^Zaa`aaa_a^aa^aa[`_aaa`]]`a[H[[Q_
@HWI-EAS443:3:58:1612:413#0/1
TACTGATCCAAAATGGAAGGCTTTGTGCGACAATGCTTCA
+HWI-EAS443:3:58:1612:413#0/1
aa`aaa`QJ^```aa[X_a¥[a_`a^`__[R`^`aQNZVY
@HWI-EAS443:3:60:273:1049#0/1
TACTGATCCAAAATGGAAGGCTTTGTGCGACAATGCTTCA
+HWI-EAS443:3:60:273:1049#0/1
a`bSRTVaV[X^a_[aX]aaTPX]bN[___]_`]UZ]WPX
@HWI-EAS443:3:62:1269:1922#0/1
TACTGATCCAAAATGGAAGGCTTTGTGCGACAATGCTTCA
+HWI-EAS443:3:62:1269:1922#0/1
]Uaaabbaabb[b`abbb^X^a]ab`aaababbab``ba`
@HWI-EAS443:3:66:1271:814#0/1
TACTGATCCAAAATGGAAGGCTTTGTGCGACAATGCTTCA
+HWI-EAS443:3:66:1271:814#0/1
]¥`aa_abaa`bba`Y^`aa¥aaaa_a¥aa_`]_a]a`^a
@HWI-EAS443:3:67:1140:804#0/1
TACTGATCCAAAATGGAAGGCTTTGTGCGACAATGCTTCA
+HWI-EAS443:3:67:1140:804#0/1
abbbbbba^HZ`aa_a^[_a__aaa_ba``aaa`ba¥a^`
@HWI-EAS443:3:69:596:1728#0/1
TACTGATCCAAAATGGAAGGCTTTGTGCGACAATGCTTCA

+HWI-EAS443:3:69:596:1728#0/1
abaa`aabaaa`aa`¥^]¥aa_a`^__`_^`aaa____aa
@HWI-EAS443:3:74:1147:1466#0/1
TACTGATCCAAAATGGAAGGCTTTGTGCGACAATGCTTCA
+HWI-EAS443:3:74:1147:1466#0/1
]aaa`aVZbbaa_^[^`aT¥TWaa_[a_`a[a_]a_R][`
@HWI-EAS443:3:74:349:151#0/1
TACTGATCCAAAATGGAAGGCTTTGTGCGACAATGCTTCA
+HWI-EAS443:3:74:349:151#0/1
a]R^a^__`a_`_^`]__`_¥^_V_Z_X]^X^^¥^Z^ZZ^
@HWI-EAS443:3:76:253:601#0/1
TACTGATCCAAAATGGAAGGCTTTGTGCGACAATGCTTCA
+HWI-EAS443:3:76:253:601#0/1
aaaa``aabaa^````a]`aaaa``]```_Z`[^¥[¥^Z_
@HWI-EAS443:3:77:329:1145#0/1
TACTGATCCAAAATGGAAGGCTTTGTGCGACAATGCTTCA
+HWI-EAS443:3:77:329:1145#0/1
_bb``Z^bb_a`Z_aaa¥aa_ab]`Vba`^`a^a]]__a`
@HWI-EAS443:3:77:705:883#0/1
TACTGATCCAAAATGGAAGGCTTTGTGCGACAATGCTTCA
+HWI-EAS443:3:77:705:883#0/1
aY`a`a_b^_X¥]Yaa^a__a_`a_]__Y_W`_a`a`_`_
@HWI-EAS443:3:78:1185:1881#0/1
TACTGATCCAAAATGGAAGGCTTTGTGCGACAATGCTTCA
+HWI-EAS443:3:78:1185:1881#0/1
``[`aab``a[babaab`a]]Za`]`ZR¥]aa`¥b_^VT`
@HWI-EAS443:3:78:67:947#0/1
TACTGATCCAAAATGGAAGGCTTTGTGCGACAATGCTTCA
+HWI-EAS443:3:78:67:947#0/1
ababb`abbbbbaaaaa_a`ba``a_aa`__aaaa`_`_`
@HWI-EAS443:3:79:1481:75#0/1
TACTGATCCAAAATGGAAGGCTTTGTGCGACAATGCTTCA
+HWI-EAS443:3:79:1481:75#0/1
[_N¥QQQRbaZ]`b]baab¥]¥U¥b[¥aaSH]aa_P_MMV
@HWI-EAS443:3:81:209:1237#0/1
TACTGATCCAAAATGGAAGGCTTTGTGCGACAATGCTTCA
+HWI-EAS443:3:81:209:1237#0/1
abba]_`bbabaaaa`a`__aaaaaW`aa]_Z``aa_]aa
@HWI-EAS443:3:85:481:153#0/1
TACTGATCCAAAATGGAAGGCTTTGTGCGACAATGCTTCA
+HWI-EAS443:3:85:481:153#0/1
aaa`aaaa`aaaaa_a`ZV]]aa`aX_Ya_]`_]_]_]]^
@HWI-EAS443:3:86:1790:566#0/1
TACTGATCCAAAATGGAAGGCTTTGTGCGACAATGCTTCA
+HWI-EAS443:3:86:1790:566#0/1
]b`]ba_¥Y]bab^_V_aRJ]aaa_]_aWb^a``__`ZS]
@HWI-EAS443:3:87:1044:1024#0/1
TACTGATCCAAAATGGAAGGCTTTGTGCGACAATGCTTCA
+HWI-EAS443:3:87:1044:1024#0/1
a_aa`_a`aa]`aaaa[`]a_`aW]M_^__`__``^`_Za

```
@HWI-EAS443:3:92:1109:1480#0/1
TACTGATCCAAAATGGAAGGCTTTGTGCGACAATGCTTCA
+HWI-EAS443:3:92:1109:1480#0/1
]b¥a^[_````_¥¥b_aXZb^ba]_Yb`aa_aa`a`aa[_
@HWI-EAS443:3:95:1143:1162#0/1
TACTGATCCAAAATGGAAGGCTTTGTGCGACAATGCTTCA
+HWI-EAS443:3:95:1143:1162#0/1
WZ_UaZ¥_S_[___``_¥_b[`U_b^^_¥YFY^¥¥`_N[¥
@HWI-EAS443:3:99:620:1054#0/1
TACTGATCCAAAATGGAAGGCTTTGTGCGACAATGCTTCA
+HWI-EAS443:3:99:620:1054#0/1
aaWZa^a¥aa`^aab`a_aa[a_X_[a_aa_aa^a``a_a
@HWI-EAS443:3:69:1301:1515#0/1
CAAAATGGAAGGCTTTGTGCGACAANGCTTCAATCCAATG
+HWI-EAS443:3:69:1301:1515#0/1
aYa[ab¥^_b`bbbaa`V`[`aba[DWaababbbb`baa`
@HWI-EAS443:3:21:1138:913#0/1
ACTGATCCAAAATGGAAGGCTTTGTGCGACAATGCTTCAA
+HWI-EAS443:3:21:1138:913#0/1
a]XaaZa`]`]]`ZW_a¥ZZ^``_]¥O¥[^^^`_XZZ^a¥
@HWI-EAS443:3:31:1441:1615#0/1
ACTGATCCAAAATGGAAGGCTTTGTGCGACAATGCTTCAA
+HWI-EAS443:3:31:1441:1615#0/1
aaaaaa``aaaaaaaaa`a^_aaa]a^a_]_``aZ`^[^^
@HWI-EAS443:3:33:1466:876#0/1
ACTGATCCAAAATGGAAGGCTTTGTGCGACAATGCTTCAA
+HWI-EAS443:3:33:1466:876#0/1
aaaa`baaaaaaaaa`a`a_aaaaYaWa^_`_V`]^^T_]
@HWI-EAS443:3:42:513:1339#0/1
ACTGATCCAAAATGGAAGGCTTTGTGCGACAATGCTTCAA
+HWI-EAS443:3:42:513:1339#0/1
aaVQ]Pba_¥[^YRIR^V¥]_^__O]`a]Z_]_^Y]]_]_
@HWI-EAS443:3:48:472:1323#0/1
ACTGATCCAAAATGGAAGGCTTTGTGCGACAATGCTTCAA
+HWI-EAS443:3:48:472:1323#0/1
a¥baaabaaba`abaaaaa`aaa`_a]`aaaa_]_`a__`
@HWI-EAS443:3:50:1067:1421#0/1
ACTGATCCAAAATGGAAGGCTTTGTGCGACAATGCTTCAA
+HWI-EAS443:3:50:1067:1421#0/1
a¥aa_``X_`a`TN[aaTTTaaa¥SaYbZL_``aO_YFVY
@HWI-EAS443:3:59:351:1806#0/1
ACTGATCCAAAATGGAAGGCTTTGTGCGACAATGCTTCAA
+HWI-EAS443:3:59:351:1806#0/1
`^¥OVZ_]_YY_QU^_RD[NVPZXZ[aV``]Y]SWSU__Y
@HWI-EAS443:3:66:696:586#0/1
ACTGATCCAAAATGGAAGGCTTTGTGCGACAATGCTTCAA
+HWI-EAS443:3:66:696:586#0/1
]a¥_ZX]`XWWZWa¥ZXPX¥TT_S[`a`_[YLWY]P]SXU
@HWI-EAS443:3:67:1369:1262#0/1
ACTGATCCAAAATGGAAGGCTTTGTGCGACAATGCTTCAA
```

```
+HWI-EAS443:3:67:1369:1262#0/1
a`aaaaaaaa`a`aaa]_`_aa`_¥a``¥^`_a__`^V__
@HWI-EAS443:3:69:53:2036#0/1
ACTGATCCAAAATGGAAGGCTTTGTGCGACAATGCTTCAA
+HWI-EAS443:3:69:53:2036#0/1
_aUT^¥`_Z¥LK_^__[aZa`]^`PXWNV[^Z^]_]O_[N
@HWI-EAS443:3:6:454:1836#0/1
ACTGATCCAAAATGGAAGGCTTTGTGCGACAATGCTTCAA
+HWI-EAS443:3:6:454:1836#0/1
ab¥b]_baaaaZX]__aRZYV¥]YR`]^a^_]ZZ¥RQ¥¥_
@HWI-EAS443:3:70:243:173#0/1
ACTGATCCAAAATGGAAGGCTTTGTGCGACAATGCTTCAA
+HWI-EAS443:3:70:243:173#0/1
aa`a``aa`a]]`aa``_a__^_`^_]`_^[V]]]V^]][
@HWI-EAS443:3:78:678:502#0/1
ACTGATCCAAAATGGAAGGCTTTGTGCGACAATGCTTCAA
+HWI-EAS443:3:78:678:502#0/1
a^`b`aaa]a¥a_a`aaaZ^`a__^`_^ZS`]____¥[__
@HWI-EAS443:3:79:1073:90#0/1
ACTGATCCAAAATGGAAGGCTTTGTGCGACAATGCTTCAA
+HWI-EAS443:3:79:1073:90#0/1
]abbbbbabab`abaaaba_aaa_Wb`a`_^_aa^¥¥U`a
@HWI-EAS443:3:81:351:795#0/1
ACTGATCCAAAATGGAAGGCTTTGTGCGACAATGCTTCAA
+HWI-EAS443:3:81:351:795#0/1
aa]aaaaa^a^]Z`TR]`_^_`^`Sa_a`_X[¥`a^^`VU
@HWI-EAS443:3:97:881:186#0/1
ACTGATCCAAAATGGAAGGCTTTGTGCGACAATGCTTCAA
+HWI-EAS443:3:97:881:186#0/1
a_abaaaaa_`_aa`^[]_]a`aa^_Z`W^_^^aU`^X_^
@HWI-EAS443:3:98:1332:925#0/1
ACTGATCCAAAATGGAAGGCTTTGTGCGACAATGCTTCAA
+HWI-EAS443:3:98:1332:925#0/1
``ab_b__aaa`aaXaaaVJabba^a``]¥a``a¥]^Ga^
@HWI-EAS443:3:99:367:1081#0/1
ACTGATCCAAAATGGAAGGCTTTGTGCGACAATGCTTCAA
+HWI-EAS443:3:99:367:1081#0/1
a^Z__a_[_]O[_a_`[`^¥`_Y_ZY[[[NWZZ_X_^Q__
@HWI-EAS443:3:24:866:1889#0/1
GTGATCCAAAATGGAAGGCTTTGTGCGACAATGCTTCAAT
+HWI-EAS443:3:24:866:1889#0/1
IVa¥W[¥]``UY[^a`^aVZMI[KWa_]UK__aJONLWa`
@HWI-EAS443:3:28:1320:1203#0/1
CTGATCCAAAATGGAAGGCTTTGTGCGACAATGCTTCAAT
+HWI-EAS443:3:28:1320:1203#0/1
W`_aaaaabbbbba^```aaba_`_]__Uabb_YZ`]_^`
@HWI-EAS443:3:51:1015:314#0/1
CTGATCCAAAATGGAAGGCTTTGTGCGACAATGCTTCAAT
+HWI-EAS443:3:51:1015:314#0/1
abbbbabbabbbbbbabbbbbbbababbaabbbabbabbb
```

@HWI-EAS443:3:58:1122:259#0/1
CTGATCCAAAATGGAAGGCTTTGTGCGACAATGCTTCAAT
+HWI-EAS443:3:58:1122:259#0/1
abbabbba`aabbbbbababaab]a`baUaaab_a`aa`a
@HWI-EAS443:3:58:1629:674#0/1
CTGATCCAAAATGGAAGGCTTTGTGCGACAATGCTTCAAT
+HWI-EAS443:3:58:1629:674#0/1
abbababbbaabbbbaabaaaaa`a_aa_baaa`aaaaaa
@HWI-EAS443:3:66:971:213#0/1
CAAATGGAAGGCTTTGTGCGACAATGCTTCAATCCAATGA
+HWI-EAS443:3:66:971:213#0/1
aabaabaaababaXa_abba^abbbb_aa`ab`a]a`baa
@HWI-EAS443:3:73:1318:951#0/1
CTGATCCAAAATGGAAGGCTTTGTGCGACAATGCTTCAAT
+HWI-EAS443:3:73:1318:951#0/1
aaaababababaaaa`ba_a_abbb`b`aaabaab_aaaaa`
@HWI-EAS443:3:76:446:1210#0/1
CTGATCCAAAATGGAAGGCTTTGTGCGACAATGCTTCAAT
+HWI-EAS443:3:76:446:1210#0/1
ab`Tabbb```bbb`ababbbbbaaababbba`ba`aaaa
@HWI-EAS443:3:78:582:1000#0/1
CTGATCCAAAATGGAAGGCTTTGTGCGACAATGCTTCAAT
+HWI-EAS443:3:78:582:1000#0/1
aVX`[aa_[^V]ZT¥¥RW[V]]_]]]`_^]RU¥_]Z^^_¥
@HWI-EAS443:3:81:431:1219#0/1
TGCTTTGTGCGACAATGCTTCAATCCAATGATCGTCGAGC
+HWI-EAS443:3:81:431:1219#0/1
¥Zaaaa¥_ababaa_`aaaaa`]_aaaaaa`aba_`Y^^¥
@HWI-EAS443:3:89:1005:1007#0/1
CTGATCCAAAATGGAAGGCTTTGTGCGACAATGCTTCAAT
+HWI-EAS443:3:89:1005:1007#0/1
abb`b`baYb_a^baaaabab_a_aa`a]bbaaa`aaaa`
@HWI-EAS443:3:11:1276:1882#0/1
CAAAATGGAAGGCTTTGTGCGACAATGCTTCAATCCAATG
+HWI-EAS443:3:11:1276:1882#0/1
abb`abbab`W¥`a_b`Z_¥`aU`__YK[]Y`¥_¥XaaTa
@HWI-EAS443:3:15:648:296#0/1
GATCCAAAATGGAAGGCTTTGTGCGACAATGCTTCAATCC
+HWI-EAS443:3:15:648:296#0/1
ab_X^babaaab^a_]]aa¥__aaaaZa`a^`a`_Z]_^_
@HWI-EAS443:3:15:728:165#0/1
CAAAATGGAAGGCTTTGTGCGACAATGCTTCAATCCAATG
+HWI-EAS443:3:15:728:165#0/1
`bbaaaa`a^^XX_aa`]^U¥a_a_]_[^¥__a^R^_[]W
@HWI-EAS443:3:21:1391:1817#0/1
CAAAATGGAAGGCTTTGTGCGACAATGCTTCAATCCAATG
+HWI-EAS443:3:21:1391:1817#0/1
ab¥`^Zaaa_]Z¥QPZTORF¥_Q]__aUSUVa[]_^LRWZ
@HWI-EAS443:3:25:931:910#0/1
CCAAAATGGAAGGCTTTGTGCGACAATGCTTCAATCCAAT

```
+HWI-EAS443:3:25:931:910#0/1
abbb_bbabaaaabbba`aba```aaabaaa`aa_`aaa`
@HWI-EAS443:3:2:1726:1768#0/1
AGGCTTTGTGCGACAATGCTTCAATCCAATGATCGTCGAG
+HWI-EAS443:3:2:1726:1768#0/1
`aaMaaaaX`_aaU`_[a[_^^TY^TS_¥^]^[V_WN¥YU
@HWI-EAS443:3:30:1023:317#0/1
GATCCAAAATGGAAGGCTTTGTGCGACAATGCTTCAATCC
+HWI-EAS443:3:30:1023:317#0/1
aab]¥aaaaabaaaa`aaaaa^_Va`^a`^a_``^^`^R`
@HWI-EAS443:3:30:942:883#0/1
ATGGAAGGCTTTGTGCGACAATGCTTCAATCCAATGATCG
+HWI-EAS443:3:30:942:883#0/1
¥__^RZ_UZ_b_]Uaaaaaa`^aa_W]aaaa_]^][XHPa
@HWI-EAS443:3:31:434:233#0/1
AAAATGGAAGGCTTTGTGCGACAATGCTTCAATCCAATGA
+HWI-EAS443:3:31:434:233#0/1
a`WZ[aaaaab`[]`a``aa``aa_aaa_a`ZZ_¥`Y¥`_
@HWI-EAS443:3:34:727:922#0/1
AAATGGAAGGCTTTGTGCGACAATGCTTCAATCCAATGAT
+HWI-EAS443:3:34:727:922#0/1
^¥OHa_ZU`a__aa]Z`]T`_[P`TPY]]U¥[[[ZHQYBB
@HWI-EAS443:3:37:100:1253#0/1
CCAAAATGGAAGGCTTTGTGCGACAATGCTTCAATCCAAT
+HWI-EAS443:3:37:100:1253#0/1
ab`_]]X[W`¥V¥_a^`][ab`X`ZXS`a^R]Z`¥aa¥`^
@HWI-EAS443:3:38:1193:963#0/1
AAGGCTTTGTGCGACAATGCTTCAATCCAATGATCGTCGA
+HWI-EAS443:3:38:1193:963#0/1
aaa]aaaa`_a_`_aaaaa[`^`_a_a[ZWW¥¥¥Y^¥¥^W
@HWI-EAS443:3:39:1522:614#0/1
AAATGGAAGGCTTTGTGCGACAATGCTTCAATCCAATGAT
+HWI-EAS443:3:39:1522:614#0/1
``_aa]ab_[a`babaa^a[S`^¥`^`_a_UX[X``^[]W
@HWI-EAS443:3:40:1055:1653#0/1
ATGGAAGGCTTTGTGCGACAATGCTTCAATCCAATGATCG
+HWI-EAS443:3:40:1055:1653#0/1
a_`^`a^^`aa``]_`_aaa`^^_^]a_Y]^]__ZY¥Y_]
@HWI-EAS443:3:40:458:106#0/1
GGCTTTGTGCGACAATGCTTCAATCCAATGATCGTCGAGC
+HWI-EAS443:3:40:458:106#0/1
aba``ab^ba`a_`__a_¥^_[V]]]X^Y]]]]]Y]^]YR
@HWI-EAS443:3:45:431:578#0/1
CCAAAATGGAAGGCTTTGTGCGACAATGCTTCAATCCAAT
+HWI-EAS443:3:45:431:578#0/1
a`aTJ`^O_b_P^aaa]]VZ[__[`__¥Ua`[aV¥X^`^[
@HWI-EAS443:3:48:1185:1274#0/1
AGGCTTTGTGCGACAATGCTTCAATCCAATGATCGTCGAG
+HWI-EAS443:3:48:1185:1274#0/1
aaaaaabb`aaaaaaabaaaaaaaa`aaaaa``_a]^a_`
```

```
@HWI-EAS443:3:48:1734:1959#0/1
CCAAAATGGAAGGCTTTGTGCGACAATGCTTCAATCCAAT
+HWI-EAS443:3:48:1734:1959#0/1
`_LW^PV[QW]SXQZ^aa^`]a__a_``_]]_LKYRUVQ^
@HWI-EAS443:3:53:1143:1787#0/1
AGGCTTTGTGCGACAATGCTTCAATCCAATGATCGTCGAG
+HWI-EAS443:3:53:1143:1787#0/1
a`WO`ab_W]Zb_^aWaa_`^_a`aYaaaa``_`a_``_a
@HWI-EAS443:3:55:100:352#0/1
CAAAATGGAAGGCTTTGTGCGACAATGCTTCAATCCAATG
+HWI-EAS443:3:55:100:352#0/1
aaXUSU`]_Xa^Wa[aWSZ_[S[[_aa¥a][ZLR]TRNV`
@HWI-EAS443:3:55:1671:1424#0/1
GCTTTGTGCGACAATGCTTCAATCCAATGATCGTCGAGCT
+HWI-EAS443:3:55:1671:1424#0/1
aaaa_]_a]aa_aaaaYaa^`__]Z¥`aa`___YZ^^[W^
@HWI-EAS443:3:60:1443:777#0/1
GCTTTGTGCGACAATGCTTCAATCCAATGATCGTCGAGCT
+HWI-EAS443:3:60:1443:777#0/1
`]aa]b`a_a``aaa]_aa`a`_]_¥]^`_^_`^SaZaZ^
@HWI-EAS443:3:60:852:982#0/1
AAATGGAAGGCTTTGTGCGACAATGCTTCAATCCAATGAT
+HWI-EAS443:3:60:852:982#0/1
aaaaaaaaaa`aaaa_a_a_]aaaa_```a_a¥V¥```_`
@HWI-EAS443:3:62:55:820#0/1
CGATCATTGGATTGAAGCATTGTCGCACAAAGCCTTCCAT
+HWI-EAS443:3:62:55:820#0/1
ab]aaaaaaaaaaa_aaaaa`aZ^```_a`P¥``^^]_[^
@HWI-EAS443:3:63:1539:619#0/1
GGAAGGCTTTGTGCGACAATGCTTCAATCCAATGATCGTC
+HWI-EAS443:3:63:1539:619#0/1
`bb_¥Y`bb^¥]bbbabbbabbabaa[`[a^aaaa`aa[`
@HWI-EAS443:3:70:537:236#0/1
GCTTTGTGCGACAATGCTTCAATCCAATGATCGTCGAGCT
+HWI-EAS443:3:70:537:236#0/1
a`[^V`G]XVVMYTKPUS^¥SXV[R_SUQ[UTYNHST[MN
@HWI-EAS443:3:71:1290:123#0/1
CCAAAATGGAAGGCTTTGTGCGACAATGCTTCAATCCAAT
+HWI-EAS443:3:71:1290:123#0/1
^^b`_``b[[ba¥S_bbaVbaa_¥¥Z_abb``__bbbabb
@HWI-EAS443:3:7:930:1756#0/1
ATCCAAAATGGAAGGCTTTGTGCGACAATGCTTCAATCCA
+HWI-EAS443:3:7:930:1756#0/1
a`J^aaabaa_HWT`aaaV_Saa`a_a^`a_a_Ja_XP^B
@HWI-EAS443:3:82:1009:773#0/1
AGGCTTTGTGCGACAATGCTTCAATCCAATGATCGTCGAG
+HWI-EAS443:3:82:1009:773#0/1
aaaaaaaa`baaaaaaaaaaaaaaa``_^ab`a`a]^^_a
@HWI-EAS443:3:83:121:1718#0/1
AAAATGGAAGGCTTTGTGCGACAATGCTTCAATCCAATGA
```

```
+HWI-EAS443:3:83:121:1718#0/1
a]`a]TRWaa`_aa[][Y_Q]W]___a`^`a]¥Ua[QXVV
@HWI-EAS443:3:8:1141:544#0/1
CCAAAATGGAAGGCTTTGTGCGACAATGCTTCAATCCAAT
+HWI-EAS443:3:8:1141:544#0/1
`bbbbbbbbbaababbab`a[`a]b`ba_abaaabY]a`a
@HWI-EAS443:3:8:1200:247#0/1
AAAATGGAAGGCTTTGTGCGACAATGCTTCAATCCAATGA
+HWI-EAS443:3:8:1200:247#0/1
WW¥aabaUaa__baa_XY¥]aX`]`PRO]UX^_YN]aU_^
@HWI-EAS443:3:91:453:448#0/1
AAGGCTTTGTGCGACAATGCTTCAATCCAATGATCGTCGA
+HWI-EAS443:3:91:453:448#0/1
aaaaaaaaa_b^aa_aaa`aa_`_Y^`a_``a¥^__Y^a]
@HWI-EAS443:3:92:865:401#0/1
ATGGAAGGCTTTGTGCGACAATGCTTCAATCCAATGATCG
+HWI-EAS443:3:92:865:401#0/1
aa``aaaaa`aaa_a`aa[baaa_`a^a_a¥aa`aa¥^¥a
@HWI-EAS443:3:94:1369:956#0/1
AAATGGAAGGCTTTGTGCGACAATGCTTCAATCCAATGAT
+HWI-EAS443:3:94:1369:956#0/1
^`]abbaabaS`a_V`aIba[abaaSaa_aaa_^a``aaa
@HWI-EAS443:3:95:527:92#0/1
GGCTTTGTGCGACAATGCTTCAATCCAATGATCGTCGAGC
+HWI-EAS443:3:95:527:92#0/1
abbbbab^baababababb`abbbbbbbbbabbbabbbbab^
@HWI-EAS443:3:98:110:1290#0/1
CAAAATGGAAGGCTTTGTGCGACAATGCTTCAATCCAATG
+HWI-EAS443:3:98:110:1290#0/1
aababababbbbbaaaaa``[a__`aaaaa`aa^^`Y_a]__
@HWI-EAS443:3:98:853:1617#0/1
AGGCTTTGTGCGACAATGCTTCAATCCAATGATCGTCGAG
+HWI-EAS443:3:98:853:1617#0/1
_Z`aaaba]a_aa`aaa`b_aaa_a`a_a_```Wa`QY`^
@HWI-EAS443:3:9:469:29#0/1
GCTTTGTGCGACAATGCTTCAATCCAATGATCGTCGAGCT
+HWI-EAS443:3:9:469:29#0/1
a`a_ZJVa_[^]][^_[[Y]¥^RFTXXY^Y]U]XUVNYYB
@HWI-EAS443:3:9:864:1330#0/1
TTGGATTGAAGCATTGTCGCACAAAGCCTTCCATTTTGGA
+HWI-EAS443:3:9:864:1330#0/1
¥Z¥¥]bb]`¥WKaYX]¥X`ZJ_`a¥XW]`ZNPYT^[__[_
@HWI-EAS443:3:16:239:500#0/1
CCAAAATTGAACCATTCTTGAGGACGACACCACACACTCC
+HWI-EAS443:3:16:239:500#0/1
abZZa_OO`_¥a__[V¥X_`^a_``S¥`a_][`SX`¥TBB
@HWI-EAS443:3:24:813:315#0/1
GAGAAGCCTGAGGCGCTGCCTTCTTCAGTCTCTTCAGCAG
+HWI-EAS443:3:24:813:315#0/1
Z`abZbabWX_QYab_a^UaU¥Q`_¥_b[`Q__RG_[KKX
```

@HWI-EAS443:3:12:955:1399#0/1
CATCCGACGATTTTGCTCTCATAGTGAATGCACCAGACCA
+HWI-EAS443:3:12:955:1399#0/1
T¥b[`aU`bNaUbba]`ab[abbb[`ababb[aNbK`¥_`
@HWI-EAS443:3:44:387:160#0/1
CACTATGAGAGCAAAATCGTCAGATGATTGGAGCCCATCC
+HWI-EAS443:3:44:387:160#0/1
`aababb`aaaa`aa`aaa^[F^^`a]_`a_Y^]¥VMYWX
@HWI-EAS443:3:68:91:807#0/1
GGGTTGTTACCTCTATTTCCTCTTTGTTCATTGATTCCAT
+HWI-EAS443:3:68:91:807#0/1
^aa`¥^VXVI`a]a[[ba¥b]a``¥Z]__aaY^`^_^T_a
@HWI-EAS443:3:87:1081:1420#0/1
CGCTGCATCTGCACTCCCATTCGTTTCTGGGAGGCCTGAA
+HWI-EAS443:3:87:1081:1420#0/1
ab[^`_XV]OYM][HP^BBBBBBBBBBBBBBBBBBBBBBB
@HWI-EAS443:3:46:1402:507#0/1
CAAAATGACTATCGTCAACATCCACAGCACTCTGCTGTTC
+HWI-EAS443:3:46:1402:507#0/1
a[[_aaa]`bab[Z¥`aa]__`a[U¥VZ¥___a[Z`__`]
@HWI-EAS443:3:45:1114:1567#0/1
GCGAGCGAAAGCAGGTACTGATCCAAAATGGAAGGCTTTG
+HWI-EAS443:3:45:1114:1567#0/1
YG¥aU^bWa[aYYF^_WNNaaZ[YUYNNPXX_Z¥WQ[__]
@HWI-EAS443:3:97:841:2033#0/1
GGACCAGAGCGAAAGCAGGTCAAATATATTCAATATGGAG
+HWI-EAS443:3:97:841:2033#0/1
YLV¥MQa[YZ_a^aaXXZXNa¥^[aa¥^a`¥XW`aaaX_¥
@HWI-EAS443:3:18:926:288#0/1
CTCTCCATATTGAATATATTTGACCTGCTTTCGCTCTGGT
+HWI-EAS443:3:18:926:288#0/1
ababaabbbbbbbbbbbbbbaaaaaaaaaba`aa____a`¥
@HWI-EAS443:3:42:525:1264#0/1
ACCAGAGCGAAAGCAGGTCAAATATATTCAATATGGAGAG
+HWI-EAS443:3:42:525:1264#0/1
aa`aaa`_`a_aa``aa^^aa_a^`___]__`¥`_^Z`[`
@HWI-EAS443:3:5:162:275#0/1
CAGCGAGCGAAAGCAGGTCAAATATATTCAATATGGAGAG
+HWI-EAS443:3:5:162:275#0/1
abaaba`abba]aa_aa_^aa`aaa`aa_^`^a`aa^a^a
@HWI-EAS443:3:85:575:88#0/1
GTCTGGAGCGAAAGCAGGTCAAATATATTCAATATGGAGA
+HWI-EAS443:3:85:575:88#0/1
aa^aaa]a_a_`_a__`aW]__`______^_]_[___W`Z
@HWI-EAS443:3:35:751:215#0/1
CGAGAGCGAAAGCAGGTCAAATATATTCAATATGGAGAGA
+HWI-EAS443:3:35:751:215#0/1
aa¥aaaa[^^^^a[^aZba`[_P[```_^¥Z_```_`G¥P
@HWI-EAS443:3:93:454:1448#0/1
CGCGAGCGAAAGCAGGTCAAATATATTCAATATGGAGAGA

```
+HWI-EAS443:3:93:454:1448#0/1
abaa`abba_aaaaaa^bbaaaa`aaa_^^a`a``^a_aa
@HWI-EAS443:3:39:359:89#0/1
CAGGAGCGAAAGCAGGTCAAATATATTCAATATGGAGAGA
+HWI-EAS443:3:39:359:89#0/1
aabb`bbbaa`baaaa_a`_aa_a``a`GXa]^a_]a[aY
@HWI-EAS443:3:7:1481:863#0/1
GCGAGCGAAAGCAGGTCAAATATATTCAATATGGAGAGAA
+HWI-EAS443:3:7:1481:863#0/1
a_b_a^aa``a^aa_[^``_a^a^a`_`^a````[_]a[]
@HWI-EAS443:3:50:919:1843#0/1
GAGCGAAAGCAGGTCAAATATATTCAATATGGAGAGAATA
+HWI-EAS443:3:50:919:1843#0/1
`abaaabb`W`aaab`a^Y`abaa_`¥b_aa¥Ta_`^aab
@HWI-EAS443:3:61:1639:1608#0/1
AGCAGGTCAAATATATTCAATATGGAGAGAATAAAAGAAT
+HWI-EAS443:3:61:1639:1608#0/1
S]ab`[^_a___b]`^Q_UXaV¥_TGTXRYVbb``Z_VXV
@HWI-EAS443:3:74:174:1790#0/1
GAGCGAAAGCAGGTCAAATATATTCAATATGGAGAGAATA
+HWI-EAS443:3:74:174:1790#0/1
`ab`a^bbabbaa^bbbbbababababaaaa`]`aaaaaa
@HWI-EAS443:3:81:487:437#0/1
GGAGCGAAAGCAGGTCAAATATATTCAATATGGAGAGAAT
+HWI-EAS443:3:81:487:437#0/1
aa_a]baaaa`aaa]`aa`a`aaaa`^_a_`^`[[]a]__
@HWI-EAS443:3:100:263:1135#0/1
AATGGGACTTATGACTACCCAAAATACTCAGAGGAAACTT
+HWI-EAS443:3:100:263:1135#0/1
a``^YOU]¥_a`aa__ZHPS^[^]^[TWS]`^_]]]^^^B
@HWI-EAS443:3:37:1309:465#0/1
AGTGGGGACATCAAGATACGGCAAGAAGTTCAAGCCGGAA
+HWI-EAS443:3:37:1309:465#0/1
IZ`X¥`¥U_U_aZ__ba_¥H¥`a¥b¥¥`WF_V_ZVW_^_Z
@HWI-EAS443:3:56:1546:87#0/1
CGTTTCCTCTGAGTATTTTGGGTAGTCATAAGTCCCATTT
+HWI-EAS443:3:56:1546:87#0/1
`_MT`VJ``bbab]U[Y_ba`aW¥VYR`^RLY[QFJaaaa
@HWI-EAS443:3:73:1693:1805#0/1
AAAATGGGACTTATGACTACCCAAAATACTCAGAGGAAAC
+HWI-EAS443:3:73:1693:1805#0/1
a_a¥[a]`^_]``_``__^]Z_^^^¥_^[^^[]]YQQ^^V
@HWI-EAS443:3:75:998:262#0/1
GTGGGGACATCAAGATACGGCAAGAAGTTCAAGCCGGAAA
+HWI-EAS443:3:75:998:262#0/1
a_baaa[_aa_`aa`__YT_U_a[^^a]]Y_]¥MV``Z]^
@HWI-EAS443:3:76:1295:1843#0/1
GTTTCCTCTGAGTATTTTGGGTAGTCATAAGTCCCATTTT
+HWI-EAS443:3:76:1295:1843#0/1
^GY^[TY]][_[JY^RUYPRRK]ZYW]XZZ]R[Z]XZRXR
```

```
@HWI-EAS443:3:9:531:1678#0/1
GTCATGGGGAATATCTCAAACCTTTCAAATGATGACACTG
+HWI-EAS443:3:9:531:1678#0/1
`¥bNa`bba_Y¥^a¥a^abaQ`aba_aZ_aYaaRaab`¥_
@HWI-EAS443:3:62:401:1953#0/1
CGCCCTTTGGGCAGCTGTTTGAAATTTTCCTTTGAGGATA
+HWI-EAS443:3:62:401:1953#0/1
a^^a__QZU`a`Vab^^XO¥^XVXNXYaaa```_^Y`_¥X
@HWI-EAS443:3:72:1197:1127#0/1
GAAGGACGGACATCCGACATGCGGACAGAAGTTATAAGAA
+HWI-EAS443:3:72:1197:1127#0/1
aaa`aa`bb]]`a]_aU]a_a_ba`O_]Y`^G^L_]]a_^
@HWI-EAS443:3:20:1676:1746#0/1
CGCCAGACTTATGATTGGACATTAAATAGAAATCAACAGG
+HWI-EAS443:3:20:1676:1746#0/1
^b]aXb`]a_WI¥a`_bb`bbXXXbHGTW_``QR[_^I]a
@HWI-EAS443:3:3:361:613#0/1
ATGGACATAAAGAAGACAAGAGATATGGCCCAGCATTAAG
+HWI-EAS443:3:3:361:613#0/1
a``b^Vab]aabaab^`aa__a]Y_Wa__V[a__¥^]`Y_
@HWI-EAS443:3:71:101:1327#0/1
TAGACATAAAGAAGACAAGAGATATGGCCCAGCATTAAGC
+HWI-EAS443:3:71:101:1327#0/1
UI_baa¥XX¥Z]`]`aa^___a[a``¥aab`W[O^^Z^_`
@HWI-EAS443:3:30:1660:880#0/1
GACATAAAGAAGACAAGAGATATGGCCCAGCATTAAGCAT
+HWI-EAS443:3:30:1660:880#0/1
abaa`ab`aa^bbaaab`b¥^^a_Z]Xaa¥Y`__`__^]]
@HWI-EAS443:3:40:854:307#0/1
CATAAAGAAGACAAGAGATATGGCCCAGCATTAAGCATCA
+HWI-EAS443:3:40:854:307#0/1
abbabbbbabaaaaaabaab_baa_[H¥a`a`_U]_`_`_
@HWI-EAS443:3:48:1275:1511#0/1
ATAAAGAAGACAAGAGATATGGCCCAGCATTAAGCATCAA
+HWI-EAS443:3:48:1275:1511#0/1
abaaaba_aa`a]^^_[]`_a__]ZVYU^_^_W_X^_^_^
@HWI-EAS443:3:13:1144:186#0/1
CGCACTCAGAATGAAGTGGATGATGGCAATGAAATACCCA
+HWI-EAS443:3:13:1144:186#0/1
abaaaaaab``aaa_a]b`Q]a_^]a[Y^`___XW]VWR_
@HWI-EAS443:3:19:998:1726#0/1
TGCCACCATCAAGGGAGCAATTTTACAATCCTGGAGCTCT
+HWI-EAS443:3:19:998:1726#0/1
[X¥a`b`b]V`]_¥]]Xbb`ab^[^[b_a^Y^a_ZMJ__[
@HWI-EAS443:3:4:1087:2005#0/1
GTGGCCCACTTCAATTGGCTTGTTCTCTTCATGTTCTTTG
+HWI-EAS443:3:4:1087:2005#0/1
a¥`aZ]_a`[PUaaQX^]`XR[Z`MMURVUV¥VGXPN]SB
@HWI-EAS443:3:24:1685:1139#0/1
TGGCCCACTTCAATTGGCTTGTTCTCTTCATGTTCTTTGT
```

```
+HWI-EAS443:3:24:1685:1139#0/1
aaaabbaaaaababaa]_aaYY¥FZ]`bY]aV]aYbaa_^
@HWI-EAS443:3:69:616:377#0/1
CGGCCCACTTCAATTGGCTTGTTCTCTTCATGTTCTTTGT
+HWI-EAS443:3:69:616:377#0/1
abaaaaa`aaYa_]__`_`aRU^Y[VaSJQ¥[W]RZ__YZ
@HWI-EAS443:3:35:108:197#0/1
GGCCCACTTCAATTGGCTTGTTCTCTTCATGTTCTTTGTC
+HWI-EAS443:3:35:108:197#0/1
aaabaZaa``Z`a`aa`a_UW`¥^Z¥^[X¥[V^^_a`ZZ[
@HWI-EAS443:3:50:1237:706#0/1
GGCCCACTTCAATTGGCTTGTTCTCTTCATGTTCTTTGTC
+HWI-EAS443:3:50:1237:706#0/1
¥aa`aaaaa]X_aaaaaa^T[^T]U^^RW`X^¥O]aaZXO
@HWI-EAS443:3:67:130:807#0/1
GGCCCACTTCAATTGGCTTGTTCTCTTCATGTTCTTTGTC
+HWI-EAS443:3:67:130:807#0/1
Z^`baY^bbaVU`Y^_a^^GM_[``aa_T__Z_aaaZIX[
@HWI-EAS443:3:82:493:598#0/1
TGAGTCTTCTGTCAAAGAGAAAGACATGACCAAGGAATTC
+HWI-EAS443:3:82:493:598#0/1
^Z¥_^`abbabaa^I_b_[`_bbYabab`bb`a`]ab`aa
@HWI-EAS443:3:20:1580:1766#0/1
CTCAAAGGAAAATTTCAAACAGCTGCCCAAAGGGCAAAGG
+HWI-EAS443:3:20:1580:1766#0/1
aZ]VV¥ab^^`bbaYX^a^^`^`¥]`_[`[_a`]U]__^Y
@HWI-EAS443:3:91:622:1128#0/1
GAGGACTTGAATGGAATGGTAACACGGTTCGAGGCTCTGA
+HWI-EAS443:3:91:622:1128#0/1
ZIP[a[babZS^ZaTV¥a_VaaYRR[aZ[GVGZKV_Y[XZ
@HWI-EAS443:3:5:301:1315#0/1
TAGGGCGTGGATTGTCTCCGAAAATCCCACTGCATATATA
+HWI-EAS443:3:5:301:1315#0/1
[aba`]aabaaa`_[_¥b_`I][aaWP_a_aa]aa^^K_a
@HWI-EAS443:3:43:335:1344#0/1
CAAGGAAATTGGAAACGGCTGCTTTTAATTTTATCACAAA
+HWI-EAS443:3:43:335:1344#0/1
ab`babbabaaa_bbbba_b^YUb`IH¥Y_ba[FWbaba]
@HWI-EAS443:3:98:1587:881#0/1
CAAGGAAATTGGAAACGGCTGCTTTGAATTTTATAACAAA
+HWI-EAS443:3:98:1587:881#0/1
^bbbb^bbbb``aab`aabb`NZbb¥P_¥babHDFaUabb
@HWI-EAS443:3:15:861:693#0/1
AAGGAAATTGGAAACGGCTGCTTTGAATTTTATCACAAAA
+HWI-EAS443:3:15:861:693#0/1
_Y^abaT^_^_YV`R_XQXbRGWa__XV[YUFFRQWNBBB
@HWI-EAS443:3:25:1630:1629#0/1
AGGAAATTGGAAACGGCTGCTTTGAATTTTATCACAAATG
+HWI-EAS443:3:25:1630:1629#0/1
¥`[a^ab_¥[_`^^a_Y^_OO¥_HURW¥`¥RJY[][Y]BB
```

@HWI-EAS443:3:6:1271:1802#0/1
AGGAAATTGGAAACGGCTGCTTTGAATTTTATCACAAATG
+HWI-EAS443:3:6:1271:1802#0/1
`ba¥`^`^``bbb¥`¥Z^¥S]X_OXVU_Y_PPG`UBBBBB
@HWI-EAS443:3:58:858:1059#0/1
ATTTGTCCTTCCAGGGGCGGGGGGTCTTCGGGCTCTCGGA
+HWI-EAS443:3:58:858:1059#0/1
`a`b]Zbaaab]^baa```a][PYKPa[UODZWMTQN¥TL
@HWI-EAS443:3:48:1647:835#0/1
TGCAAAGCCAGAAGATTTGTCCTTCCAGGGGCGGGGGGTG
+HWI-EAS443:3:48:1647:835#0/1
OX`_^aab`X]NFZX¥_^baRRZ__```Z¥__Y¥Z[BBBB
@HWI-EAS443:3:95:1142:912#0/1
CGGGGGTCTTCGAGCTCTCGGACGAAAAGGCAACGAACCC
+HWI-EAS443:3:95:1142:912#0/1
`aa[ZR]V``[``a__`^Xaa`aaX^a__```a_[UYPFY
@HWI-EAS443:3:43:1254:146#0/1
GCAAAGCCAGAAGATTTGTCCTTCCAGGGGCGGGGGGTCT
+HWI-EAS443:3:43:1254:146#0/1
`_a^]^^^¥^^^aS^_V^W¥¥[V^U[_X[_BBBBBBBBBB
@HWI-EAS443:3:4:246:327#0/1
AGAAGATTTGTCCTTCCAGGGGCGGGGGGTCTTCGAGCTC
+HWI-EAS443:3:4:246:327#0/1
Za`^a`ababZ^__aaaabaaQ`]]T_FYYS]Y¥_TVNBB
@HWI-EAS443:3:38:953:1014#0/1
TCCGGCTTTCGTTTCATTACCAACACTACGTCCCCTTGCC
+HWI-EAS443:3:38:953:1014#0/1
aaba¥]Zaaaa¥__[^¥aa]^_a`a_[R`^UVTY¥[RR^W
@HWI-EAS443:3:73:805:763#0/1
GTGGACAGGGCCGAAGGTACCATGTTTCAATCTTTCAACC
+HWI-EAS443:3:73:805:763#0/1
a]`X`_````a`¥H[OYUZYaZU___`[aPDNTa¥ON]QX
@HWI-EAS443:3:15:1783:1240#0/1
CCCGCTTTCGTTTCATTACCAACACTACGTCACCTTGCCC
+HWI-EAS443:3:15:1783:1240#0/1
a_W^E^baRXQY`Uaa¥_SNa`]S]baZaWYFZPOPTWLY
@HWI-EAS443:3:59:170:662#0/1
CCCGCTTTCGGTTCATTACCAACACTACGTCCCCTTGCCC
+HWI-EAS443:3:59:170:662#0/1
¥H[WHJ]__OLYU¥WW^aZZ[J^X[OYYYWJFRNUPJWUW
@HWI-EAS443:3:28:125:148#0/1
GGCTTTCGTTTCATTACCAACACTACGTCCCCTTGCCCAA
+HWI-EAS443:3:28:125:148#0/1
aaa`aaaa]aa_``aa_``_`_`_[__YX__^¥XU_X]YY
@HWI-EAS443:3:13:1529:1177#0/1
GTCCCGCTTTCGTTTCATTACCAACACTACGTCCCCTTGC
+HWI-EAS443:3:13:1529:1177#0/1
^^aR[```UaRY[¥`_PS^aZ_^XH]¥_[R^JGPNPVVWN
@HWI-EAS443:3:17:217:1726#0/1
CGCTTTCGTTTCATTACCAACACTACGTCCCCTTGCCCAA

```
+HWI-EAS443:3:17:217:1726#0/1
abU[aYabJMZXXXPab`Sa]bTPaRG[_`ba[SNTPWTN
@HWI-EAS443:3:75:626:467#0/1
CCCGCTTTCGTTTCATTACCAACACTACGTCCCCTTGCCC
+HWI-EAS443:3:75:626:467#0/1
^Pa_FO`b`_V¥``SZ]X_^]__^WTa^aG[V[]US¥WS]
@HWI-EAS443:3:91:415:378#0/1
GTCCCGCTTTCGTTTCATTACCAACACTACGTCCCCTTGC
+HWI-EAS443:3:91:415:378#0/1
aY^U]_GQUZX`U¥]XT]X_NVGQUUGHYBBBBBBBBBBB
@HWI-EAS443:3:100:1780:1972#0/1
CATCGACGAACGGGGTGAATCAATAATTGTAGAATCTGGC
+HWI-EAS443:3:100:1780:1972#0/1
aWUb_a_b`a]a``UDZa[^[SS¥YVP[[OVWPHRBBBBB
@HWI-EAS443:3:49:339:301#0/1
CAATAATTGTAGAATCTGGCGACCCGAATGCACTATTGAA
+HWI-EAS443:3:49:339:301#0/1
_[Ubbbb¥`_¥^[URZbb`I^b¥babbb_[H_abbb¥X¥T
@HWI-EAS443:3:52:873:1065#0/1
TATTGCAGCAAACTTGTTAGTTTCGATTTTTGGATCTTCC
+HWI-EAS443:3:52:873:1065#0/1
aa__WWU`R^X`[a``N`[a^``U^U¥``[FRYHTON¥LP
@HWI-EAS443:3:94:195:1826#0/1
AGAAGATCCCAAGGACAAAGAACATGAAAAGAACAAGCCA
+HWI-EAS443:3:94:195:1826#0/1
`VaaWaaaa`a`]¥`aa]`a`_aa___`_`¥aaa]P[QQ[
@HWI-EAS443:3:47:1165:267#0/1
GATCGGAATACTGCCCGACATGACCCCAAGCATTAAGGAA
+HWI-EAS443:3:47:1165:267#0/1
a`aaa`aaa_`aa_^_a___a``W_ZZ^^_Q¥¥^UU__¥W
@HWI-EAS443:3:100:419:1525#0/1
ATTGGGCAAGGGGACGTAGTGCTGGGAATGAAACGAAAAC
+HWI-EAS443:3:100:419:1525#0/1
_baZ^`baaba_V__a`Z]V]FT`aN^[[a]a`GaVBBBB
@HWI-EAS443:3:62:767:1581#0/1
GTGCTGGTAATGAAACGAAAACGGGACTCTAGCATACTTA
+HWI-EAS443:3:62:767:1581#0/1
XVaWa`bZabbabbab_`^^aX__Xbabb_¥¥_baa¥__b
@HWI-EAS443:3:31:63:831#0/1
CGGGTGGGATTCTTCAAGGAAAGCCATAGCCTCTAGAACA
+HWI-EAS443:3:31:63:831#0/1
aaZ[S[]XO¥a`_]__`^_]_Z_`_Z]`a`]_]_Z¥V¥_`
@HWI-EAS443:3:37:689:143#0/1
AATAACAGACACTATCAAGAGTTGGAGACACAATATATTG
+HWI-EAS443:3:37:689:143#0/1
abaaaaaaaaaa`a]```a]a¥`a`Z]ZHQa¥X[`¥V[[^
@HWI-EAS443:3:60:1087:1685#0/1
CAGGGCAGAGAGAGGAATTGCCCGCTAATTTCACGGGAAC
+HWI-EAS443:3:60:1087:1685#0/1
TXVZTW`bb_aZ^`^^aTbaUba`P`a`babaZX`^Gab`
```

```
@HWI-EAS443:3:94:1638:1714#0/1
GTTGAAAGATACCCACGGTCGATTCGAGCCATGCCAGTTA
+HWI-EAS443:3:94:1638:1714#0/1
a_a[_[``^N`]¥S_T`[PFUS¥VS¥XTRNPPWNQV¥WWO
@HWI-EAS443:3:15:490:1530#0/1
AGATAACATGGTGGAATAGGAATGGCCCAACAACAAGTAC
+HWI-EAS443:3:15:490:1530#0/1
_bba¥aaabba_aaa`b]a]aaaaab[`aa__a[__`_a`
@HWI-EAS443:3:48:497:1068#0/1
TGGGGCTATTAGTATCAGATGGAGGACCAAACTTATACAA
+HWI-EAS443:3:48:497:1068#0/1
a]]`ba^ba_`aPWZ]X_[_``ab¥K¥RY_YG`aY_Z^_]
@HWI-EAS443:3:54:606:528#0/1
GGGGCTATTAGTATCAGATGGAGGACCAAACTTATACAAT
+HWI-EAS443:3:54:606:528#0/1
a`abaabaaabS_aa_a__aa^aa^_]_a_^aa``^]^`_
@HWI-EAS443:3:79:687:1536#0/1
ACAAAAGCAACAAAAATGAAGGCAATACTAGTAGTTTGTG
+HWI-EAS443:3:79:687:1536#0/1
abaU`[]aa````a`aXY`Y]]a][^^]_]^U¥XO]]]XU
@HWI-EAS443:3:14:1281:135#0/1
CGCAGCAAAAGCAGGGGAAAACAAAAGCAACAAAAATGAA
+HWI-EAS443:3:14:1281:135#0/1
a`_aa`aaa`a]]_aaa_____a```a_[KR_]_^^__]Z
@HWI-EAS443:3:40:517:57#0/1
AGCAAAAGCAGGGGAAAACAAAAGCAACAAAAANGAAGGC
+HWI-EAS443:3:40:517:57#0/1
¥WX[]¥YaX]a^]aY^a``__`Z]Z¥G[YY¥UVDXX]_¥_
@HWI-EAS443:3:35:1219:574#0/1
GGCAGGGGAAAACAAAAGCAACAAAAATGAAGGCAATACT
+HWI-EAS443:3:35:1219:574#0/1
aaaababba`aa_aaa¥a¥`a_a^__a`a`U`_aa_^_S]
@HWI-EAS443:3:74:95:379#0/1
GGCAAAAGCAGGGGAAAACAAAAGCAACAAAAATGAAGGC
+HWI-EAS443:3:74:95:379#0/1
[[¥[`SZZ__]YR`]P¥aWY_a¥^`_a^``WSV__[F[ZZ
@HWI-EAS443:3:94:365:1811#0/1
CAGCAAAAGCAGGGGAAAACAAAAGCAACAAAAATGAAGG
+HWI-EAS443:3:94:365:1811#0/1
aa`aaa^a_``a_aa_`aa`aaa`_aaa_aa_``a``^^¥
@HWI-EAS443:3:100:464:130#0/1
AGCAAAAGCAGGGGAAAACAAAAGCAACAAAAATGAAGGC
+HWI-EAS443:3:100:464:130#0/1
aaaba_aa]_aa`aaa_a_aa``aY`_^``¥___`____^
@HWI-EAS443:3:10:1266:1198#0/1
CAAAAGCAGGGGAAAACAAAAGCAACAAAAATGAAGGCAA
+HWI-EAS443:3:10:1266:1198#0/1
abbbbbbbbbbbbbabbbbaabaaa`aaaaaab_a`a^aa
@HWI-EAS443:3:10:437:1837#0/1
AAGCAGGGGAAAACAAAAGCAACAAAAATGAAGGCAATAC
```

```
+HWI-EAS443:3:10:437:1837#0/1
a`ZX_^W`aababaaa^^`__a^a`a`a_``__¥_a_[_[
@HWI-EAS443:3:13:983:531#0/1
GCAAAAGCAGGGGAAAACAAAAGCAACAAAAATGAAGGCA
+HWI-EAS443:3:13:983:531#0/1
aaaababaabbba```a_aaaaaa`aaa_]`_aba¥b`^a
@HWI-EAS443:3:14:39:556#0/1
CAGGGGAAAACAAAAGCAACAAAAATGAAGGCAATACTAG
+HWI-EAS443:3:14:39:556#0/1
aaa_aa[`_aaaaaaaaaaa`^aa]aaa`aa`_``a`_aa
@HWI-EAS443:3:14:515:1791#0/1
GGGGAAAACAAAAGCAACAAAAATGAAGGCAATACTAGTA
+HWI-EAS443:3:14:515:1791#0/1
aa_]babb`bbbaaabbabbabaaaaaaaabaaaaab`[b
@HWI-EAS443:3:15:765:142#0/1
AACAAAAGCAACAAAAATGAAGGCAATACTAGTAGTTCTG
+HWI-EAS443:3:15:765:142#0/1
aab`b`W`aa`aaa^`aa_`aa`]aaa`S`a^^a`Z`_^^
@HWI-EAS443:3:16:824:571#0/1
GGGAAAACAAAAGCAACAAAAATGAAGGCAATACTAGTAG
+HWI-EAS443:3:16:824:571#0/1
aaa`aaa`aa_aaa`_aa`_`_aa`__]_`[_`__`_^`_
@HWI-EAS443:3:18:1436:1097#0/1
AACAAAAGCAACAAAAATGAAGGCAATACTAGTAGTTCTG
+HWI-EAS443:3:18:1436:1097#0/1
SYST`[Yb`[a^^Zaaaa^]ab^V[[aY^[^][]aaa[Ta
@HWI-EAS443:3:19:919:275#0/1
GCAGGGGAAAACAAAAGCAACAAAAATGAAGGCAATACTA
+HWI-EAS443:3:19:919:275#0/1
_b^RY^^^_Z]ba`^bb[bba`aaaa`[ba_aXa[XaW``
@HWI-EAS443:3:21:1000:108#0/1
GGGGAAAACAAAAGCAACAAAAATGAAGGCAATACTAGTA
+HWI-EAS443:3:21:1000:108#0/1
a`^`aabaabbaaaaa]a]¥`^aa``aaa[aa¥TYY_]S_
@HWI-EAS443:3:21:1414:1685#0/1
AACAAAAGCAACAAAAATGAAGGCAATACTAGTAGTTCTG
+HWI-EAS443:3:21:1414:1685#0/1
_bbb[a_^aZ```aaa]babaab]aaa__]a__Y^¥`UaU
@HWI-EAS443:3:21:1569:1493#0/1
GCAGGGGAAAACAAAAGCAACAAAAATGAAGGCAATACTA
+HWI-EAS443:3:21:1569:1493#0/1
]¥`abVbZO]_YY_aaaa`a``[YZ^^]__^[Z^_a^M_a
@HWI-EAS443:3:22:1220:264#0/1
GAAAACAAAAGCAACAAAAATGAAGGCAATACTAGTAGTT
+HWI-EAS443:3:22:1220:264#0/1
^][``aaaY¥`a^^Xa``a_aa^_`a^___`Y¥`aY`a__
@HWI-EAS443:3:23:359:1045#0/1
GCCGGCTTCATTGAAGGGGGGTGGACAGGGATGGTAGATG
+HWI-EAS443:3:23:359:1045#0/1
`ba`^EY^aabb^Y]_a_¥M_M``]^^a``_¥a`W``U[_
```

```
@HWI-EAS443:3:25:364:996#0/1
CAAAAGCAGGGGAAAACAAAAGCAACAAAAATGAAGGCAA
+HWI-EAS443:3:25:364:996#0/1
V]``^Va_``Z_URR`babaaY`¥bbbb_a[^bbb`_bba
@HWI-EAS443:3:26:1255:1440#0/1
ACAAAAGCAACAAAAATGAAGGCAATACTAGTAGTTCTGC
+HWI-EAS443:3:26:1255:1440#0/1
a`ababa`ab`ba_aaaaaaaaaa``a^aa`]baZ__``_
@HWI-EAS443:3:27:1205:1614#0/1
CAAAAGCAGGGGAAAACAAAAGCAACAAAAATGAAGGCAA
+HWI-EAS443:3:27:1205:1614#0/1
SY_IYa`_`¥UZaXaaa^ISbb_^`abbbabaaaba]Lab
@HWI-EAS443:3:2:528:907#0/1
GGAAAACAAAAGCAACAAAAATGAAGGCAATACTAGTAGT
+HWI-EAS443:3:2:528:907#0/1
`aZSS^W_^YRF[aa_aa`a`]^`Z]TMT__``aRR_aa¥
@HWI-EAS443:3:32:1305:997#0/1
GCAGGGGAAAACAAAAGCAACAAAAATGAAGGCAATACTA
+HWI-EAS443:3:32:1305:997#0/1
`^]a^``[`^]`a`a_a_aa^`__]````_``_]]Z`_aa
@HWI-EAS443:3:35:1331:1717#0/1
CAAAAGCAGGGGAAAACAAAAGCAACAAAAATGAAGGCAA
+HWI-EAS443:3:35:1331:1717#0/1
abbaaaaaaaaaabaaabaa``_aa^a``ba`aa`a^^`a
@HWI-EAS443:3:38:670:1839#0/1
GCAGGGGAAAACAAAAGCAACAAAAATGAAGGCAATACTA
+HWI-EAS443:3:38:670:1839#0/1
abbb^¥]`bbaab_¥```aaabbabba]_¥_YP``abUa`
@HWI-EAS443:3:3:593:839#0/1
AACAAAAGCAACAAAAATGAAGGCAATACTAGTAGTTCTG
+HWI-EAS443:3:3:593:839#0/1
abbaa`aaabb`ab`aabbaaaba`aa`^aaa[aa``_``
@HWI-EAS443:3:42:1095:1506#0/1
GCAAAAGCAGGGGAAAACAAAAGCAACAAAAATGAAGGCA
+HWI-EAS443:3:42:1095:1506#0/1
`aaaaaaaa`a`__`ab_aa^``_a`_`a_`^___a____
@HWI-EAS443:3:46:1647:801#0/1
GAAAACAAAAGCAACAAAAATGAAGGCAATACTAGTAGTT
+HWI-EAS443:3:46:1647:801#0/1
a`a_aaaaaaa_aa_```_`aa]¥aa___a^]`_a]^_[_
@HWI-EAS443:3:46:50:1459#0/1
AACAAAAGCAACAAAAATGAAGGCAATACTAGTAGTTCTG
+HWI-EAS443:3:46:50:1459#0/1
_]aX``a`]__V¥aaa``a]a`]]`¥_¥¥a_SL¥][`^[Y
@HWI-EAS443:3:48:1388:1345#0/1
GGAAAACAAAAGCAACAAAAATGAAGGCAATACTAGTAGT
+HWI-EAS443:3:48:1388:1345#0/1
`a`aaaaaaaaa``a`a`a`_aa`_a```_``¥`a_^`_[
@HWI-EAS443:3:4:205:1064#0/1
GCAGGGGAAAACAAAAGCAACAAAAATGAAGGCAATACTA
```

```
+HWI-EAS443:3:4:205:1064#0/1
`ba]S]_¥¥VTD¥_aJ`aaZF¥WXXZ^a¥]a_L`Xa`_¥[
@HWI-EAS443:3:4:750:1049#0/1
AGCAGGGGAAAACAAAAGCAACAAAAATGAAGGCAATACT
+HWI-EAS443:3:4:750:1049#0/1
aa`aaabaaa`aYY__aa`a``a_aa_a__``_a`^_`Y^
@HWI-EAS443:3:50:1696:1668#0/1
AACAAAAGCAACAAAAATGAAGGCAATACTAGTAGTTCTG
+HWI-EAS443:3:50:1696:1668#0/1
abababa^`bba^aababaaaaaaaaaa_a_a_aa__UZY
@HWI-EAS443:3:50:1778:715#0/1
GGGGAAAACAAAAGCAACAAAAATGAAGGCAATACTAGTA
+HWI-EAS443:3:50:1778:715#0/1
ababaaaaaaaabb_ab_aaaaaba`aaaaaaaa_aaa]a
@HWI-EAS443:3:52:370:1130#0/1
GCAGGGGAAAACAAAAGCAACAAAAATGAAGGCAATACTA
+HWI-EAS443:3:52:370:1130#0/1
abab`a¥¥_aa^_U_`aaaa`aa`_a`a`]a`^`^a`]_`
@HWI-EAS443:3:53:1276:371#0/1
CAAAAGCAGGGGAAAACAAAAGCAACAAAAATGAAGGCAA
+HWI-EAS443:3:53:1276:371#0/1
^babbbabb`b`abbb`bbbba`bbbbabbbbbbbbb`a_
@HWI-EAS443:3:53:665:985#0/1
ACAAAAGCAACAAAAATGAAGGCAATACTAGTAGTTCTGC
+HWI-EAS443:3:53:665:985#0/1
aabbaa`baaaa`]aaabaabaaaaaa`aba^`a_a`aa^
@HWI-EAS443:3:55:757:1353#0/1
AAGCAGGGGAAAACAAAAGCAACAAAAATGAAGGCAATAC
+HWI-EAS443:3:55:757:1353#0/1
a^b`X^¥Z^aa`aaaaa]aaaaa`a_a_aa`aaaaaa`a`
@HWI-EAS443:3:58:1667:676#0/1
GAAAACAAAAGCAACAAAAATGAAGGCAATACTAGTAGTT
+HWI-EAS443:3:58:1667:676#0/1
a_a`a^````a^]a___a___a___a_a_aa^``a]`_^`
@HWI-EAS443:3:5:811:201#0/1
GCAAAAGCAGGGGAAAACAAAAGCAACAAAAATGAAGGCA
+HWI-EAS443:3:5:811:201#0/1
aa_Pa]__`¥WZ[^]Q_]S[`^^]¥]^]VW[Z]^XW]]BB
@HWI-EAS443:3:60:775:669#0/1
AAAAGCAGGGGAAAACAAAAGCAACAAAAATGAAGGCAAT
+HWI-EAS443:3:60:775:669#0/1
_]a``a^ba`baaaa`a^^aa]aa`a`Y`_aa``aa_`aa
@HWI-EAS443:3:61:812:334#0/1
GGGAAAACAAAAGCAACAAAAATGAAGGCAATACTAGTAG
+HWI-EAS443:3:61:812:334#0/1
aaa___`_a```___`_`¥[]IY^YZ^]]_^^][]ZUV^Y
@HWI-EAS443:3:62:212:1261#0/1
GGGGAAAACAAAAGCAACAAAAATGAAGGCAATACTAGTA
+HWI-EAS443:3:62:212:1261#0/1
`¥V^]¥_[T`]X_aZ_aa^^VUYYY¥L]TS`¥Y][Y`^WY
```

@HWI-EAS443:3:63:323:1840#0/1
GAAAACAAAAGCAACAAAAATGAAGGCAATACTAGTAGTT
+HWI-EAS443:3:63:323:1840#0/1
_bbaabbbabbbababa`baabb`aabbaaa`aaa^a[S`
@HWI-EAS443:3:69:268:1958#0/1
GGAAAACAAAAGCAACAAAAATGAAGGCAATACTAGTAGT
+HWI-EAS443:3:69:268:1958#0/1
a[a`U_`b¥Xa__T¥baaaaa__aa[_aa__```b_Ua^Y
@HWI-EAS443:3:70:685:250#0/1
GGGAAAACAAAAGCAACAAAAATGAAGGCAATACTAGTAG
+HWI-EAS443:3:70:685:250#0/1
abababbbbb`aabbbabbaabbba`aaaaaba`aaa_``
@HWI-EAS443:3:73:1287:1434#0/1
ACAAAAGCAACAAAAATGAAGGCAATACTAGTAGTTCTGC
+HWI-EAS443:3:73:1287:1434#0/1
a^aaaaaaa_¥__[a^`a_^aaa`¥`^]`¥^[a_Y__¥^[
@HWI-EAS443:3:76:367:831#0/1
AAAACAAAAGCAACAAAAATGAAGGCAATACTAGTAGTTC
+HWI-EAS443:3:76:367:831#0/1
ab¥bbbaabbbbbabbabaaaa_aaaaaa`aaaa]aa^_a
@HWI-EAS443:3:77:869:434#0/1
GCCGGCTTCATTGAAGGGGGGTGGACAGGGATGGTAGATG
+HWI-EAS443:3:77:869:434#0/1
a^[^XG¥_]aa`aS^Y`^]_a¥`]W`Qa_aGO_`P^PUSU
@HWI-EAS443:3:81:1278:84#0/1
AAGCAGGGGAAAACAAAAGCAACAAAAATGAAGGCAATAC
+HWI-EAS443:3:81:1278:84#0/1
_ab``^abb]Zaa`Uaba_X[W^`]`¥``_aXaaa_S]aG
@HWI-EAS443:3:81:670:1415#0/1
CAGGGGAAAACAAAAGCAACAAAAATGAAGGCAATACTAG
+HWI-EAS443:3:81:670:1415#0/1
aa^``aa^¥^aX¥^`aaaa`b¥^Y__`baaa`aa`aaa]`
@HWI-EAS443:3:84:1490:786#0/1
ACAAAAGCAACAAAAATGAAGGCAATACTAGTAGTTCTGC
+HWI-EAS443:3:84:1490:786#0/1
_[_O¥abaa^_`aa__aa`[aba``a__a^^^a_S]P]VZ
@HWI-EAS443:3:84:203:1732#0/1
AGCAAAAGCAGGGGAAAACAAAAGCAACAAAAATGAAGGC
+HWI-EAS443:3:84:203:1732#0/1
a```Za`^[Y_Z`]W_`a`Y¥_a]```Z__[_¥]^_^]O`
@HWI-EAS443:3:86:303:859#0/1
GCAAAAGCAGGGGAAAACAAAAGCAACAAAAATGAAGGCA
+HWI-EAS443:3:86:303:859#0/1
UURUNH[^^Yab`Y¥`Y`aSU[PZYYST__ZV^¥]abaVa
@HWI-EAS443:3:87:644:1860#0/1
GAAAACAAAAGCAACAAAAATGAAGGCAATACTAGTAGTT
+HWI-EAS443:3:87:644:1860#0/1
abbbbbbZ]aaabbabb¥`aaba_]abaaaa]aaa^aa_a
@HWI-EAS443:3:8:1665:15#0/1
GGAAAACAAAAGCAACAAAAATGAAGGCAATACTAGTAGT

```
+HWI-EAS443:3:8:1665:15#0/1
]a_`a`QT^a_a`QTGPZZRYXa`T¥aV¥^_YS^_[YZ_X
@HWI-EAS443:3:8:428:1649#0/1
ACAAAAGCAACAAAAATGAAGGCAATACTAGTAGTTCTGC
+HWI-EAS443:3:8:428:1649#0/1
aaaaaaa``a_a``a`a`^_____^_a[`a^[`_[]S^]Z
@HWI-EAS443:3:95:214:53#0/1
AACAAAAGCAACAAAAATGAAGGCAATACTAGTAGTTCTG
+HWI-EAS443:3:95:214:53#0/1
aa_aaaYaaaaY`a___aaY_ba_a`aa^_aaZ_`¥_W__
@HWI-EAS443:3:48:1218:196#0/1
GCAAAAGCAGGAGTTTAAAATGAATCCAAACCAAAAGATA
+HWI-EAS443:3:48:1218:196#0/1
a`a`_`a__aa¥a]aa]Y__``_^_^[¥^^YY¥]]Z]]]^
@HWI-EAS443:3:55:564:1421#0/1
CAAAAGCAGGAGTTTAAAATGAATCCAAACCAAAAGATAA
+HWI-EAS443:3:55:564:1421#0/1
aababb^abbaa[`aa`aaaaaaa``aaa`aa__aa^aa`
@HWI-EAS443:3:85:117:557#0/1
GCAAAAGCAGGAGTTTAAAATGAATCCAAACCAAAAGATA
+HWI-EAS443:3:85:117:557#0/1
aa^``U^aa_a`b_aa``_``aaa``^a``````^J`_`a
@HWI-EAS443:3:29:804:264#0/1
AAAGAGGGCCTTCTACAGAAGGAGTGCCTGAGTCCATGAG
+HWI-EAS443:3:29:804:264#0/1
`aaaaaaaa_`a__^]¥`_¥]a_aWa¥U`_¥aU¥^¥__U¥
@HWI-EAS443:3:88:824:1262#0/1
TGTTGATATTCTTCCCTCATGGACTCAGGCACTCCTTCTG
+HWI-EAS443:3:88:824:1262#0/1
IY[[_X^ZRQOXa]]R_¥UV¥]]]QLO_^[]_QIWH]¥HY
@HWI-EAS443:3:57:114:678#0/1
GGGGCCCTCTTTGCGTGCGATTGGACCAGGCGGGCATGGG
+HWI-EAS443:3:57:114:678#0/1
^bbb^b_V_^aa_a_Y^aaa]^``_`^^]BBBBBBBBBBB
@HWI-EAS443:3:18:809:1385#0/1
GCAGTTGCAACGGCATCATATTCCATGCTTTTGGCTGGAC
+HWI-EAS443:3:18:809:1385#0/1
¥FS_RZ`a^¥¥a_WTRYXX[ZZZZUJVTVWTUTYVUWSWN
@HWI-EAS443:3:92:797:154#0/1
CCAGTTGCTTCGAATGTTATTTTGTCTCCCGGCTCTATTA
+HWI-EAS443:3:92:797:154#0/1
aabb_bbbbaabaaababaaaaaa__`a_a``¥``_RIXS
@HWI-EAS443:3:81:1551:1521#0/1
GATTGCTTCAAATGAGAATGTGGAAACCATGGGCTCCAAT
+HWI-EAS443:3:81:1551:1521#0/1
a`V¥V¥`]_a_a`aabaaaa^aa__ZGXZ_aVJLPXYQZ^
@HWI-EAS443:3:51:1236:437#0/1
AGATCGGCTGCATATCCTGACCCCTGCTCATTTTGATGGT
+HWI-EAS443:3:51:1236:437#0/1
aaaa`abaa`a¥J^GUa`Y^ZT[[WNV__VNT_`[BBBBB
```

@HWI-EAS443:3:94:131:126#0/1
ATTCCCCAAGACAAGTTCATGGCCCAATCATGAATCGAAC
+HWI-EAS443:3:94:131:126#0/1
a¥aaa]^XabZY_`a_aaJY]PXSPKPTULRUVGYF[QQN
@HWI-EAS443:3:17:267:788#0/1
CAAAAGCAACAAAAATGAAGGCAATACTAGTAGTTCTGCT
+HWI-EAS443:3:17:267:788#0/1
aab`aaaaaa]aaaa_aaaaaba`aaa`aa]aa^`^_a__
@HWI-EAS443:3:18:1081:418#0/1
CAAAAATGAAGGCAATACTAGTAGTTCTGCTATATACATT
+HWI-EAS443:3:18:1081:418#0/1
aaSTX^a^WR¥XRV]aZWVaPER``[]_YNZ^W]WS``^T
@HWI-EAS443:3:1:1791:1583#0/1
AAGCAACAAAAATGAAGGCAATACTAGTAGTTCTGCTATA
+HWI-EAS443:3:1:1791:1583#0/1
¥a`PUaaaabV]`[a_]a_a_a[LFJX_a]_OL`ZQSWBB
@HWI-EAS443:3:36:985:747#0/1
CAAAAGCAACAAAAATGAAGGCAATACTAGTAGTTCTGCT
+HWI-EAS443:3:36:985:747#0/1
aababbbaaabbbaabbaabaabbbbaaba`aaaa`aaaa
@HWI-EAS443:3:52:332:1544#0/1
CAAAAGCAACAAAAATGAAGGCAATACTAGTAGTTCTGCT
+HWI-EAS443:3:52:332:1544#0/1
abbb`abbaabaaaaab`b`aaaaaa`aaa^aa^`_a_``
@HWI-EAS443:3:53:743:1379#0/1
AAGCAACAAAAATGAAGGCAATACTAGTAGTTCTGCTATA
+HWI-EAS443:3:53:743:1379#0/1
aU¥ab¥bbaVaaaa`Z_a__aa]¥aaa_aa`_a`__a```
@HWI-EAS443:3:92:504:1939#0/1
CAAAAGCAACAAAAATGAAGGCAATACTAGTAGTTCTGCT
+HWI-EAS443:3:92:504:1939#0/1
abbab¥bba_bbabaa`aa^a¥aaaa_`aa[a]_aa`___
@HWI-EAS443:3:61:1119:450#0/1
GTCCCGTTTTCGTTTCATTACCAACACTACATCCCCTTGG
+HWI-EAS443:3:61:1119:450#0/1
aXVWZaOZ]^^¥WX^XX^YW^YZX]_^XXTXXXUUSZPOG
@HWI-EAS443:3:33:1788:1324#0/1
AAAGCTAATGTGCTAATTGGGCAAGGGGATGTAGTGTTGG
+HWI-EAS443:3:33:1788:1324#0/1
aaababaaba_a_a_`b[aa`]`^aa^`]]aXZ]U_^¥_^
@HWI-EAS443:3:72:375:335#0/1
CCCGTTTTCGTTTCATTACCAACACTACATCCCCTTGCCC
+HWI-EAS443:3:72:375:335#0/1
aa`b_aabbb_aaaabaaaaaaaaaaa[FWaaa`]^W^]^
@HWI-EAS443:3:81:89:604#0/1
TGTAGTGTTGGTAATGAAACGAAAACGGGACTCTAGCATA
+HWI-EAS443:3:81:89:604#0/1
`_U]a]`^aa[QPP[WQ[_]XSJ¥`GSYLTUWVIPPW¥G]
@HWI-EAS443:3:91:1580:1603#0/1
AGCTAATGTGCTAATTGGGCAAGGGGATGTAGTGTTGGTA

```
+HWI-EAS443:3:91:1580:1603#0/1
Z[¥a[abb^[VZQX_`^^¥^V[`_bSPEZR`b[aI¥b]]_
@HWI-EAS443:3:59:768:1312#0/1
GAAATCTCCCTTTCGAAAGAGCAACCGTTATGGCAGCATT
+HWI-EAS443:3:59:768:1312#0/1
ab[``^`]a`UbVDUa_[_U¥`¥aa`a[]``SP]¥SHGUU
@HWI-EAS443:3:22:1408:956#0/1
AATGAATTCAATAAGGCATGTGAATTGACTGATTCAAGTT
+HWI-EAS443:3:22:1408:956#0/1
S_`YXX_a_a¥YZTFOS`a_XXT]`]SS]`VZ[XTM[[D[
@HWI-EAS443:3:90:718:601#0/1
GCTCAGTTCATTGATGCTTAATGCTGGGCCATCTCTTGTC
+HWI-EAS443:3:90:718:601#0/1
abbaa`Ya^`a`_``a`aa_aa^a__a^_^`a`^^__aZ_
@HWI-EAS443:3:21:1281:1242#0/1
CAAGAGATGGCCCAGCATTAAGCATCAATGAACTGAGCAA
+HWI-EAS443:3:21:1281:1242#0/1
a_O¥`aZTb][WZa^`Y````¥`WZ__^__Y_OR¥¥¥__^
@HWI-EAS443:3:22:912:662#0/1
CAAGAGATGGCCCAGCATTAAGCATCAATGAACTGAGCAA
+HWI-EAS443:3:22:912:662#0/1
_ZQ``^WaaaaX```_a`aa]__a_¥^``aY_W]^___ZX
@HWI-EAS443:3:2:1446:1024#0/1
CAAGAGATGGCCCAGCATTAAGCATCAATGAACTGAGCAA
+HWI-EAS443:3:2:1446:1024#0/1
abaaaa_a`aa`¥aa¥_aab``_`_^_aa___]^]__Y^Y
@HWI-EAS443:3:2:1603:1165#0/1
CAAGAGATGGCCCAGCATTAAGCATCAATGAACTGAGCAA
+HWI-EAS443:3:2:1603:1165#0/1
aa^aaa_aa]a^^a^¥a``a___^__X`aY[_]aZ^^G[^
@HWI-EAS443:3:3:53:1988#0/1
CAAGAGATGGCCCAGCATTAAGCATCAATGAACTGAGCAA
+HWI-EAS443:3:3:53:1988#0/1
]VbZ_]b__U_bO[aO[b_ab]]b`bbbba_`aW¥[_W^]
@HWI-EAS443:3:46:1320:1094#0/1
CAAGAGATGGCCCAGCATTAAGCATCAATGAACTGAGCAA
+HWI-EAS443:3:46:1320:1094#0/1
a`_`Ya`aab_Z__a`aaaa_a__a_`_``[[Z^`^`¥`_
@HWI-EAS443:3:49:1231:1129#0/1
CAAGAGATGGCCCAGCATTAAGCATCAATGAACTGAGCAA
+HWI-EAS443:3:49:1231:1129#0/1
abaaab`_abaaaaa_aa_a`__^``ZV_a_[Va`__Z`W
@HWI-EAS443:3:69:1071:400#0/1
CAAGAGATGGCCCAGCATTAAGCATCAATGAACTGAGCAA
+HWI-EAS443:3:69:1071:400#0/1
a`]a``aaba^b]`a`¥^aa``[`aa__aa___a¥XW_`a
@HWI-EAS443:3:89:925:45#0/1
TTGCTCAGTTCATTGATGCTTAATGCTGGGCCATCTCTTG
+HWI-EAS443:3:89:925:45#0/1
abb`[`aa_a]ababaaa^^_a^Wa]`aa`^Xa``¥_¥`a
```

```
@HWI-EAS443:3:1:554:751#0/1
AAGAGATGGCCCAGCATTAAGCATCAATGAACTGAGCAAT
+HWI-EAS443:3:1:554:751#0/1
aaa`b___a_Sa`a]a_`a`__``X````Z_Z]`^`W`BB
@HWI-EAS443:3:54:160:839#0/1
ATTGCTCAGTTCATTGATGCTTAATGCTGGGCCATCTCTT
+HWI-EAS443:3:54:160:839#0/1
aaababaaa^baabaaaaa_`a^aaaa^a]a`_````_`_
@HWI-EAS443:3:69:702:83#0/1
AAGAGATGGCCCAGCATTAAGCATCAATGAACTGAGCAAT
+HWI-EAS443:3:69:702:83#0/1
`]aaa`aaa_]_^a]aa`_¥a`_]W¥]a_^]Y__]^PZ^^
@HWI-EAS443:3:98:87:1876#0/1
ATTGCTCAGTTCATTGATGCTTAATGCTGGGCCATGTCTT
+HWI-EAS443:3:98:87:1876#0/1
Q_]HY__VXO]_W¥Y_[OY_^Y_T^^¥IO¥ZS¥ZRG¥¥ZM
@HWI-EAS443:3:54:153:1043#0/1
CAGGGCTATTAGTATCAGATGGAGGACCAAACTTATACAA
+HWI-EAS443:3:54:153:1043#0/1
a]¥b_]a_P]Y_Yaaa_¥Y`a_P_`Y¥R]`_¥_`^__]_a
@HWI-EAS443:3:95:426:1246#0/1
TGGCAATGAGATACCCAATTACAGCGGACAAGAGAAAGCT
+HWI-EAS443:3:95:426:1246#0/1
aa`_a`abab_abaaaa`a_a`aaQDXa^_Y`]a_^¥aZa
@HWI-EAS443:3:3:1497:1732#0/1
AGGTAATATATGTGGACTTCCCCCCGTGTTACTCCAATTT
+HWI-EAS443:3:3:1497:1732#0/1
^_^S_baW^aa[Ra_XF_a]aaVZ[``bPL__a_a^¥aU]
@HWI-EAS443:3:97:597:869#0/1
CACGGAGGGAAGTCCACATATATTACCTATAGAAAGCCAA
+HWI-EAS443:3:97:597:869#0/1
aa__b^bbbY```aa``aaa_Y``a]`_aa`aZ__a_`S_
@HWI-EAS443:3:12:1057:1212#0/1
ATATATTACCTAGAGAAAGCCAACAAAATAAAATCTGAGA
+HWI-EAS443:3:12:1057:1212#0/1
`a_Y^^[aaYaXU`¥X`Z`]a_`_a``_`a^_``_^___`
@HWI-EAS443:3:15:1243:793#0/1
GTTGGCTTTCTCTAGGTAATATATGTGGACTTCCCTCCGT
+HWI-EAS443:3:15:1243:793#0/1
`_babab`aa`_Y```ZY_a_a_aa¥aa_Z]_R¥[a_Za^
@HWI-EAS443:3:15:391:93#0/1
GAAGTCCACATATATTACCTAGAGAAAGCCAACAAAATAA
+HWI-EAS443:3:15:391:93#0/1
abba``abaabab`ba¥aaaaaaba`aa_`aaZ`T_^a``
@HWI-EAS443:3:15:753:609#0/1
GAGGGAAGTCCACATATATTACCTAGAGAAAGCCAACAAA
+HWI-EAS443:3:15:753:609#0/1
aba`baab_a`a_ba_aaaaaaaaaaa__aa`]_`a_a^a
@HWI-EAS443:3:17:1688:321#0/1
AGTCCACATATATTACCTAGAGAAAGCCAACAAAATAAAA
```

+HWI-EAS443:3:17:1688:321#0/1
a`WVP_]I````Y`aYR[¥`WaX^_^RW¥¥[]_]Y]Y¥^^
@HWI-EAS443:3:17:314:1036#0/1
AGGTAATATATGTGGACTTCCCTCCGTGTTACTCCAATTT
+HWI-EAS443:3:17:314:1036#0/1
[a^Z^`aaaa____QX_a]_Z_a`a`Za^Z_[__¥_P¥W_
@HWI-EAS443:3:17:380:1028#0/1
TCCACATATATTACCTAGAGAAAGCCAACAAAATAAAATC
+HWI-EAS443:3:17:380:1028#0/1
_baaa¥bb`_a`ba`¥`b`a^b`aV`a¥G`[ababWb_a_
@HWI-EAS443:3:19:641:1745#0/1
GTCCACATATATTACCTAGAGAAAGCCAACAAAATAAAAT
+HWI-EAS443:3:19:641:1745#0/1
aW]MY[X¥]SS_RTZY_YOSX^¥[¥aa^^]``OVZ[ZW¥X
@HWI-EAS443:3:1:208:549#0/1
ACATATATTACCTAGAGAAAGCCAACAAAATAAAATCTGA
+HWI-EAS443:3:1:208:549#0/1
aaa`a``aaa__aaaa`aa_a^``^``_^^a^a_^_YaaB
@HWI-EAS443:3:22:378:1664#0/1
GTCCACATATATTACCTAGAGAAAGCCAACAAAATAAAAT
+HWI-EAS443:3:22:378:1664#0/1
`]```a`aV_ZWP]ba`¥^`_^^`Y]_``^S]W`^aW_a_
@HWI-EAS443:3:23:1014:228#0/1
GAAGTCCACATATATTACCTAGAGAAAGCCAACAAAATAA
+HWI-EAS443:3:23:1014:228#0/1
a_aaa`Ya_aaaaaaaaa_a_`aa_`]``_`__`^__``_
@HWI-EAS443:3:23:127:721#0/1
GGCTTTCTCTAGGTAATATATGTGGACTTCCCTCCGTGTT
+HWI-EAS443:3:23:127:721#0/1
aaaaaaabaaaaaNa`aaa_a`[]a_a`_a_`^aa`S_]_
@HWI-EAS443:3:24:606:1610#0/1
GGTAATATATGTGGACTTCCCTCCGTGTTACTCCAATTTC
+HWI-EAS443:3:24:606:1610#0/1
`b^b]babbaa_¥a`aabaab_`___``a``_`aaL[aaa
@HWI-EAS443:3:26:356:1823#0/1
CATATATTACCTAGAGAAAGCCAACAAAATAAAATCTGAG
+HWI-EAS443:3:26:356:1823#0/1
``[N]Waa]abbaVY]V_^Qa¥aab``YY^`X^^V¥_a_]
@HWI-EAS443:3:26:639:854#0/1
AACACGGAGGGAAGTCCACATATATTACCTAGAGAAAGCC
+HWI-EAS443:3:26:639:854#0/1
abba_abaa^a¥^`Ua[]aaa`a`^_a]_aaa___]_a¥Y
@HWI-EAS443:3:28:1634:427#0/1
GAGGGAAGTCCACATATATTACCTAGAGAAAGCCAACAAA
+HWI-EAS443:3:28:1634:427#0/1
aZY_`Z^Z¥RV]`]]_`_a`[]]aT_]`]^[aVX^^V^^]
@HWI-EAS443:3:37:699:993#0/1
CTAGGTAATATATGTGGACTTCCCTCCGTGTTACTCCAAT
+HWI-EAS443:3:37:699:993#0/1
abVabP^]Z`aba]Va^YMG]bb`^a__V_VWW_[^ZUXR

```
@HWI-EAS443:3:3:108:81#0/1
AGGTAATATATGTGGACTTCCCTCCGTGTTACTCCAATTT
+HWI-EAS443:3:3:108:81#0/1
aaa`abbaaaaa__]_aaaa]`aa_a_a``_¥a`^^¥``^
@HWI-EAS443:3:40:942:1318#0/1
ACGGAGGGAAGTCCACATATATTACCTAGAGAAAGCCAAC
+HWI-EAS443:3:40:942:1318#0/1
`¥bb^YO^XY¥U``S[Y[X[`^X`^_V`__[_a`¥[^W^¥
@HWI-EAS443:3:45:1196:1618#0/1
TGGCTTTCTCTAGGTAATATATGTGGACTTCCCTCCGTGT
+HWI-EAS443:3:45:1196:1618#0/1
aaa_abb`a_b_a`¥aaa`a`a__`_`]a_[^_a_X`]`^
@HWI-EAS443:3:45:358:265#0/1
GCTTTCTCTAGGTAATATATGTGGACTTCCCTCCGTGTTA
+HWI-EAS443:3:45:358:265#0/1
a[aaa`b_aa[QSa`aabaa]`aaY]aa__`___a^aX__
@HWI-EAS443:3:45:474:303#0/1
GTTGGCTTTCTCTAGGTAATATATGTGGACTTCCCTCCGT
+HWI-EAS443:3:45:474:303#0/1
a¥aaaaaaa``_`_aaW__^_`]__^`a[^___[]^`X_Y
@HWI-EAS443:3:46:684:2008#0/1
GGTAATATATGTGGACTTCCCTCCGTGTTACTCCAATTTC
+HWI-EAS443:3:46:684:2008#0/1
a`¥__^aaaa`]a`]aa]_¥]`_X[W^^^]R[Y___]]¥U
@HWI-EAS443:3:47:1628:543#0/1
GAAGTCCACATATATTACCTAGAGAAAGCCAACAAAATAA
+HWI-EAS443:3:47:1628:543#0/1
a`aa`a`a_aa``_a_a`Y__`[`___]^MX^]^]U_^^^
@HWI-EAS443:3:48:958:49#0/1
CATATATTACCTAGAGAAAGCCAACAAAATAAAATCTGAG
+HWI-EAS443:3:48:958:49#0/1
ab_bbbab`^Z`bbba_Yab]`bRK__abba¥aa`H^b_a
@HWI-EAS443:3:51:1248:1055#0/1
CACATATATTACCTAGAGAAAGCCAACAAAATAAAATCTG
+HWI-EAS443:3:51:1248:1055#0/1
]`bbbabbbaba]abbaaaaa`¥a`aababbbaabbb_ba
@HWI-EAS443:3:54:283:1906#0/1
AACACGGAGGGAAGTCCACATATATTACCTAGAGAAAGCC
+HWI-EAS443:3:54:283:1906#0/1
ab^a`¥[¥]aaXU_U[]a^a`a^]a¥_^_a[S___`^UP`
@HWI-EAS443:3:56:1239:385#0/1
GTTGGCTTTCTCTAGGTAATATATGTGGACTTCCCTCCGT
+HWI-EAS443:3:56:1239:385#0/1
a`bbaaabb`aab_aaYaaaaaaa``a`_^aa_¥`a`¥`_
@HWI-EAS443:3:56:217:2024#0/1
GTTGGCTTTCTCTAGGTAATATATGTGGACTTCCCTCCGT
+HWI-EAS443:3:56:217:2024#0/1
aaabaabbbaaaa_^_[bbaaaaa^^aa__aaaa_`a_aY
@HWI-EAS443:3:57:1515:1753#0/1
GTCCACATATATTACCTAGAGAAAGCCAACAAAATAAAAT
```

```
+HWI-EAS443:3:57:1515:1753#0/1
]H[`Y[_b¥a`a`_XW^]a[]]]Y¥JOY_^`][Va^^_Z^
@HWI-EAS443:3:57:803:620#0/1
CACGGAGGGAAGTCCACATATATTACCTAGAGAAAGCCAA
+HWI-EAS443:3:57:803:620#0/1
a_Y_XUX`aaaa¥¥]```aaaaa`__^a`aaa`^a`X_a`
@HWI-EAS443:3:57:999:1524#0/1
ACACGGAGGGAAGTCCACATATATTACCTAGAGAAAGCCA
+HWI-EAS443:3:57:999:1524#0/1
aUa``aa`bb__bYXZa__a`[a`_a`_aaa__^a_`^^_
@HWI-EAS443:3:60:860:619#0/1
TCCACATATATTACCTAGAGAAAGCCAACAAAATAAAATC
+HWI-EAS443:3:60:860:619#0/1
]XTH[X`baaaaba`^a_abaaa^`a`^^`aa_a¥¥aaaZ
@HWI-EAS443:3:61:470:132#0/1
TCCACATATATTACCTAGAGAAAGCCAACAAAATAAAATC
+HWI-EAS443:3:61:470:132#0/1
aaaaaaaaa`baaaa`¥aa`a_]_a`aZ¥``_``__]^^W
@HWI-EAS443:3:63:1123:1492#0/1
GAGGGAAGTCCACATATATTACCTAGAGAAAGCCAACAAA
+HWI-EAS443:3:63:1123:1492#0/1
`_¥S[¥¥[HNaaR]¥¥^a^a]_Y`a`]baa_a^`¥^]ZZY
@HWI-EAS443:3:66:680:101#0/1
CACATATATTACCTAGAGAAAGCCAACAAAATAAAATCTG
+HWI-EAS443:3:66:680:101#0/1
abbbbbbbbababbabaaba^ba`aa_aaa^aaaaaa]aa
@HWI-EAS443:3:6:569:1490#0/1
CACATATATTACCTAGAGAAAGCCAACAAAATAAAATCTG
+HWI-EAS443:3:6:569:1490#0/1
XM`US_a_a_a`^b`b^¥aX`aaaaa_aa_b`Yaba`T_`
@HWI-EAS443:3:72:1201:895#0/1
CACATATATTACCTAGAGAAAGCCAACAAAATAAAATCTG
+HWI-EAS443:3:72:1201:895#0/1
abbb_a^_`aba`bab[baaa_[aba_X_a_bbaa_aY^_
@HWI-EAS443:3:76:451:847#0/1
CTCAGATTTTATTTTGTTGGCTTTCTCTAGGTAATATATG
+HWI-EAS443:3:76:451:847#0/1
ab`^`a]`aaZ^aa`_aZ__a__[¥^_[I^aX]W[___^`
@HWI-EAS443:3:78:1370:154#0/1
AGTCCACATATATTACCTAGAGAAAGCCAACAAAATAAAA
+HWI-EAS443:3:78:1370:154#0/1
a_[Q___aa_aa_`_ZZa_a_`]_]]][[VN^_^¥][^][
@HWI-EAS443:3:82:1778:912#0/1
GTCCACATATATTACCTAGAGAAAGCCAACAAAATAAAAT
+HWI-EAS443:3:82:1778:912#0/1
Y_^^``a^a`baabaY_bba^aX`aabaaa_aaaa`aaa`
@HWI-EAS443:3:83:1101:209#0/1
CTAGGTAATATATGTGGACTTCCCTCCGTGTTACTCCAAT
+HWI-EAS443:3:83:1101:209#0/1
a`XZ`_`]^`b_a_]``USa`^¥Ya[Ya^``_]P¥Y^`^`
```

```
@HWI-EAS443:3:88:1307:220#0/1
AGGTAATATATGTGGACTTCCCTCCGTGTTACTCCAATTT
+HWI-EAS443:3:88:1307:220#0/1
]a¥^babbbabb]b_aabb_P_`aabaaab¥Q_[Xb``a_
@HWI-EAS443:3:88:533:913#0/1
CACATATATTACCTAGAGAAAGCCAACAAAATAAAATCTG
+HWI-EAS443:3:88:533:913#0/1
Ua`^a`¥Xa¥aab`¥`a^abbba_`_Y_`_¥a_XM_bb^a
@HWI-EAS443:3:92:330:1336#0/1
GAAGTCCACATATATTACCTAGAGAAAGCCAACAAAATAA
+HWI-EAS443:3:92:330:1336#0/1
aba`_abbaaa`aabbaa`aababbbaa``a_`aaaaa`a
@HWI-EAS443:3:92:384:1188#0/1
CACGGAGGGAAGTCCACATATATTACCTAGAGAAAGCCAA
+HWI-EAS443:3:92:384:1188#0/1
aMaaaa¥aW_aaOaaa``a``__`_`^`a^```aZ]^_`]
@HWI-EAS443:3:93:1486:1443#0/1
ATTGGAGTAACACGGAGGGAAGTCCACATATATTACCTAG
+HWI-EAS443:3:93:1486:1443#0/1
X__Y_^b¥a`^VU^ba_aaW^aV¥]]`^`_aaaa`OJ[[a
@HWI-EAS443:3:77:867:1207#0/1
AAAATGAAGGCAATACTAGTAGTTCTGCTATATACATTTG
+HWI-EAS443:3:77:867:1207#0/1
aa]a^SZaVa`X`[_baaZNXa__`_```a_aa_a_^_^^
@HWI-EAS443:3:38:339:501#0/1
CGAATGTTATTTTGTCTCCCGGCTCTACCAGTTTCCAGTA
+HWI-EAS443:3:38:339:501#0/1
aa[HUVPSOU^`^[ORYOT]^__BBBBBBBBBBBBBBBBB
@HWI-EAS443:3:31:613:294#0/1
GCCAGTTGCTTCGAATGTTATTTTGTCTCCCGGCTCTACC
+HWI-EAS443:3:31:613:294#0/1
aa^[[I[[^[aaXVYX_LYQ]a[¥MFV¥]X^S_BBBBBBB
@HWI-EAS443:3:30:1291:2019#0/1
ATGGGTGTCTTTCAACCAGAATCTGGAATATCAGATAGGA
+HWI-EAS443:3:30:1291:2019#0/1
a¥_`_¥Z^aa__aaa¥][__Za^`a`^][]_Z_][WY_P]
@HWI-EAS443:3:25:1589:1238#0/1
GGGATGCAATTTTCCTCATTGACTGTGAATGTGAGAGGAT
+HWI-EAS443:3:25:1589:1238#0/1
aa`Y`]X_^]]aZXaaZD^]`X]]^XaU^Xa[_^XFW_¥R
@HWI-EAS443:3:77:1243:288#0/1
GAAAATTGCATCCCACTCTGTTCTGGTGGAGCAGCAGCAA
+HWI-EAS443:3:77:1243:288#0/1
a`]]aaaa`aaUMFW]a[``^`X``_V__]VUY]]]ZNTV
@HWI-EAS443:3:35:583:798#0/1
GAAAAGAGAAAGCCAACAAAATAAAATCTGAGAAGACACA
+HWI-EAS443:3:35:583:798#0/1
_^OZaa`aXXT`__R__a`^[aaa`Z^`_`_`¥_^_``U_
@HWI-EAS443:3:14:1636:1898#0/1
GGGGTCTTCTCAGATTTTATTTTGTTGGCTTTCTCTAGGT
```

```
+HWI-EAS443:3:14:1636:1898#0/1
V_b`UPK`a``X¥Z_R¥ba`ZX¥bY`]ROX``O[GRT^IS
@HWI-EAS443:3:27:1702:824#0/1
GTCTTCTCAGATTTTATTTTGTTGGNTTTCTCTAGGTAAT
+HWI-EAS443:3:27:1702:824#0/1
aa_baaaaaba`aaaabaaaa__a[D[a`[`^`]¥aY_^a
@HWI-EAS443:3:67:441:184#0/1
ATGAAAGCCAACAAAATAAAATCTGAGAAGACACACATTC
+HWI-EAS443:3:67:441:184#0/1
ababV^`aa^_a`^aaaaaaaa^a_Wa_]a`_`_`^^]TU
@HWI-EAS443:3:25:216:2013#0/1
AGGGAGTGCCTGATTAATGATCCCTGGGTTTTGCTTAATG
+HWI-EAS443:3:25:216:2013#0/1
a`aa__[`aa`^_a__a_]_^]___S`[X_][^__]`^W¥
@HWI-EAS443:3:12:1773:1563#0/1
CTAGAGAAAGCCAACAAAATAAAATCTGAGAAGACACACA
+HWI-EAS443:3:12:1773:1563#0/1
¥`aa`bb^X`bbb_Y_bbbbbbabbbbbabb¥bb`bbbaa
@HWI-EAS443:3:12:221:1578#0/1
GTCTTCTCAGATTTTATTTTGTTGGCTTTCTCTAGGTAAT
+HWI-EAS443:3:12:221:1578#0/1
`Xaa`^]_[]¥X`YW`_ZZ__N¥^]_PQP_^`WW[¥NX¥X
@HWI-EAS443:3:18:1431:1257#0/1
AAAGCCAACAAAATAAAATCTGAGAAGACACACATTCACA
+HWI-EAS443:3:18:1431:1257#0/1
^``WY¥ZXZb¥`aaa``][_aa¥T_aa`W_]_Wa^WX_Z_
@HWI-EAS443:3:27:1095:1132#0/1
GTCTTCTCAGATTTTATTTTGTTGGCTTTCTCTAGGTAAT
+HWI-EAS443:3:27:1095:1132#0/1
a_aaaab`Y_¥aaaa^a`_^a]_``Ua_^_`X^_`^V^]_
@HWI-EAS443:3:4:1788:652#0/1
GTCTTCTCAGATTTTATTTTGTTGGCTTTCTCTAGGTAAT
+HWI-EAS443:3:4:1788:652#0/1
aRVWZRTXU`RW_`^ZW]a^_UX^WNNT^SZUY[]^USZB
@HWI-EAS443:3:67:319:101#0/1
GTCTTCTCAGATTTTATTTTGTTGGCTTTCTCTAGGTAAT
+HWI-EAS443:3:67:319:101#0/1
a[__^W_YR`]¥aaa`aaa]Y]a_aa_``_`_^T_`U]X_
@HWI-EAS443:3:75:862:1342#0/1
GAAAGCCAACAAAATAAAATCTGAGAAGACACACATTCAC
+HWI-EAS443:3:75:862:1342#0/1
^`aZ`bbaabbb`a`ba``aba`¥aa`abbaaabaaa_ab
@HWI-EAS443:3:81:1311:666#0/1
GTCTTCTCAGATTTTATTTTGTTGGCTTTCTCTAGGTAAT
+HWI-EAS443:3:81:1311:666#0/1
a_`aa`aaaa`aaaaabaaab_a__Xaa`Za]a_a`^__a
@HWI-EAS443:3:97:619:1468#0/1
ATTACCTAGAGAAAGCCAACAAAATAAAATCTGAGAAGAC
+HWI-EAS443:3:97:619:1468#0/1
aa`aaaab`a`aaaa``aa¥aaaaaaa``a_`a^a`^___
```

```
@HWI-EAS443:3:18:141:1722#0/1
GCAAAAGCAGGGGAAAACAAAAGCAACAAAAATAACGGTA
+HWI-EAS443:3:18:141:1722#0/1
abbbbbbababaabbbbbabbb`bbbbbbbbbbbabaWU]
@HWI-EAS443:3:48:1066:129#0/1
GGGCAGCAAAAGCAGGGGAAAACAAAAGCAACAAAAATAC
+HWI-EAS443:3:48:1066:129#0/1
a_aa_baa`aaa_`a`a___`^]_^^_^Z¥___^^W^]ZW
@HWI-EAS443:3:35:1460:1167#0/1
GAAATCCTNATGTTAAATACATATTCTACACTGTAAAGAC
+HWI-EAS443:3:35:1460:1167#0/1
¥_TVa_`ODY^¥W]]X^`b`aa¥aaPb_ba_]a_WHH¥V`
@HWI-EAS443:3:39:548:29#0/1
AAATCCTAATCTTAAATACATATTCTACACTGTAGAGACC
+HWI-EAS443:3:39:548:29#0/1
]_`a`aa¥W]GZaa]]_Z¥[]^^¥__[][`^ZTRT_`¥UM
@HWI-EAS443:3:44:41:678#0/1
GAAATCCTAATCTTAAATACATATTCTACACTGTAGAGAC
+HWI-EAS443:3:44:41:678#0/1
`_a_a`a`aaXEYaa`^aa_`aaa`_a`Y_^^^X_[]^_X
@HWI-EAS443:3:56:1026:622#0/1
TTCTCATTCTTCTGAAATCCTAATGTTAAATACATATTCT
+HWI-EAS443:3:56:1026:622#0/1
¥RFURPQFRP]UP_]`¥`[TPTV]^¥QVRaWRUXFKXODR
@HWI-EAS443:3:19:1227:1092#0/1
GAAATCCTAATGTTAAATACATATTCTACACTGTAGANAC
+HWI-EAS443:3:19:1227:1092#0/1
_RQZ`SPJ^¥YZ]P]_PSa¥S[^aYL[`^YTZ^_ZPODLG
@HWI-EAS443:3:89:493:1357#0/1
CGGGCTTCTGAAATCCTAATGTTAAATACATATTCTACAC
+HWI-EAS443:3:89:493:1357#0/1
abb`babbbabaabbababbbabbXabbabbababbbaba
@HWI-EAS443:3:12:1463:1385#0/1
GAAATCCTAATGTTAAATACATATTCTACACTGTAGAGAC
+HWI-EAS443:3:12:1463:1385#0/1
a`Y¥`¥Q]¥`_`_]Y]]a`a_^```[`aW_[^a¥_`^_]X
@HWI-EAS443:3:13:958:441#0/1
ATGCTTCTGAAATCCTAATGTTAAATACATATTCTACACT
+HWI-EAS443:3:13:958:441#0/1
abZRY^TZ`ab_`TLZ¥a`^^¥_U^[X¥`^]`]``¥^_L]
@HWI-EAS443:3:14:668:1612#0/1
GAAATCCTAATGTTAAATACATATTCTACACTGTAGAGAC
+HWI-EAS443:3:14:668:1612#0/1
`bb`a_aaabaaaaaaaaaaaaaaaaaa``_```baaa_`
@HWI-EAS443:3:16:91:2014#0/1
GCTTCTGAAATCCTAATGTTAAATACATATTCTACACTGT
+HWI-EAS443:3:16:91:2014#0/1
aa`^`ab_R`Ybbabbbaaa¥Vaa_`¥aba_aabaab_aW
@HWI-EAS443:3:25:31:1284#0/1
GAAATCCTAATGTTAAATACATATTCTACACTGTAGAGAC
```

```
+HWI-EAS443:3:25:31:1284#0/1
¥J¥_aba`[_`a_a^]]^YaV`aaaaa]aaa`a_aa```_
@HWI-EAS443:3:26:1381:1235#0/1
TGAAATCCTAATGTTAAATACATATTCTACACTGTAGAGA
+HWI-EAS443:3:26:1381:1235#0/1
abaaaaaaaa`aaaaaaaaaaaaaaaaa_aa`a^``^^_
@HWI-EAS443:3:33:641:24#0/1
TGCTTCTGAAATCCTAATGTTAAATACATATTCTACACTG
+HWI-EAS443:3:33:641:24#0/1
aaa[___WQZS_Y]Y]TX]T__ZW¥[X^[W¥TWXXX¥YY[
@HWI-EAS443:3:34:164:1845#0/1
TTTTTCTCATGCTTCTGAAATCCTAATGTTAAATACATAT
+HWI-EAS443:3:34:164:1845#0/1
aaba_bab_aabaabaa¥¥aaaba`a``_aa`aaaa````
@HWI-EAS443:3:35:880:784#0/1
CTGAAATCCTAATGTTAAATACATATTCTACACTGTAGAG
+HWI-EAS443:3:35:880:784#0/1
abbbbbbbbba`baabbaaabbbbbbb`b`abbbaaa^`^
@HWI-EAS443:3:37:1228:1238#0/1
TGCTTCTGAAATCCTAATGTTAAATACATATTCTACACTG
+HWI-EAS443:3:37:1228:1238#0/1
`bba`aab[aaaab]T^abaaaaaaa`a`[aab`a_aa`_
@HWI-EAS443:3:37:844:484#0/1
GAAATCCTAATGTTAAATACATATTCTACACTGTAGAGAC
+HWI-EAS443:3:37:844:484#0/1
aaaaaaaa`aaaaaa`abaabaa``aa_a^a`a]a^```]
@HWI-EAS443:3:39:1601:102#0/1
CTAATGTTAAATACATATTCTACACTGTAGAGACCCATTA
+HWI-EAS443:3:39:1601:102#0/1
abbabbbbbbbbbaabbbbabbbabbbabbaX`_^Wa_]^
@HWI-EAS443:3:39:293:382#0/1
CTCATGCTTCTGAAATCCTAATGTTAAATACATATTCTAC
+HWI-EAS443:3:39:293:382#0/1
_ba^`bbbbaaa_ZZ`b]abb`_]`a`_a`aa___aaaa_
@HWI-EAS443:3:40:243:561#0/1
TTCTCATGCTTCTGAAATCCTAATGTTAAATACATATTCT
+HWI-EAS443:3:40:243:561#0/1
aaaabaaaaaaa`aa`X`aaa_aaa^[aXaaa```W_^a`
@HWI-EAS443:3:46:1372:777#0/1
TGCTTCTGAAATCCTAATGTTAAATACATATTCTACACTG
+HWI-EAS443:3:46:1372:777#0/1
a[`^`_^a]Y[Z¥^__^^aV]^T^W]]_^]]^[^]W^WU]
@HWI-EAS443:3:53:1215:540#0/1
AAATCCTAATGTTAAATACATATTCTACACTGTAGAGACC
+HWI-EAS443:3:53:1215:540#0/1
aaabaababbbaaabbbbaabbbaaba`aaba`aaa_a`Y
@HWI-EAS443:3:54:88:757#0/1
ATGCTTCTGAAATCCTAATGTTAAATACATATTCTACACT
+HWI-EAS443:3:54:88:757#0/1
`aababbbb_aababaabbbab`aabaaababbaaa`a`a
```

@HWI-EAS443:3:54:961:1128#0/1
TTTTCTCATGCTTCTGAAATCCTAATGTTAAATACATATT
+HWI-EAS443:3:54:961:1128#0/1
aa_`aa`[a^[^_T^V¥VZ¥__^bbab`aa^_]__b_Y]_
@HWI-EAS443:3:55:97:829#0/1
ATGCTTCTGAAATCCTAATGTTAAATACATATTCTACACT
+HWI-EAS443:3:55:97:829#0/1
`^^_``]¥Y]`__[__`^`a^_Z_`_WQV¥Z`ZX^]]_Y]
@HWI-EAS443:3:58:973:940#0/1
CTCATGCTTCTGAAATCCTAATGTTAAATACATATTCTAC
+HWI-EAS443:3:58:973:940#0/1
abbbbabbbabaaaaaaabaabbaaa__aaaab`baaaaa
@HWI-EAS443:3:59:1602:831#0/1
GCTTCTGAAATCCTAATGTTAAATACATATTCTACACTGT
+HWI-EAS443:3:59:1602:831#0/1
U¥¥V^aa__Ya`¥`¥a^aZ[`a`b`ba^`aUF_]TM[a]G
@HWI-EAS443:3:65:1486:210#0/1
GAAATCCTAATGTTAAATACATATTCTACACTGTAGAGAC
+HWI-EAS443:3:65:1486:210#0/1
a`]_`_^_`___`_`X_`_[^___]___[^Y[`^]ZYWUT
@HWI-EAS443:3:67:585:1011#0/1
TTTTTCTCATGCTTCTGAAATCCTAATGTTAAATACATAT
+HWI-EAS443:3:67:585:1011#0/1
aaaaabaa_aaa`aaa`^]_aaaaaa_a``a`a`a_````
@HWI-EAS443:3:69:787:1946#0/1
AAATCCTAATGTTAAATACATATTCTACACTGTAGAGACC
+HWI-EAS443:3:69:787:1946#0/1
`VPX`bW]]Y`UZ^¥aV`a__a__a^aa`_Z[V]WRM`YG
@HWI-EAS443:3:70:1429:1261#0/1
CTGAAATCCTAATGTTAAATACATATTCTACACTGTAGAG
+HWI-EAS443:3:70:1429:1261#0/1
abba[^abbbb`bb`bab`bbbbbbbaabbaaaabaaa__
@HWI-EAS443:3:75:188:190#0/1
GAAATCCTAATGTTAAATACATATTCTACACTGTAGAGAC
+HWI-EAS443:3:75:188:190#0/1
aaaaaaaaaaa`_aa`aa_``a`a`aa_a`_`aX___^_]
@HWI-EAS443:3:75:405:1305#0/1
GAAATCCTAATGTTAAATACATATTCTACACTGTAGAGAC
+HWI-EAS443:3:75:405:1305#0/1
^V¥¥`bb```bbabb`abbabb`baaba^Xaaa_b]`_aa
@HWI-EAS443:3:76:1234:693#0/1
TGAAATCCTAATGTTAAATACATATTCTACACTGTAGAGA
+HWI-EAS443:3:76:1234:693#0/1
Ta_`ababb_bW^`b^^aabb`bab]Wab`¥W_bbabaab
@HWI-EAS443:3:76:747:1028#0/1
CTCATGCTTCTGAAATCCTAATGTTAAATACATATTCTAC
+HWI-EAS443:3:76:747:1028#0/1
aaaaa`baaaaaaa_a_aaab`a¥a`aa`a`_`a``_aa_
@HWI-EAS443:3:76:764:1451#0/1
TTTTTCTCATGCTTCTGAAATCCTAATGTTAAATACATAT

+HWI-EAS443:3:76:764:1451#0/1
aaaa``_aaa__`_``_`]`^__`a_^`^^¥_```^_^__
@HWI-EAS443:3:82:1478:1214#0/1
GAAATCCTAATGTTAAATACATATTCTACACTGTAGAGAC
+HWI-EAS443:3:82:1478:1214#0/1
a_Y`^¥^baaa_aa^¥_aa_^a`aYaa`^a¥`a]_]a]`U
@HWI-EAS443:3:83:437:580#0/1
GAAATCCTAATGTTAAATACATATTCTACACTGTAGAGAC
+HWI-EAS443:3:83:437:580#0/1
`__Y¥_WWTXZUU`][WX`]¥aaa```aSaaaa``]ZTVY
@HWI-EAS443:3:84:839:229#0/1
GAAATCCTAATGTTAAATACATATTCTACACTGTAGAGAC
+HWI-EAS443:3:84:839:229#0/1
aa]^a`¥^a¥`a__a`a_[Y`_`a___`[Y_``[`^¥Z_Y
@HWI-EAS443:3:87:345:578#0/1
GCTTCTGAAATCCTAATGTTAAATACATATTCTACACTGT
+HWI-EAS443:3:87:345:578#0/1
aa_^`a`[`__R``¥`a[XX_aaa_`a`a__`aX`_`_`¥
@HWI-EAS443:3:93:362:1878#0/1
GCTTCTGAAATCCTAATGTTAAATACATATTCTACACTGT
+HWI-EAS443:3:93:362:1878#0/1
aabaab¥aa_aaaabaaa`^aaa`a]a`a`V___Sa^^¥W
@HWI-EAS443:3:93:934:388#0/1
GCTTCTGAAATCCTAATGTTAAATACATATTCTACACTGT
+HWI-EAS443:3:93:934:388#0/1
aOabaaaabaaaaabaabaaaaaaa``aaa`aaa_a[``_
@HWI-EAS443:3:94:100:1007#0/1
GAAATCCTAATGTTAAATACATATTCTACACTGTAGAGAC
+HWI-EAS443:3:94:100:1007#0/1
¥^``bbbba]baaab``aaabbab`a`b^a^^aYVa_V¥Y
@HWI-EAS443:3:95:475:1567#0/1
GAAATCCTAATGTTAAATACATATTCTACACTGTAGAGAC
+HWI-EAS443:3:95:475:1567#0/1
`¥_S`Z¥^_^T```]`baa]a`]`]VZa_aO`a`a__``[
@HWI-EAS443:3:24:1775:1702#0/1
GGGAGTTTTTCCTCAACTGTCATACTCCTCTGCATTGTCT
+HWI-EAS443:3:24:1775:1702#0/1
_`bI^ZQ¥`[`[a^_b``XQ[MHR`UUYDNW]XILOaW_G
@HWI-EAS443:3:51:489:447#0/1
TTCGGAGACAATGCAGAGGAGTATGACAGTTGAGGGGCTA
+HWI-EAS443:3:51:489:447#0/1
]ZXabaa_ab]ab[F_[b`Ra_¥a`PPQaX^_Y^WH¥HX^
@HWI-EAS443:3:17:806:304#0/1
GGGCATTTTTCCTCAACTGTCATACTCCTCTGCATTGTCT
+HWI-EAS443:3:17:806:304#0/1
aa]G_`a___aa`_a_^aaZ_`^`]_`_`U`_^_^^]]^^
@HWI-EAS443:3:47:1488:878#0/1
GGGCATTTTTCCTCAACTGTCATACTCCTCTGCATTGTCT
+HWI-EAS443:3:47:1488:878#0/1
]aaHYb^aaa`[a``¥__[V[`a__[^WT]U^^_`^UU]]

```
@HWI-EAS443:3:50:1734:942#0/1
CTCAACTGTCATACTCCTCTGCATTGTCTCCGAAGAGATA
+HWI-EAS443:3:50:1734:942#0/1
`¥`a`^_]¥][X¥_UZ^¥`aa^^_`^^`b_`aZWaLFYYa
@HWI-EAS443:3:77:145:1477#0/1
GGGTATTTTTCCTCAACTGTCATACTCCTCTGCATTTTCT
+HWI-EAS443:3:77:145:1477#0/1
S`UOUP_]]_aTZZa_`USLa^PaUR¥L¥UU¥MRPBBBBB
@HWI-EAS443:3:7:1403:1752#0/1
GGGCATTTTTCCTCAACTGTCATACTCCTCTGCATTGTCT
+HWI-EAS443:3:7:1403:1752#0/1
abRO¥___aa`[^]R]WSaO___VW^VV_^`XV_]X_G[B
@HWI-EAS443:3:87:247:1319#0/1
GGGCATTTTTCCTCAACTGTCATACTCCTCTGCATTGTCT
+HWI-EAS443:3:87:247:1319#0/1
]a_V`YZTX[`_HSZYX`SX^Tab[`]a]`ZXYX_ZYRa¥
@HWI-EAS443:3:92:813:851#0/1
GGGCATTTTTCCTCAACTGTCATACTCCTCTGCATTGTCT
+HWI-EAS443:3:92:813:851#0/1
``¥FY]`]T]Q^_`_a`VPO^TSTXNOV^[T^^UWYYQJV
@HWI-EAS443:3:25:72:237#0/1
TTTTCCTCAACTGTCATACTCCCCTGCATTGTCTCCGAAG
+HWI-EAS443:3:25:72:237#0/1
`bbaa`]a`[aaa`bb_S^_`ab_`[^Y^a`¥``[``YS¥
@HWI-EAS443:3:45:150:1108#0/1
GGGTATTTTTCCTCACCTGTCATACTCCTCTGCATTGTCT
+HWI-EAS443:3:45:150:1108#0/1
aaWH¥a^_T¥]^^`^VX^ZRYT_`YYU[_^VQ[^[V¥VZT
@HWI-EAS443:3:47:1373:1458#0/1
TTTTCCTCAACTGTCATACTTCTCTGCATTGTCTCCGAAG
+HWI-EAS443:3:47:1373:1458#0/1
]_^bbbba_baY_¥¥^Y¥^`T_[^Z`Xb`Y_¥¥NGT`]S`
@HWI-EAS443:3:50:551:239#0/1
GTATTTTTCCTCAACTGTCATACTCCTCTGCATTGGCTCC
+HWI-EAS443:3:50:551:239#0/1
a¥S^a```[X]a]`][`]P¥[PNX_`Z^aZSHZ]YJ[ZPX
@HWI-EAS443:3:61:1450:604#0/1
ATTTTTCCTCAACTCTCATACTCCTCTGCATTGTCTCCGA
+HWI-EAS443:3:61:1450:604#0/1
abbbbba`a^_aa^XUaa_X`abaaaaa^¥aWTTUVNLSQ
@HWI-EAS443:3:62:1668:579#0/1
CAAGGGGATTTTTCCTCAACTGTCATACTCCTCTGCATTG
+HWI-EAS443:3:62:1668:579#0/1
aabbbbYZ[bbbb^`aaVZb`aabbaaaaa[a^ab_`aab
@HWI-EAS443:3:76:1379:128#0/1
GGTATTTTTCCTCAACTGTCATACCCCTCTGCATTGTCTC
+HWI-EAS443:3:76:1379:128#0/1
abVa`b`baY]aY__Waa^__aaR[RYWMW^W]`_^ZW¥W
@HWI-EAS443:3:7:385:497#0/1
GTATTTTTCCTCAACTGTCATATTCCTCTGCATTGTCTCC
```

```
+HWI-EAS443:3:7:385:497#0/1
^FUa[XTZV`YO[___`OMS_^XQ]Y]IUWU`]SSHLG^B
@HWI-EAS443:3:7:838:767#0/1
GGTATTTTTCCTCAACTGTCTTACTCCTCTGCATTGTCTC
+HWI-EAS443:3:7:838:767#0/1
abG]Yaba_¥]_aa]^_a^`^^¥Xa^__W]_^¥^^^XX^W
@HWI-EAS443:3:82:1741:1709#0/1
TCCTCAACTGTCATACTCCTGTGCATTGTCTCCGAAGAAA
+HWI-EAS443:3:82:1741:1709#0/1
YZ]XZ_S^P¥^P]^_UZWP[DYZU]b¥YNHW]TPFI`aYS
@HWI-EAS443:3:91:86:1930#0/1
TTTTCCTCAACTGTCATACTCCTATGCATTGTCTCCGAAG
+HWI-EAS443:3:91:86:1930#0/1
¥baXa`^¥XX¥^_]Z]]X]`a`UZY^¥X_¥YR]ZSJTVSO
@HWI-EAS443:3:98:1468:1500#0/1
GGGTATTTTTCCTCAACTGTCATACTCCTATGCATTGTCT
+HWI-EAS443:3:98:1468:1500#0/1
aba_^aaV^aS^a^_`^aaS__``S___SGZ^O`^_aIX`
@HWI-EAS443:3:14:932:674#0/1
GGGTATTTTTCCTCAACTGTCATACTCCTCTGCATTGCAT
+HWI-EAS443:3:14:932:674#0/1
aabU^``a]aa``_``]``]``[YW`]^[W^_Y`__[]HV
@HWI-EAS443:3:83:340:1134#0/1
ATNTTTCCTCAACTGTCATACTCCTCTGCATTGTCTCCGA
+HWI-EAS443:3:83:340:1134#0/1
`^D¥``baabaaaaa_`aaa`a`a`a_`aaa]`_`````_
@HWI-EAS443:3:88:921:1060#0/1
GGGATTTTTCCTCAACTGTCATACTCCTCTGCATTGTCTC
+HWI-EAS443:3:88:921:1060#0/1
^ZFY]a`X_W_]][]a_QY¥aaYW[UJ^Q[[WYaa`X¥[Z
@HWI-EAS443:3:19:1562:1268#0/1
TATTTNTCCTCAACTGTCATACTCCTCTGCATTGTCTCCG
+HWI-EAS443:3:19:1562:1268#0/1
aabaYD¥]`^¥`a_a^¥Za``^^RS^__`¥^___]]_XU¥
@HWI-EAS443:3:39:1132:787#0/1
GGTATTTTTCCTCAACTGTCATACTCNTCTGCATTGTCTC
+HWI-EAS443:3:39:1132:787#0/1
abZaaaaaaaaa_`]a`aY`WDX^`¥DX_`¥SY_^^Y¥^X
@HWI-EAS443:3:49:1336:1630#0/1
GGGTATTTTTCCTCAACTNTCATACTCCTCTGCATTGTCT
+HWI-EAS443:3:49:1336:1630#0/1
aabX`aaaaa_¥a^``XPDR¥^W]]`]P[[_^]]_]_¥X`
@HWI-EAS443:3:65:134:1811#0/1
GGGTANTTTTCCTCAACTGTCATACTCCTCTGCATTGTCT
+HWI-EAS443:3:65:134:1811#0/1
abbZ]D¥`bbabaaaa`a`[_aa`a`aa_a`aa`aa_``_
@HWI-EAS443:3:87:977:611#0/1
GGTATTTTTCCTCAACTGTCATACTCCTCTGCATTGNCTC
+HWI-EAS443:3:87:977:611#0/1
¥b_`X¥^_[GTb`XaTabaaaY]aa^]baV]`]b_WDL``
```

```
@HWI-EAS443:3:95:224:706#0/1
GGGTATTNTTCCTCAACTGTCATACTCCTCTGCATTGTCT
+HWI-EAS443:3:95:224:706#0/1
aaaZ]V¥D¥a_]__`]^a_QR`¥^^^`WZ`ZVQ[`W¥Z^¥
@HWI-EAS443:3:56:220:288#0/1
GCATTTTTCCTCAACTGTCATACTCCTCTGCATTGTCTCC
+HWI-EAS443:3:56:220:288#0/1
`FU]`aaa^a__¥SXXaW¥_`[WW]]`__^Z¥^^]]]X^X
@HWI-EAS443:3:27:1411:1635#0/1
ATTTTCCTCAACTGTCATACTCCTCTGCATTGTCTCCGAA
+HWI-EAS443:3:27:1411:1635#0/1
`URP]aXUZ`aUaVN^```¥`]][__a]`_^ZZ__]]^]]
@HWI-EAS443:3:46:1750:1428#0/1
NGTATTTTTCCTCAACTGTCATACTCCTCTGCATTGTCTC
+HWI-EAS443:3:46:1750:1428#0/1
DMUXXZZYYXXYYXZZYYYXZZZZZZXZZYYWZZZZZXZV
@HWI-EAS443:3:69:1584:1470#0/1
ATTTCTTCGGAGACAATGCAGAGGAGTATGACAGTTGAGC
+HWI-EAS443:3:69:1584:1470#0/1
aaaaabaaba`a``aaaa_^`]`a^^[__a_]_aZ_^¥^T
@HWI-EAS443:3:78:153:1250#0/1
CCCTCAACTGTCATACTCCTCTGCATTGTCTCCGAAGAAA
+HWI-EAS443:3:78:153:1250#0/1
abbaba`aaa^baaa``a¥^a_¥a``_]]`__a____^__
@HWI-EAS443:3:92:1697:856#0/1
TGGTATTTTTCCTCAACTGTCATACTCCTCTGCATTGTCT
+HWI-EAS443:3:92:1697:856#0/1
Z`¥L_bb]a`W`a_aa_``^Y````aWWa[^aXaaa_NYa
@HWI-EAS443:3:100:1152:1227#0/1
GGTATTTTTCCTCAACTGTCATACTCCTCTGCATTGTCTC
+HWI-EAS443:3:100:1152:1227#0/1
aaHa[[KZ^TU¥Z[`^UPWPXXa^`GH]¥_`¥]_U^^BBB
@HWI-EAS443:3:100:475:260#0/1
ATTTTTCCTCAACTGTCATACTCCTCTGCATTGTCTCCGA
+HWI-EAS443:3:100:475:260#0/1
¥_bbb_babababa_b]_b_`aVaa`__aa[a]¥`_Oa^
@HWI-EAS443:3:11:1168:377#0/1
ATTTTTCCTCAACTGTCATACTCCTCTGCATTGTCTCCGA
+HWI-EAS443:3:11:1168:377#0/1
abbaaa`aaaaaa]`]`aa`aa_`a_aa_aa`a_]_`^a`
@HWI-EAS443:3:11:1480:977#0/1
TTTTCCTCAACTGTCATACTCCTCTGCATTGTCTCCGAAG
+HWI-EAS443:3:11:1480:977#0/1
^`_``^X^¥bb¥_WG[aa`]ZU^^]a¥a``a[YYTV`_`_
@HWI-EAS443:3:12:1105:1683#0/1
GGTATTTTTCCTCAACTGTCATACTCCTCTGCATTGTCTC
+HWI-EAS443:3:12:1105:1683#0/1
aaZaaa`aa^Z_Yaa¥`a``aa]W]a`a_`_^_a_`_X_[
@HWI-EAS443:3:12:1505:1392#0/1
TTTTCCTCAACTGTCATACTCCTCTGCATTGTCTCCGAAG
```

```
+HWI-EAS443:3:12:1505:1392#0/1
¥_`^]RT`baa^]U_bb^ZWTZaaa^[`a`aYF¥aT^^_[
@HWI-EAS443:3:13:972:253#0/1
GGTATTTTTCCTCAACTGTCATACTCCTCTGCATTGTCTC
+HWI-EAS443:3:13:972:253#0/1
a`P[`^[__¥¥^`W]]]`V]]X^LW[¥Y]^_VW]]^WUWT
@HWI-EAS443:3:14:1237:301#0/1
TTTTCCTCAACTGTCATACTCCTCTGCATTGTCTCCGAAG
+HWI-EAS443:3:14:1237:301#0/1
aaaa`_a^__]_`]__``___W`Y_`^^__`]U_]W_]Z_
@HWI-EAS443:3:15:1050:1870#0/1
AGGGTATTTTTCCTCAACTGTCATACTCCTCTGCATTGTC
+HWI-EAS443:3:15:1050:1870#0/1
__aaU_^^aaa^[`a``aaa^aa`a`a^^`aa`Y```]^_
@HWI-EAS443:3:15:114:1458#0/1
TTTTCCTCAACTGTCATACTCCTCTGCATTGTCTCCGAAG
+HWI-EAS443:3:15:114:1458#0/1
abbaaaaaabaaa_aaaaaa^a_a`a]_aa¥]``aa___`
@HWI-EAS443:3:15:341:43#0/1
GTATTTTTCCTCAACTGTCATACTCCTCTGCATTGTCTCC
+HWI-EAS443:3:15:341:43#0/1
a]__a`a``]_U^Y^a`YQ`[^WZYW^_^^Z¥^^_]V]^U
@HWI-EAS443:3:17:109:806#0/1
GTATTTTTCCTCAACTGTCATACTCCTCTGCATTGTCTCC
+HWI-EAS443:3:17:109:806#0/1
aZXaaa_`aaaaa_a^a_`^`__a___aa__`_^_^___]
@HWI-EAS443:3:17:1194:692#0/1
GGTATTTTTCCTCAACTGTCATACTCCTCTGCATTGTCTC
+HWI-EAS443:3:17:1194:692#0/1
aaVaa`aa```a__`]_a]¥¥^_]^^__X^_]__^_[X[]
@HWI-EAS443:3:17:416:891#0/1
GGGTATTTTTCCTCAACTGTCATACTCCTCTGCATTGTCT
+HWI-EAS443:3:17:416:891#0/1
aaaYaaaaaa`````a```_`a`a__a__`^__`__^^_^
@HWI-EAS443:3:18:1527:1526#0/1
GGGTATTTTTCCTCAACTGTCATACTCCTCTGCATTGTCT
+HWI-EAS443:3:18:1527:1526#0/1
a``X`abaaaaaaaaa_aa_^aa`_aa^`^_a__`_]]_`
@HWI-EAS443:3:18:215:1484#0/1
TTTTCCTCAACTGTCATACTCCTCTGCATTGTCTCCGAAG
+HWI-EAS443:3:18:215:1484#0/1
aa`bababbba`a``aa]aa___``^^_aaaZY[_X__]]
@HWI-EAS443:3:19:127:921#0/1
GTATTTTTCCTCAACTGTCATACTCCTCTGCATTGTCTCC
+HWI-EAS443:3:19:127:921#0/1
aU`]^a_`a__^¥]a^^U]YWa`_`a]__^__¥]_Z_]__
@HWI-EAS443:3:19:1393:667#0/1
GGGTATTTTTCCTCAACTGTCATACTCCTCTGCATTGTCT
+HWI-EAS443:3:19:1393:667#0/1
aY^TX¥`]]_[LZS]XOMSPSOZRP^MU^VTTWYYSWWZ¥
```

```
@HWI-EAS443:3:19:211:830#0/1
GTATTTTTCCTCAACTGTCATACTCCTCTGCATTGTCTCC
+HWI-EAS443:3:19:211:830#0/1
VTJ`¥`baa`]baa¥R[]`]`baYa¥]`ab_`^aaa`aa_
@HWI-EAS443:3:1:1307:567#0/1
GTATTTTTCCTCAACTGTCATACTCCTCTGCATTGTCTCC
+HWI-EAS443:3:1:1307:567#0/1
`SW^`__]ZX¥]__Y]^¥X¥^¥X¥UX]U]^ZW]]^ZXBBB
@HWI-EAS443:3:1:341:648#0/1
GGGTATTTTTCCTCAACTGTCATACTCCTCTGCATTGTCT
+HWI-EAS443:3:1:341:648#0/1
abaYaaaaaaZWa__`]]_W¥```^`O_]W][S`^U]BBB
@HWI-EAS443:3:21:109:962#0/1
CTCAACTGTCATACTCCTCTGCATTGTCTCCGAAGAAATA
+HWI-EAS443:3:21:109:962#0/1
aaa`aaaa_aaaaa`aa`_```_`_^^_``____`^_`]`
@HWI-EAS443:3:21:1268:1414#0/1
TCCTCAACTGTCATACTCCTCTGCATTGTCTCCGAAGAAA
+HWI-EAS443:3:21:1268:1414#0/1
[ab]a]``ab_a^[^_a``aa`a]__`a]^___aaYVW][
@HWI-EAS443:3:21:1275:1885#0/1
GGTATTTTTCCTCAACTGTCATACTCCTCTGCATTGTCTC
+HWI-EAS443:3:21:1275:1885#0/1
ab_a``[TX_aa_T`VV_]ZaaXHWHS[F^]Ua¥`_G`^W
@HWI-EAS443:3:21:840:1296#0/1
GGTATTTTTCCTCAACTGTCATACTCCTCTGCATTGTCTC
+HWI-EAS443:3:21:840:1296#0/1
a`XaaaaaaaaaaabaYa]_a``a^_U__a^^`a^_`_¥¥
@HWI-EAS443:3:22:1386:1527#0/1
GGGTATTTTTCCTCAACTGTCATACTCCTCTGCATTGTCT
+HWI-EAS443:3:22:1386:1527#0/1
XaYMb_[bb`]I`WX^^aa_`aa_W`]XUW_^R]]`bWQ]
@HWI-EAS443:3:22:37:662#0/1
GTATTTTTCCTCAACTGTCATACTCCTCTGCATTGTCTCC
+HWI-EAS443:3:22:37:662#0/1
aJ¥a``a^_X`[T¥^[WOTX¥[L[VHWLYPSSRQOS_¥UU
@HWI-EAS443:3:22:441:1798#0/1
TTTTCCTCAACTGTCATACTCCTCTGCATTGTCTCCGAAG
+HWI-EAS443:3:22:441:1798#0/1
a_a__a_abaa_`^aaaaa^``[__^`a___^`_``^^`^
@HWI-EAS443:3:22:442:899#0/1
TTTTCCTCAACTGTCATACTCCTCTGCATTGTCTCCGAAG
+HWI-EAS443:3:22:442:899#0/1
ab_aabbbbaU^O[_¥`ba``_ba^aaaa`X_^`^]_Y^_
@HWI-EAS443:3:24:1303:1313#0/1
TTTTCCTCAACTGTCATACTCCTCTGCATTGTCTCCGAAG
+HWI-EAS443:3:24:1303:1313#0/1
]``¥T``b__KZUWGP_]¥bXOU_`b[a]V^HP]US`Wb`
@HWI-EAS443:3:24:694:75#0/1
GGTATTTTTCCTCAACTGTCATACTCCTCTGCATTGTCTC
```

+HWI-EAS443:3:24:694:75#0/1
`YIU`^UU_]XZ]SVXV^GZRX_OGXMPKZ¥OZBBBBBBB
@HWI-EAS443:3:25:1425:1146#0/1
GGGTATTTTTCCTCAACTGTCATACTCCTCTGCATTGTCT
+HWI-EAS443:3:25:1425:1146#0/1
abaH_bbbb`_ab_aa_``_``aa`a`_`^_a[a`a]]U_
@HWI-EAS443:3:26:1240:1551#0/1
TTTTCCTCAACTGTCATACTCCTCTGCATTGTCTCCGAAG
+HWI-EAS443:3:26:1240:1551#0/1
abbaaaaaaaaaa_`baa``a`aaaaaaa_a`a`aaa__a
@HWI-EAS443:3:26:703:1190#0/1
GTATTTTTCCTCAACTGTCATACTCCTCTGCATTGTCTCC
+HWI-EAS443:3:26:703:1190#0/1
a]]bbabbaaabbaaaaa`aa]aaaaa`aaaa``__a_a`
@HWI-EAS443:3:26:891:198#0/1
GTATTTTTCCTCAACTGTCATACTCCTCTGCATTGTCTCC
+HWI-EAS443:3:26:891:198#0/1
]NT]Z[UPX^P_Y[LYYGXRNXY^YLO]]_XUMSSZZWSW
@HWI-EAS443:3:27:957:990#0/1
GGTATTTTTCCTCAACTGTCATACTCCTCTGCATTGTCTC
+HWI-EAS443:3:27:957:990#0/1
abJaaaa]_b]a`^a_ZaX`_[[Ua_X__]_^``a__RX^
@HWI-EAS443:3:27:976:833#0/1
TTTTCCTCAACTGTCATACTCCTCTGCATTGTCTCCGAAG
+HWI-EAS443:3:27:976:833#0/1
a^T^`a_¥`aa^_J_¥_RPW`Va``a^ZU_[`X]X^`XT¥
@HWI-EAS443:3:28:1062:765#0/1
GGGTATTTTTCCTCAACTGTCATACTCCTCTGCATTGTCT
+HWI-EAS443:3:28:1062:765#0/1
``b]abbbbba`b¥aaaaa_aaaa_b__aaaa]`aaa_`_
@HWI-EAS443:3:28:34:864#0/1
TTTTCCTCAACTGTCATACTCCTCTGCATTGTCTCCGAAG
+HWI-EAS443:3:28:34:864#0/1
abbbabbbbabaa`aaaaaabaaaabaaaaaaaa`a`¥`¥
@HWI-EAS443:3:28:557:1062#0/1
GGGTATTTTTCCTCAACTGTCATACTCCTCTGCATTGTCT
+HWI-EAS443:3:28:557:1062#0/1
ab_L^ababbaaaaaaaaaa¥aaa`aaa_aa__`aa`___`
@HWI-EAS443:3:28:935:1213#0/1
GGTATTTTTCCTCAACTGTCATACTCCTCTGCATTGTCTC
+HWI-EAS443:3:28:935:1213#0/1
abXaaaaa^^YX_aba`a_^a_aa_^`a_[_`_^^^_a__
@HWI-EAS443:3:28:938:891#0/1
GGTATTTTTCCTCAACTGTCATACTCCTCTGCATTGTCTC
+HWI-EAS443:3:28:938:891#0/1
a^X^a`aa``a[¥``X^]RZV¥[__Z^`_`a]`^_U¥Z_Y
@HWI-EAS443:3:29:342:1327#0/1
GGTATTTTTCCTCAACTGTCATACTCCTCTGCATTGTCTC
+HWI-EAS443:3:29:342:1327#0/1
TYZXHXXX_`a_XX^b]V_baS[a`ab^aab_aaa¥R¥]`

```
@HWI-EAS443:3:29:469:2038#0/1
GGGTATTTTTCCTCAACTGTCATACTCCTCTGCATTGTCT
+HWI-EAS443:3:29:469:2038#0/1
abbRbabbbabbbabba``Ybbaba`baaa``bba_a^a^
@HWI-EAS443:3:2:1439:651#0/1
GGGTATTTTTCCTCAACTGTCATACTCCTCTGCATTGTCT
+HWI-EAS443:3:2:1439:651#0/1
aaaXaaa_aa_^^]^]^_`Z]]Y]V_^]]¥_]W]^]VZZZ
@HWI-EAS443:3:2:517:1803#0/1
ATTTTTCCTCAACTGTCATACTCCTCTGCATTGTCTCCGA
+HWI-EAS443:3:2:517:1803#0/1
`[¥_`YYM`¥bbH_a^¥¥a`aa___`_a_`a_a_Y^STaa
@HWI-EAS443:3:30:1401:1078#0/1
TTTTCCTCAACTGTCATACTCCTCTGCATTGTCTCCGAAG
+HWI-EAS443:3:30:1401:1078#0/1
ab_XS`bbaaR_]_aaaaaYaaa^aX]a`Y_¥_aOO`Z[a
@HWI-EAS443:3:31:461:1420#0/1
GGTATTTTTCCTCAACTGTCATACTCCTCTGCATTGTCTC
+HWI-EAS443:3:31:461:1420#0/1
aaPa[[`]aaa¥Z[T¥[_Waa^[¥¥_```__[`a^^^^^^
@HWI-EAS443:3:32:1147:1315#0/1
GGTATTTTTCCTCAACTGTCATACTCCTCTGCATTGTCTC
+HWI-EAS443:3:32:1147:1315#0/1
]¥]bbbbbbabbabb`aa`aab_aaaaab]aaaaa^^bab
@HWI-EAS443:3:32:17:183#0/1
GGTATTTTTCCTCAACTGTCATACTCCTCTGCATTGTCTC
+HWI-EAS443:3:32:17:183#0/1
a`H[¥``_aa]`_YX]X`Z^_ZY^^XW]]Y]YZXXYYYYZ
@HWI-EAS443:3:33:1154:1044#0/1
GGTATTTTTCCTCAACTGTCATACTCCTCTGCATTGTCTC
+HWI-EAS443:3:33:1154:1044#0/1
a`V¥`ab`baaaaa`aabXaa`a`ab^aaa_][a_¥_Wa_
@HWI-EAS443:3:33:1163:413#0/1
ATTTTTCCTCAACTGTCATACTCCTCTGCATTGTCTCCGA
+HWI-EAS443:3:33:1163:413#0/1
abaaabaaaaaaaaaabaaa`a[]aa`a_aa`¥^]_[Ua^
@HWI-EAS443:3:33:963:787#0/1
GGTATTTTTCCTCAACTGTCATACTCCTCTGCATTGTCTC
+HWI-EAS443:3:33:963:787#0/1
ab¥S_b`b``ab`Xa_aa__a_aaa`[]__[_]_^_^W`^
@HWI-EAS443:3:34:1423:594#0/1
GGTATTTTTCCTCAACTGTCATACTCCTCTGCATTGTCTC
+HWI-EAS443:3:34:1423:594#0/1
Z¥[¥`^X`ba[`aXX_VYQ¥`aZ_a]¥_X`UJ¥aYWYWBB
@HWI-EAS443:3:35:486:1638#0/1
CCTCAACTGTCATACTCCTCTGCATTGTCTCCGAAGAAAT
+HWI-EAS443:3:35:486:1638#0/1
a`]_abbT]Q]]^Y]SaZGWY_a_]UNVaX`^___^[J^S
@HWI-EAS443:3:36:1079:176#0/1
GGTATTTTTCCTCAACTGTCATACTCCTCTGCATTGTCTC
```

```
+HWI-EAS443:3:36:1079:176#0/1
_`[[`baa_aaaa`a`ab_aaaaaa_``a__R``Z`X`_a
@HWI-EAS443:3:36:1218:1232#0/1
GGGTATTTTTCCTCAACTGTCATACTCCTCTGCATTGTCT
+HWI-EAS443:3:36:1218:1232#0/1
¥ba¥¥baaaa``aZ`abaWaaaaa¥`a_aaYa_aaaaa[`
@HWI-EAS443:3:36:1573:1660#0/1
CTCAACTGTCATACTCCTCTGCATTGTCTCCGAAGAAATA
+HWI-EAS443:3:36:1573:1660#0/1
SZba_ab`XX``bTZP¥_`a`UX^_^ZN^`aW__^¥Z`]¥
@HWI-EAS443:3:37:1303:1183#0/1
GGTATTTTTCCTCAACTGTCATACTCCTCTGCATTGTCTC
+HWI-EAS443:3:37:1303:1183#0/1
^bZaababaaaaaaab`a[_aaaabbaaaXa_aaa`_ba_
@HWI-EAS443:3:37:1313:314#0/1
GTATTTTTCCTCAACTGTCATACTCCTCTGCATTGTCTCC
+HWI-EAS443:3:37:1313:314#0/1
`Q^_`aba`^`_¥ZXa]Z^]_`X_^`XRVSa^`]`[¥]]T
@HWI-EAS443:3:37:1341:702#0/1
GGGTATTTTTCCTCAACTGTCATACTCCTCTGCATTGTCT
+HWI-EAS443:3:37:1341:702#0/1
abbYaabaaaa_aaaa_`a]aaaa^a_]_W`aaaa_a]`_
@HWI-EAS443:3:38:506:505#0/1
GGGTATTTTTCCTCAACTGTCATACTCCTCTGCATTGTCT
+HWI-EAS443:3:38:506:505#0/1
a`aS_`aa_[^^^^`_Y^_]__]_`]_Y]WY]]]X]_Y]Y
@HWI-EAS443:3:39:1421:960#0/1
ATTTTTCCTCAACTGTCATACTCCTCTGCATTGTCTCCGA
+HWI-EAS443:3:39:1421:960#0/1
`abbbbaabaaaaaa`aaaa^b^_aa`aaa`^`_a`aWa]
@HWI-EAS443:3:3:770:680#0/1
TTTTCCTCAACTGTCATACTCCTCTGCATTGTCTCCGAAG
+HWI-EAS443:3:3:770:680#0/1
aabba_a_^a`aa`Za`a^a`_``aa^]``a[Xa]]a¥]`
@HWI-EAS443:3:3:825:895#0/1
GTATTTTTCCTCAACTGTCATACTCCTCTGCATTGTCTCC
+HWI-EAS443:3:3:825:895#0/1
`M_`Xba¥^aba`aYa^a_Y_a`a`^[``a[aa`aSY¥XF
@HWI-EAS443:3:42:1080:460#0/1
TTTTCCTCAACTGTCATACTCCTCTGCATTGTCTCCGAAG
+HWI-EAS443:3:42:1080:460#0/1
^SXYFY¥SZZ_^`X¥¥Z_WQWPP]]_UV_NFRZSPHM¥TY
@HWI-EAS443:3:42:149:1975#0/1
CTCAACTGTCATACTCCTCTGCATTGTCTCCGAAGAAATA
+HWI-EAS443:3:42:149:1975#0/1
aa```a]^R_``aa]``W_`X_Z]`^O`^aa¥¥]_`]`Z^
@HWI-EAS443:3:44:1208:1533#0/1
GGTATTTTTCCTCAACTGTCATACTCCTCTGCATTGTCTC
+HWI-EAS443:3:44:1208:1533#0/1
a[FV^_¥XT¥Z`ZY]]_[W[]_[UZZQY]T¥VQXTWTXPM
```

```
@HWI-EAS443:3:44:1476:889#0/1
GGTATTTTTCCTCAACTGTCATACTCCTCTGCATTGTCTC
+HWI-EAS443:3:44:1476:889#0/1
ab^aabaaaa`a``a^a`_`aa__a`^`__a__a``^]_Z
@HWI-EAS443:3:44:789:1208#0/1
GGTATTTTTCCTCAACTGTCATACTCCTCTGCATTGTCTC
+HWI-EAS443:3:44:789:1208#0/1
aYS^aa`_^RVT`W`_X[MS_YSR¥WX[^T[^]^]_SMVY
@HWI-EAS443:3:45:257:2038#0/1
CTCAACTGTCATACTCCTCTGCATTGTCTCCGAAGAAATA
+HWI-EAS443:3:45:257:2038#0/1
Q_`b`UaVaZ`b]K`ZQ_abbVV_bb_¥¥`L_ba_ab`Ya
@HWI-EAS443:3:45:61:493#0/1
TTCCTCAACTGTCATACTCCTCTGCATTGTCTCCGAAGAA
+HWI-EAS443:3:45:61:493#0/1
a^Va`_VS```^_]_]^UXSYXT]ZTV`a^]W_Z_Y^ZNZ
@HWI-EAS443:3:45:80:1177#0/1
GGTATTTTTCCTCAACTGTCATACTCCTCTGCATTGTCTC
+HWI-EAS443:3:45:80:1177#0/1
]_LX¥a``__]__YU¥`_SY¥`_Y]`_]¥_[W¥_`¥YW_[
@HWI-EAS443:3:45:81:1647#0/1
GGGTATTTTTCCTCAACTGTCATACTCCTCTGCATTGTCT
+HWI-EAS443:3:45:81:1647#0/1
abZT[b`abb^ababbaa`_aaaa``^`bSaaT^aaa`aa
@HWI-EAS443:3:47:190:455#0/1
GGTATTTTTCCTCAACTGTCATACTCCTCTGCATTGTCTC
+HWI-EAS443:3:47:190:455#0/1
aaW`abaaa]_a_`a]a`_]__`W_^Y_¥`_S_`_]ZY^¥
@HWI-EAS443:3:48:1702:115#0/1
GGTATTTTTCCTCAACTGTCATACTCCTCTGCATTGTCTC
+HWI-EAS443:3:48:1702:115#0/1
abIaa^a`aXYb_^aWaba^W`¥]_UU`¥`¥RN_`_¥T_R
@HWI-EAS443:3:48:588:2045#0/1
GGGTATTTTTCCTCAACTGTCATACTCCTCTGCATTGTCT
+HWI-EAS443:3:48:588:2045#0/1
H_¥^`bbVbb¥^bba_XXba]b_a¥bbaISVZXaX`Y]T^
@HWI-EAS443:3:48:635:1066#0/1
GGTATTTTTCCTCAACTGTCATACTCCTCTGCATTGTCTC
+HWI-EAS443:3:48:635:1066#0/1
abZaabaaaa`aaa``a_[``````_`aaa__`a_`__`_
@HWI-EAS443:3:48:660:1446#0/1
GGGTATTTTTCCTCAACTGTCATACTCCTCTGCATTGTCT
+HWI-EAS443:3:48:660:1446#0/1
aa`T`aa````a``aa^`a^_`a^[`¥YZ^`_^__`_¥V]
@HWI-EAS443:3:49:1111:654#0/1
GGTATTTTTCCTCAACTGTCATACTCCTCTGCATTGTCTC
+HWI-EAS443:3:49:1111:654#0/1
¥_Ua¥_bbabab`aa_]b`Vbaa_]^Ta`^aa¥_aba_aa
@HWI-EAS443:3:49:1609:259#0/1
GGGTATTTTTCCTCAACTGTCATACTCCTCTGCATTGTCT
```

+HWI-EAS443:3:49:1609:259#0/1
aabR_aaab]`]aS`__aaZN[`_^_`Y]Y]Z^_^__X¥^
@HWI-EAS443:3:4:348:1034#0/1
GGTATTTTTCCTCAACTGTCATACTCCTCTGCATTGTCTC
+HWI-EAS443:3:4:348:1034#0/1
aaYaaaaaaa`aaaa_aaW````^`^^_^____a_a_]_Z
@HWI-EAS443:3:50:1328:1762#0/1
TTTTCCTCAACTGTCATACTCCTCTGCATTGTCTCCGAAG
+HWI-EAS443:3:50:1328:1762#0/1
_^_```baaaaa`^^_b^Ua^^__`a¥abaa[a`][`]`a
@HWI-EAS443:3:51:1356:1204#0/1
GGGTATTTTTCCTCAACTGTCATACTCCTCTGCATTGTCT
+HWI-EAS443:3:51:1356:1204#0/1
abaY]`baa```^aa`aaaZ]a`__a_a`^```^```Z^_
@HWI-EAS443:3:51:404:592#0/1
GGTATTTTTCCTCAACTGTCATACTCCTCTGCATTGTCTC
+HWI-EAS443:3:51:404:592#0/1
aa¥Xaaaba__a¥a_``a_]`a_]_]^a_aa^__``^]`^
@HWI-EAS443:3:51:630:732#0/1
GGGTATTTTTCCTCAACTGTCATACTCCTCTGCATTGTCT
+HWI-EAS443:3:51:630:732#0/1
ab]Q[baaaa`_aa]Y_a`_Ya_`]`Y[]^a`_[_`_]_`
@HWI-EAS443:3:51:980:1109#0/1
TTCCTCAACTGTCATACTCCTCTGCATTGTCTCCGAAGAA
+HWI-EAS443:3:51:980:1109#0/1
abbaaabaaab_aba`aba_b_ba_aabba__a^b__a_^
@HWI-EAS443:3:52:1096:253#0/1
TTTCCTCAACTGTCATACTCCTCTGCATTGTCTCCGAAGA
+HWI-EAS443:3:52:1096:253#0/1
ab`Z]^a^_[`a`]`a`aa¥[_^``Z^a_a`^a]Y_^V^Z
@HWI-EAS443:3:52:1211:628#0/1
GGTATTTTTCCTCAACTGTCATACTCCTCTGCATTGTCTC
+HWI-EAS443:3:52:1211:628#0/1
ab[`aaaaa``a`a__`a^_`a__`]^`Xaa^`_``_W`]
@HWI-EAS443:3:52:1407:808#0/1
GGTATTTTTCCTCAACTGTCATACTCCTCTGCATTGTCTC
+HWI-EAS443:3:52:1407:808#0/1
]]_ZTYb`]TV]``T`¥_TJWXSJOJR`Sa^T`¥`^^GYO
@HWI-EAS443:3:52:842:624#0/1
TTTTCCTCAACTGTCATACTCCTCTGCATTGTCTCCGAAG
+HWI-EAS443:3:52:842:624#0/1
]`a_Z¥a¥_^¥^`¥^Z_a`aa__Z__¥`ba`_]ZV]a]]_
@HWI-EAS443:3:53:820:1079#0/1
TCCTCAACTGTCATACTCCTCTGCATTGTCTCCGAAGAAA
+HWI-EAS443:3:53:820:1079#0/1
^__`_bab_^aaaa`^a`]`aa_aa`_b^_a¥]__]_¥_[
@HWI-EAS443:3:55:150:905#0/1
CTCAACTGTCATACTCCTCTGCATTGTCTCCGAAGAAATA
+HWI-EAS443:3:55:150:905#0/1
a[OXV]a_VZ¥Z]YZ_YY^¥_YPYYZSYV¥^ZPQXTOYXY

```
@HWI-EAS443:3:56:1077:379#0/1
ATTTTTCCTCAACTGTCATACTCCTCTGCATTGTCTCCGA
+HWI-EAS443:3:56:1077:379#0/1
`^`a^¥^b`_^_]^¥RQ^a]V¥^a`^¥b¥aa]]Ua``aa`
@HWI-EAS443:3:56:557:1952#0/1
GGGTATTTTTCCTCAACTGTCATACTCCTCTGCATTGTCT
+HWI-EAS443:3:56:557:1952#0/1
¥ZYGX_¥`aa`````]Y]]YV¥T^^]WW[^¥^_`^¥WV¥Z
@HWI-EAS443:3:57:1293:899#0/1
TTTTCCTCAACTGTCATACTCCTCTGCATTGTCTCCGAAG
+HWI-EAS443:3:57:1293:899#0/1
aba`[¥aa`a_aa``aaa_a`]_]a`¥`a`a___]Y_]_`
@HWI-EAS443:3:57:1414:358#0/1
GGGTATTTTTCCTCAACTGTCATACTCCTCTGCATTGTCT
+HWI-EAS443:3:57:1414:358#0/1
]baWaababb[ab_aa_aaYS`a^_a[]a_aa^Vba`^_a
@HWI-EAS443:3:57:1481:598#0/1
GTATTTTTCCTCAACTGTCATACTCCTCTGCATTGTCTCC
+HWI-EAS443:3:57:1481:598#0/1
_T_aaaa``¥a^_a_]^TX`aa]_Z^`^``Q_^Z^]]_]X
@HWI-EAS443:3:57:677:563#0/1
TTTTTCCTCAACTGTCATACTCCTCTGCATTGTCTCCGAA
+HWI-EAS443:3:57:677:563#0/1
]ba]^]b_Q]b_[]Y^Vb^Pa]_a_^[`a^`a_`a_L^__
@HWI-EAS443:3:58:176:1850#0/1
GGGTATTTTTCCTCAACTGTCATACTCCTCTGCATTGTCT
+HWI-EAS443:3:58:176:1850#0/1
`b`Uab`aaa]aa_aa_``^_``a^`_]^^`]_^_^^]O]
@HWI-EAS443:3:59:1211:736#0/1
TTTTCCTCAACTGTCATACTCCTCTGCATTGTCTCCGAAG
+HWI-EAS443:3:59:1211:736#0/1
abaaaababaaaa_`aaaWa_aa`aaaabaa^```ZR^[[
@HWI-EAS443:3:59:312:24#0/1
GGGTATTTTTCCTCAACTGTCATACTCCTCTGCATTGTCT
+HWI-EAS443:3:59:312:24#0/1
abbO`a`a_a`_]_a__^`^^¥^_]`_]^]_Y]^^ZWYY]
@HWI-EAS443:3:59:347:621#0/1
TATTTTTCCTCAACTGTCATACTCCTCTGCATTGTCTCCG
+HWI-EAS443:3:59:347:621#0/1
abab_aV`bbaYaa_`]aYaa`aaba]¥aba_^aPMK¥[[
@HWI-EAS443:3:5:739:110#0/1
TTTTCCTCAACTGTCATACTCCTCTGCATTGTCTCCGAAG
+HWI-EAS443:3:5:739:110#0/1
abaaa_a^`aZaaZSaaa]a_]aZa_W_a`a^]_][a^_B
@HWI-EAS443:3:60:783:1620#0/1
GGGTATTTTTCCTCAACTGTCATACTCCTCTGCATTGTCT
+HWI-EAS443:3:60:783:1620#0/1
]a`Xabbb`a``babbaba_ababaaa`aaaa^`a^^`a`
@HWI-EAS443:3:61:1006:1028#0/1
ATTTTTCCTCAACTGTCATACTCCTCTGCATTGTCTCCGA
```

+HWI-EAS443:3:61:1006:1028#0/1
TaabaX_^RZ_¥aaVLWaV¥babaa^RZaba`¥XZ¥RX]Q
@HWI-EAS443:3:61:543:1515#0/1
GGTATTTTTCCTCAACTGTCATACTCCTCTGCATTGTCTC
+HWI-EAS443:3:61:543:1515#0/1
`]YX_aaaa`````a`__]`a]_a]aa]_`___]^¥Z]ZZ
@HWI-EAS443:3:63:767:850#0/1
GTATTTTTCCTCAACTGTCATACTCCTCTGCATTGTCTCC
+HWI-EAS443:3:63:767:850#0/1
a]`aaaabab`aa`aaa_ab`ba`aaa``a`aaaa````^
@HWI-EAS443:3:63:853:104#0/1
GTATTTTTCCTCAACTGTCATACTCCTCTGCATTGTCTCC
+HWI-EAS443:3:63:853:104#0/1
a]``a`_a__`_____`]]``_^_X^a]_`Y]_^`]Z^Z]
@HWI-EAS443:3:64:1040:263#0/1
GGTATTTTTCCTCAACTGTCATACTCCTCTGCATTGTCTC
+HWI-EAS443:3:64:1040:263#0/1
]bOb¥HT]ZVba`b][YTRZT``ZZ__`Waa_a``__Z_^
@HWI-EAS443:3:64:1769:395#0/1
GGGTATTTTTCCTCAACTGTCATACTCCTCTGCATTGTCT
+HWI-EAS443:3:64:1769:395#0/1
aba]aabbbba`ba_a_ba¥aa^_^a¥`aaa_a_a`^`_a
@HWI-EAS443:3:65:1129:1932#0/1
GGGTATTTTTCCTCAACTGTCATACTCCTCTGCATTGTCT
+HWI-EAS443:3:65:1129:1932#0/1
a`aS`a_`__¥Wa`^_Z_[V__^]QQVW]X¥X^^VXZXOV
@HWI-EAS443:3:65:726:471#0/1
ATTTTTCCTCAACTGTCATACTCCTCTGCATTGTCTCCGA
+HWI-EAS443:3:65:726:471#0/1
aabbaaabbaaaaaa``aaa`aa``aa`a¥a_a_`aa[a]
@HWI-EAS443:3:66:1182:259#0/1
TTTTCCTCAACTGTCATACTCCTCTGCATTGTCTCCGAAG
+HWI-EAS443:3:66:1182:259#0/1
aaaa`aaaaa^aa^``a``a_^a^`a_a`^_]``^_`]Y_
@HWI-EAS443:3:66:1757:277#0/1
GTATTTTTCCTCAACTGTCATACTCCTCTGCATTGTCTCC
+HWI-EAS443:3:66:1757:277#0/1
a_aaaaaa``a`aa^a`^_aa`_`^__^`_^`^``^^^^Y
@HWI-EAS443:3:66:343:966#0/1
GGGTATTTTTCCTCAACTGTCATACTCCTCTGCATTGTCT
+HWI-EAS443:3:66:343:966#0/1
_abW[abaabbaa`aaaaa_aaaa`aaaaa`aa]`aa_aa
@HWI-EAS443:3:68:1230:1135#0/1
TTTTCCTCAACTGTCATACTCCTCTGCATTGTCTCCGAAG
+HWI-EAS443:3:68:1230:1135#0/1
_`¥XZV^a`Y[`_WUa`Ya¥¥`Za¥_a^_¥JP]XYVKUXY
@HWI-EAS443:3:69:1258:1955#0/1
CCTCAACTGTCATACTCCTCTGCATTGTCTCCGAAGAAAT
+HWI-EAS443:3:69:1258:1955#0/1
^a`[`a¥¥`Kbb``S^¥a_`]ab__bVb_]SbX``ZW¥`_

@HWI-EAS443:3:6:171:1144#0/1
GGTATTTTTCCTCAACTGTCATACTCCTCTGCATTGTCTC
+HWI-EAS443:3:6:171:1144#0/1
aaH`aaaaaaaa_aa_aaY`aa____^`_`a^`^`a]X]a
@HWI-EAS443:3:6:541:1331#0/1
TATTTTTCCTCAACTGTCATACTCCTCTGCATTGTCTCCG
+HWI-EAS443:3:6:541:1331#0/1
Taa_H]^bbba_ZVb`R_bZX[X^`_NYb`b[Y]^OWEN^
@HWI-EAS443:3:6:907:1527#0/1
TTTTCCTCAACTGTCATACTCCTCTGCATTGTCTCCGAAG
+HWI-EAS443:3:6:907:1527#0/1
ababa]baab_aa_`aa^V__^]^aa_a`aa_^aZ]a__`
@HWI-EAS443:3:70:612:1421#0/1
GGGTATTTTTCCTCAACTGTCATACTCCTCTGCATTGTCT
+HWI-EAS443:3:70:612:1421#0/1
`a`Waaa`a_a]aaa_S]`_```aa^]a`a]_]``^^Z^^
@HWI-EAS443:3:72:1154:232#0/1
TTTTCCTCAACTGTCATACTCCTCTGCATTGTCTCCGAAG
+HWI-EAS443:3:72:1154:232#0/1
¥¥``UWN]_a[H]JRW^UYY[[U[`a[_]VR¥U]YY^^VZ
@HWI-EAS443:3:72:239:494#0/1
GGTATTTTTCCTCAACTGTCATACTCCTCTGCATTGTCTC
+HWI-EAS443:3:72:239:494#0/1
aaXaaaaaa`a`^``_`_[`a`a^`__``_____`]^___
@HWI-EAS443:3:72:362:1231#0/1
GTATTTTTCCTCAACTGTCATACTCCTCTGCATTGTCTCC
+HWI-EAS443:3:72:362:1231#0/1
^^W_aaaa_¥Z^VSUaW]`]_]aaa]Tb]¥aY^a^[`Z`a
@HWI-EAS443:3:72:488:706#0/1
GGGTATTTTTCCTCAACTGTCATACTCCTCTGCATTGTCT
+HWI-EAS443:3:72:488:706#0/1
`bbTa_aaa`]aaa^`^]aWaa]W``^_`aa``_]a`^`_
@HWI-EAS443:3:72:736:1781#0/1
ATTTTTCCTCAACTGTCATACTCCTCTGCATTGTCTCCGA
+HWI-EAS443:3:72:736:1781#0/1
[aaaaaab`aa]b``^b]`aa`abaaa_`aa_`]`__`__
@HWI-EAS443:3:72:809:1751#0/1
GGTATTTTTCCTCAACTGTCATACTCCTCTGCATTGTCTC
+HWI-EAS443:3:72:809:1751#0/1
a]Paaabb^abaa^_[aa_aaaa`^`]``a`^_W]_Ya]a
@HWI-EAS443:3:73:636:1970#0/1
GGGTATTTTTCCTCAACTGTCATACTCCTCTGCATTGTCT
+HWI-EAS443:3:73:636:1970#0/1
aa]Ua¥GZ`Saa_aYS`^U[Y]Y``^`YS]_^a_]¥TN[Z
@HWI-EAS443:3:73:979:545#0/1
GTATTTTTCCTCAACTGTCATACTCCTCTGCATTGTCTCC
+HWI-EAS443:3:73:979:545#0/1
`[]aaaa`a__¥_`¥__X¥^`__^¥Z^_^`^_a__^^_]]
@HWI-EAS443:3:74:876:1388#0/1
TTCCTCAACTGTCATACTCCTCTGCATTGTCTCCGAAGAA

```
+HWI-EAS443:3:74:876:1388#0/1
]][RRZLS]WaW^¥_Z_¥]a][a^aY`OGI¥`]`Y]L^]_
@HWI-EAS443:3:75:1335:1022#0/1
ATTTTTCCTCAACTGTCATACTCCTCTGCATTGTCTCCGA
+HWI-EAS443:3:75:1335:1022#0/1
a^`XH¥Y_bb``Z_¥N¥aaa[Z^^_]`VZ[GUbOK][ZbY
@HWI-EAS443:3:76:940:1094#0/1
TTTTCCTCAACTGTCATACTCCTCTGCATTGTCTCCGAAG
+HWI-EAS443:3:76:940:1094#0/1
aaabaa^b_aa_aT`_a`_aaaa_a^_]`¥_^^^`^a¥__
@HWI-EAS443:3:77:1416:1630#0/1
TTTTCCTCAACTGTCATACTCCTCTGCATTGTCTCCGAAG
+HWI-EAS443:3:77:1416:1630#0/1
Ua[bUbbaabb`bYbbb¥`a[`b`^]U[aaaaaaa_``_a
@HWI-EAS443:3:77:751:835#0/1
GGTATTTTTCCTCAACTGTCATACTCCTCTGCATTGTCTC
+HWI-EAS443:3:77:751:835#0/1
aaSaaaaa_[]`aa]^`a`aaaa_aa^`_][_`a`_`__]
@HWI-EAS443:3:77:843:261#0/1
GGTATTTTTCCTCAACTGTCATACTCCTCTGCATTGTCTC
+HWI-EAS443:3:77:843:261#0/1
aaWOT[a[XRX`Y_[XZ_WW^^_X¥]ZUQ]^Z^Z^_VZZ^
@HWI-EAS443:3:78:1704:1965#0/1
TTTTCCTCAACTGTCATACTCCTCTGCATTGTCTCCGAAG
+HWI-EAS443:3:78:1704:1965#0/1
a^aYMY_]_aP¥WUW^`_Y]XWZY_]VW^X_]XWMDXX[]
@HWI-EAS443:3:78:236:112#0/1
GGGTATTTTTCCTCAACTGTCATACTCCTCTGCATTGTCT
+HWI-EAS443:3:78:236:112#0/1
aaaZ^a_`aa`]RYa`]]^]]]__]_^]^^^^^^^^^XW]
@HWI-EAS443:3:78:284:915#0/1
TTTTCCTCAACTGTCATACTCCTCTGCATTGTCTCCGAAG
+HWI-EAS443:3:78:284:915#0/1
abaaaaaabaa_a`b¥_aaa``_aa_]aa_aZ`__^`^_`
@HWI-EAS443:3:78:379:1155#0/1
CTCAACTGTCATACTCCTCTGCATTGTCTCCGAAGAAATA
+HWI-EAS443:3:78:379:1155#0/1
a_¥X_W[XWP[Y¥S_X_aaa^ZZ¥O¥Z]]`_YZY[ORYY`
@HWI-EAS443:3:78:707:1012#0/1
GGGTATTTTTCCTCAACTGTCATACTCCTCTGCATTGTCT
+HWI-EAS443:3:78:707:1012#0/1
_S_Ga`U`¥`]_aSa_¥__I]VRZVWZ_]_X_]¥VQTSU[
@HWI-EAS443:3:79:869:232#0/1
GGGTATTTTTCCTCAACTGTCATACTCCTCTGCATTGTCT
+HWI-EAS443:3:79:869:232#0/1
a``W`_Z[Z[^^a_TZMXZTT^X_¥^YH]VSW_^^QZ[X^
@HWI-EAS443:3:80:1760:71#0/1
GGGTATTTTTCCTCAACTGTCATACTCCTCTGCATTGTCT
+HWI-EAS443:3:80:1760:71#0/1
abbYaabaab`_a^a`_aa_Zaa`]`T¥`]``W_`__]X[
```

@HWI-EAS443:3:81:1185:948#0/1
CCTCAACTGTCATACTCCTCTGCATTGTCTCCGAAGAAAT
+HWI-EAS443:3:81:1185:948#0/1
aaa`¥`_aa^`^Zaaa_`a`__aZX_aW^_T¥a]_^OSSS
@HWI-EAS443:3:81:1627:1099#0/1
GGGTATTTTTCCTCAACTGTCATACTCCTCTGCATTGTCT
+HWI-EAS443:3:81:1627:1099#0/1
`aYD[bbabaaaa^`a_a`_``a^_a`_a_aa_`baa[]`
@HWI-EAS443:3:81:964:1672#0/1
TTTTCCTCAACTGTCATACTCCTCTGCATTGTCTCCGAAG
+HWI-EAS443:3:81:964:1672#0/1
aaa]R_`aaa`Y]V[`_¥Ya^R_```]¥¥`_Z``a¥][_Z
@HWI-EAS443:3:82:1694:1489#0/1
TTTTCCTCAACTGTCATACTCCTCTGCATTGTCTCCGAAG
+HWI-EAS443:3:82:1694:1489#0/1
abbba`bbabbbbaabbb`b¥ab`b^]abbaaaa^`a`aa
@HWI-EAS443:3:83:1375:707#0/1
TTTTCCTCAACTGTCATACTCCTCTGCATTGTCTCCGAAG
+HWI-EAS443:3:83:1375:707#0/1
a`b_`aaYb`abQN]a`aS_^Y]O`_]`a¥TXSaaGaZ^]
@HWI-EAS443:3:85:1132:241#0/1
GGGTATTTTTCCTCAACTGTCATACTCCTCTGCATTGTCT
+HWI-EAS443:3:85:1132:241#0/1
aaaXabbaaa__a_aa^aa_^aa^^]UYa^__^_a^_Y]^
@HWI-EAS443:3:85:1394:533#0/1
TCCTCAACTGTCATACTCCTCTGCATTGTCTCCGAAGAAA
+HWI-EAS443:3:85:1394:533#0/1
`]]^a]a¥a`XYa]]¥a][_Y]`Y[YU¥¥_`[`¥][a]__
@HWI-EAS443:3:85:59:1009#0/1
TTTTCCTCAACTGTCATACTCCTCTGCATTGTCTCCGAAG
+HWI-EAS443:3:85:59:1009#0/1
aaaaaaa`ab__a_`aaaa`aa```^__a_^]_^a_`^[^
@HWI-EAS443:3:86:107:1992#0/1
TTTCTTCGGAGACAATGCAGAGGAGTATGACAGTTGAGGA
+HWI-EAS443:3:86:107:1992#0/1
ababaaa¥^`¥aaaaaaa^[¥[Y`]SaY^¥WLSZ[HUNZZ
@HWI-EAS443:3:86:499:1082#0/1
GGTATTTTTCCTCAACTGTCATACTCCTCTGCATTGTCTC
+HWI-EAS443:3:86:499:1082#0/1
^aQSZab^Y^aaWWWa`a]a`^^]__`^a_`aaaa`_a``
@HWI-EAS443:3:86:731:1074#0/1
TATTTTTCCTCAACTGTCATACTCCTCTGCATTGTCTCCG
+HWI-EAS443:3:86:731:1074#0/1
`a`_W^a__aaa`[_aGaaa^__a_¥PT__a`O_ZVLS]_
@HWI-EAS443:3:87:657:723#0/1
TATTTTTCCTCAACTGTCATACTCCTCTGCATTGTCTCCG
+HWI-EAS443:3:87:657:723#0/1
[^a¥Z`a¥]__L[[XY_V_`W¥a^`^T_baa]]YX_P[UY
@HWI-EAS443:3:88:1078:247#0/1
GGTATTTTTCCTCAACTGTCATACTCCTCTGCATTGTCTC

+HWI-EAS443:3:88:1078:247#0/1
`aS^babab_aaaa¥aa`TR_aa_`_]a^]IR_`_`^]^]
@HWI-EAS443:3:89:955:1793#0/1
TTTTCCTCAACTGTCATACTCCTCTGCATTGTCTCCGAAG
+HWI-EAS443:3:89:955:1793#0/1
aaabYbaa^`P_^`aaab`ab]_`ab_`^aa^_a_]aZ`_
@HWI-EAS443:3:8:279:358#0/1
CCTCAACTGTCATACTCCTCTGCATTGTCTCCGAAGAAAT
+HWI-EAS443:3:8:279:358#0/1
aaVa]aa^`_YY`aa¥`]`_^_X]_``ZXW]^_YX_]Z][
@HWI-EAS443:3:90:1727:389#0/1
GGGTATTTTTCCTCAACTGTCATACTCCTCTGCATTGTCT
+HWI-EAS443:3:90:1727:389#0/1
abb]abbbbba`a__a`aa`_aaa^a__a_aaa^aaa`^`
@HWI-EAS443:3:90:913:1280#0/1
GGTATTTTTCCTCAACTGTCATACTCCTCTGCATTGTCTC
+HWI-EAS443:3:90:913:1280#0/1
ab¥abbbbbbbaaabaaaXaaba`a`[``_a_b`aa_^`Y
@HWI-EAS443:3:92:1001:1770#0/1
TATTTTTCCTCAACTGTCATACTCCTCTGCATTGTCTCCG
+HWI-EAS443:3:92:1001:1770#0/1
aaaaa`_`a``a_`a`X]a^`aa[]a_aa`a_^_YV[^^_
@HWI-EAS443:3:92:1641:180#0/1
GGTATTTTTCCTCAACTGTCATACTCCTCTGCATTGTCTC
+HWI-EAS443:3:92:1641:180#0/1
`^WI___^YSQ]ZU^Z]]VVZZZGVTUQSUVXWZVXXGZX
@HWI-EAS443:3:92:530:1424#0/1
TTCCTCAACTGTCATACTCCTCTGCATTGTCTCCGAAGAA
+HWI-EAS443:3:92:530:1424#0/1
`bbbba^aa`aTaa`WX¥bb_K^^bba_`^aaa`]a_aaa
@HWI-EAS443:3:93:533:1667#0/1
TTTTCCTCAACTGTCATACTCCTCTGCATTGTCTCCGAAG
+HWI-EAS443:3:93:533:1667#0/1
Ybbb^Y_Z¥`_]NY]^Ya`X^_Za_aaa¥WaZXR¥_b`Q^
@HWI-EAS443:3:94:148:1433#0/1
GGTATTTTTCCTCAACTGTCATACTCCTCTGCATTGTCTC
+HWI-EAS443:3:94:148:1433#0/1
ab[abba`aaaa`bbaab_a`ab`a`a`aa`a`aaa^_``
@HWI-EAS443:3:94:230:1711#0/1
GGTATTTTTCCTCAACTGTCATACTCCTCTGCATTGTCTC
+HWI-EAS443:3:94:230:1711#0/1
`a¥a¥b`aaaaYaa`bZP¥aa_`a_``a``X^aa]X^`_Z
@HWI-EAS443:3:95:60:1018#0/1
GGTATTTTTCCTCAACTGTCATACTCCTCTGCATTGTCTC
+HWI-EAS443:3:95:60:1018#0/1
a_QOW[HRU_W]_a^[X¥VZVTZW^ZNZ__]TW]^PPLRW
@HWI-EAS443:3:95:907:708#0/1
TTTCCTCAACTGTCATACTCCTCTGCATTGTCTCCGAAGA
+HWI-EAS443:3:95:907:708#0/1
abb`aaaa_`aa^^`aa]a^aa_a`[aaaa^^a^_b^_a]

```
@HWI-EAS443:3:95:955:317#0/1
TTTCCTCAACTGTCATACTCCTCTGCATTGTCTCCGAAGA
+HWI-EAS443:3:95:955:317#0/1
abbaab`abaaba_abb_b__aaab^abaa__`N^``^a_
@HWI-EAS443:3:96:1241:1622#0/1
GGGTATTTTTCCTCAACTGTCATACTCCTCTGCATTGTCT
+HWI-EAS443:3:96:1241:1622#0/1
aa]X`aaaab___`aa`aa^__`_``ZW`__^_``^_^]_
@HWI-EAS443:3:96:258:1454#0/1
TTCCTCAACTGTCATACTCCTCTGCATTGTCTCCGAAGAA
+HWI-EAS443:3:96:258:1454#0/1
aabba_YW¥^WNU_S[a^]`¥]````]`aOMZaXZJW`N[
@HWI-EAS443:3:96:539:984#0/1
TTTCCTCAACTGTCATACTCCTCTGCATTGTCTCCGAAGA
+HWI-EAS443:3:96:539:984#0/1
_VRXa`^^a``a_^`W`a[GYY[_XRZ]^]V_`^^a`[[]
@HWI-EAS443:3:97:1175:399#0/1
ATTTTTCCTCAACTGTCATACTCCTCTGCATTGTCTCCGA
+HWI-EAS443:3:97:1175:399#0/1
abbbabaabX_aaab]baba__¥U¥`ab]aaaa`Xa_Xa_
@HWI-EAS443:3:97:1448:1014#0/1
GTATTTTTCCTCAACTGTCATACTCCTCTGCATTGTCTCC
+HWI-EAS443:3:97:1448:1014#0/1
`a`_aaba¥¥_W`_]_a]V___XaX¥_Z``XX_^]^Z^ZX
@HWI-EAS443:3:97:412:1747#0/1
GGGTATTTTTCCTCAACTGTCATACTCCTCTGCATTGTCT
+HWI-EAS443:3:97:412:1747#0/1
^`aYabbbbbbaa¥baabaWba`aaaaa[aaa``a__¥aa
@HWI-EAS443:3:98:1007:1787#0/1
TTTCCTCAACTGTCATACTCCTCTGCATTGTCTCCGAAGA
+HWI-EAS443:3:98:1007:1787#0/1
`[V^]aa`b_aaX_aaaa^^¥__``_aa_a^¥^WY`_[a_
@HWI-EAS443:3:98:230:243#0/1
TTTTCCTCAACTGTCATACTCCTCTGCATTGTCTCCGAAG
+HWI-EAS443:3:98:230:243#0/1
_¥¥ZS`ba¥bVaST_]¥a_]_Zba_aYa`¥b__aa_a]Na
@HWI-EAS443:3:98:433:691#0/1
GGTATTTTTCCTCAACTGTCATACTCCTCTGCATTGTCTC
+HWI-EAS443:3:98:433:691#0/1
aaP_a`[`Z`[^__aW`a[_a___^[^¥Z``^¥_ZZ]^]_
@HWI-EAS443:3:98:790:913#0/1
GGGTATTTTTCCTCAACTGTCATACTCCTCTGCATTGTCT
+HWI-EAS443:3:98:790:913#0/1
ab]Q`_a`YYRTa[YS``YP^X[Xa[M^Y¥]SW]]V^MRW
@HWI-EAS443:3:99:1063:1023#0/1
GTATTTTTCCTCAACTGTCATACTCCTCTGCATTGTCTCC
+HWI-EAS443:3:99:1063:1023#0/1
`aaabbbb_aa_aaaaaa^aab_a`_a_aa_aaaa```_^
@HWI-EAS443:3:76:1012:1971#0/1
GAGGGTGACAAAAACATAATGGACTCCAACAGCATGTCAA
```

+HWI-EAS443:3:76:1012:1971#0/1
TYJYbOO___`bbbabaaa_]XT¥SHJ_]Y¥DLa_V__aa
@HWI-EAS443:3:67:657:1363#0/1
GCAGGGCGACAAAAACATAATGGACTCCAACACCATGTCA
+HWI-EAS443:3:67:657:1363#0/1
abba`[DWabYab`_`babaa_`aaaa``a`a]aaaa^aa
@HWI-EAS443:3:75:687:413#0/1
GCAGGGCGACAAAAACATAATGGACTCCAACACCATGTCA
+HWI-EAS443:3:75:687:413#0/1
````^]GY^]b`_a^_^`_Z]aa]aaa_^¥Z_aa_aa_]^
@HWI-EAS443:3:98:494:863#0/1
GGGTGACAAAAACATAATGGACTCCAACACCANGTCAAGC
+HWI-EAS443:3:98:494:863#0/1
`YY^_Y`^¥`[^`U`_``_T¥^`Z_^^][__XDT[]_Y_]
@HWI-EAS443:3:20:1382:511#0/1
TGGGTGACAAAAACATAATGGACTCCAACACCATGTCAAG
+HWI-EAS443:3:20:1382:511#0/1
abbb_bbababaa`bbaaaaaa_ba_aaaa__aaa_X```
@HWI-EAS443:3:54:419:1217#0/1
TGCAGGGTGACAAAAACATAATGGACTCCAACACCATGTC
+HWI-EAS443:3:54:419:1217#0/1
abaaaa^¥_aba^`a`abbbaaa``aaaaa``aa^aaa`a
@HWI-EAS443:3:68:236:732#0/1
GGGGTGACAAAAACATAATGGACTCCAACACCATGTCAAG
+HWI-EAS443:3:68:236:732#0/1
aaZYZbbaa]Q_`bb_ab_a_a^]a_`a`aa`a_a¥__^`
@HWI-EAS443:3:11:1087:23#0/1
AGGGTGACAAAAACATAATGGACTCCAACACCATGTCAAG
+HWI-EAS443:3:11:1087:23#0/1
abbb]baabba`ab_a]N^__^]]]]]_Z]ZZ]]^ZU]Z^
@HWI-EAS443:3:12:1488:855#0/1
AGCAGGGTGACAAAAACATAATGGACTCCAACACCATGTC
+HWI-EAS443:3:12:1488:855#0/1
`aaaabbWbb__a^aa_`aaa_a¥a^a_^`a_`R_aaa_]
@HWI-EAS443:3:13:41:391#0/1
GGGTGACAAAAACATAATGGACTCCAACACCATGTCAAGC
+HWI-EAS443:3:13:41:391#0/1
aabHa]a`VVT__V_`__aaaa_a[^^a`a]^__Y``P_^
@HWI-EAS443:3:13:974:277#0/1
CAGGGTGACAAAAACATAATGGACTCCAACACCATGTCAA
+HWI-EAS443:3:13:974:277#0/1
aaa_OF_aaaa¥V]a_a[`a^`a^aaaZXXaYWa^aZZ¥X
@HWI-EAS443:3:14:1241:767#0/1
CAGGGTGACAAAAACATAATGGACTCCAACACCATGTCAA
+HWI-EAS443:3:14:1241:767#0/1
aabba^ababaabaaaaa`aaaaaaaa`^F^`^aaa_`a_
@HWI-EAS443:3:14:181:1693#0/1
CAGGGTGACAAAAACATAATGGACTCCAACACCATGTCAA
+HWI-EAS443:3:14:181:1693#0/1
aa_Y_V[`aa`aaa_a`a]`¥^a`__``a`a_Za_]]__^
````

```
@HWI-EAS443:3:15:1112:1631#0/1
CAGGGTGACAAAAACATAATGGACTCCAACACCATGTCAA
+HWI-EAS443:3:15:1112:1631#0/1
_a`a_RYab`ba_bbbaa^^W^baa`^bbbb_Y¥b`R__U
@HWI-EAS443:3:17:192:641#0/1
AGCAGGGTGACAAAAACATAATGGACTCCAACACCATGTC
+HWI-EAS443:3:17:192:641#0/1
aaaa`a`Waa_a][aa_a`a`a_Z__^``^a_a_¥_`_Z_
@HWI-EAS443:3:19:739:1168#0/1
AGCAGGGTGACAAAAACATAATGGACTCCAACACCATGTC
+HWI-EAS443:3:19:739:1168#0/1
```Y`aaVa``aa^`baaaa_aa_`aaaaa`aa_^a`aZ^
@HWI-EAS443:3:19:845:759#0/1
GGGTGACAAAAACATAATGGACTCCAACACCATGTCAAGC
+HWI-EAS443:3:19:845:759#0/1
`¥YU``][a__]_]_a``a^_^]X_]`Q^XQ_^_WT]]^O
@HWI-EAS443:3:22:1449:1633#0/1
GGGTGACAAAAACATAATGGACTCCAACACCATGTCAAGC
+HWI-EAS443:3:22:1449:1633#0/1
`b`Oba_a_Y`_`]¥_`a_`_^`a_``Z`^^`^_]^[X[X
@HWI-EAS443:3:23:1179:945#0/1
AAAAGCAGGGTGACAAAAACATAATGGACTCCAACACCAT
+HWI-EAS443:3:23:1179:945#0/1
```abbbbba¥baaaaaabaaaa_aa`a_aa]a``aa]aa
@HWI-EAS443:3:23:1587:361#0/1
GGGTGACAAAAACATAATGGACTCCAACACCATGTCAAGC
+HWI-EAS443:3:23:1587:361#0/1
ab`^_aabbaba`baaabbb__a__aa_b^`aaa`_a_a]
@HWI-EAS443:3:25:925:121#0/1
CAGGGTGACAAAAACATAATGGACTCCAACACCATGTCAA
+HWI-EAS443:3:25:925:121#0/1
^bbbb_bbbbbbabbbbbbbbaaba`abab`Ybab_Uaa
@HWI-EAS443:3:26:116:1514#0/1
GGGTGACAAAAACATAATGGACTCCAACACCATGTCAAGC
+HWI-EAS443:3:26:116:1514#0/1
¥Y¥PY_aM[baU[aX¥a]`R`_`a^Ra]`a_a`aNXaKWW
@HWI-EAS443:3:26:139:206#0/1
GCAGGGTGACAAAAACATAATGGACTCCAACACCATGTCA
+HWI-EAS443:3:26:139:206#0/1
`ababbVbabaa`aa`a`aaaaa`_`a`[`_a__a`a^__
@HWI-EAS443:3:27:1473:1815#0/1
AGGGTGACAAAAACATAATGGACTCCAACACCATGTCAAG
+HWI-EAS443:3:27:1473:1815#0/1
_¥^¥Haa``bb_bb`bbb_bb`aa_a_a]``_¥bb`_aaa
@HWI-EAS443:3:27:226:1146#0/1
AAAGCAGGGTGACAAAAACATAATGGACTCCAACACCATG
+HWI-EAS443:3:27:226:1146#0/1
YR]`a``TSPb]b]`KL^`Z`W`¥¥`ZY`aa]W]XY`_^`
@HWI-EAS443:3:28:160:1434#0/1
AGCAGGGTGACAAAAACATAATGGACTCCAACACCATGTC
```

```
+HWI-EAS443:3:28:160:1434#0/1
a]a_[aY]a`bbaaaaab_`T^a`abaaa_Q_aaaa]`U^
@HWI-EAS443:3:29:348:186#0/1
GGGTGACAAAAACATAATGGACTCCAACACCATGTCAAGC
+HWI-EAS443:3:29:348:186#0/1
ab`_a_bbbaabaaaaaaaaaaaaaaaaa`a`a^__]`^
@HWI-EAS443:3:2:598:1187#0/1
CAGGGTGACAAAAACATAATGGACTCCAACACCATGTCAA
+HWI-EAS443:3:2:598:1187#0/1
Y``abHbb`V`a`¥a`b`baba[a]]¥``aaa_aabUZ__
@HWI-EAS443:3:32:219:132#0/1
GCAGGGTGACAAAAACATAATGGACTCCAACACCATGTCA
+HWI-EAS443:3:32:219:132#0/1
aaa_Y]G``Y__[_¥]^^^V]^XXX^^]XXXX]YX]XXXV
@HWI-EAS443:3:32:392:956#0/1
GCAGGGTGACAAAAACATAATGGACTCCAACACCATGTCA
+HWI-EAS443:3:32:392:956#0/1
]abb`b_b_ba`¥b`abaUYUab]aabbabaZbaYaa_bb
@HWI-EAS443:3:32:438:532#0/1
AAGCAGGGTGACAAAAACATAATGGACTCCAACACCATGT
+HWI-EAS443:3:32:438:532#0/1
a`a````^S^Y^`X¥___`_a_^_]]Y]`]_`]^]]]]]Z
@HWI-EAS443:3:32:528:1404#0/1
AAGCAGGGTGACAAAAACATAATGGACTCCAACACCATGT
+HWI-EAS443:3:32:528:1404#0/1
aa`aaaa^Vaaa_[a___^`[__XY`a____`a`_]_OTM
@HWI-EAS443:3:32:713:500#0/1
GCAGGGTGACAAAAACATAATGGACTCCAACACCATGTCA
+HWI-EAS443:3:32:713:500#0/1
`bb`_¥Vab_b``aa]a_bZ^`[^__`]``]aa_aXaT`R
@HWI-EAS443:3:33:491:602#0/1
GGGTGACAAAAACATAATGGACTCCAACACCATGTCAAGC
+HWI-EAS443:3:33:491:602#0/1
^^aZaZa^^aaaba`¥[¥aabbbbb`_abbbbab`abaa`
@HWI-EAS443:3:45:935:376#0/1
AGCAGGGTGACAAAAACATAATGGACTCCAACACCATGTC
+HWI-EAS443:3:45:935:376#0/1
`baaabb^bbaaaa_aa_baaabaa`a^_aa_``a`aa__
@HWI-EAS443:3:47:1430:953#0/1
GCAAAAGCAGGGTGACAAAAACATAATGGACTCCAACACC
+HWI-EAS443:3:47:1430:953#0/1
^a_¥]Vaaab`aX``¥a_[XWKa_T_`aa¥^XS¥¥HR¥ZH
@HWI-EAS443:3:47:1618:1616#0/1
CAAAAGCAGGGTGACAAAAACATAATGGACTCCAACACCA
+HWI-EAS443:3:47:1618:1616#0/1
`^XaUa``b¥R[_``__P_a_``_^`aa[_`Y_`WSaWX]
@HWI-EAS443:3:47:212:1830#0/1
AGCAGGGTGACAAAAACATAATGGACTCCAACACCATGTC
+HWI-EAS443:3:47:212:1830#0/1
a]Y_Y[aUS`a`ZXba`baaZ]`M`[a]aW__a`a`aa^^
```

@HWI-EAS443:3:49:338:723#0/1
AAAGCAGGGTGACAAAAACATAATGGACTCCAACACCATG
+HWI-EAS443:3:49:338:723#0/1
aaaaa]^baX```a`a`a^aaaa[a`a]a]_`_``]_`ba
@HWI-EAS443:3:50:1528:500#0/1
GGGTGACAAAAACATAATGGACTCCAACACCATGTCAAGC
+HWI-EAS443:3:50:1528:500#0/1
abb]`babaabbabbaab]aa_aa_aa`a__aaa]_```_
@HWI-EAS443:3:52:1189:1685#0/1
GGGTGACAAAAACATAATGGACTCCAACACCATGTCAAGC
+HWI-EAS443:3:52:1189:1685#0/1
abaWa_aab_aa_aa^`a_`___]^_a¥``__aa_^`]__
@HWI-EAS443:3:52:966:75#0/1
AGGGTGACAAAAACATAATGGACTCCAACACCATGTCAAG
+HWI-EAS443:3:52:966:75#0/1
abbaG[¥¥a¥^_a^_a_`ba_`^__¥__X]¥_aaa[_[X`
@HWI-EAS443:3:53:859:1043#0/1
GCAGGGTGACAAAAACATAATGGACTCCAACACCATGTCA
+HWI-EAS443:3:53:859:1043#0/1
aaaaabWaaaa```a_``a]aa`^aa_^_`^`__``aZ^_
@HWI-EAS443:3:55:353:1146#0/1
CAGGGTGACAAAAACATAATGGACTCCAACACCATGTCAA
+HWI-EAS443:3:55:353:1146#0/1
`^SZ``bbb`Z`_bb]bbbabaa`baaabab`^aabZa`a
@HWI-EAS443:3:61:1754:188#0/1
GCAGGGTGACAAAAACATAATGGACTCCAACACCATGTCA
+HWI-EAS443:3:61:1754:188#0/1
aaaa`aWa_¥__¥_^^^a__`a__``U[]^V^SQ^_]VUW
@HWI-EAS443:3:61:588:1109#0/1
GGGTGACAAAAACATAATGGACTCCAACACCATGTCAAGC
+HWI-EAS443:3:61:588:1109#0/1
aX]Z_abbbaaaaa`[a_X[a_^aaW^``X¥Z_]V]XXY]
@HWI-EAS443:3:67:1433:1169#0/1
CAGGGTGACAAAAACATAATGGACTCCAACACCATGTCAA
+HWI-EAS443:3:67:1433:1169#0/1
a]b_[FY^a]Zaaa__aZWa`^^`_`^^^`a``_aa¥X]_
@HWI-EAS443:3:69:1602:1947#0/1
CAGGGTGACAAAAACATAATGGACTCCAACACCATGTCAA
+HWI-EAS443:3:69:1602:1947#0/1
`abba_bbaaa]ababaaa[a[`babaa`aaaaaaa^aa[
@HWI-EAS443:3:69:385:691#0/1
GGGTGACAAAAACATAATGGACTCCAACACCATGTCAAGC
+HWI-EAS443:3:69:385:691#0/1
a`aS^aa_^`]a`aaa]``_a``aaa[]aaaaaa^aa_aW
@HWI-EAS443:3:69:800:214#0/1
GCAGGGTGACAAAAACATAATGGACTCCAACACCATGTCA
+HWI-EAS443:3:69:800:214#0/1
ab`^aaYbaaaaa_Q[aa_]^_^`]_]^`_Y^YY_^`XX]
@HWI-EAS443:3:6:410:1318#0/1
GGGTGACAAAAACATAATGGACTCCAACACCATGTCAAGC

```
+HWI-EAS443:3:6:410:1318#0/1
abUV__bbbYaa]`abb`a`aa]`[[Za`[babaXaV_aR
@HWI-EAS443:3:70:350:1781#0/1
AGCAGGGTGACAAAAACATAATGGACTCCAACACCATGTC
+HWI-EAS443:3:70:350:1781#0/1
_a_¥¥a[F^``a_]Z[Y`]Z[U[_aa^_WZX_`]]_Z[[]
@HWI-EAS443:3:71:1473:734#0/1
CAGGGTGACAAAAACATAATGGACTCCAACACCATGTCAA
+HWI-EAS443:3:71:1473:734#0/1
abbbbabbbabbbbbbbbbbbaaabbbabbabbbbbbaaa
@HWI-EAS443:3:72:1016:479#0/1
CAAAAGCAGGGTGACAAAAACATAATGGACTCCAACACCA
+HWI-EAS443:3:72:1016:479#0/1
ababaaabaaa]ba_aabaaababaababaaTaaa`aa_a
@HWI-EAS443:3:72:1344:958#0/1
AGCAGGGTGACAAAAACATAATGGACTCCAACACCATGTC
+HWI-EAS443:3:72:1344:958#0/1
aa`a`[_Gaa`a_a`a^aaa_`a^````]``]aY_^^`Y_
@HWI-EAS443:3:81:243:2025#0/1
AAAGCAGGGTGACAAAAACATAATGGACTCCAACACCATG
+HWI-EAS443:3:81:243:2025#0/1
[_`]]__`XHX^aaZJR__]]^YRVY[^YXY^^^S_^V]B
@HWI-EAS443:3:81:393:1330#0/1
AGCAGGGTGACAAAAACATAATGGACTCCAACACCATGTC
+HWI-EAS443:3:81:393:1330#0/1
a`¥^``ZG_b_[`a]_aaaaY[a¥``_aaa_aa^a`_`X]
@HWI-EAS443:3:81:962:1224#0/1
CAGGGTGACAAAAACATAATGGACTCCAACACCATGTCAA
+HWI-EAS443:3:81:962:1224#0/1
a_`¥_]bba`¥Z_b```ba`a[aa`a`a_abaaaab[[a_
@HWI-EAS443:3:83:1015:730#0/1
GGGTGACAAAAACATAATGGACTCCAACACCATGTCAAGC
+HWI-EAS443:3:83:1015:730#0/1
``aR_aaa^]aaaab^]aaaaa^_aaa_b[_`aa__a^a_
@HWI-EAS443:3:83:914:1329#0/1
CAAAAGCAGGGTGACAAAAACATAATGGACTCCAACACCA
+HWI-EAS443:3:83:914:1329#0/1
``bab]a`Qa`¥[[`_T^¥aab^WJ^ZGX``___aXaW``
@HWI-EAS443:3:85:106:1235#0/1
GGGTGACAAAAACATAATGGACTCCAACACCATGTCAAGC
+HWI-EAS443:3:85:106:1235#0/1
Ybb``bbZbZNZab_`¥`[R`b_`ab_UY`Y[`_]aaXR_
@HWI-EAS443:3:85:933:1153#0/1
GCAGGGTGACAAAAACATAATGGACTCCAACACCATGTCA
+HWI-EAS443:3:85:933:1153#0/1
a`^a^^U__[ZUZZa_^¥^aYWN¥[_a_¥[XW``U^¥LX^
@HWI-EAS443:3:86:760:689#0/1
GCAGGGTGACAAAAACATAATGGACTCCAACACCATGTCA
+HWI-EAS443:3:86:760:689#0/1
aaabb_SZa``[a``_`aa`aaa_^`_a`]RZaa`a`SY_
```

```
@HWI-EAS443:3:87:891:381#0/1
GGGTGACAAAAACATAATGGACTCCAACACCATGTCAAGC
+HWI-EAS443:3:87:891:381#0/1
]b^Zb`ba`^``]H¥bZ`aaa``[abbabaaabaSaa``W
@HWI-EAS443:3:8:942:1175#0/1
GCAAAAGCAGGGTGACAAAAACATAATGGACTCCAACACC
+HWI-EAS443:3:8:942:1175#0/1
T_]_ab_`a¥_aSba[`Z^`Y`a`aXYba^_`a]___[XZ
@HWI-EAS443:3:90:777:1423#0/1
CAGGGTGACAAAAACATAATGGACTCCAACACCATGTCAA
+HWI-EAS443:3:90:777:1423#0/1
``^``WXaa]`_^`^^[]ZW``YZ_`TY_PX``a__Qa^V
@HWI-EAS443:3:92:1118:442#0/1
GGGTGACAAAAACATAATGGACTCCAACACCATGTCAAGC
+HWI-EAS443:3:92:1118:442#0/1
J_¥Sa]bb¥T¥^X_a¥a^a``a^^baaM¥aa¥aa`a`]`a
@HWI-EAS443:3:93:1674:611#0/1
AGCAGGGTGACAAAAACATAATGGACTCCAACACCATGTC
+HWI-EAS443:3:93:1674:611#0/1
a`^aZ`aYaa``_]aa_`a``a``^Y_^^]___XU^__XU
@HWI-EAS443:3:94:1295:968#0/1
CAGGGTGACAAAAACATAATGGACTCCAACACCATGTCAA
+HWI-EAS443:3:94:1295:968#0/1
IV`bbY`aa[a¥¥`bbbb__bbbabbabbab]Uabba_b`
@HWI-EAS443:3:98:1220:850#0/1
CAGGGTGACAAAAACATAATGGACTCCAACACCATGTCAA
+HWI-EAS443:3:98:1220:850#0/1
]bbbb`bbabbbbbbbbbbbbbbabbaabab]^abba^ba
@HWI-EAS443:3:99:807:436#0/1
GGGTGACAAAAACATAATGGACTCCAACACCATGTCAAGC
+HWI-EAS443:3:99:807:436#0/1
abaSb`babbaa_bbaabaaaaa_aaaaa`]aaba_a`_a
@HWI-EAS443:3:52:1356:1798#0/1
CAATATGGGGAGAATAAAAGAACTGAGAGATCTAATGTCG
+HWI-EAS443:3:52:1356:1798#0/1
¥¥b``b]W¥¥[Ubbbbbb]][ab`^`[_a`aaWab^^`aa
@HWI-EAS443:3:81:91:1074#0/1
CAATATGGAGAGAATAAAAGAACTGACAGATCTAATGTCG
+HWI-EAS443:3:81:91:1074#0/1
a]`]U^ba`a_[_^aaaaa`_`__aY¥O`]]^__X^_]]^
@HWI-EAS443:3:53:485:765#0/1
CAATATGGAGAGAATAAAAGAACTNAGAGATCTAATGTCG
+HWI-EAS443:3:53:485:765#0/1
abab`b`babbababbbabaaba^D^babab`baabbaa_
@HWI-EAS443:3:10:1044:1875#0/1
TATGGAGAGAATAAAAGAACTGAGAGATCTAATGTCGCAG
+HWI-EAS443:3:10:1044:1875#0/1
_]a[``bX^W`bbaaaa]a_^[a_a[a`[`b`abYQ[^aa
@HWI-EAS443:3:12:286:741#0/1
ATATGGAGAGAATAAAAGAACTGAGAGATCTAATGTCGCA
```

+HWI-EAS443:3:12:286:741#0/1
ababbb_b`bbbabb_^_aaaaab`baa`_aaaab]]a`a
@HWI-EAS443:3:13:1189:1196#0/1
CAATATGGAGAGAATAAAAGAACTGAGAGATCTAATGTCG
+HWI-EAS443:3:13:1189:1196#0/1
aa`babababaaaabaabaaa`aaaa`aaaa_a``aa___
@HWI-EAS443:3:21:1458:802#0/1
CAATATGGAGAGAATAAAAGAACTGAGAGATCTAATGTCG
+HWI-EAS443:3:21:1458:802#0/1
a`aa`aaa^a^_]aa`_a_`]_]`__`__`_WaY^_a]W^
@HWI-EAS443:3:27:1321:908#0/1
CAATATGGAGAGAATAAAAGAACTGAGAGATCTAATGTCG
+HWI-EAS443:3:27:1321:908#0/1
a^UbU``^`b`aK`^a[Xabb`_a¥XXX]Ya`[JY_]¥R[
@HWI-EAS443:3:2:1042:693#0/1
TATGGAGAGAATAAAAGAACTGAGAGATCTAATGTCGCAG
+HWI-EAS443:3:2:1042:693#0/1
`aab][b`aaa`aaTX``YXabaa`a_`^aa``aRY`V_B
@HWI-EAS443:3:2:336:688#0/1
AATATGGAGAGAATAAAAGAACTGAGAGATCTAATGTCGC
+HWI-EAS443:3:2:336:688#0/1
aa`Z^_`Y_]_¥^`aYU¥a]a_aa`aaaa[]_`^^aY_`X
@HWI-EAS443:3:33:1372:68#0/1
TATGGAGAGAATAAAAGAACTGAGAGATCTAATGTCGCAG
+HWI-EAS443:3:33:1372:68#0/1
_aaab[ZTbaUa]ba]``a``U_YZZY_WaaY^`SSWUQ_
@HWI-EAS443:3:35:1121:1896#0/1
TATGGAGAGAATAAAAGAACTGAGAGATCTAATGTCGCAG
+HWI-EAS443:3:35:1121:1896#0/1
aa___S`a`a`aaabaaabaaa`^_]_¥a`aa`aH`Za_¥
@HWI-EAS443:3:38:727:352#0/1
ATATGGAGAGAATAAAAGAACTGAGAGATCTAATGTCGCA
+HWI-EAS443:3:38:727:352#0/1
ababbb`^^ba_`_`aabaaaaaaa`aa^_`_`a^_¥__
@HWI-EAS443:3:40:401:1108#0/1
CGACATTAGATCTCTCAGTTCTTTTATTCTCTCCATATTG
+HWI-EAS443:3:40:401:1108#0/1
aaabaabab_a`aaaa¥a¥`aaaaa^]_aaaaa`]___HV
@HWI-EAS443:3:40:588:1704#0/1
AATATGGAGAGAATAAAAGAACTGAGAGATCTAATGTCGC
+HWI-EAS443:3:40:588:1704#0/1
`baabba``aa`_aaaaa_aaaa_a[H]__`aaS``]`]^
@HWI-EAS443:3:46:192:351#0/1
TATGGAGAGAATAAAAGAACTGAGAGATCTAATGTCGCAG
+HWI-EAS443:3:46:192:351#0/1
aabb``a_aaaa`a_a_`a^aa_^_`_`_a_^a`^]^¥]Y
@HWI-EAS443:3:47:1274:366#0/1
TATGGAGAGAATAAAAGAACTGAGAGATCTAATGTCGCAG
+HWI-EAS443:3:47:1274:366#0/1
aS]a`[`Ya]_a_^_X`^^]_^]_]aY^V_XX_`XV_VY]

```
@HWI-EAS443:3:49:788:1182#0/1
CAATATGGAGAGAATAAAAGAACTGAGAGATCTAATGTCG
+HWI-EAS443:3:49:788:1182#0/1
`U^YZQX^^_[¥PX`_UN_]Wa`]`[Y`a_Ya`aZabX^]
@HWI-EAS443:3:50:1241:1724#0/1
CAATATGGAGAGAATAAAAGAACTGAGAGATCTAATGTCG
+HWI-EAS443:3:50:1241:1724#0/1
W[a¥¥^¥`Xa``_`bb``Wab_bbbbbbbZ_ab¥bbXS`
@HWI-EAS443:3:50:1475:1511#0/1
CAATATGGAGAGAATAAAAGAACTGAGAGATCTAATGTCG
+HWI-EAS443:3:50:1475:1511#0/1
aaa`ZabbUbaa`^ab__`aaaa`aaS_ab``aa`abYV_
@HWI-EAS443:3:52:1323:1174#0/1
TATGGAGAGAATAAAAGAACTGAGAGATCTAATGTCGCAG
+HWI-EAS443:3:52:1323:1174#0/1
`aa[aaa¥b`aaba_aa`¥¥`a[aaa^a_a_^aa[_a]_]
@HWI-EAS443:3:53:1402:320#0/1
CAATATGGAGAGAATAAAAGAACTGAGAGATCTAATGTCG
+HWI-EAS443:3:53:1402:320#0/1
SSTaa_¥`aS^b`abbbbbabYM_aab[_`a[b_Waaaaa
@HWI-EAS443:3:54:1054:891#0/1
AATATGGAGAGAATAAAAGAACTGAGAGATCTAATGTCGC
+HWI-EAS443:3:54:1054:891#0/1
aab_bbbababaabbbaabaa`bbbbbaababbabb__ba
@HWI-EAS443:3:5:590:556#0/1
AATATGGAGAGAATAAAAGAACTGAGAGATCTAATGTCGC
+HWI-EAS443:3:5:590:556#0/1
aabaabb`bab`_aaa```aa]bbaaabaa_a`_aa_`_X
@HWI-EAS443:3:62:1529:109#0/1
CAATATGGAGAGAATAAAAGAACTGAGAGATCTAATGTCG
+HWI-EAS443:3:62:1529:109#0/1
[bababbb_b_baa^_`abb`__aaa`Xaa``ab]^a^`_
@HWI-EAS443:3:63:714:1576#0/1
CAATATGGAGAGAATAAAAGAACTGAGAGATCTAATGTCG
+HWI-EAS443:3:63:714:1576#0/1
aaaa[`ab_`PaU````M_P]aaaaa]a__]___a_aU]a
@HWI-EAS443:3:65:528:917#0/1
CAATATGGAGAGAATAAAAGAACTGAGAGATCTAATGTCG
+HWI-EAS443:3:65:528:917#0/1
aba`Z_]S_aaZ¥VYabaQ]`Ta]]U`__U[_`ZZ^aWZZ
@HWI-EAS443:3:68:1183:995#0/1
CAATATGGAGAGAATAAAAGAACTGAGAGATCTAATGTCG
+HWI-EAS443:3:68:1183:995#0/1
abbbabbbabab^aaaabbbaabbbbabbbaabb¥ab^a`
@HWI-EAS443:3:75:444:565#0/1
TGCGACATTAGATCTCTCAGTTCTTTTATTCTCTCCATAT
+HWI-EAS443:3:75:444:565#0/1
aabbab¥FZ¥`]aaa`aa^a_a`a`aaaaaaa_`aa_a``
@HWI-EAS443:3:76:935:653#0/1
AATATGGAGAGAATAAAAGAACTGAGAGATCTAATGTCGC
```

```
+HWI-EAS443:3:76:935:653#0/1
_aZU_ab^¥]`babbb]baaa[bbbbaaa_`aa]ab_`^Y
@HWI-EAS443:3:81:1760:1620#0/1
AATATGGAGAGAATAAAAGAACTGAGAGATCTAATGTCGC
+HWI-EAS443:3:81:1760:1620#0/1
a]a__abYbR_`Oaa¥`^RI]^``_]a`¥^R^¥]_aW]¥X
@HWI-EAS443:3:87:242:888#0/1
TATGGAGAGAATAAAAGAACTGAGAGATCTAATGTCGCAG
+HWI-EAS443:3:87:242:888#0/1
aabaX[]ab¥aa_^[a^a_`aa_`[]__``]Y_a^a__``
@HWI-EAS443:3:89:1706:26#0/1
CAATATGGAGAGAATAAAAGAACTGAGAGATCTAATGTCG
+HWI-EAS443:3:89:1706:26#0/1
^bbbbbbabbabbbbbabbbbabbbbabbbbVbbabba^b
@HWI-EAS443:3:8:345:1024#0/1
TATGGAGAGAATAAAAGAACTGAGAGATCTAATGTCGCAG
+HWI-EAS443:3:8:345:1024#0/1
aaa_RR^ZQ¥aa`__aa[aUa¥aaaa___^a^abPL]S_`
@HWI-EAS443:3:90:112:944#0/1
CAATATGGAGAGAATAAAAGAACTGAGAGATCTAATGTCG
+HWI-EAS443:3:90:112:944#0/1
^¥¥baba`T`a^QR¥aZ^ba`TZaT^aaa^W[aa``b[`_
@HWI-EAS443:3:92:170:157#0/1
AATATGGAGAGAATAAAAGAACTGAGAGATCTAATGTCGC
+HWI-EAS443:3:92:170:157#0/1
TQ_N_bbbaa^`[a¥bb`bbb``baaa`[`aaa^abNZ`T
@HWI-EAS443:3:95:617:299#0/1
CAATATGGAGAGAATAAAAGAACTGAGAGATCTAATGTCG
+HWI-EAS443:3:95:617:299#0/1
]baa_`¥`abba_babbbbaaa[bbaabbaa]ababb_^a
@HWI-EAS443:3:97:1612:1381#0/1
CAATATGGAGAGAATAAAAGAACTGAGAGATCTAATGTCG
+HWI-EAS443:3:97:1612:1381#0/1
ab_bK^^b`bX`^abab^a`aaaaba`b`_[F[babaMI¥
@HWI-EAS443:3:9:1589:990#0/1
TATGGAGAGAATAAAAGAACTGAGAGATCTAATGTCGCAG
+HWI-EAS443:3:9:1589:990#0/1
aUa_a_`[aY]b_`]¥a`RR_XX_a`_^T^]a]^^YLTBB
@HWI-EAS443:3:9:516:686#0/1
CAATATGGAGAGAATAAAAGAACTGAGAGATCTAATGTCG
+HWI-EAS443:3:9:516:686#0/1
]]^`aabbbaYa^Y`b`¥_Z_a`bbbbbbaa`_baaba`^
@HWI-EAS443:3:11:1063:1477#0/1
ATTTGAATGGATGTCAATCCGGCTCTACTTTTCCTAAAAA
+HWI-EAS443:3:11:1063:1477#0/1
abbabbbbbab]^`abbaaaaa_b`aaaaaaa^a`aaaaa
@HWI-EAS443:3:51:1036:870#0/1
GATTGACATCCATTCAAATGGTTTGCCTGCTTTTCCTAAA
+HWI-EAS443:3:51:1036:870#0/1
``a_aaaab`]aba_^`a_Z^¥a`^¥¥^_^_`aa^^^aa`
```

@HWI-EAS443:3:68:1642:1395#0/1
GATTGACATCCATTCAAATGGTTTGCCTGCTTTTCCTAAA
+HWI-EAS443:3:68:1642:1395#0/1
a``aaa`ba_a`aa_aa`_]VV`a``_`a^aa`_]Y``a`
@HWI-EAS443:3:89:1392:292#0/1
GATTGACATCCATTCAAATGGTTTGCCTGCTTTTCCTAAA
+HWI-EAS443:3:89:1392:292#0/1
``babbaab`_aaaaaa_ab`¥]^aV_baQ__]b[Rbba`
@HWI-EAS443:3:16:1180:1596#0/1
ATTTGAATGGATGTCAATCCGACTCTACNTTTCCTAAAAA
+HWI-EAS443:3:16:1180:1596#0/1
¥abaaaba`b`^]YZ_^Ya^PVYa^V]XD[`__`aaaaaa
@HWI-EAS443:3:100:388:1112#0/1
ATTTGAATGGATGTCAATCCGACTCTACTTTTCCTAAAAA
+HWI-EAS443:3:100:388:1112#0/1
abbaabaabaaabaabb^_Xa__a`a`_`aa`_Zaaa`a`
@HWI-EAS443:3:10:966:1673#0/1
ATTTGAATGGATGTCAATCCGACTCTACTTTTCCTAAAAA
+HWI-EAS443:3:10:966:1673#0/1
aabaaaa_baaaa_`aa`_Y_^Y`[`_Y`__aW^]aa``_
@HWI-EAS443:3:13:463:931#0/1
ATTTGAATGGATGTCAATCCGACTCTACTTTTCCTAAAAA
+HWI-EAS443:3:13:463:931#0/1
`baabaa_b_a`a^__aaa``_]``_a__a`a]^`aaa``
@HWI-EAS443:3:1:1491:1173#0/1
TTTGAATGGATGTCAATCCGACTCTACTTTTCCTAAAAAT
+HWI-EAS443:3:1:1491:1173#0/1
abba_a_`^_aaWY__a^¥aZ^^[]^]^___VX``_`_BB
@HWI-EAS443:3:24:470:1290#0/1
ATTTGAATGGATGTCAATCCGACTCTACTTTTCCTAAAAA
+HWI-EAS443:3:24:470:1290#0/1
ababb[_abaaa`_a_a¥`aa_aaaaa`aaaa_a_abaaa
@HWI-EAS443:3:33:1076:71#0/1
ATTTGAATGGATGTCAATCCGACTCTACTTTTCCTAAAAA
+HWI-EAS443:3:33:1076:71#0/1
aabbaaaabaaab`_`aa`_a^¥`_a]^`a`aUX__`_a^
@HWI-EAS443:3:34:1225:1144#0/1
ATTTGAATGGATGTCAATCCGACTCTACTTTTCCTAAAAA
+HWI-EAS443:3:34:1225:1144#0/1
aaaaaaba`[a_`_``a``^XV¥aa¥TUaaa_[^_`_^_Z
@HWI-EAS443:3:38:1501:1392#0/1
ATTTGAATGGATGTCAATCCGACTCTACTTTTCCTAAAAA
+HWI-EAS443:3:38:1501:1392#0/1
Va_N_`a`^`_Z_NPZ^_`XPRNZ_`^¥aZ¥XPX^__[_P
@HWI-EAS443:3:44:622:1406#0/1
ATTTGAATGGATGTCAATCCGACTCTACTTTTCCTAAAAA
+HWI-EAS443:3:44:622:1406#0/1
aabaa_a][^aaa¥`^a__a_]_a[a_`_`aa[^_aaaa`
@HWI-EAS443:3:45:1693:1217#0/1
ATTTGAATGGATGTCAATCCGACTCTACTTTTCCTAAAAA

```
+HWI-EAS443:3:45:1693:1217#0/1
abbb^abbbababa_aaaa`a]_aaba_aabaYaaaaaa_
@HWI-EAS443:3:49:661:2024#0/1
GGAATTTTTAGGAAAAGTAGAGTCGGATTGACATCCATTC
+HWI-EAS443:3:49:661:2024#0/1
`b^ZX_abb_bb_`¥S[J[a_TVL^a[QJ[VKWHKZ_Y¥T
@HWI-EAS443:3:51:1241:1120#0/1
GAATGGATGTCAATCCGACTCTACTTTTCCTAAAAATTCC
+HWI-EAS443:3:51:1241:1120#0/1
_`bbbbabb_[_a`Z¥b]_a``¥Za^`aY[aababaaa[X
@HWI-EAS443:3:53:1423:1899#0/1
AATGGATGTCAATCCGACTCTACTTTTCCTAAAAATTCCA
+HWI-EAS443:3:53:1423:1899#0/1
ababba_bXV_a]R[a¥R¥Y_`U^a`b^¥a^aaa`^_]U_
@HWI-EAS443:3:54:103:371#0/1
TTTGAATGGATGTCAATCCGACTCTACTTTTCCTAAAAAT
+HWI-EAS443:3:54:103:371#0/1
a_¥a^`abbabba`aaaa_a_`_``_Za`aa^_aaaaaa_
@HWI-EAS443:3:55:43:126#0/1
ATTTGAATGGATGTCAATCCGACTCTACTTTTCCTAAAAA
+HWI-EAS443:3:55:43:126#0/1
aaa^aaaabb_`a^`_[___`Y]__a^]^_]_]^__W]`U
@HWI-EAS443:3:5:304:1130#0/1
TTTGAATGGATGTCAATCCGACTCTACTTTTCCTAAAAAT
+HWI-EAS443:3:5:304:1130#0/1
abbba_b```ba`_bbbaaa^Zaaaa``abaa_aaaab_a
@HWI-EAS443:3:61:901:610#0/1
ATTTGAATGGATGTCAATCCGACTCTACTTTTCCTAAAAA
+HWI-EAS443:3:61:901:610#0/1
aababa`aaa`ab[``aaa`_W__a``_aaa¥S[a`a``]
@HWI-EAS443:3:62:1261:736#0/1
CATTTGAATGGATGTCAATCCGACTCTACTTTTCCTAAAA
+HWI-EAS443:3:62:1261:736#0/1
aaabbabbbbabbbaabababbaababb_bbbaa`abbbb
@HWI-EAS443:3:68:1562:48#0/1
ATTTGAATGGATGTCAATCCGACTCTACTTTTCCTAAAAA
+HWI-EAS443:3:68:1562:48#0/1
abbbbbbbbbbbba]aab]]a__a]ba`aaab¥Yaaaaa`
@HWI-EAS443:3:71:805:1270#0/1
ATTTGAATGGATGTCAATCCGACTCTACTTTTCCTAAAAA
+HWI-EAS443:3:71:805:1270#0/1
aa`abab__aa`a^aa``aa¥U^`a_]]_____``a_aa`
@HWI-EAS443:3:75:965:2015#0/1
ATTTGAATGGATGTCAATCCGACTCTACTTTTCCTAAAAA
+HWI-EAS443:3:75:965:2015#0/1
aaaaaaba`Yaaa¥^aa_a__¥M¥]^_¥_¥_]_`_aaaaa
@HWI-EAS443:3:76:1586:1332#0/1
ATTTGAATGGATGTCAATCCGACTCTACTTTTCCTAAAAA
+HWI-EAS443:3:76:1586:1332#0/1
abbbbaabbaaab_`aba_aa]_a_a`[aaaaXaaaaaa`
```

```
@HWI-EAS443:3:77:1476:57#0/1
ATTTGAATGGATGTCAATCCGACTCTACTTTTCCTAAAAA
+HWI-EAS443:3:77:1476:57#0/1
V`bbb¥¥abbbbb^U`¥RWP`X^a``TT_bbbb_bbabab
@HWI-EAS443:3:77:772:1792#0/1
ATTTGAATGGATGTCAATCCGACTCTACTTTTCCTAAAAA
+HWI-EAS443:3:77:772:1792#0/1
abab`abaaaabaaabb`aa¥aaaaaa``aaaa_`baaaa
@HWI-EAS443:3:7:605:1077#0/1
ATTTGAATGGATGTCAATCCGACTCTACTTTTCCTAAAAA
+HWI-EAS443:3:7:605:1077#0/1
aaaaaa`[N[]aa_^_aa_^aVU_[_^]^`^_]X^`a`aY
@HWI-EAS443:3:83:871:1365#0/1
ATTTGAATGGATGTCAATCCGACTCTACTTTTCCTAAAAA
+HWI-EAS443:3:83:871:1365#0/1
aaaaa^¥^aaaa`W``a`^]a^[___^```_`__`a_``^
@HWI-EAS443:3:86:394:860#0/1
TTTGAATGGATGTCAATCCGACTCTACTTTTCCTAAAAAT
+HWI-EAS443:3:86:394:860#0/1
`baaaa`aa``a^aaa``a_[_]a`X]```a``a`a_^^`
@HWI-EAS443:3:89:755:511#0/1
ATTTGAATGGATGTCAATCCGACTCTACTTTTCCTAAAAA
+HWI-EAS443:3:89:755:511#0/1
ababaa[ab`aa`_bbaaa`a`_a`a¥^aaa`a_a`a`a`
@HWI-EAS443:3:91:1600:1507#0/1
TTTGAATGGATGTCAATCCGACTCTACTTTTCCTAAAAAT
+HWI-EAS443:3:91:1600:1507#0/1
abbaabababaaab`a`aa_[``aa`a_O_aaa^aabaaa`a
@HWI-EAS443:3:91:937:613#0/1
ATTTGAATGGATGTCAATCCGACTCTACTTTTCCTAAAAA
+HWI-EAS443:3:91:937:613#0/1
ab^``bbbbbbaaZX_`X]¥¥Z¥aX_^S]`aaYN_`a_``
@HWI-EAS443:3:95:1679:1324#0/1
ATTTGAATGGATGTCAATCCGACTCTACTTTTCCTAAAAA
+HWI-EAS443:3:95:1679:1324#0/1
`bbbbbbbbbbaa]Sb`_]``aSa`aaaaaab_aaaaaaa
@HWI-EAS443:3:97:428:1076#0/1
ATTTGAATGGATGTCAATCCGACTCTACTTTTCCTAAAAA
+HWI-EAS443:3:97:428:1076#0/1
aa[`_bbabb]¥_Y`a``[Sa¥[aWa`]^aaa`[aa_]]a
@HWI-EAS443:3:97:881:1542#0/1
ATTTGAATGGATGTCAATCCGACTCTACTTTTCCTAAAAA
+HWI-EAS443:3:97:881:1542#0/1
aabbbbaaaaabb_aaaa`_a`Ya_a^]aaaa]]aaaaaa
@HWI-EAS443:3:17:1331:1843#0/1
GGACACGTTAAATTCATTATTTTTGCCGTCTGAGTTCTTC
+HWI-EAS443:3:17:1331:1843#0/1
R]¥b_VV`b`^`]ababK^b]b`Y^a`aaaab`b_]WaJP
@HWI-EAS443:3:17:1397:584#0/1
GGCATTTTTTCATGAAGGACAGGTTAAATTCATTATTTTT
```

+HWI-EAS443:3:17:1397:584#0/1
a^^`]SX``TL[_a_¥¥¥U]V]]Y_TZ]]_[¥^S[`^]__
@HWI-EAS443:3:37:1255:1055#0/1
GTTAAATTCATTATTTTTGCCGTCTGAGTTCTCCAATGGT
+HWI-EAS443:3:37:1255:1055#0/1
]HXYSU_¥_S[YTW[T[LRMN^M_ZS^a[YaY_FW^VBBB
@HWI-EAS443:3:44:1084:829#0/1
GTTAAATCCATTATTTTTGCCGTCTGAGTTCTTCAATGGT
+HWI-EAS443:3:44:1084:829#0/1
a`a^`^aa_aaa`aaa`a``]a^^`aZ`_`]a`¥^^_`¥Y
@HWI-EAS443:3:49:490:219#0/1
GACAAGTTAAATTCATTATTTTTGCAGTCTGAGTTCTTCA
+HWI-EAS443:3:49:490:219#0/1
^`b¥S[Z¥^Xaba^J¥b^b^^^`Z[Hb¥Ta]]a^aJa¥F¥
@HWI-EAS443:3:57:672:841#0/1
GAAGGACAAGTTAAATTCATTATTTTTGCCGTCTGCGTTC
+HWI-EAS443:3:57:672:841#0/1
aaa^^^aaZ__a]T[aaa`aaaa`a`_a_`aX^^]HX^__
@HWI-EAS443:3:62:1551:105#0/1
GGCATTTTTTCATGAAGGATAAGTTAAATTCATTATTTTT
+HWI-EAS443:3:62:1551:105#0/1
aa_Q^Y_a^^^U__XNa]JFL^_W]UWY^¥VMUY[WWY^¥
@HWI-EAS443:3:62:222:266#0/1
TTTTTCATGAAGGACAAGTTAAATCCATTATTTTTGCCGT
+HWI-EAS443:3:62:222:266#0/1
ababaaaaaaaaa`a``a_`_a_]G¥`][¥____]__^_X
@HWI-EAS443:3:69:1298:1589#0/1
TTTTTCATGAAGGACAAGTTAAATTCATTATTTTTACCGT
+HWI-EAS443:3:69:1298:1589#0/1
baaX`__aa`b`_`[^U_`aa`^_]]XX^```[``[a_
@HWI-EAS443:3:73:538:557#0/1
GACACGTTAAATTCATTATTTTTGCCGTCTGAGTTCTTCA
+HWI-EAS443:3:73:538:557#0/1
`a_XHPUXW_a^SW¥`X[WTRLU_[_aZSSb¥¥ETZ[¥XV
@HWI-EAS443:3:74:265:754#0/1
GGCATTTTTTCATGAAGGACAAGTTAAACTCATTATTTTT
+HWI-EAS443:3:74:265:754#0/1
a¥b`^V``abb`^¥b````ab^^]]`aa``baabaaa`
@HWI-EAS443:3:76:1404:1454#0/1
GGCATTTTTTCATGAGGGACAAGTTAAATTCATTATTTTT
+HWI-EAS443:3:76:1404:1454#0/1
aa^^^_aa`a^_`[[__`[^¥_[]`Z^^a_Y]_^_a_]_]
@HWI-EAS443:3:85:1073:482#0/1
GGACAAGTTAAATTCATTATTTTTGCCGTCCGAGTTCTTC
+HWI-EAS443:3:85:1073:482#0/1
^b¥b]^aTYS]aba¥aaa[aa^a_a`ab`]O]a`_aX¥ZL
@HWI-EAS443:3:87:506:381#0/1
GGACAAGTTAAATTCATTATTTTTGCCGTCCGAGTTCTTC
+HWI-EAS443:3:87:506:381#0/1
abbbbababb`abaaabaabbbbabaabababab_aaaaa

```
@HWI-EAS443:3:91:1307:951#0/1
CATGAAGGACGAGTTAAATTCATTATTTTTGCCGTCTGAG
+HWI-EAS443:3:91:1307:951#0/1
Y_bbbbbbaa^`b`b`aabbbbbbbbbbbbb^b¥bbbb_`
@HWI-EAS443:3:95:1231:710#0/1
GGACAAGTTAAGTTCATTATTTTTGCCGTCTGAGTTCTTC
+HWI-EAS443:3:95:1231:710#0/1
ab__a¥`^abaPaba`ba_a`aaabR[aa_[a_b`¥[a`_
@HWI-EAS443:3:98:1170:1718#0/1
GGCATTTTTTCATGAAGGACAAGTTTAATTCATTATTTTT
+HWI-EAS443:3:98:1170:1718#0/1
Xba```b`Xbab_bbY^baVaabY_¥babaUYY`aY_b`[
@HWI-EAS443:3:14:1490:1696#0/1
ATNGATGCCAGGGTCGACTTCGAGTCTGGAAGGATCAAGA
+HWI-EAS443:3:14:1490:1696#0/1
a[DUaS`^aaa^aR_Y_^a`__RaYN`^O^MPaNQN]_NX
@HWI-EAS443:3:86:1021:1712#0/1
CAAAAATAATGAATTTAACTTGTCCTTCATGAAAAAAAAA
+HWI-EAS443:3:86:1021:1712#0/1
abbbbbbabbbbbabbabb_aaba¥`aabbbbaaaaaaa¥a
@HWI-EAS443:3:24:1685:1401#0/1
GGCANTTTTTCATGAAGGACAAGTTAAATTCATTATTTTT
+HWI-EAS443:3:24:1685:1401#0/1
]_^PD[aaabVaa__^U`X_XT__b_``abTQaa_b_aa_
@HWI-EAS443:3:2:1416:1557#0/1
GGCATNTTTTCATGAAGGACAAGTTAAATTCATTATTTTT
+HWI-EAS443:3:2:1416:1557#0/1
aa_a¥D^aaa_``__^aa^]¥[`]`_]]`_Y^a_^``_^_
@HWI-EAS443:3:30:1056:2045#0/1
AGTTAAATTCATTATTTTTGCCGTCTGAGTNCTTCAATGG
+HWI-EAS443:3:30:1056:2045#0/1
aa`¥babaaabbbaaa_baaaaa`a_b_b[DYaaaa`_`^
@HWI-EAS443:3:70:86:1505#0/1
CATTTNTTCATGAAGGACAAGTTAAATTCATTATTTTTGC
+HWI-EAS443:3:70:86:1505#0/1
a¥ab^DWaa_`a`^¥_a_XZa]aaaaa_a_a````_____
@HWI-EAS443:3:81:413:505#0/1
TTTTTCNTGAAGGACAAGTTAAATTCATTATTTTTGCCGT
+HWI-EAS443:3:81:413:505#0/1
[N]aWUD]SaVabTO_`__aaXaa]Y`^X_^]¥ZX_`XWL
@HWI-EAS443:3:81:675:663#0/1
GACAAGTTAAATNCATTATTTTTGCCGTCTGAGTTCTTCA
+HWI-EAS443:3:81:675:663#0/1
abaY^a_^_Zb]DZaaa`aaaaaa__`_`aaaaP`^a^``
@HWI-EAS443:3:84:1404:762#0/1
GACAAGTTAAANTCATTATTTTTGCCGTCTGAGTTCTTCA
+HWI-EAS443:3:84:1404:762#0/1
aabYab]aa_[D[]^aa]_aaaaa[^¥]_`a]a[_]``XX
@HWI-EAS443:3:93:1575:1081#0/1
ATGANGGACAAGTTAAATTCATTATTTTTGCCGTCTGAGT
```

```
+HWI-EAS443:3:93:1575:1081#0/1
a]X¥DY__`_Wa[a^[_aa[U`]`aaaaa___`RSa_QaP
@HWI-EAS443:3:49:169:1355#0/1
GGCATTTTTTCATGAAGGACAAGTTAAATTCATTATTTNT
+HWI-EAS443:3:49:169:1355#0/1
¥baaaaa`_aa`a`aaa`]`SYaVaa`P_[__a^_a_[DY
@HWI-EAS443:3:22:1139:276#0/1
GGCATTTTTTCATGAAGGACAAGTTAAATTCATTATTTTC
+HWI-EAS443:3:22:1139:276#0/1
aa]W`aaaaaa``^a^_a]`¥_YW_a¥¥``^^_`__^`^V
@HWI-EAS443:3:100:49:162#0/1
AAGTTAAATTCATTATTTTTGCCGTCTGAGTTCTTCAATG
+HWI-EAS443:3:100:49:162#0/1
R`[OYaaS[U_bX¥[bbbb[O_aaVP[¥V^RJF`XYXBBB
@HWI-EAS443:3:10:341:1534#0/1
CATTTTTTCATGAAGGACAAGTTAAATTCATTATTTTTGC
+HWI-EAS443:3:10:341:1534#0/1
abbbbbbbbbbbbbaaaba^a_aaaaaaa`aaaaaa`^`a
@HWI-EAS443:3:10:660:1961#0/1
AAGTTAAATTCATTATTTTTGCCGTCTGAGTTCTTCAATG
+HWI-EAS443:3:10:660:1961#0/1
aZ^Uaa¥aaa_X__]`aa_`U]_]¥`]a^^XY^]W_^^_]
@HWI-EAS443:3:10:725:305#0/1
GCATTTTTTCATGAAGGACAAGTTAAATTCATTATTTTTG
+HWI-EAS443:3:10:725:305#0/1
a^R[TQW¥XMY^RYQLTNITZZSPR[TOTQTYYSXTPU[X
@HWI-EAS443:3:11:1121:984#0/1
GTTAAATTCATTATTTTTGCCGTCTGAGTTCTTCAATGGT
+HWI-EAS443:3:11:1121:984#0/1
`XWRYV¥]]X[WWXTYS^SQW][ZJ^XXDRTQPS^[S^aV
@HWI-EAS443:3:11:1571:1371#0/1
ATTTTTTCATGAAGGACAAGTTAAATTCATTATTTTTGCC
+HWI-EAS443:3:11:1571:1371#0/1
abaaabbbabbaaaU_`_[aXa`a^`a_aa`Ya_a_^__V
@HWI-EAS443:3:11:845:1842#0/1
AGGACAAGTTAAATTCATTATTTTTGCCGTCTGAGTTCTT
+HWI-EAS443:3:11:845:1842#0/1
aaaX¥K¥_S_aWL[]W[[`b`^`_SaZR^UUZ``aJYI]_
@HWI-EAS443:3:11:999:107#0/1
GGACAAGTTAAATTCATTATTTTTGCCGTCTGAGTTCTTC
+HWI-EAS443:3:11:999:107#0/1
abaaa`b_aa``aa]_b`aabba`a^_a^]aa^a^_Z``]
@HWI-EAS443:3:12:171:1340#0/1
TTTTCATGAAGGACAAGTTAAATTCATTATTTTTGCCGTC
+HWI-EAS443:3:12:171:1340#0/1
[a¥[_¥WY_WY]]]_]a¥¥_ZP]aa``^Y`a``^]ZW[[`
@HWI-EAS443:3:12:656:653#0/1
GGCATTTTTTCATGAAGGACAAGTTAAATTCATTATTTTT
+HWI-EAS443:3:12:656:653#0/1
aa_]_``]`a`Z_a_T`_a^]_`Y`__^]Y[``__^_[[_
```

```
@HWI-EAS443:3:12:901:736#0/1
TTCATGAAGGACAAGTTAAATTCATTATTTTTGCCGTCTG
+HWI-EAS443:3:12:901:736#0/1
a]¥X]Z][MSTa]abWb]][a_TMZ[¥^a^__aYT`X]__
@HWI-EAS443:3:13:1052:1517#0/1
TGAAGGACAAGTTAAATTCATTATTTTTGCCGTCTGAGTT
+HWI-EAS443:3:13:1052:1517#0/1
`ba_a¥_^abaRaa^`aa__aaaaaa`aa`SbZU`ba`X[
@HWI-EAS443:3:13:1160:1319#0/1
GGACAAGTTAAATTCATTATTTTTGCCGTCTGAGTTCTTC
+HWI-EAS443:3:13:1160:1319#0/1
aa__`]aS_`a_`_a¥_[`____a___`][__[OOZR^[]
@HWI-EAS443:3:13:1657:1710#0/1
GGCATTTTTTCATGAAGGACAAGTTAAATTCATTATTTTT
+HWI-EAS443:3:13:1657:1710#0/1
a]UY`a_]^RPV_^T^[YYYUQ`^¥^_[^YFR^]W]¥VV]
@HWI-EAS443:3:13:477:989#0/1
ATTGAAGAACTCAGACGGCAAAAATAATGAATTTAACTTG
+HWI-EAS443:3:13:477:989#0/1
a```aaa_X_aa`]```¥`a]Z_aX¥`a`_¥___a``[_¥
@HWI-EAS443:3:13:893:1529#0/1
GGCATTTTTTCATGAAGGACAAGTTAAATTCATTATTTTT
+HWI-EAS443:3:13:893:1529#0/1
a^]Z¥_^^ab``]]__]QW¥^_a_a[`_a¥Z]`UYa`__`
@HWI-EAS443:3:14:1600:160#0/1
GACAAGTTAAATTCATTATTTTTGCCGTCTGAGTTCTTCA
+HWI-EAS443:3:14:1600:160#0/1
aab]X_]a_]aa]a_aa`b`aaW`PV`X^_`]a]¥P`YL[
@HWI-EAS443:3:14:983:1713#0/1
GGCATTTTTTCATGAAGGACAAGTTAAATTCATTATTTTT
+HWI-EAS443:3:14:983:1713#0/1
a[^aaa_ba``aaa___¥]^___^a_Z_`_^_aU___^^_
@HWI-EAS443:3:15:1006:1454#0/1
GGCATTTTTTCATGAAGGACAAGTTAAATTCATTATTTTT
+HWI-EAS443:3:15:1006:1454#0/1
`Y`^aaa_`_[_`^aaa`]V][a__^]`__W[¥]^a__^¥
@HWI-EAS443:3:15:1370:1102#0/1
TTTTTCATGAAGGACAAGTTAAATTCATTATTTTTGCCGT
+HWI-EAS443:3:15:1370:1102#0/1
`a¥`^S¥_]aY¥[JRW¥_U`YT]Z_HSYa]ba`WX]a[`T
@HWI-EAS443:3:15:779:1875#0/1
ATGAAGGACAAGTTAAATTCATTATTTTTGCCGTCTGAGT
+HWI-EAS443:3:15:779:1875#0/1
a_¥baaaba^^a^_a[a``_`_a_`_`]]__]_Z_W_X][
@HWI-EAS443:3:16:1713:1805#0/1
CATTTTTTCATGAAGGACAAGTTAAATTCATTATTTTTGC
+HWI-EAS443:3:16:1713:1805#0/1
`U`b¥W`aa`]`^^`a^][aa_¥aa`_a_`a[ab```a__
@HWI-EAS443:3:16:448:361#0/1
TTTTCATGAAGGACAAGTTAAATTCATTATTTTTGCCGTC
```

```
+HWI-EAS443:3:16:448:361#0/1
^aa¥_Q_ba`Z¥Za¥aaa_`_aba_a``bb^baaaa[bP`
@HWI-EAS443:3:16:72:1301#0/1
ATGAAGGACAAGTTAAATTCATTATTTTTGCCGTCTGAGT
+HWI-EAS443:3:16:72:1301#0/1
aab`V_a_a`^a_```^_```_`]_`_a``_`a^`^a]aY
@HWI-EAS443:3:16:941:1107#0/1
ATGAAGGACAAGTTAAATTCATTATTTTTGCCGTCTGAGT
+HWI-EAS443:3:16:941:1107#0/1
abaaabaaaaaa_aaa_abaaaaaaaaaa`a`a_]aa`a^
@HWI-EAS443:3:17:1117:1021#0/1
GGCATTTTTTCATGAAGGACAAGTTAAATTCATTATTTTT
+HWI-EAS443:3:17:1117:1021#0/1
```R[bbbaaaaab]Y]baaa]a^a`^aa^a`_aaa^_`^
@HWI-EAS443:3:17:552:75#0/1
AAGGACAAGTTAAATTCATTATTTTTGCCGTCTGAGTTCT
+HWI-EAS443:3:17:552:75#0/1
¥^bbVa^`aR`_¥_a`^J¥]Y^[¥WN^a¥aW[`a[aZV^a
@HWI-EAS443:3:17:930:1836#0/1
CATTTTTTCATGAAGGACAAGTTAAATTCATTATTTTTGC
+HWI-EAS443:3:17:930:1836#0/1
a`baaabbbbaabb`a`bba``abaabbaababbba_a``
@HWI-EAS443:3:18:1256:460#0/1
GGACAAGTTAAATTCATTATTTTTGCCGTCTGAGTTCTTC
+HWI-EAS443:3:18:1256:460#0/1
aba^`^b`a```aaaa`aaaaa^_a[ab^[ab]a[`__`^
@HWI-EAS443:3:18:1513:1381#0/1
GGCATTTTTTCATGAAGGACAAGTTAAATTCATTATTTTT
+HWI-EAS443:3:18:1513:1381#0/1
aaaaabbaab`aa`^abb]W]a]`a`^`aa``b_`aa``a
@HWI-EAS443:3:18:1774:1310#0/1
CATTTTTTCATGAAGGACAAGTTAAATTCATTATTTTTGC
+HWI-EAS443:3:18:1774:1310#0/1
ab_V`^`ZQ[bb`Z]^¥S¥a^V`]_Zaa_R^¥[`¥[`WP[
@HWI-EAS443:3:19:1083:1179#0/1
GAAGGACAAGTTAAATTCATTATTTTTGCCGTCTGAGTTC
+HWI-EAS443:3:19:1083:1179#0/1
a``_`[^aZ[]baaX`a]]a`aaa__^aYFY^]`a__¥_V
@HWI-EAS443:3:19:1143:426#0/1
GTTAAATTCATTATTTTTGCCGTCTGAGTTCTTCAATGGT
+HWI-EAS443:3:19:1143:426#0/1
a[aa_]aaYaaa_`a¥_a_]__]Sa`_aY_Y]__^__[]Y
@HWI-EAS443:3:1:1480:1158#0/1
GTTAAATTCATTATTTTTGCCGTCTGAGTTCTTCAATGGT
+HWI-EAS443:3:1:1480:1158#0/1
a[`NN[`a^Z`¥^_`^^Y_WU_ZM`]L]XUT]^Y¥[_[BB
@HWI-EAS443:3:1:1611:620#0/1
GCATTTTTTCATGAAGGACAAGTTAAATTCATTATTTTTG
+HWI-EAS443:3:1:1611:620#0/1
aaaba`abbTabba`aa_`^_`_ba_aba]`aaaaa`a`_
```
```

```
@HWI-EAS443:3:20:1394:504#0/1
GTTAAATTCATTATTTTTGCCGTCTGAGTTCTTCAATGGT
+HWI-EAS443:3:20:1394:504#0/1
aUaWYY_`¥W[aYaa][¥S`]a]`_`__W¥M_UNX]SVW[
@HWI-EAS443:3:20:687:1294#0/1
GCATTTTTTCATGAAGGACAAGTTAAATTCATTATTTTTG
+HWI-EAS443:3:20:687:1294#0/1
_aabbabbbabaabaaaabbaa`aaaaaabaa_`aaa__a
@HWI-EAS443:3:20:951:373#0/1
CAAGTTAAATTCATTATTTTTGCCGTCTGAGTTCTTCAAT
+HWI-EAS443:3:20:951:373#0/1
Z^`_W_[a___W`a^Za```aY_U_¥_^baa`_Za_aXW[
@HWI-EAS443:3:21:101:1086#0/1
ATTTTTTCATGAAGGACAAGTTAAATTCATTATTTTTGCC
+HWI-EAS443:3:21:101:1086#0/1
aaaa`aa`aaaaaa]``a_a_aa^``aaaa`_a_a_``_]
@HWI-EAS443:3:21:1039:312#0/1
GAAGGACAAGTTAAATTCATTATTTTTGCCGTCTGAGTTC
+HWI-EAS443:3:21:1039:312#0/1
aYW^_]]Z¥a]`a_W_^_]^^^_][^¥^W]^U^YY^][XY
@HWI-EAS443:3:21:1214:1923#0/1
GGACAAGTTAAATTCATTATTTTTGCCGTCTGAGTTCTTC
+HWI-EAS443:3:21:1214:1923#0/1
a_NQWWP¥Y^[^[X^[NSYVNNRXSNTYWWL]]NQNWWST
@HWI-EAS443:3:21:718:1530#0/1
GACAAGTTAAATTCATTATTTTTGCCGTCTGAGTTCTTCA
+HWI-EAS443:3:21:718:1530#0/1
^abb`aabaaabbbbbaabbba_aa`_a`abab``a`aab
@HWI-EAS443:3:22:188:1392#0/1
TGAAGGACAAGTTAAATTCATTATTTTTGCCGTCTGAGTT
+HWI-EAS443:3:22:188:1392#0/1
a_`_aaaaa^b]a`a^aaaaaa^b_aa_[a``Za`aa_]_
@HWI-EAS443:3:22:417:501#0/1
GGCATTTTTTCATGAAGGACAAGTTAAATTCATTATTTTT
+HWI-EAS443:3:22:417:501#0/1
a_a`a^P]X`aa^¥a]_a[]UW]S^_[]_]^]_XX_]^YY
@HWI-EAS443:3:23:1074:657#0/1
ATTTTTTCATGAAGGACAAGTTAAATTCATTATTTTTGCC
+HWI-EAS443:3:23:1074:657#0/1
abbbbbb`baaaaaaa_aaaa`aa_ba`_baabaaa_`__
@HWI-EAS443:3:23:422:932#0/1
GGACAAGTTAAATTCATTATTTTTGCCGTCTGAGTTCTTC
+HWI-EAS443:3:23:422:932#0/1
`bba`V_`abaababa^aZbaabaaaa]aaabab¥aaaa_
@HWI-EAS443:3:23:509:1607#0/1
GACAAGTTAAATTCATTATTTTTGCCGTCTGAGTTCTTCA
+HWI-EAS443:3:23:509:1607#0/1
abb¥bb`baabbbbaabbbbbb`aabRT]bb^[JRWab_b
@HWI-EAS443:3:23:683:1310#0/1
TTTTTTCATGAAGGACAAGTTAAATTCATTATTTTTGCCG
```

+HWI-EAS443:3:23:683:1310#0/1
^^_a^Za`¥^ZZaaYaa_¥____M]__ab^aaa`X`a`_a
@HWI-EAS443:3:24:1415:2013#0/1
GGCATTTTTTCATGAAGGACAAGTTAAATTCATTATTTTT
+HWI-EAS443:3:24:1415:2013#0/1
aa[Z¥]V__[N[_`a_a_X_`][P^`[`^TV__]]_^[]_
@HWI-EAS443:3:24:1571:1100#0/1
AGGACAAGTTAAATTCATTATTTTTGCCGTCTGAGTTCTT
+HWI-EAS443:3:24:1571:1100#0/1
abb`aa`b_bababaaabaabaaaaa_ab^Yaaaa`_¥a_
@HWI-EAS443:3:24:1758:114#0/1
TTTTCATGAAGGACAAGTTAAATTCATTATTTTTGCCGTC
+HWI-EAS443:3:24:1758:114#0/1
aaba_aa`_a^b_]^[`a``_^a_^]aa^`a```^^Y^^[
@HWI-EAS443:3:24:730:1125#0/1
GGCATTTTTTCATGAAGGACAAGTTAAATTCATTATTTTT
+HWI-EAS443:3:24:730:1125#0/1
`baaabaaaaaaaaa`a``aa`a^aaa_a``aa`_`_a``
@HWI-EAS443:3:25:1049:265#0/1
TTTTTTCATGAAGGACAAGTTAAATTCATTATTTTTGCCG
+HWI-EAS443:3:25:1049:265#0/1
abbaaababbaaba`aaaa_aa`_aa_`aaaa``_aa_]`
@HWI-EAS443:3:25:1147:1121#0/1
AAGTTAAATTCATTATTTTTGCCGTCTGAGTTCTTCAATG
+HWI-EAS443:3:25:1147:1121#0/1
¥`a[`ba^`a```b`R`bb`a]aaaabba`^aaaaaa`aa
@HWI-EAS443:3:25:1591:1397#0/1
ATGAAGGACAAGTTAAATTCATTATTTTTGCCGTCTGAGT
+HWI-EAS443:3:25:1591:1397#0/1
``a`bbbaa``_aba]aba_`b_abaaaaaa_a`]aa^^^
@HWI-EAS443:3:25:1613:707#0/1
CATTTTTTCATGAAGGACAAGTTAAATTCATTATTTTTGC
+HWI-EAS443:3:25:1613:707#0/1
_^abbbbbbbbbbbababb_bbbbbbbbbbbbbabbbbba
@HWI-EAS443:3:25:1686:502#0/1
TTTTCATGAAGGACAAGTTAAATTCATTATTTTTGCCGTC
+HWI-EAS443:3:25:1686:502#0/1
abbaaaaaaabb_^a_`_a___aa__a_`aa`__]^]`[Z
@HWI-EAS443:3:25:216:1862#0/1
TGAAGGACAAGTTAAATTCATTATTTTTGCCGTCTGAGTT
+HWI-EAS443:3:25:216:1862#0/1
`ba^b^ab][URYaVaaY¥ba]H[]^a`Wab^R_^a`^¥¥
@HWI-EAS443:3:26:1173:842#0/1
AGAACTCAGACGGCAAAAATAATGAATTTAACTTGTCCTT
+HWI-EAS443:3:26:1173:842#0/1
aaaaabaabaaaaaaaaa`aaaaaa]aaa``_``a]_^``
@HWI-EAS443:3:27:38:634#0/1
GACAAGTTAAATTCATTATTTTTGCCGTCTGAGTTCTTCA
+HWI-EAS443:3:27:38:634#0/1
aaaaaa`baaabaaaaa`aaaaaaa`a_aaa[`^a_aaaa

@HWI-EAS443:3:27:475:206#0/1
GGCATTTTTTCATGAAGGACAAGTTAAATTCATTATTTTT
+HWI-EAS443:3:27:475:206#0/1
a``]aaaaaaaaW]^^aa]``[`Z^__^``___^^`__^Y
@HWI-EAS443:3:28:1064:1335#0/1
GAAGGACAAGTTAAATTCATTATTTTTGCCGTCTGAGTTC
+HWI-EAS443:3:28:1064:1335#0/1
a^aST¥^^Uaa_X_`aXG¥`bbaa]aW¥`^aW`V`_aGSV
@HWI-EAS443:3:28:440:218#0/1
ATGAAGGACAAGTTAAATTCATTATTTTTGCCGTCTGAGT
+HWI-EAS443:3:28:440:218#0/1
`a_aa_aU]__b_a_¥¥aZVPa]``aaaaaTF¥PU[b[a`
@HWI-EAS443:3:28:49:714#0/1
GGCATTTTTTCATGAAGGACAAGTTAAATTCATTATTTTT
+HWI-EAS443:3:28:49:714#0/1
aa_UY`^ZU]bb_ZVMYa``]_b[[WI[^_¥`a`a`a^`_
@HWI-EAS443:3:29:1242:739#0/1
GTTAAATTCATTATTTTTGCCGTCTGAGTTCTTCAATGGT
+HWI-EAS443:3:29:1242:739#0/1
ZIXabbabab_VYba¥]`a¥][¥ZO__b[aaa`]`a^WNR
@HWI-EAS443:3:29:1748:1146#0/1
GGCATTTTTTCATGAAGGACAAGTTAAATTCATTATTTTT
+HWI-EAS443:3:29:1748:1146#0/1
a`]_a_a`aa__^``^`_^V^[_^^]][_]X_`^^`_`^^
@HWI-EAS443:3:2:1051:1299#0/1
GAAGGACAAGTTAAATTCATTATTTTTGCCGTCTGAGTTC
+HWI-EAS443:3:2:1051:1299#0/1
YZ^]ZW__Za[a``Uaaaaaa`aa_aaa__a^]^a`_^aP
@HWI-EAS443:3:2:429:1517#0/1
GGCATTTTTTCATGAAGGACAAGTTAAATTCATTATTTTT
+HWI-EAS443:3:2:429:1517#0/1
abW_^a__ba[¥^a¥Ya`¥_aX_^W^]`_]S`_Y`_]^X^
@HWI-EAS443:3:30:830:1200#0/1
GTTAAATTCATTATTTTTGCCGTCTGAGTTCTTCAATGGT
+HWI-EAS443:3:30:830:1200#0/1
a^`^¥]``^^aaaaa`__[``^¥^_`_^]ZZ_`a___]][
@HWI-EAS443:3:31:1036:654#0/1
AGTTAAATTCATTATTTTTGCCGTCTGAGTTCTTCAATGG
+HWI-EAS443:3:31:1036:654#0/1
abaa_¥aba`Z_bbbaXaa``^aS`a]]a^a]`]__V[_^
@HWI-EAS443:3:31:1346:1069#0/1
GGACAAGTTAAATTCATTATTTTTGCCGTCTGAGTTCTTC
+HWI-EAS443:3:31:1346:1069#0/1
Za¥_P¥`bba`_aba^aa^abab`a__aaab`ab_a^``]
@HWI-EAS443:3:31:1361:1477#0/1
GCATTTTTTCATGAAGGACAAGTTAAATTCATTATTTTTG
+HWI-EAS443:3:31:1361:1477#0/1
`aV[YNZ[`VSYb`a¥U`aZTaU`aa`a_`^____``]_^
@HWI-EAS443:3:31:180:712#0/1
GGCATTTTTTCATGAAGGACAAGTTAAATTCATTATTTTT

```
+HWI-EAS443:3:31:180:712#0/1
^baPbbbbbab`abbaaaabaa`abaaaaaabb_abaa`T
@HWI-EAS443:3:31:672:324#0/1
GGACAAGTTAAATTCATTATTTTTGCCGTCTGAGTTCTTC
+HWI-EAS443:3:31:672:324#0/1
`]``a]a[``a`__`_`[`a¥^`^^^_`Q_^^Z^Y__^]X
@HWI-EAS443:3:31:877:66#0/1
GACAAGTTAAATTCATTATTTTTGCCGTCTGAGTTCTTCA
+HWI-EAS443:3:31:877:66#0/1
`WZZ^a^b`K¥aa]_aaYaa````J[a]_``¥`_^]aZ]Z
@HWI-EAS443:3:32:1443:1675#0/1
GGCATTTTTTCATGAAGGACAAGTTAAATTCATTATTTTT
+HWI-EAS443:3:32:1443:1675#0/1
V_^Y``]U[PZZ¥]^[a_Z^_U]V^Z¥U¥V[]aZ`[[^X_
@HWI-EAS443:3:32:737:1064#0/1
GGCATTTTTTCATGAAGGACAAGTTAAATTCATTATTTTT
+HWI-EAS443:3:32:737:1064#0/1
`bbbbab_aab`a`¥Z_`^a`^`^a`a`aaaaa]X_`a``
@HWI-EAS443:3:32:958:531#0/1
GGCATTTTTTCATGAAGGACAAGTTAAATTCATTATTTTT
+HWI-EAS443:3:32:958:531#0/1
[aZZXV^a_¥b`a^VXYUP^XY¥T`Z[P]Z¥V`]a`[_]U
@HWI-EAS443:3:33:158:280#0/1
GGACAAGTTAAATTCATTATTTTTGCCGTCTGAGTTCTTC
+HWI-EAS443:3:33:158:280#0/1
]Y¥_X[^_]aaYaabbaaaaa^aaab_a`a_a]aU_aa¥a
@HWI-EAS443:3:33:378:647#0/1
GGCATTTTTTCATGAAGGACAAGTTAAATTCATTATTTTT
+HWI-EAS443:3:33:378:647#0/1
ab`Z]_aa`aaa```aaa]a]_aW_`__`_a`_^`a^_¥^
@HWI-EAS443:3:34:1354:1298#0/1
CATTTTTTCATGAAGGACAAGTTAAATTCATTATTTTTGC
+HWI-EAS443:3:34:1354:1298#0/1
aabbbbaaaaabaa`a_aaaa`aaa`aaaaaa`a``]_]^
@HWI-EAS443:3:34:1670:673#0/1
GCATTTTTTCATGAAGGACAAGTTAAATTCATTATTTTTG
+HWI-EAS443:3:34:1670:673#0/1
a^```bbab`aaa`Wba^a_`aaa]__aaWaa^__`[]WR
@HWI-EAS443:3:34:443:1675#0/1
GTTAAATTCATTATTTTTGCCGTCTGAGTTCTTCAATGGT
+HWI-EAS443:3:34:443:1675#0/1
a`^``abb`^``ba`aba¥aa_]a_Y`]^_a__aa___QY
@HWI-EAS443:3:35:1591:847#0/1
ATGAAGGACAAGTTAAATTCATTATTTTTGCCGTCTGAGT
+HWI-EAS443:3:35:1591:847#0/1
aaaaabaa`aa`_aa`_aa[_aaaa`b_aaa^a`^a`^^_
@HWI-EAS443:3:35:663:1918#0/1
CATTTTTTCATGAAGGACAAGTTAAATTCATTATTTTTGC
+HWI-EAS443:3:35:663:1918#0/1
`¥`aababbb_abaa`abaa`_aaaaa`aaa``aaa``^a
```

```
@HWI-EAS443:3:35:697:996#0/1
GCATTTTTTCATGAAGGACAAGTTAAATTCATTATTTTTG
+HWI-EAS443:3:35:697:996#0/1
abaaaaaaaab^_aa`a`a`]a_`a``_`a``_`a__[__
@HWI-EAS443:3:36:1492:60#0/1
GGCATTTTTTCATGAAGGACAAGTTAAATTCATTATTTTT
+HWI-EAS443:3:36:1492:60#0/1
abbabbbbbb`abb`abb_`aabaaab_baaaba`aaaab
@HWI-EAS443:3:36:418:790#0/1
CATTTTTTCATGAAGGACAAGTTAAATTCATTATTTTTGC
+HWI-EAS443:3:36:418:790#0/1
abbbbaabbbabaabaab[`b`aa_`a`a¥aa[a`a^aaa
@HWI-EAS443:3:37:1259:1198#0/1
GGACAAGTTAAATTCATTATTTTTGCCGTCTGAGTTCTTC
+HWI-EAS443:3:37:1259:1198#0/1
a`U__^`aa_a^ab`^a``aa`a^``^UNK`_K¥O]a^_R
@HWI-EAS443:3:37:1506:467#0/1
GGCATTTTTTCATGAAGGACAAGTTAAATTCATTATTTTT
+HWI-EAS443:3:37:1506:467#0/1
aa__aaaaaa^a_a_`a__[_``_`a]]a[``_^__``^^
@HWI-EAS443:3:37:1603:585#0/1
TTTCATGAAGGACAAGTTAAATTCATTATTTTTGCCGTCT
+HWI-EAS443:3:37:1603:585#0/1
`b`aaaba`aa__`__¥a^_¥aa^ab`_aQa`aaa^a^_`
@HWI-EAS443:3:37:1640:1319#0/1
GCATTTTTTCATGAAGGACAAGTTAAATTCATTATTTTTG
+HWI-EAS443:3:37:1640:1319#0/1
aa_`aaaaaaaabaaaaa__`a¥aa^_aaOY_aaa^aa_]
@HWI-EAS443:3:37:379:136#0/1
TGAAGGACAAGTTAAATTCATTATTTTTGCCGTCTGAGTT
+HWI-EAS443:3:37:379:136#0/1
aabaabaa``a_a[a_aaa`aaabaa``_Y`a[`]`_a^_
@HWI-EAS443:3:37:738:1713#0/1
GGCATTTTTTCATGAAGGACAAGTTAAATTCATTATTTTT
+HWI-EAS443:3:37:738:1713#0/1
]aaa_[_^VWY`a`^``Z^^[_a_aaab¥XaYY_Z`aXP_
@HWI-EAS443:3:37:785:1211#0/1
GACAAGTTAAATTCATTATTTTTGCCGTCTGAGTTCTTCA
+HWI-EAS443:3:37:785:1211#0/1
aa^`_aaaaaaaa`a_aVaaa`a_aa`^aY`]]]a__^a_
@HWI-EAS443:3:37:988:990#0/1
GAAGGACAAGTTAAATTCATTATTTTTGCCGTCTGAGTTC
+HWI-EAS443:3:37:988:990#0/1
a]]]]a]`[]_a_`Y]`a`a`a`_`]a_aG_^a^_^`S¥_
@HWI-EAS443:3:38:1149:1879#0/1
AGGACAAGTTAAATTCATTATTTTTGCCGTCTGAGTTCTT
+HWI-EAS443:3:38:1149:1879#0/1
a_`a[a`aYa_`_`aaaa`_`aaa_]aZ`WIZ]__Y]Z^[
@HWI-EAS443:3:38:1495:574#0/1
GAAGGACAAGTTAAATTCATTATTTTTGCCGTCTGAGTTC
```

+HWI-EAS443:3:38:1495:574#0/1
a`_aa^__`a_^`XR_a_`_`_a`___^_XWZY]_ZaZZV
@HWI-EAS443:3:39:124:230#0/1
ATGAAGGACAAGTTAAATTCATTATTTTTGCCGTCTGAGT
+HWI-EAS443:3:39:124:230#0/1
^`a_X_^R_YQ[RX^QV^_`^aXYY]`^_`_]`Z``WP[N
@HWI-EAS443:3:39:1386:1012#0/1
GACAAGTTAAATTCATTATTTTTGCCGTCTGAGTTCTTCA
+HWI-EAS443:3:39:1386:1012#0/1
aabaaa`baa`baaabaaaa`aaaaaa¥`aa[`[_[a]`[
@HWI-EAS443:3:39:1547:703#0/1
GGACAAGTTAAATTCATTATTTTTGCCGTCTGAGTTCTTC
+HWI-EAS443:3:39:1547:703#0/1
aa``a`a`b`a`aa_aa`_a_aaa__W`^`^Y]_^_]__[
@HWI-EAS443:3:39:1639:1369#0/1
GGACAAGTTAAATTCATTATTTTTGCCGTCTGAGTTCTTC
+HWI-EAS443:3:39:1639:1369#0/1
abaJa_b^aaa_b_abaa`aaaaaaaYa`aa^Y`___a`Y
@HWI-EAS443:3:3:320:852#0/1
GGCATTTTTTCATGAAGGACAAGTTAAATTCATTATTTTT
+HWI-EAS443:3:3:320:852#0/1
S_`S[b¥^`baZab_P]a_`bab^b_abbba]aabbabb^
@HWI-EAS443:3:3:664:619#0/1
GAAGGACAAGTTAAATTCATTATTTTTGCCGTCTGAGTTC
+HWI-EAS443:3:3:664:619#0/1
a[^[TV¥^`aZ__`_a^a_``^``Y[__NU`[U^[Xa[[T
@HWI-EAS443:3:40:40:2024#0/1
TTTTCATGAAGGACAAGTTAAATTCATTATTTTTGCCGTC
+HWI-EAS443:3:40:40:2024#0/1
aaaabaaaab_``aa^`[`aaaa`a_Z¥Taa`_^`a_`R`
@HWI-EAS443:3:40:681:356#0/1
GGACAAGTTAAATTCATTATTTTTGCCGTCTGAGTTCTTC
+HWI-EAS443:3:40:681:356#0/1
`a^_¥O_X_X]X_^^X_`^_^]^^^XZTKO__Y]ZZ]_XZ
@HWI-EAS443:3:42:1018:1114#0/1
GCATTTTTTCATGAAGGACAAGTTAAATTCATTATTTTTG
+HWI-EAS443:3:42:1018:1114#0/1
``^aa``baZ``¥X^_^_]^[_`^``]`aa```Y`ZDUY^
@HWI-EAS443:3:42:138:410#0/1
GTTAAATTCATTATTTTTGCCGTCTGAGTTCTTCAATGGT
+HWI-EAS443:3:42:138:410#0/1
a_`U^_^_a]``]`_]______]]__]_[^^_Z]_]^_YX
@HWI-EAS443:3:42:277:352#0/1
TTTCATGAAGGACAAGTTAAATTCATTATTTTTGCCGTCT
+HWI-EAS443:3:42:277:352#0/1
aaaaa`aaaa`_^_`a^a_¥Wa__]a_¥a`^a[`^aaU__
@HWI-EAS443:3:42:433:1654#0/1
GGCATTTTTTCATGAAGGACAAGTTAAATTCATTATTTTT
+HWI-EAS443:3:42:433:1654#0/1
`Wa`[a^ba``a¥aaa]__aa]_Wa¥W]a_a`a_aaa_[_

@HWI-EAS443:3:42:977:1481#0/1
GGCATTTTTTCATGAAGGACAAGTTAAATTCATTATTTTT
+HWI-EAS443:3:42:977:1481#0/1
abaabbbbbbabaaaabYaa^]]`aa^aab_aa`aaaaa_
@HWI-EAS443:3:43:1220:1070#0/1
GACAAGTTAAATTCATTATTTTTGCCGTCTGAGTTCTTCA
+HWI-EAS443:3:43:1220:1070#0/1
^SVX[HXWU¥b]`aa`b`[aT`a`^L]ab`aZ^^a``]
@HWI-EAS443:3:43:1338:1738#0/1
CATTTTTTCATGAAGGACAAGTTAAATTCATTATTTTTGC
+HWI-EAS443:3:43:1338:1738#0/1
ZY__T_`a`abZVZ`^Zb`U_UYYa``YZa`[YXabaa`Y
@HWI-EAS443:3:43:504:1685#0/1
CATGAAGGACAAGTTAAATTCATTATTTTTGCCGTCTGAG
+HWI-EAS443:3:43:504:1685#0/1
aa¥^]Zb___`]`Y¥^^HZ¥aaaaaaaabaa_[_MXT`aY
@HWI-EAS443:3:44:1202:601#0/1
GGCATTTTTTCATGAAGGACAAGTTAAATTCATTATTTTT
+HWI-EAS443:3:44:1202:601#0/1
a``^`a``aa¥`aa_aa^¥```a_]_]``aS¥a`_a__`^
@HWI-EAS443:3:44:1322:1157#0/1
TGAAGGACAAGTTAAATTCATTATTTTTGCCGTCTGAGTT
+HWI-EAS443:3:44:1322:1157#0/1
¥]XTV[Z_]a`MU_¥]]^Z`baaaaa_a`][`_]]aYaWN
@HWI-EAS443:3:44:403:957#0/1
GGCATTTTTTCATGAAGGACAAGTTAAATTCATTATTTTT
+HWI-EAS443:3:44:403:957#0/1
]`WU`UTUaUVX`bYF]aWX^`SR]VNP]a^M_ZL]aa^a
@HWI-EAS443:3:45:1188:532#0/1
TGAAGGACAAGTTAAATTCATTATTTTTGCCGTCTGAGTT
+HWI-EAS443:3:45:1188:532#0/1
aa`a_U__``a`a`a^aa__a`aaaaaaa_[a]¥`a_a[_
@HWI-EAS443:3:45:891:815#0/1
GACAAGTTAAATTCATTATTTTTGCCGTCTGAGTTCTTCA
+HWI-EAS443:3:45:891:815#0/1
aa`__``a````a_Ya_`_aa`_a___]S`_^_]^^a[^]
@HWI-EAS443:3:46:1021:1030#0/1
TGAAGGACAAGTTAAATTCATTATTTTTGCCGTCTGAGTT
+HWI-EAS443:3:46:1021:1030#0/1
ab_`bb`a`_a]a`aabaaaaaaaaaaaa`^a_aaa_aZa
@HWI-EAS443:3:46:1210:727#0/1
GCATTTTTTCATGAAGGACAAGTTAAATTCATTATTTTTG
+HWI-EAS443:3:46:1210:727#0/1
aa^_aaaaaaaaa`]a`_`__a^a`[]aa`_a__a`a_^_
@HWI-EAS443:3:46:1234:972#0/1
GTTAAATTCATTATTTTTGCCGTCTGAGTTCTTCAATGGT
+HWI-EAS443:3:46:1234:972#0/1
a`[GM]`YW¥__X]a`aaa_¥VT]ZVX^VVY[¥^]L]^YF
@HWI-EAS443:3:46:445:1119#0/1
TTTTTTCATGAAGGACAAGTTAAATTCATTATTTTTGCCG

```
+HWI-EAS443:3:46:445:1119#0/1
ababaaaaaa^a```a``b_aaa^````aaaaaaa_a]¥a
@HWI-EAS443:3:46:980:1189#0/1
GGCATTTTTTCATGAAGGACAAGTTAAATTCATTATTTTT
+HWI-EAS443:3:46:980:1189#0/1
aaaaaaaaaa`a`aa`aa```_a^`_^^``]`a``a`^`^
@HWI-EAS443:3:47:1749:360#0/1
GGCATTTTTTCATGAAGGACAAGTTAAATTCATTATTTTT
+HWI-EAS443:3:47:1749:360#0/1
aa`Y^][]^ZV[¥XTT]]]MM[[R^[_T^^PS_VW¥US¥X
@HWI-EAS443:3:47:523:1611#0/1
CATTTTTTCATGAAGGACAAGTTAAATTCATTATTTTTGC
+HWI-EAS443:3:47:523:1611#0/1
`Yaba_aaa`^]^^aa]TVVb__`]X`^X^_^V^__^ZWO
@HWI-EAS443:3:47:864:459#0/1
GGACAAGTTAAATTCATTATTTTTGCCGTCTGAGTTCTTC
+HWI-EAS443:3:47:864:459#0/1
ab__¥P¥[aaaaba^]b``aaaa]]WU^^[^a_a^`[`_[
@HWI-EAS443:3:48:1003:1597#0/1
GGACAAGTTAAATTCATTATTTTTGCCGTCTGAGTTCTTC
+HWI-EAS443:3:48:1003:1597#0/1
aZZ`^Y^TX[`__^__a^¥`_Y`^^^^Z_`]WG¥[YW[^[
@HWI-EAS443:3:48:686:1765#0/1
GGACAAGTTAAATTCATTATTTTTGCCGTCTGAGTTCTTC
+HWI-EAS443:3:48:686:1765#0/1
¥[ab`X_^U^`¥bb`b`bXb_a`bbT`bZZZ¥`b_a_^`[
@HWI-EAS443:3:49:1104:1065#0/1
CATTTTTTCATGAAGGACAAGTTAAATTCATTATTTTTGC
+HWI-EAS443:3:49:1104:1065#0/1
ab`bab_b`bbbaabbaaaababba¥baaabbaaabaab_
@HWI-EAS443:3:49:1547:326#0/1
GACAAGTTAAATTCATTATTTTTGCCGTCTGAGTTCTTCA
+HWI-EAS443:3:49:1547:326#0/1
``^a`a`b`aabaaaabbbba`a_[^b]aaaabaaabaa_
@HWI-EAS443:3:4:1690:1506#0/1
GTTAAATTCATTATTTTTGCCGTCTGAGTTCTTCAATGGT
+HWI-EAS443:3:4:1690:1506#0/1
a`aZZZ`Y_[a[[[¥`[_^VS_Z[S_YaT[[][][S[^[[
@HWI-EAS443:3:4:736:1601#0/1
TGAAGGACAAGTTAAATTCATTATTTTTGCCGTCTGAGTT
+HWI-EAS443:3:4:736:1601#0/1
`bbabbbbabbaba``bbabbaabbbaaa_aaa_abab`a
@HWI-EAS443:3:4:798:1388#0/1
GTTAAATTCATTATTTTTGCCGTCTGAGTTCTTCAATGGT
+HWI-EAS443:3:4:798:1388#0/1
aaaaaabbaaaa`aaaaaa`_a^]aa`a^`_aa____`[[
@HWI-EAS443:3:51:1049:1469#0/1
CATGAAGGACAAGTTAAATTCATTATTTTTGCCGTCTGAG
+HWI-EAS443:3:51:1049:1469#0/1
abbbbabbabb_b`bbbbabbbbbabbbbaa`aab`bb`b
```

```
@HWI-EAS443:3:51:1102:812#0/1
GTTAAATTCATTATTTTTGCCGTCTGAGTTCTTCAATGGT
+HWI-EAS443:3:51:1102:812#0/1
]_b]aaa`]`]````[_aa`^a_^_a]aTZ¥`_[`_]_^T
@HWI-EAS443:3:51:128:784#0/1
GTTAAATTCATTATTTTTGCCGTCTGAGTTCTTCAATGGT
+HWI-EAS443:3:51:128:784#0/1
`¥aa`aabaaba`baaba^[]`¥_`a`a_^_a^_ZOYa`R
@HWI-EAS443:3:51:1602:770#0/1
GGCATTTTTTCATGAAGGACAAGTTAAATTCATTATTTTT
+HWI-EAS443:3:51:1602:770#0/1
``X_b¥I`abb`b_bbaaT`]^_^bb``bYR^bbbbba`b
@HWI-EAS443:3:51:464:1048#0/1
TTTTTCATGAAGGACAAGTTAAATTCATTATTTTTGCCGT
+HWI-EAS443:3:51:464:1048#0/1
aaabababaaa]¥`a^`a_a^``_W_^_^a_Wa`a`a[^]^Y
@HWI-EAS443:3:51:572:1774#0/1
GGCATTTTTTCATGAAGGACAAGTTAAATTCATTATTTTT
+HWI-EAS443:3:51:572:1774#0/1
¥`bbbbbb`]^baa^aa^[a_Za_babaa`a_aaaba[a`
@HWI-EAS443:3:51:631:1032#0/1
GCATTTTTTCATGAAGGACAAGTTAAATTCATTATTTTTG
+HWI-EAS443:3:51:631:1032#0/1
aa`aaaaa___aaaa_a^_`^a_``]Yaa_]aa`aaa_`_
@HWI-EAS443:3:51:83:1214#0/1
TGAAGGACAAGTTAAATTCATTATTTTTGCCGTCTGAGTT
+HWI-EAS443:3:51:83:1214#0/1
abababa_aaabbaa`bbaabbababaabaaa_aaa]a[`
@HWI-EAS443:3:51:988:1284#0/1
GAAGGACAAGTTAAATTCATTATTTTTGCCGTCTGAGTTC
+HWI-EAS443:3:51:988:1284#0/1
a^abaa_`]aaaaYU]aYW_`^a`aaaa^_a__`a[a^^]
@HWI-EAS443:3:52:1375:660#0/1
GGCATTTTTTCATGAAGGACAAGTTAAATTCATTATTTTT
+HWI-EAS443:3:52:1375:660#0/1
`^aaaababaab_aaabb```¥_`a```aa_`baaaaa[a
@HWI-EAS443:3:52:357:683#0/1
GGACAAGTTAAATTCATTATTTTTGCCGTCTGAGTTCTTC
+HWI-EAS443:3:52:357:683#0/1
`WX__^a^^__Yaa[]_`X__^]Y_^^`V]]_]`WYZ^]X
@HWI-EAS443:3:52:643:1880#0/1
GACAAGTTAAATTCATTATTTTTGCCGTCTGAGTTCTTCA
+HWI-EAS443:3:52:643:1880#0/1
^b_X__[__[]]`aR]¥`[L[XS`_[]U_¥a¥WQX``¥[_
@HWI-EAS443:3:52:677:1468#0/1
GTTAAATTCATTATTTTTGCCGTCTGAGTTCTTCAATGGT
+HWI-EAS443:3:52:677:1468#0/1
`_bbaaba`abaaaba¥aaa¥a[_ab_^_`aaaaa`aa^R
@HWI-EAS443:3:52:735:1009#0/1
GGCATTTTTTCATGAAGGACAAGTTAAATTCATTATTTTT
```

```
+HWI-EAS443:3:52:735:1009#0/1
abb`baaaaabaa^^`a^T`a[b^`a]a`a`_aa_aaa``
@HWI-EAS443:3:53:50:643#0/1
ATTTTTTCATGAAGGACAAGTTAAATTCATTATTTTTGCC
+HWI-EAS443:3:53:50:643#0/1
abbb_^_a^U_aab``¥`^a^a___a__]a`_a`_`_a_T
@HWI-EAS443:3:54:1056:646#0/1
GCATTTTTTCATGAAGGACAAGTTAAATTCATTATTTTTG
+HWI-EAS443:3:54:1056:646#0/1
aa`aaaaaa[aaa_]aa]_^_a^``^]a_^^a^_a``__^
@HWI-EAS443:3:54:131:1994#0/1
GACAAGTTAAATTCATTATTTTTGCCGTCTGAGTTCTTCA
+HWI-EAS443:3:54:131:1994#0/1
KZa^TXaba`¥`__b`Zabbabb`a]aVabaa¥Z^^]^_a
@HWI-EAS443:3:54:1551:1467#0/1
GACAAGTTAAATTCATTATTTTTGCCGTCTGAGTTCTTCA
+HWI-EAS443:3:54:1551:1467#0/1
a]W`^]M¥`aL^_W`]__YW[[aTLJ¥MFTa^a¥L]_^[[
@HWI-EAS443:3:54:519:306#0/1
GTTAAATTCATTATTTTTGCCGTCTGAGTTCTTCAATGGT
+HWI-EAS443:3:54:519:306#0/1
a_aa`_ba^aaaaaaa```]_a]^a`_a[`^`_]_`^`_Y
@HWI-EAS443:3:54:656:1419#0/1
GTTAAATTCATTATTTTTGCCGTCTGAGTTCTTCAATGGT
+HWI-EAS443:3:54:656:1419#0/1
`[_WUUY^¥a[]Q]aa`QQQ_ZK¥X[QXY_UZ^PQWWUPQ
@HWI-EAS443:3:54:708:1476#0/1
GGCATTTTTTCATGAAGGACAAGTTAAATTCATTATTTTT
+HWI-EAS443:3:54:708:1476#0/1
a^a]bbaaaaa`_]_W_a`]^¥aZa_a`a___`aaa````
@HWI-EAS443:3:55:243:695#0/1
GCATTTTTTCATGAAGGACAAGTTAAATTCATTATTTTTG
+HWI-EAS443:3:55:243:695#0/1
a`a`Zaaa`_`aa``a_^`_Qa^_`¥]a__``_]__`^__
@HWI-EAS443:3:55:538:1377#0/1
GGCATTTTTTCATGAAGGACAAGTTAAATTCATTATTTTT
+HWI-EAS443:3:55:538:1377#0/1
^aa^a`¥```aa^¥aa`a]a_]^]__¥UZb_`_aaaaa]`
@HWI-EAS443:3:56:1529:1751#0/1
GGCATTTTTTCATGAAGGACAAGTTAAATTCATTATTTTT
+HWI-EAS443:3:56:1529:1751#0/1
abaabbbbaaa`bbaabbaS`ab_aaaaab^_aaaaaaaa
@HWI-EAS443:3:56:1709:1542#0/1
GCATTTTTTCATGAAGGACAAGTTAAATTCATTATTTTTG
+HWI-EAS443:3:56:1709:1542#0/1
aX[_aa`a^H]^]_^`]VQS^_X_]]^`YTX`__`^a__V
@HWI-EAS443:3:56:1764:138#0/1
GGCATTTTTTCATGAAGGACAAGTTAAATTCATTATTTTT
+HWI-EAS443:3:56:1764:138#0/1
X__WYW_Y_¥[Y¥_W¥¥^TV^`¥QZ_U_]_RZa]Z````[
```

```
@HWI-EAS443:3:56:666:1037#0/1
AAGTTAAATTCATTATTTTTGCCGTCTGAGTTCTTCAATG
+HWI-EAS443:3:56:666:1037#0/1
^^aabbb`bb``aabbabbaa]`a`aababaaaaaaa^a`
@HWI-EAS443:3:56:996:2009#0/1
GGACAAGTTAAATTCATTATTTTTGCCGTCTGAGTTCTTC
+HWI-EAS443:3:56:996:2009#0/1
aba`aab_aaaaaa`a`_aa`aba_]¥a^_aa``_^_a`¥
@HWI-EAS443:3:57:1120:544#0/1
GGACAAGTTAAATTCATTATTTTTGCCGTCTGAGTTCTTC
+HWI-EAS443:3:57:1120:544#0/1
aa`aaaaababababaaaababab^aa``a_aaaaa]a^a``
@HWI-EAS443:3:57:1217:200#0/1
CATTTTTTCATGAAGGACAAGTTAAATTCATTATTTTTGC
+HWI-EAS443:3:57:1217:200#0/1
aabbbabbabbbaabbaba`baba_aaaaabbabbabaaa
@HWI-EAS443:3:57:529:561#0/1
CATTTTTTCATGAAGGACAAGTTAAATTCATTATTTTTGC
+HWI-EAS443:3:57:529:561#0/1
abbbbaaa`babaaaa`aa`aYaaaaaa^`aaabaa_a`W
@HWI-EAS443:3:57:823:1849#0/1
GGCATTTTTTCATGAAGGACAAGTTAAATTCATTATTTTT
+HWI-EAS443:3:57:823:1849#0/1
Y`a`^`b¥¥a```abb`¥PX`X`¥]^^bb``]]LZbb^aZ
@HWI-EAS443:3:58:1214:867#0/1
GGCATTTTTTCATGAAGGACAAGTTAAATTCATTATTTTT
+HWI-EAS443:3:58:1214:867#0/1
a`^_aabaaaX_a_`Y¥`^^^^`]^_]^__^W`_Y_`_^_
@HWI-EAS443:3:58:214:390#0/1
GTTAAATTCATTATTTTTGCCGTCTGAGTTCTTCAATGGT
+HWI-EAS443:3:58:214:390#0/1
a_aaa_aa_`aaaaa_`_]`U_^_a][_W`¥_^^]Y__^[
@HWI-EAS443:3:58:79:1320#0/1
GGACAAGTTAAATTCATTATTTTTGCCGTCTGAGTTCTTC
+HWI-EAS443:3:58:79:1320#0/1
`[Va]WWM]¥[YZ]Wa^Q¥][[¥^aYV^____X^S[YY__
@HWI-EAS443:3:59:1247:1955#0/1
TTCATGAAGGACAAGTTAAATTCATTATTTTTGCCGTCTG
+HWI-EAS443:3:59:1247:1955#0/1
^]_babbbbaa`bab]`aaa`aaaaabaaab_aaP[[]a]
@HWI-EAS443:3:59:667:359#0/1
GTTAAATTCATTATTTTTGCCGTCTGAGTTCTTCAATGGT
+HWI-EAS443:3:59:667:359#0/1
aabaaaba`abbaaaaabaaa^`aaaaa^`a`__`Y]``[
@HWI-EAS443:3:59:863:270#0/1
GGCATTTTTTCATGAAGGACAAGTTAAATTCATTATTTTT
+HWI-EAS443:3:59:863:270#0/1
aa`]``abaa_``^^ab`^^`¥Q^^`_^``_^``^UX`^^
@HWI-EAS443:3:5:1239:1807#0/1
GGACAAGTTAAATTCATTATTTTTGCCGTCTGAGTTCTTC
```

```
+HWI-EAS443:3:5:1239:1807#0/1
¥`_PR¥```a```^babab¥_`b¥Y]S^_^abba[babaU
@HWI-EAS443:3:60:1725:1861#0/1
ATGAAGGACAAGTTAAATTCATTATTTTTGCCGTCTGAGT
+HWI-EAS443:3:60:1725:1861#0/1
a]aZ_aaL_TVYZ`_]_[_]^¥T[_^`_^_HUZMM_]]^V
@HWI-EAS443:3:60:413:208#0/1
TTTCATGAAGGACAAGTTAAATTCATTATTTTTGCCGTCT
+HWI-EAS443:3:60:413:208#0/1
aa`_^aa^aa_`_Q^a__[`^``]Ya^]``_¥^_[Z^W]^
@HWI-EAS443:3:61:1585:317#0/1
GACAAGTTAAATTCATTATTTTTGCCGTCTGAGTTCTTCA
+HWI-EAS443:3:61:1585:317#0/1
aaa___aa`_`aa_^a^`aa^_a``]]_``_]_[^Y^^HU
@HWI-EAS443:3:61:278:1834#0/1
CATGAAGGACAAGTTAAATTCATTATTTTTGCCGTCTGAG
+HWI-EAS443:3:61:278:1834#0/1
abbbbbbbbabbbaabbbaa`S]a^baaaaabZa^^___`
@HWI-EAS443:3:61:447:466#0/1
GCATTTTTTCATGAAGGACAAGTTAAATTCATTATTTTTG
+HWI-EAS443:3:61:447:466#0/1
a_T¥]S[SU]]STSU`VTWSXYVUQPROY]Y]TVVYWZWZ
@HWI-EAS443:3:61:506:1037#0/1
CATTTTTTCATGAAGGACAAGTTAAATTCATTATTTTTGC
+HWI-EAS443:3:61:506:1037#0/1
aababbbbbbabbaabbbbabaa¥[`a``ba]aaa_a_UY
@HWI-EAS443:3:63:167:877#0/1
GGCATTTTTTCATGAAGGACAAGTTAAATTCATTATTTTT
+HWI-EAS443:3:63:167:877#0/1
``babaabaaaaaa]aaa__`_a`a[`_a```a``a_a_^
@HWI-EAS443:3:63:332:38#0/1
GGCATTTTTTCATGAAGGACAAGTTAAATTCATTATTTTT
+HWI-EAS443:3:63:332:38#0/1
abb]bbbbabaaab__aa````baaaYaa`__a`_a`a__
@HWI-EAS443:3:63:346:868#0/1
GGACAAGTTAAATTCATTATTTTTGCCGTCTGAGTTCTTC
+HWI-EAS443:3:63:346:868#0/1
`^bba`bbabbaaaa`aaaaaaa`_`aa__aaaa^`ba^b
@HWI-EAS443:3:63:586:1096#0/1
GGACAAGTTAAATTCATTATTTTTGCCGTCTGAGTTCTTC
+HWI-EAS443:3:63:586:1096#0/1
`aba`T`abaVbb`a^b`[abaaa`bb`¥aaa``]__aaa
@HWI-EAS443:3:64:701:140#0/1
GTTAAATTCATTATTTTTGCCGTCTGAGTTCTTCAATGGT
+HWI-EAS443:3:64:701:140#0/1
aa``aaaa_¥`_¥`_```_[^_]¥^a]^[][_^]W[]^[Z
@HWI-EAS443:3:65:1359:918#0/1
AGGACAAGTTAAATTCATTATTTTTGCCGTCTGAGTTCTT
+HWI-EAS443:3:65:1359:918#0/1
abaaa^```aaaaaaaaaaaaaaaa``a`aaa^a^aZa`
```

@HWI-EAS443:3:65:197:456#0/1
GGACAAGTTAAATTCATTATTTTTGCCGTCTGAGTTCTTC
+HWI-EAS443:3:65:197:456#0/1
abbaaaaaaaaaa`abbaabbaa`aaab_aaaab[`aaa_
@HWI-EAS443:3:65:600:503#0/1
ATGAAGGACAAGTTAAATTCATTATTTTTGCCGTCTGAGT
+HWI-EAS443:3:65:600:503#0/1
aaaaa`]¥a`aaZaaba`^_a`X`aaaY[aa_b[]a`T`U
@HWI-EAS443:3:65:825:570#0/1
GAAGGACAAGTTAAATTCATTATTTTTGCCGTCTGAGTTC
+HWI-EAS443:3:65:825:570#0/1
a¥bY[V_Z]b_aa]aaab__aaaaaa^aa`_Y^^`N_U]¥
@HWI-EAS443:3:66:1000:1447#0/1
GGACAAGTTAAATTCATTATTTTTGCCGTCTGAGTTCTTC
+HWI-EAS443:3:66:1000:1447#0/1
aaaa^X`_aaaaa`a_a`aaa_a_`a`a_``a__^^`_^^
@HWI-EAS443:3:66:1286:24#0/1
ATTTTTTCATGAAGGACAAGTTAAATTCATTATTTTTGCC
+HWI-EAS443:3:66:1286:24#0/1
abbbab`aba``aba_^a¥b__a`^^VHZbbabaaaa_[_
@HWI-EAS443:3:66:1468:1495#0/1
GGACAAGTTAAATTCATTATTTTTGCCGTCTGAGTTCTTC
+HWI-EAS443:3:66:1468:1495#0/1
_SQWY[[^`PX_a^]_¥__[_][^IZ¥SWS]`]SZX][ZW
@HWI-EAS443:3:67:1127:625#0/1
CATTTTTTCATGAAGGACAAGTTAAATTCATTATTTTTGC
+HWI-EAS443:3:67:1127:625#0/1
a^`[`_Z]a__a[`_`X]]aa__a¥`_a_]^`]`aaa_`U
@HWI-EAS443:3:67:1249:202#0/1
TTGAAGAACTCAGACGGCAAAAATAATGAATTTAACTTGT
+HWI-EAS443:3:67:1249:202#0/1
Xbba_aabaaaaaZJ_ba`ba_abbbbbaaa_a_a]aa__
@HWI-EAS443:3:68:112:1247#0/1
GGACAAGTTAAATTCATTATTTTTGCCGTCTGAGTTCTTC
+HWI-EAS443:3:68:112:1247#0/1
bbb`X`abbbabbaaaaabbbabaaa^`aab``aaaab
@HWI-EAS443:3:68:1395:1434#0/1
GTTAAATTCATTATTTTTGCCGTCTGAGTTCTTCAATGGT
+HWI-EAS443:3:68:1395:1434#0/1
a]_R^X`___aYU^_[^I]UU^[¥]YZSIYYZ¥YW^XUWI
@HWI-EAS443:3:68:1690:135#0/1
GGACAAGTTAAATTCATTATTTTTGCCGTCTGAGTTCTTC
+HWI-EAS443:3:68:1690:135#0/1
aa`]YZHMZa]aaaaaaaaaaaa`^^_`^___X`¥_Y`_V
@HWI-EAS443:3:68:1781:679#0/1
GGCATTTTTTCATGAAGGACAAGTTAAATTCATTATTTTT
+HWI-EAS443:3:68:1781:679#0/1
aa]abaab```aaaaaaY^^__`_`X`]aa`]`_```a`_
@HWI-EAS443:3:68:405:1583#0/1
ATGAAGGACAAGTTAAATTCATTATTTTTGCCGTCTGAGT

```
+HWI-EAS443:3:68:405:1583#0/1
`¥]VYW¥U_^_aQa]NZ]a`^]`aaaaaaaba]^a``a`V
@HWI-EAS443:3:68:778:1012#0/1
GTTAAATTCATTATTTTTGCCGTCTGAGTTCTTCAATGGT
+HWI-EAS443:3:68:778:1012#0/1
a`aZ^_aaa`a`[aaa``a_[_^`_`^aZ^__`_a[__[Y
@HWI-EAS443:3:68:840:1430#0/1
GGCATTTTTTCATGAAGGACAAGTTAAATTCATTATTTTT
+HWI-EAS443:3:68:840:1430#0/1
a_`[abbba`bb]b¥[^`aaa`S]ab`aaa`a`[^``a`a
@HWI-EAS443:3:69:1053:1713#0/1
GGACAAGTTAAATTCATTATTTTTGCCGTCTGAGTTCTTC
+HWI-EAS443:3:69:1053:1713#0/1
Y[a¥T``Kbb[Y¥b^^a`ba^ba]aa^`_aaaab^a`aa_
@HWI-EAS443:3:69:1789:894#0/1
GGCATTTTTTCATGAAGGACAAGTTAAATTCATTATTTTT
+HWI-EAS443:3:69:1789:894#0/1
aaa`aaaaaa`aa`a`b`Y^^Z`¥a___`a]_a^_`____
@HWI-EAS443:3:69:292:1854#0/1
GCATTTTTTCATGAAGGACAAGTTAAATTCATTATTTTTG
+HWI-EAS443:3:69:292:1854#0/1
aY_W]¥]¥]Za¥T_a`¥]Y]__RZ`¥WY]a^^]__]¥^]_
@HWI-EAS443:3:69:842:654#0/1
CATTTTTTCATGAAGGACAAGTTAAATTCATTATTTTTGC
+HWI-EAS443:3:69:842:654#0/1
aaabbbbbbababababaaaa_b_bba`ba`]aaabb`aaaa
@HWI-EAS443:3:69:879:1081#0/1
GGACAAGTTAAATTCATTATTTTTGCCGTCTGAGTTCTTC
+HWI-EAS443:3:69:879:1081#0/1
^]`YSW¥[a^[O]`a]]a^`^a^`^`^]]``¥V_X¥_Y[^
@HWI-EAS443:3:69:929:1429#0/1
GACAAGTTAAATTCATTATTTTTGCCGTCTGAGTTCTTCA
+HWI-EAS443:3:69:929:1429#0/1
a]aWO[[aaaaaaaa`^aa[F[_Y`a_]_[X]][_a^¥^a
@HWI-EAS443:3:6:1146:1819#0/1
GTTAAATTCATTATTTTTGCCGTCTGAGTTCTTCAATGGT
+HWI-EAS443:3:6:1146:1819#0/1
aa^¥IR¥VV]Z][]]¥^___^`USX_Z`PQW^VZ_X[¥YR
@HWI-EAS443:3:6:77:1764#0/1
GGCATTTTTTCATGAAGGACAAGTTAAATTCATTATTTTT
+HWI-EAS443:3:6:77:1764#0/1
^baabbbbbbabbbbbb_abaababaaabbbabaabaaaa
@HWI-EAS443:3:6:945:1633#0/1
CAAGTTAAATTCATTATTTTTGCCGTCTGAGTTCTTCAAT
+HWI-EAS443:3:6:945:1633#0/1
aa`b`baa`ab_bb`^bb`aa`a[b`^aaa`^``a_aa]_
@HWI-EAS443:3:71:1036:1609#0/1
GAAGGACAAGTTAAATTCATTATTTTTGCCGTCTGAGTTC
+HWI-EAS443:3:71:1036:1609#0/1
a_aa_a`baW¥Y```aaaaaaaa`a`_^b_`_a```_V``
```

@HWI-EAS443:3:71:1256:695#0/1
GACAAGTTAAATTCATTATTTTTGCCGTCTGAGTTCTTCA
+HWI-EAS443:3:71:1256:695#0/1
a[aaVa[_[JM[XOVZV¥Q¥Q`WNLVWL[Z^^Y¥P[R[XR
@HWI-EAS443:3:71:1264:1670#0/1
GGACAAGTTAAATTCATTATTTTTGCCGTCTGAGTTCTTC
+HWI-EAS443:3:71:1264:1670#0/1
aa¥D[YW[a`¥NOQR`a]Y]YW[UT¥PPXFW__]TJLOTV
@HWI-EAS443:3:71:1305:1793#0/1
TGAAGGACAAGTTAAATTCATTATTTTTGCCGTCTGAGTT
+HWI-EAS443:3:71:1305:1793#0/1
aaaaa`aaa^a_aaa_a`_aaa_aaa^b_aWa]aa¥^^]¥
@HWI-EAS443:3:71:768:501#0/1
AGGACAAGTTAAATTCATTATTTTTGCCGTCTGAGTTCTT
+HWI-EAS443:3:71:768:501#0/1
^¥YRX_R^RaYLUaaZT`^Z`_aW_a_[`X`_ZL]VTMSO
@HWI-EAS443:3:72:1245:168#0/1
GTTAAATTCATTATTTTTGCCGTCTGAGTTCTTCAATGGT
+HWI-EAS443:3:72:1245:168#0/1
a````]aaYaa`_a`a_a_Y_aZ]_`^`[^[^^Y^[^_^[
@HWI-EAS443:3:72:394:796#0/1
CATTTTTTCATGAAGGACAAGTTAAATTCATTATTTTTGC
+HWI-EAS443:3:72:394:796#0/1
_`bba_WZaaa`¥J¥^V_a_a_a_Z_aZU]a[H[aa_a`Z
@HWI-EAS443:3:72:759:329#0/1
GACAAGTTAAATTCATTATTTTTGCCGTCTGAGTTCTTCA
+HWI-EAS443:3:72:759:329#0/1
`a``]a_a`aaaaa`ba^aaa_aa`]a`^_a__U`[a_¥a
@HWI-EAS443:3:72:822:1663#0/1
GACAAGTTAAATTCATTATTTTTGCCGTCTGAGTTCTTCA
+HWI-EAS443:3:72:822:1663#0/1
_bbabbabb`¥babbbbaabaabaaba`bbbaaa`aa`aa
@HWI-EAS443:3:73:1704:1551#0/1
GGCATTTTTTCATGAAGGACAAGTTAAATTCATTATTTTT
+HWI-EAS443:3:73:1704:1551#0/1
aa_``ba``a]_`a^^`aW]¥¥a]_____`[X_YZ_`¥^_
@HWI-EAS443:3:73:692:43#0/1
GGCATTTTTTCATGAAGGACAAGTTAAATTCATTATTTTT
+HWI-EAS443:3:73:692:43#0/1
a]R^¥`TW[`]^]a[]]^]YSSYSUSUX]ZXY^W[_]VUZ
@HWI-EAS443:3:73:842:446#0/1
AGTTAAATTCATTATTTTTGCCGTCTGAGTTCTTCAATGG
+HWI-EAS443:3:73:842:446#0/1
```baa^abb^]babbabaaab^Y_baab_aaaaaa_aaZ
@HWI-EAS443:3:73:933:536#0/1
GGCATTTTTTCATGAAGGACAAGTTAAATTCATTATTTTT
+HWI-EAS443:3:73:933:536#0/1
aaa^aabbbaaaaaaaba`_`_a_a``aaa_abaaa``aa
@HWI-EAS443:3:74:1095:18#0/1
GACAAGTTAAATTCATTATTTTTGCCGTCTGAGTTCTTCA
```

+HWI-EAS443:3:74:1095:18#0/1
``baWa`baaaaab`aa`aaa^aaa`b__bb`b```a_]_
@HWI-EAS443:3:74:1731:569#0/1
TGAAGGACAAGTTAAATTCATTATTTTTGCCGTCTGAGTT
+HWI-EAS443:3:74:1731:569#0/1
aaaaaaaaaabaaaaabaaabaabaaaa`a_aaaab`a^a
@HWI-EAS443:3:74:413:755#0/1
GGCATTTTTTCATGAAGGACAAGTTAAATTCATTATTTTT
+HWI-EAS443:3:74:413:755#0/1
ababbbabbabaabaaaaaaa`a`aaa_aaaabaaaaaaa
@HWI-EAS443:3:74:712:1942#0/1
GGCATTTTTTCATGAAGGACAAGTTAAATTCATTATTTTT
+HWI-EAS443:3:74:712:1942#0/1
abaXS`^¥a`bbb`ba¥aaa``W_^a`XLa`_b[[a`aa`
@HWI-EAS443:3:75:1015:1137#0/1
ACAAGTTAAATTCATTATTTTTGCCGTCTGAGTTCTTCAA
+HWI-EAS443:3:75:1015:1137#0/1
aaa`b[aabaaa`aaaabaa``aa`a[aaaaa`a_aaa_`
@HWI-EAS443:3:75:1024:168#0/1
GACAAGTTAAATTCATTATTTTTGCCGTCTGAGTTCTTCA
+HWI-EAS443:3:75:1024:168#0/1
^`_aaba_`a`bbaaabab^`aa^¥^b`_bbab^``aa^a
@HWI-EAS443:3:75:1381:1382#0/1
GGCATTTTTTCATGAAGGACAAGTTAAATTCATTATTTTT
+HWI-EAS443:3:75:1381:1382#0/1
YS]POW`WSQ]X`_SS]]]_FIUT`XM[a`R^`V^Ia_`]
@HWI-EAS443:3:75:1454:971#0/1
TGAAGGACAAGTTAAATTCATTATTTTTGCCGTCTGAGTT
+HWI-EAS443:3:75:1454:971#0/1
Q_b^bbbab]_abbaa_b`abbbb``¥_YY`aU`]b[a`a
@HWI-EAS443:3:75:1579:1008#0/1
GTTAAATTCATTATTTTTGCCGTCTGAGTTCTTCAATGGT
+HWI-EAS443:3:75:1579:1008#0/1
``^VY`a_QU¥Z_aa`[_Z]ZWX^a]V^Y`^a^^¥^Y¥`P
@HWI-EAS443:3:75:1638:1951#0/1
GTTAAATTCATTATTTTTGCCGTCTGAGTTCTTCAATGGT
+HWI-EAS443:3:75:1638:1951#0/1
abba[^baba_¥baab_b`bab_`aa`b]aabaZ`]aaa[
@HWI-EAS443:3:75:1641:515#0/1
CATTTTTTCATGAAGGACAAGTTAAATTCATTATTTTTGC
+HWI-EAS443:3:75:1641:515#0/1
abbbbbbbabbbbabbaabaabbbbabbabbbbbbbbbba
@HWI-EAS443:3:76:74:1412#0/1
GACAAGTTAAATTCATTATTTTTGCCGTCTGAGTTCTTCA
+HWI-EAS443:3:76:74:1412#0/1
a`a¥]_H_`S_^Y^_`_¥]^__^Y]XTT_Y_S¥Y[[[^]Z
@HWI-EAS443:3:77:1223:791#0/1
GTTAAATTCATTATTTTTGCCGTCTGAGTTCTTCAATGGT
+HWI-EAS443:3:77:1223:791#0/1
aaaa`abaaaba_aaaaaa__a]^a__a]_^`_Y``aa_]

@HWI-EAS443:3:77:1534:1897#0/1
CATTTTTTCATGAAGGACAAGTTAAATTCATTATTTTTGC
+HWI-EAS443:3:77:1534:1897#0/1
aaaabbbaaaa`bbaaa``aa`aaaabaaaaaaaaaaaaW
@HWI-EAS443:3:77:406:1851#0/1
GGCATTTTTTCATGAAGGACAAGTTAAATTCATTATTTTT
+HWI-EAS443:3:77:406:1851#0/1
a]¥`]Z_`Z¥^aYYaYUYZNVWVQPRZ`__`¥`__a[N[[
@HWI-EAS443:3:77:452:953#0/1
GGCATTTTTTCATGAAGGACAAGTTAAATTCATTATTTTT
+HWI-EAS443:3:77:452:953#0/1
aab`aabababaaaaaa`aa___a``aaaa```aaa`a_
@HWI-EAS443:3:77:792:836#0/1
GACAAGTTAAATTCATTATTTTTGCCGTCTGAGTTCTTCA
+HWI-EAS443:3:77:792:836#0/1
aabaab`a`baaaaabaaaaaaa`a_a_aaa_a]`aa_aa
@HWI-EAS443:3:78:1000:1986#0/1
GTTAAATTCATTATTTTTGCCGTCTGAGTTCTTCAATGGT
+HWI-EAS443:3:78:1000:1986#0/1
^Zb``abbabaaaaabaaba_a````_a_aab`a`a^a`Y
@HWI-EAS443:3:78:1280:804#0/1
ATTTTTTCATGAAGGACAAGTTAAATTCATTATTTTTGCC
+HWI-EAS443:3:78:1280:804#0/1
abba``b``aa^Taa^``Sa___T`a___a`[aa`a^¥RR
@HWI-EAS443:3:78:537:754#0/1
ATGAAGGACAAGTTAAATTCATTATTTTTGCCGTCTGAGT
+HWI-EAS443:3:78:537:754#0/1
aaa``aa_a`]a^``a`a_a``__``__^`_^_]Z__]_[
@HWI-EAS443:3:78:785:1116#0/1
GTTAAATTCATTATTTTTGCCGTCTGAGTTCTTCAATGGT
+HWI-EAS443:3:78:785:1116#0/1
^P]^TX`ZG[aa_`a_a_`a_aX^_`[a^[¥`^[Z^]^]X
@HWI-EAS443:3:79:69:1380#0/1
GGCATTTTTTCATGAAGGACAAGTTAAATTCATTATTTTT
+HWI-EAS443:3:79:69:1380#0/1
`a_SS`aaa_V]VYQZ``__SS[I`XF[T¥a`PTM^```U
@HWI-EAS443:3:79:759:1444#0/1
CAAGTTAAATTCATTATTTTTGCCGTCTGAGTTCTTCAAT
+HWI-EAS443:3:79:759:1444#0/1
aabaabba¥bbbabbababaa`bbb`abbaa`a`b`aa`a
@HWI-EAS443:3:7:1264:1967#0/1
TTTTTTCATGAAGGACAAGTTAAATTCATTATTTTTGCCG
+HWI-EAS443:3:7:1264:1967#0/1
¥`aa^]¥W_aMS¥_Y`[aaXa¥^Y^[¥aZ_`a]_`RT]^¥
@HWI-EAS443:3:7:1279:755#0/1
TGAAGGACAAGTTAAATTCATTATTTTTGCCGTCTGAGTT
+HWI-EAS443:3:7:1279:755#0/1
`b`[^Y^]^¥YSbaaba`aa^aVY`W]b^PJa_J_]aaNN
@HWI-EAS443:3:7:172:426#0/1
TTTTTTCATGAAGGACAAGTTAAATTCATTATTTTTGCCG

```
+HWI-EAS443:3:7:172:426#0/1
abbabbbaabbabbaa``babaaaba`ab`aaaaa`a`_b
@HWI-EAS443:3:7:388:289#0/1
GGCATTTTTTCATGAAGGACAAGTTAAATTCATTATTTTT
+HWI-EAS443:3:7:388:289#0/1
a[¥R`__a____]`VP[W¥[]Y_Y_W]__¥]]^][^[S]Y
@HWI-EAS443:3:7:612:1399#0/1
GTTAAATTCATTATTTTTGCCGTCTGAGTTCTTCAATGGT
+HWI-EAS443:3:7:612:1399#0/1
a__a_`aa^aa`aaa_`¥_^R]PPYRX_[[X`ZP_VV^BB
@HWI-EAS443:3:80:160:171#0/1
CATTTTTTCATGAAGGACAAGTTAAATTCATTATTTTTGC
+HWI-EAS443:3:80:160:171#0/1
UU``a`a`_`bb_P¥W_]`a_a[HR]b_abbba`¥Z`aba
@HWI-EAS443:3:81:1159:1840#0/1
GACAAGTTAAATTCATTATTTTTGCCGTCTGAGTTCTTCA
+HWI-EAS443:3:81:1159:1840#0/1
ab``ZY¥a`^Ybbaa`]]a_a__b``bU[^a_aZ[X`XY`
@HWI-EAS443:3:81:1717:1440#0/1
GACAAGTTAAATTCATTATTTTTGCCGTCTGAGTTCTTCA
+HWI-EAS443:3:81:1717:1440#0/1
abX``b^aab_aaaaabaaaaaa`_¥a__`__a^a]aa]^
@HWI-EAS443:3:81:500:1135#0/1
TTTTTTCATGAAGGACAAGTTAAATTCATTATTTTTGCCG
+HWI-EAS443:3:81:500:1135#0/1
ababbaaa_]_aaaaaaaa_aaaaa_a```^a`a_aa_``
@HWI-EAS443:3:82:168:76#0/1
GGCATTTTTTCATGAAGGACAAGTTAAATTCATTATTTTT
+HWI-EAS443:3:82:168:76#0/1
ab[_[_U^_a¥Ra^RR`^NY`L`N_`___^¥^__WYV¥_]
@HWI-EAS443:3:82:337:1061#0/1
GGCATTTTTTCATGAAGGACAAGTTAAATTCATTATTTTT
+HWI-EAS443:3:82:337:1061#0/1
aa^U__XY`[_bba`X`b[]`^_F¥_`_^H_]a`_a___`
@HWI-EAS443:3:82:344:165#0/1
TGAAGGACAAGTTAAATTCATTATTTTTGCCGTCTGAGTT
+HWI-EAS443:3:82:344:165#0/1
aba^```V_a^¥a^``aaaab```aaaaaa`b[X`a^a¥`
@HWI-EAS443:3:83:584:276#0/1
CATTTTTTCATGAAGGACAAGTTAAATTCATTATTTTTGC
+HWI-EAS443:3:83:584:276#0/1
^]a_`b_a``a_a`a`Xaaaa_``_aa_]ababaaa¥`b_
@HWI-EAS443:3:84:1087:488#0/1
GACAAGTTAAATTCATTATTTTTGCCGTCTGAGTTCTTCA
+HWI-EAS443:3:84:1087:488#0/1
aaaa`b^baaaab¥_ba`bbaaba`_a`_aa`a_a]aa_`
@HWI-EAS443:3:85:1432:901#0/1
GGCATTTTTTCATGAAGGACAAGTTAAATTCATTATTTTT
+HWI-EAS443:3:85:1432:901#0/1
aa^``aaaa`T¥aaa`a`]YX¥a]a^^]a_]_`_`a___^
```

~~~
@HWI-EAS443:3:86:1371:408#0/1
GTTAAATTCATTATTTTTGCCGTCTGAGTTCTTCAATGGT
+HWI-EAS443:3:86:1371:408#0/1
^UR`WOWOX]ZHUbbb¥a]TZaaV^]a`M¥XYZI_VQNPG
@HWI-EAS443:3:86:617:819#0/1
GGCATTTTTTCATGAAGGACAAGTTAAATTCATTATTTTT
+HWI-EAS443:3:86:617:819#0/1
`baaaaaaaaaa_a`^aa``aaa]^_][a^``a``_a``^
@HWI-EAS443:3:86:772:962#0/1
TTAAATTCATTATTTTTGCCGTCTGAGTTCTTCAATGGTG
+HWI-EAS443:3:86:772:962#0/1
`a^]^a`^¥a__`ba^a`]`^`a`_]`¥^ab`]`[Y_]P^
@HWI-EAS443:3:86:817:685#0/1
GGCATTTTTTCATGAAGGACAAGTTAAATTCATTATTTTT
+HWI-EAS443:3:86:817:685#0/1
_¥bb¥`_a_Ybabbb_ab`_bVb^aa[[aabbaabaaab_
@HWI-EAS443:3:87:1493:790#0/1
GGCATTTTTTCATGAAGGACAAGTTAAATTCATTATTTTT
+HWI-EAS443:3:87:1493:790#0/1
`aa`bbab`baaab``aaa``ab_ba``aaa`ba^a_aaa
@HWI-EAS443:3:87:651:47#0/1
GACAAGTTAAATTCATTATTTTTGCCGTCTGAGTTCTTCA
+HWI-EAS443:3:87:651:47#0/1
aaa]_aaa^Y¥a_^^_`___`aaa^[a^^a_Va[_^`_`¥
@HWI-EAS443:3:88:359:1637#0/1
GGACAAGTTAAATTCATTATTTTTGCCGTCTGAGTTCTTC
+HWI-EAS443:3:88:359:1637#0/1
_aSaYZG[a^VR_[SGWa`^a[WUMM`^XM^TWRR[_a]]
@HWI-EAS443:3:88:67:838#0/1
AAGTTAAATTCATTATTTTTGCCGTCTGAGTTCTTCAATG
+HWI-EAS443:3:88:67:838#0/1
V¥^bb`T]_`aba`babaaabVa[`^^abaUaa```X^a¥
@HWI-EAS443:3:88:685:941#0/1
GGACAAGTTAAATTCATTATTTTTGCCGTCTGAGTTCTTC
+HWI-EAS443:3:88:685:941#0/1
aaab][``a`_Q_`a`aa¥`aaa_`a]a]a`a_a]__`_`
@HWI-EAS443:3:89:1216:1597#0/1
GGACAAGTTAAATTCATTATTTTTGCCGTCTGAGTTCTTC
+HWI-EAS443:3:89:1216:1597#0/1
`JR^[]QN`¥[Y^____]_`SY_WWMW[]_¥VIaS[S¥GO
@HWI-EAS443:3:89:1347:239#0/1
GTTAAATTCATTATTTTTGCCGTCTGAGTTCTTCAATGGT
+HWI-EAS443:3:89:1347:239#0/1
YYY_`Q]QPb_`W¥`aaabYO^Y_b¥Yb[YQ[`_QEWaaQ
@HWI-EAS443:3:89:159:1267#0/1
GGCATTTTTTCATGAAGGACAAGTTAAATTCATTATTTTT
+HWI-EAS443:3:89:159:1267#0/1
aa`^``XaY[OXSSHT¥NW[¥¥]LNXQY¥WV[[Y[RHRPS
@HWI-EAS443:3:8:1080:2010#0/1
CATTTTTTCATGAAGGACAAGTTAAATTCATTATTTTTGC
~~~

+HWI-EAS443:3:8:1080:2010#0/1
¥`¥bab`a``bbbabb`abab`bb^Ybaaaab`^^`_`ba
@HWI-EAS443:3:8:1742:442#0/1
GGCATTTTTTCATGAAGGACAAGTTAAATTCATTATTTTT
+HWI-EAS443:3:8:1742:442#0/1
ab`bbbbbbb`aabaab`a_`_a_aa`¥aaU`a_`aaaaa
@HWI-EAS443:3:8:283:1359#0/1
GGCATTTTTTCATGAAGGACAAGTTAAATTCATTATTTTT
+HWI-EAS443:3:8:283:1359#0/1
aaaaaaaaaa_a`aaaaaa_``a^_```a¥W`_¥_`^¥S]
@HWI-EAS443:3:8:978:611#0/1
GGCATTTTTTCATGAAGGACAAGTTAAATTCATTATTTTT
+HWI-EAS443:3:8:978:611#0/1
aX¥_aaa_aa__¥¥^^aa]W_]a_]^_]``W^`^[`_`_]
@HWI-EAS443:3:90:1055:1132#0/1
GACAAGTTAAATTCATTATTTTTGCCGTCTGAGTTCTTCA
+HWI-EAS443:3:90:1055:1132#0/1
aa_¥_a`a``_aa_^a_aa`a`_`_¥`^_`a_a[^_`_]Z
@HWI-EAS443:3:90:639:516#0/1
TGAAGGACAAGTTAAATTCATTATTTTTGCCGTCTGAGTT
+HWI-EAS443:3:90:639:516#0/1
^JSW¥`U^__VVX]]Y^aaZ^aYa¥Z]W^¥T]WX`___W_
@HWI-EAS443:3:90:898:1376#0/1
GGCATTTTTTCATGAAGGACAAGTTAAATTCATTATTTTT
+HWI-EAS443:3:90:898:1376#0/1
aaa^`]¥U``[[]_Z[aa[[]Y`XV¥PR[___^[Y[Y]Y^
@HWI-EAS443:3:91:462:325#0/1
ATGAAGGACAAGTTAAATTCATTATTTTTGCCGTCTGAGT
+HWI-EAS443:3:91:462:325#0/1
`TO[a``]`TUb]¥aZW]a^__`^`_^_^¥UY_YX]`Z`[
@HWI-EAS443:3:92:1623:613#0/1
TGAAGGACAAGTTAAATTCATTATTTTTGCCGTCTGAGTT
+HWI-EAS443:3:92:1623:613#0/1
aba__^`a_ababa`aabX^aaaaabaa`aXS]YaV¥_VZ
@HWI-EAS443:3:92:607:77#0/1
GGCATTTTTTCATGAAGGACAAGTTAAATTCATTATTTTT
+HWI-EAS443:3:92:607:77#0/1
aa^]aaaaa___]_]^a`W]_[a]``[Z__ZZa^^`Z]^_
@HWI-EAS443:3:93:319:1089#0/1
TTTTCATGAAGGACAAGTTAAATTCATTATTTTTGCCGTC
+HWI-EAS443:3:93:319:1089#0/1
aaa^a]¥aaa`¥_aZQX_a`__``__a]^a`_[_`_Y_VX
@HWI-EAS443:3:93:744:345#0/1
TTTTCATGAAGGACAAGTTAAATTCATTATTTTTGCCGTC
+HWI-EAS443:3:93:744:345#0/1
aab^¥`bab_ba`]a_]_aa`_`a__a_``^`a`__^`]_
@HWI-EAS443:3:93:913:1237#0/1
GTTAAATTCATTATTTTTGCCGTCTGAGTTCTTCAATGGT
+HWI-EAS443:3:93:913:1237#0/1
`_aaa``b`abaaaabaaaa`aa``aaa^aaaa_aaaa^^

@HWI-EAS443:3:94:1138:53#0/1
GACAAGTTAAATTCATTATTTTTGCCGTCTGAGTTCTTCA
+HWI-EAS443:3:94:1138:53#0/1
`^_``bab``aaa[_aa`b`aaa`^¥a__aa_a]_^_^W^
@HWI-EAS443:3:94:1170:1106#0/1
GGCATTTTTTCATGAAGGACAAGTTAAATTCATTATTTTT
+HWI-EAS443:3:94:1170:1106#0/1
aaaa`bbb`__`aaa`a]ZP[¥b_``_`a_¥_a__aaaa_
@HWI-EAS443:3:94:1437:245#0/1
CATTTTTTCATGAAGGACAAGTTAAATTCATTATTTTTGC
+HWI-EAS443:3:94:1437:245#0/1
`abbbbbb`_bbbbbb`a`a`_aa_bba_ab`abaaab__
@HWI-EAS443:3:94:1529:811#0/1
CAAGTTAAATTCATTATTTTTGCCGTCTGAGTTCTTCAAT
+HWI-EAS443:3:94:1529:811#0/1
`_P_bbaaaa¥`_`aU`aabba¥L¥aaaaba_baba`aab
@HWI-EAS443:3:94:873:236#0/1
GCATTTTTTCATGAAGGACAAGTTAAATTCATTATTTTTG
+HWI-EAS443:3:94:873:236#0/1
_Y`aa¥¥a`P_`baYb``^]¥`]`¥N]aa_`a_YaU`^_`
@HWI-EAS443:3:95:1540:416#0/1
GGACAAGTTAAATTCATTATTTTTGCCGTCTGAGTTCTTC
+HWI-EAS443:3:95:1540:416#0/1
`b``a`b[`baSS]Saa_a¥a]¥`aV_aSWZ`YbMJL]^S
@HWI-EAS443:3:95:1670:1771#0/1
GGACAAGTTAAATTCATTATTTTTGCCGTCTGAGTTCTTC
+HWI-EAS443:3:95:1670:1771#0/1
aa``aaa`aaaaba^ab`ababa`bUVb^__a_b`_]`a[
@HWI-EAS443:3:95:82:1512#0/1
GGCATTTTTTCATGAAGGACAAGTTAAATTCATTATTTTT
+HWI-EAS443:3:95:82:1512#0/1
¥bbb_Tab_bVaaV^^_¥ZRab_aba_^b``ab_^`ba[a
@HWI-EAS443:3:97:1120:480#0/1
GGCATTTTTTCATGAAGGACAAGTTAAATTCATTATTTTT
+HWI-EAS443:3:97:1120:480#0/1
aa]_aabbb`Waaa`aaa__`]b_a___aa]¥a__a`___
@HWI-EAS443:3:97:920:209#0/1
AGGACAAGTTAAATTCATTATTTTTGCCGTCTGAGTTCTT
+HWI-EAS443:3:97:920:209#0/1
aba``aab`aaa`bb_abaabbaaaaW_a_]`a_a`_Xa^
@HWI-EAS443:3:98:1560:576#0/1
GGCATTTTTTCATGAAGGACAAGTTAAATTCATTATTTTT
+HWI-EAS443:3:98:1560:576#0/1
_aPa_bbbbaIYbb`bba``]P`a]b`bab`aabab¥^_b
@HWI-EAS443:3:98:328:1637#0/1
ATTTTTTCATGAAGGACAAGTTAAATTCATTATTTTTGCC
+HWI-EAS443:3:98:328:1637#0/1
U`b¥bb[bb_`Wa]`aaa¥baaaa`[`^aaa`a¥SSa^¥¥
@HWI-EAS443:3:98:480:2032#0/1
TTTTTCATGAAGGACAAGTTAAATTCATTATTTTTGCCGT

```
+HWI-EAS443:3:98:480:2032#0/1
a`aa`¥aa`]`ba_aaaa_aa^`^`^^a_aa]]^^^__aU
@HWI-EAS443:3:98:587:2010#0/1
GGACAAGTTAAATTCATTATTTTTGCCGTCTGAGTTCTTC
+HWI-EAS443:3:98:587:2010#0/1
aaab¥]a]aa_aaa^]`Y]ba`WZaX`a[[`aaa_¥T_a_
@HWI-EAS443:3:98:927:1163#0/1
AGGACAAGTTAAATTCATTATTTTTGCCGTCTGAGTTCTT
+HWI-EAS443:3:98:927:1163#0/1
ab`_]]aaUa[`ab^`bb]baa`aaaa`b_aaa_bZ`[`a
@HWI-EAS443:3:99:388:1834#0/1
GGACAAGTTAAATTCATTATTTTTGCCGTCTGAGTTCTTC
+HWI-EAS443:3:99:388:1834#0/1
`_YOY]`PQ_PYQWZYWQP]WPWZ_`URNLXXV_QPU^X^
@HWI-EAS443:3:99:988:74#0/1
GCATTTTTTCATGAAGGACAAGTTAAATTCATTATTTTTG
+HWI-EAS443:3:99:988:74#0/1
`S^aa]^_JVS]bZWa]NX^^a]_T¥W`_[]__]`^YY__
@HWI-EAS443:3:9:114:1650#0/1
GGCATTTTTTCATGAAGGACAAGTTAAATTCATTATTTTT
+HWI-EAS443:3:9:114:1650#0/1
aaaaaaaaaaaaaaaa_a^a^_a]aaa^a`a`a`^aaa__
@HWI-EAS443:3:9:1605:1699#0/1
GGACAAGTTAAATTCATTATTTTTGCCGTCTGAGTTCTTC
+HWI-EAS443:3:9:1605:1699#0/1
abW``V¥Wb[XUa`[^a`^aa]_^aTGaMG]a__W_]^^Y
@HWI-EAS443:3:9:215:55#0/1
GCATTTTTTCATGAAGGACAAGTTAAATTCATTATTTTTG
+HWI-EAS443:3:9:215:55#0/1
a`O^[YZ]^W^V_Y^^][]YV]Z^V[[W[Z[^[[^][[^Z
@HWI-EAS443:3:9:721:1238#0/1
GGCATTTTTTCATGAAGGACAAGTTAAATTCATTATTTTT
+HWI-EAS443:3:9:721:1238#0/1
ab`bbbbbbbbbbbbbaaababbaabb_bbabaabab
@HWI-EAS443:3:17:1490:1983#0/1
CTGAAAGTCATCCATGGCTGCACAGGATGCATTAACCAAG
+HWI-EAS443:3:17:1490:1983#0/1
`]S¥V_TQW_]WU[ZTYYN^BBBBBBBBBBBBBBBBBBBB
@HWI-EAS443:3:22:481:333#0/1
GAGACCGATATGCGTCTCCACAACTTGAGGGGTTTTCGGC
+HWI-EAS443:3:22:481:333#0/1
¥_bb]]baaabbaaXaba``a^a`bbbaabaaH`ab_^a_
@HWI-EAS443:3:24:1364:642#0/1
AGCGGGGCCAAAATGAATTCAATAAGGCATGTGAATTGAC
+HWI-EAS443:3:24:1364:642#0/1
aa`WUYF¥a_PY[^YS`aSP]][X¥[ON^`aZa`a[_a^J
@HWI-EAS443:3:2:512:1023#0/1
CGGTCCGTCTTCCTTCTTTGGTCCTACATTTGCTTACCAT
+HWI-EAS443:3:2:512:1023#0/1
_bbaba_^^[aaab]a`bbbba```aabaaa`a`aaaQZa
```

@HWI-EAS443:3:39:227:1379#0/1
GAGAAGCCTGGAGAGGAGATGGCCACCAAAGCGGACTACA
+HWI-EAS443:3:39:227:1379#0/1
aZbababbaZa`aab^a_a]S`abab`b`¥_W¥]`_^aa^
@HWI-EAS443:3:47:819:84#0/1
AGAAGACACACATTCACATCTTTTCATTCACTGAAAAATA
+HWI-EAS443:3:47:819:84#0/1
aa^ba¥ba]_^aa``]]abWaa`aY_a`Q^^aa_^¥¥``_
@HWI-EAS443:3:70:977:1980#0/1
CATAAATCCCAATTACCTCATGGCTTGGAAGCAAGGCCTA
+HWI-EAS443:3:70:977:1980#0/1
aaab__aYaaaa_[aaV¥_TZX]aa]U[__BBBBBBBBBB
@HWI-EAS443:3:71:1496:945#0/1
TGGTCCGTCTTCCTTCTTTGGTCCTACATTTGCTTACCAT
+HWI-EAS443:3:71:1496:945#0/1
aaaXaab_aa`M]aaaaaaaa__^`aa`aaaa_a_¥^YV`
@HWI-EAS443:3:88:932:57#0/1
ACGAACGGCACTATTGAAGCACCGATTTGAGATAATTGAA
+HWI-EAS443:3:88:932:57#0/1
aabaa]abaa_b`bb`¥[aV]`[`Na`aa]a^a``aaa__
@HWI-EAS443:3:90:1430:227#0/1
GTCGACTTTGATGACTGCAAAGATGTTGGAGACCTTGAAC
+HWI-EAS443:3:90:1430:227#0/1
a¥H]a_aba``a`[^_aZ_aaa_^aR`a]_`^ZO_`_^_U
@HWI-EAS443:3:92:607:244#0/1
AGTTCCTGCTGATGGCAAAGAGGCCCATCAGGCAATCTGA
+HWI-EAS443:3:92:607:244#0/1
`_`aa`Zaa^`_`a^¥¥]_^¥Za^¥Z^]¥ZTZRRZXRXZB
@HWI-EAS443:3:93:1290:726#0/1
GAGACCGATATGCGTCTCCACAACTTGAGGGGTTTTCGGC
+HWI-EAS443:3:93:1290:726#0/1
aabb`_bab`ba^a_aa__aa__`aaa`a``aQ__`¥__[
@HWI-EAS443:3:93:1645:103#0/1
CTCCAGTCTCGGGTCAGTGAGTGAGAACTCCATCTGGGAG
+HWI-EAS443:3:93:1645:103#0/1
`bb`^¥`__V`ab^_ab_bXb`a`¥X[FYTPY`U`_^YBB
@HWI-EAS443:3:94:517:1453#0/1
CAAAGATGTTGGAGACCTTAAACAGTATGACAGCGGTGAG
+HWI-EAS443:3:94:517:1453#0/1
R_ab]bUbJaab¥RZ^bbb^bbabba`_abab^DMMa``a
@HWI-EAS443:3:29:590:1285#0/1
GACCTTGGTCGGCAAGCTTGCGCATAGTTCCTNTAATCTC
+HWI-EAS443:3:29:590:1285#0/1
aaababa^ab`¥^a_V[X_a`Vab_a^^_^XTDEO__T`[
@HWI-EAS443:3:15:594:1717#0/1
TGCGGGGCTATATGAAGCAATCGAGGGGTGCCTGATTAAT
+HWI-EAS443:3:15:594:1717#0/1
aaOP]I_aa_]UVaaQQU_ONXVWOYGXFWa[LV]V_[Z_
@HWI-EAS443:3:19:612:1970#0/1
GAGGATCCCAAGGACAAAGAACATGAAGAGAAAAAGCCAA

```
+HWI-EAS443:3:19:612:1970#0/1
aUYNI[_`b`S``^`Y_aaWa_`Y_Wa`]X^UGY_HGKVS
@HWI-EAS443:3:26:892:86#0/1
AGGGGGGCTTTCGGCTGAATCGAGAAAATTGCTTCTCATT
+HWI-EAS443:3:26:892:86#0/1
a^^b¥SUHaab]X^aba[ZaXPWRPJa]]`bPY]]aQ^PT
@HWI-EAS443:3:34:774:36#0/1
AGCCCCCCAAGATCGAAGGCTCCAGGTTCCAGGTTGTGCC
+HWI-EAS443:3:34:774:36#0/1
¥¥``_W_[O¥__VYTVRZYDTZ]YVWBBBBBBBBBBBBBB
@HWI-EAS443:3:46:184:953#0/1
CATCCATGGCTGCACAGGGTGCATTGAGCAAGGCCGTGTT
+HWI-EAS443:3:46:184:953#0/1
MX`ababbbb_`b^W¥`_K`bV]`^]U¥IU]]]aa[PDSb
@HWI-EAS443:3:52:1474:1682#0/1
TACCATTGGGATCAGCTGAAAGGCATCCATGGCTGCACAG
+HWI-EAS443:3:52:1474:1682#0/1
Y^`UOSW_XWHSX_]ZU```XTN[V[NRBBBBBBBBBBBB
@HWI-EAS443:3:5:1735:1773#0/1
CATATTTTCACCGAGTGCCCAATTCAATTGGCTTGTTGTC
+HWI-EAS443:3:5:1735:1773#0/1
aaaaa`aabbaaVa]VaaXJaZbbaaabbW_a`_VS]GZB
@HWI-EAS443:3:61:1773:1530#0/1
TCGGGGGGTTTCGGCTGAATCGAGAAAATTGCTTCTCATT
+HWI-EAS443:3:61:1773:1530#0/1
a^YUWabG`a`]NT^aa_`¥^^X^_^`a¥¥[ZYZR[QUWU
@HWI-EAS443:3:67:1601:707#0/1
TTCCCACAACTACTTCAATGCATGTGTGAGGAAGGAGTTG
+HWI-EAS443:3:67:1601:707#0/1
a_I]ababb_bbaaa]baaaaaabPZWa_W_`[aa`b`_a
@HWI-EAS443:3:82:318:911#0/1
GGCCTATCGCAGTCCCCAAGAGCATGTCTCCTATTTCTAG
+HWI-EAS443:3:82:318:911#0/1
`¥aaT`¥bbTQZZ`YFQNU¥V¥`[O^NU]a^MJYW¥UTa`
@HWI-EAS443:3:88:317:137#0/1
ACGGCATTGCCACAACTACTTCAATGCATGTGTGAGGAAG
+HWI-EAS443:3:88:317:137#0/1
aabbaa`a_a`P^aaU`a_``VU[`_]X]`UaXaU^a^Z`
@HWI-EAS443:3:89:1603:719#0/1
CACACATTCACATCTTTTCATTCACTTGAGAGGAGATAGC
+HWI-EAS443:3:89:1603:719#0/1
SXbb`bObbab_a`a^ba¥bbaOb`aKUb`aabb[baLOY
@HWI-EAS443:3:96:1008:868#0/1
TGGGTCTTCCTTCTTTGGTCCTACATTTGCTTACCATTGG
+HWI-EAS443:3:96:1008:868#0/1
^Y_a^`b¥[¥_][^aa`Z]ZS^]¥``a¥]SZ`_SV]aZ``
@HWI-EAS443:3:10:1732:262#0/1
CTCAAGTTGTGGAGACGCATATAGACNGTTGAATAAAGAT
+HWI-EAS443:3:10:1732:262#0/1
G]b¥¥¥`Za_^X[b¥[_ZRX¥bbb]HDTMTY]SY]HZ^^S
```

```
@HWI-EAS443:3:12:1055:433#0/1
AGCANTGCCACAACTACTTCAATGCATGTGTGAGGAAGGA
+HWI-EAS443:3:12:1055:433#0/1
`b`RD[¥]Ta]`¥`_][_a_[_¥`]]_aVa¥_Va¥TM^_V
@HWI-EAS443:3:16:740:1372#0/1
CAAATAGTAGCATTGCCACAACTACTTNAATGCATGTGTG
+HWI-EAS443:3:16:740:1372#0/1
abbbaabYbbba[b^aaa`a]Qb_aa¥D]aaaWaaYOXX^
@HWI-EAS443:3:46:336:1755#0/1
CATTGCCACAACTACTTCNATGCATGTGTGAGGAAGGAGT
+HWI-EAS443:3:46:336:1755#0/1
a_b^abX^Zbbaa`_a`UD^`^Vaa[[a[`_aa`__Y``Y
@HWI-EAS443:3:57:1063:1609#0/1
CACATTCACATCTTTTCATTCACTGGAGAAGAGANGGCCA
+HWI-EAS443:3:57:1063:1609#0/1
USYS``[`V[baaaT[[ZY`^^`ab`b_`b[baMD¥]LX^
@HWI-EAS443:3:57:760:1892#0/1
CGATTGNTTCATATAGCCCCCCAAGATCGAAGGCTCCAGG
+HWI-EAS443:3:57:760:1892#0/1
aX`TH^D¥abb^aa`aba^aT^ba_ba``bba`KWW_TDF
@HWI-EAS443:3:76:992:1922#0/1
AGCAGGTGCTAGTAGAGCTACAGGACATTGNAAATGAAGA
+HWI-EAS443:3:76:992:1922#0/1
a][]O_^a`ab_FZ^^``^a^a___^`_aVDW_[]`^¥G_
@HWI-EAS443:3:85:963:687#0/1
CTTCCTCACACATGCATTGAAGTAGTTGTGGCNATGCTAC
+HWI-EAS443:3:85:963:687#0/1
a`Y`a^GYV_RZ]``V¥]YTS_V`U¥``a_T[DR¥¥TNT]
@HWI-EAS443:3:93:880:1793#0/1
CATTTGCTTACCATTGGGATCNGCTGAAAGTCATCCATGG
+HWI-EAS443:3:93:880:1793#0/1
_^b[R¥a`aa``a]aa__WYMDYaX_[]a`M¥¥]TZ^[¥X
@HWI-EAS443:3:10:154:139#0/1
CACATGAGAAAGGCATAATCCCAATTACCTCATGGCTTGG
+HWI-EAS443:3:10:154:139#0/1
a_bb`babUb`aaaabbaXU`aZ_Y^UL]QSGX`_Q_]BB
@HWI-EAS443:3:53:918:95#0/1
GCATGGAAATGAGGCGCTGCCTCTTCAGTCTCTTCAGCAG
+HWI-EAS443:3:53:918:95#0/1
Tb`_abaaabbaab]b^abXZb^a___b`X`Z`aV^aU[`
@HWI-EAS443:3:67:335:1602#0/1
CGGGTCTTCAATACTTAATTTCAGAGCATCATCAGCAGGA
+HWI-EAS443:3:67:335:1602#0/1
aaaaQbaaaa`a¥``aaaaaaaaY`aa`aBBBBBBBBBBB
@HWI-EAS443:3:69:780:946#0/1
ATCAAAGTCTACTTTTTCTGGTGCCATATTTCACCGAGTG
+HWI-EAS443:3:69:780:946#0/1
a`X^_Z`Y_baabaa`]^aa¥Za`]¥`Y``a`baV_``_^
@HWI-EAS443:3:97:1354:198#0/1
GGGATTTATGCCTTCTCATGTGGTTTGACTATGTTAGGCT
```

```
+HWI-EAS443:3:97:1354:198#0/1
^^^aaaaba`X_aa`a_`aaX`^]`a`_^a``aZ_`_[W_
@HWI-EAS443:3:16:1249:543#0/1
GTCTTCTACAAATCAGGAAGAAATTTAGGCTTCTCTACCC
+HWI-EAS443:3:16:1249:543#0/1
a^a_a_Y^P`][^`Z[_]aaY¥`__]`^^^`_G_X[VT]T
@HWI-EAS443:3:16:698:1451#0/1
GGNCCGCGACACTTGGCCTATCGCAGTCCTCAAGAGCATG
+HWI-EAS443:3:16:698:1451#0/1
a[D[__aQ`^TQ_VRPa^[a`__ZRUS^UZ[_R[_¥¥¥WB
@HWI-EAS443:3:25:648:1396#0/1
ATGGCTTGGAAGCAGGTGCTAGCAGAGCTACAGGCGATTG
+HWI-EAS443:3:25:648:1396#0/1
^Z_¥`aaR[U`_ZPXPP_]^YQ¥[TX^^UZZ^PX_GSJML
@HWI-EAS443:3:26:514:570#0/1
ATTGGTTCTCACATATAGGAACATGGGCCTCGACCGGTTC
+HWI-EAS443:3:26:514:570#0/1
_Z][aabb_R]ab_WQa]_`b^]``[¥¥H_^T`¥Za_ZX^
@HWI-EAS443:3:30:1554:1136#0/1
GATGATTGAAGGAAGAGACCGAATCATGGCCTGGACAGGG
+HWI-EAS443:3:30:1554:1136#0/1
aaXF]XaRY¥`[XXY^a^^^aNT]]_`UUUV^W^OIMSBB
@HWI-EAS443:3:42:1704:1587#0/1
AAGAAATTTAGGCTTCTCTACCCCTGTTGTGTTAAAATTA
+HWI-EAS443:3:42:1704:1587#0/1
aa_O¥`babaVW_baHa^]aa[b_a]`_Z_a[aPFUFLOM
@HWI-EAS443:3:43:1122:497#0/1
ATAAATCCCAATTACCTCATGGCTTGGAAGCAGGGGCGAG
+HWI-EAS443:3:43:1122:497#0/1
a_HUY]^_]]`a_`Y_ZR^VZ`Z¥¥XWBBBBBBBBBBBBB
@HWI-EAS443:3:44:1662:215#0/1
CACCCGGTATTCAACAGTCTATATGCGTCTCCACAACTTG
+HWI-EAS443:3:44:1662:215#0/1
abbabbb]bb_baaa`^aabaa`¥^^b`]aaa¥aa_^a^]
@HWI-EAS443:3:46:68:896#0/1
CTGGTGATATGCGTCTCCACAACTTGAGGGGTTTTCGGCT
+HWI-EAS443:3:46:68:896#0/1
aa`Y`aY``WRZWZ¥Qab`N`XXbba]]a`[TOZZXRW`^
@HWI-EAS443:3:51:1371:315#0/1
CAGGCACTCCTCGATTGCTTCATATAGCCCCCCAGTTTCG
+HWI-EAS443:3:51:1371:315#0/1
`aaa`abbbab`babbaTab[`babab`a_`a`abbaaab
@HWI-EAS443:3:70:1211:844#0/1
CCGGTTCATTGAAATTGGAGTAACACGGAGGGAACCCACA
+HWI-EAS443:3:70:1211:844#0/1
abYb`¥¥^babbbb_¥[PY`[`a^^¥a`aa]a__UN][¥a
@HWI-EAS443:3:81:155:1551#0/1
AAGAGACCGAATCATGGCCTGGACAGTGGTGAACCAGTCC
+HWI-EAS443:3:81:155:1551#0/1
¥WVHPW]]Y`PR^ST¥UV`UMVPRQSGUUSM[XBBBBBBB
```

@HWI-EAS443:3:15:313:1180#0/1
TGCGTCTTCCTTCTTTGGTCCTACATTTGCTTACCATTGG
+HWI-EAS443:3:15:313:1180#0/1
aa``_bb_a]Z¥WX¥[`^`]bbbTOTa^R_[Q`aS¥[Y[a
@HWI-EAS443:3:21:199:1185#0/1
CTTCCTCACACATGCATTGAAGTAGTTGTGGCAATGCTCC
+HWI-EAS443:3:21:199:1185#0/1
aa_aY``a[_a]`^_`a_NWZ[[_aR[¥X_UZYV`SXRIH
@HWI-EAS443:3:26:1703:1683#0/1
GGGGCATGGAAATGAGGCGCTGCCTTCTTTAGTCTCTTGA
+HWI-EAS443:3:26:1703:1683#0/1
a]U_X¥^aZ_aaa`[NLG¥^_aYS]BBBBBBBBBBBBBBB
@HWI-EAS443:3:27:1381:1522#0/1
AGGGGGGTTTCGGCTGAATCGAGAAAATTGCTTCTCATTG
+HWI-EAS443:3:27:1381:1522#0/1
aab`_^¥_baZ`a`bbb`a]aaaaaaa`a¥^_a_a_aa`a
@HWI-EAS443:3:37:547:787#0/1
GCGAAAAATACTGTGTTCTAGAAATAGGAGACATGCTCTT
+HWI-EAS443:3:37:547:787#0/1
a^_T_YTG[Y`¥YW¥^_`a^]W^¥^P]ZHZO[SO___aZZ
@HWI-EAS443:3:40:549:1680#0/1
ANTTCCTTGGTCATGACTTTCTCTTTGACAGAAGACTCGG
+HWI-EAS443:3:40:549:1680#0/1
¥DYZ`bMLX]R]`]]Y[_¥a`^`Z_[[`a`__a]``]__¥
@HWI-EAS443:3:45:1048:1280#0/1
GAGAAAGACATGACCAAGGAATTCTTTGAAATCAAATCNG
+HWI-EAS443:3:45:1048:1280#0/1
a`a^``aaaaaa^_Ya^aa_aaaaaaa_aa^a`_a`aPDZ
@HWI-EAS443:3:54:1087:729#0/1
GGGGCCGTCTCCTCTCCAGTGAATGAAAAGATGTGAATGT
+HWI-EAS443:3:54:1087:729#0/1
``aaZUa¥``aY`YaaVX_^`]]`[YZaVZR_a[X^¥¥`¥
@HWI-EAS443:3:54:228:1726#0/1
GTCCCGATTGAACATATCACAAGCATGAGGAGGAACTATT
+HWI-EAS443:3:54:228:1726#0/1
aZaZaa`_`[^aYX__]^X_`U^RX^`^a][`^^Z[^X]]
@HWI-EAS443:3:54:972:1369#0/1
GGTCCGTCTTCCTTCTTTGGTCCTACATTTGCTTACCATT
+HWI-EAS443:3:54:972:1369#0/1
`aa`]aa_b`aaaa_aaaa_Y]Xaa`aa`_a_a`a_]``_
@HWI-EAS443:3:57:584:1877#0/1
CACAACTACTTCAATGCATGTGTGAGGAAGGAGTTGAAAC
+HWI-EAS443:3:57:584:1877#0/1
ZY[_a]GN¥]¥T][_baY`SXa]`X^[_HHIPIYZ¥a¥HM
@HWI-EAS443:3:62:573:1326#0/1
AGGGCGTAGTCCGCTTTGGTGGCCATCTCCTCTCCAGTCC
+HWI-EAS443:3:62:573:1326#0/1
^__[GVYa`a`__¥XXSPWU_X¥_]TQV]_Y[R[XYBBBB
@HWI-EAS443:3:64:530:1208#0/1
GGTCCGTCTTCCTTCTTTGGTCCTACATTTGCTTACCATT

```
+HWI-EAS443:3:64:530:1208#0/1
`aaa[^^ab_`aa`^bab`b]ababab``[_aba`baa`b
@HWI-EAS443:3:70:1755:1727#0/1
ATGCCGAGTCTTCTGTCAAAGAGAAATACATGACCAAGGA
+HWI-EAS443:3:70:1755:1727#0/1
WDMPOY¥^S_b`^aOUSa^^_¥__a`I_]a`^WFP_`YWW
@HWI-EAS443:3:72:1379:1024#0/1
GATCCGTCTTCCTTCTTTGGTCCTACATTTGCTTACCATT
+HWI-EAS443:3:72:1379:1024#0/1
a[`a^a_V`a`aa`_aab__]_^__][^_``]`]`WU^^_
@HWI-EAS443:3:76:340:1685#0/1
AGTCCGTCTTCCTTCTTTGGTCCTACATTTGCTTACCATT
+HWI-EAS443:3:76:340:1685#0/1
]b_bb`abaaabaaa`aa[]`baaaaaaa`_aa`aa_a`_
@HWI-EAS443:3:78:864:377#0/1
GTCCTACATTTGCTTACCATTGGGATCAGCTGAAAGTCGT
+HWI-EAS443:3:78:864:377#0/1
aZ][`a[a^]`a^``a^]`a`a`_^[^_a^_`^]]_RRS¥
@HWI-EAS443:3:7:1257:138#0/1
GAGGAACTATTTTACAGCAGAAGTGTTCCACTGCAGGGAT
+HWI-EAS443:3:7:1257:138#0/1
`_¥`]VZ_Z¥YZ¥[FNY^U^¥XYVSBBBBBBBBBBBBBBB
@HWI-EAS443:3:82:1715:1166#0/1
GAGGTTTTCGACTGAATCGAGAAAATTGCTTCTCATTGTT
+HWI-EAS443:3:82:1715:1166#0/1
WQa[Q``ba_ZVbbabb`babababa_Yab^b¥aa`b`_
@HWI-EAS443:3:83:717:1528#0/1
GGTCCGTCTTCCTTCTTTGGTCCTACATTTGCTTACCATT
+HWI-EAS443:3:83:717:1528#0/1
WYMXQ¥H_]¥``aVaaaa^`U_¥ab_^XO^^`baaaa]]Z
@HWI-EAS443:3:92:1033:1095#0/1
GGTCCGTCTTCCTTCTTTGGTCCTACATTTGCTTACCATT
+HWI-EAS443:3:92:1033:1095#0/1
¥b^bb¥XH`V]aa_Q`a_bbY]_```aa__a`abaZ^a_`
@HWI-EAS443:3:99:62:657#0/1
AGTCCGTCTTCCTTCTTTGGTCCTACATTTGCTTACCATT
+HWI-EAS443:3:99:62:657#0/1
`b¥`aaaKZabWT^aa¥X_aR`[^^aa¥[aa]a¥a¥WOaa
@HWI-EAS443:3:96:1768:1069#0/1
ACGGCCTTGCTNAATGCATCCTGTGCNGCCATGGATGACT
+HWI-EAS443:3:96:1768:1069#0/1
a¥``[_a`a_XDZa``_aa[R``_]KDXPU^W]]V]]UQB
@HWI-EAS443:3:12:1792:562#0/1
AATTGAAGGAAGAGACCGAATCATGGCCTGGACAGGTGGT
+HWI-EAS443:3:12:1792:562#0/1
X_``^^`_^abba[VSOV`aaaY__`WNYZaUTIPFS¥QL
@HWI-EAS443:3:13:1145:60#0/1
CGTGGGAGGGGTTTTCGGCTGAATCGAGAAAATTGCTTCT
+HWI-EAS443:3:13:1145:60#0/1
abbbb``babb]a`b¥_b^aaaaZT`_`_a``Z_bZa[N_
```

@HWI-EAS443:3:19:1108:247#0/1
GGCCGGTTTCCGATTTGTTTTCAAAGAATTCCTTGGTCAT
+HWI-EAS443:3:19:1108:247#0/1
aaaaba[ab^ab]aaab_aaa_a]aa_^a`YV_`aa]X_`
@HWI-EAS443:3:22:1343:1176#0/1
GCTGGCCCGAATGCACTATTGAAGCACCGATTTGAGATAA
+HWI-EAS443:3:22:1343:1176#0/1
_^_aaaa`]_aa]``^Y`a`_`_][_ZT`U^a_`U`___`
@HWI-EAS443:3:22:386:604#0/1
GGGGGTGGGGGGCTATATGAAGCAATCGAGGAGTGCCTGA
+HWI-EAS443:3:22:386:604#0/1
aaaaaXaaaaa]a^`aa`aa]_aa___a¥_ZU_W`YW_aZ
@HWI-EAS443:3:25:296:1354#0/1
TGTGGCCCGAATGCACTATTGAAGCACCGATTTGAGATAA
+HWI-EAS443:3:25:296:1354#0/1
abS[`aaXT_aQP`_WTa`Z¥[aWGYFTaZS_`^¥_S^Y`
@HWI-EAS443:3:26:1773:162#0/1
AGAAAGGCATAAATCCCAATTACCTCATGGCTTGGTCCTT
+HWI-EAS443:3:26:1773:162#0/1
aa^Y^bb_XO_aa¥ZXHYX]VY_TQLZW_RJVGRSLNWWV
@HWI-EAS443:3:27:1313:884#0/1
AGAGCCTATGTAGATGGATTCGAGCCGAACGGCTGGAATG
+HWI-EAS443:3:27:1313:884#0/1
`ba`S^¥X_`[`]_aaaaba]T^a`V^__PY[]BBBBBBB
@HWI-EAS443:3:44:658:1163#0/1
GCGGGGGAAGGCTCTATTGGGAAAGTGTGCAGGACCTTAC
+HWI-EAS443:3:44:658:1163#0/1
_[`ZW][RV[Y__Za^^`¥`_ZRZ^WRP_¥WYUZ¥V[ZZT
@HWI-EAS443:3:44:938:863#0/1
GATTTTACATGAAACAAACTTCCAAATGTGTGCATATTGC
+HWI-EAS443:3:44:938:863#0/1
aaYY`WQSSYU¥TV]Z^Y]a`a`PaUZ^NXLQVPVYY¥XR
@HWI-EAS443:3:46:1460:1420#0/1
GCCGGTCATTGAAATTGGAGTAACACGGAGGGAAGTCCAC
+HWI-EAS443:3:46:1460:1420#0/1
a`aaXZR`___SZ`_[^]NXMYW^Z`_¥MY_aXV¥LLZSW
@HWI-EAS443:3:49:1181:18#0/1
CGCCCTCAATGCAGCCGTTCGGCTCGAATCCATCTAAATA
+HWI-EAS443:3:49:1181:18#0/1
abababbabba_ab^_b]_Zb___^¥[Y^_W]]U]UGYVS
@HWI-EAS443:3:50:1752:549#0/1
GGAAGATCCGAAAATCGAAACTAACAAGTTTGCTGAATCG
+HWI-EAS443:3:50:1752:549#0/1
aa_bbabb`ababbabaaaaV^ba_a`aab`aaaaaaa^a
@HWI-EAS443:3:59:1005:1805#0/1
GTCCGCTTTGGTGGCCATCTCCTCTCCAGTGAATGGTAAG
+HWI-EAS443:3:59:1005:1805#0/1
aU[`WFPUQR[OX]_UZZYYNVTXL[BBBBBBBBBBBBBB
@HWI-EAS443:3:60:1401:246#0/1
ATTGAAGCACCGATTTGAGATAATTGAAGGAAGAGCAGGA

```
+HWI-EAS443:3:60:1401:246#0/1
aba^aaa_a_]baabab_a``a^aaa__a`__`X``UaZZ
@HWI-EAS443:3:68:159:1879#0/1
CCAAGGACAAAGAACATGAAGAGAACAAGCCAATTTTTAA
+HWI-EAS443:3:68:159:1879#0/1
abb^aaabaaaabbab`a`_aa_aabaa`a`b[a_^a_a`
@HWI-EAS443:3:71:201:1381#0/1
GGGGTTCTTCTCTTCATTTTCAATGTCCTGTAGCTCTGCT
+HWI-EAS443:3:71:201:1381#0/1
a_]_S_`aa`aba``aaaaaaW]a^Yaa__Y]_a_X[¥¥P
@HWI-EAS443:3:78:1506:450#0/1
GGGGGCTCCAACATCTTTGCAGTCATCAAAGTCTACTTTT
+HWI-EAS443:3:78:1506:450#0/1
aaYVMTaR]OW`[WS__W`aSJMTNTFU[WRFTZ^U¥L¥L
@HWI-EAS443:3:89:1566:320#0/1
GTGAATACATAATGAAGGGAGTGTACATAAATACGGACTT
+HWI-EAS443:3:89:1566:320#0/1
SaSWXb]a¥a¥X`bbb__b^bba__ISb`abXaR`VH]a`
@HWI-EAS443:3:10:1793:251#0/1
NTGAAATGGAAATCCGAATACATGAAACAAACTTCAAAAT
+HWI-EAS443:3:10:1793:251#0/1
DM[W[Y[[[[[[UT[[[[VYWY[[[YXNT[[W[VTI[Z[[
@HWI-EAS443:3:11:1289:2022#0/1
CAGGGGTTTTCGTCTGAATCGAGAAAATTGCTTCTCATTG
+HWI-EAS443:3:11:1289:2022#0/1
aaabbbVabb`]G¥`aaa_aa_a`S`a]_^T__X_]^`_`
@HWI-EAS443:3:13:1271:128#0/1
ACCGTCTTCCTTCTTTGGTCCTACATTTGCTTACCATTGG
+HWI-EAS443:3:13:1271:128#0/1
a_aa__``_¥^`X`^__^_^Y]^Y]_`__V_^^]VV_]^^
@HWI-EAS443:3:15:958:2047#0/1
NTCGCAGTCCTCAAGAGCATGTCTCCTATTTCTAGAACAC
+HWI-EAS443:3:15:958:2047#0/1
DKRLQOPKMVVVUVTTTVTSQTQQNNTUXTTVXPXXXXWT
@HWI-EAS443:3:23:693:1678#0/1
GGGGGGTTTCGGCTGAATCGAGAAAATTGCTTCTCATTGT
+HWI-EAS443:3:23:693:1678#0/1
_aI^I`Y__a`a_a]a^P[`NK[_[[__U]_¥_Z^^^YKO
@HWI-EAS443:3:26:1740:124#0/1
GAGAAAGGCATAAATCACAATTACCTCATGGCTTGGAAGG
+HWI-EAS443:3:26:1740:124#0/1
`a`^a[b`_[aa_`_PGZ`_[ZaOV¥V^¥a_Y_^ZZGO]G
@HWI-EAS443:3:29:91:539#0/1
GCCGTCTTCCTTCTTTGGTCCTACATTTGCTTACCATTGG
+HWI-EAS443:3:29:91:539#0/1
aa`bUb__[]Xb`a`a`NX_aaa]P]a_aa_``T`NY^^_
@HWI-EAS443:3:32:18:1241#0/1
ACCGTCTTCCTTCTTTGGTCCTACATTTGCTTACCATTGG
+HWI-EAS443:3:32:18:1241#0/1
aaaa]a``aaa``¥¥][YYaa¥X]`[__][_ZPY`]]_^]
```

```
@HWI-EAS443:3:35:680:1452#0/1
CGCATGGAAATGAGGCGCTGCCTTCTTCCGTCTCTTCAGC
+HWI-EAS443:3:35:680:1452#0/1
HZ[][_QY_[_a[¥^¥_`Z¥Z^^_X__LIYTbaa]`_XZ`
@HWI-EAS443:3:36:1063:1896#0/1
AGAGGGGCTTTCGGCTGAATCGAGAAAATTGCTTCTCATT
+HWI-EAS443:3:36:1063:1896#0/1
aaa`U¥¥H`aYWWYW_H`__]]]]][aYYJYQV¥`U`_[T
@HWI-EAS443:3:38:1615:2038#0/1
CATTGCCACAACTACTTCAATGCATGTGTGAGGAAGGAGA
+HWI-EAS443:3:38:1615:2038#0/1
I[S^^ba`T^_Vabbb]`a]bba_b`ab``X^SaTWRIYG
@HWI-EAS443:3:39:198:894#0/1
GTCCGTCTTCCTTCTTTGGTCCTACATTTGCTTACCATTG
+HWI-EAS443:3:39:198:894#0/1
_Taa`[b]a`_]ab_aa`_ZT`^`^a`]```[]]_a^`¥X
@HWI-EAS443:3:43:893:149#0/1
TATCGGAGAGTCACCCAGGGGAGTGGAGGAGGGCTCTATT
+HWI-EAS443:3:43:893:149#0/1
_IZ^]aUZX`V]aZ_`O`^`STa¥YZT[_OEQ[UVSOGUY
@HWI-EAS443:3:46:66:569#0/1
ATAAATCCCAATTACCTCATGGCTTTGAAGCAGGTGCTAT
+HWI-EAS443:3:46:66:569#0/1
`S¥a`_aa`a`a^^__`]_a_`^a^BBBBBBBBBBBBBBB
@HWI-EAS443:3:49:1315:784#0/1
CGCCCACTTCAATTGGCTTGTTCTCTTCATGTTTTTTGTC
+HWI-EAS443:3:49:1315:784#0/1
aabb^Z]b¥_a_[aaaabba[¥GVN``T]aa]RD[a`HXD
@HWI-EAS443:3:4:588:103#0/1
GGTCTCTTCAGCAGATTGATAGCATGATTGAGGCCGAGTC
+HWI-EAS443:3:4:588:103#0/1
]bTT`_¥aY^``^a___aa```¥]____[^]`]VV¥Y`WS
@HWI-EAS443:3:51:90:552#0/1
GTCCGTCTTCCTTCTTTGGTCCTACATTTGCTTACCATTG
+HWI-EAS443:3:51:90:552#0/1
a¥^a]_`aa^^aa_aa`¥¥Z_^`__`aa``^`^¥T_]___
@HWI-EAS443:3:54:219:1863#0/1
GTCCGTCTTCCTTCTTTGGTCCTACATTTGCTTACCATTG
+HWI-EAS443:3:54:219:1863#0/1
aa_^a_a¥G]``___a^``_^`]Y_aaU___[]_S_^YV]
@HWI-EAS443:3:55:535:995#0/1
GTCCGTCTTCCTTCTTTGGTCCTACATTTGCTTACCATTG
+HWI-EAS443:3:55:535:995#0/1
aaaaababbab`a`a`ab`_a_aa`aa`baaa``a[J]aa
@HWI-EAS443:3:59:523:1830#0/1
GGGTTTTCGGCTGAAACGAGAAAATTGCTTCTCATTGTTA
+HWI-EAS443:3:59:523:1830#0/1
^¥[Qa`a¥SYa`¥aa¥_Y^___¥____`_[QWZ[^]Z[]G
@HWI-EAS443:3:63:1016:1153#0/1
GTCCGTCTTCCTTCTTTGGTCCTACATTTGCTTACCATTG
```

+HWI-EAS443:3:63:1016:1153#0/1
aaba`aaaaaaba`aaaa``aaaa`aaaaa`_]`_[_]`¥
@HWI-EAS443:3:64:52:939#0/1
TTGCTTGCGATATGTTCAACCGGGGCAACATCTTCTCCTA
+HWI-EAS443:3:64:52:939#0/1
X^aYW¥Za[XaVU_I^`¥NF[]V`a`YVa]]a¥W¥Z¥`W¥
@HWI-EAS443:3:65:795:1112#0/1
GTCCGTCTTCCTTCTTTGGTCCTACATTTGCTTACCATTG
+HWI-EAS443:3:65:795:1112#0/1
`^baa^aabaa`aaaaa``_`aaa__aaa_aa_a^`a`aa
@HWI-EAS443:3:6:1729:1241#0/1
GAGCATTGCCACAACTACTTCAATGCATGTGTGAGGAAGG
+HWI-EAS443:3:6:1729:1241#0/1
Mabb_aa[ZVbab_`ab^aZZaabaabbab[U[baba_bY
@HWI-EAS443:3:70:758:429#0/1
CTTCCGTCTTCCTTCTTTGGTCCTACATTTGCTTACCATT
+HWI-EAS443:3:70:758:429#0/1
ababaaa_abaabaabaaaa^aaaa_`a`aaabaa``aaa
@HWI-EAS443:3:72:1216:844#0/1
CCCGTCTTCCTTCTTTGGTCCTACATTTGCTTACCATTGG
+HWI-EAS443:3:72:1216:844#0/1
``aa`bab]bbbabbbbb^baab`bababbbaa`]aaaa]
@HWI-EAS443:3:72:1759:419#0/1
GCCGTCTTCCTTCTTTGGTCCTACATTTGCTTACCATTGG
+HWI-EAS443:3:72:1759:419#0/1
a[ab^abaaaba`aaaa^[``a__^aaaSU]`aZa_aaZ_
@HWI-EAS443:3:75:37:1051#0/1
GGGGGGTTTCGGCTGAATCGAGAAAATTGCTTCTCATTGT
+HWI-EAS443:3:75:37:1051#0/1
V_W^W]`W^^]¥`¥¥[X___XTWZY[_`]`¥¥_]X`a_^R
@HWI-EAS443:3:77:1680:549#0/1
GGGGGGTTTCGGCTGAATCGAGAAAATTGCTTCTCATTGT
+HWI-EAS443:3:77:1680:549#0/1
abb`baXab¥aa`bbba[_a_ba[a`a[b`aa]__]a[aa
@HWI-EAS443:3:7:855:745#0/1
TGAGCCTATGTAGATGGATTCGAGCGGAACGGCTGCATTG
+HWI-EAS443:3:7:855:745#0/1
ab`aZNZSaa_]]¥]JZYX_O]XNYG__ZVBBBBBBBBBB
@HWI-EAS443:3:88:1077:1350#0/1
ATCCGTCTTCCTTCTTTGGTCCTACATTTGCTTACCATTG
+HWI-EAS443:3:88:1077:1350#0/1
^XY_ZQW¥¥`b``^a``a`TZ_`]abba^`^^]`]YZWX^
@HWI-EAS443:3:96:1723:1482#0/1
GAAATAGTAGCATTGCCACAACTACTTCAATGCATGTGTG
+HWI-EAS443:3:96:1723:1482#0/1
G^abbbbaba¥`bbbaabbbbabbbbbabbbb`_ababbb
@HWI-EAS443:3:97:1004:622#0/1
GGGGGGTTTCGGCTGAATCGAGAAAATTGCTTCTCATTGT
+HWI-EAS443:3:97:1004:622#0/1
abaaaa^ab_aa_aabaa_a_aa_`_`a__``W`_````]

```
@HWI-EAS443:3:9:534:63#0/1
GTCCGTCTTCCTTCTTTGGTCCTACATTTGCTTACCATTG
+HWI-EAS443:3:9:534:63#0/1
a]Y]`VX_]UV^^VX]^]^WWWYXYY]YYXXX^YXYXXY^
@HWI-EAS443:3:15:1225:351#0/1
TGTGCAGCCATGGATGACTTTCAGCTGATCCCAATGGTAA
+HWI-EAS443:3:15:1225:351#0/1
`a^aaaa_aa_a`_`_aXa_a]_aY]`[^[X^__^a¥Y^U
@HWI-EAS443:3:17:1261:273#0/1
TCGGGGGGGGCTATATGAAGCAATCGAGGAGTGCCTGATT
+HWI-EAS443:3:17:1261:273#0/1
]aaa^baa``[`]`¥^]^[^Q¥_^PVF¥]TVR]NP_^U__
@HWI-EAS443:3:19:1013:981#0/1
GCCATATTTTCACCGAGTGCCCACTTCAATTGGCTTTTTA
+HWI-EAS443:3:19:1013:981#0/1
Q_^Y^V`¥S`bbZ^_`]L]Z]W]¥¥Y_a`SH_[GSTBBBB
@HWI-EAS443:3:1:482:1327#0/1
AGCGCCCCAAGATCGAAGGTTCCAGGTTCCAGGTTGTCCC
+HWI-EAS443:3:1:482:1327#0/1
a]ZG`¥_[^_`a^¥]^_a^P`]^_a_U¥^]¥¥_W^BBBBB
@HWI-EAS443:3:20:1419:1220#0/1
CGCCGTGTGCATATTGCAGCAAACTTGTTAGTTTCGATTT
+HWI-EAS443:3:20:1419:1220#0/1
L_ba_^^aY¥T`bb_Zb_W`aabWa`ML^bb^b]GQaa`a
@HWI-EAS443:3:20:177:1274#0/1
CCGCGACTGGAGCCACACAAATGGGAAAAATACTGTGTTC
+HWI-EAS443:3:20:177:1274#0/1
`X[HR[aZSUZW[ZWZ`YNQRZUP¥MZNUZ_XV]XU]UUU
@HWI-EAS443:3:21:1635:1258#0/1
TGTGCAGCCATGGATGACTTTCAGCTGATCCCAATGGTAA
+HWI-EAS443:3:21:1635:1258#0/1
aa^___a[Y^___[__]^__]^^^UV¥YUV[]]RZZVRUX
@HWI-EAS443:3:23:785:681#0/1
GCCCACTGCAGGGCTACTGAATACATAATGAAGGGAGAGT
+HWI-EAS443:3:23:785:681#0/1
a``[K_[V`SMR[_N_]¥aUR¥RY]ZNSW]U[Z^]N¥BBB
@HWI-EAS443:3:24:1118:1167#0/1
TTCGGGGATCTTCTCTTCATTTTCAATGTCCTGTAGCTCT
+HWI-EAS443:3:24:1118:1167#0/1
`aabaa_[_^abba`aa``aaaaaaa`a_a_aa_``___]
@HWI-EAS443:3:25:956:2014#0/1
CAACTTCTATCCAGCTTGAATCAGTCAATTCACATGCCTT
+HWI-EAS443:3:25:956:2014#0/1
_¥]Y¥`aaXQ_b^_^]^OR_X[¥XMX¥b^^aaR¥GN¥[G[
@HWI-EAS443:3:29:399:211#0/1
CCTTAAGTTGTGGAGACGCATATAGACTGTTGAATACAGA
+HWI-EAS443:3:29:399:211#0/1
ab^`aZ^]]b¥b`_^Y`¥SZa¥^]a]PV]N_]][BBBBBB
@HWI-EAS443:3:29:459:748#0/1
ATAAAATGAAGGGAGTGTACATAAATACGGCCTTGCTCAA
```

```
+HWI-EAS443:3:29:459:748#0/1
aaaZa`_a`_a¥`__X_W[Y__¥Za`__a_aY^^]^V]_^
@HWI-EAS443:3:30:1011:2030#0/1
GACGAAAGGAATCCCATAGACTCCTACTGGCCATTTTTTT
+HWI-EAS443:3:30:1011:2030#0/1
¥__¥ab¥]a`a^`]T_`[UWYXQ__¥FPN[WQSMSBBBBB
@HWI-EAS443:3:30:114:903#0/1
GAACTTTAGGCTTCTCTACCCCTGTTGTGTTACATATACT
+HWI-EAS443:3:30:114:903#0/1
ZVVHX^`^a`baa]_^``¥¥aaWXP^ZZ_a_aa]_aZ_SQ
@HWI-EAS443:3:32:283:1062#0/1
CAAGGACAAAGAACATGAAGAGAACAAGCCAATTGACGTG
+HWI-EAS443:3:32:283:1062#0/1
aaa]`aa_a^_Y_aaaaaaaa_W_aa``^Z``_^[[GYX[
@HWI-EAS443:3:37:692:266#0/1
AGGTGTTCACTTCTTTTGACATTTGGGAAAGCTTGCCCTC
+HWI-EAS443:3:37:692:266#0/1
`^^GU]ZS_`_aX_a_a`aRaa_X[``[HFQJF[[TVZ]X
@HWI-EAS443:3:38:771:432#0/1
GTGGAGTCCGCTTTGGTGGCCATCTCCTCTCCAGTGAATG
+HWI-EAS443:3:38:771:432#0/1
`^`PT_aa^]]_XUY_V^_^^___[U]YaTGZN[W[[U¥¥
@HWI-EAS443:3:48:427:215#0/1
CTCAGATTGCCTGATGGGCCTCTTTGCCATCAGCGGGCAA
+HWI-EAS443:3:48:427:215#0/1
`X`a`¥HV^^HQaWVa__MVOVZ]U^]SKT¥BBBBBBBBB
@HWI-EAS443:3:4:335:870#0/1
GGCCATGTTTCCGATTTGTTTTCAAAGAATTCCTTGATTC
+HWI-EAS443:3:4:335:870#0/1
a_][YSPZ^XN^aPPP¥]TYX]¥TW[aUS`QRYXT]VYQB
@HWI-EAS443:3:52:544:1353#0/1
CGCGAGCTTGCCGACCAAAGTCTCCCACCGAACTTCTCCA
+HWI-EAS443:3:52:544:1353#0/1
ab]_UUZ¥Q_aS``]HYP]VMZ[¥Qa_YWaPVS]aRYG]¥
@HWI-EAS443:3:55:1607:25#0/1
CGGGGAGTCACCCAGGGGAGTGGAGGAAGGCTCTATTGGG
+HWI-EAS443:3:55:1607:25#0/1
`b_¥__baaba_]^bb_b_baba^baZT]`X`^`[]UX]_
@HWI-EAS443:3:56:1104:1114#0/1
GAGAAAGGCATAAATCCCAATTACCTCATGGCTTGGGAGC
+HWI-EAS443:3:56:1104:1114#0/1
aaa]_¥[ZTaaab[^ZSTZP^`^¥YTKZ^`XR]BBBBBBB
@HWI-EAS443:3:57:200:465#0/1
TTACCATTGGGATCAGCTGAAAGTCATCCATGGCTGCACA
+HWI-EAS443:3:57:200:465#0/1
aaaa_Z`aabaa`]]`_aaaaaaZ^^_]VW_aaW^_]VLL
@HWI-EAS443:3:57:726:1142#0/1
ATTTCATCGACGAACGGGGTGAATCAATAATTGTAGTATC
+HWI-EAS443:3:57:726:1142#0/1
abaaabaaaaaa``¥``a`U`_[^¥_``^]`a`^a[X^`^
```

```
@HWI-EAS443:3:59:451:244#0/1
GCCATATTTTCACCGAGTGCCCACTTCAATTGGCTTTTTC
+HWI-EAS443:3:59:451:244#0/1
aa¥¥a¥aaa`abaZ^X¥W]_Z^X`a``^Z^^SSY]RFPUN
@HWI-EAS443:3:59:695:1441#0/1
CACTGCAGGGCTACTGAATACATAATGAAGGGAGTGAAAA
+HWI-EAS443:3:59:695:1441#0/1
[``a_^]V_aX_aWa_`a`^aa`_PN^¥F]BBBBBBBBBB
@HWI-EAS443:3:5:1608:759#0/1
GCCATGCTCAATGCATCCTGTGCAGCCATGGATGACTTTC
+HWI-EAS443:3:5:1608:759#0/1
abba¥bb^QY]aa¥ab]a]bababba`aaaa^[__^_a`B
@HWI-EAS443:3:60:1208:1393#0/1
GAGAAAGGCATAAATCCCAATTACCTCATGGCTTGGGAGC
+HWI-EAS443:3:60:1208:1393#0/1
_^F^aaa_`aX¥b_`]`Z`Taa[P]XQWXa[ZTFHSF¥KF
@HWI-EAS443:3:64:717:88#0/1
AATCCCTTGGTCATGTCTTTCTCTTTGACAGAAGACTCGG
+HWI-EAS443:3:64:717:88#0/1
]``^^aa^a[__`a`PY^a__^¥``__W^[_W]VXX^V^]
@HWI-EAS443:3:67:1662:1725#0/1
GGCCATGTTTCCGATTTGTTTTCAAAGAATTCCTTGATTC
+HWI-EAS443:3:67:1662:1725#0/1
`a^_``__aa_aa`aaaa_`_aa_aaa`_aa_T`_a_a`¥
@HWI-EAS443:3:68:531:219#0/1
CGCCCTCCACAACTTGAGGGGTTTTCGGCTGAATCGAGAA
+HWI-EAS443:3:68:531:219#0/1
``a^`_¥¥U`W¥_^^_¥___[L^_]]`]]aa`ZV[`¥ZUG
@HWI-EAS443:3:69:1143:1045#0/1
CGGGGCTTCAATAGTGCATTCGGGTCACCAGATTCTACAA
+HWI-EAS443:3:69:1143:1045#0/1
_W_O_a`Z¥_`VO^[WSa¥VX`_XY^XaXT]¥`a`^XXZX
@HWI-EAS443:3:69:789:381#0/1
CTCAAGAATGGTTCAATTTTGGCGTTCACTTCTTTTTACA
+HWI-EAS443:3:69:789:381#0/1
abbbabb^aab`aababbbbV`abaZUY]W_Y`aa^HYO¥
@HWI-EAS443:3:69:846:106#0/1
CGGCTGCATCCTGTGCAGCCATGGATGACTTTCAGCTGAT
+HWI-EAS443:3:69:846:106#0/1
abbbbab`]VZ`b]`^a^SY[]``X_`X¥``^X_¥FV][X
@HWI-EAS443:3:6:24:359#0/1
TCATGCTCTGCTAGCACCTGCTTCCAAGCCATGAGGTAAT
+HWI-EAS443:3:6:24:359#0/1
abbaaaaa`aa_W_¥]]a`aa___`]Q_]W__a]`_Y]^a
@HWI-EAS443:3:72:1371:881#0/1
GGGCAAGATCCGAAAATCGAAACTAACAAGTTTGCTGCAA
+HWI-EAS443:3:72:1371:881#0/1
R_QJ¥PRXabaY_a__IY`YR¥^_SSZ_¥aZ`Z`]Vba^T
@HWI-EAS443:3:80:1209:1131#0/1
CACCCCTGTTGTGTTACATATACTGTTCACCACTGTCGAG
```

~~~
+HWI-EAS443:3:80:1209:1131#0/1
V_aaSV_Ma[S__ab][Saaba]aa`bb`UYabba_VHRa
@HWI-EAS443:3:81:1197:1162#0/1
GGGCTTTCGGCTGAATCGAGAAAATTGCTTCTCATTGTTC
+HWI-EAS443:3:81:1197:1162#0/1
V_^JXaa`_]]`baba`bbbbabbaaaabb^abUbUbSaa
@HWI-EAS443:3:81:568:598#0/1
CTGAAGAGGAGATGGCCACCAAAGCGGACTACACCCTTGA
+HWI-EAS443:3:81:568:598#0/1
abbbbbbaabbbaa`aaaa`ba``aa^a^`aaaa]^_]`a
@HWI-EAS443:3:84:326:152#0/1
AGCAGGTGCTAGCAGAGCTACAGGACATTGAAAATGTAGA
+HWI-EAS443:3:84:326:152#0/1
aaa_aa^aaaaaa`a_aa_a_a¥^`^_^``]¥Y¥^]U_`^
@HWI-EAS443:3:85:1279:713#0/1
GGGCTTTCGGCTGAATCGAGAAAATTGCTTCTCATTGTTC
+HWI-EAS443:3:85:1279:713#0/1
Z`WFZW[a_XQ`[YbZbaV¥R_V^`aYVXZR_`[b`b`^F
@HWI-EAS443:3:93:1220:789#0/1
CGTGACTCGGGTCTTCAATACTTAATTTCAGAGCATGCAT
+HWI-EAS443:3:93:1220:789#0/1
]T¥^VM^]Z_aYQLWRY`¥aZJL]VNWOFVWLRVBBBBBB
@HWI-EAS443:3:94:1603:190#0/1
CCCGTGTTGTGTTACATATACTGTTCACCACTGTCCAGGC
+HWI-EAS443:3:94:1603:190#0/1
a_`aXaa^[`_[a_`aaaa`aaa_`V`¥R`[`_VUV¥MSP
@HWI-EAS443:3:94:460:425#0/1
GGGGTTTCGGCTGAATCGAGAAAATTGCTTCTCATTGTTC
+HWI-EAS443:3:94:460:425#0/1
aaab[aaaa_aa`_``]a_`_]_____^`_]a_^_`_]`Y
@HWI-EAS443:3:95:1603:1232#0/1
GGGGTTTCGGCTGAATCGAGAAAATTGCTTCTCATTGTTC
+HWI-EAS443:3:95:1603:1232#0/1
ab[b`bbKbabbbabbabbbbba]bb`bbbab_bbbb]bX
@HWI-EAS443:3:97:1536:448#0/1
ACTGGAGCCACACAAATGGGAAAAATACTGTGTTCTAGAA
+HWI-EAS443:3:97:1536:448#0/1
a¥aHT_a``WDX__]¥a]_KRUZTX^TMJWV^^ZOXXRZB
@HWI-EAS443:3:98:96:779#0/1
CGGTTCTCTTTGTAATCATACAAATCAGGAAGAAATCTAG
+HWI-EAS443:3:98:96:779#0/1
aa`¥Yab`^`aaabbb_``ZU`a`^a`aa¥V¥_`X`_`Y^
@HWI-EAS443:3:26:644:1500#0/1
AGGGCGTGGTGTCNTCCTCAAGAATGGTTCAATTTTGGCG
+HWI-EAS443:3:26:644:1500#0/1
```bb_a`aX`[¥DR`aa]¥]aa`_]_Wa_a__^_^]G`_
@HWI-EAS443:3:34:1557:1729#0/1
TTGGCCTATCGCAGTCCTCAAGAGCANGTCTCCTATTTCT
+HWI-EAS443:3:34:1557:1729#0/1
UXX_`Xbab`¥aa``b_bbbaa¥]¥[D^¥_^^Ya__^XHU
~~~

```
@HWI-EAS443:3:63:425:132#0/1
CTATTTTACAGCAGAAGTGTCCCACTGCAGGGCTNCTGAC
+HWI-EAS443:3:63:425:132#0/1
a]X]^a`_a`b`_a]Y]]aT_]`a^S¥VV_`Y]MDT^^QF
@HWI-EAS443:3:74:479:1166#0/1
CGGCCAGTAGGAGTCTATGGGATTCCTTNCGTCAGTCCGA
+HWI-EAS443:3:74:479:1166#0/1
G¥]]^]]¥YWQ`_W`Z`^^baXa¥`X`ZD^baa```bbaX
@HWI-EAS443:3:100:388:1119#0/1
CACTATAAGACAAGAAATGGCCAGTAGGAGTGTATGGGAT
+HWI-EAS443:3:100:388:1119#0/1
aa^__Q_a]ZZ__V¥YYR^W_¥T]MI¥PTVVFH]_^_BBB
@HWI-EAS443:3:100:482:211#0/1
GTCTTCCTTCTTTGGTCCTACATTTGCTTACCATTGGGAT
+HWI-EAS443:3:100:482:211#0/1
a`^ab_`ab^bbbba`W_a`_a``a_¥a`^X___`_`^^^
@HWI-EAS443:3:100:646:407#0/1
GGCGTTCACTTATTTTGACATTTGGGAAAGCTTGCCCTCA
+HWI-EAS443:3:100:646:407#0/1
ZaS`[aSTNa`I^a`ZNP]Sa^WT_a^`¥YX[[aS__XVB
@HWI-EAS443:3:100:742:679#0/1
CATTTGCTTACCATTGGGATCAGCTGAAAGTCATCCATGG
+HWI-EAS443:3:100:742:679#0/1
Waba^``b`b_QZabb`]`aabaababaaa`aaa]_``ba
@HWI-EAS443:3:100:795:382#0/1
CAAATAGTAGCATTGCCACAACTACTTCAATGCATGTGTG
+HWI-EAS443:3:100:795:382#0/1
`bb]baa]ababaaaa`a_aa^baaaa`aa``_aaa^b]b
@HWI-EAS443:3:100:809:1484#0/1
TCCGTCTTCCTTCTTTGGTCCTACATTTGCTTACCATTGG
+HWI-EAS443:3:100:809:1484#0/1
`[Qb_^aYTaa_]_`[¥XUH¥a`__a``Y___a_RW_`_B
@HWI-EAS443:3:10:1282:1206#0/1
CAAATAGTAGCATTGCCACAACTACTTCAATGCATGTGTG
+HWI-EAS443:3:10:1282:1206#0/1
[ba`[aRI_¥a^aab^bbba_Z¥`aaba]abb]ab`ab¥b
@HWI-EAS443:3:10:1602:1595#0/1
CAAATAGTAGCATTGCCACAACTACTTCAATGCATGTGTG
+HWI-EAS443:3:10:1602:1595#0/1
^bbbbab]ababba[bbb_bbabbabbbabbb^aab]`ba
@HWI-EAS443:3:10:1602:2044#0/1
ATCAGCAGGAAGTTTGACCGCTGATGGCAAAGAGGCCCAT
+HWI-EAS443:3:10:1602:2044#0/1
aa`a]¥aa¥a_GH_¥Z_ZZRXUTYUVXNY¥¥NWS¥QGNUS
@HWI-EAS443:3:10:1676:1348#0/1
ATTTGCTTACCATTGGGATCAGCTGAAAGTCATCCATGGC
+HWI-EAS443:3:10:1676:1348#0/1
^`bbb`bba_]_abb]baa_`a¥a`aa`_QRaa¥Z_a``W
@HWI-EAS443:3:10:1766:1490#0/1
ATCGCAGTCCTCAAGAGCATGTCTCCTATTTCTAGAACAC
```

```
+HWI-EAS443:3:10:1766:1490#0/1
]ba_``a_][_WY]a`^¥^_]P¥`WWZ¥`Z_]_]X[YLYY
@HWI-EAS443:3:10:253:1434#0/1
CACAACTACTTCAATGCATGTGTGAGGAAGGAGTTGAACC
+HWI-EAS443:3:10:253:1434#0/1
ababbbbbbbb_bbbbbabbab`bbb`abb[bbabbaaa^
@HWI-EAS443:3:10:356:433#0/1
CTTCCTTCTTTGGTCCTACATTTGCTTACCATTGGGATCA
+HWI-EAS443:3:10:356:433#0/1
]abbba`abbbbaabbbbabbbbbbbaba`aab`Waaa`a
@HWI-EAS443:3:10:751:1887#0/1
CATTGCCACAACTACTTCAATGCATGTGTGAGGAAGGAGT
+HWI-EAS443:3:10:751:1887#0/1
¥_b¥X^bbbb^O^bbbb_ba`T^a`a¥b`bbb`aa_ab`_
@HWI-EAS443:3:10:837:106#0/1
GGGGCTATATGAAGCAATCGAGGAGGGCCTGATTAATGAT
+HWI-EAS443:3:10:837:106#0/1
aZ_[VVU]OQTLRRTR]VV]Y]YVVHVYYYXVXYVVXYVV
@HWI-EAS443:3:10:910:366#0/1
GCAAGAATCAAAACTAGGCTTTTCACTATAAGAGAAGAAA
+HWI-EAS443:3:10:910:366#0/1
aX[Z^QYYZ¥XQSQSZW¥RT^Z^YWPNYX¥BBBBBBBBBB
@HWI-EAS443:3:11:1033:1506#0/1
CAAATAGTAGCATTGCCACAACTACTTCAATGCATGTGTG
+HWI-EAS443:3:11:1033:1506#0/1
aaV`aabT[^bab`a[aaaba[ba_a_`aaa`^`aa_a^`
@HWI-EAS443:3:11:130:1290#0/1
ATTGCCACAACTACTTCAATGCATGTGTGAGGAAGGAGTT
+HWI-EAS443:3:11:130:1290#0/1
`baabaabbaaabaa_baY`aaaaa_a]a_a`_a`__`^_
@HWI-EAS443:3:11:1348:980#0/1
GGGGGGCTATATGAAGCAATCGAGGAGCGCCTGATTAATG
+HWI-EAS443:3:11:1348:980#0/1
aaa_`aaa`aaaa`_a___a_a_a_Wa`ZUW^¥W¥YWWBB
@HWI-EAS443:3:11:1405:1956#0/1
CAAATAGTAGCATTGCCACAACTACTTCAATGCATGTGTG
+HWI-EAS443:3:11:1405:1956#0/1
abbbabb_aaaaba`abbaba`ababaab_baa_a^X]V[
@HWI-EAS443:3:11:165:1573#0/1
CCGAAAGAGGCGAAGAGACAATTGACGAAAAATTTGAGAT
+HWI-EAS443:3:11:165:1573#0/1
]X_VZXa[Z^aR`YFZ__YV[YVMIEO[YXVLTYNBBBBB
@HWI-EAS443:3:11:384:73#0/1
AAATAGTAGCATTGCCACAACTACTTCAATGCATGTGTGA
+HWI-EAS443:3:11:384:73#0/1
ababaaZabb`aa_a_a`aa_a___a_a__a_`_aZaZa[
@HWI-EAS443:3:11:420:1636#0/1
CTCAAGAGCATGTCTCCTATTTCTAGAACACAGTATTTTT
+HWI-EAS443:3:11:420:1636#0/1
aaaa^_]a`aaa`b``aaTaaa__¥aY]_`aaUMY^_^^S
```

```
@HWI-EAS443:3:11:447:1936#0/1
TTTCCGTCTTCCTTCTTTGGTCCTACATTTGCTTACCATT
+HWI-EAS443:3:11:447:1936#0/1
aaaaba_ba`aaa`a```_]Z_a`a`a__^__`_`_^a``
@HWI-EAS443:3:11:477:1391#0/1
ATCGACGAACGGGGGGAATCAATAATTGTAGAATCTGGTG
+HWI-EAS443:3:11:477:1391#0/1
a[b`aW`aaaba_aO[MYa]PT]`_a_]_aa_a¥X_^_V_
@HWI-EAS443:3:11:725:401#0/1
AAAATACTGTGTTCTAGAAATAGGAGACATGCTCTTGAGG
+HWI-EAS443:3:11:725:401#0/1
a`aaa]abbab^aabaaa``a```_a^``aa_``a_aa``
@HWI-EAS443:3:11:995:321#0/1
AAATAGTAGCATTGCCACAACTACTTCAATGCATGTGTGA
+HWI-EAS443:3:11:995:321#0/1
a`¥``Y``baaab_][[aa`a_aW_`]_]_`W¥]^]`W_^
@HWI-EAS443:3:12:1022:873#0/1
TCCCTTCATTATGTATTCAGTAGCCCTGCAGAGGGACACT
+HWI-EAS443:3:12:1022:873#0/1
[]SZT`Q``^a_¥¥]^Y`¥]PQPa]GULR^XFOLGK[_XB
@HWI-EAS443:3:12:1108:1296#0/1
CATTTGCTTACCATTGGGATCAGCTGAAAGTCATCCATGG
+HWI-EAS443:3:12:1108:1296#0/1
aa[`aaZba[_^bbb_¥Za_Z_Vbbaaa`aX[baa^aba`
@HWI-EAS443:3:12:1233:145#0/1
CATTTGCTTACCATTGGGATCAGCTGAAAGTCATCCATGG
+HWI-EAS443:3:12:1233:145#0/1
VU_a_IV__XV]Vba_O[^TT¥a¥W_^``¥U_``XRQa¥[
@HWI-EAS443:3:12:1344:1530#0/1
TGAAAAGCCTAGTTTTGATTCTTGCCCCGCTCTCTTCGTC
+HWI-EAS443:3:12:1344:1530#0/1
`b_QT_`a^`UUPW_[b`^WOHZTZWU]b`]SZaHS`aYa
@HWI-EAS443:3:12:135:1825#0/1
CATGACCAAGGTATTCTTTGAAAACAAATCGGAAACATGG
+HWI-EAS443:3:12:135:1825#0/1
¥V__[aaa^^]Va`_^^`_X_W_^`a¥ZUDWGXXaZ_]W]
@HWI-EAS443:3:12:1513:39#0/1
AAATAGTAGCATTGCCACAACTACTTCAATGCATGTGTGA
+HWI-EAS443:3:12:1513:39#0/1
¥aa_aaL`¥]_ab`a__^`aOa_T^a`_^`a_X^aXaW]S
@HWI-EAS443:3:12:1523:190#0/1
GCAGGAGCTAGCAGAGCTACAGGACATTGAAAATGAAGAG
+HWI-EAS443:3:12:1523:190#0/1
`^^`^ZZ`a``Y^a[Y¥]]Y__[[S]Z_XV_Z]^ZW]_]]
@HWI-EAS443:3:12:273:811#0/1
CACAACTACTTCAATGCATGTGTGAGGAAGGAGTTGAACC
+HWI-EAS443:3:12:273:811#0/1
aabaa`abbaa¥a[aa`Xaa_a^a^`a^¥¥`_a_a[___Q
@HWI-EAS443:3:12:521:703#0/1
CATTTGCTTACCATTGGGATCAGCTGAAAGTCATCCATGG
```

+HWI-EAS443:3:12:521:703#0/1
abbabbba`___¥a_R_baabbaab]Z_]``Q_a¥QO^aa
@HWI-EAS443:3:13:1055:1457#0/1
GGGGGGCTATATGAAGCAATCGAGGAGTGCGTGATTAATG
+HWI-EAS443:3:13:1055:1457#0/1
S_ab][a¥Z]b]YY]baabYa]aU^TROYUFN_a_¥[X_a
@HWI-EAS443:3:13:1077:312#0/1
ACATAATGAAGGGAGTGTACATAAATACGGCCTTGGTCAA
+HWI-EAS443:3:13:1077:312#0/1
ZZ¥WaZ`¥a_^Q¥WRP`^a`abaab]aab`_[XXXFW_`_
@HWI-EAS443:3:13:1133:824#0/1
ACTTTTTCTGGTGCCATATTTTCACCGACTGCCCACTTCA
+HWI-EAS443:3:13:1133:824#0/1
a]b_babaaab`baaaaa```]]baaa¥XVa_`_`]`a][
@HWI-EAS443:3:13:1509:785#0/1
CCTGGGTGACTCTCAGATTGGCCATGTTTCCGATTTGTTT
+HWI-EAS443:3:13:1509:785#0/1
a`abbbb[^ba`baabaaab__a`aYZbb_TPP_baaaba
@HWI-EAS443:3:13:556:675#0/1
CAAATAGTAGCATTGCCACAACTACTTCAATGCATGTGTG
+HWI-EAS443:3:13:556:675#0/1
aa[UR^GP¥ZQ_a_NR`TT^_^aaa^SZ`YL^XX`¥KX`a
@HWI-EAS443:3:14:1724:327#0/1
GCTTACCATTGGGATCAGCTGAAAGTCATCCATGGCTGCA
+HWI-EAS443:3:14:1724:327#0/1
_XZ¥ZYYXaZTXVRQV^_R[¥ZU^]WTVVSUVZZZTUZRB
@HWI-EAS443:3:14:369:2045#0/1
CTCAAGAATGGTTCAATTTTGGCGTTCACGTCTTTTGACA
+HWI-EAS443:3:14:369:2045#0/1
`NYW]ZWRJM]GRRO[WXR_[PU]HGZBBBBBBBBBBBBB
@HWI-EAS443:3:14:379:1499#0/1
CAAATAGTAGCATTGCCACAACTACTTCAATGCATGTGTG
+HWI-EAS443:3:14:379:1499#0/1
aa`aa¥O¥`ZU[[[a]S`Y_aZPY`[^a^Y^^^[Y^VZX`
@HWI-EAS443:3:14:454:1868#0/1
ATTGCCACAACTACTTCAATGCATGTGTGAGGAAGGAGTT
+HWI-EAS443:3:14:454:1868#0/1
VSYVb¥UaUbZZS_aab`aSRQT_]RMMaZY`]¥_``a__
@HWI-EAS443:3:14:484:607#0/1
GCCACAACTACTTCAATGCATGTGTGAGGAAGGAGTTGAA
+HWI-EAS443:3:14:484:607#0/1
abaPX_^[]aaa]]¥R¥Z]]]XZ_P`¥J`SP^ZU_GS¥UE
@HWI-EAS443:3:14:686:210#0/1
GTTCGGTGGGAGACTTTGGTCGGCAAGCTTGCGCACAGTT
+HWI-EAS443:3:14:686:210#0/1
a[[`a_SS`^Y[ONU¥^^ZRY¥¥Z¥[WUWQZQYRNFRWUB
@HWI-EAS443:3:15:1026:947#0/1
CAAATAGTAGCATTGCCACAACTACTTCAATGCATGTGTG
+HWI-EAS443:3:15:1026:947#0/1
Y^XXa`b¥¥¥```b`_a^a`^babba^V_Z`bb`abab^a

```
@HWI-EAS443:3:15:1394:538#0/1
AGCATGTCTCCTATTTCTAGAACACAGTATTTTTCCCATT
+HWI-EAS443:3:15:1394:538#0/1
ab`Va`Xb``^a¥aa[Ya`__a¥]a^bVS`a`aa_¥___`
@HWI-EAS443:3:15:1501:1510#0/1
CAAATAGTAGCATTGCCACAACTACTTCAATGCATGTGTG
+HWI-EAS443:3:15:1501:1510#0/1
^^a``bbaa_a`[bb``¥^abbbbaba^_a]a`[ab_bXb
@HWI-EAS443:3:15:154:1329#0/1
CTCACGTTGTGGAGACGCATATAGACTGTTGAATACAGAT
+HWI-EAS443:3:15:154:1329#0/1
aI`XHHYUORaY`Y``N[NZ_Z]_XU¥BBBBBBBBBBBBB
@HWI-EAS443:3:15:290:984#0/1
CATTTGCTTACCATTGGGATCAGCTGAAAGTCATCCATGG
+HWI-EAS443:3:15:290:984#0/1
abbbabbbaaba```bZU_aaabaaaabaa_ab_`aa`a^
@HWI-EAS443:3:15:423:1987#0/1
GCTTGCGATATGTTCAATCGGGGCAAAATCTTCTCCTATT
+HWI-EAS443:3:15:423:1987#0/1
]Xab¥^`¥^^a`H¥a_aa^]N¥QZV]JaaaaSG]ab``Wa
@HWI-EAS443:3:15:979:1115#0/1
CATTGCCACAACTACTTCAATGCATGTGTGAGGAAGGAGT
+HWI-EAS443:3:15:979:1115#0/1
abbbabbaaaaabaabbbaaab``aaaa¥aaaaaaaa`_^
@HWI-EAS443:3:15:988:1992#0/1
AACCGGTTCTCTTTGTAATCATACAAATCAGGACGAAATT
+HWI-EAS443:3:15:988:1992#0/1
abaaab^a]aaaaaaa``][a`aa_a_ZQ__`WIYa_^VR
@HWI-EAS443:3:16:1044:1863#0/1
CATGGAAATGAGGCGCTGCCTTCTTCAGTCTCTTTAGCAG
+HWI-EAS443:3:16:1044:1863#0/1
a`[aa`]ab`aaV`T[_``¥__¥a_a_]M]¥`]_GXHTHN
@HWI-EAS443:3:16:105:1704#0/1
GTAGCATTGCCACAACTACTTCAATGCATGTGTGAGGAAG
+HWI-EAS443:3:16:105:1704#0/1
[]bZbbb`a`a]aabbbbbbabbaa`baab^^[a`aaaaa
@HWI-EAS443:3:16:1110:267#0/1
TGACTTTCAGCTGATCCCAATGGTAAGCAAATGTAGGACC
+HWI-EAS443:3:16:1110:267#0/1
aa¥S¥W^¥`^^VaX[_V_¥ZY¥ZOQ^aWTYJ_aRZ_``^P
@HWI-EAS443:3:16:1153:2013#0/1
AAATAGTAGCATTGCCACAACTACTTCAATGCATGTGTGA
+HWI-EAS443:3:16:1153:2013#0/1
aaaaa]Ua__``_^^^_^`^Z_a]`X_`^^`^_`^ZXLX¥
@HWI-EAS443:3:16:1297:477#0/1
ATCGCAGTCCTCAAGAGCATGTCTCCTATTTCTAGAACAC
+HWI-EAS443:3:16:1297:477#0/1
aaba_S]_a]a`aa_`]¥`]Y]Ta_[`_[``]a`_¥_U__
@HWI-EAS443:3:16:1423:1931#0/1
AAAGAATTCCTTGGACATGTCTTTCTCTTTGACAGAAGAC
```

```
+HWI-EAS443:3:16:1423:1931#0/1
_^¥^___[RaXI[WHFa^`^L]U¥HT^ZY¥_XRXZ^_^BB
@HWI-EAS443:3:16:1720:1080#0/1
CAAATCAGGAAGAAATTTAGGCTTCGCTACCCCTGTTGTG
+HWI-EAS443:3:16:1720:1080#0/1
_XHV_Q¥RW__`[LQ^UMSXO`]ZQFULJVXLR¥X^BBBB
@HWI-EAS443:3:16:687:1324#0/1
GCCACAACTACTTCAATGCATGTGTGAGGAAGGAGTTGAA
+HWI-EAS443:3:16:687:1324#0/1
ab`babbabbabbbbab`aabba`[aaa`aaa`a``a^`a
@HWI-EAS443:3:17:1113:2016#0/1
AGGGGGTTTCGGCTGAATCGAGAAAATTGCTTCTCATTGT
+HWI-EAS443:3:17:1113:2016#0/1
abaaaa^a`aaa¥a`ba]`a__aa``^___Z^Z_^a^^`X
@HWI-EAS443:3:17:151:1466#0/1
AATAATTCTAGAATCTGGTGACCCGAATGCACTATTGAAG
+HWI-EAS443:3:17:151:1466#0/1
aaaaaa¥N[[a`_^__a_^`__`a]___`_`__`_^_^_¥
@HWI-EAS443:3:17:1523:815#0/1
ATCGCAAGCATGAGGAGTAACTATTTTACAGCAGAAGTGT
+HWI-EAS443:3:17:1523:815#0/1
`_`^WWZ[YQ[UF^Z_]DUTRX^RXXU[YZ[[¥^V_[RZV
@HWI-EAS443:3:17:1550:1354#0/1
CTACTGGCCATTTCTTGTCTTATAGTGAAAAGCGTAGTTT
+HWI-EAS443:3:17:1550:1354#0/1
_YYVW`^O]UPSSWYY`OL[VO^OOR^VS[¥¥SDRSX^Y^
@HWI-EAS443:3:17:1737:1034#0/1
AGCATTGCCACAACTACTTCAATGCATGTGTGAGGAAGGA
+HWI-EAS443:3:17:1737:1034#0/1
a^XU]¥_]_U^U_Q[XXZ`]_XZ^ZY¥_XaX_[¥YSY[YL
@HWI-EAS443:3:17:418:629#0/1
GCCATGGATGACTTTCAGCTGATCCCAATGGTAAGCAAAT
+HWI-EAS443:3:17:418:629#0/1
abaaaba``a``a`_aa`a]a^`]_a_Y^a_V_X^_^^¥^
@HWI-EAS443:3:17:554:562#0/1
GTGAATTGACTGATTCAAGCTGGATAGAAGTTGATGAAAT
+HWI-EAS443:3:17:554:562#0/1
aM¥]a]¥`S[^`Y[_^[¥QY[[aSU`¥_]PX_^Y^`V^S]
@HWI-EAS443:3:18:1018:1023#0/1
AAGAGCATGTCTCCTATTTCTAGAACACAGTATTTTTCCC
+HWI-EAS443:3:18:1018:1023#0/1
a[`aaaaJaU¥`aa_T_`_]__`__V_X`aV[¥_`^`]¥X
@HWI-EAS443:3:18:1301:1406#0/1
CAAATAGTAGCATTGCCACAACTACTTCAATGCATGTGTG
+HWI-EAS443:3:18:1301:1406#0/1
abbbbbbabb`abbababbba`bbaabaaaab`abbaa`b
@HWI-EAS443:3:18:1658:1425#0/1
ATAGGAACATGGGCCTCGACACTTGGCATATCGCAGTCCT
+HWI-EAS443:3:18:1658:1425#0/1
XX¥b^Ya_a`aSNOa_S]_¥QR¥V^U]G]_Z_aW¥SUR[^
```

```
@HWI-EAS443:3:18:423:1083#0/1
CTCATCACTGTCATACTGTTTAAGGTCTGCAACATCTTTG
+HWI-EAS443:3:18:423:1083#0/1
a¥TILXHZ_`¥YY[YQ[`LOWSV]QFSQFRVXWUWVSYZ]
@HWI-EAS443:3:19:1119:182#0/1
CGAATACATGAAACAAACTTCAAAATGTGTGCATATTGCA
+HWI-EAS443:3:19:1119:182#0/1
aabaaa`baaaaaaaaa^aaaHa``aa¥a`a_^a``__^`
@HWI-EAS443:3:19:1139:1996#0/1
AGCAGGTGCTAGCAGAGCTACAGGACATTGAATATGAAGA
+HWI-EAS443:3:19:1139:1996#0/1
aa`b^bQ_aP_GX`a_a_`aaaa_`[`_^__`_`[^__¥_
@HWI-EAS443:3:19:1617:365#0/1
TTTCCGTCTTCCTTCTTTGGTCCTACATTTGCTTACCATT
+HWI-EAS443:3:19:1617:365#0/1
``a_ZW_]]]OS_ROYa_]][¥VQ[W¥¥Z^ZW]¥XLT][B
@HWI-EAS443:3:19:223:984#0/1
CAAATAGTAGCATTGCCACAACTACTTCAATGCATGTGTG
+HWI-EAS443:3:19:223:984#0/1
ab`]`b`_`aa`aa``_`_aa``a^b_^a_``a_aaXa¥a
@HWI-EAS443:3:19:435:940#0/1
ATGGCTTTGAAGCAGGTGCTAGCAGAGCTACAGGACATTG
+HWI-EAS443:3:19:435:940#0/1
^_`]^aWFXN_RVY]_SORLYSSJ[S_XPZS_Z^WUULWW
@HWI-EAS443:3:19:946:1583#0/1
CTTCCTTCTTTGGTCCTACATTTGCTTACCATTGGGATCA
+HWI-EAS443:3:19:946:1583#0/1
abbbabbbaaaaa^_aaabaaabaaaaaaaabaaZKOYBB
@HWI-EAS443:3:1:1078:1124#0/1
ACTCGGAGAAAATATGGCACCAGAAAAAGTAGACTTTGAT
+HWI-EAS443:3:1:1078:1124#0/1
]`ZU^^D[Z_ST^Z]a_]]QP¥`¥``^YaU]]`_[GSa[B
@HWI-EAS443:3:1:1094:651#0/1
CTTGGAAGCAGGTGGTAGCAGAGCTACAGGACATTGAAAA
+HWI-EAS443:3:1:1094:651#0/1
_W]]^YV]`^``_XDWaaWQPUa^aa_]a`[GRU¥ZUSBB
@HWI-EAS443:3:1:328:1716#0/1
ATTGCCACAACTACTTCAATGCATGTGTGAGGAAGGAGTT
+HWI-EAS443:3:1:328:1716#0/1
^aaa`YO__b_aaW`¥¥bbbba`aba^U]YWb_aa]BBBB
@HWI-EAS443:3:1:585:291#0/1
CAAATAGTAGCATTGCCACAACTACTTCAATGCATGTGTG
+HWI-EAS443:3:1:585:291#0/1
aVb¥a_TP¥_YZba¥QZ`Saa[TYOX`]_^`_^]aa_aBB
@HWI-EAS443:3:20:1247:432#0/1
TTGCCACAACTACTTCAATGCATGTGTGAGGAAGGAGTTG
+HWI-EAS443:3:20:1247:432#0/1
]aaV_a¥a_VaaYaa[aa]_[^aa]`]b]_UX¥`¥W`]^_
@HWI-EAS443:3:20:1358:353#0/1
TACATTTGCTTACCATTGGGATCAGCTGAAAGTCATCCAT
```

```
+HWI-EAS443:3:20:1358:353#0/1
aaaababaab^`]^`aab`R`__aaY`a`aaa^_aa]PX_
@HWI-EAS443:3:20:1461:391#0/1
CAAATAGTAGCATTGCCACAACTACTTCAATGCATGTGTG
+HWI-EAS443:3:20:1461:391#0/1
abbbbbbabbbbbbba_`aaa_baaaa`aaaaaaabab`a
@HWI-EAS443:3:20:151:1401#0/1
GGGGTTTTCGGCTGAATCGAGAAAATTGCTTCTCTTTGTT
+HWI-EAS443:3:20:151:1401#0/1
¥ZTZ]ab]]WGXZ_]`bb]]]]`a__aY_a`^a¥]_aa[_
@HWI-EAS443:3:20:1775:1881#0/1
GTAGCATTGCCACAACTACTTCAATGCATGTGTGAGGAAG
+HWI-EAS443:3:20:1775:1881#0/1
a^a_Y__a_]^_V`^V``_`_^`_^a]]_`Y_]_X`Z]Z^
@HWI-EAS443:3:20:216:236#0/1
CAAATAGTAGCATTGCCACAACTACTTCAATGCATGTGTG
+HWI-EAS443:3:20:216:236#0/1
`bbab[a_`ababaaaababa_aaaaab``aaa_aa_a]a
@HWI-EAS443:3:20:345:1809#0/1
CAAATAGTAGCATTGCCACAACTACTTCAATGCATGTGTG
+HWI-EAS443:3:20:345:1809#0/1
¥bbbbb[Q`bbbbbabbbb^`ab_bbabbaa_bbaa]bSb
@HWI-EAS443:3:20:79:740#0/1
CAAATAGTAGCATTGCCACAACTACTTCAATGCATGTGTG
+HWI-EAS443:3:20:79:740#0/1
abba```]abaaaaaaaaaaaaaaaaaa`_aa``a^a]_
@HWI-EAS443:3:20:987:647#0/1
GCCATGGATGACTTTCAGCTGATCCCAATGGTAAGCAAAT
+HWI-EAS443:3:20:987:647#0/1
aaa`````a_aU`a`_`__Y_]^^^][_]__ZXZUNP^YY
@HWI-EAS443:3:21:1183:1927#0/1
ATTGCCACAACTACTTCAATGCATGTGTGAGGAAGGAGTT
+HWI-EAS443:3:21:1183:1927#0/1
^]TU¥^W__^¥Z`a^]]a]Z_Y]]XX]P¥¥¥[[UZXSOVF
@HWI-EAS443:3:21:1440:1575#0/1
TCCGTCTTCCTTCTTTGGTCCTACATTTGCTTACCATTGG
+HWI-EAS443:3:21:1440:1575#0/1
aaa^U[aaa__a__a`a_^ZY__Q_`^aZR_^aVZ[]^X¥
@HWI-EAS443:3:21:185:1321#0/1
CAAATAGTAGCATTGCCACAACTACTTCAATGCATGTGTG
+HWI-EAS443:3:21:185:1321#0/1
ab_bab[^baaa_a`aabba]aaa^a^a`a^_aa`a]`]_
@HWI-EAS443:3:21:36:675#0/1
GTCCTACATTTGCTTACCATTGGGATCAGCTGAAAGTCAT
+HWI-EAS443:3:21:36:675#0/1
a]`aa`aaaaa`a`[[^``_``^^^VTW`__`__Z^Y__]
@HWI-EAS443:3:21:479:358#0/1
CTTGGGGGGCTATATGAAGCAATCGAGGAGCGCCTGATTA
+HWI-EAS443:3:21:479:358#0/1
^_¥__YOPW_baba^aZ[W^_`]`a_a]_[FZa_`W``[_
```

```
@HWI-EAS443:3:21:522:503#0/1
CATAAATCCCAATTACCTCATGGCTTGGAAGCAGGCGCTA
+HWI-EAS443:3:21:522:503#0/1
^`a`baVV¥T^[^]`aba^`[aaa]_BBBBBBBBBBBBBB
@HWI-EAS443:3:21:62:1559#0/1
GGGGCTATATGACGCAATCGAGGAGTGCCTGATTAATGAT
+HWI-EAS443:3:21:62:1559#0/1
]_TR[_^a`^ZQHT`baabbabSVa[b]F[ba¥QaXS]XV
@HWI-EAS443:3:21:837:1287#0/1
AAATAGTAGCATTGCCACAACTACTTCAATGCATGTGTGA
+HWI-EAS443:3:21:837:1287#0/1
aaaaaa^aaYaaaaa_a```_aa^`^___^_^_^`Z`Z`^
@HWI-EAS443:3:22:1296:1491#0/1
GGAACTATTTTACAGCAGAAGTGTCACACTGCAGGGCTAC
+HWI-EAS443:3:22:1296:1491#0/1
_POWFQVPU[¥]L]X]_a[UU¥^BBBBBBBBBBBBBBBBB
@HWI-EAS443:3:22:1462:1971#0/1
ACTGGTCACCACTGTCCAGGCCATGATTCGGTCTCTTCCT
+HWI-EAS443:3:22:1462:1971#0/1
`a``HVZaa`b`¥PS[H_UL]^¥U`YS¥XGXPWYG]¥]XX
@HWI-EAS443:3:22:1784:148#0/1
CATTGCCACAACTACTTCAATGCATGTGTGAGGAAGGAGT
+HWI-EAS443:3:22:1784:148#0/1
[^abb¥U_^a_I_`Z`_U_a_bMaaa]a[`_aSa``T`Y[
@HWI-EAS443:3:22:276:452#0/1
TGACTTTCAGCTGATCCCAATGGTAAGCAAATGTAGGACC
+HWI-EAS443:3:22:276:452#0/1
abaaaaaaa_aa_`_aaba`_a_Z`a]`____aW_a`_^¥
@HWI-EAS443:3:22:794:1623#0/1
CAAATAGTAGCATTGCCACAACTACTTCAATGCATGTGTG
+HWI-EAS443:3:22:794:1623#0/1
[`Z¥aab]bZbb``^]aabba`aaa`aaa`a]`aa_^_O^
@HWI-EAS443:3:22:880:714#0/1
CAAATAGTAGCATTGCCACAACTACTTCAATGCATGTGTG
+HWI-EAS443:3:22:880:714#0/1
abaa``^a``aabaaY^aaa`abaabaaa`aa]aabWb]a
@HWI-EAS443:3:23:1069:801#0/1
CAAATAGTAGCATTGCCACAACTACTTCAATGCATGTGTG
+HWI-EAS443:3:23:1069:801#0/1
abb`bbaaaaaab`aaababa`aabaaaa`_aa`aaZ`Wb
@HWI-EAS443:3:23:633:196#0/1
ATCGCAGTCCTCAAGAGCATGTCTCCTATTTCTAGAACAC
+HWI-EAS443:3:23:633:196#0/1
ab`SUabR¥V_]a__`aZV^_YSSZXZ]__]Z_V]ZVQWR
@HWI-EAS443:3:23:888:1666#0/1
GGCATGGAAATGAGGCGCTGCCTTCTTCAGTATCTTCAGC
+HWI-EAS443:3:23:888:1666#0/1
```T`a_^ab]aaZNXb^¥Z[`VSR]¥¥_YFH^^N]TTEP
@HWI-EAS443:3:23:996:875#0/1
CAAATAGTAGCATTGCCACAACTACTTCAATGCATGTGTG
```
```

+HWI-EAS443:3:23:996:875#0/1
`bbbabV[aa`W^aaa`aaaYZaa`a`[```Ya^a_XaT¥
@HWI-EAS443:3:24:1294:1214#0/1
AAGAGCATGTCTCCTATTTCTAGAACACAGTATTTTTCCC
+HWI-EAS443:3:24:1294:1214#0/1
]_]baababaX`b`a`aa``a___aaa_aaX^aaa_a[__
@HWI-EAS443:3:24:1342:255#0/1
CTTCCTTCTTTGGTCCTACATTTGCTTACCATTGGGATCA
+HWI-EAS443:3:24:1342:255#0/1
abbbbbbbbabbaabbbbabbbbbabbaaaabbb^a`a_a
@HWI-EAS443:3:24:301:701#0/1
CAAATAGTAGCATTGCCACAACTACTTCAATGCATGTGTG
+HWI-EAS443:3:24:301:701#0/1
abbabbb^bba]aaababababbabb`babbabb`aaa¥a_b
@HWI-EAS443:3:24:679:273#0/1
ATGAAATGGAAATCCGAACACATGAAACAAACTTCCAAAT
+HWI-EAS443:3:24:679:273#0/1
aababaaaaaa`aa¥aaa`a`a_`aa`_aa```^^^____
@HWI-EAS443:3:24:773:1817#0/1
AAATGGGAAAAATACTGTGTTCTAGAAATAGGAGACATGC
+HWI-EAS443:3:24:773:1817#0/1
U]aZ`_]_bbb_¥[]a^^^¥^Yaa^```ab]^Ybabaab`
@HWI-EAS443:3:24:96:943#0/1
CAAATAGTAGCATTGCCACAACTACTTCAATGCATGTGTG
+HWI-EAS443:3:24:96:943#0/1
abbbab_¥aaabaaaaaaaaaabbaaaaaaaaaaaa^a¥a
@HWI-EAS443:3:25:1245:441#0/1
AGGAACTTTGACCGCTGATGGCAAAGAGGCCCATGAGGCA
+HWI-EAS443:3:25:1245:441#0/1
_Z¥VX[_X]a^^_¥_¥T]RUVMZ^XTY¥TQJRTQEX[]SW
@HWI-EAS443:3:25:1282:670#0/1
CGCAGTCCTCAAGAGCATGTCTCCTATTTCTAGAACACAG
+HWI-EAS443:3:25:1282:670#0/1
__]V^¥`_aaa^aabba`^X`aY``¥```^`_a^^[_¥_`
@HWI-EAS443:3:25:164:1111#0/1
CAAATAGTAGCATTGCCACAACTACTTCAATGCATGTGTG
+HWI-EAS443:3:25:164:1111#0/1
abbbaa^]¥`_S_]abba^_a`aaa`aa`Z^aaa``_a¥^
@HWI-EAS443:3:25:186:762#0/1
CAAATAGTAGCATTGCCACAACTACTTCAATGCATGTGTG
+HWI-EAS443:3:25:186:762#0/1
[_Z[[¥`¥V_^Y[XY__``Va^^PaYaa¥¥_¥OQRZ¥R_Z
@HWI-EAS443:3:25:464:490#0/1
CAAATAGTAGCATTGCCACAACTACTTCAATGCATGTGTG
+HWI-EAS443:3:25:464:490#0/1
[`babbbaaabbbbbbbbbbbbbabbabbabbabbbabab
@HWI-EAS443:3:25:482:436#0/1
CAAATAGTAGCATTGCCACAACTACTTCAATGCATGTGTG
+HWI-EAS443:3:25:482:436#0/1
abbbbb_baba_bbbbbbabbbbbbbbabbbaabbb_b_`

```
@HWI-EAS443:3:25:514:1038#0/1
GTCCTACATTTGCTTACCATTGGGATCAGCTGAAAGTCAT
+HWI-EAS443:3:25:514:1038#0/1
aZ[`V¥a__VUY¥_UX^_¥¥¥[PR^U^VUR^V¥ZTUV¥¥W
@HWI-EAS443:3:25:544:355#0/1
CAAATAGTAGCATTGCCACAACTACTTCAATGCATGTGTG
+HWI-EAS443:3:25:544:355#0/1
_```bba^a`a^a`aZ_X^aa¥_a^ab`bbba`_`bbb_`
@HWI-EAS443:3:25:559:775#0/1
CAAATAGTAGCATTGCCACAACTACTTCAATGCATGTGTG
+HWI-EAS443:3:25:559:775#0/1
abb`¥]¥_`b]N]aaaa`bbba¥Z`babbaabaaaaabYa
@HWI-EAS443:3:25:615:1180#0/1
CAAATAGTAGCATTGCCACAACTACTTCAATGCATGTGTG
+HWI-EAS443:3:25:615:1180#0/1
G]baZ``_`bbbabaa`abb``babbbabbbbbbbbabbb
@HWI-EAS443:3:25:724:849#0/1
GGATCTCCTCTTCATTTTCAATGTCCTGTAGCTCTGCTAG
+HWI-EAS443:3:25:724:849#0/1
aaV^Z]HPKXN_^]_¥N^[¥S¥]QSWU[N¥WWYSWWWWVV
@HWI-EAS443:3:25:845:837#0/1
CGAGACTGGAGACACACAAATGGGAAAAATACTGTGTTCT
+HWI-EAS443:3:25:845:837#0/1
aZO_XZab[a]DWa`V¥^`]`]¥UGOST¥aa`[aW¥`UZ_
@HWI-EAS443:3:26:1006:158#0/1
GGGATTTATGCCTTTCTCATGTGGTTTGAGTATGTTAGGC
+HWI-EAS443:3:26:1006:158#0/1
_Z¥UJR[QQXSVHYTU`YT]¥Y[TW[MYOETPRWQQKUBB
@HWI-EAS443:3:26:1234:420#0/1
GCCACACAAATGGGAAAAATACTGTGTTCTAGAAATAGGA
+HWI-EAS443:3:26:1234:420#0/1
_Y_`_aa^¥`¥a``^¥`_[a__``Z_Z¥¥aaa`^^`_a^^
@HWI-EAS443:3:26:1611:1480#0/1
CAAATAGTAGCATTGCCACAACTACTTCAATGCATGTGTG
+HWI-EAS443:3:26:1611:1480#0/1
^¥aa_abJ_ba[bV_a_aaab`abaaa^__``O`aa^bO_
@HWI-EAS443:3:26:242:648#0/1
CGGCTGAATCGAGAAAATTGCTTCTCATTTTTCAGGCACT
+HWI-EAS443:3:26:242:648#0/1
`]M_K[a_``aaUM`]U_¥U`Z__`^_^PHY_ZFRMFOW]
@HWI-EAS443:3:26:322:163#0/1
AAATAGTAGCATTGCCACAACTACTTCAATGCATGTGTGA
+HWI-EAS443:3:26:322:163#0/1
abbbba_baaaaaabaaabbaaaaa`aa_`aa``aZa]a_
@HWI-EAS443:3:26:619:1559#0/1
CACTGGAGAGGAGATGGCCACCAAAGCGCACTACACCCTT
+HWI-EAS443:3:26:619:1559#0/1
ab]X]SNTW¥YPDWZYPM_`_¥[RXBBBBBBBBBBBBBBB
@HWI-EAS443:3:26:670:244#0/1
ATCGCAGTCCTCAAGAGCATGTCTCCTATTTCTAGAACAC
```

```
+HWI-EAS443:3:26:670:244#0/1
a_``___ZY_¥`__^Z`_^_]]]Z]]ZV^^ZZ^^ZZZZZZ
@HWI-EAS443:3:27:1290:921#0/1
GTCCGGGCCATGATTCGGTCTCTTCCTTCAATTATCTCAA
+HWI-EAS443:3:27:1290:921#0/1
a^```^^]^a``^¥`^^^[[^Y[`]]]`^Z___Y^V^X_^
@HWI-EAS443:3:27:1409:1610#0/1
CATTGCCACAACTACTTCAATGCATGTGTGAGGAAGGAGT
+HWI-EAS443:3:27:1409:1610#0/1
aab_``b^`b``bb^aX``_`a]aba]]_aZ^_a_`a_a_
@HWI-EAS443:3:27:1557:711#0/1
ATATAGCCCCCCAAGATCGATGGTTCCAGGTTCCAGGTTG
+HWI-EAS443:3:27:1557:711#0/1
ab`aaa_`aa`[a`]``````a_Z]P[ZV¥V]UYSY_T^]
@HWI-EAS443:3:27:226:560#0/1
CATTGCCACAACTACTTCAATGCATGTGTGAGGAAGGAGT
+HWI-EAS443:3:27:226:560#0/1
a[bbbbb`bbabbbabbbbaaba_ab`babaaa_abaab`
@HWI-EAS443:3:28:1025:998#0/1
CAAATAGTAGCATTGCCACAACTACTTCAATGCATGTGTG
+HWI-EAS443:3:28:1025:998#0/1
abbbbb`¥bbbabbaabbbbbbbbbba`abbaaabbabab
@HWI-EAS443:3:28:1094:1345#0/1
CAGCTTGCTACAGATCTGGGCTCTGGCTCATCACTGTCAT
+HWI-EAS443:3:28:1094:1345#0/1
_S^abbab]ZD^`ZLUaaY`aaaaba]ab]_V`TT`]R[`
@HWI-EAS443:3:28:1113:1049#0/1
GCCACAACTACTTCAATGCATGTGTGAGGAAGGAGTTGAA
+HWI-EAS443:3:28:1113:1049#0/1
a`aaabaaab`a_¥`aaa`_ba¥a^aaa_^aa_]^W_Z¥`
@HWI-EAS443:3:28:1450:688#0/1
CAAATAGTAGCATTGCCACAACTACTTCAATGCATGTGTG
+HWI-EAS443:3:28:1450:688#0/1
abbabbbabaabbbb_bbbbb_babbbabbbb`ababb_b
@HWI-EAS443:3:28:1590:674#0/1
CAAATAGTAGCATTGCCACAACTACTTCAATGCATGTGTG
+HWI-EAS443:3:28:1590:674#0/1
Y`bbbbbba`_aba__bbbbbbbbbbabbbbb`bbbab_b
@HWI-EAS443:3:28:1624:449#0/1
CAAATAGTAGCATTGCCACAACTACTTCAATGCATGTGTG
+HWI-EAS443:3:28:1624:449#0/1
a_babbba`bb`bbaX¥b^bb`bbab_Zaaabaa`b_b_b
@HWI-EAS443:3:28:1744:129#0/1
ATTGCAGCAAACTTGTTAGTTTCGTTTTTCGGATCTTCCC
+HWI-EAS443:3:28:1744:129#0/1
abbbabb^aa`abb]`bUa_baY`G_`aa^ZIFPLZaXGT
@HWI-EAS443:3:28:586:315#0/1
CAAATAGTAGCATTGCCACAACTACTTCAATGCATGTGTG
+HWI-EAS443:3:28:586:315#0/1
abbb`ba_baabaaaaSbaaaaabaa_aa]_a_`aa^a]a
```

```
@HWI-EAS443:3:28:624:1686#0/1
CAAGAGCATGTCTCCTATTTCTAGAACACAGTATTTTTCC
+HWI-EAS443:3:28:624:1686#0/1
a`a_Waba_[_a__a^``]`a_a]^aa[_`XLT]Z^_YWO
@HWI-EAS443:3:28:724:537#0/1
CCCCAAGATCGAAGGTTCCAGGTTCCAGGTTGTTCCTAAG
+HWI-EAS443:3:28:724:537#0/1
abbb_Za]]babbbb^_]`WZ^a`^^ZXa[`a[X_[```¥
@HWI-EAS443:3:28:957:571#0/1
CAAATAGTAGCATTGCCACAACTACTTCAATGCATGTGTG
+HWI-EAS443:3:28:957:571#0/1
`aabbbb¥Zbbaaaaaa]bb`aabaa`bba`bV^_aZaXb
@HWI-EAS443:3:29:1641:1609#0/1
AGAGAACAAGCCAATTGAAGTGGGCACTCGGGGAAAATAT
+HWI-EAS443:3:29:1641:1609#0/1
_`_aNYKabUbSUX^_WRQXMT[OPTPRNMVMWBBBBBBB
@HWI-EAS443:3:29:176:1788#0/1
CCCACTTCAATTGGCTTGTTCTCTTCATTTTCTTTGTCCT
+HWI-EAS443:3:29:176:1788#0/1
a`a¥^_U]_`PYOPT^WDESY¥XZYYVSJOVTW^THNBBB
@HWI-EAS443:3:29:340:1906#0/1
AGCAGGTGCTAGCAGAGCTACAGGACATTGAAAAGGAAGA
+HWI-EAS443:3:29:340:1906#0/1
a^aaaa^ab^`_`aaa_a_aa_[X`aaZ¥Z_^Y¥]W]`^`
@HWI-EAS443:3:29:369:1965#0/1
ATGAAATGGAAATCCGAATACATGAAACAAACCTCCAAAT
+HWI-EAS443:3:29:369:1965#0/1
a__aaa]^[__^]_`]_a_a`a^]`_`aaa_^WKZZ_S^^
@HWI-EAS443:3:29:417:483#0/1
GCTGGAAATAGCCTAACATAGTCAAACCACATGAGAAAGG
+HWI-EAS443:3:29:417:483#0/1
a_ab`abaaa`aaaaa_aa`a^aa_a`]_]a_``^_a`^]
@HWI-EAS443:3:29:442:1694#0/1
GAAAAATACTGTGTTCTAGAAATAGGAGACATGCTCTTGA
+HWI-EAS443:3:29:442:1694#0/1
aa^a`baab`a_aaabbb_a`aaaaWaYaba__b`_`_^`
@HWI-EAS443:3:2:1049:850#0/1
ATTGCCACAACTACTTCAATGCATGTGTGAGGAAGGAGTT
+HWI-EAS443:3:2:1049:850#0/1
TZaba^¥_bbbba]bba`]^aaVbbab``_ba_a]aYa¥]
@HWI-EAS443:3:2:1438:1083#0/1
GTGCAGCCATGGATGACTTTCAGCTGATCCCAATGGTAAG
+HWI-EAS443:3:2:1438:1083#0/1
a¥¥`]_UWY[XLR]LUT_V[PNQSPUWIDHVVTVUWUBBB
@HWI-EAS443:3:2:1579:375#0/1
GCCGAGTCTTCTGACAAAGAGAAAGACATGACCAAGGAAT
+HWI-EAS443:3:2:1579:375#0/1
[`aa^[`Z¥_Vb_FUaaaa_aa_aa]`aX[_TN_]_Z]Y_
@HWI-EAS443:3:2:244:1748#0/1
AGCCGAACGGCTGCATTGAGGGCAAGATTTCCCAAATGTC
```

+HWI-EAS443:3:2:244:1748#0/1
`^TQ_^a^T¥]`]BBBBBBBBBBBBBBBBBBBBBBBBBBB
@HWI-EAS443:3:2:316:2039#0/1
GGGGTTTTCGGCTGAATCGAGAAAATTGCTTGTCATTGTT
+HWI-EAS443:3:2:316:2039#0/1
``baa[a_a]aV^a``a`aba_]¥Ya`aIXPG`Ha¥^a]_
@HWI-EAS443:3:2:631:829#0/1
CAAAGATGTTGGAGACCTTAAACAGTATGACAGAGATGAG
+HWI-EAS443:3:2:631:829#0/1
ab`[ZR[b`bbRZa^_[`_aa^_ba[Taa^[_aP__O[`Y
@HWI-EAS443:3:2:689:1739#0/1
AGAAGCAATTTTCTCGATTCAGCCGAAAACACCTCAAGTT
+HWI-EAS443:3:2:689:1739#0/1
¥TZaRZa`baaYG_V^_a___[VVa^¥__FFG¥S_`__XQ
@HWI-EAS443:3:2:758:1699#0/1
GCCTTTCTCATGTGGTTTGAGTATGTTAGGCTCTTTCCAG
+HWI-EAS443:3:2:758:1699#0/1
aYL¥HZ^N_^SbO`^UXV]YGW^V_OVUV]YXX]BBBBBB
@HWI-EAS443:3:30:1065:753#0/1
AAGAGCATGTCTCCTATTTCTAGAACACAGTATTTTTCCC
+HWI-EAS443:3:30:1065:753#0/1
a`aa`a^a`aaa_aa^a`a_a``S^_aaa`Waaa`_]SSU
@HWI-EAS443:3:30:1328:445#0/1
GCAGGGGCTAGCAGAGCTACAGGACATTGAAAATGAAGAG
+HWI-EAS443:3:30:1328:445#0/1
aaU__O_b`UaaU[XP]_baQaaa_aa^_U]_aa`[[__a
@HWI-EAS443:3:30:212:454#0/1
AGTGGTATTCCCTCCCCCTCGTGACTCGGGCCTTCAATAC
+HWI-EAS443:3:30:212:454#0/1
a^Q^UFOaX[`_`__a_¥¥¥_[¥Y¥TXR]RFU¥STTPRRW
@HWI-EAS443:3:30:338:1898#0/1
CATTGCCACAACTACTTCAATGCATGTGTGAGGAAGGAGT
+HWI-EAS443:3:30:338:1898#0/1
aaa¥T]baa_b[^_`]¥aaaXXa_[XT[Q[aPS[__ZWRU
@HWI-EAS443:3:30:406:1289#0/1
TCGCAGTCCTCAAGAGCATGTCTCCTATTTCTAGAACACA
+HWI-EAS443:3:30:406:1289#0/1
¥``a`Zabb_baba`^_]_a_aaaa^`]_aa_a]U_a__a
@HWI-EAS443:3:30:586:554#0/1
GTTTCCGATTTGTTTTCAAAGAATTCCGTGGTCATGTCTT
+HWI-EAS443:3:30:586:554#0/1
_QY^¥QW[QW]]]]W^_YYW]XZ`]WMDP^[R``Z^VLX[
@HWI-EAS443:3:30:598:1616#0/1
CAAATAGTAGCATTGCCACAACTACTTCAATGCATGTGTG
+HWI-EAS443:3:30:598:1616#0/1
_bbbaba¥b]abaaWS^bab]abbaa^abaabaaa_R_^`
@HWI-EAS443:3:30:744:971#0/1
AGTAGCATTGCCACAACTACTTCAATGCATGTGTGAGGAA
+HWI-EAS443:3:30:744:971#0/1
V¥YV]^XX]`_a¥a`W`^]¥_¥¥^_[S^W[YNaY[¥Y¥Z^

@HWI-EAS443:3:31:1042:511#0/1
CTTCCTTCTTTGGTCCTACATTTGCTTACCATTGGGATCA
+HWI-EAS443:3:31:1042:511#0/1
^_]aaaa`^a_aa`ab`aa`a``aab```aa_]][``^^]
@HWI-EAS443:3:31:1460:541#0/1
GCTCAATGCATCCTGTGCAGCCATGGATGATTTTCAGCTG
+HWI-EAS443:3:31:1460:541#0/1
¥a¥_a]^^¥`_b_bb`¥babYLabb``abWD^_^¥`^¥`]
@HWI-EAS443:3:31:1506:908#0/1
CATTGCCACAACTACTTCAATGCATGTGTGAGGAAGGAGT
+HWI-EAS443:3:31:1506:908#0/1
a]bbbaab_abaaa^baaaaaa_aaaab_bXba`_aa^aZ
@HWI-EAS443:3:31:1752:630#0/1
CTCGATTGCTTCATATAGCCCCCCAAGATCGAAGGATCCA
+HWI-EAS443:3:31:1752:630#0/1
aabZbbbbbbaaaaba]aaa]`aab`bba_aaa_SE[PZN
@HWI-EAS443:3:31:344:1360#0/1
TCGCAGTCCTCAAGAGCATGTCTCCTATTTCTAGAACACA
+HWI-EAS443:3:31:344:1360#0/1
aaab`aW^``ZW^XVW_KRa`a_Za[MS_Y`]]`T]a`_Q
@HWI-EAS443:3:31:502:1591#0/1
CAAATAGTAGCATTGCCACAACTACTTCAATGCATGTGTG
+HWI-EAS443:3:31:502:1591#0/1
abba]_bXb_bbaaQabbba`bbbbabba``aabb_`[T[
@HWI-EAS443:3:32:1003:90#0/1
CAAATAGTAGCATTGCCACAACTACTTCAATGCATGTGTG
+HWI-EAS443:3:32:1003:90#0/1
abbbbbbabbabbbb_aaaba`bb`bbabaaba`bbabab
@HWI-EAS443:3:32:1171:797#0/1
CCGTCTTCCTTCTTTGGTCCTACATTTGCTTACCATTGGG
+HWI-EAS443:3:32:1171:797#0/1
ababaaabbbbbbabbaa`abbbbaabaabbaaababaaa
@HWI-EAS443:3:32:1421:106#0/1
ATGACTTTCAGCTGATCCCAATGGTAAGCAAATGTAGGAC
+HWI-EAS443:3:32:1421:106#0/1
a`bI__b`VY][]]R^XPTSS¥_PUV][UXY]_]^M¥]^¥
@HWI-EAS443:3:32:1649:2034#0/1
AGCATTGCCACAACTACTTCAATGCATGTGTGAGGAAGGA
+HWI-EAS443:3:32:1649:2034#0/1
OMU^]]X]`]FX]¥ba`aaa¥aaaI¥_ZLYF¥Y`YNOR¥^
@HWI-EAS443:3:32:255:1700#0/1
CAAGAGCATGTCTCCTATTTCTAGAACACAGTATTTTTCC
+HWI-EAS443:3:32:255:1700#0/1
aa_R][a^SVYZQ_a¥O¥^__][¥X]``aa[Z`___ZZ_a
@HWI-EAS443:3:32:978:1022#0/1
AGAAGACCCCAAGGACAAAGAACATGAAGAGAACAAGCCA
+HWI-EAS443:3:32:978:1022#0/1
ZbbYZZH_`ZZb_a_PTY[][`a_ZZ_a`a`]YSZ_[Q¥`
@HWI-EAS443:3:33:1027:214#0/1
AAATAGTAGCATTGCCACAACTACTTCAATGCATGTGTGA

```
+HWI-EAS443:3:33:1027:214#0/1
aaaaaaWaa_`_``^¥_]`_]^__``^_^_^MKU^Z`X^Z
@HWI-EAS443:3:33:1139:515#0/1
GGGCATGGAAATGAGGCGCTGCCTTCTTAAGTCTCTTCAG
+HWI-EAS443:3:33:1139:515#0/1
_^a¥^]b^X]Za_W_X`aX¥a[VSBBBBBBBBBBBBBBBB
@HWI-EAS443:3:33:1163:85#0/1
GAAAGGCATAAATCCCAATTACCTCATGGCATGGAAGCAG
+HWI-EAS443:3:33:1163:85#0/1
a_a``aT]`V`aaaY^XNY][[PWX_W_TZXYT^XUWQSX
@HWI-EAS443:3:33:1305:1886#0/1
GTTTTCGGCTGAATCGTGAAAATTGCTTCTCATTGTTCAG
+HWI-EAS443:3:33:1305:1886#0/1
a_`[`^a_``]P^`Y_Z_a`__`^Z^W^]]^[YUPSSNV]
@HWI-EAS443:3:33:1749:809#0/1
CGATTGGCCGTGTTTCCGATTTGTTTTCAAAGAATTCCTT
+HWI-EAS443:3:33:1749:809#0/1
aaZ`VT_[[KY`a]]YY¥`_aa`UYaXOX_Y[W[YJNQ`W
@HWI-EAS443:3:33:455:1101#0/1
CTTGGAAGCAGGTGCTAGCAGGGCTACAGGACATTGAAAA
+HWI-EAS443:3:33:455:1101#0/1
aa_YT`a]a`a`W¥`¥]^aXZ^¥¥¥¥PVaZ^_TW¥RIR__
@HWI-EAS443:3:33:515:61#0/1
CGATTTGTTTTCAAAGAATTCCTTGGTCATGTTTTTCTCT
+HWI-EAS443:3:33:515:61#0/1
_Y```W`_abab^ZZa`aaaaRaaa^R```aVHY_^]_[_
@HWI-EAS443:3:33:756:1844#0/1
ATTCGAGCCGAACGGCTGCATTGAGGGCAAGCTTTTCCAA
+HWI-EAS443:3:33:756:1844#0/1
a^^a^a_aa__]MVY_[]^[U^GVVWR^XWQIIIRROXZ]
@HWI-EAS443:3:33:83:1568#0/1
ATCGCAGTCCTCAAGAGCATGTCTCCTATTTCTAGAACAC
+HWI-EAS443:3:33:83:1568#0/1
_V¥U^YSY``W`[WOW¥aYWVRXT¥¥QOQYW^YZWZ^W^[
@HWI-EAS443:3:33:87:1066#0/1
CAAATAGTAGCATTGCCACAACTACTTCAATGCATGTGTG
+HWI-EAS443:3:33:87:1066#0/1
ab_U`Z]Y`bbba``abb_]V_^ba`ab`V`]X`aa_aY`
@HWI-EAS443:3:33:889:1480#0/1
AATGCATCCTGTGCAGCCATGGATGACTTTCAGCGGATCC
+HWI-EAS443:3:33:889:1480#0/1
a^UJM^UW__Z[HUQTZ[aVZQPRR_P^X[]¥_SGPM__]
@HWI-EAS443:3:34:1161:401#0/1
GGCGTGGTGTCGTCCTCAAGAATGGTTCAATTTTTGCGTT
+HWI-EAS443:3:34:1161:401#0/1
a`a_N_TZ]QS`^R^RZ_a``_`^]¥¥¥V^ZBBBBBBBBB
@HWI-EAS443:3:34:1610:630#0/1
ACAACTACTTCAATGCATGTGTGAGGAAGGAGTTGAACCA
+HWI-EAS443:3:34:1610:630#0/1
aaabaaa]a_Z`baa[aba_`¥a`aaX^`a_^Z^¥^ZVHV
```

@HWI-EAS443:3:34:1681:272#0/1
CTCCTTCCTCACACATGCATTGAAGTAGTTGTGGCAATGC
+HWI-EAS443:3:34:1681:272#0/1
ab`bbbaabbaabbbbb`bbbbaab_ab`ab^aaX]^XPM
@HWI-EAS443:3:34:172:1069#0/1
CTCAAGTTGTGGAGACGCATATAGACTGTTTAATACAGAT
+HWI-EAS443:3:34:172:1069#0/1
aaa__``aa_a_aaa__aa`Y`a_`a`_SYGYYWY^ZLTV
@HWI-EAS443:3:34:225:543#0/1
CAAGAGCATGTCTCCTATTTCTAGAACACAGTATTTTTCC
+HWI-EAS443:3:34:225:543#0/1
aaaa``aa_a^b`aa^Ya_```_^V^_aaaaW__`__]^U
@HWI-EAS443:3:34:256:1483#0/1
CAAATAGTAGCATTGCCACAACTACTTCAATGCATGTGTG
+HWI-EAS443:3:34:256:1483#0/1
ab`bbba_a_baaa[abba_baba`aabaN]aaaaa^¥T[
@HWI-EAS443:3:34:291:174#0/1
TCCGTCTTCCTTCTTTGGTCCTACATTTGCTTACCATTGG
+HWI-EAS443:3:34:291:174#0/1
^`b_NZ_aabaa``a`S__aa`a_a`_`^`a_a^^_^[[Z
@HWI-EAS443:3:34:391:1939#0/1
CGCAGTCCTCAAGAGCATGTCTCCTATTTCTAGAACACAG
+HWI-EAS443:3:34:391:1939#0/1
^^`_YR`a]a]^¥¥_^TYTWaYab`[V¥`b_aX^`aaaaJ
@HWI-EAS443:3:34:47:894#0/1
GAACCGGTTCTCTTTGTAATCATACAAACCAGGAAGAAAT
+HWI-EAS443:3:34:47:894#0/1
T_`````_[[^`bb``¥`aab_[¥_¥V`¥a^_a¥NZaY^[
@HWI-EAS443:3:34:482:1980#0/1
CATAAATCCCAATTACCTCATGGCTTGGAAGCAGGGGCTA
+HWI-EAS443:3:34:482:1980#0/1
``^^RZ^bbbaZY_ab`]`a[ZYaU`HPU^VBBBBBBBBB
@HWI-EAS443:3:34:572:1302#0/1
CATTGCCACAACTACTTCAATGCATGTGTGAGGAAGGAGT
+HWI-EAS443:3:34:572:1302#0/1
_¥^^Y_a^_Y_^Sb¥¥^^_aZT_`_a^]W__a_^W^Z^Z]
@HWI-EAS443:3:34:606:382#0/1
CAAATAGTAGCATTGCCACAACTACTTCAATGCATGTGTG
+HWI-EAS443:3:34:606:382#0/1
¥`_]abbU¥bb]baababa]aa_Z`aba`¥Ia]_abW]_a
@HWI-EAS443:3:34:850:1364#0/1
ATAAGACAAGAAATGGCCAGTAGGAGGCTATGGGATTCCT
+HWI-EAS443:3:34:850:1364#0/1
aaS`_¥`aYRXZS[X`aa¥][Y_SSSG]^``]^ZYBBBBB
@HWI-EAS443:3:35:1160:601#0/1
CCTACATTTGCTTACCATTGGGATCAGCTGAAAGTCATCC
+HWI-EAS443:3:35:1160:601#0/1
a_`^Qaa¥]`Y`_¥U¥Y`[``US^]_`V_aSV]¥SSWVL¥
@HWI-EAS443:3:35:1215:1877#0/1
GAGCATGTCTCCTATTTCTAGAACACAGTATTTTTCCCAT

+HWI-EAS443:3:35:1215:1877#0/1
`b_```aabaabaaa```aaaa`¥_`b_[aabaaaa_aZa
@HWI-EAS443:3:35:1217:1004#0/1
GACCCGAATGCACTATTGAGGCACCGATTTGAGATAATTG
+HWI-EAS443:3:35:1217:1004#0/1
^Ya_WU^M[]Q^TYLXU]UG¥LYYHPRP[Y[HY`_VMTUV
@HWI-EAS443:3:35:1345:426#0/1
CAAATAGTAGCATTGCCACAACTACTTCAATGCATGTGTG
+HWI-EAS443:3:35:1345:426#0/1
Z¥Z_V[_I_`[¥Y¥SPYZ^VZ]a^Z_W]¥YY¥^U`a`^DY
@HWI-EAS443:3:35:174:460#0/1
CACACATTCACATCTTTTCATTCACTGGAGAAGAGATGGC
+HWI-EAS443:3:35:174:460#0/1
ab`_TZZ]^a_ZZa_`¥¥aa]^_Z_^XZI¥X[`VX`YN]_
@HWI-EAS443:3:35:182:348#0/1
CAAATAGTAGCATTGCCACAACTACTTCAATGCATGTGTG
+HWI-EAS443:3:35:182:348#0/1
aa]a^``__bbaaaaaaaaaaaaaaa`a^W^aa]aaZa]`
@HWI-EAS443:3:35:248:475#0/1
GCCACAACTACTTCAATGCATGTGTGAGGAAGGAGTTGAA
+HWI-EAS443:3:35:248:475#0/1
`bb_abbbabbaabaaaaa]ab_b_`K``XP_a_a_```Z
@HWI-EAS443:3:35:292:1837#0/1
TTTCCGTCTTCCTTCTTTGGTCCTACATTTGCTTACCATT
+HWI-EAS443:3:35:292:1837#0/1
aaabb^S`aabbaaaa`_SZXaa^a`a`_^^a_]a`_`^Y
@HWI-EAS443:3:35:38:456#0/1
GCTTCCAAGCCATGAGGCAATTGGGATTTATGCCTTTCTC
+HWI-EAS443:3:35:38:456#0/1
`a^`baM`abbbaa]baGaaaaaa¥a`a_aY^`b_a[a``
@HWI-EAS443:3:35:521:1752#0/1
TCCGTCTTCCTTCTTTGGTCCTACATTTGCTTACCATTGG
+HWI-EAS443:3:35:521:1752#0/1
`aaZM]`abba``V_¥ZSX^b`]aa`]]ab]OZaaa_`¥R
@HWI-EAS443:3:35:688:1954#0/1
CAAATAGTAGCATTGCCACAACTACTTCAATGCATGTGTG
+HWI-EAS443:3:35:688:1954#0/1
abaZabWG[¥b_aa_aa]`^Y_`a``aUa^__a```Y_Y_
@HWI-EAS443:3:36:1111:1264#0/1
GTAGCATTGCCACAACTACTTCAATGCATGTGTGAGGAAG
+HWI-EAS443:3:36:1111:1264#0/1
UZPYa`¥ZV_WY]R_¥aW¥a_`^abb`^[^W]]aZW_]]`
@HWI-EAS443:3:36:1142:1465#0/1
CAAATAGTAGCATTGCCACAACTACTTCAATGCATGTGTG
+HWI-EAS443:3:36:1142:1465#0/1
abbbbbbab]baba__b`bbbaababb`bbaaa_ba`a_a
@HWI-EAS443:3:36:139:34#0/1
CAAATAGTAGCATTGCCACAACTACTTCAATGCATGTGTG
+HWI-EAS443:3:36:139:34#0/1
aba_aabU[baaaaaaa`aa]aa````aaX^aa^_`V`¥_

```
@HWI-EAS443:3:36:1435:270#0/1
AGGAGAAGATGTTGCCCCGATTGAATATATCGCAAGCATG
+HWI-EAS443:3:36:1435:270#0/1
abbXV_bb]]baa¥a`abba]XaaXO¥]aa_aXU]`[_Xa
@HWI-EAS443:3:36:1486:480#0/1
ATCGCAGTCCTCAAGAGCATGTCTCCTATTTCTAGAACAC
+HWI-EAS443:3:36:1486:480#0/1
a¥a``^`[aZ]^^^`^_YY^^]^^ZY¥XZ_[JVU_Y¥TT^
@HWI-EAS443:3:36:1506:495#0/1
CAAATAGTAGCATTGCCACAACTACTTCAATGCATGTGTG
+HWI-EAS443:3:36:1506:495#0/1
abbabbb^baaabbaabbbaaabbabb_baaa]`ba_b^a
@HWI-EAS443:3:36:1506:765#0/1
CCTTCTTTGGTCCTACATTTGCTTACCATTGGGATCAGCT
+HWI-EAS443:3:36:1506:765#0/1
ab]¥_S^Zb`aaaa]_Ua`aaaaTa`aRPYa¥Z^Z[_a¥_
@HWI-EAS443:3:36:497:1039#0/1
CGATTTGAGATAATTGAAGGAAGAGACCCAATCATGGCCT
+HWI-EAS443:3:36:497:1039#0/1
a¥Q]]aba_¥`ababaa`bTYa`aaa`[GV[`_`__Y`_]
@HWI-EAS443:3:37:1210:188#0/1
ACATTTGCTTACCATTGGGATCAGCTGAAAGTCATCCATG
+HWI-EAS443:3:37:1210:188#0/1
aba`aba`aaa__aaaaa`^`_aa`aa^aT^]aa`_¥___
@HWI-EAS443:3:37:1470:1641#0/1
CAAATAGTAGCATTGCCACAACTACTTCAATGCATGTGTG
+HWI-EAS443:3:37:1470:1641#0/1
abbaaaaWaa`_`b`_aaaaabababa`Z^bab`aa^a]`
@HWI-EAS443:3:37:1512:25#0/1
AAATAGTAGCATTGCCACAACTACTTCAATGCATGTGTGA
+HWI-EAS443:3:37:1512:25#0/1
a`a_a`X`baabaa_aa`aaaaa`aa`aSTa_`aa^a^a]
@HWI-EAS443:3:37:1653:1888#0/1
GGGTTTTCGGCTGAATCGCGAAAATTGCTTCTCATTGTTC
+HWI-EAS443:3:37:1653:1888#0/1
aa_U[S[[[____Z^Z`VFY_[SRY_`T]U_¥^WZ_^X^X
@HWI-EAS443:3:37:1740:253#0/1
ATAAATACGGCCTTGCTCAATGCATCCTGTGCCGCCATGG
+HWI-EAS443:3:37:1740:253#0/1
[a^]_b`bbb`WX_ZUVaa]_X]]SU]Q¥RR]HS_R^^[Z
@HWI-EAS443:3:37:303:1090#0/1
AAATAGTAGCATTGCCACAACTACTTCAATGCATGTGTGA
+HWI-EAS443:3:37:303:1090#0/1
a^ababX`aaabaa[aaba`aaa_a`b_H[aaa`a_aZa^
@HWI-EAS443:3:37:408:1804#0/1
CAGTGATGAGCCAGAGCCCAGATCTCTAGAAAGCTGGGTC
+HWI-EAS443:3:37:408:1804#0/1
`¥]JWba`baa^]_a_bb_aaaaaaa_aUFOU¥`_`_UX_
@HWI-EAS443:3:37:504:926#0/1
TGTCGTCCTCAAGAATGGTTCAATTTTGGAGTTCACTTCT
```

```
+HWI-EAS443:3:37:504:926#0/1
`]a_a_Zb^X^`^``VXTVH`]Y^Y^WMXMZa[SQYXTLT
@HWI-EAS443:3:37:586:1318#0/1
GCCACAACTACTTCAATGCATGTGTGAGGAAGGAGTTGAA
+HWI-EAS443:3:37:586:1318#0/1
abbbab_baTa[bbbababbb`_`aa``ZVY¥a_a^aab_
@HWI-EAS443:3:37:604:155#0/1
GCATTGCCACAACTACTTCAATGCATGTGTGAGGAAGGAG
+HWI-EAS443:3:37:604:155#0/1
ab]a^`a`aaaa````a_____a^¥``Y¥TVYYV¥^_X]]
@HWI-EAS443:3:37:694:1324#0/1
ATTGCCACAACTACTTCAATGCATGTGTGAGGAAGGAGTT
+HWI-EAS443:3:37:694:1324#0/1
abb`abb`bb_aaaaaaaa_aaaaa]`]`W`_[a``_a]_
@HWI-EAS443:3:37:694:1628#0/1
GGGGGCTATATGAACCAATCGAGGAGTGCCTGATTAATGA
+HWI-EAS443:3:37:694:1628#0/1
a^XN_b_Y^^^aaRHZb`¥```¥aK]GSbM¥_OT[]_Zaa
@HWI-EAS443:3:37:86:1668#0/1
GGGGCATGGTAATGAGGCGCTGCCTTCTTCAGTCTCTTCA
+HWI-EAS443:3:37:86:1668#0/1
YKY^aba`]_bba``]^aaaaaab^^a`YQJYPbaa`^a^
@HWI-EAS443:3:38:1583:1328#0/1
ATAAATACGGCCTTGCTCAATGCATCCTGTGAAGCCATGG
+HWI-EAS443:3:38:1583:1328#0/1
a_b`a``_baa`_R_ZXS¥a[`a``]SU^MRVGSWWSVGM
@HWI-EAS443:3:38:502:705#0/1
TTTTACAGCAGAAGTCTCCCACTGCAGGGCTACTGAATAC
+HWI-EAS443:3:38:502:705#0/1
_baaY^aa^W]aa`ZN¥`^ZY]Y[`___^MZXU^a_]__]
@HWI-EAS443:3:38:755:1812#0/1
CATTTGAGAAATGACACTGATGTGGTGAACTTTGTAAGTA
+HWI-EAS443:3:38:755:1812#0/1
_^bbb``bbbba`¥Q[`aaa`aZ¥VY``bQW```S^a]V[
@HWI-EAS443:3:38:863:1525#0/1
ATTTTCACCAAGTGCCCACTTCAATTGGCTTGTTCTCTTC
+HWI-EAS443:3:38:863:1525#0/1
__a`aa`aaa`a^`baaaa`a^aa___Va_[OTXJVSOTI
@HWI-EAS443:3:39:1053:233#0/1
GTAGCATTGCCACAACTACTTCAATGCATGTGTGAGGAAG
+HWI-EAS443:3:39:1053:233#0/1
a[``[_`_a][a_a```_Y^]^`^^_UY``Y]W^Z__ZV^
@HWI-EAS443:3:39:1340:335#0/1
ATTTGTGTGGCTCCAGTCTCGGGTCAGTGAGTGAGGACTC
+HWI-EAS443:3:39:1340:335#0/1
abbbb]¥¥baabaa`^S¥VXZ_[BBBBBBBBBBBBBBBBB
@HWI-EAS443:3:39:1727:747#0/1
AATCAGGCACTCCTCGATTGCTTCATATAGCACCCCAAGA
+HWI-EAS443:3:39:1727:747#0/1
aabaaaaa_``a`aaaaZ__¥_`T_`^`a[VFLT^Y_WUW
```

```
@HWI-EAS443:3:39:554:382#0/1
AATACATAATGAAGGGAGTGTACATATATACGGCCTTGCT
+HWI-EAS443:3:39:554:382#0/1
aa`]aa_PU_aZV[a`[]TaWX^_a¥XY]_¥^___¥U^]_
@HWI-EAS443:3:39:791:936#0/1
CACAACTACTTCAATGCATGTGTGAGGAAGGAGTTGAACC
+HWI-EAS443:3:39:791:936#0/1
__a_`_¥a_`]a^]aaaXaaabaa_`ZY`aaX¥^`Y_`[W
@HWI-EAS443:3:3:1638:658#0/1
AATAGTAGCATTGCCACAACTACTTCAATGCATGTGTGAG
+HWI-EAS443:3:3:1638:658#0/1
aaaab_bbaabaa_`a_aaaaa^aa`__``[_aa]aX`]a
@HWI-EAS443:3:3:221:1722#0/1
CATAAATCCCAATTACCTCATGGCTTGGAAGTAGGTGCTA
+HWI-EAS443:3:3:221:1722#0/1
abRO]]a_]X`]¥]US]]Y__¥_]X^¥_BBBBBBBBBBBB
@HWI-EAS443:3:3:279:952#0/1
CATATAGCCCCCCAAGATCGAAGGGTCCAGGTTCCAGGTT
+HWI-EAS443:3:3:279:952#0/1
a_]baaa`a^a_``]_Z__^^]_^GYSVYR[OJNU^BBBB
@HWI-EAS443:3:3:321:1548#0/1
AAATAGTAGCATTGCCACAACTACTTCAATGCATGTGTGA
+HWI-EAS443:3:3:321:1548#0/1
a[Vaaa__a``WWXTYZ`a¥W^``_^^a^[`Z_Y¥[aG_Y
@HWI-EAS443:3:3:516:1044#0/1
ACGGCTGCATTGAGGGCAAGCTTTTCCAAATGTCAAAAGA
+HWI-EAS443:3:3:516:1044#0/1
^YaaRY`U^`]X]a_`VX¥QY^STFVU`_]U¥_^Ya^Y_O
@HWI-EAS443:3:3:679:517#0/1
CATTGCCACAACTACTTCAATGCATGTGTGAGGAAGGAGT
+HWI-EAS443:3:3:679:517#0/1
a``]a]_bab__baaabbbaaaaaab_bXaab[a`aaa`]
@HWI-EAS443:3:3:737:226#0/1
AAATAGTAGCATTGCCACAACTACTTCAATGCATGTGTGA
+HWI-EAS443:3:3:737:226#0/1
a`a_aaWaaa`_^_^`]`a_]a`__`]_¥`X¥^]aU`V`]
@HWI-EAS443:3:3:819:440#0/1
CCTCCGTGTTACTCCAATTTCAATGAACAGGTTCTCTTTG
+HWI-EAS443:3:3:819:440#0/1
abbbbbaaabbaba`babbbaaabbaa[D]b_a`a_aaa`
@HWI-EAS443:3:3:835:1616#0/1
GTTCTCTTCATTTTCTTTGTCCTTGGGATCTTCTCTTCAT
+HWI-EAS443:3:3:835:1616#0/1
]`baaYaabbbbbbababbaa`bba`a_a_aa_aabaaab
@HWI-EAS443:3:3:860:952#0/1
AACCACATGAGAAAGGGATAAATCCCAATTACCTCATGGC
+HWI-EAS443:3:3:860:952#0/1
`Xb`ZV_]Zb`ZbZ`aJ`b¥abb`Qa`baa[Rab¥`¥a¥B
@HWI-EAS443:3:3:961:927#0/1
CATGAAAATGAGGCGCTGCCTTCTTCAGTCTCTTCAGCAG
```

+HWI-EAS443:3:3:961:927#0/1
_Z]_T_T`babab_baa`a_Vaaaa`^a]_aa^_^Y_N^Z
@HWI-EAS443:3:40:1111:419#0/1
CAAATAGTAGCATTGCCACAACTACTTCAATGCATGTGTG
+HWI-EAS443:3:40:1111:419#0/1
abbabbbaabbabbbbbbbbbbaa`a]_S_`bb`ab^b^b
@HWI-EAS443:3:40:1188:411#0/1
GGCTTCTCTACCCCTGTTGTGTTACACATACTGTTCACCA
+HWI-EAS443:3:40:1188:411#0/1
aaXaaaa_a]^][^U_YX¥U[Z`XLUH[WVRU^R_^WU_^
@HWI-EAS443:3:40:1247:667#0/1
TGTGGTTTGACTATGTTAGGCTCTTTCCCGCCAAAGAATG
+HWI-EAS443:3:40:1247:667#0/1
[`abV]b^`¥_U`b_N¥VVZ_`a_aa^YFPY`¥PXUR¥_¥
@HWI-EAS443:3:40:1286:1452#0/1
TATAGCCCCCCAAGATCGAAGGTTCCCGGTTCCAGGTTGT
+HWI-EAS443:3:40:1286:1452#0/1
aaa_Y¥__¥^^`]R]YMVX`VUW[ZIDMXUWZRUPTPU[V
@HWI-EAS443:3:40:1589:117#0/1
AGCTGAAAGTCATCCATGGCTGCACAGGATGCCTTGAGCA
+HWI-EAS443:3:40:1589:117#0/1
aa`_]aXD[¥]_a¥^^aa`^Y_YYTS[[NRVIDVVSMQLO
@HWI-EAS443:3:40:425:1188#0/1
CTTCCTTCTTTGGTCCTACATTTGCTTACCATTGGGATCA
+HWI-EAS443:3:40:425:1188#0/1
abb^bbb`[_ZQTL[ab¥¥a_aa^[TFX[_[Z[OFRTU^Y
@HWI-EAS443:3:40:52:713#0/1
GTTCAATCGGGGCAACATCTTCTCCTCTTTCATCAAGTTC
+HWI-EAS443:3:40:52:713#0/1
aWWXXUSX^¥^VUTY_R[WRVZY][SGVYSTOWYPTOLUT
@HWI-EAS443:3:42:1023:1781#0/1
CATTCTTTGGCTGGAAAGAGCCTGACATAGTCAAACCACA
+HWI-EAS443:3:42:1023:1781#0/1
]VRYPW[S`_¥XJWT``QL[[XLJMN]^^[SV`_`WVXN]
@HWI-EAS443:3:42:1029:1503#0/1
GGGGGAGGGAATACCACTATATGATACAATCAAATGCATG
+HWI-EAS443:3:42:1029:1503#0/1
aaYUaYbVXVVQa^`bS_Vaa_a``¥N]]R¥`__^[`a_S
@HWI-EAS443:3:42:1146:1276#0/1
ATCCTGGGCAGCCATGGATGACTTTCAGCTGATCCCAATG
+HWI-EAS443:3:42:1146:1276#0/1
a_`a^XLS`^a__a¥a]a^]^P[_UYWZLW`[MRUTYLUX
@HWI-EAS443:3:42:1147:912#0/1
CAAATAGTAGCATTGCCACAACTACTTCAATGCATGTGTG
+HWI-EAS443:3:42:1147:912#0/1
abbbbbbabbbbbbbbabbbbbbbbbbabbbb`abbab_b
@HWI-EAS443:3:42:1312:1283#0/1
CAAATAGTAGCATTGCCACAACTACTTCAATGCATGTGTG
+HWI-EAS443:3:42:1312:1283#0/1
a__¥`U¥___¥S__]VZa`_Y]_``aa`_^¥X¥_]aZaM^

@HWI-EAS443:3:42:1472:1771#0/1
CAAATAGTAGCATTGCCACAACTACTTCAATGCATGTGTG
+HWI-EAS443:3:42:1472:1771#0/1
R]WS[Z_aZ_`^bZa¥^abbb_a¥b_^T_abbb^aaa`]]
@HWI-EAS443:3:42:1767:524#0/1
ATACTGTGTTCTAGAAATAGGAGACATGCTCTTGAGGACT
+HWI-EAS443:3:42:1767:524#0/1
[ba]``U`V_]ZbbaUU_¥RUHaa¥aZaaa_aa]Pa[`Y`
@HWI-EAS443:3:42:241:1133#0/1
CATTTGCTTACCATTGGGATCAGCTGAAAGTCATCCATGG
+HWI-EAS443:3:42:241:1133#0/1
`bZK``a[babb]`a_b[_T¥b][^_U_YRZ``aa``bUa
@HWI-EAS443:3:42:343:1674#0/1
GACTTTCAGCTGATCCCAATGGTAAGCAAATGTAGGACCA
+HWI-EAS443:3:42:343:1674#0/1
]aab_`bbbaabbaaaaaaaaa[aa]aaaaaa_a`a```a
@HWI-EAS443:3:42:460:2043#0/1
CAAATAGTAGCATTGCCACAACTACTTCAATGCATGTGTG
+HWI-EAS443:3:42:460:2043#0/1
^bbb`MYPY`a^aQYba¥aaa^_a`¥]a``¥_ZZ^VMVIR
@HWI-EAS443:3:42:701:1941#0/1
AGTAGCATTGCCACAACTACTTCAATGCATGTGTGAGGAA
+HWI-EAS443:3:42:701:1941#0/1
a`Yaaaa_a_a^aaaa_``_`_`a__```_`_[MXa`_a_
@HWI-EAS443:3:42:901:802#0/1
CAAATAGTAGCATTGCCACAACTACTTCAATGCATGTGTG
+HWI-EAS443:3:42:901:802#0/1
`bbabbaZaaa`a`b`aaaaa_ba`baaaa`a_`aa^a^a
@HWI-EAS443:3:43:1035:1670#0/1
CAAATAGTAGCATTGCCACAACTACTTCAATGCATGTGTG
+HWI-EAS443:3:43:1035:1670#0/1
X``^aa`a¥`^¥`T¥¥_Y¥bbKbbbbb`bbbaX¥```^^_
@HWI-EAS443:3:43:1099:339#0/1
CATTGCCACAACTACTTCAATGCATGTGTGAGGAAGGAGT
+HWI-EAS443:3:43:1099:339#0/1
abab]`abbbb`bbabbaaabbaabb_bab]^_``ba`a_
@HWI-EAS443:3:43:1428:281#0/1
CAAATAGTAGCATTGCCACAACTACTTCAATGCATGTGTG
+HWI-EAS443:3:43:1428:281#0/1
abab_^[^bb`M`baba^^X^_bb[bYVSa]`]SR^Za]b
@HWI-EAS443:3:43:1516:1532#0/1
ATAAATCCCAATTACCTCATGGATTGGAAGCAGGTGCTAG
+HWI-EAS443:3:43:1516:1532#0/1
^TZbaaaa`bbb`a_aUaaaP`Iaa`¥R^TKRW^QSV]X_
@HWI-EAS443:3:43:1647:1396#0/1
CGCAGTCCTCAAGAGCATGTCTCCTATTTCTAGAACACAG
+HWI-EAS443:3:43:1647:1396#0/1
abbbb]b`]a`bbabaaaa]`aa`a_bba`b_a]a__`aa
@HWI-EAS443:3:43:223:18#0/1
CATTTGCTTACCATTGGGATCAGCTGAAAGTCATCCATGG

```
+HWI-EAS443:3:43:223:18#0/1
`bbb`babbbbbb_abaaaa`ab[ba`aab_aaaa``aba
@HWI-EAS443:3:43:480:372#0/1
CAAATAGTAGCATTGCCACAACTACTTCAATGCATGTGTG
+HWI-EAS443:3:43:480:372#0/1
abaaaab_ab_]aaaa^]aaa^abaaa_`a_a``aa^bWa
@HWI-EAS443:3:43:516:94#0/1
GCCACAACTACTTCAATGCATGTGTGAGGAAGGAGTTGAA
+HWI-EAS443:3:43:516:94#0/1
`ab_aaaaa`a]a^aa[b__ab`b[bVaR]_ba_aUaa`a
@HWI-EAS443:3:43:709:1798#0/1
GGGGCATGGAAATGAGGCGGTGCCTTCTTCAGTCTCTTCA
+HWI-EAS443:3:43:709:1798#0/1
aa````XZ_[_]_XZ^_aYFO[VSYNDRNUW[NMWSTXNR
@HWI-EAS443:3:44:1459:967#0/1
AAATAGTAGCATTGCCACAACTACTTCAATGCATGTGTGA
+HWI-EAS443:3:44:1459:967#0/1
a_baab_`ba¥aZ¥a[__a]_aa_aaVa_aY¥_`W[a_aY
@HWI-EAS443:3:44:1678:1727#0/1
ACGGCCCTGCTCAATGCATCCTGTGCAGCCATGGATGACT
+HWI-EAS443:3:44:1678:1727#0/1
[SNaGSW]Ya]^UUTMMS_U]_TBBBBBBBBBBBBBBBBB
@HWI-EAS443:3:44:420:1172#0/1
ATTGCCACAACTACTTCAATGCATGTGTGAGGAAGGAGTT
+HWI-EAS443:3:44:420:1172#0/1
abba]Xaa`a`aaaba]`aabaaaa`_^a_`_^__a]aY_
@HWI-EAS443:3:44:607:592#0/1
CAGCCATGGATGACTTTCAGCTGATCCCAATGGTAAGCAA
+HWI-EAS443:3:44:607:592#0/1
[`a¥^_U[aa^a]^Z[OQZ_ZRJWQW]aa¥]]`S]VVSRH
@HWI-EAS443:3:44:644:191#0/1
GTCCTCAAGAGCATGTCTCCTATTTCTAGAACACAGTATT
+HWI-EAS443:3:44:644:191#0/1
a``aYS^M[R^¥XU_QTWZXXYZYUVOSYGVUSUYVMSPQ
@HWI-EAS443:3:44:705:879#0/1
CGTCTCCACAACTTGAGGGGTTTTCGGCTGAACCGAGAAA
+HWI-EAS443:3:44:705:879#0/1
`aa`aaa^`aaaaab_aaaa_aa`_a_aa_`_Z_`^^^__
@HWI-EAS443:3:45:1060:897#0/1
GCCCACTTCAATTGGCTTGTTCTCTTCATGGTCTTTGTCC
+HWI-EAS443:3:45:1060:897#0/1
a^`[^Ya][]][X]_Y_WQVUVUTX[OLXVM[SSXVJMQQ
@HWI-EAS443:3:45:123:321#0/1
GTGCAGCCATGGATGACTTTCAGCTGATCCCAATGGTAAG
+HWI-EAS443:3:45:123:321#0/1
^`a`a`_Xbab[a^a___a[a_a`_U^_]_]_¥`^PU^^]
@HWI-EAS443:3:45:1589:709#0/1
CCGTCTTCCTTCTTTGGTCCTACATTTGCTTACCATTGGG
+HWI-EAS443:3:45:1589:709#0/1
aabaabaaabbab_abb`aaa`ab`aabaaaaaaaaaa_`
```

```
@HWI-EAS443:3:45:1706:1340#0/1
GATTCGAGCCGAACGGCTGCATTTAGGGCAAGCTTTCCCA
+HWI-EAS443:3:45:1706:1340#0/1
a_b`XX`YPXTSWU`YVVSBBBBBBBBBBBBBBBBBBBBB
@HWI-EAS443:3:45:430:864#0/1
TGCCACAACTACTTCAATGCATGTGTGAGGAAGGAGTTGA
+HWI-EAS443:3:45:430:864#0/1
¥]]aabbaab`babaaaaaaabb¥``^^aa_`aa```_`[
@HWI-EAS443:3:46:1351:685#0/1
AGAGAACAAGCCAATTGAAGTGGGCACTCGGGGAAAATAT
+HWI-EAS443:3:46:1351:685#0/1
Z`Q¥¥UaWV_RT_V[aMUOOYa_aWTQJ__VF_P¥SFFYS
@HWI-EAS443:3:46:1602:1861#0/1
CATATAGCCCCCCAAGATCGAAGGGTCCAGGTTCCAGGTT
+HWI-EAS443:3:46:1602:1861#0/1
_abbbba_¥a````^baa¥_]aa[F^[^^^^HZU^_`¥M^
@HWI-EAS443:3:46:1685:762#0/1
CAATTACCTCATGGCTTGGAAGCAGGGGCTAGCAGAGCTA
+HWI-EAS443:3:46:1685:762#0/1
aa`b`aaaaaa`a`aaa¥a[¥_Y^_`GW^YP¥ZQXKVUPT
@HWI-EAS443:3:46:215:227#0/1
ATCCCAATTACCTCATGGCTTGGAAGGAGGTGCTAGCAGA
+HWI-EAS443:3:46:215:227#0/1
a```_bba_a`a^]__aa__`S¥UUYDHUaQ_SQZBBBBB
@HWI-EAS443:3:46:378:524#0/1
GGCTCTATTGGGAAAGTGTGAAGGACCTTACTGGCAAAAT
+HWI-EAS443:3:46:378:524#0/1
abbbaa`aa`b_T]_b¥a``aaaa]aaaaa`_X`^aa_aa
@HWI-EAS443:3:46:713:1907#0/1
CATGAAGACATTCTTTGGCTGGAAAGGGCCTAACATAGTC
+HWI-EAS443:3:46:713:1907#0/1
UU[¥_Yb_`¥_]a¥WHXST_`ZX_]SHS¥[Q¥ONYGX`VF
@HWI-EAS443:3:47:1113:709#0/1
GAAAAATACTGTGTTCTAGAAATAGGAGACATGCTCTTGA
+HWI-EAS443:3:47:1113:709#0/1
aaaaa`aa]aa]a^`^aaa``_````^_^^```^^]^_^]
@HWI-EAS443:3:47:385:471#0/1
CAAATAGTAGCATTGCCACAACTACTTCAATGCATGTGTG
+HWI-EAS443:3:47:385:471#0/1
aaaababba¥^``abba¥_ba]`_[aa]VZ``[`bbab`b
@HWI-EAS443:3:47:583:636#0/1
ATAAATCCCAATTACCTCATGGCTTGGAAGCAGGGGCTAG
+HWI-EAS443:3:47:583:636#0/1
aa^ZY]`_a^Xaa`a]`]`_¥V[^^]_[_[NT¥RFK^OOO
@HWI-EAS443:3:47:672:105#0/1
CGGGTCTTCAATACTTAATTTCAGACCATCCATCAGCAGG
+HWI-EAS443:3:47:672:105#0/1
_bab_I¥``W_TXZ^]bbaabTa^]HTaaYWR]`[^H]a_
@HWI-EAS443:3:47:751:96#0/1
AAATAGTAGCATTGCCACAACTACTTCAATGCATGTGTGA
```

```
+HWI-EAS443:3:47:751:96#0/1
aaabaaaab``bba^_aaaa_aa^a`_`__a]^aa_`^aZ
@HWI-EAS443:3:48:1108:1721#0/1
CCGTCTTCCTTCTTTGGTCCTACATTTGCTTACCATTGGG
+HWI-EAS443:3:48:1108:1721#0/1
abRFXYXZQSS¥]`TaYD¥^UZa`HVQ]``[K_^`VP^__
@HWI-EAS443:3:48:1786:396#0/1
CAAATAGTAGCATTGCCACAACTACTTCAATGCATGTGTG
+HWI-EAS443:3:48:1786:396#0/1
Xa]_bba^`a^`^^a]_[`a[I]ba`ZUa^aba[aaabP`
@HWI-EAS443:3:48:1790:126#0/1
CATTGCCACAACTACTTCAATGCATGTGTGAGGAAGGAGT
+HWI-EAS443:3:48:1790:126#0/1
aabbb_aaabbabb^aaaa`a_a_bb_a^a^a__]aa]aX
@HWI-EAS443:3:48:272:1370#0/1
GCTTGCCCTCAATGCAGCCGTTCGGCTCGGATCCATCTAC
+HWI-EAS443:3:48:272:1370#0/1
_^`_[^WaQZZ[[UVQBBBBBBBBBBBBBBBBBBBBBBBB
@HWI-EAS443:3:48:27:308#0/1
CAAATAGTAGCATTGCCACAACTACTTCAATGCATGTGTG
+HWI-EAS443:3:48:27:308#0/1
_`_abba^U^b]_a^^a`a^VW`aRaba`a`VT`^YZaZ[
@HWI-EAS443:3:48:422:733#0/1
CAAATAGTAGCATTGCCACAACTACTTCAATGCATGTGTG
+HWI-EAS443:3:48:422:733#0/1
R_¥aZbbbX`Zabbab_b_ba_ab__baaaab]aabbba`
@HWI-EAS443:3:48:574:774#0/1
GCCACAACTACTTCAATGCATGTGTGAGGAAGGAGTTGAA
+HWI-EAS443:3:48:574:774#0/1
]¥`a[_NL]`]^`aa``b_SXa^]^a_`]YX^Z^_QQa_]
@HWI-EAS443:3:48:882:379#0/1
CATTAATCAGGCACTCCTCGATTGCTTGATATAGCCCCCC
+HWI-EAS443:3:48:882:379#0/1
U_a[aa`ab`ab``b[¥Wbb`]_bZ[XH]aa^]_V[FXOR
@HWI-EAS443:3:48:94:710#0/1
AAGACATTCTTTGGCCGGAAAGAGCCTAACATAGTCAAAC
+HWI-EAS443:3:48:94:710#0/1
a^¥a^a`baaaa``]`^`X]¥X[]W¥]][W[^¥XR[][^Z
@HWI-EAS443:3:49:1374:124#0/1
CAAATAGTAGCATTGCCACAACTACTTCAATGCATGTGTG
+HWI-EAS443:3:49:1374:124#0/1
^ba`aaa`ba`bbbbaaaaaa^aa^aa]a`aa^_aa_a_b
@HWI-EAS443:3:49:1423:119#0/1
CACACATGCATTGAAGTAGTTGTGGCAATGCTACTATTTG
+HWI-EAS443:3:49:1423:119#0/1
abbb[bbbbbbbabbbabbbbbabb^b_a]P^_aaa`bba
@HWI-EAS443:3:49:1493:444#0/1
CCGGTTCTCTTTGTAGTCATACAAATCAGGAAGAAATTTA
+HWI-EAS443:3:49:1493:444#0/1
a`LVQ_^Z_ZabaUYUUT]`]Z¥Z¥]_``b^]aaa`^aaa
```

```
@HWI-EAS443:3:49:1584:1258#0/1
GCCACAACTACTTCAATGCATGTGTGAGGAAGGAGTTGAA
+HWI-EAS443:3:49:1584:1258#0/1
a_aaaaa¥b`_ba_aaa___^a^aZa^_^^`__^`Z_X_^
@HWI-EAS443:3:49:1646:1396#0/1
TACTGTATACATAATGAAGGGAGTGTACATAAATACGGCC
+HWI-EAS443:3:49:1646:1396#0/1
a``a]VYbaaabaabb_¥^`a`aWbaa_aa_[[a_^¥X¥Z
@HWI-EAS443:3:49:266:352#0/1
AGCCTATGTAGATGGATTCGAGCCGAACGGCTGGATTGAG
+HWI-EAS443:3:49:266:352#0/1
aa]¥baba_[a^``abbbabba^^ba__ab]a[FVU^]YZ
@HWI-EAS443:3:49:385:422#0/1
TTCAATTACCTCAAATCGGTGCTTCAATAGTGCATTCGGG
+HWI-EAS443:3:49:385:422#0/1
ababbbaaaaaaa`b`aaaW`]aa_aaaaa_b`aaa^`[`
@HWI-EAS443:3:49:863:1881#0/1
CTTGGAATCTTCTCTTCATTTTCAATGTCCTGTAGCTCTG
+HWI-EAS443:3:49:863:1881#0/1
U_Vbbab]]abW_b]ab]baa`bbbbbaaY`bX[Z_abSJ
@HWI-EAS443:3:49:91:1637#0/1
ATGGCTTTGAAGCAGGTGCTAGCAGAGCTACAGGACATTG
+HWI-EAS443:3:49:91:1637#0/1
`a``bb^GZ]a__WaYR]Z__ZY`][__aa]aaaNSZa`I
@HWI-EAS443:3:4:1063:88#0/1
TGGCACCAGAAAAAGGAGACTTTGATGACTGCAAAGATGT
+HWI-EAS443:3:4:1063:88#0/1
Za`R`aa^b`a`_¥PFYaVNaaaaOVa_U`^[QWH[Y_]]
@HWI-EAS443:3:4:1114:1809#0/1
CATAAATACGGCCTCGCTCAATGCATCCTGTGCAGCCATG
+HWI-EAS443:3:4:1114:1809#0/1
]Y]`baabbbaaaTD¥QVT_a_b[`aZRW_R_H`HRGaOP
@HWI-EAS443:3:4:1291:1884#0/1
CAAATAGTAGCATTGCCACAACTACTTCAATGCATGTGTG
+HWI-EAS443:3:4:1291:1884#0/1
aa_aba]D^aababa`_]N`_Zab_a_aa_a^X_`_¥a[¥
@HWI-EAS443:3:4:132:1997#0/1
TTGGGGGGCTATATGAAGCAAACGAGGAGTGCCTGATTAA
+HWI-EAS443:3:4:132:1997#0/1
_[a^^XP¥R¥[bVQH][J^UPSaVK[FTPF^_^a_Na`XY
@HWI-EAS443:3:4:1491:197#0/1
CAAATAGTAGCATTGCCACAACTACTTCAATGCATGTGTG
+HWI-EAS443:3:4:1491:197#0/1
abbbaa`aabaaaa`_W`aaa__aXaa_`_`aS_Z]^aZ_
@HWI-EAS443:3:4:1584:1883#0/1
ATCCCAAGGAGAAAGAACATGAAGAGAACAAGCCAATTGA
+HWI-EAS443:3:4:1584:1883#0/1
_a[Q_^[``¥LU_aa`_Z^_¥¥__ZX]_Z`¥RGNZ^_^ZX
@HWI-EAS443:3:4:503:895#0/1
TTTCCGTCTTCCTTCTTTGGTCCTACATTTGCTTACCATT
```

+HWI-EAS443:3:4:503:895#0/1
aabaaaa_aa``aa`a^a^]]]Z]a_`a_a_`___]^`^^
@HWI-EAS443:3:4:874:1507#0/1
GCCACAACTACTTCAATGCATGTGTGAGGAAGGAGTTGAA
+HWI-EAS443:3:4:874:1507#0/1
aU]bLbbaab^aa]aaaa^`^a_a_`^a^^_a_`aX]_`_
@HWI-EAS443:3:50:131:852#0/1
CAAATAGTAGCATTGCCACAACTACTTCAATGCATGTGTG
+HWI-EAS443:3:50:131:852#0/1
`bbbbbbabbaabbbbababbabbabbabbab_abb`b_b
@HWI-EAS443:3:50:1712:1227#0/1
TCCTGCTGATGGATGGTCTGAAATTAAGTATTGAAGACCC
+HWI-EAS443:3:50:1712:1227#0/1
]_T`babba^a`aa]H]a`][`aba_^`[aa_VXaa___X
@HWI-EAS443:3:50:179:163#0/1
GTGCAGCCATGGATGACTTTCAGCTGATCCCAATGGTAAG
+HWI-EAS443:3:50:179:163#0/1
`bbabbabbbabbbbbbbbbabb_bbabbb`bbba``b`b
@HWI-EAS443:3:50:212:843#0/1
TGGCCATGTTTCCGATTTGTTTTCAAAGTATTCCTTGGTC
+HWI-EAS443:3:50:212:843#0/1
aaW`a`baabba`babbaa`ba`aa`aaa_aa`abba_]`
@HWI-EAS443:3:50:212:866#0/1
CAAATAGTAGCATTGCCACAACTACTTCAATGCATGTGTG
+HWI-EAS443:3:50:212:866#0/1
Y_aa_aa^bbbaba]``aaaabbbaabababbbaa`^aab
@HWI-EAS443:3:50:278:1115#0/1
GCATTGCCACAACTACTTCAATGCATGTGTGAGGAAGGAG
+HWI-EAS443:3:50:278:1115#0/1
aaaaa`a_a_`a]abaaa`aaa`__aaXbaa]b^`aa_^`
@HWI-EAS443:3:50:370:350#0/1
CATTGCCACAACTACTTCAATGCATGTGTGAGGAAGGAGT
+HWI-EAS443:3:50:370:350#0/1
`bbb_bbbbbbbbaabb`bb_bbabbbabbabbaabbaa`
@HWI-EAS443:3:50:482:218#0/1
GATGGCCACCAAAGCGGTCTACACCCTTGACGAAGAGAGC
+HWI-EAS443:3:50:482:218#0/1
¥Sbaa``[_`aba^URVT[baZb^b]]`ba[bXab`b`a_
@HWI-EAS443:3:50:558:972#0/1
GTAGCATTGCCACAACTACTTCAATGCATGTGTGAGGAAG
+HWI-EAS443:3:50:558:972#0/1
a_H]]_bbaa`YNURV^a[]ZW_X¥`]Taa`a]¥_a¥O[W
@HWI-EAS443:3:50:612:598#0/1
CATTTGCTTACCATTGGGATCAGCTGAAAGTCATCCATGG
+HWI-EAS443:3:50:612:598#0/1
aaaaa`baa]]b`bb]bYZ``bbSaab`b_a`bbaaa``b
@HWI-EAS443:3:50:652:62#0/1
ACACAAATGGGAAAAATACTGTGTTCTAGAAATAGGAGAC
+HWI-EAS443:3:50:652:62#0/1
a`a`b]babbaVbab¥aaaaa_ba__a]_aaab_`a[aaa

@HWI-EAS443:3:50:736:633#0/1
GTCTCCTATTTCTAGAACACAGTATTTTTCCCATTTGTGT
+HWI-EAS443:3:50:736:633#0/1
aa`aaaa`babababaa^baaabb^abbaaa_`aa`aaa_a^
@HWI-EAS443:3:50:914:1039#0/1
CCGAAAATCGAAACTAACAAGTTTGCTGCAACATGCACAC
+HWI-EAS443:3:50:914:1039#0/1
a`a`ab_^¥V[^aNb__aab[PS`aW]_]a`[T_a_^_¥Q
@HWI-EAS443:3:50:993:205#0/1
GCTCTCTTCGTCAAGGGTGGAGTCCGCTTTGGTGGCCATC
+HWI-EAS443:3:50:993:205#0/1
`aab_Zbbbbaa`a`bb__GW^a_`aQZ``HYS``[^XTY
@HWI-EAS443:3:51:1199:393#0/1
CCGTCTTCCTTCTTTGGTCCTACATTTGCTTACCATTGGG
+HWI-EAS443:3:51:1199:393#0/1
a`aYaa]bUbb][a]_a]_W`aaba_ab^`b`aXab^aaa
@HWI-EAS443:3:51:1227:451#0/1
CAAATAGTAGCATTGCCACAACTACTTCAATGCATGTGTG
+HWI-EAS443:3:51:1227:451#0/1
aaaabbbaba¥bab^Vababaabaaba`baabaaaa^aXb
@HWI-EAS443:3:51:1548:36#0/1
CAAATAGTAGCATTGCCACAACTACTTCAATGCATGTGTG
+HWI-EAS443:3:51:1548:36#0/1
a`]`_¥¥S[M^`bb`_``abaa`a``a`b`b``abb]Y]_
@HWI-EAS443:3:51:366:1207#0/1
CAAGAGCATGTCTCCTATTTCTAGAACACAGTATTTTTCC
+HWI-EAS443:3:51:366:1207#0/1
abababbaaa`aaaaaabaa`aaa`aaa_aa]`a`aa`_Z
@HWI-EAS443:3:51:384:1814#0/1
CACAACTACTTCAATGCATGTGTGAGGAAGGAGTTGAACC
+HWI-EAS443:3:51:384:1814#0/1
`aVb]_a_Ya`_b`_a¥aa`Z`^¥``__^]`a````_aY^
@HWI-EAS443:3:51:42:202#0/1
AGAGCATGTCTCCTATTTCTAGAACACAGTATTTTTCCCA
+HWI-EAS443:3:51:42:202#0/1
XTVa_ZZa]a`PY¥R]baS][a`_X]^^`RV¥]`Y`_FOZ
@HWI-EAS443:3:51:558:774#0/1
AATAGTAGCATTGCCACAACTACTTCAATGCATGTGTGAG
+HWI-EAS443:3:51:558:774#0/1
``a`b`baa`bba^]aabaaaaaaaaaaaa``aaaa`a`a
@HWI-EAS443:3:52:1107:326#0/1
AAATAGTAGCATTGCCACAACTACTTCAATGCATGTGTGA
+HWI-EAS443:3:52:1107:326#0/1
aaabaa_aa`aaa¥`a_aaa^aa_aa^a_`a```a`aY__
@HWI-EAS443:3:52:112:557#0/1
GATTCGAGCCGAACGGCTGCATTGAGGGGAAGCTTTCCCA
+HWI-EAS443:3:52:112:557#0/1
aaaa^`aaaa`VV]^[[`[NRR¥HPIU[DOTOISYUNSUZ
@HWI-EAS443:3:52:1220:42#0/1
CGATTAGCCATGTTTCCGATTTGTTTTCAAAGAATTCCTT

```
+HWI-EAS443:3:52:1220:42#0/1
```b_O¥^^`bbbb`_¥a``b`babbaPZaaba``a¥Tab
@HWI-EAS443:3:52:1249:625#0/1
CCGTCTTCCTTCTTTGGTCCTACATTTGCTTACCATTGGG
+HWI-EAS443:3:52:1249:625#0/1
a`aa_aaaaaa_baaa``_`aa`_aaa]¥aaaa[aaa___
@HWI-EAS443:3:52:1322:1880#0/1
CAAATAGTAGCATTGCCACAACTACTTCAATGCATGTGTG
+HWI-EAS443:3:52:1322:1880#0/1
¥aa¥baXaabb¥aba_Y_Vba_ab`a_W¥baaXabb`bX^
@HWI-EAS443:3:52:1479:772#0/1
TGTCCGGGCCATGATTCGGTCTCTTCCTTCAATTATCTCA
+HWI-EAS443:3:52:1479:772#0/1
_a[`¥a¥N_Ya`a¥`^^^^VU^UZ]TXX^V¥S][Z`[YUT
@HWI-EAS443:3:52:1729:1087#0/1
TTTCCGTCTTCCTTCTTTGGTCCTACATTTGCTTACCATT
+HWI-EAS443:3:52:1729:1087#0/1
ab`^Z^__b`^Zaa^a`aV`Y_]``^`a_`_^a__¥X¥_`
@HWI-EAS443:3:52:340:1386#0/1
CAAATAGTAGCATTGCCACAACTACTTCAATGCATGTGTG
+HWI-EAS443:3:52:340:1386#0/1
abaaaaaRaaaaa`[`a__aa_a`aa__a`aaa_aaY]Xa
@HWI-EAS443:3:52:442:1025#0/1
CAAATAGTAGCATTGCCACAACTACTTCAATGCATGTGTG
+HWI-EAS443:3:52:442:1025#0/1
abaab`aXbaaaaaaa`a`aaaaaaaaaaa`aa`aa]aZa
@HWI-EAS443:3:52:687:1709#0/1
CAAATAGTAGCATTGCCACAACTACTTCAATGCATGTGTG
+HWI-EAS443:3:52:687:1709#0/1
a`_aYK¥^bb_W`]__aaa_^`aaaaa_a`aa^Z``Q¥[[
@HWI-EAS443:3:53:1205:1342#0/1
GTAGCATTGCCACAACTACTTCAATGCATGTGTGAGGAAG
+HWI-EAS443:3:53:1205:1342#0/1
a[[¥VSaaaa¥_WYa_ZaY_aZ¥S_X[_a[U]_`Ta_^T[
@HWI-EAS443:3:53:189:550#0/1
CCATGGATGACTTTCAGCTGATCCCAATGGTAAGCAAATG
+HWI-EAS443:3:53:189:550#0/1
aa_bbbabababbaabbaab_aa`aY`ab^R^`a_``]aa
@HWI-EAS443:3:53:195:772#0/1
CAAATAGTAGCATTGCCACAACTACTTCAATGCATGTGTG
+HWI-EAS443:3:53:195:772#0/1
ababbbbababababbbbaabbaababbb`baab`abbab`b
@HWI-EAS443:3:53:373:946#0/1
GCCACAACTACTTCAATGCATGTGTGAGGAAGGAGTTGAA
+HWI-EAS443:3:53:373:946#0/1
aaa[bbaabbbbaaabab``bb`b_aab^Vaba`a^aa`a
@HWI-EAS443:3:53:784:948#0/1
GAGAAGATGTTGCCCCGATTGAACATATCCCAAGCATGAG
+HWI-EAS443:3:53:784:948#0/1
`abbbb_babbbbb`bbbaababbbbbb_T_`]aaaabaa
```
```

```
@HWI-EAS443:3:54:1418:1346#0/1
ATCGCAGTCCTCAAGAGCATGTCTCCTATTTCTAGAACAC
+HWI-EAS443:3:54:1418:1346#0/1
_YPaaV_a`YZaa]_NNS_UQN`WN]]``aa]a`]Z_W`R
@HWI-EAS443:3:54:1455:665#0/1
TGGGGGGCTATATGAAGCAATCGAGGAGTGCCTTATTAAT
+HWI-EAS443:3:54:1455:665#0/1
a`]¥_XYU_]`Z`[¥]TNR__R]V_ZOSS[VZaUZ`^][¥
@HWI-EAS443:3:54:1716:406#0/1
CAAATAGTAGCATTGCCACAACTACTTCAATGCATGTGTG
+HWI-EAS443:3:54:1716:406#0/1
a`^[`SaaV`aa_OP[__ZZaaa_^`a[b`^[Y]]a[a]`
@HWI-EAS443:3:54:216:897#0/1
TGGTCCTACATTTGCTTACCATTGGGATCAGCTGAAAGTC
+HWI-EAS443:3:54:216:897#0/1
aaa_a`abaaaaaaaa`baa_aabaa```aaaaa`a``^_
@HWI-EAS443:3:54:257:759#0/1
CAAATAGTAGCATTGCCACAACTACTTCAATGCATGTGTG
+HWI-EAS443:3:54:257:759#0/1
abbbbbb]bbbababbabb`aabbaababaaa`aab]b`a
@HWI-EAS443:3:54:367:833#0/1
AAATAGTAGCATTGCCACAACTACTTCAATGCATGTGTGA
+HWI-EAS443:3:54:367:833#0/1
abab^Yab_a_baaaaa`aaaab`aaaaa`b^_aa_a]a_
@HWI-EAS443:3:54:645:335#0/1
TAGCATTGCCACAACTACTTCAATGCATGTGTGAGGAAGG
+HWI-EAS443:3:54:645:335#0/1
aba^]`aaaaa_aa^aaaba^aa_aaX`b]`[b^aa_^aa
@HWI-EAS443:3:55:102:1335#0/1
TGCAGTGGGACACTTCTGCTGTAGAATAGTTCCTCCTCAT
+HWI-EAS443:3:55:102:1335#0/1
a]_aaaabaaabaaaabbabaaabbaaa____a`^a``aa
@HWI-EAS443:3:55:1134:1354#0/1
AAATAGTAGCATTGCCACAACTACTTCAATGCATGTGTGA
+HWI-EAS443:3:55:1134:1354#0/1
aa¥X]_Yba]T^¥[YQJS^WPaa^Ua_^a_aUZY¥[_Y``
@HWI-EAS443:3:55:1229:1984#0/1
CCAGGAAGGTCCTGCACACTTTCCCAATAGAGCCTTCCTC
+HWI-EAS443:3:55:1229:1984#0/1
aSabG`]_a]YTa`K__a¥a`]_^Paaa^``_¥Wa^HU^W
@HWI-EAS443:3:55:1250:199#0/1
GCAGCCGTTCGGCTCGAATCCATCTACATAGTCTCTAAAG
+HWI-EAS443:3:55:1250:199#0/1
abbbabba`a_b`a``U^XJ[]ZS]UR]YU[[Z_V_QU_W
@HWI-EAS443:3:55:1302:694#0/1
GCCACAACTACTTCAATGCATGTGTGAGGAAGGAGTTGAA
+HWI-EAS443:3:55:1302:694#0/1
``]`abaW`_X_`¥_Z_`U¥ba]bY_]`a```a[`U¥Y_^
@HWI-EAS443:3:55:161:1743#0/1
CCGTCTTCCTTCTTTGGTCCTACATTTGCTTACCATTGGG
```

+HWI-EAS443:3:55:161:1743#0/1
aba_aaa`aaa`aaa`_^_`aaaaa``````___``^_Z]
@HWI-EAS443:3:55:271:1815#0/1
GCCAAAATTGAACAATTCTTGAGGACGACACCACGCCCCC
+HWI-EAS443:3:55:271:1815#0/1
]U¥WM]aa¥[a[]I¥a``_`V_RHPZZUTWTP`¥HR_FKK
@HWI-EAS443:3:55:390:979#0/1
GGCTTCTCTACCACTGTTGTGTTACATATACTGTTCACCA
+HWI-EAS443:3:55:390:979#0/1
`]bba`abab`a```a_`ZZ`^^]XOX`````aV¥X^]a[
@HWI-EAS443:3:56:1229:583#0/1
CAAATAGTAGCATTGCCACAACTACTTCAATGCATGTGTG
+HWI-EAS443:3:56:1229:583#0/1
ababbbb_bbaabbaa_aaaaaab`ba`a`a`^aaa[b_a
@HWI-EAS443:3:56:1628:163#0/1
CAAATAGTAGCATTGCCACAACTACTTCAATGCATGTGTG
+HWI-EAS443:3:56:1628:163#0/1
S`bbbbab]``b`ZaXaWZaX``^W`¥Xa`abbaba]b`b
@HWI-EAS443:3:56:200:1389#0/1
ATTTGCTTACCATTGGGATCAGCTGAAAGTCATCCATGGC
+HWI-EAS443:3:56:200:1389#0/1
abbaaaaaa``^aa_]^¥[_`a``_^a`_^aaZY_^[[¥`
@HWI-EAS443:3:56:443:200#0/1
CAAATAGTAGCATTGCCACAACTACTTCAATGCATGTGTG
+HWI-EAS443:3:56:443:200#0/1
ab`abab`abaabbbaaaaabaaaaba_a^ab`_bb]a`b
@HWI-EAS443:3:56:462:178#0/1
CTGGAGAAGTTCGGTGGGAGGCTTTGGTCGGCAAGCTTGC
+HWI-EAS443:3:56:462:178#0/1
aaab`ba_bU]_]WZa__a¥GN_`[LRUQUBBBBBBBBBB
@HWI-EAS443:3:56:553:872#0/1
GTCACGAGGGGGAGGGAATACCTCTATATGATGCAATCAA
+HWI-EAS443:3:56:553:872#0/1
`_`bab¥baaaaNZb[]]^]¥a`W_Y^___^aa``^____
@HWI-EAS443:3:56:984:1594#0/1
CAAATAGTAGCATTGCCACAACTACTTCAATGCATGTGTG
+HWI-EAS443:3:56:984:1594#0/1
abbabb¥abaabbab`ba[[T^bb_baabbbb``baabab
@HWI-EAS443:3:57:1032:649#0/1
CAAATAGTAGCATTGCCACAACTACTTCAATGCATGTGTG
+HWI-EAS443:3:57:1032:649#0/1
^abaa¥¥Paa¥a_bbbVb^bbab^]bbXbabb``ababa`
@HWI-EAS443:3:57:1359:483#0/1
GTCCTACATTTGCTTACCATTGGGATCAGCTGAAAGTCAT
+HWI-EAS443:3:57:1359:483#0/1
a``abbabbbba`bbbaabb`baa`a`a`_bba_`a[Ua`
@HWI-EAS443:3:57:1425:265#0/1
ATCGCAGTCCTCAAGAGCATGTCTCCTATTTCTAGAACAC
+HWI-EAS443:3:57:1425:265#0/1
`_[aZ`a_TU_]_]`^[¥^^]]Z_W]^]___]_]]]YW¥Z

```
@HWI-EAS443:3:57:1443:675#0/1
GGGGGCTATATGGAGCAATCGAGGAGTGCCTGATTAATGA
+HWI-EAS443:3:57:1443:675#0/1
abaaabbbbbbbaaaaaaa_`^a`^a_a_``a_`aa__a`
@HWI-EAS443:3:57:44:489#0/1
CCAATGGTAAGCAAATGTAGGACCAAAGAAGGAAGACGGA
+HWI-EAS443:3:57:44:489#0/1
aba``ba__Saaaa``a^`a``^^_^Ya`^`¥U^_WY_^Y
@HWI-EAS443:3:57:495:1579#0/1
AATGCATCCTGCGCAGCCATGGATGACTTTCAGCTGATCC
+HWI-EAS443:3:57:495:1579#0/1
¥Ua[S^¥aaXVHN_XN_ZPXROT^`aW^^¥`¥RLTPL_BB
@HWI-EAS443:3:57:598:632#0/1
CAAATAGTAGCATTGCCACAACTACTTCAATGCATGTGTG
+HWI-EAS443:3:57:598:632#0/1
`a`aabbbbbb_bbbY_bbaaa`aaaaaaaab```b`aXb
@HWI-EAS443:3:57:933:676#0/1
CAAATAGTAGCATTGCCACAACTACTTCAATGCATGTGTG
+HWI-EAS443:3:57:933:676#0/1
Z``b`Za[`a^Va`a_aaYaba_U]]aXY_aa¥_a[_^`b
@HWI-EAS443:3:58:1085:883#0/1
ATAAATCCCAATTACCTCATGGCTTGAAAGCAGGTGCTAG
+HWI-EAS443:3:58:1085:883#0/1
a```aaaa`aaaaa`_aa``a``a[OVY`ZLV[YNUSYY[
@HWI-EAS443:3:58:1106:393#0/1
CAAATAGTAGCATTGCCACAACTACTTCAATGCATGTGTG
+HWI-EAS443:3:58:1106:393#0/1
aaaaabaaab_aaaaa_a^aa`aa_aaaaaaa__ab^a^a
@HWI-EAS443:3:58:1233:487#0/1
GCCACAACTACTTCAATGCATGTGTGAGGAAGGAGTTGAA
+HWI-EAS443:3:58:1233:487#0/1
aa[X`¥a``aV]aSa__a_[[aV_Ya_a¥RVa_V_Z^^]`
@HWI-EAS443:3:58:1586:184#0/1
GTTCAATCGGGGCAACATCTTCTCCTATTTCATCAGGTTC
+HWI-EAS443:3:58:1586:184#0/1
aT[ZNXZQVX`_SJPXQQPTYOSRILM[XOPBBBBBBBBB
@HWI-EAS443:3:58:1652:582#0/1
TTTCCGTCTTCCTTCTTTGGTCCTACATTTGCTTACCATT
+HWI-EAS443:3:58:1652:582#0/1
a^¥Y_a]T_a__`aNa``R[_OW__^^__aa[__WHLV``
@HWI-EAS443:3:58:1686:938#0/1
TCGCAGTCCTCAAGAGCATGTCTCCTATTTCTAGAACACA
+HWI-EAS443:3:58:1686:938#0/1
`Y¥_[aZP`YXRTYaXLMKZTV¥V¥]___[[`SPX]V¥^]
@HWI-EAS443:3:58:1722:1459#0/1
GTAGCATTGCCACAACTACTTCAATGCATGTGTGAGGAAG
+HWI-EAS443:3:58:1722:1459#0/1
`V¥^[[Y[_Q[OZ_Y]a][`^UQYUaRX[^M[X^V`¥U[`
@HWI-EAS443:3:58:189:1410#0/1
CAGGCCATGATTCCGTCTCTTCCTTCAATTATCTCAAATC
```

```
+HWI-EAS443:3:58:189:1410#0/1
QXbO[U]V_S`baabT_aa`_[a[a¥R_ab_b_a``aa_a
@HWI-EAS443:3:58:209:2002#0/1
AGTCAAACCACATGAGAAAGGCATAAATCCCAAGTACCTC
+HWI-EAS443:3:58:209:2002#0/1
a`Rb¥YY¥SJZ`^GN`]QZLVU_^¥¥¥JVQO[WHLZU[¥W
@HWI-EAS443:3:58:431:151#0/1
CCGTCTTCCTTCTTTGGTCCTACATTTGCTTACCATTGGG
+HWI-EAS443:3:58:431:151#0/1
abbaabbaabbaaabbbaaaaaaabaaaaabaa^^aaa^a
@HWI-EAS443:3:58:520:1503#0/1
CATTTGCTTACCATTGGGATCAGCTGAAAGTCATCCATGG
+HWI-EAS443:3:58:520:1503#0/1
abbbaaaa`a^]^``^R___]`a_aa``a`V`__[Z```^
@HWI-EAS443:3:58:586:1206#0/1
ATGAAATGTAAATCCGAATACATGAAACAAACTTCCAAAT
+HWI-EAS443:3:58:586:1206#0/1
^Ya¥_]a¥IW]aba^]^a^``_[¥^^ZZ_V_[a`][`¥aa
@HWI-EAS443:3:58:755:1490#0/1
CAAATAGTAGCATTGCCACAACTACTTCAATGCATGTGTG
+HWI-EAS443:3:58:755:1490#0/1
Y`aabaa_b_`b_`^`Zab`bbbbbbb_ab`babba`a_b
@HWI-EAS443:3:58:766:1036#0/1
CACAACTACTTCAATGCATGTGTGAGGAAGGAGTTGAACC
+HWI-EAS443:3:58:766:1036#0/1
`U`]Y]^]V_U_[X``][]a[`¥aYb^Y¥]¥Q¥L][¥]ZV
@HWI-EAS443:3:58:976:1021#0/1
TGCCACAACTACTTCAATGCATGTGTGAGGAAGGAGTTGA
+HWI-EAS443:3:58:976:1021#0/1
_`^^¥a`Yab`Y__Z___]T^`aY`V`_a_]]ZYP]X_[Y
@HWI-EAS443:3:59:1040:578#0/1
GTCCTACATTTGCTTACCATTGGGATCAGCTGAAAGTCAT
+HWI-EAS443:3:59:1040:578#0/1
aaZWXSUS]^XRWPUR_Q¥U¥T[aZK[a^X_LL_VVY_GF
@HWI-EAS443:3:59:1141:213#0/1
ATGACTTTCAGCTGATCCCAATGGTAAGCAAATGTAGGAC
+HWI-EAS443:3:59:1141:213#0/1
a`a__aaa_`a`aaa__]_^]a`_Y__a`__V^_YOV^YY
@HWI-EAS443:3:59:1358:1227#0/1
CATTGCCACAACTACTTCAATGCATGTGTGAGGAAGGAGT
+HWI-EAS443:3:59:1358:1227#0/1
[bbbbVbb```aababbbbb`ba[aa`baba__`aX^``^
@HWI-EAS443:3:59:1674:193#0/1
CTACATTTGCTTACCATTGGGATCAGCTGAAAGTCATCCA
+HWI-EAS443:3:59:1674:193#0/1
aabaabbbbbbbbbabbbabbaaaabbabbb_b_a_YaQ[
@HWI-EAS443:3:59:230:1032#0/1
CGGTGGGAGACTTTGGTCGGCAAGCTTGCGCTTAGTTCCT
+HWI-EAS443:3:59:230:1032#0/1
ab`^aaaa¥Q[aaa¥`^^[^aaaaa`a```ZN_]^UY¥T[
```

@HWI-EAS443:3:59:386:584#0/1
CAAATAGTAGCATTGCCACAACTACTTCAATGCATGTGTG
+HWI-EAS443:3:59:386:584#0/1
T¥`ZaQHVa^^¥_TTUZ¥aXa[aZ^aa[`¥YZZb^]F¥[^
@HWI-EAS443:3:59:524:1331#0/1
GCAGTCCTCAAGAGCATGTCTCCTATTTCTAGAACACAGT
+HWI-EAS443:3:59:524:1331#0/1
a]XQO_]a^`_a^]Y^^bV`]``¥V_`aa_]ZXUGTHZPQ
@HWI-EAS443:3:59:525:1604#0/1
GAGAAAGGCATAAATCCCAATTACCTCATGTCTTGGAAGC
+HWI-EAS443:3:59:525:1604#0/1
_QY¥ab`aaa`_baX`V`_¥aaa_Z]_BBBBBBBBBBBBB
@HWI-EAS443:3:59:67:1965#0/1
AAGGCATGTGAATTGACTGATTCAAGCCGGATAGAACTTG
+HWI-EAS443:3:59:67:1965#0/1
aaaa]bab^¥b`]^_aaa__S^a``_``][[X^Z]XS_XZ
@HWI-EAS443:3:59:925:512#0/1
CAAATAGTAGCATTGCCACAACTACTTCAATGCATGTGTG
+HWI-EAS443:3:59:925:512#0/1
abbbba`aababbbaaababaabbbbabb`ba_aa]Xb^_
@HWI-EAS443:3:5:1012:1089#0/1
CAAATAGTAGCATTGCCACAACTACTTCAATGCATGTGTG
+HWI-EAS443:3:5:1012:1089#0/1
aab`a``Ia[aS_][aWOZ_`]```a_^Y^`bI`b]H[M[
@HWI-EAS443:3:5:1059:773#0/1
AAACTTTAGAGCCTATGTAGATGGATTCGAGCAGAACGGC
+HWI-EAS443:3:5:1059:773#0/1
``aaabaababSXaabb[^`_`a__aa``_a¥G`__Ra^B
@HWI-EAS443:3:5:1368:390#0/1
AGAGAGCAGGGCAGGAATCAAAACTAGGCTTTTCACTATA
+HWI-EAS443:3:5:1368:390#0/1
X_`Y^baWXYP^WGX]_[]abPaUa_`^X`a_a_bH`_[B
@HWI-EAS443:3:5:1390:362#0/1
GGGGCATGGAAATGAGGCGCTGCCTTGTTCAGTCTCTTCA
+HWI-EAS443:3:5:1390:362#0/1
a¥TSG¥X]]Y_]_aV]`PYQ][OGVVG[¥PTPNNBBBBBB
@HWI-EAS443:3:5:1411:1668#0/1
GACCCAGCTTGCTAGAGATCTGGGCTCTGGCTGATCACTG
+HWI-EAS443:3:5:1411:1668#0/1
aa[_HQbU`¥[Va`Yab``bUWY]TS_^``QZFZ^F]^BB
@HWI-EAS443:3:5:1420:1264#0/1
GGTTCTCACATATAGGAACATGGGCGTCGACACTTGGCCT
+HWI-EAS443:3:5:1420:1264#0/1
ab`bbba`]bbabbabab]bba`¥`U^Saa^a]`_```_`
@HWI-EAS443:3:5:1434:376#0/1
GTCTTATAGTGAAAAGCCTAGTTTTGATTCTTCCCCTGCT
+HWI-EAS443:3:5:1434:376#0/1
aTYRYNNUWOYNU¥W_Z¥]O_^WZYXWXYZBBBBBBBBBB
@HWI-EAS443:3:5:1521:1759#0/1
ACCCCGGTCGTCGATGAAATGGAAATCCGAATACATGAAA

```
+HWI-EAS443:3:5:1521:1759#0/1
^W¥abZDYL_]W`^`a_UM[]a^YS]UH____XW^]`__B
@HWI-EAS443:3:5:328:1757#0/1
CCGTCTTCCTTCTTTGGTCCTACATTTGCTTACCATTGGG
+HWI-EAS443:3:5:328:1757#0/1
[`aab`MaTa_bb`V``[Y`__Vbbba]Zb[bU___a¥TJ
@HWI-EAS443:3:5:958:719#0/1
CGGGGCACCAGATTCTACAATTATTGATTCACCCCGTTCG
+HWI-EAS443:3:5:958:719#0/1
aa¥_HO[b__aa[^_]]QU_`b`baaaa¥T_SLJT¥UaZT
@HWI-EAS443:3:5:993:577#0/1
GGCCAGTATGAGTCTATGGGATTCCTTTCGTCAGTCCGAA
+HWI-EAS443:3:5:993:577#0/1
ab^]¥baabbab_^abbbbbaab_a`baYba_aa]¥^a`_
@HWI-EAS443:3:60:1360:1821#0/1
ATCGCAGTCCTCAAGAGCATGTCTCCTATTTCTAGAACAC
+HWI-EAS443:3:60:1360:1821#0/1
a`¥`_a`]_^a_```_`_`_[_^__Z^^X`]^`Z_^ZT^Z
@HWI-EAS443:3:60:294:1498#0/1
TAGCATTGCCACAACTACTTCAATGCATGTGTGAGGAAGG
+HWI-EAS443:3:60:294:1498#0/1
abbbabb_abaaba``baa`aa`a`aa`a__Wa`a^^`S_
@HWI-EAS443:3:60:34:1078#0/1
CAAATAGTAGCATTGCCACAACTACTTCAATGCATGTGTG
+HWI-EAS443:3:60:34:1078#0/1
aba_abaXbbaaababaaaaW`aa_a`aaa^^_^_aUa^_
@HWI-EAS443:3:60:928:1994#0/1
AGAAATTTAGGCTTCTCTACCCCTGTTGTGTTATATATAC
+HWI-EAS443:3:60:928:1994#0/1
a[X`Z^_Z``_UY]PaZW_^VXXRZZV`XWM`MER^_^_V
@HWI-EAS443:3:61:1035:1060#0/1
CAAATAGTAGCATTGCCACAACTACTTCAATGCATGTGTG
+HWI-EAS443:3:61:1035:1060#0/1
ab`^baa^ababbbbbabbbba`baa_baaa]_aa^`_a
@HWI-EAS443:3:61:1496:679#0/1
AAGAATTCCTTGGTCACGTCTTTCTCTTTGACAGAAGACT
+HWI-EAS443:3:61:1496:679#0/1
abbaabb_aabb`[`abbaabbbaabaaaa`a¥___V[T[
@HWI-EAS443:3:61:1530:321#0/1
GACATTCTTTGGCTGGAAAGAGCCTAACATACTCAAACCA
+HWI-EAS443:3:61:1530:321#0/1
a`_aaaa`a`^a_aaa^a_`U^`a_``]_`]YLH]]XWKO
@HWI-EAS443:3:61:244:296#0/1
TGGCTCCAGTCTCGGGTCAGTGAGTGAGAAATCCATACTT
+HWI-EAS443:3:61:244:296#0/1
abaa`¥`X`Y`_^__¥RVSQXVNXKVNVVZFRNTRTTLQO
@HWI-EAS443:3:61:526:820#0/1
TACATAAATACGGCCTTGCTCAATGCATCATGTGCAGCCA
+HWI-EAS443:3:61:526:820#0/1
ab`abb`__ba`a_``aaa^`aa]^aaa^G``]_]]^[VY
```

```
@HWI-EAS443:3:61:745:1820#0/1
GTTCTCTTCATGTTCTTTGTCCTTGGAATCTTCTCTTCAT
+HWI-EAS443:3:61:745:1820#0/1
aSZ^WWO[ZS¥DXR_U_¥^O_^X^BBBBBBBBBBBBBBBB
@HWI-EAS443:3:61:901:1472#0/1
CAAATAGTAGCATTGCCACAACTACTTCAATGCATGTGTG
+HWI-EAS443:3:61:901:1472#0/1
a`bbbbaababababbabbbba¥abbbabbabba[aab_a^a
@HWI-EAS443:3:61:90:966#0/1
GTAGCATTGCCACAACTACTTCAATGCATGTGTGAGGAAG
+HWI-EAS443:3:61:90:966#0/1
^LUaVV]YG¥¥O^_a]_X^_]¥PQO[_XPVT¥OWOT¥TWP
@HWI-EAS443:3:62:1008:1156#0/1
GAAAACTTCAGAGCCTATGTAGATGGATTCGAGCCGAACG
+HWI-EAS443:3:62:1008:1156#0/1
¥_`W_Y`^b^^Y`a¥a]aZV_b`_ZZ]``aaa]`^a^aaa
@HWI-EAS443:3:62:1168:125#0/1
CAAATAGTAGCATTGCCACAACTACTTCAATGCATGTGTG
+HWI-EAS443:3:62:1168:125#0/1
`a_abba]bbaabbbb`aaababba`a_aZbba]aa_b]a
@HWI-EAS443:3:62:1185:1556#0/1
AACTTGAGGGGTTTTCGGCTGAATGGAGAAAATTGCTTCT
+HWI-EAS443:3:62:1185:1556#0/1
^aZa¥_bb^^_Vaba__¥W`bbb[FV_`_a`[ba_P_a``
@HWI-EAS443:3:62:172:302#0/1
GCCCCATTTCATCTTGATCTTGGAGGTTCCATTGTTTCTC
+HWI-EAS443:3:62:172:302#0/1
a__Y_¥YaPT_UG^¥a]XU[PZLFQ^UBBBBBBBBBBBBB
@HWI-EAS443:3:62:195:780#0/1
TATTTTTCCCATTTGTGTGGCTCCAGTCCCGGGTCAGTGA
+HWI-EAS443:3:62:195:780#0/1
aab`aa_ba^[¥b`^`[^aaa`b`[YY^BBBBBBBBBBBB
@HWI-EAS443:3:62:27:1109#0/1
CGCAGTCCTCAAGAGCATGTCTCCTATTTCTAGAACACAG
+HWI-EAS443:3:62:27:1109#0/1
aaab_`aa_aaa]P^aa__`a``a`^___a`_Y]a^_a¥_
@HWI-EAS443:3:62:34:695#0/1
CAAATAGTAGCATTGCCACAACTACTTCAATGCATGTGTG
+HWI-EAS443:3:62:34:695#0/1
aaaabab¥bbbaaaaaaaaaaaaaaaaaa__`^^¥XaZ`
@HWI-EAS443:3:62:553:20#0/1
GGGCATGGAAATGAGGCACTGCCTTCTTCAGTCTCTTCAG
+HWI-EAS443:3:62:553:20#0/1
abaaaaaaT]aaa_`a_`^_`¥`^^`_``Za¥¥^¥_¥Y¥V
@HWI-EAS443:3:63:1346:978#0/1
GCATTGCCACAACTACTTCAATGCATGTGTGAGGAAGGAG
+HWI-EAS443:3:63:1346:978#0/1
a]S_`Wa_^_a_¥Xa___a]]``a]Y]Y_YWY__X]¥NQ]
@HWI-EAS443:3:63:1362:535#0/1
TGGGGCACGGAAATGAGGCGCTGCCTTCTTCAGTCTCTTC
```

```
+HWI-EAS443:3:63:1362:535#0/1
aaaaaaa_a`_aa`aYZ`_a¥`a__a_¥__^¥`YW^]^BB
@HWI-EAS443:3:63:864:1333#0/1
AAATAGTAGCATTGCCACAACTACTTCAATGCATGTGTGA
+HWI-EAS443:3:63:864:1333#0/1
a`[a`]Ya`^]^_^a`abaa__aa¥^aa_¥^[___[`[¥^
@HWI-EAS443:3:64:1107:448#0/1
AGCATTGCCACAACTACTTCAATGCATGTGTGAGGAAGGA
+HWI-EAS443:3:64:1107:448#0/1
`a`Yaaaaaa`_a`_`_`_]a^`_^_`a^_Z____ZZ^^^
@HWI-EAS443:3:64:1482:591#0/1
ATCGCAGTCCTCAAGAGCATGTCTCCTATTTCTAGAACAC
+HWI-EAS443:3:64:1482:591#0/1
]Vaaa]b[baVZ]aaRa]U]aYX_XVZRYZ`]aZ_T^Y^U
@HWI-EAS443:3:64:154:1265#0/1
GTCGTCCTCAAGAATAGTTCAATTTTGGCGTTCACTTCTT
+HWI-EAS443:3:64:154:1265#0/1
¥J_``b_X_^¥_ab_S_X`aa^a``a]V]_Za`X]^^[_a
@HWI-EAS443:3:64:16:811#0/1
CTTCCTTCTTTGGTCCTACATTTGCTTACCATTGGGATCA
+HWI-EAS443:3:64:16:811#0/1
a¥^`ZXX¥a`]S][OSLW_____`_VU^Z]BBBBBBBBBB
@HWI-EAS443:3:64:373:1492#0/1
ATCGCAGTCCTCAAGAGCATGTCTCCTATTTCTAGAACAC
+HWI-EAS443:3:64:373:1492#0/1
a^¥XZ_`^aa__]YIRT]`VSYa^Y]][¥WX_]ZX]_WY^
@HWI-EAS443:3:64:387:963#0/1
CTTTGGTCCTACATTTGCTTACCATTGGGATCAGCTGAAA
+HWI-EAS443:3:64:387:963#0/1
`Z_``^^bbaa`]^a^[``¥aaa`]a`¥`a`aa]a__^]Y
@HWI-EAS443:3:64:448:1253#0/1
CAAATAGTAGCATTGCCACAACTACTTCAATGCATGTGTG
+HWI-EAS443:3:64:448:1253#0/1
Z_`Z_Y_LYa^baSX[`]YX`ZVa¥]]ZaSUW_M_]X¥Xa
@HWI-EAS443:3:64:567:766#0/1
GACCCAGCTTGCTAGAGATCTGGGCTATGGCTCATCACTG
+HWI-EAS443:3:64:567:766#0/1
]T[_UW¥]__Z_RZS]¥VOVLUVTXX`Q¥¥YR]VL[¥BBB
@HWI-EAS443:3:64:613:883#0/1
CATAAATCCCAATTACCTCATGGCTTGGACGCAGGTGCTA
+HWI-EAS443:3:64:613:883#0/1
KU_WaYaba^Va``a`U`^T[X]^aBBBBBBBBBBBBBBB
@HWI-EAS443:3:64:68:1276#0/1
TACATTTGCTTACCATTGGGATCAGCTGAAAGTCATCCAT
+HWI-EAS443:3:64:68:1276#0/1
aaababaabba``aaaa`a`aa_aaaaaa_P]^aa_aV¥^
@HWI-EAS443:3:64:778:1655#0/1
CCTTCTTTGGTCCTACATTTGCTTACCATTGGGATCAGCT
+HWI-EAS443:3:64:778:1655#0/1
Z_ZZWRRO_W]_^TWGW]^XMOU^MYOTM¥FRPLVU^_YL
```

```
@HWI-EAS443:3:65:1085:1010#0/1
AATAGTAGCATTGCCACAACTACTTCAATGCATGTGTGAG
+HWI-EAS443:3:65:1085:1010#0/1
aabba_abaaaaaa`_a`aa`aaaaaaaa_`_`a_a^a__
@HWI-EAS443:3:65:110:238#0/1
CAAATAGTAGCATTGCCACAACTACTTCAATGCATGTGTG
+HWI-EAS443:3:65:110:238#0/1
abaaa``Xaabaa`aaa``^^`_a``_`a___`[]¥VaY_
@HWI-EAS443:3:65:1257:1208#0/1
ATTCAGTAGCCCTGCAGTGGGACACTTCTGCTATAAAATA
+HWI-EAS443:3:65:1257:1208#0/1
ab]Z^]__V__bS[aa^```]`]]H]_`[[a``_`abZ^[
@HWI-EAS443:3:65:1432:504#0/1
TTCCTTCTTTGGTCCTACATTTGCTTACCATTGGGATCAG
+HWI-EAS443:3:65:1432:504#0/1
aaaabaaba^X`_Y_a`¥aa`aaM_``[_]a`_Z[W_Z_]
@HWI-EAS443:3:65:1617:952#0/1
CTTTGGTCCTACATTTGCTTACCATTGGGATCAGCTGAAA
+HWI-EAS443:3:65:1617:952#0/1
_bb_baabbb`bbbbbbabbbbbbbbbbabb_ba`a`bbb
@HWI-EAS443:3:65:753:2027#0/1
CAAATAGTAGCATTGCCACAACTACTTCAATGCATGTGTG
+HWI-EAS443:3:65:753:2027#0/1
a^^`a`[GY^a`a]__^``ab`a^[aaaaaa`aZ]Z[_X`
@HWI-EAS443:3:65:79:655#0/1
TCGCCTCTTTCGGGCTGACGAAAGGAATCCCATAGACTCC
+HWI-EAS443:3:65:79:655#0/1
aaabaabaaXaa`aa^aaaa`^^```a__¥a^^US_^¥_¥
@HWI-EAS443:3:66:1016:420#0/1
CAAATAGTAGCATTGCCACAACTACTTCAATGCATGTGTG
+HWI-EAS443:3:66:1016:420#0/1
aba`aab_aa^]b^]aaa`aaa_aaa`aaaa`YaaaX^_a
@HWI-EAS443:3:66:128:2028#0/1
CAAATAGTAGCATTGCCACAACTACTTCAATGCATGTGTG
+HWI-EAS443:3:66:128:2028#0/1
aba_`^WUa[aa__^a``aa`__a_]_`]TY]`^[[U¥X]
@HWI-EAS443:3:66:134:1615#0/1
GGGGCAATATGAAGCAATCGAGGAGTGCCTGATTAATGAT
+HWI-EAS443:3:66:134:1615#0/1
`b`^a`^^ab`]X^`]aaa¥¥`aa^Vaaa``¥]ZR_[[`^
@HWI-EAS443:3:66:1443:1558#0/1
GTAGCATTGCCACAACTACTTCAATGCATGTGTGAGGAAG
+HWI-EAS443:3:66:1443:1558#0/1
R_[`W^b¥b`ba¥abaa¥`bb`a^R`aabb^aababbab^
@HWI-EAS443:3:66:1476:75#0/1
GTCCTACATTTGCTTACCATTGGGATCAGCTGAAAGTCAT
+HWI-EAS443:3:66:1476:75#0/1
aSb_R]H¥bba^_¥X¥Qaa¥_aaa]___aQ`_^Z_^PZVZ
@HWI-EAS443:3:66:1540:1454#0/1
CTCAAGAGCATGTCTCCTATTTCTAGAACACAGTATTTTT
```

```
+HWI-EAS443:3:66:1540:1454#0/1
ab^[`^^`bba`^Uaa^^aaaa`aaaaa_a`bb]_a_aa¥
@HWI-EAS443:3:66:236:1421#0/1
AGCATGTCTCCTATTTCTAGAACACAGTATTTTTCCCATT
+HWI-EAS443:3:66:236:1421#0/1
aaaa_`_aaa`a]`a`a_a__a``aa_VR]`_____`^`^
@HWI-EAS443:3:66:539:1001#0/1
CAAATAGTAGCATTGCCACAACTACTTCAATGCATGTGTG
+HWI-EAS443:3:66:539:1001#0/1
abaaba]Ua_^a`ZY^aa`a`^ab``_aY]^^^^`aXaY_
@HWI-EAS443:3:66:550:1981#0/1
CCACAACTTGAGGGGCTTTCGGCTGAATCGAGAAAATTGC
+HWI-EAS443:3:66:550:1981#0/1
¥Z_ROXU^`bb`UUUDYaa`TL_^Z^¥]¥]a_]__aX_L^
@HWI-EAS443:3:66:560:116#0/1
CAAATAGTAGCATTGCCACAACTACTTCAATGCATGTGTG
+HWI-EAS443:3:66:560:116#0/1
aba`aa`]bbaaa_`a_`a``_aa_aa_`_`a__aaX`]`
@HWI-EAS443:3:66:709:2013#0/1
CATTAATCCGGCACTCCTCGATTGCTTCATATAGCCCCCC
+HWI-EAS443:3:66:709:2013#0/1
a_UW]X^SIS[ba]V^[Oa¥KMK`VYR__X[XXW[NGQVL
@HWI-EAS443:3:66:768:496#0/1
TCCGTCTTCCTTCTTTGGTCCTACATTTGCTTACCATTGG
+HWI-EAS443:3:66:768:496#0/1
aaba_aaaa`aaaaaaa_Zaa[[[[a`PRHZUU^XU`]]]
@HWI-EAS443:3:67:1111:1207#0/1
CAAATAGTAGCATTGCCACAACTACTTCAATGCATGTGTG
+HWI-EAS443:3:67:1111:1207#0/1
¥`b``__]bbaa_a^abb^abaabaaa_aaa`]aaaWaYa
@HWI-EAS443:3:67:1124:1963#0/1
TTCCGTCTTCCTTCTTTGGTCCTACATTTGCTTACCATTG
+HWI-EAS443:3:67:1124:1963#0/1
]`aba_b`b¥¥__a_`a`WF]aaaaa]]`aa[a_a_aa_a
@HWI-EAS443:3:67:1152:1810#0/1
CACAAATGGGAAAAATACTGTGTTCTAGAAATAGGAGACA
+HWI-EAS443:3:67:1152:1810#0/1
a`a]_b```bab^abbba_`[baaabaab`Zaaa`aaa^`
@HWI-EAS443:3:67:1186:1874#0/1
AGCATTGCCACAACTACTTCAATGCATGTGTGAGGAAGGA
+HWI-EAS443:3:67:1186:1874#0/1
a``a``_aaa_aa^`aa``_a`a^]_``]a]]__^^`_Z^
@HWI-EAS443:3:67:1388:203#0/1
ATTGCCACAACTACTTCAATGCATGTGTGAGGAAGGAGTT
+HWI-EAS443:3:67:1388:203#0/1
a`aabbbaabaabaabbbb]_Vabb]b`aaba`ab`[b_`
@HWI-EAS443:3:67:1785:592#0/1
GCCATGGATGACTTTCAGCTGATCCCAATGGTAAGCAAAT
+HWI-EAS443:3:67:1785:592#0/1
aa__a`b_aa^`a`¥[aa``a_`[Z¥`_`_`Y_[^YX__Y
```

```
@HWI-EAS443:3:67:265:895#0/1
CTTCCTTCTTTGGTCCTACATTTGCTTACCATTGGGATCA
+HWI-EAS443:3:67:265:895#0/1
`bb¥a`aa_a^aaRX`_`ba__^^a``][__aaa]``U^_
@HWI-EAS443:3:67:322:1289#0/1
GTAGCATTGCCACAACTACTTCAATGCATGTGTGAGGAAG
+HWI-EAS443:3:67:322:1289#0/1
_`bb`ba``_abb`aabbbbabbaaaaaba`a_a^`]``Z
@HWI-EAS443:3:67:408:873#0/1
CAAATAGTAGCATTGCCACAACTACTTCAATGCATGTGTG
+HWI-EAS443:3:67:408:873#0/1
abba_aa_aaabaaabaaaaaaabaaaaa_aa``aa^a]a
@HWI-EAS443:3:67:55:608#0/1
CAAATAGTAGCATTGCCACAACTACTTCAATGCATGTGTG
+HWI-EAS443:3:67:55:608#0/1
abbbaZT__aaaaaaaaa`aa_aba``WP¥aa^a_a]aZ`
@HWI-EAS443:3:67:909:734#0/1
ATGGCTTGGAAGCAGGTGCTAGCAGAGCTACAGGAAATTG
+HWI-EAS443:3:67:909:734#0/1
V___XQ[[]WZWGQWWT][RVYXPV_]][ZTS¥ZYFPUUZ
@HWI-EAS443:3:67:921:559#0/1
ATCCCAATGGTAAGCAAATGTAGGACCAAAGAAGGAAGAC
+HWI-EAS443:3:67:921:559#0/1
a]F¥V`aXa¥PWW]_STaa[L`VSST][]Z`VVZ¥WV¥P¥
@HWI-EAS443:3:68:1037:500#0/1
GGATGACTTTCAGCTGATCCCAATGGTAAGCAAATGTAGG
+HWI-EAS443:3:68:1037:500#0/1
^aaba]ab`aa``^aaaaaa``ababZaaa]`X`_¥Zaa_
@HWI-EAS443:3:68:1147:91#0/1
CATTGCCACAACTACTTCAATGCATGTGTGAGGAAGGAGT
+HWI-EAS443:3:68:1147:91#0/1
`abbaaaaabb`bbabaaaabb_`ab`a_a_a`H^a`_a^
@HWI-EAS443:3:68:20:1069#0/1
ATGACTTTCAGCTGATCCCAATGGTAAGCAAATGTAGGAC
+HWI-EAS443:3:68:20:1069#0/1
a_ZH¥`a``]^^[¥`_a¥^[X^`XQT___YXXW]NU[V[]
@HWI-EAS443:3:68:371:144#0/1
GTAGCATTGCCACAACTACTTCAATGCATGTGTGAGGAAG
+HWI-EAS443:3:68:371:144#0/1
`__a][_]a_a]aaa``a___]a_]`_]]aV`Z_]a_^¥_
@HWI-EAS443:3:68:877:526#0/1
CAAATAGTAGCATTGCCACAACTACTTCAATGCATGTGTG
+HWI-EAS443:3:68:877:526#0/1
`b_abba^baaabbbabbaabaab_aaaaaaaaaba_a_`
@HWI-EAS443:3:68:901:1424#0/1
CAAATAGTAGCATTGCCACAACTACTTCAATGCATGTGTG
+HWI-EAS443:3:68:901:1424#0/1
abbbb`_U`ab`^^^baZabaaabaaaa_aaaaaaa_b_a
@HWI-EAS443:3:69:1010:1061#0/1
CAAATAGTAGCATTGCCACAACTACTTCAATGCATGTGTG
```

+HWI-EAS443:3:69:1010:1061#0/1
aa^Ya_Y]^XSX``ba¥]_Y]Ya]_Y[^ZVa`]U^_Ra¥Z
@HWI-EAS443:3:69:1238:1788#0/1
TTTCCGTCTTCCTTCTTTGGTCCTACATTTGCTTACCATT
+HWI-EAS443:3:69:1238:1788#0/1
abbbaa]aaa_abaaaa`a`_aa`baba_a`aa`aa[a`_
@HWI-EAS443:3:69:1244:1283#0/1
CGATTGGCCATGTTTCCGCTTTGTTTTCAAAGAATTCCTT
+HWI-EAS443:3:69:1244:1283#0/1
abb`abbbZY¥JP]_^b^ON_`_T]`aaW]Ta__aa^__a
@HWI-EAS443:3:69:1371:1808#0/1
CAGGGGAGTGGAGGAAGGCTCTATTGGGAAAGGGTGCAGG
+HWI-EAS443:3:69:1371:1808#0/1
_¥_`aaSbS^]UbaVXba[QXU[HSX`[Q`Z_N`]_ZVVY
@HWI-EAS443:3:69:1429:1488#0/1
CAAATAGTAGCATTGCCACAACTACTTCAATGCATGTGTG
+HWI-EAS443:3:69:1429:1488#0/1
abbbaabZ`baTaaba`^_a^a]bab`_baaabaaa_aZa
@HWI-EAS443:3:69:1492:1443#0/1
CAAATAGTAGCATTGCCACAACTACTTCAATGCATGTGTG
+HWI-EAS443:3:69:1492:1443#0/1
ababbb`Xabaabaab^`aaa_ab`aaaaaabaaab_a_a
@HWI-EAS443:3:69:1748:643#0/1
CATGGAAATGAGGCGCTGCCTTCTTCAGCCTCTTCAGCAG
+HWI-EAS443:3:69:1748:643#0/1
aaZ]^UQYUX]ZY^¥HS_`^ZYS^BBBBBBBBBBBBBBBB
@HWI-EAS443:3:69:315:1263#0/1
TGACTTTCAGCTGATCCCAATGGTAAGCAAATGTAGGACC
+HWI-EAS443:3:69:315:1263#0/1
ababa[ab¥T_[¥]]aaaPY]]VLRRW_¥U[¥UWY[XY^_
@HWI-EAS443:3:69:330:197#0/1
TAAATCCCAATTACCTCATGGCTTGGAAGCAGGGGCTAGC
+HWI-EAS443:3:69:330:197#0/1
```Y`_[_J[^a^U[aaa^]¥]^[RRLTTETXBBBBBBBB
@HWI-EAS443:3:69:501:512#0/1
ATTAATCAGTCACTCCTCGATTGCTTCATATAGCCCCCCA
+HWI-EAS443:3:69:501:512#0/1
aab^_aa]a`a^aaaaaaaa_a_aa`aaaaa``_a^``_`
@HWI-EAS443:3:69:51:412#0/1
CAAATAGTAGCATTGCCACAACTACTTCAATGCATGTGTG
+HWI-EAS443:3:69:51:412#0/1
a`OR^aaU`aba`a`aa```_a`aa`___R^a]^``]ZY`
@HWI-EAS443:3:69:543:289#0/1
CGGGCCTTCAATACTTAATTTCAGAGCATCCATCAGCAGG
+HWI-EAS443:3:69:543:289#0/1
``b]MaZ¥bb_`ab```Z``XWaabaaaaaaaZaaa`^^V
@HWI-EAS443:3:69:564:26#0/1
GTGCAGCCATGGATGACTTTCAGCTGATCCCAATGGTAAG
+HWI-EAS443:3:69:564:26#0/1
^[^``^^¥`aa]]`_¥Y____`OLV`_XQJRNSZYYUUQZ
```

@HWI-EAS443:3:69:674:1662#0/1
TGCCCTGTTCTCTTCGTCAAGGGTGTAGTCCGCTTTGGTG
+HWI-EAS443:3:69:674:1662#0/1
aaaaaVZ`bZba`aS_`aK^RaRIKGYMZa^Q`ZV¥SDJ]
@HWI-EAS443:3:69:680:1041#0/1
GTTTCCGATTTGTTTTCAAAGAATTCCTTGGACATGTCTT
+HWI-EAS443:3:69:680:1041#0/1
aa^``aa¥PY^aWVTT[`I[Y_a`SXR[SMWG[W`]UVPS
@HWI-EAS443:3:69:85:807#0/1
TGAAGAGACTGAAGAAGGCAGCGCCCCATTTCCATGCCCC
+HWI-EAS443:3:69:85:807#0/1
aba_b]aaa`aa^`X^]_`¥``_`a`__`__^^Z[¥^_^`
@HWI-EAS443:3:6:1048:662#0/1
ATCGCAGTCCTCAAGAGCATGTCTCCTATTTCTAGAACAC
+HWI-EAS443:3:6:1048:662#0/1
abZa`ab¥a`aaaaa^¥Y`aaYV`PV__``^^^_`]_X]^
@HWI-EAS443:3:6:143:1904#0/1
CACATTTGGAAGTTTGTTTCATGTATTAGGATTTCCATTT
+HWI-EAS443:3:6:143:1904#0/1
¥_ZTT__RR```¥^_QXa`aWW]``I_FF^X^^QJ_VZ_¥
@HWI-EAS443:3:6:1473:2030#0/1
TTTCCGATTTGTTTTCAAAGAATTCCTTGGTCTTGTCTTT
+HWI-EAS443:3:6:1473:2030#0/1
`b__LS_VY]_ZY`VHTG_bb[aYFG`Y`_`OHJZBBBBB
@HWI-EAS443:3:6:1483:1109#0/1
GTCCGCTTTGGTGGCCATCTCCACTCCAGTGAATGAAAAG
+HWI-EAS443:3:6:1483:1109#0/1
a_^aa]`aaaa]`a^^_aU`[UHU]]Z^aX`U__`UXXXB
@HWI-EAS443:3:6:1529:956#0/1
GACCGAATCATGGCCTGGACAGTGGTGAAAAGTATATGTA
+HWI-EAS443:3:6:1529:956#0/1
aa¥X]^a[OYab_XVZb¥WG¥OO_YJGY[JS^VV]^¥BBB
@HWI-EAS443:3:6:1539:488#0/1
CAAATAGTAGCATTGCCACAACTACTTCAATGCATGTGTG
+HWI-EAS443:3:6:1539:488#0/1
I_bbabb_a`_bbbb`aab_aO`b_bbabbbb_bbaab[]
@HWI-EAS443:3:6:552:1038#0/1
CAAATAGTAGCATTGCCACAACTACTTCAATGCATGTGTG
+HWI-EAS443:3:6:552:1038#0/1
a`[`````]ba`b`]Za]a_MaaUY_[abb^_U^_]N_Y[
@HWI-EAS443:3:6:599:639#0/1
CAGAGCCCAGATCTCTAGCAAGCTGTGTCCAAAATGAATT
+HWI-EAS443:3:6:599:639#0/1
]a[UaSS_a_GSU¥__SY__U^^BBBBBBBBBBBBBBBBB
@HWI-EAS443:3:6:730:1439#0/1
GCAGTCCTCAAGAGCATGTCTCCTATTTCTAGAACACAGT
+HWI-EAS443:3:6:730:1439#0/1
a__[GSN¥`_^[UaZa`[]RZXT^]]__SX¥]_^Z[X_`B
@HWI-EAS443:3:6:732:1374#0/1
CAGCCATGGATGACTTTCAGCTGATCCCAATGGTAAGCAA

+HWI-EAS443:3:6:732:1374#0/1
aa``^`_`UWQb_R]¥¥¥a^X`]`PJGZa`aaa[PX]L]B
@HWI-EAS443:3:6:826:565#0/1
CCGTCTTCCTTCTTTGGTCCTACATTTGCTTACCATTGGG
+HWI-EAS443:3:6:826:565#0/1
a]_b`[`[aab_`a_abZ`abbbabb^[ababYT``aUaY
@HWI-EAS443:3:70:1200:1585#0/1
CTCCAGTCTCGGGTCAGTGAGTGAGAACTGCATACTTACA
+HWI-EAS443:3:70:1200:1585#0/1
ab_babaa`^X`aHN¥`J]]MD[a``L]YKPbaU`¥^¥`a
@HWI-EAS443:3:70:1618:1940#0/1
CATTTGCTTAGCATTGGGATCAGCTGAAAGTCATCCATGG
+HWI-EAS443:3:70:1618:1940#0/1
aba`aabbURHVUaaZUZaa`_a[]NY_^YV_`___a^ZU
@HWI-EAS443:3:70:1656:1144#0/1
CGTCTTCCTTCTTTGGTCCTACATTTGCTTACCATTGGGA
+HWI-EAS443:3:70:1656:1144#0/1
__`Y^YUV`ba_Z^a[]P_PTTKRRSVFSP_^RU[YU¥_V
@HWI-EAS443:3:70:1741:228#0/1
CGTGGTGCCGTCCTCAAGAATGGTTCAATTTTGGCGTTCA
+HWI-EAS443:3:70:1741:228#0/1
`b``b_aaaaa``[¥^a]aaa_a_^X^_`aabVSS_TYNY
@HWI-EAS443:3:70:430:1566#0/1
AAATAGTAGCATTGCCACAACTACTTCAATGCATGTGTGA
+HWI-EAS443:3:70:430:1566#0/1
aaaaa_Vaab`_^]aa_aaaa]a[__a`a^¥a^_SLOY^^
@HWI-EAS443:3:70:466:1965#0/1
GAATTCCTTGGTCATGTCTTTCTTTTTGACAGAAGACTCG
+HWI-EAS443:3:70:466:1965#0/1
¥WPU¥YY_VVSP^KZaY``__]Z_[][S`_ZZ_XZX[RTR
@HWI-EAS443:3:70:523:1160#0/1
AAATAGTAGCATTGCCACAACTACTTCAATGCATGTGTGA
+HWI-EAS443:3:70:523:1160#0/1
a`a``¥LY]a]`__a`_a¥]Z`]a`S^]]`a_`¥^¥^U__
@HWI-EAS443:3:70:555:233#0/1
GCCCGCTTCAATTGGCTTGTTCTCTTCATGTTCTTTGTCC
+HWI-EAS443:3:70:555:233#0/1
aaaab`aaa`a``a``_¥X[_N^T^ZVS^^^_]__`VX]Z
@HWI-EAS443:3:70:561:1757#0/1
AGCCCCTCAAGATCGAAGGTTCCAGGTTCCAGGTTGTCCC
+HWI-EAS443:3:70:561:1757#0/1
`]baW```YW]`V_XR¥_YT^]_WTTPXX^SY]¥¥GQNWW
@HWI-EAS443:3:70:759:1710#0/1
CCTACATTTGCTTACCATTGGGATCAGCTGAAAGTCATCC
+HWI-EAS443:3:70:759:1710#0/1
abbbababbabbbbbbbbbbaabaabaababbaaabaa_a
@HWI-EAS443:3:70:885:1005#0/1
CAAATAGTAGCATTGCCACAACTACTTCAATGCATGTGTG
+HWI-EAS443:3:70:885:1005#0/1
a`ZRO``]aYaY_]]`_^a``aaa`_`aa_^a___^Y_X^

```
@HWI-EAS443:3:71:1759:63#0/1
CACAACTACTTCAATGCATGTGTGAGGAAGGAGTTGAACC
+HWI-EAS443:3:71:1759:63#0/1
aa^a_Xaaabb_aaa__b`bab¥¥]baP`_W]^````a¥R
@HWI-EAS443:3:71:699:1021#0/1
CAAATAGTAGCATTGCCACAACTACTTCAATGCATGTGTG
+HWI-EAS443:3:71:699:1021#0/1
abbaaba`aaa_aa_abababaaaaaaa_aa_aaaaa^a_a
@HWI-EAS443:3:71:982:1864#0/1
ATTGCCACAACTACTTCAATGCATGTGTGAGGAAGGAGTT
+HWI-EAS443:3:71:982:1864#0/1
Y¥VO_^W_`b_[^T_X`a^¥[V`^XWa[a]UZ]¥]_aJ]Q
@HWI-EAS443:3:72:1013:659#0/1
AAATAGTAGCATTGCCACAACTACTTCAATGCATGTGTGA
+HWI-EAS443:3:72:1013:659#0/1
¥X^__^NY^W^`TW]abaaa]¥S_Q[b^V^]Z]]]F_H__
@HWI-EAS443:3:72:1156:1954#0/1
CAAATAGTAGCATTGCCACAACTACTTCAATGCATGTGTG
+HWI-EAS443:3:72:1156:1954#0/1
abbbbbaababbabbbbbbbbbabbababababbbbabaa
@HWI-EAS443:3:72:1271:813#0/1
ATGGATGACTTTCAGCTGATCCCAATGGTAAGCAAATGTA
+HWI-EAS443:3:72:1271:813#0/1
aabb`ba`[`aaaaa[aba__``a__aaY`___``__aWa
@HWI-EAS443:3:72:36:955#0/1
CACTCCCCTGGGTGACTCTCCGATTGGCCATGATTCCGAT
+HWI-EAS443:3:72:36:955#0/1
aaa`aab``aa_WZQ[^`]`___X]PUZ[UUVSSZUTWWU
@HWI-EAS443:3:72:409:397#0/1
CAAATAGTAGCATTGCCACAACTACTTCAATGCATGTGTG
+HWI-EAS443:3:72:409:397#0/1
abaabbb_`ababZ]a]bbb`abb`babb`abaa]a_b^b
@HWI-EAS443:3:72:496:1626#0/1
CTTGCGCATAGCTCCTGTAATCTCAAATTTTTCTTCAATT
+HWI-EAS443:3:72:496:1626#0/1
]MYX^Q]WS_ZGZ^ZQMSYLU[Y_YRMVKWGM[XXZYOYO
@HWI-EAS443:3:72:622:1984#0/1
CAAATAGTAGCATTGCCACAACTACTTCAATGCATGTGTG
+HWI-EAS443:3:72:622:1984#0/1
^_Z]^_UF¥`_Z]T]a_V_aa_]^`[¥X_V¥Z]ZZZRZTT
@HWI-EAS443:3:72:975:1929#0/1
CAAGAGCATGTCTCCTATTTCTAGAACACAGTATTTTTCC
+HWI-EAS443:3:72:975:1929#0/1
___V`Ybba`^aa_a_]b¥b`_a^aXa`ab`_a¥_^a__]
@HWI-EAS443:3:73:101:1634#0/1
CAAATAGTAGCATTGCCACAACTACTTCAATGCATGTGTG
+HWI-EAS443:3:73:101:1634#0/1
aa``¥V¥T`¥[YYQ¥^``^[X]^__^^[_[¥]_^^]X]YY
@HWI-EAS443:3:73:1064:983#0/1
GTGTTCTAGAAATAGGAGACATGCTCTTGAGGACTGCGAT
```

```
+HWI-EAS443:3:73:1064:983#0/1
``aaa^a`bZaaaaaaaaXaabba```baaaX`_¥_`_`H
@HWI-EAS443:3:73:1786:633#0/1
TTCATCGACGAACGGGGGGAATCAATAATTGTAGAATCTG
+HWI-EAS443:3:73:1786:633#0/1
ab`^a`^_``[^X_aaaP^_WX]ZZY^TT^]YR_^UJQUZ
@HWI-EAS443:3:73:271:1780#0/1
ATTGCCACAACTACTTCAATGCATGTGTGAGGAAGGAGTT
+HWI-EAS443:3:73:271:1780#0/1
aXDT^]O^]]X^a__a`a__Y____OSSX^^XWPN^^UUX
@HWI-EAS443:3:73:645:701#0/1
GGCATGGGAATGAGGCGCTGCCTTCTTCAGTCTCTTCAGC
+HWI-EAS443:3:73:645:701#0/1
a_aa`a¥X[_aa^```^a`a^^_a]`a^SaW^a^a`¥^]^
@HWI-EAS443:3:73:738:702#0/1
ATAGTAGCATTGCCACAACTACTTCAATGCATGTGTGAGG
+HWI-EAS443:3:73:738:702#0/1
ababaabaabaa`^aaaa`aaaaaaaa_aaaab_aZa_aa
@HWI-EAS443:3:73:944:1304#0/1
GGGGTTTTCGGCTGAATCGAGAGAATTGCTTCTCATTGTT
+HWI-EAS443:3:73:944:1304#0/1
aaaaUaa`baa_`aaa``a^`^a`a]¥`]]`__``^^^_]
@HWI-EAS443:3:74:1016:1093#0/1
AGCCCCTCAAGATCGAAGGTTCCAGGTTCCAGGTTGTCCC
+HWI-EAS443:3:74:1016:1093#0/1
a`a`aaa_a`a`_`^^^`_V_Z^^^_W[[a^¥ZRU_V^VW
@HWI-EAS443:3:74:1682:1286#0/1
CACAAATGGGAAAAATACTGTGTTCTAGAAATAGGAGACA
+HWI-EAS443:3:74:1682:1286#0/1
abbbbbbbbbabbbbbbaabab`babbbbaaabaaaaaaa
@HWI-EAS443:3:74:196:1763#0/1
CGAAAATCGAAACTAACAAGCTTGCTGCAATATGCACACA
+HWI-EAS443:3:74:196:1763#0/1
a]¥X`aY]``ZS[W]XZ`VXTRPYTLQQ[TN_XX____]]
@HWI-EAS443:3:75:1339:453#0/1
GAAAGGAATCCCATAGACTCCTACTGGCCATTTATTGTCT
+HWI-EAS443:3:75:1339:453#0/1
][`Sa]__b_VL_b^b^X^aZ^¥N_`¥abb`T[F]__GXF
@HWI-EAS443:3:75:1508:1858#0/1
GGCCTTGCTCAATGCATCCTGTGCAGCGATGGATGACTTT
+HWI-EAS443:3:75:1508:1858#0/1
aa¥]``^a¥`aba_T```]`aX[DYaTJ^]aa]a__`[aY
@HWI-EAS443:3:75:1707:1733#0/1
CAAATAGTAGCATTGCCACAACTACTTCAATGCATGTGTG
+HWI-EAS443:3:75:1707:1733#0/1
aabbbbb`bbabbbaabbbbbbbbabb`bbbb`bbbab_b
@HWI-EAS443:3:75:1745:1077#0/1
TGTCCTGTAGCTCTGATAGCACCTGCTTCCAAGCCATGAG
+HWI-EAS443:3:75:1745:1077#0/1
`]`babbabb`^aaXGXaa`_]a_]V¥__^PY^W]_X]O_
```

```
@HWI-EAS443:3:75:200:1973#0/1
ATCGCAGTCCTCAAGAGCATGTCTCCTATTTCTAGAACAC
+HWI-EAS443:3:75:200:1973#0/1
a`_^a]^V_[Z_^_]a¥]WV]W]]_^XYYZ]]W¥X¥]^]Z
@HWI-EAS443:3:75:559:348#0/1
TAGCATTGCCACAACTACTTCAATGCATGTGTGAGGAAGG
+HWI-EAS443:3:75:559:348#0/1
abbaababa`babaabbaabbb_aa_abb`baaabaaaaa
@HWI-EAS443:3:75:570:754#0/1
CAAATAGTAGCATTGCCACAACTACTTCAATGCATGTGTG
+HWI-EAS443:3:75:570:754#0/1
_a`b`aZabbabbbbabbabbbbbbab]bbbbbabbbb_b
@HWI-EAS443:3:75:687:109#0/1
GGCCAGTAAGAGTCTATGGGATTCCTTTCGTCAGTCCGAA
+HWI-EAS443:3:75:687:109#0/1
aababbaabbab`aaabaa]Z]b¥`aaa`a^`_a^ZZa^Y
@HWI-EAS443:3:76:1060:972#0/1
CACTCCCCTGGGTGACTCTCCGATTGGCCATGTTTGCGAT
+HWI-EAS443:3:76:1060:972#0/1
]`a[Y[a`__b`Q]V_]`]Z¥XQW^VOVYPNTNZZDVNQT
@HWI-EAS443:3:76:1071:1071#0/1
ACAGATTTTACCAGTAAGGTCCTGCACACTTTCCCAATAG
+HWI-EAS443:3:76:1071:1071#0/1
aa_`aababaaaab_aaa`Zaa`a`a`a`aa`aaab_`a`
@HWI-EAS443:3:76:124:256#0/1
CATTGCCACAACTACTTCAATGCATGTGTGAGGAAGGAGT
+HWI-EAS443:3:76:124:256#0/1
abbabbbabbbabbbbb`b^aabaaaaa]babbaaaa`a_
@HWI-EAS443:3:76:1503:1053#0/1
CAAATAGTAGCATTGCCACAACTACTTCAATGCATGTGTG
+HWI-EAS443:3:76:1503:1053#0/1
`bbbbbbbbaabbabaabbbbbbbbbbbbaabbbbbabab
@HWI-EAS443:3:76:1772:1110#0/1
CAAATAGTAGCATTGCCACAACTACTTCAATGCATGTGTG
+HWI-EAS443:3:76:1772:1110#0/1
aab_Z_aab¥a`]aMaaaabaabaaaa_aa[]``bb`aY`
@HWI-EAS443:3:76:356:656#0/1
CGAAAACCCCTCAAATTGTGGAGACGCATATAGACTGTTG
+HWI-EAS443:3:76:356:656#0/1
^```b`bb`a`aa`^a_a_[_`b`]a`b`^^^b^V^]JZN
@HWI-EAS443:3:76:552:1329#0/1
CATTGCCACAACTACTTCAATGCATGTGTGAGGAAGGAGT
+HWI-EAS443:3:76:552:1329#0/1
OY¥¥¥```bbbbbbaa¥¥`aabbbbb`b[`Sa```^`ba_
@HWI-EAS443:3:76:638:91#0/1
CATTGCCACAACTACTTCAATGCATGTGTGAGGAAGGAGT
+HWI-EAS443:3:76:638:91#0/1
`bbbabbbbbbabbabbaaabb`bbbab^b`baaabaaa`
@HWI-EAS443:3:76:738:592#0/1
TCATATAGCCCCCCAAGATCGAAGGGTCCAGGTTCCAGGT
```

+HWI-EAS443:3:76:738:592#0/1
]baaa^_WYT]`[RZ`a`YS_^VX_FMN[STSIMSPU`QV
@HWI-EAS443:3:77:1114:911#0/1
GAGAAAGGCATAAATCCCAATTACCTCACGGCTTGGAAGC
+HWI-EAS443:3:77:1114:911#0/1
a_babbbaababbaaaa_babbb`aaab`Z^_aaX^W[[L
@HWI-EAS443:3:77:137:1652#0/1
CAAATAGTAGCATTGCCACAACTACTTCAATGCATGTGTG
+HWI-EAS443:3:77:137:1652#0/1
a__``_^Oaa[^`UY^abaab_`b`]ZaaW_`¥U[a]PWa
@HWI-EAS443:3:77:204:854#0/1
AGTAGCATTGCCACAACTACTTCAATGCATGTGTGAGGAA
+HWI-EAS443:3:77:204:854#0/1
a]`]]Y`[`XTVW]`aaW_a_][TQR[T_RUQZHY¥[¥O_
@HWI-EAS443:3:77:382:570#0/1
TGACTTTCAGCTGATCCCAATGGTAAGCAAATGTAGGACC
+HWI-EAS443:3:77:382:570#0/1
aaaaaaaaaa]J]_^^^YX]]`^U¥^___[^]^W[^^¥YU
@HWI-EAS443:3:77:551:1414#0/1
GACTTTGGTCGGCAAGCTTGCGCGTAGTTCCTGTAATCTC
+HWI-EAS443:3:77:551:1414#0/1
a``a]bUFZaIWTXSUb`_]a[a^LNHLaY]_X^^[aa_a
@HWI-EAS443:3:77:597:1648#0/1
ACTCCCCTGGGTGACTCTCCGATTGGTCATGTTTCCGATT
+HWI-EAS443:3:77:597:1648#0/1
aa[abaaS`aaV`b`_a^_`[LU]^`X]W^_¥¥_^`¥]Z]
@HWI-EAS443:3:77:683:638#0/1
ACCCCTATTGTGTTACATATACTGTTCACCACTGTCCAGG
+HWI-EAS443:3:77:683:638#0/1
aaaa`aaa_a`^]`aaaa____aa_^`a_a`_`^]_^__Y
@HWI-EAS443:3:77:720:1270#0/1
CATTTGCTTACCATTGGGATCAGCTGAAAGTCATCCATGG
+HWI-EAS443:3:77:720:1270#0/1
`_`aaababbabaaaa]aa_aba`aaaaa__aa`a```a_
@HWI-EAS443:3:77:735:1928#0/1
ATTGCCACAACTACTTCAATGCATGTGTGAGGAAGGAGTT
+HWI-EAS443:3:77:735:1928#0/1
`_`^a`¥```_`_TR^a^¥X[aU`_S`R`T]`a¥^__aY`
@HWI-EAS443:3:77:868:703#0/1
AGACGTTAAACAGTATGACAGTGATGAGCCAGAGCCCAGA
+HWI-EAS443:3:77:868:703#0/1
ab`_HWb``aaa]___^aaa__a`^`¥aa`Z[Z]aa_aaZ
@HWI-EAS443:3:78:1220:740#0/1
GGGGCTATATGAAGCAATCGAGGAGTGACTGATTAATGAT
+HWI-EAS443:3:78:1220:740#0/1
_Z_NP_[T¥aWT^^J[_^L¥GZ`VNUMFFXa_`YU]BBBB
@HWI-EAS443:3:78:391:1885#0/1
AAATCCCAATTACCTCATGGCTTTGAAGCAGGTGCTAGCA
+HWI-EAS443:3:78:391:1885#0/1
a`[]_a_a^_^ZVZ]_^^]]^^XFRVZTQ[STQTWPVGLR

@HWI-EAS443:3:78:566:791#0/1
TACATTTGCTTACCATTGGGATCAGCTGAAAGTCATCCAT
+HWI-EAS443:3:78:566:791#0/1
Z]`_``^WaaaaXZ`__```]Z[`]^`^`a]]_a`_`_`Y
@HWI-EAS443:3:78:569:486#0/1
TTCGGATTTCCATTTCATCGACGAACGGGGCGAATCAATA
+HWI-EAS443:3:78:569:486#0/1
`W``Z_]a^]aa^aa`a^``^_¥N]^`__]M^aa[[aYTZ
@HWI-EAS443:3:78:718:280#0/1
GCTTCATATAGCCCCCCAAGATCGAAGGTTCCAGGGTCCA
+HWI-EAS443:3:78:718:280#0/1
aaaaaa`XVaU_]NUaa``XOMJY`XVVFPTRUZ_HZBBB
@HWI-EAS443:3:78:960:1839#0/1
CAGTCCTCAAGAGCATGTCTCCTATTTCTAGAACACAGTA
+HWI-EAS443:3:78:960:1839#0/1
a`^[^^aaaa^a_]T_a[]^```____^`__````^a]W`
@HWI-EAS443:3:79:1301:2015#0/1
CAAATAGTAGCATTGCCACAACTACTTCAATGCATGTGTG
+HWI-EAS443:3:79:1301:2015#0/1
Z_bbbb_Xabbbb```ba^`bbbbbbaabbabb¥bbab_b
@HWI-EAS443:3:79:1324:1586#0/1
TGTTGCCCCGATTGAACATATCGCAAGAATGAGGAGGAAC
+HWI-EAS443:3:79:1324:1586#0/1
Y_Q_VZ_¥J_¥Sa]a]]]¥WU]_Y[^¥F[^_HS¥OY^_QW
@HWI-EAS443:3:79:1444:1177#0/1
CAAATAGTAGCATTGCCACAACTACTTCAATGCATGTGTG
+HWI-EAS443:3:79:1444:1177#0/1
abababb`aaaaababaaabbbbbaab`baaba`ba_a^`
@HWI-EAS443:3:79:1565:1088#0/1
GCAGCAAACTTGTTAGTTTCGATTTTCGGATCTTCGCCAT
+HWI-EAS443:3:79:1565:1088#0/1
aaaaaaa``babaa¥b_baY`U[a``X_^FSR[¥ZGXXY_
@HWI-EAS443:3:79:1749:1341#0/1
ATTGCAGCAAACTTGTTAGTTTCGATTTTCGGACCTTCCC
+HWI-EAS443:3:79:1749:1341#0/1
aa_¥bba`b_b_aba^]T]UbaHXN`_a`WHWIIERaW[[
@HWI-EAS443:3:79:414:736#0/1
CAAATAGTAGCATTGCCACAACTACTTCAATGCATGTGTG
+HWI-EAS443:3:79:414:736#0/1
abbaaba[baaaaaaa`aaaaa`aaa`_a]aaa_aa^a]a
@HWI-EAS443:3:79:522:125#0/1
GGAAAAATACTGTGTTCTAGAAATAGGAGACATGCTCTTG
+HWI-EAS443:3:79:522:125#0/1
U_^`¥_¥_U¥¥Q_XY[Sa_^X¥¥aa]]bbaaaa_^`_baa
@HWI-EAS443:3:79:604:1813#0/1
GGGGCATGGAAATGAGGCGCTGCCTTCTTCAGCCTCTTCA
+HWI-EAS443:3:79:604:1813#0/1
aUWGTX¥V`U]aQa]]RXZUQ`TNU_aBBBBBBBBBBBBB
@HWI-EAS443:3:79:765:1658#0/1
TTCGGATTTCCATTTCATCGACGAACGGGGGGAATCAATA

```
+HWI-EAS443:3:79:765:1658#0/1
`bb]aTb_bbbZ_a_bbbbaa[b`b_aaaaHb`U`YYa`]
@HWI-EAS443:3:79:80:113#0/1
CAAATAGTAGCATTGCCACAACTACTTCAATGCATGTGTG
+HWI-EAS443:3:79:80:113#0/1
¥aaabba__aabaaaa]Mbb`a`_a_baaaabaYba_`ab
@HWI-EAS443:3:7:1013:73#0/1
ATTCCTTGGACATGTCTTTCTCTTTGACAGAAGACTCGGC
+HWI-EAS443:3:7:1013:73#0/1
abbbabbabW`bbba¥ba`]b^bbaa___a]^a_R_]a_S
@HWI-EAS443:3:7:1017:125#0/1
ATGGCTTGGAAGCAGGTGCTAGCAGAGCTACAGGAAATTG
+HWI-EAS443:3:7:1017:125#0/1
`aa`[_aaa]_ZG[¥_TX[V¥NO¥]SZLY^HZU[TGRRWB
@HWI-EAS443:3:7:1035:1053#0/1
CAAATAGTAGCATTGCCACAACTACTTCAATGCATGTGTG
+HWI-EAS443:3:7:1035:1053#0/1
abb_baba`ba`baaaaa`aa¥b`_aa^aaaa¥aaaXa]b
@HWI-EAS443:3:7:1515:651#0/1
CATTTGCTTACCATTGGGATCAGCTGAAAGTCATCCATGG
+HWI-EAS443:3:7:1515:651#0/1
abbbbb`bbbbbbbbbaaab`ba_bbbaabaabaZ_baa`
@HWI-EAS443:3:7:204:803#0/1
CAAATAGTAGCATTGCCACAACTACTTCAATGCATGTGTG
+HWI-EAS443:3:7:204:803#0/1
aaaabaa^bb_`aaaaaa`aa_aaaa`_aa`a__aa^a^a
@HWI-EAS443:3:7:394:57#0/1
TTCTTTGGCTGGAAAGAGCCTAATATAGTCAAACCACATG
+HWI-EAS443:3:7:394:57#0/1
aaa^a^baaabbaaaa`a^[a_¥G`_``Z^__`]W____^
@HWI-EAS443:3:7:651:1644#0/1
GGGGGCTAGATGAAGCAATCGAGGAGTGCCTGATTAATGA
+HWI-EAS443:3:7:651:1644#0/1
`RZ_YFR`X_¥aYT_Q[X]L_N`¥N_NZQYMUGQ¥T^QBB
@HWI-EAS443:3:7:873:307#0/1
CAAATAGTAGCATTGCCACAACTACTTCAATGCATGTGTG
+HWI-EAS443:3:7:873:307#0/1
abbbbbbaba`abbbaab`aa`bbaaa_baaaYaaa_b^b
@HWI-EAS443:3:80:1067:681#0/1
GTAGCATTGCCACAACTACTTCAATGCATGTGTGAGGAAG
+HWI-EAS443:3:80:1067:681#0/1
aabb`abaaaaaaaaaba`baaaaab_`abaa_a`b_aaa
@HWI-EAS443:3:80:1308:850#0/1
TAGATGGATTCGAGCCGAACGGCTGCATTTAGGGCAAGCT
+HWI-EAS443:3:80:1308:850#0/1
^Xa^`a`¥abaaa`_aa`_]_a_[NLPY¥FS¥^RVLY[RM
@HWI-EAS443:3:80:1372:1460#0/1
GATGAAATGGGGCATGGAAATGAGGCGCTGCCTTATTCAG
+HWI-EAS443:3:80:1372:1460#0/1
aaaabaa[aa```U_a__``a`a__R`Y_aQG_SD[_V^Y
```

@HWI-EAS443:3:80:194:1109#0/1
CATTTGCTTACCATTGGGATCAGCTGAAAGTCATCCATGG
+HWI-EAS443:3:80:194:1109#0/1
a^^a`_a_`a`aX`_`^Z_]`a^___]W]WW¥Z[`Z^^XN
@HWI-EAS443:3:80:247:1234#0/1
TACAGATTTTCCCAGTAAGGTCCTGCACACTTTCCCAATA
+HWI-EAS443:3:80:247:1234#0/1
``bbb^]_[YD[bab_aaba¥`aaaaaaa`a`¥aa¥^a`_
@HWI-EAS443:3:80:454:1680#0/1
AGAATATGGGGAAGACCCGAAAATCGAAACTAACAAGTTT
+HWI-EAS443:3:80:454:1680#0/1
^]¥`ab¥QJX]``T_ab`aa_aa`a`¥^aa`a``aa_W]_
@HWI-EAS443:3:80:64:94#0/1
CATTAATCAGGCACTCCTCGATTGCTTCCTATAGCCCCCC
+HWI-EAS443:3:80:64:94#0/1
abbbbabaabaaaab_aa_a`_`aZa``H``]Z_U^WUBB
@HWI-EAS443:3:81:1002:375#0/1
CAAATAGTAGCATTGCCACAACTACTTCAATGCATGTGTG
+HWI-EAS443:3:81:1002:375#0/1
^`bbba`Sb``bbaaa^b`ba`abaaaaaaaaaaaa¥aUa
@HWI-EAS443:3:81:1303:73#0/1
TTCGGCTGAATTGAGAAAATTGCTTCTCATTGTTCAGGCA
+HWI-EAS443:3:81:1303:73#0/1
ab_aa[aa`aaaX[_a`a_aaa]a_V`Z]```]_ZZ`_]]
@HWI-EAS443:3:81:1577:590#0/1
CAAATAGTAGCATTGCCACAACTACTTCAATGCATGTGTG
+HWI-EAS443:3:81:1577:590#0/1
`bab`^XY^^[`bbb`Ubaa`Yaa¥b]P_ab`[`b__`]b
@HWI-EAS443:3:81:1598:339#0/1
ATTGCCACAACTACTTCAATGCATGTGTGAGGAAGGAGTT
+HWI-EAS443:3:81:1598:339#0/1
abaa]aaabb_abaab`bbabaaab`a]]`b¥`ab_`___
@HWI-EAS443:3:81:957:1956#0/1
AGCCTATGTAGACGGATTCGAGCCGAACGGCTGCATTGAG
+HWI-EAS443:3:81:957:1956#0/1
aW`ba]_a``b]X_b`Va]U[U¥`Z[^V^¥`R¥KZNTJLX
@HWI-EAS443:3:82:1207:511#0/1
ACCCGAGTCACGAGGGGGAGGGAATACAACTATATGATGC
+HWI-EAS443:3:82:1207:511#0/1
¥¥¥Z¥__W_a¥_OW^_^WSa][UZ_^YHTOX¥]X]bK[aZ
@HWI-EAS443:3:82:1247:364#0/1
CAAATAGTAGCATTGCCACAACTACTTCAATGCATGTGTG
+HWI-EAS443:3:82:1247:364#0/1
abbbbbbbbbbbbbbbababaabbbbbababbbbbbaabb
@HWI-EAS443:3:82:1408:927#0/1
CACAACTACTTCAATGCATGTGTGAGGAAGGAGTTGAACC
+HWI-EAS443:3:82:1408:927#0/1
abaa_U`aY`baba`b¥Y___aYaaaaa_`b`b`aba^_^
@HWI-EAS443:3:82:1579:674#0/1
GGGCACTCGGTGAAAATATGGCACCAGAAAATGTAGACTT

+HWI-EAS443:3:82:1579:674#0/1
Ybb_b]a[ab_]aab]aa]_aab_¥ab^_aaaaU`bOLZ_
@HWI-EAS443:3:82:1737:1499#0/1
CAAATAGTAGCATTGCCACAACTACTTCAATGCATGTGTG
+HWI-EAS443:3:82:1737:1499#0/1
_`[``¥Y_¥^`Z^S_NF^b¥```¥_^^^a[_¥_^ab]¥¥b
@HWI-EAS443:3:82:681:1421#0/1
TTGCTCAATGCAACCTGTGCAGCCATGGATGACTTTCAGC
+HWI-EAS443:3:82:681:1421#0/1
aa_a`aab_aa`S¥_aaRR`a_a_`a_Ua_]]__¥a¥_ZS
@HWI-EAS443:3:83:128:1545#0/1
TCCGTCTTCCTTCTTTGGTCCTACATTTGCTTACCATTGG
+HWI-EAS443:3:83:128:1545#0/1
abba_babaaaaaaaabY^aaaaaa`_a`a`_aaaa__aX
@HWI-EAS443:3:83:1750:350#0/1
CATTTGCTTACCATTGGGATCAGCTGAAAGTCATCCATGG
+HWI-EAS443:3:83:1750:350#0/1
Y_``_Z]aY_ZbbYb_^_a¥Q`_aabbbZ_S¥¥Yb^abaa
@HWI-EAS443:3:83:1776:1081#0/1
GTAGCATTGCCACAACTACTTCAATGCATGTGTGAGGAAG
+HWI-EAS443:3:83:1776:1081#0/1
a_]¥WU_aX]¥`¥`ZZ_`Z[]U]X^[SW]__^U^]`]WYZ
@HWI-EAS443:3:84:487:372#0/1
AGCCCCTCAAGATCGAAGGTTCCAGGTTCCAGGTTGTCCC
+HWI-EAS443:3:84:487:372#0/1
T`_[__aa`]`]a`UZU^``]¥TYOWPK]VXV`HXSXKV_
@HWI-EAS443:3:84:563:1456#0/1
ATCGCAGTCCTCAAGAGCATGTCTCCTATTTCTAGAACAC
+HWI-EAS443:3:84:563:1456#0/1
aY¥O¥[`X^__`T¥MX^`_^¥]_]_`¥_X]UW¥WWU^]ZZ
@HWI-EAS443:3:84:661:2021#0/1
CTCCTATTTCTAGAACACAGTATTTTTCCCATTTGTGTGG
+HWI-EAS443:3:84:661:2021#0/1
^_¥SZOXX]Z_Z]a[Za^aWNU]]]Y__T^]`G¥ZX[``^
@HWI-EAS443:3:84:747:240#0/1
GCCACAACTACTTCAATGCATGTGTGAGGAAGGAGTTGAA
+HWI-EAS443:3:84:747:240#0/1
X^ZZbbb`a[`aTZabbbX[bb]b]ab^¥`Ua``bUb``a
@HWI-EAS443:3:84:760:457#0/1
CAAAGAAGCCCATCAGGCAATCTGAGGGGGCGTGGTGTCG
+HWI-EAS443:3:84:760:457#0/1
aabbbbaaabba_bbbb`a_bbbbbb`aaa`baab_a``_
@HWI-EAS443:3:84:853:1111#0/1
CAAATAGTAGCATTGCCACAACTACTTCAATGCATGTGTG
+HWI-EAS443:3:84:853:1111#0/1
aa`_aa_Xaa[_aT_`aa_a_^`a___Za`Za`^_aX`X`
@HWI-EAS443:3:85:1004:1755#0/1
CATTAATCAGGCACTCCTCGATTGCTTTATATAGCCCCCC
+HWI-EAS443:3:85:1004:1755#0/1
abaabbaab_¥`baaaaa_aaaaaaaaaaabaa_]a_^_[

```
@HWI-EAS443:3:85:1200:292#0/1
CAAATAGTAGCATTGCCACAACTACTTCAATGCATGTGTG
+HWI-EAS443:3:85:1200:292#0/1
``R_bbbabbbbbbbaaaabaabbaab^baaba`bbabab
@HWI-EAS443:3:85:1260:908#0/1
GGAGAAGGTCGGTGGGAGACTTTGGTCGGCAAGCTTGCGC
+HWI-EAS443:3:85:1260:908#0/1
abMba]ZGaZba]^a^`ZS]`abZZQ¥^]_``a^`^Z¥_¥
@HWI-EAS443:3:85:1483:129#0/1
AAGGGAGTGTACATGAATACGGCCTTGCTCAATGCATCCT
+HWI-EAS443:3:85:1483:129#0/1
a^baa]a]`NYNa`Ha[a__a¥Z^_]]S_U_S]¥H`TYW¥
@HWI-EAS443:3:85:1738:1389#0/1
AAATAGTAGCATTGCCACAACTACTTCAATGCATGTGTGA
+HWI-EAS443:3:85:1738:1389#0/1
aaa`aa]ba_¥_a__[`_`a_a_Wa^_`_a`U]^a^aZ`]
@HWI-EAS443:3:85:521:873#0/1
AAATAGTAGCATTGCCACAACTACTTCAATGCATGTGTGA
+HWI-EAS443:3:85:521:873#0/1
aaaa`aQ_`a``aaaaa`_]_`a``aa`^`aa`a^[a¥`^
@HWI-EAS443:3:85:579:1418#0/1
GCATTGCCACAACTACTTCAATGCATGTGTGAGGAAGGAG
+HWI-EAS443:3:85:579:1418#0/1
aa_aa_aa`_`aaa`aaa_`]`a`aa`_aZa_a_`]__`]
@HWI-EAS443:3:85:888:888#0/1
CAAATAGTAGCATTGCCACAACTACTTCAATGCATGTGTG
+HWI-EAS443:3:85:888:888#0/1
ab`Y_b^a^`¥¥]`bbaaabZabaaba_a^aaba^babTa
@HWI-EAS443:3:86:326:17#0/1
CTTGGGGGGCTATATGAAGCAATCGATGAGTGCCTGATTA
+HWI-EAS443:3:86:326:17#0/1
^^UX¥Z[T_]^]MMUT¥[`UQGONR[HQLG[HZQ¥¥Wa_[
@HWI-EAS443:3:86:556:1538#0/1
GCATTGCCACAACTACTTCAATGCATGTGTGAGGAAGGAG
+HWI-EAS443:3:86:556:1538#0/1
__[O[R`]WSWZXP_aTWaa_XQ__Q_P`Y¥_`XMZ¥¥UY
@HWI-EAS443:3:86:691:219#0/1
CGTTCGTCGATGAAATGGAAATCCGCATACATGAAACAAA
+HWI-EAS443:3:86:691:219#0/1
ababb```bbabaaaabbabba``]I]aaaaaaa_N``_
@HWI-EAS443:3:86:783:143#0/1
CATTTGCTTACCATTGGGATCAGCTGAAAGTCATCCATGG
+HWI-EAS443:3:86:783:143#0/1
aaaaababba^aa__`]`a_a¥aVabaZYXU[[]W`^``_
@HWI-EAS443:3:86:827:1064#0/1
GAAGAAATTTAGGCTTCTCTACCCCTGTTGCGTTACATAT
+HWI-EAS443:3:86:827:1064#0/1
`Z_aa`]baaa``_aa]_]aa]Z[]^_]_aZ`R`]W]^`[
@HWI-EAS443:3:87:1349:908#0/1
CAAATAGTAGCATTGCCACAACTACTTCAATGCATGTGTG
```

```
+HWI-EAS443:3:87:1349:908#0/1
`babbbb^bbaabbbabbabbaababbabaa`aabaab_b
@HWI-EAS443:3:87:1360:1734#0/1
CAAATAGTAGCATTGCCACAACTACTTCAATGCATGTGTG
+HWI-EAS443:3:87:1360:1734#0/1
ab_[___¥b[MW^[_GOTaaaaa`Ta[aaa_a`SabOa^b
@HWI-EAS443:3:87:1736:523#0/1
GCATTGCCACAACTACTTCAATGCATGTGTGAGGAAGGAG
+HWI-EAS443:3:87:1736:523#0/1
[[`aab`Zaaaa`ab_ab````a[^aa_`Ra_aaa_a_^a
@HWI-EAS443:3:87:1782:596#0/1
GAACAAGCCAATAGAAGTGGGCACTCGGTGAAAATATGGC
+HWI-EAS443:3:87:1782:596#0/1
aab]bbbbabbabbbab`baa`b^a[aa_b__ba`aa^`Z
@HWI-EAS443:3:87:702:1813#0/1
CACATTCACATCTTTTCATTCACTGGAGAGTAGATGGCCA
+HWI-EAS443:3:87:702:1813#0/1
aaaa[aab_b]Ta_`a_aaa_a]`a__W_[G^[RMW`US^
@HWI-EAS443:3:88:1041:1267#0/1
ATTGCCACAACTACTTCAATGCATGTGTGAGGAAGGAGTT
+HWI-EAS443:3:88:1041:1267#0/1
aa^_[b]bba`aba¥a]a_^bTaaaUaX_`a_a_]]ZaW^
@HWI-EAS443:3:88:1192:879#0/1
CATTGCCACAACTACTTCAATGCATGTGTGAGGAAGGAGT
+HWI-EAS443:3:88:1192:879#0/1
aab^^[S]`]``Vb]aaYa[bbT_ab`b_bYa`S]bb]aZ
@HWI-EAS443:3:88:1209:1054#0/1
CATTGCCACAACTACTTCAATGCATGTGTGAGGAAGGAGT
+HWI-EAS443:3:88:1209:1054#0/1
_`[P_HZW]a`_U^^`^^¥SX`aa_YU¥O]`_X_a]]]aa
@HWI-EAS443:3:88:135:1759#0/1
GAAGCCTAAATTTCTTCCTGATTAGTATGATTACAAAGAG
+HWI-EAS443:3:88:135:1759#0/1
aXa`a[¥P[]`a]ba`aaaa__aPaTaa_`aaaa_^[M]Z
@HWI-EAS443:3:88:1512:1233#0/1
AGCCGAACGGCTGGATTGAGGGCAAGCTTTCCCAAATGTC
+HWI-EAS443:3:88:1512:1233#0/1
`aX^babZ¥_UbHDM[¥OQVZYLPUOVX`]Q[_`a¥`a¥R
@HWI-EAS443:3:88:1528:259#0/1
GAACGGGGTGAATCAATAATTGTAGAATCTGGTGAACCGA
+HWI-EAS443:3:88:1528:259#0/1
Y`a^¥^`UDY`_`I_aa`Ra]a`_]¥^_W[a^WaXDHG_O
@HWI-EAS443:3:88:1587:619#0/1
ATACTGTTCACCACTGTCCAGGCCATGATTCGGGCTCTTC
+HWI-EAS443:3:88:1587:619#0/1
^TUaaa]_]¥`¥`_aa_`_``¥[^]X[ZW]UZ[NQ[T[¥¥
@HWI-EAS443:3:88:1665:1033#0/1
TGGGCCTCGACACTTGGCCTATCGCAGTCCACAAGAGCAT
+HWI-EAS443:3:88:1665:1033#0/1
a¥bb__T[PXTJ_a`¥TWVD[]a_`]ZZRFLV¥¥_WaH__
```

@HWI-EAS443:3:88:1733:1181#0/1
CAAGAGCATGTCTCCTATTTCTAGAACACAGTATTTTTCC
+HWI-EAS443:3:88:1733:1181#0/1
aabbabbbbbaab`aa[abb[baaabRaabbWabaa`aP`
@HWI-EAS443:3:88:326:497#0/1
ATTGCAGCAAACTTGTTAGTTTCGATTTTCGGCTCTTCCC
+HWI-EAS443:3:88:326:497#0/1
]^abababba`aa`b`__bX``a_``a`X]]XDT]_]_[Y
@HWI-EAS443:3:88:348:1603#0/1
TAGATGGATTCGAGCCGAACGGCTGCATTTAGGGCAAGCT
+HWI-EAS443:3:88:348:1603#0/1
]aaaL_^bZ¥bR``aaXaa_baYSaSSV_I_`aaX^`PDI
@HWI-EAS443:3:88:352:129#0/1
CTGGGTGACTCTCCGATTGGCCATGCTTCCGATTTGTTTT
+HWI-EAS443:3:88:352:129#0/1
abbbb]bbaaab`aa`aabaa`[a]b]`U``U]`_aY`]`
@HWI-EAS443:3:88:384:738#0/1
CAGCAAACTTGTTAGTTTCGATTTTCGGATATTCCCCATA
+HWI-EAS443:3:88:384:738#0/1
a_[aab`]abb^babUaaaYHGQ__a`]MMG^[TPI`[[X
@HWI-EAS443:3:88:640:428#0/1
CATTTGCTTACCATTGGGATCAGCTGAAAGTCATCCATGG
+HWI-EAS443:3:88:640:428#0/1
abbb`b`b_aaaa`abaaaa`]aaaa_aa`^_a`a¥]aaa
@HWI-EAS443:3:88:816:1936#0/1
ATGAAATGGAAATCCGAATACATGAAATAAACTTCCAAAT
+HWI-EAS443:3:88:816:1936#0/1
`a_]aa_a_Y_`a_]`aaa`_a__aaaaba`____^`_`_
@HWI-EAS443:3:88:858:1230#0/1
ACTGCCGGGCTACTGAATACATAATGAAGGGAGTGTACAT
+HWI-EAS443:3:88:858:1230#0/1
aabb¥F]abaabbbbaaaaaabaaaa`aaaa_a_a^a__`
@HWI-EAS443:3:89:1045:1722#0/1
ATACTGTGTTCTAGAAATAGGAGACATGCTCTTGAGGACT
+HWI-EAS443:3:89:1045:1722#0/1
abba^aabaaabbbba]b``aba_aa_a____aV`^aa`_
@HWI-EAS443:3:89:1240:1715#0/1
CGGCTGCATTGAGGGCAATCTTTCCCAAATGTCAAAAGAA
+HWI-EAS443:3:89:1240:1715#0/1
a[babY`b[b_[a_`Z¥`H_b_b[SLZaa][aWMbb`]`a
@HWI-EAS443:3:89:1300:815#0/1
AAATAGTAGCATTGCCACAACTACTTCAATGCATGTGTGA
+HWI-EAS443:3:89:1300:815#0/1
abaaaa_aba_aaaa``_aa[ab^aa_a`aa_a`a_aZ`_
@HWI-EAS443:3:89:147:488#0/1
CGGTGGGAGACCTTGGTCGGCAAGCTTGCGCATAGTTCCT
+HWI-EAS443:3:89:147:488#0/1
aWZW^Wa]PP¥a``¥ZJO^``^W¥^Y`^```WVQ`LNPYV
@HWI-EAS443:3:89:1667:1530#0/1
GGGGTCTATATGAAGCAATCGAGGAGTGCCTGATTAATGA

```
+HWI-EAS443:3:89:1667:1530#0/1
[`b`Rbbabbabbba]bbb^abbaaa`a]abb`baaa`a`
@HWI-EAS443:3:89:996:1462#0/1
CTTTCAGCTGATCCCAATGGTAAGCAAATGTAGGACCAAA
+HWI-EAS443:3:89:996:1462#0/1
abbabb`[babaaababbbb]b_`[babaa_ba`b`_b`b
@HWI-EAS443:3:8:1042:804#0/1
TCCGTCTTCCTTCTTTGGTCCTACATTTGCTTACCATTGG
+HWI-EAS443:3:8:1042:804#0/1
aa_a`a`ba`ba_baaX`_`aa_¥`aaaa]a`aW__`]__
@HWI-EAS443:3:8:1236:685#0/1
CAGTCCTCAAGAGCATGTCTCCTATTTCTAGAACACAGTA
+HWI-EAS443:3:8:1236:685#0/1
aab`^`bbbbbaaabbbaabaaaab`babbaaa_aaba^a
@HWI-EAS443:3:8:1591:168#0/1
CAAATAGTAGCATTGCCACAACTACTTCAATGCATGTGTG
+HWI-EAS443:3:8:1591:168#0/1
aabbabba`baabbbabbaba_ab^aaaaU^b_^bbaa_a
@HWI-EAS443:3:8:1689:1134#0/1
AGAGCATGTCTCCTATTTCTAGAACACAGTATTTTTCCCA
+HWI-EAS443:3:8:1689:1134#0/1
ababaabb_abaaa_aaa^aaa_``_]aaW`a_`_`WZ_^
@HWI-EAS443:3:8:1759:845#0/1
CATTTGCTTACCATTGGGATCAGCTGAAAGTCATCCATGG
+HWI-EAS443:3:8:1759:845#0/1
aaaabbaabaa_aab_^ba¥DW`_aa`aaa__a`Z]__a_
@HWI-EAS443:3:8:411:1503#0/1
CAAATAGTAGCATTGCCACAACTACTTCAATGCATGTGTG
+HWI-EAS443:3:8:411:1503#0/1
aaa`aa`Xa_`aY_]W]`a`_`^a`___`_]__[``^]U]
@HWI-EAS443:3:8:564:1634#0/1
ATTGACTGATTCAAGCTGGATAGAAATTGATGAAATAGGA
+HWI-EAS443:3:8:564:1634#0/1
aa_¥T_Za_^_a`b`Yab`YYa_G`Ha`^]V`¥`Z¥_U^_
@HWI-EAS443:3:90:1005:1248#0/1
CAAATAGTAGCATTGCCACAACTACTTCAATGCATGTGTG
+HWI-EAS443:3:90:1005:1248#0/1
aaYR]`_Z_aa``XRR¥]PT¥baaa]bab¥]_X]¥`H`]`
@HWI-EAS443:3:90:1131:1698#0/1
CAGTCCTCAAGAGCATGTCTCCTATTTCTAGAACACAGTA
+HWI-EAS443:3:90:1131:1698#0/1
ab`aabbabbababbaabbbbbbbbbb`bbbbb`bab]V]
@HWI-EAS443:3:90:1420:171#0/1
ACCAGATTCTACAATTATTGATTCACCCCGTTCGTGGATG
+HWI-EAS443:3:90:1420:171#0/1
^¥`_¥Ub`W__V[[MV_b]]]Pa`UXFV]¥Q[QS]GaSPZ
@HWI-EAS443:3:90:1490:1326#0/1
AGCATTGCCACAACTACTTCAATGCATGTGTGAGGAAGGA
+HWI-EAS443:3:90:1490:1326#0/1
abb`bb]a]baa``aaaba_aaab_`ab_b]aaa__aa^`
```

```
@HWI-EAS443:3:90:1750:277#0/1
CAAATAGTAGCATTGCCACAACTACTTCAATGCATGTGTG
+HWI-EAS443:3:90:1750:277#0/1
abbbbbbabaabbbabbbabbbbb_baabbbbaabb`bZb
@HWI-EAS443:3:90:363:816#0/1
CAAATAGTAGCATTGCCACAACTACTTCAATGCATGTGTG
+HWI-EAS443:3:90:363:816#0/1
ab]aa`aUaaaaaa``aba``a_a``a_`__aa]_`¥a]a
@HWI-EAS443:3:90:47:1050#0/1
ATTGCCACAACTACTTCAATGCATGTGTGAGGAAGGAGTT
+HWI-EAS443:3:90:47:1050#0/1
``Ja¥¥``a`aaaa^a¥a][XXW¥a^a^a`a[^aa^__G_
@HWI-EAS443:3:90:735:1132#0/1
AAATAGTAGCATTGCCACAACTACTTCAATGCATGTGTGA
+HWI-EAS443:3:90:735:1132#0/1
[^__ab`a``aaaa¥`aaaaaaaaaa_a^aaaaaa_aWa]
@HWI-EAS443:3:91:1085:204#0/1
ATTGCTTTATATAGCCCCCCAAGATCGAAGGTTCCAGGTT
+HWI-EAS443:3:91:1085:204#0/1
ab`a`b_H^baaaa`]____aaaaa_a^_aaG_PST`^V^
@HWI-EAS443:3:91:1277:335#0/1
AGCATTGCCACAACTACTTCAATGCATGTGTGAGGAAGGA
+HWI-EAS443:3:91:1277:335#0/1
``^aba_Y]aYa[aab[baab_ab_[aa^a_a_a_[`a_`
@HWI-EAS443:3:91:1282:320#0/1
GAAGTTCGGTGGGAGACTTTGGTCGGAAAGCTTGCGCATA
+HWI-EAS443:3:91:1282:320#0/1
a``_Oaa`YR`a`^[¥_]^_`aWV^ZG[]YOXXXYZFNYO
@HWI-EAS443:3:91:1380:294#0/1
AGCAGGGCAAGGATCAAAACTAGGCTTTTCACTATAAGAC
+HWI-EAS443:3:91:1380:294#0/1
abIa¥`]^_a^D[aa`[b^L^aa`YR`a`M_F]Ta[aIW[
@HWI-EAS443:3:91:1570:896#0/1
CATTGCCACAACTACTTCAATGCATGTGTGAGGAAGGAGT
+HWI-EAS443:3:91:1570:896#0/1
R`a^Vb[``_b¥b¥¥Va```aa`bba_b``JbXbb`bba]
@HWI-EAS443:3:91:1717:173#0/1
CTTCCTTCTTTGGTCCTACATTTGCTTACCATTGGGATCA
+HWI-EAS443:3:91:1717:173#0/1
aaa¥__b`abba¥`a`bbabbabb]bbaa^bbbb`^ab_b
@HWI-EAS443:3:91:298:574#0/1
GCACCAGAAAAAGTAGACTTTGATGACTGCGAAGATGTTG
+HWI-EAS443:3:91:298:574#0/1
aZKX^SNL¥¥YOWG^_^SZYW]WIXWWWWXIHMMWUOIHZ
@HWI-EAS443:3:91:316:1913#0/1
CATTGCCACAACTACTTCAATGCATGTGTGAGGAAGGAGT
+HWI-EAS443:3:91:316:1913#0/1
abbb`ababbbb`bbbabbbbabbbaababa^`aaa_a^]
@HWI-EAS443:3:91:860:954#0/1
CCTACATTTGCTTACCATTGGGATCAGCTGAAAGTCATCC
```

+HWI-EAS443:3:91:860:954#0/1
abbbbabbbbbbbbbbbbbbbbbbbabbabbabaa`aba`a
@HWI-EAS443:3:92:1157:719#0/1
CGATTTGAGTTAATTGAAGGAAGAGACCGAATCATGGCCT
+HWI-EAS443:3:92:1157:719#0/1
S`_¥aW[aVI^bbaab_O__b_S[baIabZ_N]aaba]X]
@HWI-EAS443:3:92:1493:1745#0/1
GAGATCTGGGCTCTGGCTCATCACTGTAATACTGTTTAAG
+HWI-EAS443:3:92:1493:1745#0/1
aaaa`aa]]a``[_`a`aaa¥QZ]aaWG```_`a^_`a__
@HWI-EAS443:3:92:1525:922#0/1
CATTGCCACAACTACTTCAATGCATGTGTGAGGAAGGAGT
+HWI-EAS443:3:92:1525:922#0/1
_`ba¥Ybbabb`bbbbbaabbbaabbaaabbabaaabab`
@HWI-EAS443:3:92:1616:175#0/1
CTCTACCCCTGTTGTGGTACATATACTGTTCACCACTGTC
+HWI-EAS443:3:92:1616:175#0/1
abTY]X`RHUa_N``]J¥RR[_aZa`^^P¥R[^Q[UN_]B
@HWI-EAS443:3:92:865:1433#0/1
CATTTGCTTACCATTGGGATCAGCTGAAAGTCATCCATGG
+HWI-EAS443:3:92:865:1433#0/1
``b_Y[Ya^ababbbaaa_aaaaa`bb``a`a_aUa_a][
@HWI-EAS443:3:92:899:1961#0/1
AAGGAATTCTTTGGAAACAAATCGGAAACATGGCCAATCG
+HWI-EAS443:3:92:899:1961#0/1
[_UW¥aaabb_b[H[^ba]b`b][aSaaaX`VYW__SaKZ
@HWI-EAS443:3:93:1079:1118#0/1
CAAATAGTAGCATTGCCACAACTACTTCAATGCATGTGTG
+HWI-EAS443:3:93:1079:1118#0/1
`bb`aba]bb``baa`aaaaaaabaaaaa`ab^aaa]a^a
@HWI-EAS443:3:93:1091:1874#0/1
GGGGAGGGGAGGAAGGCTCTATTGGGAAAGTGTGCAGGAC
+HWI-EAS443:3:93:1091:1874#0/1
``WTQ¥QYFObaS`VXXabSZWa`XbGaabMaHXNVVT_N
@HWI-EAS443:3:93:1339:378#0/1
TAGAACTTGATGAAATAGGAGAAGATGTTGCCACGATTGA
+HWI-EAS443:3:93:1339:378#0/1
_b`Zaabb`bbb¥_ab_``TYa]`ZZbY``YTG^aZ_a_^
@HWI-EAS443:3:93:1568:1875#0/1
CAAATAGTAGCATTGCCACAACTACTTCAATGCATGTGTG
+HWI-EAS443:3:93:1568:1875#0/1
abbbbb^]b`_a^bbaaa`abaabaaa_a_ab^aaa`a]b
@HWI-EAS443:3:93:1604:796#0/1
CAAATAGTAGCATTGCCACAACTACTTCAATGCATGTGTG
+HWI-EAS443:3:93:1604:796#0/1
aa[_`_Ya``Uaa`a[Za_T`]baaaaKZ^aa[a`TGaPa
@HWI-EAS443:3:93:354:1375#0/1
GCATTGCCACAACTACTTCAATGCATGTGTGAGGAAGGAG
+HWI-EAS443:3:93:354:1375#0/1
abaaa`baaa]aaaaaaa`a_a`a[a`_a____^^Y_^^a

```
@HWI-EAS443:3:93:482:1678#0/1
ATGACTTTCAGCTGATCCCAATGGTAAGCAAATGTAGGAC
+HWI-EAS443:3:93:482:1678#0/1
```Z^aa^aaaa_`a``_`_`a``Za`]¥_aa`_X``V_^
@HWI-EAS443:3:93:511:1831#0/1
AGCATTGCCACAACTACTTCAATGCATGTGTGAGGAAGGA
+HWI-EAS443:3:93:511:1831#0/1
a]WQ[aZ_`aa`aYN^a]^`aZ__]^_^]]Y^_^]__¥Y]
@HWI-EAS443:3:93:721:749#0/1
AAATAGTAGCATTGCCACAACTACTTCAATGCATGTGTGA
+HWI-EAS443:3:93:721:749#0/1
YUU_aWD^``^Z_a^[R^_`^`]a^^__][a`]]b^`XaT
@HWI-EAS443:3:94:1093:641#0/1
TCCGTCTTCCTTCTTTGGTCCTACATTTGCTTACCATTGG
+HWI-EAS443:3:94:1093:641#0/1
a_aa`W_a`¥`b[`aa_a`aaaaZ[_aaa_``a¥W[__`^
@HWI-EAS443:3:94:1143:927#0/1
CTCCAGTCTCGGGTCAGTGAGTGAGAAGTCCATACTTACA
+HWI-EAS443:3:94:1143:927#0/1
`VTO]YTb`][ab_R¥_[]WY[]]XFMG[TNFPZV]]ZT¥
@HWI-EAS443:3:94:1636:127#0/1
ATGCCCCATTTCATCTTGATCTTGGAGGTTCTATTGGTTC
+HWI-EAS443:3:94:1636:127#0/1
¥YaNSI[_¥aU^LGN_`aZ`^aXXHBBBBBBBBBBBBBBB
@HWI-EAS443:3:94:1670:235#0/1
CATAAATCCCAATTACCTCATGGCTTGGAAACAGGTGCTA
+HWI-EAS443:3:94:1670:235#0/1
V`bab_bW`TbbbabW`bbbbbbUbb[SaZGQa]`Hb[^Y
@HWI-EAS443:3:94:236:1022#0/1
CAAATAGTAGCATTGCCACAACTACTTCAATGCATGTGTG
+HWI-EAS443:3:94:236:1022#0/1
a`^`bbbabbb`baaba_a_a`aabaa_W¥a`aa`b`a_b
@HWI-EAS443:3:94:765:1558#0/1
ACAAAATAAAATCTGAGAAGACATACATTCACATCTTTTC
+HWI-EAS443:3:94:765:1558#0/1
aVTZV¥ba`a]aQ`ba`¥a¥a[bI_a_`Vaaa_baaa^YU
@HWI-EAS443:3:94:893:1089#0/1
CAAATAGTAGCATTGCCACAACTACTTCAATGCATGTGTG
+HWI-EAS443:3:94:893:1089#0/1
aaaaa``¥aaa``aa^_a`a``ab^aa]`Y`^¥_aa^a]a
@HWI-EAS443:3:95:1040:240#0/1
CTTCCTTCTTTGGTCCTACATTTGCTTACCATTGGGATCA
+HWI-EAS443:3:95:1040:240#0/1
I^a^_b__a`a__``a`]_bbb```bbbbaZbbb_babaa
@HWI-EAS443:3:95:1215:1123#0/1
CTCGATTGCTTCATATAGCCCCCCAAGATCGAAGGGTCCA
+HWI-EAS443:3:95:1215:1123#0/1
``W¥_abb]bbbaba_K_b`Zb]Zabbaaabb^baIa_aN
@HWI-EAS443:3:95:134:887#0/1
GCCACAACTACTTCAATGCATGTGTGAGGAAGGAGTTGAA
```
```

+HWI-EAS443:3:95:134:887#0/1
^abbbbbabbbbbbbaXabbabb`babbb`]ba`abaabaa
@HWI-EAS443:3:95:1555:231#0/1
CAGCAAACTTGTTAGTTTCGATTTTCGGTTCTTCCCCATA
+HWI-EAS443:3:95:1555:231#0/1
O^[_ab_]¥ababbbbb_W`^_bbb^baG`Na`]VWS]]L
@HWI-EAS443:3:95:51:202#0/1
GTGAATCACTAATTGTAGAATCTGGTGACCCGAATGCACT
+HWI-EAS443:3:95:51:202#0/1
a_a^U¥a]UTVZXZ^QQQVZXXYZ^ZZVMVUZTLTZVVVR
@HWI-EAS443:3:95:57:1548#0/1
AACTCCTTCCTCACACATGCATTGAAGTAGTTGTGGCAAT
+HWI-EAS443:3:95:57:1548#0/1
`aabb]abaabab]ba`aa¥`aaaaaa^aaTW¥^^^^a`_
@HWI-EAS443:3:96:1066:1320#0/1
CAGGTGCTAGCAGAGCTACAGGACATTGGAAATGAAGAGA
+HWI-EAS443:3:96:1066:1320#0/1
aaaaU_ba¥Z^[]_`¥aS¥a`^_N_T_RHW¥^a`¥¥QU]_
@HWI-EAS443:3:96:1371:2042#0/1
CACTCCCCTGGGTGACTCTCCGATTGGGCATGTTTCCGAT
+HWI-EAS443:3:96:1371:2042#0/1
^Z`]Sa]GabbaHb`N_`RMVaS`[b_QG_]aVXXQZ`RB
@HWI-EAS443:3:96:1543:633#0/1
CAATTACCTCATGGCTTGGAAGCAGGGGCTAGCAGAGCTA
+HWI-EAS443:3:96:1543:633#0/1
aaWJVbY`aSa¥_Y]a_¥_[[TYN]^HW_]YRDXSZZMNV
@HWI-EAS443:3:96:1561:1859#0/1
CAAATAGTAGCATTGCCACAACTACTTCAATGCATGTGTG
+HWI-EAS443:3:96:1561:1859#0/1
`a_`b``Xba`bbbaaaa]_a¥b`aaa¥abab``ab¥aab
@HWI-EAS443:3:96:293:22#0/1
AGCATTGCCACAACTACTTCAATGCATGTGTGAGGAAGGA
+HWI-EAS443:3:96:293:22#0/1
^a¥^^_]Za_Waa`b^S^`RbY`YYaaaVa_`_aZ_]_[P
@HWI-EAS443:3:96:421:588#0/1
CGTCTCCACAACTTGAGGGGTTTTCGGCTGAATGGAGAAA
+HWI-EAS443:3:96:421:588#0/1
abab¥YW[^Y_]_]ba_]^_HSIZP]V^^VV`NM[[Y`BB
@HWI-EAS443:3:96:826:1253#0/1
GTAGGAGTCTATGGGATTACTTTCGTCAGTCCGAAAGAGG
+HWI-EAS443:3:96:826:1253#0/1
a[_aaab`ab`bba`aa[KV`ba`b[Z^a_[_a`_``^_`
@HWI-EAS443:3:96:845:1052#0/1
CAAGAGCATGTCTCCTATTTCTAGAACACAGTATTTTTCC
+HWI-EAS443:3:96:845:1052#0/1
]Y¥`_aaZ^HVR¥ba]Z^]aaaa`VX¥aaV_R¥`aVa`_^
@HWI-EAS443:3:97:101:107#0/1
ATAAATCCCAATTACCTGATGGCTTGGAAGCAGGTGCTAG
+HWI-EAS443:3:97:101:107#0/1
a`aaU]^U___a`_]]ZVY^`_W]`[^SP^WP^^XVUVVB

```
@HWI-EAS443:3:97:1363:652#0/1
CATAAATACGGCCTTGCTCAATGCATCCTGTGCAGACATG
+HWI-EAS443:3:97:1363:652#0/1
Yb¥Saba___b_[a¥U_^Uaaaaba¥W_T`QYQT_HZ]Vb
@HWI-EAS443:3:97:144:1862#0/1
GCAGTCCTCAAGAGCATGTCTCCTATTTCTAGAACACAGT
+HWI-EAS443:3:97:144:1862#0/1
```[TMY__YXZ_``aT_QSZT_]W^VLW¥a¥SWU]¥^YN
@HWI-EAS443:3:97:253:1582#0/1
CATTTGCTTACCATTGGGATCAGCTGAAAGTCATCCATGG
+HWI-EAS443:3:97:253:1582#0/1
^bbaa^_b``]baaa`]ba]bbba]aaaabXaaa]_a`_`
@HWI-EAS443:3:97:301:1349#0/1
CACCGAGTGCCCACTTCAATTGGCTTGTTCTCCTCATGTT
+HWI-EAS443:3:97:301:1349#0/1
T^bba^aR``XT^`aa^aVT]U^T_[PZ[ZSMH[^W]TOZ
@HWI-EAS443:3:97:649:918#0/1
CACAACTTGAGGGGTTTTCGGCTGAATCGCGAAAATTGCT
+HWI-EAS443:3:97:649:918#0/1
a`a_abbbbabbaaabbb_b`aabaaa`UO¥[UTR``_¥_
@HWI-EAS443:3:97:874:1491#0/1
CAGTCCTCAAGAGCATGTCTCCTATTTCTAGAACACAGTA
+HWI-EAS443:3:97:874:1491#0/1
abXa`abab_bab¥bab`abaaa`aa_`aaabb]aab]R[
@HWI-EAS443:3:98:378:1224#0/1
CTTTCCCATTAGAGCCTTCCTCCACTCCCCTGGGTGACTC
+HWI-EAS443:3:98:378:1224#0/1
abbbbbbbbbbbbbaabbaabaaaabaaaaaaaa^ba_a`
@HWI-EAS443:3:98:422:1790#0/1
CACACATGCATTGAAGTAGTTGTGGCAATGCTACTATTTG
+HWI-EAS443:3:98:422:1790#0/1
abbb_bbbbbab`bbbabaaba`_aabb`b_ba_aaaa[[
@HWI-EAS443:3:98:507:771#0/1
GGGGGGTATATGAAGCAATCGAGGAGTGCCTGATTAATGA
+HWI-EAS443:3:98:507:771#0/1
SX_bYF[US¥^]XWRTTZXWaUR]^_¥[H[aa[aV[Z`_^
@HWI-EAS443:3:98:557:1522#0/1
CATTTGCTTACCATTGGGATCAGCTGAAAGTCATCCATGG
+HWI-EAS443:3:98:557:1522#0/1
abaaba_a^aa`b_abbaa`babaaaaaZUba_aab`a`
@HWI-EAS443:3:98:863:1174#0/1
ATTGCCACAACTACTTCAATGCATGTGTGAGGAAGGAGTT
+HWI-EAS443:3:98:863:1174#0/1
ZQQ¥````__a¥¥Sa¥L`a¥X¥`^aa_QaX[`^¥_Z]]R[
@HWI-EAS443:3:99:1338:92#0/1
GACCCGAGTCACGAGGGGGAGGGAATAGCACTATATGATG
+HWI-EAS443:3:99:1338:92#0/1
aaXX¥b`Zb]a]a`a]aba__b_T][_GHSZ_Qa`YTZ^`
@HWI-EAS443:3:99:146:47#0/1
AGCATTGCCACAACTACTTCAATGCATGTGTGAGGAAGGA
```
```

```
+HWI-EAS443:3:99:146:47#0/1
ab_]`aa_a][`a___^a_¥aSXa^_aaZaRa]a`]Qa^X
@HWI-EAS443:3:99:1566:405#0/1
AGGAAATTGAAAATGAAGAGAAGATCCCAAGGACAAAGAA
+HWI-EAS443:3:99:1566:405#0/1
`^^]H`aXPPaaa]`a__`baba^¥VW[`_aJ_Ga_a_¥a
@HWI-EAS443:3:9:1095:1800#0/1
GGGGCTATATGAAGCAATCGAGGAGGGCCTGATTAATGAT
+HWI-EAS443:3:9:1095:1800#0/1
aZba^L[a_X]Ub[Sb]b_b_a_¥SHTV_aa¥`_Y^_a_N
@HWI-EAS443:3:9:1108:182#0/1
CAAATAGTAGCATTGCCACAACTACTTCAATGCATGTGTG
+HWI-EAS443:3:9:1108:182#0/1
¥^bbb`b^b`abbb`[a_`_babb^b`Zbbab¥_abab_b
@HWI-EAS443:3:9:1445:1695#0/1
ACCCGAATGCAATATTGAAGCACCGATTTGAGATAATTGA
+HWI-EAS443:3:9:1445:1695#0/1
aLXYaTWaa_¥F^[`_`aab_¥ZMY__a_a``W^___^_^
@HWI-EAS443:3:9:1468:307#0/1
AGCCAAGTGAAGTGGGCACTCGGTGAAAATATGGCACCAG
+HWI-EAS443:3:9:1468:307#0/1
`ba¥aaaQa¥¥b_aaa___a_a`SWQ_[__X]_`Y[STV]
@HWI-EAS443:3:9:1567:1605#0/1
CATTGCCACAACTACTTCAATGCATGTGTGAGGAAGGAGT
+HWI-EAS443:3:9:1567:1605#0/1
^bbOaOab`¥¥baa`aaab`YR^ab`ZZF__bT]a`a^a]
@HWI-EAS443:3:9:1699:1783#0/1
ATTGCAGCAAACTTGTTAGTTTCGATTTTCGGAGCTTCCC
+HWI-EAS443:3:9:1699:1783#0/1
`bV^a_YZ`_X[b]_PQ`¥¥_[_WDZ_]^^GUDHIP_USW
@HWI-EAS443:3:9:737:242#0/1
CATAAATCCCAATTACCTCATGGCTTGGAAGCAGGGGCTA
+HWI-EAS443:3:9:737:242#0/1
abbbaabaaabaa_aaab`aa]`]_^P]]`XXUZ^HUXXU
@HWI-EAS443:3:9:779:1462#0/1
CAAATAGTAGCATTGCCACAACTACTTCAATGCATGTGTG
+HWI-EAS443:3:9:779:1462#0/1
a`Z[]_¥UbZbaba]a`aa_]a^aZ_]M``]a]_a`UaR`
@HWI-EAS443:3:9:995:1342#0/1
CAAATAGTAGCATTGCCACAACTACTTCAATGCATGTGTG
+HWI-EAS443:3:9:995:1342#0/1
aa]abbbSb[[abbb`aaa`a`bbaa`ab``a]_abWa[a
@HWI-EAS443:3:12:63:1049#0/1
CGCGGGATCTTCTCTTCATTTTCAATGTCCTGTAGCTCTG
+HWI-EAS443:3:12:63:1049#0/1
a`a^`Z¥]`^_[`````````__W^__W¥`__[`]___]Y
@HWI-EAS443:3:17:58:2004#0/1
ACACCGAGTGCCCACTTCAATTGGCTTGTTCTCTTCATTT
+HWI-EAS443:3:17:58:2004#0/1
abbba_W]HY_b_]YO]`]RTOQBBBBBBBBBBBBBBBBB
```

@HWI-EAS443:3:18:325:1774#0/1
AATAATTCCTTGGTCATGTCTTTCTCTTTGACAGAAGACT
+HWI-EAS443:3:18:325:1774#0/1
¥TGRYaaaXaa``U^aaaL¥]`Z`__^W`_][`WY_W^_X
@HWI-EAS443:3:21:1655:1603#0/1
GCGAGTAGGAGTCTATGGGATTCCTTTCGTCAGTCCGAAA
+HWI-EAS443:3:21:1655:1603#0/1
aQFV_W_`_¥TSSMYWXZUUVZ¥XUWWWUUUW¥WWVWVNS
@HWI-EAS443:3:24:1566:1893#0/1
GGGGAAGATCCGAAAATCGAAACTAACAAGTTTGCTGAAT
+HWI-EAS443:3:24:1566:1893#0/1
aab]baaaa`^Z_baa^ab_^VSa`a¥b_`X``RLSaI^^
@HWI-EAS443:3:25:744:181#0/1
CCTCGATTGCTTCATATAGCCCCCCAAGATCGAAGGTGCC
+HWI-EAS443:3:25:744:181#0/1
aa^bb_aaba`a^RTb^^¥__¥aaZQX]^M¥STU[_MGJE
@HWI-EAS443:3:33:793:580#0/1
TGACGAAAGGAATCCCATAGACTCCTACTGGCCATTTTTT
+HWI-EAS443:3:33:793:580#0/1
aaabbb]aa`aaaaaab__aa_a`a`__^`^SSRY]]LU`
@HWI-EAS443:3:34:1372:1475#0/1
CCTCCCCGTTCGTCGATGAAATGGAAATCCGAATACATGA
+HWI-EAS443:3:34:1372:1475#0/1
aabbbba`[___Q_b``a_`b^[`aaa__a^a__`Z_]R^
@HWI-EAS443:3:42:741:875#0/1
ATCGCAAGCATGAGGAGGAACTATTTTACAGCAGAAGCCA
+HWI-EAS443:3:42:741:875#0/1
aaa`a``a`^aa`b`_a``_]aaaaa`___a__]a_]``^
@HWI-EAS443:3:48:1581:237#0/1
AGCCTATGTAGATGGATTCGAGCCGAACGGCTGCATTTAG
+HWI-EAS443:3:48:1581:237#0/1
¥``aY^_aXaaabbb]_^[^[PZIOT]P]BBBBBBBBBBB
@HWI-EAS443:3:49:1002:1354#0/1
GGCATAAATCCCAATTACCTCATGGCTTGGAAGCAGGGGC
+HWI-EAS443:3:49:1002:1354#0/1
aaaa^]`]ba``a_aaa`^_``_ab_a_[^¥^_T[S`BBB
@HWI-EAS443:3:49:1757:819#0/1
TAGATGGATTCGAGCCGAACGGCTGCATTGAGGGCAAAGT
+HWI-EAS443:3:49:1757:819#0/1
a^P_bbbab`_`aZ¥W¥[RR_a[_¥GF¥^_OBBBBBBBBB
@HWI-EAS443:3:4:1467:1965#0/1
CATGGAAATGAGGCGCTGCCTTCTTCAGTCTCTTCAGGAG
+HWI-EAS443:3:4:1467:1965#0/1
VbabbJU[abb^]WRXab]a`^_ab_]aPR_HX`MaZG]^
@HWI-EAS443:3:4:350:235#0/1
ATGAAGGGAGTGTACATAAATACGGCCTTGCTCAATGAAT
+HWI-EAS443:3:4:350:235#0/1
a`¥^aa^_Xa`YUZ]aVYY`a_]``Y^`]]Q¥R[¥^XLOV
@HWI-EAS443:3:4:652:363#0/1
GGGTCTTCTCTTCATTTTCAATGTCCTGTAGCTCTGCTAG

```
+HWI-EAS443:3:4:652:363#0/1
aba]_a```]aa_``a^a__]`__NZa_X``Z_^]YY]^T
@HWI-EAS443:3:51:581:46#0/1
GGTCACCAAAGCGGACTACACCCTTGACGAAGAGAGCAGG
+HWI-EAS443:3:51:581:46#0/1
T_a]Y`bbU¥b`bWI_Yaabaaa^__`a`R_a_W_a`]`a
@HWI-EAS443:3:51:75:1320#0/1
TCCGGCTTCTCTACCCCTGTTGTGTTACATATACTGTTCA
+HWI-EAS443:3:51:75:1320#0/1
aaaab``]^^]RXV_`a`RP¥QU`V[`Q_^U^ZY`U^W¥_
@HWI-EAS443:3:53:1117:653#0/1
CGGGGTCTCATTTGAGAAATGATACTGATGTGGTGAACTT
+HWI-EAS443:3:53:1117:653#0/1
aa[[b_baaa_bab`^`aaab`aaabb`ab][__a¥VYaa
@HWI-EAS443:3:53:1517:454#0/1
GTCACCCAGGGGAGTGGAGGAAGGCTCTATTGGGAAACAG
+HWI-EAS443:3:53:1517:454#0/1
RR`a`XZX]a^¥TIZ`ZY¥^_ZWWPXPPP`ZVK_¥X^FFE
@HWI-EAS443:3:55:1140:644#0/1
GATTGCTTCATATAGCCCCCCAAGATCGAAGGTTCCAAGT
+HWI-EAS443:3:55:1140:644#0/1
ab`ZXXaaaaaa`aV^a_P¥``_[X_Y_¥¥_YMVQN^VXH
@HWI-EAS443:3:55:649:1942#0/1
GAGAAGCCTAAATTTCTTCCTGATTTGTATGATTACAGAG
+HWI-EAS443:3:55:649:1942#0/1
¥VV^JWY^baUYX`SW_WVQY¥YS]PS`^aQXV¥_PBBBB
@HWI-EAS443:3:62:21:551#0/1
TCCGGGGGCTATATGAAGCAATCGAGGAGTGCCTGATTAA
+HWI-EAS443:3:62:21:551#0/1
a__aa^aabbaaaa_``_Z¥a_`^Raa_a¥`]Z_a``_[¥
@HWI-EAS443:3:62:354:410#0/1
TACTTGAAGGAAGAGACCGAATCATGGCCTGGACAGTGGT
+HWI-EAS443:3:62:354:410#0/1
RVI_V¥ZZO^VO^aba]^`aYY^ZY_QPPX^`P]RTP^WT
@HWI-EAS443:3:65:1636:1230#0/1
TGTTGTAAAATAGTTCCTCCTCATGCTTGCGATATGTTCA
+HWI-EAS443:3:65:1636:1230#0/1
aa_baaabaaaaa^a^_a`_a_aaa_aaa^a`a_a___^¥
@HWI-EAS443:3:65:687:1805#0/1
GTTTTCCGTCTTCCTTCTTTGGTCCTACATTTGCTTACCA
+HWI-EAS443:3:65:687:1805#0/1
a¥aaaaaaYaaaa`aab`a_a`^a_abaaa_a`ba^aaaa
@HWI-EAS443:3:68:1212:460#0/1
GCTCCACTTCAATTGGCTTGTTCTCTTCATGTTCTTTGTC
+HWI-EAS443:3:68:1212:460#0/1
aba`ababb``babaaaaaRZaZa^aa]_a]aa`aaaa`_
@HWI-EAS443:3:69:734:268#0/1
ACCCGGCTGAATCGAGAAAATTGCTTCTCATTGTTCAGGC
+HWI-EAS443:3:69:734:268#0/1
a[b_W_[ZI¥aTUYI¥[_`aa`ZX_aU¥``aa¥HaG^_^F
```

```
@HWI-EAS443:3:6:464:57#0/1
GTGAATCAATAATTGTAGAATCTGGTGACCCGAATGCGCT
+HWI-EAS443:3:6:464:57#0/1
a_aa`_a^¥a_`aaa__a^__Y]a_Z_^]]W`Z^^_VaZ[
@HWI-EAS443:3:72:426:1412#0/1
ATAAATCCCAATTACCTCATGGCTTGGAAGCAGGTGCCAG
+HWI-EAS443:3:72:426:1412#0/1
_```U]Z]]``^Q^a__a¥Y[X^¥UURQ^BBBBBBBBBBB
@HWI-EAS443:3:72:491:788#0/1
GCGGTCTCCACAACTTGAGGGGTTTTCGGCTGAATCGAGA
+HWI-EAS443:3:72:491:788#0/1
ab^`_`aaaaa`_Va^]^Z_aaVZa_a^a_aa`a[[a_`^
@HWI-EAS443:3:74:1404:985#0/1
GACGCATTGAGCAAGGCCGTATTTATGTACACTCCCTTCA
+HWI-EAS443:3:74:1404:985#0/1
aaIbaaabaaaab`b`a_b__aaa_aa¥aaaSa`X¥_aR_
@HWI-EAS443:3:78:984:258#0/1
GAAAGGCATAAATCCCAATTACCTCATGGCTTGGAAGAAG
+HWI-EAS443:3:78:984:258#0/1
a^a^RSS¥aa`_^XT^a____Z^ZL¥X^ZP_[NBBBBBBB
@HWI-EAS443:3:80:798:1577#0/1
GGGGGCTATATGAAGCAATCGAGGAGTGCCTGATTAANGA
+HWI-EAS443:3:80:798:1577#0/1
aUYXaba``ZY``a^^^][_][WU¥[QZYQYTZVR_ZDFY
@HWI-EAS443:3:81:90:1495#0/1
TGACCAAGGAATTCTTTGAAAACAAATCGGAAACATGNCC
+HWI-EAS443:3:81:90:1495#0/1
a[Qabab_abaaabbaaababbabbaaaa`aS_a`_WD¥a
@HWI-EAS443:3:83:1042:1153#0/1
CAGTGGGAAAGTGTGCAGGACCTTACTGGCAAAATCTGTA
+HWI-EAS443:3:83:1042:1153#0/1
aaaQaabaa_aZa`baabaaa`aa]a`^aaa`^^a_ab]a
@HWI-EAS443:3:83:70:2024#0/1
GGCCTATCGCAGTCCTCAAGAGCATGTCTCCTATTTCTAG
+HWI-EAS443:3:83:70:2024#0/1
`_ba_`_aZb_`G`^¥aa`_a[PR]_S`_aa[H[]`a_`¥
@HWI-EAS443:3:84:822:851#0/1
AGAAGTTCGGTGGGAGACTTTGGTCGGCAAGCTTGCGAGA
+HWI-EAS443:3:84:822:851#0/1
aa^TbN`a`UYa_a^`Z`^]Z`¥Qa_^[W]VYV^_G`^^U
@HWI-EAS443:3:85:986:593#0/1
CCTCGATTGAACATATCGCAAGCATGAGGAGGAACTATTT
+HWI-EAS443:3:85:986:593#0/1
]aabbbbbbbabbbbabba`babbbbbbababaababab
@HWI-EAS443:3:86:1209:63#0/1
AGGGTACATAAATACGGCCTTGCTCAATGCATCCTGTGCA
+HWI-EAS443:3:86:1209:63#0/1
^ZKaWJ^bba][bbaa_¥VR^a`a__``aaa_YY`]R]FW
@HWI-EAS443:3:88:485:1887#0/1
ACCGAATCATGGCCTGGACAGTGGTGAACAGTATATGGAA
```

```
+HWI-EAS443:3:88:485:1887#0/1
a]`^]^^`X`R]`_aa_¥V_¥VaZUX[YU[¥T`_^Q^X^_
@HWI-EAS443:3:89:630:1833#0/1
AGCCTATGTAGATGGATTCGAGCCGAACGGCTGCATTTAC
+HWI-EAS443:3:89:630:1833#0/1
_W^a_bY]^b`]a^`Z^YF]]T[`aRUQBBBBBBBBBBBB
@HWI-EAS443:3:8:773:1316#0/1
TGCATATTGCAGCAAACTTGTTAGTTTCGATTTTCGGGTG
+HWI-EAS443:3:8:773:1316#0/1
aabaab_abbaXU^a^aaaa^a_`_aaa`]`^`_SOZUZB
@HWI-EAS443:3:91:980:888#0/1
GCCCTGCTCTCTTCGTCAAGGGTGTAGTCCGCTTTGGGGG
+HWI-EAS443:3:91:980:888#0/1
aRDQRZZ[¥[`¥VMOUPNU`XMK`R¥YYPMPOFX¥¥XBBB
@HWI-EAS443:3:94:212:710#0/1
ACACAGCTTGCTAGAGATCTGGGCTCTGGCTCATCACTGT
+HWI-EAS443:3:94:212:710#0/1
a_G`]a`a`a`a_a_`_^_`^]_]__^^]]^Y_^X]^Y]W
@HWI-EAS443:3:95:243:1580#0/1
GGCCTATCGCAGTCCTCAAGAGCATGTCTCCTATTTCTAG
+HWI-EAS443:3:95:243:1580#0/1
abbabbbb]bbaabababa`babaaaa`ba_aaaaa`aa`
@HWI-EAS443:3:96:1311:726#0/1
ATGCATCCTGTGCAGCCATGGATGACTTTCAGCTGATACC
+HWI-EAS443:3:96:1311:726#0/1
__KZb[UYYaOa_abUX^_bQa_b[Ua__GZbWUPa]GNB
@HWI-EAS443:3:96:942:999#0/1
ACCGAATCATGGCCTGGACAGTGGTGAACAGTATATGGAA
+HWI-EAS443:3:96:942:999#0/1
a`X¥aaaaa`aaRTXa`_XZ[H]¥[_V^QZ¥¥`¥VG`^_B
@HWI-EAS443:3:97:938:1686#0/1
GAGGAAGAGAGCAGGGCAAGAATCAAAACTAGGCTTTTCA
+HWI-EAS443:3:97:938:1686#0/1
_¥N`[]TPY[S]HNP`a[aaaX]Wa^_]YFZOHVK]Y[^[
@HWI-EAS443:3:98:1429:1313#0/1
GGGCACTCGGTGAAAATATGGCACCAGAAAAAGTAGAGTT
+HWI-EAS443:3:98:1429:1313#0/1
aa``]¥[¥bbYbaa_Za`aa_[_VYa``_]Q_`G__TQYY
@HWI-EAS443:3:98:828:1156#0/1
GAAAGGCATAAATCCCAATTACCTCATGGCTTGGAAGAAG
+HWI-EAS443:3:98:828:1156#0/1
^b]b]_a`aaaa`]aY_¥aaaX¥a¥a_a`_aZWU_[BBBB
@HWI-EAS443:3:99:1198:1006#0/1
AGATGTTGGAGACCTTAAACAGTATGACAGTGATGAGACA
+HWI-EAS443:3:99:1198:1006#0/1
¥^_bbabbb[aaSY¥`¥aaaaaaa_`Q[T]U^_`a]XDNZ
@HWI-EAS443:3:10:560:1558#0/1
CGAATGCACTATTGAAGCACCGATTTGAGNTAATTGAAGG
+HWI-EAS443:3:10:560:1558#0/1
`bba`bbaa_b[Vb^`aba_^_YT_YYY[D[a_aaa^``_
```

@HWI-EAS443:3:15:1073:274#0/1
GGTGAACTTTGTAAGTATGGAGNTCTCACTCACTGACCCG
+HWI-EAS443:3:15:1073:274#0/1
aa¥aa¥`aaaa[``aVa`a^VWD[[`_a^`_a¥``a_ZN`
@HWI-EAS443:3:15:1414:220#0/1
CAAATAGTAGCATTGCCACAACTACTTCANTGCATGTGTG
+HWI-EAS443:3:15:1414:220#0/1
ab_abaa`ababbbaaYabba`bbabaa¥D¥bYaab`b_a
@HWI-EAS443:3:16:1116:2006#0/1
CTTGGGGGGCTATATGAAGCAATCGAGGAGTGCCNGATTA
+HWI-EAS443:3:16:1116:2006#0/1
``J_[I`ba^bb_baaZaaaabaa_`Z^aaSV`SDX`_``
@HWI-EAS443:3:16:220:1132#0/1
AAGAGCCTAACATAGTCAAACCACNTGAGAAAGGCATAAA
+HWI-EAS443:3:16:220:1132#0/1
abaaaaa`abaaaaa_aaaaa``YDWa]__`^`^[`^`_a
@HWI-EAS443:3:17:521:1261#0/1
AATTGANTGATTCAAGCTGGATAGAACTTGATGAAATAGG
+HWI-EAS443:3:17:521:1261#0/1
aab^D^aaaaaaaa```a``aa_aaa`a_aa`_a`a__
@HWI-EAS443:3:17:538:864#0/1
CATTCTTTGGCNGGAAAGAGCCTAACATAGTCAAACCACA
+HWI-EAS443:3:17:538:864#0/1
aa`ZY`Z¥a`^D[b_a`__]W¥^a`]_^a_W^^`]_[_]^
@HWI-EAS443:3:17:541:1924#0/1
CAAACTTGTTANTTTCGATTTTCGGATCTTCCCCATATTC
+HWI-EAS443:3:17:541:1924#0/1
aaaa`aabaa[DUaaaWMM````_XKPMXNPN_ZZ_Wa^V
@HWI-EAS443:3:22:1311:1531#0/1
CAAAATGAATTCAATAAGNCATGTGAATTGACTGATTCAA
+HWI-EAS443:3:22:1311:1531#0/1
]`a^`b¥[`ba_aabab]D¥abaSbaba^aaaabaaa]ba
@HWI-EAS443:3:22:1744:1347#0/1
GGGGGCTATATGAAGCAATCGAGGAGTGCCTGATTNATGA
+HWI-EAS443:3:22:1744:1347#0/1
aaa[aabababa_a`VZa`^XZa`[`^a_UaaX`YDT__Z
@HWI-EAS443:3:23:1337:727#0/1
CTCTGCTNGCACCTGCTTCCAAGCCATGAGGTAATTGGGA
+HWI-EAS443:3:23:1337:727#0/1
a`_[aaSD[aa___aST__W_¥[^ZZ¥a¥^_UWS]_`U¥W
@HWI-EAS443:3:23:1776:1538#0/1
CACCTGCTTCCAAGCCATGAGGTAATTGGGNTTTATGCCT
+HWI-EAS443:3:23:1776:1538#0/1
a`Y_babb]]`aK`ULU]babaHWXaabaUDM_XXa]XL]
@HWI-EAS443:3:25:1601:980#0/1
GAGGGGTTTTCGGCTGAATCGAGAAAATTGNTTCTCATTG
+HWI-EAS443:3:25:1601:980#0/1
aaaa_bUa`a``_]a_b_¥SX[__`_a`_KDZMH]Y_YR_
@HWI-EAS443:3:26:221:1886#0/1
TACATAGGCTCTAAAGTTTTCAAGGCTGGAGANGTTCGGT

```
+HWI-EAS443:3:26:221:1886#0/1
abbbbaaabaaMab_ZVa]]¥L]a_a_a[GWPDLV_]PZL
@HWI-EAS443:3:27:245:1750#0/1
CCCGAATGCACTATTGAAGCACCGNTTTGAGATAATTGAA
+HWI-EAS443:3:27:245:1750#0/1
abaaY_`]aVaZ^_Z]]_XaaaaXDX`^[]^W_``^__[I
@HWI-EAS443:3:28:13:161#0/1
GCACCAGANAAAGTAGACTTTGATGACTGCAAAGATGTTG
+HWI-EAS443:3:28:13:161#0/1
_abbb`]^DUZ^aYb`aaa`a]^aa¥``a`¥`¥`__a]^_
@HWI-EAS443:3:29:445:1915#0/1
ATATTTTCACCGAGTGCCCACTTCAATNGGCTTGTTCTCT
+HWI-EAS443:3:29:445:1915#0/1
a`a]___aaaaY`VPZ__¥_^X^a_XRDSJXZ]UOZZXBB
@HWI-EAS443:3:31:383:1239#0/1
GTCCCACTGCAGGGCTACTGAATACATAATGAAGGGNGTG
+HWI-EAS443:3:31:383:1239#0/1
^H_b`V`Y`_¥UZ^``Y`Ua]_Z_]^^RY`_YXXQTDSY]
@HWI-EAS443:3:32:1230:1714#0/1
CAGTCANTTCACATGCCTTATTGAATTCATTTTGGACCCA
+HWI-EAS443:3:32:1230:1714#0/1
a_Z¥a^D[¥aba_aQJ¥a`]aaZ]_aa`[][_TMVXa_a`
@HWI-EAS443:3:32:1443:624#0/1
GGAGACCTTAAACAGTATGANAGTGATGAGCCAGAGCCCA
+HWI-EAS443:3:32:1443:624#0/1
a_`^`a`ab]``_a]^a_`ZDY`^`¥]]_]]X_Y]Z]^_]
@HWI-EAS443:3:35:504:1695#0/1
CATTTGATTGCATCATNTAGTGGTATTCCCTCCCCCTCGT
+HWI-EAS443:3:35:504:1695#0/1
aba_`a]``aa``aa¥DY`]^¥[[^a_`a`_aaa]`^`]X
@HWI-EAS443:3:36:546:1951#0/1
AAGAATTCCTTGGTCATGTCNTTCTCTTTGACAGAAGACT
+HWI-EAS443:3:36:546:1951#0/1
aaaaT_abb_a]YGaa`^]UDT``_a`Y`]^^`___]_`X
@HWI-EAS443:3:37:1324:411#0/1
ATTTGTTTTNAAAGAATTCCTTGGTCATGTCTTTCTCTTT
+HWI-EAS443:3:37:1324:411#0/1
^_``a^`a^D^aa`¥`bba``b__S`_abGba``_`aaa_
@HWI-EAS443:3:37:261:670#0/1
CAGGCCATGATTCGGTNTCTTCCTTCAATTATCTCAAATC
+HWI-EAS443:3:37:261:670#0/1
`Y^¥SYZU`^U_Z[SKDO¥RV_Z_`^VVOOLT^PXXUWMP
@HWI-EAS443:3:39:1305:517#0/1
ATCAGCNGGAACTTTGACCGCTGATGGCAAAGAGGCCCAT
+HWI-EAS443:3:39:1305:517#0/1
`¥a^^YD^¥Ta``a`V_`¥¥¥`YU¥]]^^^SUUZSSUPVW
@HWI-EAS443:3:3:1169:1364#0/1
TGAAGNGAAGATCCCAAGGACAAAGAACATGAAGAGAACA
+HWI-EAS443:3:3:1169:1364#0/1
a`^a^DU¥]^^``^`[]a]WV___`_]^_]a_^a]_^^X_
```

@HWI-EAS443:3:40:1048:1050#0/1
CATTAATCAGGCACTNCTCGATTGCTTCATATAGCCCCCC
+HWI-EAS443:3:40:1048:1050#0/1
`Ya[^U^`XWa`]_UDUaa`X`_`MaMZPZZZ[U^`XQ¥U
@HWI-EAS443:3:42:815:643#0/1
ATACTGTTCACCACTGTCCAGGCCANGATTCGGTCTCTTC
+HWI-EAS443:3:42:815:643#0/1
ab`Zab^]aaa]^X_`]aa_a][_¥DX__`a¥[SW_SP]]
@HWI-EAS443:3:46:15:620#0/1
TTTCAAAGAATTCCTTGGTCATGTCTTNCTCTTTGACAGA
+HWI-EAS443:3:46:15:620#0/1
aaa``aabaa`a_``bY`^^_aaZ^a[DH_^a```]]__^
@HWI-EAS443:3:47:201:1561#0/1
GGGGCANGGAAATGAGGCGCTGCCTTCTTCAGTCTCTTCA
+HWI-EAS443:3:47:201:1561#0/1
aa`]a[D[a_a^abbaaaa__`STX_W[a¥_`[V_¥_`¥^
@HWI-EAS443:3:49:1255:1697#0/1
AAAATAGTTCCTCCTCANGCTTGCGATATGTTCAATCGGG
+HWI-EAS443:3:49:1255:1697#0/1
ababba]Y``¥a¥_``[DXW[_a]``_^`XT[SY_`TT_Z
@HWI-EAS443:3:4:1051:1433#0/1
AACAAAATAAAANCTGAGAAGACACACATTCACATCTTTT
+HWI-EAS443:3:4:1051:1433#0/1
aa^aaaabaa`YD¥a¥¥aa`a`aa]a_]]a[aZa_]_^_`
@HWI-EAS443:3:50:1268:1472#0/1
ATTTTCTCGATTCAGCCGAAANCCCCTCAAGTTGTGGAGA
+HWI-EAS443:3:50:1268:1472#0/1
abbabbb]`b`aba]ab^``YDW^ZUTXXM^Z`XRb`aa`
@HWI-EAS443:3:50:17:636#0/1
GAAATTTAGGCTTCTCTACCCCTGTTGTNTTACATATACT
+HWI-EAS443:3:50:17:636#0/1
^aababbb_aabbaaaab`baa`]]^QXDX`a]``aa`_a
@HWI-EAS443:3:50:872:1701#0/1
ATCCCAATTACCTCATGGCTTNGAAGCAGGTGCTAGCAGA
+HWI-EAS443:3:50:872:1701#0/1
aa[`_aaaaa[_`^_aa^_a[DPQ]ZW]TVWQQ]^[PUZV
@HWI-EAS443:3:51:676:949#0/1
TGGCACCAGAAAAAGTAGACTTTNATGACTGCAAAGATGT
+HWI-EAS443:3:51:676:949#0/1
baaaa]`PZ]^aUOa_P`S`WDXab_Z_`__```][^[
@HWI-EAS443:3:52:1071:1218#0/1
CGTCCTCAAGAATGGTTCAATTTTGGCGTTCANTTCTTTT
+HWI-EAS443:3:52:1071:1218#0/1
aaa`bbb_bbbbbbbaab]Z`bb_^][`][¥GDU¥N`aa`
@HWI-EAS443:3:53:1303:648#0/1
AGCNTAAATTTCTTCCTGATTTGTATGATTACAAAGAGAA
+HWI-EAS443:3:53:1303:648#0/1
aa[D]baabbbaabaaba`ababaabb`aaa_aaaaa`a_
@HWI-EAS443:3:53:914:1242#0/1
AGAGAACAAGCCAATTGAAGTGGGCACTCNGTGAAAATAT

```
+HWI-EAS443:3:53:914:1242#0/1
a``_aaabaa_a_aaaa___]aaa`a``XD]X]__````X
@HWI-EAS443:3:55:308:628#0/1
AAGAGGCGAAGAGACAATTGAAGAAAAANTTGAGATTACA
+HWI-EAS443:3:55:308:628#0/1
aaa]ababa^`aa_a`_aaa`aa^``aXD[aaU```a__[
@HWI-EAS443:3:56:1386:1042#0/1
GCATGGAAATGAGGCGCTGCCTTCTTCAGTCTCTNCAGCA
+HWI-EAS443:3:56:1386:1042#0/1
aa¥]Z^NUa_]XUa_a¥a_^P[ZPUUW_`ZL_YZDDXSUU
@HWI-EAS443:3:56:689:11#0/1
ATCGACGAACGGGGTGAATCAATAATTGTAGAATNTGGTG
+HWI-EAS443:3:56:689:11#0/1
_¥]`Z_`]XV_ab_L_RY`YX¥^V[a^Y[W[UaZDZW^U`
@HWI-EAS443:3:56:75:441#0/1
AAGATGTTGGAGACCTTAAACAGNATGACAGTGATGAGCC
+HWI-EAS443:3:56:75:441#0/1
aabXabaaba`aa`_`a```_`YDZ`a_^^`S_^[^^^W^
@HWI-EAS443:3:58:1409:502#0/1
GGGATCTTCTCTTCATNTTCAATGTCCTGTAGCTCTGCTA
+HWI-EAS443:3:58:1409:502#0/1
aabZ_`baaaaaaa`[D[a``_a___`a`a``_[W`a^_]
@HWI-EAS443:3:5:183:967#0/1
AAAGAATTCCTTGGTCATGTCTTTCTNTTTGACAGAAGAC
+HWI-EAS443:3:5:183:967#0/1
abbb__bb`abbaa]aabaaaaaba^D[aaa^^`a_aa]B
@HWI-EAS443:3:60:1128:902#0/1
CCACTTCAATTGGCTTGNTCTCTTCATGTTCTTTGTCCTT
+HWI-EAS443:3:60:1128:902#0/1
aaaaa````^Z^]O`]WD¥MXU^``Y`]RZ_a``VPXY^[
@HWI-EAS443:3:61:565:530#0/1
CGATTGGNCATGTTTCCGATTTGTTTTCAAAGAATTCCTT
+HWI-EAS443:3:61:565:530#0/1
^abbba^D]_¥`]bb^_ZX^^abUaaab_¥¥a`X[a]Z`[
@HWI-EAS443:3:63:1116:1572#0/1
ATTCGGATTTCCATTTCATCGACGAACGGGGTGAANCAAT
+HWI-EAS443:3:63:1116:1572#0/1
abba`¥_```ZYaaabbb]ZZ`^_``_N[[][a`VDSXW_
@HWI-EAS443:3:64:1221:1796#0/1
CAAATAGTAGCATTGCCACAACTACTTCANTGCATGTGTG
+HWI-EAS443:3:64:1221:1796#0/1
aa`babb^abaabaaaab`bbabb^aaa^D[aaa`a_aSa
@HWI-EAS443:3:65:550:1146#0/1
GCTTGCGATATGTTCAATCGGGGCAACATNTTCTCCTATT
+HWI-EAS443:3:65:550:1146#0/1
abbbabbbbabb_abb``aa`a`aa]aaWDYaaaaaaaba
@HWI-EAS443:3:69:1451:1421#0/1
ACTTCCCTCCGTGTTACTCNAATTTCAATGAACCGGTTCT
+HWI-EAS443:3:69:1451:1421#0/1
aaaa^aT^aYa[V]¥Y]_WDWX`a``a^]]a^`_`¥X[[Z
```

```
@HWI-EAS443:3:71:1169:285#0/1
AAATNGGAGACATGCTCTTGAGGACTGCGATAGGCCAAGT
+HWI-EAS443:3:71:1169:285#0/1
^X^ZD^¥¥Z]a`aa`aaa_b^b`¥VS¥Ua_^__¥T¥V^^M
@HWI-EAS443:3:71:844:1994#0/1
CATATAGCCCCCNAAGATCGAAGGTTCCAGGTTCCAGGTT
+HWI-EAS443:3:71:844:1994#0/1
`b`babababbZD^a]b`b_TY^_^abb¥FHY^aa`FVW^
@HWI-EAS443:3:72:1573:628#0/1
GAACAAGCCAATTGNAGTGGGCACTCGGTGAAAATATGGC
+HWI-EAS443:3:72:1573:628#0/1
aaaaaabaabaaa¥D[b`aaa_a_``aa]a`aa_`````Z
@HWI-EAS443:3:72:733:1480#0/1
CAGATTTTGCCAGTAAGGTCCTGCNCACTTTCCCAATAGA
+HWI-EAS443:3:72:733:1480#0/1
`W^_X]aaababa¥ab`aZab`a[D[a`aa]baa_]Xa^¥
@HWI-EAS443:3:75:1115:735#0/1
ACCTTCGATCTTGGGGGGCTATATGAAGCAATCGANGAGT
+HWI-EAS443:3:75:1115:735#0/1
ab``aaa[_S__[ba[`]U]O]`ZN¥XY_``U`_UD]T_H
@HWI-EAS443:3:75:1771:988#0/1
CCGAGTCTTCTGTCAAAGAGAANGACATGACCAAGGAATT
+HWI-EAS443:3:75:1771:988#0/1
abaa`abbbabb_``aab`^a^D[aaaaaa`a`aaa_`_a
@HWI-EAS443:3:76:1757:477#0/1
CAGGCACTCCTCGATTGNTTCATATAGCCCCCCAAGATCG
+HWI-EAS443:3:76:1757:477#0/1
__bbbbbb^T``__``[D^```baa[^]b]Z¥ba`a__Ub
@HWI-EAS443:3:79:960:1334#0/1
GGCAAGCTTTCCCAAATGTCAAAAGAAGTGAACNCCAAAA
+HWI-EAS443:3:79:960:1334#0/1
a_`ZI_a_aa¥`aa`Y`b_W]Y[^^FZ[FXaa[D[S_XZ]
@HWI-EAS443:3:83:87:1980#0/1
TCCACTCCCCTGGGTGACTCTNCGATTGGCCATGTTTCCG
+HWI-EAS443:3:83:87:1980#0/1
abbbaabaab`___R[aa_`UDZFL[^_Z_aXYSWY^U_R
@HWI-EAS443:3:85:1419:602#0/1
AAGAATTCCTTGGTCATGTCNTTCTCTTTGACAGAAGACT
+HWI-EAS443:3:85:1419:602#0/1
abbb`b^X`baaa^_aaa¥TD^a]bYbaabWN_aa_`_^_
@HWI-EAS443:3:86:1158:1360#0/1
CACCGAGTGCCCACTTCAATTGGCTTGTNCTCTTCATGTT
+HWI-EAS443:3:86:1158:1360#0/1
a``babbbba`abbabaabbabbNb`¥¥DNbHba_a`Z^a
@HWI-EAS443:3:88:1081:635#0/1
GGCCTTGCTCAATGCATCCTGTGCAGCCATGGANGACTTT
+HWI-EAS443:3:88:1081:635#0/1
Zb`a]aa`b`T`bbbb_[``bbab`a^aaZbaUD¥`¥b`a
@HWI-EAS443:3:88:1250:1472#0/1
ATTTNAGAAATGATACTGATGTGGTGAACTTTGTAAGTAT
```

```
+HWI-EAS443:3:88:1250:1472#0/1
^b`¥DWbbab_O`abab_`bbaVb^a`ZJaaababaa^¥^
@HWI-EAS443:3:8:943:680#0/1
CATGGGCCTCGACACTTGGNCTATCGCAGTCCTCAAGAGC
+HWI-EAS443:3:8:943:680#0/1
aba_a][^`aa^_^ZVaaVDX``a`___aU_¥_]_¥a^`Q
@HWI-EAS443:3:90:744:190#0/1
GGGACAANCTGGAACCTGGAACCTTCGATCTTGGGGGGCT
+HWI-EAS443:3:90:744:190#0/1
__Tbb`[D[a`X_b`a_ba`aab^Zaa^a`bab``^a`[[
@HWI-EAS443:3:91:1726:1919#0/1
CAGGCCATGATTNGGTCTCTTCCTTCAATTATCTCAAATC
+HWI-EAS443:3:91:1726:1919#0/1
aaaaabbabba^DV`aaaaaa^_bbXaa`aab___`aaW¥
@HWI-EAS443:3:91:238:1708#0/1
GAGNAAGGCATAAATCCCAATTACCTCATGGCTTGGAAGC
+HWI-EAS443:3:91:238:1708#0/1
ZU^D[]_ZZ``b][M[Y]^^N_a_X¥W`[WBBBBBBBBBB
@HWI-EAS443:3:94:1269:1794#0/1
AACTTCTCCAGCCTTGAAAACTTTAGAGCCNATGTAGATG
+HWI-EAS443:3:94:1269:1794#0/1
``_b]KZa``a__a]`_``Y__[`_^]a_ODN_`X^__`_
@HWI-EAS443:3:94:1677:1734#0/1
CAGCAAACTTGTTAGTTTCGATTTTCGGATCTTCCCNATA
+HWI-EAS443:3:94:1677:1734#0/1
^aaabbb`aababbbYbbVaPKaab_YUKWDX`PP[DD_H
@HWI-EAS443:3:94:467:132#0/1
AATTCCTTGGTCATGTCTTTNTCTTTGACAGAAGACTCGG
+HWI-EAS443:3:94:467:132#0/1
a__``^a_aa^^]_aYZ_`XDW___^_]Y[_^^X^XYV^^
@HWI-EAS443:3:94:83:860#0/1
AGTGTACATAAATACNGCCTTGCTCAATGCATCCTGTGCA
+HWI-EAS443:3:94:83:860#0/1
aa]b]`^aaaS]a_[DP___[_`_^[^_`Z]___U^^_^W
@HWI-EAS443:3:97:1558:15#0/1
ACTGTTTAAGGTCTCCAACATCTTTGCAGTCATCNAAGTC
+HWI-EAS443:3:97:1558:15#0/1
a_abSbaba`SKLaWW`aZ`¥H[``^Y_BBBBBBBBBBBB
@HWI-EAS443:3:97:448:1380#0/1
CCGATTTGNGATAATTGAAGGAAGAGACCGAATCATGGCC
+HWI-EAS443:3:97:448:1380#0/1
^b_Y`a_[D]abbbbbb`ba]`b¥bbbaab_ababba]a`
@HWI-EAS443:3:9:1471:1368#0/1
GGCTGGANAAGTTCGGTGGGAGACTTTGGTCGGCAAGCTT
+HWI-EAS443:3:9:1471:1368#0/1
Y`TW_b¥DVX__^`¥^T¥_XaaY¥V]X]VYJEWTZ[]TRX
@HWI-EAS443:3:9:960:992#0/1
GGGGCATGGAANTGAGGCGCTGCCTTCTTCAGTCTCTTCA
+HWI-EAS443:3:9:960:992#0/1
aYa][a`[^RXDX]]a[¥ZZ_`¥Y[^S[^^Y`QUZYV[UU
```

```
@HWI-EAS443:3:100:332:640#0/1
CGGGGGGGCTATATGAAGCAATCGAGGAGTGCCTGATTAA
+HWI-EAS443:3:100:332:640#0/1
ab```bbaabababbaaaab]a_a`aa__SaZ_aa__`_B
@HWI-EAS443:3:10:497:1225#0/1
CGGGGGGGCTATATGAAGCAATCGAGGAGTGCCTGATTAA
+HWI-EAS443:3:10:497:1225#0/1
Zb`W¥_HQQZ_a¥Waaaa`X¥]RSKYXPXRRSN__^¥¥^¥
@HWI-EAS443:3:12:1330:1040#0/1
GATTGAGGGGTTTTCGGCTGAATCGAGAAAATTGCTTCTC
+HWI-EAS443:3:12:1330:1040#0/1
a``aaa``]aZa`[[aa]a^___¥¥YVa`^^¥¥`W^¥X^M
@HWI-EAS443:3:12:1767:148#0/1
CAGCTGAAAGTCATCCATGGCTGCACAGGATGCATTGAAA
+HWI-EAS443:3:12:1767:148#0/1
a_bT````TaG]SXTSLWa¥[__YBBBBBBBBBBBBBBBB
@HWI-EAS443:3:13:1581:1737#0/1
AATGAAGGGAGTGTACATAAATACGGCCTTGCTCAATGAA
+HWI-EAS443:3:13:1581:1737#0/1
`ba_Z¥W^^YaQb`]Ga_GXaaa_[`_UXWFJ__a`G]BB
@HWI-EAS443:3:15:1784:803#0/1
ATGAGAAAGGCATAAATCCCAATTACCTCATGGCTTGGGA
+HWI-EAS443:3:15:1784:803#0/1
a¥aV¥YW¥O]baaY¥[`[Yaa][P``UaRaa¥]ZBBBBBB
@HWI-EAS443:3:16:1619:288#0/1
AGCCTGCACACTTTCCCAATAGAGCCTTCCTCCACTCCCC
+HWI-EAS443:3:16:1619:288#0/1
abZT^``aXa]`_a[^`aa`]YY`YG`[MR_SU¥KXWMBB
@HWI-EAS443:3:17:725:394#0/1
ACCGACGAACGGGGTGAATCAATAATTGTAGAATCTGGTG
+HWI-EAS443:3:17:725:394#0/1
[¥_[QXaI`¥`^_]Q]`_]X_[^PT`X^QZ]GX`¥_THT^
@HWI-EAS443:3:18:646:1377#0/1
CGGGGATCTTCTCTTCATTTTCAATGTCCTGTAGCTCTGC
+HWI-EAS443:3:18:646:1377#0/1
a_`[`Kaba]abbT^abaaVS^YU^bQ`Q_aYaa`_a_`^
@HWI-EAS443:3:19:331:1583#0/1
GTCCCGATTGAACATATCGCAAGCATGAGGAGGAACTATT
+HWI-EAS443:3:19:331:1583#0/1
^GQZUSQ`]]]bXa_`^`a¥¥YVU___^_O¥^^S_^Y[V]
@HWI-EAS443:3:20:233:380#0/1
CTAGAACACAGTATTTTTCCCATTTGTGTGGCTCCAGTCT
+HWI-EAS443:3:20:233:380#0/1
abbaaa``abb¥b`aabaa`a_aaaa_a¥`Z___a^UM^W
@HWI-EAS443:3:21:1189:1750#0/1
TGGAAAGAGCCTAACATAGTCAAACCACATGAGAAAGGAA
+HWI-EAS443:3:21:1189:1750#0/1
abb`_X_]aSHU^b[_W¥VS^baV`Q¥SXWX_`aaJWYGU
@HWI-EAS443:3:22:1491:966#0/1
CGGGGGGGCTATATGAAGCAATCGAGGAGTGCCTGATTAA
```

```
+HWI-EAS443:3:22:1491:966#0/1
^abab`X_a_¥_R[¥¥T¥¥aWa``S_ZZZT¥OMY[EY]UQ
@HWI-EAS443:3:23:616:589#0/1
TCGCTGGATAGAACTTGATGAAATAGGAGAAGATGTTGCC
+HWI-EAS443:3:23:616:589#0/1
Uab]a^a^XS_X^^aabX`_FYY]_aaa[]P]`_a__^JW
@HWI-EAS443:3:24:1128:781#0/1
CGGGTGAACAGTATATGTAACACAACAGGGGTAGAGAAGC
+HWI-EAS443:3:24:1128:781#0/1
abba_bb_abbabbbbbabbbbababababbbb`bbabaaa^
@HWI-EAS443:3:24:185:1550#0/1
AGGAGGAGGAACTATTTTACAGCAGAAGTGTCCCACTGCA
+HWI-EAS443:3:24:185:1550#0/1
¥a^a`_`aaaaaabbaaabaaaabaaaa¥`_aaaaa`a_a
@HWI-EAS443:3:25:16:1694#0/1
GACTCCACAACTTGAGGGGTTTTCGGCTGAATCGAGAAAA
+HWI-EAS443:3:25:16:1694#0/1
`Vb`bb_a^Zaa`aa_aa`W`_``a[``_aa^^____``_
@HWI-EAS443:3:25:985:987#0/1
TCCCAGGCCATGATTCGGTCTCTTCCTTCAATTATCTCAA
+HWI-EAS443:3:25:985:987#0/1
aa`aaa`aaaaaa```__]^^___`__`_````__Z_^_`
@HWI-EAS443:3:26:1728:340#0/1
ATGCGGACTACACCCTTGACGAAGAGAGCAGGGCAAGAAT
+HWI-EAS443:3:26:1728:340#0/1
a__Zab``aaZaZ[aa¥`_[`Z[`^`¥_^Y]`Y[N¥aUZW
@HWI-EAS443:3:27:1449:1116#0/1
CTCAAGTTGTGGAGACGCATATAGACTGTTGAATACAGGT
+HWI-EAS443:3:27:1449:1116#0/1
YXVRab]`[S¥Zab_`[¥[a`¥HUZ¥]]G^RPYGZV[BBB
@HWI-EAS443:3:27:231:1822#0/1
AACAAGTTTGCTGCAATATGCACACATTTGGAAGTTTGCT
+HWI-EAS443:3:27:231:1822#0/1
^U^]baXa`ab¥]^aabZ`[^aaa`b^¥¥_aZRXW__R``
@HWI-EAS443:3:27:322:1195#0/1
GCTCTCTTCATGTTCTTTGTCCTTGGGATCTTCTCTTCAT
+HWI-EAS443:3:27:322:1195#0/1
`a`ba`babb__Saa^Y¥U^baa^a^a]a`a`a``a_aa`
@HWI-EAS443:3:28:1340:705#0/1
GGGGCATGGAAATGAGGCGCTGCCTTCTTCAGTCTCTTAA
+HWI-EAS443:3:28:1340:705#0/1
_ZGWY^HZZ`TPX^YRVU¥``_[WS¥BBBBBBBBBBBBBB
@HWI-EAS443:3:29:860:1734#0/1
GACGCCTCTTTCGGACTGACGAAAGGAATCCCATAGACTC
+HWI-EAS443:3:29:860:1734#0/1
Q^^FWbbbabb]]]b`a]aba`bb^`bbbbabbbaaaa__
@HWI-EAS443:3:30:1467:1994#0/1
AAATCCCAATTACCTCATGGCTTGGAAGCAGGTGCTAGAA
+HWI-EAS443:3:30:1467:1994#0/1
_aaa_^`aa`_```__a___`^YRUU¥VR[R[BBBBBBBB
```

```
@HWI-EAS443:3:30:978:536#0/1
CCGGGCTCTGGCTCATCACTGTCATACTGTTTAAGGTCTC
+HWI-EAS443:3:30:978:536#0/1
ab`abaaababa_aa__``aa]^aaa_aaaaaa___TT_]
@HWI-EAS443:3:31:100:488#0/1
TACAAAGATGTTGGAGACCTTAAACAGTATGACAGTGATG
+HWI-EAS443:3:31:100:488#0/1
ababa^aaaa`aaaaa`aa__aa`aaaXa_`Z``XDZZ¥Z
@HWI-EAS443:3:31:1040:1397#0/1
TGCGTCTCCACAACTTGAGGGGTTTTCGGCTGAATCGACA
+HWI-EAS443:3:31:1040:1397#0/1
Wab_`[]W^`WQW_`[`ZXUa^UaU_HSab^JT¥P]QYKZ
@HWI-EAS443:3:31:131:957#0/1
ACATATCGCAAGCATGAGGAGGAACTATTTTACAGCAGCA
+HWI-EAS443:3:31:131:957#0/1
aaVDU^^_a_^¥]P¥XV_^YWURV^¥^^WWU^¥^_^^NFV
@HWI-EAS443:3:31:29:1680#0/1
TTACTTCTTTTGACATTTGGGAAAGCTTGCCCTCAATGCA
+HWI-EAS443:3:31:29:1680#0/1
aabbaaaaaaa¥W`a`¥ZO[^`[]]a`^^_^a__RZ^]a`
@HWI-EAS443:3:32:474:934#0/1
CCGGGGCTATATGAAGCAATCGAGGAGTGCCTGATTAATG
+HWI-EAS443:3:32:474:934#0/1
¥DP__[RLJ^ZZ^NRV^¥`QY[N¥^Y¥U^VWT¥URRBBBB
@HWI-EAS443:3:34:1678:1563#0/1
CACAACTTGAGGGGTTTTCGGCTGAATCGAGAAAATTGGT
+HWI-EAS443:3:34:1678:1563#0/1
`¥Y^_YaWVW_Z_^Z]WaROULT[Z`¥BBBBBBBBBBBBB
@HWI-EAS443:3:36:1327:873#0/1
CTCTTCCTTCAATTATCTCAAATCGGTGCTTCAATAGTAC
+HWI-EAS443:3:36:1327:873#0/1
abbbbbabbbbaaaaabbaa^`baaa_``aa_aaaab¥aa
@HWI-EAS443:3:36:1352:210#0/1
TAGGGGGGCTATATGAAGCAATCGAGGAGTGCCTGATTAA
+HWI-EAS443:3:36:1352:210#0/1
aabbaaabaaa````¥aaZa``_```ZNa]aSP[aZ^Y¥¥
@HWI-EAS443:3:36:63:457#0/1
GCATTCCTTGGTCATGTCTTTCTCTTTGACAGAAGACTCG
+HWI-EAS443:3:36:63:457#0/1
]GP_Z``a^__S[O¥ZW`a_]YZYZ[WJ[[[U[]V]¥RYX
@HWI-EAS443:3:36:815:902#0/1
AGGAATTCCTTGGTCATGTCTTTCTCTTTGACAGAAGACT
+HWI-EAS443:3:36:815:902#0/1
Z`S^T¥Y`b_KU__bbb`¥`b¥V_a_a]_a]P_]Z]_`aX
@HWI-EAS443:3:38:1485:532#0/1
CCGGGGGGCTATATGAAGCAATCGAGGAGTGCCTGATTAA
+HWI-EAS443:3:38:1485:532#0/1
`ba^__]`aaaaa`a`ab```aaa^`a_bRWUN^¥Za__V
@HWI-EAS443:3:38:414:997#0/1
CCGGGGGCTATATGAAGCAATCGAGGAGTGCCTGATTAAT
```

+HWI-EAS443:3:38:414:997#0/1
a``a`_ZaZa_`V_[YRY`YR`PNRYXXGTUYVUUZ¥V[Q
@HWI-EAS443:3:3:1390:320#0/1
GCCCTCCTCATGCTTGCGATATGTTCAATCGGGGCAACAT
+HWI-EAS443:3:3:1390:320#0/1
a]a¥a¥`a_aaZ¥aaaZaaa`a`[_^_`_T^[[`T¥¥U¥[
@HWI-EAS443:3:3:646:760#0/1
ACGGGGGGCTATATGAAGCAATCGAGGAGTGCCTGATTAA
+HWI-EAS443:3:3:646:760#0/1
a`a_a[ab`abaaba_[]`___Za``a¥a¥aVT]`^U_^^
@HWI-EAS443:3:40:26:1598#0/1
AGCCCGTTCGTCGATGAAATGGAAATCCGAATACATGAAA
+HWI-EAS443:3:40:26:1598#0/1
a]`aba_`aa^aa_`^a_a``^]a_`_`^_`_`_`ZT^G^
@HWI-EAS443:3:42:425:703#0/1
GCCGAAGAGACAATTGAAGAAAAATTTGAGATTACAGGAA
+HWI-EAS443:3:42:425:703#0/1
[G^^P_YVRVXW`a`Y`U`_a[YPaV]X_^[[W¥U^VYYa
@HWI-EAS443:3:43:1220:255#0/1
CTAGAACACAGTATTTTTCCCATTTGTGTGGCTCCAGTCT
+HWI-EAS443:3:43:1220:255#0/1
`bba_aaba`a`abbbaaaaa`bab`^a^`a]`]Y¥_XZ]
@HWI-EAS443:3:43:323:466#0/1
GTCATGGAAATGAGGCGCTGCCTTCTTCAGTCTCTTCAGC
+HWI-EAS443:3:43:323:466#0/1
aYS`_aa```_```aaa``_^_`____ZZ^WX[[___[¥U
@HWI-EAS443:3:45:1466:211#0/1
TAGAAATAGGAGACATGCTCTTGAGGACTGCGATAGGCCA
+HWI-EAS443:3:45:1466:211#0/1
a`baaaa`aa`a_^`aa`_``_a^``^Z^_[a[V[[[QSU
@HWI-EAS443:3:47:1346:481#0/1
GNTCAATCGGGGCAACATCTTCTCCTATTTCATCAAGTTC
+HWI-EAS443:3:47:1346:481#0/1
¥D^aaaaaaaa`]`a_aa_aa_a__a`aaa__`¥_Za_`]
@HWI-EAS443:3:48:299:835#0/1
AAGAATTCCTTGGTCATGTCTTTCTCTTTGACAGAAGAAT
+HWI-EAS443:3:48:299:835#0/1
a_``]b]ba`bb_U`aab_^]ZbaZ_aa``UUV^[YaQH]
@HWI-EAS443:3:48:349:526#0/1
TGGGGGGGCTATATGAAGCAATCGAGGAGTGCCTGATTAA
+HWI-EAS443:3:48:349:526#0/1
U¥__Y¥_]PR]XYXZ¥^[M¥]¥X^Y^]X_N_XLT[MUZRR
@HWI-EAS443:3:4:1295:717#0/1
GGGAGGGGTTTTCGGCTGAATCGAGAAAATTGCTTCTCAT
+HWI-EAS443:3:4:1295:717#0/1
`baYab``S`a_ZU_][aa^VUaPX¥]¥]^V_RV[Y[M^]
@HWI-EAS443:3:4:26:180#0/1
ACCATTGGGATCAGCTGAAAGTCATCCATGGCTGCACAGG
+HWI-EAS443:3:4:26:180#0/1
aa]`^aa^]_¥_YXY`Z_`ZURQV^__^Z_YYP]BBBBBB

```
@HWI-EAS443:3:4:527:281#0/1
CGCCAGAAAAAGTAGACTTTGATGACTGCAAAGATGTTGG
+HWI-EAS443:3:4:527:281#0/1
a```_^^aaaaa^aaa_aaa^__a__a^[`^_[^`a^_^Y
@HWI-EAS443:3:53:212:1828#0/1
GGGGAGGAACTATTTTACAGCAGAAGTGTCCCACTGCAGG
+HWI-EAS443:3:53:212:1828#0/1
¥^^^_ba`Yaa`aaaaba^¥[_`^¥¥ZZX^]^]UUaZ`VT
@HWI-EAS443:3:55:1114:327#0/1
GTCTGGACAGTGGTGAACAGTATATGTAACACAACAGGGG
+HWI-EAS443:3:55:1114:327#0/1
a__aaa`_aa^a_X``___aZ____aZ__^`]_^Z^a_``
@HWI-EAS443:3:56:431:595#0/1
CNCCTCTTTCGGACTGACGAAAGGAATCCCATAGACTCCT
+HWI-EAS443:3:56:431:595#0/1
VD[__`_`aaa`_`_^_`a__^a¥N^__Y`_Z_]__]^]]
@HWI-EAS443:3:57:304:989#0/1
GGACTGGAGAGGAGATGGCCACCAAAGCGGACTACACCCT
+HWI-EAS443:3:57:304:989#0/1
abaabaaaaaaa]aaa`[__a]aZaa^aaaa^__^`YSU_
@HWI-EAS443:3:58:717:76#0/1
CACCTATTTCATCAAGTTCTATCCAGCTTGAATCAGTCAA
+HWI-EAS443:3:58:717:76#0/1
abaababbbaaaabaaaaaaaaa`aaababa¥a_`a^_`a
@HWI-EAS443:3:59:1353:1044#0/1
TCGGGGCTATATGAAGCAATCGAGGAGTGCCTGATTAATG
+HWI-EAS443:3:59:1353:1044#0/1
`aaaab`abaaaba``[[b`_``^[ZaU`ba[_VaW¥aYT
@HWI-EAS443:3:5:214:341#0/1
GAGATGGCCACCAAAGCGGACTACACCCTTGACGAAGAAG
+HWI-EAS443:3:5:214:341#0/1
`Uaa`_````¥_W^[a^`[_`a`Z``[]¥`¥Y¥`¥X`¥`B
@HWI-EAS443:3:60:1329:1434#0/1
CGTAAATACGGCCTTGCTCAATGCATCCTGTGCAGCCATG
+HWI-EAS443:3:60:1329:1434#0/1
YHXbbbabb`a^aabbaaabaa`abaaa```a`aaa]aaa
@HWI-EAS443:3:65:1009:1931#0/1
CTAGAACACAGTATTTTTCCCATTTGTGTGGCTCCAGTCT
+HWI-EAS443:3:65:1009:1931#0/1
a[ZaXa_aa_UT][_a]X[^]X`_a¥P^^`W][[TBBBBB
@HWI-EAS443:3:68:277:743#0/1
GTCCCGATTGAACATATCGCAAGCATGAGGAGGAACTATT
+HWI-EAS443:3:68:277:743#0/1
a`abba`aaaab_a_a^¥`a__`_[`[^]^Z`_^ZS^^`]
@HWI-EAS443:3:71:1603:232#0/1
CTATGTTAGGCTCTTTCCAGCCAAAGAATGTCTTCATGNA
+HWI-EAS443:3:71:1603:232#0/1
abb`bbbab``a`aabba`baaaaabaab```bb_aa¥D[
@HWI-EAS443:3:72:1243:1864#0/1
AAACCACATGAGAAAGGCATAAATCCCAATTACCTCATTG
```

+HWI-EAS443:3:72:1243:1864#0/1
_¥ba`[_aS^^RX`P[``_Z_]aX`R`a[_`aZY]Z__X_
@HWI-EAS443:3:74:1130:1080#0/1
ACGGGGGCGTGGTGTCGTCCTCAAGAATGGTTCAATTTTG
+HWI-EAS443:3:74:1130:1080#0/1
`__]¥`]`_JZZVa¥a[NY_X[PUZSJY]]N[UWHW¥BBB
@HWI-EAS443:3:77:1650:1999#0/1
AGCCGGTTCATTGAAATTGGAGTAACACGGAGGGAAGTCC
+HWI-EAS443:3:77:1650:1999#0/1
aba_ZW^¥G`]__I^¥TZ`UT[S_SZW__[V[Z_[SQI[G
@HWI-EAS443:3:79:471:1365#0/1
AGGGCCGTATTTATGTACACTCCCTTCATTATGTATTCAG
+HWI-EAS443:3:79:471:1365#0/1
WX[[]baY__a¥¥_YVab_WO[Z_¥T_]_TQ¥[VXVT]a^
@HWI-EAS443:3:80:361:1202#0/1
CATGAAGACATTCTTTGGCTGGAAAGAGCCTAACATAGAC
+HWI-EAS443:3:80:361:1202#0/1
aY]aaa_aaaaZ]¥[XT¥aVXZUJ_¥^[Q[_a]VYX`VHX
@HWI-EAS443:3:80:618:1976#0/1
CCACAACTTGAGGGGTTTTCGGCTGAATCGAGAAAATTCC
+HWI-EAS443:3:80:618:1976#0/1
`b¥]Q]a`ba]^`aaYW_aaaS¥YZ^RLSSQ_``]H]SBB
@HWI-EAS443:3:83:1084:1389#0/1
GATGGGCTCTGGCTCATCACTGTCATACTGTTTAAGGTCT
+HWI-EAS443:3:83:1084:1389#0/1
a`a_aaa``aaaaaaaaaa_aa_^aaaa_a^a_`^[_]`_
@HWI-EAS443:3:83:244:143#0/1
GGCTTTCTCATGTGGTTTGACTATGTTAGGCTCTTTCCAG
+HWI-EAS443:3:83:244:143#0/1
^HQV¥Y`[^[]`P__]]TVTRV¥TYXYSSPFRSJW[TQLJ
@HWI-EAS443:3:85:1372:1416#0/1
TNTGGGCACTCGGTGAAAATATGGCACCAGAAAAAGTAGA
+HWI-EAS443:3:85:1372:1416#0/1
WD[baa]`a]ab_O^^aaaa_aaa^_`XUaaaa[]aXaa[
@HWI-EAS443:3:87:795:605#0/1
GGGGCTTTTCACTATAAGACAAGAAATGGCCAGTAGGAGT
+HWI-EAS443:3:87:795:605#0/1
G^_XbY¥`_Waa_[[`Vbb__`Ubb[bba]UbaJM[bb[[
@HWI-EAS443:3:8:1322:57#0/1
TCATTTTGGACCCAGCTTGCTAGAGATCTGGGCTCTGGAT
+HWI-EAS443:3:8:1322:57#0/1
_aaaaaab`b¥P[P[Za¥_]TR[^W[RGV^`BBBBBBBBB
@HWI-EAS443:3:8:436:1054#0/1
CGCAAGCTTGCCGACCAAAGTCTCCCACCGAACTTCTCAA
+HWI-EAS443:3:8:436:1054#0/1
aa`Z]aa_T_]^aa`^]^¥_^___``ZV_`TTZZWT¥TDL
@HWI-EAS443:3:90:1444:1362#0/1
CATTAATCAGGCACTCCTCGATTGCTTCATATAGCCCCTC
+HWI-EAS443:3:90:1444:1362#0/1
aY_][VV[YO[a_X__a¥Z[a¥VW]a`[^Zaa^SFQVYKY

```
@HWI-EAS443:3:93:953:640#0/1
CTGTAGCTCTGCTAGCACCTGCTTCCAAGCCATGAGGTGA
+HWI-EAS443:3:93:953:640#0/1
abbabbbabab¥aaaaa_aaa_aa¥`a`aN__aa[a`MDX
@HWI-EAS443:3:94:884:877#0/1
TCCATTTCATCGACGAACGGGGTGAATCAATAATTGTATG
+HWI-EAS443:3:94:884:877#0/1
aabbaaaaa`a`a`]____]aZ^___`___`[S^`_O__¥
@HWI-EAS443:3:98:460:98#0/1
CATTTGTGTGGCTCCAGTCTCGGGTCAGTGAGTGAGAAAT
+HWI-EAS443:3:98:460:98#0/1
abba[_R__b]_b_``b¥_¥_PbbGHY^YSGaGaJXG_QW
@HWI-EAS443:3:99:412:2005#0/1
TACATATACTGTTCACCACTGTCCAGGCCATGATTCGGCC
+HWI-EAS443:3:99:412:2005#0/1
_a`bab_bb_USa`abVbaa[XUU`aaJJ_P]a]a[aZBB
@HWI-EAS443:3:100:875:1337#0/1
ACCCGTTCGTCGATGAAATGGAAATCCGAATACATGAAAC
+HWI-EAS443:3:100:875:1337#0/1
aa`OWQ[YXVY_Z`aW_TRZ_]aUXJOYV¥X`]`XUWXBB
@HWI-EAS443:3:10:79:2046#0/1
NCCGAATGCACTATTGAAGCACCGATTTGAGATAATTGAA
+HWI-EAS443:3:10:79:2046#0/1
DNVVSSYWNTUUUURUTQTVUVWRVVVTQRWWUSSVRUVV
@HWI-EAS443:3:11:367:597#0/1
CGCCATATTTTCACCGAGTGCCCACTTCAATTGGCTTGTT
+HWI-EAS443:3:11:367:597#0/1
abab_a`abbaaaaab`]S`]]a`aa`aa__Z__`]_WUU
@HWI-EAS443:3:12:1081:942#0/1
TGGGTGTAGTCCGCTTTGGTGGCCATCTCCTCTCCAGTGA
+HWI-EAS443:3:12:1081:942#0/1
aaaaJ]Y]aTaa¥ZYUa__Y``RV_Y_¥VXW_¥[NT[T_V
@HWI-EAS443:3:12:1346:185#0/1
CGGCGTTCACTTCTTTTGACATTTGGGAAAGCTTGCCCTC
+HWI-EAS443:3:12:1346:185#0/1
aaa_b_aaa^aaS]aa]X¥R`_`a^`a¥_^a¥_`U[T¥^W
@HWI-EAS443:3:12:1720:1109#0/1
NTGAGAAGCAATTTTCTCGATTCAGCCGAAAACCCCTCAA
+HWI-EAS443:3:12:1720:1109#0/1
DLVQVVXYXVYYXVXRYUXRRYXVOGWXXUYVXUXYYXBB
@HWI-EAS443:3:13:735:793#0/1
ATGGGGGGCTATATGAAGCAATCGAGGAGTGCCTGATTAA
+HWI-EAS443:3:13:735:793#0/1
aabaa^`aaabaaa]a`aaa_a_`[a_]a^aU^`a_^]__
@HWI-EAS443:3:13:891:1359#0/1
TGCCCCCCAAGATCGAAGGTTCCAGGTTCCAGGTTGTCCC
+HWI-EAS443:3:13:891:1359#0/1
WW_b__XW^]Q]a_YQVSTYZ¥Y¥ZWMTZSNX^TUZTONE
@HWI-EAS443:3:14:1138:282#0/1
GCTCCCTTCATTATGTATTCAGTAGCCCTGCAGTGGGACA
```

+HWI-EAS443:3:14:1138:282#0/1
a[_`a`_``¥`_^`a^_`_^]`U__^ZU]a]Y_Y_V^USY
@HWI-EAS443:3:14:1561:1533#0/1
CGGTCTCCAACATCTTTGCAGTCATCAAAGTCTACTTTTT
+HWI-EAS443:3:14:1561:1533#0/1
a`^Sa]^UU¥M[¥T¥¥OZL^b``U`^a_WbWJYaZY][Ya
@HWI-EAS443:3:15:1499:1027#0/1
AGGGGGGCTATATGAAGCAATCGAGGAGTGCCTGATTAAT
+HWI-EAS443:3:15:1499:1027#0/1
_¥a_^a_T_abbb_]]abbab^ZX``U``^Z__aab`][a
@HWI-EAS443:3:15:1647:1829#0/1
GTGAAATTAAGTATTGAAGACCCGAGTCACGAGGGGGAGG
+HWI-EAS443:3:15:1647:1829#0/1
G[¥_¥abb`baba`¥`]¥`YRXS__aOGZ``bbbba_aba
@HWI-EAS443:3:16:1159:231#0/1
CCCCATTTGTGTGGCTCCAGTCTCGGGTCAGTGAGTGAGA
+HWI-EAS443:3:16:1159:231#0/1
ab``aaaaa^a]aa__^XU_W^¥^^_aWZTXK^UWS^UVQ
@HWI-EAS443:3:16:319:1530#0/1
CAGTGGGCACTCGGTGAAAATATGGCACCAGAAAAAGTAG
+HWI-EAS443:3:16:319:1530#0/1
^RXRZSRa[PXTOPFMXRaa_a^^^_]¥a[`W]^_VPX^W
@HWI-EAS443:3:16:736:650#0/1
CCCCTGTTGTGTTACATATACTGTTCACCACTGTCCAGGA
+HWI-EAS443:3:16:736:650#0/1
`a_^M^]V[UbIT^]abZa_¥ba_aXaa`a[`a¥¥^Z`^G
@HWI-EAS443:3:17:1178:733#0/1
CGGGGGGCTATATGAAGCAATCGAGGAGTGCCTGATTAAT
+HWI-EAS443:3:17:1178:733#0/1
]`bbbX`abbabaaa_ZW_aabaa`ZJ¥T^¥aaa___a__
@HWI-EAS443:3:17:1292:349#0/1
CCCGCTTTGGTGGCCATCTCCTCTCCAGTGAATGAAAAGA
+HWI-EAS443:3:17:1292:349#0/1
ababZabaab_ba``___a_[^^a[[`a_a`a``¥_ZX`¥
@HWI-EAS443:3:17:1624:676#0/1
CGTCCTTGGGATCTTCTCTTCATTTTCAATGTCCTGTAGC
+HWI-EAS443:3:17:1624:676#0/1
_bbbabbbba_aabbbbabbabbbbabbbaba__aaaaa`
@HWI-EAS443:3:17:1774:546#0/1
ACGGGGCAACATCTTCTCCTATTTCATCAAGTTCTATCCA
+HWI-EAS443:3:17:1774:546#0/1
`]a¥a_W^`SVa^]]Ta^[a_a___`a[`^`]__a``XZ`
@HWI-EAS443:3:17:233:314#0/1
CGGGGGGCTATATGAAGCAATCGAGGAGTGCCTGATTAAT
+HWI-EAS443:3:17:233:314#0/1
a_I__¥H¥T`_Sa_aW¥Q_a^O]STFR_Na[X_[P¥YPY`
@HWI-EAS443:3:17:384:60#0/1
CGGGGGGCTATATGAAGCAATCGAGGAGTGCCTGATTAAT
+HWI-EAS443:3:17:384:60#0/1
ab_aaa`a`a_^a`a_a]]__^_¥_`^^W`]]¥_UWHP¥¥

```
@HWI-EAS443:3:17:442:146#0/1
GGTGGTGAACTTTGTAAGTATGGAGTTCTCACTCACTGAC
+HWI-EAS443:3:17:442:146#0/1
ab]ba]a_``aaa`_`a`Za``__^^^^__`_^^^_¥__¥
@HWI-EAS443:3:17:667:592#0/1
CGGCTTCTCTACCCCTGTTGTGTTACATATACTGTTCACC
+HWI-EAS443:3:17:667:592#0/1
ab`aaa`Q]Waa`b¥VPS^[J^Z_TKVRZZ_^Z^^WR[[R
@HWI-EAS443:3:18:1372:969#0/1
TTCCCAATTACCTCATGGCTTGGAAGCAGGTGCTAGCAGA
+HWI-EAS443:3:18:1372:969#0/1
aa``V]aa`a`[aa__a^^__^]V[ZVNNSLSHU[[LR_V
@HWI-EAS443:3:18:1464:787#0/1
CGGCCATGTTTCCGATTTGTTTTCAAAGAATTCCTTGGTC
+HWI-EAS443:3:18:1464:787#0/1
aaaa`aaaaabaaa_aaab_aaaa`_a`^^`a``aaa_^^
@HWI-EAS443:3:18:1525:508#0/1
AACAAACTTCCAAATGTGTGCATATTGCAGCAAACTTGTA
+HWI-EAS443:3:18:1525:508#0/1
aaaaaaaaaY_a`_a`[a_`]_a^_`a]a_Y``_]`_^^]
@HWI-EAS443:3:19:1072:754#0/1
GCCCGTTCGTCGATGAAATGGAAATCCGAATACATGAAAC
+HWI-EAS443:3:19:1072:754#0/1
aaYaaaZRTN_bVVWFLVXY]OUYW]`ZKX^_____YU^U
@HWI-EAS443:3:19:1412:1889#0/1
AGGGGGGCTATATGAAGCAATCGAGGAGTGCCTGATTAAT
+HWI-EAS443:3:19:1412:1889#0/1
a^V^_X`¥aaabab aaa_a`[`]Za^^`U^Ya___aaZ``
@HWI-EAS443:3:19:263:1173#0/1
CGCCGAGTCTTCTGTCAAAGAGAAAGACATGACCAAGGAA
+HWI-EAS443:3:19:263:1173#0/1
a`_]`a_O¥¥`_Y[U___Y_V_Z]_^^[QU[_XZ__]]^^
@HWI-EAS443:3:20:1008:374#0/1
GCGATTTGTTTTCAAAGAATTCCTTGGTCATGTCTTTCTC
+HWI-EAS443:3:20:1008:374#0/1
I_`^Z__]Y_VZ^aYa[bb`Z`baZ_JJ¥bab`^]ab^``
@HWI-EAS443:3:20:1274:228#0/1
TGGGGTTTTCGGCTGAATCGAGAAAATTGCTTCTCATTGT
+HWI-EAS443:3:20:1274:228#0/1
abbbb_a¥b_aa_aaaaa^a_a^_`_aaaUaa^`_^`a_]
@HWI-EAS443:3:20:823:584#0/1
CGTTCTCTTCATGTTCTTTGTCCTTGGGATCTTCTCTTCA
+HWI-EAS443:3:20:823:584#0/1
aabbbbbbbb`bb`babbbba`abbbbaababbababaaa
@HWI-EAS443:3:20:83:349#0/1
CGGCTTCTCTACCCCTGTTGTGTTACATATACTGTTCACC
+HWI-EAS443:3:20:83:349#0/1
_OZba`TF¥^VZ¥`^NZNX`TTUVZPY[^Q^TXUUU]NLZ
@HWI-EAS443:3:21:100:1431#0/1
ATCAGCTGAAAGTCATCCATGGCTGCACAGGATGCATTGG
```

+HWI-EAS443:3:21:100:1431#0/1
aXbMa_aT¥]``U`aa^`_Xaa`PQT_XK]SGP`]X]_SF
@HWI-EAS443:3:21:423:976#0/1
CGCTAGCAGAGCTACAGGACATTGAAAATGAAGAGAAGAT
+HWI-EAS443:3:21:423:976#0/1
`^b_a`][`^]aa`aaaa`aaZ_`_a__ZIY[``a__^_]
@HWI-EAS443:3:22:479:489#0/1
GGCATGTGAATTGACTGATTCAAGCTGGATAGAACTTGAA
+HWI-EAS443:3:22:479:489#0/1
abaaaa¥baaaaaa`ba``a_a_aaaaa_aaa_a]a_a_G
@HWI-EAS443:3:23:450:117#0/1
CGTCCTGTAGCTCTGCTAGCACCTGCTTCCAAGCCATGAG
+HWI-EAS443:3:23:450:117#0/1
abba``[Zbbaa`J`_U]aa`a^^aXVO__XX[^_V^_U`
@HWI-EAS443:3:23:917:1121#0/1
TAGGTCTCCAACATCTTTGCAGTCATCAAAGTCTACTTTT
+HWI-EAS443:3:23:917:1121#0/1
Y[]_S¥WSS]X_¥SFY_ZP___W___ZZS¥ZV__VOZR]U
@HWI-EAS443:3:24:236:1236#0/1
CGGGGCATGGAAATGAGGCGCTGCCTTCTTCAGTCTCTTC
+HWI-EAS443:3:24:236:1236#0/1
aaa`bbXbaaa`[aaZ^_[aa`a_aaaaaaaba]aaaaa^
@HWI-EAS443:3:24:48:952#0/1
CGTCCTTGGGATCTTCTCTTCATTTTCAATGTCCTGTAGC
+HWI-EAS443:3:24:48:952#0/1
aa_aa__aaa^]aa`_]a__a¥a_``_a`[_W``¥^X_Ua
@HWI-EAS443:3:25:181:1034#0/1
GGCCTATGTAGATGGATTCGAGCCGAACGGCTGCATTGAG
+HWI-EAS443:3:25:181:1034#0/1
`bbabba_abbabbbabaababbabbbbbaaaa_aYbUaa
@HWI-EAS443:3:25:705:381#0/1
NGAGCCAGAGCCCAGATCTCTAGCAAGCTGGGTCCAAAAT
+HWI-EAS443:3:25:705:381#0/1
DPWVVXWWXXXWWWVYYUXXYYXUYUXXXXXXXTWVWYXY
@HWI-EAS443:3:26:1251:60#0/1
TCCCTGCTCTCTTCGTCAAGGGTGTAGTCCGCTTTGGTGG
+HWI-EAS443:3:26:1251:60#0/1
a^``aa^]YG`a`^aZ^]^_`]U`P^^U¥U`W^¥¥]^U^]
@HWI-EAS443:3:26:149:710#0/1
GCGGGTCTTCAATACTTAATTTCAGAGCATCCATCAGCAG
+HWI-EAS443:3:26:149:710#0/1
abab`Gbaaa_aaaaaaaaaaa`aa^]aa_aa_`aa_aa_
@HWI-EAS443:3:26:1621:253#0/1
GAATAATTGTAGAATCTGGTGACCCGAATGCACTATTGAA
+HWI-EAS443:3:26:1621:253#0/1
I_bbVab```bbaVbaabbb¥aY^Vb¥bbbXSJ`¥_b¥^`
@HWI-EAS443:3:27:981:1676#0/1
CGTCCTGCACACTTTCCCAATAGAGCCTTCCTCCACTCCC
+HWI-EAS443:3:27:981:1676#0/1
`^¥__aZ__^a`a_`_a`aaaaR```[[^`_X¥^`^`^]X

@HWI-EAS443:3:28:192:595#0/1
CATCGGGGCAACATCTTCTCCTATTTCATCAAGTTCTATC
+HWI-EAS443:3:28:192:595#0/1
aa]a``bbbaaa_`abaaa`aaabbabb_a^XV]ab`aab
@HWI-EAS443:3:28:252:1225#0/1
GACCCAGGGGAGTGGAGGAAGGCTCTATTGGGAAAGTGTG
+HWI-EAS443:3:28:252:1225#0/1
HXabbbb]`b¥babbbbb``b]bbba^`ba_bbbab]b^]
@HWI-EAS443:3:28:349:738#0/1
GGCTGGGTCCAAAATGAATTCAATAAGGCATGTGAATTGA
+HWI-EAS443:3:28:349:738#0/1
a_baabaYbababaa]^abbaaaaaa`aaaaa_a``aaaa
@HWI-EAS443:3:28:535:2029#0/1
CGGGGGGCTATATGAAGCAATCGAGGAGTGCCTGATTAAT
+HWI-EAS443:3:28:535:2029#0/1
ab`^aaab`baaaaaa_aaa`a_`_]``_`a^__^__aZR
@HWI-EAS443:3:29:883:979#0/1
GCCCGTTCGTCGATGAAATGGAAATCCGAATACATGAAAC
+HWI-EAS443:3:29:883:979#0/1
aabab[abbabbaaa`abaaaabaa`aaaaab`a_a_aa_
@HWI-EAS443:3:2:1549:1088#0/1
CGGCCAGTAGGAGTCTATGGGATTCCTTTCGTCAGTCCGA
+HWI-EAS443:3:2:1549:1088#0/1
abbabab[]bbaba_b_baa__aaaZaabNa_Z_b^RZaZ
@HWI-EAS443:3:2:360:857#0/1
CGGGGCATGGAAATGAGGCGCTGCCTTCTTCAGTCTCTTC
+HWI-EAS443:3:2:360:857#0/1
`b`[ab_aaba^a__F¥_^a^UaXT`aU]_S]`W_`VZVS
@HWI-EAS443:3:30:1444:1125#0/1
GACCGGTTCATTGAAATTGGAGTAACACGGAGGGAAGTCC
+HWI-EAS443:3:30:1444:1125#0/1
ababbaababbb]aaabbb^ab]aa`aaaaa`aa_a`]^]
@HWI-EAS443:3:30:247:2020#0/1
TTAGTGGTATTCCCTCCCCCTCGTGACTCGGGTCTTCAAT
+HWI-EAS443:3:30:247:2020#0/1
_Za]X_]YS_abba`bbbba^a]]]`a^_NRZYa^ZX^`V
@HWI-EAS443:3:31:1708:640#0/1
CATGAGAAAGGCATAAATCCCAATTACCTCATGGCTTGGG
+HWI-EAS443:3:31:1708:640#0/1
aaT[Tb[``^_Ib`UaabaTZaabaa_T`Xa[ZYPV]aBB
@HWI-EAS443:3:31:406:1710#0/1
ACCCGTTCGTCGATGAAATGGAAATCCGAATACATGAAAC
+HWI-EAS443:3:31:406:1710#0/1
aaab`Y__RP__]^ZZ¥^X¥XZ][U_`[^]W_NTUS___N
@HWI-EAS443:3:32:1118:232#0/1
GGGGGCATGGAAATGAGGCGCTGCCTTCTTCAGTCTCTTC
+HWI-EAS443:3:32:1118:232#0/1
abb^aababa`]aaaaa`_aa^aW^`___^_¥`¥V``¥¥W
@HWI-EAS443:3:33:1351:961#0/1
CGCCAATCGGAGAGTCACCCAGGGGAGTGGAGGAAGGCTC

```
+HWI-EAS443:3:33:1351:961#0/1
aaaaaaa_``aaaaaaa``a``aaa`¥T`a¥`WTT¥^R]Y
@HWI-EAS443:3:33:1677:491#0/1
AGGTGCTTCAATAGTGCATTCGGGTCACCAGATTCTACAA
+HWI-EAS443:3:33:1677:491#0/1
aab_ba]b_aab`a_a¥`a`^aa_V`__¥`a]VVV_^R]]
@HWI-EAS443:3:33:889:1074#0/1
GCGGCCTTGCTCAATGCATCCTGTGCAGCCATGGATGACT
+HWI-EAS443:3:33:889:1074#0/1
ab¥]]aaaaa_aa`_aaaa_``a^a`_¥Za_aZ_¥]_VW`
@HWI-EAS443:3:34:725:2042#0/1
GGTCCTTGGGATCTTCTCTTCATTTTCAATGTCCTGTAGC
+HWI-EAS443:3:34:725:2042#0/1
aa_b`^aa`¥a_`a`aaa`a`aa`a`_a_`a^¥]¥_X_XL
@HWI-EAS443:3:34:949:304#0/1
TGTCTCTTCAGCAGATTGAGAGCATGATTGAGGCCGAGTC
+HWI-EAS443:3:34:949:304#0/1
aa_`aaaaaaaaa_`aa_`_a`^``a_`^`_Y]V]X^WV
@HWI-EAS443:3:36:1293:1817#0/1
AGCCCTGCTCTCTTCGTCAAGGGTGTAGTCCGCTTTGGTG
+HWI-EAS443:3:36:1293:1817#0/1
abbb_]_aaabaa_aaaaa_]¥]G`]aYY^a^T¥¥_][TY
@HWI-EAS443:3:36:340:250#0/1
CGGGGTTTTCGGCTGAATCGAGAAAATTGCTTCTCATTGT
+HWI-EAS443:3:36:340:250#0/1
abbbaVababb`baabbbbb^aaa`_aaaa_aaaaa_aaW
@HWI-EAS443:3:36:599:587#0/1
CGGGGCTATATGAAGCAATCGAGGAGTGCCTGATTAATGA
+HWI-EAS443:3:36:599:587#0/1
`a`b`babaaaa_`a`aaaaa`a`^aX^aa^a]_a^`_]_
@HWI-EAS443:3:37:1078:758#0/1
GGGCTGGAGAAGTTCGGTGGGAGACTTTGGTCGGCAAGCT
+HWI-EAS443:3:37:1078:758#0/1
aa_^_aa¥a^]a¥^^`]STEYY^U^^^XNSW[QU^TNTUV
@HWI-EAS443:3:37:536:1909#0/1
CACGGGGTGAATCAATAATTGTAGAATCTGGTGACCCGAA
+HWI-EAS443:3:37:536:1909#0/1
`__]PXXW``]aaa_^¥__``Za`aa`^UGZU^__``¥a_
@HWI-EAS443:3:38:1298:1299#0/1
GGATTCGAGCCGAACGGCTGCATTGAGGGCAAGCTTTCCA
+HWI-EAS443:3:38:1298:1299#0/1
ab`abaa]]aa``]aa`aa¥___^GUXZ^RR¥[PZ]^RRN
@HWI-EAS443:3:38:1753:615#0/1
ATTTCATCGACGAACGGGGTGAATCAATAATTGTAGAATG
+HWI-EAS443:3:38:1753:615#0/1
`a`X¥`_a`U_b`X^LP_`[a^O]RY¥[YZTTTLU_JYPD
@HWI-EAS443:3:38:188:687#0/1
AGGCCAATCGGAGAGTCACCCAGGGGAGTGGAGGAAGGCT
+HWI-EAS443:3:38:188:687#0/1
a[^``````a¥`]¥^Y``^`aX`]¥]K¥S^SPX¥R¥¥^SK
```

@HWI-EAS443:3:38:569:146#0/1
CGGGGCTATATGAAGCAATCGAGGAGTGCCTGATTAATGA
+HWI-EAS443:3:38:569:146#0/1
a_baaab`bbaaa`aaa`_ab[aa[aX_`VZa[^___]`]
@HWI-EAS443:3:38:610:431#0/1
GCCCGTTCGTCGATGAAATGGAAATCCGAATACATGAAAC
+HWI-EAS443:3:38:610:431#0/1
aa`Q_aa_`U`_[a¥]a`aa_a^Y¥aa`_]VaS`XW``_U
@HWI-EAS443:3:38:806:1568#0/1
CGGGGGGCTATATGAAGCAATCGAGGAGTGCCTGATTAAT
+HWI-EAS443:3:38:806:1568#0/1
ababaaaaaaaaaa``aaaaaaa``^__Y^WW_`^__¥_¥
@HWI-EAS443:3:39:766:446#0/1
TCCCGTTCGTCGATGAAATGGAAATCCGAATACATGAAAC
+HWI-EAS443:3:39:766:446#0/1
a```aZ]````VS¥VSYUUZYT_W]YQ[`¥WMQV^^_[YV
@HWI-EAS443:3:3:658:491#0/1
CGGGGGGCTATATGAAGCAATCGAGGAGTGCCTGATTAAT
+HWI-EAS443:3:3:658:491#0/1
aabbbbaabbaaa^¥ababaa`b_a`_a¥a¥X`a_`aa``
@HWI-EAS443:3:40:1241:358#0/1
GGGTGAATCAATAATTGTAGAATCTGGTGACCCGAATGCC
+HWI-EAS443:3:40:1241:358#0/1
a_¥U^ZJ_[RW^]^^^_ZY`ZXZZZ¥ZWZYYX]ZXWZZWR
@HWI-EAS443:3:42:1422:585#0/1
GGTGGCTCCAGTCTCGGGTCAGTGAGTGAGAACTCCATAC
+HWI-EAS443:3:42:1422:585#0/1
_b`a_aaaa^_Wa_[Z``L¥X]^_^[F^S^ZYGUU]YR]S
@HWI-EAS443:3:42:840:1990#0/1
TGGTGCTAGCAGAGCTACAGGACATTGAAAATGAAGAGAA
+HWI-EAS443:3:42:840:1990#0/1
aaX¥`bbaaab_`^^aaaa`YMa`aaa_aaaa¥aaaaXaa
@HWI-EAS443:3:42:869:883#0/1
GCTCCCTTCATTATGTATTCAGTAGCCCTGCAGTGGGACA
+HWI-EAS443:3:42:869:883#0/1
¥abbbbbb`_`bbbbabbabbbabbb`_baabb^baa^aa
@HWI-EAS443:3:43:1560:1051#0/1
CGTCATACTGTTTAAGGTCTCCAACATCTTTGCAGTCATC
+HWI-EAS443:3:43:1560:1051#0/1
aa¥X^__`¥a`aba`aaZZaSWX_V`^O^^]`Y[_[]Y`X
@HWI-EAS443:3:45:1650:1715#0/1
AACAAACTTCCAAATGTGTGCATATTGCAGCAAACTTGTA
+HWI-EAS443:3:45:1650:1715#0/1
`_`¥aa]abPMU`^ba__]_W[T[aY__^NL]_]O]ZPTX
@HWI-EAS443:3:45:532:1060#0/1
CAGGGGGCGTGGTGTCGTCCTCAAGAATGGTTCAATTTTG
+HWI-EAS443:3:45:532:1060#0/1
T^^¥aababab]T^a¥`a]¥[aa``aaaUV^`¥^[_``
@HWI-EAS443:3:45:599:396#0/1
CGGGGCATGGAAATGAGGCGCTGCCTTCTTCAGTCTCTTC

```
+HWI-EAS443:3:45:599:396#0/1
ababa¥`a`^Xaba]]ba`abb`Waa`]`__a`Y¥`]``]
@HWI-EAS443:3:46:1023:1872#0/1
TCGGCCTTGCTCAATGCATCCTGTGCAGCCATGGATGACT
+HWI-EAS443:3:46:1023:1872#0/1
aa^aaaa^]^[`^]aSRYaa¥X¥W¥^X]__`[`[__`¥U¥
@HWI-EAS443:3:46:1168:23#0/1
ATTTTCACCGAGTGCCCACTTCAATTGGCTTGTTCTCTTT
+HWI-EAS443:3:46:1168:23#0/1
abab`ab__bab`baaaa`aa^]`_M_a[`]VUUSUFWVJ
@HWI-EAS443:3:46:1792:1373#0/1
NCGAAAGAGGCGAAGAGACAATTGAAGAAAAATTTGAGAT
+HWI-EAS443:3:46:1792:1373#0/1
DNXZXZZWWXXZZZZZZZVZXZZZZZXWYZZZXZZWZZZZ
@HWI-EAS443:3:46:553:744#0/1
ACCCGTTCGTCGATGAAATGGAAATCCGAATACATGAAAC
+HWI-EAS443:3:46:553:744#0/1
¥^bab_a_a`a^HQYXZZ^S_[Y]_YL¥Y_YVS[O¥__[H
@HWI-EAS443:3:47:1132:1857#0/1
AGGGGCTATATGAAGCAATCGAGGAGTGCCTGATTAATGA
+HWI-EAS443:3:47:1132:1857#0/1
abbaabaaaa¥aaa`_aa^^_V`_]aV_]Z]__WV___U^
@HWI-EAS443:3:47:830:1366#0/1
GTTGTCTTATAGTGAAAAGCCTAGTTTTGATTCTTGCCCT
+HWI-EAS443:3:47:830:1366#0/1
K_`[Z[b``abbYV¥`_Y^[YZ^`^bbb¥_`]^bSYIYSW
@HWI-EAS443:3:48:1716:1587#0/1
ACTCCGTGTTACTCCAATTTCAATGAACCGGTTCTCTTTG
+HWI-EAS443:3:48:1716:1587#0/1
]abSa`S`I_`¥`a_`]^a__Z[_a__W]_a^XPVS^[_T
@HWI-EAS443:3:49:1222:1452#0/1
GGGGGGGCTATATGAAGCAATCGAGGAGTGCCTGATTAAT
+HWI-EAS443:3:49:1222:1452#0/1
a[PIUHTY^WH¥X]YY^U¥UTYUV[aXVRSFOUYRR^UXX
@HWI-EAS443:3:49:792:424#0/1
GGGCTCCAGTCTCGGGTCAGTGAGTGAGAACTCCATACTT
+HWI-EAS443:3:49:792:424#0/1
``a`aa]a^X`__a`aQ_a[F[XYS`U[__X]^¥_`_¥``
@HWI-EAS443:3:4:1015:712#0/1
TGGAGGAACTATTTTACAGCAGAAGTGTCCCACTGCAGGG
+HWI-EAS443:3:4:1015:712#0/1
abbabbbaaaaaaaaa_a`^aaa``_a__]¥`Y`a^]a`_
@HWI-EAS443:3:4:1034:365#0/1
AGGGGAGGGAATACCACTATATGATGCAATCAAATGCATG
+HWI-EAS443:3:4:1034:365#0/1
abbbbabbb`aaa``a_baaaaaa_a`aaaXa_a`a_a``
@HWI-EAS443:3:4:456:274#0/1
TCCCGAGTCACGAGGGGGAGGGAATACCACTATATGATGC
+HWI-EAS443:3:4:456:274#0/1
a`aabab`baab`babababaa``_a``W^a¥`_`a_a`^_¥
```

@HWI-EAS443:3:4:571:698#0/1
CTGGGGCATGGAAATGAGGCGCTGCCTTCTTCAGTCTCTT
+HWI-EAS443:3:4:571:698#0/1
abb_ba`ba`]ab`bbbbaaaaabaaaaaba`ab_]a`aa
@HWI-EAS443:3:4:923:938#0/1
ACTCTTCCTTCAATTATCTCAAATCGGTGCTTCAATAGTG
+HWI-EAS443:3:4:923:938#0/1
ab`ababb`baaaa`b]`a]aaa_`_[[aPYa_a_`^PX_
@HWI-EAS443:3:50:1163:612#0/1
CGGGCATGGAAATGAGGCGCTGCCTTCTTCAGTCTCTTCA
+HWI-EAS443:3:50:1163:612#0/1
`bab_bbababab bbabaaabbbaaba`abaaba^baaa_a
@HWI-EAS443:3:50:751:1898#0/1
AGGGCATGGAAATGAGGCGCTGCCTTCTTCAGTCTCTTCA
+HWI-EAS443:3:50:751:1898#0/1
`a]b`babbaaaaba``aaaaaa`_`[bb`aa¥[`_a`_a
@HWI-EAS443:3:51:1010:627#0/1
AGGCACCAGAAAAAGTAGACTTTGATGACTGCAAAGATGT
+HWI-EAS443:3:51:1010:627#0/1
abb_ababbaaaaaa_aba`baaaaaaa`aa`a`aa``a_
@HWI-EAS443:3:52:1452:1115#0/1
CTTTCCCAAATGTCAAAAGAAGTGAACGCCAAAATTGAAG
+HWI-EAS443:3:52:1452:1115#0/1
Sa``¥baaabaZXXU¥¥aa`aaab_KXabaa^`bba``bJ
@HWI-EAS443:3:52:1462:1140#0/1
GGGTCCTGCACACTTTCCCAATAGAGCCTTCCTCCACTCC
+HWI-EAS443:3:52:1462:1140#0/1
__¥U]Z__T¥X¥S`[_¥^¥Z¥`[NVNXZ[ZVPXNRTQTZZ
@HWI-EAS443:3:52:1692:1481#0/1
CGGGGGGCTATATGAAGCAATCGAGGAGTGCCTGATTAAT
+HWI-EAS443:3:52:1692:1481#0/1
a_]_aab]aababaa`T^a`aaa_`[Z`P^^Uaa_aa_^a
@HWI-EAS443:3:53:315:949#0/1
GGGGGGGCTATATGAAGCAATCGAGGAGTGCCTGATTAAT
+HWI-EAS443:3:53:315:949#0/1
abaababaaaaaabaaa`a`a`¥`aa¥a¥aa¥aa^`__`_
@HWI-EAS443:3:54:1014:1449#0/1
CGAGGGGTTTTCGGCTGAATCGAGAAAATTGCTTCTCATT
+HWI-EAS443:3:54:1014:1449#0/1
a_Qa_baW`ba`aa]a`aaa`aaaa`aaa`¥_a¥Pa¥[`[
@HWI-EAS443:3:54:555:1380#0/1
GCTTTGTCCTTGGGATCTTCTCTTCATTTTCAATGTCCTG
+HWI-EAS443:3:54:555:1380#0/1
abbbbbaa`abb`a_babbaa`abaabaabaabaaa`^a_
@HWI-EAS443:3:55:1284:1212#0/1
CGGCACCAGAAAAAGTAGACTTTGATGACTGCAAAGATGT
+HWI-EAS443:3:55:1284:1212#0/1
ab`a`_`]`_``^[_]``Z^bbba]ZVY^a_a``¥ba[ba
@HWI-EAS443:3:55:74:657#0/1
CGGCCATTTCTTGTCTTATAGTGAAAAGCCTAGTTTTGAT

```
+HWI-EAS443:3:55:74:657#0/1
`bbb`abbb_bbbaabb_babababaaaaaa^ba`a`a`a
@HWI-EAS443:3:56:507:1201#0/1
TGGGGTGAATCAATAATTGTAGAATCTGGTGACCCGAATG
+HWI-EAS443:3:56:507:1201#0/1
a`]^bZbbbaabbb`bbabaab`baa`ab^a`a__`aa`a
@HWI-EAS443:3:57:107:467#0/1
AGGGGGGCTATATGAAGCAATCGAGGAGTGCCTGATTAAT
+HWI-EAS443:3:57:107:467#0/1
a`aaabaa_]aZaa^Y_`aa``a_a_ZaZa`_`aZ___¥_
@HWI-EAS443:3:57:238:1378#0/1
ATGGGGGGCTATATGAAGCAATCGAGGAGTGCCTGATTAA
+HWI-EAS443:3:57:238:1378#0/1
`baba``[^aaab]P]PXa]aaa`¥a[[a¥a`]]``_`¥^
@HWI-EAS443:3:57:513:633#0/1
GGAGGGGTTTTCGGCTGAATCGAGAAAATTGCTTCTCATT
+HWI-EAS443:3:57:513:633#0/1
aa[`aa_U_a`]^`_aaa_U_a_`_`__^^_`____]_]_
@HWI-EAS443:3:58:1159:1017#0/1
CTTCAATTGGCTTGTTCTCTTCATGTTCTTTGTCCTTGGA
+HWI-EAS443:3:58:1159:1017#0/1
aa`aaaa¥b`]`aYV[_¥OOWSN_]Z^XZ`_]ZV¥[``¥O
@HWI-EAS443:3:58:1334:871#0/1
CGGGCATGGAAATGAGGCGCTGCCTTCTTCAGTCTCTTCA
+HWI-EAS443:3:58:1334:871#0/1
a_`^^[aaYDXY`_^a¥P``aaa^]b¥a]_]`WW]_a][L
@HWI-EAS443:3:58:336:34#0/1
GACAACTTGAGGGGTTTTCGGCTGAATCGAGAAAATTGCT
+HWI-EAS443:3:58:336:34#0/1
I_]¥]`bbbaba^_a`¥[¥^[]]`¥Y¥Z]W^`_]X_Y_Z]
@HWI-EAS443:3:58:501:660#0/1
CGGGGCATGGAAATGAGGCGCTGCCTTCTTCAGTCTCTTC
+HWI-EAS443:3:58:501:660#0/1
aabaaaaaaaaaabaabaaaaaa`_aa_aa`aa__`__[M
@HWI-EAS443:3:59:1570:876#0/1
GCCCGAATGCACTATTGAAGCACCGATTTGAGATAATTGA
+HWI-EAS443:3:59:1570:876#0/1
aa_X]]aaa`_aa_aaa_Xa]``]a]_aa`]X]]]YX]XL
@HWI-EAS443:3:59:262:747#0/1
CGGCATGTGAATTGACTGATTCAAGCTGGATAGAACTTGA
+HWI-EAS443:3:59:262:747#0/1
abaaabb[bbabbbaabaabaaaaababb_baa`bZ`aa^
@HWI-EAS443:3:59:386:693#0/1
TGGGGTTTTCGGCTGAATCGAGAAAATTGCTTCTCATTGT
+HWI-EAS443:3:59:386:693#0/1
aaa_a]a____a``aa`]_a]_^]_J_^`_^^`_^YW]XY
@HWI-EAS443:3:59:982:1307#0/1
CCACAACTTGAGGGGTTTTCGGCTGAATCGAGAAAATTGA
+HWI-EAS443:3:59:982:1307#0/1
abbaababbbaabaa_bba_aabbabaaaaaZ`aaXXaUD
```

```
@HWI-EAS443:3:5:1216:78#0/1
AGGGCCTCGACACTTGGCCTATCGCAGTCCTCAAGAGCAT
+HWI-EAS443:3:5:1216:78#0/1
Z¥]aaa_Z`HZ``baba`]baba`¥aa`P¥aZaabaa_aB
@HWI-EAS443:3:5:1349:1893#0/1
GGGGGGGCTATATGAAGCAATCGAGGAGTGCCTGATTAAT
+HWI-EAS443:3:5:1349:1893#0/1
abbbb[X[babbabaa_Y]b]¥baa``a`a^^aa_aaa^_
@HWI-EAS443:3:5:1477:1622#0/1
ATCTTCGTCAAGGGTGTAGTCCGCTTTGGTGGCCATCTCC
+HWI-EAS443:3:5:1477:1622#0/1
HV]Z¥^bbaTbbbaaa_b_aWH]R]`baQK`ZHI]^aBBB
@HWI-EAS443:3:5:1748:889#0/1
CGGGGGGCTATATGAAGCAATCGAGGAGTGCCTGATTAAT
+HWI-EAS443:3:5:1748:889#0/1
aaab```[aZabbbaababbaab`b]ab¥b_Xbb`abaaa
@HWI-EAS443:3:60:1170:664#0/1
TCCCCGTTCGTCGATGAAATGGAAATCCGAATACATGAAA
+HWI-EAS443:3:60:1170:664#0/1
aab`abaa_`_^[]aa`aa_``^_a_`a`]Y`_]`]T¥``
@HWI-EAS443:3:60:1335:1739#0/1
AGACCCGAATGCACTATTGAAGCACCGATTTGAGATAATT
+HWI-EAS443:3:60:1335:1739#0/1
[]`QX_UUa[¥HZ¥b``_OPY_YI[Xb[aa¥¥V]_`¥^a[
@HWI-EAS443:3:60:412:150#0/1
AACCGGTTCATTGAAATTGGAGTAACACGGAGGGAAGTCA
+HWI-EAS443:3:60:412:150#0/1
aa`abb[]YY_aaaa`a__a_a_`_¥``a_^a^_YX_WWM
@HWI-EAS443:3:60:972:210#0/1
AAAATGGCCAGTAGGAGTCTATGGGATTCCTTTCGTCAGT
+HWI-EAS443:3:60:972:210#0/1
`Z_ba`_[[]aVV__V_R]^]]_]ZNZ¥V]T_YOVQOQXT
@HWI-EAS443:3:61:1190:252#0/1
GAGAAAGGCATAAATCCCAATTACCTCATGGCTTGGAAGA
+HWI-EAS443:3:61:1190:252#0/1
a]^PZ_`^[^¥Z]^[XU¥a^_SLY_]^ZZ[ZVBBBBBBBB
@HWI-EAS443:3:61:295:909#0/1
TCGGGGTGAATCAATAATTGTAGAATCTGGTGACCCGAAT
+HWI-EAS443:3:61:295:909#0/1
ZabbaaWbba`¥U_aSX_^aTV^PV¥^aa[NSOU`YV^[_
@HWI-EAS443:3:62:703:1226#0/1
AGGGGGGCTATATGAAGCAATCGAGGAGTGCCTGATTAAT
+HWI-EAS443:3:62:703:1226#0/1
a`Z`]Z¥¥[_ba[aYVaWS_a^Y]^XPZV]¥T^a__^`a[
@HWI-EAS443:3:63:1793:1144#0/1
NGGGCATGGAAATGAGGCGCTGCCTTCTTCAGTCTCTTCA
+HWI-EAS443:3:63:1793:1144#0/1
BBBBBBBBBBBBBBBBBBBBBBBBBBBBBBBBBBBBBBBB
@HWI-EAS443:3:64:1411:999#0/1
GCAGGGGTAGAGAAGCCTAAATTTCTTCCTGATTTGTATG
```

+HWI-EAS443:3:64:1411:999#0/1
^Zab`b^¥[aTVSa`aa¥a_aabZPR[WJ]W_S[aa__ab
@HWI-EAS443:3:64:580:1218#0/1
CGGGATTTATGCCTTTCTCATGTGGTTTGACTATGTTAGG
+HWI-EAS443:3:64:580:1218#0/1
a`]^]^`aa¥¥_`aa``aa_a^^`W]_`^`_aa[]V^YU¥
@HWI-EAS443:3:65:1011:39#0/1
ACGGTCTCTTCCTTCAATTATCTCAAATCGGTGCTTCAAT
+HWI-EAS443:3:65:1011:39#0/1
a``]O]Z^``^YZ^ZZWZ`]YYZVQZ`X`_]Q]VN_ZNUU
@HWI-EAS443:3:65:236:352#0/1
CAGGCATGTGAATTGACTGATTCAAGCTGGATAGAACTTG
+HWI-EAS443:3:65:236:352#0/1
[bbbbbbabbbXabba_abb`]a`aa``b``Yb]S^`_a`
@HWI-EAS443:3:66:464:308#0/1
CACCCCTGTTGTGTTACATATACTGTTCACCACTGTCCAG
+HWI-EAS443:3:66:464:308#0/1
``abbaaa]ab`a¥`aaaa``]`aa[[aaaa`a``V__^Z
@HWI-EAS443:3:67:600:1686#0/1
AGACACTTGGCCTATCGCAGTCCTCAAGAGCATGTCTCCT
+HWI-EAS443:3:67:600:1686#0/1
_NY[[^IWPM[`^aX__^YROW[XZSZNZ_ZW]R¥YZU^Z
@HWI-EAS443:3:67:689:1554#0/1
GAGGGAGTGTACATAAATACGGCCTTGCTCAATGCATCCT
+HWI-EAS443:3:67:689:1554#0/1
]_S^aa[_b``ba```aPa``¥¥aV_W[Z]_RWR_[YX_N
@HWI-EAS443:3:68:1231:1487#0/1
CGTGGGCACTCGGTGAAAATATGGCACCAGAAAAAGTAGA
+HWI-EAS443:3:68:1231:1487#0/1
[b`b`bbbb^_]bHba`bbabbb_a_^aa`baaaabG`_a
@HWI-EAS443:3:68:1480:810#0/1
ACCTCCTCATGCTTGCGATATGTTCAATCGGGGCAACATC
+HWI-EAS443:3:68:1480:810#0/1
aabbbbababb]bbb_baba_bab_baaabaaaU[a^ab`
@HWI-EAS443:3:68:1544:194#0/1
GGGTGCCATATTTTCACCGAGTGCCCACTTCAATTGGCTT
+HWI-EAS443:3:68:1544:194#0/1
`_`O^T^`X`baaZVaaa^V^¥bbZ_]¥]YSS]`^^`X_F
@HWI-EAS443:3:68:169:1210#0/1
TCCTGTGCAGCCATGGATGACTTTCAGCTGATCCCAATGG
+HWI-EAS443:3:68:169:1210#0/1
abbb[`bbb]bbaaaaaaaaaabaaaaa``a`aa]U_^^a
@HWI-EAS443:3:68:246:279#0/1
CGCAGGGCAAGAATCAAAACTAGGCTTTTCACTATAAGAC
+HWI-EAS443:3:68:246:279#0/1
a`abbbbaaababbababbabbaa``aa`a¥a`a`aa]aS
@HWI-EAS443:3:68:92:443#0/1
TCAGGTGCTAGCAGAGCTACAGGACATTGAAAATGAAGAG
+HWI-EAS443:3:68:92:443#0/1
a`a`a__a`aaa_``a`^aa^a`]__]^_XZ^^]_^_]`]

```
@HWI-EAS443:3:69:512:575#0/1
AGGGTCACCAGATTCTACAATTATTGATTCACCCCGTTCG
+HWI-EAS443:3:69:512:575#0/1
a]G]R[[Y``[ZU^][[X[N_]`YaZYLTP[__R__WY¥_
@HWI-EAS443:3:70:1441:1518#0/1
AGGGGCTATATGAAGCAATCGAGGAGTGCCTGATTAATGA
+HWI-EAS443:3:70:1441:1518#0/1
aa_`_aab`^``aaa`aba_a_`_^_O_O^_`^_`Z]X__
@HWI-EAS443:3:70:1690:230#0/1
CGGGGTTTTCGGCTGAATCGAGAAAATTGCTTCTCATTGT
+HWI-EAS443:3:70:1690:230#0/1
ab_aaQa^a¥XRM`a`XV[a]__`a]]¥Y_bb``T^```^
@HWI-EAS443:3:70:527:384#0/1
GGCGTCTCCACAACTTGAGGGGTTTTCGGCTGAATCGAGA
+HWI-EAS443:3:70:527:384#0/1
abaa_a`aa`a__¥a`a_a`_a^`a_T`ZZ^Y`_U¥_W[X
@HWI-EAS443:3:70:718:155#0/1
CTTCCCTCCGTGTTACTCCAATTTCAATGAACCGGTTCTT
+HWI-EAS443:3:70:718:155#0/1
]`bbbbbbaZ_babbbabbbbbbbaaa`S_bW`bb_a`ba
@HWI-EAS443:3:71:1356:286#0/1
GGCATGGAAATGAGGCGCTGCCTTCTTCAGTCTCTTCAGA
+HWI-EAS443:3:71:1356:286#0/1
aabR^ab`[a`a`^R]a`a]UXa]_a]Q]_^X_W`__V]G
@HWI-EAS443:3:72:1554:956#0/1
GACGGGGTGAATCAATAATTGTAGAATCTGGTGACCCGAA
+HWI-EAS443:3:72:1554:956#0/1
aaaaaaZ¥abaa`a`a``aab_aa_aa_aaaVa__¥^a_^
@HWI-EAS443:3:72:1558:2021#0/1
CGGGGCTATATGAAGCAATCGAGGAGTGCCTGATTAATGA
+HWI-EAS443:3:72:1558:2021#0/1
`a^^`^b]b`b^^^Ja__aaa_ba_]_a_aabaaaa__]Z
@HWI-EAS443:3:72:515:908#0/1
GGGCTCTATTGGGAAAGTGTGCAGGACCTTACTGGCAAAA
+HWI-EAS443:3:72:515:908#0/1
``Ra`^NO^ZVWTJX``PRYZ[Z[]^YTRNWZP¥UQ[_VR
@HWI-EAS443:3:72:692:366#0/1
TGGGGCGTGGTGTCGTCCTCAAGAATGGTTCAATTTTGGC
+HWI-EAS443:3:72:692:366#0/1
a_QWa]_RTa]^SaY¥aa`a¥Y__R[a¥XX[V]``a¥VVZ
@HWI-EAS443:3:72:813:680#0/1
TGTCTCTTCAGCAGATTGAGAGCATGATTGAGGCCGAGTC
+HWI-EAS443:3:72:813:680#0/1
`_Z]a`bb^``[``T`¥ZXZ[`]a]^a[]YOZ[``___S¥
@HWI-EAS443:3:73:1031:798#0/1
GGGGGGGCTATATGAAGCAATCGAGGAGTGCCTGATTAAT
+HWI-EAS443:3:73:1031:798#0/1
abaa`a_aabb`aa]_a_aa`_a_aa`a^`^]`aaa``__
@HWI-EAS443:3:73:1079:202#0/1
GGCGTCTCCACAACTTGAGGGGTTTTCGGCTGAATCGAGA
```

+HWI-EAS443:3:73:1079:202#0/1
WaSa^_ZXSba]aYabaP_X¥`^]aSJZ_^Va_]XL_L_V
@HWI-EAS443:3:73:344:684#0/1
AGGGCACTCGGTGAAAATATGGCACCAGAAAAAGTAGACT
+HWI-EAS443:3:73:344:684#0/1
a``^abaaaaa^]][Z^_Y]a]¥¥^^^a__¥]P[PY^^¥¥
@HWI-EAS443:3:74:379:545#0/1
GCCTTGGTCATGTCTTTCTCTTTGACAGAAGACTCGGCCT
+HWI-EAS443:3:74:379:545#0/1
a`a``ba_`aa[`aaa^YZ_aaaX`[a_``_]___`a]Y[
@HWI-EAS443:3:74:579:1033#0/1
GCCTCCTCATGCTTGCGATATGTTCAATCGGGGCAACATC
+HWI-EAS443:3:74:579:1033#0/1
aZb¥bbb`Z^_aabaaa_bbaaVYa^^_``a_aab]^^]a
@HWI-EAS443:3:74:792:415#0/1
CTCTCTTCATGTTCTTTGTCCTTGGGATCTTCTCTTCATT
+HWI-EAS443:3:74:792:415#0/1
`aabbbbbabb¥_bbabaababb_W^N`Yba_aabaa_bb
@HWI-EAS443:3:75:1176:1122#0/1
TGGGCGTGGTGTCGTCCTCAAGAATGGTTCAATTTTGGCG
+HWI-EAS443:3:75:1176:1122#0/1
a`Z_^aab`P_a¥a_^[QT`[K``Y]`aWQOHXVU_^TLT
@HWI-EAS443:3:75:754:1906#0/1
CGGCCAGTAGGAGTCTATGGGATTCCTTTCGTCAGTCCGA
+HWI-EAS443:3:75:754:1906#0/1
a[¥baaa^`¥aa^Z¥aa`_Z``_a^T¥aa_YU_aYS^`[_
@HWI-EAS443:3:76:167:962#0/1
CGCCCTGCTCTCTTCGTCAAGGGTGTAGTCCGCTTTGGTG
+HWI-EAS443:3:76:167:962#0/1
_aa^^aa`aaaaa_aa`a_````PaX`X[`a``^a^`^T`
@HWI-EAS443:3:76:1793:1451#0/1
NGTCGATGAAATGGAAATCCGAATACATGAAACAAACTTC
+HWI-EAS443:3:76:1793:1451#0/1
DMXWSUWPUXXZVYWYUXWXZZZZZZZUXYZXXZXZXZZT
@HWI-EAS443:3:77:1287:73#0/1
TTGCGTCTCCACAACTTGAGGGGTTTTCGGCTGAATCGAG
+HWI-EAS443:3:77:1287:73#0/1
]_aSaa[^ZO^Y^Y]]ab]bb`_T^X[Xa`S_^¥¥FQ]N_
@HWI-EAS443:3:77:1314:1067#0/1
CGGGGGGCTATATGAAGCAATCGAGGAGTGCCTGATTAAT
+HWI-EAS443:3:77:1314:1067#0/1
^_I_a^`_b^b[^_]abaaabab`[¥YaQ^a_aaa¥S_a`
@HWI-EAS443:3:77:272:750#0/1
CGGGGCATGGAAATGAGGCGCTGCCTTCTTCAGTCTCTTC
+HWI-EAS443:3:77:272:750#0/1
abb_P^_¥b][T^X¥aaaaXX`]YTX``X[VY]]XT]^_Y
@HWI-EAS443:3:77:859:1270#0/1
AGAAGTTCGGTGGGAGACTTTGGTCGGCAAGCTTGCGCAG
+HWI-EAS443:3:77:859:1270#0/1
a`W^XZ`Z¥b¥`a``U_`_ba`_^aXabbb_N_^]a`_aa

@HWI-EAS443:3:77:907:249#0/1
CTGCGATATGTTCAATCGGGGCAACATCTTCTCCTATTTC
+HWI-EAS443:3:77:907:249#0/1
abbabbbbbababababbbbabbabbabbabbabb`babbba
@HWI-EAS443:3:79:147:834#0/1
AGCCCACTTCAATTGGCTTGTTCTCTTCATGTTCTTTGTC
+HWI-EAS443:3:79:147:834#0/1
a_``a``_`aZ_]]^]_^RU]YRWX¥]]X]RXWMW`]]YX
@HWI-EAS443:3:79:193:1530#0/1
TGCCACCAAAGCGGACTACACCCTTGACGAAGAGAGCAGG
+HWI-EAS443:3:79:193:1530#0/1
ab`b`ZW_a_b]ZY_babbbbabaUU`baP__aa]aab^O
@HWI-EAS443:3:79:360:54#0/1
AGCCCCATTTCATCTTGATCTTGGAGGTTCCATTGGTTCT
+HWI-EAS443:3:79:360:54#0/1
abaab^¥ab^^aaaba`aaZaaaa_baT``XXTV¥WFYWT
@HWI-EAS443:3:7:1057:879#0/1
ACCCGATTGAACATATCGCAAGCATGAGGAGGAACTATTT
+HWI-EAS443:3:7:1057:879#0/1
^Yb`[]`^¥Ha]b]QUR¥SZ__OVTQTZa_`^[WG^Z^SP
@HWI-EAS443:3:7:120:1451#0/1
CGGGGCATGGAAATGAGGCGCTGCCTTCTTCAGTCTCTTC
+HWI-EAS443:3:7:120:1451#0/1
XZOIaZaaa_Xaaa`W]^aaaaa__a`¥_`__XZ]]^`]a
@HWI-EAS443:3:7:1517:631#0/1
CGGGGCTATATGAAGCAATCGAGGAGTGCCTGATTAATGA
+HWI-EAS443:3:7:1517:631#0/1
]a_^_R_[a[^abbb^[a]P_^]`Z^a^UM^``aaaU`ba
@HWI-EAS443:3:80:1377:609#0/1
GGCAGGGCAAGAATCAAAACTAGGCTTTTCACTATAAGAC
+HWI-EAS443:3:80:1377:609#0/1
a`aaaa`[_````]Z_aaa]_`]_[[_`a¥aT__`^``[Z
@HWI-EAS443:3:80:1710:863#0/1
AGGGGCTATATGAAGCAATCGAGGAGTGCCTGATTAATGA
+HWI-EAS443:3:80:1710:863#0/1
abaa``ababaW``Yaaa_ZZRVY¥aS__R`VY¥a`a_S`
@HWI-EAS443:3:80:595:18#0/1
CGGGGCTATATGAAGCAATCGAGGAGTGCCTGATTAATGA
+HWI-EAS443:3:80:595:18#0/1
``a__baa_aa``aaba`]a]a`¥`[a¥]]a¥Z^¥___
@HWI-EAS443:3:81:1039:526#0/1
GGAGGGGTTTTCGGCTGAATCGAGAAAATTGCTTCTCATT
+HWI-EAS443:3:81:1039:526#0/1
`a_aaa`Y``_^`aZaaa_]]a[`^^`_`_a_````__]^
@HWI-EAS443:3:82:1731:1148#0/1
GCCGAATGCACTATTGAAGCACCGATTTGAGATAATTGAA
+HWI-EAS443:3:82:1731:1148#0/1
Q_Za_abb[abbbbabba`a`a`X`bbbbbbbbbabbXb_
@HWI-EAS443:3:82:1747:1078#0/1
GATTAATCAGGCACTCCTCGATTGCTTCATATAGCCCCCC

```
+HWI-EAS443:3:82:1747:1078#0/1
[aa¥_]]a_^^SbabbVb¥_abZR]ab^^^_`b`a`XXa_
@HWI-EAS443:3:82:691:217#0/1
TAGAACACAGTATTTTTCCCATTTGTGTGGCTCCAGTCTC
+HWI-EAS443:3:82:691:217#0/1
ab`abaabbb]abaaa_`^¥Y_``a]`¥a]V[]_X[VWTZ
@HWI-EAS443:3:83:1183:1982#0/1
CGGGGCTATATGAAGCAATCGAGGAGTGCCTGATTAATGA
+HWI-EAS443:3:83:1183:1982#0/1
aba[_bbbaabba`aabbaLaaaaGaXaa_aaa_a]a_a]
@HWI-EAS443:3:83:309:1592#0/1
AGCGCAAGCTTGCCGACCAAAGTCTCCCACCGAACTTCTC
+HWI-EAS443:3:83:309:1592#0/1
]]_WW¥]K___N¥]aa`]Z¥]YF¥Y_XZ¥]R_XX[R[U[V
@HWI-EAS443:3:83:997:63#0/1
CGGCATGTGAATTGACTGATTCAAGCTGGATAGAACTTGA
+HWI-EAS443:3:83:997:63#0/1
abbaabbaba`aab`]b¥Y`aa``b`ab^S^__N_G_aa_
@HWI-EAS443:3:85:1010:490#0/1
CGGGCACTCGGTGAAAATATGGCACCAGAAAAAGTAGACT
+HWI-EAS443:3:85:1010:490#0/1
a]bb`bUbbb`Ha]U__`abbaaa[_baS_[]aa^aaaU_
@HWI-EAS443:3:85:449:735#0/1
CGGGGGGCTATATGAAGCAATCGAGGAGTGCCTGATTAAT
+HWI-EAS443:3:85:449:735#0/1
aaba_TWab_a]a_[^`____aaa`ZQ_W_`^^a¥`aa¥¥
@HWI-EAS443:3:86:1221:1259#0/1
AGGCACCAGAAAAAGTAGACTTTGATGACTGCAAAGATGT
+HWI-EAS443:3:86:1221:1259#0/1
_`aaa^a^```b^¥bYbba^aaababaa^`b^aaaa``a_
@HWI-EAS443:3:87:1003:1015#0/1
CGGGGGGCTATATGAAGCAATCGAGGAGTGCCTGATTAAT
+HWI-EAS443:3:87:1003:1015#0/1
`_[_¥___bbbb`b`bbba``aa^bab`Ua]P_b`Y__`b
@HWI-EAS443:3:87:1483:178#0/1
CGGGGTTTTCGGCTGAATCGAGAAAATTGCTTCTCATTGT
+HWI-EAS443:3:87:1483:178#0/1
[_bbaa]¥`[a^_UUW¥W]HYaYaa`RHVIOKS]J[a_QP
@HWI-EAS443:3:87:545:444#0/1
GACCGGTTCTCTTTGTAATCATACAAATCAGGAAGAAATT
+HWI-EAS443:3:87:545:444#0/1
a``W_RYXPZS^aaa___aWL_[TH^]_¥a`a[SaPP__Z
@HWI-EAS443:3:87:816:443#0/1
GGAGGGGTTTTCGGCTGAATCGAGAAAATTGCTTCTCATT
+HWI-EAS443:3:87:816:443#0/1
J_^ab`a¥¥`_baUKbbb`Uaaabb`¥`aTGS`_baaW¥a
@HWI-EAS443:3:87:916:689#0/1
CGCCCCATTTCATCTTGATCTTGGAGGTTCCATTGGTTCT
+HWI-EAS443:3:87:916:689#0/1
aa`baaabbaa_b_babaaabbab__`Xa^ZVR]^aRZL]
```

```
@HWI-EAS443:3:88:20:299#0/1
CGCGCATAGTTCCTGTAATCTCAAATTTTTCTTCAATTGT
+HWI-EAS443:3:88:20:299#0/1
aaaba¥`aa_`a```¥^[_a`a[ZG]_``^aa``_S^___
@HWI-EAS443:3:88:882:1302#0/1
CGGGGCATGGAAATGAGGCGCTGCCTTCTTCAGTCTCTTC
+HWI-EAS443:3:88:882:1302#0/1
ab`baaabbbaaa`aaaaaaabaa``a_aa¥aa^`aa`]a
@HWI-EAS443:3:89:1392:1033#0/1
ATACTGTTCACCACTGTCCAGGCCATGATTCGGTCTCTTG
+HWI-EAS443:3:89:1392:1033#0/1
^V_b_^_]Ta^aVS_bababa_^Q__V_a^N¥VFJJM[YM
@HWI-EAS443:3:89:1444:453#0/1
ACTTGGTCATGTCTTTCTCTTTGACAGAAGACTCGGCCTC
+HWI-EAS443:3:89:1444:453#0/1
aab¥_____ab_abba`b¥ba_aaab^aaba_a]a]RW][
@HWI-EAS443:3:8:290:382#0/1
ATTGCAGCAAACTTGTTAGTTTCGATTTTCGGATCTTCCG
+HWI-EAS443:3:8:290:382#0/1
Z]]^a_JW¥O[Ya]^aZ_a_a¥V¥PSZa_YPXUYY][VBB
@HWI-EAS443:3:8:763:1211#0/1
AGTCTCCACAACTTGAGGGGTTTTCGGCTGAATCGAGAAA
+HWI-EAS443:3:8:763:1211#0/1
ab``a`a_`aa]`abab`a`]a`a``]``a_``VZ[^[^`
@HWI-EAS443:3:8:913:1605#0/1
CTCTCTTCATGTTCTTTGTCCTTGGGATCTTCTCTTCATT
+HWI-EAS443:3:8:913:1605#0/1
ZZba`bbaab¥¥a_bb_bUYab`a]a^a_b^I_`Xaba__
@HWI-EAS443:3:90:1277:1616#0/1
CGAGGGGTTTTCGGCTGAATCGAGAAAATTGCTTCTCATT
+HWI-EAS443:3:90:1277:1616#0/1
ab``aYaababbb`^[¥ba`bb_`[Za`aa^R_`a``aba
@HWI-EAS443:3:90:392:588#0/1
GCCCGAATGCACTATTGAAGCACCGATTTGAGATAATTGA
+HWI-EAS443:3:90:392:588#0/1
aaaYa]a[_ZS]`Xaa^]]aaa`_a]_`a`_a`_¥Z^___
@HWI-EAS443:3:90:753:1357#0/1
GGGGGGGCTATATGAAGCAATCGAGGAGTGCCTGATTAAT
+HWI-EAS443:3:90:753:1357#0/1
YFRZ_a]VUa`_S_[]^ZXaX`_[a^¥_G`P[_XPK¥U^Z
@HWI-EAS443:3:91:1197:825#0/1
CACCCGAATGCACTATTGAAGCACCGATTTGAGATAATTG
+HWI-EAS443:3:91:1197:825#0/1
abbabbbbbbaaababbbabbbaaaa`aabbab_aaab`a
@HWI-EAS443:3:91:1519:1262#0/1
CGGGGTGAATCAATAATTGTAGAATCTGGTGACCCGAATN
+HWI-EAS443:3:91:1519:1262#0/1
aaa^aHa¥K`_a^b`aaa]W[a¥Ta^^a_]a_[ZS_`XZD
@HWI-EAS443:3:91:831:1411#0/1
TGCCCCCCAAGATCGAAGGTTCCAGGTTCCAGGTTGTCCC
```

```
+HWI-EAS443:3:91:831:1411#0/1
`Z¥aa`a`¥`_a_a]aaa`U_[__XNSZZZ_¥¥XV_OJMV
@HWI-EAS443:3:92:210:1161#0/1
AGCCAGTAAGGTCCTGCACACTTTCCCAATAGAGCCTTCC
+HWI-EAS443:3:92:210:1161#0/1
^`_b]¥]aaaa`a_a¥]a`aZa`^aaXa]X``¥P¥¥`P^¥
@HWI-EAS443:3:92:515:1510#0/1
CACATTCTTTGGCTGGAAAGAGCCTAACATAGTCAAACCA
+HWI-EAS443:3:92:515:1510#0/1
GP[^VabbUZaX_W`b``a¥a`HQYbKbbYbbbaab¥G_b
@HWI-EAS443:3:92:569:361#0/1
CGGGGGCTATATGAAGCAATCGAGGAGTGCCTGATTAATG
+HWI-EAS443:3:92:569:361#0/1
abbbb`abbbbb`b_b^_aaabaaaaa`b_]abaaa``a`
@HWI-EAS443:3:92:872:154#0/1
CGGGTCCAAAATGAATTCAATAAGGCATGTGAATTGACTG
+HWI-EAS443:3:92:872:154#0/1
_`a^^a`_abbbbbabbabbbabb^aabb``_ababa_aa
@HWI-EAS443:3:93:1196:894#0/1
GGCGTCTCCACAACTTGAGGGGTTTTCGGCTGAATCGAGA
+HWI-EAS443:3:93:1196:894#0/1
aab`a`a`a`aaa`abb`b_``[`aa_aa`_aaa`RZ¥Za
@HWI-EAS443:3:93:1345:415#0/1
AGGCGTTCACTTCTTTTGACATTTGGGAAAGCTTGCCCTC
+HWI-EAS443:3:93:1345:415#0/1
`[`V[V[SZZa_T]`aaY¥]_``^U^]]^Y^[[¥TZXWWU
@HWI-EAS443:3:93:925:805#0/1
CACCCGAGACTGGAGCCACACAAATGGGAAAAATACTGTG
+HWI-EAS443:3:93:925:805#0/1
_Z``Z`^^¥abbba_a`a^]a^``aa^a_^``aaaZaaZa
@HWI-EAS443:3:94:1280:797#0/1
GAGGCTTCTCTACCCCTGTTGTGTTACATATACTGTTCAC
+HWI-EAS443:3:94:1280:797#0/1
`_bba`___]`bVaaa[a_a[¥ZZ`ZV_aaa^¥ab¥__¥G
@HWI-EAS443:3:94:1606:1393#0/1
ACTCGGTGAAAATATGGCACCAGAAAAAGTAGACTTTGAN
+HWI-EAS443:3:94:1606:1393#0/1
`aa_b`G¥`Q]a[_aaa_`W^_a[U_^`aU_¥`XXa^aPD
@HWI-EAS443:3:94:501:399#0/1
CGGGGGGCTATATGAAGCAATCGAGGAGTGCCTGATTAAT
+HWI-EAS443:3:94:501:399#0/1
abbababaaabbbbaa`aaaa`baaa`a_b__bb`aaa`a
@HWI-EAS443:3:94:803:504#0/1
AGCGTCTCCACAACTTGAGGGGTTTTCGGCTGAATCGAGA
+HWI-EAS443:3:94:803:504#0/1
aaabaa`a¥_`XWNZ`a]aaa]Za`a[^__`aa`_N___]
@HWI-EAS443:3:95:1634:617#0/1
GACGAAAGGAATCCCATAGACTCCTACTGGCCATTTCTTT
+HWI-EAS443:3:95:1634:617#0/1
`b`baX_aXb_a`^b_a_aYa`aaa``^bbaaa_bbR^_N
```

```
@HWI-EAS443:3:95:674:397#0/1
TACAAGTTTGCTGCAATATGCACACATTTGGAAGTTTGTT
+HWI-EAS443:3:95:674:397#0/1
]`abbb^bbabbba`]a``aabbb`abbbbbaVb[aabaa
@HWI-EAS443:3:96:1300:829#0/1
ACGGCCTCAATCATGCTCTCAATCTGCTGAAGAGACTGAA
+HWI-EAS443:3:96:1300:829#0/1
¥]b__WYQ[_WXYZ_^aa`aaa]T`abaa]_`]a]_X_P`
@HWI-EAS443:3:96:315:418#0/1
CGGCCATGTTTCCGATTTGTTTTCAAAGAATTCCTTGGTC
+HWI-EAS443:3:96:315:418#0/1
abbbaabb_aaaaaaabab^aa`aa_aa`aaa`_a`aaY]
@HWI-EAS443:3:97:1008:1771#0/1
TGCTAGCAGAGCTACAGGACATTGAAAATGAAGAGAAGAA
+HWI-EAS443:3:97:1008:1771#0/1
abaa`[T_WaQ_aa`_b[a^P`]a^a]^^`S^a¥]`U^aY
@HWI-EAS443:3:97:1500:723#0/1
GCCATATTTTCACCGAGTGCCCACTTCAATTGGCTTGTTA
+HWI-EAS443:3:97:1500:723#0/1
a`V¥¥a`a¥DM_HZZVbU¥NN¥`PRaYaWYSb]¥BBBBBB
@HWI-EAS443:3:97:926:984#0/1
ACCTCCTCATGCTTGCGATATGTTCAATCGGGGCAACATC
+HWI-EAS443:3:97:926:984#0/1
aa`babbababababababaabbb`aaa`a_a`_aaaa_aa]
@HWI-EAS443:3:98:1117:700#0/1
ATCCCTTCATTATGTATTCAGTAGCCCTGCAGTGGGACAC
+HWI-EAS443:3:98:1117:700#0/1
aba]`abaaaaabb_aaaaaa_aa^¥_`a``a¥aa`^[__
@HWI-EAS443:3:9:1206:872#0/1
CGGCATGTGAATTGACTGATTCAAGCTGGATAGAACTTGA
+HWI-EAS443:3:9:1206:872#0/1
aba^``bWaaV__a`_`aX_a__Q_]a__^]^TZ^]__a]
@HWI-EAS443:3:9:334:769#0/1
ACCCGTTCGTCGATGAAATGGAAATCCGAATACATGAAAC
+HWI-EAS443:3:9:334:769#0/1
aa_`a¥S__WW]___XXT_¥a_a_[P^TT]WRR^¥YY^_W
@HWI-EAS443:3:100:1034:542#0/1
CTCCAGCCTTGAAAACTTTAGAGCCTATGTAGATGGATTC
+HWI-EAS443:3:100:1034:542#0/1
[bb_``_Xabbbbba_aabbbab__baba]]V_[a]_ba^
@HWI-EAS443:3:100:1097:798#0/1
AGCTGGGTCCAAAATGAATTCAATAAGGCATGTGAATTGA
+HWI-EAS443:3:100:1097:798#0/1
_`^^_¥¥K_Xbaaabbabaaaa_ab_aaYa`^_a_a`XXa
@HWI-EAS443:3:100:109:365#0/1
ACCGGTTCTCTTTGTAATCATACAAATCAGGAAGAAATTT
+HWI-EAS443:3:100:109:365#0/1
aaa_a]`__Z`a]`VZ[`_a_^_a_`__^___^a^[^_[B
@HWI-EAS443:3:100:1483:419#0/1
CCCCGATTGAACATATCGCAAGCATGAGGAGGAACTATTT
```

+HWI-EAS443:3:100:1483:419#0/1
abbabab_``a^abba_baaaa^`ba_b_ab`aaa_aa_a
@HWI-EAS443:3:100:1564:1375#0/1
CTCATGGCTTGGAAGCAGGTGCTAGCAGAGCTACAGGACA
+HWI-EAS443:3:100:1564:1375#0/1
ab`b``a`b`K`U_][aaa_GQa]^Y`a_RS_`Qa_aYQ`
@HWI-EAS443:3:100:1635:1390#0/1
GGGGGCTATATGAAGCAATCGAGGAGTGCCTGATTAATGA
+HWI-EAS443:3:100:1635:1390#0/1
aa_`aaaaaaba_aaa^aa[¥VV[UXTYTH¥^U¥XWS_BB
@HWI-EAS443:3:100:1654:856#0/1
CGGGTCACCAGATTCTACAATTATTGATTCACCCCGTTCG
+HWI-EAS443:3:100:1654:856#0/1
ababaab`aababb_ba_aabaabbaabb^a`[_[aYa`a
@HWI-EAS443:3:100:284:685#0/1
GGGATCTTCTCTTCATTTTCAATGTCCTGTAGCTCTGCTA
+HWI-EAS443:3:100:284:685#0/1
aa¥]Y]a_`a`__`^^^aa^aVa`^Y`^a]a_U_¥[_V__
@HWI-EAS443:3:100:284:927#0/1
AGGCAATCTGAGGGGGCGTGGTGTCGTCCTCAAGAATGGT
+HWI-EAS443:3:100:284:927#0/1
aaa`baa_aaaXaa]`aaa_`W^Z_a_VQ^_````^[``¥
@HWI-EAS443:3:100:306:1541#0/1
AATGAAGGGAGTGTACATAAATACGGCCTTGCTCAATGCA
+HWI-EAS443:3:100:306:1541#0/1
]b`bbbaa^aba¥P`abaab^^`abaZaa^¥_aa_^YYUB
@HWI-EAS443:3:100:314:1130#0/1
AAATGGCCAGTAGGAGTCTATGGGATTCCTTTCGTCAGTC
+HWI-EAS443:3:100:314:1130#0/1
ab`aaaaaaa^ab`ab]aa`aa^a_`aa_`a`_aX_V^U_
@HWI-EAS443:3:100:333:1902#0/1
CACCGATTTGAGATAATTGAAGGAAGAGACCGAATCATGG
+HWI-EAS443:3:100:333:1902#0/1
[aVa][I^aGbWb`aa]_U`a^b_a^_`[OON`aa[BBBB
@HWI-EAS443:3:100:365:625#0/1
GGTTTGACTATGTTAGGCTCTTTCCAGCCAAAGAATGTCT
+HWI-EAS443:3:100:365:625#0/1
_a`a_ba¥abbbW^`aa_`_a`b`_a`Sa[__Z^a¥aRBB
@HWI-EAS443:3:100:404:1287#0/1
CGATTGGCCATGTTTCCGATTTGTTTTCAAAGAATTCCTT
+HWI-EAS443:3:100:404:1287#0/1
abXZVYR`aWXU[__GW]PX`W[X]`X`Y`a]Y]ZZPTPB
@HWI-EAS443:3:100:537:1206#0/1
AGATGTTGCCCCGATTGAACATATCGCAAGCATGAGGAGG
+HWI-EAS443:3:100:537:1206#0/1
abaa`aa`]`^_aaab^`b]a_`]_a_^_a]X``__aZ_B
@HWI-EAS443:3:100:562:1600#0/1
CATTAATCAGGCACTCCTCGATTGCTTCATATAGCCCCCC
+HWI-EAS443:3:100:562:1600#0/1
aa^__ba__^_Xabbab`bbb[U`abba_ab^b^a`a`^B

```
@HWI-EAS443:3:100:586:1375#0/1
CTGAATACATAATGAAGGGAGTGTACATAAATACGGCCTT
+HWI-EAS443:3:100:586:1375#0/1
`ab`abb`bbbbbb`bbb]`a`abbb`babbabab`aYaa
@HWI-EAS443:3:100:610:782#0/1
CAGCTTGCTAGAGATCTGGGCTCTGGCTCATCACTGTCAT
+HWI-EAS443:3:100:610:782#0/1
aab`bbbabbbbbbb_bbbb`bbbbbababa_babba`ab
@HWI-EAS443:3:100:647:1136#0/1
AATGGTTCAATTTTGGCGTTCACTTCTTTTGACATTTGGG
+HWI-EAS443:3:100:647:1136#0/1
aa`ab_a_a¥aa`a`__a^_^_Y``]``_`S[]_¥]^P_Y
@HWI-EAS443:3:100:728:537#0/1
CATGTGGTTTGACTATGTTAGGCTCTTTCCAGCCAAAGAA
+HWI-EAS443:3:100:728:537#0/1
a^ab_abaaH[UUX[UaZ]¥S¥¥__ZUaPH[SYPY^`aBB
@HWI-EAS443:3:100:749:88#0/1
GGCATGGAAATGAGGCGCTGCCTTCTTCAGTCTCTTCAGC
+HWI-EAS443:3:100:749:88#0/1
aa[YT_a¥_¥_`^ZRUaY¥_U¥_¥¥¥_UW_¥¥^W¥^SWBB
@HWI-EAS443:3:100:757:1816#0/1
ATCCAGCTTGAATCAGTCAATTCACATGCCTTATTGAATT
+HWI-EAS443:3:100:757:1816#0/1
aa`Zaa^`aba^aYaa]__^__^a]_^_¥[^^`_^¥¥P`^
@HWI-EAS443:3:100:782:205#0/1
GGCGTTCACTTCTTTTGACATTTGGGAAAGCTTGCCCTCA
+HWI-EAS443:3:100:782:205#0/1
_Q¥a_WPIR]_NX_``[]UU¥WUQ`_GU]XGZ_¥MVBBBB
@HWI-EAS443:3:100:820:1317#0/1
GTATTCAGTAGCCCTGCAGTGGGACACTTCTGCTGTAAAA
+HWI-EAS443:3:100:820:1317#0/1
^ababa]`]bb`b^`aabb_abb[``_aaaa`[a`^`U`B
@HWI-EAS443:3:100:821:1807#0/1
GGGGGCTATATGAAGCAATCGAGGAGTGCCTGATTAATGA
+HWI-EAS443:3:100:821:1807#0/1
aaaa__aa_``a^L`^b`aG`¥^`_QT^G]aa`_^_Y_aB
@HWI-EAS443:3:100:849:428#0/1
ATATGTTCAATCGGGGCAACATCTTCTCCTATTTCATCAA
+HWI-EAS443:3:100:849:428#0/1
abaab_^_``b]aa`XU__W_a^aa[`_^a`_`a]`a]a^
@HWI-EAS443:3:100:902:833#0/1
GTCAAACCACATGAGAAAGGCATAAATCCCAATTACCTCA
+HWI-EAS443:3:100:902:833#0/1
a]Yabb`_aZbab]aa^Y^a]babaaa`]_aaa`_X]^]_
@HWI-EAS443:3:100:929:712#0/1
CATCTTTGCAGTCATCAAAGTCTACTTTTTCTGGTGCCAT
+HWI-EAS443:3:100:929:712#0/1
abaa_¥V^babaabbUb__a`P`_[a_ba`[R`aaaK[ab
@HWI-EAS443:3:100:975:718#0/1
CAAAGATGTTGGAGACCTTAAACAGTATGACAGTGATGAG
```

```
+HWI-EAS443:3:100:975:718#0/1
aabbbabbbbbbbbbaa`bbbaaabb``bab_`b`a_`_]_
@HWI-EAS443:3:100:97:1490#0/1
GACATTCTTTGGCTGGAAAGAGCCTAACATAGTCAAACCA
+HWI-EAS443:3:100:97:1490#0/1
abbabbabbbaaaba^_^`S^`aa_aa_Vaa__aaaaOU^
@HWI-EAS443:3:100:995:262#0/1
CCTTGACGAAGAGAGCAGGGCAAGAATCAAAACTAGGCTT
+HWI-EAS443:3:100:995:262#0/1
abbbba¥`VV`a]a`S¥_[VS[aa^aaY``^a_aaa_G_B
@HWI-EAS443:3:10:1121:711#0/1
CATTAATCAGGCACTCCTCGATTGCTTCATATAGCCCCCC
+HWI-EAS443:3:10:1121:711#0/1
Z[`¥`[aTSaaaU[NaZbababW¥]`SaVU^]a`¥YHZa_
@HWI-EAS443:3:10:1170:539#0/1
CATAAATACGGCCTTGCTCAATGCATCCTGTGCAGCCATG
+HWI-EAS443:3:10:1170:539#0/1
G^bbbbb_a¥U¥``aaabZaaba¥¥b``bb`]FZaZZ__a
@HWI-EAS443:3:10:1173:992#0/1
AGGCAATCTGAGGGGGCGTGGTGTCGTCCTCAAGAATGGT
+HWI-EAS443:3:10:1173:992#0/1
`aZO_ba`a`aaaaXaP`Paa]_YU^^]SV_S_a]QP_a_
@HWI-EAS443:3:10:1224:1011#0/1
GCACTATTGAAGCACCGATTTGAGATAATTGAAGGAAGAG
+HWI-EAS443:3:10:1224:1011#0/1
a]^X_¥``aV^_V^YY`X`__]V¥U]]X]]^XW]UXV^XY
@HWI-EAS443:3:10:1275:1033#0/1
CATTAATCAGGCACTCCTCGATTGCTTCATATAGCCCCCC
+HWI-EAS443:3:10:1275:1033#0/1
OT__[W_T_¥^[bb_Q^___WZ^_^]]Zbb^`^_SW[ZLS
@HWI-EAS443:3:10:1282:1801#0/1
CACCGATTTGAGATAATTGAAGGAAGAGACCGAATCATGG
+HWI-EAS443:3:10:1282:1801#0/1
aWUbbaa]baa_a^`a¥_^``aa¥_abbb¥Jb`aSIaa¥`
@HWI-EAS443:3:10:1364:1595#0/1
AGAATTCCTTGGTCATGTCTTTCTCTTTGACAGAAGACTC
+HWI-EAS443:3:10:1364:1595#0/1
abbaba_Zaaaa¥`a¥]^a_`_]`Zaa^`]X`_]W[^W_R
@HWI-EAS443:3:10:1380:1024#0/1
AGCAGGTGCTAGCAGAGCTACAGGACATTGAAAATGAAGA
+HWI-EAS443:3:10:1380:1024#0/1
ab`_`ba^`abb]aa_aXba`aaa_Yaa]__¥`a^``_a`
@HWI-EAS443:3:10:1429:609#0/1
ATAAGACAAGAAATGGCCAGTAGGAGTCTATGGGATTCCT
+HWI-EAS443:3:10:1429:609#0/1
aaaaaa[_aa_`]``¥SSW^V^__X^YU]Y^_]]VX¥RV]
@HWI-EAS443:3:10:1433:1062#0/1
AGACATTCTTTGGCTGGAAAGAGCCTAACATAGTCAAACC
+HWI-EAS443:3:10:1433:1062#0/1
_]]`a`bR`abba`a^`¥a_[]_[_`aa^``__^]_a`UR
```

@HWI-EAS443:3:10:1440:1339#0/1
TCCGATTGGCCATGTTTCCGATTTGTTTTCAAAGAATTCC
+HWI-EAS443:3:10:1440:1339#0/1
a`aa``a_`^^___^`_XY_X]_`]]]__W]]_]]X^]YY
@HWI-EAS443:3:10:1518:1866#0/1
GGGGCATGGAAATGAGGCGCTGCCTTCTTCAGTCTCTTCA
+HWI-EAS443:3:10:1518:1866#0/1
a_aaN___[W_VW¥VU_¥SPSYSUSTUX¥HVWVQWVVVBB
@HWI-EAS443:3:10:1577:1182#0/1
AATGGAACCTCCAAGATCAAGATGAAATGGGGCATGGAAA
+HWI-EAS443:3:10:1577:1182#0/1
a_aa]aa¥]aZ_^]^]_Z`¥_¥^`W¥¥_`¥_¥WW¥^SWWW
@HWI-EAS443:3:10:1609:1608#0/1
TGTCATACTGTTTAAGGTCTCCAACATCTTTGCAGTCATC
+HWI-EAS443:3:10:1609:1608#0/1
aba`abb_baaaaa`aa__a_¥``]_a]a]_`]_`]]^a¥
@HWI-EAS443:3:10:1619:1243#0/1
TAGCAAGCTGGGTCCAAAATGAATTCAATAAGGCATGTGA
+HWI-EAS443:3:10:1619:1243#0/1
a_aa`^¥R_a¥ZNTY]aa_``Z^a`W`¥``Y__OY]UV`Y
@HWI-EAS443:3:10:190:648#0/1
AGTAGCCCTGCAGTGGGACACTTCTGCTGTAAAATAGTTC
+HWI-EAS443:3:10:190:648#0/1
T[aaV]aaaa`a`Ua``^a`^^a_aaa_a`aaa`U[^W¥¥
@HWI-EAS443:3:10:203:441#0/1
AACAATGAGAAGCAATTTTCTCGATTCAGCCGAAAACCCC
+HWI-EAS443:3:10:203:441#0/1
aaaaaaa`b`aa_b_aaa``_aba``a_a``a^`__^]__
@HWI-EAS443:3:10:226:1486#0/1
CTAAATTTCTTCCTGATTTGTATGATTACAAAGAGAACCG
+HWI-EAS443:3:10:226:1486#0/1
aaa_^`baaabbaaa`aa_aQa`a]__a`a_^X`¥Z__[[
@HWI-EAS443:3:10:284:845#0/1
CCCCCAAGATCGAAGGTTCCAGGTTCCAGGTTGTCCCTAA
+HWI-EAS443:3:10:284:845#0/1
abbbab¥ba]a`a`aa_a]]_aa_a_`a_a^_`[U`YW¥_
@HWI-EAS443:3:10:307:1828#0/1
TCGGCTGAATCGAGAAAATTGCTTCTCATTGTTCAGGCAC
+HWI-EAS443:3:10:307:1828#0/1
V^Z¥Y``¥`V^[[X``b_]_VN^XY^[]_`_]X¥[UZ[`S
@HWI-EAS443:3:10:406:838#0/1
AATGGCCAGTAGGAGTCTATGGGATTCCTTTCGTCAGTCC
+HWI-EAS443:3:10:406:838#0/1
^Yaaaab``Xa]_V¥T^_`^``a^X^[_^____VX¥XRYV
@HWI-EAS443:3:10:454:44#0/1
TGTTTCATGTATTCGGATTTCCATTTCATCGACGAACGGG
+HWI-EAS443:3:10:454:44#0/1
abaa_]_aaa`aa_a`^`__^Z^`a[_a`_]]R_¥]V¥]^
@HWI-EAS443:3:10:480:1689#0/1
TGCCTGAACAATGAGAAGCAATTTTCTCGATTCAGCCGAA

```
+HWI-EAS443:3:10:480:1689#0/1
]aaba]]abZ__bbabbababba`aababaaaaaaa`aaa
@HWI-EAS443:3:10:486:739#0/1
ACTATGTTAGGCTCTTTCCAGCCAAAGAATGTCTTCATGC
+HWI-EAS443:3:10:486:739#0/1
ab¥[`a``^Y]GPLX^ZSQab^V_[aaa[_`RZ`__[_¥Q
@HWI-EAS443:3:10:533:1016#0/1
AGACATGACCAAGGAATTCTTTGAAAACAAATCGGAAACA
+HWI-EAS443:3:10:533:1016#0/1
^`a`aabaa]aaaaaaaaaaaaaa_aa_aa^`aa___aa`
@HWI-EAS443:3:10:695:355#0/1
CACACATTTGGAAGTTTGTTTCATGTATTCGGATTTCCAT
+HWI-EAS443:3:10:695:355#0/1
abbbb`abbbaa`b_abbabaaaaa`baba^aaaaa]`aa
@HWI-EAS443:3:10:720:482#0/1
GTCCTTGGGATCTTCTCTTCATTTTCAATGTCCTGTAGCT
+HWI-EAS443:3:10:720:482#0/1
a_a]a_]X¥DX[a__a¥a_[[_`_]¥_¥¥^WWS¥ZRUXUU
@HWI-EAS443:3:10:721:1426#0/1
ATCGACGAACGGGGTGAATCAATAATTGTAGAATCTGGTG
+HWI-EAS443:3:10:721:1426#0/1
a`aaa_a_a]^___G^`a^V]_a_[__XY^^`^ZR]¥VX¥
@HWI-EAS443:3:10:743:860#0/1
AGGCACTCCTCGATTGCTTCATATAGCCCCCCAAGATCGA
+HWI-EAS443:3:10:743:860#0/1
]babbZ`_]X`a¥¥_W^aa`aaa_a^^ZV^T]^X^^XY]`
@HWI-EAS443:3:10:784:1570#0/1
ATATGGCACCAGAAAAAGTAGACTTTGATGACTGCAAAGA
+HWI-EAS443:3:10:784:1570#0/1
`a````_^¥^^^ab]_b`G`b^[a`[b```__]a]___R`
@HWI-EAS443:3:10:86:1199#0/1
CTTGGGGGGCTATATGAAGCAATCGAGGAGTGCCTGATTA
+HWI-EAS443:3:10:86:1199#0/1
abbb`¥^babababba]bb`aaabb^aa`a_aa``a_aaa
@HWI-EAS443:3:10:919:127#0/1
TAGCCCTGCAGTGGGACACTTCTGCTGTAAAATAGTTCCT
+HWI-EAS443:3:10:919:127#0/1
a`¥[_Zaa_ab_`__[RWY_^¥]^S¥aRWX]V]OYXXLNR
@HWI-EAS443:3:10:966:1580#0/1
ATGAATTCAATAAGGCATGTGAATTGACTGATTCAAGCTG
+HWI-EAS443:3:10:966:1580#0/1
aaaa`aa_aaaa```]aaa_a`aa```^`a``_____¥_`
@HWI-EAS443:3:10:970:1258#0/1
CTCCCTTCATTATGTATTCAGTAGCCCTGCAGTGGGACAC
+HWI-EAS443:3:10:970:1258#0/1
aababbbabbbbbaaaabbbbabbaaaabaaaVa```_a_
@HWI-EAS443:3:11:1034:1402#0/1
CTCCACTCCCCTGGGTGACTCTCCGATTGGCCATGTTTCC
+HWI-EAS443:3:11:1034:1402#0/1
aab_b^aaaaa`aa^Raaaa¥_`aa`VY[US[^[VPY¥U[
```

```
@HWI-EAS443:3:11:1113:491#0/1
CGATTGGCCATGTTTCCGATTTGTTTTCAAAGAATTCCTT
+HWI-EAS443:3:11:1113:491#0/1
a`Zabba_a`aaaab_`^^aaab_aaa^``aa_^``]¥aa
@HWI-EAS443:3:11:1249:1132#0/1
CGATAGGCCAAGTGTCGAGGCCCATGTTCCTATATGTGAG
+HWI-EAS443:3:11:1249:1132#0/1
Va`V[_ZZ_`aXZ¥^``[^a`[L[X`^UTT[a`a_b[¥T]
@HWI-EAS443:3:11:1343:436#0/1
CAAGAATGGTTCAATTTTGGCGTTCACTTCTTTTGACATT
+HWI-EAS443:3:11:1343:436#0/1
aababaabb`a`aa`baaaa^a[a¥^Ya¥S¥a`__]^_¥Y
@HWI-EAS443:3:11:1358:1280#0/1
CGGCTGAATCGAGAAAATTGCTTCTCATTGTTCAGGCACT
+HWI-EAS443:3:11:1358:1280#0/1
aba_`b`^Zabab`^`aaaa]`a]]W]aaa[`]^]_Z^Z`
@HWI-EAS443:3:11:1403:1990#0/1
GAAAGGCATAAATCCCAATTACCTCATGGCTTGGAAGCAG
+HWI-EAS443:3:11:1403:1990#0/1
a_Y`aa_ba`baa^__aaaaa`WaUaa`TIX^UQQBBBBB
@HWI-EAS443:3:11:1406:1464#0/1
GGACATTGAAAATGAAGAGAAGATCCCAAGGACAAAGAAC
+HWI-EAS443:3:11:1406:1464#0/1
aa`[`a`aaaaaaa___a_aa___VWT]^_^]Z_]_^^]W
@HWI-EAS443:3:11:1431:551#0/1
TGAGAAAGGCATAAATCCCAATTACCTCATGGCTTGGAAG
+HWI-EAS443:3:11:1431:551#0/1
abbabbabbaaab`_a]aaaaaa`_[aaa`a_[aa¥a¥]B
@HWI-EAS443:3:11:1590:399#0/1
TTTGTTTTCAAAGAATTCCTTGGTCATGTCTTTCTCTTTG
+HWI-EAS443:3:11:1590:399#0/1
¥`aaYb_babb¥`^Z^aa¥a``a_^Uab]`aaaW`]^_a`
@HWI-EAS443:3:11:1601:473#0/1
AAAGAATTCCTTGGTCATGTCTTTCTCTTTGACAGAAGAC
+HWI-EAS443:3:11:1601:473#0/1
`_aaaZba]aaaabU_aab_Za`aY`_a_aa¥X`__]`_X
@HWI-EAS443:3:11:162:909#0/1
GGGGGCTATATGAAGCAATCGAGGAGTGCCTGATTAATGA
+HWI-EAS443:3:11:162:909#0/1
^aaaaaa]`a`aa]abaY`aaaa_`aW``a``_`_``^__
@HWI-EAS443:3:11:1737:673#0/1
GCGCATAGTTCCTGTAATCTCAAATTTTTCTTCAATTGTC
+HWI-EAS443:3:11:1737:673#0/1
a`ba_bab`baabb__ababa`b`babbb_bb_aaaaa__
@HWI-EAS443:3:11:1748:932#0/1
GAAGGAAGAGACCGAATCATGGCCTGGACAGTGGTGAACA
+HWI-EAS443:3:11:1748:932#0/1
[[a_V^aba_a_¥a___``aba]^`^YXR]`V`_RZ]_R_
@HWI-EAS443:3:11:1765:327#0/1
TAGCCCCCCAAGATCGAAGGTTCCAGGTTCCAGGTTGTCC
```

```
+HWI-EAS443:3:11:1765:327#0/1
abbaaZ`^aa_a`a¥]`a`^UZRX[``V^GbRU[SUUTTB
@HWI-EAS443:3:11:178:1215#0/1
CAAGGACAAAGAACATGAAGAGAACAAGCCAATTGAAGTG
+HWI-EAS443:3:11:178:1215#0/1
a^L_aaa^a^`aaaaaa_`^b`VZ`aa_`aaa``a^aaU_
@HWI-EAS443:3:11:188:611#0/1
AAAGAGCCTAACATAGTCAAACCACATGAGAAAGGCATAA
+HWI-EAS443:3:11:188:611#0/1
aab`X`aa`aa]a`aa`aa]``_`aa__a``ZR__[`a``
@HWI-EAS443:3:11:24:733#0/1
CCCAAGATCGAAGGTTCCAGGTTCCAGGTTGTCCCTAAGT
+HWI-EAS443:3:11:24:733#0/1
aaa¥U__]a`¥Q]`L]_[PV_WW_]VUVR¥[W^ZVZZRZU
@HWI-EAS443:3:11:264:854#0/1
CCCCGATTGAACATATCGCAAGCATGAGGAGGAACTATTT
+HWI-EAS443:3:11:264:854#0/1
abb_a`_`Ya`Q`b`bbbb[T¥]]aaYYa^a]`_`H`[``
@HWI-EAS443:3:11:268:334#0/1
GTTTCCGATTTGTTTTCAAAGAATTCCTTGGTCATGTCTT
+HWI-EAS443:3:11:268:334#0/1
aZaaa_a_`_aa^^a]a]`_``^^]^^_`__[`^``Y___
@HWI-EAS443:3:11:303:1903#0/1
CAATAGTGCATTCGGGTCACCAGATTCTACAATTATTGAT
+HWI-EAS443:3:11:303:1903#0/1
aba_[aZ_aaa¥a___Waaaaa¥[_]_]^^_^^^`^_^^Z
@HWI-EAS443:3:11:305:632#0/1
AGAATTCCTTGGTCATGTCTTTCTCTTTGACAGAAGACTC
+HWI-EAS443:3:11:305:632#0/1
VZKW]¥ba_aU]_bTbb`a`aaa`a]WT`Q`a`a]]^a[]
@HWI-EAS443:3:11:329:1700#0/1
CAGGAACTTTGACCGCTGATGGCAAAGAGGCCCATCAGGC
+HWI-EAS443:3:11:329:1700#0/1
abaa_aa^aa^ZabTbRY^X_`b]^`]^MX`_U_`a^RBB
@HWI-EAS443:3:11:330:1586#0/1
GGCATGGAAATGAGGCGCTGCCTTCTTCAGTCTCTTCAGC
+HWI-EAS443:3:11:330:1586#0/1
aabb`aZabbaaaa___a]_a``__aa__`Z_`V^a^`¥`
@HWI-EAS443:3:11:360:992#0/1
CGGTCTCTTCCTTCAATTATCTCAAATCGGTGCTTCAATA
+HWI-EAS443:3:11:360:992#0/1
aa`Yaa`a_aa`aaa^a`aa`aaa_a_aaXNX¥^^[`__^
@HWI-EAS443:3:11:526:1574#0/1
ATGGCTTGGAAGCAGGTGCTAGCAGAGCTACAGGACATTG
+HWI-EAS443:3:11:526:1574#0/1
a_[`a`^_Y_`ZWXPXVV¥V[RQLQWGPZVV^¥XZX^VYR
@HWI-EAS443:3:11:572:349#0/1
CAAGGACAAAGAACATGAAGAGAACAAGCCAATTGAAGTG
+HWI-EAS443:3:11:572:349#0/1
`aaa[a``_Y_`__`_a_``__``_____¥^]^]`^^_V^
```

```
@HWI-EAS443:3:11:594:1174#0/1
ATATACTGTTCACCACTGTCCAGGCCATGATTCGGTCTCT
+HWI-EAS443:3:11:594:1174#0/1
[`S¥T¥a^_a`_bbaaaaY^a`ba_[U^XT^¥NU^JYWL¥
@HWI-EAS443:3:11:620:1711#0/1
TGCCTTTCTCATGTGGTTTGACTATGTTAGGCTCTTTCCA
+HWI-EAS443:3:11:620:1711#0/1
```¥]YUT_a¥U_QYbR``[¥]_aaaF`_X_V^aHU_`aa
@HWI-EAS443:3:11:700:1714#0/1
GGGGGAGGGAATACCACTATATGATGCAATCAAATGCATG
+HWI-EAS443:3:11:700:1714#0/1
T_`[¥a__U]_XbXabb¥_aaabbabab[UHY`bUa^`UM
@HWI-EAS443:3:11:709:468#0/1
GGGGCATGGAAATGAGGCGCTGCCTTCTTCAGTCTCTTCA
+HWI-EAS443:3:11:709:468#0/1
a_¥J``Sa`^`ZWY]OV¥_Q^Z[SZZ¥__¥¥¥VS¥SUZUU
@HWI-EAS443:3:11:712:1969#0/1
AGCAGGTGCTAGCAGAGCTACAGGACATTGAAAATGAAGA
+HWI-EAS443:3:11:712:1969#0/1
`aa[`aZa]_a__^aaW`]_¥aa]^_^^^_^__]^_]a__
@HWI-EAS443:3:11:718:1430#0/1
GGGGCATGGAAATGAGGCGCTGCCTTCTTCAGTCTCTTCA
+HWI-EAS443:3:11:718:1430#0/1
`aT]`Q`aaabbbab`a`bXaaa``a`a_aa^^_`YaaZa
@HWI-EAS443:3:11:735:1328#0/1
GGAATTCTTTGAAAACAAATCGGAAACATGGCCAATCGGA
+HWI-EAS443:3:11:735:1328#0/1
ababbbabbbbbaa_ab¥bb``baababa]U]_aa__^Z_
@HWI-EAS443:3:11:836:262#0/1
AATCAGTCAATTCACATGCCTTATTGAATTCATTTTGGAC
+HWI-EAS443:3:11:836:262#0/1
```_a_FW^^[^R]aa¥]aa]P``^a`a^^]___X]a_^U
@HWI-EAS443:3:11:853:952#0/1
ATCCGAAAATCGAAACTAACAAGTTTGCTGCAATATGCAC
+HWI-EAS443:3:11:853:952#0/1
aabaa_abaaab__`_aa_]`_a^__`^_a_^]^^_^¥_]
@HWI-EAS443:3:11:862:1592#0/1
GGGGGCTATATGAAGCAATCGAGGAGTGCCTGATTAATGA
+HWI-EAS443:3:11:862:1592#0/1
Wabb_XZb]a``a^_U¥W`a[_b`X`¥aaT^a_][`aaa_
@HWI-EAS443:3:11:896:1567#0/1
CGGGGTGAATCAATAATTGTAGAATCTGGTGACCCGAATG
+HWI-EAS443:3:11:896:1567#0/1
```ab]abbaaaab`ab_b_a`a`aaaaaWa___`_a`a]
@HWI-EAS443:3:11:944:926#0/1
CTTGGGATCTTCTCTTCATTTTCAATGTCCTGTAGCTCTG
+HWI-EAS443:3:11:944:926#0/1
abbbba``abbaabbbaaabbbaa`baa_`ba`baaaaaa
@HWI-EAS443:3:11:950:1609#0/1
ATTTCATCGACGAACGGGGTGAATCAATAATTGTAGAATC
```
```

```
+HWI-EAS443:3:11:950:1609#0/1
aaaaaa_a_a`_`__^__WOX_^`Z^^_^_a_][__]Z[Z
@HWI-EAS443:3:11:955:259#0/1
ATTTTCACCGAGTGCCCACTTCAATTGGCTTGTTCTCTTC
+HWI-EAS443:3:11:955:259#0/1
abbaaaaaaa`a^a`_`__a`]``__a_Z^aS^VDTX__R
@HWI-EAS443:3:12:1068:1861#0/1
CAGTCATCAAAGTCTACTTTTTCTGGTGCCATATTTTCAC
+HWI-EAS443:3:12:1068:1861#0/1
aaa¥^aaa```Y[``¥]_a__a^`a`W^S]``aba_`]a_
@HWI-EAS443:3:12:1121:1363#0/1
GGGGGCTATATGAAGCAATCGAGGAGTGCCTGATTAATGA
+HWI-EAS443:3:12:1121:1363#0/1
aaa`a`aaa¥aa]aa_aaa¥a`^Z_aZ_`¥^a_aa__^_`
@HWI-EAS443:3:12:1180:215#0/1
ATCAATAATTGTAGAATCTGGTGACCCGAATGCACTATTG
+HWI-EAS443:3:12:1180:215#0/1
`YU`__a]¥T_U]QWPX¥W_aW¥XZWV^UX¥]O¥^P¥YTQ
@HWI-EAS443:3:12:1197:1206#0/1
AGCCAACAAAATAAAATCTGAGAAGACACACATTCACATC
+HWI-EAS443:3:12:1197:1206#0/1
`aba``abb`_^baba]aa][`^[W`^a_a``a`aSY`aZ
@HWI-EAS443:3:12:1274:960#0/1
CGATTGGCCATGTTTCCGATTTGTTTTCAAAGAATTCCTT
+HWI-EAS443:3:12:1274:960#0/1
aaab_bbb``b`Y_`N`_aa`ba`aaa`a`a¥a``aS_aa
@HWI-EAS443:3:12:1359:563#0/1
CCTGCTCTCTTCGTCAAGGGTGTAGTCCGCTTTGGTGGCC
+HWI-EAS443:3:12:1359:563#0/1
¥]bbabbbb`babb]abb_ZDYSWbab_b]abb`b]_baa
@HWI-EAS443:3:12:1382:94#0/1
AGCAGGTGCTAGCAGAGCTACAGGACATTGAAAATGAAGA
+HWI-EAS443:3:12:1382:94#0/1
`a^_a`P¥`aa`Q__Y_^_`¥_]_WU¥aaTP[^__Z¥]__
@HWI-EAS443:3:12:1426:1082#0/1
ATAAAATCTGAGAAGACACACATTCACATCTTTTCATTCA
+HWI-EAS443:3:12:1426:1082#0/1
a¥`_ZZaaaa^^][¥^aa¥^]^`__`aa`^`__Z¥a____
@HWI-EAS443:3:12:1435:73#0/1
ATGAAGACATTCTTTGGCTGGAAAGAGCCTAACATAGTCA
+HWI-EAS443:3:12:1435:73#0/1
aabbaaaaaaa^]Y_`a_ba`_^]`_`RM^^^S^_YWR^_
@HWI-EAS443:3:12:1454:779#0/1
CATTCACATCTTTTCATTCACTGGAGAGGAGATGGCCACC
+HWI-EAS443:3:12:1454:779#0/1
a[_TYaa_bb`bbbabab_X^ba]Z_ba¥`bZaXaX^SUS
@HWI-EAS443:3:12:1497:1055#0/1
ATTCCTTGGTCATGTCTTTCTCTTTGACAGAAGACTCGGC
+HWI-EAS443:3:12:1497:1055#0/1
a_]^_bab^RY¥a`_Y^bbY`Z^`]]]W]aa][X^]R]XB
```

@HWI-EAS443:3:12:1509:555#0/1
CATGTTTCCGATTTGTTTTCAAAGAATTCCTTGGTCATGT
+HWI-EAS443:3:12:1509:555#0/1
``bbbbbbbbbbbbbabbbabaabaabb_Vabb_`¥^aba
@HWI-EAS443:3:12:154:884#0/1
TTGGGATTTATGCCTTTCTCATGTGGTTTGACTATGTTAG
+HWI-EAS443:3:12:154:884#0/1
abbaababababaaaaaaaaaaa_aa_aaa`a`aaa]_`_
@HWI-EAS443:3:12:1606:1107#0/1
CAGTCATCAAAGTCTACTTTTTCTGGTGCCATATTTTCAC
+HWI-EAS443:3:12:1606:1107#0/1
a`b_aaa_^_`aa`aaabbbab`b_b___]aa_b`b`_`a
@HWI-EAS443:3:12:160:602#0/1
ATTACAAAGAGAACCGGTTCATTGAAATTGGAGTAACACG
+HWI-EAS443:3:12:160:602#0/1
_a``aaaaaaa_aaa`a^```a`a^Z_a``__aZ`]`a``
@HWI-EAS443:3:12:1712:511#0/1
CAGCAAACTTGTTAGTTTCGATTTTCGGATCTTCCCCATA
+HWI-EAS443:3:12:1712:511#0/1
¥^b_b`a]ba`_bb]Xa¥Z`U][^aI__PIZ`ZGYLSS[O
@HWI-EAS443:3:12:1721:1367#0/1
GGGGGCTATATGAAGCAATCGAGGAGTGCCTGATTAATGA
+HWI-EAS443:3:12:1721:1367#0/1
abbbaaabbabaaab^___Rb_aa_a¥aV¥aa^`a__``_
@HWI-EAS443:3:12:1785:778#0/1
GAAGCAGGTGCTAGCAGAGCTACAGGACATTGAAAATGAA
+HWI-EAS443:3:12:1785:778#0/1
aa][Ya`aQZ_[^a^aa^^W_XY___]]W_¥¥WZ]`a^]Z
@HWI-EAS443:3:12:294:771#0/1
AAGAATTCCTTGGTCATGTCTTTCTCTTTGACAGAAGACT
+HWI-EAS443:3:12:294:771#0/1
aa_Z`aaa_aaaaX``aa^a`aaaa```a__aa_`S_^^_
@HWI-EAS443:3:12:296:707#0/1
GGGGCATGGAAATGAGGCGCTGCCTTCTTCAGTCTCTTCA
+HWI-EAS443:3:12:296:707#0/1
abbabb`baabbbbbbabbaaaaaabaaa_^a_aa`aa]a
@HWI-EAS443:3:12:313:280#0/1
GGGGGCTATATGAAGCAATCGAGGAGTGCCTGATTAATGA
+HWI-EAS443:3:12:313:280#0/1
a_Za¥aaW[`_a¥`[V[Z^]]]a_[TSZ_^¥^TW[ZQZ¥Y
@HWI-EAS443:3:12:313:369#0/1
AGAATTCCTTGGTCATGTCTTTCTCTTTGACAGAAGACTC
+HWI-EAS443:3:12:313:369#0/1
a_U¥aV``]a_^U_U]__baa^Z^¥`aa^T``¥Y_X_a¥Z
@HWI-EAS443:3:12:367:1367#0/1
AGCTTGCTAGAGATCTGGGCTCTGGCTCATCACTGTCATA
+HWI-EAS443:3:12:367:1367#0/1
U`bbbabbaa_¥Wbba_a````aaa^abba]aaaaaVaaa
@HWI-EAS443:3:12:377:667#0/1
ATCCTGTGCAGCCATGGATGACTTTCAGCTGATCCCAATG

```
+HWI-EAS443:3:12:377:667#0/1
`]b`_]L^_aa_N__`PW_aQZ`aJZ]Y]^^YVYZ]]^_`
@HWI-EAS443:3:12:394:917#0/1
TGTCCAGGCCATGATTCGGTCTCTTCCTTCAATTATCTCA
+HWI-EAS443:3:12:394:917#0/1
aa^ab`_^aa^_^]_a_V_X^¥]Z`]Z__¥]]^^Y_O[V`
@HWI-EAS443:3:12:461:1167#0/1
ACCGAACTTCTCCAGCCTTGAAAACTTTAGAGCCTATGTA
+HWI-EAS443:3:12:461:1167#0/1
^`¥X[a¥¥X_``¥¥TRaYRaYUNHV_^`]_]_S^`[^`R_
@HWI-EAS443:3:12:483:101#0/1
AGGCTGGAGAAGTTCGGTGGGAGACTTTGGTCGGCAAGCT
+HWI-EAS443:3:12:483:101#0/1
ab]bba`bba]a`aa]ZQba`[aMX__[L[QW¥]XU_¥ZB
@HWI-EAS443:3:12:484:1955#0/1
TTCGGTCTCTTCCTTCAATTATCTCAAATCGGTGCTTCAA
+HWI-EAS443:3:12:484:1955#0/1
aaZWVTUNL^VOSVU`ZT]]__^]`___WSX`J^]MZ[BB
@HWI-EAS443:3:12:501:691#0/1
GGGGCTATATGAAGCAATCGAGGAGTGCCTGATTAATGAT
+HWI-EAS443:3:12:501:691#0/1
ab`_``_¥VRa`Y`[_^P¥[T_ZG[GZU^T¥NQSSSUZTT
@HWI-EAS443:3:12:507:1062#0/1
AAGGAAGAGACCGAATCATGGCCTGGACAGTGGTGAACAG
+HWI-EAS443:3:12:507:1062#0/1
a_U_Ua][aaa``J`a`M`^W_`__`_[RTU`^Z_[^_Z^
@HWI-EAS443:3:12:555:711#0/1
GGCATGGAAATGAGGCGCTGCCTTCTTCAGTCTCTTCAGC
+HWI-EAS443:3:12:555:711#0/1
`b`a`_WI_T¥aa`_Raaaaaaaa`aX[VaTaaZ_a_XaW
@HWI-EAS443:3:12:634:354#0/1
GGGGGCTATATGAAGCAATCGAGGAGTGCCTGATTAATGA
+HWI-EAS443:3:12:634:354#0/1
`^¥OY^`a_aaa__^_``__`¥____W_Q¥V`¥^^¥¥¥_¥
@HWI-EAS443:3:12:682:1692#0/1
GGGTTTTCGGCTGAATCGAGAAAATTGCTTCTCATTGTTC
+HWI-EAS443:3:12:682:1692#0/1
a`a]a_`b``a`_b_^`Y`__`a`]_^`_]__`^WW_Z]]
@HWI-EAS443:3:12:733:781#0/1
CCACTATATGATGCAATCAAATGCATGAAGACATTCTTTG
+HWI-EAS443:3:12:733:781#0/1
a_P[aaa`YU¥XYQ][Z^aaa]a]Zaa`[WW¥``_Y]^_¥
@HWI-EAS443:3:12:775:1928#0/1
GTGAATTGACTGATTCAAGCTGGATAGAACTTGATGAAAT
+HWI-EAS443:3:12:775:1928#0/1
a`babbaab^aa`a`_bba`aa`a`a_`b^`aaaaa_aa_
@HWI-EAS443:3:12:825:257#0/1
CGATTGGCCATGTTTCCGATTTGTTTTCAAAGAATTCCTT
+HWI-EAS443:3:12:825:257#0/1
abaa`baaa_ab]aa_aa]`aaa__aa__`a_`_^aXW__
```

```
@HWI-EAS443:3:12:826:1104#0/1
AGTAGGAGTCTATGGGATTCCTTTCGTCAGTCCGAAAGAG
+HWI-EAS443:3:12:826:1104#0/1
a]¥a[_``ab`ababaaaa`_``aTa__]`¥_]__^__]_
@HWI-EAS443:3:12:842:422#0/1
GGCTGAATCGAGAAAATTGCTTCTCATTGTTCAGGCACTT
+HWI-EAS443:3:12:842:422#0/1
abab^X¥a``YaU`aa``]a]][`N``_T¥Y^]``R¥^`]
@HWI-EAS443:3:12:873:807#0/1
TTGAAAACTTTAGAGCCTATGTAGATGGATTCGAGCCGAA
+HWI-EAS443:3:12:873:807#0/1
aaa`a`aabababaaaababb`T```ab_`a`ba`_`a`_
@HWI-EAS443:3:12:98:1933#0/1
TTCCAAATGTGTGCATATTGCAGCAAACTTGTTAGTTTCG
+HWI-EAS443:3:12:98:1933#0/1
`aa_bbaa`]`Z`aaaa^__aa¥aa¥_a`^`^``_W_][Y
@HWI-EAS443:3:13:1061:1106#0/1
GGTGAACTTTGTAAGTATGGAGTTCTCACTCACTGACCCG
+HWI-EAS443:3:13:1061:1106#0/1
aa_bbbaabaa_b]aWaaaa^a_aaaa``a_``aa```]a
@HWI-EAS443:3:13:1092:301#0/1
GTCCAGGCCATGATTCGGTCTCTTCCTTCAATTATCTCAA
+HWI-EAS443:3:13:1092:301#0/1
`[[`]T[^¥aaaZ[^]``WO_WR`^]_^]_^]^^^O^¥Z^
@HWI-EAS443:3:13:1177:264#0/1
CACCTGCTTCCAAGCCATGAGGTAATTGGGATTTATGCCT
+HWI-EAS443:3:13:1177:264#0/1
ab`abbbabbbaaaa_aba`aa[`_bbaaa``aaaaaVYa
@HWI-EAS443:3:13:1180:1887#0/1
CTGAAATTAAGTATTGAAGACCCGAGTCACGAGGGGGAGG
+HWI-EAS443:3:13:1180:1887#0/1
a_bbb`b`Ybb[bba_aa^b[W¥`Zaaa[]^ba]aZ]]``
@HWI-EAS443:3:13:1181:689#0/1
CGTCTCCACAACTTGAGGGGTTTTCGGCTGAATCGAGAAA
+HWI-EAS443:3:13:1181:689#0/1
a`¥`aaa¥¥aaaYaaVaXM]Paa_``a_a]^YXXaV]aa`
@HWI-EAS443:3:13:1201:173#0/1
CAGCCTTGAAAACTTTAGAGCCTATGTAGATGGATTCGAG
+HWI-EAS443:3:13:1201:173#0/1
abbaa_aZWba][ba`abSba_a`aa^aa[`aaaX`^__¥
@HWI-EAS443:3:13:1258:181#0/1
GGCCTGGACAGTGGTGAACAGTATATGTAACACAACAGGG
+HWI-EAS443:3:13:1258:181#0/1
`aZUa¥SUbbb`X^I]`a``¥PX¥N_aQT[¥_`ZVW¥a__
@HWI-EAS443:3:13:126:286#0/1
GGGGCATGGAAATGAGGCGCTGCCTTCTTCAGTCTCTTCA
+HWI-EAS443:3:13:126:286#0/1
`[`¥ba]¥`UHOb_a`Uaba]Z¥Z]a^^aa`aYV]^a[[^
@HWI-EAS443:3:13:1371:1814#0/1
CGGGTCACCAGATTCTACAATTATTGATTCACCCCGTTCG
```

+HWI-EAS443:3:13:1371:1814#0/1
abSZ]^_`X_R_b`W`a]a]a]aa¥ZX`a¥aS]`aaQHFa
@HWI-EAS443:3:13:1519:1071#0/1
GGGGCATGGAAATGAGGCGCTGCCTTCTTCAGTCTCTTCA
+HWI-EAS443:3:13:1519:1071#0/1
aa`a_``^¥Z``^^`aZ_a_`_U`[aX_¥`U_RQYUZ^RW
@HWI-EAS443:3:13:1591:550#0/1
ATCAGCAGGAACTTTGACCGCTGATGGCAAAGAGGCCCAT
+HWI-EAS443:3:13:1591:550#0/1
a^F]a¥¥P^V_G[¥VLW_UXFT¥XLXUUWRUUVV[RUGUB
@HWI-EAS443:3:13:1619:1464#0/1
AGCGGACTACACCCTTGACGAAGAGAGCAGGGCAAGAATC
+HWI-EAS443:3:13:1619:1464#0/1
aaaa``ababTV`aaa_X¥Z_a_`]aZ`_`Q___Z_VM
@HWI-EAS443:3:13:1655:918#0/1
ACCGATTTGAGATAATTGAAGGAAGAGACCGAATCATGGC
+HWI-EAS443:3:13:1655:918#0/1
^]a`N_babaaa_`]aaa^_`a__``a_]¥_]Z^[^__VS
@HWI-EAS443:3:13:165:985#0/1
AGCCAACAAAATAAAATCTGAGAAGACACACATTCACATC
+HWI-EAS443:3:13:165:985#0/1
a_ba^aa`Ya¥a`aaa`aaa_aaa`^^^_a`aaX___^`_
@HWI-EAS443:3:13:166:405#0/1
GGGGCATGGAAATGAGGCGCTGCCTTCTTCAGTCTCTTCA
+HWI-EAS443:3:13:166:405#0/1
_``ab]aabbababba`ab¥ab``aaaaa`__^aa]`a`]
@HWI-EAS443:3:13:1692:834#0/1
ATGCACTATTGAAGCACCGATTTGAGATAATTGAAGGAAG
+HWI-EAS443:3:13:1692:834#0/1
`¥a`b`baba``^`¥[^aaaaaaa^ba`a`aa_`^`X`^`
@HWI-EAS443:3:13:1742:912#0/1
AATGAATTCAATAAGGCATGTGAATTGACTGATTCAAGCT
+HWI-EAS443:3:13:1742:912#0/1
abbaab`b`baaaa]a^aaa]]FW_ab_Ya`_`aO[_a¥_
@HWI-EAS443:3:13:1755:105#0/1
AAACCCCTCAAGTTGTGGAGACGCATATAGACTGTTGAAT
+HWI-EAS443:3:13:1755:105#0/1
][b^¥R¥_[`V`Z^[U_a]aWFUYLa``W[YO^YZ¥BBBB
@HWI-EAS443:3:13:1766:208#0/1
GTGGGCACTCGGTGAAAATATGGCACCAGAAAAAGTAGAC
+HWI-EAS443:3:13:1766:208#0/1
aaaabYWV¥^¥OT^ZXMY_N^[WRTTLTZTWXWUBBBBBB
@HWI-EAS443:3:13:1784:1398#0/1
CGACGAACGGGGTGAATCAATAATTGTAGAATCTGGTGAC
+HWI-EAS443:3:13:1784:1398#0/1
`baXa`a^`aaaNa_^a]aaa_[aaa_`a___^_`_X`[T
@HWI-EAS443:3:13:191:410#0/1
CAATGAGAAGCAATTTTCTCGATTCAGCCGAAAACCCCTC
+HWI-EAS443:3:13:191:410#0/1
abaababaaa`a`aaaaaa`a`a__aaaaa^^aa_^`^]_

```
@HWI-EAS443:3:13:214:782#0/1
ATCCCAAGGACAAAGAACATGAAGAGAACAAGCCAATTGA
+HWI-EAS443:3:13:214:782#0/1
a[`bZVQa__`S¥Xa_^[U¥a]V^[_¥P¥^YQ^¥^RZ^`]
@HWI-EAS443:3:13:346:1905#0/1
GGAAGAAATTTAGGCTTCTCTACCCCTGTTGTGTTACATA
+HWI-EAS443:3:13:346:1905#0/1
`bb^¥Sabaa_b_`baa``^abaY```aX`_Wa^^a_a_a
@HWI-EAS443:3:13:351:1853#0/1
AGTTCCTGTAATCTCAAATTTTTCTTCAATTGTCTCTTCG
+HWI-EAS443:3:13:351:1853#0/1
a¥L^¥b^¥¥_b]a¥ab_]¥Za¥baabbb_aa^Xb_]aa^V
@HWI-EAS443:3:13:36:184#0/1
GAAAGGCATAAATCCCAATTACCTCATGGCTTGGAAGCAG
+HWI-EAS443:3:13:36:184#0/1
abb¥a`aaaaaaaaaa`W_aa`a_`¥``_`a[VYQVWSZB
@HWI-EAS443:3:13:385:794#0/1
GGGGCATGGAAATGAGGCGCTGCCTTCTTCAGTCTCTTCA
+HWI-EAS443:3:13:385:794#0/1
abaa_U_aa_aaa`a^aa`aaa_[aa_a`_``_]a_a_Z_
@HWI-EAS443:3:13:395:1337#0/1
TTCGGATTTCCATTTCATCGACGAACGGGGTGAATCAATA
+HWI-EAS443:3:13:395:1337#0/1
abb`abaa`aaabbabaaa_aaa`a^^^_VK¥a_^``_a_
@HWI-EAS443:3:13:460:105#0/1
GATCCGAAAATCGAAACTAACAAGTTTGCTGCAATATGCA
+HWI-EAS443:3:13:460:105#0/1
][XS¥]ZbVTZP¥VK¥_a_RWaPY`¥^Z[]_^S_^VMRUV
@HWI-EAS443:3:13:465:618#0/1
CATTTGAGAAATGATACTGATGTGGTGAACTTTGTAAGTA
+HWI-EAS443:3:13:465:618#0/1
aaT[^]UUQa^_[[^a`Y^^__W_TUSOJPX^^UY^Y]XW
@HWI-EAS443:3:13:549:1496#0/1
GCCGAGTCTTCTGTCAAAGAGAAAGACATGACCAAGGAAT
+HWI-EAS443:3:13:549:1496#0/1
aab`aZL¥_a]Q]__`_[[^a¥^¥_`a^^``_^_¥T]^YW
@HWI-EAS443:3:13:644:1651#0/1
TGGGGGGCTATATGAAGCAATCGAGGAGTGCCTGATTAAT
+HWI-EAS443:3:13:644:1651#0/1
abaaa`__aaaaa`_`_`aaa_^_`¥__Qa]U_`_^^`_]
@HWI-EAS443:3:13:685:1209#0/1
TAATCAGGCACTCCTCGATTGCTTCATATAGCCCCCCAAG
+HWI-EAS443:3:13:685:1209#0/1
ab]a`a_aabaaaaaYaa`aaa^a[_aa`a[^W[]aQ^__
@HWI-EAS443:3:13:69:1850#0/1
GCCGTATTTATGTACACTCCCTTCATTATGTATTCAGTAG
+HWI-EAS443:3:13:69:1850#0/1
```_Za__T`_aN_]]PYbb]¥Y`YRaSK^GS^ZXa_G_]
@HWI-EAS443:3:13:75:227#0/1
TCATTTTCAATGTCCTGTAGCTCTGCTAGCACCTGCTTCC
```
```

+HWI-EAS443:3:13:75:227#0/1
abaaaa_aa_RaRa¥_a¥aSa]aUZ]][]]__[ZM^^_]_
@HWI-EAS443:3:13:761:283#0/1
AGAACCAATGGAACCTCCAAGATCAAGATGAAATGGGGCA
+HWI-EAS443:3:13:761:283#0/1
a``baaa`aa^`a___a___a]_[[Z`^^`]]]^_]]]X]
@HWI-EAS443:3:13:784:686#0/1
TGAATTCAATAAGGCATGTGAATTGACTGATTCAAGCTGG
+HWI-EAS443:3:13:784:686#0/1
¥ZaWaPVS¥aa^Y__WYYJY^^]TR¥Z[_^[WV_`_IP__
@HWI-EAS443:3:13:814:731#0/1
CGTCTCCACAACTTGAGGGGTTTTCGGCTGAATCGAGAAA
+HWI-EAS443:3:13:814:731#0/1
aaZ``a_¥_aaabaa`bbaa^aaaa`a`baa_``a_a_aa
@HWI-EAS443:3:13:889:2021#0/1
GGGGGCTATATGAAGCAATCGAGGAGTGCCTGATTAATGA
+HWI-EAS443:3:13:889:2021#0/1
abaaaaa^_bX_]aa_`aVa__a_]_^Z¥UQ_¥M[]___[
@HWI-EAS443:3:13:916:600#0/1
GGGTGAATCAATAATTGTAGAATCTGGTGACCCGAATGCA
+HWI-EAS443:3:13:916:600#0/1
XabPXZa^T_U_QW^T`a_]a`baab^a`_ZWY¥^WU¥H]
@HWI-EAS443:3:13:917:1848#0/1
GGAGGAACTATTTTACAGCAGAAGTGTCCCACTGCAGGGC
+HWI-EAS443:3:13:917:1848#0/1
abaaabb`]abaaaaaaaaa`aaa_a`^_a``_`Y_a^a_
@HWI-EAS443:3:13:93:539#0/1
CGGGTCTTCAATACTTAATTTCAGAGCATCCATCAGCAGG
+HWI-EAS443:3:13:93:539#0/1
aa``R`aa_¥][T]a`^]aa___``]WHYZPUX[^_VL¥_
@HWI-EAS443:3:13:955:1222#0/1
ATTTGTGTGGCTCCAGTCTCGGGTCAGTGAGTGAGAACTC
+HWI-EAS443:3:13:955:1222#0/1
abbbaaa`b_abaaa_aaaa_aaUa``_]_aX`^__`Z]_
@HWI-EAS443:3:13:970:914#0/1
AAGCAATTTTCTCGATTCAGCCGAAAACCCCTCAAGTTGT
+HWI-EAS443:3:13:970:914#0/1
^`]`XWVX¥`Z¥Q¥Y``¥`aabaWZ[YOZ¥¥ZX_PTY_^N
@HWI-EAS443:3:14:1016:1086#0/1
CGTTCGTCGATGAAATGGAAATCCGAATACATGAAACAAA
+HWI-EAS443:3:14:1016:1086#0/1
aaabba__baaaaa`_aa`_^``_a_a`a_``_`]`¥a`^
@HWI-EAS443:3:14:1065:555#0/1
CAGATTTTGCCAGTAAGGTCCTGCACACTTTCCCAATAGA
+HWI-EAS443:3:14:1065:555#0/1
`ba`ab]aSb^]aY`^aaaa`_aaaa`_^^a[_¥`a`]^a
@HWI-EAS443:3:14:1094:375#0/1
ACTGAATACATAATGAAGGGAGTGTACATAAATACGGCCT
+HWI-EAS443:3:14:1094:375#0/1
¥abZUZaYaa``XX^L]aWY[]JZX_^Z^___`_RWYUTa

```
@HWI-EAS443:3:14:1128:851#0/1
GGGGGCTATATGAAGCAATCGAGGAGTGCCTGATTAATGA
+HWI-EAS443:3:14:1128:851#0/1
a_```[`aaaa__^_]``a_a]a`Z_V^V[`a]^`__]^W
@HWI-EAS443:3:14:1160:61#0/1
AAGAATTCCTTGGTCATGTCTTTCTCTTTGACAGAAGACT
+HWI-EAS443:3:14:1160:61#0/1
abb_aabX]babaX_]abaab``]a^aaab___a_a`^W_
@HWI-EAS443:3:14:1200:1177#0/1
ACGAACGGGGTGAATCAATAATTGTAGAATCTGGTGACCC
+HWI-EAS443:3:14:1200:1177#0/1
^^_^Z``_a_OZSHT[QWaZVa]ZYU__a¥SZWRY^¥XN[
@HWI-EAS443:3:14:1223:963#0/1
GCCTTTCTCATGTGGTTTGACTATGTTAGGCTCTTTCCAG
+HWI-EAS443:3:14:1223:963#0/1
a`^^aTUXaaba[a^S`aa][aa^aV`[_^U¥VS`_V`YL
@HWI-EAS443:3:14:1255:670#0/1
GGCATGGAAATGAGGCGCTGCCTTCTTCAGTCTCTTCAGC
+HWI-EAS443:3:14:1255:670#0/1
a_aa_a``a_aa`^___``___¥^V``X¥_¥¥_VW[VZWW
@HWI-EAS443:3:14:1271:605#0/1
GATCCCAAGGACAAAGAACATGAAGAGAACAAGCCAATTG
+HWI-EAS443:3:14:1271:605#0/1
a_a^a`]_aY`^]__]__]aa`]_a`a^^]^_`R^_]_U]
@HWI-EAS443:3:14:1276:1039#0/1
ATCAAAGTCTACTTTTTCTGGTGCCATATTTTCACCGAGT
+HWI-EAS443:3:14:1276:1039#0/1
a^aaaa`]a`aZ`aaaaaa`a_Z_`a^^a`_a_a`U]V^X
@HWI-EAS443:3:14:1284:2032#0/1
GGGGCTATATGAAGCAATCGAGGAGTGCCTGATTAATGAT
+HWI-EAS443:3:14:1284:2032#0/1
a_`a_aaab`_aaa_aa_^a_a`_^V_YU_Z]^_`^_^]^
@HWI-EAS443:3:14:1289:278#0/1
ACATGAGAAAGGCATAAATCCCAATTACCTCATGGCTTGG
+HWI-EAS443:3:14:1289:278#0/1
aaa``Z`aaa_^_aaaa_aa[V__`¥^^R`MX[^aY_^[B
@HWI-EAS443:3:14:1298:1021#0/1
GCACACATTTGGAAGTTTGTTTCATGTATTCGGATTTCCA
+HWI-EAS443:3:14:1298:1021#0/1
a_baa¥_`a^__aSa]__a_aa_`a[]a`][_^Z`P`¥¥_
@HWI-EAS443:3:14:1312:1225#0/1
GCAAATGTAGGACCAAAGAAGGAAGACGGAAAACAAACCT
+HWI-EAS443:3:14:1312:1225#0/1
aaaaaaa]aaa`__a`_a__a`]````__a^_^^`]]_V_
@HWI-EAS443:3:14:1320:1485#0/1
GACGAAAGGAATCCCATAGACTCCTACTGGCCATTTCTTG
+HWI-EAS443:3:14:1320:1485#0/1
abbabbb_bbbaaaSaab_aaaaa_aaa¥a_Xa¥_aXa_I
@HWI-EAS443:3:14:1391:874#0/1
CACTATATGATGCAATCAAATGCATGAAGACATTCTTTGG
```

```
+HWI-EAS443:3:14:1391:874#0/1
aaabaaaab]abaa`a`aaaaa]a`aa`a_^aa``a^`_¥
@HWI-EAS443:3:14:1413:119#0/1
GCCTATGTAGATGGATTCGAGCCGAACGGCTGCATTGAGG
+HWI-EAS443:3:14:1413:119#0/1
aaaa`aa¥¥a__a`^^^_`___S^S^¥¥^ZVRGRUWMPBB
@HWI-EAS443:3:14:142:1360#0/1
CAAATCAGGAAGAAATTTAGGCTTCTCTACCCCTGTTGTG
+HWI-EAS443:3:14:142:1360#0/1
aabaaaa`a`aa^aa`^`a^]`_`^_a_aa]a__`Y__X_
@HWI-EAS443:3:14:1509:649#0/1
TCCACTCCCCTGGGTGACTCTCCGATTGGCCATGTTTCCG
+HWI-EAS443:3:14:1509:649#0/1
aaaZTY[_`_`[[[Y__]][_XY[U__Z]]X]]]T¥ZWU¥
@HWI-EAS443:3:14:150:516#0/1
TGGCGTTCACTTCTTTTGACATTTGGGAAAGCTTGCCCTC
+HWI-EAS443:3:14:150:516#0/1
aab`b`T^[_`_a`a`a¥`a[ZYYGW`_`_`Z¥_V^^Y^`
@HWI-EAS443:3:14:1521:1663#0/1
ATGAAATGGGGCATGGAAATGAGGCGCTGCCTTCTTCAGT
+HWI-EAS443:3:14:1521:1663#0/1
_a_[R]aV_¥a]aQ^aa_`a]aa`GXaab`HaX^¥[H`YS
@HWI-EAS443:3:14:1523:549#0/1
GTGAATCAATAATTGTAGAATCTGGTGACCCGAATGCACT
+HWI-EAS443:3:14:1523:549#0/1
``baa_``_```]`__aa]`_a^a_Ya_[¥U]`_[_T^Y_
@HWI-EAS443:3:14:1534:619#0/1
GCGCATAGTTCCTGTAATCTCAAATTTTTCTTCAATTGTC
+HWI-EAS443:3:14:1534:619#0/1
aaa[`a`a_a_Wa`X^[a^`_``[_a``a_`^]``__`IQ
@HWI-EAS443:3:14:1606:1093#0/1
ATTCAGTAGCCCTGCAGTGGGACACTTCTGCTGTAAAATA
+HWI-EAS443:3:14:1606:1093#0/1
aba`ba`a_`_aa`[``^a`a`^a_aa__aVaa^_aba`_
@HWI-EAS443:3:14:1651:1080#0/1
GAAAGGCATAAATCCCAATTACCTCATGGCTTGGAAGCAG
+HWI-EAS443:3:14:1651:1080#0/1
aa[aaY_¥[^S]_¥STWTW]]YQWR]T__YO]BBBBBBBB
@HWI-EAS443:3:14:224:334#0/1
GGGGGCTATATGAAGCAATCGAGGAGTGCCTGATTAATGA
+HWI-EAS443:3:14:224:334#0/1
abb]`aaabaabaaa`aa`aa_a__a^``_aa_a`a__`_
@HWI-EAS443:3:14:248:182#0/1
CCACAACTTGAGGGGTTTTCGGCTGAATCGAGAAAATTGC
+HWI-EAS443:3:14:248:182#0/1
a_Y`]a^aaaa¥aaa_a`__QX^^_a`__`W[Y]^[_^`T
@HWI-EAS443:3:14:364:1779#0/1
TATTGAAGCACCGATTTGAGATAATTGAAGGAAGAGACCG
+HWI-EAS443:3:14:364:1779#0/1
``ba_abbabbb_aaaa^abaaaaaaaba__aa`a`a¥__
```

```
@HWI-EAS443:3:14:368:1927#0/1
GGCATGGAAATGAGGCGCTGCCTTCTTCAGTCTCTTCAGC
+HWI-EAS443:3:14:368:1927#0/1
a```_aSaaS_aaWN^TPPVV]O^PVRZ¥XSPJRFR`¥UW
@HWI-EAS443:3:14:464:1241#0/1
GGCATGGAAATGAGGCGCTGCCTTCTTCAGTCTCTTCAGC
+HWI-EAS443:3:14:464:1241#0/1
abbbaaabbbbbbbab`bbabaabbbbaba`babaa_aXa
@HWI-EAS443:3:14:471:1008#0/1
TTCCGATTTGTTTTCAAAGAATTCCTTGGTCATGTCTTTC
+HWI-EAS443:3:14:471:1008#0/1
a`bbb`V`ab^a`_^aaY`a`a``aaaaY^aaaaW]ZR^^
@HWI-EAS443:3:14:510:807#0/1
GGGGTTTTCGGCTGAATCGAGAAAATTGCTTCTCATTGTT
+HWI-EAS443:3:14:510:807#0/1
a`a^Paa`b_aa`aa_[^a``^S^`__a`____`__^`_^
@HWI-EAS443:3:14:524:1090#0/1
GGGGCATGGAAATGAGGCGCTGCCTTCTTCAGTCTCTTCA
+HWI-EAS443:3:14:524:1090#0/1
`aaaaaabb_bbbbababba`b`a[b_aaba_a]a__a_a
@HWI-EAS443:3:14:56:733#0/1
TGTTAGGCTCTTTCCAGCCAAAGAATGTCTTCATGCATTT
+HWI-EAS443:3:14:56:733#0/1
ab^_baaa`a`_aa__[aa`__a_[`a^__Y]__^]]``^
@HWI-EAS443:3:14:59:1352#0/1
GCGTGGTGTCGTCCTCAAGAATGGTTCAATTTTGGCGTTC
+HWI-EAS443:3:14:59:1352#0/1
aab_``_baababaaaab`aaaaa``a__^aabJa]a_]P
@HWI-EAS443:3:14:664:1137#0/1
GGGGCATGGAAATGAGGCGCTGCCTTCTTCAGTCTCTTCA
+HWI-EAS443:3:14:664:1137#0/1
a_V_¥___``¥[Y^`PYXY_Z`¥V¥^U[^]¥¥UMZU]VWS
@HWI-EAS443:3:14:792:1791#0/1
AGTGTACATAAATACGGCCTTGCTCAATGCATCCTGTGCA
+HWI-EAS443:3:14:792:1791#0/1
`_]_]aaa```¥O[][`^Z]`_`][``¥¥`a^]Z^ZZ^Z^
@HWI-EAS443:3:14:854:1294#0/1
GGGGGCTATATGAAGCAATCGAGGAGTGCCTGATTAATGA
+HWI-EAS443:3:14:854:1294#0/1
^aaWX_ab¥b¥ba¥`aa^]a]_]b_a^¥Y^Z]]``_``a`
@HWI-EAS443:3:14:862:1734#0/1
TTCTTGAGGACGACACCACGCCCCCTCAGATTGCCTGATG
+HWI-EAS443:3:14:862:1734#0/1
_Ua`X¥_a_¥Z_W¥``[_``¥YJU]UXZ¥XFX¥XUXPPXB
@HWI-EAS443:3:14:889:844#0/1
GAATCAGTCAATTCACATGCCTTATTGAATTCATTTTGGA
+HWI-EAS443:3:14:889:844#0/1
a_`a`ab`b`[Va¥Z`¥]`_^[_`^aa]]_YGX[`¥_][^
@HWI-EAS443:3:14:903:133#0/1
ATTCGGATTTCCATTTCATCGACGAACGGGGTGAATCAAT
```

```
+HWI-EAS443:3:14:903:133#0/1
abaaaa_`aa_[`a`a_`a^a_^aX_^a___Za_^`UY]`
@HWI-EAS443:3:14:91:1921#0/1
ATGGAACCTCCAAGATCAAGATGAAATGGGGCATGGAAAT
+HWI-EAS443:3:14:91:1921#0/1
aaa_aaaaaaaa]`aQa``a__`a``¥]TWYZ_]XNQ_Z_
@HWI-EAS443:3:14:923:656#0/1
AGGTGCTAGCAGAGCTACAGGACATTGAAAATGAAGAGAA
+HWI-EAS443:3:14:923:656#0/1
aaa]^_aabaaa``_aa__a]_^_a`a`[___a^_`_a__
@HWI-EAS443:3:14:929:1559#0/1
CAAGAAATGGCCAGTAGGAGTCTATGGGATTCCTTTCGTC
+HWI-EAS443:3:14:929:1559#0/1
aa``Y`a]aa]``]NXa¥K[X_ZX¥``XTUS^`S__^[PN
@HWI-EAS443:3:14:965:1853#0/1
CTAAATTTCTTCCTGATTTGTATGATTACAAAGAGAACCG
+HWI-EAS443:3:14:965:1853#0/1
aa`a___XYbL]V_aWY_`V[aa^G]]a_^_R]_SZU`P]
@HWI-EAS443:3:15:1067:366#0/1
GGTCGGCAAGCTTGCGCATAGTTCCTGTAATCTCAAATTT
+HWI-EAS443:3:15:1067:366#0/1
a]Xa]a^aaaa_`¥^`¥]a`a`a`¥_aX[Z__aVP[___a
@HWI-EAS443:3:15:1069:1994#0/1
ACCCGAATGCACTATTGAAGCACCGATTTGAGATAATTGA
+HWI-EAS443:3:15:1069:1994#0/1
a``aa_a_aaRT_a^`^X]_Z_Y___X]Y_a^YW_]]]_^
@HWI-EAS443:3:15:1083:138#0/1
CTTCAATTGGCTTGTTCTCTTCATGTTCTTTGTCCTTGGG
+HWI-EAS443:3:15:1083:138#0/1
aaaaabaaba_aa_`[P[[^a_`a_¥[DYaa_^]Yaa^^a
@HWI-EAS443:3:15:110:1024#0/1
AAAGAATTCCTTGGTCATGTCTTTCTCTTTGACAGAAGAC
+HWI-EAS443:3:15:110:1024#0/1
`aaaZX`a`U^aaa_]^^a]``^XYa`a`__]``_a```[
@HWI-EAS443:3:15:1204:1766#0/1
GCCTGGACAGTGGTGAACAGTATATGTAACACAACAGGGG
+HWI-EAS443:3:15:1204:1766#0/1
aaabbabaaa¥aa¥aaaaba_aaaaa]a``_aa_^`^`¥_
@HWI-EAS443:3:15:1298:602#0/1
AGGACATTGAAAATGAAGAGAAGATCCCAAGGACAAAGAA
+HWI-EAS443:3:15:1298:602#0/1
a¥a¥]a^^^]^^YaaaabVSX¥aaaZWFSbYN¥[QO^a_`
@HWI-EAS443:3:15:137:1639#0/1
GAAGTTCGGTGGGAGACTTTGGTCGGCAAGCTTGCGCATA
+HWI-EAS443:3:15:137:1639#0/1
aZZ]S[^T[Oa^¥aaa`a]`ZUUaS_^``[a[^[^_aa][
@HWI-EAS443:3:15:1394:48#0/1
ATGGGCCTCGACACTTGGCCTATCGCAGTCCTCAAGAGCA
+HWI-EAS443:3:15:1394:48#0/1
a^ba^ab¥``X¥^_a¥_`[N____`Q^_U_XS_¥Z]_`H¥
```

```
@HWI-EAS443:3:15:1396:22#0/1
CTCCACAACTTGAGGGGTTTTCGGCTGAATCGAGAAAATT
+HWI-EAS443:3:15:1396:22#0/1
aTa_T¥UWbb_bTZ]Z_S^`¥]`a_``XZ]X`XWVYWUWY
@HWI-EAS443:3:15:1517:1652#0/1
GAGGAACTATTTTACAGCAGAAGTGTCCCACTGCAGGGCT
+HWI-EAS443:3:15:1517:1652#0/1
^_ZYVa`baba]a`aaa_aaW`^]b^[VXa`^a]]a]_V[
@HWI-EAS443:3:15:1542:736#0/1
ATTCTTTGGCTGGAAAGAGCCTAACATAGTCAAACCACAT
+HWI-EAS443:3:15:1542:736#0/1
aaa]`_S`^^``[X__RWZ^Z`S`U[^[Z[_`¥ZSNTN_X
@HWI-EAS443:3:15:154:224#0/1
CCCGAATGCACTATTGAAGCACCGATTTGAGATAATTGAA
+HWI-EAS443:3:15:154:224#0/1
abbaabaaaaaaaaaa``aa_`a__``a^a_```````^
@HWI-EAS443:3:15:1741:1180#0/1
TGGCGTTCACTTCTTTTGACATTTGGGAAAGCTTGCCCTC
+HWI-EAS443:3:15:1741:1180#0/1
abbbbaaTaYa_V`ba`N^W``_`U]a__`SZ`_ZR^S_^
@HWI-EAS443:3:15:245:1166#0/1
GCGTCTCCACAACTTGAGGGGTTTTCGGCTGAATCGAGAA
+HWI-EAS443:3:15:245:1166#0/1
abbYaabbab`aabaaabaaa]abaaa[aaaab`aaaa`a
@HWI-EAS443:3:15:246:488#0/1
AGCCAACAAAATAAAATCTGAGAAGACACACATTCACATC
+HWI-EAS443:3:15:246:488#0/1
abbaQY^¥`aa^abaaa_aaaaa[[_```bU_aaaa_aa¥
@HWI-EAS443:3:15:259:726#0/1
GGGGCATGGAAATGAGGCGCTGCCTTCTTCAGTCTCTTCA
+HWI-EAS443:3:15:259:726#0/1
^]__aa_aaaa_aaaa[a``_a`U_`]`]_YNM_¥_^ZFY
@HWI-EAS443:3:15:27:1366#0/1
GGGGGCTATATGAAGCAATCGAGGAGTGCCTGATTAATGA
+HWI-EAS443:3:15:27:1366#0/1
]bbabbaabbbaabbbbbbaaabbab^baabb`bbbbaaa
@HWI-EAS443:3:15:368:286#0/1
GTTTCCGATTTGTTTTCAAAGAATTCCTTGGTCATGTCTT
+HWI-EAS443:3:15:368:286#0/1
aY____aWZ[W_T``[]``^_ZN[Z_[]^^]WUT[]Z^^^
@HWI-EAS443:3:15:504:629#0/1
GCAACATCTTCTCCTATTTCATCAAGTTCTATCCAGCTTG
+HWI-EAS443:3:15:504:629#0/1
aaaaaaaaaaaaaaa^aa``aa_a__^___a_`_a_a`_a
@HWI-EAS443:3:15:538:1315#0/1
AACCCATACAGGTTTGTTTTCCGTCTTCCTTCTTTGGTCC
+HWI-EAS443:3:15:538:1315#0/1
abaaaa_`aaaaU_^a[_``__WW]__W_¥^^¥__Z¥XX^
@HWI-EAS443:3:15:548:601#0/1
CTTGAATCAGTCAATTCACATGCCTTATTGAATTCATTTT
```

```
+HWI-EAS443:3:15:548:601#0/1
abbb`ba````bbaaabbaaaa`aaa`aaa`_`aaaaa^`
@HWI-EAS443:3:15:594:574#0/1
GGGTTTTCGGCTGAATCGAGAAAATTGCTTCTCATTGTTC
+HWI-EAS443:3:15:594:574#0/1
a`b_aaa`^``Xbba``a]a¥`[`a`¥`^^``¥[^`a^]_
@HWI-EAS443:3:15:609:1961#0/1
CAGGCCATGATTCGGTCTCTTCCTTCAATTATCTCAAATC
+HWI-EAS443:3:15:609:1961#0/1
abbbabbbababbaa_a__abab_aaaaaaaaa_abbSU
@HWI-EAS443:3:15:650:374#0/1
CATGTTTCCGATTTGTTTTCAAAGAATTCCTTGGTCATGT
+HWI-EAS443:3:15:650:374#0/1
abbbbbbbbaabbbbaabbababba^babab bba`a`ab_
@HWI-EAS443:3:15:74:1386#0/1
GATCTCTAGCAAGCTGGGTCCAAAATGAATTCAATAAGGC
+HWI-EAS443:3:15:74:1386#0/1
S]^[Y[````_[[^`TPPa`¥]Z`[`^`Y^Z`b_a`^a^`
@HWI-EAS443:3:15:812:1396#0/1
AATTGAAGGAAGAGACCGAATCATGGCCTGGACAGTGGTG
+HWI-EAS443:3:15:812:1396#0/1
`babbbaa_abb``a^`a`a_`aaaa_Q`¥U][`_O_aW`
@HWI-EAS443:3:15:880:1889#0/1
GAAGAGACCGAATCATGGCCTGGACAGTGGTGAACAGTAT
+HWI-EAS443:3:15:880:1889#0/1
]RU^Va`O``a_WV]_``YZQ]I_U`_]]]N[^^Z__X_¥
@HWI-EAS443:3:15:997:1033#0/1
CGAATGCACTATTGAAGCACCGATTTGAGATAATTGAAGG
+HWI-EAS443:3:15:997:1033#0/1
¥`bbbaaababb`bababbaababbbbababaababaaaa
@HWI-EAS443:3:16:1019:461#0/1
GGAGGAAGGCTCTATTGGGAAAGTGTGCAGGACCTTACTG
+HWI-EAS443:3:16:1019:461#0/1
aa_aaHP]_`^`_¥X]a`a[U[[T_WaU^]VSYU]]]ST]
@HWI-EAS443:3:16:103:1768#0/1
TGAGAAAGGCATAAATCCCAATTACCTCATGGCTTGGAAG
+HWI-EAS443:3:16:103:1768#0/1
`V`ababaabb`a_a`a`aaaaaaa___aaa]_`_J`X`B
@HWI-EAS443:3:16:1153:408#0/1
CAGAGCCCAGATCTCTAGCAAGCTGGGTCCAAAATGAATT
+HWI-EAS443:3:16:1153:408#0/1
aba`b`[`aaabaa`aaaaaaa``aaa^aaa`a^aa`_^`
@HWI-EAS443:3:16:1161:1005#0/1
GGCTCTGGCTCATCACTGTCATACTGTTTAAGGTCTCCAA
+HWI-EAS443:3:16:1161:1005#0/1
`X]Q]`YRY_¥QUUVNX¥NY^ZVGSPKZXSXVZTUNWRY^
@HWI-EAS443:3:16:1172:304#0/1
TATTTTCACCGAGTGCCCACTTCAATTGGCTTGTTCTCTT
+HWI-EAS443:3:16:1172:304#0/1
`]bb`aabaaaa`GaX_a^_a_YZ__Z¥XNVaJUZMRFZU
```

```
@HWI-EAS443:3:16:1243:212#0/1
GAACTTTGTAAGTATGGAGTTCTCACTCACTGACCCGAGA
+HWI-EAS443:3:16:1243:212#0/1
ab[`H`]¥F]a]`V¥TaUV^Z[a[UMUR_^R`BBBBBBBB
@HWI-EAS443:3:16:1301:541#0/1
GAAAAAGTAGACTTTGATGACTGCAAAGATGTTGGAGACC
+HWI-EAS443:3:16:1301:541#0/1
a`aaa`a_ba`aaa`aaaa___a__````_a_`a__a]]Y
@HWI-EAS443:3:16:1360:1457#0/1
GACTGAAGAAGGCAGCGCCTCATTTCCATGCCCCATTTCA
+HWI-EAS443:3:16:1360:1457#0/1
_ba`aaaaa¥X¥_b_^aPX^Q]_Y`QX[Q_XLN]¥`_[Y¥
@HWI-EAS443:3:16:136:160#0/1
CCAATTTCAATGAACCGGTTCTCTTTGTAATCATACAAAT
+HWI-EAS443:3:16:136:160#0/1
aaaab^^aa[^[`aaaaa]`a_``^_aZ]__]_¥__]^_^
@HWI-EAS443:3:16:1377:342#0/1
GTCCGCTTTGGTGGCCATCTCCTCTCCAGTGAATGAAAAG
+HWI-EAS443:3:16:1377:342#0/1
a_aaa`aabaaY_aa^^[Ya]]_^¥V][^U`a_^^Y^_Y_
@HWI-EAS443:3:16:1446:1273#0/1
ACTTGAGGGGTTTTCGGCTGAATCGAGAAAATTGCTTCTC
+HWI-EAS443:3:16:1446:1273#0/1
ab`ZS``bba_b]Z`T[]b_a[`_`S]P[_aa_^R^[TZ_
@HWI-EAS443:3:16:146:460#0/1
ATTTTACAGCAGAAGTGTCCCACTGCAGGGCTACTGAATA
+HWI-EAS443:3:16:146:460#0/1
a`bbabaabbb]ab`G`_a`aaa^a`[aXXa__`_a^___
@HWI-EAS443:3:16:1528:1044#0/1
TGGGGGGCTATATGAAGCAATCGAGGAGTGCCTGATTAAT
+HWI-EAS443:3:16:1528:1044#0/1
URR]IOQ_`PQ`aUTQ_VRYT]`KZYQVLXMLUaW_¥]ZS
@HWI-EAS443:3:16:1601:1979#0/1
CACTCACTGACCCGAGACTGGAGCCACACAAATGGGAAAA
+HWI-EAS443:3:16:1601:1979#0/1
ababbbbb`b`SX¥`_Ka`a`ZV_S^`[UbaaY¥a``]`a
@HWI-EAS443:3:16:1613:348#0/1
GCCATATTTTCACCGAGTGCCCACTTCAATTGGCTTGTTC
+HWI-EAS443:3:16:1613:348#0/1
a`ZZ[]]`a]a_KV^QYYaUOT^]ZWZ`ZZ[_LQ[[MTBB
@HWI-EAS443:3:16:1619:552#0/1
ACATAATGAAGGGAGTGTACATAAATACGGCCTTGCTCAA
+HWI-EAS443:3:16:1619:552#0/1
aaabaabb`aaaa`a]a]a^_`a``_a^a`^^]^^^]ZX^
@HWI-EAS443:3:16:1654:382#0/1
GGCATGGAAATGAGGCGCTGCCTTCTTCAGTCTCTTCAGC
+HWI-EAS443:3:16:1654:382#0/1
aa_aa`Y¥```_¥a`Wa_aaWQ_`Y]]W¥_[Q^¥¥WUWYB
@HWI-EAS443:3:16:1673:794#0/1
ACATAATGAAGGGAGTGTACATAAATACGGCCTTGCTCAA
```

```
+HWI-EAS443:3:16:1673:794#0/1
ababaabbaab`a^a_b_a`_aa`a`__`^`Xaa^]_¥¥_
@HWI-EAS443:3:16:1718:333#0/1
GCATAAATCCCAATTACCTCATGGCTTGGAAGCAGGTGCT
+HWI-EAS443:3:16:1718:333#0/1
aa_ab`a`S^_``a__ZV_^^^a_____]V_[VYU[IVBB
@HWI-EAS443:3:16:1721:1483#0/1
ATTCAGTAGCCCTGCAGTGGGACACTTCTGCTGTAAAATA
+HWI-EAS443:3:16:1721:1483#0/1
aa_QZ`^^a^`WP]P^_G_¥`^_^_`_^aa^``]Z]^^O_
@HWI-EAS443:3:16:290:707#0/1
CCTCTTTCGGACTGACGAAAGGAATCCCATAGACTCCTAC
+HWI-EAS443:3:16:290:707#0/1
ab`aa_¥¥`^aaXZ]aa`Z_a]`a¥`X`a`_V^`Y`__`_
@HWI-EAS443:3:16:343:1481#0/1
GGGGCATGGAAATGAGGCGCTGCCTTCTTCAGTCTCTTCA
+HWI-EAS443:3:16:343:1481#0/1
aa`]aa]a````aaaa_a]`]`a`_¥`]a^]ZSa¥`^¥S¥
@HWI-EAS443:3:16:35:111#0/1
GCTGAAAGTCATCCATGGCTGCACAGGATGCATTGAGCAA
+HWI-EAS443:3:16:35:111#0/1
a_a`Sa]a_aa`[a`_a`^_]NU¥^¥[¥SYSTQ[TTUS[B
@HWI-EAS443:3:16:371:1124#0/1
CCCTGCTCTCTTCGTCAAGGGTGTAGTCCGCTTTGGTGGC
+HWI-EAS443:3:16:371:1124#0/1
aaaa`]]aaaaa`aV]S]`V[Qa[`^U^]X][^YK]W^UY
@HWI-EAS443:3:16:378:106#0/1
CATTCTTTGGCTGGAAAGAGCCTAACATAGTCAAACCACA
+HWI-EAS443:3:16:378:106#0/1
abbbababababbb`aaaaa]Yaaa^aaaa^``_a`____
@HWI-EAS443:3:16:379:1741#0/1
TTTCGGCTGAATCGAGAAAATTGCTTCTCATTGTTCAGGC
+HWI-EAS443:3:16:379:1741#0/1
[KYa`a`]a[_]_aa`_aa`__Y_[¥_YW`_¥¥XY_]]]]
@HWI-EAS443:3:16:413:152#0/1
CTCCAGCCTTGAAAACTTTAGAGCCTATGTAGATGGATTC
+HWI-EAS443:3:16:413:152#0/1
abba_bbababaa`aaababbaaa`aabb`abaaa`a`a`
@HWI-EAS443:3:16:431:1092#0/1
AGTCATCAAAGTCTACTTTTTCTGGTGCCATATTTTCACC
+HWI-EAS443:3:16:431:1092#0/1
aa¥baa`[aZZYaa``aaaa`aa``Za`a`aaa``¥aa_^
@HWI-EAS443:3:16:501:1398#0/1
AGAATTCCTTGGTCATGTCTTTCTCTTTGACAGAAGACTC
+HWI-EAS443:3:16:501:1398#0/1
aaa`ab]baaa_Xaaaa_ba_a_aaa__a[`aa_a^`]__
@HWI-EAS443:3:16:548:528#0/1
CAGATCTCTAGCAAGCTGGGTCCAAAATGAATTCAATAAG
+HWI-EAS443:3:16:548:528#0/1
a_a`a`ab`ab`b`aa_`baUaa_b_^aa`_a__a`aaaa
```

```
@HWI-EAS443:3:16:627:496#0/1
ACAAGAAATGGCCAGTAGGAGTCTATGGGATTCCTTTCGT
+HWI-EAS443:3:16:627:496#0/1
`b`bba^[`a[_[]a]aaaaa__aaa¥aa__^V]__`Y[Y
@HWI-EAS443:3:16:62:251#0/1
GCCCACTTCAATTGGCTTGTTCTCTTCATGTTCTTTGTCC
+HWI-EAS443:3:16:62:251#0/1
abaaa_aaa¥`__a`a_¥XQ]X`]]_V_]RY^W[__¥WYZ
@HWI-EAS443:3:16:707:1357#0/1
GGGGTTTTCGGCTGAATCGAGAAAATTGCTTCTCATTGTT
+HWI-EAS443:3:16:707:1357#0/1
aaaVOa_a`a_a`aa_¥_^___a_`][_^^_`__`^]_ZZ
@HWI-EAS443:3:16:769:1764#0/1
GAGATAATTGAAGGAAGAGACCGAATCATGGCCTGGACAG
+HWI-EAS443:3:16:769:1764#0/1
`Zaaaaaaaaaaaaaaaaa`aa_a`aa____]__Z^R`]
@HWI-EAS443:3:16:786:738#0/1
AACATCTTTGCAGTCATCAAAGTCTACTTTTTCTGGTGCC
+HWI-EAS443:3:16:786:738#0/1
`aabaaabaa`aa`aaa`a^¥a]`aa`aaaaa`a`_Y``_
@HWI-EAS443:3:16:796:871#0/1
AAGACATTCTTTGGCTGGAAAGAGCCTAACATAGTCAAAC
+HWI-EAS443:3:16:796:871#0/1
a^]]^^[^]`____^_a_^__]Y`_]^]_]_^^_T]^_`U
@HWI-EAS443:3:16:935:204#0/1
CGATTTGAGATAATTGAAGGAAGAGACCGAATCATGGCCT
+HWI-EAS443:3:16:935:204#0/1
ab¥aaba_a_aa`a][^_`aV_`_a_`[_]_^_`^`_WV^
@HWI-EAS443:3:17:1065:1055#0/1
ATTTTACAGCAGAAGTGTCCCACTGCAGGGCTACTGAATA
+HWI-EAS443:3:17:1065:1055#0/1
aaabababaaaaaaaaVa`a_`a``a_`_a_X_`¥`a__`_
@HWI-EAS443:3:17:1116:1691#0/1
GGTGAACTTTGTAAGTATGGAGTTCTCACTCACTGACCCG
+HWI-EAS443:3:17:1116:1691#0/1
T_[``[ab`ba`ba``b`bba`aababbbabbabaaaaaa
@HWI-EAS443:3:17:1130:1337#0/1
CTTGCTAGAGATCTGGGCTCTGGCTCATCACTGTCATACT
+HWI-EAS443:3:17:1130:1337#0/1
aaa``aaa`aa`_`¥`aaaaa`Z^U_`a[`_aaUY`_]_a
@HWI-EAS443:3:17:1131:302#0/1
AGACAATTGAAGAAAAATTTGAGATTACAGGAACTATGCG
+HWI-EAS443:3:17:1131:302#0/1
ab``aabaaaabaa___aaaa_a```a_a`]_a^`a_^_`
@HWI-EAS443:3:17:122:114#0/1
ATCGGAAACATGGCCAATCGGAGAGTCACCCAGGGGAGTG
+HWI-EAS443:3:17:122:114#0/1
Zbbaaa]Zaaaabaa]^`]¥^^a]`Wa^Z^]S^Y_aU`T`
@HWI-EAS443:3:17:132:1431#0/1
CATAAATACGGCCTTGCTCAATGCATCCTGTGCAGCCATG
```

+HWI-EAS443:3:17:132:1431#0/1
a`abbb]`aaaUaaaab_aYaa[]`aaaa^^^[K]a_[GU
@HWI-EAS443:3:17:1330:728#0/1
GAGCCTTCCTCCACTCCCCTGGGTGACTCTCCGATTGGCC
+HWI-EAS443:3:17:1330:728#0/1
^R```a`a]_W]W]SX[SZ¥U]aP_[FXOQ¥S^KUJYVT¥
@HWI-EAS443:3:17:1365:771#0/1
ATCTTTTCATTCACTGGAGAGGAGATGGCCACCAAAGCGG
+HWI-EAS443:3:17:1365:771#0/1
a[aba`Vaa`aaa¥]Z]aaa]V^[a¥]a`_][^aLRTX^X
@HWI-EAS443:3:17:137:331#0/1
GAAGATCCCAAGGACAAAGAACATGAAGAGAACAAGCCAA
+HWI-EAS443:3:17:137:331#0/1
aabaaaabaaX`_abaa_aaaaaaaaa`aa`a`aa`a_a_
@HWI-EAS443:3:17:1419:669#0/1
CTTGCTCAATGCATCCTGTGCAGCCATGGATGACTTTCAG
+HWI-EAS443:3:17:1419:669#0/1
`bbbabaabbabbbb_ba_b^aaa_aaaaaab`Wa``¥`a
@HWI-EAS443:3:17:1430:1839#0/1
CATTAATCAGGCACTCCTCGATTGCTTCATATAGCCCCCC
+HWI-EAS443:3:17:1430:1839#0/1
`baabbaabba`abba`aa^b`bb_abaabaUa_`aQ_aa
@HWI-EAS443:3:17:1439:618#0/1
CAATGCATCCTGTGCAGCCATGGATGACTTTCAGCTGATC
+HWI-EAS443:3:17:1439:618#0/1
V__¥a``bb`]bUX[aaV^ab_`[__^^baaZ[QZ^XX_a
@HWI-EAS443:3:17:1453:444#0/1
ATCCCAATTACCTCATGGCTTGGAAGCAGGTGCTAGCAGA
+HWI-EAS443:3:17:1453:444#0/1
aa[a_ab``a]¥aTRaa[_aaY_¥_V^`U^HYVW¥`UZ_Q
@HWI-EAS443:3:17:1474:411#0/1
ATTCGGATTTCCATTTCATCGACGAACGGGGTGAATCAAT
+HWI-EAS443:3:17:1474:411#0/1
aba_^JRa_`¥aabaaX_a`aa_`_`]]aa_]a`_ZZ^^_
@HWI-EAS443:3:17:1750:372#0/1
CATGGAAATGAGGCGCTGCCTTCTTCAGTCTCTTCAGCAG
+HWI-EAS443:3:17:1750:372#0/1
aabbbb_ba]bbbb`[`b``a_¥__Y^^`]a_aa[_`Z^`
@HWI-EAS443:3:17:1772:279#0/1
CCACTCCCCTGGGTGACTCTCCGATTGGCCATGTTTCCGA
+HWI-EAS443:3:17:1772:279#0/1
aaaabbbaaaabb^b`___a_[`¥_^a___^^XW^aWW]U
@HWI-EAS443:3:17:207:1592#0/1
GAGCCTATGTAGATGGATTCGAGCCGAACGGCTGCATTGA
+HWI-EAS443:3:17:207:1592#0/1
aaYGW¥a_bV¥^V]NPPT]^_aXUYRS¥¥]WVBBBBBBBB
@HWI-EAS443:3:17:218:1150#0/1
GGGGCATGGAAATGAGGCGCTGCCTTCTTCAGTCTCTTCA
+HWI-EAS443:3:17:218:1150#0/1
R][TVV`Q[XYbba^^abab¥ZabaabW]`W`S```¥aZ`

```
@HWI-EAS443:3:17:219:1305#0/1
CATTAATCAGGCACTCCTCGATTGCTTCATATAGCCCCCC
+HWI-EAS443:3:17:219:1305#0/1
]]Y_¥Y^^_N]a_b¥^WW_[a`a]a_abZ]]NK_aIZU]`
@HWI-EAS443:3:17:270:1860#0/1
ACCGAACTTCTCCAGCCTTGAAAACTTTAGAGCCTATGTA
+HWI-EAS443:3:17:270:1860#0/1
^baa`aaaaaaaaa_aa`_`aaaa``__a``]a^aaa`^a
@HWI-EAS443:3:17:271:563#0/1
CTGGGCTCTGGCTCATCACTGTCATACTGTTTAAGGTCTC
+HWI-EAS443:3:17:271:563#0/1
aa_aa_a`a`aaaaa`a^`_aUaaa¥_aa^_a____XR^¥
@HWI-EAS443:3:17:27:138#0/1
TCAAAACTAGGCTTTTCACTATAAGACAAGAAATGGCCAG
+HWI-EAS443:3:17:27:138#0/1
Y¥^TT_`a¥_¥¥VX`S```]T`aa]`TJ[XYOXT]X[RRY
@HWI-EAS443:3:17:340:1040#0/1
GAGACCTTAAACAGTATGACAGTGATGAGCCAGAGCCCAG
+HWI-EAS443:3:17:340:1040#0/1
`a`aZ^_[]a`[`b]]^aaa]]]YW`V`WN[Z^^`RTQVL
@HWI-EAS443:3:17:410:1975#0/1
GGGGGCTATATGAAGCAATCGAGGAGTGCCTGATTAATGA
+HWI-EAS443:3:17:410:1975#0/1
aa`YJ`a[]`VZba[`abS_aa¥T]aW`^a`_aS_a_``_
@HWI-EAS443:3:17:419:799#0/1
CAGGGGTAGAGAAGCCTAAATTTCTTCCTGATTTGTATGA
+HWI-EAS443:3:17:419:799#0/1
`[_`UY[Y¥Za_a_`aYaW_a_``^[__[[MZ]^_Y[^a]
@HWI-EAS443:3:17:421:1237#0/1
GCCCACTTCAATTGGCTTGTTCTCTTCATGTTCTTTGTCC
+HWI-EAS443:3:17:421:1237#0/1
abZb`a`a_b]]`]PU]]Q¥]RPXa`[Y^SQ[QW`XDNW`
@HWI-EAS443:3:17:463:1563#0/1
AGACATTCTTTGGCTGGAAAGAGCCTAACATAGTCAAACC
+HWI-EAS443:3:17:463:1563#0/1
_[a`a`aaa^_`Yaa^^[a`ZXZW^^_a``¥__YX``_^^
@HWI-EAS443:3:17:490:2001#0/1
GGGGGCTATATGAAGCAATCGAGGAGTGCCTGATTAATGA
+HWI-EAS443:3:17:490:2001#0/1
aa]PNZ`_a_¥Y[_ZH_a^V]NTNNNLZVTSSLXR_]TW_
@HWI-EAS443:3:17:502:1357#0/1
TGAACTTTGTAAGTATGGAGTTCTCACTCACTGACCCGAG
+HWI-EAS443:3:17:502:1357#0/1
M[VQ¥bbbbaaaWJa]_W_abab_YWbab_S`W¥]`aa`a
@HWI-EAS443:3:17:554:92#0/1
ATAGCCCCCCAAGATCGAAGGTTCCAGGTTCCAGGTTGTC
+HWI-EAS443:3:17:554:92#0/1
abaa`^aaa`]aa^¥¥]Z_^XIRQSG¥^U¥T¥¥¥^PW^WW
@HWI-EAS443:3:17:556:160#0/1
GGACAGTGGTGAACAGTATATGTAACACAACAGGGGTAGA
```

```
+HWI-EAS443:3:17:556:160#0/1
ab¥abbabbab`a`ab]aaaaa`aa_a_a`_`aa_`Z__^
@HWI-EAS443:3:17:607:79#0/1
ATCCGAAAATCGAAACTAACAAGTTTGCTGCAATATGCAC
+HWI-EAS443:3:17:607:79#0/1
_```ba`baaa^S``_``_¥__aX^___^a]__`__¥X^]
@HWI-EAS443:3:17:64:42#0/1
TTCTTGTCTTATAGTGAAAAGCCTAGTTTTGATTCTTGCC
+HWI-EAS443:3:17:64:42#0/1
aaaaaa_aaaa``a]a_`_`___^`a^_^__]_^^]^_]_
@HWI-EAS443:3:17:693:1742#0/1
CACCACGCCCCCTCAGATTGCCTGATGGGCCTCTTTGCCA
+HWI-EAS443:3:17:693:1742#0/1
`a^_aa``_a_`_`a`PFF¥P^HZ_F^TPUaF_DMI]BBB
@HWI-EAS443:3:17:719:1545#0/1
CAATAATTGTAGAATCTGGTGACCCGAATGCACTATTGAA
+HWI-EAS443:3:17:719:1545#0/1
aabba`abb_ba`bab_aa[aaaa^aaa_`Xa_``a_]a]
@HWI-EAS443:3:17:782:264#0/1
GTTTTGATTCTTGCCCTGCTCTCTTCGTCAAGGGTGTAGT
+HWI-EAS443:3:17:782:264#0/1
a¥^aaa_aa`_`^^`[[_``¥`__¥_a_`_Uaa^VaV¥^U
@HWI-EAS443:3:17:788:1293#0/1
ACCGAATCATGGCCTGGACAGTGGTGAACAGTATATGTAA
+HWI-EAS443:3:17:788:1293#0/1
abb`]`a`[`_`a``___a`_ZW^V[Z¥`_]W]^]]_V]]
@HWI-EAS443:3:17:795:1493#0/1
TGGCGTTCACTTCTTTTGACATTTGGGAAAGCTTGCCCTC
+HWI-EAS443:3:17:795:1493#0/1
aab`b_a¥TL^_U_a_`S¥a[[aaWa[[a_^^]a_^_aT^
@HWI-EAS443:3:17:79:907#0/1
CAAGAAATGGCCAGTAGGAGTCTATGGGATTCCTTTCGTC
+HWI-EAS443:3:17:79:907#0/1
aaaa_aa_a_`a``Z^WT]^V^`____^^Y_^]¥_^[YPZ
@HWI-EAS443:3:17:809:905#0/1
GGGGCATGGAAATGAGGCGCTGCCTTCTTCAGTCTCTTCA
+HWI-EAS443:3:17:809:905#0/1
aa_a`aaaa_aaaaaa]aa_`a^____a¥^_ZMM_¥__W^
@HWI-EAS443:3:17:82:189#0/1
CACTGTCATACTGTTTAAGGTCTCCAACATCTTTGCAGTC
+HWI-EAS443:3:17:82:189#0/1
abaab`bbabaaaabaa]^aZaaa_a```__a__a^``X_
@HWI-EAS443:3:17:971:921#0/1
AGACAATTGAAGAAAAATTTGAGATTACAGGAACTATGCG
+HWI-EAS443:3:17:971:921#0/1
]Y¥Y`_aa]Z¥b_a__`___a__`_^]LX__][Z`P^¥X_
@HWI-EAS443:3:17:98:601#0/1
TGGGATTTATGCCTTTCTCATGTGGTTTGACTATGTTAGG
+HWI-EAS443:3:17:98:601#0/1
ab¥]]aaa[]¥_`aa`aa__`a_`___^T^[]^]_YZ[^^
```

@HWI-EAS443:3:18:1023:1426#0/1
AAGCAATTTTCTCGATTCAGCCGAAAACCCCTCAAGTTGT
+HWI-EAS443:3:18:1023:1426#0/1
abaa^`aa_^`baa]aa_aaaaa`a`a_`_a_aaZ`U¥`W
@HWI-EAS443:3:18:1094:130#0/1
CGAACTTCTCCAGCCTTGAAAACTTTAGAGCCTATGTAGA
+HWI-EAS443:3:18:1094:130#0/1
abaaaaaaaaaa`a`a`aaa_a^`a`_aRa]`a_`aY^_¥
@HWI-EAS443:3:18:1164:767#0/1
GGGGGCTATATGAAGCAATCGAGGAGTGCCTGATTAATGA
+HWI-EAS443:3:18:1164:767#0/1
_ba[`]aa]_]bb``^aa]Y`Zaa^a_`]``a__aZV¥``
@HWI-EAS443:3:18:1166:1242#0/1
TCAAGTTCTATCCAGCTTGAATCAGTCAATTCACATGCCT
+HWI-EAS443:3:18:1166:1242#0/1
aaab`_aabba__aaaa`baaaa_`__a`a`aa_`_^Y[`
@HWI-EAS443:3:18:1177:1233#0/1
TCCGATTGGCCATGTTTCCGATTTGTTTTCAAAGAATTCC
+HWI-EAS443:3:18:1177:1233#0/1
aabbaaa`aaaaa`[ab____a`aa]aa`__aa___^`^[
@HWI-EAS443:3:18:1231:446#0/1
AATGCATCCTGTGCAGCCATGGATGACTTTCAGCTGATCC
+HWI-EAS443:3:18:1231:446#0/1
a`ba[_aa^]a]aa_^_Y^¥^`^X_YN_YVY]^VY]VXXV
@HWI-EAS443:3:18:1265:158#0/1
GGCCATGTTTCCGATTTGTTTTCAAAGAATTCCTTGGTCA
+HWI-EAS443:3:18:1265:158#0/1
aaaa`bb_aa`aaaaaab````____a`Z``__a`a_Z_^
@HWI-EAS443:3:18:1399:610#0/1
GGGTGAATCAATAATTGTAGAATCTGGTGACCCGAATGCA
+HWI-EAS443:3:18:1399:610#0/1
aaaT_¥aa`¥X^__a`^¥^`Xa[¥_a`Za^W]W`^^^^^]
@HWI-EAS443:3:18:1403:1142#0/1
CGGCTGAATCGAGAAAATTGCTTCTCATTGTTCAGGCACT
+HWI-EAS443:3:18:1403:1142#0/1
a`aaabab`a`_b_`_aaaa]aa_a`aaaa``_`aaY^_`
@HWI-EAS443:3:18:1447:276#0/1
ATCCCAAGGACAAAGAACATGAAGAGAACAAGCCAATTGA
+HWI-EAS443:3:18:1447:276#0/1
aaaa[^^aa¥_a`aa`_^`a^_``_`_^[`_aVNZ^_V^]
@HWI-EAS443:3:18:1447:412#0/1
GTCTCCACAACTTGAGGGGTTTTCGGCTGAATCGAGAAAA
+HWI-EAS443:3:18:1447:412#0/1
a_]]Z[a]^a^aab_`aa`Pa_]Xa_¥`_a^]W]]_]^^[
@HWI-EAS443:3:18:1499:1072#0/1
GTGCTTCAATAGTGCATTCGGGTCACCAGATTCTACAATT
+HWI-EAS443:3:18:1499:1072#0/1
aaaaaa`aaaaa¥bXaa`a[Za¥_a^^¥a_aa¥a`U^Ya`
@HWI-EAS443:3:18:1502:1009#0/1
AACCAATGGAACCTCCAAGATCAAGATGAAATGGGGCATG

+HWI-EAS443:3:18:1502:1009#0/1
¥V^`abaU_Z`K`b`S^aZYTa^Q`V``_SUa_QV^Z_XV
@HWI-EAS443:3:18:158:1022#0/1
CCGATTTGAGATAATTGAAGGAAGAGACCGAATCATGGCC
+HWI-EAS443:3:18:158:1022#0/1
aa^_aaa``a``a_a_a_`a_Y[___a``[^_^__^^]aY
@HWI-EAS443:3:18:1665:1870#0/1
GTCCTGCACACTTTCCCAATAGAGCCTTCCTCCACTCCCC
+HWI-EAS443:3:18:1665:1870#0/1
ZLYa``^`aa[]]^¥YUbXQZY¥^`YYYUUM_UU[UUUUW
@HWI-EAS443:3:18:1693:598#0/1
CCTGATTTGTATGATTACAAAGAGAACCGGTTCATTGAAA
+HWI-EAS443:3:18:1693:598#0/1
abbbbbbbabbaabbbbaa`ababaaa^`b`a^ababaaa
@HWI-EAS443:3:18:170:1591#0/1
CCCCTGTTGTGTTACATATACTGTTCACCACTGTCCAGGC
+HWI-EAS443:3:18:170:1591#0/1
abaa_`W^aQ__``a``aa]_aa^_`aaa`a_¥[R_¥¥Z]
@HWI-EAS443:3:18:1732:1903#0/1
GAGCCTTCCTCCACTCCCCTGGGTGACTCTCCGATTGGCC
+HWI-EAS443:3:18:1732:1903#0/1
`^^a_[^[a]X¥aS`Iaa]a_^`UZab¥¥a^_a]a]`¥MQ
@HWI-EAS443:3:18:1755:1069#0/1
AAGAGACCGAATCATGGCCTGGACAGTGGTGAACAGTATA
+HWI-EAS443:3:18:1755:1069#0/1
[^]¥RZ_^¥]]¥]Y^_^Y¥]WRR[^¥VV¥[`YTTQRDKVY
@HWI-EAS443:3:18:211:1069#0/1
CGAAAATCGAAACTAACAAGTTTGCTGCAATATGCACACA
+HWI-EAS443:3:18:211:1069#0/1
aa_ab^aa^]`]aa`^`aaa]````__aa_`_a_aa`^`_
@HWI-EAS443:3:18:268:320#0/1
GAAGATCCCAAGGACAAAGAACATGAAGAGAACAAGCCAA
+HWI-EAS443:3:18:268:320#0/1
]_TZ`Z`[KZbb`]ab¥N^`a[`ab`]Y^aaa]`^`aa_X
@HWI-EAS443:3:18:300:356#0/1
AGGACATTGAAAATGAAGAGAAGATCCCAAGGACAAAGAA
+HWI-EAS443:3:18:300:356#0/1
a`_[^`^aaa]¥_aba_a^¥¥````_a_`__¥_^__^a`_
@HWI-EAS443:3:18:367:1694#0/1
AAGAATTCCTTGGTCATGTCTTTCTCTTTGACAGAAGACT
+HWI-EAS443:3:18:367:1694#0/1
aa^^_^S`^aa]UWaYaaPY`__[a__^aS_`_W¥a]`^_
@HWI-EAS443:3:18:406:1177#0/1
AAACAAACTTCCAAATGTGTGCATATTGCAGCAAACTTGT
+HWI-EAS443:3:18:406:1177#0/1
_ba_bbbaaa_aaa``a^`Y_a_a_`_^_a^aa¥^_^__^
@HWI-EAS443:3:18:415:239#0/1
CCGAGACTGGAGCCACACAAATGGGAAAAATACTGTGTTC
+HWI-EAS443:3:18:415:239#0/1
aa`aa``_``aaY`__^_]^__`^`Y¥]]^^_W^^Y_^UL

```
@HWI-EAS443:3:18:448:1118#0/1
TCCACTCCCCTGGGTGACTCTCCGATTGGCCATGTTTCCG
+HWI-EAS443:3:18:448:1118#0/1
aaabaaaaa__a^aUa_[`a^^`_[U¥`^Z[¥T¥T¥^V[Q
@HWI-EAS443:3:18:471:268#0/1
GCATTGAGCAAGGCCGTATTTATGTACACTCCCTTCATTA
+HWI-EAS443:3:18:471:268#0/1
a```_``_aa]`[]^_V_[^¥^^]Z^]¥UZZ]YWXZZWZZ
@HWI-EAS443:3:18:607:753#0/1
GGTGACCCGAATGCACTATTGAAGCACCGATTTGAGATAA
+HWI-EAS443:3:18:607:753#0/1
abOb`a[^aYaXVW_^^aa``_^__^¥Z`[_[TORRV^^_
@HWI-EAS443:3:18:639:614#0/1
ATCAAATGCATGAAGACATTCTTTGGCTGGAAAGAGCCTA
+HWI-EAS443:3:18:639:614#0/1
]aa`baa_ZYaaaba`aaa_X_`__a``a^^aa_`_WP`_
@HWI-EAS443:3:18:703:628#0/1
CGTCTCCACAACTTGAGGGGTTTTCGGCTGAATCGAGAAA
+HWI-EAS443:3:18:703:628#0/1
aaabaa_]_aa_`bb`bab`G^aa_aa`]Xaa[[^`a`Ya
@HWI-EAS443:3:18:789:1534#0/1
CATTGAAAATGAAGAGAAGATCCCAAGGACAAAGAACATG
+HWI-EAS443:3:18:789:1534#0/1
abbabbbabaabaaa`bbaaaa_aa`a_a_aaaaa_`aaa
@HWI-EAS443:3:18:842:564#0/1
CCCGAATGCACTATTGAAGCACCGATTTGAGATAATTGAA
+HWI-EAS443:3:18:842:564#0/1
aZb_IYYa[`a[aaa_a[X^___`__Y__W_]]_]__¥_^
@HWI-EAS443:3:18:848:365#0/1
CTCCAACATCTTTGCAGTCATCAAAGTCTACTTTTTCTGG
+HWI-EAS443:3:18:848:365#0/1
abbaababbbbaaabab`abaaaaaa¥aaa_aa`_```aa
@HWI-EAS443:3:18:856:315#0/1
TTGGGGGGCTATATGAAGCAATCGAGGAGTGCCTGATTAA
+HWI-EAS443:3:18:856:315#0/1
^abba_aab``bbab`aaaa_a¥a``a_aY`__``¥`_`^
@HWI-EAS443:3:18:875:1581#0/1
TGTGGTTTGACTATGTTAGGCTCTTTCCAGCCAAAGAATG
+HWI-EAS443:3:18:875:1581#0/1
a`_baYb``a_^`b¥^aaa]VYP_aa_Wa`_aa_aU^`^O
@HWI-EAS443:3:18:895:771#0/1
AAGAGCCTAACATAGTCAAACCACATGAGAAAGGCATAAA
+HWI-EAS443:3:18:895:771#0/1
^``]baba_`baa]a_]aaa^`a_`aa`^_¥X]___`_a`
@HWI-EAS443:3:18:983:45#0/1
GCCCACTTCAATTGGCTTGTTCTCTTCATGTTCTTTGTCC
+HWI-EAS443:3:18:983:45#0/1
a``U`]^]_`]^_`_R_W[U¥SMWWVMTT]WWHW^WWYWY
@HWI-EAS443:3:19:1000:554#0/1
GTGCCATATTTTCACCGAGTGCCCACTTCAATTGGCTTGT
```

```
+HWI-EAS443:3:19:1000:554#0/1
aaba`ab`bbaaabaaa`a_aa_^``aa__aa_`a_a_P¥
@HWI-EAS443:3:19:1006:622#0/1
CACCTGCTTCCAAGCCATGAGGTAATTGGGATTTATGCCT
+HWI-EAS443:3:19:1006:622#0/1
]W_a^_^S_a¥]a¥[Y`^ZWaXPXP]`_`aX`a[`]^UY[
@HWI-EAS443:3:19:1008:857#0/1
GCCAAGTGTCGAGGCCCATGTTCCTATATGTGAGAACCAA
+HWI-EAS443:3:19:1008:857#0/1
```]_b^a_abbaaaa]aa`^aa_a]N]_a_a``a^UV``
@HWI-EAS443:3:19:1061:550#0/1
ATTAATCAGGCACTCCTCGATTGCTTCATATAGCCCCCCA
+HWI-EAS443:3:19:1061:550#0/1
a``_W¥ab`J¥baaa_V^]^bbb`aXU¥`aaaa`P^PXa_
@HWI-EAS443:3:19:1075:100#0/1
ATGAAATGGAAATCCGAATACATGAAACAAACTTCCAAAT
+HWI-EAS443:3:19:1075:100#0/1
aa_b_aaa`___aa^a`_a_^_a`__`^a_^^a_^¥_]^`
@HWI-EAS443:3:19:107:185#0/1
GAGAAAGGCATAAATCCCAATTACCTCATGGCTTGGAAGC
+HWI-EAS443:3:19:107:185#0/1
V]`baa_X_`Y``ba__aa^^Zaaa`]]P``aYMT^BBBB
@HWI-EAS443:3:19:1291:1040#0/1
AATTACCTCATGGCTTGGAAGCAGGTGCTAGCAGAGCTAC
+HWI-EAS443:3:19:1291:1040#0/1
ab^```a_`a^a`aa`U¥S^[SWZWMN_QRVQQVRVBBBB
@HWI-EAS443:3:19:1317:79#0/1
CGGGGTGAATCAATAATTGTAGAATCTGGTGACCCGAATG
+HWI-EAS443:3:19:1317:79#0/1
aaa¥VFZ¥Xa^RRa]¥`baNPaX¥]LZ¥]S_^PST[]R^^
@HWI-EAS443:3:19:1365:1037#0/1
TGCTAGCAGAGCTACAGGACATTGAAAATGAAGAGAAGAT
+HWI-EAS443:3:19:1365:1037#0/1
_V¥_bbbba¥[P]b`aa_aaaaaa_`aaa^]```a____]
@HWI-EAS443:3:19:1513:88#0/1
GGGGCATGGAAATGAGGCGCTGCCTTCTTCAGTCTCTTCA
+HWI-EAS443:3:19:1513:88#0/1
aaaa``aba^_^abaa^Ya^_`WW¥_U_`VU^US^WWWWS
@HWI-EAS443:3:19:152:1121#0/1
GCAGGGCAAGAATCAAAACTAGGCTTTTCACTATAAGACA
+HWI-EAS443:3:19:152:1121#0/1
[__¥U_`_aULU^¥aa[]aa¥OTVDRX^G`WU]VZ[EPX^
@HWI-EAS443:3:19:1582:1499#0/1
TTCCGATTTGTTTTCAAAGAATTCCTTGGTCATGTCTTTC
+HWI-EAS443:3:19:1582:1499#0/1
aaaaa_`¥Yaaaa__a^]`_R_`^_a`a_Z^_]Y¥]`^_V
@HWI-EAS443:3:19:1640:184#0/1
TGGGGCATGGAAATGAGGCGCTGCCTTCTTCAGTCTCTTC
+HWI-EAS443:3:19:1640:184#0/1
a_^aa_`aba`a`aa_a_¥___]NYW_W`¥WV_UQZWY¥B
```
```

@HWI-EAS443:3:19:169:473#0/1
GGGCACTCGGTGAAAATATGGCACCAGAAAAAGTAGACTT
+HWI-EAS443:3:19:169:473#0/1
_WS]¥]W[Y]NQV]a]WBBBBBBBBBBBBBBBBBBBBBBB
@HWI-EAS443:3:19:1711:1250#0/1
CATTAATCAGGCACTCCTCGATTGCTTCATATAGCCCCCC
+HWI-EAS443:3:19:1711:1250#0/1
aabbaaaaababb_b`ZbaaaaaaUaa_aaaaaaWLQ^_¥
@HWI-EAS443:3:19:1717:1106#0/1
GGGGGCTATATGAAGCAATCGAGGAGTGCCTGATTAATGA
+HWI-EAS443:3:19:1717:1106#0/1
aabbaababababbaaa_aaa``^ba_`_a[P`aUaa_V__Y
@HWI-EAS443:3:19:1780:1424#0/1
GGGGCATGGAAATGAGGCGCTGCCTTCTTCAGTCTCTTCA
+HWI-EAS443:3:19:1780:1424#0/1
ab_aabbab`baba_^`ab`ba^_a_aa`Q¥aQ`a_aa_`
@HWI-EAS443:3:19:230:1075#0/1
CTCATTTGAGAAATGATACTGATGTGGTGAACTTTGTAAG
+HWI-EAS443:3:19:230:1075#0/1
¥R^X`aaRL]`a`Y`a[_^^_VYWY^NN`RUT`^__]WL^
@HWI-EAS443:3:19:23:770#0/1
GAAAATCGAAACTAACAAGTTTGCTGCAATATGCACACAT
+HWI-EAS443:3:19:23:770#0/1
abbbba^a`ba`aaa``_a`a`^__aaaaa`aa_`````_
@HWI-EAS443:3:19:263:130#0/1
ATAAATACGGCCTTGCTCAATGCATCCTGTGCAGCCATGG
+HWI-EAS443:3:19:263:130#0/1
abab`aaaa_``_`__^^_`X```^_]^_Y^^^^]^YY]^
@HWI-EAS443:3:19:286:220#0/1
AATTCCTTGGTCATGTCTTTCTCTTTGACAGAAGACTCGG
+HWI-EAS443:3:19:286:220#0/1
Y]_`]W]Zb_MZ_bb^b_`bb__b¥ba``abaa]abaab_
@HWI-EAS443:3:19:310:1160#0/1
CAATAATTGTAGAATCTGGTGACCCGAATGCACTATTGAA
+HWI-EAS443:3:19:310:1160#0/1
a¥[^aZaaa`___aaaaaa]aaaaaa```a[]aa_`a`[`
@HWI-EAS443:3:19:319:1602#0/1
CGGCCTTGCTCAATGCATCCTGTGCAGCCATGGATGACTT
+HWI-EAS443:3:19:319:1602#0/1
a¥¥_a_abaababa]bb]aaa^RZaa^ba```]_]Y`^`^
@HWI-EAS443:3:19:352:147#0/1
GAGAGGAGATGGCCACCAAAGCGGACTACACCCTTGACGA
+HWI-EAS443:3:19:352:147#0/1
aY^X`^¥`Z[PYUU_[UXYUaU¥_WPUSWTMWSWSTUS¥W
@HWI-EAS443:3:19:361:1706#0/1
GGGGGCTATATGAAGCAATCGAGGAGTGCCTGATTAATGA
+HWI-EAS443:3:19:361:1706#0/1
UUW_a_[X]aaVRVV_aa]_TY]JZWP¥Y`bba^b^aVW`
@HWI-EAS443:3:19:393:1532#0/1
GGGTTTTCGGCTGAATCGAGAAAATTGCTTCTCATTGTTC

```
+HWI-EAS443:3:19:393:1532#0/1
aaaIb]ababba_`bba`a__b]a]a]aaaaaa[aa__a_
@HWI-EAS443:3:19:401:1566#0/1
GTGCCATATTTTCACCGAGTGCCCACTTCAATTGGCTTGT
+HWI-EAS443:3:19:401:1566#0/1
`_]_Z`ab`aaZT``Za`]N¥a_¥XY¥Z]^¥U¥^O_^]]X
@HWI-EAS443:3:19:40:675#0/1
TGGAGAAGTTCGGTGGGAGACTTTGGTCGGCAAGCTTGCG
+HWI-EAS443:3:19:40:675#0/1
ab_a`aabaaaaaZa`_T_]¥^a_W^^a_ZQ^a__^^Y^`
@HWI-EAS443:3:19:422:520#0/1
AAATTTAGGCTTCTCTACCCCTGTTGTGTTACATATACTG
+HWI-EAS443:3:19:422:520#0/1
`bba[¥bbabbabX]ab`aaZ^aO____]S``_aa`aY[_
@HWI-EAS443:3:19:436:513#0/1
GAACAGTATATGTAACACAACAGGGGTAGAGAAGCCTAAA
+HWI-EAS443:3:19:436:513#0/1
aaaaaaX]_^R¥]a`a_aP[_`_a__W^_]`UGY_Z]^^_
@HWI-EAS443:3:19:475:501#0/1
TTTTCGGCTGAATCGAGAAAATTGCTTCTCATTGTTCAGG
+HWI-EAS443:3:19:475:501#0/1
`b`]]Z^[Yba`_]a_]U_aaaaa`¥]`_`^[X]R_Z[``
@HWI-EAS443:3:19:510:1533#0/1
CACCCTTGACGAAGAGAGCAGGGCAAGAATCAAAACTAGG
+HWI-EAS443:3:19:510:1533#0/1
aba`_a[aaa^```_^]_`^][¥a]a`a`_^a_^_]Za]X
@HWI-EAS443:3:19:597:1425#0/1
GAAAACCCCTCAAGTTGTGGAGACGCATATAGACTGTTGA
+HWI-EAS443:3:19:597:1425#0/1
aaaaabbbaaaa```_a^aXa`a`a__`a^`_``_`X[GP
@HWI-EAS443:3:19:625:230#0/1
ATCGAAACTAACAAGTTTGCTGCAATATGCACACATTTGG
+HWI-EAS443:3:19:625:230#0/1
a`_aaaa_aba``W]^``a`¥a``X___`_`_`^__][a^
@HWI-EAS443:3:19:69:575#0/1
CACATCTTTTCATTCACTGGAGAGGAGATGGCCACCAAAG
+HWI-EAS443:3:19:69:575#0/1
___Yb_T^bbabba_[a_`ab^U_V]]XL]Z`^```^`a`
@HWI-EAS443:3:19:729:1306#0/1
GAACGCCAAAATTGAACCATTCTTGAGGACGACACCACGC
+HWI-EAS443:3:19:729:1306#0/1
a^aaaa_PFMX_WVZ]TXO`T`[[[[]SSS_]W_S¥_RBB
@HWI-EAS443:3:19:742:89#0/1
AGAAGATCCCAAGGACAAAGAACATGAAGAGAACAAGCCA
+HWI-EAS443:3:19:742:89#0/1
abaaaX`[`aa]__`_[[`a[^^_aYRW__a^_X]^`^^^
@HWI-EAS443:3:19:769:229#0/1
CTTCTGTCAAAGAGAAAGACATGACCAAGGAATTCTTTGA
+HWI-EAS443:3:19:769:229#0/1
abbaaaabaaababaaabaaaaaaaZ`J^_`ZaVP^aITa
```

@HWI-EAS443:3:19:827:1598#0/1
GGACATTGAAAATGAAGAGAAGATCCCAAGGACAAAGAAC
+HWI-EAS443:3:19:827:1598#0/1
aYL¥[YXa_`W[ZY^W¥U][SXS[[Y`ZSR^`^__^a_[W
@HWI-EAS443:3:19:831:1300#0/1
GGGGCATGGAAATGAGGCGCTGCCTTCTTCAGTCTCTTCA
+HWI-EAS443:3:19:831:1300#0/1
aaa`]a`aaaaa`[^¥aa¥`__ZY[__`]U__[P``^^S^
@HWI-EAS443:3:19:848:883#0/1
ACGGCCTTGCTCAATGCATCCTGTGCAGCCATGGATGACT
+HWI-EAS443:3:19:848:883#0/1
a_aa¥¥_`aUXa^]]V[_^`_[YRX[Z[^VSQaX¥^_UUU
@HWI-EAS443:3:19:855:1271#0/1
ATGAAGGGAGTGTACATAAATACGGCCTTGCTCAATGCAT
+HWI-EAS443:3:19:855:1271#0/1
aaba¥ba`aa¥a]baa`aaa`aaa_]___[TW_^_^]^_]
@HWI-EAS443:3:19:890:905#0/1
ATCAGGCACTCCTCGATTGCTTCATATAGCCCCCCAAGAT
+HWI-EAS443:3:19:890:905#0/1
__aaa[`^abaa_]aaXV_U_aaa_a`a^YU]`¥a`¥^__
@HWI-EAS443:3:19:936:995#0/1
CATTAATCAGGCACTCCTCGATTGCTTCATATAGCCCCCC
+HWI-EAS443:3:19:936:995#0/1
abbbbabbbbbbbabbabbbbabbabbabbbababa`aaa
@HWI-EAS443:3:19:945:471#0/1
ATAAATACGGCCTTGCTCAATGCATCCTGTGCAGCCATGG
+HWI-EAS443:3:19:945:471#0/1
aaaa`aa_a`a_a`_GW_a`__`a__U``X]S¥__O^___
@HWI-EAS443:3:19:971:1212#0/1
ACCGAATCATGGCCTGGACAGTGGTGAACAGTATATGTAA
+HWI-EAS443:3:19:971:1212#0/1
a`[a`[a^]`aa``aX[[___S¥a¥`[a_`^U^``¥VX__
@HWI-EAS443:3:19:988:213#0/1
GACCCGAATGCACTATTGAAGCACCGATTTGAGATAATTG
+HWI-EAS443:3:19:988:213#0/1
_¥```_Y¥]]J`¥aX`¥``^]Y[S]]^a`_]Q_][¥Z]¥_
@HWI-EAS443:3:1:1019:1502#0/1
TACAAAGAGAACCGGTTCATTGAAATTGGAGTAACACGGA
+HWI-EAS443:3:1:1019:1502#0/1
`b^[a`aabbb`abb_]]ababa_^aabS^b^aba^a`BB
@HWI-EAS443:3:1:1019:464#0/1
TGAGAAAGGCATAAATCCCAATTACCTCATGGCTTGGAAG
+HWI-EAS443:3:1:1019:464#0/1
aaa_a`_`aXaaaa_a]]]__a_`W[_]``__W`^T^BBB
@HWI-EAS443:3:1:1112:296#0/1
TGGGCACTCGGTGAAAATATGGCACCAGAAAAAGTAGACT
+HWI-EAS443:3:1:1112:296#0/1
]QZOU]ZTOXYL]TQWPY¥ZP[BBBBBBBBBBBBBBBBBB
@HWI-EAS443:3:1:1137:1236#0/1
GGTGAACTTTGTAAGTATGGAGTTCTCACTCACTGACCCG

+HWI-EAS443:3:1:1137:1236#0/1
`b_``aUa_aa^b_bWa_[aaa_aYa_`^`Z_¥a[`BBBB
@HWI-EAS443:3:1:1152:202#0/1
CTTTGACCGCTGATGGCAAAGAGGCCCATCAGGCAATCTG
+HWI-EAS443:3:1:1152:202#0/1
aabba`[aUba_aZb_RZa_baaabaUaJJb`aa`_aBBB
@HWI-EAS443:3:1:1273:140#0/1
GCTCATCACTGTCATACTGTTTAAGGTCTCCAACATCTTT
+HWI-EAS443:3:1:1273:140#0/1
a_aUa]]V[aa_^`]Y]``Z_`__W_SSZW_]U^^_XBBB
@HWI-EAS443:3:1:1317:616#0/1
CGACGAACGGGGTGAATCAATAATTGTAGAATCTGGTGAC
+HWI-EAS443:3:1:1317:616#0/1
ab`abba_b_ab^bbabaaba_abbbaabba_]a`aZb``
@HWI-EAS443:3:1:1338:174#0/1
GTTTGTTTTCCGTCTTCCTTCTTTGGTCCTACATTTGCTT
+HWI-EAS443:3:1:1338:174#0/1
aa[^VP¥W`M[^S[]UKP¥^Z_¥ZXXRUXVWZZ]BBBBBB
@HWI-EAS443:3:1:1347:1663#0/1
TGTGAATTGACTGATTCAAGCTGGATAGAACTTGATGAAA
+HWI-EAS443:3:1:1347:1663#0/1
ab¥a^a`aa_^aaaaaOa[¥Xaa[_a^`^aSaaa]a_^^B
@HWI-EAS443:3:1:1445:661#0/1
TAAGCAAATGTAGGACCAAAGAAGGAAGACGGAAAACAAA
+HWI-EAS443:3:1:1445:661#0/1
`]X[RVN[`X¥][[GP¥a_W``Y`Y[_^WS`^`]SBBBBB
@HWI-EAS443:3:1:1449:36#0/1
ACGGCCTTGCTCAATGCATCCTGTGCAGCCATGGATGACT
+HWI-EAS443:3:1:1449:36#0/1
]a^VYUUa[D¥[^YaXR[aEKZLYWN^WPFTBBBBBBBBB
@HWI-EAS443:3:1:1553:261#0/1
ACATAATGAAGGGAGTGTACATAAATACGGCCTTGCTCAA
+HWI-EAS443:3:1:1553:261#0/1
aWQ^a¥^`[`aa`VWU`[^¥R[¥`_a_]YVRPV`BBBBBB
@HWI-EAS443:3:1:1563:1150#0/1
TGGTGAACTTTGTAAGTATGGAGTTCTCACTCACTGACCC
+HWI-EAS443:3:1:1563:1150#0/1
`b_K_]]]bbab_aaaZ]aaa_a`XZ`_`^a^¥U_`^VBB
@HWI-EAS443:3:1:162:1242#0/1
AAGAATTCCTTGGTCATGTCTTTCTCTTTGACAGAAGACT
+HWI-EAS443:3:1:162:1242#0/1
¥a¥`a¥b]`abbaPY]ab`Z^[Y^_^ba`^`^aa^a`]BB
@HWI-EAS443:3:1:1678:1457#0/1
CTCATTTGAGAAATGATACTGATGTGGTGAACTTTGTAAG
+HWI-EAS443:3:1:1678:1457#0/1
ab`bbbbbba`bbbbbb`bbbab¥ababaa_aaaa_aab
@HWI-EAS443:3:1:338:1503#0/1
GGGTTTTCGGCTGAATCGAGAAAATTGCTTCTCATTGTTC
+HWI-EAS443:3:1:338:1503#0/1
`bbG^baYZRV^a^aY]^YZ]`Y^`O_O¥__^^_^Z_BBB

```
@HWI-EAS443:3:1:384:247#0/1
GGGGGGCTATATGAAGCAATCGAGGAGTGCCTGATTAATG
+HWI-EAS443:3:1:384:247#0/1
aaa_a_`a`a``_[¥Y^__¥¥_¥__¥]W`WY¥_Z^¥¥TBB
@HWI-EAS443:3:1:441:600#0/1
GTGAATCAATAATTGTAGAATCTGGTGACCCGAATGCACT
+HWI-EAS443:3:1:441:600#0/1
a¥aaY_`^^__Y]ab]UaZ_`¥_aaXZ`SW¥`^_`^XBBB
@HWI-EAS443:3:1:445:130#0/1
CCACATGAGAAAGGCATAAATCCCAATTACCTCATGGCTT
+HWI-EAS443:3:1:445:130#0/1
aaaG`¥aa`W]Ub`^SZYYV]YS``XY_^S[]]`SBBBBB
@HWI-EAS443:3:1:452:526#0/1
GGGAGTGTACATAAATACGGCCTTGCTCAATGCATCCTGT
+HWI-EAS443:3:1:452:526#0/1
a`_^`V_¥_Q_`¥`__^W¥¥TT¥T¥T¥Y_¥Y¥U^¥USBBB
@HWI-EAS443:3:1:520:563#0/1
CCTGTTGTGTTACATATACTGTTCACCACTGTCCAGGCCA
+HWI-EAS443:3:1:520:563#0/1
`V¥VGTW[`Q^¥URZW]aUb¥W^¥aQGVZ_a_[TPBBBBB
@HWI-EAS443:3:1:584:1016#0/1
AATTGACTGATTCAAGCTGGATAGAACTTGATGAAATAGG
+HWI-EAS443:3:1:584:1016#0/1
aUa_b`¥`````_a__[P^`^_``¥^¥a_`¥^a¥^¥`^BB
@HWI-EAS443:3:1:704:792#0/1
TTCCGATTTGTTTTCAAAGAATTCCTTGGTCATGTCTTTC
+HWI-EAS443:3:1:704:792#0/1
[[¥¥``babbWaaaV¥```b^b`YXYJ_aaaWab_Zaaa_
@HWI-EAS443:3:1:743:912#0/1
GATTTGTTTTCAAAGAATTCCTTGGTCATGTCTTTCTCTT
+HWI-EAS443:3:1:743:912#0/1
]¥b``a`abba¥X`a``bba_aba¥Z[_ab[W`aaGa_BB
@HWI-EAS443:3:1:88:1071#0/1
AGAAGCCTAAATTTCTTCCTGATTTGTATGATTACAAAGA
+HWI-EAS443:3:1:88:1071#0/1
aaaa__^aaaaaaaa```_a_¥_a`a]_aa``_`__`__B
@HWI-EAS443:3:1:925:1497#0/1
AAGAATTCCTTGGTCATGTCTTTCTCTTTGACAGAAGACT
+HWI-EAS443:3:1:925:1497#0/1
aaa`_bb_abbbaaaabbaab`a_baabab`Zaaa]^[]B
@HWI-EAS443:3:1:997:1285#0/1
ATAAATACGGCCTTGCTCAATGCATCCTGTGCAGCCATGG
+HWI-EAS443:3:1:997:1285#0/1
a`aa```Z``^]aaaZ^Ya`_¥^a^LUaa_YTY_]]]_BB
@HWI-EAS443:3:20:100:437#0/1
GGTGAACTTTGTAAGTATGGAGTTCTCACTCACTGACCCG
+HWI-EAS443:3:20:100:437#0/1
abbabb¥_XabUb`a]`bab[aY_baabb_aba_ba``aa
@HWI-EAS443:3:20:1010:671#0/1
AGCTTGCCGACCAAAGTCTCCCACCGAACTTCTCCAGCCT
```

```
+HWI-EAS443:3:20:1010:671#0/1
^aaaa`a`aaa_aa^¥Y`a`X^_ZaaZL]`_^¥_¥U_WUW
@HWI-EAS443:3:20:1052:881#0/1
CCAGATTCTACAATTATTGATTCACCCCGTTCGTCGATGA
+HWI-EAS443:3:20:1052:881#0/1
a`ab`aabbaaaaba`aaaaa__aa_`]a_a_a^_[^¥¥^
@HWI-EAS443:3:20:1069:301#0/1
CATAATGAAGGGAGTGTACATAAATACGGCCTTGCTCAAT
+HWI-EAS443:3:20:1069:301#0/1
^_b¥W[bbaabbaa]b_a`aaaaaaa`aaXa`aaa^]`^`
@HWI-EAS443:3:20:1070:1948#0/1
GCCAGTAAGGTCCTGCACACTTTCCCAATAGAGCCTTCCT
+HWI-EAS443:3:20:1070:1948#0/1
aaab`Y``a]`ab]WZ`aa`aa]^^`^W_b`aMN]]aZS`
@HWI-EAS443:3:20:1078:1270#0/1
TTCGGCTGAATCGAGAAAATTGCTTCTCATTGTTCAGGCA
+HWI-EAS443:3:20:1078:1270#0/1
ZUN]`QR_RW]_`ZQX_a¥S^`aa`aVa`]O¥VT_WTYQM
@HWI-EAS443:3:20:1183:1010#0/1
TTTGGACCCAGCTTGCTAGAGATCTGGGCTCTGGCTCATC
+HWI-EAS443:3:20:1183:1010#0/1
^a``_`baaab_baaa_a_`a`^¥`aaa_aaa`^a_]a`[
@HWI-EAS443:3:20:1367:601#0/1
GGGATTTATGCCTTTCTCATGTGGTTTGACTATGTTAGGC
+HWI-EAS443:3:20:1367:601#0/1
aaaaaaaaaaa`aaa`a``aa]aa_aaa`]`aaa]`_`VO
@HWI-EAS443:3:20:1394:1375#0/1
AGAGAACCGGTTCATTGAAATTGGAGTAACACGGAGGGAA
+HWI-EAS443:3:20:1394:1375#0/1
[`[```aYb`Xaabaaba_`a`b`aa`aa_^U_P[]_^[^
@HWI-EAS443:3:20:1399:1029#0/1
GAGGGGGAGGGAATACCACTATATGATGCAATCAAATGCA
+HWI-EAS443:3:20:1399:1029#0/1
a`baa`aaa_a]`a`^`a^a__a`a__`^a_a]`^`]^Z^
@HWI-EAS443:3:20:1443:624#0/1
GGGGCTATATGAAGCAATCGAGGAGTGCCTGATTAATGAT
+HWI-EAS443:3:20:1443:624#0/1
a_TOVPQUSR¥Z]YLS^^Z`Y¥¥Z^U`ZTKVSYSUTW^XZ
@HWI-EAS443:3:20:1449:901#0/1
GGGGGCTATATGAAGCAATCGAGGAGTGCCTGATTAATGA
+HWI-EAS443:3:20:1449:901#0/1
[a]```bbbbbbba`]abbab`b`^a]aZ^ba[``_`ab`
@HWI-EAS443:3:20:1487:304#0/1
CCACTCCCCTGGGTGACTCTCCGATTGGCCATGTTTCCGA
+HWI-EAS443:3:20:1487:304#0/1
aaaaabaaa`bab^aa¥a]`Z_a_`a^[_XZ^aU[^MZYH
@HWI-EAS443:3:20:1584:606#0/1
GGCTGCATTGAGGGCAAGCTTTCCCAAATGTCAAAAGAAG
+HWI-EAS443:3:20:1584:606#0/1
a`_`_¥a_aW_``aW]^aV_a`YWV]^__a^V^^]^__¥V
```

```
@HWI-EAS443:3:20:159:174#0/1
GGGGGCTATATGAAGCAATCGAGGAGTGCCTGATTAATGA
+HWI-EAS443:3:20:159:174#0/1
ab^Xa``a_a¥¥aaa_a`_`a]_^PXU_]Y_]XX^^XU_Y
@HWI-EAS443:3:20:1607:1424#0/1
GAGAAATGATACTGATGTGGTGAACTTTGTAAGTATGGAG
+HWI-EAS443:3:20:1607:1424#0/1
a]TUa``YY__]__]^`][ZX[_¥Z_^__^]]_O^]]ZUR
@HWI-EAS443:3:20:1609:278#0/1
TGCTCAATGCATCCTGTGCAGCCATGGATGACTTTCAGCT
+HWI-EAS443:3:20:1609:278#0/1
_S`aJbabba`a¥GZaYaS[a^UXVU`S]¥__^Y^N[^U[
@HWI-EAS443:3:20:1614:911#0/1
ATCCGAATACATGAAACAAACTTCCAAATGTGTGCATATT
+HWI-EAS443:3:20:1614:911#0/1
`baaaabbb`baaaab_bb``aa__`Yaaa`b_`_aa_a`
@HWI-EAS443:3:20:1622:138#0/1
CACCGATTTGAGATAATTGAAGGAAGAGACCGAATCATGG
+HWI-EAS443:3:20:1622:138#0/1
__^_¥_``_bababbabb`ba^^ababbaaabaaa``aa_
@HWI-EAS443:3:20:1622:1587#0/1
AGAAGTTCGGTGGGAGACTTTGGTCGGCAAGCTTGCGCAT
+HWI-EAS443:3:20:1622:1587#0/1
aa`aa¥babW_b^ST`^_¥^^__O^_`Z^^]OYRYO^UXY
@HWI-EAS443:3:20:1624:613#0/1
AGCAGGTGCTAGCAGAGCTACAGGACATTGAAAATGAAGA
+HWI-EAS443:3:20:1624:613#0/1
a`^a_ZX^aa_aS¥_Z^T]`X``a^Z_`__]^_^__]Z]Z
@HWI-EAS443:3:20:1683:143#0/1
GCCCTGCAGTGGGACACTTCTGCTGTAAAATAGTTCCTCC
+HWI-EAS443:3:20:1683:143#0/1
aa_[_a_ababa`_[`¥aaY``¥`a`[`bV`__W[LFYPU
@HWI-EAS443:3:20:1683:1755#0/1
ATCGACGAACGGGGTGAATCAATAATTGTAGAATCTGGTG
+HWI-EAS443:3:20:1683:1755#0/1
`a_a`aa^_W``aaX`V_YJV^_V__]_X]_¥V^X^]]OY
@HWI-EAS443:3:20:1727:855#0/1
TGAAATTGGAGTAACACGGAGGGAAGTCCACATATATTAC
+HWI-EAS443:3:20:1727:855#0/1
aaV[Wa^Y[Xa^``]_]a_]_^]XZ^]YU][]^W^]^]YY
@HWI-EAS443:3:20:1738:795#0/1
TAGCCCCCCAAGATCGAAGGTTCCAGGTTCCAGGTTGTCC
+HWI-EAS443:3:20:1738:795#0/1
aba`Z_a_aaaa`a[`_aa¥W^¥U^^¥^¥¥W¥^¥W^¥VWW
@HWI-EAS443:3:20:245:531#0/1
AGCCTATGTAGATGGATTCGAGCCGAACGGCTGCATTGAG
+HWI-EAS443:3:20:245:531#0/1
`bba``ab^aaaabb]`aa`aa`[]_^_a`]aaX_^`R^S
@HWI-EAS443:3:20:308:1549#0/1
CATAAATACGGCCTTGCTCAATGCATCCTGTGCAGCCATG
```

+HWI-EAS443:3:20:308:1549#0/1
ababb`aabaabaa_`]aabb_[a^_a`^]V_``a__a]Z
@HWI-EAS443:3:20:314:1210#0/1
CCCAAGGACAAAGAACATGAAGAGAACAAGCCAATTGAAG
+HWI-EAS443:3:20:314:1210#0/1
ab^]_a_aa_aaaaa`aa_Y`aa_`__a[]`_`____]^`
@HWI-EAS443:3:20:355:1129#0/1
CAATTATTGATTCACCCCGTTCGTCGATGAAATGGAAATC
+HWI-EAS443:3:20:355:1129#0/1
aaaaa_aabaa[aa```aa]_]a___`¥a_a`___]_`¥U
@HWI-EAS443:3:20:391:1599#0/1
GGCTCATCACTGTCATACTGTTTAAGGTCTCCAACATCTT
+HWI-EAS443:3:20:391:1599#0/1
U`abaY¥bbaaaaaabbabbaaabaaa_b`ababa_aaaa
@HWI-EAS443:3:20:466:1053#0/1
AGAAGTTCGGTGGGAGACTTTGGTCGGCAAGCTTGCGCAT
+HWI-EAS443:3:20:466:1053#0/1
ab``]GZba`X]_``a_a```^¥[a`a``X]`_^^_Y^^_
@HWI-EAS443:3:20:572:1613#0/1
GGGTGAATCAATAATTGTAGAATCTGGTGACCCGAATGCA
+HWI-EAS443:3:20:572:1613#0/1
]`__baaabbbbbababVbaabba_b]Y]bbb_b`b`baa
@HWI-EAS443:3:20:61:115#0/1
GGCATGTGAATTGACTGATTCAAGCTGGATAGAACTTGAT
+HWI-EAS443:3:20:61:115#0/1
aaaabb^aaabbba_aaaaaaa_baaba_`aa`a_a_a_`
@HWI-EAS443:3:20:629:416#0/1
GGCATGGAAATGAGGCGCTGCCTTCTTCAGTCTCTTCAGC
+HWI-EAS443:3:20:629:416#0/1
`a`_`b_¥``aaa`_`a`_a]¥_^¥``¥Z_W]_¥¥WTW¥Q
@HWI-EAS443:3:20:635:1279#0/1
GAAAGGCATAAATCCCAATTACCTCATGGCTTGGAAGCAG
+HWI-EAS443:3:20:635:1279#0/1
^a]aa^a_```]]_P__`a`aa`_`__a_a_[JOZ`YYWB
@HWI-EAS443:3:20:649:22#0/1
GGGGCTATATGAAGCAATCGAGGAGTGCCTGATTAATGAT
+HWI-EAS443:3:20:649:22#0/1
a`aaa`Z[V_a^¥¥WT]]]`W^^Q[LYUYW^SV]ZUWWWT
@HWI-EAS443:3:20:685:553#0/1
GGAGTCTATGGGATTCCTTTCGTCAGTCCGAAAGAGGCGA
+HWI-EAS443:3:20:685:553#0/1
aa^a`[][aa`_][_]a``[^`]__`Z_]_[]]`Z_^M]Z
@HWI-EAS443:3:20:717:109#0/1
CAGCTTGCTAGAGATCTGGGCTCTGGCTCATCACTGTCAT
+HWI-EAS443:3:20:717:109#0/1
aababbbabaaaaaa_`a_`^`_`a¥__```¥`]``^]``
@HWI-EAS443:3:20:739:129#0/1
GAAATGGAAATCCGAATACATGAAACAAACTTCCAAATGT
+HWI-EAS443:3:20:739:129#0/1
^bb`a`__^]`aa_`aaa_aa``aa¥aa_`a_^Y__^_^Z

~~~
@HWI-EAS443:3:20:82:249#0/1
GAATTCCTTGGTCATGTCTTTCTCTTTGACAGAAGACTCG
+HWI-EAS443:3:20:82:249#0/1
aa_`aaaa]_`S]^_Y]a__b_Y]a`a`]``a``¥^_]^X
@HWI-EAS443:3:20:834:1620#0/1
CATTAATCAGGCACTCCTCGATTGCTTCATATAGCCCCCC
+HWI-EAS443:3:20:834:1620#0/1
V]bb`]bbb`a_b]_`_a^`a[[a_`TU`bb^^^`]W`[^
@HWI-EAS443:3:20:876:2043#0/1
CATGCCTTATTGAATTCATTTTGGACCCAGCTTGCTAGAG
+HWI-EAS443:3:20:876:2043#0/1
abaaaab__aZa`a__aa`^`[_[a^_a__^__R__``__
@HWI-EAS443:3:20:893:587#0/1
CAGCTTGCTAGAGATCTGGGCTCTGGCTCATCACTGTCAT
+HWI-EAS443:3:20:893:587#0/1
a¥`b¥Xa^^bbb_abaa_¥]`^`_aaaaaaa]aaaa_[^X
@HWI-EAS443:3:20:956:585#0/1
AACAAGTTTGCTGCAATATGCACACATTTGGAAGTTTGTT
+HWI-EAS443:3:20:956:585#0/1
Y¥^YWXJ¥b_^baYUabaa_W]TX`abaabba[^V[``NN
@HWI-EAS443:3:20:99:773#0/1
CAGCAAACTTGTTAGTTTCGATTTTCGGATCTTCCCCATA
+HWI-EAS443:3:20:99:773#0/1
ab[bba`¥aa`[`Z_^`a^__``^JW`[I_^]aIa`WP¥^
@HWI-EAS443:3:21:1000:870#0/1
TAGGAGTCTATGGGATTCCTTTCGTCAGTCCGAAAGAGGC
+HWI-EAS443:3:21:1000:870#0/1
a]b¥ZXa_``aa¥¥`abba]_]a`[]¥]`_a_^ZZ]Ya[Y
@HWI-EAS443:3:21:1013:912#0/1
AAGAATTCCTTGGTCATGTCTTTCTCTTTGACAGAAGACT
+HWI-EAS443:3:21:1013:912#0/1
]abb_abaabba^^a`bb`abaaaaabb^``aa__a_a^_
@HWI-EAS443:3:21:1014:1448#0/1
TGGGGCATGGAAATGAGGCGCTGCCTTCTTCAGTCTCTTC
+HWI-EAS443:3:21:1014:1448#0/1
abbaaabababbba_aaaaaaaa`_aaaaa_``U`a```W
@HWI-EAS443:3:21:101:683#0/1
ATTTAGGCTTCTCTACCCCTGTTGTGTTACATATACTGTT
+HWI-EAS443:3:21:101:683#0/1
abba`]aaaaa``aaaaaa_`a`]``¥_YFY`Y¥UQW]__
@HWI-EAS443:3:21:1027:768#0/1
ATTTTTCCCATTTGTGTGGCTCCAGTCTCGGGTCAGTGAG
+HWI-EAS443:3:21:1027:768#0/1
_bbabaaaa`baaa[aW```````[NV]^^_aPPZYFVU]
@HWI-EAS443:3:21:1030:296#0/1
GAGACCGAATCATGGCCTGGACAGTGGTGAACAGTATATG
+HWI-EAS443:3:21:1030:296#0/1
`^`]]ZZS¥ZTYaZ^U[]]]SO[YR^¥L¥SSKTYQUGTBB
@HWI-EAS443:3:21:1043:424#0/1
CACATTTGGAAGTTTGTTTCATGTATTCGGATTTCCATTT
~~~

```
+HWI-EAS443:3:21:1043:424#0/1
`aaaabbba`aa]aa_[aa][a`¥]Z`aaa¥Y^^[`]``Z
@HWI-EAS443:3:21:1067:1272#0/1
CAAAGATGTTGGAGACCTTAAACAGTATGACAGTGATGAG
+HWI-EAS443:3:21:1067:1272#0/1
`¥¥U_Xabaa`]]b__a`a]aYaY`[a[]_a__R^¥LQS`
@HWI-EAS443:3:21:1088:1073#0/1
GGGGCATGGAAATGAGGCGCTGCCTTCTTCAGTCTCTTCA
+HWI-EAS443:3:21:1088:1073#0/1
a_R`_Za^_S]Y]`]]Va[¥Va___Z[¥^X^[PZ`ZZ_¥Y
@HWI-EAS443:3:21:1092:1793#0/1
CGATTTGTTTTCAAAGAATTCCTTGGTCATGTCTTTCTCT
+HWI-EAS443:3:21:1092:1793#0/1
H[b_¥aaVXb_``_[_babb^ba`baa_baba^babab_a
@HWI-EAS443:3:21:1179:477#0/1
GCTGCATTGAGGGCAAGCTTTCCCAAATGTCAAAAGAAGT
+HWI-EAS443:3:21:1179:477#0/1
aaaa^aaa_`a`a]`aa_``a_`_`__^`Z¥`__``^__Z
@HWI-EAS443:3:21:1181:1811#0/1
CAAATGTCAAAAGAAGTGAACGCCAAAATTGAACCATTCT
+HWI-EAS443:3:21:1181:1811#0/1
aaa_YWYVQ^V_]`_[RX^aYaIDV¥QUX`[¥`XZ¥]XG[
@HWI-EAS443:3:21:1189:1825#0/1
ATAAATACGGCCTTGCTCAATGCATCCTGTGCAGCCATGG
+HWI-EAS443:3:21:1189:1825#0/1
a_]a^_a``aa_^_YY_[a[OMW_YPZ]^U]VR_WT_]]U
@HWI-EAS443:3:21:1213:1626#0/1
AATTGAAGGAAGAGACCGAATCATGGCCTGGACAGTGGTG
+HWI-EAS443:3:21:1213:1626#0/1
UUZ]UXX[[W[``_`_V^]VQ¥]P[SGVWTSNX_`ZVYYB
@HWI-EAS443:3:21:1263:1632#0/1
GGCATAAATCCCAATTACCTCATGGCTTGGAAGCAGGTGC
+HWI-EAS443:3:21:1263:1632#0/1
a][VXWZ_YPS`XY_^`]LP¥[¥a^BBBBBBBBBBBBBBB
@HWI-EAS443:3:21:1263:708#0/1
TGCCCACTTCAATTGGCTTGTTCTCTTCATGTTCTTTGTC
+HWI-EAS443:3:21:1263:708#0/1
aba__aaa`¥_aaa`Y___[Y_O_U`_UUZZY`M¥``¥WS
@HWI-EAS443:3:21:1278:938#0/1
CTCGATTGCTTCATATAGCCCCCCAAGATCGAAGGTTCCA
+HWI-EAS443:3:21:1278:938#0/1
abaaaaaaabbaabaaaaa`a`aZa_a__`a`V`YN`Y¥¥
@HWI-EAS443:3:21:1303:1133#0/1
TCCGATTGGCCATGTTTCCGATTTGTTTTCAAAGAATTCC
+HWI-EAS443:3:21:1303:1133#0/1
aaaa_aaaa_`_`a^aa^__Z^a``^_aaX_a__^]^`SV
@HWI-EAS443:3:21:1324:534#0/1
GACATTCTTTGGCTGGAAAGAGCCTAACATAGTCAAACCA
+HWI-EAS443:3:21:1324:534#0/1
aa[_[a`a`_a``aaa^a_Y__[_^^_[]]__]^^`_XU]
```

```
@HWI-EAS443:3:21:136:1143#0/1
CATAAATACGGCCTTGCTCAATGCATCCTGTGCAGCCATG
+HWI-EAS443:3:21:136:1143#0/1
aba`S]`X_a[T`[^[]^`a¥[[^]T^a]TTS¥^^_^[U^
@HWI-EAS443:3:21:1424:491#0/1
CACCGATTTGAGATAATTGAAGGAAGAGACCGAATCATGG
+HWI-EAS443:3:21:1424:491#0/1
aa`ab`bbbb_a`aa`abb`aaa_^a``_^]a_^a[_a`_
@HWI-EAS443:3:21:1454:1627#0/1
CAGAAAAAGTAGACTTTGATGACTGCAAAGATGTTGGAGA
+HWI-EAS443:3:21:1454:1627#0/1
Y_^XM¥__`Tb^VOQ]W``^aX_¥aa`[]^_a_NP``[``
@HWI-EAS443:3:21:1482:1744#0/1
TCGGGTCAGTGAGTGAGAACTCCATACTTACAAAGTTCAC
+HWI-EAS443:3:21:1482:1744#0/1
_a¥^[U^[_W¥¥_Qa`¥¥^]Z^aa]][`a`O`a`_`]Y]Z
@HWI-EAS443:3:21:1483:277#0/1
AGCAGGTGCTAGCAGAGCTACAGGACATTGAAAATGAAGA
+HWI-EAS443:3:21:1483:277#0/1
`WW^[^V]`ZV]]]_UW[YXYX_YVXVWQ]YVYVY[YYVX
@HWI-EAS443:3:21:1513:819#0/1
GCCTATGTAGATGGATTCGAGCCGAACGGCTGCATTGAGG
+HWI-EAS443:3:21:1513:819#0/1
a^_aa`^`aaaaa`^aa[]```U¥V]U_UUZSTZS¥BBBB
@HWI-EAS443:3:21:1517:1399#0/1
GAAAGAGGCGAAGAGACAATTGAAGAAAAATTTGAGATTA
+HWI-EAS443:3:21:1517:1399#0/1
aaa]a_baa`aaa`aSZ_a`aa]___`]__aaa_]a[___
@HWI-EAS443:3:21:1539:1783#0/1
AAGAATTCCTTGGTCATGTCTTTCTCTTTGACAGAAGACT
+HWI-EAS443:3:21:1539:1783#0/1
[XaYZ`XZ`]aa`_`K`aa^_`_P[^a`]a_^aa`_]_VP
@HWI-EAS443:3:21:1562:586#0/1
TGGGCCTCGACACTTGGCCTATCGCAGTCCTCAAGAGCAT
+HWI-EAS443:3:21:1562:586#0/1
^T]Z`aaJ^^`X`¥abaa__aX]_¥`aaUX_Y[WaZ^SY`
@HWI-EAS443:3:21:1658:619#0/1
GGGGGCTATATGAAGCAATCGAGGAGTGCCTGATTAATGA
+HWI-EAS443:3:21:1658:619#0/1
aa`Y`_`_aaaa__`]a`_Ua_a`U`VTVU]`X]^XXXYY
@HWI-EAS443:3:21:166:1027#0/1
GGGCATGGAAATGAGGCGCTGCCTTCTTCAGTCTCTTCAG
+HWI-EAS443:3:21:166:1027#0/1
abaabab`_b`aaa__a`aaaa]¥`[a__^a__a``a]`Y
@HWI-EAS443:3:21:1733:1583#0/1
GAAAGGCATAAATCCCAATTACCTCATGGCTTGGAAGCAG
+HWI-EAS443:3:21:1733:1583#0/1
a`aa]ba_aaa`a[^_a_a``Wa]G`TQ[¥a_S¥NXQTSB
@HWI-EAS443:3:21:1774:1355#0/1
ATTTTGGACCCAGCTTGCTAGAGATCTGGGCTCTGGCTCA
```

```
+HWI-EAS443:3:21:1774:1355#0/1
]b`b`T[`aVa`R[a^^_aaaa`X__Z[T[N___a[Q`¥U
@HWI-EAS443:3:21:201:568#0/1
ATCTTGATCTTGGAGGTTCCATTGGTTCTCACATATAGGA
+HWI-EAS443:3:21:201:568#0/1
aaaaab`abaa]]aaa_`aa_`¥¥VN`]``__]`QX¥GZ¥
@HWI-EAS443:3:21:205:749#0/1
CGACGAACGGGGTGAATCAATAATTGTAGAATCTGGTGAC
+HWI-EAS443:3:21:205:749#0/1
abb``Q]^¥aabYaY_]_a`a`Z``aV¥`[a_^^aWV^__
@HWI-EAS443:3:21:21:1173#0/1
AATTGAAGGAAGAGACCGAATCATGGCCTGGACAGTGGTG
+HWI-EAS443:3:21:21:1173#0/1
^`abba^`aa``aaaaaba`aaaaa`a^a`a`a``^a`[¥
@HWI-EAS443:3:21:222:1633#0/1
CCTCGATTGCTTCATATAGCCCCCCAAGATCGAAGGTTCC
+HWI-EAS443:3:21:222:1633#0/1
``_^_a[¥¥¥Z^Z]XZ^a^Z¥VZa_SNZXWSYSTQNS¥NU
@HWI-EAS443:3:21:223:1243#0/1
GGCTGAATCGAGAAAATTGCTTCTCATTGTTCAGGCACTT
+HWI-EAS443:3:21:223:1243#0/1
`Sa`baaSa_]]_]¥]^^_a__]_O_^^XY][]`X^_[G^
@HWI-EAS443:3:21:237:1862#0/1
CATGATTCGGTCTCTTCCTTCAATTATCTCAAATCGGTGC
+HWI-EAS443:3:21:237:1862#0/1
Y`PYY¥ba``]Y`_OI_ba]a_Z^a``¥U__^Z]^YVW^]
@HWI-EAS443:3:21:25:463#0/1
GGGGCATGGAAATGAGGCGCTGCCTTCTTCAGTCTCTTCA
+HWI-EAS443:3:21:25:463#0/1
a_a]a`_a`^_`_`_[^```^`__¥_V`¥¥¥_W¥_^¥¥W¥
@HWI-EAS443:3:21:274:327#0/1
TAATCAGGCACTCCTCGATTGCTTCATATAGCCCCCCAAG
+HWI-EAS443:3:21:274:327#0/1
aabaaaaaa`aaa`a`]¥`_`__^___`_a]_`___YWV_
@HWI-EAS443:3:21:329:184#0/1
ACCCGAGTCACGAGGGGGAGGGAATACCACTATATGATGC
+HWI-EAS443:3:21:329:184#0/1
abaaaaa`ba`_Uaaaa`_aaaX`^_]^_¥]`]]^_^]ZW
@HWI-EAS443:3:21:338:1706#0/1
ATGCATCCTGTGCAGCCATGGATGACTTTCAGCTGATCCC
+HWI-EAS443:3:21:338:1706#0/1
a¥^abaa¥^[UMY___S_W][`_`__[__[`^_¥_Y]X^[
@HWI-EAS443:3:21:398:225#0/1
CATGGCCTGGACAGTGGTGAACAGTATATGTAACACAACA
+HWI-EAS443:3:21:398:225#0/1
[]_bK_abb[[bV[bbaHaa¥Z[bQaa_aaH¥]aaa_]GH
@HWI-EAS443:3:21:399:1778#0/1
AAAGAGCCTAACATAGTCAAACCACATGAGAAAGGCATAA
+HWI-EAS443:3:21:399:1778#0/1
_b¥L[W^ZYLabT^[[T_^[a`[P`_]__W¥``^_SX`aa
```

```
@HWI-EAS443:3:21:415:1141#0/1
CGTCTCCACAACTTGAGGGGTTTTCGGCTGAATCGAGAAA
+HWI-EAS443:3:21:415:1141#0/1
abab`abbbaaaaba]a]_`_aaa`_a_aa`a__a__^aa
@HWI-EAS443:3:21:417:210#0/1
CCTTTCTCATGTGGTTTGACTATGTTAGGCTCTTTCCAGC
+HWI-EAS443:3:21:417:210#0/1
abbbaaabaab_ba__ab_`aa`a_``_][]__a_Y^_^]
@HWI-EAS443:3:21:426:255#0/1
GGAAGAGACCGAATCATGGCCTGGACAGTGGTGAACAGTA
+HWI-EAS443:3:21:426:255#0/1
aa``_aba_]```aaaaaaa]]a_aaa_[aa[a__U_aV`
@HWI-EAS443:3:21:431:347#0/1
AAGAATTCCTTGGTCATGTCTTTCTCTTTGACAGAAGACT
+HWI-EAS443:3:21:431:347#0/1
_[```[_X`aaaa¥[[`]___a^VVR`T¥a^X_aX_X_Y]
@HWI-EAS443:3:21:470:1373#0/1
TTGGGGGGCTATATGAAGCAATCGAGGAGTGCCTGATTAA
+HWI-EAS443:3:21:470:1373#0/1
ZWUTbaaa`_`^¥¥`]Q]T]a_`_^H[aVM^[_a___^Y¥
@HWI-EAS443:3:21:492:322#0/1
CCACTCCCCTGGGTGACTCTCCGATTGGCCATGTTTCCGA
+HWI-EAS443:3:21:492:322#0/1
`]]]¥]_]]NW_aT[VN¥[_Z[U[¥T^XFXUUXRKPQLSM
@HWI-EAS443:3:21:540:32#0/1
CAAAGAGAAAGACATGACCAAGGAATTCTTTGAAAACAAA
+HWI-EAS443:3:21:540:32#0/1
aaaab`b`aaaaa`_aaa```a`___`_]^_a__a_^^__
@HWI-EAS443:3:21:542:580#0/1
GGGGCATGGAAATGAGGCGCTGCCTTCTTCAGTCTCTTCA
+HWI-EAS443:3:21:542:580#0/1
ab^Z_`aabXaaaaaa_]^_aa`_`aaa_aaaSWZ__a_]
@HWI-EAS443:3:21:569:461#0/1
CTTTGGTCGGCAAGCTTGCGCATAGTTCCTGTAATCTCAA
+HWI-EAS443:3:21:569:461#0/1
abbbbb`a__¥_^[¥aaa]][``_^[`^^aa^^]aT^__`
@HWI-EAS443:3:21:705:1766#0/1
CAAGAATCAAAACTAGGCTTTTCACTATAAGACAAGAAAT
+HWI-EAS443:3:21:705:1766#0/1
a`a`___a`^_a¥Y__Z_]]^_^^]]^_[]XZZ_Z]ZZZW
@HWI-EAS443:3:21:711:833#0/1
TGTGAATTGACTGATTCAAGCTGGATAGAACTTGATGAAA
+HWI-EAS443:3:21:711:833#0/1
aaabbbbb_aabbabaa`a`aaaaaaa_^`_aaa__a__`
@HWI-EAS443:3:21:736:682#0/1
CCCTGTTGTGTTACATATACTGTTCACCACTGTCCAGGCC
+HWI-EAS443:3:21:736:682#0/1
aa`¥`YY_¥]^``_a_``___]Z^V_X]__^WT]W__UT^
@HWI-EAS443:3:21:749:1462#0/1
CATGTTTCCGATTTGTTTTCAAAGAATTCCTTGGTCATGT
```

```
+HWI-EAS443:3:21:749:1462#0/1
aaaa¥a__baaaaaa]aaaaa`[``_a^^^a___[[_]`¥
@HWI-EAS443:3:21:753:192#0/1
GCGCATAGTTCCTGTAATCTCAAATTTTTCTTCAATTGTC
+HWI-EAS443:3:21:753:192#0/1
`abbbbba_bbabb_bababbb__bbbbb_bbabbbab`a
@HWI-EAS443:3:21:765:899#0/1
TAATCAGGCACTCCTCGATTGCTTCATATAGCCCCCCAAG
+HWI-EAS443:3:21:765:899#0/1
``aa`aaab]aZaaa_a``a`^`a^^a`__a_[__[__X]
@HWI-EAS443:3:21:773:855#0/1
GGTGAACTTTGTAAGTATGGAGTTCTCACTCACTGACCCG
+HWI-EAS443:3:21:773:855#0/1
a`L¥_]_a`_`W``¥RUVaa¥`_`____]_Z__^`_¥[W^
@HWI-EAS443:3:21:787:631#0/1
CGGCTGCATTGAGGGCAAGCTTTCCCAAATGTCAAAAGAA
+HWI-EAS443:3:21:787:631#0/1
a]V`_aaa_^X^`_b^]X¥Z[a^Z^]`^a``Y]`a]`^_a
@HWI-EAS443:3:21:794:1214#0/1
TTCGCCTCTTTCGGACTGACGAAAGGAATCCCATAGACTC
+HWI-EAS443:3:21:794:1214#0/1
aa^aa`aaaaaa`[`aa```a^^aa__a^U`_a__`_a__
@HWI-EAS443:3:21:796:477#0/1
AAGAGACCGAATCATGGCCTGGACAGTGGTGAACAGTATA
+HWI-EAS443:3:21:796:477#0/1
aba`a`_`a``__`^_Z]^_[_]^]]U[_X_X]R^_UU^]
@HWI-EAS443:3:21:815:1228#0/1
CGGCTGAATCGAGAAAATTGCTTCTCATTGTTCAGGCACT
+HWI-EAS443:3:21:815:1228#0/1
aa`baabb`aa`^`^^_`a`_```^^^`_Z^`]^__^^Y]
@HWI-EAS443:3:21:835:1941#0/1
GCTTGGAAGCAGGTGCTAGCAGAGCTACAGGACATTGAAA
+HWI-EAS443:3:21:835:1941#0/1
abaaa]a`a^a_]X]aaa[¥a_]^`_a_`_^_^_`_¥__`
@HWI-EAS443:3:21:88:1380#0/1
ATAAATACGGCCTTGCTCAATGCATCCTGTGCAGCCATGG
+HWI-EAS443:3:21:88:1380#0/1
a`aaaaaa]T]aa_a`_a^¥^`aa``_YV^Z_ZW`Y]^_^
@HWI-EAS443:3:21:893:638#0/1
ACGGGGTGAATCAATAATTGTAGAATCTGGTGACCCGAAT
+HWI-EAS443:3:21:893:638#0/1
aaZ_abX_]]a^`]`_`aab_aaY_[_`_TTa`TZ`ZSZ`
@HWI-EAS443:3:21:947:1722#0/1
GGGGCATGGAAATGAGGCGCTGCCTTCTTCAGTCTCTTCA
+HWI-EAS443:3:21:947:1722#0/1
_]Z`aa`_aaaaaa`][a`a_`_¥[a¥_`[a^_¥`__`¥_
@HWI-EAS443:3:21:997:131#0/1
GTATTTTTCCCATTTGTGTGGCTCCAGTCTCGGGTCAGTG
+HWI-EAS443:3:21:997:131#0/1
a_J]a¥```¥^¥__^YNZQYZ¥[WBBBBBBBBBBBBBBBB
```

@HWI-EAS443:3:22:1025:493#0/1
CAAGAATCAAAACTAGGCTTTTCACTATAAGACAAGAAAT
+HWI-EAS443:3:22:1025:493#0/1
__`a¥`U`b`a`SS_aa_bbbabaabbb]``_¥b_Y¥```
@HWI-EAS443:3:22:1065:377#0/1
CTCGGTGAAAATATGGCACCAGAAAAAGTAGACTTTGATG
+HWI-EAS443:3:22:1065:377#0/1
a`_aa_a_aaaaaaaaaa__aa_aa`^`V`aa]]`_a_`a
@HWI-EAS443:3:22:1079:566#0/1
ACCGGTTCTCTTTGTAATCATACAAATCAGGAAGAAATTT
+HWI-EAS443:3:22:1079:566#0/1
a_`aa^aaaaa`_a[_`a¥``^_a^`^Y_a_][_[_]_^^
@HWI-EAS443:3:22:115:1717#0/1
ATACAAATCAGGAAGAAATTTAGGCTTCTCTACCCCTGTT
+HWI-EAS443:3:22:115:1717#0/1
aaaabaa`aa_]^``_a__^_a^]a`^`^`^_^`a_]]X]
@HWI-EAS443:3:22:116:549#0/1
CTTGCGCATAGTTCCTGTAATCTCAAATTTTTCTTCAATT
+HWI-EAS443:3:22:116:549#0/1
a_`aaa_aa¥Y[[Z`__¥TW`a``V_``]`_`[`_]_]__
@HWI-EAS443:3:22:1172:1853#0/1
GGTGAATCAATAATTGTAGAATCTGGTGACCCGAATGCAC
+HWI-EAS443:3:22:1172:1853#0/1
aaZ```a``_`WS_a_WWa`a`[]^^Xa`_`Z`¥Y]`_^^
@HWI-EAS443:3:22:1185:110#0/1
GAAAGGCATAAATCCCAATTACCTCATGGCTTGGAAGCAG
+HWI-EAS443:3:22:1185:110#0/1
aa`__^_aa[__aQTZ_¥G`_[WYWY¥aWW]WQ[NUYQTB
@HWI-EAS443:3:22:1217:427#0/1
CTTGGTCATGTCTTTCTCTTTGACAGAAGACTCGGCCTCA
+HWI-EAS443:3:22:1217:427#0/1
ab_bba`baaaaabbbabbaab_abbbaaa^b[bb]_a_b
@HWI-EAS443:3:22:121:1134#0/1
TGTCCTTGGGATCTTCTCTTCATTTTCAATGTCCTGTAGC
+HWI-EAS443:3:22:121:1134#0/1
aaP`V_aa`[_`_aaaabaaa_a``_aaa_`]aa`_^`YZ
@HWI-EAS443:3:22:1248:1015#0/1
GAAATAGGAGACATGCTCTTGAGGACTGCGATAGGCCAAG
+HWI-EAS443:3:22:1248:1015#0/1
aa`]aaaaa``¥^``a`^``a`aa^Z^^Va^__^[[VZ[^
@HWI-EAS443:3:22:1283:1713#0/1
CAGTATTTTTCCCATTTGTGTGGCTCCAGTCTCGGGTCAG
+HWI-EAS443:3:22:1283:1713#0/1
aa]Q]aaaaaaa__aaaa]a^aR]a_^[^W[WU[_]LY]V
@HWI-EAS443:3:22:133:1696#0/1
GGAAGAAATTTAGGCTTCTCTACCCCTGTTGTGTTACATA
+HWI-EAS443:3:22:133:1696#0/1
a`bbbbabaaaaV^baa`abababbbUVZ__[aa_T_aaa
@HWI-EAS443:3:22:1367:903#0/1
CAGCTTGCTAGAGATCTGGGCTCTGGCTCATCACTGTCAT

+HWI-EAS443:3:22:1367:903#0/1
_ab_bbaabaaaaaa_ab`aXa]^a`_a^aa[a_`a^_`_
@HWI-EAS443:3:22:1385:1249#0/1
AGGAGAAGATGTTGCCCCGATTGAACATATCGCAAGCATG
+HWI-EAS443:3:22:1385:1249#0/1
^bbWba``[]baaaaaJ[]NWaPU`a``____XW_aW[Ua
@HWI-EAS443:3:22:138:1965#0/1
GGGGTTTTCGGCTGAATCGAGAAAATTGCTTCTCATTGTT
+HWI-EAS443:3:22:138:1965#0/1
aaa^]bbaba_`aabbab`bab^aaaa]aa_aaa`_aVQV
@HWI-EAS443:3:22:141:289#0/1
ATTCCCTCCCCCTCGTGACTCGGGTCTTCAATACTTAATT
+HWI-EAS443:3:22:141:289#0/1
a__abbaab`aa`aa_]Y¥aa_Z^T^`_`Z_^VW¥_`G^_
@HWI-EAS443:3:22:1449:205#0/1
GAGAAGATCCCAAGGACAAAGAACATGAAGAGAACAAGCC
+HWI-EAS443:3:22:1449:205#0/1
a^]^aa^RPT¥P^_¥Y[U__[^RPY__^^[^a_WSZ]`LH
@HWI-EAS443:3:22:1475:484#0/1
TACATAAATACGGCCTTGCTCAATGCATCCTGTGCAGCCA
+HWI-EAS443:3:22:1475:484#0/1
aa_``]`baa`___Y_^a__]_]ZWZ__ST_`WYV]`¥ZZ
@HWI-EAS443:3:22:1568:1452#0/1
TTTCAAAGAATTCCTTGGTCATGTCTTTCTCTTTGACAGA
+HWI-EAS443:3:22:1568:1452#0/1
ab^_`a```Va``_baab___a^^_aaaO_^aaa`¥YQ__
@HWI-EAS443:3:22:1602:528#0/1
GTTACATATACTGTTCACCACTGTCCAGGCCATGATTCGG
+HWI-EAS443:3:22:1602:528#0/1
a_^U^aa_a`¥`a[__`^W¥Va^W¥Z][UUV]Z_Z]¥YZR
@HWI-EAS443:3:22:1609:28#0/1
GCAGGTGCTAGCAGAGCTACAGGACATTGAAAATGAAGAG
+HWI-EAS443:3:22:1609:28#0/1
aZ_aaM[PaX]YXa]_QXWNVa[[VS]__YZ]XYUZ]_^^
@HWI-EAS443:3:22:1613:1740#0/1
CCTCATGCTTGCGATATGTTCAATCGGGGCAACATCTTCT
+HWI-EAS443:3:22:1613:1740#0/1
`]YH_b_H^a``X^¥b```]U`^W``b`^LT[WW`[Wa]Z
@HWI-EAS443:3:22:1615:1885#0/1
GAAAGGCATAAATCCCAATTACCTCATGGCTTGGAAGCAG
+HWI-EAS443:3:22:1615:1885#0/1
¥¥¥U^a_b`_`bbb¥WVbbbb`aa`b]]a``_NSOZX[BB
@HWI-EAS443:3:22:1670:1498#0/1
CGATTGGCCATGTTTCCGATTTGTTTTCAAAGAATTCCTT
+HWI-EAS443:3:22:1670:1498#0/1
`^M^b`Za_T^^^bb[X¥S¥`YZaab_¥[aaba`ZaX^aa
@HWI-EAS443:3:22:183:1115#0/1
GGCATGGAAATGAGGCGCTGCCTTCTTCAGTCTCTTCAGC
+HWI-EAS443:3:22:183:1115#0/1
¥`baa_`aaabab`aa]bb`ab`aaa_aaa`aabaaaa_a

```
@HWI-EAS443:3:22:184:1387#0/1
ATCTTTGCAGTCATCAAAGTCTACTTTTTCTGGTGCCATA
+HWI-EAS443:3:22:184:1387#0/1
aa`a``aaa__aaa__[_a^a``_`_```___¥W___`__
@HWI-EAS443:3:22:184:560#0/1
CATAAATCCCAATTACCTCATGGCTTGGAAGCAGGTGCTA
+HWI-EAS443:3:22:184:560#0/1
ab`a`a`ab`Z]`aba`aaa]aZYaa[XYZVTYSTHV]¥P
@HWI-EAS443:3:22:197:75#0/1
GTTTTCAAAGAATTCCTTGGTCATGTCTTTCTCTTTGACA
+HWI-EAS443:3:22:197:75#0/1
a__^a_a^__OP]__X_XZROPZYVMW]^UX]^_Q][XXV
@HWI-EAS443:3:22:249:1549#0/1
GCTTGCTAGAGATCTGGGCTCTGGCTCATCACTGTCATAC
+HWI-EAS443:3:22:249:1549#0/1
a`]`I`_¥W¥_`]OXWLX`ZZ``TXNR^¥V_`[`U]Z]^[
@HWI-EAS443:3:22:286:1169#0/1
GGGGCTATATGAAGCAATCGAGGAGTGCCTGATTAATGAT
+HWI-EAS443:3:22:286:1169#0/1
aaa_aaba^a``aaabaa_a_aa`a^aa]aa_aa`a`aa`
@HWI-EAS443:3:22:323:821#0/1
CATGGGCCTCGACACTTGGCCTATCGCAGTCCTCAAGAGC
+HWI-EAS443:3:22:323:821#0/1
aaaaa_aaa¥[V`aa``__a_^a_L]``_T____¥_`]]Z
@HWI-EAS443:3:22:339:682#0/1
AAGAGCCTAACATAGTCAAACCACATGAGAAAGGCATAAA
+HWI-EAS443:3:22:339:682#0/1
`a_Y^`aaaaXaaaa_aaaa``a¥a`a`_`a¥__^a`a`a
@HWI-EAS443:3:22:341:104#0/1
GGCCTGGACAGTGGTGAACAGTATATGTAACACAACAGGG
+HWI-EAS443:3:22:341:104#0/1
abaaaaa`_aa¥]a^a_a_aa^aa`aa__a^`¥``__`¥a
@HWI-EAS443:3:22:368:1256#0/1
TTGGGGGGCTATATGAAGCAATCGAGGAGTGCCTGATTAA
+HWI-EAS443:3:22:368:1256#0/1
a¥¥_^_^`a`babaa]]Zaaaaaa_a__a]^`_aa_``_X
@HWI-EAS443:3:22:396:1546#0/1
CGAAAATCGAAACTAACAAGTTTGCTGCAATATGCACACA
+HWI-EAS443:3:22:396:1546#0/1
a^ZZaVOZZVU][T]FWaRWW¥SXXTX_X``]QLZ__^^_
@HWI-EAS443:3:22:458:1570#0/1
GCCATATTTTCACCGAGTGCCCACTTCAATTGGCTTGTTC
+HWI-EAS443:3:22:458:1570#0/1
a``¥_aV`aabba]aa[_a^Z``_`Ya`QS`Y__¥YRRWV
@HWI-EAS443:3:22:511:195#0/1
CGCATAGTTCCTGTAATCTCAAATTTTTCTTCAATTGTCT
+HWI-EAS443:3:22:511:195#0/1
_ab^b]_]_Z[a_ZVU^_`Y]_XX_]`aZ^__`]^^`ZT¥
@HWI-EAS443:3:22:519:1241#0/1
AATTCCTTGGTCATGTCTTTCTCTTTGACAGAAGACTCGG
```

```
+HWI-EAS443:3:22:519:1241#0/1
aaa^T¥`YbaZa_baXaaaaa`_a`___X_`aa_`__[^_
@HWI-EAS443:3:22:525:124#0/1
AGTGTACATAAATACGGCCTTGCTCAATGCATCCTGTGCA
+HWI-EAS443:3:22:525:124#0/1
_[MZaba¥]WK`bb¥_¥`Y¥QX^VUVYV^YQPVJQ¥FZ^[
@HWI-EAS443:3:22:552:188#0/1
CTCCTCGATTGCTTCATATAGCCCCCCAAGATCGAAGGTT
+HWI-EAS443:3:22:552:188#0/1
aa¥aSY]UXUaZ`ZRS¥Y[YVZY_SaZ`^S¥¥UQUBBBBB
@HWI-EAS443:3:22:609:1730#0/1
GGGATCTTCTCTTCATTTTCAATGTCCTGTAGCTCTGCTA
+HWI-EAS443:3:22:609:1730#0/1
`b`ababbbbbbbbbbbbbbbba_bbb``baa_b`aa`a
@HWI-EAS443:3:22:628:1057#0/1
CGAGTCACGAGGGGGAGGGAATACCACTATATGATGCAAT
+HWI-EAS443:3:22:628:1057#0/1
abbb`ababa_`aaa]bab`]`a_abaaaaa___`a`a]_
@HWI-EAS443:3:22:636:424#0/1
CTGGCTCATCACTGTCATACTGTTTAAGGTCTCCAACATC
+HWI-EAS443:3:22:636:424#0/1
abbababbbabaab`aaaa`ab_aaaaaa]`a_aaa`aa^
@HWI-EAS443:3:22:688:793#0/1
CTTGTCTTATAGTGAAAAGCCTAGTTTTGATTCTTGCCCT
+HWI-EAS443:3:22:688:793#0/1
aa`_R]a__SYaW^`_a`a¥_][_V^]`_]``]^ZSL_^X
@HWI-EAS443:3:22:757:203#0/1
GCCATGTTTCCGATTTGTTTTCAAAGAATTCCTTGGTCAT
+HWI-EAS443:3:22:757:203#0/1
aaa`aaXaaaaaa`__`S`[YY`aa]a^__`^]_a_Y^_^
@HWI-EAS443:3:22:757:345#0/1
GAATCAATAATTGTAGAATCTGGTGACCCGAATGCACTAT
+HWI-EAS443:3:22:757:345#0/1
T[ZUZ¥`¥`Y`a[__baababb_`^aa_aVbabaSa_`a_
@HWI-EAS443:3:22:770:924#0/1
CACCAGAAAAAGTAGACTTTGATGACTGCAAAGATGTTGG
+HWI-EAS443:3:22:770:924#0/1
^bbabbb]^b`babbb^babbaabaaba_bba`^ab¥^`a
@HWI-EAS443:3:22:795:1345#0/1
ATTGCAGCAAACTTGTTAGTTTCGATTTTCGGATCTTCCC
+HWI-EAS443:3:22:795:1345#0/1
a``_aa_ab_`````^^`_[__]_VW]¥^Y]NLO[^YY[W
@HWI-EAS443:3:22:799:95#0/1
TCATTATGTATTCAGTAGCCCTGCAGTGGGACACTTCTGC
+HWI-EAS443:3:22:799:95#0/1
aZX_aS[a^aaa`_P_YX^^¥_`_^_Ga]_XZ^VXV^^ZV
@HWI-EAS443:3:22:824:1932#0/1
GGGGAGTGGAGGAAGGCTCTATTGGGAAAGTGTGCAGGAC
+HWI-EAS443:3:22:824:1932#0/1
aaaaabZb^a`a`aa`aaa`a`a^a^_``^QKLX^^__^¥
```

@HWI-EAS443:3:22:882:1688#0/1
GGGGCATGGAAATGAGGCGCTGCCTTCTTCAGTCTCTTCA
+HWI-EAS443:3:22:882:1688#0/1
a_]`[`¥^]]baSab`VaabW_¥a^`¥¥_¥b]VX^a[^_]
@HWI-EAS443:3:22:939:1038#0/1
TATAGCCCCCCAAGATCGAAGGTTCCAGGTTCCAGGTTGT
+HWI-EAS443:3:22:939:1038#0/1
abab`]aaa^`aa]aa`]V]`aV`Z^]¥Z¥¥PTZZRJ[[^
@HWI-EAS443:3:23:1135:741#0/1
GGGGGCTATATGAAGCAATCGAGGAGTGCCTGATTAATGA
+HWI-EAS443:3:23:1135:741#0/1
abbbabbaaaaaa]aaaba_a_a]^a_`_``a^Za```a`
@HWI-EAS443:3:23:1139:933#0/1
GGGGCATGGAAATGAGGCGCTGCCTTCTTCAGTCTCTTCA
+HWI-EAS443:3:23:1139:933#0/1
ababbbbba_a`ab`aaabaaa_`aa]aa`aa__a``a^^
@HWI-EAS443:3:23:1145:1021#0/1
AACTTGATGAAATAGGAGAAGATGTTGCCCCGATTGAACA
+HWI-EAS443:3:23:1145:1021#0/1
Q_bb`¥Y``¥^^bababbabaaaa_ab`Y```aaaaaa^b
@HWI-EAS443:3:23:1157:238#0/1
GGTGAACTTTGTAAGTATGGAGTTCTCACTCACTGACCCG
+HWI-EAS443:3:23:1157:238#0/1
a`aababaababaa_baabaaa`aa]a``aa`aba¥Ya
@HWI-EAS443:3:23:1179:401#0/1
CGAAAATCGAAACTAACAAGTTTGCTGCAATATGCACACA
+HWI-EAS443:3:23:1179:401#0/1
aababaaaba`a_aaa^ba`[a___aa__aa`a`Ua__]_
@HWI-EAS443:3:23:1220:286#0/1
GTTTGTTTCATGTATTCGGATTTCCATTTCATCGACGAAC
+HWI-EAS443:3:23:1220:286#0/1
a_aaa¥`a_``]X`___aa_^_`X[^_^`^_^]_^Z^ZWU
@HWI-EAS443:3:23:1359:369#0/1
GAGATGGCCACCAAAGCGGACTACACCCTTGACGAAGAGA
+HWI-EAS443:3:23:1359:369#0/1
`bbabbba_a]Qabaa]baaUaaWaU[¥__^^W`¥_aZ__
@HWI-EAS443:3:23:1369:1429#0/1
GGGCCTCGACACTTGGCCTATCGCAGTCCTCAAGAGCATG
+HWI-EAS443:3:23:1369:1429#0/1
Zaabaabbbab`bb`b^baaaa^ZZY_`¥a¥¥YY¥Za^YV
@HWI-EAS443:3:23:1371:1792#0/1
CAGTCATCAAAGTCTACTTTTTCTGGTGCCATATTTTCAC
+HWI-EAS443:3:23:1371:1792#0/1
abb^¥b````^`_`^a_bbaa`a___Waa`a_aaaaa_`^
@HWI-EAS443:3:23:1388:40#0/1
AATAGGAGAAGATGTTGCCCCGATTGAACATATCGCAAGC
+HWI-EAS443:3:23:1388:40#0/1
a¥b[`a]_Ya`Z^`P[a¥U^Z`]VTUYaW_[XYSPWHWQU
@HWI-EAS443:3:23:1433:1173#0/1
GGGGTTTTCGGCTGAATCGAGAAAATTGCTTCTCATTGTT

+HWI-EAS443:3:23:1433:1173#0/1
abab^abaaa``abaaa`a_a^____a_Zaa[`^_`a_V`
@HWI-EAS443:3:23:1498:1297#0/1
TACATATACTGTTCACCACTGTCCAGGCCATGATTCGGTC
+HWI-EAS443:3:23:1498:1297#0/1
^__babb_aab`b]aa_aaaa_WK`[`__aYPX^_S^^[Z
@HWI-EAS443:3:23:1534:1616#0/1
CATAAATCCCAATTACCTCATGGCTTGGAAGCAGGTGCTA
+HWI-EAS443:3:23:1534:1616#0/1
abbbbbba¥T`b`ab_a]aa`a_^baLYQaZYYQ^G]XY_
@HWI-EAS443:3:23:1541:913#0/1
CTTGGGATCTTCTCTTCATTTTCAATGTCCTGTAGCTCTG
+HWI-EAS443:3:23:1541:913#0/1
abbbbbW¥abbbbbbbaaab_baaa`aaa_`ba`aa`_Z`
@HWI-EAS443:3:23:1593:1911#0/1
CATGGAAATGAGGCGCTGCCTTCTTCAGTCTCTTCAGCAG
+HWI-EAS443:3:23:1593:1911#0/1
aab¥babbbVbV]_QQVaSaa[Ubb_b`]^¥[]a`aaX[]
@HWI-EAS443:3:23:1626:1033#0/1
GGGGGCTATATGAAGCAATCGAGGAGTGCCTGATTAATGA
+HWI-EAS443:3:23:1626:1033#0/1
Yabb`[_b¥^baWa]]]_]N]P_S[_[aVK`a¥ZVSY^`^
@HWI-EAS443:3:23:168:1675#0/1
AACGGGGTGAATCAATAATTGTAGAATCTGGTGACCCGAA
+HWI-EAS443:3:23:168:1675#0/1
a`ZTHZSZa]OVbaa^]aaRaRaa`a_OXXRND``V]]_Z
@HWI-EAS443:3:23:16:1556#0/1
GCCTAACATAGTCAAACCACATGAGAAAGGCATAAATCCC
+HWI-EAS443:3:23:16:1556#0/1
abaaaaabaaV¥a^^_``aa``^^[_]__¥^a`aa_a_¥_
@HWI-EAS443:3:23:1710:1979#0/1
GAACTATTTTACAGCAGAAGTGTCCCACTGCAGGGCTACT
+HWI-EAS443:3:23:1710:1979#0/1
aabbabbbbbb`bbab]bb_]_^a[`aa¥a`aaa^^a_X`
@HWI-EAS443:3:23:1774:924#0/1
GATATGTTCAATCGGGGCAACATCTTCTCCTATTTCATCA
+HWI-EAS443:3:23:1774:924#0/1
aaa_abY^LP`YY`SX__]ZV¥XRX^ZWQV¥^¥^_]]]S]
@HWI-EAS443:3:23:212:1178#0/1
CTTGGGATCTTCTCTTCATTTTCAATGTCCTGTAGCTCTG
+HWI-EAS443:3:23:212:1178#0/1
aabbabTaabbaabaababbabaaaaa`aaa__a_a_aV[
@HWI-EAS443:3:23:222:264#0/1
CTCGATTGCTTCATATAGCCCCCCAAGATCGAAGGTTCCA
+HWI-EAS443:3:23:222:264#0/1
`__Z`baa`a`bbbabbb__T_a_`aaa`aa]]_¥V]]ZX
@HWI-EAS443:3:23:294:617#0/1
CACCGATTTGAGATAATTGAAGGAAGAGACCGAATCATGG
+HWI-EAS443:3:23:294:617#0/1
ababb`ababaa`aaaa^aa_a[`a__``a_`a_¥]_`_Z

```
@HWI-EAS443:3:23:29:99#0/1
CGATTGGCCATGTTTCCGATTTGTTTTCAAAGAATTCCTT
+HWI-EAS443:3:23:29:99#0/1
abb`XaU``VYaa¥a_¥J]aa`Vaaab]^Y_^O¥aa[T^^
@HWI-EAS443:3:23:310:1148#0/1
GAAAATGAAGAGAAGATCCCAAGGACAAAGAACATGAAGA
+HWI-EAS443:3:23:310:1148#0/1
^^aa^¥W]``[``^`a`Yba^R¥SV^aaYa`__aaa^`_]
@HWI-EAS443:3:23:322:912#0/1
CATGTTTCCGATTTGTTTTCAAAGAATTCCTTGGTCATGT
+HWI-EAS443:3:23:322:912#0/1
aaaa_aaaaa__aaa^aa^_UYa_^]aXZ_``__Z]__a]
@HWI-EAS443:3:23:324:1527#0/1
CATTCTTTGGCTGGAAAGAGCCTAACATAGTCAAACCACA
+HWI-EAS443:3:23:324:1527#0/1
ab]`abaa_¥X`baaab`V^a__^^aa_baZ]Y`MY`aS]
@HWI-EAS443:3:23:326:453#0/1
CCCAGATCTCTAGCAAGCTGGGTCCAAAATGAATTCAATA
+HWI-EAS443:3:23:326:453#0/1
abbaaaaa_aaaaaa`aa``a`X`a`a`Y`a`_`]^`_^a
@HWI-EAS443:3:23:400:310#0/1
GTGAACTTTGTAAGTATGGAGTTCTCACTCACTGACCCGA
+HWI-EAS443:3:23:400:310#0/1
a[_ZNQ^P]VJUTVPUXP¥UVX_`VNV¥Y]^WZ^Z^¥VUW
@HWI-EAS443:3:23:401:741#0/1
GGAAATCCGAATACATGAAACAAACTTCCAAATGTGTGCA
+HWI-EAS443:3:23:401:741#0/1
aaL__V]Y`a`T]Ybbbbaba`[Ya[]`bY]aa__a`XOX
@HWI-EAS443:3:23:453:1985#0/1
AGGGAATACCACTATATGATGCAATCAAATGCATGAAGAC
+HWI-EAS443:3:23:453:1985#0/1
a]^[X__aabaaYa`bXaa`_a`aa`aaa^Za`_^aa^`_
@HWI-EAS443:3:23:467:323#0/1
AGATCTCTAGCAAGCTGGGTCCAAAATGAATTCAATAAGG
+HWI-EAS443:3:23:467:323#0/1
abaaaaaaa]a``b_a`aa]_aaa`a_`]^`Y_a_`_^_^
@HWI-EAS443:3:23:500:919#0/1
CTTTCCCAATAGAGCCTTCCTCCACTCCCCTGGGTGACTC
+HWI-EAS443:3:23:500:919#0/1
abbbbabbabb`a]Zbaa]]]a`Q`^`a^_^`a_S]`_`_
@HWI-EAS443:3:23:508:1787#0/1
GCCGAACGGCTGCATTGAGGGCAAGCTTTCCCAAATGTCA
+HWI-EAS443:3:23:508:1787#0/1
]aabbbab[baaaba`K`T__abbb[aaaa^¥L`bYU[aa
@HWI-EAS443:3:23:580:1672#0/1
CGGCTGAATCGAGAAAATTGCTTCTCATTGTTCAGGCACT
+HWI-EAS443:3:23:580:1672#0/1
`__[b]baaNFU[`aabaX]SVS[a_b`aaN]_]Gab_W_
@HWI-EAS443:3:23:588:143#0/1
AATGAATTCAATAAGGCATGTGAATTGACTGATTCAAGCT
```

```
+HWI-EAS443:3:23:588:143#0/1
a¥aaa`````_]U^aPVU¥_]aYU_^[ZLZW[_XY`]VTY
@HWI-EAS443:3:23:608:887#0/1
TGCATGAAGACATTCTTTGGCTGGAAAGAGCCTAACATAG
+HWI-EAS443:3:23:608:887#0/1
VV[Z`^`a`aab_]¥`_`¥YT__QH^¥YT[WMQVZM`_X]
@HWI-EAS443:3:23:627:1857#0/1
CTTGGGGGGCTATATGAAGCAATCGAGGAGTGCCTGATTA
+HWI-EAS443:3:23:627:1857#0/1
abb``aab`aaaabaXaa`_aaa_a_a``a]`a]`aa¥¥a
@HWI-EAS443:3:23:656:1484#0/1
GTTTCCGATTTGTTTTCAAAGAATTCCTTGGTCATGTCTT
+HWI-EAS443:3:23:656:1484#0/1
`aa_b`^^_aaa_abaa`_aa__a`a^aa_a[aa``_^a`
@HWI-EAS443:3:23:657:1109#0/1
TTTTCAAAGAATTCCTTGGTCATGTCTTTCTCTTTGACAG
+HWI-EAS443:3:23:657:1109#0/1
¥b]`a]aa]T]a]`]_ab¥Raaab^^a`_RaY[[_a_]a¥
@HWI-EAS443:3:23:739:1214#0/1
GGGGCTATATGAAGCAATCGAGGAGTGCCTGATTAATGAT
+HWI-EAS443:3:23:739:1214#0/1
abaabababab`aaaa``aa`aa`a]aY_X__`__``_^___
@HWI-EAS443:3:23:835:380#0/1
GGGGCATGGAAATGAGGCGCTGCCTTCTTCAGTCTCTTCA
+HWI-EAS443:3:23:835:380#0/1
aa``_aaba_a``a`^_^a^`a]`_Y_Z^¥¥a¥S_^`¥¥V
@HWI-EAS443:3:23:854:1762#0/1
CCGAAAGAGGCGAAGAGACAATTGAAGAAAAATTTGAGAT
+HWI-EAS443:3:23:854:1762#0/1
ababbababbbbabbbbaabbaaabbbabaaaab`aaaa^
@HWI-EAS443:3:23:86:749#0/1
CGGCTGAATCGAGAAAATTGCTTCTCATTGTTCAGGCACT
+HWI-EAS443:3:23:86:749#0/1
ababa`baaaaabaabaabYa_aa`a]aaa_a`a_`a`__
@HWI-EAS443:3:23:872:1863#0/1
CTTGGGGGGCTATATGAAGCAATCGAGGAGTGCCTGATTA
+HWI-EAS443:3:23:872:1863#0/1
aba`a`[L`bab_`X]aWT`aXaa^^`__]W¥^`]a¥^__
@HWI-EAS443:3:23:899:1364#0/1
ATGGCCAGTAGGAGTCTATGGGATTCCTTTCGTCAGTCCG
+HWI-EAS443:3:23:899:1364#0/1
ab^aa]WUN^aZXWGVaU`[^Xa^VTV```RTQNNWJJZZ
@HWI-EAS443:3:23:942:475#0/1
AGATAATTGAAGGAAGAGACCGAATCATGGCCTGGACAGT
+HWI-EAS443:3:23:942:475#0/1
^bba]abbbbbbababababaaaba_a`aaaaa`aaa]^a_X
@HWI-EAS443:3:24:1035:237#0/1
CGTCTCCACAACTTGAGGGGTTTTCGGCTGAATCGAGAAA
+HWI-EAS443:3:24:1035:237#0/1
ab_Za^W_`a``b_baa]ba_`_`Z`[N[``_____`U^X
```

```
@HWI-EAS443:3:24:1049:482#0/1
GCGATATGTTCAATCGGGGCAACATCTTCTCCTATTTCAT
+HWI-EAS443:3:24:1049:482#0/1
aabaaaab```aaa``_`__^^^_a_aa]a__```a`_]`
@HWI-EAS443:3:24:1072:1484#0/1
GACGAAAGGAATCCCATAGACTCCTACTGGCCATTTCTTG
+HWI-EAS443:3:24:1072:1484#0/1
abab[b`bbb]ba]aabaababab_aaa`baa`T_aX_a]
@HWI-EAS443:3:24:1112:391#0/1
CATTAATCAGGCACTCCTCGATTGCTTCATATAGCCCCCC
+HWI-EAS443:3:24:1112:391#0/1
`¥[_a_ba_a_¥__YVZaXY_UY¥¥UH¥^]¥[QYFMGOLD
@HWI-EAS443:3:24:1117:1067#0/1
GAGGGGTTTTCGGCTGAATCGAGAAAATTGCTTCTCATTG
+HWI-EAS443:3:24:1117:1067#0/1
aaaaab_a`^^a``__aa^`_^a]`_Za_`V[`¥]````_
@HWI-EAS443:3:24:1120:501#0/1
GGGCATGGAAATGAGGCGCTGCCTTCTTCAGTCTCTTCAG
+HWI-EAS443:3:24:1120:501#0/1
a`¥`Z^b[Za`b^]`TTaWTZMT]]W]_ZQ]NP_T_Z[^¥
@HWI-EAS443:3:24:116:1706#0/1
AACTTGATGAAATAGGAGAAGATGTTGCCCCGATTGAACA
+HWI-EAS443:3:24:116:1706#0/1
U¥b¥b]bb`]]bb_ba`^_S__]_]XUWa`ZV_a[NZa_a
@HWI-EAS443:3:24:1202:1945#0/1
GATGAGCCAGAGCCCAGATCTCTAGCAAGCTGGGTCCAAA
+HWI-EAS443:3:24:1202:1945#0/1
a]b_b^aab[bb]`aab_]`^TZab_aabYSa_¥PFV`^`
@HWI-EAS443:3:24:1248:1040#0/1
GCAGAGCTACAGGACATTGAAAATGAAGAGAAGATCCCAA
+HWI-EAS443:3:24:1248:1040#0/1
aaR]X¥`a_a]a_LR__a_^^]`_a]Ya__][`¥ZUURUS
@HWI-EAS443:3:24:1249:857#0/1
GAGAAGACACACATTCACATCTTTTCATTCACTGGAGAGG
+HWI-EAS443:3:24:1249:857#0/1
`_Z_bbba_baa`bbababbaabab_baaaa`ba`a```a
@HWI-EAS443:3:24:1263:1886#0/1
CCAGCTTGCTAGAGATCTGGGCTCTGGCTCATCACTGTCA
+HWI-EAS443:3:24:1263:1886#0/1
aba`UaX`abbabab``Ya`aa`aabaa_`_a^aTaaY^b
@HWI-EAS443:3:24:1285:339#0/1
ATTGAGCAAGGCCGTATTTATGTACACTCCCTTCATTATG
+HWI-EAS443:3:24:1285:339#0/1
]b`bababbabbab_aaaaa`aaa_`Sa_Y_``__aa`a`
@HWI-EAS443:3:24:1376:1940#0/1
ATTCTTTGGCTGGAAAGAGCCTAACATAGTCAAACCACAT
+HWI-EAS443:3:24:1376:1940#0/1
aaa_aa_aa``aa¥`aaaa_^a__U_a`_]¥aaaZa_[a]
@HWI-EAS443:3:24:1379:1493#0/1
TATTGGGAAAGTGTGCAGGACCTTACTGGCAAAATCTGTA
```

```
+HWI-EAS443:3:24:1379:1493#0/1
`abbaaa`ba^W`a`aab`]_abbaJH^``a_aa`]aa^`
@HWI-EAS443:3:24:1504:241#0/1
TGGCTCCAGTCTCGGGTCAGTGAGTGAGAACTCCATACTT
+HWI-EAS443:3:24:1504:241#0/1
aaaa``a`Z_^a``^^U[U`G[^_Z`[aW_LYUR¥[TPWZ
@HWI-EAS443:3:24:1620:519#0/1
GGGGCGTGGTGTCGTCCTCAAGAATGGTTCAATTTTGGCG
+HWI-EAS443:3:24:1620:519#0/1
aa_X¥^V]`U`_^[^^Z_V[_^^V¥[XSWS][^^X`][U¥
@HWI-EAS443:3:24:1625:92#0/1
ATTCTTTGGCTGGAAAGAGCCTAACATAGTCAAACCACAT
+HWI-EAS443:3:24:1625:92#0/1
abb`bbabbabbbaaaa`a^^``aY_`_a_^^aaSU]Z^]
@HWI-EAS443:3:24:1636:264#0/1
GAGAAGATCCCAAGGACAAAGAACATGAAGAGAACAAGCC
+HWI-EAS443:3:24:1636:264#0/1
aaaaa_¥[``Xa_`__]^^_^]_X]__]]_]^]_Z^]]RZ
@HWI-EAS443:3:24:203:687#0/1
CTCGATTGCTTCATATAGCCCCCCAAGATCGAAGGTTCCA
+HWI-EAS443:3:24:203:687#0/1
ab_¥`aa^`aaaaaaaa¥a`aaaaaa`aZ^``_^^R^¥PQ
@HWI-EAS443:3:24:227:1285#0/1
CCCGTTCGTCGATGAAATGGAAATCCGAATACATGAAACA
+HWI-EAS443:3:24:227:1285#0/1
aba`W`_SW]_Q]aaa__]YZaa^``^a_^a``aQ`[`]a
@HWI-EAS443:3:24:246:1548#0/1
CAAAGTCTACTTTTTCTGGTGCCATATTTTCACCGAGTGC
+HWI-EAS443:3:24:246:1548#0/1
abaaa]b`baaaaaaaaa`[^aaaa`aa``bbbaa¥¥]aa
@HWI-EAS443:3:24:257:617#0/1
GGCCATGTTTCCGATTTGTTTTCAAAGAATTCCTTGGTCA
+HWI-EAS443:3:24:257:617#0/1
`^ababb^aba`aa]aab`aaaa]aaaaZaaa_aa_aW_a
@HWI-EAS443:3:24:262:917#0/1
GGGGCATGGAAATGAGGCGCTGCCTTCTTCAGTCTCTTCA
+HWI-EAS443:3:24:262:917#0/1
aaa`aa^aaa```a__^`````¥¥_¥___VLX¥___S¥U_
@HWI-EAS443:3:24:270:989#0/1
CTCCAGTCTCGGGTCAGTGAGTGAGAACTCCATACTTACA
+HWI-EAS443:3:24:270:989#0/1
abaabbabab`b`Ya`_Z]``^aaa`b`_aabaa[aaa^`
@HWI-EAS443:3:24:276:151#0/1
AGTGTACATAAATACGGCCTTGCTCAATGCATCCTGTGCA
+HWI-EAS443:3:24:276:151#0/1
]`]a¥_Z`ba`b_b`baa`^`aaa`^[`_aa````aU___
@HWI-EAS443:3:24:321:1434#0/1
GAGATAATTGAAGGAAGAGACCGAATCATGGCCTGGACAG
+HWI-EAS443:3:24:321:1434#0/1
``a[aaabaaaaaa_a]aaa``_a_a_aaa__]a^[`¥`_
```

```
@HWI-EAS443:3:24:353:698#0/1
CAAACTTGTTAGTTTCGATTTTCGGATCTTCCCCATATTC
+HWI-EAS443:3:24:353:698#0/1
abba`bbaabba`babb^aaa__a`H[Sa`X]`aa`_aa`
@HWI-EAS443:3:24:384:2027#0/1
GAGGAGGAACTATTTTACAGCAGAAGTGTCCCACTGCAGG
+HWI-EAS443:3:24:384:2027#0/1
aaa`]^_baa_``a_abaaX`aaa__XXXa_`aaX_]``]
@HWI-EAS443:3:24:38:1865#0/1
GGGGTTTTCGGCTGAATCGAGAAAATTGCTTCTCATTGTT
+HWI-EAS443:3:24:38:1865#0/1
aabaWababbaba]ab_a_ba`ba_aa_aa`a`aaa`_V_
@HWI-EAS443:3:24:412:205#0/1
AACACAACAGGGGTAGAGAAGCCTAAATTTCTTCCTGATT
+HWI-EAS443:3:24:412:205#0/1
[_[abb`babb`[K`bbbbbaba¥^`^`_V_`ab[a__aa
@HWI-EAS443:3:24:478:1476#0/1
AGAATTCCTTGGTCATGTCTTTCTCTTTGACAGAAGACTC
+HWI-EAS443:3:24:478:1476#0/1
aaabaa[``]`^H_X^`Y`aaaa`a`_a¥W_`NQ¥__WWP
@HWI-EAS443:3:24:569:184#0/1
CAATAATTGTAGAATCTGGTGACCCGAATGCACTATTGAA
+HWI-EAS443:3:24:569:184#0/1
abbbb`b_b`bbbbaaaba_bb`_aba`ba`a`aaa`a`_
@HWI-EAS443:3:24:587:860#0/1
AATTGACTGATTCAAGCTGGATAGAACTTGATGAAATAGG
+HWI-EAS443:3:24:587:860#0/1
abbb]_U_b`aa`]`aaaba`aaaaaaaaaaaa`_`a^a`
@HWI-EAS443:3:24:596:181#0/1
GAGCAGGGCAAGAATCAAAACTAGGCTTTTCACTATAAGA
+HWI-EAS443:3:24:596:181#0/1
aZVWTaaa¥b`a¥`aa`aaa_a```_____¥a_X^[¥Z[]
@HWI-EAS443:3:24:59:407#0/1
GCCTTATTGAATTCATTTTGGACCCAGCTTGCTAGAGATC
+HWI-EAS443:3:24:59:407#0/1
abbbbabbbbaabb`aaaaaaaaa```aaaaaa^aaa^_a
@HWI-EAS443:3:24:613:1312#0/1
GAAATAGGAGAAGATGTTGCCCCGATTGAACATATCGCAA
+HWI-EAS443:3:24:613:1312#0/1
aaaaaaaaa]aaa`_`¥aaa`¥]aZ_]_`b`a_`]_`W]_
@HWI-EAS443:3:24:640:545#0/1
ATGTTCTTTGTCCTTGGGATCTTCTCTTCATTTTCAATGT
+HWI-EAS443:3:24:640:545#0/1
aa`abbbbbbab^aa`Zb_a^a`ababbaaaba`]baaa`
@HWI-EAS443:3:24:699:1969#0/1
ACACCCTTGACGAAGAGAGCAGGGCAAGAATCAAAACTAG
+HWI-EAS443:3:24:699:1969#0/1
aab][`[aba]U^a_aZIJ`^__W_]a_]`^^S¥_UNJ_O
@HWI-EAS443:3:24:702:542#0/1
AAGAAATTTAGGCTTCTCTACCCCTGTTGTGTTACATATA
```

+HWI-EAS443:3:24:702:542#0/1
aaaaabba`aba`aa`a^aaa_a[`¥[`a^a_`a^aaa`a
@HWI-EAS443:3:24:726:1092#0/1
GGCCGAGTCTTCTGTCAAAGAGAAAGACATGACCAAGGAA
+HWI-EAS443:3:24:726:1092#0/1
abbba]baaaababaa`aab`baaab`aaaaa`aa`aa`a
@HWI-EAS443:3:24:734:917#0/1
GCATGGAAATGAGGCGCTGCCTTCTTCAGTCTCTTCAGCA
+HWI-EAS443:3:24:734:917#0/1
abbabbababa`b`aaaaaaaabaaaaaa_`aaa``__`_
@HWI-EAS443:3:24:748:1871#0/1
TAATCAGGCACTCCTCGATTGCTTCATATAGCCCCCCAAG
+HWI-EAS443:3:24:748:1871#0/1
[_Ubb`]WZ_]``Xa_UaUa]_aHWa_aaaQST^XUX^a_
@HWI-EAS443:3:24:78:1258#0/1
AATGGAAATCCGAATACATGAAACAAACTTCCAAATGTGT
+HWI-EAS443:3:24:78:1258#0/1
[^VYWZVU]```^U[`_[]]`^`¥`^`a¥^^Y`L¥ZTZOT
@HWI-EAS443:3:24:795:455#0/1
AGGACATTGAAAATGAAGAGAAGATCCCAAGGACAAAGAA
+HWI-EAS443:3:24:795:455#0/1
a`aaaabbbaaaaaaa`baa`aa`^`__a^a``_`__a``
@HWI-EAS443:3:24:803:391#0/1
CATTCTTTGGCTGGAAAGAGCCTAACATAGTCAAACCACA
+HWI-EAS443:3:24:803:391#0/1
abbb^baY]`]abaaa]a`aa^__a^_a_a^`aaa[__X_
@HWI-EAS443:3:24:826:971#0/1
ATCAAAACTAGGCTTTTCACTATAAGACAAGAAATGGCCA
+HWI-EAS443:3:24:826:971#0/1
UY`a^aaaa`^`a`¥_V__VV^__¥_T]]_]_Z[V_RFS[
@HWI-EAS443:3:24:861:1570#0/1
GGACAACCTGGAACCTGGAACCTTCGATCTTGGGGGGCTA
+HWI-EAS443:3:24:861:1570#0/1
a___a^P_a_[M``[^HUVa`W_Ta_^NU__^TZ_QZ]¥¥
@HWI-EAS443:3:24:867:57#0/1
ATTGAAAATGAAGAGAAGATCCCAAGGACAAAGAACATGA
+HWI-EAS443:3:24:867:57#0/1
abbbaababb_bbbbaabaaa]aa_baa^aaaaa_a_`a`
@HWI-EAS443:3:24:86:277#0/1
GGGTTTTCGGCTGAATCGAGAAAATTGCTTCTCATTGTTC
+HWI-EAS443:3:24:86:277#0/1
aaaX```a`_`_a``^[`^__^VZ^^___]_]X^^^[WX^
@HWI-EAS443:3:24:987:997#0/1
GCCAACAAAATAAAATCTGAGAAGACACACATTCACATCT
+HWI-EAS443:3:24:987:997#0/1
abaa_b¥P]Y^¥V^a^a]][a`]`Y[¥``^_`_`_a_^Z`
@HWI-EAS443:3:25:1034:1303#0/1
TGAAGACATTCTTTGGCTGGAAAGAGCCTAACATAGTCAA
+HWI-EAS443:3:25:1034:1303#0/1
]bbbbbbbbbaaaaaaaaaaaaa`a¥¥`a^``aa``[Z_a

```
@HWI-EAS443:3:25:1101:1395#0/1
TGGAAGCAGGTGCTAGCAGAGCTACAGGACATTGAAAATG
+HWI-EAS443:3:25:1101:1395#0/1
a`a`bba_baUaaaaa``a`a`aa_aa_a_a_``__``_`
@HWI-EAS443:3:25:1116:1567#0/1
CTTGGGGGGCTATATGAAGCAATCGAGGAGTGCCTGATTA
+HWI-EAS443:3:25:1116:1567#0/1
aaa_`_¥a[^abb`b_ba^a``ababab`bVbab¥b^ba_
@HWI-EAS443:3:25:1121:1628#0/1
GGGGGCTATATGAAGCAATCGAGGAGTGCCTGATTAATGA
+HWI-EAS443:3:25:1121:1628#0/1
aaWPa_a[```U`^]````VXZ^]R¥U¥]Y_^XX_]¥]XY
@HWI-EAS443:3:25:1134:1768#0/1
AAGAACATGAAGAGAACAAGCCAATTGAAGTGGGCACTCG
+HWI-EAS443:3:25:1134:1768#0/1
[`YS¥Za_Z^a`bbbaaba^`¥a¥U]XZUYL_T]T]LT`a
@HWI-EAS443:3:25:1163:755#0/1
CCCAGATCTCTAGCAAGCTGGGTCCAAAATGAATTCAATA
+HWI-EAS443:3:25:1163:755#0/1
a`¥baababbbbabb`baa`b]]Ta``¥^baaaa]`a`^a
@HWI-EAS443:3:25:121:986#0/1
CCACTCCCCTGGGTGACTCTCCGATTGGCCATGTTTCCGA
+HWI-EAS443:3:25:121:986#0/1
a`bbbabbba_aZHWb`aaaaaW`Z¥``a`S_^Ta_^aYL
@HWI-EAS443:3:25:1349:1139#0/1
TTCCCATTTGTGTGGCTCCAGTCTCGGGTCAGTGAGTGAG
+HWI-EAS443:3:25:1349:1139#0/1
aa[_aabaaa_b]b_]a_OZ[WV[]_¥_OV]XGVY]GZPP
@HWI-EAS443:3:25:1395:2030#0/1
CTCCTATTTCATCAAGTTCTATCCAGCTTGAATCAGTCAA
+HWI-EAS443:3:25:1395:2030#0/1
O]aa``abb`a`bb_`^`bbb`a`a_`bb`bbb_b_a_`b
@HWI-EAS443:3:25:1492:869#0/1
ATGGCTTGGAAGCAGGTGCTAGCAGAGCTACAGGACATTG
+HWI-EAS443:3:25:1492:869#0/1
aba`ab_JZ^a¥VR]aQaUT¥VTTY[XSZSL[¥_KKZQ]Z
@HWI-EAS443:3:25:1539:652#0/1
CTTGCTAGAGATCTGGGCTCTGGCTCATCACTGTCATACT
+HWI-EAS443:3:25:1539:652#0/1
J[bbbbb]bbab`bbabbabbba_babaaabaabbbbb[b
@HWI-EAS443:3:25:153:835#0/1
AAGAATTCCTTGGTCATGTCTTTCTCTTTGACAGAAGACT
+HWI-EAS443:3:25:153:835#0/1
^^`aabaabbbba_aZab`aaa``aabaaa_aaaaS^_a_
@HWI-EAS443:3:25:1553:1072#0/1
CCCCGTTCGTCGATGAAATGGAAATCCGAATACATGAAAC
+HWI-EAS443:3:25:1553:1072#0/1
_`ab_Y[¥`Sa¥bbaXHTabbabb`TY_aaaZW¥_`aVZV
@HWI-EAS443:3:25:1583:1866#0/1
AGGGAATACCACTATATGATGCAATCAAATGCATGAAGAC
```

+HWI-EAS443:3:25:1583:1866#0/1
]T_^^babaaabaaaaaba_YZb`a_a_]a[]a`Za`_aS
@HWI-EAS443:3:25:1789:1760#0/1
TATTTTCACCGAGTGCCCACTTCAATTGGCTTGTTCTCTT
+HWI-EAS443:3:25:1789:1760#0/1
abbabbbbbabbaabaaaa_aaa`a_```]`_HX]QYO__
@HWI-EAS443:3:25:191:488#0/1
AATGAACCCATACAGGTTTGTTTTCCGTCTTCCTTCTTTG
+HWI-EAS443:3:25:191:488#0/1
_Va``bbabaaa_W]]Xa`a_a`a`__^`a_`[_````^_
@HWI-EAS443:3:25:219:321#0/1
CTTTGGTCGGCAAGCTTGCGCATAGTTCCTGTAATCTCAA
+HWI-EAS443:3:25:219:321#0/1
S`ab`aaaaabaa`bbbb`bbbaa]a¥^]`__^aab``b]
@HWI-EAS443:3:25:240:1062#0/1
CCCCTGTTGTGTTACATATACTGTTCACCACTGTCCAGGC
+HWI-EAS443:3:25:240:1062#0/1
abbbbbbababababababbbbaaba`aaa`baa__a`a`]_
@HWI-EAS443:3:25:283:1660#0/1
CTGATTCAAGCTGGATAGAACTTGATGAAATAGGAGAAGA
+HWI-EAS443:3:25:283:1660#0/1
I_WT_`a_Y`a__`_Z_S^`b`bbb`babbaX`bbbb_^¥
@HWI-EAS443:3:25:31:1865#0/1
ACTCGGTGAAAATATGGCACCAGAAAAAGTAGACTTTGAT
+HWI-EAS443:3:25:31:1865#0/1
^aabaX]]^Y^aaZY[`baaaa_P_`]N__¥aUY][ZZ
@HWI-EAS443:3:25:334:906#0/1
CCGATTGGCCATGTTTCCGATTTGTTTTCAAAGAATTCCT
+HWI-EAS443:3:25:334:906#0/1
abbbbbbbb`[^``bbaaa_aaaa^aa`^_aa`^`a`a``
@HWI-EAS443:3:25:409:356#0/1
CTCGGGTCTTCAATACTTAATTTCAGAGCATCCATCAGCA
+HWI-EAS443:3:25:409:356#0/1
aababaZabbabbab`a`babbaaaaa^aaaaaa_`a_`a
@HWI-EAS443:3:25:410:72#0/1
TGTGGACTTCCCTCCGTGTTACTCCAATTTCAATGAACCG
+HWI-EAS443:3:25:410:72#0/1
¥_V`Za_[UHZZa_Z_H__ZS^[V¥YP[Za^PT[PJ]Y]^
@HWI-EAS443:3:25:443:1312#0/1
ACTTGATGAAATAGGAGAAGATGTTGCCCCGATTGAACAT
+HWI-EAS443:3:25:443:1312#0/1
aaaaaaaaaaaaaaa`aaa]a`a^__a_a``__^`^b_a^
@HWI-EAS443:3:25:450:936#0/1
ACCGATTTGAGATAATTGAAGGAAGAGACCGAATCATGGC
+HWI-EAS443:3:25:450:936#0/1
abbbaabbbabaababbbaaaaaaaaaa``aaa_a^aaa^`
@HWI-EAS443:3:25:452:643#0/1
CAGTAAGGTCCTGCACACTTTCCCAATAGAGCCTTCCTCC
+HWI-EAS443:3:25:452:643#0/1
aabbbabaaaabbbb_bbbbbbabbaaaaba`_aab`aaa

@HWI-EAS443:3:25:462:1485#0/1
TGGGGGGCTATATGAAGCAATCGAGGAGTGCCTGATTAAT
+HWI-EAS443:3:25:462:1485#0/1
aaa``aaaaaaaaaaa_aaa`a]``^Z[Z^a`__^`_`__
@HWI-EAS443:3:25:539:1663#0/1
CTGGACAGTGGTGAACAGTATATGTAACACAACAGGGGTA
+HWI-EAS443:3:25:539:1663#0/1
Xababbbba`^bab`aba`ab`b_abbabbbbb^^aa`]`
@HWI-EAS443:3:25:541:29#0/1
ATCAAAGTCTACTTTTTCTGGTGCCATATTTTCACCGAGT
+HWI-EAS443:3:25:541:29#0/1
abbbbab`aa_abbbaaabba_aaaaaabaaaaaaaaaaZ
@HWI-EAS443:3:25:57:1625#0/1
CATGCTTGCGATATGTTCAATCGGGGCAACATCTTCTCCT
+HWI-EAS443:3:25:57:1625#0/1
H_`^`````bbb^ba`Zabbbbb`b_a¥bbbbabbb``bb
@HWI-EAS443:3:25:655:1425#0/1
AGAGATCTGGGCTCTGGCTCATCACTGTCATACTGTTTAA
+HWI-EAS443:3:25:655:1425#0/1
aaaaa[b_`[`b`aa_a```a_``_]_]`_``¥^^]_^a_
@HWI-EAS443:3:25:728:623#0/1
GATTCAGCCGAAAACCCCTCAAGTTGTGGAGACGCATATA
+HWI-EAS443:3:25:728:623#0/1
`¥^`]__¥a`]G^[VYVVU]RU_VUYR]WY^YU^OYXXUV
@HWI-EAS443:3:25:757:455#0/1
CTCAAGTTGTGGAGACGCATATAGACTGTTGAATACAGAT
+HWI-EAS443:3:25:757:455#0/1
a_W_[¥]`K__¥^b_a^`_a^[Y^XYY]RXYa`_]J[aRJ
@HWI-EAS443:3:25:772:1766#0/1
GTATTCAGTAGCCCTGCAGTGGGACACTTCTGCTGTAAAA
+HWI-EAS443:3:25:772:1766#0/1
`Xaa[`a`¥a_`[a_`aa_X`^_¥VUS]O]]WRU]V¥_a^
@HWI-EAS443:3:25:838:1680#0/1
TCAATACTTAATTTCAGAGCATCCATCAGCAGGAACTTTG
+HWI-EAS443:3:25:838:1680#0/1
`O[___[X_^Z]Y^aaZ_Y]¥YX^aZSZX^`VQW¥ZZVXR
@HWI-EAS443:3:25:846:1221#0/1
TGCCCTGCTCTCTTCGTCAAGGGTGTAGTCCGCTTTGGTG
+HWI-EAS443:3:25:846:1221#0/1
abbZ^a]_¥W`a^[```]a]a`a[a]XXQS_]UW_¥¥VXV
@HWI-EAS443:3:25:886:1598#0/1
CAATAATTGTAGAATCTGGTGACCCGAATGCACTATTGAA
+HWI-EAS443:3:25:886:1598#0/1
abbbbabab_bba`a`baa^abaabab`aaa``ba`aa`a
@HWI-EAS443:3:25:932:1449#0/1
CATAAATCCCAATTACCTCATGGCTTGGAAGCAGGTGCTA
+HWI-EAS443:3:25:932:1449#0/1
abbb`bbbbbbaaaabbaaaaaaaa`[__a[QYZ_V[X¥]
@HWI-EAS443:3:25:965:831#0/1
TTGACTGATTCAAGCTGGATAGAACTTGATGAAATAGGAG

```
+HWI-EAS443:3:25:965:831#0/1
a`a^PZ¥aaaaXV¥X__^_a¥``a_aa]_a_^ZZ`aa[Va
@HWI-EAS443:3:25:970:1573#0/1
ACCGATTTGAGATAATTGAAGGAAGAGACCGAATCATGGC
+HWI-EAS443:3:25:970:1573#0/1
aaabaa_baaaaaa`aaaba__aaaa^a_aaaa`_a``¥_
@HWI-EAS443:3:25:970:1897#0/1
TTCGGATTTCCATTTCATCGACGAACGGGGTGAATCAATA
+HWI-EAS443:3:25:970:1897#0/1
a^^b`¥X^^[^]][X`__¥_¥Za`aWUYYZX`^XXW`]_]
@HWI-EAS443:3:26:1022:1777#0/1
CGGTTCATTGAAATTGGAGTAACACGGAGGGAAGTCCACA
+HWI-EAS443:3:26:1022:1777#0/1
_]aW_YXa^¥^`[¥aYR__[^aaa_aa[]`]][`Y_^___
@HWI-EAS443:3:26:1267:1350#0/1
GGAAATCCGAATACATGAAACAAACTTCCAAATGTGTGCA
+HWI-EAS443:3:26:1267:1350#0/1
ab``abbb_bba^ab``X`a^^a[``a`_`^aa[_a¥`_a
@HWI-EAS443:3:26:1298:296#0/1
GGAGTGTACATAAATACGGCCTTGCTCAATGCATCCTGTG
+HWI-EAS443:3:26:1298:296#0/1
a__b¥b_`_`a]__a^V^_]¥___UZU_¥S_¥¥YYQYWQZ
@HWI-EAS443:3:26:1353:1029#0/1
CATTAATCAGGCACTCCTCGATTGCTTCATATAGCCCCCC
+HWI-EAS443:3:26:1353:1029#0/1
`bbabZbabb_baabb_babaaababba_`aa_b¥_``]_
@HWI-EAS443:3:26:1415:769#0/1
CTGTAGCTCTGCTAGCACCTGCTTCCAAGCCATGAGGTAA
+HWI-EAS443:3:26:1415:769#0/1
YabN^T_b`baS_````Ub[b^`X¥KXVW_ZbW¥Hab`¥_
@HWI-EAS443:3:26:1445:1076#0/1
TGGAGTTCTCACTCACTGACCCGAGACTGGAGCCACACAA
+HWI-EAS443:3:26:1445:1076#0/1
Ma``¥ZbU[`Y¥Y]bVU]``YZ]Tb[aa`ab`Zaaa[QZR
@HWI-EAS443:3:26:1447:704#0/1
ACTGTTCACCACTGTCCAGGCCATGATTCGGTCTCTTCCT
+HWI-EAS443:3:26:1447:704#0/1
_T]b_]aYZZZI[]L^M[RWW¥a]]TSONVVJJSGRZUZY
@HWI-EAS443:3:26:1520:1612#0/1
GGCTTCTCTACCCCTGTTGTGTTACATATACTGTTCACCA
+HWI-EAS443:3:26:1520:1612#0/1
aabb¥_U¥P_Zab``_^¥[¥`[a`[^aaa_[abU]Y_XY_
@HWI-EAS443:3:26:153:1299#0/1
ATCCGAAAATCGAAACTAACAAGTTTGCTGCAATATGCAC
+HWI-EAS443:3:26:153:1299#0/1
aaab[`ab`aa^]aa_`aa````Z_^¥a_`a```_^_`a_
@HWI-EAS443:3:26:1545:1015#0/1
GCCATGATTCGGTCTCTTCCTTCAATTATCTCAAATCGGT
+HWI-EAS443:3:26:1545:1015#0/1
a`aabbbab_bba]a]`aU¥`a_aaa``b_aa`aa]¥[^Z
```

```
@HWI-EAS443:3:26:1568:483#0/1
AAACTTTAGAGCCTATGTAGATGGATTCGAGCCGAACGGC
+HWI-EAS443:3:26:1568:483#0/1
aabbaaa_b`bbYbabb_aaa`ZX]``]a`aaZaU`[_]Q
@HWI-EAS443:3:26:1610:1155#0/1
ATTTTGGCGTTCACTTCTTTTGACATTTGGGAAAGCTTGC
+HWI-EAS443:3:26:1610:1155#0/1
abbbbab^b_a^a¥_aaaaaaK_^``a_K`a^^a]W`]XX
@HWI-EAS443:3:26:175:1730#0/1
GGGGCATGGAAATGAGGCGCTGCCTTCTTCAGTCTCTTCA
+HWI-EAS443:3:26:175:1730#0/1
aba`abaaabbababa_babaabbababbaaa^aaaaaaa
@HWI-EAS443:3:26:210:176#0/1
GCCATGTTTCCGATTTGTTTTCAAAGAATTCCTTGGTCAT
+HWI-EAS443:3:26:210:176#0/1
abb^bb`aaab_Waaab`aaaaaaaaa`aa_aa`a`]``a
@HWI-EAS443:3:26:216:1025#0/1
CTGGAGAGGAGATGGCCACCAAAGCGGACTACACCCTTGA
+HWI-EAS443:3:26:216:1025#0/1
aaab_aba__TbX]a_aa`b`b^_aa_Zaa¥`b_`b`Za`
@HWI-EAS443:3:26:229:211#0/1
TTCAGTAGCCCTGCAGTGGGACACTTCTGCTGTAAAATAG
+HWI-EAS443:3:26:229:211#0/1
aabba`aaaaa__a]a_`__a`aa`__aa```__`b__]^
@HWI-EAS443:3:26:244:1806#0/1
GAGGGAATACCACTATATGATGCAATCAAATGCATGAAGA
+HWI-EAS443:3:26:244:1806#0/1
^^a`[^I^bbbbbbbbb_b^Sbbbb_baa`aaa[_a`ab
@HWI-EAS443:3:26:283:800#0/1
TTGGGGGGCTATATGAAGCAATCGAGGAGTGCCTGATTAA
+HWI-EAS443:3:26:283:800#0/1
`aba_UOG[`Z^`VW`RRV_Q]TaV¥¥JPY_[WV`_QFRU
@HWI-EAS443:3:26:298:1571#0/1
GAAGTTCGGTGGGAGACTTTGGTCGGCAAGCTTGCGCATA
+HWI-EAS443:3:26:298:1571#0/1
MXbaaabUb_]XSaaaaaaaa`Za_Xa¥KW_a`Uaa_]__
@HWI-EAS443:3:26:303:644#0/1
CACTCGGTGAAAATATGGCACCAGAAAAAGTAGACTTTGA
+HWI-EAS443:3:26:303:644#0/1
abbbbbaQ`bbbbbbbbbaababbabaa`aZb`baaaaba
@HWI-EAS443:3:26:328:558#0/1
GTTTCCGATTTGTTTTCAAAGAATTCCTTGGTCATGTCTT
+HWI-EAS443:3:26:328:558#0/1
_Xaa`bb`ba`b¥___`aaaba]a]`S`aa]N__aa^aa_
@HWI-EAS443:3:26:337:1878#0/1
AAAATAGTTCCTCCTCATGCTTGCGATATGTTCAATCGGG
+HWI-EAS443:3:26:337:1878#0/1
abb_aa_]`aa_`a_`a]¥__^_`]`^`_QKL¥_]]]XVZ
@HWI-EAS443:3:26:355:1938#0/1
CAGGTGCTAGCAGAGCTACAGGACATTGAAAATGAAGAGA
```

```
+HWI-EAS443:3:26:355:1938#0/1
ab__][b`a_aa``___a_a_]`a__`_```^_^_a^`^¥
@HWI-EAS443:3:26:364:1305#0/1
GGGGGCTATATGAAGCAATCGAGGAGTGCCTGATTAATGA
+HWI-EAS443:3:26:364:1305#0/1
`a]a_a`aaaa]`a_`a``a`^___`X`````^`_`__[]
@HWI-EAS443:3:26:399:950#0/1
ACCGGTTCATTGAAATTGGAGTAACACGGAGGGAAGTCCA
+HWI-EAS443:3:26:399:950#0/1
Z___a`a_]_Vaaa^_W]`SVNa^aab```]a¥YaaT[TZ
@HWI-EAS443:3:26:411:292#0/1
CGTCTCCACAACTTGAGGGGTTTTCGGCTGAATCGAGAAA
+HWI-EAS443:3:26:411:292#0/1
aNbbab_b]`_N_]ba_b¥[PaaaZUXZX`aaa`a_aaaU
@HWI-EAS443:3:26:487:765#0/1
CACTGTCATACTGTTTAAGGTCTCCAACATCTTTGCAGTC
+HWI-EAS443:3:26:487:765#0/1
abaZ[Ya]Vb___U^``U_UZ_aa`X`]``_``]_a_]OW
@HWI-EAS443:3:26:495:696#0/1
CTTGATGAAATAGGAGAAGATGTTGCCCCGATTGAACATA
+HWI-EAS443:3:26:495:696#0/1
abbba`ba`]a[]`[aaa`a___a`aa[Y_^`_Zab^`^`
@HWI-EAS443:3:26:532:2012#0/1
GAGATAATTGAAGGAAGAGACCGAATCATGGCCTGGACAG
+HWI-EAS443:3:26:532:2012#0/1
HZaabbbbaa]`bbbaab[bb``Ubaaba`abab`ababb
@HWI-EAS443:3:26:545:603#0/1
ATCTGGGCTCTGGCTCATCACTGTCATACTGTTTAAGGTC
+HWI-EAS443:3:26:545:603#0/1
aab^a``a`b_`Sa`Y_aaa_W`Z`^a`_`aV_^^_^]V_
@HWI-EAS443:3:26:557:677#0/1
GGTGAACAGTATATGTAACACAACAGGGGTAGAGAAGCCT
+HWI-EAS443:3:26:557:677#0/1
abXa`b_^[`¥a`a_¥aaa__`a`_a[__Ia¥Wa`a^]_Y
@HWI-EAS443:3:26:584:1405#0/1
GTTTTCGGCTGAATCGAGAAAATTGCTTCTCATTGTTCAG
+HWI-EAS443:3:26:584:1405#0/1
a[^`a````X^[^¥]Ya`a]¥[^¥Y]ZZ]¥`[YU[UZVY¥
@HWI-EAS443:3:26:585:1946#0/1
CACACATTCACATCTTTTCATTCACTGGAGAGGAGATGGC
+HWI-EAS443:3:26:585:1946#0/1
[`babbaabbbbababababbbaaabaaa`aaa__a_aa_`]
@HWI-EAS443:3:26:587:1279#0/1
TTTCTTGTCTTATAGTGAAAAGCCTAGTTTTGATTCTTGC
+HWI-EAS443:3:26:587:1279#0/1
aaVbbb]U`a`Y`V`a`][a`]aaa^ZVa_a]aaaYa_a_
@HWI-EAS443:3:26:620:176#0/1
TGGAAATCCGAATACATGAAACAAACTTCCAAATGTGTGC
+HWI-EAS443:3:26:620:176#0/1
]`_YbVR^bbaabbababbb¥]_ab_aaZZa[K]a`a`_^
```

```
@HWI-EAS443:3:26:636:1521#0/1
AAGAGCCTAACATAGTCAAACCACATGAGAAAGGCATAAA
+HWI-EAS443:3:26:636:1521#0/1
`a]aaaa`abba_a`_aa¥baaaaa```_aaa___a_ba`
@HWI-EAS443:3:26:674:1073#0/1
AATTACCTCATGGCTTGGAAGCAGGTGCTAGCAGAGCTAC
+HWI-EAS443:3:26:674:1073#0/1
_aabaaaaaaaaaa`_W_X^[UOX]VV]ZZXUXYRTPTXR
@HWI-EAS443:3:26:706:205#0/1
TTCATCGACGAACGGGGTGAATCAATAATTGTAGAATCTG
+HWI-EAS443:3:26:706:205#0/1
aaabaabaaaaa__`a_O[_`[U[`_`^`__]`_^_UYV^
@HWI-EAS443:3:26:729:1917#0/1
ACGGCCTTGCTCAATGCATCCTGTGCAGCCATGGATGACT
+HWI-EAS443:3:26:729:1917#0/1
aaU]`a]SS`^aT`_U[`¥]_X¥WUS^U^UZWSWWU¥UWS
@HWI-EAS443:3:26:769:1301#0/1
AACCGGTTCATTGAAATTGGAGTAACACGGAGGGAAGTCC
+HWI-EAS443:3:26:769:1301#0/1
__``b_¥Ya`^bbb_S_a`ZS¥X`baY_aaaaaa¥O¥¥_[
@HWI-EAS443:3:26:773:953#0/1
GGCCAGTAGGAGTCTATGGGATTCCTTTCGTCAGTCCGAA
+HWI-EAS443:3:26:773:953#0/1
`ab_`__aba`a]¥aaaab_QXWT_X`_R¥WSS`Z[^]¥`
@HWI-EAS443:3:26:779:1394#0/1
GCTGGGTCCAAAATGAATTCAATAAGGCATGTGAATTGAC
+HWI-EAS443:3:26:779:1394#0/1
abbbbb¥^bab_S¥V`a`_aaaaa_a`^a¥T¥``a`W¥`_
@HWI-EAS443:3:26:830:1436#0/1
CACAACTTGAGGGGTTTTCGGCTGAATCGAGAAAATTGCT
+HWI-EAS443:3:26:830:1436#0/1
a`]¥WaaWX^`X[]_`XZ[a_aX___^Z`UY_^X_WRXWW
@HWI-EAS443:3:26:932:902#0/1
CATGTTTCCGATTTGTTTTCAAAGAATTCCTTGGTCATGT
+HWI-EAS443:3:26:932:902#0/1
_aabaabbb``U`bb`abb_bbbbbabaaab^``abbbb`
@HWI-EAS443:3:26:933:1448#0/1
GCAGGTGCTAGCAGAGCTACAGGACATTGAAAATGAAGAG
+HWI-EAS443:3:26:933:1448#0/1
``baa`aaab_baaabaaaaba`a_aaa`_aaaaaaa`aa
@HWI-EAS443:3:26:967:851#0/1
CGTCTCCACAACTTGAGGGGTTTTCGGCTGAATCGAGAAA
+HWI-EAS443:3:26:967:851#0/1
a`QZ^¥^[^`a_a`ZLU_ZS[GPWSDIHYaYRQaT]]Z_]
@HWI-EAS443:3:26:982:1052#0/1
GGTGAACTTTGTAAGTATGGAGTTCTCACTCACTGACCCG
+HWI-EAS443:3:26:982:1052#0/1
`^Xba_J]a^_Zaaa_aa]]U^`__¥`a__`aa[¥¥^XQ]
@HWI-EAS443:3:26:998:180#0/1
GAAATTTAGGCTTCTCTACCCCTGTTGTGTTACATATACT
```

+HWI-EAS443:3:26:998:180#0/1
a_aaaa_`aa`aa¥_]^`^]^Z]]Y^]X^W]¥YZ]]ZRSX
@HWI-EAS443:3:27:1017:121#0/1
CAGGAACTTTGACCGCTGATGGCAAAGAGGCCCATCAGGC
+HWI-EAS443:3:27:1017:121#0/1
`aa`_aaaaaaa`_a_aaaaaa``_^`^aa[TN^WGYa^Z
@HWI-EAS443:3:27:1021:726#0/1
GAGTGTACATAAATACGGCCTTGCTCAATGCATCCTGTGC
+HWI-EAS443:3:27:1021:726#0/1
a_aZa]a_``Z_a`a_aaYY__UT¥X_^]RT__[Z^_]^Z
@HWI-EAS443:3:27:1252:1015#0/1
CAACCTGGAACCTGGAACCTTCGATCTTGGGGGGCTATAT
+HWI-EAS443:3:27:1252:1015#0/1
a`Z_]Za^^`aaaa^``aV`a]aZ_]a]`]`¥[¥^^_`_`
@HWI-EAS443:3:27:1269:215#0/1
TTGGGATCTTCTCTTCATTTTCAATGTCCTGTAGCTCTGC
+HWI-EAS443:3:27:1269:215#0/1
```_[Ya`aaaa_aa__baaaa``aa[aW`a]_`_`U__U
@HWI-EAS443:3:27:135:920#0/1
CAGGACATTGAAAATGAAGAGAAGATCCCAAGGACAAAGA
+HWI-EAS443:3:27:135:920#0/1
abaabababab``baba^abaaa]``abaaaa_aaaaY_a
@HWI-EAS443:3:27:1361:1302#0/1
GGTGAACTTTGTAAGTATGGAGTTCTCACTCACTGACCCG
+HWI-EAS443:3:27:1361:1302#0/1
aa_`Yaaaa__[`__Ya_a`a`^__`a_¥^S^^_`_¥UW_
@HWI-EAS443:3:27:1410:589#0/1
GTCCTTGGGATCTTCTCTTCATTTTCAATGTCCTGTAGCT
+HWI-EAS443:3:27:1410:589#0/1
aU]¥[S`]aU]Y_]]_N_[]`__`X^_T[YZUYZ_Y_ZUU
@HWI-EAS443:3:27:1419:1473#0/1
AAAACTTTAGAGCCTATGTAGATGGATTCGAGCCGAACGG
+HWI-EAS443:3:27:1419:1473#0/1
`a`_abba_aaaY_aa`a__a_^¥^`a[]]_U`ZY_ZYYZ
@HWI-EAS443:3:27:142:1211#0/1
GCCCTGCAGTGGGACACTTCTGCTGTAAAATAGTTCCTCC
+HWI-EAS443:3:27:142:1211#0/1
`a^_`]a¥XR`b`bbbb[^ba[``]RQ¥ba]^^X`ba^`b
@HWI-EAS443:3:27:1443:213#0/1
AGAGAACAAGCCAATTGAAGTGGGCACTCGGTGAAAATAT
+HWI-EAS443:3:27:1443:213#0/1
ab`a`^aaba`_^__^a_¥aW_][a¥]ZWa[X^X]_^X[O
@HWI-EAS443:3:27:1460:1207#0/1
GGCATGGAAATGAGGCGCTGCCTTCTTCAGTCTCTTCAGC
+HWI-EAS443:3:27:1460:1207#0/1
a][Vaba¥aaa[[XaUaaPH`a_a¥a`[aa_Y]__[X`^W
@HWI-EAS443:3:27:1612:378#0/1
GAACCGGTTCTCTTTGTAATCATACAAATCAGGAAGAAAT
+HWI-EAS443:3:27:1612:378#0/1
aaaaabb`baa_aaaaWa_a]a_a_a`aa__a___a_``_
```

```
@HWI-EAS443:3:27:1651:860#0/1
AAGGGAGTGTACATAAATACGGCCTTGCTCAATGCATCCT
+HWI-EAS443:3:27:1651:860#0/1
ab_^VYbUa_XL[aaa_b`^^[WN`aZabaabaa[¥]]S^
@HWI-EAS443:3:27:1658:401#0/1
CATTAATCAGGCACTCCTCGATTGCTTCATATAGCCCCCC
+HWI-EAS443:3:27:1658:401#0/1
a`bbbbbb[`a`_¥^¥^aT_^`_¥Z_a]aaaa_XPL`]V]
@HWI-EAS443:3:27:1690:1079#0/1
CTCCTCATGCTTGCGATATGTTCAATCGGGGCAACATCTT
+HWI-EAS443:3:27:1690:1079#0/1
abbaaaW_babbb¥aabbaaaaa_aa]abab_a`]aa^aa
@HWI-EAS443:3:27:211:794#0/1
GGGGTTTTCGGCTGAATCGAGAAAATTGCTTCTCATTGTT
+HWI-EAS443:3:27:211:794#0/1
aX^_R_a]_]``^a^a_`^O]V[ZWZ]¥]^¥^_]S]^_[^
@HWI-EAS443:3:27:242:1665#0/1
GAAAGGCATAAATCCCAATTACCTCATGGCTTGGAAGCAG
+HWI-EAS443:3:27:242:1665#0/1
`bYbabbbbbbbabbba``abaaabb`a_aa_W]_a]N][
@HWI-EAS443:3:27:291:650#0/1
ACCGATTTGAGATAATTGAAGGAAGAGACCGAATCATGGC
+HWI-EAS443:3:27:291:650#0/1
^aab[aa]a`^]aaaa`a^_aa``a`^aaa`a__```__^
@HWI-EAS443:3:27:357:763#0/1
AGTCTATATGCGTCTCCACAACTTGAGGGGTTTTCGGCTG
+HWI-EAS443:3:27:357:763#0/1
abababababaab`_b_ba``S_aaaa_`aaaZaaZ_¥`_¥^
@HWI-EAS443:3:27:379:748#0/1
CTCCTCATGCTTGCGATATGTTCAATCGGGGCAACATCTT
+HWI-EAS443:3:27:379:748#0/1
a``_`ba`_Y¥`¥```]]¥b[[Z]^`ZZN]ZVXW]]W¥_`
@HWI-EAS443:3:27:440:725#0/1
CCGAAAGAGGCGAAGAGACAATTGAAGAAAAATTTGAGAT
+HWI-EAS443:3:27:440:725#0/1
abTQ[`XU[`a``Y¥SMY[Q^][VLZa_``__]_SP`__Z
@HWI-EAS443:3:27:486:1382#0/1
AGACAAGAAATGGCCAGTAGGAGTCTATGGGATTCCTTTC
+HWI-EAS443:3:27:486:1382#0/1
^abaa^baM¥aa^`aaa¥b_[`_¥``aa_`___```a_]a
@HWI-EAS443:3:27:526:1022#0/1
AGGCTTCTCTACCCCTGTTGTGTTACATATACTGTTCACC
+HWI-EAS443:3:27:526:1022#0/1
^T_SZZ`]`]a_^baU_UXMD[W_Z]LWaaa`X^Z^[]P`
@HWI-EAS443:3:27:557:1541#0/1
GCCTAACATAGTCAAACCACATGAGAAAGGCATAAATCCC
+HWI-EAS443:3:27:557:1541#0/1
`bbaabb`aba]abbb`baabbababba_aaaabab````
@HWI-EAS443:3:27:705:1068#0/1
ATGGCACCAGAAAAAGTAGACTTTGATGACTGCAAAGATG
```

```
+HWI-EAS443:3:27:705:1068#0/1
a`]aaabbbba_``aa_a^aaaaaaaaaaa¥EZa_aaa_a
@HWI-EAS443:3:27:777:1598#0/1
CTCTTCATGTTCTTTGTCCTTGGGATCTTCTCTTCATTTT
+HWI-EAS443:3:27:777:1598#0/1
a`_Xa`Y[T]X`a`Z^L¥Ya__OTXZ[]^_```X]__]]X
@HWI-EAS443:3:27:816:668#0/1
CCGATTGGCCATGTTTCCGATTTGTTTTCAAAGAATTCCT
+HWI-EAS443:3:27:816:668#0/1
`aa`baaaaaa_a`ab_a]Z_aa___aa]__``_`a_[¥_
@HWI-EAS443:3:27:86:1101#0/1
GGCATGTGAATTGACTGATTCAAGCTGGATAGAACTTGAT
+HWI-EAS443:3:27:86:1101#0/1
`bbbbb^bb`aabbbabaaabb^aaab_aabaabaa``aa
@HWI-EAS443:3:27:874:1199#0/1
GCAATATGCACACATTTGGAAGTTTGTTTCATGTATTCGG
+HWI-EAS443:3:27:874:1199#0/1
aaaa`aaaaa_a_`aa`a``^aY__`]a_a___]_aZ^__
@HWI-EAS443:3:27:880:447#0/1
GCCTGGACAGTGGTGAACAGTATATGTAACACAACAGGGG
+HWI-EAS443:3:27:880:447#0/1
aa_aaaa``a]`¥ZaW]a^¥]`a_a`__^Y`^``]]___`
@HWI-EAS443:3:27:900:475#0/1
CACCCAGGGGAGTGGAGGAAGGCTCTATTGGGAAAGTGTG
+HWI-EAS443:3:27:900:475#0/1
aaabaabaaaaa^aa`aa^`a_^`_`_aaaa`Y`_¥U_Y^
@HWI-EAS443:3:27:945:453#0/1
AGCTGAAAGTCATCCATGGCTGCACAGGATGCATTGAGCA
+HWI-EAS443:3:27:945:453#0/1
aaabbbbbaaaba`_aab`aaa_`_`a``_`¥`_`]__¥a
@HWI-EAS443:3:27:979:1920#0/1
GGAGGGAATACCACTATATGATGCAATCAAATGCATGAAG
+HWI-EAS443:3:27:979:1920#0/1
_baaaabbbbaaaaabbbaaaaa`b`aaaabaa_baaa``
@HWI-EAS443:3:27:989:1537#0/1
GAGAAGATCCCAAGGACAAAGAACATGAAGAGAACAAGCC
+HWI-EAS443:3:27:989:1537#0/1
abbbbbbabbaaaa`babaaaaaaaaaa`aa`aaab`a_^
@HWI-EAS443:3:28:1011:1069#0/1
ACTTCTCCAGCCTTGAAAACTTTAGAGCCTATGTAGATGG
+HWI-EAS443:3:28:1011:1069#0/1
aabbaabaaaaabaa`aaa_a^aaaa___a`aa^``_aa`
@HWI-EAS443:3:28:1076:254#0/1
ATAAATACGGCCTTGCTCAATGCATCCTGTGCAGCCATGG
+HWI-EAS443:3:28:1076:254#0/1
a]_]a_^Y````NFQTT^X][[X_QR`_YNPNU¥GP¥U]]
@HWI-EAS443:3:28:1092:1860#0/1
AGCAGGTGCTAGCAGAGCTACAGGACATTGAAAATGAAGA
+HWI-EAS443:3:28:1092:1860#0/1
Y^a¥X`I`_Q_PQ`a_`a]a`^¥]__]``YV`__aaY_^^
```

```
@HWI-EAS443:3:28:1146:381#0/1
AGGACATTGAAAATGAAGAGAAGATCCCAAGGACAAAGAA
+HWI-EAS443:3:28:1146:381#0/1
abaaaabbbaa`aaaaaaaa`aaa_a`_`aa__^a`a`_`
@HWI-EAS443:3:28:1147:364#0/1
CGATTGGCCATGTTTCCGATTTGTTTTCAAAGAATTCCTT
+HWI-EAS443:3:28:1147:364#0/1
ababbbba`aa``ba^abaab`¥_abb_`aaba`aa_^aa
@HWI-EAS443:3:28:1155:1523#0/1
CTGGAACCTTCGATCTTGGGGGGCTATATGAAGCAATCGA
+HWI-EAS443:3:28:1155:1523#0/1
_ba]abbabb]_aa_bbba_aba[ba`aZSa`b_aaaZ_[
@HWI-EAS443:3:28:1179:353#0/1
GGGGCATGGAAATGAGGCGCTGCCTTCTTCAGTCTCTTCA
+HWI-EAS443:3:28:1179:353#0/1
aaba¥aabaaaaaa`aa_b`aa^__aWa`Z_`YP`W_aW¥
@HWI-EAS443:3:28:1188:1088#0/1
CAAAATGAATTCAATAAGGCATGTGAATTGACTGATTCAA
+HWI-EAS443:3:28:1188:1088#0/1
[`¥a¥^^QIZ[S`¥b`a`^^^a]aaababVaa^]^a[_a_
@HWI-EAS443:3:28:1192:1292#0/1
GTGTGCATATTGCAGCAAACTTGTTAGTTTCGATTTTCGG
+HWI-EAS443:3:28:1192:1292#0/1
_V`[¥XHZ`]_]`aa_¥[ZX`^`_a``¥[_T__]a^^a`T
@HWI-EAS443:3:28:1200:1442#0/1
AGCCCCCCAAGATCGAAGGTTCCAGGTTCCAGGTTGTCCC
+HWI-EAS443:3:28:1200:1442#0/1
a_¥`^aaaaa]_`Va]^_ZS^¥W^][T¥SU]¥^MYQSWUW
@HWI-EAS443:3:28:1201:295#0/1
ACGGCCTTGCTCAATGCATCCTGTGCAGCCATGGATGACT
+HWI-EAS443:3:28:1201:295#0/1
¥`_^_[^Z¥a^_a[RS^]S[]W[DZZW`WTN]__ZO`MKP
@HWI-EAS443:3:28:1222:263#0/1
GGCTGCATTGAGGGCAAGCTTTCCCAAATGTCAAAAGAAG
+HWI-EAS443:3:28:1222:263#0/1
a_aa¥[`]_QZ_aa_]_[LW`]TSW^]_a`T^^W_X]TOV
@HWI-EAS443:3:28:1224:71#0/1
CAAGGACAAAGAACATGAAGAGAACAAGCCAATTGAAGTG
+HWI-EAS443:3:28:1224:71#0/1
aa_b`¥ababba_^aab_`b``_a`aa_SW__a`¥ZVXNX
@HWI-EAS443:3:28:1282:256#0/1
GATCTTGGGGGGCTATATGAAGCAATCGAGGAGTGCCTGA
+HWI-EAS443:3:28:1282:256#0/1
abbaaab`baaaaaab`abaaa_aaa[a__aU[NZXU_^T
@HWI-EAS443:3:28:1282:496#0/1
GCCCCCCAAGATCGAAGGTTCCAGGTTCCAGGTTGTCCCT
+HWI-EAS443:3:28:1282:496#0/1
`aa`aa¥aaa`a`aaaa`S`^a`a[T^Z¥_^_U]]^]SU¥
@HWI-EAS443:3:28:1293:1527#0/1
CATTTCTTGTCTTATAGTGAAAAGCCTAGTTTTGATTCTT
```

+HWI-EAS443:3:28:1293:1527#0/1
`aa^¥babaI``^Y[__Xb]S[`bYaI_aLF[abaY^T_a
@HWI-EAS443:3:28:1302:1829#0/1
ACCGAACTTCTCCAGCCTTGAAAACTTTAGAGCCTATGTA
+HWI-EAS443:3:28:1302:1829#0/1
`a`[^a^¥Q¥Z_aa^UYY`aX`a`X¥a^`^^_ZV¥¥^_U]
@HWI-EAS443:3:28:1365:1727#0/1
GACACACATTCACATCTTTTCATTCACTGGAGAGGAGATG
+HWI-EAS443:3:28:1365:1727#0/1
aW^U^]^]_Y`bbaa^]N]aa`[`Qb[aaaXLa[UaZ_H^
@HWI-EAS443:3:28:1402:1406#0/1
CCCGAATGCACTATTGAAGCACCGATTTGAGATAATTGAA
+HWI-EAS443:3:28:1402:1406#0/1
ab^`]^aZ^Y^aa¥aa¥a]`WW¥aVU[aa[a[]__X_a]]
@HWI-EAS443:3:28:1457:1507#0/1
TTCGGATTTCCATTTCATCGACGAACGGGGTGAATCAATA
+HWI-EAS443:3:28:1457:1507#0/1
TVa^_ZaYa_`_a__aa_¥a^^aa__`_]aR^_]aW`Z_V
@HWI-EAS443:3:28:1504:1129#0/1
GAAATGGCCAGTAGGAGTCTATGGGATTCCTTTCGTCAGT
+HWI-EAS443:3:28:1504:1129#0/1
`UGS]```]__P¥`[WVW[a[[a`^SPa¥¥_^_^]¥WYUK
@HWI-EAS443:3:28:1516:712#0/1
CCCTGTTGTGTTACATATACTGTTCACCACTGTCCAGGCC
+HWI-EAS443:3:28:1516:712#0/1
a`b_aXbYH_``babb`bb`ba`bbba_aa`baZaaa_`U
@HWI-EAS443:3:28:1518:204#0/1
AGAAAAAGTAGACTTTGATGACTGCAAAGATGTTGGAGAC
+HWI-EAS443:3:28:1518:204#0/1
aba`¥^¥b[ab¥[^N_aa`^J^a_L[KQ[¥`_TV_[¥__Q
@HWI-EAS443:3:28:151:834#0/1
GCCAGTAGGAGTCTATGGGATTCCTTTCGTCAGTCCGAAA
+HWI-EAS443:3:28:151:834#0/1
abaUaabb```_abaaaaaa`aabaaa`a^aa`¥^`]Y`a
@HWI-EAS443:3:28:1533:975#0/1
GGGGGCTATATGAAGCAATCGAGGAGTGCCTGATTAATGA
+HWI-EAS443:3:28:1533:975#0/1
aa`_a`bbbabb_aaaaa_`a`aa^a^a^Z`[_`_```[_
@HWI-EAS443:3:28:1547:1169#0/1
AACTTCTCCAGCCTTGAAAACTTTAGAGCCTATGTAGATG
+HWI-EAS443:3:28:1547:1169#0/1
ab`ababaaaaZ_aaa`a``^aaa_b_a¥_a_a`__]¥`]
@HWI-EAS443:3:28:1604:601#0/1
GTCGTCCTCAAGAATGGTTCAATTTTGGCGTTCACTTCTT
+HWI-EAS443:3:28:1604:601#0/1
a_``^`_a`aaaa_`aa^a^_]aa`a_`U`^_Y^]_`T`^
@HWI-EAS443:3:28:1609:1192#0/1
CCCCGTTCGTCGATGAAATGGAAATCCGAATACATGAAAC
+HWI-EAS443:3:28:1609:1192#0/1
a```bba]a]_I[b[¥aaYWQ^a_UPUYTa`_``__`]U

@HWI-EAS443:3:28:1614:1895#0/1
CAGTCATCAAAGTCTACTTTTTCTGGTGCCATATTTTCAC
+HWI-EAS443:3:28:1614:1895#0/1
a¥WQ]`a^ab^^_¥ba_baaa[]`^`V_QD¥aaa`a_]aU
@HWI-EAS443:3:28:1644:1598#0/1
GACATTCTTTGGCTGGAAAGAGCCTAACATAGTCAAACCA
+HWI-EAS443:3:28:1644:1598#0/1
ababaa]ba¥a^I]aaUb_aaa^_]a^R_aa]]]a_`]__
@HWI-EAS443:3:28:1722:1822#0/1
AAGAATTCCTTGGTCATGTCTTTCTCTTTGACAGAAGACT
+HWI-EAS443:3:28:1722:1822#0/1
YRR__`^VM[^X_`Y]^_KO]R^¥]^VXNZ]UI^^VLZ`Y
@HWI-EAS443:3:28:1789:906#0/1
TAAATCCCAATTACCTCATGGCTTGGAAGCAGGTGCTAGC
+HWI-EAS443:3:28:1789:906#0/1
[a`a`aa_a][^¥ZZaaa_a`V`ZO¥¥[WNUQ[VVBBBBB
@HWI-EAS443:3:28:21:300#0/1
GCTTCATATAGCCCCCCAAGATCGAAGGTTCCAGGTTCCA
+HWI-EAS443:3:28:21:300#0/1
abaa`bbb`a_aaaa^a[X_`^]aWMWYNYY¥VVVNYX^Y
@HWI-EAS443:3:28:32:161#0/1
CTCCAACATCTTTGCAGTCATCAAAGTCTACTTTTTCTGG
+HWI-EAS443:3:28:32:161#0/1
abbb`Va^^``^aaaaa```aba`S`^aa^``aaaaa`^_
@HWI-EAS443:3:28:410:1560#0/1
GATGGCCACCAAAGCGGACTACACCCTTGACGAAGAGAGC
+HWI-EAS443:3:28:410:1560#0/1
YW`^``b`b]W]`]]ab```b^baababaab]abaaa`Z_
@HWI-EAS443:3:28:454:1631#0/1
ATCATTAATCAGGCACTCCTCGATTGCTTCATATAGCCCC
+HWI-EAS443:3:28:454:1631#0/1
a^_a``__¥^b¥Sa_a^`^¥SU^SO_]^^__^aXaU^X^_
@HWI-EAS443:3:28:489:734#0/1
ATCTTTGCAGTCATCAAAGTCTACTTTTTCTGGTGCCATA
+HWI-EAS443:3:28:489:734#0/1
_Xa]_ZX_¥¥X^Z[FYY^[]]a_W_¥^`_¥^XV]Z¥¥]]X
@HWI-EAS443:3:28:509:195#0/1
GATGAAATGGAAATCCGAATACATGAAACAAACTTCCAAA
+HWI-EAS443:3:28:509:195#0/1
^^]^^¥Xa`^W]¥]aa][]`a___aaaaaa__]a^ZZa[^
@HWI-EAS443:3:28:530:1139#0/1
GGGGGGCTATATGAAGCAATCGAGGAGTGCCTGATTAATG
+HWI-EAS443:3:28:530:1139#0/1
aaaaaaaaba_N^_[Z`]_[a^Y_^UWW¥^XZ_YZV^RS]
@HWI-EAS443:3:28:603:147#0/1
AAACTTTAGAGCCTATGTAGATGGATTCGAGCCGAACGGC
+HWI-EAS443:3:28:603:147#0/1
^_^a¥Zab[UQWaaRWUHSaaVaa¥VQ^[^UVGZ[VBBBB
@HWI-EAS443:3:28:66:1267#0/1
TGAGGGGTTTTCGGCTGAATCGAGAAAATTGCTTCTCATT

```
+HWI-EAS443:3:28:66:1267#0/1
ab`[aaZ[aaaa`R¥`aba_baa_aaaa^¥`a__`_a_^^
@HWI-EAS443:3:28:693:567#0/1
CGGTGGGAGACTTTGGTCGGCAAGCTTGCGCATAGTTCCT
+HWI-EAS443:3:28:693:567#0/1
abb`bb_`^Z_ab``U[babbbaaaa]_]^^[a^a_a[^^
@HWI-EAS443:3:28:694:1686#0/1
TCAAAACTAGGCTTTTCACTATAAGACAAGAAATGGCCAG
+HWI-EAS443:3:28:694:1686#0/1
a__aZ_Z]^UOZWH¥¥_aXM[Y^YQZ_YSEXVBBBBBBBB
@HWI-EAS443:3:28:702:274#0/1
GTCCTGCACACTTTCCCAATAGAGCCTTCCTCCACTCCCC
+HWI-EAS443:3:28:702:274#0/1
^a`]`````aa[aaa_W`aaaaa¥¥`^^[a`_^a¥[¥^PS
@HWI-EAS443:3:28:725:470#0/1
GGGGAAGATCCGAAAATCGAAACTAACAAGTTTGCTGCAA
+HWI-EAS443:3:28:725:470#0/1
ab`bbbbabaaababbabaaaa`bababaa`aa`_ab_aa
@HWI-EAS443:3:28:76:1744#0/1
TCGGCTGAATCGAGAAAATTGCTTCTCATTGTTCAGGCAC
+HWI-EAS443:3:28:76:1744#0/1
ab_``YZab_aV_]]Yb¥]XF]__`P_[]a]_`aa]Y^`U
@HWI-EAS443:3:28:834:874#0/1
GTGCATTCGGGTCACCAGATTCTACAATTATTGATTCACC
+HWI-EAS443:3:28:834:874#0/1
a`aaaaa`aaa__a_`^`T_`_aa``_`a``a`_`]_`ZZ
@HWI-EAS443:3:28:871:777#0/1
CGAAGAGACAATTGAAGAAAAATTTGAGATTACAGGAACT
+HWI-EAS443:3:28:871:777#0/1
`aLV^QVXTaK`Z`^^aZZZ]aZaabWZ^X]¥IQ[VQ`[`
@HWI-EAS443:3:28:919:789#0/1
CCCGAATGCACTATTGAAGCACCGATTTGAGATAATTGAA
+HWI-EAS443:3:28:919:789#0/1
`aabaaaa_b¥b_abYbbabbbbaWbb_b_bab`bbaaaa
@HWI-EAS443:3:28:941:1235#0/1
TTTCGGCTGAATCGAGAAAATTGCTTCTCATTGTTCAGGC
+HWI-EAS443:3:28:941:1235#0/1
aaa_Z`a`¥aa]``[_a``a^____`[`¥¥[^_]^]^^UV
@HWI-EAS443:3:28:954:1756#0/1
ATAAGACAAGAAATGGCCAGTAGGAGTCTATGGGATTCCT
+HWI-EAS443:3:28:954:1756#0/1
a_aaaaa_a`__`__]QZ__V_]_V[WW[^^]_]]XVVZV
@HWI-EAS443:3:28:976:563#0/1
TGGCGTTCACTTCTTTTGACATTTGGGAAAGCTTGCCCTC
+HWI-EAS443:3:28:976:563#0/1
_baabb_[¥[Z]X_ab_OY¥]P_^I[¥FY_UX_ZOO[Y¥Z
@HWI-EAS443:3:28:984:1008#0/1
TCCTGTAATCTCAAATTTTTCTTCAATTGTCTCTTCGCCT
+HWI-EAS443:3:28:984:1008#0/1
aaaaa_a`baaaba`bbbaaaaaaaaa_a`_aaa__``^`
```

```
@HWI-EAS443:3:28:991:778#0/1
AGGAGGAACTATTTTACAGCAGAAGTGTCCCACTGCAGGG
+HWI-EAS443:3:28:991:778#0/1
]b`_bba^ababbbbbaba`aaaaa_a_a__a_ab[__a^
@HWI-EAS443:3:29:1017:655#0/1
CACCGATTTGAGATAATTGAAGGAAGAGACCGAATCATGG
+HWI-EAS443:3:29:1017:655#0/1
`abababaabbb_`abbab`bbb]bb[bbabaabbbb_b`
@HWI-EAS443:3:29:1035:1193#0/1
AGCAGGTGCTAGCAGAGCTACAGGACATTGAAAATGAAGA
+HWI-EAS443:3:29:1035:1193#0/1
¥YabaYWQYaXYO^N]¥XWa¥R]XXZ¥ZX¥UR¥`_`U¥Z_
@HWI-EAS443:3:29:1137:1176#0/1
CATTAATCAGGCACTCCTCGATTGCTTCATATAGCCCCCC
+HWI-EAS443:3:29:1137:1176#0/1
aba^a]a`a^R^`_a`_aaba`¥¥]`a[``aaa_^^^^^B
@HWI-EAS443:3:29:1152:1879#0/1
CCAGGGGAGTGGAGGAAGGCTCTATTGGGAAAGTGTGCAG
+HWI-EAS443:3:29:1152:1879#0/1
ab_]SR^TaX[¥X`VSZYY]^[[X_`aSUNWa^KVQKXZX
@HWI-EAS443:3:29:1164:1629#0/1
AACTTCTCCAGCCTTGAAAACTTTAGAGCCTATGTAGATG
+HWI-EAS443:3:29:1164:1629#0/1
``]Z`VXaaU_]¥a¥MQW¥[W__^TTVSLSO`_`X`]_VB
@HWI-EAS443:3:29:1227:719#0/1
GTTTGTTTCATGTATTCGGATTTCCATTTCATCGACGAAC
+HWI-EAS443:3:29:1227:719#0/1
_LZ^aPaaaWRWT[_`]`_X__Y^^_^]]X^¥^Q[U^WBB
@HWI-EAS443:3:29:1266:821#0/1
GTTTTCGGCTGAATCGAGAAAATTGCTTCTCATTGTTCAG
+HWI-EAS443:3:29:1266:821#0/1
a_`aa`XR_a_`__]a¥ZWa¥_`a¥]^]W]]]]_`^_X]]
@HWI-EAS443:3:29:1280:953#0/1
GCCAAGTGTCGAGGCCCATGTTCCTATATGTGAGAACCAA
+HWI-EAS443:3:29:1280:953#0/1
aabbba`baabaaaa]aaa]`aa``]``aa¥aaaaaaPaa
@HWI-EAS443:3:29:1303:1562#0/1
AAGACATTCTTTGGCTGGAAAGAGCCTAACATAGTCAAAC
+HWI-EAS443:3:29:1303:1562#0/1
_ZaS[_^Z]YZaZa^_aX¥]¥RT^]Y^¥`[[VY[VPa]BB
@HWI-EAS443:3:29:1317:1031#0/1
GGGGGGCTATATGAAGCAATCGAGGAGTGCCTGATTAATG
+HWI-EAS443:3:29:1317:1031#0/1
_aaa^aaaaaaaa``aZa``_`[a^[_W_^[_`[a^a¥¥^
@HWI-EAS443:3:29:1336:553#0/1
TCGGCTGAATCGAGAAAATTGCTTCTCATTGTTCAGGCAC
+HWI-EAS443:3:29:1336:553#0/1
aaaa__a`a__a_a]__``__^a`^_]^_`[]_Y__^¥_Y
@HWI-EAS443:3:29:133:797#0/1
GGCGTGGTGTCGTCCTCAAGAATGGTTCAATTTTGGCGTT
```

+HWI-EAS443:3:29:133:797#0/1
aab_Q]]YWU`XT_]U_]``^]TYQT^PZS][^YTU`BBB
@HWI-EAS443:3:29:1515:1495#0/1
GAGAACAAGCCAATTGAAGTGGGCACTCGGTGAAAATATG
+HWI-EAS443:3:29:1515:1495#0/1
abaabaaabbbbab]ba^VTaaaaaa[a_aX`R``aa_`_
@HWI-EAS443:3:29:152:1451#0/1
GAAAGGCATAAATCCCAATTACCTCATGGCTTGGAAGCAG
+HWI-EAS443:3:29:152:1451#0/1
`ba``^`aa`¥`aabb_baaaaaabbaaaba^[SV¥Y]ZB
@HWI-EAS443:3:29:156:759#0/1
GGCCAATCGGAGAGTCACCCAGGGGAGTGGAGGAAGGCTC
+HWI-EAS443:3:29:156:759#0/1
`aa]XX___]OVa`[__]¥Z`_]_]X`X`XX[¥SY]XYRT
@HWI-EAS443:3:29:157:967#0/1
GTCTTCATGCATTTGATTGCATCATATAGTGGTATTCCCT
+HWI-EAS443:3:29:157:967#0/1
_R^`^]Z]`[_`YX^V]`]]^[SV]¥]_[W¥QMX]V[[YT
@HWI-EAS443:3:29:158:1979#0/1
GAACGGCTGCATTGAGGGCAAGCTTTCCCAAATGTCAAAA
+HWI-EAS443:3:29:158:1979#0/1
]bbaaabaaa]]`^aSQWaU^aa_a`aa`J_aU^Xa``Xb
@HWI-EAS443:3:29:160:49#0/1
CATTAATCAGGCACTCCTCGATTGCTTCATATAGCCCCCC
+HWI-EAS443:3:29:160:49#0/1
abbaaabababaaaaaaaaaaa_`__a_```a`a^U``___^
@HWI-EAS443:3:29:1666:515#0/1
TTGCCAGTAAGGTCCTGCACACTTTCCCAATAGAGCCTTC
+HWI-EAS443:3:29:1666:515#0/1
a`^^M_b_aab`UU¥_`UaU_Wa``UU¥_`¥^_¥WQVBBB
@HWI-EAS443:3:29:1684:339#0/1
CACAACTTGAGGGGTTTTCGGCTGAATCGAGAAAATTGCT
+HWI-EAS443:3:29:1684:339#0/1
PTZXZQVTUaXX`N[PPLFLX[[UTWVW[X¥^OXWBBB
@HWI-EAS443:3:29:188:1656#0/1
GGCTTGTTCTCTTCATGTTCTTTGTCCTTGGGATCTTCTC
+HWI-EAS443:3:29:188:1656#0/1
aaaa^L^aZaaaaa^^`_aa]a``Waaa`aaKU[[_`[¥_
@HWI-EAS443:3:29:18:463#0/1
TTCCAAATGTGTGCATATTGCAGCAAACTTGTTAGTTTCG
+HWI-EAS443:3:29:18:463#0/1
a`¥_]TP_`_aL[]___[TZ¥¥^[`ZW_^X`[ZZZW^][B
@HWI-EAS443:3:29:199:292#0/1
CCACATCAGTATCATTTCTCAAATGAGACCTTCCTTTTAT
+HWI-EAS443:3:29:199:292#0/1
abababaaaaa_baaaa`aaa_aa[`aa`[_``[```___]a
@HWI-EAS443:3:29:233:334#0/1
GCTCAATGCATCCTGTGCAGCCATGGATGACTTTCAGCTG
+HWI-EAS443:3:29:233:334#0/1
]_Q_^`XLa]]aaXWLYPT¥a_P]a``_aa`WYYRWYZYB

```
@HWI-EAS443:3:29:233:847#0/1
AGAGATCTGGGCTCTGGCTCATCACTGTCATACTGTTTAA
+HWI-EAS443:3:29:233:847#0/1
``_RWO^YSY[`YNXXN][[[[]]]^VTXV_WQY¥V^WBB
@HWI-EAS443:3:29:36:962#0/1
ATTTCATCGACGAACGGGGTGAATCAATAATTGTAGAATC
+HWI-EAS443:3:29:36:962#0/1
aaa_a`]a`W`_^______V___^_^^^___]]XX¥^]XX
@HWI-EAS443:3:29:432:348#0/1
ACCGAATCATGGCCTGGACAGTGGTGAACAGTATATGTAA
+HWI-EAS443:3:29:432:348#0/1
a^b_a[aaUb__aa_aa`[SNLRMR¥GR_SYHX^aW]G_B
@HWI-EAS443:3:29:475:966#0/1
TGGGGCATGGAAATGAGGCGCTGCCTTCTTCAGTCTCTTC
+HWI-EAS443:3:29:475:966#0/1
ababaa[aaaaa¥aaa[^````aa[``_`]YPY¥__`H^¥
@HWI-EAS443:3:29:51:1194#0/1
GATGCATTGAGCAAGGCCGTATTTATGTACACTCCCTTCA
+HWI-EAS443:3:29:51:1194#0/1
```abbaaa^]baZ]`aa^^aaaaa`^X[Za_¥aaa¥_`]
@HWI-EAS443:3:29:542:776#0/1
TGGGGGGCTATATGAAGCAATCGAGGAGTGCCTGATTAAT
+HWI-EAS443:3:29:542:776#0/1
aaaa```aabaa`aaa_^[^V_]M¥¥¥`¥`_¥¥__^W`¥¥
@HWI-EAS443:3:29:590:771#0/1
ATTTTACAGCAGAAGTGTCCCACTGCAGGGCTACTGAATA
+HWI-EAS443:3:29:590:771#0/1
ab]a`a`aaaba`]`ZX[a_a`^_a``a`[`Ya`[_`W^`
@HWI-EAS443:3:29:619:422#0/1
GAGGAACTATTTTACAGCAGAAGTGTCCCACTGCAGGGCT
+HWI-EAS443:3:29:619:422#0/1
¥RRY[]]][MX``[XS¥VSZVXZY^Z[[XUZRV[[^ORWY
@HWI-EAS443:3:29:627:1893#0/1
GAGAAAGGCATAAATCCCAATTACCTCATGGCTTGGAAGC
+HWI-EAS443:3:29:627:1893#0/1
`]U¥`b^`ba__b`aaa`^ba_aabaTb``Uaa`YWBBBB
@HWI-EAS443:3:29:629:1883#0/1
TTTCATCGACGAACGGGGTGAATCAATAATTGTAGAATCT
+HWI-EAS443:3:29:629:1883#0/1
aaabbab`bba]a[^¥a`OV`Y_ZU]_aa]`VYa]a_Q_¥
@HWI-EAS443:3:29:696:659#0/1
CACTCCTCGATTGCTTCATATAGCCCCCCAAGATCGAAGG
+HWI-EAS443:3:29:696:659#0/1
`aaaaW[a````aaa^_aa``^]]PX`^__S`_`__`__B
@HWI-EAS443:3:29:732:1235#0/1
ATCTGGTGACCCGAATGCACTATTGAAGCACCGATTTGAG
+HWI-EAS443:3:29:732:1235#0/1
[[ZH^¥NVb^_^YW`aaaa`_Z^¥[Y^R^`__]S[`_`[a
@HWI-EAS443:3:29:740:1197#0/1
GAGCCTATGTAGATGGATTCGAGCCGAACGGCTGCATTGA
```
```

```
+HWI-EAS443:3:29:740:1197#0/1
aa]ZZ`¥`b]a^`ZV[^^`Yaa^^^^V[^Y`^Y¥BBBBBB
@HWI-EAS443:3:29:749:1561#0/1
AGAATTCCTTGGTCATGTCTTTCTCTTTGACAGAAGACTC
+HWI-EAS443:3:29:749:1561#0/1
]_a^a`a`_a]_^aa`a]`a_`a_^]a]_¥]`]a^]]_X_
@HWI-EAS443:3:29:762:805#0/1
GGGATCTTCTCTTCATTTTCAATGTCCTGTAGCTCTGCTA
+HWI-EAS443:3:29:762:805#0/1
`[]`_P__a`a_^_``a^______¥^^__V_`¥W_W^^WB
@HWI-EAS443:3:29:785:547#0/1
GAGGGGTTTTCGGCTGAATCGAGAAAATTGCTTCTCATTG
+HWI-EAS443:3:29:785:547#0/1
abba`]`_baa[a`aabaa_```_Y_`]a_X]a_aV_a[`
@HWI-EAS443:3:29:80:1284#0/1
CCACAACTTGAGGGGTTTTCGGCTGAATCGAGAAAATTGC
+HWI-EAS443:3:29:80:1284#0/1
abba]`_^_W]a¥]_]]]__JNX`W[[WWU]UQS¥ZSXZW
@HWI-EAS443:3:29:842:1605#0/1
CGAGGGGGAGGGAATACCACTATATGATGCAATCAAATGC
+HWI-EAS443:3:29:842:1605#0/1
abbaabbabbabbaabbabaaababab``abaabbaa`ab
@HWI-EAS443:3:29:844:528#0/1
GGGGCATGGAAATGAGGCGCTGCCTTCTTCAGTCTCTTCA
+HWI-EAS443:3:29:844:528#0/1
`TZOX]aa_TYa^a``aaa]^aa`a_Ya^_¥_```___[U
@HWI-EAS443:3:29:859:678#0/1
ACCCCGTTCGTCGATGAAATGGAAATCCGAATACATGAAA
+HWI-EAS443:3:29:859:678#0/1
abbYZ¥_^a^Z__VZb]aY^XH[`SXQ[a_]¥KVZ_a_[^
@HWI-EAS443:3:29:904:31#0/1
AAGACATTCTTTGGCTGGAAAGAGCCTAACATAGTCAAAC
+HWI-EAS443:3:29:904:31#0/1
a¥baaW`a_a`aaa`aaa`aa_`a_^_``^_``_]____X
@HWI-EAS443:3:29:918:728#0/1
AGCAGGTGCTAGCAGAGCTACAGGACATTGAAAATGAAGA
+HWI-EAS443:3:29:918:728#0/1
aa```UV^`aa`]X__VZX_S__Z]YW^_RUXYXV]a]Z^
@HWI-EAS443:3:29:963:1320#0/1
AAGCAGGTGCTAGCAGAGCTACAGGACATTGAAAATGAAG
+HWI-EAS443:3:29:963:1320#0/1
`a^aaaa^_aaaa``a__a_`_aa___a____```^_^`¥
@HWI-EAS443:3:29:97:1454#0/1
CTTCCCTCCGTGTTACTCCAATTTCAATGAACCGGTTCTC
+HWI-EAS443:3:29:97:1454#0/1
`¥`bb_Tab^N]W]¥_Y[¥[_^HWX__Y_`ZR][_LWYVa
@HWI-EAS443:3:2:1016:1357#0/1
GATTGGCCATGTTTCCGATTTGTTTTCAAAGAATTCCTTG
+HWI-EAS443:3:2:1016:1357#0/1
a_a¥UYXV[Z`¥¥]WS[X¥_Z^X^^^]^WZ_]UYXSMZ]]
```

```
@HWI-EAS443:3:2:1046:813#0/1
TACATATACTGTTCACCACTGTCCAGGCCATGATTCGGTC
+HWI-EAS443:3:2:1046:813#0/1
abaabaaaaabaaaa_aaaaaa```^[[Ya`a``W^^_]O
@HWI-EAS443:3:2:1172:1115#0/1
ATCTACATAGGCTCTAAAGTTTTCAAGGCTGGAGAAGTTC
+HWI-EAS443:3:2:1172:1115#0/1
`_Z__]`U^`ba_GYUZ[`TIZ][P^[YRXV^LRDUTHWB
@HWI-EAS443:3:2:1238:282#0/1
AGGAGTCTATGGGATTCCTTTCGTCAGTCCGAAAGAGGCG
+HWI-EAS443:3:2:1238:282#0/1
```]b[X`]]aaa[`]P^aa^``¥V__^^¥¥LZVRT^[SB
@HWI-EAS443:3:2:1246:1846#0/1
GGAAGCAGGTGCTAGCAGAGCTACAGGACATTGAAAATGA
+HWI-EAS443:3:2:1246:1846#0/1
ab`[ba`baHa_¥aa[abaa]_^_aa¥`]^``_U_[__aX
@HWI-EAS443:3:2:1249:372#0/1
CTTGGGGGGCTATATGAAGCAATCGAGGAGTGCCTGATTA
+HWI-EAS443:3:2:1249:372#0/1
aabbba``abbabaaba`_Q][`]a_b_^aW_¥Vba[_]S
@HWI-EAS443:3:2:1333:1356#0/1
CAAAGAATTCCTTGGTCATGTCTTTCTCTTTGACAGAAGA
+HWI-EAS443:3:2:1333:1356#0/1
aa`ab__aa__aab_^_aaa^_a_a¥`_aa_a^]_W^_[_
@HWI-EAS443:3:2:1368:1266#0/1
GGGAGACTTTGGTCGGCAAGCTTGCGCATAGTTCCTGTAA
+HWI-EAS443:3:2:1368:1266#0/1
]NWUWU]`a_]VR]WXR]]VV_¥]S[]XVGMSPRRGPQWU
@HWI-EAS443:3:2:1585:779#0/1
CTCTTGAGGACTGCGATAGGCCAAGTGTCGAGGCCCATGT
+HWI-EAS443:3:2:1585:779#0/1
_WaabbZbb`a¥b[Wa]aaaWSMRT_b`[a__ZTRZ¥`^T
@HWI-EAS443:3:2:1585:813#0/1
ATCAGGCAATCTGAGGGGGCGTGGTGTCGTCCTCAAGAAT
+HWI-EAS443:3:2:1585:813#0/1
`aY`ba`b^baabR`a_aa_a`a^Z_¥¥_¥SU_LZU¥aVB
@HWI-EAS443:3:2:1616:523#0/1
GGTTTTCGGCTGAATCGAGAAAATTGCTTCTCATTGTTCA
+HWI-EAS443:3:2:1616:523#0/1
ab]```Zaa_aaaa_S_[`]^__Y_`[_^UUOZ`]_Z^RX
@HWI-EAS443:3:2:1663:536#0/1
GGCTTGGAAGCAGGTGCTAGCAGAGCTACAGGACATTGAA
+HWI-EAS443:3:2:1663:536#0/1
aYRLW[`ZWFNMU^QTUWLSMKHUUQNNYUUUYSUMBBBB
@HWI-EAS443:3:2:1775:1801#0/1
GCTAGCACCTGCTTCCAAGCCATGAGGTAATTGGGATTTA
+HWI-EAS443:3:2:1775:1801#0/1
_`b]ab`^_WaaaYW]`b`¥]aaa[``Y[Z`a]a`¥a¥X_
@HWI-EAS443:3:2:192:373#0/1
CGGTTCATTGAAATTGGAGTAACACGGAGGGAAGTCCACA
```
```

+HWI-EAS443:3:2:192:373#0/1
abbTa`aaa_aaa`_ba_a[Y``^]aa_ba`a_a]YY^`¥
@HWI-EAS443:3:2:193:1102#0/1
AATGAAGGGAGTGTACATAAATACGGCCTTGCTCAATGCA
+HWI-EAS443:3:2:193:1102#0/1
ab`b`abb_abO`O_Y_aaaa]``a_YHW_`^_`a_YXU_
@HWI-EAS443:3:2:263:811#0/1
CACGAGGGGGAGGGAATACCACTATATGATGCAATCAAAT
+HWI-EAS443:3:2:263:811#0/1
`YaaPabab_¥Z^_S[S[UHTUS``T]a^aaZ[Z[DSZNB
@HWI-EAS443:3:2:279:879#0/1
GGGGGCTATATGAAGCAATCGAGGAGTGCCTGATTAATGA
+HWI-EAS443:3:2:279:879#0/1
aaaaa`a^a`aa`_`YZa_[a^a^[_N^SS^a[^[^Y[Y[
@HWI-EAS443:3:2:347:426#0/1
AAGAATTCCTTGGTCATGTCTTTCTCTTTGACAGAAGACT
+HWI-EAS443:3:2:347:426#0/1
YUVVW¥`YZ¥¥b_Xa]W`WVX^]aa¥Yaa[^_P[V__XBB
@HWI-EAS443:3:2:354:1656#0/1
GGGGCATGGAAATGAGGCGCTGCCTTCTTCAGTCTCTTCA
+HWI-EAS443:3:2:354:1656#0/1
aa^b_ZZab]aZ`a][`U]U^a^TSQJY`TY^PX^^¥]U¥
@HWI-EAS443:3:2:403:868#0/1
AGAGCCTAACATAGTCAAACCACATGAGAAAGGCATAAAT
+HWI-EAS443:3:2:403:868#0/1
aa_^a^aaa____`W___aX^¥^_]__`]]]`X^_[_X]B
@HWI-EAS443:3:2:421:520#0/1
GAGAGGAGATGGCCACCAAAGCGGACTACACCCTTGACGA
+HWI-EAS443:3:2:421:520#0/1
a`aa^``[`_aa^¥[KZa``a]a¥^_]_¥^UYGY¥__¥^¥
@HWI-EAS443:3:2:555:430#0/1
CGAAAATCGAAACTAACAAGTTTGCTGCAATATGCACACA
+HWI-EAS443:3:2:555:430#0/1
aabbbbaba_`b`aabababYbbaUabb`ababb_aaaSa
@HWI-EAS443:3:2:628:1476#0/1
CAATAATTGTAGAATCTGGTGACCCGAATGCACTATTGAA
+HWI-EAS443:3:2:628:1476#0/1
aaabaabab`bbaaaa]^`¥aa^¥]a`a_aa_¥aa``a__
@HWI-EAS443:3:2:678:1529#0/1
CAAGAAATGGCCAGTAGGAGTCTATGGGATTCCTTTCGTC
+HWI-EAS443:3:2:678:1529#0/1
abaabbbbb`a`a_M`]aab_aaa_b_aaaa`^`a__a¥_
@HWI-EAS443:3:2:720:1323#0/1
TGAACTTTGTAAGTATGGAGTTCTCACTCACTGACCCGAG
+HWI-EAS443:3:2:720:1323#0/1
abaab[^]^aa`aGaa]b[aY^_`V`^`_^__`^XWD[^^
@HWI-EAS443:3:2:738:1545#0/1
GGCCTGGACAGTGGTGAACAGTATATGTAACACAACAGGG
+HWI-EAS443:3:2:738:1545#0/1
abab_aba]ba`Ub`Za_]b]`]^b^_U¥aQXFW`__``^

@HWI-EAS443:3:2:751:628#0/1
ATATCGCAAGCATGAGGAGGAACTATTTTACAGCAGAAGT
+HWI-EAS443:3:2:751:628#0/1
^_]^`^ZYPWO_`^YZ]X`a]`¥W_^[b`[Y_^UYVZTTL
@HWI-EAS443:3:2:86:1187#0/1
GAAAGGCATAAATCCCAATTACCTCATGGCTTGGAAGCAG
+HWI-EAS443:3:2:86:1187#0/1
]_]VbU_a[`babW]Wabb[abbaSb_aaZ]`[aX``_BB
@HWI-EAS443:3:2:87:1163#0/1
CACTGCAGGGCTACTGAATACATAATGAAGGGAGTGTACA
+HWI-EAS443:3:2:87:1163#0/1
`JGQ`W[XaTJWWT^¥ZJ[RVP]SO__]WQ_[LFL]BBBB
@HWI-EAS443:3:2:974:259#0/1
CGGTGAAAATATGGCACCAGAAAAAGTAGACTTTGATGAC
+HWI-EAS443:3:2:974:259#0/1
aab_bba_aaabWa^_[a`aa`a__a_aa`_abab`a`_^
@HWI-EAS443:3:2:997:415#0/1
GAAGGGAGTGTACATAAATACGGCCTTGCTCAATGCATCC
+HWI-EAS443:3:2:997:415#0/1
a]a`aZUUXa¥_¥Z_¥Wa^_Y_¥W¥¥¥[WX¥WW¥¥S¥¥YY
@HWI-EAS443:3:30:1027:1138#0/1
GGTTTTCGGCTGAATCGAGAAAATTGCTTCTCATTGTTCA
+HWI-EAS443:3:30:1027:1138#0/1
aa]aaaaa`aaaaa``a`a`aa`_`__`__`]___^XZX^
@HWI-EAS443:3:30:1076:1113#0/1
GGGGGCTATATGAAGCAATCGAGGAGTGCCTGATTAATGA
+HWI-EAS443:3:30:1076:1113#0/1
a`]`aabb`abbaa``aaaaaaa`_aX`aaaa^```__U^
@HWI-EAS443:3:30:1099:1493#0/1
GCTGAAAGTCATCCATGGCTGCACAGGATGCATTGAGCAA
+HWI-EAS443:3:30:1099:1493#0/1
ab`b`bba¥`a`bbbabaaaaa_aU]Xa_a]U`^VX^^QX
@HWI-EAS443:3:30:1146:2030#0/1
TGCGTCTCCACAACTTGAGGGGTTTTCGGCTGAATCGAGA
+HWI-EAS443:3:30:1146:2030#0/1
_`V_``a`^b_`aaaVXZUa`aV[`_¥X_a^_aaXYZQa]
@HWI-EAS443:3:30:1180:409#0/1
GAACTTCTCCAGCCTTGAAAACTTTAGAGCCTATGTAGAT
+HWI-EAS443:3:30:1180:409#0/1
_aaabaT]^UZb]W]U``aaS____`aJXTV`U`aYQaRY
@HWI-EAS443:3:30:1193:1536#0/1
ACCCCTGTTGTGTTACATATACTGTTCACCACTGTCCAGG
+HWI-EAS443:3:30:1193:1536#0/1
`ba_[]`__b[``aaYaa]aa_`VM^a__[S`__]_GZZ]
@HWI-EAS443:3:30:1207:224#0/1
CTGCTGTAAAATAGTTCCTCCTCATGCTTGCGATATGTTC
+HWI-EAS443:3:30:1207:224#0/1
aabbabbbbbbbbbabaabbaaab__]bbbabb_bbbX_`
@HWI-EAS443:3:30:1241:1509#0/1
GAAGTTCGGTGGGAGACTTTGGTCGGCAAGCTTGCGCATA

```
+HWI-EAS443:3:30:1241:1509#0/1
a^W`¥aa^]¥^`_]`¥_`]YVQU^NS]^PY[Y¥VRTSSUT
@HWI-EAS443:3:30:1311:1941#0/1
ATCCCAAGGACAAAGAACATGAAGAGAACAAGCCAATTGA
+HWI-EAS443:3:30:1311:1941#0/1
aZTPON¥ZPZYZa_aa][WW¥ZW^[Z^_Va`USNSRRRUZ
@HWI-EAS443:3:30:1337:866#0/1
ATTGCAGCAAACTTGTTAGTTTCGATTTTCGGATCTTCCC
+HWI-EAS443:3:30:1337:866#0/1
^a`babb_aa__]J_abb_`ab`ZW^`b^^a_G[S][DIN
@HWI-EAS443:3:30:1559:505#0/1
CAAATGCATGAAGACATTCTTTGGCTGGAAAGAGCCTAAC
+HWI-EAS443:3:30:1559:505#0/1
^abbbbbbbbbbababbbbbababba``]a`a^_F[baaa
@HWI-EAS443:3:30:1563:1906#0/1
GGGGCATGGAAATGAGGCGCTGCCTTCTTCAGTCTCTTCA
+HWI-EAS443:3:30:1563:1906#0/1
_VX`S`^`a_aYX``a]N]_¥aRNKZUZ_¥¥WLKZWS¥WV
@HWI-EAS443:3:30:1606:446#0/1
ACCACATGAGAAAGGCATAAATCCCAATTACCTCATGGCT
+HWI-EAS443:3:30:1606:446#0/1
a¥YX¥a`b_`aaT]aXb^L^P[U`_`a_^aVaa_^YY^G[
@HWI-EAS443:3:30:1649:427#0/1
ATAAATACGGCCTTGCTCAATGCATCCTGTGCAGCCATGG
+HWI-EAS443:3:30:1649:427#0/1
abababbY[^`aa`ZU`a`b_a]a^¥Y``¥a__`VR__`]
@HWI-EAS443:3:30:1678:1359#0/1
AAATAGTTCCTCCTCATGCTTGCGATATGTTCAATCGGGG
+HWI-EAS443:3:30:1678:1359#0/1
aa`__]Wa__a¥]_]X__]_]`W[^^Z_[Y]V]^^XOZZX
@HWI-EAS443:3:30:1685:498#0/1
GGCATGGAAATGAGGCGCTGCCTTCTTCAGTCTCTTCAGC
+HWI-EAS443:3:30:1685:498#0/1
abbbabbaaaaba`a_aaaa¥_`aS`a_[aW¥`___Q[¥W
@HWI-EAS443:3:30:16:256#0/1
GGGGCATGGAAATGAGGCGCTGCCTTCTTCAGTCTCTTCA
+HWI-EAS443:3:30:16:256#0/1
_¥^`^]^b`ZZ_`a]``aaa`a^]_^^^^_YZZ`^^[YTP
@HWI-EAS443:3:30:1711:243#0/1
CAGGAACTTTGACCGCTGATGGCAAAGAGGCCCATCAGGC
+HWI-EAS443:3:30:1711:243#0/1
`b_b`b]bb^a_`¥b]abba`bVa¥`aa`OQUY`^UPa¥S
@HWI-EAS443:3:30:184:1155#0/1
GGGGGCTATATGAAGCAATCGAGGAGTGCCTGATTAATGA
+HWI-EAS443:3:30:184:1155#0/1
aabbababbb`abb^b[bbb`_``Ub]`a`a`aaaa[[a^
@HWI-EAS443:3:30:184:422#0/1
AATAGTTCCTCCTCATGCTTGCGATATGTTCAATCGGGGC
+HWI-EAS443:3:30:184:422#0/1
`XUU^RRY^VTYN[TU[WY¥W¥ZQQTRVTRQUSNRSSBBB
```

```
@HWI-EAS443:3:30:221:1898#0/1
CCAACATCTTTGCAGTCATCAAAGTCTACTTTTTCTGGTG
+HWI-EAS443:3:30:221:1898#0/1
ab`]aaaba``_ba¥Za`_aa`^¥^a`_a`__```_UTX_
@HWI-EAS443:3:30:382:640#0/1
ATGCCTTTCTCATGTGGTTTGACTATGTTAGGCTCTTTCC
+HWI-EAS443:3:30:382:640#0/1
a]_a^`]^`_a_`aW[`Z_Y][^XXW`V]YYWXXSW[YUB
@HWI-EAS443:3:30:395:399#0/1
TGCCCCGATTGAACATATCGCAAGCATGAGGAGGAACTAT
+HWI-EAS443:3:30:395:399#0/1
ab_U]R_V¥X]]aU^`Z^V]`XL^_OZ¥^]QLX_YYR¥W[
@HWI-EAS443:3:30:483:875#0/1
TTCGGCTGAATCGAGAAAATTGCTTCTCATTGTTCAGGCA
+HWI-EAS443:3:30:483:875#0/1
aaaaaaaaaaa``P[]`_aa`_``]`_`]`_[Z]`__¥]_
@HWI-EAS443:3:30:490:605#0/1
TGCGTCTCCACAACTTGAGGGGTTTTCGGCTGAATCGAGA
+HWI-EAS443:3:30:490:605#0/1
¥¥`a_bb_JQ_`aYa_a_a]_`[_baaZ¥Z]]¥]ZWRV^a
@HWI-EAS443:3:30:50:591#0/1
GATTACAGGAACTATGCGCAAGCTTGCCGACCAAAGTCTC
+HWI-EAS443:3:30:50:591#0/1
Xba][ZT¥]^bXaa_a]aXa_`aQL]`a¥RV^ZHXIPTU^
@HWI-EAS443:3:30:52:458#0/1
GGGGCATGGAAATGAGGCGCTGCCTTCTTCAGTCTCTTCA
+HWI-EAS443:3:30:52:458#0/1
]ba`abab_]bZbbZbaabaaaaaaa`b`aa`Na```aX_
@HWI-EAS443:3:30:584:959#0/1
GATTGGCCATGTTTCCGATTTGTTTTCAAAGAATTCCTTG
+HWI-EAS443:3:30:584:959#0/1
`aMa``bbbababbaba¥_b¥b[bab`¥`baaaW]]`_a`
@HWI-EAS443:3:30:624:836#0/1
ACCCGAGTCACGAGGGGGAGGGAATACCACTATATGATGC
+HWI-EAS443:3:30:624:836#0/1
aaaaa_a^a``aZa_^``______[`_¥_XZ`^__^]]YZ
@HWI-EAS443:3:30:635:854#0/1
GTTCTTTGTCCTTGGGATCTTCTCTTCATTTTCAATGTCC
+HWI-EAS443:3:30:635:854#0/1
a_``a`_`P^`_____Y]]__a^`__^^`^^^__][]ZZ]
@HWI-EAS443:3:30:679:1153#0/1
GGTGAACTTTGTAAGTATGGAGTTCTCACTCACTGACCCG
+HWI-EAS443:3:30:679:1153#0/1
a_R___aa_]^F_Z]`_aaaQbQUaa]^a`aX`]_aaG__
@HWI-EAS443:3:30:711:939#0/1
AGCTTGCTAGAGATCTGGGCTCTGGCTCATCACTGTCATA
+HWI-EAS443:3:30:711:939#0/1
Z¥X`^^WRVG]Z_^^YXa^^a_a[P^_ba__¥a_`]^XZZ
@HWI-EAS443:3:30:777:1819#0/1
CGAAAATCGAAACTAACAAGTTTGCTGCAATATGCACACA
```

```
+HWI-EAS443:3:30:777:1819#0/1
a_`^bb`_``aaaa`aaa_]^¥^^`_a[aZa_^]_a^_^_
@HWI-EAS443:3:30:782:1885#0/1
GTTACATATACTGTTCACCACTGTCCAGGCCATGATTCGG
+HWI-EAS443:3:30:782:1885#0/1
^bbbabbbbbbab`bbbbbbaa`_abb^aabaa`a_a]]Q
@HWI-EAS443:3:30:798:520#0/1
GATGAAATGGAAATCCGAATACATGAAACAAACTTCCAAA
+HWI-EAS443:3:30:798:520#0/1
]¥^_a`a_VZ[Z_aa`a`aaa_```aaabb_R_a_]W`a`
@HWI-EAS443:3:30:834:1534#0/1
GGAAAGAGCCTAACATAGTCAAACCACATGAGAAAGGCAT
+HWI-EAS443:3:30:834:1534#0/1
aabbb`abbaaabaaaba`babaa__aa``aaaa`^]]aa
@HWI-EAS443:3:30:867:1962#0/1
GAAAGAGGCGAAGAGACAATTGAAGAAAAATTTGAGATTA
+HWI-EAS443:3:30:867:1962#0/1
``a``_aaa_b``a`abbaab^aaaaaaaaaa`a^`a]a
@HWI-EAS443:3:30:869:1524#0/1
CATGACCAAGGAATTCTTTGAAAACAAATCGGAAACATGG
+HWI-EAS443:3:30:869:1524#0/1
aaaaaaaaa]aa`aaa```_a`¥``a^____`_a^_^]W
@HWI-EAS443:3:30:909:1048#0/1
CGATGAAATGGAAATCCGAATACATGAAACAAACTTCCAA
+HWI-EAS443:3:30:909:1048#0/1
¥bbabaYab``ba`babaaabb¥bbabbbabbbbbb`_ab
@HWI-EAS443:3:30:909:1301#0/1
CATTTTGGACCCAGCTTGCTAGAGATCTGGGCTCTGGCTC
+HWI-EAS443:3:30:909:1301#0/1
a^aaaaaabaaaaaaaa_a_a_```¥___S_b_a^_Z_]a
@HWI-EAS443:3:30:969:1494#0/1
GCCATGTTTCCGATTTGTTTTCAAAGAATTCCTTGGTCAT
+HWI-EAS443:3:30:969:1494#0/1
aaaaaaUaaaaa_aaabU`aaa``aa_^```¥`]a¥W_¥_
@HWI-EAS443:3:30:96:678#0/1
GAAAGGCATAAATCCCAATTACCTCATGGCTTGGAAGCAG
+HWI-EAS443:3:30:96:678#0/1
`_^^aaabababaaa^aaaaaa]a`a`a_]Y]`_`WV_^[[B
@HWI-EAS443:3:31:1022:1583#0/1
CAAATGCATGAAGACATTCTTTGGCTGGAAAGAGCCTAAC
+HWI-EAS443:3:31:1022:1583#0/1
abbbbaaba]bbababbaaa`ab_aaababa_a`U[abb^
@HWI-EAS443:3:31:1043:1646#0/1
GAAAAGATGTGAATGTGTGTCTTCTCAGATTTTATTTTGT
+HWI-EAS443:3:31:1043:1646#0/1
a``a^a`_bOa_`aa__]a]]^`a`¥^`Z_``]`[a[aWY
@HWI-EAS443:3:31:1061:510#0/1
TCTCATTTGAGAAATGATACTGATGTGGTGAACTTTGTAA
+HWI-EAS443:3:31:1061:510#0/1
Z_b]bbbbbbb_bbbb`baab`ba^Vbbaabbab^bbaT^
```

@HWI-EAS443:3:31:1129:1327#0/1
GAGCCTTCCTCCACTCCCCTGGGTGACTCTCCGATTGGCC
+HWI-EAS443:3:31:1129:1327#0/1
Zaabbabbbbbbb`bba`abbb[TTabaaaabbU``aa[[
@HWI-EAS443:3:31:1135:1620#0/1
CATAGTCAAACCACATGAGAAAGGCATAAATCCCAATTAC
+HWI-EAS443:3:31:1135:1620#0/1
Vbabb]b_bbabaab[abbbbbbbaaaabbba__bba`aa
@HWI-EAS443:3:31:1194:1030#0/1
ATTTCATCGACGAACGGGGTGAATCAATAATTGTAGAATC
+HWI-EAS443:3:31:1194:1030#0/1
_a_aa`aaaaa[^^Ya`]¥L]`¥XOW_¥___^_]``]`XU
@HWI-EAS443:3:31:1328:1888#0/1
AGCCAACAAAATAAAATCTGAGAAGACACACATTCACATC
+HWI-EAS443:3:31:1328:1888#0/1
T_^R[T¥UYMPZa¥`^`^VW^aaaQ¥aWUZUX]UJZG[XD
@HWI-EAS443:3:31:1431:912#0/1
CTTGCTAGAGATCTGGGCTCTGGCTCATCACTGTCATACT
+HWI-EAS443:3:31:1431:912#0/1
^abbab_bbbbbab`bbab_baba__bb`b]bbaabbbbb
@HWI-EAS443:3:31:1455:588#0/1
GGCCACCAAAGCGGACTACACCCTTGACGAAGAGAGCAGG
+HWI-EAS443:3:31:1455:588#0/1
abbbbaabbbbab^`^abab`aaa`^a``aaaaa_a``a_
@HWI-EAS443:3:31:1510:1750#0/1
AAGAAATGGCCAGTAGGAGTCTATGGGATTCCTTTCGTCA
+HWI-EAS443:3:31:1510:1750#0/1
a_bab_bbaab^aRXaa^XPV_aa_[_`^[[^`__L¥TVZ
@HWI-EAS443:3:31:1536:1412#0/1
CATATCGCAAGCATGAGGAGGAACTATTTTACAGCAGAAG
+HWI-EAS443:3:31:1536:1412#0/1
`bbabba`abb_]bbaabbbabaabbbbabba`]^a`aaa
@HWI-EAS443:3:31:1566:1137#0/1
TGCGTCTCCACAACTTGAGGGGTTTTCGGCTGAATCGAGA
+HWI-EAS443:3:31:1566:1137#0/1
abab__aa`a]aa_bbb_b_`b_aa^a^aXaaba_¥a¥a¥
@HWI-EAS443:3:31:157:180#0/1
GGCTGAATCGAGAAAATTGCTTCTCATTGTTCAGGCACTT
+HWI-EAS443:3:31:157:180#0/1
^bbb_ba``¥N]baUab][aa[a^baQab^Ua^P`a`aY[
@HWI-EAS443:3:31:1580:1787#0/1
GAGAAAGGCATAAATCCCAATTACCTCATGGCTTGGAAGC
+HWI-EAS443:3:31:1580:1787#0/1
M`bab`Z¥WababbaW]_`^``b^W^[`aba]aaW]H_VF
@HWI-EAS443:3:31:17:754#0/1
CCAACATCTTTGCAGTCATCAAAGTCTACTTTTTCTGGTG
+HWI-EAS443:3:31:17:754#0/1
`^`¥^_Z`¥N¥`a`aR_]_STTMYT[¥_Q]VW[U`]LMEH
@HWI-EAS443:3:31:186:1760#0/1
GGGGGCTATATGAAGCAATCGAGGAGTGCCTGATTAATGA

+HWI-EAS443:3:31:186:1760#0/1
[a_``bbbaaa`baabbb^aaaa_a`W`a`_`^_aaU^¥`
@HWI-EAS443:3:31:247:135#0/1
CAGCCTTGAAAACTTTAGAGCCTATGTAGATGGATTCGAG
+HWI-EAS443:3:31:247:135#0/1
aa^^]Z¥]_b___]^ab]a¥b]¥aaaHZ`[]¥_]`_a__^
@HWI-EAS443:3:31:278:1966#0/1
GGGCAAGAATCAAAACTAGGCTTTTCACTATAAGACAAGA
+HWI-EAS443:3:31:278:1966#0/1
`bbaYaaaZ`bbb[abaa`aba`a`bba_b_aa__aaa``
@HWI-EAS443:3:31:293:1947#0/1
ACAGTCTATATGCGTCTCCACAACTTGAGGGGTTTTCGGC
+HWI-EAS443:3:31:293:1947#0/1
aa]ZZa]__`_Sa_Y_[a`W____^^SW[][ZGZVZ]OUY
@HWI-EAS443:3:31:309:128#0/1
ATCATTAATCAGGCACTCCTCGATTGCTTCATATAGCCCC
+HWI-EAS443:3:31:309:128#0/1
aabb¥[V¥][`Y^Y`^]_X^_[[VX]]¥V^X]_]_SXVVP
@HWI-EAS443:3:31:309:1906#0/1
CTCGGTGAAAATATGGCACCAGAAAAAGTAGACTTTGATG
+HWI-EAS443:3:31:309:1906#0/1
aYaaaZa`aa_aa___``a`_aaa``__Ua[Y`[^]^_^X
@HWI-EAS443:3:31:372:1210#0/1
AGAGCCTAACATAGTCAAACCACATGAGAAAGGCATAAAT
+HWI-EAS443:3:31:372:1210#0/1
_ZWV`a]RS^^Y]QTaaa``_V``[Y[[YGOVU``]`]^X
@HWI-EAS443:3:31:403:1824#0/1
CGAAAGAGGCGAAGAGACAATTGAAGAAAAATTTGAGATT
+HWI-EAS443:3:31:403:1824#0/1
aab_SX]a_b__aZa`[P_a_ZXV]¥_`Xa^¥_WU_V^]Y
@HWI-EAS443:3:31:403:377#0/1
GAATATGGGGAAGATCCGAAAATCGAAACTAACAAGTTTG
+HWI-EAS443:3:31:403:377#0/1
a[a^bba__aTO^aYa`a^]ba_`^ZYa`^[_]a_aO][^
@HWI-EAS443:3:31:425:197#0/1
CAATAATTGTAGAATCTGGTGACCCGAATGCACTATTGAA
+HWI-EAS443:3:31:425:197#0/1
aa`ba¥XVb_b`_b`a_bbW_aaaYa`a^[`]S`_``ZFS
@HWI-EAS443:3:31:45:682#0/1
CGTCTCCACAACTTGAGGGGTTTTCGGCTGAATCGAGAAA
+HWI-EAS443:3:31:45:682#0/1
aa[a_b_W`¥Y`aa^`aS`aY]YUa_aa`aaa_]TO[¥U`
@HWI-EAS443:3:31:491:500#0/1
ATGGCTTGGAAGCAGGTGCTAGCAGAGCTACAGGACATTG
+HWI-EAS443:3:31:491:500#0/1
``ba`a`¥OW_]UN_¥ORZWY_[OXG[GXKX_TQUX_ZTV
@HWI-EAS443:3:31:498:1191#0/1
AATCAGGCACTCCTCGATTGCTTCATATAGCCCCCCAAGA
+HWI-EAS443:3:31:498:1191#0/1
aaaab]`aaaaaaaaa][^a`^]_a[aZLZ`_Z__¥_^¥^

```
@HWI-EAS443:3:31:541:469#0/1
ATATACTGTTCACCACTGTCCAGGCCATGATTCGGTCTCT
+HWI-EAS443:3:31:541:469#0/1
aaaaaaab^aaaaaa_a`^`__``^^```_^^Z^`W__^]
@HWI-EAS443:3:31:610:228#0/1
AAAGTCTACTTTTTCTGGTGCCATATTTTCACCGAGTGCC
+HWI-EAS443:3:31:610:228#0/1
`bab[abaaaaa`a`a`VOXaaaa`a`a`a`_^a`TTY`]
@HWI-EAS443:3:31:633:1516#0/1
AAGACATTCTTTGGCTGGAAAGAGCCTAACATAGTCAAAC
+HWI-EAS443:3:31:633:1516#0/1
a`¥`¥L]`MT]¥_ZRZ^]¥T_LTSUMUYa_`^^NYRY__I
@HWI-EAS443:3:31:635:58#0/1
AGAAAAAGTAGACTTTGATGACTGCAAAGATGTTGGAGAC
+HWI-EAS443:3:31:635:58#0/1
abaaa`ab]aaaabaabaaa__``_a`aa``a^aa_]^`[
@HWI-EAS443:3:31:671:870#0/1
AGCCGAAAACCCCTCAAGTTGTGGAGACGCATATAGACTG
+HWI-EAS443:3:31:671:870#0/1
ZZabb__[bb_ab`a_Ya^aa]bb_aaaaaaa^`aa`a`a
@HWI-EAS443:3:31:721:647#0/1
CAAACTTCCAAATGTGTGCATATTGCAGCAAACTTGTTAG
+HWI-EAS443:3:31:721:647#0/1
abbabbbaaaaaba_a_aaaaaa`aab^baa`aa`aa`aX
@HWI-EAS443:3:31:810:772#0/1
CTGAAATTAAGTATTGAAGACCCGAGTCACGAGGGGGAGG
+HWI-EAS443:3:31:810:772#0/1
abbZX^]_aba_baZXZ]_a`Y`a`[^Z^a`_`TQ[^¥a^
@HWI-EAS443:3:31:813:1182#0/1
CATGACCAAGGAATTCTTTGAAAACAAATCGGAAACATGG
+HWI-EAS443:3:31:813:1182#0/1
abbbbbba``bbbbbabbbbabaaaa¥`aa`_`_aabaaa
@HWI-EAS443:3:31:833:61#0/1
AACAAGTTTGCTGCAATATGCACACATTTGGAAGTTTGTT
+HWI-EAS443:3:31:833:61#0/1
[^TVbaa`^ZVabbXbb`_a^aaaa_Z¥_ba_aaV¥``]_
@HWI-EAS443:3:31:854:619#0/1
GACGAAAGGAATCCCATAGACTCCTACTGGCCATTTCTTG
+HWI-EAS443:3:31:854:619#0/1
_^bb¥a`T^b^bb`___a^_`b`aaa_a¥`¥^[W_¥GYYF
@HWI-EAS443:3:31:890:265#0/1
GTTTCCGATTTGTTTTCAAAGAATTCCTTGGTCATGTCTT
+HWI-EAS443:3:31:890:265#0/1
aYZ^Za_VW_]YX`a^]^^`¥XR^_S``a`^W^^^]Z]_]
@HWI-EAS443:3:31:894:1310#0/1
CAAATCGGTGCTTCAATAGTGCATTCGGGTCACCAGATTC
+HWI-EAS443:3:31:894:1310#0/1
ab_`aba`W`aaaa_aaa_X`a`_`a___Xa```¥_^TZP
@HWI-EAS443:3:31:935:1174#0/1
ATCAGTATCATTTCTCAAATGAGACCTTCCTTTTATAATG
```

```
+HWI-EAS443:3:31:935:1174#0/1
¥a`___a_^a¥`__X`aaaa`_aa^`_aa_aaa_``¥P]a
@HWI-EAS443:3:31:939:810#0/1
GCCCTGCTCTCTTCGTCAAGGGTGTAGTCCGCTTTGGTGG
+HWI-EAS443:3:31:939:810#0/1
`aaaa_a__a`a``_^_a`a__[`]`[Z``]aaaa`^]`^
@HWI-EAS443:3:31:948:423#0/1
GTAAAATAGTTCCTCCTCATGCTTGCGATATGTTCAATCG
+HWI-EAS443:3:31:948:423#0/1
S`babZ```Ya]S``X`b]a^[_U]]aaa`ab]_a]bV^^
@HWI-EAS443:3:31:969:1015#0/1
GTGTTACATATACTGTTCACCACTGTCCAGGCCATGATTC
+HWI-EAS443:3:31:969:1015#0/1
aab_aaaaaaaa`a```aa`__a_[Yaa[ZQ[WX¥^¥^UU
@HWI-EAS443:3:31:96:392#0/1
CACTCGGTGAAAATATGGCACCAGAAAAAGTAGACTTTGA
+HWI-EAS443:3:31:96:392#0/1
aaa`aaa]]Y]VO¥T[[_]V][V^[[_¥[]W[_^__X]^]
@HWI-EAS443:3:32:1003:663#0/1
TCCGTGTTACTCCAATTTCAATGAACCGGTTCTCTTTGTA
+HWI-EAS443:3:32:1003:663#0/1
abaa`a`a]aa`aa_`a``a``_]`_`a_]_]`[`^^]Z]
@HWI-EAS443:3:32:1024:463#0/1
CTTGGGATCTTCTCTTCATTTTCAATGTCCTGTAGCTCTG
+HWI-EAS443:3:32:1024:463#0/1
^a_W_¥V¥bbbbb[bbaa`_abVa]`_X_]aaa^W_`b^`
@HWI-EAS443:3:32:1046:2022#0/1
GCACACATTTGGAAGTTTGTTTCATGTATTCGGATTTCCA
+HWI-EAS443:3:32:1046:2022#0/1
Ya`^``aaabab_`baba¥T¥a`bb`Waa``ZYa]W`aba
@HWI-EAS443:3:32:1066:267#0/1
GGCATGGAAATGAGGCGCTGCCTTCTTCAGTCTCTTCAGC
+HWI-EAS443:3:32:1066:267#0/1
a`a`__a_a]`a_a_]aY[V[WW[¥¥[VW¥WU[TVV^Z[W
@HWI-EAS443:3:32:1088:1077#0/1
ATTCCTTGGTCATGTCTTTCTCTTTGACAGAAGACTCGGC
+HWI-EAS443:3:32:1088:1077#0/1
__^RXU^bb_Y___V]]T^Z_a``[Z]W_a__¥]YNaWS]
@HWI-EAS443:3:32:1104:453#0/1
CCTGCTTCCAAGCCATGAGGTAATTGGGATTTATGCCTTT
+HWI-EAS443:3:32:1104:453#0/1
abbab`a^bbabbbaaa_a`[Uaaa[`baaaaaaa]aaaa
@HWI-EAS443:3:32:1145:904#0/1
CATTAATCAGGCACTCCTCGATTGCTTCATATAGCCCCCC
+HWI-EAS443:3:32:1145:904#0/1
a^]S]]a]_V_JQ^]YQWV`^UVZ¥ZS][Z^TX¥^¥_¥[U
@HWI-EAS443:3:32:114:1885#0/1
AGAAGATGTTGCCCCGATTGAACATATCGCAAGCATGAGG
+HWI-EAS443:3:32:114:1885#0/1
a_`]T_[VW___¥¥ZW¥__Uab_`^aUXUXSYPW^WYY]X
```

```
@HWI-EAS443:3:32:1296:1425#0/1
GAAATTTAGGCTTCTCTACCCCTGTTGTGTTACATATACT
+HWI-EAS443:3:32:1296:1425#0/1
aaaabbabb`baaaa_aa^aaaaa_`__a[aa_^a`__a_
@HWI-EAS443:3:32:1304:1633#0/1
ACCGAATCATGGCCTGGACAGTGGTGAACAGTATATGTAA
+HWI-EAS443:3:32:1304:1633#0/1
]^b`¥¥[¥__[S]a`W^_ab`a¥]]X]ZZ`XRX[`[]Sa_
@HWI-EAS443:3:32:1310:1449#0/1
AAGCGGACTACACCCTTGACGAAGAGAGCAGGGCAAGAAT
+HWI-EAS443:3:32:1310:1449#0/1
QR__XVZZZS_`ZXXYSZU¥_[XaR[Z^NX¥ZZFYU¥V]Y
@HWI-EAS443:3:32:1339:20#0/1
GCCTGGACAGTGGTGAACAGTATATGTAACACAACAGGGG
+HWI-EAS443:3:32:1339:20#0/1
a``_a^^_`[J]_S_]XV[aUX_VX_]]YVTV^WPWa]__
@HWI-EAS443:3:32:139:1149#0/1
CTCCCTTCATTATGTATTCAGTAGCCCTGCAGTGGGACAC
+HWI-EAS443:3:32:139:1149#0/1
aabaaaaaU_``a][a```_V]`]___^__]]Y_^ZN[Z[
@HWI-EAS443:3:32:1417:1786#0/1
ATGAAATGGGGCATGGAAATGAGGCGCTGCCTTCTTCAGT
+HWI-EAS443:3:32:1417:1786#0/1
`aa`]aa`aaaYa^^Va``^Za_VY[_^^M][]W_Ua__W
@HWI-EAS443:3:32:1450:1645#0/1
AACATAGTCAAACCACATGAGAAAGGCATAAATCCCAATT
+HWI-EAS443:3:32:1450:1645#0/1
abaaaaa`b`ab_]`^aaaa_aaa_`^a`aaa`P_Uaa`_
@HWI-EAS443:3:32:1453:274#0/1
GGGGCATGGAAATGAGGCGCTGCCTTCTTCAGTCTCTTCA
+HWI-EAS443:3:32:1453:274#0/1
`W_b]RR_WMYWW]]^¥P[L¥ZXV_BBBBBBBBBBBBBBB
@HWI-EAS443:3:32:145:803#0/1
GCCTTATTGAATTCATTTTGGACCCAGCTTGCTAGAGATC
+HWI-EAS443:3:32:145:803#0/1
abbbbbaaab`bbb_aaabaaaaaa_`aa``a`a¥`_`¥a
@HWI-EAS443:3:32:1470:1309#0/1
CTTCATTATGTATTCAGTAGCCCTGCAGTGGGACACTTCT
+HWI-EAS443:3:32:1470:1309#0/1
ab```abbb`bbbbab`^a`a`abbabb¥ab^][b_b_aa
@HWI-EAS443:3:32:1472:116#0/1
AATTCATTTTGGACCCAGCTTGCTAGAGATCTGGGCTCTG
+HWI-EAS443:3:32:1472:116#0/1
`bbbaaabbaab_aRNQYZ`a`Va_^]a^_^aa^[F¥]]a
@HWI-EAS443:3:32:1476:54#0/1
AGCTTTCCCAAATGTCAAAAGAAGTGAACGCCAAAATTGA
+HWI-EAS443:3:32:1476:54#0/1
`b]aba``aababa`^aa`aaa_]_aaa]_`Y___¥aaaa
@HWI-EAS443:3:32:148:251#0/1
GCCATGTTTCCGATTTGTTTTCAAAGAATTCCTTGGTCAT
```

+HWI-EAS443:3:32:148:251#0/1
abaOV^_¥[a_]JQ¥_aXV_`aT¥[U¥WTZ]Y[R][X[S[
@HWI-EAS443:3:32:1529:1030#0/1
GACGAAAGGAATCCCATAGACTCCTACTGGCCATTTCTTG
+HWI-EAS443:3:32:1529:1030#0/1
¥_abaaabb`baa`aaa`aa_a`Z`__]YY¥_TJ^]W[aY
@HWI-EAS443:3:32:1574:1062#0/1
CAGGTTTGTTTTCCGTCTTCCTTCTTTGGTCCTACATTTG
+HWI-EAS443:3:32:1574:1062#0/1
aab`Xb`babababaaZa[ba`YaaRbabb^^`Sa_`_bab_
@HWI-EAS443:3:32:1673:673#0/1
AGCCTATGTAGATGGATTCGAGCCGAACGGCTGCATTGAG
+HWI-EAS443:3:32:1673:673#0/1
aaY^baabaaaa`]a^aa`a_a`¥a¥a¥a^]¥`G^W]HZ]
@HWI-EAS443:3:32:194:1825#0/1
GTGACCCGAATGCACTATTGAAGCACCGATTTGAGATAAT
+HWI-EAS443:3:32:194:1825#0/1
a`Wbbbba]bbabbbabbbabbbbbbb`aaaaaaba^aa`
@HWI-EAS443:3:32:196:654#0/1
GGGGCATGGAAATGAGGCGCTGCCTTCTTCAGTCTCTTCA
+HWI-EAS443:3:32:196:654#0/1
`babb`abbaa`bbababbbaaaaa`a`aaaaWa_a¥_]_
@HWI-EAS443:3:32:208:1002#0/1
TAATCAGGCACTCCTCGATTGCTTCATATAGCCCCCCAAG
+HWI-EAS443:3:32:208:1002#0/1
``aabb^W`aaaa]```V_Y¥a]``^```a_a``b^b_NT
@HWI-EAS443:3:32:264:1570#0/1
CACGAGGGGGAGGGAATACCACTATATGATGCAATCAAAT
+HWI-EAS443:3:32:264:1570#0/1
a`a`^]Y_a_`Y_Y`^U]a]¥]^_`_^^^¥^__Y^MZ[]]
@HWI-EAS443:3:32:278:98#0/1
GGACTTCCCTCCGTGTTACTCCAATTTCAATGAACCGGTT
+HWI-EAS443:3:32:278:98#0/1
aaa_aaaaaaa`aZa__a_^__^^^¥^^^^]]]]^X]TON
@HWI-EAS443:3:32:324:1256#0/1
CGAAGAGACAATTGAAGAAAAATTTGAGATTACAGGAACT
+HWI-EAS443:3:32:324:1256#0/1
aaaa`[[HQUX^^ZZRO^X^RZ][X_]^^^]^]^_ZXZ¥^
@HWI-EAS443:3:32:324:1878#0/1
ATCATTAATCAGGCACTCCTCGATTGCTTCATATAGCCCC
+HWI-EAS443:3:32:324:1878#0/1
]Yb^Z_WS_b^W^bb`_aaHaZ]XWQa_[X`U^[aY_]`_
@HWI-EAS443:3:32:329:1139#0/1
GGCCAGTAGGAGTCTATGGGATTCCTTTCGTCAGTCCGAA
+HWI-EAS443:3:32:329:1139#0/1
a]bbba¥bab`^_aaaa`]a`a`a`aa`aa^`a`[`]]¥`
@HWI-EAS443:3:32:376:1457#0/1
CTCATTTGAGAAATGATACTGATGTGGTGAACTTTGTAAG
+HWI-EAS443:3:32:376:1457#0/1
a_a^aaaa`a`Y````¥`a_____W¥THX__[_^Z^Y]LQ

@HWI-EAS443:3:32:394:414#0/1
GAACTATGCGCAAGCTTGCCGACCAAAGTCTCCCACCGAA
+HWI-EAS443:3:32:394:414#0/1
a`^`aZ_a_a_`Z_^`XX`__[Z_¥Z`_PUUVSZVVVWU[
@HWI-EAS443:3:32:394:713#0/1
ATTCAGTAGCCCTGCAGTGGGACACTTCTGCTGTAAAATA
+HWI-EAS443:3:32:394:713#0/1
a``a[Z_a`aaa___a_W_WZ[`_]^`]ZZ[^^TVQFSWT
@HWI-EAS443:3:32:412:1040#0/1
GTCCAAAATGAATTCAATAAGGCATGTGAATTGACTGATT
+HWI-EAS443:3:32:412:1040#0/1
`abab```b_`bbabaaaa^^aaaaa^aa`aaa`aaa``_
@HWI-EAS443:3:32:466:1788#0/1
GAAAGGCATAAATCCCAATTACCTCATGGCTTGGAAGCAG
+HWI-EAS443:3:32:466:1788#0/1
aaab^^bbbbababbbbbaaaaaa[b`a]Y`^R¥_`W¥QP
@HWI-EAS443:3:32:485:1683#0/1
TTCCGATTTGTTTTCAAAGAATTCCTTGGTCATGTCTTTC
+HWI-EAS443:3:32:485:1683#0/1
aabba^Ta]`]aaaba`¥Za__^]aaY]YPa^_N[]_a__
@HWI-EAS443:3:32:488:852#0/1
CTCATTTGAGAAATGATACTGATGTGGTGAACTTTGTAAG
+HWI-EAS443:3:32:488:852#0/1
`ab__b`¥^bV^babaabb_^`^a`Y[Y`^Y]`a_aP`^]
@HWI-EAS443:3:32:518:1316#0/1
ATCAGGCACTCCTCGATTGCTTCATATAGCCCCCCAAGAT
+HWI-EAS443:3:32:518:1316#0/1
aab_NR`baaabaaaaaa]a[_a`aa_aY`_Ya[``UV]R
@HWI-EAS443:3:32:520:1683#0/1
ATCAGCAGGAACTTTGACCGCTGATGGCAAAGAGGCCCAT
+HWI-EAS443:3:32:520:1683#0/1
`abba¥T`a^T^^]Z`R[^^]QSYPIKa_]ZPM]UV^YUB
@HWI-EAS443:3:32:557:1219#0/1
ATTGCAGCAAACTTGTTAGTTTCGATTTTCGGATCTTCCC
+HWI-EAS443:3:32:557:1219#0/1
a`a¥ba[b`VV]aa^__a`Y_^a`W¥V^^VR]I[YXYRV^
@HWI-EAS443:3:32:701:992#0/1
CCCGAATGCACTATTGAAGCACCGATTTGAGATAATTGAA
+HWI-EAS443:3:32:701:992#0/1
aba`baa`aa`aaaa_a`aaa`_a^_a___`_``_^^`_U
@HWI-EAS443:3:32:858:1944#0/1
CGGAGAGTCACCCAGGGGAGTGGAGGAAGGCTCTATTGGG
+HWI-EAS443:3:32:858:1944#0/1
aXa`^TUYba^^`¥VMP`^[QVYSVGUHVYVSMBBBBBBB
@HWI-EAS443:3:32:874:1251#0/1
GCTCTCTTCGTCAAGGGTGTAGTCCGCTTTGGTGGCCATC
+HWI-EAS443:3:32:874:1251#0/1
]_]_R`bba`^]Z[V`a^bT^XWZXTZ^aaZ¥^¥aT_RMZ
@HWI-EAS443:3:32:981:1636#0/1
AACATCTTTGCAGTCATCAAAGTCTACTTTTTCTGGTGCC

```
+HWI-EAS443:3:32:981:1636#0/1
aa`aaaaaR`a`a_`aa____]O_`_`a^___`]_^Z^a`
@HWI-EAS443:3:33:1005:1110#0/1
AGAAGTTCGGTGGGAGACTTTGGTCGGCAAGCTTGCGCAT
+HWI-EAS443:3:33:1005:1110#0/1
aaa_aHaaaa^a^a``__a_`Y[^`][[_YLWVX^X]V^X
@HWI-EAS443:3:33:1125:1563#0/1
CAGGGGTAGAGAAGCCTAAATTTCTTCCTGATTTGTATGA
+HWI-EAS443:3:33:1125:1563#0/1
a^]^V_JbaaUJXS_¥^`Z¥OU¥aXFKR]XLDDZ¥V¥VZU
@HWI-EAS443:3:33:1134:1132#0/1
CAATCTGAGGGGGCGTGGTGTCGTCCTCAAGAATGGTTCA
+HWI-EAS443:3:33:1134:1132#0/1
aa_aabba^`Vb¥bb[^R_aY¥b_]XT¥_U`a¥^YaVZ_Q
@HWI-EAS443:3:33:113:1277#0/1
ATCGACGAACGGGGTGAATCAATAATTGTAGAATCTGGTG
+HWI-EAS443:3:33:113:1277#0/1
a_a^aaa_aVU[Y]VVUWZ_TTZ[UY^][VUZUV][Z[TT
@HWI-EAS443:3:33:1176:1086#0/1
GGCCATCTCCTCTCCAGTGAATGAAAAGATGTGAATGTGT
+HWI-EAS443:3:33:1176:1086#0/1
aba`Z_Z^a]]^]`]]]H]_VZ]_`]¥_^Y_[Y¥WU¥PRQ
@HWI-EAS443:3:33:1303:1505#0/1
CCCCGATTGAACATATCGCAAGCATGAGGAGGAACTATTT
+HWI-EAS443:3:33:1303:1505#0/1
[bbbb`b^WXa]]bbXXaI_^_aaaU[^QQb`Z`G[SXaa
@HWI-EAS443:3:33:1327:918#0/1
GGTGAACTTTGTAAGTATGGAGTTCTCACTCACTGACCCG
+HWI-EAS443:3:33:1327:918#0/1
]MQYT`¥ZaY^TZM]KVVYQQTRVZ[GT^^^MHPU_PPF¥
@HWI-EAS443:3:33:1351:1192#0/1
AGCAGAGCTACAGGACATTGAAAATGAAGAGAAGATCCCA
+HWI-EAS443:3:33:1351:1192#0/1
aaa`_^b`ab_a_`a_aaaa``_aa`]```aZO^^]ZWW¥
@HWI-EAS443:3:33:1412:1706#0/1
ATTCACATGCCTTATTGAATTCATTTTGGACCCAGCTTGC
+HWI-EAS443:3:33:1412:1706#0/1
aaaaa]]]a`aaa_a_a__a_`__`_^^^_YSMY]X^WVY
@HWI-EAS443:3:33:141:1330#0/1
GGGTTTTCGGCTGAATCGAGAAAATTGCTTCTCATTGTTC
+HWI-EAS443:3:33:141:1330#0/1
aa^]aaaba]a__S_aaa^`a_QZ``_a``a_Va_`_U_a
@HWI-EAS443:3:33:1446:122#0/1
GGGGGCTATATGAAGCAATCGAGGAGTGCCTGATTAATGA
+HWI-EAS443:3:33:1446:122#0/1
abab`bbaabbabba_aa_`a_a`_aXa__a¥U_]]Q]_U
@HWI-EAS443:3:33:1463:748#0/1
AGAGAAGATCCCAAGGACAAAGAACATGAAGAGAACAAGC
+HWI-EAS443:3:33:1463:748#0/1
aabaabaaaaaS^O^`¥^aa_¥a_¥a_]^V_¥T¥RN¥S^[
```

```
@HWI-EAS443:3:33:1467:1074#0/1
AACCCATACAGGTTTGTTTTCCGTCTTCCTTCTTTGGTCC
+HWI-EAS443:3:33:1467:1074#0/1
_aaaabaaaa]]Xa`a^aaa]^YT¥_^Z___OW__¥^VXX
@HWI-EAS443:3:33:1550:894#0/1
ATGAAGACATTCTTTGGCTGGAAAGAGCCTAACATAGTCA
+HWI-EAS443:3:33:1550:894#0/1
abaaaba^abaaa`aaa_aaa^`a__^_^_]TKT^]WXX_
@HWI-EAS443:3:33:1587:169#0/1
ACCGGTTCTCTTTGTAATCATACAAATCAGGAAGAAATTT
+HWI-EAS443:3:33:1587:169#0/1
____`_a_`_`a__¥]_aY`a_]`^a]^]`^NIVZ]ZZ^X
@HWI-EAS443:3:33:1599:56#0/1
GGTGAATCAATAATTGTAGAATCTGGTGACCCGAATGCAC
+HWI-EAS443:3:33:1599:56#0/1
`aXb^_^aK_abWaa`aX_^a^aabbJaaa^MOIW`bWUR
@HWI-EAS443:3:33:15:627#0/1
AGGAGACATGCTCTTGAGGACTGCGATAGGCCAAGTGTCG
+HWI-EAS443:3:33:15:627#0/1
abb`babaaa```a`a__a_a``_`_Z^_^`_W^_USN__
@HWI-EAS443:3:33:188:1410#0/1
AGAAAAAGTAGACTTTGATGACTGCAAAGATGTTGGAGAC
+HWI-EAS443:3:33:188:1410#0/1
aaa`WZa`WaaYa_aa_^^aaa__a__a_]_¥W_^^]X`_
@HWI-EAS443:3:33:277:794#0/1
CAGCTTGCTAGAGATCTGGGCTCTGGCTCATCACTGTCAT
+HWI-EAS443:3:33:277:794#0/1
[WTaaabb`a^^^aba_aabbbba`aba`a`a_a__¥^`a
@HWI-EAS443:3:33:368:1903#0/1
GGGGGGCTATATGAAGCAATCGAGGAGTGCCTGATTAATG
+HWI-EAS443:3:33:368:1903#0/1
ab``_[`ababa_ababa____YaZ`ZW_a_XRR]_a_¥¥
@HWI-EAS443:3:33:509:1121#0/1
GGGGCTATATGAAGCAATCGAGGAGTGCCTGATTAATGAT
+HWI-EAS443:3:33:509:1121#0/1
aUaa`a`]^X`YPNaaJ]Y]¥K__aU_VX[TIV]_]]X_V
@HWI-EAS443:3:33:520:244#0/1
GGGGGCTATATGAAGCAATCGAGGAGTGCCTGATTAATGA
+HWI-EAS443:3:33:520:244#0/1
aaaaaaa``aaa`aa_`_]_a_`__`Y_^[Q_U¥^]¥¥WY
@HWI-EAS443:3:33:52:324#0/1
GGCCAATCGGAGAGTCACCCAGGGGAGTGGAGGAAGGCTC
+HWI-EAS443:3:33:52:324#0/1
``aa`aaa__Z_]`¥a`a`_U_¥Xa]`S¥`V¥TRZWZXXW
@HWI-EAS443:3:33:538:1751#0/1
CCCCCCAAGATCGAAGGTTCCAGGTTCCAGGTTGTCCCTA
+HWI-EAS443:3:33:538:1751#0/1
abababaa_a`a_aaTWO`___¥IKWU^_¥¥NKKKNWUW_
@HWI-EAS443:3:33:570:503#0/1
GCTAGCAGAGCTACAGGACATTGAAAATGAAGAGAAGATC
```

+HWI-EAS443:3:33:570:503#0/1
abba^abbbb``aaa`a^baa``_^Z^a^```PZa]Xa_R
@HWI-EAS443:3:33:647:319#0/1
GATTGGCCATGTTTCCGATTTGTTTTCAAAGAATTCCTTG
+HWI-EAS443:3:33:647:319#0/1
`[^`]^``^`_U]aaXY¥^]``___]S[[]^VWYYST[¥V
@HWI-EAS443:3:33:662:1005#0/1
ATTCCTTGGTCATGTCTTTCTCTTTGACAGAAGACTCGGC
+HWI-EAS443:3:33:662:1005#0/1
a_aaaaaaa[bb`]`aa__aaaaaaaUaa``_Z^^``VSR
@HWI-EAS443:3:33:740:438#0/1
GTGGGAGACTTTGGTCGGCAAGCTTGCGCATAGTTCCTGT
+HWI-EAS443:3:33:740:438#0/1
a`aaa]b]aaaba_ZaX`_`_^_^^]U_]XHUWZZXX]_X
@HWI-EAS443:3:33:742:1809#0/1
AGCCCCCCAAGATCGAAGGTTCCAGGTTCCAGGTTGTCCC
+HWI-EAS443:3:33:742:1809#0/1
a_aaaaaa`a_`¥_^aZZWM^W_^VWQVWW^WUWUVSUWW
@HWI-EAS443:3:33:748:1272#0/1
TGAAAATGAAGAGAAGATCCCAAGGACAAAGAACATGAAG
+HWI-EAS443:3:33:748:1272#0/1
_`¥[`_`^Zaaaaaa_a^aa`a`_a`O__`]¥]S_]]_`_
@HWI-EAS443:3:33:778:1820#0/1
GGGGTTTTCGGCTGAATCGAGAAAATTGCTTCTCATTGTT
+HWI-EAS443:3:33:778:1820#0/1
aaab¥`aaaaabaaba]`_`^a^_`^^``Y¥W]`^]_]__
@HWI-EAS443:3:33:803:1376#0/1
AGTCAATTCACATGCCTTATTGAATTCATTTTGGACCCAG
+HWI-EAS443:3:33:803:1376#0/1
^`^b[__[`a`X[Zaaa`__¥Z`^aZ^`_`]_UU¥^^R]]
@HWI-EAS443:3:33:826:1804#0/1
GTCAATTCACATGCCTTATTGAATTCATTTTGGACCCAGC
+HWI-EAS443:3:33:826:1804#0/1
a`bbbbbababaabababaaaaaaababaaa_X`Y]^a__
@HWI-EAS443:3:33:857:970#0/1
AGCAGGTGCTAGCAGAGCTACAGGACATTGAAAATGAAGA
+HWI-EAS443:3:33:857:970#0/1
aaaa_a^^aaaa``_`a`a__`^`V^^_`_W]V]_^]]]_
@HWI-EAS443:3:33:861:1067#0/1
CGTCTCCACAACTTGAGGGGTTTTCGGCTGAATCGAGAAA
+HWI-EAS443:3:33:861:1067#0/1
a`T`Xa```a]S_aXM]¥FZG¥`_¥`X`^`PVDX[SWMXX
@HWI-EAS443:3:33:882:108#0/1
GGCATGGAAATGAGGCGCTGCCTTCTTCAGTCTCTTCAGC
+HWI-EAS443:3:33:882:108#0/1
abaaaaaaaaab`a`_a__a_V^_^`__Y`YYY¥¥¥YW¥Y
@HWI-EAS443:3:33:888:107#0/1
TATGTTAGGCTCTTTCCAGCCAAAGAATGTCTTCATGCAT
+HWI-EAS443:3:33:888:107#0/1
aXbbab¥OTbV[_aa_a¥¥`VXS^XFY[[K^_TVVZ^U_^

@HWI-EAS443:3:33:892:1817#0/1
GGGGGCTATATGAAGCAATCGAGGAGTGCCTGATTAATGA
+HWI-EAS443:3:33:892:1817#0/1
IRbU_Y`]U_bba_^¥_ba__bbbaVZ]_^a[YaLVa_ab
@HWI-EAS443:3:33:892:820#0/1
GGGGGCTATATGAAGCAATCGAGGAGTGCCTGATTAATGA
+HWI-EAS443:3:33:892:820#0/1
a`aa`abbbabaaaabb`bab`_a`aVa_aaa^a_a```a
@HWI-EAS443:3:33:975:1607#0/1
GAATCATGGCCTGGACAGTGGTGAACAGTATATGTAACAC
+HWI-EAS443:3:33:975:1607#0/1
a`_abbaaaaa`¥`__a`_]aY_``^^_^a`[U_N`_U`S
@HWI-EAS443:3:33:97:1689#0/1
GGAGTCTATGGGATTCCTTTCGTCAGTCCGAAAGAGGCGA
+HWI-EAS443:3:33:97:1689#0/1
]VaX`ba^_aa_aaaaa_a^a_W]][Waa^WTY^_^Z[[¥
@HWI-EAS443:3:34:1029:732#0/1
ATTGGTTCTCACATATAGGAACATGGGCCTCGACACTTGG
+HWI-EAS443:3:34:1029:732#0/1
aaa`¥Q]a_]a____`_``]`_]]_QV^`Z¥YWUWYW¥YW
@HWI-EAS443:3:34:1040:806#0/1
CAAGAATCAAAACTAGGCTTTTCACTATAAGACAAGAAAT
+HWI-EAS443:3:34:1040:806#0/1
ab_baabbab]aab]aaaaabaabaaaaaaa`]a_`^^^^
@HWI-EAS443:3:34:1102:1503#0/1
CTCGATTGCTTCATATAGCCCCCCAAGATCGAAGGTTCCA
+HWI-EAS443:3:34:1102:1503#0/1
`bbbbababbbbbbbab``aabbabaabaaaaa_]U_WQ]
@HWI-EAS443:3:34:1120:1497#0/1
GATTCTACAATTATTGATTCACCCCGTTCGTCGATGAAAT
+HWI-EAS443:3:34:1120:1497#0/1
aaaaaaa_a`aaaaaaaaa_aaa__`_^a`]`_Z]_^^^Z
@HWI-EAS443:3:34:1121:1777#0/1
GACCGAATCATGGCCTGGACAGTGGTGAACAGTATATGTA
+HWI-EAS443:3:34:1121:1777#0/1
ababbaababababbbbbbbaaaba^aRa_a`b[TVaa_aHZ
@HWI-EAS443:3:34:1181:237#0/1
CTGGAAAGAGCCTAACATAGTCAAACCACATGAGAAAGGC
+HWI-EAS443:3:34:1181:237#0/1
abbbbbbb`bbbb^a_aa`baabaaa¥aaaaa`a]^^_^]
@HWI-EAS443:3:34:1238:1017#0/1
CATTAATCAGGCACTCCTCGATTGCTTCATATAGCCCCCC
+HWI-EAS443:3:34:1238:1017#0/1
abaaa_aa]`aaaaa^``_`aV_`^^]Y_a_][_UUQQVB
@HWI-EAS443:3:34:1265:60#0/1
AAGAATTCCTTGGTCATGTCTTTCTCTTTGACAGAAGACT
+HWI-EAS443:3:34:1265:60#0/1
Y`baa`a^``baa^`XV^¥]]a`abWa`_X_^aa¥W]]BB
@HWI-EAS443:3:34:1281:1897#0/1
CAAAGAGGCCCATCAGGCAATCTGAGGGGGCGTGGTGTCG

+HWI-EAS443:3:34:1281:1897#0/1
Xaa]JR^^_EXa_aaa_X]`aa¥``aBBBBBBBBBBBBBB
@HWI-EAS443:3:34:1288:442#0/1
CATGACCAAGGAATTCTTTGAAAACAAATCGGAAACATGG
+HWI-EAS443:3:34:1288:442#0/1
WTW_TY_¥X__RW¥a^```__^aaa_]¥_^___a``WY¥B
@HWI-EAS443:3:34:1319:1472#0/1
CCTCCGTGTTACTCCAATTTCAATGAACCGGTTCTCTTTG
+HWI-EAS443:3:34:1319:1472#0/1
`a^abYYa]ZV^^`^YZa``a_^__aa`ab]^Z¥YRTU^R
@HWI-EAS443:3:34:1389:1171#0/1
GCGTCTCCACAACTTGAGGGGTTTTCGGCTGAATCGAGAA
+HWI-EAS443:3:34:1389:1171#0/1
]`[¥b^_Wa[^Z^aabZbb``UZ`a][a`aZ__`XWMUSZ
@HWI-EAS443:3:34:1403:1171#0/1
AAGAAATTTAGGCTTCTCTACCCCTGTTGTGTTACATATA
+HWI-EAS443:3:34:1403:1171#0/1
abbabVb`_`_V¥aa`aa]ba``^P]_]a`a¥a[S_BBBB
@HWI-EAS443:3:34:1417:1497#0/1
TGGCCATGTTTCCGATTTGTTTTCAAAGAATTCCTTGGTC
+HWI-EAS443:3:34:1417:1497#0/1
`baa`¥bbbbbaabaabbaabbbaaa^aaaaaU]_`_`WZ
@HWI-EAS443:3:34:1432:183#0/1
GTTCGTCGATGAAATGGAAATCCGAATACATGAAACAAAC
+HWI-EAS443:3:34:1432:183#0/1
aaaaaaaa_abaaa`a`a__`_`a__`__``^`_[Y]^XW
@HWI-EAS443:3:34:1461:918#0/1
CCAAGGACAAAGAACATGAAGAGAACAAGCCAATTGAAGT
+HWI-EAS443:3:34:1461:918#0/1
_UaabYLVT^ab`__aabaQ`aa^`]__aXUN]`U¥NSZB
@HWI-EAS443:3:34:1465:1843#0/1
GCTTGCTAGAGATCTGGGCTCTGGCTCATCACTGTCATAC
+HWI-EAS443:3:34:1465:1843#0/1
aaaaa`]^b_a¥`_``aa]a`_aa[_W___`T__ZWW_¥B
@HWI-EAS443:3:34:151:1255#0/1
AGCTTGAATCAGTCAATTCACATGCCTTATTGAATTCATT
+HWI-EAS443:3:34:151:1255#0/1
^]a_[_Sa^aaaZ```^XVaa¥W]`Z`XW[S]TZX[UZWZ
@HWI-EAS443:3:34:1566:1624#0/1
GGCATAAATCCCAATTACCTCATGGCTTGGAAGCAGGTGC
+HWI-EAS443:3:34:1566:1624#0/1
abbbbbbbbbbaabaaaaaaYaaa]^aa¥YBBBBBBBBBB
@HWI-EAS443:3:34:1591:898#0/1
GGTGTCGTCCTCAAGAATGGTTCAATTTTGGCGTTCACTT
+HWI-EAS443:3:34:1591:898#0/1
ab[b_]a``X`[¥[¥]aXO[Q__ZS¥aa`WSOVQ¥UTNTZ
@HWI-EAS443:3:34:1609:567#0/1
AGAGCCTAACATAGTCAAACCACATGAGAAAGGCATAAAT
+HWI-EAS443:3:34:1609:567#0/1
aXX_YPWZ[````a[U]NVGMWZ`[`[aBBBBBBBBBBBB

@HWI-EAS443:3:34:1624:1913#0/1
GAGGGGTTTTCGGCTGAATCGAGAAAATTGCTTCTCATTG
+HWI-EAS443:3:34:1624:1913#0/1
a`¥`aa_^VWYYU_YV]]OF[^`^S¥VMVVBBBBBBBBBB
@HWI-EAS443:3:34:227:1520#0/1
CAATAATTGTAGAATCTGGTGACCCGAATGCACTATTGAA
+HWI-EAS443:3:34:227:1520#0/1
_O¥a^a^_`^b`_aa__¥aRWa]aa¥]_]_`_Y`____^]
@HWI-EAS443:3:34:252:2034#0/1
AAGGGAGTGTACATAAATACGGCCTTGCTCAATGCATCCT
+HWI-EAS443:3:34:252:2034#0/1
UVWZW^S[[Y_a]]S_U^^`_[aaa_`X[^`]¥`Q]GFFN
@HWI-EAS443:3:34:256:324#0/1
CAAGTGTCGAGGCCCATGTTCCTATATGTGAGAACCAATG
+HWI-EAS443:3:34:256:324#0/1
aa[^POJ]]``aaa`[_¥Z^_[XTZ_^XR[V]X_WV]VVB
@HWI-EAS443:3:34:261:988#0/1
ATCTTGGGGGGCTATATGAAGCAATCGAGGAGTGCCTGAT
+HWI-EAS443:3:34:261:988#0/1
abbbba^`a`abaaa``aZUYaaX`^^a`[]_W[[^]^Z_
@HWI-EAS443:3:34:262:1565#0/1
CATCAGCAGGAACTTTGACCGCTGATGGCAAAGAGGCCCA
+HWI-EAS443:3:34:262:1565#0/1
^W]`bab¥aZX^b`¥`¥aaa_a_`]RR__S_`^aRY_[R[
@HWI-EAS443:3:34:29:2023#0/1
GGACCAAAGAAGGAAGACGGAAAACAAACCTGTATGGGTT
+HWI-EAS443:3:34:29:2023#0/1
aabaa^^Y_ZW___^`]]__``aa`aZ]^]V¥O¥¥Z][U]
@HWI-EAS443:3:34:328:807#0/1
GGGTTTTCGGCTGAATCGAGAAAATTGCTTCTCATTGTTC
+HWI-EAS443:3:34:328:807#0/1
`aa`abbbbababbbaababa``_`a__aaaa`aa`a_`^
@HWI-EAS443:3:34:36:1853#0/1
GTCCCACTGCAGGGCTACTGAATACATAATGAAGGGAGTG
+HWI-EAS443:3:34:36:1853#0/1
aabbbabaaabbaababbb_aabbbabb``]^aaY`Y_V[
@HWI-EAS443:3:34:402:549#0/1
GCGTCTCCACAACTTGAGGGGTTTTCGGCTGAATCGAGAA
+HWI-EAS443:3:34:402:549#0/1
abbabaab`__[`a```baa_Ra`aaa_a_Y]__YWV___
@HWI-EAS443:3:34:530:869#0/1
GGCATAAATCCCAATTACCTCATGGCTTGGAAGCAGGTGC
+HWI-EAS443:3:34:530:869#0/1
ababbbb[babba_bbaabaabba_aa_Z`¥a[GTYVFRX
@HWI-EAS443:3:34:589:108#0/1
GATAATTGAAGGAAGAGACCGAATCATGGCCTGGACAGTG
+HWI-EAS443:3:34:589:108#0/1
aaabaaaaaaaaaa``aaaa`__aaa_a``^`_]]]Y]Y^
@HWI-EAS443:3:34:606:918#0/1
ATTCGGATTTCCATTTCATCGACGAACGGGGTGAATCAAT

```
+HWI-EAS443:3:34:606:918#0/1
a_`a`_RY__aaaaa``a_a]__a__]^^_]XYTV]SYVZ
@HWI-EAS443:3:34:656:1072#0/1
ATTTCATCGACGAACGGGGTGAATCAATAATTGTAGAATC
+HWI-EAS443:3:34:656:1072#0/1
ababbba]a^¥]`a__Ra¥RaSa_^XUaaV`¥FX¥WX^RP
@HWI-EAS443:3:34:711:743#0/1
AGCAGGGCAAGAATCAAAACTAGGCTTTTCACTATAAGAC
+HWI-EAS443:3:34:711:743#0/1
ab[YS`bb^_b`aba¥_I__aaa`Yaaaaab¥aYZ`__]^
@HWI-EAS443:3:34:744:1491#0/1
GGGGGCTATATGAAGCAATCGAGGAGTGCCTGATTAATGA
+HWI-EAS443:3:34:744:1491#0/1
aa_a_b_aa```_¥]_aZ_`[^YY]U[`ZZ[^^^_[TU¥B
@HWI-EAS443:3:34:846:1914#0/1
GGGGGCTATATGAAGCAATCGAGGAGTGCCTGATTAATGA
+HWI-EAS443:3:34:846:1914#0/1
`b^Xaa_bba`aba`aaa_aa___`_¥_a`^a`W¥_[U¥_
@HWI-EAS443:3:34:859:476#0/1
GAGTCTATGGGATTCCTTTCGTCAGTCCGAAAGAGGCGAA
+HWI-EAS443:3:34:859:476#0/1
aab`abaaabaaaaaaabaaa[¥a`^_`a`__^]`_R[^^
@HWI-EAS443:3:34:876:982#0/1
AGTCAAACCACATGAGAAAGGCATAAATCCCAATTACCTC
+HWI-EAS443:3:34:876:982#0/1
aa_bba]`aaaaaaaa`a]`__`aa_a`_`_`¥^^]WY]S
@HWI-EAS443:3:34:900:478#0/1
CTATGCGCAAGCTTGCCGACCAAAGTCTCCCACCGAACTT
+HWI-EAS443:3:34:900:478#0/1
ababaabababbaaa]^`a`^aaa_^Yaa^_`UR¥¥¥S^¥
@HWI-EAS443:3:34:908:686#0/1
GAGCAGGGCAAGAATCAAAACTAGGCTTTTCACTATAAGA
+HWI-EAS443:3:34:908:686#0/1
a___``aaaaaaZ_aaaaab``_a`_`__``a]`__V^][
@HWI-EAS443:3:34:996:198#0/1
CATGACCAAGGAATTCTTTGAAAACAAATCGGAAACATGG
+HWI-EAS443:3:34:996:198#0/1
^a[`bZ^V`a]ZZ]`Z`[``a^^`^¥[[aaaa]_]]Z]^B
@HWI-EAS443:3:34:997:415#0/1
ACCGATTTGAGATAATTGAAGGAAGAGACCGAATCATGGC
+HWI-EAS443:3:34:997:415#0/1
aaaaaabab_`aa_X`abaaaa``a`aa___^]_W`^_ZW
@HWI-EAS443:3:34:998:858#0/1
CAATAATTGTAGAATCTGGTGACCCGAATGCACTATTGAA
+HWI-EAS443:3:34:998:858#0/1
aa^baaabb`aba`aaa`aZa_a``a````]_Za_a]]YW
@HWI-EAS443:3:35:1005:498#0/1
CCCAGCTTGCTAGAGATCTGGGCTCTGGCTCATCACTGTC
+HWI-EAS443:3:35:1005:498#0/1
aaaaa```aaaa]aa^`aa`^___`_a_a_`]_``Z_`¥`
```

@HWI-EAS443:3:35:1024:307#0/1
GGCATGGAAATGAGGCGCTGCCTTCTTCAGTCTCTTCAGC
+HWI-EAS443:3:35:1024:307#0/1
]baaaa^`[aaa`a¥aaa[¥]___^^_PS]Z^Z^^YQYR^
@HWI-EAS443:3:35:1056:881#0/1
GTTCGTCGATGAAATGGAAATCCGAATACATGAAACAAAC
+HWI-EAS443:3:35:1056:881#0/1
_^aa][_a``a^^]WZ¥a^``__``_`[_Y[`]T¥^^R¥`
@HWI-EAS443:3:35:1094:344#0/1
TTTAGAGCCTATGTAGATGGATTCGAGCCGAACGGCTGCA
+HWI-EAS443:3:35:1094:344#0/1
abbaaaaaaaabb¥aaaaaaaaaaa`a¥`a_`_a^^¥]PZ
@HWI-EAS443:3:35:1121:1562#0/1
GAACTATGCGCAAGCTTGCCGACCAAAGTCTCCCACCGAA
+HWI-EAS443:3:35:1121:1562#0/1
__¥^_bZSab`aa^^¥]^Y`[`aX``a`[aa^]`X^[a]`
@HWI-EAS443:3:35:1136:1074#0/1
AAACCCCTCAAGTTGTGGAGACGCATATAGACTGTTGAAT
+HWI-EAS443:3:35:1136:1074#0/1
aa``]aaZa^]b^ab^aaaa`]a]^_`^¥^_`^_Z]PZWN
@HWI-EAS443:3:35:1159:1132#0/1
ACCCCTCAAGTTGTGGAGACGCATATAGACTGTTGAATAC
+HWI-EAS443:3:35:1159:1132#0/1
aaa`a`aa_X¥aaXa_aa____`[[_TU``aaR^RWYWUZ
@HWI-EAS443:3:35:1248:481#0/1
GGCCATGTTTCCGATTTGTTTTCAAAGAATTCCTTGGTCA
+HWI-EAS443:3:35:1248:481#0/1
a_aa]`UR`bba__aaab`ab^_aaaa_Y^__`]aa^P[^
@HWI-EAS443:3:35:1273:879#0/1
TTCGGATTTCCATTTCATCGACGAACGGGGTGAATCAATA
+HWI-EAS443:3:35:1273:879#0/1
aaabaaaa`aaaaaa_aa__`___`Y^^`ZGZX[WYZ^SX
@HWI-EAS443:3:35:1330:803#0/1
ATGAGGAGGAACTATTTTACAGCAGAAGTGTCCCACTGCA
+HWI-EAS443:3:35:1330:803#0/1
abbaZ``ababa]abbabaaaaaa`a`a`babaaa^`a_a
@HWI-EAS443:3:35:1341:640#0/1
GGTTCTCACATATAGGAACATGGGCCTCGACACTTGGCCT
+HWI-EAS443:3:35:1341:640#0/1
[X[abbbbbbbbabaaabaa`a^`[V`Yabba`a_[`aWa
@HWI-EAS443:3:35:1382:1307#0/1
CATTAATCAGGCACTCCTCGATTGCTTCATATAGCCCCCC
+HWI-EAS443:3:35:1382:1307#0/1
`bbaaaabb`aba`a`_a`aaaaaa`_Q^aaaa]`_]aS_
@HWI-EAS443:3:35:1382:808#0/1
GAGCCTTCCTCCACTCCCCTGGGTGACTCTCCGATTGGCC
+HWI-EAS443:3:35:1382:808#0/1
]UT[]`W]^_bba]a_YWQUPZTHZXT¥]_H__UXX[^F^
@HWI-EAS443:3:35:1402:124#0/1
GTGAACTTTGTAAGTATGGAGTTCTCACTCACTGACCCGA

+HWI-EAS443:3:35:1402:124#0/1
`Z]UZaZUSZTW_YDS¥Y¥Ua¥aaa`_Q`__^^_^]T¥[R
@HWI-EAS443:3:35:1409:512#0/1
ATTGAATTCATTTTGGACCCAGCTTGCTAGAGATCTGGGC
+HWI-EAS443:3:35:1409:512#0/1
¥`VRHOaa_ab``¥WTbV`¥aNaXQNOJSRKUF^PX¥WXa
@HWI-EAS443:3:35:1454:912#0/1
AAGCAGGTGCTAGCAGAGCTACAGGACATTGAAAATGAAG
+HWI-EAS443:3:35:1454:912#0/1
__```^P[¥^``a^__X__a`_a_a`a[aa_V¥_¥`^_Ta
@HWI-EAS443:3:35:1523:852#0/1
TAGCAAGCTGGGTCCAAAATGAATTCAATAAGGCATGTGA
+HWI-EAS443:3:35:1523:852#0/1
abbb]R]ab_]VSaababa_baaaa_b¥ab^ba_aaa`a_
@HWI-EAS443:3:35:1639:1610#0/1
CTTGACGAAGAGAGCAGGGCAAGAATCAAAACTAGGCTTT
+HWI-EAS443:3:35:1639:1610#0/1
`aab`_`ababbaaaaaa`_`a__`_a_`ba`ab`_^_W`
@HWI-EAS443:3:35:1661:1525#0/1
AGGCACTCCTCGATTGCTTCATATAGCCCCCCAAGATCGA
+HWI-EAS443:3:35:1661:1525#0/1
ab`_a`baaaab`[a``aa¥a`a`_¥aS`a_aaY^^U`S`
@HWI-EAS443:3:35:1701:589#0/1
ATAAAATCTGAGAAGACACACATTCACATCTTTTCATTCA
+HWI-EAS443:3:35:1701:589#0/1
ababbab^b``bb_a`[`_`[_aaZ^`Yaaaaaa¥`aaLZ
@HWI-EAS443:3:35:1707:274#0/1
GAGGCGAAGAGACAATTGAAGAAAAATTTGAGATTACAGG
+HWI-EAS443:3:35:1707:274#0/1
aaa^aaaaa_^____`aa^]a^_^]^a]aa_`_`^^Z_Za
@HWI-EAS443:3:35:268:1437#0/1
GCAGAAGTGTCCCACTGCAGGGCTACTGAATACATAATGA
+HWI-EAS443:3:35:268:1437#0/1
`aa`a_¥P_Sbaaa`_`abZNXa_YXX^][[¥`Y`_a][V
@HWI-EAS443:3:35:312:2023#0/1
ACCGATTTGAGATAATTGAAGGAAGAGACCGAATCATGGC
+HWI-EAS443:3:35:312:2023#0/1
abaX_a^a]ZXZ^¥___[T¥Z___ZT¥`a__^¥[_`V]Ua
@HWI-EAS443:3:35:347:1557#0/1
GAAATGGCCAGTAGGAGTCTATGGGATTCCTTTCGTCAGT
+HWI-EAS443:3:35:347:1557#0/1
^aYLX`Za__]XOVTU]LW[`R[Z__UX``[^ZYURV[LU
@HWI-EAS443:3:35:412:1430#0/1
CCAGCTTGCTAGAGATCTGGGCTCTGGCTCATCACTGTCA
+HWI-EAS443:3:35:412:1430#0/1
a^QU^[]TaO`_QYVZ_UNNTWU[NLULV_XUZNWU^VQN
@HWI-EAS443:3:35:439:520#0/1
CATGGAAATGAGGCGCTGCCTTCTTCAGTCTCTTCAGCAG
+HWI-EAS443:3:35:439:520#0/1
abb_aaaaaaababaaaaa`a`a`a_`a¥__a^_a``a[`

```
@HWI-EAS443:3:35:446:2030#0/1
ACATCTTCTCCTATTTCATCAAGTTCTATCCAGCTTGAAT
+HWI-EAS443:3:35:446:2030#0/1
¥`U_`[_a¥a_W]^SY`b^baYPF]a`_¥bbaWa`]Z]^X
@HWI-EAS443:3:35:461:1393#0/1
TAATCAGGCACTCCTCGATTGCTTCATATAGCCCCCCAAG
+HWI-EAS443:3:35:461:1393#0/1
a]a`a`_^b`a`aa_aaaZZ^U^^`]__VJSb`_`a[I¥W
@HWI-EAS443:3:35:470:501#0/1
CCGAACTTCTCCAGCCTTGAAAACTTTAGAGCCTATGTAG
+HWI-EAS443:3:35:470:501#0/1
abbabaa^baa`]a`aaaa^W_I¥aaXa_[aaa`W^]P_^
@HWI-EAS443:3:35:517:475#0/1
AGACATTCTTTGGCTGGAAAGAGCCTAACATAGTCAAACC
+HWI-EAS443:3:35:517:475#0/1
abbabaaaaaY^Wa``U_^^¥QQ[X_WaXY`____Q_YG^
@HWI-EAS443:3:35:537:1821#0/1
GGGGCATGGAAATGAGGCGCTGCCTTCTTCAGTCTCTTCA
+HWI-EAS443:3:35:537:1821#0/1
_]bbbbb_bbbbbbb`abbbbbabaabaab`Zaaa^ab`a
@HWI-EAS443:3:35:623:728#0/1
CTTCAGTCTCTTCAGCAGATTGAGAGCATGATTGAGGCCG
+HWI-EAS443:3:35:623:728#0/1
aa`[DX_`]`__]OU[Za^``Y__UZ_LT__Y^UV_^_^U
@HWI-EAS443:3:35:639:418#0/1
GGCATGGAAATGAGGCGCTGCCTTCTTCAGTCTCTTCAGC
+HWI-EAS443:3:35:639:418#0/1
aaaa]a`^_¥aa`_aaa__aa`a`^__^W^^_a``]US[_
@HWI-EAS443:3:35:710:133#0/1
GGTGAACTTTGTAAGTATGGAGTTCTCACTCACTGACCCG
+HWI-EAS443:3:35:710:133#0/1
a_U_aaaa_aa¥__aT_``___¥^___¥_¥___¥^`¥_Y^
@HWI-EAS443:3:35:72:514#0/1
AATTCCTTGGTCATGTCTTTCTCTTTGACAGAAGACTCGG
+HWI-EAS443:3:35:72:514#0/1
[_L`aa`a^[Raaaa[]aa_a`aa_a__]U]P__Z`^`a¥
@HWI-EAS443:3:35:73:214#0/1
GGGGCATGGAAATGAGGCGCTGCCTTCTTCAGTCTCTTCA
+HWI-EAS443:3:35:73:214#0/1
a`__a`_`_[_`__Z____`____¥¥^¥VZO¥WYY^¥W[T
@HWI-EAS443:3:35:747:1000#0/1
CATAGTTCCTGTAATCTCAAATTTTTCTTCAATTGTCTCT
+HWI-EAS443:3:35:747:1000#0/1
_^_VZ_abaaba_]aaaa^`abaaaaa`aba__`__`__X
@HWI-EAS443:3:35:75:479#0/1
GCCCACTTCAATTGGCTTGTTCTCTTCATGTTCTTTGTCC
+HWI-EAS443:3:35:75:479#0/1
aba_VZZ[a`]ZPZYaZTTG[[R]TLYZRNMX_]_^WFVO
@HWI-EAS443:3:35:765:1245#0/1
AGAACACAGTATTTTTCCCATTTGTGTGGCTCCAGTCTCG
```

```
+HWI-EAS443:3:35:765:1245#0/1
abaababbbZ¥bbbaaab`[aa`^Za`a_a`aaT]SVOMR
@HWI-EAS443:3:35:786:2030#0/1
GAATTCCTTGGTCATGTCTTTCTCTTTGACAGAAGACTCG
+HWI-EAS443:3:35:786:2030#0/1
]S[ba`b`_Y``_b^YI^_aabV¥][U_X^aa`[¥`XVT`
@HWI-EAS443:3:35:814:1323#0/1
ACCGATTTGAGATAATTGAAGGAAGAGACCGAATCATGGC
+HWI-EAS443:3:35:814:1323#0/1
aab]a`aaa]a]a_a¥R^b_VNZaW[_W_a`U_ZaaX_Va
@HWI-EAS443:3:35:822:1337#0/1
GATGGCAAAGAGGCCCATCAGGCAATCTGAGGGGGCGTGG
+HWI-EAS443:3:35:822:1337#0/1
```]]bb^aa]¥]a]a]Xa^¥¥`^`¥a^¥[X]^VYU^QTV
@HWI-EAS443:3:35:939:1763#0/1
CCAGATTCTACAATTATTGATTCACCCCGTTCGTCGATGA
+HWI-EAS443:3:35:939:1763#0/1
ab_I]aabaaab]bab`a]]a^¥b`aa_a^`a__a``_^`
@HWI-EAS443:3:35:942:712#0/1
GATGCATTGAGCAAGGCCGTATTTATGTACACTCCCTTCA
+HWI-EAS443:3:35:942:712#0/1
_aaabbba]]abaaaaaba^`_aaaaaZ_Z`a`a_Y__^Z
@HWI-EAS443:3:35:951:63#0/1
CTTCAGTCTCTTCAGCAGATTGAGAGCATGATTGAGGCCG
+HWI-EAS443:3:35:951:63#0/1
abbbbbaababbbaaaababbbaaaab_aabaaa``aaV_
@HWI-EAS443:3:35:96:59#0/1
GGGCACTCGGTGAAAATATGGCACCAGAAAAAGTAGACTT
+HWI-EAS443:3:35:96:59#0/1
abaaa_]]a`V```_``_`a_]]Y___^RWPZ_U`__Z_Z
@HWI-EAS443:3:36:103:1921#0/1
TGGCCAATCGGAGAGTCACCCAGGGGAGTGGAGGAAGGCT
+HWI-EAS443:3:36:103:1921#0/1
X`aa`]JVaXa`SS[`a_Xa_PVX[`aXO¥]EY`WZ¥PTI
@HWI-EAS443:3:36:1079:1404#0/1
TCCGATTTGTTTTCAAAGAATTCCTTGGTCATGTCTTTCT
+HWI-EAS443:3:36:1079:1404#0/1
`ab_X_a_`_aa^Z_aa```aaaaa``_¥_Y_aZa`^a``
@HWI-EAS443:3:36:1098:510#0/1
ATGGCCAGTAGGAGTCTATGGGATTCCTTTCGTCAGTCCG
+HWI-EAS443:3:36:1098:510#0/1
W^_b`]]`Wa^¥P¥TF]WZa^]]`a__V__[TV[_XP¥YV
@HWI-EAS443:3:36:1138:1428#0/1
GGGGAAGATCCGAAAATCGAAACTAACAAGTTTGCTGCAA
+HWI-EAS443:3:36:1138:1428#0/1
aaaa``^ba`aaabbaaaa^aaaaaab`aa__`_a`aaaa
@HWI-EAS443:3:36:1172:559#0/1
TCGGCTGAATCGAGAAAATTGCTTCTCATTGTTCAGGCAC
+HWI-EAS443:3:36:1172:559#0/1
^aabbb_]_``__`_aa¥`a`_¥a`aa_]Sa¥^```a_]a
```
```

```
@HWI-EAS443:3:36:1181:1410#0/1
CTCCGTGTTACTCCAATTTCAATGAACCGGTTCTCTTTGT
+HWI-EAS443:3:36:1181:1410#0/1
abbbbababbbbabb`bbaaaba`bbbaa`_a`abbaa`_
@HWI-EAS443:3:36:1207:288#0/1
GTGAATCAATAATTGTAGAATCTGGTGACCCGAATGCACT
+HWI-EAS443:3:36:1207:288#0/1
aab^aaba^aa`aab^ab`_a`_a`]`_^``__^__a_Y_
@HWI-EAS443:3:36:1294:905#0/1
GTGTACATAAATACGGCCTTGCTCAATGCATCCTGTGCAG
+HWI-EAS443:3:36:1294:905#0/1
a[aa]`]`IZa]]`a`ba`_a^__a``]`[YZUSYW__YO
@HWI-EAS443:3:36:1326:1256#0/1
CATTAATCAGGCACTCCTCGATTGCTTCATATAGCCCCCC
+HWI-EAS443:3:36:1326:1256#0/1
aba``aabbbabb`babbbbab`baaa[aaba]a``aa`b
@HWI-EAS443:3:36:1393:1840#0/1
ACCGGTTCATTGAAATTGGAGTAACACGGAGGGAAGTCCA
+HWI-EAS443:3:36:1393:1840#0/1
`a``b`ba`abaa^aabaaaa]aa`aa^aa^W__`_]_Ra
@HWI-EAS443:3:36:1414:752#0/1
ATTTTGGACCCAGCTTGCTAGAGATCTGGGCTCTGGCTCA
+HWI-EAS443:3:36:1414:752#0/1
Q^¥_^_`a__WX_ba¥_¥¥babb¥]Z¥Z_W_Q^abbbb]`
@HWI-EAS443:3:36:1418:619#0/1
CTTGGGATCTTCTCTTCATTTTCAATGTCCTGTAGCTCTG
+HWI-EAS443:3:36:1418:619#0/1
`U¥ZWWP`][aa[[Y_`___aaZ¥^_Y[Z]_YQ_¥`b]¥W
@HWI-EAS443:3:36:1428:688#0/1
CAGAGCTACAGGACATTGAAAATGAAGAGAAGATCCCAAG
+HWI-EAS443:3:36:1428:688#0/1
`ababbbbbbbaaaabba_aaaabaaa_aaa]^`¥U_a]a
@HWI-EAS443:3:36:1467:34#0/1
ATTCGGATTTCCATTTCATCGACGAACGGGGTGAATCAAT
+HWI-EAS443:3:36:1467:34#0/1
`bbbbbbb__a^`abaa`aab`a`]]aa`a^T__^`aZV`
@HWI-EAS443:3:36:1477:84#0/1
TGCTTCAATAGTGCATTCGGGTCACCAGATTCTACAATTA
+HWI-EAS443:3:36:1477:84#0/1
aaSS_^b]bY[HWX_]_ZXZ_Y]]¥_Q][`XY]¥ZTV¥XI
@HWI-EAS443:3:36:153:1838#0/1
GAGGAGATGGCCACCAAAGCGGACTACACCCTTGACGAAG
+HWI-EAS443:3:36:153:1838#0/1
V__VZ¥^_Ub`a`a`ZKWaa`abbabbbbba_^aabXL_S
@HWI-EAS443:3:36:1553:1340#0/1
ACACCCTTGACGAAGAGAGCAGGGCAAGAATCAAAACTAG
+HWI-EAS443:3:36:1553:1340#0/1
a```aab^^_`ZV]]aa_a_Y``___`]YR]L[R_S[_]_
@HWI-EAS443:3:36:1555:341#0/1
CGGGGCAACATCTTCTCCTATTTCATCAAGTTCTATCCAG
```

```
+HWI-EAS443:3:36:1555:341#0/1
aaS¥^ba__aa`aa__`aaaaa__a`X``a``^`aaa`_`
@HWI-EAS443:3:36:1560:1039#0/1
CGATTGGCCATGTTTCCGATTTGTTTTCAAAGAATTCCTT
+HWI-EAS443:3:36:1560:1039#0/1
¥`U`a`¥b`Y`_YWVZ_a^`abZPWa`RNLTYVRZ_ba_`
@HWI-EAS443:3:36:1573:653#0/1
ATCAGGCACTCCTCGATTGCTTCATATAGCCCCCCAAGAT
+HWI-EAS443:3:36:1573:653#0/1
abb¥Z_a``]_Q`a_``[aa^`a¥abaXZ]_TVJ¥]ba]`
@HWI-EAS443:3:36:1674:1754#0/1
AATGGTTCAATTTTGGCGTTCACTTCTTTTGACATTTGGG
+HWI-EAS443:3:36:1674:1754#0/1
aaaa[Xb[b_^^_YPX_a^SG[MPQJYWZ[SQGUTNYLL[
@HWI-EAS443:3:36:1684:1216#0/1
GCCAAGTGTCGAGGCCCATGTTCCTATATGTGAGAACCAA
+HWI-EAS443:3:36:1684:1216#0/1
aaa__[OZJY]ZUNYNZXXVNX¥WP[_TTVQQONRV^`[T
@HWI-EAS443:3:36:1788:1165#0/1
TTCCGATTTGTTTTCAAAGAATTCCTTGGTCATGTCTTTC
+HWI-EAS443:3:36:1788:1165#0/1
aabababbbb_aaaa`aaaa_aa]Zaa_`¥`^a``[aa_a
@HWI-EAS443:3:36:195:1252#0/1
CGTCCTCAAGAATGGTTCAATTTTGGCGTTCACTTCTTTT
+HWI-EAS443:3:36:195:1252#0/1
a`[bb_a_S^a^aa_`_a]```a``X^TUROMY^YP]^_S
@HWI-EAS443:3:36:277:1049#0/1
GGAGTGGAGGAAGGCTCTATTGGGAAAGTGTGCAGGACCT
+HWI-EAS443:3:36:277:1049#0/1
a`¥aNW_Q¥^]OSRaaXT[]_WUUL^ZMLUWVV¥]NFXQQ
@HWI-EAS443:3:36:289:787#0/1
GCAGGTGCTAGCAGAGCTACAGGACATTGAAAATGAAGAG
+HWI-EAS443:3:36:289:787#0/1
abbaa^Wba_aa`_abb]aabba¥`a`b`a`aaaaa`^a_
@HWI-EAS443:3:36:358:1678#0/1
GAAATTTAGGCTTCTCTACCCCTGTTGTGTTACATATACT
+HWI-EAS443:3:36:358:1678#0/1
aW_bbbbabaaabaaaUaab_ab¥UU[`[_aaaaab`a^_
@HWI-EAS443:3:36:38:1207#0/1
ACCGATTTGAGATAATTGAAGGAAGAGACCGAATCATGGC
+HWI-EAS443:3:36:38:1207#0/1
a`baaaa`a`a`a_Va`aa`a[[^aa¥_a_`_`^`_]__`
@HWI-EAS443:3:36:39:1437#0/1
TAGCCCCCCAAGATCGAAGGTTCCAGGTTCCAGGTTGTCC
+HWI-EAS443:3:36:39:1437#0/1
ababbbbbaaaa_`a`]T[a¥`_[HRZN]`aPY¥T_¥RW`
@HWI-EAS443:3:36:45:1079#0/1
AAGAATTCCTTGGTCATGTCTTTCTCTTTGACAGAAGACT
+HWI-EAS443:3:36:45:1079#0/1
abaaaa_ab_`_aYaa_a¥a``_``a^___]aa_¥W___^
```

@HWI-EAS443:3:36:499:259#0/1
AGTCATCAAAGTCTACTTTTTCTGGTGCCATATTTTCACC
+HWI-EAS443:3:36:499:259#0/1
a`X^R]]¥¥`aO`[Y^`___[¥___Za_`_]^¥a^]__]]
@HWI-EAS443:3:36:519:1556#0/1
CTTCATGTTCTTTGTCCTTGGGATCTTCTCTTCATTTTCA
+HWI-EAS443:3:36:519:1556#0/1
a_b_W¥`TZa_YZT[ba¥V_¥MH[¥aP]_a`aa^Sa]]¥]
@HWI-EAS443:3:36:51:1453#0/1
GAACTTCTCCAGCCTTGAAAACTTTAGAGCCTATGTAGAT
+HWI-EAS443:3:36:51:1453#0/1
a`^a`ababbaabaa``aaXS_ba`aa`_`a``aaZ[]__
@HWI-EAS443:3:36:525:964#0/1
CATTAATCAGGCACTCCTCGATTGCTTCATATAGCCCCCC
+HWI-EAS443:3:36:525:964#0/1
abba`]abbbabaaaba`aaaaaaaa`aa`aa`_a```[a
@HWI-EAS443:3:36:530:1273#0/1
CATGAAGACATTCTTTGGCTGGAAAGAGCCTAACATAGTC
+HWI-EAS443:3:36:530:1273#0/1
[`SXTY__`^_X¥^¥^¥YY``R_``R[a`]XT_VJ¥S¥W_
@HWI-EAS443:3:36:544:1584#0/1
AGCCCCCCAAGATCGAAGGTTCCAGGTTCCAGGTTGTCCC
+HWI-EAS443:3:36:544:1584#0/1
a^ababaa_`¥a^a`__Z[QU^_^^[QS^ZPPSUWPHM¥^
@HWI-EAS443:3:36:576:455#0/1
GGATCTTCTCTTCATTTTCAATGTCCTGTAGCTCTGCTAG
+HWI-EAS443:3:36:576:455#0/1
abbababbabaabbbbbb`aabaaaa`^^aa``_aa`^a_
@HWI-EAS443:3:36:57:821#0/1
GTCCTGCACACTTTCCCAATAGAGCCTTCCTCCACTCCCC
+HWI-EAS443:3:36:57:821#0/1
a`bb`_baaZaaaaaaa]YZ_]^^`a`a`a`__XZ```¥^
@HWI-EAS443:3:36:66:1436#0/1
CCCGAATGCACTATTGAAGCACCGATTTGAGATAATTGAA
+HWI-EAS443:3:36:66:1436#0/1
abbaa`]ab^aaaaaa]a[_a`aa__`a`_`^_¥V_^_¥R
@HWI-EAS443:3:36:71:637#0/1
AATCAGGCACTCCTCGATTGCTTCATATAGCCCCCCAAGA
+HWI-EAS443:3:36:71:637#0/1
_Z]]^`a`P_aaa`aaa_``a__a`^¥][¥a^_``_VW]O
@HWI-EAS443:3:36:783:1547#0/1
CTCTTGAGGACTGCGATAGGCCAAGTGTCGAGGCCCATGT
+HWI-EAS443:3:36:783:1547#0/1
aWTVW`V¥__ZUZY_¥XNNVURVT_U¥VZYHG]UTUNSUL
@HWI-EAS443:3:36:784:642#0/1
GGGGGCTATATGAAGCAATCGAGGAGTGCCTGATTAATGA
+HWI-EAS443:3:36:784:642#0/1
aa_¥_baaabba`aaabaa`aaa``a]^`_``^`_``]__
@HWI-EAS443:3:36:822:849#0/1
ATGAGAAAGGCATAAATCCCAATTACCTCATGGCTTGGAA

```
+HWI-EAS443:3:36:822:849#0/1
a`a_bb_^``ba_`ba^a`_`aa`baaa`__a[_a^]YVZ
@HWI-EAS443:3:36:835:1001#0/1
GGCTCCAGTCTCGGGTCAGTGAGTGAGAACTCCATACTTA
+HWI-EAS443:3:36:835:1001#0/1
a`a`bb`a]a^`aaaRaS_X_ZaR_¥`Za_^[`X^`a[^S
@HWI-EAS443:3:36:906:147#0/1
CTACTGAATACATAATGAAGGGAGTGTACATAAATACGGC
+HWI-EAS443:3:36:906:147#0/1
a_]Za]ZWa_R^YXT_`NY__ZZ_X`]^__¥WTVXa_]X_
@HWI-EAS443:3:36:909:408#0/1
GGGGTTTTCGGCTGAATCGAGAAAATTGCTTCTCATTGTT
+HWI-EAS443:3:36:909:408#0/1
a`]aQXaRIOYLWaT_[TSFMXR_[H[V_TYL][SOJ^NN
@HWI-EAS443:3:36:924:1798#0/1
CATGTTTCCGATTTGTTTTCAAAGAATTCCTTGGTCATGT
+HWI-EAS443:3:36:924:1798#0/1
aaa][aabbaa_```X`__¥`aa`__````_]^Y]_`^¥W
@HWI-EAS443:3:36:952:1002#0/1
CACTTTCCCAATAGAGCCTTCCTCCACTCCCCTGGGTGAC
+HWI-EAS443:3:36:952:1002#0/1
a]`^[a`bbba]¥Qaaaaa]_¥]a_`a_``a^[X^^V_[`
@HWI-EAS443:3:36:960:760#0/1
GTCCGCTTTGGTGGCCATCTCCTCTCCAGTGAATGAAAAG
+HWI-EAS443:3:36:960:760#0/1
`_b_]a__a`aU]`Y_XUY¥__YX¥Y`G[U_U¥¥ZVZ_`X
@HWI-EAS443:3:37:1033:162#0/1
CAAAATAAAATCTGAGAAGACACACATTCACATCTTTTCA
+HWI-EAS443:3:37:1033:162#0/1
aba_`b]]b^WZ]a_aa_aa[]a`]_]XZO[___]NUULT
@HWI-EAS443:3:37:1065:961#0/1
AATGAGAAGCAATTTTCTCGATTCAGCCGAAAACCCCTCA
+HWI-EAS443:3:37:1065:961#0/1
ab¥_Z_bbb^_T`aab`aa^^a`[^``_aQN]V^`^`WaZ
@HWI-EAS443:3:37:1113:1273#0/1
CGGCCTTGCTCAATGCATCCTGTGCAGCCATGGATGACTT
+HWI-EAS443:3:37:1113:1273#0/1
aa`aabaababaababbaa_aba`ba`_aU_a_aa__a`Y
@HWI-EAS443:3:37:1149:1181#0/1
GGGGGCTATATGAAGCAATCGAGGAGTGCCTGATTAATGA
+HWI-EAS443:3:37:1149:1181#0/1
ab_`]baababa`¥b`_ba]a^b^aa_SbM`^[`aa``a`
@HWI-EAS443:3:37:1198:914#0/1
GCGCATAGTTCCTGTAATCTCAAATTTTTCTTCAATTGTC
+HWI-EAS443:3:37:1198:914#0/1
aba`aaabZa`_ab]][a`a`aaUaaaaXDX`aa``a`^_
@HWI-EAS443:3:37:11:699#0/1
CAACAAAATAAAATCTGAGAAGACACACATTCACATCTTT
+HWI-EAS443:3:37:11:699#0/1
`V]aa`^aaa_V[_aa`V^^[aZ¥]aa¥V`Wa_a___`]a
```

```
@HWI-EAS443:3:37:1236:108#0/1
CCAGATTCTACAATTATTGATTCACCCCGTTCGTCGATGA
+HWI-EAS443:3:37:1236:108#0/1
abb`aababa`aabaababaaaaaab^abS_`a_aa_aaY
@HWI-EAS443:3:37:1250:122#0/1
GGGGGCTATATGAAGCAATCGAGGAGTGCCTGATTAATGA
+HWI-EAS443:3:37:1250:122#0/1
abaaaabaaaaaaaaa`aa_a_aa_a^aaN____`[a``^
@HWI-EAS443:3:37:1279:1449#0/1
AGCAAACTTGTTAGTTTCGATTTTCGGATCTTCCCCATAT
+HWI-EAS443:3:37:1279:1449#0/1
^```^^ab^YYa`_Rba^]W``aa^^OJSDZ^^`_^^]X]
@HWI-EAS443:3:37:127:502#0/1
TGTGGTTTGACTATGTTAGGCTCTTTCCAGCCAAAGAATG
+HWI-EAS443:3:37:127:502#0/1
aaXaa_a_XV]a`a`^`a_]¥¥`_`^^^S[_ZHU]TXXV_
@HWI-EAS443:3:37:130:708#0/1
AAAGGCATAAATCCCAATTACCTCATGGCTTGGAAGCAGG
+HWI-EAS443:3:37:130:708#0/1
a`^¥_aa]`^`aZa__`__a_[_aaZ_W__WPYWW¥ULX]
@HWI-EAS443:3:37:1340:321#0/1
GGGGCATGGAAATGAGGCGCTGCCTTCTTCAGTCTCTTCA
+HWI-EAS443:3:37:1340:321#0/1
aa`[__`aa_¥_`aYa¥__W^`^¥¥_V`¥PS]ZZ^¥¥XWV
@HWI-EAS443:3:37:1349:997#0/1
GGGGGAGGGAATACCACTATATGATGCAATCAAATGCATG
+HWI-EAS443:3:37:1349:997#0/1
ab_Yaaaa]VZa]___`a`]X]`^_a`]UXaaa]^]_a]_
@HWI-EAS443:3:37:1352:904#0/1
TGCCCTGCTCTCTTCGTCAAGGGTGTAGTCCGCTTTGGTG
+HWI-EAS443:3:37:1352:904#0/1
abaa`]__bbaabbYZXY^_aabSb_a¥_Gba`^a``VY^
@HWI-EAS443:3:37:1365:1570#0/1
CATTAATCAGGCACTCCTCGATTGCTTCATATAGCCCCCC
+HWI-EAS443:3:37:1365:1570#0/1
a__¥]__ab`_`a^Za]b``]^_]Z`_WaN]][[]^`a^X
@HWI-EAS443:3:37:1376:1026#0/1
GGCACTCGGTGAAAATATGGCACCAGAAAAAGTAGACTTT
+HWI-EAS443:3:37:1376:1026#0/1
``aaaabbaRb]abaaabaa``aa`a[]¥NS¥N¥_]XXa`
@HWI-EAS443:3:37:1393:981#0/1
AAGAATTCCTTGGTCATGTCTTTCTCTTTGACAGAAGACT
+HWI-EAS443:3:37:1393:981#0/1
abaa`aaabbbba``abbaabbbabaaaaXZabaa`aa`_
@HWI-EAS443:3:37:1413:556#0/1
CGAACGGGGTGAATCAATAATTGTAGAATCTGGTGACCCG
+HWI-EAS443:3:37:1413:556#0/1
abbbbbbbbabbbbababbabbbbbbabbNbba^baabba
@HWI-EAS443:3:37:1433:1388#0/1
GGCATGGAAATGAGGCGCTGCCTTCTTCAGTCTCTTCAGC
```

+HWI-EAS443:3:37:1433:1388#0/1
]a]¥_a`Y`a]]_^^T^]¥aU_a^Waa¥[R`Xb_aa_a^Q
@HWI-EAS443:3:37:1462:1399#0/1
CAGCTTGCTAGAGATCTGGGCTCTGGCTCATCACTGTCAT
+HWI-EAS443:3:37:1462:1399#0/1
_[ba__]Wb_[Xaab^I][``V]^¥UZD[ZbYY_UZQ¥_X
@HWI-EAS443:3:37:150:1232#0/1
GGCATAAATCCCAATTACCTCATGGCTTGGAAGCAGGTGC
+HWI-EAS443:3:37:150:1232#0/1
`^bbbb`abbbbbb`bbbbbabaa]ba^GM¥_P[¥_a¥X`
@HWI-EAS443:3:37:1531:557#0/1
GCACCGATTTGAGATAATTGAAGGAAGAGACCGAATCATG
+HWI-EAS443:3:37:1531:557#0/1
_ba^ababbbb`baaaaaaaaaaaaaa`¥I¥^aaa`Zaa`
@HWI-EAS443:3:37:1551:964#0/1
CACTTCTGCTGTAAAATAGTTCCTCCTCATGCTTGCGATA
+HWI-EAS443:3:37:1551:964#0/1
^^_babbb[bbbabbabbb`bbabbbb`_Zbbbbbbb_bb
@HWI-EAS443:3:37:1601:913#0/1
AGCCAACAAAATAAAATCTGAGAAGACACACATTCACATC
+HWI-EAS443:3:37:1601:913#0/1
aaaaabaab`aaabaaaaaa_a_aab_a[N[_aaa`aaa_
@HWI-EAS443:3:37:1662:339#0/1
ACAGATTTTGCCAGTAAGGTCCTGCACACTTTCCCAATAG
+HWI-EAS443:3:37:1662:339#0/1
aaaa`baabaa__`¥aaaa[_aaaab_`aU`_aa`b`_`¥
@HWI-EAS443:3:37:173:732#0/1
CATAAATCCCAATTACCTCATGGCTTGGAAGCAGGTGCTA
+HWI-EAS443:3:37:173:732#0/1
aaaa``aaaba`a`aaaaa`_[R¥a¥PZQYPZQNWMW^RS
@HWI-EAS443:3:37:184:577#0/1
TTGGGGGGCTATATGAAGCAATCGAGGAGTGCCTGATTAA
+HWI-EAS443:3:37:184:577#0/1
¥¥¥`aaXI`__a]]_Ya]aaa]a`^¥^¥a^`aa¥_]_V`Z
@HWI-EAS443:3:37:208:32#0/1
GCTTGAATCAGTCAATTCACATGCCTTATTGAATTCATTT
+HWI-EAS443:3:37:208:32#0/1
abaaaaaaa[b_baaaaaa`a_aaa_a]Z_^_H^`_`_``
@HWI-EAS443:3:37:20:1397#0/1
CTGCTGTAAAATAGTTCCTCCTCATGCTTGCGATATGTTC
+HWI-EAS443:3:37:20:1397#0/1
a]]``aabbbba_`Yaaa¥]`[a`ZY]]XV_aXXZ_aZ^]
@HWI-EAS443:3:37:241:1190#0/1
GGGGCTATATGAAGCAATCGAGGAGTGCCTGATTAATGAT
+HWI-EAS443:3:37:241:1190#0/1
a`]``^aZ__¥WQ[^baaaXU]`_`Ya_a]T]^S`a_^]Y
@HWI-EAS443:3:37:251:265#0/1
GGGGGCTATATGAAGCAATCGAGGAGTGCCTGATTAATGA
+HWI-EAS443:3:37:251:265#0/1
aaaa]aaaaaa]aaaaaa_]]P]ZX^X__LS^Z^[¥UW^_

@HWI-EAS443:3:37:273:1779#0/1
GATATGTTCAATCGGGGCAACATCTTCTCCTATTTCATCA
+HWI-EAS443:3:37:273:1779#0/1
T¥^aX]]aa^^a_J]_Y`a`[a¥^_^ba_FXb^aaabab_
@HWI-EAS443:3:37:306:33#0/1
GGGGTTTTCGGCTGAATCGAGAAAATTGCTTCTCATTGTT
+HWI-EAS443:3:37:306:33#0/1
abaa]aaa`aa__`__]^_[`__]_]^^[XTUT_U]X]TW
@HWI-EAS443:3:37:370:751#0/1
CCTCCTCATGCTTGCGATATGTTCAATCGGGGCAACATCT
+HWI-EAS443:3:37:370:751#0/1
abaaaab]aaaaa_aaa`]aa]_a`V^`^ZZ_¥HZ^`__`
@HWI-EAS443:3:37:391:1130#0/1
ATCTTCTCTTCATTTTCAATGTCCTGTAGCTCTGCTAGCA
+HWI-EAS443:3:37:391:1130#0/1
abbabbabbbbbbbbbbbabaabbaaab[U`b__a¥R`a`
@HWI-EAS443:3:37:430:950#0/1
CCAGATTCTACAATTATTGATTCACCCCGTTCGTCGATGA
+HWI-EAS443:3:37:430:950#0/1
ab¥a_U_aa[[^V_]Ta_`]aaaaaaaa_]Wa_^aa^Z`¥
@HWI-EAS443:3:37:447:571#0/1
CGAAAATCGAAACTAACAAGTTTGCTGCAATATGCACACA
+HWI-EAS443:3:37:447:571#0/1
abaab`a`aa]a_aaYaaa`^`_`a``__]^___aa_`a`
@HWI-EAS443:3:37:514:440#0/1
GAACAATGAGAAGCAATTTTCTCGATTCAGCCGAAAACCC
+HWI-EAS443:3:37:514:440#0/1
a`aaaaaaaaaa`_a`b_aabaaba__`a^]¥]X¥aa`__
@HWI-EAS443:3:37:576:1479#0/1
GGGGCATGGAAATGAGGCGCTGCCTTCTTCAGTCTCTTCA
+HWI-EAS443:3:37:576:1479#0/1
[aTV__a¥¥`bb`[`^]_`a_[`b_a`^`MJX`baa`aa_
@HWI-EAS443:3:37:583:1912#0/1
GGAAAGAGCCTAACATAGTCAAACCACATGAGAAAGGCAT
+HWI-EAS443:3:37:583:1912#0/1
R¥I[baaaabbbbbbbba`[`bbb`^`abaZZS[^X``ba
@HWI-EAS443:3:37:611:135#0/1
GGGGTTTTCGGCTGAATCGAGAAAATTGCTTCTCATTGTT
+HWI-EAS443:3:37:611:135#0/1
ab`^Zaa^a[V_V``_]]_¥_][XSZ][_XX]Y^^XY]YX
@HWI-EAS443:3:37:696:1113#0/1
AAGAGACCGAATCATGGCCTGGACAGTGGTGAACAGTATA
+HWI-EAS443:3:37:696:1113#0/1
baaaaaaa`^_`[`a```a¥^`__W__X][N¥__TN]^
@HWI-EAS443:3:37:716:842#0/1
TAATCAGGCACTCCTCGATTGCTTCATATAGCCCCCCAAG
+HWI-EAS443:3:37:716:842#0/1
````_¥Z`aa`aaaab_^aaa`aaaaaa_XXaaaa`[`[_
@HWI-EAS443:3:37:754:561#0/1
TTCACTTCTTTTGACATTTGGGAAAGCTTGCCCTCAATGC
````

```
+HWI-EAS443:3:37:754:561#0/1
_`ba_ababaZabba_`^¥]SZ``[a`^Z[_ab_bTT^Z_
@HWI-EAS443:3:37:757:444#0/1
AGAGAACAAGCCAATTGAAGTGGGCACTCGGTGAAAATAT
+HWI-EAS443:3:37:757:444#0/1
aababbaabaa^aaaaa`aa^aaa`a__aR_G[]_]a_]`
@HWI-EAS443:3:37:762:366#0/1
GTGCTAGCAGAGCTACAGGACATTGAAAATGAAGAGAAGA
+HWI-EAS443:3:37:762:366#0/1
X`ZX¥¥a__ba`^`^`a]a]¥_]_`[][ZXY_I_Z]]`^`
@HWI-EAS443:3:37:77:854#0/1
TTTTCACCGAGTGCCCACTTCAATTGGCTTGTTCTCTTCA
+HWI-EAS443:3:37:77:854#0/1
aaa`abba`Ya_aaaaR`aaaaa^_^^^Y^Y^WX¥T^WRD
@HWI-EAS443:3:37:786:182#0/1
GACTCGGCCTCAATCATGCTCTCAATCTGCTGAAGAGACT
+HWI-EAS443:3:37:786:182#0/1
__^Y`b]]Y¥Z^ba]VV[¥¥a^ZXa_a^aG¥^__`a^Z^S
@HWI-EAS443:3:37:791:837#0/1
GCCGAACGGCTGCATTGAGGGCAAGCTTTCCCAAATGTCA
+HWI-EAS443:3:37:791:837#0/1
ab`bbbbbaaaa`a`a_]_``UH^`X[`_S_``Z^[][_[
@HWI-EAS443:3:37:82:332#0/1
AGCAGGTGCTAGCAGAGCTACAGGACATTGAAAATGAAGA
+HWI-EAS443:3:37:82:332#0/1
]aaab`_baaa^aa]_aaa^^^_aaa_aVGDHU_a_G]YY
@HWI-EAS443:3:37:852:2015#0/1
AAGCTTGCGCATAGTTCCTGTAATCTCAAATTTTTCTTCA
+HWI-EAS443:3:37:852:2015#0/1
aba```_b_aaa``Zaa^aa_```¥__`OMU`^a`__^_a
@HWI-EAS443:3:37:903:1682#0/1
CTTGGTCATGTCTTTCTCTTTGACAGAAGACTCGGCCTCA
+HWI-EAS443:3:37:903:1682#0/1
_ba^[J¥a]a`a`^[aaa`a_`_a_`aa^Xa_a_]V]Y_^
@HWI-EAS443:3:37:913:817#0/1
AGCCAACAAAATAAAATCTGAGAAGACACACATTCACATC
+HWI-EAS443:3:37:913:817#0/1
aaaaaaababaaaaa`aaaa]aaa_a`aaVb`_`aa`¥aa
@HWI-EAS443:3:37:962:1151#0/1
GGCTTGGAAGCAGGTGCTAGCAGAGCTACAGGACATTGAA
+HWI-EAS443:3:37:962:1151#0/1
U^`T_[bbb`Ua_^`_b_Z_]Y^¥a^`[b]ZTG_]GXMF^
@HWI-EAS443:3:37:998:1573#0/1
GTTACATATACTGTTCACCACTGTCCAGGCCATGATTCGG
+HWI-EAS443:3:37:998:1573#0/1
a`]¥YUM¥Z_]`¥¥V]]aaaa[_Wab]GYO¥aW¥^`^`ZM
@HWI-EAS443:3:38:1008:904#0/1
GTCCTGCACACTTTCCCAATAGAGCCTTCCTCCACTCCCC
+HWI-EAS443:3:38:1008:904#0/1
a[^WRaaa[¥`^^U_]VUXU]¥OYY][SVLQWUUPW^UXU
```

```
@HWI-EAS443:3:38:1022:1494#0/1
GTGGGCACTCGGTGAAAATATGGCACCAGAAAAAGTAGAC
+HWI-EAS443:3:38:1022:1494#0/1
_SUZa_¥a__¥UZaa¥]]]`ba`[`abXZLTUV¥PNYP^P
@HWI-EAS443:3:38:109:970#0/1
CTTTGTAATCATACAAATCAGGAAGAAATTTAGGCTTCTC
+HWI-EAS443:3:38:109:970#0/1
_^b^^_b_bbaaaa^¥`aa``^``__S_`a]aa¥aa`¥`^
@HWI-EAS443:3:38:1282:94#0/1
GAACTATTTTACAGCAGAAGTGTCCCACTGCAGGGCTACT
+HWI-EAS443:3:38:1282:94#0/1
aababaaabba_aaa`b`]^^a`a``a^a_X[_`]W`^^U
@HWI-EAS443:3:38:1340:1316#0/1
GAACCCATACAGGTTTGTTTTCCGTCTTCCTTCTTTGGTC
+HWI-EAS443:3:38:1340:1316#0/1
`abaaaaaba^¥aOa__[aa`_a]]_aaaQ_Z]a]__]PG
@HWI-EAS443:3:38:1345:140#0/1
GCATCCTGTGCAGCCATGGATGACTTTCAGCTGATCCCAA
+HWI-EAS443:3:38:1345:140#0/1
aaba``a`aaaa`XZ`Vaa¥¥a^^^]XV`RDVZK¥UUQYW
@HWI-EAS443:3:38:1358:162#0/1
ATCCCAATTACCTCATGGCTTGGAAGCAGGTGCTAGCAGA
+HWI-EAS443:3:38:1358:162#0/1
a`a`ababbaa_aZaaaaa__T]UWXZT]WLPNRVTVTRL
@HWI-EAS443:3:38:1407:247#0/1
GCGTCTCCACAACTTGAGGGGTTTTCGGCTGAATCGAGAA
+HWI-EAS443:3:38:1407:247#0/1
aab`_aaaa__]Y_ab^aa_a¥`a`^`_^XYVXVV¥X^^Y
@HWI-EAS443:3:38:140:1552#0/1
GAAAGTCATCCATGGCTGCACAGGATGCATTGAGCAAGGC
+HWI-EAS443:3:38:140:1552#0/1
a][_]_b¥`bbaaaaba`a^aa`_ZX_`^]]¥¥¥ZZ_[]U
@HWI-EAS443:3:38:1440:1984#0/1
CAGGCACTCCTCGATTGCTTCATATAGCCCCCCAAGATCG
+HWI-EAS443:3:38:1440:1984#0/1
WZY_]bb]_][^a_]a]Zba]bbaaZa_^DKGN¥^V^GVH
@HWI-EAS443:3:38:1468:381#0/1
GGGCACTCGGTGAAAATATGGCACCAGAAAAAGTAGACTT
+HWI-EAS443:3:38:1468:381#0/1
abb`¥__aab_aWRY]]`ZWYR_]__`VOSO]WNW¥]WUS
@HWI-EAS443:3:38:153:763#0/1
GATTGCTTCATATAGCCCCCCAAGATCGAAGGTTCCAGGT
+HWI-EAS443:3:38:153:763#0/1
abaaabaa_^`aaaa````aaaZ]a`aZPXT¥NL[Z[]VR
@HWI-EAS443:3:38:1556:99#0/1
CAAATGTAGGACCAAAGAAGGAAGACGGAAAACAAACCTG
+HWI-EAS443:3:38:1556:99#0/1
`bbbabababba`abbba`a`_aa_abaa_¥]]^``YV__
@HWI-EAS443:3:38:1574:1291#0/1
TTTGTAATCATACAAATCAGGAAGAAATTTAGGCTTCTCT
```

```
+HWI-EAS443:3:38:1574:1291#0/1
¥b¥`WX[Yaba¥¥ZV]a___]]]a_]H]_¥YYYY¥YYYVS
@HWI-EAS443:3:38:1576:981#0/1
ACAGGACATTGAAAATGAAGAGAAGATCCCAAGGACAAAG
+HWI-EAS443:3:38:1576:981#0/1
aaaaa¥a_aaa_U¥_a_`a_]a_aa_]]aUZXSSWW^ZZX
@HWI-EAS443:3:38:158:1869#0/1
CCCTGGGTGACTCTCCGATTGGCCATGTTTCCGATTTGTT
+HWI-EAS443:3:38:158:1869#0/1
abbbbbb_bbbbbaabbUaa[`bba]W¥aa¥`]a_aa`a`
@HWI-EAS443:3:38:1652:111#0/1
ATCTTCTCTTCATTTTCAATGTCCTGTAGCTCTGCTAGCA
+HWI-EAS443:3:38:1652:111#0/1
a_bbbababb]]bbb`_`bbbababbbab][V_]U[`^XU
@HWI-EAS443:3:38:169:741#0/1
GTGGGCACTCGGTGAAAATATGGCACCAGAAAAAGTAGAC
+HWI-EAS443:3:38:169:741#0/1
a_`aa`[aaaZaU_^¥`]_____`U``_¥^¥]N¥[S_[ZS
@HWI-EAS443:3:38:1784:2016#0/1
AGCCGAACGGCTGCATTGAGGGCAAGCTTTCCCAAATGTC
+HWI-EAS443:3:38:1784:2016#0/1
aV`_`aaN^Y¥U¥N^X^GNTU^GS¥WTV`UWHYTYYSMFR
@HWI-EAS443:3:38:183:664#0/1
CCCAATAGAGCCTTCCTCCACTCCCCTGGGTGACTCTCCG
+HWI-EAS443:3:38:183:664#0/1
aaabaa`aaaaaaa_aaaaaa`aaaa^^¥^W^_`a^¥^¥^
@HWI-EAS443:3:38:18:1382#0/1
TACAAATCAGGAAGAAATTTAGGCTTCTCTACCCCTGTTG
+HWI-EAS443:3:38:18:1382#0/1
^aa`^`aa]`^^``_aa`_[^``a``a_b_a`^`a^_U_[
@HWI-EAS443:3:38:190:1927#0/1
GTCCTTGGGATCTTCTCTTCATTTTCAATGTCCTGTAGCT
+HWI-EAS443:3:38:190:1927#0/1
a_a]``Pa¥XSU¥aa`a`_`[_____[X¥LMRV¥P^XX`_
@HWI-EAS443:3:38:248:487#0/1
TATGTAGATGGATTCGAGCCGAACGGCTGCATTGAGGGCA
+HWI-EAS443:3:38:248:487#0/1
aaa_]a_]abb`aaaaaaaaaa]a_¥a]¥U¥ZYSS__]U[
@HWI-EAS443:3:38:263:1095#0/1
TGAAATTGGAGTAACACGGAGGGAAGTCCACATATATTAC
+HWI-EAS443:3:38:263:1095#0/1
aabaa`aa^aa[abaaa[```_^`R_X_a^Z`__``^¥^X
@HWI-EAS443:3:38:319:1777#0/1
CCAATTTCAATGAACCGGTTCTCTTTGTAATCATACAAAT
+HWI-EAS443:3:38:319:1777#0/1
a``_aa`aa]__`aaa``_^__`_[YWX_^]P_]___]`[
@HWI-EAS443:3:38:356:704#0/1
AATATGGCACCAGAAAAAGTAGACTTTGATGACTGCAAAG
+HWI-EAS443:3:38:356:704#0/1
aa[aaa`a_a__a`a_a_`X__[``^^_]_]][][]X_UX
```

```
@HWI-EAS443:3:38:428:1482#0/1
CATATCGCAAGCATGAGGAGGAACTATTTTACAGCAGAAG
+HWI-EAS443:3:38:428:1482#0/1
^`bbba^^a^Z_aaaa]a_a]Waa_`]]__`_a[[]^a`V
@HWI-EAS443:3:38:458:861#0/1
GTCCCACTGCAGGGCTACTGAATACATAATGAAGGGAGTG
+HWI-EAS443:3:38:458:861#0/1
a_bbbaaabaaa_`baaaabaaba`]`aa___]a___a^_
@HWI-EAS443:3:38:462:190#0/1
CTCGGGTCAGTGAGTGAGAACTCCATACTTACAAAGTTCA
+HWI-EAS443:3:38:462:190#0/1
aaba_ZZa]`Z^^a^a`]T^¥U^aYVQ[_]]^__a_X[^]
@HWI-EAS443:3:38:483:302#0/1
CATTAATCAGGCACTCCTCGATTGCTTCATATAGCCCCCC
+HWI-EAS443:3:38:483:302#0/1
aaabZabbbaaaaabaaaab`_``a`_aa```a___a]`X
@HWI-EAS443:3:38:500:1017#0/1
TTGATTCTTGCCCTGCTCTCTTCGTCAAGGGTGTAGTCCG
+HWI-EAS443:3:38:500:1017#0/1
a`WX]WV]]``[¥QOX^aa^]¥`ZNW^^^QKKRTT^VU`[
@HWI-EAS443:3:38:522:780#0/1
CTTGGAAGCAGGTGCTAGCAGAGCTACAGGACATTGAAAA
+HWI-EAS443:3:38:522:780#0/1
abb`b`bbaaV_Uababababab_aaa_aab`^a]_aZZZ__
@HWI-EAS443:3:38:582:266#0/1
CCTGAACAATGAGAAGCAATTTTCTCGATTCAGCCGAAAA
+HWI-EAS443:3:38:582:266#0/1
a_`a_a``]Z`¥`^a`]`__a^_`a__]]YXV_][[S[MU
@HWI-EAS443:3:38:60:95#0/1
CATTAATCAGGCACTCCTCGATTGCTTCATATAGCCCCCC
+HWI-EAS443:3:38:60:95#0/1
aaaa^[^``a_`X^_a¥^_a]_^______¥[TRZZ¥¥¥¥V
@HWI-EAS443:3:38:623:142#0/1
GAAATCCGAATACATGAAACAAACTTCCAAATGTGTGCAT
+HWI-EAS443:3:38:623:142#0/1
^W¥^_``]^[W``^aaa¥aY^¥O¥a`a_a]ZY_VaZa__X
@HWI-EAS443:3:38:658:1133#0/1
AGCAGGTGCTAGCAGAGCTACAGGACATTGAAAATGAAGA
+HWI-EAS443:3:38:658:1133#0/1
]a`Y]a]_a`a___a_``^aaaa`_`Y_`_Y^]___`__^
@HWI-EAS443:3:38:665:877#0/1
GGGGAAGATCCGAAAATCGAAACTAACAAGTTTGCTGCAA
+HWI-EAS443:3:38:665:877#0/1
aZaa_^``¥`¥``^aa_aa_Y_`_WRW¥I[U[Y_^^_^YW
@HWI-EAS443:3:38:679:1747#0/1
GGGGCATGGAAATGAGGCGCTGCCTTCTTCAGTCTCTTCA
+HWI-EAS443:3:38:679:1747#0/1
W¥_VU`_]Y^Z_]`b¥Y_¥_^___^Z__aaW`V`^`a]a_
@HWI-EAS443:3:38:691:463#0/1
CGGCCTCAATCATGCTCTCAATCTGCTGAAGAGACTGAAG
```

```
+HWI-EAS443:3:38:691:463#0/1
_b¥bbbba`Zb[WW`[W¥__X`aa^WZ¥XZ_aa]T`baaZ
@HWI-EAS443:3:38:717:351#0/1
GGGGCATGGAAATGAGGCGCTGCCTTCTTCAGTCTCTTCA
+HWI-EAS443:3:38:717:351#0/1
aba`aaabbaaaaaa``aa`aaa``_a`aWW`X__¥__^¥
@HWI-EAS443:3:38:721:371#0/1
TGTATTCGGATTTCCATTTCATCGACGAACGGGGTGAATC
+HWI-EAS443:3:38:721:371#0/1
ab^bba`bb`^``__^aaa^aa```aaa_T¥^_`Qa`_¥S
@HWI-EAS443:3:38:756:404#0/1
ACAATTGAAGAAAAATTTGAGATTACAGGAACTATGCGCA
+HWI-EAS443:3:38:756:404#0/1
_b`Na^_b`^]]a_abaa¥^b`bbab`aaa`_ab_aZa__
@HWI-EAS443:3:38:764:74#0/1
GAGAAATGATACTGATGTGGTGAACTTTGTAAGTATGGAG
+HWI-EAS443:3:38:764:74#0/1
a^a]__a_^¥^]]_]¥_Z`^U`]ZZ^XZZXOZ]ZZX¥ZXZ
@HWI-EAS443:3:38:860:266#0/1
CAAATGCATGAAGACATTCTTTGGCTGGAAAGAGCCTAAC
+HWI-EAS443:3:38:860:266#0/1
abbbabbbabaaaaaa_Y`aa[aaaaaa_Y]]]`ZZ_^ZG
@HWI-EAS443:3:38:88:1760#0/1
GTTCGTCGATGAAATGGAAATCCGAATACATGAAACAAAC
+HWI-EAS443:3:38:88:1760#0/1
]]aa^PY¥`H^_]RYa]bbb_bbTba`bbaT^aaba_ba^
@HWI-EAS443:3:38:902:63#0/1
CAACAAAATAAAATCTGAGAAGACACACATTCACATCTTT
+HWI-EAS443:3:38:902:63#0/1
Ybbbbababbbbbbbabbbbbabaabbb`aaaaaabbabaa
@HWI-EAS443:3:38:913:1228#0/1
AGCAGGGCAAGAATCAAAACTAGGCTTTTCACTATAAGAC
+HWI-EAS443:3:38:913:1228#0/1
aaaaaaababaaaabbaab``aa`a___`]_]```_`_`W
@HWI-EAS443:3:38:944:812#0/1
GTGGGAGACTTTGGTCGGCAAGCTTGCGCATAGTTCCTGT
+HWI-EAS443:3:38:944:812#0/1
a_aaa_a¥MZa_H¥G¥_]Y[YQ`][_Z^^RUVQLTXZXWM
@HWI-EAS443:3:38:960:447#0/1
GTGGGAGACTTTGGTCGGCAAGCTTGCGCATAGTTCCTGT
+HWI-EAS443:3:38:960:447#0/1
a]aaaaa_`a_`_]]`^][]]_`^]^¥`ZXXXYR]YY^^X
@HWI-EAS443:3:39:100:1590#0/1
GCCCACTTCAATTGGCTTGTTCTCTTCATGTTCTTTGTCC
+HWI-EAS443:3:39:100:1590#0/1
`bbbbbbbbabaaaabaa^[_^`aaaaaaa_`_aabR]a]
@HWI-EAS443:3:39:102:1082#0/1
GTTTGTTTCATGTATTCGGATTTCCATTTCATCGACGAAC
+HWI-EAS443:3:39:102:1082#0/1
`]``a_``aa`__aaa``a¥^^_a]P`_^`_]]^]^^_[Y
```

```
@HWI-EAS443:3:39:1053:816#0/1
CAGGAACTATGCGCAAGCTTGCCGACCAAAGTCTCCCACC
+HWI-EAS443:3:39:1053:816#0/1
a_ab¥baaaaaabaaa_```_aa_aa^a][^W¥_a¥__^Q
@HWI-EAS443:3:39:1063:498#0/1
ACCAGAAAAAGTAGACTTTGATGACTGCAAAGATGTTGGA
+HWI-EAS443:3:39:1063:498#0/1
aaa_b¥^a^`a_aa`aaaaa`aa_¥a_`a``_¥_aZ_``]
@HWI-EAS443:3:39:10:1487#0/1
CATGGAAATGAGGCGCTGCCTTCTTCAGTCTCTTCAGCAG
+HWI-EAS443:3:39:10:1487#0/1
a`a`Z¥U__]^``aaa_``a^^```a`][a`U^_a¥X[NX
@HWI-EAS443:3:39:1103:1806#0/1
GGAAATCCGAATACATGAAACAAACTTCCAAATGTGTGCA
+HWI-EAS443:3:39:1103:1806#0/1
aaa_a_bbabaabaaa`a`aZbaaa`__aaXOT`YVLV_Y
@HWI-EAS443:3:39:1111:1174#0/1
GAATCAGTCAATTCACATGCCTTATTGAATTCATTTTGGA
+HWI-EAS443:3:39:1111:1174#0/1
abba``aY_]_aaaaa¥`aa_[aa_^_aa`[FZ_``__a]
@HWI-EAS443:3:39:111:116#0/1
CACTCCTCGATTGCTTCATATAGCCCCCCAAGATCGAAGG
+HWI-EAS443:3:39:111:116#0/1
aabbbbaaaa]Z]a__]VW]¥W]Xa¥X`__X¥¥RZ[NZ[Z
@HWI-EAS443:3:39:1129:274#0/1
ATTGCAGCAAACTTGTTAGTTTCGATTTTCGGATCTTCCC
+HWI-EAS443:3:39:1129:274#0/1
abbabbbbbb_`bbbaaaa^aa`aU``a``]OGRV_YWWW
@HWI-EAS443:3:39:1139:1489#0/1
AGGCAATCTGAGGGGGCGTGGTGTCGTCCTCAAGAATGGT
+HWI-EAS443:3:39:1139:1489#0/1
Q_bb``_`a][QX¥bbZWP__TWN]aPQ_aa_W_aa`^`V
@HWI-EAS443:3:39:1169:858#0/1
CACTGCAGGGCTACTGAATACATAATGAAGGGAGTGTACA
+HWI-EAS443:3:39:1169:858#0/1
aaaabaaaaaaaa^bba`aa^`aa_a`a_a_]]`^aZ_]Z
@HWI-EAS443:3:39:1190:206#0/1
CTCATTTGAGAAATGATACTGATGTGGTGAACTTTGTAAG
+HWI-EAS443:3:39:1190:206#0/1
abb_ba_a`a_bbbbbbb_aaaaa^a[M^TZX¥a]ZZ__^
@HWI-EAS443:3:39:1199:390#0/1
CTTTGTAAGTATGGAGTTCTCACTCACTGACCCGAGACTG
+HWI-EAS443:3:39:1199:390#0/1
[babaaaabababb[_`a]baba`bbabbbbV`b`ba_aa
@HWI-EAS443:3:39:1422:601#0/1
GAGGAACTATTTTACAGCAGAAGTGTCCCACTGCAGGGCT
+HWI-EAS443:3:39:1422:601#0/1
ab`aa`b_baabbbbabaa`_aWVa^Va`a`¥_Xa[aV^]
@HWI-EAS443:3:39:144:1754#0/1
AAAGAGGCGAAGAGACAATTGAAGAAAAATTTGAGATTAC
```

+HWI-EAS443:3:39:144:1754#0/1
aba^a__b`abaaa]`a`_`[¥]Y]aa_``_^¥a_a`_a^
@HWI-EAS443:3:39:1463:1715#0/1
ACGGGGTGAATCAATAATTGTAGAATCTGGTGACCCGAAT
+HWI-EAS443:3:39:1463:1715#0/1
[_Z``a^YVa]O^T_^W^_]O]^[ZVZ]XVFR¥[`[U]RT
@HWI-EAS443:3:39:1499:167#0/1
TGTATTCAACAGTCTATATGCGTCTCCACAACTTGAGGGG
+HWI-EAS443:3:39:1499:167#0/1
aa]a``aa``]`]]__^^[TS[WXX¥TZUXXHXVWUYWTV
@HWI-EAS443:3:39:1507:673#0/1
GGGCACTCGGTGAAAATATGGCACCAGAAAAAGTAGACTT
+HWI-EAS443:3:39:1507:673#0/1
a¥aa`]^aa_]a]Z_`_¥Z_¥_]Z]_^¥^]TLTZ_^XX]Z
@HWI-EAS443:3:39:1518:890#0/1
CCTGATTTGTATGATTACAAAGAGAACCGGTTCATTGAAA
+HWI-EAS443:3:39:1518:890#0/1
abb¥bbbbbabbbbbbbbaaaaaaaZV_bb[`_`aaa^`Z
@HWI-EAS443:3:39:1564:1705#0/1
GGGGGCTATATGAAGCAATCGAGGAGTGCCTGATTAATGA
+HWI-EAS443:3:39:1564:1705#0/1
`VZUTXV[_[a[OZTXY]LNPPUZUXYJTY^LGR[RQBBB
@HWI-EAS443:3:39:156:195#0/1
CAGGGCAAGAATCAAAACTAGGCTTTTCACTATAAGACAA
+HWI-EAS443:3:39:156:195#0/1
abbabbaaa`bbbbXbbababaaa`aaaaaaaaa``_¥a
@HWI-EAS443:3:39:168:123#0/1
AATTCACATGCCTTATTGAATTCATTTTGGACCCAGCTTG
+HWI-EAS443:3:39:168:123#0/1
aV`bbaaVa_¥Z`aaaaaaaaa___^a``Z]_^aUa`_]_
@HWI-EAS443:3:39:1724:102#0/1
TGGGGGGCTATATGAAGCAATCGAGGAGTGCCTGATTAAT
+HWI-EAS443:3:39:1724:102#0/1
]ab`aZaaa_aZZ`^[[MY[^TXU`^G]R[PKRXKVVVVV
@HWI-EAS443:3:39:1728:968#0/1
AAATCCCAATTACCTCATGGCTTGGAAGCAGGTGCTAGCA
+HWI-EAS443:3:39:1728:968#0/1
ab_a`a`[Z`]Za___a_`ZRaXT_YBBBBBBBBBBBBBB
@HWI-EAS443:3:39:1742:180#0/1
ATGAGAAGCAATTTTCTCGATTCAGCCGAAAACCCCTCAA
+HWI-EAS443:3:39:1742:180#0/1
abbaaaab`aaababaa_a^`a[[a_X`^a^XSP[YYWZY
@HWI-EAS443:3:39:1749:251#0/1
AGTAGGAGTCTATGGGATTCCTTTCGTCAGTCCGAAAGAG
+HWI-EAS443:3:39:1749:251#0/1
abaaabab^``^_baa```_]`a``aY___^VY]_^]^YY
@HWI-EAS443:3:39:1761:681#0/1
CTAACATAGTCAAACCACATGAGAAAGGCATAAATCCCAA
+HWI-EAS443:3:39:1761:681#0/1
^`aXTH_VUY_¥NS_ZWVZa`UN]YYTaSQY_^XaWPN][

```
@HWI-EAS443:3:39:1781:1887#0/1
GTTTGTTTCATGTATTCGGATTTCCATTTCATCGACGAAC
+HWI-EAS443:3:39:1781:1887#0/1
_`aaa]aa_a`a[`aa`aZ^__^]_a_aa`YQUZ^W]]VR
@HWI-EAS443:3:39:179:576#0/1
GCCAGTAGGAGTCTATGGGATTCCTTTCGTCAGTCCGAAA
+HWI-EAS443:3:39:179:576#0/1
aaaaa^aa]_a¥a`_^_^^____`_¥^`_W_[^P^^^[[[
@HWI-EAS443:3:39:228:347#0/1
CAACAGTCTATATGCGTCTCCACAACTTGAGGGGTTTTCG
+HWI-EAS443:3:39:228:347#0/1
`W¥aS_WbYb`aabbb¥a]a]UaX_ZYIYX^¥aSGa___X
@HWI-EAS443:3:39:228:939#0/1
GGGGTTTTCGGCTGAATCGAGAAAATTGCTTCTCATTGTT
+HWI-EAS443:3:39:228:939#0/1
`_b¥Q_`a^a_`a``[]a`Zaa[a`]a_a^aV[^T^__UR
@HWI-EAS443:3:39:281:787#0/1
AAGAATTCCTTGGTCATGTCTTTCTCTTTGACAGAAGACT
+HWI-EAS443:3:39:281:787#0/1
a_b`W_bbaabbaZ]S`aaaaaaa`aa`^a_]`_a`__][
@HWI-EAS443:3:39:303:962#0/1
CATGTTTCCGATTTGTTTTCAAAGAATTCCTTGGTCATGT
+HWI-EAS443:3:39:303:962#0/1
^WX`X]`ba_Q]baa]`a^^_`¥Z_H^][b`[¥PIZaa[X
@HWI-EAS443:3:39:367:384#0/1
AATTGACTGATTCAAGCTGGATAGAACTTGATGAAATAGG
+HWI-EAS443:3:39:367:384#0/1
a`baaa_abaaaaaaa`aaa_``a`a^a``_`a__^`_a_
@HWI-EAS443:3:39:367:461#0/1
GTGGACTTCCCTCCGTGTTACTCCAATTTCAATGAACCGG
+HWI-EAS443:3:39:367:461#0/1
a¥aTQ¥`abaaaa^¥P]]aa__a____]^`[_XFS_YZ`]
@HWI-EAS443:3:39:371:588#0/1
GGGCACTCGGTGAAAATATGGCACCAGAAAAAGTAGACTT
+HWI-EAS443:3:39:371:588#0/1
aaaaba`bbb[]¥a]Ya¥`aaaaWaS_aa^^]`Taa_¥__
@HWI-EAS443:3:39:372:1428#0/1
GGGGCATGGAAATGAGGCGCTGCCTTCTTCAGTCTCTTCA
+HWI-EAS443:3:39:372:1428#0/1
aa_ab_aaa^`aa_aa`a_a`a_`_]¥_a__N¥_`aT`VW
@HWI-EAS443:3:39:448:859#0/1
GAAATGGCCAGTAGGAGTCTATGGGATTCCTTTCGTCAGT
+HWI-EAS443:3:39:448:859#0/1
WTZ¥]a`aaa^^]``Z]¥`a[]^`_`^^¥`Z¥]ZYYU[[U
@HWI-EAS443:3:39:483:366#0/1
ATAAATACGGCCTTGCTCAATGCATCCTGTGCAGCCATGG
+HWI-EAS443:3:39:483:366#0/1
_b¥__bbaa`bbVaa`aba_^[aaYa[__YUN[Za¥_U_¥
@HWI-EAS443:3:39:608:1626#0/1
GGGGTTTTCGGCTGAATCGAGAAAATTGCTTCTCATTGTT
```

```
+HWI-EAS443:3:39:608:1626#0/1
¥_¥`U_Z_b[Wb`[aSQab`aa_aa``aba^¥[aa_X^]]
@HWI-EAS443:3:39:644:160#0/1
ATAGTTCCTCCTCATGCTTGCGATATGTTCAATCGGGGCA
+HWI-EAS443:3:39:644:160#0/1
]babaabaabbaaa___``a_ba]]]aW^aa]^¥]Y¥_^^
@HWI-EAS443:3:39:697:1030#0/1
TTGCCCCGATTGAACATATCGCAAGCATGAGGAGGAACTA
+HWI-EAS443:3:39:697:1030#0/1
aababaaa_a^aaaabaa`_a`_^`a^a^X`_Xa_^UZ_X
@HWI-EAS443:3:39:697:479#0/1
CGGCTGAATCGAGAAAATTGCTTCTCATTGTTCAGGCACT
+HWI-EAS443:3:39:697:479#0/1
ab`aabababb`b`Ubaaaa^aa`aaa_a_ZU^`aa_`]_
@HWI-EAS443:3:39:710:1299#0/1
CGGTTCATTGAAATTGGAGTAACACGGAGGGAAGTCCACA
+HWI-EAS443:3:39:710:1299#0/1
a`YX^```^[``^^`a]`aU_a¥][_[R^TRT]_XZY]^]
@HWI-EAS443:3:39:770:541#0/1
TACTGTTTAAGGTCTCCAACATCTTTGCAGTCATCAAAGT
+HWI-EAS443:3:39:770:541#0/1
aababaabaa``^a`aaa`_``aa`aaa``¥W_`a`_[`Z
@HWI-EAS443:3:39:800:1810#0/1
ATCCGAAAATCGAAACTAACAAGTTTGCTGCAATATGCAC
+HWI-EAS443:3:39:800:1810#0/1
aVa``]aba`a___aaaa^`^_U]a^Y_]_WTL]_`_^`W
@HWI-EAS443:3:39:827:1876#0/1
AAGAAATTTAGGCTTCTCTACCCCTGTTGTGTTACATATA
+HWI-EAS443:3:39:827:1876#0/1
``a_bba_`b`[_a__]^R`Oa__O¥Z^]RPDN[]WMVOZ
@HWI-EAS443:3:39:851:735#0/1
CTCTTCATGTTCTTTGTCCTTGGGATCTTCTCTTCATTTT
+HWI-EAS443:3:39:851:735#0/1
a¥aabb_¥_Z`_abbb`aba_bX[a`ab¥[`_abb_^a`b
@HWI-EAS443:3:39:897:321#0/1
CTCGTGACTCGGGTCTTCAATACTTAATTTCAGAGCATCC
+HWI-EAS443:3:39:897:321#0/1
a_aaZa]`aa_``O___^^`Y__^a_]_`]PY]XWFQS]X
@HWI-EAS443:3:39:901:1349#0/1
GCAAATGTAGGACCAAAGAAGGAAGACGGAAAACAAACCT
+HWI-EAS443:3:39:901:1349#0/1
abb_abb`aabaaaabbaaaa__aaa_babaaa_baa^__
@HWI-EAS443:3:39:933:1251#0/1
GAAAGGCATAAATCCCAATTACCTCATGGCTTGGAAGCAG
+HWI-EAS443:3:39:933:1251#0/1
`a`__^`¥`bbb_[]__`^`^[IX``Z]`BBBBBBBBBBB
@HWI-EAS443:3:3:1026:220#0/1
GGTGAACTTTGTAAGTATGGAGTTCTCACTCACTGACCCG
+HWI-EAS443:3:3:1026:220#0/1
aa]a`__a`aaY_^_X_``^^_^^U^X^X^Y^X^_YYYXX
```

@HWI-EAS443:3:3:1033:496#0/1
GAACTTTGTAAGTATGGAGTTCTCACTCACTGACCCGAGA
+HWI-EAS443:3:3:1033:496#0/1
a_XY`RSYGXW`_XT[US]]`Z]]ZQ`XU^¥_XW]Z]YXU
@HWI-EAS443:3:3:1102:1815#0/1
TTCCTCCACTCCCCTGGGTGACTCTCCGATTGGCCATGTT
+HWI-EAS443:3:3:1102:1815#0/1
a^Z_a]a`¥__`YUY]_aO``MTY__M^T¥¥^¥SSV]¥G¥
@HWI-EAS443:3:3:1256:1254#0/1
AGGCATGTGAATTGACTGATTCAAGCTGGATAGAACTTGA
+HWI-EAS443:3:3:1256:1254#0/1
a_aVOYWTa[b[_a_a`b`bb¥a_`_`__^_aaKa_aTa^
@HWI-EAS443:3:3:1383:356#0/1
GAGACATGCTCTTGAGGACTGCGATAGGCCAAGTGTCGAG
+HWI-EAS443:3:3:1383:356#0/1
^a``_]bb`bab^bZP`bTaa]ba`ba_ZZ``¥Z_NN``a
@HWI-EAS443:3:3:141:326#0/1
CGAGGGGGAGGGAATACCACTATATGATGCAATCAAATGC
+HWI-EAS443:3:3:141:326#0/1
ababbbbb`bbba[aaa^aaa_aaabaaaOaaaYa`^``_
@HWI-EAS443:3:3:1490:1258#0/1
CTTGGGGGGCTATATGAAGCAATCGAGGAGTGCCTGATTA
+HWI-EAS443:3:3:1490:1258#0/1
abb[b^¥bbba^Q^``S_YG[a[R__a]PZabUU[WTZ¥]
@HWI-EAS443:3:3:1546:834#0/1
CATTAATCAGGCACTCCTCGATTGCTTCATATAGCCCCCC
+HWI-EAS443:3:3:1546:834#0/1
a]_`aa__`^^a_J[Xa[Waaa``X`a]^aa`a`Q`¥[_R
@HWI-EAS443:3:3:162:1612#0/1
CACAACTTGAGGGGTTTTCGGCTGAATCGAGAAAATTGCT
+HWI-EAS443:3:3:162:1612#0/1
abaaa^baba`¥_`Vaaa`_`_aa``^¥]¥X]_a`__[WX
@HWI-EAS443:3:3:1644:519#0/1
TTCGTCGATGAAATGGAAATCCGAATACATGAAACAAACT
+HWI-EAS443:3:3:1644:519#0/1
aa_``_a^```^^^_Y[^^]^¥_^^`ZZ[^]^]^Y]][W[
@HWI-EAS443:3:3:1699:336#0/1
AGAAATTTAGGCTTCTCTACCCCTGTTGTGTTACATATAC
+HWI-EAS443:3:3:1699:336#0/1
ab`_a`a_aa`_aa_`Zaa]]Z__^^_`Z[Z_^¥]^^X_Z
@HWI-EAS443:3:3:1721:514#0/1
GAAAGGCATAAATCCCAATTACCTCATGGCTTGGAAGCAG
+HWI-EAS443:3:3:1721:514#0/1
`_b`b_aaaaaaa_]Za_a_`_W_]¥_a]X`a[]Y]]VBB
@HWI-EAS443:3:3:1762:1221#0/1
ATAATGAAGGGAGTGTACATAAATACGGCCTTGCTCAATG
+HWI-EAS443:3:3:1762:1221#0/1
`b^`aa^_aa[J[I¥TXT`_YYVRUIYZTWR^]BBBBBBB
@HWI-EAS443:3:3:1762:820#0/1
TGGGGCATGGAAATGAGGCGCTGCCTTCTTCAGTCTCTTC

+HWI-EAS443:3:3:1762:820#0/1
a_YXUFZR_a[¥[]]^__KYNK]NNXSPYZPT[ZP^¥ZTB
@HWI-EAS443:3:3:190:1306#0/1
AGAAAAATTTGAGATTACAGGAACTATGCGCAAGCTTGCC
+HWI-EAS443:3:3:190:1306#0/1
ababaa^abba`aa_aaaaa__a`_a`a``_a^_[_^^X[
@HWI-EAS443:3:3:221:99#0/1
AGAAAGGCATAAATCCCAATTACCTCATGGCTTGGAAGCA
+HWI-EAS443:3:3:221:99#0/1
`a`aaaa¥_a`a`a¥]Y___]^]_^¥___^]¥_¥_XYXBB
@HWI-EAS443:3:3:241:1884#0/1
CAAGGACAAAGAACATGAAGAGAACAAGCCAATTGAAGTG
+HWI-EAS443:3:3:241:1884#0/1
]¥^T¥]¥¥_abb_[[X¥__Ua`b`b_`bXJaab¥`ba¥MY
@HWI-EAS443:3:3:266:1599#0/1
ATCCCAATTACCTCATGGCTTGGAAGCAGGTGCTAGCAGA
+HWI-EAS443:3:3:266:1599#0/1
aaaa``aaa`^a`¥a_¥_^`_V`__]VYZ_GQW¥VBBBBB
@HWI-EAS443:3:3:359:222#0/1
TCGGCCTCAATCATGCTCTCAATCTGCTGAAGAGACTGAA
+HWI-EAS443:3:3:359:222#0/1
[^`aXaXFQ_aR]`a_`Z¥aa`_a_a_]X¥SaX`ZGW`[Q
@HWI-EAS443:3:3:439:1015#0/1
CTGTAATCTCAAATTTTTCTTCAATTGTCTCTTCGCCTCT
+HWI-EAS443:3:3:439:1015#0/1
aa`Z¥¥baV[ab¥baaab_aaaaba`aa]_baabba`a¥a
@HWI-EAS443:3:3:484:1755#0/1
CAGGCACTCCTCGATTGCTTCATATAGCCCCCCAAGATCG
+HWI-EAS443:3:3:484:1755#0/1
aaab`ba_`N`^`b]Zb_`b¥`a_^a_VQXHG]_¥aZBBB
@HWI-EAS443:3:3:538:233#0/1
GGGGTTTTCGGCTGAATCGAGAAAATTGCTTCTCATTGTT
+HWI-EAS443:3:3:538:233#0/1
ababV_a[Zaa^_a__¥_aX`]__]V]`^Y_Y¥]]XY_YY
@HWI-EAS443:3:3:599:69#0/1
AGGGAATACCACTATATGATGCAATCAAATGCATGAAGAC
+HWI-EAS443:3:3:599:69#0/1
^baab^aaaaaH`^baaa``b^aa`a``a]a^`aa`aa^G
@HWI-EAS443:3:3:609:181#0/1
GAAGATCCCAAGGACAAAGAACATGAAGAGAACAAGCCAA
+HWI-EAS443:3:3:609:181#0/1
aaa]]^`_a`^aa_^^__b]_]_``^^a_``^Z`^`[V^^
@HWI-EAS443:3:3:62:590#0/1
ATGGCTTGGAAGCAGGTGCTAGCAGAGCTACAGGACATTG
+HWI-EAS443:3:3:62:590#0/1
`Ya`a_aZJV_KXT__UNRUQNLTQXXUGVXTRXYNUBBB
@HWI-EAS443:3:3:684:1732#0/1
GCATAGTTCCTGTAATCTCAAATTTTTCTTCAATTGTCTC
+HWI-EAS443:3:3:684:1732#0/1
```Xa``a_S_aI^WTO]¥____X`__W¥]_`YXLXKI¥Z
```

@HWI-EAS443:3:3:689:1198#0/1
CTGCAAAGATGTTGGAGACCTTAAACAGTATGACAGTGAT
+HWI-EAS443:3:3:689:1198#0/1
abbbb¥¥aabb]_ab``aa^aaabbaabYbaaaa`b^a``
@HWI-EAS443:3:3:700:749#0/1
ATCAGCAGGAACTTTGACCGCTGATGGCAAAGAGGCCCAT
+HWI-EAS443:3:3:700:749#0/1
a_TaaaZbbZaV_]_J`ab^¥_]¥[aWU^_¥`Q`aPGF^]
@HWI-EAS443:3:3:709:1385#0/1
TACCCCTGTTGTGTTACATATACTGTTCACCACTGTCCAG
+HWI-EAS443:3:3:709:1385#0/1
aa_Yb^abaabY``a¥aabaa^^aaa_Oa`[a_a_¥L]a`
@HWI-EAS443:3:3:724:673#0/1
CATGTTTCCGATTTGTTTTCAAAGAATTCCTTGGTCATGT
+HWI-EAS443:3:3:724:673#0/1
P[a``¥bb`aaa``b^bb^`bbbb¥ab`V`aa``]aaab`
@HWI-EAS443:3:3:734:652#0/1
GGCCAAGTGTCGAGGCCCATGTTCCTATATGTGAGAACCA
+HWI-EAS443:3:3:734:652#0/1
a`a^X^a_a^_`_]]WV[a_ZR_WT¥`__¥_Y_^`¥^WG_
@HWI-EAS443:3:3:73:1857#0/1
ACCGAACTTCTCCAGCCTTGAAAACTTTAGAGCCTATGTA
+HWI-EAS443:3:3:73:1857#0/1
a`_`^aaaaaa_aa_[_aa_aa__R__`aW^_^]_`__W^
@HWI-EAS443:3:3:903:1577#0/1
GGCCAATCGGAGAGTCACCCAGGGGAGTGGAGGAAGGCTC
+HWI-EAS443:3:3:903:1577#0/1
`a`Z]XXP[YM]^^¥Z¥^Y¥Z]X¥¥S`K^ZN`TVPBBBBB
@HWI-EAS443:3:3:958:704#0/1
AAAGAATTCCTTGGTCATGTCTTTCTCTTTGACAGAAGAC
+HWI-EAS443:3:3:958:704#0/1
abaa_^ba_`bab`¥_^bba`aab_b^abb[ZNa`____B
@HWI-EAS443:3:40:1066:761#0/1
ACCACTATATGATGCAATCAAATGCATGAAGACATTCTTT
+HWI-EAS443:3:40:1066:761#0/1
aaaa`bababa_ab`a`a``aaaa_a^_P]``^Z`^a_^_
@HWI-EAS443:3:40:1070:1521#0/1
CTGGACAGTGGTGAACAGTATATGTAACACAACAGGGGTA
+HWI-EAS443:3:40:1070:1521#0/1
_`¥aabbb```Z¥]`ab]a__¥a¥Y_RXWX``_ba¥_¥^[
@HWI-EAS443:3:40:1072:444#0/1
CAAGGACAAAGAACATGAAGAGAACAAGCCAATTGAAGTG
+HWI-EAS443:3:40:1072:444#0/1
abbbbbbabbbaaabaaaaaaaaaa`Ya__`_aUa`_aZ_
@HWI-EAS443:3:40:1077:1961#0/1
CGATTGGCCATGTTTCCGATTTGTTTTCAAAGAATTCCTT
+HWI-EAS443:3:40:1077:1961#0/1
`ab_aba_aaab]baa_a_^ba````__Q_^`^aa__Ya_
@HWI-EAS443:3:40:1184:788#0/1
CACATATAGGAACATGGGCCTCGACACTTGGCCTATCGCA

```
+HWI-EAS443:3:40:1184:788#0/1
I[_a`aa^¥a[abb[]a^_`aaaaa``__`¥____^bba¥
@HWI-EAS443:3:40:1230:407#0/1
ATGGCCAGTAGGAGTCTATGGGATTCCTTTCGTCAGTCCG
+HWI-EAS443:3:40:1230:407#0/1
abbb```a[^aa_a[`aa`ba`^_^R_^^aVXZ`]_]Y__
@HWI-EAS443:3:40:1261:1601#0/1
AAATACGGCCTTGCTCAATGCATCCTGTGCAGCCATGGAT
+HWI-EAS443:3:40:1261:1601#0/1
^bbababaaa`a^a[_`a^``ba_`¥^W]`_`aa`^`_Z[
@HWI-EAS443:3:40:1288:92#0/1
ACCGATTTGAGATAATTGAAGGAAGAGACCGAATCATGGC
+HWI-EAS443:3:40:1288:92#0/1
a_abaaaaa`b_aaaaaaa_aa_^`]]]W]_VZ]]YZ¥ZW
@HWI-EAS443:3:40:1296:1863#0/1
CCCGAATGCACTATTGAAGCACCGATTTGAGATAATTGAA
+HWI-EAS443:3:40:1296:1863#0/1
aaababaabaaaaaa`aaaaa]aa___`a__a[aaa¥]a`
@HWI-EAS443:3:40:1328:1976#0/1
ACCCGAGTCACGAGGGGGAGGGAATACCACTATATGATGC
+HWI-EAS443:3:40:1328:1976#0/1
ab`aaaa`ba_aV`aaaaYa_aaa_]a]aZ¥^W¥[¥][`V
@HWI-EAS443:3:40:1373:1280#0/1
CCCCGTTCGTCGATGAAATGGAAATCCGAATACATGAAAC
+HWI-EAS443:3:40:1373:1280#0/1
a_bbb_`a`YO_abaTTaa``a`a_W`b_a_`ab_^`aZR
@HWI-EAS443:3:40:1376:739#0/1
ACATGAGAAAGGCATAAATCCCAATTACCTCATGGCTTGG
+HWI-EAS443:3:40:1376:739#0/1
_¥^ba]babb`a`baaa_a^T_aa`_Zaaa_a___a_a_Y
@HWI-EAS443:3:40:139:669#0/1
GGGGCATGGAAATGAGGCGCTGCCTTCTTCAGTCTCTTCA
+HWI-EAS443:3:40:139:669#0/1
a¥WTZX`XZQR¥[^UQZ`¥`Ya`^U[U[U^ZZU[W]NLUS
@HWI-EAS443:3:40:1439:1821#0/1
GGGGGCTATATGAAGCAATCGAGGAGTGCCTGATTAATGA
+HWI-EAS443:3:40:1439:1821#0/1
a_`aa`_aaaaa`_a]aa^_a^a_]ZU`aTZ_^```_^_¥
@HWI-EAS443:3:40:148:1078#0/1
TGGGGGGCTATATGAAGCAATCGAGGAGTGCCTGATTAAT
+HWI-EAS443:3:40:148:1078#0/1
`aa¥aa_`aaaabaaaabaU_aa_¥[M^Xa_`_`_[]a__
@HWI-EAS443:3:40:1517:1913#0/1
TGGGAAAGCTTGCCCTCAATGCAGCCGTTCGGCTCGAATC
+HWI-EAS443:3:40:1517:1913#0/1
[aab`bbaaa[Za_]_JW¥__aaaJU]`[]R^^^ZUYVLJ
@HWI-EAS443:3:40:1579:572#0/1
CTTTGTAAGTATGGAGTTCTCACTCACTGACCCGAGACTG
+HWI-EAS443:3:40:1579:572#0/1
`bbbabbbbbbbbbbbbbbbbbbb_bbbbaaabb`baa`a
```

```
@HWI-EAS443:3:40:1596:169#0/1
CATATCGCAAGCATGAGGAGGAACTATTTTACAGCAGAAG
+HWI-EAS443:3:40:1596:169#0/1
abbbbabaabbaabbabb`babba`a¥^_aaa`^`aaaaa
@HWI-EAS443:3:40:1649:469#0/1
GGTGAACTTTGTAAGTATGGAGTTCTCACTCACTGACCCG
+HWI-EAS443:3:40:1649:469#0/1
aaNa^`__aa_Y]_aT_aa^¥a[¥W_^```WSb¥`¥¥UQZ
@HWI-EAS443:3:40:1669:1438#0/1
ATTCTTTGGCTGGAAAGAGCCTAACATAGTCAAACCACAT
+HWI-EAS443:3:40:1669:1438#0/1
¥^aX^baY¥Z¥ba]^^TV¥^KaZZ`ROZaRV^][`baXZ]
@HWI-EAS443:3:40:1692:973#0/1
TAAATCCCAATTACCTCATGGCTTGGAAGCAGGTGCTAGC
+HWI-EAS443:3:40:1692:973#0/1
aaa_aaa_a`a``]U____`a^``WPMS[RTY¥XX[TUQL
@HWI-EAS443:3:40:1775:1585#0/1
CACTGCAGGGCTACTGAATACATAATGAAGGGAGTGTACA
+HWI-EAS443:3:40:1775:1585#0/1
`bab`b`ZX_^bb[bbbbbb_bbaa`aa``a_`b_bV^aa
@HWI-EAS443:3:40:186:1618#0/1
AACAAATCGGAAACATGGCCAATCGGAGAGTCACCCAGGG
+HWI-EAS443:3:40:186:1618#0/1
¥T_abab`RPW_``a_ZW¥¥`a_a`UKZM[SZ]_baYYVP
@HWI-EAS443:3:40:192:1785#0/1
ATCATGGCCTGGACAGTGGTGAACAGTATATGTAACACAA
+HWI-EAS443:3:40:192:1785#0/1
a_baa^`aa`¥P]¥a_[Z_Y¥_Z]¥[Wa¥a][Y`a`V]``
@HWI-EAS443:3:40:229:1966#0/1
TGTAGCTCTGCTAGCACCTGCTTCCAAGCCATGAGGTAAT
+HWI-EAS443:3:40:229:1966#0/1
Z^^¥I`_^RV][R_a__aY¥[Y[_YHLWa`_[YZ^_TYRV
@HWI-EAS443:3:40:22:1750#0/1
GGGGGCTATATGAAGCAATCGAGGAGTGCCTGATTAATGA
+HWI-EAS443:3:40:22:1750#0/1
ZH^T^a[^a_XX`_V]^]¥^][Z][PGXZ`^XXYXXYYY¥
@HWI-EAS443:3:40:244:470#0/1
GCAGGTGCTAGCAGAGCTACAGGACATTGAAAATGAAGAG
+HWI-EAS443:3:40:244:470#0/1
a_V]]Y^^_Y_ZQY]_]]^]¥]Y]XYV]YU]Y]XVVRW]Z
@HWI-EAS443:3:40:254:625#0/1
CACACATTTGGAAGTTTGTTTCATGTATTCGGATTTCCAT
+HWI-EAS443:3:40:254:625#0/1
a_`]]Y`a^^^¥GZU¥aa___a[`YXL¥XY^`_YVV]YHY
@HWI-EAS443:3:40:314:924#0/1
CAAGAAATGGCCAGTAGGAGTCTATGGGATTCCTTTCGTC
+HWI-EAS443:3:40:314:924#0/1
a_bba[[_abbabbX__`bb`bbaba_^RY`ab_]`a_T]
@HWI-EAS443:3:40:391:361#0/1
GGGGGCTATATGAAGCAATCGAGGAGTGCCTGATTAATGA
```

```
+HWI-EAS443:3:40:391:361#0/1
ab`aaa`a`a`a`aa_`Z¥aa_aa^`X`_^W`]]Y_^]_]
@HWI-EAS443:3:40:400:1353#0/1
CCCCTGTTGTGTTACATATACTGTTCACCACTGTCCAGGC
+HWI-EAS443:3:40:400:1353#0/1
aababb``_a`aa`aaaY_^``^^_¥H^ba[a`Wba^[R_
@HWI-EAS443:3:40:424:1821#0/1
GCCTGGACAGTGGTGAACAGTATATGTAACACAACAGGGG
+HWI-EAS443:3:40:424:1821#0/1
a^MW[TY_¥]VPVYa`a^¥a]a]Z[[U[O¥[^ZZY[^^[Q
@HWI-EAS443:3:40:427:1466#0/1
ATTTGGGAAAGCTTGCCCTCAATGCAGCCGTTCGGCTCGA
+HWI-EAS443:3:40:427:1466#0/1
aaaa`aa`aa`a^^aa_`^a`W¥_a`¥a`_¥X^¥¥_X¥WS
@HWI-EAS443:3:40:452:719#0/1
GTGGGAGACTTTGGTCGGCAAGCTTGCGCATAGTTCCTGT
+HWI-EAS443:3:40:452:719#0/1
`Ra][]`G`¥_^UOUOZO_Y^[aZZXXOTZRWYUZS]U]U
@HWI-EAS443:3:40:479:1729#0/1
AGAACACAGTATTTTTCCCATTTGTGTGGCTCCAGTCTCG
+HWI-EAS443:3:40:479:1729#0/1
^aTabba`PYaa`aa^aaa]^^]_¥[Y`Vaa`^ORK^Q]N
@HWI-EAS443:3:40:494:650#0/1
GGTGCTTCAATAGTGCATTCGGGTCACCAGATTCTACAAT
+HWI-EAS443:3:40:494:650#0/1
abHa`a`^¥YY¥XQ_`a_Y]Qa_O__^¥GTRR][YPY]GX
@HWI-EAS443:3:40:539:1464#0/1
GACTGACGAAAGGAATCCCATAGACTCCTACTGGCCATTT
+HWI-EAS443:3:40:539:1464#0/1
a]a``_a^aaa[`^]`a¥aa_a^^__``]a^^_]a_XY¥Y
@HWI-EAS443:3:40:740:479#0/1
CGACACTTGGCCTATCGCAGTCCTCAAGAGCATGTCTCCT
+HWI-EAS443:3:40:740:479#0/1
a`baabaaab_`^baabaaa]a``a_P_I`_^``^a^``¥
@HWI-EAS443:3:40:757:79#0/1
GCGCATAGTTCCTGTAATCTCAAATTTTTCTTCAATTGTC
+HWI-EAS443:3:40:757:79#0/1
a][[^a[`Z_WX__ZZ]^X]]ZSS]^Z_UW¥WWWUXYXXW
@HWI-EAS443:3:40:782:589#0/1
ATTCTTTGGCTGGAAAGAGCCTAACATAGTCAAACCACAT
+HWI-EAS443:3:40:782:589#0/1
aaaabaaaaaabaaaaa`aaaaabXaXa__`a`Raa_[P]
@HWI-EAS443:3:40:786:526#0/1
AAGAGACCGAATCATGGCCTGGACAGTGGTGAACAGTATA
+HWI-EAS443:3:40:786:526#0/1
``baaaaaba`a]]``a_aaa`__^]R]_]_^[`__Y_^_
@HWI-EAS443:3:40:80:338#0/1
GCCGAAAACCCCTCAAGTTGTGGAGACGCATATAGACTGT
+HWI-EAS443:3:40:80:338#0/1
aaa`¥IXZ__^`^][^^Q[[R][__¥ZRXZZNXQSPSZSN
```

```
@HWI-EAS443:3:40:814:1233#0/1
GACCGAATCATGGCCTGGACAGTGGTGAACAGTATATGTA
+HWI-EAS443:3:40:814:1233#0/1
_aaZJV]``a`a]_a__`[`_UZZRNZRKSTVNT^^T¥UX
@HWI-EAS443:3:40:867:1212#0/1
CGAACTTCTCCAGCCTTGAAAACTTTAGAGCCTATGTAGA
+HWI-EAS443:3:40:867:1212#0/1
aaa_bab^][W]]aaaa_T^ZaU`YXZ]Va_`^___UZ[`
@HWI-EAS443:3:40:886:64#0/1
GGCTGCACAGGATGCATTGAGCAAGGCCGTATTTATGTAC
+HWI-EAS443:3:40:886:64#0/1
a^^]_]XXX]YYX^¥XYYXYYYXXYYYXQSPWYWVWVUWQ
@HWI-EAS443:3:40:93:1274#0/1
TTTCGGCTGAATCGAGAAAATTGCTTCTCATTGTTCAGGC
+HWI-EAS443:3:40:93:1274#0/1
_V]¥¥`babbbab_K`_XYUVZR]]`__[JUUWFV`_Y¥Z
@HWI-EAS443:3:40:974:514#0/1
CAATAATTGTAGAATCTGGTGACCCGAATGCACTATTGAA
+HWI-EAS443:3:40:974:514#0/1
aaaa]^a_b`^`_V^`[^b_baba``N_WVZa^X_SZbaa
@HWI-EAS443:3:42:1015:1292#0/1
AGCAGGTGCTAGCAGAGCTACAGGACATTGAAAATGAAGA
+HWI-EAS443:3:42:1015:1292#0/1
a`````_^aabaaaa_`_aa`aa^]^`¥_a_^__`a^``_
@HWI-EAS443:3:42:101:1133#0/1
GCATGGAAATGAGGCGCTGCCTTCTTCAGTCTCTTCAGCA
+HWI-EAS443:3:42:101:1133#0/1
a_[Q_]]^W]^VVTa[aY`¥_T]^X]`Y[VU[¥V_]Z]]R
@HWI-EAS443:3:42:101:947#0/1
CACGAGGGGGAGGGAATACCACTATATGATGCAATCAAAT
+HWI-EAS443:3:42:101:947#0/1
abb_[^bab`Yaaaa``a`abaa`aaaba`a`aaaa¥O^`
@HWI-EAS443:3:42:1059:395#0/1
GCGCATAGTTCCTGTAATCTCAAATTTTTCTTCAATTGTC
+HWI-EAS443:3:42:1059:395#0/1
aabaaa`b^aaaab``ababaa`abbabaaaa``^aaa`a
@HWI-EAS443:3:42:1086:1778#0/1
CAAGGACAAAGAACATGAAGAGAACAAGCCAATTGAAGTG
+HWI-EAS443:3:42:1086:1778#0/1
`bb``baaaaaaa`aab`abb_ba`baa]`a```_aaaU¥
@HWI-EAS443:3:42:1095:1297#0/1
AATGGCCAGTAGGAGTCTATGGGATTCCTTTCGTCAGTCC
+HWI-EAS443:3:42:1095:1297#0/1
_abVabb`^Yb`_`aHa`abVZaaabY[abb^^Z^[RR]]
@HWI-EAS443:3:42:1097:1186#0/1
GGCATGGAAATGAGGCGCTGCCTTCTTCAGTCTCTTCAGC
+HWI-EAS443:3:42:1097:1186#0/1
aa`aabaaaaabaaa`b`aa`aaaaaaa`aZ]a_`aZ[¥¥
@HWI-EAS443:3:42:1127:1288#0/1
ATTTCATCGACGAACGGGGTGAATCAATAATTGTAGAATC
```

```
+HWI-EAS443:3:42:1127:1288#0/1
R¥^aabbbbb[`V^`_a_`TUZQ_]_W_UU^]_Z___`^X
@HWI-EAS443:3:42:1210:323#0/1
CTCTTCATGTTCTTTGTCCTTGGGATCTTCTCTTCATTTT
+HWI-EAS443:3:42:1210:323#0/1
a```bZ`bbaa^ab`bbabbbbbbab_baab_bbb`bbab
@HWI-EAS443:3:42:1252:1799#0/1
CATTCTTTGGCTGGAAAGAGCCTAACATAGTCAAACCACA
+HWI-EAS443:3:42:1252:1799#0/1
_aaa`^aa`U`aXIaZ`b`aa]baa^¥b___``aba[^`a
@HWI-EAS443:3:42:1304:1329#0/1
GTCAATTCACATGCCTTATTGAATTCATTTTGGACCCAGC
+HWI-EAS443:3:42:1304:1329#0/1
a`aaabaaa_ba`aaba`aaa`aaa^aabaa_Z_`a^__¥
@HWI-EAS443:3:42:1311:191#0/1
CTCCCCTGGGTGACTCTCCGATTGGCCATGTTTCCGATTT
+HWI-EAS443:3:42:1311:191#0/1
abaabbbbba]aaaaaa]^]__`a`_a__aW`a_Z^P```
@HWI-EAS443:3:42:1342:1597#0/1
AGCAGGTGCTAGCAGAGCTACAGGACATTGAAAATGAAGA
+HWI-EAS443:3:42:1342:1597#0/1
SS`^a_SY_¥¥aaXM^`XabaaZZ[^]]Z_TX_aaaa`a^
@HWI-EAS443:3:42:1381:1143#0/1
GGTGAACTTTGTAAGTATGGAGTTCTCACTCACTGACCCG
+HWI-EAS443:3:42:1381:1143#0/1
aaY][`_a``a]__]U__^_[ZY__`]Z`_]]Y^`]_^WW
@HWI-EAS443:3:42:1440:876#0/1
GACATTCTTTGGCTGGAAAGAGCCTAACATAGTCAAACCA
+HWI-EAS443:3:42:1440:876#0/1
aa^_`a_a_`_`Yaa_Z^_^ZX¥Z_]^]]^^_XX^__^XY
@HWI-EAS443:3:42:1455:14#0/1
GGCATGTGAATTGACTGATTCAAGCTGGATAGAACTTGAT
+HWI-EAS443:3:42:1455:14#0/1
a`¥LW`W`Z^```]Z_aX¥^¥VW¥U]__]^¥]VXOZ^]Y[
@HWI-EAS443:3:42:1483:84#0/1
TGAGAAGCAATTTTCTCGATTCAGCCGAAAACCCCTCAAG
+HWI-EAS443:3:42:1483:84#0/1
abababaaaaabab`aaa_aa_aa_`b_`_`^__¥a__^a
@HWI-EAS443:3:42:1529:988#0/1
GAATTCAATAAGGCATGTGAATTGACTGATTCAAGCTGGA
+HWI-EAS443:3:42:1529:988#0/1
a_^ab]baa_aba`aaa_``_aaa`_aaaaa_a^a_aaaX
@HWI-EAS443:3:42:1541:1556#0/1
GTTTGTTTTCCGTCTTCCTTCTTTGGTCCTACATTTGCTT
+HWI-EAS443:3:42:1541:1556#0/1
^H^UONZYYSUV[SSZQU[XRU¥SSONSSVRZWTWY[[¥T
@HWI-EAS443:3:42:1598:529#0/1
CCTCAAGAATGGTTCAATTTTGGCGTTCACTTCTTTTGAC
+HWI-EAS443:3:42:1598:529#0/1
H_``ZZb```_[Y¥V_¥_````aabaa`aa[]G]bbaXJG
```

```
@HWI-EAS443:3:42:1660:638#0/1
GGGAGACTTTGGTCGGCAAGCTTGCGCATAGTTCCTGTAA
+HWI-EAS443:3:42:1660:638#0/1
abb`b`aabab]aa`baaaa`aaaUaa``aa```^aaZZ_
@HWI-EAS443:3:42:1668:1645#0/1
GGCACTCGGTGAAAATATGGCACCAGAAAAAGTAGACTTT
+HWI-EAS443:3:42:1668:1645#0/1
a_U_bba`[^QX^¥aa_Y`Z¥MTL[_¥^`¥[_N]YZ[S[^
@HWI-EAS443:3:42:1722:232#0/1
GGGGTAGAGAAGCCTAAATTTCTTCCTGATTTGTATGATT
+HWI-EAS443:3:42:1722:232#0/1
ab_]V`a_a```YY`^^^`__X^_UU]¥Y____Z]`_Z^X
@HWI-EAS443:3:42:1778:1616#0/1
ATTTGAGAAATGATACTGATGTGGTGAACTTTGTAAGTAT
+HWI-EAS443:3:42:1778:1616#0/1
abaX^`^a`aaaaaa_`a_a`Wa[^aa^¥_aaR^_^___[
@HWI-EAS443:3:42:235:349#0/1
AACTAGGCTTTTCACTATAAGACAAGAAATGGCCAGTAGG
+HWI-EAS443:3:42:235:349#0/1
abaY___]Y`__X]WXRZ```WNQYZWM]ZXZNYS¥OKVT
@HWI-EAS443:3:42:266:925#0/1
CGATTGGCCATGTTTCCGATTTGTTTTCAAAGAATTCCTT
+HWI-EAS443:3:42:266:925#0/1
abaa_aaaa¥a`^`^Va_V_a`aX`_Z[_`¥]`^a_^Z`^
@HWI-EAS443:3:42:269:1408#0/1
TGCATCCTGTGCAGCCATGGATGACTTTCAGCTGATCCCA
+HWI-EAS443:3:42:269:1408#0/1
ab¥_abaa[aabab[]aab_]]b¥b`_aaaaaU``aba``
@HWI-EAS443:3:42:286:1081#0/1
GGGGGCTATATGAAGCAATCGAGGAGTGCCTGATTAATGA
+HWI-EAS443:3:42:286:1081#0/1
T_````aY`Wababb_Xa`abaa`a`]abbabba]a`a^`
@HWI-EAS443:3:42:383:1668#0/1
CTTGCTAGAGATCTGGGCTCTGGCTCATCACTGTCATACT
+HWI-EAS443:3:42:383:1668#0/1
abbbbbbbbabbbb``bbabbbaabbbabbbab`__abbb
@HWI-EAS443:3:42:452:789#0/1
CAATGAGAAGCAATTTTCTCGATTCAGCCGAAAACCCCTC
+HWI-EAS443:3:42:452:789#0/1
^abbbbbbbba_[abbbababbbabbbaabbb_ba`¥bba
@HWI-EAS443:3:42:456:1611#0/1
GGGGGCTATATGAAGCAATCGAGGAGTGCCTGATTAATGA
+HWI-EAS443:3:42:456:1611#0/1
`QSZ]a]a``^a```¥aa_[`O`¥UU¥_a_[[Z_]UZV`]
@HWI-EAS443:3:42:490:954#0/1
AATGAAGGGAGTGTACATAAATACGGCCTTGCTCAATGCA
+HWI-EAS443:3:42:490:954#0/1
WR`¥I_b```_]a]`a_`R_a_`]__aaX]^a]^`V]`a^
@HWI-EAS443:3:42:630:1078#0/1
AGCAGAGCTACAGGACATTGAAAATGAAGAGAAGATCCCA
```

```
+HWI-EAS443:3:42:630:1078#0/1
aaa[a_T_`ZXQaa]aa`aXT¥[T^aa`a__U[][PZ^Y`
@HWI-EAS443:3:42:675:817#0/1
GGGGCATGGAAATGAGGCGCTGCCTTCTTCAGTCTCTTCA
+HWI-EAS443:3:42:675:817#0/1
abbabbaabaabbbbaabbaaaa`ab`ba`]aa_aaaaa`
@HWI-EAS443:3:42:786:429#0/1
ATCGACGAACGGGGTGAATCAATAATTGTAGAATCTGGTG
+HWI-EAS443:3:42:786:429#0/1
abb¥Q]aa`]_```Ua]ZYJ]`Y`[a`¥]```VYFT`aG_
@HWI-EAS443:3:42:863:444#0/1
GGCATGGAAATGAGGCGCTGCCTTCTTCAGTCTCTTCAGC
+HWI-EAS443:3:42:863:444#0/1
abaaabb``_abaaaaa_`aa_¥_^aa^^`^_`__a]_a¥
@HWI-EAS443:3:42:879:1136#0/1
GGCTCCAGTCTCGGGTCAGTGAGTGAGAACTCCATACTTA
+HWI-EAS443:3:42:879:1136#0/1
abaaa`_¥S_]`[ZSM[XXQ]W[PUX^]`[`ZNQ¥¥X`[J
@HWI-EAS443:3:42:889:777#0/1
TCTTTGCAGTCATCAAAGTCTACTTTTTCTGGTGCCATAT
+HWI-EAS443:3:42:889:777#0/1
aabaaa]_¥[a`a_a__`UYaZa``aaa_a]YXa]`a`Ya
@HWI-EAS443:3:42:912:620#0/1
TGTTCACCACTGTCCAGGCCATGATTCGGTCTCTTCCTTC
+HWI-EAS443:3:42:912:620#0/1
abM`ababa`_[[[`¥`[_Y]]_Y^¥X]]XYMHO[GQ[_X
@HWI-EAS443:3:42:942:448#0/1
CCTCAAGTTGTGGAGACGCATATAGACTGTTGAATACAGA
+HWI-EAS443:3:42:942:448#0/1
aa]_XN`[^VO^Y``]aa_^`_ZVUS^_[ITVDVVQOQMN
@HWI-EAS443:3:43:1000:1234#0/1
GAACTTTGTAAGTATGGAGTTCTCACTCACTGACCCGAGA
+HWI-EAS443:3:43:1000:1234#0/1
`Ub`b`^aT`_]_b^ba`[aaaabbaba_aba_a_^b_aa
@HWI-EAS443:3:43:1144:1131#0/1
CTGATTCAAGCTGGATAGAACTTGATGAAATAGGAGAAGA
+HWI-EAS443:3:43:1144:1131#0/1
abb_bba`aa^ba]_ab`a¥O_abaab_`aba_a`a_``_
@HWI-EAS443:3:43:1158:1395#0/1
CACCTGCTTCCAAGCCATGAGGTAATTGGGATTTATGCCT
+HWI-EAS443:3:43:1158:1395#0/1
a[bbbbababbbbb`bab`¥``][_aa_aababbaaa_Wa
@HWI-EAS443:3:43:1188:833#0/1
CTTCCTCCACTCCCCTGGGTGACTCTCCGATTGGCCATGT
+HWI-EAS443:3:43:1188:833#0/1
abbbbbabbbbbb^bbbbb]bbbbababbaaaab_aabaa
@HWI-EAS443:3:43:11:1242#0/1
GGGCACTCGGTGAAAATATGGCACCAGAAAAAGTAGACTT
+HWI-EAS443:3:43:11:1242#0/1
ababaaabaaX`YW]aa`¥XZ^¥a`X_¥][^]XQ`Z^[[Z
```

@HWI-EAS443:3:43:1209:365#0/1
ATTTCATCGACGAACGGGGTGAATCAATAATTGTAGAATC
+HWI-EAS443:3:43:1209:365#0/1
]baaabbbbb^b__[]aa`U__`a`a`a_aaa^__a`a_X
@HWI-EAS443:3:43:1258:43#0/1
CATTAATCAGGCACTCCTCGATTGCTTCATATAGCCCCCC
+HWI-EAS443:3:43:1258:43#0/1
abbbbbbbbbbbaI_bb`a_baaaa`_a__a`a``[Z_``_
@HWI-EAS443:3:43:1283:563#0/1
CAGGAACTTTGACCGCTGATGGCAAAGAGGCCCATCAGGC
+HWI-EAS443:3:43:1283:563#0/1
abbbabbbbbab``bbabbabaaabaaaaaa^^aa_aa``
@HWI-EAS443:3:43:1317:747#0/1
CCTCTTTCGGACTGACGAAAGGAATCCCATAGACTCCTAC
+HWI-EAS443:3:43:1317:747#0/1
abbabbabbababaabbbaabababababaaaaaaaaa__^[
@HWI-EAS443:3:43:1365:1049#0/1
AGACATTCTTTGGCTGGAAAGAGCCTAACATAGTCAAACC
+HWI-EAS443:3:43:1365:1049#0/1
abba`abaaaaaaaaab_aaa_`_^`_a_`_a^^^`_`¥¥
@HWI-EAS443:3:43:1401:1821#0/1
GTAGGAGTCTATGGGATTCCTTTCGTCAGTCCGAAAGAGG
+HWI-EAS443:3:43:1401:1821#0/1
a_a``aa[¥a```aa_``]]`_`W^^]^_^[V_X]_]W`^
@HWI-EAS443:3:43:1463:502#0/1
ACCCTTGACGAAGAGAGCAGGGCAAGAATCAAAACTAGGC
+HWI-EAS443:3:43:1463:502#0/1
]b`abbbaX^Vaba`aaaaa`¥Yaaa__a[[``X¥Za^`_
@HWI-EAS443:3:43:1492:383#0/1
AGAACACAGTATTTTTCCCATTTGTGTGGCTCCAGTCTCG
+HWI-EAS443:3:43:1492:383#0/1
Ya`a`a``¥M_aa`^^¥_aZa``baa`ZV]¥TLI¥S¥WVY
@HWI-EAS443:3:43:151:2030#0/1
CTCCACTCCCCTGGGTGACTCTCCGATTGGCCATGTTTCC
+HWI-EAS443:3:43:151:2030#0/1
aabbababaa__bbb_aba`_P^aS```a_``_HFQXaU`
@HWI-EAS443:3:43:1540:513#0/1
CCCCGTTCGTCGATGAAATGGAAATCCGAATACATGAAAC
+HWI-EAS443:3:43:1540:513#0/1
a_ab`abab``baabaaaaaa]^```]`a]`a_aaaaa_Y
@HWI-EAS443:3:43:1541:2012#0/1
TGGGGGGCTATATGAAGCAATCGAGGAGTGCCTGATTAAT
+HWI-EAS443:3:43:1541:2012#0/1
J_Zabb_]Y`Yaa_aa¥S¥a`Q[``b¥_W¥[RM_[[Y]Z^
@HWI-EAS443:3:43:1592:129#0/1
TTCCGATTTGTTTTCAAAGAATTCCTTGGTCATGTCTTTC
+HWI-EAS443:3:43:1592:129#0/1
a^[^`Zaa`bWaaaY^`_a]^a_ZT][aW¥^TZ`VQ]¥_Y
@HWI-EAS443:3:43:1643:1148#0/1
GAAATTTAGGCTTCTCTACCCCTGTTGTGTTACATATACT

```
+HWI-EAS443:3:43:1643:1148#0/1
aaa`b^bb`a]aa^_aaa___^^a___¥``a^]`^]^`ZW
@HWI-EAS443:3:43:1650:1317#0/1
AATTGAAGGAAGAGACCGAATCATGGCCTGGACAGTGGTG
+HWI-EAS443:3:43:1650:1317#0/1
^IaabaaYaaa[_`a]__X^RGNSXKPOXLSFO¥ZQT]VU
@HWI-EAS443:3:43:1657:275#0/1
CATCGACGAACGGGGTGAATCAATAATTGTAGAATCTGGT
+HWI-EAS443:3:43:1657:275#0/1
abb]aa`aaa`ba¥`S]Z_]XZ_`¥]`aa]Z[[`^R[``Y
@HWI-EAS443:3:43:1735:971#0/1
CAGTCATCAAAGTCTACTTTTTCTGGTGCCATATTTTCAC
+HWI-EAS443:3:43:1735:971#0/1
a^XX^_bb`WGUJZ`Z¥_`]YT^¥_WQ_]a`^Ua`]_X¥[
@HWI-EAS443:3:43:1748:1761#0/1
CAGAGCCCAGATCTCTAGCAAGCTGGGTCCAAAATGAATT
+HWI-EAS443:3:43:1748:1761#0/1
a`]a`aaaaaa`R_¥_aaaZ]a^`a`_XT_W`^__]`_]]
@HWI-EAS443:3:43:1754:553#0/1
ATTCGAGCCGAACGGCTGCATTGAGGGCAAGCTTTCCCAA
+HWI-EAS443:3:43:1754:553#0/1
a`]a^a```aaaYa`_`¥T]Z]RXX]XIN]XRU[[LTQVY
@HWI-EAS443:3:43:227:1071#0/1
TGTCCAGGCCATGATTCGGTCTCTTCCTTCAATTATCTCA
+HWI-EAS443:3:43:227:1071#0/1
aa[b`aaaaa]]aYQ[_aSHU¥__`__[```[[]V^[]Y]
@HWI-EAS443:3:43:286:84#0/1
CATGATTCGGTCTCTTCCTTCAATTATCTCAAATCGGTGC
+HWI-EAS443:3:43:286:84#0/1
_bbbabbaab^a_aab`_`a^_[aa_`¥]]Z``S]]XO_W
@HWI-EAS443:3:43:293:1891#0/1
CCCGAATGCACTATTGAAGCACCGATTTGAGATAATTGAA
+HWI-EAS443:3:43:293:1891#0/1
]b`aY`aa]baaaba[`b]aaaaaaaaa`YZaaa`aaaa_
@HWI-EAS443:3:43:360:1153#0/1
GGGCACTCGGTGAAAATATGGCACCAGAAAAAGTAGACTT
+HWI-EAS443:3:43:360:1153#0/1
abb`aabbbbR`]aa``a`U`[a^a_``¥GNaWS_[YW^R
@HWI-EAS443:3:43:409:813#0/1
CATGAAGACATTCTTTGGCTGGAAAGAGCCTAACATAGTC
+HWI-EAS443:3:43:409:813#0/1
abbbbbbbabbbaaabbbabb`abbaaaaaaab`aaaa`a
@HWI-EAS443:3:43:429:806#0/1
GAACTTTGTAAGTATGGAGTTCTCACTCACTGACCCGAGA
+HWI-EAS443:3:43:429:806#0/1
aab_aaab[aab_a¥`a]a_a`_`a_a_]Ya^aa^_`[a_
@HWI-EAS443:3:43:452:1202#0/1
CACTGTCATACTGTTTAAGGTCTCCAACATCTTTGCAGTC
+HWI-EAS443:3:43:452:1202#0/1
^¥a``¥`^aba`a`b``]`aSbb`b_b_babbbaW_aa`b
```

```
@HWI-EAS443:3:43:517:1312#0/1
GGGGCATGGAAATGAGGCGCTGCCTTCTTCAGTCTCTTCA
+HWI-EAS443:3:43:517:1312#0/1
aJVa[a`a^W___aQ¥^H^¥N_^VV]YV`¥`YSZUSWNS¥
@HWI-EAS443:3:43:524:1363#0/1
AAAAGGAAGGTCTCATTTGAGAAATGATACTGATGTGGTG
+HWI-EAS443:3:43:524:1363#0/1
]¥Y^_aaa`]¥`^ab``aa_aabaaa`ba```bb¥S_a¥^
@HWI-EAS443:3:43:569:1705#0/1
CGATTGGCCATGTTTCCGATTTGTTTTCAAAGAATTCCTT
+HWI-EAS443:3:43:569:1705#0/1
`aaa`Zaa`aa]_abaab`ababWaab`aab`a`aa]`aa
@HWI-EAS443:3:43:590:1555#0/1
GGGCACTCGGTGAAAATATGGCACCAGAAAAAGTAGACTT
+HWI-EAS443:3:43:590:1555#0/1
aa`ba`b_b]^TU_]a`^_aa_aaa^¥Yaa`_^^`a_`_¥
@HWI-EAS443:3:43:595:142#0/1
TGTGGACTTCCCTCCGTGTTACTCCAATTTCAATGAACCG
+HWI-EAS443:3:43:595:142#0/1
ab]baa``aaa`_aab]a^_a]_]_a¥¥a_^^^Z_`_¥V`
@HWI-EAS443:3:43:622:460#0/1
CCCTGTTGTGTTACATATACTGTTCACCACTGTCCAGGCC
+HWI-EAS443:3:43:622:460#0/1
`bbbbabb`b_baa``bbaabb_aaaaaaaa_aa`a_a^
@HWI-EAS443:3:43:648:554#0/1
CAATAATTGTAGAATCTGGTGACCCGAATGCACTATTGAA
+HWI-EAS443:3:43:648:554#0/1
`Y`YRK^ab_``ZXaZ^]VY]``]Ya_VR]_[]^ZW¥ZR^
@HWI-EAS443:3:43:685:460#0/1
AGCAGGTGCTAGCAGAGCTACAGGACATTGAAAATGAAGA
+HWI-EAS443:3:43:685:460#0/1
aaaa`a]aaaaa`ab_aaa``aa``_aaaa__````__a`
@HWI-EAS443:3:43:702:156#0/1
ATTTCATCGACGAACGGGGTGAATCAATAATTGTAGAATC
+HWI-EAS443:3:43:702:156#0/1
aabaaaaabaaa``W^_a_Y__`_YZ[_]__``UT_^_]U
@HWI-EAS443:3:43:887:61#0/1
ATCTACATAGGCTCTAAAGTTTTCAAGGCTGGAGAAGTTC
+HWI-EAS443:3:43:887:61#0/1
ab`baa[aZ`abbabZ¥Z^V`a^UXZ^`Q[_¥D¥HZ[RWM
@HWI-EAS443:3:43:990:987#0/1
TAGAGATCTGGGCTCTGGCTCATCACTGTCATACTGTTTA
+HWI-EAS443:3:43:990:987#0/1
aabaaaaabaaa`a`aa```aa`aaa_`__`a]^_aY]__
@HWI-EAS443:3:44:1035:888#0/1
AATTCACATGCCTTATTGAATTCATTTTGGACCCAGCTTG
+HWI-EAS443:3:44:1035:888#0/1
aabaab`aaaabbbaa`a`]aa``aa`a`_^a__]````_
@HWI-EAS443:3:44:105:1953#0/1
ATCGCAAGCATGAGGAGGAACTATTTTACAGCAGAAGTGT
```

+HWI-EAS443:3:44:105:1953#0/1
_a`^``T¥X¥`b[a¥¥_[abP^a_¥b`_`aY__N^_[]U_
@HWI-EAS443:3:44:1102:1444#0/1
CTCCAGCCTTGAAAACTTTAGAGCCTATGTAGATGGATTC
+HWI-EAS443:3:44:1102:1444#0/1
I_¥_bbaTa`b`¥¥a`_[__U_ZVX`__`Xab[M[bZG_Y
@HWI-EAS443:3:44:1129:613#0/1
GTCCAGGCCATGATTCGGTCTCTTCCTTCAATTATCTCAA
+HWI-EAS443:3:44:1129:613#0/1
a``_aaaaaa^aaaa[^a]aa_a`__aa_[_aa`aa`]__
@HWI-EAS443:3:44:1160:583#0/1
GGGCAAGCTTTCCCAAATGTCAAAAGAAGTGAACGCCAAA
+HWI-EAS443:3:44:1160:583#0/1
a`a`_ZZ]`a`a^^a[^`b^_a¥^Xa]a_Y¥X¥U`]^]]^
@HWI-EAS443:3:44:1174:589#0/1
GGGGCATGGAAATGAGGCGCTGCCTTCTTCAGTCTCTTCA
+HWI-EAS443:3:44:1174:589#0/1
aaba_a`bbaa_ab``_]a`aa]_`a_aa_`a^¥___`UZ
@HWI-EAS443:3:44:1209:1935#0/1
GCGTCTCCACAACTTGAGGGGTTTTCGGCTGAATCGAGAA
+HWI-EAS443:3:44:1209:1935#0/1
`aba`aa_a^_a_aaa_`aaaT___Z[a]__a`_YXY___
@HWI-EAS443:3:44:1256:1423#0/1
GTTTCCGATTTGTTTTCAAAGAATTCCTTGGTCATGTCTT
+HWI-EAS443:3:44:1256:1423#0/1
_^aaa`b`Zaaba`baaa]aa^Raa][aab`YS[_a]_a`
@HWI-EAS443:3:44:1269:825#0/1
GGGGGGCTATATGAAGCAATCGAGGAGTGCCTGATTAATG
+HWI-EAS443:3:44:1269:825#0/1
a`^V[X]`]^^^`_[RU]^^^^U]ZT^N_Z[ZRTYWUUWW
@HWI-EAS443:3:44:1280:1057#0/1
GATGCTCTGAAATTAAGTATTGAAGACCCGAGTCACGAGG
+HWI-EAS443:3:44:1280:1057#0/1
a`_Yaa`]Z_Z[_^a`]Naaaaaa^^X`^_Z_UZ__a]_`
@HWI-EAS443:3:44:1332:830#0/1
ACCGATTTGAGATAATTGAAGGAAGAGACCGAATCATGGC
+HWI-EAS443:3:44:1332:830#0/1
a_aa^aaaa^aa_aaa``a[¥`_^````^^a]^_]_^`^W
@HWI-EAS443:3:44:1352:1025#0/1
CACAACTTGAGGGGTTTTCGGCTGAATCGAGAAAATTGCT
+HWI-EAS443:3:44:1352:1025#0/1
a``aaabaaababa^baaa_a``aaa`Z`_`_^`^^_^R`
@HWI-EAS443:3:44:1531:952#0/1
CATAAATACGGCCTTGCTCAATGCATCCTGTGCAGCCATG
+HWI-EAS443:3:44:1531:952#0/1
`Va_a]a_`]UYa_`a¥_^ZRRYVY^^¥__V^WUY¥[[V¥
@HWI-EAS443:3:44:1566:1881#0/1
TGCAGTGGGACACTTCTGCTGTAAAATAGTTCCTCCTCAT
+HWI-EAS443:3:44:1566:1881#0/1
[^^^X¥¥WQR`abbbabZaaS^]a`¥aa_a]SNYTS__a_

```
@HWI-EAS443:3:44:1571:67#0/1
AATGCATCCTGTGCAGCCATGGATGACTTTCAGCTGATCC
+HWI-EAS443:3:44:1571:67#0/1
aa^^``^a[`¥Ua]_YUUXY`^P¥]^S^^^^¥aYU`¥[QT
@HWI-EAS443:3:44:1585:119#0/1
CTGAAAGTCATCCATGGCTGCACAGGATGCATTGAGCAAG
+HWI-EAS443:3:44:1585:119#0/1
aa``babaab`aaab¥^^aZa¥S_V¥ZUQVZY_ZT]OZ[L
@HWI-EAS443:3:44:1593:1975#0/1
GCCTTCCTCCACTCCCCTGGGTGACTCTCCGATTGGCCAT
+HWI-EAS443:3:44:1593:1975#0/1
aZa`aVZ_`^]_`[RU¥S[VQJVSMRWULSVBBBBBBBBB
@HWI-EAS443:3:44:159:1028#0/1
CATGGAAATGAGGCGCTGCCTTCTTCAGTCTCTTCAGCAG
+HWI-EAS443:3:44:159:1028#0/1
aaabbaaabaaa[aa`a``aa`^aa`aa_^__`aU`^[[_
@HWI-EAS443:3:44:1702:971#0/1
GGGGCATGGAAATGAGGCGCTGCCTTCTTCAGTCTCTTCA
+HWI-EAS443:3:44:1702:971#0/1
aXa_^UT_TXTQ]`G_¥^a¥W`W¥V¥VX[WM^TSSQWVMG
@HWI-EAS443:3:44:1703:255#0/1
GAGGGGTTTTCGGCTGAATCGAGAAAATTGCTTCTCATTG
+HWI-EAS443:3:44:1703:255#0/1
_¥_UUTRaa`Ga^`ab^TaXa]]]]___`_U[NFY]]]]^
@HWI-EAS443:3:44:269:293#0/1
CTCCTCGATTGCTTCATATAGCCCCCCAAGATCGAAGGTT
+HWI-EAS443:3:44:269:293#0/1
`a`ababaaaaaaa_aaaaba`^]^`[a]_]ZV[_aa`SY
@HWI-EAS443:3:44:298:242#0/1
GAATACCACTATATGATGCAATCAAATGCATGAAGACATT
+HWI-EAS443:3:44:298:242#0/1
aaaaaaaa_aaaaab_`a_a_`_aa^_a`a_aa_a`_``_
@HWI-EAS443:3:44:319:395#0/1
GTGGGAGACTTTGGTCGGCAAGCTTGCGCATAGTTCCTGT
+HWI-EAS443:3:44:319:395#0/1
a`ba`^b`^aaa`]¥`¥¥aa`___``^a_`_^_W__^^_Y
@HWI-EAS443:3:44:470:749#0/1
AAAGAATTCCTTGGTCATGTCTTTCTCTTTGACAGAAGAC
+HWI-EAS443:3:44:470:749#0/1
^__aa_baba`bab[`aaaP`aa_a_]`a`]¥__`a`XDM
@HWI-EAS443:3:44:495:355#0/1
CAGATTTTGCCAGTAAGGTCCTGCACACTTTCCCAATAGA
+HWI-EAS443:3:44:495:355#0/1
abbbabaaabaaa_babaTa`aaaaaaaa`aa_`a`_aa_
@HWI-EAS443:3:44:517:1276#0/1
AAGAAATGGCCAGTAGGAGTCTATGGGATTCCTTTCGTCA
+HWI-EAS443:3:44:517:1276#0/1
`a^]^[b`bVTV^N_]RRV_a`T^_¥]]]aY[Ya¥WZPQY
@HWI-EAS443:3:44:537:201#0/1
AATTCCTTGGTCATGTCTTTCTCTTTGACAGAAGACTCGG
```

```
+HWI-EAS443:3:44:537:201#0/1
aababababaabNb`bbaabbbab`bbab[a`b`aaaa`a^a
@HWI-EAS443:3:44:563:1897#0/1
GAATTCCTTGGTCATGTCTTTCTCTTTGACAGAAGACTCG
+HWI-EAS443:3:44:563:1897#0/1
aaaaa`aaa^a[aaa]`_baaaa_aaaa`_aaa__`^a]Z
@HWI-EAS443:3:44:619:1931#0/1
CAAAGAGAACCGGTTCATTGAAATTGGAGTAACACGGAGG
+HWI-EAS443:3:44:619:1931#0/1
aaaaaaaaa`_aa_a__a`a_aa_^[U__Z_`Y_Z_^^_Q
@HWI-EAS443:3:44:633:26#0/1
GCCTTCCTCCACTCCCCTGGGTGACTCTCCGATTGGCCAT
+HWI-EAS443:3:44:633:26#0/1
a_`a_]Z]_a`_^`_^¥¥___W^¥V¥Y¥WW¥VYY¥¥UVUW
@HWI-EAS443:3:44:648:562#0/1
AAAATAAAATCTGAGAAGACACACATTCACATCTTTTCAT
+HWI-EAS443:3:44:648:562#0/1
`aV¥^¥^^a_Z¥Ub¥bYaa`b`[YXa[[aX`aYb_^b¥¥¥
@HWI-EAS443:3:44:699:983#0/1
CCCTCCGTGTTACTCCAATTTCAATGAACCGGTTCTCTTT
+HWI-EAS443:3:44:699:983#0/1
_R][b`b`a^_XSa]WYP^^_KX`Q__]WGa]O_P`P^^_
@HWI-EAS443:3:44:69:970#0/1
TTGGAAGCAGGTGCTAGCAGAGCTACAGGACATTGAAAAT
+HWI-EAS443:3:44:69:970#0/1
aaa`aaaaaa_X^a^a_^_`__`^``_`aT[_^__]__^_
@HWI-EAS443:3:44:700:145#0/1
GAAAGGCATAAATCCCAATTACCTCATGGCTTGGAAGCAG
+HWI-EAS443:3:44:700:145#0/1
^aXaa`abbbbbbabababbbb`a_`_aaabaaa`^X[XNV]
@HWI-EAS443:3:44:700:585#0/1
ATGAAATGGAAATCCGAATACATGAAACAAACTTCCAAAT
+HWI-EAS443:3:44:700:585#0/1
a_b`[^bbaRa^a``aaa`baaaaa_aaba__aaa_a_]a
@HWI-EAS443:3:44:708:412#0/1
AAGAATTCCTTGGTCATGTCTTTCTCTTTGACAGAAGACT
+HWI-EAS443:3:44:708:412#0/1
aa`]^aaaY¥babYa`b`Taaa^T^^a_``X^`aZaa^ZZ
@HWI-EAS443:3:44:724:393#0/1
GCCTTTCTCATGTGGTTTGACTATGTTAGGCTCTTTCCAG
+HWI-EAS443:3:44:724:393#0/1
abaaaaa`^]`b^baX]YbY_`]_a[[[]]T[WX]`RX^X
@HWI-EAS443:3:44:85:671#0/1
GCCTTCCTCCACTCCCCTGGGTGACTCTCCGATTGGCCAT
+HWI-EAS443:3:44:85:671#0/1
abbabaaaabaabbaaaabaaNa__a¥`_aZU^_aU^_^V
@HWI-EAS443:3:44:914:1120#0/1
TGGTGAACAGTATATGTAACACAACAGGGGTAGAGAAGCC
+HWI-EAS443:3:44:914:1120#0/1
a`a`aaa`aa]aaaaa_``aa``_]^__`^[`___`¥`[S
```

@HWI-EAS443:3:44:926:843#0/1
CCGAACTTCTCCAGCCTTGAAAACTTTAGAGCCTATGTAG
+HWI-EAS443:3:44:926:843#0/1
aab_aaaaabab_aaaaab`a```aaa`aaa`¥aaaa^`a
@HWI-EAS443:3:44:946:641#0/1
CTTGGGGGGCTATATGAAGCAATCGAGGAGTGCCTGATTA
+HWI-EAS443:3:44:946:641#0/1
__¥N[aaba`a¥_bbbb````_a`a^_aa`X`^`ba`aa_
@HWI-EAS443:3:45:1133:1630#0/1
ACTCGGTGAAAATATGGCACCAGAAAAAGTAGACTTTGAT
+HWI-EAS443:3:45:1133:1630#0/1
__baabW`_aaaa_aa_Y`Z^^a¥¥ZVW_O``Z]aZ_`^_
@HWI-EAS443:3:45:1192:111#0/1
AGAGACCGAATCATGGCCTGGACAGTGGTGAACAGTATAT
+HWI-EAS443:3:45:1192:111#0/1
_bab^`aaVV][__abaZabUVY[_¥_aU_U¥[[XDT¥ZW
@HWI-EAS443:3:45:1193:947#0/1
AGAATATGGGGAAGATCCGAAAATCGAAACTAACAAGTTT
+HWI-EAS443:3:45:1193:947#0/1
a_abababa¥a][`abbaa`abaa`a_aa`aaaa`_a]`_
@HWI-EAS443:3:45:120:870#0/1
GGATCTTCTCTTCATTTTCAATGTCCTGTAGCTCTGCTAG
+HWI-EAS443:3:45:120:870#0/1
aaaa_bbaaabbaabaaaaaa``____[_a`]`_VYZ^`_
@HWI-EAS443:3:45:1288:384#0/1
CCTCGATTGCTTCATATAGCCCCCCAAGATCGAAGGTTCC
+HWI-EAS443:3:45:1288:384#0/1
_`bab_RZ`¥abb_a[`b`aR`bTXa[`a]^_Z_bZN^¥]
@HWI-EAS443:3:45:1309:912#0/1
CAGGCACTCCTCGATTGCTTCATATAGCCCCCCAAGATCG
+HWI-EAS443:3:45:1309:912#0/1
Z¥`XTbbbZX`b`b_]``bb`_`b]]a^^`^``a]aTPVa
@HWI-EAS443:3:45:1328:1132#0/1
TGAAGGAAGAGACCGAATCATGGCCTGGACAGTGGTGAAC
+HWI-EAS443:3:45:1328:1132#0/1
aa`aaZZ_Y_``¥YV¥T^a`aa_YZ][_YRXYT_^U_^_S
@HWI-EAS443:3:45:1334:962#0/1
CATCCATCAGCAGGAACTTTGACCGCTGATGGCAAAGAGG
+HWI-EAS443:3:45:1334:962#0/1
aa`[R]]XW`MT`_H]Pa`¥aa]`_PHX[XS[V[¥TPKKT
@HWI-EAS443:3:45:1344:972#0/1
AATATGGGGAAGATCCGAAAATCGAAACTAACAAGTTTGC
+HWI-EAS443:3:45:1344:972#0/1
VPHN[[a]W¥]UYa¥ZaZZU¥_]bZZ_`aX``^^_Y]]a^
@HWI-EAS443:3:45:1374:1336#0/1
TGCCCCATTTCATCTTGATCTTGGAGGTTCCATTGGTTCT
+HWI-EAS443:3:45:1374:1336#0/1
aaaa`aabbb_ab^aaaaa_aaba_baX`__`_^]_X]UU
@HWI-EAS443:3:45:1395:78#0/1
CAGTGATGAGCCAGAGCCCAGATCTCTAGCAAGCTGGGTC

+HWI-EAS443:3:45:1395:78#0/1
`abababbbbaabaab`aa`baa_a`aaa_a^a[_`ba^X
@HWI-EAS443:3:45:1433:1253#0/1
TCGAGCCGAACGGCTGCATTGAGGGCAAGCTTTCCCAAAT
+HWI-EAS443:3:45:1433:1253#0/1
[a[U_a_]_b``¥_^^DOV¥^S]]]R]QVPN¥¥ZSVNV]W
@HWI-EAS443:3:45:1444:19#0/1
ATTGAATTCATTTTGGACCCAGCTTGCTAGAGATCTGGGC
+HWI-EAS443:3:45:1444:19#0/1
aaabaaaaaa_a]aaaa[_UZ]T]^]LTZ^][VTTS^^ZS
@HWI-EAS443:3:45:1465:638#0/1
GGGGCATGGAAATGAGGCGCTGCCTTCTTCAGTCTCTTCA
+HWI-EAS443:3:45:1465:638#0/1
a`aaa_`aa^_]aa`a¥`aZ`a[_a`Z`__XXSWYW^[YY
@HWI-EAS443:3:45:1478:1290#0/1
ATGAAATGGAAATCCGAATACATGAAACAAACTTCCAAAT
+HWI-EAS443:3:45:1478:1290#0/1
a``aVaa`_¥P]aU^^aaa^T^`^`a`aab]W_aSVX_X¥
@HWI-EAS443:3:45:1504:526#0/1
ACGGGGTGAATCAATAATTGTAGAATCTGGTGACCCGAAT
+HWI-EAS443:3:45:1504:526#0/1
]a^Zab_b¥a^Y_a`a]bab]Za`^¥`^aa[``]ZW^Y`Y
@HWI-EAS443:3:45:1523:1490#0/1
ATGAGAAGCAATTTTCTCGATTCAGCCGAAAACCCCTCAA
+HWI-EAS443:3:45:1523:1490#0/1
`a__abba]_ba`a`_b`b_aa_a^aaba`_a_a^^_]`]
@HWI-EAS443:3:45:1526:1103#0/1
GGGGGCTATATGAAGCAATCGAGGAGTGCCTGATTAATGA
+HWI-EAS443:3:45:1526:1103#0/1
V_[Z]U¥TWU¥]PUUMQZ^Y_X[YYXOUOLVY]¥YQUY`B
@HWI-EAS443:3:45:1574:756#0/1
CTCCCCTGGGTGACTCTCCGATTGGCCATGTTTCCGATTT
+HWI-EAS443:3:45:1574:756#0/1
ababaa`bba[ba_aaaaaa__``aTT^Y[^a`V[`W^_`
@HWI-EAS443:3:45:1605:1597#0/1
GGGGCATGGAAATGAGGCGCTGCCTTCTTCAGTCTCTTCA
+HWI-EAS443:3:45:1605:1597#0/1
aaabX`^_aa_`a]^a_`___]YS¥_^^_ZYYYZ_V^YU[
@HWI-EAS443:3:45:1644:1766#0/1
AAAGAGCCTAACATAGTCAAACCACATGAGAAAGGCATAA
+HWI-EAS443:3:45:1644:1766#0/1
a^ba`b`bbbV[¥X``]¥b¥^V`aSa`a¥a^XX^[__``a
@HWI-EAS443:3:45:1681:1336#0/1
GCCATGTTTCCGATTTGTTTTCAAAGAATTCCTTGGTCAT
+HWI-EAS443:3:45:1681:1336#0/1
aa_abaaab`_a_aabbaaba_`a`aa_aa^]aab``Zaa
@HWI-EAS443:3:45:169:1620#0/1
AGAAATGGCCAGTAGGAGTCTATGGGATTCCTTTCGTCAG
+HWI-EAS443:3:45:169:1620#0/1
a]]]a__`aUY_[aa]X_LS]_]aa_U`¥JT]`¥[ZODSQ

@HWI-EAS443:3:45:1714:117#0/1
TGGGTCCAAAATGAATTCAATAAGGCATGTGAATTGACTG
+HWI-EAS443:3:45:1714:117#0/1
aaaa¥`_`aa_ab^]aa^`^`_^aa^^_a[_^[__]Y[^`
@HWI-EAS443:3:45:1757:961#0/1
AGAAATTTAGGCTTCTCTACCCCTGTTGTGTTACATATAC
+HWI-EAS443:3:45:1757:961#0/1
XY_R[_¥a_V`X__[¥]aWW_a[H]X]][¥U__Z__YT^_
@HWI-EAS443:3:45:1771:1901#0/1
AGCTCTGCTAGCACCTGCTTCCAAGCCATGAGGTAATTGG
+HWI-EAS443:3:45:1771:1901#0/1
¥]`_UW^[R__Xa`XX___`[^]]^XZS`a^`^R[VZZ_U
@HWI-EAS443:3:45:1791:83#0/1
AAGTGCCTGAACAATGAGAAGCAATTTTCTCGATTCAGCC
+HWI-EAS443:3:45:1791:83#0/1
`^_J_TT`baW^]Hab`b``aa]I`aba`aXa_aa_Y_SQ
@HWI-EAS443:3:45:201:1603#0/1
CAACAGTCTATATGCGTCTCCACAACTTGAGGGGTTTTCG
+HWI-EAS443:3:45:201:1603#0/1
[U_babaT^_¥¥¥UW_^_^UXPP]]XV_VRGRWQW¥`BBB
@HWI-EAS443:3:45:216:260#0/1
CAATGCAGCCGTTCGGCTCGAATCCATCTACATAGGCTCT
+HWI-EAS443:3:45:216:260#0/1
abbbaabb__a`__a`]a]ZT^WV_URU[¥PaUWBBBBBB
@HWI-EAS443:3:45:234:75#0/1
GGCATGGAAATGAGGCGCTGCCTTCTTCAGTCTCTTCAGC
+HWI-EAS443:3:45:234:75#0/1
aa]aaaaa``aaa`__a__aWW`_V_[MW`XU_¥__WW[U
@HWI-EAS443:3:45:260:205#0/1
GCAGGTGCTAGCAGAGCTACAGGACATTGAAAATGAAGAG
+HWI-EAS443:3:45:260:205#0/1
aabab^aaaaa`aa`aaaa_aaa___`aa]_a`aa`````
@HWI-EAS443:3:45:28:475#0/1
CTTGAGGACTGCGATAGGCCAAGTGTCGAGGCCCATGTTC
+HWI-EAS443:3:45:28:475#0/1
aabbaaaaa`_`ba___aa_^T_FRJ[^T¥_][¥ZRURNY
@HWI-EAS443:3:45:32:240#0/1
CTTGGGGGGCTATATGAAGCAATCGAGGAGTGCCTGATTA
+HWI-EAS443:3:45:32:240#0/1
G]bb```b^Ta_SXaba_O_Y_¥W`¥a[S¥Q_[abb^¥W`
@HWI-EAS443:3:45:351:1891#0/1
GGGGGCTATATGAAGCAATCGAGGAGTGCCTGATTAATGA
+HWI-EAS443:3:45:351:1891#0/1
Y`ZW`babbbbba_¥¥aba_a`b`^aZ`a_aa_`aa^aa_
@HWI-EAS443:3:45:459:966#0/1
TGAAGACCCGAGTCACGAGGGGGAGGGAATACCACTATAT
+HWI-EAS443:3:45:459:966#0/1
ZbYWaZSZY`aaPJ`V``a]aa`_a_P`a`]O[ZR_a]a]
@HWI-EAS443:3:45:487:789#0/1
AGGAACTTTGACCGCTGATGGCAAAGAGGCCCATCAGGCA

```
+HWI-EAS443:3:45:487:789#0/1
^¥^aaabbbaaaaa`ba``a¥aa`a_`^a_^^aa_]YY`a
@HWI-EAS443:3:45:576:705#0/1
GGTGAACTTTGTAAGTATGGAGTTCTCACTCACTGACCCG
+HWI-EAS443:3:45:576:705#0/1
_a^a[aaabbb[``aZX`a`^^aa]a_a_aYa^`b`UP`]
@HWI-EAS443:3:45:656:1710#0/1
GAGCCCAGATCTCTAGCAAGCTGGGTCCAAAATGAATTCA
+HWI-EAS443:3:45:656:1710#0/1
Z[aUK`¥bb_¥b_Xb_a_bX¥baVb_¥abbbbabbaba_b
@HWI-EAS443:3:45:743:2017#0/1
AACTTTAGAGCCTATGTAGATGGATTCGAGCCGAACGGCT
+HWI-EAS443:3:45:743:2017#0/1
Tb`Z¥QQ_ZTHZbTbZQR¥`V¥`MT]UaMZXMa`^YR_aa
@HWI-EAS443:3:45:773:988#0/1
CCTTCATTATGTATTCAGTAGCCCTGCAGTGGGACACTTC
+HWI-EAS443:3:45:773:988#0/1
abb¥S_^`V[bbaa`aa`[aaa^`^a`XZWaS`YTVUZSS
@HWI-EAS443:3:45:800:845#0/1
ATTCGGATTTCCATTTCATCGACGAACGGGGTGAATCAAT
+HWI-EAS443:3:45:800:845#0/1
aaaaa`a_aaa_^a``__a_a_Xa[_[]_`_Z_^^]]ZZ_
@HWI-EAS443:3:45:840:240#0/1
GATTTGAGATAATTGAAGGAAGAGACCGAATCATGGCCTG
+HWI-EAS443:3:45:840:240#0/1
[SU_TVN__]WQW]ZYRZ[RT[ZaR]T]USZ[RZ]VMMQQ
@HWI-EAS443:3:45:919:1563#0/1
AGAAGCAATTTTCTCGATTCAGCCGAAAACCCCTCAAGTT
+HWI-EAS443:3:45:919:1563#0/1
^ZZaaR^[```¥^_N_aa__Y`M[a][]¥[YTXV¥UV]W]
@HWI-EAS443:3:45:979:1275#0/1
GGGGCATGGAAATGAGGCGCTGCCTTCTTCAGTCTCTTCA
+HWI-EAS443:3:45:979:1275#0/1
aaaaababa`aabaaaaaaaaa__aa_aa]]a_a`_aU^_
@HWI-EAS443:3:46:1039:982#0/1
AAGAGGCGAAGAGACAATTGAAGAAAAATTTGAGATTACA
+HWI-EAS443:3:46:1039:982#0/1
aba]a`bbba`¥a^__`aaaZ¥¥[`a__`aaa_a_```¥_
@HWI-EAS443:3:46:1122:457#0/1
CGTCTCCACAACTTGAGGGGTTTTCGGCTGAATCGAGAAA
+HWI-EAS443:3:46:1122:457#0/1
abbbbbbabbbabbbbbbbb_bbaabbabbbaa_bbbbbb
@HWI-EAS443:3:46:1140:363#0/1
CGTCTCCACAACTTGAGGGGTTTTCGGCTGAATCGAGAAA
+HWI-EAS443:3:46:1140:363#0/1
a`S`aabZ¥Z]_W¥b[bbbb_a]WHaaa_a][X`a]a_aY
@HWI-EAS443:3:46:115:284#0/1
GCAGGTGCTAGCAGAGCTACAGGACATTGAAAATGAAGAG
+HWI-EAS443:3:46:115:284#0/1
ab^_`R^aaaa``a_aaaa_aaa¥W`]aa_[_¥``^`[``
```

```
@HWI-EAS443:3:46:1223:1732#0/1
CATTAATCAGGCACTCCTCGATTGCTTCATATAGCCCCCC
+HWI-EAS443:3:46:1223:1732#0/1
[_`aa]bbbba_b`b``bbbb]bb_baaabaabbb_``WY
@HWI-EAS443:3:46:1279:1822#0/1
CTTGGGGGGCTATATGAAGCAATCGAGGAGTGCCTGATTA
+HWI-EAS443:3:46:1279:1822#0/1
_ba_V_aZ[XWbb¥JV`^`Wab`^_abaaZXZZX]aaaaT
@HWI-EAS443:3:46:1296:1478#0/1
GAGGGGTTTTCGGCTGAATCGAGAAAATTGCTTCTCATTG
+HWI-EAS443:3:46:1296:1478#0/1
a^``ab_abaa___abaa_Za_aaaa`a`a__aZ`_``_a
@HWI-EAS443:3:46:1320:41#0/1
TCCACAACTTGAGGGGTTTTCGGCTGAATCGAGAAAATTG
+HWI-EAS443:3:46:1320:41#0/1
[aa`abb`aa`]bb`b``¥b`bba`[[a_TOYba`a`Y`¥
@HWI-EAS443:3:46:1347:971#0/1
GGGGCATGGAAATGAGGCGCTGCCTTCTTCAGTCTCTTCA
+HWI-EAS443:3:46:1347:971#0/1
`[`^```a`T]`^a`a``a^`_SZ_`Y``[^^YP]T]^UX
@HWI-EAS443:3:46:1373:302#0/1
CATCACTGTCATACTGTTTAAGGTCTCCAACATCTTTGCA
+HWI-EAS443:3:46:1373:302#0/1
a^b`babbaabba_a`aabbabbaab_aa_W`b`aaab_a
@HWI-EAS443:3:46:1432:1136#0/1
CTTGGGGGGCTATATGAAGCAATCGAGGAGTGCCTGATTA
+HWI-EAS443:3:46:1432:1136#0/1
abbbb_aaabaaaaab_aa_aaa`a]b`_a^a__aa]aa_
@HWI-EAS443:3:46:1444:1540#0/1
CGCTGATGGCAAAGAGGCCCATCAGGCAATCTGAGGGGGC
+HWI-EAS443:3:46:1444:1540#0/1
aaa^VXaa]^a``]V`_Y]^Z¥]SGUX]^¥¥¥MSTU[^BB
@HWI-EAS443:3:46:1478:38#0/1
GGGGTTTTCGGCTGAATCGAGAAAATTGCTTCTCATTGTT
+HWI-EAS443:3:46:1478:38#0/1
aa¥aI_a¥``_UV`U]LR¥T_NQUV¥^ZR^¥¥ZUZV^^TZ
@HWI-EAS443:3:46:1482:1012#0/1
GAAAGGCATAAATCCCAATTACCTCATGGCTTGGAAGCAG
+HWI-EAS443:3:46:1482:1012#0/1
_X[]a_WXXXZ]VYVSX[UV¥XNV^YV¥¥BBBBBBBBBBB
@HWI-EAS443:3:46:1543:68#0/1
CATTAATCAGGCACTCCTCGATTGCTTCATATAGCCCCCC
+HWI-EAS443:3:46:1543:68#0/1
_abb`abbabbbaaaa]a_ba`aaaa_``a__a`^_ZZa¥
@HWI-EAS443:3:46:1588:936#0/1
GTAGATGGATTCGAGCCGAACGGCTGCATTGAGGGCAAGC
+HWI-EAS443:3:46:1588:936#0/1
aW_`__b^¥`a_aY¥^W_W¥Y¥WYWYSPPQFQVW¥NTUPU
@HWI-EAS443:3:46:15:2033#0/1
CTTCATTTTCAATGTCCTGTAGCTCTGCTAGCACCTGCTT
```

```
+HWI-EAS443:3:46:15:2033#0/1
a¥^Z_Z`Va`]GQWW¥W[[XBBBBBBBBBBBBBBBBBBBB
@HWI-EAS443:3:46:1647:681#0/1
GAACAAGCCAATTGAAGTGGGCACTCGGTGAAAATATGGC
+HWI-EAS443:3:46:1647:681#0/1
aYVV``]]XXa_`[¥¥[[_X_ZRT[]__L_QQU^^U]VZP
@HWI-EAS443:3:46:1655:167#0/1
GGGGTTTTCGGCTGAATCGAGAAAATTGCTTCTCATTGTT
+HWI-EAS443:3:46:1655:167#0/1
`N`^R`_^W`¥V^Y`Y^W_VYSQUW¥¥¥T¥WWWTNWWXVS
@HWI-EAS443:3:46:1736:1497#0/1
GAGAAATGATACTGATGTGGTGAACTTTGTAAGTATGGAG
+HWI-EAS443:3:46:1736:1497#0/1
a_`^`_T`¥[__TXR[]ZXETMQVUW]¥[TWLTEUGQ_YZ
@HWI-EAS443:3:46:1763:320#0/1
GAGAAGATGTTGCCCCGATTGAACATATCGCAAGCATGAG
+HWI-EAS443:3:46:1763:320#0/1
a`a_^``aa]`^^]¥^a¥__`^aY__^¥¥`YWY[VW¥¥W^
@HWI-EAS443:3:46:211:1953#0/1
GGACATTGAAAATGAAGAGAAGATCCCAAGGACAAAGAAC
+HWI-EAS443:3:46:211:1953#0/1
a`aaaaaa__aaaaaaaaa`^_a__[[aa`_`^```_`aZ
@HWI-EAS443:3:46:236:1700#0/1
AATCAAAACTAGGCTTTTCACTATAAGACAAGAAATGGCC
+HWI-EAS443:3:46:236:1700#0/1
a^¥a`a``XWba_^aa``^^R¥aa`a`[H``Q_aaQQNUP
@HWI-EAS443:3:46:296:237#0/1
GCTGAAAGTCATCCATGGCTGCACAGGATGCATTGAGCAA
+HWI-EAS443:3:46:296:237#0/1
aaba_aaaSaa¥_^`[_baHa¥U^^^aPU_NV^¥LSLTX¥
@HWI-EAS443:3:46:299:1214#0/1
GACAAGAAATGGCCAGTAGGAGTCTATGGGATTCCTTTCG
+HWI-EAS443:3:46:299:1214#0/1
aaa]]ba`aaba`_`a[]_`O`__^Z`_Y`[_a_Y___[[
@HWI-EAS443:3:46:310:1191#0/1
CCCGAATGCACTATTGAAGCACCGATTTGAGATAATTGAA
+HWI-EAS443:3:46:310:1191#0/1
_]H[Z_TZ]¥]]_^_Z[¥`SW¥Y[TRa__]_^`_¥Y^a]Z
@HWI-EAS443:3:46:311:237#0/1
CGAAAATCGAAACTAACAAGTTTGCTGCAATATGCACACA
+HWI-EAS443:3:46:311:237#0/1
ababbbbabaaaab_b^b`a`aaaaabaaaa`baaaaaaa
@HWI-EAS443:3:46:396:794#0/1
ACATCTTCTCCTATTTCATCAAGTTCTATCCAGCTTGAAT
+HWI-EAS443:3:46:396:794#0/1
aabbb^aabbabbb`babbabbbaaabaaaaababbaaaa
@HWI-EAS443:3:46:413:1933#0/1
CAGTGATGAGCCAGAGCCCAGATCTCTAGCAAGCTGGGTC
+HWI-EAS443:3:46:413:1933#0/1
aUaJHP[ZQVR[aVFSTV_[JVZ¥S^VTT^ZUMPWTSUGM
```

@HWI-EAS443:3:46:421:1413#0/1
ACAGATTTTGCCAGTAAGGTCCTGCACACTTTCCCAATAG
+HWI-EAS443:3:46:421:1413#0/1
aXbb_`a]]_`aa_J^a`^Y`Z]aa]`a`aa``¥¥]_¥a_
@HWI-EAS443:3:46:436:357#0/1
AAAGAGCCTAACATAGTCAAACCACATGAGAAAGGCATAA
+HWI-EAS443:3:46:436:357#0/1
a`bbaaaaaaaaababaaaabaaa`aaaaba`aa__aaaa
@HWI-EAS443:3:46:476:2013#0/1
CTTCTCCTATTTCATCAAGTTCTATCCAGCTTGAATCAGT
+HWI-EAS443:3:46:476:2013#0/1
ab[OQ^TTa[a^`a_]_GX]]X^`a]^aaUV`a]WSQ¥aS
@HWI-EAS443:3:46:500:1000#0/1
TTCGAGCCGAACGGCTGCATTGAGGGCAAGCTTTCCCAAA
+HWI-EAS443:3:46:500:1000#0/1
a`a`a``aaabaa``__]]¥[R[Z^[HX^YVVW^X[^]`¥
@HWI-EAS443:3:46:509:135#0/1
CTTGGGGGGCTATATGAAGCAATCGAGGAGTGCCTGATTA
+HWI-EAS443:3:46:509:135#0/1
abbbbbb_babbbabbbababb`abbbaab^ba`aba__a
@HWI-EAS443:3:46:567:762#0/1
CATGGAAATGAGGCGCTGCCTTCTTCAGTCTCTTCAGCAG
+HWI-EAS443:3:46:567:762#0/1
a_bba`a`a_^a`¥_aa`]^aa`a_Z[_Z]X^^YXY_SY`
@HWI-EAS443:3:46:595:1832#0/1
GGGGCATGGAAATGAGGCGCTGCCTTCTTCAGTCTCTTCA
+HWI-EAS443:3:46:595:1832#0/1
^JYT^`^`]ZW]Xa_`^`a]`^MU¥_^Z^_^]KU[QOZUT
@HWI-EAS443:3:46:637:905#0/1
GGGGCTATATGAAGCAATCGAGGAGTGCCTGATTAATGAT
+HWI-EAS443:3:46:637:905#0/1
`bbbbbbbbbaaa^baaabaaaaa^aa```_aa``aa`_
@HWI-EAS443:3:46:696:1076#0/1
GGGCACTCGGTGAAAATATGGCACCAGAAAAAGTAGACTT
+HWI-EAS443:3:46:696:1076#0/1
a_UXQXaaabKPNO[V]X[_aZ_ZU[[P_PZU¥H¥]]V[]
@HWI-EAS443:3:46:715:484#0/1
GTTCGGTGGGAGACTTTGGTCGGCAAGCTTGCGCATAGTT
+HWI-EAS443:3:46:715:484#0/1
UZ^V_aTabaXZM]aa`aa[`^[J[`a`^V[[^PT[Y]YY
@HWI-EAS443:3:46:719:237#0/1
CAGGAACTATGCGCAAGCTTGCCGACCAAAGTCTCCCACC
+HWI-EAS443:3:46:719:237#0/1
a¥_a`_``Yaa_`aaaaa^Y^^a`ZY_aa`^QVY¥Y]`XP
@HWI-EAS443:3:46:744:765#0/1
CAAAGAGGCCCATCAGGCAATCTGAGGGGGCGTGGTGTCG
+HWI-EAS443:3:46:744:765#0/1
ab]bbabbaaa^aaaabaaaa^`^]`aaaaXa¥aaYYXN_
@HWI-EAS443:3:46:811:963#0/1
GGGGCATGGAAATGAGGCGCTGCCTTCTTCAGTCTCTTCA

+HWI-EAS443:3:46:811:963#0/1
^WXaaa¥bbaa¥```aa`a`_`V¥`^S^a]S_P¥`^^XU[
@HWI-EAS443:3:46:838:499#0/1
CGGCTGAATCGAGAAAATTGCTTCTCATTGTTCAGGCACT
+HWI-EAS443:3:46:838:499#0/1
abb`bbbaa`baa`]aa_`_`aaaaa`aaa`¥¥¥a[_^¥X
@HWI-EAS443:3:46:839:504#0/1
GGCTTGGAAGCAGGTGCTAGCAGAGCTACAGGACATTGAA
+HWI-EAS443:3:46:839:504#0/1
`SSR]^a[`QFVX]UVXY][JX^RRLMKL_SVYYLU[UNT
@HWI-EAS443:3:46:926:248#0/1
GAGGAAGGCTCTATTGGGAAAGTGTGCAGGACCTTACTGG
+HWI-EAS443:3:46:926:248#0/1
^V[VJS^]¥`_aV_Z¥_^WQM¥TTS¥QQZ¥XTTY¥ZXKRV
@HWI-EAS443:3:46:928:1365#0/1
ATGAATTCAATAAGGCATGTGAATTGACTGATTCAAGCTG
+HWI-EAS443:3:46:928:1365#0/1
a`bSLSbaaab]I^¥S^a`Z]a^a`]ZR[___Z_a^aW``
@HWI-EAS443:3:46:930:88#0/1
GGGGGCTATATGAAGCAATCGAGGAGTGCCTGATTAATGA
+HWI-EAS443:3:46:930:88#0/1
abaaa[]_a^aa_^aZ[Z___]aa^_X_]^_ZRZ^]YS_Y
@HWI-EAS443:3:46:935:168#0/1
CGTTCGTCGATGAAATGGAAATCCGAATACATGAAACAAA
+HWI-EAS443:3:46:935:168#0/1
`abbbbabbbbbaaaabbaaaa`]aaaaa`aaaaa`_aaa
@HWI-EAS443:3:47:1040:974#0/1
CAATGAGAAGCAATTTTCTCGATTCAGCCGAAAACCCCTC
+HWI-EAS443:3:47:1040:974#0/1
Xaaaa¥]ZQ¥_aabaaabb¥b_]baaJVX`RWLbaXT]]K
@HWI-EAS443:3:47:1048:1233#0/1
CAGCTTGCTAGAGATCTGGGCTCTGGCTCATCACTGTCAT
+HWI-EAS443:3:47:1048:1233#0/1
^_`Zaba_`aabbaaa]bP_[aa^abab^_]^`S`_F_ab
@HWI-EAS443:3:47:1125:1229#0/1
CATTAATCAGGCACTCCTCGATTGCTTCATATAGCCCCCC
+HWI-EAS443:3:47:1125:1229#0/1
^__^_Z_^aaa`]]_[Y_``a__X_a`[`baaab][XOU[
@HWI-EAS443:3:47:1161:1748#0/1
AGAGCCTAACATAGTCAAACCACATGAGAAAGGCATAAAT
+HWI-EAS443:3:47:1161:1748#0/1
aa```aa`a_aa`^^^`aa^¥___[[¥_`_^]_X]^``]_
@HWI-EAS443:3:47:1192:927#0/1
GAGAAAGGCATAAATCCCAATTACCTCATGGCTTGGAAGC
+HWI-EAS443:3:47:1192:927#0/1
aa_]V^baabbaaaba^``aaa``^__``aa_a_TZLSTT
@HWI-EAS443:3:47:1346:1281#0/1
AGAAATTTAGGCTTCTCTACCCCTGTTGTGTTACATATAC
+HWI-EAS443:3:47:1346:1281#0/1
`ba`^bbabb`^aaa_WY_V]`^VVY`ZNW¥^_^_¥¥¥[T

```
@HWI-EAS443:3:47:1388:160#0/1
GGTTCTCTTTGTAATCATACAAATCAGGAAGAAATTTAGG
+HWI-EAS443:3:47:1388:160#0/1
a_FT_¥`a¥]aZ[`]Y_]`W_a`Y¥S]ZVSWLRY^Y]][S
@HWI-EAS443:3:47:1425:1064#0/1
TTCGGATTTCCATTTCATCGACGAACGGGGTGAATCAATA
+HWI-EAS443:3:47:1425:1064#0/1
_¥^_a^a_V¥^_^`_UY_]a[^a]][]¥`[Q[`¥]XS[^¥
@HWI-EAS443:3:47:1425:1750#0/1
GACCGAATCATGGCCTGGACAGTGGTGAACAGTATATGTA
+HWI-EAS443:3:47:1425:1750#0/1
a`_^aa`_]^_`_[_`_^TX__T^¥WZYQW¥_T¥WW^XWT
@HWI-EAS443:3:47:1436:542#0/1
GGGGGCTATATGAAGCAATCGAGGAGTGCCTGATTAATGA
+HWI-EAS443:3:47:1436:542#0/1
abaaaaaababa_``_a`a^_^aa]___]Y_aY__Y__`X
@HWI-EAS443:3:47:1458:1615#0/1
CATATAGCCCCCCAAGATCGAAGGTTCCAGGTTCCAGGTT
+HWI-EAS443:3:47:1458:1615#0/1
abbbbbbbb``aaaaaaa`aaaaa^a^^¥_a¥aWNU``W¥
@HWI-EAS443:3:47:145:1429#0/1
TCCACTCCCCTGGGTGACTCTCCGATTGGCCATGTTTCCG
+HWI-EAS443:3:47:145:1429#0/1
Kabab[H_b[¥¥W_W_bYaIa]aUL¥aHYW_V`WZa]TMG
@HWI-EAS443:3:47:1464:468#0/1
TATGTGGACTTCCCTCCGTGTTACTCCAATTTCAATGAAC
+HWI-EAS443:3:47:1464:468#0/1
a`aa]ba`_aa`]`a__a]aXZ¥^`ZZ^]___]^]^`XXW
@HWI-EAS443:3:47:1482:1383#0/1
GTGGTGAACTTTGTAAGTATGGAGTTCTCACTCACTGACC
+HWI-EAS443:3:47:1482:1383#0/1
aPVJ^¥OX¥`[P¥[`ZZS¥X^S]UTVMYZ¥US¥WS_¥WWS
@HWI-EAS443:3:47:1529:103#0/1
GGCTTGGAAGCAGGTGCTAGCAGAGCTACAGGACATTGAA
+HWI-EAS443:3:47:1529:103#0/1
ababbab^ab^_aa¥__[^a_¥`T_Z¥¥W^^^WWWVWUWT
@HWI-EAS443:3:47:1535:896#0/1
CACCCCGTTCGTCGATGAAATGGAAATCCGAATACATGAA
+HWI-EAS443:3:47:1535:896#0/1
[`b^^``^aX^I`bbabaa]R]``¥Z¥aa^]aa`[]Za`]
@HWI-EAS443:3:47:1540:1656#0/1
CATGGAAATGAGGCGCTGCCTTCTTCAGTCTCTTCAGCAG
+HWI-EAS443:3:47:1540:1656#0/1
a`a^``^Q__UZVS^^XUX¥¥]Y_WFV¥LV[PX`VU^QRU
@HWI-EAS443:3:47:157:347#0/1
CGGTCTCTTCCTTCAATTATCTCAAATCGGTGCTTCAATA
+HWI-EAS443:3:47:157:347#0/1
`aa^bbbbbbbbb_bbbbbbababaab_ba_baabaa`aa
@HWI-EAS443:3:47:1589:1683#0/1
AGAATTCCTTGGTCATGTCTTTCTCTTTGACAGAAGACTC
```

```
+HWI-EAS443:3:47:1589:1683#0/1
aa_WY`_^a`[_Y^^__V_a_`]_¥_^_]]Y^XTLV_XXX
@HWI-EAS443:3:47:1681:1329#0/1
TTTCCGATTTGTTTTCAAAGAATTCCTTGGTCATGTCTTT
+HWI-EAS443:3:47:1681:1329#0/1
abbaaa]abbb`aaba`````^aa__aaaa^_`aa__a``
@HWI-EAS443:3:47:1720:490#0/1
CAAATCGGTGCTTCAATAGTGCATTCGGGTCACCAGATTC
+HWI-EAS443:3:47:1720:490#0/1
abbabbbb_bbbbababa[[aabbaaaaa^`a__abaaa_
@HWI-EAS443:3:47:190:449#0/1
CTCAAGTTGTGGAGACGCATATAGACTGTTGAATACAGAT
+HWI-EAS443:3:47:190:449#0/1
abba``Sba_bbab__baaaa^_aa_^aS[TUaR]_`[GZ
@HWI-EAS443:3:47:277:1152#0/1
CCGAACTTCTCCAGCCTTGAAAACTTTAGAGCCTATGTAG
+HWI-EAS443:3:47:277:1152#0/1
ab_VX`aaa`Z_]Y]`b^`^Y`_Y]]`R]aLXTa_aa^Pa
@HWI-EAS443:3:47:277:525#0/1
GGGGCATGGAAATGAGGCGCTGCCTTCTTCAGTCTCTTCA
+HWI-EAS443:3:47:277:525#0/1
aaaaaaaaa`a``aaaa_a_^a¥_^a]_^^_`[T^¥¥¥¥¥
@HWI-EAS443:3:47:288:1754#0/1
GGGCACTCGGTGAAAATATGGCACCAGAAAAAGTAGACTT
+HWI-EAS443:3:47:288:1754#0/1
_ba^bbbabbZa`abaaaa`[aa__a^¥`a__aSaaa^`a
@HWI-EAS443:3:47:336:629#0/1
GGGGGCTATATGAAGCAATCGAGGAGTGCCTGATTAATGA
+HWI-EAS443:3:47:336:629#0/1
a`aaaa`a`a_a_`U^[_U]`U`a[`V][]V_UY]^[V`¥
@HWI-EAS443:3:47:346:502#0/1
AACCCATACAGGTTTGTTTTCCGTCTTCCTTCTTTGGTCC
+HWI-EAS443:3:47:346:502#0/1
aa_aa_¥¥[^`a_``_Yaa_VW`OK¥_YU_RJ[__PZOZS
@HWI-EAS443:3:47:35:1666#0/1
GATGGCCACCAAAGCGGACTACACCCTTGACGAAGAGAGC
+HWI-EAS443:3:47:35:1666#0/1
]`bbbaa```bb`_^_^_aaa`a]```a[`]^`Z^^[¥]]
@HWI-EAS443:3:47:440:1268#0/1
CACACATTCACATCTTTTCATTCACTGGAGAGGAGATGGC
+HWI-EAS443:3:47:440:1268#0/1
]Vba``_bb_abbbaba_Ta`b^aUaX¥ZaYP¥a`[Z]`Q
@HWI-EAS443:3:47:466:1686#0/1
GGGGGCTATATGAAGCAATCGAGGAGTGCCTGATTAATGA
+HWI-EAS443:3:47:466:1686#0/1
Yababbbababb_]babbb`b[ba_a^UaZbbababaa_^
@HWI-EAS443:3:47:473:1790#0/1
GAGAAAGGCATAAATCCCAATTACCTCATGGCTTGGAAGC
+HWI-EAS443:3:47:473:1790#0/1
`bb`b¥`^`bbb`aaZ_W`aaa]XR]Xa^aWU`¥T]_]SQ
```

```
@HWI-EAS443:3:47:508:130#0/1
TTCGAGCCGAACGGCTGCATTGAGGGCAAGCTTTCCCAAA
+HWI-EAS443:3:47:508:130#0/1
a^aa`a_aba`aa__¥^RU[^¥¥S]_TNY¥QUUQGTSSYS
@HWI-EAS443:3:47:571:451#0/1
CAAATCAGGAAGAAATTTAGGCTTCTCTACCCCTGTTGTG
+HWI-EAS443:3:47:571:451#0/1
¥bbbababbbbb`ba^bba^Yabaabb^aaaaa]aZaba`
@HWI-EAS443:3:47:595:1750#0/1
CTTAAACAGTATGACAGTGATGAGCCAGAGCCCAGATCTC
+HWI-EAS443:3:47:595:1750#0/1
`ba_RR[]Wababaa`aX¥V`_PZT]a¥WXV][Y[Y_U_[
@HWI-EAS443:3:47:605:836#0/1
CATGTTTCCGATTTGTTTTCAAAGAATTCCTTGGTCATGT
+HWI-EAS443:3:47:605:836#0/1
aabaabbabbabbbbabbbaabbb`bbb_ababa^aabba
@HWI-EAS443:3:47:674:100#0/1
CTCCAACATCTTTGCAGTCATCAAAGTCTACTTTTTCTGG
+HWI-EAS443:3:47:674:100#0/1
ab[``babbab`aababaaba]aaabSTW¥^aaaba^a`¥
@HWI-EAS443:3:47:687:993#0/1
GAAAAGATGTGAATGTGTGTCTTCTCAGATTTTATTTTGT
+HWI-EAS443:3:47:687:993#0/1
a^ba_b`Vb_baaba_bYb__aa]a_`^R_`aa[aaa`Z_
@HWI-EAS443:3:47:690:1245#0/1
AGCTCTGCTAGCACCTGCTTCCAAGCCATGAGGTAATTGG
+HWI-EAS443:3:47:690:1245#0/1
_ZZ`X`ba_X¥ORT[`a`]`^^_WQUTV]_ZXYNUZ_X¥`
@HWI-EAS443:3:47:716:245#0/1
GGGGCTATATGAAGCAATCGAGGAGTGCCTGATTAATGAT
+HWI-EAS443:3:47:716:245#0/1
ZGMZW¥`¥_¥a`^[]_ZY]a_a`YU[`][`aZ]¥^U¥`^Z
@HWI-EAS443:3:47:764:884#0/1
GTCCTTGGGATCTTCTCTTCATTTTCAATGTCCTGTAGCT
+HWI-EAS443:3:47:764:884#0/1
a`a``abaa¥a[a``aaaaUVaaaa``_a`__^`_^____
@HWI-EAS443:3:47:840:630#0/1
AGGGCAAGAATCAAAACTAGGCTTTTCACTATAAGACAAG
+HWI-EAS443:3:47:840:630#0/1
```b_]_`^VVSa`aa[Y¥b`YYaRPQQHPQQ_JZ[Z¥][
@HWI-EAS443:3:47:845:936#0/1
CAGTGATGAGCCAGAGCCCAGATCTCTAGCAAGCTGGGTC
+HWI-EAS443:3:47:845:936#0/1
```^]¥aa_^ab`]^bba_baaaabaaaaaX]b_a[]`XY
@HWI-EAS443:3:47:849:809#0/1
GCGTCTCCACAACTTGAGGGGTTTTCGGCTGAATCGAGAA
+HWI-EAS443:3:47:849:809#0/1
aab`aaaaaa``_aaaabaaaU_`a^a`^```^^Z_]`]^
@HWI-EAS443:3:47:85:1415#0/1
AGCAGGTGCTAGCAGAGCTACAGGACATTGAAAATGAAGA
```

```
+HWI-EAS443:3:47:85:1415#0/1
abaaab`^abba_a`_baaa_a`_`_`aa_^`a`a`_``_
@HWI-EAS443:3:47:930:571#0/1
GTATTCAGTAGCCCTGCAGTGGGACACTTCTGCTGTAAAA
+HWI-EAS443:3:47:930:571#0/1
`Z¥a`U_[[_[QWUQR[Z`Y`[XUUSSS¥]^PUZ_U¥^WN
@HWI-EAS443:3:47:931:75#0/1
CATTCTTTGGCTGGAAAGAGCCTAACATAGTCAAACCACA
+HWI-EAS443:3:47:931:75#0/1
Zabbabbbbbbbbbbbbbb`^aabbbbabaabbb`aa`a
@HWI-EAS443:3:47:949:1196#0/1
TGCTAGCAGAGCTACAGGACATTGAAAATGAAGAGAAGAT
+HWI-EAS443:3:47:949:1196#0/1
]M^^¥a]¥_]baaaa¥_a^M^aaa_^^a_^¥`aaa]]^¥_
@HWI-EAS443:3:47:97:1310#0/1
GAAGATGTTGCCCCGATTGAACATATCGCAAGCATGAGGA
+HWI-EAS443:3:47:97:1310#0/1
a_aaaba]a`a_`aa_aaa`a¥aaa_^¥¥`[`[_`^]X¥^
@HWI-EAS443:3:47:997:651#0/1
GGGGGCTATATGAAGCAATCGAGGAGTGCCTGATTAATGA
+HWI-EAS443:3:47:997:651#0/1
abbababbbabbaaaaaaa`a_b`_a_a__aa^aa``a``
@HWI-EAS443:3:48:1021:214#0/1
TGAAATTGGAGTAACACGGAGGGAAGTCCACATATATTAC
+HWI-EAS443:3:48:1021:214#0/1
_``a_aaa^^b^`__a^a`^`_`^^`WRQ¥]]___]a]`V
@HWI-EAS443:3:48:1041:599#0/1
GTACATAAATACGGCCTTGCTCAATGCATCCTGTGCAGCC
+HWI-EAS443:3:48:1041:599#0/1
a`aaaaaaaaa_aa^`aa¥Y^]Z`_`^__XS¥_UZV^_W]
@HWI-EAS443:3:48:1084:654#0/1
CATATAGCCCCCCAAGATCGAAGGTTCCAGGTTCCAGGTT
+HWI-EAS443:3:48:1084:654#0/1
```_aaaba``b`[_aZ_a_]_``R_Z``[`Y[YQO``^_
@HWI-EAS443:3:48:1102:1468#0/1
GGAGACTTTGGTCGGCAAGCTTGCGCATAGTTCCTGTAAT
+HWI-EAS443:3:48:1102:1468#0/1
aa^a¥abb^a``]Wbaaa_[`_YW]`Z[]_XZ^[__Z]XW
@HWI-EAS443:3:48:1246:92#0/1
GCCGTATTTATGTACACTCCCTTCATTATGTATTCAGTAG
+HWI-EAS443:3:48:1246:92#0/1
aa¥b_abbbaaaaaa^Wa___aa]_aa`aa__a`X]a^_¥
@HWI-EAS443:3:48:1274:1144#0/1
GCAGGTGCTAGCAGAGCTACAGGACATTGAAAATGAAGAG
+HWI-EAS443:3:48:1274:1144#0/1
abbab_abbabaab^baaaaaaaaaaaaaaaa`aa`aa``
@HWI-EAS443:3:48:1314:257#0/1
CATCTTGATCTTGGAGGTTCCATTGGTTCTCACATATAGG
+HWI-EAS443:3:48:1314:257#0/1
aab^_ab_`aba_`^b¥G__`^aUM]]THQW^WNKX_ZYY
```
```

```
@HWI-EAS443:3:48:1355:1236#0/1
GGTGACCCGAATGCACTATTGAAGCACCGATTTGAGATAA
+HWI-EAS443:3:48:1355:1236#0/1
aa_b_aaaa_aa_R¥Waaaa`¥``TX]]a^a``aY_^`^]
@HWI-EAS443:3:48:1364:429#0/1
CACCCCGTTCGTCGATGAAATGGAAATCCGAATACATGAA
+HWI-EAS443:3:48:1364:429#0/1
aaba^bbbbbb`^baababa`_a`__^`_baaa`^a`aaa
@HWI-EAS443:3:48:1377:1426#0/1
CAGTCATCAAAGTCTACTTTTTCTGGTGCCATATTTTCAC
+HWI-EAS443:3:48:1377:1426#0/1
abba`bb]bb`^``bb¥bbbaaab^U¥]`_aa[bbaaPa`
@HWI-EAS443:3:48:1382:1487#0/1
CACTGTCATACTGTTTAAGGTCTCCAACATCTTTGCAGTC
+HWI-EAS443:3:48:1382:1487#0/1
ab]a```aa_abba^bbaba`aaaabbaabab`^a_ab`Z
@HWI-EAS443:3:48:1399:15#0/1
CGAAAGAGGCGAAGAGACAATTGAAGAAAAATTTGAGATT
+HWI-EAS443:3:48:1399:15#0/1
aa`aaaaa`abb``_a`aaab_a`ab``]`_`a`_Z_``^
@HWI-EAS443:3:48:1420:1575#0/1
ATTCTTTGGCTGGAAAGAGCCTAACATAGTCAAACCACAT
+HWI-EAS443:3:48:1420:1575#0/1
aab_aa``a`aaa_aa```^^a`aZ___`XT__`XX]P^]
@HWI-EAS443:3:48:1446:1634#0/1
AAGAATTCCTTGGTCATGTCTTTCTCTTTGACAGAAGACT
+HWI-EAS443:3:48:1446:1634#0/1
aaa_¥aaY¥_abb_`a[b__aaa¥a[`_`a^ZY_^V]]Z^
@HWI-EAS443:3:48:1493:778#0/1
CGAGGGGGAGGGAATACCACTATATGATGCAATCAAATGC
+HWI-EAS443:3:48:1493:778#0/1
ababbbbaabbba[baaab`aababbaaaaaaa^aaaaa^
@HWI-EAS443:3:48:1511:540#0/1
ATCCTGTGCAGCCATGGATGACTTTCAGCTGATCCCAATG
+HWI-EAS443:3:48:1511:540#0/1
aZ¥Z[bZ¥N_b[[`]]_a``]SY¥ZYVZU]_¥¥Z^Z_SY¥
@HWI-EAS443:3:48:1537:1684#0/1
ATAGAGCCTTCCTCCACTCCCCTGGGTGACTCTCCGATTG
+HWI-EAS443:3:48:1537:1684#0/1
a`b``abaaa``__``_[aXNYaa[TY[¥^_Z_¥W^T[`¥
@HWI-EAS443:3:48:1556:866#0/1
GGGGGCTATATGAAGCAATCGAGGAGTGCCTGATTAATGA
+HWI-EAS443:3:48:1556:866#0/1
aaaa`]`aaaa`]]_^``a]aYa`T`U][¥_aZ^`]^__^
@HWI-EAS443:3:48:1560:716#0/1
AATTGACTGATTCAAGCTGGATAGAACTTGATGAAATAGG
+HWI-EAS443:3:48:1560:716#0/1
TNbbbabbb``¥bb_a_U_a`[`bba]`bba_babbbab`
@HWI-EAS443:3:48:1582:1747#0/1
AATGAATTCAATAAGGCATGTGAATTGACTGATTCAAGCT
```

```
+HWI-EAS443:3:48:1582:1747#0/1
aaba`aaa_aaaa`aa`_a_]a`[aa__[a`^]_Y`[_U^
@HWI-EAS443:3:48:1583:1038#0/1
GGTGAACTTTGTAAGTATGGAGTTCTCACTCACTGACCCG
+HWI-EAS443:3:48:1583:1038#0/1
aa[a`]_a_ab___a^[a]a]_]`_`]^^a__^]a_WWY_
@HWI-EAS443:3:48:1615:145#0/1
TGGCTCATCACTGTCATACTGTTTAAGGTCTCCAACATCT
+HWI-EAS443:3:48:1615:145#0/1
abaaa`aa¥`^a_^^``^_aa^aa__``[Z^¥[[]U^[V¥
@HWI-EAS443:3:48:1663:1232#0/1
GGCTCATCACTGTCATACTGTTTAAGGTCTCCAACATCTT
+HWI-EAS443:3:48:1663:1232#0/1
a``^aaa`_a^b]XZ`]¥``¥ZYX__`TP]S]]_]Y]W`^
@HWI-EAS443:3:48:1703:1795#0/1
CACTGCAGGGCTACTGAATACATAATGAAGGGAGTGTACA
+HWI-EAS443:3:48:1703:1795#0/1
aa`^_[_ab_aa``abaa`a¥aa_^aa`¥`aa_a]a^a__
@HWI-EAS443:3:48:1708:267#0/1
ATCAGGCACTCCTCGATTGCTTCATATAGCCCCCCAAGAT
+HWI-EAS443:3:48:1708:267#0/1
`a_a^`]baaaaaaba`aaQ__`a_aa```W__¥[^WY^^
@HWI-EAS443:3:48:1737:945#0/1
CACTTCAATTGGCTTGTTCTCTTCATGTTCTTTGTCCTTG
+HWI-EAS443:3:48:1737:945#0/1
aaaaba_]aYaa^baSV¥V`Ia_Z]_¥R`Z¥abb¥¥]a^P
@HWI-EAS443:3:48:1769:288#0/1
TGTGAATTGACTGATTCAAGCTGGATAGAACTTGATGAAA
+HWI-EAS443:3:48:1769:288#0/1
_bX_JV``a¥^]RO``]`]aR__^PZ_`U_Wa_aVX^OX_
@HWI-EAS443:3:48:1785:1209#0/1
CATGACCAAGGAATTCTTTGAAAACAAATCGGAAACATGG
+HWI-EAS443:3:48:1785:1209#0/1
_bbbbbbbbab`bbbabbbbba_aaabbbabaaaaaaaaa
@HWI-EAS443:3:48:1788:1199#0/1
GAAGATCCCAAGGACAAAGAACATGAAGAGAACAAGCCAA
+HWI-EAS443:3:48:1788:1199#0/1
aT`_¥YaV¥[^^_Y_[]_a¥]YSOT]W_Z`^TVYU]OZ]Z
@HWI-EAS443:3:48:188:459#0/1
GCGTGGTGTCGTCCTCAAGAATGGTTCAATTTTGGCGTTC
+HWI-EAS443:3:48:188:459#0/1
aaaa`^Yaa]aaaaaa]aaaaa_U¥a_][`aaaW]¥][[Z
@HWI-EAS443:3:48:234:1109#0/1
ATAAATACGGCCTTGCTCAATGCATCCTGTGCAGCCATGG
+HWI-EAS443:3:48:234:1109#0/1
aa_^Zaa_^_``^_^^]__^]__^Y[Z_^Y¥]V]VV_]Y_
@HWI-EAS443:3:48:278:804#0/1
ATCATGGCCTGGACAGTGGTGAACAGTATATGTAACACAA
+HWI-EAS443:3:48:278:804#0/1
aa_b^```aaa___]a]`aX__`V`aV_^`^_]_a]_[``
```

```
@HWI-EAS443:3:48:288:178#0/1
GGGGGCTATATGAAGCAATCGAGGAGTGCCTGATTAATGA
+HWI-EAS443:3:48:288:178#0/1
ab^bbabaaabbabbabbbaaaba_a^a¥]aa_`a__``^
@HWI-EAS443:3:48:325:878#0/1
GAAGATCCCAAGGACAAAGAACATGAAGAGAACAAGCCAA
+HWI-EAS443:3:48:325:878#0/1
aaU^[U]a]aa^Y^`_aaaaaV`aaa`_]a`^]a[_US_Z
@HWI-EAS443:3:48:342:1014#0/1
CTTCTGTCAAAGAGAAAGACATGACCAAGGAATTCTTTGA
+HWI-EAS443:3:48:342:1014#0/1
`bbbaabbb_`b`abaabaaaab_a_aaaa^^`aYaaba`
@HWI-EAS443:3:48:380:1290#0/1
GGCCACCAAAGCGGACTACACCCTTGACGAAGAGAGCAGG
+HWI-EAS443:3:48:380:1290#0/1
Z`ZW_Wb]_WW____`]R[_]R_Y[[`Y`a]aaa]X]Xaa
@HWI-EAS443:3:48:417:116#0/1
GAAAAAGTAGACTTTGATGACTGCAAAGATGTTGGAGACC
+HWI-EAS443:3:48:417:116#0/1
aaa^Z^a_aaa^aaaa`aa_^`a]___a``a]___]`^ZV
@HWI-EAS443:3:48:493:1577#0/1
AGACTTTGGTCGGCAAGCTTGCGCATAGTTCCTGTAATCT
+HWI-EAS443:3:48:493:1577#0/1
`ba`aaa¥MNb`[Yaaaaa`_V[¥¥a`a[_RW_aUXY`X[
@HWI-EAS443:3:48:498:615#0/1
CAGCTTGCTAGAGATCTGGGCTCTGGCTCATCACTGTCAT
+HWI-EAS443:3:48:498:615#0/1
`aa`a_U]`aaabaaa_]_a^aaa_aaa_aa_a`a_N]_a
@HWI-EAS443:3:48:516:1746#0/1
GAAGTTCGGTGGGAGACTTTGGTCGGCAAGCTTGCGCATA
+HWI-EAS443:3:48:516:1746#0/1
abaa^baa`¥_a_`a_aaba_¥^_W`aa]O]^`[U¥ZZ`^
@HWI-EAS443:3:48:604:1237#0/1
CTCCCCTGGGTGACTCTCCGATTGGCCATGTTTCCGATTT
+HWI-EAS443:3:48:604:1237#0/1
abbb`b]bbb_Yabbbaaab`[abaa`a]_[abZZ___aa
@HWI-EAS443:3:48:66:924#0/1
GCTAGCAGAGCTACAGGACATTGAAAATGAAGAGAAGATC
+HWI-EAS443:3:48:66:924#0/1
abab`bbaabbbbbabbaaabb`_ababaaaaab`Y`_[
@HWI-EAS443:3:48:688:627#0/1
GGCTTGGAAGCAGGTGCTAGCAGAGCTACAGGACATTGAA
+HWI-EAS443:3:48:688:627#0/1
aaaZ^a`_aa`U¥[FO`U[]V]a`_]`ZNY_Y[R^_¥X[`
@HWI-EAS443:3:48:754:198#0/1
GGGGCATGGAAATGAGGCGCTGCCTTCTTCAGTCTCTTCA
+HWI-EAS443:3:48:754:198#0/1
abaa_a_``_aaa¥`a``a`_a¥Y^_¥``ZW]TS¥V^WQV
@HWI-EAS443:3:48:80:539#0/1
CCCGTTCGTCGATGAAATGGAAATCCGAATACATGAAACA
```

+HWI-EAS443:3:48:80:539#0/1
aaab`aab`ab`aa_a`a_aaa`a][_`___U__a`aa_a
@HWI-EAS443:3:48:882:1602#0/1
AGAGATCTGGGCTCTGGCTCATCACTGTCATACTGTTTAA
+HWI-EAS443:3:48:882:1602#0/1
`QZa_`a[_^]YTR_aY[Y^_]^_Ra_ZWS_^X^^]_ZWY
@HWI-EAS443:3:48:906:118#0/1
CGGTGGGAGACTTTGGTCGGCAAGCTTGCGCATAGTTCCT
+HWI-EAS443:3:48:906:118#0/1
`bbabbb^b__aaaaa_aab```b_^aaYb_Xa^aW^_]_
@HWI-EAS443:3:48:916:864#0/1
GGAACTATTTTACAGCAGAAGTGTCCCACTGCAGGGCTAC
+HWI-EAS443:3:48:916:864#0/1
`a]Yaa^abZaa^^`Y_aaa__a_][X`U_``_b_]N[UK
@HWI-EAS443:3:48:91:1025#0/1
ATCAAAACTAGGCTTTTCACTATAAGACAAGAAATGGCCA
+HWI-EAS443:3:48:91:1025#0/1
``_a`a_abaaa_ab`a`aaa`a_aa]Za``_aY_^_YVW
@HWI-EAS443:3:48:932:1450#0/1
CATAAATACGGCCTTGCTCAATGCATCCTGTGCAGCCATG
+HWI-EAS443:3:48:932:1450#0/1
aabbaabaabaa``aX`^`aa`a`a^aa```_Zaa¥`_^]
@HWI-EAS443:3:48:942:566#0/1
GCGTCTCCACAACTTGAGGGGTTTTCGGCTGAATCGAGAA
+HWI-EAS443:3:48:942:566#0/1
a^][^^`a_]^[Z^`UVaa``R]^WQW_SXaXVR[VPY]R
@HWI-EAS443:3:48:943:1007#0/1
TCCGATTTGTTTTCAAAGAATTCCTTGGTCATGTCTTTCT
+HWI-EAS443:3:48:943:1007#0/1
aaaa]T`b^F¥^aYOT_a¥_`_aW¥X`VX^]Y]_]_a`¥¥
@HWI-EAS443:3:48:956:1197#0/1
CTGAAATTAAGTATTGAAGACCCGAGTCACGAGGGGGAGG
+HWI-EAS443:3:48:956:1197#0/1
abbbbbbbbbbaaaaaabbbaaab_babaab_abaab`aa
@HWI-EAS443:3:48:963:691#0/1
CGGTGGGAGACTTTGGTCGGCAAGCTTGCGCATAGTTCCT
+HWI-EAS443:3:48:963:691#0/1
aab]abb`b^`bbbaaaa^b`aaa`a`a`aa_a_a^a`_a
@HWI-EAS443:3:48:974:1356#0/1
GAGCAGGGCAAGAATCAAAACTAGGCTTTTCACTATAAGA
+HWI-EAS443:3:48:974:1356#0/1
`a¥_`WUS`IY]Z^aXO^]_ZW^PI^_YXX[aHVH[L]LL
@HWI-EAS443:3:48:990:258#0/1
TAATCAGGCACTCCTCGATTGCTTCATATAGCCCCCCAAG
+HWI-EAS443:3:48:990:258#0/1
^^¥S^b_W[_T`IQ_`[aaaa^]a¥aa`a^YJQ]^V¥]^_
@HWI-EAS443:3:49:103:90#0/1
CTTGCTCAATGCATCCTGTGCAGCCATGGATGACTTTCAG
+HWI-EAS443:3:49:103:90#0/1
a`abbaababaaba`aaaaa`a`a_aaa`¥[``_aaa_aa

```
@HWI-EAS443:3:49:1047:1352#0/1
CATGTTTCCGATTTGTTTTCAAAGAATTCCTTGGTCATGT
+HWI-EAS443:3:49:1047:1352#0/1
aa`baababb_bbba`bbbbaabaaabababbb__abbba
@HWI-EAS443:3:49:1055:1491#0/1
GGGGCATGGAAATGAGGCGCTGCCTTCTTCAGTCTCTTCA
+HWI-EAS443:3:49:1055:1491#0/1
W_aUb]``a`X`^_bb^]b_a¥I¥b^a```QG]UTbYaSa
@HWI-EAS443:3:49:106:891#0/1
CTTCTCTACCCCTGTTGTGTTACATATACTGTTCACCACT
+HWI-EAS443:3:49:106:891#0/1
^_¥____`_Wa[¥X_`a]`bb[Zab_a_O_¥¥_[[YZ^b_
@HWI-EAS443:3:49:1108:1316#0/1
CACTGTCCAGGCCATGATTCGGTCTCTTCCTTCAATTATC
+HWI-EAS443:3:49:1108:1316#0/1
_b`bbbabba`^abababba``]a`Z¥baaba¥_aaaab_
@HWI-EAS443:3:49:1115:271#0/1
AGAACTTGATGAAATAGGAGAAGATGTTGCCCCGATTGAA
+HWI-EAS443:3:49:1115:271#0/1
abbbabbb_b]a_ba`bb_baabaabaabY`_aaa`_aaa
@HWI-EAS443:3:49:1187:838#0/1
GGCATGGAAATGAGGCGCTGCCTTCTTCAGTCTCTTCAGC
+HWI-EAS443:3:49:1187:838#0/1
abaaabaababbZ[U`b`aa[^aaRaaQ^][[[Z_¥H^[R
@HWI-EAS443:3:49:1209:1411#0/1
ATTTTTCCCATTTGTGTGGCTCCAGTCTCGGGTCAGTGAG
+HWI-EAS443:3:49:1209:1411#0/1
]ab_babba_a_baab`^VN¥Za¥_]]W¥^[aYZ[XNMDW
@HWI-EAS443:3:49:1209:424#0/1
GTGGGCACTCGGTGAAAATATGGCACCAGAAAAAGTAGAC
+HWI-EAS443:3:49:1209:424#0/1
a]W^_``_aZa`VXTTa`^W]¥_U]W[[]Z¥^[Z^U[^^U
@HWI-EAS443:3:49:1226:1111#0/1
GTGCTAGCAGAGCTACAGGACATTGAAAATGAAGAGAAGA
+HWI-EAS443:3:49:1226:1111#0/1
`a`__aaaaaVa`_^[_a`]Y[`_[X¥^_[`^`^^^_^__
@HWI-EAS443:3:49:1227:1262#0/1
TCCACTCCCCTGGGTGACTCTCCGATTGGCCATGTTTCCG
+HWI-EAS443:3:49:1227:1262#0/1
abab`ba_`aaaa]T_[XYa^`ZaW^^M`_[P^UU_]]V[
@HWI-EAS443:3:49:1269:1479#0/1
GTGCTAGCAGAGCTACAGGACATTGAAAATGAAGAGAAGA
+HWI-EAS443:3:49:1269:1479#0/1
abbbbbbbbaabbbbbbbba`bbaaaaabbbaaabbaaaa
@HWI-EAS443:3:49:1280:1298#0/1
AGAAATTTAGGCTTCTCTACCCCTGTTGTGTTACATATAC
+HWI-EAS443:3:49:1280:1298#0/1
aba`bb`¥ab_abb`b_abY_`]`a^aaaa`a__a_a``^
@HWI-EAS443:3:49:1294:127#0/1
GTGGGAGACTTTGGTCGGCAAGCTTGCGCATAGTTCCTGT
```

```
+HWI-EAS443:3:49:1294:127#0/1
a]a]W[a^Waaa``]^W`^]X]XZ^]O[WX_YXX^ZU_]X
@HWI-EAS443:3:49:1298:1449#0/1
GGGGCATGGAAATGAGGCGCTGCCTTCTTCAGTCTCTTCA
+HWI-EAS443:3:49:1298:1449#0/1
a`a][b`a]_aaaa``aaa[``_[_aH[_X^`ZH^V[_U_
@HWI-EAS443:3:49:1317:11#0/1
GGGGCATGGAAATGAGGCGCTGCCTTCTTCAGTCTCTTCA
+HWI-EAS443:3:49:1317:11#0/1
aaa`TZ`a]WY__`_aa[a[YV]SVVUZRRUUNPRUU^SR
@HWI-EAS443:3:49:1411:422#0/1
CCTTGGGATCTTCTCTTCATTTTCAATGTCCTGTAGCTCT
+HWI-EAS443:3:49:1411:422#0/1
abbbabb`a^aaaabbbbbbbbabab]aaabbaaaa_^_
@HWI-EAS443:3:49:1446:1484#0/1
GGGGGCTATATGAAGCAATCGAGGAGTGCCTGATTAATGA
+HWI-EAS443:3:49:1446:1484#0/1
ab_TWab`baaa[`_aaba`__a`[a_`][`^`aaaYa_a
@HWI-EAS443:3:49:1450:1309#0/1
GGCCGAGTCTTCTGTCAAAGAGAAAGACATGACCAAGGAA
+HWI-EAS443:3:49:1450:1309#0/1
`bbb`ab^bbbabbY]Z_aa_baaa_]_aaaa__aaba__
@HWI-EAS443:3:49:1463:1754#0/1
ATAAGACAAGAAATGGCCAGTAGGAGTCTATGGGATTCCT
+HWI-EAS443:3:49:1463:1754#0/1
abaaaa`aba`aaa`a`__`¥_`__`WU_¥`_`^^¥_V[`
@HWI-EAS443:3:49:1465:1469#0/1
ATGAAATGGAAATCCGAATACATGAAACAAACTTCCAAAT
+HWI-EAS443:3:49:1465:1469#0/1
__b`a¥`Z__[__babaaaa¥`a_`T__baYTYa_Q_^X`
@HWI-EAS443:3:49:1534:799#0/1
TGAACGCCAAAATTGAACCATTCTTGAGGACGACACCACG
+HWI-EAS443:3:49:1534:799#0/1
`_aaZa__]a_abaa`a_Qaaa^aa^]__^Wa[U_RV_V_
@HWI-EAS443:3:49:1663:248#0/1
CTTGGGGGGCTATATGAAGCAATCGAGGAGTGCCTGATTA
+HWI-EAS443:3:49:1663:248#0/1
Q¥_a_¥^a__a`_Z¥¥_WY[b[[[_]]__¥R¥^O_`]a__
@HWI-EAS443:3:49:1680:1217#0/1
ATGGCTTGGAAGCAGGTGCTAGCAGAGCTACAGGACATTG
+HWI-EAS443:3:49:1680:1217#0/1
T¥a_¥a^OY[^^Y^a^RYV]^RYX]P[JS¥¥N¥¥H[UPYW
@HWI-EAS443:3:49:1762:1054#0/1
GCATGTGAATTGACTGATTCAAGCTGGATAGAACTTGATG
+HWI-EAS443:3:49:1762:1054#0/1
]W]a`^`Z¥abaa_]_a_¥_[T¥``bb_^a`S[D[_``]_
@HWI-EAS443:3:49:1775:133#0/1
AGAGAAGCCTAAATTTCTTCCTGATTTGTATGATTACAAA
+HWI-EAS443:3:49:1775:133#0/1
Z¥Z]][PY_VSW]b_Z_`^ZQa]X^_[TY¥aa_a_]`_¥]
```

```
@HWI-EAS443:3:49:180:1034#0/1
TCCAATTTCAATGAACCGGTTCTCTTTGTAATCATACAAA
+HWI-EAS443:3:49:180:1034#0/1
`[_a[babaa``aabaaaa^^Ya_aa`¥¥```U^aa¥a__
@HWI-EAS443:3:49:192:1612#0/1
ATGGCTTGGAAGCAGGTGCTAGCAGAGCTACAGGACATTG
+HWI-EAS443:3:49:192:1612#0/1
`^bb^b_S^¥`¥Y_a_HT¥^^PUXTMXLW^_^¥¥UJW]WF
@HWI-EAS443:3:49:19:1102#0/1
CACAACTTGAGGGGTTTTCGGCTGAATCGAGAAAATTGCT
+HWI-EAS443:3:49:19:1102#0/1
a^bbZbbbbZa¥^aQ¥b_QYa[__`a]¥a_^_U]a¥_ZW_
@HWI-EAS443:3:49:248:1983#0/1
CGGGGTGAATCAATAATTGTAGAATCTGGTGACCCGAATG
+HWI-EAS443:3:49:248:1983#0/1
abbba[bbbbabab_bbbbab`bab_ab_aaa`Z_[aaaa
@HWI-EAS443:3:49:279:1080#0/1
CATCAGCAGGAACTTTGACCGCTGATGGCAAAGAGGCCCA
+HWI-EAS443:3:49:279:1080#0/1
`baabbbbaaab`bbbbaa]a`aba`aa``aaaa```__a
@HWI-EAS443:3:49:337:1110#0/1
GAAAGGCATAAATCCCAATTACCTCATGGCTTGGAAGCAG
+HWI-EAS443:3:49:337:1110#0/1
a^aa`__`_`]`aXTZ]`aa_XY^_a`a`aa^U`VXTTVV
@HWI-EAS443:3:49:374:1258#0/1
AGAATTCCTTGGTCATGTCTTTCTCTTTGACAGAAGACTC
+HWI-EAS443:3:49:374:1258#0/1
^YT[a`[¥`^`¥UVO¥_`a][aH]P¥a]P_`aa`Y``_ZK
@HWI-EAS443:3:49:399:1764#0/1
TAAGACAAGAAATGGCCAGTAGGAGTCTATGGGATTCCTT
+HWI-EAS443:3:49:399:1764#0/1
`aaaaaa_W`aa`aaaa`a¥`a`__RX___a__R__YZ^_
@HWI-EAS443:3:49:407:1007#0/1
GGGGCATGGAAATGAGGCGCTGCCTTCTTCAGTCTCTTCA
+HWI-EAS443:3:49:407:1007#0/1
aba]_baa`aaaaaa_`aaaZa`[`___¥^^ZWYYU^¥NW
@HWI-EAS443:3:49:413:1553#0/1
AGCCCCCCAAGATCGAAGGTTCCAGGTTCCAGGTTGTCCC
+HWI-EAS443:3:49:413:1553#0/1
_V]`aaaabaZ`UZ]]Xa`[YV_[¥`U__¥]`^¥`¥¥Z_¥
@HWI-EAS443:3:49:458:1273#0/1
GGGGCATGGAAATGAGGCGCTGCCTTCTTCAGTCTCTTCA
+HWI-EAS443:3:49:458:1273#0/1
a_X[aaaba_a_aaba[abaa^V¥`a_aa__a[¥`T_a`a
@HWI-EAS443:3:49:492:457#0/1
CTCCAACATCTTTGCAGTCATCAAAGTCTACTTTTTCTGG
+HWI-EAS443:3:49:492:457#0/1
`aTSab^bbba_ba^bbV`a`a`]a_R`a__bbb`b`a_`
@HWI-EAS443:3:49:532:1768#0/1
CACCGATTTGAGATAATTGAAGGAAGAGACCGAATCATGG
```

```
+HWI-EAS443:3:49:532:1768#0/1
ab__b[abaaab`^aab]JWabb_a_aXaaYa_a^^`aba
@HWI-EAS443:3:49:548:1207#0/1
GAAATCCGAATACATGAAACAAACTTCCAAATGTGTGCAT
+HWI-EAS443:3:49:548:1207#0/1
a_`_^_ab^aaa`a`aaa`_a___a`Y`a``aa[_SZ[`_
@HWI-EAS443:3:49:577:167#0/1
TGGGGCATGGAAATGAGGCGCTGCCTTCTTCAGTCTCTTC
+HWI-EAS443:3:49:577:167#0/1
ab`b``bbbbababaabaabaaa[]`_X_`^[a[__V`_]
@HWI-EAS443:3:49:608:1262#0/1
CACAACTTGAGGGGTTTTCGGCTGAATCGAGAAAATTGCT
+HWI-EAS443:3:49:608:1262#0/1
]`Q_abbb_abaababb]`bb`bbbaaabbaa_`]^abab
@HWI-EAS443:3:49:645:665#0/1
GCGCAAGCTTGCCGACCAAAGTCTCCCACCGAACTTCTCC
+HWI-EAS443:3:49:645:665#0/1
a_QQ_]]a`a_TXbW_P¥ZXUP[^]aaa]X^WZ_`]MK[T
@HWI-EAS443:3:49:664:664#0/1
CCCCGTTCGTCGATGAAATGGAAATCCGAATACATGAAAC
+HWI-EAS443:3:49:664:664#0/1
abbbb`babaabbabbab``ba_aa```baa`_aaaaaa_
@HWI-EAS443:3:49:670:62#0/1
GGGGAGGGAATACCACTATATGATGCAATCAAATGCATGA
+HWI-EAS443:3:49:670:62#0/1
`aa]_b`Y[Zba¥S_[a`a_____aR_]a^__`_aU_]__
@HWI-EAS443:3:49:738:1438#0/1
CAGCTTGCTAGAGATCTGGGCTCTGGCTCATCACTGTCAT
+HWI-EAS443:3:49:738:1438#0/1
a¥bUbbb_bab¥¥aaab`Y^aaaaab_a_baaa^`b__aa
@HWI-EAS443:3:49:73:349#0/1
GGGGGCTATATGAAGCAATCGAGGAGTGCCTGATTAATGA
+HWI-EAS443:3:49:73:349#0/1
a]]aaabbbbbaabaabaaa`aba^`^aa`ababaaaaaa
@HWI-EAS443:3:49:757:424#0/1
GGCATGTGAATTGACTGATTCAAGCTGGATAGAACTTGAT
+HWI-EAS443:3:49:757:424#0/1
aaaabbab]Z]aa^aab__aa_aa]aa]_`aa_a__^_[a
@HWI-EAS443:3:49:781:157#0/1
CTCATGCTTGCGATATGTTCAATCGGGGCAACATCTTCTC
+HWI-EAS443:3:49:781:157#0/1
aabbabbbabaaa`_bb`babbaaab_aY`a_aa^`a_a]
@HWI-EAS443:3:49:835:563#0/1
GAAAAAGTAGACTTTGATGACTGCAAAGATGTTGGAGACC
+HWI-EAS443:3:49:835:563#0/1
a`aa`ab_ba``aabaaab`aaa_aa`_aaa^aZa]aa_¥
@HWI-EAS443:3:49:837:223#0/1
CAAAACTAGGCTTTTCACTATAAGACAAGAAATGGCCAGT
+HWI-EAS443:3:49:837:223#0/1
`abaaababbbbbaabb^bbbabba¥babbbbbbb_aaa_
```

@HWI-EAS443:3:49:84:853#0/1
CAGCTTGCTAGAGATCTGGGCTCTGGCTCATCACTGTCAT
+HWI-EAS443:3:49:84:853#0/1
a_U`b````ba]ba``aa`^ba`¥]`^ZTa`U_`aa]S_]
@HWI-EAS443:3:49:861:57#0/1
GACATTCTTTGGCTGGAAAGAGCCTAACATAGTCAAACCA
+HWI-EAS443:3:49:861:57#0/1
`¥a[bbbbb`_b¥`bbaa`]Za]Y`]a¥aaaa¥``^_F[]
@HWI-EAS443:3:49:884:1619#0/1
GGGGCATGGAAATGAGGCGCTGCCTTCTTCAGTCTCTTCA
+HWI-EAS443:3:49:884:1619#0/1
abaaaaabb_abbaabbabaaaaZab`aa]aa_]a_aaN_
@HWI-EAS443:3:49:895:163#0/1
CTTCAATTGGCTTGTTCTCTTCATGTTCTTTGTCCTTGGG
+HWI-EAS443:3:49:895:163#0/1
aba`abb`bb_bb_bb`_R_bVbaa[b]bbb_b]¥ab¥`a
@HWI-EAS443:3:49:970:1345#0/1
GTTTGTTTTCCGTCTTCCTTCTTTGGTCCTACATTTGCTT
+HWI-EAS443:3:49:970:1345#0/1
aaaab`bbb_[VVYaa``aaUaa_a`X]_`__aa`a`]a`
@HWI-EAS443:3:49:972:1961#0/1
AAAGAGCCTAACATAGTCAAACCACATGAGAAAGGCATAA
+HWI-EAS443:3:49:972:1961#0/1
S^__[YW_aZY[a_SZP¥baa^`]Y]a`^aa_]]aa`a^U
@HWI-EAS443:3:49:976:961#0/1
ACCCGAATGCACTATTGAAGCACCGATTTGAGATAATTGA
+HWI-EAS443:3:49:976:961#0/1
aa¥]aaa^a_a^`¥aaaa¥]^`_]a]a_aa^a^`__a_a^
@HWI-EAS443:3:4:1052:1978#0/1
CAAAGAATTCCTTGGTCATGTCTTTCTCTTTGACAGAAGA
+HWI-EAS443:3:4:1052:1978#0/1
a`bbbaabaV_b`bba]bbaabaab^baab_babbbbbba
@HWI-EAS443:3:4:1082:294#0/1
GTTCCTGTAATCTCAAATTTTTCTTCAATTGTCTCTTCGC
+HWI-EAS443:3:4:1082:294#0/1
a[]PZN[JJX_UPLS]TU_^^^X]PSV][X]VU_VVSPBB
@HWI-EAS443:3:4:1094:144#0/1
CAAGCCATGAGGTAATTGGGATTTATGCCTTTCTCATGTG
+HWI-EAS443:3:4:1094:144#0/1
`aba``aab`aaZ`_a`aa`]_``a_`_Xa]_Z^X¥_`X`
@HWI-EAS443:3:4:1112:1039#0/1
GCTGGGTCCAAAATGAATTCAATAAGGCATGTGAATTGAC
+HWI-EAS443:3:4:1112:1039#0/1
aa__a`__``]`U]`[SPXY]]]YW_^]¥^^X[VXZW_WZ
@HWI-EAS443:3:4:1114:1106#0/1
TAGGAGTCTATGGGATTCCTTTCGTCAGTCCGAAAGAGGC
+HWI-EAS443:3:4:1114:1106#0/1
aaabbb_ababbaU]aaaaaaaJ`]__aZaYa^_]a_`_]
@HWI-EAS443:3:4:1180:652#0/1
ACGGCCTTGCTCAATGCATCCTGTGCAGCCATGGATGACT

+HWI-EAS443:3:4:1180:652#0/1
aaba`¥baYaa`aaa`^ba_Z_a¥`¥`_¥^_`aa^^]¥W¥
@HWI-EAS443:3:4:1240:1836#0/1
GATCTGGGCTCTGGCTCATCACTGTCATACTGTTTAAGGT
+HWI-EAS443:3:4:1240:1836#0/1
a^aa¥b_]Zba[aX[a`baa``aa`aa_aYXaXa__X[aS
@HWI-EAS443:3:4:1246:1650#0/1
GCTTGTTCTCTTCATGTTCTTTGTCCTTGGGATCTTCTCT
+HWI-EAS443:3:4:1246:1650#0/1
a^_^T`aZUSaaXabbV[]aa_a]`aa¥a_^_WO]a_b^^
@HWI-EAS443:3:4:1259:1016#0/1
TAATCAGGCACTCCTCGATTGCTTCATATAGCCCCCCAAG
+HWI-EAS443:3:4:1259:1016#0/1
`]abaabaab```]a]aab`a_aa``aaaaaR[[_Za`[`
@HWI-EAS443:3:4:1265:2027#0/1
GAGCCTTCCTCCACTCCCCTGGGTGACTCTCCGATTGGCC
+HWI-EAS443:3:4:1265:2027#0/1
aaaa_aa_aaa`a`a_Za`aa``[`_¥_^a^`^^¥^`XTT
@HWI-EAS443:3:4:1299:1653#0/1
GAGACTTTGGTCGGCAAGCTTGCGCATAGTTCCTGTAATC
+HWI-EAS443:3:4:1299:1653#0/1
¥XaLY^`b_``^¥^a]^_Y_XY[]``aP[F¥XZ^_NTNYG
@HWI-EAS443:3:4:1331:233#0/1
ATGCTCTTGAGGACTGCGATAGGCCAAGTGTCGAGGCCCA
+HWI-EAS443:3:4:1331:233#0/1
ab^U¥Q__^Zab[JTaQa`¥`aa_Y¥V`¥aX____`UUBB
@HWI-EAS443:3:4:1348:1192#0/1
TAACACAACAGGGGTAGAGAAGCCTAAATTTCTTCCTGAT
+HWI-EAS443:3:4:1348:1192#0/1
a]aXYY]```aa_]L`a^ba`^[]`__`Y^^`__¥P[aS^
@HWI-EAS443:3:4:1423:112#0/1
GGGGCTATATGAAGCAATCGAGGAGTGCCTGATTAATGAT
+HWI-EAS443:3:4:1423:112#0/1
aa_¥Z_Z`Za`]`_W___W_W_`Y^T¥WW¥^S¥_WWY¥WY
@HWI-EAS443:3:4:1569:1077#0/1
GTGGTGTCGTCCTCAAGAATGGTTCAATTTTGGCGTTCAC
+HWI-EAS443:3:4:1569:1077#0/1
]JPQQZR`^N]_YMTIP[[^_TPZLTY]]¥[]WZMVMMLZ
@HWI-EAS443:3:4:1571:1427#0/1
CATCAGGCAATCTGAGGGGGCGTGGTGTCGTCCTCAAGAA
+HWI-EAS443:3:4:1571:1427#0/1
ab`abbb`baaaa`aa_ba[¥_]a_VLaVZ^¥N`U_¥XaW
@HWI-EAS443:3:4:1691:850#0/1
TGGCCATGTTTCCGATTTGTTTTCAAAGAATTCCTTGGTC
+HWI-EAS443:3:4:1691:850#0/1
^^aX__^^Ua`[`a_aaab_[a`_^_`_]W_]RR`X[¥T^
@HWI-EAS443:3:4:1714:630#0/1
GGGGCATGGAAATGAGGCGCTGCCTTCTTCAGTCTCTTCA
+HWI-EAS443:3:4:1714:630#0/1
a`aUKWa`_^VSZTXNS^^MPKTRPUW^WSSSNMPWBBBB

```
@HWI-EAS443:3:4:189:297#0/1
GGGGTTTTCGGCTGAATCGAGAAAATTGCTTCTCATTGTT
+HWI-EAS443:3:4:189:297#0/1
aa`YF[YV[]Y[]XPY¥Z`VYTU]]]]UW[ZVYVV]Z¥TB
@HWI-EAS443:3:4:195:1317#0/1
GTCCGCTTTGGTGGCCATCTCCTCTCCAGTGAATGAAAAG
+HWI-EAS443:3:4:195:1317#0/1
aa_aaaaaYb[N__`Y_]`_a_]^]`a``Ua__X_]WKBB
@HWI-EAS443:3:4:228:749#0/1
GTGAATCAATAATTGTAGAATCTGGTGACCCGAATGCACT
+HWI-EAS443:3:4:228:749#0/1
a¥aaa`a_aa^_`a_]]a`a_[_`_Xa_¥__aX_]_[^]]
@HWI-EAS443:3:4:233:988#0/1
GGAGAAGTTCGGTGGGAGACTTTGGTCGGCAAGCTTGCGC
+HWI-EAS443:3:4:233:988#0/1
ab_baaa^`]`a¥a]aZa^P`_aaa¥]¥`]Y]a^Y_`^]¥
@HWI-EAS443:3:4:243:1565#0/1
GCCATGTTTCCGATTTGTTTTCAAAGAATTCCTTGGTCAT
+HWI-EAS443:3:4:243:1565#0/1
aX^ZaaY__[aa[`_aaP^^[_[`]]^[]]]``^a_SS__
@HWI-EAS443:3:4:272:991#0/1
CGATGAAATGGAAATCCGAATACATGAAACAAACTTCCAA
+HWI-EAS443:3:4:272:991#0/1
`¥Z¥bbb^]abaaa^¥Z_`Y^abaa_aaa`bb^aa`^H`]
@HWI-EAS443:3:4:340:1375#0/1
CCAAGGACAAAGAACATGAAGAGAACAAGCCAATTGAAGT
+HWI-EAS443:3:4:340:1375#0/1
aaabbaabaaabaa`aabaabaaaa`aaaX_`a``a_aaS
@HWI-EAS443:3:4:347:146#0/1
ATATGCGTCTCCACAACTTGAGGGGTTTTCGGCTGAATCG
+HWI-EAS443:3:4:347:146#0/1
`aaab`b`_a___^¥`Xaaa_aaaaY]__X_^X^_^¥WVY
@HWI-EAS443:3:4:424:1507#0/1
CGTCTCCACAACTTGAGGGGTTTTCGGCTGAATCGAGAAA
+HWI-EAS443:3:4:424:1507#0/1
`b`bbaabaabZaaa_aaa¥M^aa`aa_`aa^]_aa_]a`
@HWI-EAS443:3:4:471:883#0/1
AACAAAATAAAATCTGAGAAGACACACATTCACATCTTTT
+HWI-EAS443:3:4:471:883#0/1
ab`aa]`bbbabaaab`aaaaa_a_a_aaaaa¥a_¥a`_`
@HWI-EAS443:3:4:506:334#0/1
GCACCAGAAAAAGTAGACTTTGATGACTGCAAAGATGTTG
+HWI-EAS443:3:4:506:334#0/1
a]WZOX^VZY]Xa^aaYW]^[_[¥aYOY]ZRXY_[V_WBB
@HWI-EAS443:3:4:539:415#0/1
AAACCACATGAGAAAGGCATAAATCCCAATTACCTCATGG
+HWI-EAS443:3:4:539:415#0/1
¥¥¥^b_babab`aYY^_Rabaab]Q^Zaaba]LX¥Waaa¥
@HWI-EAS443:3:4:585:1004#0/1
GGCATGTGAATTGACTGATTCAAGCTGGATAGAACTTGAT
```

```
+HWI-EAS443:3:4:585:1004#0/1
]ba`b^V^O`bVa``aa^aa^[`bOab__aaa]`Uaaa^_
@HWI-EAS443:3:4:588:1128#0/1
CCTCGATTGCTTCATATAGCCCCCCAAGATCGAAGGTTCC
+HWI-EAS443:3:4:588:1128#0/1
aYMX_]^Ha]_a^a^[¥TYZOTU]ZFQ[[BBBBBBBBBBB
@HWI-EAS443:3:4:625:753#0/1
ATTTTCACCGAGTGCCCACTTCAATTGGCTTGTTCTCTTC
+HWI-EAS443:3:4:625:753#0/1
]bbab]bbabaa]ba_abaaa]`aa^b__``M_[HWX]]N
@HWI-EAS443:3:4:685:1318#0/1
CAGATTTTGCCAGTAAGGTCCTGCACACTTTCCCAATAGA
+HWI-EAS443:3:4:685:1318#0/1
aaa]`bbaababb``ab`_¥`aaabba_a_a^]abaaaa`
@HWI-EAS443:3:4:715:151#0/1
CGATTGGCCATGTTTCCGATTTGTTTTCAAAGAATTCCTT
+HWI-EAS443:3:4:715:151#0/1
abaaabaaaaab`aa`¥b]^_`a^aaa^_``a^X_`]X`_
@HWI-EAS443:3:4:744:1500#0/1
ATTGCTTCATATAGCCCCCCAAGATCGAAGGTTCCAGGTT
+HWI-EAS443:3:4:744:1500#0/1
a^¥¥T¥T_^ab`¥XYSX]XP]`P[SGTP[aBBBBBBBBBB
@HWI-EAS443:3:4:770:1744#0/1
GCCTGAACAATGAGAAGCAATTTTCTCGATTCAGCCGAAA
+HWI-EAS443:3:4:770:1744#0/1
aaaaaaa`aa`a_aaaaaa_ba`a^a_a__a^`aR]`__`
@HWI-EAS443:3:4:770:883#0/1
CAGTAGGAGTCTATGGGATTCCTTTCGTCAGTCCGAAAGA
+HWI-EAS443:3:4:770:883#0/1
aabaa_bab_`aabbbaaaa`aaa`aba¥aa¥Z_a`¥a^¥
@HWI-EAS443:3:4:774:950#0/1
GGCCATTTCTTGTCTTATAGTGAAAAGCCTAGTTTTGATT
+HWI-EAS443:3:4:774:950#0/1
ab``aaba`baaa_baaaaa_aaaaaa_`a`b^aaaa_`_
@HWI-EAS443:3:4:822:34#0/1
TGAGAAAGGCATAAATCCCAATTACCTCATGGCTTGGAAG
+HWI-EAS443:3:4:822:34#0/1
ab^a_`bZa`aaY_QVX[[a`QHX]^`]WU_WNX]]BBBB
@HWI-EAS443:3:4:828:1550#0/1
GGGCACTCGGTGAAAATATGGCACCAGAAAAAGTAGACTT
+HWI-EAS443:3:4:828:1550#0/1
aab_b_babbXba`aaaaaaa``^aaa``^``^]__`Xa`
@HWI-EAS443:3:4:875:712#0/1
CATGATTCGGTCTCTTCCTTCAATTATCTCAAATCGGTGC
+HWI-EAS443:3:4:875:712#0/1
a^Y`YZXZ_]P_XT``G`T]^aa`a_^GZR[X]¥HVXY^S
@HWI-EAS443:3:4:964:1483#0/1
CTTCCCTCCGTGTTACTCCAATTTCAATGAACCGGTTCTC
+HWI-EAS443:3:4:964:1483#0/1
`W^a^VZ`YZ]TMY_`aaY^^a]Y^Y¥U^Z_]¥][QR^`^
```

```
@HWI-EAS443:3:50:1018:672#0/1
GGACAACCTGGAACCTGGAACCTTCGATCTTGGGGGGCTA
+HWI-EAS443:3:50:1018:672#0/1
abaaaaaaabaaa``baa^a^^a`_a¥`^aaaa_a``¥a^
@HWI-EAS443:3:50:104:1464#0/1
TTGAAGCACCGATTTGAGATAATTGAAGGAAGAGACCGAA
+HWI-EAS443:3:50:104:1464#0/1
abaa`a¥_V]b`aaabaaab``aaa_a]aa`aaXa]R]``
@HWI-EAS443:3:50:1089:1345#0/1
ATCATTAATCAGGCACTCCTCGATTGCTTCATATAGCCCC
+HWI-EAS443:3:50:1089:1345#0/1
¥_Zbba`a_aa`Y¥_bbaa`¥bb]``¥a^Uabab_Z]UWW
@HWI-EAS443:3:50:1116:1597#0/1
GAATTCCTTGGTCATGTCTTTCTCTTTGACAGAAGACTCG
+HWI-EAS443:3:50:1116:1597#0/1
a`abbaabbab`_aabaaaba]aaaaab_aabaaa__^``
@HWI-EAS443:3:50:116:412#0/1
ATCCTGTGCAGCCATGGATGACTTTCAGCTGATCCCAATG
+HWI-EAS443:3:50:116:412#0/1
ab``aZS^bbb_ab]`Yaaaa`^]Ua`WJ[b]XUSN`ZUT
@HWI-EAS443:3:50:1279:564#0/1
AACCAATGGAACCTCCAAGATCAAGATGAAATGGGGCATG
+HWI-EAS443:3:50:1279:564#0/1
]_babbbbbbb`a_aba_Xbaaaaa`aba_a`a`ab__V_
@HWI-EAS443:3:50:1312:1442#0/1
CACTCCCCTGGGTGACTCTCCGATTGGCCATGTTTCCGAT
+HWI-EAS443:3:50:1312:1442#0/1
abaabbabaab`_aa_b`aaaa_``_`a_``a^`b]`[GX
@HWI-EAS443:3:50:1328:1600#0/1
GGCCTGGACAGTGGTGAACAGTATATGTAACACAACAGGG
+HWI-EAS443:3:50:1328:1600#0/1
aa`ba_`aab_¥ba`_ba]`aZaa`a]Zaa_a^]aU¥`Va
@HWI-EAS443:3:50:1388:565#0/1
ATGCTCTTGAGGACTGCGATAGGCCAAGTGTCGAGGCCCA
+HWI-EAS443:3:50:1388:565#0/1
`babaabaaaaaaU_aab`][aa¥]W_aZa__¥__a_WX_
@HWI-EAS443:3:50:1395:881#0/1
ATTGGTTCTCACATATAGGAACATGGGCCTCGACACTTGG
+HWI-EAS443:3:50:1395:881#0/1
aaaaa_aa_]_`_`_a`a`_`]¥Z_`_Z_^¥`^_¥^¥__]
@HWI-EAS443:3:50:1408:98#0/1
GGGGCATGGAAATGAGGCGCTGCCTTCTTCAGTCTCTTCA
+HWI-EAS443:3:50:1408:98#0/1
a_a]a`bb`aa_bbab_`aaaa__a`V`a`Za_R``a_^¥
@HWI-EAS443:3:50:1488:1094#0/1
TGCATTGAGGGCAAGCTTTCCCAAATGTCAAAAGAAGTGA
+HWI-EAS443:3:50:1488:1094#0/1
aaabS_aV`bbaaab_bbYT`^_]Z^`^]Z__``Zaa^a_
@HWI-EAS443:3:50:148:775#0/1
GCAGGGCAAGAATCAAAACTAGGCTTTTCACTATAAGACA
```

```
+HWI-EAS443:3:50:148:775#0/1
T^bb¥^bbab`abWabaWabWLW`]bXNWa[aSa``Y]Y]
@HWI-EAS443:3:50:1492:1118#0/1
ATCCTGTGCAGCCATGGATGACTTTCAGCTGATCCCAATG
+HWI-EAS443:3:50:1492:1118#0/1
a`U^``Z¥S_a¥]^`a¥]__Z[^a¥V^`[aa``XTTTU__
@HWI-EAS443:3:50:1509:1240#0/1
CTGGGTGACTCTCCGATTGGCCATGTTTCCGATTTGTTTT
+HWI-EAS443:3:50:1509:1240#0/1
abbbbabbbbbbbabbbbbba^aa`ab`Y¥b`abb`¥ab`
@HWI-EAS443:3:50:1559:1339#0/1
ATGAAATGGAAATCCGAATACATGAAACAAACTTCCAAAT
+HWI-EAS443:3:50:1559:1339#0/1
aaaab¥`ab`a`aaZbabaa¥^aaaaa`aaa_aa`_a`_b
@HWI-EAS443:3:50:1584:1559#0/1
TGCCCTGCTCTCTTCGTCAAGGGTGTAGTCCGCTTTGGTG
+HWI-EAS443:3:50:1584:1559#0/1
abbaabbabab`bb]bb^a``aa[a`a¥`Z¥a^abaaY[]
@HWI-EAS443:3:50:159:126#0/1
CAGTCATCAAAGTCTACTTTTTCTGGTGCCATATTTTCAC
+HWI-EAS443:3:50:159:126#0/1
_[aababab^_bJa¥_aZbb`W_`U`[b`U`^[b^b`aaa
@HWI-EAS443:3:50:166:692#0/1
GGGGGCTATATGAAGCAATCGAGGAGTGCCTGATTAATGA
+HWI-EAS443:3:50:166:692#0/1
]b_a`^bbbabbbbabbaaaba^_aa_a[M_aabab]a`a
@HWI-EAS443:3:50:1686:1499#0/1
GATGTTGCCCCGATTGAACATATCGCAAGCATGAGGAGGA
+HWI-EAS443:3:50:1686:1499#0/1
^W_a_a_T¥VSXX[Z]¥aU__[VQVRXRRSZ[WMW^VRVT
@HWI-EAS443:3:50:169:1603#0/1
GGGAGTGTACATAAATACGGCCTTGCTCAATGCATCCTGT
+HWI-EAS443:3:50:169:1603#0/1
`ba`¥U_a`^b_aa]aba````_`a]^]`^Z_[__]^_X]
@HWI-EAS443:3:50:1708:1321#0/1
TGTGAATTGACTGATTCAAGCTGGATAGAACTTGATGAAA
+HWI-EAS443:3:50:1708:1321#0/1
a`_baabaa`_aaaabaa`a_aa]^aa`^a_a`^^`a_^_
@HWI-EAS443:3:50:171:66#0/1
CATTAATCAGGCACTCCTCGATTGCTTCATATAGCCCCCC
+HWI-EAS443:3:50:171:66#0/1
`abb[_`babaa_^aabab`a`aaababbaabbabaab`a
@HWI-EAS443:3:50:1745:918#0/1
CAAGAAATGGCCAGTAGGAGTCTATGGGATTCCTTTCGTC
+HWI-EAS443:3:50:1745:918#0/1
Ubaabbbaba`aabbba_``a_babb`baKa[[bbaUb_[
@HWI-EAS443:3:50:210:577#0/1
GGGGGCTATATGAAGCAATCGAGGAGTGCCTGATTAATGA
+HWI-EAS443:3:50:210:577#0/1
^`bbbabbbbbbbbbabaaaabaaa`a`abaaabaaaaa
```

```
@HWI-EAS443:3:50:235:1541#0/1
CTGAACAATGAGAAGCAATTTTCTCGATTCAGCCGAAAAC
+HWI-EAS443:3:50:235:1541#0/1
abbbbaabababb]aaa]]bbb`aaaaaa`aaa_aa^`a^
@HWI-EAS443:3:50:356:394#0/1
TTCTCCTATTTCATCAAGTTCTATCCAGCTTGAATCAGTC
+HWI-EAS443:3:50:356:394#0/1
ab_baababbbbbaabbaabaaba`Y`a]bbba_a^`a`a
@HWI-EAS443:3:50:379:904#0/1
GCTTCCAAGCCATGAGGTAATTGGGATTTATGCCTTTCTC
+HWI-EAS443:3:50:379:904#0/1
K_]bab`bb[bbbbabbIaabbb`^`ab_a^bbaaaYH]a
@HWI-EAS443:3:50:394:1355#0/1
GCCGAGTCTTCTGTCAAAGAGAAAGACATGACCAAGGAAT
+HWI-EAS443:3:50:394:1355#0/1
a^^aX`U[aa_aXO¥a`XW^PTJTV_U`_^_¥Y_aa`^ZP
@HWI-EAS443:3:50:443:1040#0/1
TGTGAATTGACTGATTCAAGCTGGATAGAACTTGATGAAA
+HWI-EAS443:3:50:443:1040#0/1
abX¥a_abba]`¥`ab_a]`Raab`T¥a_[Taaa^_a]¥_
@HWI-EAS443:3:50:495:694#0/1
GCTGTAAAATAGTTCCTCCTCATGCTTGCGATATGTTCAA
+HWI-EAS443:3:50:495:694#0/1
aaababb``aaa_a_aaa_a`a`a^_aa_`_aaaa__Z_`
@HWI-EAS443:3:50:529:772#0/1
AGCTTGCTAGAGATCTGGGCTCTGGCTCATCACTGTCATA
+HWI-EAS443:3:50:529:772#0/1
`aaabbbabbb_baaabbbbbbbbabaabbabaab^abab
@HWI-EAS443:3:50:628:1472#0/1
ATTGAAGCACCGATTTGAGATAATTGAAGGAAGAGACCGA
+HWI-EAS443:3:50:628:1472#0/1
`bbb`bbba[aba^bbbaa`baabaaaaba`abaaa^[`a
@HWI-EAS443:3:50:658:1868#0/1
CACTATATGATGCAATCAAATGCATGAAGACATTCTTTGG
+HWI-EAS443:3:50:658:1868#0/1
a]H¥bb_b___SU¥`a`^abbaa``Waa`]G]aPV^]TY_
@HWI-EAS443:3:50:670:1267#0/1
GGGGGGCTATATGAAGCAATCGAGGAGTGCCTGATTAATG
+HWI-EAS443:3:50:670:1267#0/1
a``aaa`bab_^`a__aa`a¥`_`a__]_]^_a__a^_``
@HWI-EAS443:3:50:692:1655#0/1
ATCGACGAACGGGGTGAATCAATAATTGTAGAATCTGGTG
+HWI-EAS443:3:50:692:1655#0/1
a¥`baabGWJWaaa]aa_`¥WP[^]^XPLWa^_]O_ZVDT
@HWI-EAS443:3:50:727:1035#0/1
CTCCACAACTTGAGGGGTTTTCGGCTGAATCGAGAAAATT
+HWI-EAS443:3:50:727:1035#0/1
Z¥[`a_[^__abbbbba^¥`¥^^¥`_``¥a¥a_aaa_ab`
@HWI-EAS443:3:50:734:1277#0/1
CTCGATTGCTTCATATAGCCCCCCAAGATCGAAGGTTCCA
```

```
+HWI-EAS443:3:50:734:1277#0/1
a_^b`b_bab`_^a_bba_aaab]a_baaT`aaba[b_[Z
@HWI-EAS443:3:50:823:730#0/1
TTGAGATAATTGAAGGAAGAGACCGAATCATGGCCTGGAC
+HWI-EAS443:3:50:823:730#0/1
aaa`b_a^aaab__aa_a^```__a__a_``a][X`a_]^
@HWI-EAS443:3:50:841:1152#0/1
CGATGAAATGGAAATCCGAATACATGAAACAAACTTCCAA
+HWI-EAS443:3:50:841:1152#0/1
ab_`aa`_a`aa_^_`a`aba^aaa```__a``^___V_`
@HWI-EAS443:3:50:84:1617#0/1
CCAGATTCTACAATTATTGATTCACCCCGTTCGTCGATGA
+HWI-EAS443:3:50:84:1617#0/1
]X`^T_aaa]_a__aaaaaaa][`RHY¥¥a^XSXXVT^`_
@HWI-EAS443:3:50:901:779#0/1
AACATCTTCTCCTATTTCATCAAGTTCTATCCAGCTTGAA
+HWI-EAS443:3:50:901:779#0/1
]KYVabba¥ba`[U`ba^a`U`ab]a_a_`a_¥TYabbaa
@HWI-EAS443:3:50:907:1364#0/1
CATGGAAATGAGGCGCTGCCTTCTTCAGTCTCTTCAGCAG
+HWI-EAS443:3:50:907:1364#0/1
ab`^Xabbbabbaabbba¥^b_[ababaa`bbbaaaaaab
@HWI-EAS443:3:51:1046:1764#0/1
CACCACTGTCCAGGCCATGATTCGGTCTCTTCCTTCAATT
+HWI-EAS443:3:51:1046:1764#0/1
ab_bbabaa_aab_]`a_a`a¥^_`]^^¥a_U]`aY^___
@HWI-EAS443:3:51:1192:1218#0/1
CAACATCTTTGCAGTCATCAAAGTCTACTTTTTCTGGTGC
+HWI-EAS443:3:51:1192:1218#0/1
H]¥a`ZZ`a``^abbbbbaaab`Z¥_¥Z^¥a`X`a¥¥X`T
@HWI-EAS443:3:51:1285:611#0/1
GCGAAGAGACAATTGAAGAAAAATTTGAGATTACAGGAAC
+HWI-EAS443:3:51:1285:611#0/1
aaa[_baa^_aabaZ]aa_`_`_`aaa^`_aaa]`a[^_[
@HWI-EAS443:3:51:1290:2023#0/1
CGATTGGCCATGTTTCCGATTTGTTTTCAAAGAATTCCTT
+HWI-EAS443:3:51:1290:2023#0/1
abbb_baaaa`a[ab[bb_`aaaaaaaa`aaa`aa`XWaa
@HWI-EAS443:3:51:1298:1393#0/1
AAGAATTCCTTGGTCATGTCTTTCTCTTTGACAGAAGACT
+HWI-EAS443:3:51:1298:1393#0/1
`abbR`aa]]`baX`_a]`aab]Z`]L^`[U[`^a_SY[`
@HWI-EAS443:3:51:12:805#0/1
AAGAATTCCTTGGTCATGTCTTTCTCTTTGACAGAAGACT
+HWI-EAS443:3:51:12:805#0/1
a`aXT__]_]a`aS_]aaY_a^¥^`_aa`^RSZa]^a^^]
@HWI-EAS443:3:51:1320:193#0/1
CATTAATCAGGCACTCCTCGATTGCTTCATATAGCCCCCC
+HWI-EAS443:3:51:1320:193#0/1
abbaVa_]_aaaa]_^Ya_b^]¥_`abb```¥`_aaa`X`
```

```
@HWI-EAS443:3:51:1359:1823#0/1
ATTGGTTCTCACATATAGGAACATGGGCCTCGACACTTGG
+HWI-EAS443:3:51:1359:1823#0/1
aab`a_aa`__[[a]b`a_[_Z`¥^^_Z^[¥_^]`Z___Q
@HWI-EAS443:3:51:1515:806#0/1
GATGAAATGGAAATCCGAATACATGAAACAAACTTCCAAA
+HWI-EAS443:3:51:1515:806#0/1
ababbbabbaababa¥baabba`abaabaaa``baaYa``
@HWI-EAS443:3:51:153:1159#0/1
ATCAGGCACTCCTCGATTGCTTCATATAGCCCCCCAAGAT
+HWI-EAS443:3:51:153:1159#0/1
[_```^a]baaaaa_]_¥aa`aa`aaaa]]X¥a]`V^`_^
@HWI-EAS443:3:51:1574:738#0/1
AGAAGTTCGGTGGGAGACTTTGGTCGGCAAGCTTGCGCAT
+HWI-EAS443:3:51:1574:738#0/1
^`a_¥V[]V]]¥aaZ[_^]`¥P¥VZX¥`]]`¥`¥^Y_U¥]
@HWI-EAS443:3:51:1673:1760#0/1
GGCATGGAAATGAGGCGCTGCCTTCTTCAGTCTCTTCAGC
+HWI-EAS443:3:51:1673:1760#0/1
`_[]_b_`^]`[][_¥`VX_Y¥¥¥N¥^_[^_W_W`_QVVG
@HWI-EAS443:3:51:1705:1410#0/1
TGCGTCTCCACAACTTGAGGGGTTTTCGGCTGAATCGAGA
+HWI-EAS443:3:51:1705:1410#0/1
_Z`aW`aab^`_a]abaY^aba[_a`Ta_R_a]_[V`S`^
@HWI-EAS443:3:51:1711:856#0/1
GAATTCCTTGGTCATGTCTTTCTCTTTGACAGAAGACTCG
+HWI-EAS443:3:51:1711:856#0/1
^abab_bbbYb`aabbaabbbabbbabb`baa`abba]aI
@HWI-EAS443:3:51:172:572#0/1
GGCTTCTCTACCCCTGTTGTGTTACATATACTGTTCACCA
+HWI-EAS443:3:51:172:572#0/1
aaaa_]]aaa_````a``[_`^_ZZ___^^U^a^[U^W[¥
@HWI-EAS443:3:51:22:395#0/1
CCTCCTCATGCTTGCGATATGTTCAATCGGGGCAACATCT
+HWI-EAS443:3:51:22:395#0/1
abaabbbbbb^bbb^bbaa```aaaaaaaa``]`a^^`Z`
@HWI-EAS443:3:51:309:1753#0/1
GTCCCACTGCAGGGCTACTGAATACATAATGAAGGGAGTG
+HWI-EAS443:3:51:309:1753#0/1
_S_Y¥]a]_LZRQ[]]_XN`[T¥^TZPTTWa`VZ^`QQJW
@HWI-EAS443:3:51:324:1002#0/1
GGGGTTTTCGGCTGAATCGAGAAAATTGCTTCTCATTGTT
+HWI-EAS443:3:51:324:1002#0/1
^¥`a_aabbbbabbabbabUbaa]abbbaaba`_aaba`a
@HWI-EAS443:3:51:476:725#0/1
ACATGGCCAATCGGAGAGTCACCCAGGGGAGTGGAGGAAG
+HWI-EAS443:3:51:476:725#0/1
a`aaab]V`_a_a]VXY`¥^^`]^^aaa]T^Sa`^aaU¥¥
@HWI-EAS443:3:51:494:1748#0/1
CGTCTCCACAACTTGAGGGGTTTTCGGCTGAATCGAGAAA
```

```
+HWI-EAS443:3:51:494:1748#0/1
a]ababa^YUY_aba_a¥baU^]a^V^[[¥^`]]a__]YZ
@HWI-EAS443:3:51:501:347#0/1
GGGCACTCGGTGAAAATATGGCACCAGAAAAAGTAGACTT
+HWI-EAS443:3:51:501:347#0/1
]`_`a]_`¥_X^O^`a^]]]Z]WX^_[X`a`_]O^a`Z[`
@HWI-EAS443:3:51:619:822#0/1
CATGTTTCCGATTTGTTTTCAAAGAATTCCTTGGTCATGT
+HWI-EAS443:3:51:619:822#0/1
_ba`bbbbbaabbbb`bbbbbaaa_abbaabb`aaab`ba
@HWI-EAS443:3:51:678:370#0/1
AGCCCCCCAAGATCGAAGGTTCCAGGTTCCAGGTTGTCCC
+HWI-EAS443:3:51:678:370#0/1
^_]SPY_a]R¥PWPa_^a¥LOQLG__G_`STU^F[XG[¥¥
@HWI-EAS443:3:51:697:161#0/1
ATCATTAATCAGGCACTCCTCGATTGCTTCATATAGCCCC
+HWI-EAS443:3:51:697:161#0/1
`aa^bbbbba_ba]b`b__b]b`a_a^bbaaaa`aa`TUa
@HWI-EAS443:3:51:705:1002#0/1
GTAAGTATGGAGTTCTCACTCACTGACCCGAGACTGGAGC
+HWI-EAS443:3:51:705:1002#0/1
^^bba`a`baa_ab]aabab[__aaaaa`aaaa`ba`_a`
@HWI-EAS443:3:51:735:1954#0/1
ATGTGGTGAACTTTGTAAGTATGGAGTTCTCACTCACTGA
+HWI-EAS443:3:51:735:1954#0/1
^``Ya````a_aaa``bba]aaaaaa^___^a_`^_]__a
@HWI-EAS443:3:51:810:1855#0/1
GGGGGCTATATGAAGCAATCGAGGAGTGCCTGATTAATGA
+HWI-EAS443:3:51:810:1855#0/1
`a_QN_X¥^aa`a]YXa__¥WZa_Y¥V_aY``¥^X`¥NZ^
@HWI-EAS443:3:51:821:456#0/1
GGGGTTTTCGGCTGAATCGAGAAAATTGCTTCTCATTGTT
+HWI-EAS443:3:51:821:456#0/1
a`[a_`aaa^_aaaaab`a_a__```aa``__a^^aaaV`
@HWI-EAS443:3:51:85:1080#0/1
AAGAGCCTAACATAGTCAAACCACATGAGAAAGGCATAAA
+HWI-EAS443:3:51:85:1080#0/1
aaaVNWaS[^GY_T_N`a`]___YRZZa]LJV``V_[`BB
@HWI-EAS443:3:51:85:507#0/1
GAAGCCTAAATTTCTTCCTGATTTGTATGATTACAAAGAG
+HWI-EAS443:3:51:85:507#0/1
aaWXGU`^PXa^¥_aa`a`_OZ]XX^[aa¥aY__[Y__SS
@HWI-EAS443:3:51:88:1684#0/1
GGGGGCTATATGAAGCAATCGAGGAGTGCCTGATTAATGA
+HWI-EAS443:3:51:88:1684#0/1
aaaaabaaaabaaaa`baaa]_a`_`]`_^aa_aaaaa__
@HWI-EAS443:3:51:924:1767#0/1
GAGACTTTGGTCGGCAAGCTTGCGCATAGTTCCTGTAATC
+HWI-EAS443:3:51:924:1767#0/1
`baaZ^^a_]FVSZZ¥]`aa]VM[KUaYaTXGY]]VWXZM
```

@HWI-EAS443:3:51:966:1475#0/1
GGCTTGGAAGCAGGTGCTAGCAGAGCTACAGGACATTGAA
+HWI-EAS443:3:51:966:1475#0/1
aa¥`_aa`abZ_]_Q_a```Y_`X^¥VaZ_`_]¥_[aYV]
@HWI-EAS443:3:52:1106:1445#0/1
ATTCATTTTGGACCCAGCTTGCTAGAGATCTGGGCTCTGG
+HWI-EAS443:3:52:1106:1445#0/1
_ba_aabbaa^aa¥W]_a^`a`aaa_¥YZUa`^]_`Saaa
@HWI-EAS443:3:52:1141:2035#0/1
CTCGATTGCTTCATATAGCCCCCCAAGATCGAAGGTTCCA
+HWI-EAS443:3:52:1141:2035#0/1
aaXR^a_ab``¥_b_a`a`U`X`_]Ya`^Z¥`]P]P[GU¥
@HWI-EAS443:3:52:1204:647#0/1
GCGAAGAGACAATTGAAGAAAAATTTGAGATTACAGGAAC
+HWI-EAS443:3:52:1204:647#0/1
`Z`a`a_a]a`Z]]¥RWa]_^¥_]_a_¥T¥^__¥_`_W]U
@HWI-EAS443:3:52:1227:95#0/1
CATGGAAATGAGGCGCTGCCTTCTTCAGTCTCTTCAGCAG
+HWI-EAS443:3:52:1227:95#0/1
a]]``¥^abaaaa_bX¥b[^``_aa[__¥¥`Qa^NUXJQa
@HWI-EAS443:3:52:1270:1188#0/1
CACATGCCTTATTGAATTCATTTTGGACCCAGCTTGCTAG
+HWI-EAS443:3:52:1270:1188#0/1
[aaab[bab`Z`bb`aaa_abba`a^a_ZZa`_bb¥_aaa
@HWI-EAS443:3:52:1276:1309#0/1
ACCAGATTCTACAATTATTGATTCACCCCGTTCGTCGATG
+HWI-EAS443:3:52:1276:1309#0/1
a^aba_aaaa`_aaabab aaa_a_`W][^a^_Ya_`a___
@HWI-EAS443:3:52:1306:368#0/1
GGAGTCTATGGGATTCCTTTCGTCAGTCCGAAAGAGGCGA
+HWI-EAS443:3:52:1306:368#0/1
]]_`S]``bbbaa``[Waaa_b_¥_`¥]T_[_``]aaYaX
@HWI-EAS443:3:52:1318:30#0/1
TCATCGACGAACGGGGTGAATCAATAATTGTAGAATCTGG
+HWI-EAS443:3:52:1318:30#0/1
aaab]aa_a_`]a__`Xa`_`^_^a^^a_¥¥_a^_^]]__
@HWI-EAS443:3:52:1335:1211#0/1
TACAGGAACTATGCGCAAGCTTGCCGACCAAAGTCTCCCA
+HWI-EAS443:3:52:1335:1211#0/1
]ba]¥IS_]^`ZX]XY_STW`R`U]`¥YH]a[WRIX^UZU
@HWI-EAS443:3:52:1401:72#0/1
GGGGCATGGAAATGAGGCGCTGCCTTCTTCAGTCTCTTCA
+HWI-EAS443:3:52:1401:72#0/1
_bbbabbbabbbbbbaababb__bb^bb``ab]b_ba]a
@HWI-EAS443:3:52:1431:646#0/1
ACCCCTGTTGTGTTACATATACTGTTCACCACTGTCCAGG
+HWI-EAS443:3:52:1431:646#0/1
a`a^_`a_a`___a`_`aa`_]_`^_]_ZY^]_UZ]V]Y]
@HWI-EAS443:3:52:1458:1540#0/1
GAGGAACTATTTTACAGCAGAAGTGTCCCACTGCAGGGCT

```
+HWI-EAS443:3:52:1458:1540#0/1
`aaa_a_baaabbaZaa^aa``]]`_Y]Xa^``]_aa_U`
@HWI-EAS443:3:52:1503:835#0/1
CTGGTGACCCGAATGCACTATTGAAGCACCGATTTGAGAT
+HWI-EAS443:3:52:1503:835#0/1
abba[abaa_aab_Yaaaaaab_aa`aa]^]^baaaa__a
@HWI-EAS443:3:52:1526:1317#0/1
GGCGTTCACTTCTTTTGACATTTGGGAAAGCTTGCCCTCA
+HWI-EAS443:3:52:1526:1317#0/1
aaab__ba^abXbbbb_a[aaa_Ua¥__^`Vaa]_¥¥`^_
@HWI-EAS443:3:52:1624:233#0/1
CCTTGACGAAGAGAGCAGGGCAAGAATCAAAACTAGGCTT
+HWI-EAS443:3:52:1624:233#0/1
]I`ba_`baa]_`aa_^bX`X_]R]_a^]a_`Xa`a]X]]
@HWI-EAS443:3:52:1658:1184#0/1
GGAGTGTACATAAATACGGCCTTGCTCAATGCATCCTGTG
+HWI-EAS443:3:52:1658:1184#0/1
abab[a_bbaa`aaaaZb`^]aa`_a¥`^`_Ya``[_R[¥
@HWI-EAS443:3:52:1690:996#0/1
CTCCACTCCCCTGGGTGACTCTCCGATTGGCCATGTTTCC
+HWI-EAS443:3:52:1690:996#0/1
__bbabbb`__`aaaabbaa]a^[_`aaZ¥__`^Z]b`¥]
@HWI-EAS443:3:52:1760:446#0/1
TCCGCTTTGGTGGCCATCTCCTCTCCAGTGAATGAAAAGA
+HWI-EAS443:3:52:1760:446#0/1
L_abab_a`]M¥bb^^J_RTab_TO^WH¥^¥a`ZH[a`I^
@HWI-EAS443:3:52:1783:439#0/1
CGGTGGGAGACTTTGGTCGGCAAGCTTGCGCATAGTTCCT
+HWI-EAS443:3:52:1783:439#0/1
`b^U^`aY``]_`a^RYXEP¥PXTXP][TTZZVLSS^WTW
@HWI-EAS443:3:52:181:1885#0/1
CATTCGGGTCACCAGATTCTACAATTATTGATTCACCCCG
+HWI-EAS443:3:52:181:1885#0/1
abbbb_bb```_abb_bbbbaababaaabbaaaa`a_`]a
@HWI-EAS443:3:52:218:1700#0/1
GGCCATGTTTCCGATTTGTTTTCAAAGAATTCCTTGGTCA
+HWI-EAS443:3:52:218:1700#0/1
aabbabb`aababaaaaba`aa_aba``aaaaaabaa_aa
@HWI-EAS443:3:52:251:185#0/1
GGGGGCTATATGAAGCAATCGAGGAGTGCCTGATTAATGA
+HWI-EAS443:3:52:251:185#0/1
a^]``]`_`_a`_^¥[]^]R]¥`^W^WYRW¥_Y]YUTQWW
@HWI-EAS443:3:52:252:591#0/1
GGGGCAACATCTTCTCCTATTTCATCAAGTTCTATCCAGC
+HWI-EAS443:3:52:252:591#0/1
`¥aa`_aa^a_a`b`_`a_baa``aa_[__```a`a__aa
@HWI-EAS443:3:52:295:1658#0/1
CACCAGAAAAAGTAGACTTTGATGACTGCAAAGATGTTGG
+HWI-EAS443:3:52:295:1658#0/1
aabbbb`bbbbb`bb`aaaba_H]aabb_bbaabbb_aba
```

```
@HWI-EAS443:3:52:349:1757#0/1
CATGGAAATGAGGCGCTGCCTTCTTCAGTCTCTTCAGCAG
+HWI-EAS443:3:52:349:1757#0/1
abbababbabaaaaabbbaaabaaaaa__aaa`a_aa__a
@HWI-EAS443:3:52:354:163#0/1
AGTGTACATAAATACGGCCTTGCTCAATGCATCCTGTGCA
+HWI-EAS443:3:52:354:163#0/1
_`^aT¥baaZ^_b`_`a_Z_aa^``]aaa`]`¥]_b``[^
@HWI-EAS443:3:52:354:2031#0/1
ATGGCTTGGAAGCAGGTGCTAGCAGAGCTACAGGACATTG
+HWI-EAS443:3:52:354:2031#0/1
a[^a^a`Y`[¥[PZY_OLLQTVZZVHYNQVSVNSRVZVRX
@HWI-EAS443:3:52:365:1040#0/1
GGGGCTATATGAAGCAATCGAGGAGTGCCTGATTAATGAT
+HWI-EAS443:3:52:365:1040#0/1
ab`aYa]¥GXY]¥ZXXZQPYZ^`S`W`^^`[R[_¥UZJV[
@HWI-EAS443:3:52:374:1144#0/1
GGGGGCTATATGAAGCAATCGAGGAGTGCCTGATTAATGA
+HWI-EAS443:3:52:374:1144#0/1
aaaaa]a`a_aaaa_ZRS[YZ[_¥W[TL[W[`Z^ZZZX^]
@HWI-EAS443:3:52:377:1287#0/1
GAGGGGTTTTCGGCTGAATCGAGAAAATTGCTTCTCATTG
+HWI-EAS443:3:52:377:1287#0/1
_[``YaO```aaZLY`ZL[_a[aUZV^_Z¥]X¥¥_^[__^
@HWI-EAS443:3:52:403:1369#0/1
CAAGAAATGGCCAGTAGGAGTCTATGGGATTCCTTTCGTC
+HWI-EAS443:3:52:403:1369#0/1
aa`_aababba¥]a]aabVX]]a``W¥`_a]QX_aaVGXX
@HWI-EAS443:3:52:432:1870#0/1
ACACCCTTGACGAAGAGAGCAGGGCAAGAATCAAAACTAG
+HWI-EAS443:3:52:432:1870#0/1
a`^[¥¥a_`__^_`[]`^__^XXY]]_]]]^W_WX_¥W_Z
@HWI-EAS443:3:52:441:872#0/1
TTTGTTTTCAAAGAATTCCTTGGTCATGTCTTTCTCTTTG
+HWI-EAS443:3:52:441:872#0/1
a^¥baaaa_`]]a__aaaZ^a_`SR`aa_Za`[[a_a`[P
@HWI-EAS443:3:52:456:1658#0/1
GAGATAATTGAAGGAAGAGACCGAATCATGGCCTGGACAG
+HWI-EAS443:3:52:456:1658#0/1
S¥^^]^bab¥bbba^bbbba`K[a^bbbaba^[[][¥T^Y
@HWI-EAS443:3:52:485:1347#0/1
GGGTTTTCGGCTGAATCGAGAAAATTGCTTCTCATTGTTC
+HWI-EAS443:3:52:485:1347#0/1
ab`Ibaa_b][TaaXZYT`]Y`RUYPZa`bT`aV_]aR[U
@HWI-EAS443:3:52:513:2002#0/1
CCCTGCTCTCTTCGTCAAGGGTGTAGTCCGCTTTGGTGGC
+HWI-EAS443:3:52:513:2002#0/1
abaa¥Y]bba]a[^`aa]aaZT_[^`KaT_]Y`[[^PDR]
@HWI-EAS443:3:52:515:820#0/1
ATCCTGTGCAGCCATGGATGACTTTCAGCTGATCCCAATG
```

```
+HWI-EAS443:3:52:515:820#0/1
X]_`ab`bYaaa]^a^]`aa[`aaZ`Z¥__]]W¥][]Y^`
@HWI-EAS443:3:52:553:1792#0/1
AGGAGATGGCCACCAAAGCGGACTACACCCTTGACGAAGA
+HWI-EAS443:3:52:553:1792#0/1
a]^_`aV_`_aa^`__`_UKVaVU¥U_^YXRZ^¥W]NZU[
@HWI-EAS443:3:52:576:830#0/1
GTGTGGCTCCAGTCTCGGGTCAGTGAGTGAGAACTCCATA
+HWI-EAS443:3:52:576:830#0/1
`]aZZ^XaZ^_YY[WU]]XVMDPS^X[DZY]P¥QNPBBBB
@HWI-EAS443:3:52:581:822#0/1
ACGGCCTTGCTCAATGCATCCTGTGCAGCCATGGATGACT
+HWI-EAS443:3:52:581:822#0/1
aYb`_a_aS_`_`^^_^a_¥U`_Y`_¥a¥]^_`^^_`YTW
@HWI-EAS443:3:52:665:369#0/1
GGCATGGAAATGAGGCGCTGCCTTCTTCAGTCTCTTCAGC
+HWI-EAS443:3:52:665:369#0/1
]X__L_`U^``ba]`Qa_PZ]^]ZZ¥`X]a]]_]a_][[[
@HWI-EAS443:3:52:687:632#0/1
AAAATTGAACCATTCTTGAGGACGACACCACGCCCCCTCA
+HWI-EAS443:3:52:687:632#0/1
a¥S¥^`a_aaa`]b_``a]`_]]a_^a`X^^U¥`T_[[LY
@HWI-EAS443:3:52:697:1579#0/1
AAGCAATTTTCTCGATTCAGCCGAAAACCCCTCAAGTTGT
+HWI-EAS443:3:52:697:1579#0/1
a_`aa_aaaaaa`a_a__`__``]``a`Z]^`_^^`Z`^^
@HWI-EAS443:3:52:762:109#0/1
GGGGCTATATGAAGCAATCGAGGAGTGCCTGATTAATGAT
+HWI-EAS443:3:52:762:109#0/1
a`aa]_]a[Q_U_a^_]]T]N¥YPYTWWU]^WWVY]VZTX
@HWI-EAS443:3:52:816:1749#0/1
TGAACCCATACAGGTTTGTTTTCCGTCTTCCTTCTTTGGT
+HWI-EAS443:3:52:816:1749#0/1
aba_a``aZaaS_aG`_b__aa^][[[¥_`_^TVa^]¥aW
@HWI-EAS443:3:52:84:238#0/1
GGGGCATGGAAATGAGGCGCTGCCTTCTTCAGTCTCTTCA
+HWI-EAS443:3:52:84:238#0/1
aaa```Xab_a`_a_a_``Z_a¥_Z^U^_Z[_U[[[[ZWS
@HWI-EAS443:3:52:883:375#0/1
CCCCTGTTGTGTTACATATACTGTTCACCACTGTCCAGGC
+HWI-EAS443:3:52:883:375#0/1
abbb`aabb_baaaa`aabbabb¥a[aa_`aaa`]__a_Z
@HWI-EAS443:3:52:907:338#0/1
GCCCACTTCAATTGGCTTGTTCTCTTCATGTTCTTTGTCC
+HWI-EAS443:3:52:907:338#0/1
`aba`_ba`aaaaaa__a_XYSTDW`^¥][Z`^a_`XWVR
@HWI-EAS443:3:52:942:710#0/1
GGGCACTCGGTGAAAATATGGCACCAGAAAAAGTAGACTT
+HWI-EAS443:3:52:942:710#0/1
abaab`baababaaaabababaa_[aba_aa`b]aaa]aa
```

```
@HWI-EAS443:3:53:1040:955#0/1
CAGTCATCAAAGTCTACTTTTTCTGGTGCCATATTTTCAC
+HWI-EAS443:3:53:1040:955#0/1
`bbaabbab`aaV`bbabbbbbaba`_`a`baabbba]a`
@HWI-EAS443:3:53:1083:1235#0/1
CGTTCGTCGATGAAATGGAAATCCGAATACATGAAACAAA
+HWI-EAS443:3:53:1083:1235#0/1
abababaab`baaaababaaaaa_aaaaaaaaaaa`]`a`
@HWI-EAS443:3:53:1102:478#0/1
CGGCTGAATCGAGAAAATTGCTTCTCATTGTTCAGGCACT
+HWI-EAS443:3:53:1102:478#0/1
abb_bbbababaaaaaabaa_ab_b``bbaa]]aa`^_`a
@HWI-EAS443:3:53:1117:446#0/1
CCCGAATGCACTATTGAAGCACCGATTTGAGATAATTGAA
+HWI-EAS443:3:53:1117:446#0/1
[b`¥`bbbabbbbbbbabaabaaabbbbbbbabbabbbba
@HWI-EAS443:3:53:1190:1293#0/1
GGGGGCTATATGAAGCAATCGAGGAGTGCCTGATTAATGA
+HWI-EAS443:3:53:1190:1293#0/1
a_a[K^a`_`baVaPX`_^UbXa^¥_[[_U^^RaYT`^]V
@HWI-EAS443:3:53:1233:209#0/1
ATTTCTTCCTGATTTGTATGATTACAAAGAGAACCGGTTC
+HWI-EAS443:3:53:1233:209#0/1
_bbb`NaYP[baa_`b`b¥Z¥^b_ab^`V_aaa[JbVSZG
@HWI-EAS443:3:53:1285:920#0/1
ATCCGAAAATCGAAACTAACAAGTTTGCTGCAATATGCAC
+HWI-EAS443:3:53:1285:920#0/1
]¥W^`_abbaZa^a¥Wbb^H]a_Xaa¥]^a^abbaa_Y__
@HWI-EAS443:3:53:1348:521#0/1
CACCAGAAAAAGTAGACTTTGATGACTGCAAAGATGTTGG
+HWI-EAS443:3:53:1348:521#0/1
ZZa^``b^````a_R[bbbb`ab_`^¥^`baabbbbab`V
@HWI-EAS443:3:53:1351:1994#0/1
ATTGGTTCTCACATATAGGAACATGGGCCTCGACACTTGG
+HWI-EAS443:3:53:1351:1994#0/1
aba[_Z]_UQVZa__a`_aWaG`¥_¥^YUZQWZNQSS¥^R
@HWI-EAS443:3:53:141:600#0/1
AGAGACCGAATCATGGCCTGGACAGTGGTGAACAGTATAT
+HWI-EAS443:3:53:141:600#0/1
aa]a]aaaaa`ba]aa`¥aS```aaUa_Ya``SQYUX[_¥
@HWI-EAS443:3:53:1426:216#0/1
AGAGCCTAACATAGTCAAACCACATGAGAAAGGCATAAAT
+HWI-EAS443:3:53:1426:216#0/1
]`W]`aaa_^ab[aaa__^GY¥`a`bU]aa`X```a_`aO
@HWI-EAS443:3:53:1443:777#0/1
ACCGATTTGAGATAATTGAAGGAAGAGACCGAATCATGGC
+HWI-EAS443:3:53:1443:777#0/1
aSSbabbbaab`aa_a``[_aa`aaaa`_Y`W_a^aa]VX
@HWI-EAS443:3:53:1538:2002#0/1
GAAATGGGGCATGGAAATGAGGCGCTGCCTTCTTCAGTCT
```

```
+HWI-EAS443:3:53:1538:2002#0/1
a_ZPRYV^Y[a_^LLWTTbX¥`^`HX¥FR_XP[DO[RPGR
@HWI-EAS443:3:53:154:1338#0/1
GTTCTTTGTCCTTGGGATCTTCTCTTCATTTTCAATGTCC
+HWI-EAS443:3:53:154:1338#0/1
a_]a_`aa]_``]_R_U]^__[_]__[^`__¥`YY¥_Z[Q
@HWI-EAS443:3:53:158:1367#0/1
AATTTAGGCTTCTCTACCCCTGTTGTGTTACATATACTGT
+HWI-EAS443:3:53:158:1367#0/1
aabbbbbb_bb`a¥`babb[_¥_ab]aaaa_^a`aa``a]
@HWI-EAS443:3:53:166:475#0/1
GCTTGTTCTCTTCATGTTCTTTGTCCTTGGGATCTTCTCT
+HWI-EAS443:3:53:166:475#0/1
a]Yaa`aVa[bbaa_]Y___[aaYUH]_b]^Va_aa___a
@HWI-EAS443:3:53:1739:16#0/1
ACCGGTTCTCTTTGTAATCATACAAATCAGGAAGAAATTT
+HWI-EAS443:3:53:1739:16#0/1
a`VS_aaab`ba`¥Y]``X[a__aaaaU^`YX`V_a_``^
@HWI-EAS443:3:53:1748:1145#0/1
GGGCATGGAAATGAGGCGCTGCCTTCTTCAGTCTCTTCAG
+HWI-EAS443:3:53:1748:1145#0/1
_^TV[Z_b`baa__]_¥RZ`a^ab__]b^^`^]aab`]`¥
@HWI-EAS443:3:53:1751:1319#0/1
GCAGGTGCTAGCAGAGCTACAGGACATTGAAAATGAAGAG
+HWI-EAS443:3:53:1751:1319#0/1
aa`bb_aaa`a[abU^¥aaVaaa_¥aaaa``_aaa`_a__
@HWI-EAS443:3:53:1770:319#0/1
AGGGAGTGTACATAAATACGGCCTTGCTCAATGCATCCTG
+HWI-EAS443:3:53:1770:319#0/1
``W^aaNaabab`aaabaaVa¥_¥`]^[S_b__`a`X`VF
@HWI-EAS443:3:53:202:224#0/1
AGAATTCCTTGGTCATGTCTTTCTCTTTGACAGAAGACTC
+HWI-EAS443:3:53:202:224#0/1
¥_b]`bbbbbbb`bbbb`aaababbbbbbaaab``baa`¥
@HWI-EAS443:3:53:244:947#0/1
CAAAGAATTCCTTGGTCATGTCTTTCTCTTTGACAGAAGA
+HWI-EAS443:3:53:244:947#0/1
abbbbbabbaababbababbabbaaabaabaa`aaaaaa`
@HWI-EAS443:3:53:260:43#0/1
GAGGAGGAACTATTTTACAGCAGAAGTGTCCCACTGCAGG
+HWI-EAS443:3:53:260:43#0/1
aabb`ba]``aaaaaa_[_aa_a^`a]a_YYXY^_`RU¥_
@HWI-EAS443:3:53:26:1897#0/1
AGGGGTTTTCGGCTGAATCGAGAAAATTGCTTCTCATTGT
+HWI-EAS443:3:53:26:1897#0/1
ab`b_a_a_ab`a_abaab]WP_[ab_aa__`a`_aaZ_X
@HWI-EAS443:3:53:270:1137#0/1
GTGCCATATTTTCACCGAGTGCCCACTTCAATTGGCTTGT
+HWI-EAS443:3:53:270:1137#0/1
aaa]`a^`bab`¥ba]aa_R_a^_```a`a]X[aU]`X`Z
```

```
@HWI-EAS443:3:53:273:1336#0/1
CATTAATCAGGCACTCCTCGATTGCTTCATATAGCCCCCC
+HWI-EAS443:3:53:273:1336#0/1
abbb`^abbbbbabbabbbba_aaba`aaabaaa_`a]a_
@HWI-EAS443:3:53:304:1113#0/1
AGAACATGAAGAGAACAAGCCAATTGAAGTGGGCACTCGG
+HWI-EAS443:3:53:304:1113#0/1
abaaaabaaabaa`Z`b`a]aa`aaa^`a_a]```_^___
@HWI-EAS443:3:53:359:104#0/1
GGCGTGGTGTCGTCCTCAAGAATGGTTCAATTTTGGCGTT
+HWI-EAS443:3:53:359:104#0/1
ab`b^bb_a]_a__]___^__^_`aZ^UU^_^a`TZT]WW
@HWI-EAS443:3:53:376:1994#0/1
GAACAATGAGAAGCAATTTTCTCGATTCAGCCGAAAACCC
+HWI-EAS443:3:53:376:1994#0/1
Y`abbaba_abbKb^baaba_bbb[bb_a[bYaaab`_a^
@HWI-EAS443:3:53:394:707#0/1
AGGGAGTGTACATAAATACGGCCTTGCTCAATGCATCCTG
+HWI-EAS443:3:53:394:707#0/1
`babababa`abaabbaabaa``aaa`aaaaaa`aa__aa
@HWI-EAS443:3:53:404:618#0/1
CGGCTGAATCGAGAAAATTGCTTCTCATTGTTCAGGCACT
+HWI-EAS443:3:53:404:618#0/1
a`bbbbb_^a_]a^aa]b_]baaabaaabb`aa`aa_Y]a
@HWI-EAS443:3:53:410:1129#0/1
GGGGTTTTCGGCTGAATCGAGAAAATTGCTTCTCATTGTT
+HWI-EAS443:3:53:410:1129#0/1
ab`_]aaaaaa`a`]]]`a_a¥Z^_``¥^a`Z__`_^aZ^
@HWI-EAS443:3:53:471:1895#0/1
ATTTGAGATTACAGGAACTATGCGCAAGCTTGCCGACCAA
+HWI-EAS443:3:53:471:1895#0/1
aabb`abaabaaabaa_`aaa`a][a`__aa_a[a`_Z_¥
@HWI-EAS443:3:53:51:1741#0/1
GATCCGAAAATCGAAACTAACAAGTTTGCTGCAATATGCA
+HWI-EAS443:3:53:51:1741#0/1
``V^a^^_bTb]aa`]`aU]¥U¥aX``SaaZ[`a^LXUHZ
@HWI-EAS443:3:53:529:550#0/1
CCCTTGACGAAGAGAGCAGGGCAAGAATCAAAACTAGGCT
+HWI-EAS443:3:53:529:550#0/1
ababaaaab`abaa`b`abaaa]abaaa`aa_`_``aaaa
@HWI-EAS443:3:53:565:824#0/1
AGGACATTGAAAATGAAGAGAAGATCCCAAGGACAAAGAA
+HWI-EAS443:3:53:565:824#0/1
^¥XR[YX]aYZQT]`___YWVY`_¥W_X¥O[SZTL[^^WW
@HWI-EAS443:3:53:589:780#0/1
CGGGTCACCAGATTCTACAATTATTGATTCACCCCGTTCG
+HWI-EAS443:3:53:589:780#0/1
aa`Z`aaa_S^Yaaa``WYQ_aaaa`X__]__¥U¥^^^]]
@HWI-EAS443:3:53:603:782#0/1
AGGACATTGAAAATGAAGAGAAGATCCCAAGGACAAAGAA
```

+HWI-EAS443:3:53:603:782#0/1
ab`aa_a`b_a_aab`_baaa^aaa`a^_ab_^^_aaaa^
@HWI-EAS443:3:53:625:264#0/1
GTTACATATACTGTTCACCACTGTCCAGGCCATGATTCGG
+HWI-EAS443:3:53:625:264#0/1
¥_^a`a_UYaR`_]_`YZ`aaaa`a`aa`_Z]T][¥]ZWR
@HWI-EAS443:3:53:663:339#0/1
CCCGAATGCACTATTGAAGCACCGATTTGAGATAATTGAA
+HWI-EAS443:3:53:663:339#0/1
¥ab¥H_T`¥_a`ab`bUU`X¥SWa`a¥¥_b``_Z]abaY[
@HWI-EAS443:3:53:672:377#0/1
AAGACAAGAAATGGCCAGTAGGAGTCTATGGGATTCCTTT
+HWI-EAS443:3:53:672:377#0/1
`abbb`a__ab]`bb]]b^¥_a]aZaaUabaa[[_``¥`_
@HWI-EAS443:3:53:683:1224#0/1
CGGCTGAATCGAGAAAATTGCTTCTCATTGTTCAGGCACT
+HWI-EAS443:3:53:683:1224#0/1
abaa_ba]X`[`_`a_SZ_aaaaa_`aaab`[aaa_]]Z]
@HWI-EAS443:3:53:725:973#0/1
GGCATGGAAATGAGGCGCTGCCTTCTTCAGTCTCTTCAGC
+HWI-EAS443:3:53:725:973#0/1
aaaaabbaaab`_aabbaa_a_ba_bb`ab]^a`ab``a^
@HWI-EAS443:3:53:735:1016#0/1
AAAAGTAGACTTTGATGACTGCAAAGATGTTGGAGACCTT
+HWI-EAS443:3:53:735:1016#0/1
`^`bb^¥`aaababbbaaaaaaaaabaab`aaa_a`__a`
@HWI-EAS443:3:53:738:1072#0/1
TGCCTTTCTCATGTGGTTTGACTATGTTAGGCTCTTTCCA
+HWI-EAS443:3:53:738:1072#0/1
ababbabbba^abababa^a`b^abb[P]¥TX_ba`[_¥
@HWI-EAS443:3:53:756:1416#0/1
GGGGGCTATATGAAGCAATCGAGGAGTGCCTGATTAATGA
+HWI-EAS443:3:53:756:1416#0/1
abaab]`ba`a^`aYaa_SJ__aYV_^`]`_R]]^`[`aa
@HWI-EAS443:3:53:939:347#0/1
GTCACGAGGGGGAGGGAATACCACTATATGATGCAATCAA
+HWI-EAS443:3:53:939:347#0/1
^abbbbabbbbb`bbbabbaa^a`bababbb_baaaa_aa
@HWI-EAS443:3:53:948:808#0/1
GGGTTTTCGGCTGAATCGAGAAAATTGCTTCTCATTGTTC
+HWI-EAS443:3:53:948:808#0/1
ab]aababab_bab`aaa¥aaaaaaabaaba`_aaa__`[
@HWI-EAS443:3:53:968:604#0/1
GAGAAAGGCATAAATCCCAATTACCTCATGGCTTGGAAGC
+HWI-EAS443:3:53:968:604#0/1
aaabbbbbaabbbaa`_aa`baba`a``abbZba_aZ`ZQ
@HWI-EAS443:3:53:986:1785#0/1
AGCATCCATCAGCAGGAACTTTGACCGCTGATGGCAAAGA
+HWI-EAS443:3:53:986:1785#0/1
TTQYTOYTQX_¥FY_`¥W`^___]_[¥U¥WPTb`¥]W^ZX

@HWI-EAS443:3:54:1015:202#0/1
GAGACTTTGGTCGGCAAGCTTGCGCATAGTTCCTGTAATC
+HWI-EAS443:3:54:1015:202#0/1
`bba`bbbb`a_aa^a`````a^]UZ`^a]a[]`a]W^_Z
@HWI-EAS443:3:54:1017:998#0/1
CAGTCATCAAAGTCTACTTTTTCTGGTGCCATATTTTCAC
+HWI-EAS443:3:54:1017:998#0/1
aababbbbbbbbabbbabbbbbabb`Zbbbabababbbaaba
@HWI-EAS443:3:54:1047:1718#0/1
ATGAGAAGCAATTTTCTCGATTCAGCCGAAAACCCCTCAA
+HWI-EAS443:3:54:1047:1718#0/1
aaa]aaba`aa_^_``a¥a_`_a¥_¥_aa_^UINIS]XXM
@HWI-EAS443:3:54:105:1266#0/1
AGCCAGAGCCCAGATCTCTAGCAAGCTGGGTCCAAAATGA
+HWI-EAS443:3:54:105:1266#0/1
¥DY_X_`_b`aWW[^aZ^`a]X^]^W_``_WVUM[NRV_N
@HWI-EAS443:3:54:1094:1209#0/1
AAAGAGCCTAACATAGTCAAACCACATGAGAAAGGCATAA
+HWI-EAS443:3:54:1094:1209#0/1
_^¥]_Ya^_aaT_^`^]``a``X[`aaaa`aabaaa^`a]
@HWI-EAS443:3:54:112:2042#0/1
AACAAAATAAAATCTGAGAAGACACACATTCACATCTTTT
+HWI-EAS443:3:54:112:2042#0/1
J[`b`ZW`b`¥`_P`b_a_aaaaaTa__a_`a_aaZWZa_
@HWI-EAS443:3:54:1147:1890#0/1
CTAGCAAGCTGGGTCCAAAATGAATTCAATAAGGCATGTG
+HWI-EAS443:3:54:1147:1890#0/1
abbaababbabba^__babbbbbbbba``^bbbbabbbbb
@HWI-EAS443:3:54:1219:521#0/1
ACCAAAGCGGACTACACCCTTGACGAAGAGAGCAGGGCAA
+HWI-EAS443:3:54:1219:521#0/1
aaaa_a`_¥]]¥_][_`ZV`aa_Y__¥_[_]]FZa`R]aa
@HWI-EAS443:3:54:1221:155#0/1
CATCAGTATCATTTCTCAAATGAGACCTTCCTTTTATAAT
+HWI-EAS443:3:54:1221:155#0/1
`ba`bbabbbbbbbaaaababbaba``baaabbbbbbbaa
@HWI-EAS443:3:54:1234:915#0/1
TGCCTTTCTCATGTGGTTTGACTATGTTAGGCTCTTTCCA
+HWI-EAS443:3:54:1234:915#0/1
a_]_bab``Z`bb`aa_a`a`ab`aaaa`¥aV_R_a`[^_
@HWI-EAS443:3:54:124:841#0/1
CGATTGGCCATGTTTCCGATTTGTTTTCAAAGAATTCCTT
+HWI-EAS443:3:54:124:841#0/1
abaaabbbaa``^aaa``Z^aaaZaaa^`a`_¥¥]YW^``
@HWI-EAS443:3:54:1296:1168#0/1
TGCAAAGATGTTGGAGACCTTAAACAGTATGACAGTGATG
+HWI-EAS443:3:54:1296:1168#0/1
SaY¥¥`¥a_VVX^bbba[b_WaT`U^_Ibb`_bbWIT_^`
@HWI-EAS443:3:54:132:1248#0/1
GGGGGCTATATGAAGCAATCGAGGAGTGCCTGATTAATGA

+HWI-EAS443:3:54:132:1248#0/1
aaaaaababaaaa`[aaaa`a[aa`a]a`__`^`_Y]_a_
@HWI-EAS443:3:54:1360:522#0/1
AAGACATTCTTTGGCTGGAAAGAGCCTAACATAGTCAAAC
+HWI-EAS443:3:54:1360:522#0/1
`b_]_abbM]a[H¥I]X_[Z^`_W]^``a`aaa]Z`Yaa[
@HWI-EAS443:3:54:1456:1534#0/1
GGGGCATGGAAATGAGGCGCTGCCTTCTTCAGTCTCTTCA
+HWI-EAS443:3:54:1456:1534#0/1
`ZTZLX]]a`aXX^[`¥]^YY`¥W`_¥_^UZ^TSYWTYSQ
@HWI-EAS443:3:54:1496:453#0/1
ATACTGTTCACCACTGTCCAGGCCATGATTCGGTCTCTTC
+HWI-EAS443:3:54:1496:453#0/1
_baabbaba`baaab``a`[`a____¥Y_`[`[¥PTFP]Q
@HWI-EAS443:3:54:155:237#0/1
CATGGAAATGAGGCGCTGCCTTCTTCAGTCTCTTCAGCAG
+HWI-EAS443:3:54:155:237#0/1
a_`_`]TTW`¥UR¥¥^Ua[`_[VKTY[ULQQO^^HS_LT[
@HWI-EAS443:3:54:1759:112#0/1
CATTATGTATTCAGTAGCCCTGCAGTGGGACACTTCTGCT
+HWI-EAS443:3:54:1759:112#0/1
abbbbbbaaabaabab_a`^`¥]]¥ZQZ`_^a]a`^aUW]
@HWI-EAS443:3:54:239:1241#0/1
CAATAAGGCATGTGAATTGACTGATTCAAGCTGGATAGAA
+HWI-EAS443:3:54:239:1241#0/1
`b¥baaba`abb_baaaabaaababa`bSa]^aa]``_^^
@HWI-EAS443:3:54:259:330#0/1
CAGATTTTGCCAGTAAGGTCCTGCACACTTTCCCAATAGA
+HWI-EAS443:3:54:259:330#0/1
aa_ababbabb`b`b`ba_a`baaaaaaba_aa`bTa^`a
@HWI-EAS443:3:54:281:728#0/1
CACCACGCCCCCTCAGATTGCCTGATGGGCCTCTTTGCCA
+HWI-EAS443:3:54:281:728#0/1
abab`X]_]Z`bVS]a]_Z_aaUHQUa¥VQ]THXZRSFXX
@HWI-EAS443:3:54:282:1072#0/1
ATTCGGATTTCCATTTCATCGACGAACGGGGTGAATCAAT
+HWI-EAS443:3:54:282:1072#0/1
a`bbb`aa`]a_aaaaabaaa¥`a`^[]__aV¥XX_VQY]
@HWI-EAS443:3:54:315:1001#0/1
CTCGGTGAAAATATGGCACCAGAAAAAGTAGACTTTGATG
+HWI-EAS443:3:54:315:1001#0/1
^R[]QN^U```¥¥¥aX¥[XPUXR][TVLXZUW_S`¥UVOT
@HWI-EAS443:3:54:316:605#0/1
GAACTTTGACCGCTGATGGCAAAGAGGCCCATCAGGCAAT
+HWI-EAS443:3:54:316:605#0/1
a^a``aa^`_`_V_a_¥aa^___YONY¥¥YUSQ¥TTPYX¥
@HWI-EAS443:3:54:367:1084#0/1
GAAAGGCATAAATCCCAATTACCTCATGGCTTGGAAGCAG
+HWI-EAS443:3:54:367:1084#0/1
`]_`a_a`Y^[^W^`aZ_^aUa_`Saa_[]`^_BBBBBBB

```
@HWI-EAS443:3:54:369:1479#0/1
AGACAATTGAAGAAAAATTTGAGATTACAGGAACTATGCG
+HWI-EAS443:3:54:369:1479#0/1
XT]_UU¥_¥YYUJYWY__a`]Y[]`]W]UOUVTXW[QBBB
@HWI-EAS443:3:54:395:1205#0/1
ATAAATCCCAATTACCTCATGGCTTGGAAGCAGGTGCTAG
+HWI-EAS443:3:54:395:1205#0/1
abbb`babaaaaaa`aaaaaaaaa¥_Y_YWYQZQYY[][
@HWI-EAS443:3:54:465:672#0/1
CACTTTCCCAATAGAGCCTTCCTCCACTCCCCTGGGTGAC
+HWI-EAS443:3:54:465:672#0/1
a``bbbbbabab__aaaaaaa`aa`aaa`a`aaaaa_`Y¥
@HWI-EAS443:3:54:511:954#0/1
TATGCGCAAGCTTGCCGACCAAAGTCTCCCACCGAACTTC
+HWI-EAS443:3:54:511:954#0/1
aba`__]aa`a_^a^aaa__aa_`¥^^_¥``U¥__`^_¥Z
@HWI-EAS443:3:54:604:679#0/1
CTTGGGGGGCTATATGAAGCAATCGAGGAGTGCCTGATTA
+HWI-EAS443:3:54:604:679#0/1
aa`_[abbb_`bba`_RSbabba]bbbbaaX`¥]a^Q^a`
@HWI-EAS443:3:54:612:1145#0/1
AAAGGAAGGTCTCATTTGAGAAATGATACTGATGTGGTGA
+HWI-EAS443:3:54:612:1145#0/1
aaaaaaaaa^aa`_aaaa_a^_``a`a``aa_aa^``]a^
@HWI-EAS443:3:54:61:391#0/1
GTGTCGTCCTCAAGAATGGTTCAATTTTGGCGTTCACTTC
+HWI-EAS443:3:54:61:391#0/1
_X]T`^¥`Y]X`¥]ZX]aaXYYY[^^_ZVYT_ZOQQRUZQ
@HWI-EAS443:3:54:639:1607#0/1
GGCCAGTAGGAGTCTATGGGATTCCTTTCGTCAGTCCGAA
+HWI-EAS443:3:54:639:1607#0/1
abbaab^bbbTT]ba]aaa^`ab`]a``XW````a^H^a`
@HWI-EAS443:3:54:719:1130#0/1
GGGTTTTCGGCTGAATCGAGAAAATTGCTTCTCATTGTTC
+HWI-EAS443:3:54:719:1130#0/1
a¥Z]bbba`bababbaaba`aa`aa`aaba`aaaaaa_a^
@HWI-EAS443:3:54:723:890#0/1
GGAGGAACTATTTTACAGCAGAAGTGTCCCACTGCAGGGC
+HWI-EAS443:3:54:723:890#0/1
_SZ^P]]_T[a____`_^]¥Y¥VRK_[]Z[W[]`SVZW_P
@HWI-EAS443:3:54:740:1669#0/1
CTCTATTGGGAAAGTGTGCAGGACCTTACTGGCAAAATCT
+HWI-EAS443:3:54:740:1669#0/1
abaabbbbbbbbbb`b_bba_bb`abbbabaabbbaab_b
@HWI-EAS443:3:54:744:1434#0/1
AAGAATTCCTTGGTCATGTCTTTCTCTTTGACAGAAGACT
+HWI-EAS443:3:54:744:1434#0/1
`]^aW___abba^MZaaa_`a`]Z`_aaa_`a`a__`^`^
@HWI-EAS443:3:54:796:1609#0/1
TTGGAGACCTTAAACAGTATGACAGTGATGAGCCAGAGCC
```

+HWI-EAS443:3:54:796:1609#0/1
aaaabaaaaabbaaabb`baab`aa[`a[aa^a_`a``S[
@HWI-EAS443:3:54:815:486#0/1
CATGTTTCCGATTTGTTTTCAAAGAATTCCTTGGTCATGT
+HWI-EAS443:3:54:815:486#0/1
^b`b_`b^``_`a`¥bZaa`ab`bbb`a_¥aba^`¥V_a^
@HWI-EAS443:3:54:843:1897#0/1
GGAGGAACTATTTTACAGCAGAAGTGTCCCACTGCAGGGC
+HWI-EAS443:3:54:843:1897#0/1
ab``¥b`Y^`aa^_``abaa``^¥U¥SZ]XaI^`]_]T[Z
@HWI-EAS443:3:54:881:680#0/1
CTTGGGATCTTCTCTTCATTTTCAATGTCCTGTAGCTCTG
+HWI-EAS443:3:54:881:680#0/1
`bbbbb`b_bbababbbbbbbabababbaaabbabbbaaab
@HWI-EAS443:3:54:982:450#0/1
CAGTAGCCCTGCAGTGGGACACTTCTGCTGTAAAATAGTT
+HWI-EAS443:3:54:982:450#0/1
Za``aUa^^a_J^bb`babaaKbaabbabb]K]_a`a¥Y]
@HWI-EAS443:3:54:98:909#0/1
ACCGATTTGAGATAATTGAAGGAAGAGACCGAATCATGGC
+HWI-EAS443:3:54:98:909#0/1
aaa^[]_aa]a¥aa`a]a¥X__Y^``Z]__a`__`a_^X_
@HWI-EAS443:3:55:1089:1217#0/1
GGAGTCTATGGGATTCCTTTCGTCAGTCCGAAAGAGGCGA
+HWI-EAS443:3:55:1089:1217#0/1
_]¥aU^^R_a]]]VYV][¥[VY^STY[XNTT¥]]WU_[YZ
@HWI-EAS443:3:55:1176:187#0/1
GTGGGAGACTTTGGTCGGCAAGCTTGCGCATAGTTCCTGT
+HWI-EAS443:3:55:1176:187#0/1
a_aa``]ZYa`_`_U]ZYJY^_WZZ^X[XZYXZUWRRX]Y
@HWI-EAS443:3:55:1177:198#0/1
AAGAGCCTAACATAGTCAAACCACATGAGAAAGGCATAAA
+HWI-EAS443:3:55:1177:198#0/1
`baaaYZaaa[aaa`]_aaa`^`^`aa`a^__a`^^a^_a
@HWI-EAS443:3:55:1179:185#0/1
CGGCTGAATCGAGAAAATTGCTTCTCATTGTTCAGGCACT
+HWI-EAS443:3:55:1179:185#0/1
abaaaabaab_``bbbaabbaaa^abaa^]¥a_aa`__[a
@HWI-EAS443:3:55:1205:12#0/1
CAGGAACTTTGACCGCTGATGGCAAAGAGGCCCATCAGGC
+HWI-EAS443:3:55:1205:12#0/1
``aa_a`aaaaa``a`aa`aaaaa``a`]a``]¥^]```U
@HWI-EAS443:3:55:1224:1209#0/1
GGCTCATCACTGTCATACTGTTTAAGGTCTCCAACATCTT
+HWI-EAS443:3:55:1224:1209#0/1
^`a`R`bb]]ba`a]aa^^^a]^aa`bX_b`_aa_aaOa_
@HWI-EAS443:3:55:1237:180#0/1
AGGGGTTTTCGGCTGAATCGAGAAAATTGCTTCTCATTGT
+HWI-EAS443:3:55:1237:180#0/1
I_a¥¥Wa``__a_____¥XWZb_Z¥a_ba^Z`_RVX`]_`

```
@HWI-EAS443:3:55:1266:1628#0/1
CATAAATACGGCCTTGCTCAATGCATCCTGTGCAGCCATG
+HWI-EAS443:3:55:1266:1628#0/1
__b]abb`Yaa¥YPa_L_XS_YU[^R[UX_`]^[[T^^VY
@HWI-EAS443:3:55:130:1531#0/1
GAGGAACTATTTTACAGCAGAAGTGTCCCACTGCAGGGCT
+HWI-EAS443:3:55:130:1531#0/1
Zaab¥a][]``aabaa`]^^____`]]^aaa____[[UX^
@HWI-EAS443:3:55:1319:1050#0/1
GAGATCTGGGCTCTGGCTCATCACTGTCATACTGTTTAAG
+HWI-EAS443:3:55:1319:1050#0/1
aab`Z_b``a`a[aa_aa`a_[]_a`¥Za_`_aa_`a__a
@HWI-EAS443:3:55:1322:1190#0/1
CATTGGGATCAGCTGAAAGTCATCCATGGCTGCACAGGAT
+HWI-EAS443:3:55:1322:1190#0/1
abb_bba_b`bbabbbbbb]abaaaabbaaa`X__aaXa_
@HWI-EAS443:3:55:1403:1376#0/1
TGTGAATTGACTGATTCAAGCTGGATAGAACTTGATGAAA
+HWI-EAS443:3:55:1403:1376#0/1
aaY^¥Yaaa_]^`W`^^][`[¥aa^__`_[N`^_[_`YV¥
@HWI-EAS443:3:55:1426:950#0/1
CACTTTCCCAATAGAGCCTTCCTCCACTCCCCTGGGTGAC
+HWI-EAS443:3:55:1426:950#0/1
aZ_W¥¥Z[[`aXaaZbS^`a¥¥`VESZQX]_YW[¥YOXV[
@HWI-EAS443:3:55:1443:779#0/1
GCTCAATGCATCCTGTGCAGCCATGGATGACTTTCAGCTG
+HWI-EAS443:3:55:1443:779#0/1
abbabbb]ba_¥]`ZZ``aa_aaabaa]`^X`a]J[YG_[
@HWI-EAS443:3:55:1501:414#0/1
GGGGGAGGGAATACCACTATATGATGCAATCAAATGCATG
+HWI-EAS443:3:55:1501:414#0/1
aa_ba^b_S]]`a[Y`_aaaaaaY_a¥_`XZ`__[X]`_a
@HWI-EAS443:3:55:1513:915#0/1
TCCAAAATGAATTCAATAAGGCATGTGAATTGACTGATTC
+HWI-EAS443:3:55:1513:915#0/1
YYSaYaa`X`a]]W]`]__VL¥aaaa_WZ]¥I[FZ¥aaZX
@HWI-EAS443:3:55:1530:1301#0/1
AAACTTCCAAATGTGTGCATATTGCAGCAAACTTGTTAGT
+HWI-EAS443:3:55:1530:1301#0/1
aa[UX^^_^aaba_¥W]`¥_`a[`R_ZR`]UW]```¥_Y_
@HWI-EAS443:3:55:1569:1366#0/1
CAATGGAACCTCCAAGATCAAGATGAAATGGGGCATGGAA
+HWI-EAS443:3:55:1569:1366#0/1
aaab_SUbV][NZaSaa___P_`^b_J_a`aa_`a¥a¥_[
@HWI-EAS443:3:55:15:1051#0/1
GCATAAATCCCAATTACCTCATGGCTTGGAAGCAGGTGCT
+HWI-EAS443:3:55:15:1051#0/1
`aaaaaaaaa_a_aa_aaa`__¥a_¥_¥]^ZUSUZVHPQY
@HWI-EAS443:3:55:1606:199#0/1
CATAAATACGGCCTTGCTCAATGCATCCTGTGCAGCCATG
```

+HWI-EAS443:3:55:1606:199#0/1
abbba`bbZ_^aa_]R¥[_a_]^`a_]T]RZ`XZ^N^UBB
@HWI-EAS443:3:55:1613:352#0/1
AGTATATGTAACACAACAGGGGTAGAGAAGCCTAAATTTC
+HWI-EAS443:3:55:1613:352#0/1
^_X_U`^^^a___[W_Z_a`a]V^]W^X^]XM[__`^Y¥^
@HWI-EAS443:3:55:1714:875#0/1
CATTAATCAGGCACTCCTCGATTGCTTCATATAGCCCCCC
+HWI-EAS443:3:55:1714:875#0/1
[aabbbaaaZT_ba]^bbb[Vb_]bbab`[aabaabaabb
@HWI-EAS443:3:55:176:792#0/1
GCTTGCTAGAGATCTGGGCTCTGGCTCATCACTGTCATAC
+HWI-EAS443:3:55:176:792#0/1
abbbaabba^^a`a`a`aaaaaa_aa_aa]`_a`_]X]`Y
@HWI-EAS443:3:55:1778:1897#0/1
GAGGAACTATTTTACAGCAGAAGTGTCCCACTGCAGGGCT
+HWI-EAS443:3:55:1778:1897#0/1
aaaaaaaaabaa`a_aaaaaaa^^`^_^__^``[^^[¥Z[
@HWI-EAS443:3:55:17:225#0/1
CCATGATTCGGTCTCTTCCTTCAATTATCTCAAATCGGTG
+HWI-EAS443:3:55:17:225#0/1
aaWZ]P[Y_OLKSRST¥V[^WORWV][YN[[[]]RLBBBB
@HWI-EAS443:3:55:214:54#0/1
TCCTCCTCATGCTTGCGATATGTTCAATCGGGGCAACATC
+HWI-EAS443:3:55:214:54#0/1
aa_a`_aaaab^aab^a``_aa____¥_^`_`^]]]Y]]V
@HWI-EAS443:3:55:224:12#0/1
ATTGAAGGAAGAGACCGAATCATGGCCTGGACAGTGGTGA
+HWI-EAS443:3:55:224:12#0/1
aaabaabb`]baa`__a]Oa`_`aa_]^_`]]_^Y^¥N]]
@HWI-EAS443:3:55:256:101#0/1
GGGGTTTTCGGCTGAATCGAGAAAATTGCTTCTCATTGTT
+HWI-EAS443:3:55:256:101#0/1
aaSPN_WFLW[U^_WVNVVMZVKIMVUPMTUNURSTNRTR
@HWI-EAS443:3:55:284:89#0/1
GACCGAATCATGGCCTGGACAGTGGTGAACAGTATATGTA
+HWI-EAS443:3:55:284:89#0/1
aa[ZaYZaZ^a`aX^`___U__]T`GaU]UX_U^UXYUYX
@HWI-EAS443:3:55:302:571#0/1
CATATTTTCACCGAGTGCCCACTTCAATTGGCTTGTTCTC
+HWI-EAS443:3:55:302:571#0/1
ab`aa]baabbaa_a_aa_]__a`__`__`]]_¥NNWM[P
@HWI-EAS443:3:55:329:1005#0/1
GCGAAGAGACAATTGAAGAAAAATTTGAGATTACAGGAAC
+HWI-EAS443:3:55:329:1005#0/1
aaa`aaa`¥`aaaaa`_a^[__]__a`]a__`_X^`_^_^
@HWI-EAS443:3:55:382:1875#0/1
GTGAATTGACTGATTCAAGCTGGATAGAACTTGATGAAAT
+HWI-EAS443:3:55:382:1875#0/1
`]XVXa]_ROUPSaa``[R¥¥XRXRUZ¥`N_`R^_QSVT[

```
@HWI-EAS443:3:55:401:1456#0/1
GGGGTTTTCGGCTGAATCGAGAAAATTGCTTCTCATTGTT
+HWI-EAS443:3:55:401:1456#0/1
¥¥Y¥H¥aWYb¥SWJ[]]aaY^XWY_X_bYX`HPXRVXW[_
@HWI-EAS443:3:55:43:1025#0/1
CCCGTTCGTCGATGAAATGGAAATCCGAATACATGAAACA
+HWI-EAS443:3:55:43:1025#0/1
_`bbbbabaabaa``ba^`aaabaabaaaaaa`aa``aab
@HWI-EAS443:3:55:460:316#0/1
CATGGAAATGAGGCGCTGCCTTCTTCAGTCTCTTCAGCAG
+HWI-EAS443:3:55:460:316#0/1
`a`b_`a[aa```aa`a`^^XX[_aZY]WY]__]SXVQQZ
@HWI-EAS443:3:55:498:1553#0/1
CGTTCACTTCTTTTGACATTTGGGAAAGCTTGCCCTCAAT
+HWI-EAS443:3:55:498:1553#0/1
ab`bbbbaabbbbb_aabbab_a`a`aa`abaa]¥aaaaa
@HWI-EAS443:3:55:509:904#0/1
GGGGCATGGAAATGAGGCGCTGCCTTCTTCAGTCTCTTCA
+HWI-EAS443:3:55:509:904#0/1
aaaaaa`aa_`aaa``]aaaaa_```_```_a¥[`_`_^_
@HWI-EAS443:3:55:567:972#0/1
CAAAGTCTACTTTTTCTGGTGCCATATTTTCACCGAGTGC
+HWI-EAS443:3:55:567:972#0/1
a`XN__]a^[``¥ZYZa^aR``a`[W`_``abaaa]aUaa
@HWI-EAS443:3:55:623:1062#0/1
CATGGAAATGAGGCGCTGCCTTCTTCAGTCTCTTCAGCAG
+HWI-EAS443:3:55:623:1062#0/1
aaXW]]aZ]^Z_T[`ZVR__]]^]¥XR¥TL¥TYZEO[PST
@HWI-EAS443:3:55:625:679#0/1
GAACTATTTTACAGCAGAAGTGTCCCACTGCAGGGCTACT
+HWI-EAS443:3:55:625:679#0/1
`aa__ba^Z_JRT]^`WSRX^__Z]UXXRTSV`Y`U¥`^¥
@HWI-EAS443:3:55:660:439#0/1
TTGGGATCTTCTCTTCATTTTCAATGTCCTGTAGCTCTGC
+HWI-EAS443:3:55:660:439#0/1
ababb]bbbbabaaaaabbabaaaab]aaab`a``a]a^[
@HWI-EAS443:3:55:720:1735#0/1
GGGGGCTATATGAAGCAATCGAGGAGTGCCTGATTAATGA
+HWI-EAS443:3:55:720:1735#0/1
aaa`_aaaaba```_aaaa``a]_aY___aa`aa`a```
@HWI-EAS443:3:55:834:995#0/1
CGATTGCTTCATATAGCCCCCCAAGATCGAAGGTTCCAGG
+HWI-EAS443:3:55:834:995#0/1
`bbabbaaabbababaaa_aaaaaaaa^a_aaaW_`_Z`_
@HWI-EAS443:3:55:860:950#0/1
CTGGTGACCCGAATGCACTATTGAAGCACCGATTTGAGAT
+HWI-EAS443:3:55:860:950#0/1
a_¥SZ]NZH[XX]Z¥W¥`a`_``^MW_ZWMXO¥[_a[^V_
@HWI-EAS443:3:55:866:1222#0/1
AAAGAACATGAAGAGAACAAGCCAATTGAAGTGGGCACTC
```

```
+HWI-EAS443:3:55:866:1222#0/1
a```a^b``]_baaaa`abaaY^aaaaba__^a_a_a^``
@HWI-EAS443:3:55:873:69#0/1
GTTTGTTTCATGTATTCGGATTTCCATTTCATCGACGAAC
+HWI-EAS443:3:55:873:69#0/1
a``aa^^^S[aaW]``Ya`]_`¥]X^^`]PVVY]XX_XWV
@HWI-EAS443:3:55:887:1381#0/1
GGGGGCTATATGAAGCAATCGAGGAGTGCCTGATTAATGA
+HWI-EAS443:3:55:887:1381#0/1
`aa`¥`a`baaaaa_aaa_^a_`a`_]a__aa^a`_``__
@HWI-EAS443:3:56:1010:589#0/1
CCCCTGTTGTGTTACATATACTGTTCACCACTGTCCAGGC
+HWI-EAS443:3:56:1010:589#0/1
a`Za¥WZW`N¥Y_Y[a^Y]UQ]][NLQTUXXBBBBBBBBB
@HWI-EAS443:3:56:1048:1159#0/1
TTCGGATTTCCATTTCATCGACGAACGGGGTGAATCAATA
+HWI-EAS443:3:56:1048:1159#0/1
aa`aa`__N__aaa``_`^a_X_^]V_`_YY__^_WU^`_
@HWI-EAS443:3:56:1098:1097#0/1
GCTTGGAAGCAGGTGCTAGCAGAGCTACAGGACATTGAAA
+HWI-EAS443:3:56:1098:1097#0/1
aaaaab]aaa`aa¥]`_`a]_`___a_]a___^`_`[^_]
@HWI-EAS443:3:56:1112:1861#0/1
CATGTTTCCGATTTGTTTTCAAAGAATTCCTTGGTCATGT
+HWI-EAS443:3:56:1112:1861#0/1
abb`aabbbbabbbbaabbabaaba_aa^_babb_^bbb`
@HWI-EAS443:3:56:1132:618#0/1
GAGGAGATGGCCACCAAAGCGGACTACACCCTTGACGAAG
+HWI-EAS443:3:56:1132:618#0/1
aaa``ba`aa`aa¥V`]Z_[a``_`^[_[^^a^¥^[a^__
@HWI-EAS443:3:56:1137:498#0/1
CGAAAATCGAAACTAACAAGTTTGCTGCAATATGCACACA
+HWI-EAS443:3:56:1137:498#0/1
X_bbbb__b_]¥_^`a``^``_`^¥abaabbabbaaaaab
@HWI-EAS443:3:56:1189:363#0/1
GGGGTTTTCGGCTGAATCGAGAAAATTGCTTCTCATTGTT
+HWI-EAS443:3:56:1189:363#0/1
_baa[aa`^`a_abaa__`Xa_^__`_]U[a]`R]^`a^_
@HWI-EAS443:3:56:1295:1633#0/1
TGCCTTATTGAATTCATTTTGGACCCAGCTTGCTAGAGAT
+HWI-EAS443:3:56:1295:1633#0/1
a[[aaaaaba]^ba`_aaaaa__X[RRZV___RaX^^YY[
@HWI-EAS443:3:56:1298:1278#0/1
GCCCACTTCAATTGGCTTGTTCTCTTCATGTTCTTTGTCC
+HWI-EAS443:3:56:1298:1278#0/1
a__a]W_a_`a^aa`^^`YHSUUX]]V]W[V]M^]]VFRY
@HWI-EAS443:3:56:1381:416#0/1
GGCTTGGAAGCAGGTGCTAGCAGAGCTACAGGACATTGAA
+HWI-EAS443:3:56:1381:416#0/1
_b`^_SaabbaYU`W``aaYHZZa`X`a`¥SXQXXaaY]Q
```

```
@HWI-EAS443:3:56:1411:923#0/1
ATTTTCTCGATTCAGCCGAAAACCCCTCAAGTTGTGGAGA
+HWI-EAS443:3:56:1411:923#0/1
abbbaab^baaa_`a]U_a``a]T¥^_`_T`Zaa]`a__^
@HWI-EAS443:3:56:144:931#0/1
CACACATTCACATCTTTTCATTCACTGGAGAGGAGATGGC
+HWI-EAS443:3:56:144:931#0/1
`¥a`aba_^Za[aaaab`ba``aaa^[``ba`Xaa`^`a_
@HWI-EAS443:3:56:1503:78#0/1
CATGAAGACATTCTTTGGCTGGAAAGAGCCTAACATAGTC
+HWI-EAS443:3:56:1503:78#0/1
`bba_b_baabb^baa]`]bbb]a^a_b¥[a_]K¥_`^WO
@HWI-EAS443:3:56:1538:995#0/1
GGGCACTCGGTGAAAATATGGCACCAGAAAAAGTAGACTT
+HWI-EAS443:3:56:1538:995#0/1
a`Y]a``_bbWb_``_S`aP¥^^WW]]___^]_R_[¥Z_¥
@HWI-EAS443:3:56:1542:963#0/1
CATAAATACGGCCTTGCTCAATGCATCCTGTGCAGCCATG
+HWI-EAS443:3:56:1542:963#0/1
`a_QRV_b¥__U[__YN¥ZZ¥_ZG^_^YO^MVX]VTQ_]Y
@HWI-EAS443:3:56:15:1042#0/1
CAGATCTCTAGCAAGCTGGGTCCAAAATGAATTCAATAAG
+HWI-EAS443:3:56:15:1042#0/1
`aa^`aaaa_aa[__`aa_^]_^¥__]^^_]^_]Y]_^]¥
@HWI-EAS443:3:56:1650:1725#0/1
GACACACATTCACATCTTTTCATTCACTGGAGAGGAGATG
+HWI-EAS443:3:56:1650:1725#0/1
`bab`babbbbbab`b[^bb`abbbbbbbbabbbbbba_b
@HWI-EAS443:3:56:1650:229#0/1
AGAGAACAAGCCAATTGAAGTGGGCACTCGGTGAAAATAT
+HWI-EAS443:3:56:1650:229#0/1
aaaa_bab`b¥[a`aaaaa^]][^Z`[a]_a^a]^^`_]U
@HWI-EAS443:3:56:166:614#0/1
ATAAATCCCAATTACCTCATGGCTTGGAAGCAGGTGCTAG
+HWI-EAS443:3:56:166:614#0/1
aaaaa_a`_``a``_``__`a[[_]LYS[SPSTUUUUNSU
@HWI-EAS443:3:56:169:1141#0/1
CACTGTCATACTGTTTAAGGTCTCCAACATCTTTGCAGTC
+HWI-EAS443:3:56:169:1141#0/1
`ba_a[```¥U¥b^aW`¥LW^aY_^`Zaaaaaa_a_S]W]
@HWI-EAS443:3:56:1770:1773#0/1
CTGGGTCCAAAATGAATTCAATAAGGCATGTGAATTGACT
+HWI-EAS443:3:56:1770:1773#0/1
_abbbabb``^bbbaaabaaabaaba`aba_aaaa``a[b
@HWI-EAS443:3:56:223:1509#0/1
ATCTCCTCTCCAGTGAATGAAAAGATGTGAATGTGTGTCT
+HWI-EAS443:3:56:223:1509#0/1
`aa^_`bbba_^Yaab`aa`a`aba`_Y¥]_^TX[WZ^^a
@HWI-EAS443:3:56:278:990#0/1
CGATTGAACATATCGCAAGCATGAGGAGGAACTATTTTAC
```

```
+HWI-EAS443:3:56:278:990#0/1
_`Zabbbbabbba_b```^__`a[a]Z`a`]]_aaaa``¥
@HWI-EAS443:3:56:289:677#0/1
CCGCTTTGGTGGCCATCTCCTCTCCAGTGAATGAAAAGAT
+HWI-EAS443:3:56:289:677#0/1
`a_¥]Y¥]YD]V_YNQM¥V]Z`PU[PXFXQHX`a`aWBBB
@HWI-EAS443:3:56:329:1473#0/1
CATGAAGACATTCTTTGGCTGGAAAGAGCCTAACATAGTC
+HWI-EAS443:3:56:329:1473#0/1
aa`a^¥^^`aaZPZ¥XU]^VZ]J¥PSO¥T_VWZM[¥[TU[
@HWI-EAS443:3:56:368:247#0/1
AGCAAATGTAGGACCAAAGAAGGAAGACGGAAAACAAACC
+HWI-EAS443:3:56:368:247#0/1
_bbb`bbbabbbaaaa^T_aabb`^ba[Z¥]`_^Z¥G¥]U
@HWI-EAS443:3:56:369:701#0/1
CATTAATCAGGCACTCCTCGATTGCTTCATATAGCCCCCC
+HWI-EAS443:3:56:369:701#0/1
abbbb`babaabaaabaaabaaaa`a`aaaaa_a_a___[
@HWI-EAS443:3:56:418:1321#0/1
GGGGTTTTCGGCTGAATCGAGAAAATTGCTTCTCATTGTT
+HWI-EAS443:3:56:418:1321#0/1
R[RT^ZX_UUTW_[X¥aU¥^VR_ZZaZZ_WT`a[_`__QO
@HWI-EAS443:3:56:441:471#0/1
GAACAAGCCAATTGAAGTGGGCACTCGGTGAAAATATGGC
+HWI-EAS443:3:56:441:471#0/1
`W``^^T`a[[a¥UXWXG``a_a`^^bbQ^V[YZ[X_¥Z¥
@HWI-EAS443:3:56:493:588#0/1
AAAAGGAAGGTCTCATTTGAGAAATGATACTGATGTGGTG
+HWI-EAS443:3:56:493:588#0/1
_VW_b^Vba``Za`a``baabbaabaZaY`abbbbbb`[a
@HWI-EAS443:3:56:507:340#0/1
ATCTGGTGACCCGAATGCACTATTGAAGCACCGATTTGAG
+HWI-EAS443:3:56:507:340#0/1
abaaaa`_aaaaabaaaaaabaaaa`a^[a__b^a`aa_a
@HWI-EAS443:3:56:531:783#0/1
GGGGCATGGAAATGAGGCGCTGCCTTCTTCAGTCTCTTCA
+HWI-EAS443:3:56:531:783#0/1
aaaa`a_`a_`¥`¥ZaU`a___V^_¥¥``^U[U¥___^W^
@HWI-EAS443:3:56:544:790#0/1
CTCCCTTCATTATGTATTCAGTAGCCCTGCAGTGGGACAC
+HWI-EAS443:3:56:544:790#0/1
^baa`bba^bbaa`^aaba_b_aa_a`_`VPX¥V¥^YY_]
@HWI-EAS443:3:56:651:1216#0/1
TGGTGAACTTTGTAAGTATGGAGTTCTCACTCACTGACCC
+HWI-EAS443:3:56:651:1216#0/1
aaaP_`WU_Y^`QTYZR¥V]XZXT]^`b¥Z[Y]Z^PRVSU
@HWI-EAS443:3:56:652:168#0/1
GCTGGGTCCAAAATGAATTCAATAAGGCATGTGAATTGAC
+HWI-EAS443:3:56:652:168#0/1
a`aaaaXa``¥`_aa¥Z[^___`¥¥^[^]_`]`^]^]_^X
```

@HWI-EAS443:3:56:660:818#0/1
GGGGGCTATATGAAGCAATCGAGGAGTGCCTGATTAATGA
+HWI-EAS443:3:56:660:818#0/1
a__aaabaaaaaaa`a_aa_a_a`^_Y``_^a_`_```a_
@HWI-EAS443:3:56:713:892#0/1
ACTCGGTGAAAATATGGCACCAGAAAAAGTAGACTTTGAT
+HWI-EAS443:3:56:713:892#0/1
abWUW`aba^`Taa``a]`[[Z¥¥RP]^VW__H]aaa_[Z
@HWI-EAS443:3:56:732:991#0/1
GGGCACTCGGTGAAAATATGGCACCAGAAAAAGTAGACTT
+HWI-EAS443:3:56:732:991#0/1
abaabbbaaaab`[aabbaaaaa[``b`aaZaaUba`Xab
@HWI-EAS443:3:56:755:1524#0/1
GATGAAATGGAAATCCGAATACATGAAACAAACTTCCAAA
+HWI-EAS443:3:56:755:1524#0/1
a`V¥aaY[a_[_^]]UXa^XXY_[]_`^^_aGYaU]U`XY
@HWI-EAS443:3:56:804:463#0/1
CATGAAGACATTCTTTGGCTGGAAAGAGCCTAACATAGTC
+HWI-EAS443:3:56:804:463#0/1
abbbabbba`baaa`aaa`ab__`a`^aa^aabX`aa_^_
@HWI-EAS443:3:56:851:503#0/1
CAAAGTTCACCACATCAGTATCATTTCTCAAATGAGACCT
+HWI-EAS443:3:56:851:503#0/1
`b¥]¥I]b_a¥`[`¥¥¥bZaaY]`_X^a_a_Y`a[a[Z^^
@HWI-EAS443:3:56:882:455#0/1
CGAAAGGAATCCCATAGACTCCTACTGGCCATTTCTTGTC
+HWI-EAS443:3:56:882:455#0/1
ab``Z`a_baaZX_b_Ra`aY^^YYaa]_[a`a`X^RUUH
@HWI-EAS443:3:56:89:1585#0/1
CAGGACATTGAAAATGAAGAGAAGATCCCAAGGACAAAGA
+HWI-EAS443:3:56:89:1585#0/1
a^^¥Y_``¥U¥UOU`a``^aaaa^`[`^`^¥[LQY[T]_¥
@HWI-EAS443:3:57:1059:961#0/1
GGGCATGGAAATGAGGCGCTGCCTTCTTCAGTCTCTTCAG
+HWI-EAS443:3:57:1059:961#0/1
a_aXYWY]_`R`bba_R_a]b`a`ba]^[_]KIX¥]¥PT^
@HWI-EAS443:3:57:1101:37#0/1
GGGGGCTATATGAAGCAATCGAGGAGTGCCTGATTAATGA
+HWI-EAS443:3:57:1101:37#0/1
ab_`babbbbb_^bbaaaaaa`bbab_a`[abaaa]]ba^
@HWI-EAS443:3:57:1105:128#0/1
GGCCTTGCTCAATGCATCCTGTGCAGCCATGGATGACTTT
+HWI-EAS443:3:57:1105:128#0/1
Z_ZWWQ_VRH^`PW]Z¥`¥`aT¥MW^Y¥Z[^_M¥]YZ[ZB
@HWI-EAS443:3:57:1164:244#0/1
CGGCTGAATCGAGAAAATTGCTTCTCATTGTTCAGGCACT
+HWI-EAS443:3:57:1164:244#0/1
abaabbbbbababababaaaabababaaaaaaabaa_aab_a]`
@HWI-EAS443:3:57:1228:340#0/1
ACATTCTTTGGCTGGAAAGAGCCTAACATAGTCAAACCAC

+HWI-EAS443:3:57:1228:340#0/1
a_abb`abYba_`aaaaaa___`aaa¥aaaa``a¥`X`a]
@HWI-EAS443:3:57:1247:1614#0/1
TGCCTTATTGAATTCATTTTGGACCCAGCTTGCTAGAGAT
+HWI-EAS443:3:57:1247:1614#0/1
[a__^]aaaaH[b_U_`]UWaaIUH[OTW]V^Za[]M`ZB
@HWI-EAS443:3:57:1264:1206#0/1
GCAGGTGCTAGCAGAGCTACAGGACATTGAAAATGAAGAG
+HWI-EAS443:3:57:1264:1206#0/1
a`aaa[`a__¥a`a_baaa`baa^^`aaa[`]^`aa___a
@HWI-EAS443:3:57:1298:1302#0/1
CTCGGGTCTTCAATACTTAATTTCAGAGCATCCATCAGCA
+HWI-EAS443:3:57:1298:1302#0/1
ababab`aabaabba`bbb`bba_bbaaabaa`a`P_a`a
@HWI-EAS443:3:57:132:675#0/1
CGATTGCTTCATATAGCCCCCCAAGATCGAAGGTTCCAGG
+HWI-EAS443:3:57:132:675#0/1
abaaa`aaaaaaaaZ`aa`_aZ^]a_``_[_a`V^[ZXQT
@HWI-EAS443:3:57:1392:292#0/1
AAGAGCCTAACATAGTCAAACCACATGAGAAAGGCATAAA
+HWI-EAS443:3:57:1392:292#0/1
aab¥[GYb`bX`bbaa_`abY^a`a_aaaaa]]Z^a`_`_
@HWI-EAS443:3:57:1483:1517#0/1
ATACTGTTCACCACTGTCCAGGCCATGATTCGGTCTCTTC
+HWI-EAS443:3:57:1483:1517#0/1
abb`^bSV`b^^a[`¥]^Z`¥][a`YTYVTHX]LQYY¥WB
@HWI-EAS443:3:57:1570:561#0/1
AGCCTAAATTTCTTCCTGATTTGTATGATTACAAAGAGAA
+HWI-EAS443:3:57:1570:561#0/1
aaaabab`bba`bb¥aabaaaaa_aba_abbaa_aa_aa_
@HWI-EAS443:3:57:1579:694#0/1
GGTGAACTTTGTAAGTATGGAGTTCTCACTCACTGACCCG
+HWI-EAS443:3:57:1579:694#0/1
aa¥`aa`baba[_aa^aaaaaa¥`_a]a^a_a`_a_U¥¥a
@HWI-EAS443:3:57:1602:1896#0/1
AATTTAGGCTTCTCTACCCCTGTTGTGTTACATATACTGT
+HWI-EAS443:3:57:1602:1896#0/1
^ab`_``a^a[S[J[¥S¥T[___¥GUO¥SZ`^¥W_¥^SaB
@HWI-EAS443:3:57:1638:1667#0/1
CAGTAGGAGTCTATGGGATTCCTTTCGTCAGTCCGAAAGA
+HWI-EAS443:3:57:1638:1667#0/1
^Xa[bbbb_aaabbbbba``[]aaaab`aabaa`b¥`aa`
@HWI-EAS443:3:57:1709:126#0/1
AGCCAATTGAAGTGGGCACTCGGTGAAAATATGGCACCAG
+HWI-EAS443:3:57:1709:126#0/1
````aababaaa`babaaV^^aa]a¥]_`___`_XVSS[a
@HWI-EAS443:3:57:1717:103#0/1
GGGGTTTTCGGCTGAATCGAGAAAATTGCTTCTCATTGTT
+HWI-EAS443:3:57:1717:103#0/1
aaab[bbb¥bb[aaa`a¥`_aa`__aa_Zaa[`R]`_X[`
````

```
@HWI-EAS443:3:57:1786:1107#0/1
GTGCTAGCAGAGCTACAGGACATTGAAAATGAAGAGAAGA
+HWI-EAS443:3:57:1786:1107#0/1
a_Y_aabaab``aaa``^_]Y^aa````aaa`]Za¥X][_
@HWI-EAS443:3:57:211:1241#0/1
GCCGAAAACCCCTCAAGTTGTGGAGACGCATATAGACTGT
+HWI-EAS443:3:57:211:1241#0/1
aa^```Y`_a^_a`_^a]aaY``__^___^_^`^`_^]_B
@HWI-EAS443:3:57:230:418#0/1
GGAAACATGGCCAATCGGAGAGTCACCCAGGGGAGTGGAG
+HWI-EAS443:3:57:230:418#0/1
aa`a]]]`a^]`¥^`^^`_^Y^U[^[[YV_¥]UU[U^YSY
@HWI-EAS443:3:57:281:311#0/1
GTCAATTCACATGCCTTATTGAATTCATTTTGGACCCAGC
+HWI-EAS443:3:57:281:311#0/1
aaaaaaaabaaa`a_`aaa`a][a___a`__`___]]__]
@HWI-EAS443:3:57:364:179#0/1
CATAAATCCCAATTACCTCATGGCTTGGAAGCAGGTGCTA
+HWI-EAS443:3:57:364:179#0/1
aaaabaaa]```a``_^^Z``aaX`[R¥QZYMMZTNLBBB
@HWI-EAS443:3:57:423:148#0/1
CACTTTCCCAATAGAGCCTTCCTCCACTCCCCTGGGTGAC
+HWI-EAS443:3:57:423:148#0/1
_b`abbabbb_ab_`bUaa]`^a_a]``_a]a^ZaZSa_^
@HWI-EAS443:3:57:431:1114#0/1
AAATTTAGGCTTCTCTACCCCTGTTGTGTTACATATACTG
+HWI-EAS443:3:57:431:1114#0/1
a_abaaab_aba_aaaaa_`aaa_`aY¥W``^¥_a``[_`
@HWI-EAS443:3:57:457:26#0/1
TGGGAAAGTGTGCAGGACCTTACTGGCAAAATCTGTATTC
+HWI-EAS443:3:57:457:26#0/1
abba^`Wa_b_a^_a`^^`___]^__]YU¥^^]a`W]`^^
@HWI-EAS443:3:57:48:395#0/1
ACCGAACTTCTCCAGCCTTGAAAACTTTAGAGCCTATGTA
+HWI-EAS443:3:57:48:395#0/1
aaaa[]aaaaaa``a`]a`a``¥J[__a`a¥aV__``aZ¥
@HWI-EAS443:3:57:502:736#0/1
CAAAGAATTCCTTGGTCATGTCTTTCTCTTTGACAGAAGA
+HWI-EAS443:3:57:502:736#0/1
aaabb]aaaaaabbb`aabba`aaaaa`aaaa_aaaaaaa
@HWI-EAS443:3:57:540:1665#0/1
GGGGGCTATATGAAGCAATCGAGGAGTGCCTGATTAATGA
+HWI-EAS443:3:57:540:1665#0/1
M^ZSabbaaa___bR[_Y[[Vab_b``baaZ^]abbbbYb
@HWI-EAS443:3:57:542:923#0/1
TCCAAAATGAATTCAATAAGGCATGTGAATTGACTGATTC
+HWI-EAS443:3:57:542:923#0/1
Y¥Y`_`¥¥b`[`[a`¥b^``b_Y__N¥_``a`YT_`]¥]`
@HWI-EAS443:3:57:563:1173#0/1
CAATAATTGTAGAATCTGGTGACCCGAATGCACTATTGAA
```

+HWI-EAS443:3:57:563:1173#0/1
abbbbbbbabab_bbbbbaabbaababbbb`aabbbbbab
@HWI-EAS443:3:57:597:901#0/1
ATCAGGAAGAAATTTAGGCTTCTCTACCCCTGTTGTGTTA
+HWI-EAS443:3:57:597:901#0/1
aaaaaa`aa^aaaaaaaa``a___^Y^_aa_`]^_^^[^_
@HWI-EAS443:3:57:602:1322#0/1
GGGAGACTTTGGTCGGCAAGCTTGCGCATAGTTCCTGTAA
+HWI-EAS443:3:57:602:1322#0/1
YZY^^```[^W¥S¥[^[[¥Z[]YZWWV]YV]VW[[VTU
@HWI-EAS443:3:57:621:1663#0/1
CTGAAATTAAGTATTGAAGACCCGAGTCACGAGGGGGAGG
+HWI-EAS443:3:57:621:1663#0/1
UV_b_KV_QX_Y_V_[__XOT¥VXXWOWXXVXaa`X][¥]
@HWI-EAS443:3:57:629:574#0/1
GAGAAAGGCATAAATCCCAATTACCTCATGGCTTGGAAGC
+HWI-EAS443:3:57:629:574#0/1
aaba^Taaaabaaa```aaaaa``a___`a_``_[ZT^VT
@HWI-EAS443:3:57:65:111#0/1
CGTCTCCACAACTTGAGGGGTTTTCGGCTGAATCGAGAAA
+HWI-EAS443:3:57:65:111#0/1
[bbabbb][b_`abbabbbb_abaXa`_abba_^aaa[_a
@HWI-EAS443:3:57:680:1111#0/1
GGGTGAATCAATAATTGTAGAATCTGGTGACCCGAATGCA
+HWI-EAS443:3:57:680:1111#0/1
abbababbaa`bbbbbb`ababaabbb_bbababa`bbaa
@HWI-EAS443:3:57:701:945#0/1
CATAAATCCCAATTACCTCATGGCTTGGAAGCAGGTGCTA
+HWI-EAS443:3:57:701:945#0/1
]_b_^_bab__bbbbbbbaba^aab_O_¥¥¥Z^[[ROZ]Z
@HWI-EAS443:3:57:705:191#0/1
GGCAAGAATCAAAACTAGGCTTTTCACTATAAGACAAGAA
+HWI-EAS443:3:57:705:191#0/1
aaaaaa^aaaa``a_aaa``__`a^a`a``_`_]__]_^^
@HWI-EAS443:3:57:721:346#0/1
AGCTCTGCTAGCACCTGCTTCCAAGCCATGAGGTAATTGG
+HWI-EAS443:3:57:721:346#0/1
aa``aaaaa`a``_`aa`_`__aa_`^^``^aaWW¥`^^^
@HWI-EAS443:3:57:736:265#0/1
GGGGCATGGAAATGAGGCGCTGCCTTCTTCAGTCTCTTCA
+HWI-EAS443:3:57:736:265#0/1
aaaaaaaba```aaaaa`b^`aYS__Y_a][]YY][`^VU
@HWI-EAS443:3:57:739:1404#0/1
GGGGAGGGAATACCACTATATGATGCAATCAAATGCATGA
+HWI-EAS443:3:57:739:1404#0/1
`^_^^__]`^``^^`¥aaa^a¥^]^S^^^_a]_Z_Z`^Z_
@HWI-EAS443:3:57:821:1269#0/1
CATGGAAATGAGGCGCTGCCTTCTTCAGTCTCTTCAGCAG
+HWI-EAS443:3:57:821:1269#0/1
a_ab`bX`_ba[W_[aa`]_a_][a^`_¥a__a¥PTFNZ`

```
@HWI-EAS443:3:57:844:627#0/1
GCAGTCATCAAAGTCTACTTTTTCTGGTGCCATATTTTCA
+HWI-EAS443:3:57:844:627#0/1
ab`b`babaaaa__abaabababab^aba_baaab`bbaaaa
@HWI-EAS443:3:57:872:534#0/1
TTGGGATCTTCTCTTCATTTTCAATGTCCTGTAGCTCTGC
+HWI-EAS443:3:57:872:534#0/1
aaaaa^aabaaa`baaabaaaaa`aa__[`a`aaa``_^]
@HWI-EAS443:3:57:945:828#0/1
GTCCAGGCCATGATTCGGTCTCTTCCTTCAATTATCTCAA
+HWI-EAS443:3:57:945:828#0/1
aa`^^`aaaaa_V_`aaa_^`Z``^^``__```[`[^]]a
@HWI-EAS443:3:57:953:113#0/1
AAAGGCATAAATCCCAATTACCTCATGGCTTGGAAGCAGG
+HWI-EAS443:3:57:953:113#0/1
a`bbaabbb`aaa_a^^b]aa_a`a`aa`aa]^^a`X^`B
@HWI-EAS443:3:57:979:1580#0/1
ACGGGGTGAATCAATAATTGTAGAATCTGGTGACCCGAAT
+HWI-EAS443:3:57:979:1580#0/1
X_bVO_¥¥¥_^Vabb^Z¥[Za^T`baa¥]_TbbZa_aab_
@HWI-EAS443:3:58:1039:389#0/1
CAGAAAAAGTAGACTTTGATGACTGCAAAGATGTTGGAGA
+HWI-EAS443:3:58:1039:389#0/1
`bbab]abb^bb_babbabbbUabba`baa_ba_bbaaaa
@HWI-EAS443:3:58:107:283#0/1
CTGGAAAGAGCCTAACATAGTCAAACCACATGAGAAAGGC
+HWI-EAS443:3:58:107:283#0/1
abbaa^¥abababbbbbaaaaabababaabaa`a[]`aaa
@HWI-EAS443:3:58:117:1252#0/1
GATGAAATGGAAATCCGAATACATGAAACAAACTTCCAAA
+HWI-EAS443:3:58:117:1252#0/1
¥[_^_aba`aX``aa^`]¥`a`a_]Z`aaaa_¥_^`^___
@HWI-EAS443:3:58:1248:459#0/1
ATTGGTTCTCACATATAGGAACATGGGCCTCGACACTTGG
+HWI-EAS443:3:58:1248:459#0/1
abbba^a_`U]¥^`[¥[aa_ZR^_`__¥_`_``Y_T[^]^
@HWI-EAS443:3:58:1361:291#0/1
CTTGGGGGGCTATATGAAGCAATCGAGGAGTGCCTGATTA
+HWI-EAS443:3:58:1361:291#0/1
abbbbabbbabbbabba```aab_a`ba_b__R_abaaaa
@HWI-EAS443:3:58:1438:840#0/1
GTACATAAATACGGCCTTGCTCAATGCATCCTGTGCAGCC
+HWI-EAS443:3:58:1438:840#0/1
a]a__baa`aaaa_]]Z`RZN^]Z__]^_¥^]`X_NU^OX
@HWI-EAS443:3:58:145:267#0/1
GACATTCTTTGGCTGGAAAGAGCCTAACATAGTCAAACCA
+HWI-EAS443:3:58:145:267#0/1
aa_¥_^aa^__``[a^Y[^^P[XTO[UVWSSVVOKX]UUT
@HWI-EAS443:3:58:1462:1941#0/1
ATTCCTTGGTCATGTCTTTCTCTTTGACAGAAGACTCGGC
```

```
+HWI-EAS443:3:58:1462:1941#0/1
_]aa¥b`^Y^a[`a_Waaa`a]aaX¥``Yaaa]_a`_WUS
@HWI-EAS443:3:58:1479:1232#0/1
AGACTTTGGTCGGCAAGCTTGCGCATAGTTCCTGTAATCT
+HWI-EAS443:3:58:1479:1232#0/1
]`M^Taa_a_a[```aa^]]__[XXa^_Z`][_^[Z¥_Za
@HWI-EAS443:3:58:1488:1734#0/1
ATCAGGCACTCCTCGATTGCTTCATATAGCCCCCCAAGAT
+HWI-EAS443:3:58:1488:1734#0/1
aaaaab`b_a^aaaaa```N````aaaa__`¥`¥__`_`^
@HWI-EAS443:3:58:14:1387#0/1
CTGCAGGGCTACTGAATACATAATGAAGGGAGTGTACATA
+HWI-EAS443:3:58:14:1387#0/1
`_]¥b]]aa`Y^Q¥]a^a`Z`aaa`]`XYQ^aR`¥[`_¥^
@HWI-EAS443:3:58:1540:423#0/1
ATTGGGATCAGCTGAAAGTCATCCATGGCTGCACAGGATG
+HWI-EAS443:3:58:1540:423#0/1
`Z_`W]¥¥a`a]¥¥`]_a¥X[¥UW¥]ZJVYBBBBBBBBBB
@HWI-EAS443:3:58:1617:909#0/1
CTGGTGCCATATTTTCACCGAGTGCCCACTTCAATTGGCT
+HWI-EAS443:3:58:1617:909#0/1
`¥bbH¥^S¥VO¥`aaab`aaTaY`RJV`¥^__`[`K]TX_
@HWI-EAS443:3:58:1638:1324#0/1
CTCTTTGTAATCATACAAATCAGGAAGAAATTTAGGCTTC
+HWI-EAS443:3:58:1638:1324#0/1
ababbbaabababbb`bbba^bba`abaaabbaaaa`ba`
@HWI-EAS443:3:58:1770:1027#0/1
CTCCAACATCTTTGCAGTCATCAAAGTCTACTTTTTCTGG
+HWI-EAS443:3:58:1770:1027#0/1
a¥XUV¥^QWPOPY[ZTTKXYRLROUWHZRMWXY[YNXYYV
@HWI-EAS443:3:58:1777:545#0/1
GCTAGCAGAGCTACAGGACATTGAAAATGAAGAGAAGATC
+HWI-EAS443:3:58:1777:545#0/1
^`baaa``aa`abbbbbbbba`_aaaa`a`aabba`aa_
@HWI-EAS443:3:58:189:1735#0/1
ATCCGAAAATCGAAACTAACAAGTTTGCTGCAATATGCAC
+HWI-EAS443:3:58:189:1735#0/1
¥__aZU`b¥YS¥Y^a_aa``_^_Z`]WYV`_aa^a]W[__
@HWI-EAS443:3:58:454:773#0/1
GCCATATTTTCACCGAGTGCCCACTTCAATTGGCTTGTTC
+HWI-EAS443:3:58:454:773#0/1
`^]aaZaa¥``aa_a_aYa____^`^_`_^]__¥ZXESVV
@HWI-EAS443:3:58:477:1698#0/1
CTAGTTTTGATTCTTGCCCTGCTCTCTTCGTCAAGGGTGT
+HWI-EAS443:3:58:477:1698#0/1
abbbababababbbbbbabbaabaaa]F]aaba`a^`a]Ya_
@HWI-EAS443:3:58:502:988#0/1
ATCCCAATTACCTCATGGCTTGGAAGCAGGTGCTAGCAGA
+HWI-EAS443:3:58:502:988#0/1
aaa_¥aW^aaa_aaa`a`_``[¥¥_RXDOZRRXYOWQVQN
```

@HWI-EAS443:3:58:506:1687#0/1
CATTAATCAGGCACTCCTCGATTGCTTCATATAGCCCCCC
+HWI-EAS443:3:58:506:1687#0/1
a`XZ]OSU¥V_Y]`¥U^_RVWW`QFZ``aQY`][¥G¥VBB
@HWI-EAS443:3:58:540:1097#0/1
AATTCCTTGGTCATGTCTTTCTCTTTGACAGAAGACTCGG
+HWI-EAS443:3:58:540:1097#0/1
`YY_^GPU_bZRWZ[T[RRX__```]TZ¥^_PTYWH[VU[
@HWI-EAS443:3:58:544:1365#0/1
GTCAAACCACATGAGAAAGGCATAAATCCCAATTACCTCA
+HWI-EAS443:3:58:544:1365#0/1
^Xbabbbb¥^_Z`^`¥]¥Z_^aabbU``babab^`]Z_ab
@HWI-EAS443:3:58:60:1140#0/1
CAGCAAACTTGTTAGTTTCGATTTTCGGATCTTCCCCATA
+HWI-EAS443:3:58:60:1140#0/1
aaa_`Ya`aaaaa``W_`_aR^[^_UTZUZUSYUW]ZXVV
@HWI-EAS443:3:58:621:1591#0/1
CGGTGGGAGACTTTGGTCGGCAAGCTTGCGCATAGTTCCT
+HWI-EAS443:3:58:621:1591#0/1
a`MSa]X[a]]]a`HRLVRZMP]UYPF¥TV_QZPUEX¥YU
@HWI-EAS443:3:58:691:1614#0/1
GGTGAATCAATAATTGTAGAATCTGGTGACCCGAATGCAC
+HWI-EAS443:3:58:691:1614#0/1
`bJaab``__bba`abaaaaaaaaaa]aaaa`a_aaaa`_
@HWI-EAS443:3:58:790:1257#0/1
GGGGGAGGGAATACCACTATATGATGCAATCAAATGCATG
+HWI-EAS443:3:58:790:1257#0/1
¥O___[``a¥__[X[^]`_`Y¥X¥Z^¥XZ^U_¥¥¥VZ_Z¥
@HWI-EAS443:3:58:790:823#0/1
ATTGAAGCACCGATTTGAGATAATTGAAGGAAGAGACCGA
+HWI-EAS443:3:58:790:823#0/1
a`aaYaa`_````aabaab`a`_a`a_`_aa_baa_`^_a
@HWI-EAS443:3:58:791:1066#0/1
ATCGACGAACGGGGTGAATCAATAATTGTAGAATCTGGTG
+HWI-EAS443:3:58:791:1066#0/1
aa]aa]_aWa[a_`Raaaa^_^`_`aaYa_a___TY¥aVa
@HWI-EAS443:3:58:812:1995#0/1
GAGAAAGGCATAAATCCCAATTACCTCATGGCTTGGAAGC
+HWI-EAS443:3:58:812:1995#0/1
aI[NPS[]¥a¥Zaa`V¥O^NY_YBBBBBBBBBBBBBBBBB
@HWI-EAS443:3:58:822:860#0/1
CAATGAACCGGTTCTCTTTGTAATCATACAAATCAGGAAG
+HWI-EAS443:3:58:822:860#0/1
a`a_a`aaaa`U^`aaa`_aZZ^a[_a__`a]_]a¥Z[R_
@HWI-EAS443:3:58:889:14#0/1
GGGTTTTCGGCTGAATCGAGAAAATTGCTTCTCATTGTTC
+HWI-EAS443:3:58:889:14#0/1
`a`W`aa]a`_`]T_[X^¥_^^`^¥V¥X^]]_W^^[^UVR
@HWI-EAS443:3:58:897:1054#0/1
CGAAAATCGAAACTAACAAGTTTGCTGCAATATGCACACA

```
+HWI-EAS443:3:58:897:1054#0/1
aabbbbbababbabaaabba^abaabbaaabaaa_baa_a
@HWI-EAS443:3:58:903:805#0/1
ATTTGTTTTCAAAGAATTCCTTGGTCATGTCTTTCTCTTT
+HWI-EAS443:3:58:903:805#0/1
`bb]b`aba_aYaba_aaaaaa_¥^¥X_baaaaa_a[aa`
@HWI-EAS443:3:58:913:821#0/1
ACGGCCTTGCTCAATGCATCCTGTGCAGCCATGGATGACT
+HWI-EAS443:3:58:913:821#0/1
a`aaaaaa`^a_aaaa__`Z¥aa^¥``aUa``a¥[]X[^a
@HWI-EAS443:3:59:1063:1483#0/1
CATTAATCAGGCACTCCTCGATTGCTTCATATAGCCCCCC
+HWI-EAS443:3:59:1063:1483#0/1
_[b_S_ab¥¥a__``ba[Yab¥bbb`bb_b`aa]ZTGN_Y
@HWI-EAS443:3:59:1128:785#0/1
GGCCAATCGGAGAGTCACCCAGGGGAGTGGAGGAAGGCTC
+HWI-EAS443:3:59:1128:785#0/1
abaaa`_`aa^`^`_`a`^`]aaaaXaUaa¥U^WZPST¥V
@HWI-EAS443:3:59:1164:1075#0/1
GCCGAGTCTTCTGTCAAAGAGAAAGACATGACCAAGGAAT
+HWI-EAS443:3:59:1164:1075#0/1
a`a^ab`aaba`b^_a``a`a`^`aa^^```X__¥UW^¥T
@HWI-EAS443:3:59:1243:321#0/1
GGGGGGCTATATGAAGCAATCGAGGAGTGCCTGATTAATG
+HWI-EAS443:3:59:1243:321#0/1
a`RN`XabHaa`ZVZ`YYT__[RX`TYFRTRHVPRUQGQU
@HWI-EAS443:3:59:1284:259#0/1
CACTTCTGCTGTAAAATAGTTCCTCCTCATGCTTGCGATA
+HWI-EAS443:3:59:1284:259#0/1
aaabaaaaaabaabbaaaa`aa_a__aaaaaXa`aYY]]]
@HWI-EAS443:3:59:1313:77#0/1
TCGGCCTCAATCATGCTCTCAATCTGCTGAAGAGACTGAA
+HWI-EAS443:3:59:1313:77#0/1
aaaa``aZaaa^_aa_a____``¥_a_aa]MXZ[WWQ_TT
@HWI-EAS443:3:59:1335:206#0/1
CAAGGACAAAGAACATGAAGAGAACAAGCCAATTGAAGTG
+HWI-EAS443:3:59:1335:206#0/1
`b`bba`babaabbbabaabbbbbbb_bbab_ba^TM^[`
@HWI-EAS443:3:59:1392:722#0/1
ATTGCATCATATAGTGGTATTCCCTCCCCCTCGTGACTCG
+HWI-EAS443:3:59:1392:722#0/1
abaa`abaa`ab`a¥`a_`aaa]aabbb`a_Za^^VX_X`
@HWI-EAS443:3:59:1409:1188#0/1
CAATGGAACCTCCAAGATCAAGATGAAATGGGGCATGGAA
+HWI-EAS443:3:59:1409:1188#0/1
`bab_¥`aa]aa_baab_ba`babaa_babbXaa_X¥`__
@HWI-EAS443:3:59:1414:1607#0/1
CACTCCCCTGGGTGACTCTCCGATTGGCCATGTTTCCGAT
+HWI-EAS443:3:59:1414:1607#0/1
_`]aV]`ab_bbUba^b_aPWS^aaX_¥a¥YZTa`RTQSV
```

```
@HWI-EAS443:3:59:1478:321#0/1
ATATGCACACATTTGGAAGTTTGTTTCATGTATTCGGATT
+HWI-EAS443:3:59:1478:321#0/1
aaabb¥`a_^abaaaaa`a_aaa`aba^bbYTZa_XZ_`^
@HWI-EAS443:3:59:1551:972#0/1
CTGGAACCTTCGATCTTGGGGGGCTATATGAAGCAATCGA
+HWI-EAS443:3:59:1551:972#0/1
aaWaZ^`a`_W_RXX^Xa[aaaaY_^¥a```M]Y]UQY¥S
@HWI-EAS443:3:59:1595:1899#0/1
ATCGAAACTAACAAGTTTGCTGCAATATGCACACATTTGG
+HWI-EAS443:3:59:1595:1899#0/1
a`ab^^ZR¥^_a_PY_`aY^X]`Y`^baa_aQ^XYYZaSO
@HWI-EAS443:3:59:1616:618#0/1
CAATTTTGGCGTTCACTTCTTTTGACATTTGGGAAAGCTT
+HWI-EAS443:3:59:1616:618#0/1
abbbbbbb``bba`a``aZabbb¥^a_abbbTb_aYNV^[
@HWI-EAS443:3:59:1634:565#0/1
GATTTGAGATAATTGAAGGAAGAGACCGAATCATGGCCTG
+HWI-EAS443:3:59:1634:565#0/1
``bbbb_bbbbabbbbabbbaababababbbab^abbOO^aI
@HWI-EAS443:3:59:1783:795#0/1
ACAGATTTTGCCAGTAAGGTCCTGCACACTTTCCCAATAG
+HWI-EAS443:3:59:1783:795#0/1
``___aaaa]baaaZ`aaUR_aaa]Z¥]aa`¥a^_YKYYP
@HWI-EAS443:3:59:208:85#0/1
ATTGAGGGCAAGCTTTCCCAAATGTCAAAAGAAGTGAACG
+HWI-EAS443:3:59:208:85#0/1
aaXWV_¥b`aTaaXZZRLLQ`HVaZYJWHN^V]`WXQNTX
@HWI-EAS443:3:59:22:924#0/1
ATCAAAACTAGGCTTTTCACTATAAGACAAGAAATGGCCA
+HWI-EAS443:3:59:22:924#0/1
aabaaaba]^`aa^a_a^b_aa`aa`aaaaa^_^_^]_YQ
@HWI-EAS443:3:59:269:1102#0/1
GGGGGCTATATGAAGCAATCGAGGAGTGCCTGATTAATGA
+HWI-EAS443:3:59:269:1102#0/1
a¥_`aaa`aaa`¥`__aW¥`¥_a`^_V__``¥^`^WWV]V
@HWI-EAS443:3:59:314:839#0/1
CAACCTGGAACCTGGAACCTTCGATCTTGGGGGGCTATAT
+HWI-EAS443:3:59:314:839#0/1
aaaa___a]`a`Qa`_^__`aaaW[`^VX[_WWZSPUV¥W
@HWI-EAS443:3:59:317:1143#0/1
CATGGAAATGAGGCGCTGCCTTCTTCAGTCTCTTCAGCAG
+HWI-EAS443:3:59:317:1143#0/1
`¥]__O]ZY_aRQZ]aaZT]]`[a__¥^W¥XBBBBBBBBB
@HWI-EAS443:3:59:346:1400#0/1
TGCCCCGATTGAACATATCGCAAGCATGAGGAGGAACTAT
+HWI-EAS443:3:59:346:1400#0/1
_]aabb_X_[``abaS`_HUZRT[Z]a_R¥_YO[SJLQZY
@HWI-EAS443:3:59:359:2008#0/1
GAAATGGAAATCCGAATACATGAAACAAACTTCCAAATGT
```

```
+HWI-EAS443:3:59:359:2008#0/1
aa`aa_^_aZa`__aa^a__`^`aaaa`aa_Y`^_HIU[Z
@HWI-EAS443:3:59:408:1459#0/1
CAAGCTTGCGCATAGTTCCTGTAATCTCAAATTTTTCTTC
+HWI-EAS443:3:59:408:1459#0/1
aa`aaa^``aaaa_[^aY^a`^aa_``baa_```a^O_^Z
@HWI-EAS443:3:59:435:1425#0/1
GAACGGGGTGAATCAATAATTGTAGAATCTGGTGACCCGA
+HWI-EAS443:3:59:435:1425#0/1
`abaa_]aWaaa``aaba`baa^aa¥b`b_`_U__IHRZU
@HWI-EAS443:3:59:531:1109#0/1
CCCCGTTCGTCGATGAAATGGAAATCCGAATACATGAAAC
+HWI-EAS443:3:59:531:1109#0/1
aaaabaaaaYaaa__a`a^`_``^a`a`a__`_a¥Z_^_S
@HWI-EAS443:3:59:583:1184#0/1
GGCTTGTTCTCTTCATGTTCTTTGTCCTTGGGATCTTCTC
+HWI-EAS443:3:59:583:1184#0/1
aa_bb_¥a`__`aaZXa[__aa_`Zaa_aaa_XXZZ^¥`Z
@HWI-EAS443:3:59:613:644#0/1
GGCCCATGTTCCTATATGTGAGAACCAATGGAACCTCCAA
+HWI-EAS443:3:59:613:644#0/1
a__[_`YR_baabaaaZ_]a``aab`Uaaa```_`PH`a`
@HWI-EAS443:3:59:67:1074#0/1
GAAGGAAGAGACCGAATCATGGCCTGGACAGTGGTGAACA
+HWI-EAS443:3:59:67:1074#0/1
`¥Waaaa]¥¥a]aaaa``a`aaaa¥]^Za]¥H_TLUU^_[
@HWI-EAS443:3:59:688:1147#0/1
ACGGCCTTGCTCAATGCATCCTGTGCAGCCATGGATGACT
+HWI-EAS443:3:59:688:1147#0/1
abaaaaaa]aaaaba_aba]a`````^__`^¥_^¥SU_^^
@HWI-EAS443:3:59:704:894#0/1
CAGAGCCCAGATCTCTAGCAAGCTGGGTCCAAAATGAATT
+HWI-EAS443:3:59:704:894#0/1
aa_T_W¥bb¥QY]`bbbUY`Z`aa[P^^`^_¥WVYJVaaY
@HWI-EAS443:3:59:771:659#0/1
GAGACCGAATCATGGCCTGGACAGTGGTGAACAGTATATG
+HWI-EAS443:3:59:771:659#0/1
a¥]Zaabaaaaa`¥a`a_a¥¥_aYI[_R_[aW^^PNU_ZN
@HWI-EAS443:3:59:780:1310#0/1
GAAAAGCCTAGTTTTGATTCTTGCCCTGCTCTCTTCGTCA
+HWI-EAS443:3:59:780:1310#0/1
aaa`^a^aaaa_aaa`aaa`a_aaa^aaa`a¥_``UP[^X
@HWI-EAS443:3:59:790:1547#0/1
GACATTCTTTGGCTGGAAAGAGCCTAACATAGTCAAACCA
+HWI-EAS443:3:59:790:1547#0/1
ababbaababaaabaa`aa[aa_ababaaab^`__^ZZX_
@HWI-EAS443:3:59:863:1275#0/1
CTCCAGTCTCGGGTCAGTGAGTGAGAACTCCATACTTACA
+HWI-EAS443:3:59:863:1275#0/1
``_[`aUWVVaa^KWHYa_XX]U[P]YH^aa[[]TN]a`Y
```

@HWI-EAS443:3:59:962:550#0/1
TGGGTCCAAAATGAATTCAATAAGGCATGTGAATTGACTG
+HWI-EAS443:3:59:962:550#0/1
abaa_aaaaaaaaaaa``a`aa`aa``aa_a]^a`[Z_`_
@HWI-EAS443:3:5:1033:1610#0/1
GAAGATCCCAAGGACAAAGAACATGAAGAGAACAAGCCAA
+HWI-EAS443:3:5:1033:1610#0/1
aab``aa__aaaaa_`_`a__Zaaa^_a`_`_O^_a¥Y`_
@HWI-EAS443:3:5:1077:1693#0/1
GAAGTTCGGTGGGAGACTTTGGTCGGCAAGCTTGCGCATA
+HWI-EAS443:3:5:1077:1693#0/1
]`b`]`]_]`_b_W`]Vaaa`[]]aa[[V`aa`WM`^_^B
@HWI-EAS443:3:5:1133:556#0/1
GAGTGTACATAAATACGGCCTTGCTCAATGCATCCTGTGC
+HWI-EAS443:3:5:1133:556#0/1
a``^`Za¥`a_`]`[L^`Uaa¥¥Y`X^`¥¥V¥_Z[Z`Y_B
@HWI-EAS443:3:5:1152:980#0/1
CATTCTTTGGCTGGAAAGAGCCTAACATAGTCAAACCACA
+HWI-EAS443:3:5:1152:980#0/1
Y¥`X__YZ^VO¥aZ]^Za_[`b`¥¥MVaabU[¥^^ZV¥`a
@HWI-EAS443:3:5:115:891#0/1
GCCCACTTCAATTGGCTTGTTCTCTTCATGTTCTTTGTCC
+HWI-EAS443:3:5:115:891#0/1
`abaaaaaaaaa`^[U_YNR__^_^_Ua_NO_W_^__]_B
@HWI-EAS443:3:5:1169:1357#0/1
AGCAGGTGCTAGCAGAGCTACAGGACATTGAAAATGAAGA
+HWI-EAS443:3:5:1169:1357#0/1
a_aaa`VS__a[^a_]a]_a^`a_¥W`_`W]^__]^]_]B
@HWI-EAS443:3:5:116:1112#0/1
CAATTTTCTCGATTCAGCCGAAAACCCCTCAAGTTGTGGA
+HWI-EAS443:3:5:116:1112#0/1
ab_bbbbababbababbaabaaaaaaaaaa``b_ab^baa
@HWI-EAS443:3:5:1204:1463#0/1
GGGCAAGCTTTCCCAAATGTCAAAAGAAGTGAACGCCAAA
+HWI-EAS443:3:5:1204:1463#0/1
aaaabbbbbbaa¥abbbba`bababbaababba_aaaaaa
@HWI-EAS443:3:5:1227:979#0/1
GGGATCTTCTCTTCATTTTCAATGTCCTGTAGCTCTGCTA
+HWI-EAS443:3:5:1227:979#0/1
XabU_abbZ]]_bT¥`[bbb`aaab¥X`babaaa_b_aab
@HWI-EAS443:3:5:1246:288#0/1
GGCCAGTAGGAGTCTATGGGATTCCTTTCGTCAGTCCGAA
+HWI-EAS443:3:5:1246:288#0/1
`bb`bbababab^`aaab_`aa`Y_aab]b`^¥Y¥]``^^
@HWI-EAS443:3:5:1342:1868#0/1
CATAGTCAAACCACATGAGAAAGGCATAAATCCCAATTAC
+HWI-EAS443:3:5:1342:1868#0/1
^babbbb_bbS_abba`aabaaab^bbbbbbVHZ_bbbaa
@HWI-EAS443:3:5:1342:1905#0/1
CGATTGGCCATGTTTCCGATTTGTTTTCAAAGAATTCCTT

+HWI-EAS443:3:5:1342:1905#0/1
abbab^_`baab_bb^^bSbbbb^abb¥abbba_b___ab
@HWI-EAS443:3:5:1350:739#0/1
AGACAATTGAAGAAAAATTTGAGATTACAGGAACTATGCG
+HWI-EAS443:3:5:1350:739#0/1
a`a[ba`^^_a`a_a_U_abb[¥Xa_a`a^RSa_a_`^M_
@HWI-EAS443:3:5:1394:648#0/1
GAATTCCTTGGTCATGTCTTTCTCTTTGACAGAAGACTCG
+HWI-EAS443:3:5:1394:648#0/1
ab`aa__aaba_^aTa_^aaa__^a__a_[[[a__`__[B
@HWI-EAS443:3:5:1406:776#0/1
GCCCACTTCAATTGGCTTGTTCTCTTCATGTTCTTTGTCC
+HWI-EAS443:3:5:1406:776#0/1
aaaa``ab_aaaaa__aaP[a[_Xa`RP]_``Ua`_H[RO
@HWI-EAS443:3:5:1421:876#0/1
GAGAAAGGCATAAATCCCAATTACCTCATGGCTTGGAAGC
+HWI-EAS443:3:5:1421:876#0/1
abaaaabbaabaaaaaa`aaa`a¥a_Va___NaBBBBBBB
@HWI-EAS443:3:5:1466:1933#0/1
AAATAGTTCCTCCTCATGCTTGCGATATGTTCAATCGGGG
+HWI-EAS443:3:5:1466:1933#0/1
aa]HWSS[T]^N]_L_Ya[aaa¥a]^a]XRV¥T[^VaUBB
@HWI-EAS443:3:5:1535:1617#0/1
AAGCAGGTGCTAGCAGAGCTACAGGACATTGAAAATGAAG
+HWI-EAS443:3:5:1535:1617#0/1
U]bba[[Y`a`b^T_bZb_a_T_X]]aa[`^Ya```b]_a
@HWI-EAS443:3:5:161:1189#0/1
ATTCATTTTGGACCCAGCTTGCTAGAGATCTGGGCTCTGG
+HWI-EAS443:3:5:161:1189#0/1
`R¥``^b__]Q[^_O¥W]]]S¥¥`[JV^UV_[FOLP]_BB
@HWI-EAS443:3:5:1741:1616#0/1
GCCTAACATAGTCAAACCACATGAGAAAGGCATAAATCCC
+HWI-EAS443:3:5:1741:1616#0/1
a_X¥aaaa[a`[^^``X_]Z_¥Z^a_`YY[¥Y__a]BBBB
@HWI-EAS443:3:5:232:963#0/1
GGCATAAATCCCAATTACCTCATGGCTTGGAAGCAGGTGC
+HWI-EAS443:3:5:232:963#0/1
abbbbbaba`_^abaabaabaaaaaaaa^a_`___ZaBBB
@HWI-EAS443:3:5:235:118#0/1
GGGGCATGGAAATGAGGCGCTGCCTTCTTCAGTCTCTTCA
+HWI-EAS443:3:5:235:118#0/1
a_a`_]^`_X¥^^`^a^^^_Ya¥^^YSWWW¥WTP¥¥BBBB
@HWI-EAS443:3:5:23:1764#0/1
GTCCTGTAGCTCTGCTAGCACCTGCTTCCAAGCCATGAGG
+HWI-EAS443:3:5:23:1764#0/1
a`aV_TW``[ULVS[U^XV_]URUY[SQVBBBBBBBBBBB
@HWI-EAS443:3:5:248:2037#0/1
ATCCTGTGCAGCCATGGATGACTTTCAGCTGATCCCAATG
+HWI-EAS443:3:5:248:2037#0/1
a[O^__¥a`Y[^V_¥V^ZZ`^Y]¥_Ua_¥T_¥¥^W¥WTBB

@HWI-EAS443:3:5:269:557#0/1
ATGGCACCAGAAAAAGTAGACTTTGATGACTGCAAAGATG
+HWI-EAS443:3:5:269:557#0/1
ababbabaab]`_¥`a¥_baaaaab`aaaaab__b^b_ab
@HWI-EAS443:3:5:270:710#0/1
ACCGGTTCTCTTTGTAATCATACAAATCAGGAAGAAATTT
+HWI-EAS443:3:5:270:710#0/1
a`^^a]aaa`aa¥aT``a_`____YPX^_aa¥_a^[]_`B
@HWI-EAS443:3:5:286:1430#0/1
AGCTTTCCCAAATGTCAAAAGAAGTGAACGCCAAAATTGA
+HWI-EAS443:3:5:286:1430#0/1
]P]W¥Y^NYa[[YaHHYYSSRS__Y]`a_`WLJBBBBBBB
@HWI-EAS443:3:5:288:220#0/1
AGGGAGTGTACATAAATACGGCCTTGCTCAATGCATCCTG
+HWI-EAS443:3:5:288:220#0/1
abbbabaaaaa_b`aabaa_aW[a`Z_a__`a_Za_]W¥B
@HWI-EAS443:3:5:413:1134#0/1
TGGGGGGCTATATGAAGCAATCGAGGAGTGCCTGATTAAT
+HWI-EAS443:3:5:413:1134#0/1
aaaaa^aa`abaaa_`aaaaa]a_``_a_____a___``B
@HWI-EAS443:3:5:432:435#0/1
TGGGGGGCTATATGAAGCAATCGAGGAGTGCCTGATTAAT
+HWI-EAS443:3:5:432:435#0/1
abbabaaaabbaabaa`abab¥bab^`a^b^_a`_````_
@HWI-EAS443:3:5:442:1612#0/1
GTTACATATACTGTTCACCACTGTCCAGGCCATGATTCGG
+HWI-EAS443:3:5:442:1612#0/1
^S^_`ab`T¥H¥[_aa[`S_aaa_`_a^`[Ua`X¥]NV^_
@HWI-EAS443:3:5:442:620#0/1
TGTTTTCAAAGAATTCCTTGGTCATGTCTTTCTCTTTGAC
+HWI-EAS443:3:5:442:620#0/1
ab^abaaaaaba_ba``aabbY`aab_^`aa_a^aa___Z
@HWI-EAS443:3:5:443:680#0/1
AGCAGGTGCTAGCAGAGCTACAGGACATTGAAAATGAAGA
+HWI-EAS443:3:5:443:680#0/1
a]aabb[a`aba[aaaaaaaaabaa``aaaa_a`aa`aaa
@HWI-EAS443:3:5:470:993#0/1
CCCAAATGTCAAAAGAAGTGAACGCCAAAATTGAACCATT
+HWI-EAS443:3:5:470:993#0/1
abbb``aa^ba_X``aaa¥aaaaa_a_a`_``_`a`H`aB
@HWI-EAS443:3:5:689:206#0/1
GCTGAAAGTCATCCATGGCTGCACAGGATGCATTGAGCAA
+HWI-EAS443:3:5:689:206#0/1
a`aaaaaa__a`__`aa][X`V¥QY__[UXLYRBBBBBBB
@HWI-EAS443:3:5:690:1313#0/1
TTTCCGATTTGTTTTCAAAGAATTCCTTGGTCATGTCTTT
+HWI-EAS443:3:5:690:1313#0/1
a]_abbY]aQ_J¥aX¥aZ]a^_b`VPZ]aa¥W_`aT_a`B
@HWI-EAS443:3:5:708:802#0/1
CACTCCCCTGGGTGACTCTCCGATTGGCCATGTTTCCGAT

```
+HWI-EAS443:3:5:708:802#0/1
aa``Ya`WHU`bSaa[aa[`¥Y[a`_¥`Y¥ZXVaSU_`ZB
@HWI-EAS443:3:5:716:662#0/1
CATTCTTTGGCTGGAAAGAGCCTAACATAGTCAAACCACA
+HWI-EAS443:3:5:716:662#0/1
`bb`bababaa`aba`¥^ab^Z_[bS`a_b¥aaaa¥]aX^
@HWI-EAS443:3:5:752:1659#0/1
ATTAATCAGGCACTCCTCGATTGCTTCATATAGCCCCCCA
+HWI-EAS443:3:5:752:1659#0/1
aab```b`aaabY^a_`Sa^`_`ZHZWa`_Sa_^WTSQBB
@HWI-EAS443:3:5:77:482#0/1
GGGGGGCTATATGAAGCAATCGAGGAGTGCCTGATTAATG
+HWI-EAS443:3:5:77:482#0/1
aaaaaaaaaaaab`aa`baa`a`aa_aYa__aa__a`¥_`
@HWI-EAS443:3:5:795:1692#0/1
GGGGGCTATATGAAGCAATCGAGGAGTGCCTGATTAATGA
+HWI-EAS443:3:5:795:1692#0/1
a^a_¥]ab_`aa]_aaaaUaaQ__aaZYMX_aa__Ya¥a_
@HWI-EAS443:3:5:868:955#0/1
ATAATGAAGGGAGTGTACATAAATACGGCCTTGCTCAATG
+HWI-EAS443:3:5:868:955#0/1
aaababbbb_a`aab_]`bbabaabaab^YaaaaaX`aba
@HWI-EAS443:3:5:956:860#0/1
GCCCTGCTCTCTTCGTCAAGGGTGTAGTCCGCTTTGGTGG
+HWI-EAS443:3:5:956:860#0/1
a^a`_``a``^`][a¥]¥Xaa`S`ZT¥SYX^S^^¥Y]BBB
@HWI-EAS443:3:5:958:1456#0/1
GCCCTGCTCTCTTCGTCAAGGGTGTAGTCCGCTTTGGTGG
+HWI-EAS443:3:5:958:1456#0/1
`a``¥`^^_a__a`aa[TaW_a¥_^aS^```Z^`^XXUQ`
@HWI-EAS443:3:60:1066:1325#0/1
GGGGAGGGAATACCACTATATGATGCAATCAAATGCATGA
+HWI-EAS443:3:60:1066:1325#0/1
Y_a^[a`bbba_^abbbb`a`a^bba``abbbaaa^aa`a
@HWI-EAS443:3:60:1086:616#0/1
ATATACTGTTCACCACTGTCCAGGCCATGATTCGGTCTCT
+HWI-EAS443:3:60:1086:616#0/1
abaaaabb``aaa`^aaa_a]aa_a_`_`_`a^¥`][`]V
@HWI-EAS443:3:60:1100:27#0/1
GGCATGGAAATGAGGCGCTGCCTTCTTCAGTCTCTTCAGC
+HWI-EAS443:3:60:1100:27#0/1
abb`a`baabab`a_¥a`aa¥¥_`__a[^_^Z_S_^VZWW
@HWI-EAS443:3:60:1103:1252#0/1
CAAGCTTGCCGACCAAAGTCTCCCACCGAACTTCTCCAGC
+HWI-EAS443:3:60:1103:1252#0/1
_X]_¥aba¥_W¥a_^[b¥[XYXNY_QVVYTXUSRXZYSOX
@HWI-EAS443:3:60:1153:435#0/1
GAACCTGGAACCTTCGATCTTGGGGGGCTATATGAAGCAA
+HWI-EAS443:3:60:1153:435#0/1
aaa__aaa`a`_a__a__Z_`[`_`__^_[`[[¥^^VS]_
```

@HWI-EAS443:3:60:1194:868#0/1
GGGGTTTTCGGCTGAATCGAGAAAATTGCTTCTCATTGTT
+HWI-EAS443:3:60:1194:868#0/1
^^[a¥bbbabaa`b]a^Xa`aaaaaaaa_^]aaVaaaa_a
@HWI-EAS443:3:60:1289:998#0/1
AGCAGGTGCTAGCAGAGCTACAGGACATTGAAAATGAAGA
+HWI-EAS443:3:60:1289:998#0/1
aZ_aa^[`]`^Z[X]¥]_^`X_aa]¥[_a^^^^[aa][]_
@HWI-EAS443:3:60:1303:1512#0/1
GATTCAAGCTGGATAGAACTTGATGAAATAGGAGAAGATG
+HWI-EAS443:3:60:1303:1512#0/1
a¥Y`]^WaJ_]`GMZW`^^]_`_`_Z]`_^^^W`^^Y^Y_
@HWI-EAS443:3:60:1390:535#0/1
TTGGGGGGCTATATGAAGCAATCGAGGAGTGCCTGATTAA
+HWI-EAS443:3:60:1390:535#0/1
abbaaaaaaaabaaa``aa`a`^a_aa_`]a^]_`]____
@HWI-EAS443:3:60:1476:1071#0/1
GCCTTATTGAATTCATTTTGGACCCAGCTTGCTAGAGATC
+HWI-EAS443:3:60:1476:1071#0/1
a]P^a`X`aa`bbRJU`[`T¥^VGXP]TX_XWZRVXZFVN
@HWI-EAS443:3:60:1515:54#0/1
GGGCATGGAAATGAGGCGCTGCCTTCTTCAGTCTCTTCAG
+HWI-EAS443:3:60:1515:54#0/1
aaZ`TUZ_LQ`V^^a_[]UY^SSZTFZVHN`PJQSQUUPT
@HWI-EAS443:3:60:1528:629#0/1
CACATTCACATCTTTTCATTCACTGGAGAGGAGATGGCCA
+HWI-EAS443:3:60:1528:629#0/1
ab`ab`bbaaaabbabaabaaa`a``aaaa__a__a_RXa
@HWI-EAS443:3:60:1532:149#0/1
GGGGCATGGAAATGAGGCGCTGCCTTCTTCAGTCTCTTCA
+HWI-EAS443:3:60:1532:149#0/1
aa``^`aa`^a``aaa`_a[_`ZUZ_Z`_^__WT_Z¥[W[
@HWI-EAS443:3:60:1544:942#0/1
ACAAAATAAAATCTGAGAAGACACACATTCACATCTTTTC
+HWI-EAS443:3:60:1544:942#0/1
aaaaaaaaaaaa_aaaa``a_``]_]_aa`____¥a```¥
@HWI-EAS443:3:60:1546:979#0/1
AATTCCTTGGTCATGTCTTTCTCTTTGACAGAAGACTCGG
+HWI-EAS443:3:60:1546:979#0/1
a`ba^aab`]Zaaaa¥`aaa^a_ab`a^`a_`a_a___^`
@HWI-EAS443:3:60:1575:928#0/1
CCCTGCTCTCTTCGTCAAGGGTGTAGTCCGCTTTGGTGGC
+HWI-EAS443:3:60:1575:928#0/1
aaabbababbbb`b^aaaabb¥b^aa`a_a¥aaaaa^]aZ
@HWI-EAS443:3:60:1611:1602#0/1
ATGATTCGGTCTCTTCCTTCAATTATCTCAAATCGGTGCT
+HWI-EAS443:3:60:1611:1602#0/1
aaaaaa``[¥_a^`a`_a``__a__^Z_]`^]ZS`aX¥VY
@HWI-EAS443:3:60:1633:396#0/1
GGAGTTCTCACTCACTGACCCGAGACTGGAGCCACACAAA

```
+HWI-EAS443:3:60:1633:396#0/1
a^`a`a`a^a`a_a_aaa^^^a_a`]aa_`aU[Z_^Y_^`
@HWI-EAS443:3:60:1677:1626#0/1
CATTAATCAGGCACTCCTCGATTGCTTCATATAGCCCCCC
+HWI-EAS443:3:60:1677:1626#0/1
aaabaab_a__[]`aZ]`_aaaaaa``a^a`a[ZTUW_ZZ
@HWI-EAS443:3:60:1712:11#0/1
GGAACTATTTTACAGCAGAAGTGTCCCACTGCAGGGCTAC
+HWI-EAS443:3:60:1712:11#0/1
aa`__a[aaaaa]___``]__Y`Y]Z]_Y][U[VVZW]^X
@HWI-EAS443:3:60:1753:1908#0/1
CAAGAAATGGCCAGTAGGAGTCTATGGGATTCCTTTCGTC
+HWI-EAS443:3:60:1753:1908#0/1
abb_ababaaaa`ZVa_a`¥ZXa¥[JUaX_]UU[aaGXZN
@HWI-EAS443:3:60:183:57#0/1
CATAAATCCCAATTACCTCATGGCTTGGAAGCAGGTGCTA
+HWI-EAS443:3:60:183:57#0/1
abbbb_baaaa_baaaaaaaaaaaa`[YOY^YV¥¥MXQVQ
@HWI-EAS443:3:60:253:1582#0/1
TGTGAATTGACTGATTCAAGCTGGATAGAACTTGATGAAA
+HWI-EAS443:3:60:253:1582#0/1
]aXbaaa`¥Ob^^a_`aaaa`a`___`_`a__a`_^a^`S
@HWI-EAS443:3:60:299:813#0/1
GAGCCCAGATCTCTAGCAAGCTGGGTCCAAAATGAATTCA
+HWI-EAS443:3:60:299:813#0/1
^b]`aaba[aaab_X``a`baaaaa_``^`]_`_a`aa`a
@HWI-EAS443:3:60:339:208#0/1
CATAAATCCCAATTACCTCATGGCTTGGAAGCAGGTGCTA
+HWI-EAS443:3:60:339:208#0/1
U`¥`_S¥¥babbbbabaaa`^aaaa_O_HRZBBBBBBBBB
@HWI-EAS443:3:60:363:283#0/1
AATTCACATGCCTTATTGAATTCATTTTGGACCCAGCTTG
+HWI-EAS443:3:60:363:283#0/1
aa_aaaaa`_aa`aa_aa_]aa_]`_`^_][_¥PTY]Z]Y
@HWI-EAS443:3:60:421:1207#0/1
AAAGAGCCTAACATAGTCAAACCACATGAGAAAGGCATAA
+HWI-EAS443:3:60:421:1207#0/1
abba`]`aabbaaaaa_baaaaaa`a`a__a_`___`_a`
@HWI-EAS443:3:60:441:541#0/1
CTTGGAAGCAGGTGCTAGCAGAGCTACAGGACATTGAAAA
+HWI-EAS443:3:60:441:541#0/1
a_abbaaaaaa`]_aaaaaa`_aa_a_aaa^____a]___
@HWI-EAS443:3:60:474:946#0/1
TGGTGACCCGAATGCACTATTGAAGCACCGATTTGAGATA
+HWI-EAS443:3:60:474:946#0/1
abaabbbbbbaababbab[bbbbaa^ba]a`aabba`aaa
@HWI-EAS443:3:60:486:658#0/1
TACGGCCTTGCTCAATGCATCCTGTGCAGCCATGGATGAC
+HWI-EAS443:3:60:486:658#0/1
abaaaaaa_Z]_a_[¥aaa__`a`T]^a^]a¥_`__[_^Z
```

```
@HWI-EAS443:3:60:519:958#0/1
CACCCTTGACGAAGAGAGCAGGGCAAGAATCAAAACTAGG
+HWI-EAS443:3:60:519:958#0/1
Y[¥Z^``ab_`a¥[W`b_T¥XZaYY`^`^b¥VY`bW]a`b
@HWI-EAS443:3:60:556:1318#0/1
ACCGAACTTCTCCAGCCTTGAAAACTTTAGAGCCTATGTA
+HWI-EAS443:3:60:556:1318#0/1
]]ab]PH__^XWSSaa]_WWM_^Z^NPWT][X_Y]_TLXZ
@HWI-EAS443:3:60:610:1915#0/1
GGCCTGGACAGTGGTGAACAGTATATGTAACACAACAGGG
+HWI-EAS443:3:60:610:1915#0/1
a_a`a_a`^a`X^_V`aa_^a]a___^X_a^a^``^^__`
@HWI-EAS443:3:60:64:351#0/1
GGGGTTTTCGGCTGAATCGAGAAAATTGCTTCTCATTGTT
+HWI-EAS443:3:60:64:351#0/1
a]``R[[]]POLQZXNXJMNUT]SPNPX[RPRQZSVVWSM
@HWI-EAS443:3:60:679:1308#0/1
GAAAGGCATAAATCCCAATTACCTCATGGCTTGGAAGCAG
+HWI-EAS443:3:60:679:1308#0/1
^a[^``babbbaaaaaa^aaa]aa`a¥`]^a_RL^ZLSKV
@HWI-EAS443:3:60:680:221#0/1
AGAAGCCTAAATTTCTTCCTGATTTGTATGATTACAAAGA
+HWI-EAS443:3:60:680:221#0/1
``URWJU¥¥b¥¥[_Z^aSR]_R]^]]Xa`a__¥SUY`[W¥
@HWI-EAS443:3:60:694:683#0/1
TGAGAAAGGCATAAATCCCAATTACCTCATGGCTTGGAAG
+HWI-EAS443:3:60:694:683#0/1
abaaabb_aabaaabaa````aaaaaaa_```]^aQ__^^
@HWI-EAS443:3:60:773:1712#0/1
GGCACCAGAAAAAGTAGACTTTGATGACTGCAAAGATGTT
+HWI-EAS443:3:60:773:1712#0/1
`aaa_abaa_a_WaOa`a^a_``_``_a_`^`]^`_]_X^
@HWI-EAS443:3:60:802:143#0/1
ACTGTTTAAGGTCTCCAACATCTTTGCAGTCATCAAAGTC
+HWI-EAS443:3:60:802:143#0/1
aa`]]a`aa``Z_a_`aa`_a___``___Z_^_¥__]]X^
@HWI-EAS443:3:60:809:1731#0/1
AACGCCAAAATTGAACCATTCTTGAGGACGACACCACGCC
+HWI-EAS443:3:60:809:1731#0/1
_aaZ[UX]_^[`T]^]^Y_a^¥__YZX^[`^¥¥T]`ZTSN
@HWI-EAS443:3:60:815:745#0/1
GGAAGATCCGAAAATCGAAACTAACAAGTTTGCTGCAATA
+HWI-EAS443:3:60:815:745#0/1
a``UZ[a`b¥^ab`aa`_¥Ua`¥[a^V]_aa``aa__[a`
@HWI-EAS443:3:60:830:522#0/1
GTTCTCACATATAGGAACATGGGCCTCGACACTTGGCCTA
+HWI-EAS443:3:60:830:522#0/1
aaaaaaaaaaab`aa`baa`aa`_```a_¥a`__a___`a
@HWI-EAS443:3:60:847:2024#0/1
GTACATAAATACGGCCTTGCTCAATGCATCCTGTGCAGCC
```

```
+HWI-EAS443:3:60:847:2024#0/1
a]]G]`aY__a_`]URYUTXT]_]SPPYPVNNNVUTVWUL
@HWI-EAS443:3:60:849:1293#0/1
GGGGCATGGAAATGAGGCGCTGCCTTCTTCAGTCTCTTCA
+HWI-EAS443:3:60:849:1293#0/1
Y^`_X_^`bWX^¥`b`a_`]QZ_`^a_a_S_TUZ_Z`_X¥
@HWI-EAS443:3:60:872:1459#0/1
ATCAGGCACTCCTCGATTGCTTCATATAGCCCCCCAAGAT
+HWI-EAS443:3:60:872:1459#0/1
aabaabababbb`[a]¥^[[aa``[]_]W[[¥aX`TOVRP
@HWI-EAS443:3:60:911:426#0/1
ATTTTCACCGAGTGCCCACTTCAATTGGCTTGTTCTCTTC
+HWI-EAS443:3:60:911:426#0/1
abbaa`a``a_a]a__aa`aa`a_^_^__`^GR]N¥W_aR
@HWI-EAS443:3:60:934:524#0/1
GTGCTAGCAGAGCTACAGGACATTGAAAATGAAGAGAAGA
+HWI-EAS443:3:60:934:524#0/1
aZV_a¥¥``_[¥]]]¥`aa]_^aaa^]X[_`]Y_^ZZ[_]
@HWI-EAS443:3:60:961:254#0/1
AGAAGATGTTGCCCCGATTGAACATATCGCAAGCATGAGG
+HWI-EAS443:3:60:961:254#0/1
aa]`aa_a[aa__]a`]_`_^a^`_^^^Z^WP[RXVZV[]
@HWI-EAS443:3:61:1041:59#0/1
ACGGCCTTGCTCAATGCATCCTGTGCAGCCATGGATGACT
+HWI-EAS443:3:61:1041:59#0/1
aaaaaa_aa`a_aa`a_aa_a_a¥aaaa[[¥_^^¥`¥VN¥
@HWI-EAS443:3:61:1050:1095#0/1
CCCGAATGCACTATTGAAGCACCGATTTGAGATAATTGAA
+HWI-EAS443:3:61:1050:1095#0/1
abba¥`__```baa```XZ^aaba_`aa_U_a_^Y¥¥`[R
@HWI-EAS443:3:61:105:1574#0/1
CCCACTTCAATTGGCTTGTTCTCTTCATGTTCTTTGTCCT
+HWI-EAS443:3:61:105:1574#0/1
abba`a^ZX^`U]_^aa`^ZQFY_]TRTHWZR`¥`SSPQO
@HWI-EAS443:3:61:1068:231#0/1
GCCGAGTCTTCTGTCAAAGAGAAAGACATGACCAAGGAAT
+HWI-EAS443:3:61:1068:231#0/1
a^_]_a]¥^_^]`]][]__]]Y]]X]U]Z^XWXWZWZZZX
@HWI-EAS443:3:61:1121:225#0/1
TGCGTCTCCACAACTTGAGGGGTTTTCGGCTGAATCGAGA
+HWI-EAS443:3:61:1121:225#0/1
abaaaaaaa`a`aXabbaaabaVa```a`_``^^^V^]^]
@HWI-EAS443:3:61:1184:1046#0/1
GGGGCATGGAAATGAGGCGCTGCCTTCTTCAGTCTCTTCA
+HWI-EAS443:3:61:1184:1046#0/1
`¥X¥U_[W[JY¥]a^PXZ][Z^YQJVVNVUBBBBBBBBBB
@HWI-EAS443:3:61:1187:945#0/1
AATATGGGGAAGATCCGAAAATCGAAACTAACAAGTTTGC
+HWI-EAS443:3:61:1187:945#0/1
ab`a`b¥ab`bb``^`babbaabaa`_Yaa``_]_X_][Y
```

```
@HWI-EAS443:3:61:124:38#0/1
AGCAGGTGCTAGCAGAGCTACAGGACATTGAAAATGAAGA
+HWI-EAS443:3:61:124:38#0/1
_abaab]aaa]``_a_a`___]]¥LXU^_^Y]^^[^V^_]
@HWI-EAS443:3:61:1260:708#0/1
CGTCTCCACAACTTGAGGGGTTTTCGGCTGAATCGAGAAA
+HWI-EAS443:3:61:1260:708#0/1
a``b_``¥b¥¥abbb_bbab`baba_aaaabb^Za_a¥P^
@HWI-EAS443:3:61:1338:1132#0/1
ACGGCCTTGCTCAATGCATCCTGTGCAGCCATGGATGACT
+HWI-EAS443:3:61:1338:1132#0/1
aaba¥`bbaaa```aa`[`aa`aaaa`^`[`^Y_¥[RUN¥
@HWI-EAS443:3:61:1343:1117#0/1
CAACAAAATAAAATCTGAGAAGACACACATTCACATCTTT
+HWI-EAS443:3:61:1343:1117#0/1
¥¥b¥bbbababb_bb`babaabbbbb`]Yba¥a`ab¥aaa
@HWI-EAS443:3:61:1358:1085#0/1
TCTTGATCTTGGAGGTTCCATTGGTTCTCACATATAGGAA
+HWI-EAS443:3:61:1358:1085#0/1
S_a_`ababXS¥T_][]`[R^a_`QFP][XPTS¥X^YVLL
@HWI-EAS443:3:61:135:748#0/1
CAGCTTGCTAGAGATCTGGGCTCTGGCTCATCACTGTCAT
+HWI-EAS443:3:61:135:748#0/1
abbbbbbaa^abbbabaaa`b`baaabaabaaa``a__``
@HWI-EAS443:3:61:1403:441#0/1
GCGTGGTGTCGTCCTCAAGAATGGTTCAATTTTGGCGTTC
+HWI-EAS443:3:61:1403:441#0/1
abb`b_LW`^a_a^_¥^aaaaa`a^`_a^aa`ZMZ]U^YO
@HWI-EAS443:3:61:1409:176#0/1
CAACTTGAGGGGTTTTCGGCTGAATCGAGAAAATTGCTTC
+HWI-EAS443:3:61:1409:176#0/1
aaabbbbabbaa_bbbbaabbbbbab_abaaaabaaV_]V
@HWI-EAS443:3:61:1409:1914#0/1
TTCCGATTTGTTTTCAAAGAATTCCTTGGTCATGTCTTTC
+HWI-EAS443:3:61:1409:1914#0/1
aaVa^U`^]a_a^ZY][``_``^`^`^`aUX_]aXSU_VQ
@HWI-EAS443:3:61:1484:1662#0/1
TTGGGATCTTCTCTTCATTTTCAATGTCCTGTAGCTCTGC
+HWI-EAS443:3:61:1484:1662#0/1
a_a^]^^^W`^ba_]aaabaabbaaa¥a`a`_]_^_TWZX
@HWI-EAS443:3:61:1541:1301#0/1
GGGGCATGGAAATGAGGCGCTGCCTTCTTCAGTCTCTTCA
+HWI-EAS443:3:61:1541:1301#0/1
aaa`Z`aaa_a^a]`aaaa^_a``aa_`_V[^UV[SWYUW
@HWI-EAS443:3:61:1561:194#0/1
CAGTGGTGAACAGTATATGTAACACAACAGGGGTAGAGAA
+HWI-EAS443:3:61:1561:194#0/1
abbbbbbbbbbbbabbabbbbbbbbbbaabbba]aa¥a__
@HWI-EAS443:3:61:1581:733#0/1
CCCTGGGTGACTCTCCGATTGGCCATGTTTCCGATTTGTT
```

```
+HWI-EAS443:3:61:1581:733#0/1
aabbb_bX¥aaaaa]ZZaa^^[a^HYV[aaTU¥Z¥_]]RZ
@HWI-EAS443:3:61:1600:184#0/1
CCGAACTTCTCCAGCCTTGAAAACTTTAGAGCCTATGTAG
+HWI-EAS443:3:61:1600:184#0/1
_bbbbbbbaabbababbbbbbbbbbbbbbaba`abba`_a
@HWI-EAS443:3:61:1672:1171#0/1
AAATAGTTCCTCCTCATGCTTGCGATATGTTCAATCGGGG
+HWI-EAS443:3:61:1672:1171#0/1
`b`]X^]aa[aY`__aaaaaaaa`Y_U_]W^ZU]^RQOYY
@HWI-EAS443:3:61:1712:561#0/1
ATAAATACGGCCTTGCTCAATGCATCCTGTGCAGCCATGG
+HWI-EAS443:3:61:1712:561#0/1
abbbaaaTaba``[]^`_¥Z`¥__W_]]ZV]VQ^[YS^^U
@HWI-EAS443:3:61:1723:882#0/1
TGTGGACTTCCCTCCGTGTTACTCCAATTTCAATGAACCG
+HWI-EAS443:3:61:1723:882#0/1
`aaa]`bba__aaa]^_baaaaaaa`aaa`_^T][^_[OM
@HWI-EAS443:3:61:1756:25#0/1
GTCCTGTAGCTCTGCTAGCACCTGCTTCCAAGCCATGAGG
+HWI-EAS443:3:61:1756:25#0/1
aa````¥aa``^XPZ_[]YY¥_U]YX_VXL¥XQRQWQJWY
@HWI-EAS443:3:61:194:1125#0/1
CATAAATCCCAATTACCTCATGGCTTGGAAGCAGGTGCTA
+HWI-EAS443:3:61:194:1125#0/1
ab_Sabb^a`bbabaabab``a`aa`X^YW][]X¥SF[__
@HWI-EAS443:3:61:248:761#0/1
GGGGCATGGAAATGAGGCGCTGCCTTCTTCAGTCTCTTCA
+HWI-EAS443:3:61:248:761#0/1
______U[a^¥ZX`__[¥[[¥[[[U¥`¥^[WZT¥WWYUWU
@HWI-EAS443:3:61:254:70#0/1
ATTGAATTCATTTTGGACCCAGCTTGCTAGAGATCTGGGC
+HWI-EAS443:3:61:254:70#0/1
`abb_abbaabaaabaaaaa`aa`]`___^[`]^]YYY¥V
@HWI-EAS443:3:61:283:622#0/1
ATTCGGATTTCCATTTCATCGACGAACGGGGTGAATCAAT
+HWI-EAS443:3:61:283:622#0/1
abbbbbaaaabbaababa`aa^a]S_a_a^ZW__]][^_^
@HWI-EAS443:3:61:286:1137#0/1
GGGGGCTATATGAAGCAATCGAGGAGTGCCTGATTAATGA
+HWI-EAS443:3:61:286:1137#0/1
aaa`]`aa]`aa[]]¥]_a_]W[_[`N___Y]TU¥XVWP[
@HWI-EAS443:3:61:286:117#0/1
GCTTGTTCTCTTCATGTTCTTTGTCCTTGGGATCTTCTCT
+HWI-EAS443:3:61:286:117#0/1
a]a___^_`^__^]]]]]^^_XYXXXXZ^]]Z^X_]U]ZZ
@HWI-EAS443:3:61:288:881#0/1
CTCCTATTTCATCAAGTTCTATCCAGCTTGAATCAGTCAA
+HWI-EAS443:3:61:288:881#0/1
abb```bbababaaa_aabab`aa]`a`_a____aaa]][
```

```
@HWI-EAS443:3:61:307:1328#0/1
CCCTGGGTGACTCTCCGATTGGCCATGTTTCCGATTTGTT
+HWI-EAS443:3:61:307:1328#0/1
a`]`LYZ^`]¥¥P]^`SPWRIM[`VDPQOMPORV[Z[BBB
@HWI-EAS443:3:61:309:1393#0/1
CTGAAAGTCATCCATGGCTGCACAGGATGCATTGAGCAAG
+HWI-EAS443:3:61:309:1393#0/1
aa`_[___bb_UZaa^]¥SZ^H[OW]SBBBBBBBBBBBBB
@HWI-EAS443:3:61:360:1049#0/1
CCATTTCATCGACGAACGGGGTGAATCAATAATTGTAGAA
+HWI-EAS443:3:61:360:1049#0/1
Y`bbbaabbbbbbbabbb[bb]a¥S_aabbabaaba`baa
@HWI-EAS443:3:61:37:1366#0/1
TTGGGATTTATGCCTTTCTCATGTGGTTTGACTATGTTAG
+HWI-EAS443:3:61:37:1366#0/1
]]b_QYZaab[[_a`aab`^YYa]aaX`_a¥U^]_`Y[QP
@HWI-EAS443:3:61:421:1612#0/1
AGAGAACAAGCCAATTGAAGTGGGCACTCGGTGAAAATAT
+HWI-EAS443:3:61:421:1612#0/1
L_W]Xba]``bZV]bb^`ba[a^aaaa`U¥NOG_¥`H^G^
@HWI-EAS443:3:61:434:877#0/1
AGCAGGTGCTAGCAGAGCTACAGGACATTGAAAATGAAGA
+HWI-EAS443:3:61:434:877#0/1
abbba`N_ba__a`aaaaaaa`¥ZS_`_aa]`Z_a__¥[]
@HWI-EAS443:3:61:438:854#0/1
GGGGCATGGAAATGAGGCGCTGCCTTCTTCAGTCTCTTCA
+HWI-EAS443:3:61:438:854#0/1
a_`_a`ab_[`aa^^aaa``_`_a_^_`__^[WX[¥¥^YY
@HWI-EAS443:3:61:446:659#0/1
GAACCGGTTCTCTTTGTAATCATACAAATCAGGAAGAAAT
+HWI-EAS443:3:61:446:659#0/1
a^baaab^a`aa`aaa_a]_aZ]]aY`^`a^``T¥`^]]^
@HWI-EAS443:3:61:486:560#0/1
AGCAGGTGCTAGCAGAGCTACAGGACATTGAAAATGAAGA
+HWI-EAS443:3:61:486:560#0/1
_a_W^`aa_Qa_ba_R_b^_aZ_`S_OX^`V¥¥¥^^YPTY
@HWI-EAS443:3:61:498:965#0/1
TACTGTTTAAGGTCTCCAACATCTTTGCAGTCATCAAAGT
+HWI-EAS443:3:61:498:965#0/1
a_^abaab`aaaO_abaaa`^¥a_[_``^_R___YU¥Z]X
@HWI-EAS443:3:61:49:83#0/1
TCCAGGCCATGATTCGGTCTCTTCCTTCAATTATCTCAAA
+HWI-EAS443:3:61:49:83#0/1
_`_aba]a]R¥T_]_]¥G[Y¥JJQZXVSTU¥YR]XXXZYT
@HWI-EAS443:3:61:510:266#0/1
CAAGGACAAAGAACATGAAGAGAACAAGCCAATTGAAGTG
+HWI-EAS443:3:61:510:266#0/1
aaababbbbbababba`ba`b]a`ba]^baaaaa`a__`Q_
@HWI-EAS443:3:61:512:270#0/1
ATTCGGGTCACCAGATTCTACAATTATTGATTCACCCCGT
```

+HWI-EAS443:3:61:512:270#0/1
abaaaaa[aaaaab``aaa_aYJ^^X^`a]__]a_^XX^X
@HWI-EAS443:3:61:549:1453#0/1
GGGGGCTATATGAAGCAATCGAGGAGTGCCTGATTAATGA
+HWI-EAS443:3:61:549:1453#0/1
]]][^]Z]_``aaa``^_]_]HZ¥QYTV¥_Z`SY]YZ]YV
@HWI-EAS443:3:61:563:1062#0/1
GAAAGGCATAAATCCCAATTACCTCATGGCTTGGAAGCAG
+HWI-EAS443:3:61:563:1062#0/1
aT__Z]a`a__[a___`a__a`^]¥QZP¥U]]GVURUXYV
@HWI-EAS443:3:61:573:1109#0/1
AGCAGGTGCTAGCAGAGCTACAGGACATTGAAAATGAAGA
+HWI-EAS443:3:61:573:1109#0/1
a]a`¥]Vaa^]]]`a`_a`a`a^`X]_^^YVYNV[TT]Y]
@HWI-EAS443:3:61:585:556#0/1
GGGGGCTATATGAAGCAATCGAGGAGTGCCTGATTAATGA
+HWI-EAS443:3:61:585:556#0/1
aaaaaaaaa`aaa_a`]`_`a_aa_aVa_`_`^`_^]^YV
@HWI-EAS443:3:61:586:395#0/1
AGCAGGTGCTAGCAGAGCTACAGGACATTGAAAATGAAGA
+HWI-EAS443:3:61:586:395#0/1
a^_`aa_aa`aa`_a`aa_a`a``T^`[^[[^^][¥[WVS
@HWI-EAS443:3:61:645:1670#0/1
TAATCAGGCACTCCTCGATTGCTTCATATAGCCCCCCAAG
+HWI-EAS443:3:61:645:1670#0/1
aaaaaaaaabaaa__aaa``ab_aaaaa`a_aZW^ZV^VU
@HWI-EAS443:3:61:682:409#0/1
AAAGAAGTGAACGCCAAAATTGAACCATTCTTGAGGACGA
+HWI-EAS443:3:61:682:409#0/1
¥¥WW__aZ_Z___^^ZQZ_]`bbbb_¥aaaa``[ZZWY`^
@HWI-EAS443:3:61:685:509#0/1
TGGGGGGCTATATGAAGCAATCGAGGAGTGCCTGATTAAT
+HWI-EAS443:3:61:685:509#0/1
`_Z_````aaaaaa]a``a`]^_V_`V_]a_]`____^WZ
@HWI-EAS443:3:61:712:367#0/1
ATTCGGGTCACCAGATTCTACAATTATTGATTCACCCCGT
+HWI-EAS443:3:61:712:367#0/1
a`aaa]bH_aa`^__aaaa`¥`]a^___a^__U[]]WU]W
@HWI-EAS443:3:61:755:718#0/1
TTCGGTGGGAGACTTTGGTCGGCAAGCTTGCGCATAGTTC
+HWI-EAS443:3:61:755:718#0/1
aa_UOTZNN``¥F[]ZKZMVNNQWWaBBBBBBBBBBBBBB
@HWI-EAS443:3:61:766:524#0/1
CACATTCACATCTTTTCATTCACTGGAGAGGAGATGGCCA
+HWI-EAS443:3:61:766:524#0/1
aWaba`abab_]bbaaa[b`ZH_bb_Z_Tba^`Q[`¥U¥¥
@HWI-EAS443:3:61:792:375#0/1
TACAGGTTTGTTTTCCGTCTTCCTTCTTTGGTCCTACATT
+HWI-EAS443:3:61:792:375#0/1
ababbb^aaa]baa`aaaaaabbaaaaa`aa]¥Y_`]]_^

@HWI-EAS443:3:61:792:867#0/1
ATAAAAGGAAGGTCTCATTTGAGAAATGATACTGATGTGG
+HWI-EAS443:3:61:792:867#0/1
]`_ZZa]``a_¥Y¥aab`a`aS_XW¥_]_a¥_a`a`]VLX
@HWI-EAS443:3:61:793:1042#0/1
ACATAATGAAGGGAGTGTACATAAATACGGCCTTGCTCAA
+HWI-EAS443:3:61:793:1042#0/1
abaaa_aaa`aaa_a]aXaaa]K`_`a`____]VQ]VYY]
@HWI-EAS443:3:61:875:574#0/1
TGCCCCATTTCATCTTGATCTTGGAGGTTCCATTGGTTCT
+HWI-EAS443:3:61:875:574#0/1
abba`bbbbbbaa`a_baaaaaa¥S¥a^_^[]`XTPYVDN
@HWI-EAS443:3:61:926:422#0/1
CCAGATTCTACAATTATTGATTCACCCCGTTCGTCGATGA
+HWI-EAS443:3:61:926:422#0/1
abbbbbbbbbbbbbabbbbbaabbbbaabaaaaa`a__a`
@HWI-EAS443:3:61:928:633#0/1
GGCTTCTCTACCCCTGTTGTGTTACATATACTGTTCACCA
+HWI-EAS443:3:61:928:633#0/1
a]]``]X]¥_[_[X¥^LWZ^aVYW_S¥a^`ZZVP¥ZUZVW
@HWI-EAS443:3:61:939:360#0/1
GCCGAACGGCTGCATTGAGGGCAAGCTTTCCCAAATGTCA
+HWI-EAS443:3:61:939:360#0/1
_`a_U]_aZ__¥MZ¥ZMZV__QFR[^N^¥SW¥VVNRRPQS
@HWI-EAS443:3:61:944:1570#0/1
CTGTCATACTGTTTAAGGTCTCCAACATCTTTGCAGTCAT
+HWI-EAS443:3:61:944:1570#0/1
abba_a``ab_`aab`aaYaabb¥ba¥^aa[`ZU_`W`UT
@HWI-EAS443:3:61:963:2044#0/1
AGCAGAGCTACAGGACATTGAAAATGAAGAGAAGATCCCA
+HWI-EAS443:3:61:963:2044#0/1
]`_aaa^aa`aa_aaaaaaaa¥¥aaaaaaa`a]^^]ZWXZ
@HWI-EAS443:3:61:980:1496#0/1
ATCATTAATCAGGCACTCCTCGATTGCTTCATATAGCCCC
+HWI-EAS443:3:61:980:1496#0/1
abba`ab¥babbaaabababaaY`aaba`aa`_aa`__UV
@HWI-EAS443:3:62:1017:994#0/1
GGGGGCTATATGAAGCAATCGAGGAGTGCCTGATTAATGA
+HWI-EAS443:3:62:1017:994#0/1
aaa`aaa`aaaaaa`a`a`aa_a`]_]_]_^`]_a^W^^_
@HWI-EAS443:3:62:1049:245#0/1
GACATTCTTTGGCTGGAAAGAGCCTAACATAGTCAAACCA
+HWI-EAS443:3:62:1049:245#0/1
aYU^__X]^]__]^_ZOP^]XXYXYXZZUYW]XYYZZZ]Z
@HWI-EAS443:3:62:1081:1117#0/1
TGGGGCATGGAAATGAGGCGCTGCCTTCTTCAGTCTCTTC
+HWI-EAS443:3:62:1081:1117#0/1
ab__aaaZ^`a_`a`aa``aaaa`_a¥^_aa[a__`¥_`a
@HWI-EAS443:3:62:1122:110#0/1
GTTTTCGGCTGAATCGAGAAAATTGCTTCTCATTGTTCAG

```
+HWI-EAS443:3:62:1122:110#0/1
a____]_]W_`^^]Y_X`XXYZ]ZWQYZZXYXZZZZVWYW
@HWI-EAS443:3:62:1320:755#0/1
CTTGGGGGGCTATATGAAGCAATCGAGGAGTGCCTGATTA
+HWI-EAS443:3:62:1320:755#0/1
aabbbabaaabaabab`Z_abaa_a_ab`a^a`_aa_a`a
@HWI-EAS443:3:62:1326:1915#0/1
GGCCACCAAAGCGGACTACACCCTTGACGAAGAGAGCAGG
+HWI-EAS443:3:62:1326:1915#0/1
abababbbbabbaabaaaaab^a`aaa_baaaabaa_ab_
@HWI-EAS443:3:62:1363:1529#0/1
AAGAATTCCTTGGTCATGTCTTTCTCTTTGACAGAAGACT
+HWI-EAS443:3:62:1363:1529#0/1
`_YS`a[JY`VWOS^a^`R]b^^a`R]aaaYX`Y[[`U`a
@HWI-EAS443:3:62:1372:1243#0/1
CGAGACTGGAGCCACACAAATGGGAAAAATACTGTGTTCT
+HWI-EAS443:3:62:1372:1243#0/1
aba`aaabaaaa__`a`aaaaa`a``aaaaa_a_]a_aaa
@HWI-EAS443:3:62:1374:438#0/1
AACAAAATAAAATCTGAGAAGACACACATTCACATCTTTT
+HWI-EAS443:3:62:1374:438#0/1
ababbabbabbababbaba_aaaa`a`aaa`aaaa`aaa`
@HWI-EAS443:3:62:1387:94#0/1
CCCTCCGTGTTACTCCAATTTCAATGAACCGGTTCTCTTT
+HWI-EAS443:3:62:1387:94#0/1
aaabaab`aab`Uaaa_[aaa_a``Z_^_Ya¥[_[^X_^`
@HWI-EAS443:3:62:1469:1753#0/1
CACCGATTTGAGATAATTGAAGGAAGAGACCGAATCATGG
+HWI-EAS443:3:62:1469:1753#0/1
aaa_baa`bbaba]abbbaa_aab_`_a`a_aa`_Ia^aa
@HWI-EAS443:3:62:1520:188#0/1
CTTGGTCATGTCTTTCTCTTTGACAGAAGACTCGGCCTCA
+HWI-EAS443:3:62:1520:188#0/1
```¥]PV`[bb]_Y_aba¥]]Z`a]]``UZb_VaBBBBBB
@HWI-EAS443:3:62:1520:743#0/1
TAGAGATCTGGGCTCTGGCTCATCACTGTCATACTGTTTA
+HWI-EAS443:3:62:1520:743#0/1
aab`a`a_`aa_Y`_`aaaa_``^_^^a__a__]`_]_aa
@HWI-EAS443:3:62:1532:892#0/1
CGTCTCCACAACTTGAGGGGTTTTCGGCTGAATCGAGAAA
+HWI-EAS443:3:62:1532:892#0/1
ababbaaaaab`bbababbb`bab`ab`b`b^X]a¥a¥aa
@HWI-EAS443:3:62:1641:1799#0/1
GGGGTTTTCGGCTGAATCGAGAAAATTGCTTCTCATTGTT
+HWI-EAS443:3:62:1641:1799#0/1
_ab__ab`_V_^^bab[aabb_a`_aYI_abTb_aa`VW]
@HWI-EAS443:3:62:1647:1626#0/1
ACTTCTGCTGTAAAATAGTTCCTCCTCATGCTTGCGATAT
+HWI-EAS443:3:62:1647:1626#0/1
^G]^FVa`a`ZabbY]_ZO^a_Y]`ZaVK¥[`¥ZR¥TN^Y
```
```

@HWI-EAS443:3:62:1709:341#0/1
GGGGGGCTATATGAAGCAATCGAGGAGTGCCTGATTAATG
+HWI-EAS443:3:62:1709:341#0/1
aaaa``^aaaaa^_aaaa``_a¥`_[`W`Z^¥`[UY`__`
@HWI-EAS443:3:62:1747:530#0/1
CCTTCATTATGTATTCAGTAGCCCTGCAGTGGGACACTTC
+HWI-EAS443:3:62:1747:530#0/1
aaba``bbaba`^`baaa]aa]a`aa_aa^```ZW]S]^^
@HWI-EAS443:3:62:221:1403#0/1
CATATTTTCACCGAGTGCCCACTTCAATTGGCTTGTTCTC
+HWI-EAS443:3:62:221:1403#0/1
aaaaaaaaabaaa[`Vaaa`V_a`a_]___]_^[PSYTWL
@HWI-EAS443:3:62:279:1301#0/1
CTTCTCTTCATTTTCAATGTCCTGTAGCTCTGCTAGCACC
+HWI-EAS443:3:62:279:1301#0/1
aab`Sabaabaa]`a]`_]^][Z^ZUQYVPLSVVSNFFU^
@HWI-EAS443:3:62:285:526#0/1
AATAGTTCCTCCTCATGCTTGCGATATGTTCAATCGGGGC
+HWI-EAS443:3:62:285:526#0/1
a]`YVYRYWQ^`_YVY[X^^__[WXX[XNW[XSSPZSQWU
@HWI-EAS443:3:62:302:1266#0/1
ATCAGGCACTCCTCGATTGCTTCATATAGCCCCCCAAGAT
+HWI-EAS443:3:62:302:1266#0/1
T^_Y^`^_`Zab]a^bXRUW^`ab`^W_X^XW]WX^^V`^
@HWI-EAS443:3:62:397:2037#0/1
GGACACTTCTGCTGTAAAATAGTTCCTCCTCATGCTTGCG
+HWI-EAS443:3:62:397:2037#0/1
]_aaa`aaaabaaa`bbbbabUU[R_`aaabb__]`a_a_
@HWI-EAS443:3:62:464:168#0/1
CAAATGCATGAAGACATTCTTTGGCTGGAAAGAGCCTAAC
+HWI-EAS443:3:62:464:168#0/1
a`b__bb`]aa[aababaaaa```aaa_V^][¥^¥Y¥^`a
@HWI-EAS443:3:62:475:316#0/1
GAAGTTCGGTGGGAGACTTTGGTCGGCAAGCTTGCGCATA
+HWI-EAS443:3:62:475:316#0/1
¥_[aZa¥_^L^a`]a]]`[_]^R¥V¥``[^^^Z^VZ[^[W
@HWI-EAS443:3:62:532:1138#0/1
ATTTCATCGACGAACGGGGTGAATCAATAATTGTAGAATC
+HWI-EAS443:3:62:532:1138#0/1
bbbaabb]`bb`^a`aaa¥aaaaaaba^Yaa`Xa[]aa
@HWI-EAS443:3:62:535:1052#0/1
GAAAGAGCCTAACATAGTCAAACCACATGAGAAAGGCATA
+HWI-EAS443:3:62:535:1052#0/1
`bbbabababbbabbaa`aa_baab`aaa`]aY`a¥aaa`
@HWI-EAS443:3:62:541:864#0/1
CTGAACAATGAGAAGCAATTTTCTCGATTCAGCCGAAAAC
+HWI-EAS443:3:62:541:864#0/1
aaa]`¥aX]baa^T_[[]^__aa`_]_`_^[VZVWXXYXZ
@HWI-EAS443:3:62:578:1464#0/1
ATACGGCCTTGCTCAATGCATCCTGTGCAGCCATGGATGA

```
+HWI-EAS443:3:62:578:1464#0/1
__aba`b^X_Z_U_U_W_VWXV]XUX[VP]a¥XPTRGOXQ
@HWI-EAS443:3:62:600:596#0/1
AGAGAAAGACATGACCAAGGAATTCTTTGAAAACAAATCG
+HWI-EAS443:3:62:600:596#0/1
aba_aa^a__b_aabab^aa`aa_]a]^ba[_aaa`_aU]
@HWI-EAS443:3:62:663:1510#0/1
CGATTGGCCATGTTTCCGATTTGTTTTCAAAGAATTCCTT
+HWI-EAS443:3:62:663:1510#0/1
aZH¥O]a`^[WTVQT¥[^MT^]_VZ¥V_]S¥UZ¥ZXTZVV
@HWI-EAS443:3:62:697:370#0/1
ATTTCTTCCTGATTTGTATGATTACAAAGAGAACCGGTTC
+HWI-EAS443:3:62:697:370#0/1
]¥_^^abba`a¥_a^`a`aa^aaab^[YY_Z_a^H_aW^^
@HWI-EAS443:3:62:708:193#0/1
AGGCTTTTCACTATAAGACAAGAAATGGCCAGTAGGAGTC
+HWI-EAS443:3:62:708:193#0/1
aaaaaaaaab`aaaa`a``a``_^__]a___]W^]^Z]WX
@HWI-EAS443:3:62:714:818#0/1
GAGAAGCCTAAATTTCTTCCTGATTTGTATGATTACAAAG
+HWI-EAS443:3:62:714:818#0/1
`a_`ab_`b¥a_babaaaaaa`¥aaba[abbaa]a`aaa`
@HWI-EAS443:3:62:742:609#0/1
TGAACATATCGCAAGCATGAGGAGGAACTATTTTACAGCA
+HWI-EAS443:3:62:742:609#0/1
`aabX[aa_aa_^^Z^^`^P^aW_a][^Y_[[_^a]_`_P
@HWI-EAS443:3:62:754:1342#0/1
TAATCAGGCACTCCTCGATTGCTTCATATAGCCCCCCAAG
+HWI-EAS443:3:62:754:1342#0/1
XZZ_`_T[Zab[U_^^^_a`^WU]ba]]Z¥QaaaaMY_SN
@HWI-EAS443:3:62:754:2045#0/1
CAGGTGCTAGCAGAGCTACAGGACATTGAAAATGAAGAGA
+HWI-EAS443:3:62:754:2045#0/1
`bY``Sbbbbbbaab_ababa`a_aaa`aabaa`a_`b_a
@HWI-EAS443:3:62:813:858#0/1
AAAGAGCCTAACATAGTCAAACCACATGAGAAAGGCATAA
+HWI-EAS443:3:62:813:858#0/1
abba]abaaaaa_a]a_aaab_aa_a`^_`__a^^^__`_
@HWI-EAS443:3:62:885:591#0/1
CACTGGAGAGGAGATGGCCACCAAAGCGGACTACACCCTT
+HWI-EAS443:3:62:885:591#0/1
abbaa_^`a]YZaaaa`aabaa^aa`^`a`_`a^aYY`_]
@HWI-EAS443:3:62:908:100#0/1
TGGGATCTTCTCTTCATTTTCAATGTCCTGTAGCTCTGCT
+HWI-EAS443:3:62:908:100#0/1
abab]a`aa]a_aa^_aaaa_`_`a_]_aa___U^X^`^W
@HWI-EAS443:3:62:934:140#0/1
ATTCTTTGGCTGGAAAGAGCCTAACATAGTCAAACCACAT
+HWI-EAS443:3:62:934:140#0/1
_abb_b^^`]_`aa_`b_a^]_aa`a_aaX`a^`XFX^`¥
```

```
@HWI-EAS443:3:62:978:995#0/1
GTCACGAGGGGGAGGGAATACCACTATATGATGCAATCAA
+HWI-EAS443:3:62:978:995#0/1
a``a]WX`a`aa``aaa_Z^_aa[a`aa`_^^`aa^`Y`^
@HWI-EAS443:3:63:1024:2010#0/1
ATTCGGATTTCCATTTCATCGACGAACGGGGTGAATCAAT
+HWI-EAS443:3:63:1024:2010#0/1
aaaaaZW``_[`a¥a[_^Y_`Z`^[[_YTT¥Taa¥[_R¥a
@HWI-EAS443:3:63:1070:438#0/1
GAGACCGAATCATGGCCTGGACAGTGGTGAACAGTATATG
+HWI-EAS443:3:63:1070:438#0/1
abaa`aa_`a]___aWa_`[[a`_¥_aUa__]_`V^____
@HWI-EAS443:3:63:1073:716#0/1
AGCAGAGCTACAGGACATTGAAAATGAAGAGAAGATCCCA
+HWI-EAS443:3:63:1073:716#0/1
XW¥V_[a_TY`N¥Q¥U^¥ZX]]JXY`HP_YXWVU]VXRZN
@HWI-EAS443:3:63:1074:1166#0/1
GTTCAATCGGGGCAACATCTTCTCCTATTTCATCAAGTTC
+HWI-EAS443:3:63:1074:1166#0/1
^GP¥¥]]]YMWV[P```a]`aaa¥`^YZ`YXPR¥¥W[¥W^
@HWI-EAS443:3:63:1116:730#0/1
GGCGTGGTGTCGTCCTCAAGAATGGTTCAATTTTGGCGTT
+HWI-EAS443:3:63:1116:730#0/1
a¥_`^`aO_]a^^a]^`_`[]_a_aO_]^X^__``]___]
@HWI-EAS443:3:63:115:426#0/1
ATCAAATGCATGAAGACATTCTTTGGCTGGAAAGAGCCTA
+HWI-EAS443:3:63:115:426#0/1
_]a`^`U^````^^^a]WY```[[X``]][^Z^][_[¥WS
@HWI-EAS443:3:63:1189:511#0/1
CAAGAATCAAAACTAGGCTTTTCACTATAAGACAAGAAAT
+HWI-EAS443:3:63:1189:511#0/1
`babbbbbbbbbabbbbbbbbbbbabbba`aaaaaa`aaa
@HWI-EAS443:3:63:1197:79#0/1
GCTTGCGCATAGTTCCTGTAATCTCAAATTTTTCTTCAAT
+HWI-EAS443:3:63:1197:79#0/1
aabaaYbaaa`a_a``ab^^^a]^``U¥aaaaa_aa`_`a
@HWI-EAS443:3:63:1221:914#0/1
TGGGGGGCTATATGAAGCAATCGAGGAGTGCCTGATTAAT
+HWI-EAS443:3:63:1221:914#0/1
]Z^bQ`]¥^Xa¥W[a]]b¥a_Yb_a]L]M`__Z_Q]PT^V
@HWI-EAS443:3:63:1233:1910#0/1
GGTGAACTTTGTAAGTATGGAGTTCTCACTCACTGACCCG
+HWI-EAS443:3:63:1233:1910#0/1
`[VZabab^a`H`TaYaaaY[YTHOZaTLW^ZL_`abUY]
@HWI-EAS443:3:63:1280:847#0/1
GAGAAGCCTAAATTTCTTCCTGATTTGTATGATTACAAAG
+HWI-EAS443:3:63:1280:847#0/1
Yab_I[b¥¥T__[^ba_b`ab_R__aba^aa^^Z`aaaaa
@HWI-EAS443:3:63:1288:851#0/1
GGCACTCGGTGAAAATATGGCACCAGAAAAAGTAGACTTT
```

+HWI-EAS443:3:63:1288:851#0/1
Q__V`¥Z``bb^¥__Xabaa`b^][_V]a`T_[`Y_aa`^
@HWI-EAS443:3:63:1329:613#0/1
CATATCGCAAGCATGAGGAGGAACTATTTTACAGCAGAAG
+HWI-EAS443:3:63:1329:613#0/1
[aabb```babaabba]bbbZbabaababbbUaaaaaaaa
@HWI-EAS443:3:63:135:974#0/1
GCAGGTGCTAGCAGAGCTACAGGACATTGAAAATGAAGAG
+HWI-EAS443:3:63:135:974#0/1
aaaaa]`ba_aa_a^aa_a`aa^^¥_`^^Z^]]``^^`^_
@HWI-EAS443:3:63:1369:209#0/1
ATAAATCCCAATTACCTCATGGCTTGGAAGCAGGTGCTAG
+HWI-EAS443:3:63:1369:209#0/1
aaab`a`a[^`aa`a`_aa^_`_a`T`]YWQTTBBBBBBB
@HWI-EAS443:3:63:1369:394#0/1
TACATAAATACGGCCTTGCTCAATGCATCCTGTGCAGCCA
+HWI-EAS443:3:63:1369:394#0/1
aaaaaa`_aaa^a``aaaa_aaa`_aa`QaZ__a_^`^a_
@HWI-EAS443:3:63:1372:726#0/1
CCCGAGTCACGAGGGGGAGGGAATACCACTATATGATGCA
+HWI-EAS443:3:63:1372:726#0/1
`X^_Q_SY]G^]aYaab^XP[_Y_a`a_[¥^]_aa]a^a]
@HWI-EAS443:3:63:1407:859#0/1
ACAAATCAGGAAGAAATTTAGGCTTCTCTACCCCTGTTGT
+HWI-EAS443:3:63:1407:859#0/1
_Y¥ZTYZ]V_GS¥T]VZ_Y^aa^`aa`¥]UZWa^PTJS¥K
@HWI-EAS443:3:63:1419:938#0/1
AAGTTGTGGAGACGCATATAGACTGTTGAATACAGATTTT
+HWI-EAS443:3:63:1419:938#0/1
a¥ba_b`a`ab`aa`ba^_``^aa]G[`Z¥QNQ¥_[[_BB
@HWI-EAS443:3:63:1446:1359#0/1
TGGAAATCCGAATACATGAAACAAACTTCCAAATGTGTGC
+HWI-EAS443:3:63:1446:1359#0/1
aaaaa`aaabaaaa`aa`aaaaab_aa`_]`_aaaX`¥aa
@HWI-EAS443:3:63:1456:1831#0/1
AAGTGCCTGAACAATGAGAAGCAATTTTCTCGATTCAGCC
+HWI-EAS443:3:63:1456:1831#0/1
VH_aaVa`Ya^V`WYa¥S¥_``^^`K[a_SHbbJRS_BBB
@HWI-EAS443:3:63:1479:272#0/1
CAAGAAATGGCCAGTAGGAGTCTATGGGATTCCTTTCGTC
+HWI-EAS443:3:63:1479:272#0/1
aaab^`aaaaa_aa_aa^[a¥``aaaa`X^^Z[__^U^[^
@HWI-EAS443:3:63:1510:1446#0/1
AGCAGAGCTACAGGACATTGAAAATGAAGAGAAGATCCCA
+HWI-EAS443:3:63:1510:1446#0/1
ab^]aabaaa[`]Z^aabaJX[ab`Y_a¥aaaa`_][FZa
@HWI-EAS443:3:63:151:1406#0/1
CGGCTGAATCGAGAAAATTGCTTCTCATTGTTCAGGCACT
+HWI-EAS443:3:63:151:1406#0/1
aabbbbbaab^aababbab]abbaa_aaba`aa`^aaa``

@HWI-EAS443:3:63:1529:542#0/1
AGAATTCCTTGGTCATGTCTTTCTCTTTGACAGAAGACTC
+HWI-EAS443:3:63:1529:542#0/1
aa`aaaa`aaba_`aaa`aaaaaa`aaa__^`aaaaa]a_
@HWI-EAS443:3:63:1558:1577#0/1
GGGCACTCGGTGAAAATATGGCACCAGAAAAAGTAGACTT
+HWI-EAS443:3:63:1558:1577#0/1
_^``bbaXaa[baabbbbbaa¥b`U`_aXa_aa[aVZDZa
@HWI-EAS443:3:63:1558:444#0/1
CCAGCTTGCTAGAGATCTGGGCTCTGGCTCATCACTGTCA
+HWI-EAS443:3:63:1558:444#0/1
a`_bbaabaabaabaaaaa]aabaaaa`a_aaaaaaa^aa
@HWI-EAS443:3:63:1605:436#0/1
CCTCGATTGCTTCATATAGCCCCCCAAGATCGAAGGTTCC
+HWI-EAS443:3:63:1605:436#0/1
ababbabb_a``abbbbbbaaabaaa¥aaaaaaab_S]`_
@HWI-EAS443:3:63:162:1358#0/1
GGTTTGACTATGTTAGGCTCTTTCCAGCCAAAGAATGTCT
+HWI-EAS443:3:63:162:1358#0/1
`b]aaa``_^a¥`_a]^Y^aaa`a[[S¥a`aa^aa]]S[Y
@HWI-EAS443:3:63:1630:1662#0/1
AAGAATTCCTTGGTCATGTCTTTCTCTTTGACAGAAGACT
+HWI-EAS443:3:63:1630:1662#0/1
`¥`^a_`¥]aaa¥¥_aaaWaaaa_a`aa`_W^aa[Z]^^_
@HWI-EAS443:3:63:1786:76#0/1
GCATCCTGTGCAGCCATGGATGACTTTCAGCTGATCCCAA
+HWI-EAS443:3:63:1786:76#0/1
`abb``a`aaa`^```a`a`ab_]aaa`_a_]^]]]_[^W
@HWI-EAS443:3:63:20:522#0/1
CACTGGAGAGGAGATGGCCACCAAAGCGGACTACACCCTT
+HWI-EAS443:3:63:20:522#0/1
aaabaa`aabaa]]^``aa¥a`¥``_`aY¥YYSV[^^[[V
@HWI-EAS443:3:63:215:406#0/1
AACTTTAGAGCCTATGTAGATGGATTCGAGCCGAACGGCT
+HWI-EAS443:3:63:215:406#0/1
_¥a`a`[T¥__^_YK]LZ_SZ_^aZ]_a]¥Z_]BBBBBBB
@HWI-EAS443:3:63:227:871#0/1
GCCCACTTCAATTGGCTTGTTCTCTTCATGTTCTTTGTCC
+HWI-EAS443:3:63:227:871#0/1
aaabaaaaaab`aaa^```Y^XXU``_^ZIQ_Z``^UVY^
@HWI-EAS443:3:63:263:1054#0/1
TGGGGGGCTATATGAAGCAATCGAGGAGTGCCTGATTAAT
+HWI-EAS443:3:63:263:1054#0/1
aa_a``a_ab`aaaaaa`a^`a_Z__Y^T`_``a^]_a_^
@HWI-EAS443:3:63:324:659#0/1
AGACATTCTTTGGCTGGAAAGAGCCTAACATAGTCAAACC
+HWI-EAS443:3:63:324:659#0/1
aabaaaaaaaaaa``aaY^a`¥`_a`aa___`_]a`_``a
@HWI-EAS443:3:63:337:178#0/1
CTATTTTACAGCAGAAGTGTCCCACTGCAGGGCTACTGAA

+HWI-EAS443:3:63:337:178#0/1
ab_bbabbababbbaba¥a``aaaa_a`aa^__`___`_]
@HWI-EAS443:3:63:392:179#0/1
CCAGAAAAAGTAGACTTTGATGACTGCAAAGATGTTGGAG
+HWI-EAS443:3:63:392:179#0/1
aaa^aa`a`^Pa^``a```^[`_`_`__]^_X]_[]_ZS[
@HWI-EAS443:3:63:39:1061#0/1
GGGGGCTATATGAAGCAATCGAGGAGTGCCTGATTAATGA
+HWI-EAS443:3:63:39:1061#0/1
W`_`ab¥Z]bba``bbbaaababbab_bb`bbaaa`Maa]
@HWI-EAS443:3:63:408:1841#0/1
GAGGGGGAGGGAATACCACTATATGATGCAATCAAATGCA
+HWI-EAS443:3:63:408:1841#0/1
ab_aaaaabaaaabaaaaaaba`a`a_`aaa``aaa_Yaa
@HWI-EAS443:3:63:496:601#0/1
GCTGGGTCCAAAATGAATTCAATAAGGCATGTGAATTGAC
+HWI-EAS443:3:63:496:601#0/1
aaaaaaOaaa`aaaa`]`a``Sa`aa____a]a`]____`
@HWI-EAS443:3:63:502:677#0/1
TGGGATCTTCTCTTCATTTTCAATGTCCTGTAGCTCTGCT
+HWI-EAS443:3:63:502:677#0/1
abaYX_^a[[W```]a````a```¥Z[^[WV_¥_]a]X[Y
@HWI-EAS443:3:63:606:1408#0/1
TGAAAATGAAGAGAAGATCCCAAGGACAAAGAACATGAAG
+HWI-EAS443:3:63:606:1408#0/1
aa¥Ya_a`aa`a[`aaa[`aX]^¥[```a`^aXU^^]^`Z
@HWI-EAS443:3:63:624:1544#0/1
AATAGTTCCTCCTCATGCTTGCGATATGTTCAATCGGGGC
+HWI-EAS443:3:63:624:1544#0/1
a_¥^XY^Y]XZZZ^^]V_^ZXX]YY]WVVZZ]ZYYXYXVV
@HWI-EAS443:3:63:671:1215#0/1
AGCGGACTACACCCTTGACGAAGAGAGCAGGGCAAGAATC
+HWI-EAS443:3:63:671:1215#0/1
`[b¥YWZZ`a^X[_`aabaaaaaUZU][_a]Uaa_¥¥XZ_
@HWI-EAS443:3:63:675:1491#0/1
AAGAATCAAAACTAGGCTTTTCACTATAAGACAAGAAATG
+HWI-EAS443:3:63:675:1491#0/1
WT¥]^I^VZ¥bbba`YWY^ZUYaa_S```¥`BBBBBBBBB
@HWI-EAS443:3:63:708:468#0/1
CGGGGTGAATCAATAATTGTAGAATCTGGTGACCCGAATG
+HWI-EAS443:3:63:708:468#0/1
^^aaU^``ababbaabbb]b`^ababbQ_[`Z_aY]aa
@HWI-EAS443:3:63:730:646#0/1
GGAACCTTCGATCTTGGGGGGCTATATGAAGCAATCGAGG
+HWI-EAS443:3:63:730:646#0/1
[aabb_bb[b]a`ab]_aaa]``bab```baaa`QZ]Vaa
@HWI-EAS443:3:63:753:231#0/1
AGAAGACACACATTCACATCTTTTCATTCACTGGAGAGGA
+HWI-EAS443:3:63:753:231#0/1
Z_ZVa__¥]b_^_b]_^¥aY_a_a`a_`WaVaa^`a_¥MZ

```
@HWI-EAS443:3:63:763:764#0/1
ACACCCTTGACGAAGAGAGCAGGGCAAGAATCAAAACTAG
+HWI-EAS443:3:63:763:764#0/1
aabba`_`aa`aaZ_Z]^_^^aa`_Q_`[^^¥_]W]____
@HWI-EAS443:3:63:869:558#0/1
CATGGAAATGAGGCGCTGCCTTCTTCAGTCTCTTCAGCAG
+HWI-EAS443:3:63:869:558#0/1
a`bb`bbbab`¥_abba`baabbaababXaaa`a`X`]]_
@HWI-EAS443:3:63:941:1034#0/1
ACTGAATACATAATGAAGGGAGTGTACATAAATACGGCCT
+HWI-EAS443:3:63:941:1034#0/1
a`baaaa``^a]`aa``aa_``^a_a[`aaaa_`^^`Wa]
@HWI-EAS443:3:64:1009:1161#0/1
GGGGGCTATATGAAGCAATCGAGGAGTGCCTGATTAATGA
+HWI-EAS443:3:64:1009:1161#0/1
¥_bb_bb`bbb`ababbaaaa``aa__b][abababa``a
@HWI-EAS443:3:64:103:701#0/1
CGATTGGCCATGTTTCCGATTTGTTTTCAAAGAATTCCTT
+HWI-EAS443:3:64:103:701#0/1
a_LZabab_U_``a`[``]_aa^¥aaa_`^``^]`]Y^_`
@HWI-EAS443:3:64:1069:599#0/1
GTTAGGCTCTTTCCAGCCAAAGAATGTCTTCATGCATTTG
+HWI-EAS443:3:64:1069:599#0/1
aaaaaaaaaa`a_Wa___a_a`_``a^_aa^_^[]^_^^_
@HWI-EAS443:3:64:1103:549#0/1
GTAGGAGTCTATGGGATTCCTTTCGTCAGTCCGAAAGAGG
+HWI-EAS443:3:64:1103:549#0/1
a`abaab_aaaaaaa_a__]aa___]`_^]^¥a^```]Z_
@HWI-EAS443:3:64:1132:1575#0/1
CAGCTTGCTAGAGATCTGGGCTCTGGCTCATCACTGTCAT
+HWI-EAS443:3:64:1132:1575#0/1
`abbbbabbbbaabbbbb^U¥bbbbabbabba¥[[bbaab
@HWI-EAS443:3:64:1147:1009#0/1
ACCACATGAGAAAGGCATAAATCCCAATTACCTCATGGCT
+HWI-EAS443:3:64:1147:1009#0/1
`a^a¥¥a^`_`¥¥^W_a`[]a`bbT`bbbaa¥NF¥^a`Xa
@HWI-EAS443:3:64:1155:298#0/1
GTGGTGAACTTTGTAAGTATGGAGTTCTCACTCACTGACC
+HWI-EAS443:3:64:1155:298#0/1
a]ab^baa¥aaaa`a^`^`Y``^]^_Z`U^¥_a__`_ZVZ
@HWI-EAS443:3:64:1156:1317#0/1
CCCACTTCAATTGGCTTGTTCTCTTCATGTTCTTTGTCCT
+HWI-EAS443:3:64:1156:1317#0/1
aa`_a_ab^aa]`][_]PW`XX¥[]XV`¥^[X]_]JVZ[Z
@HWI-EAS443:3:64:1187:2001#0/1
AACATAGTCAAACCACATGAGAAAGGCATAAATCCCAATT
+HWI-EAS443:3:64:1187:2001#0/1
ab_baba_aaab^_b]a```a`aaa`^`aaa`a[Z_a`_N
@HWI-EAS443:3:64:1201:906#0/1
GCCATATTTTCACCGAGTGCCCACTTCAATTGGCTTGTTC
```

```
+HWI-EAS443:3:64:1201:906#0/1
`aaa`^abbbabaaaaaab_a_a`aa`a]aaaa]a^IU]G
@HWI-EAS443:3:64:1248:1750#0/1
GAAGTTCGGTGGGAGACTTTGGTCGGCAAGCTTGCGCATA
+HWI-EAS443:3:64:1248:1750#0/1
aba]U`aab^aa]¥^]_aaaa[PNP[[``Q]YYZ][]__[
@HWI-EAS443:3:64:128:1638#0/1
GGGGGCTATATGAAGCAATCGAGGAGTGCCTGATTAATGA
+HWI-EAS443:3:64:128:1638#0/1
`b_```b^bbb_bbabbaaaaa`a`a_abaa`aabbaa_a
@HWI-EAS443:3:64:1307:1867#0/1
AGAAGATCCCAAGGACAAAGAACATGAAGAGAACAAGCCA
+HWI-EAS443:3:64:1307:1867#0/1
_`_aa_`aaa]`]aZU```]``_^¥__ZVY_^^Wa^^Z_^
@HWI-EAS443:3:64:1379:468#0/1
TCAAAGTCTACTTTTTCTGGTGCCATATTTTCACCGAGTG
+HWI-EAS443:3:64:1379:468#0/1
a_`aaa]aaaaaaa`aaa`X]`_```_aaa__a__a__Za
@HWI-EAS443:3:64:1397:172#0/1
CCTTGGGATCTTCTCTTCATTTTCAATGTCCTGTAGCTCT
+HWI-EAS443:3:64:1397:172#0/1
aabbaaaababbababbababbb`baaaaa_bbaa``aa`
@HWI-EAS443:3:64:1410:211#0/1
GGGGCATGGAAATGAGGCGCTGCCTTCTTCAGTCTCTTCA
+HWI-EAS443:3:64:1410:211#0/1
aabaaT`ba_aaaaaa``aWa`^S_^U_a¥^_VQ`U__¥Z
@HWI-EAS443:3:64:1488:1757#0/1
CAATGAGAAGCAATTTTCTCGATTCAGCCGAAAACCCCTC
+HWI-EAS443:3:64:1488:1757#0/1
aTabb``bbb]ba`^¥abb]`^[]^baaaa`a``X___¥S
@HWI-EAS443:3:64:1638:1695#0/1
GTGAACTTTGTAAGTATGGAGTTCTCACTCACTGACCCGA
+HWI-EAS443:3:64:1638:1695#0/1
aa^a__a`aa^Z[]]`Y``T`¥`]]X¥U`N_]__^¥]^_Y
@HWI-EAS443:3:64:1654:671#0/1
CTCGATTGCTTCATATAGCCCCCCAAGATCGAAGGTTCCA
+HWI-EAS443:3:64:1654:671#0/1
aba_^babaaabbaaaa``X^`aa]`]aa``]^Y_UZTUS
@HWI-EAS443:3:64:165:1733#0/1
GACGAAAGGAATCCCATAGACTCCTACTGGCCATTTCTTG
+HWI-EAS443:3:64:165:1733#0/1
`abbbbb`bbbbbbbbbbaababaaa`a__aaaYa_T]aW
@HWI-EAS443:3:64:1676:1005#0/1
TGAATCAGTCAATTCACATGCCTTATTGAATTCATTTTGG
+HWI-EAS443:3:64:1676:1005#0/1
a`aabbab^`a`aa¥a_`a_`^aa_a_a`_``_]`_a_^]
@HWI-EAS443:3:64:1683:441#0/1
TGTTACTCCAATTTCAATGAACCGGTTCTCTTTGTAATCA
+HWI-EAS443:3:64:1683:441#0/1
aaaaaaa`abaaaa^aa`_`a^`a`]`[`V_aa_]`]`^_
```

```
@HWI-EAS443:3:64:1687:1268#0/1
GAACAATGAGAAGCAATTTTCTCGATTCAGCCGAAAACCC
+HWI-EAS443:3:64:1687:1268#0/1
_]_]a``abab`^`S_a`^]ababa]XG]¥WT__aaaW[L
@HWI-EAS443:3:64:1727:810#0/1
CAGGTGCTAGCAGAGCTACAGGACATTGAAAATGAAGAGA
+HWI-EAS443:3:64:1727:810#0/1
abbZabab`aabaab^``^abaaaa]`¥UaaaaT^`^^]a
@HWI-EAS443:3:64:1753:50#0/1
TACAAAGAGAACCGGTTCATTGAAATTGGAGTAACACGGA
+HWI-EAS443:3:64:1753:50#0/1
]aY^_bbYa_V^`baV¥^]`[_][[][__^a[[____^ZW
@HWI-EAS443:3:64:1761:1399#0/1
CTTGGAAGCAGGTGCTAGCAGAGCTACAGGACATTGAAAA
+HWI-EAS443:3:64:1761:1399#0/1
¥```¥[[V]___Q[`¥¥^T[]HXOU¥^^a_WHXZ]¥W`W¥
@HWI-EAS443:3:64:1772:1792#0/1
GATGTTGCCCCGATTGAACATATCGCAAGCATGAGGAGGA
+HWI-EAS443:3:64:1772:1792#0/1
a_a]]`]a^^``a`a[Va^]_`_WW]Z_[I^`YR^[U^YW
@HWI-EAS443:3:64:221:1457#0/1
GGGGCATGGAAATGAGGCGCTGCCTTCTTCAGTCTCTTCA
+HWI-EAS443:3:64:221:1457#0/1
R_^G[__XYZ_[_`W_ZQ_Z]X_Z¥¥Y_X¥XXYVYZ¥Z`¥
@HWI-EAS443:3:64:257:474#0/1
AGCAGAGCTACAGGACATTGAAAATGAAGAGAAGATCCCA
+HWI-EAS443:3:64:257:474#0/1
aaaa``ab_aaaaa_a``aa__a__`_]_``_X_]^_^^X
@HWI-EAS443:3:64:306:205#0/1
GTGAACTTTGTAAGTATGGAGTTCTCACTCACTGACCCGA
+HWI-EAS443:3:64:306:205#0/1
`Xaaa_aa_b_`aa_a]aaaa[_a_]¥[Ya`_`aa[YU¥[
@HWI-EAS443:3:64:312:1958#0/1
ACCAAAGAAGGAAGACGGAAAACAAACCTGTATGGGTTCA
+HWI-EAS443:3:64:312:1958#0/1
]Z]¥a_TMR¥`Za[YUTVZY[_¥_]T[NZTLWOJP^YTZ]
@HWI-EAS443:3:64:31:944#0/1
AGAATTCCTTGGTCATGTCTTTCTCTTTGACAGAAGACTC
+HWI-EAS443:3:64:31:944#0/1
a`a`````_]]¥T[`][T[PON[SV^X^VZ^[YQX¥V[XY
@HWI-EAS443:3:64:399:23#0/1
TACATAAATACGGCCTTGCTCAATGCATCCTGTGCAGCCA
+HWI-EAS443:3:64:399:23#0/1
a^a^_Raaaaa^¥__]a[[¥``___[a^ZYU[V`[RZQ¥]
@HWI-EAS443:3:64:414:1510#0/1
GAGAACAAGCCAATTGAAGTGGGCACTCGGTGAAAATATG
+HWI-EAS443:3:64:414:1510#0/1
abbaaba_^bbaaaaaaaa_aaaaa_aaaa¥^```aaa_R
@HWI-EAS443:3:64:445:263#0/1
GGGGCATGGAAATGAGGCGCTGCCTTCTTCAGTCTCTTCA
```

+HWI-EAS443:3:64:445:263#0/1
ab_`a¥`]aZW`aaY^`_`Z[_`_[__Q¥^X¥U_WW^¥UW
@HWI-EAS443:3:64:474:1754#0/1
GGGGCATGGAAATGAGGCGCTGCCTTCTTCAGTCTCTTCA
+HWI-EAS443:3:64:474:1754#0/1
`b`a`Xab``bbbbbaabaabbab`a^b`aab_Saaaa_a
@HWI-EAS443:3:64:527:904#0/1
GCGTCTCCACAACTTGAGGGGTTTTCGGCTGAATCGAGAA
+HWI-EAS443:3:64:527:904#0/1
aa^abba_`_¥aVaaYa]]TU¥`aYYH^UaYX[`^LONQT
@HWI-EAS443:3:64:528:244#0/1
GTGGGCACTCGGTGAAAATATGGCACCAGAAAAAGTAGAC
+HWI-EAS443:3:64:528:244#0/1
aHP_ZW[MPUYPL_QVWMOQ[QTPRFKQNPXXZUUFGSBB
@HWI-EAS443:3:64:535:291#0/1
CTCTTCGTCAAGGGTGTAGTCCGCTTTGGTGGCCATCTCC
+HWI-EAS443:3:64:535:291#0/1
``bbba¥abbbbbbVba_¥_¥`b`aba^a¥`_`]_`Z[]_
@HWI-EAS443:3:64:538:281#0/1
GGGGCATGGAAATGAGGCGCTGCCTTCTTCAGTCTCTTCA
+HWI-EAS443:3:64:538:281#0/1
abaa_a__a`a```_`[``W^_W_¥_W]_^YSXTX[_^TV
@HWI-EAS443:3:64:562:992#0/1
AGGACATTGAAAATGAAGAGAAGATCCCAAGGACAAAGAA
+HWI-EAS443:3:64:562:992#0/1
PUWUY[a[¥PJ[[Z_XT_^W_`_Y_``¥ZaYNH]_]T^]_
@HWI-EAS443:3:64:572:1012#0/1
GCTGGGTCCAAAATGAATTCAATAAGGCATGTGAATTGAC
+HWI-EAS443:3:64:572:1012#0/1
abaa^XT`aaaa¥`]W_a`[a]Z`Y^_a_`a[__^``Y[_
@HWI-EAS443:3:64:588:2043#0/1
CTGCTGTAAAATAGTTCCTCCTCATGCTTGCGATATGTTC
+HWI-EAS443:3:64:588:2043#0/1
a_ba^b]abbab]VXa`_XaaYa`YNR_]_`aY^`^Z[_^
@HWI-EAS443:3:64:65:401#0/1
AGACATTCTTTGGCTGGAAAGAGCCTAACATAGTCAAACC
+HWI-EAS443:3:64:65:401#0/1
aaaaaaaaa_`a]aa`a_a`___[^__a¥¥YVY^____¥_
@HWI-EAS443:3:64:681:1389#0/1
GTATTCAGTAGCCCTGCAGTGGGACACTTCTGCTGTAAAA
+HWI-EAS443:3:64:681:1389#0/1
¥¥bbZ`b_QRaa]`bZW¥QRbabbVb[[[b[ab_b`bbab
@HWI-EAS443:3:64:775:254#0/1
CGAAGAGAGCAGGGCAAGAATCAAAACTAGGCTTTTCACT
+HWI-EAS443:3:64:775:254#0/1
_]a¥a]]_``][M¥RY`Z_a^YP]YRU`YbR¥WY_Y[ZQW
@HWI-EAS443:3:64:77:215#0/1
CCCGAATGCACTATTGAAGCACCGATTTGAGATAATTGAA
+HWI-EAS443:3:64:77:215#0/1
Z__LQa_R¥_ba[`_¥_PR¥b^a_]^V__X`a]¥`U`aWQ

@HWI-EAS443:3:64:787:1204#0/1
TGGTTTGACTATGTTAGGCTCTTTCCAGCCAAAGAATGTC
+HWI-EAS443:3:64:787:1204#0/1
]`U[baba_a_V_^]_b`JX^a^aa_`_Ia^`aa[¥^]Q^
@HWI-EAS443:3:64:834:1331#0/1
CTTCTGTCAAAGAGAAAGACATGACCAAGGAATTCTTTGA
+HWI-EAS443:3:64:834:1331#0/1
`bbaababbbabaaaaaababaaba`aa_aaaa_]`aaaT
@HWI-EAS443:3:64:834:1634#0/1
GACGAAAGGAATCCCATAGACTCCTACTGGCCATTTCTTG
+HWI-EAS443:3:64:834:1634#0/1
Z^aa^ba¥¥¥`babaabbaaabaaaa]¥]¥aaa]aaY_`_
@HWI-EAS443:3:64:859:600#0/1
CTTGGGGGGCTATATGAAGCAATCGAGGAGTGCCTGATTA
+HWI-EAS443:3:64:859:600#0/1
aabbbbaabbbbbabaaba_aaa`a]aa_b]aa_aa`a``
@HWI-EAS443:3:64:961:1368#0/1
CTGGGTCCAAAATGAATTCAATAAGGCATGTGAATTGACT
+HWI-EAS443:3:64:961:1368#0/1
Ja¥]aab¥``_babbbaababbb`ba_bababba``ab`¥
@HWI-EAS443:3:64:984:544#0/1
CTTTGGTCGGCAAGCTTGCGCATAGTTCCTGTAATCTCAA
+HWI-EAS443:3:64:984:544#0/1
abbbbbaaaaababaaa`aba_baa^a¥_aa_^^aWaaa]
@HWI-EAS443:3:65:1031:477#0/1
AATTGACTGATTCAAGCTGGATAGAACTTGATGAAATAGG
+HWI-EAS443:3:65:1031:477#0/1
``aabaabbaba`babaabb_abb`baaab`aba`aa_`a
@HWI-EAS443:3:65:104:512#0/1
GCTGAAAGTCATCCATGGCTGCACAGGATGCATTGAGCAA
+HWI-EAS443:3:65:104:512#0/1
a`^aa_U¥VZZ¥__[_a`a¥[¥VVY[XXN[NYZBBBBBBB
@HWI-EAS443:3:65:1054:90#0/1
CATGAAGACATTCTTTGGCTGGAAAGAGCCTAACATAGTC
+HWI-EAS443:3:65:1054:90#0/1
abab`IZ``ababbb_YZ^aa][Y`^[YW[a^`[X]``[^
@HWI-EAS443:3:65:1071:874#0/1
CGGTGCTTCAATAGTGCATTCGGGTCACCAGATTCTACAA
+HWI-EAS443:3:65:1071:874#0/1
aab``baababa_SYaa_aaaaba[^]`aa__`a_aa[aZ
@HWI-EAS443:3:65:1133:532#0/1
CCTTGGGATCTTCTCTTCATTTTCAATGTCCTGTAGCTCT
+HWI-EAS443:3:65:1133:532#0/1
aa_bbbaaa_bab`aba_`baWXaZ_aa`^M¥`Q__`Y__
@HWI-EAS443:3:65:1149:749#0/1
GGGGTTTTCGGCTGAATCGAGAAAATTGCTTCTCATTGTT
+HWI-EAS443:3:65:1149:749#0/1
ab`]N]a¥¥_¥_a`aa_¥¥[¥]]```[^_[¥ZX_WXZ`Z[
@HWI-EAS443:3:65:1195:1435#0/1
GGGGCATGGAAATGAGGCGCTGCCTTCTTCAGTCTCTTCA

+HWI-EAS443:3:65:1195:1435#0/1
a`^_ab_[[aaaa^`a]_`X`__`a_¥_a___X^_¥`_ZY
@HWI-EAS443:3:65:1207:846#0/1
ATTTCATCGACGAACGGGGTGAATCAATAATTGTAGAATC
+HWI-EAS443:3:65:1207:846#0/1
a^ba[abbb^[aa]¥_¥[_F_[a`[]a`R`aab]RY_`YR
@HWI-EAS443:3:65:1246:98#0/1
GTCCGCTTTGGTGGCCATCTCCTCTCCAGTGAATGAAAAG
+HWI-EAS443:3:65:1246:98#0/1
a[_¥_W_`__aX]SZBBBBBBBBBBBBBBBBBBBBBBBBB
@HWI-EAS443:3:65:1279:706#0/1
AATTCATTTTGGACCCAGCTTGCTAGAGATCTGGGCTCTG
+HWI-EAS443:3:65:1279:706#0/1
]Z[bb`aab]abaaa^[W_b`b^a]b`a`]`aa_``a`_Z
@HWI-EAS443:3:65:1287:1123#0/1
AGATGTTGCCCCGATTGAACATATCGCAAGCATGAGGAGG
+HWI-EAS443:3:65:1287:1123#0/1
abbbaa`b`b`aaaaaaaaaaa_Y``^`a^`aa^a__a`
@HWI-EAS443:3:65:1296:24#0/1
TTCCGATTTGTTTTCAAAGAATTCCTTGGTCATGTCTTTC
+HWI-EAS443:3:65:1296:24#0/1
aaaaa[`aabaaaa]a_ab``baV_aaba]_aaa_^a``U
@HWI-EAS443:3:65:1346:352#0/1
ATTAATCAGGCACTCCTCGATTGCTTCATATAGCCCCCCA
+HWI-EAS443:3:65:1346:352#0/1
aaaaaa^aa`aa`a_^^^a___a¥_a___a¥`[[W¥]_¥_
@HWI-EAS443:3:65:1376:890#0/1
CTCTGGCTCATCACTGTCATACTGTTTAAGGTCTCCAACA
+HWI-EAS443:3:65:1376:890#0/1
ab]bbbbaaba`b]a]`aaa`abaaaab`ba__a`¥b`aa
@HWI-EAS443:3:65:1411:616#0/1
CATGGAAATGAGGCGCTGCCTTCTTCAGTCTCTTCAGCAG
+HWI-EAS443:3:65:1411:616#0/1
abb`bbbbbabba^`aaaaaaaaaaaaa[``ab_Z^`V`a
@HWI-EAS443:3:65:1412:666#0/1
CACTGTCATACTGTTTAAGGTCTCCAACATCTTTGCAGTC
+HWI-EAS443:3:65:1412:666#0/1
ababba`bababb_abbaaa^`baaba`baaa`aa]aa^^
@HWI-EAS443:3:65:1442:1585#0/1
ACGGCCTTGCTCAATGCATCCTGTGCAGCCATGGATGACT
+HWI-EAS443:3:65:1442:1585#0/1
aa`^_aaa¥^__aa`a^a_XU_VX_^[_`¥_^¥_Z¥_YW_
@HWI-EAS443:3:65:1448:96#0/1
CACTTCAATTGGCTTGTTCTCTTCATGTTCTTTGTCCTTG
+HWI-EAS443:3:65:1448:96#0/1
]bJbb¥babbba`a`^ZaK`JbaJ[abab``bb^aaabaa
@HWI-EAS443:3:65:1467:1923#0/1
ATAGTTCCTCCTCATGCTTGCGATATGTTCAATCGGGGCA
+HWI-EAS443:3:65:1467:1923#0/1
a^_a]`Ya^[`]a`a]O¥_`^`a`X`a_^X_a`[X^]QNV

```
@HWI-EAS443:3:65:1632:1516#0/1
CTTAAACAGTATGACAGTGATGAGCCAGAGCCCAGATCTC
+HWI-EAS443:3:65:1632:1516#0/1
`abbbbb_O_bbbbaabZ[__¥_babaa[aaaaa[__aaa
@HWI-EAS443:3:65:182:918#0/1
CAGGCACTCCTCGATTGCTTCATATAGCCCCCCAAGATCG
+HWI-EAS443:3:65:182:918#0/1
a`W`a`b^¥X_aa_`a^a_a`V`aaa]a^]^a^]]^^W¥Y
@HWI-EAS443:3:65:187:1166#0/1
CCCCGTTCGTCGATGAAATGGAAATCCGAATACATGAAAC
+HWI-EAS443:3:65:187:1166#0/1
abbb`aa_T_aa_`_`aaa¥VT`a^]a`aa]aaa_W``a_
@HWI-EAS443:3:65:225:1212#0/1
GAATTCCTTGGTCATGTCTTTCTCTTTGACAGAAGACTCG
+HWI-EAS443:3:65:225:1212#0/1
^PLQU`XJZbbX]_Z]R`]]]YP]]a]W[a]ZRXXQYU¥`
@HWI-EAS443:3:65:226:283#0/1
CTCCAATTTCAATGAACCGGTTCTCTTTGTAATCATACAA
+HWI-EAS443:3:65:226:283#0/1
abbb^^aaaaaaaaab``ba_`aaaa``a^a[_]`___`_
@HWI-EAS443:3:65:277:1211#0/1
GGCATGGAAATGAGGCGCTGCCTTCTTCAGTCTCTTCAGC
+HWI-EAS443:3:65:277:1211#0/1
`X`UPNZF[YS]MHJV]WKVP[MMRKYIDRN[UVRZYZL[
@HWI-EAS443:3:65:395:1482#0/1
AGCCCCCCAAGATCGAAGGTTCCAGGTTCCAGGTTGTCCC
+HWI-EAS443:3:65:395:1482#0/1
aV```^a`VV[][XU^^PUMYU¥¥QSKPOPYJQRUQVTVQ
@HWI-EAS443:3:65:508:1785#0/1
GACCCGAATGCACTATTGAAGCACCGATTTGAGATAATTG
+HWI-EAS443:3:65:508:1785#0/1
aaba_a`^`_aaaaaa_aaa]aa_`[][`aa^Y__¥Z^`_
@HWI-EAS443:3:65:515:911#0/1
AAGAGGCGAAGAGACAATTGAAGAAAAATTTGAGATTACA
+HWI-EAS443:3:65:515:911#0/1
a`aaaa__aa__`__``aa____^Y^__`a__`^`^^_``
@HWI-EAS443:3:65:522:573#0/1
CTGGAGAGGAGATGGCCACCAAAGCGGACTACACCCTTGA
+HWI-EAS443:3:65:522:573#0/1
a_^[]X`[U]abZT[`Waa[a`b¥Wbabb`^b`]`a]aZ]
@HWI-EAS443:3:65:543:850#0/1
GAATACATAATGAAGGGAGTGTACATAAATACGGCCTTGC
+HWI-EAS443:3:65:543:850#0/1
`abb[b]aabba`a`aaab`b_Y_aa[aaaabaa_a__`_
@HWI-EAS443:3:65:555:513#0/1
GCTGGGTCCAAAATGAATTCAATAAGGCATGTGAATTGAC
+HWI-EAS443:3:65:555:513#0/1
abbaab¥bbbbb`bbaaaaaaaaaabaaaab_b_Zaaaaa
@HWI-EAS443:3:65:566:572#0/1
GAAGTTCGGTGGGAGACTTTGGTCGGCAAGCTTGCGCATA
```

```
+HWI-EAS443:3:65:566:572#0/1
S`aa`abbb]]Wa]aYaaaaab_a]X`a]^``aa]``[]_
@HWI-EAS443:3:65:578:233#0/1
GGAAATCCGAATACATGAAACAAACTTCCAAATGTGTGCA
+HWI-EAS443:3:65:578:233#0/1
_`aa`aaaa_aabaaaabaa`baa`aaaaa_`aa_`W_``
@HWI-EAS443:3:65:599:532#0/1
AAGAATTCCTTGGTCATGTCTTTCTCTTTGACAGAAGACT
+HWI-EAS443:3:65:599:532#0/1
abba_aabaaaaa^`aaa`aaa`_``a``^[`a_a___``
@HWI-EAS443:3:65:618:1403#0/1
GGTGAACTTTGTAAGTATGGAGTTCTCACTCACTGACCCG
+HWI-EAS443:3:65:618:1403#0/1
ZaH]_X¥`_^__b_babbVa]U^aaa_bbabaaX`_`[`[
@HWI-EAS443:3:65:623:1043#0/1
CAGAGCTACAGGACATTGAAAATGAAGAGAAGATCCCAAG
+HWI-EAS443:3:65:623:1043#0/1
ab``abb`ab`aa`aaba^^aaaaaa``a`_`a`¥¥a`]`
@HWI-EAS443:3:65:66:684#0/1
CATTAATCAGGCACTCCTCGATTGCTTCATATAGCCCCCC
+HWI-EAS443:3:65:66:684#0/1
^RR`]a^^aaXbaZ_Q^UVaUUXOWUQF[Z_[XSRPWR_B
@HWI-EAS443:3:65:698:743#0/1
GGGGGCTATATGAAGCAATCGAGGAGTGCCTGATTAATGA
+HWI-EAS443:3:65:698:743#0/1
aaa`a]_X_abY`[aab]__a[]^[`]aZ[aa[a^]`_^^
@HWI-EAS443:3:65:69:839#0/1
TTTGGCTGGAAAGAGCCTAACATAGTCAAACCACATGAGA
+HWI-EAS443:3:65:69:839#0/1
abababababaZ`ba`a`a_aaaaa`]^`_`]N^]^_____]
@HWI-EAS443:3:65:761:1349#0/1
CATTCTTTGGCTGGAAAGAGCCTAACATAGTCAAACCACA
+HWI-EAS443:3:65:761:1349#0/1
`aaaaaa_`aa_aaa^a_¥WRaa_`_`[^]_`a`_^a^aa
@HWI-EAS443:3:65:785:455#0/1
GGAAATCCGAATACATGAAACAAACTTCCAAATGTGTGCA
+HWI-EAS443:3:65:785:455#0/1
aabbbbbabbbbbbbbbbaabbababababaabbab`bab
@HWI-EAS443:3:65:788:22#0/1
GCCAGAGCCCAGATCTCTAGCAAGCTGGGTCCAAAATGAA
+HWI-EAS443:3:65:788:22#0/1
aYaa^`]_^Z[¥ZY^V^¥U^Y¥VXNX^Y^V[W^Z[V_^VX
@HWI-EAS443:3:65:791:309#0/1
CAGCTTGAATCAGTCAATTCACATGCCTTATTGAATTCAT
+HWI-EAS443:3:65:791:309#0/1
[``a_`bbabbb`aabbbabbaba]babbababaaab_ab
@HWI-EAS443:3:65:793:1895#0/1
ACTCGGTGAAAATATGGCACCAGAAAAAGTAGACTTTGAT
+HWI-EAS443:3:65:793:1895#0/1
a`Y`_[Xa^```[__UOW_^_^UT[[`^^XaV_X^^`Z^^
```

@HWI-EAS443:3:65:804:832#0/1
CTTGGGATCTTCTCTTCATTTTCAATGTCCTGTAGCTCTG
+HWI-EAS443:3:65:804:832#0/1
`^`aQ_SOZaabba_^^W¥^aab`¥`aX^a_a]^]XV¥_W
@HWI-EAS443:3:65:810:1354#0/1
ATACTGTTCACCACTGTCCAGGCCATGATTCGGTCTCTTC
+HWI-EAS443:3:65:810:1354#0/1
`aaaaa`aaa`aa`a_^aaa^^a`a[V^___]]Y]YY]^U
@HWI-EAS443:3:65:841:507#0/1
GGCCAATCGGAGAGTCACCCAGGGGAGTGGAGGAAGGCTC
+HWI-EAS443:3:65:841:507#0/1
[a]`a`a`aa`_`Z`a_`a¥```Z`Z_¥`¥T^YR¥¥¥¥ZZ
@HWI-EAS443:3:65:873:535#0/1
GCCAGTAGGAGTCTATGGGATTCCTTTCGTCAGTCCGAAA
+HWI-EAS443:3:65:873:535#0/1
a```aZ`]`_^Y_a¥¥aa`¥¥_aY¥^¥[]U¥^`PUY_[ZZ
@HWI-EAS443:3:65:884:974#0/1
GGGGGCTATATGAAGCAATCGAGGAGTGCCTGATTAATGA
+HWI-EAS443:3:65:884:974#0/1
a^Z_R^X]`]`¥`V]¥]^¥_]]]aQOKT______ZUR¥^Z
@HWI-EAS443:3:65:900:691#0/1
GGGGGCTATATGAAGCAATCGAGGAGTGCCTGATTAATGA
+HWI-EAS443:3:65:900:691#0/1
a^¥aaaa`_]aaaaaab^^_a_]_^a^`_[aaV¥W^^Y`_
@HWI-EAS443:3:65:908:2032#0/1
ATTGAAGCACCGATTTGAGATAATTGAAGGAAGAGACCGA
+HWI-EAS443:3:65:908:2032#0/1
]_ba]aa_`]^aaZ_YbU]¥Wa_a``baZ¥_]a`_a`__a
@HWI-EAS443:3:65:925:1248#0/1
TGAAAATGAAGAGAAGATCCCAAGGACAAAGAACATGAAG
+HWI-EAS443:3:65:925:1248#0/1
aa`aa`aba^aa_aa``^aaaaaaaaaaaaaaaa`_b__a
@HWI-EAS443:3:65:93:975#0/1
GGGGCATGGAAATGAGGCGCTGCCTTCTTCAGTCTCTTCA
+HWI-EAS443:3:65:93:975#0/1
aba``bbab``Yaaaaabaaa``a_a````W`Z___^_`_
@HWI-EAS443:3:65:96:1411#0/1
CGAAAATCGAAACTAACAAGTTTGCTGCAATATGCACACA
+HWI-EAS443:3:65:96:1411#0/1
aa``bbaaa`¥aa`a`aa^`]a`_`^````__``````]^
@HWI-EAS443:3:65:974:398#0/1
GCTTGCTAGAGATCTGGGCTCTGGCTCATCACTGTCATAC
+HWI-EAS443:3:65:974:398#0/1
aaabbabaaaa`aaaaaaaaaaa`_aaa_``_aa¥^`__^
@HWI-EAS443:3:65:981:309#0/1
AAAACCCCTCAAGTTGTGGAGACGCATATAGACTGTTGAA
+HWI-EAS443:3:65:981:309#0/1
aabaaa_`````a^`a^a`aa`_a^`a`aa`_^_`Z_PNY
@HWI-EAS443:3:65:997:1034#0/1
GGGGGAGGGAATACCACTATATGATGCAATCAAATGCATG

```
+HWI-EAS443:3:65:997:1034#0/1
ab`_``¥ba¥N_^_QN¥__aa`[`_O_a¥aaa_`Y^_`^_
@HWI-EAS443:3:66:1003:1919#0/1
TGAAAATGAAGAGAAGATCCCAAGGACAAAGAACATGAAG
+HWI-EAS443:3:66:1003:1919#0/1
a]]_aaaaaaaaaa^`a¥_a__a`[a_Y`a`_^^`^`^_`
@HWI-EAS443:3:66:1022:1701#0/1
GAAAGGCATAAATCCCAATTACCTCATGGCTTGGAAGCAG
+HWI-EAS443:3:66:1022:1701#0/1
abbbbbbbbbbba^^]_bbbba_¥abaa_aab^`UaXV^^
@HWI-EAS443:3:66:1051:1913#0/1
CCGATTGGCCATGTTTCCGATTTGTTTTCAAAGAATTCCT
+HWI-EAS443:3:66:1051:1913#0/1
ab`^]`a`__[a^]_^^^`___`]U^a____`]]]__UW]
@HWI-EAS443:3:66:1097:1113#0/1
GGTGAATCAATAATTGTAGAATCTGGTGACCCGAATGCAC
+HWI-EAS443:3:66:1097:1113#0/1
aaWa```baab_a`ab__`aaaaaa`_ab``^a`_aa_^`
@HWI-EAS443:3:66:109:1053#0/1
GCTCATCACTGTCATACTGTTTAAGGTCTCCAACATCTTT
+HWI-EAS443:3:66:109:1053#0/1
aba_R_aaaaa¥baaaa_a`_a[a][T``a^a[`aa_a^¥
@HWI-EAS443:3:66:1158:1754#0/1
CTTTCCCAATAGAGCCTTCCTCCACTCCCCTGGGTGACTC
+HWI-EAS443:3:66:1158:1754#0/1
T^`W[`_bba¥X¥]_Z[aW[]Xabba^Q¥¥VaZXG_a[JV
@HWI-EAS443:3:66:1219:1168#0/1
CAGTGATGAGCCAGAGCCCAGATCTCTAGCAAGCTGGGTC
+HWI-EAS443:3:66:1219:1168#0/1
abaabbbaaa`bbaaabaaaaaaaaaaa`aaaa_a``]]]
@HWI-EAS443:3:66:1228:393#0/1
CATTAATCAGGCACTCCTCGATTGCTTCATATAGCCCCCC
+HWI-EAS443:3:66:1228:393#0/1
abbb`abbba]WY`¥bX`XZR_XNPaYFHXPQMX`_a_^X
@HWI-EAS443:3:66:1280:1467#0/1
ATCAGGCACTCCTCGATTGCTTCATATAGCCCCCCAAGAT
+HWI-EAS443:3:66:1280:1467#0/1
WT_`]`][`aab_T[Ua]_ba_ab`bb_X_a`aaa`[¥Z_
@HWI-EAS443:3:66:1288:326#0/1
CACAACTTGAGGGGTTTTCGGCTGAATCGAGAAAATTGCT
+HWI-EAS443:3:66:1288:326#0/1
ababaaabb]abab_bbaaaaaabba`[baaaaaa`a`Ya
@HWI-EAS443:3:66:1338:523#0/1
AGAAGATCCCAAGGACAAAGAACATGAAGAGAACAAGCCA
+HWI-EAS443:3:66:1338:523#0/1
aabbb`a`[a_`aa_aaaa`a`a`aa_`aa`a__a`a]^`
@HWI-EAS443:3:66:134:1243#0/1
CCATGATTCGGTCTCTTCCTTCAATTATCTCAAATCGGTG
+HWI-EAS443:3:66:134:1243#0/1
abaa`a`_aa^X_¥^`___`^`___a``___`]WX_]ZV^
```

```
@HWI-EAS443:3:66:1436:58#0/1
CACTCCCCTGGGTGACTCTCCGATTGGCCATGTTTCCGAT
+HWI-EAS443:3:66:1436:58#0/1
aa[[b`bbba^XV__Raab`abaaaaaZZPZaP¥[HJ[G]
@HWI-EAS443:3:66:1438:978#0/1
AAGAGCCTAACATAGTCAAACCACATGAGAAAGGCATAAA
+HWI-EAS443:3:66:1438:978#0/1
abaa_aaaaa_aa`aaaaaa`_`_aa``_a`_`_a`aaa`
@HWI-EAS443:3:66:1470:323#0/1
GAGTGGAGGAAGGCTCTATTGGGAAAGTGTGCAGGACCTT
+HWI-EAS443:3:66:1470:323#0/1
]`babb_a`_aaaaaYa_aaaabaaaaSbH`a¥aa_`_ab
@HWI-EAS443:3:66:153:716#0/1
GAAAGGCATAAATCCCAATTACCTCATGGCTTGGAAGCAG
+HWI-EAS443:3:66:153:716#0/1
aa¥S``abaaaaaaaabaY[^a`_aY_[Ya`XZ^USJXOM
@HWI-EAS443:3:66:1606:617#0/1
AGAACAAGCCAATTGAAGTGGGCACTCGGTGAAAATATGG
+HWI-EAS443:3:66:1606:617#0/1
IPQ__^`aa¥V_¥`_SQ_^^[`U``ab`aW^^URQRLT_M
@HWI-EAS443:3:66:1627:1395#0/1
GAGAAAGGCATAAATCCCAATTACCTCATGGCTTGGAAGC
+HWI-EAS443:3:66:1627:1395#0/1
]ab^ZT_baa_ba_a^_[`aaaa_abaabbaaa``TFT[T
@HWI-EAS443:3:66:1629:1688#0/1
GGAAGATCCGAAAATCGAAACTAACAAGTTTGCTGCAATA
+HWI-EAS443:3:66:1629:1688#0/1
a`SSYZ`_`a]aba_Y]_¥aQ^Y^L]ZNS_YRYZaUW___
@HWI-EAS443:3:66:1661:1683#0/1
CAGTGGGACACTTCTGCTGTAAAATAGTTCCTCCTCATGC
+HWI-EAS443:3:66:1661:1683#0/1
aba`bbbbaba`^^baab`abba`ba`a`[]ba`a_aa_^
@HWI-EAS443:3:66:1699:891#0/1
TGGGGCATGGAAATGAGGCGCTGCCTTCTTCAGTCTCTTC
+HWI-EAS443:3:66:1699:891#0/1
aa^Z^aaaaaaaaa`aa``___aU`_`]aaU`_[___^`Y
@HWI-EAS443:3:66:1740:77#0/1
GAAGATCCCAAGGACAAAGAACATGAAGAGAACAAGCCAA
+HWI-EAS443:3:66:1740:77#0/1
a¥_aX_YJERXYa¥UXT^]R^ZVY_PR^¥YSPHRU^MNUU
@HWI-EAS443:3:66:201:899#0/1
TGGCCATGTTTCCGATTTGTTTTCAAAGAATTCCTTGGTC
+HWI-EAS443:3:66:201:899#0/1
aaabbab_aabbbb`aab_`abaaabaabaaaa`aaba]a
@HWI-EAS443:3:66:210:1479#0/1
GGGGGCTATATGAAGCAATCGAGGAGTGCCTGATTAATGA
+HWI-EAS443:3:66:210:1479#0/1
aa¥`_aabaaa_a_]`aa_a`^`Z__Y``_`^__^__]¥_
@HWI-EAS443:3:66:240:1031#0/1
CTTCAATTGGCTTGTTCTCTTCATGTTCTTTGTCCTTGGG
```

```
+HWI-EAS443:3:66:240:1031#0/1
aaaa``aaa_`a_HVaS`¥`a_`^[Ya_a`a¥Z__`__]]
@HWI-EAS443:3:66:260:1827#0/1
GAAATGGGGCATGGAAATGAGGCGCTGCCTTCTTCAGTCT
+HWI-EAS443:3:66:260:1827#0/1
`UORXOGPK¥aYGSYRUXQZQPWZ[XNYYBBBBBBBBBBB
@HWI-EAS443:3:66:314:187#0/1
GGGTTTTCGGCTGAATCGAGAAAATTGCTTCTCATTGTTC
+HWI-EAS443:3:66:314:187#0/1
abb_aaaaaaaa_aa__a_`__]`__[]]___V]]Y]V^[
@HWI-EAS443:3:66:316:690#0/1
GAGCCAGAGCCCAGATCTCTAGCAAGCTGGGTCCAAAATG
+HWI-EAS443:3:66:316:690#0/1
abbbbbbabbbabaaabaaaaaba]`````a^a_`[```a
@HWI-EAS443:3:66:347:2036#0/1
AAAGAGCCTAACATAGTCAAACCACATGAGAAAGGCATAA
+HWI-EAS443:3:66:347:2036#0/1
aab¥a[aaaaa^a_a^¥aa__```YWZRZR_Y`OJY]Yaa
@HWI-EAS443:3:66:380:745#0/1
GGGGCATGGAAATGAGGCGCTGCCTTCTTCAGTCTCTTCA
+HWI-EAS443:3:66:380:745#0/1
]]]^[a^Xa]X]]ba_¥aabXaVUM^a^]aa`U¥]a]VZ[
@HWI-EAS443:3:66:389:1639#0/1
AGCTCTGCTAGCACCTGCTTCCAAGCCATGAGGTAATTGG
+HWI-EAS443:3:66:389:1639#0/1
`Z_]aU^`Y]_`_ZZXQ]^[^Z[^Y^V^RPQ[WMW[VUUT
@HWI-EAS443:3:66:398:640#0/1
CACCGATTTGAGATAATTGAAGGAAGAGACCGAATCATGG
+HWI-EAS443:3:66:398:640#0/1
aaa``Vbaaa_b`aaaaaa]__``¥`U_`a_a_^_aa[__
@HWI-EAS443:3:66:40:223#0/1
GGGGGCTATATGAAGCAATCGAGGAGTGCCTGATTAATGA
+HWI-EAS443:3:66:40:223#0/1
abbbbabbb^aba`aa``_aa`ba_aZ_`^`a^`_]]^__
@HWI-EAS443:3:66:416:120#0/1
ACGGCCTTGCTCAATGCATCCTGTGCAGCCATGGATGACT
+HWI-EAS443:3:66:416:120#0/1
_`^aaaa]_`aa`ZQ``a]^^V]Z[ZWU]X[[]YUTXNRY
@HWI-EAS443:3:66:446:416#0/1
CATCAGGCAATCTGAGGGGGCGTGGTGTCGTCCTCAAGAA
+HWI-EAS443:3:66:446:416#0/1
X`W_X^[_VYbb`_¥¥]XZ___]bb]_Z``X__X`X]]Z`
@HWI-EAS443:3:66:44:1315#0/1
GGCATGGAAATGAGGCGCTGCCTTCTTCAGTCTCTTCAGC
+HWI-EAS443:3:66:44:1315#0/1
`_bb_^a`Wa_aaa`aabaaaa`a]¥aa_a[aaa`_a_^a
@HWI-EAS443:3:66:475:1482#0/1
TGTCCTTGGGATCTTCTCTTCATTTTCAATGTCCTGTAGC
+HWI-EAS443:3:66:475:1482#0/1
a`_aaaa_`a]`aa`aaaaaaaaaa`aaaa_]a_`^]a]_
```

```
@HWI-EAS443:3:66:501:1245#0/1
CCATGTTTCCGATTTGTTTTCAAAGAATTCCTTGGTCATG
+HWI-EAS443:3:66:501:1245#0/1
aa¥T[```_]_^a^]`[]_X__]ZUY¥_R[YZ¥S^T_Y_]
@HWI-EAS443:3:66:52:233#0/1
AAATCCCAATTACCTCATGGCTTGGAAGCAGGTGCTAGCA
+HWI-EAS443:3:66:52:233#0/1
ab`a`_a^^a_`_a_a_]`_^_^W^][TTYTWNUUWWSQS
@HWI-EAS443:3:66:573:219#0/1
AAACTTTAGAGCCTATGTAGATGGATTCGAGCCGAACGGC
+HWI-EAS443:3:66:573:219#0/1
a__H_b``ba`SZaba__abaaaaaaa¥`_aaaa___`_^
@HWI-EAS443:3:66:615:449#0/1
CACCGATTTGAGATAATTGAAGGAAGAGACCGAATCATGG
+HWI-EAS443:3:66:615:449#0/1
aaaab_aaab^aaa`aaaa^`aaa_a_`a_`a___`^_`¥
@HWI-EAS443:3:66:624:262#0/1
TGGAAATCCGAATACATGAAACAAACTTCCAAATGTGTGC
+HWI-EAS443:3:66:624:262#0/1
abbbbabbaabbbbababbbabaaaabaaaaaaaa^a]a_
@HWI-EAS443:3:66:625:1048#0/1
GGTGAACTTTGTAAGTATGGAGTTCTCACTCACTGACCCG
+HWI-EAS443:3:66:625:1048#0/1
aa_aaaaaaaa¥aaa_aaa`_`]^aaaa_`a__aa`__`a
@HWI-EAS443:3:66:625:1245#0/1
GGCATGGAAATGAGGCGCTGCCTTCTTCAGTCTCTTCAGC
+HWI-EAS443:3:66:625:1245#0/1
a`bb`a``aaaaaaRa`^___a`_Ta__]Z[_]X]¥V`^R
@HWI-EAS443:3:66:642:378#0/1
GAGCTACAGGACATTGAAAATGAAGAGAAGATCCCAAGGA
+HWI-EAS443:3:66:642:378#0/1
aaaaaa`aaaa_a__`_`_^aa`__aaa_a`_^X`¥W___
@HWI-EAS443:3:66:651:15#0/1
CACTGTCATACTGTTTAAGGTCTCCAACATCTTTGCAGTC
+HWI-EAS443:3:66:651:15#0/1
ab^`b```a¥W_`Z^`X^^WT^aaa`aa^a^¥```___¥^
@HWI-EAS443:3:66:657:829#0/1
CATAAATACGGCCTTGCTCAATGCATCCTGTGCAGCCATG
+HWI-EAS443:3:66:657:829#0/1
aaaa`aaaabaaaVa^aaa`_``aaUa``_X]a_`]_W_^
@HWI-EAS443:3:66:709:310#0/1
CCTCGATTGCTTCATATAGCCCCCCAAGATCGAAGGTTCC
+HWI-EAS443:3:66:709:310#0/1
abaaaaa_aaaaa^aaa```_`a_`a[```^_XU¥^U¥ZZ
@HWI-EAS443:3:66:739:1809#0/1
TACGGCCTTGCTCAATGCATCCTGTGCAGCCATGGATGAC
+HWI-EAS443:3:66:739:1809#0/1
¥`ba^__`^Za[aY[TY_b`^^a¥N¥]a]a`^X]_XR_[P
@HWI-EAS443:3:66:794:1525#0/1
GAAGATCCGAAAATCGAAACTAACAAGTTTGCTGCAATAT
```

```
+HWI-EAS443:3:66:794:1525#0/1
`b`b`_`abbbbbaaaa```aababba_aaaaab`aaaaa
@HWI-EAS443:3:66:848:331#0/1
GCTTCTCTACCCCTGTTGTGTTACATATACTGTTCACCAC
+HWI-EAS443:3:66:848:331#0/1
`a`_aaba`]a`a¥QT¥O[W_``X`a_a`a``Y_^`]R]_
@HWI-EAS443:3:66:856:1183#0/1
ATCAGGCACTCCTCGATTGCTTCATATAGCCCCCCAAGAT
+HWI-EAS443:3:66:856:1183#0/1
_]X_`Z``[^a¥¥LVWTQ]¥_a_`]_a]]]]_^``a[^a[
@HWI-EAS443:3:66:857:543#0/1
TGATTCAAGCTGGATAGAACTTGATGAAATAGGAGAAGAT
+HWI-EAS443:3:66:857:543#0/1
a`aaaaab`aaba^_aa^b^a`aZ_aa¥a`_a`_a_]a^_
@HWI-EAS443:3:66:866:1484#0/1
GGGGGCTATATGAAGCAATCGAGGAGTGCCTGATTAATGA
+HWI-EAS443:3:66:866:1484#0/1
aaa¥`b`aaaaaaaabbaaaa`a__a^aa`_a^``a_X`¥
@HWI-EAS443:3:66:873:1003#0/1
CATTTGTGTGGCTCCAGTCTCGGGTCAGTGAGTGAGAACT
+HWI-EAS443:3:66:873:1003#0/1
_¥`abaa`_a___`_¥YZZX```ZNXX¥[`Y[USZXDUUV
@HWI-EAS443:3:66:956:1065#0/1
GTTTCCGATTTGTTTTCAAAGAATTCCTTGGTCATGTCTT
+HWI-EAS443:3:66:956:1065#0/1
aP^aaab`¥]``^``a]Ta`___a]P^aa`^UaX_a^_aa
@HWI-EAS443:3:66:963:1578#0/1
AATGCATCCTGTGCAGCCATGGATGACTTTCAGCTGATCC
+HWI-EAS443:3:66:963:1578#0/1
_XYSZ]Vb_`[DRb¥aba]Y`Q_Y][T`aRV]WUV^YOOX
@HWI-EAS443:3:66:963:540#0/1
GCTCAATGCATCCTGTGCAGCCATGGATGACTTTCAGCTG
+HWI-EAS443:3:66:963:540#0/1
aaaa_^aaaaa`aaa`aaaa_``a`]¥a`¥[aa`_^_Y_a
@HWI-EAS443:3:66:963:809#0/1
CTTGTTCTCTTCATGTTCTTTGTCCTTGGGATCTTCTCTT
+HWI-EAS443:3:66:963:809#0/1
aba``aababbbbbaabbbbbbaaaaaa_aabbba`baba
@HWI-EAS443:3:67:1002:1245#0/1
GGGGGCTATATGAAGCAATCGAGGAGTGCCTGATTAATGA
+HWI-EAS443:3:67:1002:1245#0/1
aa``abab`_aa]```aaa_a]`a_aYa`_`a_aa``^aa
@HWI-EAS443:3:67:1015:992#0/1
TATAAGACAAGAAATGGCCAGTAGGAGTCTATGGGATTCC
+HWI-EAS443:3:67:1015:992#0/1
_bbaaY^`aa^X]`a^^aa_UQX]_WSR]¥`aU¥_GZ^_R
@HWI-EAS443:3:67:1174:480#0/1
GTGAACAGTATATGTAACACAACAGGGGTAGAGAAGCCTA
+HWI-EAS443:3:67:1174:480#0/1
]M]UDXZa]¥`_^a¥^YY]T[[W[^__XW[[YTSYYRMX]
```

```
@HWI-EAS443:3:67:1185:342#0/1
CATCCATGGCTGCACAGGATGCATTGAGCAAGGCCGTATT
+HWI-EAS443:3:67:1185:342#0/1
abb_`b`a_aa``¥T¥aa]¥¥X¥aa]`a`aaa^W_aYU^_
@HWI-EAS443:3:67:1210:254#0/1
CAAGAAATGGCCAGTAGGAGTCTATGGGATTCCTTTCGTC
+HWI-EAS443:3:67:1210:254#0/1
abab`aa`ab`aaba^^_W_U^a```aa__a__`aaZ^SP
@HWI-EAS443:3:67:1226:188#0/1
ATCAAAACTAGGCTTTTCACTATAAGACAAGAAATGGCCA
+HWI-EAS443:3:67:1226:188#0/1
aba`aaa`aaa`aaaaa_a__`aa`]_^a`_`_^^_][R^
@HWI-EAS443:3:67:1234:85#0/1
GAGACCGAATCATGGCCTGGACAGTGGTGAACAGTATATG
+HWI-EAS443:3:67:1234:85#0/1
```_^aa_`_]_`a`_V_]]]X^_X]^Y]YXX^^XU^X__
@HWI-EAS443:3:67:1286:1638#0/1
CCCCGTTCGTCGATGAAATGGAAATCCGAATACATGAAAC
+HWI-EAS443:3:67:1286:1638#0/1
ab`ba]a]_``_Xb`aba`ab`abaVaTa_aa[aa`a`aY
@HWI-EAS443:3:67:1340:1860#0/1
GAACACAGTATTTTTCCCATTTGTGTGGCTCCAGTCTCGG
+HWI-EAS443:3:67:1340:1860#0/1
[abaZbbZVb^babb`_abaaa`a`^_aT__[ZFWJTQFU
@HWI-EAS443:3:67:1366:682#0/1
GGGGGGCTATATGAAGCAATCGAGGAGTGCCTGATTAATG
+HWI-EAS443:3:67:1366:682#0/1
abaabaa`aaabaaaaaa`a^b`aa]aT``VaaZ`^`]]a
@HWI-EAS443:3:67:1442:1559#0/1
GGGCACTCGGTGAAAATATGGCACCAGAAAAAGTAGACTT
+HWI-EAS443:3:67:1442:1559#0/1
ab`abbbbb`_aaaabbbb``_ba^baaaaaa`Wa_a^aa
@HWI-EAS443:3:67:1480:388#0/1
CACTTTCCCAATAGAGCCTTCCTCCACTCCCCTGGGTGAC
+HWI-EAS443:3:67:1480:388#0/1
a^T¥`a_aaba^ab_]a`]`Z`aa`aXaaY`^`^`aXZ^U
@HWI-EAS443:3:67:1485:1874#0/1
GCGTTCACTTCTTTTGACATTTGGGAAAGCTTGCCCTCAA
+HWI-EAS443:3:67:1485:1874#0/1
aaaaa]a_aa]aaaa^aW`a¥_`Z_a``¥U`¥^¥WU^U`_
@HWI-EAS443:3:67:1488:92#0/1
CAAAGTCTACTTTTTCTGGTGCCATATTTTCACCGAGTGC
+HWI-EAS443:3:67:1488:92#0/1
aXZPWW^aW]aaaaa`_¥TOLSPV[_^¥]^]_^U^X[U^U
@HWI-EAS443:3:67:156:754#0/1
GGGGGCTATATGAAGCAATCGAGGAGTGCCTGATTAATGA
+HWI-EAS443:3:67:156:754#0/1
aa__[``aa]aa_`]¥]_^^__a[DWYaa_[a[^_`^^_U
@HWI-EAS443:3:67:1572:1587#0/1
CGATTGGCCATGTTTCCGATTTGTTTTCAAAGAATTCCTT
```
```

```
+HWI-EAS443:3:67:1572:1587#0/1
a``a_Z]ababb[ba][baaa^b`aa_``aa^]]aa]X`a
@HWI-EAS443:3:67:1673:1225#0/1
ACTCGGTGAAAATATGGCACCAGAAAAAGTAGACTTTGAT
+HWI-EAS443:3:67:1673:1225#0/1
aa_```abba^abaaaaU]^^^``````^`Saa``aa_aa`
@HWI-EAS443:3:67:1691:1411#0/1
GGAGGGAATACCACTATATGATGCAATCAAATGCATGAAG
+HWI-EAS443:3:67:1691:1411#0/1
¥_^`a[¥bbbabbaa`aabaaabaaa``baa_Y_aaaaa]
@HWI-EAS443:3:67:170:257#0/1
GTGGGCACTCGGTGAAAATATGGCACCAGAAAAAGTAGAC
+HWI-EAS443:3:67:170:257#0/1
ZYaaaa`a___]P_YO[`Y^^___^^ZZGX_Z_][SWTQY
@HWI-EAS443:3:67:1727:1289#0/1
CCGATTTGTTTTCAAAGAATTCCTTGGTCATGTCTTTCTC
+HWI-EAS443:3:67:1727:1289#0/1
`aa_ab_b^b_babaaabbbaaababb`W`b¥a]abb¥b¥
@HWI-EAS443:3:67:175:515#0/1
AAACAAACTTCCAAATGTGTGCATATTGCAGCAAACTTGT
+HWI-EAS443:3:67:175:515#0/1
`a]aaaW__^a_a`]_`¥`W^a`^^_]^``]]^[]W^Y^Y
@HWI-EAS443:3:67:229:873#0/1
AAGCCAACAAAATAAAATCTGAGAAGACACACATTCACAT
+HWI-EAS443:3:67:229:873#0/1
aV_ab`aa[a`_`aaa^a_a`_a__````^`^_`^`_]`^
@HWI-EAS443:3:67:274:1738#0/1
GGGGGCTATATGAAGCAATCGAGGAGTGCCTGATTAATGA
+HWI-EAS443:3:67:274:1738#0/1
VYS`a_S_SSNL^ZTa``[``[]¥[[P¥b^YWTPTP`IT¥
@HWI-EAS443:3:67:285:775#0/1
CGAAAATCGAAACTAACAAGTTTGCTGCAATATGCACACA
+HWI-EAS443:3:67:285:775#0/1
aaa`baabba^aa_aa_a__^_``a`a_`^`__``a`aa`
@HWI-EAS443:3:67:344:1964#0/1
CCTGCTTCCAAGCCATGAGGTAATTGGGATTTATGCCTTT
+HWI-EAS443:3:67:344:1964#0/1
abaaa]`aba][a_a``]]^X____^[V_]]]a]_[UY]^
@HWI-EAS443:3:67:557:821#0/1
GGGGGCTATATGAAGCAATCGAGGAGTGCCTGATTAATGA
+HWI-EAS443:3:67:557:821#0/1
^[`XY_aZaba``a`abb_¥`_a``a¥^a_`[`a^_`^`_
@HWI-EAS443:3:67:709:873#0/1
CATAAATCCCAATTACCTCATGGCTTGGAAGCAGGTGCTA
+HWI-EAS443:3:67:709:873#0/1
ab`bbb_babaab`[```aa_^``a_]_VZ¥UUX[JWS¥]
@HWI-EAS443:3:67:725:904#0/1
CTGGTGACCCGAATGCACTATTGAAGCACCGATTTGAGAT
+HWI-EAS443:3:67:725:904#0/1
aaaa_aaaaaaa`aa^a`a_aa``aaaaa`aZ_`_``a__
```

@HWI-EAS443:3:67:746:343#0/1
GTGGGCACTCGGTGAAAATATGGCACCAGAAAAAGTAGAC
+HWI-EAS443:3:67:746:343#0/1
aabbaaaaaa`aWaaaaaaaaaaaa`__a_`]__`Wa__W
@HWI-EAS443:3:67:769:1046#0/1
CGGCTGAATCGAGAAAATTGCTTCTCATTGTTCAGGCACT
+HWI-EAS443:3:67:769:1046#0/1
M``Y[]¥_]¥VQZT¥X`Y_^]^Z`_aZaab`a_¥]¥Q¥¥b
@HWI-EAS443:3:67:809:275#0/1
AATACGGCCTTGCTCAATGCATCCTGTGCAGCCATGGATG
+HWI-EAS443:3:67:809:275#0/1
T^aa`bb_bVa]]b`abaaaaaa_aaa¥a`b_[`abaa`a
@HWI-EAS443:3:67:868:1704#0/1
AGCAGGGCAAGAATCAAAACTAGGCTTTTCACTATAAGAC
+HWI-EAS443:3:67:868:1704#0/1
a`]aZ^^[`a``aa`aaa___^FY`ZP_a`a_`__a`SWD
@HWI-EAS443:3:67:896:912#0/1
CGGGGCAACATCTTCTCCTATTTCATCAAGTTCTATCCAG
+HWI-EAS443:3:67:896:912#0/1
a`_`abaaabbabb`baaabbbaabaababaaaaaaa`ba
@HWI-EAS443:3:67:89:614#0/1
GGGCACTCGGTGAAAATATGGCACCAGAAAAAGTAGACTT
+HWI-EAS443:3:67:89:614#0/1
aaabaaabab[baabaa`a[^`Z`_aa_]_T``Va^__^^
@HWI-EAS443:3:67:959:687#0/1
GGGGCATGGAAATGAGGCGCTGCCTTCTTCAGTCTCTTCA
+HWI-EAS443:3:67:959:687#0/1
aa``aa[aa__`^a_^^]a_``Z^¥`W_`^_a¥Z_^¥_WV
@HWI-EAS443:3:67:967:554#0/1
AACAAAATAAAATCTGAGAAGACACACATTCACATCTTTT
+HWI-EAS443:3:67:967:554#0/1
aaabab`babaab`ba^aaaaaa`aaaabaaa``a_aaa`
@HWI-EAS443:3:68:1037:869#0/1
CGTCTCCACAACTTGAGGGGTTTTCGGCTGAATCGAGAAA
+HWI-EAS443:3:68:1037:869#0/1
Q_```bbb`babbab```¥¥O_bbba[aabbab`b_`b`a
@HWI-EAS443:3:68:1046:1716#0/1
TTCTTGCCCTGCTCTCTTCGTCAAGGGTGTAGTCCGCTTT
+HWI-EAS443:3:68:1046:1716#0/1
a``^X_¥b`aaaab`a`a]a^ab`a`XX¥LZ`YT]`X_`[
@HWI-EAS443:3:68:1050:958#0/1
ATGAAATGGAAATCCGAATACATGAAACAAACTTCCAAAT
+HWI-EAS443:3:68:1050:958#0/1
aaaa[a^a`Y_]``_a__``__]`a__^`^]]`^_[_]W`
@HWI-EAS443:3:68:1099:16#0/1
ATTCCTTGGTCATGTCTTTCTCTTTGACAGAAGACTCGGC
+HWI-EAS443:3:68:1099:16#0/1
aabaabbba[ababaabaa[b^bbab``aaaaa``_Yb_Z
@HWI-EAS443:3:68:1100:288#0/1
TCGGAGAGTCACCCAGGGGAGTGGAGGAAGGCTCTATTGG

+HWI-EAS443:3:68:1100:288#0/1
`ab`Y`]¥`^Xaa¥^ab__^aU[_P_^U^^WPYUYU[]YV
@HWI-EAS443:3:68:1148:214#0/1
GTCCGCTTTGGTGGCCATCTCCTCTCCAGTGAATGAAAAG
+HWI-EAS443:3:68:1148:214#0/1
a^a`a_aaa_aWba``aa^^]^[X^Z^T_¥a^¥X]S]__X
@HWI-EAS443:3:68:1163:1499#0/1
CGTCTCCACAACTTGAGGGGTTTTCGGCTGAATCGAGAAA
+HWI-EAS443:3:68:1163:1499#0/1
ab^baabbbba```¥^`bb_`__baa_abbaaa^``a¥aa
@HWI-EAS443:3:68:1194:113#0/1
CATTAATCAGGCACTCCTCGATTGCTTCATATAGCCCCCC
+HWI-EAS443:3:68:1194:113#0/1
aabbbabbbbbbb`b`bbababbbabaaabaaaa[a]]]]
@HWI-EAS443:3:68:1259:100#0/1
ATGAGAAAGGCATAAATCCCAATTACCTCATGGCTTGGAA
+HWI-EAS443:3:68:1259:100#0/1
abaaaaaaa__aaaa`a__[``a`a_]`__`^_X`[NZVY
@HWI-EAS443:3:68:1274:108#0/1
GGCCACCAAAGCGGACTACACCCTTGACGAAGAGAGCAGG
+HWI-EAS443:3:68:1274:108#0/1
_aaaaaaaa^[¥aaa__a^_^`_aa`a`a]_ZVZYYO[_R
@HWI-EAS443:3:68:1277:327#0/1
GCCTTATTGAATTCATTTTGGACCCAGCTTGCTAGAGATC
+HWI-EAS443:3:68:1277:327#0/1
¥`bbb]bab`_bbbbbbbab_bb`aaabb_abbbbbab`
@HWI-EAS443:3:68:1314:742#0/1
CTTCTGCTGTAAAATAGTTCCTCCTCATGCTTGCGATATG
+HWI-EAS443:3:68:1314:742#0/1
`abbbaabaabbbbbab`baaabababaaaaab`bbaaaa
@HWI-EAS443:3:68:1375:1374#0/1
CCGATTTGAGATAATTGAAGGAAGAGACCGAATCATGGCC
+HWI-EAS443:3:68:1375:1374#0/1
_aaabbbbabbb`abbbaba_bbb`aba`baaa^b`aa^^
@HWI-EAS443:3:68:1458:583#0/1
GGTTCTCTTTGTAATCATACAAATCAGGAAGAAATTTAGG
+HWI-EAS443:3:68:1458:583#0/1
abXXQ_Q`^^`XXRZX^GST_XRWW_a¥]VPR_^¥¥XQ_]
@HWI-EAS443:3:68:1533:425#0/1
GGGACAACCTGGAACCTGGAACCTTCGATCTTGGGGGGCT
+HWI-EAS443:3:68:1533:425#0/1
aa`YS]`_¥_`R][Y[^¥]Y¥SSZ[^^VYU[^]PZNSUVV
@HWI-EAS443:3:68:1633:752#0/1
GGGGCAACATCTTCTCCTATTTCATCAAGTTCTATCCAGC
+HWI-EAS443:3:68:1633:752#0/1
a`a`Va`a]Y`___a_`a`a_^^aaa_a`^]^_``WW_`_
@HWI-EAS443:3:68:163:464#0/1
GAGACCTTAAACAGTATGACAGTGATGAGCCAGAGCCCAG
+HWI-EAS443:3:68:163:464#0/1
^_ababbb_[``aaX^abbb``Ga¥[`_a`aZ_¥[^]XXT

@HWI-EAS443:3:68:1704:559#0/1
CTTTGGCTGGAAAGAGCCTAACATAGTCAAACCACATGAG
+HWI-EAS443:3:68:1704:559#0/1
aaaZabab`SS_`TW^GVa`_`^[^¥H^aab`^WR``b^Y
@HWI-EAS443:3:68:1709:51#0/1
TAACATAGTCAAACCACATGAGAAAGGCATAAATCCCAAT
+HWI-EAS443:3:68:1709:51#0/1
aabababbaaaaba`a`aba_a`aaaaY`b`aa_YWO_`_
@HWI-EAS443:3:68:1727:298#0/1
TTTTACAGCAGAAGTGTCCCACTGCAGGGCTACTGAATAC
+HWI-EAS443:3:68:1727:298#0/1
]abY^bZaa``[`¥R`ZS`XXZaa[Sa]`_[¥`Y`YX_YN
@HWI-EAS443:3:68:199:594#0/1
TACATATACTGTTCACCACTGTCCAGGCCATGATTCGGTC
+HWI-EAS443:3:68:199:594#0/1
`[a_¥ab`a`a`Wa``_YUaaX`¥`^_a[V^RY^^RKTEN
@HWI-EAS443:3:68:216:1353#0/1
CAGGTTTGTTTTCCGTCTTCCTTCTTTGGTCCTACATTTG
+HWI-EAS443:3:68:216:1353#0/1
aa`UF¥aaP]__]¥YH]OX_a`]_XR[SWVa`W__S^^[F
@HWI-EAS443:3:68:252:1316#0/1
TTCAAAGAATTCCTTGGTCATGTCTTTCTCTTTGACAGAA
+HWI-EAS443:3:68:252:1316#0/1
aba^¥Y^ZZb`abaabaZ_aaa_a_abaa_aa`a]aa_a`
@HWI-EAS443:3:68:256:112#0/1
GGGGGCTATATGAAGCAATCGAGGAGTGCCTGATTAATGA
+HWI-EAS443:3:68:256:112#0/1
VV_a_Y¥¥¥a¥_Z]a]]]`_UZZ`_V_SSUZaV[_OZUNS
@HWI-EAS443:3:68:286:307#0/1
TTAATCAGGCACTCCTCGATTGCTTCATATAGCCCCCCAA
+HWI-EAS443:3:68:286:307#0/1
a^W^`^UUT_Y_a`]_a[X^]XaZaZ]ZaUWWb]a^_]TJ
@HWI-EAS443:3:68:311:1079#0/1
CATTAATCAGGCACTCCTCGATTGCTTCATATAGCCCCCC
+HWI-EAS443:3:68:311:1079#0/1
a``b_bbbb_]aaaaaaaaa`aabaaaaaab`a`^a_^_]
@HWI-EAS443:3:68:329:1780#0/1
GGGGGCTATATGAAGCAATCGAGGAGTGCCTGATTAATGA
+HWI-EAS443:3:68:329:1780#0/1
aa]]aaaaabaaaaabbaa_aaaa``¥[]_]`_`_aa`_a
@HWI-EAS443:3:68:32:1555#0/1
CTGTATTCAACAGTCTATATGCGTCTCCACAACTTGAGGG
+HWI-EAS443:3:68:32:1555#0/1
aaT[aaa_aaa_Y]a`aaa^V_^^a__a__^`]_[]^^^¥
@HWI-EAS443:3:68:32:894#0/1
CGGCTGAATCGAGAAAATTGCTTCTCATTGTTCAGGCACT
+HWI-EAS443:3:68:32:894#0/1
aaaaZ`V]]a]`a^X``]^¥¥^^^`_TV^`ZZ`__[__¥¥
@HWI-EAS443:3:68:364:925#0/1
CGAATGCACTATTGAAGCACCGATTTGAGATAATTGAAGG

~~~
+HWI-EAS443:3:68:364:925#0/1
a^O]_``abbbaabaaaaaaa]W]aaa_``_a`a`¥[`_`
@HWI-EAS443:3:68:418:290#0/1
TGTAATCTCAAATTTTTCTTCAATTGTCTCTTCGCCTCTT
+HWI-EAS443:3:68:418:290#0/1
¥^^_[_ZJ_Y^aabbab^b^[aX]W___^a^]^Y]`]Z¥`
@HWI-EAS443:3:68:472:911#0/1
AGATGTTGCCCCGATTGAACATATCGCAAGCATGAGGAGG
+HWI-EAS443:3:68:472:911#0/1
[aaWb¥`_aa`]_^^a`abaa^_Ya]a_]YTW_W[][_^V
@HWI-EAS443:3:68:482:1556#0/1
GACCCGAATGCACTATTGAAGCACCGATTTGAGATAATTG
+HWI-EAS443:3:68:482:1556#0/1
abbbaa_aaabaa`aa``^_Z^aa````a_]]a`_a_a_`
@HWI-EAS443:3:68:518:13#0/1
GTTCACCACTGTCCAGGCCATGATTCGGTCTCTTCCTTCA
+HWI-EAS443:3:68:518:13#0/1
`Y][`````aa___¥^`¥]]W^[^ZT[ZRW[UW[RSVVVW
@HWI-EAS443:3:68:571:617#0/1
AGATTCTACAATTATTGATTCACCCCGTTCGTCGATGAAA
+HWI-EAS443:3:68:571:617#0/1
abaaaa^aaaaaaaaaa`aaaaaa_]a^_¥a_aaaaa^``
@HWI-EAS443:3:68:583:421#0/1
AGATAATTGAAGGAAGAGACCGAATCATGGCCTGGACAGT
+HWI-EAS443:3:68:583:421#0/1
`aabababbabaaaaaaaaa`a`^a_`aa`a_a`_`¥a^^
@HWI-EAS443:3:68:585:1672#0/1
GGGAGTGTACATAAATACGGCCTTGCTCAATGCATCCTGT
+HWI-EAS443:3:68:585:1672#0/1
^¥I¥aYU``V`a`[¥X¥`a^b`aY``S^_]S^XSUX^HUH
@HWI-EAS443:3:68:58:1277#0/1
CAAGCCAATTGAAGTGGGCACTCGGTGAAAATATGGCACC
+HWI-EAS443:3:68:58:1277#0/1
aaY]_aa`]aa`_ZO]^_`^`]`^^RYU^__Y[[]X^[]S
@HWI-EAS443:3:68:626:1159#0/1
CTGAATACATAATGAAGGGAGTGTACATAAATACGGCCTT
+HWI-EAS443:3:68:626:1159#0/1
aa_TXa^b`b¥Zab`[`Vb_a`b`aaaa^`aaaab`¥Z`a
@HWI-EAS443:3:68:718:872#0/1
CCACTCCCCTGGGTGACTCTCCGATTGGCCATGTTTCCGA
+HWI-EAS443:3:68:718:872#0/1
a_Waaab``U_ZRNZ]¥a`_`^]YU^]a^^[[UVa¥LXZN
@HWI-EAS443:3:68:71:885#0/1
GCCTTGCTCAATGCATCCTGTGCAGCCATGGATGACTTTC
+HWI-EAS443:3:68:71:885#0/1
abbabbbbaaXZabbaa`aZ_abaaaaaaa`aa_R[a__a
@HWI-EAS443:3:68:781:744#0/1
GAGAAGCAATTTTCTCGATTCAGCCGAAAACCCCTCAAGT
+HWI-EAS443:3:68:781:744#0/1
aaaabaabab`a`ababX`aaa```U`_aa_]W`a_a]`W
~~~

@HWI-EAS443:3:68:795:2027#0/1
TCCACTCCCCTGGGTGACTCTCCGATTGGCCATGTTTCCG
+HWI-EAS443:3:68:795:2027#0/1
abbbaaaba_R^`[NYaL¥]_`^`YS_GQUaaOFJ_G_ZD
@HWI-EAS443:3:68:824:277#0/1
GCATGGAAATGAGGCGCTGCCTTCTTCAGTCTCTTCAGCA
+HWI-EAS443:3:68:824:277#0/1
aa`_a]_a_`^`a_a`a__¥¥___`___aY`__¥_[V^]_
@HWI-EAS443:3:68:855:1322#0/1
GAAATAGGAGACATGCTCTTGAGGACTGCGATAGGCCAAG
+HWI-EAS443:3:68:855:1322#0/1
[baabaa¥]`bbbaabaaaaaaaaaaaaaaa^a_a_`_]_
@HWI-EAS443:3:68:867:62#0/1
CACACATTCACATCTTTTCATTCACTGGAGAGGAGATGGC
+HWI-EAS443:3:68:867:62#0/1
a]b`V[baUb^W_¥`a[``bb`[b_Z_b]a]baaaSb^NV
@HWI-EAS443:3:68:873:700#0/1
CAAACTTCCAAATGTGTGCATATTGCAGCAAACTTGTTAG
+HWI-EAS443:3:68:873:700#0/1
abbaabbabbbbbb_babbbb_bbbbbabb`aabbbaaab
@HWI-EAS443:3:68:933:1445#0/1
GGGCAACATCTTCTCCTATTTCATCAAGTTCTATCCAGCT
+HWI-EAS443:3:68:933:1445#0/1
aa`^bYU_^_aaaHLaWTYQ]SX^T¥¥Z]]Y_aW]`IG_^
@HWI-EAS443:3:68:93:70#0/1
CAAAGAGAACCGGTTCATTGAAATTGGAGTAACACGGAGG
+HWI-EAS443:3:68:93:70#0/1
abaab]aaV`aaa^^_S_]aaaaaaa^¥`W_`a_____`]
@HWI-EAS443:3:68:980:463#0/1
GGCATGGAAATGAGGCGCTGCCTTCTTCAGTCTCTTCAGC
+HWI-EAS443:3:68:980:463#0/1
a`Za_a`_`X¥`_¥^``___^_]_Y^^^Y_Z[_^`^W¥X¥
@HWI-EAS443:3:69:1037:1880#0/1
AAGAGCCTAACATAGTCAAACCACATGAGAAAGGCATAAA
+HWI-EAS443:3:69:1037:1880#0/1
ab_[T`__abaaYaXXa`aaWX]^a``_XaaP[_¥a^_a_
@HWI-EAS443:3:69:1045:629#0/1
ATTTTGGACCCAGCTTGCTAGAGATCTGGGCTCTGGCTCA
+HWI-EAS443:3:69:1045:629#0/1
[a^_]Z¥aa`WD^^`X_¥X]a_][]a`ZP]]]]a¥Y]___
@HWI-EAS443:3:69:1072:505#0/1
GGGGGCTATATGAAGCAATCGAGGAGTGCCTGATTAATGA
+HWI-EAS443:3:69:1072:505#0/1
ababbabbbbbbbbbabaa`a`ba`a_ba_aaaaaaaa`a
@HWI-EAS443:3:69:1081:275#0/1
CGTCTCCACAACTTGAGGGGTTTTCGGCTGAATCGAGAAA
+HWI-EAS443:3:69:1081:275#0/1
`abbbabbbababbabbbbb_abbabaaa]baa_a`a`aa
@HWI-EAS443:3:69:1092:823#0/1
TTTGAGATTACAGGAACTATGCGCAAGCTTGCCGACCAAA

+HWI-EAS443:3:69:1092:823#0/1
¥[UZW^T_Z¥Z¥[FYaR__^ZT¥Z[Y]aQ`]aZHQYaa[Y
@HWI-EAS443:3:69:1100:1506#0/1
GGGGGCTATATGAAGCAATCGAGGAGTGCCTGATTAATGA
+HWI-EAS443:3:69:1100:1506#0/1
a¥V[^a¥aaa_¥`aaaaa__a]]YY^M^___``_____`_
@HWI-EAS443:3:69:1112:1507#0/1
AGGACATTGAAAATGAAGAGAAGATCCCAAGGACAAAGAA
+HWI-EAS443:3:69:1112:1507#0/1
a__````^`X^ab_a_abaaa__]__^aa]¥``_`¥``aa
@HWI-EAS443:3:69:1161:1162#0/1
GAAGTGGGCACTCGGTGAAAATATGGCACCAGAAAAAGTA
+HWI-EAS443:3:69:1161:1162#0/1
aa`a[a`aaa__¥]TS]^`^a_a_[`X__Y^_¥__X_¥V_
@HWI-EAS443:3:69:117:1077#0/1
AAGAATTCCTTGGTCATGTCTTTCTCTTTGACAGAAGACT
+HWI-EAS443:3:69:117:1077#0/1
`_`a_a_^_a]^aY```a¥`aa`aaa``XZVa[_Z][_``
@HWI-EAS443:3:69:1203:60#0/1
GGGGCATGGAAATGAGGCGCTGCCTTCTTCAGTCTCTTCA
+HWI-EAS443:3:69:1203:60#0/1
_V^_¥^^¥`^U[[¥^¥_Y¥YYZNLVWWWVUTYWPUSTVXT
@HWI-EAS443:3:69:1240:383#0/1
CCCCGTTCGTCGATGAAATGGAAATCCGAATACATGAAAC
+HWI-EAS443:3:69:1240:383#0/1
^bbbbbb[_Z`babaaba_aa_bb`_ab`a``]`a`aaa^
@HWI-EAS443:3:69:1254:251#0/1
ATAATTGTAGAATCTGGTGACCCGAATGCACTATTGAAGC
+HWI-EAS443:3:69:1254:251#0/1
`baabbb[ab`aaaaba_a__Z`a_aaaY`^`aa_a]_a]
@HWI-EAS443:3:69:1258:1277#0/1
CAGAGCTACAGGACATTGAAAATGAAGAGAAGATCCCAAG
+HWI-EAS443:3:69:1258:1277#0/1
`¥]X``aa]a_XW_^b[`[`_Yaa`aa^]`aa`¥¥T`]¥_
@HWI-EAS443:3:69:1304:217#0/1
GCTTGCTAGAGATCTGGGCTCTGGCTCATCACTGTCATAC
+HWI-EAS443:3:69:1304:217#0/1
aaaa]aaaa`_ba`aa^a¥`U^`_Y`_`_Z_^``_OU``]
@HWI-EAS443:3:69:1477:634#0/1
CTCCTATTTCATCAAGTTCTATCCAGCTTGAATCAGTCAA
+HWI-EAS443:3:69:1477:634#0/1
abaabbabbaaaaba_`aaabaa_aa`a`aaaa`a`_¥a_
@HWI-EAS443:3:69:1526:957#0/1
GCCCCCCAAGATCGAAGGTTCCAGGTTCCAGGTTGTCCCT
+HWI-EAS443:3:69:1526:957#0/1
a`¥_`¥^_X_`^__¥]ZYEY_ZY_[Q^]][`XVZVLITV¥
@HWI-EAS443:3:69:1619:1560#0/1
TACATATACTGTTCACCACTGTCCAGGCCATGATTCGGTC
+HWI-EAS443:3:69:1619:1560#0/1
aa¥babab`aa_a^a¥`a^aZ¥a_aa^`_a``_a_WO^_R

@HWI-EAS443:3:69:164:1353#0/1
GAAAGAGCCTAACATAGTCAAACCACATGAGAAAGGCATA
+HWI-EAS443:3:69:164:1353#0/1
ab¥`abbbba^bab]b_]abbb^aaaaaa^a`aa__aa``
@HWI-EAS443:3:69:1658:242#0/1
GTCCAGGCCATGATTCGGTCTCTTCCTTCAATTATCTCAA
+HWI-EAS443:3:69:1658:242#0/1
aa_]ab`aaaba`aa`ba^¥_W`_ZYa`T^]aa__Q[M¥`
@HWI-EAS443:3:69:1662:926#0/1
CATTCTTTGGCTGGAAAGAGCCTAACATAGTCAAACCACA
+HWI-EAS443:3:69:1662:926#0/1
aabbbbbbbbabbaabbabbaabbbabbbba`bab``aaa
@HWI-EAS443:3:69:170:307#0/1
CTGCACAGGATGCATTGAGCAAGGCCGTATTTATGTACAC
+HWI-EAS443:3:69:170:307#0/1
aaaba`aa]a_¥a``_[¥`_`__]_`aWW¥a]^_`V^Z[T
@HWI-EAS443:3:69:197:798#0/1
CGGCTGAATCGAGAAAATTGCTTCTCATTGTTCAGGCACT
+HWI-EAS443:3:69:197:798#0/1
aZZa`]^YNN_[`a`Y__[OV_^`]XFXW[IOY¥VX_^V^
@HWI-EAS443:3:69:229:787#0/1
AAAGAGCCTAACATAGTCAAACCACATGAGAAAGGCATAA
+HWI-EAS443:3:69:229:787#0/1
S[Zaab^babababba]baba_abbb`a`_a_a_aaaaab
@HWI-EAS443:3:69:287:415#0/1
GGCAACATCTTCTCCTATTTCATCAAGTTCTATCCAGCTT
+HWI-EAS443:3:69:287:415#0/1
aaaaaa_aaaaaaaaaaaaaaa`a_ZZ^_a_a`_`]a__]
@HWI-EAS443:3:69:302:853#0/1
GGGCACTCGGTGAAAATATGGCACCAGAAAAAGTAGACTT
+HWI-EAS443:3:69:302:853#0/1
aaabbbaaab_ab`bbaaaaaaaaaaaaaa```^aaa_a`
@HWI-EAS443:3:69:441:331#0/1
CGAATGCACTATTGAAGCACCGATTTGAGATAATTGAAGG
+HWI-EAS443:3:69:441:331#0/1
a`_`abb_bbabbaaaaaabbbaaa]a`baa`aa`a`aaa
@HWI-EAS443:3:69:46:905#0/1
CGATTGGCCATGTTTCCGATTTGTTTTCAAAGAATTCCTT
+HWI-EAS443:3:69:46:905#0/1
abaaaaabba`a[a`aba_]aaaZa_```X`_XR`a`^``
@HWI-EAS443:3:69:519:632#0/1
GAGACATGCTCTTGAGGACTGCGATAGGCCAAGTGTCGAG
+HWI-EAS443:3:69:519:632#0/1
^O¥_]Z`TWP_aaYVR__NJ^Ya`UQYFWW]P[POGSX[U
@HWI-EAS443:3:69:606:866#0/1
CTGCACACTTTCCCAATAGAGCCTTCCTCCACTCCCCTGG
+HWI-EAS443:3:69:606:866#0/1
abab]`b`][^a`^baa]`Q^a_aaaaa`W]]aab`b_`Z
@HWI-EAS443:3:69:610:1540#0/1
CGTCTCCACAACTTGAGGGGTTTTCGGCTGAATCGAGAAA

```
+HWI-EAS443:3:69:610:1540#0/1
a__baaaY`]Z_`a¥`_`_`P`[¥a`^a_`a`]`_^_^^a
@HWI-EAS443:3:69:616:1295#0/1
CACAACTTGAGGGGTTTTCGGCTGAATCGAGAAAATTGCT
+HWI-EAS443:3:69:616:1295#0/1
`a`]__`a___RZTIR__XOP_^]`]^]V^G¥^_]Y^VXY
@HWI-EAS443:3:69:619:1320#0/1
TTTTGGACCCAGCTTGCTAGAGATCTGGGCTCTGGCTCAT
+HWI-EAS443:3:69:619:1320#0/1
aa¥I¥_¥aa_`¥b`VY_S`NX]XP`^Y¥^_[a¥__[V^ZP
@HWI-EAS443:3:69:631:1571#0/1
CACTCCCCTGGGTGACTCTCCGATTGGCCATGTTTCCGAT
+HWI-EAS443:3:69:631:1571#0/1
abbabbbb`aa]^aabab`aa`¥`_VZ`^__^P__X][SX
@HWI-EAS443:3:69:636:376#0/1
CATTAATCAGGCACTCCTCGATTGCTTCATATAGCCCCCC
+HWI-EAS443:3:69:636:376#0/1
`[abbbbbb`bbbabaaaabbaaaaaaaaabaa`_¥aaaa
@HWI-EAS443:3:69:695:618#0/1
GGTCTCCAACATCTTTGCAGTCATCAAAGTCTACTTTTTC
+HWI-EAS443:3:69:695:618#0/1
__PZVS_S__aY`aKYbZ_^Q^TaYQTT¥VY¥aRJaaTaa
@HWI-EAS443:3:69:743:586#0/1
GGCTTGGAAGCAGGTGCTAGCAGAGCTACAGGACATTGAA
+HWI-EAS443:3:69:743:586#0/1
a^baabaaaaa^Wa]]aaa`aaa^`a`a]aa¥Z[Z]a[`^
@HWI-EAS443:3:69:768:1844#0/1
GGTGAACTTTGTAAGTATGGAGTTCTCACTCACTGACCCG
+HWI-EAS443:3:69:768:1844#0/1
aaXZ¥aaa`_aZ`V`Qa¥[^_`^¥^^aa``_a`X¥aS_`a
@HWI-EAS443:3:69:787:450#0/1
CACCTGCTTCCAAGCCATGAGGTAATTGGGATTTATGCCT
+HWI-EAS443:3:69:787:450#0/1
`V[X¥`aa```_W]^[[``VXYJW_aa¥_aT¥_[`a_YX^
@HWI-EAS443:3:69:924:976#0/1
AGCATCCATCAGCAGGAACTTTGACCGCTGATGGCAAAGA
+HWI-EAS443:3:69:924:976#0/1
aabbabbbaaaaaaaa```aaa`aaaa`a``_`^`a_`_^
@HWI-EAS443:3:69:958:409#0/1
TGAATACATAATGAAGGGAGTGTACATAAATACGGCCTTG
+HWI-EAS443:3:69:958:409#0/1
aa`aaaaab`_aba_a`_]aXa_a_aaaa`aa_`]^^_ZY
@HWI-EAS443:3:6:1009:627#0/1
CTGGTGCCATATTTTCACCGAGTGCCCACTTCAATTGGCT
+HWI-EAS443:3:6:1009:627#0/1
abbbabbbbbbbbab`babbab`b`_aaabbabbbabb_b
@HWI-EAS443:3:6:1010:363#0/1
CGAACGGGGTGAATCAATAATTGTAGAATCTGGTGACCCG
+HWI-EAS443:3:6:1010:363#0/1
R^S`b_bbaUaabb_aW_b__¥b_`¥aabbVabUbbaV_b
```

@HWI-EAS443:3:6:1016:653#0/1
GTTTTCGGCTGAATCGAGAAAATTGCTTCTCATTGTTCAG
+HWI-EAS443:3:6:1016:653#0/1
aS^^QVY¥Y¥]_^SM¥VLFOWYZRXSTTUYY]]^XYZRBB
@HWI-EAS443:3:6:1025:375#0/1
GGAGTGTACATAAATACGGCCTTGCTCAATGCATCCTGTG
+HWI-EAS443:3:6:1025:375#0/1
abU^Xb_`aaaa`a`b¥[ZT[]____¥``VYX¥_¥Y_¥Q_
@HWI-EAS443:3:6:1067:1067#0/1
ACACCCTTGACGAAGAGAGCAGGGCAAGAATCAAAACTAG
+HWI-EAS443:3:6:1067:1067#0/1
aaS]aa¥aX^¥bbXYZbb]`bWGV¥WU]a_a^^_Raab
@HWI-EAS443:3:6:1131:1164#0/1
GGGAGACTTTGGTCGGCAAGCTTGCGCATAGTTCCTGTAA
+HWI-EAS443:3:6:1131:1164#0/1
X_ba_MYW``ab]¥`_TYLWXBBBBBBBBBBBBBBBBBBB
@HWI-EAS443:3:6:1213:1534#0/1
GCCGAACGGCTGCATTGAGGGCAAGCTTTCCCAAATGTCA
+HWI-EAS443:3:6:1213:1534#0/1
abb^a`^b_X__ZZ^_U^Q`aU[]]R[``^^____¥]UV¥
@HWI-EAS443:3:6:1263:1319#0/1
GGGGGCTATATGAAGCAATCGAGGAGTGCCTGATTAATGA
+HWI-EAS443:3:6:1263:1319#0/1
YabbaJ_bbababab bbW__b_ab[a`b`Y[a_aaaaab^
@HWI-EAS443:3:6:1309:998#0/1
CGTCTCCACAACTTGAGGGGTTTTCGGCTGAATCGAGAAA
+HWI-EAS443:3:6:1309:998#0/1
abaaaabb_aa`abaaaabaYaaa_`a_`aaaa_a_a```
@HWI-EAS443:3:6:1390:696#0/1
CACTTTCCCAATAGAGCCTTCCTCCACTCCCCTGGGTGAC
+HWI-EAS443:3:6:1390:696#0/1
^aa``^^`_a````ab``^`¥Y`Z]]]a^aU]^baa[b[]
@HWI-EAS443:3:6:1403:1042#0/1
CAAAGATGTTGGAGACCTTAAACAGTATGACAGTGATGAG
+HWI-EAS443:3:6:1403:1042#0/1
abbbbXa``bab[ba``aa_`a`a__ab`a_a`Z]``a_B
@HWI-EAS443:3:6:1413:1132#0/1
GTGGTGTCGTCCTCAAGAATGGTTCAATTTTGGCGTTCAC
+HWI-EAS443:3:6:1413:1132#0/1
a¥_a]^¥_][W¥PXW^]ZN`a_]OX^R_a^¥[X]aXXYSB
@HWI-EAS443:3:6:1464:658#0/1
GTAAAATAGTTCCTCCTCATGCTTGCGATATGTTCAATCG
+HWI-EAS443:3:6:1464:658#0/1
`NWbaa^]b]``^_OFYW^O`]XX_Z`PX___WWX]WBBB
@HWI-EAS443:3:6:1499:480#0/1
CATAATGAAGGGAGTGTACATAAATACGGCCTTGCTCAAT
+HWI-EAS443:3:6:1499:480#0/1
a¥_babba_b`b`aabTbabaa¥abbaaa^Ua`b^_Ya_a
@HWI-EAS443:3:6:1501:1737#0/1
CACTGCAGGGCTACTGAATACATAATGAAGGGAGTGTACA

```
+HWI-EAS443:3:6:1501:1737#0/1
[bGbbWbbbb¥ab_abbaa`_[a```baaaabZa_a`aV`
@HWI-EAS443:3:6:1564:1657#0/1
CATGGAAATGAGGCGCTGCCTTCTTCAGTCTCTTCAGCAG
+HWI-EAS443:3:6:1564:1657#0/1
aab`baab`abba¥a_aa_`ab[`^JZb]^a¥aa]_aV^]
@HWI-EAS443:3:6:156:403#0/1
ATTTGAGAAATGATACTGATGTGGTGAACTTTGTAAGTAT
+HWI-EAS443:3:6:156:403#0/1
aa`ab^a``_```b^ab`aabW]aYa^aYa`aa^_Wa^`^
@HWI-EAS443:3:6:1579:1864#0/1
AAATCCGAATACATGAAACAAACTTCCAAATGTGTGCATA
+HWI-EAS443:3:6:1579:1864#0/1
aaaa_`b`_aa]ababa``aaaO¥_`^a``^a^a`a¥¥^`
@HWI-EAS443:3:6:1629:1210#0/1
GGAGAGGAGATGGCCACCAAAGCGGACTACACCCTTGACG
+HWI-EAS443:3:6:1629:1210#0/1
`baaaabbaaaba_V`Q^aaZaUa`aV]`PaMX_X_`RLU
@HWI-EAS443:3:6:163:1127#0/1
CTCATCACTGTCATACTGTTTAAGGTCTCCAACATCTTTG
+HWI-EAS443:3:6:163:1127#0/1
SSabQMZbabaT`XbbbXI_abba`X¥aabX]ba``a`¥b
@HWI-EAS443:3:6:166:2007#0/1
CCCCAAGATCGAAGGTTCCAGGTTCCAGGTTGTCCCTAAG
+HWI-EAS443:3:6:166:2007#0/1
ab]bbbbaaaaababXaaaa_U]aa^aa`Wa[Y`¥`aaa^
@HWI-EAS443:3:6:1695:1159#0/1
ACCTTCGATCTTGGGGGGCTATATGAAGCAATCGAGGAGT
+HWI-EAS443:3:6:1695:1159#0/1
a``aaab_]Pabaaa``_Ra^___[W_VR¥_YM[[__^_B
@HWI-EAS443:3:6:1703:1239#0/1
ATCTGTATTCAACAGTCTATATGCGTCTCCACAACTTGAG
+HWI-EAS443:3:6:1703:1239#0/1
J¥_ab¥bbbU^XPY[TU`a`[a^aa``a```X[[R^`a_[
@HWI-EAS443:3:6:1708:87#0/1
CATAAATCCCAATTACCTCATGGCTTGGAAGCAGGTGCTA
+HWI-EAS443:3:6:1708:87#0/1
U^bbb_][[U]``ba¥U[Wab_b`bbK`ZPDT^[W_BBBB
@HWI-EAS443:3:6:1725:821#0/1
AGCAATTTTCTCGATTCAGCCGAAAACCCCTCAAGTTGTG
+HWI-EAS443:3:6:1725:821#0/1
]a[`_W_aa`¥[`¥Wa``ZN[Z__T]WVDJYIHX`S_ZU]
@HWI-EAS443:3:6:219:624#0/1
CACTCGGTGAAAATATGGCACCAGAAAAAGTAGACTTTGA
+HWI-EAS443:3:6:219:624#0/1
S_X[aXa]`ZZR^¥^IYa__¥^Y]YVJ]Q_Y[]aZWa[NB
@HWI-EAS443:3:6:24:966#0/1
TGCTTCAATAGTGCATTCGGGTCACCAGATTCTACAATTA
+HWI-EAS443:3:6:24:966#0/1
`b`baabaa]b[aabb^^bab^abaa_aa_a_`__``aa`
```

```
@HWI-EAS443:3:6:25:835#0/1
GAGAAAGGCATAAATCCCAATTACCTCATGGCTTGGAAGC
+HWI-EAS443:3:6:25:835#0/1
^__^^]_aa``____^ZY¥]`]`]¥_]W¥Z]X_¥YYBBBB
@HWI-EAS443:3:6:291:261#0/1
ATAAGACAAGAAATGGCCAGTAGGAGTCTATGGGATTCCT
+HWI-EAS443:3:6:291:261#0/1
aaaaa^¥aaa`_¥`aa_T]]L[Y`^a]_`_U___^^^VW]
@HWI-EAS443:3:6:325:277#0/1
CTCCACAACTTGAGGGGTTTTCGGCTGAATCGAGAAAATT
+HWI-EAS443:3:6:325:277#0/1
abaab`_bYbbbaaaabRaaa_a_^`aaa^`a]a__``__
@HWI-EAS443:3:6:336:1977#0/1
CATTCTTTGGCTGGAAAGAGCCTAACATAGTCAAACCACA
+HWI-EAS443:3:6:336:1977#0/1
abbaabbabbaabbbaba`abbbbbbaab`abbbaaaa`b
@HWI-EAS443:3:6:392:1148#0/1
GGGATCTTCTCTTCATTTTCAATGTCCTGTAGCTCTGCTA
+HWI-EAS443:3:6:392:1148#0/1
¥bb_babbbbaba_bbbbabbabb`aaabaaaa`a_a_`a
@HWI-EAS443:3:6:566:1879#0/1
AGAAGACACACATTCACATCTTTTCATTCACTGGAGAGGA
+HWI-EAS443:3:6:566:1879#0/1
^YSX_]baZ¥^bZV^aPVaPbaTbbba[``N^^][¥¥bBB
@HWI-EAS443:3:6:58:1044#0/1
GCGCAAGCTTGCCGACCAAAGTCTCCCACCGAACTTCTCC
+HWI-EAS443:3:6:58:1044#0/1
`bbaaaaaa``aaaa`aaaa^U``__a`_¥]]PZ^`^_¥_
@HWI-EAS443:3:6:666:1683#0/1
AACTTTAGAGCCTATGTAGATGGATTCGAGCCGAACGGCT
+HWI-EAS443:3:6:666:1683#0/1
aaIUbaVbU[P_`aa`]Z`aOLX_SV[`^BBBBBBBBBBB
@HWI-EAS443:3:6:692:1362#0/1
AGAATGTCTTCATGCATTTGATTGCATCATATAGTGGTAT
+HWI-EAS443:3:6:692:1362#0/1
abaa`a_aaaaa[__ba```_¥aa_`^a````a_W_]Z^_
@HWI-EAS443:3:6:705:1244#0/1
GGGGGCTATATGAAGCAATCGAGGAGTGCCTGATTAATGA
+HWI-EAS443:3:6:705:1244#0/1
[HTU¥a_aY`¥]MXU`]S_OO^ZUZZX]VE]Q¥`PRW_HB
@HWI-EAS443:3:6:711:85#0/1
CAACAAAATAAAATCTGAGAAGACACACATTCACATCTTT
+HWI-EAS443:3:6:711:85#0/1
^abaaabbbbbbaaabbaaaabaaa_a_aba^a_aaaaaa
@HWI-EAS443:3:6:712:114#0/1
GCCAATTGAAGTGGGCACTCGGTGAAAATATGGCACCAGA
+HWI-EAS443:3:6:712:114#0/1
a_^[`^aYX]`]_[_]_XZP¥`V^OUOWXXZXNLPKUBBB
@HWI-EAS443:3:6:765:837#0/1
GGTGAACTTTGTAAGTATGGAGTTCTCACTCACTGACCCG
```

```
+HWI-EAS443:3:6:765:837#0/1
aaWaa`_aaa`__`a¥_a_`_`¥_[`__`__^¥`__¥YUa
@HWI-EAS443:3:6:856:324#0/1
GGGTTTTCGGCTGAATCGAGAAAATTGCTTCTCATTGTTC
+HWI-EAS443:3:6:856:324#0/1
abaX`M¥[a_Na`TXLL^V`^]V]]X]]_^¥]Q][¥a¥VB
@HWI-EAS443:3:6:857:410#0/1
GTTCAATCGGGGCAACATCTTCTCCTATTTCATCAAGTTC
+HWI-EAS443:3:6:857:410#0/1
]JOXY^^W_YV¥¥Z¥WX^X]¥PYUS[^X][S¥XOXQ[WBB
@HWI-EAS443:3:6:88:284#0/1
CCCGAATGCACTATTGAAGCACCGATTTGAGATAATTGAA
+HWI-EAS443:3:6:88:284#0/1
abbbababbbabababa`a`a``a``aaa_baaa`aaa``
@HWI-EAS443:3:6:982:1076#0/1
GACATTCTTTGGCTGGAAAGAGCCTAACATAGTCAAACCA
+HWI-EAS443:3:6:982:1076#0/1
ab_abaaaaaaa`aaa`aa`]a¥]```^``_a]^_``UX_
@HWI-EAS443:3:6:98:1283#0/1
TTTTCACCGAGTGCCCACTTCAATTGGCTTGTTCTCTTCA
+HWI-EAS443:3:6:98:1283#0/1
aa__baaa[]bXa``b``aa_a_]``aaYS[ZVW`^___B
@HWI-EAS443:3:70:1026:39#0/1
TGGGGGGCTATATGAAGCAATCGAGGAGTGCCTGATTAAT
+HWI-EAS443:3:70:1026:39#0/1
_bb`a_aaa_]`aba`a]aaa_a_aa^_Ua_]_a^`a]__
@HWI-EAS443:3:70:1106:1759#0/1
GAACGCCAAAATTGAACCATTCTTGAGGACGACACCACGC
+HWI-EAS443:3:70:1106:1759#0/1
H^ababbabba_bbbbbabaabbbbbbbbbabbbbaba`b
@HWI-EAS443:3:70:1143:729#0/1
GCACCAGAAAAAGTAGACTTTGATGACTGCAAAGATGTTG
+HWI-EAS443:3:70:1143:729#0/1
aaab`ba¥_aa]`_a`abbbabb`aa`aaaaaYaa_aZ_`
@HWI-EAS443:3:70:1177:588#0/1
CTCGATTGCTTCATATAGCCCCCCAAGATCGAAGGTTCCA
+HWI-EAS443:3:70:1177:588#0/1
_WX`^]abaa_UTaaZ_R]_HW_]XVZV`]U_BBBBBBBB
@HWI-EAS443:3:70:1193:211#0/1
ACCGATTTGAGATAATTGAAGGAAGAGACCGAATCATGGC
+HWI-EAS443:3:70:1193:211#0/1
_TQZOX¥bb_UQWU`a]aVQR_W]`][¥UQXVT¥[¥a¥WF
@HWI-EAS443:3:70:1226:1302#0/1
GGGGGCTATATGAAGCAATCGAGGAGTGCCTGATTAATGA
+HWI-EAS443:3:70:1226:1302#0/1
a`UM^WaS]^VXYU_]a`a`]NTQRQF¥]U[UPUX]XY[[
@HWI-EAS443:3:70:1226:1876#0/1
GCCTTGCTCAATGCATCCTGTGCAGCCATGGATGACTTTC
+HWI-EAS443:3:70:1226:1876#0/1
a¥`URI¥_¥a`a_aaRQ[[XH_``_^__S]_aN^VUQUZQ
```

@HWI-EAS443:3:70:1237:522#0/1
GTCCAAAATGAATTCAATAAGGCATGTGAATTGACTGATT
+HWI-EAS443:3:70:1237:522#0/1
a`a`a__[`aaaaa]a_aa`a^^`a`Xa__a^^]]W_Y^]
@HWI-EAS443:3:70:1250:457#0/1
TCCAGCCTTGAAAACTTTAGAGCCTATGTAGATGGATTCG
+HWI-EAS443:3:70:1250:457#0/1
aaaaaaabbbaa`a_aaaba`aa_a``a^aa_`aa__^__
@HWI-EAS443:3:70:1252:250#0/1
AGGCCGTATTTATGTACACTCCCTTCATTATGTATTCAGT
+HWI-EAS443:3:70:1252:250#0/1
abbaabaaabbbbbabRb`a`aaaa^_a_aaa`aaa[`a`
@HWI-EAS443:3:70:127:1205#0/1
TCCGATTTGTTTTCAAAGAATTCCTTGGTCATGTCTTTCT
+HWI-EAS443:3:70:127:1205#0/1
^a`Z`Z[ab^¥^``_Z^a``ba`ba^^a_aba`¥_aa^aa
@HWI-EAS443:3:70:138:806#0/1
GGGGGCTATATGAAGCAATCGAGGAGTGCCTGATTAATGA
+HWI-EAS443:3:70:138:806#0/1
aaZ]WW`_`a__UUVR___^^[Z[DZFZYX[`[`^V¥^VV
@HWI-EAS443:3:70:1433:361#0/1
GTCAATTCACATGCCTTATTGAATTCATTTTGGACCCAGC
+HWI-EAS443:3:70:1433:361#0/1
^W^TX^SS_ab]RPN¥XO_Z_RRUYNQ¥`^aXURX^`Z]Q
@HWI-EAS443:3:70:1557:2024#0/1
CTTCAGTCTCTTCAGCAGATTGAGAGCATGATTGAGGCCG
+HWI-EAS443:3:70:1557:2024#0/1
`_^ZZ[F[ZW¥¥[^^¥W`^`_`_`¥TUX^]¥ZW`aV_VRZ
@HWI-EAS443:3:70:1603:843#0/1
AGGGGTTTTCGGCTGAATCGAGAAAATTGCTTCTCATTGT
+HWI-EAS443:3:70:1603:843#0/1
abbba]aaaaa`Zaabaaaa_b____a`__aa_`]_a`_¥
@HWI-EAS443:3:70:1637:1755#0/1
AGACAATTGAAGAAAAATTTGAGATTACAGGAACTATGCG
+HWI-EAS443:3:70:1637:1755#0/1
aa¥``abaW[aaaaaa`aaa_aaa_a`^`a]^a^_aaV__
@HWI-EAS443:3:70:1734:1001#0/1
GAACGCCAAAATTGAACCATTCTTGAGGACGACACCACGC
+HWI-EAS443:3:70:1734:1001#0/1
a]]U``W_a_`_`ZM`ZZ¥aaZa]YUY^[H]UY[Q¥aNVP
@HWI-EAS443:3:70:1789:163#0/1
GCCATGTTTCCGATTTGTTTTCAAAGAATTCCTTGGTCAT
+HWI-EAS443:3:70:1789:163#0/1
`a``bbaab_ab`aaaa^`a```^`a`^a`WW_```¥U¥_
@HWI-EAS443:3:70:265:1317#0/1
CTGGGTGACTCTCCGATTGGCCATGTTTCCGATTTGTTTT
+HWI-EAS443:3:70:265:1317#0/1
aab_aWaaa^baaa_]a`__abYYX[_a_aZQ[```X```
@HWI-EAS443:3:70:273:1377#0/1
GAAAGAGCCTAACATAGTCAAACCACATGAGAAAGGCATA

```
+HWI-EAS443:3:70:273:1377#0/1
aaX¥ababbabbababa_aa_baa_aaa_]a^aa`Zaaaa
@HWI-EAS443:3:70:327:1918#0/1
ATCAGTCAATTCACATGCCTTATTGAATTCATTTTGGACC
+HWI-EAS443:3:70:327:1918#0/1
aab`^]aaa__baa_S¥aaa_a^^U[T^_a_^XZSWS^_Z
@HWI-EAS443:3:70:407:1343#0/1
GTTGGAGACCTTAAACAGTATGACAGTGATGAGCCAGAGC
+HWI-EAS443:3:70:407:1343#0/1
aa`]_aWRXb[_`YWbVaP`VUaZUSWP^WZPFG[a^a__
@HWI-EAS443:3:70:436:994#0/1
GAGGGGTTTTCGGCTGAATCGAGAAAATTGCTTCTCATTG
+HWI-EAS443:3:70:436:994#0/1
aWXX¥_Y```_QQ^]`a`¥a[_T_W[[¥[WY[[YTNT[SY
@HWI-EAS443:3:70:446:277#0/1
CCGAACTTCTCCAGCCTTGAAAACTTTAGAGCCTATGTAG
+HWI-EAS443:3:70:446:277#0/1
`bbVbbbba]abbbababb_aaaaaa`aaab`aaaaaYbb
@HWI-EAS443:3:70:479:1605#0/1
GACCGAATCATGGCCTGGACAGTGGTGAACAGTATATGTA
+HWI-EAS443:3:70:479:1605#0/1
^abbba`bbbbbbbbbaaaab`[[bZabbaba_abaa[[a
@HWI-EAS443:3:70:548:54#0/1
GGGCATGGAAATGAGGCGCTGCCTTCTTCAGTCTCTTCAG
+HWI-EAS443:3:70:548:54#0/1
_W[YWSX`ZTTYXQ[_YYYV^SSUUYYYMXURVVNPQMRP
@HWI-EAS443:3:70:569:1284#0/1
GGGGTTTTCGGCTGAATCGAGAAAATTGCTTCTCATTGTT
+HWI-EAS443:3:70:569:1284#0/1
aa_`P``Ua_]`a`_Y]`[]Z_]`X^`_a``a`[¥_[^ZZ
@HWI-EAS443:3:70:56:515#0/1
GTCGTCCTCAAGAATGGTTCAATTTTGGCGTTCACTTCTT
+HWI-EAS443:3:70:56:515#0/1
`¥`b[aa_a^aa`Z_ba^`[`K[a_aQV¥`¥¥¥DT]Q[^a
@HWI-EAS443:3:70:598:732#0/1
TTTCAAAGAATTCCTTGGTCATGTCTTTCTCTTTGACAGA
+HWI-EAS443:3:70:598:732#0/1
aa^[a`R`_Ya]OY`_aVX_X_aZ___[_^^Z_a_]_^[^
@HWI-EAS443:3:70:603:770#0/1
GGGCACTCGGTGAAAATATGGCACCAGAAAAAGTAGACTT
+HWI-EAS443:3:70:603:770#0/1
Zaaaaa``¥a]a^U[a`[Y_^___`X¥X_`a`]M^¥ZYa`
@HWI-EAS443:3:70:604:1356#0/1
ATGAGAAGCAATTTTCTCGATTCAGCCGAAAACCCCTCAA
+HWI-EAS443:3:70:604:1356#0/1
a`aa`abaa^aaaaaaaaa`aaaa_a_``Z[aa_`a^`_[
@HWI-EAS443:3:70:668:1170#0/1
AAAGATGTTGGAGACCTTAAACAGTATGACAGTGATGAGC
+HWI-EAS443:3:70:668:1170#0/1
^ZW¥PMPL¥``S^_`a[[TQVZ]^X[UUU_`]QUQPYNQL
```

@HWI-EAS443:3:70:740:967#0/1
GGGGGCTATATGAAGCAATCGAGGAGTGCCTGATTAATGA
+HWI-EAS443:3:70:740:967#0/1
aba`ababaZ^aaa`aa_a_aaa`Z¥Q`]^`a__`^a___
@HWI-EAS443:3:70:742:33#0/1
GGCCATGTTTCCGATTTGTTTTCAAAGAATTCCTTGGTCA
+HWI-EAS443:3:70:742:33#0/1
abaaabb`^abab^abababaaaaaa`a_baa_aabb]aa
@HWI-EAS443:3:70:752:1224#0/1
ATTGCAGCAAACTTGTTAGTTTCGATTTTCGGATCTTCCC
+HWI-EAS443:3:70:752:1224#0/1
_abXab]`b`a]aaa`a]a]`a^`X^_]XVYWDMI¥`G^]
@HWI-EAS443:3:70:763:1244#0/1
GCTCAATGCATCCTGTGCAGCCATGGATGACTTTCAGCTG
+HWI-EAS443:3:70:763:1244#0/1
aba^UW_ZS¥R`b]RSaa`[`a]^_a_]a_^YS[T]ZUP¥
@HWI-EAS443:3:70:787:338#0/1
GTATGGAGTTCTCACTCACTGACCCGAGACTGGAGCCACA
+HWI-EAS443:3:70:787:338#0/1
aaaaaaaaaaaaaa`aaaaaaaaa_a`^_a``]^a]`^Y_
@HWI-EAS443:3:70:811:1285#0/1
ATAAATACGGCCTTGCTCAATGCATCCTGTGCAGCCATGG
+HWI-EAS443:3:70:811:1285#0/1
VbU^]_Y^][aa``_a]aaaa¥bZSZ_]¥N_``]¥¥`Z`T
@HWI-EAS443:3:70:829:25#0/1
GGGGCATGGAAATGAGGCGCTGCCTTCTTCAGTCTCTTCA
+HWI-EAS443:3:70:829:25#0/1
^a^a^a`bbZ]Z]XVUV_`_`baa`[[```Z`RV_¥[Y]W
@HWI-EAS443:3:70:857:1234#0/1
GCCCCATTTCATCTTGATCTTGGAGGTTCCATTGGTTCTC
+HWI-EAS443:3:70:857:1234#0/1
]abb`R^aabbb_`b^babbbbbbbMab_H_`V^S`R]a
@HWI-EAS443:3:70:888:220#0/1
CAGATCTCTAGCAAGCTGGGTCCAAAATGAATTCAATAAG
+HWI-EAS443:3:70:888:220#0/1
a_bYa^aaabaaa```aaaaX`^^_aa`a^[`a_[[^a^a
@HWI-EAS443:3:70:926:106#0/1
TATAGGAACATGGGCCTCGACACTTGGCCTATCGCAGTCC
+HWI-EAS443:3:70:926:106#0/1
aaa`W`__``]`¥FOaY_aV___^_`_YN___¥__Y¥WYV
@HWI-EAS443:3:70:93:2042#0/1
GGGGTTTTCGGCTGAATCGAGAAAATTGCTTCTCATTGTT
+HWI-EAS443:3:70:93:2042#0/1
Z__^H^aab]`ba]_VS_aa¥RWaa`aZ_^_a¥`[^^YLQ
@HWI-EAS443:3:70:98:1040#0/1
AGTAGGAGTCTATGGGATTCCTTTCGTCAGTCCGAAAGAG
+HWI-EAS443:3:70:98:1040#0/1
aa^^aaaa_baaa`¥a_a_aa_a`aa]`]_^a_`^[`__^
@HWI-EAS443:3:71:1021:308#0/1
AGAAAAAGTAGACTTTGATGACTGCAAAGATGTTGGAGAC

```
+HWI-EAS443:3:71:1021:308#0/1
_`]]___X]b``_a^^b¥ab`^a¥PQ[[S]aaY_]UW^XF
@HWI-EAS443:3:71:1070:380#0/1
ATCATTAATCAGGCACTCCTCGATTGCTTCATATAGCCCC
+HWI-EAS443:3:71:1070:380#0/1
abaaaa`aaaba`aaaa_`aabaaabaaa_aaaa``¥N_]
@HWI-EAS443:3:71:1104:358#0/1
TCCTCCTCATGCTTGCGATATGTTCAATCGGGGCAACATC
+HWI-EAS443:3:71:1104:358#0/1
``aaaaVY^[OZ^YbbbaWK^^P_Xa`Y[WXH``_aY^ZP
@HWI-EAS443:3:71:114:1928#0/1
ACCAGAAAAAGTAGACTTTGATGACTGCAAAGATGTTGGA
+HWI-EAS443:3:71:114:1928#0/1
¥`¥R_T¥XYTDW]QZU]YR¥`P_XYUJ[OGXVWTVTV_Za
@HWI-EAS443:3:71:1153:1559#0/1
GCCTAACATAGTCAAACCACATGAGAAAGGCATAAATCCC
+HWI-EAS443:3:71:1153:1559#0/1
aaaaaa`¥`[ZX_a`a^YY[SY[_^`__YRZ`__a__UOU
@HWI-EAS443:3:71:1162:1577#0/1
ATTGACTGATTCAAGCTGGATAGAACTTGATGAAATAGGA
+HWI-EAS443:3:71:1162:1577#0/1
``ZY^aaba¥S¥__[_a`b`Y_]`a^a^`]a_^`_`]_]W
@HWI-EAS443:3:71:117:861#0/1
GGGAGACTTTGGTCGGCAAGCTTGCGCATAGTTCCTGTAA
+HWI-EAS443:3:71:117:861#0/1
__ZZ]M^Z]]`aW^aa^aZ_a_]a_aaWZU^QX^^]^OSV
@HWI-EAS443:3:71:1205:1373#0/1
ATGAAATGGAAATCCGAATACATGAAACAAACTTCCAAAT
+HWI-EAS443:3:71:1205:1373#0/1
aba`_X`_OS[Oaaaaa]Z[_a]__aY``a_[^^_]___^
@HWI-EAS443:3:71:1246:1314#0/1
GCCTGAACAATGAGAAGCAATTTTCTCGATTCAGCCGAAA
+HWI-EAS443:3:71:1246:1314#0/1
_W¥^]aaaa¥]_[`_aaaaYaa_`a_a```W`aa[[_UQW
@HWI-EAS443:3:71:1247:1281#0/1
AGAAATTTAGGCTTCTCTACCCCTGTTGTGTTACATATAC
+HWI-EAS443:3:71:1247:1281#0/1
`bababbabba`aa`aaaa_aa_`a^aa___aa]_aa_aX
@HWI-EAS443:3:71:1247:1790#0/1
GATTGGCCATGTTTCCGATTTGTTTTCAAAGAATTCCTTG
+HWI-EAS443:3:71:1247:1790#0/1
Zabab`bb_``abbab`X`bbbabbbabbb_bbbabbaaa
@HWI-EAS443:3:71:1266:333#0/1
CATGGCCAATCGGAGAGTCACCCAGGGGAGTGGAGGAAGG
+HWI-EAS443:3:71:1266:333#0/1
aabbb[Q`]a`a^DQ_`¥aU__`¥`_X]E]Vaa_a^SPIV
@HWI-EAS443:3:71:1275:633#0/1
GGGCGTGGTGTCGTCCTCAAGAATGGTTCAATTTTGGCGT
+HWI-EAS443:3:71:1275:633#0/1
abaab`bb_a__a__`a`a`¥aaa_a_``__aaaa_`Ya^
```

```
@HWI-EAS443:3:71:1306:276#0/1
ATCGACGAACGGGGTGAATCAATAATTGTAGAATCTGGTG
+HWI-EAS443:3:71:1306:276#0/1
ababa`ba`X``a`Ma]]`_`¥`W^a]SX__¥]VR^_]Q¥
@HWI-EAS443:3:71:1307:190#0/1
AAGAATTCCTTGGTCATGTCTTTCTCTTTGACAGAAGACT
+HWI-EAS443:3:71:1307:190#0/1
``ba_aaa`aa]aTa`ab^aaaaYaabaa`Z^`a`_a^^^
@HWI-EAS443:3:71:1310:253#0/1
CTGGGTCCAAAATGAATTCAATAAGGCATGTGAATTGACT
+HWI-EAS443:3:71:1310:253#0/1
`bbbb_ababbbabbbba`bbbbbbbabbbbbbbbabbab
@HWI-EAS443:3:71:1353:1994#0/1
GGGGGCTATATGAAGCAATCGAGGAGTGCCTGATTAATGA
+HWI-EAS443:3:71:1353:1994#0/1
]FUX^TQ_T`RS^[^aa`]a^Pa`]^TU]]ZYZ]RG^G]N
@HWI-EAS443:3:71:1375:610#0/1
GAAATGGAAATCCGAATACATGAAACAAACTTCCAAATGT
+HWI-EAS443:3:71:1375:610#0/1
aba_Z``LYa``[a`a_a^a_`aa^`a``_``___^_`[Y
@HWI-EAS443:3:71:1376:840#0/1
GGCCTTGCTCAATGCATCCTGTGCAGCCATGGATGACTTT
+HWI-EAS443:3:71:1376:840#0/1
ababab`abbbbabaabaaab_a_`bbabaaaaaa_aaa
@HWI-EAS443:3:71:146:1828#0/1
GATCCCAAGGACAAAGAACATGAAGAGAACAAGCCAATTG
+HWI-EAS443:3:71:146:1828#0/1
U^V[]_XP_Yb]^X_[`_bb]``bbb¥X_b```¥`abaW]
@HWI-EAS443:3:71:14:783#0/1
CAAGAAATGGCCAGTAGGAGTCTATGGGATTCCTTTCGTC
+HWI-EAS443:3:71:14:783#0/1
a`aaaZQ^]_b^`[Y`a_X`_aa^`^`^T[^^a__`YSSN
@HWI-EAS443:3:71:1513:1369#0/1
TTTCATTCACTGGAGAGGAGATGGCCACCAAAGCGGACTA
+HWI-EAS443:3:71:1513:1369#0/1
aaa_ababa^`ZV`a`aaX[__^_Y_YV__¥_¥Y_¥¥GZZ
@HWI-EAS443:3:71:153:1672#0/1
GGGGGCTATATGAAGCAATCGAGGAGTGCCTGATTAATGA
+HWI-EAS443:3:71:153:1672#0/1
aaaaaba¥[L[_Wa]aa_``¥N¥^X^Na^¥^¥NUR[TW^[
@HWI-EAS443:3:71:1560:1721#0/1
GGCCAATCGGAGAGTCACCCAGGGGAGTGGAGGAAGGCTC
+HWI-EAS443:3:71:1560:1721#0/1
aaSSa```X`_OR]Ya]]VGP]Y]`XaUZRGUU^SW]SBB
@HWI-EAS443:3:71:1584:1628#0/1
GGTGAACTTTGTAAGTATGGAGTTCTCACTCACTGACCCG
+HWI-EAS443:3:71:1584:1628#0/1
]YU_¥S_a^^_`R`^R```[aa_a`a`¥aaaaaa^aa_Ya
@HWI-EAS443:3:71:1618:1489#0/1
ACTCCCCTGGGTGACTCTCCGATTGGCCATGTTTCCGATT
```

```
+HWI-EAS443:3:71:1618:1489#0/1
URba`^b]Q^ZIb``baaUJR`a``YIN_JDPbaHG]GWa
@HWI-EAS443:3:71:162:1486#0/1
CATTAATCAGGCACTCCTCGATTGCTTCATATAGCCCCCC
+HWI-EAS443:3:71:162:1486#0/1
_a^``]¥aa`Z``aaaa__aa¥^_`[VWa^]V`]_V[^__
@HWI-EAS443:3:71:1666:68#0/1
GCTCATCACTGTCATACTGTTTAAGGTCTCCAACATCTTT
+HWI-EAS443:3:71:1666:68#0/1
abb^VR]a_X]TV_a_[^``a[_]`aR^`]X]_SX_N]^S
@HWI-EAS443:3:71:1691:951#0/1
GGAGTGTACATAAATACGGCCTTGCTCAATGCATCCTGTG
+HWI-EAS443:3:71:1691:951#0/1
_`ab^b_`Zaaaa`aaaa`_aa`^U^_aa`^^aaZ[a_[_
@HWI-EAS443:3:71:1714:1886#0/1
GTCTCCAACATCTTTGCAGTCATCAAAGTCTACTTTTTCT
+HWI-EAS443:3:71:1714:1886#0/1
aPZ`Y`^MTYWYWQ___][N[V`^^[U_Z^YOP¥^`]UUX
@HWI-EAS443:3:71:1757:978#0/1
CTGAAAGTCATCCATGGCTGCACAGGATGCATTGAGCAAG
+HWI-EAS443:3:71:1757:978#0/1
aab¥^``T_aa`aa___``]^`¥[Q[[]_W]_a_UZPT]Z
@HWI-EAS443:3:71:197:376#0/1
CATTGGTTCTCACATATAGGAACATGGGCCTCGACACTTG
+HWI-EAS443:3:71:197:376#0/1
ab`ab^S_aa`X`aa]a¥Z¥O^_`[`_[^_¥`XV^aZ^__
@HWI-EAS443:3:71:206:288#0/1
GGGTTTTCGGCTGAATCGAGAAAATTGCTTCTCATTGTTC
+HWI-EAS443:3:71:206:288#0/1
aaaX_aaaa^aa]^^_a`]a^`]]`__^^^_]]]]^]Y^]
@HWI-EAS443:3:71:251:996#0/1
GACGAAAGGAATCCCATAGACTCCTACTGGCCATTTCTTG
+HWI-EAS443:3:71:251:996#0/1
aabbbb_`abbabba]`^aabaaaaaaa¥H¥aZV^`]`]Y
@HWI-EAS443:3:71:290:982#0/1
CGATTGGCCATGTTTCCGATTTGTTTTCAAAGAATTCCTT
+HWI-EAS443:3:71:290:982#0/1
ab¥bbbbba_aaSabab_]Zbb¥aabb`^ba¥`a_a]T`a
@HWI-EAS443:3:71:297:173#0/1
AAAGGAATCCCATAGACTCCTACTGGCCATTTCTTGTCTT
+HWI-EAS443:3:71:297:173#0/1
a^Z`baa``aaaa`aaba__`_X`^[[_[PY¥UZ¥XTQT^
@HWI-EAS443:3:71:326:1806#0/1
CGATTGGCCATGTTTCCGATTTGTTTTCAAAGAATTCCTT
+HWI-EAS443:3:71:326:1806#0/1
Z_bba__`a¥[[WY_aa]`Q_XZXa_]`_^a``YX`a`^a
@HWI-EAS443:3:71:340:269#0/1
GGGGTTTTCGGCTGAATCGAGAAAATTGCTTCTCATTGTT
+HWI-EAS443:3:71:340:269#0/1
aba¥T]ab_aa`b_`][ab`aZZ^`a^Z^__`aaaa`^¥_
```

```
@HWI-EAS443:3:71:362:1684#0/1
TGGCCACCAAAGCGGACTACACCCTTGACGAAGAGAGCAG
+HWI-EAS443:3:71:362:1684#0/1
`bbbbaabbaabba`_a`baaaabaa^aa___aa___]`_
@HWI-EAS443:3:71:390:292#0/1
GATTTGTTTTCAAAGAATTCCTTGGTCATGTCTTTCTCTT
+HWI-EAS443:3:71:390:292#0/1
a_aaaa``aaaa_aa`[]`__a```Za``a^__]___^_`
@HWI-EAS443:3:71:427:648#0/1
TGTAGCTCTGCTAGCACCTGCTTCCAAGCCATGAGGTAAT
+HWI-EAS443:3:71:427:648#0/1
abT_¥`aa`W¥GS__`b_TZYZZa_R¥^`S[¥ZU[[OOU`
@HWI-EAS443:3:71:433:1608#0/1
ATGCACTATTGAAGCACCGATTTGAGATAATTGAAGGAAG
+HWI-EAS443:3:71:433:1608#0/1
aVVaa`WZaSS[¥T^_a___``_WZa_^`_`^_VVWX^[_
@HWI-EAS443:3:71:440:1381#0/1
CGATTGGCCATGTTTCCGATTTGTTTTCAAAGAATTCCTT
+HWI-EAS443:3:71:440:1381#0/1
aaaa``_baa`Y^``aaa__``^^^_^____^_Z^_^`^_
@HWI-EAS443:3:71:488:1093#0/1
GAGACCGAATCATGGCCTGGACAGTGGTGAACAGTATATG
+HWI-EAS443:3:71:488:1093#0/1
a¥YZba^¥Ya`aa`_aa_^`]]a]N^_N_aa]V`W_`_ZS
@HWI-EAS443:3:71:512:911#0/1
GAGAAAGGCATAAATCCCAATTACCTCATGGCTTGGAAGC
+HWI-EAS443:3:71:512:911#0/1
ab`^`]^abbbba]aa`aaba`baa¥aa`a_aa`T¥X[S_
@HWI-EAS443:3:71:52:1093#0/1
ACGGGGTGAATCAATAATTGTAGAATCTGGTGACCCGAAT
+HWI-EAS443:3:71:52:1093#0/1
aba`¥a^abaa`aaaaaaa_a`_`]`___X_aa``Ua_`
@HWI-EAS443:3:71:531:1258#0/1
GTTCGGTGGGAGACTTTGGTCGGCAAGCTTGCGCATAGTT
+HWI-EAS443:3:71:531:1258#0/1
_Zbb^^^][NQXZ`aaaRY^aX¥aa¥aa_^Va`_]`a^U_
@HWI-EAS443:3:71:566:1180#0/1
GAGAGGAGATGGCCACCAAAGCGGACTACACCCTTGACGA
+HWI-EAS443:3:71:566:1180#0/1
`ZZTZ^L^^``[`aaaa^T`a``]```aab]R_`^¥`¥[Y
@HWI-EAS443:3:71:56:1196#0/1
GACATTCTTTGGCTGGAAAGAGCCTAACATAGTCAAACCA
+HWI-EAS443:3:71:56:1196#0/1
`baa`bbaaabbaab^T]ba```aaabaaaba_aX^ba^a
@HWI-EAS443:3:71:585:682#0/1
GGTGAACTTTGTAAGTATGGAGTTCTCACTCACTGACCCG
+HWI-EAS443:3:71:585:682#0/1
`bU]X[¥`_U_Z_S[ab[[_P¥T¥aS`^`aab`X¥[¥¥a[
@HWI-EAS443:3:71:59:801#0/1
GGGGCTATATGAAGCAATCGAGGAGTGCCTGATTAATGAT
```

```
+HWI-EAS443:3:71:59:801#0/1
aa[`aaZ¥a_``^¥`_U_``V^^]_U[_`^VUW]]R[V^]
@HWI-EAS443:3:71:641:268#0/1
GCCCTGCTCTCTTCGTCAAGGGTGTAGTCCGCTTTGGTGG
+HWI-EAS443:3:71:641:268#0/1
a`aW`aa^_]¥]a]``a`U_aZJZ[a_Y_¥]^_]¥PUBBB
@HWI-EAS443:3:71:655:1351#0/1
GGCATGGAAATGAGGCGCTGCCTTCTTCAGTCTCTTCAGC
+HWI-EAS443:3:71:655:1351#0/1
UQaWX`_]¥abbaaab_baaaba[`a_^V__LUb``Za`]
@HWI-EAS443:3:71:670:481#0/1
CATCTTTGCAGTCATCAAAGTCTACTTTTTCTGGTGCCAT
+HWI-EAS443:3:71:670:481#0/1
aabbabbbabbZbbabbabb`bbaabbbbaaba]_ba`bb
@HWI-EAS443:3:71:714:1679#0/1
AGGAATTCTTTGAAAACAAATCGGAAACATGGCCAATCGG
+HWI-EAS443:3:71:714:1679#0/1
a^W__`Yba^`^¥`¥a`a__^^[W^_]Y_X^¥QX^_YVQY
@HWI-EAS443:3:71:847:841#0/1
CACTGCAGGGCTACTGAATACATAATGAAGGGAGTGTACA
+HWI-EAS443:3:71:847:841#0/1
a¥__a^¥Z`bbaZX]aS_ZUYa_]¥`b¥O^a__^_a^``^
@HWI-EAS443:3:71:849:1011#0/1
TTGAGATTACAGGAACTATGCGCAAGCTTGCCGACCAAAG
+HWI-EAS443:3:71:849:1011#0/1
aa]ZX][ab__b_NU]b^Z^aa_aZP^SX`[]a__^`_`^
@HWI-EAS443:3:71:878:88#0/1
GGGGGCTATATGAAGCAATCGAGGAGTGCCTGATTAATGA
+HWI-EAS443:3:71:878:88#0/1
abbaa`^`b_aa_L]S`ab]a^b]]aXa_]`a_a_`_`_X
@HWI-EAS443:3:71:883:1540#0/1
CATCAAAGTCTACTTTTTCTGGTGCCATATTTTCACCGAG
+HWI-EAS443:3:71:883:1540#0/1
_^W`^`S^^b^Z_a]^aaa¥MNZS¥aa¥a¥`^`````^[a
@HWI-EAS443:3:71:949:168#0/1
TGTGAATTGACTGATTCAAGCTGGATAGAACTTGATGAAA
+HWI-EAS443:3:71:949:168#0/1
aa^a¥`aba_``a_a``a^__aa`[_``^aY``a__`W_^
@HWI-EAS443:3:71:96:53#0/1
AGGCTTTTCACTATAAGACAAGAAATGGCCAGTAGGAGTC
+HWI-EAS443:3:71:96:53#0/1
aaYaa`a¥a_a_^__S_^X^___Y`ZU^_^]ZXVSVWWXW
@HWI-EAS443:3:71:977:1882#0/1
GGGGGCTATATGAAGCAATCGAGGAGTGCCTGATTAATGA
+HWI-EAS443:3:71:977:1882#0/1
_Z]ZMV_baaaa``aa`[U]W]a^Z[Z_a]VMJQY¥_^Z[
@HWI-EAS443:3:71:981:549#0/1
GGCACTCCTCGATTGCTTCATATAGCCCCCCAAGATCGAA
+HWI-EAS443:3:71:981:549#0/1
abbbbb_ab_b_aa__aa`a]``_¥`a^``_TWa[aVaV[
```

```
@HWI-EAS443:3:72:1048:481#0/1
CGTCTCCACAACTTGAGGGGTTTTCGGCTGAATCGAGAAA
+HWI-EAS443:3:72:1048:481#0/1
a`[T]`_P]¥_ZV¥`X^¥_YU_aW_Q¥aNT_]VJ¥Ra_¥_
@HWI-EAS443:3:72:1067:687#0/1
CATAAATACGGCCTTGCTCAATGCATCCTGTGCAGCCATG
+HWI-EAS443:3:72:1067:687#0/1
a`baa_aaaba`aab[`a_``_a```a`_a_aa`a`¥a`a
@HWI-EAS443:3:72:1111:1156#0/1
GTCTCTTCCTTCAATTATCTCAAATCGGTGCTTCAATAGT
+HWI-EAS443:3:72:1111:1156#0/1
a_ababbbaaaaaaabaa`aaaaa``aaZaaaa]a_`[]^
@HWI-EAS443:3:72:1118:1178#0/1
TCAAAGTCTACTTTTTCTGGTGCCATATTTTCACCGAGTG
+HWI-EAS443:3:72:1118:1178#0/1
abab]__a`b`aaaaaaaa__`aaaa`aaaa`a_aa`__a
@HWI-EAS443:3:72:1133:1052#0/1
GCGCATAGTTCCTGTAATCTCAAATTTTTCTTCAATTGTC
+HWI-EAS443:3:72:1133:1052#0/1
abaaba`¥[aaaa`X]ab_a`_^Waabaaaaa^`aaaa__
@HWI-EAS443:3:72:1152:1602#0/1
ACTGTTTAAGGTCTCCAACATCTTTGCAGTCATCAAAGTC
+HWI-EAS443:3:72:1152:1602#0/1
aaa__a`aaa_Ya``_aa]a_^`^___a_]_a_^_^`_X_
@HWI-EAS443:3:72:1171:1230#0/1
GAAAGGCATAAATCCCAATTACCTCATGGCTTGGAAGCAG
+HWI-EAS443:3:72:1171:1230#0/1
a_b^ababbbbaaaa`abbaa_aaa``a`aa^N[Y^^PZZ
@HWI-EAS443:3:72:1240:1251#0/1
AGACTTTGGTCGGCAAGCTTGCGCATAGTTCCTGTAATCT
+HWI-EAS443:3:72:1240:1251#0/1
aaa`_[`__Wa_a]a`_aX`_____`_¥^__]_¥TWZ_]^
@HWI-EAS443:3:72:1249:259#0/1
GAGAAAGGCATAAATCCCAATTACCTCATGGCTTGGAAGC
+HWI-EAS443:3:72:1249:259#0/1
a[baaaa__aa_`aaQ_^_^aa`^_`_`]^_^_^ZVU]ZM
@HWI-EAS443:3:72:1259:1025#0/1
TTCGGCTGAATCGAGAAAATTGCTTCTCATTGTTCAGGCA
+HWI-EAS443:3:72:1259:1025#0/1
`a`bbX`_PV``bYSSa^U]WDMYa__`[I_aZ¥_M^^Y]
@HWI-EAS443:3:72:1302:467#0/1
ATAAATACGGCCTTGCTCAATGCATCCTGTGCAGCCATGG
+HWI-EAS443:3:72:1302:467#0/1
`aYaa¥]H[bWW__XZ]aba`Z__aaaa^G^[Zaa[`^``
@HWI-EAS443:3:72:1422:1245#0/1
CTGATTCAAGCTGGATAGAACTTGATGAAATAGGAGAAGA
+HWI-EAS443:3:72:1422:1245#0/1
a`bbababababaabb¥`b`¥b`aba¥a```aaab]aaaaaa
@HWI-EAS443:3:72:1450:334#0/1
CAATGAGAAGCAATTTTCTCGATTCAGCCGAAAACCCCTC
```

```
+HWI-EAS443:3:72:1450:334#0/1
``bba`ba_babbbbbb`bababb_aa^_bbbb`_Y_Ya_
@HWI-EAS443:3:72:1454:793#0/1
GGCATGGAAATGAGGCGCTGCCTTCTTCAGTCTCTTCAGC
+HWI-EAS443:3:72:1454:793#0/1
abaabbbabbb^`aa`baaba`a`Va`Z^b_Va^aaU^_S
@HWI-EAS443:3:72:1524:781#0/1
GGCGAAGAGACAATTGAAGAAAAATTTGAGATTACAGGAA
+HWI-EAS443:3:72:1524:781#0/1
aa¥aZ¥_QX`]]aY¥]Q``¥_TNUSa[`]`S]WVRK¥RPY
@HWI-EAS443:3:72:1568:1291#0/1
GTTTCCGATTTGTTTTCAAAGAATTCCTTGGTCATGTCTT
+HWI-EAS443:3:72:1568:1291#0/1
a__a_`aa`a`aX`a`aa`_`_^`_][__a`Y`^``]^__
@HWI-EAS443:3:72:1585:69#0/1
GGGGGCTATATGAAGCAATCGAGGAGTGCCTGATTAATGA
+HWI-EAS443:3:72:1585:69#0/1
aaa`_Ya`_X`_]^_]aa]Y[N¥`P^KTQS`aNT^WW__R
@HWI-EAS443:3:72:1589:250#0/1
GGCCGAGTCTTCTGTCAAAGAGAAAGACATGACCAAGGAA
+HWI-EAS443:3:72:1589:250#0/1
abSYY[YTNaUFS`^Y[_[aXZU][_[_Y_¥UVV^WS]WU
@HWI-EAS443:3:72:160:840#0/1
CACTTGGCCTATCGCAGTCCTCAAGAGCATGTCTCCTATT
+HWI-EAS443:3:72:160:840#0/1
a`ZPGZX]Z]`V]^XY_U][Z]]```a`_Wa^]¥X[^TX[
@HWI-EAS443:3:72:161:66#0/1
TTGCGATATGTTCAATCGGGGCAACATCTTCTCCTATTTC
+HWI-EAS443:3:72:161:66#0/1
¥aaabaaabb_Yaaa_aa__a]a`^^aOa_¥a_]`^a[`]
@HWI-EAS443:3:72:163:577#0/1
GTTTCCGATTTGTTTTCAAAGAATTCCTTGGTCATGTCTT
+HWI-EAS443:3:72:163:577#0/1
a`aaaaa[`a`_[`a__a_a_]X^a__`__^]^_^`]_^]
@HWI-EAS443:3:72:1678:1801#0/1
CATTCTTTGGCTGGAAAGAGCCTAACATAGTCAAACCACA
+HWI-EAS443:3:72:1678:1801#0/1
ababbbbb`bab`__bbbbbX[aaabbaa`^Y_bbaaa_a
@HWI-EAS443:3:72:1722:194#0/1
GGGGGCTATATGAAGCAATCGAGGAGTGCCTGATTAATGA
+HWI-EAS443:3:72:1722:194#0/1
]bab]`bbb_bb`^`abba]b]a`Ra^aX`aa`__`a_aa
@HWI-EAS443:3:72:1730:834#0/1
CGATTGGCCATGTTTCCGATTTGTTTTCAAAGAATTCCTT
+HWI-EAS443:3:72:1730:834#0/1
ababba``^a`ZUbb_b__`aab`bbb`ab^^^`aba_aa
@HWI-EAS443:3:72:174:270#0/1
CAAAGAGAACCGGTTCATTGAAATTGGAGTAACACGGAGG
+HWI-EAS443:3:72:174:270#0/1
a]^``[`_]a`aa]^Z[```[___Z]Z^_W___^]]^Z_]
```

@HWI-EAS443:3:72:1773:1501#0/1
GGGGGCTATATGAAGCAATCGAGGAGTGCCTGATTAATGA
+HWI-EAS443:3:72:1773:1501#0/1
a___babbaaa^_aaaaa`[a`aa^a_`__aa_`a__`]a
@HWI-EAS443:3:72:1780:1375#0/1
ACCGAACTTCTCCAGCCTTGAAAACTTTAGAGCCTATGTA
+HWI-EAS443:3:72:1780:1375#0/1
aaaaaaaaaaaa_a``aa`a```_^a_`_a`a__a_a`V]
@HWI-EAS443:3:72:205:1801#0/1
TCTCATTTGAGAAATGATACTGATGTGGTGAACTTTGTAA
+HWI-EAS443:3:72:205:1801#0/1
ababb`baaaab`aaababbaaa`a¥____aa`a_a_^aY
@HWI-EAS443:3:72:216:1962#0/1
CACTTTCCCAATAGAGCCTTCCTCCACTCCCCTGGGTGAC
+HWI-EAS443:3:72:216:1962#0/1
abaa`[baaba^aVaWaa]`a`^]`a`¥]`^]S¥UWW^¥W
@HWI-EAS443:3:72:234:1059#0/1
GCCCTGCTCTCTTCGTCAAGGGTGTAGTCCGCTTTGGTGG
+HWI-EAS443:3:72:234:1059#0/1
abbbaaaabababaa¥`]_aaaSa^^`^aaaa_a_a]W`^
@HWI-EAS443:3:72:237:1135#0/1
GAATTCAATAAGGCATGTGAATTGACTGATTCAAGCTGGA
+HWI-EAS443:3:72:237:1135#0/1
^a`aa_HY^aa^R^XaaTa_aa_aa_a``_¥¥]Y^Z[__^
@HWI-EAS443:3:72:266:616#0/1
ATTGAATTCATTTTGGACCCAGCTTGCTAGAGATCTGGGC
+HWI-EAS443:3:72:266:616#0/1
abbaaabaaabaaaa_aa`a```_`a`_`Y_`_^_^^]V_
@HWI-EAS443:3:72:338:1118#0/1
ACTCCCTTCATTATGTATTCAGTAGCCCTGCAGTGGGACA
+HWI-EAS443:3:72:338:1118#0/1
abR`aa`_`a¥a`a`V^]W^^¥^`_]a_^WZW¥W^]Y]]_
@HWI-EAS443:3:72:364:289#0/1
CATCGACGAACGGGGTGAATCAATAATTGTAGAATCTGGT
+HWI-EAS443:3:72:364:289#0/1
ababbaaa`bX^aaaGb]U[aaaaa__aa_b`a`Z__a_V
@HWI-EAS443:3:72:37:89#0/1
ATGAAACAAACTTCCAAATGTGTGCATATTGCAGCAAACT
+HWI-EAS443:3:72:37:89#0/1
^¥bbYIZ¥YFORVYV^W_aa[^YZYaXaa`aY[¥JOTSFR
@HWI-EAS443:3:72:389:1090#0/1
GGGGGCTATATGAAGCAATCGAGGAGTGCCTGATTAATGA
+HWI-EAS443:3:72:389:1090#0/1
aaababbabab_a]`bbba```a_aa_aaaaaaaa_aaa
@HWI-EAS443:3:72:390:1810#0/1
ATGAAATGGAAATCCGAATACATGAAACAAACTTCCAAAT
+HWI-EAS443:3:72:390:1810#0/1
_^a_`a[_]``]_aa^_a]`^a]]^_a_a__]_[`[^U]^
@HWI-EAS443:3:72:452:197#0/1
CATGGAAATGAGGCGCTGCCTTCTTCAGTCTCTTCAGCAG

```
+HWI-EAS443:3:72:452:197#0/1
a]a]aX_aaaa[Z^aaaaVXUa`_`^_¥Q____YRRL_¥¥
@HWI-EAS443:3:72:472:387#0/1
GGGGCATGGAAATGAGGCGCTGCCTTCTTCAGTCTCTTCA
+HWI-EAS443:3:72:472:387#0/1
aaa`aaba`_aaa``__a_``a¥`_`_aa_`_^[a__^V^
@HWI-EAS443:3:72:485:189#0/1
GGCATGGAAATGAGGCGCTGCCTTCTTCAGTCTCTTCAGC
+HWI-EAS443:3:72:485:189#0/1
aaaa`bba`¥^a^`a`[]Z`__Y_T_aZU]W[UY[[TR[¥
@HWI-EAS443:3:72:494:1351#0/1
GGCTCCAGTCTCGGGTCAGTGAGTGAGAACTCCATACTTA
+HWI-EAS443:3:72:494:1351#0/1
`ab`b_Waabaa]_a_`WWX_a_T[`^`aaaaaaaa`aaa
@HWI-EAS443:3:72:538:237#0/1
GGGGGCTATATGAAGCAATCGAGGAGTGCCTGATTAATGA
+HWI-EAS443:3:72:538:237#0/1
a_Ra]^a_]aabaaaaaaa_a[][X`¥^^^_a]¥^`^ZV¥
@HWI-EAS443:3:72:562:593#0/1
TCACTTCTTTTGACATTTGGGAAAGCTTGCCCTCAATGCA
+HWI-EAS443:3:72:562:593#0/1
]bbba`_`]_Y``aab`aa]baabaaa_aaa`a¥aa```a
@HWI-EAS443:3:72:571:311#0/1
CAGCTTGCTAGAGATCTGGGCTCTGGCTCATCACTGTCAT
+HWI-EAS443:3:72:571:311#0/1
abaXWROX_Ta_SXHPQQZNP]WS[`^[X_SRX[ZYPU[V
@HWI-EAS443:3:72:571:326#0/1
GACCCGAGTCACGAGGGGGAGGGAATACCACTATATGATG
+HWI-EAS443:3:72:571:326#0/1
`bbbababababbababab``abba_aabaababbabaaaaa
@HWI-EAS443:3:72:581:567#0/1
CCTCCTCATGCTTGCGATATGTTCAATCGGGGCAACATCT
+HWI-EAS443:3:72:581:567#0/1
`a^R_^a``a^aaW^``aa`^aaXaaaa]X``_`]a^Z]]
@HWI-EAS443:3:72:609:1985#0/1
GTGAACTTTGTAAGTATGGAGTTCTCACTCACTGACCCGA
+HWI-EAS443:3:72:609:1985#0/1
^`]bU`ba^`X`ba[¥a`X¥aV_a_`abaaaa`U`__aa^
@HWI-EAS443:3:72:644:592#0/1
TATAGCCCCCCAAGATCGAAGGTTCCAGGTTCCAGGTTGT
+HWI-EAS443:3:72:644:592#0/1
ababaaaaaaaa]`_``aa_`_Q_Z^[^_W^[^¥`_W^S[
@HWI-EAS443:3:72:674:942#0/1
GGTGAACTTTGTAAGTATGGAGTTCTCACTCACTGACCCG
+HWI-EAS443:3:72:674:942#0/1
a`a`Ybabbbb_bba_baa`aaaabaabaaaaaaaa`aaa
@HWI-EAS443:3:72:676:330#0/1
GGCACTCCTCGATTGCTTCATATAGCCCCCCAAGATCGAA
+HWI-EAS443:3:72:676:330#0/1
]^^Z`Ka`^[XZMXa]QT`PYWZbR¥`aaaaaPZMPWXYP
```

```
@HWI-EAS443:3:72:697:924#0/1
GGTGAACTTTGTAAGTATGGAGTTCTCACTCACTGACCCG
+HWI-EAS443:3:72:697:924#0/1
a^[aaa_aaab]a_a¥`aa``a]aaa`____`a`aaaZ^`
@HWI-EAS443:3:72:75:120#0/1
TTTGGACCCAGCTTGCTAGAGATCTGGGCTCTGGCTCATC
+HWI-EAS443:3:72:75:120#0/1
aabbaaaaaaaaa]^a`a``a``_^`__¥X^^^^_WYS[¥
@HWI-EAS443:3:72:777:470#0/1
CAGCTTGCTAGAGATCTGGGCTCTGGCTCATCACTGTCAT
+HWI-EAS443:3:72:777:470#0/1
aabaababbbaaaa_aaa^aaaaaaaa``aaaa¥aa__a`
@HWI-EAS443:3:72:799:408#0/1
AGGAACTATGCGCAAGCTTGCCGACCAAAGTCTCCCACCG
+HWI-EAS443:3:72:799:408#0/1
a``^b_aaba]^`aaaaaaa`^a`a¥`^`aZ^a¥Z__^¥_
@HWI-EAS443:3:72:819:273#0/1
ATTTCATCGACGAACGGGGTGAATCAATAATTGTAGAATC
+HWI-EAS443:3:72:819:273#0/1
V_ababa_¥ababb`aa]bU^YYb__aaaabab_`_`aa_
@HWI-EAS443:3:72:832:1649#0/1
AGCCTATGTAGATGGATTCGAGCCGAACGGCTGCATTGAG
+HWI-EAS443:3:72:832:1649#0/1
^`bbaba``[[a`]Z^]`aa[]aa`aaaaZa`^LW[QJVO
@HWI-EAS443:3:72:846:516#0/1
GCCAGTAGGAGTCTATGGGATTCCTTTCGTCAGTCCGAAA
+HWI-EAS443:3:72:846:516#0/1
aabaa``aa`b¥aa]aaa]`^___aaa]aV`a_¥`_____
@HWI-EAS443:3:72:91:1900#0/1
GGGGTTTTCGGCTGAATCGAGAAAATTGCTTCTCATTGTT
+HWI-EAS443:3:72:91:1900#0/1
[b[`^aba_Z]`U`a_Ta`aVU¥L`_`Va`]R[]`[[^ZZ
@HWI-EAS443:3:72:954:993#0/1
GGGGCGTGGTGTCGTCCTCAAGAATGGTTCAATTTTGGCG
+HWI-EAS443:3:72:954:993#0/1
abab]bY[aZ][abZaaaa¥¥^aaaba]``a_a_ab[`_^
@HWI-EAS443:3:72:967:482#0/1
GATTGGCCATGTTTCCGATTTGTTTTCAAAGAATTCCTTG
+HWI-EAS443:3:72:967:482#0/1
aZ¥__a[[]]XJY`a`¥SR]_`U^¥[^_^^^U¥_¥Y[¥[]
@HWI-EAS443:3:72:977:233#0/1
GGGTTTTCGGCTGAATCGAGAAAATTGCTTCTCATTGTTC
+HWI-EAS443:3:72:977:233#0/1
a`b]_aaaaa__aaa___]a``]^_`^^`_¥__^a]_Y^X
@HWI-EAS443:3:72:991:64#0/1
GGGGCATGGAAATGAGGCGCTGCCTTCTTCAGTCTCTTCA
+HWI-EAS443:3:72:991:64#0/1
aa_aXa_ba__^aa¥`__`S_`¥U__S¥_VZ`WN¥V^YFQ
@HWI-EAS443:3:73:1061:587#0/1
GACATGCTCTTGAGGACTGCGATAGGCCAAGTGTCGAGGC
```

```
+HWI-EAS443:3:73:1061:587#0/1
¥^aYbb`bbbbb````abba`aab_aaYaVaXb_````XU
@HWI-EAS443:3:73:1072:203#0/1
ATTGCAGCAAACTTGTTAGTTTCGATTTTCGGATCTTCCC
+HWI-EAS443:3:73:1072:203#0/1
a`ba¥aba`ba__O_aaaa``bab_aaaaV[¥VYU^]ONS
@HWI-EAS443:3:73:1167:1934#0/1
GGCCAATCGGAGAGTCACCCAGGGGAGTGGAGGAAGGCTC
+HWI-EAS443:3:73:1167:1934#0/1
abbbbaabab`aaaZaaaaZ``_¥___W]^U_Y¥Z_¥¥¥^
@HWI-EAS443:3:73:1167:556#0/1
ATGAAATGGGGCATGGAAATGAGGCGCTGCCTTCTTCAGT
+HWI-EAS443:3:73:1167:556#0/1
``_YR^^_aa]O_X`^`ULYba¥TZ^RR_XZ^]]``Q``]
@HWI-EAS443:3:73:1188:1269#0/1
ATACATGAAACAAACTTCCAAATGTGTGCATATTGCAGCA
+HWI-EAS443:3:73:1188:1269#0/1
^K[YRTZU^``_Y_`_N[a`_]ZZXGTW[¥WN¥]PTYSIW
@HWI-EAS443:3:73:1196:1824#0/1
CAAAGAGGCCCATCAGGCAATCTGAGGGGGCGTGGTGTCG
+HWI-EAS443:3:73:1196:1824#0/1
abab`aaaaaba]aba^aaa¥^aa`a^`aaZaUZXWQPI^
@HWI-EAS443:3:73:1198:1716#0/1
GGAGAGTCACCCAGGGGAGTGGAGGAAGGCTCTATTGGGA
+HWI-EAS443:3:73:1198:1716#0/1
aba`aa¥baa_Ua]Z`¥]`MaY^_aMY]SFTWQ`_¥Z^V^
@HWI-EAS443:3:73:1217:1782#0/1
ATCAGGCACTCCTCGATTGCTTCATATAGCCCCCCAAGAT
+HWI-EAS443:3:73:1217:1782#0/1
ZWT]¥`bbb_a^b__Z]Tb`aaT]]ba`_aR^bTa]I__K
@HWI-EAS443:3:73:1255:1536#0/1
GTGACCCGAATGCACTATTGAAGCACCGATTTGAGATAAT
+HWI-EAS443:3:73:1255:1536#0/1
a^aaaa`baa``_aaaaaaa`aa^a`aa```aa_a``_``
@HWI-EAS443:3:73:1296:681#0/1
ACGGGGTGAATCAATAATTGTAGAATCTGGTGACCCGAAT
+HWI-EAS443:3:73:1296:681#0/1
^a`a`b_babbaaabaabaa_bb`a`a`ba_aa^`^a``a
@HWI-EAS443:3:73:1519:1709#0/1
TTTTCGGCTGAATCGAGAAAATTGCTTCTCATTGTTCAGG
+HWI-EAS443:3:73:1519:1709#0/1
abaabbaababa`_a_a`aaaaa__]aZa_a``__`_^_^
@HWI-EAS443:3:73:1541:1280#0/1
GCCTTGCTCAATGCATCCTGTGCAGCCATGGATGACTTTC
+HWI-EAS443:3:73:1541:1280#0/1
aaaZW]`¥XZZY[¥[ZULOYJXWQ[ZTPQXPRZVVGZZR_
@HWI-EAS443:3:73:1581:403#0/1
CCCCCAAGATCGAAGGTTCCAGGTTCCAGGTTGTCCCTAA
+HWI-EAS443:3:73:1581:403#0/1
abbbabbbbbababb`_b_aa^a[b^^aabWbba¥^]aaa
```

```
@HWI-EAS443:3:73:1598:871#0/1
GGGAGTGTACATAAATACGGCCTTGCTCAATGCATCCTGT
+HWI-EAS443:3:73:1598:871#0/1
aaa^Z¥a_aa]a_`aa``^`U]`__]_UY_^]^_^WW^[U
@HWI-EAS443:3:73:1610:1119#0/1
GGGCATGGAAATGAGGCGCTGCCTTCTTCAGTCTCTTCAG
+HWI-EAS443:3:73:1610:1119#0/1
abaaabbbabaababa_a¥aa`aaa¥aa___^``_`a_¥_
@HWI-EAS443:3:73:1621:1605#0/1
AGCCTTCCTCCACTCCCCTGGGTGACTCTCCGATTGGCCA
+HWI-EAS443:3:73:1621:1605#0/1
a¥``aa__a`_^_aa]_¥¥^__V[_¥_V_W¥¥S^]¥¥S¥W
@HWI-EAS443:3:73:1734:637#0/1
GGGGCTATATGAAGCAATCGAGGAGTGCCTGATTAATGAT
+HWI-EAS443:3:73:1734:637#0/1
a`^_¥[abaab]aa]a_TR`Z`aPaUaRU`aZ__ZX¥[ZT
@HWI-EAS443:3:73:175:854#0/1
CAAGAAATGGCCAGTAGGAGTCTATGGGATTCCTTTCGTC
+HWI-EAS443:3:73:175:854#0/1
aa`aa]a_a`a_`aZ`aa]_¥]_V_`___U^__]^^Z^WU
@HWI-EAS443:3:73:201:392#0/1
CCTCCACTCCCCTGGGTGACTCTCCGATTGGCCATGTTTC
+HWI-EAS443:3:73:201:392#0/1
aa]a`]SS^a`UV`RUMRP¥]^NW^WRTWUTWVUBBBBBB
@HWI-EAS443:3:73:255:1812#0/1
TGGTGCCATATTTTCACCGAGTGCCCACTTCAATTGGCTT
+HWI-EAS443:3:73:255:1812#0/1
T¥TZ_^`_aaa]`ababbaaa¥abaaaaa_`a]Y^[^aa¥
@HWI-EAS443:3:73:262:923#0/1
AACATGGCCAATCGGAGAGTCACCCAGGGGAGTGGAGGAA
+HWI-EAS443:3:73:262:923#0/1
aaaaaaaa_a_]_`_]`^ZX¥_^`YS[^_^S¥U^¥UW¥WW
@HWI-EAS443:3:73:366:1880#0/1
CGAGGCCCATGTTCCTATATGTGAGAACCAATGGAACCTC
+HWI-EAS443:3:73:366:1880#0/1
a^a_abb`WZaYZ^a][_a[VLY¥[]`a`aa[^Z¥`^_X`
@HWI-EAS443:3:73:432:1059#0/1
GCTTTTCACTATAAGACAAGAAATGGCCAGTAGGAGTCTA
+HWI-EAS443:3:73:432:1059#0/1
abababbbbabbbbabaabbb[baaaa_a]`a__b^__T
@HWI-EAS443:3:73:441:1430#0/1
GAGCCTAACATAGTCAAACCACATGAGAAAGGCATAAATC
+HWI-EAS443:3:73:441:1430#0/1
_babb^`bbbaa_abbbbbb]baaaZ_baab`bb_H_baa
@HWI-EAS443:3:73:533:371#0/1
CACTGCAGGGCTACTGAATACATAATGAAGGGAGTGTACA
+HWI-EAS443:3:73:533:371#0/1
a]]b^bb```_baabbaab`aaa`abbaaaa`Za_a_aa`
@HWI-EAS443:3:73:540:1660#0/1
CGGGGCAACATCTTCTCCTATTTCATCAAGTTCTATCCAG
```

+HWI-EAS443:3:73:540:1660#0/1
a[P^Za_`_```^]```a__[_^_a^``]_ZZ_]_^_^`^
@HWI-EAS443:3:73:578:108#0/1
GCTTCATATAGCCCCCCAAGATCGAAGGTTCCAGGTTCCA
+HWI-EAS443:3:73:578:108#0/1
aaa_]`a_U`]_[`[_a`¥¥_S`ZS]]_N¥¥WS^¥NUWWW
@HWI-EAS443:3:73:581:1554#0/1
GGCATGGAAATGAGGCGCTGCCTTCTTCAGTCTCTTCAGC
+HWI-EAS443:3:73:581:1554#0/1
[`bZX^¥I__TZ¥Z`bbb`babUbaaa`aba_]aVaa_Tb
@HWI-EAS443:3:73:594:1306#0/1
GTTACATATACTGTTCACCACTGTCCAGGCCATGATTCGG
+HWI-EAS443:3:73:594:1306#0/1
``bb`bbbabbbb`abbabbaaa^K`ba^aaaa^aaa_``
@HWI-EAS443:3:73:595:1853#0/1
CGAAAATCGAAACTAACAAGTTTGCTGCAATATGCACACA
+HWI-EAS443:3:73:595:1853#0/1
a¥babbaa¥```aabbZ^_¥¥a``a]aaaa`ba`_aaaaa
@HWI-EAS443:3:73:708:388#0/1
AGCAATTTTCTCGATTCAGCCGAAAACCCCTCAAGTTGTG
+HWI-EAS443:3:73:708:388#0/1
aa_`aa`aaa`_a``a__`^^a__`_]]]]_]_W_Z__X`
@HWI-EAS443:3:73:717:51#0/1
TAAATACGGCCTTGCTCAATGCATCCTGTGCAGCCATGGA
+HWI-EAS443:3:73:717:51#0/1
aaaaaaaba]^_``_``_```__^¥_^_¥^WZ`¥T¥^[^Z
@HWI-EAS443:3:73:733:1389#0/1
CCTTGGTCATGTCTTTCTCTTTGACAGAAGACTCGGCCTC
+HWI-EAS443:3:73:733:1389#0/1
aaaaaa[aaaa^````a`^````¥`^^a``___`Y[``¥¥
@HWI-EAS443:3:73:803:766#0/1
GAGGAACTATTTTACAGCAGAAGTGTCCCACTGCAGGGCT
+HWI-EAS443:3:73:803:766#0/1
a`aaaaaabbbaaaaaaaaaaaa_a_a_a`__aaaa]`_`
@HWI-EAS443:3:73:810:715#0/1
GCAGGTGCTAGCAGAGCTACAGGACATTGAAAATGAAGAG
+HWI-EAS443:3:73:810:715#0/1
ab_Z_[S]Y^U^YWTZ`_]]`]X`¥¥a_XPPW¥YY^ZFV[
@HWI-EAS443:3:73:817:333#0/1
GGTCCTGCACACTTTCCCAATAGAGCCTTCCTCCACTCCC
+HWI-EAS443:3:73:817:333#0/1
ab_aabbbbaa`aaaaa`baaaaaaa]aa`_aa¥a^`^`S
@HWI-EAS443:3:73:899:1554#0/1
TTCTGCTGTAAAATAGTTCCTCCTCATGCTTGCGATATGT
+HWI-EAS443:3:73:899:1554#0/1
aa`aaa`aa`bbaa`_aa_aaaaaaa]`a_a`aa__a_`]
@HWI-EAS443:3:73:942:304#0/1
GTTCGGTGGGAGACTTTGGTCGGCAAGCTTGCGCATAGTT
+HWI-EAS443:3:73:942:304#0/1
_LPN^YH_P[[`NNX[ZXQUSN^L[[UQVYRRYUTYU^WU

```
@HWI-EAS443:3:73:951:17#0/1
TAAATCCCAATTACCTCATGGCTTGGAAGCAGGTGCTAGC
+HWI-EAS443:3:73:951:17#0/1
ababaa``_aaaa__````aa`aaZ`V[^PV[]NTQTQTO
@HWI-EAS443:3:73:973:684#0/1
AGCAGGTGCTAGCAGAGCTACAGGACATTGAAAATGAAGA
+HWI-EAS443:3:73:973:684#0/1
ZZXX`ZHV`ab¥__¥¥¥a`YT¥b]a`]^`a]]`aa]Y]ZZ
@HWI-EAS443:3:73:977:1104#0/1
GACATTCTTTGGCTGGAAAGAGCCTAACATAGTCAAACCA
+HWI-EAS443:3:73:977:1104#0/1
PUUMY`¥abaa``bbaa]PXZ`_a¥__]aa]T^aaab]]a
@HWI-EAS443:3:73:98:1124#0/1
CCCCGATTGAACATATCGCAAGCATGAGGAGGAACTATTT
+HWI-EAS443:3:73:98:1124#0/1
abbb]M¥IOU`WZZS_]]]RYS^U]]R]_`WWW[WNPXTL
@HWI-EAS443:3:73:994:227#0/1
AGTGGGCACTCGGTGAAAATATGGCACCAGAAAAAGTAGA
+HWI-EAS443:3:73:994:227#0/1
aaabaaaa]a_aa]a`aaaa_aaa]_R^_`^_``__W_YY
@HWI-EAS443:3:74:1023:700#0/1
GAAGATCCCAAGGACAAAGAACATGAAGAGAACAAGCCAA
+HWI-EAS443:3:74:1023:700#0/1
aaaaaab`a_`aaa_`aa^Y`^abaZ`a`aa__a^a]¥`^
@HWI-EAS443:3:74:106:1674#0/1
CCCCGTTCGTCGATGAAATGGAAATCCGAATACATGAAAC
+HWI-EAS443:3:74:106:1674#0/1
a_a`]P[a`Sa[[WZaZ]^T[`XY]`¥V^]Z`^¥T]^__]
@HWI-EAS443:3:74:1117:175#0/1
ACCCCGTTCGTCGATGAAATGGAAATCCGAATACATGAAA
+HWI-EAS443:3:74:1117:175#0/1
a]aa_a`aaaV`a`_aa`__a__]_`YJ[a_^__^^^]_^
@HWI-EAS443:3:74:1203:1299#0/1
GGGGTTTTCGGCTGAATCGAGAAAATTGCTTCTCATTGTT
+HWI-EAS443:3:74:1203:1299#0/1
abbbT[`a_Zaaaba_aabaa`X`a_a`_[`Y`a^`¥`U_
@HWI-EAS443:3:74:1206:1030#0/1
ATTCGGATTTCCATTTCATCGACGAACGGGGTGAATCAAT
+HWI-EAS443:3:74:1206:1030#0/1
_bb``b`bbbaaabaaaaaabaab^aaaaba]aa_a]__`
@HWI-EAS443:3:74:1215:1265#0/1
GAAATGGCCAGTAGGAGTCTATGGGATTCCTTTCGTCAGT
+HWI-EAS443:3:74:1215:1265#0/1
aa¥a```a`a^RT]XS`VL¥__`_¥S^_P[^`__¥PUZZW
@HWI-EAS443:3:74:1281:1405#0/1
AGCAGGTGCTAGCAGAGCTACAGGACATTGAAAATGAAGA
+HWI-EAS443:3:74:1281:1405#0/1
abbaY^F[]]^Y_``aaaab`aaYTW_^a^``[a_a__^`
@HWI-EAS443:3:74:1316:1665#0/1
CATGTTTCCGATTTGTTTTCAAAGAATTCCTTGGTCATGT
```

```
+HWI-EAS443:3:74:1316:1665#0/1
N`^a`abbabbbaabbbbbbaa`bba`a_aabbb_¥aabb
@HWI-EAS443:3:74:1388:400#0/1
GGGGGGCTATATGAAGCAATCGAGGAGTGCCTGATTAATG
+HWI-EAS443:3:74:1388:400#0/1
a]_T¥SXTaa__a]YNR]_^Z^^_[WUNZ_¥U`T[VVVQW
@HWI-EAS443:3:74:145:1829#0/1
CAAGGACAAAGAACATGAAGAGAACAAGCCAATTGAAGTG
+HWI-EAS443:3:74:145:1829#0/1
Z^_a``bbb`bbbbbbbbZ_b^Zbbb_bbbb_aababaWa
@HWI-EAS443:3:74:1535:1790#0/1
TGTGGTTTGACTATGTTAGGCTCTTTCCAGCCAAAGAATG
+HWI-EAS443:3:74:1535:1790#0/1
aa]a¥`a¥UY]`aa_¥¥TZaZ¥XZaa¥¥WYPO`a]]W_¥X
@HWI-EAS443:3:74:161:696#0/1
AACTTTGTAAGTATGGAGTTCTCACTCACTGACCCGAGAC
+HWI-EAS443:3:74:161:696#0/1
WS_``_`U¥_]T`¥X¥¥]WT]Ybaa``Za]RT^`[`Y`¥V
@HWI-EAS443:3:74:1627:1401#0/1
CAGCTTGCTAGAGATCTGGGCTCTGGCTCATCACTGTCAT
+HWI-EAS443:3:74:1627:1401#0/1
a__`aabb]babbaabb¥¥Za^a``Q^a_[¥_a_aa_aaa
@HWI-EAS443:3:74:1680:1915#0/1
AGTCAATTCACATGCCTTATTGAATTCATTTTGGACCCAG
+HWI-EAS443:3:74:1680:1915#0/1
¥`]¥ba`bbbaaa`_¥abaaabaa`aa`aaa_aa`¥XUZY
@HWI-EAS443:3:74:1756:1310#0/1
CATAAATCCCAATTACCTCATGGCTTGGAAGCAGGTGCTA
+HWI-EAS443:3:74:1756:1310#0/1
abbbbabbabbbbbbabbbbba`aaaT[¥ba¥`^`Y¥¥[^
@HWI-EAS443:3:74:1770:711#0/1
CAGGGCAAGAATCAAAACTAGGCTTTTCACTATAAGACAA
+HWI-EAS443:3:74:1770:711#0/1
ab¥¥bbaaba`bbb`aa`baa`Iabbb^a¥bbaaa```ba
@HWI-EAS443:3:74:198:164#0/1
AGTCAATTCACATGCCTTATTGAATTCATTTTGGACCCAG
+HWI-EAS443:3:74:198:164#0/1
aaa_H]bZ`bbZbb_`aaZXaa_YWa[_aa__a_Z`^[ZZ
@HWI-EAS443:3:74:259:1920#0/1
CAGCAAACTTGTTAGTTTCGATTTTCGGATCTTCCCCATA
+HWI-EAS443:3:74:259:1920#0/1
aaUaa¥U]_a____T[^^aXU]]^^_POGUVV[VSZ[[Y[
@HWI-EAS443:3:74:268:692#0/1
GGGCAACATCTTCTCCTATTTCATCAAGTTCTATCCAGCT
+HWI-EAS443:3:74:268:692#0/1
_bbb`bbababbbbbbb`baabbaba]b`aa`]aaaaaaa
@HWI-EAS443:3:74:276:614#0/1
GAGAAAGGCATAAATCCCAATTACCTCATGGCTTGGAAGC
+HWI-EAS443:3:74:276:614#0/1
aaa`aaa`aaaa__```_aa`aa___^`__`__]H]U^TW
```

```
@HWI-EAS443:3:74:276:775#0/1
ACTCGGTGAAAATATGGCACCAGAAAAAGTAGACTTTGAT
+HWI-EAS443:3:74:276:775#0/1
Z¥Z``bTY^TabXMXYW[`a]a^V^`¥]R[Y^^[[^][^Z
@HWI-EAS443:3:74:359:996#0/1
GAGAAAGGCATAAATCCCAATTACCTCATGGCTTGGAAGC
+HWI-EAS443:3:74:359:996#0/1
aa`aba_a`babaa``a_]aaa``]_aaa¥¥a]_WMU__R
@HWI-EAS443:3:74:385:394#0/1
AATAATTGTAGAATCTGGTGACCCGAATGCACTATTGAAG
+HWI-EAS443:3:74:385:394#0/1
aabb`bbaabbbaaabba^baa`aaaaaaaa`baaa`a_a
@HWI-EAS443:3:74:397:1724#0/1
CAGCTTGCTAGAGATCTGGGCTCTGGCTCATCACTGTCAT
+HWI-EAS443:3:74:397:1724#0/1
a`Z__]a___]VZ__`[PW`b¥X^`]b¥¥[]```Y]V_X]
@HWI-EAS443:3:74:449:847#0/1
ATAAGGCATGTGAATTGACTGATTCAAGCTGGATAGAACT
+HWI-EAS443:3:74:449:847#0/1
a`^`aaa_aa`aaa_aaaaaaa__``_a_`a`^^_a^a^`
@HWI-EAS443:3:74:598:1237#0/1
CTCATTTGAGAAATGATACTGATGTGGTGAACTTTGTAAG
+HWI-EAS443:3:74:598:1237#0/1
aa`¥`^b^aa`^`baaabaa`Z`b][FXa```a__^_]a`
@HWI-EAS443:3:74:599:1207#0/1
CAAGCCATGAGGTAATTGGGATTTATGCCTTTCTCATGTG
+HWI-EAS443:3:74:599:1207#0/1
`a^a]`[```__QY[^```__Z__`Ya`_``[^^^_``]_
@HWI-EAS443:3:74:610:625#0/1
GAATGGTTCAATTTTGGCGTTCACTTCTTTTGACATTTGG
+HWI-EAS443:3:74:610:625#0/1
abbaba]bb`bbaa`aZ_Z^G¥UYYQJabaaY[a^``¥P[
@HWI-EAS443:3:74:644:128#0/1
TCCTGTAGCTCTGCTAGCACCTGCTTCCAAGCCATGAGGT
+HWI-EAS443:3:74:644:128#0/1
abb`_`aa```_][[V`a_]__`^__VUUXYPUQQ`X`^R
@HWI-EAS443:3:74:738:1063#0/1
GGCATGGAAATGAGGCGCTGCCTTCTTCAGTCTCTTCAGC
+HWI-EAS443:3:74:738:1063#0/1
`abba_Z_`bbbbaabbbbb`aaaaaa`aa```baa`^]a
@HWI-EAS443:3:74:774:671#0/1
CAAAGAATTCCTTGGTCATGTCTTTCTCTTTGACAGAAGA
+HWI-EAS443:3:74:774:671#0/1
aa`abbaaababbba_baabaababab`aaaa`aaaaaaa
@HWI-EAS443:3:74:790:619#0/1
GGCCAGTAGGAGTCTATGGGATTCCTTTCGTCAGTCCGAA
+HWI-EAS443:3:74:790:619#0/1
abb`ab`bba]a`ab`bbbb^`_QZaba`b_Xa`a¥X^aa
@HWI-EAS443:3:74:818:462#0/1
CATTCTTTGGCTGGAAAGAGCCTAACATAGTCAAACCACA
```

```
+HWI-EAS443:3:74:818:462#0/1
abbbbbbaa`babbaaba`aaabaa_baaba`aaa`[`]a
@HWI-EAS443:3:74:822:1459#0/1
GGCCAATCGGAGAGTCACCCAGGGGAGTGGAGGAAGGCTC
+HWI-EAS443:3:74:822:1459#0/1
a`aaabbaa]Za_aO]aaaaZXRW[SaW^a¥`_^]^^¥¥W
@HWI-EAS443:3:74:830:873#0/1
TGGGGCATGGAAATGAGGCGCTGCCTTCTTCAGTCTCTTC
+HWI-EAS443:3:74:830:873#0/1
abab_a_`_a[]`aaa`_baa`a``aa_a__[a¥__`___
@HWI-EAS443:3:74:895:676#0/1
GAAGTTCGGTGGGAGACTTTGGTCGGCAAGCTTGCGCATA
+HWI-EAS443:3:74:895:676#0/1
aaab``Z`aUaa`]a[a```aaVa`a`a_``_[_^a¥¥¥^
@HWI-EAS443:3:74:915:1195#0/1
ATTGAAAATGAAGAGAAGATCCCAAGGACAAAGAACATGA
+HWI-EAS443:3:74:915:1195#0/1
aa`^W¥a]a`aaa^]`_``[aa_aa`Y^Z^aa_`a[_`]]
@HWI-EAS443:3:74:923:1527#0/1
GACTGATTCAAGCTGGATAGAACTTGATGAAATAGGAGAA
+HWI-EAS443:3:74:923:1527#0/1
abbba[aabaabaaa_Yaabababaaaaaaaaaa`aaaaa
@HWI-EAS443:3:74:937:696#0/1
CCGATTTGTTTTCAAAGAATTCCTTGGTCATGTCTTTCTC
+HWI-EAS443:3:74:937:696#0/1
aabbbbbb_bbbabbbbbabababbbaabbbbbabbb_b`
@HWI-EAS443:3:74:970:492#0/1
CGAACTTCTCCAGCCTTGAAAACTTTAGAGCCTATGTAGA
+HWI-EAS443:3:74:970:492#0/1
ababaab`babbababbbabab`bbbaaaaa^babb_aaa
@HWI-EAS443:3:75:1116:802#0/1
CAGGAACTATGCGCAAGCTTGCCGACCAAAGTCTCCCACC
+HWI-EAS443:3:75:1116:802#0/1
abbab]Zbabbabaaabbbbaaaab_a_a_ba`aa^aa^a
@HWI-EAS443:3:75:1127:686#0/1
ATTTTCACCGAGTGCCCACTTCAATTGGCTTGTTCTCTTC
+HWI-EAS443:3:75:1127:686#0/1
_bb_[`b_^bb`WbaabaRabab]a`ab_a_WYX]_^aWP
@HWI-EAS443:3:75:1408:843#0/1
GCAAGAATCAAAACTAGGCTTTTCACTATAAGACAAGAAA
+HWI-EAS443:3:75:1408:843#0/1
`abbb`bbbbbabab``abbabbabaabb`abaabaaaab
@HWI-EAS443:3:75:151:1663#0/1
ATAAATCCCAATTACCTCATGGCTTGGAAGCAGGTGCTAG
+HWI-EAS443:3:75:151:1663#0/1
aa¥Q_aaa]Qa¥a`^¥`_U^`^___NZI[Z^]]¥OUUVVB
@HWI-EAS443:3:75:1574:42#0/1
GAATGCACTATTGAAGCACCGATTTGAGATAATTGAAGGA
+HWI-EAS443:3:75:1574:42#0/1
aababab_b``a`^Ybb¥][a^bbbb¥aWbaaaabaabab
```

@HWI-EAS443:3:75:1602:574#0/1
GGCATGGAAATGAGGCGCTGCCTTCTTCAGTCTCTTCAGC
+HWI-EAS443:3:75:1602:574#0/1
`b[QTab]``aa_aYU_____¥a_QY`_]a`VYQ_¥NYXP
@HWI-EAS443:3:75:1605:798#0/1
CCTGGGTGACTCTCCGATTGGCCATGTTTCCGATTTGTTT
+HWI-EAS443:3:75:1605:798#0/1
``]babIa`H_SbaWaOSZZO¥[¥`]_^aUT_H]]^_NZ`
@HWI-EAS443:3:75:1693:775#0/1
GAACACAGTATTTTTCCCATTTGTGTGGCTCCAGTCTCGG
+HWI-EAS443:3:75:1693:775#0/1
[b_bbabbabbabbba[aababba_aa[_b___baS``_b
@HWI-EAS443:3:75:1695:176#0/1
ATTTCATCGACGAACGGGGTGAATCAATAATTGTAGAATC
+HWI-EAS443:3:75:1695:176#0/1
_b_bab`]``Laa`aa`a_S[Z]]G[_a``baa^_VV`_Z
@HWI-EAS443:3:75:1715:956#0/1
GTTCTCACTCACTGACCCGAGACTGGAGCCACACAAATGG
+HWI-EAS443:3:75:1715:956#0/1
aPYXT`TJ_LQH]UZ``a_BBBBBBBBBBBBBBBBBBBBB
@HWI-EAS443:3:75:1718:277#0/1
CTGCACACTTTCCCAATAGAGCCTTCCTCCACTCCCCTGG
+HWI-EAS443:3:75:1718:277#0/1
]`a^a```bbb^ZZ^bbbbb^Z__b_X_a_aa^_^ZR[ab
@HWI-EAS443:3:75:239:577#0/1
TACATATACTGTTCACCACTGTCCAGGCCATGATTCGGTC
+HWI-EAS443:3:75:239:577#0/1
`_aaa¥XJ¥Za^aa`¥^YX]a]`a^QW_`[¥YV]VVQNOY
@HWI-EAS443:3:75:277:840#0/1
AATCAGGCACTCCTCGATTGCTTCATATAGCCCCCCAAGA
+HWI-EAS443:3:75:277:840#0/1
aZZaa[OZL]aUPT_Y__[a[___[]_¥Y¥[]RNU^¥YYU
@HWI-EAS443:3:75:27:559#0/1
CACTGTCATACTGTTTAAGGTCTCCAACATCTTTGCAGTC
+HWI-EAS443:3:75:27:559#0/1
abbbbabbbaabb`ab¥¥^b_abbababbb¥b`aaaab`a
@HWI-EAS443:3:75:29:1813#0/1
CCACTCCCCTGGGTGACTCTCCGATTGGCCATGTTTCCGA
+HWI-EAS443:3:75:29:1813#0/1
abaa_aaaa]_URFX¥`^a^__WUW¥VW_`VUSUSUTWUT
@HWI-EAS443:3:75:31:1385#0/1
GCTTGCGATATGTTCAATCGGGGCAACATCTTCTCCTATT
+HWI-EAS443:3:75:31:1385#0/1
aba`Y^baa`aa`aaa^^a^¥``aa_a``aaaaa_a`__a
@HWI-EAS443:3:75:389:583#0/1
CATGTTTCCGATTTGTTTTCAAAGAATTCCTTGGTCATGT
+HWI-EAS443:3:75:389:583#0/1
abbabbbabbabbbbbbbbbbbbbbbabbbbbbba`bbb`a
@HWI-EAS443:3:75:393:1069#0/1
TGGAGACCTTAAACAGTATGACAGTGATGAGCCAGAGCCC

+HWI-EAS443:3:75:393:1069#0/1
`babaZabaW__X_aa¥baaa__a_a¥_a`[¥_aVIWJU`
@HWI-EAS443:3:75:397:1049#0/1
GCACACTTTCCCAATAGAGCCTTCCTCCACTCCCCTGGGT
+HWI-EAS443:3:75:397:1049#0/1
]bbbba`L¥``bb^]aa_``a`aX``a_Z`]aa_aR`__G
@HWI-EAS443:3:75:437:1142#0/1
CAGCAGGAACTTTGACCGCTGATGGCAAAGAGGCCCATCA
+HWI-EAS443:3:75:437:1142#0/1
`aaaV``_a`aa``aa``a^`^^``^a]`Y^¥]^```¥[Z
@HWI-EAS443:3:75:496:1199#0/1
GTCAAACCACATGAGAAAGGCATAAATCCCAATTACCTCA
+HWI-EAS443:3:75:496:1199#0/1
a]a`^aa_`]aa^^_`VT^aabb`_U^a`aaaa]aaa``a
@HWI-EAS443:3:75:508:70#0/1
TCCACAACTTGAGGGGTTTTCGGCTGAATCGAGAAAATTG
+HWI-EAS443:3:75:508:70#0/1
ab]Za`U`bb]SZbbbaa[baZb_T``H][abY``aaa]X
@HWI-EAS443:3:75:549:904#0/1
GGGGCATGGAAATGAGGCGCTGCCTTCTTCAGTCTCTTCA
+HWI-EAS443:3:75:549:904#0/1
a_]aZVbbb¥X]Za¥O^aa``aaa__`]_a^_```¥^]¥[
@HWI-EAS443:3:75:583:500#0/1
CATTCTTTGGCTGGAAAGAGCCTAACATAGTCAAACCACA
+HWI-EAS443:3:75:583:500#0/1
a^bbabb]abbba^K_b¥baZJa]`a^baZbbbbb`aaab
@HWI-EAS443:3:75:590:1007#0/1
CTTGGGATCTTCTCTTCATTTTCAATGTCCTGTAGCTCTG
+HWI-EAS443:3:75:590:1007#0/1
aaabab^]_bbabbbb_^_baabbbbb^bb`¥`_abba`a
@HWI-EAS443:3:75:636:1018#0/1
CTCATTTGAGAAATGATACTGATGTGGTGAACTTTGTAAG
+HWI-EAS443:3:75:636:1018#0/1
aa_a¥^_]W___X^a_^¥^_ZaaX`a][a`][aaa¥¥`^]
@HWI-EAS443:3:75:725:815#0/1
GGGTTTTCGGCTGAATCGAGAAAATTGCTTCTCATTGTTC
+HWI-EAS443:3:75:725:815#0/1
a`bIbbaaaabbaaababaa_aaaaabaaaaaaaaaa^`a
@HWI-EAS443:3:75:764:1367#0/1
GTTTCCGATTTGTTTTCAAAGAATTCCTTGGTCATGTCTT
+HWI-EAS443:3:75:764:1367#0/1
aaabaaba^bab`b`baabaa]_aa_]aaa¥^``a`_W`a
@HWI-EAS443:3:75:767:310#0/1
AATTGAAGGAAGAGACCGAATCATGGCCTGGACAGTGGTG
+HWI-EAS443:3:75:767:310#0/1
a`bbab^V`Zaaaa]]ab`^`^a`aa_Za```Y`a^`_Z`
@HWI-EAS443:3:75:780:1064#0/1
GGGGGCTATATGAAGCAATCGAGGAGTGCCTGATTAATGA
+HWI-EAS443:3:75:780:1064#0/1
][_`a`a¥Z_```]`ba^]^`W``R`X[`a`a]_aa`^a^

```
@HWI-EAS443:3:75:803:524#0/1
CAAGCCAATTGAAGTGGGCACTCGGTGAAAATATGGCACC
+HWI-EAS443:3:75:803:524#0/1
ab^b_a^aaabaa]Sb__¥b_a`b]W`_aaa][Ya`_Y¥]
@HWI-EAS443:3:75:863:1499#0/1
CGATTGGCCATGTTTCCGATTTGTTTTCAAAGAATTCCTT
+HWI-EAS443:3:75:863:1499#0/1
Zaa¥^]¥a^b_]]a`^a``U`^``bba¥^`a```^YS¥aa
@HWI-EAS443:3:75:967:1414#0/1
AAAGAATTCCTTGGTCATGTCTTTCTCTTTGACAGAAGAC
+HWI-EAS443:3:75:967:1414#0/1
aX^`^a¥ZY^b`]aYaaaa_`baaaa_baaa_`aaa_aaa
@HWI-EAS443:3:76:102:112#0/1
TGGTGCCATATTTTCACCGAGTGCCCACTTCAATTGGCTT
+HWI-EAS443:3:76:102:112#0/1
¥Z`FZ]^ababb`aab`[a_aZ`YU`_^^_a^]^^__Z¥_
@HWI-EAS443:3:76:1042:1510#0/1
AACTAACAAGTTTGCTGCAATATGCACACATTTGGAAGTT
+HWI-EAS443:3:76:1042:1510#0/1
`_a¥b¥^`^WFXaab`W`^^aaaa¥_a]`a`aWaY`¥aO_
@HWI-EAS443:3:76:1108:1734#0/1
CGATTGGCCATGTTTCCGATTTGTTTTCAAAGAATTCCTT
+HWI-EAS443:3:76:1108:1734#0/1
ab^baaY[aa`XQZ`VD[¥]`_]L[`VV¥aVZ_[Y¥[_N^
@HWI-EAS443:3:76:1164:1442#0/1
CTGGGTCCAAAATGAATTCAATAAGGCATGTGAATTGACT
+HWI-EAS443:3:76:1164:1442#0/1
^_a`^RO^a^^a`_`b_b_bbbb```^abbababab b¥ab
@HWI-EAS443:3:76:1268:769#0/1
TTGGGGGGCTATATGAAGCAATCGAGGAGTGCCTGATTAA
+HWI-EAS443:3:76:1268:769#0/1
Y`b_^ZW`]b]a_[`^]aab^`]`TSQW]UXVJTa¥_GT[
@HWI-EAS443:3:76:1276:884#0/1
GAGGGGTTTTCGGCTGAATCGAGAAAATTGCTTCTCATTG
+HWI-EAS443:3:76:1276:884#0/1
ZQ[a``Y_aa__¥]a_a_]_[[ZX^]___]]X_¥^X^^]]
@HWI-EAS443:3:76:1283:1196#0/1
CCTCCTCATGCTTGCGATATGTTCAATCGGGGCAACATCT
+HWI-EAS443:3:76:1283:1196#0/1
`_Z`]S_V_a_Y]]WYQHSX]QT]ZOMTW`WGY^YW¥UNM
@HWI-EAS443:3:76:1283:1326#0/1
AAGAATTCCTTGGTCATGTCTTTCTCTTTGACAGAAGACT
+HWI-EAS443:3:76:1283:1326#0/1
abba`aaaaaaaa^aaaa`aaaa`a_aa`a`]aa`__^_`
@HWI-EAS443:3:76:1288:1134#0/1
TGGCCAATCGGAGAGTCACCCAGGGGAGTGGAGGAAGGCT
+HWI-EAS443:3:76:1288:1134#0/1
aa`a]aaaaaa[`^^^^`^a_]]_¥^^`[`^V``¥^^YS[
@HWI-EAS443:3:76:1352:369#0/1
ACACCCTTGACGAAGAGAGCAGGGCAAGAATCAAAACTAG
```

```
+HWI-EAS443:3:76:1352:369#0/1
a_b]X]bbb__ba`^ab`aa]a`a___a^a`O^``_Zaa_
@HWI-EAS443:3:76:1408:991#0/1
ATTCAGTAGCCCTGCAGTGGGACACTTCTGCTGTAAAATA
+HWI-EAS443:3:76:1408:991#0/1
U`_bbba``bY^¥TG_b^bbba_bb¥`Ua`aSb`bbba[_
@HWI-EAS443:3:76:143:1789#0/1
ATCAGGCACTCCTCGATTGCTTCATATAGCCCCCCAAGAT
+HWI-EAS443:3:76:143:1789#0/1
[]`ba]_Z_a```aa`^]`a_aaa_a__U_¥]`a_^^¥YT
@HWI-EAS443:3:76:1470:1546#0/1
AGACAATTGAAGAAAAATTTGAGATTACAGGAACTATGCG
+HWI-EAS443:3:76:1470:1546#0/1
a[[`[I]aYZNKON[UZaa]aWYZaYVX__XXZW[^XSG¥
@HWI-EAS443:3:76:154:2014#0/1
TTTGAGATTACAGGAACTATGCGCAAGCTTGCCGACCAAA
+HWI-EAS443:3:76:154:2014#0/1
aaaaa`a_`aaba^aba_``^a]``a^a^^W]`]_^^___
@HWI-EAS443:3:76:1585:731#0/1
GGGGGCTATATGAAGCAATCGAGGAGTGCCTGATTAATGA
+HWI-EAS443:3:76:1585:731#0/1
a__^_UTIY]¥]U^TN[ZWG_]_VQUU]RXX]N^XYYXRU
@HWI-EAS443:3:76:1590:1116#0/1
AATGAATTCAATAAGGCATGTGAATTGACTGATTCAAGCT
+HWI-EAS443:3:76:1590:1116#0/1
abbba`bb^`aaaaaaaaab`a_aaaaa_aa_aa``]^¥`
@HWI-EAS443:3:76:1601:1694#0/1
ATCAAGTTCTATCCAGCTTGAATCAGTCAATTCACATGCC
+HWI-EAS443:3:76:1601:1694#0/1
a``baa`baabaW^aa`aabaaa_aa^]_____a]a^^T[
@HWI-EAS443:3:76:1618:397#0/1
GGGGTTTTCGGCTGAATCGAGAAAATTGCTTCTCATTGTT
+HWI-EAS443:3:76:1618:397#0/1
aaabZaaaX`aX`a^__Ua]`]^^__^^O_ZV_Z^_^^^^
@HWI-EAS443:3:76:1665:568#0/1
ATTCGGATTTCCATTTCATCGACGAACGGGGTGAATCAAT
+HWI-EAS443:3:76:1665:568#0/1
abbb`baba``aabaaaaa`baaaa_^__aaXaa_`____
@HWI-EAS443:3:76:1684:1560#0/1
CTGGAACCTGGAACCTTCGATCTTGGGGGGCTATATGAAG
+HWI-EAS443:3:76:1684:1560#0/1
`]¥]_`]¥]``a`X]Za]`^UR_]]QHW^]Y¥^a]^X¥Z¥
@HWI-EAS443:3:76:1684:599#0/1
CTCCAGTCTCGGGTCAGTGAGTGAGAACTCCATACTTACA
+HWI-EAS443:3:76:1684:599#0/1
ab¥[_bZab_^T¥V`abbb_`^¥_¥XaM]Y`aaa[``a_b
@HWI-EAS443:3:76:179:1203#0/1
GAAGAGACAATTGAAGAAAAATTTGAGATTACAGGAACTA
+HWI-EAS443:3:76:179:1203#0/1
_a^^^ZX]__U^¥]U¥_`]^TUMLHMS^^BBBBBBBBBBB
```

```
@HWI-EAS443:3:76:17:1194#0/1
CTTTCCCAATAGAGCCTTCCTCCACTCCCCTGGGTGACTC
+HWI-EAS443:3:76:17:1194#0/1
`bbabbbbbabb^Za```]babbbb]aaaaV`[XE]a^¥]
@HWI-EAS443:3:76:192:573#0/1
AAAGGAATCCCATAGACTCCTACTGGCCATTTCTTGTCTT
+HWI-EAS443:3:76:192:573#0/1
`a`b`¥^_[^aa`_aaa____]_¥_^]_[RRXXLUVTUUT
@HWI-EAS443:3:76:233:52#0/1
CCCAGATCTCTAGCAAGCTGGGTCCAAAATGAATTCAATA
+HWI-EAS443:3:76:233:52#0/1
abbaa]aaaaaa`Y`_]_`aa_]^Y¥^Y^^^Z]_ZZ]W][
@HWI-EAS443:3:76:234:751#0/1
TAATTGTAGAATCTGGTGACCCGAATGCACTATTGAAGCA
+HWI-EAS443:3:76:234:751#0/1
aWI_Z[Naaaa]__aaMPYUY__XNVXY]_TUV¥]XPW^T
@HWI-EAS443:3:76:28:1787#0/1
ACGGCCTTGCTCAATGCATCCTGTGCAGCCATGGATGACT
+HWI-EAS443:3:76:28:1787#0/1
__a`ab`_[aZ`aa_Yaa_aa[VXRUIV`^OVUTY[U[W[
@HWI-EAS443:3:76:29:1199#0/1
GGGGCTATATGAAGCAATCGAGGAGTGCCTGATTAATGAT
+HWI-EAS443:3:76:29:1199#0/1
a__`aY^]_a```[Z¥_]_^VV^X^G]V_]_V[^[YRTVX
@HWI-EAS443:3:76:322:1435#0/1
CATTAATCAGGCACTCCTCGATTGCTTCATATAGCCCCCC
+HWI-EAS443:3:76:322:1435#0/1
abbb_U_bba``aaaa_`^[]aaa``aaa_U]a``_][Z^
@HWI-EAS443:3:76:347:101#0/1
CACTTTCCCAATAGAGCCTTCCTCCACTCCCCTGGGTGAC
+HWI-EAS443:3:76:347:101#0/1
abbbbbab`b`abb_a_`^a_`aa_]X`]^`XX]^UR^WS
@HWI-EAS443:3:76:399:958#0/1
TGGCCACCAAAGCGGACTACACCCTTGACGAAGAGAGCAG
+HWI-EAS443:3:76:399:958#0/1
X¥¥b`¥_¥bb_V_a]a``a``aaZWZ¥¥abYZaaa`a]`¥
@HWI-EAS443:3:76:420:351#0/1
TTTCGGACTGACGAAAGGAATCCCATAGACTCCTACTGGC
+HWI-EAS443:3:76:420:351#0/1
aabbaab`babbaaabaaaa`a_¥a``a]_aaR_a_^`__
@HWI-EAS443:3:76:509:1961#0/1
ATCCCAATTACCTCATGGCTTGGAAGCAGGTGCTAGCAGA
+HWI-EAS443:3:76:509:1961#0/1
aaababaaaaaa_aa_U¥`__]Z¥`TUQUZNR_VYWZZW[
@HWI-EAS443:3:76:51:1195#0/1
GGGGGCTATATGAAGCAATCGAGGAGTGCCTGATTAATGA
+HWI-EAS443:3:76:51:1195#0/1
a[`_^a_a_b^XW_¥aaa^__^Z^^_W]__U]^]]_^^RZ
@HWI-EAS443:3:76:534:1501#0/1
CCTCCTCATGCTTGCGATATGTTCAATCGGGGCAACATCT
```

```
+HWI-EAS443:3:76:534:1501#0/1
aaT`_Z¥TabbaaaY]`¥O^^TXZ^a_a`a]]^_^^`]N¥
@HWI-EAS443:3:76:562:806#0/1
AAGAATTCCTTGGTCATGTCTTTCTCTTTGACAGAAGACT
+HWI-EAS443:3:76:562:806#0/1
Q]_`¥`bbbaaba_aaab_aaabaaaaaa``aaaa_a_a^
@HWI-EAS443:3:76:57:1711#0/1
AGAGCCTAACATAGTCAAACCACATGAGAAAGGCATAAAT
+HWI-EAS443:3:76:57:1711#0/1
a^_`]baabbaaaTZaaaaaaaaa_^__[K]_]_a_`aa_
@HWI-EAS443:3:76:584:1912#0/1
GGGTGAATCAATAATTGTAGAATCTGGTGACCCGAATGCA
+HWI-EAS443:3:76:584:1912#0/1
aaQK¥_b`a_aa_`a`^¥a^^_aa^_QRUa__``_a`UWa
@HWI-EAS443:3:76:615:1931#0/1
CATTAATCAGGCACTCCTCGATTGCTTCATATAGCCCCCC
+HWI-EAS443:3:76:615:1931#0/1
aaaaaW`ab^^aaa```]a`a][UY^^_`^`]`U_¥^]]U
@HWI-EAS443:3:76:622:1403#0/1
ATAAATCCCAATTACCTCATGGCTTGGAAGCAGGTGCTAG
+HWI-EAS443:3:76:622:1403#0/1
aaa_Zaaaaaaaaa`a_`a[YV_a_¥[W[SUY[TKXZV^V
@HWI-EAS443:3:76:640:740#0/1
GCCAGAGCCCAGATCTCTAGCAAGCTGGGTCCAAAATGAA
+HWI-EAS443:3:76:640:740#0/1
`W`PYVZ_¥]YVPVGU^_Y_VYRSGVUZIIPU¥]VVTQVV
@HWI-EAS443:3:76:658:959#0/1
GGGGCATGGAAATGAGGCGCTGCCTTCTTCAGTCTCTTCA
+HWI-EAS443:3:76:658:959#0/1
a`_Z^Y¥a[R__]__[W`__^TDR^^¥__Z__¥__^XUV¥
@HWI-EAS443:3:76:680:1781#0/1
TCGACACTTGGCCTATCGCAGTCCTCAAGAGCATGTCTCC
+HWI-EAS443:3:76:680:1781#0/1
ab`bbbaa``a__abaaaaaa_aa`aaa_a_aa_`_¥```
@HWI-EAS443:3:76:683:1446#0/1
CCCACTTCAATTGGCTTGTTCTCTTCATGTTCTTTGTCCT
+HWI-EAS443:3:76:683:1446#0/1
abaaaaaaba`aa_baaJ_`_a_a`a_``X^^__a_^^_^
@HWI-EAS443:3:76:711:1146#0/1
CATGACCAAGGAATTCTTTGAAAACAAATCGGAAACATGG
+HWI-EAS443:3:76:711:1146#0/1
`abbbb`Y`aaaababbaabb_`_abaa`aba`]aa`aa_
@HWI-EAS443:3:76:727:1599#0/1
GAAAATCGAAACTAACAAGTTTGCTGCAATATGCACACAT
+HWI-EAS443:3:76:727:1599#0/1
`^Yb_W^¥V¥Ya``YXPXZY_U[W¥U_`¥S`[__X_W]__
@HWI-EAS443:3:76:784:1625#0/1
CATGGAAATGAGGCGCTGCCTTCTTCAGTCTCTTCAGCAG
+HWI-EAS443:3:76:784:1625#0/1
a`a_Y_^Z^R^¥Y^LZZ[[]W¥Y``X]WNPT_^V¥ULNX^
```

```
@HWI-EAS443:3:76:818:96#0/1
ACCGATTTGAGATAATTGAAGGAAGAGACCGAATCATGGC
+HWI-EAS443:3:76:818:96#0/1
a`^b`bbbbaa]baaaaba`baa`b_b`Z^a^``^__a`]
@HWI-EAS443:3:76:849:1465#0/1
TGAAAATGAAGAGAAGATCCCAAGGACAAAGAACATGAAG
+HWI-EAS443:3:76:849:1465#0/1
a`^^`a_`aaa`_]Z_]____`^^]]_`]_]]]^^]^]]]
@HWI-EAS443:3:76:859:1883#0/1
TTGTCCTTGGGATCTTCTCTTCATTTTCAATGTCCTGTAG
+HWI-EAS443:3:76:859:1883#0/1
`a]]aaaaaaaX__a```aa`aa`__^aa___^a`¥_Za^
@HWI-EAS443:3:76:85:1005#0/1
CATGGAAATGAGGCGCTGCCTTCTTCAGTCTCTTCAGCAG
+HWI-EAS443:3:76:85:1005#0/1
a]VaWIW¥]`^_V]``¥`U^ZVa[_`¥[R[¥_TU¥U[QVY
@HWI-EAS443:3:76:88:86#0/1
GGGAGACTTTGGTCGGCAAGCTTGCGCATAGTTCCTGTAA
+HWI-EAS443:3:76:88:86#0/1
¥aaab_OY¥aaaY_aX]a]QZU]^U¥U[WQZXZU¥WZGGV
@HWI-EAS443:3:76:894:1493#0/1
GGGTTTTCGGCTGAATCGAGAAAATTGCTTCTCATTGTTC
+HWI-EAS443:3:76:894:1493#0/1
a]¥L¥¥^aaa_T^a`TTVR_`ZU_LP^ZVLFJSSR¥SROR
@HWI-EAS443:3:76:905:1189#0/1
AGAATTCCTTGGTCATGTCTTTCTCTTTGACAGAAGACTC
+HWI-EAS443:3:76:905:1189#0/1
ab```aaaa`a`]`a`aZaaa^`^Za`_a_V_a_¥WWW]]
@HWI-EAS443:3:76:90:2001#0/1
ATTTGAGATTACAGGAACTATGCGCAAGCTTGCCGACCAA
+HWI-EAS443:3:76:90:2001#0/1
W¥¥U^]]O]`baa¥Zabb¥Z_`a`b]RL]_RL]_XWY^`]
@HWI-EAS443:3:76:926:1292#0/1
AGCAGGGCAAGAATCAAAACTAGGCTTTTCACTATAAGAC
+HWI-EAS443:3:76:926:1292#0/1
_]`aaa`aaaa__a`aa_a`aa`_a_a``_a¥aa`a`_`¥
@HWI-EAS443:3:76:942:1415#0/1
AGAAGACACACATTCACATCTTTTCATTCACTGGAGAGGA
+HWI-EAS443:3:76:942:1415#0/1
TT__[bSbTbUb_U]bab¥`Wa^`a^`a__YLIRaa_WW`
@HWI-EAS443:3:76:949:578#0/1
CATTAATCAGGCACTCCTCGATTGCTTCATATAGCCCCCC
+HWI-EAS443:3:76:949:578#0/1
abbb_bbab`bIba`[bbaba``]abaX_ab`aa[VNPS`
@HWI-EAS443:3:76:950:327#0/1
CCTTGACGAAGAGAGCAGGGCAAGAATCAAAACTAGGCTT
+HWI-EAS443:3:76:950:327#0/1
]aabbbbbb`bbbbaababbbbbaabbabbababbba_aa
@HWI-EAS443:3:76:960:140#0/1
GGCATGGAAATGAGGCGCTGCCTTCTTCAGTCTCTTCAGC
```

+HWI-EAS443:3:76:960:140#0/1
aUX^W^]¥U^Q^V_¥S¥WQ¥UVWUWWWNWUVNUSVWGQGU
@HWI-EAS443:3:76:990:1663#0/1
CATTAATCAGGCACTCCTCGATTGCTTCATATAGCCCCCC
+HWI-EAS443:3:76:990:1663#0/1
abbbbbbbb`abbaabbbabbaaaaaabbabaa`a``___
@HWI-EAS443:3:77:1000:1641#0/1
ATCCGAAAATCGAAACTAACAAGTTTGCTGCAATATGCAC
+HWI-EAS443:3:77:1000:1641#0/1
^a¥XZ`¥aa]¥`_bba^W`]`¥`L`]UY[`¥a_aaW_Ra_
@HWI-EAS443:3:77:1013:1261#0/1
AGCAGGAACTTTGACCGCTGATGGCAAAGAGGCCCATCAG
+HWI-EAS443:3:77:1013:1261#0/1
aZ_L_WXaaaW`X`_ba_UT`aa`^^TX_VG_Y¥XZZ_Z`
@HWI-EAS443:3:77:1014:1209#0/1
GAAGATCCCAAGGACAAAGAACATGAAGAGAACAAGCCAA
+HWI-EAS443:3:77:1014:1209#0/1
a]¥_aa_a_XZ]V^aaaYb[aa]`a_`ba``__`aa_Xaa
@HWI-EAS443:3:77:1019:137#0/1
CTGGGTGACTCTCCGATTGGCCATGTTTCCGATTTGTTTT
+HWI-EAS443:3:77:1019:137#0/1
abbbbaabbbababbabbab¥^aaaabba_aVUabbUaab
@HWI-EAS443:3:77:1040:564#0/1
GGGGCATGGAAATGAGGCGCTGCCTTCTTCAGTCTCTTCA
+HWI-EAS443:3:77:1040:564#0/1
aabaabaa`aa^aaaaaaa_a`^]aa``a_aa`_a_`__`
@HWI-EAS443:3:77:1059:888#0/1
GCAGGTGCTAGCAGAGCTACAGGACATTGAAAATGAAGAG
+HWI-EAS443:3:77:1059:888#0/1
`bbba`ababbabaababa]babaaaabba`abaaaaaaa
@HWI-EAS443:3:77:105:295#0/1
GTCCTGCACACTTTCCCAATAGAGCCTTCCTCCACTCCCC
+HWI-EAS443:3:77:105:295#0/1
aRTZPUURGR¥T]YOZP`ZTXYPXRRTRTTTRPORRTRPB
@HWI-EAS443:3:77:1264:1396#0/1
TTCGAGCCGAACGGCTGCATTGAGGGCAAGCTTTCCCAAA
+HWI-EAS443:3:77:1264:1396#0/1
`aab`ab]bab`b¥aaaaX]]WT¥_VFHPXTU`Y^¥_T`]
@HWI-EAS443:3:77:1280:1596#0/1
GGGGGCTATATGAAGCAATCGAGGAGTGCCTGATTAATGA
+HWI-EAS443:3:77:1280:1596#0/1
SW_b]``_babaa_`aab`^^¥¥`^^]ba^babaab^`aa
@HWI-EAS443:3:77:1374:1242#0/1
CAGGCCATGATTCGGTCTCTTCCTTCAATTATCTCAAATC
+HWI-EAS443:3:77:1374:1242#0/1
abb``^bbbabbb`babbaaabbbb]bbbbb`aaababa]
@HWI-EAS443:3:77:1386:1658#0/1
CCCCAAGATCGAAGGTTCCAGGTTCCAGGTTGTCCCTAAG
+HWI-EAS443:3:77:1386:1658#0/1
¥bab`^^^]a_^[¥_Q`[]abbaTXUab¥DZ]H[_b`ZaU

```
@HWI-EAS443:3:77:1405:398#0/1
CATGACCAAGGAATTCTTTGAAAACAAATCGGAAACATGG
+HWI-EAS443:3:77:1405:398#0/1
`abba`bbaaabbbb¥bbbb`babaaabb`abbbbabbb`
@HWI-EAS443:3:77:1435:1327#0/1
CTGGGCTCTGGCTCATCACTGTCATACTGTTTAAGGTCTC
+HWI-EAS443:3:77:1435:1327#0/1
a`bbabb_babababbbbababb^bbbbabbabbbabba_ba
@HWI-EAS443:3:77:1488:508#0/1
ATTGCAGCAAACTTGTTAGTTTCGATTTTCGGATCTTCCC
+HWI-EAS443:3:77:1488:508#0/1
_bbb`bbabb``ba`abababbabW`aba`^^N^[a`Y[_
@HWI-EAS443:3:77:1508:1884#0/1
AGAACACAGTATTTTTCCCATTTGTGTGGCTCCAGTCTCG
+HWI-EAS443:3:77:1508:1884#0/1
aa`b_¥b]babbbbb^W^``baa[`aaaVEUV^[aBBBBB
@HWI-EAS443:3:77:1595:47#0/1
GGTTTGTTTTCCGTCTTCCTTCTTTGGTCCTACATTTGCT
+HWI-EAS443:3:77:1595:47#0/1
]aIa`ba`b`_aa`^aa[__a`aaa__`]^_`Z]aaa_V^
@HWI-EAS443:3:77:1598:511#0/1
GTCCAAAATGAATTCAATAAGGCATGTGAATTGACTGATT
+HWI-EAS443:3:77:1598:511#0/1
a_a`aaaabbaab[_b`a^^ba_aaa_a_`aaaa`aa^a`
@HWI-EAS443:3:77:1642:676#0/1
GAGAAAGGCATAAATCCCAATTACCTCATGGCTTGGAAGC
+HWI-EAS443:3:77:1642:676#0/1
ababbbab`bbbbab__aa`bb``^a`aaba_b`aa]]ZQ
@HWI-EAS443:3:77:1655:1153#0/1
TGTGGTTTGACTATGTTAGGCTCTTTCCAGCCAAAGAATG
+HWI-EAS443:3:77:1655:1153#0/1
abPbb¥a[bKa[abaaa¥`a[]a^`aXSW^J¥Z¥¥RI___
@HWI-EAS443:3:77:1660:1671#0/1
ATAAAAGGAAGGTCTCATTTGAGAAATGATACTGATGTGG
+HWI-EAS443:3:77:1660:1671#0/1
¥^]a`^aab`baaa`_aabbb`baU`ba[aaTaaab¥`aa
@HWI-EAS443:3:77:1667:1314#0/1
CGGCCTTGCTCAATGCATCCTGTGCAGCCATGGATGACTT
+HWI-EAS443:3:77:1667:1314#0/1
Q_b^bb[La`abbbT[ZZ__TZ`__b]Zabbb¥T¥b^]`a
@HWI-EAS443:3:77:1678:936#0/1
CAAGAAATGGCCAGTAGGAGTCTATGGGATTCCTTTCGTC
+HWI-EAS443:3:77:1678:936#0/1
Wabb`a^`a¥]UVb__XU_]Vab`a^`¥¥b_RI`baH_^]
@HWI-EAS443:3:77:1708:994#0/1
TAGCAGAGCTACAGGACATTGAAAATGAAGAGAAGATCCC
+HWI-EAS443:3:77:1708:994#0/1
aaa_¥aaZQ_^U_¥_^[_^`¥Z`^_aa__a^^^Xa_VGU¥
@HWI-EAS443:3:77:1721:1259#0/1
GGCATGGAAATGAGGCGCTGCCTTCTTCAGTCTCTTCAGC
```

+HWI-EAS443:3:77:1721:1259#0/1
abaaabb`aaaaaaaaa`a`_`aa_aa^¥a_Y`_`]Z^^W
@HWI-EAS443:3:77:1723:1262#0/1
ATCTTCTCCTATTTCATCAAGTTCTATCCAGCTTGAATCA
+HWI-EAS443:3:77:1723:1262#0/1
_S_Vaa¥]`¥]aaXW_aYaGXa]`_W_`Xaa]`_a]]_V¥
@HWI-EAS443:3:77:26:1520#0/1
GCTCAATGCATCCTGTGCAGCCATGGATGACTTTCAGCTG
+HWI-EAS443:3:77:26:1520#0/1
[babbbabbbbbaa_[abb]`ab`_``_]]``]_aa]aX]
@HWI-EAS443:3:77:329:50#0/1
GGGGGCTATATGAAGCAATCGAGGAGTGCCTGATTAATGA
+HWI-EAS443:3:77:329:50#0/1
aaa`a``_a^`a_^_Y¥^[^]U_^R]W^VV^_VZVV[U]Y
@HWI-EAS443:3:77:330:713#0/1
GGGCACTCGGTGAAAATATGGCACCAGAAAAAGTAGACTT
+HWI-EAS443:3:77:330:713#0/1
ababba_b^`Gb`a^V`aa``ab`aaa_Y___`Ua`_^``
@HWI-EAS443:3:77:375:663#0/1
GGGCACTCGGTGAAAATATGGCACCAGAAAAAGTAGACTT
+HWI-EAS443:3:77:375:663#0/1
Z^`b``abaa`aXZ[`baababbba_a]bbaaa[abaaaa
@HWI-EAS443:3:77:37:1317#0/1
GGGGCATGGAAATGAGGCGCTGCCTTCTTCAGTCTCTTCA
+HWI-EAS443:3:77:37:1317#0/1
abababa_``bba`bbabbbaab`a`aaabaaW``a[UZa
@HWI-EAS443:3:77:389:447#0/1
GGTGTCGTCCTCAAGAATGGTTCAATTTTGGCGTTCACTT
+HWI-EAS443:3:77:389:447#0/1
ab¥b_bbaa_``aa`^`^Z_X__WQ^Ya_UW^`[USTR[]
@HWI-EAS443:3:77:396:802#0/1
GGACAACCTGGAACCTGGAACCTTCGATCTTGGGGGGCTA
+HWI-EAS443:3:77:396:802#0/1
aba`__baa_Waaaa`a]Xaaa_aaa__`_`_M¥Z__^__
@HWI-EAS443:3:77:418:245#0/1
ACTGTTTAAGGTCTCCAACATCTTTGCAGTCATCAAAGTC
+HWI-EAS443:3:77:418:245#0/1
[GW¥^¥]¥_aYOM¥]X]^a`_Z¥^```_aZGM¥U`]MRSV
@HWI-EAS443:3:77:446:1651#0/1
AGAGCCTTCCTCCACTCCCCTGGGTGACTCTCCGATTGGC
+HWI-EAS443:3:77:446:1651#0/1
a`a_`a`^aa¥aaa`]`a^_¥¥SWU`^W]_¥^`^SS¥SWU
@HWI-EAS443:3:77:494:1585#0/1
GCTGGAAAGAGCCTAACATAGTCAAACCACATGAGAAAGG
+HWI-EAS443:3:77:494:1585#0/1
`ba`abbbaba`aa]abbbba`abbbaaaaba`a`a`aa`
@HWI-EAS443:3:77:51:804#0/1
AGAGACCGAATCATGGCCTGGACAGTGGTGAACAGTATAT
+HWI-EAS443:3:77:51:804#0/1
a_a`___[V`Z¥_¥_WW`¥_Y¥^W_QW¥N¥UW¥W¥SYWWU

```
@HWI-EAS443:3:77:528:613#0/1
TGGGGCATGGAAATGAGGCGCTGCCTTCTTCAGTCTCTTC
+HWI-EAS443:3:77:528:613#0/1
`bbaa`bababb_bbbbababa`baaaaaaa`_^``_`aa
@HWI-EAS443:3:77:605:1202#0/1
AGACATTCTTTGGCTGGAAAGAGCCTAACATAGTCAAACC
+HWI-EAS443:3:77:605:1202#0/1
aabaaaabbaaa_baaaa`a`aa__aaa`_a`a_`aa_`_
@HWI-EAS443:3:77:661:1821#0/1
AGATGGCCACCAAAGCGGACTACACCCTTGACGAAGAGAG
+HWI-EAS443:3:77:661:1821#0/1
aaaaa`abbbbbaaaba`aa`bbbb`a_[_b^_^a_a__Z
@HWI-EAS443:3:77:665:26#0/1
CCCGAATGCACTATTGAAGCACCGATTTGAGATAATTGAA
+HWI-EAS443:3:77:665:26#0/1
`bbbabbbbabbbbbbbbbabbababbbb^baaaababab
@HWI-EAS443:3:77:687:690#0/1
ACATAATGAAGGGAGTGTACATAAATACGGCCTTGCTCAA
+HWI-EAS443:3:77:687:690#0/1
a^Ub_W^T[XR_Q]UJWZ^aab]aZU^aba_aaa¥a_Yaa
@HWI-EAS443:3:77:732:1368#0/1
ACACCCTTGACGAAGAGAGCAGGGCAAGAATCAAAACTAG
+HWI-EAS443:3:77:732:1368#0/1
aabaabaa`ba_a`_a__`_a`_¥a`_`_`^_``^a`aa]
@HWI-EAS443:3:77:75:930#0/1
GAACGGGGTGAATCAATAATTGTAGAATCTGGTGACCCGA
+HWI-EAS443:3:77:75:930#0/1
aa_baaaa_bb`aba`a^]``a`a`a^^`_a]Y`a``a``
@HWI-EAS443:3:77:763:265#0/1
AAGAATTCCTTGGTCATGTCTTTCTCTTTGACAGAAGACT
+HWI-EAS443:3:77:763:265#0/1
a`aaabb^abab`T`aab_abaaaa^aaaa__aaa_``^a
@HWI-EAS443:3:77:772:673#0/1
CGACGAACGGGGTGAATCAATAATTGTAGAATCTGGTGAC
+HWI-EAS443:3:77:772:673#0/1
a]bbbab``abb_b_bab`Za_bbabaab]`U`bbbYb`b
@HWI-EAS443:3:77:782:1853#0/1
CTGGAGAAGTTCGGTGGGAGACTTTGGTCGGCAAGCTTGC
+HWI-EAS443:3:77:782:1853#0/1
a```bbaabbb`a`Y[Uaa`aabaab`W^]¥Xbb`]ZQS`
@HWI-EAS443:3:77:813:139#0/1
CTTGGGGGGCTATATGAAGCAATCGAGGAGTGCCTGATTA
+HWI-EAS443:3:77:813:139#0/1
ab`XO_aY_bba`aabSa^T[b`Xa_bba_XaaX_a__]_
@HWI-EAS443:3:77:834:1694#0/1
TTCGGATTTCCATTTCATCGACGAACGGGGTGAATCAATA
+HWI-EAS443:3:77:834:1694#0/1
]¥X¥Zbbaab_abXaabU¥Y]^J[bWM_a`_V`_ZPaa``
@HWI-EAS443:3:77:955:1740#0/1
GCCGAAAACCCCTCAAGTTGTGGAGACGCATATAGACTGT
```

+HWI-EAS443:3:77:955:1740#0/1
aaZ^X¥¥¥]GOa_]aYb]]`_]aa`]TY¥^`]^``_]^]G
@HWI-EAS443:3:78:1036:1642#0/1
ATCCCAATTACCTCATGGCTTGGAAGCAGGTGCTAGCAGA
+HWI-EAS443:3:78:1036:1642#0/1
`a`[a_aaaa^a``a__[`a]OO^`NX^ZUXTNZUVVXVU
@HWI-EAS443:3:78:108:1867#0/1
AAGAATTCCTTGGTCATGTCTTTCTCTTTGACAGAAGACT
+HWI-EAS443:3:78:108:1867#0/1
Y`]Z`aaaaa`_`XaaaaNa`__a_a_^`_]aa_a`X`a^
@HWI-EAS443:3:78:1090:966#0/1
GCTTGCTAGAGATCTGGGCTCTGGCTCATCACTGTCATAC
+HWI-EAS443:3:78:1090:966#0/1
a^X[WMO¥`[ZXPQY`a^X]X]X]]VWU[R_U]]UU^YZY
@HWI-EAS443:3:78:1094:1571#0/1
AATCCCAATTACCTCATGGCTTGGAAGCAGGTGCTAGCAG
+HWI-EAS443:3:78:1094:1571#0/1
aaa`[`_a`a`_`_a`^^Za_a_WPU_]MHWNUZUUVLXZ
@HWI-EAS443:3:78:1135:1066#0/1
GTCTCTTCAGCAGATTGAGAGCATGATTGAGGCCGAGTCT
+HWI-EAS443:3:78:1135:1066#0/1
``aa¥aa``a[aa^_^a_a¥a^a^`a_^_]a_[GHRSSBB
@HWI-EAS443:3:78:1214:649#0/1
ATTCATTTTGGACCCAGCTTGCTAGAGATCTGGGCTCTGG
+HWI-EAS443:3:78:1214:649#0/1
aa^`]aaa```a[^[`a_aZVHPY¥T[_^VV[[UY`][VY
@HWI-EAS443:3:78:1235:1344#0/1
CTCGATTGCTTCATATAGCCCCCCAAGATCGAAGGTTCCA
+HWI-EAS443:3:78:1235:1344#0/1
abb_R_Za`a`abbaab`X`T^^_`aaab¥aabaU]^]^`
@HWI-EAS443:3:78:124:1143#0/1
TCGGCTGAATCGAGAAAATTGCTTCTCATTGTTCAGGCAC
+HWI-EAS443:3:78:124:1143#0/1
aa`a`aa```bZX_¥aaaa`a`aaa``[aaaW_aa`_X_^
@HWI-EAS443:3:78:1275:807#0/1
AACAATGAGAAGCAATTTTCTCGATTCAGCCGAAAACCCC
+HWI-EAS443:3:78:1275:807#0/1
aa^TSbb_bT^`¥OHR^X_aa]]YX`OFVL^^[QYU[T[W
@HWI-EAS443:3:78:134:989#0/1
TGTGAATTGACTGATTCAAGCTGGATAGAACTTGATGAAA
+HWI-EAS443:3:78:134:989#0/1
abXa``aaaaaaaaaaaa^aaaa`^``a^a_a`a`[[¥`_
@HWI-EAS443:3:78:1384:418#0/1
TCCCAATTACCTCATGGCTTGGAAGCAGGTGCTAGCAGAG
+HWI-EAS443:3:78:1384:418#0/1
aZa^aaaaaa]`_a_`a_aa]_¥[YHSRRJUTR[QQBBBB
@HWI-EAS443:3:78:1537:1591#0/1
GAACAATGAGAAGCAATTTTCTCGATTCAGCCGAAAACCC
+HWI-EAS443:3:78:1537:1591#0/1
J¥a]_[bbbb`bUYbbbbb`Ibab_aaSb`T]`¥_abZPP

```
@HWI-EAS443:3:78:154:1278#0/1
TTCCGATTTGTTTTCAAAGAATTCCTTGGTCATGTCTTTC
+HWI-EAS443:3:78:154:1278#0/1
abab]O^aa`^aa`aaaa^Z`aa_^aaa^]a_aa^aa^U`
@HWI-EAS443:3:78:1567:218#0/1
ATCGGAGAGTCACCCAGGGGAGTGGAGGAAGGCTCTATTG
+HWI-EAS443:3:78:1567:218#0/1
a`Z`bab^aYYaMYTU`aaaG¥U][G^_PW`TE[UVVXBB
@HWI-EAS443:3:78:1570:1295#0/1
CTGCTGTAAAATAGTTCCTCCTCATGCTTGCGATATGTTC
+HWI-EAS443:3:78:1570:1295#0/1
aabZZaaabbaaaaZaaa_aXTZ_`a^a_`_a`[`]_P^^
@HWI-EAS443:3:78:1593:654#0/1
ACATGACCAAGGAATTCTTTGAAAACAAATCGGAAACATG
+HWI-EAS443:3:78:1593:654#0/1
`ZbaaaGaaaaaa`aa]a_ba[[__^_`]]]a``aaV_^`
@HWI-EAS443:3:78:1614:806#0/1
GGACTTCCCTCCGTGTTACTCCAATTTCAATGAACCGGTT
+HWI-EAS443:3:78:1614:806#0/1
aaa_ab_a`aaaa`baba]a_Za¥aaa^`^aaaaVZ_a¥`
@HWI-EAS443:3:78:1639:1784#0/1
AGCTGAAAGTCATCCATGGCTGCACAGGATGCATTGAGCA
+HWI-EAS443:3:78:1639:1784#0/1
_ZZa]]aa`aaaa_^a`a^¥^`N[OT¥Y[ZYNX_ZU[TNS
@HWI-EAS443:3:78:1645:1199#0/1
GGCATGGAAATGAGGCGCTGCCTTCTTCAGTCTCTTCAGC
+HWI-EAS443:3:78:1645:1199#0/1
aRF¥XXVQPZXaMR¥KX]UORTX`VPGRFZU¥¥^_U¥SPG
@HWI-EAS443:3:78:1659:965#0/1
GCAGGGCAAGAATCAAAACTAGGCTTTTCACTATAAGACA
+HWI-EAS443:3:78:1659:965#0/1
a`¥^¥^a]a`Ya`_aaaaY]]aVZa_``Y___a___YW^¥
@HWI-EAS443:3:78:1780:1936#0/1
AAAGAATTCCTTGGTCATGTCTTTCTCTTTGACAGAAGAC
+HWI-EAS443:3:78:1780:1936#0/1
aa¥b`K^]aW_[]aX]W^^NX_Y_S[G[YT[WX^_^`QQZ
@HWI-EAS443:3:78:216:804#0/1
GGCCAATCGGAGAGTCACCCAGGGGAGTGGAGGAAGGCTC
+HWI-EAS443:3:78:216:804#0/1
a`a`¥¥`]a¥^`]¥S¥^^][E¥]`[RYR¥TPPYSPXXNBB
@HWI-EAS443:3:78:219:1561#0/1
GCCTGAACAATGAGAAGCAATTTTCTCGATTCAGCCGAAA
+HWI-EAS443:3:78:219:1561#0/1
abbZW]ZZ¥a¥N[QZ_J][VV[a`a``¥[S_b]I``__a]
@HWI-EAS443:3:78:237:1434#0/1
ACATAATGAAGGGAGTGTACATAAATACGGCCTTGCTCAA
+HWI-EAS443:3:78:237:1434#0/1
`_XXQ_Z]]Q_a^L_Ra]W_ZYU`ZUZ`Z^^UP]XZX`_¥
@HWI-EAS443:3:78:238:1835#0/1
ATGAAATGGGGCATGGAAATGAGGCGCTGCCTTCTTCAGT
```

```
+HWI-EAS443:3:78:238:1835#0/1
a¥Q]NZSOU]Z^`Z]OW_¥`_a_Za[`Z^_^P[b_Z`¥LD
@HWI-EAS443:3:78:275:14#0/1
AAAGAGCCTAACATAGTCAAACCACATGAGAAAGGCATAA
+HWI-EAS443:3:78:275:14#0/1
X^ZaY^aa_¥_a]`^_M[¥S^`¥`_a^`¥`_Y__Z__`¥^
@HWI-EAS443:3:78:294:338#0/1
TAAATCCCAATTACCTCATGGCTTGGAAGCAGGTGCTAGC
+HWI-EAS443:3:78:294:338#0/1
abbaaaaaaaaba_aaaaaaa`ab``^a[[^V^NUYWOV]
@HWI-EAS443:3:78:295:1329#0/1
GACAACCTGGAACCTGGAACCTTCGATCTTGGGGGGCTAT
+HWI-EAS443:3:78:295:1329#0/1
a`bab`a`]`¥V_`^`]]aa``_a`___```^TZ[][___
@HWI-EAS443:3:78:323:1876#0/1
GGGTTTTCGGCTGAATCGAGAAAATTGCTTCTCATTGTTC
+HWI-EAS443:3:78:323:1876#0/1
]_`Xbbaaaab`_a`aR^a`a_baa___[a][aa`_UORQ
@HWI-EAS443:3:78:326:427#0/1
GGTGAACTTTGTAAGTATGGAGTTCTCACTCACTGACCCG
+HWI-EAS443:3:78:326:427#0/1
ab_`ababb``aba``bbbbbb_aaa^abaaabaaa`]Za
@HWI-EAS443:3:78:350:515#0/1
AAAATAGTTCCTCCTCATGCTTGCGATATGTTCAATCGGG
+HWI-EAS443:3:78:350:515#0/1
ab`a`aa]a`_`^_^_a[_^_`_^a^^^_`XZ^_^^^_Z]
@HWI-EAS443:3:78:393:247#0/1
AAAGAGCCTAACATAGTCAAACCACATGAGAAAGGCATAA
+HWI-EAS443:3:78:393:247#0/1
aabbababbbbaaaaaaabab``a_aaa````[a^aaaa`
@HWI-EAS443:3:78:453:256#0/1
AAATCCCAATTACCTCATGGCTTGGAAGCAGGTGCTAGCA
+HWI-EAS443:3:78:453:256#0/1
aaaaa``aabaa`aaa`]``_]`[XR¥TYMU_NZUWVZUV
@HWI-EAS443:3:78:484:213#0/1
ACTTTCCCAATAGAGCCTTCCTCCACTCCCCTGGGTGACT
+HWI-EAS443:3:78:484:213#0/1
T¥TMTGT_b`ZTPP]VNZNWSR¥¥¥RT`¥RYEY_RJYUXB
@HWI-EAS443:3:78:509:114#0/1
ATGCATCCTGTGCAGCCATGGATGACTTTCAGCTGATCCC
+HWI-EAS443:3:78:509:114#0/1
a`a^a`_aab^a_`a^`__`____]VY_]]__]]_Y]ZVZ
@HWI-EAS443:3:78:524:318#0/1
ATCGAAACTAACAAGTTTGCTGCAATATGCACACATTTGG
+HWI-EAS443:3:78:524:318#0/1
a`aa_aa`aa]`aaa__a__^a^^^_`_`_`^__]_^^__
@HWI-EAS443:3:78:590:1909#0/1
CTTGGGGGGCTATATGAAGCAATCGAGGAGTGCCTGATTA
+HWI-EAS443:3:78:590:1909#0/1
aaa_VSa¥^b^babab^]¥aa`¥ZU`a_]aY`a`[____^
```

```
@HWI-EAS443:3:78:620:661#0/1
AACTTCTCCAGCCTTGAAAACTTTAGAGCCTATGTAGATG
+HWI-EAS443:3:78:620:661#0/1
_XZUPF[`[]V¥XWYZ]aU]`[S_[¥Za]Y^P]aVZaYTa
@HWI-EAS443:3:78:755:1144#0/1
CAGATCTCTAGCAAGCTGGGTCCAAAATGAATTCAATAAG
+HWI-EAS443:3:78:755:1144#0/1
][[]]]aba^]__aa`_XXUY`Y]]]Y_aVJ[X]_Z[YYZ
@HWI-EAS443:3:78:783:305#0/1
TGCCCCGATTGAACATATCGCAAGCATGAGGAGGAACTAT
+HWI-EAS443:3:78:783:305#0/1
aa`aaaaa_`aabaa`b``a`__a]__`]``_a`__]^_`
@HWI-EAS443:3:78:816:78#0/1
AGCCAATTGAAGTGGGCACTCGGTGAAAATATGGCACCAG
+HWI-EAS443:3:78:816:78#0/1
ab`_aabba`aaZ`ab_`Xa^a`Wa]___^^aaaWZRSV`
@HWI-EAS443:3:78:818:579#0/1
TTCCGATTTGTTTTCAAAGAATTCCTTGGTCATGTCTTTC
+HWI-EAS443:3:78:818:579#0/1
aaa`a^aaaa^aa_]a^_`[Z`a]]a`a_^¥^aa_¥`_]¥
@HWI-EAS443:3:78:820:79#0/1
CTGCACAGGATGCATTGAGCAAGGCCGTATTTATGTACAC
+HWI-EAS443:3:78:820:79#0/1
a_Y_`aaaa[`a¥````Y]_`_``XY]VU¥__]``XYTVN
@HWI-EAS443:3:78:852:1088#0/1
ATCCGAAAATCGAAACTAACAAGTTTGCTGCAATATGCAC
+HWI-EAS443:3:78:852:1088#0/1
a`a``aaa^aaa_[`]`a__`^YU`__``a^]Z__`^Z^^
@HWI-EAS443:3:78:890:340#0/1
CTTGGTCATGTCTTTCTCTTTGACAGAAGACTCGGCCTCA
+HWI-EAS443:3:78:890:340#0/1
``bb_T¥ba`X_a_aVa_b`ba_S^bbaba``abbY]_X`
@HWI-EAS443:3:78:951:240#0/1
GGGGTTTTCGGCTGAATCGAGAAAATTGCTTCTCATTGTT
+HWI-EAS443:3:78:951:240#0/1
aaabTaa`Y``a`a___Z]^`^^]__[¥Z^____^^__Y^
@HWI-EAS443:3:78:965:944#0/1
CATGGAAATGAGGCGCTGCCTTCTTCAGTCTCTTCAGCAG
+HWI-EAS443:3:78:965:944#0/1
^aaaba[bbbbbbbaaab``abaaa`bb__a]ba]`aa`b
@HWI-EAS443:3:78:999:461#0/1
GGAGTCTATGGGATTCCTTTCGTCAGTCCGAAAGAGGCGA
+HWI-EAS443:3:78:999:461#0/1
_a]babb[a_¥b¥`[V¥b^^U¥¥`aaWS__W_¥aZ`a]a]
@HWI-EAS443:3:79:1036:1076#0/1
AGCGGACTACACCCTTGACGAAGAGAGCAGGGCAAGAATC
+HWI-EAS443:3:79:1036:1076#0/1
a`aaa``_aaaaaaaaaa`aaaaa`_`aaaTaa``_^_a_
@HWI-EAS443:3:79:1086:551#0/1
GTTTCCGATTTGTTTTCAAAGAATTCCTTGGTCATGTCTT
```

+HWI-EAS443:3:79:1086:551#0/1
a_aa```_Z`aa^aaa^aa_``_aa__aaa_^a_a_^Waa
@HWI-EAS443:3:79:1172:945#0/1
GCTTGCTAGAGATCTGGGCTCTGGCTCATCACTGTCATAC
+HWI-EAS443:3:79:1172:945#0/1
abbaa``^baa_aaaaaaaaa`aaaaaa`_a^aa`Yaa`_
@HWI-EAS443:3:79:1237:987#0/1
CGATTGGCCATGTTTCCGATTTGTTTTCAAAGAATTCCTT
+HWI-EAS443:3:79:1237:987#0/1
abbbbbbbab`_abbaaa_abbb`bbb_H_aab_b`_^bb
@HWI-EAS443:3:79:1238:1630#0/1
GAAAGGCATAAATCCCAATTACCTCATGGCTTGGAAGCAG
+HWI-EAS443:3:79:1238:1630#0/1
aUY`babaab`¥baabab]ab`Y``aaa`V¥a``_]V[__
@HWI-EAS443:3:79:1397:717#0/1
ATCGAAACTAACAAGTTTGCTGCAATATGCACACATTTGG
+HWI-EAS443:3:79:1397:717#0/1
aa_aaab^aa`Sa`Xa`__Z_`P__`_^^¥^XYU^_a_`_
@HWI-EAS443:3:79:1409:980#0/1
ACCGGTTCTCTTTGTAATCATACAAATCAGGAAGAAATTT
+HWI-EAS443:3:79:1409:980#0/1
a_``_a__Y^^]ab_aaa`a¥Z]aaabV`_WW^X^TX`a`
@HWI-EAS443:3:79:141:613#0/1
AAATGAATTCAATAAGGCATGTGAATTGACTGATTCAAGC
+HWI-EAS443:3:79:141:613#0/1
_abaaa]aaaaa`a`a_a_aa^a^Z``__]`aY_]¥`]``
@HWI-EAS443:3:79:1428:1139#0/1
AGAATATGGGGAAGATCCGAAAATCGAAACTAACAAGTTT
+HWI-EAS443:3:79:1428:1139#0/1
¥a¥¥`aa`aba]V[a`Y`b``^aa`b[ZZY``][aYVSa`
@HWI-EAS443:3:79:1557:1443#0/1
GGCCATTTCTTGTCTTATAGTGAAAAGCCTAGTTTTGATT
+HWI-EAS443:3:79:1557:1443#0/1
`]]a_a^aZ^a`[a^aZaaa_¥__``a¥]a_a_Z_Xa[a`
@HWI-EAS443:3:79:1579:314#0/1
AATATGGGGAAGATCCGAAAATCGAAACTAACAAGTTTGC
+HWI-EAS443:3:79:1579:314#0/1
abbb`baZ[aabb_GZbbbbaaZa`aa_aaa`b_bZ`¥`^
@HWI-EAS443:3:79:1660:858#0/1
GAATTCAATAAGGCATGTGAATTGACTGATTCAAGCTGGA
+HWI-EAS443:3:79:1660:858#0/1
``bbbbbabbbab`abaaa_abbaa_baaba`b_bPaaaa
@HWI-EAS443:3:79:1662:1330#0/1
TTTCGGCTGAATCGAGAAAATTGCTTCTCATTGTTCAGGC
+HWI-EAS443:3:79:1662:1330#0/1
aba`aa_a]ba_[a^aZ_`_aa^`a_¥`_¥`^¥UaSVR_U
@HWI-EAS443:3:79:1702:1904#0/1
ACCAGAAAAAGTAGACTTTGATGACTGCAAAGATGTTGGA
+HWI-EAS443:3:79:1702:1904#0/1
[Y^a^^baaabNaaa_aaa__¥¥`aaa_aaWaa_aW^aa_

@HWI-EAS443:3:79:171:1643#0/1
GATGAAATGGGGCATGGAAATGAGGCGCTGCCTTCTTCAG
+HWI-EAS443:3:79:171:1643#0/1
aaZ^ZQ_¥a`aaba`^^]YX¥]`WN]¥a¥[`^[[^[^Y]Z
@HWI-EAS443:3:79:1739:1691#0/1
GGGGGCTATATGAAGCAATCGAGGAGTGCCTGATTAATGA
+HWI-EAS443:3:79:1739:1691#0/1
_bbbbabaabbb```ab`a`baaaaa]aaU_a_aaa``_`
@HWI-EAS443:3:79:1758:168#0/1
GGGGCATGGAAATGAGGCGCTGCCTTCTTCAGTCTCTTCA
+HWI-EAS443:3:79:1758:168#0/1
S_PT[_bbb_aaaba`aa`Ua[]b^K_bbU`bb^babaZ_
@HWI-EAS443:3:79:1780:588#0/1
AATCAATAATTGTAGAATCTGGTGACCCGAATGCACTATT
+HWI-EAS443:3:79:1780:588#0/1
ab]aabb^abba¥baaaaa__][[`]__a`^^_^]Z__a`
@HWI-EAS443:3:79:205:306#0/1
GGGCACTCGGTGAAAATATGGCACCAGAAAAAGTAGACTT
+HWI-EAS443:3:79:205:306#0/1
abbbbbaab¥VaaaaaaaaaZ^]_aa`]X``aaW``_]_^
@HWI-EAS443:3:79:318:1068#0/1
GTTTCCGATTTGTTTTCAAAGAATTCCTTGGTCATGTCTT
+HWI-EAS443:3:79:318:1068#0/1
a[a`ab¥P¥¥`aU]a]aZT]VSZ^^¥`¥[`_W`_¥^YY¥W
@HWI-EAS443:3:79:336:1457#0/1
TGTGAATTGACTGATTCAAGCTGGATAGAACTTGATGAAA
+HWI-EAS443:3:79:336:1457#0/1
ab]a_abaa`aaabaaaa``aa¥a[aa`aa_aa``aa`_`
@HWI-EAS443:3:79:354:1766#0/1
ATTTTCACCGAGTGCCCACTTCAATTGGCTTGTTCTCTTC
+HWI-EAS443:3:79:354:1766#0/1
Z¥VWRZ`aa^Z[FYZ]U]__^_X]VRXZ`BBBBBBBBBBB
@HWI-EAS443:3:79:409:1172#0/1
TGTGGTTTGACTATGTTAGGCTCTTTCCAGCCAAAGAATG
+HWI-EAS443:3:79:409:1172#0/1
ab`aa`aaaaabaa`a`[aaa_`a`a`a`__[O¥a`¥VXY
@HWI-EAS443:3:79:409:1197#0/1
GGGGCATGGAAATGAGGCGCTGCCTTCTTCAGTCTCTTCA
+HWI-EAS443:3:79:409:1197#0/1
`^]_aW¥b[JTX[aa^T^¥__a^¥]a`^YUXTWV^[`ZU]
@HWI-EAS443:3:79:426:1600#0/1
GAGAACCGGTTCATTGAAATTGGAGTAACACGGAGGGAAG
+HWI-EAS443:3:79:426:1600#0/1
``aabbbbb¥^bb`aaa``b```abYX_baa``aaaaaa`
@HWI-EAS443:3:79:426:841#0/1
CCACTCCCCTGGGTGACTCTCCGATTGGCCATGTTTCCGA
+HWI-EAS443:3:79:426:841#0/1
ab`a[aaaaXX`_UaaZ[[^_]^¥^¥WR]VOM^V¥^TWXQ
@HWI-EAS443:3:79:432:399#0/1
CACCCTTGACGAAGAGAGCAGGGCAAGAATCAAAACTAGG

```
+HWI-EAS443:3:79:432:399#0/1
abaaaaaaaab`_a`a`a_`a_]_a`a`_a^`_`_^`_a]
@HWI-EAS443:3:79:43:1904#0/1
GGTTTGACTATGTTAGGCTCTTTCCAGCCAAAGAATGTCT
+HWI-EAS443:3:79:43:1904#0/1
`¥L¥_WM``aa]M[^_]`]__^aa^^[aaaaaa`a`^QZ¥
@HWI-EAS443:3:79:442:1282#0/1
GGTTTTCGGCTGAATCGAGAAAATTGCTTCTCATTGTTCA
+HWI-EAS443:3:79:442:1282#0/1
][Rbbbba`a___b_aZQ^^a¥a``^aaaab_X`^^``^`
@HWI-EAS443:3:79:465:1483#0/1
TGGTGCCATATTTTCACCGAGTGCCCACTTCAATTGGCTT
+HWI-EAS443:3:79:465:1483#0/1
a^PYb]H^¥R¥]`]]bb`¥]]V_abaZa``b^YZZZZ¥ZU
@HWI-EAS443:3:79:522:1869#0/1
ATAAAAGGAAGGTCTCATTTGAGAAATGATACTGATGTGG
+HWI-EAS443:3:79:522:1869#0/1
_VYZK^YOU¥W[Y^aa`a`_`V¥X^`_```a``Wa_`]UY
@HWI-EAS443:3:79:56:758#0/1
AAAGAATTCCTTGGTCATGTCTTTCTCTTTGACAGAAGAC
+HWI-EAS443:3:79:56:758#0/1
aY`_TU`aa`aaa_]aaa`Y```___a``a`Sb]^ZZX[]
@HWI-EAS443:3:79:596:1023#0/1
TACTGTTTAAGGTCTCCAACATCTTTGCAGTCATCAAAGT
+HWI-EAS443:3:79:596:1023#0/1
`¥`^a]aa]]¥^S]`]_TX__aa`_^aa`^^`^`_aa[[¥
@HWI-EAS443:3:79:640:1100#0/1
GTCCTTGGGATCTTCTCTTCATTTTCAATGTCCTGTAGCT
+HWI-EAS443:3:79:640:1100#0/1
aYa_QW[OTRYa_^[W[X[TTQYP^^_[_]Za^YXM^¥`R
@HWI-EAS443:3:79:664:1343#0/1
ATCATTAATCAGGCACTCCTCGATTGCTTCATATAGCCCC
+HWI-EAS443:3:79:664:1343#0/1
]bbb]aaaabaaabaaabaaa_aa^aa_aaaa``a¥[`_a
@HWI-EAS443:3:79:664:854#0/1
GGGGGCTATATGAAGCAATCGAGGAGTGCCTGATTAATGA
+HWI-EAS443:3:79:664:854#0/1
aa_baaabababaaaaaabaa`aa`a_a_a_`aaa^a_`_a_
@HWI-EAS443:3:79:677:1449#0/1
CATGGGCCTCGACACTTGGCCTATCGCAGTCCTCAAGAGC
+HWI-EAS443:3:79:677:1449#0/1
[a^¥]^ab_`_¥a¥_]_``abababababa]`aa]a``_X_b
@HWI-EAS443:3:79:753:1913#0/1
CACCACTGTCCAGGCCATGATTCGGTCTCTTCCTTCAATT
+HWI-EAS443:3:79:753:1913#0/1
a_aaUaaa¥baa`aaba[NVS]_[YGVLR[_]]J]¥¥Y[¥
@HWI-EAS443:3:79:861:868#0/1
CCCCGTTCGTCGATGAAATGGAAATCCGAATACATGAAAC
+HWI-EAS443:3:79:861:868#0/1
aba``X_]ZHY¥^_[]^^]_Z__^_]¥_]¥Q_``[V[]^¥
```

```
@HWI-EAS443:3:79:865:1772#0/1
CAAGGCCGTATTTATGTACACTCCCTTCATTATGTATTCA
+HWI-EAS443:3:79:865:1772#0/1
Yb_Q]`b[Rbb_P`]YXS_]^^_aaR^X`a_T]__[_^aU
@HWI-EAS443:3:79:950:1340#0/1
ACGAGGGGGAGGGAATACCACTATATGATGCAATCAAATG
+HWI-EAS443:3:79:950:1340#0/1
_Y`_UQVSV]X_``aP¥_a]_P¥]`_ZWU^¥¥^`__X¥ZU
@HWI-EAS443:3:79:968:1942#0/1
AGAGCCTTCCTCCACTCCCCTGGGTGACTCTCCGATTGGC
+HWI-EAS443:3:79:968:1942#0/1
aaaaaaaaaaa¥`a_`aaa_`a^^[_a¥``^[_Y`¥¥¥W_
@HWI-EAS443:3:7:1006:765#0/1
CATGGAAATGAGGCGCTGCCTTCTTCAGTCTCTTCAGCAG
+HWI-EAS443:3:7:1006:765#0/1
abbabaaa`aaab^bbaa¥^a`Waa]``^_]]a_WN_Y_a
@HWI-EAS443:3:7:1020:1645#0/1
GAATACCACTATATGATGCAATCAAATGCATGAAGACATT
+HWI-EAS443:3:7:1020:1645#0/1
a]a`ZP]_]aaaa]``]a`aY^__`¥aa__^a`__^¥_]_
@HWI-EAS443:3:7:103:1939#0/1
GGACACTTCTGCTGTAAAATAGTTCCTCCTCATGCTTGCG
+HWI-EAS443:3:7:103:1939#0/1
^a[`S[¥H¥_^¥b`b¥¥abab]Z¥`_aa]W]a[_]Ua]¥a
@HWI-EAS443:3:7:104:823#0/1
AGCCAACAAAATAAAATCTGAGAAGACACACATTCACATC
+HWI-EAS443:3:7:104:823#0/1
[abbabab^a_ab`^_aaaa_`^_a```_aYU`aaaaaa`
@HWI-EAS443:3:7:1222:1885#0/1
CAATAATTGTAGAATCTGGTGACCCGAATGCACTATTGAA
+HWI-EAS443:3:7:1222:1885#0/1
abbbaabbbSabb`_babaWabaURaa`a`R^`ab_`[aa
@HWI-EAS443:3:7:1229:527#0/1
CCTGGGTGACTCTCCGATTGGCCATGTTTCCGATTTGTTT
+HWI-EAS443:3:7:1229:527#0/1
Z_[a_¥`a_¥¥^^^Va`Y[a__a`^_X_a^[_Uaa]_H¥_
@HWI-EAS443:3:7:1333:1235#0/1
TGGGGGGCTATATGAAGCAATCGAGGAGTGCCTGATTAAT
+HWI-EAS443:3:7:1333:1235#0/1
aaa`aa``aaaaaa_``_aaa]______¥`SW__¥_`^_`
@HWI-EAS443:3:7:1356:700#0/1
ATGTATTCAGTAGCCCTGCAGTGGGACACTTCTGCTGTAA
+HWI-EAS443:3:7:1356:700#0/1
ab_Z_b_abbaaa]ZV`a_aa¥^^``Ua_a`V`aX_a]aa
@HWI-EAS443:3:7:1401:1050#0/1
AATCAGGCACTCCTCGATTGCTTCATATAGCCCCCCAAGA
+HWI-EAS443:3:7:1401:1050#0/1
aab`a`aba_aaaa`ba_aa_aaUaaa`a_YW[WV]_^_^
@HWI-EAS443:3:7:1402:1103#0/1
GAAAAAGTAGACTTTGATGACTGCAAAGATGTTGGAGACC
```

```
+HWI-EAS443:3:7:1402:1103#0/1
a``^a¥b`a_a_aaa``a`_^_aYa]_a__a_^`[W][ZT
@HWI-EAS443:3:7:1403:323#0/1
GGGGTTTTCGGCTGAATCGAGAAAATTGCTTCTCATTGTT
+HWI-EAS443:3:7:1403:323#0/1
^aab]]a[¥aa__¥RZZXYN]^RVVZW[LHV]^YG^^VBB
@HWI-EAS443:3:7:1448:975#0/1
AGTAGGAGTCTATGGGATTCCTTTCGTCAGTCCGAAAGAG
+HWI-EAS443:3:7:1448:975#0/1
a_S_bb_^¥ZaU_baa]ZZUU¥^a_a[X^`RZX^VW_`¥`
@HWI-EAS443:3:7:1477:1352#0/1
CATGGAAATGAGGCGCTGCCTTCTTCAGTCTCTTCAGCAG
+HWI-EAS443:3:7:1477:1352#0/1
abb`^a]^`aaba``bb_Va_^`aa^_aU^`]aa¥¥aW_a
@HWI-EAS443:3:7:149:192#0/1
CATGGAAATGAGGCGCTGCCTTCTTCAGTCTCTTCAGCAG
+HWI-EAS443:3:7:149:192#0/1
aa`a```a`aaaaaaaab_^a_`_`¥^¥¥¥_^_UQ¥`Q¥_
@HWI-EAS443:3:7:1505:1009#0/1
CAGATTTTGCCAGTAAGGTCCTGCACACTTTCCCAATAGA
+HWI-EAS443:3:7:1505:1009#0/1
abbbbbbbbaaabaabab``¥ba_aaaaab`_Y_a^aaaa
@HWI-EAS443:3:7:158:597#0/1
ATTTGTGTGGCTCCAGTCTCGGGTCAGTGAGTGAGAACTC
+HWI-EAS443:3:7:158:597#0/1
ababa`b]aab`a]V_L]TY^a`QUZUR]NVQ_XVP`HSZ
@HWI-EAS443:3:7:1742:442#0/1
CTTGGGGGGCTATATGAAGCAATCGAGGAGTGCCTGATTA
+HWI-EAS443:3:7:1742:442#0/1
Z_^`bbbb[abbba[baaaa[aa¥OQ]a[X^aVK[_]a`_
@HWI-EAS443:3:7:1746:693#0/1
GCATATTGCAGCAAACTTGTTAGTTTCGATTTTCGGATCT
+HWI-EAS443:3:7:1746:693#0/1
aa_`Z]__QVTX[^[M]`_Z`QVS[XQWL]WYWSMBBBBB
@HWI-EAS443:3:7:1776:102#0/1
GGGGGCTATATGAAGCAATCGAGGAGTGCCTGATTAATGA
+HWI-EAS443:3:7:1776:102#0/1
R_bb_T[a_b]baVbabba¥b`b][bVb[]b`ab`]Z_b`
@HWI-EAS443:3:7:1788:965#0/1
ACCGATTTGAGATAATTGAAGGAAGAGACCGAATCATGGC
+HWI-EAS443:3:7:1788:965#0/1
YVXZ_aabb_b_b`]aaaa`a]_a__^_Y]a]_^G_``BB
@HWI-EAS443:3:7:189:774#0/1
GGCATGGAAATGAGGCGCTGCCTTCTTCAGTCTCTTCAGC
+HWI-EAS443:3:7:189:774#0/1
abaaaaa^aaabaaaaaa]aa[aa_aa`a`W`a¥a]¥_^V
@HWI-EAS443:3:7:196:680#0/1
GGAAGATCCGAAAATCGAAACTAACAAGTTTGCTGCAATA
+HWI-EAS443:3:7:196:680#0/1
[[Y¥__a`_aY`bb_aa`_^aaa][`P_[Z^`_Xaaa]^]
```

```
@HWI-EAS443:3:7:252:1609#0/1
TTTCACTATAAGACAAGAAATGGCCAGTAGGAGTCTATGG
+HWI-EAS443:3:7:252:1609#0/1
abbbaaabbabbaabaaa_aaa_Oa`a¥a`aaa_^__aa^
@HWI-EAS443:3:7:366:908#0/1
AGAACACAGTATTTTTCCCATTTGTGTGGCTCCAGTCTCG
+HWI-EAS443:3:7:366:908#0/1
___bab^a`Zaaaa]a_`bbaaa^J_W``_]]YXWVaaBB
@HWI-EAS443:3:7:397:424#0/1
CACATGCCTTATTGAATTCATTTTGGACCCAGCTTGCTAG
+HWI-EAS443:3:7:397:424#0/1
ab]_`a`_aa[aa^¥_a`V^aW^a`__Y`¥`aZ_aa^a`a
@HWI-EAS443:3:7:415:1599#0/1
CCACAACTTGAGGGGTTTTCGGCTGAATCGAGAAAATTGC
+HWI-EAS443:3:7:415:1599#0/1
S_]b[KM`b^bbaaba`ab[b_ababa`Va_^^`SaW^^`
@HWI-EAS443:3:7:433:1964#0/1
CAATAATTGTAGAATCTGGTGACCCGAATGCACTATTGAA
+HWI-EAS443:3:7:433:1964#0/1
[`Xb^Va¥`ZJ_a]VT_b^]RTbaa`a]_^_^R__aJIaa
@HWI-EAS443:3:7:461:1286#0/1
GAGTGTACATAAATACGGCCTTGCTCAATGCATCCTGTGC
+HWI-EAS443:3:7:461:1286#0/1
ab[_bUaabbbbbabaaaa^aaaa``baaaaaaa]aZWT[
@HWI-EAS443:3:7:521:1800#0/1
TTTCACCGAGTGCCCACTTCAATTGGCTTGTTCTCTTCAT
+HWI-EAS443:3:7:521:1800#0/1
a]aabaa_[]a_Y`^`a`a^``[W_QV_TJF¥^]O]VFVV
@HWI-EAS443:3:7:52:526#0/1
GGCTTTTCACTATAAGACAAGAAATGGCCAGTAGGAGTCT
+HWI-EAS443:3:7:52:526#0/1
a`a_a]`^``_a___a_a__`[_^___]TR[FU__X^UR¥
@HWI-EAS443:3:7:61:167#0/1
ATCAGGCACTCCTCGATTGCTTCATATAGCCCCCCAAGAT
+HWI-EAS443:3:7:61:167#0/1
`ab`a_]aa^baaa`Y¥aa¥[¥Z^^U`_¥`^^^ZRYZZ¥W
@HWI-EAS443:3:7:65:226#0/1
GGGGCATGGAAATGAGGCGCTGCCTTCTTCAGTCTCTTCA
+HWI-EAS443:3:7:65:226#0/1
O__^_^_b_¥V]`ba`aa_aab_]`WT_a``¥VT]¥^VL^
@HWI-EAS443:3:7:70:584#0/1
GTGTACATAAATACGGCCTTGCTCAATGCATCCTGTGCAG
+HWI-EAS443:3:7:70:584#0/1
aVa_a`]``^a^a`_``^]_^^¥UX]¥¥¥_^TSUVNZSY^
@HWI-EAS443:3:7:713:736#0/1
GTCCAAAATGAATTCAATAAGGCATGTGAATTGACTGATT
+HWI-EAS443:3:7:713:736#0/1
aY_WT`aa``]Y]_R]V_R_`_TVU`Sa^O[J_`[__^¥X
@HWI-EAS443:3:7:754:1008#0/1
GCTTGCTAGAGATCTGGGCTCTGGCTCATCACTGTCATAC
```

+HWI-EAS443:3:7:754:1008#0/1
a_aaa_`_aaa[^_`aaa`a_^__]_¥`______^U_^_X
@HWI-EAS443:3:7:792:720#0/1
GGCCAGTAGGAGTCTATGGGATTCCTTTCGTCAGTCCGAA
+HWI-EAS443:3:7:792:720#0/1
abbabaabba`a__aaabaa_aa__aaa]`¥¥_`NW¥a__
@HWI-EAS443:3:7:869:1958#0/1
CGATAGGCCAAGTGTCGAGGCCCATGTTCCTATATGTGAG
+HWI-EAS443:3:7:869:1958#0/1
aaaab^babbbaa[YZbbbaa_^b__^a¥[`aaaZTKY¥B
@HWI-EAS443:3:80:1015:96#0/1
GTTCTCTTCATGTTCTTTGTCCTTGGGATCTTCTCTTCAT
+HWI-EAS443:3:80:1015:96#0/1
a¥a_a_aaaRb_`a_aaab¥`_aaa]¥Y`_`_]a¥a^Q[a
@HWI-EAS443:3:80:1016:1296#0/1
TTGGGGGGCTATATGAAGCAATCGAGGAGTGCCTGATTAA
+HWI-EAS443:3:80:1016:1296#0/1
_b^``abb_ba_``a`a`aaZ`_bXb[Q__bbaabZ]ba]
@HWI-EAS443:3:80:1079:1297#0/1
TAATCAGGCACTCCTCGATTGCTTCATATAGCCCCCCAAG
+HWI-EAS443:3:80:1079:1297#0/1
aab``b_`aa_``^_`a`___a`_```__a^]_]]]Z_¥_
@HWI-EAS443:3:80:1126:698#0/1
GGTGAATCAATAATTGTAGAATCTGGTGACCCGAATGCAC
+HWI-EAS443:3:80:1126:698#0/1
ab]a¥Y``¥]a^[^¥`XX`[__]L]^W^¥ZXS][[T^]RL
@HWI-EAS443:3:80:1170:1654#0/1
GAAGATCCCAAGGACAAAGAACATGAAGAGAACAAGCCAA
+HWI-EAS443:3:80:1170:1654#0/1
abbba`[[T]a_b[Vbbbba`^aabaVSbba_H¥`]VZ__
@HWI-EAS443:3:80:1186:302#0/1
ATGAGAAAGGCATAAATCCCAATTACCTCATGGCTTGGAA
+HWI-EAS443:3:80:1186:302#0/1
abaabaa_baaa`aaab^RY^aa`__]`````_]aaZ[SS
@HWI-EAS443:3:80:1246:422#0/1
GGCATAAATCCCAATTACCTCATGGCTTGGAAGCAGGTGC
+HWI-EAS443:3:80:1246:422#0/1
abaabababab^aaaabaa__a_aaa`]a_L^Ma[P¥T`SLV
@HWI-EAS443:3:80:1254:235#0/1
GTTTGTTTCATGTATTCGGATTTCCATTTCATCGACGAAC
+HWI-EAS443:3:80:1254:235#0/1
aaaaa_aa]aaa]_aa_aa^a``^^[`aaY_^^a_Y___V
@HWI-EAS443:3:80:125:331#0/1
CACTTTCCCAATAGAGCCTTCCTCCACTCCCCTGGGTGAC
+HWI-EAS443:3:80:125:331#0/1
a^a``Vaaabaa`_`_`^^¥^^^^^^¥Z`^]^¥[S[UVVU
@HWI-EAS443:3:80:1272:1087#0/1
CTTCTTTTGACATTTGGGAAAGCTTGCCCTCAATGCAGCC
+HWI-EAS443:3:80:1272:1087#0/1
aaZ¥```]bYY¥_b[`Taa`¥_Y¥ZZOZKaOW]b]L]NDD

```
@HWI-EAS443:3:80:1309:1950#0/1
CTGTAATCTCAAATTTTTCTTCAATTGTCTCTTCGCCTCT
+HWI-EAS443:3:80:1309:1950#0/1
a`b_b_baabbbba`bbbaabbbaaabaabbbabba`_aa
@HWI-EAS443:3:80:1316:1453#0/1
CCTTTCTCATGTGGTTTGACTATGTTAGGCTCTTTCCAGC
+HWI-EAS443:3:80:1316:1453#0/1
^_ba¥^babb`]`baab[¥`a`¥`aaaa_X_[[a`¥^aZF
@HWI-EAS443:3:80:1327:785#0/1
CTTCAATAGTGCATTCGGGTCACCAGATTCTACAATTATT
+HWI-EAS443:3:80:1327:785#0/1
`bbbbbbb`]b`baa^`bb_bbaaabaa^Z``aaabaabb
@HWI-EAS443:3:80:1341:1630#0/1
ATCAGGCACTCCTCGATTGCTTCATATAGCCCCCCAAGAT
+HWI-EAS443:3:80:1341:1630#0/1
J^[b`_`aa^¥GXY^^a`Q]a_aa`ba`ST_[aSNY]RV]
@HWI-EAS443:3:80:1493:239#0/1
GCTGGAAAGAGCCTAACATAGTCAAACCACATGAGAAAGG
+HWI-EAS443:3:80:1493:239#0/1
aaa^_a¥aaa`^X`_a^_a`a_Z_¥_R]`Z__a]a^Z^]^
@HWI-EAS443:3:80:1506:955#0/1
GAGAAAGGCATAAATCCCAATTACCTCATGGCTTGGAAGC
+HWI-EAS443:3:80:1506:955#0/1
aZ_`aaa``aabaaaa_`aabba[TWZ[a__^b_WX][UB
@HWI-EAS443:3:80:1527:518#0/1
GGGGTTTTCGGCTGAATCGAGAAAATTGCTTCTCATTGTT
+HWI-EAS443:3:80:1527:518#0/1
aaV¥DUbXU_``[]^]T]a_]aTa_`aJ[_aX`_Z_aV[]
@HWI-EAS443:3:80:1543:1463#0/1
ATTCGGATTTCCATTTCATCGACGAACGGGGTGAATCAAT
+HWI-EAS443:3:80:1543:1463#0/1
abbbbb_aaaba`ba`_a_aabaa[UR_aaa]a`aaXZ]a
@HWI-EAS443:3:80:1672:1255#0/1
CGATTGGCCATGTTTCCGATTTGTTTTCAAAGAATTCCTT
+HWI-EAS443:3:80:1672:1255#0/1
¥`_a`ba_abbb`bbabbbbbaaabbbabbbbbbbbb]ab
@HWI-EAS443:3:80:190:106#0/1
AATGGTTCAATTTTGGCGTTCACTTCTTTTGACATTTGGG
+HWI-EAS443:3:80:190:106#0/1
a^``_V_`__`__`_X¥`Y¥[SX^ZT[__]TWWUY]XMX]
@HWI-EAS443:3:80:20:1760#0/1
GGGGCATGGAAATGAGGCGCTGCCTTCTTCAGTCTCTTCA
+HWI-EAS443:3:80:20:1760#0/1
_S]X_[`ZXU]_]ZZY[```^]_a_YM[R[HYL]`^R[YL
@HWI-EAS443:3:80:295:217#0/1
ATCTACATAGGCTCTAAAGTTTTCAAGGCTGGAGAAGTTC
+HWI-EAS443:3:80:295:217#0/1
`ab^baaababa^a¥UUabS`^`¥[T[`Z^X_V¥RZWMUL
@HWI-EAS443:3:80:315:1018#0/1
TGGGGCATGGAAATGAGGCGCTGCCTTCTTCAGTCTCTTC
```

+HWI-EAS443:3:80:315:1018#0/1
``a`aaW`¥_``a_baaaabb`_``Z_]aaaa`]`a_^`^
@HWI-EAS443:3:80:362:397#0/1
AGGGGTTTTCGGCTGAATCGAGAAAATTGCTTCTCATTGT
+HWI-EAS443:3:80:362:397#0/1
ZabbbRUUSa_X_[`a^¥abb`_V`KZ[`V_``aZYZ]¥_
@HWI-EAS443:3:80:379:657#0/1
ATCAGGCAATCTGAGGGGGCGTGGTGTCGTCCTCAAGAAT
+HWI-EAS443:3:80:379:657#0/1
``aa[`_[[aa`V]Y[__`__^a_UXU__T¥¥^`^__`¥^
@HWI-EAS443:3:80:386:747#0/1
CATTAATCAGGCACTCCTCGATTGCTTCATATAGCCCCCC
+HWI-EAS443:3:80:386:747#0/1
¥_b_aa__]ba__Z[]_`_¥U_Z_]]aa^¥_]]ZYSWa``
@HWI-EAS443:3:80:431:511#0/1
AGGCAATCTGAGGGGGCGTGGTGTCGTCCTCAAGAATGGT
+HWI-EAS443:3:80:431:511#0/1
aa`¥]]]¥X]U^_Y]P_aH__W^T^TP_¥W[XN]a[[ZUU
@HWI-EAS443:3:80:43:946#0/1
CAAATGTAGGACCAAAGAAGGAAGACGGAAAACAAACCTG
+HWI-EAS443:3:80:43:946#0/1
aaYaaa]aa_aaaa`aa]]_^]^a¥^`___`__`__^^]`
@HWI-EAS443:3:80:440:661#0/1
GCATCCTGTGCAGCCATGGATGACTTTCAGCTGATCCCAA
+HWI-EAS443:3:80:440:661#0/1
a]UXba]O¥`]XZ¥¥`]Y_US]YZ_ZZV]__¥]SRX^T`[
@HWI-EAS443:3:80:486:1416#0/1
CATCGACGAACGGGGTGAATCAATAATTGTAGAATCTGGT
+HWI-EAS443:3:80:486:1416#0/1
]Q]a_]_¥¥_^XY_]K¥_ZV_WV_X]XW`K`XXZT_WX[]
@HWI-EAS443:3:80:565:52#0/1
AGAAATTTAGGCTTCTCTACCCCTGTTGTGTTACATATAC
+HWI-EAS443:3:80:565:52#0/1
abaaaaa`a`a``a¥a_`a]_^_[`X_`]^]_`ZX^__^^
@HWI-EAS443:3:80:577:1704#0/1
AATTTAGGCTTCTCTACCCCTGTTGTGTTACATATACTGT
+HWI-EAS443:3:80:577:1704#0/1
X[Ya_aaab_aa`a_a`Za^]aO_`I`]__]a`_XaU_[[
@HWI-EAS443:3:80:578:1040#0/1
TCCGAAAATCGAAACTAACAAGTTTGCTGCAATATGCACA
+HWI-EAS443:3:80:578:1040#0/1
aaaa_^bbaaa``_aa`aaaa`^``_``aaaa`aa_aa``
@HWI-EAS443:3:80:590:1492#0/1
GGGGGCTATATGAAGCAATCGAGGAGTGCCTGATTAATGA
+HWI-EAS443:3:80:590:1492#0/1
a_a``aaaabaa`a_bba`a_``a_aX_``aa_a`a`a_]
@HWI-EAS443:3:80:768:1658#0/1
GGGGCATGGAAATGAGGCGCTGCCTTCTTCAGTCTCTTCA
+HWI-EAS443:3:80:768:1658#0/1
aba_bbab`_babaa``a`baaaaa`__`_aZa_a__`_^

```
@HWI-EAS443:3:80:794:133#0/1
CATGTATTCGGATTTCCATTTCATCGACGAACGGGGTGAA
+HWI-EAS443:3:80:794:133#0/1
a``bLZb__aaU____`_````_^[a^Y`VS¥]^U_X^^]
@HWI-EAS443:3:80:814:912#0/1
CCTTGGGATCTTCTCTTCATTTTCAATGTCCTGTAGCTCT
+HWI-EAS443:3:80:814:912#0/1
K_a]`a_Naa`b^b]`a`bbbaaaaaa_`b_aaaa^aa``
@HWI-EAS443:3:80:843:206#0/1
AAATCCCAATTACCTCATGGCTTGGAAGCAGGTGCTAGCA
+HWI-EAS443:3:80:843:206#0/1
aaZ_aa^Z]a``][aa`^]Y]a^`_[_ZFVU¥BBBBBBBB
@HWI-EAS443:3:80:848:234#0/1
CTCCACAACTTGAGGGGTTTTCGGCTGAATCGAGAAAATT
+HWI-EAS443:3:80:848:234#0/1
aaaabaaaababaaaaa^aaaaaa_a`aa^Xa_a_a__`[
@HWI-EAS443:3:80:885:1309#0/1
ATTCGAGCCGAACGGCTGCATTGAGGGCAAGCTTTCCCAA
+HWI-EAS443:3:80:885:1309#0/1
aaaaaa`ba_a_`Z^`_a_aZYGY[_^ZVZQRRZ_ZXWZZ
@HWI-EAS443:3:80:891:1213#0/1
AGCAGGTGCTAGCAGAGCTACAGGACATTGAAAATGAAGA
+HWI-EAS443:3:80:891:1213#0/1
a`_`aaVW_aa¥`_]^a``_`a^Z]¥a_``^[W[]Z¥[]_
@HWI-EAS443:3:80:955:1819#0/1
CTCCCTTCATTATGTATTCAGTAGCCCTGCAGTGGGACAC
+HWI-EAS443:3:80:955:1819#0/1
ab`babbbaabba`abbbaaa^ba_^_aa_aa_a`__aa]
@HWI-EAS443:3:81:1036:1499#0/1
CATATTTTCACCGAGTGCCCACTTCAATTGGCTTGTTCTC
+HWI-EAS443:3:81:1036:1499#0/1
abbXabaaababb`a^baaa_a`baa_a`aa`a`S[[MRO
@HWI-EAS443:3:81:1037:1151#0/1
CCTTGGGATCTTCTCTTCATTTTCAATGTCCTGTAGCTCT
+HWI-EAS443:3:81:1037:1151#0/1
abaa¥¥^a_¥a_[`bbb¥^_`a`aaaaa`abbaa`^`ZaX
@HWI-EAS443:3:81:1049:987#0/1
GGTGAACTTTGTAAGTATGGAGTTCTCACTCACTGACCCG
+HWI-EAS443:3:81:1049:987#0/1
_]Z`aZG^^]^T[¥¥N`aaaaaaa`[X`_aaaaa__[R`a
@HWI-EAS443:3:81:1060:1853#0/1
CAGTATTTTTCCCATTTGTGTGGCTCCAGTCTCGGGTCAG
+HWI-EAS443:3:81:1060:1853#0/1
`b`^ab]abaaaaaaaaa]``¥F_``^^^¥Y¥[YX^NV]T
@HWI-EAS443:3:81:1062:973#0/1
GGCTTGTTCTCTTCATGTTCTTTGTCCTTGGGATCTTCTC
+HWI-EAS443:3:81:1062:973#0/1
aa_aaa_a_a^aa_aa^_``aaa^Z¥^`aaa`Z__aaWa^
@HWI-EAS443:3:81:107:1452#0/1
GGGGCATGGAAATGAGGCGCTGCCTTCTTCAGTCTCTTCA
```

````
+HWI-EAS443:3:81:107:1452#0/1
`X`_``]]]_^]__][Q^``^]``YXYY^^^XU^^^^¥^^
@HWI-EAS443:3:81:1093:1202#0/1
CAGAAAAAGTAGACTTTGATGACTGCAAAGATGTTGGAGA
+HWI-EAS443:3:81:1093:1202#0/1
aabab_`aa_`aaaaabaaba`a``a`a`aaa`_a_a`a]
@HWI-EAS443:3:81:1116:40#0/1
CATTCTTTGGCTGGAAAGAGCCTAACATAGTCAAACCACA
+HWI-EAS443:3:81:1116:40#0/1
Z¥``Za`_bbaab__^^a]``]^XaW`]`a`U^aaZ]Y]^
@HWI-EAS443:3:81:1133:1303#0/1
AACCTGGAACCTTCGATCTTGGGGGGCTATATGAAGCAAT
+HWI-EAS443:3:81:1133:1303#0/1
__¥_Xa[^a[^baba`aab_`a]__a``aaaab^]YX]¥a
@HWI-EAS443:3:81:113:87#0/1
GTCCAGGCCATGATTCGGTCTCTTCCTTCAATTATCTCAA
+HWI-EAS443:3:81:113:87#0/1
a_ab^aaaa`aa`__¥[`W¥`YN^WV`]^^[^¥Y[Q]]ZZ
@HWI-EAS443:3:81:1162:525#0/1
ATTAATCAGGCACTCCTCGATTGCTTCATATAGCCCCCCA
+HWI-EAS443:3:81:1162:525#0/1
[W``_ba`aR[_aaaaa_aa`_a_a[S`aaaa`^XT^a¥a
@HWI-EAS443:3:81:1216:1719#0/1
CGAAAATCGAAACTAACAAGTTTGCTGCAATATGCACACA
+HWI-EAS443:3:81:1216:1719#0/1
^aa¥bbaYbab_ZW`aY]a^T_^V_a^H]a_`ab__QRX]
@HWI-EAS443:3:81:127:1829#0/1
GGGGGCTATATGAAGCAATCGAGGAGTGCCTGATTAATGA
+HWI-EAS443:3:81:127:1829#0/1
^aaa```bbbbbbbbbbbbaa``aaa`_aa`a_a`ba_`a
@HWI-EAS443:3:81:1302:425#0/1
ATTAATCAGGCACTCCTCGATTGCTTCATATAGCCCCCCA
+HWI-EAS443:3:81:1302:425#0/1
abbbab_baa`b`b^_baaaaaa`aa_aaaaaaZ`V_V[a
@HWI-EAS443:3:81:1311:180#0/1
GGAAAGAGCCTAACATAGTCAAACCACATGAGAAAGGCAT
+HWI-EAS443:3:81:1311:180#0/1
a`¥a`]^`]]a_aV_aaa^^`__ZX_W]__^____^_U^]
@HWI-EAS443:3:81:1406:1708#0/1
AGGATGCATTGAGCAAGGCCGTATTTATGTACACTCCCTT
+HWI-EAS443:3:81:1406:1708#0/1
aa`a_a]ab`^aaY`b``^[a__```__X__R_O`^Q]__
@HWI-EAS443:3:81:1440:1889#0/1
CTGGGTCCAAAATGAATTCAATAAGGCATGTGAATTGACT
+HWI-EAS443:3:81:1440:1889#0/1
Ob_b_babbbbbb_bbbbbbbabbbaabbbb`bababa`a
@HWI-EAS443:3:81:1503:1428#0/1
GCAAGCTGGGTCCAAAATGAATTCAATAAGGCATGTGAAT
+HWI-EAS443:3:81:1503:1428#0/1
a__``aaa_a]][_]__aa__a_]^_^`_`QW_`aZ_]^^
````

```
@HWI-EAS443:3:81:151:694#0/1
CAGTCATCAAAGTCTACTTTTTCTGGTGCCATATTTTCAC
+HWI-EAS443:3:81:151:694#0/1
aaa]``V`_a`^P¥Z``aaX¥]`]¥`[RXa_ZJV^]__aa
@HWI-EAS443:3:81:1578:450#0/1
ATCGAAACTAACAAGTTTGCTGCAATATGCACACATTTGG
+HWI-EAS443:3:81:1578:450#0/1
a]V¥^``ab_ba`W^]baabaa¥`Ta`[aUa^a_aaaSR`
@HWI-EAS443:3:81:1629:412#0/1
AGAACTTGATGAAATAGGAGAAGATGTTGCCCCGATTGAA
+HWI-EAS443:3:81:1629:412#0/1
[aabX`ab`aba^¥b]^`a`a`b`aa_a_X¥_X__`a_¥a
@HWI-EAS443:3:81:1668:1455#0/1
ATTCGGATTTCCATTTCATCGACGAACGGGGTGAATCAAT
+HWI-EAS443:3:81:1668:1455#0/1
`abbbb]V_ab]S_Zaa``]aY_^X`Z`aa^Xa_a_Y^]_
@HWI-EAS443:3:81:1673:384#0/1
AGCAATTTTCTCGATTCAGCCGAAAACCCCTCAAGTTGTG
+HWI-EAS443:3:81:1673:384#0/1
aVL`_aaba¥[¥a``a^a^W_a`^^_XSJV_^^[aS__[_
@HWI-EAS443:3:81:170:513#0/1
ACCGATTTGAGATAATTGAAGGAAGAGACCGAATCATGGC
+HWI-EAS443:3:81:170:513#0/1
_aa`]a`ab^a^``aaa_]]aa_aa_a^`aa_Y^_¥^`_`
@HWI-EAS443:3:81:1711:774#0/1
GACATTCTTTGGCTGGAAAGAGCCTAACATAGTCAAACCA
+HWI-EAS443:3:81:1711:774#0/1
a]WX[ZO`VN`HOX[P]X`P]TOXT¥_Y]aYLSKYPRGRU
@HWI-EAS443:3:81:1726:767#0/1
ATTCAGTAGCCCTGCAGTGGGACACTTCTGCTGTAAAATA
+HWI-EAS443:3:81:1726:767#0/1
^W]TXa^^QL[a^V`^[XFX[]W][``^`aM`___^`¥_^
@HWI-EAS443:3:81:199:946#0/1
GGCATAAATCCCAATTACCTCATGGCTTGGAAGCAGGTGC
+HWI-EAS443:3:81:199:946#0/1
abbabbU[babbbbabbbbab_ababbbM]_`YZP_aN__
@HWI-EAS443:3:81:303:669#0/1
AGCTTTCCCAAATGTCAAAAGAAGTGAACGCCAAAATTGA
+HWI-EAS443:3:81:303:669#0/1
abaaaab`a^UY_aZba[_a`Z``V]aaaaa^]YPY[Z][
@HWI-EAS443:3:81:315:1237#0/1
TGGAGACCTTAAACAGTATGACAGTGATGAGCCAGAGCCC
+HWI-EAS443:3:81:315:1237#0/1
aaaaa¥¥baabaaaaa[]a^aaa`[__[^¥¥__QRU__`^
@HWI-EAS443:3:81:415:750#0/1
ACCGAACTTCTCCAGCCTTGAAAACTTTAGAGCCTATGTA
+HWI-EAS443:3:81:415:750#0/1
`ba]aaZab``aa`_`ba_aX]`WZ_aaaaaT]_a`aa][
@HWI-EAS443:3:81:426:1326#0/1
GGCTTGTTCTCTTCATGTTCTTTGTCCTTGGGATCTTCTC
```

```
+HWI-EAS443:3:81:426:1326#0/1
a_aaaa]ababbbaabaabaabaaaa``^Y]¥Y`^aaaa_
@HWI-EAS443:3:81:462:1059#0/1
GAGGGGTTTTCGGCTGAATCGAGAAAATTGCTTCTCATTG
+HWI-EAS443:3:81:462:1059#0/1
]QYa`ba_b`]_]`abaaaa]``Wa_R`¥_aaW]_ba]]V
@HWI-EAS443:3:81:470:1742#0/1
TGGGACACTTCTGCTGTAAAATAGTTCCTCCTCATGCTTG
+HWI-EAS443:3:81:470:1742#0/1
`^`_abbbaabbaaaa_bbbaa`]¥a`a`aaabaa`````
@HWI-EAS443:3:81:477:1448#0/1
GACCCGAATGCACTATTGAAGCACCGATTTGAGATAATTG
+HWI-EAS443:3:81:477:1448#0/1
¥`aa`aa_[``_`aaaa``_abb``^aaa``_^]]]]_¥^
@HWI-EAS443:3:81:641:411#0/1
ATGTCAAAAGAAGTGAACGCCAAAATTGAACCATTCTTGA
+HWI-EAS443:3:81:641:411#0/1
`^aZY^_bbaaa`[`bbaaaaaaaaa`a`^```aaa`¥a_
@HWI-EAS443:3:81:710:1652#0/1
CATTAATCAGGCACTCCTCGATTGCTTCATATAGCCCCCC
+HWI-EAS443:3:81:710:1652#0/1
_bZ_``_VZ_aY[a[[abaa`Qa^a`aaa^a_a]]R]]b[
@HWI-EAS443:3:81:718:554#0/1
ATGAGAAAGGCATAAATCCCAATTACCTCATGGCTTGGAA
+HWI-EAS443:3:81:718:554#0/1
^_a```^W¥¥__ababbaa^]Zabbab_aaaaa_a``a_`
@HWI-EAS443:3:81:727:770#0/1
ATCACTGTCATACTGTTTAAGGTCTCCAACATCTTTGCAG
+HWI-EAS443:3:81:727:770#0/1
X`]LTZ_`b^]aa`b[aaa`ba_a_`[`a`V`aY_Yb^]a
@HWI-EAS443:3:81:779:53#0/1
CACCCCGTTCGTCGATGAAATGGAAATCCGAATACATGAA
+HWI-EAS443:3:81:779:53#0/1
a_a]aba¥_a``_a^T^_`ZX`V¥`_`]__Z^_[]]]__T
@HWI-EAS443:3:81:841:385#0/1
CACAACTTGAGGGGTTTTCGGCTGAATCGAGAAAATTGCT
+HWI-EAS443:3:81:841:385#0/1
`b¥aa]ab`__bbbWa`aaabaaabaa`aaa_aaaaaaXa
@HWI-EAS443:3:81:844:744#0/1
TGGAGACCTTAAACAGTATGACAGTGATGAGCCAGAGCCC
+HWI-EAS443:3:81:844:744#0/1
a`aaaaaabb_a[`ab`abaaaaa^a_`]aa^a`^`a^^_
@HWI-EAS443:3:81:856:1532#0/1
ATCGACGAACGGGGTGAATCAATAATTGTAGAATCTGGTG
+HWI-EAS443:3:81:856:1532#0/1
a_ab``a`a_[_``Vaa_`__``^^`]`Wa```__]^^X¥
@HWI-EAS443:3:81:884:1595#0/1
GAACCGGTTCATTGAAATTGGAGTAACACGGAGGGAAGTC
+HWI-EAS443:3:81:884:1595#0/1
_`aa]]`]]]Zb]b_aabaa`Z^`bba`_aa`baaaa_^W
```

```
@HWI-EAS443:3:81:915:1754#0/1
ATCAAGTTCTATCCAGCTTGAATCAGTCAATTCACATGCC
+HWI-EAS443:3:81:915:1754#0/1
¥^[]¥UVUb`bbb``_]^VY_a^_[V[`aY_]YaTaN¥¥^
@HWI-EAS443:3:81:933:84#0/1
CGTCTCCACAACTTGAGGGGTTTTCGGCTGAATCGAGAAA
+HWI-EAS443:3:81:933:84#0/1
abbbb`a`aaa^bbaabbbb_bbaaaaaaaaa`_a_a`__
@HWI-EAS443:3:82:1055:1491#0/1
GGGATCTTCTCTTCATTTTCAATGTCCTGTAGCTCTGCTA
+HWI-EAS443:3:82:1055:1491#0/1
UbbZWUba_`bb^R¥bba```a`Y^__abVbaa_Va]`aa
@HWI-EAS443:3:82:1100:1377#0/1
TCCCAATTACCTCATGGCTTGGAAGCAGGTGCTAGCAGAG
+HWI-EAS443:3:82:1100:1377#0/1
abbb`Waa_][a¥XXXMU¥[Y[]]RJBBBBBBBBBBBBBB
@HWI-EAS443:3:82:1102:721#0/1
CCGAACTTCTCCAGCCTTGAAAACTTTAGAGCCTATGTAG
+HWI-EAS443:3:82:1102:721#0/1
`babbabbbbaabababbbb`aaabbbbbaaabbbbb`aa
@HWI-EAS443:3:82:1119:1073#0/1
ATCGAAACTAACAAGTTTGCTGCAATATGCACACATTTGG
+HWI-EAS443:3:82:1119:1073#0/1
_W[aZa`b`]¥`a`a]``ZZ]aa`]]aaaaa`a`¥Z]aa_
@HWI-EAS443:3:82:1119:1630#0/1
AGGTAATTGGGATTTATGCCTTTCTCATGTGGTTTGACTA
+HWI-EAS443:3:82:1119:1630#0/1
aabRaabbbab`bbaaabaaabaabaabbaaaaaaa`a`a
@HWI-EAS443:3:82:1175:1744#0/1
GGCCAGTAGGAGTCTATGGGATTCCTTTCGTCAGTCCGAA
+HWI-EAS443:3:82:1175:1744#0/1
S_^W¥ZL[a]QW^ab^abba_`P``baVNaI_WJU]RZYa
@HWI-EAS443:3:82:1210:803#0/1
GAAAACTTTAGAGCCTATGTAGATGGATTCGAGCCGAACG
+HWI-EAS443:3:82:1210:803#0/1
a`ZX_¥baaaa^a``a^ab_[a`aaa__`X^aa¥N^¥^[[
@HWI-EAS443:3:82:122:722#0/1
GCGTCTCCACAACTTGAGGGGTTTTCGGCTGAATCGAGAA
+HWI-EAS443:3:82:122:722#0/1
aabaabbb_abaabababbab[aaabaaaa`YaaaaWa`a
@HWI-EAS443:3:82:1249:1577#0/1
CATGGAAATGAGGCGCTGCCTTCTTCAGTCTCTTCAGCAG
+HWI-EAS443:3:82:1249:1577#0/1
aa`Yaab`aba`__aaa`JZ`aS_a^^]V¥aaa`]a¥a`^
@HWI-EAS443:3:82:1251:1136#0/1
ATTGAAGCACCGATTTGAGATAATTGAAGGAAGAGACCGA
+HWI-EAS443:3:82:1251:1136#0/1
aa^baabbaWWX_aTUNW¥T¥aW^a]TR^]X]a`___Ua^
@HWI-EAS443:3:82:1259:203#0/1
GCCATCTCCTCTCCAGTGAATGAAAAGATGTGAATGTGTG
```

```
+HWI-EAS443:3:82:1259:203#0/1
aa_aa]a`]a^`¥]_a]a`__a__^_ZX__]`^]^`Z^]^
@HWI-EAS443:3:82:1385:1109#0/1
GCTGCACAGGATGCATTGAGCAAGGCCGTATTTATGTACA
+HWI-EAS443:3:82:1385:1109#0/1
]aa_^_aa^]`_]Y__`[_¥R_a``_^VV^`aaYXYVYT]
@HWI-EAS443:3:82:1454:169#0/1
CACTTGGCCTATCGCAGTCCTCAAGAGCATGTCTCCTATT
+HWI-EAS443:3:82:1454:169#0/1
ab¥`````bbbba_`_Vb`babab`b¥[`abbba¥^b_ba
@HWI-EAS443:3:82:1463:226#0/1
GGGGGCTATATGAAGCAATCGAGGAGTGCCTGATTAATGA
+HWI-EAS443:3:82:1463:226#0/1
^_¥Y__]ab``S`¥`Ub_S[[G¥YV[XUTUa`Q¥U¥UXa_
@HWI-EAS443:3:82:1525:1172#0/1
CACACATTCACATCTTTTCATTCACTGGAGAGGAGATGGC
+HWI-EAS443:3:82:1525:1172#0/1
XYK_PY]bbbb`a[abaabbb_]bb_aaa_a__bba[b_U
@HWI-EAS443:3:82:1732:1933#0/1
CAAAACTAGGCTTTTCACTATAAGACAAGAAATGGCCAGT
+HWI-EAS443:3:82:1732:1933#0/1
¥b_``aabbb__a_b`baaa_K¥bXa`_¥[X_¥aa_RYWE
@HWI-EAS443:3:82:216:1998#0/1
CCACTCCCCTGGGTGACTCTCCGATTGGCCATGTTTCCGA
+HWI-EAS443:3:82:216:1998#0/1
abbbababba¥^XRV_aab`ab_¥^^Z[a^L[PV__^^QQ
@HWI-EAS443:3:82:283:1074#0/1
TCATCGACGAACGGGGTGAATCAATAATTGTAGAATCTGG
+HWI-EAS443:3:82:283:1074#0/1
aaaaba`aa_¥aaa_a]aaaaaa`aa`aaa_aa_```_a`
@HWI-EAS443:3:82:295:1034#0/1
ACGGGGTGAATCAATAATTGTAGAATCTGGTGACCCGAAT
+HWI-EAS443:3:82:295:1034#0/1
`aZ_ab`XX`ba^Z¥^[`aba``ababb_aWa`aa`a`a`
@HWI-EAS443:3:82:334:179#0/1
GAAGGGAGTGTACATAAATACGGCCTTGCTCAATGCATCC
+HWI-EAS443:3:82:334:179#0/1
_`a_ab`a`¥`aa_`aa_ab^]]_`[¥`_`a`[UX¥_[JY
@HWI-EAS443:3:82:352:1861#0/1
ACTTTCCCAATAGAGCCTTCCTCCACTCCCCTGGGTGACT
+HWI-EAS443:3:82:352:1861#0/1
abaa_a`aba_aZaXaa_¥^a¥_a``^a``aX¥]UWZY[W
@HWI-EAS443:3:82:354:1704#0/1
GGGGCATGGAAATGAGGCGCTGCCTTCTTCAGTCTCTTCA
+HWI-EAS443:3:82:354:1704#0/1
`baabbaaabbbabb`_abbaabaa_baaaa`_a`baaa`
@HWI-EAS443:3:82:411:1677#0/1
CATATAGCCCCCCAAGATCGAAGGTTCCAGGTTCCAGGTT
+HWI-EAS443:3:82:411:1677#0/1
`bababababbbbaaa_^a`a`aa_HV^¥`X^^T_¥^aV[U_
```

@HWI-EAS443:3:82:411:1940#0/1
AGAAATTTAGGCTTCTCTACCCCTGTTGTGTTACATATAC
+HWI-EAS443:3:82:411:1940#0/1
a`¥U^a¥Va]X`a`b_]Z`aaaaX`T¥]^_]^aa`_aZaa
@HWI-EAS443:3:82:419:701#0/1
GACATTCTTTGGCTGGAAAGAGCCTAACATAGTCAAACCA
+HWI-EAS443:3:82:419:701#0/1
abb¥`aba_a_aaaa_VYYV^Yaa]Y`][`X`[_`X``Y_
@HWI-EAS443:3:82:471:958#0/1
ACCGATTTGAGATAATTGAAGGAAGAGACCGAATCATGGC
+HWI-EAS443:3:82:471:958#0/1
[U¥¥Ubb_Zab^UaaaQ_¥_]aSY^_aaaabaaRW^]a[^
@HWI-EAS443:3:82:523:138#0/1
ACGGCCTTGCTCAATGCATCCTGTGCAGCCATGGATGACT
+HWI-EAS443:3:82:523:138#0/1
`Waa^aaaaaZ]^]^a¥a^]XSVSU[XYUXNY`]TXUSST
@HWI-EAS443:3:82:588:555#0/1
GGGGCTATATGAAGCAATCGAGGAGTGCCTGATTAATGAT
+HWI-EAS443:3:82:588:555#0/1
aa___`^_aabaaaaaaa_a`aa_a^`][`a``a``^^`_
@HWI-EAS443:3:82:657:1803#0/1
TGGGGCATGGAAATGAGGCGCTGCCTTCTTCAGTCTCTTC
+HWI-EAS443:3:82:657:1803#0/1
```¥_bba`aaaabab`__baaaaaaaaaaa_`]^aaa``
@HWI-EAS443:3:82:666:1559#0/1
TTCGGCTGAATCGAGAAAATTGCTTCTCATTGTTCAGGCA
+HWI-EAS443:3:82:666:1559#0/1
a`a^Tb¥W^_^V]YEYa¥TYUY__aaa_a__b_`_a[Ia`
@HWI-EAS443:3:82:673:1862#0/1
GTCTCCAACATCTTTGCAGTCATCAAAGTCTACTTTTTCT
+HWI-EAS443:3:82:673:1862#0/1
aYaW`ba`aaab]`_[a[Z_aa_baZ`_Ya_a¥___`¥^¥
@HWI-EAS443:3:82:67:1651#0/1
TAATCAGGCACTCCTCGATTGCTTCATATAGCCCCCCAAG
+HWI-EAS443:3:82:67:1651#0/1
`[aaa_H_`aaSaa`aRO[^T^__`a]a_aZaaY_``XZZ
@HWI-EAS443:3:82:685:840#0/1
TACATAATGAAGGGAGTGTACATAAATACGGCCTTGCTCA
+HWI-EAS443:3:82:685:840#0/1
aababaaabaa`X^aa_a_babab_aaaaaaaa_aaa`aa
@HWI-EAS443:3:82:823:41#0/1
AGCCCCCCAAGATCGAAGGTTCCAGGTTCCAGGTTGTCCC
+HWI-EAS443:3:82:823:41#0/1
abaaaaaaaaba`ab__`aV_`Y_`a__ZYQaaW`aVU¥W
@HWI-EAS443:3:82:855:190#0/1
CAAGAAATGGCCAGTAGGAGTCTATGGGATTCCTTTCGTC
+HWI-EAS443:3:82:855:190#0/1
ab_X_Z_bb`]RabZ`aab`H_]`^_Y`aa^VG^``__]R
@HWI-EAS443:3:82:883:1159#0/1
TTTGATTCTTGCCCTGCTCTCTTCGTCAAGGGTGTAGTCC
```

+HWI-EAS443:3:82:883:1159#0/1
aa`baaaa`a^^a`a_`a```aaa`Z_a`a`_Wa¥`a¥__
@HWI-EAS443:3:82:902:1444#0/1
GCCACCAAAGCGGACTACACCCTTGACGAAGAGAGCAGGG
+HWI-EAS443:3:82:902:1444#0/1
aba^`bbaabbbabaaa^baab`abaaaWZ_aaaaaa_[`
@HWI-EAS443:3:82:91:465#0/1
AATGCATCCTGTGCAGCCATGGATGACTTTCAGCTGATCC
+HWI-EAS443:3:82:91:465#0/1
`ab```a]¥Z_[``[a]baa`_XV^I_`^]a^]`[a[GS^
@HWI-EAS443:3:82:945:1509#0/1
TAAAATCTGAGAAGACACACATTCACATCTTTTCATTCAC
+HWI-EAS443:3:82:945:1509#0/1
aaaVa_a__`¥V`b^aa[aaa]a``Rab¥bba`_aaaYba
@HWI-EAS443:3:83:1025:846#0/1
CTCATGGCTTGGAAGCAGGTGCTAGCAGAGCTACAGGACA
+HWI-EAS443:3:83:1025:846#0/1
]abbaa¥ba_¥TM]_`Z¥_XM]_¥_Z^_V_]^_]_[b¥R¥
@HWI-EAS443:3:83:1089:818#0/1
TTCATTTTGGACCCAGCTTGCTAGAGATCTGGGCTCTGGC
+HWI-EAS443:3:83:1089:818#0/1
aa`][aaaa`a``^`_V_^UVVFYYSJUZVS[XLXLXQ^[
@HWI-EAS443:3:83:1118:1689#0/1
CGATTGCTTCATATAGCCCCCCAAGATCGAAGGTTCCAGG
+HWI-EAS443:3:83:1118:1689#0/1
aaaaa`aaaaaabaa`aa_]`aa`aa_`a```]H_UXX]]
@HWI-EAS443:3:83:1138:359#0/1
GGCATGGAAATGAGGCGCTGCCTTCTTCAGTCTCTTCAGC
+HWI-EAS443:3:83:1138:359#0/1
``WHI_`P``T_aa]S`UP`[^_ZL¥XX`^IDQMYRPU`R
@HWI-EAS443:3:83:113:1387#0/1
GGGCATGGAAATGAGGCGCTGCCTTCTTCAGTCTCTTCAG
+HWI-EAS443:3:83:113:1387#0/1
```bb_baa_Taaba_aa``aaa_`aa__ZU]RY]^_^_Z
@HWI-EAS443:3:83:1154:830#0/1
ATTTTACAGCAGAAGTGTCCCACTGCAGGGCTACTGAATA
+HWI-EAS443:3:83:1154:830#0/1
abb`a_`aaaa]YGYUa^¥^Z`Z^`^]^SY^__^a_^_ZZ
@HWI-EAS443:3:83:1163:1173#0/1
GGCATGGAAATGAGGCGCTGCCTTCTTCAGTCTCTTCAGC
+HWI-EAS443:3:83:1163:1173#0/1
aaaa]`__aaaaaa_a_aaSVX_]^`_`_]NOXM`WTP]T
@HWI-EAS443:3:83:1186:973#0/1
ATGCATCCTGTGCAGCCATGGATGACTTTCAGCTGATCCC
+HWI-EAS443:3:83:1186:973#0/1
aa`abbaaaba_abba`babaaaa`abaa_a```a_`_Y^
@HWI-EAS443:3:83:1312:745#0/1
GCAGGGCAAGAATCAAAACTAGGCTTTTCACTATAAGACA
+HWI-EAS443:3:83:1312:745#0/1
``bbaabbabaaaabaab`aaa``__aa]aa`aaaaaa_a
```

```
@HWI-EAS443:3:83:1364:288#0/1
CATCAGGCAATCTGAGGGGGCGTGGTGTCGTCCTCAAGAA
+HWI-EAS443:3:83:1364:288#0/1
abbZbbb^bab`b`bbbbaa]ababa`ZH]aS[aa_a`b_
@HWI-EAS443:3:83:1436:860#0/1
CGAAAATCGAAACTAACAAGTTTGCTGCAATATGCACACA
+HWI-EAS443:3:83:1436:860#0/1
abbab`a`_Xbaabbb^a_`YbXXaGa^a]aUa`MOQY_U
@HWI-EAS443:3:83:1462:166#0/1
CTTGGGGGGCTATATGAAGCAATCGAGGAGTGCCTGATTA
+HWI-EAS443:3:83:1462:166#0/1
aba¥¥baS_`baa`aa_aa``a_QaYaa_a[a¥S``^¥_`
@HWI-EAS443:3:83:1466:800#0/1
TTTTACAGCAGAAGTGTCCCACTGCAGGGCTACTGAATAC
+HWI-EAS443:3:83:1466:800#0/1
SVVY`]baZ¥bS^L[bYb`[aS]X_]_aW]a^__aYL`_V
@HWI-EAS443:3:83:1483:123#0/1
CGGAAACATGGCCAATCGGAGAGTCACCCAGGGGAGTGGA
+HWI-EAS443:3:83:1483:123#0/1
abbbbbbbbabXRYabZaZZX[LJR¥IRNWZbbZTUJYVJ
@HWI-EAS443:3:83:1500:1263#0/1
GCAGTGGGACACTTCTGCTGTAAAATAGTTCCTCCTCATG
+HWI-EAS443:3:83:1500:1263#0/1
a`bb_abaaba``a`baaaa^a`baaa^^aX]a]¥YP_`_
@HWI-EAS443:3:83:1622:677#0/1
AAGAGAACAAGCCAATTGAAGTGGGCACTCGGTGAAAATA
+HWI-EAS443:3:83:1622:677#0/1
[b_a_^[¥b[^babab`bYUZaaaaR^Z^^a`F¥QPRWR]
@HWI-EAS443:3:83:1662:1154#0/1
TGCACACATTTGGAAGTTTGTTTCATGTATTCGGATTTCC
+HWI-EAS443:3:83:1662:1154#0/1
aaX]_aa[a^bbbJabIa]_Saa]^Ta`_a[Gb`_^U_ZW
@HWI-EAS443:3:83:1703:1441#0/1
GATTGGCCATGTTTCCGATTTGTTTTCAAAGAATTCCTTG
+HWI-EAS443:3:83:1703:1441#0/1
aaaaaaa_aaaaaaaa_VVaab^`a`¥`_aa`^`a]_aa`
@HWI-EAS443:3:83:1730:1344#0/1
GGCATGGAAATGAGGCGCTGCCTTCTTCAGTCTCTTCAGC
+HWI-EAS443:3:83:1730:1344#0/1
aba_RZ`a[a]aab`]b_]UNSXXX___aaJZb_aXBBBB
@HWI-EAS443:3:83:1767:1791#0/1
ATACTTAATTTCAGAGCATCCATCAGCAGGAACTTTGACC
+HWI-EAS443:3:83:1767:1791#0/1
V]_Y¥^a`_b_X_`aaX^YH_¥`QZOM¥`V¥U¥XZW]WKN
@HWI-EAS443:3:83:1771:578#0/1
GAATTCATTTTGGACCCAGCTTGCTAGAGATCTGGGCTCT
+HWI-EAS443:3:83:1771:578#0/1
aaaaaa`bbaaaaa`a`_`¥^aW[^_Z___]]^]¥YV]^R
@HWI-EAS443:3:83:209:1658#0/1
GGGAATACCACTATATGATGCAATCAAATGCATGAAGACA
```

+HWI-EAS443:3:83:209:1658#0/1
abaabaaab`abbaZab^aa`bab`ba^_abba^Yaa`aa
@HWI-EAS443:3:83:236:1458#0/1
TGCCTTTCTCATGTGGTTTGACTATGTTAGGCTCTTTCCA
+HWI-EAS443:3:83:236:1458#0/1
O_bbbba`a_U`XZ_ZIY_baba]ab`ZZ__^_Z`aaaa¥
@HWI-EAS443:3:83:244:198#0/1
GGACAAAGAACATGAAGAGAACAAGCCAATTGAAGTGGGC
+HWI-EAS443:3:83:244:198#0/1
aaa`aaaa`a```a_aaa`_`^`__[Z_]^]_]]_U_^ZZ
@HWI-EAS443:3:83:248:469#0/1
AGCAAACTTGTTAGTTTCGATTTTCGGATCTTCCCCATAT
+HWI-EAS443:3:83:248:469#0/1
a]ba__aa`a_`__^``aaZ]^__^]ZGZX]XWZ_SRVV_
@HWI-EAS443:3:83:266:15#0/1
CAGGCACTCCTCGATTGCTTCATATAGCCCCCCAAGATCG
+HWI-EAS443:3:83:266:15#0/1
_^_^^]_a``a_`_W__a_Z]]¥[¥_[YVY¥Z^UV_]SV^
@HWI-EAS443:3:83:382:1869#0/1
CAGCAAACTTGTTAGTTTCGATTTTCGGATCTTCCCCATA
+HWI-EAS443:3:83:382:1869#0/1
`^`^_```]a]PQ_ZOUHMTLQRRW¥LHNOWTL¥^[[GOJ
@HWI-EAS443:3:83:431:178#0/1
CTGGAGAGGAGATGGCCACCAAAGCGGACTACACCCTTGA
+HWI-EAS443:3:83:431:178#0/1
abab_babb]baabb``a_aaaaa```aY_a`^S]¥^^``
@HWI-EAS443:3:83:450:1160#0/1
GGTGAACTTTGTAAGTATGGAGTTCTCACTCACTGACCCG
+HWI-EAS443:3:83:450:1160#0/1
TXT`babbbbb]¥V`_abba_aabab]bbabbbba]`aab
@HWI-EAS443:3:83:532:1196#0/1
GGGGCATGGAAATGAGGCGCTGCCTTCTTCAGTCTCTTCA
+HWI-EAS443:3:83:532:1196#0/1
Z__W`bbbbbaX^b_^a`aaababaabaabba`baaaa``
@HWI-EAS443:3:83:537:1569#0/1
TACCCCTGTTGTGTTACATATACTGTTCACCACTGTCCAG
+HWI-EAS443:3:83:537:1569#0/1
abbbbbb`^bbb^ab]bbbbbbabbabbbbababa_`a`^
@HWI-EAS443:3:83:564:823#0/1
AGCAATTTTCTCGATTCAGCCGAAAACCCCTCAAGTTGTG
+HWI-EAS443:3:83:564:823#0/1
¥¥V`^XS]]a^`bZUa¥^YYZ]^O[Y]aa[¥ZLGWU`aZ]
@HWI-EAS443:3:83:577:342#0/1
AGCCAGAGCCCAGATCTCTAGCAAGCTGGGTCCAAAATGA
+HWI-EAS443:3:83:577:342#0/1
^``aaa`baaa`baaaaaaaaa`¥`a`a`^Y_`^J]_``_
@HWI-EAS443:3:83:66:1118#0/1
AGGGGTTTTCGGCTGAATCGAGAAAATTGCTTCTCATTGT
+HWI-EAS443:3:83:66:1118#0/1
^a`_aZ^_R^`X__`¥``aa`Z_`a`[_¥_Z¥¥`a`_``[

```
@HWI-EAS443:3:83:682:1166#0/1
AATAATTGTAGAATCTGGTGACCCGAATGCACTATTGAAG
+HWI-EAS443:3:83:682:1166#0/1
a`ba`aaa`ab^^``aaa]_aaaa`a```___aa`aa^__
@HWI-EAS443:3:83:759:122#0/1
CAATAATTGTAGAATCTGGTGACCCGAATGCACTATTGAA
+HWI-EAS443:3:83:759:122#0/1
abbbbabbbbbbabaabba^bbaa`bbbabbbbbbb`b_b
@HWI-EAS443:3:83:817:1649#0/1
GCAGGTGCTAGCAGAGCTACAGGACATTGAAAATGAAGAG
+HWI-EAS443:3:83:817:1649#0/1
`aYa[¥]a¥_]_^Z¥`aNU]`aY_aa____a]`ZQVa[^]
@HWI-EAS443:3:83:837:72#0/1
GATGTGGTGAACTTTGTAAGTATGGAGTTCTCACTCACTG
+HWI-EAS443:3:83:837:72#0/1
a]¥TZb]R^]H¥¥aaa`ZZ]T_``]_a^[Y_X]W_V]^__
@HWI-EAS443:3:83:87:693#0/1
GGCCAGTAGGAGTCTATGGGATTCCTTTCGTCAGTCCGAA
+HWI-EAS443:3:83:87:693#0/1
^_bb_b]^a_J`^a````Zaa`a^^_aa``XZWaW^`a``
@HWI-EAS443:3:83:953:1153#0/1
GAGGGGTTTTCGGCTGAATCGAGAAAATTGCTTCTCATTG
+HWI-EAS443:3:83:953:1153#0/1
aa`aaaYaaaa`__aab`^_a_U_``aa_¥]`_V]V___`
@HWI-EAS443:3:83:959:67#0/1
CTTGGTCATGTCTTTCTCTTTGACAGAAGACTCGGCCTCA
+HWI-EAS443:3:83:959:67#0/1
[^_aaab_bbbabbabbbbbYb¥S`b_ab_[babbR¥`]V
@HWI-EAS443:3:83:963:1072#0/1
GCGAAGAGACAATTGAAGAAAAATTTGAGATTACAGGAAC
+HWI-EAS443:3:83:963:1072#0/1
aa_G^_abaaa`aa`a][^W¥]T¥aaa_a`]`a_a_`^aW
@HWI-EAS443:3:83:963:1312#0/1
AAGAATTCCTTGGTCATGTCTTTCTCTTTGACAGAAGACT
+HWI-EAS443:3:83:963:1312#0/1
YY_SW_`]R]_^RMX¥TXYY[^Z_WWWYY]SY^[a¥U`UW
@HWI-EAS443:3:83:965:1688#0/1
GGAGTCTATGGGATTCCTTTCGTCAGTCCGAAAGAGGCGA
+HWI-EAS443:3:83:965:1688#0/1
ab`aabbbbbaabbbbbbaaabaabaabbbaabaaaaaa`
@HWI-EAS443:3:84:1031:186#0/1
GTCCTTGGGATCTTCTCTTCATTTTCAATGTCCTGTAGCT
+HWI-EAS443:3:84:1031:186#0/1
aY[_`aaa][_]a]UY[_[¥^a^[a__^_`]XV]_]_]]U
@HWI-EAS443:3:84:1144:2040#0/1
GGGCACTCGGTGAAAATATGGCACCAGAAAAAGTAGACTT
+HWI-EAS443:3:84:1144:2040#0/1
Wbb___ab_b__V]U`aVa[¥H¥aL`aI_aa]UNa`a]``
@HWI-EAS443:3:84:1174:398#0/1
ATTTGTTTTCAAAGAATTCCTTGGTCATGTCTTTCTCTTT
```

```
+HWI-EAS443:3:84:1174:398#0/1
abb_baabba]^_aa`aaaaaaaZ]`aab_aaa`ab`aaa
@HWI-EAS443:3:84:1193:537#0/1
GATCCGAAAATCGAAACTAACAAGTTTGCTGCAATATGCA
+HWI-EAS443:3:84:1193:537#0/1
a_]`^`]_b``aa^_`_`][[``WZ___]^`__¥a_a`]_
@HWI-EAS443:3:84:1206:1686#0/1
ATGAGAAAGGCATAAATCCCAATTACCTCATGGCTTGGAA
+HWI-EAS443:3:84:1206:1686#0/1
aZ[aa`aa___a`__`a_VU____]]O^V_V]^X_YVVYZ
@HWI-EAS443:3:84:1211:361#0/1
TTGCGATATGTTCAATCGGGGCAACATCTTCTCCTATTTC
+HWI-EAS443:3:84:1211:361#0/1
``b`bbaabb`aaaaa```aaa``]`a_a`[a`_aaaaa_
@HWI-EAS443:3:84:124:1324#0/1
GGTGGCCATCTCCTCTCCAGTGAATGAAAAGATGTGAATG
+HWI-EAS443:3:84:124:1324#0/1
aa^`_aaa`a_aa_aWa`_aWZ`^^_^[_`^^^_W^]VY_
@HWI-EAS443:3:84:1284:886#0/1
CACTGCAGGGCTACTGAATACATAATGAAGGGAGTGTACA
+HWI-EAS443:3:84:1284:886#0/1
ababbbbbbaabbabbaaab`bbaaabaabaaaaaa`a]a
@HWI-EAS443:3:84:1331:971#0/1
GAGACCGAATCATGGCCTGGACAGTGGTGAACAGTATATG
+HWI-EAS443:3:84:1331:971#0/1
aaba`_UTG[XTH¥bZP_QXPW`¥TU¥P[RQVF]YX`V``
@HWI-EAS443:3:84:1353:1555#0/1
GTTCACCACTGTCCAGGCCATGATTCGGTCTCTTCCTTCA
+HWI-EAS443:3:84:1353:1555#0/1
a_a]ZU[^_`aa¥X_Ta¥XX_a__a_[`X__W`_]V``[_
@HWI-EAS443:3:84:1376:1428#0/1
CATTAATCAGGCACTCCTCGATTGCTTCATATAGCCCCCC
+HWI-EAS443:3:84:1376:1428#0/1
Y`Ybb_babaW_a]b]abNab]bb_a_aKbabbV[[aa¥¥
@HWI-EAS443:3:84:1417:116#0/1
AAACTTTAGAGCCTATGTAGATGGATTCGAGCCGAACGGC
+HWI-EAS443:3:84:1417:116#0/1
aa`Zbbaaa¥`_Ya`ab^_a]ab`^aaW`_`¥¥`Y^U^^U
@HWI-EAS443:3:84:141:423#0/1
GAAAACTTTAGAGCCTATGTAGATGGATTCGAGCCGAACG
+HWI-EAS443:3:84:141:423#0/1
^XS^S^]`a[]Zababaa`X]b¥H_b]a`a`V]^UX]``^
@HWI-EAS443:3:84:1528:677#0/1
CCTCCGTGTTACTCCAATTTCAATGAACCGGTTCTCTTTG
+HWI-EAS443:3:84:1528:677#0/1
aab`bbabbbbbb`aa_`abbbb`baba[bb^a_babaaa
@HWI-EAS443:3:84:1540:1135#0/1
GTAATCTCAAATTTTTCTTCAATTGTCTCTTCGCCTCTTT
+HWI-EAS443:3:84:1540:1135#0/1
^Y_aaaaaaaaaaaaaaa_`abaaa_a`a_a`__a]a``
```

```
@HWI-EAS443:3:84:1641:2015#0/1
GAAAATGAAGAGAAGATCCCAAGGACAAAGAACATGAAGA
+HWI-EAS443:3:84:1641:2015#0/1
V[QYQ[^`]IaX__Q][]VV`aa_aaa_``a¥]WZW___Y
@HWI-EAS443:3:84:167:1594#0/1
CAAAGAATTCCTTGGTCATGTCTTTCTCTTTGACAGAAGA
+HWI-EAS443:3:84:167:1594#0/1
a^[a`]__^^aaZ[VF^[`a[a_a^`_]`^__[`_^__]^
@HWI-EAS443:3:84:1703:1245#0/1
CACTTCTGCTGTAAAATAGTTCCTCCTCATGCTTGCGATA
+HWI-EAS443:3:84:1703:1245#0/1
ababbabbbbaabaaab^b`ba`baaaaaab]aaa`aaaa
@HWI-EAS443:3:84:1739:1324#0/1
CCGAAAATCGAAACTAACAAGTTTGCTGCAATATGCACAC
+HWI-EAS443:3:84:1739:1324#0/1
`^`¥bbb``_TWU^b_]`aa^^^HYZP_]^QZ`_a__`^L
@HWI-EAS443:3:84:1757:1227#0/1
GGGGCATGGAAATGAGGCGCTGCCTTCTTCAGTCTCTTCA
+HWI-EAS443:3:84:1757:1227#0/1
¥aWV[``baZ_bbb``¥ab`abUDU``ab`aa^Xa^```Q
@HWI-EAS443:3:84:1783:116#0/1
CCTGGAACCTTCGATCTTGGGGGGCTATATGAAGCAATCG
+HWI-EAS443:3:84:1783:116#0/1
aabbbbaaaaabbaa_aaa]abaa_a_a`aX]``[```Z_
@HWI-EAS443:3:84:253:299#0/1
CACTCCCCTGGGTGACTCTCCGATTGGCCATGTTTCCGAT
+HWI-EAS443:3:84:253:299#0/1
a`bab^[^T`b¥¥YZ]a¥YaPRPJ¥_^_XDD]N[_BBBBB
@HWI-EAS443:3:84:262:569#0/1
ACTTGAGGGGTTTTCGGCTGAATCGAGAAAATTGCTTCTC
+HWI-EAS443:3:84:262:569#0/1
]VI[RT^`[XXY_SZ_[XY]`[VZ¥¥_WYVY[[_]ZZ^Y`
@HWI-EAS443:3:84:289:722#0/1
TGTTACTCCAATTTCAATGAACCGGTTCTCTTTGTAATCA
+HWI-EAS443:3:84:289:722#0/1
aaaabaaaaaaaa_aa¥```aa_][V_`__a_a]¥_^`__
@HWI-EAS443:3:84:316:1054#0/1
CCCCTGTTGTGTTACATATACTGTTCACCACTGTCCAGGC
+HWI-EAS443:3:84:316:1054#0/1
aab`TPTQZYQVXZNXURO]UT]QL[`_¥^^¥^RZQSINS
@HWI-EAS443:3:84:319:1607#0/1
GCCTTCCTCCACTCCCCTGGGTGACTCTCCGATTGGCCAT
+HWI-EAS443:3:84:319:1607#0/1
_bb]`bb]`Y]aaaaa]_a¥^Q`ab_X]`aSJJP_a_¥U^
@HWI-EAS443:3:84:325:1091#0/1
AAATCCCAATTACCTCATGGCTTGGAAGCAGGTGCTAGCA
+HWI-EAS443:3:84:325:1091#0/1
a`_aaaa¥^aaaa`aaaaaaaaaWYY^ZVGS^UNRRWRU[
@HWI-EAS443:3:84:378:748#0/1
GTCTCCACAACTTGAGGGGTTTTCGGCTGAATCGAGAAAA
```

```
+HWI-EAS443:3:84:378:748#0/1
a_b¥baYba_aaaaaaaba`aa`_a^aaaa`_aa`a_^^`
@HWI-EAS443:3:84:381:827#0/1
TGGCACCAGAAAAAGTAGACTTTGATGACTGCAAAGATGT
+HWI-EAS443:3:84:381:827#0/1
`a_baa```aa`[`aYaa]`aaaYaa`V`_a_¥`]a__a_
@HWI-EAS443:3:84:38:58#0/1
TTGGGGGGCTATATGAAGCAATCGAGGAGTGCCTGATTAA
+HWI-EAS443:3:84:38:58#0/1
`aaWa^S^XZ[[SXaYR^`_Q^__]_[F[X]UTX^UWVRV
@HWI-EAS443:3:84:457:764#0/1
GGCCACCAAAGCGGACTACACCCTTGACGAAGAGAGCAGG
+HWI-EAS443:3:84:457:764#0/1
abbbb`bQW¥baaaY]aaaa_aaa¥a¥_ba[a^_[a]``X
@HWI-EAS443:3:84:488:1608#0/1
GAAAGGCATAAATCCCAATTACCTCATGGCTTGGAAGCAG
+HWI-EAS443:3:84:488:1608#0/1
a_¥HY]V`aa`bZRa_¥XXa¥W_`Taa¥Qa¥]_MFMLRLY
@HWI-EAS443:3:84:48:1818#0/1
GGGGCATGGAAATGAGGCGCTGCCTTCTTCAGTCTCTTCA
+HWI-EAS443:3:84:48:1818#0/1
^ZZ¥]V_`a]bba_a]a`aa_¥a``_a^`a__^[``a_a`
@HWI-EAS443:3:84:495:1150#0/1
ATTTTCACCGAGTGCCCACTTCAATTGGCTTGTTCTCTTC
+HWI-EAS443:3:84:495:1150#0/1
Y`aa]bbbbb`XVbb`TYa_aaaa`_a__a]¥_a`a]__R
@HWI-EAS443:3:84:508:1198#0/1
GTGGGCACTCGGTGAAAATATGGCACCAGAAAAAGTAGAC
+HWI-EAS443:3:84:508:1198#0/1
`[`b_a`aW]a^X_Qa`]_a`a_^Z`YP[P¥[NYQX_O_Z
@HWI-EAS443:3:84:564:27#0/1
CTTTGTAAGTATGGAGTTCTCACTCACTGACCCGAGACTG
+HWI-EAS443:3:84:564:27#0/1
abbbb`bbb¥aaba`aaaaaaa``^`babaa`aa_b```a
@HWI-EAS443:3:84:588:772#0/1
GAGACTTTGGTCGGCAAGCTTGCGCATAGTTCCTGTAATC
+HWI-EAS443:3:84:588:772#0/1
aaaaaaaaa]`b_ab_Y^a`_a_a``a__Ya```a^^_`_
@HWI-EAS443:3:84:615:576#0/1
CTTTGGTCGGCAAGCTTGCGCATAGTTCCTGTAATCTCAA
+HWI-EAS443:3:84:615:576#0/1
abbb^b_a```aY_aaa]```aa`__`a`^a¥Y``aaa^`
@HWI-EAS443:3:84:617:554#0/1
GAAGAGAGCAGGGCAAGAATCAAAACTAGGCTTTTCACTA
+HWI-EAS443:3:84:617:554#0/1
aaa^aa_aaa_]_aaaa_`_]aa`a^_a__[____]b`_`
@HWI-EAS443:3:84:625:1696#0/1
GAAATGGAAATCCGAATACATGAAACAAACTTCCAAATGT
+HWI-EAS443:3:84:625:1696#0/1
abbaaa`abaabbabbabbb`abb¥bbbba`a`ab`aaa]
```

@HWI-EAS443:3:84:675:1079#0/1
CTCATGCTTGCGATATGTTCAATCGGGGCAACATCTTCTC
+HWI-EAS443:3:84:675:1079#0/1
a_aaa`aaaa]baaaab_aaaaaaa`_`]_`_`a``a_`_
@HWI-EAS443:3:84:726:1200#0/1
CATTAATCAGGCACTCCTCGATTGCTTCATATAGCCCCCC
+HWI-EAS443:3:84:726:1200#0/1
``aa^``_[]_ba`aaaaaaa__aa`_`a`a_a_`_]`aZ
@HWI-EAS443:3:84:734:766#0/1
GAACCGGTTCTCTTTGTAATCATACAAATCAGGAAGAAAT
+HWI-EAS443:3:84:734:766#0/1
aa_¥^Y_XYVaaa¥aa_`^^a`a`]]aaa`WaV]]WR¥WZ
@HWI-EAS443:3:84:756:1154#0/1
CCTCCGTGTTACTCCAATTTCAATGAACCGGTTCTCTTTG
+HWI-EAS443:3:84:756:1154#0/1
`[^a`Z[^_`]^[`a_U^[a_`VZYN_aa]aWRUaa^^¥_
@HWI-EAS443:3:84:791:787#0/1
GCATGGAAATGAGGCGCTGCCTTCTTCAGTCTCTTCAGCA
+HWI-EAS443:3:84:791:787#0/1
a_aaaa^[`_a^a__`_aa¥_`_`a_¥_`S]_`a¥MX^R^
@HWI-EAS443:3:84:819:1594#0/1
AATCGGGGCAACATCTTCTCCTATTTCATCAAGTTCTATC
+HWI-EAS443:3:84:819:1594#0/1
a`aa[]`^^Y`_```^__``_a^```__a__¥_S_Z^__]
@HWI-EAS443:3:84:872:1896#0/1
AGCAGGAACTTTGACCGCTGATGGCAAAGAGGCCCATCAG
+HWI-EAS443:3:84:872:1896#0/1
XH]¥_¥aa^`_`^aa¥X_`Y¥¥`X`a^a¥`¥Z`P^]`Z[K
@HWI-EAS443:3:84:932:1880#0/1
GGGATCTTCTCTTCATTTTCAATGTCCTGTAGCTCTGCTA
+HWI-EAS443:3:84:932:1880#0/1
`abaa`bbabbbbbbbabbbbbabababbbbabbbab`aa`b
@HWI-EAS443:3:84:935:1615#0/1
CATAAATACGGCCTTGCTCAATGCATCCTGTGCAGCCATG
+HWI-EAS443:3:84:935:1615#0/1
a`a^_aaa[[[aa_a]a`_a`aa_`_]`XEM[`_Z^`a`¥
@HWI-EAS443:3:84:953:1932#0/1
ATAAATCCCAATTACCTCATGGCTTGGAAGCAGGTGCTAG
+HWI-EAS443:3:84:953:1932#0/1
aabbaaabaaaa`a_aa`a`_`aa`_`[XXTSW[KR[VYR
@HWI-EAS443:3:84:997:1913#0/1
AAGAATTCCTTGGTCATGTCTTTCTCTTTGACAGAAGACT
+HWI-EAS443:3:84:997:1913#0/1
UNU_VXU_¥ZXZWJLYZ[Za_X[^Z]`TZ_Y`a^¥___ZT
@HWI-EAS443:3:85:1038:1095#0/1
AGCAATTTTCTCGATTCAGCCGAAAACCCCTCAAGTTGTG
+HWI-EAS443:3:85:1038:1095#0/1
]]USZRW¥`]a[``S`]R]aPZ[XPTZ[]^ZS]N]^SV
@HWI-EAS443:3:85:1049:1479#0/1
CCTTGGGATCTTCTCTTCATTTTCAATGTCCTGTAGCTCT

```
+HWI-EAS443:3:85:1049:1479#0/1
`abbb`babababbbbbaaabbbababababaaaaaa`baa[a`
@HWI-EAS443:3:85:1077:144#0/1
AAAGAATTCCTTGGTCATGTCTTTCTCTTTGACAGAAGAC
+HWI-EAS443:3:85:1077:144#0/1
a¥a^_aab`]`Z^_[a`aa^Z_`W]^___¥_^]`_a[W^[
@HWI-EAS443:3:85:1091:35#0/1
CTTCCTTCAATTATCTCAAATCGGTGCTTCAATAGTGCAT
+HWI-EAS443:3:85:1091:35#0/1
aabbb`bbaaa^`bab]`aa]]aa`b]aa^[aab`_]L[`
@HWI-EAS443:3:85:1146:303#0/1
GAATTCCTTGGTCATGTCTTTCTCTTTGACAGAAGACTCG
+HWI-EAS443:3:85:1146:303#0/1
`aa````baaaY]¥Y`Yaa`aaaZ`_aaa[[^`_`a^^TN
@HWI-EAS443:3:85:1186:1801#0/1
ACCGAATCATGGCCTGGACAGTGGTGAACAGTATATGTAA
+HWI-EAS443:3:85:1186:1801#0/1
_¥`W_aaa`a]^^a`¥^UaaYYX`_S_^Wa¥Ya^a^Y[``
@HWI-EAS443:3:85:1187:1889#0/1
GCCTGGACAGTGGTGAACAGTATATGTAACACAACAGGGG
+HWI-EAS443:3:85:1187:1889#0/1
Z_bbb`bbb]]ab[``baba^bb`ba``aaaaaa````a_
@HWI-EAS443:3:85:1209:575#0/1
GTGGTTTGACTATGTTAGGCTCTTTCCAGCCAAAGAATGT
+HWI-EAS443:3:85:1209:575#0/1
aXaa^aaa`^a`_aX`__[]^^```YW]_^^^____]_TX
@HWI-EAS443:3:85:120:1427#0/1
CTTGGGATCTTCTCTTCATTTTCAATGTCCTGTAGCTCTG
+HWI-EAS443:3:85:120:1427#0/1
aababa]ababaabaab`aaaaa]aaa`a``_``Ya]a^^
@HWI-EAS443:3:85:1210:60#0/1
TGGGATCTTCTCTTCATTTTCAATGTCCTGTAGCTCTGCT
+HWI-EAS443:3:85:1210:60#0/1
abba_a`aa_a`ba_`aaaa`aa_a___aa_``¥a_^`_]
@HWI-EAS443:3:85:1260:1826#0/1
CTCAATGCATCCTGTGCAGCCATGGATGACTTTCAGCTGA
+HWI-EAS443:3:85:1260:1826#0/1
]]a_a]aaaaaabb`b[_aaV_aabaaba_`b^Wbb_abZ
@HWI-EAS443:3:85:1270:807#0/1
TGAATACATAATGAAGGGAGTGTACATAAATACGGCCTTG
+HWI-EAS443:3:85:1270:807#0/1
aa`a``^^`a]aaa]`aa]`^a`a`_a`^aaa_``_[_`]
@HWI-EAS443:3:85:128:992#0/1
CACTGTCATACTGTTTAAGGTCTCCAACATCTTTGCAGTC
+HWI-EAS443:3:85:128:992#0/1
aTabaababb`_`abba^a¥[aaaaaa``aaa_`a`Sa[a
@HWI-EAS443:3:85:1352:1787#0/1
GAGATCTGGGCTCTGGCTCATCACTGTCATACTGTTTAAG
+HWI-EAS443:3:85:1352:1787#0/1
``aab`U`]^WaY]]`W]]___aSaa_¥a_Y¥VYZ]L¥]^
```

```
@HWI-EAS443:3:85:1421:185#0/1
CTGGGTCCAAAATGAATTCAATAAGGCATGTGAATTGACT
+HWI-EAS443:3:85:1421:185#0/1
``]¥PG^RWXY[a][[aX[a__TZaUUU]]ZYJUa``]U[
@HWI-EAS443:3:85:1463:81#0/1
AACATCTTCTCCTATTTCATCAAGTTCTATCCAGCTTGAA
+HWI-EAS443:3:85:1463:81#0/1
aa_[b`abaa`_babbb_ab¥aa`aa`aaa`Zaa¥aaa_^
@HWI-EAS443:3:85:1477:912#0/1
CATTAATCAGGCACTCCTCGATTGCTTCATATAGCCCCCC
+HWI-EAS443:3:85:1477:912#0/1
`babbaaab`a^_G`ab`Maaaa_`^[N_baa`Ua_G_`_
@HWI-EAS443:3:85:1518:39#0/1
CACCGATTTGAGATAATTGAAGGAAGAGACCGAATCATGG
+HWI-EAS443:3:85:1518:39#0/1
U¥bbbbababbbbbbbbbababbb^bb`ba`aaab_abba
@HWI-EAS443:3:85:159:1410#0/1
ATTGTAGAATCTGGTGACCCGAATGCACTATTGAAGCACC
+HWI-EAS443:3:85:159:1410#0/1
aaba_]b^[`aaaa¥a^aaaa[`aaa```aa_`a`a¥___
@HWI-EAS443:3:85:185:195#0/1
GAACTTGATGAAATAGGAGAAGATGTTGCCCCGATTGAAC
+HWI-EAS443:3:85:185:195#0/1
a`b_aabaaa]__a_a`^a^_a_a`Z]]^_X^_W_V_^aZ
@HWI-EAS443:3:85:212:940#0/1
CAGTGGGACACTTCTGCTGTAAAATAGTTCCTCCTCATGC
+HWI-EAS443:3:85:212:940#0/1
aaaXS__S]aaX`]XR]ZZ¥_aaWVOZ¥[¥Y¥¥¥XQTZ^W
@HWI-EAS443:3:85:23:1751#0/1
AAGAATTCCTTGGTCATGTCTTTCTCTTTGACAGAAGACT
+HWI-EAS443:3:85:23:1751#0/1
a`O¥S¥aaaaa]X[```YTaa_`aaa_`__]Z_X¥¥_`aW
@HWI-EAS443:3:85:250:759#0/1
GGGTTTTCGGCTGAATCGAGAAAATTGCTTCTCATTGTTC
+HWI-EAS443:3:85:250:759#0/1
aaa[aa`a`^aa]ZS¥ba_`[V_S]a`^[]a```¥Q_V^`
@HWI-EAS443:3:85:263:325#0/1
CACTTTCCCAATAGAGCCTTCCTCCACTCCCCTGGGTGAC
+HWI-EAS443:3:85:263:325#0/1
^aababbbbb]b_bbbb`b`baaabba_abaaY`^`Ta`U
@HWI-EAS443:3:85:318:1499#0/1
AGGTTCCATTGGTTCTCACATATAGGAACATGGGCCTCGA
+HWI-EAS443:3:85:318:1499#0/1
_]`]aab¥^a_]_aa`a[aaaaaa`¥]Z_aY^Y^`a_^V^
@HWI-EAS443:3:85:342:1630#0/1
GGGTTTTCGGCTGAATCGAGAAAATTGCTTCTCATTGTTC
+HWI-EAS443:3:85:342:1630#0/1
a`b_bbbb`_bbbbbaa`^abba``aaa`aaa`baaa]aa
@HWI-EAS443:3:85:363:871#0/1
CTCATTTGAGAAATGATACTGATGTGGTGAACTTTGTAAG
```

```
+HWI-EAS443:3:85:363:871#0/1
aaaa`bab_`a`b]`]abaaa`_^W_`Oa_`aa^_a]^LY
@HWI-EAS443:3:85:369:817#0/1
GTGCCCACTTCAATTGGCTTGTTCTCTTCATGTTCTTTGT
+HWI-EAS443:3:85:369:817#0/1
aaababa_aab_``[]_U_¥HUUPZH`Y[[GHP_S]aZHV
@HWI-EAS443:3:85:409:1642#0/1
TTTTGGACCCAGCTTGCTAGAGATCTGGGCTCTGGCTCAT
+HWI-EAS443:3:85:409:1642#0/1
abbba`bbbaaaa_a^aaa`aaa_a_Q_]`^a_`Za_aa`
@HWI-EAS443:3:85:542:132#0/1
AAACTTTAGAGCCTATGTAGATGGATTCGAGCCGAACGGC
+HWI-EAS443:3:85:542:132#0/1
a`aabaa_a_a_aa_aa]]a_aa_^^_X__`]]_^_][]U
@HWI-EAS443:3:85:574:326#0/1
GGGCATGGAAATGAGGCGCTGCCTTCTTCAGTCTCTTCAG
+HWI-EAS443:3:85:574:326#0/1
abaaaab_aaaaaaaaaa`aa``aa_`a`aaW_a``_`a`
@HWI-EAS443:3:85:631:1934#0/1
AAATACGGCCTTGCTCAATGCATCCTGTGCAGCCATGGAT
+HWI-EAS443:3:85:631:1934#0/1
a``Wabaa`[a``__`a``aa__`a]_U`_¥V¥[_U[[_X
@HWI-EAS443:3:85:66:1821#0/1
GAACTATGCGCAAGCTTGCCGACCAAAGTCTCCCACCGAA
+HWI-EAS443:3:85:66:1821#0/1
`a`b[__bbb]aW¥¥a__``Wbab_T`aS[^baaaa_ZZW
@HWI-EAS443:3:85:673:164#0/1
GTTTGTTTCATGTATTCGGATTTCCATTTCATCGACGAAC
+HWI-EAS443:3:85:673:164#0/1
a^`aa]_[XZaa^_a_^`a^_`_XX^_^]`^_¥_WZ__^X
@HWI-EAS443:3:85:676:173#0/1
GTGCCATATTTTCACCGAGTGCCCACTTCAATTGGCTTGT
+HWI-EAS443:3:85:676:173#0/1
^V``^aa]baaaaa^`_Y_^a`]¥ZY_^a]`¥¥¥]U_XHL
@HWI-EAS443:3:85:705:903#0/1
CATATAGCCCCCCAAGATCGAAGGTTCCAGGTTCCAGGTT
+HWI-EAS443:3:85:705:903#0/1
abbbbbbbbbabbb`abba_a^`aXaaX[`a^a]aZa`Za
@HWI-EAS443:3:85:716:534#0/1
GGTTTGACTATGTTAGGCTCTTTCCAGCCAAAGAATGTCT
+HWI-EAS443:3:85:716:534#0/1
a`T`ba`aaba_V[aaa¥`^aaa_a`a`^Z_aaV``a[¥^
@HWI-EAS443:3:85:719:1297#0/1
GAGGGGTTTTCGGCTGAATCGAGAAAATTGCTTCTCATTG
+HWI-EAS443:3:85:719:1297#0/1
a_`aa`]aaaaaaaaabaa``aaaaaaa`_[a``aa``a`
@HWI-EAS443:3:85:770:926#0/1
CGGTGGGAGACTTTGGTCGGCAAGCTTGCGCATAGTTCCT
+HWI-EAS443:3:85:770:926#0/1
a`[Taaa`babb`abb¥_FZWbaabaa[a_a`_`a_a__a
```

```
@HWI-EAS443:3:85:838:88#0/1
CTCCCTTCATTATGTATTCAGTAGCCCTGCAGTGGGACAC
+HWI-EAS443:3:85:838:88#0/1
`bbbabbbabbabbaaabaa_[a_W]`aa`aa^ba`^Z]U
@HWI-EAS443:3:85:971:110#0/1
CTTATTGAATTCATTTTGGACCCAGCTTGCTAGAGATCTG
+HWI-EAS443:3:85:971:110#0/1
`aaWaaba__ba[babbabbab`aa_aaa`baa`baYP`_
@HWI-EAS443:3:86:1021:227#0/1
GACCCAGCTTGCTAGAGATCTGGGCTCTGGCTCATCACTG
+HWI-EAS443:3:86:1021:227#0/1
VabbbZ[X[bbaab`bbbb_aa`aUbaaaa`a_aa^a¥__
@HWI-EAS443:3:86:1074:870#0/1
CACTGACCCGAGACTGGAGCCACACAAATGGGAAAAATAC
+HWI-EAS443:3:86:1074:870#0/1
`^[_aZ_Za`ab^W`ab¥^ab`b]W`Ubbbaa^`[[H`IV
@HWI-EAS443:3:86:1079:1231#0/1
TCCACTCCCCTGGGTGACTCTCCGATTGGCCATGTTTCCG
+HWI-EAS443:3:86:1079:1231#0/1
aaa_`Xa`aaX``aQ__^`O¥`¥RXKWZP``MYYRZXNTY
@HWI-EAS443:3:86:1083:1457#0/1
AGAAGATGTTGCCCCGATTGAACATATCGCAAGCATGAGG
+HWI-EAS443:3:86:1083:1457#0/1
ababbbaa]aa^___a``_aab]a`aa____[YV]a^]a`
@HWI-EAS443:3:86:1239:783#0/1
CAGCAAACTTGTTAGTTTCGATTTTCGGATCTTCCCCATA
+HWI-EAS443:3:86:1239:783#0/1
ababaababbabba`abb`_Sbbba^¥ZEZGab¥¥`U_aX
@HWI-EAS443:3:86:1448:786#0/1
CAAGAAATGGCCAGTAGGAGTCTATGGGATTCCTTTCGTC
+HWI-EAS443:3:86:1448:786#0/1
abVbbabbbbb`_¥^abb_b¥aba_V_a^`a[]^a`H`a¥
@HWI-EAS443:3:86:1505:334#0/1
GATTGGCCATGTTTCCGATTTGTTTTCAAAGAATTCCTTG
+HWI-EAS443:3:86:1505:334#0/1
]abbb`b`bba]bbaabbbbbbabbbYbabaaaaaSJ^ba
@HWI-EAS443:3:86:1558:1163#0/1
CGGCCTTGCTCAATGCATCCTGTGCAGCCATGGATGACTT
+HWI-EAS443:3:86:1558:1163#0/1
`ab_bbaabab_bbbabbbbbbabab_aRbab]aZb_Zaa
@HWI-EAS443:3:86:178:248#0/1
TCCCAATAGAGCCTTCCTCCACTCCCCTGGGTGACTCTCC
+HWI-EAS443:3:86:178:248#0/1
```^aa```^Xa`^^Z`]`T¥¥`¥`]`RZNZR^RZSTPST
@HWI-EAS443:3:86:190:1412#0/1
TACATATACTGTTCACCACTGTCCAGGCCATGATTCGGTC
+HWI-EAS443:3:86:190:1412#0/1
abaaababa`a_aaaaaaaa`]a^_]Y_[a`]_^^`WYZ]
@HWI-EAS443:3:86:195:390#0/1
AGAGCCTAACATAGTCAAACCACATGAGAAAGGCATAAAT
```
```

```
+HWI-EAS443:3:86:195:390#0/1
UZNW^_b¥`a^_¥¥ZV_bX¥X``¥^Ya]aV_GG^aa_`a^
@HWI-EAS443:3:86:205:1260#0/1
GTGCTAGCAGAGCTACAGGACATTGAAAATGAAGAGAAGA
+HWI-EAS443:3:86:205:1260#0/1
_YQ_ab`ab]aaa`aZV````a^[Z[a[`YVV]]_`_a¥`
@HWI-EAS443:3:86:223:1148#0/1
TGGGGCATGGAAATGAGGCGCTGCCTTCTTCAGTCTCTTC
+HWI-EAS443:3:86:223:1148#0/1
`b¥b`¥b_bbX`a_b__aaaa`a``a`aa^aaa_VaXY[Q
@HWI-EAS443:3:86:417:1837#0/1
CATGGAAATGAGGCGCTGCCTTCTTCAGTCTCTTCAGCAG
+HWI-EAS443:3:86:417:1837#0/1
S¥[b][`[Uba¥ZaSV`UR_OY[X]X^YR_X¥XUY`UGYX
@HWI-EAS443:3:86:417:606#0/1
CAGGCACTCCTCGATTGCTTCATATAGCCCCCCAAGATCG
+HWI-EAS443:3:86:417:606#0/1
aabaa`abbababababaaaa`_^^a`aa`X_X^aZO]W^R[
@HWI-EAS443:3:86:444:332#0/1
GGGGGCTATATGAAGCAATCGAGGAGTGCCTGATTAATGA
+HWI-EAS443:3:86:444:332#0/1
aaaab`baa]``aaaa`ba_a]a`^`^a^`aa``____`]
@HWI-EAS443:3:86:488:1417#0/1
GGGGCATGGAAATGAGGCGCTGCCTTCTTCAGTCTCTTCA
+HWI-EAS443:3:86:488:1417#0/1
a_ab[baa`Z^aa`bYaa_b^`Z_]¥NWRZV`YXW¥_¥[_
@HWI-EAS443:3:86:501:684#0/1
ACCCGAATGCACTATTGAAGCACCGATTTGAGATAATTGA
+HWI-EAS443:3:86:501:684#0/1
S¥S¥_Z]Zaa[```bb`_a¥¥_aaaa_`Y_Y_a_aZ]]bV
@HWI-EAS443:3:86:530:77#0/1
GGGGTTTTCGGCTGAATCGAGAAAATTGCTTCTCATTGTT
+HWI-EAS443:3:86:530:77#0/1
abbb`aaaaaaZaaa``^b_a_```a`a^__Ya]__^_Y_
@HWI-EAS443:3:86:532:546#0/1
CTTTGTAATCATACAAATCAGGAAGAAATTTAGGCTTCTC
+HWI-EAS443:3:86:532:546#0/1
aaa^^_babVbabaaSY]aa_a_aaY`^`__]`Za`]RLR
@HWI-EAS443:3:86:561:333#0/1
GGGGGCTATATGAAGCAATCGAGGAGTGCCTGATTAATGA
+HWI-EAS443:3:86:561:333#0/1
XX`b`Q_^[U^aWZXT__[`__bYV^PZW[aaV[Z^XX`^
@HWI-EAS443:3:86:568:1499#0/1
GGCATGGAAATGAGGCGCTGCCTTCTTCAGTCTCTTCAGC
+HWI-EAS443:3:86:568:1499#0/1
`ab_]`b^T`aaababaaa_P¥`a_`a_aa``]aaa^a[Z
@HWI-EAS443:3:86:650:859#0/1
TTCCGATTTGTTTTCAAAGAATTCCTTGGTCATGTCTTTC
+HWI-EAS443:3:86:650:859#0/1
aaa_b`aaa`_`_a`aa^a`_a``_aaa_Y_`aa_aaa`]
```

```
@HWI-EAS443:3:86:67:426#0/1
CAATGAGAAGCAATTTTCTCGATTCAGCCGAAAACCCCTC
+HWI-EAS443:3:86:67:426#0/1
a```bbbaXa_aVabbabbbbbaa^^aaab``a`aW_a`a
@HWI-EAS443:3:86:716:1666#0/1
GTGAACTTTGTAAGTATGGAGTTCTCACTCACTGACCCGA
+HWI-EAS443:3:86:716:1666#0/1
__W_bbbbbbabbbabbb`aaab^babbbbbbabaaXaa^
@HWI-EAS443:3:86:721:326#0/1
AAACTTTAGAGCCTATGTAGATGGATTCGAGCCGAACGGC
+HWI-EAS443:3:86:721:326#0/1
aaa^aaaaa_a__aaaaY`_`_`_^`aN[`_UZ^Z_W_¥W
@HWI-EAS443:3:86:721:548#0/1
GGGGGCTATATGAAGCAATCGAGGAGTGCCTGATTAATGA
+HWI-EAS443:3:86:721:548#0/1
ababbbabbbbbbabaaabaab`baaa_baaaaaaa_aaaa
@HWI-EAS443:3:86:746:1315#0/1
GCAGGTGCTAGCAGAGCTACAGGACATTGAAAATGAAGAG
+HWI-EAS443:3:86:746:1315#0/1
aaa^a^_aZ¥__a__a_``aaa__]`^`^]^__`]^^]_^
@HWI-EAS443:3:86:761:86#0/1
GAAAGGCATAAATCCCAATTACCTCATGGCTTGGAAGCAG
+HWI-EAS443:3:86:761:86#0/1
abaabaaabbaabaaababaa_Za_`^a__a`PXU¥VWZW
@HWI-EAS443:3:86:805:1687#0/1
TATGCGTCTCCACAACTTGAGGGGTTTTCGGCTGAATCGA
+HWI-EAS443:3:86:805:1687#0/1
]a]a`aa`Z__¥`aaabaV¥aYUb`a``J`Y`a`b`Z¥aa
@HWI-EAS443:3:86:807:1161#0/1
ATTGAATTCATTTTGGACCCAGCTTGCTAGAGATCTGGGC
+HWI-EAS443:3:86:807:1161#0/1
aaabaabaaaaaaa`aaa`_``aaa`_aaaa_a¥``ZX]¥
@HWI-EAS443:3:86:811:1308#0/1
CTGAAAGTCATCCATGGCTGCACAGGATGCATTGAGCAAG
+HWI-EAS443:3:86:811:1308#0/1
ababbbb`baaaabaaaaa]]_X[aaa[__aYa___V`__
@HWI-EAS443:3:86:827:499#0/1
CCCCTGGGTGACTCTCCGATTGGCCATGTTTCCGATTTGT
+HWI-EAS443:3:86:827:499#0/1
aaaa_baa[a`aa`__aa_`]a_`_¥Y`^`a^^¥RZa^¥T
@HWI-EAS443:3:86:839:1129#0/1
ATCCGAATACATGAAACAAACTTCCAAATGTGTGCATATT
+HWI-EAS443:3:86:839:1129#0/1
abbbabbababa`b`bbbababbbab```a^b^aaaaba`
@HWI-EAS443:3:86:928:720#0/1
GAAGGAAGAGACCGAATCATGGCCTGGACAGTGGTGAACA
+HWI-EAS443:3:86:928:720#0/1
_^XaaXM]^^]_aa]aa]_`_`[]``_YS^`]a]Waa]_Q
@HWI-EAS443:3:86:933:1131#0/1
CCCGAGACTGGAGCCACACAAATGGGAAAAATACTGTGTT
```

```
+HWI-EAS443:3:86:933:1131#0/1
^bbbbbbbbbbabab_bbb`aa`abb^_ababaabaaaab
@HWI-EAS443:3:86:955:207#0/1
CTTGGTCATGTCTTTCTCTTTGACAGAAGACTCGGCCTCA
+HWI-EAS443:3:86:955:207#0/1
abbbbbbbbbbabba^aabbbbaabbbabb`baa`¥`a^_
@HWI-EAS443:3:86:963:1715#0/1
GGGGGGCTATATGAAGCAATCGAGGAGTGCCTGATTAATG
+HWI-EAS443:3:86:963:1715#0/1
[`b_G_UZU[`a`b]abaKaNb`bbJ¥]^```a^``^aa^
@HWI-EAS443:3:87:109:1446#0/1
ATGGCTTGGAAGCAGGTGCTAGCAGAGCTACAGGACATTG
+HWI-EAS443:3:87:109:1446#0/1
aa^`baaUbVS_[]WYM]^Y¥XRP¥]^VDSR[U]VZZLSU
@HWI-EAS443:3:87:1130:436#0/1
GGGAATACCACTATATGATGCAATCAAATGCATGAAGACA
+HWI-EAS443:3:87:1130:436#0/1
abaaabbaaaaabbaabaaa_baaaaaaaaaaaaaaaa]a
@HWI-EAS443:3:87:1199:925#0/1
CTTCTGTCAAAGAGAAAGACATGACCAAGGAATTCTTTGA
+HWI-EAS443:3:87:1199:925#0/1
aa`a`aab¥^T``X]``_Y¥]a]Wb__[V[I_a^baZUaY
@HWI-EAS443:3:87:1202:901#0/1
CCTGCTTCCAAGCCATGAGGTAATTGGGATTTATGCCTTT
+HWI-EAS443:3:87:1202:901#0/1
abb`bbbabbbaaaabbab]`babbbbbabb_babaabaa
@HWI-EAS443:3:87:1237:1037#0/1
GCTCTCTTCGTCAAGGGTGTAGTCCGCTTTGGTGGCCATC
+HWI-EAS443:3:87:1237:1037#0/1
aaa`a]]_]b¥^a^aaa^a^_Z_[]]Y``aa]^]¥YNQXN
@HWI-EAS443:3:87:1297:1200#0/1
ACATGCTCTTGAGGACTGCGATAGGCCAAGTGTCGAGGCC
+HWI-EAS443:3:87:1297:1200#0/1
``baabaaabb`abaaa``a`a`a`U_aaaZ_ZZ`_¥`¥U
@HWI-EAS443:3:87:1322:770#0/1
ATTCTTTGGCTGGAAAGAGCCTAACATAGTCAAACCACAT
+HWI-EAS443:3:87:1322:770#0/1
abbaabbbabba^aabaaa`^`[a]`aa`_¥`ba^Ma^aa
@HWI-EAS443:3:87:1323:840#0/1
CGAAACTAACAAGTTTGCTGCAATATGCACACATTTGGAA
+HWI-EAS443:3:87:1323:840#0/1
``a¥a^^Ya`^^`_abb]_b_b¥bbaZSa_b¥baaaaU`a
@HWI-EAS443:3:87:1337:876#0/1
ATCGACGAACGGGGTGAATCAATAATTGTAGAATCTGGTG
+HWI-EAS443:3:87:1337:876#0/1
_OG^`a^_a_[]abH`^S_`OR]UUV]aRP[VaZW^¥OT[
@HWI-EAS443:3:87:1444:1673#0/1
AAAGGAATCCCATAGACTCCTACTGGCCATTTCTTGTCTT
+HWI-EAS443:3:87:1444:1673#0/1
abU_XaaT][]a__a^K]]]aXSIV¥T__P_UFX¥QLJL_
```

```
@HWI-EAS443:3:87:1454:1239#0/1
CACGAGGGGGAGGGAATACCACTATATGATGCAATCAAAT
+HWI-EAS443:3:87:1454:1239#0/1
_bababbbbbabbaaaaa`_a¥baaaaaaab]aaa_a`aa
@HWI-EAS443:3:87:145:1020#0/1
TGAAGGGAGTGTACATAAATACGGCCTTGCTCAATGCATC
+HWI-EAS443:3:87:145:1020#0/1
aa[M[`a^a¥aZaaa^Qa[]___Sa¥¥UZRZX^¥^¥__W^
@HWI-EAS443:3:87:1509:424#0/1
CAGCTTGCTAGAGATCTGGGCTCTGGCTCATCACTGTCAT
+HWI-EAS443:3:87:1509:424#0/1
aaa`aa[abb`bbbaa_aS]Ia``_b_b_`bYba`b_^`a
@HWI-EAS443:3:87:1554:656#0/1
CATGGGCCTCGACACTTGGCCTATCGCAGTCCTCAAGAGC
+HWI-EAS443:3:87:1554:656#0/1
abab^bb_bbbb_bababaaabbbaaba``]Ya[aaa_a`
@HWI-EAS443:3:87:1600:526#0/1
CATTAATCAGGCACTCCTCGATTGCTTCATATAGCCCCCC
+HWI-EAS443:3:87:1600:526#0/1
aabbabbba`[`bbbb`bb[bbabbbbbbbb[bbaZ_KVa
@HWI-EAS443:3:87:1617:1193#0/1
ATCAGGCACTCCTCGATTGCTTCATATAGCCCCCCAAGAT
+HWI-EAS443:3:87:1617:1193#0/1
L^a]_X`bab_baab^aaaab_^Zbb^bb`QRWWbbbaXa
@HWI-EAS443:3:87:1663:792#0/1
GGGGTTTTCGGCTGAATCGAGAAAATTGCTTCTCATTGTT
+HWI-EAS443:3:87:1663:792#0/1
abaaHa`a¥b`M_a``_[a_aZY_]``aUa[PVN_]ZaX`
@HWI-EAS443:3:87:1685:91#0/1
CATTGAGCAAGGCCGTATTTATGTACACTCCCTTCATTAT
+HWI-EAS443:3:87:1685:91#0/1
a```[bbaabb__abWbaa]^]abb_babaaab[WUabab
@HWI-EAS443:3:87:171:411#0/1
GGGGCATGGAAATGAGGCGCTGCCTTCTTCAGTCTCTTCA
+HWI-EAS443:3:87:171:411#0/1
ab`a`a`aa^^]_^_`__a¥^_¥¥_^¥¥W[ZZVUVU[WWW
@HWI-EAS443:3:87:1791:472#0/1
AGCAGGTGCTAGCAGAGCTACAGGACATTGAAAATGAAGA
+HWI-EAS443:3:87:1791:472#0/1
aaaaa`^_aaa`_aa`a``a_aaaa__a`a^`a`a_````
@HWI-EAS443:3:87:265:352#0/1
ATAAAATCTGAGAAGACACACATTCACATCTTTTCATTCA
+HWI-EAS443:3:87:265:352#0/1
a``_[^aaab_aW__``_]`]`a__`__`_^^_____^^^
@HWI-EAS443:3:87:28:1430#0/1
GGCATGTGAATTGACTGATTCAAGCTGGATAGAACTTGAT
+HWI-EAS443:3:87:28:1430#0/1
_`ba¥_TSS_`^YYVYXRR^_YGYZ^]USZZW]a^WQS¥¥
@HWI-EAS443:3:87:294:770#0/1
AGAATATGGGGAAGATCCGAAAATCGAAACTAACAAGTTT
```

```
+HWI-EAS443:3:87:294:770#0/1
a`V¥baaaab`V¥b_^abb¥ab__^aa`a_aaT`a`a_^^
@HWI-EAS443:3:87:324:1865#0/1
GGCCAATCGGAGAGTCACCCAGGGGAGTGGAGGAAGGCTC
+HWI-EAS443:3:87:324:1865#0/1
^_a_¥`^b``[Saa_aaaaX^`¥¥X¥]V`¥NNZ`X¥SXX^
@HWI-EAS443:3:87:328:1931#0/1
GATTTGTTTTCAAAGAATTCCTTGGTCATGTCTTTCTCTT
+HWI-EAS443:3:87:328:1931#0/1
Xb``a¥^Xab``ba¥a¥aaaaaa_J``a`bYaabaa^abY
@HWI-EAS443:3:87:339:413#0/1
GGGGGCTATATGAAGCAATCGAGGAGTGCCTGATTAATGA
+HWI-EAS443:3:87:339:413#0/1
aaa]¥^`aa`^a_a_a_a_aa_a_]]]aa]]`]_]^___^
@HWI-EAS443:3:87:427:973#0/1
ATGAGAAGCAATTTTCTCGATTCAGCCGAAAACCCCTCAA
+HWI-EAS443:3:87:427:973#0/1
abaa`abbaaRaaaaaaaaaa_aaa_a_ZR``_Y```_^Z
@HWI-EAS443:3:87:463:1023#0/1
AATGAATTCAATAAGGCATGTGAATTGACTGATTCAAGCT
+HWI-EAS443:3:87:463:1023#0/1
aaaaaaaa_aa`a`_aa_aa]a`_`_``_aa__```__^^
@HWI-EAS443:3:87:483:938#0/1
GGGGGAGGGAATACCACTATATGATGCAATCAAATGCATG
+HWI-EAS443:3:87:483:938#0/1
aba`b_¥]_^`a__`aa`a_aaa_```a`_a_[[^`_a`a
@HWI-EAS443:3:87:558:184#0/1
GGCATGGAAATGAGGCGCTGCCTTCTTCAGTCTCTTCAGC
+HWI-EAS443:3:87:558:184#0/1
a_]aabba`aaa_b[W]]_Y_a[`RXTX^RQR[UZ^XZTL
@HWI-EAS443:3:87:572:856#0/1
GAAATGGCCAGTAGGAGTCTATGGGATTCCTTTCGTCAGT
+HWI-EAS443:3:87:572:856#0/1
ab__abaa[_a`Yab[SUHR[PZ_a[XHY`¥Y]SZMW__V
@HWI-EAS443:3:87:596:771#0/1
GCTTCATATAGCCCCCCAAGATCGAAGGTTCCAGGTTCCA
+HWI-EAS443:3:87:596:771#0/1
aaaaaa`aaa^`^aaa_`]``_a__YY^PY[[R¥^UYV[V
@HWI-EAS443:3:87:607:394#0/1
CCTGGGTGACTCTCCGATTGGCCATGTTTCCGATTTGTTT
+HWI-EAS443:3:87:607:394#0/1
a`^^^`[_[_¥ZXaaaT_^_X¥`__[JYSS¥`Y¥a^^Y__
@HWI-EAS443:3:87:633:709#0/1
CACTCGGTGAAAATATGGCACCAGAAAAAGTAGACTTTGA
+HWI-EAS443:3:87:633:709#0/1
a_a`aab^_b_]a^_b_`^^`a`aS]`]__[`ba^aaaa]
@HWI-EAS443:3:87:649:257#0/1
GTCTTCATGCATTTGATTGCATCATATAGTGGTATTCCCT
+HWI-EAS443:3:87:649:257#0/1
a__ba]`ab`aaa`a`a_a__`a_a_``aWa`W^__¥^W_
```

```
@HWI-EAS443:3:87:69:1441#0/1
CAAAGTCTACTTTTTCTGGTGCCATATTTTCACCGAGTGC
+HWI-EAS443:3:87:69:1441#0/1
a^Y]][a`][]`a`aa_`_T_``a_`_^^^_`a_[__]^^
@HWI-EAS443:3:87:739:689#0/1
TGGGGGGCTATATGAAGCAATCGAGGAGTGCCTGATTAAT
+HWI-EAS443:3:87:739:689#0/1
aba[``_bbbaaaa]_`baaaaa_a^^aTa```a]aaaaa
@HWI-EAS443:3:87:800:256#0/1
GGGGGCTATATGAAGCAATCGAGGAGTGCCTGATTAATGA
+HWI-EAS443:3:87:800:256#0/1
aa_aa``aa_aa_[^__`]V_]`aW_Y`VU`_X]]]]X^]
@HWI-EAS443:3:87:803:1425#0/1
ATTTTGGCGTTCACTTCTTTTGACATTTGGGAAAGCTTGC
+HWI-EAS443:3:87:803:1425#0/1
aa]aba]]a`¥H]bUaST`a_SRLVT[a[_^ZaUaYFO_¥
@HWI-EAS443:3:87:817:1598#0/1
GGGGGCTATATGAAGCAATCGAGGAGTGCCTGATTAATGA
+HWI-EAS443:3:87:817:1598#0/1
`QNW]`XI[TQXaX]TVGW]]b^W][Q¥OLWZ[V¥`WLTQ
@HWI-EAS443:3:87:821:850#0/1
CACCACTGTCCAGGCCATGATTCGGTCTCTTCCTTCAATT
+HWI-EAS443:3:87:821:850#0/1
aaa`aa`b]aa``Sa_a[Z`_`_`aR[XZV_]__[ZU]__
@HWI-EAS443:3:87:832:1130#0/1
GAAGCAGGTGCTAGCAGAGCTACAGGACATTGAAAATGAA
+HWI-EAS443:3:87:832:1130#0/1
aaaa¥_Y^X]a^___``[^_¥a^`^[¥__`][][S[]^[]
@HWI-EAS443:3:87:845:1810#0/1
ACAGATTTTGCCAGTAAGGTCCTGCACACTTTCCCAATAG
+HWI-EAS443:3:87:845:1810#0/1
aa_XOGZ]aR_¥`¥PYQPNTa_X[P^¥_Y^__[]¥b^ZTW
@HWI-EAS443:3:87:86:1311#0/1
CAGTCATCAAAGTCTACTTTTTCTGGTGCCATATTTTCAC
+HWI-EAS443:3:87:86:1311#0/1
aa¥F`__b_[`aV]`R`_aaa_a`_]Ja_a]a__^_`^a]
@HWI-EAS443:3:87:906:1688#0/1
GGCATGTGAATTGACTGATTCAAGCTGGATAGAACTTGAT
+HWI-EAS443:3:87:906:1688#0/1
^bXbbbab^V`aabbabbbbabb`babb^bbb`a¥b[a_a
@HWI-EAS443:3:87:923:241#0/1
GCGGACTACACCCTTGACGAAGAGAGCAGGGCAAGAATCA
+HWI-EAS443:3:87:923:241#0/1
aaaL`^ZX]bZ`SN¥]]P^[`]Zb^[[`^aa^T^aWU`X_
@HWI-EAS443:3:87:930:481#0/1
CACATTTGGAAGTTTGTTTCATGTATTCGGATTTCCATTT
+HWI-EAS443:3:87:930:481#0/1
abaaabbbba_aabab`abaaab_aaaaaaaa``a_aab`
@HWI-EAS443:3:87:931:1799#0/1
CCCCTGTTGTGTTACATATACTGTTCACCACTGTCCAGGC
```

+HWI-EAS443:3:87:931:1799#0/1
aaabbaabbbbabbabbbbbababbbbbabb`__a`b`aa
@HWI-EAS443:3:88:1022:171#0/1
CGTCTCCACAACTTGAGGGGTTTTCGGCTGAATCGAGAAA
+HWI-EAS443:3:88:1022:171#0/1
]bbabbba_bb`abb`bbba[_ba_`aabbaa`U`aba_a
@HWI-EAS443:3:88:1041:14#0/1
GTCCGAAAGAGGCGAAGAGACAATTGAAGAAAAATTTGAG
+HWI-EAS443:3:88:1041:14#0/1
a_]W_^_Z`^_YM^¥¥¥¥VRWYY]__YYQYZSXUVWXSY]
@HWI-EAS443:3:88:1043:1494#0/1
AGCCAATTGAAGTGGGCACTCGGTGAAAATATGGCACCAG
+HWI-EAS443:3:88:1043:1494#0/1
abYb`a`[a`aa^b_V_`aXaaZ[a__]a^aaab`ZX_X¥
@HWI-EAS443:3:88:1048:1595#0/1
GAATTCATTTTGGACCCAGCTTGCTAGAGATCTGGGCTCT
+HWI-EAS443:3:88:1048:1595#0/1
I_U_a[¥MNY_VWVW^[¥_W_Z``ZI^VW_HV`a]]O[_a
@HWI-EAS443:3:88:1068:1573#0/1
GGGGGCTATATGAAGCAATCGAGGAGTGCCTGATTAATGA
+HWI-EAS443:3:88:1068:1573#0/1
a`U][UQQWTWTSVPN_R[ITYRNL]Y]JGXXRX`¥RPO]
@HWI-EAS443:3:88:1069:126#0/1
ATTTGAGAAATGATACTGATGTGGTGAACTTTGTAAGTAT
+HWI-EAS443:3:88:1069:126#0/1
]b`aba`YYabb`bbaab_ab^bb^a]aT`aaa_a_a___
@HWI-EAS443:3:88:1097:396#0/1
CAGCTTGCTAGAGATCTGGGCTCTGGCTCATCACTGTCAT
+HWI-EAS443:3:88:1097:396#0/1
¥W_^]aa`]bbabbbbb_^[abbb_^^bbbaW]Xbbaa`b
@HWI-EAS443:3:88:1121:268#0/1
CTGCAATATGCACACATTTGGAAGTTTGTTTCATGTATTC
+HWI-EAS443:3:88:1121:268#0/1
`bbbbbbbbbbbbbabbbbbbbbb`bbbabb`abbaabba
@HWI-EAS443:3:88:1156:430#0/1
AGAAGATGTTGCCCCGATTGAACATATCGCAAGCATGAGG
+HWI-EAS443:3:88:1156:430#0/1
¥`_S[¥aN¥HX¥^XWNYH^_baab`[_aTIWR]]]]aZa`
@HWI-EAS443:3:88:115:1670#0/1
ATGTACACTCCCTTCATTATGTATTCAGTAGCCCTGCAGT
+HWI-EAS443:3:88:115:1670#0/1
`¥I¥`aa¥]_Z^`aaa^`aaa_aa``a_¥a_]Y]X_`_SY
@HWI-EAS443:3:88:1171:585#0/1
ATCGACGAACGGGGTGAATCAATAATTGTAGAATCTGGTG
+HWI-EAS443:3:88:1171:585#0/1
ab`b^aba_a`aa`]aaaa_a_]_`a``Y^b^`_]`a`Oa
@HWI-EAS443:3:88:1183:741#0/1
GGCATGGAAATGAGGCGCTGCCTTCTTCAGTCTCTTCAGC
+HWI-EAS443:3:88:1183:741#0/1
aaab`a`aababaa_abab`a`aaaaaa_aXZa¥`a_aaY

```
@HWI-EAS443:3:88:1186:180#0/1
TGAGAAAGGCATAAATCCCAATTACCTCATGGCTTGGAAG
+HWI-EAS443:3:88:1186:180#0/1
abaa_aabbaabaaaa_a`aabaa`_a`aaaa_aa`_O]X
@HWI-EAS443:3:88:1217:361#0/1
AGGCTCTTTCCAGCCAAAGAATGTCTTCATGCATTTGATT
+HWI-EAS443:3:88:1217:361#0/1
ab`aba^abaaa¥_`WX`bb`aYJPaa]aa_LXb^`]aV`
@HWI-EAS443:3:88:1268:1711#0/1
CATTAATCAGGCACTCCTCGATTGCTTCATATAGCCCCCC
+HWI-EAS443:3:88:1268:1711#0/1
abbbbbaaabaabbaaababbababbbbbbbbb``aaQ_^
@HWI-EAS443:3:88:1335:951#0/1
CAGAAAAAGTAGACTTTGATGACTGCAAAGATGTTGGAGA
+HWI-EAS443:3:88:1335:951#0/1
]bbbbbabbabbbbbbbbaaabbbbabbbabbbbbbabaa
@HWI-EAS443:3:88:1362:448#0/1
GAACTTTGTAAGTATGGAGTTCTCACTCACTGACCCGAGA
+HWI-EAS443:3:88:1362:448#0/1
`]b[baba_baa_b`ba_a``_b^aaa_a_aaaQQ^a_a_
@HWI-EAS443:3:88:139:282#0/1
GTTTGTTTTCCGTCTTCCTTCTTTGGTCCTACATTTGCTT
+HWI-EAS443:3:88:139:282#0/1
_P]V]¥[X``¥^FO¥XZa__`_[_`YXQX^[[^_[_ZZZ^
@HWI-EAS443:3:88:1460:945#0/1
ATGAGAAGCAATTTTCTCGATTCAGCCGAAAACCCCTCAA
+HWI-EAS443:3:88:1460:945#0/1
abaabbbb`bababbabababb^abY^babaaY¥^aa_^`
@HWI-EAS443:3:88:1533:127#0/1
CGATTGGCCATGTTTCCGATTTGTTTTCAAAGAATTCCTT
+HWI-EAS443:3:88:1533:127#0/1
a¥V`abaZV`bbaaa¥`[P__aaTa_YTY^aa````_S_a
@HWI-EAS443:3:88:1566:659#0/1
ATTTGTTTTCAAAGAATTCCTTGGTCATGTCTTTCTCTTT
+HWI-EAS443:3:88:1566:659#0/1
__[abU[a`TXbab`b``P_a[`SHWXaabaab¥^b`abb
@HWI-EAS443:3:88:1572:1338#0/1
CTGAAATTAAGTATTGAAGACCCGAGTCACGAGGGGGAGG
+HWI-EAS443:3:88:1572:1338#0/1
aba[bbbbbbbabbbabbaaa_abbb__baabbbaababb
@HWI-EAS443:3:88:1582:603#0/1
ATTGAATTCATTTTGGACCCAGCTTGCTAGAGATCTGGGC
+HWI-EAS443:3:88:1582:603#0/1
_¥YbYa^[`bba]¥Z^OGU^`baa¥URa_X`XI_GO]FOF
@HWI-EAS443:3:88:1731:782#0/1
GAAAGGCATAAATCCCAATTACCTCATGGCTTGGAAGCAG
+HWI-EAS443:3:88:1731:782#0/1
`R`_]`[]]aa`¥XX_a`[S[^]S_[TTX[]YYVL[TQBB
@HWI-EAS443:3:88:1744:319#0/1
TGGCTCATCACTGTCATACTGTTTAAGGTCTCCAACATCT
```

```
+HWI-EAS443:3:88:1744:319#0/1
SS^b_X[a[_`b`a]aaa]¥baaaaa¥```aUa`b_aa[a
@HWI-EAS443:3:88:1757:1145#0/1
GGGGCATGGAAATGAGGCGCTGCCTTCTTCAGTCTCTTCA
+HWI-EAS443:3:88:1757:1145#0/1
aba]W_Zbb]a__`[`]aa^``Z^[^U^_ZV_VJUSZ^W[
@HWI-EAS443:3:88:1779:420#0/1
ATAGCCCCCCAAGATCGAAGGTTCCAGGTTCCAGGTTGTC
+HWI-EAS443:3:88:1779:420#0/1
`ba^`a]]aa`^]``Y]``_a¥¥S¥[Y^U_ZTZ[YY^TP[
@HWI-EAS443:3:88:228:821#0/1
GGGGCATGGAAATGAGGCGCTGCCTTCTTCAGTCTCTTCA
+HWI-EAS443:3:88:228:821#0/1
aaaa_Z^aa_`X`a_aa_aa`a`_^_¥aa`^_W[``__¥Z
@HWI-EAS443:3:88:431:760#0/1
CGAAGAGAGCAGGGCAAGAATCAAAACTAGGCTTTTCACT
+HWI-EAS443:3:88:431:760#0/1
a`X_][¥`^`aa`a__aa`¥````_`^`a^^^___^_a[^
@HWI-EAS443:3:88:516:1194#0/1
GGCACCAGAAAAAGTAGACTTTGATGACTGCAAAGATGTT
+HWI-EAS443:3:88:516:1194#0/1
Zabbbbbb_aT^`a[bb¥b^P`b`ab]bab^a[M^aab^¥
@HWI-EAS443:3:88:525:1847#0/1
GCCAGTAAGGTCCTGCACACTTTCCCAATAGAGCCTTCCT
+HWI-EAS443:3:88:525:1847#0/1
aa_ab_abbYUYbaa`a`_^a_^baaba]a^a`aWa^Z`¥
@HWI-EAS443:3:88:565:382#0/1
TTTGTAATCATACAAATCAGGAAGAAATTTAGGCTTCTCT
+HWI-EAS443:3:88:565:382#0/1
aa`ZP¥baaaaaaa^_`aaaaYR^^a`]TR^a^__¥P¥[_
@HWI-EAS443:3:88:609:448#0/1
GGGGCTATATGAAGCAATCGAGGAGTGCCTGATTAATGAT
+HWI-EAS443:3:88:609:448#0/1
abababbaaab_aaabbaab`ba]`_aa]aa_aaa`aa`a
@HWI-EAS443:3:88:677:920#0/1
GAAAGGCATAAATCCCAATTACCTCATGGCTTGGAAGCAG
+HWI-EAS443:3:88:677:920#0/1
aba_``ab`Uba^bb[aaa`Uaaaaaaa]aaaZ__^`YQR
@HWI-EAS443:3:88:684:731#0/1
CAAAGAATTCCTTGGTCATGTCTTTCTCTTTGACAGAAGA
+HWI-EAS443:3:88:684:731#0/1
Sa`[¥_bbaZaaaaa___bbYbbb_bb]ba¥`bbbbbbb`
@HWI-EAS443:3:88:773:864#0/1
CAGGGCAAGAATCAAAACTAGGCTTTTCACTATAAGACAA
+HWI-EAS443:3:88:773:864#0/1
^Q¥^b`^[_Tabb_H_``b`_QXbaba__Z_ba_^^¥^a^
@HWI-EAS443:3:88:809:918#0/1
TACTGAATACATAATGAAGGGAGTGTACATAAATACGGCC
+HWI-EAS443:3:88:809:918#0/1
`b`]aaab^aaaaY_aa^__`¥¥O[GZ_`X¥^_aa]^`^_
```

```
@HWI-EAS443:3:88:815:376#0/1
GGCTCATCACTGTCATACTGTTTAAGGTCTCCAACATCTT
+HWI-EAS443:3:88:815:376#0/1
ab_`bbaababaaaaa_aa^abaa]_a]`aaaaba`a`a]
@HWI-EAS443:3:88:923:215#0/1
CCGGTTCTCTTTGTAATCATACAAATCAGGAAGAAATTTA
+HWI-EAS443:3:88:923:215#0/1
abbaabbbbbbbaabababbbababb_bbaaabababbab
@HWI-EAS443:3:88:934:429#0/1
GGTTTGTTTTCCGTCTTCCTTCTTTGGTCCTACATTTGCT
+HWI-EAS443:3:88:934:429#0/1
abI`Q`[[_b_¥b]P_]a_]^`aaaaa^_`aaVa_aaaQ¥
@HWI-EAS443:3:88:951:1699#0/1
GACACACATTCACATCTTTTCATTCACTGGAGAGGAGATG
+HWI-EAS443:3:88:951:1699#0/1
aaP`Zb_`bbXbbb```b`aababba_b[U`[ab``baa`
@HWI-EAS443:3:88:995:1766#0/1
CATTAATCAGGCACTCCTCGATTGCTTCATATAGCCCCCC
+HWI-EAS443:3:88:995:1766#0/1
aaa`a`Z_aa^`aabU`aab`Va^^]aaaa_ab`a_a^`[
@HWI-EAS443:3:88:997:1143#0/1
GGCCTTGCTCAATGCATCCTGTGCAGCCATGGATGACTTT
+HWI-EAS443:3:88:997:1143#0/1
T```^W_]RQM¥Ra``_a`^`Q_^`b`abL¥[¥ba]]b``
@HWI-EAS443:3:88:99:2007#0/1
CGGCTGAATCGAGAAAATTGCTTCTCATTGTTCAGGCACT
+HWI-EAS443:3:88:99:2007#0/1
aaaa_a`_^`_Va[a_`_ZX_____`^_^_Y^X_^_^[V]
@HWI-EAS443:3:89:1012:1276#0/1
TGGCCATCTCCTCTCCAGTGAATGAAAAGATGTGAATGTG
+HWI-EAS443:3:89:1012:1276#0/1
[I_^Zbb_O^aaaaaaab^ba]^V]a`]]`ab]a__aa_a
@HWI-EAS443:3:89:1076:1044#0/1
GGGGCATGGAAATGAGGCGCTGCCTTCTTCAGTCTCTTCA
+HWI-EAS443:3:89:1076:1044#0/1
aaa[^__a`aa^aaaa`aa^`aa_``_aaa__^_``a__Z
@HWI-EAS443:3:89:109:1605#0/1
GCCGTATTTATGTACACTCCCTTCATTATGTATTCAGTAG
+HWI-EAS443:3:89:109:1605#0/1
^ab``aabbbbb¥bbbbabbbaaa^aabab]aaaaaa]^`
@HWI-EAS443:3:89:112:1205#0/1
GCATAAATCCCAATTACCTCATGGCTTGGAAGCAGGTGCT
+HWI-EAS443:3:89:112:1205#0/1
abaab``aaa^_ba`abba_aa^_aa^Y__`¥[ZZ^HHW¥
@HWI-EAS443:3:89:116:526#0/1
AAGATGTGAATGTGTGTCTTCTCAGATTTTATTTTGTTGG
+HWI-EAS443:3:89:116:526#0/1
aaa`X__¥`U]]KUSbWT`]ZPZM[OY_``LQ__`PG_[_
@HWI-EAS443:3:89:1203:777#0/1
GGGGTTTTCGGCTGAATCGAGAAAATTGCTTCTCATTGTT
```

```
+HWI-EAS443:3:89:1203:777#0/1
a¥Q_OZO^VbXRXX]SGWRTXaTRTTM]GLVU`HR_SYUP
@HWI-EAS443:3:89:1249:1156#0/1
ACATAATGAAGGGAGTGTACATAAATACGGCCTTGCTCAA
+HWI-EAS443:3:89:1249:1156#0/1
a¥Ub^bbb`]`a`Z`¥^baaaaWa`a_O[¥aaa_`Ua_a`
@HWI-EAS443:3:89:1275:72#0/1
CATGGAAATGAGGCGCTGCCTTCTTCAGTCTCTTCAGCAG
+HWI-EAS443:3:89:1275:72#0/1
aa`abbbbbbabbaa^_ba]bb]abaabaaa¥aaVZ`XRa
@HWI-EAS443:3:89:1417:1094#0/1
AGCAGGTGCTAGCAGAGCTACAGGACATTGAAAATGAAGA
+HWI-EAS443:3:89:1417:1094#0/1
a`aa``Xa]¥`Y¥`b`aV]_a`a_Z^Yaa_V__`^``[^Y
@HWI-EAS443:3:89:1541:782#0/1
GTTTTCAAAGAATTCCTTGGTCATGTCTTTCTCTTTGACA
+HWI-EAS443:3:89:1541:782#0/1
a`abaaaaab^¥aaa^aaa_^_a``]¥a``R]Za^__^^^
@HWI-EAS443:3:89:1546:47#0/1
ATCGACGAACGGGGTGAATCAATAATTGTAGAATCTGGTG
+HWI-EAS443:3:89:1546:47#0/1
aa_^^aba¥V_```X_`aaR^aa`_aaa_a^_a_M_]^Y[
@HWI-EAS443:3:89:1592:1145#0/1
GGCTGGAAAGAGCCTAACATAGTCAAACCACATGAGAAAG
+HWI-EAS443:3:89:1592:1145#0/1
^Ta_bb^^a`Zb¥SYa_bb¥¥baV¥XaKI`b]S¥U_Z^ZW
@HWI-EAS443:3:89:1630:617#0/1
AGCCAATTGAAGTGGGCACTCGGTGAAAATATGGCACCAG
+HWI-EAS443:3:89:1630:617#0/1
aa]_aaabbaaaY_aaa_^`X^a_a_^_`_]`_TY_^V^_
@HWI-EAS443:3:89:1703:399#0/1
GGGGTTTTCGGCTGAATCGAGAAAATTGCTTCTCATTGTT
+HWI-EAS443:3:89:1703:399#0/1
aaa`]aaa_b`U_a`a`Z`^`_R``^__]a_UaR]^_ZLV
@HWI-EAS443:3:89:1749:67#0/1
GGGCACTCGGTGAAAATATGGCACCAGAAAAAGTAGACTT
+HWI-EAS443:3:89:1749:67#0/1
_`b_`bbbbbaabbbbbbbaa¥b[`baaba`ab_aa`X`b
@HWI-EAS443:3:89:1760:237#0/1
ACACCCTTGACGAAGAGAGCAGGGCAAGAATCAAAACTAG
+HWI-EAS443:3:89:1760:237#0/1
aabaa`aaaa¥`¥`b^^]aTU``Y^a]a^__Z__^_Za_]
@HWI-EAS443:3:89:210:625#0/1
ATGGCTTGGAAGCAGGTGCTAGCAGAGCTACAGGACATTG
+HWI-EAS443:3:89:210:625#0/1
a]ab[aaTa`^LOa[UP`VSUQ_P¥]^QGN`P[Y]SNY_D
@HWI-EAS443:3:89:228:1047#0/1
CACACATTCACATCTTTTCATTCACTGGAGAGGAGATGGC
+HWI-EAS443:3:89:228:1047#0/1
aZ]a`[`^`X`__WZ¥[]__`_a`___ZL[__[^`W¥V[T
```

```
@HWI-EAS443:3:89:234:1836#0/1
CAATTATCTCAAATCGGTGCTTCAATAGTGCATTCGGGTC
+HWI-EAS443:3:89:234:1836#0/1
a_Ta¥aLT^`T_T^bTV`bRYV`QZSa__I¥a__`P]XG`
@HWI-EAS443:3:89:250:1882#0/1
AATGGAACCTCCAAGATCAAGATGAAATGGGGCATGGAAA
+HWI-EAS443:3:89:250:1882#0/1
a`a_`aaaa`aaa``a`aa``[^`a_`_][^[_`^_^_^^
@HWI-EAS443:3:89:250:983#0/1
CGGCTGAATCGAGAAAATTGCTTCTCATTGTTCAGGCACT
+HWI-EAS443:3:89:250:983#0/1
aaabbbbbaab_baaabbaabaaaaababa`aaaa`Z`^`
@HWI-EAS443:3:89:285:1748#0/1
GGGGCATGGAAATGAGGCGCTGCCTTCTTCAGTCTCTTCA
+HWI-EAS443:3:89:285:1748#0/1
]_`_`b`bbbbbbb_b`bbbabb```aaabb`¥aaaaba]
@HWI-EAS443:3:89:30:29#0/1
GGGACACTTCTGCTGTAAAATAGTTCCTCCTCATGCTTGC
+HWI-EAS443:3:89:30:29#0/1
a[`¥_¥]`__^^^_`[_^b^^U`X_YZY[[YZVV_]UU]X
@HWI-EAS443:3:89:314:1971#0/1
GGGGCATGGAAATGAGGCGCTGCCTTCTTCAGTCTCTTCA
+HWI-EAS443:3:89:314:1971#0/1
`aa[`a_`_]``aaa___a`_^___[¥__S¥PPJ_¥S_N¥
@HWI-EAS443:3:89:328:526#0/1
CTATGTAGATGGATTCGAGCCGAACGGCTGCATTGAGGGC
+HWI-EAS443:3:89:328:526#0/1
a_¥___`XXZ_]ZZ¥X]ZW__^]W^¥HX_[TMDVYXV_RD
@HWI-EAS443:3:89:337:1693#0/1
CACTGGAGAGGAGATGGCCACCAAAGCGGACTACACCCTT
+HWI-EAS443:3:89:337:1693#0/1
ab`aa`aaaa¥]a¥a¥a]¥_^_¥aW`¥W¥__Pa¥__Z]UU
@HWI-EAS443:3:89:352:255#0/1
AGCAGAGCTACAGGACATTGAAAATGAAGAGAAGATCCCA
+HWI-EAS443:3:89:352:255#0/1
a``aa`baaa_aba`_aaaa`Ya^aa`V]`a_a``_]W]_
@HWI-EAS443:3:89:567:208#0/1
GATCCCAAGGACAAAGAACATGAAGAGAACAAGCCAATTG
+HWI-EAS443:3:89:567:208#0/1
abbaaaaabb`_a_aa`a_`aaaaaaa^`_aaaZ^__`]`
@HWI-EAS443:3:89:589:1706#0/1
GAAGATCCCAAGGACAAAGAACATGAAGAGAACAAGCCAA
+HWI-EAS443:3:89:589:1706#0/1
abbZa`_aaaa`aaa_^aaaaaaaaaaaa```aaWFLY^^
@HWI-EAS443:3:89:591:286#0/1
TAAGACAAGAAATGGCCAGTAGGAGTCTATGGGATTCCTT
+HWI-EAS443:3:89:591:286#0/1
abbaaaa`a`]¥¥_````a^a_^W`V__^___YT^]YYY_
@HWI-EAS443:3:89:606:175#0/1
CGAGGGGGAGGGAATACCACTATATGATGCAATCAAATGC
```

+HWI-EAS443:3:89:606:175#0/1
aba``]¥^_^]``_``U^]O¥```a`aaaXaa`W_]_^__
@HWI-EAS443:3:89:614:716#0/1
GAAAGGCATAAATCCCAATTACCTCATGGCTTGGAAGCAG
+HWI-EAS443:3:89:614:716#0/1
aaaa`a`aaaaa_a``^aa`a`]__`¥_`^`^U^WT[TV¥
@HWI-EAS443:3:89:643:1683#0/1
CGTTCGTCGATGAAATGGAAATCCGAATACATGAAACAAA
+HWI-EAS443:3:89:643:1683#0/1
aa^aaVYaaa``a_a_^S`_^a^a_```a^_[Q]__Xa`]
@HWI-EAS443:3:89:646:561#0/1
GGAGGAACTATTTTACAGCAGAAGTGTCCCACTGCAGGGC
+HWI-EAS443:3:89:646:561#0/1
ab`bbabbbbbaba`ababaa_a_a^a`abaabaaaaa`
@HWI-EAS443:3:89:727:551#0/1
AGACAAGAAATGGCCAGTAGGAGTCTATGGGATTCCTTTC
+HWI-EAS443:3:89:727:551#0/1
aaaaabaaaaaaa`aaaX``_U_^`a`_Z_a`_a^¥¥a`¥
@HWI-EAS443:3:89:748:910#0/1
GCGTCTCCACAACTTGAGGGGTTTTCGGCTGAATCGAGAA
+HWI-EAS443:3:89:748:910#0/1
aa[^aa`aa```_abaWa¥ab]`a_aaY_`__`__`_a]_
@HWI-EAS443:3:89:826:997#0/1
CCCCGTTCGTCGATGAAATGGAAATCCGAATACATGAAAC
+HWI-EAS443:3:89:826:997#0/1
R_bab`]b`bU_ab_^`bb`¥^a^`^^^_a^_a¥aX_Y_T
@HWI-EAS443:3:89:85:786#0/1
GGGGCATGGAAATGAGGCGCTGCCTTCTTCAGTCTCTTCA
+HWI-EAS443:3:89:85:786#0/1
[`^``_W¥a^QFYY_Z`a_a]ba¥Z_N^a¥¥]aZaa`¥LV
@HWI-EAS443:3:89:912:1204#0/1
GGTGCTTCAATAGTGCATTCGGGTCACCAGATTCTACAAT
+HWI-EAS443:3:89:912:1204#0/1
aaYbabbaaaa¥^^aabaa__b`^aaa_abaab`ba_``a
@HWI-EAS443:3:89:931:88#0/1
TGCCATATTTTCACCGAGTGCCCACTTCAATTGGCTTGTT
+HWI-EAS443:3:89:931:88#0/1
aba^ab^aaba]a`_bab¥b_]a^_a^_``a_a`Ya`PZZ
@HWI-EAS443:3:89:966:54#0/1
AAACTTTAGAGCCTATGTAGATGGATTCGAGCCGAACGGC
+HWI-EAS443:3:89:966:54#0/1
`U`X`ab]b]b`_K`bb_Waa]b^_aaJaabZTb_`_baT
@HWI-EAS443:3:8:1007:402#0/1
AGAGAAGATCCCAAGGACAAAGAACATGAAGAGAACAAGC
+HWI-EAS443:3:8:1007:402#0/1
abbaabb``a`_a_a_`_aa`a`a¥__```a`___UaaaU
@HWI-EAS443:3:8:1023:31#0/1
CACGAGGGGGAGGGAATACCACTATATGATGCAATCAAAT
+HWI-EAS443:3:8:1023:31#0/1
aaababbaaa`aaa^`a`^]`X``a^`__^`V^]_R^^__

```
@HWI-EAS443:3:8:1132:1275#0/1
GGCATGGAAATGAGGCGCTGCCTTCTTCAGTCTCTTCAGC
+HWI-EAS443:3:8:1132:1275#0/1
`b__W_b]babb`aa[`a_`]¥_aaaUSU¥NU_P`^_a]U
@HWI-EAS443:3:8:1159:275#0/1
GGGCACTCGGTGAAAATATGGCACCAGAAAAAGTAGACTT
+HWI-EAS443:3:8:1159:275#0/1
aa``b_a]baZa__a`a^`¥`_^¥_]a^]]^_`R¥_^X_[
@HWI-EAS443:3:8:1175:158#0/1
ATAGGCCAAGTGTCGAGGCCCATGTTCCTATATGTGAGAA
+HWI-EAS443:3:8:1175:158#0/1
aaaaa`aa¥b^aY_a_]a^]__]`]XYU]^]¥]_U^UY]]
@HWI-EAS443:3:8:1334:732#0/1
GAAGATCCCAAGGACAAAGAACATGAAGAGAACAAGCCAA
+HWI-EAS443:3:8:1334:732#0/1
abbaaaaaV`_aa_Y`aaa`^^^aaZ_a`a`_Z`]`^O^]
@HWI-EAS443:3:8:1342:814#0/1
TGATGAAATAGGAGAAGATGTTGCCCCGATTGAACATATC
+HWI-EAS443:3:8:1342:814#0/1
^^YWaY¥]V]aa`a__a`¥aZ_¥MOKMZ^ZZ¥U^V]`^BB
@HWI-EAS443:3:8:1373:670#0/1
AGCAGAGCTACAGGACATTGAAAATGAAGAGAAGATCCCA
+HWI-EAS443:3:8:1373:670#0/1
aaaababaab^aaa```aba````aa`Sb``_`__^ZZW_
@HWI-EAS443:3:8:1442:1202#0/1
AATCAGGAAGAAATTTAGGCTTCTCTACCCCTGTTGTGTT
+HWI-EAS443:3:8:1442:1202#0/1
``a]`¥bb¥¥X^Raab^`TQ^aa^Y^a¥XYF^`GZ_]¥_a
@HWI-EAS443:3:8:1527:1155#0/1
CGTCTCCACAACTTGAGGGGTTTTCGGCTGAATCGAGAAA
+HWI-EAS443:3:8:1527:1155#0/1
^_]bb`aWVab`bbbbabb_abaa¥_aJabbabZaaa[aW
@HWI-EAS443:3:8:1559:1637#0/1
TGCTTGCGATATGTTCAATCGGGGCAACATCTTCTCCTAT
+HWI-EAS443:3:8:1559:1637#0/1
a`Q`^Y]_[`]`bXaS__]W]FX_TO_^aUPX`V[JF[__
@HWI-EAS443:3:8:1576:837#0/1
CTTGGGGGGCTATATGAAGCAATCGAGGAGTGCCTGATTA
+HWI-EAS443:3:8:1576:837#0/1
abbbbb``babbbabbaababbb`a_baaa¥a_Zaaaaaa
@HWI-EAS443:3:8:1595:300#0/1
GGGGTTTTCGGCTGAATCGAGAAAATTGCTTCTCATTGTT
+HWI-EAS443:3:8:1595:300#0/1
aZZFJTS[WY¥Y]V[UZR¥VZLLF¥MWMROWUTPOXXBBB
@HWI-EAS443:3:8:1614:515#0/1
GGGGGCTATATGAAGCAATCGAGGAGTGCCTGATTAATGA
+HWI-EAS443:3:8:1614:515#0/1
aaUaa^XI___`U]_YXV¥YaW¥¥T^T_WNW¥TWYYUW¥B
@HWI-EAS443:3:8:1620:1265#0/1
ATCGGGGCAACATCTTCTCCTATTTCATCAAGTTCTATCC
```

```
+HWI-EAS443:3:8:1620:1265#0/1
abbZHZPQ^bZ`a¥aaaa¥]aaa_aX_aS]_X]R¥]a`RU
@HWI-EAS443:3:8:1639:237#0/1
CTTGGGGGGCTATATGAAGCAATCGAGGAGTGCCTGATTA
+HWI-EAS443:3:8:1639:237#0/1
`bbbabbba_bababbaaa`aaa^b]a__a¥aYYaa__a_
@HWI-EAS443:3:8:1660:1878#0/1
CGATTGGCCATGTTTCCGATTTGTTTTCAAAGAATTCCTT
+HWI-EAS443:3:8:1660:1878#0/1
abaaYbaY^aU¥TKYP^`_]a`aSW_]P¥WUHLPT[HS_B
@HWI-EAS443:3:8:1683:1537#0/1
TGGGGGGCTATATGAAGCAATCGAGGAGTGCCTGATTAAT
+HWI-EAS443:3:8:1683:1537#0/1
aba]b_X]a`]abXa^a_b``G_S_[¥b^¥FJ^`Z[aNBB
@HWI-EAS443:3:8:1721:1944#0/1
AGCAGGTGCTAGCAGAGCTACAGGACATTGAAAATGAAGA
+HWI-EAS443:3:8:1721:1944#0/1
a¥FZb]OX`_[a]_aSaWaa]aa__V^`aaSJ¥Ta_^^Y_
@HWI-EAS443:3:8:199:372#0/1
GGGGCATGGAAATGAGGCGCTGCCTTCTTCAGTCTCTTCA
+HWI-EAS443:3:8:199:372#0/1
`b`]¥]Yaa`a]¥`a^Y[]^J¥NPNNUXZYY]XUZFMXNN
@HWI-EAS443:3:8:257:802#0/1
TTAGGCTTCTCTACCCCTGTTGTGTTACATATACTGTTCA
+HWI-EAS443:3:8:257:802#0/1
GZW_aY[_XUXTOO_`X_`[__URS^`^R_U][MZ¥RTXY
@HWI-EAS443:3:8:258:368#0/1
CATGGAAATGAGGCGCTGCCTTCTTCAGTCTCTTCAGCAG
+HWI-EAS443:3:8:258:368#0/1
a`¥`baabaZ¥a`aa`aa_a_a``_¥_a[M_`Va¥_`W__
@HWI-EAS443:3:8:259:472#0/1
AGAAGATCCCAAGGACAAAGAACATGAAGAGAACAAGCCA
+HWI-EAS443:3:8:259:472#0/1
aa`aa``_`_aa```_``aa___`aa]^a_`_]Z^^_XX^
@HWI-EAS443:3:8:275:278#0/1
AACGGGGTGAATCAATAATTGTAGAATCTGGTGACCCGAA
+HWI-EAS443:3:8:275:278#0/1
aaaaaaa¥aa_a__`aa_aaa]_a`_a_]a`Va_]]]_]]
@HWI-EAS443:3:8:293:125#0/1
GGTGAACTTTGTAAGTATGGAGTTCTCACTCACTGACCCG
+HWI-EAS443:3:8:293:125#0/1
aa_]aaaaaab^a`a^aaaa_a_a¥`__]`_a^_a`¥QZ_
@HWI-EAS443:3:8:349:456#0/1
GACAACCTGGAACCTGGAACCTTCGATCTTGGGGGGCTAT
+HWI-EAS443:3:8:349:456#0/1
^R¥QW]VZQVMYRQVZRX^J[S]V^TUVQQ[R_¥_^W¥_¥
@HWI-EAS443:3:8:357:1776#0/1
GGGTGTAGTCCGCTTTGGTGGCCATCTCCTCTCCAGTGAA
+HWI-EAS443:3:8:357:1776#0/1
`ab[babbaaba^bbbZZ¥^b_`aaHbb^a^`^`Z^WXaa
```

```
@HWI-EAS443:3:8:454:240#0/1
GGGGGCTATATGAAGCAATCGAGGAGTGCCTGATTAATGA
+HWI-EAS443:3:8:454:240#0/1
aa`^X]a``]aa____a``Z`[__¥`U_YW^^¥VW^¥Y^^
@HWI-EAS443:3:8:469:1180#0/1
GAGCCAGAGCCCAGATCTCTAGCAAGCTGGGTCCAAAATG
+HWI-EAS443:3:8:469:1180#0/1
`abaaUU`aa__[P]`_`Z^]]X__^UQZ]RIKN]_X¥`N
@HWI-EAS443:3:8:470:427#0/1
AAACTTTAGAGCCTATGTAGATGGATTCGAGCCGAACGGC
+HWI-EAS443:3:8:470:427#0/1
_V`Wab^S^]`[]a`a`UYa`aa__a]¥`a^NN_[¥Y``Y
@HWI-EAS443:3:8:623:32#0/1
ACCAGATTCTACAATTATTGATTCACCCCGTTCGTCGATG
+HWI-EAS443:3:8:623:32#0/1
aa^Xa]_a`a`¥`___`_a`^WWV]__]^`Z^Z^YY_ZZ_
@HWI-EAS443:3:8:669:1448#0/1
ATTTCATCGACGAACGGGGTGAATCAATAATTGTAGAATC
+HWI-EAS443:3:8:669:1448#0/1
`aba``b]^^XYX^¥V¥_YL^¥T[PX[aYZ^U`W^a__ZG
@HWI-EAS443:3:8:68:846#0/1
CATTAATCAGGCACTCCTCGATTGCTTCATATAGCCCCCC
+HWI-EAS443:3:8:68:846#0/1
K_ab_]Y]bba[^[[[[ZU[[`baW[[YZ[]W^TTWDMP[
@HWI-EAS443:3:8:703:491#0/1
GAAGACATTCTTTGGCTGGAAAGAGCCTAACATAGTCAAA
+HWI-EAS443:3:8:703:491#0/1
a_T`a`baaabaaaa`aab_aaa_aV^aSa^_¥`a]``a_
@HWI-EAS443:3:8:718:1685#0/1
AACTTTGACCGCTGATGGCAAAGAGGCCCATCAGGCAATC
+HWI-EAS443:3:8:718:1685#0/1
aaaa`^aba`a]`¥`¥aX_a`_^]U]^U`_ZG^¥YMUZ¥W
@HWI-EAS443:3:8:755:1713#0/1
AAGGAAGGTCTCATTTGAGAAATGATACTGATGTGGTGAA
+HWI-EAS443:3:8:755:1713#0/1
abb`aba_Uaa_`aa``abaaba`a_a_a```a[]TKW_a
@HWI-EAS443:3:8:78:540#0/1
GGGGCATGGAAATGAGGCGCTGCCTTCTTCAGTCTCTTCA
+HWI-EAS443:3:8:78:540#0/1
a_a[`X¥^TGYWU]]_aaa`]X¥M¥^ZYRVU^QYWQOVVZ
@HWI-EAS443:3:8:820:1915#0/1
CATAAATACGGCCTTGCTCAATGCATCCTGTGCAGCCATG
+HWI-EAS443:3:8:820:1915#0/1
aa]aaa`a[WP[``¥]R]`a`__ZaNQ]¥_TSUP^G_ZUT
@HWI-EAS443:3:8:83:62#0/1
GTGCCATATTTTCACCGAGTGCCCACTTCAATTGGCTTGT
+HWI-EAS443:3:8:83:62#0/1
`TaaaX_W^_```a`__[`^__^^Z^_]]]X_]`]Y]^UB
@HWI-EAS443:3:8:903:907#0/1
GAAATTAAGTATTGAAGACCCGAGTCACGAGGGGGAGGGA
```

+HWI-EAS443:3:8:903:907#0/1
a]aa^`_ZVY`a]U_Y¥`]IZaXV[Ua^a]a^aa_Y`¥]_
@HWI-EAS443:3:8:972:955#0/1
GGGGGCTATATGAAGCAATCGAGGAGTGCCTGATTAATGA
+HWI-EAS443:3:8:972:955#0/1
aaa_aaaaaaab_`_aa`_W`¥a`¥_W_W^``Y]_¥¥^]^
@HWI-EAS443:3:8:974:1862#0/1
GGCATGGAAATGAGGCGCTGCCTTCTTCAGTCTCTTCAGC
+HWI-EAS443:3:8:974:1862#0/1
`aKaYZSaaaa`aZ_]ZQWaaU_Y__`^]XMM¥H¥ZaYZU
@HWI-EAS443:3:90:1041:789#0/1
CATAAATACGGCCTTGCTCAATGCATCCTGTGCAGCCATG
+HWI-EAS443:3:90:1041:789#0/1
aa`U_Q[Za`ba`b^aZ[]__bb`a``Y`aUab_aY``a¥
@HWI-EAS443:3:90:1104:1153#0/1
ATCGCAAGCATGAGGAGGAACTATTTTACAGCAGAAGTGT
+HWI-EAS443:3:90:1104:1153#0/1
aaTU^a`b`¥]`aaa`b`[a`a_aa``a`a_YaQa_[QYG
@HWI-EAS443:3:90:1133:462#0/1
ATGCTTGCGATATGTTCAATCGGGGCAACATCTTCTCCTA
+HWI-EAS443:3:90:1133:462#0/1
aaa`bbaabaaaba[`_aa``a`_`¥__``aZa_Y`____
@HWI-EAS443:3:90:1158:212#0/1
CTTGCCCTGCTCTCTTCGTCAAGGGTGTAGTCCGCTTTGG
+HWI-EAS443:3:90:1158:212#0/1
`bbbbbaaaabaaaa`_]_^aaba`Sb^a^_]_a^``a¥Z
@HWI-EAS443:3:90:1211:131#0/1
GAAAATGAAGAGAAGATCCCAAGGACAAAGAACATGAAGA
+HWI-EAS443:3:90:1211:131#0/1
a__^^`a__a^_¥^^_`W]Z]]a]ZZ^Z^`]]XZ]]XZ]Z
@HWI-EAS443:3:90:1213:1237#0/1
AGCCAACAAAATAAAATCTGAGAAGACACACATTCACATC
+HWI-EAS443:3:90:1213:1237#0/1
``_aa¥aba^a`bba^b__`a`_Y]]`a]ba`baO`aa_¥
@HWI-EAS443:3:90:1219:338#0/1
ACATGGCCAATCGGAGAGTCACCCAGGGGAGTGGAGGAAG
+HWI-EAS443:3:90:1219:338#0/1
a^Z`__a`_¥SN¥_TZ`_RVXYZMT^¥_QZ]N]TJ[¥PX¥
@HWI-EAS443:3:90:1256:1167#0/1
CTGAAAGTCATCCATGGCTGCACAGGATGCATTGAGCAAG
+HWI-EAS443:3:90:1256:1167#0/1
abaaabbabbbaabbbbbabbaaaaaaa^^aaaVX`[`aa
@HWI-EAS443:3:90:1278:683#0/1
GCCGAACGGCTGCATTGAGGGCAAGCTTTCCCAAATGTCA
+HWI-EAS443:3:90:1278:683#0/1
a`^aaaaa_a_YY]U_N[[U_Y¥`VQYZ]US_`^`[_QW_
@HWI-EAS443:3:90:1288:1114#0/1
AAGAGACCGAATCATGGCCTGGACAGTGGTGAACAGTATA
+HWI-EAS443:3:90:1288:1114#0/1
Z_X``¥a[aa``]IT__Z]aa``_aa^a_T_XZNZ_¥`¥`

```
@HWI-EAS443:3:90:1322:1406#0/1
CAGCTTGCTAGAGATCTGGGCTCTGGCTCATCACTGTCAT
+HWI-EAS443:3:90:1322:1406#0/1
```b¥a_babba__MU`a[aVabb]baaa[aaa_¥baa`_
@HWI-EAS443:3:90:1380:454#0/1
GCCCTGCTCTCTTCGTCAAGGGTGTAGTCCGCTTTGGTGG
+HWI-EAS443:3:90:1380:454#0/1
aaaaabababaaaaa^^a`abbSa_`a¥^QaUa``aa¥a`
@HWI-EAS443:3:90:1398:1144#0/1
GGCATGGAAATGAGGCGCTGCCTTCTTCAGTCTCTTCAGC
+HWI-EAS443:3:90:1398:1144#0/1
a`baabbaaaaa`a`_b`aa_[aa¥`a_aa¥¥a¥_`]__N
@HWI-EAS443:3:90:1421:1597#0/1
GGGGGCTATATGAAGCAATCGAGGAGTGCCTGATTAATGA
+HWI-EAS443:3:90:1421:1597#0/1
`bbWWWZ]abU`bbbaba`abYVa_``b_b```L]ZY[ba
@HWI-EAS443:3:90:144:375#0/1
ACCAGAAAAAGTAGACTTTGATGACTGCAAAGATGTTGGA
+HWI-EAS443:3:90:144:375#0/1
V_`[aYXVY^_GX`^Y__[Y[_a¥]_`_PW_`[V`W^]S]
@HWI-EAS443:3:90:146:1114#0/1
CTGTAGCTCTGCTAGCACCTGCTTCCAAGCCATGAGGTAA
+HWI-EAS443:3:90:146:1114#0/1
^U``Z``_`Y¥bQ`Ub[][baaT``aSI_aaZ[X^baWUG
@HWI-EAS443:3:90:1543:342#0/1
TGGGCCTCGACACTTGGCCTATCGCAGTCCTCAAGAGCAT
+HWI-EAS443:3:90:1543:342#0/1
a_WIR_aa_XQ_VbW^¥^Q^aa`bU``^Ra_R__aaa¥a^
@HWI-EAS443:3:90:154:1844#0/1
CTCTGGCTCATCACTGTCATACTGTTTAAGGTCTCCAACA
+HWI-EAS443:3:90:154:1844#0/1
aab[LT¥[aa`_S]W]M`ZXUZZ¥YV_¥HGKKWWW^]Y_¥
@HWI-EAS443:3:90:1563:676#0/1
CGAAAACCCCTCAAGTTGTGGAGACGCATATAGACTGTTG
+HWI-EAS443:3:90:1563:676#0/1
abbbbb¥_ba^]aSbbbb_aa]`_U[Uaaabaa¥M¥ZM[I
@HWI-EAS443:3:90:1572:1173#0/1
AAATGGAAATCCGAATACATGAAACAAACTTCCAAATGTG
+HWI-EAS443:3:90:1572:1173#0/1
aaa`aa`]`aa[aa]aa`aa_`a`]a]PT`_][]X]_^Y_
@HWI-EAS443:3:90:1616:31#0/1
CATGGAAATGAGGCGCTGCCTTCTTCAGTCTCTTCAGCAG
+HWI-EAS443:3:90:1616:31#0/1
abbbbbabbbbbaabbab_abb`bb]aaa`b_ba[aa[_a
@HWI-EAS443:3:90:1666:1012#0/1
ACACCCTTGACGAAGAGAGCAGGGCAAGAATCAAAACTAG
+HWI-EAS443:3:90:1666:1012#0/1
¥W`_RWa_U_Y¥`bb_`Z`a[a[YYZa`aaaW]X[Z[_aa
@HWI-EAS443:3:90:1666:293#0/1
CGTCTCCACAACTTGAGGGGTTTTCGGCTGAATCGAGAAA
```
```

+HWI-EAS443:3:90:1666:293#0/1
abbabaa^aab^babababbb^bbaZa`]b`aabYbabaab
@HWI-EAS443:3:90:1693:64#0/1
CCAGTAAGGTCCTGCACACTTTCCCAATAGAGCCTTCCTC
+HWI-EAS443:3:90:1693:64#0/1
]W¥`a[ZbaNXQ`a^XXX_Waa``Zb`]]Y]_XS]UQSXS
@HWI-EAS443:3:90:1709:1921#0/1
CAAAGAATTCCTTGGTCATGTCTTTCTCTTTGACAGAAGA
+HWI-EAS443:3:90:1709:1921#0/1
GYb`b`bb`Taa_aa^_baba[^b]ab`bbb_b^`aaaaa
@HWI-EAS443:3:90:199:1145#0/1
CAGCTTGCTAGAGATCTGGGCTCTGGCTCATCACTGTCAT
+HWI-EAS443:3:90:199:1145#0/1
`bbbabbaa[`b`a`a`[¥_`aaa^Y``aaaZaU__Y[aa
@HWI-EAS443:3:90:289:1543#0/1
TGGCCATGTTTCCGATTTGTTTTCAAAGAATTCCTTGGTC
+HWI-EAS443:3:90:289:1543#0/1
abbbbaababaab^T`abbaaaaabaabb`aaa`a`a_Va
@HWI-EAS443:3:90:362:1893#0/1
CATGCTTGCGATATGTTCAATCGGGGCAACATCTTCTCCT
+HWI-EAS443:3:90:362:1893#0/1
abbbaab___aaa_]WT___[``Y¥¥aa`[a`a^_aY`[[
@HWI-EAS443:3:90:373:17#0/1
GGGTTTTCGGCTGAATCGAGAAAATTGCTTCTCATTGTTC
+HWI-EAS443:3:90:373:17#0/1
aaaW``Za`a_aa]`¥]^W`XRX¥]T^Q_WQTOMUT[ZWV
@HWI-EAS443:3:90:498:310#0/1
CGGCTGAATCGAGAAAATTGCTTCTCATTGTTCAGGCACT
+HWI-EAS443:3:90:498:310#0/1
aaW[b`a`a_^]¥Y_^U`b[`_]¥`a_aa`¥`_aabP`__
@HWI-EAS443:3:90:598:1169#0/1
GTCATCAAAGTCTACTTTTTCTGGTGCCATATTTTCACCG
+HWI-EAS443:3:90:598:1169#0/1
[[aZaa_XW^_`aaUbaab`__^_Saaaaaaaaaaabb^b
@HWI-EAS443:3:90:632:396#0/1
AGAGACCGAATCATGGCCTGGACAGTGGTGAACAGTATAT
+HWI-EAS443:3:90:632:396#0/1
abaaaa`aaaaaaba_aYaa¥__a__`a]aa```aWa_aa
@HWI-EAS443:3:90:661:657#0/1
AGCCAATTGAAGTGGGCACTCGGTGAAAATATGGCACCAG
+HWI-EAS443:3:90:661:657#0/1
aaaaa`aaaa_a^a_aaa__`b`W^_^a_````_W^O¥__
@HWI-EAS443:3:90:663:1736#0/1
GGGGGCTATATGAAGCAATCGAGGAGTGCCTGATTAATGA
+HWI-EAS443:3:90:663:1736#0/1
ab`aabbbbababbbaabaa`bab_a__abaaaaabaaaa
@HWI-EAS443:3:90:701:729#0/1
GGGGCATGGAAATGAGGCGCTGCCTTCTTCAGTCTCTTCA
+HWI-EAS443:3:90:701:729#0/1
ababbbaba^b]`aababbaabb```a`aaaa`^aaaaG`

```
@HWI-EAS443:3:90:757:1927#0/1
TGAAGGGAGTGTACATAAATACGGCCTTGCTCAATGCATC
+HWI-EAS443:3:90:757:1927#0/1
a`_bbbb`[_YVab_aaaaab_T`__a_Y^]]aa_Z]b]V
@HWI-EAS443:3:90:832:1285#0/1
AGGACATTGAAAATGAAGAGAAGATCCCAAGGACAAAGAA
+HWI-EAS443:3:90:832:1285#0/1
`b_baa_[`¥QWXab_``U^a_¥_¥¥^Waa`R_]aXa``_
@HWI-EAS443:3:90:846:354#0/1
GTTTCCGATTTGTTTTCAAAGAATTCCTTGGTCATGTCTT
+HWI-EAS443:3:90:846:354#0/1
`Y]a^_]W`___R_`_¥¥X``TT_YY[^¥¥]V`W^`VV[`
@HWI-EAS443:3:90:855:1447#0/1
GGGTGAATCAATAATTGTAGAATCTGGTGACCCGAATGCA
+HWI-EAS443:3:90:855:1447#0/1
[Y[YS^abbb_`bbabaaababab_`[`aa`]aaabaa^a
@HWI-EAS443:3:90:863:1739#0/1
GAAAGAGGCGAAGAGACAATTGAAGAAAAATTTGAGATTA
+HWI-EAS443:3:90:863:1739#0/1
ab_a_`bbbaabbabb`bbbbbaa^abbaba`aaa__a_a
@HWI-EAS443:3:90:88:984#0/1
ATTGGTTCTCACATATAGGAACATGGGCCTCGACACTTGG
+HWI-EAS443:3:90:88:984#0/1
`__[HYY¥^¥^[¥V¥YVZWY_WWYUYQWTQWNQWIIIYVT
@HWI-EAS443:3:90:929:998#0/1
CAAGCTTGCGCATAGTTCCTGTAATCTCAAATTTTTCTTC
+HWI-EAS443:3:90:929:998#0/1
Sab__QW_^[¥¥_K[__[]a`VK_]]a[^J]`^ab[N_ZW
@HWI-EAS443:3:90:932:71#0/1
TTTCAAAGAATTCCTTGGTCATGTCTTTCTCTTTGACAGA
+HWI-EAS443:3:90:932:71#0/1
abbaa`ab`]aa_^`aba_^`ab]¥aaa]a]aa^`¥^_aZ
@HWI-EAS443:3:90:937:477#0/1
ATCTCAAATTTTTCTTCAATTGTCTCTTCGCCTCTTTCGG
+HWI-EAS443:3:90:937:477#0/1
U`bbab¥`aaabbba`````b`a^aabbab``a^ab_a`a
@HWI-EAS443:3:90:984:1956#0/1
CACTGGAGAGGAGATGGCCACCAAAGCGGACTACACCCTT
+HWI-EAS443:3:90:984:1956#0/1
aaab_aabb^`_T_SV_aab`aaa]^a__`aaa_a`aY_a
@HWI-EAS443:3:90:988:68#0/1
CTTCAATAGTGCATTCGGGTCACCAGATTCTACAATTATT
+HWI-EAS443:3:90:988:68#0/1
abbbbbbababababbbabba^[baaab_bbab_`^]bbaba
@HWI-EAS443:3:91:1008:1033#0/1
CTCCTATTTCATCAAGTTCTATCCAGCTTGAATCAGTCAA
+HWI-EAS443:3:91:1008:1033#0/1
``b_T^bVbb_XZbbb_ZV_abaa`ba``bbba_aa`Yaa
@HWI-EAS443:3:91:1009:802#0/1
GGGATTTATGCCTTTCTCATGTGGTTTGACTATGTTAGGC
```

```
+HWI-EAS443:3:91:1009:802#0/1
aWa[¥[Z^YIXP¥_]^XWW]TONWV^WUW_]X]_GGZ]QO
@HWI-EAS443:3:91:1165:472#0/1
GGGGGCTATATGAAGCAATCGAGGAGTGCCTGATTAATGA
+HWI-EAS443:3:91:1165:472#0/1
a]_[Z_¥T^[[_Z_¥P`^VU^[^^YYV[RZ^`YY^]][UY
@HWI-EAS443:3:91:1219:553#0/1
GGCATGGAAATGAGGCGCTGCCTTCTTCAGTCTCTTCAGC
+HWI-EAS443:3:91:1219:553#0/1
][[¥^XaZ¥R_RRU^YWTW^VWZN[¥¥WNXSQ¥NU¥USWU
@HWI-EAS443:3:91:132:1500#0/1
GAAGATCCCAAGGACAAAGAACATGAAGAGAACAAGCCAA
+HWI-EAS443:3:91:132:1500#0/1
a¥UZaYaa_aa__a`a`a`^`a`_^^]_`_^_X_]]^V^]
@HWI-EAS443:3:91:1377:1585#0/1
CTTTGGTCGGCAAGCTTGCGCATAGTTCCTGTAATCTCAA
+HWI-EAS443:3:91:1377:1585#0/1
K`bbaaaaa_abb]_bbbbbabbbb`babbbb`ba¥baba
@HWI-EAS443:3:91:1519:1626#0/1
AGGAACATGGGCCTCGACACTTGGCCTATCGCAGTCCTCA
+HWI-EAS443:3:91:1519:1626#0/1
abaabaaaa``VZa__``aZa`aaZUaba__¥a`__Z_W_
@HWI-EAS443:3:91:151:571#0/1
GAGCTACAGGACATTGAAAATGAAGAGAAGATCCCAAGGA
+HWI-EAS443:3:91:151:571#0/1
`QVXU_MWY[ZSWXSGXOZSPURSPTLMXPZWNXTRZRQX
@HWI-EAS443:3:91:1535:1537#0/1
CAACAGTCTATATGCGTCTCCACAACTTGAGGGGTTTTCG
+HWI-EAS443:3:91:1535:1537#0/1
[`bb`aUH_bb`bbab¥bbab`WbaTa`aUbbb`R¥¥Y]a
@HWI-EAS443:3:91:1653:1376#0/1
GAATACCACTATATGATGCAATCAAATGCATGAAGACATT
+HWI-EAS443:3:91:1653:1376#0/1
aa_^_]]WF_``^``a¥^¥_a^[a__]^Y`a`SOXUM_Z^
@HWI-EAS443:3:91:1700:1213#0/1
GGGGTTTTCGGCTGAATCGAGAAAATTGCTTCTCATTGTT
+HWI-EAS443:3:91:1700:1213#0/1
a^ZaW]b_X`_U`b```¥_I_]a_`aa_Y]aTa]__a¥W`
@HWI-EAS443:3:91:1756:1183#0/1
AGAAATTTAGGCTTCTCTACCCCTGTTGTGTTACATATAC
+HWI-EAS443:3:91:1756:1183#0/1
aba]_babbababaa]aaaaaaaaa_`Y``aa^Z_`a``X
@HWI-EAS443:3:91:1757:648#0/1
AAGAATTCCTTGGTCATGTCTTTCTCTTTGACAGAAGACT
+HWI-EAS443:3:91:1757:648#0/1
abbaabbaabbba``bbba_bbb^b`abbb``aa`a`aW_
@HWI-EAS443:3:91:1790:900#0/1
AATGCATCCTGTGCAGCCATGGATGACTTTCAGCTGATCC
+HWI-EAS443:3:91:1790:900#0/1
abbaabbaaba^a`^_aaa]]aabaa_aba][aX`a`^YF
```

```
@HWI-EAS443:3:91:184:917#0/1
GCCGAACGGCTGCATTGAGGGCAAGCTTTCCCAAATGTCA
+HWI-EAS443:3:91:184:917#0/1
aa¥a^a¥^U_^aa^OYPRY__YUM^Z]_[GP]H]OZRORO
@HWI-EAS443:3:91:206:423#0/1
TCCGATTTGTTTTCAAAGAATTCCTTGGTCATGTCTTTCT
+HWI-EAS443:3:91:206:423#0/1
YS]aa^a`bYaa__WUPXX^a____`__TZR^`]_^]V^_
@HWI-EAS443:3:91:228:762#0/1
CGAGTCTTCTGTCAAAGAGAAAGACATGACCAAGGAATTC
+HWI-EAS443:3:91:228:762#0/1
Y`aY`bbbbab¥aabXbbab¥abba`abbbbaaba^J^a`
@HWI-EAS443:3:91:236:1022#0/1
ATTCGGATTTCCATTTCATCGACGAACGGGGTGAATCAAT
+HWI-EAS443:3:91:236:1022#0/1
^QW_`aXS]a^]RUZX^a]Y`_``]Z_Y]¥]V^^X^YUY¥
@HWI-EAS443:3:91:297:537#0/1
AAAGAAGTGAACGCCAAAATTGAACCATTCTTGAGGACGA
+HWI-EAS443:3:91:297:537#0/1
a]baaabVbbbaaa`aaaaaababa]aaa`aaa``_a^aa
@HWI-EAS443:3:91:307:738#0/1
GACCGAATCATGGCCTGGACAGTGGTGAACAGTATATGTA
+HWI-EAS443:3:91:307:738#0/1
a]N]Z[P^ZUZ]UWQYZTW[Z_WW^N¥UURUZMVSWWUTV
@HWI-EAS443:3:91:333:990#0/1
GCTTGGAAGCAGGTGCTAGCAGAGCTACAGGACATTGAAA
+HWI-EAS443:3:91:333:990#0/1
`^_`aaS___`P`LTYVXXDRZPN]U___^TXY]¥]YT^U
@HWI-EAS443:3:91:345:666#0/1
GGGGGGCTATATGAAGCAATCGAGGAGTGCCTGATTAATG
+HWI-EAS443:3:91:345:666#0/1
a^^aa`P]¥`U_`ZT_Y¥a_]X`Z[]`U]`]R_X[_YU¥Y
@HWI-EAS443:3:91:362:397#0/1
ACGGGGTGAATCAATAATTGTAGAATCTGGTGACCCGAAT
+HWI-EAS443:3:91:362:397#0/1
[abbbb]b`bbaaa`[`a``^bbaaaaabaYba_¥V`a__
@HWI-EAS443:3:91:377:23#0/1
TGGGGCATGGAAATGAGGCGCTGCCTTCTTCAGTCTCTTC
+HWI-EAS443:3:91:377:23#0/1
abb¥a]aaaa]``aaaa`Wa^_`_¥[_P[a_[¥W]_ZZ_S
@HWI-EAS443:3:91:446:894#0/1
CCACTGCAGGGCTACTGAATACATAATGAAGGGAGTGTAC
+HWI-EAS443:3:91:446:894#0/1
``KXZ¥¥Z^_``¥RZSaa^¥¥]YWXL¥ZRGZPYUZ`[ESN
@HWI-EAS443:3:91:459:1522#0/1
CGAAAATCGAAACTAACAAGTTTGCTGCAATATGCACACA
+HWI-EAS443:3:91:459:1522#0/1
aabbb^bbbabbababbb`bababaaabaabbbaababba
@HWI-EAS443:3:91:464:336#0/1
CGGCTGAATCGAGAAAATTGCTTCTCATTGTTCAGGCACT
```

```
+HWI-EAS443:3:91:464:336#0/1
OXaa¥]¥¥a__Xabb[```_Xa_`b`babbb^[bb`[_Z_
@HWI-EAS443:3:91:492:827#0/1
CAATGAGAAGCAATTTTCTCGATTCAGCCGAAAACCCCTC
+HWI-EAS443:3:91:492:827#0/1
I_`abbbabbb``ababVba`¥_a``baabb]aba^abba
@HWI-EAS443:3:91:522:515#0/1
AGCAGGTGCTAGCAGAGCTACAGGACATTGAAAATGAAGA
+HWI-EAS443:3:91:522:515#0/1
ab``[a`bbbbb^ba¥Za`b[ZabbIV]Tba¥`a_N_`[`
@HWI-EAS443:3:91:523:198#0/1
AGTCCGCTTTGGTGGCCATCTCCTCTCCAGTGAATGAAAA
+HWI-EAS443:3:91:523:198#0/1
`_[aX_aaabb__b`XZ^aa_¥Wa_Z]_S¥Ua`_aaYX_U
@HWI-EAS443:3:91:535:647#0/1
CTCAAATTTTTCTTCAATTGTCTCTTCGCCTCTTTCGGAC
+HWI-EAS443:3:91:535:647#0/1
abbabbbbbbbbbbbbbbbbbbbbbababababbaaaaaaaa
@HWI-EAS443:3:91:543:1163#0/1
ATCCATCAGCAGGAACTTTGACCGCTGATGGCAAAGAGGC
+HWI-EAS443:3:91:543:1163#0/1
aaaaaaaa¥a```]`aa`_^aa`_¥^`^^``U^^¥¥^¥WT
@HWI-EAS443:3:91:617:543#0/1
ATTTCATCGACGAACGGGGTGAATCAATAATTGTAGAATC
+HWI-EAS443:3:91:617:543#0/1
abaaaaa`baab`aaaa`aWa_aa`__a__aaa^aaa`^_
@HWI-EAS443:3:91:649:1297#0/1
CTGGTGCCATATTTTCACCGAGTGCCCACTTCAATTGGCT
+HWI-EAS443:3:91:649:1297#0/1
`ba[U_ba¥_Saa`abbaa^ba``^`baaaa^Z_a¥TW`^
@HWI-EAS443:3:91:65:997#0/1
ATCAGGCACTCCTCGATTGCTTCATATAGCCCCCCAAGAT
+HWI-EAS443:3:91:65:997#0/1
abab^bbabaaaaaa```_``aaa_a_aa_[___Z__^^¥
@HWI-EAS443:3:91:668:443#0/1
TGAGAAGCAATTTTCTCGATTCAGCCGAAAACCCCTCAAG
+HWI-EAS443:3:91:668:443#0/1
Y_V__aa^¥ab_bbabbbaab`[^]Yba_^`^Z]`]V`¥b
@HWI-EAS443:3:91:696:2005#0/1
ATCTGGGCTCTGGCTCATCACTGTCATACTGTTTAAGGTC
+HWI-EAS443:3:91:696:2005#0/1
[`b_baa_Z^]UZ[aba`^`_`][^^`^`_aQ`aa``XYY
@HWI-EAS443:3:91:764:1378#0/1
CCAAAGAAGGAAGACGGAAAACAAACCTGTATGGGTTCAT
+HWI-EAS443:3:91:764:1378#0/1
a^aY`^b¥`bb^VX]bbbabbaa`b^¥a_aabbba]aaab
@HWI-EAS443:3:91:764:1976#0/1
CAATAATTGTAGAATCTGGTGACCCGAATGCACTATTGAA
+HWI-EAS443:3:91:764:1976#0/1
]_ab^`baaabb`bbbbbba`abbba`b`ab¥bbbaabab
```

```
@HWI-EAS443:3:91:811:216#0/1
GCGTCTCCACAACTTGAGGGGTTTTCGGCTGAATCGAGAA
+HWI-EAS443:3:91:811:216#0/1
aa[[__^_]X]_TS¥`^]V_aXX`YX]^X]_]UUR]X]YX
@HWI-EAS443:3:91:844:512#0/1
CTCTTCGTCAAGGGTGTAGTCCGCTTTGGTGGCCATCTCC
+HWI-EAS443:3:91:844:512#0/1
V¥bb_Q[b`_bZ_aWZUab¥¥_bab``baH_abSaab`aa
@HWI-EAS443:3:91:849:1525#0/1
AGAAGTTCGGTGGGAGACTTTGGTCGGCAAGCTTGCGCAT
+HWI-EAS443:3:91:849:1525#0/1
```]baaba`[`^a`aZ^a`aaa`aY`¥baa__aWH¥`]`
@HWI-EAS443:3:91:89:140#0/1
CGACGAACGGGGTGAATCAATAATTGTAGAATCTGGTGAC
+HWI-EAS443:3:91:89:140#0/1
ab¥Z`b^a]]`aNaXHQZZaa_[`a_Z`a__`WR¥a_a^L
@HWI-EAS443:3:91:977:520#0/1
ATTTCATCGACGAACGGGGTGAATCAATAATTGTAGAATC
+HWI-EAS443:3:91:977:520#0/1
^`a``¥X_bbabaaab^`aZbaba_`]`babbaaab^`]V
@HWI-EAS443:3:91:979:1452#0/1
CACTGCAGGGCTACTGAATACATAATGAAGGGAGTGTACA
+HWI-EAS443:3:91:979:1452#0/1
ab`T`aa¥aaba`]]aaa¥a]Y_Y``_Ya_aT^TQVQVW^
@HWI-EAS443:3:91:997:1413#0/1
CGGCTGAATCGAGAAAATTGCTTCTCATTGTTCAGGCACT
+HWI-EAS443:3:91:997:1413#0/1
_¥a__aa`_aaa__¥aaaab`bb`a`aab^a`bbab]_a`
@HWI-EAS443:3:91:998:1808#0/1
CAAAGATGTTGGAGACCTTAAACAGTATGACAGTGATGAG
+HWI-EAS443:3:91:998:1808#0/1
X`Y]a`[bbb_Z^M``X`b¥XT`a[ababbbbaV`¥Z_aX
@HWI-EAS443:3:92:1103:1033#0/1
GGGGCTATATGAAGCAATCGAGGAGTGCCTGATTAATGAT
+HWI-EAS443:3:92:1103:1033#0/1
a`a``aa`a`_aa`[`_^^`]a¥]_Y`_]__]__`^__]Y
@HWI-EAS443:3:92:1103:1856#0/1
ATCATTAATCAGGCACTCCTCGATTGCTTCATATAGCCCC
+HWI-EAS443:3:92:1103:1856#0/1
a]I_[]Wa``UZW]b_a[Ha_`^^___a^Ya_a`_R^_RQ
@HWI-EAS443:3:92:112:164#0/1
CATGGAAATGAGGCGCTGCCTTCTTCAGTCTCTTCAGCAG
+HWI-EAS443:3:92:112:164#0/1
GW_VVbb[bb__bba``ba[b[bbbNbb^baabba^bQZb
@HWI-EAS443:3:92:1175:822#0/1
TGGGGGGCTATATGAAGCAATCGAGGAGTGCCTGATTAAT
+HWI-EAS443:3:92:1175:822#0/1
abaaab`abababbb`ba_aaa_b`aa`b[bZ`abaa``aa
@HWI-EAS443:3:92:1178:1531#0/1
AACAAAATAAAATCTGAGAAGACACACATTCACATCTTTT
```
```

```
+HWI-EAS443:3:92:1178:1531#0/1
aa^babaa`abaa^aa`_aaa_```bW_a__[_aa_``aa
@HWI-EAS443:3:92:1190:416#0/1
GCCCATCAGGCAATCTGAGGGGGCGTGGTGTCGTCCTCAA
+HWI-EAS443:3:92:1190:416#0/1
aa^`aa^`a]`a`_N_^Y[[`^_N_[^aUaY[`YUPLO_X
@HWI-EAS443:3:92:123:1432#0/1
AGCCCCCCAAGATCGAAGGTTCCAGGTTCCAGGTTGTCCC
+HWI-EAS443:3:92:123:1432#0/1
aaaaabaaaa]`a]a`]``Q[^^OY[PZY[¥Y_U^_NTS_
@HWI-EAS443:3:92:1268:618#0/1
ATCAGGCACTCCTCGATTGCTTCATATAGCCCCCCAAGAT
+HWI-EAS443:3:92:1268:618#0/1
aaaaba`a____a[`_a```a_¥`aa_a`TKWXS^`¥_`_
@HWI-EAS443:3:92:127:533#0/1
CATAAATACGGCCTTGCTCAATGCATCCTGTGCAGCCATG
+HWI-EAS443:3:92:127:533#0/1
aabaabbbaaaaaaa[aa`aaaaaaaaa`b`a``a____`
@HWI-EAS443:3:92:1341:1272#0/1
ATCTTTGCAGTCATCAAAGTCTACTTTTTCTGGTGCCATA
+HWI-EAS443:3:92:1341:1272#0/1
a^`aVZ[aZ_Ua`_Z_¥`a[PW_YZ`_P_aaba¥b[YZ`a
@HWI-EAS443:3:92:1352:1239#0/1
GAACTATTTTACAGCAGAAGTGTCCCACTGCAGGGCTACT
+HWI-EAS443:3:92:1352:1239#0/1
]abaaaa`aaba`a^ab`aa_`^]¥¥a^___TaU^_^^__
@HWI-EAS443:3:92:1378:991#0/1
GCCCTGCAGTGGGACACTTCTGCTGTAAAATAGTTCCTCC
+HWI-EAS443:3:92:1378:991#0/1
a`_Xa`]ZaS`a_U_a_a^_aaW_a_¥_b^___W_¥S¥[N
@HWI-EAS443:3:92:1402:1247#0/1
CGAACGGCTGCATTGAGGGCAAGCTTTCCCAAATGTCAAA
+HWI-EAS443:3:92:1402:1247#0/1
abaa`b`aa`X^_^MZUa^WXa^Y]aYPN]`__`¥P[__`
@HWI-EAS443:3:92:141:1899#0/1
GGGTTTTCGGCTGAATCGAGAAAATTGCTTCTCATTGTTC
+HWI-EAS443:3:92:141:1899#0/1
aba__aaa_aaSabb^baa_¥]a`[`_¥_¥a`ba¥W`X^`
@HWI-EAS443:3:92:1440:1082#0/1
CTCATTTGAGAAATGATACTGATGTGGTGAACTTTGTAAG
+HWI-EAS443:3:92:1440:1082#0/1
ababbbbbbbbaabbbbaabbbbbab`_ba^O]aaa`aa¥
@HWI-EAS443:3:92:1468:118#0/1
GAAAGTCATCCATGGCTGCACAGGATGCATTGAGCAAGGC
+HWI-EAS443:3:92:1468:118#0/1
aa_a^HMaaZ`b_aaY`^V¥J^]_VRVXRY¥^[HUBBBBB
@HWI-EAS443:3:92:1484:1114#0/1
CACCCTTGACGAAGAGAGCAGGGCAAGAATCAAAACTAGG
+HWI-EAS443:3:92:1484:1114#0/1
a``]XQQ``Sba]`_bab[]`abZ¥_a`_[a`a__]_`ZH
```

```
@HWI-EAS443:3:92:1536:546#0/1
CAGCAAACTTGTTAGTTTCGATTTTCGGATCTTCCCCATA
+HWI-EAS443:3:92:1536:546#0/1
abaabaa_baaab`aaaa_aSaaa`_`_O]W^`WXXZ]`X
@HWI-EAS443:3:92:1719:582#0/1
TGTGAATTGACTGATTCAAGCTGGATAGAACTTGATGAAA
+HWI-EAS443:3:92:1719:582#0/1
ab_aaa`[^__a`]aa^___T`X]Ua_a_aXaa`Xa_]]^
@HWI-EAS443:3:92:1728:933#0/1
GGGGGCTATATGAAGCAATCGAGGAGTGCCTGATTAATGA
+HWI-EAS443:3:92:1728:933#0/1
aa[U`^_][Z_`_VWO[Y_SYNYYVQVNGTYTTK[]VV^B
@HWI-EAS443:3:92:1742:304#0/1
ATAAGACAAGAAATGGCCAGTAGGAGTCTATGGGATTCCT
+HWI-EAS443:3:92:1742:304#0/1
a^a¥]Z_Yaa`_]`_`RZU`TZ¥_U_OW`YY`a]W^]QGR
@HWI-EAS443:3:92:1745:1240#0/1
CGTCTCCACAACTTGAGGGGTTTTCGGCTGAATCGAGAAA
+HWI-EAS443:3:92:1745:1240#0/1
ab_aaa`[`_a_aa¥]baabVa`a[a_^aaa_`¥^Za__^
@HWI-EAS443:3:92:1751:1026#0/1
GGCTTGGAAGCAGGTGCTAGCAGAGCTACAGGACATTGAA
+HWI-EAS443:3:92:1751:1026#0/1
ab`abba`aaV¥``]]___^Z^aYaZ_`Waa_]^Y[^XZX
@HWI-EAS443:3:92:24:777#0/1
GAACAATGAGAAGCAATTTTCTCGATTCAGCCGAAAACCC
+HWI-EAS443:3:92:24:777#0/1
`_`_]__b`^]Z``]Z]a`aa_`a``^_]_]Y_¥¥Y¥]WR
@HWI-EAS443:3:92:258:976#0/1
AGAACATGAAGAGAACAAGCCAATTGAAGTGGGCACTCGG
+HWI-EAS443:3:92:258:976#0/1
aaa]aY^aaaa]V[VaZDVaa^a^^a`RWW_a_]__¥Wa^
@HWI-EAS443:3:92:318:1485#0/1
ATTTCATCGACGAACGGGGTGAATCAATAATTGTAGAATC
+HWI-EAS443:3:92:318:1485#0/1
aaa`aa_`^a^_`_^Y^`_X_`^¥_^_^`_Z__U_[]_WU
@HWI-EAS443:3:92:321:1906#0/1
ATCAGGCACTCCTCGATTGCTTCATATAGCCCCCCAAGAT
+HWI-EAS443:3:92:321:1906#0/1
`bbabbb_]H_bYQ``Y_^aaY`[XaaaYaaa_][_XaZG
@HWI-EAS443:3:92:327:1640#0/1
CGGGGTGAATCAATAATTGTAGAATCTGGTGACCCGAATG
+HWI-EAS443:3:92:327:1640#0/1
a^`^`]^`bbbbbbbabaaaabababaaa_aabaaaaa_`
@HWI-EAS443:3:92:335:1085#0/1
ATTCGGATTTCCATTTCATCGACGAACGGGGTGAATCAAT
+HWI-EAS443:3:92:335:1085#0/1
^`b```JVZZ``]`aa_¥`aaab`aa[^`aaOa`aa___a
@HWI-EAS443:3:92:341:192#0/1
ATCTTGATCTTGGAGGTTCCATTGGTTCTCACATATAGGA
```

+HWI-EAS443:3:92:341:192#0/1
`babababaaaba`aa[aa`a``aa]__`_`Z_`__^¥_]
@HWI-EAS443:3:92:397:1448#0/1
CAAAGAAGGAAGACGGAAAACAAACCTGTATGGGTTCATT
+HWI-EAS443:3:92:397:1448#0/1
YJPM_TY]ZWZZ¥]^`¥Z_VLJRP¥XQSZ[[VIZNOMS]]
@HWI-EAS443:3:92:440:120#0/1
GCTCTATTGGGAAAGTGTGCAGGACCTTACTGGCAAAATC
+HWI-EAS443:3:92:440:120#0/1
aaa_aX`aaba¥[aa]a]a```_^^^__^]ZZ]^_^`^^R
@HWI-EAS443:3:92:476:387#0/1
CATCGACGAACGGGGTGAATCAATAATTGTAGAATCTGGT
+HWI-EAS443:3:92:476:387#0/1
``a¥¥_][^SJ_a^][b_Z]Za]`UN[[_T`a_`YQY_`V
@HWI-EAS443:3:92:492:1280#0/1
GGGTTTTCGGCTGAATCGAGAAAATTGCTTCTCATTGTTC
+HWI-EAS443:3:92:492:1280#0/1
_bb^babb_abaabb`K`_bbabaaba^a`_a_baaaa_a
@HWI-EAS443:3:92:554:1220#0/1
AGCAGGTGCTAGCAGAGCTACAGGACATTGAAAATGAAGA
+HWI-EAS443:3:92:554:1220#0/1
a]aRY]_]a]a`]a^MXaS_Y_`_aV]]^_^]^^^¥]^¥^
@HWI-EAS443:3:92:633:928#0/1
ACGCCAAAATTGAACCATTCTTGAGGACGACACCACGCCC
+HWI-EAS443:3:92:633:928#0/1
ab`H[Wa]_^`a_aVYZaaa`I]^`__^aVW¥_Qa_N¥_N
@HWI-EAS443:3:92:640:670#0/1
TCCACAACTTGAGGGGTTTTCGGCTGAATCGAGAAAATTG
+HWI-EAS443:3:92:640:670#0/1
abaa`]`_baa_a`aa]`]HZ[`^aaa]_a__a`X`^_`_
@HWI-EAS443:3:92:663:874#0/1
AGAACACAGTATTTTTCCCATTTGTGTGGCTCCAGTCTCG
+HWI-EAS443:3:92:663:874#0/1
``^baa_ba_aaaa`aa`_aaaaa_a`aa`a_aS`¥¥ZS^
@HWI-EAS443:3:92:668:917#0/1
AGAATTCCTTGGTCATGTCTTTCTCTTTGACAGAAGACTC
+HWI-EAS443:3:92:668:917#0/1
^VGRYaSa_]b]ZQ_]^R]PXZ[`¥]a`aI]]^_aY_^^^
@HWI-EAS443:3:92:775:477#0/1
TTCCGATTTGTTTTCAAAGAATTCCTTGGTCATGTCTTTC
+HWI-EAS443:3:92:775:477#0/1
ab_ab`abab`aaaaa`a``]aa`_`aaa^_`aa__a`__
@HWI-EAS443:3:92:815:284#0/1
ATGGCTTGGAAGCAGGTGCTAGCAGAGCTACAGGACATTG
+HWI-EAS443:3:92:815:284#0/1
aaaaaba^_V]`¥``¥FZQRY_T¥_[]NT[P[``ZVS^]P
@HWI-EAS443:3:92:845:678#0/1
GACGAAAGGAATCCCATAGACTCCTACTGGCCATTTCTTG
+HWI-EAS443:3:92:845:678#0/1
ab`b]aXbXWJ`aU`_a¥a¥Y[a_ZLY_a__]__^^MY`Z

```
@HWI-EAS443:3:92:897:130#0/1
ATTCAACAGTCTATATGCGTCTCCACAACTTGAGGGGTTT
+HWI-EAS443:3:92:897:130#0/1
abbaba`ab`abaaaba`a````_a]_`_baa_aaaaX`_
@HWI-EAS443:3:92:925:1219#0/1
TATGTATTCAGTAGCCCTGCAGTGGGACACTTCTGCTGTA
+HWI-EAS443:3:92:925:1219#0/1
aaab[aaaaaa__X``a`___`[^__]W]]`___]]a_]_
@HWI-EAS443:3:92:950:1980#0/1
TGGGATTTATGCCTTTCTCATGTGGTTTGACTATGTTAGG
+HWI-EAS443:3:92:950:1980#0/1
abaaa_Z_b`aa_`aaa_aaa__aa^`_aa`a__`]_a^G
@HWI-EAS443:3:92:961:1201#0/1
CGTCTCCACAACTTGAGGGGTTTTCGGCTGAATCGAGAAA
+HWI-EAS443:3:92:961:1201#0/1
[_aba`b]a`aab_b^]abaYa`aa^]^bab_`^a]a_Q]
@HWI-EAS443:3:92:978:895#0/1
AAGAATTCCTTGGTCATGTCTTTCTCTTTGACAGAAGACT
+HWI-EAS443:3:92:978:895#0/1
aaa_`a_^`aaaa^aaaa]a``aa```_`a__`a__`_Z]
@HWI-EAS443:3:93:1000:1017#0/1
GAGTCTATGGGATTCCTTTCGTCAGTCCGAAAGAGGCGAA
+HWI-EAS443:3:93:1000:1017#0/1
aZb__aa¥_¥^Ia``_^``PaZ_Y[F^__R_^^^PS^[N^
@HWI-EAS443:3:93:1039:1622#0/1
GGGATCTTCTCTTCATTTTCAATGTCCTGTAGCTCTGCTA
+HWI-EAS443:3:93:1039:1622#0/1
aa_Ra_aaa`_aa_a^___`a_a`R_]a`^_a^a`^]___
@HWI-EAS443:3:93:1055:394#0/1
AGTGTACATAAATACGGCCTTGCTCAATGCATCCTGTGCA
+HWI-EAS443:3:93:1055:394#0/1
ab^`^`abbabaaaa`][a__`aaY__aa¥aa¥¥`aV`Ra
@HWI-EAS443:3:93:1081:1091#0/1
TTCATTTTCAATGTCCTGTAGCTCTGCTAGCACCTGCTTC
+HWI-EAS443:3:93:1081:1091#0/1
a]baa_bbba]aa[aZaa_a]]aa[_RUQa]V[HZ`GZYB
@HWI-EAS443:3:93:1111:1530#0/1
GTGCCATATTTTCACCGAGTGCCCACTTCAATTGGCTTGT
+HWI-EAS443:3:93:1111:1530#0/1
_R_¥Qaabaaaa`b`X``aPa^``a^^UT[[][VYZ]]XZ
@HWI-EAS443:3:93:1148:628#0/1
GGGGCTATATGAAGCAATCGAGGAGTGCCTGATTAATGAT
+HWI-EAS443:3:93:1148:628#0/1
_NNT]VR^P^a]Y[^`¥QV_QZNR`PHSN¥QW^`Z_]_U^
@HWI-EAS443:3:93:1327:666#0/1
GTTCCATTGGTTCTCACATATAGGAACATGGGCCTCGACA
+HWI-EAS443:3:93:1327:666#0/1
a¥^VY_T[aaP]^]U`GU]``_U_ZZJXTLMTOOYJTBBB
@HWI-EAS443:3:93:1364:1761#0/1
AATGGAAATCCGAATACATGAAACAAACTTCCAAATGTGT
```

+HWI-EAS443:3:93:1364:1761#0/1
aaa_a_`_aY_aa_aaS``]`_`_`_`[_^W_a_^_[P]]
@HWI-EAS443:3:93:1401:317#0/1
TGACTGCAAAGATGTTGGAGACCTTAAACAGTATGACAGT
+HWI-EAS443:3:93:1401:317#0/1
^aaaab_aa`b_`a_b]aa``__ba```Z]a``aa_X__W
@HWI-EAS443:3:93:1416:2011#0/1
TGGGGCATGGAAATGAGGCGCTGCCTTCTTCAGTCTCTTC
+HWI-EAS443:3:93:1416:2011#0/1
`]¥bH`X_Sa`aZUaZa¥]a^__ZXZXMV¥¥__JNVKP¥]
@HWI-EAS443:3:93:1482:850#0/1
CTATAAGACAAGAAATGGCCAGTAGGAGTCTATGGGATTC
+HWI-EAS443:3:93:1482:850#0/1
`ab`RX]^b_]b_aa_b[U_Xa`ZWaZaX^__a__`XY]U
@HWI-EAS443:3:93:1537:994#0/1
GGGGTTTTCGGCTGAATCGAGAAAATTGCTTCTCATTGTT
+HWI-EAS443:3:93:1537:994#0/1
aa`]QPQWaY`_a_¥_SQ_^[XY_Y_X[YY^R[SV^PMRJ
@HWI-EAS443:3:93:1564:574#0/1
TGGCGTTCACTTCTTTTGACATTTGGGAAAGCTTGCCCTC
+HWI-EAS443:3:93:1564:574#0/1
`[^^`VX^WGT`UH]_aTVLXP_`SW^U_TTP^XTGTXNB
@HWI-EAS443:3:93:1744:803#0/1
ATCCCAATTACCTCATGGCTTGGAAGCAGGTGCTAGCAGA
+HWI-EAS443:3:93:1744:803#0/1
`[`XY__`aa_YY]_¥UY```Z¥ZRRUBBBBBBBBBBBBB
@HWI-EAS443:3:93:229:988#0/1
AAAGAGCCTAACATAGTCAAACCACATGAGAAAGGCATAA
+HWI-EAS443:3:93:229:988#0/1
a`¥a]`baaab`a`aa_aa]aaa``a_aa`a____``__`
@HWI-EAS443:3:93:235:295#0/1
AAATCCCAATTACCTCATGGCTTGGAAGCAGGTGCTAGCA
+HWI-EAS443:3:93:235:295#0/1
a`X_R[a`]]_`PY___^`]Y_¥R]RY_RUU¥HPUVSRBB
@HWI-EAS443:3:93:263:797#0/1
CTTTGACCGCTGATGGCAAAGAGGCCCATCAGGCAATCTG
+HWI-EAS443:3:93:263:797#0/1
a^_`[^``_a¥`X[]`_¥``¥[]`¥Z^XFF¥`¥F¥NVZZ`
@HWI-EAS443:3:93:301:1633#0/1
AAACTTTAGAGCCTATGTAGATGGATTCGAGCCGAACGGC
+HWI-EAS443:3:93:301:1633#0/1
Ta_^aY``]P]¥¥¥aa_P^__a]_RNS¥QX_¥a]Y^P^BB
@HWI-EAS443:3:93:357:1744#0/1
CCGAGACTGGAGCCACACAAATGGGAAAAATACTGTGTTC
+HWI-EAS443:3:93:357:1744#0/1
]^bbaX``Z]^bbaaabaa`Q^a__]`]b[V`][[W_¥]R
@HWI-EAS443:3:93:377:1921#0/1
CTGGAACCTGGAACCTTCGATCTTGGGGGGCTATATGAAG
+HWI-EAS443:3:93:377:1921#0/1
aY]^`a`a¥_^`^Z^V¥_[Y^^¥^Z]WW]X¥^]^__W¥^¥

@HWI-EAS443:3:93:399:798#0/1
AGTCATCAAAGTCTACTTTTTCTGGTGCCATATTTTCACC
+HWI-EAS443:3:93:399:798#0/1
aa_aaaaWTV]_a_SZaa`a`aaa^U`Wa``_``___`]S
@HWI-EAS443:3:93:411:454#0/1
GACTTTGGTCGGCAAGCTTGCGCATAGTTCCTGTAATCTC
+HWI-EAS443:3:93:411:454#0/1
aa^`]a`WW_^__`]_`^]_[`_`_X_W_YUZ`TXXZW]X
@HWI-EAS443:3:93:426:336#0/1
GTTCAATCGGGGCAACATCTTCTCCTATTTCATCAAGTTC
+HWI-EAS443:3:93:426:336#0/1
a_``[¥Z___`__]Z]__[__^^^W^]__]]^_]]]YY^Z
@HWI-EAS443:3:93:443:1158#0/1
GAAGAAATTTAGGCTTCTCTACCCCTGTTGTGTTACATAT
+HWI-EAS443:3:93:443:1158#0/1
a^^aZaabaaaaaYaa^aa`^_aa`]]Y`aY_^`_Z_`^_
@HWI-EAS443:3:93:490:101#0/1
GGCTGGAGAAGTTCGGTGGGAGACTTTGGTCGGCAAGCTT
+HWI-EAS443:3:93:490:101#0/1
aa_aaa_a``b^a¥abZa`aY^W^¥_`]]WWSYTYWUPUB
@HWI-EAS443:3:93:496:1946#0/1
GATTCGGTCTCTTCCTTCAATTATCTCAAATCGGTGCTTC
+HWI-EAS443:3:93:496:1946#0/1
W^ZX¥a¥]`_X^`bb^a^`a^^a`¥_`_^b¥Zb[HZ^_]Y
@HWI-EAS443:3:93:563:484#0/1
ATGGAGTTCTCACTCACTGACCCGAGACTGGAGCCACACA
+HWI-EAS443:3:93:563:484#0/1
^Y[a`_]a^_]_]^]Yaaaa`a`a`a_aaaa_a[[`Y¥XX
@HWI-EAS443:3:93:635:1638#0/1
CATGGAAATGAGGCGCTGCCTTCTTCAGTCTCTTCAGCAG
+HWI-EAS443:3:93:635:1638#0/1
abab_baba`bb[aab_baaaaaaaaa`^_a`a`Za_N^a
@HWI-EAS443:3:93:67:120#0/1
GCCGTATTTATGTACACTCCCTTCATTATGTATTCAGTAG
+HWI-EAS443:3:93:67:120#0/1
a^a`L_bZP^aa_``a¥___Y_[^U^[[^_V^^^UZ][RB
@HWI-EAS443:3:93:680:38#0/1
CATTAATCAGGCACTCCTCGATTGCTTCATATAGCCCCCC
+HWI-EAS443:3:93:680:38#0/1
[aSXaa`a]aU]a[]^SXYaZQ[a__`Z_^]_a__¥[N[`
@HWI-EAS443:3:93:715:863#0/1
CAATTTCAATGAACCGGTTCTCTTTGTAATCATACAAATC
+HWI-EAS443:3:93:715:863#0/1
a`¥baaabaaaa`a`aa`aaaaaaaa_aa^_aaa_`]`^_
@HWI-EAS443:3:93:730:942#0/1
CATATTTTCACCGAGTGCCCACTTCAATTGGCTTGTTCTC
+HWI-EAS443:3:93:730:942#0/1
abaabbaa`bbaa_a^a_``a`aaa````^]_aa[]^O^^
@HWI-EAS443:3:93:739:473#0/1
GGCATGGAAATGAGGCGCTGCCTTCTTCAGTCTCTTCAGC

```
+HWI-EAS443:3:93:739:473#0/1
aVa`H_ZVXT[aXY¥_a[_MN¥V`Y]^FHHZXa__¥_ZHU
@HWI-EAS443:3:93:820:1011#0/1
CGTGTTACTCCAATTTCAATGAACCGGTTCTCTTTGTAAT
+HWI-EAS443:3:93:820:1011#0/1
ab_aa_baaaab^aaaaaa`aaa``a`[`]``a``a__^a
@HWI-EAS443:3:93:829:1484#0/1
AGGACATTGAAAATGAAGAGAAGATCCCAAGGACAAAGAA
+HWI-EAS443:3:93:829:1484#0/1
_aaYa`ba`aababb__bba`aaa`a_`a_b`aaa__a``
@HWI-EAS443:3:93:865:578#0/1
CGAAAATCGAAACTAACAAGTTTGCTGCAATATGCACACA
+HWI-EAS443:3:93:865:578#0/1
a`aba[]`a_a`_aaa`a`aZ`a___a_`_a_a`¥_]_]Z
@HWI-EAS443:3:93:902:1330#0/1
ATGCGCAAGCTTGCCGACCAAAGTCTCCCACCGAACTTCT
+HWI-EAS443:3:93:902:1330#0/1
`aabaa_`a¥^``_aaa^_`Z]`¥Y]_Y]_¥a_^^¥^¥S_
@HWI-EAS443:3:93:937:345#0/1
GGCCAGTAGGAGTCTATGGGATTCCTTTCGTCAGTCCGAA
+HWI-EAS443:3:93:937:345#0/1
¥_aabbabbbbaa_abbaabbab``bbbXaaXa`a`G][`
@HWI-EAS443:3:93:942:110#0/1
CAACTTGAGGGGTTTTCGGCTGAATCGAGAAAATTGCTTC
+HWI-EAS443:3:93:942:110#0/1
aaa`abbabaa_[aba[^b_aaaa__a_a]Xa]_a`T]__
@HWI-EAS443:3:93:978:139#0/1
CTTGGGGGGCTATATGAAGCAATCGAGGAGTGCCTGATTA
+HWI-EAS443:3:93:978:139#0/1
`_aaabbababbabbb`ab`bbbaa`b[aa]a[Sab¥`^a
@HWI-EAS443:3:94:1117:413#0/1
GGGCACTCGGTGAAAATATGGCACCAGAAAAAGTAGACTT
+HWI-EAS443:3:94:1117:413#0/1
aa__^__``aVa]Xa`_^`a]Z_ZW_`Z`_^^aW_^^RX`
@HWI-EAS443:3:94:1184:1411#0/1
ATGAAACAAACTTCCAAATGTGTGCATATTGCAGCAAACT
+HWI-EAS443:3:94:1184:1411#0/1
_abb`ab_¥_bbab``aa`ba`[aaaaaaa_Yb_¥a__^a
@HWI-EAS443:3:94:1216:363#0/1
GTGAACTTTGTAAGTATGGAGTTCTCACTCACTGACCCGA
+HWI-EAS443:3:94:1216:363#0/1
a_aMQVb`ab_a]b^aaa_^a¥a¥_¥]¥aU_W_``YN¥_¥
@HWI-EAS443:3:94:1412:217#0/1
AGAATTCCTTGGTCATGTCTTTCTCTTTGACAGAAGACTC
+HWI-EAS443:3:94:1412:217#0/1
abaV`_Q¥aba]TIbaab_abb]`Zaa]aOIXa[`_^U^X
@HWI-EAS443:3:94:1421:734#0/1
AGATCCCAAGGACAAAGAACATGAAGAGAACAAGCCAATT
+HWI-EAS443:3:94:1421:734#0/1
a^^^^]aUV]`PJPY¥SUa^a¥Y]aa`]VU¥^¥_YGSR^G
```

@HWI-EAS443:3:94:1502:1586#0/1
ATCTTCTCCTATTTCATCAAGTTCTATCCAGCTTGAATCA
+HWI-EAS443:3:94:1502:1586#0/1
ab^ab`ababbbaaba`_b`a¥b__ba_^a]XWaa`aa^b
@HWI-EAS443:3:94:1507:336#0/1
GAGGCGAAGAGACAATTGAAGAAAAATTTGAGATTACAGG
+HWI-EAS443:3:94:1507:336#0/1
¥[ba^aa^a`b`^``a¥XYaaaba_`a_aa_^`__`Z_`_
@HWI-EAS443:3:94:1508:557#0/1
AAGAATTCCTTGGTCATGTCTTTCTCTTTGACAGAAGACT
+HWI-EAS443:3:94:1508:557#0/1
``b`I`abJaaa_`_aaaa`b¥a`SSa```SR`__]a__^
@HWI-EAS443:3:94:154:427#0/1
ATCAGGCACTCCTCGATTGCTTCATATAGCCCCCCAAGAT
+HWI-EAS443:3:94:154:427#0/1
aaa_]^`aa`a`a_aa_[SYa`a``_`XR`Y^Y`Z^ZZXW
@HWI-EAS443:3:94:1597:1024#0/1
CGAAAATCGAAACTAACAAGTTTGCTGCAATATGCACACA
+HWI-EAS443:3:94:1597:1024#0/1
aabbabba_a``Ybb`]aaV^`aXJZaRV^a^aaM]_X]`
@HWI-EAS443:3:94:1642:800#0/1
GGGAGACTTTGGTCGGCAAGCTTGCGCATAGTTCCTGTAA
+HWI-EAS443:3:94:1642:800#0/1
a_a`a`_baa`a^^_[]a]^[^a`R`]``^a^a_Z`aXX]
@HWI-EAS443:3:94:178:1050#0/1
GATTTGTTTTCAAAGAATTCCTTGGTCATGTCTTTCTCTT
+HWI-EAS443:3:94:178:1050#0/1
a[_^ab_`X_a_[`]J^__]___^ZZ_]¥_XY¥]]^]U^^
@HWI-EAS443:3:94:23:340#0/1
AAAGAGCCTAACATAGTCAAACCACATGAGAAAGGCATAA
+HWI-EAS443:3:94:23:340#0/1
ab]`Wb`a]`a¥TJYaX]Tb^UQZ_b[[¥N]¥^_aO^_Z[
@HWI-EAS443:3:94:258:340#0/1
CTGTTGTGTTACATATACTGTTCACCACTGTCCAGGCCAT
+HWI-EAS443:3:94:258:340#0/1
abaabbabaababababbbabbabbaabbaaaa__aba`Z``
@HWI-EAS443:3:94:277:1304#0/1
GACGAAAGGAATCCCATAGACTCCTACTGGCCATTTCTTG
+HWI-EAS443:3:94:277:1304#0/1
aaa`aa`aaa[aa_¥abaaaaa_`_``ZY___]ZX_W^ZZ
@HWI-EAS443:3:94:302:65#0/1
TCCGAAAATCGAAACTAACAAGTTTGCTGCAATATGCACA
+HWI-EAS443:3:94:302:65#0/1
``]b`^b^]Ya^^YY_`Y__WaYa_`__a_]]`^Y`[]]¥
@HWI-EAS443:3:94:361:131#0/1
CTCTAGCAAGCTGGGTCCAAAATGAATTCAATAAGGCATG
+HWI-EAS443:3:94:361:131#0/1
aVb^S_Y_Q`XXQPJ]OF]_Y¥_a^__[^QF^]U^MUQ`]
@HWI-EAS443:3:94:378:18#0/1
ACCGAACTTCTCCAGCCTTGAAAACTTTAGAGCCTATGTA

```
+HWI-EAS443:3:94:378:18#0/1
aa]b]`Q_aab``_`_Ua`b]a`_U_a``a_aY_`__a[_
@HWI-EAS443:3:94:447:729#0/1
GGGGCATGGAAATGAGGCGCTGCCTTCTTCAGTCTCTTCA
+HWI-EAS443:3:94:447:729#0/1
a`¥`]]aaa`_a``a`____^_^^¥YQX][¥¥SOZ^¥_UU
@HWI-EAS443:3:94:52:1670#0/1
GGGGAAGATCCGAAAATCGAAACTAACAAGTTTGCTGCAA
+HWI-EAS443:3:94:52:1670#0/1
V_P_``bbabbb``bbaba_^`baa_b`^a`_]aaaa_ba
@HWI-EAS443:3:94:535:1897#0/1
GACCGAATCATGGCCTGGACAGTGGTGAACAGTATATGTA
+HWI-EAS443:3:94:535:1897#0/1
O_bbabbababbabab_a^_abab_XbabbaaYbbba[[b
@HWI-EAS443:3:94:562:414#0/1
CATAAATACGGCCTTGCTCAATGCATCCTGTGCAGCCATG
+HWI-EAS443:3:94:562:414#0/1
SZbbbbb¥¥bbba`_a_`abb]bVbaaabbQ_]`ba¥aab
@HWI-EAS443:3:94:572:1717#0/1
GAACTTTGTAAGTATGGAGTTCTCACTCACTGACCCGAGA
+HWI-EAS443:3:94:572:1717#0/1
a]abaaaa[bb]]a_aaba`aaaaaaa`aaaaa`a_aaaa
@HWI-EAS443:3:94:589:2036#0/1
TTCTGCTGTAAAATAGTTCCTCCTCATGCTTGCGATATGT
+HWI-EAS443:3:94:589:2036#0/1
`W`WZW`bYYabYSYXUT_a``b_RV^^`WXU_`[PVW]P
@HWI-EAS443:3:94:591:314#0/1
ATCCTGTGCAGCCATGGATGACTTTCAGCTGATCCCAATG
+HWI-EAS443:3:94:591:314#0/1
`Z``a]_Y_S]LQPW]X]___¥`¥_[Z^WZZWZ[^Y_^¥`
@HWI-EAS443:3:94:639:1188#0/1
GGGGTTTTCGGCTGAATCGAGAAAATTGCTTCTCATTGTT
+HWI-EAS443:3:94:639:1188#0/1
ab`a¥aaa_`MaaZP^YS[``^a`a_^__`_¥^a``_`_`
@HWI-EAS443:3:94:669:1606#0/1
AAGAGCCTAACATAGTCAAACCACATGAGAAAGGCATAAA
+HWI-EAS443:3:94:669:1606#0/1
_a^a`aaaaa`a_^a_aa]Z]`aa``aaaa`__`_a_a^`
@HWI-EAS443:3:94:707:1253#0/1
GATCTTGGAGGTTCCATTGGTTCTCACATATAGGAACATG
+HWI-EAS443:3:94:707:1253#0/1
a___]aaaSbQP¥^`¥a¥^^^_]]TZ_`[[`OFM_^U]T¥
@HWI-EAS443:3:94:783:635#0/1
GTATATGTAACACAACAGGGGTAGAGAAGCCTAAATTTCT
+HWI-EAS443:3:94:783:635#0/1
a^aaaab`aaaaaaa_aaaaa^`a_aa`a__```_```^`
@HWI-EAS443:3:94:822:1270#0/1
TAGCCCCCCAAGATCGAAGGTTCCAGGTTCCAGGTTGTCC
+HWI-EAS443:3:94:822:1270#0/1
L_VX]_b_`__X¥]^^`TT[TZWVRXQJMFSY[YG^_VDX
```

```
@HWI-EAS443:3:94:825:1797#0/1
GGGGGCTATATGAAGCAATCGAGGAGTGCCTGATTAATGA
+HWI-EAS443:3:94:825:1797#0/1
^aabaaaabbababbabaaaaaba]a_aa]aaa`a`a_aa
@HWI-EAS443:3:94:870:1817#0/1
GGTGACCCGAATGCACTATTGAAGCACCGATTTGAGATAA
+HWI-EAS443:3:94:870:1817#0/1
`aXab]bbba``aabbbababaabaa__ba`aaa_a`Zaa
@HWI-EAS443:3:94:898:177#0/1
GGGCACTCGGTGAAAATATGGCACCAGAAAAAGTAGACTT
+HWI-EAS443:3:94:898:177#0/1
aaa__`a[a]Xa]_^^_^a_`^_YWW`^]XZZ`Z]^WY]V
@HWI-EAS443:3:94:898:367#0/1
GTTTCCGATTTGTTTTCAAAGAATTCCTTGGTCATGTCTT
+HWI-EAS443:3:94:898:367#0/1
a^a`_^b^`a`a^`a`___aa`^a^]]`_a[XX]aa]Y__
@HWI-EAS443:3:94:931:1072#0/1
GCATAAATCCCAATTACCTCATGGCTTGGAAGCAGGTGCT
+HWI-EAS443:3:94:931:1072#0/1
a`ba^``baa`[ab_]aa`]]abbaaa`a_XY^W__U[Z[
@HWI-EAS443:3:94:997:1208#0/1
CACAACTTGAGGGGTTTTCGGCTGAATCGAGAAAATTGCT
+HWI-EAS443:3:94:997:1208#0/1
aaab`^baaaa^_^]aaaaabab`aa`^ba`[``_a_W__
@HWI-EAS443:3:95:1037:1336#0/1
GGGGGCTATATGAAGCAATCGAGGAGTGCCTGATTAATGA
+HWI-EAS443:3:95:1037:1336#0/1
abbab`bab_aabbbaab`Pbabbabaa]``b`abaaaaa
@HWI-EAS443:3:95:109:1485#0/1
GATTCGAGCCGAACGGCTGCATTGAGGGCAAGCTTTCCCA
+HWI-EAS443:3:95:109:1485#0/1
a_bbbbbb_aba[aaaa^a[`[`S^T_bSXZ`YW__V_¥U
@HWI-EAS443:3:95:1116:1263#0/1
GGCTCCAGTCTCGGGTCAGTGAGTGAGAACTCCATACTTA
+HWI-EAS443:3:95:1116:1263#0/1
aa]`]XZYWa_¥]_YMQY][aa[VZVX[]_YNVa[[SRUP
@HWI-EAS443:3:95:1119:1384#0/1
ACTATTTTACAGCAGAAGTGTCCCACTGCAGGGCTACTGA
+HWI-EAS443:3:95:1119:1384#0/1
aaaaaabaaaaaaaaa_`]a___^a^_a^___`_`^Za`]
@HWI-EAS443:3:95:117:1613#0/1
GTGCTAGCAGAGCTACAGGACATTGAAAATGAAGAGAAGA
+HWI-EAS443:3:95:117:1613#0/1
`b`bbbba¥bbbbbaba[aabbabaabb^aaaaab_abaa
@HWI-EAS443:3:95:1190:724#0/1
ATGTATTCGGATTTCCATTTCATCGACGAACGGGGTGAAT
+HWI-EAS443:3:95:1190:724#0/1
aab`bbaaaba`aa_aa`aa`aa`b_`a`]Wa[aaY]a_`
@HWI-EAS443:3:95:1198:1209#0/1
GTTCTATCCAGCTTGAATCAGTCAATTCACATGCCTTATT
```

```
+HWI-EAS443:3:95:1198:1209#0/1
aabaaaa_`ba^baaa_a^`a]a`abaaa_a___¥a__`a
@HWI-EAS443:3:95:1245:1266#0/1
TTCGAGCCGAACGGCTGCATTGAGGGCAAGCTTTCCCAAA
+HWI-EAS443:3:95:1245:1266#0/1
`baa`ba`bbb`b`abaP¥a_Y`_a_____[_``[_^```
@HWI-EAS443:3:95:1422:692#0/1
CACCACTGTCCAGGCCATGATTCGGTCTCTTCCTTCAATT
+HWI-EAS443:3:95:1422:692#0/1
aaY`a`b`ab_bb``_bba_``Z___a¥Z__FNbb`ba`a
@HWI-EAS443:3:95:1440:838#0/1
GGGGAGTGGAGGAAGGCTCTATTGGGAAAGTGTGCAGGAC
+HWI-EAS443:3:95:1440:838#0/1
^bbbba_b`abbaaaaVa^a`aa]aY_aaa¥]Va__a_YN
@HWI-EAS443:3:95:1487:854#0/1
CACCACTGTCCAGGCCATGATTCGGTCTCTTCCTTCAATT
+HWI-EAS443:3:95:1487:854#0/1
ab`_aT`bbb_babbbbbbabbab___babbaabbaa`bb
@HWI-EAS443:3:95:1587:1421#0/1
CAGGATGCATTGAGCAAGGCCGTATTTATGTACACTCCCT
+HWI-EAS443:3:95:1587:1421#0/1
`bbbaa¥_bababa¥bb_ba_a_abaab_``aWbaa¥_`^
@HWI-EAS443:3:95:1657:58#0/1
CAATAATTGTAGAATCTGGTGACCCGAATGCACTATTGAA
+HWI-EAS443:3:95:1657:58#0/1
abb`Laa`bbaabba]aaUPP¥aaab`ab`YbXbaaba`W
@HWI-EAS443:3:95:1703:325#0/1
TTCCGATTTGTTTTCAAAGAATTCCTTGGTCATGTCTTTC
+HWI-EAS443:3:95:1703:325#0/1
ab[D¥]XZO_LYaU]aaa`TRa`T^^_b_`]J``XP¥_XD
@HWI-EAS443:3:95:239:1420#0/1
ATTTTGGCGTTCACTTCTTTTGACATTTGGGAAAGCTTGC
+HWI-EAS443:3:95:239:1420#0/1
_abbbbbbbbb_S_abaaab`aa`[aa``aa_a``¥[`_`
@HWI-EAS443:3:95:366:1775#0/1
GGAGTGTACATAAATACGGCCTTGCTCAATGCATCCTGTG
+HWI-EAS443:3:95:366:1775#0/1
]VUV_UP¥_]S`^Z¥`YZ¥Q_X^]]_]`[^YK[Y¥V[TT¥
@HWI-EAS443:3:95:428:1096#0/1
TGCATATTGCAGCAAACTTGTTAGTTTCGATTTTCGGATC
+HWI-EAS443:3:95:428:1096#0/1
`baU¥ab]RY`RJS_HTbUaaa]bY][bYS]_QTZHW^[^
@HWI-EAS443:3:95:42:1241#0/1
ACCGATTTGAGATAATTGAAGGAAGAGACCGAATCATGGC
+HWI-EAS443:3:95:42:1241#0/1
abbbaaaba]__aa`a^`^]`][]a_`V`aaa_^a]`_^U
@HWI-EAS443:3:95:455:553#0/1
CACTGCAGGGCTACTGAATACATAATGAAGGGAGTGTACA
+HWI-EAS443:3:95:455:553#0/1
aaabbbbbbbbbbbbbbabbabba^bbaabaaabXa`^_a
```

```
@HWI-EAS443:3:95:474:1370#0/1
GCATGGAAATGAGGCGCTGCCTTCTTCAGTCTCTTCAGCA
+HWI-EAS443:3:95:474:1370#0/1
^aa^aa_]a````__aaaa_``aQa^YY][`_¥``V_[WZ
@HWI-EAS443:3:95:487:62#0/1
GAAATGGAAATCCGAATACATGAAACAAACTTCCAAATGT
+HWI-EAS443:3:95:487:62#0/1
a_aa`]^a_^b_`a^P`aYaaa`a_^a`_]a__¥__]_`Z
@HWI-EAS443:3:95:512:652#0/1
GGGTGAATCAATAATTGTAGAATCTGGTGACCCGAATGCA
+HWI-EAS443:3:95:512:652#0/1
abbababbababbbbbaaaabbb`bbbabbaaa`baaa`a
@HWI-EAS443:3:95:520:1303#0/1
CAAGTTTGCTGCAATATGCACACATTTGGAAGTTTGTTTC
+HWI-EAS443:3:95:520:1303#0/1
abb`babbbbbabbbbbbbbbbbbbbaa_`aabb_abb`
@HWI-EAS443:3:95:563:450#0/1
AGAAAGGCATAAATCCCAATTACCTCATGGCTTGGAAGCA
+HWI-EAS443:3:95:563:450#0/1
aaaababaaabbba`a_`aa`a`_aa``aa`baaaZ_`OV
@HWI-EAS443:3:95:610:66#0/1
ATTTCATCGACGAACGGGGTGAATCAATAATTGTAGAATC
+HWI-EAS443:3:95:610:66#0/1
abbbaaa_aa_baaVaa[aW__]`^_^a_^``a]__¥_]]
@HWI-EAS443:3:95:611:1709#0/1
CGTCTCCACAACTTGAGGGGTTTTCGGCTGAATCGAGAAA
+HWI-EAS443:3:95:611:1709#0/1
`¥V`a_bba]b_aaa[aR¥aT[a^aa_]^]`__`aRba[`
@HWI-EAS443:3:95:647:1661#0/1
TCTGAAATTAAGTATTGAAGACCCGAGTCACGAGGGGGAG
+HWI-EAS443:3:95:647:1661#0/1
abb`aaabbaab`aaaabbaaaa¥a`a¥`a^a^a__^`]`
@HWI-EAS443:3:95:655:937#0/1
AATTGAAGGAAGAGACCGAATCATGGCCTGGACAGTGGTG
+HWI-EAS443:3:95:655:937#0/1
abbbba`bbabaabaaabYaaaaaaaaaa_a_baZa_^a
@HWI-EAS443:3:95:657:39#0/1
GGTTTTCGGCTGAATCGAGAAAATTGCTTCTCATTGTTCA
+HWI-EAS443:3:95:657:39#0/1
a]Q[¥VDR¥QK_SVRQ¥W_]UTNNQQQUYYWVVV]]UYUY
@HWI-EAS443:3:95:697:82#0/1
GGCGAAGAGACAATTGAAGAAAAATTTGAGATTACAGGAA
+HWI-EAS443:3:95:697:82#0/1
aaT¥GSbYa]SN_``a]XaGY¥X^_^]NS`ZNHRSSY]WU
@HWI-EAS443:3:95:710:486#0/1
GACGAAAGGAATCCCATAGACTCCTACTGGCCATTTCTTG
+HWI-EAS443:3:95:710:486#0/1
`aY`bab_Ybaa_aTbbbbaaba[a_aaa_aW]]a¥^Ra^
@HWI-EAS443:3:95:755:1698#0/1
CACCCCGTTCGTCGATGAAATGGAAATCCGAATACATGAA
```

```
+HWI-EAS443:3:95:755:1698#0/1
`X_b`bbaba]`bbabbabbb_^`bbba^abbababaaaa
@HWI-EAS443:3:95:794:973#0/1
GATTGGCCATGTTTCCGATTTGTTTTCAAAGAATTCCTTG
+HWI-EAS443:3:95:794:973#0/1
aa_a``a`_a¥[aaRY_aRU]a``a`_X¥`a]_`^]__`a
@HWI-EAS443:3:95:802:290#0/1
GATCCGAAAATCGAAACTAACAAGTTTGCTGCAATATGCA
+HWI-EAS443:3:95:802:290#0/1
aaa_aaaabb_^ba_aaaa_`aa^VW¥`^`a_aa__``X_
@HWI-EAS443:3:95:905:460#0/1
GAACCCATACAGGTTTGTTTTCCGTCTTCCTTCTTTGGTC
+HWI-EAS443:3:95:905:460#0/1
a]aa```aaaaaa¥aa]`aaa^Y¥`_aaZYa]¥a__`XUZ
@HWI-EAS443:3:95:997:1428#0/1
CACTGCAGGGCTACTGAATACATAATGAAGGGAGTGTACA
+HWI-EAS443:3:95:997:1428#0/1
aaabaaaabaabb`babbb``abbbbbaabaaab_a^a_a
@HWI-EAS443:3:96:1034:1757#0/1
AGAATGGTTCAATTTTGGCGTTCACTTCTTTTGACATTTG
+HWI-EAS443:3:96:1034:1757#0/1
aabbaaa__a[_aaab¥^`a[^]¥U_`^__a__VNY^^_^
@HWI-EAS443:3:96:1113:244#0/1
ACATAATGAAGGGAGTGTACATAAATACGGCCTTGCTCAA
+HWI-EAS443:3:96:1113:244#0/1
`_abaabaaabaaab_a`aV`aaaaa`]aaVYaa`V_V__
@HWI-EAS443:3:96:1120:1211#0/1
ATGCCCCATTTCATCTTGATCTTGGAGGTTCCATTGGTTC
+HWI-EAS443:3:96:1120:1211#0/1
a¥GV_Z[Yb``M^a_]____][__a]`MFQXLOTTOXO_B
@HWI-EAS443:3:96:1175:1459#0/1
ATCCAGCTTGAATCAGTCAATTCACATGCCTTATTGAATT
+HWI-EAS443:3:96:1175:1459#0/1
aV_Za][]abbabTba_``V`_`b__]`¥___^aZ``_`_
@HWI-EAS443:3:96:1202:490#0/1
AGCTCTGCTAGCACCTGCTTCCAAGCCATGAGGTAATTGG
+HWI-EAS443:3:96:1202:490#0/1
`[Y_^`aW`___^TX_`^¥^]UWZaUV[_^X`_QXO]V^^
@HWI-EAS443:3:96:131:1899#0/1
CAAGGACAAAGAACATGAAGAGAACAAGCCAATTGAAGTG
+HWI-EAS443:3:96:131:1899#0/1
`a_M]aa[WX[aaa[aa[aaaa__`a_`_^a[_`__a^X^
@HWI-EAS443:3:96:1350:1609#0/1
TTCTGCTGTAAAATAGTTCCTCCTCATGCTTGCGATATGT
+HWI-EAS443:3:96:1350:1609#0/1
a`]^aaa^_abaaab_[a`¥a¥`^_aa`[a`a`b`aaaa¥
@HWI-EAS443:3:96:1460:627#0/1
CAAGTTGTGGAGACGCATATAGACTGTTGAATACAGATTT
+HWI-EAS443:3:96:1460:627#0/1
ab[baababb_`bVaabb_Wbab_aaIJMZX_`¥aJKQYB
```

```
@HWI-EAS443:3:96:1506:1065#0/1
GAGTGTACATAAATACGGCCTTGCTCAATGCATCCTGTGC
+HWI-EAS443:3:96:1506:1065#0/1
]Z`]¥SZQ^`aabaaP]^TU]`aS¥T_[WWa¥a¥]^XZRB
@HWI-EAS443:3:96:1508:960#0/1
GTTTGCTGCAATATGCACACATTTGGAAGTTTGTTTCATG
+HWI-EAS443:3:96:1508:960#0/1
a`a_U_aaaa_ba`X¥aaaTaaa]`_^_a_aaa^^_W[`a
@HWI-EAS443:3:96:1522:801#0/1
ATGCTCTGAAATTAAGTATTGAAGACCCGAGTCACGAGGG
+HWI-EAS443:3:96:1522:801#0/1
```aaNaba_Yb`abbabbabaaaaQ_¥aY[Ta`_a]_Z_
@HWI-EAS443:3:96:1625:113#0/1
GGTAATTGGGATTTATGCCTTTCTCATGTGGTTTGACTAT
+HWI-EAS443:3:96:1625:113#0/1
a¥S^S]X`a¥GIXXWWUGWZ^SPUMO]^XZZZUBBBBBBB
@HWI-EAS443:3:96:164:392#0/1
AGATAATTGAAGGAAGAGACCGAATCATGGCCTGGACAGT
+HWI-EAS443:3:96:164:392#0/1
aaa_LXT``^¥a^a]`]_aP¥Y¥]^^`___[YXMDEa[FO
@HWI-EAS443:3:96:1653:1113#0/1
CACCACTGTCCAGGCCATGATTCGGTCTCTTCCTTCAATT
+HWI-EAS443:3:96:1653:1113#0/1
abaababbaT¥bbb^^abb_abaaaZ_a]a_V^ba[aaaa
@HWI-EAS443:3:96:202:1431#0/1
TTTAGGCTTCTCTACCCCTGTTGTGTTACATATACTGTTC
+HWI-EAS443:3:96:202:1431#0/1
^TZb[Y_abb]aaba_RaUa`_^Ya^Z¥[]`_][P^^O`a
@HWI-EAS443:3:96:217:445#0/1
CTTGGGGGGCTATATGAAGCAATCGAGGAGTGCCTGATTA
+HWI-EAS443:3:96:217:445#0/1
aaaa[Zaaa``a`aaa^aa__^`a`]__Y¥S^_Y_`Y^T¥
@HWI-EAS443:3:96:253:2003#0/1
ATGAAATGGAAATCCGAATACATGAAACAAACTTCCAAAT
+HWI-EAS443:3:96:253:2003#0/1
aaaa_a_M``]`_aa```^_]aXX__¥[a__Y]]X_^^^^
@HWI-EAS443:3:96:346:286#0/1
ATTGAGCAAGGCCGTATTTATGTACACTCCCTTCATTATG
+HWI-EAS443:3:96:346:286#0/1
_]aaa`a`aaa^]aY__``^`_Y^W^^W]XY^^X]^^]^^
@HWI-EAS443:3:96:35:462#0/1
GAAGATCCCAAGGACAAAGAACATGAAGAGAACAAGCCAA
+HWI-EAS443:3:96:35:462#0/1
a_¥a¥^a]]`^`a_`WWaa^_¥``a]]`^Z¥¥_a`aYV^¥
@HWI-EAS443:3:96:382:415#0/1
ACATGAGAAAGGCATAAATCCCAATTACCTCATGGCTTGG
+HWI-EAS443:3:96:382:415#0/1
aaaaaaaaaa]a_aa]]]a_^¥`_a__Y[]X[]_^V_UPZ
@HWI-EAS443:3:96:410:984#0/1
TGTGAATTGACTGATTCAAGCTGGATAGAACTTGATGAAA
```
```

+HWI-EAS443:3:96:410:984#0/1
aa_aaaaa`a`aaaa``a``¥``a_``]Za_a`_^`a__^
@HWI-EAS443:3:96:415:1651#0/1
GATGAAATGGAAATCCGAATACATGAAACAAACTTCCAAA
+HWI-EAS443:3:96:415:1651#0/1
a`[^````_`_``ab`]aaaa`a``aa``aa_````_`^Y
@HWI-EAS443:3:96:470:489#0/1
CATGACCAAGGAATTCTTTGAAAACAAATCGGAAACATGG
+HWI-EAS443:3:96:470:489#0/1
abaaaa`aa^bZZZa[aaba_Y^_a___a`aa_¥]a^aa`
@HWI-EAS443:3:96:497:255#0/1
GTTCGGTGGGAGACTTTGGTCGGCAAGCTTGCGCATAGTT
+HWI-EAS443:3:96:497:255#0/1
a_aaabQaaa_`W^aa`a_W___Y_¥_Z¥^_U_S[¥¥^W¥
@HWI-EAS443:3:96:58:1850#0/1
GGGGTTTTCGGCTGAATCGAGAAAATTGCTTCTCATTGTT
+HWI-EAS443:3:96:58:1850#0/1
]W_aUaa`_ZTLV¥b]_aa^^aa^aaYa^X]V]a^N]_WU
@HWI-EAS443:3:96:648:2029#0/1
GTGGGACACTTCTGCTGTAAAATAGTTCCTCCTCATGCTT
+HWI-EAS443:3:96:648:2029#0/1
a]aa__`a`baaaaaab_bbb``aa]___`_]`_a``_``
@HWI-EAS443:3:96:660:1211#0/1
AAAGAGCCTAACATAGTCAAACCACATGAGAAAGGCATAA
+HWI-EAS443:3:96:660:1211#0/1
abbbabb]b^babaaaaaaabaaaaaaaa`aaaaa_aaaa
@HWI-EAS443:3:96:664:816#0/1
CACACATTCACATCTTTTCATTCACTGGAGAGGAGATGGC
+HWI-EAS443:3:96:664:816#0/1
a]_`¥YT^XUTYXW_PYLXS_YU`UT¥`aV`[NQZBBBBB
@HWI-EAS443:3:96:690:1125#0/1
TGGGAGACTTTGGTCGGCAAGCTTGCGCATAGTTCCTGTA
+HWI-EAS443:3:96:690:1125#0/1
aaaaabaaabbbaa___`baaab_[Za^```a`a^aaaX]
@HWI-EAS443:3:96:707:1251#0/1
AGATAATTGAAGGAAGAGACCGAATCATGGCCTGGACAGT
+HWI-EAS443:3:96:707:1251#0/1
ab`babaa[U]`ba[b]aa_]b`_b^ab_[SM`a^a_a]S
@HWI-EAS443:3:96:754:351#0/1
CTGGAAAGAGCCTAACATAGTCAAACCACATGAGAAAGGC
+HWI-EAS443:3:96:754:351#0/1
Y`a``bbbbbbbbbbabbbbabbabaabababbbbabb_aab
@HWI-EAS443:3:96:779:801#0/1
GCAATATGCACACATTTGGAAGTTTGTTTCATGTATTCGG
+HWI-EAS443:3:96:779:801#0/1
a`aaaaabaa```aaaaba`_b_aa]^aa__ab_^``]aa
@HWI-EAS443:3:96:830:523#0/1
AGAAATTTAGGCTTCTCTACCCCTGTTGTGTTACATATAC
+HWI-EAS443:3:96:830:523#0/1
a_[_¥^a^`Z`_]`RZW]]F[KV¥]WY[L]U__]]]]]YV

```
@HWI-EAS443:3:96:860:359#0/1
TGTAATCTCAAATTTTTCTTCAATTGTCTCTTCGCCTCTT
+HWI-EAS443:3:96:860:359#0/1
aa_^¥`_baa`¥aabaaaa`_aaaaa^¥a_``__]]`W``
@HWI-EAS443:3:96:861:672#0/1
GGGGCATGGAAATGAGGCGCTGCCTTCTTCAGTCTCTTCA
+HWI-EAS443:3:96:861:672#0/1
aaaa]a_abaaaaa`a_Ua]aa_¥aa¥a___a¥Y__a`¥¥
@HWI-EAS443:3:96:916:529#0/1
ACAAATCAGGAAGAAATTTAGGCTTCTCTACCCCTGTTGT
+HWI-EAS443:3:96:916:529#0/1
a_a`W``abb_[b]V]a]]`b`Ta]LWS_`^^a`]_QZ_O
@HWI-EAS443:3:96:949:1432#0/1
ATTAATCAGGCACTCCTCGATTGCTTCATATAGCCCCCCA
+HWI-EAS443:3:96:949:1432#0/1
aaaa_`VaX[U_Z]aX``a]_U`R`]_`]`]^Z[SRQVHZ
@HWI-EAS443:3:96:950:524#0/1
GGGGTTTTCGGCTGAATCGAGAAAATTGCTTCTCATTGTT
+HWI-EAS443:3:96:950:524#0/1
aaba[baaaa¥]aabaaaaZa___a_a_¥a_YaX]``^`_
@HWI-EAS443:3:96:95:940#0/1
AGGGAGTGTACATAAATACGGCCTTGCTCAATGCATCCTG
+HWI-EAS443:3:96:95:940#0/1
^aaa¥a`^La``W¥aa`aa_Z`^P¥WQ[^^U¥¥^`¥¥Z¥¥
@HWI-EAS443:3:96:984:1243#0/1
ATTTCATCGACGAACGGGGTGAATCAATAATTGTAGAATC
+HWI-EAS443:3:96:984:1243#0/1
_[`a__YZ^]W__V___a^[a``_¥TSaX]`X^Z^``aa¥
@HWI-EAS443:3:96:98:1643#0/1
AGAGCCCAGATCTCTAGCAAGCTGGGTCCAAAATGAATTC
+HWI-EAS443:3:96:98:1643#0/1
Q_b^aab¥Y]^`[`Z_W^aW^Q¥_S]DH[_`P[¥a¥[[T`
@HWI-EAS443:3:97:1074:477#0/1
TAGGCCAAGTGTCGAGGCCCATGTTCCTATATGTGAGAAC
+HWI-EAS443:3:97:1074:477#0/1
aV`WH¥bbb]`U]a^a`]a]`^`YaaUba_baaUa``¥aQ
@HWI-EAS443:3:97:1114:1902#0/1
CTTCTCTTCATTTTCAATGTCCTGTAGCTCTGCTAGCACC
+HWI-EAS443:3:97:1114:1902#0/1
L_b[_^[¥aaaab`aaba`_b`bbbbaabaabU_bbaVWa
@HWI-EAS443:3:97:1185:1321#0/1
GGCATGGAAATGAGGCGCTGCCTTCTTCAGTCTCTTCAGC
+HWI-EAS443:3:97:1185:1321#0/1
aa^babb`^`baaab[a`a_R[ab]abU^a_KX`abU¥_U
@HWI-EAS443:3:97:1196:1504#0/1
GTTTCATGTATTCGGATTTCCATTTCATCGACGAACGGGG
+HWI-EAS443:3:97:1196:1504#0/1
aW_`[W]a[_a`_aaa__aXW]a^_]_`Z^ZZa__X^]X^
@HWI-EAS443:3:97:1266:1586#0/1
AATAGTTCCTCCTCATGCTTGCGATATGTTCAATCGGGGC
```

+HWI-EAS443:3:97:1266:1586#0/1
a¥_^YVYV[YYX^R^X^Y^X]Y_YYXV^YVXXXVRVVVXH
@HWI-EAS443:3:97:130:533#0/1
CAGTATTTTTCCCATTTGTGTGGCTCCAGTCTCGGGTCAG
+HWI-EAS443:3:97:130:533#0/1
abbaabb`aaaaaabbbb`b^b__a¥_Y_Y__W^aaSV¥]
@HWI-EAS443:3:97:1349:436#0/1
GGGGGCTATATGAAGCAATCGAGGAGTGCCTGATTAATGA
+HWI-EAS443:3:97:1349:436#0/1
`babbbbbbbb]bbaabbb_baba_a]aa_aa`aaaaaa`
@HWI-EAS443:3:97:1433:1418#0/1
TTCCGATTTGTTTTCAAAGAATTCCTTGGTCATGTCTTTC
+HWI-EAS443:3:97:1433:1418#0/1
a^`_a_^aa_Haaaa_aaa`W^aYVa^b_^][`aZW`_aG
@HWI-EAS443:3:97:1436:426#0/1
GGCTCCAGTCTCGGGTCAGTGAGTGAGAACTCCATACTTA
+HWI-EAS443:3:97:1436:426#0/1
aa`baa`baaa_aaaL[`a]_``N`_a[_¥¥Z¥`__U^``
@HWI-EAS443:3:97:1549:366#0/1
ATTAATCAGGCACTCCTCGATTGCTTCATATAGCCCCCCA
+HWI-EAS443:3:97:1549:366#0/1
abaa_`a`a`¥a]aX^a_a`__a]`]Q_`a`_[NGXYVX_
@HWI-EAS443:3:97:1602:1108#0/1
CACTCACTGACCCGAGACTGGAGCCACACAAATGGGAAAA
+HWI-EAS443:3:97:1602:1108#0/1
ab_bbaabbaaa^bbbbabbb`_a`a_a_aa_aaa[VX[Z
@HWI-EAS443:3:97:1621:514#0/1
CGGTCTCTTCCTTCAATTATCTCAAATCGGTGCTTCAATA
+HWI-EAS443:3:97:1621:514#0/1
G]]b[^Z`baabbabbba`aKa_b¥abb¥_XaVbb]`b`a
@HWI-EAS443:3:97:229:1028#0/1
GCTGGGTCCAAAATGAATTCAATAAGGCATGTGAATTGAC
+HWI-EAS443:3:97:229:1028#0/1
_Z]aa[H¥_aaaaa`XZ`aa__`Za_]___a[a__`¥_`V
@HWI-EAS443:3:97:230:616#0/1
TGCGTCTCCACAACTTGAGGGGTTTTCGGCTGAATCGAGA
+HWI-EAS443:3:97:230:616#0/1
`b_aaYTaa`a`__aa`_[aaa^aaaQaRU`_`a^T`¥Z¥
@HWI-EAS443:3:97:281:1442#0/1
AGGAGGAACTATTTTACAGCAGAAGTGTCCCACTGCAGGG
+HWI-EAS443:3:97:281:1442#0/1
a[a[_aab`]U_baa_]aa_]]aa¥LR`_`_^_a^]`^Pa
@HWI-EAS443:3:97:286:201#0/1
GGCCTTGCTCAATGCATCCTGTGCAGCCATGGATGACTTT
+HWI-EAS443:3:97:286:201#0/1
abaaaabaa^aaaa[a`_``a_a[`aY[Y^`Z¥¥`URU¥V
@HWI-EAS443:3:97:321:1119#0/1
CACTTCTGCTGTAAAATAGTTCCTCCTCATGCTTGCGATA
+HWI-EAS443:3:97:321:1119#0/1
`bbbba``baaabbaba¥b`b^^aa``_abbaaaa]aa`a

```
@HWI-EAS443:3:97:342:1140#0/1
GCCATATTTTCACCGAGTGCCCACTTCAATTGGCTTGTTC
+HWI-EAS443:3:97:342:1140#0/1
aaaaaaaaaa`aa_a_a^aUY_`]a_]`^_WZWZ_VJVZB
@HWI-EAS443:3:97:378:1616#0/1
ATTAATCAGGCACTCCTCGATTGCTTCATATAGCCCCCCA
+HWI-EAS443:3:97:378:1616#0/1
aab]RWSZQOT[aa_a`^T^`¥_Ya]_]__`__][V^NZ`
@HWI-EAS443:3:97:401:709#0/1
GGGGGCTATATGAAGCAATCGAGGAGTGCCTGATTAATGA
+HWI-EAS443:3:97:401:709#0/1
abbbaababba`_aaabaaaaaaa`aWa__aa``a`a¥_a
@HWI-EAS443:3:97:448:1975#0/1
CACACATTTGGAAGTTTGTTTCATGTATTCGGATTTCCAT
+HWI-EAS443:3:97:448:1975#0/1
a`aaa`a`ab`bb`a^aa`bba^abaYbb`_Yaaaa__``
@HWI-EAS443:3:97:479:1691#0/1
CATATACTGTTCACCACTGTCCAGGCCATGATTCGGTCTC
+HWI-EAS443:3:97:479:1691#0/1
``_W^a^ab_]ab`¥__]^]_Waa``^_`_a¥H^[JYWYX
@HWI-EAS443:3:97:499:2008#0/1
TTTCAAAGAATTCCTTGGTCATGTCTTTCTCTTTGACAGA
+HWI-EAS443:3:97:499:2008#0/1
`baa^`Zaa`a``¥`aaaY_aab^aa_^W__^a``a`___
@HWI-EAS443:3:97:544:438#0/1
GTTTGCTGCAATATGCACACATTTGGAAGTTTGTTTCATG
+HWI-EAS443:3:97:544:438#0/1
a_aa`_`a_a`a``a___`[^aa`a__`a]`_a_`_]^``
@HWI-EAS443:3:97:553:1212#0/1
AAGGAAGAGACCGAATCATGGCCTGGACAGTGGTGAACAG
+HWI-EAS443:3:97:553:1212#0/1
aaab`a^aa_a^a^aa``aaa_Z`^_^^a`^_`X`]]]`_
@HWI-EAS443:3:97:570:1221#0/1
GGCATGGAAATGAGGCGCTGCCTTCTTCAGTCTCTTCAGC
+HWI-EAS443:3:97:570:1221#0/1
abaT``__¥_aa`bbbb`aaa``a^a_X_aU_`aaa```a
@HWI-EAS443:3:97:582:774#0/1
ACAACTTGAGGGGTTTTCGGCTGAATCGAGAAAATTGCTT
+HWI-EAS443:3:97:582:774#0/1
a`ab`babab aaa^aaaaa``abaaa`a`a__aa`__^`_
@HWI-EAS443:3:97:592:44#0/1
CAGGCAATCTGAGGGGGCGTGGTGTCGTCCTCAAGAATGG
+HWI-EAS443:3:97:592:44#0/1
Z]`_^][__bbZbaaba``Gb_NUV¥aZG^a]a[ab^aaa
@HWI-EAS443:3:97:634:105#0/1
GAAGAGAACAAGCCAATTGAAGTGGGCACTCGGTGAAAAT
+HWI-EAS443:3:97:634:105#0/1
aaaaaa`aaaaa^_`aaaa`_a]a``U_Y_Y_`O`¥]]_^
@HWI-EAS443:3:97:656:1028#0/1
GGGGCTATATGAAGCAATCGAGGAGTGCCTGATTAATGAT
```

~~~
+HWI-EAS443:3:97:656:1028#0/1
aaa`_a_a`aa_^_¥`__``¥aa^`U__^_`Y__¥¥`___
@HWI-EAS443:3:97:703:922#0/1
GCAGGTGCTAGCAGAGCTACAGGACATTGAAAATGAAGAG
+HWI-EAS443:3:97:703:922#0/1
a^]]ZN_`a_¥NZXV___TFV]TU¥VVV`MU^^[_ZZ^ZU
@HWI-EAS443:3:97:71:442#0/1
AATAATTGTAGAATCTGGTGACCCGAATGCACTATTGAAG
+HWI-EAS443:3:97:71:442#0/1
[`ba^bb`ab^¥_¥VTU_¥b¥_]_a_aaa`b_[`aaa^aa
@HWI-EAS443:3:97:773:1442#0/1
GAGCCCAGATCTCTAGCAAGCTGGGTCCAAAATGAATTCA
+HWI-EAS443:3:97:773:1442#0/1
a```aaaab`L``_aa[`aJ]aaQ`NY`^``_a]a`a_Na
@HWI-EAS443:3:97:777:237#0/1
CATAAATACGGCCTTGCTCAATGCATCCTGTGCAGCCATG
+HWI-EAS443:3:97:777:237#0/1
`ab]¥TU`_¥aT_[aa_a]_a¥a^a``W¥aZb__aV__`a
@HWI-EAS443:3:97:781:104#0/1
CGTCTCCACAACTTGAGGGGTTTTCGGCTGAATCGAGAAA
+HWI-EAS443:3:97:781:104#0/1
aaa_a_ab`a_]bbb`bbbbV_ba``aW`b^_a_`___]a
@HWI-EAS443:3:97:802:1906#0/1
ACTTGATGAAATAGGAGAAGATGTTGCCCCGATTGAACAT
+HWI-EAS443:3:97:802:1906#0/1
a``¥b_aaYa]TTY]]aYU^[SXOQ_¥]G]`S]X_Ra]ZX
@HWI-EAS443:3:97:834:1899#0/1
ACCCAGCTTGCTAGAGATCTGGGCTCTGGCTCATCACTGT
+HWI-EAS443:3:97:834:1899#0/1
`¥G[]a`aaaZaa_a`a___¥W_a_[¥_^`Y^a[Q`XOUY
@HWI-EAS443:3:97:97:870#0/1
ATTTGAGAAATGATACTGATGTGGTGAACTTTGTAAGTAT
+HWI-EAS443:3:97:97:870#0/1
abb`^]]__^[^U``_^^¥_aYa`Y`^[[_Q_b^¥¥_]_]
@HWI-EAS443:3:98:1034:929#0/1
ATACTGTTCACCACTGTCCAGGCCATGATTCGGTCTCTTC
+HWI-EAS443:3:98:1034:929#0/1
_WY`aa`abba^_X_`T^W]aa_Va`ba]Z]TWVXV¥a`[
@HWI-EAS443:3:98:1059:480#0/1
ATGAAGACATTCTTTGGCTGGAAAGAGCCTAACATAGTCA
+HWI-EAS443:3:98:1059:480#0/1
aaabbaaabbbababba_abb_aaa`a^Saab¥aaaa¥`a
@HWI-EAS443:3:98:1165:1303#0/1
AGAAGATCCCAAGGACAAAGAACATGAAGAGAACAAGCCA
+HWI-EAS443:3:98:1165:1303#0/1
aabbbaaaaaaabaa_aa`a``_`baaaaaa_]``aXW`
@HWI-EAS443:3:98:126:420#0/1
CATCTTTGCAGTCATCAAAGTCTACTTTTTCTGGTGCCAT
+HWI-EAS443:3:98:126:420#0/1
`bbaaaabaaba`aaaa``a]aa``aaaaaaa``]a_]_a
~~~

```
@HWI-EAS443:3:98:1605:454#0/1
GGCATGGAAATGAGGCGCTGCCTTCTTCAGTCTCTTCAGC
+HWI-EAS443:3:98:1605:454#0/1
a¥YR^a`___¥`¥[_Ua¥Z`¥QW_N_^YU_YS¥U_¥YZLU
@HWI-EAS443:3:98:1773:1420#0/1
GAGAAAGGCATAAATCCCAATTACCTCATGGCTTGGAAGC
+HWI-EAS443:3:98:1773:1420#0/1
J_bbbab__]Kbbb```bbbbb]_T_abbbbRb]aT[a`L
@HWI-EAS443:3:98:179:695#0/1
GGGGCTATATGAAGCAATCGAGGAGTGCCTGATTAATGAT
+HWI-EAS443:3:98:179:695#0/1
a^^a``a`]``__]VT]S¥[X[RZ^W^^U[_]X]XU^^VR
@HWI-EAS443:3:98:268:1858#0/1
ATCTGGTGACCCGAATGCACTATTGAAGCACCGATTTGAG
+HWI-EAS443:3:98:268:1858#0/1
a]a^____a_a_aa`]_Y]X]]XX`U_]YXOW]OYZ]^^^
@HWI-EAS443:3:98:317:1432#0/1
GCCGAGTCTTCTGTCAAAGAGAAAGACATGACCAAGGAAT
+HWI-EAS443:3:98:317:1432#0/1
ab^Zab_bbbaababbaaaaaa]aba^baaa__bbb`^aa
@HWI-EAS443:3:98:321:317#0/1
TGGGGGGCTATATGAAGCAATCGAGGAGTGCCTGATTAAT
+HWI-EAS443:3:98:321:317#0/1
aba`_abaaaa`aaa`a_`aa_a^a`T_W`_Y_a_^``__
@HWI-EAS443:3:98:361:525#0/1
AAGCTGGATAGAACTTGATGAAATAGGAGAAGATGTTGCC
+HWI-EAS443:3:98:361:525#0/1
^U^`aa¥X`_a^]Zaaa``]^]¥a__a`_^_a[a_Y]_Z¥
@HWI-EAS443:3:98:402:1147#0/1
CACCCAGGGGAGTGGAGGAAGGCTCTATTGGGAAAGTGTG
+HWI-EAS443:3:98:402:1147#0/1
R__bb`ba__[b^abbbbabba]_ab^bbaaaba_b__a_
@HWI-EAS443:3:98:444:404#0/1
CATGGAAATGAGGCGCTGCCTTCTTCAGTCTCTTCAGCAG
+HWI-EAS443:3:98:444:404#0/1
[¥`bbaaa_bbb_bb]bbZZ^^¥^R]^_YR`Z``a``^`b
@HWI-EAS443:3:98:495:576#0/1
AGTTCCTGTAATCTCAAATTTTTCTTCAATTGTCTCTTCG
+HWI-EAS443:3:98:495:576#0/1
aaabbbbbabbb_b`^X]`bbbaZaabbbabba`a`ab`a
@HWI-EAS443:3:98:502:222#0/1
CATTAATCAGGCACTCCTCGATTGCTTCATATAGCCCCCC
+HWI-EAS443:3:98:502:222#0/1
VT[__¥_b^TPU__]_]W¥X]N^]aXVJV¥TQYHPY[Z¥_
@HWI-EAS443:3:98:506:320#0/1
ATCCGAAAATCGAAACTAACAAGTTTGCTGCAATATGCAC
+HWI-EAS443:3:98:506:320#0/1
Xa[abb_b`bab[J¥a^a]`¥¥aTb`ba]`_[_baaa^ba
@HWI-EAS443:3:98:528:879#0/1
GGGGCATGGAAATGAGGCGCTGCCTTCTTCAGTCTCTTCA
```

+HWI-EAS443:3:98:528:879#0/1
ab`a_`aaa_^`aa^_`^bZaa`]_aW``¥`_VN`^__V^
@HWI-EAS443:3:98:532:1980#0/1
AGAGAAGATCCCAAGGACAAAGAACATGAAGAGAACAAGC
+HWI-EAS443:3:98:532:1980#0/1
aXa^[baa`T]P¥WP^aa``_S¥__a``^_^a`[_a`^ZS
@HWI-EAS443:3:98:537:397#0/1
ATGAAATGGGGCATGGAAATGAGGCGCTGCCTTCTTCAGT
+HWI-EAS443:3:98:537:397#0/1
abbaaabbabaaaaaba``bba``aa`_a¥[aa¥`a^¥a`
@HWI-EAS443:3:98:547:1558#0/1
ATCCAGCTTGAATCAGTCAATTCACATGCCTTATTGAATT
+HWI-EAS443:3:98:547:1558#0/1
a]H^^]_UNW_YYaaa_`aZa]__[YUUWWZ__[]__]`^
@HWI-EAS443:3:98:561:453#0/1
GGAGTCTATGGGATTCCTTTCGTCAGTCCGAAAGAGGCGA
+HWI-EAS443:3:98:561:453#0/1
a`[][_aaa_aa]__^``a`^aZ_][V]]^VZ]_X_XX^Y
@HWI-EAS443:3:98:566:793#0/1
CAAGGACAAAGAACATGAAGAGAACAAGCCAATTGAAGTG
+HWI-EAS443:3:98:566:793#0/1
X`bbb`b_`_ba```bab[bbba`^^`baab_baa_]a¥a
@HWI-EAS443:3:98:613:1301#0/1
CTCGGGTCAGTGAGTGAGAACTCCATACTTACAAAGTTCA
+HWI-EAS443:3:98:613:1301#0/1
^`^`a^_bab^bba[b`U`abaa__a`]¥bbbaba`U``b
@HWI-EAS443:3:98:614:643#0/1
CACATATAGGAACATGGGCCTCGACACTTGGCCTATCGCA
+HWI-EAS443:3:98:614:643#0/1
aaabababbb`baa_XQ`]^a`aa^a``ZaVFZbb`T[Wa
@HWI-EAS443:3:98:629:1567#0/1
CATATCGCAAGCATGAGGAGGAACTATTTTACAGCAGAAG
+HWI-EAS443:3:98:629:1567#0/1
abbbaa_Saaaaa`aabYaa`aa_^_`aaaa_a__`a_aS
@HWI-EAS443:3:98:650:1425#0/1
AGAATTCCTTGGTCATGTCTTTCTCTTTGACAGAAGACTC
+HWI-EAS443:3:98:650:1425#0/1
`aaabaa_abbbWaab`_aaaba``aa`a`_a`aa`__a]
@HWI-EAS443:3:98:653:357#0/1
CCGATTTGAGATAATTGAAGGAAGAGACCGAATCATGGCC
+HWI-EAS443:3:98:653:357#0/1
aab`bbbbaabbbbabbbbbbabbbaaa`baaaaabbaUa
@HWI-EAS443:3:98:677:119#0/1
GCAGTCATCAAAGTCTACTTTTTCTGGTGCCATATTTTCA
+HWI-EAS443:3:98:677:119#0/1
a_aa_aaa^`a`a_]aa`aaaaa^`a`^a^]_a^a_a`^_
@HWI-EAS443:3:98:742:1404#0/1
GTCTTCTGTCAAAGAGAAAGACATGACCAAGGAATTCTTT
+HWI-EAS443:3:98:742:1404#0/1
a_abbbbb`baa^aaaaaaaaa_aaa_`ba_`a___a`a

```
@HWI-EAS443:3:98:796:1258#0/1
GGGGGCTATATGAAGCAATCGAGGAGTGCCTGATTAATGA
+HWI-EAS443:3:98:796:1258#0/1
aa^R]``ZHYVaaZTT`SV^WVZ¥_WDYURS_UFKQ^Y]]
@HWI-EAS443:3:98:80:1006#0/1
CACCGATTTGAGATAATTGAAGGAAGAGACCGAATCATGG
+HWI-EAS443:3:98:80:1006#0/1
]Mb__aa`TW^a]ba[ba_ZR]]_SZ`ba__][aa]`_a_
@HWI-EAS443:3:98:820:1231#0/1
GTTTCCGATTTGTTTTCAAAGAATTCCTTGGTCATGTCTT
+HWI-EAS443:3:98:820:1231#0/1
[J¥a_a`XS¥ab_[Za`_]`aX¥Z^^^a__]TQ_`aTS[_
@HWI-EAS443:3:98:825:353#0/1
CTTCCTTCAATTATCTCAAATCGGTGCTTCAATAGTGCAT
+HWI-EAS443:3:98:825:353#0/1
aabaabb]aabbababababaaa`a`Zaaa``_``aaZa¥_a
@HWI-EAS443:3:98:88:1603#0/1
GGAACATGGGCCTCGACACTTGGCCTATCGCAGTCCTCAA
+HWI-EAS443:3:98:88:1603#0/1
a[abX]_`]aa`aa_`]`b]^_ZZ[¥b]Z¥ZaRNR¥¥P^B
@HWI-EAS443:3:98:959:474#0/1
TGGGGGGCTATATGAAGCAATCGAGGAGTGCCTGATTAAT
+HWI-EAS443:3:98:959:474#0/1
aaaa`b``baaaaba`aaa`a_a`aa¥a¥`Z^aa^a_a^`
@HWI-EAS443:3:98:960:2024#0/1
CATAGTCAAACCACATGAGAAAGGCATAAATCCCAATTAC
+HWI-EAS443:3:98:960:2024#0/1
`bbbbbabbb¥_bbbbbaa¥abbbaabbaab¥a_bbb`ba
@HWI-EAS443:3:98:971:85#0/1
TTCGGATTTCCATTTCATCGACGAACGGGGTGAATCAATA
+HWI-EAS443:3:98:971:85#0/1
aXQ_bXK¥THX]a^ZY_`_a_[aZ`VX^`]IaYX^LTN^W
@HWI-EAS443:3:98:981:1204#0/1
ACCGAATCATGGCCTGGACAGTGGTGAACAGTATATGTAA
+HWI-EAS443:3:98:981:1204#0/1
aaaa`ababbba`aaa_a`a`^aaZ`__Y`aV`aa_aXa`
@HWI-EAS443:3:99:1024:1673#0/1
ATTCTTTGGCTGGAAAGAGCCTAACATAGTCAAACCACAT
+HWI-EAS443:3:99:1024:1673#0/1
`¥a`a`ab`^aa]aaaaa`_`aaa_`__`_Ya`_[¥a]`a
@HWI-EAS443:3:99:1068:1709#0/1
ATCCGAAAATCGAAACTAACAAGTTTGCTGCAATATGCAC
+HWI-EAS443:3:99:1068:1709#0/1
a`a`^`ba¥_Vab^aabaW]`[aUbbba_aaa_^`_a_`¥
@HWI-EAS443:3:99:1073:1249#0/1
GGCAACATCTTCTCCTATTTCATCAAGTTCTATCCAGCTT
+HWI-EAS443:3:99:1073:1249#0/1
aaabbabbbbbbbbbbbbbbabbabaaabbbbbaaabYba
@HWI-EAS443:3:99:1088:1092#0/1
GGGGTTTTCGGCTGAATCGAGAAAATTGCTTCTCATTGTT
```

```
+HWI-EAS443:3:99:1088:1092#0/1
_b_bS_baba_S_]b`_I_]`]aaababU``V`aa`a`a¥
@HWI-EAS443:3:99:1100:1374#0/1
AAACTTTAGAGCCTATGTAGATGGATTCGAGCCGAACGGC
+HWI-EAS443:3:99:1100:1374#0/1
a^ZU__^Ya]``_Z_abYaa]^T_a^UH]b`]]a]_UZP]
@HWI-EAS443:3:99:1198:1877#0/1
TAAATCCCAATTACCTCATGGCTTGGAAGCAGGTGCTAGC
+HWI-EAS443:3:99:1198:1877#0/1
aabbbUa_b`aaba_aTaaab_bbaaSb^Oa[^HVH[`XS
@HWI-EAS443:3:99:1261:755#0/1
TAACATAGTCAAACCACATGAGAAAGGCATAAATCCCAAT
+HWI-EAS443:3:99:1261:755#0/1
^a_T]``aaaba`aba^abbbaabab`aabab`abR_aaa
@HWI-EAS443:3:99:1304:1100#0/1
ATAAATACGGCCTTGCTCAATGCATCCTGTGCAGCCATGG
+HWI-EAS443:3:99:1304:1100#0/1
^a^`ab_SVVTQ[b[T`aba_X^b`NSZX``^^a]V[a]a
@HWI-EAS443:3:99:1316:233#0/1
TGGGTCCAAAATGAATTCAATAAGGCATGTGAATTGACTG
+HWI-EAS443:3:99:1316:233#0/1
]ba_YZ^_X`abbaabaWaaba`ba_`bb^ba`a_a_^_b
@HWI-EAS443:3:99:1329:929#0/1
GGAGGAACTATTTTACAGCAGAAGTGTCCCACTGCAGGGC
+HWI-EAS443:3:99:1329:929#0/1
^_ab`abababaabbaababaaaa^a`aZaa^aaZ[a`X_
@HWI-EAS443:3:99:1456:1556#0/1
AGGAGTCTATGGGATTCCTTTCGTCAGTCCGAAAGAGGCG
+HWI-EAS443:3:99:1456:1556#0/1
`b`aa^`ba^^a^Uaa][b`aRaa_aa_W[a_a`[X[a_a
@HWI-EAS443:3:99:1460:1644#0/1
GGCATGGAAATGAGGCGCTGCCTTCTTCAGTCTCTTCAGC
+HWI-EAS443:3:99:1460:1644#0/1
aba[abba`aabbb`]`ab_F^a`[aa__b^IaZO^[`a_
@HWI-EAS443:3:99:1465:909#0/1
GGGGCATGGAAATGAGGCGCTGCCTTCTTCAGTCTCTTCA
+HWI-EAS443:3:99:1465:909#0/1
ab]ba`ab_a`abba`a]b[aaW[`aPaa^`aQY]¥a_Va
@HWI-EAS443:3:99:1476:358#0/1
TGAAGAAAAATTTGAGATTACAGGAACTATGCGCAAGCTT
+HWI-EAS443:3:99:1476:358#0/1
abaab`baa_baaaaaabb`^aa_``]baabXa_^^aZ`B
@HWI-EAS443:3:99:1483:1596#0/1
CATAAATACGGCCTTGCTCAATGCATCCTGTGCAGCCATG
+HWI-EAS443:3:99:1483:1596#0/1
Jab`^bb`_a`aabbaa_ba`aaaab_b[bababaXabab
@HWI-EAS443:3:99:1517:645#0/1
ATTTCATCGACGAACGGGGTGAATCAATAATTGTAGAATC
+HWI-EAS443:3:99:1517:645#0/1
abb__b``ba`a_a`_aaaW`_¥^_a_b`_a``[aaSa_U
```

```
@HWI-EAS443:3:99:157:1624#0/1
TTGGGGGGCTATATGAAGCAATCGAGGAGTGCCTGATTAA
+HWI-EAS443:3:99:157:1624#0/1
a`^Ta_Y]aba`abaaYWaa`]a]a__[Y_^SZ`aaZ`]P
@HWI-EAS443:3:99:1592:138#0/1
ATCCGAAAATCGAAACTAACAAGTTTGCTGCAATATGCAC
+HWI-EAS443:3:99:1592:138#0/1
`_WOGZa`aaTST_aSaa__b[IZ]¥_^^_U`¥`_ZYW__
@HWI-EAS443:3:99:1601:1074#0/1
GGCATGGAAATGAGGCGCTGCCTTCTTCAGTCTCTTCAGC
+HWI-EAS443:3:99:1601:1074#0/1
ab_babbbbabbbab_babb_``a`bba`aaQa`baYYV_
@HWI-EAS443:3:99:1614:1457#0/1
CAGCCTTGAAAACTTTAGAGCCTATGTAGATGGATTCGAG
+HWI-EAS443:3:99:1614:1457#0/1
`ba[Nbbbbbb`^a`ababb`abbbbaa`aabaaa`a`ba
@HWI-EAS443:3:99:1685:1115#0/1
ACATGGGCCTCGACACTTGGCCTATCGCAGTCCTCAAGAG
+HWI-EAS443:3:99:1685:1115#0/1
a]```I__abR^aXa_`QIT_Rbab_]ZVa_[¥a¥_Xa]_
@HWI-EAS443:3:99:1692:869#0/1
GAAAACTTTAGAGCCTATGTAGATGGATTCGAGCCGAACG
+HWI-EAS443:3:99:1692:869#0/1
_b_aa[bbbaaaa`bbbbaN_bbbabaab_baa_IaaZDW
@HWI-EAS443:3:99:1741:997#0/1
AGAAGTTCGGTGGGAGACTTTGGTCGGCAAGCTTGCGCAT
+HWI-EAS443:3:99:1741:997#0/1
VbaYZI[`]_[a__abUUW¥HZ^PQYOG^¥TVaaQD[RBB
@HWI-EAS443:3:99:1744:1229#0/1
ACCGAGTGCCCACTTCAATTGGCTTGTTCTCTTCATGTTC
+HWI-EAS443:3:99:1744:1229#0/1
`aZa^_^[`H_aI¥Z]]a`Z_a__[HO]GRGU¥BBBBBBB
@HWI-EAS443:3:99:214:1884#0/1
CATAAATACGGCCTTGCTCAATGCATCCTGTGCAGCCATG
+HWI-EAS443:3:99:214:1884#0/1
_bab]baa_HZO]baZ_^b[b]^YYXOLX^_XY`UX]aY]
@HWI-EAS443:3:99:219:1572#0/1
GGTGAATCAATAATTGTAGAATCTGGTGACCCGAATGCAC
+HWI-EAS443:3:99:219:1572#0/1
`a]abbbabbbabaababbbbabab_bbaaaabbababa
@HWI-EAS443:3:99:229:1374#0/1
CAACATCTTCTCCTATTTCATCAAGTTCTATCCAGCTTGA
+HWI-EAS443:3:99:229:1374#0/1
a^Yaaaa`a`ba]R`ba__][_`a[ZTDPa`aS^UYa^``
@HWI-EAS443:3:99:235:50#0/1
AGGAACTATGCGCAAGCTTGCCGACCAAAGTCTCCCACCG
+HWI-EAS443:3:99:235:50#0/1
abbaaabaaa[a^`_a___a¥^`_U¥^^^`¥¥]UTW^TU_
@HWI-EAS443:3:99:237:460#0/1
GGGGGGCTATATGAAGCAATCGAGGAGTGCCTGATTAATG
```

```
+HWI-EAS443:3:99:237:460#0/1
abaa`aaaaaaaaa`a`aa`]a``_¥aWa_¥_a^a_`__`
@HWI-EAS443:3:99:280:835#0/1
AGCAGGTGCTAGCAGAGCTACAGGACATTGAAAATGAAGA
+HWI-EAS443:3:99:280:835#0/1
^U]FWbZYb[U]aZa]]a]_ab[ba]IX]^OGUZ]ZH]`Q
@HWI-EAS443:3:99:300:981#0/1
TTCGGATTTCCATTTCATCGACGAACGGGGTGAATCAATA
+HWI-EAS443:3:99:300:981#0/1
aa`aaa_``]_^aa``__a`¥_a^_¥_]__Va_^]S_^`]
@HWI-EAS443:3:99:306:1343#0/1
GGATTCGAGCCGAACGGCTGCATTGAGGGCAAGCTTTCCC
+HWI-EAS443:3:99:306:1343#0/1
`aY`_U]aVQS__¥¥a^`ZZ[WHUZMJPY^MDIBBBBBBB
@HWI-EAS443:3:99:31:1274#0/1
CTCTTCCTTCAATTATCTCAAATCGGTGCTTCAATAGTGC
+HWI-EAS443:3:99:31:1274#0/1
`[Y_¥YZ¥^`b`baaa`aa_a]`a]_Za_aaa^Vaaa]aa
@HWI-EAS443:3:99:36:353#0/1
GTTCGGTGGGAGACTTTGGTCGGCAAGCTTGCGCATAGTT
+HWI-EAS443:3:99:36:353#0/1
aaaabb_baa`a[_aaaaa¥a^ZX``aaX^_W___Z¥ZNR
@HWI-EAS443:3:99:378:406#0/1
ATTGGTTCTCACATATAGGAACATGGGCCTCGACACTTGG
+HWI-EAS443:3:99:378:406#0/1
^]_``X``^ZY[aaaaaaaW^^Y^a¥_W_^``[^ZXZ^^U
@HWI-EAS443:3:99:38:467#0/1
TCTCATTTGAGAAATGATACTGATGTGGTGAACTTTGTAA
+HWI-EAS443:3:99:38:467#0/1
aaa¥Z_aaa_a`aa`b``a_aaa_a_`_V`]_]`__a^]V
@HWI-EAS443:3:99:504:1556#0/1
CGGAAACATGGCCAATCGGAGAGTCACCCAGGGGAGTGGA
+HWI-EAS443:3:99:504:1556#0/1
^a¥W¥bbbb^]`[abb¥bbababababba]]b_ZTa¥^JTba
@HWI-EAS443:3:99:517:797#0/1
ACATCAGTATCATTTCTCAAATGAGACCTTCCTTTTATAA
+HWI-EAS443:3:99:517:797#0/1
]¥aa`^¥YV¥a^a`Z`^]_¥^``_V`][`a_]aaaa__`_
@HWI-EAS443:3:99:530:1010#0/1
AGAGCCTAACATAGTCAAACCACATGAGAAAGGCATAAAT
+HWI-EAS443:3:99:530:1010#0/1
a`aaa`aZba^aaaaaaaaaaa`aaaa_aaa__aaaaa¥_
@HWI-EAS443:3:99:534:298#0/1
ATGAGAAGCAATTTTCTCGATTCAGCCGAAAACCCCTCAA
+HWI-EAS443:3:99:534:298#0/1
aabY^V]a_]ZWa`a```^W¥__`__^aZ^^]UQVVVV]X
@HWI-EAS443:3:99:553:845#0/1
CCACTCCCCTGGGTGACTCTCCGATTGGCCATGTTTCCGA
+HWI-EAS443:3:99:553:845#0/1
abbb`bbbbaabb_bbabbaaaaaa`a^`]_aaZaa¥^a¥
```

```
@HWI-EAS443:3:99:575:207#0/1
GGGTTTTCGGCTGAATCGAGAAAATTGCTTCTCATTGTTC
+HWI-EAS443:3:99:575:207#0/1
aba¥aaa`b`_aaaa`_a``a`__`aa^`_T^Y^_]`¥^X
@HWI-EAS443:3:99:623:680#0/1
GGTGAACTTTGTAAGTATGGAGTTCTCACTCACTGACCCG
+HWI-EAS443:3:99:623:680#0/1
a_RWS]aaa`a[`]a_a`^VJZ_Z[]_a`___^P`_ZHSa
@HWI-EAS443:3:99:667:1567#0/1
GGGGGCTATATGAAGCAATCGAGGAGTGCCTGATTAATGA
+HWI-EAS443:3:99:667:1567#0/1
a[``T]U`__a[aa___RZ``Z]`X`W¥[Y_`S___[P¥_
@HWI-EAS443:3:99:684:1225#0/1
GGCATGGAAATGAGGCGCTGCCTTCTTCAGTCTCTTCAGC
+HWI-EAS443:3:99:684:1225#0/1
a¥`b^`^`]`^_ab_`aaa^P¥_aVaaU^]^¥a``[¥W_¥
@HWI-EAS443:3:99:717:1138#0/1
ATGTATTCGGATTTCCATTTCATCGACGAACGGGGTGAAT
+HWI-EAS443:3:99:717:1138#0/1
`bb_`_baaabbba_aababa``[`W[baa¥bZ`a^baaa
@HWI-EAS443:3:99:730:251#0/1
CAAGGACAAAGAACATGAAGAGAACAAGCCAATTGAAGTG
+HWI-EAS443:3:99:730:251#0/1
aaababbbbabaa¥abbaabbba_aaab_abaaab`_b_b
@HWI-EAS443:3:99:762:719#0/1
GACCCGAATGCACTATTGAAGCACCGATTTGAGATAATTG
+HWI-EAS443:3:99:762:719#0/1
abababbbb`_`Y`abbaa]__¥Na]aaaa[Z`a`_[aab
@HWI-EAS443:3:99:773:2027#0/1
GGGGCATGGAAATGAGGCGCTGCCTTCTTCAGTCTCTTCA
+HWI-EAS443:3:99:773:2027#0/1
ZbabZa]``Tbabbabbbb_ab]a`¥WaZO]baabXab_Z
@HWI-EAS443:3:99:786:1432#0/1
CGTCAAGGGTGTAGTCCGCTTTGGTGGCCATCTCCTCTCC
+HWI-EAS443:3:99:786:1432#0/1
ababaaabb^babbabaa^aaa`a_aaa_aa]`a]`^_¥¥
@HWI-EAS443:3:99:813:2014#0/1
CATGTATTCGGATTTCCATTTCATCGACGAACGGGGTGAA
+HWI-EAS443:3:99:813:2014#0/1
abaaXabbabaaa```aaaaa`b_aaa_`a``a``ZMV_a
@HWI-EAS443:3:99:815:479#0/1
AGAAGTTCGGTGGGAGACTTTGGTCGGCAAGCTTGCGCAT
+HWI-EAS443:3:99:815:479#0/1
ab_a_]aabb^b_a`aaaaaab]Raaaa^`aa``a¥aV_`
@HWI-EAS443:3:99:844:1352#0/1
GCTTGCTAGAGATCTGGGCTCTGGCTCATCACTGTCATAC
+HWI-EAS443:3:99:844:1352#0/1
a_aabaaabab_`Z_aab`a`_abaa_a__aa_a_]`a`]
@HWI-EAS443:3:99:883:901#0/1
GGGCACTCGGTGAAAATATGGCACCAGAAAAAGTAGACTT
```

```
+HWI-EAS443:3:99:883:901#0/1
_bbbab```¥T[`Z__aba_]Za`a`bX[``]aH`a`a_a
@HWI-EAS443:3:99:915:1033#0/1
GCTGGGTCCAAAATGAATTCAATAAGGCATGTGAATTGAC
+HWI-EAS443:3:99:915:1033#0/1
`[U^ZYG_]a_Yaaa¥V_`U]X^___ZY^^`W__¥^Q_^]
@HWI-EAS443:3:99:973:1585#0/1
CATTAATCAGGCACTCCTCGATTGCTTCATATAGCCCCCC
+HWI-EAS443:3:99:973:1585#0/1
abbbabbabbabb`aa`ab`abbbabb_bbbbba[N_`Za
@HWI-EAS443:3:99:995:1012#0/1
AAATGAATTCAATAAGGCATGTGAATTGACTGATTCAAGC
+HWI-EAS443:3:99:995:1012#0/1
aa^`aba`aaa`aa`_SY[a^Y^`Ub_`]Vaaaa`XaYaH
@HWI-EAS443:3:9:1102:95#0/1
CACCCAGGGGAGTGGAGGAAGGCTCTATTGGGAAAGTGTG
+HWI-EAS443:3:9:1102:95#0/1
^¥aY`_bbbb^aab^P_a¥b`bX¥PXSVa_^]`_aaV]Xa
@HWI-EAS443:3:9:1105:388#0/1
AAATAAAATCTGAGAAGACACACATTCACATCTTTTCATT
+HWI-EAS443:3:9:1105:388#0/1
aaabaab`a_aa``_a``_a[a]^``^a^aa_aaa_]``_
@HWI-EAS443:3:9:1111:727#0/1
CACATCAGTATCATTTCTCAAATGAGACCTTCCTTTTATA
+HWI-EAS443:3:9:1111:727#0/1
_b``_^``]^a_`bba^__a`aabaaa___b__aabaaaZ
@HWI-EAS443:3:9:1127:1410#0/1
AGGGAGTGTACATAAATACGGCCTTGCTCAATGCATCCTG
+HWI-EAS443:3:9:1127:1410#0/1
Yb``]b`_R_`ab^b``_`bbYaaaaH[T_aaa]ba^ZZZ
@HWI-EAS443:3:9:1216:1558#0/1
ATCCCAATTACCTCATGGCTTGGAAGCAGGTGCTAGCAGA
+HWI-EAS443:3:9:1216:1558#0/1
aabbab`abb^abb`_aaaab[b_ZON[`^WRVZ¥Y¥¥_U
@HWI-EAS443:3:9:123:723#0/1
GGGGCATGGAAATGAGGCGCTGCCTTCTTCAGTCTCTTCA
+HWI-EAS443:3:9:123:723#0/1
ab`Y]_VGUZVT^THIX¥a_[`a]GYVBBBBBBBBBBBBB
@HWI-EAS443:3:9:1283:1252#0/1
AGAGATCTGGGCTCTGGCTCATCACTGTCATACTGTTTAA
+HWI-EAS443:3:9:1283:1252#0/1
`bXHRLX^N`PYQQV_RZaZSMOT¥UTQT[JXQFXYa]`¥
@HWI-EAS443:3:9:1284:1550#0/1
ATAGTCAAACCACATGAGAAAGGCATAAATCCCAATTACC
+HWI-EAS443:3:9:1284:1550#0/1
aa_]_aab^DEaI^a``a`]a_¥`aYaW`a_BBBBBBBBB
@HWI-EAS443:3:9:1468:57#0/1
CGAAAATCGAAACTAACAAGTTTGCTGCAATATGCACACA
+HWI-EAS443:3:9:1468:57#0/1
aabbbbb]aa`b_`baabab_bbaUabSaaaaab¥a^_^^
```

```
@HWI-EAS443:3:9:1495:1350#0/1
GACCCGAGTCACGAGGGGGAGGGAATACCACTATATGATG
+HWI-EAS443:3:9:1495:1350#0/1
aa_a_baa_aaaaX_aaaa_a^`Y__aUYXHX___`_^_^
@HWI-EAS443:3:9:1609:1764#0/1
CAGTCATCAAAGTCTACTTTTTCTGGTGCCATATTTTCAC
+HWI-EAS443:3:9:1609:1764#0/1
aY^¥Z]_`aa]ZNVa^W`aa`a^XVZR_YY____`__U_U
@HWI-EAS443:3:9:1651:1785#0/1
GGGGGCTATATGAAGCAATCGAGGAGTGCCTGATTAATGA
+HWI-EAS443:3:9:1651:1785#0/1
aa^`^abbbabaaa_]`aaQaa``_aZ`__[a_``a_¥a`
@HWI-EAS443:3:9:1682:1307#0/1
AATTGAAGGAAGAGACCGAATCATGGCCTGGACAGTGGTG
+HWI-EAS443:3:9:1682:1307#0/1
^`bbabbbbaaa^abaU_bbaY_a``__`_^Y^`^]_^O]
@HWI-EAS443:3:9:1687:118#0/1
AAGAATTCCTTGGTCATGTCTTTCTCTTTGACAGAAGACT
+HWI-EAS443:3:9:1687:118#0/1
a]`a_bb¥^baab__`aa`]aa`WaWaaaa^^_a^__^W^
@HWI-EAS443:3:9:1694:1746#0/1
ATTTCATCGACGAACGGGGTGAATCAATAATTGTAGAATC
+HWI-EAS443:3:9:1694:1746#0/1
a`ab_aXRb¥O__^¥^`Z_V``YTHXO_¥_¥^¥G_]^_¥Z
@HWI-EAS443:3:9:1718:984#0/1
CTTCAATAGTGCATTCGGGTCACCAGATTCTACAATTATT
+HWI-EAS443:3:9:1718:984#0/1
a_^aaTa_`S_RZ^`Pa`_¥U[[YX[W__^__]_]__]``
@HWI-EAS443:3:9:182:1810#0/1
GGTGAACTTTGTAAGTATGGAGTTCTCACTCACTGACCCG
+HWI-EAS443:3:9:182:1810#0/1
`aabbbbbabaab`bababaa`abbbbbbbbbaabaaabb
@HWI-EAS443:3:9:209:1205#0/1
ATGTAACACAACAGGGGTAGAGAAGCCTAAATTTCTTCCT
+HWI-EAS443:3:9:209:1205#0/1
_aaa_ba¥````a`^_a^¥``aa^`^^abaa`aaa^_`[`
@HWI-EAS443:3:9:263:1674#0/1
ATCATTAATCAGGCACTCCTCGATTGCTTCATATAGCCCC
+HWI-EAS443:3:9:263:1674#0/1
a[`^_X]a¥_[Z]_a¥^YaYUT_GT__][_¥H¥S^]_^]]
@HWI-EAS443:3:9:351:1201#0/1
GTTTCCGATTTGTTTTCAAAGAATTCCTTGGTCATGTCTT
+HWI-EAS443:3:9:351:1201#0/1
a``aabbaaaab_`aabaa^a__`_a_a_aa^^]^aY__`
@HWI-EAS443:3:9:398:543#0/1
TTGGGGGGCTATATGAAGCAATCGAGGAGTGCCTGATTAA
+HWI-EAS443:3:9:398:543#0/1
aabba`^aaaaaaaa]__aaa_^_```^_¥_`¥`aW__]¥
@HWI-EAS443:3:9:425:60#0/1
CGAAAATCGAAACTAACAAGTTTGCTGCAATATGCACACA
```

+HWI-EAS443:3:9:425:60#0/1
`bbbbbbbbabbbabbababababa`babaaaabaaaaaa
@HWI-EAS443:3:9:515:983#0/1
CAAGGAATTCTTTGAAAACAAATCGGAAACATGGCCAATC
+HWI-EAS443:3:9:515:983#0/1
aabaaaaab]bbaa_baabaaaaaa`_aaaa___a]a_]¥
@HWI-EAS443:3:9:517:699#0/1
ACGGCCTTGCTCAATGCATCCTGTGCAGCCATGGATGACT
+HWI-EAS443:3:9:517:699#0/1
`ab_ab[_ZQ]aa_V^[a]_¥_QU`X^`UZV__`ZV[N^Q
@HWI-EAS443:3:9:679:787#0/1
CATGTTTCCGATTTGTTTTCAAAGAATTCCTTGGTCATGT
+HWI-EAS443:3:9:679:787#0/1
`abb_UUPWb][P`b^`_`TU`_^_[``WY_``]O¥¥]¥P
@HWI-EAS443:3:9:693:255#0/1
GGGGGCTATATGAAGCAATCGAGGAGTGCCTGATTAATGA
+HWI-EAS443:3:9:693:255#0/1
aaY][]X`aa`a`_^W`_^W^Z`_X_[Z¥V`TZ`_X]]_]
@HWI-EAS443:3:9:708:62#0/1
AGGGGTTTTCGGCTGAATCGAGAAAATTGCTTCTCATTGT
+HWI-EAS443:3:9:708:62#0/1
a`VabNaa`aaa]aaaa__a^_^¥^a_¥_^¥_]_]__]aW
@HWI-EAS443:3:9:724:259#0/1
AGTTCCTCCTCATGCTTGCGATATGTTCAATCGGGGCAAC
+HWI-EAS443:3:9:724:259#0/1
abaaaab^a`baaaaaab`baaa`a^a^`a^]__``¥V_Y
@HWI-EAS443:3:9:750:521#0/1
CAACAAAATAAAATCTGAGAAGACACACATTCACATCTTT
+HWI-EAS443:3:9:750:521#0/1
P_]_Xb^`baaZ`a`a^ba¥a]a]Yaa^aaaVb_¥b^bab
@HWI-EAS443:3:9:809:740#0/1
AGAAATTTAGGCTTCTCTACCCCTGTTGTGTTACATATAC
+HWI-EAS443:3:9:809:740#0/1
aaaaaaaaaaaaaa_```a___^^`_^_S`X__^__`__V
@HWI-EAS443:3:9:911:874#0/1
GGCGTGGTGTCGTCCTCAAGAATGGTTCAATTTTGGCGTT
+HWI-EAS443:3:9:911:874#0/1
ab```ba_b`aba`]aaaaaa`ab_^_`a`a`a`_¥[a_`
@HWI-EAS443:3:9:916:1165#0/1
CATGTTTCCGATTTGTTTTCAAAGAATTCCTTGGTCATGT
+HWI-EAS443:3:9:916:1165#0/1
aa^a^aab¥`G]`_b_a_bba`_b_S`a]Z`aaa[a__b^
@HWI-EAS443:3:77:522:1148#0/1
CGTCCCTCTTAGTTTGCATAGTTTCCCGTTATGCTTGTCT
+HWI-EAS443:3:77:522:1148#0/1
aaaabbbbbbbaa`aaaabTbaaaaba_V`a_aa`aa`aa
@HWI-EAS443:3:1:583:205#0/1
GTTAAATACATATTCTACACTGTAGAGACCCATTAGAGCA
+HWI-EAS443:3:1:583:205#0/1
`W]W^¥_[ZZ_[aZY]^][M[XQQ¥UXXMFTYWVV[BBBB

```
@HWI-EAS443:3:25:696:360#0/1
GCTGGCTGGATCCTGGGAAATCCAGAATGTGAATCACTCT
+HWI-EAS443:3:25:696:360#0/1
aY_]]_a]N_Ua]LVZY_a`V¥Y[VVFF[G]_`_TX¥UN¥
@HWI-EAS443:3:63:1192:95#0/1
GTTAAATACATATTCTACACTGTAGAGACCCATTAGAGCA
+HWI-EAS443:3:63:1192:95#0/1
aab``abaaabaa`_`_`_`aa^^aW_`[]^__^^`___]
@HWI-EAS443:3:71:588:317#0/1
GTTAAATACATATTCTACACTGTAGAGACCCATTAGAGCA
+HWI-EAS443:3:71:588:317#0/1
a_¥Z[]aXV`R[^`aYa_VLI[QX[]Y__^NNT¥`WWV¥Z
@HWI-EAS443:3:71:870:1673#0/1
GTTAAATACATATTCTACACTGTAGAGACCCATTAGAGCA
+HWI-EAS443:3:71:870:1673#0/1
`aababbbaaaaa_babaaa_aWa]^_`aa`a`]`^`^_a
@HWI-EAS443:3:87:1770:90#0/1
GTTAAATACATATTCTACACTGTAGAGACCCATTAGAGCA
+HWI-EAS443:3:87:1770:90#0/1
a`S_QPaJNY_aaaP`YT^Pa_LPXR^]GRGWT[PM[XP^
@HWI-EAS443:3:99:1136:937#0/1
GTTAAATACATATTCTACACTGTAGAGACCCATTAGAGCA
+HWI-EAS443:3:99:1136:937#0/1
aaaaaaaaa^a_a_`a_[a^aa¥``_]_XX^__¥`W_]S`
@HWI-EAS443:3:52:81:200#0/1
GATGGATCAAGTAAGAGAGAGTCGAAACCCAGGAAACGCT
+HWI-EAS443:3:52:81:200#0/1
aa_aa`_]_Za]W_a^`^`_aZ^]_]^O¥Z^]^R[[MTUZ
@HWI-EAS443:3:86:791:1848#0/1
ATATTAGGCCCTCTTTGCGTGCGATTGGACCAGGCGGGCA
+HWI-EAS443:3:86:791:1848#0/1
abb`XZ]W_[Za__¥aa^_O`YaaXLQU`aaW[BBBBBBB
@HWI-EAS443:3:88:1147:182#0/1
GGCCCTCTTTGCGTGCGATTGGACCAGGCGGTCGTGGGGA
+HWI-EAS443:3:88:1147:182#0/1
¥_¥Z_`^_^`^a_Z``b_Z^^aYPW]aaBBBBBBBBBBBB
@HWI-EAS443:3:22:1631:402#0/1
AGGCCCTCTTTGCGTGCGATTGGACCAGGCGGNGATGGAA
+HWI-EAS443:3:22:1631:402#0/1
`a^Z¥Z¥^][`YYa¥X[bY]^a^]`U^`BBBBBBBBBBBB
@HWI-EAS443:3:13:1081:1774#0/1
ACCAGCGCGGTCATGGAAAAGAACATAGTACTGAAAGCGA
+HWI-EAS443:3:13:1081:1774#0/1
a_[W]VY[QV]Y¥PMJWWPSSUWSY^^U]BBBBBBBBBBB
@HWI-EAS443:3:18:698:648#0/1
GATGGCCCTCTTTGCGTGCGATTGGACCAGGCGGTCATCG
+HWI-EAS443:3:18:698:648#0/1
aW`¥a[a_UW``_a]`Ra`aYNY_a[^a^`ZaBBBBBBBB
@HWI-EAS443:3:35:1026:1326#0/1
CGCCCTCTTTGCGTGCGATTGGACCAGGCGGGCATGGAAA
```

```
+HWI-EAS443:3:35:1026:1326#0/1
abbbbabaaaabaaabaab`a`aa¥a__bbBBBBBBBBBB
@HWI-EAS443:3:19:903:2044#0/1
GAGCTTTCTCGTTTCAGCTTATTTAATGATAAAAAAAAAA
+HWI-EAS443:3:19:903:2044#0/1
abaabb`bbab`aabbbaabbaaaaabaaabbbabababa
@HWI-EAS443:3:42:612:202#0/1
GTGGTTTTTATCATTAAATAAGCTGAAACGAGAAAGCTCT
+HWI-EAS443:3:42:612:202#0/1
_S[DP_WaaUKa_]_]Z_V^¥__X__a^_a¥_¥Y¥a¥]U]
@HWI-EAS443:3:10:240:889#0/1
GTTCTTTTCCATTACCGCCTGGTCCAATCGCACGCAAAGA
+HWI-EAS443:3:10:240:889#0/1
aWV[[RL_`[V]NWLWF^BBBBBBBBBBBBBBBBBBBBBB
@HWI-EAS443:3:14:168:867#0/1
GTGACAAAAACATAATGGACTCCAACACCATGTCATGCTT
+HWI-EAS443:3:14:168:867#0/1
`¥_S_aa[¥aa`^Q]`aaaa_aaa_a^`a_`aS__^a_`]
@HWI-EAS443:3:27:1503:104#0/1
GACCGCCTGGACCAATCGCACGCAAAGAGGGCCTATTATC
+HWI-EAS443:3:27:1503:104#0/1
ZW][_Z__ORFRT¥a]H[[aXbU_Q_Z[bZXDD¥RYOQOJ
@HWI-EAS443:3:40:401:786#0/1
TTTTTATCATTAAATAAGCTGAACCGAGAAAGCTCTTATC
+HWI-EAS443:3:40:401:786#0/1
_a]_a[`_```PXY^RY_aW`_ZGX`W`G^X^^QZXUVZU
@HWI-EAS443:3:42:52:691#0/1
GTTTTTTACCATTAAATAAGCTGAAACGAGAAAGCTCTTA
+HWI-EAS443:3:42:52:691#0/1
a_]^]_]X^W_```__[^¥a__a_^^`[^`PM[_¥]¥__X
@HWI-EAS443:3:56:1463:1626#0/1
GACAAAAACATAATGGACTCCAACACCATGTAAAGCTTTC
+HWI-EAS443:3:56:1463:1626#0/1
ab_`_OUaP_]Waab`P[`GNWa`_ZK_a_UGQX`P¥XY_
@HWI-EAS443:3:5:1142:966#0/1
GCGGGCATGGAAAAGAACATAGTACTGAAAGCGAACTTCA
+HWI-EAS443:3:5:1142:966#0/1
`_]]HWSU_]R]XV]WX]V`Y`VWS¥]QU¥OT¥BBBBBBB
@HWI-EAS443:3:89:1200:1284#0/1
TTCGCTTTCAGTACTATGTTCTTTTCCATGACCGTCTGGT
+HWI-EAS443:3:89:1200:1284#0/1
aa`aaa`b`aa__`aaab_aaabaaa_aaaa^¥`_^]__U
@HWI-EAS443:3:99:849:1304#0/1
GAAGGTCGCTTTCAGTACTATGTTCTTTTCCATGACCGCC
+HWI-EAS443:3:99:849:1304#0/1
[bT[Ia[`bbU^]Sbaaaba``b``_a`_HZU`ba]HY]`
@HWI-EAS443:3:55:855:1022#0/1
GGCGTTTTTTATCATTAAATAAGCTGAAACGAGAAAGCTC
+HWI-EAS443:3:55:855:1022#0/1
aaG[^``_[R`[_NY]XQNSSa`YY_a]aa`X[^¥UaXW]
```

```
@HWI-EAS443:3:88:633:1723#0/1
TTTGCGTGCGATTGGACCAGGCGGTCATGGAAAAGAAAAT
+HWI-EAS443:3:88:633:1723#0/1
_^a^]Y]abba`ababbbaa^^aYDMRTYXLSBBBBBBBB
@HWI-EAS443:3:37:1201:964#0/1
CCGCCTGGTCCAATCGNACGCAAAGAGGGCCTATTATCTT
+HWI-EAS443:3:37:1201:964#0/1
`b`a`_a^W[`aa`_ZD[a_a`a_[_`__Ga¥^a_¥aa^`
@HWI-EAS443:3:76:801:886#0/1
ATTAAATAAGCTGAAACGAGAAAGCTCTTATCTCNTGTTC
+HWI-EAS443:3:76:801:886#0/1
^a[¥¥R`_^YS^RW¥^_V_aa`V`]]aba_^¥a]D[aaa_
@HWI-EAS443:3:20:406:1853#0/1
GCGTTTTTTATCATTAAATAAGCTGAAACGAGAAAGCTCT
+HWI-EAS443:3:20:406:1853#0/1
¥IXSVP_ZNOLYXXUXSQ^Z_ZV^QX`XTXLMTQZZVVLS
@HWI-EAS443:3:28:1127:1248#0/1
GCGTGACAAAAACATAATGGACTCCAACACCATGTCAAGC
+HWI-EAS443:3:28:1127:1248#0/1
^P_SWZ¥][TL¥]^VU¥X`¥YQX_OXRU_UW[XaUZLX^Q
@HWI-EAS443:3:65:1629:1121#0/1
GGCAAAAACATAATGGACTCCAACACCATGTCAAGCTTTC
+HWI-EAS443:3:65:1629:1121#0/1
a`a`aa`baabaaaabab_`aa`aa_aab^aa__Va``_
@HWI-EAS443:3:39:1556:917#0/1
TTTTTATCATTAAATAAGCTGAAACGAGAAAGCTCTTATN
+HWI-EAS443:3:39:1556:917#0/1
]bb]aXaab`bb¥¥_[]¥O^a`W]Kaa`NH¥_Wb¥`SH]D
@HWI-EAS443:3:47:595:144#0/1
GACCGCCTGGTCCAATCGCACGCAAAGAGGGCCTATTATA
+HWI-EAS443:3:47:595:144#0/1
_[aaU``a`^`[X^`__ba_¥a_]_^UY`¥`[[_¥_TZWG
@HWI-EAS443:3:10:607:50#0/1
CACTGAAGTTCGCTTTCAGTACTATGTTCTTTTCCATGAC
+HWI-EAS443:3:10:607:50#0/1
a^`aZaabaaab^_``[¥bTaaa_^`R^V_a`_aZ_¥U``
@HWI-EAS443:3:11:566:864#0/1
TGAAGTTCGCTTTCAGTACTATGTTCTTTTCCATGACCGC
+HWI-EAS443:3:11:566:864#0/1
aba`a__ba^a^aaaaX_`a_aa]`____a`¥__```^`X
@HWI-EAS443:3:11:876:48#0/1
GTTTTTTATCATTAAATAAGCTGAAACGAGAAAGCTCTTA
+HWI-EAS443:3:11:876:48#0/1
a^__`_][_]]_^¥¥[^]^^Z[^V^[^^[^XXY[Z[Z[[[
@HWI-EAS443:3:12:211:322#0/1
GTGTTTTTTATCATTAAATAAGCTGAAACGAGAAAGCTCT
+HWI-EAS443:3:12:211:322#0/1
aabaabbaaabaababaaaaaaa`aaaaaaaa_[]a_a^`
@HWI-EAS443:3:12:719:756#0/1
GTTCTTTTCCATGACCGCCTGGTCCAATCGCACGCAAAGA
```

```
+HWI-EAS443:3:12:719:756#0/1
a_a`aaaaaaaa``^U__¥_^[U[_`__¥_¥_¥¥_[PY^U
@HWI-EAS443:3:13:104:1958#0/1
CTTTCAGTACTATGTTCTTTTCCATGACCGCCTGGTCCAA
+HWI-EAS443:3:13:104:1958#0/1
a`_[^ZXGaa]`aa[a^[U]aaP]_Y^a^`a^[`PRZ¥``
@HWI-EAS443:3:13:1180:549#0/1
GGTGTTTTTTATCATTAAATAAGCTGAAACGAGAAAGCTC
+HWI-EAS443:3:13:1180:549#0/1
`YXa_a`]`Y^`]]_`]WZa]^_^Y_`]O[_¥`¥^[^[¥W
@HWI-EAS443:3:13:82:303#0/1
CAAAAACATAATGGACTCCAACACCATGTCAAGCTTTCAG
+HWI-EAS443:3:13:82:303#0/1
aaaaababaa``a_aaabaaaaaaaaaaYaa``a`¥_][]
@HWI-EAS443:3:13:971:166#0/1
CAAAAACATAATGGACTCCAACACCATGTCAAGCTTTCAG
+HWI-EAS443:3:13:971:166#0/1
abbbabaabaaab`a^a`^aaa`^_`a_Z``^`^`Z_^^_
@HWI-EAS443:3:14:976:1106#0/1
TTTTTATCATTAAATAAGCTGAAACGAGAAAGCTCTTATC
+HWI-EAS443:3:14:976:1106#0/1
`[Y[[Z¥¥SZ`ZS^`^V[]X¥V_[O^ZZURY_X_[_¥[]_
@HWI-EAS443:3:15:1737:1093#0/1
ATTAAATAAGCTGAAACGAGAAAGCTCTTATCTCTTGTTC
+HWI-EAS443:3:15:1737:1093#0/1
abbbbbbbaaabbbbaab_ba`ab_babaab`b_bbbaa_
@HWI-EAS443:3:15:429:601#0/1
GACCGCCTGGTCCAATCGCACGCAAAGAGGGCCTATTATC
+HWI-EAS443:3:15:429:601#0/1
aaaabbb_ba]aabaaaaaa_a_`¥[`]__`_^`___a_¥
@HWI-EAS443:3:15:589:1916#0/1
CAAAAACATAATGGACTCCAACACCATGTCAAGCTTTCAG
+HWI-EAS443:3:15:589:1916#0/1
abZbbbbbbU]]b¥X¥`abb^^bV`a_YP`b^Z[aY```a
@HWI-EAS443:3:15:668:655#0/1
TTTTATCATTAAATAAGCTGAAACGAGAAAGCTCTTATCT
+HWI-EAS443:3:15:668:655#0/1
aba_aabbbaaa¥ba`¥bab`baaaaaaaZaaaaa¥PZ_a
@HWI-EAS443:3:15:769:634#0/1
ATTAAATAAGCTGAAACGAGAAAGCTCTTATCTCTTGTTC
+HWI-EAS443:3:15:769:634#0/1
abbbabbbbaaba_bbabaaaa`baaaaaaaaaabaa_aa
@HWI-EAS443:3:15:912:1315#0/1
CATTAAATAAGCTGAAACGAGAAAGCTCTTATCTCTTGTT
+HWI-EAS443:3:15:912:1315#0/1
aabbbbbbaabbbbabbbbbbb`babbb_abbb^babbaaa
@HWI-EAS443:3:16:128:951#0/1
GTACTATGTTCTTTTCCATGACCGCCTGGTCCAATCGCAC
+HWI-EAS443:3:16:128:951#0/1
a¥aa^_`b_aaaaa``aa_``a`_a[`a_¥^¥^X[`_V`[
```

```
@HWI-EAS443:3:16:1568:1707#0/1
CCTGTCGCTTTCAATCTGTGCCGCATTTCTTCAATTAACC
+HWI-EAS443:3:16:1568:1707#0/1
`bb``abbabb_b``ZZb_a`aaQ_^bbabaaaaaaa`XY
@HWI-EAS443:3:16:629:829#0/1
CGGTCATGGAAAAGAACATAGTACTGAAAGCGAACTTCAG
+HWI-EAS443:3:16:629:829#0/1
abb^ab`aaaaa`b_aaaaaa¥aaaa``_aaaaa^_a__`
@HWI-EAS443:3:17:1134:142#0/1
TTTCAGTACTATGTTCTTTTCCATGACCGCCTGGTCCAAT
+HWI-EAS443:3:17:1134:142#0/1
^aaaa`XR_^^_a[YV¥`]`^X`¥]^¥Z]U]^]aVYY__]
@HWI-EAS443:3:17:1189:232#0/1
GGTGACAAAAACATAATGGACTCCAACACCATGTCAAGCT
+HWI-EAS443:3:17:1189:232#0/1
a_R__abaa```a^W]^a`¥]]_^``_a`UZaaYS`U`]]
@HWI-EAS443:3:17:1198:255#0/1
GGTGTTTTTTATCATTAAATAAGCTGAAACGAGAAAGCTC
+HWI-EAS443:3:17:1198:255#0/1
^a__Xab^bbbbX¥baZ¥K`a_`¥baTXZ]^a^W_a_`^M
@HWI-EAS443:3:17:1715:423#0/1
ATTAAATAAGCTGAAACGAGAAAGCTCTTATCTCTTGTTC
+HWI-EAS443:3:17:1715:423#0/1
a`b]aaa_ab_ab_a`aa`b__]a`__aa_a_a[a_a]`]
@HWI-EAS443:3:17:452:122#0/1
GAAGTTCGCTTTCAGTACTATGTTCTTTTCCATGACCGCC
+HWI-EAS443:3:17:452:122#0/1
aa`aYaaaa```[^_X_^_U_a]__`]_^^__^_]]_]]^
@HWI-EAS443:3:17:486:1566#0/1
GTTCTTTTCCATGACCGCCTGGTCCAATCGCACGCAAAGA
+HWI-EAS443:3:17:486:1566#0/1
a_bbbaaaaaab`]`aa`aa`aZ]K¥a`a__a]`[`a¥`a
@HWI-EAS443:3:18:880:324#0/1
TTCAGTACTATGTTCTTTTCCATGACCGCCTGGTCCAATC
+HWI-EAS443:3:18:880:324#0/1
aaa]aS[aaaabZ__aaa[__aYS`N¥aUZ]¥¥VUMXS¥Y
@HWI-EAS443:3:19:957:1330#0/1
CAAAAACATAATGGACTCCAACACCATGTCAAGCTTTCAG
+HWI-EAS443:3:19:957:1330#0/1
U`b_babbbbba`ababbab`ababba`][baaaa`_aa^
@HWI-EAS443:3:1:1383:776#0/1
CAAAAACATAATGGACTCCAACACCATGTCAAGCTTTCAG
+HWI-EAS443:3:1:1383:776#0/1
a`aa[_]_b[_`^S[`YSX]`W^[[aab]U``]]^BBBBB
@HWI-EAS443:3:20:1263:236#0/1
GACAAAAACATAATGGACTCCAACACCATGTCAAGCTTTC
+HWI-EAS443:3:20:1263:236#0/1
aa^aaa]a_`aa``^[`a`^`^^_`]]a^a^_`^`^^^^V
@HWI-EAS443:3:20:1290:1942#0/1
TTCTTTTCCATGACCGCCTGGTCCAATCGCACGCAAAGAG
```

```
+HWI-EAS443:3:20:1290:1942#0/1
U[babbbaaabWQZU[a`abVX[aaZSaa_baa_`_`a_`
@HWI-EAS443:3:20:712:1080#0/1
GTGTTTTTTATCATTAAATAAGCTGAAACGAGAAAGCTCT
+HWI-EAS443:3:20:712:1080#0/1
aU_Z]a¥[^U_Ta¥Y]____ZY¥Z[__a[]¥¥^_Z]_¥W^
@HWI-EAS443:3:21:371:1342#0/1
AAAACATAATGGACTCCAACACCATGTCAAGCTTTCAGGT
+HWI-EAS443:3:21:371:1342#0/1
_bbbababaa`a`aa``^``aaaaaa^aa`````__`_¥X
@HWI-EAS443:3:21:524:204#0/1
ATGTTCTTTTCCATGACCGCCTGGTCCAATCGCACGCAAA
+HWI-EAS443:3:21:524:204#0/1
aba`a_aabaaaa`^`_`_^_``_W^___^Z_[ZW[R[¥¥
@HWI-EAS443:3:21:548:1195#0/1
GGTGTTTTTTATCATTAAATAAGCTGAAACGAGAAAGCTC
+HWI-EAS443:3:21:548:1195#0/1
¥bIb]abb`^SY`_aY^a`aaa`_¥a^a[a^``_^^__`W
@HWI-EAS443:3:22:1209:981#0/1
CATTAAATAAGCTGAAACGAGAAAGCTCTTATCTCTTGTT
+HWI-EAS443:3:22:1209:981#0/1
^^^_bab_^a``^a]aaa^[^]_^¥¥`YaTZ_V`_`_a[_
@HWI-EAS443:3:23:798:231#0/1
GTGTTTTTTATCATTAAATAAGCTGAAACGAGAAAGCTCT
+HWI-EAS443:3:23:798:231#0/1
M_a^^¥^_`aVab¥^¥^Wb]_a_Y]]_WOaJb]Y_aVYWQ
@HWI-EAS443:3:24:1127:801#0/1
GTGTTTTTTATCATTAAATAAGCTGAAACGAGAAAGCTCT
+HWI-EAS443:3:24:1127:801#0/1
Q[[_[`^a`bbbaba^_aa_W[¥aa_^]`_]`¥¥[_Z`Wa
@HWI-EAS443:3:24:540:765#0/1
TTTTTATCATTAAATAAGCTGAAACGAGAAAGCTCTTATC
+HWI-EAS443:3:24:540:765#0/1
^``_bb_b`babbaaa`a¥`b¥baa`a_`a_aa`aaa`a`
@HWI-EAS443:3:24:638:1363#0/1
GTGTTTTTTATCATTAAATAAGCTGAAACGAGAAAGCTCT
+HWI-EAS443:3:24:638:1363#0/1
aH_PW`aaa`abaaYYaaaaaaa`_aaYaXaa¥ZV`Z`__
@HWI-EAS443:3:25:1308:514#0/1
AGATAATAGGCCCTCTTTGCGTGCGATTGGACCAGGCGGT
+HWI-EAS443:3:25:1308:514#0/1
ababbabbbbba[`aaa_aR^_a_a_`_a`^^U__aBBBB
@HWI-EAS443:3:25:1506:1635#0/1
TTTTATCATTAAATAAGCTGAAACGAGAAAGCTCTTATCT
+HWI-EAS443:3:25:1506:1635#0/1
aabbbaababaaaaa_`_a```a`]^]]Y¥`[_V]`^_Wa
@HWI-EAS443:3:25:761:442#0/1
AAAACATAATGGACTCCAACACCATGTCAAGCTTTCAGGT
+HWI-EAS443:3:25:761:442#0/1
aabbaaa`aabaa_`aaa`_a__aa`]_a_`__^^__`^W
```

```
@HWI-EAS443:3:25:856:666#0/1
ATTAAATAAGCTGAAACGAGAAAGCTCTTATCTCTTGTTC
+HWI-EAS443:3:25:856:666#0/1
aab`ababaaaabZa_ab_aa_^a^a``a^a^`¥a]a_`_
@HWI-EAS443:3:26:1554:1161#0/1
CATTAAATAAGCTGAAACGAGAAAGCTCTTATCTCTTGTT
+HWI-EAS443:3:26:1554:1161#0/1
`Lbb`ab`ab`bbbbb`bbba_^aaaa¥b`ab`b`bbb_a
@HWI-EAS443:3:28:1260:1384#0/1
CATTAAATAAGCTGAAACGAGAAAGCTCTTATCTCTTGTT
+HWI-EAS443:3:28:1260:1384#0/1
```bbb`_ba^R[^`aW`abXMUV¥]]_a]a]X]_aaa]_
@HWI-EAS443:3:28:603:734#0/1
CCGCCTGGTCCAATCGCACGCAAAGAGGGCCTATTATCTT
+HWI-EAS443:3:28:603:734#0/1
aa`aa`ba`a`aaaaaab¥a^^a```^_^`^``a```¥a_
@HWI-EAS443:3:29:1036:1458#0/1
ACTGAAGTTCGCTTTCAGTACTATGTTCTTTTCCATGACC
+HWI-EAS443:3:29:1036:1458#0/1
`^aUW_VSaa]Saaa__aGLVZ¥Q__^[ZYV]L[a]U_^P
@HWI-EAS443:3:29:645:1472#0/1
TTTTATCATTAAATAAGCTGAAACGAGAAAGCTCTTATCT
+HWI-EAS443:3:29:645:1472#0/1
aaa^_Za``]_aaa]_¥`a`a_`^YZ_]Xa`__`_^`]__
@HWI-EAS443:3:2:320:251#0/1
CATTAAATAAGCTGAAACGAGAAAGCTCTTATCTCTTGTT
+HWI-EAS443:3:2:320:251#0/1
abbbbabbaabaab`aaaaaba`aaaa_aa_aYa^a__aa
@HWI-EAS443:3:30:188:835#0/1
GTGACAAAAACATAATGGACTCCAACACCATGTCAAGCTT
+HWI-EAS443:3:30:188:835#0/1
a¥aaa`Y^¥¥bbb]baa`aaabaaYa`aaaaa^aa`____
@HWI-EAS443:3:30:525:797#0/1
GACAAAAACATAATGGACTCCAACACCATGTCAAGCTTTC
+HWI-EAS443:3:30:525:797#0/1
][a`¥^a`a_baba`_¥``babb_^_aa``^bb^a^`_Z_
@HWI-EAS443:3:31:1171:623#0/1
CAAAAACATAATGGACTCCAACACCATGTCAAGCTTTCAG
+HWI-EAS443:3:31:1171:623#0/1
abb]Zaaa^_aaaY_aaa^a_`b`aaa`a_aab¥¥Ya`a_
@HWI-EAS443:3:31:1326:1009#0/1
TGACAAAAACATAATGGACTCCAACACCATGTCAAGCTTT
+HWI-EAS443:3:31:1326:1009#0/1
`bbbbaabaabb`ab`Z``aaaaa_aaaaaa_`a_aaa`^
@HWI-EAS443:3:31:318:1181#0/1
CATTAAATAAGCTGAAACGAGAAAGCTCTTATCTCTTGTT
+HWI-EAS443:3:31:318:1181#0/1
aa``^aaa_a_a_Y]aaaX¥ZWJPX¥U`]_W^[__XN[S]
@HWI-EAS443:3:31:672:1015#0/1
GTGTTTTTTATCATTAAATAAGCTGAAACGAGAAAGCTCT
```
```

+HWI-EAS443:3:31:672:1015#0/1
a^`^a`aa`aaa`aaaa[_a`_`_a`a`a``_^XZ^_^__
@HWI-EAS443:3:31:699:1366#0/1
TTCCATGACCGCCTGGTCCAATCGCACGCAAAGAGGGCCT
+HWI-EAS443:3:31:699:1366#0/1
^bbbba]bbaabbaaa^baaa_ba`aaaaaa]aa__``a_
@HWI-EAS443:3:31:758:488#0/1
GTGTTTTTTATCATTAAATAAGCTGAAACGAGAAAGCTCT
+HWI-EAS443:3:31:758:488#0/1
ZSZHY``[ZZUV[S[`]]]¥RV_[``a_a]Y_XXT^¥^^X
@HWI-EAS443:3:32:1346:1723#0/1
TTTTTATCATTAAATAAGCTGAAACGAGAAAGCTCTTATC
+HWI-EAS443:3:32:1346:1723#0/1
aaabaaaaabaaaaa``a`aaaaa]_a``_^a____a``_
@HWI-EAS443:3:32:1443:2010#0/1
GTTTTTTATCATTAAATAAGCTGAAACGAGAAAGCTCTTA
+HWI-EAS443:3:32:1443:2010#0/1
aaaa`b_¥]K_bbb_TY__bI]S[_a[`a_^`a`_¥^a`¥
@HWI-EAS443:3:32:1547:299#0/1
CAAAAACATAATGGACTCCAACACCATGTCAAGCTTTCAG
+HWI-EAS443:3:32:1547:299#0/1
aa`[`a^_baY[aZ^[a`Y¥[^_VUS^]MDX¥MHY[aY[`
@HWI-EAS443:3:32:700:1450#0/1
GTGTTTTTTATCATTAAATAAGCTGAAACGAGAAAGCTCT
+HWI-EAS443:3:32:700:1450#0/1
_T]JLRa[__Y]¥[XVZX[WRN¥XY[[¥^¥[¥_WSVYYYR
@HWI-EAS443:3:33:1190:93#0/1
CATTAAATAAGCTGAAACGAGAAAGCTCTTATCTCTTGTT
+HWI-EAS443:3:33:1190:93#0/1
abbbbbbbbbbbbbbababbabaaab`aaaa`aVa]aaa_a
@HWI-EAS443:3:33:1343:1329#0/1
GTGTTTTTTATCATTAAATAAGCTGAAACGAGAAAGCTCT
+HWI-EAS443:3:33:1343:1329#0/1
a_a``aa_a`aa^aa_a``a___^____XZ¥VMT[¥[^Y_
@HWI-EAS443:3:33:296:1873#0/1
GTGTTTTTTATCATTAAATAAGCTGAAACGAGAAAGCTCT
+HWI-EAS443:3:33:296:1873#0/1
^UZQ[X_aaaa`[a]a^^Q¥UPZRHX`^TVTKOZ[SWR¥Y
@HWI-EAS443:3:33:325:58#0/1
GTGTTTTTTATCATTAAATAAGCTGAAACGAGAAAGCTCT
+HWI-EAS443:3:33:325:58#0/1
a^^V_`__Y^_`_`¥_]X^M¥^[^_¥]XZ_Z^Y[Z]O]]Y
@HWI-EAS443:3:33:834:1131#0/1
CTTTCAGTACTATGTTCTTTTCCATGACCGCCTGGTCCAA
+HWI-EAS443:3:33:834:1131#0/1
abbbbba`Zabbbbbbabbbabbbbaabbabaaa_¥]J]b
@HWI-EAS443:3:34:1037:856#0/1
TTTTATCATTAAATAAGCTGAAACGAGAAAGCTCTTATCT
+HWI-EAS443:3:34:1037:856#0/1
ababbbabbbaaaa]aaaaaabaaaaa`^]`]a_aa^aP`

```
@HWI-EAS443:3:34:1413:27#0/1
CATTAAATAAGCTGAAACGAGAAAGCTCTTATCTCTTGTT
+HWI-EAS443:3:34:1413:27#0/1
K¥`bb^¥aa_b`bba`_XbbaaaabV``aba][baa^a_a
@HWI-EAS443:3:34:588:612#0/1
GTGTTTTTTATCATTAAATAAGCTGAAACGAGAAAGCTCT
+HWI-EAS443:3:34:588:612#0/1
a^a_`_^^`_`a]a_]```_`___`_a__`Z`W^]_¥^Y]
@HWI-EAS443:3:36:293:1085#0/1
ATTAAATAAGCTGAAACGAGAAAGCTCTTATCTCTTGTTC
+HWI-EAS443:3:36:293:1085#0/1
aaaa`R`bV`a_aa_`aaa`_aU__^`a¥___^_`_^Y¥^
@HWI-EAS443:3:37:1694:1509#0/1
GTGTTTTTTATCATTAAATAAGCTGAAACGAGAAAGCTCT
+HWI-EAS443:3:37:1694:1509#0/1
a_b^aaaaa``¥`a`a__`_^¥¥__`_]aX^Z``X]S`]]
@HWI-EAS443:3:38:1069:914#0/1
CAAAAACATAATGGACTCCAACACCATGTCAAGCTTTCAG
+HWI-EAS443:3:38:1069:914#0/1
a^aaa^ab`aba`¥¥aaa_`]^aa_aa`X[POW]W^_¥_^
@HWI-EAS443:3:38:63:1212#0/1
CATTAAATAAGCTGAAACGAGAAAGCTCTTATCTCTTGTT
+HWI-EAS443:3:38:63:1212#0/1
aU]W¥¥X[^¥Y__`¥]¥``_S¥XU^_Y`X]Z^Y¥¥^[[VU
@HWI-EAS443:3:38:893:52#0/1
GTGTTTTTTATCATTAAATAAGCTGAAACGAGAAAGCTCT
+HWI-EAS443:3:38:893:52#0/1
aZa__aaaa__Z^aa_]^_¥^_¥]_^¥X^]U]YV[XYYZZ
@HWI-EAS443:3:39:1391:1435#0/1
ACTATGTTCTTTTCCATGACCGCCTGGTCCAATCGCACGC
+HWI-EAS443:3:39:1391:1435#0/1
aa`aab`a_baab_ab[a^`_b`[SXYI``aS^aXLUH^[
@HWI-EAS443:3:39:756:375#0/1
GTGTTTTTTATCATTAAATAAGCTGAAACGAGAAAGCTCT
+HWI-EAS443:3:39:756:375#0/1
a]b_]aaaa`[^`_`a]`_a__`]_a`U¥`¥X¥UX_¥_XV
@HWI-EAS443:3:3:1225:950#0/1
GGTGTTTTTTATCATTAAATAAGCTGAAACGAGAAAGCTC
+HWI-EAS443:3:3:1225:950#0/1
aa[b[aa^baaa^aaa_`aa^PW]`a_``Z__`^_^_¥_P
@HWI-EAS443:3:3:1230:1699#0/1
GGTGTTTTTTATCATTAAATAAGCTGAAACGAGAAAGCTC
+HWI-EAS443:3:3:1230:1699#0/1
^ZZ`^_aa^aa¥Y[_`___a_`_[^_]``]_]`]]]]^_U
@HWI-EAS443:3:3:723:780#0/1
GTGTTTTTTATCATTAAATAAGCTGAAACGAGAAAGCTCT
+HWI-EAS443:3:3:723:780#0/1
`H]JMUOT[`]^a]UU^]PWLWS¥a_`^S[WYSX^_XUTZ
@HWI-EAS443:3:42:301:1738#0/1
CAGTACTATGTTCTTTTCCATGACCGCCTGGTCCAATCGC
```

```
+HWI-EAS443:3:42:301:1738#0/1
_aXWbbb]_V^UR_aZ^^a^aaaa`^bb``[[a`aa_a_a
@HWI-EAS443:3:43:1184:1219#0/1
GGTGTTTTTTATCATTAAATAAGCTGAAACGAGAAAGCTC
+HWI-EAS443:3:43:1184:1219#0/1
a`Vb]Z_HXZa_¥_`YRZZ[MSYTV`WUVWYZS[NRPKPP
@HWI-EAS443:3:43:1324:600#0/1
TTTTTATCATTAAATAAGCTGAAACGAGAAAGCTCTTATC
+HWI-EAS443:3:43:1324:600#0/1
X^SYZYPS^Z]T¥[QW^_S^Z^`]__a`ZWSWM^Q¥Y¥¥T
@HWI-EAS443:3:45:558:655#0/1
CAAAAACATAATGGACTCCAACACCATGTCAAGCTTTCAG
+HWI-EAS443:3:45:558:655#0/1
aa_a`baab_^`aaa_aa]aa]`_`aa`[`a``_a____a
@HWI-EAS443:3:45:940:682#0/1
CATTAAATAAGCTGAAACGAGAAAGCTCTTATCTCTTGTT
+HWI-EAS443:3:45:940:682#0/1
_bbbaabbaYa`aa`_baaaba``baa_aa`aab^aabaa
@HWI-EAS443:3:46:117:743#0/1
GTTCTTTTCCATGACCGCCTGGTCCAATCGCACGCAAAGA
+HWI-EAS443:3:46:117:743#0/1
^^a]aaa]_aaaWa¥``^Y¥VNGZYY]]¥a¥`TVV][^YQ
@HWI-EAS443:3:46:1722:1942#0/1
CAAAAACATAATGGACTCCAACACCATGTCAAGCTTTCAG
+HWI-EAS443:3:46:1722:1942#0/1
abbbbaabbaa`b``aaaaa`abaaa_b_aaaaaaa_O¥`
@HWI-EAS443:3:46:1744:1291#0/1
TTATCATTAAATAAGCTGAAACGAGAAAGCTCTTATCTCT
+HWI-EAS443:3:46:1744:1291#0/1
a``aa^a`_aaa]`_``aaa^aa^__^__^_]__]a^_Y^
@HWI-EAS443:3:46:504:461#0/1
ACCGCCTGGTCCAATCGCACGCAAAGAGGGCCTATTATCT
+HWI-EAS443:3:46:504:461#0/1
`_a_Wa__`U`_`___aW_^a__S__^_^¥^]_W_¥Z^W_
@HWI-EAS443:3:46:57:1643#0/1
GGTGTTTTTTATCATTAAATAAGCTGAAACGAGAAAGCTC
+HWI-EAS443:3:46:57:1643#0/1
a]]b[aaaaa_a``a_`[`aa¥]_`aa`aa_^`__^Z¥_T
@HWI-EAS443:3:46:693:479#0/1
ATTAAATAAGCTGAAACGAGAAAGCTCTTATCTCTTGTTC
+HWI-EAS443:3:46:693:479#0/1
¥`bab`bbbbbbbbabbbaa¥``aababbaaaaabbaaaa
@HWI-EAS443:3:47:1759:197#0/1
ACTGAAGTTCGCTTTCAGTACTATGTTCTTTTCCATGACC
+HWI-EAS443:3:47:1759:197#0/1
aaabHTP[```__`aaYaX¥Sa]a_¥^Y``_]^NV]]RYR
@HWI-EAS443:3:47:684:524#0/1
TTTTTATCATTAAATAAGCTGAAACGAGAAAGCTCTTATC
+HWI-EAS443:3:47:684:524#0/1
[]_a`¥b_aba¥]Z]]¥Z¥`a_a__a]][Z^_]__`UV^]
```

```
@HWI-EAS443:3:48:105:1377#0/1
GGTGTTTTTTATCATTAAATAAGCTGAAACGAGAAAGCTC
+HWI-EAS443:3:48:105:1377#0/1
_ZR`T`_aa¥YYZaZ[U]a_`_`PRX[`Y]Z¥_[WYY[UZ
@HWI-EAS443:3:48:346:1224#0/1
CGGTCATGGAAAAGAACATAGTACTGAAAGCGAACTTCAG
+HWI-EAS443:3:48:346:1224#0/1
[^VJQV^a¥X_aYY^^Z`b`WS[a_Y^^aY`_`_Xab`_K
@HWI-EAS443:3:49:148:1774#0/1
CATTAAATAAGCTGAAACGAGAAAGCTCTTATCTCTTGTT
+HWI-EAS443:3:49:148:1774#0/1
HYaaba_aa^a^¥`¥baS^`_ba¥¥^b`a¥^bbbbbab]Y
@HWI-EAS443:3:4:1592:198#0/1
ATTAAATAAGCTGAAACGAGAAAGCTCTTATCTCTTGTTC
+HWI-EAS443:3:4:1592:198#0/1
Xbbaaa]^Z`X^[YYa^baa__X_Va_``]YF[[^__W[B
@HWI-EAS443:3:4:475:1369#0/1
CATTAAATAAGCTGAAACGAGAAAGCTCTTATCTCTTGTT
+HWI-EAS443:3:4:475:1369#0/1
aaabbbba`abaabababbabaaaaa`_baab_b_babaa
@HWI-EAS443:3:51:1262:1690#0/1
CAAAAACATAATGGACTCCAACACCATGTCAAGCTTTCAG
+HWI-EAS443:3:51:1262:1690#0/1
_aaaab]`_bbabbab_Q_bb¥aVbb^b_]XabJb`bTbb
@HWI-EAS443:3:51:1453:293#0/1
CATTAAATAAGCTGAAACGAGAAAGCTCTTATCTCTTGTT
+HWI-EAS443:3:51:1453:293#0/1
]abbbbbbbab_bbbbbbbbab`bababbabbbabbb`b
@HWI-EAS443:3:52:363:1009#0/1
ATTAAATAAGCTGAAACGAGAAAGCTCTTATCTCTTGTTC
+HWI-EAS443:3:52:363:1009#0/1
aabaaaba`aa_a`aa`a`aY`[`_a`a`_```]a_a[__
@HWI-EAS443:3:52:576:1723#0/1
GTGTTTTTTATCATTAAATAAGCTGAAACGAGAAAGCTCT
+HWI-EAS443:3:52:576:1723#0/1
aOWGR¥VV[]]YX¥_[]XX[[YVR¥]]XO[WXYSVZWYSY
@HWI-EAS443:3:52:689:1758#0/1
CATTAAATAAGCTGAAACGAGAAAGCTCTTATCTCTTGTT
+HWI-EAS443:3:52:689:1758#0/1
abbaaaba`aaaaa^b`aaaaa]`a_a__a_a`a_a`a]¥
@HWI-EAS443:3:53:1783:808#0/1
GTGTTTTTTATCATTAAATAAGCTGAAACGAGAAAGCTCT
+HWI-EAS443:3:53:1783:808#0/1
_^¥_aaa]`a_WV_aaaVaZ^X¥[a`a^WZZ[^T[]RVWQ
@HWI-EAS443:3:54:216:865#0/1
GTGTTTTTTATCATTAAATAAGCTGAAACGAGAAAGCTCT
+HWI-EAS443:3:54:216:865#0/1
a^a^aabbaaaaaaa`a_aa_a^aa]aa`a^`^^Z`Y[`_
@HWI-EAS443:3:54:805:1057#0/1
GGTGACAAAAACATAATGGACTCCAACACCATGTCAAGCT
```

```
+HWI-EAS443:3:54:805:1057#0/1
abIbaabbabb^abbaaaaa`ab_aaaa`]aba^_`^a`a
@HWI-EAS443:3:55:1122:1139#0/1
GACCGCCTGGTCCAATCGCACGCAAAGAGGGCCTATTATC
+HWI-EAS443:3:55:1122:1139#0/1
aabbabbabaZ_aa`X]ba]ab``a`aaa`a_`aaa`a`¥
@HWI-EAS443:3:55:1439:1814#0/1
TTTATCATTAAATAAGCTGAAACGAGAAAGCTCTTATCTC
+HWI-EAS443:3:55:1439:1814#0/1
a^a^`U_`___^^a^aT¥``¥`V]^^__YYVIWW^[[[Z[
@HWI-EAS443:3:55:1505:426#0/1
GTTTTTTATCATTAAATAAGCTGAAACGAGAAAGCTCTTA
+HWI-EAS443:3:55:1505:426#0/1
a__a`aa_`_`aa^___a__]__^__]__^[]]_W_Z^]]
@HWI-EAS443:3:55:1730:955#0/1
GGTGACAAAAACATAATGGACTCCAACACCATGTCAAGCT
+HWI-EAS443:3:55:1730:955#0/1
[JR_`S^Z_a`aXbaa]ba__a_``Z¥a`_`a__T_aYT_
@HWI-EAS443:3:55:1779:973#0/1
GACCGCCTGGTCCAATCGCACGCAAAGAGGGCCTATTATC
+HWI-EAS443:3:55:1779:973#0/1
__a_T`b_TO^W_b_]V``Zb^WZ_TZ[_]ZbW`abaaaa
@HWI-EAS443:3:55:941:842#0/1
CAAAAACATAATGGACTCCAACACCATGTCAAGCTTTCAG
+HWI-EAS443:3:55:941:842#0/1
abbabb_aaa_bbb_`bbabbabaabbbaab`a_ab_Q`V
@HWI-EAS443:3:56:1432:1175#0/1
GTTCGCTTTCAGTACTATGTTCTTTTCCATGACCGCCTGG
+HWI-EAS443:3:56:1432:1175#0/1
^^a`aabbb_aa^]_babba^]bab_V]]aa]`Xa`]`a`
@HWI-EAS443:3:56:821:1074#0/1
ACCGCCTGGTCCAATCGCACGCAAAGAGGGCCTATTATCT
+HWI-EAS443:3:56:821:1074#0/1
a`aa_aaaaW_a``a`__a^``^[__[``_XV`¥^Z^a__
@HWI-EAS443:3:57:1243:1487#0/1
ATTAAATAAGCTGAAACGAGAAAGCTCTTATCTCTTGTTC
+HWI-EAS443:3:57:1243:1487#0/1
abb`]IVa`aR¥a`]a_¥Y__`_¥][P]``__^^`__X[R
@HWI-EAS443:3:57:354:1168#0/1
CACTGAAGTTCGCTTTCAGTACTATGTTCTTTTCCATGAC
+HWI-EAS443:3:57:354:1168#0/1
ab`b¥W]aabaaaabba[b_aaaabb_`abaaa_]a```a
@HWI-EAS443:3:57:911:666#0/1
GGTGTTTTTTATCATTAAATAAGCTGAAACGAGAAAGCTC
+HWI-EAS443:3:57:911:666#0/1
abX_V`bbbbba^`aa`aaa`¥][`a]a_aa[a_[Za_``
@HWI-EAS443:3:58:1430:389#0/1
CAAAAACATAATGGACTCCAACACCATGTCAAGCTTTCAG
+HWI-EAS443:3:58:1430:389#0/1
abaaabaabaaababaaba```a``aaa``a_a^``a_`a
```

@HWI-EAS443:3:58:697:57#0/1
AAAACATAATGGACTCCAACACCATGTCAAGCTTTCAGGT
+HWI-EAS443:3:58:697:57#0/1
a_¥`a[aS``a]PUYa¥`a_`Z[_aa]_a^a[```Y[a_O
@HWI-EAS443:3:58:804:797#0/1
ATTAAATAAGCTGAAACGAGAAAGCTCTTATCTCTTGTTC
+HWI-EAS443:3:58:804:797#0/1
a`baab_¥aaaaa`aaa__b_^W__a^aa`a[`a_`aYa`
@HWI-EAS443:3:58:836:1720#0/1
GTGTTTTTTATCATTAAATAAGCTGAAACGAGAAAGCTCT
+HWI-EAS443:3:58:836:1720#0/1
a¥`T_¥Y[___^_`_W]]^]VTVV^^^ZVYV^[XXXXXZ]
@HWI-EAS443:3:59:531:695#0/1
GTGTTTTTTATCATTAAATAAGCTGAAACGAGAAAGCTCT
+HWI-EAS443:3:59:531:695#0/1
a_a_aaaa]X]SX_``¥^`]Y[Y[`]¥WX[^ZXUOULYV¥
@HWI-EAS443:3:5:35:1254#0/1
GGTGTTTTTTATCATTAAATAAGCTGAAACGAGAAAGCTC
+HWI-EAS443:3:5:35:1254#0/1
^^OSU_aXaaaaY`Ya^Yaaa[a^Saa]]V[QZTHK¥¥_B
@HWI-EAS443:3:60:341:1918#0/1
GTTTTTTATCATTAAATAAGCTGAAACGAGAAAGCTCTTA
+HWI-EAS443:3:60:341:1918#0/1
a]_X^]Z¥Z_`[UW^a_^YU[¥U¥]OYSZ][]WY[WLSWU
@HWI-EAS443:3:61:1162:638#0/1
TTTTTATCATTAAATAAGCTGAAACGAGAAAGCTCTTATC
+HWI-EAS443:3:61:1162:638#0/1
abbbaaaaabaaaaa_aaaabY`^aaaa_a^a[`^a_^_U
@HWI-EAS443:3:61:587:686#0/1
TTTCAGTACTATGTTCTTTTCCATGACCGCCTGGTCCAAT
+HWI-EAS443:3:61:587:686#0/1
a`a`]_[__]Uaa^a`a__^`a_]`¥`__`^_^]U_X_YY
@HWI-EAS443:3:61:692:988#0/1
GGTGTTTTTTATCATTAAATAAGCTGAAACGAGAAAGCTC
+HWI-EAS443:3:61:692:988#0/1
a`Y¥]`___`__^``^¥P¥__TX_WY]_`_]^[Z][^[[W
@HWI-EAS443:3:62:145:1999#0/1
GTTTTTTATCATTAAATAAGCTGAAACGAGAAAGCTCTTA
+HWI-EAS443:3:62:145:1999#0/1
NN[aTa[D[]VO`X[^RYVU[QX]X¥W^SQLP¥QGOMW
@HWI-EAS443:3:62:798:1743#0/1
AACATAATGGACTCCAACACCATGTCAAGCTTTCAGGTAG
+HWI-EAS443:3:62:798:1743#0/1
abaaaa`¥`aaaab¥b`aaaaa`a¥aaaa``Z`Y_`ZWa]
@HWI-EAS443:3:63:58:138#0/1
GTGTTTTTTATCATTAAATAAGCTGAAACGAGAAAGCTCT
+HWI-EAS443:3:63:58:138#0/1
aVaZSY__^^][[aZORH¥XUT^Z_^]Y]VY^UUUWWU[[
@HWI-EAS443:3:64:1081:1185#0/1
TTTTATCATTAAATAAGCTGAAACGAGAAAGCTCTTATCT

+HWI-EAS443:3:64:1081:1185#0/1
aaaa_^__`a`a]a___a]]aaa`aaYU]^Y^]Y``_`X`
@HWI-EAS443:3:64:1733:1031#0/1
GTTTTTTATCATTAAATAAGCTGAAACGAGAAAGCTCTTA
+HWI-EAS443:3:64:1733:1031#0/1
a`Z[[T]Y¥FJPRYJP^a`ZMZ¥UWFW`^W[ZRRDDSY]Q
@HWI-EAS443:3:65:125:698#0/1
GGTGTTTTTTATCATTAAATAAGCTGAAACGAGAAAGCTC
+HWI-EAS443:3:65:125:698#0/1
a]La[_`_TQ[[`[_[¥VXTUY^^]^VXHXXRX_WTZ[SZ
@HWI-EAS443:3:65:504:1603#0/1
ACATAATGGACTCCAACACCATGTCAAGCTTTCAGGTAGA
+HWI-EAS443:3:65:504:1603#0/1
a`^ab_Wa]_a^a`_W`_a`a^^Z^`]__ZZ__^^[G_¥[
@HWI-EAS443:3:65:749:384#0/1
GGTGTTTTTTATCATTAAATAAGCTGAAACGAGAAAGCTC
+HWI-EAS443:3:65:749:384#0/1
abY`Vaabaaaa_[aaa^`aa_a^_a_`__a^`Z]Y`__]
@HWI-EAS443:3:65:800:356#0/1
ATCATTAAATAAGCTGAAACGAGAAAGCTCTTATCTCTTG
+HWI-EAS443:3:65:800:356#0/1
aa^`aa]___[_¥_¥_¥`MYW¥¥]^^a¥]X^Y`^Y_Y_]^
@HWI-EAS443:3:66:1380:1256#0/1
GTGTTTTTTATCATTAAATAAGCTGAAACGAGAAAGCTCT
+HWI-EAS443:3:66:1380:1256#0/1
YN^VZ^^^O^¥Za]X]VW[[¥aYP_]Z]¥ZY^VNT^¥[]`
@HWI-EAS443:3:66:269:2028#0/1
GGTGTTTTTTATCATTAAATAAGCTGAAACGAGAAAGCTC
+HWI-EAS443:3:66:269:2028#0/1
^[[b]]ab`]Y`aaV`aa^a`aaaaa^b^aa_aaYQZ`Z_
@HWI-EAS443:3:66:550:1282#0/1
CATTAAATAAGCTGAAACGAGAAAGCTCTTATCTCTTGTT
+HWI-EAS443:3:66:550:1282#0/1
abab_a¥`a_aa__`a`aa`^¥`^aa`_`^Za]a_a]^[¥
@HWI-EAS443:3:66:77:274#0/1
TTTCCATGACCGCCTGGTCCAATCGCACGCAAAGAGGGCC
+HWI-EAS443:3:66:77:274#0/1
aaabbba^[aa`aa`_¥Z^`aa_aaa_[a^¥^[¥[^Z^]U
@HWI-EAS443:3:67:1394:489#0/1
CAAAAACATAATGGACTCCAACACCATGTCAAGCTTTCAG
+HWI-EAS443:3:67:1394:489#0/1
abb``abbbbbbaaaabababababaabbb`^baa_a`_]a`
@HWI-EAS443:3:6:1300:537#0/1
ATTAAATAAGCTGAAACGAGAAAGCTCTTATCTCTTGTTC
+HWI-EAS443:3:6:1300:537#0/1
abbbbaba_]_a`a`a`aab`]^a]`Wb^aa]`Yaaa_a`
@HWI-EAS443:3:6:1614:650#0/1
GGTGTTTTTTATCATTAAATAAGCTGAAACGAGAAAGCTC
+HWI-EAS443:3:6:1614:650#0/1
aaVa`abbab_aaabbaa`bbab__b`b_`]aa__`a_`Y

```
@HWI-EAS443:3:6:1669:2018#0/1
GTGTTTTTTATCATTAAATAAGCTGAAACGAGAAAGCTCT
+HWI-EAS443:3:6:1669:2018#0/1
]O_W[`a_Z]^_Y]aZ]__¥¥aa`a¥X]`a¥`PQQ^]_T_
@HWI-EAS443:3:6:195:1856#0/1
GACCGCCTGGTCCAATCGCACGCAAAGAGGGCCTATTATC
+HWI-EAS443:3:6:195:1856#0/1
_`bb_ZabaZ]bTabZa`abZ`^Xaa_`ab`¥]baaUa_Z
@HWI-EAS443:3:70:1551:117#0/1
GTGTTTTTTATCATTAAATAAGCTGAAACGAGAAAGCTCT
+HWI-EAS443:3:70:1551:117#0/1
a`a`aaaaaaa_`aaa_aa`_`_`a__¥Y`_`^[Z_]_Z`
@HWI-EAS443:3:70:858:1434#0/1
ATTAAATAAGCTGAAACGAGAAAGCTCTTATCTCTTGTTC
+HWI-EAS443:3:70:858:1434#0/1
aaabbaabbabaaa``babaaa^_aaaaaaaaa`aaa__a
@HWI-EAS443:3:71:1348:1489#0/1
GTGTTTTTTATCATTAAATAAGCTGAAACGAGAAAGCTCT
+HWI-EAS443:3:71:1348:1489#0/1
`^b_`babaaa^aa`aa`aa__¥aa`a```_``_^_¥_¥_
@HWI-EAS443:3:72:347:1157#0/1
GGTGTTTTTTATCATTAAATAAGCTGAAACGAGAAAGCTC
+HWI-EAS443:3:72:347:1157#0/1
T`Gb[a`abW_a^bba¥b__]X]b`b`ba_baaaVVa]_]
@HWI-EAS443:3:72:646:820#0/1
GTTTTTTATCATTAAATAAGCTGAAACGAGAAAGCTCTTA
+HWI-EAS443:3:72:646:820#0/1
a_aaaaaa`_``aaa_``_a`___a_`_`^_]^^^^^_^Y
@HWI-EAS443:3:72:864:918#0/1
CAAAAACATAATGGACTCCAACACCATGTCAAGCTTTCAG
+HWI-EAS443:3:72:864:918#0/1
^]Z¥Y¥_S`Z]`_¥¥Y_a_W¥W^aa^`^Wa^U]_VZ^__^
@HWI-EAS443:3:73:1363:1630#0/1
CATTAAATAAGCTGAAACGAGAAAGCTCTTATCTCTTGTT
+HWI-EAS443:3:73:1363:1630#0/1
`bbbbbbbababaa`a`_`^`^W_^]_^^^¥_¥_¥_^_[^
@HWI-EAS443:3:73:343:1302#0/1
GTTTTTTATCATTAAATAAGCTGAAACGAGAAAGCTCTTA
+HWI-EAS443:3:73:343:1302#0/1
aaaaaaaaabaaaaaaaaa¥_a`aaa_`a``__^`^^a__
@HWI-EAS443:3:73:435:710#0/1
CAAAAACATAATGGACTCCAACACCATGTCAAGCTTTCAG
+HWI-EAS443:3:73:435:710#0/1
`bbbb_bbb_bbbababbabbbbbb`ab]bbaaaa`aaaa
@HWI-EAS443:3:74:193:202#0/1
ATCATTAAATAAGCTGAAACGAGAAAGCTCTTATCTCTTG
+HWI-EAS443:3:74:193:202#0/1
`bbabba_ab`aaaaaaaaba`a___aa`a``^a^`__^_
@HWI-EAS443:3:75:1235:1081#0/1
ACAAAAACATAATGGACTCCAACACCATGTCAAGCTTTCA
```

```
+HWI-EAS443:3:75:1235:1081#0/1
``babab`^`bab`__^aaaabb_X`aa¥__`_^___`S`
@HWI-EAS443:3:75:1464:440#0/1
GGTGACAAAAACATAATGGACTCCAACACCATGTCAAGCT
+HWI-EAS443:3:75:1464:440#0/1
aaY^bZbbab`Z`a``^`aR`]^MVaaaa`a`aQ]aTa_a
@HWI-EAS443:3:76:161:1068#0/1
CATTAAATAAGCTGAAACGAGAAAGCTCTTATCTCTTGTT
+HWI-EAS443:3:76:161:1068#0/1
aabbabba^`abababababbaa[a`aaaaa_aa`aaa`a``
@HWI-EAS443:3:76:69:813#0/1
TGACCGCCTGGTCCAATCGCACGCAAAGAGGGCCTATTAT
+HWI-EAS443:3:76:69:813#0/1
__`a`aa_[`aS`__`_¥_a_`__^^O¥NW¥¥¥WV¥¥U^W
@HWI-EAS443:3:77:315:1545#0/1
GTGTTTTTTATCATTAAATAAGCTGAAACGAGAAAGCTCT
+HWI-EAS443:3:77:315:1545#0/1
a_a``a``aaaaaaa`aa`^aX_^[[``a``_^[¥_`]__
@HWI-EAS443:3:77:680:1202#0/1
CTGAAGTTCGCTTTCAGTACTATGTTCTTTTCCATGACCG
+HWI-EAS443:3:77:680:1202#0/1
aa`bbaabbabbbabba_aababbabbbbbbaaba¥bbaa
@HWI-EAS443:3:79:1059:969#0/1
GTTCGCTTTCAGTACTATGTTCTTTTCCATGACCGCCTGG
+HWI-EAS443:3:79:1059:969#0/1
`UV_^_`_ba[X]aYW]`_[_`aaa__]`a_YYSQVJZVN
@HWI-EAS443:3:79:1358:1581#0/1
GGTGTTTTTTATCATTAAATAAGCTGAAACGAGAAAGCTC
+HWI-EAS443:3:79:1358:1581#0/1
`b_aTY¥a]]¥`^]b¥`a_a`^Z]¥Y`bbZW]]`¥^`]YT
@HWI-EAS443:3:79:306:631#0/1
TAGGCCCTCTTTGCGTGCGATTGGACCAGGCGGTCATGGA
+HWI-EAS443:3:79:306:631#0/1
aba``_babbbaabbabb_aa`aab`bbbbba`JFUZVRR
@HWI-EAS443:3:79:992:1589#0/1
GTGACAAAAACATAATGGACTCCAACACCATGTCAAGCTT
+HWI-EAS443:3:79:992:1589#0/1
`]_aaabbaaaa_baa`YaZ_a^aa^_`_`__YR`W^^¥Y
@HWI-EAS443:3:80:1153:665#0/1
GTTCGCTTTCAGTACTATGTTCTTTTCCATGACCGCCTGG
+HWI-EAS443:3:80:1153:665#0/1
a^a`a_a____`T`Va`_a_[^_`__¥^__`[[Y^VW^`^
@HWI-EAS443:3:80:527:1736#0/1
GACCGCCTGGTCCAATCGCACGCAAAGAGGGCCTATTATC
+HWI-EAS443:3:80:527:1736#0/1
_`bbabbb`_R]bbbaa]`b_a¥``_a_a_a``_baaaaa
@HWI-EAS443:3:80:559:689#0/1
GACCGCCTGGTCCAATCGCACGCAAAGAGGGCCTATTATC
+HWI-EAS443:3:80:559:689#0/1
¥]_``^_Z^XZ^___`¥_^]Z_abaaaa¥_XX]¥`bba`]
```

```
@HWI-EAS443:3:80:670:285#0/1
AGATAATAGGCCCTCTTTGCGTGCGATTGGACCAGGCGGT
+HWI-EAS443:3:80:670:285#0/1
_`a`Z`aaaaaY`a_`^_aZ]^a_`]X]a_VQSZ^BBBBB
@HWI-EAS443:3:81:1266:1578#0/1
TTATCATTAAATAAGCTGAAACGAGAAAGCTCTTATCTCT
+HWI-EAS443:3:81:1266:1578#0/1
aaaa_`baaa``aa_`_S^a__Y]¥`^Y_]__`_`_¥^]^
@HWI-EAS443:3:81:1339:1221#0/1
TTTTTATCATTAAATAAGCTGAAACGAGAAAGCTCTTATC
+HWI-EAS443:3:81:1339:1221#0/1
W^YX^ZP^Q^b_][¥^^X][a_aKX¥```OG`]]^^`ZV¥
@HWI-EAS443:3:83:538:1882#0/1
GAAGTTCGCTTTCAGTACTATGTTCTTTTCCATGACCGCC
+HWI-EAS443:3:83:538:1882#0/1
^^_XT]a_abaaa`a_baaba`]aabaaaaa`a[a]b_a_
@HWI-EAS443:3:83:921:575#0/1
ACCGCCTGGTCCAATCGCACGCAAAGAGGGCCTATTATCT
+HWI-EAS443:3:83:921:575#0/1
T^`aabaabUaaaaa_ab`Vaba^Z`¥`^a_[¥__a_``_
@HWI-EAS443:3:84:1742:1563#0/1
CATTAAATAAGCTGAAACGAGAAAGCTCTTATCTCTTGTT
+HWI-EAS443:3:84:1742:1563#0/1
P_¥R]QQ^R[`¥bUUU¥Z^bb¥babb`¥_Z`K`b[_`__a
@HWI-EAS443:3:85:193:1858#0/1
TGACCGCCTGGTCCAATCGCACGCAAAGAGGGCCTATTAT
+HWI-EAS443:3:85:193:1858#0/1
^abbbbba^`_OIQ``U`Xbaa`aaaVQVXXVa[[a¥RZX
@HWI-EAS443:3:85:640:1534#0/1
GTGTTTTTTATCATTAAATAAGCTGAAACGAGAAAGCTCT
+HWI-EAS443:3:85:640:1534#0/1
aY^]aa`aaaa_aaaabaaaa_`_`a`]a^`]__^_a`W`
@HWI-EAS443:3:86:1262:392#0/1
ACTGAAGTTCGCTTTCAGTACTATGTTCTTTTCCATGACC
+HWI-EAS443:3:86:1262:392#0/1
]]ba¥Z_N^¥`ZWW¥ZM_Y]``^a]^a¥[[a^QSZ^a_VS
@HWI-EAS443:3:86:650:676#0/1
CATTAAATAAGCTGAAACGAGAAAGCTCTTATCTCTTGTT
+HWI-EAS443:3:86:650:676#0/1
aabbbbbbbbaaababab_bb_aab_babbabababbbaa
@HWI-EAS443:3:86:672:80#0/1
GTGTTTTTTATCATTAAATAAGCTGAAACGAGAAAGCTCT
+HWI-EAS443:3:86:672:80#0/1
a^bV`^a]aa`¥aaa]W]aYL_Y]aU`_Y___YNR_U]Y`
@HWI-EAS443:3:87:1339:183#0/1
GGTGACAAAAACATAATGGACTCCAACACCATGTCAAGCT
+HWI-EAS443:3:87:1339:183#0/1
`ZL]`WOQO^]]YV^SV]`ZVYOD[¥S_KN¥P¥QQPYVNS
@HWI-EAS443:3:89:161:787#0/1
CATTAAATAAGCTGAAACGAGAAAGCTCTTATCTCTTGTT
```

```
+HWI-EAS443:3:89:161:787#0/1
abaabbbb^babbbabbbbbabaababbbbab`aab`bba
@HWI-EAS443:3:89:970:2036#0/1
ATTAAATAAGCTGAAACGAGAAAGCTCTTATCTCTTGTTC
+HWI-EAS443:3:89:970:2036#0/1
`bbbaabbbaaa`aa`bbaaaa_a^a¥aHa`_aYa`QXW^
@HWI-EAS443:3:90:220:605#0/1
CAAAAACATAATGGACTCCAACACCATGTCAAGCTTTCAG
+HWI-EAS443:3:90:220:605#0/1
]`b`bbbbbbbbababbbbbbbbbbbbbabbabbaaabab
@HWI-EAS443:3:90:932:393#0/1
CTTTTCCATGACCGCCTGGTCCAATCGCACGCAAAGAGGG
+HWI-EAS443:3:90:932:393#0/1
`bbababbbaYa`bbaabb`abaabab[bb_a`a^bba^b
@HWI-EAS443:3:91:1170:817#0/1
ACTATGTTCTTTTCCATGACCGCCTGGTCCAATCGCACGC
+HWI-EAS443:3:91:1170:817#0/1
a`b_abaaaabaaa`aaa^aaa_a_aaT_____[_]`^`W
@HWI-EAS443:3:91:467:1679#0/1
TGTTCTTTTCCATGACCGCCTGGTCCAATCGCACGCAAAG
+HWI-EAS443:3:91:467:1679#0/1
a`aaaabbaaabaa¥baaaa`abZa`aa`_``a_`¥_`_]
@HWI-EAS443:3:91:566:1217#0/1
TTTTATCATTAAATAAGCTGAAACGAGAAAGCTCTTATCT
+HWI-EAS443:3:91:566:1217#0/1
abbb_ababaaaabaaaaaaaa`aa_``_^a_a_a``a_`
@HWI-EAS443:3:91:765:144#0/1
GTTTTTTATCATTAAATAAGCTGAAACGAGAAAGCTCTTA
+HWI-EAS443:3:91:765:144#0/1
aQJS^¥YTXYXXYNXW[XXXNXW[MMTUYV[YWSXUYVXX
@HWI-EAS443:3:92:1160:1605#0/1
CAAAAACATAATGGACTCCAACACCATGTCAAGCTTTCAG
+HWI-EAS443:3:92:1160:1605#0/1
abbbbbbbbbbbbabbbbabbbbabbaba`bbba`bbaba
@HWI-EAS443:3:94:675:766#0/1
CACTGAAGTTCGCTTTCAGTACTATGTTCTTTTCCATGAC
+HWI-EAS443:3:94:675:766#0/1
a^`bb^^_[bbaa`abaa``a`aaba_aabaaaaSaa_a_
@HWI-EAS443:3:97:973:1847#0/1
GACCGCCTGGTCCAATCGCACGCAAAGAGGGCCTATTATC
+HWI-EAS443:3:97:973:1847#0/1
a`aa¥a^^_`ZS_aa^]a_a¥`X`_a¥¥`_¥MS¥Z¥`¥OB
@HWI-EAS443:3:9:1579:897#0/1
ATTAAATAAGCTGAAACGAGAAAGCTCTTATCTCTTGTTC
+HWI-EAS443:3:9:1579:897#0/1
abb`aaaaaaaab`aaab`a`_`a[_]aa`aYa^a_a_a^
@HWI-EAS443:3:9:770:764#0/1
ATTAAATAAGCTGAAACGAGAAAGCTCTTATCTCTTGTTC
+HWI-EAS443:3:9:770:764#0/1
a``TZa[Y_¥X`baa`Z_a_a_aa_aYa[Y`S^Qaa_[^O
```

```
@HWI-EAS443:3:8:220:374#0/1
CGGTTACCACCATCAAAATGAGCAGGGGTCAGGATATGCA
+HWI-EAS443:3:8:220:374#0/1
^R]U¥YDV`^¥Q^`QQ]¥]Y[OZU]TVRTSSYJPSY¥[TB
@HWI-EAS443:3:94:598:184#0/1
GGGGCATTCATTTCGACTGATTTGACTATTTTTCCCTTTT
+HWI-EAS443:3:94:598:184#0/1
a_XNX^[IMZ¥Q]QPHZO[OV`SWP[VPOFPS^GNYYWYB
@HWI-EAS443:3:87:665:1536#0/1
AACAAAGCCTCTATCAGAATGCAGATGCATATGTTTTTGT
+HWI-EAS443:3:87:665:1536#0/1
`_RUZWHDWSYY__T¥]^URV`[]PYWVUJ_`a]``WVPF
@HWI-EAS443:3:88:1540:1827#0/1
CAAAATTAAACAGAGAAGAGATAGATGGGGTAAAGCAGGA
+HWI-EAS443:3:88:1540:1827#0/1
aa_QY_bb¥`aZU^b[b_]ZOaW[`bb_JbIK_¥aLaa^`
@HWI-EAS443:3:12:697:410#0/1
GCCTATGGCACCTATACGTGTATCTGTAGTCTTTGATGAA
+HWI-EAS443:3:12:697:410#0/1
S^_¥aZaVa_X_^L]]Z_]`_VXbbba`_S¥]Z^bb`_HZ
@HWI-EAS443:3:100:117:1925#0/1
AATGGACATGAACTGAGCAATCTTGCAAAAGGAGAGAAAG
+HWI-EAS443:3:100:117:1925#0/1
a`b^``aaa_¥aa^^a_`_aa^`a__^^`_`___^^__BB
@HWI-EAS443:3:1:637:18#0/1
ACGGCCCCCTTGCCCAATTAGCACATTAGCTTTCTCTCTT
+HWI-EAS443:3:1:637:18#0/1
a¥aaVOOX`_R]MR]V`_^_O[UX^^^YLIV^XXBBBBBB
@HWI-EAS443:3:1:792:1504#0/1
GCAGACAAGAGAAAGCTAATGTGCTAATTGGGCAAGGGGA
+HWI-EAS443:3:1:792:1504#0/1
aa`[^YY`^^aa_``¥a``ZDT__`^^__`_^NZ¥`¥aBB
@HWI-EAS443:3:27:327:1749#0/1
CCAGACAAGAGAAAGCTAATGTGCTAATTGGGCAAGGGGA
+HWI-EAS443:3:27:327:1749#0/1
S¥abbaba`aabaSU[]bab¥XWbbabaaab¥^`baaa`a
@HWI-EAS443:3:28:1265:1320#0/1
GCAGACAAGAGAAAGCTAATGTGCTAATTGGGCAAGGGGA
+HWI-EAS443:3:28:1265:1320#0/1
]Y[a``]SZ]`VYU_S__``a[]_]]¥a`a``^X]]``YR
@HWI-EAS443:3:36:799:778#0/1
GCAGACAAGAGAAAGCTAATGTGCTAATTGGGCAAGGGGA
+HWI-EAS443:3:36:799:778#0/1
`a_aa_S^aaaaaaa_aa`_a^a``[T]^`__^^___`_[
@HWI-EAS443:3:45:202:176#0/1
GCAGACAAGAGAAAGCTAATGTGCTAATTGGGCAAGGGGA
+HWI-EAS443:3:45:202:176#0/1
a`aaaaaaabaaaaaaa`_aa_a_aaaaaaaa___aaaa]
@HWI-EAS443:3:62:245:438#0/1
GCAGACAAGAGAAAGCTAATGTGCTAATTGGGCAAGGGGA
```

+HWI-EAS443:3:62:245:438#0/1
aaaa]aaaaaa`WX`a_R_`a]aa`Z_`____`[¥_[Z^R
@HWI-EAS443:3:71:54:520#0/1
TCCCCTTGCCCAATTAGCACATTAGCTTTCTCTTGTCTGC
+HWI-EAS443:3:71:54:520#0/1
`bbbaVT^ab``_Y¥¥[¥[_O_``]`]``aa`a``¥]_]]
@HWI-EAS443:3:89:860:351#0/1
GCAGACAAGAGAAAGCTAATGTGCTAATTGGGCAAGGGGA
+HWI-EAS443:3:89:860:351#0/1
`UX_WTX][_^^_`^PPPVS¥NLTYF¥V_TBBBBBBBBBB
@HWI-EAS443:3:92:1688:517#0/1
AAGAGATAAAAGGAGAGAAAGCTAATGTGCTAATTGGGCA
+HWI-EAS443:3:92:1688:517#0/1
^K`^bab[`U¥^^_b]a^YS`Sa_VY]NZa`HV^[a_]U^
@HWI-EAS443:3:48:1096:1598#0/1
GACAAGAGAAAGCTAATGTGCTAATNGGGCAAGGGGACGT
+HWI-EAS443:3:48:1096:1598#0/1
aVU]]baaaXNT`QWTaa]]^[^]QDU¥`S[^_Z`ZXH`Z
@HWI-EAS443:3:100:1355:1612#0/1
TACGTCCCCTTGCCCAATTAGCACATTAGGTTTCTCTTGT
+HWI-EAS443:3:100:1355:1612#0/1
aa__]T¥JYa`__USYU^¥YVUT¥a]^`OFU``X_H^]]B
@HWI-EAS443:3:23:1063:1963#0/1
GAGAAAGCTAATGTGCTAATCGGGCAAGGGGACGTAGAGT
+HWI-EAS443:3:23:1063:1963#0/1
`a`_b`]^`[aab`baIWb¥`a_`_aSa`JYTXaRZXDI^
@HWI-EAS443:3:24:1140:1918#0/1
GCTCCCTTGCCCAATTAGCACATTCGCTTTCTCTCCTTTT
+HWI-EAS443:3:24:1140:1918#0/1
`HW`Z`PUU]a`baUXVS[`__V_[KZ_[¥_^¥aT_¥¥TF
@HWI-EAS443:3:38:1406:1755#0/1
GTTCCCTTGGCCAATTAGCACATTAGCTTTCTCTCCTTTT
+HWI-EAS443:3:38:1406:1755#0/1
a_`aaaaaaa`___aaa`__``a`___a`^X^^`X]``_Z
@HWI-EAS443:3:63:1158:1544#0/1
AAAGGAGAGAAAGCTAATGTGCTAATTGGNAAAGGGGACG
+HWI-EAS443:3:63:1158:1544#0/1
]`ab__aX`a``_¥_¥_¥¥OYZba^]NR¥DF[^Ya``Ya`
@HWI-EAS443:3:94:286:1785#0/1
CTTGCCCAATTAGCACATTAGCTTTCTCTCGTTTNGCAAG
+HWI-EAS443:3:94:286:1785#0/1
¥bb]ab^[`_`URUa]¥Y¥]aaa]a]P¥¥HG`aYDY¥`aa
@HWI-EAS443:3:19:20:1232#0/1
GCATCAATGAACTGAGCAATCTTGCAAAGGAGAGAAAGCT
+HWI-EAS443:3:19:20:1232#0/1
a`abaa]baabaa_aaaa`baaaaaa`_aa`aa`a]`a``
@HWI-EAS443:3:29:1124:199#0/1
CAATCTTGCAAAGGAGAGAAAGCTAATGTGCTAATTGGGC
+HWI-EAS443:3:29:1124:199#0/1
`b¥`a`bab`bbaa`aabbaababb`abYbaaaabb`aa_

```
@HWI-EAS443:3:35:1612:534#0/1
CAAAAGGAGAGAAAGCTATGTGCTAATTGGGCAAGGGGAC
+HWI-EAS443:3:35:1612:534#0/1
a¥_`]__`_]babba]a``aXaaa^ab_baaa^bbb^^X^
@HWI-EAS443:3:51:1373:985#0/1
GAGAAAGCTAATGTGCTAATTGGGCAGGGGACGTAGTGTT
+HWI-EAS443:3:51:1373:985#0/1
SaW¥^I¥aab[[`Ua]bba_b]aZa]¥_I__bba]b^^_Y
@HWI-EAS443:3:62:1050:1155#0/1
GCATCAATGAACTGAGCAATCTTGCAAAGGAGAGAAAGCT
+HWI-EAS443:3:62:1050:1155#0/1
``aaaa`^a_a_aaaaaaaa_a`a``__a`_``[`]_`a_
@HWI-EAS443:3:76:151:555#0/1
AAAGGAGAGAAAGCTATGTGCTAATTGGGCAAGGGGACGT
+HWI-EAS443:3:76:151:555#0/1
_XZabab`bbabaaaaab^baa_aa`aa`a`_a`a`]__Z
@HWI-EAS443:3:10:604:1159#0/1
TGTCCCCTTGCCCAATTAGCACATTAGCTTTCTCTTGTCT
+HWI-EAS443:3:10:604:1159#0/1
```aa`_aaaa`]YY[V]a^]^^_^]]]^X_]_]_X^¥Y^
@HWI-EAS443:3:10:651:1547#0/1
CAGACAAGAGAAAGCTAATGTGCTAATTGGGCAAGGGGAC
+HWI-EAS443:3:10:651:1547#0/1
ababbabababbaaabaaaa_aaaaabaaa``aaa`_a`^
@HWI-EAS443:3:12:1061:1880#0/1
GAACTGAGCAATCTTGCAAAAGGAGAGAAAGCTAGGAAGA
+HWI-EAS443:3:12:1061:1880#0/1
aaaa¥]aa^bba_`_a``X]a_¥aa_``aa_Z``a^__¥^
@HWI-EAS443:3:13:579:775#0/1
TGTCCCCTTGCCCAATTAGCACATTAGCTTTCTCTTGTCT
+HWI-EAS443:3:13:579:775#0/1
aa_aaaa_`aY]a__^^``a``__]`_`___]^^`_`Z^^
@HWI-EAS443:3:15:141:987#0/1
AGACAAGAGAAAGCTAATGTGCTAATTGGGCAAGGGGACA
+HWI-EAS443:3:15:141:987#0/1
_Y¥aM^UYZ``VX`]¥`ZVNYZ^Z`_`]_¥^]ZR_`a`Ya
@HWI-EAS443:3:19:1265:702#0/1
GTCCCCTTGCCCAATTAGCACATTAGCTTTCTCTTGTCTG
+HWI-EAS443:3:19:1265:702#0/1
aabb^¥a```aaabaaaa[baaaaa]_aa`_a`a`a[¥aa
@HWI-EAS443:3:21:18:27#0/1
CAGACAAGAGAAAGCTAATGTGCTAATTGGGCAAGGGGAC
+HWI-EAS443:3:21:18:27#0/1
a`abbabbabbbabaaaaab^aa`_a``aaaa``a`a``]
@HWI-EAS443:3:22:570:1284#0/1
GTCCCCTTGCCCAATTAGCACATTAGCTTTCTCTTGTCTG
+HWI-EAS443:3:22:570:1284#0/1
a_b`aaaabaabaa]¥`aaaaa_aa__aaa_`_a[]]Ya`
@HWI-EAS443:3:24:530:542#0/1
AGTCCCCTTGCCCAATTAGCACATTAGCTTTCTCTTGTCT
```
```

```
+HWI-EAS443:3:24:530:542#0/1
aa`aaaa`aaaaaaa__`aaa_`aaaaaa`aaaa`aa^_^
@HWI-EAS443:3:24:729:1004#0/1
GTCCCCTTGCCCAATTAGCACATTAGCTTTCTCTTGTCTG
+HWI-EAS443:3:24:729:1004#0/1
aaaaa`a_``a`X]_Z_```aZ__a^a^__aa[`_^]]]¥
@HWI-EAS443:3:26:1326:93#0/1
GTCCCCTTGCCCAATTAGCACATTAGCTTTCTCTTGTCTG
+HWI-EAS443:3:26:1326:93#0/1
a`W``aa_a_¥__`_^``¥`V]a_^_V`a`V_Vaa__X__
@HWI-EAS443:3:29:1360:1595#0/1
CAGACAAGAGAAAGCTAATGTGCTAATTGGGCAAGGGGAC
+HWI-EAS443:3:29:1360:1595#0/1
M`bbaaa_bbbbab`ba]Uaabbb`bbaba_a`W¥ab__a
@HWI-EAS443:3:32:680:255#0/1
GTCCCCTTGCCCAATTAGCACATTAGCTTTCTCTTGTCTG
+HWI-EAS443:3:32:680:255#0/1
_Q]T^aa`[aT¥_QUNSV[_YY`W¥YY^_Z^Z_ZVZR^^[
@HWI-EAS443:3:34:299:280#0/1
CAGACAAGAGAAAGCTAATGTGCTAATTGGGCAAGGGGAC
+HWI-EAS443:3:34:299:280#0/1
abbbbbbabaaabbbbaaaa^aaaaa`aa`__]`a```_^
@HWI-EAS443:3:40:707:1931#0/1
CAGACAAGAGAAAGCTAATGTGCTAATTGGGCAAGGGGAC
+HWI-EAS443:3:40:707:1931#0/1
abbbabbabbbb_`aaaba`]aaaa`_a_]^aaa`__``V
@HWI-EAS443:3:46:445:396#0/1
CAGACAAGAGAAAGCTAATGTGCTAATTGGGCAAGGGGAC
+HWI-EAS443:3:46:445:396#0/1
¥__``aZaabb``aMb_Z^`_babbabb^baXQ]``b`Q`
@HWI-EAS443:3:47:592:558#0/1
CAGACAAGAGAAAGCTAATGTGCTAATTGGGCAAGGGGAC
+HWI-EAS443:3:47:592:558#0/1
aab_T]abbbaa^Y¥]^[ba]¥_a__aaaZa_RZ`]`_]Y
@HWI-EAS443:3:49:622:334#0/1
GTCCCCTTGCCCAATTAGCACATTAGCTTTCTCTTGTCTG
+HWI-EAS443:3:49:622:334#0/1
aX[X^_Uaa_^_^[]G`Z^__^___[]__^__[Z]_^X][
@HWI-EAS443:3:50:52:177#0/1
GTCCCCTTGCCCAATTAGCACATTAGCTTTCTCTTGTCTG
+HWI-EAS443:3:50:52:177#0/1
^`]bbbbW¥_ba`^Ua_OV¥_[aa]GVaa`¥`aaa[[`]Z
@HWI-EAS443:3:51:1526:848#0/1
GTCCCCTTGCCCAATTAGCACATTAGCTTTCTCTTGTCTG
+HWI-EAS443:3:51:1526:848#0/1
`][[_`_aa___]^QY_^_`V^``_^]```¥a]^`_^X^]
@HWI-EAS443:3:51:658:1900#0/1
CAGACAAGAGAAAGCTAATGTGCTAATTGGGCAAGGGGAC
+HWI-EAS443:3:51:658:1900#0/1
a``abb`bba`^`b¥`¥`ba]babbaa¥¥_V`aabbbaaa
```

```
@HWI-EAS443:3:52:257:728#0/1
GTCCCCTTGCCCAATTAGCACATTAGCTTTCTCTTGTCTG
+HWI-EAS443:3:52:257:728#0/1
]SWO_RYRXEOZSL[NZZV¥RMUURZOYQUSYPWUVYR[[
@HWI-EAS443:3:59:1043:130#0/1
CAGACAAGAGAAAGCTAATGTGCTAATTGGGCAAGGGGAC
+HWI-EAS443:3:59:1043:130#0/1
¥a^abbbbbbaabb``b_aaabbababbbbb`aab[Wa_[
@HWI-EAS443:3:5:184:765#0/1
CAGACAAGAGAAAGCTAATGTGCTAATTGGGCAAGGGGAC
+HWI-EAS443:3:5:184:765#0/1
abbbabbbab_`bbabaabba``ababbbba¥a`abab_¥
@HWI-EAS443:3:62:1390:93#0/1
GTCCCCTTGCCCAATTAGCACATTAGCTTTCTCTTGTCTG
+HWI-EAS443:3:62:1390:93#0/1
aWWVRUaNJSV^VOYUL^RWVX]]SQYZ¥^U¥V]WVUYVX
@HWI-EAS443:3:70:304:1618#0/1
GTCCCCTTGCCCAATTAGCACATTAGCTTTCTCTTGTCTG
+HWI-EAS443:3:70:304:1618#0/1
Y`bb_baYW]bb_a_`¥W_a`]]a¥O]`^_a_a`_aYTX_
@HWI-EAS443:3:72:1361:1714#0/1
TGTCCCCTTGCCCAATTAGCACATTAGCTTTCTCTTGTCT
+HWI-EAS443:3:72:1361:1714#0/1
aba`^`aaaa_`aaa_^b``a_aa_aa___a^aXa`a[__
@HWI-EAS443:3:72:329:912#0/1
GTCCCCTTGCCCAATTAGCACATTAGCTTTCTCTTGTCTG
+HWI-EAS443:3:72:329:912#0/1
Yabbbb_¥¥bbba_X]¥Z_`b`abT_]a`a]a`a_^___Y
@HWI-EAS443:3:74:1735:109#0/1
CAGACAAGAGAAAGCTAATGTGCTAATTGGGCAAGGGGAC
+HWI-EAS443:3:74:1735:109#0/1
aabaab```bb]`bYb[aababab]_^abbb^_Z]aa]TH
@HWI-EAS443:3:78:148:2003#0/1
CAGACAAGAGAAAGCTAATGTGCTAATTGGGCAAGGGGAC
+HWI-EAS443:3:78:148:2003#0/1
_Y_Xa]]aaaa^aUX]U^]¥L¥^¥]U[`]`Z``[Z[XNT_
@HWI-EAS443:3:79:622:876#0/1
CAGACAAGAGAAAGCTAATGTGCTAATTGGGCAAGGGGAC
+HWI-EAS443:3:79:622:876#0/1
ababbbbbab^]aaaaaaa]¥aaaa`aaaa[^``baaaZ^
@HWI-EAS443:3:81:1364:961#0/1
AGGAGAGAAAGCTAATGTGCTAATTGGGCAAGGGACGTAG
+HWI-EAS443:3:81:1364:961#0/1
_``abbbbbbaabaabaababbabaaaY[aaba]``a__a
@HWI-EAS443:3:84:990:1522#0/1
CAGACAAGAGAAAGCTAATGTGCTAATTGGGCAAGGGGAC
+HWI-EAS443:3:84:990:1522#0/1
]Z_T``_`^bbbaa^UL`Z¥^baa]S¥P¥`aY]TZ`P¥S_
@HWI-EAS443:3:88:1595:158#0/1
GTCCCCTTGCCCAATTAGCACATTAGCTTTCTCTTGTCTG
```

```
+HWI-EAS443:3:88:1595:158#0/1
_babbbbb`a_a`aa`baab`abab_Nbaaababbba[aa
@HWI-EAS443:3:88:1690:1854#0/1
CTGACNAATTAGCACATTAGCTTTCTCTCCTTTTGCAAGA
+HWI-EAS443:3:88:1690:1854#0/1
[^[`]D]bbba``aabbbbaa`bbab`baa`bb`Y`_a]b
@HWI-EAS443:3:91:1433:1521#0/1
GTCCCCTTGCCCAATTAGCACATTAGCTTTCTCTTGTCTG
+HWI-EAS443:3:91:1433:1521#0/1
]FZ_`]`VS]``^`]_^_UYMZ`__^VX_`^YTYa_^]__
@HWI-EAS443:3:97:1148:1695#0/1
CAGACAAGAGAAAGCTAATGTGCTAATTGGGCAAGGGGAC
+HWI-EAS443:3:97:1148:1695#0/1
K```[ba`bbb`abbbabba_babbbabbbbbaaa[bTUY
@HWI-EAS443:3:98:742:347#0/1
GTCCCCTTGCCCAATTAGCACATTAGCTTTCTCTTGTCTG
+HWI-EAS443:3:98:742:347#0/1
`aa`[abab_^_`_a`aa^_`_``^_]_``V_^__^^^`a
@HWI-EAS443:3:11:1340:1443#0/1
AGCTAATGTGCTAATTGGGCAAGGGGGCGTAGTGTTGGGA
+HWI-EAS443:3:11:1340:1443#0/1
¥Z_U_[SbXX¥Ub_Z`a[]aU`b[a[HMaUV_Z_U_aZFV
@HWI-EAS443:3:20:1362:956#0/1
GAGCAATCTTGCAAAAGGAGAGAAAGCTAATGTCCTAAGG
+HWI-EAS443:3:20:1362:956#0/1
abb_b`bba`b]bb¥abb`b^b_a`a]aaabaaS``_aa`
@HWI-EAS443:3:40:771:617#0/1
AGCTAATGTGCTAATTGGGCAAGGGGCCGTAGTGTTGGGA
+HWI-EAS443:3:40:771:617#0/1
W[ba^¥Q`_`_^Q_¥¥__Y^U]¥Z]TF^`QX`]VZY]V`R
@HWI-EAS443:3:52:499:1745#0/1
GCATCAATGAACTGAGCAATCTTGAAAAAGGAGAGAAAAA
+HWI-EAS443:3:52:499:1745#0/1
abba`a`¥]L]a]]YaV`aa^aaa]VXa_`^Y_[`^UUUT
@HWI-EAS443:3:92:666:1170#0/1
AGCTAATGTGCTAATTGGGCAAGGGGAGGTAGTGTTGGGA
+HWI-EAS443:3:92:666:1170#0/1
ab_]GZP]¥aaaaa`Y`_a_^_`_^_WG_RU^W_^_Z_G^
@HWI-EAS443:3:11:1015:920#0/1
CGTCCCCTTGCCCAATTAGCACATTAGCTTTCTCTTGTCT
+HWI-EAS443:3:11:1015:920#0/1
__Za_Y_aa`^aaa¥a_a``]¥X]__Z[^`_[[Z_¥`TNX
@HWI-EAS443:3:12:842:1160#0/1
AGACAAGAGAAAGCTAATGTGCTAATTGGGCAAGGGGACG
+HWI-EAS443:3:12:842:1160#0/1
a]a_]Z^ab`ba`aa]¥ab__a`a`a_`_]^__a]a_^_a
@HWI-EAS443:3:35:1221:458#0/1
GCAAAAGGAGAGAAAGCTAATGTGCTAATTGGGCAGGACG
+HWI-EAS443:3:35:1221:458#0/1
aa`[`[aa[[`aa`_a¥a_^`aY__`^]a_a__`_a_^W`
```

```
@HWI-EAS443:3:37:1029:643#0/1
AAACAAGAGAAAGCTAATGTGCTAATTGGGCAAGGGGACG
+HWI-EAS443:3:37:1029:643#0/1
a^^]aabaa`_Z¥a`Z`aa_aaa_¥^aaaVa_Za``_Z[a
@HWI-EAS443:3:44:498:263#0/1
AGACAAGAGAAAGCTAATGTGCTAATTGGGCAAGGGGACG
+HWI-EAS443:3:44:498:263#0/1
`ZS]]``Z]b`V`a]b¥bb_ba]aQY_aba`__Xb`a_¥a
@HWI-EAS443:3:46:1309:1370#0/1
CGTCCCCTTGCCCAATTAGCACATTAGCTTTCTCTTGTCT
+HWI-EAS443:3:46:1309:1370#0/1
]__aaaaabbbaa`aaaaa`aa_b[_a`aa``a_aaa__a
@HWI-EAS443:3:50:647:417#0/1
AGACAAGAGAAAGCTAATGTGCTAATTGGGCAAGGGGACG
+HWI-EAS443:3:50:647:417#0/1
aba`abba`bbbbabbab`ab]bbbbbbbbaaa`bbaaab
@HWI-EAS443:3:53:315:136#0/1
AGACAAGAGAAAGCTAATGTGCTAATTGGGCAAGGGGACG
+HWI-EAS443:3:53:315:136#0/1
ab]aaababaaaaaaa]ab_b_a_aaa_aa_^^aa_a^¥a
@HWI-EAS443:3:57:289:1658#0/1
AGACAAGAGAAAGCTAATGTGCTAATTGGGCAAGGGGACG
+HWI-EAS443:3:57:289:1658#0/1
¥¥[¥]^_`Zaa`Z_a_a``]`^````[`]_VY`^`^^YP]
@HWI-EAS443:3:61:917:1756#0/1
AGACAAGAGAAAGCTAATGTGCTAATTGGGCAAGGGGACG
+HWI-EAS443:3:61:917:1756#0/1
¥aa`a^¥bbbba``a_bbb_bb`_]aa^`¥[^T¥Y_^¥TZ
@HWI-EAS443:3:71:857:1646#0/1
AGACAAGAGAAAGCTAATGTGCTAATTGGGCAAGGGGACG
+HWI-EAS443:3:71:857:1646#0/1
^X_TO¥`^O¥bTKZO_V_aG^a`Y[^_]^[ZN^¥_Z^^¥[
@HWI-EAS443:3:72:1618:656#0/1
AGACAAGAGAAAGCTAATGTGCTAATTGGGCAAGGGGACG
+HWI-EAS443:3:72:1618:656#0/1
abba`aab`ababZ^V`_Z]`a`_`aaba_[]a^Z^aXSZ
@HWI-EAS443:3:76:836:1588#0/1
AGACAAGAGAAAGCTAATGTGCTAATTGGGCAAGGGGACG
+HWI-EAS443:3:76:836:1588#0/1
U_¥aaba_a``^`]aaaa[¥`aa_a`a`a__`^a_`_]_]
@HWI-EAS443:3:77:855:108#0/1
CTTGCAAAGGAGAGAAAGCTAATGTGCTAATTGGGCAAGG
+HWI-EAS443:3:77:855:108#0/1
abbabbabbbbbabbb`bbaa_ab]aababbab``aaabb
@HWI-EAS443:3:77:910:288#0/1
CGTCCCCTTGCCCAATTAGCACATTAGCTTTCTCTTGTCT
+HWI-EAS443:3:77:910:288#0/1
abbabbababbbbbbbbbbbabaababaabbaababaaaaa
@HWI-EAS443:3:7:1066:1066#0/1
AGACAAGAGAAAGCTAATGTGCTAATTGGGCAAGGGGACG
```

+HWI-EAS443:3:7:1066:1066#0/1
ababb`bbbbbbbUbaaab¥a^abababab_aaaaaaYXb
@HWI-EAS443:3:87:1654:264#0/1
ACAAGAGAAAGCTAATGTGCTAATTGGGCAAGGGGACGAA
+HWI-EAS443:3:87:1654:264#0/1
a`J[``a^bW^U`a¥¥`aaS]]_¥aa`b^a`ababZY[DS
@HWI-EAS443:3:92:1410:1914#0/1
AGACAAGAGAAAGCTAATGTGCTAATTGGGCAAGGGGACG
+HWI-EAS443:3:92:1410:1914#0/1
aVaabLU]_Sa`bHRP_b_``ab^a^a`[b]a_a`baZGa
@HWI-EAS443:3:8:321:284#0/1
AGCTAATGNGCTAATTGGGCAAGGGGACGTAGTGTTGGGA
+HWI-EAS443:3:8:321:284#0/1
[`a]a]b]D[b`]`bYYaaaaX]ba`Xaa]]b^aS^aaGa
@HWI-EAS443:3:21:400:819#0/1
CACGTTCCCTTGCCCAATTAGCACATTAGCTTTCTCTCCT
+HWI-EAS443:3:21:400:819#0/1
a^^`[[aa]LW^Y``[_¥^ZW]_`a¥XZU[_`_Z¥_^_]Z
@HWI-EAS443:3:90:179:1994#0/1
GGTCCCCTTGCCCAATTAGCACATTAGCTTTCTGTCCTTT
+HWI-EAS443:3:90:179:1994#0/1
R_K[[`a]a_baaabb]`^`aa_¥^bbb```aaVV]ba[b
@HWI-EAS443:3:92:147:1672#0/1
GAAAGCTAATGTGCTAATTGGGCAAGGTGACGTAGTGTTN
+HWI-EAS443:3:92:147:1672#0/1
PXZZV^bZXaNJZXaVSZ[^`_bY¥b[HOK_b__b_`¥OD
@HWI-EAS443:3:10:606:1848#0/1
CAAGCTTGCAAAAGGAGAGAAAGCTAATGTGCTAATTGGG
+HWI-EAS443:3:10:606:1848#0/1
W__HV`[abb`bbbY^baabb_baabb`a^a[bbaa`bbX
@HWI-EAS443:3:12:1742:953#0/1
GACAAGAGAAAGCTAATGTGCTAATTGGGCAAGGGGACGT
+HWI-EAS443:3:12:1742:953#0/1
a[_``a``_a¥`P¥Z_a_T`Z`UW]_]S^H[VXZ¥[YS[Y
@HWI-EAS443:3:13:1236:804#0/1
CAAACTTGCAAAAGGAGAGAAAGCTAATGTGCTAATTGGG
+HWI-EAS443:3:13:1236:804#0/1
aabaaabaaa_]]ab_a`ba_]a_]aXaa^```__^`a_`
@HWI-EAS443:3:17:1311:377#0/1
GACAAGAGAAAGCTAATGTGCTAATTGGGCAAGGGGACGT
+HWI-EAS443:3:17:1311:377#0/1
_O¥]ZXV¥ZTTaZ`W^__R]W_OTWZ`a`Y^]`[__YO_Z
@HWI-EAS443:3:26:287:1559#0/1
GACAAGAGAAAGCTAATGTGCTAATTGGGCAAGGGGACGT
+HWI-EAS443:3:26:287:1559#0/1
H_bZ[abb`_a_bb`¥_`¥Yabb`aa``aa]a`ZUU]`b`
@HWI-EAS443:3:30:1680:1179#0/1
GACAAGAGAAAGCTAATGTGCTAATTGGGCAAGGGGACGT
+HWI-EAS443:3:30:1680:1179#0/1
aaQ¥a__``a`a]b^]`a^^]baaabaa]U`]b_UZTV]]

@HWI-EAS443:3:31:1153:1628#0/1
GACAAGAGAAAGCTAATGTGCTAATTGGGCAAGGGGACGT
+HWI-EAS443:3:31:1153:1628#0/1
aabbbbbb`bbaaaaaab`a^aaa`a`a[a`_aa`a]`b]
@HWI-EAS443:3:32:14:982#0/1
TGCAAAAGGAGAGAAAGCTAATGTGCTAATTGGGCACGGG
+HWI-EAS443:3:32:14:982#0/1
a__TWabbaab_b_[a`aa`aaa_a`aa``aa`¥baJ^`a
@HWI-EAS443:3:33:268:1842#0/1
GCACCAATGAACTGAGCAATCTTGCAAAAGGAGAGAAAGC
+HWI-EAS443:3:33:268:1842#0/1
a^YNY`YZ``baYVSX[URW[]__aaXaYSIES`PaZ[KF
@HWI-EAS443:3:34:1294:262#0/1
AATGAACTGAGCAATCTTGCAAAAGGAGAGAAAGCTGATG
+HWI-EAS443:3:34:1294:262#0/1
aRZ_[ILWU_]]``a_J^aYWLFMP^ZGZS[GW_ZZGZUB
@HWI-EAS443:3:34:1353:783#0/1
GAAAGCTAATGTGCTAATTGGGCAAGGGGACGTAGTTTTG
+HWI-EAS443:3:34:1353:783#0/1
a]]ZaIRNX_[OZ[[XZ_XWRPSFV_^]`YL¥NN[BBBBB
@HWI-EAS443:3:39:585:1248#0/1
GACAAGAGAAAGCTAATGTGCTAATTGGGCAAGGGGACGT
+HWI-EAS443:3:39:585:1248#0/1
``b`T`baaZ¥``aa^aa_`_aaaaaa_¥aa``a__``_]
@HWI-EAS443:3:54:375:787#0/1
AGCTAATGTGCTAATTGGGCAAGGGGACGTAGTGTTCGAT
+HWI-EAS443:3:54:375:787#0/1
ababaU`a`aa_Xabaaa_`__ba_a¥^a¥_a¥a_`_a]`
@HWI-EAS443:3:54:9:298#0/1
GACAAGAGAAAGCTAATGTGCTAATTGGGCAAGGGGACGT
+HWI-EAS443:3:54:9:298#0/1
a`a`_aaaaa``^a]_aaZ_``R_^_`^]_^X`___ZZ_X
@HWI-EAS443:3:55:1507:767#0/1
ACGTCCCCTTGCCCAATTAGCACATTAGCTTTCTCTTGTC
+HWI-EAS443:3:55:1507:767#0/1
a__]_`a`¥`a]]]]aZ`_ZZYU`^_[_Za`]V`Z¥]L[Y
@HWI-EAS443:3:59:1196:1811#0/1
GACAAGAGAAAGCTAATGTGCTAATTGGGCAAGGGGACGT
+HWI-EAS443:3:59:1196:1811#0/1
^bbab`bbbaaaaabaaabb`baabba``_]W[aaVMIUI
@HWI-EAS443:3:59:702:1534#0/1
GACAAGAGAAAGCTAATGTGCTAATTGGGCAAGGGGACGT
+HWI-EAS443:3:59:702:1534#0/1
`a`aababbbaaaab]abab¥`bbababaa]R¥__LNVSX
@HWI-EAS443:3:62:200:236#0/1
GCACCAATGAACTGAGCAATCTTGCAAAAGGAGAGAAAGC
+HWI-EAS443:3:62:200:236#0/1
a]QG¥VVRXLNXXYYNWLT]]^V_YRGGXYYVYUXUWYVX
@HWI-EAS443:3:63:397:1171#0/1
GACAAGAGAAAGCTAATGTGCTAATTGGGCAAGGGGACGT

```
+HWI-EAS443:3:63:397:1171#0/1
a``b`bZa_Y`_aa_¥]_Ta]_UZ`]``]_¥]Z`a_UWZU
@HWI-EAS443:3:67:800:1030#0/1
GACAAGAGAAAGCTAATGTGCTAATTGGGCAAGGGGACGT
+HWI-EAS443:3:67:800:1030#0/1
aaa]ababb_N`aabaaa_a_a]a`aaaaaaaa^aa`_a^
@HWI-EAS443:3:68:988:1586#0/1
GACAAGAGAAAGCTAATGTGCTAATTGGGCAAGGGGACGT
+HWI-EAS443:3:68:988:1586#0/1
aaaZYXbabbaaaa`]^a^_a`^¥`aaa]a`_a``¥V^_X
@HWI-EAS443:3:70:722:1469#0/1
TGCAAAAGGAGAGAAAGCTAATGTGCTAATTGGGCAGGGC
+HWI-EAS443:3:70:722:1469#0/1
abb^baabaa_baa^b`babaaaYaaa`a``a_`aa`^[a
@HWI-EAS443:3:80:1475:216#0/1
TGCGAAAGGAGAGAAAGCTAATGTGCTAATTGGGCAAGGG
+HWI-EAS443:3:80:1475:216#0/1
ZaWF[`^^a]bbb]]a`X`a`abZ`U[_aa`aa¥¥[__V]
@HWI-EAS443:3:8:1192:565#0/1
GCAGCAATGAACTGAGCAATCTTGCAAAAGGAGAGAAAGC
+HWI-EAS443:3:8:1192:565#0/1
`ONJPYS`]XWJTMVU_`O_NS^XZZRVUXZW¥V¥XUG_W
@HWI-EAS443:3:8:839:790#0/1
GACAAGAGAAAGCTAATGTGCTAATTGGGCAAGGGGACGT
+HWI-EAS443:3:8:839:790#0/1
]OR_X]V¥ZYH]RY[]]XQVV^O¥XNQFTZ[U`U_QQUBB
@HWI-EAS443:3:8:918:1950#0/1
GACAAGAGAAAGCTAATGTGCTAATTGGGCAAGGGGACGT
+HWI-EAS443:3:8:918:1950#0/1
^b[bbbbbbbaa_abb`[U[aaaaa`_aa^a`a_aZXLTB
@HWI-EAS443:3:61:669:572#0/1
GACGTCCCCTTGCNCAATTAGCACATTAGCTTTCTCTCCT
+HWI-EAS443:3:61:669:572#0/1
a[`a¥`^_^YPYPDY_a``^^ba`a¥`_a`_^^]`^¥]W^
@HWI-EAS443:3:100:120:258#0/1
AATGGACTGAGCAATCTTGCAAAAGGAGAGAAAGCTAATG
+HWI-EAS443:3:100:120:258#0/1
T__bY¥_bZY_VW]bbab]^^V]PabKY^bZGN[¥`a^BB
@HWI-EAS443:3:100:145:1416#0/1
GAGCAATCTTGCAAAAGTAGAGAAAGCTAATGTGCTAATT
+HWI-EAS443:3:100:145:1416#0/1
RY_¥T[abb_`bb_XH]TU^[`_bab¥baWaX[_]baaBB
@HWI-EAS443:3:100:1494:548#0/1
AAGCTAATGTGCTAATTGGGCAAGGGGAGGTAGTGTTGGT
+HWI-EAS443:3:100:1494:548#0/1
``[FUQQ]ZWR]Y`aSV]_T`^I`]aaXH¥PW]L]SBBBB
@HWI-EAS443:3:100:1585:64#0/1
AGCTAATGTGATAATTGGGCAAGGGGACGTAGTGTTGGTA
+HWI-EAS443:3:100:1585:64#0/1
`_]¥a^_babGa_Yaaaa_O__`^aWTLU][^X_Z¥YBBB
```

```
@HWI-EAS443:3:100:175:828#0/1
CTTGCAAAAGGAGAGAAAGCTAATGTGATAATTGGGCAAG
+HWI-EAS443:3:100:175:828#0/1
abZb]_J_T_`a^Z¥^a]_JJXaaaaaHWb¥bZ[N]aBBB
@HWI-EAS443:3:100:460:242#0/1
GAAAGCTAATGTGCTAATTGGGCAAGGGGAGGTAGTGTTG
+HWI-EAS443:3:100:460:242#0/1
[YXO]YU][Y[[`^Z`_]TaX[_PKZ]__VG^WV_ZBBBB
@HWI-EAS443:3:100:65:1941#0/1
GAAAGATAATGTGCTAATTGGGCAAGGGGACGTAGTGTTG
+HWI-EAS443:3:100:65:1941#0/1
`^`¥_H^OYVS]_IPa`^a[R`YZ`IZ[]`RU¥MBBBBBB
@HWI-EAS443:3:10:1383:116#0/1
AGCTAATGTGCTAATTGGGCAAGGGGAGGTAGTGTTGGTA
+HWI-EAS443:3:10:1383:116#0/1
`a¥]VVa_O]Y[MZ`T_`ZYMH[]¥SRIZURZBBBBBBBB
@HWI-EAS443:3:11:1550:1215#0/1
GAAAGCTAATGTGCTAATTGGGCAAGGGGACGTAGGGTTG
+HWI-EAS443:3:11:1550:1215#0/1
`X_]`¥`]^`aX_TX][`aa¥QXYW^XR[BBBBBBBBBBB
@HWI-EAS443:3:12:1026:958#0/1
CAATGAACTGAGCAATCTTGCAAAGGGAGAGAAAGCTAAT
+HWI-EAS443:3:12:1026:958#0/1
aa`_a[M]Z¥¥aWX`aa`¥`[]`[L[[L`P¥[]¥`X]_QB
@HWI-EAS443:3:12:1761:1848#0/1
AGCACATTAGCTTTCTCTCCTTTTGCAAGATTGCTAAGTT
+HWI-EAS443:3:12:1761:1848#0/1
ab`bba`]_`a^¥`U`¥aa_a_`_^aYXYX_]VSaI`^]Z
@HWI-EAS443:3:12:439:1418#0/1
AGGAGAGAAAGCTAATGTGCTAATTGGGAAAGGGGACGTA
+HWI-EAS443:3:12:439:1418#0/1
ZbbUb^b]aYaa`aba]]`Ub``baaa_FV]ba`a``[`Y
@HWI-EAS443:3:13:285:711#0/1
GAAAGCTAATGTGCGAATTGGGCAAGGGGACGTAGTGTTG
+HWI-EAS443:3:13:285:711#0/1
``V¥^aZY`Z[T¥HDXaTaa¥OXZ^aZ]^U]_NU¥S^UUZ
@HWI-EAS443:3:14:1619:1821#0/1
GAGCAATCTTGCAAAAGGAGAGAAAGCTAATGTGGTAATT
+HWI-EAS443:3:14:1619:1821#0/1
^^_HTbbK¥]_ba_b¥``_W_^bbb`a]aab`YRMO^aR]
@HWI-EAS443:3:15:1563:1572#0/1
GAAAGCTAATGTGATAATTGGGCAAGGGGACGTAGTGTTG
+HWI-EAS443:3:15:1563:1572#0/1
Y`ababbaXbb_QHZ¥aXbaab¥¥^bbXVXa_¥__S_JXa
@HWI-EAS443:3:16:159:1346#0/1
GAAAGCTAATGTGCTAATTGGGCAAGAGGACGTAGTGTTG
+HWI-EAS443:3:16:159:1346#0/1
]S[b^a^]__a`baaabaa`a^aa`QDXa_aU]`aUYT^[
@HWI-EAS443:3:16:1648:1978#0/1
CAAAAGGAGAGAAAGGTAATGTGCTAATTGGGCAAGGGGA
```

```
+HWI-EAS443:3:16:1648:1978#0/1
abbaV___babbaa`J`_aaY[^Z__aba_`Q¥a_ZZ``a
@HWI-EAS443:3:16:879:619#0/1
GAGCAATCCTGCAAAAGGAGAGAAAGCTAATGTGCTAATT
+HWI-EAS443:3:16:879:619#0/1
^[`][[_^O_bba`_abb]bbb_Z_b_`[`ab_a¥a¥`a`
@HWI-EAS443:3:19:1482:1148#0/1
GCATGAATGAACTGAGCAATCTTGCAAAAGGAGAGAAAGC
+HWI-EAS443:3:19:1482:1148#0/1
aaaZG[_aa`a]RZ]a_^__`aWbS[ZXNa`Z_^a_J_]Z
@HWI-EAS443:3:1:1714:52#0/1
ATTAGCTTTGTCTCCTTTTGCAAGATTGCTCAGTTCATTG
+HWI-EAS443:3:1:1714:52#0/1
`¥`[UVbV^^aU¥Q[Y^UaYESZ``__G¥WL[`_^BBBBB
@HWI-EAS443:3:1:540:219#0/1
ATTAGCTTTCTCTCCTTTTGCAAGATTGGTCAGTTCATTG
+HWI-EAS443:3:1:540:219#0/1
abaa¥aZKb_¥aYY¥¥U]XaUXaa¥``UG^_aaSaYZ¥`B
@HWI-EAS443:3:1:88:1191#0/1
TGCCCAATTAGCAGATTAGCTTTCTCTCCTTTTGCAAGAT
+HWI-EAS443:3:1:88:1191#0/1
`baW`¥¥_bVV__GYa_baM¥¥^b`aa`a]]``ITW_BBB
@HWI-EAS443:3:20:1014:39#0/1
GAAAGCTAATGAGCTAATTGGGCAAGGGGACGTAGTGTTG
+HWI-EAS443:3:20:1014:39#0/1
a[[X^P^IPS¥JWMOULZMZ^SZUUUZ¥TQQ]XVSPYUY]
@HWI-EAS443:3:20:89:249#0/1
CAATCTTGCAAAAGGAGAGAAAGCTAATGCGCTAATTGGG
+HWI-EAS443:3:20:89:249#0/1
aaaY`aa¥Pa[XZ^XWXQ`¥FL_WTVU^SFS^ZTT]]^VW
@HWI-EAS443:3:22:1530:1475#0/1
GAAAGCTAATGTGCTAATTGGGCAAGGGGACGGAGTGTTG
+HWI-EAS443:3:22:1530:1475#0/1
aaa`]U_[WWYPa^X]aa`aY`SV[`V]ZW^]BBBBBBBB
@HWI-EAS443:3:23:236:602#0/1
GAGAAAGCTAATGTGCTAATCGGGCAAGGGGACGTAGTGT
+HWI-EAS443:3:23:236:602#0/1
ZU`WO[¥a_YZQ^¥a_W[^]_XUY]Z¥ZUT]ZYUR^]R`U
@HWI-EAS443:3:23:874:274#0/1
GAGCAATCTTCCAAAAGGAGAGAAAGCTAATGTGCTAATT
+HWI-EAS443:3:23:874:274#0/1
_S``¥`_`a^`[¥UVQ¥`R[YY_]_`S¥[^¥YV_V]V[YX
@HWI-EAS443:3:24:1463:589#0/1
GAGCAATTTTGCAAAAGGAGAGAAAGCTAATGTGCTAATT
+HWI-EAS443:3:24:1463:589#0/1
aaaaaaab`abaa`_a___a_b__`a^a`_aa]bXa____
@HWI-EAS443:3:24:1671:1855#0/1
AGCTAATGTGCTAATTGGGCAAGGGGACGTAATGTTGGTA
+HWI-EAS443:3:24:1671:1855#0/1
`WF¥ba``T¥H_a`W[TG^^UGa[__[^_ZW¥__VZ^¥XZ
```

```
@HWI-EAS443:3:24:608:585#0/1
GAGCAATCTTGCAAGAGGAGAGAAAGCTAATGTGCTAATT
+HWI-EAS443:3:24:608:585#0/1
abbbbabaab`baab`aaaaaaa¥aa`aaaaa^b_a``aa
@HWI-EAS443:3:25:168:314#0/1
GAGCAATCTTGCAAAAGGCGAGAAAGCTAATGTGCTAATT
+HWI-EAS443:3:25:168:314#0/1
aaabaaabaabaa`_aa_`_¥__^][R[¥_VZV_¥W¥NT¥
@HWI-EAS443:3:27:1530:105#0/1
AGCTAATGCGCTAATTGGGCAAGGGGACGTAGTGTTGGTA
+HWI-EAS443:3:27:1530:105#0/1
VaVbb`¥_OZ_b^^TXSa_aZ`ZW_S^D]Raa_Va`b^QH
@HWI-EAS443:3:27:190:671#0/1
AATGAACTGGGCAATCTTGCAAAAGGAGAGAAAGCTAATG
+HWI-EAS443:3:27:190:671#0/1
WW`b[_`a_Y^`baa`a¥__Z_a_a`[baa]a_a___`_a
@HWI-EAS443:3:27:86:304#0/1
GAGCAATCTTGCAAAAGGAGAGAAAGCTACTGTGCTAATT
+HWI-EAS443:3:27:86:304#0/1
W_[[_H_bbbba_`aa[OObabbbbaZb_H_bYaa[`Y]`
@HWI-EAS443:3:28:121:1560#0/1
CATCAATGAACTGAGCAATCCTGCAAAAGGAGAGAAAGCT
+HWI-EAS443:3:28:121:1560#0/1
abaaaaa`aaa`]a_aaZ]a`__aa``a]^__a_`a__a_
@HWI-EAS443:3:28:1311:13#0/1
GAGCAATCTTGCAAAAGGAGAGAAAGATAATGTGCTAATT
+HWI-EAS443:3:28:1311:13#0/1
``_U_¥_Y`[[J[`X`a_ZV]aa¥¥¥FW^[Z_T¥VV^X[_
@HWI-EAS443:3:28:428:1522#0/1
CATCAATGAACTGAGCAATCTTGCAAACGGAGAGAAAGCT
+HWI-EAS443:3:28:428:1522#0/1
Y[Ua`XZ^__aYUHQ^`¥¥`YWT`]R¥HZ[ZZ¥a¥L[`[^
@HWI-EAS443:3:30:292:1466#0/1
AGCTAATGTGCTAATTGGGCAAGGGGACGCAGTGTTGGTA
+HWI-EAS443:3:30:292:1466#0/1
TSXV]a_HNYa¥aYYVLN___VNRSVWIXGQWFXV^ZUX^
@HWI-EAS443:3:30:417:171#0/1
AGCAATCTTGCAAAAGGAGACAAAGCTAATGTGCTAATTG
+HWI-EAS443:3:30:417:171#0/1
aaabaaaaabaaaa^aa^aa`a]aaa`^W]a^a_a__`__
@HWI-EAS443:3:32:332:1507#0/1
GAAAGCTAATGTGCCAATTGGGCAAGGGGACGTAGTGTTG
+HWI-EAS443:3:32:332:1507#0/1
QY¥a`b_W[^aX^`a`a`ba]]aa`ZQVT_b_]aa^`[__
@HWI-EAS443:3:34:524:1076#0/1
AGGAGATAAAGCTAATGTGCTAATTGGGCAAGGGGACGTA
+HWI-EAS443:3:34:524:1076#0/1
]¥TT]`K`M[b_]U¥WabW^abX[ab`Xaaa``aS_V^X`
@HWI-EAS443:3:34:569:391#0/1
GCATCAATGAACTGCGCAATCTTGCAAAAGGAGAGAAAGC
```

```
+HWI-EAS443:3:34:569:391#0/1
^aa_bbba`ab]_XKa]Vaa^aa`b`a]VW]^_`a`U^_Z
@HWI-EAS443:3:34:82:1638#0/1
AAGCTAATGTGCTAACTGGGCAAGGGGACGTAGTGTTGGT
+HWI-EAS443:3:34:82:1638#0/1
aaabaaa[a^`b`aa]``[¥_`_]]QT]`_¥^_XaV^^XX
@HWI-EAS443:3:35:879:1214#0/1
GAAAGCTAATGCGCTAATTGGGCAAGGGGACGTAGTGTTG
+HWI-EAS443:3:35:879:1214#0/1
VZb¥b_b`b`bI_b[bbb_a_¥]a``ab[J``TMbaa`b`
@HWI-EAS443:3:37:1005:351#0/1
ACTGAGCAATCTTGCAAAAGGAGAGAAAGGTAATGTGCTA
+HWI-EAS443:3:37:1005:351#0/1
`_`¥_`]Z`]¥[]`X`¥]]`aXa`a]QXNFVUQY`W`_ZV
@HWI-EAS443:3:38:129:1743#0/1
GCATCAATGAACTGAGCAATCTTGCAAAAGGATAGAAAGC
+HWI-EAS443:3:38:129:1743#0/1
[^_bbbb_b_b^^[[]JT`[a`a`bb``a`GYH^`aZHZW
@HWI-EAS443:3:38:480:882#0/1
GCAATCTTGCAAAAGGAGAGAAAGCTAATGAGCTAATTGG
+HWI-EAS443:3:38:480:882#0/1
a^RQ]]]RMX]^GR[VR¥V]V]^Q^RSK[MDIMJ[U[T[N
@HWI-EAS443:3:39:1064:1215#0/1
ATCTTGCAATAGGAGAGAAAGCTAATGTGCTAATTGGGCA
+HWI-EAS443:3:39:1064:1215#0/1
K^U^bb`WZ_[`aK^Z[a_YSWUY]aZSI¥aYY`W`[b`P
@HWI-EAS443:3:39:1131:480#0/1
AAGCTAATGTGCTAATTGGGCAAGGGGACGTAGGGTTGGT
+HWI-EAS443:3:39:1131:480#0/1
[ZI¥_LPaa[]_b__b``OX]PJa]aUSS`U]SQaZZ`XP
@HWI-EAS443:3:39:1144:1808#0/1
AAAGCTAATGTGCTAATTGGGCAAGGGGACGAAGTGTTGG
+HWI-EAS443:3:39:1144:1808#0/1
_]^a`b_b`YK¥¥aa¥a``UU_^`_aXPZ_YG[]V¥QV]W
@HWI-EAS443:3:40:1266:1249#0/1
GAAAGCTAATGTGTTAATTGGGCAAGGGGACGTAGTGTTG
+HWI-EAS443:3:40:1266:1249#0/1
aT^_aaaYT`aM[Q]RV^Z^Z]_ZZ_]_^UW_QY]V_OW^
@HWI-EAS443:3:40:197:383#0/1
CAATCTTGCAAAAGGAGAGAAAGCTACTGTGCTAATTGGG
+HWI-EAS443:3:40:197:383#0/1
_```aabba^_aSaa_aaa^`aba^ZH[`¥aaaWSY¥a[a
@HWI-EAS443:3:40:604:1460#0/1
GAGAAAGCTAATGGGCTAATTGGGCAAGGGGACGTAGTGT
+HWI-EAS443:3:40:604:1460#0/1
a]Va¥G¥]^Ha¥YR]OWLT]^_`Y[VFW_R_H]SBBBBBB
@HWI-EAS443:3:40:644:687#0/1
TGCAACAGGAGAGAAAGCTAATGTGCTAATTGGGCAAGGG
+HWI-EAS443:3:40:644:687#0/1
abaXZHM]RQaUWZ]TW[^UXZSNYSJQINXY¥ZULGMQV
```

```
@HWI-EAS443:3:40:70:1543#0/1
CAATCTTGCAAAAGGAGAGAAAGCTACTGTGCTAATTGGG
+HWI-EAS443:3:40:70:1543#0/1
a_aab`a`^]^]aa__Va^]^`___[HS[S`_^WOZ^[TX
@HWI-EAS443:3:40:937:721#0/1
AGCTAATGTGCTAATTGGGCAAGGGGCCGTAGTGTTGGTA
+HWI-EAS443:3:40:937:721#0/1
W[]_V_¥`]Wa__Z_^`b^¥a__abTG^aa]^`_VP_[YM
@HWI-EAS443:3:44:1194:1644#0/1
TGAGCAATCTTGCAAAAGGAGAGTAAGCTAATGTGCTAAT
+HWI-EAS443:3:44:1194:1644#0/1
a`aY]`[__aaaa`aH___aa_Y^_^_^_a]__Y_V`_]_
@HWI-EAS443:3:44:1203:1330#0/1
GTCCCCTTGCCCAATTAGCACATTAGCTATCTCTCCTTTT
+HWI-EAS443:3:44:1203:1330#0/1
a_aabaaaba_abbbbaaaa`aaa`a_aaa`a_`___aa`
@HWI-EAS443:3:46:1106:120#0/1
GAAAGCTAATGCGCTAATTGGGCAAGGGGACGTAGTGTTG
+HWI-EAS443:3:46:1106:120#0/1
a^ZP`Q¥YQ^]]^YRV]V]a`[LVL]MQ[UFWNRSYBBBB
@HWI-EAS443:3:47:326:329#0/1
GAACTGAACAATCTTGCAAAAGGAGAGAAAGCTAATGTGC
+HWI-EAS443:3:47:326:329#0/1
a`b_]`]I¥aaa^[^`Wa___aa[``aa_``¥^¥```XXX
@HWI-EAS443:3:47:392:819#0/1
ATTAGCTTTATCTCCTTTTGCAAGATTGCTCAGTTCATTG
+HWI-EAS443:3:47:392:819#0/1
_Ua`]Y`ZSMX`^XQ[]¥[aaa¥^Y^[¥¥[YWaYXU`aTZ
@HWI-EAS443:3:47:700:664#0/1
GAAAGCTAATGTGCTAATTGAGCAAGGGGACGTAGTGTTG
+HWI-EAS443:3:47:700:664#0/1
aaaX`aa]aab_^Ya^]aa^`]¥Z_aa_`]¥a¥Ya]_YZ¥
@HWI-EAS443:3:48:483:1251#0/1
GAGCAATCTTGCAAAAGGAGAGAAAGCTAATGTACTAATT
+HWI-EAS443:3:48:483:1251#0/1
aaaababaaa`aaaa[ba_aaaaa`a_a`aaa_J`a`_a_
@HWI-EAS443:3:49:632:421#0/1
GAGCAATCTTGCAAAAGGAGAGAAAGCTAATGTGGTAATT
+HWI-EAS443:3:49:632:421#0/1
Q_Z_I¥[_U_b_[P^]X¥_¥¥X^FZW`aUaW[QUFU_KUW
@HWI-EAS443:3:49:819:1751#0/1
ATCTTGTAAAAGGAGAGAAAGCTAATGTGCTAATTGGGCA
+HWI-EAS443:3:49:819:1751#0/1
[bbX[`QN[`[`[_Z^bTWX¥[Za`aaSa_aa^`aa]aUT
@HWI-EAS443:3:4:137:1051#0/1
GAGCAATCTTGCAAAGGGAGAGAAAGCTAATGTGCTAATT
+HWI-EAS443:3:4:137:1051#0/1
^_^NV]aaaaaRWRPJVXUQQaP`__ZR^`¥_U[PPZ_BB
@HWI-EAS443:3:4:248:1987#0/1
AGCTAATGTGCTAATTGGGCAAGGGGACGTAGCGTTGGTA
```

+HWI-EAS443:3:4:248:1987#0/1
a`abba_a^aa___¥Y`a¥L¥_a`a]]^]X^ZF`HZ`]GO
@HWI-EAS443:3:4:531:158#0/1
ATCAATGAACTGAGCAATCTTGCAAAGGGAGAGAAAGCTA
+HWI-EAS443:3:4:531:158#0/1
Z¥YU__a¥aUWU[¥I^`__V^a[]^WG_`]aaaa_`^M[[
@HWI-EAS443:3:50:1719:471#0/1
GAAAGCTAATGTGCTAATTGGGCAAGGGCACGTAGTGTTG
+HWI-EAS443:3:50:1719:471#0/1
abaa^^`¥a_U¥aaaa`a``_]¥_U]`]ORY_X¥XT^T_a
@HWI-EAS443:3:50:938:456#0/1
GTCCCCTTGCGCAATTAGCACATTAGCTTTCTCTCCTTTT
+HWI-EAS443:3:50:938:456#0/1
aXabaUPYaWG¥^[WR^a`Ta]]a`_HK^RIR`_IRZW[`
@HWI-EAS443:3:52:380:1970#0/1
AATGAACTGAGCAATCTTGCAAAGGGAGAGAAAGCTAATG
+HWI-EAS443:3:52:380:1970#0/1
ab`a]aaaa`a_aa`aaaaa^Z^___^`__a___Y`____
@HWI-EAS443:3:52:505:1589#0/1
AAGCATCAATGAACTGAGCAATCTTGCATAAGGAGAGAAA
+HWI-EAS443:3:52:505:1589#0/1
_aaaaaaaaaaaa_a_`a_aaa_aa_`aa_`^a``aa___
@HWI-EAS443:3:53:1580:1960#0/1
GAAAGCTAATGGGCTAATTGGGCAAGGGGACGTAGTGTTG
+HWI-EAS443:3:53:1580:1960#0/1
VZ_U[Z^aZW[G]U¥U_]a`UY]`a]aaaa`aPP`]`H^_
@HWI-EAS443:3:54:121:1201#0/1
AGCTAATGTGCTAATTGGGCAAGGGGACGTAGAGTTGGTA
+HWI-EAS443:3:54:121:1201#0/1
QX`Y_b_Q__aV]]`bU_UT]R_a]baa]P__M[R_^YDU
@HWI-EAS443:3:54:1278:1652#0/1
ACTGAGCAATCTTGCAAAAGGAGAGGAAGCTAATGTGCTA
+HWI-EAS443:3:54:1278:1652#0/1
`Tab__YbbbUab`Xaababa_aab__ab_[UaaSb`[]R
@HWI-EAS443:3:55:557:1476#0/1
TTTCTCTCCTTTAGCAAGATTGCTCAGTTCATTGATGCTT
+HWI-EAS443:3:55:557:1476#0/1
abababbbabbaba`bbbab`a^aaaaaaaaaaaaaa]`a
@HWI-EAS443:3:56:784:29#0/1
AGCTAATGTGCTAATTGGGCGAGGGGACGTAGTGTTGGTA
+HWI-EAS443:3:56:784:29#0/1
^baa`bbb^baba`aaaaa[[[aaaa]]b]_aYa]_a`R]
@HWI-EAS443:3:57:1109:1275#0/1
AGCACATTAGCTTTCTCTCCTTCTGCAAGATTGCTCAGTT
+HWI-EAS443:3:57:1109:1275#0/1
aX^`_aaaaa_`aaa__`a^a___``]Z_]¥__^^[_[YZ
@HWI-EAS443:3:57:770:1322#0/1
CTTGCAAAAGGAGAGAAAGCTAATGTGGTAATTGGGCAAG
+HWI-EAS443:3:57:770:1322#0/1
aaa^Xa_b_``aUb]aa_aaa]Zba]VG]]`YaJ_R^X[_

@HWI-EAS443:3:58:1275:692#0/1
GAGCAATCTTGCAAAAGGAGAGACAGCTAATGTGCTAATT
+HWI-EAS443:3:58:1275:692#0/1
QZSWSW]Y¥Z[R[SUZa^¥aXDN`VURSX¥[[U[PR¥[
@HWI-EAS443:3:58:216:806#0/1
CAATGAACTGAGCAATCTTGCAAAAGGAGAGACAGCTAAT
+HWI-EAS443:3:58:216:806#0/1
V_WVbZNZ`bab`U¥b]aa`bbY^b¥`S`_`ZFM`ba[PX
@HWI-EAS443:3:58:89:1154#0/1
GCATCAATGAACTGAGCAATCTTGCAATAGGAGAGAAAGC
+HWI-EAS443:3:58:89:1154#0/1
aSU]^a]SaVI`[XPP]_YXZ^[]QODDK¥_QQR^`_PSY
@HWI-EAS443:3:59:94:281#0/1
CAATCTTGCGAAAGGAGAGAAAGCTAATGTGCTAATTGGG
+HWI-EAS443:3:59:94:281#0/1
aa_baabba`[ZUbb_a]]_a`aaa^_aa]aW^[^`U``^
@HWI-EAS443:3:5:993:134#0/1
CTGAGCAATCTTGCAAGAGGAGAGAAAGCTAATGTGCTAA
+HWI-EAS443:3:5:993:134#0/1
ab`S`[b[`a`a_^SHH¥a]W[L`SO^`^^_¥abUG_¥S^
@HWI-EAS443:3:60:1535:79#0/1
AGCACATTAGCTTTCTCTCCTTTTGTAAGATTGCTCAGTT
+HWI-EAS443:3:60:1535:79#0/1
ab`_aa_baaY^baabaaa[`aa¥¥IPT¥X[_a¥^U^]VR
@HWI-EAS443:3:61:1112:183#0/1
GTCCCCTCGCCCAATTAGCACATTAGCTTTCTCTCCTTTT
+HWI-EAS443:3:61:1112:183#0/1
a`YV__PDW_^_XV_Q¥_]]_QU[XX¥]V[RZU]XXYVVU
@HWI-EAS443:3:61:1506:692#0/1
CCCTTGCCCAATTAGCACATTAGCTTTCACTCCTTTTGCA
+HWI-EAS443:3:61:1506:692#0/1
bbbbbbbbbbbbbbabbbbbbbbbba`K`abXaaa`aV
@HWI-EAS443:3:62:1368:1915#0/1
GAGCAATCTTGCAAAAGGAGAGAAAGCTAATGTGGTAATT
+HWI-EAS443:3:62:1368:1915#0/1
UTM¥`bbb[aba_aababb_aba[baTbba_aI_LSZbba
@HWI-EAS443:3:62:1668:928#0/1
GAGAAAGCTAATGTGCCAATTGGGCAAGGGGACGTAGTGT
+HWI-EAS443:3:62:1668:928#0/1
`Z_^a[`aa][`^¥`a¥¥_a`]_]¥TU`a^`Z``V¥`[a¥
@HWI-EAS443:3:62:595:97#0/1
GCAAAAGGAGAGGAAGCTAATGTGCTAATTGGGCAAGGGG
+HWI-EAS443:3:62:595:97#0/1
`TLZ]]__Y_]_MUY^VYXY¥VVXY]QYVVVVUYXVYYYU
@HWI-EAS443:3:63:1047:840#0/1
CAATCTTGCAAAAGGAGAGAAAGCTAATGGGCTAATTGGG
+HWI-EAS443:3:63:1047:840#0/1
]Y]]¥aXSa_X[W_aYQ]a_^L[_SRGTPHXZ_VQXGYJ[
@HWI-EAS443:3:63:372:2031#0/1
AAGGAGAGAAAGCAAATGTGCTAATTGGGCAAGGGGACGT

```
+HWI-EAS443:3:63:372:2031#0/1
aaV`b`a`a_^U_`aa_aW`^^Y`_VYY¥`aTT]^^]]]Z
@HWI-EAS443:3:63:437:1104#0/1
TGCAAAAGGAGAGAAAGCTAATGTGCTCATTGGGCAAGGG
+HWI-EAS443:3:63:437:1104#0/1
abaOJZ[WZWX]X_a^R]]][`Z_``OI¥¥_¥OGa^T^^X
@HWI-EAS443:3:64:1519:1839#0/1
GAAAGCTAATGGGCTAATTGGGCAAGGGGACGTAGTGTTG
+HWI-EAS443:3:64:1519:1839#0/1
ab[a_a[`_b^H[aaaabS`YJa``aab_^H`_RTPaRY¥
@HWI-EAS443:3:64:942:252#0/1
AGCTAATGTGCTAATTGGGCAAGGGGAAGTAGTGTTGGTA
+HWI-EAS443:3:64:942:252#0/1
^_GR^_`[YbZUU[_X[WTXS`U`UYJD¥R`ZF]Oaa¥[S
@HWI-EAS443:3:67:1126:495#0/1
GCAAAAGGAGAGAAAGCTAATGTGCTATTTGGGCAAGGGG
+HWI-EAS443:3:67:1126:495#0/1
aab`b`b_P¥_aaa`b¥a]aaa[a^aa[a_b`a]``a_aa
@HWI-EAS443:3:67:957:1835#0/1
GAAAGCTAACGTGCTAATTGGGCAAGGGGACGTAGTGTTG
+HWI-EAS443:3:67:957:1835#0/1
¥¥bXRZ`bb_VR_X¥aab`aa`bb_]ab`RF_[_bQ`Waa
@HWI-EAS443:3:69:1688:1642#0/1
GTCCCCTTGCCCAATTAGCACATTAGCTTTCTATCCTTTT
+HWI-EAS443:3:69:1688:1642#0/1
^`W`]`babbb`aababababa^bbbbb¥a`b``I`a`bbbb
@HWI-EAS443:3:70:1364:777#0/1
GAGCAATCTTGCAAAGGGAGAGAAAGCTAATGTGCTAATT
+HWI-EAS443:3:70:1364:777#0/1
]bbabbbbbbbabaa`bb`bbbabab_babbbab]ab`aa
@HWI-EAS443:3:70:659:874#0/1
AGCTAATGAGCTAATTGGGCAAGGGGACGTAGTGTTGGTA
+HWI-EAS443:3:70:659:874#0/1
_`U_]`baSY_]_Y¥`bbYaa_Z]aaS``Ga`GaS`a^^W
@HWI-EAS443:3:75:1655:1909#0/1
ATGAACTGAGCAATCTTGCAAAAGCAGAGAAAGCTAATGT
+HWI-EAS443:3:75:1655:1909#0/1
Ub__^N`bX]O_]^¥aYU]aXYUWI¥`ab]Yaa¥]aSPPP
@HWI-EAS443:3:76:1147:25#0/1
CAAAAGGAGAGAAAGCTAATGCGCTAATTGGGCAAGGGGA
+HWI-EAS443:3:76:1147:25#0/1
`baYYbbabbba_^b]ba`a[D¥_a_aa``aaP__a``a[
@HWI-EAS443:3:76:193:973#0/1
ATCTTGCAAACGGAGAGAAAGCTAATGTGCTAATTGGGCA
+HWI-EAS443:3:76:193:973#0/1
a`aa`U^RZSRZ[``XTZT¥`^[T[[VU[R[OMVZYVYUX
@HWI-EAS443:3:76:212:618#0/1
CATCAATGAACTCAGCAATCTTGCAAAAGGAGAGAAAGCT
+HWI-EAS443:3:76:212:618#0/1
_`a^a]__bbb_K_aaba^^aZaa¥Z¥]a`a`a``WWZ]`
```

@HWI-EAS443:3:76:337:437#0/1
GCATCAATGAACTGAGCAATCTTGCAGAAGGAGAGAAAGC
+HWI-EAS443:3:76:337:437#0/1
`RW__R_I¥`¥W``S``_aW_^]a¥PGQQU[RZU___^_]
@HWI-EAS443:3:77:164:1365#0/1
GAGAAAGCTAATGTGCTAATTGAGCAAGGGGACGTAGTGT
+HWI-EAS443:3:77:164:1365#0/1
aa^a¥YXT¥abZb¥¥b_aa``_H_aaa___`S``^a_W`Q
@HWI-EAS443:3:77:399:1707#0/1
AAAGGAGAGAAAGCTAATGAGCTAATTGGGCAAGGGGACG
+HWI-EAS443:3:77:399:1707#0/1
Y^XVOXX^XZ`T¥¥YWRX]FZ][VSZV[_KZGZ¥OP¥W[V
@HWI-EAS443:3:78:442:174#0/1
AGCTAATGTGGTAATTGGGCAAGGGGACGTAGTGTTGGTA
+HWI-EAS443:3:78:442:174#0/1
Obba`_bb`aaaabaabb`a``abba^ab¥`aOa_aaaU^
@HWI-EAS443:3:78:844:1433#0/1
GAAAGCTAATGTGCTAATTGGGCAAGAGGACGTAGTGTTG
+HWI-EAS443:3:78:844:1433#0/1
]a_aa``Zab`]Zaaababa_`b```Y`^aaa^^a_b`a`
@HWI-EAS443:3:79:1030:871#0/1
AGCTAATGCGCTAATTGGGCAAGGGGACGTAGTGTTGGTA
+HWI-EAS443:3:79:1030:871#0/1
]^YaX^U^F^PQVPUV]T`^HP[]RV[``OV`G`V_^]G_
@HWI-EAS443:3:79:1297:399#0/1
GAGCAATCTTGCAAAAGGAGAGAAAGCTAGTGTGCTAATT
+HWI-EAS443:3:79:1297:399#0/1
¥F¥U]_`aa^^W¥_VO^___T¥[^]a_Y]GX^RYUPUR^S
@HWI-EAS443:3:79:248:793#0/1
AGCTCATGTGCTAATTGGGCAAGGGGACGTAGTGTTGGTA
+HWI-EAS443:3:79:248:793#0/1
]a^WJP[_[W`_TZ_UZXW`YSZbaa]a]ML^]a__U^VG
@HWI-EAS443:3:79:641:1847#0/1
GAACTGAGCAATCTTGCAAAAGGAGAGGAAGCTAATGTGC
+HWI-EAS443:3:79:641:1847#0/1
ZT^Z_a^X¥_^X[]W_^¥X¥Y_XO^``XZTZ]W]XVYMY`
@HWI-EAS443:3:7:891:981#0/1
CAATCTTGCAAAAGGAGGGAAAGCTAATGTGCTAATTGGG
+HWI-EAS443:3:7:891:981#0/1
a_baaabbbbbb]bbaabbbaabaaaaba_baaaa`_a`a
@HWI-EAS443:3:80:621:1543#0/1
GAAAGCTAATGTGCTAATTGGGCAAGGGGACGCAGTGTTG
+HWI-EAS443:3:80:621:1543#0/1
`SSVPX¥SWZYYQ]TRYX¥^T]][T¥[ZXV`PDRXZaBBB
@HWI-EAS443:3:82:456:441#0/1
GAAAGCTAATGTGCTAATTGGGCAAGGGGACGCAGTGTTG
+HWI-EAS443:3:82:456:441#0/1
aa`^ba`]VaaXa_a¥^`aaa`a]_aa¥__`a`Z¥R_]Za
@HWI-EAS443:3:83:1182:284#0/1
GAAAGCTAATGTGCTAATTGGGCAAGGGGACGCAGTGTTG

```
+HWI-EAS443:3:83:1182:284#0/1
]a^_]TWWX¥`[XDWROR[]]```Q^^XVWN_FZ[R`T¥Q
@HWI-EAS443:3:83:394:56#0/1
GAAAGCTAATGTGCTAATTGAGCAAGGGGACGTAGTGTTG
+HWI-EAS443:3:83:394:56#0/1
P]XbbabXbbb_baab[b¥_`b`¥`^`abaabW_babbaa
@HWI-EAS443:3:83:692:1327#0/1
ATCTTGCAAAAGGAGAGAAAGCTAATGTGATAATTGGGCA
+HWI-EAS443:3:83:692:1327#0/1
W_[a```[VWG^`Wa^W¥XTQ^_`]_aZZFFPVV_¥^XGF
@HWI-EAS443:3:84:1207:725#0/1
GAGAAAGCTAATGTGCTAATTGGGCAAGGTGACGTAGTGT
+HWI-EAS443:3:84:1207:725#0/1
aabbbabaaaaaa]`aaaaaaa`a```^`Ya``a^``^a^
@HWI-EAS443:3:84:388:674#0/1
ATTAGCTTTCTCTCCTTTTGCAAGATTTCTCAGTTCATTG
+HWI-EAS443:3:84:388:674#0/1
aaaaaaaaaaaaa```ab_aaa`_`¥__``_^_^a__a^`
@HWI-EAS443:3:84:474:1951#0/1
GAGAAAGCTAATGTGCTAATTGGGCAAGGGGACGCAGTGT
+HWI-EAS443:3:84:474:1951#0/1
_`a_U]X_ba]¥¥]ab]]X`abaa]ab_`X]^baG]aH]P
@HWI-EAS443:3:85:488:819#0/1
ACTGAGCAATCTTGCAAACGGAGAGAAAGCTAATGTGCTA
+HWI-EAS443:3:85:488:819#0/1
`W¥¥Za]Z¥L]¥¥aaQU[G]VR[U¥VVY]aY[VXZNSRSS
@HWI-EAS443:3:85:546:1695#0/1
ATCTTGCAAAAGGAGAGAAAGCTAATGTGCTAGTTGGGCA
+HWI-EAS443:3:85:546:1695#0/1
aaba[[`a`aa_[a`aaaaT`aaa[a__a`aYFY_a_¥_^
@HWI-EAS443:3:86:1368:1954#0/1
CATCAATGAACTGAGGAATCTTGCAAAAGGAGAGAAAGCT
+HWI-EAS443:3:86:1368:1954#0/1
_b_¥b_abb_U¥bb_SVaaJaabaW`aa`babaaa¥aZY`
@HWI-EAS443:3:86:1368:713#0/1
GAGCAATCTTGCAAAAGGAGAGAAAGCTAATGTGATAATT
+HWI-EAS443:3:86:1368:713#0/1
`_V`bb``abYM¥¥`U_`^^¥bbaaa^babb^^bObabaa
@HWI-EAS443:3:88:519:416#0/1
GAGAAAGCTAATGTGCTACTTGGGCAAGGGGACGTAGTGT
+HWI-EAS443:3:88:519:416#0/1
aaa]]``ba[T_]]^`a_H_X`¥Vaaa`]`YGab_[aP`Z
@HWI-EAS443:3:89:1289:860#0/1
GAAAGCTAATGTGCTAATTGGGCAAGGGGACCTAGTGTTG
+HWI-EAS443:3:89:1289:860#0/1
`b_^aaba^bb`baba^ba`aa[aab`aa`[H[aa^a^aa
@HWI-EAS443:3:89:990:1295#0/1
GAAAGCTAATGTGCTAATTGGGCAAGGGGAGGTAGTGTTG
+HWI-EAS443:3:89:990:1295#0/1
ab`¥bb_IWb`W^`ba^[`¥X`^`abb`b[H`_aaSa^aa
```

```
@HWI-EAS443:3:8:1270:678#0/1
GAAAGCTAATGTGCTAATTGGGCAAGGGGACGTAGAGTTG
+HWI-EAS443:3:8:1270:678#0/1
^^aaba`^`_aXaa`]``_`_aa__]X`a_GaSZ^F^N^a
@HWI-EAS443:3:8:1471:1993#0/1
GAGCAATCTTGCAAAAGGAGAGAAAGCTAATGTGGTAATT
+HWI-EAS443:3:8:1471:1993#0/1
GZa[bV]ZZVbbaaaab[_ababUb[[b`b[a_[I[_aVb
@HWI-EAS443:3:8:1485:1581#0/1
AGCTAATGGGCTAATTGGGCAAGGGGACGTAGTGTTGGTA
+HWI-EAS443:3:8:1485:1581#0/1
V^J`a_aaT^P`¥a``ZabG``a`aaWa`O__T`]_aVGW
@HWI-EAS443:3:90:1301:442#0/1
AGCAATCTTGCAAAAGGAGAGAAAGCTAATGTGGTAATTG
+HWI-EAS443:3:90:1301:442#0/1
`abbb``a`Y]`_]^bb_¥a``abaXabab`¥^F¥abaa^
@HWI-EAS443:3:90:923:1080#0/1
CCCTTGCCCAATTAGCACATTAGCTTTCTCACCTTTTGCA
+HWI-EAS443:3:90:923:1080#0/1
aa^`Xabbab^aab`aaaabaabaa]`^`^I``aa_aaaa
@HWI-EAS443:3:91:1702:466#0/1
GAAAGCTGATGTGCTAATTGGGCAAGGGGACGTAGTGTTG
+HWI-EAS443:3:91:1702:466#0/1
`ba_a`]GZ]a`VYU_[[XaUaOSKZ_a^HS[HQaZ_¥BB
@HWI-EAS443:3:92:1608:1938#0/1
GCATCAATGGACTGAGCAATCTTGCAAAAGGAGAGAAAGC
+HWI-EAS443:3:92:1608:1938#0/1
ab_a`aY`bK`P`_U`Ja`aWYab_]_aaaa^a`a`G__Z
@HWI-EAS443:3:93:641:1136#0/1
ATTAGCACATTAGCTTTCTCTCCTTTTGCAAGGTTGCTCA
+HWI-EAS443:3:93:641:1136#0/1
aaW]`_S[`aaaaa_ba_YK[`a`_^_b`[¥_]S]^_^X_
@HWI-EAS443:3:94:1168:1450#0/1
ACCAACACTACGGCCCCTTGCCCAATTAGCACATTAGCTT
+HWI-EAS443:3:94:1168:1450#0/1
aa_a^`b[abb_RJ]aa`¥^¥S^`_[¥``R_UaXM[aG__
@HWI-EAS443:3:94:1276:745#0/1
AGGAGAGAAAGCTAATGTGATAATTGGGCAAGGGGACGTA
+HWI-EAS443:3:94:1276:745#0/1
aba_]¥_a^_b````aa[ZJYRa_[][[]U^a__]WQ]]X
@HWI-EAS443:3:94:624:1853#0/1
GAGAAAGCTAATGTGCTAATTGCGCAAGGGGACGTAGTGT
+HWI-EAS443:3:94:624:1853#0/1
`¥¥]_¥Vaaaa¥TV``ab^aa¥XaW_a]RSTZ`_WY_W_Z
@HWI-EAS443:3:95:1683:1445#0/1
CAATGAACTGAGCAATCTTGCAAAAGGAGAGAAAGTTAAT
+HWI-EAS443:3:95:1683:1445#0/1
_X_[X¥ab¥aabb`_aW^¥_[bbSWZG]`a^abbYDO[a¥
@HWI-EAS443:3:95:35:499#0/1
CAACACTACGTCCCCTTGCCCAATTGGCACATTAGCTTTC
```

+HWI-EAS443:3:95:35:499#0/1
abbbbbbbab^aabbaa`]ab^a`_aaaa`aaaab¥[__[
@HWI-EAS443:3:96:1508:2044#0/1
GAGCAATCTTGAAAAAGGAGAGAAAGCTAATGTGCTAATT
+HWI-EAS443:3:96:1508:2044#0/1
ZVabb_¥S¥a_RT`a`ab`aa_ba_aSYLX`a^a¥a_R¥a
@HWI-EAS443:3:96:1597:972#0/1
AAGCTAATGTGGTAATTGGGCAAGGGGACGTAGTGTTGGT
+HWI-EAS443:3:96:1597:972#0/1
aa_L]Y¥¥a[_TXT`Z`aaaY`S__YZOSaY[b]__a`aP
@HWI-EAS443:3:96:1717:244#0/1
GAGAAAGCTAATGTGCTAATTGGGCAAGGGAACGTAGTGT
+HWI-EAS443:3:96:1717:244#0/1
V_aS]¥UPXbNUW`_Yaa[RMF[^UUVNaTNJYa_Q`BBB
@HWI-EAS443:3:96:476:943#0/1
ATCAATGAACCGAGCAATCTTGCAAAAGGAGAGAAAGCTA
+HWI-EAS443:3:96:476:943#0/1
abVbba_ab_b]_K[aX`J``S^_¥TWY_V___`^Z_V__
@HWI-EAS443:3:97:1081:1374#0/1
GAGAAAGCTAATGTGCTAATTGGGCAAGGGGAAGTAGTGT
+HWI-EAS443:3:97:1081:1374#0/1
`W]_a^UZ____aHY__^SaRYNWW¥_^PS[_GRU¥aVGQ
@HWI-EAS443:3:97:1368:865#0/1
AGCTAATGTGATAATTGGGCAAGGGGACGTAGTGTTGGTA
+HWI-EAS443:3:97:1368:865#0/1
_RQa^Z[a`^GZ]Xa__a^QWWa`_aT^^]Zb_a]Y_[SY
@HWI-EAS443:3:97:1518:1176#0/1
AATGAACTGAGCAATCTTGGAAAAGGAGAGAAAGCTAATG
+HWI-EAS443:3:97:1518:1176#0/1
abZbb`Q¥aa^TX]Va^a]_`ba`¥¥ZK^a¥W_V_aK¥]P
@HWI-EAS443:3:97:44:1039#0/1
TAGCACATTAGCTCTCTCTCCTTTTGCAAGATTGCTCAGT
+HWI-EAS443:3:97:44:1039#0/1
aa`ab`abb^b`¥N__a`_^_aa_``]__a___a¥W_Z_W
@HWI-EAS443:3:97:998:443#0/1
GAAAGCTAATGTGCTAATTGGGCAAGGGGGCGTAGTGTTG
+HWI-EAS443:3:97:998:443#0/1
aaaaa_aa`a``a]aaabaaaa^_¥aaaXaZ_¥___a^a^
@HWI-EAS443:3:98:109:1563#0/1
CATCAATGAACTGAGCAATCTTGCAAGAGGAGAGAAAGCT
+HWI-EAS443:3:98:109:1563#0/1
aa^V`bbb_QTQ^Saa_HU]Y]`W][DW_aYY``a`VR_a
@HWI-EAS443:3:98:1285:17#0/1
CAATCTTGCAAATGGAGAGAAAGCTAATGTGCTAATTGGG
+HWI-EAS443:3:98:1285:17#0/1
UW]¥UabbbbVaS`abba]ba]b`ababOEUI]bM_S`bU
@HWI-EAS443:3:98:1529:871#0/1
AGCTAATGTGGTAATTGGGCAAGGGGACGTAGTGTTGGTA
+HWI-EAS443:3:98:1529:871#0/1
a_W^bbb```L_HVb_[][aY¥abbaa]`¥SbGbWa`aZI

@HWI-EAS443:3:98:1654:1739#0/1
CAATCTTGCAAAAGGAGAGAAAGGTAATGTGCTAATTGGG
+HWI-EAS443:3:98:1654:1739#0/1
]b_^FYbbaa_[abab_[a__b_QbWbbbaWIU][a_U^b
@HWI-EAS443:3:98:627:51#0/1
AATGAACTGAGCAATATTGCAAAAGGAGAGAAAGCTAATG
+HWI-EAS443:3:98:627:51#0/1
X_`bVURV`¥`ZXb^FYa`¥R`Z_bb]baba_aaP__^ab
@HWI-EAS443:3:99:1413:1938#0/1
GAGCAATCTTGCGAAAGGAGAGAAAGCTAATGTGCTAATT
+HWI-EAS443:3:99:1413:1938#0/1
_U_[^b_Ybaa]_aa_b]M]a_b[IaNa_^_TEYD[a__a
@HWI-EAS443:3:99:1546:1031#0/1
GAGAAAGCTAATGTGCTAATTGGGAAAGGGGACGTAGTGT
+HWI-EAS443:3:99:1546:1031#0/1
abaabb`_bbbabaaT`]Zbbb¥_JYYTWbbGY]JYbGV`
@HWI-EAS443:3:9:1151:1171#0/1
TGAGCAATCTTGCAAAAGGAGAGGAAGCTAATGTGCTAAT
+HWI-EAS443:3:9:1151:1171#0/1
aaaa[aabaabaaaaaaba`b`]F^_a^aa_aa]`Y`^_a
@HWI-EAS443:3:9:1390:240#0/1
GAGCAATCTTGCAAAAGGAGAGAAAGGTAATGTGCTAATT
+HWI-EAS443:3:9:1390:240#0/1
a_^X_Y`X[^^WUV^]^aY_^]YYVXGXVRY^V]XKMLNB
@HWI-EAS443:3:9:489:1626#0/1
GAAAGCTAATGTGCTAATTGGGCAAGGGGACGTAGAGTTG
+HWI-EAS443:3:9:489:1626#0/1
aaa`a^ZY`^HP`¥_^a¥^¥WRZ^¥a]^]]G_W[YGPRUG
@HWI-EAS443:3:14:1387:1254#0/1
CATCAATGAACTGAGCAATCTTGCAAAAGGAGAGAAACAT
+HWI-EAS443:3:14:1387:1254#0/1
aaaaaabaaa_ba`__a`a`aaa``_``aa^a``_aU]KV
@HWI-EAS443:3:18:1158:1167#0/1
AGCTAATGTGCTAATTGGGCAAGGGGACGTAGTGTTGNGA
+HWI-EAS443:3:18:1158:1167#0/1
aaaWaY_OJ`QY^U`[^aZQN`^¥b]Y`^X¥aVaT]]DFU
@HWI-EAS443:3:19:716:400#0/1
AGCTAATGTGCTAATTGGGCAAGGGGACGTAGTGTTGAAA
+HWI-EAS443:3:19:716:400#0/1
_aabbbbbaa^b`abbbaaa`ababa`ab_`aXb`a`^aa
@HWI-EAS443:3:20:829:1141#0/1
ACAAGAGAAAGCTAATGTGCTAATTGGGCAAGGGGACGTA
+HWI-EAS443:3:20:829:1141#0/1
U`_Z`aaaa`]``[`aa__Za__`a__`_```a`__^_VW
@HWI-EAS443:3:23:1152:346#0/1
TGGCCCCTTGCCCAATTAGCACATTAGCTTTCTCTCCTTT
+HWI-EAS443:3:23:1152:346#0/1
ZRG[_Yaa`^a¥[aPYT^X]_]_a]WYY¥[_[_¥^_]_]^
@HWI-EAS443:3:23:52:1274#0/1
AGCTAATGTGCTAATTGGGCAAGGGGACGTAGTGTTGAAA

```
+HWI-EAS443:3:23:52:1274#0/1
Vaaaa_ba[aabbaaa``abaaa[aa`aaW``¥aaaa_aa
@HWI-EAS443:3:28:53:506#0/1
GAGCAATGAACTGAGCAATCTTGCAAAAGGAGAGAAAGCT
+HWI-EAS443:3:28:53:506#0/1
a]P_a^a`¥abZZ¥_a_V[`a_aa`_]]Z]]aa`¥]RZ``
@HWI-EAS443:3:2:860:959#0/1
AATGAACTGAGCAATCTTGCAAAAGGAGAGAAAGCTAGTG
+HWI-EAS443:3:2:860:959#0/1
R_¥¥abb`^^_H_b_bYT^^Y¥OO_a¥`^[`bXYKRYGTB
@HWI-EAS443:3:46:1198:1618#0/1
ACAAGAGAAAGCTAATGTGCTAATTGGGCAAGGGGACGTA
+HWI-EAS443:3:46:1198:1618#0/1
aab_aaYaa``ZU]``aX]¥_`aa`aaZ_^Ua`a_WX¥UV
@HWI-EAS443:3:4:1239:1581#0/1
GAGAAAGCTAATGTGCTAATTGGGCAAGGGGACGTAGAGT
+HWI-EAS443:3:4:1239:1581#0/1
_Z[IWUaSZU_L^Z`__UWW_NT_Z[Y_YNZBBBBBBBBB
@HWI-EAS443:3:4:1307:759#0/1
ACAAGAGAAAGCTAATGTGCTAATTGGGCAAGGGGACGTA
+HWI-EAS443:3:4:1307:759#0/1
a`aTY[a`Ya`___^_a]`_`_¥][`]]V^V_Z]^VX_UY
@HWI-EAS443:3:54:1627:1688#0/1
GAACTGAGCAATCTTGCAAAAGGAGAGAAAGCTAATGCGC
+HWI-EAS443:3:54:1627:1688#0/1
a_^`aaYaaa`a_aa__^^]¥aa]_`a_^_^]__]_^V_V
@HWI-EAS443:3:58:565:471#0/1
ACAAGAGAAAGCTAATGTGCTAATTGGGCAAGGGGACGTA
+HWI-EAS443:3:58:565:471#0/1
]^aZa]a¥QXa_aZZab]a[`a¥a```a^aaa``aaa`¥]
@HWI-EAS443:3:63:26:364#0/1
GANCAATCTTGCAAAAGGAGAGAAAGCTAATGTGCTAATT
+HWI-EAS443:3:63:26:364#0/1
_]D^_babaaab`aa]_aab_bUa`aaa¥S¥a]_a`_`_Z
@HWI-EAS443:3:67:1153:52#0/1
AGCTAATGTGCTAATTGGGCAAGGGGACGTAGTGTTGAAA
+HWI-EAS443:3:67:1153:52#0/1
aUYbb^b`QS[aa`a¥ab`[a_aaabZG_`[aXb]a__a`
@HWI-EAS443:3:72:1290:1982#0/1
GAGCAATCTTGCAAAAGGAGAGAAAGCTAATGTGCTATTG
+HWI-EAS443:3:72:1290:1982#0/1
_¥`^`bbbba_a`b_XW_baababb]`a]aY¥^[_^bbab
@HWI-EAS443:3:73:1128:1659#0/1
ACAAGAGAAAGCTAATGTGCTAATTGGGCAAGGGGACGTA
+HWI-EAS443:3:73:1128:1659#0/1
aaa`bbbbaa^aaaaaa^a``aaaaa_a_a_`a__]_aX_
@HWI-EAS443:3:76:452:832#0/1
ACAAGAGAAAGCTAATGTGCTAATTGGGCAAGGGGACGTA
+HWI-EAS443:3:76:452:832#0/1
^a]aaabb`aaba^__^¥``a`a`Z_^aa`aaa_a`[a^_
```

```
@HWI-EAS443:3:78:475:1206#0/1
GTCCCCTTGCCCAATTAGCACATTAGCTTTCTCTCCTCTT
+HWI-EAS443:3:78:475:1206#0/1
]^^aab_KW^bb[baabaa`W`a_a[`_¥¥b¥a_^Y`G`_
@HWI-EAS443:3:84:979:1144#0/1
GACAAAGCTAATGTGCTAATTGGGCAAGGGGACGTAGTGT
+HWI-EAS443:3:84:979:1144#0/1
`_R`_``_a`aab_aba`Y]a`^]_a`aa_`W_aZ^aX`X
@HWI-EAS443:3:88:1548:1566#0/1
GANAGCTAATGTGCTAATTGGGCAAGGGGACGTAGTGTTG
+HWI-EAS443:3:88:1548:1566#0/1
a^D]baababaa_abbabbbaa]`_b`_a_`ba`a_``ab
@HWI-EAS443:3:89:1769:1778#0/1
ACAAGAGAAAGCTAATGTGCTAATTGGGCAAGGGGACGTA
+HWI-EAS443:3:89:1769:1778#0/1
a_abb_ababaG`aa_bXba`a_a_aaa`a`aaaa^_[MS
@HWI-EAS443:3:8:914:897#0/1
AGCTAATGTGCTAATTGGGCAAGGGGACGTAGTGTTGTTA
+HWI-EAS443:3:8:914:897#0/1
]OWMV[][FR`Z__¥]]Z[Z]XU[¥XLRXPZYQTY¥PDNQ
@HWI-EAS443:3:95:1454:1207#0/1
GAAAGCTAATGTGCTAATTGGGCAAGGGGACGTAGTGNTG
+HWI-EAS443:3:95:1454:1207#0/1
UX¥T`_ba]`¥I¥[_¥¥```¥ab_baabbK]`aX`_[DZ`
@HWI-EAS443:3:96:412:243#0/1
CATCAATGAACTGAGCAATCTTGCAAAAGGAGAGAAANCT
+HWI-EAS443:3:96:412:243#0/1
[aba``bb¥babbbbb`R[babbabaaabb_aabaa]DYa
@HWI-EAS443:3:100:553:1177#0/1
CCCTTNCCCAATTAGCACATTAGCTTTCTCTCCTTTTGCA
+HWI-EAS443:3:100:553:1177#0/1
a`b][D[HYaaa`a^XY^aaa_bTaZb_aa`TVJU___`a
@HWI-EAS443:3:10:854:687#0/1
ATCAATGAACTGAGCAATCTTGCAAAAGGNGAGAAAGCTA
+HWI-EAS443:3:10:854:687#0/1
a`b`Z[_^a[ZTaaaaYaTaab_UY^_^[D^`aaYS_OW_
@HWI-EAS443:3:12:1109:1376#0/1
AGCTAATGTGCTANTTGGGCAAGGGGACGTAGTGTTGGTA
+HWI-EAS443:3:12:1109:1376#0/1
SYX_`__`aba¥YD^`a`bb[`bYba[_a¥aaJ[aa[aHI
@HWI-EAS443:3:13:629:668#0/1
ATTAGCTTTCTCTCCTTTTGCAAGATTNCTCAGTTCATTG
+HWI-EAS443:3:13:629:668#0/1
aaaaaa_a``aaaa_`aaa``a_a_]YDW__^_^_^__^_
@HWI-EAS443:3:13:740:1239#0/1
GAGCAATCTTGCNAAAGGAGAGAAAGCTAATGTGCTAATT
+HWI-EAS443:3:13:740:1239#0/1
_`^`_`a]b^a^D]]Xaa`aaaaaZ``aaX¥[T`_`_`__
@HWI-EAS443:3:16:1371:1500#0/1
GAACTGAGCAATCTTGNAAAAGGAGAGAAAGCTAATGTGC
```

```
+HWI-EAS443:3:16:1371:1500#0/1
a]a^]````a]]Y__XDW¥WU__Ya^`_X^[ZW^]__YYW
@HWI-EAS443:3:17:1148:1637#0/1
CATCAATGAACTGNGCAATCTTGCAAAAGGAGAGAAAGCT
+HWI-EAS443:3:17:1148:1637#0/1
a^[`YYbaX¥V]ZDYaa]aaa^_aaZ¥^b``a`aa`^]`_
@HWI-EAS443:3:19:1018:654#0/1
AAAGCTAATGNGCTAATTGGGCAAGGGGACGTAGTGTTGG
+HWI-EAS443:3:19:1018:654#0/1
a`¥a__^^`^D^]``^`aa``^`Z_]_`]¥a]Z_ZaZ__`
@HWI-EAS443:3:1:1006:543#0/1
GAACTGAGCAATCNTGCAAAAGGAGAGAAAGCTAATGTGC
+HWI-EAS443:3:1:1006:543#0/1
a¥^W]]YXZ_a`XDS]Y¥_¥X`XY^`a`_¥_Z`^^``YBB
@HWI-EAS443:3:23:945:1633#0/1
GAACTGAGCAATCTTGCAAAAGGAGAGAAAGCTAATNTGC
+HWI-EAS443:3:23:945:1633#0/1
``ba_aa¥]aaaaa_a``a_```^`a]a`a_^__]XDE^^
@HWI-EAS443:3:26:640:1539#0/1
GAGCAATCTTGCAAAAGGAGAGAAAGCTAATGTNCTAATT
+HWI-EAS443:3:26:640:1539#0/1
__¥aZ_^ab`ba^``aa_`aa_ab^aa`aa]]DD[^^¥_]
@HWI-EAS443:3:30:1274:1061#0/1
AGCAATCTTGCAAAAGGAGAGAAAGCTAANGTGCTAATTG
+HWI-EAS443:3:30:1274:1061#0/1
aaaa`aaa_ab^a``aa]a`_a]]a^aaUDPU_T[_U_[[
@HWI-EAS443:3:30:1557:678#0/1
GAAAGCTAATGTGCTAANTGGGCAAGGGGACGTAGTGTTG
+HWI-EAS443:3:30:1557:678#0/1
ababa_^a[ba_a`a_¥D¥a^^^`W`^aa^__]_^YXMT`
@HWI-EAS443:3:33:1679:1982#0/1
GAGCAATCTTGCAAAAGGAGAGAAAGNTAATGTGCTAATT
+HWI-EAS443:3:33:1679:1982#0/1
[abaababb`Y`aaabaa^abbbba^D[abb_Xbaa`aa_
@HWI-EAS443:3:43:1425:880#0/1
ACGTCCCCTTGCCCAATTAGCNCATTAGCTTTCTCTCCTT
+HWI-EAS443:3:43:1425:880#0/1
a`aa__aaa`_^[``aaa``WDSaaa`]__aa]a[a¥V_a
@HWI-EAS443:3:47:1378:1421#0/1
CATCAATGAACTGAGCAATCTTGCAANAGGAGAGAAAGCT
+HWI-EAS443:3:47:1378:1421#0/1
TXaS`ZQYa`abbaba__Z`aa`a^^D^`b^bbbaX^a^^
@HWI-EAS443:3:49:917:1149#0/1
GCATCAATGAACTGAGCAATCTTGCAAAAGGANAGAAAGC
+HWI-EAS443:3:49:917:1149#0/1
a`R`[_aaab`^_`J_`a```aaaa^T¥H_[[DXV^`¥`¥
@HWI-EAS443:3:51:375:1619#0/1
GAACTGAGCAATCTTGCAAAAGGNGAGAAAGCTAATGTGC
+HWI-EAS443:3:51:375:1619#0/1
^`baabaababbbbbbaba_a`ZDUa]bbbbaababa_aa
```

```
@HWI-EAS443:3:57:591:1102#0/1
GAGCAATCTTNCAAAAGGAGAGAAAGCTAATGTGCTAATT
+HWI-EAS443:3:57:591:1102#0/1
aa¥bbbabb^DWaa^aaaaaaaa`_``aa]abZa`a`_aa
@HWI-EAS443:3:60:551:1219#0/1
CATCAATGAACTGAGCAATCTTGCAAAAGGAGNGAAAGCT
+HWI-EAS443:3:60:551:1219#0/1
]babbaa^a`aba_`^a`ba`ab]]ba`a`a]D¥`ba`aa
@HWI-EAS443:3:67:1051:1464#0/1
AGCACATTAGCTTTCTCTCCTTTTGCAAGATTGCTNAGTT
+HWI-EAS443:3:67:1051:1464#0/1
aa`bb``a[[`a`a_```aa_`a^X`a[[¥_`][RDY_Z^
@HWI-EAS443:3:69:1442:762#0/1
CAATCTTGCAAAAGGAGNGAAAGCTAATGTGCTAATTGGG
+HWI-EAS443:3:69:1442:762#0/1
^¥`bb][a`]aaabba[D^aZaa¥aaa`aWaaaY`aaaaa
@HWI-EAS443:3:72:1040:1760#0/1
GAAAGCTAATGTGCTAATTGGGCAAGGGGANGTAGTGTTG
+HWI-EAS443:3:72:1040:1760#0/1
_bababb__bbQ`aaabbaa[Raa`a^XW[DZZSaVa_`a
@HWI-EAS443:3:77:596:1413#0/1
AGCTAATGTGCTAATTGGGCAAGGGGACGTANTGTTGGTA
+HWI-EAS443:3:77:596:1413#0/1
a``baaaa[`aaaaaaa_aaaaa`aa`_`_XDP__a`YS`
@HWI-EAS443:3:79:1253:860#0/1
GAGAAAGCTAATGTGCTAATTGGNCAAGGGGACGTAGTGT
+HWI-EAS443:3:79:1253:860#0/1
aW]UZN]__[U^]ST]`V]QS^XDW[[`TPUZQ^NN]V^X
@HWI-EAS443:3:79:1435:1615#0/1
AGCAATCTTNCAAAAGGAGAGAAAGCTAATGTGCTAATTG
+HWI-EAS443:3:79:1435:1615#0/1
__aaa`Ya]D¥¥Y`_bba]b`a``bWb[_a`abW`_W_[_
@HWI-EAS443:3:80:1288:1634#0/1
AGCACATTAGCTTTCTCNCCTTTTGCAAGATTGCTCAGTT
+HWI-EAS443:3:80:1288:1634#0/1
_aa__abaaa_aba_b[DW]abaa__`_a``a`_a^_`]`
@HWI-EAS443:3:81:90:1517#0/1
ATCTTGCAAAAGGAGAGAAAGCTAATGTGCTAATTGNGCA
+HWI-EAS443:3:81:90:1517#0/1
a_a````a_`____`a_`V`__^_X^ZT_^_^^^]TDR_^
@HWI-EAS443:3:85:303:1241#0/1
GTCCNCTTGCCCAATTAGCACATTAGCTTTCTCTCCTTTT
+HWI-EAS443:3:85:303:1241#0/1
^^b]D^ababb`]`abaabaZaaaaaaaaaaa]`aaa`^`
@HWI-EAS443:3:87:71:1911#0/1
GGAGAGAAAGCTAATGTGCTAATTGGGCNAGGGGACGTAG
+HWI-EAS443:3:87:71:1911#0/1
`baababaa]`a`aa_Z`^a_`_^S^_XDW¥S_]X^VLUR
@HWI-EAS443:3:88:497:1436#0/1
CAATGANCTGAGCAATCTTGCAAAAGGAGAGAAAGCTAAT
```

```
+HWI-EAS443:3:88:497:1436#0/1
_``_Y]D[]Z_¥_aW^b¥VS`]QZU¥¥`a¥``^a`aa^QX
@HWI-EAS443:3:88:531:619#0/1
CAATGAACTGAGCNATCTTGCAAAAGGAGAGAAAGCTAAT
+HWI-EAS443:3:88:531:619#0/1
aZXa`aab`_`[YDYabababaaVa_Z^aaabX]baaaaa
@HWI-EAS443:3:89:415:1127#0/1
TTAGCTTTCTCTCCTTTTGCAAGATTGCNCAGTTCATTGA
+HWI-EAS443:3:89:415:1127#0/1
abbaabbabbb`aaab`aa`ba]aaYa[D[`a_aa`aaa`
@HWI-EAS443:3:91:929:1976#0/1
GCATCAATGAACTGANCAATCTTGCAAAAGGAGAGAAAGC
+HWI-EAS443:3:91:929:1976#0/1
a``XVT[]QSUM[_RDN^_^_]_`Z]^^X[]^______VL
@HWI-EAS443:3:92:361:248#0/1
TGCNCAATTAGCACATTAGCTTTCTCTCCTTTTGCAAGAT
+HWI-EAS443:3:92:361:248#0/1
abWD]^Yaaaa`a__`a_`_`aa_`^``_`__]`¥_]`]_
@HWI-EAS443:3:93:761:1283#0/1
CATCAATGAACTGAGCAATCTTGNAAAAGGAGAGAAAGCT
+HWI-EAS443:3:93:761:1283#0/1
TV[b^__¥```b`_`b``ba^YODWa¥a¥X_`abbb^a_a
@HWI-EAS443:3:96:642:599#0/1
GAGAAAGCTAATGTGCTAATTGGGCANGGGGACGTAGTGT
+HWI-EAS443:3:96:642:599#0/1
aaaaa`aaaa`aa_aaaa[_`a``_ZDY`__^_`Z][M¥Z
@HWI-EAS443:3:9:1205:1381#0/1
GAAAGCTAATGTGCTAATTNGGCAAGGGGACGTAGTGTTG
+HWI-EAS443:3:9:1205:1381#0/1
abaaaaaaaaa_a^aaba¥D¥a]``_a```^a``a^a_aa
@HWI-EAS443:3:9:949:977#0/1
GAGCAATCTTGCAAAAGGAGAGAAAGCTAATGTGCNAATT
+HWI-EAS443:3:9:949:977#0/1
a]aaS_[Za[WXQTPP`Z¥_Xa`U[]U]]^]`X_IDK]]W
@HWI-EAS443:3:100:1012:228#0/1
CAAGAGAAAGCTAATGTGCTAATTGGGCAAGGGGACGTAG
+HWI-EAS443:3:100:1012:228#0/1
ab]aabbaabYaa`aaZ`_aaabbbaa`^]aa___¥aMBB
@HWI-EAS443:3:17:1541:1962#0/1
CATCAATGAACTGAGCAATCTTGCAAAAGGAGAGAAAGAT
+HWI-EAS443:3:17:1541:1962#0/1
`_]___aa_`X_XaP^a¥_[_^^^T]¥^_`¥___^[]LEU
@HWI-EAS443:3:21:582:1739#0/1
GNACTGAGCAATCTTGCAAAAGGAGAGAAAGCTAATGTGC
+HWI-EAS443:3:21:582:1739#0/1
[D[ba_XW`aaa`aaaaa`a_a```a_`]Y]_^`__`UOR
@HWI-EAS443:3:2:1159:1991#0/1
AGCTAATGTGCTAATTGGGCAAGGGGACGTAGTGTTGGGA
+HWI-EAS443:3:2:1159:1991#0/1
WbM]bU[b`baa[`Tb`aa`b`aa^aX^_^]aVb]_b`G^
```

```
@HWI-EAS443:3:32:481:1010#0/1
AGCTAATGTGCTAATTGGGCAAGGGGACGTAGTGTTGGCA
+HWI-EAS443:3:32:481:1010#0/1
O¥Y]¥Z[V[TVWU]Z_¥ZQ_¥H¥VZNX`YMW^VTT]`ZFY
@HWI-EAS443:3:34:1405:2043#0/1
AGCTAATGTGCTAATTGGGCAAGGGGACGTAGTGTTGGGA
+HWI-EAS443:3:34:1405:2043#0/1
a`^baa`_Ya`abba`aa__aaaaZ_^UaBBBBBBBBBBB
@HWI-EAS443:3:34:1529:1448#0/1
AGCTAATGTGCTAATTGGGCAAGGGGACGTAGTGTTGGGA
+HWI-EAS443:3:34:1529:1448#0/1
]¥``_X_¥_`_]_^^aabaa[X`]]X[]aXXaTa_`YBBB
@HWI-EAS443:3:3:688:1500#0/1
AGCTAATGTGCTAATTGGGCAAGGGGACGTAGTGTTGGGA
+HWI-EAS443:3:3:688:1500#0/1
aa`_a]^U]X^_a^_]_[aN`^WTFTW__Q`aXaHGWZBB
@HWI-EAS443:3:42:456:1513#0/1
CAAGAGAAAGCTAATGTGCTAATTGGGCAAGGGGACGTAG
+HWI-EAS443:3:42:456:1513#0/1
`aZ¥_ba^a_TV^aYWUYY__`_aba`_^_Z````aaZ^`
@HWI-EAS443:3:46:859:868#0/1
CAATCTTGCAAAAGGAGAGAAAGCTAATGTGCTAATTGAG
+HWI-EAS443:3:46:859:868#0/1
a`W_bbabaaa¥^bbabaab_¥b¥aaaab`aba]aab`¥a
@HWI-EAS443:3:48:219:162#0/1
AGCTAATGTGCTAATTGGGCAAGGGGACGTAGTGTTGGGA
+HWI-EAS443:3:48:219:162#0/1
_bbb_a`bXP_aa_ab_aZX_X_Z_^^¥aX]aVa^`U_HV
@HWI-EAS443:3:49:1017:292#0/1
CAAGAGAAAGCTAATGTGCTAATTGGGCAAGGGGACGTAG
+HWI-EAS443:3:49:1017:292#0/1
aabab`baab[bbabaababaaaabbbaaabbbb]_a^aa
@HWI-EAS443:3:54:1217:919#0/1
ATCTTGCAAAAGGAGAGAAAGCTAATGTGCTAATTGGGTA
+HWI-EAS443:3:54:1217:919#0/1
S``¥a``Y_[V^^T]a¥^¥_¥^a][¥YR^¥aQPa`b`]R¥
@HWI-EAS443:3:57:1442:35#0/1
AGCTAATGTGCTAATTGGGCAAGGGGACGTAGTGTTGGGA
+HWI-EAS443:3:57:1442:35#0/1
VZab]]a^Wb`¥``baaaa`a[a`Y`[¥[JT[IPQW^aZB
@HWI-EAS443:3:57:1489:2029#0/1
AACTGAGCAATCTTGCAAAAGGAGAGAAAGCTAATGTGAT
+HWI-EAS443:3:57:1489:2029#0/1
aaa_¥¥b_babZM_b`^M¥_bbab_bb¥bbaa¥aaaWaGa
@HWI-EAS443:3:59:1639:1472#0/1
CCAAAGGAGAGAAAGCTAATGTGCTAATTGGGCAAGGGGA
+HWI-EAS443:3:59:1639:1472#0/1
aIQYT[]_VU]^a¥¥ZaSGYZVaV[LVSV`¥BBBBBBBBB
@HWI-EAS443:3:5:1403:1409#0/1
CAAGAGAAAGCTAATGTGCTAATTGGGCAAGGGGACGTAG
```

```
+HWI-EAS443:3:5:1403:1409#0/1
ababbbbbba`aabbabb^baabbbab_aab`bba_[VTb
@HWI-EAS443:3:60:1175:1310#0/1
AGCTAATGTGCTAATTGGGCAAGGGGACGTAGTGTTGGAA
+HWI-EAS443:3:60:1175:1310#0/1
Z_ZS¥`aVTW_K`aaWXTT[a^T`bSSaSLUaRR[a_UDP
@HWI-EAS443:3:60:431:1212#0/1
CAAGAGAAAGCTAATGTGCTAATTGGGCAAGGGGACGTAG
+HWI-EAS443:3:60:431:1212#0/1
`abaa_aabbbaaabb_aabaaba``aa`a__]U]Z`¥]^
@HWI-EAS443:3:61:244:332#0/1
AGCTAATGTGCTAATTGGGCAAGGGGACGTAGTGTTGGGA
+HWI-EAS443:3:61:244:332#0/1
`bbaaab]ZbababababbabaaZ`^`aa`bZ__K`_^`[HX
@HWI-EAS443:3:63:1246:533#0/1
GCCCCCTTGCCCAATTAGCACATTAGCTTTCTCTCCTTTT
+HWI-EAS443:3:63:1246:533#0/1
aH[`ZZRVTV[YOLO`¥`^¥_^__^ZS[PXY^BBBBBBBB
@HWI-EAS443:3:63:69:1469#0/1
ATTCCCCTTGCCCAATTAGCACATTAGCTTTCTCTCCTTT
+HWI-EAS443:3:63:69:1469#0/1
¥___`bbaa`V`W¥_WZ`]]P_X_ZO_aXa][]¥[``_[V
@HWI-EAS443:3:66:556:1084#0/1
CAAGAGAAAGCTAATGTGCTAATTGGGCAAGGGGACGTAG
+HWI-EAS443:3:66:556:1084#0/1
abaaa^ab`aaaa[``^`aaaaaZaaaa`^¥__`__aY_`
@HWI-EAS443:3:68:282:1796#0/1
AGCTAATGTGCTAATTGGGCAAGGGGACGTAGTGTTGGGA
+HWI-EAS443:3:68:282:1796#0/1
aa`^aaX`X]^_a__`ZV]^^a`]__¥`¥T]XN_R]SUG^
@HWI-EAS443:3:69:1715:163#0/1
CAAGAGAAAGCTAATGTGCTAATTGGGCAAGGGGACGTAG
+HWI-EAS443:3:69:1715:163#0/1
abbabba`abbabaabab`bbbaba`_W]`¥``]aaa[a`
@HWI-EAS443:3:6:455:168#0/1
CAAGAGAAAGCTAATGTGCTAATTGGGCAAGGGGACGTAG
+HWI-EAS443:3:6:455:168#0/1
abbbbbbbab`bbabbababaaaaaaa``aaaaa``a`_a
@HWI-EAS443:3:70:530:351#0/1
AGCTAATGTGCTAATTGGGCAAGGGGACGTAGTGTTGGNA
+HWI-EAS443:3:70:530:351#0/1
abaaaa`a_aaaaaa`aa_`_`aa`a]__]ZaUa]aaXDR
@HWI-EAS443:3:73:1769:841#0/1
TGTTGCAAAAGGAGAGAAAGCTAATGTGCTAATTGGGCAA
+HWI-EAS443:3:73:1769:841#0/1
]G^`aY[¥YY]R[]`ZR]^a^_U_`_YXLa[[_]RVWVST
@HWI-EAS443:3:78:1322:327#0/1
TTAAAGGAGAGAAAGCTAATGTGCTAATTGGGCAAGGGGA
+HWI-EAS443:3:78:1322:327#0/1
WTLY```V¥O¥¥^M_][`]`Y]aU_TMN]a_TY[Z`^^_]
```

```
@HWI-EAS443:3:7:109:257#0/1
AGCTAATGTGCTAATTGGGCAAGGGGACGTAGTGTTGGGA
+HWI-EAS443:3:7:109:257#0/1
]ab¥¥W[¥¥`^_aa]b^^LY[abb]aYab^`_G__]`_P_
@HWI-EAS443:3:7:299:1072#0/1
AGCTAATGTGCTAATTGGGCAAGGGGACGTAGTGTTGGGA
+HWI-EAS443:3:7:299:1072#0/1
a]`_aKaa¥^aa_]TZ___]]aa`]a_ab_a`H`Y`aYDT
@HWI-EAS443:3:80:334:1299#0/1
GCTCCCCTTGCCCAATTAGCACATTAGCTTTCTCTCCTTT
+HWI-EAS443:3:80:334:1299#0/1
^YF^^WUL¥P`^_VMWRYY][^]¥]XV_U¥RZ]XR¥SVNV
@HWI-EAS443:3:82:1078:489#0/1
CAAGAGAAAGCTAATGTGCTAATTGGGCAAGGGGACGTAG
+HWI-EAS443:3:82:1078:489#0/1
abOb_^YZa¥XaZabbabaa__ba]¥a_J`ba_]Jaba¥Y
@HWI-EAS443:3:83:60:1269#0/1
CAAGAGAAAGCTAATGTGCTAATTGGGCAAGGGGACGTAG
+HWI-EAS443:3:83:60:1269#0/1
aYaaaaaaaaaa_G]a_a][_aa`a`_`_^a`__^`aZ_^
@HWI-EAS443:3:84:1123:238#0/1
AGCTAATGTGCTAATTGGGCAAGGGGACGTAGTGTTGGGA
+HWI-EAS443:3:84:1123:238#0/1
Zaa`babbQ^]aaba`a]Ta^aaaL¥^[XPR]ZYXTS]GY
@HWI-EAS443:3:87:1153:147#0/1
AGCAATCTTGCAAAAGGAGAGAAAGCTAATGTGCTAATCG
+HWI-EAS443:3:87:1153:147#0/1
`babbbabb``aaaabbaaaab_aa_aaaaa_b]aaaaWa
@HWI-EAS443:3:88:1122:1043#0/1
AGCTAATGTGCTAATTGGGCAAGGGGACGTAGTGTTGGGA
+HWI-EAS443:3:88:1122:1043#0/1
Vb`^OIU_SXa`_X^b_]b]V_Zb``VabUaa_b_¥aaGW
@HWI-EAS443:3:90:1241:870#0/1
AGCTAATGTGCTAATTGGGCAAGGGGACGTAGTGTTGGGA
+HWI-EAS443:3:90:1241:870#0/1
`bbbaa`aa``a]]W_aaa^[_aaaaX_b]XaU^__a]H_
@HWI-EAS443:3:92:874:1692#0/1
CAAGAGAAAGCTAATGTGCTAATTGGGCAAGGGGACGTAG
+HWI-EAS443:3:92:874:1692#0/1
ab[ba_aaaa`a]`ab]a`bbabY^a`a`aaYba`Ob^[W
@HWI-EAS443:3:96:1695:625#0/1
TACAAAAGGAGAGAAAGCTAATGTGCTAATTGGGCAAGGG
+HWI-EAS443:3:96:1695:625#0/1
^`¥OQ¥RR_V_Z]ba]]`a^[¥`Y[U`[Ta]abWG^_S`B
@HWI-EAS443:3:96:993:959#0/1
CAAGAGAAAGCTAATGTGCTAATTGGGCAAGGGGACGTAG
+HWI-EAS443:3:96:993:959#0/1
^b_[K¥¥_[_abab`b_ab]aab_U¥^Y^_^aaa]_b`aa
@HWI-EAS443:3:97:529:1154#0/1
ATCTTGCAAAAGGAGAGAAAGCTAATGTGCTAATTGGGTA
```

+HWI-EAS443:3:97:529:1154#0/1
[baabab`Obabb]ba¥aabaaa_`baWa¥a[`]`b^aW`
@HWI-EAS443:3:98:1566:813#0/1
AGCTAATGTGCTAATTGGGCAAGGGGACGTAGTGTTGGGA
+HWI-EAS443:3:98:1566:813#0/1
a`Rabbba¥babb`¥`aab]]`b`ba[^a^R¥T__[a¥GO
@HWI-EAS443:3:99:170:469#0/1
GAAAAAGGAGAGAAAGCTAATGTGCTAATTGGGCAAGGGG
+HWI-EAS443:3:99:170:469#0/1
a```__ab`a_aa__a```]aa¥a_`^```aa__^^a^`_
@HWI-EAS443:3:9:1445:941#0/1
AAGCTAATGTGCTAATTGGGCAAGGGGACGTAGTGTTGAA
+HWI-EAS443:3:9:1445:941#0/1
]¥¥^aaaaa`b]aa_baa]]X^UW^aa^W_Z__R^_aa_[
@HWI-EAS443:3:17:1253:2046#0/1
NAGCAATCTTGCAAAAGGAGAGAAAGCTAATGTGCTAATT
+HWI-EAS443:3:17:1253:2046#0/1
DKTLNWUSUTVWVNSTTSSVQTLQQNPWVWTTPTUYTBBB
@HWI-EAS443:3:19:725:338#0/1
GCATCAATGAACTGAGCAATCTTGCAAAAGGAGAGAAAGG
+HWI-EAS443:3:19:725:338#0/1
`HW¥NZ^]^]_Z[_Z_TWX`Z__UTVMYTY^^_Ya]XXRD
@HWI-EAS443:3:20:1263:353#0/1
GAATCTTGCAAAAGGAGAGAAAGCTAATGTGCTAATTGGG
+HWI-EAS443:3:20:1263:353#0/1
V_b`K``a]]ONYa]WbbaWZI_^ba]ab[aa^J`a_a`[
@HWI-EAS443:3:20:1425:1259#0/1
TAAAGCTAATGTGCTAATTGGGCAAGGGGACGTAGTGTTG
+HWI-EAS443:3:20:1425:1259#0/1
LXbbb_bb[_[_[abXaabaa`¥`Ua`Vbb_aa_bM``ab
@HWI-EAS443:3:23:1153:974#0/1
TGTCCCCTTGCCCAATTAGCACATTAGCTTTCTCTCCTTT
+HWI-EAS443:3:23:1153:974#0/1
aa_baaa_`aaaaa`aa`a``_aaa`a_a_`_aX``¥`__
@HWI-EAS443:3:24:1792:614#0/1
NAGAAAGCTAATGTGCTAATTGGGCAAGGGGACGTAGTGT
+HWI-EAS443:3:24:1792:614#0/1
DHXVQPVSUUUYSMRROLXXVJQONTBBBBBBBBBBBBBB
@HWI-EAS443:3:25:433:1539#0/1
AGCAAAAGGAGAGAAAGCTAATGTGCTAATTGGGCAAGGG
+HWI-EAS443:3:25:433:1539#0/1
a[`[a_X^baa]aa`^abaaaaa[^aaaa```[Ya`_aY_
@HWI-EAS443:3:28:1507:1397#0/1
AAGCTAATGTGCTAATTGGGCAAGGGGACGTAGTGTTGGG
+HWI-EAS443:3:28:1507:1397#0/1
aZ`b][a`aZ`ab`^`abaaaa``^^___a]_a¥aa]U_G
@HWI-EAS443:3:30:38:909#0/1
CTCCCCTTGCCCAATTAGCACATTAGCTTTCTCTCCTTTT
+HWI-EAS443:3:30:38:909#0/1
aabbba^`ba`aaaa`baaaaaaa``a___``aa_Z`__^

@HWI-EAS443:3:32:1243:1754#0/1
CAGCAATCTTGCAAAAGGAGAGAAAGCTAATGTGCTAATT
+HWI-EAS443:3:32:1243:1754#0/1
G[^b_a_a^b¥bZ_UZWZ_baY_Z_b_bba`bX_Z`_`bb
@HWI-EAS443:3:34:802:395#0/1
CCCCCTTGCCCAATTAGCACATTAGCTTTCTCTCCTTTTG
+HWI-EAS443:3:34:802:395#0/1
abbbbaabbaab^b`baaaaaaaab_bbaaa]a_Za^_`_
@HWI-EAS443:3:36:1379:176#0/1
AAGCTAATGTGCTAATTGGGCAAGGGGACGTAGTGTTGGG
+HWI-EAS443:3:36:1379:176#0/1
^]¥a^R¥a_O]X`_`_`baaaa`baa_X`]FMaR][`aTI
@HWI-EAS443:3:37:1793:2036#0/1
NAGCAATCTTGCAAAAGGAGAGAAAGCTAATGTGCTAATT
+HWI-EAS443:3:37:1793:2036#0/1
DMUUPWZWWUTHQSSSNYZXWYWVUWHUVTTSUYWYUNUY
@HWI-EAS443:3:39:1524:368#0/1
TCGTCCCCTTGCCCAATTAGCACATTAGCTTTCTCTCCTT
+HWI-EAS443:3:39:1524:368#0/1
a]_T][a^RQSZNLLHP_XLSVSZ_MYYH_]YTU¥WQLQY
@HWI-EAS443:3:42:1121:845#0/1
GCAAAAGGAGAGAAAGCTAATGTGCTAATTGGGCAAGGGN
+HWI-EAS443:3:42:1121:845#0/1
aa`[YX[]PVZ``_^a_YUK¥¥UWZ^UTZ[^__YSP__WD
@HWI-EAS443:3:43:1186:970#0/1
AAGAGAAAGCTAATGTGCTAATTGGGCAAGGGGACGTAGT
+HWI-EAS443:3:43:1186:970#0/1
a`a``bbab`baaab`a`aaaaa`aa`a^aa_`_XaX¥`U
@HWI-EAS443:3:44:512:706#0/1
AGTCCCCTTGCCCAATTAGCACATTAGCTTTCTCTCCTTT
+HWI-EAS443:3:44:512:706#0/1
a`Lab_baaba`a^bbbaaZ]ba_``]aaaa`a[`^]a`^
@HWI-EAS443:3:49:1755:1558#0/1
TCGTCCCCTTGCCCAATTAGCACATTAGCTTTCTCTCCTT
+HWI-EAS443:3:49:1755:1558#0/1
`b¥Y[XUUG[a[[`a^[W][[OMXa[Y[¥`_aR_G[^]aZ
@HWI-EAS443:3:53:1641:1200#0/1
CAGCAATCTTGCAAAAGGAGAGAAAGCTAATGTGCTAATT
+HWI-EAS443:3:53:1641:1200#0/1
GPZ_a_b_ababbUYaba¥Ta`abab]ababbab_bVabb
@HWI-EAS443:3:54:1708:469#0/1
GATCAATGAACTGAGCAATCTTGCAAAAGGAGAGAAAGCT
+HWI-EAS443:3:54:1708:469#0/1
S`[T^T^a`a`]`_aaT^ORYN_a^[^XW¥U_ab^]_[PF
@HWI-EAS443:3:55:1085:1084#0/1
AAAGCTAATGTGCTAATTGGGCAAGGGGACGTAGTGTTGA
+HWI-EAS443:3:55:1085:1084#0/1
aaaa¥a¥[]a^aTaaaaa^¥[[_aa_^[^`a__aY¥^_`_
@HWI-EAS443:3:55:1419:1977#0/1
GATCAATGAACTGAGCAATCTTGCAAAAGGAGAGAAAGCT

```
+HWI-EAS443:3:55:1419:1977#0/1
GY_YX`bbbba`bb`Vbbbbabb¥abXX`aaabbb`bbWb
@HWI-EAS443:3:56:183:800#0/1
GCGTCCCCTTGCCCAATTAGCACATTAGCTTTCTCTCCTT
+HWI-EAS443:3:56:183:800#0/1
_`¥T_aab]V]ZaVPQ]aPYQ^RX__VRUaa`_[_aZP^a
@HWI-EAS443:3:58:1248:1653#0/1
TGTCCCCTTGCCCAATTAGCACATTAGCTTTCTCTCCTTT
+HWI-EAS443:3:58:1248:1653#0/1
QM¥]¥aWXZ^]aaa]POV¥YT_^]RYJY_]]MXP`__a¥`
@HWI-EAS443:3:58:527:1090#0/1
AAGCTAATGTGCTAATTGGGCAAGGGGACGTAGTGTTGGG
+HWI-EAS443:3:58:527:1090#0/1
_¥^`_`W]WL]Zaa`]]aZ_aZY^[S[G[_NOZ¥WTY]TD
@HWI-EAS443:3:59:1597:18#0/1
ACTACGTCCCCTTGCCCAATTAGCACATTAGCTTTCTCTT
+HWI-EAS443:3:59:1597:18#0/1
a[aaabaa_T]Y``[[NN]_]]a[`¥^Z_`UHPaYQRU¥_
@HWI-EAS443:3:60:1155:1073#0/1
CCCCCTTGCCCAATTAGCACATTAGCTTTCTCTCCTTTTG
+HWI-EAS443:3:60:1155:1073#0/1
`]]]_WZ^`YF¥YTYXVYa``a¥U_U]`][][_TQ]^Z`_
@HWI-EAS443:3:61:1373:104#0/1
ATCTTGCAAAAGGAGAGAAAGCTAATGTGCTAATTGGGCG
+HWI-EAS443:3:61:1373:104#0/1
a]^Y`b]aaa]]a`bab_¥b``UVIaab_PaZU_[``VKK
@HWI-EAS443:3:63:1793:1330#0/1
NGCAAAAGGAGAGAAAGCTAATGTGCTAATTGGGCAAGGG
+HWI-EAS443:3:63:1793:1330#0/1
DLSRUSUYYYURTWWWWUWVVWWWWUWTVTNTURUUTTBB
@HWI-EAS443:3:63:362:711#0/1
AAGAGAAAGCTAATGTGCTAATTGGGCAAGGGGACGTAGT
+HWI-EAS443:3:63:362:711#0/1
O_bbaaX]^aa__a__aaa_^a_aaaaVaba`a_a`T^aW
@HWI-EAS443:3:68:955:2046#0/1
NATCAATGAACTGAGCAATCTTGCAAAAGGAGAGAAAGCT
+HWI-EAS443:3:68:955:2046#0/1
DNZZZWZZZZZZZZZWZZZZZZZZZZZZZZZXZZXZZZZZZU
@HWI-EAS443:3:70:1615:610#0/1
GGTCCCCTTGCCCAATTAGCACATTAGCTTTCTCTCCTTT
+HWI-EAS443:3:70:1615:610#0/1
ab]N¥aaa`aa``ZFY`aa^aaaaa____a`_a]a¥_`_`
@HWI-EAS443:3:72:44:1350#0/1
TGTCCCCTTGCCCAATTAGCACATTAGCTTTCTCTCCTTT
+HWI-EAS443:3:72:44:1350#0/1
a_`aa_a`]]aaa`aa_Y[```aa_a___``__``^__`_
@HWI-EAS443:3:75:1326:313#0/1
CGCAAAAGGAGAGAAAGCTAATGTGCTAATTGGGCAAGGG
+HWI-EAS443:3:75:1326:313#0/1
W¥a``babbbbZba`ab¥babYba]^`babbbb`]aab]b
```

```
@HWI-EAS443:3:79:1024:184#0/1
GTCCCCTTGCCCAATTAGCACATTAGCTTTCTCTCCTTTA
+HWI-EAS443:3:79:1024:184#0/1
_b^bbbababa^bbb`aa`a`aabab¥ba`]ab``^aaaa
@HWI-EAS443:3:79:602:113#0/1
GGCACATTAGCTTTCTCTCCTTTTGCAAGATTGCTCAGTT
+HWI-EAS443:3:79:602:113#0/1
aa`babaaaaaa`a]`]a`]`aa_`¥`_a_```^_^^`_^
@HWI-EAS443:3:80:315:264#0/1
AAGCTAATGTGCTAATTGGGCAAGGGGACGTAGTGTTGGG
+HWI-EAS443:3:80:315:264#0/1
a_aabaaab`baaaaaaa``_a^a``__]a[^aU`^a`TG
@HWI-EAS443:3:83:757:1512#0/1
TAAAAGGAGAGAAAGCTAATGTGCTAATTGGGCAAGGGGA
+HWI-EAS443:3:83:757:1512#0/1
aa[]_aa_baba]^aba_[Za[a`aa`aaaaUa`U`^Y`_
@HWI-EAS443:3:87:534:2046#0/1
NAGCAATCTTGCAAAAGGAGAGAAAGCTAATGTGCTAATT
+HWI-EAS443:3:87:534:2046#0/1
DKPLSUOWWWWWROPXWVZWZZXWUXZZXXUGWWWWVRZG
@HWI-EAS443:3:88:1060:1579#0/1
GACTACGTCCCCTTGCCCAATTAGCACATTAGCTTTCTCT
+HWI-EAS443:3:88:1060:1579#0/1
S¥RX_^¥a_PO¥ZU[QQ_SS^Z__SGD]XXSab¥_YFZZ¥
@HWI-EAS443:3:89:1411:1279#0/1
GAACTGAGCAATCTTGCAAAAGGAGAGAAAGCTAATGTGA
+HWI-EAS443:3:89:1411:1279#0/1
a`aa¥_^ba_bbb`bbaaPaabb`baabaab¥`baaaOaH
@HWI-EAS443:3:8:525:991#0/1
TCGTCCCCTTGCCCAATTAGCACATTAGCTTTCTCTCCTT
+HWI-EAS443:3:8:525:991#0/1
aaa_[baa``[aa`^b`aaaa_`a``a____````__^_^
@HWI-EAS443:3:91:652:1779#0/1
AAGCTAATGTGCTAATTGGGCAAGGGGACGTAGTGTTGGG
+HWI-EAS443:3:91:652:1779#0/1
aaY`¥_]UTF^ZMOXRY_UVa[¥_[QPU[_WJYQUU__XF
@HWI-EAS443:3:95:899:1089#0/1
AAGCTAATGTGCTAATTGGGCAAGGGGACGTAGTGTTGGA
+HWI-EAS443:3:95:899:1089#0/1
ZV¥a_aY[aJ_ab¥P^Y_¥SX`Uab]_`Xa_P_Rb_a_QF
@HWI-EAS443:3:99:791:1016#0/1
GATCAATGAACTGAGCAATCTTGCAAAAGGAGAGAAAGCT
+HWI-EAS443:3:99:791:1016#0/1
G]¥a`R`aa]b_```¥`V]^T^a``¥abaabbbbab_a[b
@HWI-EAS443:3:9:1277:308#0/1
GGTCCCCTTGCCCAATTAGCACATTAGCTTTCTCTCCTTT
+HWI-EAS443:3:9:1277:308#0/1
aYMZZWZ[^^YRV[^___¥W[V_ZUU_V]Z^V¥P[YY]^R
@HWI-EAS443:3:100:102:1121#0/1
ATCTTGCAAAAGGAGAGAAAGCTAATGTGCTAATTGGGCA
```

```
+HWI-EAS443:3:100:102:1121#0/1
aaa]__[`[_`a``a_a_aY^`_¥a_`XX``]Z_^^ZaBB
@HWI-EAS443:3:100:1284:706#0/1
AGCTAATGTGCTAATTGGGCAAGGGGACGTAGTGTTGGTA
+HWI-EAS443:3:100:1284:706#0/1
aaabaabbaa_aaaaaa`a^`aaa``__a]_a^a^`a_O^
@HWI-EAS443:3:100:130:1794#0/1
AAGCATCAATGAACTGAGCAATCTTGCAAAAGGAGAGAAA
+HWI-EAS443:3:100:130:1794#0/1
aa^`aa```a^___`^___a]_W_^`]Z]^_]Z^^^]^^B
@HWI-EAS443:3:100:1353:1423#0/1
CAATGAACTGAGCAATCTTGCAAAAGGAGAGAAAGCTAAT
+HWI-EAS443:3:100:1353:1423#0/1
Vb`abX`Xbbb_ab¥a_ab_Xabab_abaaa_KaaG^¥^b
@HWI-EAS443:3:100:147:883#0/1
CTTGCAAAAGGAGAGAAAGCTAATGTGCTAATTGGGCAAG
+HWI-EAS443:3:100:147:883#0/1
aba[a`aaab_[aaa¥T]aabaa_a[a]a_]aaa^a`_¥a
@HWI-EAS443:3:100:1519:994#0/1
CATCAATGAACTGAGCAATCTTGCAAAAGGAGAGAAAGCT
+HWI-EAS443:3:100:1519:994#0/1
Y__VZ`¥_^_^^^`XX`b_G_aba_^¥¥b]aa`aaW]WBB
@HWI-EAS443:3:100:158:503#0/1
GAGCAATCTTGCAAAAGGAGAGAAAGCTAATGTGCTAATT
+HWI-EAS443:3:100:158:503#0/1
a`bab`aaaaba``_``a^a`a_a_`Za¥^aa_a]_]__B
@HWI-EAS443:3:100:283:391#0/1
AGCAATCTTGCAAAAGGAGAGAAAGCTAATGTGCTAATTG
+HWI-EAS443:3:100:283:391#0/1
``b`b`¥`¥_TU¥H[¥_aaa^aab`Y^JU]aT_a``[^Y^
@HWI-EAS443:3:100:433:905#0/1
GAAAGCTAATGTGCTAATTGGGCAAGGGGACGTAGTGTTG
+HWI-EAS443:3:100:433:905#0/1
aaaabaaaaab_a_a`aaaaa`]^_baa`_^aZ^`ZBBBB
@HWI-EAS443:3:100:568:59#0/1
GAAAGCTAATGTGCTAATTGGGCAAGGGGACGTAGTGTTG
+HWI-EAS443:3:100:568:59#0/1
a^]TaV]]¥¥a[aZ^[]_`]PY_]]]UPXQW^OX]P]YBB
@HWI-EAS443:3:100:620:1948#0/1
CATCAATGAACTGAGCAATCTTGCAAAAGGAGAGAAAGCT
+HWI-EAS443:3:100:620:1948#0/1
abb`bbbbbbbaaaaabbaabab`baaaaaabbbbaaa]B
@HWI-EAS443:3:100:714:153#0/1
GAGCAATCTTGCAAAAGGAGAGAAAGCTAATGTGCTAATT
+HWI-EAS443:3:100:714:153#0/1
]¥`_bZY_a``Xaaaa`[]aa``]aaY`U__¥¥_U__`a`
@HWI-EAS443:3:100:739:1522#0/1
GCATCAATGAACTGAGCAATCTTGCAAAAGGAGAGAAAGC
+HWI-EAS443:3:100:739:1522#0/1
aa]]`_``VLQYa[[a]`]`_aaaa_]__aa^a_]¥PY`B
```

```
@HWI-EAS443:3:100:740:1460#0/1
GAGCAATCTTGCAAAAGGAGAGAAAGCTAATGTGCTAATT
+HWI-EAS443:3:100:740:1460#0/1
a^bbb¥``a`aR``a__b^`¥aabb_aba]]_¥a]__a]a
@HWI-EAS443:3:100:749:1947#0/1
GCAAAAGGAGAGAAAGCTAATGTGCTAATTGGGCAAGGGG
+HWI-EAS443:3:100:749:1947#0/1
abaaba_X_a`abM^_`aWW`^M¥`a[_a`_b`Y_Ha^BB
@HWI-EAS443:3:100:790:1017#0/1
CATCAATGAACTGAGCAATCTTGCAAAAGGAGAGAAAGCT
+HWI-EAS443:3:100:790:1017#0/1
`bbabbbaaaabbab^b_a]`abaaa``^b`abbaa_a[a
@HWI-EAS443:3:100:793:2016#0/1
GAGAAAGCTAATGTGCTAATTGGGCAAGGGGACGTAGTGT
+HWI-EAS443:3:100:793:2016#0/1
[b`bNab_a`aabZaaaWb^abaa^bb``_^^^a]aVBBB
@HWI-EAS443:3:100:854:966#0/1
GAGCAATCTTGCAAAAGGAGAGAAAGCTAATGTGCTAATT
+HWI-EAS443:3:100:854:966#0/1
^[aS_Za_aba`Z]`[_UWba^]a]_L_U[a[Ya]a^RBB
@HWI-EAS443:3:100:936:271#0/1
GGAGAGAAAGCTAATGTGCTAATTGGGCAAGGGGACGTAG
+HWI-EAS443:3:100:936:271#0/1
aa]ba]`^U¥G¥U^_]]a[`¥`__^XUWZZ`_¥aSW[WNa
@HWI-EAS443:3:100:965:227#0/1
GAGCAATCTTGCAAAAGGAGAGAAAGCTAATGTGCTAATT
+HWI-EAS443:3:100:965:227#0/1
aRaNTW]V^aa]_`N]¥aGZ]aaO``U^MMSXX`V_R_W]
@HWI-EAS443:3:100:984:923#0/1
AGCACATTAGCTTTCTCTCCTTTTGCAAGATTGCTCAGTT
+HWI-EAS443:3:100:984:923#0/1
aa¥_ab`aaa^aba^b`aa_aa`a^¥a`_aaaaWaW`¥_`
@HWI-EAS443:3:10:1033:258#0/1
CATCAATGAACTGAGCAATCTTGCAAAAGGAGAGAAAGCT
+HWI-EAS443:3:10:1033:258#0/1
abbbbbbbbbb`b`baa`a_aaa[a]^¥a`_aaba_^a_`
@HWI-EAS443:3:10:1139:913#0/1
CTTGCAAAAGGAGAGAAAGCTAATGTGCTAATTGGGCAAG
+HWI-EAS443:3:10:1139:913#0/1
abba^bbbbb_bbbbbbaabbbbabbbbbbbbbaa``bab
@HWI-EAS443:3:10:1254:2019#0/1
AAGCTAATGTGCTAATTGGGCAAGGGGACGTAGTGTTGGT
+HWI-EAS443:3:10:1254:2019#0/1
aaaYaaaba¥a^a`a`_``¥^_¥a^^RRT_Z¥RLRZ]ZUI
@HWI-EAS443:3:10:1257:820#0/1
GAAAGCTAATGTGCTAATTGGGCAAGGGGACGTAGTGTTG
+HWI-EAS443:3:10:1257:820#0/1
aa`__V__``a^_^]][`_WW^XZZ]]^PKMUUXKITXZZ
@HWI-EAS443:3:10:1370:1567#0/1
TGCAAAAGGAGAGAAAGCTAATGTGCTAATTGGGCAAGGG
```

+HWI-EAS443:3:10:1370:1567#0/1
a_¥Ya^^^`¥`__]]_ZSZ¥^[YOWP`]]a`a¥XOU_a__
@HWI-EAS443:3:10:1396:957#0/1
ATCTTGCAAAAGGAGAGAAAGCTAATGTGCTAATTGGGCA
+HWI-EAS443:3:10:1396:957#0/1
`aa``Z`¥_`bbb^T^`aaa]Y`a_a^S_Y__a_a^XYR`
@HWI-EAS443:3:10:1400:967#0/1
CACTACGTCCCCTTGCCCAATTAGCACATTAGCTTTCTCT
+HWI-EAS443:3:10:1400:967#0/1
aaabbabbaababba¥a`abbbbaabWbbaaa^baaN_^b
@HWI-EAS443:3:10:1456:1721#0/1
TGCAAAAGGAGAGAAAGCTAATGTGCTAATTGGGCAAGGG
+HWI-EAS443:3:10:1456:1721#0/1
ab[_b_NTbba]^[WaZQVYbaVaaG`a``a^VaZ_G^]a
@HWI-EAS443:3:10:1465:982#0/1
AGCTAATGTGCTAATTGGGCAAGGGGACGTAGTGTTGGTA
+HWI-EAS443:3:10:1465:982#0/1
``abbbb¥^_`b`aaab_a_`][__Z]]a``_]a_aaa]]
@HWI-EAS443:3:10:1478:1140#0/1
AAGCATCAATGAACTGAGCAATCTTGCAAAAGGAGAGAAA
+HWI-EAS443:3:10:1478:1140#0/1
^aba]`aaaaaaa`b`]_^a`__a`a`^[_`a`]a_a_^^
@HWI-EAS443:3:10:1495:914#0/1
TCTTGCAAAAGGAGAGAAAGCTAATGTGCTAATTGGGCAA
+HWI-EAS443:3:10:1495:914#0/1
aabba`aaaaba_a_aaaaa]``_a`^a^a__```[aX_]
@HWI-EAS443:3:10:1514:86#0/1
GAAAGCTAATGTGCTAATTGGGCAAGGGGACGTAGTGTTG
+HWI-EAS443:3:10:1514:86#0/1
a`SR^]XPW_^XYG^[Y]^^S]WXV]QBBBBBBBBBBBBB
@HWI-EAS443:3:10:1571:423#0/1
TGCAAAAGGAGAGAAAGCTAATGTGCTAATTGGGCAAGGG
+HWI-EAS443:3:10:1571:423#0/1
a`a`aaa`a_`a_a`_a^a``a`R`V`_^_^^_^Z]]_^_
@HWI-EAS443:3:10:157:952#0/1
GAAAGCTAATGTGCTAATTGGGCAAGGGGACGTAGTGTTG
+HWI-EAS443:3:10:157:952#0/1
`_S^_^Y^^`_X]^RYV^¥[^SY[V_O]`XXWHL¥V]XXY
@HWI-EAS443:3:10:1609:1075#0/1
AGCTAATGTGCTAATTGGGCAAGGGGACGTAGTGTTGGTA
+HWI-EAS443:3:10:1609:1075#0/1
a_bb`bb`aa`baaaabbb[aaaaab__b___Ya]`a_SR
@HWI-EAS443:3:10:1612:1288#0/1
ATGAACTGAGCAATCTTGCAAAAGGAGAGAAAGCTAATGT
+HWI-EAS443:3:10:1612:1288#0/1
aa¥`bbbbba``ababba_Y¥aaba[aababaaV``_ab_
@HWI-EAS443:3:10:1621:821#0/1
CTTGCAAAAGGAGAGAAAGCTAATGTGCTAATTGGGCAAG
+HWI-EAS443:3:10:1621:821#0/1
`_`baa_``abaabbbbbb`bUabbab`_`abaaR]_Y^b

```
@HWI-EAS443:3:10:1644:1790#0/1
GAGAAAGCTAATGTGCTAATTGGGCAAGGGGACGTAGTGT
+HWI-EAS443:3:10:1644:1790#0/1
Wb`bX][UU]_a_SaXa[aY[a^YRZ[RYBBBBBBBBBBB
@HWI-EAS443:3:10:1675:52#0/1
AGCTAATGTGCTAATTGGGCAAGGGGACGTAGTGTTGGTA
+HWI-EAS443:3:10:1675:52#0/1
_SZbbabb[b[aaa_[]_a^]_aa_a[G^^G_VaW_`UQT
@HWI-EAS443:3:10:1706:637#0/1
ATCTTGCAAAAGGAGAGAAAGCTAATGTGCTAATTGGGCA
+HWI-EAS443:3:10:1706:637#0/1
[V_`VW_Z¥¥_``^Y^WT^`YH[]^``]¥KZ¥_aa[`Z¥_
@HWI-EAS443:3:10:1718:823#0/1
AGCAATCTTGCAAAAGGAGAGAAAGCTAATGTGCTAATTG
+HWI-EAS443:3:10:1718:823#0/1
aa`bbb`abbaa_aaaa_baaa`aaUa`_ab_a^`___`a
@HWI-EAS443:3:10:202:1530#0/1
AGGAGAGAAAGCTAATGTGCTAATTGGGCAAGGGGACGTA
+HWI-EAS443:3:10:202:1530#0/1
ab_H¥_b^^_bbaZa]¥Ta`_ZUXW^_W``aYa`_UP`M¥
@HWI-EAS443:3:10:204:29#0/1
GCAATCTTGCAAAAGGAGAGAAAGCTAATGTGCTAATTGG
+HWI-EAS443:3:10:204:29#0/1
a_aYN[`__Z_[Z]aaZ_]_X]^_XWR^Z^QYYZZZZ¥ZV
@HWI-EAS443:3:10:219:723#0/1
CAATCTTGCAAAAGGAGAGAAAGCTAATGTGCTAATTGGG
+HWI-EAS443:3:10:219:723#0/1
^Ta_b`¥`ab`Y^b``ab_ba`a`_W``aa`a``[_]``a
@HWI-EAS443:3:10:282:1102#0/1
AAAGCTAATGTGCTAATTGGGCAAGGGGACGTAGTGTTGG
+HWI-EAS443:3:10:282:1102#0/1
_a`^ba`U¥a]aaa^aaaaa`a]Y^a`_^]^U¥_Ya]`aa
@HWI-EAS443:3:10:308:798#0/1
GAAAGCTAATGTGCTAATTGGGCAAGGGGACGTAGTGTTG
+HWI-EAS443:3:10:308:798#0/1
]X^Z]^`¥X_WGR]^XHUTGV]¥_aZ¥W`P[]NLYU[M¥Y
@HWI-EAS443:3:10:31:595#0/1
AAGCTAATGTGCTAATTGGGCAAGGGGACGTAGTGTTGGT
+HWI-EAS443:3:10:31:595#0/1
^Z_bb`V`b¥b`baaaabbaaa`aaaa_``]`aVa]_a_L
@HWI-EAS443:3:10:352:546#0/1
TGCAAAAGGAGAGAAAGCTAATGTGCTAATTGGGCAAGGG
+HWI-EAS443:3:10:352:546#0/1
aaaa^`S`b_aaaaaaa]aa_`a¥```Q]```__U]X`__
@HWI-EAS443:3:10:44:1827#0/1
GAAAGCTAATGTGCTAATTGGGCAAGGGGACGTAGTGTTG
+HWI-EAS443:3:10:44:1827#0/1
a]_Z`_aYZ_aY¥][``a__`_]¥Z`_`^X_`S^YPYW]Z
@HWI-EAS443:3:10:623:588#0/1
ATCTTGCAAAAGGAGAGAAAGCTAATGTGCTAATTGGGCA
```

```
+HWI-EAS443:3:10:623:588#0/1
]HYa_aaYR¥P`aUZ^¥[WZ¥Z_]N^_G_[UX¥UZXa^ZU
@HWI-EAS443:3:10:640:195#0/1
ATCAATGAACTGAGCAATCTTGCAAAAGGAGAGAAAGCTA
+HWI-EAS443:3:10:640:195#0/1
]ababbbababbababbaaa^a_a[]]aa_aaaa__a]aa
@HWI-EAS443:3:10:848:1520#0/1
ATGAACTGAGCAATCTTGCAAAAGGAGAGAAAGCTAATGT
+HWI-EAS443:3:10:848:1520#0/1
Ybb`^`aabbaaaaaaa_a_`[]ba_aaaaa_a_``_a^R
@HWI-EAS443:3:10:910:1300#0/1
GAGCAATCTTGCAAAAGGAGAGAAAGCTAATGTGCTAATT
+HWI-EAS443:3:10:910:1300#0/1
``a_aaa_¥ba_aa][a[[`^Z`U^YJOZ]_ZGTLQ^]RW
@HWI-EAS443:3:11:1143:333#0/1
AGCTAATGTGCTAATTGGGCAAGGGGACGTAGTGTTGGTA
+HWI-EAS443:3:11:1143:333#0/1
aaab`aab¥b_a_[aaa_a__``a__Z]`S_aRaX_`]OW
@HWI-EAS443:3:11:1172:1989#0/1
CATCAATGAACTGAGCAATCTTGCAAAAGGAGAGAAAGCT
+HWI-EAS443:3:11:1172:1989#0/1
`aaaaaaaba[aa[^`b`aTYTa]bb`[^_aa__aa__G[
@HWI-EAS443:3:11:1182:740#0/1
GAGCAATCTTGCAAAAGGAGAGAAAGCTAATGTGCTAATT
+HWI-EAS443:3:11:1182:740#0/1
^`W`K`T[P]_`a_]O^aaa]¥aS]S^`W_]PL]H¥]^a`
@HWI-EAS443:3:11:1205:319#0/1
CTTGCAAAAGGAGAGAAAGCTAATGTGCTAATTGGGCAAG
+HWI-EAS443:3:11:1205:319#0/1
abab`aa`abbaaaaaaa`_ba_aaVaaa__a]`_a¥]_a
@HWI-EAS443:3:11:1283:1259#0/1
GAAAGCTAATGTGCTAATTGGGCAAGGGGACGTAGTGTTG
+HWI-EAS443:3:11:1283:1259#0/1
a_YUX¥_[[a`Y_MLORMWXQWYVO_]]YVX_YTHT^RVB
@HWI-EAS443:3:11:1348:1979#0/1
GAGCAATCTTGCAAAAGGAGAGAAAGCTAATGTGCTAATT
+HWI-EAS443:3:11:1348:1979#0/1
abbabbbabba_babbbbbaabbbbb^ba[aaYaGa`^a`
@HWI-EAS443:3:11:1369:1663#0/1
GAGCAATCTTGCAAAAGGAGAGAAAGCTAATGTGCTAATT
+HWI-EAS443:3:11:1369:1663#0/1
aaa`bbaaab`^`aaaabababaaaaXaa_aa^aZ_````
@HWI-EAS443:3:11:1382:876#0/1
CATCAATGAACTGAGCAATCTTGCAAAAGGAGAGAAAGCT
+HWI-EAS443:3:11:1382:876#0/1
`[]HR_b_GTa][L_RHZ^]`aY[QYYV]WY^XR[TPROU
@HWI-EAS443:3:11:1396:673#0/1
GAGCAATCTTGCAAAAGGAGAGAAAGCTAATGTGCTAATT
+HWI-EAS443:3:11:1396:673#0/1
a`[aaaa`aa`_``_]aa[a_a_`Xa^__^__^_V_]]a_
```

```
@HWI-EAS443:3:11:1452:783#0/1
GAGCAATCTTGCAAAAGGAGAGAAAGCTAATGTGCTAATT
+HWI-EAS443:3:11:1452:783#0/1
Z¥b`abaabbb`bb`abbbbabab]b_b^[ba_babaaba
@HWI-EAS443:3:11:1477:817#0/1
CATCAATGAACTGAGCAATCTTGCAAAAGGAGAGAAAGCT
+HWI-EAS443:3:11:1477:817#0/1
aa`¥^_`aaabaa]```]aaa`a¥YS_¥aa[_¥_Y_[¥R_
@HWI-EAS443:3:11:1496:1946#0/1
GCAAAAGGAGAGAAAGCTAATGTGCTAATTGGGCAAGGGG
+HWI-EAS443:3:11:1496:1946#0/1
a]_aa[ab^a`aa``aR^`__`XaY^_^_^`^]U^¥_]BB
@HWI-EAS443:3:11:1520:1589#0/1
AGCAATCTTGCAAAAGGAGAGAAAGCTAATGTGCTAATTG
+HWI-EAS443:3:11:1520:1589#0/1
¥]a`ab[bbaaaaa_aa_aaaaa`_^``_^`^]_`]]_]_
@HWI-EAS443:3:11:1590:143#0/1
AAGCATCAATGAACTGAGCAATCTTGCAAAAGGAGAGAAA
+HWI-EAS443:3:11:1590:143#0/1
aabaaa_`aaaa`^___a`^__^```Z^]Z]__Z^]^^XZ
@HWI-EAS443:3:11:222:38#0/1
AAGCTAATGTGCTAATTGGGCAAGGGGACGTAGTGTTGGT
+HWI-EAS443:3:11:222:38#0/1
aXa_a__`a^aa¥_`__aa`a^^a``]X^aVW_Y_V]`¥T
@HWI-EAS443:3:11:240:353#0/1
AGCTAATGTGCTAATTGGGCAAGGGGACGTAGTGTTGGTA
+HWI-EAS443:3:11:240:353#0/1
aaa`Z_ab_`V_TV¥]a¥]Y__`Va_X``T_XS^[[_^KK
@HWI-EAS443:3:11:266:1906#0/1
GAGCAATCTTGCAAAAGGAGAGAAAGCTAATGTGCTAATT
+HWI-EAS443:3:11:266:1906#0/1
^WRUR¥_`a_^^]`¥WQP]]Z__ZN]`^YZ[UF¥RSZ¥XT
@HWI-EAS443:3:11:319:1518#0/1
GAGCAATCTTGCAAAAGGAGAGAAAGCTAATGTGCTAATT
+HWI-EAS443:3:11:319:1518#0/1
^OU^`^`aa]`ZNWYXR^_a`a_a_[LV`TRVN^X]_YYT
@HWI-EAS443:3:11:340:650#0/1
CATCAATGAACTGAGCAATCTTGCAAAAGGAGAGAAAGCT
+HWI-EAS443:3:11:340:650#0/1
abba`baaaaaaa_aaaa_aa`ab```^a``b`aa_^`__
@HWI-EAS443:3:11:364:835#0/1
AAGCATCAATGAACTGAGCAATCTTGCAAAAGGAGAGAAA
+HWI-EAS443:3:11:364:835#0/1
aaaa_Y`Z`aaa`a__Yaa`]```__`W^aY_W]aaa`^[
@HWI-EAS443:3:11:375:330#0/1
CAATGAACTGAGCAATCTTGCAAAAGGAGAGAAAGCTAAT
+HWI-EAS443:3:11:375:330#0/1
a^`bbabaaa]baaabaaabaa][aaaaaaaaa_a]aa`a
@HWI-EAS443:3:11:388:876#0/1
GCATCAATGAACTGAGCAATCTTGCAAAAGGAGAGAAAGC
```

```
+HWI-EAS443:3:11:388:876#0/1
aaa`]XX_a`^`a__aaZY^``_a_`]_Za^^_`_`[W[]
@HWI-EAS443:3:11:411:1787#0/1
AAAGGAGAGAAAGCTAATGTGCTAATTGGGCAAGGGGACG
+HWI-EAS443:3:11:411:1787#0/1
a^__¥P`ba`Z_baab[`a¥__`a_a__`Y_a_`__[`_^
@HWI-EAS443:3:11:489:1109#0/1
GAGCAATCTTGCAAAAGGAGAGAAAGCTAATGTGCTAATT
+HWI-EAS443:3:11:489:1109#0/1
aaa__aa`a[aa¥Z¥Y`XX`_aXGX`__^^]_V¥U_SXYY
@HWI-EAS443:3:11:515:262#0/1
AATGAACTGAGCAATCTTGCAAAAGGAGAGAAAGCTAATG
+HWI-EAS443:3:11:515:262#0/1
aabaaaabaabaaaa^aab_a^a¥ba_a_a_XTa_`__`_
@HWI-EAS443:3:11:53:831#0/1
AGCTAATGTGCTAATTGGGCAAGGGGACGTAGTGTTGGTA
+HWI-EAS443:3:11:53:831#0/1
`[`PQ¥]bT`Ya]a]_`a`a]_`_`a_^a^_VX_Waa`XZ
@HWI-EAS443:3:11:550:548#0/1
GCAAAAGGAGAGAAAGCTAATGTGCTAATTGGGCAAGGGG
+HWI-EAS443:3:11:550:548#0/1
a^^`YZ`aS``aa`_¥]^aI¥aW_^__T^¥¥^[UWQ_¥WB
@HWI-EAS443:3:11:735:868#0/1
GAAAGCTAATGTGCTAATTGGGCAAGGGGACGTAGTGTTG
+HWI-EAS443:3:11:735:868#0/1
aa¥X]abaaab_aaaa`aaaa`¥a_a`]_^`a^_aZa]a_
@HWI-EAS443:3:11:781:1935#0/1
GAACTGAGCAATCTTGCAAAAGGAGAGAAAGCTAATGTGC
+HWI-EAS443:3:11:781:1935#0/1
]abbbbb[bbbaabbbbb_ababbaXabbbbb`bbbb_aa
@HWI-EAS443:3:11:80:1265#0/1
AGCACATTAGCTTTCTCTCCTTTTGCAAGATTGCTCAGTT
+HWI-EAS443:3:11:80:1265#0/1
_abaaa`a]aaaaaaa``a`a`a`_aa_a`__``_a``^^
@HWI-EAS443:3:11:874:967#0/1
CAAAAGGAGAGAAAGCTAATGTGCTAATTGGGCAAGGGGA
+HWI-EAS443:3:11:874:967#0/1
X¥TZab`Z¥ZZaaab_aO[`¥Z`]__OWW[[[X``a`aa_
@HWI-EAS443:3:11:889:971#0/1
GAGAAAGCTAATGTGCTAATTGGGCAAGGGGACGTAGTGT
+HWI-EAS443:3:11:889:971#0/1
`aabbbbab¥`bb_baaabaab`b_`Xaaab_`a]ZaZa]
@HWI-EAS443:3:11:951:1474#0/1
CAATGAACTGAGCAATCTTGCAAAAGGAGAGAAAGCTAAT
+HWI-EAS443:3:11:951:1474#0/1
a[`ab`a_abaaaaab`aaa_a]_`aa_a_b_^^YZa__`
@HWI-EAS443:3:11:991:589#0/1
CAATCTTGCAAAAGGAGAGAAAGCTAATGTGCTAATTGGG
+HWI-EAS443:3:11:991:589#0/1
a¥abbbabab`X`bb`babb`abbbb`bbabbab`babba
```

@HWI-EAS443:3:12:1146:1799#0/1
AATGAACTGAGCAATCTTGCAAAAGGAGAGAAAGCTAATG
+HWI-EAS443:3:12:1146:1799#0/1
¥`ab¥V¥_]__^`V[¥``a]a`a``ba__baba`]]`¥__
@HWI-EAS443:3:12:114:1683#0/1
GAAAGCTAATGTGCTAATTGGGCAAGGGGACGTAGTGTTG
+HWI-EAS443:3:12:114:1683#0/1
ab`a[_aa[aa_abaa`aa_`abaV_]a_`aa]`a]a_a_
@HWI-EAS443:3:12:117:702#0/1
ATCTTGCAAAAGGAGAGAAAGCTAATGTGCTAATTGGGCA
+HWI-EAS443:3:12:117:702#0/1
aab``baa]¥_aaa`_aaa_aaaaaa`[aaaa^aa_a^_]
@HWI-EAS443:3:12:1203:2003#0/1
AAGCATCAATGAACTGAGCAATCTTGCAAAAGGAGAGAAA
+HWI-EAS443:3:12:1203:2003#0/1
aaa`a```b]_aa¥^_^`a_]__^__]]^^X¥^W_^__^^
@HWI-EAS443:3:12:1246:130#0/1
GAGCAATCTTGCAAAAGGAGAGAAAGCTAATGTGCTAATT
+HWI-EAS443:3:12:1246:130#0/1
aaa]a`a```aHVY__a_^]¥`VXW_Z]^UX¥Y_XXVPBB
@HWI-EAS443:3:12:1270:831#0/1
GTCCCCTTGCCCAATTAGCACATTAGCTTTCTCTCCTTTT
+HWI-EAS443:3:12:1270:831#0/1
a_`]`aa`baa_a`a_aY^a`a`a___`_¥Z__`]]`__^
@HWI-EAS443:3:12:1358:245#0/1
GAAAGCTAATGTGCTAATTGGGCAAGGGGACGTAGTGTTG
+HWI-EAS443:3:12:1358:245#0/1
aa¥_b_a`N`aYa¥`VUaaa_`QYRa_^_UT^QU_Y¥ZZB
@HWI-EAS443:3:12:1378:771#0/1
CTTGCAAAAGGAGAGAAAGCTAATGTGCTAATTGGGCAAG
+HWI-EAS443:3:12:1378:771#0/1
UW¥_J_`^`¥b^`aa`][`]`ZV_aaba]_aa]`]_`^S_
@HWI-EAS443:3:12:1479:1751#0/1
CAATGAACTGAGCAATCTTGCAAAAGGAGAGAAAGCTAAT
+HWI-EAS443:3:12:1479:1751#0/1
aaa^_aa`bab¥^ba`Ubab^a_aXaaa`aaa_aaGaaZ^
@HWI-EAS443:3:12:1506:779#0/1
AAAGCTAATGTGCTAATTGGGCAAGGGGACGTAGTGTTGG
+HWI-EAS443:3:12:1506:779#0/1
aaa[V`b`^b__G¥^`aabVSQ]__aa_¥WaW¥`Xa___V
@HWI-EAS443:3:12:1533:677#0/1
AGCTAATGTGCTAATTGGGCAAGGGGACGTAGTGTTGGTA
+HWI-EAS443:3:12:1533:677#0/1
`a``]¥b`X`^_¥Va[TN[V_X_a`^]Xa[TZS^aa_¥XY
@HWI-EAS443:3:12:1548:306#0/1
GAGCAATCTTGCAAAAGGAGAGAAAGCTAATGTGCTAATT
+HWI-EAS443:3:12:1548:306#0/1
_VVW`aa__bb]``UJ¥_SWY]abY]ZaPX_]JX]]ZZ_a
@HWI-EAS443:3:12:1570:48#0/1
AAGCTAATGTGCTAATTGGGCAAGGGGACGTAGTGTTGGT

+HWI-EAS443:3:12:1570:48#0/1
aabab_ab_[b_aaaaaaba]^^aa`aZZaWZ_Ua[``_H
@HWI-EAS443:3:12:1709:236#0/1
GCATCAATGAACTGAGCAATCTTGCAAAAGGAGAGAAAGC
+HWI-EAS443:3:12:1709:236#0/1
aa¥aW]`bb_Y`aa][U^``¥aaaV^^ZZ]_]_^`]X]^N
@HWI-EAS443:3:12:1751:407#0/1
AGCTAATGTGCTAATTGGGCAAGGGGACGTAGTGTTGGTA
+HWI-EAS443:3:12:1751:407#0/1
ababaaab_a_a_``aaab_`_]a_^_Xa]]_]a]__^XU
@HWI-EAS443:3:12:254:970#0/1
GAAAGCTAATGTGCTAATTGGGCAAGGGGACGTAGTGTTG
+HWI-EAS443:3:12:254:970#0/1
`^_`]aaZa]a_[U`XNZY`]]^_aa_`_^^a^``T_W_]
@HWI-EAS443:3:12:294:1026#0/1
CTGAGCAATCTTGCAAAAGGAGAGAAAGCTAATGTGCTAA
+HWI-EAS443:3:12:294:1026#0/1
TXY__aaW```Xaa¥_Z^X`a`[^¥^]_`]_bbb^a]_V]
@HWI-EAS443:3:12:302:670#0/1
CATCAATGAACTGAGCAATCTTGCAAAAGGAGAGAAAGCT
+HWI-EAS443:3:12:302:670#0/1
aaa]QXaaaa`aaa`a`aa^`aaa_]¥`aa__^_`a____
@HWI-EAS443:3:12:359:635#0/1
GAAAGCTAATGTGCTAATTGGGCAAGGGGACGTAGTGTTG
+HWI-EAS443:3:12:359:635#0/1
aa^aa`aa_aa]aaaa_`_a`_a`_`¥_`]^a]^aZ_X]_
@HWI-EAS443:3:12:432:1705#0/1
AAGCATCAATGAACTGAGCAATCTTGCAAAAGGAGAGAAA
+HWI-EAS443:3:12:432:1705#0/1
Y]I^YY`[^][[Z``_YHU]T__U]^a^____^]]`_^S_
@HWI-EAS443:3:12:45:245#0/1
CTTGCAAAAGGAGAGAAAGCTAATGTGCTAATTGGGCAAG
+HWI-EAS443:3:12:45:245#0/1
`Z_bab^V¥_ZT_Z`]Y``a^_T^aU`_`_]_`__a_^Xa
@HWI-EAS443:3:12:46:718#0/1
GAGAAAGCTAATGTGCTAATTGGGCAAGGGGACGTAGTGT
+HWI-EAS443:3:12:46:718#0/1
aaa`]V_aaaaaa[a__S]``_]¥^__]a_^]^aV[`U`]
@HWI-EAS443:3:12:528:1341#0/1
GCATCAATGAACTGAGCAATCTTGCAAAAGGAGAGAAAGC
+HWI-EAS443:3:12:528:1341#0/1
aa_ab``XY^^_`Y¥aaaa^aaaa```a[`[_aaaa^X`_
@HWI-EAS443:3:12:542:1883#0/1
CATCAATGAACTGAGCAATCTTGCAAAAGGAGAGAAAGCT
+HWI-EAS443:3:12:542:1883#0/1
ZZVOa_Xb_b_bab¥Vbabb`Ub¥a`a_aKabaObbba__
@HWI-EAS443:3:12:574:415#0/1
GAGCAATCTTGCAAAAGGAGAGAAAGCTAATGTGCTAATT
+HWI-EAS443:3:12:574:415#0/1
aUFWa_``a^aX_a^¥a^]__[_¥^`[_S_a_R_W¥]]¥]

```
@HWI-EAS443:3:12:623:1527#0/1
AGCTAATGTGCTAATTGGGCAAGGGGACGTAGTGTTGGTA
+HWI-EAS443:3:12:623:1527#0/1
aaa`ba`a_`aaaaaa_`]a]^_a^`^`[^^`]`W`_¥XX
@HWI-EAS443:3:12:758:1535#0/1
GAGAAAGCTAATGTGCTAATTGGGCAAGGGGACGTAGTGT
+HWI-EAS443:3:12:758:1535#0/1
aa_TaaZbb]aUPFZNY`XRYaV[XHWZZVXR_aVHYN`B
@HWI-EAS443:3:12:959:588#0/1
GAGCAATCTTGCAAAAGGAGAGAAAGCTAATGTGCTAATT
+HWI-EAS443:3:12:959:588#0/1
aa`a`abbbbb`¥aaabb]a`bba`]¥a`]aa_a[a`_a[
@HWI-EAS443:3:13:1133:637#0/1
TGCAAAAGGAGAGAAAGCTAATGTGCTAATTGGGCAAGGG
+HWI-EAS443:3:13:1133:637#0/1
aaaaa¥_ab_a`baaaa`aaaba]a`a`_a`_a[___aa`
@HWI-EAS443:3:13:1201:154#0/1
ATCAATGAACTGAGCAATCTTGCAAAAGGAGAGAAAGCTA
+HWI-EAS443:3:13:1201:154#0/1
a_`_`aaab_]¥_[LZ]a^a_a^_`^¥`___`a^_UaX_^
@HWI-EAS443:3:13:1213:359#0/1
GAGCAATCTTGCAAAAGGAGAGAAAGCTAATGTGCTAATT
+HWI-EAS443:3:13:1213:359#0/1
a`_`a`bUa```U^]_`[Waaaa_[aXZ^__`V`X_X_^]
@HWI-EAS443:3:13:1220:574#0/1
GAAAGCTAATGTGCTAATTGGGCAAGGGGACGTAGTGTTG
+HWI-EAS443:3:13:1220:574#0/1
aaaaaaa`aaa^a_a_aaaaaa^__aa__]Za^__Ya]`_
@HWI-EAS443:3:13:1230:1147#0/1
GCATCAATGAACTGAGCAATCTTGCAAAAGGAGAGAAAGC
+HWI-EAS443:3:13:1230:1147#0/1
aXaaYZX[a¥a]_^^`[a_a^a__^^¥^^¥^O]^__¥¥¥I
@HWI-EAS443:3:13:1353:1960#0/1
GCAAAAGGAGAGAAAGCTAATGTGCTAATTGGGCAAGGGG
+HWI-EAS443:3:13:1353:1960#0/1
a^_]_XWW^a^^Z¥^_W_]]]_Z_Z¥]YW^]]ZVXZ^ZXU
@HWI-EAS443:3:13:1425:857#0/1
GAGCAATCTTGCAAAAGGAGAGAAAGCTAATGTGCTAATT
+HWI-EAS443:3:13:1425:857#0/1
O_bb¥S^Q[]aY`b[JXV^^^_]_OYQRYTaa_¥F¥_aba
@HWI-EAS443:3:13:1516:1681#0/1
CAATGAACTGAGCAATCTTGCAAAAGGAGAGAAAGCTAAT
+HWI-EAS443:3:13:1516:1681#0/1
¥`b`b`¥aba_b_baaWabaaaaabba^`bab^aaVaa_a
@HWI-EAS443:3:13:1662:948#0/1
AATGAACTGAGCAATCTTGCAAAAGGAGAGAAAGCTAATG
+HWI-EAS443:3:13:1662:948#0/1
[Y[a¥aJb[IZ_`aa_aa`^_```baa^`b_]__W_]_[]
@HWI-EAS443:3:13:1682:129#0/1
GAGCAATCTTGCAAAAGGAGAGAAAGCTAATGTGCTAATT
```

+HWI-EAS443:3:13:1682:129#0/1
[XX^`a_[_a[F_a_aY^[baba[¥bZ]Z`a]^_VaZaa^
@HWI-EAS443:3:13:265:1906#0/1
AAGCTAATGTGCTAATTGGGCAAGGGGACGTAGTGTTGGT
+HWI-EAS443:3:13:265:1906#0/1
a`_aa^`aa[a`a`_a_aUSa`a_`__Za_W_XXY^_`¥R
@HWI-EAS443:3:13:288:842#0/1
CTTGCAAAAGGAGAGAAAGCTAATGTGCTAATTGGGCAAG
+HWI-EAS443:3:13:288:842#0/1
aabbb`aaaba_aabaWaaaa`aa]RZ_```aa`_a___a
@HWI-EAS443:3:13:328:952#0/1
AACTGAGCAATCTTGCAAAAGGAGAGAAAGCTAATGTGCT
+HWI-EAS443:3:13:328:952#0/1
aba^`a_`aaaaaa`aaaY`a`_a_`a`_a`_`_^^Z__^
@HWI-EAS443:3:13:397:1901#0/1
AAGCATCAATGAACTGAGCAATCTTGCAAAAGGAGAGAAA
+HWI-EAS443:3:13:397:1901#0/1
aa¥_a_```a`_`]_^]]^a`_a__^`_]_]_¥]_`_^]B
@HWI-EAS443:3:13:464:445#0/1
GAAAGCTAATGTGCTAATTGGGCAAGGGGACGTAGTGTTG
+HWI-EAS443:3:13:464:445#0/1
a`I_a_]]X_a[a]^^^`a`a__^XZaY^WX`Y^`X`Y[_
@HWI-EAS443:3:13:604:633#0/1
GCATCAATGAACTGAGCAATCTTGCAAAAGGAGAGAAAGC
+HWI-EAS443:3:13:604:633#0/1
_`¥`b`_``aababaa^`babaab`b[aab``b_bb`Za[
@HWI-EAS443:3:13:67:1771#0/1
CAAAAGGAGAGAAAGCTAATGTGCTAATTGGGCAAGGGGA
+HWI-EAS443:3:13:67:1771#0/1
`Sa`[SU[]][^`_L[^aX`_`a_``_¥¥]RN_`_WBBBB
@HWI-EAS443:3:13:769:887#0/1
GAGCAATCTTGCAAAAGGAGAGAAAGCTAATGTGCTAATT
+HWI-EAS443:3:13:769:887#0/1
`UU_UR]_]V`b¥K]Tbbaa_ba]]a_`U``b`aa]_^a`
@HWI-EAS443:3:13:770:83#0/1
GAGCAATCTTGCAAAAGGAGAGAAAGCTAATGTGCTAATT
+HWI-EAS443:3:13:770:83#0/1
^Ybb_[T``bb_a`ZV^aa`a_`^`bZ_^_]bMa_`^¥XG
@HWI-EAS443:3:14:1029:1268#0/1
TGAGCAATCTTGCAAAAGGAGAGAAAGCTAATGTGCTAAT
+HWI-EAS443:3:14:1029:1268#0/1
aba`aaaaaaaa]`Ya_aa_`aa_a^a_a_`aaW`^`^^`
@HWI-EAS443:3:14:1151:428#0/1
GAGCAATCTTGCAAAAGGAGAGAAAGCTAATGTGCTAATT
+HWI-EAS443:3:14:1151:428#0/1
aababba^bba__`aa`b_aabaa_a¥a_`aa_a]a^_`_
@HWI-EAS443:3:14:1153:1652#0/1
AGCTAATGTGCTAATTGGGCAAGGGGACGTAGTGTTGGTA
+HWI-EAS443:3:14:1153:1652#0/1
abaaba`a`baaa_aaaZa_a__aa^`Za]_a_a]_a_Z[

```
@HWI-EAS443:3:14:1157:1060#0/1
GAGCAATCTTGCAAAAGGAGAGAAAGCTAATGTGCTAATT
+HWI-EAS443:3:14:1157:1060#0/1
aa¥Q_aa`aa`¥`aa^aa[a`aa__aa___`aX`^^`U^[
@HWI-EAS443:3:14:1166:924#0/1
GAGCAATCTTGCAAAAGGAGAGAAAGCTAATGTGCTAATT
+HWI-EAS443:3:14:1166:924#0/1
aab_abbb`bbY``]abaa`^baaabaaa]bb_``aa___
@HWI-EAS443:3:14:1186:263#0/1
AGCATCAATGAACTGAGCAATCTTGCAAAAGGAGAGAAAG
+HWI-EAS443:3:14:1186:263#0/1
a`Z``Qa[aa^aY`[Waa_aaZ_`bN_Y[R`V^a`^_WXa
@HWI-EAS443:3:14:1197:569#0/1
GAGAAAGCTAATGTGCTAATTGGGCAAGGGGACGTAGTGT
+HWI-EAS443:3:14:1197:569#0/1
N]b^¥R_O¥_^`¥^b`b¥RZbbab¥`^bbaba]aTWa`b]
@HWI-EAS443:3:14:1207:797#0/1
TTGCAAAAGGAGAGAAAGCTAATGTGCTAATTGGGCAAGG
+HWI-EAS443:3:14:1207:797#0/1
aabaa^`abaaa`aa`aa`b^_aa_a_a`a`_]`a]]]aa
@HWI-EAS443:3:14:1225:1327#0/1
ATCTTGCAAAAGGAGAGAAAGCTAATGTGCTAATTGGGCA
+HWI-EAS443:3:14:1225:1327#0/1
R]a¥VJ^T¥¥b[R[aa_`V^`b[_aaaP```WRWH[`_¥_
@HWI-EAS443:3:14:1232:67#0/1
CCCTTGCCCAATTAGCACATTAGCTTTCTCTCCTTTTGCA
+HWI-EAS443:3:14:1232:67#0/1
``b]a^¥UZ`babZQUWaababa¥aaa¥aaa]U]aaabZ¥
@HWI-EAS443:3:14:1335:184#0/1
GAGCAATCTTGCAAAAGGAGAGAAAGCTAATGTGCTAATT
+HWI-EAS443:3:14:1335:184#0/1
abbbbb`aaaaaa_a_a^_baa`_^a_```aa^_Y`]_^`
@HWI-EAS443:3:14:143:688#0/1
GAGAAAGCTAATGTGCTAATTGGGCAAGGGGACGTAGTGT
+HWI-EAS443:3:14:143:688#0/1
a_Y_HOZ``Q]`[O`_[^Wa_____ZJ^Z_WS^ZVWUMYB
@HWI-EAS443:3:14:1586:75#0/1
AATGAACTGAGCAATCTTGCAAAAGGAGAGAAAGCTAATG
+HWI-EAS443:3:14:1586:75#0/1
aabaaa__]``_`_a¥_`a]_]U^___^W`_^_aX__Z^_
@HWI-EAS443:3:14:344:1213#0/1
AATGAACTGAGCAATCTTGCAAAAGGAGAGAAAGCTAATG
+HWI-EAS443:3:14:344:1213#0/1
a]ZQVFRaQSQPa¥OP_Q_YPa[`[MV`XQaU[TN]`STT
@HWI-EAS443:3:14:379:1950#0/1
GAAAGCTAATGTGCTAATTGGGCAAGGGGACGTAGTGTTG
+HWI-EAS443:3:14:379:1950#0/1
abb[ababaaaZ]aabaa_aa`baaaa`Ya]aZaa^`^_`
@HWI-EAS443:3:14:39:755#0/1
GAACTGAGCAATCTTGCAAAAGGAGAGAAAGCTAATGTGC
```

```
+HWI-EAS443:3:14:39:755#0/1
aaa[a^aa`SD[^[Xa`T_]_aUQaaPXWa]P^`^]__¥Y
@HWI-EAS443:3:14:409:552#0/1
GAGCAATCTTGCAAAAGGAGAGAAAGCTAATGTGCTAATT
+HWI-EAS443:3:14:409:552#0/1
¥W¥[¥¥`¥¥abZXW_^``]`bb_``^a_^¥]b_`]abMba
@HWI-EAS443:3:14:418:718#0/1
AATGAACTGAGCAATCTTGCAAAAGGAGAGAAAGCTAATG
+HWI-EAS443:3:14:418:718#0/1
aaaaaaaaaaaa`Z]a_`aa``__a``__```_`_^_^``
@HWI-EAS443:3:14:437:1398#0/1
TACCAACACTACGTCCCCTTGCCCAATTAGCACATTAGCT
+HWI-EAS443:3:14:437:1398#0/1
Z_ba__aU^[`aWS_bbbaaaba`U¥]ZZ`^[¥_]]Y__^
@HWI-EAS443:3:14:463:338#0/1
GAGAAAGCTAATGTGCTAATTGGGCAAGGGGACGTAGTGT
+HWI-EAS443:3:14:463:338#0/1
aaaaa`baaa`aa¥__a_aa`aa``__`_a`]]`Z^aZ`]
@HWI-EAS443:3:14:520:1000#0/1
TAGCTTTCTCTCCTTTTGCAAGATTGCTCAGTTCATTGAT
+HWI-EAS443:3:14:520:1000#0/1
aba`aaaaab`aa`a__aaa^a^_``a___aZ__a`]aa]
@HWI-EAS443:3:14:520:591#0/1
CGTCCCCTTGCCCAATTAGCACATTAGCTTTCTCTCCTTT
+HWI-EAS443:3:14:520:591#0/1
aa`]aa`[PX`¥_`aa`a`¥¥^aa__``_`_^¥X_UW^]^
@HWI-EAS443:3:14:586:1321#0/1
CAAAAGGAGAGAAAGCTAATGTGCTAATTGGGCAAGGGGA
+HWI-EAS443:3:14:586:1321#0/1
aba^````b`bba`][aa`abTaaaa`a`a`^a^Sb`_``
@HWI-EAS443:3:14:600:1088#0/1
CATCAATGAACTGAGCAATCTTGCAAAAGGAGAGAAAGCT
+HWI-EAS443:3:14:600:1088#0/1
aaaaa_aaaa```aa_a_`aa_``^[W[`^^__```_^¥_
@HWI-EAS443:3:14:618:1861#0/1
GAACTGAGCAATCTTGCAAAAGGAGAGAAAGCTAATGTGC
+HWI-EAS443:3:14:618:1861#0/1
aabbaaWabbVbbb``aab`^a^aaabbba`_abaXUK¥^
@HWI-EAS443:3:14:62:1691#0/1
GTCCCCTTGCCCAATTAGCACATTAGCTTTCTCTCCTTTT
+HWI-EAS443:3:14:62:1691#0/1
XQ_a`b]T]aa_QWR^]V_]¥¥Za[L]Z]]O]X[¥_¥W_`
@HWI-EAS443:3:14:632:1032#0/1
AATCTTGCAAAAGGAGAGAAAGCTAATGTGCTAATTGGGC
+HWI-EAS443:3:14:632:1032#0/1
¥_abaaaa`_a_aa_aaaaZS_]`^a`_Xa^_^__^a¥]¥
@HWI-EAS443:3:14:640:38#0/1
GAGAAAGCTAATGTGCTAATTGGGCAAGGGGACGTAGTGT
+HWI-EAS443:3:14:640:38#0/1
a]a`Y¥`^`_]Y_R_]^X]^X^]YXYX_Z^^ZX^WX_GZX
```

@HWI-EAS443:3:14:668:1832#0/1
CATCAATGAACTGAGCAATCTTGCAAAAGGAGAGAAAGCT
+HWI-EAS443:3:14:668:1832#0/1
_`Va¥[¥`S¥ba[U[XZ[_Y_Ua`ZWUVb^`^aba¥Z_a`
@HWI-EAS443:3:14:727:1012#0/1
ATCTTGCAAAAGGAGAGAAAGCTAATGTGCTAATTGGGCA
+HWI-EAS443:3:14:727:1012#0/1
abbba`aa`a`aa_aaaaa`^a__aa`Z`^a^_^_`^¥Z_
@HWI-EAS443:3:14:774:306#0/1
GTCCCCTTGCCCAATTAGCACATTAGCTTTCTCTCCTTTT
+HWI-EAS443:3:14:774:306#0/1
a^a[`Z¥I]¥_`_[PLWZXP[¥Y¥Z`[NYYUUJYWW^UV¥
@HWI-EAS443:3:14:798:1690#0/1
CATCAATGAACTGAGCAATCTTGCAAAAGGAGAGAAAGCT
+HWI-EAS443:3:14:798:1690#0/1
aa`aaaa`^a^`[^aa`a_]_^`__^`_a]^_``a`^^Y^
@HWI-EAS443:3:14:917:1122#0/1
GAAAGCTAATGTGCTAATTGGGCAAGGGGACGTAGTGTTG
+HWI-EAS443:3:14:917:1122#0/1
aaOR_`_T¥]`¥OZ]``aWR^[[GN]_a¥WFYGX]BBBBB
@HWI-EAS443:3:14:998:1559#0/1
ATCTTGCAAAAGGAGAGAAAGCTAATGTGCTAATTGGGCA
+HWI-EAS443:3:14:998:1559#0/1
Z`a^a[]^ZZ[^SYaV_`````]Za¥a^a_`__^`]Z[TV
@HWI-EAS443:3:15:1039:1325#0/1
GCAAAAGGAGAGAAAGCTAATGTGCTAATTGGGCAAGGGG
+HWI-EAS443:3:15:1039:1325#0/1
aaaa`_`^aaaaaa`aUaaaa`Qb```_`aaaaS`[_`^_
@HWI-EAS443:3:15:1112:986#0/1
GAGCAATCTTGCAAAAGGAGAGAAAGCTAATGTGCTAATT
+HWI-EAS443:3:15:1112:986#0/1
aa`]a``abbb``_YZb¥¥[aba]^]L]^`aa_a]__aaa
@HWI-EAS443:3:15:1142:1208#0/1
GCATCAATGAACTGAGCAATCTTGCAAAAGGAGAGAAAGC
+HWI-EAS443:3:15:1142:1208#0/1
`[[[ZZW_`]aYXX[a___]K_Q_WVWW`a[Z`_`]^_a_
@HWI-EAS443:3:15:1165:1754#0/1
GAAAGCTAATGTGCTAATTGGGCAAGGGGACGTAGTGTTG
+HWI-EAS443:3:15:1165:1754#0/1
`]R^_a^Y__`[]_VWX¥Q¥_W_^W¥Z^VY^`U[`TZSY]
@HWI-EAS443:3:15:1200:374#0/1
ATGAACTGAGCAATCTTGCAAAAGGAGAGAAAGCTAATGT
+HWI-EAS443:3:15:1200:374#0/1
Z``aab_Q^[abb`M`baaYQ[ab_ababb¥_`aa`Zab¥
@HWI-EAS443:3:15:1395:1822#0/1
AGAAAGCTAATGTGCTAATTGGGCAAGGGGACGTAGTGTT
+HWI-EAS443:3:15:1395:1822#0/1
a¥`a`¥bb¥a`_G`O`abba^PZ__^a__b`SaSYa_a_a
@HWI-EAS443:3:15:140:945#0/1
GAACTGAGCAATCTTGCAAAAGGAGAGAAAGCTAATGTGC

```
+HWI-EAS443:3:15:140:945#0/1
[`ab_]W_bbba[aabb]ab_a`]baaab_aa`a`baX`a
@HWI-EAS443:3:15:1481:1864#0/1
GAACTGAGCAATCTTGCAAAAGGAGAGAAAGCTAATGTGC
+HWI-EAS443:3:15:1481:1864#0/1
aab``a`abbbbaaab]bbabbabaabb`ab`bbbbba__
@HWI-EAS443:3:15:1534:1765#0/1
CGTCCCCTTGCCCAATTAGCACATTAGCTTTCTCTCCTTT
+HWI-EAS443:3:15:1534:1765#0/1
Ub`abaIS`JKaab`b]]UKb[YaUUababbaaaaa[`Ya
@HWI-EAS443:3:15:1623:330#0/1
AATGAACTGAGCAATCTTGCAAAAGGAGAGAAAGCTAATG
+HWI-EAS443:3:15:1623:330#0/1
a`ba_¥G¥___Y`a_X_a`YY¥^ZY[^a_a`aXaO```^_
@HWI-EAS443:3:15:1641:410#0/1
GAAAGCTAATGTGCTAATTGGGCAAGGGGACGTAGTGTTG
+HWI-EAS443:3:15:1641:410#0/1
aab_babaaab^b]baabaaab_a_baaa_^b^_b]b`ab
@HWI-EAS443:3:15:1782:592#0/1
AATCTTGCAAAAGGAGAGAAAGCTAATGTGCTAATTGGGC
+HWI-EAS443:3:15:1782:592#0/1
]]_RQb¥]_^^XbaY_¥_ba`a_bU_aaZaZZaXT^`aaH
@HWI-EAS443:3:15:1791:614#0/1
CCTTGCCCAATTAGCACATTAGCTTTCTCTCCTTTTGCAA
+HWI-EAS443:3:15:1791:614#0/1
_aa`a^]]T_abab_aWababa^^aaJaY^_^_aaa[UY¥
@HWI-EAS443:3:15:266:386#0/1
AGCTAATGTGCTAATTGGGCAAGGGGACGTAGTGTTGGTA
+HWI-EAS443:3:15:266:386#0/1
^[aaZ[`^H`abbba¥¥a^^aab^]WYaa]_a]`USaaMX
@HWI-EAS443:3:15:314:656#0/1
CATCAATGAACTGAGCAATCTTGCAAAAGGAGAGAAAGCT
+HWI-EAS443:3:15:314:656#0/1
abaaabb__baaa¥`aaaa_aaabaa^ab`_baaaa_a_`
@HWI-EAS443:3:15:383:201#0/1
GAGAAAGCTAATGTGCTAATTGGGCAAGGGGACGTAGTGT
+HWI-EAS443:3:15:383:201#0/1
a]a]`[^aa`_aa]a_^TX_`a`_^_Za_¥TX^`Z^`X]Y
@HWI-EAS443:3:15:457:1697#0/1
GAGCAATCTTGCAAAAGGAGAGAAAGCTAATGTGCTAATT
+HWI-EAS443:3:15:457:1697#0/1
^Q^a^[V^a_Y``_URa^OVbbbbaTaabY^^V]]_NaaW
@HWI-EAS443:3:15:503:1069#0/1
ACACTACGTCCCCTTGCCCAATTAGCACATTAGCTTTCTC
+HWI-EAS443:3:15:503:1069#0/1
`^bb_aba_]`]^]a_a]aZXUO^XYWZ]¥^`YWU`¥a__
@HWI-EAS443:3:15:559:44#0/1
GAAAGCTAATGTGCTAATTGGGCAAGGGGACGTAGTGTTG
+HWI-EAS443:3:15:559:44#0/1
^^a`b`aaaab¥aaaaaaa^¥a_aabaa_^^a[^`Sa]_a
```

```
@HWI-EAS443:3:15:569:360#0/1
AATGAACTGAGCAATCTTGCAAAAGGAGAGAAAGCTAATG
+HWI-EAS443:3:15:569:360#0/1
abbbabbba]bbbabbbbbabbaabaabaabaababbabb
@HWI-EAS443:3:15:595:41#0/1
TCTTGCAAAAGGAGAGAAAGCTAATGTGCTAATTGGGCAA
+HWI-EAS443:3:15:595:41#0/1
Ia¥¥a¥`a]a¥[Wbbbaaaa]Y]]_bWaa][_]Ya`_`^^
@HWI-EAS443:3:15:613:213#0/1
GAAAGCTAATGTGCTAATTGGGCAAGGGGACGTAGTGTTG
+HWI-EAS443:3:15:613:213#0/1
aa`W_]a``]aOaab_aa^`^`_a^aab_¥^aR^aZa^`a
@HWI-EAS443:3:15:701:1478#0/1
GAACTGAGCAATCTTGCAAAAGGAGAGAAAGCTAATGTGC
+HWI-EAS443:3:15:701:1478#0/1
abbbaabaaabaaaaaabbaaaaaaabaaa`_aaaaa^a_
@HWI-EAS443:3:15:804:186#0/1
TGCAAAAGGAGAGAAAGCTAATGTGCTAATTGGGCAAGGG
+HWI-EAS443:3:15:804:186#0/1
[bb_``bb`^abbaa`baa_`¥RZb``ba__`_¥T]`a_^
@HWI-EAS443:3:15:81:91#0/1
GAGCAATCTTGCAAAAGGAGAGAAAGCTAATGTGCTAATT
+HWI-EAS443:3:15:81:91#0/1
`XUZRSRV^WUV`[NYX¥QV^_]TTZ^PZ]Y[VVQNN[WN
@HWI-EAS443:3:16:1036:421#0/1
GAAAGCTAATGTGCTAATTGGGCAAGGGGACGTAGTGTTG
+HWI-EAS443:3:16:1036:421#0/1
aaa`a`a__abZa_aa`a`aaa_^_aaaa]]aZ_`Z`__`
@HWI-EAS443:3:16:131:1928#0/1
CATCAATGAACTGAGCAATCTTGCAAAAGGAGAGAAAGCT
+HWI-EAS443:3:16:131:1928#0/1
abab```a`aaa^[[a^a`a```a^`¥_^^`a`¥a_U[¥_
@HWI-EAS443:3:16:1345:935#0/1
AATCTTGCAAAAGGAGAGAAAGCTAATGTGCTAATTGGGC
+HWI-EAS443:3:16:1345:935#0/1
abb_aaaXa¥baab[aa`baY¥Y^a¥ba_b_`aX`__`aa
@HWI-EAS443:3:16:1355:1524#0/1
GAACTGAGCAATCTTGCAAAAGGAGAGAAAGCTAATGTGC
+HWI-EAS443:3:16:1355:1524#0/1
abbaba[babba`aaabaa`_ba_aaa`aaa_b`_aa]`^
@HWI-EAS443:3:16:1384:1669#0/1
GAACTGAGCAATCTTGCAAAAGGAGAGAAAGCTAATGTGC
+HWI-EAS443:3:16:1384:1669#0/1
]_aaa¥``aaaa_a``^a__^__^a_aa__a__`__`X]]
@HWI-EAS443:3:16:139:500#0/1
AGCTAATGTGCTAATTGGGCAAGGGGACGTAGTGTTGGTA
+HWI-EAS443:3:16:139:500#0/1
RR¥XaYY¥SV¥__abYUZa[]HP_^ba_bQU^R`Z_``KO
@HWI-EAS443:3:16:1472:571#0/1
GCATCAATGAACTGAGCAATCTTGCAAAAGGAGAGAAAGC
```

```
+HWI-EAS443:3:16:1472:571#0/1
``[`¥ZNZ`ba`Z^YaXaaaP`aa¥]WWX¥J¥]UT]V`a_
@HWI-EAS443:3:16:1486:671#0/1
GAGAAAGCTAATGTGCTAATTGGGCAAGGGGACGTAGTGT
+HWI-EAS443:3:16:1486:671#0/1
_¥S`[^aaaba``Za``YX^a^T]_UX_^]`X_aYWaTaX
@HWI-EAS443:3:16:1509:919#0/1
GCATCAATGAACTGAGCAATCTTGCAAAAGGAGAGAAAGC
+HWI-EAS443:3:16:1509:919#0/1
abbb`abbb^^_aaa_`aaa^aab``a_]aa_a`aa`_`]
@HWI-EAS443:3:16:1519:973#0/1
CAACACTACGTCCCCTTGCCCAATTAGCACATTAGCTTTC
+HWI-EAS443:3:16:1519:973#0/1
^`_bZbb_ab``[aa__aabbb_bbbabb_bbaa[ab`aa
@HWI-EAS443:3:16:1658:1968#0/1
AAAGGAGAGAAAGCTAATGTGCTAATTGGGCAAGGGGACG
+HWI-EAS443:3:16:1658:1968#0/1
a`aaaa`]^aaa`__Za^aU]]a^`_a_``_X^a^]QUWa
@HWI-EAS443:3:16:1666:154#0/1
GAAAGCTAATGTGCTAATTGGGCAAGGGGACGTAGTGTTG
+HWI-EAS443:3:16:1666:154#0/1
aa^H`]b[Z]]V][¥VV__`_VUTS[R][UU`UVRTUUVB
@HWI-EAS443:3:16:1740:708#0/1
ATCTTGCAAAAGGAGAGAAAGCTAATGTGCTAATTGGGCA
+HWI-EAS443:3:16:1740:708#0/1
aaaaaY^a``aaaYa_aa]`a¥aX`^a^aYa__`^X^^X_
@HWI-EAS443:3:16:1773:214#0/1
AATGAACTGAGCAATCTTGCAAAAGGAGAGAAAGCTAATG
+HWI-EAS443:3:16:1773:214#0/1
aaabaa_a`ab_aaa^aaaX_^^^a``a^_a___V^_Z__
@HWI-EAS443:3:16:328:1474#0/1
CATCAATGAACTGAGCAATCTTGCAAAAGGAGAGAAAGCT
+HWI-EAS443:3:16:328:1474#0/1
aa`__^ab^aaa^`[[Y^_]__a^¥¥]X[^`_^_``_]^Y
@HWI-EAS443:3:16:360:1132#0/1
GCAATCTTGCAAAAGGAGAGAAAGCTAATGTGCTAATTGG
+HWI-EAS443:3:16:360:1132#0/1
ab`X`bbaaaZbaab¥Y`aaaaV^_Y``_a`__aa`aWa`
@HWI-EAS443:3:16:544:241#0/1
AATGAACTGAGCAATCTTGCAAAAGGAGAGAAAGCTAATG
+HWI-EAS443:3:16:544:241#0/1
^_abbbbbbababab`aba_a_bbabaabaaa]a```a
@HWI-EAS443:3:16:593:713#0/1
ATGAACTGAGCAATCTTGCAAAAGGAGAGAAAGCTAATGT
+HWI-EAS443:3:16:593:713#0/1
aaa^aaaa_a``[aaaaa``_`aaa_aaaa`_aa_`]`a]
@HWI-EAS443:3:16:59:206#0/1
CTGAGCAATCTTGCAAAAGGAGAGAAAGCTAATGTGCTAA
+HWI-EAS443:3:16:59:206#0/1
aabababaaab`aba`a`aba```aa``a__a^aa]a_`^`
```

```
@HWI-EAS443:3:16:655:356#0/1
GAACTGAGCAATCTTGCAAAAGGAGAGAAAGCTAATGTGC
+HWI-EAS443:3:16:655:356#0/1
`__Z]]¥`aZ^aa`ab_`]^U`Y`]````aa```a[`___
@HWI-EAS443:3:16:732:297#0/1
AATTAGCACATTAGCTTTCTCTCCTTTTGCAAGATTGCTC
+HWI-EAS443:3:16:732:297#0/1
_aa`aa¥``aa``a[]a`a`]_``a__^^[__`_^__^_Y
@HWI-EAS443:3:16:771:965#0/1
CAAAAGGAGAGAAAGCTAATGTGCTAATTGGGCAAGGGGA
+HWI-EAS443:3:16:771:965#0/1
a`a`aaaaaabaa__`_aaaa^a_`a_a___`]^^`^^]]
@HWI-EAS443:3:16:872:1165#0/1
GAGCAATCTTGCAAAAGGAGAGAAAGCTAATGTGCTAATT
+HWI-EAS443:3:16:872:1165#0/1
``bababbbabb]a_baaab_babbaaaa`bb_`aba]b^
@HWI-EAS443:3:16:873:1152#0/1
AAGCTAATGTGCTAATTGGGCAAGGGGACGTAGTGTTGGT
+HWI-EAS443:3:16:873:1152#0/1
^`aa_a`^aUaa_]X`^a__[_^a__¥Y_a]^ZMU]]`SO
@HWI-EAS443:3:16:888:909#0/1
GCAAAAGGAGAGAAAGCTAATGTGCTAATTGGGCAAGGGG
+HWI-EAS443:3:16:888:909#0/1
abXY`a[a[a¥`aa_aa_¥__a]a]__]_aa_a`[]a^^`
@HWI-EAS443:3:16:895:41#0/1
GCATCAATGAACTGAGCAATCTTGCAAAAGGAGAGAAAGC
+HWI-EAS443:3:16:895:41#0/1
a^`^X]U`^`_¥_^YU^]_^Z_]aXYTVZ^Z¥^¥_]XYRV
@HWI-EAS443:3:17:1068:622#0/1
TGCAAAAGGAGAGAAAGCTAATGTGCTAATTGGGCAAGGG
+HWI-EAS443:3:17:1068:622#0/1
^abba`Zaa``bbbbab`bbabb_baaaaaabba`]`aab
@HWI-EAS443:3:17:1134:1719#0/1
AATGAACTGAGCAATCTTGCAAAAGGAGAGAAAGCTAATG
+HWI-EAS443:3:17:1134:1719#0/1
abbba`[_a`]bab^^aa_¥aZ^]^`_aaaa`___a`YY_
@HWI-EAS443:3:17:1328:743#0/1
CATCAATGAACTGAGCAATCTTGCAAAAGGAGAGAAAGCT
+HWI-EAS443:3:17:1328:743#0/1
a]a_`aaa¥_a]WTa`¥[^]^`a_^^^W_YX`^aa_O]Y]
@HWI-EAS443:3:17:1384:445#0/1
GAACTGAGCAATCTTGCAAAAGGAGAGAAAGCTAATGTGC
+HWI-EAS443:3:17:1384:445#0/1
a_a_XKQ_[]aa^`^[]__^Ta_[``a__^aX__R^UZ_Q
@HWI-EAS443:3:17:139:189#0/1
AGCATCAATGAACTGAGCAATCTTGCAAAAGGAGAGAAAG
+HWI-EAS443:3:17:139:189#0/1
Y__XVW`¥_XJ]_]b¥Z__XUa`[a_XS[Z[YS^V¥^WP¥
@HWI-EAS443:3:17:1400:531#0/1
AACTGAGCAATCTTGCAAAAGGAGAGAAAGCTAATGTGCT
```

```
+HWI-EAS443:3:17:1400:531#0/1
ababaabaaaaaaab_aa``^a_baa`_aa_a``aa_`^a
@HWI-EAS443:3:17:1427:1958#0/1
ATGAACTGAGCAATCTTGCAAAAGGAGAGAAAGCTAATGT
+HWI-EAS443:3:17:1427:1958#0/1
aa^^`I¥aR]]_Y_^ZH]aXJX[aa]a_^a__aX[[W__U
@HWI-EAS443:3:17:1527:343#0/1
GTCCCCTTGCCCAATTAGCACATTAGCTTTCTCTCCTTTT
+HWI-EAS443:3:17:1527:343#0/1
aZ]_V[`^`]XUXZ`[__[_^__¥V[_Z_TV[N[SZ^[RV
@HWI-EAS443:3:17:1572:1169#0/1
CATCAATGAACTGAGCAATCTTGCAAAAGGAGAGAAAGCT
+HWI-EAS443:3:17:1572:1169#0/1
aa`a_abb¥ba_^`_``^`__`a_¥_]^a`]`^__`W`W^
@HWI-EAS443:3:17:1626:29#0/1
CAATCTTGCAAAAGGAGAGAAAGCTAATGTGCTAATTGGG
+HWI-EAS443:3:17:1626:29#0/1
Ma¥JJabbb_bb`Vb`]b`bbbbbbaabaUbbabbb`bbb
@HWI-EAS443:3:17:1703:912#0/1
GCAAAAGGAGAGAAAGCTAATGTGCTAATTGGGCAAGGGG
+HWI-EAS443:3:17:1703:912#0/1
aaaa^aaa_aaaaZV]Ya¥]a_]_]]X]`[R[[T]¥[YR[
@HWI-EAS443:3:17:1766:85#0/1
AAGCATCAATGAACTGAGCAATCTTGCAAAAGGAGAGAAA
+HWI-EAS443:3:17:1766:85#0/1
aabaaaaa_ba__`aa]a]_^`^`_`_]X[]a^¥`__]ZZ
@HWI-EAS443:3:17:251:334#0/1
GTCCCCTTGCCCAATTAGCACATTAGCTTTCTCTCCTTTT
+HWI-EAS443:3:17:251:334#0/1
a_aaabaabaa`b`aaaaaa_aa`aa`a`_aa^`]_a^__
@HWI-EAS443:3:17:295:557#0/1
AAAGCTAATGTGCTAATTGGGCAAGGGGACGTAGTGTTGG
+HWI-EAS443:3:17:295:557#0/1
aNXa_aa`ab_aaa`_aaa`_`X`aa`a^^aZ^`WaZ`a`
@HWI-EAS443:3:17:310:582#0/1
GAACTGAGCAATCTTGCAAAAGGAGAGAAAGCTAATGTGC
+HWI-EAS443:3:17:310:582#0/1
a`a`aa`aa`[`a_`aaa_Z_a`¥a_a``_a^`]^_`Z_Z
@HWI-EAS443:3:17:408:903#0/1
GAGCAATCTTGCAAAAGGAGAGAAAGCTAATGTGCTAATT
+HWI-EAS443:3:17:408:903#0/1
TQ_aYL_a`a¥^OM[^[``a^ZS_X`b_W__a[T^]U[^^
@HWI-EAS443:3:17:415:100#0/1
GAACTGAGCAATCTTGCAAAAGGAGAGAAAGCTAATGTGC
+HWI-EAS443:3:17:415:100#0/1
aZZ]W_SaYa``[`__^[^^_a^Wa^a]¥^_U]Z[]`Z_Z
@HWI-EAS443:3:17:435:1439#0/1
GAAAGCTAATGTGCTAATTGGGCAAGGGGACGTAGTGTTG
+HWI-EAS443:3:17:435:1439#0/1
]___ab`]bb[F___^`aab``Z^aaa^_Z`aYaaVa^_a
```

@HWI-EAS443:3:17:560:896#0/1
GAAAGCTAATGTGCTAATTGGGCAAGGGGACGTAGTGTTG
+HWI-EAS443:3:17:560:896#0/1
a^Taabaaaaa[aaaaaaaa[a`¥^a_aa]_a^^aYa]a`
@HWI-EAS443:3:17:569:1684#0/1
AACTGAGCAATCTTGCAAAAGGAGAGAAAGCTAATGTGCT
+HWI-EAS443:3:17:569:1684#0/1
ZaZZMX[¥[_I^U^`Q__X_a`aT]bSXJZVVYJ^VN¥
@HWI-EAS443:3:17:677:1102#0/1
AGCACATTAGCTTTCTCTCCTTTTGCAAGATTGCTCAGTT
+HWI-EAS443:3:17:677:1102#0/1
]ON_P^S]aWQ^GS¥ZZ^Z`SN]UUX]T[_ZU]OGN¥^UO
@HWI-EAS443:3:17:679:441#0/1
CAAAAGGAGAGAAAGCTAATGTGCTAATTGGGCAAGGGGA
+HWI-EAS443:3:17:679:441#0/1
__`aab_`baa]_aa_a`[aaWaa__T__`a____aa]_]
@HWI-EAS443:3:17:819:1351#0/1
GAACTGAGCAATCTTGCAAAAGGAGAGAAAGCTAATGTGC
+HWI-EAS443:3:17:819:1351#0/1
a`[`V]S¥aaa``]_¥`[X]K]Y]a^a`_`a_`a__aW_¥
@HWI-EAS443:3:17:829:815#0/1
GCAAAAGGAGAGAAAGCTAATGTGCTAATTGGGCAAGGGG
+HWI-EAS443:3:17:829:815#0/1
_`_[XH[^_`aaXaI_a]aSab¥^__^^`W``aW_]]_]]
@HWI-EAS443:3:17:900:1397#0/1
ATCAATGAACTGAGCAATCTTGCAAAAGGAGAGAAAGCTA
+HWI-EAS443:3:17:900:1397#0/1
^Y`]VR¥b``[Ua¥T_aP`a^aV`¥]H_a_aba``a_P^]
@HWI-EAS443:3:17:956:1004#0/1
TAGCACATTAGCTTTCTCTCCTTTTGCAAGATTGCTCAGT
+HWI-EAS443:3:17:956:1004#0/1
aa`_a_ba_aa_]a_aa^aa_``_a_a`_a`a``V____^
@HWI-EAS443:3:18:1073:775#0/1
CATCAATGAACTGAGCAATCTTGCAAAAGGAGAGAAAGCT
+HWI-EAS443:3:18:1073:775#0/1
a^aa_aaa`a_`aaa`aa`_aaa__`[`a_^a`aa]^_^`
@HWI-EAS443:3:18:1274:1221#0/1
ATGAACTGAGCAATCTTGCAAAAGGAGAGAAAGCTAATGT
+HWI-EAS443:3:18:1274:1221#0/1
aa^a``aaa^abaa`aaa``Za_a_Wa``a_`_¥__[__Z
@HWI-EAS443:3:18:1300:41#0/1
CCTTGCCCAATTAGCACATTAGCTTTCTCTCCTTTTGCAA
+HWI-EAS443:3:18:1300:41#0/1
`¥aab`a¥a`aaaa¥a_`a]^a``a_Za¥aMW`]`^aW^Z
@HWI-EAS443:3:18:1448:1707#0/1
ATCAATGAACTGAGCAATCTTGCAAAAGGAGAGAAAGCTA
+HWI-EAS443:3:18:1448:1707#0/1
Y[T_`ab```a¥W``_a`O^[_Z_`[W[`]Ra_aa_[Qaa
@HWI-EAS443:3:18:1600:94#0/1
GCATCAATGAACTGAGCAATCTTGCAAAAGGAGAGAAAGC

+HWI-EAS443:3:18:1600:94#0/1
aaaa`aabb`a_aa`a`a_aZa``^_^_]`[]a_`_]]_W
@HWI-EAS443:3:18:1649:1606#0/1
CAATCTTGCAAAAGGAGAGAAAGCTAATGTGCTAATTGGG
+HWI-EAS443:3:18:1649:1606#0/1
^^^bY^T^]YZ]S^^Pb`aaZ]_RaS]_a^`X_]^^_`_]
@HWI-EAS443:3:18:1719:1767#0/1
TCTTGCAAAAGGAGAGAAAGCTAATGTGCTAATTGGGCAA
+HWI-EAS443:3:18:1719:1767#0/1
Yabb_XVXV[^SWa`ba_]a_bY``aU_[`a^`¥```Na^
@HWI-EAS443:3:18:1747:1442#0/1
GAACTGAGCAATCTTGCAAAAGGAGAGAAAGCTAATGTGC
+HWI-EAS443:3:18:1747:1442#0/1
a¥`a_aab]aaa_a`Y^`^_¥[`]_`a][]^^`UV]_V_V
@HWI-EAS443:3:18:201:274#0/1
AATGAACTGAGCAATCTTGCAAAAGGAGAGAAAGCTAATG
+HWI-EAS443:3:18:201:274#0/1
Vb_X¥_babaaba``a_ba^W^`^a`aaaaba^a`a[a^b
@HWI-EAS443:3:18:222:2009#0/1
CATCAATGAACTGAGCAATCTTGCAAAAGGAGAGAAAGCT
+HWI-EAS443:3:18:222:2009#0/1
V[¥b_aM^¥aa^[`[bb_`_bbaa¥W¥`a^aV¥_W_^]Y`
@HWI-EAS443:3:18:262:33#0/1
GAGCAATCTTGCAAAAGGAGAGAAAGCTAATGTGCTAATT
+HWI-EAS443:3:18:262:33#0/1
`a¥``_¥aaX`a``a^_^X[X``]H`_`__ZaW_UTLUZT
@HWI-EAS443:3:18:28:1019#0/1
GAAAGCTAATGTGCTAATTGGGCAAGGGGACGTAGTGTTG
+HWI-EAS443:3:18:28:1019#0/1
```baaba_bb]baaa`aa`aaaa[Zaaa]_aZ_aY¥Z`a
@HWI-EAS443:3:18:384:683#0/1
CATCAATGAACTGAGCAATCTTGCAAAAGGAGAGAAAGCT
+HWI-EAS443:3:18:384:683#0/1
`V_baaaba^bba]babaaba```aaa¥baabaabaaa_a
@HWI-EAS443:3:18:391:1155#0/1
GAGCAATCTTGCAAAAGGAGAGAAAGCTAATGTGCTAATT
+HWI-EAS443:3:18:391:1155#0/1
a_`baaab`aaba_^`b`]a`¥^_`aa````a^`_]_``_
@HWI-EAS443:3:18:409:724#0/1
CATCAATGAACTGAGCAATCTTGCAAAAGGAGAGAAAGCT
+HWI-EAS443:3:18:409:724#0/1
a_`bWQY`Ta]^^^^U`Z`a`S_X__]]`]Y`_^^^UZ¥_
@HWI-EAS443:3:18:427:1336#0/1
GAAAGCTAATGTGCTAATTGGGCAAGGGGACGTAGTGTTG
+HWI-EAS443:3:18:427:1336#0/1
abaa`aa][aX_`a_a`a`_`_``Ja___^^_TL[H`Z]^
@HWI-EAS443:3:18:454:1105#0/1
ATCTTGCAAAAGGAGAGAAAGCTAATGTGCTAATTGGGCA
+HWI-EAS443:3:18:454:1105#0/1
_b]R_]baZaaa_aaa`a_aaa`a¥a]NY^JW[_^^][_V
```

```
@HWI-EAS443:3:18:481:113#0/1
CAATCTTGCAAAAGGAGAGAAAGCTAATGTGCTAATTGGG
+HWI-EAS443:3:18:481:113#0/1
ababb_abbababababab`bbb`b`abaabab_abaaaaa_
@HWI-EAS443:3:18:529:543#0/1
CCCTTGCCCAATTAGCACATTAGCTTTCTCTCCTTTTGCA
+HWI-EAS443:3:18:529:543#0/1
U`¥J_b[R_S`b``¥aab`[abbbU`O[WaW^^S^a[aT]
@HWI-EAS443:3:18:56:908#0/1
CAAAAGGAGAGAAAGCTAATGTGCTAATTGGGCAAGGGGA
+HWI-EAS443:3:18:56:908#0/1
`P_^_a_U_a_a`___a`W__O__¥_^VR__^_]W^]]ZZ
@HWI-EAS443:3:18:570:778#0/1
GAGCAATCTTGCAAAAGGAGAGAAAGCTAATGTGCTAATT
+HWI-EAS443:3:18:570:778#0/1
aab_]aabb`````Z_aa```aa``aa`]`a`^a[___`_
@HWI-EAS443:3:18:571:1492#0/1
CAATTAGCACATTAGCTTTCTCTCCTTTTGCAAGATTGCT
+HWI-EAS443:3:18:571:1492#0/1
aaba`babaaaaaaaaaa`a`a_a_a__XM[a____]`]_
@HWI-EAS443:3:18:593:391#0/1
ATCTTGCAAAAGGAGAGAAAGCTAATGTGCTAATTGGGCA
+HWI-EAS443:3:18:593:391#0/1
a]aaaaa`_`Yaa[]aaa`^a``a[aa`[W]Z`__^]___
@HWI-EAS443:3:18:622:400#0/1
GAAAGCTAATGTGCTAATTGGGCAAGGGGACGTAGTGTTG
+HWI-EAS443:3:18:622:400#0/1
aaZ[^`_a[Xb_a_a`_a_]_a``_aaa^^^_W¥`Y`V^a
@HWI-EAS443:3:18:628:736#0/1
GAGAAAGCTAATGTGCTAATTGGGCAAGGGGACGTAGTGT
+HWI-EAS443:3:18:628:736#0/1
aabaaaaaaaaaaaWaaa`aaaa^a_`_aa``^`aZ^aZa]
@HWI-EAS443:3:18:62:1666#0/1
GAGCAATCTTGCAAAAGGAGAGAAAGCTAATGTGCTAATT
+HWI-EAS443:3:18:62:1666#0/1
_¥U_UQOZ]YUOYQQ_¥¥VY]V¥`R¥H]PP¥VR____UXV
@HWI-EAS443:3:18:643:554#0/1
CATCAATGAACTGAGCAATCTTGCAAAAGGAGAGAAAGCT
+HWI-EAS443:3:18:643:554#0/1
abaaabb_]aaaaaaaaaaa`a`a`_U`b_^a`aa^_`^_
@HWI-EAS443:3:18:69:1157#0/1
CATCAATGAACTGAGCAATCTTGCAAAAGGAGAGAAAGCT
+HWI-EAS443:3:18:69:1157#0/1
abaaaaaaaaaaaa]aaaaa_a_a``aaYa_`aaaa^`a``
@HWI-EAS443:3:18:783:1636#0/1
CATCAATGAACTGAGCAATCTTGCAAAAGGAGAGAAAGCT
+HWI-EAS443:3:18:783:1636#0/1
Y``babababbababababb```Zbaaabaaa`abbaaa`^`
@HWI-EAS443:3:18:799:199#0/1
CAAAAGGAGAGAAAGCTAATGTGCTAATTGGGCAAGGGGA
```

+HWI-EAS443:3:18:799:199#0/1
a]V[Y`]^a]__[P¥U¥FZ__TXV^]^`_ZWWYVUSUUWW
@HWI-EAS443:3:18:849:340#0/1
AATGAACTGAGCAATCTTGCAAAAGGAGAGAAAGCTAATG
+HWI-EAS443:3:18:849:340#0/1
_^ababaaa_bababa`[aa][`_aa`aaa``aa^`___`
@HWI-EAS443:3:18:859:1578#0/1
GAGCAATCTTGCAAAAGGAGAGAAAGCTAATGTGCTAATT
+HWI-EAS443:3:18:859:1578#0/1
a[`a`¥^_]``a``]]]_[^___]¥a]___]][`V`Z^_[
@HWI-EAS443:3:19:1086:2006#0/1
GCATCAATGAACTGAGCAATCTTGCAAAAGGAGAGAAAGC
+HWI-EAS443:3:19:1086:2006#0/1
¥FYOZ``T¥Q¥Z[`Xa¥UO]`YQN¥`ZYG[aX`]_a]¥YB
@HWI-EAS443:3:19:1139:1613#0/1
GCATCAATGAACTGAGCAATCTTGCAAAAGGAGAGAAAGC
+HWI-EAS443:3:19:1139:1613#0/1
aaab___baaaaa_`[`aaa_a___a`_^a__a_a_`^__
@HWI-EAS443:3:19:1155:834#0/1
GCATCAATGAACTGAGCAATCTTGCAAAAGGAGAGAAAGC
+HWI-EAS443:3:19:1155:834#0/1
a`aa``aa`a`aaa^_aaa_`a`a_a_`^a__a```]^`O
@HWI-EAS443:3:19:1170:1432#0/1
AGCTAATGTGCTAATTGGGCAAGGGGACGTAGTGTTGGTA
+HWI-EAS443:3:19:1170:1432#0/1
aaa`aaab¥^`aaaaa`aa`aa`[^`_`aV^aVa]`a]UW
@HWI-EAS443:3:19:1181:1555#0/1
GAAAGCTAATGTGCTAATTGGGCAAGGGGACGTAGTGTTG
+HWI-EAS443:3:19:1181:1555#0/1
S_a``]b`^_aZaabaa`¥[`]__aa_b¥^aa_aa^_`aa
@HWI-EAS443:3:19:1201:413#0/1
GAGAAAGCTAATGTGCTAATTGGGCAAGGGGACGTAGTGT
+HWI-EAS443:3:19:1201:413#0/1
X¥aUV_R[]¥ZUVSV]^MRZBBBBBBBBBBBBBBBBBBBB
@HWI-EAS443:3:19:1205:89#0/1
CATCAATGAACTGAGCAATCTTGCAAAAGGAGAGAAAGCT
+HWI-EAS443:3:19:1205:89#0/1
[QN]baaaa^a[a`b`Ta`b¥bbaVba^`bab`a_`aaUa
@HWI-EAS443:3:19:1215:783#0/1
GAGCAATCTTGCAAAAGGAGAGAAAGCTAATGTGCTAATT
+HWI-EAS443:3:19:1215:783#0/1
a_¥US`¥UXW`_^`¥T`aaaaa[M]_[]Z¥`a_a]Z¥___
@HWI-EAS443:3:19:1221:1692#0/1
CATCAATGAACTGAGCAATCTTGCAAAAGGAGAGAAAGCT
+HWI-EAS443:3:19:1221:1692#0/1
S`abPS_bbbabaTY^a^^b`^ba`]aa`bababbbabSa
@HWI-EAS443:3:19:1238:60#0/1
AAGCATCAATGAACTGAGCAATCTTGCAAAAGGAGAGAAA
+HWI-EAS443:3:19:1238:60#0/1
]Q]aaYHQ``aZ¥XX`]QT``[[_a_PWZOQ____RZ[[X

```
@HWI-EAS443:3:19:1241:184#0/1
AGCATCAATGAACTGAGCAATCTTGCAAAAGGAGAGAAAG
+HWI-EAS443:3:19:1241:184#0/1
aa[`^^__a`__]`_^`]__`]`¥_[^]]X`^]`^_]Z]^
@HWI-EAS443:3:19:1307:62#0/1
AATGAACTGAGCAATCTTGCAAAAGGAGAGAAAGCTAATG
+HWI-EAS443:3:19:1307:62#0/1
UY`b_P_bb¥b``VZ_]_aaa`XX``Uaaaa^`^X]]Z_a
@HWI-EAS443:3:19:1395:163#0/1
GAACTGAGCAATCTTGCAAAAGGAGAGAAAGCTAATGTGC
+HWI-EAS443:3:19:1395:163#0/1
a_a_a__a_a`aXa^`¥Y^]_``X`]a_]Z_UY_Y_]Y^P
@HWI-EAS443:3:19:1401:1648#0/1
AGCTAATGTGCTAATTGGGCAAGGGGACGTAGTGTTGGTA
+HWI-EAS443:3:19:1401:1648#0/1
a]abbba_Q__bbbbaW_a_ab^^a__aaaaa^aZ^ZbR_
@HWI-EAS443:3:19:1450:1397#0/1
GAAAGCTAATGTGCTAATTGGGCAAGGGGACGTAGTGTTG
+HWI-EAS443:3:19:1450:1397#0/1
`Y_ZW¥WOQW[]a^YSON¥X_]VYT¥X`_TF[IO]XBBBB
@HWI-EAS443:3:19:1452:552#0/1
GAAAGCTAATGTGCTAATTGGGCAAGGGGACGTAGTGTTG
+HWI-EAS443:3:19:1452:552#0/1
^¥baa`baaabaa_baabb`b`_`]¥a^V`_a^Za_a^`a
@HWI-EAS443:3:19:1584:83#0/1
AGCTAATGTGCTAATTGGGCAAGGGGACGTAGTGTTGGTA
+HWI-EAS443:3:19:1584:83#0/1
^b_bbbb_`b[aabb`bab``abQaaZ_aZUaOa_aaKPG
@HWI-EAS443:3:19:1590:1863#0/1
AACTGAGCAATCTTGCAAAAGGAGAGAAAGCTAATGTGCT
+HWI-EAS443:3:19:1590:1863#0/1
]Y]]aY[^`Va`_a¥QY``[aa^_aaa__^N_a`[`P_XP
@HWI-EAS443:3:19:1649:1920#0/1
CTTGCAAAAGGAGAGAAAGCTAATGTGCTAATTGGGCAAG
+HWI-EAS443:3:19:1649:1920#0/1
aa^Za`__U^SG¥UXQ`aS_aWU]aNWP^Ta`_`a[^GP^
@HWI-EAS443:3:19:1670:412#0/1
ACTGAGCAATCTTGCAAAAGGAGAGAAAGCTAATGTGCTA
+HWI-EAS443:3:19:1670:412#0/1
`X``__¥_``¥^b`WY_¥`a__^Sa]a_aX]aa`aOYV_`
@HWI-EAS443:3:19:1695:1358#0/1
AAGCATCAATGAACTGAGCAATCTTGCAAAAGGAGAGAAA
+HWI-EAS443:3:19:1695:1358#0/1
a]^]`a[``aaa^Za_]`_`^_Waa`T]_^^a_Xa___^]
@HWI-EAS443:3:19:181:826#0/1
GAGCAATCTTGCAAAAGGAGAGAAAGCTAATGTGCTAATT
+HWI-EAS443:3:19:181:826#0/1
^S]_U¥U_ZUTROZOWHP[[QO[NXYLT^`^XDYXQQM[^
@HWI-EAS443:3:19:208:677#0/1
AGCACATTAGCTTTCTCTCCTTTTGCAAGATTGCTCAGTT
```

```
+HWI-EAS443:3:19:208:677#0/1
a_b]baa_a`a_aa]]a_aa```aaaa`a_]]a``a_`YZ
@HWI-EAS443:3:19:293:295#0/1
CATCAATGAACTGAGCAATCTTGCAAAAGGAGAGAAAGCT
+HWI-EAS443:3:19:293:295#0/1
abaabbaaabba]ab`baaaaaba`__aa]`aaaaa`a¥a
@HWI-EAS443:3:19:380:363#0/1
CTTGCAAAAGGAGAGAAAGCTAATGTGCTAATTGGGCAAG
+HWI-EAS443:3:19:380:363#0/1
a`abaU`^X^aab_aa]aa[aa`V^Ua_[^__``_`]PZa
@HWI-EAS443:3:19:380:539#0/1
GAGCAATCTTGCAAAAGGAGAGAAAGCTAATGTGCTAATT
+HWI-EAS443:3:19:380:539#0/1
^a^abb]a`Z^a`bZbbbba_bbaY[ba`bbbaaaaaa`a
@HWI-EAS443:3:19:471:659#0/1
ATCTTGCAAAAGGAGAGAAAGCTAATGTGCTAATTGGGCA
+HWI-EAS443:3:19:471:659#0/1
a_``a_aa`a_b`a`__aa_a_```_aVa^``_``a_a`^
@HWI-EAS443:3:19:664:1127#0/1
CATCAATGAACTGAGCAATCTTGCAAAAGGAGAGAAAGCT
+HWI-EAS443:3:19:664:1127#0/1
SS^_b`ba]IVb^_ZZ¥[`abb^X[^¥baa^bab``aVLQ
@HWI-EAS443:3:19:668:688#0/1
CAATCTTGCAAAAGGAGAGAAAGCTAATGTGCTAATTGGG
+HWI-EAS443:3:19:668:688#0/1
abSWR]aaRLV¥Ua]HXT¥SPN_GWUN`aO_TTBBBBBBB
@HWI-EAS443:3:19:792:1202#0/1
CATCAATGAACTGAGCAATCTTGCAAAAGGAGAGAAAGCT
+HWI-EAS443:3:19:792:1202#0/1
abaaaabaaaa``_a`aaaaa_a`aa```a]a`aa___¥a
@HWI-EAS443:3:19:807:607#0/1
GTCCCCTTGCCCAATTAGCACATTAGCTTTCTCTCCTTTT
+HWI-EAS443:3:19:807:607#0/1
a_aa`a¥_a``_aa`aa_a^a`a```````_a^a]_`[U^
@HWI-EAS443:3:19:89:1700#0/1
TGCAAAAGGAGAGAAAGCTAATGTGCTAATTGGGCAAGGG
+HWI-EAS443:3:19:89:1700#0/1
__a`bb_`^abbbba^^aaabaa_aba_aaaa¥abaaaZ[
@HWI-EAS443:3:19:946:58#0/1
AAGCTAATGTGCTAATTGGGCAAGGGGACGTAGTGTTGGT
+HWI-EAS443:3:19:946:58#0/1
aabaaa]bb_a]a]^`aaba`_¥a`a`¥`aX_a¥`]_a_N
@HWI-EAS443:3:19:955:1799#0/1
GAACTGAGCAATCTTGCAAAAGGAGAGAAAGCTAATGTGC
+HWI-EAS443:3:19:955:1799#0/1
aaa`HSIX^bbaa[`a`_a_`a__`aa__a^_aa_¥^G¥_
@HWI-EAS443:3:1:1005:1348#0/1
GAAAGCTAATGTGCTAATTGGGCAAGGGGACGTAGTGTTG
+HWI-EAS443:3:1:1005:1348#0/1
_abbbbb¥`bbababaabbbbb_b_babaa]a_`b]b[aB
```

```
@HWI-EAS443:3:1:1009:1907#0/1
CATCAATGAACTGAGCAATCTTGCAAAAGGAGAGAAAGCT
+HWI-EAS443:3:1:1009:1907#0/1
¥bbabb_b_bYWbWG[b_aaT_a¥bb^¥aXX]aaaaSbBB
@HWI-EAS443:3:1:1223:1286#0/1
GAGCAATCTTGCAAAAGGAGAGAAAGCTAATGTGCTAATT
+HWI-EAS443:3:1:1223:1286#0/1
a`a^ba`]bbba`aa`ab^a_aaaaZG^_aaa__V`T`b_
@HWI-EAS443:3:1:1285:886#0/1
AGCTAATGTGCTAATTGGGCAAGGGGACGTAGTGTTGGTA
+HWI-EAS443:3:1:1285:886#0/1
Z^^abb_¥`b]a]a_]``a_a[abb``XaY```b¥bW`BB
@HWI-EAS443:3:1:142:1165#0/1
CAATCTTGCAAAAGGAGAGAAAGCTAATGTGCTAATTGGG
+HWI-EAS443:3:1:142:1165#0/1
ababaaab``a_¥ba^Z^_bbabXbbaba``aba`aWV`B
@HWI-EAS443:3:1:1435:1031#0/1
AGCTAATGTGCTAATTGGGCAAGGGGACGTAGTGTTGGTA
+HWI-EAS443:3:1:1435:1031#0/1
aaabbaab]]_b`[aa`aa^`Xaaa_¥F]W_[Ra]`^bBB
@HWI-EAS443:3:1:146:1255#0/1
AATGAACTGAGCAATCTTGCAAAAGGAGAGAAAGCTAATG
+HWI-EAS443:3:1:146:1255#0/1
`^]¥H[OZ`¥Z¥_]][ZY]Y[TKTa_WZW_`^R`ZQ]Z_B
@HWI-EAS443:3:1:165:601#0/1
CATCAATGAACTGAGCAATCTTGCAAAAGGAGAGAAAGCT
+HWI-EAS443:3:1:165:601#0/1
aaa_bbabbb_aaaaaa`a^a`aaa_aaaa`a`aa^aa_B
@HWI-EAS443:3:1:1712:1322#0/1
GAGCAATCTTGCAAAAGGAGAGAAAGCTAATGTGCTAATT
+HWI-EAS443:3:1:1712:1322#0/1
a`a_a``]aab`¥_^^ba^a_aa`_aR`_]^_]aYKWK_B
@HWI-EAS443:3:1:1725:1777#0/1
GAGAAAGCTAATGTGCTAATTGGGCAAGGGGACGTAGTGT
+HWI-EAS443:3:1:1725:1777#0/1
a`a`aS^U_a]aa_`G`^_^_aaSU_^^__a]G_BBBBBB
@HWI-EAS443:3:1:1741:1238#0/1
CAATTAGCACATTAGCTTTCTCTCCTTTTGCAAGATTGCT
+HWI-EAS443:3:1:1741:1238#0/1
abaab`aab_`bba_^b_b_b¥a¥abbbaaJa`aa`bb^B
@HWI-EAS443:3:1:195:354#0/1
CAATCTTGCAAAAGGAGAGAAAGCTAATGTGCTAATTGGG
+HWI-EAS443:3:1:195:354#0/1
abaaabaaaaaa`baababaa_a_aa_aa_a¥a^_a^aBB
@HWI-EAS443:3:1:199:1603#0/1
AATCTTGCAAAAGGAGAGAAAGCTAATGTGCTAATTGGGC
+HWI-EAS443:3:1:199:1603#0/1
a_[^ba_]__`¥a[_a_a`VZ`¥_a^aaXPLa`X[`_X^B
@HWI-EAS443:3:1:264:1454#0/1
ATGAACTGAGCAATCTTGCAAAAGGAGAGAAAGCTAATGT
```

```
+HWI-EAS443:3:1:264:1454#0/1
aab_aaa`]aaaaa_aa`^^_[]``[¥[`__¥^S^`WBBB
@HWI-EAS443:3:1:288:241#0/1
GAGCAATCTTGCAAAAGGAGAGAAAGCTAATGTGCTAATT
+HWI-EAS443:3:1:288:241#0/1
a^`G`aa^aYa_`_^Xaa^^_a_`_aX`__`_XaX_^_BB
@HWI-EAS443:3:1:316:50#0/1
GAGCAATCTTGCAAAAGGAGAGAAAGCTAATGTGCTAATT
+HWI-EAS443:3:1:316:50#0/1
aaaa_b`aUbba`aUWabI_a_b^ab¥b`_W^ab[`_`aB
@HWI-EAS443:3:1:459:65#0/1
AGAGAAAGCTAATGTGCTAATTGGGCAAGGGGACGTAGTG
+HWI-EAS443:3:1:459:65#0/1
aaaa`a```aa_aa_b`aa¥``aa_]^¥a_]`Y^a]XaBB
@HWI-EAS443:3:1:496:1075#0/1
CCAACACTACGTCCCCTTGCCCAATTAGCACATTAGCTTT
+HWI-EAS443:3:1:496:1075#0/1
aabb`babb_aa_XYYaab__aabaabaYa_aaa_^`a_B
@HWI-EAS443:3:1:546:1327#0/1
GAGCAATCTTGCAAAAGGAGAGAAAGCTAATGTGCTAATT
+HWI-EAS443:3:1:546:1327#0/1
`¥¥U_]aab[aaabbabba`aabbab`ba`bb_a`aaa]B
@HWI-EAS443:3:1:596:1134#0/1
ATCTTGCAAAAGGAGAGAAAGCTAATGTGCTAATTGGGCA
+HWI-EAS443:3:1:596:1134#0/1
aaaaab`aa_`b`Za`baa_`_`a^aa_a_a_^aa_aaBB
@HWI-EAS443:3:1:655:139#0/1
AGCTAATGTGCTAATTGGGCAAGGGGACGTAGTGTTGGTA
+HWI-EAS443:3:1:655:139#0/1
ab¥ba¥abW_^]_N_`^`a[SX]_ba[Z`ZRaXa^S_BBB
@HWI-EAS443:3:1:659:1040#0/1
GAAAGCTAATGTGCTAATTGGGCAAGGGGACGTAGTGTTG
+HWI-EAS443:3:1:659:1040#0/1
aa``aV_^``a^__`___`_¥`W_^`^__ZZ_XW_W^]_B
@HWI-EAS443:3:1:668:401#0/1
GAGAAAGCTAATGTGCTAATTGGGCAAGGGGACGTAGTGT
+HWI-EAS443:3:1:668:401#0/1
^_aab``[aaaba¥a^b_aa_aab_`_^aaa^W`U¥`]BB
@HWI-EAS443:3:1:732:1083#0/1
AGAAAGCTAATGTGCTAATTGGGCAAGGGGACGTAGTGTT
+HWI-EAS443:3:1:732:1083#0/1
abVJa^UYYaaa_a_aT_aaaaa_Y_ZLZaGVa[Sa]WBB
@HWI-EAS443:3:1:798:1603#0/1
GAACTGAGCAATCTTGCAAAAGGAGAGAAAGCTAATGTGC
+HWI-EAS443:3:1:798:1603#0/1
aaXIaa_abbaaU¥^¥MbaM[Ob¥a`aaba¥¥¥bb]BBBB
@HWI-EAS443:3:1:822:884#0/1
GAGCAATCTTGCAAAAGGAGAGAAAGCTAATGTGCTAATT
+HWI-EAS443:3:1:822:884#0/1
aYb]b]b_baa¥]`¥_aaa`aa^``a_aa_aaaa_aa`aB
```

```
@HWI-EAS443:3:1:868:655#0/1
GCATCAATGAACTGAGCAATCTTGCAAAAGGAGAGAAAGC
+HWI-EAS443:3:1:868:655#0/1
aa`a_``aa^`_a`^_^``aX``a]¥^]Xa]Z`^_]UBBB
@HWI-EAS443:3:1:872:599#0/1
ACTGAGCAATCTTGCAAAAGGAGAGAAAGCTAATGTGCTA
+HWI-EAS443:3:1:872:599#0/1
M`a`Z^[b`]¥bbb`XR^abb^babaa_aSa_aabX[Iaa
@HWI-EAS443:3:1:876:574#0/1
CATCAATGAACTGAGCAATCTTGCAAAAGGAGAGAAAGCT
+HWI-EAS443:3:1:876:574#0/1
ab`^^bXV]aR_b]aVR`¥]`ab_`O¥]ba^aaa]`^a`B
@HWI-EAS443:3:20:1012:466#0/1
ATCTTGCAAAAGGAGAGAAAGCTAATGTGCTAATTGGGCA
+HWI-EAS443:3:20:1012:466#0/1
ababaa_b`^I_b_aabbaab_ba_abaa_aa_a_abaXX
@HWI-EAS443:3:20:1128:293#0/1
AGCTAATGTGCTAATTGGGCAAGGGGACGTAGTGTTGGTA
+HWI-EAS443:3:20:1128:293#0/1
_a^^`^_ZZ_]__`aaa_¥T_W_^a_ZRW_]_ZSZ¥aaVP
@HWI-EAS443:3:20:1172:973#0/1
GAGAAAGCTAATGTGCTAATTGGGCAAGGGGACGTAGTGT
+HWI-EAS443:3:20:1172:973#0/1
aa_Q]a__a[M_`_b^`]_aaa¥Ya_``a`]_[aU_a]a]
@HWI-EAS443:3:20:1211:580#0/1
GAGCAATCTTGCAAAAGGAGAGAAAGCTAATGTGCTAATT
+HWI-EAS443:3:20:1211:580#0/1
[_U[ZS¥^`aaZY[[]a_Za^a`V^`Y¥[]`aNZQ[XY]^
@HWI-EAS443:3:20:1228:1744#0/1
GAACTGAGCAATCTTGCAAAAGGAGAGAAAGCTAATGTGC
+HWI-EAS443:3:20:1228:1744#0/1
aaaaa^Y¥_a^^UaZX^[aY_aa¥¥Z]aaa__`_[`aR]U
@HWI-EAS443:3:20:1361:1029#0/1
CATCAATGAACTGAGCAATCTTGCAAAAGGAGAGAAAGCT
+HWI-EAS443:3:20:1361:1029#0/1
aaaababab`abbbbabbbababababaaabbababbaaa_a
@HWI-EAS443:3:20:1363:766#0/1
TTGCAAAAGGAGAGAAAGCTAATGTGCTAATTGGGCAAGG
+HWI-EAS443:3:20:1363:766#0/1
aaaa___Ya`_a_a`_¥a_`_^`_[`WX]^`^a__Z]]]W
@HWI-EAS443:3:20:1389:1468#0/1
AAGCTAATGTGCTAATTGGGCAAGGGGACGTAGTGTTGGT
+HWI-EAS443:3:20:1389:1468#0/1
`YaZ`aa`_``]^aaa`aaaaa_aa``^[a[]ZUa^[]UL
@HWI-EAS443:3:20:1431:2041#0/1
CCTTGCCCAATTAGCACATTAGCTTTCTCTCCTTTTGCAA
+HWI-EAS443:3:20:1431:2041#0/1
`b`aa`a^aabab`[_W`aaaaa_aaYa`aa_a___aaa_
@HWI-EAS443:3:20:1495:269#0/1
TTGCAAAAGGAGAGAAAGCTAATGTGCTAATTGGGCAAGG
```

```
+HWI-EAS443:3:20:1495:269#0/1
aabb[[XL_¥__aba[^a`¥[^_a_a^`_^a^ZP`SSW_^
@HWI-EAS443:3:20:1588:16#0/1
GAACTGAGCAATCTTGCAAAAGGAGAGAAAGCTAATGTGC
+HWI-EAS443:3:20:1588:16#0/1
^W_]_^aaYaaa]`_T_a^`XWaYaYI`_S_W__^]_G_V
@HWI-EAS443:3:20:1721:539#0/1
ATGAACTGAGCAATCTTGCAAAAGGAGAGAAAGCTAATGT
+HWI-EAS443:3:20:1721:539#0/1
aaa_Y^aaa]aa¥a_a`b_a___aa_a^``a^_X^_X]_Z
@HWI-EAS443:3:20:187:282#0/1
CATCAATGAACTGAGCAATCTTGCAAAAGGAGAGAAAGCT
+HWI-EAS443:3:20:187:282#0/1
`Kb]^]a`aaaaYU¥`^aaZa_aa`Y[^aa]`__`_V`PW
@HWI-EAS443:3:20:194:656#0/1
AGGAGAGAAAGCTAATGTGCTAATTGGGCAAGGGGACGTA
+HWI-EAS443:3:20:194:656#0/1
W__V¥Z_bb¥¥bb_Z_bMbb[V¥ba_`bb`Q[baa`abRM
@HWI-EAS443:3:20:221:1609#0/1
CATCAATGAACTGAGCAATCTTGCAAAAGGAGAGAAAGCT
+HWI-EAS443:3:20:221:1609#0/1
a`ab`a``aaaaU]_`a```a¥^_`Za`WVZ_a_`__X^Y
@HWI-EAS443:3:20:314:1429#0/1
GTCCCCTTGCCCAATTAGCACATTAGCTTTCTCTCCTTTT
+HWI-EAS443:3:20:314:1429#0/1
a`baaaa`aaa`]`a_aaa_a^`aa^`]]`[`^____[__
@HWI-EAS443:3:20:327:591#0/1
GAGCAATCTTGCAAAAGGAGAGAAAGCTAATGTGCTAATT
+HWI-EAS443:3:20:327:591#0/1
^`YP_¥`ba_b`bZ`aab[`^`aaaa]a_```Z`[X_¥_`
@HWI-EAS443:3:20:400:1734#0/1
ATGAACTGAGCAATCTTGCAAAAGGAGAGAAAGCTAATGT
+HWI-EAS443:3:20:400:1734#0/1
K_]_Q^¥GRWU``_`^[Z_aS^`XVRW[`aa^R^UUZ[[_
@HWI-EAS443:3:20:503:735#0/1
ACTGAGCAATCTTGCAAAAGGAGAGAAAGCTAATGTGCTA
+HWI-EAS443:3:20:503:735#0/1
aabYUaZQ]XX^aa^a_VVY`__ba_OZb[`__a_G]R^`
@HWI-EAS443:3:20:517:601#0/1
GAGCAATCTTGCAAAAGGAGAGAAAGCTAATGTGCTAATT
+HWI-EAS443:3:20:517:601#0/1
aa`baabaabbaaaaabbabaaaa``^a`_ab^a`aa``a
@HWI-EAS443:3:20:537:1210#0/1
GAAAGCTAATGTGCTAATTGGGCAAGGGGACGTAGTGTTG
+HWI-EAS443:3:20:537:1210#0/1
^_]WaZaaTa`Ya[^`aaa¥YW_a``^a`Z`^Y]`W_V]a
@HWI-EAS443:3:20:801:1360#0/1
GAAAGCTAATGTGCTAATTGGGCAAGGGGACGTAGTGTTG
+HWI-EAS443:3:20:801:1360#0/1
T^`[¥¥X[bbab`ba`_a[`[`ab`_aaa`_`]NW__aab
```

```
@HWI-EAS443:3:20:91:925#0/1
AATGAACTGAGCAATCTTGCAAAAGGAGAGAAAGCTAATG
+HWI-EAS443:3:20:91:925#0/1
]aUIX]a[XY^SbaabaS^Z]Y^]]P_[`PV[RK¥U`__a
@HWI-EAS443:3:20:923:1991#0/1
GAACTGAGCAATCTTGCAAAAGGAGAGAAAGCTAATGTGC
+HWI-EAS443:3:20:923:1991#0/1
_PaXYS[YZ[PNZ^Za]_]`a_]_]]^¥]___WG]]_R^N
@HWI-EAS443:3:20:924:1492#0/1
ATGAACTGAGCAATCTTGCAAAAGGAGAGAAAGCTAATGT
+HWI-EAS443:3:20:924:1492#0/1
[YOQVa_[_`aa^^a`Zaa```]_`P^a`a]¥]_]`__`V
@HWI-EAS443:3:20:98:402#0/1
GCAAAAGGAGAGAAAGCTAATGTGCTAATTGGGCAAGGGG
+HWI-EAS443:3:20:98:402#0/1
`aa``]ba^baba``a_a``aaY__`]_`¥Z]_]`Y^`^]
@HWI-EAS443:3:21:1020:1108#0/1
ATCAATGAACTGAGCAATCTTGCAAAAGGAGAGAAAGCTA
+HWI-EAS443:3:21:1020:1108#0/1
_abbbaba[[WR_ba^aa_^abaVH¥_^`_[`a`__`^`^
@HWI-EAS443:3:21:104:1492#0/1
GAAAGCTAATGTGCTAATTGGGCAAGGGGACGTAGTGTTG
+HWI-EAS443:3:21:104:1492#0/1
[a¥YWb_]^aa¥¥baa_aVTX]`aZY]_[a`^U_a_a^]`
@HWI-EAS443:3:21:1139:2045#0/1
AAGCTAATGTGCTAATTGGGCAAGGGGACGTAGTGTTGGT
+HWI-EAS443:3:21:1139:2045#0/1
a`a_`_]aZ]__^_`^_`ZU[__`^^^^ZXKR¥X_ZZ^WX
@HWI-EAS443:3:21:1282:627#0/1
GAGCAATCTTGCAAAAGGAGAGAAAGCTAATGTGCTAATT
+HWI-EAS443:3:21:1282:627#0/1
a`a_]V[Za^a_¥KKF]ZR¥T__[H_Y^_XYbVa]¥¥^[X
@HWI-EAS443:3:21:1283:667#0/1
AGCTAATGTGCTAATTGGGCAAGGGGACGTAGTGTTGGTA
+HWI-EAS443:3:21:1283:667#0/1
aa`aa`aa`aaaaaaaaaa^a_a___X^aW¥YX___a_S^
@HWI-EAS443:3:21:1302:530#0/1
GAGCAATCTTGCAAAAGGAGAGAAAGCTAATGTGCTAATT
+HWI-EAS443:3:21:1302:530#0/1
`Q][W¥V_^T^W_NOXaa¥aYY_X_Y^aVV¥aVWW[[TYZ
@HWI-EAS443:3:21:1385:1120#0/1
GAGCAATCTTGCAAAAGGAGAGAAAGCTAATGTGCTAATT
+HWI-EAS443:3:21:1385:1120#0/1
`_aV_aa¥baaa_WV__a^]_a__^a^aV_a`]^]__]`]
@HWI-EAS443:3:21:1442:1313#0/1
GAACTGAGCAATCTTGCAAAAGGAGAGAAAGCTAATGTGC
+HWI-EAS443:3:21:1442:1313#0/1
__V]`aaaa]abaa^bbb^_`aa_aaaba```a`^aaS_F
@HWI-EAS443:3:21:1509:803#0/1
CCCTTGCCCAATTAGCACATTAGCTTTCTCTCCTTTTGCA
```

```
+HWI-EAS443:3:21:1509:803#0/1
a_]¥ba_`_^abbaaU^a`baab`a`aYb_b_]¥aa`^QK
@HWI-EAS443:3:21:1695:227#0/1
AAGCTAATGTGCTAATTGGGCAAGGGGACGTAGTGTTGGT
+HWI-EAS443:3:21:1695:227#0/1
aa[ZZSaS^ZaX_¥M[Zaa`¥J]aaa]OQ_]P¥LZWX`_B
@HWI-EAS443:3:21:1774:1263#0/1
GAGCAATCTTGCAAAAGGAGAGAAAGCTAATGTGCTAATT
+HWI-EAS443:3:21:1774:1263#0/1
V¥WHR]_V¥aa]b]^T`]bb_¥`Y`^_a^]^[QTHZ]`aa
@HWI-EAS443:3:21:198:1890#0/1
GAACTGAGCAATCTTGCAAAAGGAGAGAAAGCTAATGTGC
+HWI-EAS443:3:21:198:1890#0/1
RT¥^ZR]^^``a`¥S_aaYS[a`__¥_``¥bV_aa_V[`S
@HWI-EAS443:3:21:34:1424#0/1
GCATCAATGAACTGAGCAATCTTGCAAAAGGAGAGAAAGC
+HWI-EAS443:3:21:34:1424#0/1
aaYYb_a_T_[__SVS`_`_a]`][[^^¥Y¥Y^X^`^ZWW
@HWI-EAS443:3:21:432:1620#0/1
AACTGAGCAATCTTGCAAAAGGAGAGAAAGCTAATGTGCT
+HWI-EAS443:3:21:432:1620#0/1
aa^`P^¥aaaaaaa_`_``ZU_`YaWS`][Z____^ZX]^
@HWI-EAS443:3:21:452:1874#0/1
GAGCAATCTTGCAAAAGGAGAGAAAGCTAATGTGCTAATT
+HWI-EAS443:3:21:452:1874#0/1
`[[^¥RFV¥``^T[P]W_Ua]Y]^[YZWVRVXFU¥RXBBB
@HWI-EAS443:3:21:505:690#0/1
CATCAATGAACTGAGCAATCTTGCAAAAGGAGAGAAAGCT
+HWI-EAS443:3:21:505:690#0/1
abaa``aaa`_a__aa`aaaaa`_a^__a`^aa`a_^_[_
@HWI-EAS443:3:21:53:1109#0/1
CTTGCAAAAGGAGAGAAAGCTAATGTGCTAATTGGGCAAG
+HWI-EAS443:3:21:53:1109#0/1
aaaaaaa`_a`a[a`a_aaaaaa`aO_aa`_``^aYaa`_
@HWI-EAS443:3:21:552:1878#0/1
AGCAATCTTGCAAAAGGAGAGAAAGCTAATGTGCTAATTG
+HWI-EAS443:3:21:552:1878#0/1
a]bbbaabb`bbaaY]¥]aaabaaaaaaaa`^`^`a`^¥^
@HWI-EAS443:3:21:648:1421#0/1
AAAGCTAATGTGCTAATTGGGCAAGGGGACGTAGTGTTGG
+HWI-EAS443:3:21:648:1421#0/1
aa]`^[`ZY_NRW[`¥`ROXZ^X¥[Z[NY]XRTKDVP¥BB
@HWI-EAS443:3:21:689:418#0/1
TCCCCTTGCCCAATTAGCACATTAGCTTTCTCTCCTTTTG
+HWI-EAS443:3:21:689:418#0/1
`aba```ba___[`YV^aa`R^`]ZZ[JQ]]`^W__¥VXP
@HWI-EAS443:3:21:827:206#0/1
AAGCTAATGTGCTAATTGGGCAAGGGGACGTAGTGTTGGT
+HWI-EAS443:3:21:827:206#0/1
YY¥[`RFY_YW[_^baa¥P¥a```a`]XN¥YV`[ZRTYYB
```

@HWI-EAS443:3:21:897:1239#0/1
AATGAACTGAGCAATCTTGCAAAAGGAGAGAAAGCTAATG
+HWI-EAS443:3:21:897:1239#0/1
a_X^^X``[abbaaaa`_a_aab```aaaab[_aa_Y^aa
@HWI-EAS443:3:22:1121:308#0/1
AGGAGAGAAAGCTAATGTGCTAATTGGGCAAGGGGACGTA
+HWI-EAS443:3:22:1121:308#0/1
`bb]bbb`^bb_ba`abL`^`[¥`Ua]a^¥_`]aaUXaP_
@HWI-EAS443:3:22:1230:1317#0/1
CAATGAACTGAGCAATCTTGCAAAAGGAGAGAAAGCTAAT
+HWI-EAS443:3:22:1230:1317#0/1
^VX_^QU¥VNWN`¥V_US¥^]]X¥SY]N_H`a]]XMWSVO
@HWI-EAS443:3:22:1316:1361#0/1
GAGCAATCTTGCAAAAGGAGAGAAAGCTAATGTGCTAATT
+HWI-EAS443:3:22:1316:1361#0/1
Z_baba[aa`U¥a]_ZS_`ba`a]^aaaaaa`___a``]a
@HWI-EAS443:3:22:1343:2041#0/1
GAGCAATCTTGCAAAAGGAGAGAAAGCTAATGTGCTAATT
+HWI-EAS443:3:22:1343:2041#0/1
^T_`]bb__^`abb^^`babbbbbbba`a``b_aaabaaa
@HWI-EAS443:3:22:1408:1858#0/1
GAGCAATCTTGCAAAAGGAGAGAAAGCTAATGTGCTAATT
+HWI-EAS443:3:22:1408:1858#0/1
¥F[abb]U__[]_T_^TWWZ_UWZP^OZZ]_WXLR]^¥ab
@HWI-EAS443:3:22:1457:1453#0/1
CAATCTTGCAAAAGGAGAGAAAGCTAATGTGCTAATTGGG
+HWI-EAS443:3:22:1457:1453#0/1
Z¥``__abba_]T__[^¥¥a`a^]^_`¥[J¥_^Z^^]aa]
@HWI-EAS443:3:22:1558:250#0/1
AAAGGAGAGAAAGCTAATGTGCTAATTGGGCAAGGGGACG
+HWI-EAS443:3:22:1558:250#0/1
^]X__Z`Y]Y[aaaaa[`^R^`_¥W]ZX^¥T[T`^`XJJB
@HWI-EAS443:3:22:1624:916#0/1
AATGAACTGAGCAATCTTGCAAAAGGAGAGAAAGCTAATG
+HWI-EAS443:3:22:1624:916#0/1
abbbab¥baa`a`ab^bbbaaba___`aaba`]a_a``aQ
@HWI-EAS443:3:22:1673:23#0/1
GAGCAATCTTGCAAAAGGAGAGAAAGCTAATGTGCTAATT
+HWI-EAS443:3:22:1673:23#0/1
a`^L[X`¥aa`]__^_a_[a_a]]^ZPZUX¥¥V_WVVY_¥
@HWI-EAS443:3:22:1674:415#0/1
ATGAACTGAGCAATCTTGCAAAAGGAGAGAAAGCTAATGT
+HWI-EAS443:3:22:1674:415#0/1
YIOPYPa^]V¥a[bRTWaR]`WXaaUaaa`¥]_Ga¥aa¥[
@HWI-EAS443:3:22:1721:205#0/1
GAGCAATCTTGCAAAAGGAGAGAAAGCTAATGTGCTAATT
+HWI-EAS443:3:22:1721:205#0/1
aaa`baaabbb_`aa`bb`baba_`¥Va__abWa_a__a_
@HWI-EAS443:3:22:1739:241#0/1
GAAAGCTAATGTGCTAATTGGGCAAGGGGACGTAGTGTTG

```
+HWI-EAS443:3:22:1739:241#0/1
a`a^[Z`aaaa]`_`]_a`a_aW^]``ZZVX`VR_]_Z]^
@HWI-EAS443:3:22:403:443#0/1
GAAAGCTAATGTGCTAATTGGGCAAGGGGACGTAGTGTTG
+HWI-EAS443:3:22:403:443#0/1
ab_¥aaaX`abUa]_[aaa`W]]]`a]aa__aY^`Tbaa`
@HWI-EAS443:3:22:423:196#0/1
GAGCAATCTTGCAAAAGGAGAGAAAGCTAATGTGCTAATT
+HWI-EAS443:3:22:423:196#0/1
`ababbaababa`¥^abb`aaaa``a¥```aa]`^`_]^_
@HWI-EAS443:3:22:500:386#0/1
GTCCCCTTGCCCAATTAGCACATTAGCTTTCTCTCCTTTT
+HWI-EAS443:3:22:500:386#0/1
a^^aXT^Q`X[YTV`][___S¥][ZOR]W¥¥Z]]]X¥TU¥
@HWI-EAS443:3:22:55:1698#0/1
GCATCAATGAACTGAGCAATCTTGCAAAAGGAGAGAAAGC
+HWI-EAS443:3:22:55:1698#0/1
aaaaa_a`Y__P][aTYTI_``S`^Va_aIQR_a__]a`S
@HWI-EAS443:3:22:568:207#0/1
TTGCAAAAGGAGAGAAAGCTAATGTGCTAATTGGGCAAGG
+HWI-EAS443:3:22:568:207#0/1
a`ba``aa`b^[_b_`a``a`aaa_]aaa_aa```_`_a_
@HWI-EAS443:3:22:591:1993#0/1
AGCTAATGTGCTAATTGGGCAAGGGGACGTAGTGTTGGTA
+HWI-EAS443:3:22:591:1993#0/1
aaaaaa`aZaa`aa`aaa_a_aa^^__^WKW_Z_Z___O^
@HWI-EAS443:3:22:633:794#0/1
GAGCAATCTTGCAAAAGGAGAGAAAGCTAATGTGCTAATT
+HWI-EAS443:3:22:633:794#0/1
abbbabbba`_^aaaaababaab]¥b_aaaaa^a_`a^aa
@HWI-EAS443:3:22:638:1082#0/1
AAGCATCAATGAACTGAGCAATCTTGCAAAAGGAGAGAAA
+HWI-EAS443:3:22:638:1082#0/1
a`a``aa_aa_^a_a``_`a`__^]_`_]___]^``_`^_
@HWI-EAS443:3:22:655:1390#0/1
GAGCAATCTTGCAAAAGGAGAGAAAGCTAATGTGCTAATT
+HWI-EAS443:3:22:655:1390#0/1
aVZaTaW]a`a]¥P[^]_¥][W`_`_]___`aR`__]][¥
@HWI-EAS443:3:22:724:1218#0/1
GAGAAAGCTAATGTGCTAATTGGGCAAGGGGACGTAGTGT
+HWI-EAS443:3:22:724:1218#0/1
_a^^aaaaa`ab[Y^ab`baaaa`_aab_aa]Xa¥_aU`]
@HWI-EAS443:3:22:76:966#0/1
GAGAAAGCTAATGTGCTAATTGGGCAAGGGGACGTAGTGT
+HWI-EAS443:3:22:76:966#0/1
a_PYXa¥bWaZRaGaba]`a^a__^Y¥¥a_Y]`YEZaRZX
@HWI-EAS443:3:22:788:1140#0/1
GTCCCCTTGCCCAATTAGCACATTAGCTTTCTCTCCTTTT
+HWI-EAS443:3:22:788:1140#0/1
`^aZP____][`_a¥Y`U``_``__[``¥YXU]`¥]^]^X
```

```
@HWI-EAS443:3:22:789:116#0/1
GAACTGAGCAATCTTGCAAAAGGAGAGAAAGCTAATGTGC
+HWI-EAS443:3:22:789:116#0/1
aab`a`_b`b_a`aaa[a`a_aa_a`a`^_`]`__`_Z]R
@HWI-EAS443:3:22:929:1483#0/1
CCTTGCCCAATTAGCACATTAGCTTTCTCTCCTTTTGCAA
+HWI-EAS443:3:22:929:1483#0/1
``]U¥a_b__bb^^T]bbbaaa]a_``a]^baa]_aaa_Z
@HWI-EAS443:3:23:1003:1597#0/1
AGCTAATGTGCTAATTGGGCAAGGGGACGTAGTGTTGGTA
+HWI-EAS443:3:23:1003:1597#0/1
aaXJZ_``Yab[a^Z_TV_a_[`_UZS^a`Z_Z^T^a_G¥
@HWI-EAS443:3:23:1071:673#0/1
CAATGAACTGAGCAATCTTGCAAAAGGAGAGAAAGCTAAT
+HWI-EAS443:3:23:1071:673#0/1
aZa¥b¥`a^`aa^Z^aabaaa`a_I¥a_bb`aa`b]a_`a
@HWI-EAS443:3:23:1182:1600#0/1
AATTAGCACATTAGCTTTCTCTCCTTTTGCAAGATTGCTC
+HWI-EAS443:3:23:1182:1600#0/1
a^`Vbaa__aaa[]R_a]`a[U¥Ja`_aa`bW]_[^]Q`V
@HWI-EAS443:3:23:1213:1407#0/1
GGAGAGAAAGCTAATGTGCTAATTGGGCAAGGGGACGTAG
+HWI-EAS443:3:23:1213:1407#0/1
aaaaaaaaaa``aaa^aaa`aa`aa_`]_a__`¥^aWZ`
@HWI-EAS443:3:23:1259:604#0/1
GCATCAATGAACTGAGCAATCTTGCAAAAGGAGAGAAAGC
+HWI-EAS443:3:23:1259:604#0/1
aaa^]a]^a``_a_Y`^___HV__]]_^__`__^_^_YXQ
@HWI-EAS443:3:23:1331:699#0/1
ATGAACTGAGCAATCTTGCAAAAGGAGAGAAAGCTAATGT
+HWI-EAS443:3:23:1331:699#0/1
`ba¥_¥_abbabbb^baaaba`[bb`abbbaa_^aa`aa]
@HWI-EAS443:3:23:1335:112#0/1
AGCTAATGTGCTAATTGGGCAAGGGGACGTAGTGTTGGTA
+HWI-EAS443:3:23:1335:112#0/1
ababbbbbababaababba```aaaa^¥a^_aYa_ba`SZ
@HWI-EAS443:3:23:1435:228#0/1
CATCAATGAACTGAGCAATCTTGCAAAAGGAGAGAAAGCT
+HWI-EAS443:3:23:1435:228#0/1
abaaaabbbb`baaa_a`a`aaa]`a_`aa_aa`aa^_Y`
@HWI-EAS443:3:23:1472:1993#0/1
GAGAAAGCTAATGTGCTAATTGGGCAAGGGGACGTAGTGT
+HWI-EAS443:3:23:1472:1993#0/1
a___V¥a]_]_a]_``aaa`__W]___`YSaW^WY_`UUU
@HWI-EAS443:3:23:1516:845#0/1
GAAAGCTAATGTGCTAATTGGGCAAGGGGACGTAGTGTTG
+HWI-EAS443:3:23:1516:845#0/1
abaaa_a``bb^a_a``aaa]`[`_aa^a^Za^Y_Wa]__
@HWI-EAS443:3:23:1759:1247#0/1
AGCAATCTTGCAAAAGGAGAGAAAGCTAATGTGCTAATTG
```

+HWI-EAS443:3:23:1759:1247#0/1
`aZaabbabb__]]S_b]baaaa_a[a``bb^aX__``_a
@HWI-EAS443:3:23:18:235#0/1
AGCTAATGTGCTAATTGGGCAAGGGGACGTAGTGTTGGTA
+HWI-EAS443:3:23:18:235#0/1
Xabb`Yab^bbb``_ababaaababb`ab[]`Xa_``aJX
@HWI-EAS443:3:23:26:1795#0/1
AAGCTAATGTGCTAATTGGGCAAGGGGACGTAGTGTTGGT
+HWI-EAS443:3:23:26:1795#0/1
a`]`_b¥^[Pa`aaaaa`[`UT`a`^X]_`V^aR`Z_[YG
@HWI-EAS443:3:23:434:286#0/1
ATCTTGCAAAAGGAGAGAAAGCTAATGTGCTAATTGGGCA
+HWI-EAS443:3:23:434:286#0/1
aaabababbbabaaaabb`aaabaaaa]a`aaaaaaa`_^
@HWI-EAS443:3:23:502:1657#0/1
GAACTGAGCAATCTTGCAAAAGGAGAGAAAGCTAATGTGC
+HWI-EAS443:3:23:502:1657#0/1
GT``b_¥``a¥Zbaaabba^abbabaaabb¥ab¥aa¥``a
@HWI-EAS443:3:23:555:42#0/1
GAGCAATCTTGCAAAAGGAGAGAAAGCTAATGTGCTAATT
+HWI-EAS443:3:23:555:42#0/1
``bbbbbbbbbbbbaabbaabbabbb`babbb`aYaaaa_
@HWI-EAS443:3:23:563:1413#0/1
GAAAGCTAATGTGCTAATTGGGCAAGGGGACGTAGTGTTG
+HWI-EAS443:3:23:563:1413#0/1
abZ`]S_¥W¥]FXV]`_a_[]Y`¥XU_Q]aW¥^_^Y_W_`
@HWI-EAS443:3:23:660:391#0/1
GCATCAATGAACTGAGCAATCTTGCAAAAGGAGAGAAAGC
+HWI-EAS443:3:23:660:391#0/1
ab_aaaaaaaXaaa_`aa¥_]a_a__a^]aa_a]a`V[aZ
@HWI-EAS443:3:23:763:75#0/1
GAACTGAGCAATCTTGCAAAAGGAGAGAAAGCTAATGTGC
+HWI-EAS443:3:23:763:75#0/1
[bbabbbb`bba]abb__aaabb^babbbaa_a]^ab^aV
@HWI-EAS443:3:23:776:1669#0/1
CTTGCAAAAGGAGAGAAAGCTAATGTGCTAATTGGGCAAG
+HWI-EAS443:3:23:776:1669#0/1
`ba`aaaaaaa]a`]aaaa_`a_aa¥__a`aa^a_`_X]_
@HWI-EAS443:3:23:77:1412#0/1
GAAAGCTAATGTGCTAATTGGGCAAGGGGACGTAGTGTTG
+HWI-EAS443:3:23:77:1412#0/1
ZIXUYa]]baaabbVZba``W``aY`_N__aaWaaY`T``
@HWI-EAS443:3:23:854:1668#0/1
AGGAGAGAAAGCTAATGTGCTAATTGGGCAAGGGGACGTA
+HWI-EAS443:3:23:854:1668#0/1
`a`^^b``ab`_]aaaa^¥Z¥aaaaa¥[Z]]¥`[a`[¥^X
@HWI-EAS443:3:23:869:947#0/1
TACCAACACTACGTCCCCTTGCCCAATTAGCACATTAGCT
+HWI-EAS443:3:23:869:947#0/1
abaaaaaaaaa`a_^__`__`_X^U^_Z__S_^]¥¥^^V^

```
@HWI-EAS443:3:23:907:826#0/1
TGCAAAAGGAGAGAAAGCTAATGTGCTAATTGGGCAAGGG
+HWI-EAS443:3:23:907:826#0/1
]bb`XHT¥¥^```bb__bb`]__M_W_^aaaaaa`YZaaa
@HWI-EAS443:3:23:93:1186#0/1
ATTAGCACATTAGCTTTCTCTCCTTTTGCAAGATTGCTCA
+HWI-EAS443:3:23:93:1186#0/1
a^`¥]`bbba`V^a`^abaa`aa_````^W_a¥^`a_``_
@HWI-EAS443:3:24:1013:191#0/1
AGCAATCTTGCAAAAGGAGAGAAAGCTAATGTGCTAATTG
+HWI-EAS443:3:24:1013:191#0/1
_`¥bbabbbb`[S^]b]bbbb_b``Z^aabb_a``a_aaa
@HWI-EAS443:3:24:1051:834#0/1
GAAAGCTAATGTGCTAATTGGGCAAGGGGACGTAGTGTTG
+HWI-EAS443:3:24:1051:834#0/1
]_a__¥a_a_]]baaaa`Y_aa__aaaaZ^]a[W`Q_Z_]
@HWI-EAS443:3:24:1054:253#0/1
TTGCAAAAGGAGAGAAAGCTAATGTGCTAATTGGGCAAGG
+HWI-EAS443:3:24:1054:253#0/1
aabaaa`aab`aaa``_a^a_^_]Ya¥`]_``a^`^]X__
@HWI-EAS443:3:24:1129:889#0/1
GAGAAAGCTAATGTGCTAATTGGGCAAGGGGACGTAGTGT
+HWI-EAS443:3:24:1129:889#0/1
ab_b_`_]aa_^_^aa^^^aab^``_`aa`a_aa_`a[YZ
@HWI-EAS443:3:24:130:78#0/1
TGCAAAAGGAGAGAAAGCTAATGTGCTAATTGGGCAAGGG
+HWI-EAS443:3:24:130:78#0/1
aaaba`abaababaaaaaa¥`ab^b_a__aaa`a^]^a`_
@HWI-EAS443:3:24:136:794#0/1
GAGCAATCTTGCAAAAGGAGAGAAAGCTAATGTGCTAATT
+HWI-EAS443:3:24:136:794#0/1
a``bbaabaabbb¥V`baaaaa`[aaaaaaab]aaaaaa_
@HWI-EAS443:3:24:138:848#0/1
ATCTTGCAAAAGGAGAGAAAGCTAATGTGCTAATTGGGCA
+HWI-EAS443:3:24:138:848#0/1
`abaabaZ]_]_babaaba¥^`aa`]aZaa`^``[_T`aa
@HWI-EAS443:3:24:1504:1784#0/1
TGCAAAAGGAGAGAAAGCTAATGTGCTAATTGGGCAAGGG
+HWI-EAS443:3:24:1504:1784#0/1
`aV]WX_`baaa```^RFRb`aaaaR^a]`_aaa___aaa
@HWI-EAS443:3:24:1546:1738#0/1
GAGAAAGCTAATGTGCTAATTGGGCAAGGGGACGTAGTGT
+HWI-EAS443:3:24:1546:1738#0/1
`¥baaS¥Y`aa¥aNS^b^``bSPRJ¥ab[HTQaaGQYI[F
@HWI-EAS443:3:24:1551:1822#0/1
ATCAATGAACTGAGCAATCTTGCAAAAGGAGAGAAAGCTA
+HWI-EAS443:3:24:1551:1822#0/1
``aaa¥Y_I_a¥b^Wa]a^a[aY]YY]`]Xbaaa_`aGZ`
@HWI-EAS443:3:24:1560:372#0/1
CTTGCAAAAGGAGAGAAAGCTAATGTGCTAATTGGGCAAG
```

```
+HWI-EAS443:3:24:1560:372#0/1
abbbabbbbbbbbb_baaa]baaab`baaaaaabab_a`a
@HWI-EAS443:3:24:1665:756#0/1
ATCTTGCAAAAGGAGAGAAAGCTAATGTGCTAATTGGGCA
+HWI-EAS443:3:24:1665:756#0/1
^^^ZZ^XVO_aaW]`Y_a`YVKWX_YYXXSR]_UU`^a_S
@HWI-EAS443:3:24:1704:1693#0/1
GAGAAAGCTAATGTGCTAATTGGGCAAGGGGACGTAGTGT
+HWI-EAS443:3:24:1704:1693#0/1
aab^bbbbaba`aab`bb`b``b^aa_aaa¥_`aUG`Oa]
@HWI-EAS443:3:24:226:1229#0/1
TGCAAAAGGAGAGAAAGCTAATGTGCTAATTGGGCAAGGG
+HWI-EAS443:3:24:226:1229#0/1
_bba`a`b``bbbbaY`aaaZa^Q¥a`aaa`^``a_^^S[
@HWI-EAS443:3:24:286:727#0/1
AGCTAATGTGCTAATTGGGCAAGGGGACGTAGTGTTGGTA
+HWI-EAS443:3:24:286:727#0/1
_bab`babaaaa_bba`a__RWb```a^a]_^Ob`^^aGY
@HWI-EAS443:3:24:426:1741#0/1
ATCTTGCAAAAGGAGAGAAAGCTAATGTGCTAATTGGGCA
+HWI-EAS443:3:24:426:1741#0/1
a_aQ`^`a_^_a``¥a_^a`__S__¥¥X___^Z[W^UX]]
@HWI-EAS443:3:24:44:624#0/1
GCATCAATGAACTGAGCAATCTTGCAAAAGGAGAGAAAGC
+HWI-EAS443:3:24:44:624#0/1
^a¥bbbbbbb`abbbbbbbbbbbbb_[[```ababbaa`a
@HWI-EAS443:3:24:53:524#0/1
TTGCAAAAGGAGAGAAAGCTAATGTGCTAATTGGGCAAGG
+HWI-EAS443:3:24:53:524#0/1
aaaaaa`aaa_aababaa`aaaab_aa`_^a_```a^`aa
@HWI-EAS443:3:24:673:1847#0/1
AATTAGCACATTAGCTTTCTCTCCTTTTGCAAGATTGCTC
+HWI-EAS443:3:24:673:1847#0/1
^¥bZ¥`bbbb_a__`aaa]a`ba`aaaaaab_]_aa`_`^
@HWI-EAS443:3:24:789:407#0/1
GCAATCTTGCAAAAGGAGAGAAAGCTAATGTGCTAATTGG
+HWI-EAS443:3:24:789:407#0/1
aa[]Z]]]_YZ_[a`V[_``X`Z`_`VMR^WYNV]X]XZU
@HWI-EAS443:3:24:806:369#0/1
GAACTGAGCAATCTTGCAAAAGGAGAGAAAGCTAATGTGC
+HWI-EAS443:3:24:806:369#0/1
a_`^¥b^¥_`_``aab_aaa`a`_aa`a¥`a`^^[aa]`W
@HWI-EAS443:3:24:903:1266#0/1
GAAAGCTAATGTGCTAATTGGGCAAGGGGACGTAGTGTTG
+HWI-EAS443:3:24:903:1266#0/1
[]V_`a_Q_`a^aa¥aa`aaa^b¥aa¥YZX¥`^bbabS_`
@HWI-EAS443:3:25:1070:1671#0/1
GAGAAAGCTAATGTGCTAATTGGGCAAGGGGACGTAGTGT
+HWI-EAS443:3:25:1070:1671#0/1
aaaa`a``a`baa_a`_a^ba`__Y_`]]_YO¥¥LZ¥UOW
```

@HWI-EAS443:3:25:1081:70#0/1
AATGAACTGAGCAATCTTGCAAAAGGAGAGAAAGCTAATG
+HWI-EAS443:3:25:1081:70#0/1
abbbba`bbbbabbbbbbb`bababbabbbbb_babb`bb
@HWI-EAS443:3:25:1280:1590#0/1
TGCAAAAGGAGAGAAAGCTAATGTGCTAATTGGGCAAGGG
+HWI-EAS443:3:25:1280:1590#0/1
aa`aaaaa_aaaaaa`a_a`aaaXaUa_```[R¥^___Y_
@HWI-EAS443:3:25:132:431#0/1
AAGCATCAATGAACTGAGCAATCTTGCAAAAGGAGAGAAA
+HWI-EAS443:3:25:132:431#0/1
`]V_aaba]aaaaaaa`aaaaa_`^^`a_^TZ`__`aaa_
@HWI-EAS443:3:25:1418:1948#0/1
AGAAAGCTAATGTGCTAATTGGGCAAGGGGACGTAGTGTT
+HWI-EAS443:3:25:1418:1948#0/1
aaabbabbbabbab`babaabVa]a_¥]`aa_a]_a^a^`
@HWI-EAS443:3:25:1437:403#0/1
ATCTTGCAAAAGGAGAGAAAGCTAATGTGCTAATTGGGCA
+HWI-EAS443:3:25:1437:403#0/1
ababbbbabaabbabbbaaaa`aaaab`a`ba`baaaa^_
@HWI-EAS443:3:25:1456:614#0/1
AATGAACTGAGCAATCTTGCAAAAGGAGAGAAAGCTAATG
+HWI-EAS443:3:25:1456:614#0/1
PY^``^[bbbbb_`aab`^Zabbbbbb_bbbbbb]bbaaa
@HWI-EAS443:3:25:1503:320#0/1
AGCTAATGTGCTAATTGGGCAAGGGGACGTAGTGTTGGTA
+HWI-EAS443:3:25:1503:320#0/1
``bababbababbbbbaaaa``bbba`ab[_b]a`aa^PX
@HWI-EAS443:3:25:1578:1065#0/1
AAGCATCAATGAACTGAGCAATCTTGCAAAAGGAGAGAAA
+HWI-EAS443:3:25:1578:1065#0/1
ab[babbb`ab_``ba`a]babaabb_a_a]a`_aaa`a_
@HWI-EAS443:3:25:1713:745#0/1
ACCAACACTACGTCCCCTTGCCCAATTAGCACATTAGCTT
+HWI-EAS443:3:25:1713:745#0/1
[R__bab`b`[aa^OWZ]_aZ[Y``^`aa``T`^¥a`Y]¥
@HWI-EAS443:3:25:1742:1002#0/1
AGCTAATGTGCTAATTGGGCAAGGGGACGTAGTGTTGGTA
+HWI-EAS443:3:25:1742:1002#0/1
a^Z_¥`b`b_Q^ba¥`b``]XQQ_`¥X_^Ua]Z¥I`aZN^
@HWI-EAS443:3:25:343:29#0/1
TGAGCAATCTTGCAAAAGGAGAGAAAGCTAATGTGCTAAT
+HWI-EAS443:3:25:343:29#0/1
]`Yba`_^`babaa_a`a`_b_a`a_a_aa_aa^a___``
@HWI-EAS443:3:25:349:1230#0/1
AGCTAATGTGCTAATTGGGCAAGGGGACGTAGTGTTGGTA
+HWI-EAS443:3:25:349:1230#0/1
a_aaaaba[aaaa_aaa_abaaa``UO``Z``]_]aaYG[
@HWI-EAS443:3:25:359:471#0/1
AAAGCTAATGTGCTAATTGGGCAAGGGGACGTAGTGTTGG

```
+HWI-EAS443:3:25:359:471#0/1
aa^Z_`¥Z`_Za_aaaaaa`[__]Z_W^^`aZ__Y`]^__
@HWI-EAS443:3:25:399:400#0/1
GAAAGCTAATGTGCTAATTGGGCAAGGGGACGTAGTGTTG
+HWI-EAS443:3:25:399:400#0/1
aaa^aa_`aa`_aX[^_`_[S^_^_`_[¥ZXaUGaVaX^^
@HWI-EAS443:3:25:881:89#0/1
ATCTTGCAAAAGGAGAGAAAGCTAATGTGCTAATTGGGCA
+HWI-EAS443:3:25:881:89#0/1
abababbbba_ba`baba`aa_baabb`b_aa`aaaaa¥_
@HWI-EAS443:3:25:885:1814#0/1
GAGCAATCTTGCAAAAGGAGAGAAAGCTAATGTGCTAATT
+HWI-EAS443:3:25:885:1814#0/1
`baaab_baaaab`bbaaabbaab^a[ab[QVD[G¥XTYY
@HWI-EAS443:3:26:1085:380#0/1
TGCAAAAGGAGAGAAAGCTAATGTGCTAATTGGGCAAGGG
+HWI-EAS443:3:26:1085:380#0/1
abaaaaaaaabaa`¥`ba`^a`a[a^a`_a`a`a___a_]
@HWI-EAS443:3:26:1143:490#0/1
CCCTTGCCCAATTAGCACATTAGCTTTCTCTCCTTTTGCA
+HWI-EAS443:3:26:1143:490#0/1
aabbbbbabbbbbbbbbabbaab]abaab_aa`aaaaa[`
@HWI-EAS443:3:26:1146:72#0/1
AACTGAGCAATCTTGCAAAAGGAGAGAAAGCTAATGTGCT
+HWI-EAS443:3:26:1146:72#0/1
aaaa]]`aaababa__a__]aa^a`a`^[`Y``^`aV`V_
@HWI-EAS443:3:26:1176:770#0/1
ATCTTGCAAAAGGAGAGAAAGCTAATGTGCTAATTGGGCA
+HWI-EAS443:3:26:1176:770#0/1
aa]_WTQIZU[Z¥Sb]__aaVbaaRaaW^`aa¥[_]PUH[
@HWI-EAS443:3:26:1315:1852#0/1
GAGCAATCTTGCAAAAGGAGAGAAAGCTAATGTGCTAATT
+HWI-EAS443:3:26:1315:1852#0/1
^`baabbbbaabbbaba_bbbbbbbbabbbbb`a^`_aba
@HWI-EAS443:3:26:1318:898#0/1
ATTAGCTTTCTCTCCTTTTGCAAGATTGCTCAGTTCATTG
+HWI-EAS443:3:26:1318:898#0/1
`abaaaa¥a[_]]aa`aa`aNaaa__aa_`¥Ya`a_`]`[
@HWI-EAS443:3:26:1470:738#0/1
CATCAATGAACTGAGCAATCTTGCAAAAGGAGAGAAAGCT
+HWI-EAS443:3:26:1470:738#0/1
G[a```a`a``_¥X``a`a``baW_Z`b`abb_bbbb`a`
@HWI-EAS443:3:26:1472:236#0/1
ATCTTGCAAAAGGAGAGAAAGCTAATGTGCTAATTGGGCA
+HWI-EAS443:3:26:1472:236#0/1
abaabbaaaaaa_aaabaa^a__a`a_^a]a^`aaa`^[Z
@HWI-EAS443:3:26:1479:472#0/1
AGCTAATGTGCTAATTGGGCAAGGGGACGTAGTGTTGGTA
+HWI-EAS443:3:26:1479:472#0/1
XTRaaabaY`aa]aaaa_]¥^_¥Y]a]]bV¥aP]^a`^G]
```

```
@HWI-EAS443:3:26:1497:819#0/1
ATTAGCTTTCTCTCCTTTTGCAAGATTGCTCAGTTCATTG
+HWI-EAS443:3:26:1497:819#0/1
abbba`aababaaaabbaaa_aab`a``aa]`a_a]`a_a
@HWI-EAS443:3:26:1638:1803#0/1
GAACTGAGCAATCTTGCAAAAGGAGAGAAAGCTAATGTGC
+HWI-EAS443:3:26:1638:1803#0/1
a`aabbaa_bab_abb`a`aab`_aaaaaaa_a`_`a[_X
@HWI-EAS443:3:26:211:1637#0/1
GAGCAATCTTGCAAAAGGAGAGAAAGCTAATGTGCTAATT
+HWI-EAS443:3:26:211:1637#0/1
¥_aabbbbbbaaa``_¥bbbaabbbbbbaabbaabbaabb
@HWI-EAS443:3:26:289:662#0/1
TAGCACATTAGCTTTCTCTCCTTTTGCAAGATTGCTCAGT
+HWI-EAS443:3:26:289:662#0/1
a`abaaaaa`aaaaaaaaaaaaaaaaaaaa__aa`a`_^
@HWI-EAS443:3:26:436:687#0/1
CCTTGCCCAATTAGCACATTAGCTTTCTCTCCTTTTGCAA
+HWI-EAS443:3:26:436:687#0/1
`ZRR[_]a¥_a_]JX_UY`a_`aZ_¥Y_`_`_Z]]X_]U^
@HWI-EAS443:3:26:482:1235#0/1
GAAAGCTAATGTGCTAATTGGGCAAGGGGACGTAGTGTTG
+HWI-EAS443:3:26:482:1235#0/1
aba^[Za¥]__[TaaZ`]Z`[]`¥`_ZX`X`a___FYQ_]
@HWI-EAS443:3:26:503:382#0/1
GAACTGAGCAATCTTGCAAAAGGAGAGAAAGCTAATGTGC
+HWI-EAS443:3:26:503:382#0/1
a`^`V¥_aa`¥a`Vaaa^^^`___a`a^a¥`U]___aO]]
@HWI-EAS443:3:26:530:788#0/1
CAATGAACTGAGCAATCTTGCAAAAGGAGAGAAAGCTAAT
+HWI-EAS443:3:26:530:788#0/1
aaaaaaaaaaabaaaaaaaaa`a`aaa_aaaaa`a___`]
@HWI-EAS443:3:26:574:1115#0/1
CTGAGCAATCTTGCAAAAGGAGAGAAAGCTAATGTGCTAA
+HWI-EAS443:3:26:574:1115#0/1
aaa_abaaaaaaaaa`Z`aa_aa`aa_]a_aa_`X__`a^
@HWI-EAS443:3:26:580:766#0/1
GAGAAAGCTAATGTGCTAATTGGGCAAGGGGACGTAGTGT
+HWI-EAS443:3:26:580:766#0/1
aaa`_]a`a__aaWa[`[a`Y¥]___¥a^^`^^a]U^O_Z
@HWI-EAS443:3:26:676:1484#0/1
TGCAAAAGGAGAGAAAGCTAATGTGCTAATTGGGCAAGGG
+HWI-EAS443:3:26:676:1484#0/1
ZT_¥_bb``a_[^¥a_^Z^aaa`K]aab``Y`a_ZUY]¥a
@HWI-EAS443:3:26:783:617#0/1
GAGCAATCTTGCAAAAGGAGAGAAAGCTAATGTGCTAATT
+HWI-EAS443:3:26:783:617#0/1
``abbbb``¥]_¥ZU_`a`aba`]]`ab^aabaa_aaa]a
@HWI-EAS443:3:26:850:642#0/1
ATGAACTGAGCAATCTTGCAAAAGGAGAGAAAGCTAATGT
```

```
+HWI-EAS443:3:26:850:642#0/1
¥b]NZaX_bb_¥]`a`a`¥YUYTZTa`]bVbab`aWU_¥X
@HWI-EAS443:3:26:87:470#0/1
CATCAATGAACTGAGCAATCTTGCAAAAGGAGAGAAAGCT
+HWI-EAS443:3:26:87:470#0/1
^¥`b^]aYZ_a]a`^_Zaaaa]^`a`¥V`]X^`bb^LaZ^
@HWI-EAS443:3:27:1156:318#0/1
GAGAAAGCTAATGTGCTAATTGGGCAAGGGGACGTAGTGT
+HWI-EAS443:3:27:1156:318#0/1
ZSbb`]_S`_OXZQ^TVW^a]`¥`I]R`]`¥FFZJW_T__
@HWI-EAS443:3:27:1303:1836#0/1
ATCTTGCAAAAGGAGAGAAAGCTAATGTGCTAATTGGGCA
+HWI-EAS443:3:27:1303:1836#0/1
a]_aa_aaaaa`]`aa[a`a__a`___^a_a___a_U`]]
@HWI-EAS443:3:27:1339:1516#0/1
CATCAATGAACTGAGCAATCTTGCAAAAGGAGAGAAAGCT
+HWI-EAS443:3:27:1339:1516#0/1
aa^b]aab`[`aa`a_a`]aa_`__^V`a````a`_^`X_
@HWI-EAS443:3:27:1423:1540#0/1
GAGCAATCTTGCAAAAGGAGAGAAAGCTAATGTGCTAATT
+HWI-EAS443:3:27:1423:1540#0/1
a``aaab_aab]`]`__aVa`a`]`a]__a`_Xa^_^]_^
@HWI-EAS443:3:27:1423:624#0/1
CATCAATGAACTGAGCAATCTTGCAAAAGGAGAGAAAGCT
+HWI-EAS443:3:27:1423:624#0/1
abaaa`aba_`bba``[```^XQXULW`^a^V¥a`HRXQ]
@HWI-EAS443:3:27:1425:1520#0/1
GAGAGAAAGCTAATGTGCTAATTGGGCAAGGGGACGTAGT
+HWI-EAS443:3:27:1425:1520#0/1
a¥¥V_a¥ababbaaba_aa`]a`a_a]a^aaaa_`aS__[
@HWI-EAS443:3:27:1468:1174#0/1
CTTGCAAAAGGAGAGAAAGCTAATGTGCTAATTGGGCAAG
+HWI-EAS443:3:27:1468:1174#0/1
a`ab]_^X_b_^aaab`]¥_a`¥a]X[U][¥`_aa``__a
@HWI-EAS443:3:27:166:179#0/1
GAGCAATCTTGCAAAAGGAGAGAAAGCTAATGTGCTAATT
+HWI-EAS443:3:27:166:179#0/1
a`]]Z_Z]UZ^a`^YPZTQUTUWXX`]OJXRTR]¥VHTZ]
@HWI-EAS443:3:27:172:708#0/1
GAGCAATCTTGCAAAAGGAGAGAAAGCTAATGTGCTAATT
+HWI-EAS443:3:27:172:708#0/1
__`a`aYb]_aTR¥[[_aZaaa`Q_`b]SQVaV_[`¥R^]
@HWI-EAS443:3:27:1772:408#0/1
AATGAACTGAGCAATCTTGCAAAAGGAGAGAAAGCTAATG
+HWI-EAS443:3:27:1772:408#0/1
aa`bbb_bbbbaaaaaa[aaa``abaaaaabaaa]_``^a
@HWI-EAS443:3:27:321:443#0/1
CGTCCCCTTGCCCAATTAGCACATTAGCTTTCTCTCCTTT
+HWI-EAS443:3:27:321:443#0/1
ab```a`]Z`aaaaaaaa`a`aaaa``_a__aa_a``_`_
```

```
@HWI-EAS443:3:27:327:1883#0/1
AATGAACTGAGCAATCTTGCAAAAGGAGAGAAAGCTAATG
+HWI-EAS443:3:27:327:1883#0/1
`baa]baa`_`bba`ba_`aa¥a`__`_aa``a`__`_^`
@HWI-EAS443:3:27:341:97#0/1
GAGAAAGCTAATGTGCTAATTGGGCAAGGGGACGTAGTGT
+HWI-EAS443:3:27:341:97#0/1
aa_a__a_`^Y`aX_]a_]``_]X]VV___^XYaXYXY^X
@HWI-EAS443:3:27:504:1035#0/1
AATGAACTGAGCAATCTTGCAAAAGGAGAGAAAGCTAATG
+HWI-EAS443:3:27:504:1035#0/1
aa^aaabaQ_a`Za_a_`¥`aa[XY_^[`_]W[^`]]QX_
@HWI-EAS443:3:27:622:1251#0/1
CAATGAACTGAGCAATCTTGCAAAAGGAGAGAAAGCTAAT
+HWI-EAS443:3:27:622:1251#0/1
^MWZ]_Z]`S__`]]Wa[WW`NZ]G¥HYHWW^X¥[¥[RNZ
@HWI-EAS443:3:27:633:255#0/1
AGCTAATGTGCTAATTGGGCAAGGGGACGTAGTGTTGGTA
+HWI-EAS443:3:27:633:255#0/1
a`aa`[`a_aaa_^aa]aaaa`ba_a¥]b]_a__^___Z]
@HWI-EAS443:3:27:732:627#0/1
AAGCTAATGTGCTAATTGGGCAAGGGGACGTAGTGTTGGT
+HWI-EAS443:3:27:732:627#0/1
aaaa]P_b^Xa_``[aaaaa_a_aa_a_^bY_aXaX___S
@HWI-EAS443:3:27:747:1320#0/1
GCATCAATGAACTGAGCAATCTTGCAAAAGGAGAGAAAGC
+HWI-EAS443:3:27:747:1320#0/1
abaab``babbbaaaa`baaabaaaa[aaaaabaaaaaaa
@HWI-EAS443:3:27:861:1512#0/1
CATCAATGAACTGAGCAATCTTGCAAAAGGAGAGAAAGCT
+HWI-EAS443:3:27:861:1512#0/1
abaaaaaaabaaaa`abaaaaaa_a`a`a__aaaa_a_^`
@HWI-EAS443:3:27:874:1398#0/1
GTCCCCTTGCCCAATTAGCACATTAGCTTTCTCTCCTTTT
+HWI-EAS443:3:27:874:1398#0/1
a]]`__XTaaaaab¥V_[¥_aa¥_`ZYT^]^`___¥[^]^
@HWI-EAS443:3:27:946:1104#0/1
CAAAAGGAGAGAAAGCTAATGTGCTAATTGGGCAAGGGGA
+HWI-EAS443:3:27:946:1104#0/1
a`abba_aa_abbbbbbbabba`abaababaaa`_baba_
@HWI-EAS443:3:27:951:150#0/1
AGCTAATGTGCTAATTGGGCAAGGGGACGTAGTGTTGGTA
+HWI-EAS443:3:27:951:150#0/1
a^¥bbaab_ba`a_a```a^a^aaa_`¥aU__]a_``_V^
@HWI-EAS443:3:27:953:1816#0/1
GAAAGCTAATGTGCTAATTGGGCAAGGGGACGTAGTGTTG
+HWI-EAS443:3:27:953:1816#0/1
abab^¥_abaa^aaa`baa^^aaa_aa_a_L`RaaWa¥[`
@HWI-EAS443:3:28:1044:1612#0/1
ACCAACACTACGTCCCCTTGCCCAATTAGCACATTAGCTT
```

```
+HWI-EAS443:3:28:1044:1612#0/1
abb`^aaabba___YT¥^`[¥a``a_]]X_`¥^`^`ZUZ`
@HWI-EAS443:3:28:1067:1200#0/1
CATCAATGAACTGAGCAATCTTGCAAAAGGAGAGAAAGCT
+HWI-EAS443:3:28:1067:1200#0/1
a_aXVaaa`]W[_SW_`YT__^aa^`W^^`RX[_^[P¥_^
@HWI-EAS443:3:28:1175:1487#0/1
GCATCAATGAACTGAGCAATCTTGCAAAAGGAGAGAAAGC
+HWI-EAS443:3:28:1175:1487#0/1
abaabaabaab`ba`baa`aabaaaa```aa_aaaaa_a^
@HWI-EAS443:3:28:1214:358#0/1
CAATCTTGCAAAAGGAGAGAAAGCTAATGTGCTAATTGGG
+HWI-EAS443:3:28:1214:358#0/1
abbbbbbbbbbbabbabbbbbababaabb_aababbbba`
@HWI-EAS443:3:28:1303:698#0/1
TGAGCAATCTTGCAAAAGGAGAGAAAGCTAATGTGCTAAT
+HWI-EAS443:3:28:1303:698#0/1
abaaaaaaa`aaa_aa_bb`baaa`aa_aaaaa]a[a``a
@HWI-EAS443:3:28:1324:1840#0/1
GCATCAATGAACTGAGCAATCTTGCAAAAGGAGAGAAAGC
+HWI-EAS443:3:28:1324:1840#0/1
]`Y]_UXR¥¥VVT_YG¥^¥^_¥¥^_`[]WZDXa`_¥VZ[N
@HWI-EAS443:3:28:1350:1381#0/1
GAACTGAGCAATCTTGCAAAAGGAGAGAAAGCTAATGTGC
+HWI-EAS443:3:28:1350:1381#0/1
aab`baabbb`bbaabab`a_a_``__aaa`^aaaaa^_^
@HWI-EAS443:3:28:1351:706#0/1
CCCTTGCCCAATTAGCACATTAGCTTTCTCTCCTTTTGCA
+HWI-EAS443:3:28:1351:706#0/1
a``bbbbbbbbbbbbabbbbbba_abbab`baababba]b
@HWI-EAS443:3:28:1371:720#0/1
AAGGAGAGAAAGCTAATGTGCTAATTGGGCAAGGGGACGT
+HWI-EAS443:3:28:1371:720#0/1
abaaabbabaaabbaa`bababaababb`_[_aWa__^`Z
@HWI-EAS443:3:28:1417:510#0/1
TGCAAAAGGAGAGAAAGCTAATGTGCTAATTGGGCAAGGG
+HWI-EAS443:3:28:1417:510#0/1
^aX^U```a`a[_¥^^a¥`__`aTOT]_^_]`__]XR_^Z
@HWI-EAS443:3:28:143:1127#0/1
AAAGCTAATGTGCTAATTGGGCAAGGGGACGTAGTGTTGG
+HWI-EAS443:3:28:143:1127#0/1
a^aaaa^aaa^¥`a^_`a^`¥`_]][[[Y__ZWX¥^[[¥R
@HWI-EAS443:3:28:1616:530#0/1
GTCCCCTTGCCCAATTAGCACATTAGCTTTCTCTCCTTTT
+HWI-EAS443:3:28:1616:530#0/1
`X_WZ`][aZ]T``_]``[VX^a^^`WSZ^[^^[VSXY^V
@HWI-EAS443:3:28:1653:977#0/1
CATCAATGAACTGAGCAATCTTGCAAAAGGAGAGAAAGCT
+HWI-EAS443:3:28:1653:977#0/1
T`a_X_aaabb]bba¥bbbbb`b^a_b`b_bbaabV_b`b
```

@HWI-EAS443:3:28:1690:1113#0/1
GAGCAATCTTGCAAAAGGAGAGAAAGCTAATGTGCTAATT
+HWI-EAS443:3:28:1690:1113#0/1
a¥`P_ab_aabX____a`_aa_``^a]a^_aXYY¥¥VY`_
@HWI-EAS443:3:28:1723:458#0/1
CATCAATGAACTGAGCAATCTTGCAAAAGGAGAGAAAGCT
+HWI-EAS443:3:28:1723:458#0/1
T[b^`^a_H_abaa^^`aaI``_`a¥a``a`¥¥Z^^_¥H^
@HWI-EAS443:3:28:175:1532#0/1
TTGCAAAAGGAGAGAAAGCTAATGTGCTAATTGGGCAAGG
+HWI-EAS443:3:28:175:1532#0/1
`aabbabaa^aaa`bbbaaaaaa^Za_aaa`a_Ua``a_]
@HWI-EAS443:3:28:277:870#0/1
GAACTGAGCAATCTTGCAAAAGGAGAGAAAGCTAATGTGC
+HWI-EAS443:3:28:277:870#0/1
ababaa`bbbbabaabaaaa`aa`aaabSaa^a_aaa[^^
@HWI-EAS443:3:28:305:1560#0/1
TGCAAAAGGAGAGAAAGCTAATGTGCTAATTGGGCAAGGG
+HWI-EAS443:3:28:305:1560#0/1
^bbb_¥^]a_aa_baaa`^`[aaHbbaa¥a__[_aa_`VY
@HWI-EAS443:3:28:415:246#0/1
GAGCAATCTTGCAAAAGGAGAGAAAGCTAATGTGCTAATT
+HWI-EAS443:3:28:415:246#0/1
abbbbbbabbabab`aabaaaabaabaa`aaa_a_a[a`b
@HWI-EAS443:3:28:439:364#0/1
ATCTTGCAAAAGGAGAGAAAGCTAATGTGCTAATTGGGCA
+HWI-EAS443:3:28:439:364#0/1
]KZa_baZSYaab_aaa_Y^b_a[R_b___a__a]^Y_a`
@HWI-EAS443:3:28:537:1738#0/1
AGCTAATGTGCTAATTGGGCAAGGGGACGTAGTGTTGGTA
+HWI-EAS443:3:28:537:1738#0/1
a]ba`a`aUZaa_]]_^Maaaa`]a``aa]_^WXT]`]GW
@HWI-EAS443:3:28:57:1443#0/1
AGCTAATGTGCTAATTGGGCAAGGGGACGTAGTGTTGGTA
+HWI-EAS443:3:28:57:1443#0/1
aaaaaaaaZaba]aaa```a`¥^`_^X`a^[a^a]_]^RZ
@HWI-EAS443:3:28:590:1132#0/1
ATCTTGCAAAAGGAGAGAAAGCTAATGTGCTAATTGGGCA
+HWI-EAS443:3:28:590:1132#0/1
^`_¥V`aT¥¥H_]_a]Y¥aZTS[`Y[TJZ``]^[_`_Y]Z
@HWI-EAS443:3:28:602:1771#0/1
ATGAACTGAGCAATCTTGCAAAAGGAGAGAAAGCTAATGT
+HWI-EAS443:3:28:602:1771#0/1
^Y]¥aa__a]`a^Wa`__^aZP]X___`[a^¥]`___^_U
@HWI-EAS443:3:28:61:1881#0/1
GAAAGCTAATGTGCTAATTGGGCAAGGGGACGTAGTGTTG
+HWI-EAS443:3:28:61:1881#0/1
`aX`abaZbaa_`babaa`^V^baa`_[^^aa¥__^[Z`^
@HWI-EAS443:3:28:643:1950#0/1
GAGAAAGCTAATGTGCTAATTGGGCAAGGGGACGTAGTGT

```
+HWI-EAS443:3:28:643:1950#0/1
ab[bba`baaabVTVa``aa``a^_`a__¥^`_U_YUPRI
@HWI-EAS443:3:28:691:1512#0/1
GCAAAAGGAGAGAAAGCTAATGTGCTAATTGGGCAAGGGG
+HWI-EAS443:3:28:691:1512#0/1
]`aYWZ¥U^_¥_^S`aaa^SW]NX¥_S_`_[Q[¥[aa[¥O
@HWI-EAS443:3:28:739:850#0/1
GAAAGCTAATGTGCTAATTGGGCAAGGGGACGTAGTGTTG
+HWI-EAS443:3:28:739:850#0/1
aaa````Y`^a]_P`aaaaaa``a___[]```U_]TY][a
@HWI-EAS443:3:28:767:1727#0/1
GAACTGAGCAATCTTGCAAAAGGAGAGAAAGCTAATGTGC
+HWI-EAS443:3:28:767:1727#0/1
abbab``^bbbbaa`babaa]aYaaa_baaaa`aaaa___
@HWI-EAS443:3:28:806:1076#0/1
ATCTTGCAAAAGGAGAGAAAGCTAATGTGCTAATTGGGCA
+HWI-EAS443:3:28:806:1076#0/1
U¥X`[b]Y_`]V¥H_aaa¥R]V``N_]X^]Y_a¥`^`_]_
@HWI-EAS443:3:28:806:135#0/1
GAGAAAGCTAATGTGCTAATTGGGCAAGGGGACGTAGTGT
+HWI-EAS443:3:28:806:135#0/1
^``_¥V_^a_]aaKZYaWaaZa[VZWWaZXSMW[^__YXN
@HWI-EAS443:3:28:981:611#0/1
AGCTAATGTGCTAATTGGGCAAGGGGACGTAGTGTTGGTA
+HWI-EAS443:3:28:981:611#0/1
aaaaaY_a```aaaa`b]`[YWa]aa_aa^_a^a_aaaOY
@HWI-EAS443:3:28:999:1981#0/1
AATGAACTGAGCAATCTTGCAAAAGGAGAGAAAGCTAATG
+HWI-EAS443:3:28:999:1981#0/1
abbaabba`aa`bbaaaaabaaa`baaaaabaaa_`a`aa
@HWI-EAS443:3:29:1016:362#0/1
AATCTTGCAAAAGGAGAGAAAGCTAATGTGCTAATTGGGC
+HWI-EAS443:3:29:1016:362#0/1
`VUPaaU_YQOH¥a]aPZaSL]]^T^Y[GSY]YN]^^_BB
@HWI-EAS443:3:29:1017:982#0/1
AATGAACTGAGCAATCTTGCAAAAGGAGAGAAAGCTAATG
+HWI-EAS443:3:29:1017:982#0/1
abbaabbaaababaaaaab_aaaaaa`aaaaaaaaaaaaa
@HWI-EAS443:3:29:1056:1152#0/1
CATCAATGAACTGAGCAATCTTGCAAAAGGAGAGAAAGCT
+HWI-EAS443:3:29:1056:1152#0/1
aaaaaaaaaaaa`_``aaaaaaaa`^``^__aa_aaa```
@HWI-EAS443:3:29:1099:27#0/1
AGCACATTAGCTTTCTCTCCTTTTGCAAGATTGCTCAGTT
+HWI-EAS443:3:29:1099:27#0/1
aaab_abaababaaaa_a`[aaaa`_^`a``aa_a__a_a
@HWI-EAS443:3:29:1168:1475#0/1
GCATCAATGAACTGAGCAATCTTGCAAAAGGAGAGAAAGC
+HWI-EAS443:3:29:1168:1475#0/1
¥a^X`_`_baaa_]Zb[baaaaa_S_``a`Y``a]ba`a[
```

```
@HWI-EAS443:3:29:1169:1867#0/1
CAATCTTGCAAAAGGAGAGAAAGCTAATGTGCTAATTGGG
+HWI-EAS443:3:29:1169:1867#0/1
aabaa_a^babaa`a``a`aa_a_aa`a_^a`aaaa^_¥W
@HWI-EAS443:3:29:1247:978#0/1
AATGAACTGAGCAATCTTGCAAAAGGAGAGAAAGCTAATG
+HWI-EAS443:3:29:1247:978#0/1
__``bb_baabbbabXaY``^¥a¥]YO^a``ab`a`aaa[
@HWI-EAS443:3:29:1280:1263#0/1
AGCACATTAGCTTTCTCTCCTTTTGCAAGATTGCTCAGTT
+HWI-EAS443:3:29:1280:1263#0/1
abbaaabbbaaaaa_b]aa`abaaaaaaaaaaaa`aa`_
@HWI-EAS443:3:29:1368:822#0/1
CATCAATGAACTGAGCAATCTTGCAAAAGGAGAGAAAGCT
+HWI-EAS443:3:29:1368:822#0/1
abaabbbbbbabaabababaaabaaaaabaababbaaa``
@HWI-EAS443:3:29:1464:1561#0/1
AGCTAATGTGCTAATTGGGCAAGGGGACGTAGTGTTGGTA
+HWI-EAS443:3:29:1464:1561#0/1
abaaaabaabaaaaaaaaa^aab`^_G¥a]__]a_`a_V`
@HWI-EAS443:3:29:1503:1213#0/1
AGCACATTAGCTTTCTCTCCTTTTGCAAGATTGCTCAGTT
+HWI-EAS443:3:29:1503:1213#0/1
]_]]`a¥Ya`¥aaaZ[Za`_ab_^VQ^¥¥`UX_¥]`aaX`
@HWI-EAS443:3:29:1526:1760#0/1
GAGAAAGCTAATGTGCTAATTGGGCAAGGGGACGTAGTGT
+HWI-EAS443:3:29:1526:1760#0/1
^_`bba_Zaabb^`]Ya`babaaa]U[bbab__a_^b[bB
@HWI-EAS443:3:29:1545:1857#0/1
CATCAATGAACTGAGCAATCTTGCAAAAGGAGAGAAAGCT
+HWI-EAS443:3:29:1545:1857#0/1
`_`baaba`aaa[]Zaaa_^aabaa_a^^^aaaaaaa`^_
@HWI-EAS443:3:29:1598:1348#0/1
GAGCAATCTTGCAAAAGGAGAGAAAGCTAATGTGCTAATT
+HWI-EAS443:3:29:1598:1348#0/1
`]baabaabba`aa_`aX``aaa_`a]`]_aa_a_``_a`
@HWI-EAS443:3:29:15:431#0/1
CTTGCAAAAGGAGAGAAAGCTAATGTGCTAATTGGGCAAG
+HWI-EAS443:3:29:15:431#0/1
`Z`ab[]]]]a`a`aa¥Z`a_^`aa^``a_X_^_¥Z_^^`
@HWI-EAS443:3:29:167:681#0/1
CATCAATGAACTGAGCAATCTTGCAAAAGGAGAGAAAGCT
+HWI-EAS443:3:29:167:681#0/1
_X_`]Z]aa`a``]_]XTV`_¥_¥V]VTZ`U`___`TX`B
@HWI-EAS443:3:29:232:488#0/1
AGCTAATGTGCTAATTGGGCAAGGGGACGTAGTGTTGGTA
+HWI-EAS443:3:29:232:488#0/1
```Z]a]RLaaa`_a`]¥aY_aaUG^Qa_¥Z[HZY`P¥W]
@HWI-EAS443:3:29:391:1579#0/1
AACTGAGCAATCTTGCAAAAGGAGAGAAAGCTAATGTGCT
```
```

```
+HWI-EAS443:3:29:391:1579#0/1
¥^bb]_abbbabaabb]aa]a[a`aabab_a`aa`aN_`_
@HWI-EAS443:3:29:393:541#0/1
GAGAAAGCTAATGTGCTAATTGGGCAAGGGGACGTAGTGT
+HWI-EAS443:3:29:393:541#0/1
YZS_`UUXWV]W_RU]¥Q¥__R]a^SY]aaaUU^QBBBBB
@HWI-EAS443:3:29:484:391#0/1
GAAAGCTAATGTGCTAATTGGGCAAGGGGACGTAGTGTTG
+HWI-EAS443:3:29:484:391#0/1
aaaaa`aaaaa_aaaa`a`a_a``_````__a]^aGa]^_
@HWI-EAS443:3:29:578:440#0/1
ATGAACTGAGCAATCTTGCAAAAGGAGAGAAAGCTAATGT
+HWI-EAS443:3:29:578:440#0/1
abaaaaaaaabbab`a`aa``_aa``aaaa_aa^a`aaa]
@HWI-EAS443:3:29:630:1384#0/1
GAGAAAGCTAATGTGCTAATTGGGCAAGGGGACGTAGTGT
+HWI-EAS443:3:29:630:1384#0/1
ab]]ab]bababaY`^aaabaa``aa_V]Y`a_aVW¥XaY
@HWI-EAS443:3:29:681:1849#0/1
GTCCCCTTGCCCAATTAGCACATTAGCTTTCTCTCCTTTT
+HWI-EAS443:3:29:681:1849#0/1
^^bbbb`XZabbba^`^V_aa_aa]aaa]aa`a`ba____
@HWI-EAS443:3:29:698:246#0/1
GAAAGCTAATGTGCTAATTGGGCAAGGGGACGTAGTGTTG
+HWI-EAS443:3:29:698:246#0/1
`a[^¥`b]W^bT_U_]_aaa_PP¥S`aa_Y[]SWR[WBBB
@HWI-EAS443:3:29:704:1874#0/1
CATCAATGAACTGAGCAATCTTGCAAAAGGAGAGAAAGCT
+HWI-EAS443:3:29:704:1874#0/1
]]]a`^`JYZ¥WQZ[aa]Xa_aaY`a¥YZVX`a_aa_]_a
@HWI-EAS443:3:29:920:657#0/1
GAGAAAGCTAATGTGCTAATTGGGCAAGGGGACGTAGTGT
+HWI-EAS443:3:29:920:657#0/1
ababa```aaaaa_a`aa`a`aa```]_a_`RRa^^`Y__
@HWI-EAS443:3:29:941:398#0/1
GAGAAAGCTAATGTGCTAATTGGGCAAGGGGACGTAGTGT
+HWI-EAS443:3:29:941:398#0/1
abba`^b`a_^`b^a_aaaa^a`¥```a````ab_]a]aX
@HWI-EAS443:3:2:1158:1417#0/1
GAAAGCTAATGTGCTAATTGGGCAAGGGGACGTAGTGTTG
+HWI-EAS443:3:2:1158:1417#0/1
aaa]a]__aa_T_]aP¥``aa_`¥_b]a^]]a^]]Y[V`_
@HWI-EAS443:3:2:1217:2023#0/1
AATGAACTGAGCAATCTTGCAAAAGGAGAGAAAGCTAATG
+HWI-EAS443:3:2:1217:2023#0/1
a¥_a_^_`b]b__¥^S¥¥aO]Ya_a^_``aa`X`O^_^_B
@HWI-EAS443:3:2:124:735#0/1
AAGCATCAATGAACTGAGCAATCTTGCAAAAGGAGAGAAA
+HWI-EAS443:3:2:124:735#0/1
`aa_aaaaaaa_a``_`a``_a`__a_`^^_a[¥`_`a_]
```

```
@HWI-EAS443:3:2:1281:1428#0/1
GAACTGAGCAATCTTGCAAAAGGAGAGAAAGCTAATGTGC
+HWI-EAS443:3:2:1281:1428#0/1
a___a`^a`_aaaaa]]aa^^`^¥a`aa`^aW__Z]`W`W
@HWI-EAS443:3:2:1586:1742#0/1
CAATCTTGCAAAAGGAGAGAAAGCTAATGTGCTAATTGGG
+HWI-EAS443:3:2:1586:1742#0/1
_`bb`aababaa[`babababaaab[_b_a_Qa_a]__aa`_
@HWI-EAS443:3:2:1742:622#0/1
GAAAGCTAATGTGCTAATTGGGCAAGGGGACGTAGTGTTG
+HWI-EAS443:3:2:1742:622#0/1
a]__a]aZ`abRaXa[GW^```]`]`Z__XWaZO_WXX^P
@HWI-EAS443:3:2:247:357#0/1
CATCAATGAACTGAGCAATCTTGCAAAAGGAGAGAAAGCT
+HWI-EAS443:3:2:247:357#0/1
a^`]^X^_TXFPaZa]^WZVVS]_]T[¥_LSSV[`^^¥P[
@HWI-EAS443:3:2:249:1489#0/1
CATCAATGAACTGAGCAATCTTGCAAAAGGAGAGAAAGCT
+HWI-EAS443:3:2:249:1489#0/1
aab_aaaaaaaa__a¥aaa`a_a`aa_`a`^aaaa__`¥^
@HWI-EAS443:3:2:295:422#0/1
CCTTGCCCAATTAGCACATTAGCTTTCTCTCCTTTTGCAA
+HWI-EAS443:3:2:295:422#0/1
_NTV¥PZ]¥[LLUWPQQR[[W][OLRZ¥GNTWVP[WBBBB
@HWI-EAS443:3:2:29:1406#0/1
CAATCTTGCAAAAGGAGAGAAAGCTAATGTGCTAATTGGG
+HWI-EAS443:3:2:29:1406#0/1
a¥`aaXb¥R^aa[W`_a`_^aa``_a__aVa_^_Z_YaW_
@HWI-EAS443:3:2:307:900#0/1
GAAAGCTAATGTGCTAATTGGGCAAGGGGACGTAGTGTTG
+HWI-EAS443:3:2:307:900#0/1
]aabbbbb`bb_b`ababbbbb`aaba¥aaaa_aa___aa
@HWI-EAS443:3:2:320:900#0/1
GAGCAATCTTGCAAAAGGAGAGAAAGCTAATGTGCTAATT
+HWI-EAS443:3:2:320:900#0/1
aabaaaa`aba`b¥¥aaaababaa`aYa_]aa_a_`_`a`
@HWI-EAS443:3:2:347:875#0/1
AGCTAATGTGCTAATTGGGCAAGGGGACGTAGTGTTGGTA
+HWI-EAS443:3:2:347:875#0/1
_ba`_Y_aTa¥`aabaaaa^_`aa_``]b]_aZ]^_a_T]
@HWI-EAS443:3:2:351:495#0/1
GCATCAATGAACTGAGCAATCTTGCAAAAGGAGAGAAAGC
+HWI-EAS443:3:2:351:495#0/1
a`aa`aaaaa`Za[^[[```a`aaV^^X¥`a^b^a_^_aQ
@HWI-EAS443:3:2:404:801#0/1
GCATCAATGAACTGAGCAATCTTGCAAAAGGAGAGAAAGC
+HWI-EAS443:3:2:404:801#0/1
aaaa`aabb_baa``a_baa`aaX^a_`^aa^a`_a^`a^
@HWI-EAS443:3:2:422:871#0/1
GAGCAATCTTGCAAAAGGAGAGAAAGCTAATGTGCTAATT
```

```
+HWI-EAS443:3:2:422:871#0/1
UOaaa_XWXR_VUUY_baXa]a_aaZV]bK[a`__KX`XW
@HWI-EAS443:3:2:464:919#0/1
GAGCAATCTTGCAAAAGGAGAGAAAGCTAATGTGCTAATT
+HWI-EAS443:3:2:464:919#0/1
a^`baaaV`aa`_]_`ab_baaaaabUa^Xab_aZa[Y`]
@HWI-EAS443:3:2:508:389#0/1
AGCAATCTTGCAAAAGGAGAGAAAGCTAATGTGCTAATTG
+HWI-EAS443:3:2:508:389#0/1
abaabbabbbb```_ababbba`ababbabbab`baaaab
@HWI-EAS443:3:2:522:1621#0/1
AGCAATCTTGCAAAAGGAGAGAAAGCTAATGTGCTAATTG
+HWI-EAS443:3:2:522:1621#0/1
_Y]b`]aaba_`a`aabSbabba_b``aaaa]¥Zaa]a]_
@HWI-EAS443:3:2:646:463#0/1
ATGAACTGAGCAATCTTGCAAAAGGAGAGAAAGCTAATGT
+HWI-EAS443:3:2:646:463#0/1
¥b^]_a_a^`]a`¥Yaab_``W]b_a`[a`__aWaZ]abX
@HWI-EAS443:3:2:646:617#0/1
AAAGCTAATGTGCTAATTGGGCAAGGGGACGTAGTGTTGG
+HWI-EAS443:3:2:646:617#0/1
]]_`__SXSa_bY_SV`]¥]aX__``Z`_]aW^aX_X_a_
@HWI-EAS443:3:2:646:952#0/1
CATCAATGAACTGAGCAATCTTGCAAAAGGAGAGAAAGCT
+HWI-EAS443:3:2:646:952#0/1
a`a_baY^^`¥[`W]G]_a___a`^_[[aa_aaab^_`a_
@HWI-EAS443:3:2:707:1319#0/1
AGCAATCTTGCAAAAGGAGAGAAAGCTAATGTGCTAATTG
+HWI-EAS443:3:2:707:1319#0/1
`a]`baaa_U^a_`[Z¥_a`a^Y]^_aa_a`_XH]^^`^_
@HWI-EAS443:3:2:729:577#0/1
GAGCAATCTTGCAAAAGGAGAGAAAGCTAATGTGCTAATT
+HWI-EAS443:3:2:729:577#0/1
abbabbbabab]`_a`abababbaabaaaaab_a]a^```
@HWI-EAS443:3:2:815:1148#0/1
ATCTTGCAAAAGGAGAGAAAGCTAATGTGCTAATTGGGCA
+HWI-EAS443:3:2:815:1148#0/1
`aaa`aaaaaaa``ba[]S^a¥a__a`Wa[a`_aaaY___
@HWI-EAS443:3:2:880:442#0/1
CAATGAACTGAGCAATCTTGCAAAAGGAGAGAAAGCTAAT
+HWI-EAS443:3:2:880:442#0/1
aabba_Xa`__bb`W^^bab^_]K`bbab`baaRaIZ_`a
@HWI-EAS443:3:2:885:927#0/1
GCATCAATGAACTGAGCAATCTTGCAAAAGGAGAGAAAGC
+HWI-EAS443:3:2:885:927#0/1
R`^¥^U¥`b_bbbbaaa`^Y`aaba_a__ba`^]a]_a`P
@HWI-EAS443:3:30:1076:1878#0/1
CCCTTGCCCAATTAGCACATTAGCTTTCTCTCCTTTTGCA
+HWI-EAS443:3:30:1076:1878#0/1
a¥`ZYba]a^XOH_`_aab`U`^aJ`Jb``Wbba¥^ZWKX
```

@HWI-EAS443:3:30:1077:1166#0/1
ATCAATGAACTGAGCAATCTTGCAAAAGGAGAGAAAGCTA
+HWI-EAS443:3:30:1077:1166#0/1
^¥`[¥¥a^^_abaVZabbba_`^¥aY]¥_[`abaa_aa_a
@HWI-EAS443:3:30:1125:1952#0/1
ATGAACTGAGCAATCTTGCAAAAGGAGAGAAAGCTAATGT
+HWI-EAS443:3:30:1125:1952#0/1
aaaa`^b`a_aaaaaaaa``aa``]`aaaaaa__a`a_W]
@HWI-EAS443:3:30:115:974#0/1
ATGAACTGAGCAATCTTGCAAAAGGAGAGAAAGCTAATGT
+HWI-EAS443:3:30:115:974#0/1
]a]MYa¥a[¥babaaaaaaaaa`a`_a¥aaa`aaa`_aa]
@HWI-EAS443:3:30:1178:757#0/1
CATCAATGAACTGAGCAATCTTGCAAAAGGAGAGAAAGCT
+HWI-EAS443:3:30:1178:757#0/1
abbb_abaaabb`Taaa¥`ab_`aaa`]a^^bba_[_a_a
@HWI-EAS443:3:30:1248:1838#0/1
AAAGGAGAGAAAGCTAATGTGCTAATTGGGCAAGGGGACG
+HWI-EAS443:3:30:1248:1838#0/1
``aa^aaa_aa^a`^`a`_U]`__¥_]``^__¥a]V]ZU_
@HWI-EAS443:3:30:1316:1086#0/1
AATGAACTGAGCAATCTTGCAAAAGGAGAGAAAGCTAATG
+HWI-EAS443:3:30:1316:1086#0/1
¥SXa]VZa¥VYa]^]VaZ¥_aa_``]a`]aY]Va_`]]_[
@HWI-EAS443:3:30:1339:181#0/1
GCATCAATGAACTGAGCAATCTTGCAAAAGGAGAGAAAGC
+HWI-EAS443:3:30:1339:181#0/1
aaaa__abb_``aa`a__``_a`_X^^^^_]_`__]]^^X
@HWI-EAS443:3:30:1380:1791#0/1
AGCTAATGTGCTAATTGGGCAAGGGGACGTAGTGTTGGTA
+HWI-EAS443:3:30:1380:1791#0/1
a``aaabb_`¥aa^a```a_]`a[_a^_`][aW_]`a^NZ
@HWI-EAS443:3:30:1423:547#0/1
AGCATCAATGAACTGAGCAATCTTGCAAAAGGAGAGAAAG
+HWI-EAS443:3:30:1423:547#0/1
aab]a^Waabaaaba_aaaa`abaa_a]`_aa_aaa___a
@HWI-EAS443:3:30:1425:503#0/1
GAGAAAGCTAATGTGCTAATTGGGCAAGGGGACGTAGTGT
+HWI-EAS443:3:30:1425:503#0/1
a¥a__aaa_a`a^¥aWa^_`¥^¥aZW[a_`^_XaXZ]XXX
@HWI-EAS443:3:30:1500:925#0/1
AATGAACTGAGCAATCTTGCAAAAGGAGAGAAAGCTAATG
+HWI-EAS443:3:30:1500:925#0/1
[a^VTQab^Q^X`^X_XXZa]UaZa]XZ^bb^ZYUS`aa`
@HWI-EAS443:3:30:1514:1592#0/1
GAAAGCTAATGTGCTAATTGGGCAAGGGGACGTAGTGTTG
+HWI-EAS443:3:30:1514:1592#0/1
`¥K`bba_a_b]Oa]aba]a_¥W^aabbaY¥aa_^]aY`a
@HWI-EAS443:3:30:1637:1576#0/1
GAACTGAGCAATCTTGCAAAAGGAGAGAAAGCTAATGTGC

```
+HWI-EAS443:3:30:1637:1576#0/1
Ybabbabbbbaababaa^]aaabababbb`]_`aaab_a_
@HWI-EAS443:3:30:1723:1011#0/1
AATGAACTGAGCAATCTTGCAAAAGGAGAGAAAGCTAATG
+HWI-EAS443:3:30:1723:1011#0/1
aaabab``W_abbbbabbbbaa`abb_aaaaaab^aa`^a
@HWI-EAS443:3:30:180:1823#0/1
GAGCAATCTTGCAAAAGGAGAGAAAGCTAATGTGCTAATT
+HWI-EAS443:3:30:180:1823#0/1
aaabbbabaaab_`aa^Q_ababb`aaa¥aa___aa`a`^
@HWI-EAS443:3:30:231:591#0/1
CATCAATGAACTGAGCAATCTTGCAAAAGGAGAGAAAGCT
+HWI-EAS443:3:30:231:591#0/1
]Y`baaaaaaaa_aaa`TYaa_aa_¥_Y]__`_`a`__]^
@HWI-EAS443:3:30:295:46#0/1
GCATCAATGAACTGAGCAATCTTGCAAAAGGAGAGAAAGC
+HWI-EAS443:3:30:295:46#0/1
``][`[R]`_¥Y¥T^[``^XV¥[a]]YYW]_N][V[RW¥Y
@HWI-EAS443:3:30:386:345#0/1
TGCAAAAGGAGAGAAAGCTAATGTGCTAATTGGGCAAGGG
+HWI-EAS443:3:30:386:345#0/1
abaaa_Yba_aaaaa_]aaa`aa]a``a`a_a`___`_^`
@HWI-EAS443:3:30:573:465#0/1
AGCAATCTTGCAAAAGGAGAGAAAGCTAATGTGCTAATTG
+HWI-EAS443:3:30:573:465#0/1
`bb[``aaabaaa`a]aaabbbaaa_``]`a]a____`^]
@HWI-EAS443:3:30:643:358#0/1
CCCTTGCCCAATTAGCACATTAGCTTTCTCTCCTTTTGCA
+HWI-EAS443:3:30:643:358#0/1
_ab¥ZX¥abbaaa``^[baa`]`a`abaa^ab_`a`_aW^
@HWI-EAS443:3:30:80:961#0/1
AGAAAGCTAATGTGCTAATTGGGCAAGGGGACGTAGTGTT
+HWI-EAS443:3:30:80:961#0/1
`_WOHOZ_SZVa]aa_[X_aZX¥a¥WZ^^P¥aaVV`W]WP
@HWI-EAS443:3:30:818:542#0/1
AGCTAATGTGCTAATTGGGCAAGGGGACGTAGTGTTGGTA
+HWI-EAS443:3:30:818:542#0/1
aY^a^[_bXY`aa`a]`^QT__a^``S`a¥_bX`^aW_G¥
@HWI-EAS443:3:30:839:784#0/1
AAGCTAATGTGCTAATTGGGCAAGGGGACGTAGTGTTGGT
+HWI-EAS443:3:30:839:784#0/1
]`aaVUWX^VW¥¥VX[]^ZZ¥aJ``_[]_aY[`UY^_aWY
@HWI-EAS443:3:30:863:467#0/1
GAGAAAGCTAATGTGCTAATTGGGCAAGGGGACGTAGTGT
+HWI-EAS443:3:30:863:467#0/1
aa`a`aa`__U`a_a_`__a_`_][_^aZ_^Z]a]Z]O^R
@HWI-EAS443:3:30:945:1116#0/1
TGCAAAAGGAGAGAAAGCTAATGTGCTAATTGGGCAAGGG
+HWI-EAS443:3:30:945:1116#0/1
ababaab__^``_ba`aaaaaaa[`]aa`a`aa]````_a
```

```
@HWI-EAS443:3:31:1122:679#0/1
GAGCAATCTTGCAAAAGGAGAGAAAGCTAATGTGCTAATT
+HWI-EAS443:3:31:1122:679#0/1
¥Q_`a^¥_`baaa^aXbb`b_aR^^a_aaY`_]a_a__¥[
@HWI-EAS443:3:31:1228:1060#0/1
CCCTTGCCCAATTAGCACATTAGCTTTCTCTCCTTTTGCA
+HWI-EAS443:3:31:1228:1060#0/1
aaa`aaaaaaba`aaabbaaabaaaababaaaaaaaaa`a
@HWI-EAS443:3:31:1297:831#0/1
TTGCAAAAGGAGAGAAAGCTAATGTGCTAATTGGGCAAGG
+HWI-EAS443:3:31:1297:831#0/1
a`Y^`b^Waaab`]¥Q_b`baaab]baa[_``_`]¥`UaX
@HWI-EAS443:3:31:1319:1799#0/1
ATCTTGCAAAAGGAGAGAAAGCTAATGTGCTAATTGGGCA
+HWI-EAS443:3:31:1319:1799#0/1
]_bbb`RXb]bbbbb[_]aa`O[S]]bPXQ_X]a^SQIZ`
@HWI-EAS443:3:31:1327:1082#0/1
GAAAGCTAATGTGCTAATTGGGCAAGGGGACGTAGTGTTG
+HWI-EAS443:3:31:1327:1082#0/1
`baababZNaa]aaa`aaa`aaa_aaab_^_a]_]Z`^`_
@HWI-EAS443:3:31:1333:505#0/1
AATGAACTGAGCAATCTTGCAAAAGGAGAGAAAGCTAATG
+HWI-EAS443:3:31:1333:505#0/1
aabbabaaaabaaaaababaaaa`aaaba`b]ab_a[a_a
@HWI-EAS443:3:31:1390:743#0/1
GAGCAATCTTGCAAAAGGAGAGAAAGCTAATGTGCTAATT
+HWI-EAS443:3:31:1390:743#0/1
^ababbbbbbabbabbbb_bbbbababbbbbaa`aaaba
@HWI-EAS443:3:31:1398:585#0/1
CAATTAGCACATTAGCTTTCTCTCCTTTTGCAAGATTGCT
+HWI-EAS443:3:31:1398:585#0/1
aabbbba`babbbab_bbbab`bbabbbaaabbabbabaa
@HWI-EAS443:3:31:1468:1851#0/1
GAGAAAGCTAATGTGCTAATTGGGCAAGGGGACGTAGTGT
+HWI-EAS443:3:31:1468:1851#0/1
aabaaaaaaaab¥aaYabaaaa¥`Z`]a[aa]^a]WaV`]
@HWI-EAS443:3:31:1622:1620#0/1
CATCAATGAACTGAGCAATCTTGCAAAAGGAGAGAAAGCT
+HWI-EAS443:3:31:1622:1620#0/1
abbbbbaa_abbb`aabbb`ba_aaa_aaaaaaaaab_^
@HWI-EAS443:3:31:1644:1490#0/1
AAGCTAATGTGCTAATTGGGCAAGGGGACGTAGTGTTGGT
+HWI-EAS443:3:31:1644:1490#0/1
T`]_`bab``[abaaba]]_`]ab_W`_a][`_^bY^]_O
@HWI-EAS443:3:31:1716:492#0/1
GAGAAAGCTAATGTGCTAATTGGGCAAGGGGACGTAGTGT
+HWI-EAS443:3:31:1716:492#0/1
aabbaa``aa``bWaQ_^_a_aaa[^^aa``^]`Q]`XaZ
@HWI-EAS443:3:31:268:738#0/1
CATCAATGAACTGAGCAATCTTGCAAAAGGAGAGAAAGCT
```

```
+HWI-EAS443:3:31:268:738#0/1
aT_aaaba]^`aaaaaa`a```aa`^Y]a_^`aaaa_`]^
@HWI-EAS443:3:31:358:666#0/1
TGCAAAAGGAGAGAAAGCTAATGTGCTAATTGGGCAAGGG
+HWI-EAS443:3:31:358:666#0/1
aaa^aaabaaa`aa[^aa`Xaaa_aaaaaaaaa_a`a]__
@HWI-EAS443:3:31:414:457#0/1
GAGCAATCTTGCAAAAGGAGAGAAAGCTAATGTGCTAATT
+HWI-EAS443:3:31:414:457#0/1
^bbbbbbabbbbbbbabbbbbbabbabbbbbbababbaa`
@HWI-EAS443:3:31:48:494#0/1
GAAAGCTAATGTGCTAATTGGGCAAGGGGACGTAGTGTTG
+HWI-EAS443:3:31:48:494#0/1
ab^TabaaZ]a]bbaaaaaa`aba]aa`^W`aVW`X`[]¥
@HWI-EAS443:3:31:726:531#0/1
GCATCAATGAACTGAGCAATCTTGCAAAAGGAGAGAAAGC
+HWI-EAS443:3:31:726:531#0/1
`bbbbabba`bbbabbbbbabbbabaabbbbbbbbabab]
@HWI-EAS443:3:31:748:1063#0/1
ACCAACACTACGTCCCCTTGCCCAATTAGCACATTAGCTT
+HWI-EAS443:3:31:748:1063#0/1
aaaaa`aa`aa`Y^`_`VVZ^^_``^^[Z^_]^]^^¥]X]
@HWI-EAS443:3:31:790:991#0/1
GAGCAATCTTGCAAAAGGAGAGAAAGCTAATGTGCTAATT
+HWI-EAS443:3:31:790:991#0/1
`¥bbbbababbbbbbbbbbbbbbbbbabbabbabababba
@HWI-EAS443:3:31:798:1648#0/1
GAAAGCTAATGTGCTAATTGGGCAAGGGGACGTAGTGTTG
+HWI-EAS443:3:31:798:1648#0/1
_ab`_bbb]bba_aab``aa_ababbaaaaa`^aa_a]`_
@HWI-EAS443:3:31:837:187#0/1
AGCAATCTTGCAAAAGGAGAGAAAGCTAATGTGCTAATTG
+HWI-EAS443:3:31:837:187#0/1
]T_aab`a_a_aa`abaY```_a`a`aa``¥Ya^a`_]^^
@HWI-EAS443:3:31:837:854#0/1
ATCTTGCAAAAGGAGAGAAAGCTAATGTGCTAATTGGGCA
+HWI-EAS443:3:31:837:854#0/1
`abaa^___W¥a]`aYaa``Z^_]`aa_a_```_`a^```
@HWI-EAS443:3:31:848:2008#0/1
GAGCAATCTTGCAAAAGGAGAGAAAGCTAATGTGCTAATT
+HWI-EAS443:3:31:848:2008#0/1
``[bbabbabbbbbbba_bbbbbSbbabbbbbaa`aabbb
@HWI-EAS443:3:31:881:1688#0/1
AATTAGCACATTAGCTTTCTCTCCTTTTGCAAGATTGCTC
+HWI-EAS443:3:31:881:1688#0/1
abba`^`aab`bb`b^`]a`b__aa`_`aab_`a__aX`W
@HWI-EAS443:3:31:980:1983#0/1
GAACTGAGCAATCTTGCAAAAGGAGAGAAAGCTAATGTGC
+HWI-EAS443:3:31:980:1983#0/1
a`b`aaa_aaaaa`aaa`__aV__aaaaaa_`W____Y_V
```

```
@HWI-EAS443:3:32:1101:1644#0/1
GCAAAAGGAGAGAAAGCTAATGTGCTAATTGGGCAAGGGG
+HWI-EAS443:3:32:1101:1644#0/1
abaa`abaa`bbbaaaaaaaaa^a`aaaa_a__^a`aa`_
@HWI-EAS443:3:32:1107:1625#0/1
GCATCAATGAACTGAGCAATCTTGCAAAAGGAGAGAAAGC
+HWI-EAS443:3:32:1107:1625#0/1
__¥¥baaabbabb[S_`aX¥`bababbZ[`a`bab_T`aa
@HWI-EAS443:3:32:114:312#0/1
CCCTTGCCCAATTAGCACATTAGCTTTCTCTCCTTTTGCA
+HWI-EAS443:3:32:114:312#0/1
aa`[_]``ba`¥[a[aaaa_`aa`]a_a]___`_U_VY]V
@HWI-EAS443:3:32:1201:460#0/1
AGAGAAAGCTAATGTGCTAATTGGGCAAGGGGACGTAGTG
+HWI-EAS443:3:32:1201:460#0/1
`b`bZbbbb`X`bb^Y^`a`¥abaZ````_aa_^a^Tb_b
@HWI-EAS443:3:32:1217:75#0/1
GAAAGCTAATGTGCTAATTGGGCAAGGGGACGTAGTGTTG
+HWI-EAS443:3:32:1217:75#0/1
aaa`a`aa[aa_a__^_`a^a_WZ_a_a_^X`ZX`Za^`a
@HWI-EAS443:3:32:1389:1928#0/1
AGCTAATGTGCTAATTGGGCAAGGGGACGTAGTGTTGGTA
+HWI-EAS443:3:32:1389:1928#0/1
Z]abbbba_aabb_aaaY`ababb]¥`bWJXaaa_aa]Xa
@HWI-EAS443:3:32:1419:582#0/1
AGCTAATGTGCTAATTGGGCAAGGGGACGTAGTGTTGGTA
+HWI-EAS443:3:32:1419:582#0/1
`b_b_bbb]_abb¥XYb``[J^``^]`]¥O[a]_][`ZP¥
@HWI-EAS443:3:32:1427:1071#0/1
GCATCAATGAACTGAGCAATCTTGCAAAAGGAGAGAAAGC
+HWI-EAS443:3:32:1427:1071#0/1
aaaaaaaba_a`aa^^`a_[^a`a`_`_^`^Sa^a]`Zaa
@HWI-EAS443:3:32:1447:1476#0/1
GGAGAGAAAGCTAATGTGCTAATTGGGCAAGGGGACGTAG
+HWI-EAS443:3:32:1447:1476#0/1
aa_aaa]]_T]a`_``]]``_]`__X^`^]^[YU]YaRX_
@HWI-EAS443:3:32:1531:970#0/1
GCATCAATGAACTGAGCAATCTTGCAAAAGGAGAGAAAGC
+HWI-EAS443:3:32:1531:970#0/1
U]¥[`ba`aa_aa`^¥]U[bab_^U^WY_]]LaZa_aa^]
@HWI-EAS443:3:32:1585:853#0/1
ATCTTGCAAAAGGAGAGAAAGCTAATGTGCTAATTGGGCA
+HWI-EAS443:3:32:1585:853#0/1
a``^aaaa``]`^^aaaaaa`[aZ_``¥^¥^U[]a_a_a`
@HWI-EAS443:3:32:1652:930#0/1
CAATGAACTGAGCAATCTTGCAAAAGGAGAGAAAGCTAAT
+HWI-EAS443:3:32:1652:930#0/1
`a`babbbbbb`aa[^aaabaa_aXa`aa`baa`aRba`b
@HWI-EAS443:3:32:1666:470#0/1
GAACTGAGCAATCTTGCAAAAGGAGAGAAAGCTAATGTGC
```

```
+HWI-EAS443:3:32:1666:470#0/1
aJ`T]_YZT___Z`¥YM¥]UU`[Y]^_^]Z^MPR¥]`N_b
@HWI-EAS443:3:32:1728:475#0/1
GAAAGCTAATGTGCTAATTGGGCAAGGGGACGTAGTGTTG
+HWI-EAS443:3:32:1728:475#0/1
a_[_]F]ab__G_RX¥RYaX[VMP]a]__X]]GQaUaTZ`
@HWI-EAS443:3:32:22:443#0/1
GAGAAAGCTAATGTGCTAATTGGGCAAGGGGACGTAGTGT
+HWI-EAS443:3:32:22:443#0/1
aaaaaaaaa[aab_```_a`_aZ_aVV`___^_a^^_V`X
@HWI-EAS443:3:32:32:957#0/1
GAAAGCTAATGTGCTAATTGGGCAAGGGGACGTAGTGTTG
+HWI-EAS443:3:32:32:957#0/1
aa]abaabaab_aaa¥a`aaa`__`a_a_]a`UW]X`^_a
@HWI-EAS443:3:32:344:585#0/1
AAGCTAATGTGCTAATTGGGCAAGGGGACGTAGTGTTGGT
+HWI-EAS443:3:32:344:585#0/1
aaaabaaaa_babaaaaaaaaa¥aW`_```]_`Z]Z]^^W
@HWI-EAS443:3:32:348:1285#0/1
GAAAGCTAATGTGCTAATTGGGCAAGGGGACGTAGTGTTG
+HWI-EAS443:3:32:348:1285#0/1
ab`baba_abb`abab_baa_]b```_`_`aa¥``]a``a
@HWI-EAS443:3:32:508:1303#0/1
TCCCCTTGCCCAATTAGCACATTAGCTTTCTCTCCTTTTG
+HWI-EAS443:3:32:508:1303#0/1
Z]V[a¥WZ]R_¥Y_[^UZY`b_a^`_aa¥ZRUWR][¥ZRU
@HWI-EAS443:3:32:569:439#0/1
AAGCTAATGTGCTAATTGGGCAAGGGGACGTAGTGTTGGT
+HWI-EAS443:3:32:569:439#0/1
Z^aaa_^a_]aaaaaaaaaa_``aa¥`]``]U¥]a]^^`V
@HWI-EAS443:3:32:582:1841#0/1
GAACTGAGCAATCTTGCAAAAGGAGAGAAAGCTAATGTGC
+HWI-EAS443:3:32:582:1841#0/1
aba^__a_a¥K¥baabbb`baa`aaa¥ba_aa__`Y_¥_`
@HWI-EAS443:3:32:607:263#0/1
CATCAATGAACTGAGCAATCTTGCAAAAGGAGAGAAAGCT
+HWI-EAS443:3:32:607:263#0/1
ababbaaa``aaaaaaaaaaaaaa```_a`_aaaa_^a_^
@HWI-EAS443:3:32:612:140#0/1
CATCAATGAACTGAGCAATCTTGCAAAAGGAGAGAAAGCT
+HWI-EAS443:3:32:612:140#0/1
abbaabba`babaab`baa`baaa```aaaaaaaaaaa`
@HWI-EAS443:3:32:648:1823#0/1
GCATCAATGAACTGAGCAATCTTGCAAAAGGAGAGAAAGC
+HWI-EAS443:3:32:648:1823#0/1
aba]]`a`NW`a¥¥XG¥]]Y`VX`^VKRN[V_`___PTX_
@HWI-EAS443:3:32:694:1768#0/1
AATGAACTGAGCAATCTTGCAAAAGGAGAGAAAGCTAATG
+HWI-EAS443:3:32:694:1768#0/1
^a^X`baba`abbba`bbaabaaaaaaa`bb^VaVVa__
```

```
@HWI-EAS443:3:32:793:36#0/1
GAACTGAGCAATCTTGCAAAAGGAGAGAAAGCTAATGTGC
+HWI-EAS443:3:32:793:36#0/1
`]baa`]``bba[aab__a_¥ba`aabaa_b^a_a`a^aa
@HWI-EAS443:3:32:882:1907#0/1
GAAAGCTAATGTGCTAATTGGGCAAGGGGACGTAGTGTTG
+HWI-EAS443:3:32:882:1907#0/1
`bbUYb_aab_Uaaaaa`G__]a`_]_`Y]^QNUPKTNH[
@HWI-EAS443:3:32:887:1313#0/1
GAGAAAGCTAATGTGCTAATTGGGCAAGGGGACGTAGTGT
+HWI-EAS443:3:32:887:1313#0/1
a_aaaabbaaaaa]_aa^aa_a`__a_`__`_`_X__R_O
@HWI-EAS443:3:33:1103:1497#0/1
CAATCTTGCAAAAGGAGAGAAAGCTAATGTGCTAATTGGG
+HWI-EAS443:3:33:1103:1497#0/1
^ba`bWZabbabaa`VZbVab``aab^_[^]a[[ZL]aRY
@HWI-EAS443:3:33:1108:447#0/1
AGAAAGCTAATGTGCTAATTGGGCAAGGGGACGTAGTGTT
+HWI-EAS443:3:33:1108:447#0/1
ab`__]`_^`^^Ubaaaaaa``[a`a]`[ZT^XVY_]_]¥
@HWI-EAS443:3:33:1313:514#0/1
CATCAATGAACTGAGCAATCTTGCAAAAGGAGAGAAAGCT
+HWI-EAS443:3:33:1313:514#0/1
ZX^WaZ`babbbaaaabab_b^[[a`Iaba`YTW_N^`a_
@HWI-EAS443:3:33:1332:1957#0/1
AAGCTAATGTGCTAATTGGGCAAGGGGACGTAGTGTTGGT
+HWI-EAS443:3:33:1332:1957#0/1
``¥OWWWU^L]^Q``TZabZH¥Y[Za^SN¥UNTV[G[__V
@HWI-EAS443:3:33:1393:578#0/1
AATGAACTGAGCAATCTTGCAAAAGGAGAGAAAGCTAATG
+HWI-EAS443:3:33:1393:578#0/1
U`a[ZaW¥RRa]Vbbbb^b]`a`__RYa[b]_YaR_`^¥a
@HWI-EAS443:3:33:159:1746#0/1
AATGAACTGAGCAATCTTGCAAAAGGAGAGAAAGCTAATG
+HWI-EAS443:3:33:159:1746#0/1
`^[Z_U`b^¥T`]__a_a]^Y_^LYY]¥`_[^VU__[^Z[
@HWI-EAS443:3:33:1665:1096#0/1
AGAAAGCTAATGTGCTAATTGGGCAAGGGGACGTAGTGTT
+HWI-EAS443:3:33:1665:1096#0/1
`]__aaabaa`_[a```___[`^¥``_``_XUUSUVV^Y^
@HWI-EAS443:3:33:1672:449#0/1
CATCAATGAACTGAGCAATCTTGCAAAAGGAGAGAAAGCT
+HWI-EAS443:3:33:1672:449#0/1
a`baaa`T^babbab]]]a`aab^[b_abb_RPRaa^a_`
@HWI-EAS443:3:33:1700:1857#0/1
ATCTTGCAAAAGGAGAGAAAGCTAATGTGCTAATTGGGCA
+HWI-EAS443:3:33:1700:1857#0/1
]^Sa`b]]baaaaaa`^`a____a__^]¥^_^O^Z]PYW^
@HWI-EAS443:3:33:1758:1783#0/1
GAACTGAGCAATCTTGCAAAAGGAGAGAAAGCTAATGTGC
```

```
+HWI-EAS443:3:33:1758:1783#0/1
a¥aaaa^aaaaa^a_`^__^_`_^^`_^S¥[OY]Y¥XX]X
@HWI-EAS443:3:33:176:359#0/1
CATCAATGAACTGAGCAATCTTGCAAAAGGAGAGAAAGCT
+HWI-EAS443:3:33:176:359#0/1
ab``_W`]Q`_a`V_`_`aaaaaaaZYL^]¥a^_``_``]
@HWI-EAS443:3:33:209:1043#0/1
AACTGAGCAATCTTGCAAAAGGAGAGAAAGCTAATGTGCT
+HWI-EAS443:3:33:209:1043#0/1
^abaYUa_a`]a]QY]``]J__a_]^_`__``a^__X^T¥
@HWI-EAS443:3:33:229:1040#0/1
AATGAACTGAGCAATCTTGCAAAAGGAGAGAAAGCTAATG
+HWI-EAS443:3:33:229:1040#0/1
aaaa^aa__aaaaaaa`aaaa`_a`a^_``aP¥__^_``_
@HWI-EAS443:3:33:282:740#0/1
TCTTGCAAAAGGAGAGAAAGCTAATGTGCTAATTGGGCAA
+HWI-EAS443:3:33:282:740#0/1
abb_aa]aaabaabaaaa`]`aa^aa]a_`J`¥_]¥ZY__
@HWI-EAS443:3:33:336:553#0/1
GCAAAAGGAGAGAAAGCTAATGTGCTAATTGGGCAAGGGG
+HWI-EAS443:3:33:336:553#0/1
SSWZ¥TYaaaa¥aab]aaWZZb`abaaJa`]b]^a]a^¥]
@HWI-EAS443:3:33:387:769#0/1
CATCAATGAACTGAGCAATCTTGCAAAAGGAGAGAAAGCT
+HWI-EAS443:3:33:387:769#0/1
ababa`aaaaaaaYba^^aab`ba^Y^_a`^]^_a^[XZ]
@HWI-EAS443:3:33:478:1343#0/1
CATCAATGAACTGAGCAATCTTGCAAAAGGAGAGAAAGCT
+HWI-EAS443:3:33:478:1343#0/1
]SZ`U`bX^_abY[`a¥¥Yba`aaa[]^a]__W_`a^_^]
@HWI-EAS443:3:33:593:1480#0/1
CATCAATGAACTGAGCAATCTTGCAAAAGGAGAGAAAGCT
+HWI-EAS443:3:33:593:1480#0/1
a`]a_^UTZa_][W_aa_[`]Y`^[¥^`]Z]ZY^`_O¥Z]
@HWI-EAS443:3:33:690:1956#0/1
ATCTTGCAAAAGGAGAGAAAGCTAATGTGCTAATTGGGCA
+HWI-EAS443:3:33:690:1956#0/1
a_aa__a````^``_a^aa^^_^a__TNS__^^^]Y^R`_
@HWI-EAS443:3:33:696:558#0/1
GAAAGCTAATGTGCTAATTGGGCAAGGGGACGTAGTGTTG
+HWI-EAS443:3:33:696:558#0/1
TVa``^¥¥O¥bb¥_bba¥Y¥a`b``b_`b`bb``bL^Rab
@HWI-EAS443:3:33:709:464#0/1
CGTCCCCTTGCCCAATTAGCACATTAGCTTTCTCTCCTTT
+HWI-EAS443:3:33:709:464#0/1
a`^a_^aaabaaaa`aa`^`_`aa]``a^_^_VZ^UY]Z]
@HWI-EAS443:3:33:775:1782#0/1
GAGCAATCTTGCAAAAGGAGAGAAAGCTAATGTGCTAATT
+HWI-EAS443:3:33:775:1782#0/1
_`_babbbba_^_aa__^a`baa``a_aaba_Q¥H`a`a`
```

```
@HWI-EAS443:3:33:897:828#0/1
TGCAAAAGGAGAGAAAGCTAATGTGCTAATTGGGCAAGGG
+HWI-EAS443:3:33:897:828#0/1
abaaa^]`a_aaaaa`aaaaaaa_aaa_`__`W]]^_`_]
@HWI-EAS443:3:33:96:1011#0/1
AGCACATTAGCTTTCTCTCCTTTTGCAAGATTGCTCAGTT
+HWI-EAS443:3:33:96:1011#0/1
a_`[aa_^^____`a___^^_^__^_]^]^]]WZ_^]U]Z
@HWI-EAS443:3:33:979:197#0/1
CATCAATGAACTGAGCAATCTTGCAAAAGGAGAGAAAGCT
+HWI-EAS443:3:33:979:197#0/1
^Y¥_[`SZabbaa^baa__`bbbaa^aaba^_Za`]a_G`
@HWI-EAS443:3:33:999:302#0/1
CATCAATGAACTGAGCAATCTTGCAAAAGGAGAGAAAGCT
+HWI-EAS443:3:33:999:302#0/1
abbabbba_babaaa_aab_aaaaa`^`aa_`Va``Z___
@HWI-EAS443:3:34:1123:623#0/1
CATCAATGAACTGAGCAATCTTGCAAAAGGAGAGAAAGCT
+HWI-EAS443:3:34:1123:623#0/1
aa`abaa_a_[`S`[_^Ya]^a`^^[QWYb^^^[``]_Q]
@HWI-EAS443:3:34:1155:162#0/1
GAGCAATCTTGCAAAAGGAGAGAAAGCTAATGTGCTAATT
+HWI-EAS443:3:34:1155:162#0/1
a_Wabababb`ba``abaYaa_a_aa___^a`¥_S^YWYY
@HWI-EAS443:3:34:1211:1932#0/1
ATGAACTGAGCAATCTTGCAAAAGGAGAGAAAGCTAATGT
+HWI-EAS443:3:34:1211:1932#0/1
^ZY`YP[O]R^Z]^¥NRO__a]_]`Sb]F]¥_[[^^WWGB
@HWI-EAS443:3:34:1213:1065#0/1
GAGAAAGCTAATGTGCTAATTGGGCAAGGGGACGTAGTGT
+HWI-EAS443:3:34:1213:1065#0/1
TZa^_¥`Z¥Z`[QI]O]]aaa_XYYRYa[]BBBBBBBBBB
@HWI-EAS443:3:34:1219:1020#0/1
GAAAGCTAATGTGCTAATTGGGCAAGGGGACGTAGTGTTG
+HWI-EAS443:3:34:1219:1020#0/1
aa]``Y```ab¥aaa_aa`X^`__W^^a__^^X]_U_ZZ^
@HWI-EAS443:3:34:1232:1137#0/1
GAGCAATCTTGCAAAAGGAGAGAAAGCTAATGTGCTAATT
+HWI-EAS443:3:34:1232:1137#0/1
_bbab`bab``a^``abaaabababba``]`aZ^M`^__]
@HWI-EAS443:3:34:1232:571#0/1
CAATCTTGCAAAAGGAGAGAAAGCTAATGTGCTAATTGGG
+HWI-EAS443:3:34:1232:571#0/1
a]VHHU¥]OZ]_aba]b]Z]SNa]]Y¥]YH]YTRN_R[WB
@HWI-EAS443:3:34:1237:294#0/1
GAGCAATCTTGCAAAAGGAGAGAAAGCTAATGTGCTAATT
+HWI-EAS443:3:34:1237:294#0/1
a_```]a_a]^]_a^Zb_O`]_[^_]O¥]]^]V]Y^YXVY
@HWI-EAS443:3:34:1314:1139#0/1
CTTGCAAAAGGAGAGAAAGCTAATGTGCTAATTGGGCAAG
```

```
+HWI-EAS443:3:34:1314:1139#0/1
QT___]VS¥^__b_Ya¥^Y]UTWV]GWa¥QQ[Z^^aLNQ^
@HWI-EAS443:3:34:1317:999#0/1
ATCTTGCAAAAGGAGAGAAAGCTAATGTGCTAATTGGGCA
+HWI-EAS443:3:34:1317:999#0/1
R_b¥W`__¥`¥¥`aUY`a_Z__``^a_Naa[_`aaaXWXZ
@HWI-EAS443:3:34:1346:1971#0/1
AGGAGAGAAAGCTAATGTGCTAATTGGGCAAGGGGACGTA
+HWI-EAS443:3:34:1346:1971#0/1
a^``b[bbab`_abaa^P`WaaaaaaaZ``aaYYW]X_U_
@HWI-EAS443:3:34:136:85#0/1
ATCTTGCAAAAGGAGAGAAAGCTAATGTGCTAATTGGGCA
+HWI-EAS443:3:34:136:85#0/1
Y`ba^b_RT_Z`aa``baVU`_`¥W`a[`__¥^[_^VZVY
@HWI-EAS443:3:34:1414:97#0/1
CCCTTGCCCAATTAGCACATTAGCTTTCTCTCCTTTTGCA
+HWI-EAS443:3:34:1414:97#0/1
abaaababbbbbabbaaaabba`abaaab_a_Wa``_[T]
@HWI-EAS443:3:34:1489:291#0/1
GCATCAATGAACTGAGCAATCTTGCAAAAGGAGAGAAAGC
+HWI-EAS443:3:34:1489:291#0/1
aaaa``aaa^aaaa_`^a_``a`a`]_^]`_][¥Z^XYWW
@HWI-EAS443:3:34:1492:1196#0/1
CATCAATGAACTGAGCAATCTTGCAAAAGGAGAGAAAGCT
+HWI-EAS443:3:34:1492:1196#0/1
``baaabaaaaba^b_a_a`aaa_a`_aba_a^a]_Z^U^
@HWI-EAS443:3:34:1590:1295#0/1
GAGCAATCTTGCAAAAGGAGAGAAAGCTAATGTGCTAATT
+HWI-EAS443:3:34:1590:1295#0/1
`Y¥___bY¥a`bYOP¥¥¥Y]Va¥Z¥_Tabbb`][P[¥`a]
@HWI-EAS443:3:34:160:236#0/1
CTGAGCAATCTTGCAAAAGGAGAGAAAGCTAATGTGCTAA
+HWI-EAS443:3:34:160:236#0/1
aaa_bbbbabbaabaaaaaaabaaa^`aaaa`a^W`]`]¥
@HWI-EAS443:3:34:1681:33#0/1
GCATCAATGAACTGAGCAATCTTGCAAAAGGAGAGAAAGC
+HWI-EAS443:3:34:1681:33#0/1
aa_`ZWT^^PUUT_^T^LNNYRQZXUYWZ]]WUYTYIZQB
@HWI-EAS443:3:34:1726:368#0/1
GCATCAATGAACTGAGCAATCTTGCAAAAGGAGAGAAAGC
+HWI-EAS443:3:34:1726:368#0/1
abaabababbbb`¥`abaa^abb_¥aaaba_a_^_[^ZQ
@HWI-EAS443:3:34:1775:223#0/1
GAGCAATCTTGCAAAAGGAGAGAAAGCTAATGTGCTAATT
+HWI-EAS443:3:34:1775:223#0/1
aabb`a`[`bb`^aaabb`a]aa`_`Za``a`Y^V_X¥]^
@HWI-EAS443:3:34:200:484#0/1
TTAGCTTTCTCTCCTTTTGCAAGATTGCTCAGTTCATTGA
+HWI-EAS443:3:34:200:484#0/1
aaTaaaaaaaaaabaaaaaaa^a`_`aaaaaa^```a`_^
```

@HWI-EAS443:3:34:217:1380#0/1
GAAAGCTAATGTGCTAATTGGGCAAGGGGACGTAGTGTTG
+HWI-EAS443:3:34:217:1380#0/1
aaXT^aabbaa``aaaaa`ZVW`a`a`¥UP_YZ``X^U¥V
@HWI-EAS443:3:34:322:1884#0/1
AAGCATCAATGAACTGAGCAATCTTGCAAAAGGAGAGAAA
+HWI-EAS443:3:34:322:1884#0/1
ab]baX_aaa_T_`^^]T]aa`a__WX^__¥SXZ^^^a¥_
@HWI-EAS443:3:34:338:709#0/1
AGCTAATGTGCTAATTGGGCAAGGGGACGTAGTGTTGGTA
+HWI-EAS443:3:34:338:709#0/1
Zab^_O[SQW^a¥``ab_R_a`_X¥_UL]¥R`^¥[[aWFY
@HWI-EAS443:3:34:364:1633#0/1
AGAAAGCTAATGTGCTAATTGGGCAAGGGGACGTAGTGTT
+HWI-EAS443:3:34:364:1633#0/1
`Y¥_¥Y`aaa``XR``]W__P¥_a``ZX[¥_^ZO^^V]U[
@HWI-EAS443:3:34:409:1133#0/1
ATCTTGCAAAAGGAGAGAAAGCTAATGTGCTAATTGGGCA
+HWI-EAS443:3:34:409:1133#0/1
_[``XX¥^M¥a`_]a_`Z]GNU^a`^YL`a^RGU¥XUTU]
@HWI-EAS443:3:34:433:933#0/1
AAGCTAATGTGCTAATTGGGCAAGGGGACGTAGTGTTGGT
+HWI-EAS443:3:34:433:933#0/1
]babbaaa_YabbW_a`aa^`aU`[aa_aa^^aZaZ__[Z
@HWI-EAS443:3:34:441:1988#0/1
GCAAAAGGAGAGAAAGCTAATGTGCTAATTGGGCAAGGGG
+HWI-EAS443:3:34:441:1988#0/1
ab`ba]aaaabab``abaaba``_aabaa`]W_a`a][]]
@HWI-EAS443:3:34:451:1543#0/1
GAGAAAGCTAATGTGCTAATTGGGCAAGGGGACGTAGTGT
+HWI-EAS443:3:34:451:1543#0/1
¥a`ba¥bbb__a[[aaa`^`a]]]aY[W_^``]a__`XaZ
@HWI-EAS443:3:34:486:2004#0/1
ATCTTGCAAAAGGAGAGAAAGCTAATGTGCTAATTGGGCA
+HWI-EAS443:3:34:486:2004#0/1
aaba_aaTV`^_YZ^¥Zaa`a_]__]_WV]]`¥^RIWBBB
@HWI-EAS443:3:34:488:1266#0/1
GGAGAGAAAGCTAATGTGCTAATTGGGCAAGGGGACGTAG
+HWI-EAS443:3:34:488:1266#0/1
_``_`b`[S__ZSZaa_a`^W_b``aab`^a_]a`a¥R[[
@HWI-EAS443:3:34:545:37#0/1
GAGAAAGCTAATGTGCTAATTGGGCAAGGGGACGTAGTGT
+HWI-EAS443:3:34:545:37#0/1
`Xaa]W__[OSW`^URZXX_]`_Z^XYXV^^YY`PPUUUV
@HWI-EAS443:3:34:554:649#0/1
GCAAAAGGAGAGAAAGCTAATGTGCTAATTGGGCAAGGGG
+HWI-EAS443:3:34:554:649#0/1
`abaabbb`babaVQ_aa`^ab_abaabaaaa^_`aa`__
@HWI-EAS443:3:34:597:279#0/1
CGTCCCCTTGCCCAATTAGCACATTAGCTTTCTCTCCTTT

+HWI-EAS443:3:34:597:279#0/1
aaabbba^`aaabab``a^aabaaaa```_aaa``a^__^
@HWI-EAS443:3:34:629:312#0/1
CATCAATGAACTGAGCAATCTTGCAAAAGGAGAGAAAGCT
+HWI-EAS443:3:34:629:312#0/1
abaabbaaa``b`_]baaaa`aa`a__^a`_a```_^`V]
@HWI-EAS443:3:34:632:1415#0/1
CAAAAGGAGAGAAAGCTAATGTGCTAATTGGGCAAGGGGA
+HWI-EAS443:3:34:632:1415#0/1
ab]baaaaabbbb]aaa]ab`_aabaaa`a`_aa__[`]^
@HWI-EAS443:3:34:676:211#0/1
AGAGAAAGCTAATGTGCTAATTGGGCAAGGGGACGTAGTG
+HWI-EAS443:3:34:676:211#0/1
WYWbbbaa`]J]aa[X``Ya^X[]^R^_aXU^VN`BBBBB
@HWI-EAS443:3:34:834:1079#0/1
CAATCTTGCAAAAGGAGAGAAAGCTAATGTGCTAATTGGG
+HWI-EAS443:3:34:834:1079#0/1
ab^`^a^T^a_``_^^b]abWM]Z`a`]]TaY^a]^_^WR
@HWI-EAS443:3:34:864:528#0/1
GAGCAATCTTGCAAAAGGAGAGAAAGCTAATGTGCTAATT
+HWI-EAS443:3:34:864:528#0/1
a[aba_baaaaaWa``_`Ybabaa_]^`]a``S_]`^_X_
@HWI-EAS443:3:34:95:106#0/1
ATCTTGCAAAAGGAGAGAAAGCTAATGTGCTAATTGGGCA
+HWI-EAS443:3:34:95:106#0/1
¥aaaaa__aaabbaa`a[baaa``ZX`_^___[]___TSU
@HWI-EAS443:3:34:976:262#0/1
AAGCTAATGTGCTAATTGGGCAAGGGGACGTAGTGTTGGT
+HWI-EAS443:3:34:976:262#0/1
^¥_^a^X_^Yaaa]aaa`a`a_`a`]¥Y_aU[`^_WZ^ZR
@HWI-EAS443:3:35:1141:704#0/1
CATCAATGAACTGAGCAATCTTGCAAAAGGAGAGAAAGCT
+HWI-EAS443:3:35:1141:704#0/1
SSa]``¥^_V¥¥¥babbbbbbbbb__¥`^a_a^`a^`baa
@HWI-EAS443:3:35:1275:1214#0/1
AAAAGGAGAGAAAGCTAATGTGCTAATTGGGCAAGGGGAC
+HWI-EAS443:3:35:1275:1214#0/1
¥[`a`aab`abaabaa`[__R^aa_```aa`a¥a^][a]_
@HWI-EAS443:3:35:1303:260#0/1
CATCAATGAACTGAGCAATCTTGCAAAAGGAGAGAAAGCT
+HWI-EAS443:3:35:1303:260#0/1
Z¥a`bbbbb_bbbababa^`bbb]`[FXbb]b`ab]¥`T¥
@HWI-EAS443:3:35:1390:391#0/1
GAAAGCTAATGTGCTAATTGGGCAAGGGGACGTAGTGTTG
+HWI-EAS443:3:35:1390:391#0/1
aaV]aaa]aab`b]aa_b_b`aaaR^a_a[`b_`aX^WU`
@HWI-EAS443:3:35:1697:828#0/1
GTCCCCTTGCCCAATTAGCACATTAGCTTTCTCTCCTTTT
+HWI-EAS443:3:35:1697:828#0/1
``_S^bb[`_`]¥`]a¥aZ¥`a^[Za^U^_aa__¥T[^SZ

```
@HWI-EAS443:3:35:176:1961#0/1
ATCTTGCAAAAGGAGAGAAAGCTAATGTGCTAATTGGGCA
+HWI-EAS443:3:35:176:1961#0/1
a_`a]Vb_L_¥YYYYa]a_P[_`_]_]G¥a[TJQHWLLaX
@HWI-EAS443:3:35:216:1576#0/1
GTCCCCTTGCCCAATTAGCACATTAGCTTTCTCTCCTTTT
+HWI-EAS443:3:35:216:1576#0/1
aababbaaababbaaaaababaaaaaba__b`baaa`^`^
@HWI-EAS443:3:35:261:347#0/1
GAAAGCTAATGTGCTAATTGGGCAAGGGGACGTAGTGTTG
+HWI-EAS443:3:35:261:347#0/1
]_YL^Z[XUZ^M]]RHTXVX¥_[HUOM[ZU[YMDTS^VYY
@HWI-EAS443:3:35:282:1572#0/1
AACTGAGCAATCTTGCAAAAGGAGAGAAAGCTAATGTGCT
+HWI-EAS443:3:35:282:1572#0/1
abba``aa]aab_`ab`a_]`a^aa^aaR[a__`__R¥^^
@HWI-EAS443:3:35:29:355#0/1
GAGCAATCTTGCAAAAGGAGAGAAAGCTAATGTGCTAATT
+HWI-EAS443:3:35:29:355#0/1
`bbbab_bbbbb`^¥`baabbbab]aaV¥_ab^aaaaVa_
@HWI-EAS443:3:35:349:1342#0/1
CAATCTTGCAAAAGGAGAGAAAGCTAATGTGCTAATTGGG
+HWI-EAS443:3:35:349:1342#0/1
_XO^a``a`^¥HR_]¥^]]Z]YW]_]TZWJXa¥X]¥^[[M
@HWI-EAS443:3:35:433:1030#0/1
AGGAGAGAAAGCTAATGTGCTAATTGGGCAAGGGGACGTA
+HWI-EAS443:3:35:433:1030#0/1
R¥WI¥_^^``aa_X`a`X^`b¥baaYYY`^QZ]_^``WOT
@HWI-EAS443:3:35:554:1387#0/1
GTCCCCTTGCCCAATTAGCACATTAGCTTTCTCTCCTTTT
+HWI-EAS443:3:35:554:1387#0/1
a_baab]`abbb[`aaaaaaa`]ab^a`_]a`a`aa_]a]
@HWI-EAS443:3:35:561:436#0/1
TGCAAAAGGAGAGAAAGCTAATGTGCTAATTGGGCAAGGG
+HWI-EAS443:3:35:561:436#0/1
`bb_]_¥aa_abaaa_aaa[`aa[a_a^_``a``¥]^_T_
@HWI-EAS443:3:35:568:1636#0/1
GAGCAATCTTGCAAAAGGAGAGAAAGCTAATGTGCTAATT
+HWI-EAS443:3:35:568:1636#0/1
aaXaababa^`a_b_a`ab_[bbbaab_Vba^^aa_a_`_
@HWI-EAS443:3:35:56:1410#0/1
GAAAGCTAATGTGCTAATTGGGCAAGGGGACGTAGTGTTG
+HWI-EAS443:3:35:56:1410#0/1
ab```b`aaaa^a`_aaaa`a_a`Z`a_____X][T_^_Z
@HWI-EAS443:3:35:689:950#0/1
AATGAACTGAGCAATCTTGCAAAAGGAGAGAAAGCTAATG
+HWI-EAS443:3:35:689:950#0/1
aa]]SXa[[¥[^X[[___`__ZUY`VP_ZZ]U¥[[W_^T]
@HWI-EAS443:3:35:709:1286#0/1
AGCTAATGTGCTAATTGGGCAAGGGGACGTAGTGTTGGTA
```

```
+HWI-EAS443:3:35:709:1286#0/1
Z]_`^^aSXa]^Uba^^^]aZ_`_aYRR`[S]L][`_XPR
@HWI-EAS443:3:35:738:1940#0/1
CAATGAACTGAGCAATCTTGCAAAAGGAGAGAAAGCTAAT
+HWI-EAS443:3:35:738:1940#0/1
a`a][`_`]W]^`]`^]^¥Z_`Y¥]]XVZ_¥a^^ZY]]XX
@HWI-EAS443:3:35:845:580#0/1
GAGAAAGCTAATGTGCTAATTGGGCAAGGGGACGTAGTGT
+HWI-EAS443:3:35:845:580#0/1
aKaaa¥¥[a_ZaaXa][^^__^Z¥`YR]U^__^`[W¥VVW
@HWI-EAS443:3:35:877:561#0/1
TCCCCTTGCCCAATTAGCACATTAGCTTTCTCTCCTTTTG
+HWI-EAS443:3:35:877:561#0/1
`abbbaaabbbbaaabbbbaaaaa`aaa`aaaaa_aa_``
@HWI-EAS443:3:35:919:439#0/1
CTTGCAAAAGGAGAGAAAGCTAATGTGCTAATTGGGCAAG
+HWI-EAS443:3:35:919:439#0/1
Z]b`[bbba``aSS¥`aYa^aa_bb[]D¥ab`aa]a¥`]a
@HWI-EAS443:3:35:926:855#0/1
AATGAACTGAGCAATCTTGCAAAAGGAGAGAAAGCTAATG
+HWI-EAS443:3:35:926:855#0/1
]_¥UYX[¥]_a[¥RZOV^¥]Y^aaa``^`¥LO]_]]Y¥YU
@HWI-EAS443:3:35:948:1069#0/1
GAGAGAAAGCTAATGTGCTAATTGGGCAAGGGGACGTAGT
+HWI-EAS443:3:35:948:1069#0/1
a_¥___a[]^aZ¥ZVMa^____Y^^]aQ]___WNY_RT^Z
@HWI-EAS443:3:36:1007:770#0/1
GAGCAATCTTGCAAAAGGAGAGAAAGCTAATGTGCTAATT
+HWI-EAS443:3:36:1007:770#0/1
aabb`_bbbbbab`_]baaa_ab_bbaaa]`b¥bbb[bba
@HWI-EAS443:3:36:102:937#0/1
GCAAAAGGAGAGAAAGCTAATGTGCTAATTGGGCAAGGGG
+HWI-EAS443:3:36:102:937#0/1
aa__]Z]X]`^a¥S_`^_]_^_U]``_Y^^^^¥^¥[LU_Z
@HWI-EAS443:3:36:1031:838#0/1
TAGCACATTAGCTTTCTCTCCTTTTGCAAGATTGCTCAGT
+HWI-EAS443:3:36:1031:838#0/1
abbabbbbbabaabaabbbbaabbbbbaabbaa`bab`a]
@HWI-EAS443:3:36:1053:139#0/1
ATCTTGCAAAAGGAGAGAAAGCTAATGTGCTAATTGGGCA
+HWI-EAS443:3:36:1053:139#0/1
abbabbbaaaaa``b`ab`aa`a_aaaZa_a____aa_W^
@HWI-EAS443:3:36:1113:1016#0/1
GCAAAAGGAGAGAAAGCTAATGTGCTAATTGGGCAAGGGG
+HWI-EAS443:3:36:1113:1016#0/1
aaa^a_aa_^]aa[]aa`XQ`a^a_`Y¥``a^^_^__[Y[
@HWI-EAS443:3:36:1154:1954#0/1
GAGCAATCTTGCAAAAGGAGAGAAAGCTAATGTGCTAATT
+HWI-EAS443:3:36:1154:1954#0/1
aabbbbaabb__aabaabaab`ba_aa_aaaWRW``aaa_
```

```
@HWI-EAS443:3:36:1183:1437#0/1
CTTGCCCAATTAGCACATTAGCTTTCTCTCCTTTTGCAAG
+HWI-EAS443:3:36:1183:1437#0/1
abbaabbbbabbaaa`baba`aaa`ba_abb`aaa_ba`a
@HWI-EAS443:3:36:1250:1138#0/1
GCACATTAGCTTTCTCTCCTTTTGCAAGATTGCTCAGTTC
+HWI-EAS443:3:36:1250:1138#0/1
ababbb_Y^ababbbaabaaba`aaaaaaaaaaaaab^`b
@HWI-EAS443:3:36:1305:846#0/1
ATGAACTGAGCAATCTTGCAAAAGGAGAGAAAGCTAATGT
+HWI-EAS443:3:36:1305:846#0/1
¥a[Saba``ba`a]]]abaa^`aaaab_ba`^`[``__`^
@HWI-EAS443:3:36:1454:924#0/1
CAAAAGGAGAGAAAGCTAATGTGCTAATTGGGCAAGGGGA
+HWI-EAS443:3:36:1454:924#0/1
aabbabbabbbbaab_baabbabaaa`aaaa]__aaab_`
@HWI-EAS443:3:36:1546:1471#0/1
GAGAAAGCTAATGTGCTAATTGGGCAAGGGGACGTAGTGT
+HWI-EAS443:3:36:1546:1471#0/1
abbba_bbbabbb]aabaaaabaa`__a`ba]]a^Zb_a]
@HWI-EAS443:3:36:249:1999#0/1
ATCTTGCAAAAGGAGAGAAAGCTAATGTGCTAATTGGGCA
+HWI-EAS443:3:36:249:1999#0/1
aaa`_`ba[a___W]a^_aYW`_aa__Z^`]Y_¥Y[ZXZ[
@HWI-EAS443:3:36:327:1169#0/1
GAGAGAAAGCTAATGTGCTAATTGGGCAAGGGGACGTAGT
+HWI-EAS443:3:36:327:1169#0/1
aa^``^aa]aaU``a_baa^]_aba`a_Ta`^X_`aY_aV
@HWI-EAS443:3:36:393:1496#0/1
AGCTAATGTGCTAATTGGGCAAGGGGACGTAGTGTTGGTA
+HWI-EAS443:3:36:393:1496#0/1
aXbaaaZ_^aba^Zaaa`Ybaa_`a¥`aaX_YHYSaa^V[
@HWI-EAS443:3:36:406:1494#0/1
AGCTAATGTGCTAATTGGGCAAGGGGACGTAGTGTTGGTA
+HWI-EAS443:3:36:406:1494#0/1
a_¥_^a[[Raaaa]^`^a^aZRZZ___UaZ¥ZS[QX^VU_
@HWI-EAS443:3:36:509:295#0/1
GAGAAAGCTAATGTGCTAATTGGGCAAGGGGACGTAGTGT
+HWI-EAS443:3:36:509:295#0/1
a_X`ZZ^aa`aa¥V`a¥]aa_Y_¥¥SX^`_^W^`Z¥XV¥V
@HWI-EAS443:3:36:555:1990#0/1
AGCTAATGTGCTAATTGGGCAAGGGGACGTAGTGTTGGTA
+HWI-EAS443:3:36:555:1990#0/1
`aa_aa_`^_a__a``____a¥ZR^]^V^V[_UXS^^WO^
@HWI-EAS443:3:36:556:471#0/1
AATGAACTGAGCAATCTTGCAAAAGGAGAGAAAGCTAATG
+HWI-EAS443:3:36:556:471#0/1
_aa`^aaaaaaaaaaaa``^_`a`ba_¥``a_a___[_`a
@HWI-EAS443:3:36:576:369#0/1
GAGCAATCTTGCAAAAGGAGAGAAAGCTAATGTGCTAATT
```

```
+HWI-EAS443:3:36:576:369#0/1
_¥aa[W]`abbbbababa]aaab_a`a]S_aaSa`ZH¥a^
@HWI-EAS443:3:36:638:465#0/1
TAGCACATTAGCTTTCTCTCCTTTTGCAAGATTGCTCAGT
+HWI-EAS443:3:36:638:465#0/1
ab_bbaaaaaa`aba_aabaaaa_aaa`W`__`_aaa_a]
@HWI-EAS443:3:36:644:768#0/1
GAACTGAGCAATCTTGCAAAAGGAGAGAAAGCTAATGTGC
+HWI-EAS443:3:36:644:768#0/1
a_`aa]¥`aa_]a_a_a[]_Y__^^^__VWZ¥^^]¥^X^Y
@HWI-EAS443:3:36:749:639#0/1
CCCCTTGCCCAATTAGCACATTAGCTTTCTCTCCTTTTGC
+HWI-EAS443:3:36:749:639#0/1
I_b``Ub_¥bbbbb`bba`¥`a`baba^]abba]a^PYU`
@HWI-EAS443:3:36:835:692#0/1
GAGCAATCTTGCAAAAGGAGAGAAAGCTAATGTGCTAATT
+HWI-EAS443:3:36:835:692#0/1
W_R_¥P]¥RRV`b]WUa_O]V¥bUU_a^W[_ZX``XLZ[U
@HWI-EAS443:3:36:886:309#0/1
GAGCAATCTTGCAAAAGGAGAGAAAGCTAATGTGCTAATT
+HWI-EAS443:3:36:886:309#0/1
a``baba`baa_aa_abbabaaaa]a`_aa_aY__a^[^^
@HWI-EAS443:3:36:906:1873#0/1
GAAAGCTAATGTGCTAATTGGGCAAGGGGACGTAGTGTTG
+HWI-EAS443:3:36:906:1873#0/1
]ba__ba¥`abZ¥]abbbba_`aaaa_^]ab`]_][b_``
@HWI-EAS443:3:36:966:1003#0/1
GAGCAATCTTGCAAAAGGAGAGAAAGCTAATGTGCTAATT
+HWI-EAS443:3:36:966:1003#0/1
a_ababbbbb__aaabbba``ba___bb[_ba_aaa^`a`
@HWI-EAS443:3:36:990:1411#0/1
GAAAGCTAATGTGCTAATTGGGCAAGGGGACGTAGTGTTG
+HWI-EAS443:3:36:990:1411#0/1
`bab]baaab`Xaaaa`aaa``_`_aa__]`¥W^a]]^``
@HWI-EAS443:3:37:1061:1194#0/1
GAGAAAGCTAATGTGCTAATTGGGCAAGGGGACGTAGTGT
+HWI-EAS443:3:37:1061:1194#0/1
aaaaaaaaa``ba`aaaa`aa```a_[_`Z__a^X]`X^V
@HWI-EAS443:3:37:1134:1695#0/1
AAGCATCAATGAACTGAGCAATCTTGCAAAAGGAGAGAAA
+HWI-EAS443:3:37:1134:1695#0/1
aa`^`aaaaaa`__]`_¥_^``_a_a`_YKTT_``Z_b`^
@HWI-EAS443:3:37:1170:1422#0/1
CACTACGTCCCCTTGCCCAATTAGCACATTAGCTTTCTCT
+HWI-EAS443:3:37:1170:1422#0/1
Yabaa^bbba`[abYa`b`bbaaa`b_ab[b`aab^_b`a
@HWI-EAS443:3:37:1203:1955#0/1
GAGCAATCTTGCAAAAGGAGAGAAAGCTAATGTGCTAATT
+HWI-EAS443:3:37:1203:1955#0/1
[¥`Zabb`¥``[`_ab`_^¥abba`ba[^[Ub_bbbaaa`
```

```
@HWI-EAS443:3:37:1423:1045#0/1
GAAAGCTAATGTGCTAATTGGGCAAGGGGACGTAGTGTTG
+HWI-EAS443:3:37:1423:1045#0/1
`a`Z`abaUb`_a^`aaa`_`_aaab`aaZaa]`aWa^_a
@HWI-EAS443:3:37:1425:515#0/1
TGCAAAAGGAGAGAAAGCTAATGTGCTAATTGGGCAAGGG
+HWI-EAS443:3:37:1425:515#0/1
ab`_aaaaa_`aaa_`a¥a^`aa_aa`]_Wa_a[]^X`^Z
@HWI-EAS443:3:37:1448:1291#0/1
TCCCCTTGCCCAATTAGCACATTAGCTTTCTCTCCTTTTG
+HWI-EAS443:3:37:1448:1291#0/1
`^]^ZS]_]Z_a`XRV]][]_PT][^NYZU^V`a`Y¥WYY
@HWI-EAS443:3:37:1449:1101#0/1
AATGAACTGAGCAATCTTGCAAAAGGAGAGAAAGCTAATG
+HWI-EAS443:3:37:1449:1101#0/1
^`[K¥aaa^¥W`_TX^a``]YJY`a``^VT]YX]a_`^[a
@HWI-EAS443:3:37:1693:1290#0/1
GCATCAATGAACTGAGCAATCTTGCAAAAGGAGAGAAAGC
+HWI-EAS443:3:37:1693:1290#0/1
abaa^a^aaa_Va]Za_`__^aa`_`^TQMLWa``_^^^S
@HWI-EAS443:3:37:191:259#0/1
ATCTTGCAAAAGGAGAGAAAGCTAATGTGCTAATTGGGCA
+HWI-EAS443:3:37:191:259#0/1
a`aaaaa`a]`a`_a^_a___`_^`^^Y]XV_HY]_]X^Z
@HWI-EAS443:3:37:224:1786#0/1
GAGCAATCTTGCAAAAGGAGAGAAAGCTAATGTGCTAATT
+HWI-EAS443:3:37:224:1786#0/1
ababbbbbbb`bbbba_bb[b[bbbaba¥`a`^`babaaa
@HWI-EAS443:3:37:247:432#0/1
GAGAAAGCTAATGTGCTAATTGGGCAAGGGGACGTAGTGT
+HWI-EAS443:3:37:247:432#0/1
]]a``Z`aa`¥]¥X`_YH¥_]_¥__W¥`]YRV]_US^RZV
@HWI-EAS443:3:37:404:259#0/1
AAAGCTAATGTGCTAATTGGGCAAGGGGACGTAGTGTTGG
+HWI-EAS443:3:37:404:259#0/1
aa]_aa`^aa^a`_`aa`a`_`^_a__^ZR^VX`Z`Z]_X
@HWI-EAS443:3:37:500:635#0/1
CTTGCAAAAGGAGAGAAAGCTAATGTGCTAATTGGGCAAG
+HWI-EAS443:3:37:500:635#0/1
ab`ab`_aWabaabbbbaaabaaaa]aa_YP__a`]`U`a
@HWI-EAS443:3:37:548:498#0/1
AGCTAATGTGCTAATTGGGCAAGGGGACGTAGTGTTGGTA
+HWI-EAS443:3:37:548:498#0/1
`aabb]bbabbabbabb]baa[abaa¥^bWRb]a_abaX`
@HWI-EAS443:3:37:578:1660#0/1
CATCAATGAACTGAGCAATCTTGCAAAAGGAGAGAAAGCT
+HWI-EAS443:3:37:578:1660#0/1
U__X`^][¥a_¥RX_a^[`^_Z`¥¥V¥`^aa¥S¥`^_Z^J
@HWI-EAS443:3:37:780:302#0/1
GCAATCTTGCAAAAGGAGAGAAAGCTAATGTGCTAATTGG
```

```
+HWI-EAS443:3:37:780:302#0/1
aba`_`a]]a`_``a`_a¥]aX`_a``_]UU^`^^_^][^
@HWI-EAS443:3:37:806:1608#0/1
CTTGCCCAATTAGCACATTAGCTTTCTCTCCTTTTGCAAG
+HWI-EAS443:3:37:806:1608#0/1
aaa`aabbbaa`_aaaa`aaaa``ab`_aYa___``aaZ¥
@HWI-EAS443:3:38:1058:702#0/1
AAAGCTAATGTGCTAATTGGGCAAGGGGACGTAGTGTTGG
+HWI-EAS443:3:38:1058:702#0/1
^`Xaa_`__`[`¥`O_a`a`a`]_]V`]NTFUP[Q]W[_T
@HWI-EAS443:3:38:1106:96#0/1
CAATCTTGCAAAAGGAGAGAAAGCTAATGTGCTAATTGGG
+HWI-EAS443:3:38:1106:96#0/1
abbbbbbbbba^aab`baba_V``a`aabZRU``_`a`a]
@HWI-EAS443:3:38:1130:1582#0/1
ATCTTGCAAAAGGAGAGAAAGCTAATGTGCTAATTGGGCA
+HWI-EAS443:3:38:1130:1582#0/1
Va^QYb^`aa_a_Yaa_ZQUW^aa`aa_aN__^a_RV]P^
@HWI-EAS443:3:38:1135:1096#0/1
CATCAATGAACTGAGCAATCTTGCAAAAGGAGAGAAAGCT
+HWI-EAS443:3:38:1135:1096#0/1
a`aaabbabaaa`aabaaaaaab`aab_b_Za_aa_`_`[
@HWI-EAS443:3:38:1234:1155#0/1
CATCAATGAACTGAGCAATCTTGCAAAAGGAGAGAAAGCT
+HWI-EAS443:3:38:1234:1155#0/1
a_a]_a`[WWT^^SXa^S¥[Y_a[]ZZ_¥XSPNa]_¥ZQW
@HWI-EAS443:3:38:1404:1190#0/1
AGCTAATGTGCTAATTGGGCAAGGGGACGTAGTGTTGGTA
+HWI-EAS443:3:38:1404:1190#0/1
_aa`a[aa_a^aa]a`]_`]_^a_^a^ZaPKUZ]Z^_]OV
@HWI-EAS443:3:38:1516:794#0/1
CTTGCAAAAGGAGAGAAAGCTAATGTGCTAATTGGGCAAG
+HWI-EAS443:3:38:1516:794#0/1
a``¥^T_``^`[aYaaa[[VZ_aa``_P¥HQ`Y`X_YN^Y
@HWI-EAS443:3:38:1648:822#0/1
AAGCTAATGTGCTAATTGGGCAAGGGGACGTAGTGTTGGT
+HWI-EAS443:3:38:1648:822#0/1
aab`aaaaa^a_a`aa`_``a`^a[[a^`]UZ[Z_]`¥WQ
@HWI-EAS443:3:38:1765:1493#0/1
ATCTTGCAAAAGGAGAGAAAGCTAATGTGCTAATTGGGCA
+HWI-EAS443:3:38:1765:1493#0/1
a¥aa_Y__bUba¥^`¥`_ababbbb]aXaXU[[`^^bW`X
@HWI-EAS443:3:38:234:364#0/1
GAGCAATCTTGCAAAAGGAGAGAAAGCTAATGTGCTAATT
+HWI-EAS443:3:38:234:364#0/1
]__baZa¥O]baaOYO^b]aaaaaaaaa¥Y_aZ_PV[Y`]
@HWI-EAS443:3:38:260:1994#0/1
TGAGCAATCTTGCAAAAGGAGAGAAAGCTAATGTGCTAAT
+HWI-EAS443:3:38:260:1994#0/1
``^Vabaaaaa]`a`Xa¥`^^a^`a`YNX_U]XLS]_ZW_
```

```
@HWI-EAS443:3:38:287:1799#0/1
CATCAATGAACTGAGCAATCTTGCAAAAGGAGAGAAAGCT
+HWI-EAS443:3:38:287:1799#0/1
aa`aaaa_aa__]^Waaa^_`]]aa¥YZ]XZZ^X^__Z_U
@HWI-EAS443:3:38:328:318#0/1
ATCTTGCAAAAGGAGAGAAAGCTAATGTGCTAATTGGGCA
+HWI-EAS443:3:38:328:318#0/1
^XV____ZZY]^a_Y¥XaaU]¥^ZJY_VYRVUXH_XRVZZ
@HWI-EAS443:3:38:346:448#0/1
ACTGAGCAATCTTGCAAAAGGAGAGAAAGCTAATGTGCTA
+HWI-EAS443:3:38:346:448#0/1
aaXa^WQUR¥JZ_aaaa`^]``aaa¥YZQHOPYZY¥WDMQ
@HWI-EAS443:3:38:439:995#0/1
GCATCAATGAACTGAGCAATCTTGCAAAAGGAGAGAAAGC
+HWI-EAS443:3:38:439:995#0/1
a_¥`aV__¥_aa`SSa`aZ]^¥^^]¥Q_I¥Y_`^W`a]YP
@HWI-EAS443:3:38:565:1694#0/1
CATCAATGAACTGAGCAATCTTGCAAAAGGAGAGAAAGCT
+HWI-EAS443:3:38:565:1694#0/1
_bbb_aaaa_`a`aaaabb]aa]aaaaa`O``aaba`_aX
@HWI-EAS443:3:38:632:1591#0/1
GAGCAATCTTGCAAAAGGAGAGAAAGCTAATGTGCTAATT
+HWI-EAS443:3:38:632:1591#0/1
a_Z[_ZZa^WS^]^N¥YW[]Z[¥Q^a`P][Y`ZYPU^¥^L
@HWI-EAS443:3:38:666:166#0/1
GCATCAATGAACTGAGCAATCTTGCAAAAGGAGAGAAAGC
+HWI-EAS443:3:38:666:166#0/1
aaa`a``aa`a^_^__`_[__`^a^^^]Z_ZZ^_`^^Z_W
@HWI-EAS443:3:38:712:166#0/1
GAGAAAGCTAATGTGCTAATTGGGCAAGGGGACGTAGTGT
+HWI-EAS443:3:38:712:166#0/1
aaaa__`a_]¥]_X`__¥_^^^]]_]¥_]ZZZZ^XZ^X]Z
@HWI-EAS443:3:38:740:283#0/1
AGCTAATGTGCTAATTGGGCAAGGGGACGTAGTGTTGGTA
+HWI-EAS443:3:38:740:283#0/1
__]`]_ZaZIT``aX`aaa^¥_`_Z^U]]LUYQ[OYaZFN
@HWI-EAS443:3:38:744:371#0/1
CTTGCAAAAGGAGAGAAAGCTAATGTGCTAATTGGGCAAG
+HWI-EAS443:3:38:744:371#0/1
aaa`aba^aa`^a``aa`a_`aaaa]]^`_]_^__a___]
@HWI-EAS443:3:38:782:1349#0/1
GCATCAATGAACTGAGCAATCTTGCAAAAGGAGAGAAAGC
+HWI-EAS443:3:38:782:1349#0/1
Yababb`^ba]`baZ^abbababbbb¥a`a^_```a`a`X
@HWI-EAS443:3:38:843:1859#0/1
AGCTAATGTGCTAATTGGGCAAGGGGACGTAGTGTTGGTA
+HWI-EAS443:3:38:843:1859#0/1
a_baa]_Z`]a`aYb^Zb`W``a`LQUV`XXWY]Z[YT]Q
@HWI-EAS443:3:38:876:1690#0/1
GAAAGCTAATGTGCTAATTGGGCAAGGGGACGTAGTGTTG
```

```
+HWI-EAS443:3:38:876:1690#0/1
a¥baaaXVZb`a^_aaZ``RJR¥`]a`a`RZaZ`aO_O_S
@HWI-EAS443:3:38:969:647#0/1
ATGAACTGAGCAATCTTGCAAAAGGAGAGAAAGCTAATGT
+HWI-EAS443:3:38:969:647#0/1
abaX`aa¥[`aaaaaaa^aaW__]_]_]aa[]]__^ZRTM
@HWI-EAS443:3:39:1008:1326#0/1
AGCAATCTTGCAAAAGGAGAGAAAGCTAATGTGCTAATTG
+HWI-EAS443:3:39:1008:1326#0/1
aabb`abaab`aaaaaaaa`aaa^aa^_`_aR^^``_^]V
@HWI-EAS443:3:39:1017:1369#0/1
TTGCAAAAGGAGAGAAAGCTAATGTGCTAATTGGGCAAGG
+HWI-EAS443:3:39:1017:1369#0/1
aa`abaZ]baaaaabaa`^a_aa`[a]`aa_^^¥¥]_]^^
@HWI-EAS443:3:39:1114:1268#0/1
GAGCAATCTTGCAAAAGGAGAGAAAGCTAATGTGCTAATT
+HWI-EAS443:3:39:1114:1268#0/1
^__`_^_``aY`a__]a_VZ_aa^`b]`Xa]ZV¥aZ¥Z__
@HWI-EAS443:3:39:1165:315#0/1
GAACTGAGCAATCTTGCAAAAGGAGAGAAAGCTAATGTGC
+HWI-EAS443:3:39:1165:315#0/1
aabbbb`babaaaaaaaaa_^baaaaaa_a_R]a`a`]`V
@HWI-EAS443:3:39:1194:351#0/1
CTTGCAAAAGGAGAGAAAGCTAATGTGCTAATTGGGCAAG
+HWI-EAS443:3:39:1194:351#0/1
ab]G]baa``a`baba[a``_]_ab_aaa]`_^`aa`__^
@HWI-EAS443:3:39:1208:1716#0/1
GTCCCCTTGCCCAATTAGCACATTAGCTTTCTCTCCTTTT
+HWI-EAS443:3:39:1208:1716#0/1
aP`]_]Y_aa[[[_VY_[YJQ[Z^YYU^`^XSSY]VZ¥`V
@HWI-EAS443:3:39:1223:1052#0/1
CAATCTTGCAAAAGGAGAGAAAGCTAATGTGCTAATTGGG
+HWI-EAS443:3:39:1223:1052#0/1
abb_`^aab`^aa]b]baba¥]a_^`aabZ`Paa]_aaa_
@HWI-EAS443:3:39:1231:1226#0/1
CTTGCAAAAGGAGAGAAAGCTAATGTGCTAATTGGGCAAG
+HWI-EAS443:3:39:1231:1226#0/1
aa`b`ZZ^^a[]`¥[`^V`_XaUZa¥¥^`__X_[__[XV_
@HWI-EAS443:3:39:1383:640#0/1
ATGAACTGAGCAATCTTGCAAAAGGAGAGAAAGCTAATGT
+HWI-EAS443:3:39:1383:640#0/1
U[_J_a_a[`b_]`^YZ`_¥[X^Z^J[`a]S[UV_¥T__Q
@HWI-EAS443:3:39:1409:1797#0/1
AGCTAATGTGCTAATTGGGCAAGGGGACGTAGTGTTGGTA
+HWI-EAS443:3:39:1409:1797#0/1
a]¥¥``Y^ZZXaa`a_`VZ[^[QQT^O`^PVTQ]^^XUFS
@HWI-EAS443:3:39:1431:586#0/1
GCAATCTTGCAAAAGGAGAGAAAGCTAATGTGCTAATTGG
+HWI-EAS443:3:39:1431:586#0/1
aabaaaaa^^a`Z`aa^a`a____Z_^__`RVZ__]`]_Y
```

```
@HWI-EAS443:3:39:1441:1975#0/1
GAGAAAGCTAATGTGCTAATTGGGCAAGGGGACGTAGTGT
+HWI-EAS443:3:39:1441:1975#0/1
aaa^a^^V]_``aY_S]]__U`_X^`[`[_LPHSUVVXTM
@HWI-EAS443:3:39:1494:450#0/1
AACTGAGCAATCTTGCAAAAGGAGAGAAAGCTAATGTGCT
+HWI-EAS443:3:39:1494:450#0/1
abb`VS]baaaa``baa`a`aa]`aa_aYa`^Y_`¥X¥Y]
@HWI-EAS443:3:39:153:534#0/1
ATCTTGCAAAAGGAGAGAAAGCTAATGTGCTAATTGGGCA
+HWI-EAS443:3:39:153:534#0/1
]`baaaba]``aa_`_``a_a`a^Q``X`aa^_a`a`_a¥
@HWI-EAS443:3:39:1580:1113#0/1
AGCTAATGTGCTAATTGGGCAAGGGGACGTAGTGTTGGTA
+HWI-EAS443:3:39:1580:1113#0/1
aa`bbabbab]b``bbbaaaS]a[``[[_`_RYaaab]ZY
@HWI-EAS443:3:39:1618:454#0/1
TAGCTTTCTCTCCTTTTGCAAGATTGCTCAGTTCATTGAT
+HWI-EAS443:3:39:1618:454#0/1
X``W`¥Y_[^b`]^Yaa][]P_[ZaWU[]Y[NX[VS`]¥]
@HWI-EAS443:3:39:1667:269#0/1
AGGAGAGAAAGCTAATGTGCTAATTGGGCAAGGGGACGTA
+HWI-EAS443:3:39:1667:269#0/1
_aaab``ab`a`aaabbaaaaaaabb`a`a`R``b^_a]Y
@HWI-EAS443:3:39:16:1123#0/1
ATCTTGCAAAAGGAGAGAAAGCTAATGTGCTAATTGGGCA
+HWI-EAS443:3:39:16:1123#0/1
a_baaaaWW^`aa[`]aa_S`a]Z]``Uaa`RX_¥a]_a_
@HWI-EAS443:3:39:180:1566#0/1
GAGCAATCTTGCAAAAGGAGAGAAAGCTAATGTGCTAATT
+HWI-EAS443:3:39:180:1566#0/1
`¥`ba¥abbbbaV_Q_aZ__aaaaT_aaab^^T^aaaaa]
@HWI-EAS443:3:39:211:1415#0/1
CATCAATGAACTGAGCAATCTTGCAAAAGGAGAGAAAGCT
+HWI-EAS443:3:39:211:1415#0/1
`QYa^][R^`]ZZW¥``¥¥[Z___YJX__]X]Z¥R^]^ZP
@HWI-EAS443:3:39:236:594#0/1
GAAAGCTAATGTGCTAATTGGGCAAGGGGACGTAGTGTTG
+HWI-EAS443:3:39:236:594#0/1
¥Q^V]_ZOGY_V]`^[[[ZZUU¥UZ[]ZZTN[VVXQ]UU[
@HWI-EAS443:3:39:237:31#0/1
ATCTTGCAAAAGGAGAGAAAGCTAATGTGCTAATTGGGCA
+HWI-EAS443:3:39:237:31#0/1
aabbabaZ]]`ba_b¥``X]aa_^^^_]_a[[ZZU`]^_V
@HWI-EAS443:3:39:23:1464#0/1
GAAAGCTAATGTGCTAATTGGGCAAGGGGACGTAGTGTTG
+HWI-EAS443:3:39:23:1464#0/1
aa^Z`a`_]WQV[^`Y__^_U[a_TZ^UY¥¥RRZ¥WYUUL
@HWI-EAS443:3:39:309:1436#0/1
GAACTGAGCAATCTTGCAAAAGGAGAGAAAGCTAATGTGC
```

```
+HWI-EAS443:3:39:309:1436#0/1
Z[X_ZS^_b`ba[`a`a__^Z_¥```Zb_`XIV`Z__Q¥W
@HWI-EAS443:3:39:313:398#0/1
ATCTTGCAAAAGGAGAGAAAGCTAATGTGCTAATTGGGCA
+HWI-EAS443:3:39:313:398#0/1
___Y_^^¥QX^]_Y`Y`b`a_`a_¥ZaS¥]_RT_W__R_Z
@HWI-EAS443:3:39:356:1704#0/1
ATGAACTGAGCAATCTTGCAAAAGGAGAGAAAGCTAATGT
+HWI-EAS443:3:39:356:1704#0/1
^¥^_ab]aaVa]Z]a[]ZZS__^^a`^babaT[R^b]`^V
@HWI-EAS443:3:39:395:350#0/1
ATCTTGCAAAAGGAGAGAAAGCTAATGTGCTAATTGGGCA
+HWI-EAS443:3:39:395:350#0/1
aabaabb_Z`_baababa]¥aa`a_`_]a_`¥_a_`__`_
@HWI-EAS443:3:39:413:843#0/1
GAGCAATCTTGCAAAAGGAGAGAAAGCTAATGTGCTAATT
+HWI-EAS443:3:39:413:843#0/1
`bb``aabba_bab_babaaa_ba`baa`ba__abaaaa_
@HWI-EAS443:3:39:441:1583#0/1
GAGCAATCTTGCAAAAGGAGAGAAAGCTAATGTGCTAATT
+HWI-EAS443:3:39:441:1583#0/1
a^b`aa`bab^ba`ZSba`a[bZ]^YaaaaaaNab`aa^S
@HWI-EAS443:3:39:556:804#0/1
AAAAGGAGAGAAAGCTAATGTGCTAATTGGGCAAGGGGAC
+HWI-EAS443:3:39:556:804#0/1
aZ^¥_aV^abZYX`a]YZaaX]a_¥]]`Z`_WVX`^_^]Z
@HWI-EAS443:3:39:618:34#0/1
AGGAGAGAAAGCTAATGTGCTAATTGGGCAAGGGGACGTA
+HWI-EAS443:3:39:618:34#0/1
]bbb`bbb_`baab^ab_baa_``aab``_^a`a`[_`L]
@HWI-EAS443:3:39:674:400#0/1
AATGAACTGAGCAATCTTGCAAAAGGAGAGAAAGCTAATG
+HWI-EAS443:3:39:674:400#0/1
aabaa`aaaaaa`aaa_aaa__a¥a_¥a`a`^`__a__¥_
@HWI-EAS443:3:39:732:583#0/1
GAGCAATCTTGCAAAAGGAGAGAAAGCTAATGTGCTAATT
+HWI-EAS443:3:39:732:583#0/1
a`ababbbbaaaaZ__`b`aaaaaaa]a`aa_]aaa_`__
@HWI-EAS443:3:39:770:1303#0/1
GAGCAATCTTGCAAAAGGAGAGAAAGCTAATGTGCTAATT
+HWI-EAS443:3:39:770:1303#0/1
a`bbbbbbbbaaba]b`aabbbbaaaabbba`_bba`aaa
@HWI-EAS443:3:39:79:966#0/1
GAGCAATCTTGCAAAAGGAGAGAAAGCTAATGTGCTAATT
+HWI-EAS443:3:39:79:966#0/1
a¥bbbUbbbabaZ_``baa`[`a_V¥aa]`_ZXaaa[aaa
@HWI-EAS443:3:39:805:1978#0/1
GAGAGAAAGCTAATGTGCTAATTGGGCAAGGGGACGTAGT
+HWI-EAS443:3:39:805:1978#0/1
_ZU`Wa[^[V][`VVRYSV[V_UWWNW_]QLQQT¥RQWYS
```

```
@HWI-EAS443:3:39:826:1303#0/1
GAGCAATCTTGCAAAAGGAGAGAAAGCTAATGTGCTAATT
+HWI-EAS443:3:39:826:1303#0/1
[aabb`bbbbbbbabYbabbbbababababaaababaabb
@HWI-EAS443:3:3:1034:1417#0/1
GAAAGCTAATGTGCTAATTGGGCAAGGGGACGTAGTGTTG
+HWI-EAS443:3:3:1034:1417#0/1
aaaa`a`a]]a^_^b__a`a___^¥aaaaV^aOZaH_Y]_
@HWI-EAS443:3:3:1112:287#0/1
ATCTTGCAAAAGGAGAGAAAGCTAATGTGCTAATTGGGCA
+HWI-EAS443:3:3:1112:287#0/1
ab_aaaaaaa`ba_a_aa_`aU`_`aa]aX`_^`_`__YZ
@HWI-EAS443:3:3:1130:469#0/1
CATCAATGAACTGAGCAATCTTGCAAAAGGAGAGAAAGCT
+HWI-EAS443:3:3:1130:469#0/1
`b^a^`b`¥ba^aa^Z_a^^_```a[`bba`babaa]bVa
@HWI-EAS443:3:3:1141:1122#0/1
AATGAACTGAGCAATCTTGCAAAAGGAGAGAAAGCTAATG
+HWI-EAS443:3:3:1141:1122#0/1
¥b_ZXPP^ba^_¥UaSb[aa`]__a`Za_`a[QaQa__a_
@HWI-EAS443:3:3:1185:1284#0/1
GAGAAAGCTAATGTGCTAATTGGGCAAGGGGACGTAGTGT
+HWI-EAS443:3:3:1185:1284#0/1
`Z_RWXa_^SY`SYa_`[[¥Ta¥RSWFS¥`ZHU_YLPU]B
@HWI-EAS443:3:3:1392:362#0/1
AGGAGAGAAAGCTAATGTGCTAATTGGGCAAGGGGACGTA
+HWI-EAS443:3:3:1392:362#0/1
U¥VQ[ZaW¥_TO[XW]WWVDTW]_aVHNQYX]R]`]BBBB
@HWI-EAS443:3:3:1397:1388#0/1
ACCAACACTACGTCCCCTTGCCCAATTAGCACATTAGCTT
+HWI-EAS443:3:3:1397:1388#0/1
aaVa`aa_b_H__a¥``US¥^^[¥a[U__a][`^Y_ZV__
@HWI-EAS443:3:3:1521:1387#0/1
ATCTTGCAAAAGGAGAGAAAGCTAATGTGCTAATTGGGCA
+HWI-EAS443:3:3:1521:1387#0/1
[^`aa¥O]_¥XZ_^ZIa¥_]_NZ^aaa]a¥a__`]`YOO[
@HWI-EAS443:3:3:1573:1670#0/1
GAGCAATCTTGCAAAAGGAGAGAAAGCTAATGTGCTAATT
+HWI-EAS443:3:3:1573:1670#0/1
]QH¥b¥bbbY`¥TOO^OZZba]`a_b_baaab]bS_R¥`a
@HWI-EAS443:3:3:1671:649#0/1
CAATCTTGCAAAAGGAGAGAAAGCTAATGTGCTAATTGGG
+HWI-EAS443:3:3:1671:649#0/1
a`abbZabb`bVbb`_bbbbaab^baaa`Gb_a¥Yab]ZP
@HWI-EAS443:3:3:1701:1721#0/1
AAGCTAATGTGCTAATTGGGCAAGGGGACGTAGTGTTGGT
+HWI-EAS443:3:3:1701:1721#0/1
a]`ab`aab```a^`aa]`X^__`a[`_]`XT_^a__a_V
@HWI-EAS443:3:3:1730:431#0/1
AGCTAATGTGCTAATTGGGCAAGGGGACGTAGTGTTGGTA
```

+HWI-EAS443:3:3:1730:431#0/1
`baabU`_P`Xa`YaZYa__X`_a_aXX_W^[]a^`aW]V
@HWI-EAS443:3:3:1746:343#0/1
AGCTAATGTGCTAATTGGGCAAGGGGACGTAGTGTTGGTA
+HWI-EAS443:3:3:1746:343#0/1
aa[a`a]a[b_aa__`aba`_^`a]aOTaXZ`R__W_[VB
@HWI-EAS443:3:3:1789:724#0/1
GAAAGCTAATGTGCTAATTGGGCAAGGGGACGTAGTGTTG
+HWI-EAS443:3:3:1789:724#0/1
aaa¥U¥`aabb[a^]`aaa¥`¥]`[a[``_N_U]a[aUY]
@HWI-EAS443:3:3:238:150#0/1
GAACTGAGCAATCTTGCAAAAGGAGAGAAAGCTAATGTGC
+HWI-EAS443:3:3:238:150#0/1
aaTVY¥[b_a`a^`_a]^`_]aa`aaa_Y``^^_^__Q`Z
@HWI-EAS443:3:3:268:1062#0/1
CTTGCCCAATTAGCACATTAGCTTTCTCTCCTTTTGCAAG
+HWI-EAS443:3:3:268:1062#0/1
aaabaaba`bbba_`_abbaba`aaaa_a^_aabaa^aaa
@HWI-EAS443:3:3:294:1518#0/1
AGCTAATGTGCTAATTGGGCAAGGGGACGTAGTGTTGGTA
+HWI-EAS443:3:3:294:1518#0/1
¥Z^^Y]bbab[aZ`aPa`aZ`¥aa_`[^_Q__W`VYa`OO
@HWI-EAS443:3:3:300:859#0/1
CATCAATGAACTGAGCAATCTTGCAAAAGGAGAGAAAGCT
+HWI-EAS443:3:3:300:859#0/1
a¥¥`YO_NP_SS_aYFYVa_UQ[`RVX`^Y][U[¥YY]X¥
@HWI-EAS443:3:3:408:804#0/1
AGAGAAAGCTAATGTGCTAATTGGGCAAGGGGACGTAGTG
+HWI-EAS443:3:3:408:804#0/1
abaYbbaba`a`a__`ab¥]aaaaaa_`a_a`¥]a^_^^a
@HWI-EAS443:3:3:416:1726#0/1
AATGAACTGAGCAATCTTGCAAAAGGAGAGAAAGCTAATG
+HWI-EAS443:3:3:416:1726#0/1
abb`b`a_`^a_aa`^aaa`_aaa_a^aa_`a``_]_]_a
@HWI-EAS443:3:3:506:1133#0/1
CCCTTGCCCAATTAGCACATTAGCTTTCTCTCCTTTTGCA
+HWI-EAS443:3:3:506:1133#0/1
_baaabbabbaab`a^abaabaaaaabab`aa]ba`aa_a
@HWI-EAS443:3:3:613:405#0/1
TGAACTGAGCAATCTTGCAAAAGGAGAGAAAGCTAATGTG
+HWI-EAS443:3:3:613:405#0/1
_a[_aa^aaaV¥WR`aaa_¥]aabYb_b`U^a^`]^^aZa
@HWI-EAS443:3:3:628:2013#0/1
CATCAATGAACTGAGCAATCTTGCAAAAGGAGAGAAAGCT
+HWI-EAS443:3:3:628:2013#0/1
aaXP_aaa_a_R`]]Q```Q_`aX]_```a[a__[Z`_X_
@HWI-EAS443:3:3:645:545#0/1
GAGCAATCTTGCAAAAGGAGAGAAAGCTAATGTGCTAATT
+HWI-EAS443:3:3:645:545#0/1
_aa^bbaabbba_a_abbaaaab``b_aa_bb]a`a`_ba

```
@HWI-EAS443:3:3:739:750#0/1
CATCAATGAACTGAGCAATCTTGCAAAAGGAGAGAAAGCT
+HWI-EAS443:3:3:739:750#0/1
a¥ZPY_`aa`V_a_]Sa`[aaa`a``_`^]^[Yaa`]aYa
@HWI-EAS443:3:3:777:213#0/1
GAGCAATCTTGCAAAAGGAGAGAAAGCTAATGTGCTAATT
+HWI-EAS443:3:3:777:213#0/1
^^`aaabaabb]babbbbbb`abbab`aT_aa]a^_`]aa
@HWI-EAS443:3:3:813:860#0/1
GAAAGCTAATGTGCTAATTGGGCAAGGGGACGTAGTGTTG
+HWI-EAS443:3:3:813:860#0/1
Y`baaaba_bbZbaa`aaabbbaaa```a[Xa]Y]Y]^_a
@HWI-EAS443:3:3:83:275#0/1
GCATCAATGAACTGAGCAATCTTGCAAAAGGAGAGAAAGC
+HWI-EAS443:3:3:83:275#0/1
aabababababaaa`a`aaaa`ab_a`_aa`_aaaa_`a]
@HWI-EAS443:3:3:910:796#0/1
GAACTGAGCAATCTTGCAAAAGGAGAGAAAGCTAATGTGC
+HWI-EAS443:3:3:910:796#0/1
a]``^[PWaV`¥S]]a_a_]Za_Qa¥_`UZaO_WPZ¥[_V
@HWI-EAS443:3:3:935:835#0/1
GAGAAAGCTAATGTGCTAATTGGGCAAGGGGACGTAGTGT
+HWI-EAS443:3:3:935:835#0/1
¥``^`a`^Yba`[[b_a``a_a`aH__b¥a][Na^__Va]
@HWI-EAS443:3:3:992:763#0/1
GAGAAAGCTAATGTGCTAATTGGGCAAGGGGACGTAGTGT
+HWI-EAS443:3:3:992:763#0/1
a`b___aR^_Y_a¥``a_`a`a``]^_a``XTRWPOXMV]
@HWI-EAS443:3:40:1052:1380#0/1
ATCTTGCAAAAGGAGAGAAAGCTAATGTGCTAATTGGGCA
+HWI-EAS443:3:40:1052:1380#0/1
aaaabbaaaaaa`]`aaa_aaaa__`^]_`_```__a]a¥
@HWI-EAS443:3:40:1054:1745#0/1
GCCCAATTAGCACATTAGCTTTCTCTCCTTTTGCAAGATT
+HWI-EAS443:3:40:1054:1745#0/1
¥JS`RYY¥Q]¥^TTT_PW^[¥R¥J^UaaW[SXT_XN[PPM
@HWI-EAS443:3:40:1064:1678#0/1
GAGCAATCTTGCAAAAGGAGAGAAAGCTAATGTGCTAATT
+HWI-EAS443:3:40:1064:1678#0/1
^aaaaa`aaba___^`^`[Za_aa^Wa_Y¥V_^`___^_[
@HWI-EAS443:3:40:1352:509#0/1
GTCCCCTTGCCCAATTAGCACATTAGCTTTCTCTCCTTTT
+HWI-EAS443:3:40:1352:509#0/1
_BBBBBBBBBBBBBBBBBBBBBBBBBBBBBBBBBBBBBBB
@HWI-EAS443:3:40:1379:1042#0/1
GAAAGCTAATGTGCTAATTGGGCAAGGGGACGTAGTGTTG
+HWI-EAS443:3:40:1379:1042#0/1
`¥[Y][ZVTZUV¥V[VU¥]]``^XQVQWYIEZLOXBBBBB
@HWI-EAS443:3:40:1397:1101#0/1
CATCAATGAACTGAGCAATCTTGCAAAAGGAGAGAAAGCT
```

````
+HWI-EAS443:3:40:1397:1101#0/1
abbaaabb`_abaab`aaaaaaaa`__]_`^__a`^I_`^
@HWI-EAS443:3:40:1423:659#0/1
AAAGGAGAGAAAGCTAATGTGCTAATTGGGCAAGGGGACG
+HWI-EAS443:3:40:1423:659#0/1
aba`baba``W¥baa_aab`a_a`_a_a_a]aab¥Y`_aa
@HWI-EAS443:3:40:1533:1498#0/1
AGCTAATGTGCTAATTGGGCAAGGGGACGTAGTGTTGGTA
+HWI-EAS443:3:40:1533:1498#0/1
`]`aaaab_a`aa`a`a_aa]_a`_[Z`b^Y[Va[aa[FU
@HWI-EAS443:3:40:1544:206#0/1
ATCTTGCAAAAGGAGAGAAAGCTAATGTGCTAATTGGGCA
+HWI-EAS443:3:40:1544:206#0/1
aaaaa_^aaa_aa`a`aaa_a_``^`_Za^^^^`^]a]_]
@HWI-EAS443:3:40:160:352#0/1
GAAAGCTAATGTGCTAATTGGGCAAGGGGACGTAGTGTTG
+HWI-EAS443:3:40:160:352#0/1
abaaaa`a`aaQ]a^_``_`]`___`V`YWZ_YV_Y^X]`
@HWI-EAS443:3:40:1651:1849#0/1
GCATCAATGAACTGAGCAATCTTGCAAAAGGAGAGAAAGC
+HWI-EAS443:3:40:1651:1849#0/1
^VPTR[`¥]PVSV`]_Q_¥¥QG^[XSRZ]]ZUVYW^^LSL
@HWI-EAS443:3:40:1747:230#0/1
AATGAACTGAGCAATCTTGCAAAAGGAGAGAAAGCTAATG
+HWI-EAS443:3:40:1747:230#0/1
```a_^b]]Za]b`a[^]]]]PYZa]TaJ_X^_a[¥S]_`
@HWI-EAS443:3:40:1754:1307#0/1
AGCTAATGTGCTAATTGGGCAAGGGGACGTAGTGTTGGTA
+HWI-EAS443:3:40:1754:1307#0/1
ab`_bbab_aaaa_bab`aaHaa^^YG^bRRaX`^aa_XV
@HWI-EAS443:3:40:1791:585#0/1
AGCTAATGTGCTAATTGGGCAAGGGGACGTAGTGTTGGTA
+HWI-EAS443:3:40:1791:585#0/1
`a`babbb`b`ba`bab`ba`aba`_O¥b`^a]ba`b_OY
@HWI-EAS443:3:40:268:1852#0/1
CAAAAGGAGAGAAAGCTAATGTGCTAATTGGGCAAGGGGA
+HWI-EAS443:3:40:268:1852#0/1
abaab_¥Ya[RYb`b^H`YWRGWa`_IWNVGY_W]_`PaU
@HWI-EAS443:3:40:334:1082#0/1
AATGAACTGAGCAATCTTGCAAAAGGAGAGAAAGCTAATG
+HWI-EAS443:3:40:334:1082#0/1
abbabbbba_aaaaaaaaaaaa__b]P`_a`a`]a^a`_a
@HWI-EAS443:3:40:33:789#0/1
CATCAATGAACTGAGCAATCTTGCAAAAGGAGAGAAAGCT
+HWI-EAS443:3:40:33:789#0/1
abbbaaba_`aaa`^`aaabaaaa`¥GV]a]a``_¥Y^`_
@HWI-EAS443:3:40:341:795#0/1
ATCTTGCAAAAGGAGAGAAAGCTAATGTGCTAATTGGGCA
+HWI-EAS443:3:40:341:795#0/1
¥`a``a¥USW¥a_U_^]_X^^a`H[]]Wa_¥`TZ^__aa_
````
````

@HWI-EAS443:3:40:361:203#0/1
AGCAATCTTGCAAAAGGAGAGAAAGCTAATGTGCTAATTG
+HWI-EAS443:3:40:361:203#0/1
abbb[^ab[¥]`aaab]bbab_`aa`XaY_aX_`]UH]]]
@HWI-EAS443:3:40:547:227#0/1
GCATCAATGAACTGAGCAATCTTGCAAAAGGAGAGAAAGC
+HWI-EAS443:3:40:547:227#0/1
a`a`a_`_a[^^_`]^a__]^`^`]XR¥V]_Z___^]]^¥
@HWI-EAS443:3:40:886:1824#0/1
AGCAATCTTGCAAAAGGAGAGAAAGCTAATGTGCTAATTG
+HWI-EAS443:3:40:886:1824#0/1
Sab`I¥Y^[U]_UUaaTTVbU`b^SJJ[MRXL]_]¥aTQ_
@HWI-EAS443:3:40:994:1816#0/1
AAGCTAATGTGCTAATTGGGCAAGGGGACGTAGTGTTGGT
+HWI-EAS443:3:40:994:1816#0/1
a[baaaa¥aU]`a``_aa_aaa`_UO^_a_U_¥X`]^[ZG
@HWI-EAS443:3:40:995:230#0/1
GAGCAATCTTGCAAAAGGAGAGAAAGCTAATGTGCTAATT
+HWI-EAS443:3:40:995:230#0/1
Sabbaabbbaabbbbabbababbaba``L_aa^aaa^```
@HWI-EAS443:3:42:1063:1804#0/1
CAATCTTGCAAAAGGAGAGAAAGCTAATGTGCTAATTGGG
+HWI-EAS443:3:42:1063:1804#0/1
X¥¥aMa`bbbbbVTX¥_WWaba`aabb`¥^`aba`bbb]a
@HWI-EAS443:3:42:1222:1077#0/1
CATCAATGAACTGAGCAATCTTGCAAAAGGAGAGAAAGCT
+HWI-EAS443:3:42:1222:1077#0/1
aabbbbbbabbbbabbbbbbbabab`bbbaababbbbb_a
@HWI-EAS443:3:42:1315:657#0/1
AGCTAATGTGCTAATTGGGCAAGGGGACGTAGTGTTGGTA
+HWI-EAS443:3:42:1315:657#0/1
a¥b`¥[]b^``a[¥_a`_baaZbbabaab¥]a^b`Vb_G¥
@HWI-EAS443:3:42:1340:1591#0/1
AGCTAATGTGCTAATTGGGCAAGGGGACGTAGTGTTGGTA
+HWI-EAS443:3:42:1340:1591#0/1
aa`ababb`a`aaabaab_`a`ba¥a`aa_`a_^aa``[]
@HWI-EAS443:3:42:1385:1531#0/1
ATGAACTGAGCAATCTTGCAAAAGGAGAGAAAGCTAATGT
+HWI-EAS443:3:42:1385:1531#0/1
aaaa^^ba^``aaaaaaa`_`aaaaab`aa`_aZ``^aa]
@HWI-EAS443:3:42:1622:671#0/1
GAAAGCTAATGTGCTAATTGGGCAAGGGGACGTAGTGTTG
+HWI-EAS443:3:42:1622:671#0/1
abb`babaaab_b_baaaab_aa``baaa`^a]_a]aZaa
@HWI-EAS443:3:42:163:600#0/1
AGCTAATGTGCTAATTGGGCAAGGGGACGTAGTGTTGGTA
+HWI-EAS443:3:42:163:600#0/1
a_b^b]`b_Y`__X_aaa`aa`[V¥aQ^aX__PZSZ_^VS
@HWI-EAS443:3:42:168:1729#0/1
CATCAATGAACTGAGCAATCTTGCAAAAGGAGAGAAAGCT

```
+HWI-EAS443:3:42:168:1729#0/1
abbaba```bbaaa_abbaab]`a_^Y^ba`aaaaab`a_
@HWI-EAS443:3:42:1769:345#0/1
GAGCAATCTTGCAAAAGGAGAGAAAGCTAATGTGCTAATT
+HWI-EAS443:3:42:1769:345#0/1
a_bababaabbaaa`¥ba`aaaa``a_aa```]aZ__`__
@HWI-EAS443:3:42:1774:714#0/1
CATCAATGAACTGAGCAATCTTGCAAAAGGAGAGAAAGCT
+HWI-EAS443:3:42:1774:714#0/1
Y[bbba¥¥`aa`^Yabbababbbaa]a``_a``baSX^`a
@HWI-EAS443:3:42:196:218#0/1
GTCCCCTTGCCCAATTAGCACATTAGCTTTCTCTCCTTTT
+HWI-EAS443:3:42:196:218#0/1
YXbaaa¥P]^aaa___]aa`]]_VS]`TZ`]_aa____^`
@HWI-EAS443:3:42:30:263#0/1
GTCCCCTTGCCCAATTAGCACATTAGCTTTCTCTCCTTTT
+HWI-EAS443:3:42:30:263#0/1
a`baaaaaaaaaaa_¥_``_`a```a_`__`___`___¥
@HWI-EAS443:3:42:389:1797#0/1
ATGAACTGAGCAATCTTGCAAAAGGAGAGAAAGCTAATGT
+HWI-EAS443:3:42:389:1797#0/1
abaTaa[`aaaa_aa`aaaa_^a``]Yaaaa_^^a^_`_Z
@HWI-EAS443:3:42:419:848#0/1
GAAAGCTAATGTGCTAATTGGGCAAGGGGACGTAGTGTTG
+HWI-EAS443:3:42:419:848#0/1
a`RWaa_PZa`U`aaXa]X_^]¥ZL]``YJVTN][TYV^_
@HWI-EAS443:3:42:493:249#0/1
AATTAGCACATTAGCTTTCTCTCCTTTTGCAAGATTGCTC
+HWI-EAS443:3:42:493:249#0/1
aabaaba``aaa_aa_a`^a_aa^a___`_`_a_`_a_]^
@HWI-EAS443:3:42:496:1435#0/1
GCATCAATGAACTGAGCAATCTTGCAAAAGGAGAGAAAGC
+HWI-EAS443:3:42:496:1435#0/1
aa^`````_aaaZY¥`aaaa``__`a_`]a_Y_a`a_a`_
@HWI-EAS443:3:42:50:1226#0/1
GAGCAATCTTGCAAAAGGAGAGAAAGCTAATGTGCTAATT
+HWI-EAS443:3:42:50:1226#0/1
`J`bbb`bbbbbb_b¥ba`abb`W_b`bbbba`babbab`
@HWI-EAS443:3:42:73:157#0/1
AGAGAAAGCTAATGTGCTAATTGGGCAAGGGGACGTAGTG
+HWI-EAS443:3:42:73:157#0/1
`aabbaaaaa]^a¥T_`]_a`^_]^`_`¥¥¥___¥JQ¥RB
@HWI-EAS443:3:42:776:1228#0/1
GAAAGCTAATGTGCTAATTGGGCAAGGGGACGTAGTGTTG
+HWI-EAS443:3:42:776:1228#0/1
ab]bb`bbabbaabbbaabbb`bbab`b`Uab^_b`a__a
@HWI-EAS443:3:42:777:1335#0/1
ATGAACTGAGCAATCTTGCAAAAGGAGAGAAAGCTAATGT
+HWI-EAS443:3:42:777:1335#0/1
ab`]_aaaaba`[X`aa`aaaZ]a`_a_a```aa^U]`^R
```

```
@HWI-EAS443:3:42:846:1011#0/1
CATCAATGAACTGAGCAATCTTGCAAAAGGAGAGAAAGCT
+HWI-EAS443:3:42:846:1011#0/1
abbbbbbbbbaaaaaaabaaaabaa`aaaa`aaaaaaba`
@HWI-EAS443:3:42:920:1540#0/1
GAACTGAGCAATCTTGCAAAAGGAGAGAAAGCTAATGTGC
+HWI-EAS443:3:42:920:1540#0/1
aaaab``_aba`aaabbaa`abaa`aaaaaa^_`_aa`a]
@HWI-EAS443:3:43:1107:30#0/1
GAAAGCTAATGTGCTAATTGGGCAAGGGGACGTAGTGTTG
+HWI-EAS443:3:43:1107:30#0/1
abbbaaaZ_baab`aabbbbbb__`baaa`Yb_Ua^a`_a
@HWI-EAS443:3:43:1236:608#0/1
GCATCAATGAACTGAGCAATCTTGCAAAAGGAGAGAAAGC
+HWI-EAS443:3:43:1236:608#0/1
aa_a_a`baa`aa`aa_a`a_aab___]`ab_a`b`__a^
@HWI-EAS443:3:43:1455:1254#0/1
GAAAGCTAATGTGCTAATTGGGCAAGGGGACGTAGTGTTG
+HWI-EAS443:3:43:1455:1254#0/1
abaaa]ba`ba¥`¥a_`b_a`aY_``a^aV[a]_]WaO__
@HWI-EAS443:3:43:1515:425#0/1
AATGAACTGAGCAATCTTGCAAAAGGAGAGAAAGCTAATG
+HWI-EAS443:3:43:1515:425#0/1
aabbVbaaW^`bbabaaaaa__aabb`_]¥`]Z_Y^^_aa
@HWI-EAS443:3:43:151:1525#0/1
GAGCAATCTTGCAAAAGGAGAGAAAGCTAATGTGCTAATT
+HWI-EAS443:3:43:151:1525#0/1
Z[¥bbabbb__Y^__ba]abbbb`[bbaabba]bababba
@HWI-EAS443:3:43:1582:444#0/1
AGCAATCTTGCAAAAGGAGAGAAAGCTAATGTGCTAATTG
+HWI-EAS443:3:43:1582:444#0/1
`baaaaR¥^][UYU¥a`Wa[^``YYV__^_a[¥V^RZ__X
@HWI-EAS443:3:43:1591:476#0/1
AGCAATCTTGCAAAAGGAGAGAAAGCTAATGTGCTAATTG
+HWI-EAS443:3:43:1591:476#0/1
W`GXbb[abb__]a¥ba`a`aba¥a_`bTbb^b[b^a_aa
@HWI-EAS443:3:43:1679:713#0/1
GCATCAATGAACTGAGCAATCTTGCAAAAGGAGAGAAAGC
+HWI-EAS443:3:43:1679:713#0/1
aaab`a`ba^aaaa_aaaaaaaaaa``__aa_aaa_`__[
@HWI-EAS443:3:43:171:213#0/1
CATCAATGAACTGAGCAATCTTGCAAAAGGAGAGAAAGCT
+HWI-EAS443:3:43:171:213#0/1
aaabbabaaaaaaaaaaaba¥aab`__`abaaaaaa`Ya_`
@HWI-EAS443:3:43:1739:404#0/1
AGCAATCTTGCAAAAGGAGAGAAAGCTAATGTGCTAATTG
+HWI-EAS443:3:43:1739:404#0/1
abbaaaaa`¥`aa__ba^aaaa`aa`a`_aa^`^_^_`aa
@HWI-EAS443:3:43:1757:444#0/1
AAGCTAATGTGCTAATTGGGCAAGGGGACGTAGTGTTGGT
```

```
+HWI-EAS443:3:43:1757:444#0/1
aabaaaaaba_`aa`babaa^_a`aaaYXa^^b_aX[a¥I
@HWI-EAS443:3:43:1779:899#0/1
AAAGCTAATGTGCTAATTGGGCAAGGGGACGTAGTGTTGG
+HWI-EAS443:3:43:1779:899#0/1
^`X```T^`a¥`]a`]``a`Z^`__a`aZ[aVX¥Q[[[`[
@HWI-EAS443:3:43:24:985#0/1
AGCACATTAGCTTTCTCTCCTTTTGCAAGATTGCTCAGTT
+HWI-EAS443:3:43:24:985#0/1
``ababaaabaaaa_aaaa`a_``aa`[`¥___`^``a^`
@HWI-EAS443:3:43:252:932#0/1
AATGAACTGAGCAATCTTGCAAAAGGAGAGAAAGCTAATG
+HWI-EAS443:3:43:252:932#0/1
RYba[a`bb`^¥a``¥bbbbb¥_ab`baYab__b`b`bab
@HWI-EAS443:3:43:259:1058#0/1
GCATCAATGAACTGAGCAATCTTGCAAAAGGAGAGAAAGC
+HWI-EAS443:3:43:259:1058#0/1
_a_H¥Ya¥a`^OWSWa^``_X_V]_HYHY^]NVU^XUORU
@HWI-EAS443:3:43:280:306#0/1
GAGAAAGCTAATGTGCTAATTGGGCAAGGGGACGTAGTGT
+HWI-EAS443:3:43:280:306#0/1
a``aaaaa`_`aa^a`a_``_a__^¥]a]__Y^aYY`Y`Y
@HWI-EAS443:3:43:322:1711#0/1
GAGAAAGCTAATGTGCTAATTGGGCAAGGGGACGTAGTGT
+HWI-EAS443:3:43:322:1711#0/1
aXW`_``abba`_WLYaTa`_a]_`^_aUa^_YbO_`O[V
@HWI-EAS443:3:43:363:931#0/1
AATTAGCACATTAGCTTTCTCTCCTTTTGCAAGATTGCTC
+HWI-EAS443:3:43:363:931#0/1
aaaS_Iaba_a^W^U`aaa`_`[_a`aaY`_]`^___¥^^
@HWI-EAS443:3:43:369:1555#0/1
GAGCAATCTTGCAAAAGGAGAGAAAGCTAATGTGCTAATT
+HWI-EAS443:3:43:369:1555#0/1
`TU_¥bb_`_W^`_ZX¥``[ababaYR[`a`aYS`baab_
@HWI-EAS443:3:43:374:1272#0/1
CATCAATGAACTGAGCAATCTTGCAAAAGGAGAGAAAGCT
+HWI-EAS443:3:43:374:1272#0/1
abbaabbbabbbaaaababbbabaaaaabaaaaabbaa_a
@HWI-EAS443:3:43:384:564#0/1
CATCAATGAACTGAGCAATCTTGCAAAAGGAGAGAAAGCT
+HWI-EAS443:3:43:384:564#0/1
[bbabaabbabbbabbabb_bbb`baba_babbbaaabab
@HWI-EAS443:3:43:417:645#0/1
GAGCAATCTTGCAAAAGGAGAGAAAGCTAATGTGCTAATT
+HWI-EAS443:3:43:417:645#0/1
a^bbbbbbbbaaaaaabbaaaabaaaaaaaaa_a`aa_aa
@HWI-EAS443:3:43:433:1562#0/1
GAAAGCTAATGTGCTAATTGGGCAAGGGGACGTAGTGTTG
+HWI-EAS443:3:43:433:1562#0/1
`a[a][___a_¥_]`[[^_^_]_]`]_a^^]aW_]Y_]^^
```

```
@HWI-EAS443:3:43:443:1726#0/1
CATCAATGAACTGAGCAATCTTGCAAAAGGAGAGAAAGCT
+HWI-EAS443:3:43:443:1726#0/1
aabaaababaaa__aabba`_aaaa`aaaba`aaa_aaaa
@HWI-EAS443:3:43:55:194#0/1
AGAGAAAGCTAATGTGCTAATTGGGCAAGGGGACGTAGTG
+HWI-EAS443:3:43:55:194#0/1
aaabaXabaaaaab^aaaa_a`a`a`_`aaa_]]a^^aZa
@HWI-EAS443:3:43:750:1307#0/1
AGCTTTCTCTCCTTTTGCAAGATTGCTCAGTTCATTGATG
+HWI-EAS443:3:43:750:1307#0/1
aa_^__a]]a``¥`¥_^``^_]`^^U`^VaZ^¥`_X^^__
@HWI-EAS443:3:43:792:1128#0/1
AGCTAATGTGCTAATTGGGCAAGGGGACGTAGTGTTGGTA
+HWI-EAS443:3:43:792:1128#0/1
a`_a`aaa_aaa`_aaa`_]_^a`a`^_a¥^`Za^a``Z]
@HWI-EAS443:3:44:1006:2032#0/1
GAAAGCTAATGTGCTAATTGGGCAAGGGGACGTAGTGTTG
+HWI-EAS443:3:44:1006:2032#0/1
T^R[``bWb__]^`bYRZN_aa^a]aba`aabLG]]`H¥b
@HWI-EAS443:3:44:1051:395#0/1
AAGCATCAATGAACTGAGCAATCTTGCAAAAGGAGAGAAA
+HWI-EAS443:3:44:1051:395#0/1
aaa^a]a]aa`]aYY[[^`__a_^__M]^TR]`^a]a^_Z
@HWI-EAS443:3:44:1174:861#0/1
GAAAGCTAATGTGCTAATTGGGCAAGGGGACGTAGTGTTG
+HWI-EAS443:3:44:1174:861#0/1
aYS]b]_NVaPNaU`X[Raa`]MZX[a]^RT`POYTXX_[
@HWI-EAS443:3:44:1192:1352#0/1
AAGCATCAATGAACTGAGCAATCTTGCAAAAGGAGAGAAA
+HWI-EAS443:3:44:1192:1352#0/1
aaa```a`aa_``aaa`aaaa`T```__Y_^a_]a`a^^]
@HWI-EAS443:3:44:126:415#0/1
GAACTGAGCAATCTTGCAAAAGGAGAGAAAGCTAATGTGC
+HWI-EAS443:3:44:126:415#0/1
aaaaaaaaaaaaaaaa_`_`_a`^a_a``^a____a_Y`[
@HWI-EAS443:3:44:1373:539#0/1
GAACTGAGCAATCTTGCAAAAGGAGAGAAAGCTAATGTGC
+HWI-EAS443:3:44:1373:539#0/1
a`a`a^]aaa_aaa`a[`]V[a`]a```_^a^`]^_`]aV
@HWI-EAS443:3:44:1402:708#0/1
AAGCATCAATGAACTGAGCAATCTTGCAAAAGGAGAGAAA
+HWI-EAS443:3:44:1402:708#0/1
¥¥b]Zaa```aZ]]aaYa_a]a_a_a_`¥]`ba^a````]
@HWI-EAS443:3:44:1691:200#0/1
GAACTGAGCAATCTTGCAAAAGGAGAGAAAGCTAATGTGC
+HWI-EAS443:3:44:1691:200#0/1
aaaaaa[aaaabaaaaV`a]_a__a`aa`_a]`_^`_]_Y
@HWI-EAS443:3:44:1758:1065#0/1
GAACTGAGCAATCTTGCAAAAGGAGAGAAAGCTAATGTGC
```

```
+HWI-EAS443:3:44:1758:1065#0/1
]RT[^¥]¥_[Y¥Z^b`Z_ZZYa^S__bb^^ba^NPU__]U
@HWI-EAS443:3:44:231:1382#0/1
GCATCAATGAACTGAGCAATCTTGCAAAAGGAGAGAAAGC
+HWI-EAS443:3:44:231:1382#0/1
^^`a]baaZ[abYW`aa_V__a`¥aY`_W]`^`a_a_`^J
@HWI-EAS443:3:44:237:1480#0/1
ATGAACTGAGCAATCTTGCAAAAGGAGAGAAAGCTAATGT
+HWI-EAS443:3:44:237:1480#0/1
¥b`W``_Z¥`]`_aaa^Z][``^b__aaaa`¥`O_a_^^P
@HWI-EAS443:3:44:370:361#0/1
ATCTTGCAAAAGGAGAGAAAGCTAATGTGCTAATTGGGCA
+HWI-EAS443:3:44:370:361#0/1
`^baa]]a]aaabY`¥aa]aa`]W_aa`a`a``aY`a`_^
@HWI-EAS443:3:44:564:411#0/1
CTTGCAAAAGGAGAGAAAGCTAATGTGCTAATTGGGCAAG
+HWI-EAS443:3:44:564:411#0/1
abbbbbb``bb`babb]abba`abb]aaba`aaaaba^]]
@HWI-EAS443:3:44:743:465#0/1
GAGAAAGCTAATGTGCTAATTGGGCAAGGGGACGTAGTGT
+HWI-EAS443:3:44:743:465#0/1
aaba`a_``__aa_a^a^_a`a`^¥^]aa`a[Ya^Xa]`¥
@HWI-EAS443:3:44:772:610#0/1
TGCAAAAGGAGAGAAAGCTAATGTGCTAATTGGGCAAGGG
+HWI-EAS443:3:44:772:610#0/1
a`aW`aab``aaba`aa`_`_aa^a[___a`a_``_R__a
@HWI-EAS443:3:44:790:1054#0/1
GAAAGCTAATGTGCTAATTGGGCAAGGGGACGTAGTGTTG
+HWI-EAS443:3:44:790:1054#0/1
aa^YY_Z¥_a]T¥^aYW]_YZPR`Y]a[¥P[a]RYTYW_^
@HWI-EAS443:3:44:825:67#0/1
TGCAAAAGGAGAGAAAGCTAATGTGCTAATTGGGCAAGGG
+HWI-EAS443:3:44:825:67#0/1
`ba__]_ba[aaa^`^`__^^_`X`^^]^_^_^^ZZ^^^^
@HWI-EAS443:3:44:852:228#0/1
AATGAACTGAGCAATCTTGCAAAAGGAGAGAAAGCTAATG
+HWI-EAS443:3:44:852:228#0/1
aaaa_a`aWY_aa_a_aaa```__a`]a`a`^_`S[[^``
@HWI-EAS443:3:44:874:1589#0/1
GAGCAATCTTGCAAAAGGAGAGAAAGCTAATGTGCTAATT
+HWI-EAS443:3:44:874:1589#0/1
aaaaaaaaaaaaa_```_^`a_aa^aZaa`aaW`_a__^Z
@HWI-EAS443:3:44:94:1721#0/1
GAGCAATCTTGCAAAAGGAGAGAAAGCTAATGTGCTAATT
+HWI-EAS443:3:44:94:1721#0/1
WJ¥abbbaa`ab_a]aaa_aa`a`^]_b`aa`¥a_^aZ``
@HWI-EAS443:3:45:1171:700#0/1
AGCACATTAGCTTTCTCTCCTTTTGCAAGATTGCTCAGTT
+HWI-EAS443:3:45:1171:700#0/1
aaababbab`a`]aa_]aaaaaaa``_`_aaa_`_``^_
```

```
@HWI-EAS443:3:45:1261:1614#0/1
GAGCAATCTTGCAAAAGGAGAGAAAGCTAATGTGCTAATT
+HWI-EAS443:3:45:1261:1614#0/1
[a`_``[`b¥`a[aaXZ¥^a^ab]a`P_`XX``]a`````
@HWI-EAS443:3:45:1265:1434#0/1
AATGAACTGAGCAATCTTGCAAAAGGAGAGAAAGCTAATG
+HWI-EAS443:3:45:1265:1434#0/1
`Zba`bbb`]abbaa`baba_b¥bab_ba_aba`abaabb
@HWI-EAS443:3:45:1336:677#0/1
GAGCAATCTTGCAAAAGGAGAGAAAGCTAATGTGCTAATT
+HWI-EAS443:3:45:1336:677#0/1
`_baa]b`Y`b¥¥L^^]_Xa[`[VH`_`Y¥]b_a[a[[]^
@HWI-EAS443:3:45:1542:1179#0/1
AGCTAATGTGCTAATTGGGCAAGGGGACGTAGTGTTGGTA
+HWI-EAS443:3:45:1542:1179#0/1
^bU`^^XWVba¥FRa`a`aa`Ua_aUWa``Zb^W_a_RFU
@HWI-EAS443:3:45:1546:984#0/1
ATCTTGCAAAAGGAGAGAAAGCTAATGTGCTAATTGGGCA
+HWI-EAS443:3:45:1546:984#0/1
aa]a`aYa___^a]]`b_a`a[_Y`a_QXX^`^_```_a`
@HWI-EAS443:3:45:1556:1746#0/1
GAAAGCTAATGTGCTAATTGGGCAAGGGGACGTAGTGTTG
+HWI-EAS443:3:45:1556:1746#0/1
XY_aaaabaa`O¥bbaabbaaa]aabbab_abY`aZ`^a[
@HWI-EAS443:3:45:156:768#0/1
AAGCATCAATGAACTGAGCAATCTTGCAAAAGGAGAGAAA
+HWI-EAS443:3:45:156:768#0/1
aaaaaa_`aa``a^`_`a`a^__a````Z__aa_a_aa[_
@HWI-EAS443:3:45:1606:1537#0/1
GAGCAATCTTGCAAAAGGAGAGAAAGCTAATGTGCTAATT
+HWI-EAS443:3:45:1606:1537#0/1
^^b[`bbb`bbaaab`bbababZQ`b`_`T^b_b]aa`aa
@HWI-EAS443:3:45:1662:240#0/1
GAGCAATCTTGCAAAAGGAGAGAAAGCTAATGTGCTAATT
+HWI-EAS443:3:45:1662:240#0/1
Z_bbbbbbbabbb^baaaabbba`ab^babbbXb^aa`aa
@HWI-EAS443:3:45:1689:134#0/1
AACACTACGTCCCCTTGCCCAATTAGCACATTAGCTTTCT
+HWI-EAS443:3:45:1689:134#0/1
aaaa`a``aZ]¥¥Y^`]]]Y¥[VYUZ[WYY^^]^UY]]Y¥
@HWI-EAS443:3:45:1746:92#0/1
GAAAGCTAATGTGCTAATTGGGCAAGGGGACGTAGTGTTG
+HWI-EAS443:3:45:1746:92#0/1
aa^abaa`aab`b_a__aaba_]__baa`YXa]X`Ya]_`
@HWI-EAS443:3:45:183:635#0/1
AAGCATCAATGAACTGAGCAATCTTGCAAAAGGAGAGAAA
+HWI-EAS443:3:45:183:635#0/1
a`__aaaZ``^¥¥_aa_b_a`aaaaa_``a¥]S_`^Z^V¥
@HWI-EAS443:3:45:204:73#0/1
GAACTGAGCAATCTTGCAAAAGGAGAGAAAGCTAATGTGC
```

```
+HWI-EAS443:3:45:204:73#0/1
`GZRU¥P_YXY__^_`U[^UMX[U^]_]VU_VUUWYZUVJ
@HWI-EAS443:3:45:271:406#0/1
AAGCTAATGTGCTAATTGGGCAAGGGGACGTAGTGTTGGT
+HWI-EAS443:3:45:271:406#0/1
aabaa`abb`b`ababbbaaa``baaa__b__b_b^aaaR
@HWI-EAS443:3:45:279:13#0/1
AAGCATCAATGAACTGAGCAATCTTGCAAAAGGAGAGAAA
+HWI-EAS443:3:45:279:13#0/1
^^_`aa`¥`a^]a_aa]`a`]^aa__`]VV^a`S`_a][Z
@HWI-EAS443:3:45:285:898#0/1
ATGAACTGAGCAATCTTGCAAAAGGAGAGAAAGCTAATGT
+HWI-EAS443:3:45:285:898#0/1
aaa`]bb`X`aa¥X^aabaaa__b__a``aa_a_a[W`a]
@HWI-EAS443:3:45:332:1372#0/1
CAATCTTGCAAAAGGAGAGAAAGCTAATGTGCTAATTGGG
+HWI-EAS443:3:45:332:1372#0/1
__^b^b^bba¥aWb`G`abaaab``Y`baZa_a`a^^``[
@HWI-EAS443:3:45:421:424#0/1
GAAAGCTAATGTGCTAATTGGGCAAGGGGACGTAGTGTTG
+HWI-EAS443:3:45:421:424#0/1
ab_^aa]`^ab_a¥a``aa]a_^`Wa_TaRUaW]a]aW__
@HWI-EAS443:3:45:460:535#0/1
AAAAGGAGAGAAAGCTAATGTGCTAATTGGGCAAGGGGAC
+HWI-EAS443:3:45:460:535#0/1
a`^abbababba```aaaab]aaa`aaaaaa¥__aa¥_]]
@HWI-EAS443:3:45:469:1825#0/1
GAGCAATCTTGCAAAAGGAGAGAAAGCTAATGTGCTAATT
+HWI-EAS443:3:45:469:1825#0/1
¥`bab¥Mbbaaa`baaba`ab_a`_b^_abba[^a^a`b`
@HWI-EAS443:3:45:473:1154#0/1
ATCTTGCAAAAGGAGAGAAAGCTAATGTGCTAATTGGGCA
+HWI-EAS443:3:45:473:1154#0/1
aaabababaaa`aaa_aabaa`a_aa`aa^a`a_`a``_a__
@HWI-EAS443:3:45:482:441#0/1
GAAAGCTAATGTGCTAATTGGGCAAGGGGACGTAGTGTTG
+HWI-EAS443:3:45:482:441#0/1
a`[[`[`_^``WaPa[Y^_a]ZUX]a`__^]`XX`R^Z^^
@HWI-EAS443:3:45:564:318#0/1
GAAAGCTAATGTGCTAATTGGGCAAGGGGACGTAGTGTTG
+HWI-EAS443:3:45:564:318#0/1
abaaaaaaaba_a^aaaaaaa`a_aaaa`__a^_aYa__a
@HWI-EAS443:3:45:691:1886#0/1
GAACTGAGCAATCTTGCAAAAGGAGAGAAAGCTAATGTGC
+HWI-EAS443:3:45:691:1886#0/1
¥X`^^_TV]b`b`X¥XQT__`__a^aaba^T¥``^YaVYU
@HWI-EAS443:3:45:801:1573#0/1
AGCTAATGTGCTAATTGGGCAAGGGGACGTAGTGTTGGTA
+HWI-EAS443:3:45:801:1573#0/1
¥K_Y¥ZZa`a`]aab`T¥U¥`_a`]`_aaY^_]]Xa`[VV
```

```
@HWI-EAS443:3:45:836:1559#0/1
GAACTGAGCAATCTTGCAAAAGGAGAGAAAGCTAATGTGC
+HWI-EAS443:3:45:836:1559#0/1
aaba`aaaa``a`aaa_aa]_aa_a`aaa`a_`a`___aO
@HWI-EAS443:3:45:910:1257#0/1
AGCACATTAGCTTTCTCTCCTTTTGCAAGATTGCTCAGTT
+HWI-EAS443:3:45:910:1257#0/1
`a`aW_bb`a_W^^]][]Z]`]Z_X^]Z_]_X]¥`¥^_Y]
@HWI-EAS443:3:45:981:638#0/1
CTTGCAAAAGGAGAGAAAGCTAATGTGCTAATTGGGCAAG
+HWI-EAS443:3:45:981:638#0/1
aaaa__aX¥bb_b`aa`ab_`^aba¥``a``aaaYa``[a
@HWI-EAS443:3:46:1018:2012#0/1
CATCAATGAACTGAGCAATCTTGCAAAAGGAGAGAAAGCT
+HWI-EAS443:3:46:1018:2012#0/1
ababaaabaaaa__abb`aaaaa``_^aaaaaaaa`aa^a
@HWI-EAS443:3:46:1045:1069#0/1
AGCTAATGTGCTAATTGGGCAAGGGGACGTAGTGTTGGTA
+HWI-EAS443:3:46:1045:1069#0/1
abab`^abab`a_^abba`aa]aba``Za_``Ua__bXG]
@HWI-EAS443:3:46:1052:1870#0/1
GAGCAATCTTGCAAAAGGAGAGAAAGCTAATGTGCTAATT
+HWI-EAS443:3:46:1052:1870#0/1
`abbbbbbbbbaabbb_aabbbbbaa`aaaaa``aa`aa`
@HWI-EAS443:3:46:1150:1081#0/1
AGCTAATGTGCTAATTGGGCAAGGGGACGTAGTGTTGGTA
+HWI-EAS443:3:46:1150:1081#0/1
XW_SKU_¥ZQ_a__]`Z[a^^`a¥__YZ`¥Z^¥b]^a_JJ
@HWI-EAS443:3:46:1205:305#0/1
CATCAATGAACTGAGCAATCTTGCAAAAGGAGAGAAAGCT
+HWI-EAS443:3:46:1205:305#0/1
^aa_U¥abbaZ`b[`bb_babab`_ZZ]bb_bbbbaab^a
@HWI-EAS443:3:46:1270:2016#0/1
ATCTTGCAAAAGGAGAGAAAGCTAATGTGCTAATTGGGCA
+HWI-EAS443:3:46:1270:2016#0/1
a_aa]aaa`^a_¥`_^aa`_`]aa`__]^]___Z^^_Z¥_
@HWI-EAS443:3:46:1287:623#0/1
CTTGCAAAAGGAGAGAAAGCTAATGTGCTAATTGGGCAAG
+HWI-EAS443:3:46:1287:623#0/1
``a_V_R_W`aX`]^^_[_X__a_^UaY_``aaa_ZW[Za
@HWI-EAS443:3:46:1375:1369#0/1
GCATCAATGAACTGAGCAATCTTGCAAAAGGAGAGAAAGC
+HWI-EAS443:3:46:1375:1369#0/1
`]Y[V]Y_V¥]YT]]^[Y]YY[_`¥YVXSXZZ_^[SMWSF
@HWI-EAS443:3:46:1393:1659#0/1
CCCTTGCCCAATTAGCACATTAGCTTTCTCTCCTTTTGCA
+HWI-EAS443:3:46:1393:1659#0/1
`b`a]`aa`aaaaa_`aab`aba¥aba_a`a__``a`_Sa
@HWI-EAS443:3:46:145:50#0/1
GAGCAATCTTGCAAAAGGAGAGAAAGCTAATGTGCTAATT
```

```
+HWI-EAS443:3:46:145:50#0/1
aaabbYbbaabaa^aaba_aabbaabaaa`ab_aab__b_
@HWI-EAS443:3:46:164:351#0/1
GCATCAATGAACTGAGCAATCTTGCAAAAGGAGAGAAAGC
+HWI-EAS443:3:46:164:351#0/1
a`aa`aabbaaaaaaba`aaaaaa_a_]^ba_a_aa__a_
@HWI-EAS443:3:46:1748:490#0/1
GTCCCCTTGCCCAATTAGCACATTAGCTTTCTCTCCTTTT
+HWI-EAS443:3:46:1748:490#0/1
a`aaa`aaaa``a`aaaa`a^aa``a_aa`^a_a]]``a^
@HWI-EAS443:3:46:189:113#0/1
GTCCCCTTGCCCAATTAGCACATTAGCTTTCTCTCCTTTT
+HWI-EAS443:3:46:189:113#0/1
a`_``_`a_a_]``______W_`]_[U``^[_X_^T`^]]
@HWI-EAS443:3:46:443:233#0/1
GCAAAAGGAGAGAAAGCTAATGTGCTAATTGGGCAAGGGG
+HWI-EAS443:3:46:443:233#0/1
aaaYZ_b_Taa][__bY`_`_a_a^`[___aaZ__]`aa_
@HWI-EAS443:3:46:474:628#0/1
GAACTGAGCAATCTTGCAAAAGGAGAGAAAGCTAATGTGC
+HWI-EAS443:3:46:474:628#0/1
``aaaaab```aaaabaa_]_a]_aaaa``a_aa``a_a`
@HWI-EAS443:3:46:479:1360#0/1
AAAGCTAATGTGCTAATTGGGCAAGGGGACGTAGTGTTGG
+HWI-EAS443:3:46:479:1360#0/1
aaaa`aa¥^aU¥aa^aa_a_¥YWZa]`_[^[V^aTaUSaa
@HWI-EAS443:3:46:585:1868#0/1
TGCAAAAGGAGAGAAAGCTAATGTGCTAATTGGGCAAGGG
+HWI-EAS443:3:46:585:1868#0/1
]R_baZ¥a_a]_aY]a_]X^ZZ]ZWaaX`]R_aa`aZa]a
@HWI-EAS443:3:46:732:730#0/1
GAAAGCTAATGTGCTAATTGGGCAAGGGGACGTAGTGTTG
+HWI-EAS443:3:46:732:730#0/1
aa_]a`a``aa]a_```a`a_a`^^a`_`^]a^^`U`W__
@HWI-EAS443:3:46:759:467#0/1
AGCTAATGTGCTAATTGGGCAAGGGGACGTAGTGTTGGTA
+HWI-EAS443:3:46:759:467#0/1
]`__aV_`_ba`RXaabbb`a]`aa]WS`_XaSaaab`Y]
@HWI-EAS443:3:46:785:626#0/1
GTCCCCTTGCCCAATTAGCACATTAGCTTTCTCTCCTTTT
+HWI-EAS443:3:46:785:626#0/1
aaaa¥aa`[aaaa`a`[^_Z_aaa_a^a``Z`¥``[^__]
@HWI-EAS443:3:46:870:801#0/1
GTCCCCTTGCCCAATTAGCACATTAGCTTTCTCTCCTTTT
+HWI-EAS443:3:46:870:801#0/1
a`aaaaa``aaYaa``]^`a^aaa^`Zaa`X`_a`[aaa^
@HWI-EAS443:3:46:921:1966#0/1
CAATGAACTGAGCAATCTTGCAAAAGGAGAGAAAGCTAAT
+HWI-EAS443:3:46:921:1966#0/1
^Mab_]¥Z_Za```T[Q¥aaaZ`a_``_[`aa]UDV_a__
```

```
@HWI-EAS443:3:47:1030:143#0/1
TTAGCACATTAGCTTTCTCTCCTTTTGCAAGATTGCTCAG
+HWI-EAS443:3:47:1030:143#0/1
_X]]_YOSZ[W`PY`aaXQ[VFYX[XSOTP_XXT]SVQU^
@HWI-EAS443:3:47:1073:593#0/1
CAATGAACTGAGCAATCTTGCAAAAGGAGAGAAAGCTAAT
+HWI-EAS443:3:47:1073:593#0/1
abbbaabbbb`a_abbabbb`_]_aba_babaaaa^baaa
@HWI-EAS443:3:47:1171:99#0/1
GAAAGCTAATGTGCTAATTGGGCAAGGGGACGTAGTGTTG
+HWI-EAS443:3:47:1171:99#0/1
a`a_a_aa_ab]a_a__`a``]]]Va___YV_YV_Y_Y^^
@HWI-EAS443:3:47:122:1016#0/1
ATCTTGCAAAAGGAGAGAAAGCTAATGTGCTAATTGGGCA
+HWI-EAS443:3:47:122:1016#0/1
aa^Y``^ZbaUab[b`[aa`JY¥a`_^]`_a__U]¥_¥NN
@HWI-EAS443:3:47:1236:1550#0/1
ATCTTGCAAAAGGAGAGAAAGCTAATGTGCTAATTGGGCA
+HWI-EAS443:3:47:1236:1550#0/1
a_bbaa`a`a`ba^aaaaaa`_a_aaa_a^¥_``a_a`¥W
@HWI-EAS443:3:47:1256:1836#0/1
GAGCAATCTTGCAAAAGGAGAGAAAGCTAATGTGCTAATT
+HWI-EAS443:3:47:1256:1836#0/1
`[`^`b^Z``aaa_`__`abaaaa¥¥Y]`a`a^][`X^_[
@HWI-EAS443:3:47:1342:145#0/1
AACTGAGCAATCTTGCAAAAGGAGAGAAAGCTAATGTGCT
+HWI-EAS443:3:47:1342:145#0/1
aaaab`aaa`aaaaa__`_]`a_a_a_]]b]__¥_aY`Y`
@HWI-EAS443:3:47:136:1118#0/1
GTCCCCTTGCCCAATTAGCACATTAGCTTTCTCTCCTTTT
+HWI-EAS443:3:47:136:1118#0/1
aaaaabaab`a`aabab``a]aaaaa_aaa`a_a`_aaa`
@HWI-EAS443:3:47:1569:114#0/1
AATGAACTGAGCAATCTTGCAAAAGGAGAGAAAGCTAATG
+HWI-EAS443:3:47:1569:114#0/1
abbb`__bb`baa]aaba`X``a`ba_a`ba_`a¥_`¥_a
@HWI-EAS443:3:47:1647:1289#0/1
ATGAACTGAGCAATCTTGCAAAAGGAGAGAAAGCTAATGT
+HWI-EAS443:3:47:1647:1289#0/1
ab_^baaaabaaaa_aab`_V__aa^``a``_a^`_]``Y
@HWI-EAS443:3:47:165:877#0/1
GAAAGCTAATGTGCTAATTGGGCAAGGGGACGTAGTGTTG
+HWI-EAS443:3:47:165:877#0/1
abbbbbbb`babbabbbbbabbbbabbbaaa`aaaa`abb
@HWI-EAS443:3:47:1765:1084#0/1
GCATCAATGAACTGAGCAATCTTGCAAAAGGAGAGAAAGC
+HWI-EAS443:3:47:1765:1084#0/1
a`][^`¥^a__¥__]a¥¥_`^`^`YZY¥Y__Y_]_]]Y^W
@HWI-EAS443:3:47:233:356#0/1
CGTCCCCTTGCCCAATTAGCACATTAGCTTTCTCTCCTTT
```

+HWI-EAS443:3:47:233:356#0/1
abaa`baa`aa]^^`bb]]M]]^aa^`^``_V`T`[[a_`
@HWI-EAS443:3:47:255:318#0/1
GCATCAATGAACTGAGCAATCTTGCAAAAGGAGAGAAAGC
+HWI-EAS443:3:47:255:318#0/1
`TLNOQa[ZUY]^YRRVX[_[__`^_aR^a`Wa]`^SZYN
@HWI-EAS443:3:47:309:40#0/1
GAGCAATCTTGCAAAAGGAGAGAAAGCTAATGTGCTAATT
+HWI-EAS443:3:47:309:40#0/1
abb`babbbabaa`baabaaaba`aa_a``aa_a^`a_aa
@HWI-EAS443:3:47:344:1713#0/1
GAGAAAGCTAATGTGCTAATTGGGCAAGGGGACGTAGTGT
+HWI-EAS443:3:47:344:1713#0/1
]^YXa`TabbbbaIb``][a[`Q`]aaa¥X`Sab]^aR`U
@HWI-EAS443:3:47:586:252#0/1
AATGAACTGAGCAATCTTGCAAAAGGAGAGAAAGCTAATG
+HWI-EAS443:3:47:586:252#0/1
abbbKbbaabb_`[`[bbbaaaaab``aaaaaa`R[a`ab
@HWI-EAS443:3:47:638:1489#0/1
GAGCAATCTTGCAAAAGGAGAGAAAGCTAATGTGCTAATT
+HWI-EAS443:3:47:638:1489#0/1
aaaaaaa`baa`_]__aV^__`_`_`U]__`aO____^_^
@HWI-EAS443:3:47:755:914#0/1
AGCTAATGTGCTAATTGGGCAAGGGGACGTAGTGTTGGTA
+HWI-EAS443:3:47:755:914#0/1
_X`aaaabWb_aYY^¥aaY_`_a_^XS_a]ZYW_]__`UT
@HWI-EAS443:3:47:78:291#0/1
TTAGCACATTAGCTTTCTCTCCTTTTGCAAGATTGCTCAG
+HWI-EAS443:3:47:78:291#0/1
ababa¥`aba_[`a`^`a__¥Va]aYa[a^a___^[`¥]_
@HWI-EAS443:3:47:993:1551#0/1
ATCTTGCAAAAGGAGAGAAAGCTAATGTGCTAATTGGGCA
+HWI-EAS443:3:47:993:1551#0/1
`baabaaaa`U`ZJ]``````[_XZ`^Y`__¥_[W``]U^
@HWI-EAS443:3:48:1013:554#0/1
CATCAATGAACTGAGCAATCTTGCAAAAGGAGAGAAAGCT
+HWI-EAS443:3:48:1013:554#0/1
abbaaabbabbbaaaaabbaaab`aaaabb`babaaYa_a
@HWI-EAS443:3:48:1040:518#0/1
GAACTGAGCAATCTTGCAAAAGGAGAGAAAGCTAATGTGC
+HWI-EAS443:3:48:1040:518#0/1
^aaabbabaaabaabbaa`a`ba_aabaa`a_a`_aa]a^
@HWI-EAS443:3:48:1111:426#0/1
GCAAAAGGAGAGAAAGCTAATGTGCTAATTGGGCAAGGGG
+HWI-EAS443:3:48:1111:426#0/1
a_aa_`aa^a_a_]`a_``___]a]a_^```__]]]`_`_
@HWI-EAS443:3:48:1229:1311#0/1
ATCTTGCAAAAGGAGAGAAAGCTAATGTGCTAATTGGGCA
+HWI-EAS443:3:48:1229:1311#0/1
`^b¥Z]`^Y`¥_¥X``aaZa_Za`T`aW`aaa`^Yaa_`_

```
@HWI-EAS443:3:48:1254:1456#0/1
GAGCAATCTTGCAAAAGGAGAGAAAGCTAATGTGCTAATT
+HWI-EAS443:3:48:1254:1456#0/1
aababbbbbabaaab_bbababbaab`aa`ab^a`aa[a_
@HWI-EAS443:3:48:1300:1830#0/1
GAGCAATCTTGCAAAAGGAGAGAAAGCTAATGTGCTAATT
+HWI-EAS443:3:48:1300:1830#0/1
[^aaaba_`aababa[b`bbbbabWb_bbaba`[_abaab
@HWI-EAS443:3:48:1422:194#0/1
AGCAATCTTGCAAAAGGAGAGAAAGCTAATGTGCTAATTG
+HWI-EAS443:3:48:1422:194#0/1
aa_aabaa_aaa`^aaa_a_aaaZa^a_ZaaZa^a_^_]_
@HWI-EAS443:3:48:1444:1142#0/1
GAAAGCTAATGTGCTAATTGGGCAAGGGGACGTAGTGTTG
+HWI-EAS443:3:48:1444:1142#0/1
_V][QWWZXS^`_Z¥RU¥Q`Z]ZXZ^¥UUPTZX]¥L^NZR
@HWI-EAS443:3:48:1470:776#0/1
GCATCAATGAACTGAGCAATCTTGCAAAAGGAGAGAAAGC
+HWI-EAS443:3:48:1470:776#0/1
__`b_aa``Xa`a_]a¥a_`_aaa]`_¥U_`]a`a``]_]
@HWI-EAS443:3:48:1558:1153#0/1
GAGCAATCTTGCAAAAGGAGAGAAAGCTAATGTGCTAATT
+HWI-EAS443:3:48:1558:1153#0/1
WVaWa_`¥`__V[QQ[^[U[[`¥_I^X¥_UZ_X_aW]]^`
@HWI-EAS443:3:48:1612:672#0/1
TGCAAAAGGAGAGAAAGCTAATGTGCTAATTGGGCAAGGG
+HWI-EAS443:3:48:1612:672#0/1
^_WZ¥^¥¥¥X]W]_b]_WXZa_XQX_aYO]]]]aTRY``a
@HWI-EAS443:3:48:1614:737#0/1
GAGCAATCTTGCAAAAGGAGAGAAAGCTAATGTGCTAATT
+HWI-EAS443:3:48:1614:737#0/1
`^b[[aba[]bbUTQ_]`_aUUWaa_VaUb_`a_H¥`^R]
@HWI-EAS443:3:48:1636:1117#0/1
GAGCAATCTTGCAAAAGGAGAGAAAGCTAATGTGCTAATT
+HWI-EAS443:3:48:1636:1117#0/1
¥K_T¥Xbab¥`T`RWYa`b`[aa_TbaaaabbYaIa]]a¥
@HWI-EAS443:3:48:1639:252#0/1
AATGAACTGAGCAATCTTGCAAAAGGAGAGAAAGCTAATG
+HWI-EAS443:3:48:1639:252#0/1
aaaa`aaba_aaa`a^`aa__W_]^a^a¥a_¥Z_V]^Z`_
@HWI-EAS443:3:48:1645:231#0/1
GAACTGAGCAATCTTGCAAAAGGAGAGAAAGCTAATGTGC
+HWI-EAS443:3:48:1645:231#0/1
a_aaaaab`aaaa`aa^`^_^aa]a^a_¥^_[^^]^`W_X
@HWI-EAS443:3:48:177:1540#0/1
GAGCAATCTTGCAAAAGGAGAGAAAGCTAATGTGCTAATT
+HWI-EAS443:3:48:177:1540#0/1
aab[b_aa`bba``a^``_baaaXaa_`]aaa^aaa``aa
@HWI-EAS443:3:48:191:1892#0/1
GAGCAATCTTGCAAAAGGAGAGAAAGCTAATGTGCTAATT
```

```
+HWI-EAS443:3:48:191:1892#0/1
aL_IZ`YYZ`b_bWTW^^_a[aaaVa_a]YX_PRUZXa]X
@HWI-EAS443:3:48:231:1537#0/1
AATGAACTGAGCAATCTTGCAAAAGGAGAGAAAGCTAATG
+HWI-EAS443:3:48:231:1537#0/1
`[ab_^¥a`_`a__Za___¥ZZ`^_a`aaaaa¥aY_aaaa
@HWI-EAS443:3:48:559:19#0/1
GTCCCCTTGCCCAATTAGCACATTAGCTTTCTCTCCTTTT
+HWI-EAS443:3:48:559:19#0/1
a¥X`[_VRRXSQ]`¥YYZ^XV_^SY[M^_]TYPXQ¥SSSW
@HWI-EAS443:3:48:572:1055#0/1
GAGCAATCTTGCAAAAGGAGAGAAAGCTAATGTGCTAATT
+HWI-EAS443:3:48:572:1055#0/1
aaaYa`aaaaa`a`a¥W`Wa_`a¥aa]``^aaX`_^]]`_
@HWI-EAS443:3:48:707:896#0/1
CGTCCCCTTGCCCAATTAGCACATTAGCTTTCTCTCCTTT
+HWI-EAS443:3:48:707:896#0/1
aaab`bbaabbbbbbbbbb`aaabaaZaabaabaa_`bab
@HWI-EAS443:3:48:796:1466#0/1
AAGCATCAATGAACTGAGCAATCTTGCAAAAGGAGAGAAA
+HWI-EAS443:3:48:796:1466#0/1
a`^aaaaa``aaa`a````aaa_aaa``^^^aa^a__a_^
@HWI-EAS443:3:48:822:61#0/1
GAAAGCTAATGTGCTAATTGGGCAAGGGGACGTAGTGTTG
+HWI-EAS443:3:48:822:61#0/1
^]Zab^Xa¥^b]_S[¥Tb__[VW¥Y_``ZTV_VZaW^S¥^
@HWI-EAS443:3:48:91:761#0/1
AAAGCTAATGTGCTAATTGGGCAAGGGGACGTAGTGTTGG
+HWI-EAS443:3:48:91:761#0/1
aa`a_a``aa_a_`a_aaa[Y¥`Za]YZ[_a^^ZW`^_¥^
@HWI-EAS443:3:48:934:1638#0/1
GAGAAAGCTAATGTGCTAATTGGGCAAGGGGACGTAGTGT
+HWI-EAS443:3:48:934:1638#0/1
a[LY`PWaa_`_]X`TUY^UR^[^V`¥^Y_URQ]R¥_TXZ
@HWI-EAS443:3:48:971:1024#0/1
GAAAGCTAATGTGCTAATTGGGCAAGGGGACGTAGTGTTG
+HWI-EAS443:3:48:971:1024#0/1
`WLZTP¥LMZ`^`^a_`]Y^aX[_]a_`Z[]_UR^U¥[[]
@HWI-EAS443:3:48:988:1189#0/1
CATCAATGAACTGAGCAATCTTGCAAAAGGAGAGAAAGCT
+HWI-EAS443:3:48:988:1189#0/1
^X`bbaa_[^a]_IXT[¥a_ab`ZV_VVbba`ab_UO`__
@HWI-EAS443:3:49:1080:1687#0/1
AATGAACTGAGCAATCTTGCAAAAGGAGAGAAAGCTAATG
+HWI-EAS443:3:49:1080:1687#0/1
^bbbaba`_`babbbabbbaa`^]bb_abba_a`^aaaab
@HWI-EAS443:3:49:1101:1608#0/1
GTCCCCTTGCCCAATTAGCACATTAGCTTTCTCTCCTTTT
+HWI-EAS443:3:49:1101:1608#0/1
]b_a]`R`a^_a`^aaaba]^`aaaaaZaa¥[]TRU_VYa
```

```
@HWI-EAS443:3:49:1162:537#0/1
AGCTAATGTGCTAATTGGGCAAGGGGACGTAGTGTTGGTA
+HWI-EAS443:3:49:1162:537#0/1
abababbbabbbbbbb`bb`abbab``abaabab`bbaX`
@HWI-EAS443:3:49:1173:532#0/1
GAGCAATCTTGCAAAAGGAGAGAAAGCTAATGTGCTAATT
+HWI-EAS443:3:49:1173:532#0/1
`Y`ababab`_`aa^^`¥¥b`aa`_`W__`aX_bYa``aa
@HWI-EAS443:3:49:1247:1044#0/1
AGCTAATGTGCTAATTGGGCAAGGGGACGTAGTGTTGGTA
+HWI-EAS443:3:49:1247:1044#0/1
ababaab_Vaabaabaabb`a_aab^^_b_aa_b^bbaZa
@HWI-EAS443:3:49:1267:1112#0/1
TGCAAAAGGAGAGAAAGCTAATGTGCTAATTGGGCAAGGG
+HWI-EAS443:3:49:1267:1112#0/1
aaaaVa[ba`babaTaa_a_^aa_a^a^_aaa`]^]Yaa`
@HWI-EAS443:3:49:1319:345#0/1
AATGAACTGAGCAATCTTGCAAAAGGAGAGAAAGCTAATG
+HWI-EAS443:3:49:1319:345#0/1
aabbbbabbababbbabbbaaa_ab¥abab```aUa_aab
@HWI-EAS443:3:49:1386:352#0/1
CAATCTTGCAAAAGGAGAGAAAGCTAATGTGCTAATTGGG
+HWI-EAS443:3:49:1386:352#0/1
aa^^^bab]ZaVY_aab`bab[`Ibb_baYa`aX¥Z_```
@HWI-EAS443:3:49:1422:224#0/1
AGCTAATGTGCTAATTGGGCAAGGGGACGTAGTGTTGGTA
+HWI-EAS443:3:49:1422:224#0/1
abab¥Z`_aba^aYbb```aa^aa]b_Xb]XaU¥¥[`¥QO
@HWI-EAS443:3:49:1458:239#0/1
ATCTTGCAAAAGGAGAGAAAGCTAATGTGCTAATTGGGCA
+HWI-EAS443:3:49:1458:239#0/1
aa[abaaaa`a`a`ba`ab`a_]_`a`_a_a`_a^a``V_
@HWI-EAS443:3:49:1470:1485#0/1
TGAGCAATCTTGCAAAAGGAGAGAAAGCTAATGTGCTAAT
+HWI-EAS443:3:49:1470:1485#0/1
_b_aaaab`aabaaaZa`Yaaaaa_^a_a_`aa]_X_^`^
@HWI-EAS443:3:49:1546:864#0/1
AGCAATCTTGCAAAAGGAGAGAAAGCTAATGTGCTAATTG
+HWI-EAS443:3:49:1546:864#0/1
^`Y`aU[YUbaYY^```aa`ab`]b^aa_¥[[XR^LXa``
@HWI-EAS443:3:49:1666:1142#0/1
GTCCCCTTGCCCAATTAGCACATTAGCTTTCTCTCCTTTT
+HWI-EAS443:3:49:1666:1142#0/1
a`YGZUNZaZL]_Y_ZSQN[N[XSMMFY^QKUPMZPUULO
@HWI-EAS443:3:49:1712:1909#0/1
GAGAAAGCTAATGTGCTAATTGGGCAAGGGGACGTAGTGT
+HWI-EAS443:3:49:1712:1909#0/1
``aa``aaa^`a¥K[aa`a`^¥_OWPM¥V_WRKUUTPIYM
@HWI-EAS443:3:49:1725:1552#0/1
AATGAACTGAGCAATCTTGCAAAAGGAGAGAAAGCTAATG
```

```
+HWI-EAS443:3:49:1725:1552#0/1
aabbabaabaaabababab_aaa`ba`baaa`_]N]__aa
@HWI-EAS443:3:49:1778:1578#0/1
GAGCAATCTTGCAAAAGGAGAGAAAGCTAATGTGCTAATT
+HWI-EAS443:3:49:1778:1578#0/1
`^b]¥`baabbaa]a`aa]aba^`]b`bbabb[aOa`_ab
@HWI-EAS443:3:49:274:143#0/1
GAGCAATCTTGCAAAAGGAGAGAAAGCTAATGTGCTAATT
+HWI-EAS443:3:49:274:143#0/1
abbaZ¥XUXXb`aabaaa[baba``a_aa]`YYa^^`[``
@HWI-EAS443:3:49:321:460#0/1
AGCTAATGTGCTAATTGGGCAAGGGGACGTAGTGTTGGTA
+HWI-EAS443:3:49:321:460#0/1
aa¥_]ba`a`aab_bbb``]aa``ab_[a`¥R`bababYS
@HWI-EAS443:3:49:347:53#0/1
GAACTGAGCAATCTTGCAAAAGGAGAGAAAGCTAATGTGC
+HWI-EAS443:3:49:347:53#0/1
a`a¥]`¥a`aaa`aab_]_`^_a^a_a_^XaV`^]aa]_T
@HWI-EAS443:3:49:371:1551#0/1
CAAAAGGAGAGAAAGCTAATGTGCTAATTGGGCAAGGGGA
+HWI-EAS443:3:49:371:1551#0/1
_`]]¥a^b`Ybb^¥]_]_O`T¥YSaUWXS`aNJ`_Q_S`X
@HWI-EAS443:3:49:435:1686#0/1
GCATCAATGAACTGAGCAATCTTGCAAAAGGAGAGAAAGC
+HWI-EAS443:3:49:435:1686#0/1
abaa`bb_aa]abaabaaaa`aba_aaa`ba`aa`aaab`
@HWI-EAS443:3:49:442:1840#0/1
CATCAATGAACTGAGCAATCTTGCAAAAGGAGAGAAAGCT
+HWI-EAS443:3:49:442:1840#0/1
aaabb_`a^¥bbaabb`ab_babbbbb_baabbXb^_b¥X
@HWI-EAS443:3:49:455:355#0/1
CATCAATGAACTGAGCAATCTTGCAAAAGGAGAGAAAGCT
+HWI-EAS443:3:49:455:355#0/1
```]V^bbaba``_b[bb_`]a]]^H¥TbbTbabbXVbSa
@HWI-EAS443:3:49:505:318#0/1
GAACTGAGCAATCTTGCAAAAGGAGAGAAAGCTAATGTGC
+HWI-EAS443:3:49:505:318#0/1
aaa`aaaaaaab`aaaaaa`_`a]aaaaaaa_a`^aaYa^
@HWI-EAS443:3:49:507:408#0/1
AGCAATCTTGCAAAAGGAGAGAAAGCTAATGTGCTAATTG
+HWI-EAS443:3:49:507:408#0/1
`bb_Zb]b[abab^`ababb__a`]_b_`_]Hb_]aaa`a
@HWI-EAS443:3:49:670:712#0/1
CATCAATGAACTGAGCAATCTTGCAAAAGGAGAGAAAGCT
+HWI-EAS443:3:49:670:712#0/1
aabbabbbbbbabbb_bbbbbbbabaaabaabbbbbbb`b
@HWI-EAS443:3:49:696:1257#0/1
GAGAAAGCTAATGTGCTAATTGGGCAAGGGGACGTAGTGT
+HWI-EAS443:3:49:696:1257#0/1
aa`a]aZL¥XaXb_`_`aaa]V^a`^^`a_PVSaY^_UXU
```
```

```
@HWI-EAS443:3:49:751:1014#0/1
CATCAATGAACTGAGCAATCTTGCAAAAGGAGAGAAAGCT
+HWI-EAS443:3:49:751:1014#0/1
abXY_a^¥U`T^U```a_bbb¥Zbb]aa`^]b`bb`aaT]
@HWI-EAS443:3:49:786:359#0/1
GCATCAATGAACTGAGCAATCTTGCAAAAGGAGAGAAAGC
+HWI-EAS443:3:49:786:359#0/1
aaabaaabbaaabaaa`baa`aabaa```aa`a`aa`^aX
@HWI-EAS443:3:49:922:71#0/1
AATGAACTGAGCAATCTTGCAAAAGGAGAGAAAGCTAATG
+HWI-EAS443:3:49:922:71#0/1
abbbbbaabab^ba`]abb_aa_abaabbbb`abXaaaba
@HWI-EAS443:3:49:925:1112#0/1
GAACTGAGCAATCTTGCAAAAGGAGAGAAAGCTAATGTGC
+HWI-EAS443:3:49:925:1112#0/1
`U`ZX`Ya_Y][Ya_a^_]XXX_YaY_^P__Y__Y_aV_]
@HWI-EAS443:3:49:955:823#0/1
AGCTAATGTGCTAATTGGGCAAGGGGACGTAGTGTTGGTA
+HWI-EAS443:3:49:955:823#0/1
X`a^]a^¥__Y`abab^]aZ[_aabaU]aY`]^babb_T^
@HWI-EAS443:3:49:9:1991#0/1
AATGAACTGAGCAATCTTGCAAAAGGAGAGAAAGCTAATG
+HWI-EAS443:3:49:9:1991#0/1
¥¥a``¥a`baa`b_`aba`a_aaaa_¥a_a¥aaa^a___a
@HWI-EAS443:3:4:1007:1775#0/1
AGCTAATGTGCTAATTGGGCAAGGGGACGTAGTGTTGGTA
+HWI-EAS443:3:4:1007:1775#0/1
aaaa¥YP`X_]]Y^`W`_I]WX_``__WaZ[XIZ¥Y`¥BB
@HWI-EAS443:3:4:1021:1304#0/1
AAGCTAATGTGCTAATTGGGCAAGGGGACGTAGTGTTGGT
+HWI-EAS443:3:4:1021:1304#0/1
`aa_abaaa`a_aa_aaa`aaa_a``Y`_aZ`a]a]aaaR
@HWI-EAS443:3:4:1064:1509#0/1
GAAAGCTAATGTGCTAATTGGGCAAGGGGACGTAGTGTTG
+HWI-EAS443:3:4:1064:1509#0/1
`aaabab^]ab`a^a`aa_aba^aab]a[XUb^`_Wb_aa
@HWI-EAS443:3:4:1176:1706#0/1
ACTGAGCAATCTTGCAAAAGGAGAGAAAGCTAATGTGCTA
+HWI-EAS443:3:4:1176:1706#0/1
a][]`aUS^`_a_a_a^_^`a_a__`__a_^`_``]^Y^^
@HWI-EAS443:3:4:1227:1681#0/1
GAACTGAGCAATCTTGCAAAAGGAGAGAAAGCTAATGTGC
+HWI-EAS443:3:4:1227:1681#0/1
aab_aaaaaba`^aa`_a``__a```a`]`_V__`a`Z^¥
@HWI-EAS443:3:4:1276:564#0/1
AGCACATTAGCTTTCTCTCCTTTTGCAAGATTGCTCAGTT
+HWI-EAS443:3:4:1276:564#0/1
aaaa`aaa^^_aaa_a_a¥Za``a___`a___`^`]__Y_
@HWI-EAS443:3:4:1332:1713#0/1
AATTAGCACATTAGCTTTCTCTCCTTTTGCAAGATTGCTC
```

```
+HWI-EAS443:3:4:1332:1713#0/1
a^ab`aaabbbba`aaabaaaY__baaaa`aaaa`ab^a`
@HWI-EAS443:3:4:1341:198#0/1
TTGCAAAAGGAGAGAAAGCTAATGTGCTAATTGGGCAAGG
+HWI-EAS443:3:4:1341:198#0/1
aaaaaa_``a_a__a__a`_`^]_X_]`X]`_Q_^[Y]^^
@HWI-EAS443:3:4:1362:1587#0/1
CGTCCCCTTGCCCAATTAGCACATTAGCTTTCTCTCCTTT
+HWI-EAS443:3:4:1362:1587#0/1
abaaaab__b^^_`aaaaa]a]abaa`^```^`_aV^`_`
@HWI-EAS443:3:4:1498:1446#0/1
GAAAGCTAATGTGCTAATTGGGCAAGGGGACGTAGTGTTG
+HWI-EAS443:3:4:1498:1446#0/1
^_ZZ`VYP[aaXUPSSOaZ_UGWWR^XWZRX`WLITBBBB
@HWI-EAS443:3:4:1614:1067#0/1
ATGAACTGAGCAATCTTGCAAAAGGAGAGAAAGCTAATGT
+HWI-EAS443:3:4:1614:1067#0/1
SKa^a`_`]aaaab_aab`^a]Y`aa`aa_^`aQ_^_a__
@HWI-EAS443:3:4:218:1652#0/1
GAAAGCTAATGTGCTAATTGGGCAAGGGGACGTAGTGTTG
+HWI-EAS443:3:4:218:1652#0/1
_bbaa^aaaaaOa¥``aaaa`_X_¥aa_RZXa^_[LU_``
@HWI-EAS443:3:4:271:582#0/1
GAGCAATCTTGCAAAAGGAGAGAAAGCTAATGTGCTAATT
+HWI-EAS443:3:4:271:582#0/1
H_[[WSaaY`_aa`a_XZ^__U`[abbbababaa]a]Uab
@HWI-EAS443:3:4:289:1358#0/1
GAACTGAGCAATCTTGCAAAAGGAGAGAAAGCTAATGTGC
+HWI-EAS443:3:4:289:1358#0/1
ab`VK`aa[a__]]]a¥`ZZ`¥_aaa]`a^XY_]`a`G]G
@HWI-EAS443:3:4:378:199#0/1
GAGCAATCTTGCAAAAGGAGAGAAAGCTAATGTGCTAATT
+HWI-EAS443:3:4:378:199#0/1
aaX_aaZ^`¥``X`OX__U_]]¥X]_YYXUX_U_UXNKPY
@HWI-EAS443:3:4:528:684#0/1
CAACACTACGTCCCCTTGCCCAATTAGCACATTAGCTTTC
+HWI-EAS443:3:4:528:684#0/1
abbababbaa`a`__aaaa_[_``___^_¥^`a`__¥``Y
@HWI-EAS443:3:4:594:878#0/1
GAACTGAGCAATCTTGCAAAAGGAGAGAAAGCTAATGTGC
+HWI-EAS443:3:4:594:878#0/1
a_`R`a_a][^_W``a¥YY¥¥^_Ra_`__]_W]^WU^Z]W
@HWI-EAS443:3:4:603:958#0/1
GAGCAATCTTGCAAAAGGAGAGAAAGCTAATGTGCTAATT
+HWI-EAS443:3:4:603:958#0/1
aababbbababaaaaa`a_aaaaaaa`a`_aaXa_aX_a]
@HWI-EAS443:3:4:630:1355#0/1
CCCTTGCCCAATTAGCACATTAGCTTTCTCTCCTTTTGCA
+HWI-EAS443:3:4:630:1355#0/1
aba]_a_]]]`aaa`aaaaaaaaaaaa_`___Z`___a^`
```

```
@HWI-EAS443:3:4:630:868#0/1
CATCAATGAACTGAGCAATCTTGCAAAAGGAGAGAAAGCT
+HWI-EAS443:3:4:630:868#0/1
abbbbabbab_bbbaababababbbaa_aabaabaaaaaa_a
@HWI-EAS443:3:4:720:421#0/1
GAGCAATCTTGCAAAAGGAGAGAAAGCTAATGTGCTAATT
+HWI-EAS443:3:4:720:421#0/1
a^`a]Y_¥a`a_a_a¥a`¥a`_a_Z]__`^_`QaW`¥W_^
@HWI-EAS443:3:4:736:1184#0/1
AATGAACTGAGCAATCTTGCAAAAGGAGAGAAAGCTAATG
+HWI-EAS443:3:4:736:1184#0/1
aaa__``^^^¥__a^aaa`[]_aaaaa`]]aaaaWa_aaa
@HWI-EAS443:3:4:741:1250#0/1
GCATCAATGAACTGAGCAATCTTGCAAAAGGAGAGAAAGC
+HWI-EAS443:3:4:741:1250#0/1
`baaaaabaa____``_````^`a__¥_``^X__`__^a]
@HWI-EAS443:3:4:791:807#0/1
GAAAGCTAATGTGCTAATTGGGCAAGGGGACGTAGTGTTG
+HWI-EAS443:3:4:791:807#0/1
_bab^E]aabbXb^ba^abaYV¥aaa``¥__a]]VXa_`a
@HWI-EAS443:3:4:799:424#0/1
GAAAGCTAATGTGCTAATTGGGCAAGGGGACGTAGTGTTG
+HWI-EAS443:3:4:799:424#0/1
`_N_aaUU^`aaa[ZO]_[`aWXQa`XWV[¥aUaXPaa_B
@HWI-EAS443:3:4:813:179#0/1
AACTGAGCAATCTTGCAAAAGGAGAGAAAGCTAATGTGCT
+HWI-EAS443:3:4:813:179#0/1
aaaaa`aaaaa_aaa`a`__a`_a`a`^`aW^[^_`Z_]^
@HWI-EAS443:3:4:90:1366#0/1
GAAAGCTAATGTGCTAATTGGGCAAGGGGACGTAGTGTTG
+HWI-EAS443:3:4:90:1366#0/1
aaa[Q^a_a__SY__]UZ^Z¥_``^Z]NR^TSGT]QQWUZ
@HWI-EAS443:3:4:928:1641#0/1
GCATCAATGAACTGAGCAATCTTGCAAAAGGAGAGAAAGC
+HWI-EAS443:3:4:928:1641#0/1
a_aa`aaaa`a_a_`a^`a`_`_`_`_]___^`_`__^^]
@HWI-EAS443:3:4:963:214#0/1
AGCTAATGTGCTAATTGGGCAAGGGGACGTAGTGTTGGTA
+HWI-EAS443:3:4:963:214#0/1
aa^aaaab_a_aY[_a_a_]`^`_`VWOaZZ_W[RS_WHB
@HWI-EAS443:3:50:1180:1751#0/1
GTCCCCTTGCCCAATTAGCACATTAGCTTTCTCTCCTTTT
+HWI-EAS443:3:50:1180:1751#0/1
aaa``abaaaa`baaaa^[a^¥a_¥_YaaaZ_Y_`_a__[
@HWI-EAS443:3:50:1188:1849#0/1
CTTGCAAAAGGAGAGAAAGCTAATGTGCTAATTGGGCAAG
+HWI-EAS443:3:50:1188:1849#0/1
aa`bbba]b_baaaabbaaaaaaab`a_aaaaaaZ`a`a
@HWI-EAS443:3:50:1208:1690#0/1
GAGAAAGCTAATGTGCTAATTGGGCAAGGGGACGTAGTGT
```

````
+HWI-EAS443:3:50:1208:1690#0/1
abbbbbbaabbb`]bbb`aabbba_aabbbaaSa`aa^aa
@HWI-EAS443:3:50:1209:676#0/1
GAGCAATCTTGCAAAAGGAGAGAAAGCTAATGTGCTAATT
+HWI-EAS443:3:50:1209:676#0/1
_abbabbbbbbaabbbabbabbbbbbbbbbbbaa`bbbbb
@HWI-EAS443:3:50:1268:1239#0/1
CAAAAGGAGAGAAAGCTAATGTGCTAATTGGGCAAGGGGA
+HWI-EAS443:3:50:1268:1239#0/1
aaRS]bb_ba`][_bbba_ba_``]^```ba`]`_baa`¥
@HWI-EAS443:3:50:1335:1930#0/1
GCATCAATGAACTGAGCAATCTTGCAAAAGGAGAGAAAGC
+HWI-EAS443:3:50:1335:1930#0/1
`aZabb``a[`aa`_b__^`_Z_`^]aa``aa_abaa`bY
@HWI-EAS443:3:50:1423:127#0/1
AAAGCTAATGTGCTAATTGGGCAAGGGGACGTAGTGTTGG
+HWI-EAS443:3:50:1423:127#0/1
aaa``baab_`a[b`aba[`a]__a``a`_a_^a¥a_`a`
@HWI-EAS443:3:50:1516:1144#0/1
AGCTAATGTGCTAATTGGGCAAGGGGACGTAGTGTTGGTA
+HWI-EAS443:3:50:1516:1144#0/1
Y^`_R`_aSSSaTWab¥]aba`¥``a`¥ba`b^baa`^QQ
@HWI-EAS443:3:50:1535:312#0/1
GCATCAATGAACTGAGCAATCTTGCAAAAGGAGAGAAAGC
+HWI-EAS443:3:50:1535:312#0/1
aaa`aaabbaa`a`ab_aaa_aba__`_aaa_a]a___a^
@HWI-EAS443:3:50:1669:25#0/1
CTTGCAAAAGGAGAGAAAGCTAATGTGCTAATTGGGCAAG
+HWI-EAS443:3:50:1669:25#0/1
aba`aaaa_`_^[``ba`Z^_aabb`aaaaaba¥W¥`^]^
@HWI-EAS443:3:50:1739:1335#0/1
GAACTGAGCAATCTTGCAAAAGGAGAGAAAGCTAATGTGC
+HWI-EAS443:3:50:1739:1335#0/1
a`baa`Z`babb`bab_ZYWYba_a`[`a`a¥``aa```Z
@HWI-EAS443:3:50:20:75#0/1
AGCTAATGTGCTAATTGGGCAAGGGGACGTAGTGTTGGTA
+HWI-EAS443:3:50:20:75#0/1
¥[_¥^aW¥]___bba¥```_aaYX]Z¥_^^]a[][aa[Y¥
@HWI-EAS443:3:50:388:2045#0/1
GAGCAATCTTGCAAAAGGAGAGAAAGCTAATGTGCTAATT
+HWI-EAS443:3:50:388:2045#0/1
a`X`[`][bW`[Z]X^b`Z^_baa`WU^aa`aSUVP[ZP`
@HWI-EAS443:3:50:440:1485#0/1
TGCAAAAGGAGAGAAAGCTAATGTGCTAATTGGGCAAGGG
+HWI-EAS443:3:50:440:1485#0/1
ab_baaaaa_aaba`aa`aaaaa_a_a`^a`a`_Y__a]a
@HWI-EAS443:3:50:450:1051#0/1
CTTGCAAAAGGAGAGAAAGCTAATGTGCTAATTGGGCAAG
+HWI-EAS443:3:50:450:1051#0/1
abbaa`abbbabbbabbbb^b_abbab_a`bbbb_a`aab
````

```
@HWI-EAS443:3:50:681:505#0/1
GAGCAATCTTGCAAAAGGAGAGAAAGCTAATGTGCTAATT
+HWI-EAS443:3:50:681:505#0/1
aXSX_`]X^[]U__^L^_[a^P`_¥OW[]R`ZNQQYVV[¥
@HWI-EAS443:3:50:682:811#0/1
CCTTGCCCAATTAGCACATTAGCTTTCTCTCCTTTTGCAA
+HWI-EAS443:3:50:682:811#0/1
abbabbaaabbbababbbbbbbbbbb`bXbbbbbbbb_ba
@HWI-EAS443:3:50:687:528#0/1
GAACTGAGCAATCTTGCAAAAGGAGAGAAAGCTAATGTGC
+HWI-EAS443:3:50:687:528#0/1
aaa]abababababababaa¥^aba`aabaaaa`baaab`a_
@HWI-EAS443:3:50:705:1170#0/1
AAAGCTAATGTGCTAATTGGGCAAGGGGACGTAGTGTTGG
+HWI-EAS443:3:50:705:1170#0/1
`S_aU`]`^_Q^]`]^__]aaX¥_a`^aZ_^JYaP_T`a^
@HWI-EAS443:3:50:90:2020#0/1
ATCTTGCAAAAGGAGAGAAAGCTAATGTGCTAATTGGGCA
+HWI-EAS443:3:50:90:2020#0/1
`UWaYR`[b__aaaaaaaa_aa`a^``X¥Wa^a_a_¥^__
@HWI-EAS443:3:50:918:1389#0/1
GAGAAAGCTAATGTGCTAATTGGGCAAGGGGACGTAGTGT
+HWI-EAS443:3:50:918:1389#0/1
``aa`[a_a__aa[a_a`]a]`__^_Y`_a_^^`[X[Q[U
@HWI-EAS443:3:50:965:2031#0/1
AAAGCTAATGTGCTAATTGGGCAAGGGGACGTAGTGTTGG
+HWI-EAS443:3:50:965:2031#0/1
_¥¥¥¥`¥¥``]baaaba```¥`]T`aZ_X[a^`a¥a_aa^
@HWI-EAS443:3:50:968:1126#0/1
GAGAAAGCTAATGTGCTAATTGGGCAAGGGGACGTAGTGT
+HWI-EAS443:3:50:968:1126#0/1
aa¥Z_bb__^aaaXaaa_abaa]^_^_a```X]aU]aUYS
@HWI-EAS443:3:50:974:1513#0/1
ATCTTGCAAAAGGAGAGAAAGCTAATGTGCTAATTGGGCA
+HWI-EAS443:3:50:974:1513#0/1
`baabbaa`aabbaabbbaababaaab`aaa_abaab_^_
@HWI-EAS443:3:51:1076:1638#0/1
CAATCTTGCAAAAGGAGAGAAAGCTAATGTGCTAATTGGG
+HWI-EAS443:3:51:1076:1638#0/1
^aaYNW^abb^ZVaa_b_^]_S^L]`^a`^aYaZ]```a_
@HWI-EAS443:3:51:11:1432#0/1
GAGCAATCTTGCAAAAGGAGAGAAAGCTAATGTGCTAATT
+HWI-EAS443:3:51:11:1432#0/1
aaabb]bbbb]ba]aabbabaaaZWa_aaYba^aab`[_a
@HWI-EAS443:3:51:1595:152#0/1
GAGCAATCTTGCAAAAGGAGAGAAAGCTAATGTGCTAATT
+HWI-EAS443:3:51:1595:152#0/1
][^a`_bb`a`babaabbabWH]aaY]baaa]][[ba__a
@HWI-EAS443:3:51:1598:1242#0/1
ATCTTGCAAAAGGAGAGAAAGCTAATGTGCTAATTGGGCA
```

```
+HWI-EAS443:3:51:1598:1242#0/1
`b^bbbaaa`_a¥ZXQ^aaa__a_`aYW^W¥]`aaa^¥^a
@HWI-EAS443:3:51:1606:453#0/1
GAGAAAGCTAATGTGCTAATTGGGCAAGGGGACGTAGTGT
+HWI-EAS443:3:51:1606:453#0/1
abaa[`Xa`HKa``bSbabbba[]aa[MT^a^_Sa¥Q[F[
@HWI-EAS443:3:51:287:1185#0/1
AATGAACTGAGCAATCTTGCAAAAGGAGAGAAAGCTAATG
+HWI-EAS443:3:51:287:1185#0/1
abaa`aaba¥`bbbaabaaa_Z_abaaaaaaaa`]a`a`a
@HWI-EAS443:3:51:417:1008#0/1
GAAAGCTAATGTGCTAATTGGGCAAGGGGACGTAGTGTTG
+HWI-EAS443:3:51:417:1008#0/1
aZWF]a_`]ba¥VPa]]a_`Yaa`_aUVa^aaVVa¥¥S]`
@HWI-EAS443:3:51:434:1663#0/1
GAGCAATCTTGCAAAAGGAGAGAAAGCTAATGTGCTAATT
+HWI-EAS443:3:51:434:1663#0/1
a^a_bbb¥aaaa``_Y_`Ya_ab_aa`aa^aaY__`_¥_`
@HWI-EAS443:3:51:449:387#0/1
GAGCAATCTTGCAAAAGGAGAGAAAGCTAATGTGCTAATT
+HWI-EAS443:3:51:449:387#0/1
aWa¥U`[VZ[_¥[VZQ^_[`[_ZZ[[Y]YU¥]Q[W^UTYT
@HWI-EAS443:3:51:45:211#0/1
CAATCTTGCAAAAGGAGAGAAAGCTAATGTGCTAATTGGG
+HWI-EAS443:3:51:45:211#0/1
aa_baabaabaa_baababb_aa^a`_bb^aaa^_aaaa`
@HWI-EAS443:3:51:49:1715#0/1
GAAAGCTAATGTGCTAATTGGGCAAGGGGACGTAGTGTTG
+HWI-EAS443:3:51:49:1715#0/1
W_ab]b`abbbbbbb¥`abb`¥`ba`b`_`aba^¥]b^ba
@HWI-EAS443:3:51:546:304#0/1
AGCTAATGTGCTAATTGGGCAAGGGGACGTAGTGTTGGTA
+HWI-EAS443:3:51:546:304#0/1
`bab^bbb¥Z_a`aaaba_`a`aa``^ab_ab`a_a^`R_
@HWI-EAS443:3:51:552:1465#0/1
GAGCAATCTTGCAAAAGGAGAGAAAGCTAATGTGCTAATT
+HWI-EAS443:3:51:552:1465#0/1
_abaabbabbaa`aa]`baaaaaaa`[ba_bb_a`aS]`a
@HWI-EAS443:3:51:579:337#0/1
AGCAATCTTGCAAAAGGAGAGAAAGCTAATGTGCTAATTG
+HWI-EAS443:3:51:579:337#0/1
ab`bbbbbbbbbbaabba`bbaaab_a`]ab_a_baaaaa
@HWI-EAS443:3:51:655:729#0/1
CAATCTTGCAAAAGGAGAGAAAGCTAATGTGCTAATTGGG
+HWI-EAS443:3:51:655:729#0/1
`__ab^^XS`aJ_Y^bbb`¥_¥b_b_M¥^T`ab_abaX]X
@HWI-EAS443:3:51:685:1038#0/1
CAATGAACTGAGCAATCTTGCAAAAGGAGAGAAAGCTAAT
+HWI-EAS443:3:51:685:1038#0/1
`¥`bb_bbba``a_XYbbbaaab`aabbbaab```Y`baa
```

```
@HWI-EAS443:3:51:720:1081#0/1
AAAAGGAGAGAAAGCTAATGTGCTAATTGGGCAAGGGGAC
+HWI-EAS443:3:51:720:1081#0/1
aaaabbabbbbbbbabaabb_b`baabaaaaaa`abba_^
@HWI-EAS443:3:51:739:741#0/1
AGCTAATGTGCTAATTGGGCAAGGGGACGTAGTGTTGGTA
+HWI-EAS443:3:51:739:741#0/1
`aab``bbbbabbabbbbbbaabbbb]bba`babaabaZ]
@HWI-EAS443:3:51:73:893#0/1
AGCTAATGTGCTAATTGGGCAAGGGGACGTAGTGTTGGTA
+HWI-EAS443:3:51:73:893#0/1
abbab`bb_baaa`aabaaaaabaaa__bZ_aYa_aa`PV
@HWI-EAS443:3:51:884:1617#0/1
GAGCAATCTTGCAAAAGGAGAGAAAGCTAATGTGCTAATT
+HWI-EAS443:3:51:884:1617#0/1
]V_WO_W_R]a[a]WR`WQ_abb__]]_aa^][^Z^^^]_
@HWI-EAS443:3:51:963:174#0/1
GCAAAAGGAGAGAAAGCTAATGTGCTAATTGGGCAAGGGG
+HWI-EAS443:3:51:963:174#0/1
a`aa^````baba^_a`a__ab^a]a``a_a_]Z`_a^_`
@HWI-EAS443:3:52:1034:1767#0/1
AGCTAATGTGCTAATTGGGCAAGGGGACGTAGTGTTGGTA
+HWI-EAS443:3:52:1034:1767#0/1
abb^]¥¥¥V`aaa_aaab``_`Z``b_ab^¥^_b^`b^S_
@HWI-EAS443:3:52:1075:1994#0/1
GAGCAATCTTGCAAAAGGAGAGAAAGCTAATGTGCTAATT
+HWI-EAS443:3:52:1075:1994#0/1
aVY_`[_`¥X^_a`[_`a`a¥`aa[`]_`]]a]_[[^][]
@HWI-EAS443:3:52:1098:1296#0/1
AGCTAATGTGCTAATTGGGCAAGGGGACGTAGTGTTGGTA
+HWI-EAS443:3:52:1098:1296#0/1
_^Y`Y_a`RZ]_aaXa]a`^VY]S]WP¥b[Za]ZZY_aVX
@HWI-EAS443:3:52:1117:1406#0/1
TGCAAAAGGAGAGAAAGCTAATGTGCTAATTGGGCAAGGG
+HWI-EAS443:3:52:1117:1406#0/1
abaY[ZV]a`a_a`^_a__`_baZaYa_]a__^`]`_]]_
@HWI-EAS443:3:52:1133:1159#0/1
GCATCAATGAACTGAGCAATCTTGCAAAAGGAGAGAAAGC
+HWI-EAS443:3:52:1133:1159#0/1
¥abbaabbbbbabbba^^^aabbaaaaaabbababb_]b_
@HWI-EAS443:3:52:1155:824#0/1
ATCTTGCAAAAGGAGAGAAAGCTAATGTGCTAATTGGGCA
+HWI-EAS443:3:52:1155:824#0/1
^bbb`_^W`aaY`abaab^]^ab```Z[a_baaaaa_¥_a
@HWI-EAS443:3:52:1213:68#0/1
GCATCAATGAACTGAGCAATCTTGCAAAAGGAGAGAAAGC
+HWI-EAS443:3:52:1213:68#0/1
VaM¥X_O_^ZW]a¥__]¥aaOa_a[]__]aa_b[_OU_a[
@HWI-EAS443:3:52:1287:211#0/1
GCATCAATGAACTGAGCAATCTTGCAAAAGGAGAGAAAGC
```

+HWI-EAS443:3:52:1287:211#0/1
`aaa¥^aba_a[a[]`Ra`aZa`aUPX_^a`^a_a_[[]U
@HWI-EAS443:3:52:1304:390#0/1
AGCTAATGTGCTAATTGGGCAAGGGGACGTAGTGTTGGTA
+HWI-EAS443:3:52:1304:390#0/1
`bab_aaaPaR_a`aa`ba^_`aaaZ^]_]UYV]^aa]R]
@HWI-EAS443:3:52:1354:1468#0/1
AGCTAATGTGCTAATTGGGCAAGGGGACGTAGTGTTGGTA
+HWI-EAS443:3:52:1354:1468#0/1
`XOOV_aa_Z]^_`^T[S]Z¥_]`baa`]RU_G_GX_YUR
@HWI-EAS443:3:52:1396:1399#0/1
GAACTGAGCAATCTTGCAAAAGGAGAGAAAGCTAATGTGC
+HWI-EAS443:3:52:1396:1399#0/1
aabZa¥^^`baaP¥`bZ`_KXaa`ba`b]a`V]aa^aUaU
@HWI-EAS443:3:52:1514:1134#0/1
CAATTAGCACATTAGCTTTCTCTCCTTTTGCAAGATTGCT
+HWI-EAS443:3:52:1514:1134#0/1
`_abbaa`b_bbbaaabbb`a_b`_aaa_a`aaaaaaa^a
@HWI-EAS443:3:52:1606:317#0/1
TCTTGCAAAAGGAGAGAAAGCTAATGTGCTAATTGGGCAA
+HWI-EAS443:3:52:1606:317#0/1
`ab_`_aab``_ba_X]__aabaaaaYaab`ababaa_``
@HWI-EAS443:3:52:1607:1320#0/1
AATGAACTGAGCAATCTTGCAAAAGGAGAGAAAGCTAATG
+HWI-EAS443:3:52:1607:1320#0/1
Wbb`abbbaab`bbaab_bbaaaab`aa_b^`ab^``_ba
@HWI-EAS443:3:52:164:895#0/1
GAGCAATCTTGCAAAAGGAGAGAAAGCTAATGTGCTAATT
+HWI-EAS443:3:52:164:895#0/1
a`baaabbbaaaYa¥aba`bab`__b]a``aa]a_`__a_
@HWI-EAS443:3:52:1661:1060#0/1
GAGCAATCTTGCAAAAGGAGAGAAAGCTAATGTGCTAATT
+HWI-EAS443:3:52:1661:1060#0/1
_a`aba¥_b_T^aaba_b¥[]_¥aabababbbaW^_`¥`^
@HWI-EAS443:3:52:1761:1419#0/1
AACTGAGCAATCTTGCAAAAGGAGAGAAAGCTAATGTGCT
+HWI-EAS443:3:52:1761:1419#0/1
abXa_¥b`a_``aaa`]`[^^`]a``^_[`__a[^`[^Y^
@HWI-EAS443:3:52:191:2022#0/1
AGCACATTAGCTTTCTCTCCTTTTGCAAGATTGCTCAGTT
+HWI-EAS443:3:52:191:2022#0/1
Y[W]¥VZab`][a``Qaa]M[[a`Y`__[]SGXQ[``PXS
@HWI-EAS443:3:52:234:367#0/1
GAAAGCTAATGTGCTAATTGGGCAAGGGGACGTAGTGTTG
+HWI-EAS443:3:52:234:367#0/1
a`aab_aaaab_babaaaaaaaaa_aaa_aaa^aaWa__a
@HWI-EAS443:3:52:240:1416#0/1
CATCAATGAACTGAGCAATCTTGCAAAAGGAGAGAAAGCT
+HWI-EAS443:3:52:240:1416#0/1
`_U`_S]_^__b¥ZZ]aa_Y]]]Y][]¥``¥]^]_OZ¥¥Y

```
@HWI-EAS443:3:52:251:510#0/1
AGCTAATGTGCTAATTGGGCAAGGGGACGTAGTGTTGGTA
+HWI-EAS443:3:52:251:510#0/1
aabb^aba`aaaaaabaab_`]aaaaZ`b^^a]a]a`aHU
@HWI-EAS443:3:52:269:79#0/1
AGCTAATGTGCTAATTGGGCAAGGGGACGTAGTGTTGGTA
+HWI-EAS443:3:52:269:79#0/1
_bbb_ZaaXa`_`a``baa`__aaaaU_a]]aWa¥`a_TT
@HWI-EAS443:3:52:291:134#0/1
ACTGAGCAATCTTGCAAAAGGAGAGAAAGCTAATGTGCTA
+HWI-EAS443:3:52:291:134#0/1
abbaaaaa`babab`a`^_ba`a`aa__a]a__aa^a^a_
@HWI-EAS443:3:52:292:736#0/1
AGGAGAGAAAGCTAATGTGCTAATTGGGCAAGGGGACGTA
+HWI-EAS443:3:52:292:736#0/1
abbababaaaaaa]^ab_b`a__`aaaa_]¥a``aYWa^_
@HWI-EAS443:3:52:295:1161#0/1
ATCTTGCAAAAGGAGAGAAAGCTAATGTGCTAATTGGGCA
+HWI-EAS443:3:52:295:1161#0/1
OWR_[[`ZT^YV`]_abbaWbaa``^TPZ_`U¥¥b¥_TY`
@HWI-EAS443:3:52:295:1973#0/1
AGCTAATGTGCTAATTGGGCAAGGGGACGTAGTGTTGGTA
+HWI-EAS443:3:52:295:1973#0/1
aaaaaaba`aaaaa`a]``aaaa_a`__a^_aZZ^a`_W_
@HWI-EAS443:3:52:295:320#0/1
GTCCCCTTGCCCAATTAGCACATTAGCTTTCTCTCCTTTT
+HWI-EAS443:3:52:295:320#0/1
a``a`]__a_`_`aa_``_]]_`___^``_^`]`¥X¥`^`
@HWI-EAS443:3:52:366:1052#0/1
AAGCTAATGTGCTAATTGGGCAAGGGGACGTAGTGTTGGT
+HWI-EAS443:3:52:366:1052#0/1
JX]baabbb^b`a```ab]`a`^b^_a`_b^^]MZ_aaaV
@HWI-EAS443:3:52:398:735#0/1
GAGCAATCTTGCAAAAGGAGAGAAAGCTAATGTGCTAATT
+HWI-EAS443:3:52:398:735#0/1
`¥``a`b_a`aba_aaba]aabaaabaaa[a_S]^aa^]`
@HWI-EAS443:3:52:411:1097#0/1
GAAAGCTAATGTGCTAATTGGGCAAGGGGACGTAGTGTTG
+HWI-EAS443:3:52:411:1097#0/1
abbabaa]ZaaW`aba_aaaaa``Y`aaa_aa_Va^a_`a
@HWI-EAS443:3:52:462:419#0/1
GAAAGCTAATGTGCTAATTGGGCAAGGGGACGTAGTGTTG
+HWI-EAS443:3:52:462:419#0/1
a]PUHR``^¥]G^L[ZY^_`WV]RW¥V]]V]_UVXDUVU]
@HWI-EAS443:3:52:464:1012#0/1
AGGAGAGAAAGCTAATGTGCTAATTGGGCAAGGGGACGTA
+HWI-EAS443:3:52:464:1012#0/1
abbababaaabaaaaab_b_`aaaa`aa``]a`a`^`a]]
@HWI-EAS443:3:52:469:797#0/1
ACCAACACTACGTCCCCTTGCCCAATTAGCACATTAGCTT
```

```
+HWI-EAS443:3:52:469:797#0/1
¥aa¥_XWa``YWY]U¥[ZZZ[^V]]ZU[_`YS[`ZTaI¥R
@HWI-EAS443:3:52:56:246#0/1
CAATCTTGCAAAAGGAGAGAAAGCTAATGTGCTAATTGGG
+HWI-EAS443:3:52:56:246#0/1
^^Z¥``_^`^H^Yaa¥^`[]Z_a`¥WX`^Ua¥XU]`Z]`Y
@HWI-EAS443:3:52:581:1118#0/1
AGCTAATGTGCTAATTGGGCAAGGGGACGTAGTGTTGGTA
+HWI-EAS443:3:52:581:1118#0/1
a`aba`aba_baT]`aa^S]aab]``XYWLR]`b]`a¥OI
@HWI-EAS443:3:52:67:860#0/1
CATCAATGAACTGAGCAATCTTGCAAAAGGAGAGAAAGCT
+HWI-EAS443:3:52:67:860#0/1
aZOX]]b`WYTU]^aa^_a_a_a]T]_[]aVZ¥Z[KEUTX
@HWI-EAS443:3:52:719:1133#0/1
GAGAAAGCTAATGTGCTAATTGGGCAAGGGGACGTAGTGT
+HWI-EAS443:3:52:719:1133#0/1
a`aa_aaaaa``aX__aa^aaa`WY_^```_V^aW^aYaY
@HWI-EAS443:3:52:743:303#0/1
CAATCTTGCAAAAGGAGAGAAAGCTAATGTGCTAATTGGG
+HWI-EAS443:3:52:743:303#0/1
abbb`abbbbbaaba¥abaaaaaaaaaa`W_`aaaabaa`
@HWI-EAS443:3:52:784:984#0/1
GCATCAATGAACTGAGCAATCTTGCAAAAGGAGAGAAAGC
+HWI-EAS443:3:52:784:984#0/1
aaa`a__b``a_ba___b_a_aaa^___^a_¥ba_a__a^
@HWI-EAS443:3:52:798:246#0/1
GAGCAATCTTGCAAAAGGAGAGAAAGCTAATGTGCTAATT
+HWI-EAS443:3:52:798:246#0/1
aab_aababaa`aa`aaa_a`aaaa``a```a_a_a_^`_
@HWI-EAS443:3:52:813:1632#0/1
AATGAACTGAGCAATCTTGCAAAAGGAGAGAAAGCTAATG
+HWI-EAS443:3:52:813:1632#0/1
V¥a_a^`ba`aZ[ZGY`aaP_TY`a`¥b_Yb`¥a^]a```
@HWI-EAS443:3:52:813:49#0/1
ATCTTGCAAAAGGAGAGAAAGCTAATGTGCTAATTGGGCA
+HWI-EAS443:3:52:813:49#0/1
_^^_``aaaUL_aMa_aa[_X[[]_aa_aX_][__XZ^W[
@HWI-EAS443:3:52:823:1587#0/1
GCATCAATGAACTGAGCAATCTTGCAAAAGGAGAGAAAGC
+HWI-EAS443:3:52:823:1587#0/1
a^¥aa_ba`ZZU]`Q^a``]¥aZ]`SY_PbZZaSaaa[Y[
@HWI-EAS443:3:52:860:91#0/1
ATCAATGAACTGAGCAATCTTGCAAAAGGAGAGAAAGCTA
+HWI-EAS443:3:52:860:91#0/1
¥_¥`bab¥YZ`^`a_¥`a]a`_YRaa_bbabab^^^_W¥^
@HWI-EAS443:3:52:878:637#0/1
AATTAGCACATTAGCTTTCTCTCCTTTTGCAAGATTGCTC
+HWI-EAS443:3:52:878:637#0/1
ab`aaX`^`X`^aX`aaa_aT`^_`_`_a`aUaa`aaT¥]
```

```
@HWI-EAS443:3:52:905:1720#0/1
GTCCCCTTGCCCAATTAGCACATTAGCTTTCTCTCCTTTT
+HWI-EAS443:3:52:905:1720#0/1
a^a`aX^`__`_aa`__^Y`[```_a^_^_^_[_^^__`_
@HWI-EAS443:3:52:944:40#0/1
GCATCAATGAACTGAGCAATCTTGCAAAAGGAGAGAAAGC
+HWI-EAS443:3:52:944:40#0/1
aaaa_`^`b_^Xaa_b^a_a_aaaR¥Y_^a`[a`a^^]a^
@HWI-EAS443:3:52:94:1335#0/1
AATTAGCACATTAGCTTTCTCTCCTTTTGCAAGATTGCTC
+HWI-EAS443:3:52:94:1335#0/1
_¥a¥Z]`UZ^a]^^^`aaa_RZ^SY`a_T¥]_¥U[RXXPX
@HWI-EAS443:3:53:1004:1443#0/1
AGCTAATGTGCTAATTGGGCAAGGGGACGTAGTGTTGGTA
+HWI-EAS443:3:53:1004:1443#0/1
a`Pb`baba``aaaa``]aKaabab`V^b]aaaba`aaX¥
@HWI-EAS443:3:53:1017:1342#0/1
CTTGCAAAAGGAGAGAAAGCTAATGTGCTAATTGGGCAAG
+HWI-EAS443:3:53:1017:1342#0/1
ababa__b_aaWbbb_bababa`baab_b_ab_bbba`[`
@HWI-EAS443:3:53:1099:595#0/1
AGCTAATGTGCTAATTGGGCAAGGGGACGTAGTGTTGGTA
+HWI-EAS443:3:53:1099:595#0/1
`babbbbb[_ababbbbb`ab`bbb`aababaababb`QS
@HWI-EAS443:3:53:110:109#0/1
GCATCAATGAACTGAGCAATCTTGCAAAAGGAGAGAAAGC
+HWI-EAS443:3:53:110:109#0/1
a``W``[ab^^Z___a__`]T]^`]^_UZaaX`]`]]]aY
@HWI-EAS443:3:53:1173:95#0/1
CAATCTTGCAAAAGGAGAGAAAGCTAATGTGCTAATTGGG
+HWI-EAS443:3:53:1173:95#0/1
abbabb`b^b[b`^^Yb_b]a_aZabbb_]aN¥a_babba
@HWI-EAS443:3:53:1256:249#0/1
GAACTGAGCAATCTTGCAAAAGGAGAGAAAGCTAATGTGC
+HWI-EAS443:3:53:1256:249#0/1
`aa_bbab`aab_abb`_a^`abab_aaa_bVa`_aa_aX
@HWI-EAS443:3:53:1317:224#0/1
GAGCAATCTTGCAAAAGGAGAGAAAGCTAATGTGCTAATT
+HWI-EAS443:3:53:1317:224#0/1
Vababababbbaababababbb_ab_bbbbabbbbbabaaabbb
@HWI-EAS443:3:53:1386:383#0/1
AGCAATCTTGCAAAAGGAGAGAAAGCTAATGTGCTAATTG
+HWI-EAS443:3:53:1386:383#0/1
aa``aaabbbbb]abbb`b`babbbab``aa`b`b`ab^[
@HWI-EAS443:3:53:1544:263#0/1
GAAAGCTAATGTGCTAATTGGGCAAGGGGACGTAGTGTTG
+HWI-EAS443:3:53:1544:263#0/1
`ab__`a_abbab`bbbaaTaaaaabaZa`Y]_aa¥a_a`
@HWI-EAS443:3:53:1634:887#0/1
AGCTAATGTGCTAATTGGGCAAGGGGACGTAGTGTTGGTA
```

```
+HWI-EAS443:3:53:1634:887#0/1
aUT_abb[aaWbaabbb`[aaS_FWa¥`aa_[]`^bV][Y
@HWI-EAS443:3:53:1671:1261#0/1
GAACTGAGCAATCTTGCAAAAGGAGAGAAAGCTAATGTGC
+HWI-EAS443:3:53:1671:1261#0/1
`aa`bbaabb`baaabX_aa`ab[Zabab[a_a`aba_a_
@HWI-EAS443:3:53:1781:1419#0/1
CATCAATGAACTGAGCAATCTTGCAAAAGGAGAGAAAGCT
+HWI-EAS443:3:53:1781:1419#0/1
abaaaabaaa`a_[aaaaa^baa_a[_``a_``aa]_a_`
@HWI-EAS443:3:53:1789:1978#0/1
CCCTTGCCCAATTAGCACATTAGCTTTCTCTCCTTTTGCA
+HWI-EAS443:3:53:1789:1978#0/1
a``a]_aaabaaa_`^^_a`W_¥¥_aa_aaa_]]__¥_][
@HWI-EAS443:3:53:180:1184#0/1
AAGCTAATGTGCTAATTGGGCAAGGGGACGTAGTGTTGGT
+HWI-EAS443:3:53:180:1184#0/1
abaaba`ba^a`b`aa`b_`aa```a`^aa_]a]aZab^O
@HWI-EAS443:3:53:230:177#0/1
GAAAGCTAATGTGCTAATTGGGCAAGGGGACGTAGTGTTG
+HWI-EAS443:3:53:230:177#0/1
aaaab`b``ab`aab__ababa_`_ba_a^_`Y^bYa^]a
@HWI-EAS443:3:53:239:214#0/1
GAACTGAGCAATCTTGCAAAAGGAGAGAAAGCTAATGTGC
+HWI-EAS443:3:53:239:214#0/1
`[aaa``aaaa``aaa¥Z`__aa[a]a`^_a^_^Y_`V^V
@HWI-EAS443:3:53:250:1004#0/1
CAATCTTGCAAAAGGAGAGAAAGCTAATGTGCTAATTGGG
+HWI-EAS443:3:53:250:1004#0/1
ab[_`ba¥_aZ[aZb]b``ba_aaaZ¥aa^b_ba_a`^Xa
@HWI-EAS443:3:53:273:344#0/1
GAGCAATCTTGCAAAAGGAGAGAAAGCTAATGTGCTAATT
+HWI-EAS443:3:53:273:344#0/1
a^TOS^P[WUa_[_[JY^^[[X[_TUNV_[U[U]UV[UZY
@HWI-EAS443:3:53:33:550#0/1
CGTCCCCTTGCCCAATTAGCACATTAGCTTTCTCTCCTTT
+HWI-EAS443:3:53:33:550#0/1
`aababbbbbb`aaabaaaaaaaaS`aaabaaa`aa^aba
@HWI-EAS443:3:53:45:341#0/1
CATCAATGAACTGAGCAATCTTGCAAAAGGAGAGAAAGCT
+HWI-EAS443:3:53:45:341#0/1
Ybbb``abbbbbb```bbbabbbbaZXabbababa[¥a`a
@HWI-EAS443:3:53:525:759#0/1
TGCAAAAGGAGAGAAAGCTAATGTGCTAATTGGGCAAGGG
+HWI-EAS443:3:53:525:759#0/1
aa`aaa`ba_a`a]^aaaa_aa`Wa_aa_a_``a__^aa_
@HWI-EAS443:3:53:540:882#0/1
GCATCAATGAACTGAGCAATCTTGCAAAAGGAGAGAAAGC
+HWI-EAS443:3:53:540:882#0/1
`_`aaaaabaaa_a]b``___a`aa_][Yaa[b_a_[R]_
```

@HWI-EAS443:3:53:600:57#0/1
CATCAATGAACTGAGCAATCTTGCAAAAGGAGAGAAAGCT
+HWI-EAS443:3:53:600:57#0/1
abbabb`_YYV_`^aa¥`[H^aaa^`a^ab^bUabaSa^a
@HWI-EAS443:3:53:80:1875#0/1
AGCTAATGTGCTAATTGGGCAAGGGGACGTAGTGTTGGTA
+HWI-EAS443:3:53:80:1875#0/1
aSUUZH¥MG``¥¥a_S]RV_W]_O^OO]OH_RW^V[]RGX
@HWI-EAS443:3:53:89:1547#0/1
GAGCAATCTTGCAAAAGGAGAGAAAGCTAATGTGCTAATT
+HWI-EAS443:3:53:89:1547#0/1
aaaaa`¥aaab`a`[]_^aa]a`¥`a_aa_a`^a^`[_`_
@HWI-EAS443:3:54:1012:1508#0/1
GAGCAATCTTGCAAAAGGAGAGAAAGCTAATGTGCTAATT
+HWI-EAS443:3:54:1012:1508#0/1
Y]^b]bbabbaabaa[ababb`a]Z_ab_^a`_a_b]¥_a
@HWI-EAS443:3:54:1090:1774#0/1
TCCCCTTGCCCAATTAGCACATTAGCTTTCTCTCCTTTTG
+HWI-EAS443:3:54:1090:1774#0/1
WXT¥S`YY]]^Z]X]PQK`RVR_XGT]RPQUPUUR^X]WY
@HWI-EAS443:3:54:1090:273#0/1
AGCTAATGTGCTAATTGGGCAAGGGGACGTAGTGTTGGTA
+HWI-EAS443:3:54:1090:273#0/1
_bbbbbabbbbbaabbbbbaaabbbaa`a`ab_baaaa_[
@HWI-EAS443:3:54:1094:713#0/1
AGCACATTAGCTTTCTCTCCTTTTGCAAGATTGCTCAGTT
+HWI-EAS443:3:54:1094:713#0/1
a`¥Ybabbb_Z^¥`Y_a^X[bb`a`[[¥J¥^^VX[]^aa¥
@HWI-EAS443:3:54:1167:704#0/1
CATCAATGAACTGAGCAATCTTGCAAAAGGAGAGAAAGCT
+HWI-EAS443:3:54:1167:704#0/1
_¥bbbb`aa`ababbbbba[abbaaab`bbaabbababab
@HWI-EAS443:3:54:1173:1571#0/1
ATTAGCTTTCTCTCCTTTTGCAAGATTGCTCAGTTCATTG
+HWI-EAS443:3:54:1173:1571#0/1
aaba`Q`ba_b^a^`aaaa_aa_Y_aa`]a`_a_```a``
@HWI-EAS443:3:54:1210:988#0/1
AGCTAATGTGCTAATTGGGCAAGGGGACGTAGTGTTGGTA
+HWI-EAS443:3:54:1210:988#0/1
[`W`^`a`X_X_aa_a``]V^``_ZaZTa_¥¥]^Y^a`SS
@HWI-EAS443:3:54:1338:1015#0/1
GTCCCCTTGCCCAATTAGCACATTAGCTTTCTCTCCTTTT
+HWI-EAS443:3:54:1338:1015#0/1
aaaaaa``^]a]aaaaa^W]`abaaa_aaa_a_a¥``¥`¥
@HWI-EAS443:3:54:1441:1025#0/1
CAATGAACTGAGCAATCTTGCAAAAGGAGAGAAAGCTAAT
+HWI-EAS443:3:54:1441:1025#0/1
Zaabbbaa``Z`aaabbbb```b_b_U_bbbbbaba_b_a
@HWI-EAS443:3:54:1550:1252#0/1
GAAAGCTAATGTGCTAATTGGGCAAGGGGACGTAGTGTTG

```
+HWI-EAS443:3:54:1550:1252#0/1
`WJV`aaXL[]S]XYY_PYX`aPUSXRSXVZ`PRSJUFXY
@HWI-EAS443:3:54:1732:1790#0/1
AGGAGAGAAAGCTAATGTGCTAATTGGGCAAGGGGACGTA
+HWI-EAS443:3:54:1732:1790#0/1
abaabbbbbabababaabb`b_baaaaaaa_a`aaaaa]`_`
@HWI-EAS443:3:54:184:1144#0/1
GAACTGAGCAATCTTGCAAAAGGAGAGAAAGCTAATGTGC
+HWI-EAS443:3:54:184:1144#0/1
^ababb[babbababaa_aaaba]ba``aaa`aa_aa]a^
@HWI-EAS443:3:54:204:343#0/1
GAAAGCTAATGTGCTAATTGGGCAAGGGGACGTAGTGTTG
+HWI-EAS443:3:54:204:343#0/1
abaWa_`Uaab^a_a`_a_aaa_`^ab``¥^aW]aWaZ`_
@HWI-EAS443:3:54:220:1314#0/1
GAAAGCTAATGTGCTAATTGGGCAAGGGGACGTAGTGTTG
+HWI-EAS443:3:54:220:1314#0/1
``a`aI[¥[^a_¥L¥__a[]Y[¥XQ_]^UQ]`VUXFRGX_
@HWI-EAS443:3:54:239:1173#0/1
CATCAATGAACTGAGCAATCTTGCAAAAGGAGAGAAAGCT
+HWI-EAS443:3:54:239:1173#0/1
ab_a]`bbaabaaab`_abab`b`]aWaaaaaaa`XX_`¥
@HWI-EAS443:3:54:344:929#0/1
GAGCAATCTTGCAAAAGGAGAGAAAGCTAATGTGCTAATT
+HWI-EAS443:3:54:344:929#0/1
aa¥b__babbabaaaabb`bbbb_aa_aaaaa_b_aaaaa
@HWI-EAS443:3:54:360:1239#0/1
GAGCAATCTTGCAAAAGGAGAGAAAGCTAATGTGCTAATT
+HWI-EAS443:3:54:360:1239#0/1
aababb`bbbb`babbabbbbbabbbabaabaab[b`abb
@HWI-EAS443:3:54:366:961#0/1
GCATCAATGAACTGAGCAATCTTGCAAAAGGAGAGAAAGC
+HWI-EAS443:3:54:366:961#0/1
aa`ba`bbbbbabaabababababa`___aa_aaaaa_a`
@HWI-EAS443:3:54:393:464#0/1
ACGTCCCCTTGCCCAATTAGCACATTAGCTTTCTCTCCTT
+HWI-EAS443:3:54:393:464#0/1
aaaaa__a_a]^__``_`_[__Z^____¥^``__^_Y¥_]
@HWI-EAS443:3:54:39:527#0/1
AACTGAGCAATCTTGCAAAAGGAGAGAAAGCTAATGTGCT
+HWI-EAS443:3:54:39:527#0/1
a_a¥UY`bab`U[aab^XRQ``T¥`a]Z`^TW¥`]^Vb_`
@HWI-EAS443:3:54:456:1009#0/1
CTTGCAAAAGGAGAGAAAGCTAATGTGCTAATTGGGCAAG
+HWI-EAS443:3:54:456:1009#0/1
abbabbabbabaaa]bbababbbab_aabaabaaabaa``
@HWI-EAS443:3:54:577:582#0/1
GAGAAAGCTAATGTGCTAATTGGGCAAGGGGACGTAGTGT
+HWI-EAS443:3:54:577:582#0/1
abaa`aa`aa_ab^aaaaaaaaa```_aaaa]_a]_`]a]
```

```
@HWI-EAS443:3:54:585:869#0/1
AATGAACTGAGCAATCTTGCAAAAGGAGAGAAAGCTAATG
+HWI-EAS443:3:54:585:869#0/1
abaa`bbba`babababbbaa`Zaba``]abaaa¥`][`b
@HWI-EAS443:3:54:592:731#0/1
AATGAACTGAGCAATCTTGCAAAAGGAGAGAAAGCTAATG
+HWI-EAS443:3:54:592:731#0/1
]aaa`¥abbYaY^^abb^`¥UWYa`aS`]b_NZaaa¥S`b
@HWI-EAS443:3:54:776:962#0/1
AATGAACTGAGCAATCTTGCAAAAGGAGAGAAAGCTAATG
+HWI-EAS443:3:54:776:962#0/1
a^Xabbabbab`^`bab¥`Y`a_^abababa]^baa__aa
@HWI-EAS443:3:54:835:1240#0/1
GAGCAATCTTGCAAAAGGAGAGAAAGCTAATGTGCTAATT
+HWI-EAS443:3:54:835:1240#0/1
`]^^a_`¥``_`]Y]```Z^]a```a`aYY_a^a^a¥^a^
@HWI-EAS443:3:54:929:974#0/1
GTCCCCTTGCCCAATTAGCACATTAGCTTTCTCTCCTTTT
+HWI-EAS443:3:54:929:974#0/1
aaaa`]``bZa_aaaaa_`__aaaaa_aaa`a_a__aaaa
@HWI-EAS443:3:54:943:489#0/1
GAGAAAGCTAATGTGCTAATTGGGCAAGGGGACGTAGTGT
+HWI-EAS443:3:54:943:489#0/1
_XZW¥_a^aa¥`b``YWaZ`¥R_`Z[]_^aaY]_MW]VX]
@HWI-EAS443:3:54:968:863#0/1
ATCTTGCAAAAGGAGAGAAAGCTAATGTGCTAATTGGGCA
+HWI-EAS443:3:54:968:863#0/1
]U[aabba`a_`^Za`a`aaaUba`ab¥_`aaaaaba___
@HWI-EAS443:3:54:977:1830#0/1
GAGCAATCTTGCAAAAGGAGAGAAAGCTAATGTGCTAATT
+HWI-EAS443:3:54:977:1830#0/1
`baaababbbaab_VMba^ab[b_abbabbba^b]b`aa`
@HWI-EAS443:3:55:1017:236#0/1
GAGAAAGCTAATGTGCTAATTGGGCAAGGGGACGTAGTGT
+HWI-EAS443:3:55:1017:236#0/1
aaa```a`a__aaU`^_``aaa`_¥_]_`__]¥aX]aZ`X
@HWI-EAS443:3:55:1074:1415#0/1
AGGAGAGAAAGCTAATGTGCTAATTGGGCAAGGGGACGTA
+HWI-EAS443:3:55:1074:1415#0/1
ab`aabbbaaaaaaabaaa_a`aaaaaaa__ba`a^_a^_
@HWI-EAS443:3:55:1080:534#0/1
CAAAAGGAGAGAAAGCTAATGTGCTAATTGGGCAAGGGGA
+HWI-EAS443:3:55:1080:534#0/1
^¥^`[ZZ_^baa]bb]aaabbb`ZbX`bX^ba`T`^¥SaZ
@HWI-EAS443:3:55:1112:543#0/1
GAGCAATCTTGCAAAAGGAGAGAAAGCTAATGTGCTAATT
+HWI-EAS443:3:55:1112:543#0/1
aaa_aa_`aaa_]]`a_a^`aa``^`Za_^`a^_]__¥`_
@HWI-EAS443:3:55:1189:1226#0/1
ATGAACTGAGCAATCTTGCAAAAGGAGAGAAAGCTAATGT
```

```
+HWI-EAS443:3:55:1189:1226#0/1
aabaaabaa`aaaa_a_b^_`a`aa`baaa_``]a_``b^
@HWI-EAS443:3:55:1284:1652#0/1
GAGAAAGCTAATGTGCTAATTGGGCAAGGGGACGTAGTGT
+HWI-EAS443:3:55:1284:1652#0/1
Y_aaZ_a^`bYa`W]_aa`bbbb`Y^Zaaaa^]a[``X_W
@HWI-EAS443:3:55:1334:1942#0/1
GAAAGCTAATGTGCTAATTGGGCAAGGGGACGTAGTGTTG
+HWI-EAS443:3:55:1334:1942#0/1
W^aba_¥a^a[aaabbbbb`[¥^]aaZ`¥J]a^`VYa]a`
@HWI-EAS443:3:55:1335:1099#0/1
TGCAAAAGGAGAGAAAGCTAATGTGCTAATTGGGCAAGGG
+HWI-EAS443:3:55:1335:1099#0/1
S]ZS_`a`a`]`a`UYaVa_aabN¥SV¥_^_¥a_^Ja]a`
@HWI-EAS443:3:55:1344:1113#0/1
GAGAAAGCTAATGTGCTAATTGGGCAAGGGGACGTAGTGT
+HWI-EAS443:3:55:1344:1113#0/1
abbVS__G_b]aRMWTb]aab`aa]¥J_XRX`H_a_]``a
@HWI-EAS443:3:55:1346:839#0/1
AGCACATTAGCTTTCTCTCCTTTTGCAAGATTGCTCAGTT
+HWI-EAS443:3:55:1346:839#0/1
ZV_Z¥Vb^aa]^b^T^X¥HL_K``NT[R_]]K`^¥IWYaa
@HWI-EAS443:3:55:1550:415#0/1
GAGCAATCTTGCAAAAGGAGAGAAAGCTAATGTGCTAATT
+HWI-EAS443:3:55:1550:415#0/1
^baJabbbbabababa]^^bbbbbb`ab`ab`a`aa`aba
@HWI-EAS443:3:55:1643:1581#0/1
CCAATTAGCACATTAGCTTTCTCTCCTTTTGCAAGATTGC
+HWI-EAS443:3:55:1643:1581#0/1
a^ab`bb_`_Yaabab¥_`_]_T¥XZ]aaaa[aaa`aab^
@HWI-EAS443:3:55:169:594#0/1
GAGCAATCTTGCAAAAGGAGAGAAAGCTAATGTGCTAATT
+HWI-EAS443:3:55:169:594#0/1
WW`_¥``bbb`b^`^W`a_Z_ba^¥_]_]`¥b_aaa`[`_
@HWI-EAS443:3:55:170:547#0/1
GAAAGCTAATGTGCTAATTGGGCAAGGGGACGTAGTGTTG
+HWI-EAS443:3:55:170:547#0/1
abba`aaa¥bbababaaabbaaa`aaaaa^]b^_b^b_ab
@HWI-EAS443:3:55:1745:1032#0/1
GAACTGAGCAATCTTGCAAAAGGAGAGAAAGCTAATGTGC
+HWI-EAS443:3:55:1745:1032#0/1
^`babb¥^abababaa`aaaaa_]aab``aZ`a``ab]a_
@HWI-EAS443:3:55:1754:1475#0/1
GAGCAATCTTGCAAAAGGAGAGAAAGCTAATGTGCTAATT
+HWI-EAS443:3:55:1754:1475#0/1
abZbb]aO_bab¥_PVZ¥]`aa]]_¥a[aaa]V]]a]]]_
@HWI-EAS443:3:55:1765:1057#0/1
CATCAATGAACTGAGCAATCTTGCAAAAGGAGAGAAAGCT
+HWI-EAS443:3:55:1765:1057#0/1
abbbaabbababS¥Jaaaaaba`_b__]a^_[a_`_a`^a
```

```
@HWI-EAS443:3:55:178:1777#0/1
CATCAATGAACTGAGCAATCTTGCAAAAGGAGAGAAAGCT
+HWI-EAS443:3:55:178:1777#0/1
aa¥a`a^`a]aa_]_aa``_a`_bQQ]W^]X]^W¥ZZ^Z^
@HWI-EAS443:3:55:179:1592#0/1
CCCTTGCCCAATTAGCACATTAGCTTTCTCTCCTTTTGCA
+HWI-EAS443:3:55:179:1592#0/1
abb`baab^ba`baabbbabbbaaa`baaa`a`aaab^aa
@HWI-EAS443:3:55:306:1160#0/1
GAGCAATCTTGCAAAAGGAGAGAAAGCTAATGTGCTAATT
+HWI-EAS443:3:55:306:1160#0/1
Z[a¥`¥_¥a`T`]`_^^¥bba¥a___Y_^abbaaa_W_bb
@HWI-EAS443:3:55:351:243#0/1
TCCCCTTGCCCAATTAGCACATTAGCTTTCTCTCCTTTTG
+HWI-EAS443:3:55:351:243#0/1
Ya^_]`__Z¥V____V`^ZT^`a]`T][a`_[]UJ[Y]YV
@HWI-EAS443:3:55:456:1290#0/1
CATCAATGAACTGAGCAATCTTGCAAAAGGAGAGAAAGCT
+HWI-EAS443:3:55:456:1290#0/1
ababbbb]a_aa_a^a[aaaaabaaa``aa_aaa`_a`^a
@HWI-EAS443:3:55:500:952#0/1
CAATCTTGCAAAAGGAGAGAAAGCTAATGTGCTAATTGGG
+HWI-EAS443:3:55:500:952#0/1
aaaaaaaURX]HR[a`_ab][OTLbaa]a[¥XbZY_a]YN
@HWI-EAS443:3:55:516:1655#0/1
GAACTGAGCAATCTTGCAAAAGGAGAGAAAGCTAATGTGC
+HWI-EAS443:3:55:516:1655#0/1
`¥U]^¥U^`_T¥``^^__]``_U[Z^a`¥¥^``]ZZ]XY_
@HWI-EAS443:3:55:547:834#0/1
AGCTAATGTGCTAATTGGGCAAGGGGACGTAGTGTTGGTA
+HWI-EAS443:3:55:547:834#0/1
^bbbUbb]abbba^bb¥bba]abbba_ab`[a`baaa`M_
@HWI-EAS443:3:55:601:48#0/1
GCATCAATGAACTGAGCAATCTTGCAAAAGGAGAGAAAGC
+HWI-EAS443:3:55:601:48#0/1
abaaaaabb^`aaa_QZa__```a]_^¥_a`_a^`_X]aW
@HWI-EAS443:3:55:669:811#0/1
CCAATTAGCACATTAGCTTTCTCTCCTTTTGCAAGATTGC
+HWI-EAS443:3:55:669:811#0/1
NaVR`b`UWNYZbab¥^ZbZ¥`Z^_ba][aaab^a[a[`Y
@HWI-EAS443:3:55:680:608#0/1
GCATCAATGAACTGAGCAATCTTGCAAAAGGAGAGAAAGC
+HWI-EAS443:3:55:680:608#0/1
a`¥^][]``¥[¥[VXa¥_`_a__aZXUST]_]`Y_^¥¥`]
@HWI-EAS443:3:55:734:1890#0/1
GAGCAATCTTGCAAAAGGAGAGAAAGCTAATGTGCTAATT
+HWI-EAS443:3:55:734:1890#0/1
aaaa^_RK`a`R[a_aaaaa`aaa_]ZZ``_XTa_`a_`]
@HWI-EAS443:3:55:855:860#0/1
GAGCAATCTTGCAAAAGGAGAGAAAGCTAATGTGCTAATT
```

+HWI-EAS443:3:55:855:860#0/1
``baa_X]`]a¥WY]Z^``]aa_ZZaa]^[[aZaX]RR]_
@HWI-EAS443:3:55:864:797#0/1
CGTCCCCTTGCCCAATTAGCACATTAGCTTTCTCTCCTTT
+HWI-EAS443:3:55:864:797#0/1
ab__aaabbbaaabbbbab_aabbaaa_bab_b`b_abaa
@HWI-EAS443:3:55:905:683#0/1
ATCAATGAACTGAGCAATCTTGCAAAAGGAGAGAAAGCTA
+HWI-EAS443:3:55:905:683#0/1
U`R_a_MW_U¥W_[Q[aa]ZS]J`ZUabb`^abbZU`V`¥
@HWI-EAS443:3:55:936:874#0/1
GCAAAAGGAGAGAAAGCTAATGTGCTAATTGGGCAAGGGG
+HWI-EAS443:3:55:936:874#0/1
`a`a_R_a_baaaa_a^a`[ab``_a^^a`_`a]¥_a___
@HWI-EAS443:3:55:942:1707#0/1
AATGAACTGAGCAATCTTGCAAAAGGAGAGAAAGCTAATG
+HWI-EAS443:3:55:942:1707#0/1
]SSW`_`WLRaTR`X]V`bb`a_abb^aa_`]a__b`Ybb
@HWI-EAS443:3:56:1068:801#0/1
CATCAATGAACTGAGCAATCTTGCAAAAGGAGAGAAAGCT
+HWI-EAS443:3:56:1068:801#0/1
`babaa`ab]bbb`babbbabb`_ab_^`¥^bbbbabb]b
@HWI-EAS443:3:56:1089:93#0/1
CAATTAGCACATTAGCTTTCTCTCCTTTTGCAAGATTGCT
+HWI-EAS443:3:56:1089:93#0/1
abbbbababaabbababbb_a]b^^abaa_```baaaa¥a
@HWI-EAS443:3:56:1107:605#0/1
AAAGGAGAGAAAGCTAATGTGCTAATTGGGCAAGGGGACG
+HWI-EAS443:3:56:1107:605#0/1
aaaaa^a[a``]a`b[]a_[a]__K¥___`aaY`^`aS^a
@HWI-EAS443:3:56:1217:694#0/1
GCATCAATGAACTGAGCAATCTTGCAAAAGGAGAGAAAGC
+HWI-EAS443:3:56:1217:694#0/1
U`]_TT¥_b`Vb¥V_a[``aaaaaab`b[aXab`ba`]a_
@HWI-EAS443:3:56:1233:466#0/1
GAGCAATCTTGCAAAAGGAGAGAAAGCTAATGTGCTAATT
+HWI-EAS443:3:56:1233:466#0/1
a_aaabbabbbbaaaaaa_abbba`a`aaabb_a_aaaba
@HWI-EAS443:3:56:1386:1480#0/1
GAAAGCTAATGTGCTAATTGGGCAAGGGGACGTAGTGTTG
+HWI-EAS443:3:56:1386:1480#0/1
abbaa_ab`_`Z^]`b``^`ZTRVH^aa_[[_GY`Ja_^Z
@HWI-EAS443:3:56:17:1268#0/1
GAACTGAGCAATCTTGCAAAAGGAGAGAAAGCTAATGTGC
+HWI-EAS443:3:56:17:1268#0/1
`bbbbbbbb`abbbbbab_^Ya`aaaa`aaaaaa`ab_a_
@HWI-EAS443:3:56:235:573#0/1
CATCAATGAACTGAGCAATCTTGCAAAAGGAGAGAAAGCT
+HWI-EAS443:3:56:235:573#0/1
a_aaa]a__abaa_a_aa`_aaa]^^]I`_ZaaaaURa^`

```
@HWI-EAS443:3:56:331:1293#0/1
AGGAGAGAAAGCTAATGTGCTAATTGGGCAAGGGGACGTA
+HWI-EAS443:3:56:331:1293#0/1
aa`abbababa[T¥`abIb`__[baaaaaaaaaaaZ^a¥^
@HWI-EAS443:3:56:372:278#0/1
GTCCCCTTGCCCAATTAGCACATTAGCTTTCTCTCCTTTT
+HWI-EAS443:3:56:372:278#0/1
```¥aa__aa`aaaaa`[a``__``_^``]^_`___`^__
@HWI-EAS443:3:56:433:1783#0/1
GAGCAATCTTGCAAAAGGAGAGAAAGCTAATGTGCTAATT
+HWI-EAS443:3:56:433:1783#0/1
a_XZ^WQXZ`baZWP_abZW_PW^RWOaWP_XHb_aaaaX
@HWI-EAS443:3:56:582:1026#0/1
ATCTTGCAAAAGGAGAGAAAGCTAATGTGCTAATTGGGCA
+HWI-EAS443:3:56:582:1026#0/1
a`baa`a``aabaTa`aba_a`a_]aaYa_``___]a`^^
@HWI-EAS443:3:56:60:183#0/1
GAAAGCTAATGTGCTAATTGGGCAAGGGGACGTAGTGTTG
+HWI-EAS443:3:56:60:183#0/1
`baaaaaX^`a[a`a]`a`aaa^][``a_R[aTP^V`Y^`
@HWI-EAS443:3:56:650:914#0/1
AAGCATCAATGAACTGAGCAATCTTGCAAAAGGAGAGAAA
+HWI-EAS443:3:56:650:914#0/1
Y[S_ZW]V¥[[ZZ`_XQ¥Z_WXO`Z^XZYUTP¥_^¥W^[T
@HWI-EAS443:3:56:739:1393#0/1
GAGCAATCTTGCAAAAGGAGAGAAAGCTAATGTGCTAATT
+HWI-EAS443:3:56:739:1393#0/1
^X¥_^[bbaaaa¥OXa¥]R[`Ta[[_^`XaaU^]R¥_¥]_
@HWI-EAS443:3:56:794:909#0/1
GAACTGAGCAATCTTGCAAAAGGAGAGAAAGCTAATGTGC
+HWI-EAS443:3:56:794:909#0/1
aa_X`bab``aa`aaa^aa¥_aaW```aX`a¥``]`a_a¥
@HWI-EAS443:3:56:833:1882#0/1
ATCTTGCAAAAGGAGAGAAAGCTAATGTGCTAATTGGGCA
+HWI-EAS443:3:56:833:1882#0/1
T^Y[]`WbaaH`a]^a`ba_a[``IJ__ba`a_aY_a^X_
@HWI-EAS443:3:56:912:699#0/1
GAAAGCTAATGTGCTAATTGGGCAAGGGGACGTAGTGTTG
+HWI-EAS443:3:56:912:699#0/1
ab]aaab`TbbYaOaba`aaa_U__aaba_ab]Q_Va^aa
@HWI-EAS443:3:56:92:499#0/1
GAAAGCTAATGTGCTAATTGGGCAAGGGGACGTAGTGTTG
+HWI-EAS443:3:56:92:499#0/1
aaaaaaaa`aa_aaa_`a`aa`a`_aaa_[^aY^`]`]_^
@HWI-EAS443:3:56:971:1999#0/1
ATCTTGCAAAAGGAGAGAAAGCTAATGTGCTAATTGGGCA
+HWI-EAS443:3:56:971:1999#0/1
aUVWT_YaWSb_]U]^XRY[`Q_^R_`Y^__S_^X[aUGU
@HWI-EAS443:3:56:971:734#0/1
GCATCAATGAACTGAGCAATCTTGCAAAAGGAGAGAAAGC
```
```

```
+HWI-EAS443:3:56:971:734#0/1
a``a_a_``_a_aa_aX^__`a_]__^_]a_^`^``_^`]
@HWI-EAS443:3:57:100:1420#0/1
AGCAATCTTGCAAAAGGAGAGAAAGCTAATGTGCTAATTG
+HWI-EAS443:3:57:100:1420#0/1
aa]_Y[aaaaa`¥¥QW]`_`aaa_aa`V^`^U_Z``_a_`
@HWI-EAS443:3:57:1169:1239#0/1
AAGCTAATGTGCTAATTGGGCAAGGGGACGTAGTGTTGGT
+HWI-EAS443:3:57:1169:1239#0/1
aaaaaaaaaaa`aa`aaaa^____a__]_a¥_aZa^_¥`U
@HWI-EAS443:3:57:1234:449#0/1
AGAGAAAGCTAATGTGCTAATTGGGCAAGGGGACGTAGTG
+HWI-EAS443:3:57:1234:449#0/1
abbb`a`babaabb`a^baaaaaab¥_aabbaa¥a``b^b
@HWI-EAS443:3:57:1244:664#0/1
CAAAAGGAGAGAAAGCTAATGTGCTAATTGGGCAAGGGGA
+HWI-EAS443:3:57:1244:664#0/1
`bb`[aa`^_a``Z`_baaabab¥aaab`a`_TZaba``^
@HWI-EAS443:3:57:1318:1151#0/1
GAACTGAGCAATCTTGCAAAAGGAGAGAAAGCTAATGTGC
+HWI-EAS443:3:57:1318:1151#0/1
_O[U[a`a^baUQ]Z_U]YVW_ZH`W_]GO^¥_NU]]U_¥
@HWI-EAS443:3:57:1454:384#0/1
GCAAAAGGAGAGAAAGCTAATGTGCTAATTGGGCAAGGGG
+HWI-EAS443:3:57:1454:384#0/1
a[`_]]a`]a^_`^`_]]^_]aUUU]^^^^`^_Y]Y_]XY
@HWI-EAS443:3:57:150:1880#0/1
GAAAGCTAATGTGCTAATTGGGCAAGGGGACGTAGTGTTG
+HWI-EAS443:3:57:150:1880#0/1
Y[b¥MaZ^_`¥]OQ_U_Z_TLZQ]TXYBBBBBBBBBBBBB
@HWI-EAS443:3:57:1553:1528#0/1
GAGCAATCTTGCAAAAGGAGAGAAAGCTAATGTGCTAATT
+HWI-EAS443:3:57:1553:1528#0/1
Y`aabbbbbbbbbabbba`bababbbabbbbbababaabb
@HWI-EAS443:3:57:161:1891#0/1
GAAAGCTAATGTGCTAATTGGGCAAGGGGACGTAGTGTTG
+HWI-EAS443:3:57:161:1891#0/1
_baa`aab`aa_a_aaaaa¥Vaa_a^¥a[_^`]^aV`W``
@HWI-EAS443:3:57:1635:1722#0/1
GAACTGAGCAATCTTGCAAAAGGAGAGAAAGCTAATGTGC
+HWI-EAS443:3:57:1635:1722#0/1
a`b_bb¥bbaabbbabXa`a_¥babbbaababa``baUba
@HWI-EAS443:3:57:203:1578#0/1
GAACTGAGCAATCTTGCAAAAGGAGAGAAAGCTAATGTGC
+HWI-EAS443:3:57:203:1578#0/1
aabba_aabbaab``]a`aaaaa_aaaa`Z^_aR`aa]`^
@HWI-EAS443:3:57:206:69#0/1
GAGCAATCTTGCAAAAGGAGAGAAAGCTAATGTGCTAATT
+HWI-EAS443:3:57:206:69#0/1
_JZ_ZY_¥ab`YY]XVb][`PaXRaa_¥^aY^U`_Y]_][
```

```
@HWI-EAS443:3:57:237:592#0/1
CCTTGCCCAATTAGCACATTAGCTTTCTCTCCTTTTGCAA
+HWI-EAS443:3:57:237:592#0/1
abbabbbbbbbaaabbbabbbbaaabababbabaaaaa]X
@HWI-EAS443:3:57:375:1438#0/1
CATCAATGAACTGAGCAATCTTGCAAAAGGAGAGAAAGCT
+HWI-EAS443:3:57:375:1438#0/1
`ababbba_bbbbaabb`babaaabababaabbabbaa]b
@HWI-EAS443:3:57:429:84#0/1
GAAAGCTAATGTGCTAATTGGGCAAGGGGACGTAGTGTTG
+HWI-EAS443:3:57:429:84#0/1
``abbabaaab]a_a_`a`aaa[]Xabba]`aUR`Za]`a
@HWI-EAS443:3:57:558:800#0/1
AAGCATCAATGAACTGAGCAATCTTGCAAAAGGAGAGAAA
+HWI-EAS443:3:57:558:800#0/1
RZ___¥]TR^__a_`_[]VV¥¥]]S_^[¥X]_XV]]`a^`
@HWI-EAS443:3:57:620:341#0/1
CTTGCAAAAGGAGAGAAAGCTAATGTGCTAATTGGGCAAG
+HWI-EAS443:3:57:620:341#0/1
abbab_bbabb`aabbb_aabbabbab`bbababaaa_ab
@HWI-EAS443:3:57:621:1489#0/1
CTTGCAAAAGGAGAGAAAGCTAATGTGCTAATTGGGCAAG
+HWI-EAS443:3:57:621:1489#0/1
ab]RZ¥]____[U¥ab`^_]aaa`_[_a_a_^`]_a`^VZ
@HWI-EAS443:3:57:627:73#0/1
CATCAATGAACTGAGCAATCTTGCAAAAGGAGAGAAAGCT
+HWI-EAS443:3:57:627:73#0/1
aa`aa[`bX¥`ababa^¥^__Y]`_`ZWaaU`Za`[Sa]^
@HWI-EAS443:3:57:744:1301#0/1
CATCAATGAACTGAGCAATCTTGCAAAAGGAGAGAAAGCT
+HWI-EAS443:3:57:744:1301#0/1
^^_`ZUbb^b^`a_`bbaa`b`_a¥aa_bbaaaaba`a`a
@HWI-EAS443:3:57:750:1386#0/1
AGCTAATGTGCTAATTGGGCAAGGGGACGTAGTGTTGGTA
+HWI-EAS443:3:57:750:1386#0/1
````^bbb]bbb`baabb_aabbaaaaba^aa]`ab`bYa
@HWI-EAS443:3:57:751:1757#0/1
GTCCCCTTGCCCAATTAGCACATTAGCTTTCTCTCCTTTT
+HWI-EAS443:3:57:751:1757#0/1
a`U^`]a__`[[^aaZWZ`b`a`¥¥VO[a_aaYa_SX`¥`
@HWI-EAS443:3:57:853:1193#0/1
GTCCCCTTGCCCAATTAGCACATTAGCTTTCTCTCCTTTT
+HWI-EAS443:3:57:853:1193#0/1
a`bbbabaaa``aabaaa__`abaa_abba_aa`a^aaa`
@HWI-EAS443:3:57:902:149#0/1
GAAAGCTAATGTGCTAATTGGGCAAGGGGACGTAGTGTTG
+HWI-EAS443:3:57:902:149#0/1
a`bbbabaabaaaab`aaaaa`a`^aaaa__b_^b^a^aa
@HWI-EAS443:3:58:1067:1804#0/1
GAAAGCTAATGTGCTAATTGGGCAAGGGGACGTAGTGTTG
```
```

```
+HWI-EAS443:3:58:1067:1804#0/1
ababaGbba_[PYab_aaa_`XM¥XYa_[XP`WZ¥ETUT`
@HWI-EAS443:3:58:1076:1663#0/1
[truncated: 626,458 more chars]
